# ePoster

**DOI:** 10.1111/ene.70629

**Published:** 2026-06-28

## Saturday, June 27 2026

## Ageing and Dementia 1

## EPO‐0001

### Sodium channel blockers are associated with reduced dementia risk in late‐onset unexplained epilepsy

#### C. Ferreira‐Atuesta ^1^; K. Schubert^1^; X. Tai^2^; T. Hedden^3^; J. Zelano^4^; T. Marson^5^; G. Lip^6^; G. Mbizvo^7^; M. Galovic^1^

##### ^1^Department of Neurology, Clinical Neuroscience Center, University Hospital Zurich and University of Zurich, Zurich, Switzerland; ^2^Nuffield Department of Clinical Neurosciences, University of Oxford, Oxford, UK; ^3^Department of Neurology, Icahn School of Medicine at Mount Sinai, New York, USA; ^4^Department of Clinical Neuroscience, Institute of Neuroscience and Physiology, Sahlgrenska Academy, University of Gothenburg, Gothenburg, Sweden; ^5^Department of Pharmacology & Therapeutics, Institute of Systems, Molecular and Integrative Biology, Faculty of Health and Life Sciences, University of Liverpool, Liverpool, UK; ^6^Liverpool Centre for Cardiovascular Science, University of Liverpool, Liverpool, UK; ^7^Liverpool Interdisciplinary Neuroscience Centre, University of Liverpool, Liverpool, UK

**Background and aims:** Late‐onset unexplained epilepsy is associated with increased dementia risk. Network hyperexcitability, central to seizure generation, has been implicated in amyloid‐β release and tau propagation, suggesting a mechanistic link. Under this model, antiseizure medications that more effectively suppress excitability may modify dementia risk, but comparative neuroprotective effects across commonly used ASMs remain unclear.

**Methods:** We emulated a target trial using real‐world healthcare data from the TriNetX platform to compare dementia outcomes across ASM monotherapy strategies in patients with newly diagnosed LOUE. Confounding addressed with propensity‐score matching. Cox proportional hazards models estimated hazard ratios (HRs) with 95% confidence intervals (CIs) for incident all‐cause dementia and Alzheimer's disease. The cohort included 24,262 patients treated with SV2A ligands, sodium channel blockers, multi‐mechanism agents, calcium channel blockers, or GABAergic agents. Findings were externally validated in a high‐risk Down syndrome cohort and the National Alzheimer's Coordinating Center (NACC) dataset.

**Results:** Sodium channel–blocking ASMs were associated with lower dementia risk compared with SV2A ligands, including a 27% reduction in all‐cause dementia (HR 0.73, CI 0.61–0.88) and a 36% reduction in Alzheimer's disease (HR 0.66, CI 0.49–0.88). Phenytoin and carbamazepine showed the strongest inverse associations with dementia, lamotrigine was associated with lower mortality. Associations were consistent across earlier cognitive endpoints, absent for negative controls, and directionally concordant in external validation.

**Figure:**
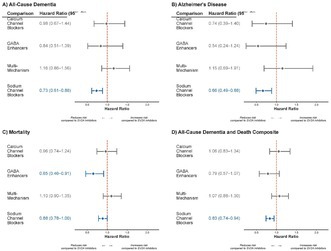
**FIGURE 1** Hazard ratios (95% CI) for dementia and mortality outcomes comparing mechanism‐based antiseizure medication groups versus SV2A inhibitors. Panels show: (A) All‐cause dementia, (B) Alzheimer's disease, (C) Mortality, (D) All‐cause dementia + death. Diamond.

**Figure:**
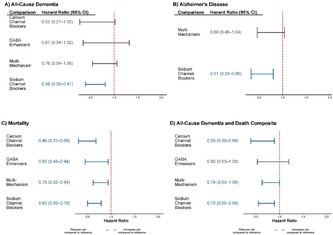
**FIGURE 2** Hazard ratios (95% CI) for dementia and mortality outcomes in the Down syndrome cohort comparing mechanism groups vs SV2A inhibitors. Panels show: (A) All‐cause dementia, (B) Alzheimer's disease, (C) Mortality, (D) All‐cause dementia + death. Diamond mark.

**Conclusion:** Sustained suppression of neuronal hyperexcitability by specific ASMs appears to modify downstream neurodegenerative risk. These findings nominate sodium channel blockers as candidates for repurposing in dementia prevention and support prospective randomized trials targeting early network excitability.

**Disclosure:** Nothing to disclose.

## EPO‐0002

### Hearing aid use and risk of dementia in adults with late‐onset epilepsy, stroke, or diabetes and comorbid hearing loss

#### C. Ferreira‐Atuesta ^1^; K. Schubert^1^; H. Jung^1^; T. Marson^2^; G. Lip^3^; G. Mbizvo^4^; M. Galovic^1^

##### ^1^Department of Neurology, Clinical Neuroscience Center, University Hospital Zurich and University of Zurich, Zurich, Switzerland; ^2^Department of Pharmacology & Therapeutics, Institute of Systems, Molecular and Integrative Biology, Faculty of Health and Life Sciences, University of Liverpool, Liverpool, UK; ^3^Liverpool Interdisciplinary Neuroscience Centre, University of Liverpool, Liverpool, UK; ^4^Liverpool Centre for Cardiovascular Science, University of Liverpool, Liverpool John Moores University and Liverpool Heart & Chest Hospital, Liverpool, UK

**Background and aims:** Hearing loss is a major modifiable risk factor for dementia, yet its impact on high‐risk neurological populations remains poorly characterized. We investigated whether hearing aid (HA) use reduces dementia incidence in adults with late‐onset epilepsy (LOE), stroke, or diabetes with comorbid hearing loss.

**Methods:** Using TriNetX, a federated electronic health record network, we conducted retrospective cohort studies emulating target trials. We analyzed propensity score‐matched cohorts of 1,093 LOE pairs, 2,363 stroke pairs, and 14,934 diabetes pairs (all aged ≥ 55 years). Primary outcomes included incident Alzheimer's disease (AD) and all‐cause dementia over a median follow‐up of 4.2–5.1 years.

**Results:** HA use was associated with significant reductions in AD across all cohorts: LOE (HR 0.52, 95% CI 0.38–0.71), stroke (HR 0.59, 0.37–0.95), and diabetes (HR 0.69, 0.53–0.90). All‐cause dementia risk was also significantly reduced in LOE (HR 0.50, 0.32–0.79) and diabetes (HR 0.81, 0.70–0.94). Negative control outcomes (glaucoma, frailty) showed no consistent protective patterns. Sensitivity analyses in LOE patients indicated that significant protective effects for AD persisted even when HA intervention was initiated after five years of hearing loss onset.

**Figure:**
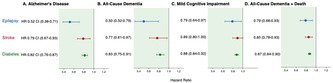
**FIGURE 1** Hazard ratios (95% CI) for outcomes across the epilepsy, stroke and diabetes cohorts.

**Conclusion:** Auditory intervention is a scalable, low‐risk strategy for dementia prevention in neurologically vulnerable populations. The protective effects in these groups may exceed those observed in the general population, likely by reducing cognitive load and potentially interrupting shared neurodegenerative pathways.

**Disclosure:** Nothing to disclose.

## EPO‐0003

### Sleep‐related experiences inflate hallucination scores across neurodegenerative disorders

#### D. Urso^1^; V. Gnoni^1^; A. Giugno^1^; M. Caccamo^1^; A. Nanni^1^; E. Rollo ^1^; G. Logroscino^1^; G. Foffani^2^

##### ^1^Center for Neurodegenerative Diseases and the Aging Brain, Department of Clinical Research in Neurology, University of Bari ‘Aldo Moro’, “Pia Fondazione Cardinale G. Panico”, Tricase, Lecce, Italy; ^2^Reina Sofia Foundation Alzheimer Center, CIEN Foundation, ISCIII, Madrid, Spain

**Background and aims:** Hallucinations are widely used to support diagnosis, prognosis, and treatment decisions in neurodegenerative disorders. However, standard clinical scales do not distinguish hallucinations occurring during full wakefulness from perceptual experiences emerging at sleep–wake transitions (hypnagogic and hypnopompic phenomena), which are non‐psychotic by definition.

**Methods:** We developed the Hypno/Awake Hallucination Assessment (HAHA), a two‐item extension of the MDS‐UPDRS hallucination item, and the Sleep‐Related Experiences Questionnaire (SREQ), a 7‐item scale capturing dream and sleep–wake boundary phenomena. These were administered in 73 patients with Parkinson's disease and in 177 consecutive patients from a tertiary neurodegenerative disorders clinic. Bayesian multiple regression tested whether sleep‐related hallucinations contributed to standard hallucination scores independently of awake hallucinations, and whether they were specifically associated with sleep‐related experiences.

**Results:** Across both cohorts, sleep‐related hallucinations showed extreme evidence of contributing to standard hallucination scores even after accounting for awake hallucinations. Sleep‐related hallucinations, but not awake hallucinations, were strongly associated with the frequency of sleep‐related experiences. Sleep‐related experiences differed across diseases with extreme evidence, being more frequent in Lewy‐body disorders than in Alzheimer's disease and frontotemporal lobar degeneration syndromes and correlated with REM sleep behavior disorder.

**Conclusion:** Standard hallucination scales conflate awake hallucinations with sleep‐related perceptual phenomena, leading to systematic inflation of hallucination estimates, particularly in Lewy‐body disorders. Disentangling these mechanisms is essential for accurate diagnosis, appropriate treatment, and mechanistic interpretation of hallucinations.

**Disclosure:** Nothing to disclose.

## EPO‐0004

### Volumetric analysis of hippocampal subfields and cognitive change in Alzheimer's dementia and mild cognitive impairment: A prospective cohort study

#### D. Jung; T. Kim

##### Department of Neurology, Incheon St. Mary's Hospital, The Catholic University of Korea, Seoul, Republic of Korea

**Background and aims:** Hippocampal subfield atrophy demonstrates differential vulnerability in Alzheimer's disease (AD), but the clinical relevance of specific subfield volume ratios for predicting cognitive decline remains unclear. This study examined associations between hippocampal subfield volume ratios and longitudinal cognitive changes in AD dementia and amnestic mild cognitive impairment (aMCI).

**Methods:** Fifty‐four participants with AD dementia or aMCI underwent baseline cognitive assessments, high‐resolution T1‐weighted MRI, and follow‐up cognitive evaluations at two years. Hippocampal subfield volumes were automatically segmented and normalized to total hippocampal volume and total intracranial volume (TIV). Linear regression analyses examined associations between standardized subfield ratios and changes in Mini‐Mental State Examination (MMSE) and Montreal Cognitive Assessment (MoCA) scores.

**Figure:**
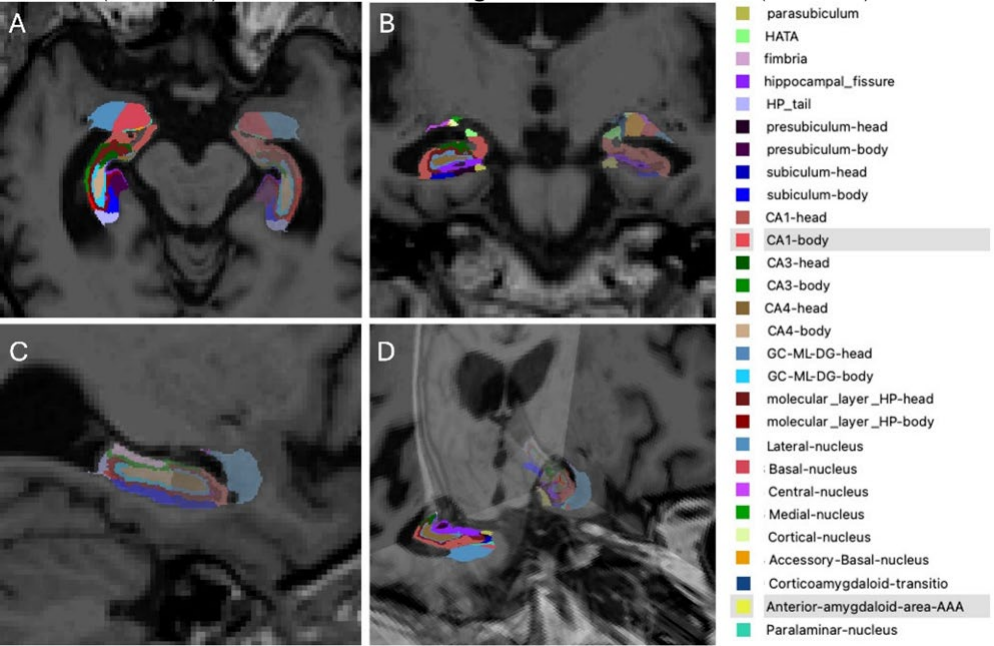
**FIGURE 1** Representative segmentation of hippocampal and amygdala subfields on high‐resolution T1‐weighted MRI. (A) Axial, (B) coronal, (C) sagittal, and (D) three‐dimensional views with color‐coded subregions.

**Results:** The volume ratios of the fimbria and cornu ammonis 3 (CA3) demonstrated the strongest associations with changes in MMSE (fimbria: B = 1.95, SE = 0.51, *p* < 0.001; CA3: B = 1.75, SE = 0.52, *p* = 0.002) and MoCA (fimbria: B = 1.818, SE = 0.559, *p* = 0.002; CA3: B = 1.58, SE = 0.57, *p* = 0.008) scores. Similar patterns emerged with TIV normalization. Associations remained significant after adjusting for age, education, baseline cognition, ApoE4 genotype, and medication use.

**Figure:**
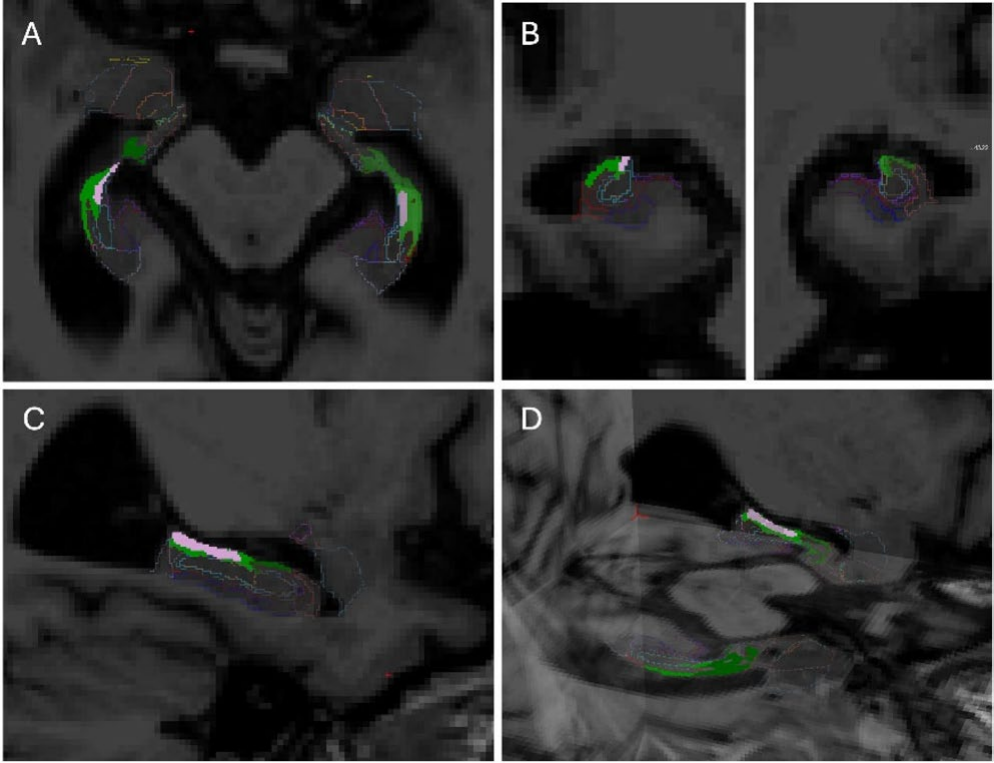
**FIGURE 2** Hippocampal subfields demonstrating associations with cognitive decline. (A‐D) Multiple viewing planes showing color‐filled regions with statistically significant associations (green for CA3, pink for fimbria).

**Figure:**
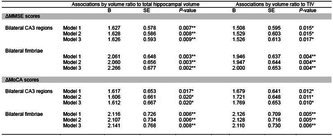
**TABLE 1** Multivariable associations of bilateral CA3 and fimbria volume ratios (per 1‐SD) with changes in cognitive scores (MMSE and MoCA).

**Conclusion:** Among hippocampal subfields, the fimbria and CA3 regions demonstrate stronger associations with longitudinal cognitive decline in AD and aMCI compared to other subfield structures. These in vivo findings may serve as useful biomarkers for risk assessment, though further validation studies are needed to confirm their clinical utility.

**Disclosure:** Nothing to disclose.

## EPO‐0005

### A 48‐month analysis of the lecanemab clarity AD open‐label extension in the ApoE ε4 non‐carriers or heterozygotes early Alzheimer's disease population

#### L. Froelich ^1^; S. Dhadda^2^; D. Li^2^; M. Kanekiyo^2^; A. Goodwin^2^; M. Hodgkinson^3^; S. Trafford^3^; S. Hersch^3^; M. Irizarry^2^; L. Kramer^2^

##### ^1^Medical Faculty Mannheim, University of Heidelberg, Central Institute of Mental Health, Mannheim, Germany; ^2^Eisai Inc, Nutley, USA; ^3^Eisai Europe Ltd, Hatfield, UK

**Background and aims:** Lecanemab, an Abeta‐directed antibody, showed meaningful delay in disease progression and biological effects consistent with disease modification in Clarity AD. Herein, we present the 48‐month results in ApoE ε4 non‐carriers or heterozygotes population from the ongoing Clarity AD open‐label extension (OLE) study.

**Methods:** Clarity AD is an 18‐month, randomized study (Core) with a subsequent OLE phase in individuals with early AD. Safety and clinical efficacy outcomes (CDR‐SB, ADAS‐Cog14, and ADCS‐MCI‐ADL) were assessed from OLE data out to 48 months. Continued lecanemab treatment in the OLE beyond the 18 months from the Core study was compared to matched historical placebo controls from Alzheimer's Disease Neuroimaging Initiative (ADNI) data and the Swedish BioFINDER1 study. Subgroup analyses were conducted for participants with no/low baseline tau.

**Results:** In the ApoE ε4 non‐carriers or heterozygotes population, 1521 participants were treated with lecanemab across the Clarity AD Core and OLE. No new safety signals were observed. ARIA rates were low and similar on placebo after 6 months. Lecanemab‐treated participants continued to accrue benefit in CDR‐SB through 48 months, with continued separation through 36 months relative to the historical controls. Results were similar for ADAS‐Cog14 and ADCS‐MCI‐ADL. Consistent rates of clinical stability or improvements were observed across assessments regardless of baseline tau levels. Lecanemab reduced relative risk of progression to next dementia stage by 55% and 62% vs ADNI and BioFINDER, respectively.

**Conclusion:** In the ApoE ε4 heterozygotes or non‐carrier subgroup of Clarity AD, lecanemab slowed decline in disease progression with expanding benefit over time.

**Disclosure:** LF reports a relationship with Anavex, Avanir/Otsuka, Biogen, BioVie, Bristol‐Myers‐Squibb, DerCampus, Eisai, Eli Lilly, FOMF, Araclon/Grifols, Janssen Cilag, Johnson&Johnson, Medical Tribune, Medfora, Medscape, Neurimmune, Neuroscios, Noselab, NovoNordisk, PharmatrophiX, Hoffmann‐LaRoche, TauRX, Schwabe, StreamUp, Vivoryon that includes: personal consulting fees. SD, DL, MK, AG, MH, ST, SH, MI, and LK are employees of Eisai.

## EPO‐0006

### Electric field modulation of amyloid‐β fibril propagation: Molecular insights supporting neuromodulation approaches in Alzheimer's disease

#### F. Toplek^1^; M. Guidetti^2^; N. Maiorana^2^; S. Marceglia ^2^; R. Capelli^1^; A. Priori^3^; C. Camilloni^1^

##### ^1^Department of Biosciences, University of Milano, Milan, Italy; ^2^”Aldo Ravelli” Center for Neurotechnology and Experimental Brain Therapeutics, Department of Health Sciences, University of Milano, Milan, Italy; ^3^”Aldo Ravelli” Center for Neurotechnology and Experimental Brain Therapeutics, Department of Health Sciences, University of Milano, Milan, Italy; Clinical Neurology Unit, “Azienda Socio‐Sanitaria Territoriale Santi Paolo E, Milan, Italy

**Background and aims:** Amyloid‐β 42 (Aβ42) fibril accumulation is a central pathological hallmark of Alzheimer's disease and a primary target of disease‐modifying therapies. Non‐invasive brain stimulation techniques, such as transcranial direct current stimulation (tDCS), are increasingly explored as complementary interventions, but their direct biological targets remain unclear. This study aimed to investigate whether electric fields, conceptually analogous to those induced by tDCS, can modulate Aβ42 fibril structure and propagation at the molecular level.

**Methods:** We performed molecular simulations on a brain‐derived Aβ42 fibril model composed of 66 monomers arranged in 33 layers, reconstructed from cryo‐EM data and including disordered N‐terminal regions. Static electric fields ranging from 0 to 0.2 V/nm were applied under different structural restraint conditions to separately assess effects on fibril surface properties and fibril elongation. Simulations were run for 1–2 μs in triplicate.

**Results:** Electric fields induced a displacement of the disordered N‐terminal regions that saturated at ~0.08 V/nm, reducing their interactions with the fibril core and modifying surface properties associated with amyloid proliferation. In addition, increased structural fluctuations were observed at fibril ends, particularly in residues involved in elongation, indicating a destabilizing effect on fibril growth. These effects were systematic and increased with field strength.

**Conclusion:** These findings provide mechanistic evidence that electric fields can influence molecular processes underlying amyloid fibril propagation. Although the applied field strengths exceed those generated by clinical tDCS, the results support the biological plausibility of neuromodulation approaches as potential modifiers of amyloid pathology and encourage further experimental and clinical investigation.

**Disclosure:** Nothing to disclose.

## EPO‐0007

### OCTARIA‐AD: Optical coherence tomography angiography as a non‐invasive biomarker for ARIA in early Alzheimer's disease

#### N. Krajnc ^1^; H. Untersteiner^1^; T. Parvizi^1^; M. Bertich^2^; S. Silvaieh^1^; E. Berger‐Sieczkowski^1^; R. Wurm^1^; B. Pemp^2^; E. Stögmann^1^; G. Bsteh^1^

##### ^1^Department of Neurology, Medical University of Vienna, Vienna, Austria; ^2^Department of Ophthalmology, Medical University of Vienna, Vienna, Austria

**Background and aims:** Alzheimer's disease (AD), the leading cause of dementia worldwide, is characterized by progressive cognitive decline driven by amyloid‐β and tau pathology. Anti‐amyloid monoclonal antibodies show clinical benefit in early AD but are limited by amyloid‐related imaging abnormalities (ARIA). Current monitoring relies on resource‐intensive MRI, which may miss early microvascular changes. Optical coherence tomography (OCT) and OCT angiography (OCT‐A) offer non‐invasive assessment of retinal neuroaxonal and microvascular integrity and may reflect cerebral small‐vessel pathology.

**Methods:** OCTARIA‐AD is a monocentric, prospective observational study enrolling 60 participants aged 50–90 years: 30 patients initiating anti‐amyloid therapy and 30 age‐ and sex‐matched controls with mild cognitive impairment or mild AD. OCT and OCT‐A are performed at baseline and four follow‐ups up to 52 weeks. Outcomes include peripapillary retinal nerve fiber layer (pRNFL) and ganglion cell‐inner plexiform layer (GCIPL) thickness, vessel density and foveal avascular zone area. ARIA is assessed using routine MRI.

**Results:** As of January 25, 2026, 8 and 7 patients receiving lecanemab and donanemab have been enrolled, respectively; two were excluded due to insufficient baseline OCT image quality. The preliminary cohort comprises 13 patients (mean age 71 years [4.7], 83.3% female, 66.7% APOE heterozygotes, median MMSE 27 [range 23–30]). Mean baseline pRNFL and GCIPL thickness were 98.8 μm (8.3) and 67.2 μm (7.1), respectively. After a median follow‐up of 33 days (range 0–50), no ARIA events were observed.

**Conclusion:** OCTARIA‐AD seeks to establish OCT/OCT‐A as accessible biomarkers for ARIA risk stratification in early AD, potentially enhancing patient safety and complementing MRI monitoring.

**Disclosure:** Nothing to disclose.

## EPO‐0008

### Higher planetary health diet index scores are associated with better executive function and processing speed in older adults

#### S. Ha

##### Department of Neurology, Veterans Health Service Medical Center, Seoul, Republic of Korea

**Background and aims:** The EAT–Lancet Planetary Health Diet integrates human health with environmental sustainability. However, evidence linking its adherence, measured by the Planetary Health Diet Index (PHDI), to domain‐specific cognitive performance remains sparse.

**Methods:** We analyzed 2,636 participants aged ≥ 60 from the National Health and Nutrition Examination Survey (2003–2010). Dietary intake was assessed via two 24‐hour recalls to calculate the PHDI. Cognitive outcomes included the CERAD battery, Animal Fluency, and the Digit Symbol Substitution Test (DSST). Survey‐weighted linear regression estimated β coefficients, adjusting for sociodemographic, lifestyle, and cardiometabolic factors.

**Results:** Higher PHDI adherence was significantly associated with superior cognitive performance, characterized by a distinct domain‐specific sensitivity. In the fully adjusted model, each 10‐point higher PHDI was associated with higher scores on the DSST (β = 0.9746; *p* = 0.0012) and Animal Fluency (β = 0.3811; *p* = 0.0003). Strong dose–response patterns were observed for both executive function (*p*‐trend = 0.0008) and semantic fluency (*p*‐trend = 0.0001). While the PHDI was positively associated with CERAD immediate recall (Trials 1–3: β = 0.1767; *p* = 0.0410), associations with delayed recall were not statistically significant.

**Figure:**
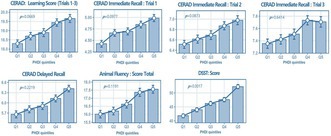
**FIGURE 1** Cognitive test scores across quintiles of the PHDI Bars show the mean cognitive test score within each PHDI quintile (Q1–Q5).

**Figure:**
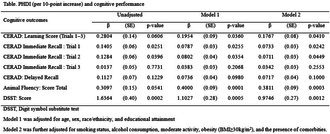
**TABLE 1** PHDI (per 10‐point increase) and cognitive performance.

**Conclusion:** Greater adherence to a planetary health‐aligned dietary pattern was associated with significantly better executive function and processing speed in older adults. These findings support the “co‐benefit” of sustainable diets for brain health, suggesting that sustainability‐aligned dietary frameworks may serve as an effective strategy for preserving cognitive resilience in an aging population.

**Disclosure:** Nothing to disclose.

## EPO‐0009

### NPTX1 differentiates idiopathic normal pressure hydrocephalus from Alzheimer's disease

#### S. Ivanovski ^1,2^; M. Kolarič^1,2^; T. Gabršček^3^; A. Emeršič^4^; R. Berlot^4,5,6^; B. Turk^1,7^; A. Sadikov^3^; D. Georgiev^3,4^; M. Fonović^1^

##### ^1^Department of Biochemistry, Molecular, and Structural Biology, Jozef Stefan Institute, Ljubljana, Slovenia; ^2^Jozef Stefan International Postgraduate School, Ljubljana, Slovenia; ^3^Artificial Intelligence Laboratory, Faculty of Computer and Information Science, University of Ljubljana, Ljubljana, Slovenia; ^4^Department of Neurology, University Medical Centre Ljubljana, Ljubljana, Slovenia; ^5^Faculty of Medicine, University of Ljubljana, Ljubljana, Slovenia; ^6^Department of Basic and Clinical Neuroscience, Institute of Psychiatry, Psychology & Neuroscience, King's College London, London, UK; ^7^Faculty of Chemistry and Chemical Technology, University of Ljubljana, Ljubljana, Slovenia

**Background and aims:** Normal pressure hydrocephalus (NPH) is a neurological syndrome characterised by dementia, gait dysfunction, urinary incontinence, and ventriculomegaly. Differentiating NPH from other dementias, including Alzheimer's disease (AD), remains challenging, and there are no cerebrospinal fluid (CSF)‐based diagnostic biomarkers specific to NPH.

**Methods:** CSF samples from 20 NPH patients, 30 AD patients, and 25 cognitively normal (CN) controls were subjected to mass spectrometry‐based proteomic analysis. Differential expression analysis was performed for all pairwise group comparisons, and differentially expressed proteins in NPH were analysed with the Gene Ontology enrichment tool. A random forest model with protein inputs was trained to assess its ability to differentiate NPH from AD patients. Feature selection algorithms were applied to identify individual and complementary candidate biomarkers. Results were evaluated with immunological testing in both discovery and verification cohorts.

**Results:** Proteomic profiling revealed upregulated immune‐related proteins, including components of the complement cascade, and many downregulated synaptic proteins in NPH patients (Figure 1). The random forest model achieved a mean classification accuracy of 92% over 150 re‐runs. Neuronal pentraxin 1 (NPTX1) and gamma‐enolase (ENO2) emerged as top biomarker candidates (Figure 2). NPTX1 concentration was lower in NPH patients than in AD patients, resulting in a diagnostic accuracy of 0.94 (95% CI 0.89–0.99) (Figure 3). Additionally, NPTX1 concentrations correlated positively with total tau, phosphorylated tau181, and amyloid β42 in NPH patients, but not in AD patients.

**Figure:**
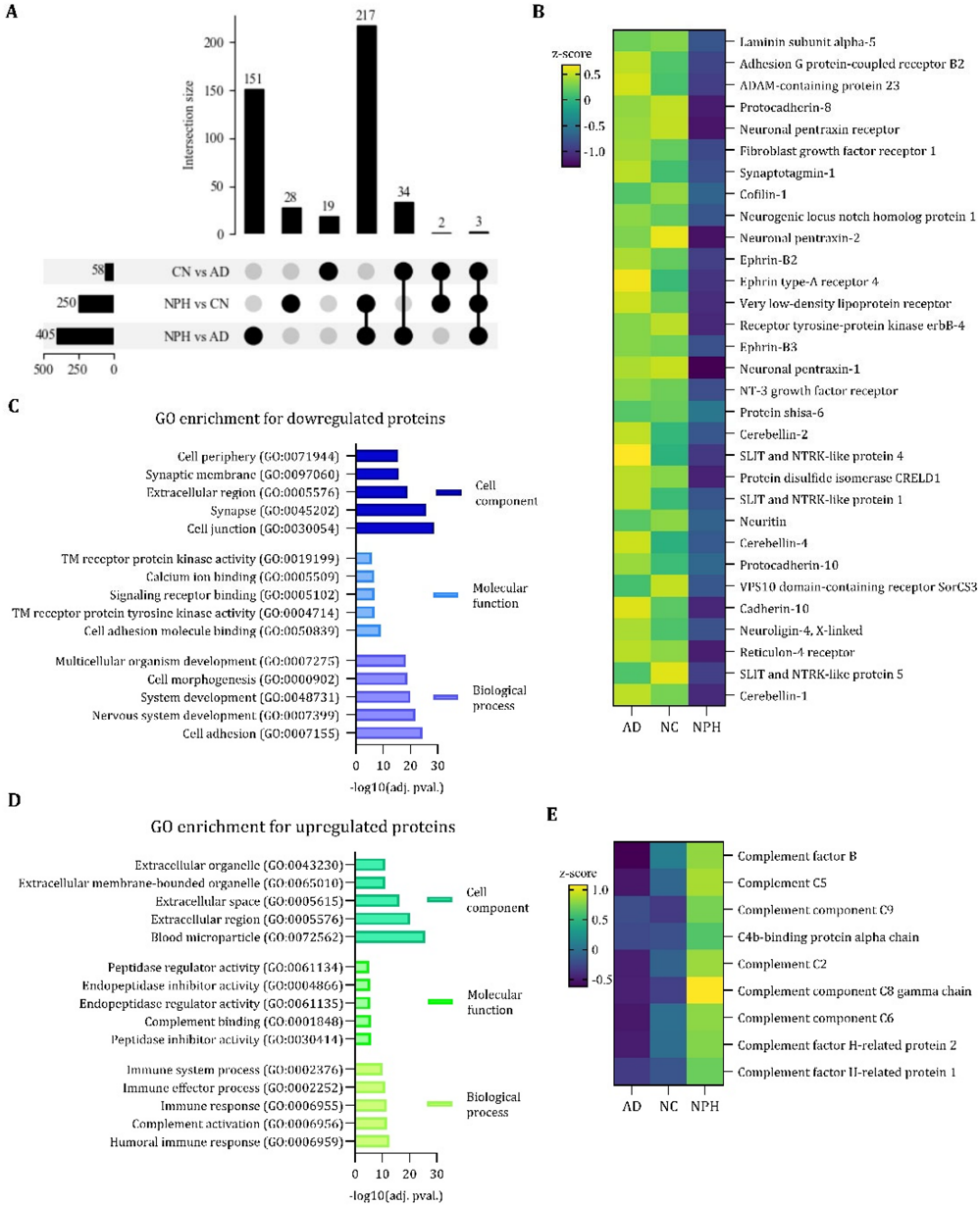
**FIGURE 1** Analysis of differentially expressed proteins specific for NPH in comparison to AD and CN.

**Figure:**
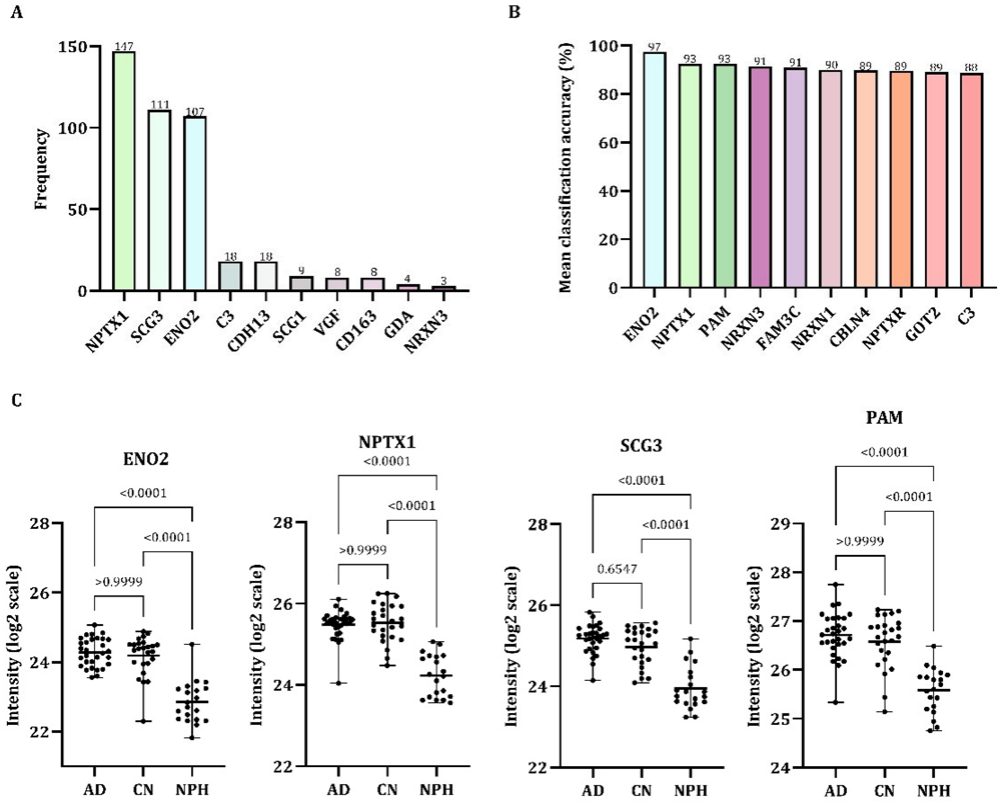
**FIGURE 2** Individual and complementary proteins identified with feature selection algorithms as potential diagnostic biomarkers.

**Figure:**
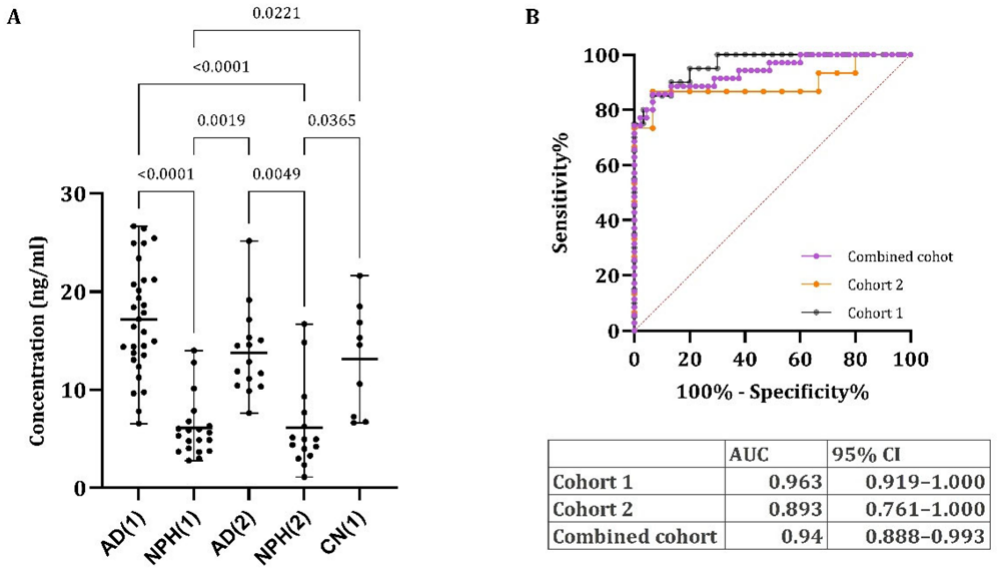
**FIGURE 3** Diagnostic performance of NPTX1 as a potential biomarker for NPH.

**Conclusion:** Together, our results highlight synaptic alterations and neuroinflammation as key characteristics of NPH and identify NPTX1 as a promising diagnostic biomarker.

**Disclosure:** Nothing to disclose.

## EPO‐0010

### CAA in Alzheimer's disease: What does CSF Aβ40 really tell us?

#### V. De Franco ^1^; F. Mazzacane^2^; C. Imbimbo^3^; E. Prodi^4^; M. Malik^4^; M. Cotta Ramusino^1^; G. Perini^1^; A. Costa^1^; A. Pichiecchio^4^

##### ^1^Unit of Behavioral Neurology and Center for Cognitive Disorders and Dementias (CDCD), IRCCS Mondino Foundation; 27100 Pavia, Italy; ^2^Department of Emergency Neurology and Stroke Unit, IRCCS Mondino Foundation; 27100 Pavia, Italy; ^3^Department of Brain and Behavioral Sciences, University of Pavia; 27100 Pavia, Italy; ^4^Advanced Imaging and Artificial Intelligence Center, IRCCS Mondino Foundation; 27100 Pavia, Italy

**Background and aims:** Cerebral amyloid angiopathy (CAA) commonly co‐occurs with Alzheimer's disease (AD), reflecting shared amyloid‐β(Aβ) pathology. Definitive CAA diagnosis requires histopathological confirmation, while in vivo evaluation uses Boston criteria. Cerebrospinal fluid (CSF) biomarkers, particularly Aβ40, support in vivo CAA diagnosis; utility in AD populations needs further study.

**Methods:** We enrolled patients with AD with available MRI and CSF data. Patients were categorized using Boston criteria versions 1.0, 1.5, and 2.0, as absent, possible, and probable CAA. CSF concentrations of Aβ40, Aβ42, total tau, and phosphorylated tau were measured using standardized chemiluminescent immunoassays. Univariate Bayesian linear regression models were used to evaluate differences in CSF biomarkers according to CAA status and specific MRI markers.

**Results:** 173 patients were included in the study. CSF Aβ40 and Aβ42 were reduced in CAA pateints when applying criteria v1.5 (posterior probabilities of reduction of 95.95% and 97.28% respectively). No significant differences between the CAA and non‐CAA groups according to the v2.0 criteria (posterior probabilities of reduction of 27.4% and 33.6%). Regarding MRI markers, the reduction in CSF Aβ40 and Aβ42 was confined to hemorrhagic markers (HMs), associated with a decrease of −12.51% (95%CrI −22.69 to −0.78) and −14.50% (−24.12 to −3.07), respectively. No significant correlations were found between CSF biomarkers and non‐hemorrhagic markers (nHMs).

**Figure:**
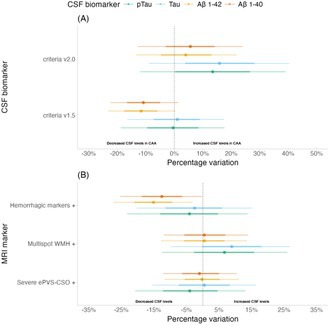
**FIGURE 1** Impact of Boston criteria versions and MRI markers on CSF biomarker levels.

**Figure:**
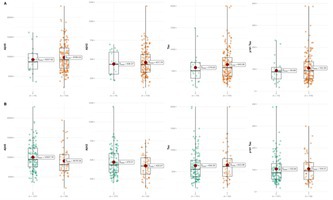
**FIGURE 2** CSF biomarker levels according to CAA diagnostic status and associations with MRI markers.

**Conclusion:** CSF β‐amyloid reduction was observed in comorbid CAA‐AD patients only with v1.5 criteria; criteria v2.0 made such association improbable. This reflects the association between CAA radiological marker and Aβ confined to HMs, while nHMS showed no association

**Disclosure:** Nothing to disclose.

## EPO‐0011

### Dual‐task walking unmasks inter‐joint coordination instability in amnestic mild cognitive impairment

#### Y. Kuan ^1^; T. Lu^2^

##### ^1^Department of Neurology, School of Medicine, College of Medicine, Taipei Medical University, Taipei, Taiwan; ^2^Department of Biomedical Engineering, National Taiwan University, Taipei, Taiwan

**Background and aims:** Gait disturbances in amnestic mild cognitive impairment (MCI) may remain subtle during normal walking and become evident only under increased cognitive load. Examining both single‐task and dual‐task walking may clarify how cognitive demands affect motor coordination and stability.

**Methods:** Older adults with amnestic MCI and cognitively normal controls performed level walking under single‐task and dual‐task conditions, including a cognitive task (serial subtraction) and a motor task (tray carrying). Three‐dimensional kinematic data were analysed to derive phase angles and continuous relative phase, characterising hip–knee and knee–ankle inter‐joint coordination. Coordination stability, pattern similarity, and temporal alignment were quantified using deviation phase, cross‐correlation coefficients, and root‐mean‐square error across key gait phases.

**Results:** During single‐task walking, spatiotemporal parameters and overall inter‐joint coordination patterns were largely comparable between groups, with only mild increases in coordination variability in amnestic MCI. Under dual‐task conditions, coordination deficits became more pronounced, particularly in knee–ankle coupling during single‐limb support and swing. Individuals with amnestic MCI showed increased variability, reduced waveform similarity, and greater phase‐position displacement, despite preserved gait timing. Cognitive dual‐task walking induced larger effects than motor dual‐task walking.

**Conclusion:** While basic gait patterns appear relatively preserved during single‐task walking, dual‐task conditions reveal impaired inter‐joint coordination in amnestic MCI. Increased instability and altered temporal organisation of knee–ankle coordination under cognitive load suggest reduced adaptability of motor control. Dual‐task gait assessment may provide a sensitive approach for detecting early cognitive–motor interference in pre‐dementia stages.

**Disclosure:** This study was supported by public research funding from the Ministry of Science and Technology, Taiwan (Grant Nos. 110‐2628‐B‐038‐025). The authors declare no commercial or financial conflicts of interest.

## EPO‐0012

### Behavioral dysexecutive syndrome at the intersection of autoimmunity and genetically predisposed frontotemporal lobar degeneration

#### Z. Chatzieleftheriou; A. Vakrakou; F. Athanasopoulos; M. Tzortzakakis; E. Zeis; P. Stathopoulos; V. Constantinides; C. Koros; S. Papageorgiou

##### First Department of Neurology, Medical School, National and Kapodistrian University of Athens, Athens, Greece

**Background and aims:** Autoimmune encephalitis can present with slowly progressive cognitive and movement disorders mimicking neurodegenerative diseases. Emerging evidence, including immunoglobulin‐like cell adhesion molecule 5 (IgLON5)‐associated disease, suggests that immune‐mediated inflammation may trigger or accelerate latent neurodegenerative processes in genetically predisposed individuals.

**Methods:** A 55‐year‐old male patient with gradual behavioral impairment characterized by apathy and compulsive behaviors underwent comprehensive clinical, neuropsychological and neurophysiological assessment, multimodal neuroimaging, extensive laboratory, including immunological and genetic testing.

**Results:** Examination revealed frontal disinhibition, asymmetric upper‐limb and truncal rigidity and REM sleep behavior disorder. Neuropsychological evaluation demonstrated executive dysfunction, mainly impaired cognitive flexibility. Neuroimaging showed frontal‐predominant cortical atrophy, electroencephalography revealed diffuse background slowing with intermittent theta activity and dopamine transporter imaging demonstrated asymmetric reduction of striatal uptake. Cerebrospinal fluid (CSF) analysis showed markedly elevated protein (330 mg/dL) without pleocytosis or intrathecal immunoglobulin synthesis. Neuronal antibody panels were negative; however, tissue‐based immunofluorescence demonstrated strong neuronal reactivity in serum and CSF. Neurodegeneration biomarkers were inconsistent with Alzheimer's disease. Whole‐body fluorodeoxyglucose positron emission tomography (FDG‐PET) excluded occult malignancy. Genetic analysis identified a pathogenic sequestosome 1 (SQSTM1) missense mutation (Pro392Leu) associated with frontotemporal dementia and a glucocerebrosidase (GBA) variant (Asn409Ser) conferring synucleinopathy risk. To target the autoimmune inflammatory response, the patient received high‐dose corticosteroids followed by plasmapheresis. Partial improvement and stabilization were observed during six months of follow‐up.

**Figure:**
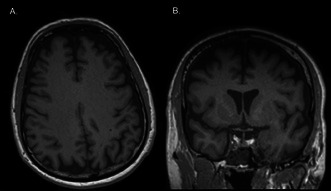
**FIGURE 1** Axial (A) and coronal (B) T1‐weighted brain MRI demonstrating frontal‐predominant cortical atrophy.

**Figure:**
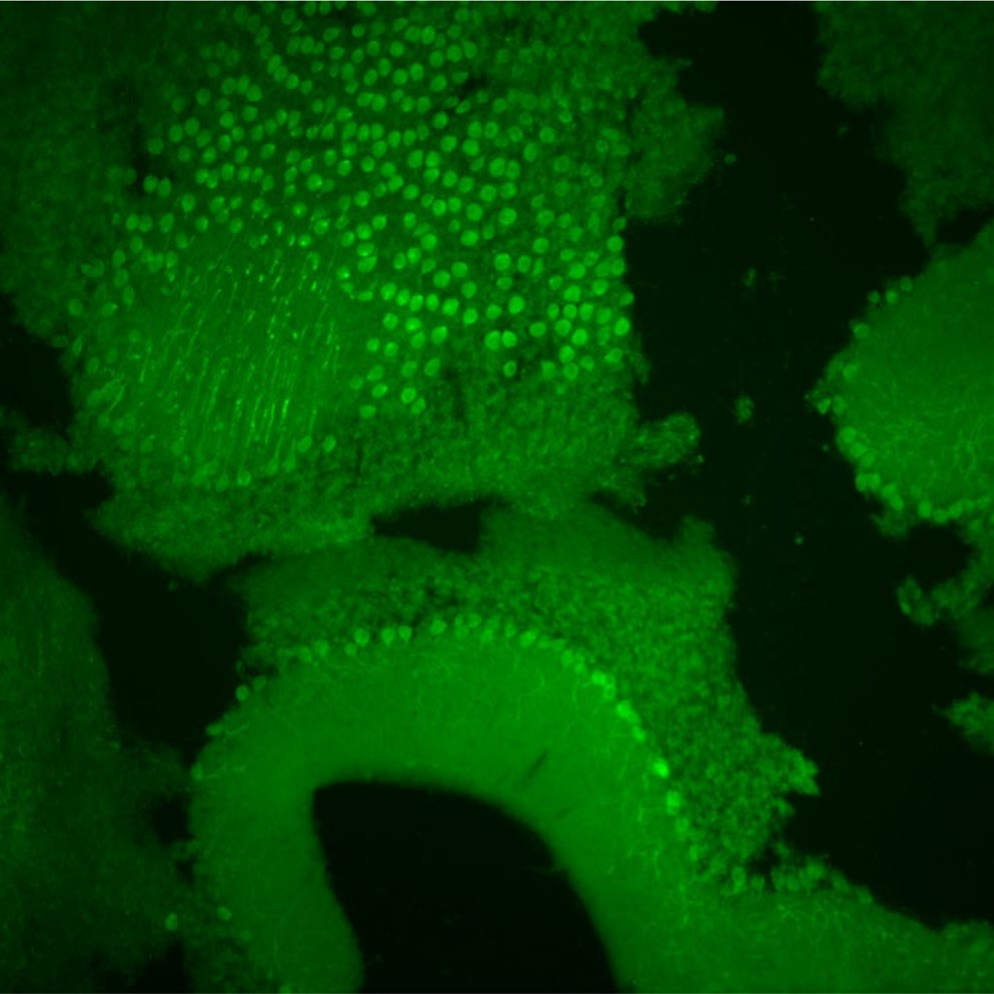
**FIGURE 2** Tissue‐based immunofluorescence on mice brain sections: patient serum demonstrating a strong reaction against cerebellar neurons.

**Conclusion:** This coexistence of neurodegeneration‐linked mutations alongside inflammatory CSF findings, neuronal immunoreactivity and response to immunotherapy suggests a bidirectional interaction between autoimmune mechanisms and genetically programmed neurodegeneration, thereby blurring diagnostic boundaries and encouraging integrated diagnostic strategies.

**Disclosure:** Nothing to disclose.

## Autonomic Nervous System Diseases

## EPO‐0013

### When symptoms outweigh criteria: Dizziness and autonomic burden across the orthostatic intolerance spectrum

#### E. Postovoi ^1^; M. Cebuc^1^; L. Rotaru^2^; A. Feodorovici^2^; G. Corcea^2^; I. Moldovanu^2^; O. Grosu^2^; M. Sangheli^1^

##### ^1^Department of Neurology No. 1, Nicolae Testemițanu State University of Medicine and Pharmacy, Chișinău, Republic of Moldova; ^2^Institute of Neurology and Neurosurgery, Chișinău, Republic of Moldova

**Background and aims:** Orthostatic dizziness is common, yet symptoms may not match hemodynamic response during the active stand‐test. This study aimed to assess whether dizziness in daily life (DDL) and during orthostatic testing (DOT) is associated with autonomic symptom burden and dizziness‐related disability.

**Methods:** In this cross‐sectional study, 21 inpatients at the Diomid Gherman Institute of Neurology and Neurosurgery underwent orthostatic testing, with blood pressure and heart rate assessed supine and serially after standing (at 0–10 minutes). Orthostatic intolerance (OI) spectrum phenotype: orthostatic hypotension (OH), orthostatic hypertension (OHT), POTS, OI. Patient‐reported outcomes: DHI, COMPASS‐31 (OI domain), BDI. Descriptive and inferential statistics were used.

**Results:** The cohort (mean age 67.9 ± 11.4 years; 61.9% male) reported frequent DDL (66.7%; provoked 64.3%, episodic 71.4%). Most had cerebral small vessel disease (81%); no χ^2^ association with DDL or DOT (*p* > 0.05). OI predominated (52.4%), whereas classical orthostatic phenotypes were less common (OH 19.0%, OHT 14.3%, POTS 4.8%); 9.5% had normal responses. Daily‐life dizziness was associated with higher total COMPASS‐31 (33.56[16.67–51.15] vs 11.27[1.78–22.55], *p* < 0.001), OI domain scores (20[10–28] vs 0[0–8], *p* < 0.001), yet showed no significant association with DOT (*p* > 0.05). DHI did not differ across OI spectrum phenotypes (*p* = 0.571), but correlated strongly with total COMPASS‐31 (ρ = 0.811, *p* < 0.001), COMPASS‐31‐OI (ρ = 0.533, *p* = 0.05) and BDI (ρ = 0.686, *p* = 0.007).

**Conclusion:** Symptoms outweighed hemodynamic criteria, as DDL tracked autonomic and depressive burden rather than orthostatic phenotype, supporting symptom‐led stratification and repeat or extended autonomic evaluation.

**Disclosure:** Nothing to disclose.

## EPO‐0014

### Heart rate entropy as a predictor of mortality after intracerebral hemorrhage: A prospective study

#### E. Abed

##### Neurology Department, Al‐Azahr University, Cairo, Egypt

**Background and aims:** Autonomic nervous system dysfunction has been linked to outcomes following intracerebral hemorrhage (ICH). Heart rate entropy (HRE) reflects autonomic complexity and may provide prognostic information. This study aimed to assess the association between HRE and mortality after ICH and to compare its predictive value with established autonomic markers and the ICH score.

**Methods:** This prospective study was conducted at Al‐Azhar University Hospitals and Cairo Fatemic Hospital and included 110 consecutive patients with spontaneous ICH admitted between October 2023 and November 2024. Sample HRE, heart rate variability parameters, and baroreflex sensitivity were assessed early after admission. Hematoma volume, intraventricular extension, infratentorial location, level of consciousness, and age were integrated into the standard ICH score. Predictive performance for mortality was evaluated using receiver operating characteristic (ROC) curve analysis and multivariable logistic regression.

**Results:** Among the studied patients (mean age 60.8 ± 11.2 years), HRE demonstrated the highest accuracy for mortality prediction (AUC 0.88), outperforming individual autonomic markers and showing comparable performance to the ICH score (AUC 0.84). Both HRE and ICH score were independently associated with mortality. Combining HRE with the ICH score significantly improved prognostic accuracy, yielding an AUC of 0.94

**Figure:**
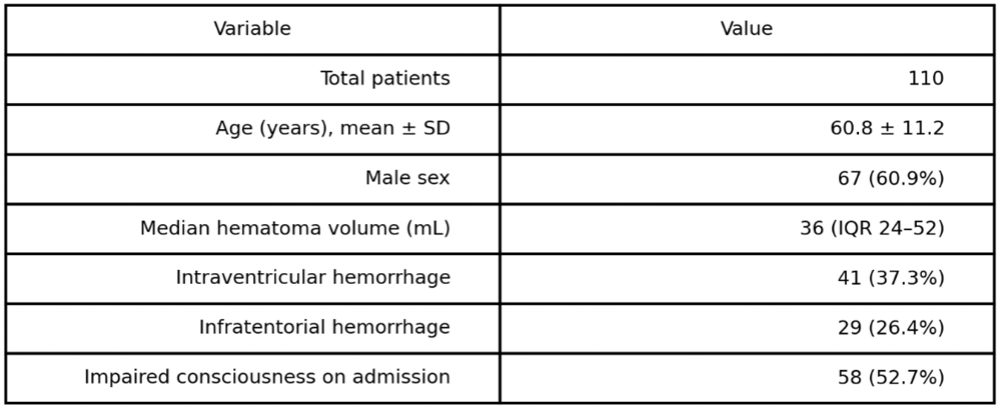
**TABLE 1** Baseline characteristics of ICH patients (*n* = 110).

**Figure:**
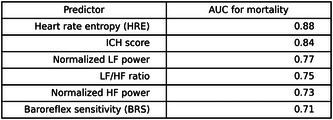
**TABLE 2** Predictive performance of autonomic markers for mortality.

**Figure:**
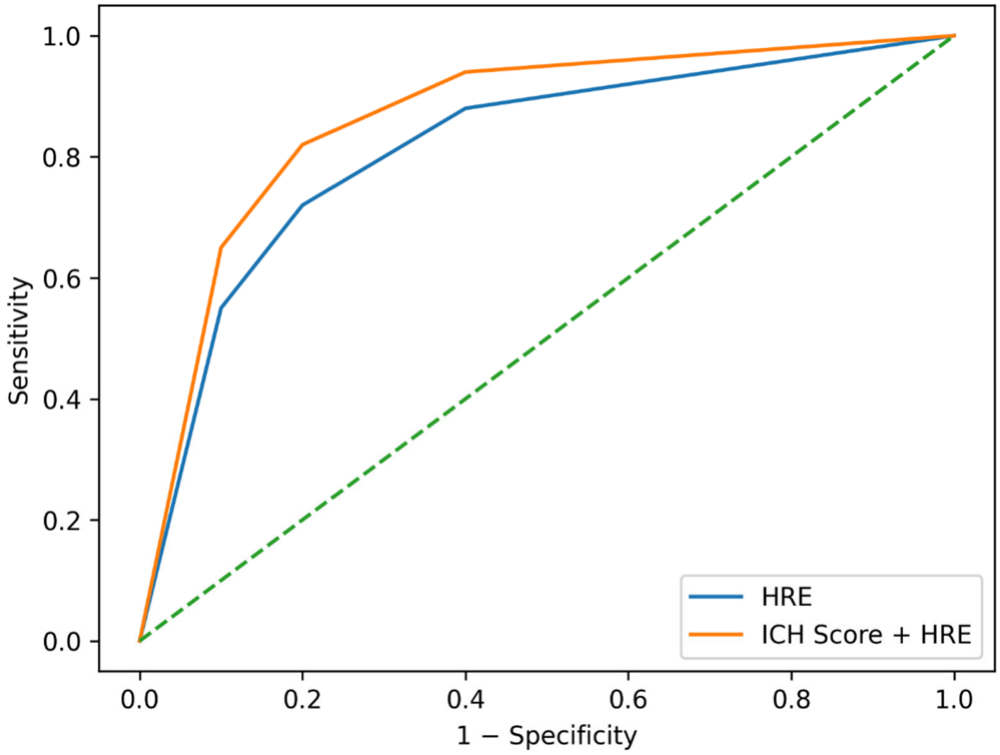
**FIGURE 1** ROC curves for mortality prediction after ICH.

**Conclusion:** Heart rate entropy provides substantial prognostic information regarding mortality after ICH and performs comparably to the ICH score. Incorporating HRE into conventional clinical scoring systems may enhance mortality prediction following intracerebral hemorrhage.

**Disclosure:** Nothing to disclose.

## EPO‐0015

### Characterization and staging of cardiovascular autonomic failure in light‐chain amyloidosis

#### F. Vitali ^1^; L. Sander^1^; G. Chiaro^1^; G. Ingle^1^; P. McNamara^1^; M. Lunn^2^; J. Gillmore^3^; V. Iodice^4^

##### ^1^National Autonomic Centre, The National Hospital for Neurology and Neurosurgery, Queen Square, London, UK; ^2^Department of Neuromuscular Diseases, UCL Queen Square Institute of Neurology, London, UK; ^3^National Amyloidosis Centre, Division of Medicine, University College London, London, UK; ^4^Department of Translational Neuroscience & Stroke, UCL Queen Square Institute of Neurology, University College London, London, UK

**Background and aims:** Light‐chain amyloidosis (AL amyloidosis) is a systemic disorder characterized by the deposition of monoclonal light‐chain as amyloid fibrils, leading to multiorgan dysfunction. Cardiovascular autonomic failure (cvAF) represents a major contributor to morbidity, yet its natural history and staging remain poorly characterized.

**Methods:** This retrospective study included 38 patients with AL amyloidosis who underwent comprehensive autonomic testing. cvAF was classified as stage 1 when mild parasympathetic and/or sympathetic impairment was present without neurogenic orthostatic hypotension (nOH), and as stage 2 when both domains were affected with nOH.

**Results:** At onset, about one‐third of patients reported autonomic symptoms. At the time of testing (median disease duration = 3 years), nearly all showed evidence of cvAF. Half were classified as stage 1, with predominant parasympathetic involvement, and 47% as stage 2. Stage 2 patients more frequently exhibited gastrointestinal (*p* = 0.032) and urogenital symptoms (*p* = 0.004), and impaired norepinephrine response during head‐up tilt (*p* < 0.001). Mortality rate and overall survival did not differ between stages. Patients with autonomic onset were more likely to have developed somatic polyneuropathy (*p* = 0.036), nOH (*p* = 0.012), and higher orthostatic intolerance ratios at T1 (*p* = 0.001). Autonomic onset was independently associated with progression to stage 2 cvAF (OR = 7.600, 95% CI 1.373–42.063, *p* = 0.020).

**Conclusion:** Cardiovascular autonomic failure is a prevalent and impactful manifestation of AL amyloidosis, often presenting early and progressing with the disease course. Our findings highlight the importance of systematic autonomic assessment and increased awareness of management strategies.

**Disclosure:** Nothing to disclose.

## EPO‐0016

### The burden of neurogenic orthostatic hypotension across autonomic disorders: Real‐world management outcomes

#### G. Winningham ^1^; S. Sajeev^1^; S. Johnstone^1^; Z. Jiang^1^; G. Chiaro^1^; V. Iodice^1^; V. Iodice^2^; V. Iodice^3^

##### ^1^National Autonomic Centre, National Hospital Neurology and Neurosurgery, London, UK; ^2^Department of Translational Neuroscience and Stroke, UCL Queen Square Institute of Neurology, Queen Square, London, UK; ^3^NIHR University College London Hospitals Biomedical Research Centre, London, UK

**Background and aims:** Neurogenic orthostatic hypotension (nOH) is a major cause of morbidity in autonomic failure, associated with falls, hospitalisations, and reduced quality of life, yet remains challenging to manage. We conducted a prospective real‐world study to evaluate symptom burden, quality of life, and treatment response in patients with established nOH across all aetiologies.

**Methods:** Patients with established nOH were prospectively followed within a tertiary referral centre management programme. All patients attended a dedicated clinic, where non‐pharmacological measures were implemented, and individualised combinations of anti‐hypotensive medications were optimised. Outcomes included validated patient‐reported measures of symptom burden and quality of life (Orthostatic Hypotension Questionnaire, OHQ; Patient Global Impression, PGI), alongside prospective recording of falls and hospital admissions. Orthostatic intolerance severity was assessed using standardised autonomic testing, including head‐up tilt, active stand, and 24‐hour blood pressure monitoring. Data are presented as mean ± SD.

**Results:** A total of 142 patients were enrolled (10 Lewy body dementia, 23 multiple system atrophy, 35 Parkinson's Disease, 62 pure autonomic failure, 12 autonomic neuropathy). Mean falls and hospital admissions were 1.6 ± 4.8 and 0.5 ± 2.6 respectively. OHQ composite‐score was 5.9 ± 2.0, and PGI score was 4.6 ± 1.5.

**Conclusion:** In real‐world tertiary‐centre cohort with long‐term follow‐up, patients with autonomic failure and nOH across all aetiologies experience high symptom burden, frequent falls, and impaired quality of life despite individualised combination therapy. These findings highlight nOH as a major contributor to morbidity, demonstrating limitations of existing management strategies.

**Disclosure:** Nothing to disclose.

## EPO‐0017

### Can SUDOSCAN replace QSART in the composite autonomic severity scale for evaluating sudomotor function in people with multiple sclerosis?

#### K. Tešija ^1^; M. Sredanović^1^; K. Jerčinović^2^; M. Krbot Skorić^1^; M. Habek^1^

##### ^1^University Hospital Center Zagreb, Zagreb, Croatia; ^2^University of Zagreb, School of Medicine, Zagreb, Croatia

**Background and aims:** The Quantitative Sudomotor Axon Reflex Test (QSART) is the gold standard for evaluating sudomotor function within the Composite Autonomic Scoring Scale (CASS). SUDOSCAN, a quick and noninvasive electrochemical test of sweat gland function, has been suggested as a potential substitute. This study investigated whether SUDOSCAN‐derived indices could replace QSART within the CASS framework and provide reliable sudomotor evaluation in people with multiple sclerosis (pwMS).

**Methods:** This cross‐sectional study involved 99 pwMS. Subjective autonomic symptoms were evaluated using the Composite Autonomic Symptom Score‐31 (COMPASS‐31), while objective autonomic testing was conducted with the CASS, which includes QSART for sudomotor assessment. Additionally, all pwMS underwent SUDOSCAN testing, and a modified CASS score was derived based on adrenergic, cardiovagal, and SUDOSCAN‐based sudomotor measures.

**Results:** A strong correlation was observed between CASS and modified CASS (*r* = 0.795, *p* < 0.001). However, no correlation was found between QSART‐based and SUDOSCAN‐based sudomotor indices (*r* = 0.043, *p* = 0.672). CASS did not correlate with COMPASS‐31 or its subdomains, whereas modified CASS showed a correlation with the gastrointestinal domain of COMPASS‐31 (*r* = 0.228, *p* = 0.027). Additionally, modified CASS correlated with age (*r* = 0.204, *p* = 0.048) and the Expanded Disability Status Scale (EDSS) score (*r* = 0.207, *p* = 0.043).

**Conclusion:** The modified CASS including SUDOSCAN showed a strong overall correlation with the standard CASS and meaningful associations with age and disability level. Although SUDOSCAN did not correlate with QSART‐based sudomotor indices, it could serve as a helpful supplement when QSART is unavailable. More long‐term studies are needed to assess its effectiveness in monitoring sudomotor dysfunction over time.

**Disclosure:** Funding: Croatian Science Foundation (IP‐2022‐10‐8200) KT: Nothing to disclose. MS: Nothing to disclose. KJ: Nothing to disclose. MKS: Received consultation and/or speaker fees from Biogen, Novartis, Roche. MH: Received consultation and/or speaker fees from Biogen, Merck, Novartis, Roche, Astra Zeneca, Amgen.

## EPO‐0018

### Cardiovascular autonomic dysfunction in rare hereditary amyloidosis

#### L. Sander ^1^; G. Chiaro^2^; J. Gillmore^4^; M. Reilly^3^; C. Mathias^5^; V. Iodice^6^

##### ^1^National Autonomic Centre, National Hospital for Neurology and Neurosurgery, London, UK; Department of Translational Neuroscience and Stroke, UCL Institute of Neurology, London; Neurologic Clinic and Policlinic, University Hospital Basel, CH; ^2^National Autonomic Centre, National Hospital for Neurology and Neurosurgery, London, UK; ^3^Centre for Neuromuscular Diseases, Department of Neuromuscular Diseases, UCL Queen Square Institute of Neurology and the National Hospital of Neurology and Neurosurgery, London, UK; ^4^National Amyloidosis Centre, Division of Medicine, University College London, London, UK; ^5^Department of Translational Neuroscience and Stroke, UCL Institute of Neurology, London; ^6^National Autonomic Centre, National Hospital for Neurology and Neurosurgery, London, UK; Department of Translational Neuroscience and Stroke, UCL Institute of Neurology, London; NIHR University College London Hospitals Biomedical Research Centre

**Background and aims:** Autonomic failure is a common feature in hereditary transthyretin amyloidosis (ATTRv), but has not been systematically described in other genetic subtypes including gelsolin (AGel), apolipoprotein A1 (AApoA1), and fibrinogen Aα‐chain amyloidosis (AFib). This study aimed to quantitatively investigate cardiovascular autonomic dysfunction in these rare entities, and to compare cardiovascular autonomic dysfunction to patients with ATTRv.

**Methods:** This retrospective study enrolled patients with genetically confirmed diagnosis of AGel, AApoA1, and AFib. Age‐ and sex‐matched control patients with ATTRv were included. All participants underwent standardised cardiovascular autonomic function testing (AFT), a subgroup had ambulatory 24 h blood pressure monitoring (ABPM) with autonomic patient diary. Stages of autonomic failure were defined according to AFT results: stage 0 (normal AFT), stage I (mild parasympathetic and/or sympathetic impairment), stage II (neurogenic orthostatic hypotension (nOH)).

**Results:** A total of 12 patients were included. Overall, 9/11 (82%) patients had abnormal AFT. 8/11 (73%) presented with stage I, and only 1/11 (9%) patient with AApoA1 had nOH. In the matched ATTRv cohort, 4/12 (33%) presented with nOH. There were no significant differences in cardiovascular autonomic parameters when comparing ATTRv versus non‐ATTRv patients. ABPM was available in two patients: one individual with AFib (stage I) had normal circadian BP rhythm, while one patient with AApoA1 (stage II) was a reverse dipper.

**Conclusion:** Cardiovascular autonomic dysfunction is very prevalent in genetic non‐TTR amyloidosis, but is usually mild. Quantitative AFT could help to monitor disease progression. Prospective studies are needed to investigate dysfunction of other autonomic domains in these patients.

**Disclosure:** LS has been supported by the Universität Basel, Switzerland, and the Freiwillige Akademische Gesellschaft Switzerland. GC, MMR and CJM: nothing to disclose. VI is supported by the National Institute for Health Research, University College London Hospitals Biomedical Research Centre and by the Autonomic Charitable Trust (ACT) (Lord Bagri) Research Award. VI has received honoraria from Theravance Biopharma US not related to this work.

## EPO‐0019

### Mucosal denervation and markers of immune activation in the gut of patients with chronic inflammatory diseases

#### M. Nolano ^1^; G. Scalise^2^; I. Borreca^2^; A. Areniello^2^; G. Caporaso^2^; B. De Conno^2^; D. Dell'Aversana^3^; F. Masciarelli^3^; G. Sarnelli^4^; D. Caprioli^5^; V. Provitera^2^

##### ^1^1. Neurology Department, Skin Biopsy Laboratory, Istituti Clinici Scientifici Maugeri IRCCS, Telese Terme, Italy. 2. Department of Neurosciences, Reproductive Sciences and Odontostomatology, University Federico II of Naples, Naples, Italy; ^2^Neurology Department, Skin Biopsy Laboratory, Istituti Clinici Scientifici Maugeri IRCCS, Telese Terme, Italy; ^3^Department of Neurosciences, Reproductive Sciences and Odontostomatology, University Federico II of Naples, Naples, Italy; ^4^Department of Clinical Medicine and Surgery, University of Naples Federico II, Naples, Italy; ^5^Department of Physiology and Pharmacology, Sapienza University, and Laboratory of Behavioral Neuropharmacology, IRCCS Fondazione Santa Lucia, Rome, Italy

**Background and aims:** Chronic intestinal inflammation is a condition characterized by immune activation, increased epithelial permeability, and altered neuro‐immune interactions. These features make it a suitable model to investigate mechanisms of mucosal denervation and impaired interoceptive signaling within the gut‐brain axis, implicated in neuropsychiatric conditions, including opioid use disorder (UOD).

**Methods:** To address this objective, we analyzed ileal biopsies from 10 patients with chronic inflammatory conditions (4M/6F; 47.3 ± 8.6) and matched healthy controls (5M/5F; 55.2 ± 10.9). Using indirect immunofluorescence, immune activation was assessed by quantifying CD68^+^ macrophages, IBA‐1^+^ activated myeloid cells, CD8^+^ lymphocytes, and IL‐24–expressing cells, while mucosal innervation was evaluated by measuring VIP+ enteric fibers.

**Results:** Compared to controls, patients with inflammatory conditions showed a marked increase in CD68^+^ macrophages (755.0 ± 338.1 vs 141.1 ± 70.5; *p* < 0.01), IBA‐1^+^ myeloid cells (862.3 ± 257.7 vs 239.9 ± 116.4; *p* < 0.01), and IL‐24–expressing cells (339.7 ± 151.4 vs 32.0 ± 44.6; *p* < 0.01). CD8^+^ lymphocytes were also increased, although the difference did not reach statistical significance (1608.6 ± 568.6 vs 1391.6 ± 392.3; *p* = 0.504). Concomitantly, a significant reduction in VIP‐positive enteric fibers was observed in inflammatory conditions compared to controls (0.036 ± 0.025 vs 0.069 ± 0.025; *p* < 0.01).

**Conclusion:** Overall, the findings reveal a distinct neuro‐immune signature characterized by chronic inflammation and enteric denervation. This pattern may underlie impaired interoceptive signaling and disrupted gut–brain axis communication in inflammatory bowel conditions, with potential relevance to neuropsychiatric disorders such as opioid use disorder.

**Disclosure:** Nothing to disclose.

## EPO‐0020

### Increased burden of gastrointestinal autonomic symptoms in multiple sclerosis: A systematic review and meta‐analysis

#### M. Sredanović ^1^; C. Hentzen^2^; I. Adamec^3^; C. Chesnel^2^; M. Krbot Skorić^4^; M. Habek^3^

##### ^1^University Hospital Center Zagreb, Department of Neurology, Referral Center for Autonomic Nervous System Disorders, Zagreb, Croatia; ^2^Sorbonne University, GRC 01 ‐ Group of Clinical research in Neuro‐urology, Hōspital Pitié Salpētrière, Paris, France; ^3^School of Medicine, University of Zagreb, Zagreb, Croatia; ^4^Faculty of Electrical Engineering and Computing, University of Zagreb, Zagreb, Croatia

**Background and aims:** Gastrointestinal autonomic symptoms are frequently reported by people with MS (pwMS) but remain insufficiently characterized, with prevalence estimates varying widely across studies. The aim of this study was to conduct a systematic review with meta‐analysis of the available scientific evidence on the prevalence of gastrointestinal autonomic symptoms in people with multiple sclerosis.

**Methods:** A systematic review of the literature (PubMed, Scopus, and Web of Science until October 2025) identified 21693 studies, of which 15 reported frequency of gastrointestinal autonomic symptoms in pwMS compared to healthy controls. Of these, 5 evaluated dysphagia, 5 evaluated bowel dysfunction in general, and a single study evaluated constipation, fecal incontinence, abnormal gastric emptying, dyspepsia, anorectal dysfunction, and intestinal symptoms.

**Results:** Meta‐analysis showed a significantly higher prevalence of gastrointestinal autonomic symptoms in pwMS than in healthy controls (0.283, IQR 0.026–0.715 in pwMS vs. 0.022, IQR 0.000–0.185 in healthy controls; OR 3.40, 95% CI 1.79–6.44; *p* = 0.0002) (Figure 1). Substantial heterogeneity was observed (I^2^ = 99%, τ^2^ = 1.37). Among symptoms with more than one study, the prevalence of dysphagia was significantly higher (OR 20.65, 95% CI 3.20–133.34; *p* = 0.001), whereas the prevalence of bowel dysfunction did not differ (OR 1.21, 95% CI 0.65‐2.24; *p* = 0.55).

**Figure:**
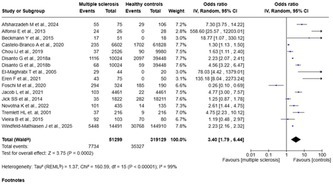
**FIGURE 1** Forest plot showing the pooled odds ratio for gastrointestinal autonomic symptoms in people with multiple sclerosis compared with healthy controls.

**Conclusion:** Gastrointestinal autonomic symptoms are significantly more frequent in people with multiple sclerosis than in healthy controls, with dysphagia showing a particularly strong association. Greater awareness and systematic screening of gastrointestinal autonomic symptoms are warranted in clinical practice to improve recognition and management of these common and impactful manifestations of MS.

**Disclosure:** MS: Nothing to disclose. CH: Consultant for Coloplast, Convatec, Bbraun. IA: Received consultation and/or speaker fees from Biogen, Merck, Novartis, Roche. CC: Nothing to disclose. MKS: Received consultation and/or speaker fees from Biogen, Novartis, Roche, Merck. MH: Received consultation and/or speaker fees from Biogen, Merck, Novartis, Roche, Astra Zeneca, Amgen.

## EPO‐0021

### Developing a pain scale for multiple system atrophy (MSA)

#### N. Campese ^1^; B. Caliò^1^; F. Leys^1^; L. Zamarian^1^; A. Schlager^1^; J. Wanschitz^1^; K. Seppi^1^; W. Poewe^1^; G. Göbel^2^; G. Höglinger^3^; J. Levine^3^; G. Calandra Buonaura^4^; P. Guaraldi^4^; R. Eleopra^5^; M. Devigili^5^; W. Meissner^6^; D. Bendetowicz^6^; M. Fabbri^7^; A. Pavy‐Le Traon^7^; A. Schrag^8^; R. Chaudhuri^9^; M. Tinazzi^10^; M. Pellecchia^11^; C. Falup‐Pecurariu^12^; S. Lorenzl^13^; V. Mylius^14^; G. Deuschl^15^; E. Coon^16^; H. Kaufmann^17^; J. Courtis^18^; R. Freeman^19^; A. Fanciulli^1^

##### ^1^Department of Neurology, Medical University of Innsbruck, Innsbruck, Austria; ^2^Institute of Clinical Epidemiology, Public Health, Health Economics, Medical Statistics and Informatics, Medical University of Innsbruck, Innsbruck, Austria; ^3^Department of Neurology, University Hospital, LMU Munich, Munich, Germany; ^4^IRCCS Istituto delle Scienze Neurologiche di Bologna, Bologna, Italy; ^5^Parkinson and Movement Disorders Unit, Fondazione IRCCS Istituto Neurologico Carlo Besta, Milan, Italy; ^6^Univ. Bordeaux, CHU Bordeaux, Service de Neurologie des Maladies Neurodégénératives, Bordeaux, France; ^7^Department of Neurology, University Hospital of Toulouse, Toulouse, France; ^8^Department of Clinical and Movement Neurosciences, University College London, London, UK; ^9^Parkinson Centre of Excellence, Kings College Hospital, London and Dubai, UK, UAE; ^10^Department of Neurosciences, Biomedicine and Movement Sciences, University of Verona, Verona, Italy; ^11^Department of Medicine, Surgery and Dentistry “Scuola Medica Salernitana,” Neuroscience Section, University of Salerno, Salerno, Italy; ^12^Department of Neurology, Faculty of Medicine, Transilvania University of Brașov, Brașov, Romania; ^13^Institute of Palliative Care, Paracelsus Medical University, Salzburg, Austria; ^14^Department of Neurology, Center for Neurorehabilitation, Valens, Switzerland; ^15^Department of Neurology, University Hospital Schleswig‐Holstein (UKSH), Christian‐Albrechts‐University Kiel, Kiel, Germany; ^16^Department of Neurology, Mayo Clinic, Rochester, USA; ^17^Department of Neurology, Dysautonomia Center, New York University Grossman School of Medicine, New York, USA; ^18^–; ^19^Department of Neurology, Beth Israel Deaconess Medical Center, Harvard Medical School, Boston, USA

**Background and aims:** Pain affects up to 87% of persons with multiple system atrophy (MSA), impacting core activities of daily living, but remains under‐recognized in clinical practice. In the absence of disease‐specific pain assessment tools, we aim at developing a patient‐reported pain scale for MSA.

**Methods:** A taskforce of movement disorder and pain experts will develop a first draft of the MSA‐pain scale. The scale development will be informed by a systematic literature review and by the results of targeted surveys addressing patients, caregivers, and healthcare professionals (ongoing) on current standards and unmet needs on pain assessment and management in MSA. The questionnaire will be developed in English and undergo a preliminary field test (*n* = 5 English‐speaking persons with MSA) with subsequent item reduction and refinement. The Delphi methodology will be followed to reach consensus on the final version of the questionnaire.

**Results:** The questionnaire development is expected to last 12 months. Once finalized, the final version of the questionnaire will be translated into other languages (German, Italian, French) and validated in a large multi‐center study.

**Conclusion:** The MSA‐pain questionnaire is expected to assist in the screening, systematic assessment and follow‐up of different types of pain in MSA. The validation of the first disease‐specific patient reported outcome measure (PROM) for pain in MSA will also hopefully pave the way for interventional trials targeting pain in MSA.

**Disclosure:** Academic study supported by a grant of the European Reference Network for Rare Neurological Diseases (ERN‐RND). The authors have no conflict of interest to report.

## EPO‐0022

### Increased infarct volume is associated with a shift towards cardiac sympathetic predominance in patients with ischemic stroke

#### R. Wang ^1^; S. Moeller^2^; A. Akhundova^3^; H. Marthol^4^; R. Kollmar^5^; M. Köhrmann^6^; A. Dörfler^7^; S. Gerner^1^; B. Kallmünzer^1^; S. Schwab^1^; M. Hilz^1^

##### ^1^Department of Neurology, University of Erlangen‐Nuremberg, Erlangen, Germany; ^2^Faculty of Health/School of Medicine, Witten/Herdecke University, Witten, Germany; ^3^Department of Neurology, Zentralklinik Bad Berka, Bad Berka, Germany; ^4^Department of Psychiatry, Klinikum am Europakanal, Erlangen, Germany; ^5^Department of Neurology, General Hospital Darmstadt, Darmstadt, Germany; ^6^Department of Neurology, University Hospital Essen, Essen, Germany; ^7^Department of Neuroradiology, University of Erlangen‐Nuremberg, Erlangen, Germany

**Background and aims:** Ischemic stroke often causes cardiovascular autonomic dysfunction. We previously showed associations between higher National Institutes of Health Stroke Scale scores and more severe cardiovascular autonomic dysfunction. This study evaluated associations between infarct‐volume and parameters of cardiac autonomic modulation in patients with acute ischemic stroke in the middle cerebral artery (MCA) territory.

**Methods:** In 48 patients with acute MCA stroke (68 ± 15 years old, 23 men and 25 women)), we monitored RR intervals (RRI), systolic, diastolic blood pressures (BPsys, BPdia), and respiration (RESP) at rest within 24 hours after stroke‐onset. We calculated parameters of total cardiac autonomic modulation (RRI‐standard‐deviation (RRI‐SD), RRI‐coefficient‐of‐variation (RRI‐CV), RRI‐total‐powers), sympathetic (RRI‐low‐frequency‐powers (RRI‐LF), normalized (nu) RRI‐LF‐powers, BPsys‐LF‐powers) and parasympathetic modulation (root‐mean‐square‐of‐successive‐RRI‐differences (RMSSD), RRI‐high‐frequency‐powers (RRI‐HF), RRI‐HFnu‐powers), sympathetic‐parasympathetic balance (RRI‐LF/HF‐ratios), and baroreflex‐sensitivity (BRS). The infarct‐volume was calculated using the Ellipsoid model ABC/2 (where A = longest dimension in axis x, B = longest perpendicular dimension to axis x (y), and C = total length in z dimension) from the diffusion‐weighted Magnetic Resonance image (DWI) within 24 hours upon stroke‐onset or Computed Tomography (CT) taken within 48 hours upon stroke‐onset.

**Results:** Values of infarct‐volumes correlated positively with RRI‐LF‐nu‐powers (correlation‐coefficient: 0.297, *p* = 0.038) and RRI‐LF/HF‐ratios (correlation‐coefficient: 0.414, *p* = 0.003), negatively with RRI‐HFnu‐powers (correlation‐coefficient: −0.297, *p* = 0.038). Patients with infarct‐volumes above 50 mL had higher RRI‐LF‐nu‐powers and RRI‐LF/HF‐ratios but lower RRI‐HF‐nu‐powers than patients with smaller infarct‐volumes.

**Conclusion:** In our patients with acute ischemic MCA‐stroke, increased infarct‐volume was associated with a shift towards increased sympathetic and reduced parasympathetic cardiac modulation. These changes bear an increased risk of secondary complications.

**Disclosure:** Nothing to disclose.

## EPO‐0023

### Lower urinary tract symptoms in Parkinson's disease: Prevalence and aetiology

#### S. Diaconu ^1^; I. Murasan^2^; R. Ilea^2^; R. Margarit^2^; A. Drujescu^2^; S. Berzunteanu^2^; D. Rusu^1^; R. Dolis^2^; C. Falup‐Pecurariu^1^

##### ^1^Department of Neurology, County Clinic Hospital, Transilvania University Brasov, Romania; ^2^Department of Neurology, County Clinic Hospital, Brasov, Romania

**Background and aims:** Lower urinary tract symptoms (LUTS) are common autonomic manifestations of Parkinson's disease (PD), frequently preceding or accompanying motor progression and significantly impairing quality of life. However, the relationship between LUTS, motor severity, and disease characteristics remains incompletely defined. The aim of this study was to assess the prevalence and pattern of LUTS in PD and to explore their associations with motor severity, disease duration, and quality of life.

**Methods:** We conducted a cross‐sectional observational study including 153 patients with idiopathic PD (mean age 67.4 ± 8.9 years, disease duration 6.1 ± 4.2 years, 58% male). LUTS were evaluated using the Overactive Bladder Questionnaire short form (OAB‐qSF) and the International Prostate Symptom Score (IPSS). Quality of life was assessed using disease‐specific patient‐reported measures. Correlation and multivariate linear regression analysis were performed.

**Results:** Overall, 107 patients (69.93%) reported at least one LUTS. Storage symptoms predominanted (66.35%) with nocturia (61.68%) and urgency (48.59%) being most frequent. Mean OAB‐q symptoms score was 38.7 ± 17.4, and mean IPSS total score was 12.4 ± 6.3. OAB‐q scores correlated significantly with UPDRS‐III (*r* = 0.35, *p* < 0.001), H&Y stage (*r* = 0.33, *p* < 0.001), age (*r* = 0.21, *p* = 0.001), and disease duration. In multivariate analysis, UPDRS‐III and age independently predicted LUTS severity. Higher LUTS burden was independently associated with poorer quality of life (*p* < 0.001).

**Conclusion:** LUTS are highly prevalent in PD, with storage symptoms predominating, and are strongly associated with motor severity and disease progression.

**Disclosure:** Nothing to disclose.

## EPO‐0024

### Sudomotor dysfunction in patients with normal and abnormal cardiac MIBG

#### Z. Jiang; G. Chiaro; V. Iodice

##### National Autonomic Centre, The National Hospital for Neurology and Neurosurgery, London, UK

**Background and aims:** While normal metaiodobenzylguanidine (MIBG) uptake on cardiac scintigraphy and sweat function on dynamic sweat test (DST) in pure autonomic failure (PAF) are predictors of phenoconversion to multiple system atrophy (MSA) over parkinson's disease (PD) or dementia with lewy bodies (DLB), they can become abnormal in late‐stage MSA, consistent with a top‐down model of disease. However, no study has evaluated the sequence of peripheral denervation across cardiovascular and sudomotor domains in MSA. We examined sweat loss in patients with normal versus abnormal MIBG uptake and considered implications for predicting phenoconversion trajectory.

**Methods:** As part of our ongoing natural history study, consecutive patients with an initial diagnosis of PAF were systematically phenotyped and followed longitudinally until phenoconversion or death. Participants underwent cardiovascular autonomic testing, cardiac MIBG scintigraphy, dynamic sweat testing (DST), and thermoregulatory sweat testing (TST).

**Results:** 24 PAF patients (median follow up 5.5 years) underwent both scintigraphy and sudomotor testing. 4/24 patients (17%) had normal MIBG uptake, of whom 1/4(25%) had pre‐ganglionic sweat loss. This patient and 2 others (75%) later phenoconverted into MSA, while the remaining patient retained a PAF diagnosis. Among the 20/24 (83%) patients with abnormal MIBG uptake, all 20/20 (100%) had evidence of post‐ganglionic sudomotor dysfunction and retained a PAF diagnosis or phenoconverted to PD/DLB.

**Figure:**
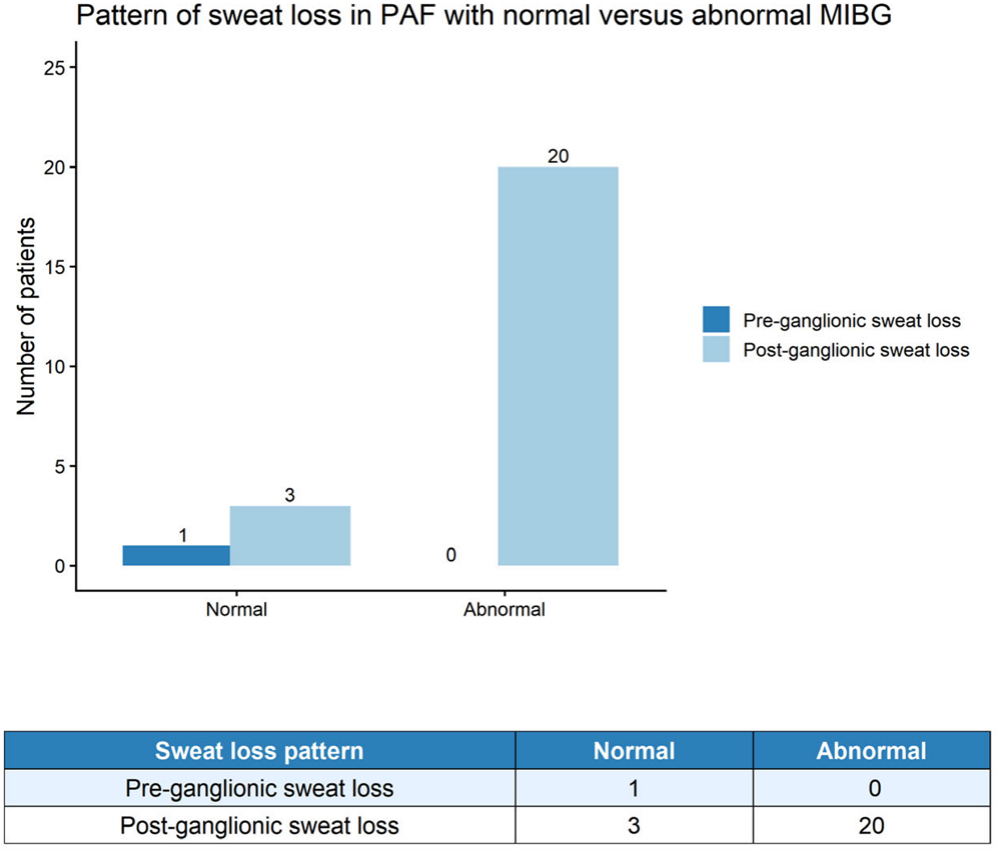
**FIGURE 1** Pattern of sweat loss in normal vs abnormal MIBG.

**Conclusion:** In PAF patients phenoconverting to MSA, cardiac sympathetic innervation may remain preserved even in established peripheral sudomotor denervation, possibly reflecting differing neuronal vulnerability to alpha‐synuclein deposition. These findings support a multimodal biological framework for PAF to improve prediction of phenoconversion trajectory across disease stages.

**Disclosure:** Nothing to disclose.

## Cerebrovascular Diseases 1

## EPO‐0025

### Ethical barriers to time‐critical stroke care: Family consent, delayed treatment, and the need for presumed consent in Armenia

#### L. Martirosyan; A. Ashughyan; A. Papoyan; K. Kosyan; R. Zhamakochyan; A. Madoyan; K. Petrosyan

##### Department of General and Vascular Neurology, Saint Gregory the Illuminator Medical Center, Yerevan, Armenia

**Background and aims:** Acute ischemic stroke is a leading cause of mortality and long‐term disability worldwide. Intravenous thrombolysis and mechanical thrombectomy are evidence‐based, time‐critical interventions that significantly improve survival and functional outcomes. Despite their established efficacy, ethical‐legal barriers may impede timely treatment in certain healthcare systems. This study examines the ethical implications of consent requirements in acute stroke care, comparing European practice with the current regulatory framework in Armenia.

**Methods:** A comparative ethical and policy analysis was conducted, reviewing European stroke guidelines and ethical frameworks (ESO, NICE) alongside Armenian Ministry of Health regulations governing informed consent in neurological emergencies.

**Results:** In most European countries, acute stroke treatment is guided by the principle of presumed consent, allowing immediate initiation of life‐saving therapy when patients lack decision‐making capacity and no prior advance directive / living will is documented. Family members serve an advisory rather than decisional role. In contrast, Armenian practice permits family members to refuse thrombolysis and thrombectomy even when the patient is conscious, and mandates written family consent when treatment is approved. This requirement, formally regulated at the ministerial level, introduces critical delays and contradicts the ethical principles of beneficence and non‐maleficence by effectively transforming time‐dependent care into a preventable source of harm.

**Conclusion:** Current consent practices in Armenia represent a significant ethical and clinical barrier to optimal stroke care. Addressing this issue requires legislative reform, implementation of presumed consent for emergency stroke treatment, and enhanced legal protection for physicians to ensure timely, ethical, and evidence‐based intervention.

**Disclosure:** Nothing to disclose.

## EPO‐0026

### Impact of inflammatory biomarkers on functional outcome in patients with acute LVO stroke undergoing mechanical thrombectomy

#### E. Ricci ^1^; G. Furlanis^1^; M. Ajčević^2^; K. Iscra^2^; M. Malesani^1^; E. Vincis^1^; G. Prandin^1^; M. Quagliotto^1^; G. Farina^1^; P. Caruso^1^; M. Naccarato^1^; P. Manganotti^1^

##### ^1^Neurology Unit, Department of Medicine, Surgery and Health Sciences, University Hospital and Health Services of Trieste ‐ ASUGI, University of Trieste, Trieste, Italy; ^2^Department of Engineering and Architecture, University of Trieste, Trieste, Italy

**Background and aims:** Despite significant advances in the acute management of ischaemic stroke, accurately monitoring response to endovascular thrombectomy (EVT) and predicting functional outcomes in patients with large vessel occlusion (LVO) remain challenging. In this context, acute‐phase inflammatory blood biomarkers may provide valuable prognostic information and enhance current prediction models. This study aimed to identify variables associated with unfavorable functional outcomes in patients with LVO stroke treated with EVT, defined as a modified Rankin Scale (mRS) score of 3–6 at 3 months, with particular emphasis on acute‐phase inflammatory biomarkers.

**Methods:** The clinical and laboratory data of 172 consecutive patients with LVO acute stroke (median age 77 years, Female 60%) treated with EVT were retrospectively analyzed. Univariate and multivariate logistic regression analyses were performed to identify variables independently associated with poor clinical outcomes (mRS 3–6 at 3 months).

**Results:** median NIHSS score at admission was 16 and successful post‐EVT recanalization (TICI 2b–3) was achieved in 73.8% of patients. Multivariate analysis demonstrated that older age (OR = 1.006, *p* = 0.038), higher NIHSS score at admission (OR = 1.027, *p* < 0.001), elevated C‐reactive protein (CRP) levels at admission (OR = 1.003, *p* = 0.020), and increased neutrophil‐to‐lymphocyte ratio (NLR) at admission (OR = 1.015, *p* = 0.004) were independently associated with an unfavorable functional outcome (mRS 3–6).

**Figure:**
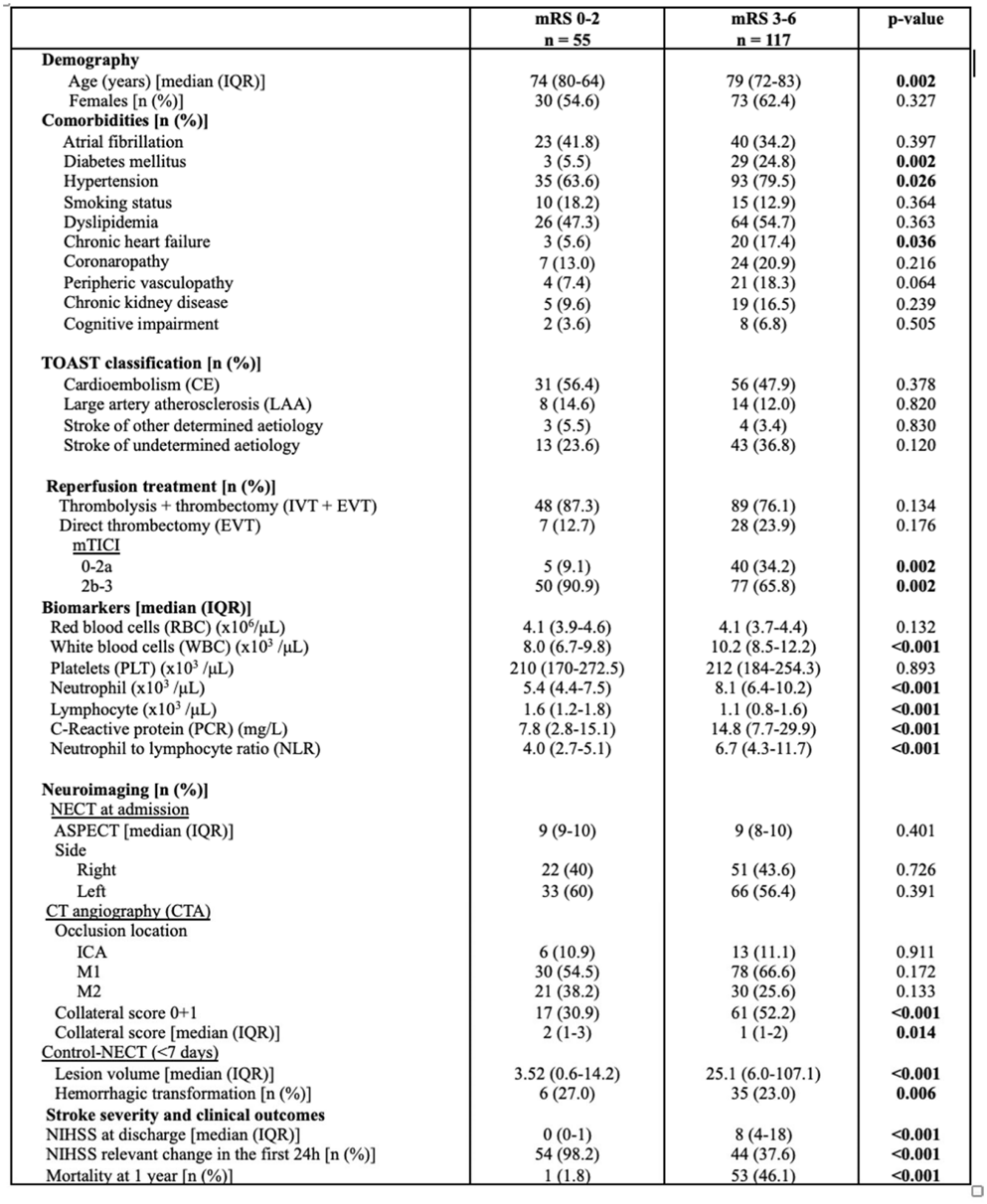
**TABLE 1** Comparison of demographics and clinical characteristics of patients with mRS 0–2 at 3 months vs mRS 3–6 at 3 months. Data are presented as medians (IQR) and frequencies.

**Figure:**
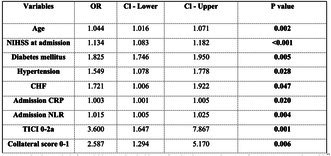
**TABLE 2** Univariate analysis for variables associated with poor functional outcome in terms of mRS 3–6 at 3 months.

**Figure:**
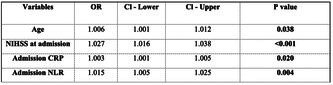
**TABLE 3** Logistic multivariate regression for variables associated with poor functional outcome in terms of mRS 3–6 at 3 months.

**Conclusion:** In a consecutive cohort of patients with LVO ischaemic stroke treated with EVT, admission levels of the inflammatory biomarkers’ NLR and CRP were independently associated with functional outcome at 3 months.

**Disclosure:** Edoardo Ricci: Nothing to disclose. Giovanni Furlanis: Nothing to disclose. Milos Ajčević: Nothing to disclose. Katerina Iscra: Nothing to disclose. Michele Malesani: Nothing to disclose. Emanuele Vincis: Nothing to disclose. Gabriele Prandin: Nothing to disclose. Magda Quagliotto: Nothing to disclose. Gianpiero Farina: Nothing to disclose. Paola Caruso: Nothing to disclose. Marcello Naccarato: Nothing to disclose. Paolo Manganotti: Nothing to disclose.

## EPO‐0027

### Impact of statin therapy on functional outcome of real‐world acute stroke cohort: An observational study

#### E. Ricci ^1^; G. Furlanis^1^; M. Ajčević^2^; K. Iscra^2^; M. Malesani^1^; E. Vincis^1^; G. Prandin^1^; M. Quagliotto^1^; G. Farina^1^; P. Caruso^1^; M. Naccarato^1^; P. Manganotti^1^

##### ^1^Neurology Unit, Department of Medicine, Surgery and Health Sciences, University Hospital and Health Services of Trieste ‐ ASUGI, University of Trieste, Strada di Fiume, Trieste, Italy; ^2^Department of Engineering and Architecture, University of Trieste, Via A. Valerio, Trieste, Italy

**Background and aims:** Growing evidence suggests that statin therapy in patients with acute ischaemic or haemorrhagic stroke may influence functional outcomes. This study aimed to identify clinical predictors of unfavorable functional outcomes, defined as a modified Rankin Scale (mRS) score of 3–6 at 3 months, with a focus on the potential impact of statin therapy.

**Methods:** The clinical data of 777 consecutive patients with acute stroke were retrospectively analyzed. Patients were stratified with favorable outcomes defined as an mRS score of 0–2 and unfavorable outcomes (mRS score of 3–6). Univariate and multivariate logistic regression analyses were then performed to identify variables independently associated with poor clinical outcomes.

**Results:** Patients in the mRS 3–6 group were older on average (80 vs. 74 years, *p* < 0.001) and had a significantly higher median NIHSS score at admission compared to those in the mRS 0–2 group (12 vs. 4, *p* < 0.001). Multivariate analysis showed that older age (OR = 1.006, *p* < 0.001), higher NIHSS scores at admission (OR = 1.036, *p* < 0.001), haemorrhagic stroke (OR = 1.101, *p* = 0.014), and the occurrence of early seizures or status epilepticus (OR = 1.142, *p* = 0.020) were independently associated with a poor functional outcome (mRS 3–6). Conversely, statin therapy was associated with a reduced risk of poor outcome (OR = 0.879, *p* = 0.001).

**Figure:**
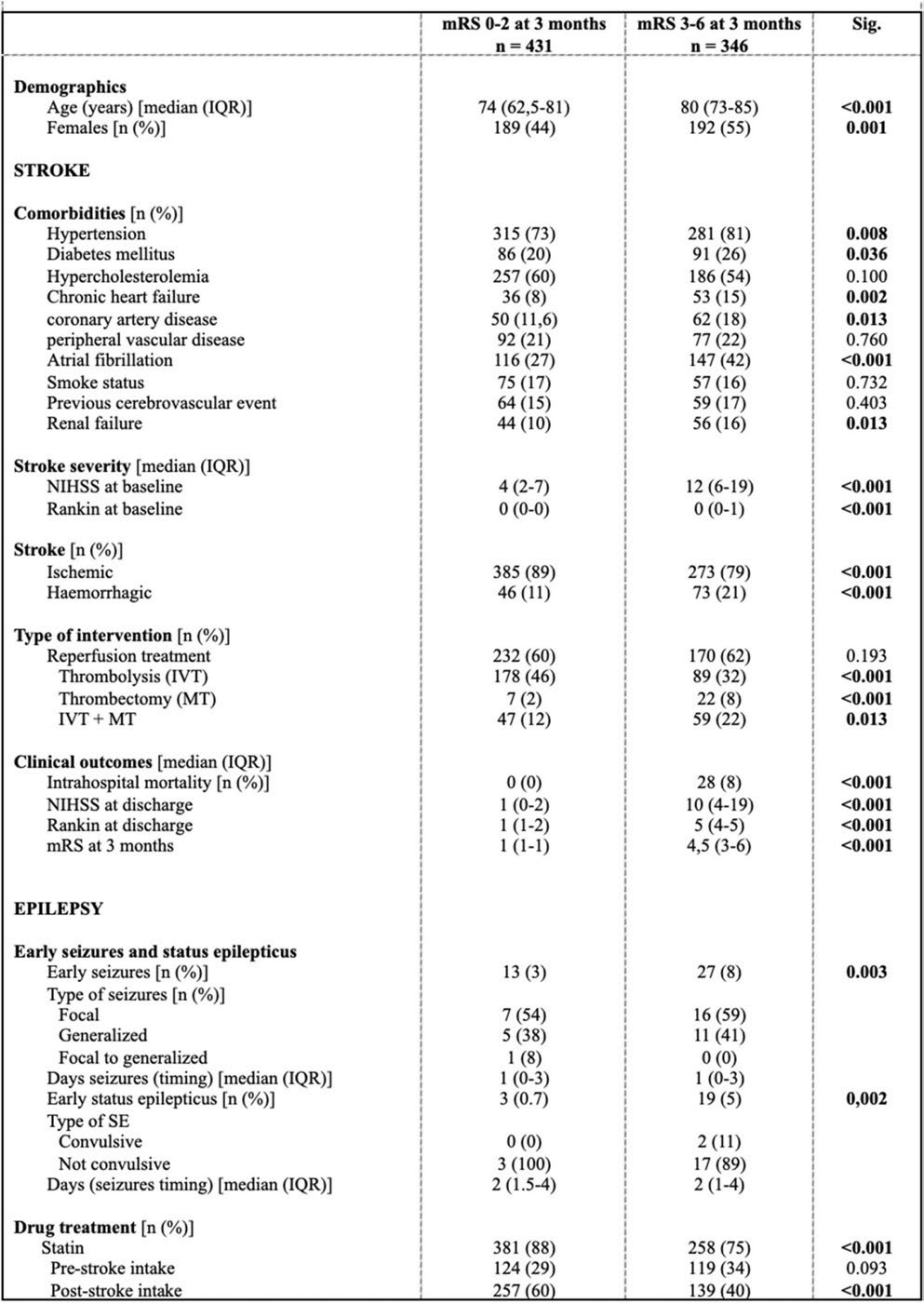
**TABLE 1** Comparison of demographics and clinical characteristics of patients with mRS 0–2 vs patients with mRS 3–6 at 3 months. Data are presented as medians (IQR) and frequencies.

**Figure:**
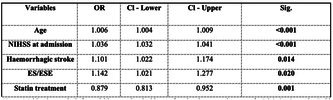
**TABLE 2** Logistic multivariate regression for variables associated with poor functional outcome in terms of mRS 3–6 at 3 months.

**Conclusion:** In a consecutive cohort of patients with acute stroke, statin therapy emerged as an independent protective factor against poor functional outcome at three months, highlighting its potential pleiotropic effects even in patients with haemorrhagic stroke. Further studies are warranted to elucidate the underlying pharmacological mechanisms.

**Disclosure:** Nothing to disclose.

## EPO‐0028

### Impact of statin therapy on post‐stroke epilepsy (PSE): An emerging anti‐epileptogenic strategy

#### E. Ricci ^1^; G. Furlanis^1^; M. Ajčević^2^; K. Iscra^2^; M. Malesani^1^; E. Vincis^1^; G. Prandin^1^; M. Quagliotto^1^; G. Farina^1^; P. Caruso^1^; M. Naccarato^1^; P. Manganotti^1^

##### ^1^Neurology Unit, Department of Medicine, Surgery and Health Sciences, University Hospital and Health Services of Trieste ‐ ASUGI, University of Trieste, Trieste, Italy; ^2^Department of Engineering and Architecture, University of Trieste, Trieste, Italy

**Background and aims:** Growing evidence support the hypothesis that statin therapy in patients with acute stroke may reduce the risk of post‐stroke epilepsy through pleiotropic mechanisms. This study aimed to evaluate factors associated with the development of post‐stroke epilepsy (PSE), with a focus on the potential role of statin therapy, in a real‐world cohort of stroke patients.

**Methods:** Clinical and radiological data of 777 consecutive patients with acute stroke were retrospectively analyzed. Data of patient with PSE and without PSE were compared. Univariate and multivariate logistic regression analysis was conducted to identify variables associated with the development of PSE.

**Results:** The two groups did not differ significantly with respect to demographic characteristics or comorbidities, the median NIHSS score at admission was significantly higher in the post‐stroke epilepsy (PSE) group than in the non‐PSE group (12 vs. 6, *p* < 0.001). Multivariate analysis showed that higher NIHSS scores at admission (OR = 1.004, 95% CI: 1.002–1.007; *p* = 0.001) and the occurrence of early seizures or early status epilepticus (ES/ESE) (OR = 1.112, 95% CI: 1.042–1.187; *p* < 0.001) were independently associated with an increased risk of PSE, while statin therapy emerged as an independent protective factor against PSE development (OR = 0.948, 95% CI: 0.915–0.999; *p* = 0.048).

**Figure:**
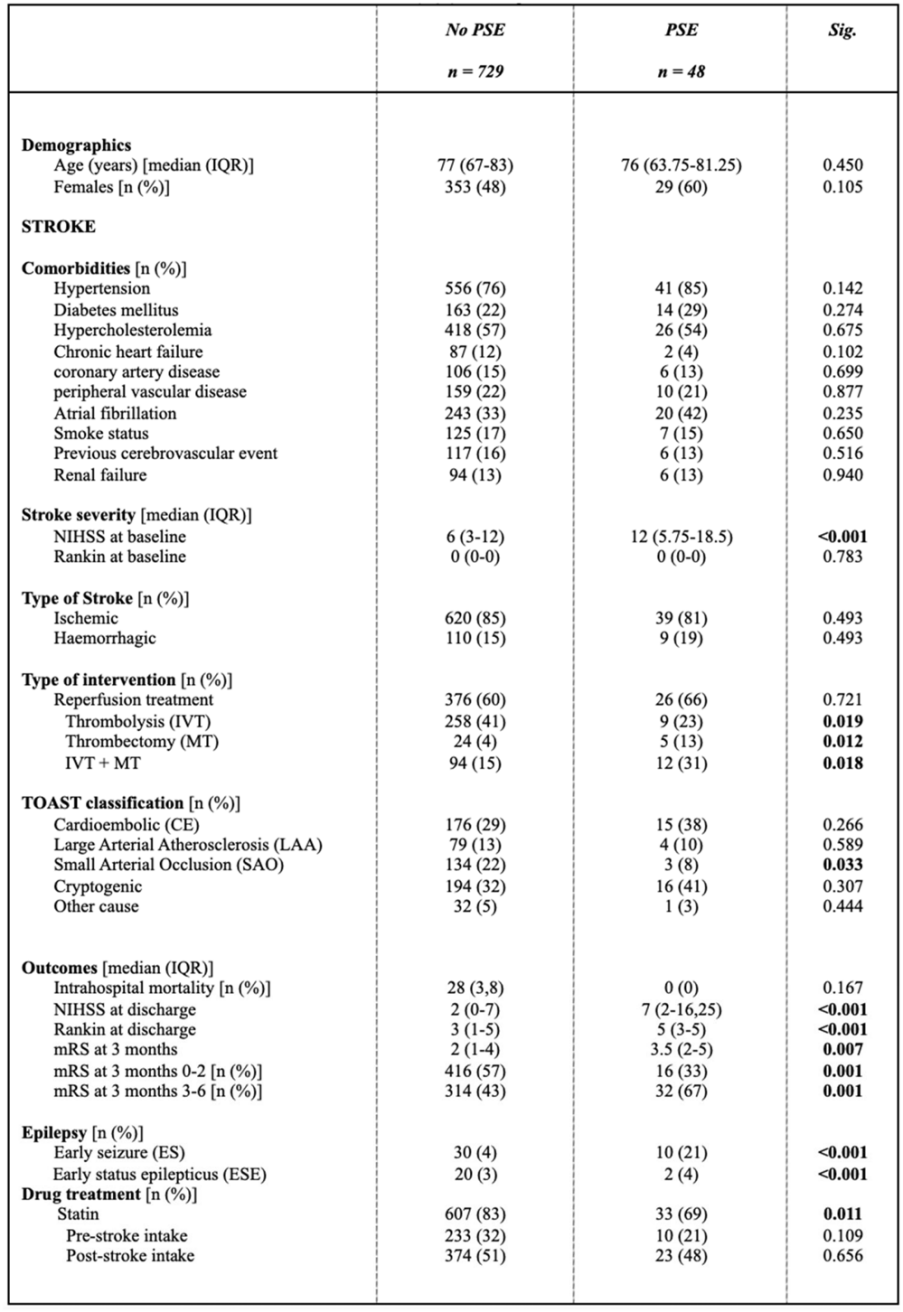
**TABLE 1** Comparison of demographics and clinical characteristics of patients with PSE vs patients without PSE. Data are presented as medians (IQR) and frequencies.

**Conclusion:** In a consecutive cohort of acute stroke patients, statin therapy emerged as an independent protective factor against the development of PSE, underscoring its potential role in antiepileptogenic processes.

**Figure:**
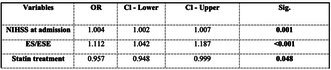
**TABLE 2** Logistic multivariate regression for variables associated with the onset of post‐stroke epilepsy.

**Disclosure:** Edoardo Ricci: Nothing to disclose. Giovanni Furlanis: Nothing to disclose. Milos Ajčević: Nothing to disclose. Katerina Iscra: Nothing to disclose. Michele Malesani: Nothing to disclose. Emanuele Vincis: Nothing to disclose. Gabriele Prandin: Nothing to disclose. Magda Quagliotto: Nothing to disclose. Gianpiero Farina: Nothing to disclose. Paola Caruso: Nothing to disclose. Marcello Naccarato: Nothing to disclose. Paolo Manganotti: Nothing to disclose.

## EPO‐0029

### Early versus late starting of direct oral anticoagulants after breakthrough ischemic stroke: A target trial analysis from the ASPERA‐R study

#### F. Gabriele ^1^; L. D'Anna^2^; M. Foschi^1^; R. Ornello^1^; A. Zini^3^; A. Cascio Rizzo^4^; L. Pantoni^5^; M. Bagnato^6^; B. Casolla^7^; B. Fuentes^8^; P. Candelaresi^9^; D. Aguiar de Sousa^10^; P. Caliandro^11^; A. Abdelalim^12^; L. Zhang^13^; A. El Bassiouny^14^; F. Ferrari^15^; M. Guarino^16^; G. Rinaldi^17^; G. Frisullo^18^; M. Mannino^19^; A. Fonseca^20^; H. Budincevic^21^; G. Viticchi^22^; L. Barba^23^; P. Lochner^24^; S. Buddha^25^; M. Piscaglia^26^; M. Zedde^27^; A. Nasreldein^28^; L. Vinciguerra^29^; A. Elsaid Elsayed^30^; M. Al Banna^31^; L. Tudisco^32^; G. Merlino^33^; F. De Santis^1^; S. Sacco^1^

##### ^1^Department of Biotechnological and Applied Clinical Sciences, University of L’Aquila, L’Aquila, Italy; ^2^Department of Stroke and Neuroscience, Charing Cross Hospital, Imperial College London NHS Healthcare Trust, London, UK (UK); ^3^IRCCS Istituto delle Scienze Neurologiche di Bologna, Department of Neurology and Stroke Center, Maggiore Hospital, Bologna, Italy; ^4^Department of Neurology and Stroke Unit, ASST Grande Ospedale Metropolitano Niguarda, Milan, Italy; ^5^Neuroscience Research Center, Department of Biomedical and Clinical Sciences, University of Milan, Italy; ^6^UOC Stroke Unit e Neurologia, Ospedale Fabrizio Spaziani, Frosinone, Italy; ^7^Stroke Unit, CHU Pasteur 2, Université Cote d'Azur, UMR2CA URRIS, Nice, France; ^8^Stroke Center and Department of Neurology. Hospital La Paz Institute for Health Research (La Paz University Hospital‐Universidad Autónoma de Madrid), Madrid, Spain; ^9^UOC Neurologia e Stroke Unit, AORN Cardarelli, Napoli, Italy; ^10^Stroke Center, Department of Neurosciences, Central Lisbon University Hospital Centre ‐ ULS São José, Faculty of Medicine, University of Lisbon, Lisbon, Portugal; ^11^Department of Neuroscience, Catholic University of the Sacred Hearth, Rome, Italy; UOC Neurology, Department of Neuroscience, Sensory Organs, and Thorax, Fondazione Policlinico Universitario A. Gemelli IRCCS, Rome, Italy; ^12^Cairo University Stroke Center, Department of Neurology, Faculty of Medicine, Cairo University, Egypt; ^13^Department of Neurology, St George's University Hospital, London, UK; ^14^Neurology Department, Faculty of Medicine, Ain Shams University, Cairo, Egypt; ^15^Department of Brain and Behavioral Sciences, University of Pavia, Pavia, Italy; ^16^IRCCS Istituto delle Scienze Neurologiche di Bologna, Italy; ^17^S.C. Neurologia, Ospedale “Di Venere”, Bari, Italy; ^18^Emergency Neurology ‐ Department of Neuroscience, Sensory Organs, and Thorax ‐ Policlinico Universitario Agostino Gemelli, IRCCS Rome, Italy; ^19^UOC Neurologia e Stroke Unit, AOOR Villa Sofia‐Cervello, Palermo, Italy; ^20^Neurology Department, Hospital de Santa Maria, Faculty of Medicine, University of Lisbon, Portugal; ^21^Department of Neurology, Sveti Duh University Hospital, Zagreb, Croatia; ^22^Neurological Clinic, Marche Polytechnic University, Ancona, Italy; ^23^Department of Neurology, Martin‐Luther‐University Halle‐Wittenberg, Germany; ^24^Department of Neurology, Saarland University Medical Center, Homburg 66421, Germany; ^25^Department of Stroke Medicine, Southmead Hospital, North Bristol NHS Trust, Bristol, UK; ^26^Department of Neurosciences, Stroke Unit – Neurology Unit, S.Maria delle Croci Hospital, AUSL Romagna, Ravenna, Italy; ^27^Neurology Unit, Stroke Unit, Azienda Unità Sanitaria Locale‐IRCCS di Reggio Emilia, Italy; ^28^Department of Neurology, Assiut University, Assiut, Egypt; ^29^Department of Neurology and Stroke Unit, ASST Crema Hospital, Italy; ^30^Neurology and Neurointervention Department, KobryElkoba Medical Complex, Cairo, Egypt; ^31^Department of Neurology, National Neuroscience Institute, King Fahad Medical City, Riyadh, Saudi Arabia; ^32^Stroke Unit, Careggi University Hospital, Florence, Italy; ^33^SOSD Stroke Unit, Udine University Hospital, Italy

**Background and aims:** Randomized trials support early initiation of direct oral anticoagulants (DOACs) after atrial fibrillation (AF)–related ischemic stroke. However, patients with breakthrough ischemic stroke occurring despite ongoing anticoagulation have been largely under‐represented. We evaluated the effectiveness and safety of early versus delayed DOAC initiation in these patients.

**Methods:** We performed a target trial emulation comparing early versus delayed DOAC initiation in patients with AF experiencing ischemic stroke on anticoagulation from the retrospective arm of the international, multicentre ASPERA study. Treatment strategies were defined using severity‐adapted timing thresholds based on baseline National Institutes of Health Stroke Scale (NIHSS) scores. A cloning–censoring–weighting approach with inverse probability weighting was applied to emulate random assignment and avoid immortal time bias. Primary outcomes were 90‐day new ischemic events and moderate‐to‐severe bleeding. Risk ratios (RRs), absolute risk differences (RDs), and hazard ratios (HRs) were estimated using weighted regression and Cox models.

**Results:** Among 833 patients (median age 81 years), 336 were assigned to early and 497 to delayed DOAC initiation. At 90 days, early initiation was associated with a lower risk of new ischemic events (RR 0.44, 95% CI 0.21–0.90; RD −3.64%, 95% CI −6.40 to −0.87; HR 0.43, 95% CI 0.21–0.91), reduced moderate‐to‐severe bleeding (RR 0.10, 95% CI 0.01–0.76) and decreased all‐cause and vascular mortality (FIGURE 1 and 2). A Net Early Benefit Score integrating ischemic and bleeding risks was positive across all NIHSS strata.

**Figure:**
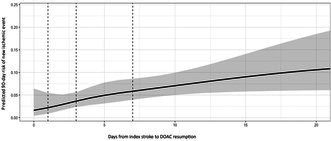
**FIGURE 1** Predicted risk of new ischemic event at 90 days based on days to DOAC resumption.

**Figure:**
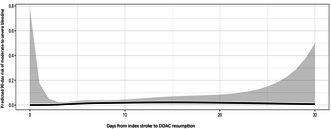
**FIGURE 2** Predicted risk of moderate‐severe bleeding at 90 days based on days to DOAC resumption.

**Conclusion:** Early severity‐adapted DOAC initiation was associated with lower risks of recurrent ischemic events and mortality at 90 days without increasing major bleeding.

**Disclosure:** AZ received consulting and speaker fees from Angels Initiative, Boehringer‐Ingelheim, Alexion, Daiichi Sankyo, Pfizer, Amgen, Aurora Biopharma, and fees for Advisory Board from Bayer, Daiichi Sankyo, Astra Zeneca, and Boehringer‐Ingelheim, none relevant to this work. The other authors report no conflicts.

## EPO‐0030

### Prophylactic induced hypertension in deep perforator infarction: Impact on early neurological deterioration and blood pressure variability

#### H. Park ^1^; J. Park^2^; J. Cha^3^

##### ^1^INHA University Hospital, Incheon Korea, Republic of; ^2^Catholic University of Korea Eunpyeong St. Mary's Hospital, Republic of Korea; ^3^Dong‐A university Hospital, Republic of Korea

**Background and aims:** Early neurological deterioration (END) is a frequent and disabling complication in acute ischemic stroke. We evaluated whether prophylactic pharmacologically induced hypertension (PPiHT) could reduce the risk of END and improve clinical outcomes in high‐risk population.

**Methods:** Among 424 patients, 118 (27.8%) received PPiHT. After PSM, 260 patients were retained (118 PPiHT, 142 non‐PPiHT). END occurred significantly less often with PPiHT. Within 3 months after stroke onset, the PPiHT group had lower mRS, lower incidence of recurrent stroke, and no increase in hemorrhagic complications. The PPiHT group had a lower incidence of END compared to patients with initial SBP ≥ 180 mmHg.

**Results:** Among 424 patients, 118 (27.8%) received PPiHT. After PSM, 260 patients were retained (118 PPiHT, 142 non‐PPiHT). END occurred significantly less often with PPiHT. Within 3 months after stroke onset, the PPiHT group had lower mRS, lower incidence of recurrent stroke, and no increase in hemorrhagic complications. The PPiHT group had a lower incidence of END compared to patients with initial SBP ≥ 180 mmHg.

**Conclusion:** PPiHT was associated with a substantial reduction in END and improved functional outcomes in lacunar infarction. Compared with the patients with initial high SBP, PPiHT had better outcomes, suggesting that it is the drug‐induced increase in SBP, rather than the SBP level itself, that is beneficial.

**Disclosure:** Nothing to disclose.

## EPO‐0031

### Machine Learning models for 3‐month functional outcome prediction after mechanical thrombectomy in acute ischaemic stroke patients

#### K. Jakubik ^1^; M. Woś^2^; A. Szewczyk^3^; P. Luchowski^4^; K. Rejdak^3^; G. Staśkiewicz^5^

##### ^1^Doctoral School, Medical University of Lublin, Lublin, Poland; ^2^Department of Information Technology and Medical Statistics with e‐Health Laboratory, Medical University of Lublin, Lublin, Poland; ^3^Chair and Department of Neurology, Medical University of Lublin, Lublin, Poland; ^4^Department of Neurology and Neurological Nursing, Medical University of Lublin, Lublin, Poland; ^5^I Department of Medical Radiology, Medical University of Lublin, Lublin, Poland

**Background and aims:** Acute ischaemic stroke (AIS) treated with mechanical thrombectomy (MT) carries variable prognosis. Early identification of patients at risk of poor prognosis is crucial for personalised management and family counselling. This study evaluates and compares the performance of various Machine Learning (ML) algorithms in predicting the 3‐month functional outcome using admission clinical and laboratory data.

**Methods:** Data from 843 AIS patients treated with MT were analysed. The primary endpoint was poor functional outcome, defined as a modified Rankin Scale (mRS) score of 3–6 at 3 months. Predictors included demographics, admission National Institutes of Health Stroke Scale (NIHSS) score, comorbidities, laboratory parameters, and recanalisation status. Five classifiers were developed and validated: Logistic Regression (LR), Random Forest (RF), Support Vector Machine (SVM), K‐Nearest Neighbors (KNN), and Multilayer Perceptron (MLP). Model performance was assessed using Accuracy, Area Under the Curve (AUC), and F1‐score.

**Results:** LR demonstrated the best overall performance (Accuracy 0.76, AUC 0.80, F1‐score 0.83). RF and SVM showed comparable results (Accuracy 0.75, AUC 0.80). Feature importance analysis in RF highlighted age and admission NIHSS score as the strongest predictors. More complex models, including MLP and KNN, achieved lower performance (Accuracy ~0.70, AUC ~0.75), indicating limited advantage over linear approaches for this tabular dataset.

**Figure:**
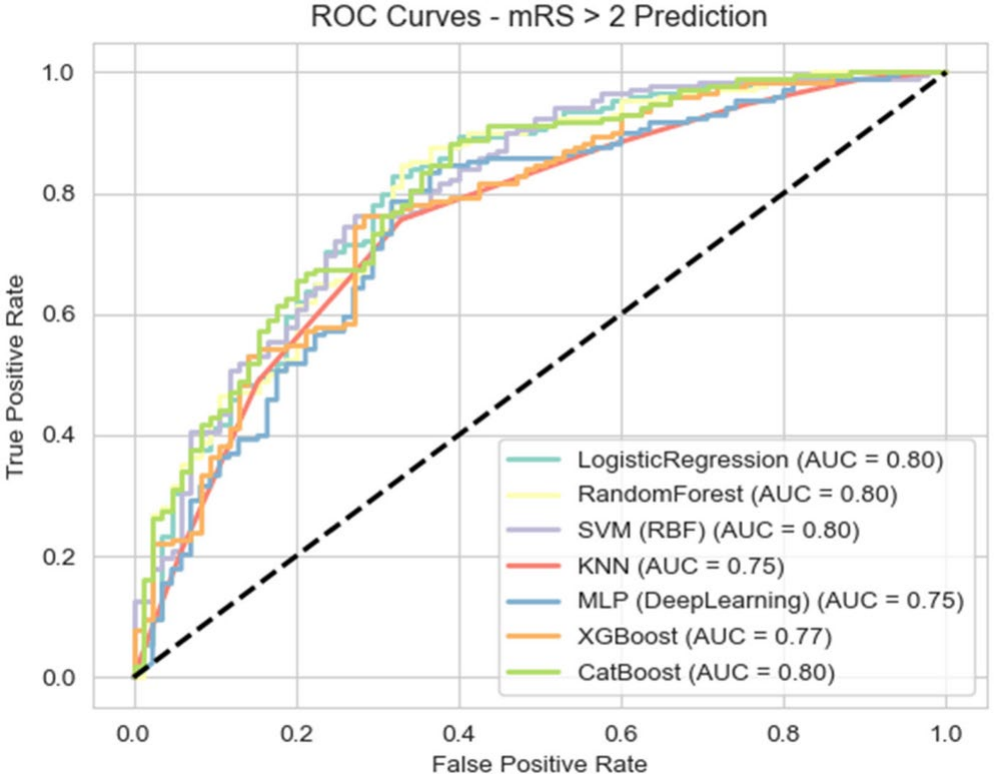
**FIGURE 1** Receiver Operating Characteristic (ROC) curves comparing the performance of seven training ML models in predicting poor functional outcome (mRS > 2) at 3 months.

**Figure:**
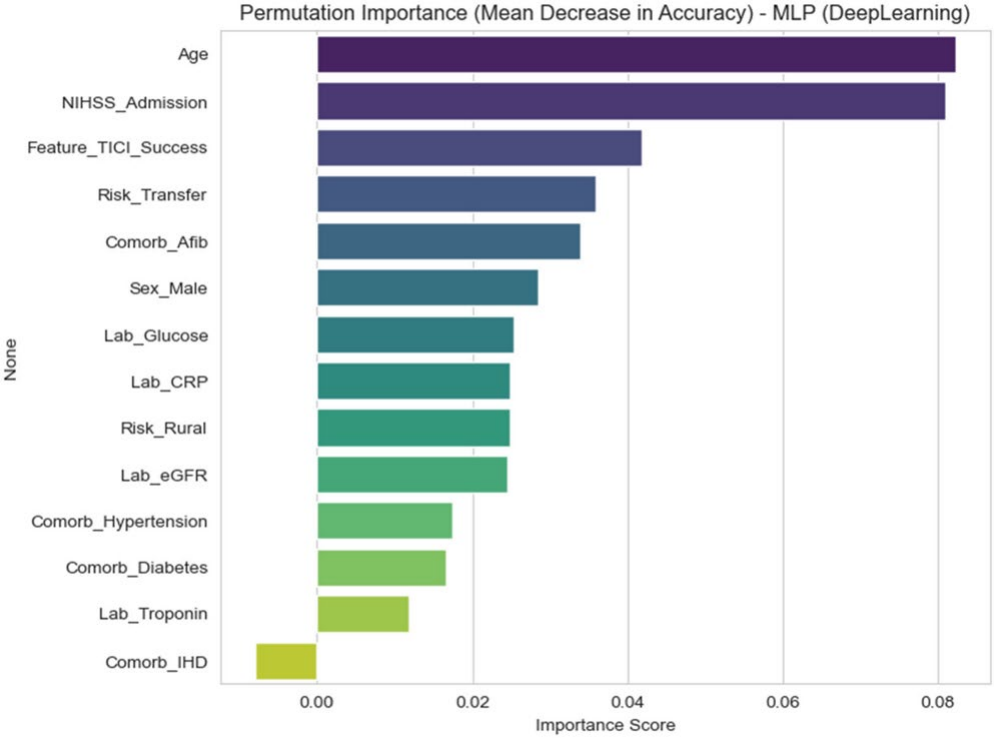
**FIGURE 2** Feature importance ranking from the MLP model, showing NIHSS score on admission and age as the strongest predictors of functional outcome, followed by successful recanalisation defined by the Thrombolysis in Cerebral Infarction (TICI) score.

**Figure:**
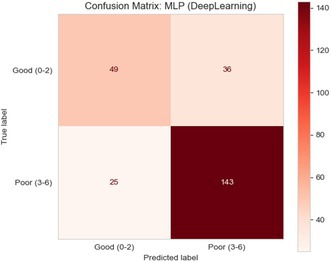
**FIGURE 3** Confusion Matrix for the MLP model on the test dataset. The matrix displays the classification performance in distinguishing between good (mRS 0–2) and poor (mRS 3–6) outcomes.

**Conclusion:** ML models based on admission data can effectively predict functional outcomes after AIS and can provide valuable prognostic support in acute clinical conditions. LR outperformed more complex algorithms, suggesting that key clinical predictors exhibit predominantly linear associations with 3‐month functional outcomes.

**Disclosure:** Nothing to disclose.

## EPO‐0032

### RNF213 variants and angiographic progression in Korean patients with Moyamoya disease and intracranial arterial stenosis

#### K. Park; S. Ahn; M. Park

##### Department of Neurology, Pusan National University Yangsan Hospital, Pusna National University School of Medicine, Yangsan, Republic of Korea

**Background and aims:** RNF213 p.R4810K is a major susceptibility variant for moyamoya disease (MMD) and has been linked to intracranial artery atherosclerosis. We investigated whether RNF213 variants are associated with angiographic progression of intracranial arterial stenosis in Korean patients with MMD and intracranial artery stenosis (ICAS).

**Methods:** We retrospectively analyzed 57 Korean patients diagnosed with MMD or ICAS who underwent RNF213 genotyping and serial angiographic follow‐up (MRA or TFCA). RNF213 p.R4810K status was classified as carrier (heterozygous) or non‐carrier. Angiographic change was categorized as 1) progression 2) no progression 3) improvement; progression was the primary outcome. Baseline clinical characteristics, vascular phenotype, and medical treatments were compared by genotype. Multivariable logistic regression adjusted for age, sex, hypertension, diabetes, dyslipidemia, smoking, and statin use.

**Results:** Among 57 patients, 31 (54.4%) were RNF213 carriers. Progression occurred in 15/31 (48.4%) carriers and 7/26 (26.9%) non‐carriers during mean 5.0 ± 2.8 years (range 2.0–14.2). The 3‐category distribution of angiographic outcomes differed by genotype (*p* = 0.017). In multivariable analysis, RNF213 carrier status was independently associated with progression (adjusted OR 4.06, 95% CI 1.05–15.66; *p* = 0.042). New stroke/TIA during follow‐up occurred in 5/57 (8.8%).

**Conclusion:** In Korean patients with MMD and intracranial artery stenosis, RNF213 p.R4810K carrier status was associated with a higher risk of angiographic progression. These findings suggest that RNF213‐associated vasculopathy may represent a progressive phenotype and warrant closer longitudinal monitoring.

**Disclosure:** Nothing to disclose

## EPO‐0033

### Nutritional risk assessed by CONUT independently predicts 90‐day outcome after mechanical thrombectomy

#### M. Molina Lopez ^1^; P. Blanco Ramírez^1^; M. Jimenez Arenas^1^; M. Mesa Hernandez^1^; J. Gomez Moreno^1^; I. Cordoba Bueno^1^; M. Garcia Falcon^1^; M. Martinez Acevedo^1^; A. Constantino Silva^1^; A. Roa Montero^1^; M. Gomez Baquero^1^; J. Ramirez‐Moreno^2^

##### ^1^Stroke Center, Department of Neurology, Badajoz University Hospital, Badajoz, Spain; ^2^Department of Biomedical Sciences, University of Extremadura; Multidisciplinary Research Group of Extremadura (GRIMEX); University Institute for Biosanitary Research of Extremadura (INUBE), Spain

**Background and aims:** Malnutrition may worsen recovery after large‐vessel occlusion stroke, yet its prognostic value in patients treated with mechanical thrombectomy remains insufficiently explored. We aimed to assess whether nutritional status measured by the Controlling Nutritional Status (CONUT) score predicts 90‐day functional outcome and mortality after mechanical thrombectomy.

**Methods:** This observational cohort study included 385 patients treated with mechanical thrombectomy. Nutritional status at admission was assessed using the CONUT score. Outcomes were poor functional outcome at 90 days (modified Rankin Scale 3–6) and 90‐day mortality. Univariable and multivariable logistic regression analyses were performed, and model discrimination was evaluated using receiver operating characteristic curves.

**Results:** Patients were distributed across CONUT categories as follows: normal 96 (21%), mild 169 (37%), moderate 99 (21%), and severe 21 (5%). Increasing CONUT scores were associated with progressively worse 90‐day modified Rankin Scale distributions. In multivariable analysis, each one‐point increase in the CONUT score was independently associated with poor functional outcome (adjusted odds ratio 1.20; 95% confidence interval 1.05–1.36; *p* = 0.008) and with 90‐day mortality (adjusted odds ratio 1.26; 95% confidence interval 1.11–1.42; *p* < 0.001). Discrimination for poor functional outcome was good (area under the curve 0.85).

**Figure:**
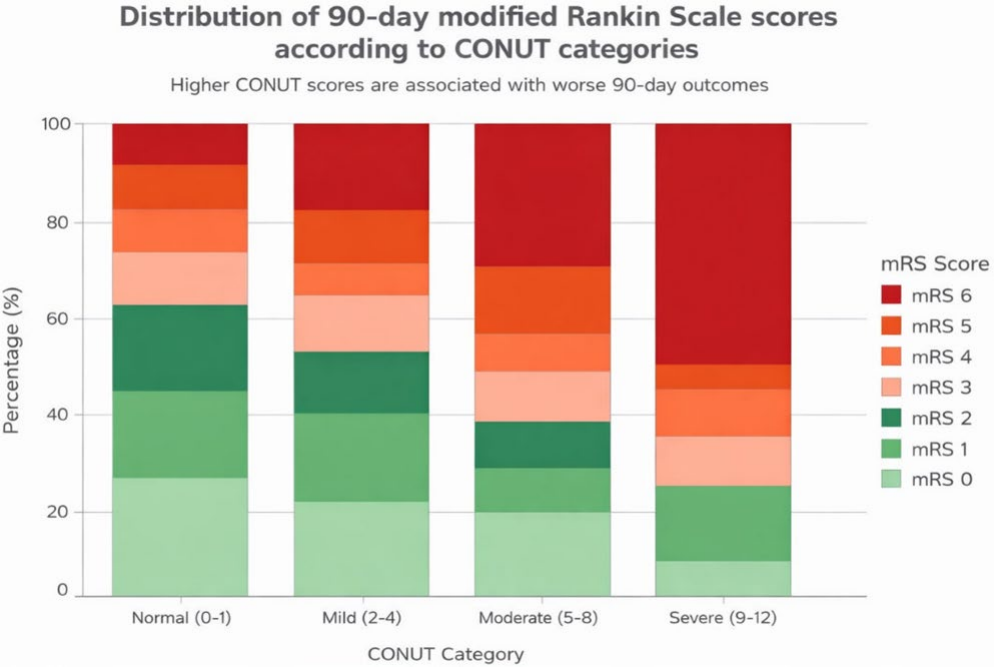
**FIGURE 1** Distribution of 90‐day modified Rankin Scale scores according to CONUT categories (normal, mild, moderate and severe malnutrition).

**Conclusion:** Nutritional risk assessed by the CONUT score independently predicts functional disability and mortality at 90 days after mechanical thrombectomy and may support early risk stratification using routine laboratory data.

**Disclosure:** Nothing to disclose.

## EPO‐0034

### The cost of acute stroke: A detailed analysis focusing on sex differences

#### M. Rodrigues ^1^; P. Pereira^2^; C. Ferreira^3^; L. Pereira^2^; J. Cerqueira^4^; M. Viana‐Baptista^5^

##### ^1^Neurology Department/Stroke Unit, ULS Almada‐Seixal, Almada; School of Medicine, University of Minho, Braga, Portugal; ^2^Neurology Department/Stroke Unit, ULS Almada‐Seixal, Almada, Portugal; ^3^Neurology Department, ULS Braga, Braga, Portugal; ^4^University of Minho, Braga; Neurology Department, ULS Braga, Braga, Portugal; ^5^Neurology Department, ULS Lisboa Ocidental, Lisbon; CEDOC ‐ NOVA Medical School, Lisbon, Portugal

**Background and aims:** Stroke has significant economic impact due to high prevalence and disability. Studies across Europe show disparities in costs among countries, underscoring the necessity for country‐specific data. Few studies have explored cost differences between sexes. Using a microcosting approach, we analysed a cohort of ischemic stroke survivors to estimate costs, comparing expenses across levels of disability and between sexes.

**Methods:** Demographic data and healthcare resource utilization information were collected from ischemic stroke patients, at two tertiary hospitals in Portugal, over two years, employing validated questionnaires. We report mean costs (euros) at 3 months and 1 year post‐stroke, per patient, with 95% confidence intervals (95% CI). Disability levels were classified by the modified Rankin Scale (mRS). Cost comparisons employed non‐parametric tests, non‐linear regression, and covariance analysis.

**Results:** We included 206 patients, median age 75 years, 52% male, and 24% employed at stroke onset. The average hospitalization cost was €11,149 (95% CI 9,970–12,328), while total outpatient costs were €4,792 (95% CI 3,970–5,614) at 3 months and €13,195 (95% CI 10,602–15,788) at 1 year. At 1 year, outpatient costs per disability level peaked at mRS 4 (€3,549 to €23,543; *p* < 0.001). Male patients exhibited average higher costs (€15,065 vs. €11,087, *p* > 0.05), driven mainly by non‐medical and indirect costs, and they increased exponentially with disability (*R* ^2^ = 0.51 in males versus 0.27 in females; *p* < 0.030).

**Conclusion:** We showed annual outpatient stroke costs amounting to 16 minimum wages. The escalation of cost with disability is notable, and consistent with prior studies. This increase was significantly more pronounced in males, emphasizing the importance of sex‐specific healthcare studies.

**Disclosure:** CUF scholarship.

## EPO‐0035

### HBOC‐mediated pre‐reperfusion oxygen delivery freezes ischemic penumbra via suppression of ferroptosis and neuroinflammation

#### Q. Li; L. Yang; Y. Jia

##### the First Affiliated Hospital of Zhengzhou University, Zhengzhou, China

**Background and aims:** Overcoming the challenges of delayed intervention and single‐target mechanisms in stroke neuroprotection, we propose a paradigm‐shifting “pre‐reperfusion” therapy using a hemoglobin‐based oxygen carrier (HBOC) for diffusive oxygen delivery. This study aims to validate the efficacy of HBOC in preserving the ischemic penumbra and elucidate its integrated mechanism involving the mitochondria‐ferroptosis‐inflammation axis.

**Methods:** In a rat transient middle cerebral artery occlusion (tMCAO) model, a single intravenous dose of HBOC or saline was administered 5 minutes post‐ischemia, followed by reperfusion at 1 hour. Assessment included: 1) safety and dose‐escalation (mortality, hemorrhagic transformation, systemic toxicity); 2) multi‐timepoint outcomes (infarct volume by MRI/TTC, neurobehavior from 4 h to 30 d); 3) mechanistic dissection (mitochondrial ultrastructure/ATP, ferroptosis markers GPX4/ACSL4/GSH/Fe^2+^, neuroinflammation via cytokine array and microglial polarization).

**Figure:**
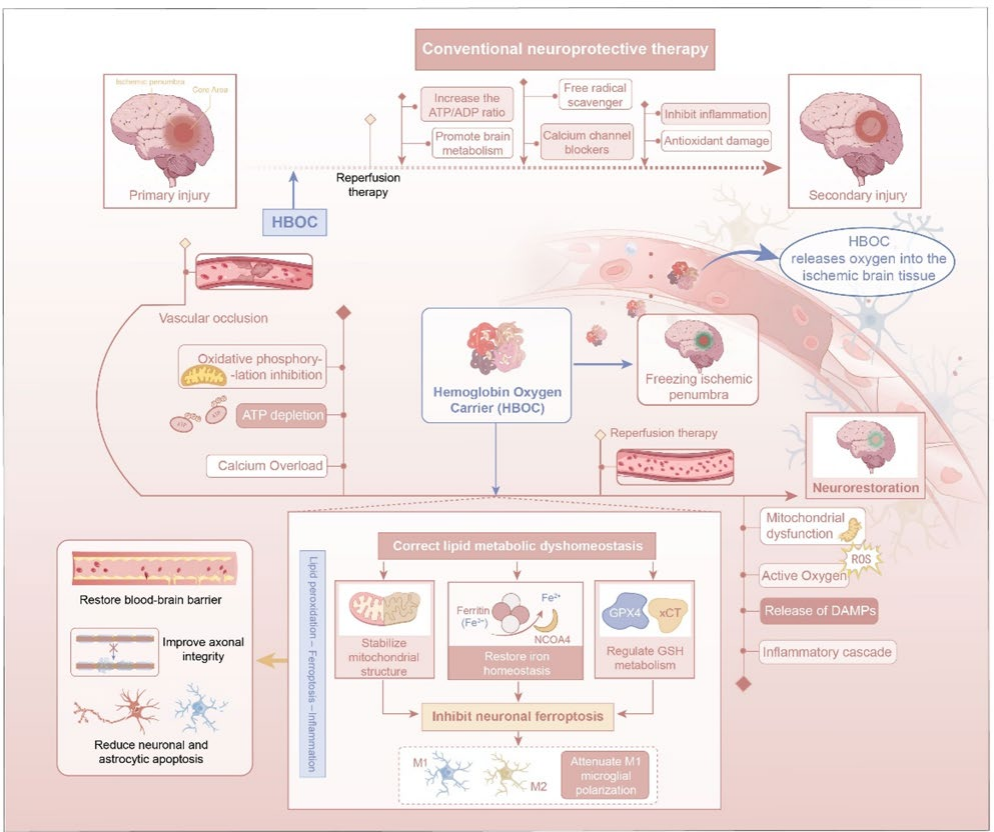
**FIGURE 1** Schematic illustrating the proposed multi‐target cytoprotective mechanism of pre‐reperfusion HBOC therapy.

**Results:** Optimal‐dose HBOC was safe, with no increased bleeding risk or systemic toxicity. It conferred dose‐dependent neuroprotection, significantly reducing infarct volume at 24 h with progressive resolution up to 30 days, alongside robust and sustained neurological improvement. Mechanistically, HBOC rapidly stabilized mitochondrial ultrastructure, suppressed lipid peroxidation, and orchestrated a coordinated anti‐ferroptotic response. This cascade drove a sustained shift in microglial polarization from pro‐inflammatory M1 to reparative M2 phenotype, attenuating neuroinflammation.

**Figure:**
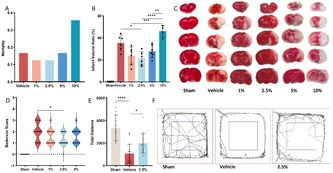
**FIGURE 2** Dose‐dependent neuroprotection and safety profile of HBOC in tMCAO rats.

**Conclusion:** Pre‐reperfusion diffusive oxygen delivery via HBOC robustly preserves the ischemic penumbra through a multi‐target mechanism centered on mitochondrial stabilization, ferroptosis inhibition, and immunomodulation. Our findings establish HBOC as a novel, practical “bridge therapy” for the hyper‐acute phase, offering a transformative strategy to extend the therapeutic window in ischemic stroke.

**Disclosure:** Nothing to disclose.

## Child Neurology/Developmental Neurology

## EPO‐0036

### Therapeutic hypothermia for neonatal hypoxic‐ischemic encephalopathy: A meta‐analysis of neurodevelopmental and mortality outcomes

#### C. Cortes Armijo^1^; D. Abouda^3^; G. Peraza^4^; H. Ramzan^5^; M. Whdan^6^; F. Aisha^7^; S. Eldesouky^8^; K. Zinhom ^9^; A. Fathallah^2^

##### ^1^Facultad de Medicina, Universidad Autónoma de Nuevo León, Nuevo León, México; ^2^Faculty of Medicine, Minya University, Minya, Egypt; ^3^Faculty of medicine, Alexandria University, Alexandria, Egypt; ^4^Institution/Organization: Facultad de Medicina, Universidad Autónoma de Nuevo León, Nuevo León, México; ^5^Dow Medical College, Dow University of Health Sciences, Karachi, Pakistan; ^6^Faculty of medicine Ain Shams University, Cairo, Egypt; ^7^Islamic International Medical College, Riphah International University; ^8^Faculty of Medicine, Modern University for Technology and Information (MTI), Cairo, Egypt; ^9^Faculty of medicine, October 6 university, Giza, Egypt

**Background and aims:** Neonatal Hypoxic‐Ischemic Encephalopathy (HIE) stills a leading cause of mortality and morbidity in newborns. While Therapeutic Hypothermia (TH) has been used widely for moderate to severe HIE management; however, uncertainty persists regarding the consistency of its benefits, particularly for long‐term neurodevelopmental outcomes. This study aimed to synthesize current evidence on the impact of TH on mortality and neurodevelopment in term and near‐term infants with HIE.

**Figure:**
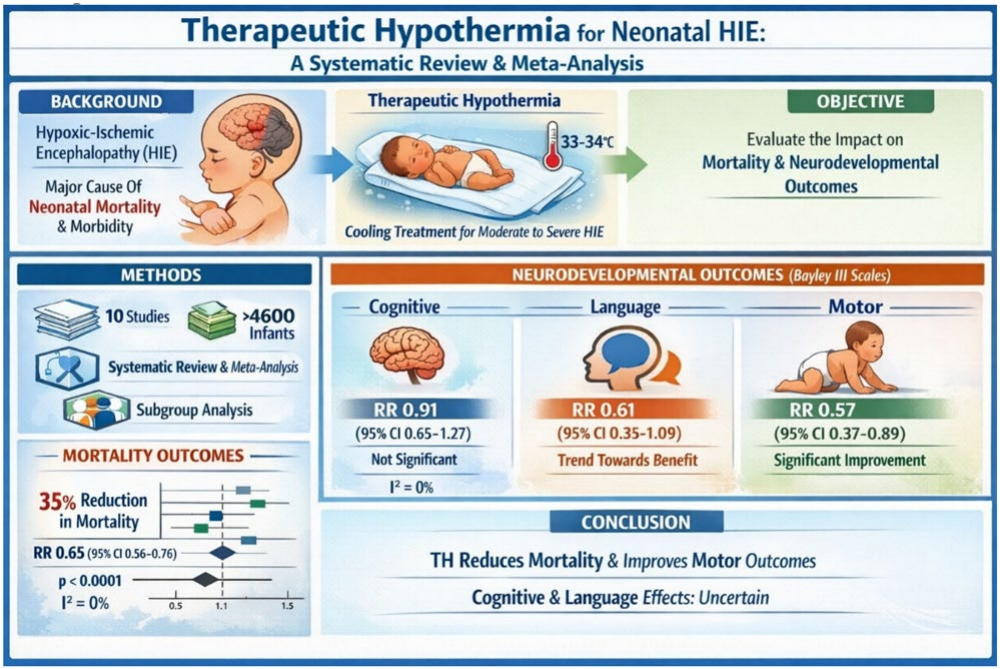
**FIGURE 1** Therapeutic hypothermia for neonatal HIE: A systematic review & meta‐analysis.

**Methods:** We conducted a meta‐analysis including 10 primary studies that compared TH with standard care in neonates diagnosed with HIE. Data from over 4,600 infants were combined for analysis. The primary outcome was all‐cause mortality, which was estimated using risk ratio. We measured Neurodevelopmental outcomes with validated assessment tools such as Bayley Scales. Additional subgroup analyses were conducted according to HIE severity and World Bank income categories.

**Results:** The combined findings showed that therapeutic hypothermia led to a meaningful reduction in all‐cause mortality, with about a 35% lower risk compared with standard treatment. The results were consistent across studies, with little variation observed. According to neurodevelopment outcomes, infants who received TH showed significantly better motor outcomes. Improvements in language and cognitive outcomes were also noted, but these effects did not always reach statistical significance and less consistent.

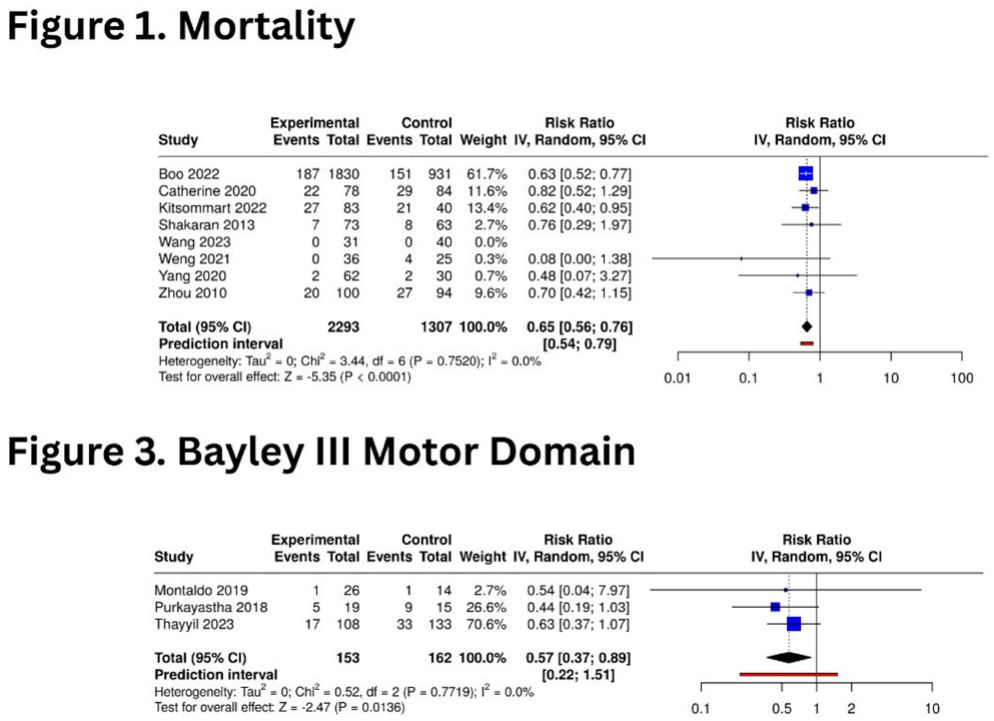

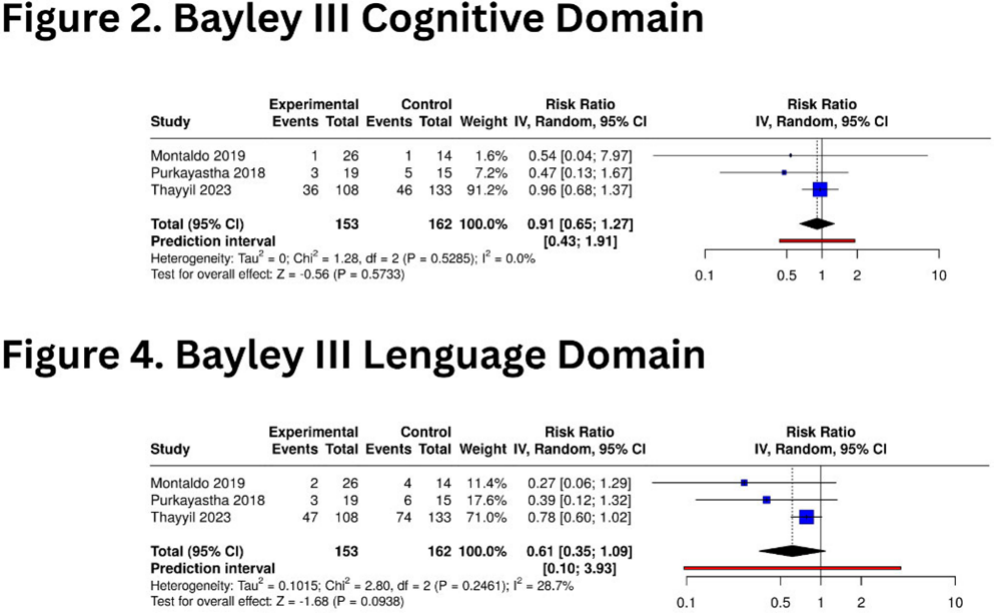

**Conclusion:** TH is effective in decreasing mortality and improving motor outcomes in infants with HIE. However language and cognitive outcomes suggest some benefit, more standardized follow‐up and long‐term studies are needed to confirm these effects.

**Disclosure:** Nothing to disclose.

## EPO‐0037

### Overcoming the “Tunnel of fear”: A systematic review of interventions for MRI‐related anxiety and phobia in pediatrics

#### Cortes Armijo^1^; D. Atef Abouda^2^; Z. Mohammed Bazbaz^3^; A. Ali^3^; B. Nawar^4^; A. Al‐Ghnaimat^5^; S. Hosny El‐Farargy^6^; A. Abdelmawgoud Sadeek^7^; A. Ahmed Radi^8^; H. Abdelbaqi^9^; A. M. Saad^2^; K. Zinhom^10^

##### ^1^Facultad de Medicina, Universidad Autónoma de Nuevo León, Nuevo León, México; ^2^Faculty of medicine, Alexandria University, Alexandria, Egypt; ^3^Najah National University, Nablus, Palestine; ^4^Institution/Organization: Faculty of Medicine, Helwan University, Cairo, Egypt; ^5^Hashemite University,Department Of Medicine, Jordan; ^6^Faculty Of Medicine Benha University, Benha, Egypt; ^7^Faculty of pharmacy, South Valley university, Egypt; ^8^Faculty of Medicine, Minia University, Minia, Egypt; ^9^Faculty of Medicine, Tanta University, Tanta, Egypt; ^10^Faculty of Medicine, October 6 University, Giza, Egypt

**Background and aims:** Pediatric MRI often fails due to anxiety‐induced motion or requires risky general anesthesia. This review synthesizes evidence on strategies to improve compliance and diagnostic quality. The study aims to evaluate the effectiveness of behavioral, digital, and pharmacological interventions in reducing anxiety in pediatric patients (ages 4–18).

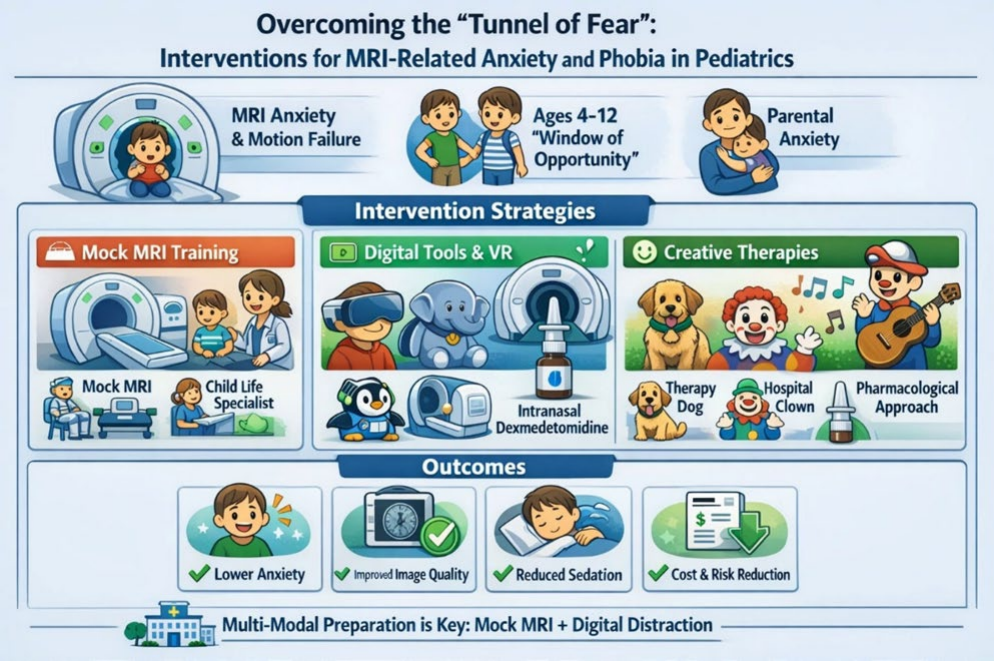

**Methods:** Following PRISMA guidelines, we analyzed 41 primary studies, including RCTs and cohorts. Outcomes included anxiety scores (e.g, mYPAS), image quality, and sedation reduction rates.

**Results:** Mock MRI sessions (30–60 minutes) led by Child Life Specialists or pediatricians successfully converted “sedation‐required” patients to “awake‐successful” status through “statue games” and sound simulation. Digital innovations, such as the “Pengunaut Trainer” VR app and in‐bore systems like “Ollie the Elephant,” provided progressive desensitization and real‐time guidance. Creative approaches, including therapy dogs, hospital clowns, and live music, significantly improved emotional status. Additionally, brief 15‐minute Patient‐Centered Communication (PCC) sessions built effective cooperation. When behavioral methods were insufficient, intranasal Dexmedetomidine (3 μg/kg) emerged as a superior, less invasive rescue sedation compared to Midazolam.

**Figure:**
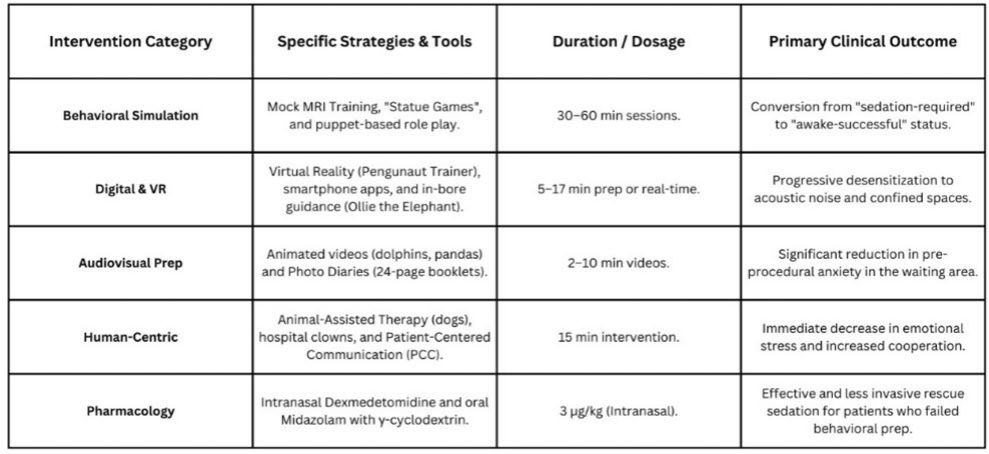
**TABLE 1** Summary of Evidence: Pediatric MRI Compliance strategies (*n* = 41).

**Conclusion:** The most effective strategy for managing pediatric MRI phobia is Multi‐modal preparation combining Mock MRI and digital distraction. Hospitals should prioritize these low‐risk protocols to decrease anesthesia reliance.

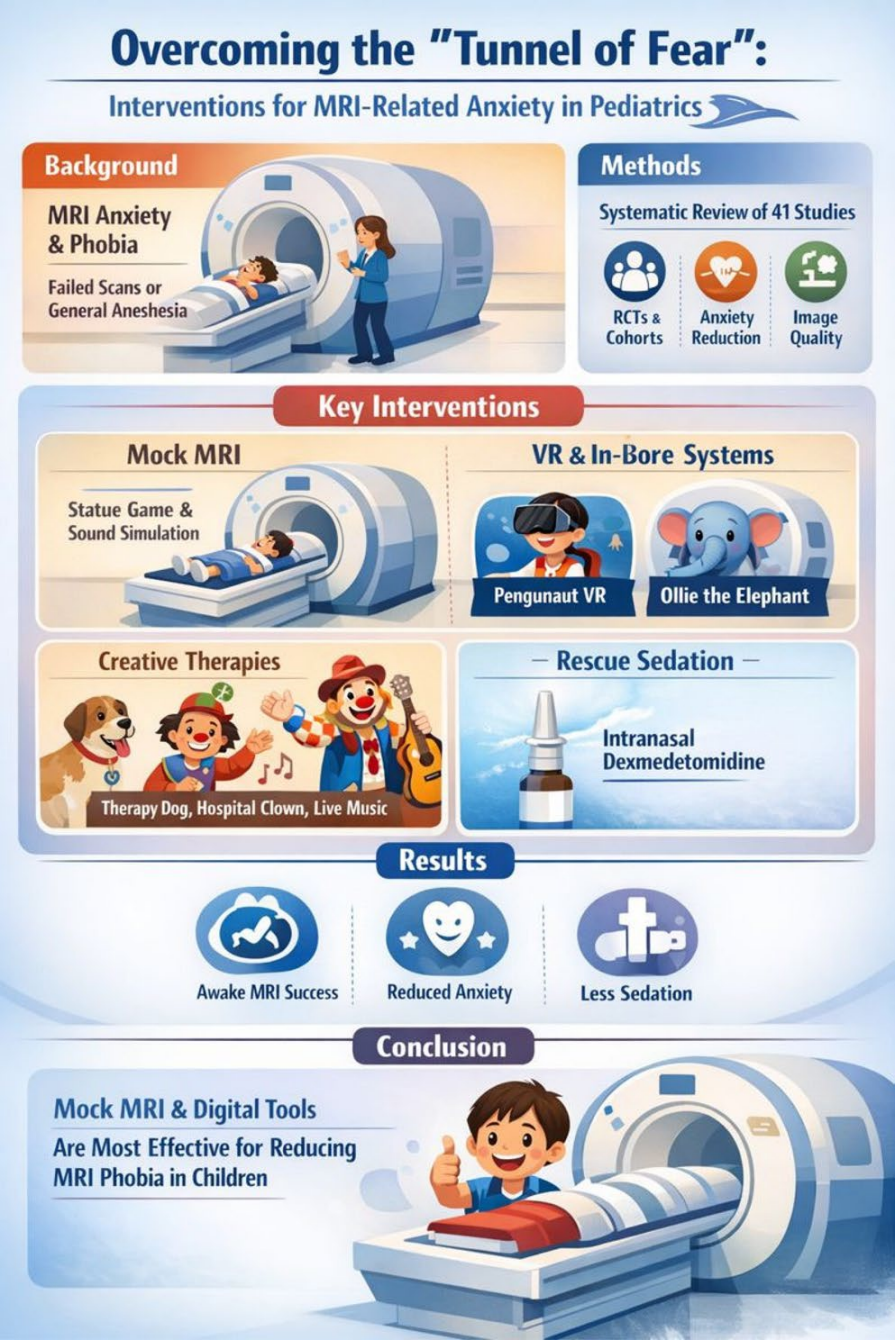

**Disclosure:** Nothing to disclose.

## EPO‐0038

### Clinical and demographical profile of pediatric MOGAD patients: Portuguese national data

#### D. Krupka ^1^; C. Silva^2^; I. Monteiro^3^; T. Painho^4^; V. Cadete^4^; C. Marques Matos^4^; R. Lopes da Silva^4^; S. Rebelo Costa^5^; J. Martins^5^; S. Figueiroa^5^; J. Fonseca^6^; C. Melo^6^; M. Sampaio^6^; R. Inácio^7^; T. Proença dos Santos^7^; S. Quintas^7^; Â. Pereira^8^; M. Matias^9^; T. Barata Silvério^9^; J. Nuno Carvalho^9^; R. Pais^10^; M. Leitão Marques^1^; C. Soares dos Santos^1^; J. Amaral^1^; J. Afonso Ribeiro^1^; C. Pereira^1^; F. Palavra^1^

##### ^1^Child Development Center – Neuropediatrics, Pediatric Hospital, Coimbra Local Health Unit, Coimbra, Portugal; ^2^Neurology Service, Santo André Hospital, Local Health Unit of the Leiria Region, Leiria, Portugal; ^3^Neurology Service, Hospitais da Universidade de Coimbra, Coimbra Local Health Unit, Coimbra, Portugal; ^4^Pediatric Neurology Unit, D. Estefânia Hospital, S. José Local Health Unit , Lisboa, Portugal; ^5^Pediatric Neurology Service, Northern Maternal and Child Center, Santo António Local Health Unit, Porto, Portugal; ^6^Neuropediatrics Unit, S. João Local Health Unit, Porto, Portugal; ^7^Neuropediatrics Unit, Santa Maria Local Health Unit, Lisboa, Portugal; ^8^Pediatric Service, Braga Local Health Unit, Braga, Portugal; ^9^Torrado da Silva Child Development Center, Hospital Garcia de Orta, Almada‐Seixal Local Health Unit, Almada, Portugal; ^10^Medical Imaging Service, Neuroradiology Unit, Coimbra Local Health Unit, Coimbra, Portugal

**Background and aims:** Myelin oligodendrocyte glycoprotein antibody–associated disease (MOGAD) is an autoimmune demyelinating disease affecting both adults and children. Despite its clinical heterogeneity, the prognosis is often favorable. This study aimed to describe demographic and clinical characteristics of pediatric MOGAD patients in Portugal and to identify potential risk factors for relapsing disease.

**Methods:** We conducted a retrospective and multicenter study including pediatric patients (age below 18 years‐old) with MOGAD diagnosed between 2011 and 2025. Data were collected from clinical records from participant centers.

**Results:** Seventy‐seven patients met the inclusion criteria; 49.7% were male and the mean age was 7.8 years‐old. A preceding infection was reported in 45.5% of cases. ADEM was the most frequent phenotype (61%) and was associated with the age at presentation (*p* = 0.005). The EDSS at onset was of 4.0 (0–9). Anti‐MOG antibodies were positive at diagnosis in 97.4% of patients with seroreversion occurring in 49.5%, which was not correlated with relapsing disease (*p* = 0.5). Most of the patients received intravenous methylprednisolone (97.4%); nine patients with more severe disease had add‐on treatment with plasmapheresis (*p* = 0.003). Complete recovery was observed in 81.6% of patients, while 26% had a relapsing disease. A univariate analysis showed a positive correlation between EDSS at presentation and at follow‐up (*p* = 0.009) and time of follow‐up and relapsing disease (*p* = 0.001).

**Conclusion:** Portuguese national data confirm previously described features of MOGAD. Therapeutic approaches appear consistent throughout centers and guided by disease severity ab initio even though standardized treatment guidelines are missing.

**Disclosure:** Nothing to disclose.

## EPO‐0039

### Transitioning from pediatric to adult care in neuromuscular diseases: A management model in the Tuscany region, Italy

#### F. Torri ^1^; M. Sacchini^2^; C. Ticci^2^; G. Astrea^3^; B. Buchignani^3^; E. Procopio^2^; R. Battini^3^; G. Ricci^1^

##### ^1^Department of Clinical and Experimental Medicine, University of Pisa, Italy; ^2^Neurometabolic disorders Unit, Meyer Hospital, Italy; ^3^IRCCS Stella Maris, Italy

**Background and aims:** The improvement of standards of care and the availability of effective, disease‐modifying treatments for neuromuscular diseases has expanded patients’ lifespan and improved their quality of life. This translates into a higher number of subjects reaching the “transition” phase to the adult care centers from the pediatric ones. With this concept in mind, the Italian region of Tuscany developed a program for transition from two pediatric reference centers for rare and neuromuscular disorders to the adult reference center.

**Methods:** This study presents retrospective data acquired longitudinally in a 2‐years period after the start of the collaborative Tuscany region program to promote the transition process to the adult care center from pediatric institutions.

**Results:** 70 patients transitioned from the pediatric centers to the adult center. Most of the patients had previously received a genetic and clinical diagnosis. All patients were firstly seen in the pediatric center in presence of the adult neurologist and pediatrician/childhood neurologist and then re‐evaluated after 6 months at the adult center; after that visit, the ordinary schedule of follow‐up was re‐established according to the disease progression and patients’ needs. Regarding multidisciplinary care, 25 patients were referred to specialized adult services for cardiology and pulmonology.

**Figure:**
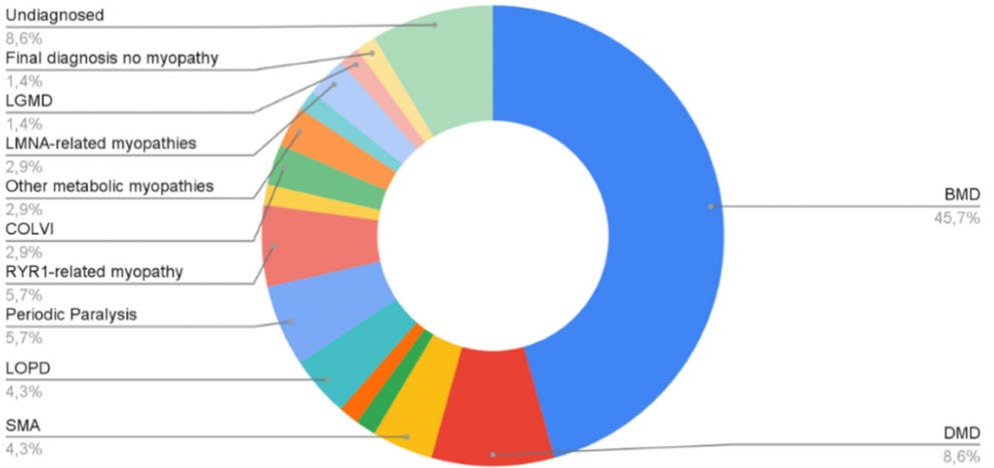
**FIGURE 1** Graphical representation of the different diagnoses included in the study cohort.

**Figure:**
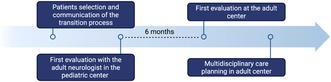
**FIGURE 2** Graphical representation of the transition process organization.

**Conclusion:** The transition passage is in most cases fragmented and is subject to data loss or even discontinuation of care. To ensure a smooth and complete transitional passage, it must be based on epidemiological awareness and an established workflow including all the necessary specialists. Tuscany region successfully provided a pre‐organized and well‐defined process for transition.

**Disclosure:** Nothing to disclose.

## EPO‐0040

### CUL1 variants underlie severe neurodevelopmental disorders: Insights from human genetics and a zebrafish microcephaly model

#### H. Xu; Q. Ye

##### Department of Neurology, Fujian Medical University Union Hospital, Fuzhou, China

**Background and aims:** Microcephaly is a neurodevelopmental anomaly defined by reduced head circumference and impaired brain growth, frequently accompanied by intellectual disability, developmental delays, and seizures. Although numerous genes have been linked to this condition, the role of CUL1—a core component of the SCF (Skp1‐Cul1‐F‐box protein) ubiquitin ligase complex—remains poorly elucidated. This study aims to explore the association between CUL1 and severe neurodevelopmental disorders (NDDs).

**Methods:** First, comprehensive clinical evaluations were performed on four unrelated affected families, confirming all patients exhibited severe microcephaly, intellectual disability, and developmental delay phenotypes. Whole‐exome sequencing (WES) was subsequently conducted to detect pathogenic variants, followed by screening and validation of heterozygous de novo and inherited variants in the CUL1 gene. To further verify the functional consequences of CUL1 loss‐of‐function, a zebrafish model with double knockdown of cul1a and cul1b was established for subsequent functional analysis to corroborate clinical findings.

**Results:** WES identified heterozygous de novo and inherited CUL1 variants in all four unrelated families, with these variants showing a clear correlation with the patients’ severe phenotypes (microcephaly, intellectual disability, and developmental delays). The cul1a&b double‐knockdown zebrafish model exhibited significant central nervous system hypoplasia and abnormal behaviors, which closely recapitulated the clinical manifestations observed in human patients.

**Figure:**
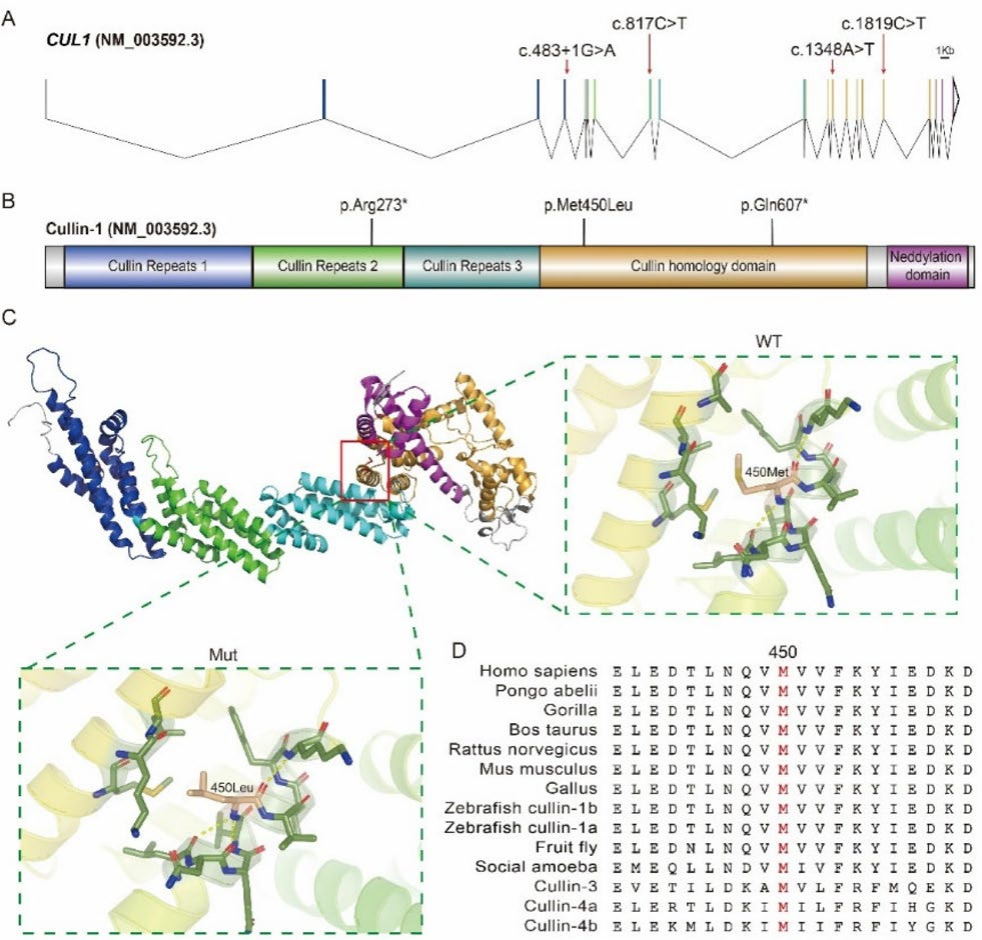
**FIGURE 1** Genetic and structural analysis of CUL1 variants.

**Figure:**
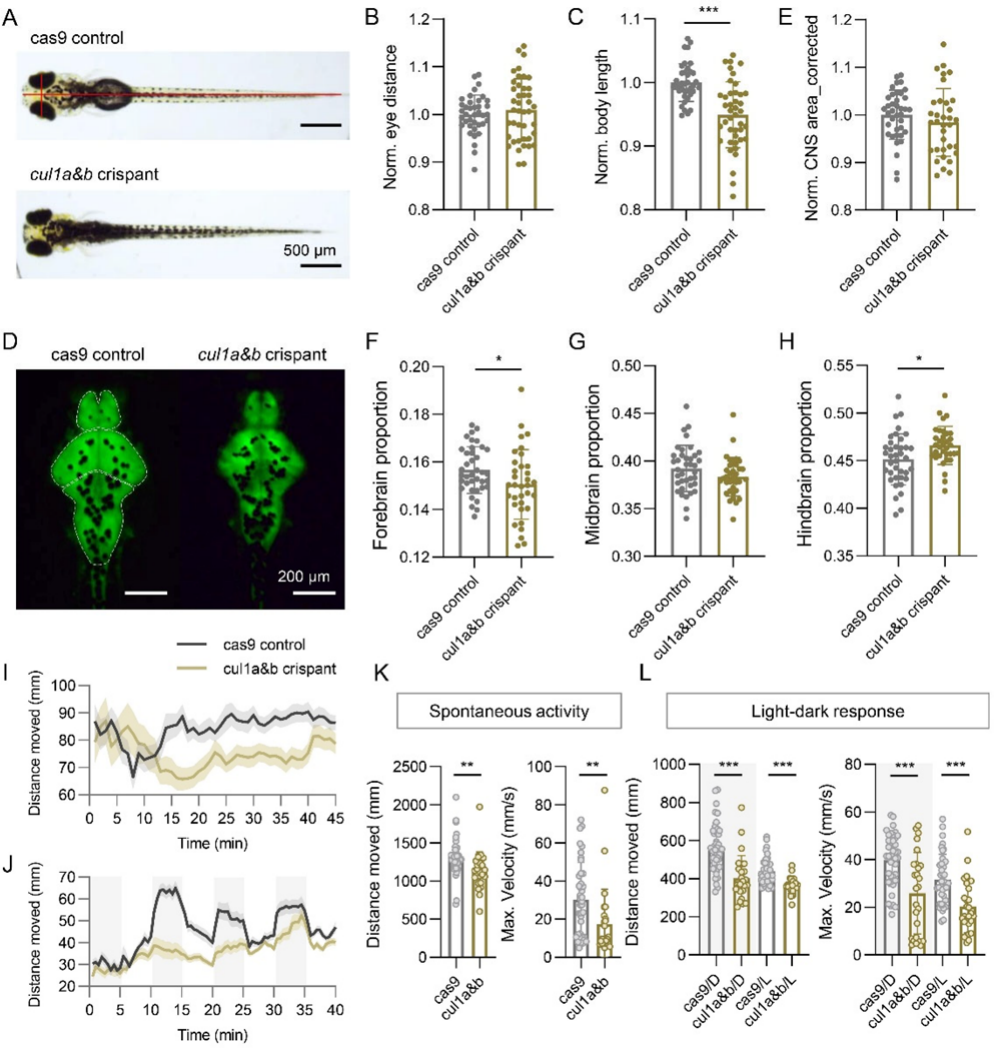
**FIGURE 2** CRISPANT knockdown of zebrafish cul1a and cul1b results in abnormal embryonic development and neurodevelopmental deficits.

**Figure:**
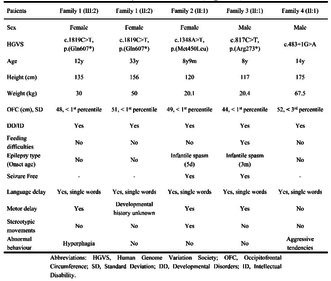
**TABLE 1** Clinical features of five patients with CUL1 variants (NM_003592.3).

**Conclusion:** These findings establish CUL1 as a novel gene associated with severe NDDs and highlight its critical role in brain development. Our study further delineates genotype‐phenotype correlations for CUL1 in NDDs, expands the genetic spectrum of disorders linked to the SCF complex, and underscores the complex's pivotal importance in neurodevelopment.

**Disclosure:** Nothing to disclose.

## EPO‐0041

### HMGB1‐TREM2 axis mediates microglial activation in neonatal sepsis‐associated encephalopathy

#### J. Lei ^1^; N. Tan^1^; L. Zhang^2^; M. Yu^1^; Z. Chen^1^; X. Zheng^1^; H. Guo^1^; H. Jiang^3^

##### ^1^Department of Neonatology, The Fifth Affiliated Hospital, Sun Yat‐sen University, Zhuhai, China; ^2^Department of Cerebrovascular Disease, The Fifth Affiliated Hospital, Sun Yat‐sen University, Zhuhai, Guangdong Province, China; ^3^Department of Rheumatology, The Fifth Affiliated Hospital, Sun Yat‐sen University, Zhuhai, China

**Background and aims:** The objective of this study is to investigate the High‐mobility group box 1 (HMGB1) ‐ Triggering Receptor Expressed on Myeloid Cells 2 (TREM2) axis in microglia during neonatal sepsis‐associated encephalopathy (nSAE).

**Methods:** An nSAE mouse model was established in 3‐day‐old C57 mice via intraperitoneal injection of lipopolysaccharide (LPS, 4 mg/kg; *n* = 3), littermates receiving saline as controls (*n* = 3). Brain tissues were collected 12 hours after modeling for analysis. Microglial activation, HMGB1 levels in brain tissue homogenates, and total HMGB1 protein expression were assessed by immunofluorescence (IF), enzyme‐linked immunosorbent assay (ELISA), and Western blot, respectively. Immunoprecipitation‐Mass Spectrometry (IP‐MS) was used to identify TREM2‐interacting proteins in microglia, and then validated by co‐immunoprecipitation (Co‐IP) and IF co‐localization.

**Results:** Compared with the control group, the number of Iba1‐positive microglia in the brain tissue of nSAE models was significantly increased, with a shift in cell morphology towards an amoeboid appearance. Both the concentration of HMGB1 in brain tissue homogenates and its total protein expression were significantly elevated in nSAE models. IP‐MS analysis indicated that HMGB1 was co‐precipitated with TREM2 antibodies in LPS‐stimulated BV2 cells. This interaction was confirmed by Co‐IP in 293T cells co‐transfected with Flag‐tagged HMGB1 and HA‐tagged TREM2 plasmids, and further validated by IF demonstrating their co‐localization.

**Figure:**
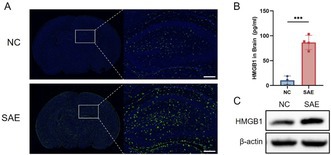
**FIGURE 1** (A) Immunofluorescence of Iba1+(green) microglia in hippocampus of nSAE mice brain slice (Scale bar, 400 μm). (B) HMGB1 levels in brain were determined by ELISA (*p* < 0.001). (C) Western blot bands of the HMGB1 expression levels in brain of mice.

**Figure:**
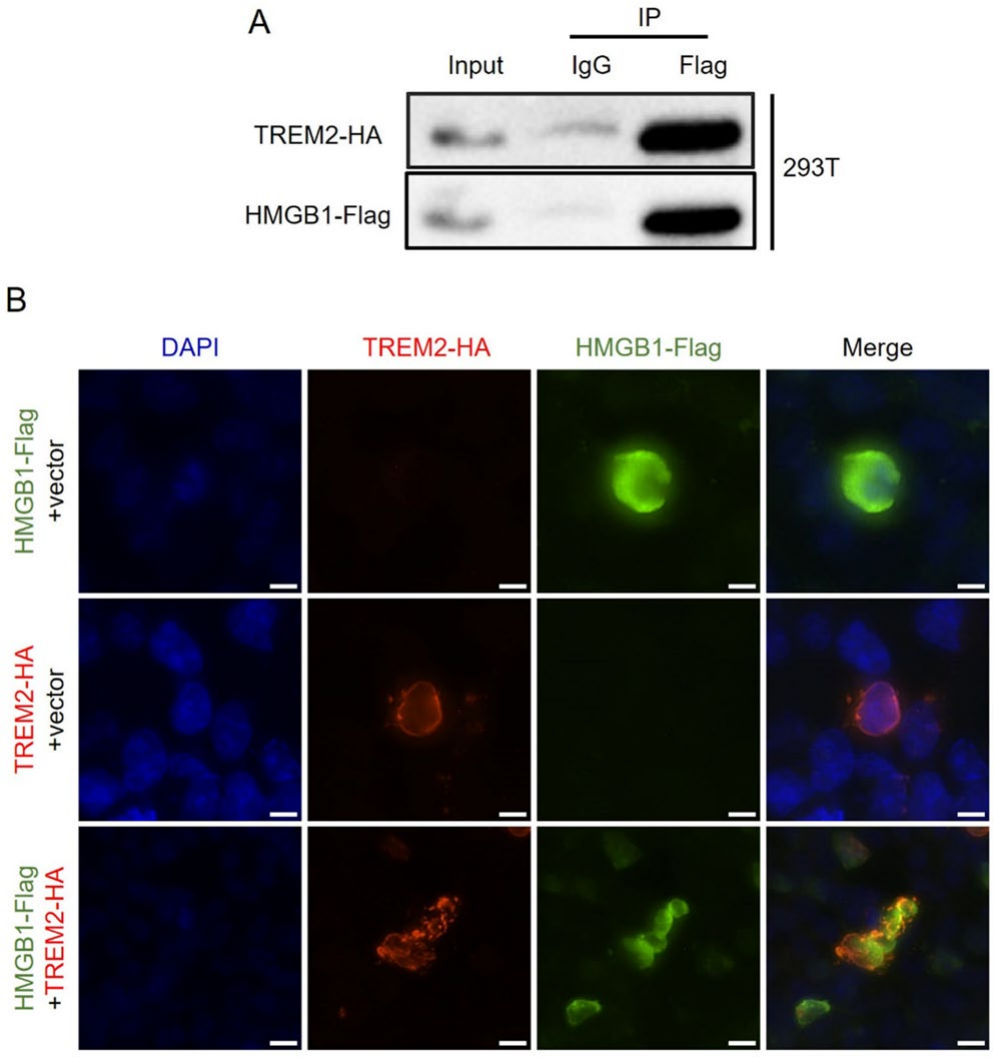
**FIGURE 2** 293T cells were transfected with pcDNA3.1‐HMGB1‐Flag and pcDNA3.1‐TREM2‐HA plasmids. (A) Anti‐Flag antibody was employed for exogenous Co‐IP experiments. (B) The co‐localization between Flag and HA was examined using confocal microscopy (Scale bar, 5 um).

**Conclusion:** This study revealed an interaction between HMGB1 and TREM2 in microglia, suggesting that HMGB1 may regulate microglial activation by binding to TREM2, thereby mediating neuroinflammation associated with nSAE. Targeting the HMGB1‐TREM2 axis may represent a novel therapeutic strategy for intervening in nSAE‐related neuroinflammation.

**Disclosure:** Nothing to disclose.

## EPO‐0042

### Speech, language, social communication, and communication assessments in Rett syndrome: A systematic review

#### L. Raniolo ^1,2^; R. Braden^1,2^; J. von Hehn^4^; J. Christodoulou^2^; D. Lieberman^5^; D. Amor^1,2,3^; A. Vogel^1,6^

##### ^1^Department of Audiology and Speech Pathology, The University of Melbourne, Melbourne, Australia; ^2^Murdoch Children's Research Institute, Melbourne, Australia; ^3^University of Melbourne Department of Paediatrics, The Royal Children's Hospital, Melbourne, Australia; ^4^Rett Syndrome Research Trust, Trumbull, Connecticut, USA; ^5^Department of Neurology, Boston Children's Hospital, Boston, Massachusetts, USA; ^6^Redenlab Ltd, Melbourne, Australia

**Background and aims:** Rett syndrome (RTT) is a neurodevelopmental disorder caused by pathogenic MECP2 variants, characterised by early regression, including loss of purposeful hand use and spoken language. Communication impairment is among its most disabling features; however, speech, language, and social communication phenotypes have not been systematically synthesised. This review aimed to (a) characterise each domain in RTT and (b) identify the most common communication measures.

**Methods:** A systematic search was conducted per PRISMA guidelines and registered on PROSPERO (CRD420251076117). PubMed, CINAHL, and ScienceDirect databases were searched from January 1985 to February 2025. Studies including individuals with genetically/clinically confirmed RTT and subjective/objective communication data were eligible. Two reviewers independently screened studies. Data were synthesised descriptively due to methodological heterogeneity. Studies were appraised using Joanna Briggs Institute tools. A supplementary search of ClinicalTrials.gov was conducted for therapeutic interventions.

**Results:** 57 studies met inclusion criteria. Communication impairment was universal but heterogeneous. Expressive language and physical speech were most severely affected, with near‐total speech loss post‐regression (median onset: 18–24 months). Pre‐regression atypicalities (e.g, high‐pitched vocalisations) were reported. Receptive abilities were relatively preserved, namely in atypical forms. Eye gaze was the predominant post‐regression communicative modality. Social motivation often persisted despite autistic features. Genetic and epigenetic factors (e.g, age) modulated severity. Most studies (63%) relied on caregiver report. No gold‐standard battery was identified.

**Figure:**
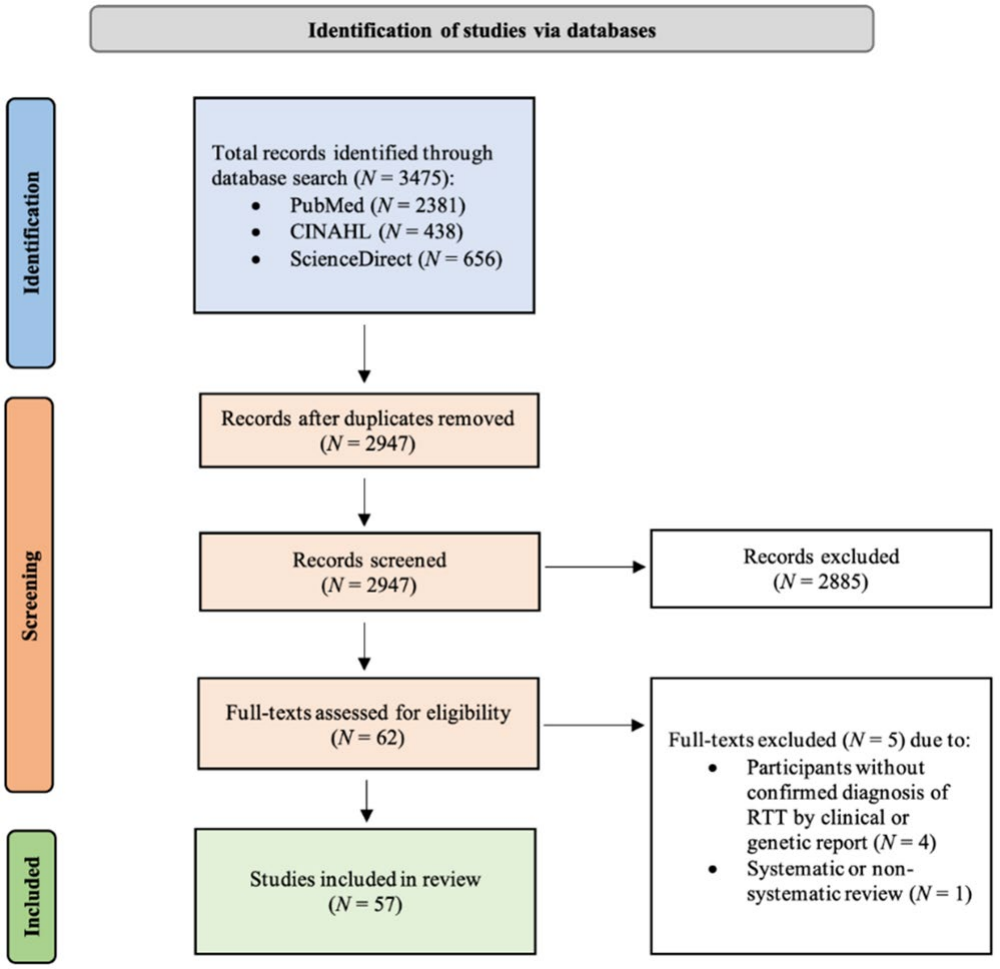
**FIGURE 1** Overview of Systematic Search Results.

**Figure:**
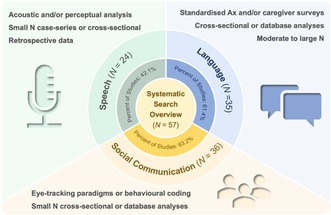
**FIGURE 2** Key Insights of the Current Systematic Review

**Conclusion:** RTT is associated with profound communication impairment, disproportionately affecting expressive language and speech. Incorporating objective and subjective tools may improve phenotype accuracy and clinical monitoring.

**Disclosure:** Adam Vogel is an employee of Redenlab Ltd, a speech neuroscience company. All other authors do not have a conflict of interest to declare.

## EPO‐0043

### Disrupted time‐varying brain connectivity associates with expressive language impairment in pediatric multiple sclerosis

#### M. Margoni^1^; M. Mercuri^2^; P. Valsasina^3^; D. Mistri ^3^; L. Moiola^4^; V. Torri Clerici^5^; M. Filippi^6^; M. Rocca^7^

##### ^1^Neuroimaging Research Unit, Division of Neuroscience, Neurology Unit, and Neurorehabilitation Unit, IRCCS San Raffaele Scientific Institute, Milan, Italy; ^2^Neuroimaging Research Unit, Division of Neuroscience, IRCCS San Raffaele Scientific Institute, Milan, Italy; and Vita‐Salute San Raffaele University, Milan, Italy; ^3^Neuroimaging Research Unit, Division of Neuroscience, IRCCS San Raffaele Scientific Institute, Milan, Italy; ^4^Neurology Unit, IRCCS San Raffaele Scientific Institute, Milan, Italy; ^5^Neuroimmunology and Neuromuscular Diseases Unit, Fondazione IRCCS Istituto Neurologico Carlo Besta, Milan, Italy; ^6^Neuroimaging Research Unit, Division of Neuroscience, Neurology Unit, Neurorehabilitation Unit, and Neurophysiology Service, IRCCS San Raffaele Scientific Institute, Milan, Italy; and Vita‐Salute San Raffaele University, Milan, Italy; ^7^Neuroimaging Research Unit, Division of Neuroscience, and Neurology Unit, IRCCS San Raffaele Scientific Institute, Milan, Italy; and Vita‐Salute San Raffaele University, Milan, Italy

**Background and aims:** Multiple sclerosis (MS) onset during developmental age makes pediatric (ped) patients vulnerable to cognitive impairment. As cognitive processes rely on dynamic reconfiguration of functional brain networks, we explored time‐varying (TV) functional connectivity (FC) in pedMS patients and its association with clinical disability and structural brain damage.

**Methods:** Ninety‐five pedMS and twenty‐four matched healthy controls (HC) underwent neurological, neuropsychological and 3.0T brain MRI assessments. Expanded Disability Status Scale (EDSS) and z‐scores of verbal and visuospatial memory, attention, receptive language, and expressive language were obtained. Brain T2 white matter (WM) lesion volume (LV) and atrophy were assessed. TVFC was assessed using sliding‐window correlation analysis; FC variability was quantified as the standard deviation of degree centrality (DC) and seed‐based correlation within basal ganglia, executive, language, sensorimotor, and default‐mode networks.

**Results:** PedMS patients showed widespread reduction of DC TVFC vs. HC, particularly involving the right middle temporal, inferior parietal and superior frontal cortices (*p* < 0.05, corrected), and extending to bilateral parahippocampal, basal ganglia, fronto‐insular and cerebellar regions (*p* < 0.001, uncorrected). PedMS patients exhibited reduced TVFC (*p* < 0.05, corrected) within basal ganglia, executive and language networks vs. HC. No associations were observed between TVFC abnormalities and EDSS score, brain T2 LV and atrophy. Reduced TVFC of the right inferior parietal lobule, right middle superior frontal gyrus and right middle temporal gyrus associated with worse expressive language z‐scores (*r* ≤ 0.30; *p* ≤ 0.04).

**Conclusion:** PedMS patients experience widespread reductions in TVFC, with selective regions showing associations with expressive language impairment, suggesting that altered brain network dynamics contribute to cognitive dysfunction.

**Disclosure:** M. Margoni reports grants and personal fees from Sanofi Genzyme, Merck Serono, Roche, Biogen, Amgen and Novartis. P. Valsasina, M. Mercuri, D. Mistri have nothing to disclose. L. Moiola received compensations for speaking activities and/or for participating to advisory board from Merck, Celgene, Biogen, Sanofi, Novartis, Roche, Alexion, and Amgen. V. Torri Clerici acted as an Advisory Board member of Novartis, Lundbeck, Amgen, Merck, Roche, Genzyme, Neuraxapharm, and Almirall and received funding for traveling and honoraria for speaking or writing from Biogen, Novartis, Genzyme, Roche, Merck, Bristol Meyer Squibb, Jansenn, and Almirall. She received support for research project by Almirall. M. Filippi is Editor‐in‐Chief of the Journal of Neurology, Associate Editor of Human Brain Mapping, Neurological Sciences, and Radiology; received compensation for consulting services from Almirall, Biogen, Bristol‐Myers Squibb, Eli Lilly, Merck, Novartis, Roche, Sanofi; speaking activities from Amgen, Bayer, Biogen, Bristol‐Myers Squibb, Celgene, Chiesi Italia SpA, Eisai, Eli Lilly, Fujirebio, Genzyme, Janssen, Merck, Neopharmed Gentili, Neuraxpharm, Novartis, Novo Nordisk, Roche, Sanofi, Takeda; participation in Advisory Boards for Alexion, Biogen, Bristol‐Myers Squibb, Eli Lilly, GE Healthcare Ltd, Merck, Neuraxpharm, Novartis, Roche, Sandoz, Sanofi, Takeda; scientific direction of educational events for Biogen, Merck, Roche, Celgene, Bristol‐Myers Squibb, Lilly, Novartis, Sanofi‐Genzyme; he receives research support from Biogen Idec, Merck‐Serono, Novartis, Roche, the Italian Ministry of Health, the Italian Ministry of University and Research, and Fondazione Italiana Sclerosi Multipla. M.A. Rocca received consulting fees from Biogen, Bristol Myers Squibb, Roche; and speaker honoraria from Alexion, Biogen, Bristol Myers Squibb, Celgene, Horizon Therapeutics Italy, Merck Serono SpA, Mitsubishi‐Tanabe Pharma, Neuraxpharm, Novartis, Roche, Sandoz, and Sanofi. She receives research support from the MS Society of Canada, the Italian Ministry of Health, the Italian Ministry of University and Research, and Fondazione Italiana Sclerosi Multipla. She is Associate Editor for Multiple Sclerosis and Related Disorders; and Associate Co‐Editor for Europe and Africa for Multiple Sclerosis Journal.

## EPO‐0044

### Non‐epileptic paroxysmal events in Rett syndrome: A systematic review of case‐based and observational evidence

#### N. Bhatti ^1^; D. Lumsden^2^

##### ^1^East Kent Hospitals University NHS Foundation Trust, Kent, UK; ^2^Complex Motor Disorder Service, Evelina London Children's Hospital, London, UK

**Background and aims:** Rett syndrome (RTT) is a severe neurodevelopmental disorder frequently complicated by epilepsy. Many individuals also experience non‐epileptic paroxysmal events that mimic seizures and risk inappropriate treatment. The spectrum and phenomenology of these events remain poorly defined. We aimed to systematically characterise non‐epileptic paroxysmal events in RTT.

**Methods:** We performed a PRISMA‐guided systematic review of observational studies from PubMed, Embase, and OVID (1962–2024). Eligible studies included case reports, case series, cohort studies, and small clinical trials describing non‐epileptic events in individuals with clinically or genetically confirmed RTT. Data were extracted on study design, participant characteristics, and event type. Events were categorised into respiratory, neuromotor, and behavioural domains. Due to heterogeneity, results were synthesised descriptively.

**Figure:**
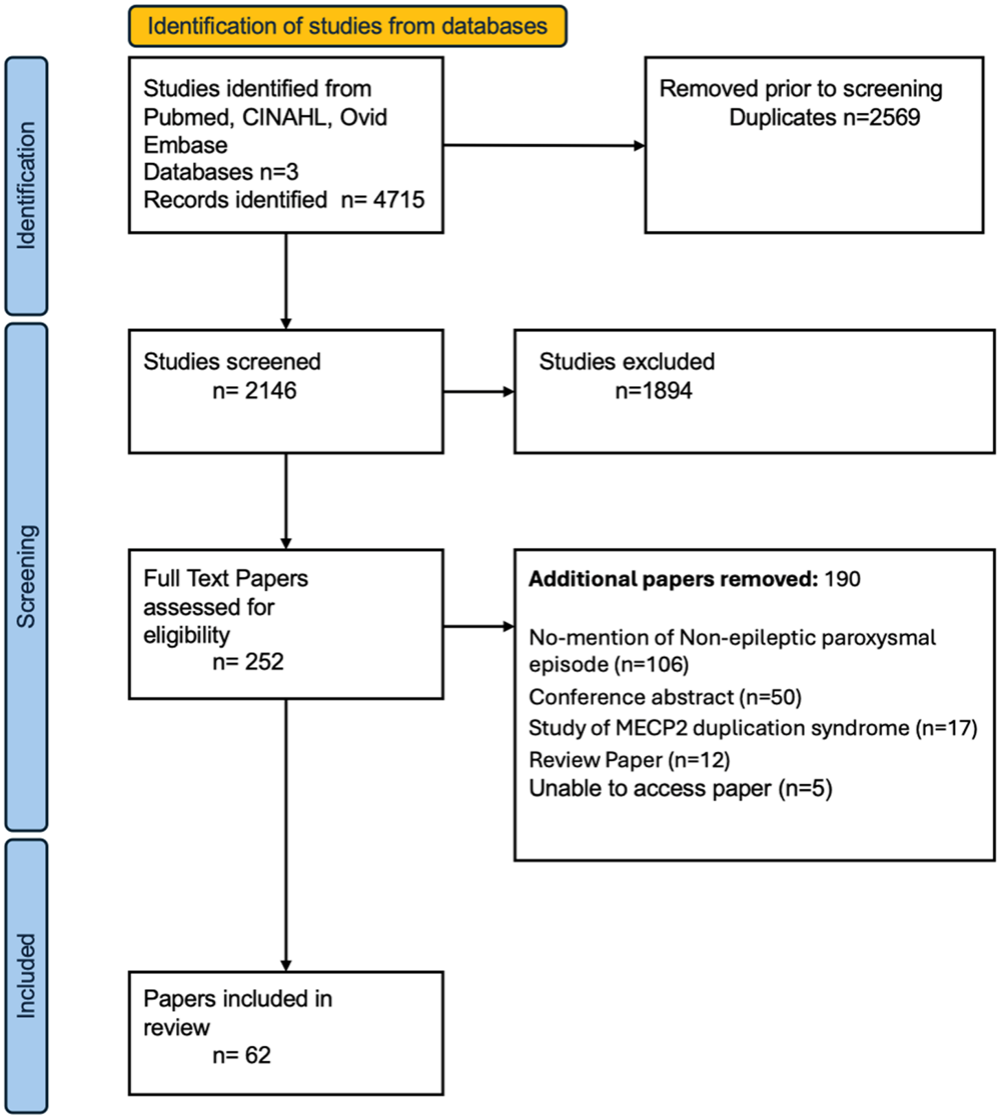
**FIGURE 1** Literature search and selection flow diagram.

**Results:** Sixty‐two studies were included. Respiratory events predominated, with apnoea reported in 1,092 clinician‐reported cases (26 studies) and 507 family‐reported cases (9 studies). Breathing abnormalities persisted into adulthood in 30/50 adults (apnoea 54%, hyperventilation 24%). Neuromotor events, including dystonia and involuntary movements, were reported in 51 patients, with three life‐threatening episodes involving airway compromise; vacant spells frequently accompanied respiratory events. Behavioural and autonomic disturbances were common. Video‐EEG and telemetry reclassified up to 80% of events labelled as seizures as non‐epileptic. The term “Rett episodes” was used inconsistently and by a minority of authors.

**Figure:**
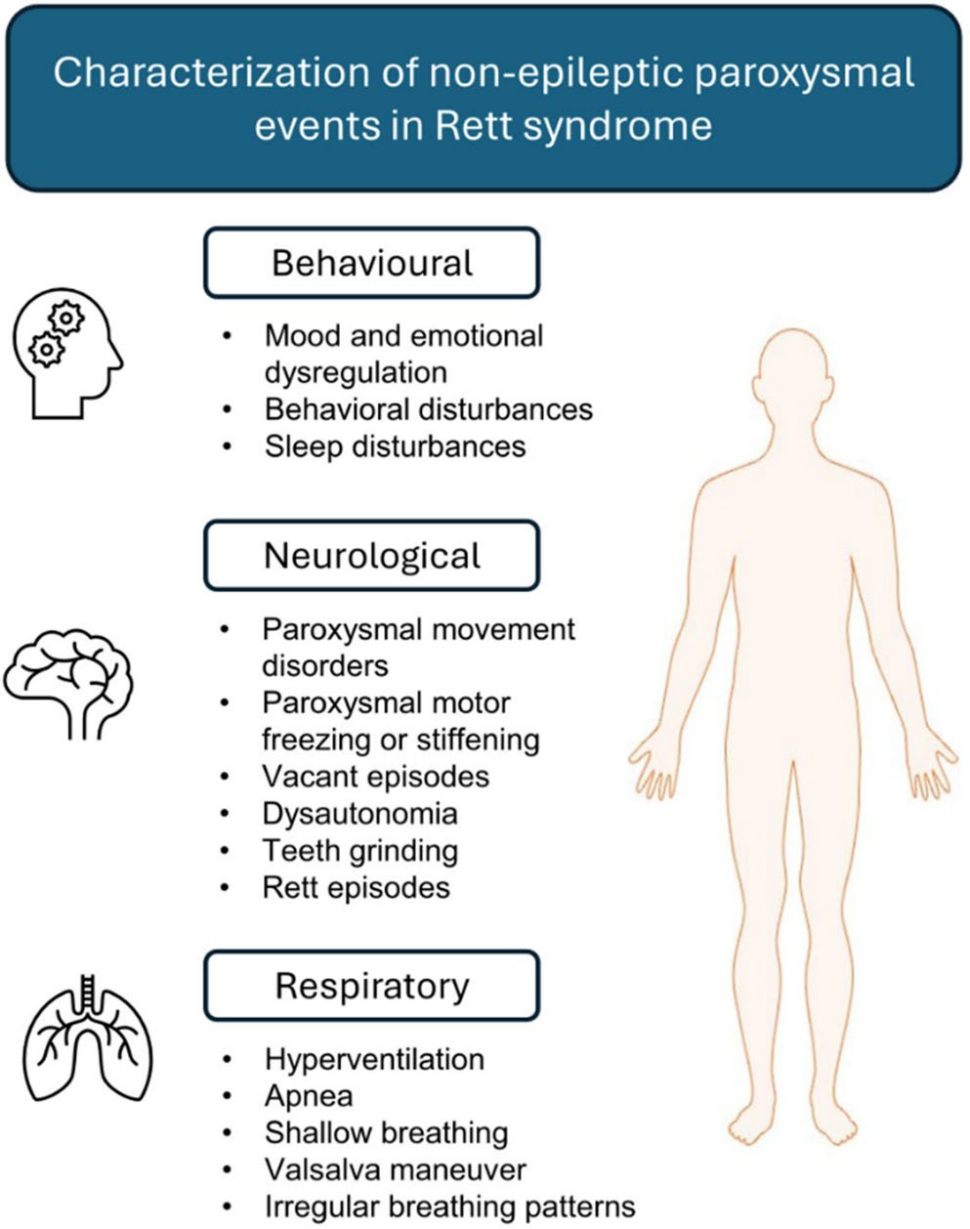
**FIGURE 2** Classification of non‐epileptic paroxysmal events in Rett syndrome.

**Figure:**
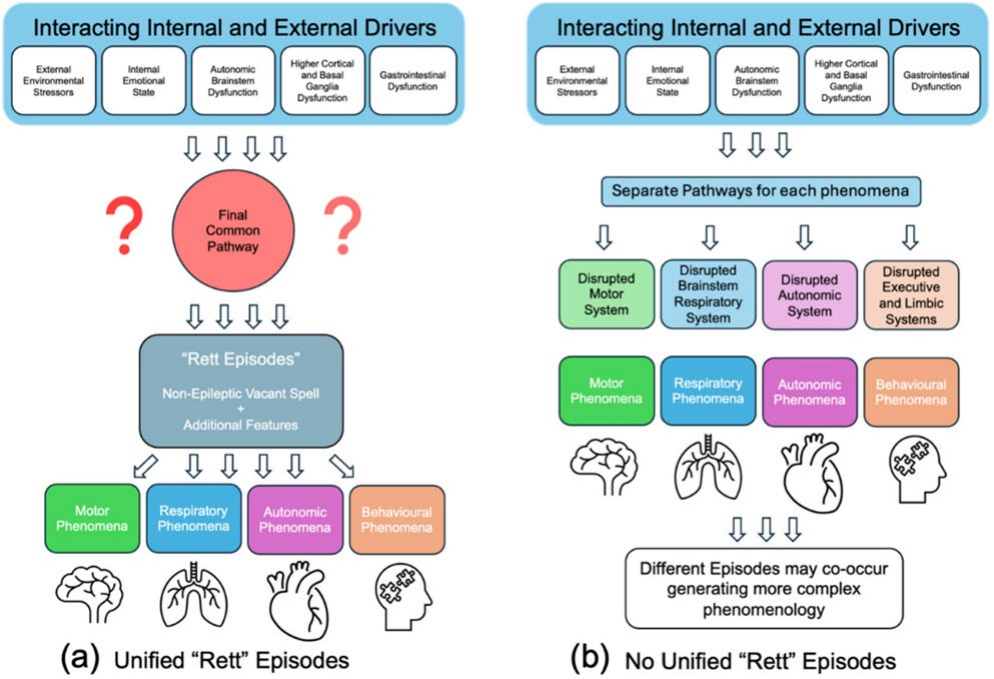
**FIGURE 3** Potential pathways for Rett episodes with an assumption that events arise from a single common pathway (a) and a model of separate pathways independently giving rise to different episodes (b), which may temporally coincide.

**Conclusion:** Non‐epileptic paroxysmal events are common and heterogeneous in RTT. Frequent misclassification as epilepsy risks unnecessary treatment and harm. Greater clinician awareness, precise phenomenological description, and objective recording are needed, alongside clearer diagnostic frameworks to optimise care and quality of life.

**Disclosure:** NB has nothing to disclose DEL has undertaken consultancy work for Acadia and Neurogene.

## EPO‐0045

### iPSC‐derived cellular models and extracellular vesicles: A translational pipeline for biomarker identification in neurodevelopmental disorders

#### R. Palumbi ^1^; M. Simone^2^; L. Margari^2^; S. Micella^1^; A. De Giacomo^1^; P. Abbrescia^1^; O. Valente^1^; A. Frigeri^1^

##### ^1^Department of Translational Biomedicine and Neurosciences (DiBraiN), University of Bari “Aldo Moro”, Bari, Italy; ^2^Department of Precision and Regenerative Medicine (DiMePRe‐J), University of Bari “Aldo Moro”, Bari, Italy

**Background and aims:** Neurodevelopmental disorders (NDDs), including Autism Spectrum Disorder (ASD) and Attention‐Deficit/Hyperactivity Disorder (ADHD), show complex etiopathogenesis and substantial clinical heterogeneity, limiting early diagnosis and personalized treatment. The identification of reliable biomarkers is therefore a major unmet clinical need. Induced pluripotent stem cell (iPSC) models and circulating extracellular vesicles (EVs) represent promising translational tools to investigate neurodevelopmental mechanisms and identify disease‐related molecular signatures.

**Methods:** A pediatric cohort (3–17 years) with NDDs diagnosed according to DSM‐5/DSM‐5‐TR criteria is being recruited, together with a typically developing control group. Participants undergo standardized neuropsychiatric, cognitive, and behavioral assessments. Peripheral blood and urine samples are collected from patients and controls. Plasma EVs are isolated by differential ultracentrifugation and characterized using immunoblotting for exosomal markers, and nanoparticle tracking analysis or transmission electron microscopy. Then plasma EVs are investigated with a multiomic approach. In parallel, urinary stem cells and peripheral blood cells are reprogrammed into patient‐specific iPSCs and differentiated into neuronal and glial precursors to generate three‐dimensional brain organoid models.

**Results:** Sixty‐one patients have been enrolled to date, including ASD, ADHD, and ASD/ADHD overlap groups, with a marked male predominance and predominantly preserved cognitive functioning. Patient‐specific iPSC lines have been successfully generated, and neuro‐glial differentiation protocols have yielded initial astrocyte cultures. Multi‐omic profiling of plasma‐derived EVs is currently ongoing.

**Conclusion:** The combined analyses of clinical phenotypes, EV molecular cargo may enable the identification of biomarker signatures supporting stratification and precision medicine approaches in NDDs. Creating diseases‐specific brain organoids models may shed a light on the underlying neurobiological pathways of these disorders.

**Disclosure:** Nothing to disclose.

## EPO‐0046

### Impact of early seizure onset on language development in genetic epilepsy with febrile seizures plus

#### S. Sager ^1^; O. Alomari^2^; E. Gurkas^1^

##### ^1^Department of Pediatric Neurology, University of Health Sciences, Kartal Dr. Lütfi Kırdar City Hospital, Istanbul, Türkiye; ^2^Hamidiye International School of Medicine, University of Health Sciences, Istanbul, Türkiye

**Background and aims:** Genetic Epilepsy with Febrile Seizures Plus (GEFS+) involves early‐onset seizures during critical neurodevelopmental windows. While seizure semiology is well‐documented, the impact on linguistic acquisition remains unexplored. This study evaluates the prevalence and characteristics of language impairments in GEFS+ using the Turkish Test of Preschool Language Development (TODIL).

**Methods:** A cohort of 51 pediatric patients (31 male, 20 female) with GEFS+ was evaluated. The mean age was 71.36 +/‐ 16.38 months. Linguistic performance was assessed across nine domains using the TODIL. Raw scores (HP) were converted to percentile ranks based on national norms. Clinical impairment was defined as a score falling below the 10th percentile.

**Results:** Impairment was most prevalent in Sound Discrimination (45.10%), Phonological Analysis (37.25%), Grammar Comprehension (33.33%), and Articulation (33.33%). Relational Vocabulary was a relative strength (1.96% impairment). Mean percentile ranks for Phonological Analysis (24.31%) and Articulation (29.97%) were significantly low. Gender‐based comparisons revealed no statistically significant differences across subtests (all *p* > 0.05), suggesting a gender‐independent linguistic phenotype.

**Figure:**
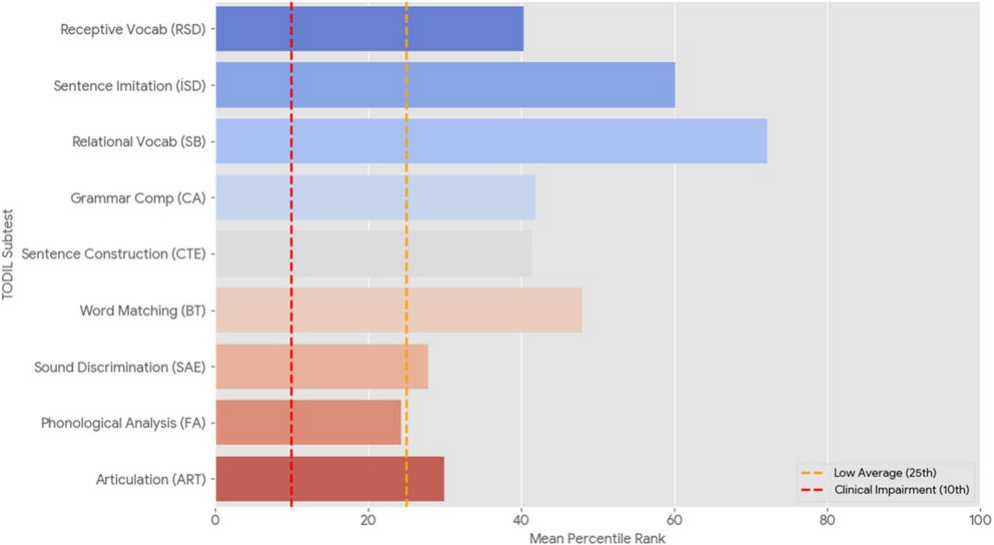
**FIGURE 1** Mean Percentile Ranks Across TODİL Subtests in Children with GEFS+ (N = 51). The dashed red line indicates the 10th percentile clinical impairment threshold, and the orange line represents the 25th percentile “at‐risk” threshold. Note the profound.

**Conclusion:** Early‐onset seizures in GEFS+ are associated with high rates of language impairment, a correlation previously under‐reported. The specific phenotype involves significant phonological and structural deficits despite preserved conceptual vocabulary. These findings underscore the urgent need for speech‐language assessment immediately following diagnosis to improve long‐term communicative outcomes.

**Disclosure:** The authors declare that they have no competing financial interests or personal relationships that could have appeared to influence the work reported in this paper. This research did not receive any specific grant from funding agencies in the public, commercial, or not‐for‐profit sectors.

## EPO‐0047

### CCL2/MCP‐1, interleukin‐8, and fractalkine/CXC3CL1: Potential biomarkers of epileptogenesis and pharmacoresistance in childhood epilepsy

#### S. Aulicka

##### Department of Neurology, St. Anne's University Hospital, Brno, Czech Republic

**Background and aims:** Growing evidence indicates that neurinflammatory pathways contribute to epileptologenesis and pharmacoresistance in epilepsy. The main impact on clinical practice is the development of new targeted therapies for epilepsy. We studied the importance of neuroinflammation in the development of epileptogenesis and pharmacoresistance in childhood epilepsy patients.

**Methods:** A cross‐sectional study conducted at two epilepsy centers in the Czech Republic compared 22 pharmacoresistant patients and 4 pharmacodependent patients to 9 controls. We analyzed the ProcartaPlex™ 9‐Plex immunoassay panel consisting of interleukin (IL)‐6, IL‐8, IL‐10, IL‐18, CXCL10/IP‐10, monocyte chemoattractant protein 1 (CCL2/MCP‐1), B lymphocyte chemoattractant (BLC), tumor necrosis factor‐alpha (TNF‐α), and chemokine (C‐X3‐X motif) ligand 1 (fractalkine/CXC3CL1) to determine their alterations in cerebrospinal fluid (CSF) and blood plasma, concurrently

**Results:** The analysis of 21 paired CSF and plasma samples in pharmacoresistant patients compared to controls revealed a significant elevation of CCL2/MCP‐1 in CSF (*p* < 0.000512) and plasma (*p* < 0.00.017). Higher levels of fractalkine/CXC3CL1 were revealed in the plasma of pharmacoresistant patients than in controls (*p* < 0.0704), and we determined an upward trend in CSF IL‐8 levels (*p* < 0.08). No significant differences in CSF and plasma levels were detected between pharmacodependent patients and controls.

**Conclusion:** Our results confirm that CCL2/MCP‐1, IL‐8, and fractalkine/CXC3CL1 are potential biomarkers of epileptogenesis and pharmacoresistance in epilepsy.These findings provide new insights into processes of epileptogenesis and development of pharmacoresistance in epilepsy with potential impact on developing disease‐modifying drugs in epilepsy.

**Disclosure:** Nothing to disclose.

## Neuropathies 1

## EPO‐0048

### Antibodies against proteolipid protein‐1 are identified in patients with inflammatory neuropathies

#### A. De Lorenzo ^1^; P. Llarch‐Cegarra^2^; M. Caballero‐Ávila^2^; G. Riesco‐Navarro^2^; E. Cortés‐Vicente^2^; A. Vesperinas^2^; C. Lleixà^3^; R. Collet‐Vidiella^2^; L. Martín‐Aguilar^2^; E. Pascual‐Goñi^2^; M. Gastaldi^4^; S. Masciocchi^4^; L. Querol^5^

##### ^1^Neuromuscular and Neuroimmunology Unit, IRCCS Humanitas Research Hospital, Rozzano, Milan, Italy and Department of Neurology, Neuromuscular Diseases Unit, Hospital de La Santa Creu I Sant Pau (IR SANT PAU); ^2^Department of Neurology, Neuromuscular Diseases Unit, Hospital de La Santa Creu I Sant Pau (IR SANT PAU); ^3^Hospital de la Santa Creu i Sant Pau, Barcelona, Spain; ^4^Neuroimmunology Laboratory and Neuroimmunology Research Section, IRCCS Mondino Foundation, Pavia, Italy; ^5^Department of Neurology, Neuromuscular Diseases Unit, Hospital de La Santa Creu I Sant Pau (IR SANT PAU) and Universitat Autònoma de Barcelona, Barcelona, Spain

**Background and aims:** Conformational antibodies to proteolipid protein‐1 (PLP1‐IgG), a major myelin protein, have been reported in CNS demyelinating disorders with peripheral involvement, but their prevalence and clinical significance in isolated inflammatory peripheral neuropathies remain unclear.

**Methods:** A validated live cell–based assay for PLP1‐IgG detection was implemented. We tested 135 patients with inflammatory neuropathies [94 chronic inflammatory demyelinating polyradiculoneuropathy (CIDP), 34 Guillain–Barré syndrome (GBS), 7 autoimmune nodopathies (AN)] and 48 controls. Clinical data were retrospectively extracted from medical records.

**Results:** PLP1‐IgG were detected in 13/135 (9.6%) inflammatory neuropathy patients and in none of the controls. Seropositivity occurred in 11/94 CIDP (11.7%), 1/72 GBS (1.4%), and 1/7 AN‐CNTN1 (14.3%). Among seropositive CIDP patients, six were male (54.5%) and mean age at onset was 53.2 ± 16.4 years. Eight (72.7%) had a typical phenotype and three (27.3%) a multifocal variant; onset was acute/subacute in eight (72.7%). Ataxia occurred in nine (81%), tremor in three (27.3%), and cranial nerve involvement in three (27.3%). Electrophysiology confirmed demyelinating neuropathy in all cases, including conduction block in seven (63.6%); one patient had GM1‐IgG. CSF analysis (8 patients) showed albuminocytological dissociation in seven (87.5%; mean protein concentration 104.7 ± 62.9 mg/dL). Treatment response was satisfactory in 7/11 to IVIg (63.6%) and in 5/9 to corticosteroids (55.6%). Two refractory patients improved partially with plasma exchange; one subsequently underwent HSCT with substantial improvement.

**Conclusion:** PLP1‐IgG identify a subset of inflammatory neuropathy patients characterized by frequent acute/subacute onset, ataxia, and generally favorable response to immunomodulatory therapy. Their pathogenic role remains unclear, particularly given co‐existing pathogenic antibodies in two cases.

**Disclosure:** No.

## EPO‐0049

### Health literacy and patient activation as modifiable determinants of perceived quality of life in adults living with ATTRv amyloidosis in Portugal

#### A. Pedro^1^; C. Simões ^2^; M. Pardal^3^; M. Lopes^3^; P. Soares^4^; I. Conceição^5^; C. Alves^5^; A. Sousa^5^; J. Silva^6^; T. Coelho^5^

##### ^1^NOVA National School of Public Health, Public Health Research Centre, Comprehensive Health Research Center, CHRC, REAL, CCAL, NOVA University Lisbon, Lisbon, Portugal; ^2^NOVA National School of Public Health, Public Health Research Centre, NOVA University Lisbon, Lisbon, Portugal; ^3^Medical department, BioPharmaceuticals, AstraZeneca, Barcarena, Portugal; ^4^Corporate Affairs and Market Access, BioPharmaceuticals, AstraZeneca, Barcarena, Portugal; ^5^Sociedade Portuguesa de Estudos de Doenças Neuromusculares, Coimbra, Portugal; ^6^Associação Portuguesa de Paramiloidose, Vila do Conde, Portugal

**Background and aims:** Hereditary transthyretin (ATTRv) amyloidosis is a progressive disease that affects daily functioning and overall wellbeing. Identifying modifiable determinants that enhance perceived quality of life (QoL) is essential for patient‐centred interventions. Health literacy and patient activation shape individuals’ ability to understand their condition and engage with care management. This study examined how these competencies relate to perceived QoL among adults with ATTRv amyloidosis.

**Methods:** A cross‐sectional analysis was conducted using data from adults (≥ 18 years) enrolled in the LANTERN study in Portugal, a national observational study of adults living with ATTRv amyloidosis in Portugal. Health literacy (HLS‐EU‐Q16), patient activation (PAM‐13), and perceived QoL (EQ‐5D VAS) were assessed. Descriptive statistics were performed, and associations between these measures were examined using Spearman correlations, with statistical significance set at *p* < 0.05.

**Results:** Among 282 participants (mean age 48 years; 42.9% male), the mean EQ‐5D VAS score was 66.31 (0–100 scale). Health literacy (0–50 scale) averaged 32.16, with 18.5% classified as inadequate, 31.1% problematic, 35.2% sufficient, and 15.2% excellent. Patient activation (0–100 scale) averaged 68.83, with most participants in level 3 (47.8%) or level 4 (40.8%). Higher EQ‐5D VAS scores were significantly associated with higher health literacy and higher patient activation (*p* < 0.001).

**Conclusion:** Despite high activation levels, nearly half of people living with ATTRv amyloidososis have suboptimal health literacy. Health literacy and patient activation were both positively associated with perceived QoL, representing modifiable intervention targets. Enhancing these competencies may support better self‐management and contribute to improved patient‐centred outcomes.

**Disclosure:** This work was supported by AstraZeneca – Produtos Farmacêuticos, Lda. NOVA National School of Public Health Research Grant: LANTERN|951114|2025).

## EPO‐0050

### Semaglutide is associated with preserved motor axon function in type 2 diabetic peripheral neuropathy

#### C. Graffe ^1^; C. Krarup^1^; M. Moldovan^2^

##### ^1^Department of Clinical Neurophysiology, Rigshospitalet, Copenhagen, Denmark; ^2^Department of Neurology, North Zealand Hospital, Hillerød, Denmark

**Background and aims:** The motor axon dysfunction reflect the peripheral neuropathy severity in patients with Type 2 Diabetes (T2DM). The aim of this cross‐sectional observational study was to investigate the effect of the Glucagon‐like peptide‐1 receptor agonist semaglutide on motor neuropathy in T2DM patients referred for electrodiagnostic investigations.

**Methods:** We compared consecutive T2DM patients treated with semaglutide and metformin (*n* = 8) with gender and age‐matched T2DM patients receiving metformin only. The dominant median nerve was stimulated at the wrist. The evoked compound motor axon potential (CMAP) was recorded from the abductor pollicis brevis muscle. Multiple measures of motor axon excitabil‐ity were obtained by threshold‐tracking. Motor unit number estimation (MUNE) was carried out by MScanFit‐2. The clinical sensory‐motor involvement was assessed by the modified Toronto Clinical Neuropathy Score (mTCNS).

**Results:** The patients (14 men and 2 women, mean age of 65.3 years) had a BMI of 30.9, a HbA1c of 53.2 (nmol/mol), and a mTCNS of 12.4. The semaglutide group had a CMAP amplitude of 7.9 mV. The metformin‐only group had a smaller CMAP of 6 mV (*p* < 0.05), and a MUNE of 73.8 which was reduced compared to normal controls (94.2, *n* = 52, *p* < 0.05). The motor axon excitability of the metformin‐only group showed depolarizing changes (*p* < 0.05). In the semaglutide group, these excitability measures did not differ from normal controls (*n* = 99).

**Conclusion:** Our data suggest that the addition of semaglutide to the treatment of T2DM preserves motor axon function, possibly by improving the hyperpolarizing Na+/K+ ATPase which is critical for maintaining the ionic homeostasis during conduction.

**Disclosure:** Nothing to disclose.

## EPO‐0051

### Toward standardized multidisciplinary assessment of Charcot‐Marie‐Tooth: Integrating MUNIX, clinical scales and plasma biomarkers

#### F. Gruosso ^1^; M. Goglia^1^; E. Frezza^1^; G. Greco^1^; G. Vietri^1^; L. Boffa^1^; D. Centonze^2^; C. Rocchi^2^; A. Petrucci^3^; R. Massa^1^

##### ^1^Neuromuscular Diseases Unit, Tor Vergata University Hospital, Rome, Italy; ^2^Department of Systems Medicine, University of Rome Tor Vergata, Rome, Italy; ^3^Center for Neuromuscular and Neurological Rare Diseases, San Camillo Forlanini Hospital, Rome, Italy

**Background and aims:** Charcot‐Marie‐Tooth disease (CMT) is a spectrum disorder with variable progression and severity, but reliable biomarkers for predicting severity are lacking. We assessed whether combining neurophysiological measures, plasma biomarkers, and clinical scales could identify predictors of severity across CMT subtypes.

**Methods:** Forty‐six genetically confirmed CMT patients performed standardized assessment at two Italian referral centres: clinical scales (CMTNSv2, NIS, ONLS, MRC, SF‐36, MAM‐16), neurophysiology (MUNIX, MUSIX, ulnar CMAP, radial SNAP), and plasma biomarkers (NfL, p‐tau181 in *n* = 22). Patients were stratified by electrophysiological phenotype (axonal 56.5%, demyelinating 43.5%). Correlations and multivariable regression identified independent predictors of clinical severity.

**Figure:**
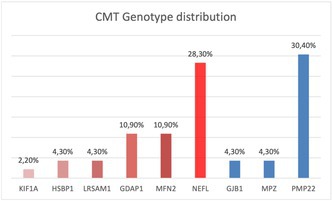
**FIGURE 1** Distribution of genetic variants in a cohort of CMT patients: red bars represent axonal genotypes (HSBP1, LRSAM1, KIF1A, GDAP1, MFN2, NEFL), while blue bars indicate demyelinating ones (GJB1, MPZ, PMP22).

**Results:** MUNIX score was significantly lower compared to normal reference (57.7 ± 34.2, *p* < 0.001) and negatively correlated with clinical disability measures. Axonal phenotype had higher MUNIX (69.7 ± 33.2) than demyelinating phenotype (40.2 ± 28.4, *p* = 0.005). NfL and p‐tau181 were elevated in 17.4% and 22.7% patients, respectively, but did not correlate with severity. Multivariable regression showed MUNIX and disease duration as independent severity predictors (*R* ^2^ = 0.650, *p* = 0.011). Phenotype‐stratified models found strong prediction in demyelinating forms (*R* ^2^ = 0.862, *p* = 0.037) in contrast to axonal ones (*R* ^2^ = 0.530, *p* = 0.450).

**Conclusion:** MUNIX emerges as the most robust objective neurophysiological predictor of clinical severity in CMT. Phenotype‐specific assessment is essential: demyelinating forms benefit from MUNIX‐based monitoring with robust predictive models, more than axonal phenotypes. Plasma biomarkers (NfL, p‐tau181), although elevated, probably represent acute rather than chronic disease pathophysiology and merit longitudinal evaluation alongside serial MUNIX measurements. This integrated phenotype‐stratified multimodal approach requires prospective validation for individualized outcome assessment and monitoring in genetically diverse CMT.

**Disclosure:** Nothing to disclose.

## EPO‐0052

### Unmasking POEMS syndrome: The diagnostic value of clinical red flags, PEST criteria and nerve conduction studies

#### I. Lopera‐Rodríguez; J. Andrada‐Moreno; L. García‐Granados; J. Ciriero‐Macías; J. Román‐Rueda; M. Salgado‐Irazabal; A. Luque‐Ambrosiani; C. Paradas; F. Gómez‐Fernández

##### Hospital Universitario Virgen del Rocío, Spain

**Background and aims:** POEMS syndrome is a rare paraneoplastic disorder frequently misdiagnosed as Chronic Inflammatory Demyelinating Polyneuropathy (CIDP). We aim to distinguish both entities by analysing PEST criteria (Papilloedema, Extravascular volume overload, Sclerotic bone lesions, Thrombocytosis), the Nerve Conduction Study (NCS) pattern and specific clinical features.

**Methods:** We conducted a descriptive retrospective study of five patients with confirmed POEMS syndrome, initially misdiagnosed as CIDP in four of them. We assessed mandatory criteria, Vascular Endothelial Growth Factor (VEGF) levels, PEST domains and NCS pattern.

**Results:** Mean age was 45.6 ± 15.8 years. All patients fulfilled mandatory criteria of polyneuropathy and monoclonal gammopathy (IgG lambda‐restricted in 80%), alongside elevated VEGF (>1000 pg/mL). Clinically, all patients showed refractoriness to immunoglobulins and 80% ultimately required autologous stem cell transplantation. Motor predominance was distinctively observed (40%) with predominant distal lower limbs weakness. NCS revealed a demyelinating background consistently associated with an axonal component, which was the predominant feature in one patient (20%). Regarding PEST criteria, papilloedema was present in 40% (Figure 1), whereas the triad of extravascular volume overload, sclerotic bone lesions (axial predominance) (Figure 2) and thrombocytosis was universally present. Hypoalbuminaemia was identified as a frequent supportive biochemical finding (40%).

**Figure:**
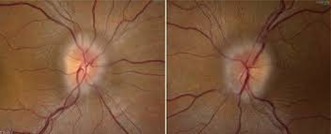
**FIGURE 1** Papilloedema.

**Figure:**
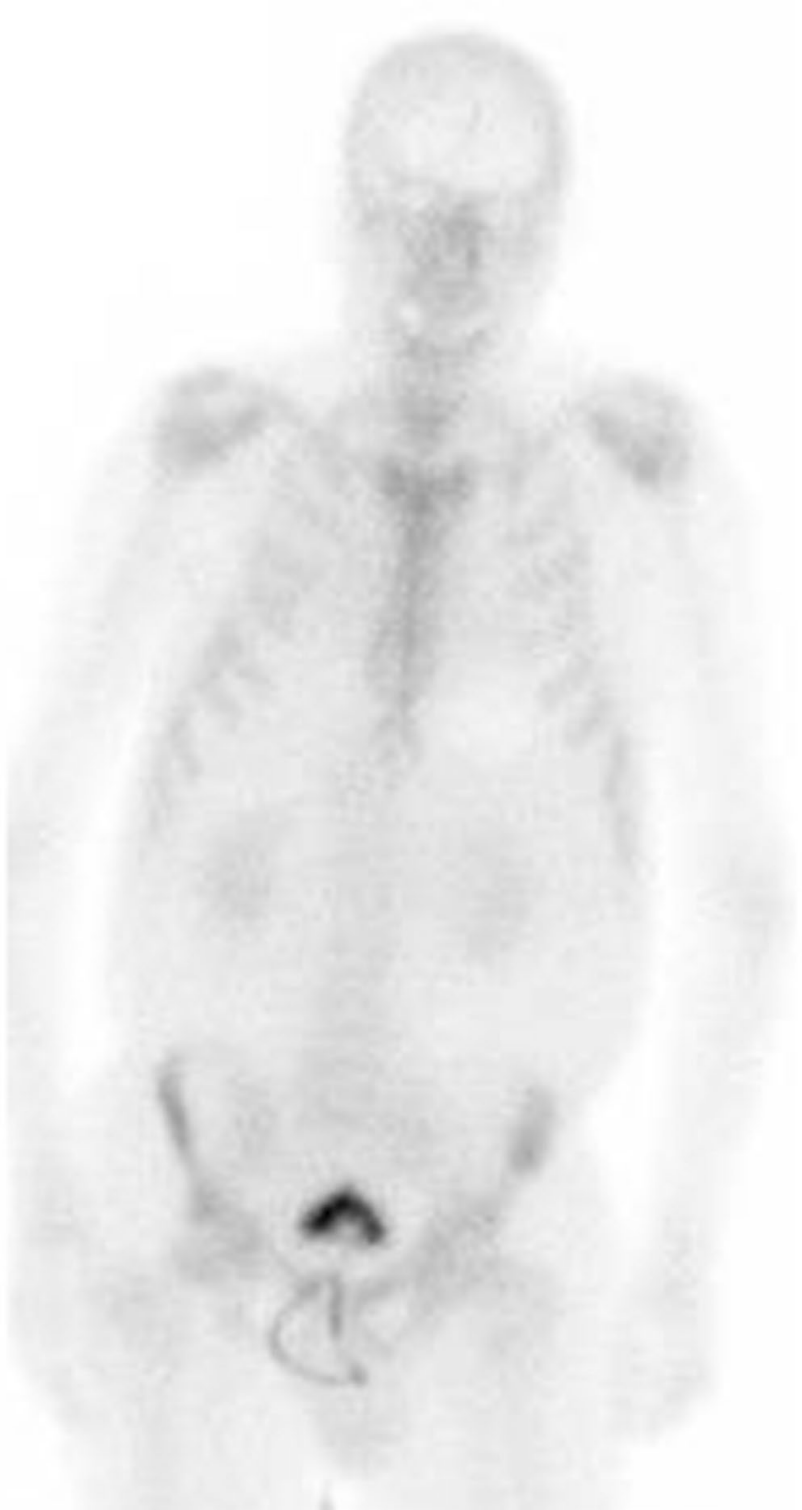
**FIGURE 2** Tc‐99m scintigraphy demonstrates axial osteoblastic lesions.

**Conclusion:** In patients with suspected CIDP, evaluation of PEST criteria is crucial, particularly in treatment‐refractory cases. Furthermore, motor‐predominant polyneuropathy or an associated axonal component on NCS should prompt VEGF testing and serum protein electrophoresis to rule out POEMS syndrome.

**Disclosure:** Nothing to disclose.

## EPO‐0053

### Distinctive characteristics of acute‐onset chronic inflammatory demyelinating polyneuropathy: A multicenter cohort study

#### I. Margarido ^1^; M. Pinto^1^; M. Henriques^2^; S. Bernardo^2^; S. Cruz^2^; C. Guerreiro^3^; B. Madureira^3^; M. Brum^3^; L. Medeiros^3^; R. Rodrigues^4^; C. Falcão Campos^4^; L. Fagundes^5^; J. Alves^5^; L. Almendra^5^; C. Vaz^6^; E. Santos^6^; S. Palma^7^; P. Pereira^7^; M. Mendes^8^; A. Veiga^8^; A. Cabral^9^; H. Costa^9^; G. Soares^10^; S. Moreira^10^; E. Alves^11^; C. Santos^11^; C. Malho^12^; J. Castelo^12^; S. Pinto^13^; G. Bonifácio^13^; L. Braz^1^

##### ^1^Department of Neurology, São João University Hospital, Porto, Portugal; ^2^Department of Neurology, Fernando da Fonseca Hospital, Amadora, Portugal; ^3^Department of Neurology, São José University Hospital, Lisboa Portugal; ^4^Department of Neurology, Santa Maria University Hospital, Lisbon, Portugal; ^5^Department of Neurology, Coimbra University Hospital, Coimbra, Portugal; ^6^Department of Neurology, Santo António University Hospital, Porto, Portugal; ^7^Department of Neurology, Almada Seixal Local Health Unit, Almada, Portugal; ^8^Department of Neurology, Trás‐s‐Montes e Alto Douro Local Health h Unit, Vila Real, Portugal; ^9^Department of Neurology, Gaia‐Espinho Local Health h Unit, Vila Nova de Gaia, Portugal; ^10^Department of Neurology, Matosinhos Local Health h Unit, Matosinhos, Portugal; ^11^Department of Neurology, Entre o Douro e Vouga Local Health h Unit, Santa Maria da Feira, Portugal; ^12^Department of Neurology, Loures/Odivelas Local Health Unit, Loures, Portugal; ^13^Department of Neurology, Arrábida Local Health Unit, Setúbal, Portugal

**Background and aims:** Approximately 16% of patients with chronic inflammatory demyelinating polyneuropathy (CIDP) present with a rapidly progressive course, reaching clinical nadir within eight weeks (acute‐onset CIDP, A‐CIDP). Limited data exists regarding the pathophysiological and clinical differences compared with classical‐onset CIDP. This study aimed to compare these two entities.

**Methods:** National, multicenter, retrospective study. Demographic, clinical, laboratory, and neurophysiological data were collected from electronic medical records of patients with A‐CIDP and CIDP and compared between groups.

**Results:** Eighty‐three patients with typical, multifocal, or motor‐predominant CIDP were included; 57.8% were male, median age was 51 (IQR 41–61) years and median follow‐up was 72.0 (IQR 40.0–138.5) months. Twenty of these patients (24%) had A‐CIDP. Median age at presentation did not differ between groups (50[30–60] vs 53[43–61] years; *p* = 0.28). A‐CIDP patients more frequently exhibited hypo/areflexia at presentation (95.0% vs 29.4%, *p* = 0.02) and fewer sensory symptoms (at presentation: 75.0% vs93.4%, *p* = 0.06; at last visit: 45.0% vs 73.8%, *p* = 0.04). At diagnosis, total leukocyte counts (8.30 × 10^9^/L [7.12–9.45] vs 6.72 × 10^9^/L [5.40–7.90], *p* = 0.01) and neutrophil counts (5.24 × 10^9^/L [4.70–6.89] vs 3.84 × 10^9^/L [3.01–5.30], *p* = 0.02) were higher in A‐CIDP. No significant differences were observed in cerebrospinal fluid protein levels or nerve conduction studies’ parameters. Immunoglobulin therapy was most frequently used in both groups, followed by oral corticosteroids and rituximab. Overall, A‐CIDP patients more often achieved an objective treatment response, which was significant for oral corticosteroid therapy (88.90% vs 50.00%, *p* = 0.04).

**Conclusion:** In our cohort A‐CIDP is characterized by more frequent hypo/areflexia, fewer sensory symptoms, higher inflammatory hematological markers, and a better treatment response, particularly to oral corticosteroids.

**Disclosure:** Nothing to disclose.

## EPO‐0054

### Phase 2 trial of Riliprubart in CIDP: Patient‐reported quality‐of‐life, pain and fatigue outcomes at week‐76

#### S. Attarian^1^; H. Hartung^2^; Y. Lu^3^; M. Alonso‐Alonso ^4^; A. Paker^4^

##### ^1^Neuromuscular Disease and ALS Reference Center, Timone University Hospital, Aix‐Marseille University, CHU Timone, Marseille Cedex 05, France; ^2^Department of Neurology, Heinrich‐Heine‐University, Germany; Brain and Mind Center, University of Sydney, Australia; Department of Neurology, Medical University of Vienna, Austria; Department of Neurology, Palacky University Olomouc, Czech Republic; ^3^Sanofi R&D, Evidence Generation and Decision Science, Cambridge, USA; ^4^Sanofi R&D, Neurology Development, Cambridge, USA

**Background and aims:** People living with chronic inflammatory demyelinating polyradiculoneuropathy(CIDP) experience fatigue, weakness and sensory abnormalities, affecting quality‐of‐life(QoL). Riliprubart, a first‐in‐class, humanized, IgG4‐monoclonal antibody, selectively inhibits activated‐C1s within the classical complement pathway and is self‐administered subcutaneously via auto‐injector. A Phase‐2 trial (NCT04658472) evaluating riliprubart in CIDP demonstrated decreased fatigue‐severity and improved QoL at week‐48. Here we assess effect of riliprubart on fatigue, pain and QoL up to week‐76.

**Methods:** Open‐label trial evaluating riliprubart across three groups: Standard‐of‐Care(SoC)‐Treated (*N* = 48), SoC‐Refractory (*N* = 18), and SoC‐Naïve (*N* = 12). Participants underwent 24‐week treatment (Part‐A), followed by optional treatment‐extension(Part‐B:52‐weeks). At week‐76, changes from baseline in health‐related QoL were descriptively analyzed using EuroQol‐Visual Analogue Scale(EQ‐VAS; range 0–100, higher score indicate better outcomes) and health utility index value(range < 0[as bad or worse than dead] to 1[full‐health]), pain through EQ‐5D‐5L Dimension(range 1[no pain/discomfort] to 5[extreme pain/discomfort]), and fatigue using Modified Rasch‐built Fatigue Severity Scale (R‐FSS; range: 0–21, higher score indicate greater fatigue‐severity).

**Results:** At week‐76, QoL, pain and fatigue were assessed in 37 SoC‐Treated, 11 SoC‐Refractory and 6 SoC‐Naïve participants. Mean[SD] change from baseline in EQ‐VAS(SoC‐Treated:12.1[18.4], SoC‐Refractory:19.1[21.8], SoC‐Naïve:8.5[16.2]) and health utility index value(SoC‐Treated:0.18[0.28], SoC‐Refractory:0.21[0.18], SoC‐Naïve:0.05[0.22]) suggested QoL improvement across all groups. Higher proportion reported no pain versus baseline(SoC‐Treated:27.0%[10/37] vs 12.8%[6/47], SoC‐Refractory:45.5%[5/11] vs 16.7%[3/18], SoC‐Naïve:33.3%[2/6] vs 16.7%[2/12]). Mean[SD] R‐FSS changes indicated reduced fatigue across SoC‐Treated (−3.7[7.6]), SoC‐Refractory (−2.2[9.5]), and SoC‐Naïve (−3.3[4.2]) groups.

**Conclusion:** Riliprubart indicated sustained decrease in fatigue and pain, and improved QoL in people with CIDP, along with clinical improvement. Two randomized, placebo‐controlled Phase‐3 studies (MOBILIZE, VITALIZE) are ongoing to confirm these findings.

**Disclosure:** Shahram Attarian: Consultant with Alexion, argenx, UCB, Janssen, Hansa Biopharma, Roche, Sanofi, Amicus, LFB, Alnylam, Astrazeneca, Pfizer and Biogen. Hans‐Peter Hartung: Consultant with Sanofi and Octapharma. He has received fees for serving on Steering and Data Monitoring Committees from Biogen, BMS Celgene, GeNeuro, Merck, Novartis, Octapharma, Roche, and TG Therapeutics. Yi Lu, Miguel Alonso‐Alonso, Asif Paker: Employees of Sanofi and may hold shares and/or stock options in the company.

## EPO‐0055

### Riliprubart in CIDP: Time and magnitude of response analysis from a phase 2 trial

#### R. A. Lewis^1^; J. Lin^2^; A. Dionne^3^; Y. Lu^4^; M. Alonso‐Alonso ^5^; A. Paker^5^; L. Querol^6^

##### ^1^Department of Neurology, Cedars Sinai Medical Center, Los Angeles, USA; ^2^Department of Neurology and Rare Disease Center, Huashan Hospital, Fudan University, Shanghai, People's Republic of China; ^3^CHU de Quebec Universite Laval, Quebec, Canada; ^4^Sanofi R&D, Evidence Generation and Decision Science, Cambridge, USA; ^5^Sanofi, Cambridge, USA; ^6^Neuromuscular Diseases Unit, Department of Neurology, Hospital de la Santa Creu i Sant Pau, Barcelona, Spain; Centro para la Investigación Biomédica en Red en Enfermedades Raras (CIBERER), Madrid, Spain

**Background and aims:** Riliprubart, a first‐in‐class humanized IgG4‐monoclonal antibody, selectively inhibits activated‐C1s in classical complement pathway and can be self‐administered subcutaneously via auto‐injector. In Phase‐2 trial (NCT04658472), riliprubart demonstrated favorable safety and encouraging efficacy in chronic inflammatory demyelinating polyradiculoneuropathy (CIDP). Here, we present time and magnitude of response analyses to characterize clinical benefits.

**Methods:** The open‐label Phase‐2 trial evaluated riliprubart across three groups: Standard‐of‐care (SoC)‐Treated, SoC‐Refractory, and SoC‐Naïve. Participants received 24‐week treatment (Part‐A), followed by an optional 52‐week treatment‐extension (Part‐B). Primary endpoint (Part‐A) evaluated percentage of participants relapsing in SoC‐Treated (≥1‐point increase in adjusted Inflammatory Neuropathy Cause and Treatment [INCAT] disability score) and percentage of participants responding in SoC‐Refractory/Naïve groups (≥1‐point decrease in adjusted INCAT score). This post‐hoc analysis focused on INCAT responders (≥1‐point decrease) in Part‐A, and evaluated time‐to‐first‐response, and proportion of participants with deep response (≥2‐point decrease), overall, and by baseline INCAT score (3–4 versus ≥ 5).

**Results:** Among 43 INCAT responders in Part‐A, first INCAT response was observed as early as Week‐2 with ≥44% of first responses achieved by Week‐4, and ≥89% by Week‐12 in all three groups. At the last INCAT assessment through Week‐24, 44% (19/43) responders achieved deep response with higher rates in participants with severe baseline impairment (INCAT ≥ 5: 69% [11/16]) versus moderate impairment (INCAT 3–4: 38% [8/21]).

**Conclusion:** Riliprubart provided rapid and substantial improvement in INCAT score with majority of responders achieving first response by Week‐12 and nearly half of responders achieving deep response (≥2‐point decrease); supporting Phase‐3 development of riliprubart as a potential treatment for people with CIDP.

**Disclosure:** Richard A. Lewis: Consultant with CSL Behring, Grifols, Pfizer, Sanofi (Steering Committee), argenx, Pharnext, Roche, Johnson & Johnson, Takeda, Boehringer Ingelheim (DSMB), and Momenta. He is also part of the scientific advisory boards Alnylam and Akcea and medical advisory board The GBS‐CIDP Foundation International. Jie Lin: Nothing to disclose. Annie Dionne: Received honoraria from argenx and Alexion for conference and ad board. Yi Lu, Miguel Alonso‐Alonso, Asif Paker: Employees of Sanofi and may hold shares and/or stock options in the company. Luis Querol: Received research grants from Instituto de Salud Carlos III – Ministry of Economy and Innovation (Spain), CIBERER, Fundació La Marató, GBS‐CIDP Foundation International, UCB and Grifols. He received speaker or expert testimony honoraria from CSL Behring, Novartis, Sanofi, Merck, Annexon, Alnylam, Biogen, Janssen, Lundbeck, argenx, UCB, Dianthus, LFB, Avilar Therapeutics, Octapharma and Roche. He serves at Clinical Trial Steering Committee for Sanofi and was Principal Investigator for UCB's CIDP01 trial.

## EPO‐0056

### GM1 neuropathies from antibody to phenotype

#### N. Dubuisson ^1^; R. Bellanti^1^; C. Bergstrom Johnson^1^; M. Misheva^1^; M. Makuch^1^; R. Prior^2^; S. Rinaldi^1^

##### ^1^University of Oxford, Oxford, UK; ^2^VIB/KULeuven, Leuven, Belgium

**Background and aims:** Multifocal motor neuropathy (MMN) is a chronic immune‐mediated, purely motor neuropathy associated with GM1 antibodies. The underlying immune mechanisms, particularly the origin, clonality, and pathogenic role of GM1‐specific B cells, remain incompletely understood. This study aims to characterize the cellular origin and functional relevance of GM1 antibodies to inform more targeted therapeutic strategies.

**Methods:** Thirty‐five MMN patients were clinically assessed using MRC‐SS, ONLS, and MMN‐RODS scores. Paraproteins and light‐chain restriction were evaluated by serum protein electrophoresis, ELISA, and high‐resolution mass spectrometry. Peripheral blood mononuclear cells were analysed by flow cytometry, followed by bulk and single‐cell sorting and in vitro culture to assess GM1‐specific IgM production. The pathogenic potential of patient‐derived antibodies was evaluated using human iPSC‐derived neuromuscular organoids.

**Results:** MMN patients showed an initial response to treatment, followed by functional decline after approximately seven years. GM1 antibodies were detected in 57% of patients, and evidence of B‐cell clonality was observed in 35%. Mass spectrometry identified additional IgM paraproteins in MMN sera. GM1 reactivity was present in both IgD^+^ and IgD^−^ memory B‐cell subsets, suggesting an early origin of autoreactivity involving defects in central and early peripheral tolerance rather than germinal‐centre–dependent somatic hypermutation. Single‐cell B‐cell receptor sequencing is ongoing to further test this hypothesis. In neuromuscular organoids, patient‐derived IgM impaired muscle contraction, supporting a direct pathogenic effect.

**Conclusion:** MMN is characterized by a clonally expanded, GM1‐reactive B‐cell response with direct pathogenic potential. These findings provide insight into disease initiation and persistence and support the development of targeted B‐cell–directed therapies.

**Disclosure:** Nothing to disclose.

## EPO‐0057

### Efficacy and safety of Tafamidis in hereditary TTR amyloidosis and mixed phenotypes‐ the effect of the highest dose in the progression of neuropathy

#### R. Alves Simões ^1^; C. Campos^1,2^; I. de Castro^1,2^; J. Castro^1,2^; I. Conceição^1,2^

##### ^1^Department of Neuroscience and Mental Health, Santa Maria Hospital, CAML, ULSSM, Lisbon, Portugal; ^2^Institute of Physiology; Egas Moniz Study Center, Faculty of Medicine, University of Lisbon, Lisbon, Portugal

**Background and aims:** Hereditary transthyretin amyloidosis (ATTRv) may present with neuropathic, cardiac, or mixed phenotypes. Tafamidis, a transthyretin stabilizer, is approved at a dose of 20 mg/day for the treatment of stage 1 ATTRv neuropathy. The 61 mg/day dose has been approved for the cardiac phenotype, although its impact on neuropathic progression in mixed phenotype has not yet been established.

**Methods:** To evaluate the rate of neuropathy progression and changes in nutritional status in patients with a mixed phenotype previously treated with Tafamidis 20 mg, after dose escalation to Tafamidis 61 mg. An ambispective observational study was conducted, including 18 patients with a mixed phenotype who were treated with Tafamidis 61 mg for at least 12 months and had previously received Tafamidis 20 mg for the same minimum period. Four patients were excluded due to temporal criteria. Neuropathy progression was assessed using the Neuropathy Impairment Scale (NIS), while nutritional status was monitored using body mass index (BMI).

**Results:** Patients treated with Tafamidis 61 mg showed slower neuropathy progression than those treated with Tafamidis 20 mg, although no statistically significant difference was reached (annual change in total NIS = 1.41 vs. 2.71; *p* = 0.55). Additionally, a potential benefit in nutritional status was observed in patients treated with Tafamidis 61 mg (annual change in BMI = 0.86 vs. −0.04; *p* = 0.02).

**Conclusion:** Dose escalation to Tafamidis 61 mg did not result in a statistically significant reduction in neuropathy progression. However, nutritional status appears to be better preserved at this dosage. Interpretation is limited by the small sample size.

**Disclosure:** Nothing to disclosure.

## EPO‐0058

### Immunological profiling of skin biopsies in patients with idiopathic small fiber neuropathy

#### T. Mederer ^1^; F. Montes^2^; P. Llarch^1^; E. Ortiz^2^; C. Lleixà^2^; M. Caballero‐Ávila^1^; R. Collet‐Vidiella^1^; A. Vesperinas^1^; Á. Carbayo^1^; L. Llansó^1^; L. Querol^1^; A. Mariscal^2^; E. Pascual‐Goñi^1^

##### ^1^Neuromuscular Disorders Unit, Department of Neurology, Hospital de la Santa Creu i Sant Pau, Barcelona, Spain; ^2^Department of Immunology, Hospital de la Santa Creu i Sant Pau, Barcelona, Spain

**Background and aims:** Small fibre neuropathy (SFN) is a heterogeneous group of disorders characterized by selective involvement of thinly myelinated A‐delta and unmyelinated C fibres. Although multiple aetiologies have been described, up to 50% of cases remain classified as idiopathic (iSFN). Immunological characterisation of skin biopsies in iSFN may help define disease subtypes and clarify underlying pathogenic mechanisms.

**Methods:** A pilot study analysed distal and proximal skin biopsies from 10 biopsy–confirmed SFN patients and distal biopsies from 5 healthy controls. Immunohistochemistry was performed for IgG, IgM, IgA, CD45, and C5b9; CD45‐positive biopsies were further stained for CD4, CD8, and CD20. Double immunofluorescence was used to assess Langerhans cells (CD207) and intraepidermal nerve fibre density (IENFD) using PGP9.5.

**Results:** Most patients were male (70%), with a mean age of 47 years (32–64). The majority had iSFN, while three patients had secondary SFN (monoclonal gammopathy, diabetes with rheumatoid arthritis, and Sjögren). All SFN patients showed reduced distal IENFD. C5b9 deposits and leukocyte infiltration, predominantly CD8+ T cells, were observed exclusively in SFN patients. Leukocyte infiltration was present in both distal and proximal skin, suggesting immune activation beyond distal sites of fibre loss. IgG deposition was significantly increased in SFN patients, whereas no differences were found for IgM, IgA, or Langerhans cell density.

**Figure:**
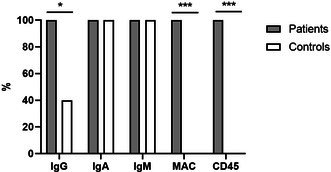
**FIGURE 1** Immunohistochemistry findings in patients and healthy controls.

**Figure:**
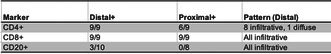
**TABLE 1** Immunohistochemistry characterisation in CD45+ patients.

**Conclusion:** Consistent immune alterations in proximal and distal skin biopsies from both idiopathic and autoimmune‐associated SFN patients support a primary role for localized immune dysregulation in SFN pathogenesis. The ongoing study includes 50 patients, and final results will be presented at the congress.

**Disclosure:** Nothing to disclose.

## Cognitive Neurology/Neuropsychology 1

## EPO‐0059

### The impact of cognitive reserve on neuropsychological profiles in different etiologies of dementia

#### A. Salvati ^1^; F. Motolese^2^; I. Anzuino^1^; G. Massa Rolandino^1^; E. Sapio^1^; D. Norata^1^; F. Capone^2^; V. Di Lazzaro^2^

##### ^1^Department of Medicine and Surgery, Unit of Neurology, Neurophysiology, Neurobiology and Psychiatry, Università Campus Bio‐Medico di Roma, Rome, Italy; ^2^Fondazione Policlinico Universitario Campus Bio‐Medico, Rome, Italy

**Background and aims:** Dementia is characterized by a progressive decline in cognitive functions. Different lifelong activities contribute to build a cognitive reserve (CR), which can influence cognitive decline trajectory. This study aims to investigate the effect of CR on neuropsychological performances in patients with dementia.

**Methods:** We enrolled 61 patients affected by by Alzheimer's disease (AD) dementia, Vascular dementia (VaD) and Mixed forms. The sample included 28 Females (46%). Mean (± SD) age was 73.96 ± 7.00 years, while mean (± SD) education years were 11.38 ± 3.75. Neuropsychological assessment included the Mini Mental State Examination (MMSE), Digit Span, Trial Making Test (TMT‐A and B), Rey Auditory Verbal Learning Test (RAVLT; Immediate and Delayed) and Verbal Fluency (Phonemic and Semantic). Cognitive reserve was assessed using the Cognitive Reserve Index Questionnaire (CRI‐q). Patients were classified as High CR (HCR) or Low CR (LCR) according to an established cut‐off value.

**Results:** Significant differences between the two groups were observed in Immediate‐RAVLT (U = 262.5; *p* = 0.043, HCR = 30.316 ± 9.417; LCR = 24.450 ± 7.891) and in Phonemic Fluency (U = 133.0; *p* = 0.044; HCR = 35.571 ± 11.501; LCR = 25.462 ± 12.231). Spearman's correlation revealed positive correlations between the total score of CRIq and MMSE, Immediate‐RAVLT and Phonemic Fluency.

**Conclusion:** HCR group showed better performances in short‐term memory and language. These findings emphasize the modulating and protective role of cognitive reserve on cognitive functioning and suggest that specific neuropsychological tests may be particularly sensitive in detecting its effects.

**Disclosure:** Nothing to disclose.

## EPO‐0060

### Predicting neurocognitive disorder development in multiple sclerosis using artificial intelligence on MRI and clinical data

#### D. Mistri ^1^; L. Storelli^1^; A. Mastropasqua^1^; M. Grosselle^1^; P. Preziosa^2^; G. Mazzetti^1^; L. Rossi^3^; M. Filippi^4^; M. Rocca^2^

##### ^1^Neuroimaging Research Unit, Division of Neuroscience, IRCCS San Raffaele Scientific Institute, Milan, Italy; ^2^Neuroimaging Research Unit, Division of Neuroscience, and Neurology Unit, IRCCS San Raffaele Scientific Institute, Milan, Italy; and Vita‐Salute San Raffaele University, Milan, Italy; ^3^Neuroimaging Research Unit, Division of Neuroscience, IRCCS San Raffaele Scientific Institute, Milan, Italy; and Vita‐Salute San Raffaele University, Milan, Italy; ^4^Neuroimaging Research Unit, Division of Neuroscience, Neurology Unit, Neurorehabilitation Unit, and Neurophysiology Service, IRCCS San Raffaele Scientific Institute, Milan, Italy; and Vita‐Salute San Raffaele University, Milan, Italy

**Background and aims:** Neurocognitive disorders (NDs) are often under‐recognized symptoms of multiple sclerosis (MS). An early prediction of ND in people with MS is crucial for implementing effective early interventions. This study aimed to develop an artificial intelligence (AI) neural network to predict ND in MS patients after at least one year, using baseline demographic, clinical, and MRI data.

**Methods:** 3D T1‐weighted brain MRIs of 223 MS patients were collected at baseline with demographics, clinical data, and cognitive reserve proxies. At baseline and after a median follow‐up of 1.5 years, each patient underwent cognitive assessment and was classified as having mild ND or major ND according to the DSM‐5‐TR manual. Patients whose cognitive status decayed over time were classified as worsened. A deep learning model used a 10‐layer ResNet to extract MRI features, combined with non‐imaging data (demographics, clinical details, lesion load, brain volumes) to classify patients as cognitively preserved or impaired. Performance was evaluated using 5‐fold cross‐validation.

**Results:** At follow‐up, 27 out of 224 MS (12%) experienced cognitive worsening. AI model achieved a mean validation accuracy of 90%, F1‐score of 81% and average AUC of 0.89. Grad‐CAM heatmaps identified the frontal, occipital, middle temporal gyri, and hippocampus as key brain regions, while important tabular features included cortical gray matter volume, age, thalamic and hippocampal volumes, T2 lesion volume, and cognitive reserve score.

**Conclusion:** AI, mainly based on MRI data, can effectively predict development of NDs, even within a short follow‐up period, enabling timely identification and management of MS patients at risk of worsening.

**Disclosure:** D. Mistri has nothing to disclose. L. Storelli received speakers’ honoraria from Biogen. A. Mastropasqua, M. Grosselle have nothing to disclose. P. Preziosa received speaker honoraria from Roche, Biogen, Novartis, Merck, Bristol Myers Squibb, Genzyme, Horizon and Sanofi. G. Mazzetti and L. Rossi have nothing to disclose. M. Filippi is Editor‐in‐Chief of the Journal of Neurology, Associate Editor of Human Brain Mapping, Neurological Sciences, and Radiology; received compensation for consulting services from Almirall, Biogen, Bristol‐Myers Squibb, Eli Lilly, Merck, Novartis, Roche, Sanofi; speaking activities from Amgen, Bayer, Biogen, Bristol‐Myers Squibb, Celgene, Chiesi Italia SpA, Eisai, Eli Lilly, Fujirebio, Genzyme, Janssen, Merck, Neopharmed Gentili, Neuraxpharm, Novartis, Novo Nordisk, Roche, Sanofi, Takeda; participation in Advisory Boards for Alexion, Biogen, Bristol‐Myers Squibb, Eli Lilly, GE Healthcare Ltd, Merck, Neuraxpharm, Novartis, Roche, Sandoz, Sanofi, Takeda; scientific direction of educational events for Biogen, Merck, Roche, Celgene, Bristol‐Myers Squibb, Lilly, Novartis, Sanofi‐Genzyme; he receives research support from Biogen Idec, Merck‐Serono, Novartis, Roche, the Italian Ministry of Health, the Italian Ministry of University and Research, and Fondazione Italiana Sclerosi Multipla. M.A. Rocca received consulting fees from Biogen, Bristol Myers Squibb, Roche; and speaker honoraria from Alexion, Biogen, Bristol Myers Squibb, Celgene, Horizon Therapeutics Italy, Merck Serono SpA, Mitsubishi‐Tanabe Pharma, Neuraxpharm, Novartis, Roche, Sandoz, and Sanofi. She receives research support from the MS Society of Canada, the Italian Ministry of Health, the Italian Ministry of University and Research, and Fondazione Italiana Sclerosi Multipla. She is Associate Editor for Multiple Sclerosis and Related Disorders; and Associate Co‐Editor for Europe and Africa for Multiple Sclerosis Journal.

## EPO‐0061

### Cognitive impairment in multiple sclerosis: Correlation between brain atrophy measures and Raos's brief repeatable battery

#### D. García Estévez ^1^; O. Álvarez de los Arcos^2^

##### ^1^Neurology Service, Complejo Hospitalario Universitario de Ourense, Ourense, Spain; ^2^Clinical Neuroscience Group, Galicia Sur Health Research Institute (IIS Galicia Sur), Vigo, Spain

**Background and aims:** Multiple sclerosis (MS) is a chronic, neurodegenerative disease of the central nervous system that progressively leads to increasing disability. The prevalence of cognitive impairment (CI) in people with MS (pwMS) ranges from 40% to 65%. Structurally, both brain atrophy and lesion load, together with disease duration, are associated with CI. Our objective was to analyze the relationship between MRI brain atrophy measures and performance on the neuropsychological tests of Rao's Brief Repeatable Battery (BRB) in pwMS.

**Methods:** We studied 95 pwMS with cognitive complaints, including both relapsing and secondary progressive MS phenotypes. Brain atrophy measures on MRI included the corpus callosum index (CCI), third ventricle width (3VW), and the bicaudate ratio (BCR). T2 lesion load was also quantified. The BRB consisted of the following tests: the Selective Reminding Test (SRT), the 10/36 Spatial Recall Test (SPART), the Symbol Digit Modalities Test (SDMT), the Paced Auditory Serial Addition Test (PASAT), and the Word List Generation Test (WLG).

**Results:** Sex: 69F/26M. Age (years): 50.1 ± 8.5. EDSS: 3.2 ± 2.1. Age was significantly correlated with the brain atrophy indices. The Table 1 shows the partial correlations, controlling for the effect of age, between the brain atrophy indices and the BRB. Brain atrophy measures were significantly higher in progressive forms. Lesion load was associated with greater atrophy levels and poorer cognitive performance (Figure 1).

**Figure:**
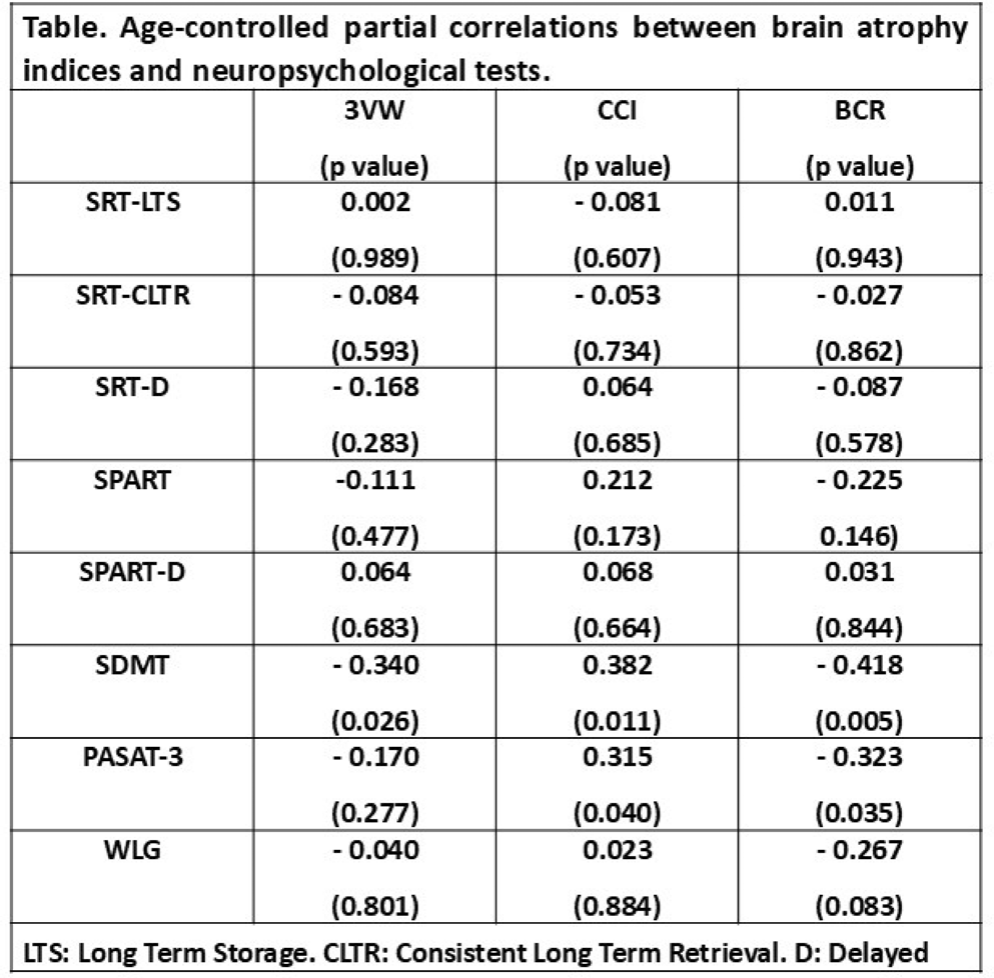
**TABLE 1** Correlations between brain atrophy indices and neuropsychological tests.

**Figure:**
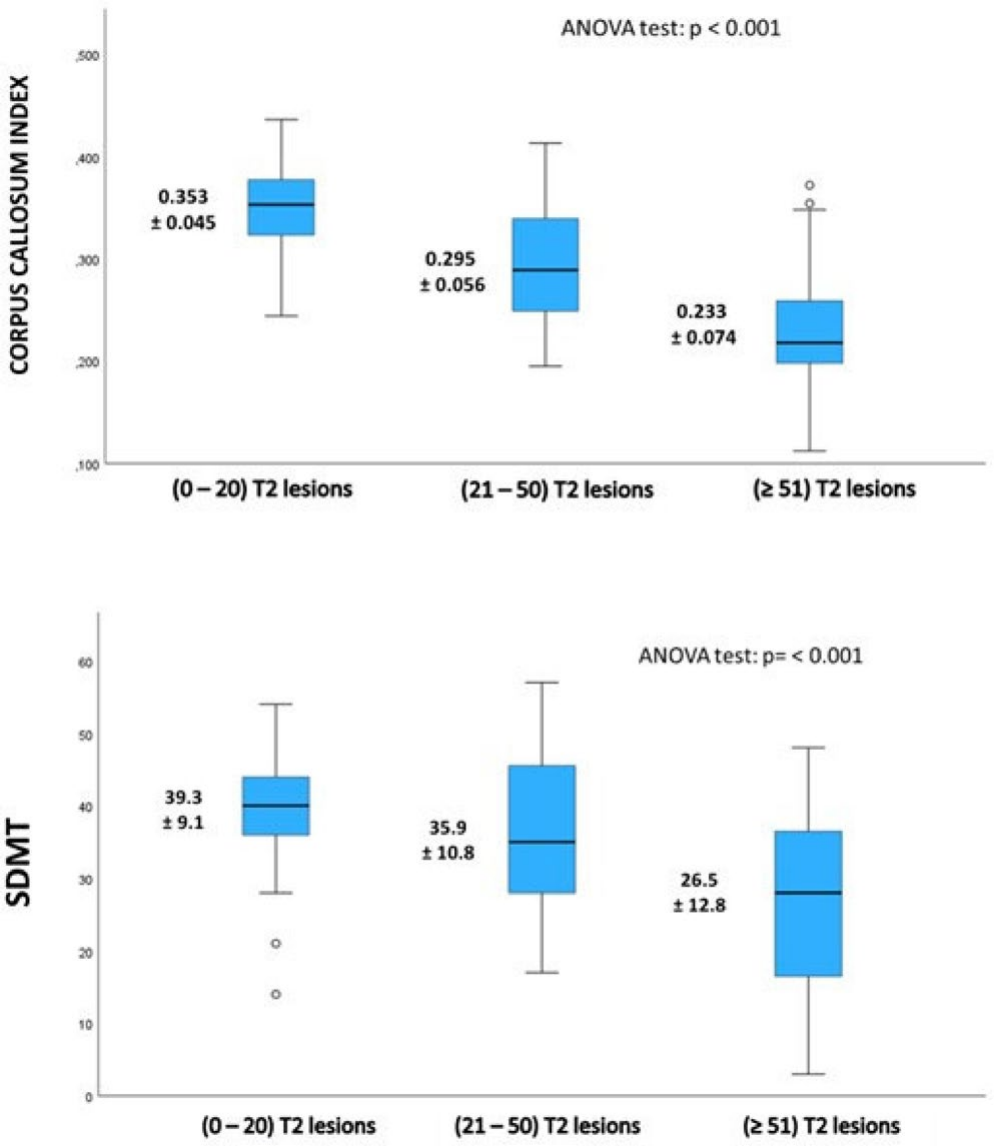
**FIGURE 1** T2 Lesion Load, brain atrophy and SDMT.

**Conclusion:** In our cohort of pwMS, brain atrophy measures were inversely correlated with performance on the SDMT and PASAT test, both reflecting information processing speed. Cognitive impairment was influenced by brain atrophy, lesion load, and progressive disease phenotypes.

**Disclosure:** Nothing to disclose.

## EPO‐0062

### Functional brain activation and reorganization before and after tailored speech language therapy in PPA: A fMRI study

#### E. Canu ^1^; L. Lumaca^2^; A. Gilioli^1^; S. Basaia^2^; A. Riva^2^; F. Freri^1^; A. Benzini^3^; R. Tomasoni^3^; P. Santacesaria^3^; G. Santi^3^; S. Aresta^3^; F. Valtorta^2^; A. Bianchi^2^; B. Lamorgese^2^; L. Zanchi^2^; J. Lanzone^4^; B. Minafra^5^; S. de Trane^5^; C. Lunetta^6^; P. Fiore^7^; B. Petronilla^3^; M. Filippi^8^; F. Agosta^9^

##### ^1^Neuroimaging Research Unit, Division of Neuroscience, and Neurology Unit, IRCCS San Raffaele Scientific Institute, Milan, Italy; and Neurotech Hub, Vita‐Salute San Raffaele University, Milan, Italy; ^2^Neuroimaging Research Unit, Division of Neuroscience, IRCCS San Raffaele Scientific Institute, Milan, Italy; and Neurotech Hub, Vita‐Salute San Raffaele University, Milan, Italy; ^3^Istituti Clinici Scientifici Maugeri IRCCS, Laboratory of Neuropsychology, Institute of Bari, Bari, Italy; ^4^Neuroimaging Research Unit, Division of Neuroscience, Neurology Unit, and Neurophysiology Service, IRCCS San Raffaele Scientific Institute, Milan, Italy; and Neurotech Hub, Vita‐Salute San Raffaele University, Milan, Italy; ^5^Istituti Clinici Scientifici Maugeri IRCCS, Neurorehabilitation Unit of Bari Institute, Bari, Italy; ^6^Istituti Clinici Scientifici Maugeri IRCCS, Department of Neurological Rehabilitation, Institute of Milan, Milan, Italy; ^7^Istituti Clinici Scientifici Maugeri IRCCS, Neurorehabilitation Unit of Bari Institute, Bari, Italy; and Department of Physical and Rehabilitation Medicine, University of Foggia, Foggia, Italy; ^8^Neuroimaging Research Unit, Division of Neuroscience, Neurology Unit, Neurorehabilitation Unit, and Neurophysiology Service, IRCCS San Raffaele Scientific Institute, Milan, Italy; and Neurotech Hub, Vita‐Salute San Raffaele University, Milan, Italy; ^9^Neuroimaging Research Unit, Division of Neuroscience, and Neurology Unit, IRCCS San Raffaele Scientific Institute, Milan, Italy; and Neurotech Hub, Vita‐Salute San Raffaele University, Milan, Italy

**Background and aims:** This study investigated functional MRI (fMRI) activation in language tasks before and after tailored speech and language therapy (SLT) targeting lexical access deficits in semantic (svPPA) and logopenic (lvPPA) variants of Primary Progressive Aphasia (PPA), and articulation deficits in nonfluent/agrammatic (nfvPPA) cases.

**Methods:** Ten PPA patients (5 lvPPA, 3 svPPA, 2 nfvPPA) underwent pre‐treatment MRI, and post‐treatment scans after five weeks of SLT. FMRI tasks were tailored to treatment: svPPA and lvPPA performed a silent confrontation naming (CN) and nfvPPA completed a speech‐observation (SO). Eight healthy controls (HC) underwent fMRI tasks at baseline only. Baseline group‐level comparisons and longitudinal analyses examined post‐treatment changes in patients were conducted.

**Results:** At baseline, during naming and compared to HC, PPA patients showed reduced activation in bilateral superior temporal regions, right supplementary motor area (SMA), and right superior frontal cortex, and increased activation in right anterior cingulate cortex and in left inferior temporal and parietal regions, mainly in lvPPA. Longitudinally, naming‐related activation increased post‐treatment in bilateral superior temporal cortex. During the SO task at baseline, compared to HC, nfvPPA cases showed reduced activation in bilateral cingulate and inferior frontal cortices, and right sensorimotor regions, alongside increased activation in right inferior occipital cortex. Qualitative single‐case analyses revealed a greater recruitment of bilateral SMA after treatment.

**Conclusion:** Findings suggest that tailored SLT in PPA induces changes in functional brain activation, reflecting treatment‐related neuroplasticity. Furthermore, SLT may enhance compensatory activation patterns already evident at baseline. Task‐based fMRI may help capture differential treatment responsiveness and monitor SLT effects in PPA.

**Disclosure:** Supported by Italian Ministry of Health (PNRR‐MCNT2‐2023‐12378220). E. Canu, S. Basaia, and B. Petronilla have received research support from the Italian Ministry of Health; L. Lumaca, A. Gilioli, A. Riva, F. Freri, A. Benzini, R. Tomasoni, P. Santacesaria, G.C. Santi, S. Aresta, F. Valtorta, A. Bianchi, B. Lamorgese, L. Zanchi, J. Lanzone, B. Minafra, S. de Trane, C. Lunetta, P. Fiore report no competing interests; M. Filippi is Editor‐in‐Chief of the Journal of Neurology, Associate Editor of Human Brain Mapping, Neurological Sciences, and Radiology; received compensation for consulting services from Almirall, Biogen, Bristol‐Myers Squibb, Eli Lilly, Merck, Novartis, Roche, Sanofi; speaking activities from Amgen, Bayer, Biogen, Bristol‐Myers Squibb, Celgene, Chiesi Italia SpA, Eisai, Eli Lilly, Fujirebio, Genzyme, Janssen, Merck, Neopharmed Gentili, Neuraxpharm, Novartis, Novo Nordisk, Roche, Sanofi, Takeda; participation in Advisory Boards for Alexion, Biogen, Bristol‐Myers Squibb, Eli Lilly, GE Healthcare Ltd, Merck, Neuraxpharm, Novartis, Roche, Sandoz, Sanofi, Takeda; scientific direction of educational events for Biogen, Merck, Roche, Celgene, Bristol‐Myers Squibb, Lilly, Novartis, Sanofi‐Genzyme; he receives research support from Biogen Idec, Merck‐Serono, Novartis, Roche, the Italian Ministry of Health, the Italian Ministry of University and Research, and Fondazione Italiana Sclerosi Multipla. F. Agosta is Associate Editor of NeuroImage: Clinical and the European Journal of Neurology; has received speaker honoraria from Biogen Idec, Bristol Myers Squibb, Eisai, Eli Lilly, GE Healthcare, Neuraxpharm, and Roche,; and receives or has received research supports from the Italian Ministry of Health, the Italian Ministry of University and Research, AriSLA (Fondazione Italiana di Ricerca per la SLA), the European Research Council (ERC), the EU Joint Programme – Neurodegenerative Disease Research (JPND), and Foundation Research on Alzheimer Disease (France).

## EPO‐0063

### Mirroring emotional faces in the developing brain: Functional connectivity of the mirror neuron system

#### F. Ferragina ^1^; K. Safar^2^; E. Broaders^3^; N. Rhodes^3^; M. Taylor^3^

##### ^1^Interdisciplinary Center for Health Sciences, Sant’Anna School of Advanced Studies, Pisa, Italy; ^2^Department of Child and Youth Studies, Trent University, Oshawa, Canada; ^3^Diagnostic & Interventional Radiology, Neurosciences & Mental Health, Hospital for Sick Children, Toronto, Canada

**Background and aims:** The Mirror Neuron System (MNS) underlies important neurocognitive functions, such as imitation learning, social cognition, and empathy, and may facilitate the recognition of emotional expressions via motor simulation. However, its development remains poorly understood. This study investigated MNS functional connectivity maturation between early childhood and adulthood during an emotional faces task.

**Figure:**
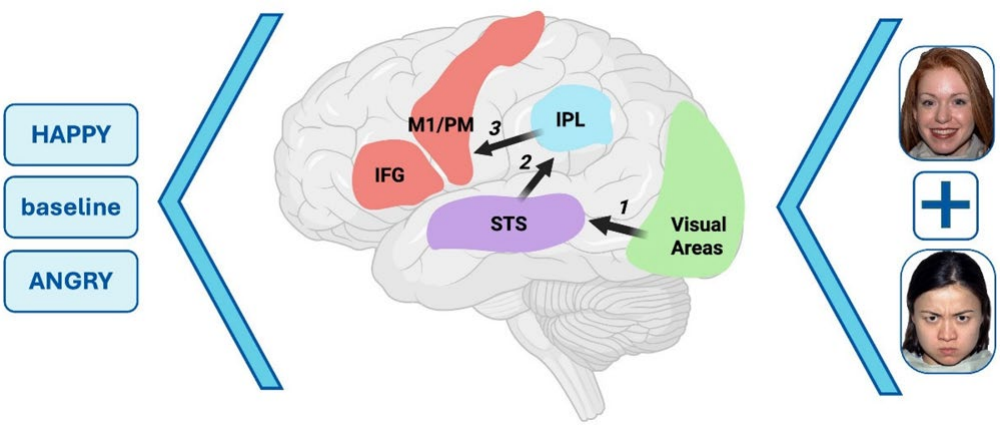
**FIGURE 1** Processing of visual inputs through the key hubs of the MNS facilitates the recognition of others' emotions via motor simulation of the observed faces. STS: superior temporal sulcus. IPL: inferior parietal lobe. M1/PM: primary motor and premotor cortices.

**Methods:** Using the innovative magnetoencephalography approach of optically pumped magnetometers (OPMs), we assessed whole‐brain connectivity of 8 bilateral regions of interest (ROIs) comprising the MNS in 57 typically developing children aged 3‐to‐5 years and 27 adults. We applied both classical network‐based statistic (NBS) and its more recent approach incorporating machine learning, NBS‐Predict.

**Results:** We observed networks of increased connectivity after face presentation in both adults and children in broadband, as well as in the alpha and beta bands; only adults recruited a theta band network. The ROIs were functionally linked to key limbic, prefrontal, parietal and occipital areas. Noteworthy, adults exhibited stronger right‐hemisphere broadband and theta connections than children. Results were validated by NBS‐Predict, with a comparable subnetwork exhibiting predictive features for classification between children and adults.

**Figure:**
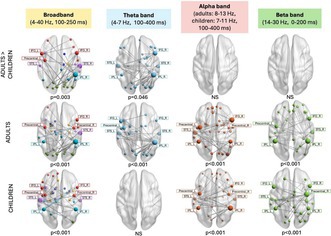
**FIGURE 2** Total NBS output. Both the within‐group analysis (Adults or Children; pcorrchildren; pcorr = 0.003 in the broadband.

**Figure:**
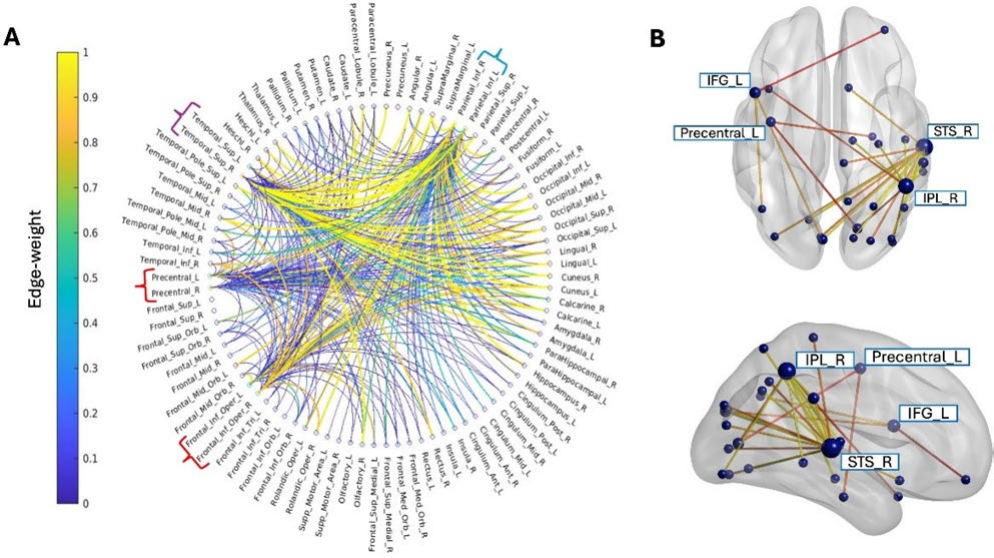
**FIGURE 3** A. Overall NBS‐Predict output (300‐edge network). Colours, from blue (=0) to yellow (=1), indicate the increasing relevance of the edge in the model prediction performance (*p* = 0.005). B. 27‐edge subnetwork with weight‐thresholded connections (>0.95), in ax.

**Conclusion:** Results suggest consolidation of right‐sided MNS connections through to adulthood, consistent with right‐hemisphere specialization in emotional processing.

**Disclosure:** The authors declare that they have no known competing financial interests or personal relationships that could have appeared to influence the work. Funding was provided by the Canadian Institutes of Health Research (PJT‐178370) and the Simons Foundation Autism Research Initiative (SFARI; 2021 Human Cognitive and Behavioural Science award). FF was supported by the Sant’Anna School of Advanced Studies, Pisa, Italy.

## EPO‐0064

### Multilingual co‐creation of aphasia topics priorities through patient and public involvement

#### J. Annoni ^1^; M. Charalambous^2^; A. Kountouri^3^

##### ^1^Department of Neurosciences, University of Fribourg, Fribourg, Switzerland; ^2^2Department of Rehabilitation Sciences, Cyprus University of Technology, Limassol, Cyprus; ^3^Cyprus Stroke Association, Limassol, Cyprus

**Background and aims:** People living with chronic aphasia face persistent challenges. it is essential to involve people with aphasia (PWAs) directly in the identification of thematic priorities concerning their quality of life. The aim is to share research agenda with PWA and health professionals across multiple countries and languages.i) evaluate the participation and adherence of strongly involved PWA and ii) collaboratively identify and prioritize topics of greatest importance to PWA through a multilingual PPI process for orienting future research.

**Methods:** The project involved more than 100 PWA from 14 countries, with 11 countries contributing to voting. The three‐phase process included (1) an online consultation phase to generate initial topics, (2) a development phase through national‐level codesign sessions to refine and rank topics and that concluded with international voting of four top priorities, and (3) a multilingual dissemination phase. Aphasia‐friendly materials and real‐time translation ensured accessibility.

**Figure:**
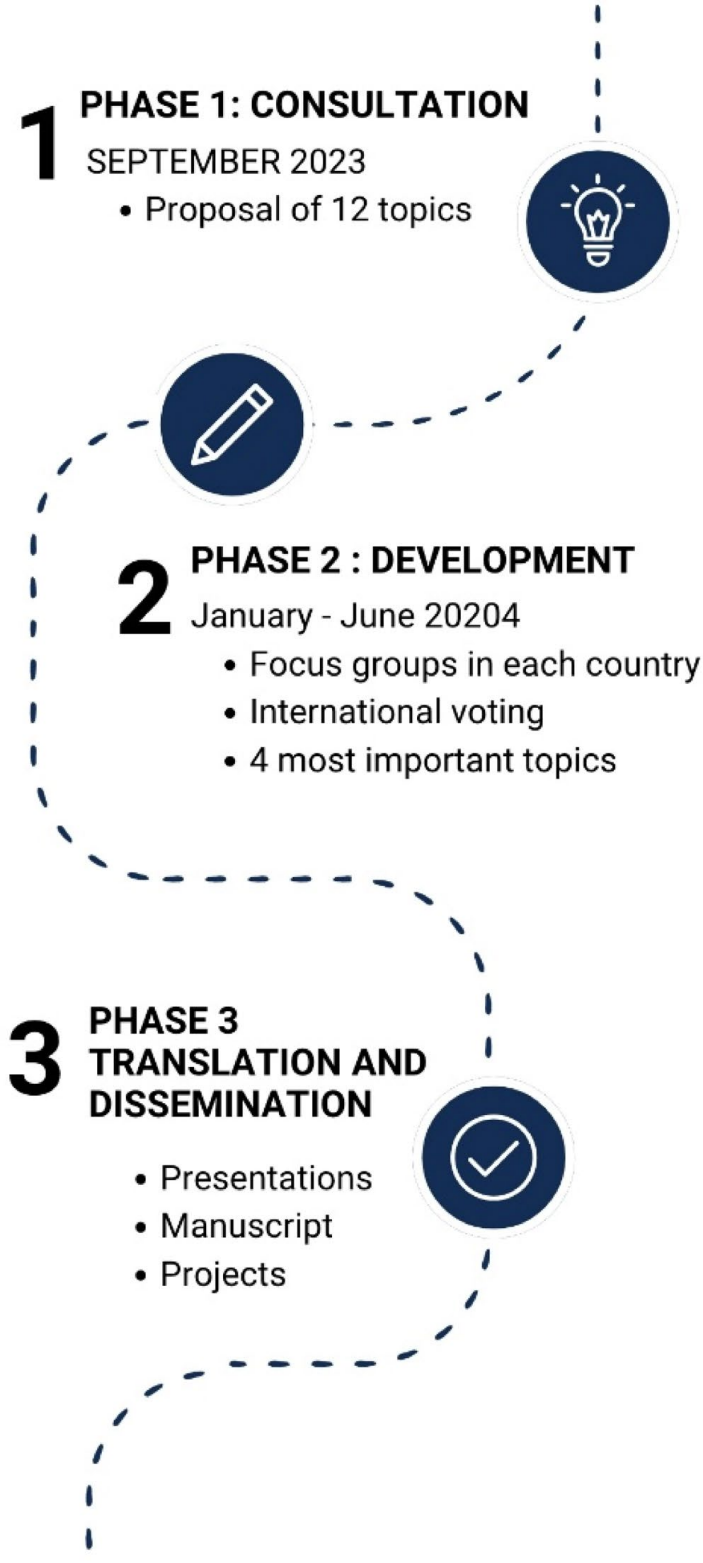
**FIGURE 1** Roadmap timeline.

**Figure:**
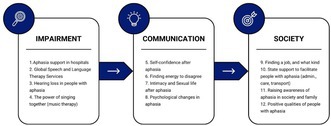
**FIGURE 2** The twelve inital topics.

**Results:** PWA played key roles in proposing topics, organizing and summarizing national and international voting and promoting dissemination. Eleven out of the 14 participating countries (78%) voted. Four priorities emerged: (1) raising awareness of aphasia among families and society; (2) psychological changes, including impacts on intimacy and relationships; (3) rebuilding self‐confidence after aphasia; and (4) improving therapy and hospital attitudes towards treatment.

**Figure:**
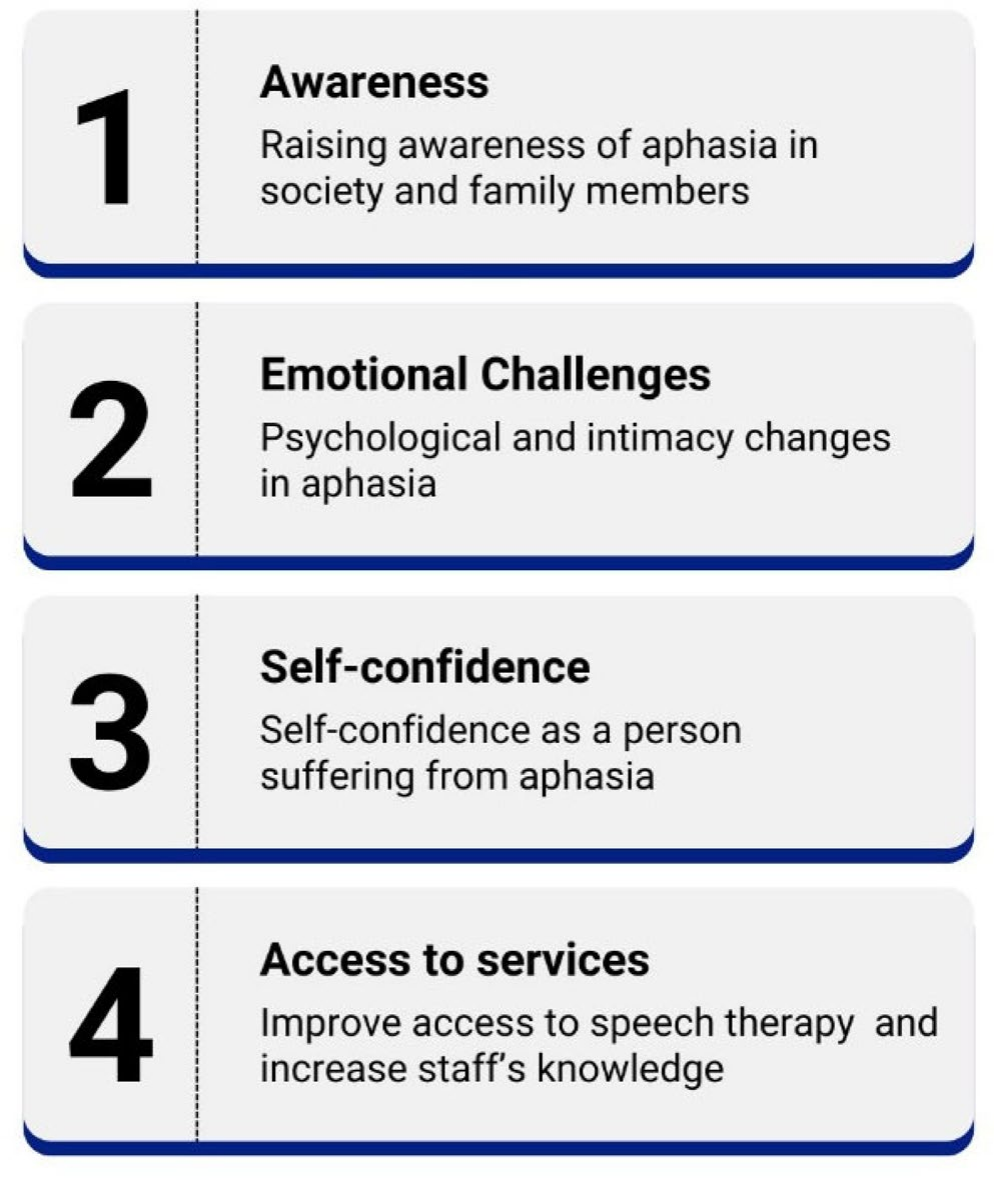
**FIGURE 3** The four important topics.

**Conclusion:** This study shows that with the right help, people with aphasia can clearly share what matters to them. Their voices highlight not only medical needs but also the full picture of living with aphasia. They can guide researchers, health professionals and policy‐makers in their decisions.

**Disclosure:** Nothing to disclose.

## EPO‐0065

### Non‐motor features: Distinguishing dementia with Lewy bodies from Alzheimer's and amyloid co‐pathology

#### J. Alves ^1^; J. Durães^1^; P. Faustino^1^; C. Bernardes^1^; M. Coelho^1^; F. Millet‐Barros^1^; M. Lima^2^; I. Baldeiras^3^; M. Tábuas‐Pereira^1^; I. Santana^1^

##### ^1^Neurology Department, Coimbra University Hospital, Coimbra Local Health Unit, Coimbra, Portugal; ^2^Faculty of Medicine of the University of Coimbra, Coimbra, Portugal; ^3^Centre for Innovative Biomedicine and Biotechnology (CIBB), University of Coimbra, Coimbra, Portugal

**Background and aims:** Dementia with Lewy bodies (DLB) presents a higher prevalence of non‐motor symptoms than Alzheimer's disease (AD). This study aimed to compare non‐motor symptoms between DLB and AD, assess the impact of comorbidity and examine correlations between AD biomarkers and symptoms.

**Methods:** This cross‐sectional study examined patients with DLB, AD and healthy controls. Data collected included demographic information, clinical characteristics, CSF AD biomarkers and results from evaluations using scales to assess non‐motor symptoms.

**Results:** A total of 134 participants were included (44.0% DLB, 40.3% AD, and 15.7% controls). After adjusting for confounders, DLB patients scored significantly higher than AD patients across all scales, except for anxiety. Logistic regression identified the following independent predictors of DLB versus AD: REM Sleep Behavior Disorder (RBD) (OR 1.574), depression (OR 1.253), photophobia (OR 1.198), and psychopathology (OR 1.174), while anxiety was associated with AD (OR 0.783). Regarding co‐pathology, it was present in 57.9% of DLB cases. Patients with co‐pathology exhibited lower depression scores (*p* = 0.029). While psychopathology predicted co‐pathology (OR 1.128), depression (OR 0.038) and RBD (OR 0.042) were more strongly associated with pure DLB. Regarding biomarkers, the Aβ42/40 ratio correlated positively with depression scores, whereas Tau and p‐Tau181 levels correlated positively with psychopathology and negatively with depression scores.

**Figure:**
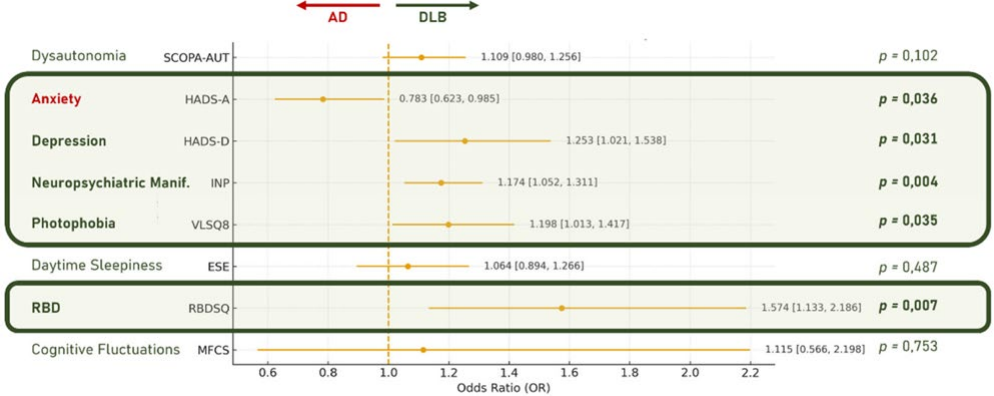
**FIGURE 1** Non‐motor clinical predictors for differentiating DLB from AD.

**Figure:**
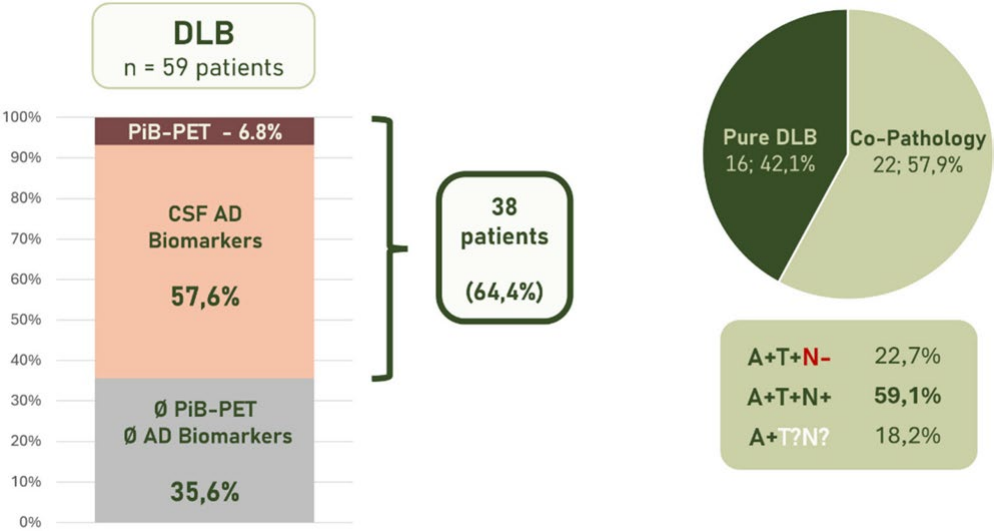
**FIGURE 2** Characterization of AD co‐pathology in a DLB cohort using CSF and PiB‐PET biomarkers.

**Figure:**
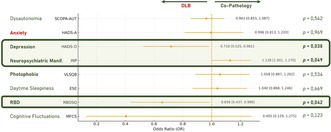
**FIGURE 3** Non‐motor clinical predictors for differentiating DLB from myloaid co‐pathology.

**Conclusion:** DLB patients present a higher burden of non‐motor symptoms compared to AD. Although photophobia is not currently a supportive clinical feature for DLB diagnosis, it emerged as a significant predictor. Amyloid co‐pathology appears to be linked to fewer depressive symptoms but increased psychiatric manifestations.

**Disclosure:** Nothing to disclose.

## EPO‐0066

### Right temporal variant frontotemporal dementia due to FTLD‐FET confirmed by neuropathology

#### N. Bennouna; J. Juncà‐Parella; P. Canasto; G. Caballero; B. Jové Comas; M. Ruiz‐Vives; J. Tutusaus; L. Molina‐Porcel; I. Aldecoa; S. Borrego‐Écija

##### Hospital Clínic de Barcelona

**Background and aims:** Frontotemporal lobar degeneration with FET protein (fused in sarcoma [FUS], Ewing sarcoma breakpoint region 1 [EWSR1], TATA‐binding protein‐associated factor 15 [TAF15]) inclusions (FTLD‐FET) is a rare subtype of frontotemporal dementia, usually presenting with early‐onset behavioural changes. Right temporal variant frontotemporal dementia (rtvFTD) is typically associated with TDP‐43 type C pathology. We report a well‐characterized case of a rtvFTD with post‐mortem confirmation of FTLD‐FET and distinctive neuroimaging features that may inform pre‐mortem diagnosis.

**Methods:** Retrospective review of the medical history, neuroimaging and neuropathological study of a 51‐year‐old brain donor with atypical frontotemporal lobar degeneration with ubiquitin‐positive inclusions (aFTLD‐U) with FET pathology (FTLD‐FET).

**Results:** A 45 odd‐year‐old man presented with early, rapidly progressive behavioural changes including apathy, disinhibition, stereotyped behaviors, altered eating habits, prosopagnosia and anosognosia with executive, language, and memory deficits. Neurological examination showed no parkinsonism or motor neuron signs. MRI revealed right‐predominant frontal and temporal atrophy. FDG‐PET showed bilateral frontal and anterior temporal hypometabolism, more extensive on the right, with marked right caudate hypometabolism. The patient died 5 years after the onset of the symptoms. Neuropathology revealed cytoplasmic inclusions positive for FET proteins (FUS, TAF15), transportin, as well as ubiquitin, consistent with atypical FTLD with ubiquitin‐positive inclusions (aFTLD‐U). Right caudate total degeneration correlated with imaging. Mild motor neuron involvement and hippocampal sclerosis were present.

**Figure:**
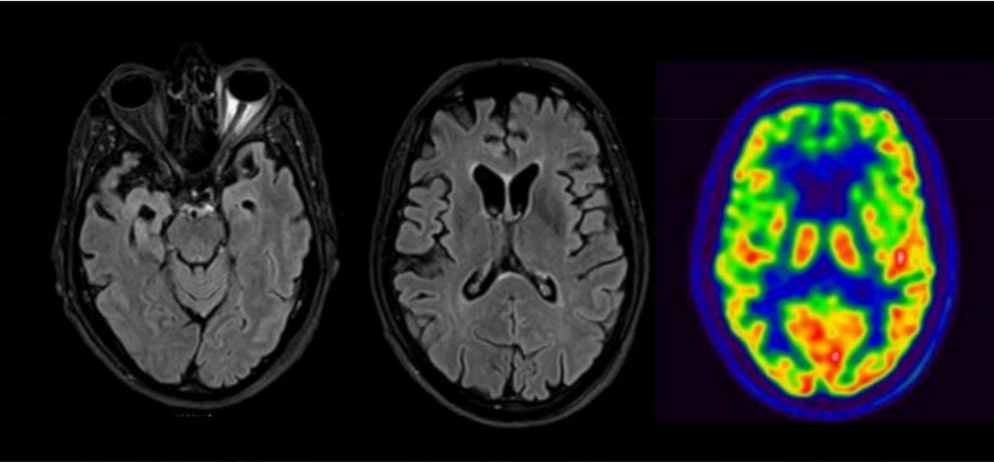
**FIGURE 1** Structural MRI and FDG‐PET reveal right‐predominant frontotemporal atrophy and corresponding hypometabolism, with additional impairment of the caudate nucleus.

**Figure:**
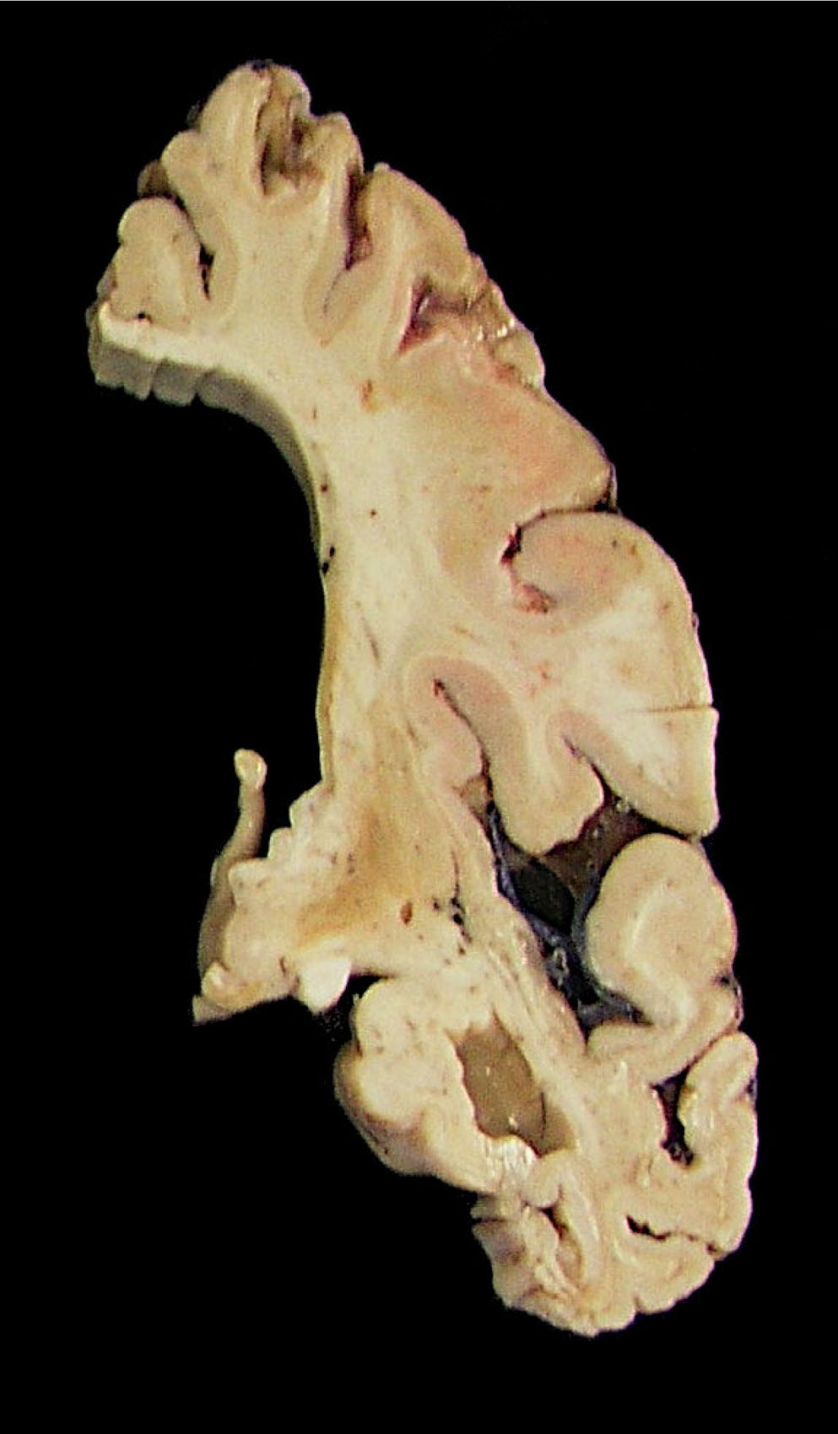
**FIGURE 2** Macroscopic brain examination showing marked right‐predominant frontotemporal atrophy, with noticeable volume loss.

**Conclusion:** This case illustrates rtvFTD with prominent striatal involvement due to FTLD‐FET. Caudate degeneration on imaging may help suggest this molecular subtype and emphasize the importance of clinicopathological correlation in early‐onset dementia.

**Disclosure:** The authors report no relevant financial or institutional conflicts of interest.

## EPO‐0067

### Alzheimer's disease (AD), mild cognitive impairment (MCI) and predictors of driving Cessation: A 7‐year longitudinal prospective study

#### P. Stamatelos ^1^; I. Beratis^2^; P. Hatzaki^1^; A. Economou^3^; N. Andronas^1^; D. Pavlou^4^; S. Fragkiadaki^1^; D. Kontaxopoulou^1^; A. Bonakis^5^; L. Stefanis^1^; G. Yannis^6^; S. Papgeorgiou^1^

##### ^1^1st Department of Neurology, Medical School, National and Kapodistrian University of Athens, Eginition Hospital, Athens, Greece; ^2^Psychology Department, The American College of Greece, Deree, Athens, Greece; ^3^Department of Psychology, National and Kapodistrian University of Athens, Athens, Greece; ^4^School of Topography and Geoinformatics, University of West Attica, Athens, Greece; ^5^2nd Department of Neurology, Medical School, National and Kapodistrian University of Athens, Attikon Hospital, Athens, Greece; ^6^Department of Transportation Planning and Engineering, School of Civil Engineering, National Technical University of Athens, Athens, Greece

**Background and aims:** Cognitive impairment has a detrimental effect on driving ability. We longitudinally assessed drivers with MCI or mild AD Dementia to identify diagnostic and prognostic factors of unsafe driving.

**Methods:** Our study sample consisted of 109 participants: 32 Controls (Mean Age 65.8 years, 57% Women), 47 MCI patients (Mean Age 69.1 years, 40% Women) and 30 AD patients (Mean Age 72.8 years, 7% Women). Baseline evaluation included neurological and neuropsychological assessment and a driving simulator test. We performed two follow‐up evaluations after a mean period of 48 and 84 months. Re‐evaluations included a structured interview with patients and caregivers. Primary endpoints were driving cessation and death. We also performed a systematic review of prospective studies regarding driving cessation among patients with cognitive impairment.

**Results:** 21/47 MCI patients (45%, Mean time to cease: 35 months) and 25/30 AD patients (83%, Mean time to cease: 15 months) ceased driving during the follow‐up period (Figure 1). 45% and 59% of the MCI and AD patients respectively had at least one dangerous driving event during follow‐up, while only 12% of NC participants had a similar event. Age (HR = 1.080, *p* = 0.007), Semantic Verbal Fluency (HR = 0.822, *p* = 0.001) and modified Tandem Walking Test (HR = 1.099, *p* = 0.043) were significant predictors of driving cessation among the cognitively impaired participants (Cox multivariate regression analysis). Our systematic review of the literature identified nine prospective studies.

**Figure:**
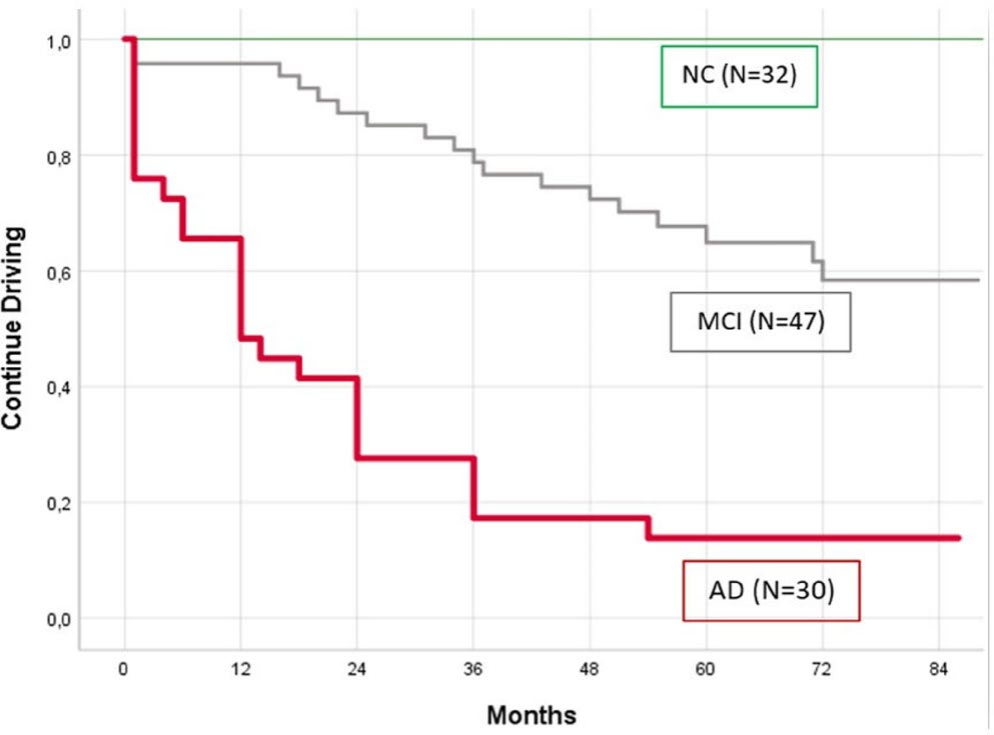
**FIGURE 1** Kaplan‐Meier plot for driving cessation among controls (green line), MCI (gray line) and AD patients (red line).

**Conclusion:** A multi‐disciplinary evaluation (neurological, neuropsychological, driving simulator test) is crucial to individualize driving risk among patients with cognitive impairment. Large‐scale, longitudinal studies are needed to establish a standard protocol of driving assessment.

**Disclosure:** This study is part of the Dr Stamatelos' PhD project with title “ Evaluation of driving behavior of patients with MCI, Dementia or Parkinson's Disease: Diagnostic and Prognostic Markers”, funded and supported by Onassis Foundation.

## EPO‐0068

### Lactoferrin‐Quercetin‐ORMOSIL nanoparticles confer neuroprotection in scopolamine‐induced cognitive deficits

#### S. Naqvi

##### Department of Pharmacology and Toxicology/Regulatory Toxicology, National Institute of Pharmaceutical Education and Research (NIPER‐R), Bijnor‐Sisendi Road, P.O. Mati, Lucknow, India

**Background and aims:** Introduction This study aimed to develop lactoferrin (LF)‐conjugated organically modified silica (ORMOSIL) nanoparticles (LF‐Q‐ORMOSIL) for brain‐targeted delivery of quercetin (Q), evaluating their ability to enhance bioavailability, cross the blood‐brain barrier (BBB), and improve cognitive outcomes in scopolamine‐induced amnesia.

**Methods:** ORMOSIL nanoparticles loaded with quercetin were synthesized and conjugated with LF, followed by physicochemical characterization (size, zeta potential, encapsulation efficiency, release profile). In vivo behavioural assessment was done using morris water maze and novel object recognition tests, biochemical assays and acetylcholinesterase (AChE) inhibition study was performed. Histological analysis for hippocampal morphology was done and in vivo imaging for nanoformulation biodistribution was performed by IVIS imager.

**Results:** Results LF‐Q‐ORMOSIL nanoparticles were synthesized with an optimal particle size (~150 nm), high encapsulation (> 80%), and sustained release. Compared to free quercetin and ORMOSIL, LF‐Q‐ORMOSIL significantly ameliorated scopolamine‐induced cognitive deficits, reduced oxidative markers (including MDA and ROS), and restored antioxidant levels (of GSH and SOD), potently inhibiting AChE and showing preserved hippocampal neurons. Biodistribution revealed many‐fold higher brain accumulation and retention at 24 h post‐administration.

**Conclusion:** Conclusion LF‐Q‐ORMOSIL nanoparticles enhance quercetin's brain delivery, neuroprotective effects, and therapeutic efficacy against cognitive impairment, and shows promise as a nanotherapeutic for Scopolamine‐induced amnesia.

**Disclosure:** Disclosures The authors declare no conflicts of interest and received no specific funding for this study.

## EPO‐0069

### Longitudinal trajectories of cognitive function in heart failure and associated risk factors: A systematic review and meta‐analysis

#### Y. Li ^1^; L. Cheng^2^; R. Feng^1^; T. Chen^3^; R. Yin^4^; C. Ma^5^; Z. Kua^3^; G. Wang^6^; Y. Dong^1^; W. Wang^1^

##### ^1^Alice Lee Centre for Nursing Studies, Yong Loo Lin School of Medicine, National University of Singapore, Singapore; ^2^National Perinatal Epidemiology Unit, Nuffield Department of Women's and Reproductive Health, University of Oxford, Oxford, UK; ^3^Yong Loo Lin School of Medicine, National University of Singapore, Singapore; ^4^Lee Kong Chian School of Medicine, Nanyang Technological University Singapore; ^5^Program in Health Services Research and Population Health, Duke‐NUS Medical School, Singapore, Singapore; ^6^Duke Global Health Institute, Duke University, Durham, North Carolina, USA

**Background and aims:** We aimed to characterise temporal trajectories of cognitive outcomes and identify factors associated with cognitive functioning in heart failure (HF).

**Methods:** We conducted a systematic review and meta‐analysis, searching four databases from inception to 6 February 2025. We included longitudinal observational studies for trajectory analyses and longitudinal observational, cross‐sectional, case‐control studies, post hoc analyses of randomised controlled trials (RCTs), and RCTs for risk‐factor analyses. Study quality was assessed using Joanna Briggs Institute checklists. We pooled prevalence and incidence of cognitive outcomes at comparable follow‐up intervals and synthesised risk estimates from cross‐sectional studies with random‐effects meta‐analysis. Risk factors from longitudinal studies were summarised using direction‐based vote counting due to limited number of studies. The protocol was registered in PROSPERO (CRD42025645235).

**Results:** We identified 35 longitudinal articles from 33 studies and 66 cross‐sectional studies. Studies with short‐term follow‐up (≤1 year) often reported cognitive improvement, whereas longer follow‐up generally showed gradual decline. Consistent modifiable risk factors across study designs included sleep problems, depression, hypertension, atrial fibrillation, inflammation, and anaemia. Other behavioural, cardiac, and systemic factors (e.g, physical capacity, perceived stress, ischaemic aetiology, cardiac index, HbA1c, serum albumin) were also associated with cognitive outcomes, although supported by limited evidence. Substantial heterogeneity was observed in quantitative analyses.

**Figure:**
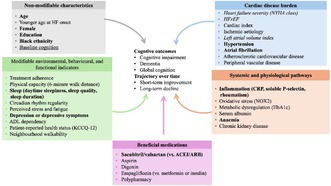
**FIGURE 1** Conceptual model of associated factors for cognitive outcomes in heart failure patients.

**Conclusion:** Our results demonstrate an increased risk of cognitive decline during follow‐up among HF patients, highlighting the need to integrate cognitive assessment into cardiac management. Sleep problems, depression, hypertension, atrial fibrillation, inflammation, and anaemia emerged as consistent modifiable risk factors and should be prioritised in interventions.

**Disclosure:** The authors declare no conflicts of interest.

## EPO‐0070

### TUSC3‐deficient intellectual disabiliy fails Mg2+ uptake for synaptic function and neurodevelopment

#### G. Park; Y. Jung

##### Department of Biological Science, Seoul National University, Seoul, Republic of Korea

**Background and aims:** Intellectual disability (ID) is characterized by deficits in cognition and adaptive behavior, with few treatment options. Tumor Suppressor Candidate 3 (TUSC3) has been genetically linked to autosomal recessive ID, but its molecular mechanism and therapeutic potential remain unclear.

**Methods:** TUSC3 knockout mice were generated and analyze for their behavior, including memory funcitn and social interactoin etc. Celluar magnesium was measured with Mag‐FRET assay. TUSC3 knockout mice were supplementated with magnesium or chelated magnesium for recovery of the pathlogy. ID‐ and Non‐ID fibroblasts were analyzed for their abiity to modulate ER magnesium. ER stress and synaptic proteins were analyzed with westrenb blot.

**Results:** We show that TUSC3 is essential for endoplasmic reticulum (ER) Mg^2+^ homeostasis and neuronal function. Using a TUSC3 knockout (KO) mouse model, we find ID‐like phenotypes including impairments in learning, memory, stress adaptation, and social behavior. Mechanistically, TUSC3 forms an ER‐localized Mg^2+^ transport complex with ERMA and its loss leads to ER Mg^2+^ depletion, PERK‐eIF2α pathway activation, synaptic dysfunction, and neuronal vulnerability. Fibroblasts from TUSC3 mutant patients similarly exhibit ER Mg^2+^ deficiency and heightened ER stress. Magnesium supplementation restores ER Mg^2+^ levels, reduces ER stress, and rescues cognitive deficits.

**Conclusion:** Our findings establish ER Mg^2+^ dysregulation as a key driver of neurodevelopmental dysfunction and a promising therapeutic target for TUSC3‐deficient ID.

**Disclosure:** Nothing to disclose.

## Epilepsy 1

## EPO‐0071

### Gut microbiota alteration in Angelman syndrome: A case–control 16S rRNA sequencing study

#### A. Pascarella ^1^; A. Bulgari^2^; D. Abelardo^1^; S. Gasparini^1^; P. Provenzano^3^; R. Roberti^4^; P. Bonanni^5^; E. Russo^4^; E. Ferlazzo^1^; C. De Caro^6^

##### ^1^Department of Medical and Surgical Sciences, MaGræcia University of Catanzaro, Catanzaro, Italy; Regional Epilepsy Centre, Great Metropolitan “Bianchi‐Melacrino‐Morelli Hospital”, Reggio Calabria, Italy; ^2^Department of Medical and Surgical Sciences, MaGræcia University of Catanzaro, Catanzaro, Italy; ^3^Research Unit of Clinical Epidemiology, CNR‐IFC, Institute of Clinical Physiology, Pisa, Italy; ^4^Department of Science of Health, Magna Græcia University of Catanzaro, Catanzaro, Italy; ^5^IRCCS E. Medea Scientific Institute, Epilepsy Unit, Conegliano, Treviso, Italy; ^6^Department of Pharmacy, University of Naples “Federico II”, Naples, Italy

**Background and aims:** Angelman syndrome (AS) is a rare neurogenetic disorder caused by impaired maternal UBE3A expression and is characterized by severe intellectual disability, motor impairment, absent speech, epilepsy and behavioral abnormalities. Evidence supports a role of the gut microbiota in various neurological disorders, particularly epilepsy, through the gut–brain axis. This study aimed to investigate gut microbiota alterations, microbial diversity and metabolic profiles in AS patients.

**Methods:** Pediatric and adult patients with AS and age‐ and sex‐matched healthy subjects (HS) were enrolled. Fecal samples were collected and analyzed using 16S rRNA gene sequencing to assess microbial composition, relative abundance and diversity. Short‐chain fatty acids (SCFAs) were quantified to evaluate metabolomic profile by gas chromatography–mass spectrometry

**Results:** Seventy‐three subjects were included, comprising 42 AS patients and 31 HS. At the phylum level, AS patients showed reduced Bacteroidota and increased Firmicutes and Proteobacteria, with a higher Firmicutes/Bacteroidota ratio. Alpha diversity was comparable between groups, whereas beta diversity differed significantly between AS and HS. At the genus level, Parasutterella, Tyzzerella, Eubacterium hallii, and UBA1819 were significantly more abundant in AS, while Eubacterium ventriosum was more abundant in HS. Venn diagram analysis revealed greater microbial heterogeneity in AS, with 2986 exclusive taxa. SCFA concentrations were significantly reduced in AS.

**Conclusion:** Angelman syndrome is associated with a distinct gut microbiota alteration characterized by different taxonomic composition, increased microbial heterogeneity and reduced SCFA production, supporting a contributory role of microbiota in AS pathophysiology and suggesting microbiota modulation as a biomarker and promising adjunctive therapeutic strategies.

**Disclosure:** This work was supported by Angelman Syndrome Alliance (ASA), 2024 Research Grant.

## EPO‐0072

### Temporal encephaloceles, epilepsy and idiopathic intracranial hypertension: Toward a pressure‐mediated pathogenetic model?

#### S. Angeloni ^1^; M. Malagoli^3^; G. Giovannini^2^; L. Taruffi^1^; M. Burani^1^; N. Orlandi^2^; A. Vaudano^1^; S. Meletti^1^; M. Pugnaghi^2^

##### ^1^Department of Biomedical, Metabolic and Neuroscience Sciences, University of Modena and Reggio Emilia, Italy; ^2^Clinical Neurophysiology and Epilepsy Center, Department of Neurosciences, University Hospital of Modena, Italy; ^3^Neuroradiology Unit, Department of Neurosciences, University Hospital of Modena, Italy

**Background and aims:** Temporal encephaloceles (TE) are often overlooked in the context of temporal lobe epilepsy (TLE). In parallel, radiological signs of idiopathic intracranial hypertension (IIH) are increasingly described in TLE, often without typical IIH symptoms. We hypothesized that chronic intracranial pressure (ICP) may contribute causally to encephalocele formation and epileptogenesis.

**Methods:** A retrospective multimodal series of consecutive TLE (*n* = 21) was conducted. High‐resolution MRI and skull‐base CT characterized TE morphology and bony defects, while scalp EEG and FDG‐PET aided in lateralization. Prespecified IIH markers included empty/partially empty sella, optic nerve sheath distension, and Meckel's cave enlargement. When serial imaging became available, volumetric analysis quantified TE progression. Primary outcome was IIH marker prevalence; secondary analyses explored correlations with TE multiplicity/bilaterality, and clinical phenotype.

**Results:** Signs of IIH were identified in 8 of 21 patients (38%). Within this subgroup, TE more frequently demonstrated multiplicity/bilaterality, with a prevalence of 62.5% among IIH+ patients (5/8) compared with 33.3% overall (7/21). One patient showed progression in TE burden, with lesion volume increasing from ~2.4 to ~6.8 cm^3^. IIH+ patients had later epilepsy onset (51.5 vs 35.0 years) and a higher proportion of epigastric/autonomic features (50% vs 38.5%). TE were predominantly left‐lateralized (88.2%).

**Conclusion:** A radiologic IIH endophenotype is frequent in TLE and correlates with lesion multiplicity, progression, and delayed seizure onset, supporting a pressure‐mediated model. TLE may represent a subclinical IIH phenotype with seizures as the main clinical expression. This hypothesis opens avenues for future research, and therapeutic ICP‐lowering strategies.

**Disclosure:** Nothing to disclose.

## EPO‐0073

### Features of endothelial dysfunction and vascular glycocalyx in patients with epilepsy

#### D. Yusupova; F. Muratov

##### Tashkent state medical university, Tashkent, Uzbekistan

**Background and aims:** In recent years, it has been established that endothelial dysfunction and damage to the blood‐brain barrier play an important role in maintaining the epileptic process. However, molecular markers of endothelial damage in epilepsy remain insufficiently studied. In this regard, the study of endothelial and stress‐associated biomarkers is of significant interest for deepening understanding of epilepsy pathogenesis and improving diagnostic approaches.

**Methods:** 51 patients with an established diagnosis of epilepsy were examined, from whom venous blood collection was carried out during the period of epileptic activity and in the early post‐sexual stage. The control group consisted of 20 healthy individuals without signs of neurological pathology. In blood serum samples using enzyme‐linked immunosorbent assay, the concentrations of endothelial damage markers and systemic stress were determined: endocane, asymmetric dimethylarginine, differentiation growth factor‐15, and syndekan‐1.

**Results:** Analysis of the obtained data showed that in patients with epilepsy, a statistically significant increase in the levels of endocane and ADMA was registered compared to the control group, which indicates a pronounced disruption of endothelial function and a decrease in the bioavailability of nitric oxide. The concentration of GDF‐15 in epilepsy patients was also significantly higher (*p* < 0.005), reflecting the activation of stress‐realizing and inflammatory mechanisms. In addition, an increase in the level of syndecane‐1 indicated a degradation of endothelial glycocalic and a disruption of the vascular barrier apparatus.

**Conclusion:** The identified changes in biomarkers allow us to consider epilepsy as a condition accompanied by systemic endothelial damage and disruption of the integrity of the vascular barrier. Increased levels of endocane.

**Disclosure:** Nothing is disclosed.

## EPO‐0074

### Induction of ecstatic phenomena via insular direct electrical stimulation: A case series in non‐ecstatic focal epilepsy

#### F. Picard ^1^; N. Mulatti^2^; J. Nilsson^3^; M. Maliia^4^

##### ^1^Department of Clinical Neurosciences, Geneva Medical School and University Hospitals, Geneva, Switzerland; ^2^DM FRCP, Consultant Clinical Neurophysiologist, Kings College Hospital, London, UK; ^3^Department of Clinical Neurophysiology, Sahlgrenska University Hospital, Gothenburg, Sweden; ^4^« Van Gogh » Epilepsy Surgery Unit, Neurology Department, CIC 1414, University Hospital, Rennes, France, and Laboratory of Signal and Image Treatment, University of Rennes, INSERM, LTSI‐U1099, Rennes, France

**Background and aims:** Stereo‐electroencephalography (SEEG) has recently advanced our understanding of ecstatic epilepsy. While the epileptogenic zone is frequently temporal, the symptomatogenic zone for ecstatic symptoms often resides in the anterior insula. However, inducing these phenomena via direct electrical stimulation (DES) in people with non‐ecstatic epilepsy is exceptionally rare, with only two cases previously reported thus far in the literature. This study presents three new cases identified across multiple centers.

**Methods:** We retrospectively analyzed data from three patients with drug‐resistant, non‐ecstatic focal epilepsy undergoing presurgical evaluation. In these patients, 50 Hz DES of the insula induced ecstatic phenomena without triggering afterdischarges.

**Results:** The identified patients had reproducible ecstatic phenomena during 50 Hz DES of the insula. Patient 1 (left anterior insula, 1–1.5 mA) described a fantastic feeling like “being with her daughter a sunny summer day on a meadow full of flowers and everything is well. Patient 2 (very anterior part of the right posterior long gyrus, 2.5–4 mA) experienced being « as one with the whole universe ». Patient 3 (right dorsal anterior insula, 2–2.5 mA) reported intense serenity and happiness. The second patient had a hippocampal sclerosis while the two others were MRI‐negative.

**Figure:**
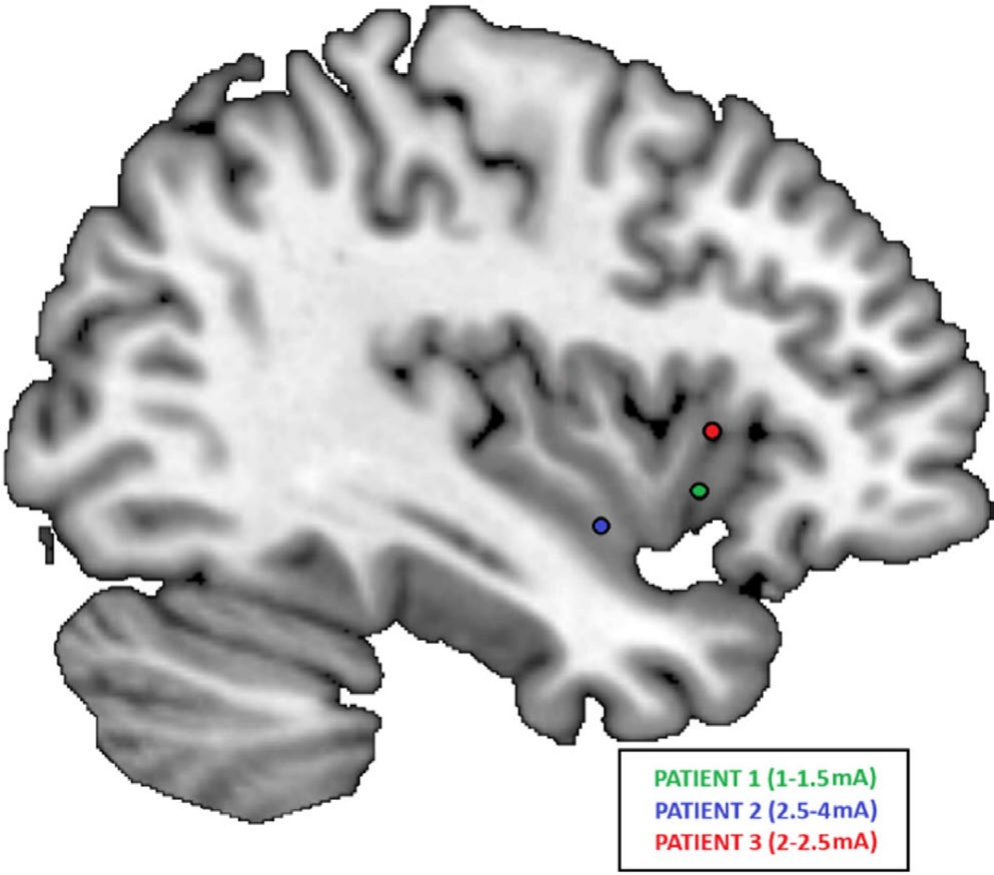
**FIGURE 1** Representation of the midway location of the bipolar SEEG electode pair evoking reproducible ecstatic symptoms for the 3 patients, projected onto a standard sagittal T1‐MRI of the MNI atlas brain.

**Conclusion:** These three additional cases provide further evidence for the insula's pivotal role in the genesis of ecstatic symptoms. The induction of these states in patients with non‐ecstatic epilepsy suggests that the insular “ecstatic network” may be a universal functional pathway rather than one unique to patients with ecstatic seizure semiology.

**Disclosure:** Nothing to disclose.

## EPO‐0075

### Predictive factors of nonadherence to antiseizure medication in epileptic patients

#### F. Hakim; S. Daoud; N. Bouattour; K. Moalla; S. Sakka; E. Smaoui; N. Charfi; M. Damak

##### Neurology Department, Habib Bourguiba Hospital Sfax, Tunisia

**Background and aims:** Poor adherence to antiseizure medications (ASM) remains a major barrier to optimal epilepsy control. Data on determinants of nonadherence in Tunisia are lacking.

**Methods:** A prospective analytical study was conducted in the Adult Neurology Department of Habib Bourguiba University Hospital, Sfax Tunisia. Adult patients meeting ILAE 2017 criteria were included. Adherence was assessed using the MMAS‐8 scale and categorized as adherent, minimally nonadherent, or majorly nonadherent. Sociodemographic, clinical, psychosocial (Stigma Scale for Epilepsy, Hospital Anxiety and Depression Scale, Acceptance of Ilness Scale), therapeutic, and healthcare‐related variables were analyzed.

**Results:** Sixty‐two patients were included (mean age 43.6 ± 11.5 years; 51.6% female). According to MMAS‐8, 37.1% were adherent, 27.4% minimally nonadherent, and 35.5% majorly nonadherent. Higher education level (*p* = 0.014) and better socioeconomic status (*p* = 0.002) were significantly associated with adherence. Seizure freedom for more than two years (*p* = 0.033) and normal EEG findings (*p* = 0.011) were also predictive of better adherence. Stigma was reported by 54.8% of patients and was markedly higher among major nonadherents (81.8%, *p* < 0.001). Depression affected 37% of the cohort, predominating in major nonadherents (50%, *p* < 0.001). Therapeutic factors associated with nonadherence included higher daily dosing frequency (*p* = 0.028) and perceived negative impact of treatment on daily life (*p* = 0.001). Medication unavailability (*p* = 0.005) and disadvantaged social coverage (*p* = 0.032) were additional contributors.

**Conclusion:** ASM nonadherence in Tunisia is frequent and multifactorial, involving socioeconomic, clinical, psychosocial, therapeutic, and health system determinants, underscoring the need for integrated, context‐adapted interventions.

**Disclosure:** Nothing to disclose.

## EPO‐0076

### Cumulative hippocampal seizure‐related burden impairs long‐term memory consolidation in focal epilepsy

#### I. Bratu ^1^; I. Lambert^1^; O. Felician^2^; S. Medina Villalon^1^; A. Trébuchon^1^; F. Bartolomei^1^

##### ^1^Department of Epileptology and Cerebral Rhythmology, Timone Hospital, Marseille, France; ^2^Department of Neurology and Neuropsychology, Timone Hospital, Marseille, France

**Background and aims:** Memory impairment is a frequent and disabling comorbidity of focal drug‐resistant epilepsy (fDRE), yet its relationship to epileptic activity is incompletely explained by seizure frequency or structural pathology alone. This study introduces the concept of cumulative hippocampal seizure‐related burden (c‐HipSZB) and examines its association with long‐term memory consolidation in patients with fDRE.

**Methods:** Twenty patients undergoing stereo‐electroencephalography (SEEG) were prospectively included. Continuous SEEG recordings covering the interval between two memory assessments (30 minutes and one week) were analysed. Hippocampal ictal involvement was identified using quantitative epileptogenicity markers and visual SEEG analysis. Permutation entropy was applied to SEEG signals to quantify postictal recovery dynamics, yielding a Postictal Alteration Time. Cumulative hippocampal ictal burden (c‐HipIC), postictal burden (c‐HipPIC) and their sum (c‐HipSZB) were computed by integrating durations across all hippocampus‐involving ictal events (clinical and subclinical). Verbal and visual memory consolidation was assessed using standardized recall and recognition paradigms. Associations were examined using correlation analyses and univariate and multivariate regression models adjusted for relevant clinical variables.

**Results:** Higher seizure‐related burden metrics in the dominant‐hemisphere hippocampus were consistently associated with poorer long‐term verbal memory performance and reduced retention between 30 minutes and one week. The strongest effects were observed for c‐HipSZB. These associations remained significant after adjustment for epilepsy duration or hippocampal lesion status. Clinical covariates were not independently associated with outcome. In contrast, seizure‐related burden metrics in the non‐dominant hippocampus showed no robust associations with visual memory outcomes.

**Conclusion:** Cumulative ictal–postictal hippocampal dysfunction emerges as a key determinant of impaired long‐term memory consolidation in fDRE.

**Disclosure:** None.

## EPO‐0077

### Newcastle's first experience with EASEE® epicranial neuromodulation for drug‐refractory focal epilepsy: A two‐patient case series

#### L. Panneerchelvam ^1^; M. Hussain^2^; M. Lai^3^; R. Thomas^1^

##### ^1^Department Of Neurology, The Newcastle Upon Tyne Hospitals, Newcastle Upon Tyne, UK; ^2^Department Of Neurosurgery, The Newcastle Upon Tyne Hospitals, Newcastle Upon Tyne, UK; ^3^Department Of Neurophysiology, The Newcastle Upon Tyne Hospitals, Newcastle Upon Tyne, UK

**Background and aims:** Many patients with focal epilepsy remain drug‐resistant and are unsuitable for resective surgery because of non‐lesional imaging, widespread epileptogenic networks, or overlap with important brain regions. The Precisis EASEE® system is an epicranial neuromodulation device delivering cortical stimulation.

**Methods:** We report the first two patients in Newcastle implanted with the EASEE® device. Both had long‐standing drug‐resistant focal epilepsy and were not candidates for resection. Patient 1 had MRI‐negative sleep‐related hypermotor epilepsy with a right parietal–frontal network. Patient 2 had left fronto‐parietal focal cortical dysplasia. Epicranial electrodes were placed over the epileptogenic zone and connected to a chest‐wall pulse generator.

**Results:** Implantation and activation were uncomplicated, taking place on the 01/04/25 and the 03/09/25 respectively. Both patients experienced mild local discomfort that resolved. Patient 1 had early improvement with variable seizures. Patient 2 showed a reduction in nocturnal seizures from 3–4 per week to 1–2 per week and aborted an aura using patient‐activated stimulation. EASEE® programming was adjusted over time to balance seizure control and tolerability.

**Conclusion:** Epicranial focal neuromodulation with the EASEE® device appears feasible and acceptable in selected patients with non‐resectable focal epilepsy and may offer a practical alternative when resection is unsafe. Further follow‐up is required to assess durability.

**Disclosure:** Nothing to disclose.

## EPO‐0078

### Structure‐function coupling of interictal epileptiform discharges to predict surgical outcomes in epilepsy

#### L. de Wouters ^1^; S. Lagarde^2^; E. Mullier^3^; N. Roehri^1^; D. Van De Ville^4^; P. Hagmann^3^; I. Rigoni^1^; S. Vulliémoz^1^

##### ^1^Epilepsy Unit, University Hospitals and Faculty of Medicine of Geneva, University of Geneva, Switzerland; ^2^APHM, Epileptology Department, Timone University Hospital, Marseille, France; ^3^Connectomics Lab, Department of Radiology, Lausanne University Hospital and University of Lausanne (CHUV‐UNIL), Lausanne, Switzerland; ^4^Neuro‐X Institute, Ecole Polytechnique Fédérale de Lausanne (EPFL), Switzerland

**Background and aims:** One third of patients with drug‐resistant epilepsy remains non‐seizure‐free after surgery. This work studies brain structure‐function coupling (SFC) during interictal epileptic discharges (IEDs) through the graph signal processing framework, to distinguish between good and poor surgical outcomes in temporal lobe epilepsy (TLE).

**Methods:** IEDs during high‐density EEG recordings of 30 TLE patients (N = 17 with good post‐surgery outcome, ILAE 1–2) were source‐reconstructed and then summarised into 118 region‐of‐interest (ROI) time‐series. Network harmonics of a template structural connectome, defined as the graph Laplacian's eigenvectors, were used to decompose the ROI time‐series. The transformed signal's energy spectrum was divided into low‐frequency (LF) content (first harmonics, more coupled to the structure) and high‐frequency (HF) one (last harmonics, more decoupled). The LF and HF signal norms along the IED were compared with a cluster‐based permutation test. SFC at the IED peak was quantified as the ratio of LF to HF signal norm and compared between outcome groups with a Wilcoxon rank‐sum test.

**Results:** Findings replicated previously published results, confirming an increased SFC during the IED (*p* < 0.05) and decreased SFC before the IED's start (*p* < 0.001). Additionally, a SFC decrease was observed after the IED (*p* < 0.05). Patients with poor surgical outcomes presented a significantly lower SFC at the IED's peak than those with good outcomes (*p* < 0.05).

**Conclusion:** SFC during IEDs differs according to the surgical outcome: IEDs of patients with good outcome are better explained by the LF network harmonics, meaning that the spatial pattern of the IED is smoother on the SC.

**Disclosure:** Nothing to disclose.

## EPO‐0079

### Efficacy of repetitive transcranial magnetic stimulation in drug‐resistant epilepsy: A systematic review and meta‐analysis

#### A. Siam^1^; O. Abbas^2^; H. Salad^3^; N. Ahmed^4^; R. haddad^5^; Mahmoud^4^; S. Waleed^6^; A. Siam^7^; M. Tahoun^8^; H. Atwan ^9^

##### ^1^Faculty of Medicine, Cairo University, Cairo, Egypt; ^2^Faculty of medicine, Al‐Azhar University, Cairo, Egypt; ^3^Faculty of Medicine and Surgery, Somali National University, Mogadishu, Somalia; ^4^Faculty of Medicine, Beni‐Suef university, Beni‐Suef, Egypt; ^5^Faculty of Medicine, October 6 university, Egypt; ^6^Alexandria faculty of medicine, Alexandria, Egypt; ^7^Faculty of science, Ain Shams University, Egypt; ^8^Warrington and Halton Hospitals NHS Foundation, UK; ^9^Department of Neurosurgery, Klinikum Rechts der Isar, School of Medicine; Technical University of Munich, Munich, Germany Faculty of Medicine, Assiut University, Assiut, Egypt

**Background and aims:** Drug‐resistant epilepsy (DRE), defined as failure of at least two anti‐seizure medications, affects one‐third of patients and poses major therapeutic challenges. Repetitive transcranial magnetic stimulation (rTMS), a non‐invasive neuromodulation technique, has been proposed for DRE. This systematic review assessed the efficacy of rTMS in reducing seizures in these patients.

**Methods:** We searched PubMed, Scopus, Web of Science, and Cochrane Central through October 31, 2025. Randomized controlled trials (RCTs) comparing low‐frequency (≤ 1 Hz) rTMS with sham stimulation in DRE patients were included. Continuous outcomes were pooled using a random‐effects model and reported as standardized mean differences (SMD) with 95% confidence intervals (CI).

**Results:** Five RCTs comprising 172 participants (88 active rTMS, 84 sham rTMS) were included. The mean participant age ranged from 8.9 to 40 years. Active rTMS significantly reduced seizure frequency at two weeks (SMD = −0.57, 95% CI: −0.96 to −0.18; *p* = 0.004), four weeks (SMD = −0.62, 95% CI: −1.03 to −0.21; *p* = 0.002), and eight weeks (SMD = −0.65, 95% CI: −0.98 to −0.32; *p* < 0.0001). A subgroup analysis based on stimulation frequency showed comparable effects for 1 Hz (SMD = −0.33, 95% CI: −0.97 to 0.31) and 0.5 Hz (SMD = −0.79, 95% CI: −1.18 to −0.4). Additionally, active rTMS showed a significant reduction in interictal epileptiform discharge (SMD = −0.44, 95% CI: −0.84 to −0.04; *p* = 0.03).

**Figure:**
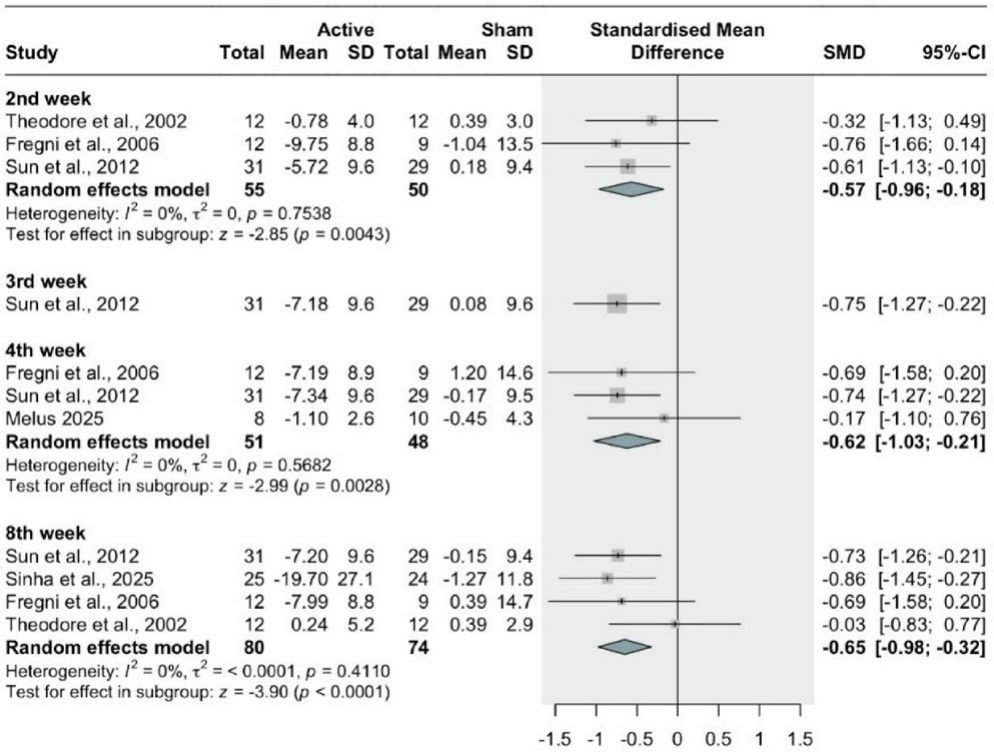
**TABLE 1** Change in seizure frequency across different time points following low‐frequency rTMS.

**Figure:**
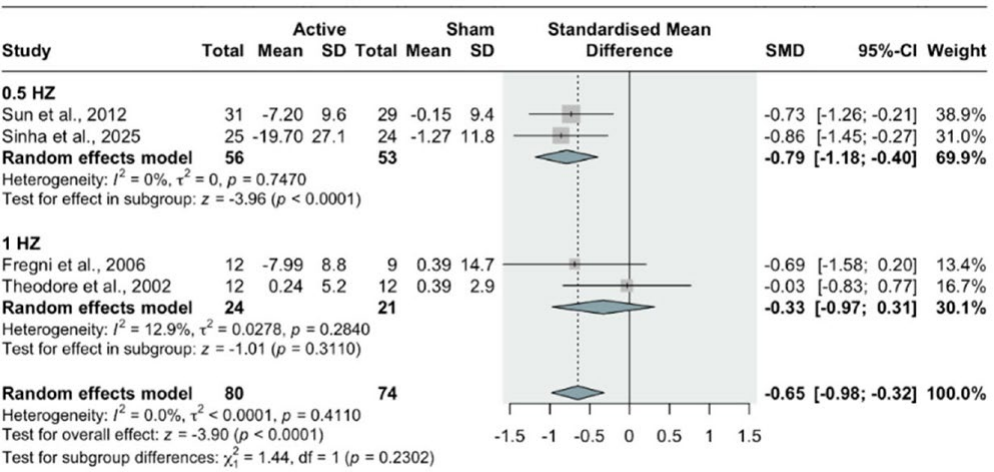
**TABLE 2** Subgroup analysis based on stimulation frequency.

**Figure:**
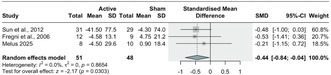
**TABLE 3** Change in interictal epileptiform discharge.

**Conclusion:** Low‐frequency rTMS is an effective therapeutic intervention for reducing seizure frequency and interictal epileptiform discharge in patients with DRE, with sustained benefits observed over 8 weeks.

**Disclosure:** Nothing to disclose.

## EPO‐0080

### Beyond visual analysis: Uncovering the electrographic signature of genetic focal epilepsy via AI‐driven gamma power quantification

#### M. Badawy ^1^; T. Voloshyn^2^

##### ^1^Neurology department, Suez medical complex (SMC), Egypt health care authority (EHA), Egypt; ^2^International Clinic of Rehabilitation, Truskavets, Ukraine

**Background and aims:** Focal cortical dysplasia (FCD) and other structural epilepsies are increasingly recognized as mTOR‐driven genetic disorders (mTORopathies). While these conditions are characterized by cellular hyper‐excitability, standard visual EEG analysis often fails to detect interictal background abnormalities. This study utilizes Machine Learning (ML) to identify quantitative EEG (qEEG) biomarkers—specifically high‐frequency activity—that correlate with this “invisible” genetic phenotype.

**Methods:** A retrospective cohort analysis was conducted using the CHB‐MIT Scalp EEG database. A total of 23,760 interictal EEG epochs (5‐second segments) were extracted from 20 pediatric patients with intractable focal epilepsy. Signal processing included a 0.5–100 Hz bandpass filter to preserve high‐frequency oscillations. A Random Forest classifier was trained to characterize patient‐specific background phenotypes, using 5‐fold cross‐validation to assess statistical reliability.

**Results:** The ML model demonstrated a mean classification accuracy of 92.91% ± 0.95% in blind testing. The results were statistically significant against random chance (*p* < 0.05), indicating the presence of distinct neurophysiological signatures in the interictal background. Crucially, Feature Importance analysis indicated that Gamma Power (30–80 Hz) was the most significant discriminator across the cohort (Importance Score > 0.28), consistently outperforming traditional biomarkers such as Delta slowing.

**Figure:**
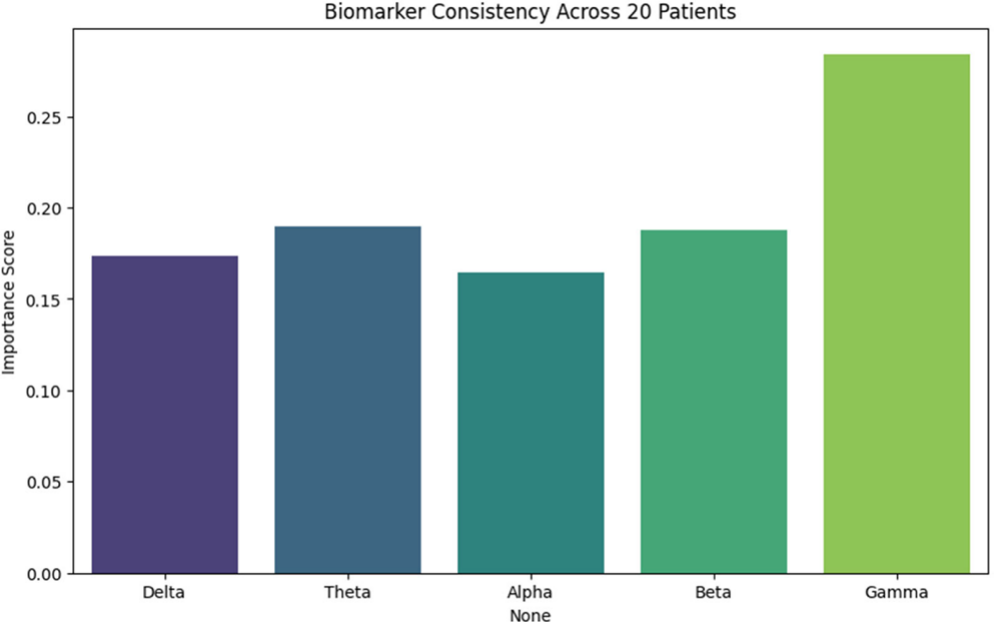
**FIGURE 1** Feature Importance analysis showing Gamma Power (30–80 Hz) as the most consistent biomarker across the 20‐patient cohort compared to other frequency bands.

**Conclusion:** These findings suggest that patients with intractable focal epilepsy exhibit a ubiquitous “Hyper‐Excitable Background Phenotype” driven by high‐frequency activity. This supports the potential of AI‐driven qEEG to serve as a non‐invasive screening tool for genetic etiologies (mTORopathies), potentially guiding early targeted therapy and genetic panel selection.

**Disclosure:** Nothing to disclose.

## EPO‐0081

### Multi‐channel vision transformer based automated sleep staging for EEG monitoring

#### N. Lin ^1^; W. Gao^1^; P. Hu^2^; Y. Dong^2^; Y. Gao^1^; H. Sun^1^; Q. Lu^1^

##### ^1^Department of Neurology, Peking Union Medical College Hospital, Beijing, China; ^2^NetEase Media Technology Co, Ltd, Beijing, China

**Background and aims:** Sleep staging is critical in EEG interpretation. However, current models are developed using polysomnography (PSG) data, which are unsuitable for standard EEG recordings. This study aims to construct a sleep staging recognition model for achieving objective and accurate sleep structure assessment.

**Methods:** The sleep staging model vEpiSleepNet consists of four modules: sleep feature extraction with transfer learning, cascaded multi‐layer dilated convolution encoding, temporal context encoding, and MLP classification (Figure 1). All signals from 19 electrodes along with A1/A2 were used for model development, distinguishing from previous models employing < 10 channels. This study used Peking Union Medical College Hospital (PUMCH) dataset, containing 117 patients and 140 h EEG data, dividing into 30‐second segments. Patient‐wise split five‐fold cross‐validation was applied.

**Figure:**
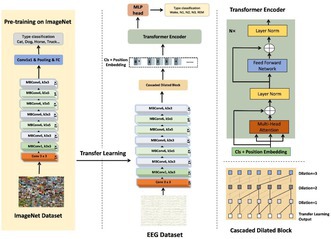
**FIGURE 1** The architecture of vEpiSleepNet.

**Results:** vEpiSleepNet achieves 70.5% accuracy and 0.700 F1‐score on PUMCH dataset, with a wake‐sleep binary classification accuracy of 87.6%. vEpiSleepNet exhibited nearly 12% improvement in F1 scores, 10% in accuracy, and 15% in Kappa, compared to the best‐performing comparative models among the five published classic models (Figure 2). On two public PSG datasets, vEpiSleepNet also showed superior performance, with 81.9% and 80.1% accuracies, respectively. Ablation experiments demonstrated the efficacy of transfer learning and cascaded dilated convolution. Additionally, the more electrodes, the better mode performance.

**Figure:**
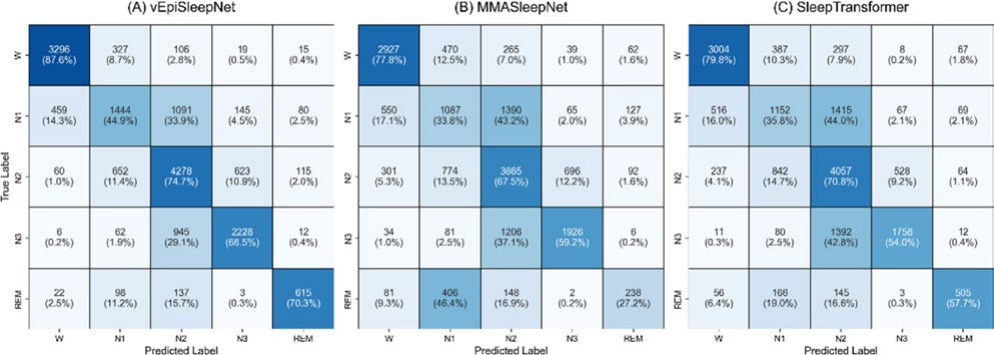
**FIGURE 2** Confusion matrix on XIEHE‐SLEEP‐16K. vEpiSleepNet showed the best performance on all wake and sleep stages, compared to the other networks, MMASleepNet and SleepTransformer.

**Conclusion:** We have developed vEpiSleepNet which is specifically designed for EEG monitoring, filling the gap in the lack of dedicated automatic sleep staging models for EEG monitoring. Accurate automated sleep staging of EEG data has the potential to advance research on epileptic discharges and improve clinical diagnostic capabilities for sleep‐related epilepsy.

**Disclosure:** Nothing to disclose.

## EPO‐0082

### VIDÉPÉ: A web‐app to interactively visualize multimodal electrophysiological and imaging data to improve epilepsy surgery evaluation

#### I. Rigoni; S. Vulliemoz; N. Roehri

##### Epilepsy Unit, University Hospitals and Faculty of Medicine, University of Geneva, Geneva, Switzerland

**Background and aims:** Drug‐resistant epilepsy affects a substantial proportion of the more than 80 million people living with epilepsy worldwide. For these patients, resective surgery offers the highest likelihood of seizure freedom, but its success critically depends on accurate delineation of the epileptogenic zone while preserving eloquent cortex. This process requires the joint interpretation of heterogeneous data, including structural, functional and nuclear imaging, and electrophysiological recordings (EEG). In current clinical practice, these modalities are typically explored in separate software environments, leading to fragmented, largely static workflows that limit interactive, multidisciplinary discussion and restrict the use of advanced analyses.

**Methods:** We developed a web‐based application for integrated visualization of multimodal presurgical epilepsy data. Volumetric neuroimaging (MRI and PET) is rendered using Niivue.js, while electrophysiological signals are displayed with Plotly.js. The application is deployed through GitHub Pages, allowing access from any modern web browser without local installation. The system supports simultaneous visualization of imaging volumes, functional maps, and time‐resolved EEG within a unified interface.

**Results:** The application is publicly available online (https://ziply.pk/2E853M) and operates across operating systems and devices. A demonstration page combining MRI, PET, and EEG is provided for interactive exploration (https://ziply.pk/Kf3H50).

**Figure:**
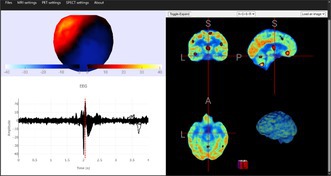
**FIGURE 1** Web‐app interface with EEG (right) and MRI/PET imaging (left). Time navigation is performed in the EEG panel, slice and view selection in the imaging panel, and a central menu bar controls independent opacity and thresholding of each volume.

**Conclusion:** This web‐based platform integrates multimodal imaging into a single interactive environment to support coherent data interpretation potentially improving clinical decision‐making in epilepsy surgery. Its browser‐based design ensures easy and broad accessibility. Ongoing work will add modalities such as intracranial EEG and will evaluate usability and clinical utility with clinicians and patients.

**Disclosure:** Nothing to disclose.

## Headache 1

## EPO‐0083

### Changes in anxiety and depression symptoms in patients with chronic migraine and medication‐overuse headache in the RESOLUTION trial

#### C. Tassorelli ^1^; R. Jensen^3^; C. Lundqvist^4^; H. Schytz^3^; F. Vernieri^6^; G. Terwindt^8^; A. Blumenfeld^9^; S. Tepper^10^; R. Lipton^11^; K. Ranc^12^; G. Jansson^12^; A. Ettrup^12^; A. Mittoux^12^; M. Lantéri‐Minet^13^

##### ^1^Department of Brain and Behavioral Sciences, University of Pavia, Pavia, Italy; ^3^Department of Neurology, Danish Headache Center, Rigshospitalet‐Glostrup, University of Copenhagen, Copenhagen, Denmark; ^4^Departments of Neurology and Health Services Research, Akershus University Hospital, Lørenskog, Norway; ^6^Unit of Headache and Neurosonology, Fondazione Policlinico Universitario Campus Bio‐Medico, Rome, Italy; ^8^Department of Neurology, Leiden University Medical Centre, Leiden, Netherlands; ^9^The San Diego Headache Center, San Diego, CA, United States; ^10^The New England Institute for Neurology and Headache, Stamford, CT, United States; ^11^H. Lundbeck A/S, Copenhagen, Denmark; ^12^Department of Neurology, Albert Einstein College of Medicine, New York, NY, United States; ^13^Pain Department and FHU InovPain, Centre Hospitalier Universitaire de Nice, Nice, France

**Background and aims:** These analyses explored the impact of eptinezumab versus placebo on anxiety and depression symptoms in adults with chronic migraine (CM) and medication‐overuse headache (MOH).

**Methods:** The phase 4 RESOLUTION (NCT05452239) trial included a 28‐day screening (baseline) period and 12‐week double‐blind, placebo‐controlled period. Adults (18–75 yrs) with CM and MOH were randomized 1:1 to IV eptinezumab 100 mg or placebo at baseline; all participants received a brief educational intervention on MOH before infusion. Secondary endpoints included the changes from baseline to Weeks 4 and 12 in Hospital Anxiety and Depression Scale (HADS) scores for anxiety (HADS‐A) and depression (HADS‐D) in the full‐analysis set, with post hoc analyses in subgroups with elevated baseline scores (> 7). *p*‐values are descriptive.

**Results:** Of 608 participants randomized, 596 (98%) completed the placebo‐controlled period. In the full‐analysis set (*n* = 602), eptinezumab resulted in greater improvements from baseline than placebo for HADS A (Week 4: between‐group difference [95% CI] −0.8 [−1.4, −0.3], *p* = 0.0017; Week 12: −1.0 [−1.5, −0.5], *p* = 0.0001) and HADS D (Week 4: −1.0 [−1.5, −0.4], *p* = 0.0006; Week 12: −1.2 [−1.8, −0.6], *p* < 0.0001). Subgroups with elevated baseline HADS‐A (226/602 [38%] participants) and HADS‐D (201/602 [33%] participants) scores showed greater reductions from baseline with eptinezumab vs placebo in the respective HADS scores, with similar between‐group differences as the full‐analysis set.

**Conclusion:** Participants with CM and MOH who also received patient education at baseline experienced greater reductions with eptinezumab than placebo in anxiety and depression scores at Weeks 4 and 12 after treatment. Similar differences between eptinezumab and placebo were seen in participants with elevated baseline anxiety and/or depression symptoms.

**Disclosure:** CT in the past 3 years has received support (financial or drugs) from AbbVie and Novartis for an investigator‐initiated trial; consulting fees for the participation in advisory boards for AbbVie, Dompé, Eli Lilly, Ipsen, Lundbeck, Organon, Pfizer, and Teva; honoraria for scientific lectures and presentations from AbbVie, Eli Lilly, Lundbeck, Pfizer, and Teva; support for attending meetings from AbbVie, Dompé, Eli Lilly, Ipsen, Lundbeck, Pfizer, and Teva; has been Principal Investigator in clinical trials sponsored by AbbVie, Biohaven, Chordate, Eli Lilly, Ipsen, Lundbeck, Pfizer, and Teva; and received grants from the European Commission, the Italian Ministry of Health, and the Italian Ministry of University. RHJ has given lectures for Allergan, ATI, Eli Lilly, Lundbeck, Merck, Novartis, Pfizer, and Teva; served as investigator in clinical trials with ATI, Eli Lilly, Lundbeck, Novartis, and Novo Nordisk; is the director of the Danish Headache Center, Lifting The Global Burden of Headache, and Founder of Master of Headache Disorders at University of Copenhagen; and has received research funding from ATI, Lundbeck Foundation, Rigshospitalet, The Medical Society in Copenhagen, Novo Nordisk Foundation, Tryg Foundation, and University of Copenhagen. CL has participated on an advisory board and received payment for lectures arranged by AbbVie, Lundbeck, Novartis, Pfizer, and Roche, and has received research sponsorship from AbbVie. HWS has received personal fees from AbbVie, Eli Lilly, Lundbeck, Novartis, and Teva, and has received research grants from Novartis and Novo Nordisk Foundation. FV has received financial support from AbbVie, Angelini, and Lundbeck for investigator‐initiated trials; consulting fees for the participation in advisory boards from AbbVie, Angelini, Eli Lilly, Lundbeck, Organon, Pfizer, and Teva; honoraria for scientific lectures and presentations from AbbVie, Eli Lilly, Lundbeck, Pfizer, and Teva; support for attending meetings from AbbVie, Eli Lilly, Lundbeck, Pfizer, and Teva; and has been Principal Investigator in clinical trials sponsored by AbbVie, Eli Lilly, Lundbeck, Pfizer, and Teva. GMT reports grants or consultancy support from AbbVie, Eli Lilly, Lundbeck, Novartis, Organon, Pfizer, and Teva, and independent support from the Clayco Foundation, Dioraphte Foundation, Dutch Research Council, Dutch Heart Foundation, Dutch Brain Foundation, IRRF, and the European Community. [Disclosures for the remaining authors will be included in the presentation.]

## EPO‐0084

### Clinical efficacy and functional outcomes of Atogepant versus Topiramate in treatment completers: Post hoc analysis from the TEMPLE trial

#### A. Blumenfeld ^1^; P. Gandhi^2^; Y. Liu^2^; K. Nagy^2^; B. Dabruzzo^2^; S. Musson^2^; J. Versijpt^3^; U. Najib^4^; A. Van Dycke^5^; U. Reuter^6^

##### ^1^The Los Angeles Headache Center, Los Angeles, USA; ^2^AbbVie Inc, North Chicago, USA; ^3^Department of Neurology, Universitair Ziekenhuis Brussel, Brussels, Belgium; ^4^Rockefeller Neuroscience Institute, West Virginia University, Morgantown, USA; ^5^Department of Neurology, General Hospital Sint‐Jan Bruges, Brugge, Belgium; ^6^Charité Universitätsmedizin Berlin, Berlin, Germany

**Background and aims:** Secondary clinical efficacy and functional endpoints from the TEMPLE trial modified‐intent‐to‐treat (mITT) population were evaluated in treatment completers.

**Methods:** TEMPLE was a phase 3b, randomized, multicenter, double‐blind, double‐dummy, parallel‐group, active‐controlled trial to evaluate tolerability, safety, and efficacy of atogepant compared with topiramate for the preventive treatment of migraine. Post‐hoc analyses in the mITT and completers populations assessed the proportion of participants with ≥50% reduction in mean monthly migraine days (MMDs) during Months 4–6 and change from baseline in Headache Impact Test‐6(HIT‐6) at Week 24 and Patient‐Reported Outcomes Measurement Information System Cognitive Function (PROMIS‐CF)–Abilities Subset–Short Form 6a(v2.0) at Week 6.

**Results:** Of 527 (atogepant = 270; topiramate = 257) mITT participants, 372 (atogepant = 209; topiramate = 163) were treatment completers. During Months 4–6, participants in the atogepant group were more likely to experience a ≥50% reduction in mean MMDs compared with topiramate in both the mITT population (64.1% vs. 39.3%; *p* < 0.0001) and in treatment completers (73.7% vs. 48.5%; nominal *p* < 0.0001) (Figure 1). At Week 24, participants in the atogepant group showed a greater improvement in HIT‐6 in both the mITT population (−11.7 vs. −7.4; *p* < 0.0001) and in treatment completers (‐13.2 vs. ‐9.2; nominal *p* < 0.0001) than in the topiramate group (Figure 2). At Week 6, atogepant demonstrated greater improvement in PROMIS‐CF in the mITT population (5.2 vs. 0.2, *p* < 0.0001) and in treatment completers (5.4 vs. 1.8, *p* ≤ 0.01) compared to topiramate (Figure 3). Results were consistent across other secondary endpoints. Atogepant was generally safe and well‐tolerated as reported previously.

**Figure:**
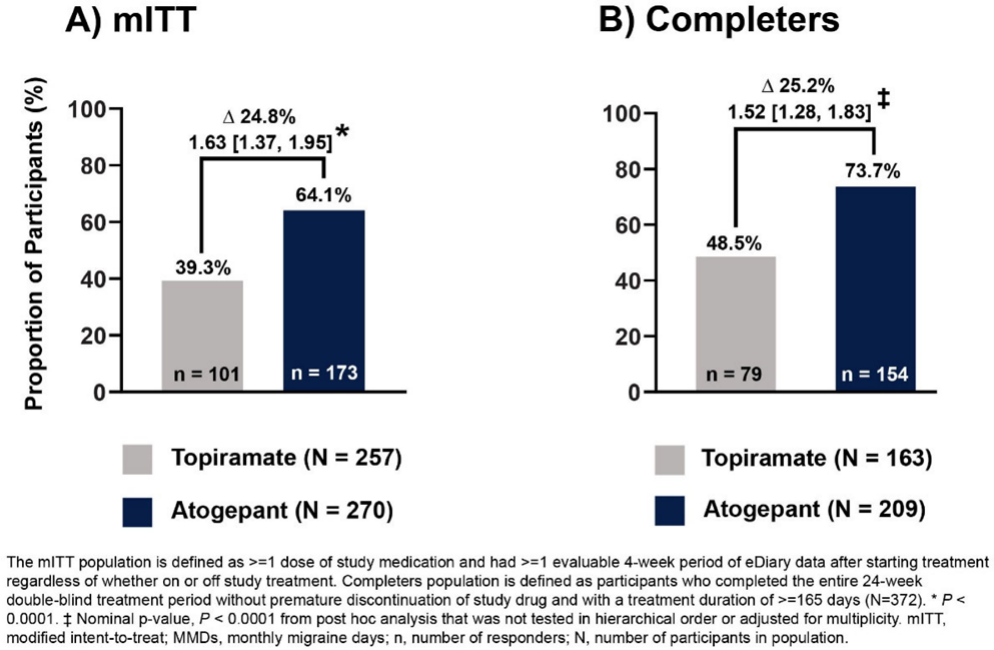
**FIGURE 1** Proportion of participants achieving ≥50% reduction in MMDs across months 4 to 6.

**Figure:**
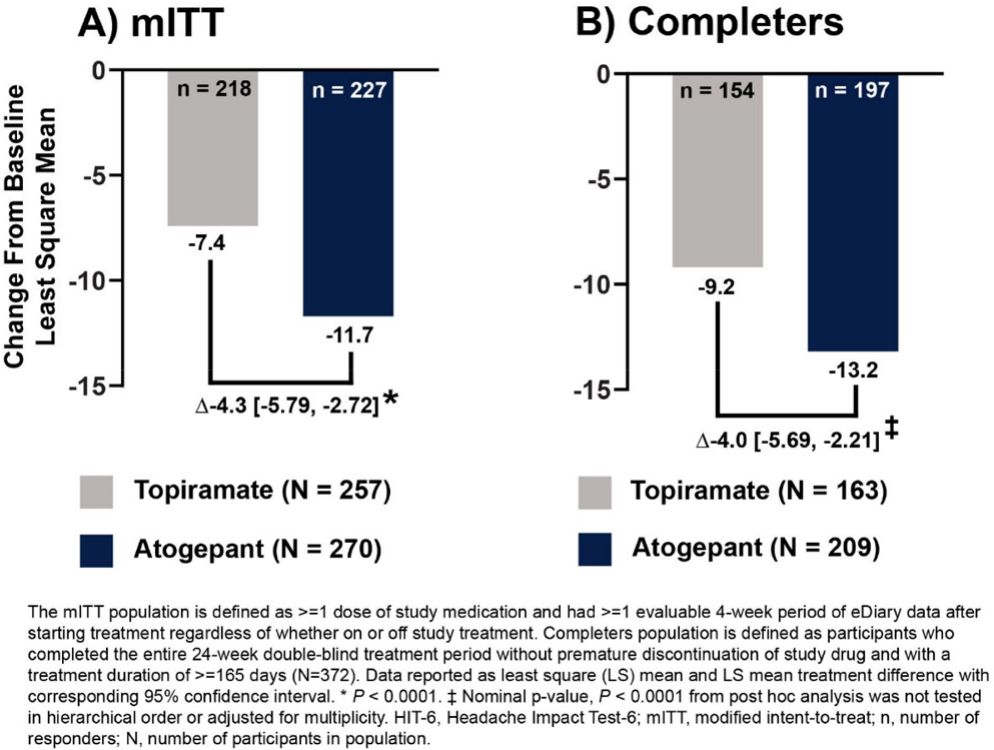
**FIGURE 2** Change from baseline in HIT‐6 total score at week 24.

**Figure:**
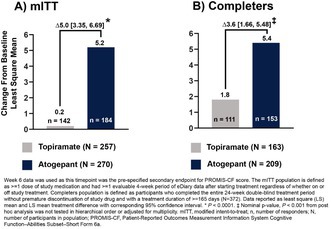
**FIGURE 3** Change from baseline in PROMIS‐CF score at week 6.

**Conclusion:** TEMPLE treatment completers randomized to atogepant demonstrated similar improvements in clinical efficacy and functional endpoints to the overall mITT population; both populations demonstrated greater improvements in clinical efficacy and functional endpoints over those randomized to topiramate.

**Disclosure:** Andrew Blumenfeld has served on advisory boards for, consulted for, and/or been a speaker or contributing author for Aeon, Allergan/AbbVie, Alder, Amgen, Axsome, BDSI, Biohaven, Eli Lilly, Impel, Lundbeck, Novartis, Revance, Teva, Theranica, and Zosano. He has received grant support from AbbVie, Amgen. Jan Versijpt received personal fees and nonfinancial support from Teva, Pfizer and Medtronic, personal fees from Novartis and Lundbeck, and grants and nonfinancial support from Allergan/AbbVie. Umer Najib has served on advisory boards for, consulted for, and received honoraria from AbbVie, Lundbeck, and Pfizer. Annelies Van Dycke has served on advisory/scientific boards and/or given lectures for AbbVie, Allergan, Angelini Pharma, Eli Lilly, Lundbeck, Neuraxpharm, Novartis, Organon, Pfizer, TEVA and UCB, for which she has received honoraria. Uwe Reuter has served on advisory boards for Amgen, Allergan, AbbVie, Eli Lilly, Lundbeck, Novartis, Pfizer, and Teva and has received institutional honoraria for lectures from Amgen, Allergan, AbbVie, Eli Lilly, Lundbeck, Novartis, electroCore, Medscape, StreaMedUp, Springer, and Teva, received institutional honoraria for consulting services from Lundbeck, Pfizer, and AbbVie; received research funding from Novartis (CHERUB01) and the German Federal Ministry of Education and Research; and is an associate editor of the Journal of Headache and Pain. PG, YL, KN, BD, and SM are employees of AbbVie and may hold AbbVie stock.

## EPO‐0085

### Efficacy of Candasertan for migraine prevention: A systematic review and meta‐analysis

#### A. Carrión Cuéllar ^1^; E. Cuellar Jáuregui^2^; T. Huiman Caro^3^

##### ^1^Universidad Autónoma de Barcelona, Barcelona, Spain; ^2^Hospital I Higos Urco, Chachapoyas, Peru; ^3^Universidad Peruana Antenor Orrego, Trujillo, Peru

**Background and aims:** Migraine is a major cause of disability, and many patients don’t tolerate or respond to first‐line preventive therapies. Candesartan has shown preventive benefit in randomized controlled trials, but its overall efficacy remains uncertain. This study aimed to evaluate the efficacy and safety of candesartan for migraine prevention.

**Methods:** A systematic review was performed according to PRISMA guidelines, using PubMed, Scopus and Web of Science for studies published until January 11,2026. Risk of bias was assessed using RoB 2.0 The primary outcome was migraine days per 4 weeks. For crossover trials, within‐participant correlation was accounted for by variance adjustment. Pooled effects were calculated using mean difference (MD) with 95% confidence intervals (CI) under a random‐effects model. Sensitivity analyses were conducted using different correlation assumptions.

**Results:** Three RCTs including 425 participants were analyzed. Mean age was 39.1 years ± 10.9 and 82.9% were females. Most studies evaluated at dose 16 mg. Candesartan significantly reduced migraine days per 4 weeks compared with placebo (MD: –1.11 days, 95% CI: –1.95 to –0.28; *p* = 0.009;). Monthly headache days showed a non‐significant reduction (MD: −1.33 days, 95% CI: –2.73 to 0.07; *p* = 0.06;). Results remained consistent across sensitivity analyses. A total of 453 adverse events were reported, occurring more frequently during candesartan exposure periods; most were mild, mainly dizziness and fatigue.

**Figure:**
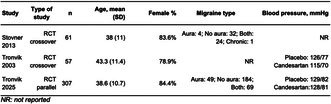
**TABLE 1** Baseline demographic and clinical characteristics.

**Figure:**
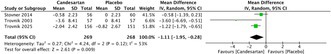
**TABLE 2** Migraine days per 4 weeks: candesartan vs placebo.

**Figure:**
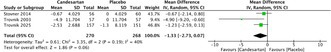
**TABLE 3** Headache days per 4 weeks: candesartan vs placebo.

**Conclusion:** Candesartan demonstrates statistically significant but modest efficacy in reducing migraine days and a trend toward reduction in headache days, with acceptable tolerability. It may represent a useful preventive option, particularly for patients who don’t respond to standard preventive therapies.

**Disclosure:** Astrid Geraldine Carrion Cuellar: Nothing to disclose Elizabeth Luisa Cuellar Jauregui: Nothing to disclose Thalia del Pilar Huiman Caro: Nothing to disclose.

## EPO‐0086

### Digital education for migraine self‐management: A Systematic review of app‐based interventions

#### C. Pace; C. Corbeiceiri

##### Federal University of Rio de Janeiro, Rio de Janeiro, Brazil

**Background and aims:** Effective migraine management requires patient education to improve self‐management, but traditional methods lack scalability. Digital tools like smartphone apps offer new educational platforms. This review evaluates their impact on clinical and self‐management outcomes.

**Methods:** A systematic review followed PRISMA guidelines. We searched PubMed, Embase, PsycINFO, Cochrane Central, and ClinicalTrials.gov up to December 2024 for randomised controlled trials (RCTs). Eligible RCTs assessed digital tools with educational or behavioural components for adult migraine. Primary outcome: change in monthly migraine days (MMD). Secondary outcomes: headache intensity, disability (MIDAS/HIT‐6), adherence. Risk of bias was assessed using the Cochrane RoB 2 tool.

**Results:** From 1,124 screened records, 16 RCTs (*n* = 2,736 participants) were included. A meta‐analysis showed digital educational interventions significantly reduced MMD by a mean of −2.3 days/month (95% CI: −3.1 to −1.5) versus control. Interventions with cognitive behavioural therapy (CBT) modules were 35% more effective than basic ones. Significant improvements occurred in headache intensity (standardised mean difference, SMD: −0.41) and disability (SMD: −0.38). Intervention groups reported high engagement and improved disease knowledge.

**Conclusion:** Digitally delivered educational interventions are effective adjuncts to standard migraine care, leading to clinically meaningful improvements. Efficacy is strongest when tools include interactive, theory‐based components like CBT. These apps represent a scalable new paradigm for patient education in neurology, directly empowering self‐management. Future development must prioritise pedagogical design.

**Disclosure:** Nothing to disclose.

## EPO‐0087

### Neurophysiological effects of ATOgepant in high‐frequency episodic migraine: The ATOM Project

#### F. Cammarota ^1^; R. De Icco^1^; V. Grillo^1^; G. Vaghi^1^; M. Corrado^2^; B. Agostini^1^; M. Giraudo^1^; D. Martinelli^2^; M. Allena^2^; G. Sances^2^; R. Greco^2^; C. Demartini^2^; M. Francavilla^1^; S. Facchetti^1^; C. Tassorelli^1^

##### ^1^Department of Brain and Behavioral Sciences, University of Pavia, Pavia, Italy; ^2^Headache Science and Neurorehabilitation Unit, IRCCS Mondino Foundation, Pavia, Italy

**Background and aims:** Atogepant is an oral CGRP receptor antagonist approved for migraine prevention. The aim of this study was to investigate the neurophysiological changes induced by atogepant in individuals with high‐frequency episodic migraine (HFEM).

**Methods:** In this prospective study, 40 participants (age 35.5 ± 21.9, 30 females) underwent interictal neurophysiological testing at baseline (T0) and after 3 months of daily atogepant 60 mg (T1). The assessment included: i) the temporal summation threshold (TST) of the nociceptive withdrawal reflex (nWR); and ii) the habituation of the nociceptive blink reflex (nBR) and of visual evoked potentials (VEPs). Habituation was assessed by means of the habituation index (HI), namely the percentage decrease of area under curve at the end of validated stimulation protocols.

**Results:** At T1, the TST increased compared to T0 (T0: 7.9 ± 4.5 mA vs. T1: 9.2 ± 4.8 mA, *p* = 0.021). At T1, the HI of the nBR decreased compared to T0 (T0: 47.4 ± 25.6 vs T1: 38.3 ± 29.5, *p* = 0.025) and the habituation deficit became more pronounced (TIME × BLOCKS: *p* = 0.045). VEPs showed a mild habituation, without changes at T1 (*p* > 0.050). At T1, 70% of participants qualified as Responders (> 50% in monthly migraine days). Being a Responder was not associated with any of the previously presented findings.

**Conclusion:** After a 3‐month atogepant treatment, we described a reduction in central sensitization and a modulation of habituation mechanisms at the brainstem level, without changes at the cortical level. These findings suggest that atogepant induced direct or indirect effects on key neurophysiological mechanisms involved in migraine pathophysiology.

**Disclosure:** The ATOM project was funded by a CORE ‐ AMD Resident Research Grant (application ID#: 1273396)

## EPO‐0088

### MIDAS and HIT‐6 questionnaires versus headache diaries for monitoring treatment response to Erenumab in migraine: A REFORM study

#### J. Thuraiaiyah ^1^; R. Christensen^1^; H. Al‐Khazali^1^; M. Ashina^2^; Z. Katsarava^3^; H. Ashina^4^

##### ^1^Department of Neurology, Danish Headache Center, Copenhagen University Hospital – Rigshospitalet, Copenhagen, Denmark; ^2^Center For Discoveries in Migraine, Danish Headache Center, Copenhagen University Hospital – Rigshospitalet, Copenhagen, Denmark; ^3^Department of Neurology, University of Duisburg‐Essen, Essen, Germany; ^4^Translational Research Center, Copenhagen University Hospital – Rigshospitalet, Copenhagen, Denmark.

**Background and aims:** Efficacy of migraine preventive treatment is often assessed based on daily headache diaries, which can be burdensome for patients. Therefore, patient‐reported outcome measures (e.g, Migraine Disability Assessment (MIDAS) and Headache Impact Test (HIT‐6)) offer alternatives for monitoring treatment response. The effectiveness of these questionnaires, however, remains uncertain. Here, we aimed to establish cut‐off scores for MIDAS and HIT‐6 corresponding with a ≥ 50% reduction in monthly migraine days (MMDs) to determine treatment responders.

**Methods:** Adults with migraine were enrolled in a prospective, open‐label study and received 140 mg erenumab for 24 weeks. A ≥50% reduction in MMD was defined as treatment success based on diary data. MIDAS and HIT‐6 were completed at baseline, Week 12, and Week 24. Optimal cut‐off values and corresponding sensitivity and specificity were determined using receiver operating characteristic curve analysis.

**Results:** Among 582 participants, 300 individuals (52%) achieved treatment success. MIDAS and HIT‐6 showed fair discriminative ability in identifying treatment responders, with area under the curve values ranging from 0.71 to 0.77. The optimal cut‐offs for MIDAS were a 16‐point absolute reduction (78% sensitivity, 60% specificity) or a 49% relative reduction (82% sensitivity, 64% specificity). For HIT‐6, optimal cut‐offs were an 8‐point reduction (53% sensitivity, 84% specificity) or a 12% relative reduction (54% sensitivity, 84% specificity). Sensitivity analyses with alternative definitions yielded consistent results.

**Conclusion:** While MIDAS and HIT‐6 provide meaningful information on treatment‐related disability reduction, they are not interchangeable with prospective headache diaries for assessing treatment response to erenumab.

**Disclosure:** JT reports receiving personal fees from Lundbeck and Pfizer, outside of the submitted work. RHC reports receiving personal fees from Pfizer, outside of the submitted work. HMA¬ reports receiving personal fees from Pfizer and Lundbeck, outside of the submitted work. ZK reports receiving honoraria from AbbVie, Lilly, Merck, Novartis, TEVA and a research grant from Novartis, outside of the submitted work. MA reports receiving personal fees from AbbVie, Amgen, Astra Zeneca, Eli Lilly, GlaxoSmithKline, Lundbeck, Novartis, Pfizer and Teva Pharmaceuticals outside of the submitted work. MA received institutional grants from Lundbeck Foundation, Novo Nordisk Foundation, Lundbeck A/S, and Novartis. MA reports serving as associate editor of The Journal of Headache and Pain, and associate editor of Brain. HA reports receiving personal fees from AbbVie, Lundbeck, Pfizer, and Teva Pharmaceuticals, outside of the submitted work. Funding: This work was supported by research grants from the Lundbeck Foundation (R403‐2022‐1352 to H.A. and R310‐2018‐3711 to M.A.).

## EPO‐0089

### PACAP‐38 is increased in the tear fluid of patients with migraine

#### M. Romozzi ^1^; L. Di Nardo^2^; L. Iannone^3^; F. Bianchi^1^; G. Cuffaro^4^; R. Turano^1^; G. Savino^4^; C. Vollono^1^; P. Calabresi^1^

##### ^1^Neurologia, Dipartimento di neuroscienze, Organi di Senso e Torace, Fondazione Policlinico Universitario Agostino Gemelli IRCCS, Rome, Italy; ^2^Università Cattolica del Sacro Cuore, Dipartimento di Medicina e Chirurgia Traslazionale, Rome, Italy; ^3^Department of Biomedical, Metabolic and Neural Sciences, University of Modena and Reggio Emilia, Modena, Italy; ^4^Oculistica, Fondazione Policlinico Universitario A. Gemelli IRCCS, Rome, Italy.

**Background and aims:** Pituitary adenylate cyclase–activating polypeptide‐38 (PACAP‐38) is a key neuropeptide in migraine pathophysiology and an emerging therapeutic target, with monoclonal antibodies currently under clinical development. PACAP‐38 has been measured mainly in blood, yielding inconsistent results. Novel, non‐invasive biological sources closely related to trigeminal activation, such as tear fluid, remain unexplored. Here, we investigated PACAP‐38 levels in tear fluid of patients with migraine compared with healthy controls (HCs) using the Schirmer test.

**Methods:** In this cross‐sectional study, we included patients with migraine fulfilling ICHD‐3 criteria and HCs. Tear fluid was collected with Schirmer strips and ELISA measured PACAP‐38 concentrations. Clinical and demographic data were recorded. Between‐group comparisons, correlation analyses, and receiver operating characteristic (ROC) analyses were performed.

**Results:** We included 26 migraine patients (76.9% female, mean age 38.0 ± 11.6 years) and 12 HCs (58.3% female, mean age 27.1 ± 5.2 years). Tear PACAP‐38 concentrations were significantly higher in patients with migraine compared with HCs (22.2 ± 33.1 vs 4.5 ± 8.4 pg/mL, *p* = 0.041). ROC analysis showed good discriminative ability (AUC = 0.71, *p* = 0.02), with an optimal cut‐off of 2.0 pg/mL (73% sensitivity, 67% specificity). PACAP‐38 levels were not associated with monthly headache days, acute medication number, ictal vs interictal sampling, or migraine subtype.

**Figure:**
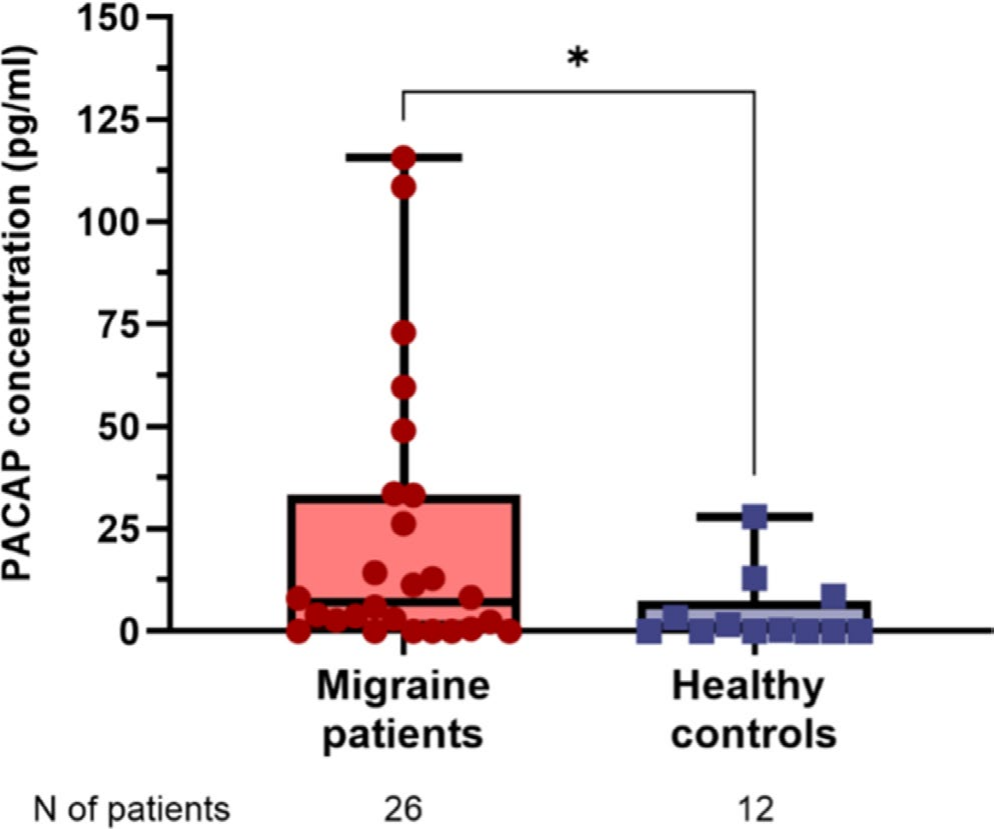
**FIGURE 1** PACAP‐38 levels in tear fluid of migraine patients compared to healthy controls.

**Figure:**
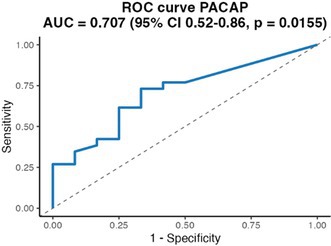
**FIGURE 2** Receiver Operating Characteristic (ROC) curve for PACAP‐38 levels in tears.

**Conclusion:** This is the first study to assess PACAP‐38 concentrations in the tear fluid of patients with migraine, demonstrating significantly elevated levels compared with HCs. Schirmer strip‐based sampling represents a rapid, non‐invasive, and patient‐friendly approach for neuropeptide detection, potentially providing a biomarker that reflects migraine‐specific mechanisms and supporting PACAP‐38 as an emerging therapeutic target.

**Disclosure:** Nothing to disclose.

## EPO‐0090

### Triple combination therapy with onabotulinumtoxinA, fremanezumab and atogepant in chronic migraine: A 52‐week prospective case series (TRIPLE study)

#### M. Romozzi ^1^; L. Iannone^2^; A. Russo^3^; I. Finkelstein^4^; T. Vaelidalo^5^; A. Ahlden^5^; A. Aamodt^6^; E. Caronna^7^; P. Pozo‐Rosich^7^; E. Tronvik^6^; C. Sundal^5^

##### ^1^Dipartimento Universitario di Neuroscienze, Università Cattolica del Sacro Cuore, Rome, Italy; Neurologia, Dipartimento di Neuroscienze, Organi di Senso e Torace, Fondazione Policlinico Universitario Agostino Gemelli IRCCS, Rome, Italy; ^2^Department of Biomedical, Metabolic and Neural Sciences, University of Modena and Reggio Emilia, Modena, Italy; ^3^Inter‐departmental Program of Headache Medicine and Primary Pain Syndromes, Department of Medical, Surgical, Neurological, Metabolic and Aging Sciences, University of Campania “Luigi Vanvitelli”, Naples, Italy; ^4^Director, Toronto Headache and Pain clinic; Pain Clinic, Toronto, Canada;; ^5^NeuroClinicNorway, Lillestrøm, Norway; ^6^NorHead, Norwegian Headache Research Centre; NTNU, Norwegian University of Science and Technology,Trondheim, Norway; ^7^Headache Clinic, Neurology Department, Vall d’Hebron Hospital, Barcelona, Spain; Headache and Neurological Pain Research Group, VHIR, Department of Medicine,Universitat Autònoma de Barcelona, Barcelona, Spain.

**Background and aims:** A substantial proportion of patients with chronic migraine (CM) persist with high disease burden despite treatment with advanced preventive monotherapies, including onabotulinumtoxinA (BoNTa) and calcitonin gene related peptide (CGRP)‐targeted drugs. Evidence supporting preventive combinations, particularly multiple regimens, is limited. This study evaluated the long‐term effectiveness and safety of combining BoNTa, fremanezumab, and atogepant in a real‐world CM population with persistent residual burden.

**Methods:** This was a 52‐week, prospective, single‐center case series including 13 consecutive adults with CM treated with BoNTa, then fremanezumab and followed by add‐on atogepant 60 mg. Monthly headache days (MHDs) and monthly migraine days (MMDs) were assessed using electronic diaries. Secondary outcomes included acute medication days, responder rates, Headache Impact Test (HIT‐6), Hospital Anxiety and Depression Scale (HADS), Patient Global Impression of Change (PGIC), and adverse events.

**Figure:**
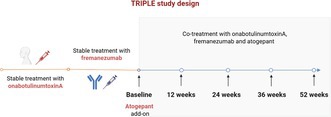
**FIGURE 1** Study design of the TRIPLE study.

**Results:** At atogepant initiation, patients had severe residual disease with mean MHDs 25.8 ± 4.8 and MMDs 19.7 ± 4.2. During triple therapy, MHDs decreased progressively to 19.1 ± 9.4 at week 52 (*p* < 0.001), and MMDs to 10.4 ± 6.2 (*p* < 0.001). A ≥ 50% MMD reduction was achieved by 30.8% of patients at week 24 and 53.8% at week 52. Acute medication days and HIT‐6 scores significantly improved, while anxiety and depression scores remained stable. PGIC consistently indicated moderate‐to‐great improvement. No serious adverse events or treatment discontinuations occurred, and all adverse events were mild.

**Figure:**
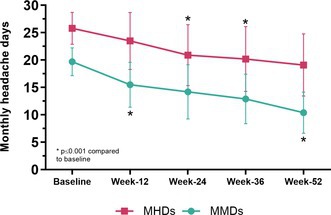
**FIGURE 2** Change in monthly headache days and migraine days during triple combination treatment.

**Figure:**
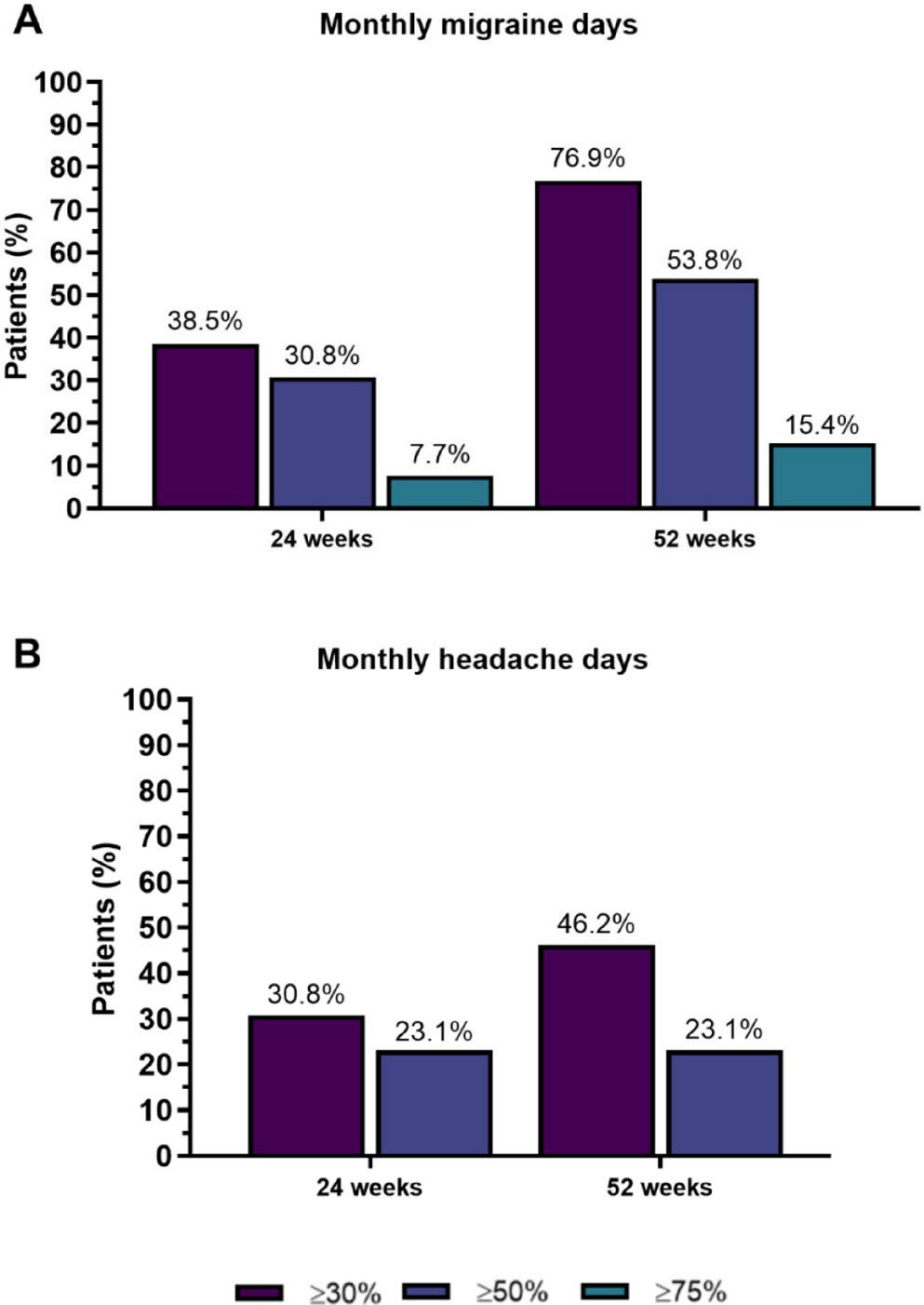
**FIGURE 3** Response rates at 24 and 52 weeks.

**Conclusion:** In this CM cohort, long‐term triple preventive therapy with BoNTa, fremanezumab, and atogepant was associated with progressive and clinically meaningful reductions in migraine frequency and disability, with favorable tolerability.

**Disclosure:** MR: received financial support, consulting fees for the participation in advisory boards and support for attending meetings from: Lundbeck, Pfizer and AbbVie. LFI received financial support, consulting fees for the participation in advisory boards and support for attending meetings from: Teva, Eli Lilly, Lundbeck, Pfizer and AbbVie; he is Associate Editor for Frontiers in Neurology and junior editor of Cephalalgia and Cephalalgia report. AR: has received speaker honoraria from Allergan, Lilly, AbbVie, Pfizer, Novartis and Teva and serves as an associate editor of Frontiers in Neurology (Headache Medicine and Facial Pain session). IF: Grants/honorarium/Consulting fees: Allergan, Amgen, Eli Lilly, J and J, Merck, Purdue, Teva, Aralez, Novartis, Lundbeck, Pfizer. AHA: has received personal fees for lectures/advisory boards: Novartis, Abbvie, TEVA, Roche, Lundbeck, Pfizer, Boehringer Ingelheim. Non‐personal research grants from several sources, including KlinBeForsk, EU, Boehringer Ingelheim, Medtronic, BMS. EC: has received honoraria from Novartis, Chiesi, Lundbeck, MedScape, Lilly; his salary has been partially funded by Río Hortega grant Acción Estratégica en Salud 2017–2020 from Instituto de Salud Carlos III (CM20/00217) and Juan Rodés fellowship, Subprograma Estatal de Incorporación de la Acción Estratégica en Salud 2023 (JR23/00065). He is a junior editor for Cephalalgia. PPR: has received, in the last 3 years, honoraria as a consultant and speaker from AbbVie, Amgen, Dr Reddy's, Eli Lilly, Lundbeck, Medscape, Novartis, Organon, Pfizer and Teva Pharmaceuticals. Her research group has received research grants from AbbVie, AGAUR, EraNet Neuron, FEDER RIS3CAT, Instituto Investigación Carlos III, MICINN, Novartis, and Teva Pharmaceuticals, and has received funding for clinical trials from AbbVie, Amgen, Biohaven, Eli Lilly, Lundbeck, Novartis, Pfizer and Teva Pharmaceuticals. She is the Honorary Secretary of the International Headache Society, is on the editorial board of Revista de Neurologia, is an associate editor for Cephalalgia, Headache, Neurologia, Frontiers of Neurology, and is an advisor of the Scientific Committee of the Editorial Board of The Journal of Headache and Pain. She is a member of the Clinical Trials Guidelines Committee and Scientific Committee of the International Headache. ET: has received personal fees for lectures/advisory boards: Novartis, Eli Lilly, Abbvie, TEVA, Roche, Lundbeck, Pfizer, Biogen, Organon. Consultant for and owner of stocks and IP in Man & Science. Stocks and IP in Nordic Brain Tech. Stocks in Keimon Medical. Non‐personal research grants from several sources, including Norwegian Research Council, KlinBeForsk, EU. Commissioned research (non‐personal): Lundbeck. CS: has received honorarium for lectures/advisory boards: Novartis, Abbvie, TEVA, Vigil NeuroScience.

## EPO‐0091

### CGRP monoclonal antibodies in adolescents with migraine: Real‐world persistence and outcomes from the German pain e‐Registry

#### M. Überall ^1^; P. Müller‐Schwefe^2^

##### ^1^IFNAP ‐ Institute of Neurological Sciences, Nürnberg, Germany; ^2^Interdisciplinary Center for Pain & Palliative Care Medicine, Göppingen, Germany

**Background and aims:** Preventive treatment options for adolescents with migraine are limited. Conventional oral preventives are often used off‐label, poorly tolerated, and frequently discontinued. Real‐world data on CGRP monoclonal antibodies (mABs) in this population are scarce. To explore real‐world treatment persistence and clinical outcomes of CGRP mABs compared with conventional oral preventives in adolescents with high‐burden migraine.

**Methods:** We analyzed depersonalized data from the German Pain e‐Registry. Adolescents (12–17 years) with ≥1 documented 6‐month episode of high‐evidence conventional oral preventives (HECP) and ≥1 subsequent CGRP mAB episode were included. The primary endpoint was a composite of 6‐month persistence and ≥50% reduction in monthly migraine days (MMD).

**Results:** In 422 adolescents (1,448 HECP; 422 CGRP mAB episodes), 6‐month persistence was 30.6% with HECP and 88.2% with CGRP mAB. A ≥ 50% MMD reduction occurred in 25.4% and 70.9%, respectively. The composite endpoint was reached in 23.7% with HECP versus 69.9% with CGRP mAB. Mean MMD decreased from 11.7 to 9.4 with HECP and from 11.6 to 4.4 with CGRP mAB. Improvements in disability and psychosocial outcomes were consistently greater with CGRP mAB.

**Conclusion:** In this high‐burden adolescent cohort, CGRP mAB treatment episodes were associated with markedly higher persistence and broader improvements than prior conventional preventive episodes. Given the non‐randomized, sequential design, these findings are hypothesis‐generating and support the need for controlled pediatric trials.

**Disclosure:** M.A. Überall is honorary board member of the German Pain Association and the German Pain League and Director of the Institute of Neurological Sciences (IFNAP) and CEO of O.Meany – Medical Data & Project Management GmbH. The German Pain e‐Registry is hosted by O.Meany – MDPM GmbH under scientific governance of IFNAP. No third‐party funding was received. The author declares no other conflicts of interest.

## EPO‐0092

### Real‐world treatment persistence in migraine prevention: A national registry comparison of CGRP monoclonal antibodies and oral preventives

#### M. Überall ^1^; P. Müller‐Schwefe^2^; M. Küster^3^; J. Jansen^4^

##### ^1^IFNAP ‐ Institute of Neurological Sciences, Nürnberg, Germany; ^2^Interdisciplinary Center for Pain & Palliative Care Medicine, Göppingen, Germany; ^3^Interdisciplinary Center for Pain & Palliative Care Medicine, Bonn, Germany; ^4^Pain Medicine Center, Berlin, Germany

**Background and aims:** Oral migraine preventive drugs are frequently discontinued in routine care. Most real‐world studies rely on prescription claims and cannot capture clinically validated reasons for treatment termination. In addition, many datasets predate the availability of monoclonal antibodies targeting calcitonin gene‐related peptide (CGRP). To compare real‐world treatment persistence and documented reasons for discontinuation among patients receiving subcutaneous CGRP monoclonal antibodies (CGRP mAB), oral high‐evidence preventive therapies (HEVP), and oral low‐evidence preventives (LEVP).

**Methods:** This observational cohort study analyzed depersonalized routine‐care data from the German Pain e‐Registry, a nationwide multicenter clinical registry. Patients with physician‐confirmed migraine receiving preventive therapy were followed for 182 days. Treatment persistence was defined as continuous use of the index therapy. Discontinuations were classified as due to adverse drug reactions (ADR) or insufficient efficacy. Persistence trajectories were assessed using Kaplan–Meier analyses and pairwise risk comparisons.

**Results:** A total of 36,465 patients were included (12,347 CGRP mAB; 23,353 HEVP; 765 LEVP). Six‐month persistence was markedly higher with CGRP mAB (89.3%) than with HEVP (32.7%) or LEVP (42.6%; *p* < 0.001). ADR‐related discontinuation occurred in 6.8% of CGRP mAB patients, compared with 45.1% in HEVP and 35.8% in LEVP. Discontinuation due to insufficient efficacy was 3.6% with CGRP mAB versus 22.2% and 21.6% with HEVP and LEVP. Sex and migraine subtype had no clinically meaningful influence. Within HEVP, tricyclic antidepressants, beta‐blockers, and valproic acid showed the lowest persistence.

**Conclusion:** CGRP monoclonal antibodies demonstrate substantially superior real‐world persistence and tolerability compared with conventional oral migraine preventives, highlighting the clinical relevance of mechanism‐based prevention.

**Disclosure:** M.A. Überall is honorary board member of the German Pain Association and the German Pain League and Director of the Institute of Neurological Sciences (IFNAP) and CEO of O.Meany – Medical Data & Project Management GmbH. The German Pain e‐Registry is hosted by O.Meany – MDPM GmbH under scientific governance of IFNAP. All other authors declare no competing interests.

## EPO‐0093

### Improving diagnostic reasoning in headache disorders through an ICHD‐3–based undergraduate curriculum intervention

#### P. Shekhawat^1^; R. Yerraballa^2^; S. Das ^2^; G. Singh^3^

##### ^1^Mass General Brigham Wentworth Douglass Hospital, Dover, USA; ^2^Armed Forces Medical College, Pune, India; ^3^SKN Medical College, Pune India

**Background and aims:** Headache disorders are among the leading causes of disability worldwide, yet undergraduate medical education in headache medicine remains variable and often lacks standardized training. Although the International Classification of Headache Disorders, 3rd edition (ICHD‐3), provides a globally accepted framework for headache diagnosis, structured instruction during medical training is inconsistent. We therefore implemented a digital ICHD‐3 instructional module and evaluated its impact.

**Methods:** Medical students from two undergraduate programs participated in a pre–post educational evaluation. Baseline knowledge and confidence were assessed using a pre‐intervention survey, followed by a 20‐minute structured teaching session focused on use of ICHD‐3, including a brief review of SNOOP red‐flag features. The post‐intervention survey repeated all assessment items. For vignette‐based questions, participants were permitted to access the digital ICHD‐3 to simulate point‐of‐care diagnostic support. Pre–post changes were analyzed using the Wilcoxon signed‐rank and McNemar's tests.

**Results:** Forty‐three students completed both assessments. Post‐intervention confidence increased significantly across all evaluated domains (all *p* < 0.001), including focused history‐taking, primary versus secondary headache disorders, SNOOP, ICHD‐3 diagnostic criteria, and communication of headache diagnoses. Perceived usefulness of headache classification increased significantly (*p* < 0.001), while interest in a quick diagnostic reference remained high and unchanged (*p* = 0.096). Diagnostic knowledge accuracy improved for key ICHD‐3 concepts, including migraine and new daily persistent headache diagnosis, typical aura, and episodic migraine‐associated syndromes (all *p* ≤ 0.019).

**Figure:**
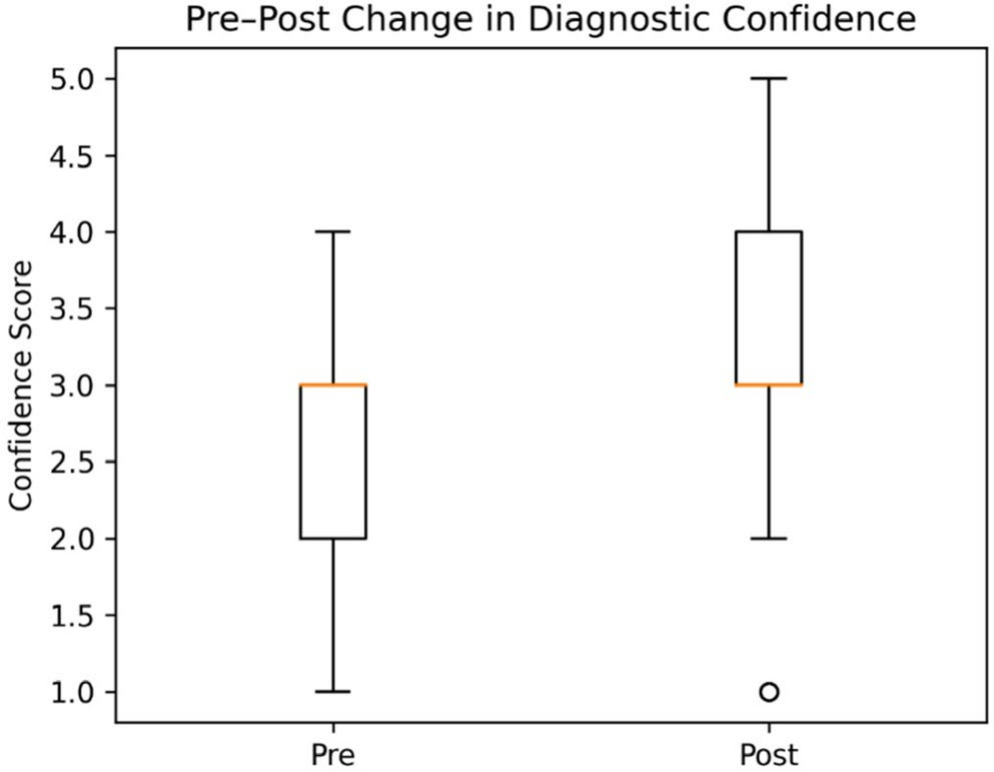
**FIGURE 1** Pre–post change in diagnostic confidence scores following the ICHD‐3 educational intervention. Post‐intervention confidence scores were significantly higher than baseline (Wilcoxon signed‐rank test, *p* < 0.001).

**Figure:**
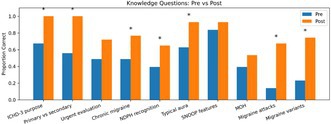
**FIGURE 2** Proportion of students correctly answering knowledge questions before and after an ICHD‐3–based educational intervention. Questions assessed core diagnostic concepts in headache disorders. Asterisk denotes *p* < 0.05 on McNemar's exact test.

**Figure:**
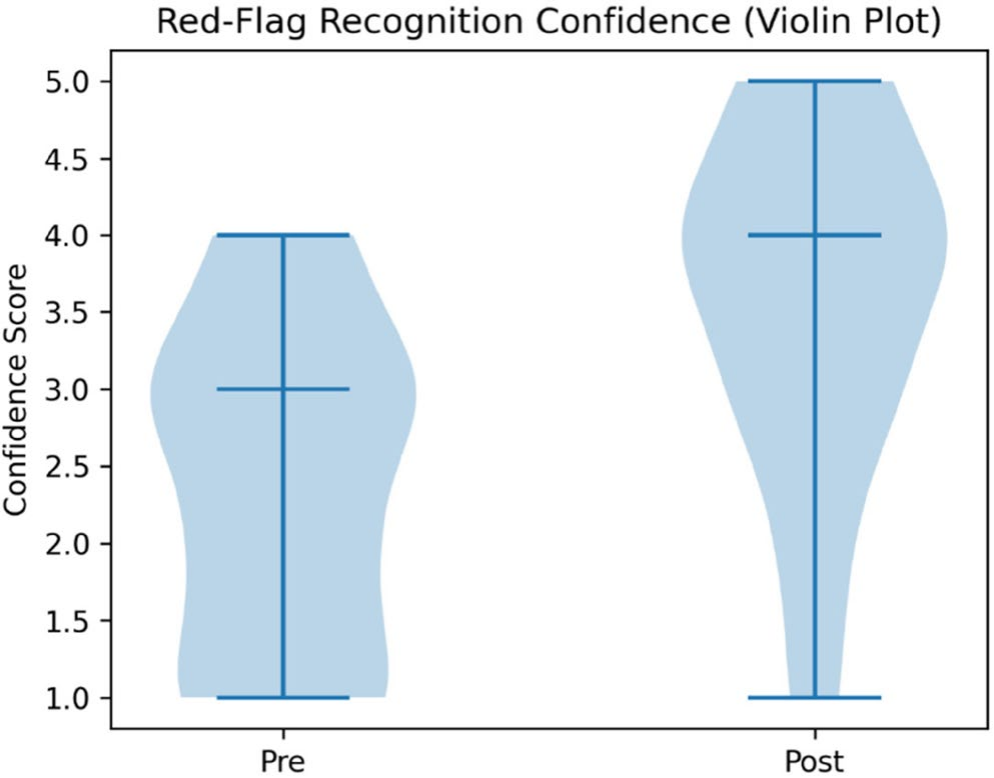
**FIGURE 3** Pre–post change in recognition of secondary headache red flags (SNOOP criteria). Post‐intervention scores demonstrated a significant improvement compared with baseline (Wilcoxon signed‐rank test, *p* < 0.001).

**Conclusion:** These findings demonstrate that a brief, digitally supported ICHD‐3–based intervention can improve diagnostic confidence and accuracy among medical students and support a scalable approach to strengthening structured headache education.

**Disclosure:** Nothing to disclose.

## EPO‐0094

### Self‐reported redosing and treatment outcomes with oral triptans for acute migraine: Results from the DREAM study

#### H. Schytz^1^; D. Hauberg^2^; U. Lønberg ^2^; L. Abraham^2^; K. Blakeman^2^; M. Spanggaard^3^; J. Olsen^3^; T. Hansen^1^

##### ^1^Danish Headache Center, Department of Neurology, Copenhagen University Hospital Rigshospitalet‐Glostrup, Copenhagen, Denmark; ^2^Pfizer; ^3^EY Parthenon

**Background and aims:** This nationwide population study evaluated self‐reported frequency of redosing after use of oral triptans, treatment effectiveness as assessed by pain relief and return to normal function, and adverse events.

**Methods:** All Danish residents ≥ 18 years with a triptan purchase or a migraine diagnosis (ICD‐10: G43) between January 2021 – December 2023 were invited to the nationwide DREAM (Danish REgistry Analyses of Migraine) study combining data from national health registers with a survey on acute migraine medication experience.

**Results:** 41,498 people reported a migraine attack within the past 3 months. Baseline characteristics are presented in Table 1 Among 21,960 individuals using oral triptans as the only or the first medication type to treat their most recent migraine attack (Figure 1) 49% reported redosing with the same or a different medication, most commonly (77%) between 2–24 h. The proportion achieving 2 h pain relief, return to normal function, and sustained pain relief was significantly lower among individuals who redosed between 2–24 h compared with those who did not redose (73% vs 88%, 32% vs 50%, 57% vs 84%, respectively; *p* < 0.01 for all comparisons). Among those redosing a significantly lower proportion experienced no adverse events vs those not redosing (Figure 2) (38% vs 50% *p* < 0.01).

**Figure:**
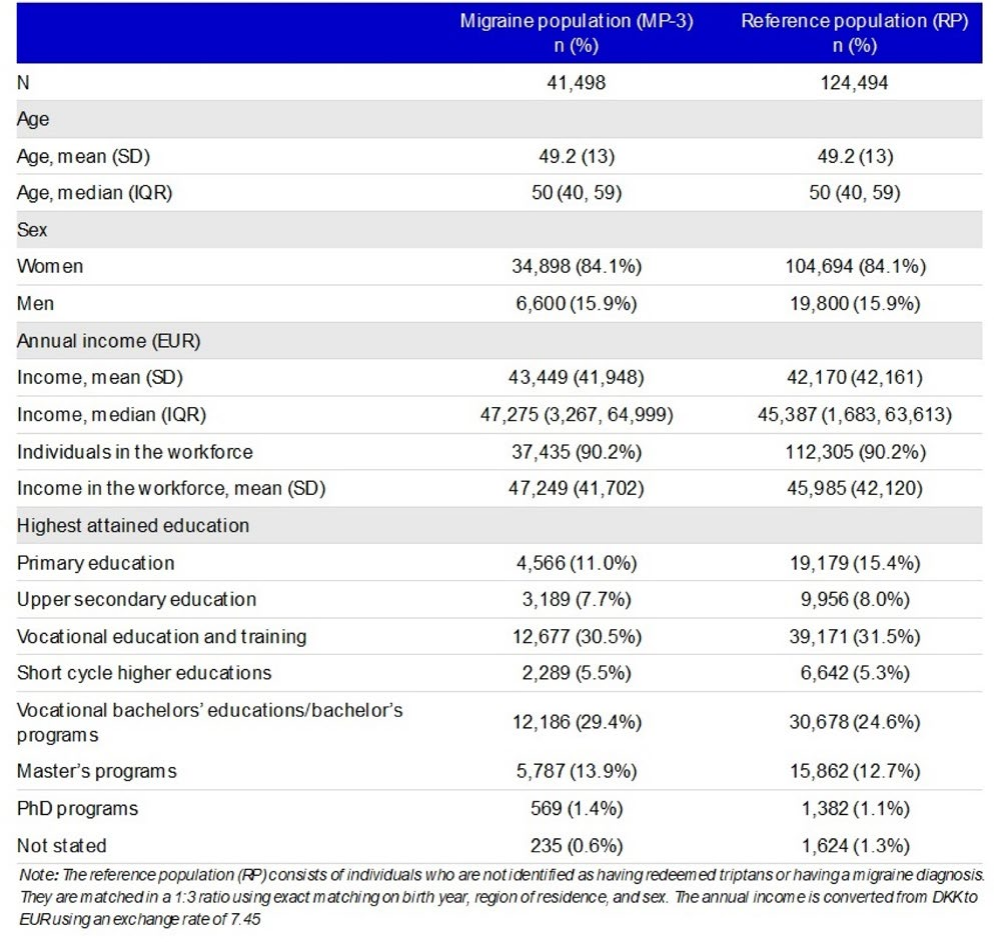
**TABLE 1** Baseline characteristics

**Figure:**
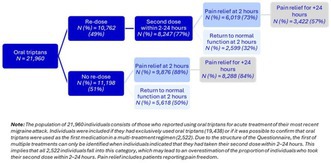
**FIGURE 1** Redosing, pain relief, and return to normal function after oral triptan use for acute treatment of the most recent migraine attack

**Figure:**
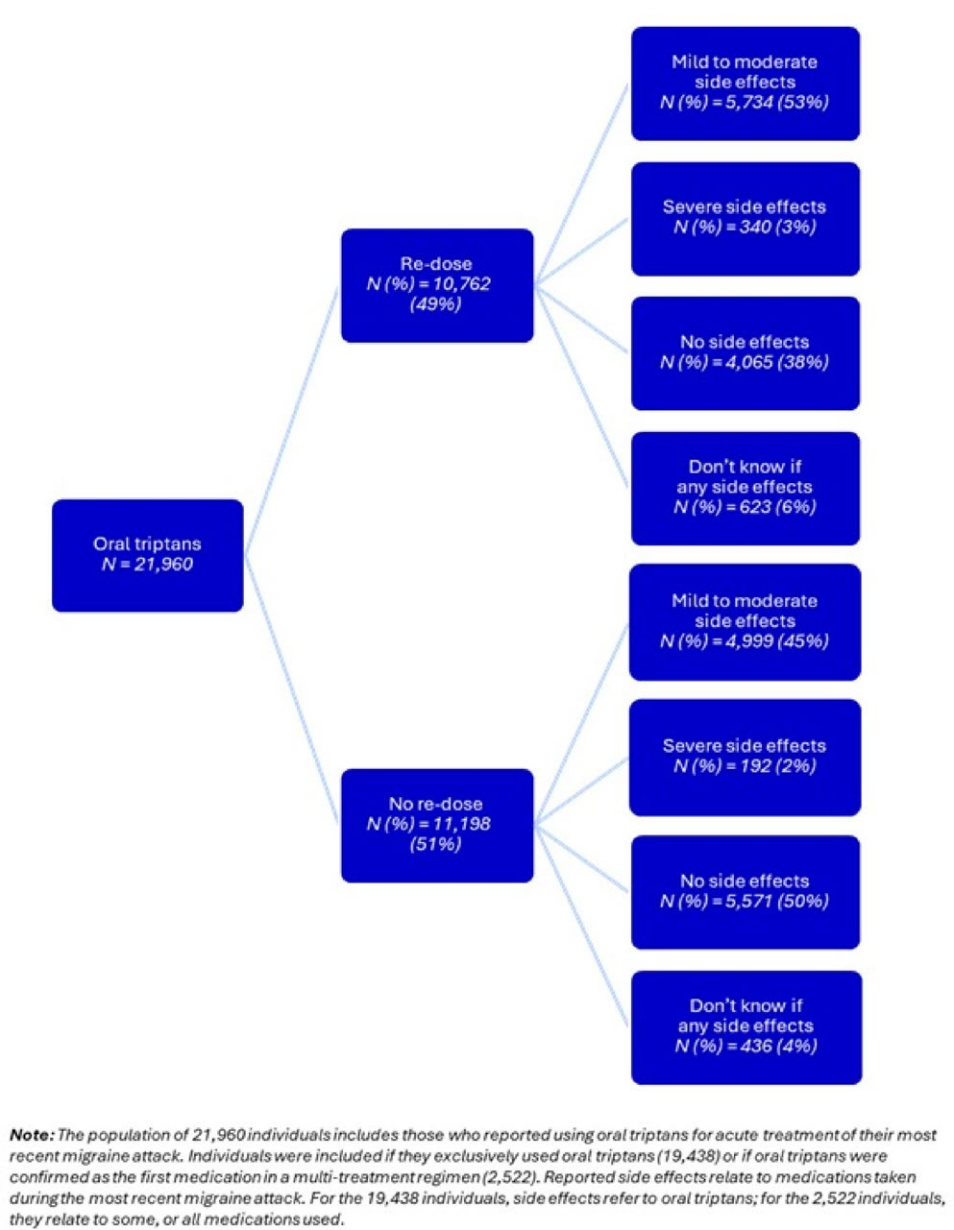
**FIGURE 2**: Redosing and side effects of medications for acute treatment of the most recent migraine attack.

**Conclusion:** In this nationwide real‐world study almost half of individuals taking oral triptans reported redosing, which was associated with poorer pain and functional outcomes and a higher frequency of adverse events. Need for redosing reflects insufficient effectiveness of the initial dose and highlights an unmet need in acute migraine treatment.

**Disclosure:** This study was sponsored by Pfizer. Medical writing support was provided by EY Parthenon, a paid vendor to Pfizer.

Infectious diseases 1

## EPO‐0095

### Inflammatory markers in tubercular meningitis and their role in predicting paradoxical reaction

#### A. Qavi; S. Deswal; A. Singh; P. Maurya; D. Kulshreshtha

##### Department of Neurology, Dr. Ram Manohar Lohia Institute of Medical Sciences, Lucknow, UP, India

**Background and aims:** Tubercular meningitis (TBM) is associated with high morbidity and mortality due to an intense inflammatory response leading to basal exudates, cranial nerve palsies, vasculitic infarcts, and hydrocephalus. Paradoxical reaction (PR) occurs in nearly 30% of patients and is an important cause of clinical deterioration during treatment. Dysregulated inflammatory mediators in serum and cerebrospinal fluid (CSF) have been implicated in PR and adverse outcomes in TBM.

**Methods:** In this prospective observational study, 151 newly diagnosed TBM patients fulfilling the Marais et al. consensus diagnostic criteria were enrolled. Baseline clinical, radiological, serum, and CSF inflammatory markers were assessed in treatment‐naïve patients and repeated at 3 months or at the time of PR or stroke. Serum markers included hsCRP, ESR, LDH, ferritin, albumin, D‐dimer, neutrophil–lymphocyte ratio (NLR), and systemic immune‐inflammation index (SII). CSF analysis included ADA, NLR, protein, sugar, and total leukocyte count.

**Results:** The mean age was 32.7 years; headache was the most common symptom (93.4%). Paradoxical reaction developed in 43 patients (28.4%), most commonly presenting with altered sensorium. Radiologically, worsening hydrocephalus was the predominant finding. On univariate analysis, serum albumin, D‐dimer, LDH, ferritin, ESR, and hsCRP were significantly associated with PR. On multivariate analysis, Serum Ferritin and ESR independently predicted the paradoxical reaction.

**Figure:**
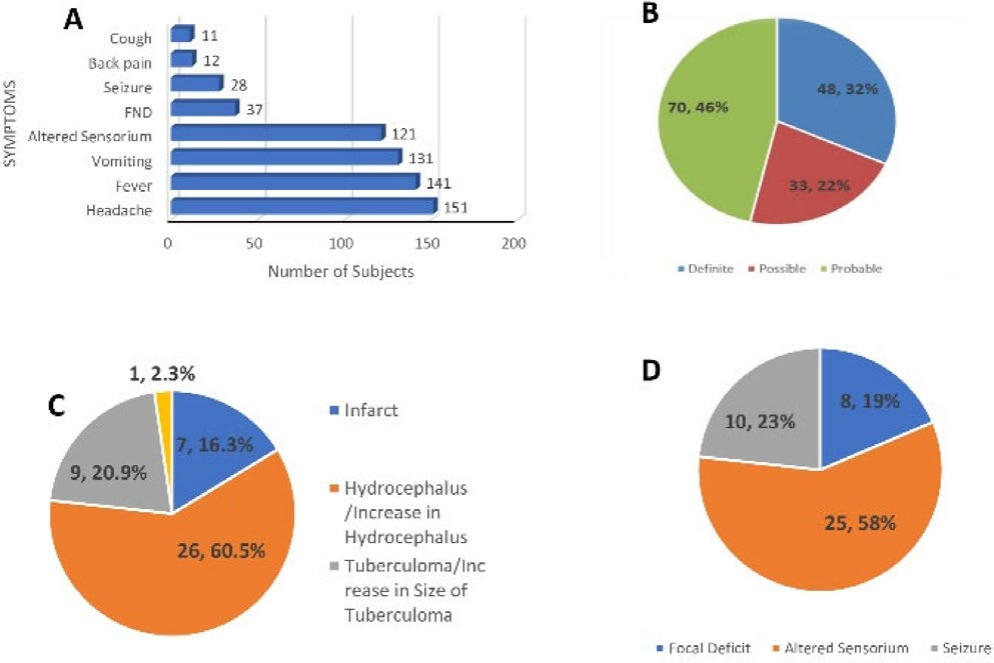
**FIGURE 1** (A)‐ Frequency of Symptoms, (B)‐ Staging of TBM, (C)‐ Radiological manifestation of paradoxical reaction, (D)‐ Clinical Manifestation of Paradoxical reaction.

**Figure:**
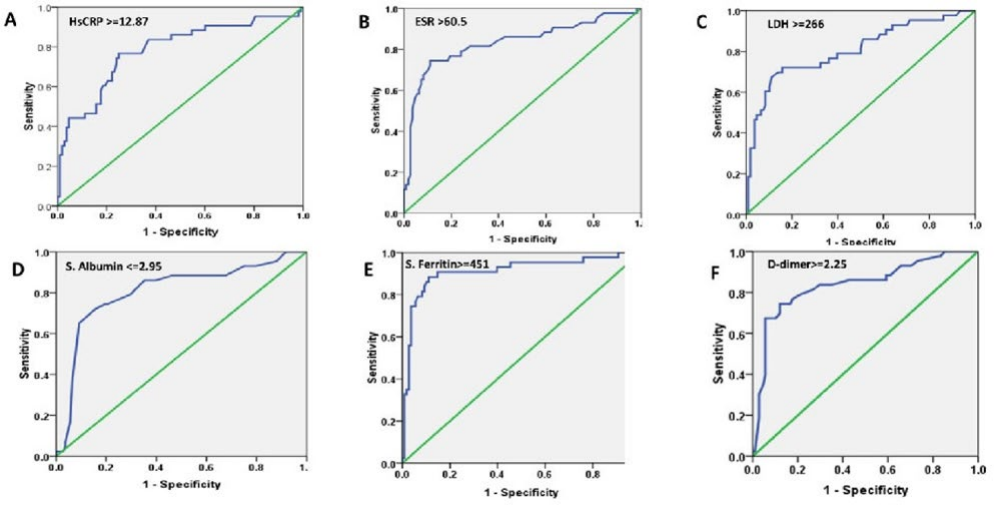
**FIGURE 2** The receiver operating characteristic curve showing the discrimination power of (A)‐ HsCRP, (B)‐ ESR, (C)‐ S. LDH, (D)‐ S. Albumin, (E)‐ S. Ferritin, (F)‐ D‐dimer between paradoxical reaction and non‐paradoxical reaction group.

**Figure:**
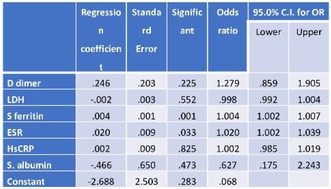
**TABLE 1** Multivariate logistic regression analysis for predictors of paradoxical reaction in TBM.

**Conclusion:** Serum inflammatory markers, particularly ferritin and ESR, may serve as useful predictors of paradoxical reaction in TBM. These findings support an immune‐mediated mechanism underlying paradoxical worsening and may help identify high‐risk patients who could benefit from early anti‐inflammatory or immunomodulatory therapy.

**Disclosure:** Nothing to disclose.

## EPO‐0096

### An emerging arboviral trigger of Guillain‐Barré syndrome: The first documented case linked to Toscana virus

#### A. Bettinelli ^1^; D. Saccomanno^2^; N. Molitierno^2^; C. Alberti^1^; D. Gagliardi^2^; D. Velardo^2^; S. Corti^1,3^; G. Comi^1,2^; M. Parisi^1,2^

##### ^1^Department of Pathophysiology and Transplantation, University of Milan, Milan, Italy; ^2^Neurology Unit, Department of Neuroscience and Mental Health, Fondazione IRCCS Ca' Granda Ospedale Maggiore Policlinico, Dino Ferrari Centre, Milan, Italy; ^3^Neuromuscular and Rare Diseases Unit, Department of Neuroscience and Mental Health, Fondazione IRCCS Ca' Granda Ospedale Maggiore Policlinico, Dino Ferrari Centre, Milan, Italy

**Background and aims:** Guillain‐Barré syndrome (GBS) may occur after various infectious triggers, including arboviral infections. Toscana virus (TOSV) is an emerging sand fly–borne phlebovirus endemic in the Mediterranean region, typically associated with neuroinvasive diseases. To date, no cases have established a clear temporal relationship between serologically confirmed acute TOSV infection and the subsequent development of GBS.

**Methods:** We report the clinical, neurophysiological, and laboratory findings of a patient diagnosed with GBS following acute TOSV infection. The diagnostic work‐up included cerebrospinal fluid (CSF) analysis, nerve conduction studies, molecular and serological testing for neurotropic pathogens and assessment of antibodies against gangliosides and sulfatides. A targeted review of the literature was conducted to identify previously reported cases of Guillain‐Barré‐like syndrome associated with TOSV infection.

**Results:** CSF findings and electrodiagnostic studies were consistent with the acute inflammatory demyelinating polyneuropathy variant of GBS. Real‐time PCR detected TOSV RNA in urine, supporting a recent TOSV infection temporally antecedent to neurological symptom onset. The patient underwent six plasma exchange sessions in addition to supportive care, resulting in progressive neurological improvement.

**Conclusion:** To our knowledge, this is the first documented case establishing a clear temporal association between TOSV acute infection and GBS onset. This report highlights the importance of considering TOSV as a potential infectious trigger of post‐infectious GBS in endemic areas.

**Disclosure:** Nothing to disclose.

## EPO‐0097

### Abstract withdrawn

## EPO‐0098

### Foodborne botulism: Signs beyond descending weakness

#### D. Dias Dinis ^1^; R. Miranda Almeida^1^; M. Fernandes^2^; D. Costa^1^; J. Lopes^1^

##### ^1^Neurology Department, Unidade Local de Saúde Santo António, Porto, Portugal; ^2^Neurophysiology Department, Unidade Local de Saúde Santo António, Porto, Portugal

**Background and aims:** Botulism neurotoxin intoxication is a rare and life‐threatening condition that typically causes a descending flaccid paralysis. However, rarely, autonomic dysfunction may be the predominant clinical manifestation and should be considered in the differential diagnosis of pure dysautonomia.

**Methods:** We describe a case series of three patients with atypical botulism presenting predominantly with dysautonomia and a favourable outcome.

**Results:** Patient 1 was a 40‐year‐old man, patient 2 a 35‐year‐old woman, and patient 3 a 70‐year‐old man, all of whom sought medical care because of blurred vision and xerostomia. Additional symptoms included gastrointestinal manifestations (abdominal pain, nausea, and vomiting in patient 1 and constipation in all patients), urinary dysfunction (urinary hesitancy in patients 1 and 3), hypotension (patient 3), mild diplopia (patient 3) and bulbar complaints with mild clinical variations among the three cases. Neurological examinations were normal in all patients, except for accommodative paresis. All three patients had consumed cured ham at the same meal. Electromyographic studies didn't reveal neuromuscular junction disfunction. Serum assays were positive for botulinum toxin type B in all patients. In two cases, toxin B‐encoding genes were detected in stool samples via PCR. Antitoxin was not administered as all patients were admitted more than two weeks after symptom onset. At a four‐month follow‐up, all patients showed nearly complete recovery.

**Conclusion:** Botulism can be a challenging diagnosis when patients do not develop the typical descending weakness or respiratory failure. Careful consideration of the full clinical presentation and epidemiological context is essential for accurate diagnosis, as timely antitoxin administration may be lifesaving.

**Disclosure:** Nothing to disclose.

## EPO‐0099

### Toscana virus meningoencephalitis, a case series

#### K. Ramírez Mora ^1^; F. Alberola Amores^1^; A. Rubio Alcantud^2^

##### ^1^Department of Neurology, General University Hospital of Elche, Elche, Spain; ^2^Department of Neurology, Vega Baja University Hospital, Orihuela, Spain

**Background and aims:** Toscana virus (TOSV) is the neurotropic arbovirus of greatest clinical relevance in the Mediterranean basin and the Middle East. Our objective is to describe a series of TOSV meningoencephalitis cases within our catchment area.

**Methods:** We describe five cases of TOSV meningoencephalitis identified between 2021 and 2024 at General University Hospital of Elche and Vega Baja University Hospital, located in the province of Alicante, Spain, in order to describe their clinical and epidemiological characteristics and relevant findings in complementary tests.

**Results:** Most patients were men in their eighth decade of life. The most frequent comorbidities included diabetes, hypertension, and prostate cancer. On admission, the predominant symptoms were fever or low‐grade fever, headache with red‐flag features, and confusion. Cerebrospinal fluid analysis most commonly showed lymphocytic pleocytosis and elevated protein levels. Neuroimaging revealed no abnormalities attributable to TOSV infection, and only one patient exhibited epileptiform activity on electroencephalography. Clinical outcomes were favorable, with all patients returning to their baseline status.

**Figure:**
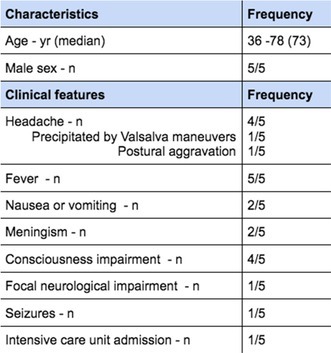
**TABLE 1** Case characteristics and clinical features (Given the small sample size, results are presented as absolute frequencies).

**Figure:**
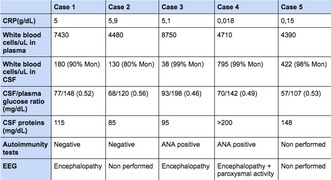
**TABLE 2** Cerebrospinal fluid (CSF) characteristics and complementary test. CRP: reactive C protein; Mon: mononuclear cells; ANA: Antinuclear antibodies; EEG: Electroencephalogram.

**Conclusion:** TOSV should be included in the differential diagnosis of aseptic meningitis and other central nervous system (CNS) infections in endemic regions. Its incidence is expected to rise in the coming years as increasing global temperatures facilitate the spread of vector‐borne diseases into new geographical areas. Identification and characterization of these cases will enhance our understanding of the disease and help determine its prognosis and management.

**Disclosure:** Nothing to disclose.

## EPO‐0100

### Otogenic meningoventriculitis due to Pseudomonas stutzeri managed with surgery and TDM‐monitored targeted antibiotic therapy: A case report

#### L. Ceccarelli; G. Virgili; I. Massari; P. Viale

##### Department of Medical and Surgical Sciences, Alma Mater Studiorum, University of Bologna, Bologna, Italy

**Background and aims:** Otogenic meningo‐ventriculitis is a challenging condition, particularly when caused by uncommon Gram‐negative pathogens and complicated by hydrocephalus. We report a rare case of Pseudomonas stutzeri meningoventriculitis managed with aggressive surgical strategy and fosfomycin‐based combination treatment with serial plasma/CSF fosfomycin monitoring.

**Methods:** Clinical, microbiological, neuroimaging, and CSF data were collected during admission. Serial CSF analyses were performed from external drains and lumbar punctures. Blood and CSF cultures guided therapy. Fosfomycin concentrations were monitored in plasma and CSF to support dosing optimization.

**Results:** A 70‐year‐old woman was admitted with acute hydrocephalus and suspected otogenic meningoventriculitis. Urgent external ventricular drainage was placed. Blood cultures grew Pseudomonas stutzeri (wild type; fosfomycin MIC 32 mg/L) and CSF microscopy showed Gram‐negative bacilli; CSF culture confirmed P. stutzeri. After initial empiric therapy with ceftriaxone plus levofloxacin, antibiotic therapy was escalated to cefepime plus fosfomycin. Neuroimaging supported meningo‐ependymal enhancement and acute otomastoiditis; ENT source control included tympanostomy and subsequent mastoid surgery. Despite persistently abnormal CSF cytochemistry, follow‐up CSF cultures remained negative. Based on adeguate source control, targeted and TDM‐guided antibiotic therapy (Figure 1), clinical/radiological improvement and sustained culture negativity, definitive ventriculo‐peritoneal shunt was placed on day 30 after admission; intraoperative CSF sampling resulted negative. Antibiotics were discontinued on day 34. At 1‐year follow‐up, no relapse occurred.

**Figure:**
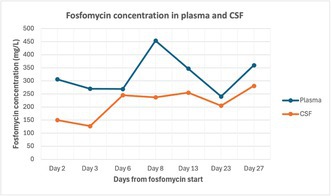
**FIGURE 1** Fosfomycin concentrations in plasma and CSF.

**Conclusion:** P. stutzeri is a rare cause of otogenic meningoventriculitis. A combining prompt source control, and fosfomycin‐based combination treatment with serial plasma/CSF fosfomycin monitoring may improve the patient's outcome.

**Disclosure:** Nothing to disclose.

## EPO‐0101

### Development of a patient reported outcome measure for infectious encephalitis: What matters most to patients

#### L. Secker ^1^; S. McKeever^1^; J. Mitchell^2^; E. Collins^3^; A. Easton^3^; B. Michael^1^

##### ^1^The Brain Infection and Inflammation Group, University of Liverpool, Liverpool, UK; ^2^The Walton Centre NHS Foundation Trust, Liverpool, UK; ^3^Encephalitis International, UK

**Background and aims:** Research on the long‐term outcomes of patients with infectious encephalitis (IE) is limited. Existing evidence from a systematic review indicates that most studies and clinicians use the modified Rankin Scale (mRS), a global disability score developed for stroke which does not capture the cognitive, behavioural, emotional and quality of life (QoL) consequences of IE. The current study aims to develop a disease‐specific patient‐reported outcome measure (PROM) to address this gap.

**Methods:** Semi‐structured interviews were developed following COSMIN guidance, with international input from clinicians and adults with lived experience of IE. Participants were recruited via Encephalitis International until data saturation was achieved (*n* = 40). Transcripts of all interviews were thematically analysed by 2 independent reviewers, to identify concepts for the initial instrument.

**Results:** 101 items were initially identified, reduced to 73 items after removing duplicates and items with < 3 responses. Item breakdown by domain included Physical (25), Health related QoL (13), Cognitive (14), Psychological (9), Behavioural (12) and were shared across multiple aetiologies. Participants with a 'good outcome' according to mRS described substantial ongoing symptoms and impact on QoL. 11 participants experienced severe initial disease, and most patients spent an extended duration (21 day mean) in hospital.

**Figure:**
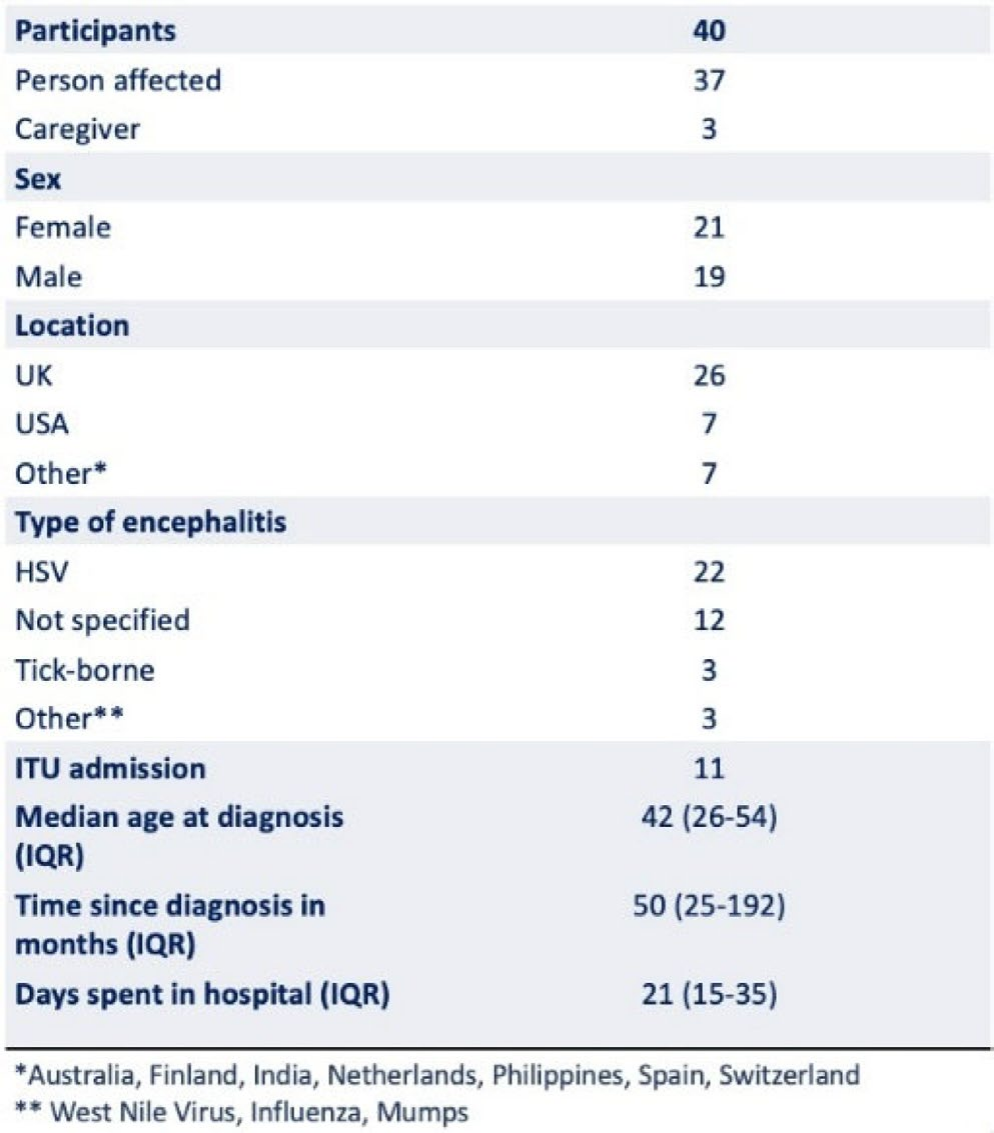
**TABLE 1** Demographics of the 40 patients who completed a semi‐structured interview.

**Figure:**
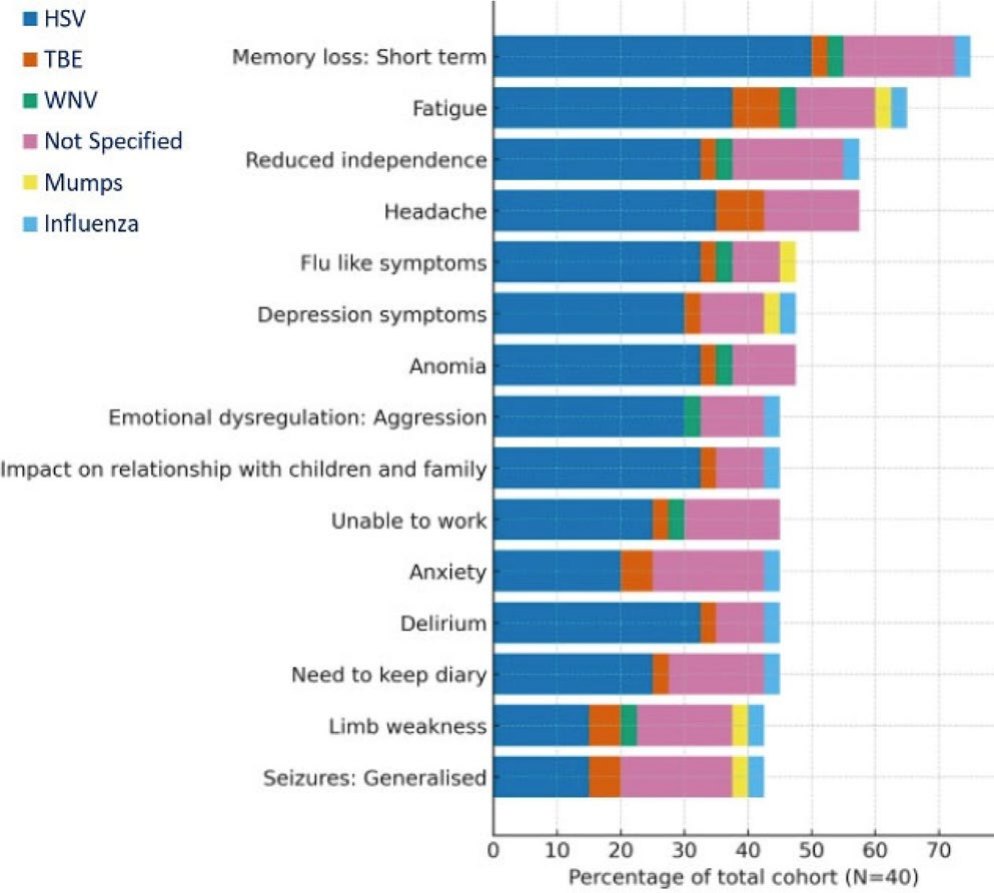
**FIGURE 1** A graph of the 20 most prevalent items identified in thematic analysis of semi‐structured interviews.

**Conclusion:** This series of semi‐structured interviews has generated 73 items, spanning across multiple domains. Common items have been shared across aetiologies, suggesting a generic PROM for IE is justifiable. The next steps in PROM development are to undertake further refinement of items and cognitive debriefing interviews to assess for content validity.

**Disclosure:** Nothing to disclose.

## EPO‐0102

### Post‐acute COVID‐19 effects on standardized cognitive and motor test scores in a longitudinal study of independent elderly subjects

#### N. Dunckley ^1^; N. Zhang^2^; C. Adler^3^; H. Shill^4^; S. Mehta^3^; E. Driver‐Dunckley^3^; C. Belden^1^; A. Atri^1^; P. Choudhury^1^; A. Kuramoto^1^; G. Serrano^1^; T. Beach^1^

##### ^1^Banner Sun Health Research Institute, Sun City, USA; ^2^Department of Quantitative Health Sciences, Mayo Clinic, Scottsdale, AZ, USA; ^3^Department of Neurology, Mayo Clinic College of Medicine, Scottsdale, USA; ^4^Department of Neurology, Barrow Neurological Institute, Phoenix, USA

**Background and aims:** There is considerable concern about long‐term consequences of COVID‐19, including cognitive and motor decline. Whether COVID‐19 adds burden beyond normal aging remains unclear, particularly in cohorts studying neurological disease. This study examined whether prior COVID‐19 illness was associated with differential cognitive or motor decline in older adults over time broadly.

**Methods:** Subjects were included if they were cognitively normal before the pandemic, completed a COVID questionnaire, and attended subsequent diagnostic conferences. All received serial evaluations with Montreal Cognitive Assessment (MoCA) and Unified Parkinson's Disease Rating Scale (UPDRS). We compared, between those reporting COVID‐19 and those denying it, conversion to cognitive impairment or dementia and changes in cognitive and motor scores longitudinally.

**Results:** 100 participants reported COVID‐19 and 71 denied infection. Illness severity was generally mild; none required ventilation. Cognitive and motor performance declined slightly without statistical significance. Conversion occurred in twenty‐six percent of COVID‐19 participants and thirty‐two percent of controls, a nonsignificant difference between groups during follow‐up across the study period. Logistic regression indicated that self‐reported COVID‐19 was not associated with diagnostic conversion, while greater age, male sex, possession of one or more apolipoprotein E‐ε4 alleles, and a diagnosis of probable Parkinson's Disease all conferred a greater likelihood of conversion.

**Figure:**
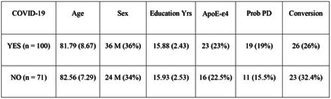
**TABLE 1** Demographic characteristics of subjects reporting having had COVID‐19 or not. Conversion refers to the percentage of subjects that converted from cognitively normal to cognitive impairment or dementia. No significant differences between groups.

**Figure:**
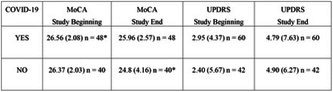
**TABLE 2** Comparison of pre‐ and post‐pandemic MoCA and UPDRS motor scores in subjects reporting having had or not having had COVID‐19.

**Figure:**
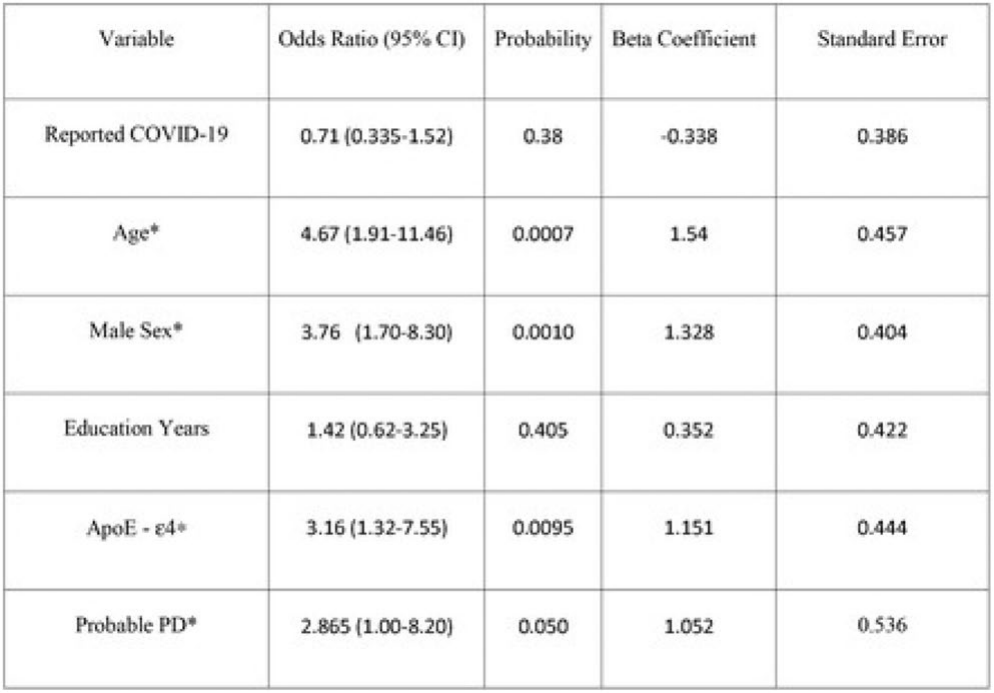
**TABLE 3** Logistic regression analysis of relationship of reported COVID‐19 on likelihood for conversion from cognitively normal to cognitively impaired or dementia, when also considering multiple covariates.

**Conclusion:** These findings suggest that mild COVID‐19 illness is not associated with greater cognitive or motor decline than expected from age‐related change alone. Limitations include modest sample size, potential misclassification of COVID‐19 status, and reliance on relatively crude screening measures that may miss subtle functional effects and longer‐term disease‐modifying consequences remain.

**Disclosure:** Nothing to disclose.

## EPO‐0103

### Temporal evolution of clinical and cognitive markers following viral encephalitis

#### N. van Andel; S. Staal; M. Brouwer; D. van de Beek; L. Ter Horst

##### Neurology, Amsterdam UMC, Amsterdam, The Netherlands

**Background and aims:** Objectives: To investigate cognitive functioning and clinical markers in patients shortly following viral encephalitis. In addition, the patients’ precepted impact of sequelae on their daily functioning.

**Methods:** Methods: Autoimmune Inflammation as cause of Relapsing Symptoms in patients with Viral Encephalitis (ARISE) was a prospective, observational, single‐centre cohort study in Amsterdam UMC, the Netherlands. All patients aged ≥ 16 years with viral encephalitis and confirmed pathogen were eligible for this study. Patients with prior cognitive impairment, recent neurosurgery or neuro‐devices were excluded. Follow‐up was at week 1, 4 and 12, and year 1 from the onset of viral encephalitis. Cognitive functioning was assessed each follow‐up. Blood samples were drawn at week 1, 4 and 12, and cerebrospinal fluid (CSF) from diagnostic lumbar puncture was collected, to be screened for neuron‐specific enolase (NSE), S100b and auto‐antibodies.

**Results:** Results: Eight patients were included between 2020 and 2024, of which 1 of 8 (13%) subjects had died during admission. Outcome at discharge was unfavourable in 7 of 8 (88%) patients. Follow‐up data was available for 7 patients. Cognitive functioning showed most pronounced deficits in short‐term memory and visuospatial/executive function. Patients reported perceiving to be most limited by physical health and energy. Daily functioning was impaired greatest in their speed, energy and attention. Median values for NSE and S100b did not exceed reference values in CSF or blood samples. No samples showed reactivity for auto‐antibodies.

**Figure:**
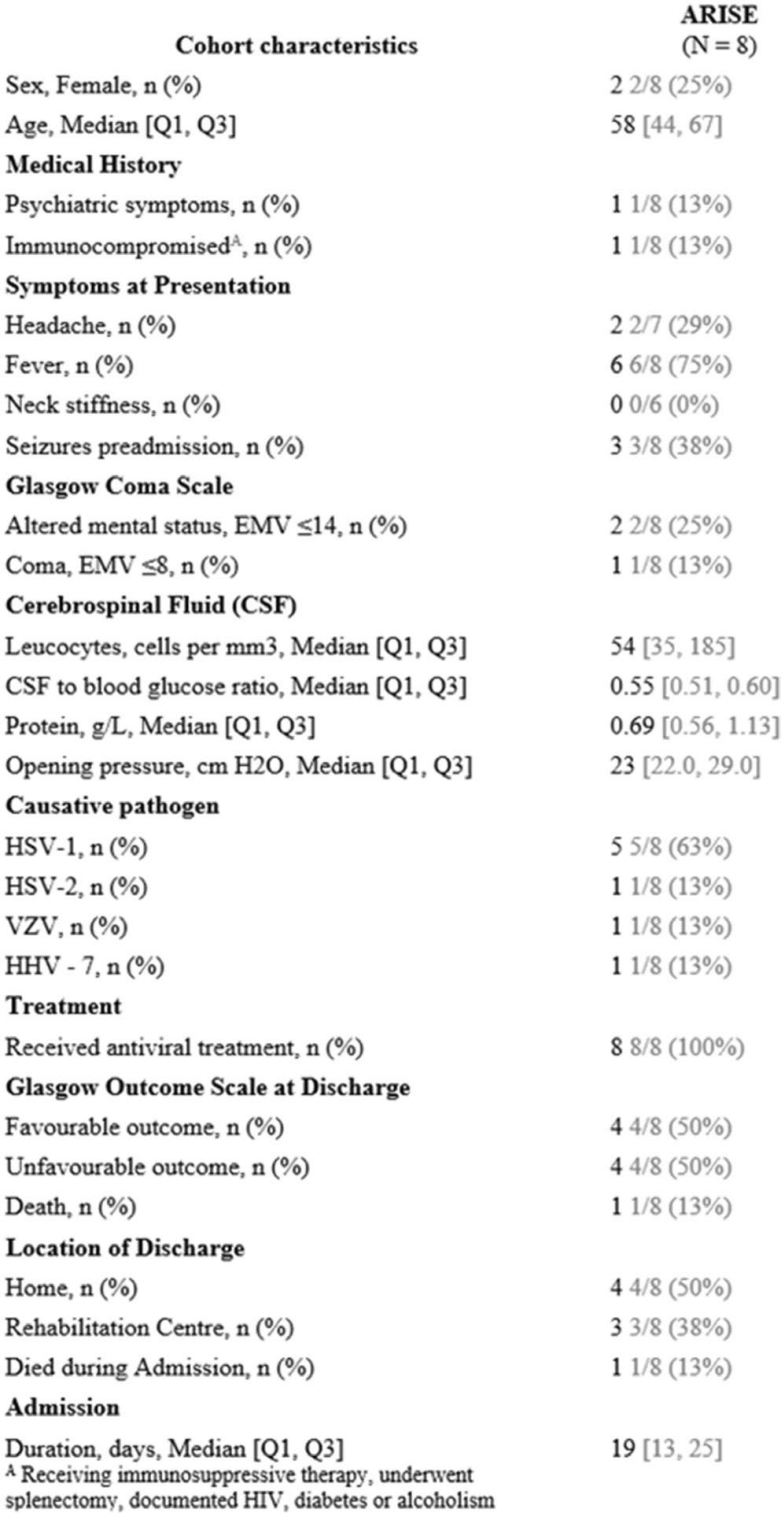
TABLE 1

**Figure:**
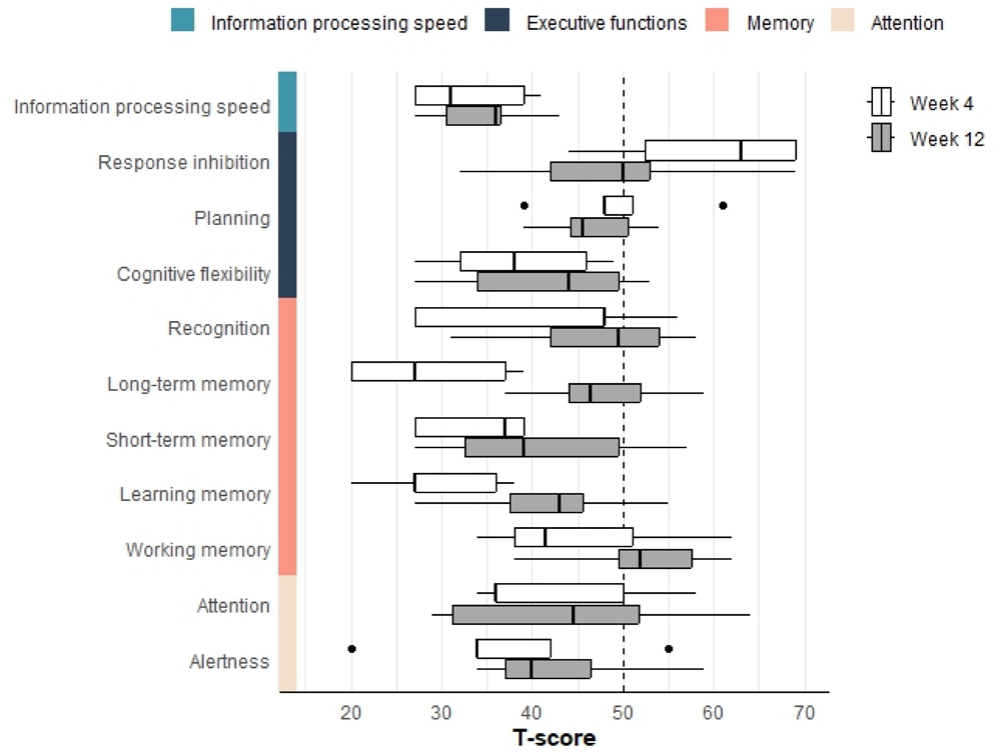
**FIGURE 1** COGBAT week 4 and week 12.

**Figure:**
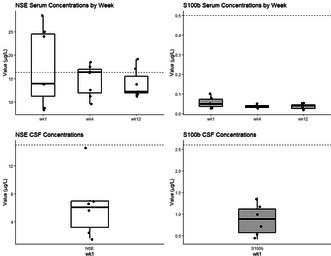
**FIGURE 2** NSE and S100b.

**Conclusion:** Conclusions: Post‐viral encephalitis results primarily in cognitive impairment of short‐term memory, speed, and attention, hindering daily functioning one year after onset.

**Disclosure:** Nothing to disclose.

## EPO‐0104

### Inkblot infarcts: A Distinctive MRI pattern of recurrent stroke in varicella zoster virus vasculopathy

#### R. Ramesh ^1^; L. Narasimhan R^1^; I. Arumugam^2^; S. Narayanasamy^3^; P. Srikanth^2^; S. Lakshmanan^3^; S. Shanmugam^1^

##### ^1^Department of Neurology, Sri Ramachandra Institute of Higher Education and Research; ^2^Department of Microbiology, Sri Ramachandra Institute of Higher Education and Research; ^3^Department of Internal Medicine, Sri Ramachandra Institute of Higher Education and Research

**Background and aims:** Varicella‐zoster virus vasculopathy (VZVV) can manifest with recurrent, antithrombotic‐refractory strokes in the absence of rash or prodrome, which presents a diagnostic and therapeutic challenge. This study aims to introduce the novel pattern in MRI brain, which we have named “Inkblot Infarct”, characterised by recurrent, slow‐expanding strokes in the same subcortical region, secondary to VZVV.

**Methods:** We prospectively analysed five adult patients presenting with recurrent ischemic strokes at a tertiary care centre. All patients underwent comprehensive vascular, cardiac, immune, and infectious evaluations, along with serial magnetic resonance imaging. VZV‐specific IgG antibody levels in cerebrospinal fluid (CSF) and serum were analysed. Patients were treated with acyclovir, and their clinical and radiological outcomes were assessed.

**Results:** All five patients demonstrated recurrent infarcts in the subcortical region, showing gradual expansion on serial imaging. None had a preceding rash or fever. Antiplatelets and anticoagulation therapy failed to prevent recurrence. Cerebrospinal fluid analysis revealed elevated anti–varicella zoster virus immunoglobulin G levels in all patients, with raised immunoglobulin G index where available. Following initiation of acyclovir, no further strokes occurred during follow‐up.

**Figure:**
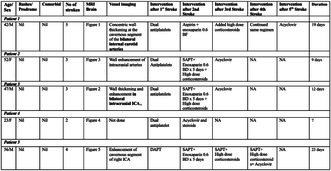
**TABLE 1** Patient Characteristics, clinical features, treatment and outcomes.

**Figure:**
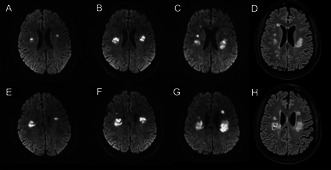
**FIGURE 1** MRI Brain diffusion‐weighted sequences, axial sections in patient 1 demonstrated restricted diffusion within the bilateral corona radiata and centrum semi‐ovale (A, E).

**Figure:**
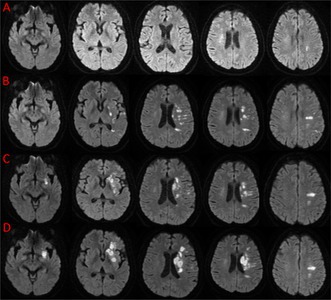
**FIGURE 2** Panels A–D show axial diffusion‐weighted MRI sequences from patient 2 over time, demonstrating the evolution of ischemic lesions in the brain at 5 anatomical levels (from left to right): the mesencephalic level, basal ganglia level, level above.

**Conclusion:** The “Inkblot Infarct” pattern represents a unique manifestation of VZVV‐related strokes, hypothesised to result from persistent viral antigen‐mediated vascular remodelling. Atypical presentations, such as absent rashes and delayed symptom progression, emphasise the need for heightened clinical suspicion. Recognition of this pattern may facilitate earlier diagnosis and targeted antiviral therapy in recurrent cryptogenic stroke.

**Disclosure:** Nothing to disclose.

## EPO‐0105

### Neurological post‐COVID condition: Cluster analysis reveals phenotypic spectrum defined by subjective symptom burden and pre‐morbid health status

#### S. Wetz; J. Walders; A. Costa; K. Reetz

##### Department of Neurology, University Hospital RWTH Aachen, Aachen, Germany

**Background and aims:** Post‐COVID‐19 condition (PCC) shows substantial heterogeneity, with primarily neurological symptoms. It is unclear whether this reflects distinct disease entities or a spectrum of a shared condition enriched for neurological manifestations.

**Methods:** We analyzed data from 509 PCC patients (353 females, 48.15 ± 12.73 years), seen at the RWTH Aachen neurological Post‐COVID‐19 outpatient clinic from 2021 to 2024. Clinical history, acute and persistent (>12 weeks) symptom profiles, patient‐reported outcome measures (PROMs) and cognitive performance were assessed. Multiple correspondence analysis followed by hierarchical clustering was used to derive data‐driven phenotypes, which were compared across symptoms, PROMs and organ involvement.

**Results:** Three clusters emerged that aligned along a pronounced severity and multisystem‐complexity continuum. Cognitive complaints were frequent across all clusters, but differed by overall symptom burden, functional impact, and extent of system involvement. Key clinical markers showed stepwise gradients from high‐to‐low‐burden phenotypes, including severe fatigue (FSMC: 61.0% vs 23.8% vs 22.5%), impaired cognition (MoCA: 25.4% vs 11.9% vs 5.6%), depressive symptoms (HADS: 44.1% vs 18.4% vs 9.9%) and arthralgia (96.6% vs 75.5% vs 19.7%). The high‐burden phenotype combined prominent neurocognitive symptoms with dyspnea, sensory/chemosensory disturbances, pain and higher psychological burden. Prior vaccination was more frequent in the lower‐burden group, while psychiatric comorbidities and higher BMI were enriched towards the high‐burden end.

**Figure:**
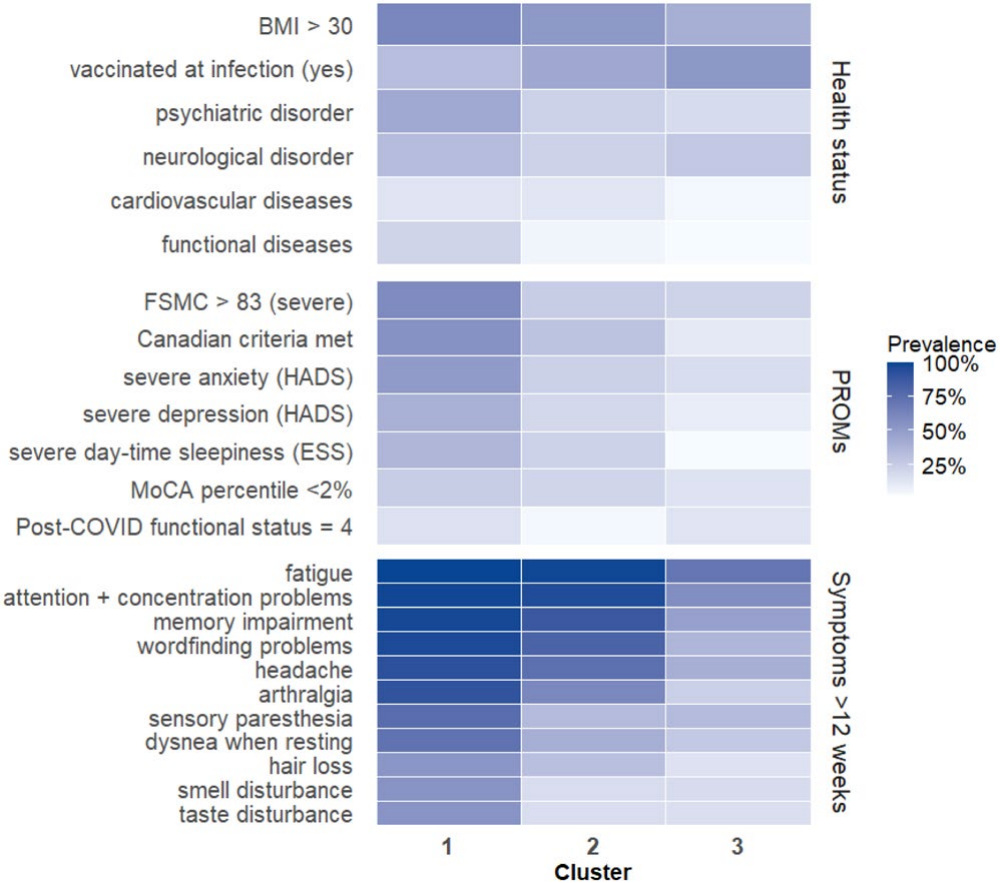
**FIGURE 1** Heatmap for health status indicators, patient‐reported outcome measures (PROMs) and symptom prevalence per cluster. Illustrated is the respective percentage of patients. Abbreviations: BMI: Body Mass Index; FSMC: Fatigue Scale for Motor and Cog.

**Figure:**
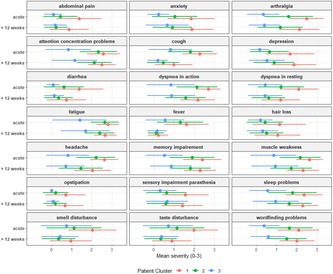
**FIGURE 2** Subjective severity rating per cluster for acute and persisting (>12 weeks) symptoms. The mean severity rating and standard deviation is shown for each patient cluster for symptoms in the acute infectious and post‐infectious stage after 12 weeks.

**Figure:**
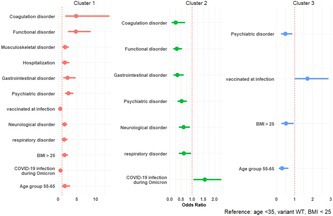
**FIGURE 3** Pre‐existing morbidities and health status associations with neurological PCC severity. The Odds Ratio is shown for neurological PCC patients to be categorized in a cluster based on their pre‐morbid health status in a One‐vs‐rest approach for each cluster. The reference for age was <35, BMI reference was <25, and COVID‐19 infection timepoint reference was during wild‐type (WT) variant wave. For the remaining factors, absence of the condition was used as reference.

**Conclusion:** Data‐driven phenotyping supports a spectrum model in which neurological symptoms are common throughout. However, severity and disease complexity is driven by non‐neurological factors (i.e. vaccination, metabolic health, comorbidities) underscoring the need of a coordinated multidisciplinary management even for patients presenting primarily with neurological symptoms.

**Disclosure:** Nothing to disclose.

## Motor Neurone Diseases 1

## EPO‐0106

### Efficacy and tolerability of β‐hydroxybutyrate in patients with ALS (KETO‐ALS): A randomised, placebo‐controlled, double‐blind study

#### C. Herrmann; J. Dorst

##### Clinic for Neurology, Ulm Neurology, Germany

**Background and aims:** Hypermetabolism and weight loss are prominent features of amyotrophic lateral sclerosis (ALS). Here, we sought to target the energy deficit in ALS by applying oral ketone bodies as high‐energetic substrates.

**Methods:** Patients with ALS were enrolled to this randomised, placebo‐controlled, double‐blind, monocentre phase 2 trial. Eligible patients had a decline of ALS functional rating scale revised (ALSFRS‐R) of at least 0·33 points/month. They were randomly assigned (1:1) to receive β‐hydroxybutyrate‐ester (βHB, 30 g/day) or placebo. Primary endpoint was change of neurofilament light chain (NfL) per month, measured as slope over 6 months. Multiple linear regressions models were applied to adjust for NfL, ALSFRS‐R, age, and slow vital capacity (SVC) at baseline. The trial is registered with clinicaltrials.gov, number NCT04820478.

**Results:** Between April 5, 2022, and February 12, 2025, 81 patients were randomly assigned to receive βHB (*n* = 40) or placebo (*n* = 41) and included in the intention‐to‐treat analysis. NfL slope was −0.5 pg/mL (IQR −3.2 to 2.0, minimum −11.2, maximum 10.4) in the ¬βHB group and 0.0 pg/mL (IQR −1.7 to 3.3, minimum −6.2, maximum 49.0) in the placebo group (*p* = 0.19). In the adjusted analysis, both NfL (*p* = 0.04) and BMI slope (*p* = 0.05) showed a benefit for the βHB group. ¬βHB was well tolerated.

**Figure:**
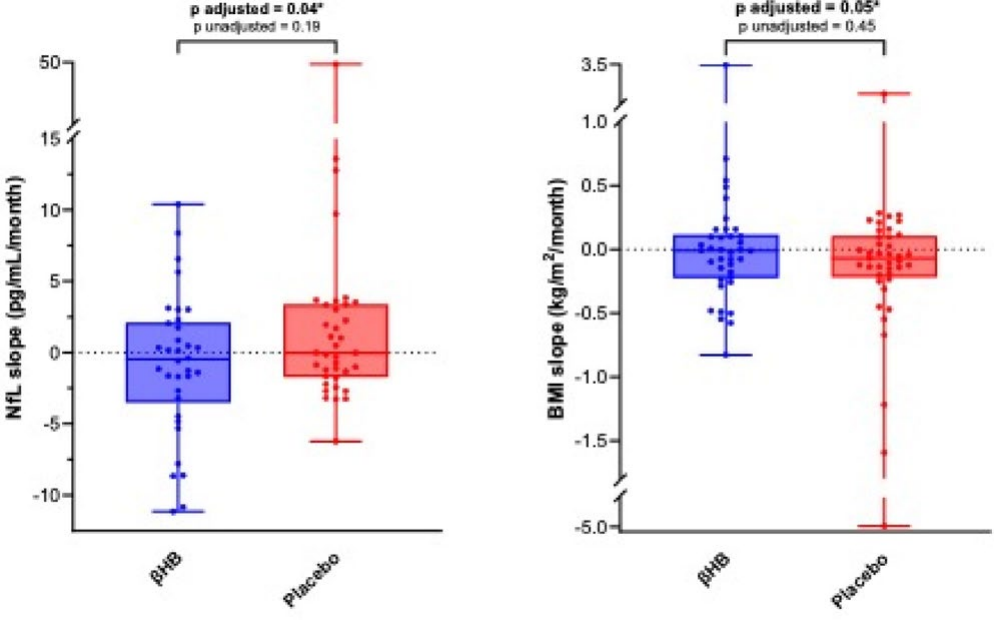
**FIGURE 1** NfL and BMI slope.

**Figure:**
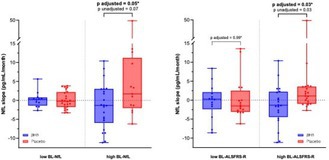
**FIGURE 2** NfL subgroups.

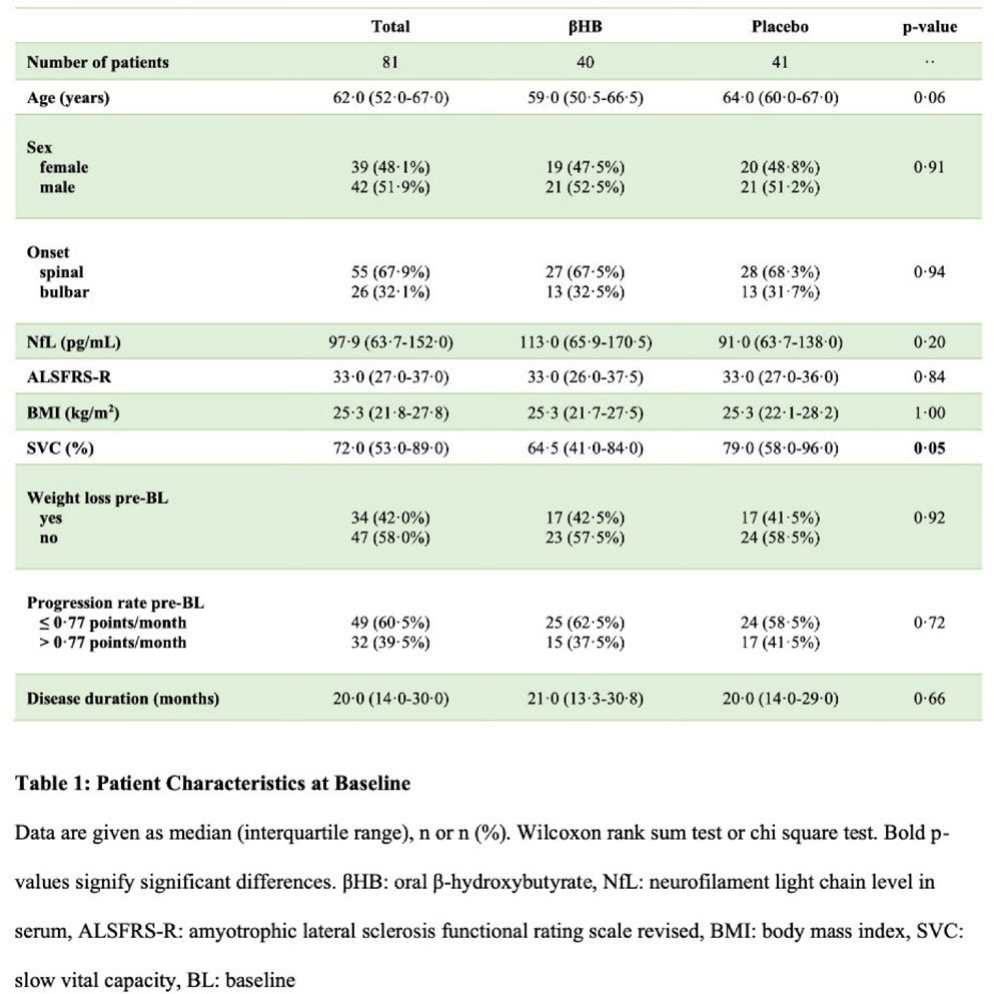

**Conclusion:** This study provides preliminary evidence for efficacy and safety of oral ketone bodies in ALS by demonstrating target engagement and a reduction of NfL. Whether these effects can be translated into meaningful clinical effects remains to be clarified by a larger phase 3 trial with extended treatment duration.

**Disclosure:** Nothing to disclose.

## EPO‐0107

### Clinical implications of retinal markers in amyotrophic lateral sclerosis: A systematic review

#### E. Assis ^1^; H. Swamy^1^; M. Reis^2^; A. Petzold^3^; H. Özdemir^4^

##### ^1^Faculty of Medicine, Immanuel Kant Baltic Federal University, Kaliningrad, Russian Federation; ^2^Faculty of Medicine, Universidad Nacional de Rosario, Rosario, Argentina; ^3^Queen Square Institute of Neurology, University College London, London, UK; ^4^Neurology Department, Medical School Hospital, Ege University, İzmir, Türkiye

**Background and aims:** Amyotrophic lateral sclerosis (ALS) is a fatal, clinically heterogeneous, multisystem neurodegenerative disease, underscoring the need for biomarkers beyond motor decline. Optical coherence tomography (OCT) enables non‐invasive retinal assessment and may identify relevant retinal biomarkers in ALS. Therefore, we systematically synthesised and evaluated the evidence on OCT‐derived retinal biomarkers in ALS and their clinical relevance.

**Methods:** A systematic review was reported in accordance with the PRISMA statement and prospectively registered in PROSPERO (CRD420251242585). We searched MEDLINE, Embase, Scopus, and the Cochrane Library for observational studies reporting OCT‐derived retinal biomarkers in adults with ALS, excluding non‐eligible designs. The primary outcome was the association between retinal OCT measures and functional severity. Risk of bias was assessed using QUIPS.

**Results:** Fifteen studies involving 1,301 patients, published between 2013 and 2025, were included, comprising predominantly cross‐sectional designs with three longitudinal studies. Average peripapillary retinal nerve fibre layer (pRNFL) thinning was reported in eight studies, while several others demonstrated thinning confined to specific quadrants. Five studies reported thinning in inner retinal layers or macular thickness. Seven studies reported significant correlations between optical coherence tomography parameters and ALSFRS‐R score. All longitudinal studies consistently demonstrated progressive pRNFL thinning over follow‐up in ALS patients. One study reported a significant correlation between RNFL thickness and respiratory function (FVC% and FEV1%), while another found no such association.

**Conclusion:** OCT‐derived retinal changes, particularly pRNFL thinning, are reported in ALS and show associations with functional severity, with longitudinal studies suggesting progressive retinal degeneration. Larger, standardised longitudinal studies are needed to define prognostic value and clinical utility.

**Disclosure:** Nothing to disclose.

## EPO‐0108

### Cortical motor mapping in ALS: Investigating hand motor representation across disease phenotypes

#### G. Fanella ^1^; B. Bardel^2^; A. Bystrup Jacobsen^1^; T. Mussigmann^3^; J. Lefaucheur^2^; H. Tankisi^4^

##### ^1^Department of Clinical Medicine, Aarhus University, Aarhus, Denmark; ^2^Department of Clinical Neurophysiology, DMU FIxIT, Henri Mondor University Hospital, APHP, Creteil, France; ^3^UR 4391, ENT Team, Faculty of Health, Paris Est Creteil University, Creteil, France; ^4^Department of Clinical Neurophysiology, Aarhus University Hospital, Aarhus, Denmark

**Background and aims:** Amyotrophic lateral sclerosis (ALS) is clinically heterogeneous, with phenotype‐dependent variation in cortical excitability. Reduced short‐interval intracortical inhibition (SICI) on transcranial magnetic stimulation (TMS) is a recognised hallmark of ALS, but its role as pathogenic driver versus compensatory plasticity remains unclear. Navigated TMS (nTMS) motor mapping quantifies cortical motor representations and may capture degeneration and plasticity. We examined whether motor map volume differs across phenotypes and relates to SICI, resting motor threshold (RMT), and lower motoneuron (LMN) involvement.

**Methods:** Twenty ALS patients were consecutively recruited and classified by phenotype. Bilateral nTMS motor mapping of first dorsal interosseous (FDI) was performed. Map volumes were derived by segmentation in 3D Slicer. Threshold‐tracking SICI was recorded at the FDI hotspot. LMN involvement was quantified using Neurophysiological Index (NI). A 6‐month follow‐up is ongoing.

**Results:** Mean age was 65.8 (±11.1) years; 17/20 were males. Mean disease duration was 21.2 months (±10.8) and ALS Functional Rating Scale‐Revised (ALSFRS‐R) was 41.1 (±3.4). Onset was spinal in 13 and bulbar in 7. Phenotypes were classical (*n* = 9), bulbar (*n* = 7), pyramidal (*n* = 4). Map volumes and SICI did not differ by onset site or phenotype. Map volume was inversely associated with RMT (*R* ^2^ = 0.22; *p* = 0.042). RMT correlated positively with disease duration (rho = 0.48; *p* = 0.039). Map volume was not associated with SICI, NI, or disease duration.

**Conclusion:** Cortical motor representation appears unrelated to SICI and LMN measures. The positive correlation between RMT and disease duration suggests a stage‐dependent increase in motor threshold that may influence map volume. Follow‐up will examine longitudinal changes.

**Disclosure:** Nothing to disclose.

## EPO‐0109

### REST inhibition as a therapeutic and prognostic target in amyotrophic lateral sclerosis: From siRNA silencing to serum biomarkers

#### G. Senerchia ^1^; N. Guida^2^; V. Valsecchi^2^; S. Anzillotti^2^; G. Pignataro^2^; L. Annunziato^2^; L. Formisano^2^; R. Dubbioso^1^

##### ^1^Clinical Neurophysiology Unit, Department of Neurosciences, Reproductive Sciences and Odontostomatology, University of Naples, Naples, Italy; ^2^Division of Pharmacology, Department of Neurosciences, Reproductive Sciences and Odontostomatology, University of Naples, Naples, Italy

**Background and aims:** The Restrictive Element‐1 Silencing Transcription factor (REST) acts as a master repressor of neuronal gene expression. Aberrant REST accumulation has been linked to several neurodegenerative conditions, but its role in amyotrophic lateral sclerosis (ALS) remains unclear.

**Methods:** REST expression was analyzed in the motor cortex, brainstem, and spinal cord of SOD1‐G93A mice at different disease stages. To assess causality, intracerebroventricular injections of a small interfering RNA targeting REST (siREST) were performed weekly for four weeks starting at the presymptomatic stage (day 90). Behavioral, histological, and molecular analyses were conducted to evaluate motor performance, neuronal survival, SOD1 aggregation, and glial activation. In parallel, REST serum levels were quantified by ELISA in ALS patients and healthy controls, and correlated with clinical outcomes.

**Results:** REST levels were markedly increased in the CNS of SOD1‐G93A mice during both early and late symptomatic phases. siREST treatment significantly reduced REST expression, prevented motor neuron loss, decreased misfolded SOD1 aggregates, and attenuated astro‐ and microgliosis. These effects translated into improved motor performance and prolonged survival (133.2 ± 1.7 vs 124.3 ± 2.1 days, *p* < 0.01). In the clinical cohort, serum REST was significantly higher in ALS patients than in controls (*p* = 0.006). Elevated REST levels independently predicted poorer tracheostomy‐free survival (multivariate HR = 13.43, 95% CI: 2.1–86.0, *p* = 0.006).

**Figure:**
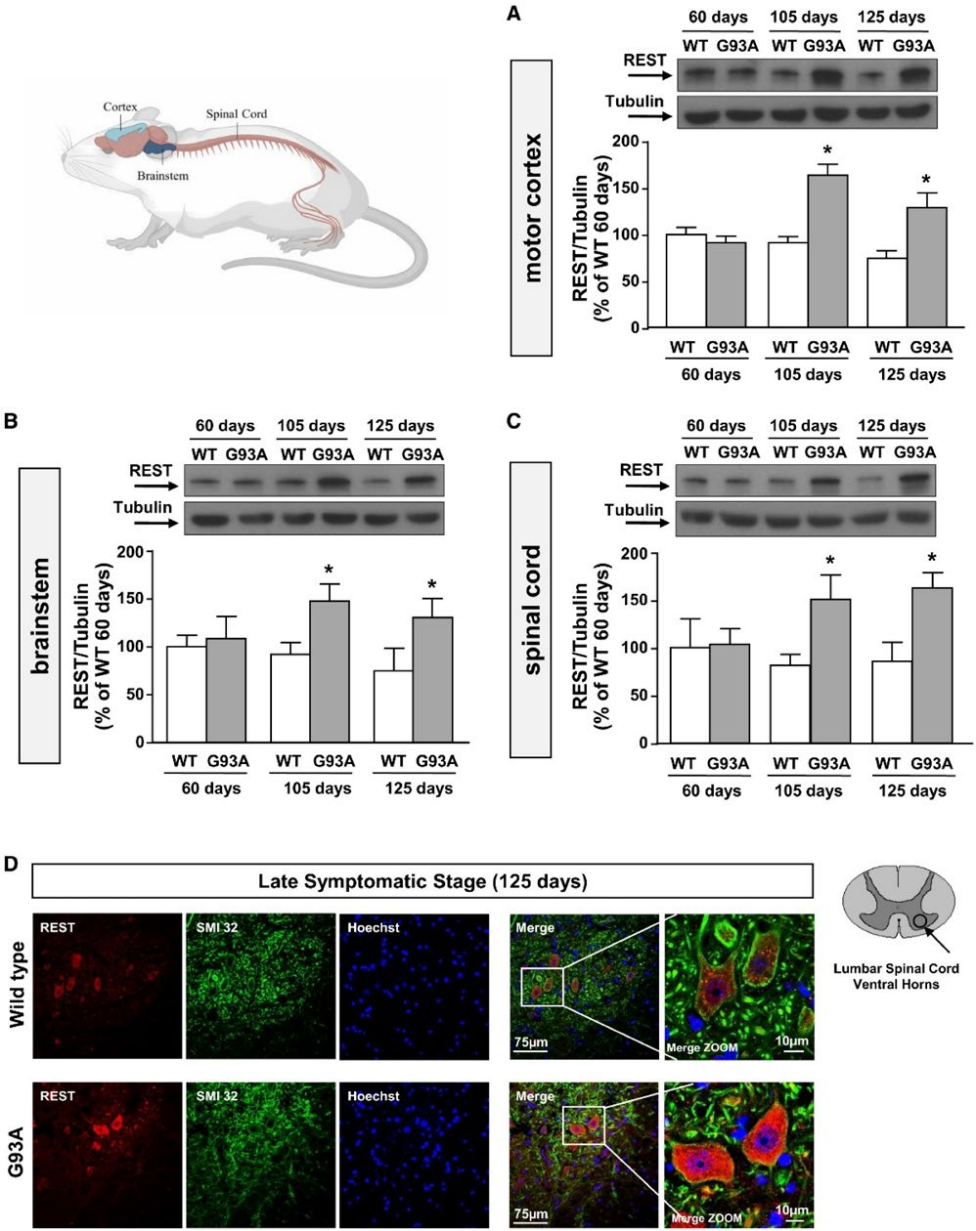
**FIGURE 1** Characterization of REST protein expression in motor cortex, brainstem, and spinal cord of G93A mice, compared with WT mice, at early symptomatic phase or late phase of the disease.

**Figure:**
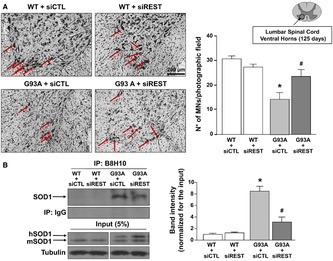
**FIGURE 2** Effect of siRNA against REST on MN survival and formation of misfolded SOD1 aggregates in fully symptomatic G93A mice compared with age‐matched WT mice.

**Figure:**
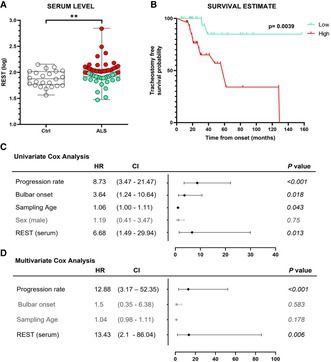
**FIGURE 3** Serum levels and prognostic value of REST protein in ALS patients.

**Conclusion:** REST upregulation contributes to ALS pathogenesis by promoting neuronal death and neuroinflammation. Its silencing through siRNA confers neuroprotection and survival benefit in ALS mice. Elevated serum REST mirrors CNS pathology and predicts disease progression, supporting REST as a novel therapeutic and prognostic target in ALS.

**Disclosure:** Nothing to disclose.

## EPO‐0110

### Fractional anisotropy of the corticospinal tract as a predictor of survival in amyotrophic lateral sclerosis: A DTI study

#### I. Bottale ^1^; A. Ghirelli^1^; F. De Mattei^2^; E. Spinelli^1^; S. Basaia^3^; M. Stanziano^4^; L. Lequio^5^; F. Denegri^5^; M. Valentini^5^; A. Calvo^2^; A. Chiò^2^; F. Agosta^1^; C. Moglia^2^; M. Filippi^6^

##### ^1^Neuroimaging Research Unit, Division of Neuroscience, and Neurology Unit, IRCCS San Raffaele Scientific Institute, Milan, Italy; and Vita‐Salute San Raffaele University, Milan, Italy; ^2^ALS Center, 'Rita Levi Montalcini' Department of Neuroscience, University of Turin, Turin, Italy; and SC Neurologia 1U, AOU Città della Salute e della Scienza di Torino, Turin, Italy; ^3^Neuroimaging Research Unit, Division of Neuroscience, IRCCS San Raffaele Scientific Institute, Milan, Italy; ^4^ALS Center, 'Rita Levi Montalcini' Department of Neuroscience, University of Turin, Turin, Italy; and Neuroradiology Unit, Fondazione IRCCS Istituto Neurologico Carlo Besta, Milan, Italy; ^5^Neuroradiology Unit, CTO Hospital, AOU Città della Salute e della Scienza di Torino, Turin, Italy; ^6^Neuroimaging Research Unit, Division of Neuroscience, Neurology Unit, Neurorehabilitation Unit, and Neurophysiology Service, IRCCS San Raffaele Scientific Institute, Milan, Italy; and Vita‐Salute San Raffaele University, Milan, Italy

**Background and aims:** Diffusion tensor imaging (DTI) metrics of key white matter (WM) tracts in amyotrophic lateral sclerosis (ALS) are crucial for assessing upper motor neuron involvement and predicting disease progression and survival. This study investigated whether major WM tracts predict survival in ALS and explored differences between spinal‐ and bulbar‐onset cases.

**Methods:** A total of 411 ALS were recruited at the Turin ALS Centre (2008–2021) and underwent 1.5 Tesla brain MRI with DTI. Fractional anisotropy (FA) was extracted from the bilateral corticospinal tracts (CST; averaged) and the corpus callosum (CC). Survival was censored in January 2024. Demographic and clinical differences were assessed. Cox proportional hazards models evaluated the association between FA and survival, adjusting for age at MRI, sex, and disease duration, in the full cohort and stratified by onset site.

**Results:** In the overall cohort, higher CST FA was significantly associated with longer survival (HR = 0.0003, 95% CI: 6.20 × 10^−6^ – 0.0158, *p* < 0.001). When stratified by onset site, CST FA remained a strong predictor of survival in spinal‐onset ALS (HR = 5.82 × 10^−5^, 95% CI: 4.66 × 10^−7^ – 0.0073, *p* < 0.001). In bulbar‐onset ALS, CST FA showed a trend toward predicting survival but did not reach statistical significance (HR = 0.0008, 95% CI: 4.15 × 10^−7^ – 1.46, *p* = 0.063). FA of the CC was not significantly associated with survival.

**Conclusion:** CST integrity is a strong predictor of survival in ALS, particularly in spinal‐onset disease, supporting CST FA as a prognostic imaging biomarker.

**Disclosure:** Supported by Italian Ministry of Health (GR‐2019‐12371291), and Next GenerationEU/National Recovery and Resilience Plan, Investment PE8‐Project Age‐It. A. Chiò serves on scientific advisory boards for Mitsubishi Tanabe, Biogen, Roche, Denali Pharma, Cytokinetics, Lilly, and Amylyx Pharmaceuticals and has received a research grant from Biogen. E.G. Spinelli and G. Cecchetti have received speaker honoraria from Eli Lilly. C. Moglia, E. Canu and S. Basaia received research support from the Italian Ministry of Health. M. Filippi is Editor‐in‐Chief of the Journal of Neurology and Associate Editor of Human Brain Mapping, Neurological Sciences, and Radiology; received compensation for consulting services from Almirall, Biogen, Bristol‐Myers Squibb, Eli Lilly, Merck, Novartis, Roche, Sanofi; speaking activities from Amgen, Bayer, Biogen, Bristol‐Myers Squibb, Celgene, Chiesi Italia SpA, Eisai, Eli Lilly, Fujirebio, Genzyme, Janssen, Merck, Neopharmed Gentili, Neuraxpharm, Novartis, Novo Nordisk, Roche, Sanofi, Takeda; participation in Advisory Boards for Alexion, Biogen, Bristol‐Myers Squibb, Eli Lilly, GE Healthcare Ltd, Merck, Neuraxpharm, Novartis, Roche, Sandoz, Sanofi, Takeda; scientific direction of educational events for Biogen, Merck, Roche, Celgene, Bristol‐Myers Squibb, Lilly, Novartis, Sanofi‐Genzyme; he receives research support from Biogen Idec, Merck‐Serono, Novartis, Roche, the Italian Ministry of Health, the Italian Ministry of University and Research, and Fondazione Italiana Sclerosi Multipla. F. Agosta is Associate Editor of NeuroImage: Clinical and the European Journal of Neurology; has received speaker honoraria from Biogen Idec, Bristol Myers Squibb, Eisai, Eli Lilly, GE Healthcare, Neuraxpharm, and Roche,; and receives or has received research supports from the Italian Ministry of Health, the Italian Ministry of University and Research, AriSLA (Fondazione Italiana di Ricerca per la SLA), the European Research Council (ERC), the EU Joint Programme – Neurodegenerative Disease Research (JPND), and Foundation Research on Alzheimer Disease (France). None of the other authors has any conflict of interest to disclose.

## EPO‐0111

### Development and validation of a classification method and personalised prediction models for patients with amyotrophic lateral sclerosis

#### J. Zhang; S. Ye; L. Chen; X. Liu; D. Fan

##### Department of Neurology, Peking University Third Hospital, Beijing, China

**Background and aims:** The heterogeneity of amyotrophic lateral sclerosis (ALS) in clinical presentations and prognosis poses a significant obstacle to the successful translation of research into therapeutic solutions. A classification method with high stability, excellent clinical applicability and strong generalizability across populations remains to be further investigated. No parametric prognostic model has yet been established for Chinese patients with ALS.

**Methods:** This retrospective cohort study involved two large cohorts: the Peking University Third Hospital(PUTH) ALS cohort and a Caucasian ALS cohort derived from Pooled Resource Open‐Access ALS Clinical Trial (PRO‐ACT) database and the Answer ALS database.

**Results:** 603 patients from PUTH ALS cohort and 3610 patients from Caucasian ALS cohort were enrolled in this study. Statistical analysis suggested that patients could be stratified into three progression groups‐‐high‐risk(HR), moderate‐risk(MR), and low‐risk (LR)‐‐based on diagnostic delay, progression rate, and FVC, namely the DRF classification method. More than 83% of patients remained in the same group 1 year after the initial assessment. The median survival time of PUTH ALS patients with HR progression is 6 months shorter than that of Caucasian ALS patients. PUTH ALS patients with HR progression are more likely to experience group transitions during follow‐up compared to Caucasian ALS patients. Our survival models enable the estimation of the survival probability for individual ALS patients.

**Conclusion:** DRF classification method is an ideal choice for stratification in future multinational clinical trials. The prediction of individual disease progression and prognosis is essential not only for personalized treatment strategies but also for the evaluation of clinical trials outcomes.

**Disclosure:** Nothing to disclose.

## EPO‐0112

### Scoliosis in patients with spinal muscular atrophy treated with disease‐modifying therapies: A pilot observational study

#### N. Ait Allali ^1^; B. Risi^2^; S. Damioli^1^; G. Giovanelli^1^; E. Franchi^1^; G. Gilberti^3^; A. Sorgente^1^; G. Garletti^1^; F. Nicolis^1^; M. Puzzi^1^; F. Garofali^1^; R. Carugati^1^; S. Pianto^1^; I. Urbano^1^; C. Colombi^1^; E. Ottelli^1^; E. Ferrari^1^; A. Padovani^4^; M. Filosto^5^

##### ^1^NeMO‐Brescia Clinical Center for Neuromuscular Diseases, Brescia, Italy; ^2^NeMO‐Brescia Clinical Center for Neuromuscular Diseases, Brescia, Italy; Department of Molecular and Translational Medicine, University of Brescia, Brescia, Italy; ^3^NeMO‐Brescia Clinical Center for Neuromuscular Diseases, Brescia, Italy; Department of Clinical and Experimental Sciences, University of Brescia, Brescia, Italy; Unit of Neurology, ERN EURO‐NMD Center ASST Spedali Civili, Brescia, Italy; ^4^NeMO‐Brescia Clinical Center for Neuromuscular Diseases, Brescia, Italy; Department of Clinical and Experimental Sciences, University of Brescia, Brescia, Italy; ^4^Department of Clinical and Experimental Sciences, University of Brescia, Brescia, Italy; Unit of Neurology, ERN EURO‐NMD Center ASST Spedali Civili, Brescia, Italy

**Background and aims:** Disease‐modifying therapies (DMTs) have modified the clinical course of spinal muscular atrophy (SMA), improving survival and motor outcomes. Nonetheless, scoliosis remains frequently observed.

**Methods:** Nine SMA patients receiving DMTs (3 SMA1, 6 SMA3; mean age 17.6 ± 13.1 years) were enrolled at baseline (T0). Scoliosis was assessed using Cobb angle. Clinical history and evaluations were collected including CHOP ‐INTEND, HFMSE, RULM, MFM‐32, forced vital capacity (FVC), SMA Independence Scale–Clinical Report (SMAIS‐CR) and SF‐36 questionnaire. Associations between Cobb angle and clinical measures were explored using Spearman's rank correlation at baseline and after 1 year follow‐up (T1).

**Results:** At baseline, scoliosis was observed in 78% of patients (*n* = 7). SMA 1 patients presented more severe scoliosis (mean Cobb angle at T0: 45.0° ± 26.0°, at T1: 48.8° ± 47.7°), with one patient undergoing stabilization intervention. SMA 3 patients showed milder curves (mean Cobb angle at T0: 18.5° ± 4.0°, at T1: 19.3° ± 5.1°). Associations with Cobb angle were found at T0 with RULM (*r* = −0.832, *p* = 0.040), at T1 with FVC in liters (*r* = 0.900, *p* = 0.037) and SMAIS‐CR (*r* = −0.900, *p* = 0.037). These associations should be interpreted cautiously, as increases in absolute FVC may reflect growth or treatment‐related effects rather than respiratory function changes per se.

**Figure:**
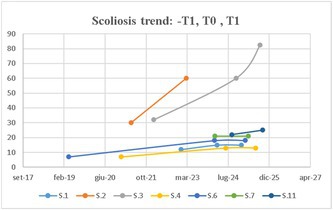
**FIGURE 1** Scoliosis progression graph. On the X‐axis, it is shown the time‐line; on the Y‐axis, Cobb angles. For patient S.1, S.3, S.4 and S.6, there are three time‐points available (restrospective, baseline and at 1‐year follow‐up).

**Figure:**
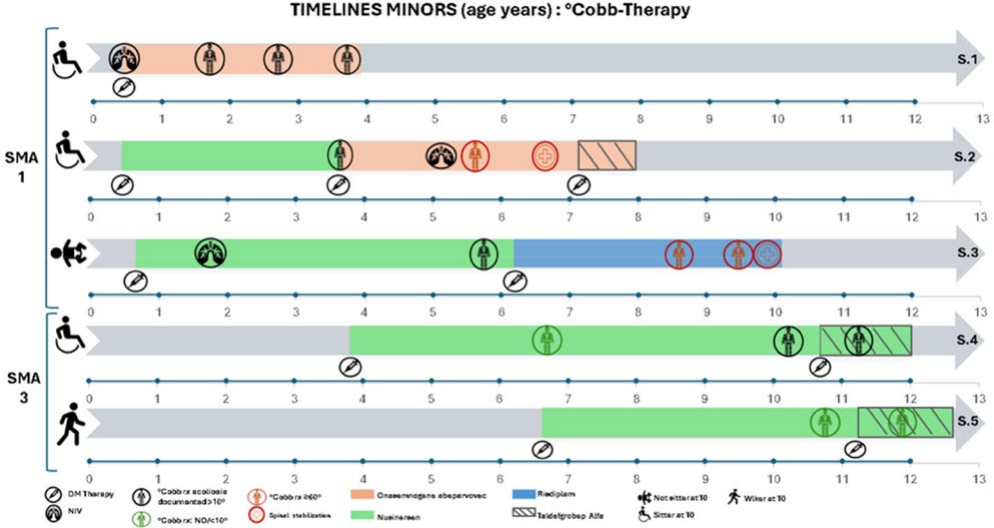
**FIGURE 2** Timeline illustrating the clinical history of individual pediatric patients by age (years), including baseline motor function (T0), phenotype, scoliosis, disease‐modifying therapies (DMTs), clinical stabilization, and respiratory support (NIV).

**Figure:**
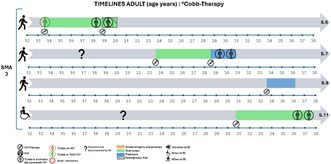
**FIGURE 3** Timeline illustrating the clinical history of individual adult patients by age (years), including baseline motor function (T0), phenotype, scoliosis, disease‐modifying therapies (DMTs), clinical stabilization, and respiratory support (NIV).

**Conclusion:** In this small exploratory‐cohort, scoliosis was frequent in SMA patients receiving DMTs and appeared to co‐occur with motor performance and daily functioning. Larger longitudinal studies are needed to better define scoliosis trajectories and clinical relevance in the DMTs‐era.

**Disclosure:** Nothing to disclose.

## EPO‐0113

### Multimodal biomarkers for early diagnosis of amyotrophic lateral sclerosis: Integration of transcranial magnetic stimulation and radiomics MRI

#### R. Tolay ^1^; B. Dik^2^; O. Kaya^2^; H. Uysal^3^; A. Koc^1^

##### ^1^Department of Neurology, Cukurova University Faculty of Medicine, Adana, Türkiye; ^2^Department of Radiology, Cukurova University Faculty of Medicine, Adana, Türkiye; ^3^Independent researcher, Adana, Türkiye

**Background and aims:** Early diagnosis of amyotrophic lateral sclerosis (ALS) remains challenging due to the limited sensitivity of conventional upper motor neuron markers. Integration of multimodal biomarkers combining neurophysiological and neuroimaging approaches may improve diagnostic accuracy, particularly in early disease stages.

**Methods:** In this prospective study, 74 patients with amyotrophic lateral sclerosis and 38 healthy controls were included. ALS patients were stratified into mild and moderate–advanced stages according to ALSFRS‐R scores. Bilateral transcranial magnetic stimulation (TMS) was applied to the abductor pollicis brevis and first dorsal interosseous muscles to assess resting motor threshold (RMT), cortical and radicular latencies, central motor conduction time (CMCT), and short‐interval intracortical inhibition (SICI). Radiomics features (*n* = 555), including texture and shape parameters, were extracted from the perirolandic cortex, posterior limb of the internal capsule, and bulbus on MRI. Feature selection was performed using the Boruta algorithm, followed by LASSO and Random Forest analyses.

**Results:** ALS patients demonstrated significant alterations in TMS parameters, including reduced SICI, altered RMT, and prolonged CMCT. Among neurophysiological measures, SICI at 3 ms ISI showed the highest diagnostic performance (AUC = 0.846). Radiomics analysis revealed increased entropy and reduced homogeneity in motor‐related regions. Multimodal models integrating TMS and radiomics MRI consistently outperformed single‐modality approaches and achieved excellent discrimination of early‐stage ALS from healthy controls (AUC = 0.984).

**Conclusion:** Multimodal integration of transcranial magnetic stimulation and radiomics MRI improves discrimination of early‐stage ALS and supports the potential value of combined functional and structural biomarkers.

**Disclosure:** Nothing to disclose.

## EPO‐0114

### Reduced nuclear TDP‐43 and cytoplasmic DLK1 as markers of motor neuron degeneration in amyotrophic lateral sclerosis

#### T. Takeda; A. Ishikawa; S. Kokubun; Y. Saito; S. Isose; K. Ito; K. Arai; K. Honda

##### Department of Neurology, National Hospital Organization Chiba Medical Center Chibahigashi National Hospital, Chiba, Japan

**Background and aims:** Loss of upper and lower motor neurons (MNs) is a defining pathological feature underlying the clinical manifestations of amyotrophic lateral sclerosis (ALS). However, the differences in MN loss and TDP‐43 pathology between these areas in ALS patients remain unknown.

**Methods:** This study included seven patients with ALS and three controls from autopsies performed. The cell density and regional density of TDP‐43‐positive inclusions in four upper MN areas (tongue, arm, trunk, and leg) and their anatomically corresponding lower MN areas were measured. The cell residual rate by comparing the cell density of each case to the average density of the controls was calculated. The numbers of large cells with loss of nuclear TDP‐43 and cytoplasmic delta‐like 1 homolog (DLK1), a MN‐related factor involved in its differentiation, were counted.

**Results:** The results showed severe MN loss in both upper and lower MN areas; however, TDP‐43‐positive inclusions differed markedly: they were rare in upper MNs but abundant in lower MN. Although in upper MN areas, TDP‐43 density was not associated with the residual rate of MNs, the density in MNs in lower MN areas was well associated with the cell residual rate. Significantly higher numbers of MNs lacking nuclear TDP‐43 and cytoplasmic DLK1 were observed in the upper and lower MN regions in ALS.

**Figure:**
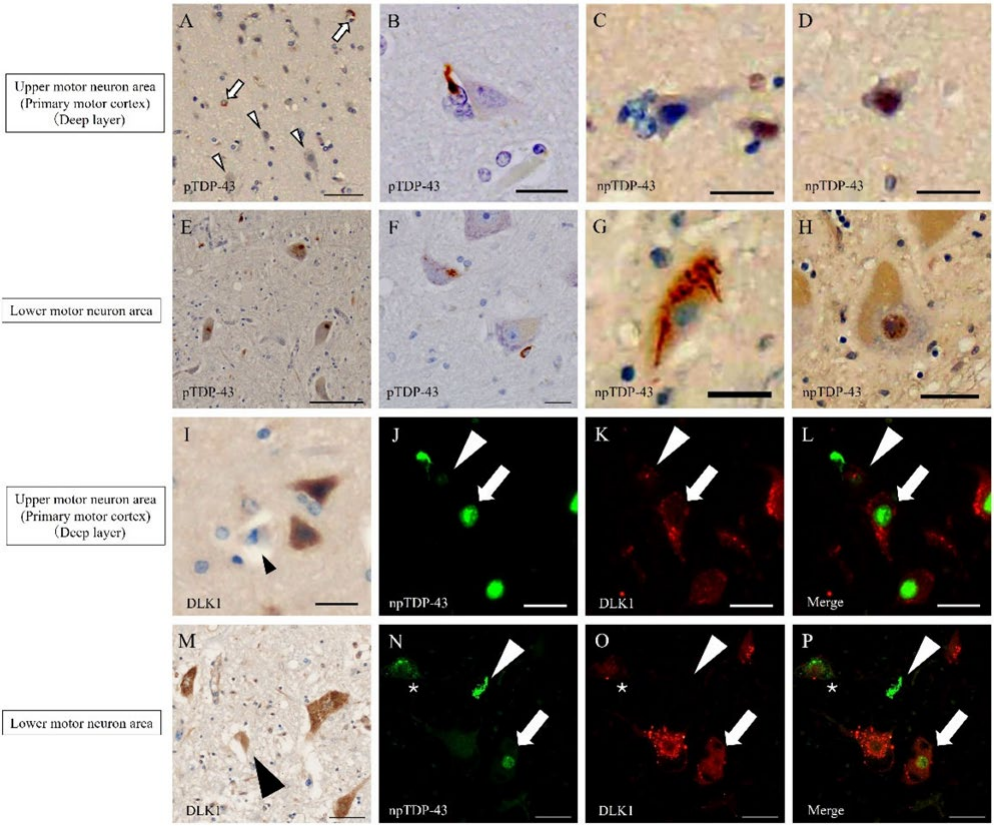
**FIGURE 1** Microscopy of TDP‐43 and DLK1 immunostaining in upper and lower motor neuron areas in samples from patients with amyotrophic lateral sclerosis.

**Figure:**
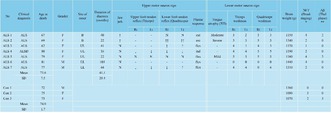
**TABLE 1** Case profile.

**Figure:**
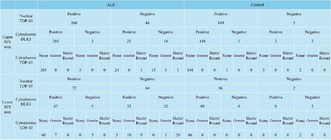
**TABLE 2** Prevalence of nuclear/cytoplasmic non‐phosphorylated TDP‐43 and cytoplasmic DLK1 in motor neurons.

**Conclusion:** These results demonstrated that the frequency and severity of abnormal cytoplasmic TDP‐43 aggregation differed significantly between the upper and lower MN regions in ALS patients, while nuclear TDP‐43 loss and cytoplasmic DLK1 reduction were consistent in both regions.

**Disclosure:** Nothing to disclose.

## EPO‐0115

### Clinical relevance of cranial motor evoked potentials in amyotrophic lateral sclerosis: A multicentre Italian study

#### V. Iuzzolino ^1^; F. Ranieri^2^; R. Dubbioso^1^; S. MND Study Group^3^; S. Neurostimulation‐Neuromodulation Study Group^4^

##### ^1^Department of Neurosciences, Reproductive Sciences, and Odontostomatology, University of Naples Federico II, Naples, Italy; ^2^Neurology Unit, Department of Neuroscience, Biomedicine, and Movement Sciences, University of Verona, Verona, Italy; ^3^Italian Neurological Society; ^4^Italian Society of Clinical Neurophysiology

**Background and aims:** Bulbar dysfunction strongly impacts prognosis in amyotrophic lateral sclerosis (ALS), but early detection remains challenging. Motor evoked potentials (MEPs) are widely used to assess corticospinal tract integrity, yet the clinical value of cranial MEPs is unclear. This study evaluated the diagnostic and prognostic significance of cranial MEPs in a real‐world ALS cohort.

**Methods:** We retrospectively analysed 510 consecutive ALS patients assessed at diagnosis with transcranial magnetic stimulation of three districts: cranial (genioglossus, *n* = 175), upper limb (first dorsal interosseous, *n* = 510), and lower limb (tibialis anterior or abductor hallucis, *n* = 479). Clinical involvement was classified using King's staging and clinical phenotypes. We assessed associations between MEP abnormalities and clinical variables and the impact of cranial MEPs on time to PEG placement.

**Results:** Cranial MEPs were more frequently abnormal than upper limb (51.4% vs 36.1%, *p* < 0.001) and similar to lower limb. They were often abnormal in patients without bulbar symptoms (27.3%) and strongly associated with bulbar involvement (*p* < 0.001). Including cranial districts increases the detection of pathological MEPs by 16% across ALS phenotypes, with rates rising along the LMN‐UMN spectrum and reaching 100% in UMN‐predominant ALS and PLS. The frequency of abnormal cranial MEPs increased across King's stages (34% at stage 1, 47% at stage 2, 64% at stage 3, *p* = 0.004). Abnormal cranial MEPs predicted shorter time to PEG (median 10 vs 18 months; *p* = 0.038).

**Figure:**
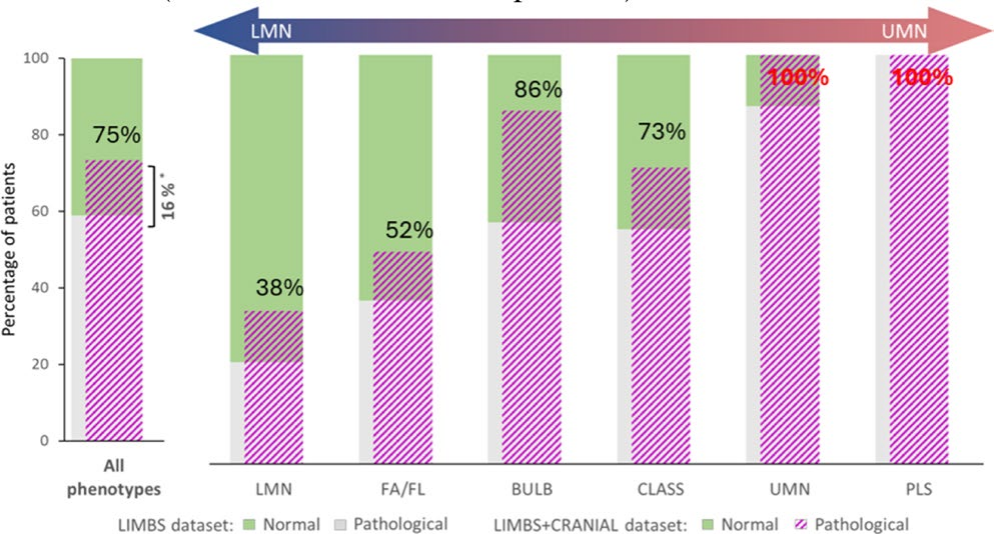
**FIGURE 1** Cranial MEPs improve diagnostic sensitivity in ALS and across the phenotypic spectrum.

**Figure:**
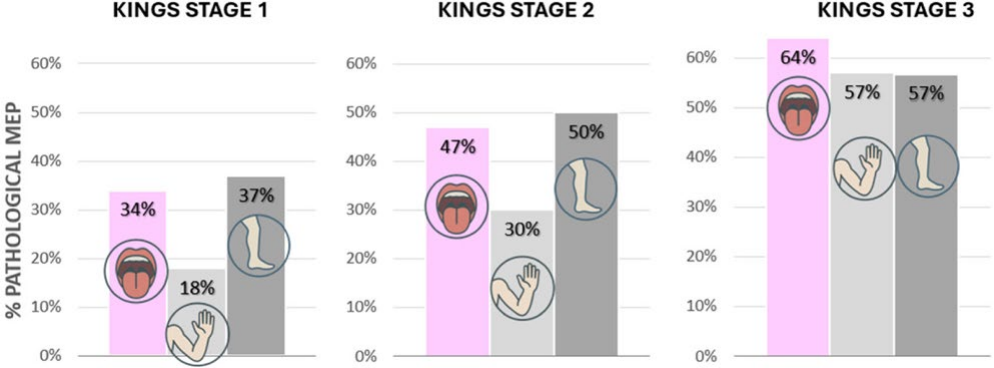
**FIGURE 2** Stage‐dependent increase of cranial MEPs involvement: In early disease stages, pathological MEPs predominantly involve the lumbosacral district, whereas cranial involvement increases across King's stages and becomes predominant at stage 3.

**Figure:**
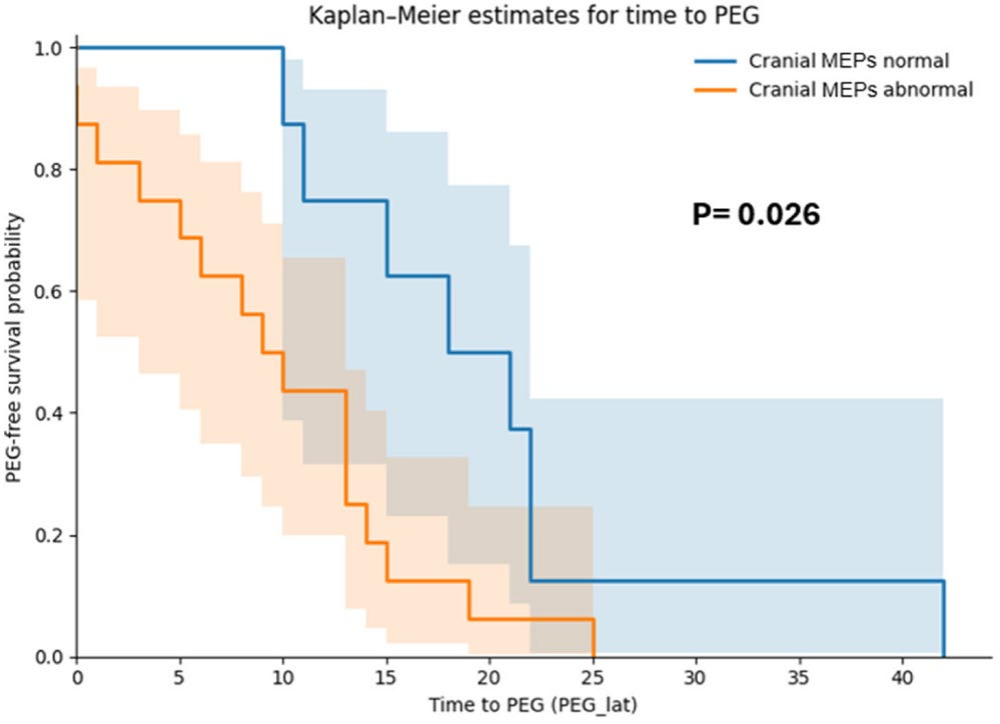
**FIGURE 3** Kaplan–Meier analysis showed that patients with abnormal cranial MEPs had a significantly shorter PEG‐free survival compared with those with normal cranial MEPs (median 10 vs 18 months; log‐rank *p* = 0.026; Cox proportional hazards HR = 0.37, *p* = 0.038).

**Conclusion:** Cranial MEPs detect subclinical bulbar involvement and predict earlier bulbar deterioration in ALS. Their integration into routine assessment may improve early stratification and prognostic evaluation in clinical practice.

**Disclosure:** Nothing to disclose.

## EPO‐0116

### The split elbow sign in amyotrophic lateral sclerosis: A systematic review and meta‐analysis

#### V. Poulidou^1^; V. Tseriotis ^2^; A. Bombaci^3^; S. Vucic^4^; N. Pavey^4^; S. Papagiannopoulos^5^; V. Kimiskidis^1^; M. Arnaoutoglou^6^

##### ^1^1st Department of Neurology, AHEPA University Hospital, Aristotle University of Thessaloniki, Thessaloniki, Greece; ^2^Department of Neurology, Agios Pavlos General Hospital of Thessaloniki, Thessaloniki, Greece; ^3^IRCSS Policlinico San Donato, San Donato Milanese, Italy; ^4^Brain and Nerve Research Centre, Concord Clinical School, The University of Sydney, Concord Hospital, Sydney, NSW 2139, Australia; ^5^3rd Department of Neurology, “G.Papanikolaou” Hospital, Aristotle University of Thessaloniki, Thessaloniki, Greece; ^6^Department of Clinical Neurophysiology, School of Medicine, AHEPA University Hospital, Aristotle University of Thessaloniki, Thessaloniki, Greece

**Background and aims:** Amyotrophic lateral sclerosis (ALS) may be associated with selective vulnerability of specific muscle groups, described as split phenomena. This study aimed to systematically evaluate the split elbow (SE) phenomenon—defined as disproportionate involvement of biceps versus triceps muscles—as a potential clinical and neurophysiological characteristic of ALS.

**Methods:** This systematic review and meta‐analysis followed PRISMA guidelines and was registered in PROSPERO (CRD42024528359). MEDLINE, Scopus, Web of Science, and grey literature were searched up to April 2025 using the terms “split elbow” and “amyotrophic lateral sclerosis.” Eligible English‐language studies included randomized, observational, diagnostic accuracy, and case–control designs. Study quality was assessed using the Joanna Briggs Institute critical appraisal tools. Random‐effects models were used to pool standardized mean differences of normalized Medical Research Council (MRC) strength scores. The diagnostic performance of the split elbow index based on compound muscle action potentials (SEI‐CMAP) was analyzed using a bivariate random‐effects model.

**Results:** Seven studies involving 1,941 patients with ALS (61.8% male) were included. There was no significant difference in strength between elbow extensors and flexors (pooled standardized mean difference [triceps–biceps] −0.17; 95% CI −1.03 to 0.69; *p* = 0.63), with substantial heterogeneity across studies (I^2^ = 97.1%). The SEI‐CMAP showed moderate accuracy in distinguishing ALS from controls, with pooled sensitivity of 0.79, specificity of 0.58, and an area under the curve of 0.66.

**Figure:**
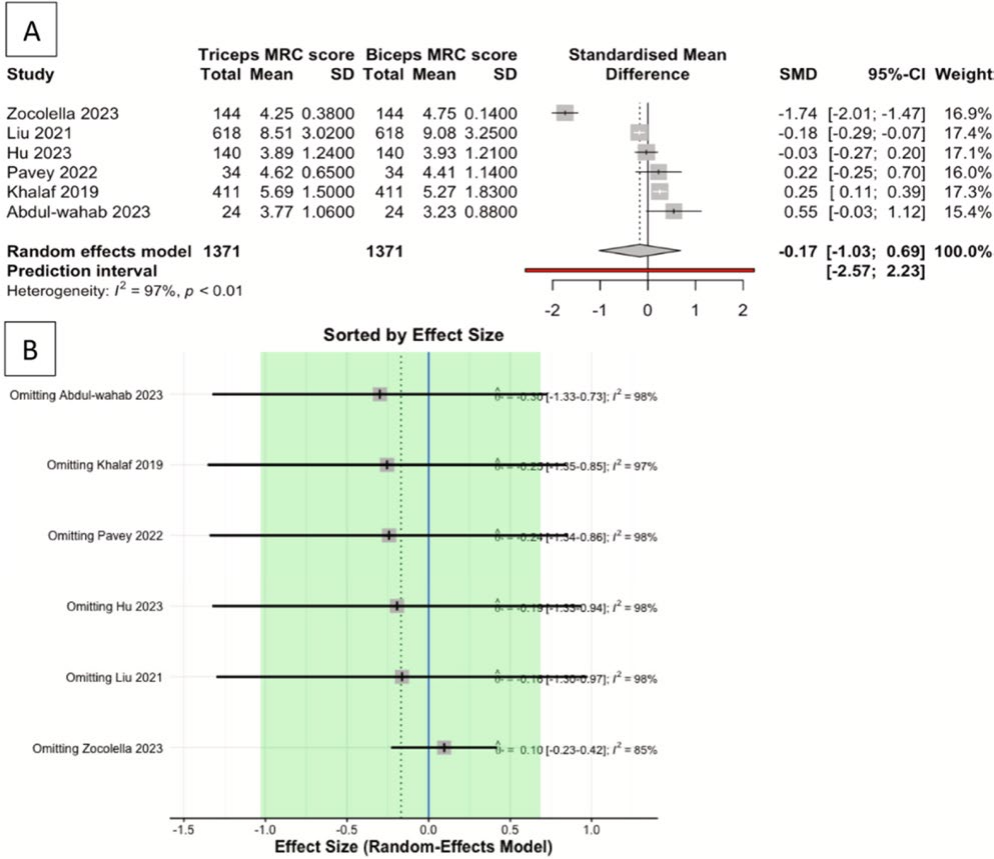
**FIGURE 1** (A) Forest plot of the meta‐analysis on the standardized mean difference (MRC triceps scores – MRC biceps scores) in the eligible studies. (B) Sensitivity analysis using the leave‐one‐out method.

**Figure:**
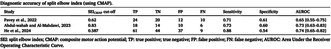
**FIGURE 2** Diagnostic accuracy of split elbow index (using CMAP).

**Figure:**
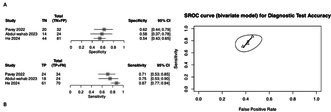
**FIGURE 3** Diagnostic accuracy of the split elbow index derived from compound muscle action potentials (SEI‐CMAP) for distinguishing amyotrophic lateral sclerosis (ALS) from controls. Forest plots show study‐level sensitivity and specificity.

**Conclusion:** Current evidence does not support a consistent split elbow pattern in ALS. High methodological heterogeneity and limited sample sizes reduce the clinical relevance of this phenomenon, suggesting limited diagnostic utility in routine practice.

**Disclosure:** Nothing to disclose.

## EPO‐0117

### Diagnostic ultrasound pattern of fasciculations in amyotrophic lateral sclerosis

#### G. Zabrodzets; Y. Rushkevich

##### Republican Scientific and Practical Center of Neurology and Neurosurgery, Minsk, Belarus

**Background and aims:** Muscle ultrasound (MUS) is an effective technique for non invasive fasciculation detection. This study aimed to characterize the fasciculation distribution pattern in amyotrophic lateral sclerosis (ALS) compared to ALS mimicking disorders.

**Methods:** This prospective study included 182 patients with suspected ALS: 149 ALS patients (median age 57.0 years, ALSFRS R 42) and 33 served as controls (median age 49.0 years), spondylotic myelopathy (*n* = 17), radiculopathy (*n* = 8), spinal muscular atrophy (*n* = 5) and multifocal motor neuropathy (*n* = 3). All participants underwent standardized symmetric MUS of 14 muscle groups across bulbar, cervical, thoracic and lumbosacral levels.

**Results:** Fasciculations were significantly more frequent in the ALS group (*p* < 0.001) and were generalized and symmetric with no significant side‐to‐side differences (*p* > 0.05). Detection rates ranged from 59.7% to 94.0%, with the highest frequencies in proximal muscles: m. biceps brachii (91.9%), posterior forearm muscle group (89.0%) and m. quadriceps femoris (92.6%). Controls showed less frequent, distally predominant fasciculations (thenar muscles 30.3–39.4%; posterior calf muscles 45.5–48.5%). The risk of fasciculation was markedly elevated in ALS: proximal muscles had high odds ratios (OR) without side to side differences (m. deltoideus OR 78–100, m. biceps brachii OR 64–89, posterior forearm group OR 113–114, m. quadriceps femoris OR 70–71), while distal muscles had lower ORs (e.g, thenar OR 11–13). A strong association (Cramer's V = 0.60–0.79) linked ALS with proximal fasciculations.

**Conclusion:** MUS identifies a distinct proximal predominant fasciculation pattern in ALS that robustly differs from mimicking disorders, supporting its value as an objective screening tool for early differential diagnosis.

**Disclosure:** Nothing to disclose.

## Movement Disorders 1

## EPO‐0118

### Platform‐based balance assessment in Parkinson's disease: A summary of tasks and parameters: A systematic review

#### C. Georgiou; S. Totou; A. Pilavas; G. Vavougyios; A. Artemiadis; P. Zis; G. Hadjigeorgiou; P. Bargiotas

##### Neurology Clinic, Movement Disorders Group, Medical School, University of Cyprus, Nicosia, Cyprus

**Background and aims:** Postural Instability (PI) is one of the cardinal motor symptoms of Parkinson's Disease (PD). Force platforms (FP) can be used as an objective balance assessment tool during both the early and advanced phases of PD. This systematic review aims to identify FP‐performed tests for PD balance assessment and summarize the most commonly calculated FP parameters.

**Methods:** We conducted a systematic review of studies published on Scopus, PubMed, and Embase from inception until September 2025. We screened 3204 articles using Rayyan, out of which 164 matched the inclusion criteria of performing FP‐based balance assessment of an idiopathic PD (iPD) group. Following eligibility assessment, 113 eligible studies were included. Study quality was assessed using JBI Critical Appraisal Tools.

**Results:** A total of 3121 iPD patients were assessed. Static balance was mainly assessed by instructing the patients to maintain a feet apart position under the eyes open (EO) or eyes closed (EC) conditions, while standing on the FP or a compliant surface [Figure 1]. Dynamic balance of PD patients was assessed by a fewer number of studies [Figure 2]. The most frequently calculated FP‐derived parameters were the Centre of Pressure (CoP) displacement, velocity, path length and the area encompassing all (total area) or 95% of all CoP points (ellipse) [Figure 3].

**Figure:**
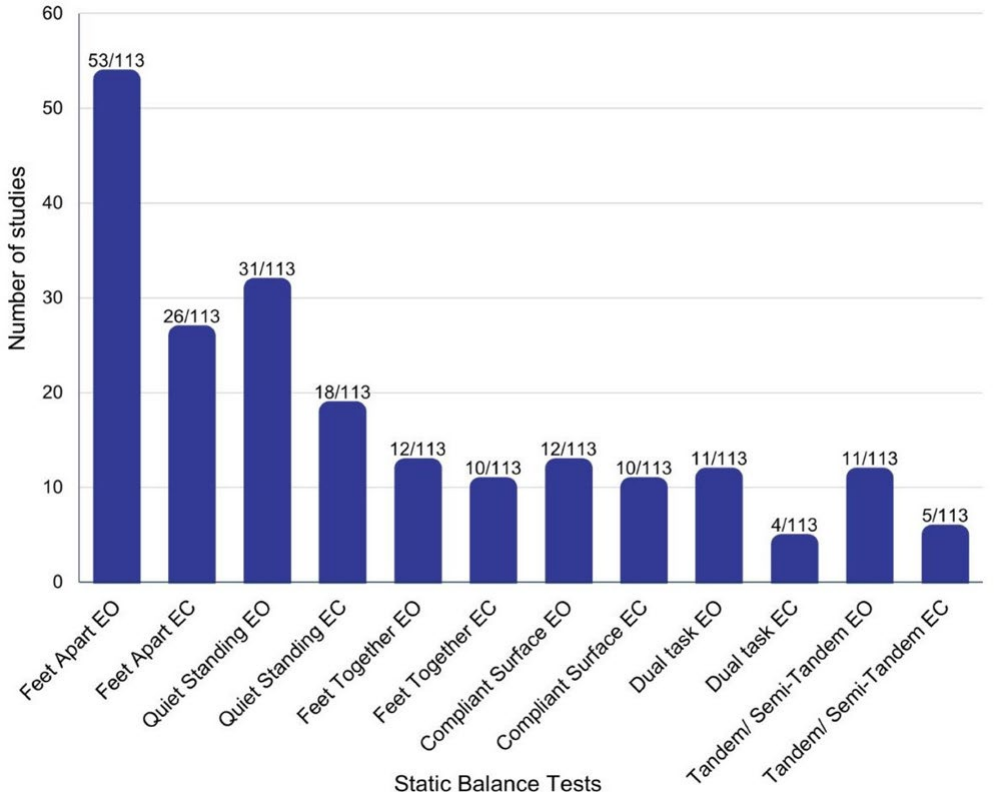
**FIGURE 1** Static balance tests.

**Figure:**
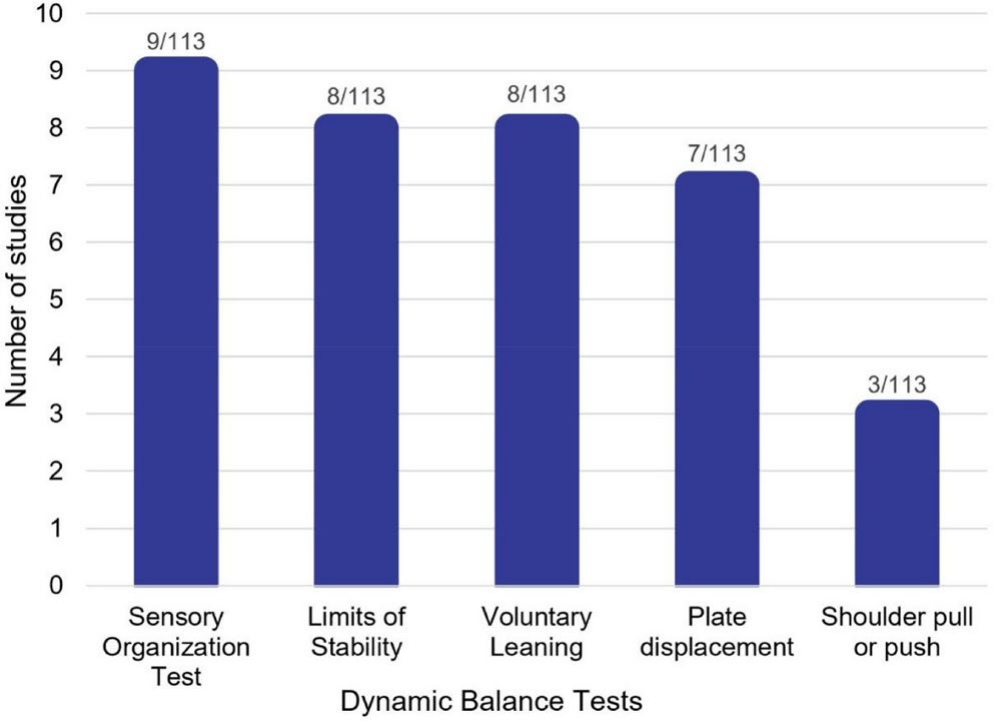
**FIGURE 2** Dynamic balance tests.

**Figure:**
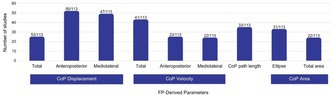
**FIGURE 3** FP‐derived parameters.

**Conclusion:** Force platforms can prove to be a valuable asset, not only for researchers, but also for clinicians. Further research is required to explore the use of FP for detecting subclinical PI in early PD, monitoring disease progression, and assessing the effectiveness of balance rehabilitation.

**Disclosure:** Nothing to disclose.

## EPO‐0119

### Efficacy of Opicapone in early motor fluctuations in Parkinson's disease: A comparison of early versus later initiation

#### J. Ferreira ^1^; F. Stocchi^2^; A. Antonini^3^; O. Rascol^4^; G. Ebersbach^5^; J. Kulisevsky^6^; M. Fonseca^7^; D. Ramos^8^; J. Rocha^8^; H. Brigas^8^; J. Holenz^8^; W. Poewe^9^

##### ^1^Laboratory of Clinical Pharmacology and Therapeutics, Faculty of Medicine, University of Lisbon, Lisbon, Portugal; ^2^Department of Neurology, IRCCS San Raffaele Pisana, Rome, Italy; ^3^Department of Neurosciences, University of Padova, Padova, Italy; ^4^University of Toulouse, University Hospital of Toulouse, INSERM, Clinical Investigation Center CIC1436 Departments of Neurosciences and Clinical Pharmacology and NS‐Park/FCRIN network, Toulouse, France; ^5^Movement Disorder Clinic Beelitz‐Heilstaetten, Beelitz‐Heilstätten, Germany; ^6^Movement Disorders Unit, Sant Pau Hospital, Barcelona, Spain; ^7^BIAL R&D Investments, S.A, Portugal; ^8^BIAL – Portela & Ca S.A, Coronado, Portugal; ^9^Department of Neurology, Medical University of Innsbruck, Innsbruck, Austria

**Background and aims:** Evidence suggests that opicapone use in Parkinson's disease patients with recent‐onset wearing‐off yields a higher magnitude of effect than that observed in later stages. However, the long‐term impact of earlier initiation is not well‐studied. This post‐hoc analysis assessed long‐term outcomes of opicapone early initiation in levodopa‐treated Parkinson's patients with early motor fluctuations.

**Methods:** Data were pooled from randomized, 3.5‐month, double‐blind, placebo‐controlled opicapone trials and their 1‐year open‐label extensions. Analyses included participants initially randomized to placebo or opicapone 50 mg who continued opicapone during the open‐label phase and had developed wearing‐off within ≤2 years prior to screening. ON/OFF status was assessed using 24‐hour patient diaries.

**Results:** Among 227 participants with recent‐onset wearing‐off (opicapone, *n* = 117; placebo, *n* = 110), opicapone 50 mg significantly reduced OFF‐time versus placebo (mean placebo‐adjusted reduction: –65.6 min [95% confidence interval (CI): –105.5 to –25.6]; *p* = 0.0014) and increased Good‐ON‐time (+88.3 min [95% CI: 47.0 to 129.6]; *p* < 0.0001) at double‐blind phase completion. Following 1‐year open‐label extension, early opicapone starters maintained numerically greater reductions in OFF‐time (–30.0 min [95% CI: –74.8 to 14.8]; *p* = 0.20) and increases in Good‐ON‐time (+38.2 min [95% CI: –6.6 to 82.9]; *p* = 0.09) compared with late starters (Figure 1, Table 1). Mean levodopa doses remained stable in both groups; no significant increase in ON‐time with troublesome dyskinesia was observed.

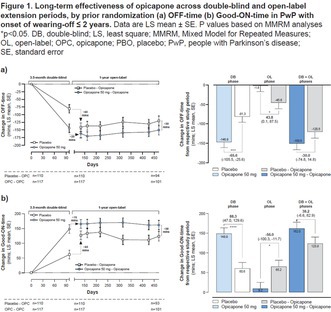

**Figure:**
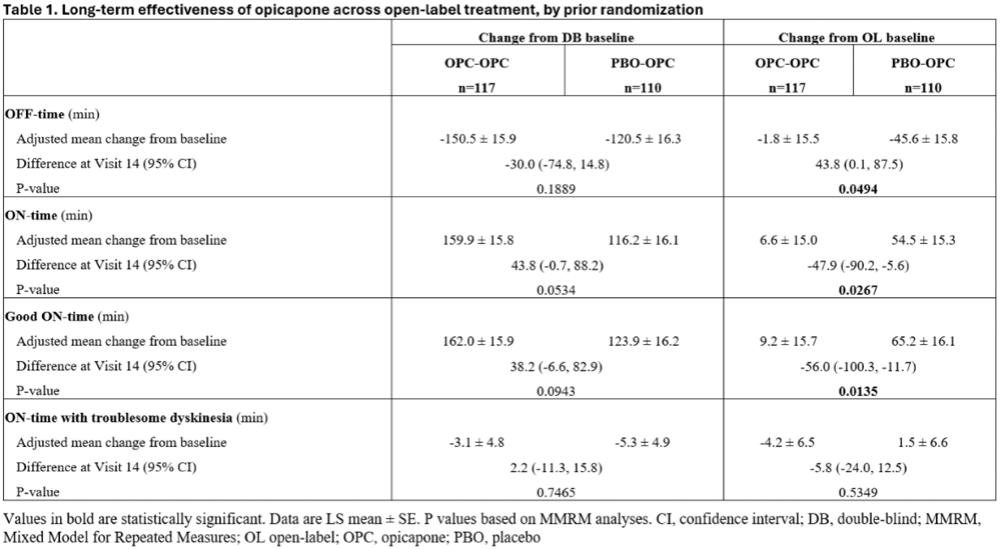
**TABLE 1** Long‐term effectiveness of opicapone across open‐label treatment, by prior randomization.

**Conclusion:** Early opicapone in PD patients with recent‐onset wearing‐off provided sustained improvements of ~2.5 hours in OFF‐time and Good‐ON‐time, without increasing dyskinesia or levodopa doses. These results support opicapone use in early wearing‐off without aggravating the risk of dyskinesia.

**Disclosure:** Supported by Bial.

## EPO‐0120

### Levodopa dose‐related risk of motor complications in early Parkinson's disease Patients treated with Opicapone: Findings from EPSILON study

#### J. Ferreira ^1^; O. Rascol^2^; F. Stocchi^3^; A. Antonini^4^; H. Brigas^5^; J. Moreira^5^; J. Rocha^5^; M. Fonseca^6^; D. Ramos^5^; J. Holenz^5^; W. Poewe^7^

##### ^1^Laboratory of Clinical Pharmacology and Therapeutics, Faculty of Medicine, University of Lisbon, Lisbon; ^2^University of Toulouse, University Hospital of Toulouse, INSERM, Clinical Investigation Center CIC1436 Departments of Neurosciences and Clinical Pharmacology and NS‐Park/FCRIN network, Toulouse, France; ^3^Department of Neurology, IRCCS San Raffaele Pisana, Rome, Italy; ^4^Department of Neurosciences, University of Padova, Padova, Italy; ^5^BIAL – Portela & Ca S.A, Coronado; ^6^BIAL R&D Investments, S.A, Portugal; ^7^Department of Neurology, Medical University of Innsbruck, Innsbruck, Austria

**Background and aims:** Opicapone (OPC) improved motor symptoms in early Parkinson's disease without increasing the risk of motor complications (MCs) in the 24‐week EPSILON study, with benefits sustained over 1 year. This post‐hoc analysis evaluated whether baseline levodopa dose and timing of OPC initiation (double‐ blind [DB] vs. open‐label extension [OLE] baseline) influenced MCs risk over 1.5 years.

**Methods:** EPSILON was a randomized, DB, placebo‐controlled study followed by a 1‐year OLE. Levodopa‐treated PD patients without MCs received OPC 50 mg or placebo during the DB phase, after which all patients received OPC 50 mg. This post‐hoc analysis assessed time to MCs, dyskinesia (MDS‐UPDRS‐IV item 4.1), and OFFs (item 4.3) over 1.5 years in patients initiating OPC early (OPC‐OPC) or after placebo (PLC‐OPC), stratified by levodopa dose (<400 vs. ≥400 mg/day). Kaplan–Meier survival analyses with log‐rank tests were used.

**Results:** Higher levodopa doses (≥400 mg/day) showed a trend for increased MCs risk, particularly in patients starting OPC later. Proportions of patients without MCs were 81.9% (<400 mg/day) and 79.0% (≥400 mg/day) in the OPC‐OPC group, vs. 73.3% (<400 mg/day) and 66.7% (≥400 mg/day) in the PLC‐OPC group (*p* = 0.04). Dyskinesia followed a similar trend (*p* = 0.06). Proportions of patients without OFFs were slightly higher in the OPC‐OPC group but independent of levodopa dose (*p* = 0.4); dystonia remained low in all groups (Figure 1a–d).

**Figure:**
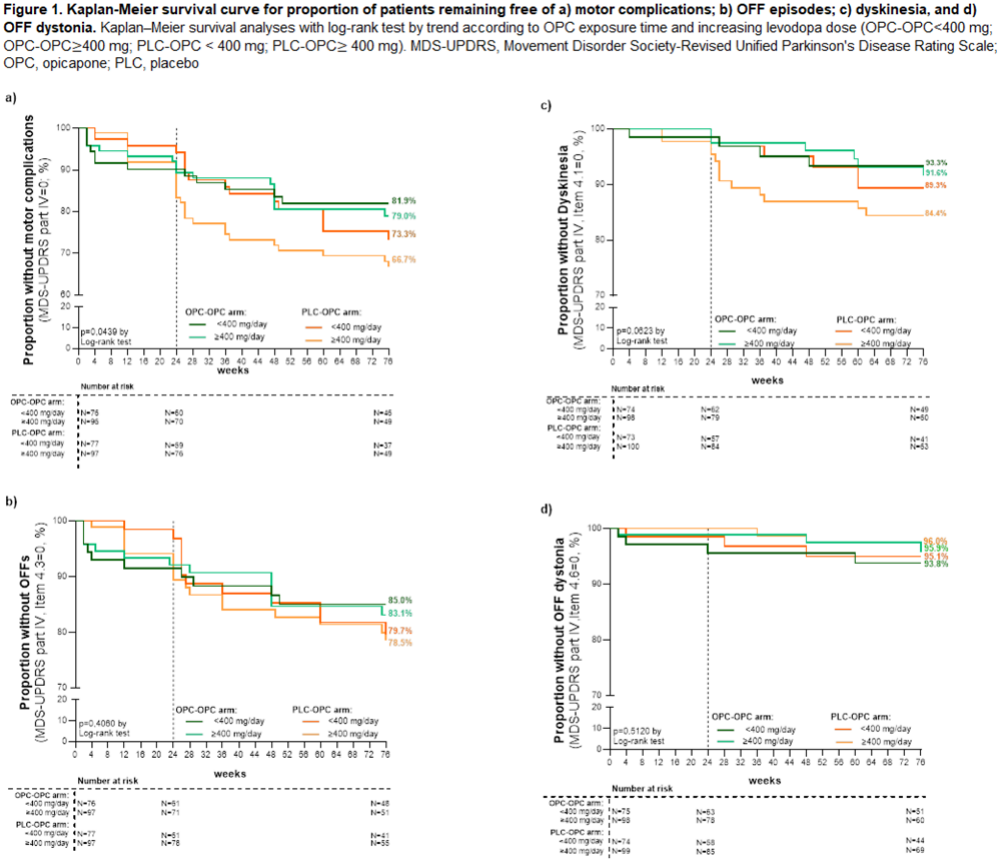
**FIGURE 1** Kaplan‐Meier survival curve for proportion of patients remaining free of a) motor complications; b) OFF episodes; c) dyskinesia, and d) OFF dystonia.

**Conclusion:** Most patients remained free of MCs with OPC; however, a trend toward higher MC risk was observed in patients on higher levodopa doses who initiated OPC later. Early OPC introduction may support motor stability, even at higher levodopa doses.

**Disclosure:** Supported by BIAL.

## EPO‐0121

### Beta and gamma local field potentials as biomarkers of motor and non‐motor outcomes in Parkinson's disease: A systematic review

#### K. Aung ^1^; S. Lane^2^; J. Osman‐Farah^3^; A. Macerollo^4^

##### ^1^Institute of Systems, Molecular and Integrative Biology, The University of Liverpool, Liverpool, UK/ The Walton Centre NHS Foundation Trust for Neurology and Neurosurgery, Liverpool, UK; ^2^Institute of Data Health Sciences, The University of Liverpool, UK; ^3^The Walton Centre NHS Foundation Trust for Neurology and Neurosurgery, Liverpool, UK; ^4^Institute of Systems, Molecular and Integrative Biology, The University of Liverpool, UK/ The Walton Centre NHS Foundation Trust for Neurology and Neurosurgery, Liverpool, UK

**Background and aims:** Deep brain stimulation (DBS) is an established therapy for idiopathic Parkinson's disease (PD). Sensing‐enabled DBS systems facilitate the recording of local field potentials (LFPs), which represent synchronised population‐level neuronal activity. Beta‐band activity, defined as 13 to 30 hertz, strongly correlates with motor symptoms including bradykinesia and rigidity. Gamma‐band oscillations of over 30 hertz have been proposed as potential complementary biomarkers. The objective of this study is to evaluate the clinical relevance of beta‐ and gamma‐band LFP biomarkers derived from postoperative recordings in people living with PD.

**Methods:** Following PRISMA 2020 guidelines, searches of PubMed, Scopus and ClinicalTrials.gov for human studies published between January 2020 and October 2025 investigating sensing‐enabled DBS of the subthalamic nucleus or globus pallidus internus were performed. Studies required quantitative beta and/or gamma analyses linked to validated motor or non‐motor outcomes. Twenty‐seven studies met the inclusion criteria. Significant methodological heterogeneity precluded quantitative meta‐analysis; therefore, the analysis will consist of a systematic narrative review mapping methodological patterns. Risk of bias was assessed using the Newcastle‐Ottawa Scale and Cochrane risk of bias tools.

**Results:** Beta burst‐derived metrics were associated with motor impairment, supporting their relevance for adaptive DBS control variables. Gamma‐related findings were heterogeneous and most commonly reported in relation to dyskinesia. Non‐motor outcomes were sparsely represented.

**Conclusion:** This review consolidates evidence on robust beta and gamma local field potential biomarkers. Findings will define methodological priorities and inform multi‐feature biomarker models for precise and objective adaptive DBS programming.

**Disclosure:** Nothing to disclose.

## EPO‐0122

### Spontaneous blink dynamics reveal distinct subtypes of isolated REM sleep behaviour disorder

#### L. Kälble ^1^; D. Skrabal^1^; J. Rusz^2^; M. Novotny^2^

##### ^1^Department of Neurology and Centre of Clinical Neuroscience, First Faculty of Medicine, Charles University and General University Hospital, Prague, Czech Republic; ^2^Department of Circuit Theory, Faculty of Electrical Engineering, Czech Technical University, Prague, Czech Republic

**Background and aims:** While distinct alterations of blinking and eyelid motor control have been described across different alpha‐synucleinopathy phenotypes, eyelid motor behavior remains poorly characterized in isolated REM sleep behavior disorder (iRBD), a recognized prodrome of alpha‐synucleinopathies. Given that the kinematics of blinking can index the function of cortical‐basal ganglia networks, subtle alterations in blink dynamics may emerge prior to phenoconversion. Here, we investigated whether spontaneous blink subtypes can be identified in iRBD and whether their patterns differ from de novo, drug‐naïve PD.

**Methods:** We analyzed single‐camera video recordings of freely spoken monologues from 101 polysomnography‐confirmed patients with iRBD and 57 patients with PD. Facial landmark tracking was used to extract nine spontaneous blink parameters, capturing: rate, amplitude, duration, opening and closing velocities, palpebral fissure width, and variability. Unsupervised k‐means clustering identified iRBD blink subtypes, which we related to clinical markers and compared to the PD cohort.

**Results:** Two stable blink clusters were identified, comprising a hyperkinetic subtype (68%) and a hypokinetic subtype (32%) with similar demographics, sex distribution, motor severity, and cognitive performance. Blink rate did not differ between subtypes; however, palpebral fissure width, blink duration, and eyelid opening and closing velocities diverged in opposite directions. Clinically, the hypokinetic subtype was associated with higher autonomic dysfunction and stronger differentiation from the PD cohort.

**Conclusion:** Distinct spontaneous blink subtypes may reflect divergent underlying pathophysiology, potentially specific to synucleinopathy phenotype. Establishing discriminating blink characteristics may aid in identifying meaningful prodromal subgroups, supporting an earlier intervention time, and informing future neuroprotective or disease‐modifying interventions.

**Disclosure:** This study received support from the Ministry of Health of the Czech Republic, grant no. NU23J‐04‐00042 and the Grant Agency of Charles University, project no. 604425.

## EPO‐0123

### Peripheral nerve safety of continuous subcutaneous foslevodopa infusion in Parkinson's disease: A prospective neurophysiological and skin biopsy study

#### M. Tosi ^1^; V. Leta^1^; R. Lombardi^2^; G. Pinola^1^; C. Leuzzi^1^; F. Colucci^1^; G. Gaudiano^1^; A. Braccia^1^; F. Pirone^1^; S. Rinaldo^1^; N. Golfrè Andreasi^1^; G. Simmini^1^; A. Luppino^1^; L. Romito^1^; R. Cilia^1^; S. Mazzetti^1^; A. Elia^1^; R. Eleopra^1^; G. Devigili^1^

##### ^1^Parkinson and Movement Disorders Unit, Department of Clinical Neurosciences, Fondazione IRCCS Istituto Neurologico Carlo Besta, Milan, Italy; ^2^Neuroalgology Unit, Fondazione IRCCS Istituto Neurologico Carlo Besta, Milan, Italy

**Background and aims:** Continuous dopaminergic infusion therapies are used in advanced Parkinson's disease (PD) to improve motor fluctuations; however, studies in patients treated with levodopa/carbidopa intestinal gel (LCIG) have reported peripheral neuropathies, including early small‐fiber involvement [1,2]. We aimed at evaluating peripheral nerve involvement during continuous subcutaneous foslevodopa infusion (SC foslevodopa) using integrated electrophysiological and morphological evaluations.

**Methods:** In this prospective observational study, we enrolled PD patients initiating SC foslevodopa who underwent baseline and 6‐month follow‐up assessments. Large‐fiber function was assessed by standard motor and sensory nerve conduction studies. Small‐fiber involvement was evaluated by validated questionnaires and distal leg skin biopsy with quantification of intraepidermal nerve fiber density (IENFD).

**Results:** 21 patients completed baseline and follow‐up nerve conduction studies: peripheral neuropathy was present in eight patients, including four sensory and four sensorimotor axonal neuropathies, at baseline. No new cases or progression of neuropathy were observed at follow‐up. Skin biopsy data at baseline and 6 months were available in 21 patients. Asymptomatic pathological IENFD was detected in 2 patients at baseline, remaining stable over time, while two additional patients developed pathological IENFD at follow‐up.

**Conclusion:** In this cohort SC foslevodopa was not associated with incident or progressive large‐fiber peripheral neuropathy over medium‐term follow‐up while skin biopsy revealed small‐fiber involvement in a subset of patients. Whether this reflects dissociated fiber involvement or disease‐related small‐fiber denervation remains to be elucidated. Combined electrophysiological and morphological monitoring may improve the detection of early peripheral nerve involvement during infusion therapies.

**Disclosure:** Nothing to disclose.

## EPO‐0124

### A Phase III, pivotal trial of staged‐bilateral MR‐guided focused ultrasound pallidothalamic tractotomy for motor complications in Parkinson's disease

#### M. Matarazzo ^1^; A. Dalvi^2^; H. Eisenberg^3^; P. Wu^4^; L. Zucker^5^; Chang^6^; H. Sarva^7^; Fishman^8^; Buch^9^; del Alamo^10^; Gonzalez‐Quarante^11^; Rodriguez‐Oroz^12^; Sani^13^; P. Ghanouni^14^; N. Patel^15^; Pourfar^16^; Mogilner^17^; J. Obeso^18^; M. Kaplitt^19^

##### ^1^HM CINAC (Centro Integral de Neurociencias Abarca Campal), Instituto de Investigación Sanitaria HM Hospitales, CIBERNED, Instituto Carlos III, Madrid, Spain; ^2^Comprehensive Movement Disorders Center, Palm Beach Neuroscience Institute, Boynton Beach, USA; ^3^Department of Neurosurgery, University of Maryland School of Medicine, Baltimore, USA; ^4^Department of Neurology, Chang Bing Show Chwan Memorial Hospital, Lukang, Taiwan; ^5^Brain and Spine Center South Florida, Delray Beach, USA; ^6^Department of Neurosurgery, Chang Bing Show Chwan Memorial Hospital, Lukang, Taiwan; ^7^Department of Neurology, Parkinson's Disease and Movement Disorders Institute, Weill Cornell Medicine, New York, USA; ^8^Department of Neurology, University of Maryland School of Medicine, Baltimore, USA; ^9^Department of Neurosurgery, Stanford School of Medicine, Palo Alto, USA; ^10^HM CINAC (Centro Integral de Neurociencias Abarca Campal), Instituto de Investigación Sanitaria HM Hospitales, Madrid, Spain; ^11^Department of Neurosurgery, Clínica Universidad de Navarra, Pamplona, Spain; ^12^Neurology Department, Clínica Universidad de Navarra, Navarra Institute for Health Research (IdiSNA), Pamplona, Spain. Centro de Investigación Biomédica en Red de Enfermedades Neurodegenerativas (CIBERNED), Instituto de Salud Carlos III, Madrid, Spain; ^13^Department of Neurosurgery, Rush University Medical Center, Chicago, USA; ^14^Department of Radiology, Stanford University School of Medicine, Palo Alto, USA; ^15^Department of Neurological Sciences, Division of Movement Disorders, RUSH University Medical Center, Chicago, USA; ^16^Department of Neurology, NYU Langone Health, New York, USA; ^17^Department of Neurosurgery, NYU Langone Health, New York, USA; ^18^HM CINAC (Centro Integral de Neurociencias Abarca Campal), Instituto de Investigación Sanitaria HM Hospitales, CIBERNED, Instituto Carlos III, Madrid, Spain; ^19^Department of Neurosurgery, Weill Cornell Medicine, New York, USA

**Background and aims:** This study evaluated the safety and efficacy of staged‐bilateral magnetic resonance‐guided focused ultrasound (MRgFUS) Pallidothalamic‐tractotomy (PTT) in patients with Parkinson's disease (PD) experiencing motor complications. MRgFUS is an incisionless technique that enables targeting of the Pallidothalamic‐tract within the subthalamic region, offering a novel alternative for PD management.

**Methods:** In this prospective, open‐label, single‐arm, multicenter phase III trial (NCT04728295), 84 participants (≥30 years) were enrolled across nine international centers. Eligible subjects underwent first‐side MRgFUS PTT followed, when appropriate, by staged‐bilateral treatment after a minimum 6‐month interval. The primary endpoint was change in MDS‐UPDRS Part III OFF bilateral upper and lower extremities (ULE) 3 months after 2nd side treatment. Secondary measures included MDS‐UPDRS Parts III total OFF and total IV scores, with safety assessed through treatment‐emergent adverse events. Nonparametric tests were used for statistical analysis.

**Results:** Fifty‐four subjects received unilateral MRgFUS PTT, and 40 underwent staged‐bilateral treatment. Following second‐side procedures, Part III OFF (ULE) improved by 32.1% [median 33 (IQR 28–40) to 21 (16–27); *p* < 0.0001] (Figure 1) , total Part III OFF improved by 34% [median 50 (44–56) to 32 (26–40); *p* < 0.0001] (Figure 2) , and Part IV improved by 67% [median 12 (9–13) to 4 (0–5); *p* < 0.0001] (Figure 3). Benefits persisted through 12 months. After unilateral treatment, 30 events occurred in 21 subjects (87% mild; non‐severe), 60% resolving within 6 months; after bilateral, 43 events in 22 subjects (70% mild; 2% severe), 40% resolving within 12 months. The most frequent events were imbalance, dysarthria, and fatigue.

**Figure:**
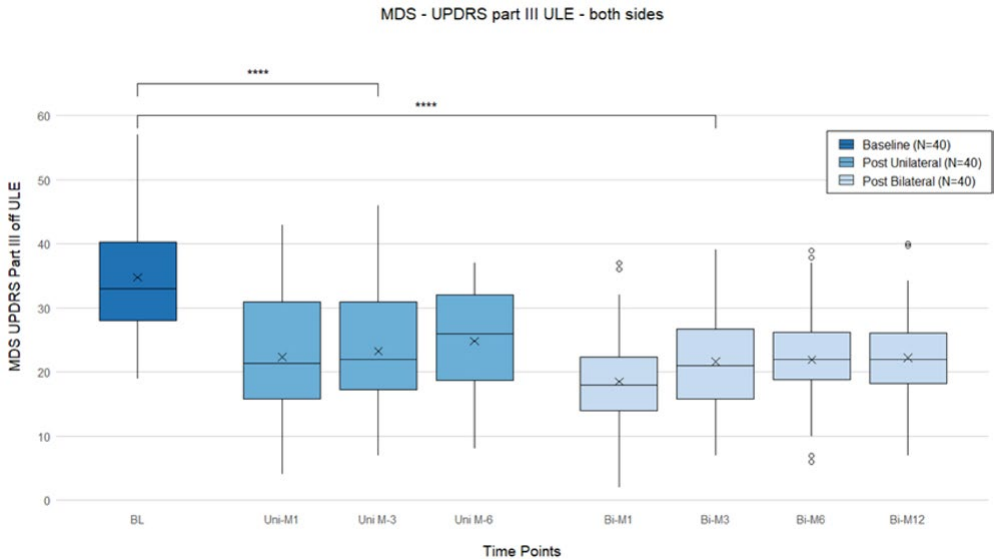
**FIGURE 1** Change from baseline in the MDS‐UPDRS Part III OFF ULE score [median (IQR)]. BL: baseline; Uni = unilateral; Bi = bilateral; M = month; **** = *p* < 0.0001.

**Figure:**
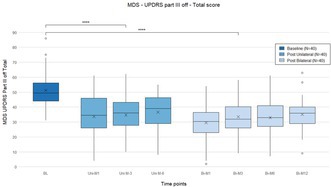
**FIGURE 2** Change from baseline in the MDS‐UPDRS Part III total score [median (IQR)]. BL: baseline; Uni = unilateral; Bi = bilateral; M = month; **** = *p* < 0.0001.

**Figure:**
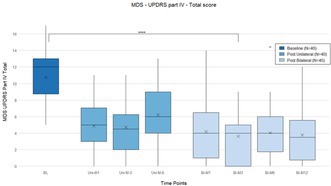
**FIGURE 3** Change from baseline in the MDS‐UPDRS Part IV score [median (IQR)]. BL: baseline; Uni = unilateral; Bi = bilateral; M = month; **** = *p* < 0.0001.

**Conclusion:** MRgFUS PTT significantly improved motor function and complications with a favorable safety profile, supporting its role as an incisionless therapy for advanced PD. The recent FDA approval enables real‐world evaluation and larger confirmatory studies. These results may also support additional regulatory approvals; the full manuscript is under review.

**Disclosure:** PW, LZ, WCC, VB, MP and MK declare no conflicts of interest. MM has received contracts or grants from the International Parkinson and Movement Disorders Society (MDS Podcast), and Michael J Fox Foundation (Grant ID: 16451); honoraria for lectures, presentations, speaker's bureaus, or educational events from Bial, Insightec, International Parkinson and Movement Disorders Society, Sociedad Española de Neurología, and Zambon. Stock: Biogen. AD has received honoraria and travel funds from Insightec and honoraria from Acadia pharmaceuticals. HME has received grants for Insightec Haifa. HS has received clinical trial support from Bial, Biogen, Bluerock, Bukwang, Cerevance, Genentech, Insightec, MeiraGTX, Neuroderm, Novo Nordisk, Prevail, Sun Pharma, and UCB. She has served as a consultant for Neuroderm and Novo Nordisk and has been a speaker for Insightec. PSF has received research funding from the FUS Foundation and Insightec. MdA is a consultant for Abbott, Boston Scientific, Insightec, and Medtronic; and has received honoraria as a speaker from Palex and reimbursment of travel expenses to attend scientific conferences from Boston Scientific, Medtronic, and Palex. LHG‐Q has received honoraria from Boston Scientific, Insightec, Medtronic, Rebrain, Siemens and for lectures, travel, and accommodation to attend scientific meetings. He had also received honoraria as a consultant from Insightec and Siemens. He is a member of the medical advisory board of Rebrain. MCR‐O has received financial support to attend scientific meetings, honoraria for lectures, advisory boards and research from Boston Scientific, Insightec, Medtronic and Palex. She has also received grants for research projects from different calls of the Spanish and Navarrian Government and from Siemens. SS has received research grant funding from Abbott, Boston Scientific, and Medtronic, NIH R01 grant funding and ad hoc paid proctorship from Insightec. PG has received honoraria for medical advisory boards for Petal Surgical, Profound Medical, and SonALAsense NP has received honoraria as a consultant for AbbVie Pharmaceuticals, Amneal Pharmaceuticals, and Supernus. AM has received grant support from Insightec. JO has received honoraria for lecturing in scientific meetings from Insightec Ltd. He has received consulting honoraria from Biogen for participating at Adverse Events Advisory Board 2023–2025, and consulting honoraria from Bayern and Roche for attending one Advisory Board for each. He also holds several non‐paid, non‐profit research grants from the Spanish Ministry of Education and Science, European Funding Agencies, Focused Ultrasound Foundation and ASAP coalition.

## EPO‐0125

### Mapping the availability of palliative care for parkinsonian syndromes in African countries: A scoping review

#### N. Kouki ^1^; L. Ali^2^; N. kouki^1^

##### ^1^University of Tunis El Manar, Faculty of Medicine of Tunis, Tunisia; ^2^University of Camerino, School of Advanced Studies, Macerata, Italy

**Background and aims:** Neurodegenerative disorders (NDDs) are progressive conditions leading to severe disability. They are affecting patients and caregivers. Neuro‐palliative care (NPC) aims to improve the quality of life of patients by addressing the multidimensional burdens of NDD, and reducing the burden on caregivers. Globally and especially in African countries, access to NPC in NDD is scarce both institutionally and professionally. This study aims to evaluate the availability and integration of NPC in the management of parkinsonian syndromes (PS) across African countries.

**Methods:** We conducted a scoping review following the Arksey.and.O’Malley framework, with Levac and JBI enhancements, to evaluate the availability and integration of palliative care in the management of parkinsonian syndromes across African countries. PubMed, Scopus, Web of Science, and Google Scholar were searched. Observational studies and grey literature were included. Two reviewers independently screened studies and extracted predefined data on degree of integration and reported barriers, with disagreements resolved by a third reviewer.

**Results:** Substantial gaps in the availability and implementation of NPC for PS across Africa were identified. NPC is rarely incorporated into NDD care pathways and remains poorly represented in national neurological policies. Services are limited, unevenly distributed, and primarily focused on oncology. Financial constraints, inadequate infrastructure, and high out‐of‐pocket costs further restrict access. Cultural beliefs, low awareness, and limited training in NPC significantly contribute to delayed referral and underutilization of palliative services.

**Conclusion:** NPC for PS in Africa remains critically insufficient. Increasing awareness and strengthening policies in sustainable palliative care models are essential to improve quality of life for affected individuals and their caregivers.

**Disclosure:** Nothing to disclose.

## EPO‐0126

### Safety and tolerability of opicapone in elderly Parkinson's disease patients: A single‐center, real‐life retrospective study

#### P. Lombardo; L. De Carolis; P. Pacilio; S. Galli; F. Garramone; A. Colucci; M. Giovannelli; D. Rinaldi

##### Department of Neuroscience, Mental Health and Sensory of Sapienza University of Rome, Sant'Andrea University Hospital, Rome, Italy

**Background and aims:** Opicapone is a Catechol‐O‐Methyltransferase inhibitor, used in patients with Parkinson's Disease (PwPD) experiencing motor fluctuations1. Registrative trials of Opicapone demonstrated its effectiveness and its favorable profile of safety and tolerability. However, these trials primarily enrolled non‐elderly patients, who represent a higher percentage of PwPD2 3. Our aim was to assess safety and tolerability of Opicapone in elderly patients in a real‐life clinical setting.

**Methods:** We retrospectively collected data from PwPD treated with Opicapone at our outpatient clinic. Patients were stratified according to age at drug initiation (60–75 vs ≥75 years). Comorbidities, time between fluctuation‐onset and drug start, clinical assessments, including Hoehn and Yahr (H&Y) stage, MDS‐UPDRS‐III, LEDD, concomitant therapy and adverse events, were evaluated at baseline (T0) and at follow‐up (T1; treatment discontinuation or last available visit).

**Results:** No statistically significant differences were observed between the two age groups regarding disease duration, H&Y stage, or total LEDD at either baseline (T0) or follow‐up (T1). Adverse events were more frequent in patients aged ≥ 75 years (50% vs 19%, *p* = 0.02), without significant difference in discontinuation rates. Subgroup analysis revealed that patients who discontinued Opicapone had higher disease severity (H&Y‐stage, *p* = 0.01; UPDRS‐III at T0 and T1, *p* = 0.02 and 0.01, respectively), a lower increase of LEDD at T1 (*p* = 0.001) and were more frequently female (*p* = 0.031). A tendency towards significance was found for differences in age at visit (*p* = 0.005).

**Conclusion:** These preliminary findings suggest that safety and tolerability were higher in patients with less advanced age and disease severity, with possible gender‐related differences. Larger, multicenter prospective studies are warranted.

**Disclosure:** Nothing to disclose.

## EPO‐0127

### Family resilience, rights utilizations, dyadic relationship and caregiver care‐efficacy in families of persons with Parkinson's disease

#### S. Freiman; V. Rozani

##### Gray Faculty of Medical and Health Sciences, The Stanley Steyer School of Health Professions, Department of Nursing Sciences, Tel Aviv University, Tel Aviv, Israel

**Background and aims:** Parkinson's disease (PD) is a chronic, progressive neurodegenerative disorder that severely affects daily functioning. Globally, an estimated 12–13 million people are currently living with PD, with numbers expected to double by 2050. Consistent with Aging in Place policies, most PDs remain at home, placing increasing physical and emotional demands on family members. Little is known about the factors that help family caregivers cope effectively with these ongoing challenges.

**Methods:** The study included 168 family caregivers of community‐dwelling individuals with PD in Israel. Data were collected online (January–June 2025), and multiple regression analyses examined associations among caregiving efficacy, family resilience, rights utilization, and dyadic relationship.

**Results:** Caregivers were mostly women (80%), partnered (95%), and retired (58%), with a mean age of 66.7 ± 10.9 years. Care recipients with PD were predominantly men (84%) with a mean age of 72.6 ± 9.7 years. On average, caregivers provided 9.7 ± 7.0 hours of care per day. Caregiving care‐efficacy was associated with family resilience (*r* = 0.68, *p* < 0.001), dyadic relationship (*r* = 0.35, *p* < 0.001), and emotional rights utilization (*r* = 0.22, *p* =0.014). Multivariate analysis showed that caregiver care‐efficacy was significantly associated with family resilience (β = 0.569, *p* < 0.001). Emotional rights utilization significantly moderated the relationship between family resilience and care‐efficacy (B = 1.99, *p* = 0.009), while dyadic relationship did not show a moderating effect. Female caregivers reported higher care‐efficacy (β = .154, *p* = 0.044). The overall model explained 50.9% of the variance (R^2^ = 0.509, *p* < 0.001).

**Conclusion:** Within the Aging in Place framework, enhancing family resilience, emotional rights utilization, and Caregiver care‐efficacy is essential to support family members in providing effective and sustainable home care for individuals with PD.

**Disclosure:** This research was partially supported by a scholarship grant from the CUENCA Institute.

## EPO‐0128

### A single‐center experience with Omaveloxolone in Friedreich ataxia

#### M. Gramaglia ^1^; A. Sarnataro^1^; G. Puorro^2^; A. Marsili^2^; A. Bonfini Rendina^1^; C. Pane^1^; F. Saccà^2^

##### ^1^1NSRO Department, Federico II University, Naples, Italy; ^2^2GENESIS Department, Federico II University, Naples, Italy

**Background and aims:** Omaveloxolone is the first approved treatment for Friedreich Ataxia (FA),an autosomal recessive neurodegenerative disorder caused by a trinucleotide GAA expansion in FXN gene encoding frataxin. In FA,reduced frataxin levels and suppression of the Nrf2 pathway lead to oxidative stress,mitochondrial dysfunction,and cellular damage.Omaveloxolone,a synthetic oleanane triterpenoid,pharmacologically activates Nrf2 and enhances antioxidant effects(1). In a phase II trial, Omaveloxolone (150 mg/die) significantly improved neurological function compared to placebo(2). This retrospective study aimed to evaluate the efficacy and safety of Omaveloxolone in FRDA patients treated in clinical practice.

**Methods:** Patients underwent baseline and follow‐up visits after 3 and 6 months. Neurological assessments included modified FA rating scale(mFARS) and FA activities of daily living(FA‐ADL) scores.Serum laboratory analyses at baseline and months 1, 2, 3, and 6 included liver enzymes and lipid profile. Clinical scale change in time, and the interaction of factors and time were assessed with a linear mixed model for repeated measures

**Results:** Fifty‐seven patients(29 female, 28 male;mean age 41.3 ± 13.6 years;disease duration 22.1 ± 10.6 years) were enrolled.Baseline mFARS was 58.6 ± 16.9, decreasing to 57.5 ± 16.7 after 3 months; 13 patients reached 6 months (59.7 ± 10.6; *p* = 0.186).FA‐ADL slightly declined from 16.2 ± 7.4 to 15.8 ± 6.8 (*p* = 0.547).Higher baseline mFARS and FA‐ADL scores predicted greater improvement (*p* < 0.001).AST/ALT increased by month 3,then decreased (*p* < 0.001).Eleven patients temporarily discontinued therapy;one permanently due to gastrointestinal side effects.

**Conclusion:** Omaveloxolone was safe and well tolerated, with mFARS and FA‐ADL improvements more evident in patients with advanced disease and higher baseline scores.

**Disclosure:** Nothing to disclose.

## EPO‐0129

### Using of remote smartphone language screening can predict clinical markers in Huntington disease

#### T. Tykalová; M. Šubert; J. Rusz

##### Department of Circuit Theory, Faculty of Electrical Engineering, Czech Technical University in Prague, Prague, Czech Republic

**Background and aims:** Screening symptoms of Huntington's disease (HD) remains limited to infrequent, clinic‐based evaluations. We evaluated whether fully automated linguistic analysis of speech tasks recorded via smartphone can remotely capture core clinical markers of HD severity.

**Methods:** In this cross‐sectional multicenter study across Czech and German sites, 53 participants including 30 HD (ranging from 0 to 3 according to Integrated Staging System) and 23 healthy controls completed smartphone‐based speech assessment, including spontaneous monologue and fairy tale retelling for 7 consequent days. Recordings were automatically transcribed and analyzed using natural language processing to derive 3 lexical and 3 syntactic features. Predictive models for clinical outcomes based on Unified Huntington's Disease Rating Scale were built using multivariate linear regression with cross‐validation.

**Results:** Language features predicted HD severity across multiple domains with high accuracy, explaining up to 47% of the variance in cognitive performance, 63% in motor impairment, and 59% in functional capacity. Retelling required only 3 days of recordings to reach 90% of maximum predictive accuracy, while monologue required 6 days. Compared to controls, HD participants showed reduced vocabulary range and increase phrase repetition in both tasks (*p* < 0.05), with additional monologue‐specific deficits in sentence length and syntactic complexity (*p* < 0.05).

**Conclusion:** Fully automated analysis of smartphone‐based speech assessment can remotely quantify cognitive, motor, and functional impairment in HD, offering a scalable, low‐burden digital biomarker for clinical trials and decentralized monitoring.

**Disclosure:** This project was supported by Czech Ministry of Health, project number NW25‐04‐00052.

## MS and Related Disorders 1

## EPO‐0130

### Shedding light on kappa free light chain index in multiple sclerosis: Experience from a Portuguese tertiary centre

#### A. Roldão Alferes ^1^; R. Martins^2^; Â. Maresch^2^; A. Mendes^2^; R. Cunha^2^; I. Monteiro^1^; R. Machado^1^; I. Correia^1^; C. Cecília Nunes^1^; C. Macário^1^; S. Batista^1^

##### ^1^Serviço de Neurologia, Unidade Local de Saúde de Coimbra, Coimbra, Portugal; ^2^Serviço de Patologia Clínica, Unidade Local de Saúde de Coimbra, Coimbra, Portugal

**Background and aims:** In the latest McDonald criteria update, Kappa Free Light Chain (k‐FLC) index was considered a proper test for the diagnosis of multiple sclerosis (MS) and could be used instead of oligoclonal bands (OCBs) when using a cut‐off point of 6.1. This study aims to describe the applications of this biomarker in MS diagnosis and to address potential correlations with disease characteristics.

**Methods:** We included all patients from our center who had an established diagnosis and underwent k‐FLC index quantification from November 2019 to June 2025. Sensitivity and specificity of k‐FLC index and OCBs were calculated. Relations with clinical, imaging and laboratory variables were addressed.

**Results:** Of the 500 patients eligible for analysis, 145 had MS, 10 CIS, 8 NMOSD, 7 MOGAD, 22 SNC infections and 308 other diagnoses. Using the cut‐off point of 6.1, k‐FLC index had higher sensitivity than OCB (86.45% vs 77.42%) but lower specificity (88.41% vs 92.46%). κ‐FLC and OCBs concordance rate was 90.8%. In the univariate analysis, k‐FLC index was significantly higher in patients with high MRI lesion load (Median 46.20 ± IQR 79.08 vs 29.82 ± 77.95; *p* = 0.015), with active lesions (53.50 ± 68.68 vs 31.55 ± 74.00 ; *p* = 0.018), with infratentorial lesions (44.27 ± 72.52 vs 28.62 ± 80.52; *p* = 0.045) and with spinal cord lesions (54.69 ± 96.65; *p* = 0.020 vs 32.03 ± 53.65).

**Conclusion:** k‐FLC index may not only be used as an OCBs substitute for MS diagnosis but also as a biomarker for prognosis.

**Disclosure:** The authors have nothing to declare.

## EPO‐0131

### Dynamics of ratios T1/T2 for evaluation of multiple sclerosis (MS) activity

#### A. Buniak ^1^; A. Mikitchuk^2^; T. Charnukha^1^; O. Pereverzeva^1^

##### ^1^Neurological Department, Republican Research and clinical center of Neurology and Neurosurgery, Minsk, Belarus; ^2^Faculty of Radiophysics and Computer Technologies, Belarusian State University, Minsk, Belarus

**Background and aims:** T1/T2 ratio is a one of the markers, which is related to microstructural integrity for the cases of MS lesions and white matter in general. Lower T1/T2 ratios are named as associated with clinical disability as well as differential impact of neuroinflammation. Note, that T1 and T2 magnetic resonance images (MRI) are divided as a result there in no necessity to convert them into the similar brightness basis.

**Methods:** MR‐data collected for 15 patients with RRMS (2023 – 2025) and 9 – with SPMS (2023 – 2025). T2FLAIR images (256 × 256 × 272) are recorded to be reference during the some consequential years as well as T1. T1 images are co‐registrated and re‐sliced with reference (SPM12) to be located in the similar spatial basis (Figure 1). Then, T1[year 1]/T2[year 1], T1[year N]/T2[year 1] are slice‐by‐slice calculated to be in the same spatial basis. Consequently, temporal differences (calculated for each pair of years) are analyzed. This allows to check differences in T1/T2 ratios qualitatively and quantitatively.

**Figure:**
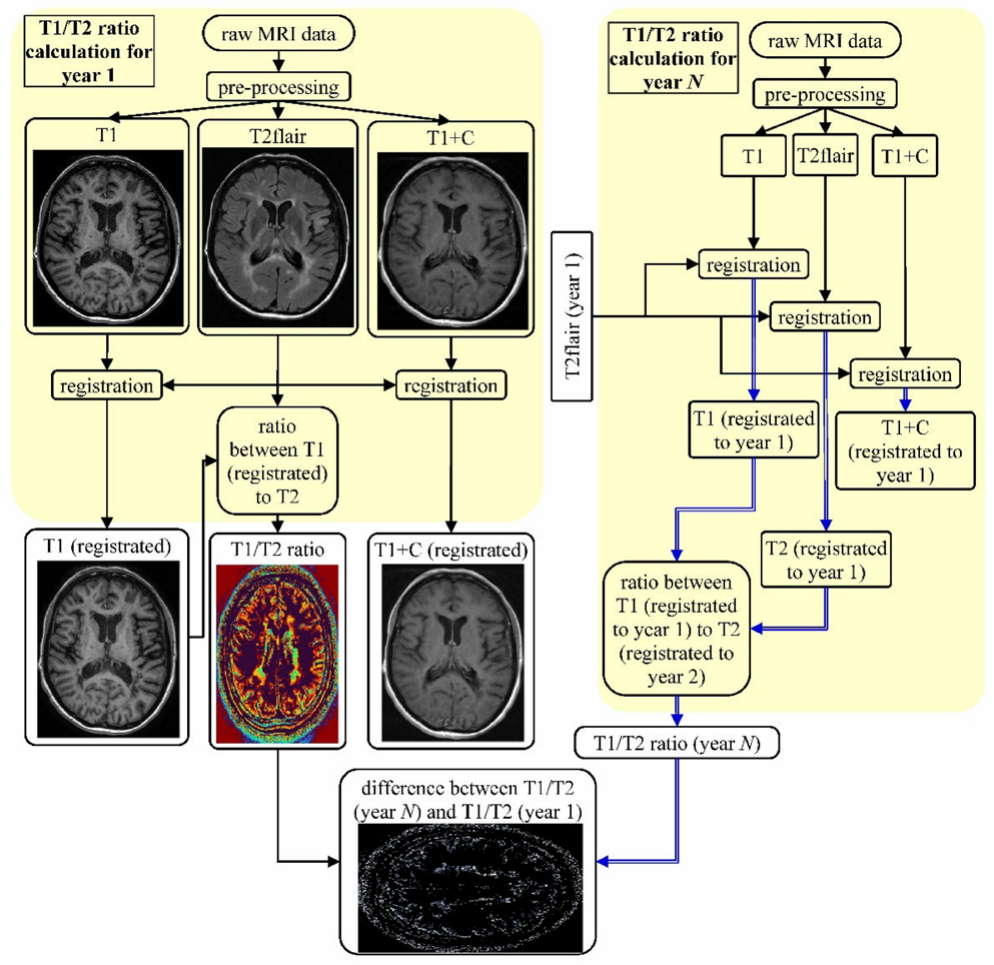
**FIGURE 1** Method of T1/T2 ratio MRI calculation.

**Results:** This approach allows to analyze both increasing of MR‐density, which corresponds to growth of T1/T2 ratio (fig. 2), and decreasing T1/T2 ratio (fig. 3) Moreover, in accordance with publications, they can be qualified as signs of partial re‐myelination and neuroinflammation correspondingly.

**Figure:**
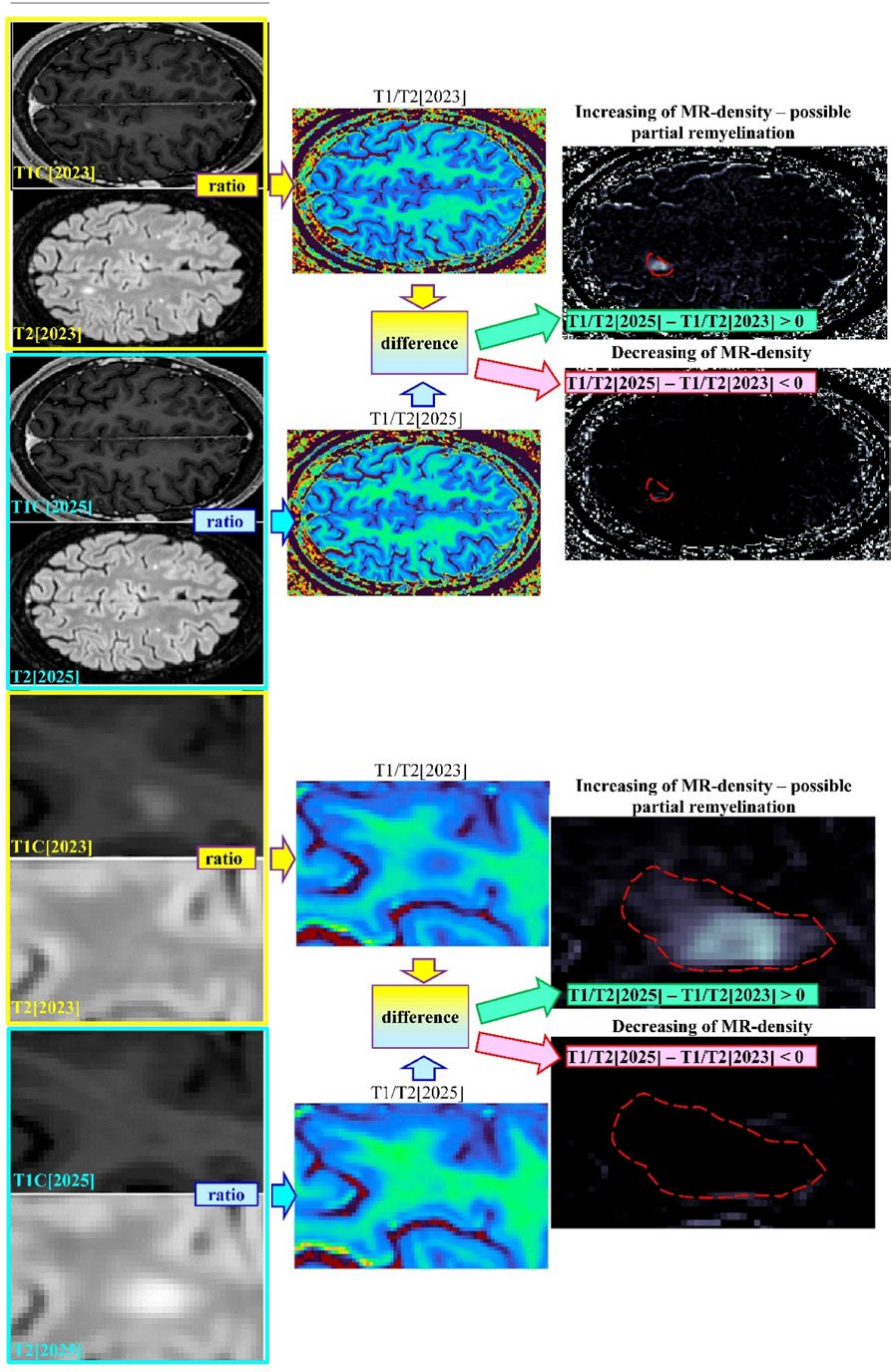
**FIGURE 2** The analyze of MR‐density with growth of T1/T2 ratio.

**Figure:**
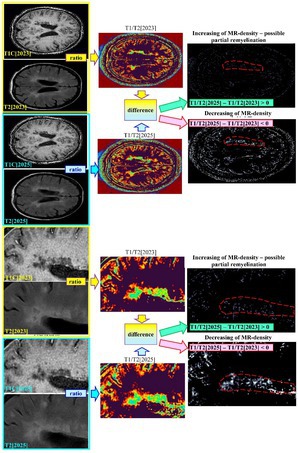
**FIGURE 3** The analyze of MR‐density with decreasing of T1/T2 ratio

**Conclusion:** Increasing of T1/T2 ratio near lesions correspond to clinically confirmed remission in RRMS, decreasing – with progression is SPMS, that correlates well with increasing of neurological symptoms.

**Disclosure:** Nothing to disclose.

## EPO‐0132

### Dynamics of statistical properties of tract density as additional confirmation of fulfillment of NEDA for MS patients

#### A. Buniak ^1^; A. Mikitchuk^2^; T. Charnukha^1^; O. Pereverzeva^1^

##### ^1^Neurological Department, Republican Research and clinicfl center of Neurology and Neurosurgery, Minsk, Belarus; ^2^Faculty of Radiophysics and Computer Technologies, Belarusian State University, Minsk, Belarus

**Background and aims:** The concept of NEDA (no evidence of disease activity) is widely used to describe multiple sclerosis (MS) patients under treatment. Radiological sustainability is one of the most important factors for this criterion. Due to a set of factors residual white matter microstructural changes can be missed on the magnetic resonance imaging (MRI). This research is dedicated to demonstrate that usage of statistical properties of tract density allows to confirm of fulfillment of NEDA for MS patients.

**Methods:** MR‐data collected for 15 patients with relapsing remitting (RR) MS (2023 – 2025), MRI is performed with GE SIGNA Pioneer (3T, 25 directions of b‐value 1000 s/mm^2^). Tractography is realized by DSI Studio with RK4 method, 10^8^ tracts are calculated. For each slice, calculation of histogram gradients and both entropy and sum of TD are performed (Figure 1)

**Figure:**
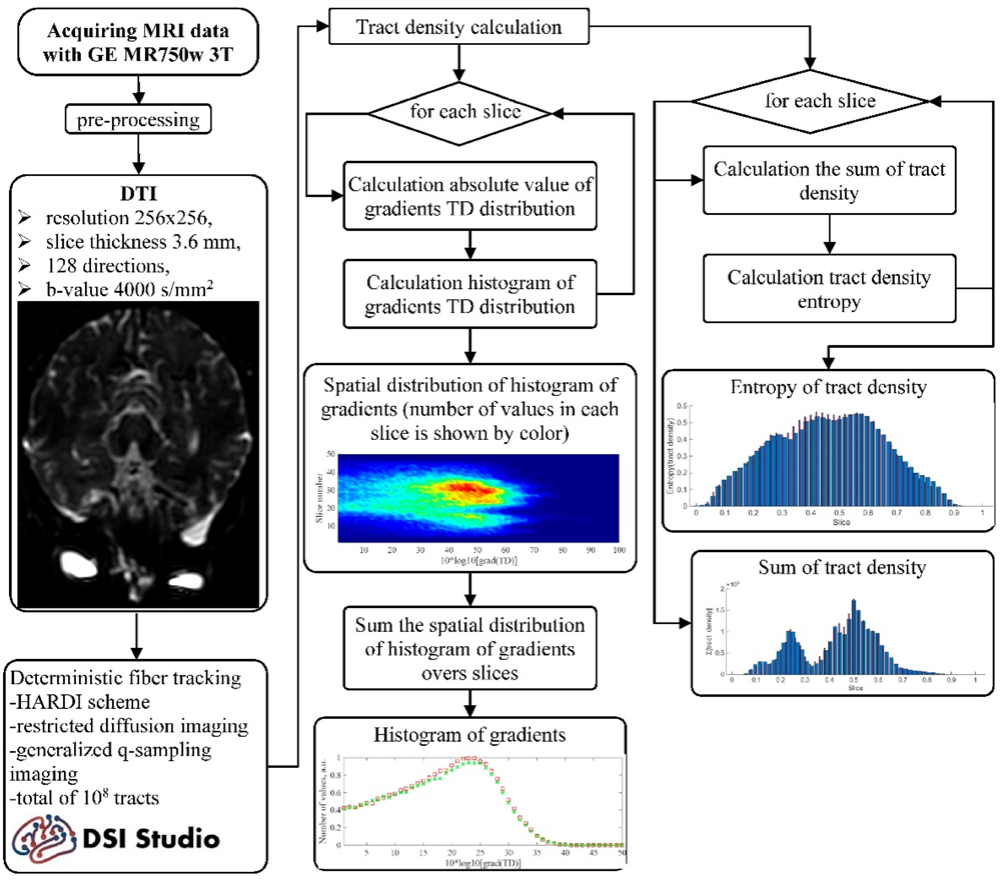
**FIGURE 1** Calculation method of histogram gradients, entropy and sum of TD.

**Results:** This approach allows to directly compare histogram of gradients spatial distribution and of statistical properties of TD (entropy and sum). Additionally, histogram difference can be visualized. In the case under study, RRMS with confirmed NEDA is characterized by sustainable histogram gradients (Fig. 2, 3). E.g, average entropy has changed negligibly on ~2.9% (from 0.69 to 0.71 [bit]), as well as sum – on 0.8% (from 2.29 to 2.27 [10^9^ tracts/voxel]) (fig. 3). Clinically all patients were stable and EDSS score didn’t change.

**Figure:**
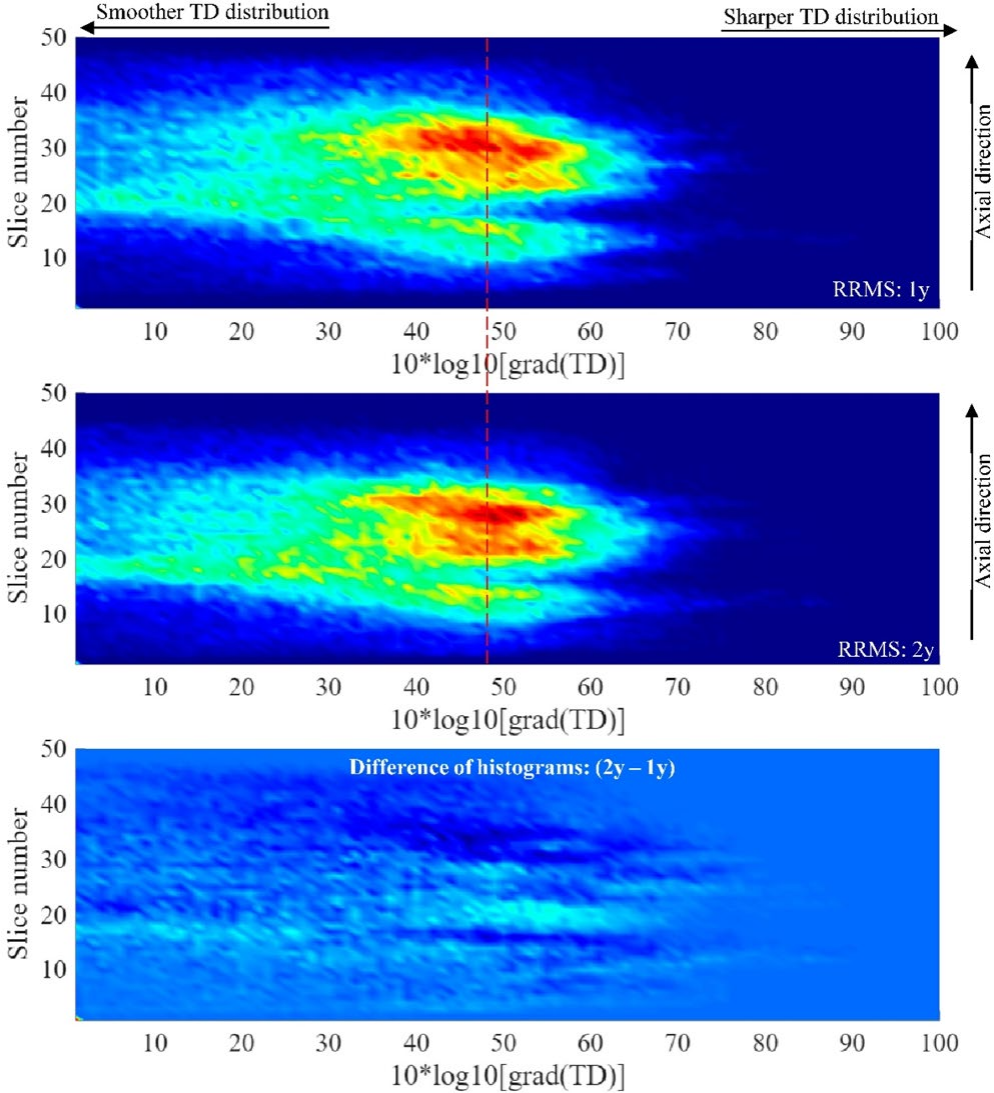
**FIGURE 2** Histogram gradients in 1 and 2 y.

**Figure:**
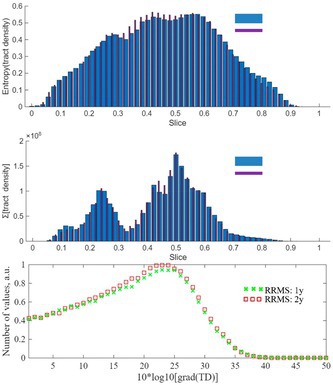
**FIGURE 3** Histograms of tract density entropy

**Conclusion:** It is shown that RRMS with confirmed NEDA also demonstrate sustainability of histogram of gradients spatial distribution, histogram difference, average entropy and sum of TD. This can be additional factor to confirm the fulfillment of NEDA

**Disclosure:** Nothing to disclose.

## EPO‐0133

### Effect of tolebrutinib on disability in relation to MRI activity: A post‐hoc analysis of HERCULES and GEMINI phase 3 trials

#### A. Traboulsee ^1^; D. Arnold^2^; R. Fox^3^; H. Wiendl^4^; A. Bar‐Or^5^; P. Vermersch^6^; B. Cree^7^; S. Syed^8^; Y. Li^9^; W. Vargas^9^; T. Turner^8^; E. Wallstroem^8^; J. Oh^10^

##### ^1^The University of British Columbia, Division of Neurology, Vancouver, Canada; ^2^McGill University and NeuroRx Research, Montréal, Canada; ^3^Mellen Center for Multiple Sclerosis, Cleveland Clinic, Cleveland, USA; ^4^Department of Neurology and Neurophysiology, University of Freiburg, Freiburg, Germany; and Brain and Mind Center, University of Sydney, Sydney, Australia; ^5^Center for Neuroinflammation and Experimental Therapeutics and Department of Neurology, University of Pennsylvania, Philadelphia, USA; ^6^University of Lille, Inserm U1172, CHU Lille, FHU Precise, Lille, France; ^7^UCSF Weill Institute for Neurosciences, University of California San Francisco, Department of Neurology, San Francisco, USA; ^8^Sanofi, Cambridge, USA; ^9^Sanofi, Bridgewater, USA; ^10^St. Michael's Hospital, University of Toronto, Toronto, Canada

**Background and aims:** Tolebrutinib is an oral, brain‐penetrant and bioactive Bruton's tyrosine kinase inhibitor that reduced the risk of disability accumulation by 31% versus placebo in non‐relapsing secondary progressive multiple sclerosis (nrSPMS) and by 29% versus teriflunomide in relapsing multiple sclerosis (RMS) in phase 3 trials. This post‐hoc analysis assessed the relationship between on‐trial lesion formation and risk of disability accumulation in the phase 3 tolebrutinib trials in nrSPMS and RMS.

**Methods:** HERCULES (NCT04411641), GEMINI 1 (NCT04410978) and GEMINI 2 (NCT04410991) were phase 3, double‐blind trials of tolebrutinib 60 mg once‐daily taken with food. In HERCULES, participants with nrSPMS were randomised 2:1 to tolebrutinib or placebo. In GEMINI, participants with RMS were randomised 1:1 to tolebrutinib or teriflunomide (14 mg). We analysed the effect of tolebrutinib on time to onset of 6‐month confirmed disability accumulation (CDA) by the presence/absence of on‐trial new gadolinium‐enhancing (Gd+) lesions and new/enlarging T2 lesions.

**Results:** In HERCULES, the formation of Gd+ or new/enlarging T2 lesions was associated with an increased risk of 6‐month CDA in the placebo group, which was mitigated with tolebrutinib, particularly for participants with new/enlarging T2 lesions (15% risk reduction in those without vs 45% in those with on‐trial lesions). In GEMINI, the risk of 6‐month CDA was similar irrespective of lesion formation in the teriflunomide group, and all analyses favoured tolebrutinib.

**Conclusion:** This post‐hoc analysis suggests that tolebrutinib decreases the risk of disability accumulation in participants with nrSPMS and RMS in both those who develop focal lesions while receiving treatment and those who do not.

**Disclosure:** AT: Consulting and/or speaking and grant/research support (Biogen, EMD Serono, Roche, Sanofi); DA: Personal compensation for serving as a Consultant (Alexion, Biogen, Celgene, Eli Lilly and Company, EMD Serono, Frequency Therapeutics, Genentech, Merck, Novartis, Roche, Sanofi, and Shionogi); equity interest (NeuroRx); RJF: Consulting (Astoria Biologica, Biogen, Bristol Myers Squibb, Cognito, EMD Serono, Galvani, Immunic, INmune Bio, Kiniksa, Novartis, Sanofi, Siemens, TG Therapeutics); advisory committees (AB Science, Biogen, Immunic, Novartis, Sanofi); clinical trial contract and research grant funding (Biogen, Novartis, and Sanofi); HW: Honoraria (AbbVie, Alexion, Argenx, Biogen, BMS, F Hoffmann‐La Roche, Janssen, Merck, Neurodiem, Novartis, Roche, Sanofi, Teva, WebMD); consulting (AbbVie, Actelion, Argenx, Biogen, BMS, EMD Serono, Fondazione Cariplo, Gossamer Bio, Idorsia, Immunic, Immunovant, Janssen, Lundbeck, Merck, NexGen, Novartis, PSI, Roche, Sanofi, Swiss Multiple Sclerosis Society, UCB, Worldwide Clinical Trials); research funding (Alexion, Amicus, Argenx, Biogen, CSL, Deutsche Forschungsgesellschaft, Deutsche Myasthenie Gesellschaft, F Hoffmann‐La Roche, German Ministry for Education & Research, Merck, Novartis, Roche, Sanofi, UCB); AB‐O: Grant support to the University of Pennsylvania (Biogen Idec, EMD Serono, Novartis, Roche Genentech); speaking and/or consulting (Accure, Atara Biotherapeutics, Biogen, Bristol Myers Squibb, EMD Serono, GlaxoSmithKline, Gossamer, Janssen, MedImmune, Novartis, Roche Genentech, Sanofi); PV: Honoraria or consulting (AB Science, Ad Scientiam, Biogen, Celgene‐BMS, Imcyse, Merck, Novartis, Roche, Sanofi); research support (Biogen, F. Hoffmann‐La Roche, Merck, Novartis, Sanofi); BACC: Consulting (Alexion, Atara, Autobahn, Avotres, Biogen, Boston Pharma, EMD Serono, Gossamer Bio, Horizon, Immunic, Neuron23, Novartis, Sandoz, Sanofi, Siemens, TG Therapeutics, Therini); research (Genentech); SS, YL, WSV, TJT, EW: Employees of Sanofi (may hold shares and/or stock options in the company); JO: Consulting and/or speaking (Amgen, Biogen, Eli Lilly and Company, EMD Serono, Novartis, Roche, Sanofi); research (Biogen, Roche).

## EPO‐0134

### Blood protein profiles are associated with brain atrophy in multiple sclerosis

#### C. Tafrali ^1^; E. Hofer^2^; M. Martinez‐Serrat^1^; M. Haindl^2^; T. Kaiser^1^; R. Demjaha^1^; S. Hechenberger^3^; B. Helmlinger^3^; D. Pinter^3^; P. Opriessnig^2^; B. Heschl^2^; A. Damulina^2^; G. Datta^4^; D. Brazel^4^; S. Ropele^2^; C. Enzinger^2^; F. Qureshi^4^; M. Khalil^1^

##### ^1^Neurology Biomarker Research Unit, Department of Neurology, Medical University of Graz, Graz, Austria; ^2^Department of Neurology, Medical University of Graz, Graz, Austria; ^3^Research Unit for Neuronal Plasticity and Repair, Medical University of Graz, Austria; ^4^Octave Bioscience, Inc, Menlo Park, USA

**Background and aims:** Brain atrophy is a key determinant of long‐term disability in multiple sclerosis (MS). While MRI‐based volumetry provides prognostic information, its limited scalability makes blood‐based biomarkers an attractive tool for monitoring neurodegeneration. The MS Disease Activity (MSDA) panel profiles 18 proteins reflecting key pathways, including neuroinflammation, immunomodulation, myelin biology, and neuroaxonal integrity. In this study, we investigate whether serum MSDA scores are predictive of subsequent brain atrophy.

**Methods:** In this retrospective study, 140 MS patients with paired serum samples and longitudinal 3T MRI (median follow‐up 2.1 years, IQR:1.2–3.2) were analyzed. Patients were stratified into high and low annualized brain volume change groups, matched for age, sex, treatment status, and disease duration (*n* = 70; 35 per group). Protein levels were measured on the Olink™ platform. Group differences were assessed using non‐parametric tests and regression models adjusted for age and sex, with Bonferroni correction for multiple testing.

**Results:** Patients in the high‐atrophy group showed greater loss of whole brain, white matter, deep gray matter, and thalamic volumes. All proteomic pathway scores, except for neuroinflammation, were significantly elevated in the high‐atrophy group compared low atrophy group. At the individual protein level, serum neurofilament light chain (NfL) was increased and CXCL9 decreased in high‐atrophy group. Higher NfL levels predicted greater brain volume loss, and combining with CXCL9 levels improved distinction between high‐ and low‐atrophy patients.

**Conclusion:** These results demonstrate that distinct serum proteomic profiles are associated with accelerated brain atrophy in MS and support using multi‐analyte blood biomarkers alongside MRI for early risk stratification and monitoring of neurodegeneration.

**Disclosure:** C. Tafrali has received travel funding and speaker honoraria from Merck and Novartis. E. Hofer has nothing to disclose. M. Martinez‐Serrat has received travel funding and speaker honoraria from Novartis. MT. Haindl has nothing to disclose. T. Kaiser has nothing to disclose. R. Demjaha has received travel funding from Janssen, Novartis and Sanofi. S. Hechenberger has received speaking honoraria from Roche, Bristol‐Myers Squibb, and Merck. B. Helmlinger has received speaking honoraria from Roche, Sanofi, and Bristol‐Myers Squibb, and travel funding from Janssen. P. Opriessnig has nothing to disclose. D. Pinter is a member of the advisory board for “Cognition and MS” for Novartis and has received speaking honoraria from Biogen, Novartis, MedAhead and Bristol‐Myers Squibb. B. Heschl has nothing to disclose. A. Damulina has participated in meetings sponsored by, received speaker honoraria or travel funding from Sanofi‐Aventis, Novartis and Janssen. G. Datta is an employee of Octave Bioscience D. Brazel is an employee of Octave Bioscience F. Qureshi is an employee of Octave Bioscience S. Ropele has nothing to disclose. C. Enzinger has received funding for traveling and speaker honoraria from Biogen Idec, Bayer Schering Pharma, Merck Serono, Novartis, Genzyme and Teva Pharmaceutical Industries Ltd./Sanofi‐Aventis, Shire; received research support from Merck Serono, Biogen Idec, and Teva Pharmaceutical Industries Ltd./Sanofi‐Aventis; and serves on scientific advisory boards for Bayer Schering Pharma, Biogen Idec, Merck Serono, Novartis, Genzyme, Roche, and Teva Pharmaceutical Industries Ltd./Sanofi‐ Aventis. M. Khalil has received travel funding and speaker honoraria from Bayer, Biogen, Novartis, Merck, Sanofi and Teva and serves on scientific advisory boards for Biogen, Bristol‐Myers Squibb, Gilead, Merck, Novartis, Alexion, Amgen and Roche. He received research grants from Biogen, Novartis and Teva.

## EPO‐0135

### Comparative efficacy and safety of disease‐modifying therapies in pediatric relapsing‐remitting multiple sclerosis: A network meta‐analysis

#### I. Kamal^1^; M. Nasser Elshabrawi^2^; D. Atef Abouda^3^; O. F Abbas^1^; M. Nabil Galal^4^; H. Tharwat Ali^5^; M. Fawzi Hemida^3^; A. Adel Mohamed^6^; K. Zinhom ^7^; M. Mamdouh^8^; A. Hashem Fathallah^9^; A. Zakarya Nourelden^1^

##### ^1^Faculty of Medicine, Al‐Azhar University, Cairo, Egypt; ^2^Faculty of Medicine, Port Said University, Port Said, Egypt; ^3^Faculty of Medicine, Alexandria University, Alexandria, Egypt; ^4^Faculty of Medicine, Menoufia University, Menoufia, Egypt; ^5^Qena Faculty of Medicine, South Valley University, Qena, Egypt; ^6^Faculty of Medicine, Zagazig University, Zagazig, Egypt; ^7^Faculty of Medicine, October 6 University, Giza, Egypt; ^8^Faculty of Medicine, Beni‐Suef University, Beni‐Suef, Egypt; ^9^Faculty of Medicine, Minya University, Minya, Egypt

**Background and aims:** Pediatric multiple sclerosis (MS) is a rare disease but increasingly reported, with higher inflammatory activity and relapses than adult‐onset MS. Available treatments include interferon beta‐1a, glatiramer acetate (GA), fingolimod, dimethyl fumarate (DMF), and teriflunomide, but comprehensive comparisons of disease‐modifying therapies (DMTs) remain limited.

**Figure:**
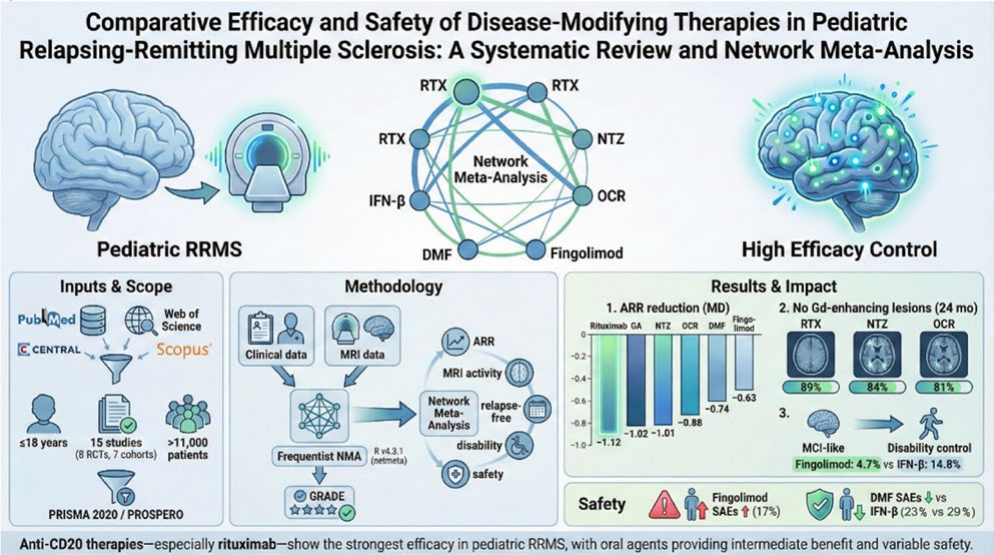
**FIGURE 1** Graphical abstract.

**Methods:** PubMed, Cochrane CENTRAL, Web of Science, and Scopus were searched for studies published up to December 2025. Outcomes assessed the efficacy of DMTs in pediatric MS, including annualized relapse rate (ARR), MRI lesion activity, safety, free relapse rate, and disability progression. All Analyses were conducted using R v4.3.1.

**Results:** Fifteen studies, including over 11,000 pediatric and young adult MS patients, were analyzed. Rituximab produced the largest ARR reduction (‐1.12), followed by GA (‐1.02) and natalizumab (−1.01), while ocrelizumab, DMF, and fingolimod also showed significant ARR reductions; interferon beta and teriflunomide were non‐significant. At 24 months, the absence of gadolinium‐enhancing lesions was most frequent with rituximab (89%), followed by natalizumab (84%) and ocrelizumab (81%). Fingolimod showed higher relapse‐free rates than low‐dose interferon beta (86% vs 39%) and ocrelizumab (77% vs 52%), and lower disability progression (4.7% vs 14.8%). Teriflunomide reduced disability progression versus placebo (11.9% vs 17.5%). Serious adverse events were more frequent with fingolimod than with low‐dose interferon beta (17% vs 7%) but less frequent with DMF (23% vs 29%).

**Figure:**
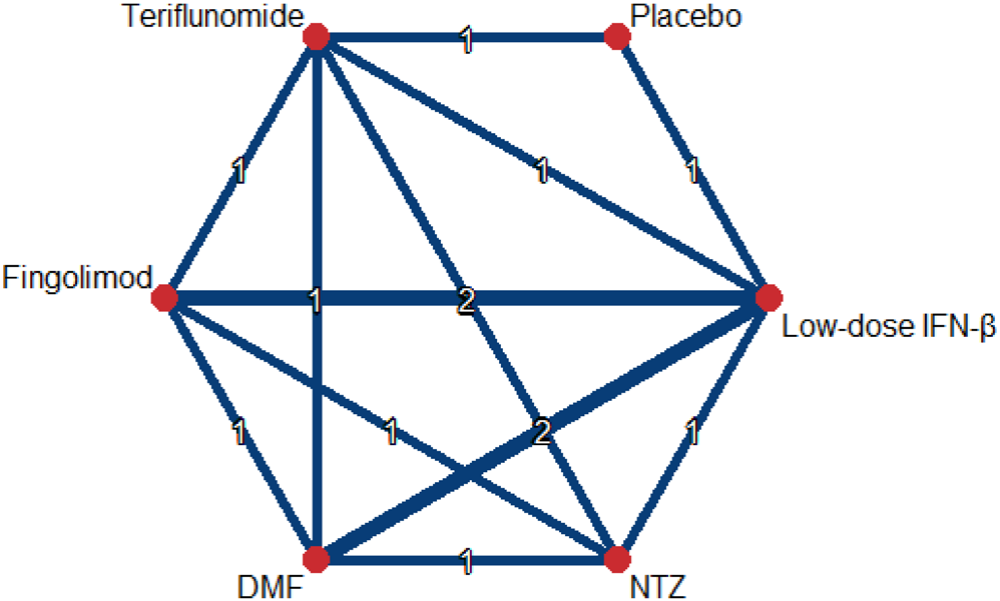
**FIGURE 2** Net graph.

**Figure:**
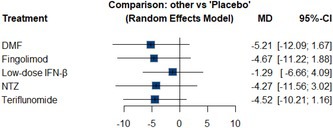
**FIGURE 3** Forest plot ARR.

**Conclusion:** Rituximab was the most effective therapy for reducing relapse rates in pediatric and young MS patients, with additional MRI, relapse‐free, and disability benefits observed across agents and variable safety profiles.

**Disclosure:** The authors declare no conflicts of interest.

## EPO‐0136

### Reproductive‐life planning, pregnancy, and peri‐ and post‐partum management in multiple sclerosis: International expert consensus

#### R. Bove^1^; C. Castelo‐Branco^2^; J. Correale^3^; K. Fink^5^; K. Hellwig^6^; M. Houtchens^7^; M. Houtchens^8^; M. Magyari^9^; M. Magyari ^10^; G. Merki‐Feld^11^; S. Montgomery^12^; S. Montgomery^13^; S. Montgomery^14^; R. Nappi^15^; R. Nappi^16^; E. Stenager^17^; E. Stenager^18^; H. Thompson^19^; Z. Tulek^20^; H. Salloukh^21^; M. Simoni^22^; J. Correalle^4^

##### ^1^University of California San Francisco Weill Institute for Neurosciences, University of California, San Francisco, USA; ^2^Department of Gynecology, Institut Clínic of Gynecology, Obstetrics and Neonatology, Hospital Clínic of Barcelona, IDIBAPS, University of Barcelona, Barcelona, Spain; ^3^Department of Neurology, Institute for Neurological Research, Fleni, Buenos Aires, Argentina; ^4^Institute of Biological Chemistry and Physicochemistry (IQUIFIB), CONICET/University of Buenos Aires, Buenos Aires, Argentina; ^5^Department of Clinical Neuroscience, Karolinska Institutet, Stockholm, Sweden; ^6^Katholisches Klinikum Bochum GmbH, Nordrhein‐Westfalen, Bochum, Germany; ^7^Harvard Medical School, Boston, USA; ^8^Brigham and Women's Hospital, Boston, USA; ^9^Danish Multiple Sclerosis Center, University Hospital Rigshospitalet, Copenhagen, Denmark; ^10^Department of Clinical Medicine, University of Copenhagen, Copenhagen, Denmark; ^11^University of Zürich, Zürich, Switzerland; ^12^Clinical Epidemiology and Biostatistics, School of Medical Sciences, Faculty of Medicine and Health, Örebro University, Örebro, Sweden; ^13^Clinical Epidemiology Division, Department of Medicine, Solna, Karolinska Institutet, Stockholm, Sweden; ^14^Department of Epidemiology and Public Health, University College London, London, UK; ^15^Department of Clinical, Surgical, Diagnostic and Pediatric Sciences, University of Pavia, Pavia, Italy; ^16^Research Center for Reproductive Medicine, Gynecological Endocrinology and Menopause, IRCCS San Matteo Foundation, Pavia, Italy; ^17^Department of Regional Research, University of Southern Denmark, Odense, Denmark; ^18^MS‐clinic of Southern Jutland (Aabenraa, Esbjerg, Kolding), Aabenraa, Denmark; ^19^Southern Health & Social Care Trust, Portadown, Northern Ireland, UK; ^20^lorence Nightingale Faculty of Nursing, Istanbul University‐Cerrahpasa, Istanbul, Türkiye; ^21^Ares Trading SA, Eysins, Switzerland, an affiliate of Merck KGaA; ^22^Unit of Endocrinology, Department of Medical Specialties, University Hospital and Department of Biomedical, Metabolic and Neural Sciences, University of Modena and Reggio Emilia, Modena, Italy

**Background and aims:** A modified Delphi consensus program was conducted to generate evidence‐based, practical clinical recommendations (CRs) for healthcare professionals (HCPs) to address gaps in the literature and guidelines relating to reproductive‐life planning, pregnancy, and peri‐ and post‐partum management for people with multiple sclerosis (PwMS).

**Methods:** A multidisciplinary steering committee (SC) of 14 international multiple sclerosis (MS) experts identified 14 key clinical questions across five themes. A total of 14 CRs were drafted, informed by evidence from a systematic literature review and expert insights. The SC and an extended faculty comprising 23 HCPs from 12 countries voted to ascertain expert agreement (≥75% scoring 7–9).

**Results:** Consensus was reached on all 14 CRs after two rounds of voting, with 13 achieving 90–100% agreement. CRs are provided across five themes: 1) General reproductive‐life planning – timing for discussions, key counseling points, and challenges when adjusting treatment; 2) Trying to conceive – factors influencing fertility and MS treatment considerations; 3) Fertility treatments – when to consider them and best practices; 4) Pregnancy – pre‐conception and early‐pregnancy care, relapse risk, and pregnancy‐related complications; and 5) Peri‐ and post‐partum – labor and delivery, relapse risk, MS treatment considerations, and breastfeeding. An overarching CR provides expert guidance on comprehensive reproductive care and effective interdisciplinary coordination.

**Conclusion:** These recommendations underscore the importance of patient‐centered reproductive‐life planning for PwMS, providing expert guidance that supports timely counseling, safe management, and informed patient‐driven clinical decisions across all reproductive stages.

**Disclosure:** This work was supported by Merck (CrossRef Funder ID: 10.13039/100009945), who provided funding for the project.

## EPO‐0137

### Rethinking annual MRI monitoring in ocrelizumab‐treated multiple sclerosis patients: A real‐world number needed to scan analysis

#### L. Schoof ^1^; R. Heijnen^1^; L. Hogenboom^1^; Z. van Kempen^1^; B. van Oosten^1^; J. Killestein^1^; F. Barkhof^2^; E. Strijbis^1^

##### ^1^MS Center Amsterdam, Neurology, Vrije Universiteit Amsterdam, Amsterdam Neuroscience, Amsterdam UMC location VUmc, the Netherlands; ^2^MS Center Amsterdam, Radiology and Nuclear Medicine, Vrije Universiteit Amsterdam, Amsterdam Neuroscience, Amsterdam UMC location VUmc, the Netherlands & UCL London, Institutes of Neurology and Healthcare Engineering, London, UK

**Background and aims:** Ocrelizumab is a widely used high‐efficacy therapy for multiple sclerosis (MS). Although current guidelines recommend annual MRI‐monitoring, the low rate of radiological disease activity during long‐term ocrelizumab therapy suggests that this recommendation should be re‐evaluated.

**Methods:** We included ocrelizumab‐treated MS patients, both relapsing‐ and primary progressive‐, from the Amsterdam MS Cohort with at least two brain MRI‐scans performed during ≥3 months of treatment. Radiological activity and significant radiological activity were defined as either ≥1 or ≥2 CELs on a MRI‐scan performed ≥ 3 months after ocrelizumab initiation or any or ≥3 new/enlarging T2 lesions on a subsequent MRI‐scan compared with a scan performed at least ≥3 months after ocrelizumab initiation. The number needed to scan (NNS) was calculated as the inverse of the predicted probability of disease activity.

**Results:** Among 1,639 MRI‐scans in 342 patients, (significant) radiological disease activity was detected in 27 scans in 23 patients (1.8% of scans; 6.8% of patients) over a median treatment duration of 49 months. All three patients in whom MRI findings prompted a treatment switch had clinical symptoms preceding the MRI scan. NNS increased with treatment duration. No new pharmacovigilance findings were identified.

**Figure:**
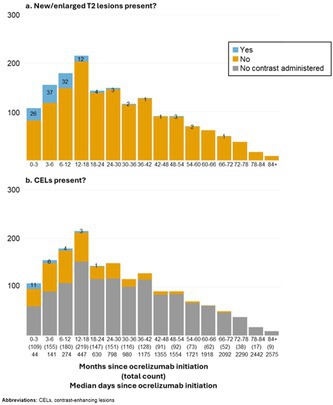
**FIGURE 1** MRI scans showing new/enlarged T2 lesions or CELs during ocrelizumab treatment.

**Figure:**
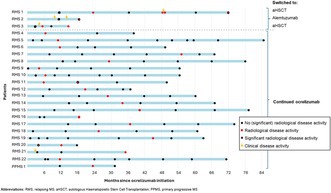
**FIGURE 2** Individual patients with (significant) radiological disease activity during ocrelizumab treatment.

**Figure:**
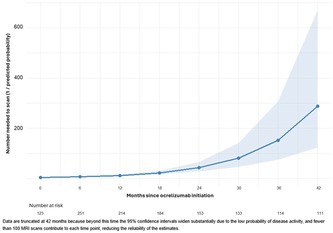
**FIGURE 3** Number needed to scan per treatment duration in months.

**Conclusion:** Routine annual MRI‐monitoring in asymptomatic patients never led to treatments changes in clinical practice. Increasing NNS over time, especially after two years of treatment, indicates that MRI intervals can be safely extended in stable patients; potentially doubling the interval from year 2 onwards. Radiological monitoring remains essential during early treatment and in clinically symptomatic patients to identify cases requiring treatment re‐evaluation.

**Disclosure:** LS: reports no disclosures. RH: reports no disclosures. LH: reports no disclosures. ZK: reports no disclosures. BO: reports no disclosures. JK: received consulting fees for F. Hoffmann‐La Roche, Biogen, Teva, Merck, Novartis and Sanofi/Genzyme (payments to institution); reports speaker relationships with F. Hoffmann‐La Roche, Biogen, Teva, Merck, Novartis and Sanofi/Genzyme (payments to institution); adjudication committee of MS clinical trials of Immunic (payments to institution). FB: is part of the steering committee or is a data safety monitoring board member for Biogen, Merck, Eisai, and Prothena; is an advisory board member for Combinostics and Scottish Brain Sciences; is a consultant for Roche, Celltrion, Rewind Therapeutics, Merck, and Bracco; has research agreements with ADDI, Merck, Biogen, GE Healthcare, and Roche; and is cofounder and shareholder of Queen Square Analytics LTD. ES: has received speaker fees from Merck and Novartis.

## EPO‐0138

### Relapse activity after ponesimod discontinuation in the OPTIMUM long‐term extension study

#### L. Kappos ^1^; C. Pozzilli^2^; E. Havrdova^3^; R. Hohlfeld^4^; R. Fox^5^; M. Freedman^6^; A. Sarfati^7^; M. Heubl^7^; S. de Guitaut^7^; A. Atallah^7^; X. Montalban^8^

##### ^1^Neurology and Research Center for Clinical Neuroimmunology and Neuroscience Basel, Clinical Research, Biomedicine and Biomedical Engineering University Hospital and University of Basel, Basel, Switzerland; ^2^Sant’Andrea Multiple Sclerosis Centre, Sapienza University of Rome, Rome, Italy; ^3^Department of Neurology, First Medical Faculty, Charles University, Prague, Czech Republic; ^4^Institute of Clinical Neuroimmunology, Ludwig Maximilians University Munich, Munich, Germany; ^5^Cleveland Clinic, Cleveland, Ohio, USA; ^6^University of Ottawa, Department of Medicine, and The Ottawa Hospital Research Institute, Ottawa, Ontario, Canada; ^7^Medical Affairs Department, Juvise Pharmaceuticals, Paris, France; ^8^Department of Neurology‐Neuroimmunology, Multiple Sclerosis Center of Catalonia, Vall d’Hebron University Hospital, Barcelona, Spain

**Background and aims:** OPTIMUM was a 2‐year phase 3 trial comparing ponesimod versus teriflunomide in relapsing multiple sclerosis. In the OPTIMUM Long‐Term Extension (LTE), all participants received ponesimod 20 mg for up to 5 additional years. In the core study analysis there were no signals of rebound or reactivation after discontinuation. We assessed whether stopping ponesimod after longer exposure was followed by clinical reactivation.

**Methods:** This post‐hoc analysis included participants who discontinued ponesimod in the OPTIMUM‐LTE, which were followed per protocol for 30 to 44 days. Confirmed relapses were counted and annualized relapse rate (ARR) was estimated for the 6 months before discontinuation (on‐treatment) and the available post‐discontinuation period.

**Results:** Among 877 participants enrolled in the OPTIMUM long‐term extension, 551 discontinued ponesimod and were included (core ponesimod: *n* = 265; core teriflunomide: *n* = 286). Median post‐discontinuation follow‐up was 38 days (IQR 31 – 48 days). There were 58 relapses during the on‐treatment reference period versus 7 relapses post‐discontinuation, corresponding to ARR 0.21 versus 0.11, respectively (RR 0.51; 95% CI [0.23 ‐ 1.12]) (Table 1). No post‐discontinuation relapse was associated with an Expanded Disability Status Scale (EDSS) increase ≥2 points (Figure 1). Subgroup analyses showed that 423 patients did not receive subsequent therapy within 30 days after discontinuation and had an ARR of 0.12 during this period, compared with 0.18 during the on‐treatment period.

**Figure:**
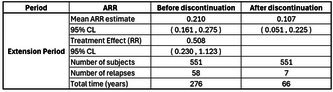
**TABLE 1** Annualized Relapse Rate (ARR) estimates before and after ponesimod discontinuation among patients that permanently discontinued ponesimod at the end of study; Negative binomial model was applied; RR: Rate Ratio, CL: Confidence Level.

**Figure:**
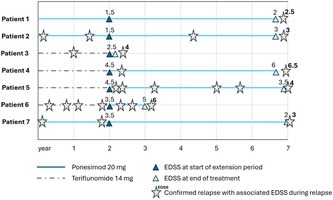
**FIGURE 1** Representative visualization of the evolution of EDSS and relapses of the 7 patients which presented relapses after discontinuation of ponesimod. EDSS: Expanded Disability Status Scale

**Conclusion:** In this analysis, ARR and relapse EDSS did not suggest a clinically meaningful reactivation or rebound after ponesimod discontinuation within the available follow‐up. Real‐world data with longer follow‐up are needed to confirm these findings.

**Disclosure:** L.K. declares no personal compensation. Over the past 3 years, the University Hospital Basel / Foundation Clinical Neuroimmunology and Neuroscience Basel received payments for steering committees, advisory boards, DSMB participation, consultancy, and educational activities from Autolus, Bayer, Biogen, BMS, Celltrion, Clene Nanomedicine, EMD Serono, Er‐KIM, Galapagos, Genentech, Idorsia, Immunic, Janssen, Laboratoires Juvise, Kiniksa, Merck Healthcare, MSD, Minoryx, Neurostatus UHB, Novartis, Roche, Sanofi, Shionogi, Wellmera and Zai Lab, as well as research support from Innosuisse, Novartis and Roche. X.M. has received speaker honoraria and travel expenses, and has served on steering committees or advisory boards for Abbvie, Actelion, Alexion, Biogen, BMS/Celgene, EMD Serono, Genzyme, Roche, Immunic, Janssen, Medday, Merck, Mylan, Nervgen, Novartis, Sandoz, Sanofi‐Genzyme, Teva, TG Therapeutics, Excemed, MSIF and NMSS. C.P. has served on advisory boards for Novartis, Merck, Biogen, Sanofi‐Genzyme, Roche, Janssen and Alexion; received travel and speaker honoraria from Merck, Serono, Biogen, Sanofi‐Genzyme, Roche, Almirall, Janssen, Alexion and Novartis; and research support from Merck, Biogen, Novartis and Almirall. R.H. has received personal fees from Novartis, Sanofi, Merck, Biogen, Teva, Janssen/J&J and Roche. E.K.H. has received grants or contracts from the Czech Ministry of Education (Cooperatio LF1 and EXCELES projects). She has received payments or honoraria for lectures, presentations, speaker bureaus, writing, or educational events from Actelion/Janssen, Biogen, Celgene/BMS, Merck, Novartis, Roche, Sanofi and Teva, and has participated in DSMBs or advisory boards for Actelion/Janssen, Biogen, Celgene/BMS, Merck, Novartis, Roche and Sanofi. R.F. has served as consultant or advisory board member for Arrowhead, Astoria Biologica, Biogen, BMS, Cognito, Eli Lilly, Roche, Galvani, Immunic, Inmune Bio, Innocare, Kiniksa, NGM Bio, Novartis, Pfizer, Population Council, Sanofi, Siemens, TG Therapeutics, Tr1X, Ventus and Viracta. His institution has received clinical trial contracts from Sanofi and Synaptogenix. He has participated in DSMBs for AB Science and has been employed by Janssen Pharmaceuticals. M.S.F. has received research or educational grants from Sanofi‐Genzyme Canada; consulting fees or honoraria from Alexion/AstraZeneca, BiogenIdec, EMD Serono/Merck Serono, Find Therapeutics, Roche, Novartis, Quanterix, Sanofi‐Genzyme and Teva Canada. He serves on advisory boards or boards of directors for Alexion/AZ, Atara, Bayer, Celestra, EMD/Merck, Find Therapeutics, Roche, Actelion/Janssen, Novartis, Sanofi‐Genzyme and Setpoint Medical, and has participated in speaker bureaus for EMD Serono and Sanofi‐Genzyme. M.H, S.G, A.A. and A.S. are employees of Juvise Pharmaceuticals.

## EPO‐0139

### Automated analysis of natural speech reveals emotional alterations in multiple sclerosis

#### P. Mašková ^1^; M. Šubert^1^; M. Novotný^1^; T. Tykalová^1^; B. Srpová^2^; L. Friedová^2^; D. Horáková^2^; T. Uher^2^; J. Rusz^1^

##### ^1^Czech Technical University in Prague, Faculty of Electrical Engineering, Department of Circuit Theory, Prague, Czech Republic; ^2^Charles University, First Faculty of Medicine, Department of Neurology and Centre of Clinical Neuroscience, Prague, Czech Republic

**Background and aims:** Speech conveys emotional states that standard clinical assessments often miss. In multiple sclerosis (MS), affective disturbances are common yet difficult to quantify objectively. This study investigated whether automated language‐based emotion analysis can identify affective alterations in MS.

**Methods:** We analyzed speech from 240 native Czech speakers, including 120 patients with MS and 120 age‐, sex‐, and education‐matched healthy controls. Participants completed two speech tasks: a spontaneous monologue and a story‐telling narration. Speech recordings were automatically transcribed using an automatic speech recognition system. Emotional content was quantified using a fine‐tuned multilingual BERT (mBERT) model adapted to recognize six emotional states: joy, surprise, love, anger, fear, and sadness.

**Results:** In spontaneous monologues, patients with MS demonstrated significantly higher levels of anger (*p* = 0.034) and lower levels of joy (*p* = 0.003) compared to controls (Figure 1). During the story‐telling task, MS patients showed reduced joy (*p* = 0.024) relative to controls. Anger was the dominant emotion in the MS group, accounting for 31% of the discourse, followed by joy (24%) and sadness (22%). In contrast, healthy controls exhibited joy as the predominant emotion (30%), followed by anger (29%) and sadness (21%). Surprise emotion was not analyzed because it was not detected in any sentence in 82% of participants.

**Figure:**
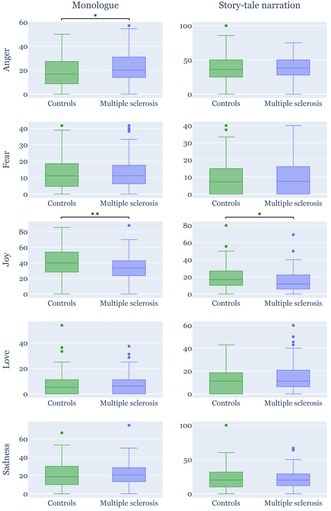
**FIGURE 1** Between‐group differences in individual emotional features. Asterisks indicate statistically significant differences based on independent‐samples *t*‐tests (*p < 0.05, **p < 0.01).

**Conclusion:** Language‐based emotion analysis revealed distinct affective alterations in individuals with MS, most notably reduced joy and increased anger. Fine‐tuned large language model enables a fully automated and objective assessment of these changes and may complement and extend conventional clinical evaluations in MS.

**Disclosure:** This project was supported by the ERC–CZ program from the Ministry of Education Youth and Sports (project no. LL2504).

## EPO‐0140

### Diffusion tensor image analysis along the perivascular space in multiple sclerosis: Microstructural influences & considerations for glymphatic function

#### R. Sharifpour^1^; S. Elands ^1^; A. Rovai^1^; X. De Tiege^1^; M. Strauss^2^

##### ^1^Laboratoire de neuroanatomie et neuroimagerie translationnelles, ULB Neuroscience Institute, Université libre de Bruxelles, Brussels, Belgium; ^2^Université libre de Bruxelles (ULB), Hôpital Universitaire de Bruxelles (H.U.B), CUB Hôpital Érasme, Service de Neurologie, Bruxelles, Belgium

**Background and aims:** The glymphatic system facilitates waste clearance through perivascular pathways and has been implicated in multiple sclerosis(MS) pathophysiology. Diffusion tensor image analysis along the perivascular space(DTI‐ALPS) has been proposed as a noninvasive marker of glymphatic function; however, its specificity in demyelinating disease remains uncertain. This study examined DTI‐ALPS in MS and its associations with structural MRI markers and indicators of disease progression.

**Methods:** Twenty‐six MS patients and 28 healthy controls (Table 1) underwent diffusion‐weighted imaging. Bilateral DTI‐ALPS indices were calculated for all participants. Structural MRI measures included lesion‐load(LL), choroid‐plexus volume(ChPV), and lateral‐ventricle volume(LVV). Clinical measures included Expanded Disability Status Scale (EDSS) and disease duration. Group comparisons were performed (Table 2), and within the MS group, correlations between DTI‐ALPS and imaging and clinical measures were assessed.

**Figure:**
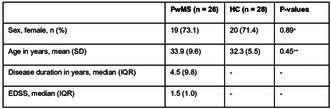
**TABLE 1** Demographics of study participants. Abbreviations: PwMS = Patient with Multiple Sclerosis; HC = Healthy Control; SD = Standard Deviation; IQR = Interquartile Range; EDSS = Expanded Disability Status Scale; *p*‐values calculated using *Chi‐Squared Te.

**Figure:**
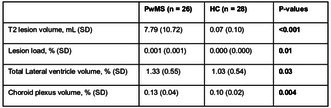
**TABLE 2** MR Imaging Characteristics describing mean normalized volumetry results.Abbreviations: PwMS = Patient with Multiple Sclerosis; HC = Healthy Control; SD = Standard Deviation. *p*‐values calculated using Independent Samples T‐Test.

**Results:** DTI‐ALPS indices did not differ significantly between MS patients and healthy controls (p‐FDR > 0.4). Within the MS group, DTI‐ALPS was not significantly associated with structural or clinical markers after correction for multiple comparisons (p_FDR > 0.76). Importantly, component analysis in patients showed that ALPS indices were strongly associated with diffusion perpendicular to the perivascular axis (the denominator of the ALPS ratio; p_FDR < 0.001). In contrast, correlations with diffusion along the perivascular direction (the numerator) were not significant (p_FDR > 0.1).

**Conclusion:** These findings suggest that DTI‐ALPS in MS may be influenced by white matter microstructural alterations rather than reflecting perivascular glymphatic function. Caution is therefore warranted when interpreting DTI‐ALPS as a marker of glymphatic function, especially in demyelinating disease, and complementary approaches may be required to assess glymphatic pathway involvement.

**Disclosure:** The authors report no conflicts of interest related to this work.

## Muscle and Neuromuscular Junction Disorder 1

## EPO‐0141

### Steroids tapering among patients with generalized myasthenia gravis (gMG) receiving efgartigimod: Interim results from the STRIVE‐gMG chart review

#### A. Habib^1^; N. Silvestri ^2^; C. Qi^2^; H. Yang^3^; A. Corse^4^; G. Smith^5^; T. Vu^6^; G. Wolfe^7^; L. Li^2^; S. Lee^3^; Y. Wu^3^; D. Ye^3^; D. Nowacek^2^; J. Aldridge^2^; A. Mahajerin^2^; G. Philips^2^; J. Howard^4^

##### ^1^University of California, Irvine, Irvine, USA; ^2^argenx US Inc, Boston, MA, USA; ^3^Analysis Group, Inc, Boston, USA; ^4^University of North Carolina at Chapel Hill School of Medicine, Chapel Hill, USA; ^5^Virginia Commonwealth University School of Medicine, Richmond, USA; ^6^University of South Florida Morsani College of Medicine, Tampa, USA; ^7^University at Buffalo Jacobs School of Medicine and Biomedical Sciences, Buffalo, USA

**Background and aims:** Long‐term steroids use among patients with gMG is associated with substantial complications. Efgartigimod, approved in the United States (US) in 2021 for AChR antibody‐positive gMG, has demonstrated clinical benefits that may facilitate steroids reduction. This study evaluated real‐world patterns of steroids tapering following efgartigimod initiation in the US.

**Methods:** STRIVE‐gMG is a retrospective chart review of patients with gMG who received efgartigimod (≥2 cycles) and concurrent steroids at efgartigimod initiation. Data are collected from neurologists in a specialty panel and from participating medical centers. This interim analysis summarizes initial data from the neurologist panel.

**Results:** As of November 10, 2025, 41 patient charts from 15 neurologists were analyzed. All patients were AChR antibody‐positive. At efgartigimod initiation, patients had a mean age of 48.9 years, mean disease duration of 3.7 years, and mean duration on current steroids of 1.1 years. Most (87.8%) had MGFA class II–III disease. Following efgartigimod initiation, all patients tapered steroids, with mean prednisone‐equivalent dose decreasing from 30.4 mg/day at efgartigimod initiation to 8.6, 4.9, and 4.0 mg/day at months 6, 12, and 18, respectively; 68.3% discontinued steroids entirely, primarily due to stable disease control. At the end of follow‐up, 92.7% remained on efgartigimod, with a median treatment duration of 17.1 months. All patients showed improvements in functional limitations and muscle weakness.

**Figure:**
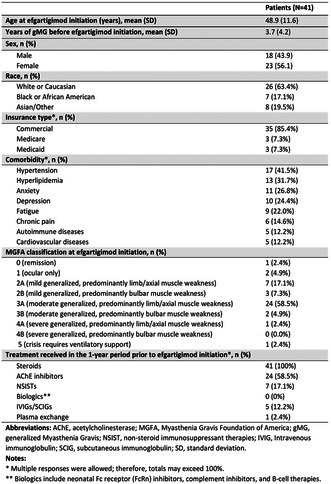
**TABLE 1** Baseline characteristics of patients.

**Figure:**
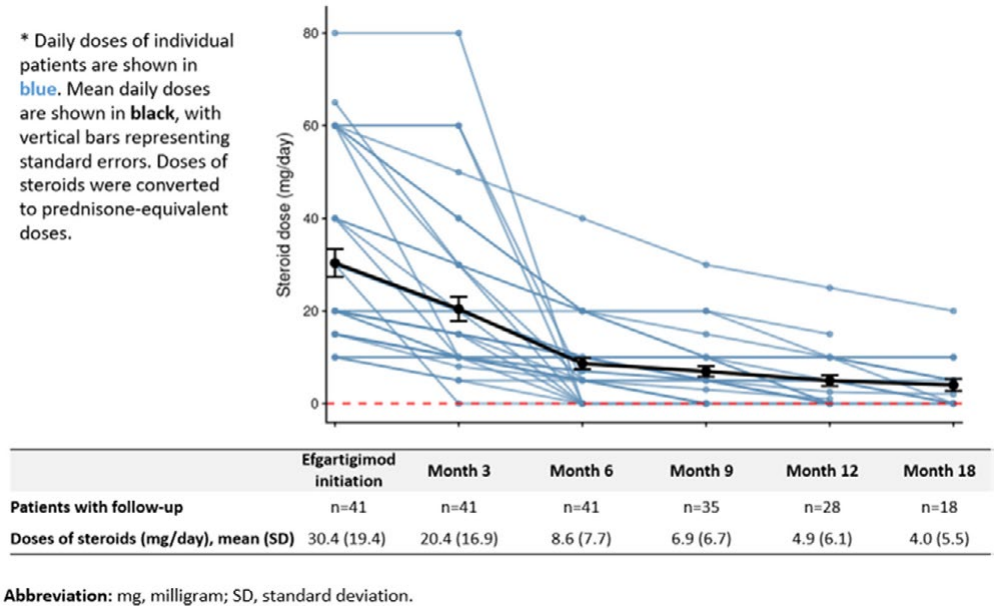
**FIGURE 1** Steroids tapering while on efgartigimod.

**Figure:**
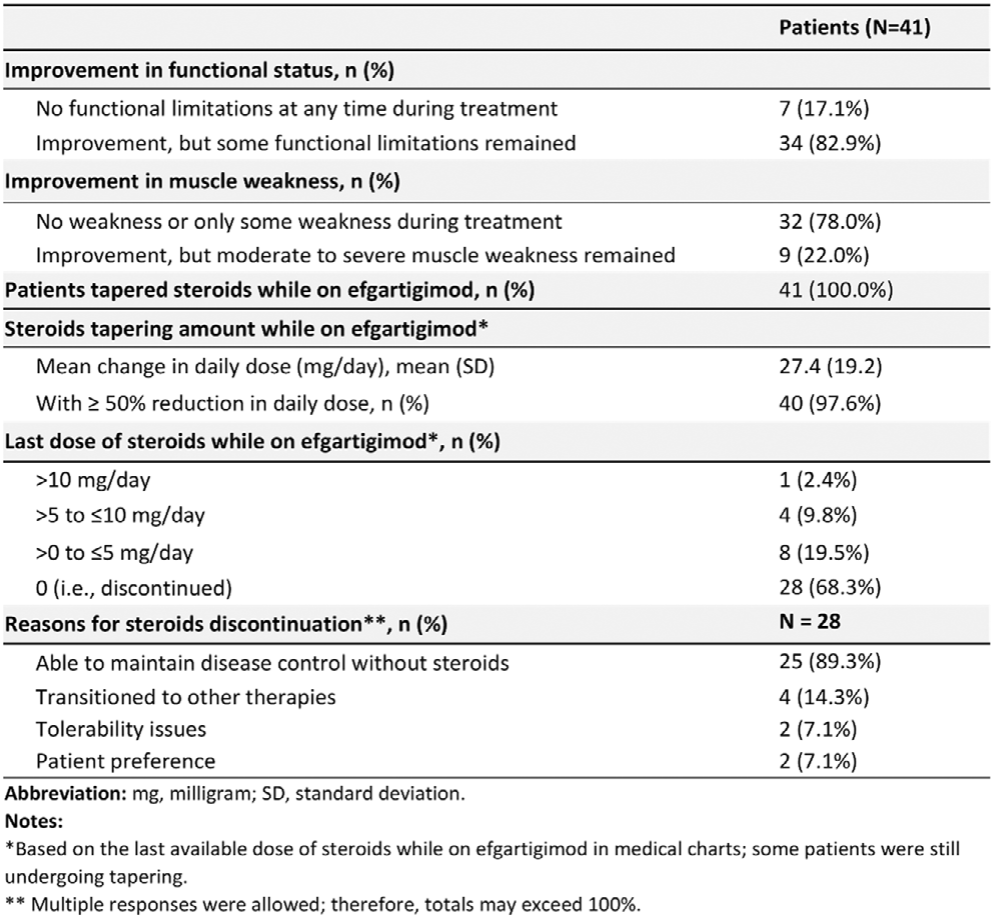
**TABLE 2** Efgartigimod treatment response and steroids tapering while on efgartigimod.

**Conclusion:** In this interim analysis, efgartigimod was associated with profound reductions in steroids dose while maintaining clinical improvement among patients with gMG. Future studies are needed to examine the impacts of steroids reduction on comorbidities and healthcare utilization.

**Disclosure:** This research study is funded by argenx US Inc. Ali A. Habib received research support/honoraria from Alexion/Astra Zeneca, argenx, UCB Pharma, Immunovant, Regeneron, CabalettaBio, Amgen, Novartis, Arcellx, Merck, Nkarta, NMDpharma, Grifols, Cartesian, Jansen/J&J, Cour pharmaceuticals, Kyverna, Vor, Vertex, and MGNet (grant number:U54NS115054) James F. Howard Jr. received research funding (paid to his institution) from Ad Scientiam, Alexion AstraZeneca Rare Disease, argenx, Cartesian Therapeutics, Centers for Disease Control and Prevention, Merck EMD Serono, MGFA, Muscular Dystrophy Association, NIH, NMD Pharma, and UCB Bioscience and received honoraria/consulting fees from AcademicCME, Alexion AstraZeneca Rare Disease, Amgen, argenx, Biohaven Ltd, Cartesian Therapeutics, CheckRare CME, CoreEvitas, Curie.bio, H. Lundbeck A/S, Japan Tobacco Company, Kyverna Therapeutics, Merck EMB Serono, NMD Pharma, Novartis Pharma, PeerView CME, Physicians' Education Resource (PER) CME, PlatformQ CME, Regeneron Pharmaceuticals, Seismic Therapeutics, TG Therapeutics, Toleranzia AB, and UCB Pharma CQ, NS, GP, LL, AM, JA, DN: Employees of argenx; HY, SL, YW, DY: Employee of Analysis Group; TV: Employee of University of South Florida Morsani College of Medicine; GS: Employee of Virginia Commonwealth University School of Medicine; GW: Employees of University at Buffalo Jacobs School of Medicine and Biomedical Sciences; JFH, AC: Employees of University of North Carolina at Chapel Hill School of Medicine; AAH: Employee of University of California, Irvine.

## EPO‐0142

### Safety, efficacy, and QoL impact of long‐term efgartigimod PH20 SC in participants with gMG: Final results of ADAPT‐SC+

#### C. Antozzi ^1^; R. Bhavaraju‐Sanka^2^; J. De Bleecker^3^; A. Meisel^4^; K. Utsugisawa^5^; W. Huang^6^; R. Jimenez^6^; F. Verhamme^6^; L. Liu^6^; D. Korobko^7^; A. Kostera‐Pruszczyk^8^; J. Verschuuren^9^; T. Vu^10^; H. Wiendl^11^; J. Howard Jr^12^

##### ^1^Neuroimmunology and Neuromuscular Diseases Unit, Fondazione IRCCS Istituto Neurologico Carlo Besta, Milan, Italy; ^2^Department of Neurology, University of Texas Health Science Center at San Antonio, San Antonio, USA; ^3^Department of Neurology, Ghent University Hospital, Ghent, Belgium; ^4^Department of Neurology, Charité – Universitätsmedizin Berlin, Berlin, Germany; ^5^Department of Neurology, Hanamaki General Hospital, Hanamaki, Japan; ^6^argenx, Ghent, Belgium; ^7^State Budgetary Healthcare Institution of Novosibirsk Region “The State Novosibirsk Regional Clinical Hospital,” Novosibirsk, Russian Federation; ^8^Department of Neurology, Medical University of Warsaw, Warsaw, Poland; ^9^Department of Neurology, Leiden University Medical Center, Leiden, The Netherlands; ^10^Department of Neurology, University of South Florida Morsani College of Medicine, Tampa, USA; ^11^Department of Neurology, Institute of Translational Neurology, University Hospital Münster, Münster, Germany; ^12^Department of Neurology, The University of North Carolina, Chapel Hill, USA

**Background and aims:** Efgartigimod is a human immunoglobulin G1 (IgG1) Fc fragment that blocks the neonatal Fc receptor. ADAPT‐SC+ (NCT04818671) was an open‐label extension study evaluating long‐term safety and efficacy of subcutaneous (SC) efgartigimod PH20 (coformulated with recombinant human hyaluronidase PH20) in participants with generalized myasthenia gravis (gMG).

**Methods:** Efgartigimod PH20 SC 1000 mg was administered in cycles of 4 once‐weekly injections. Subsequent cycles were initiated based on clinical evaluation (Year 1, ≥ 4 weeks between cycles; Year 2 and onward, ≥1 week between cycles following protocol amendment). Efficacy was assessed using Myasthenia Gravis Activities of Daily Living (MG‐ADL). Quality of life (QoL) impact was assessed during Year 1 using Myasthenia Gravis Quality of Life 15‐Item Questionnaire, Revised (MG‐QoL15r).

**Results:** During ADAPT‐SC+, 180 participants received ≥1 efgartigimod PH20 SC injection (mean [SD] study duration, 2.6 [0.9] years; 459.4 participant years of follow‐up). Adverse events were predominantly mild/moderate (Table 1). After Year 1, efgartigimod remained well tolerated in both participants who averaged <4 weeks and ≥4 weeks between treatment cycles. Among anti‐acetylcholine receptor antibody–positive (AChR‐Ab+) participants, mean MG‐ADL improvements were sustained from Week 1 through Week 163 (Figure 1). Most AChR‐Ab+ participants (59.2%) achieved minimal symptom expression (MSE; MG‐ADL score, 0–1) at least once, and 88.1% of those with MSE at least once sustained MSE during ≥4 weeks. Mean MG‐QoL15r improvements were observed by Week 1 and sustained through Week 55 (Figure 2).

**Figure:**
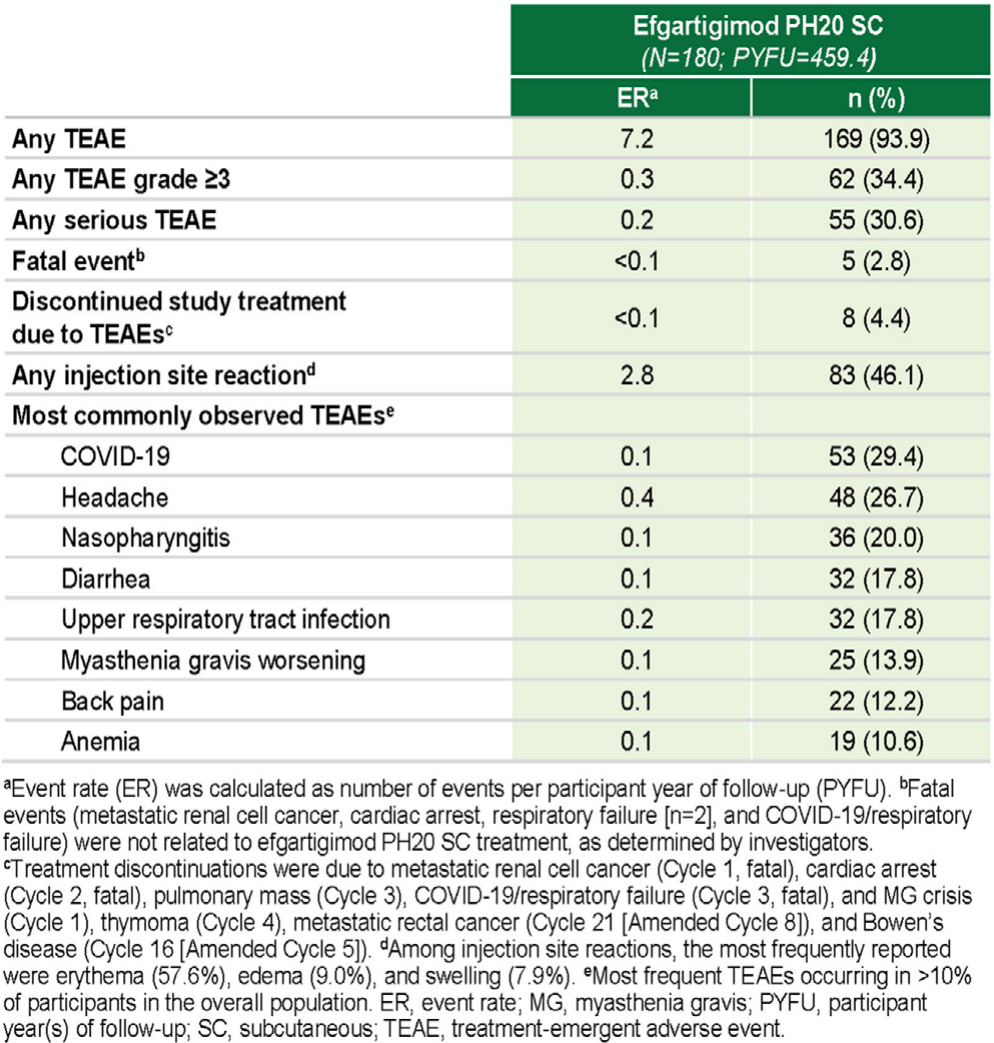
**TABLE 1** Summary of TEAEs: Overall Population.

**Figure:**
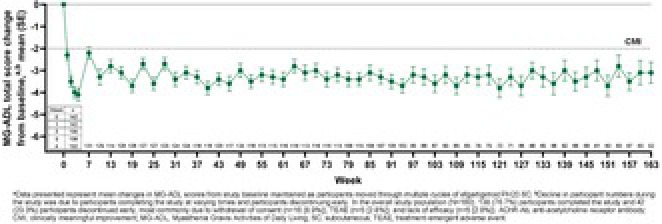
**FIGURE 1** Mean Change from Baseline in MG‐ADL Total Score Over Time: AChR‐Ab+ Population.

**Figure:**
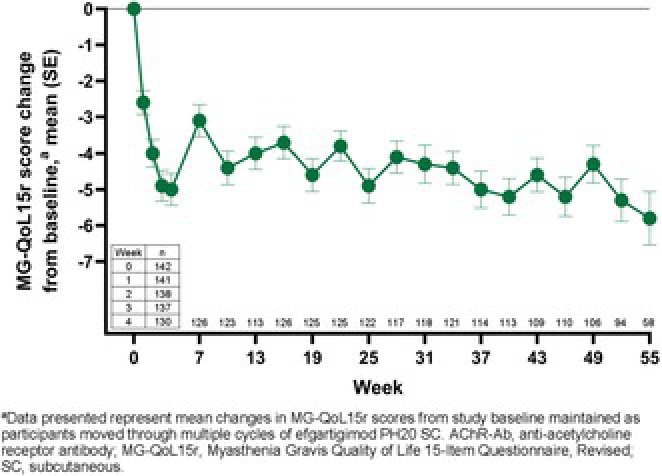
**FIGURE 2** Mean Change from Baseline in MG‐QoL15r Score Over Time: AChR‐Ab+ Population.

**Conclusion:** Long‐term efgartigimod PH20 SC treatment was well tolerated and led to sustained efficacy and QoL improvements during ADAPT‐SC+.

**Disclosure:** This study was sponsored by argenx. W‐YH, RHJ, FMV, and LL are employees of argenx. CA, RB‐S, JLDB, AM, KU, DK, AK‐P, JJGMV, TV, HW, and JFH have reported financial/nonfinancial relationships with argenx at the time of submission.

## EPO‐0143

### Efficacy of nipocalimab in patients early in their disease course of generalized myasthenia gravis: Post hoc analysis of VIVACITY‐MG3

#### C. Antozzi ^1^; W. Noel^2^; M. Fitzgibbon^3^; W. Karmous^4^; K. Gandhi^5^; I. Turkoz^5^; M. Kutch^6^; S. Ramchandren^7^; E. Cortés‐Vicente^8^

##### ^1^Immunotherapy and Apheresis Unit, Neuroimmunology and Muscle Pathology Unit, Fondazione IRCCS Istituto Neurologico C. Besta, Milan, Italy; ^2^Johnson & Johnson, Beerse, Belgium; ^3^Johnson & Johnson, Raritan, USA; ^4^Johnson & Johnson, Issy‐les‐Moulineaux, France; ^5^Johnson & Johnson, Horsham, USA; ^6^Cytel Inc, Cambridge, USA; ^7^Johnson & Johnson, Titusville, USA; ^8^Unitat Patologia Neuromuscular, Servei Neurologia, Hospital Santa Creu i Sant Pau, Barcelona, Spain

**Background and aims:** Myasthenia gravis (MG) is a chronic autoimmune condition characterized by fluctuating muscle weakness and fatigue. Nipocalimab added to standard‐of‐care (SOC) showed greater improvement than placebo (+SOC) in Myasthenia‐Gravis–Activities‐of‐Daily‐Living (MG‐ADL), and Quantitative‐Myasthenia‐Gravis (QMG) scores in VIVACITY‐MG3 study (NCT04496507). This post hoc analysis evaluated whether efficacy was achievable in patients with generalized MG (gMG) diagnosed earlier (≤5 years) before study initiation.

**Methods:** Analysis included seropositive patients treated with nipocalimab or placebo who had been diagnosed with gMG within ≤5 years (below the cohort median of 5 years). Efficacy at week (W)24 was assessed using MG‐ADL (meaningful clinical improvement [MCI]: ≥2‐point improvement; substantial clinical improvement [SCI]: ≥3‐point improvement) and QMG (MCI: ≥3‐point improvement; SCI: ≥4‐point improvement) scores in patients diagnosed with gMG ≤5 years. Two‐sample *t*‐tests compared baseline‐to‐W24 changes between groups.

**Results:** Baseline demographics were generally balanced between nipocalimab and placebo‐treated patients (Table 1). At W24, MG‐ADL reductions (mean [SD]) from baseline were greater with nipocalimab (−4.9 [2.88]) versus placebo (−2.7 [2.46]); difference: −2.22 (SE: 0.76); 95% CI (−3.74; −0.70); *p* = 0.005. Similarly, QMG reductions (mean [SD]) from baseline were greater with nipocalimab (−5.1 [4.14]) versus placebo (−2.3 [3.20]); difference: −2.85 (SE:1.08); 95% CI (−5.03; −0.66); *p* = 0.012. A greater proportion of patients receiving nipocalimab met MCI and SCI criteria for MG‐ADL and QMG at W24 versus placebo (Table 1).

**Figure:**
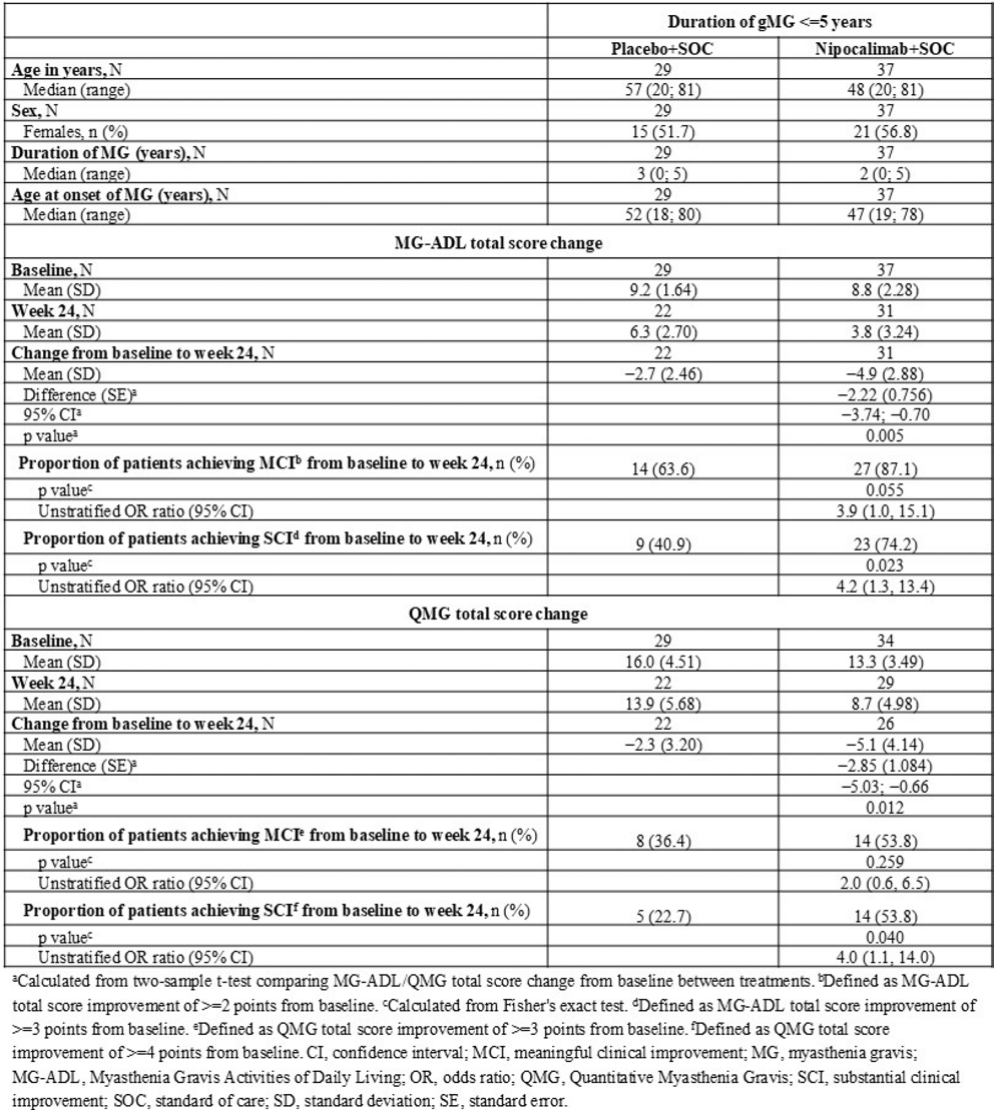
**TABLE 1** Baseline characteristics, and symptom improvement in MG‐ADL and QMG scales.

**Conclusion:** In patients diagnosed with gMG in ≤5 years before study initiation, nipocalimab demonstrated greater efficacy versus placebo, achieving MCI and SCI in MG‐ADL and QMG at W24. These findings highlight the benefit of nipocalimab in recently diagnosed patients with gMG.

**Disclosure:** Carlo Antozzi: received funding for travel, meeting attendance, and advisory board participation from Alexion, Momenta, Sanofi, Argenx, UCB, Janssen Pharmaceuticals and Johnson & Johnson. Wim Noel, Marie Fitzgibbon, Kavita Gandhi, Wisam Karmous, Ibrahim Turkoz, and Sindhu Ramchandren: are employees of Johnson & Johnson and may hold stock or stock options in the company. Michael Kutch: is an employee of Cytel Inc, which derives profits from interactions with pharmaceutical sponsors. Elena Cortés‐Vicente: received public speaking honoraria and compensation for advisory boards and/or consultation fees from Alexion Pharmaceuticals Inc, Amgen, Argenx BV, Johnson & Johnson, Lundbeck, UCB Pharma SA.

## EPO‐0144

### Safety of intravenous and subcutaneous efgartigimod reported from multiple global clinical trials in immunoglobulin g‐mediated autoimmune diseases

#### K. Gwathmey^1^; C. Broome^2^; M. Goebeler^3^; H. Murai^4^; Z. Bata‐Csörgo^5^; A. Newland^6^; J. Allen^7^; Y. Miyakawa^8^; P. Ulrichts^9^; J. Podhorna ^9^; S. Zhao^9^; S. Steeland^9^; J. Beauchamp^9^; J. Howard Jr^10^

##### ^1^Virginia Commonwealth University, Richmond, USA and Georgetown University, Washington, USA; ^2^Georgetown University, Washington, USA; ^3^University Hospital Wϋrzburg, Wϋrzburg, Germany; ^4^School of Medicine, International University of Health and Welfare, Narita, Japan; ^5^University of Szeged, Szeged, Hungary; ^6^Centre for Haematology, Barts and The London School of Medicine & Dentistry, Queen Mary University of London, London, UK; ^7^University of Minnesota, Minneapolis, USA; ^8^Saitama Medical University, Saitama, Japan; ^9^argenx, Ghent, Belgium; ^10^The University of North Carolina at Chapel Hill, Chapel Hill, USA

**Background and aims:** Efgartigimod safety has been investigated in multiple IgG‐mediated autoimmune diseases, including generalised myasthenia gravis (gMG), primary immune thrombocytopenia (ITP), and chronic inflammatory demyelinating polyradiculoneuropathy (CIDP), for which it has received regulatory approval in multiple regions. We report the long‐term safety of efgartigimod in gMG, ITP, and CIDP, administered via varying dosing regimens.

**Methods:** Data were pooled per disease from global clinical trials investigating varying efgartigimod dosing regimens and administration routes (intravenous 10 mg/kg; subcutaneous 1000 mg PH20 [coformulated with recombinant human hyaluronidase PH20]). Event rates (ER) were reported per participant year of follow‐up (PYFU).

**Results:** The analysis includes data from at least 874 patients representing ~1350 PYFU. Treatment‐emergent adverse events (TEAE) were mostly mild‐to‐moderate in severity. During controlled trials, severe infection ERs were comparable between placebo‐ and efgartigimod‐treated participants. Reported TEAE‐related treatment discontinuation rates were consistently low. Rates of discontinuation and severe and serious TEAEs did not increase with additional treatment cycles (gMG) or prolonged exposure to weekly administration (ITP, CIDP). Further analyses of participant population subgroups are ongoing. No albumin reduction or increases in low‐density lipoprotein cholesterol levels were reported in efgartigimod‐treated participants. No vaccinations, laboratory value monitoring, or pretreatment medications are required to receive efgartigimod. Efgartigimod was consistently well tolerated across trials in autoimmune diseases, including those with subcutaneous administration of efgartigimod.

**Conclusion:** Efgartigimod was well tolerated and demonstrated a similar safety profile regardless of disease state, dosing regimen, and length of exposure.

**Disclosure:** Kelly G. Gwathmey: Research or institutional support ‐ Alexion, Amgen, argenx, UCB, Xeris Pharmaceuticals Catherine M. Broome: Research or institutional support ‐ Alexion, Alpine, argenx, Electra, Novartis, Sanofi Matthias Goebeler: Research or institutional support ‐ Almirall, argenx, Biotest, GlaxoSmithKline, Janssen, Leo Pharma, Lilly, Novartis, UCB Hiroyuki Murai: Research or institutional support ‐ Alexion, argenx, AstraZeneca, Chugai, Japan Blood Products Organization, Ministry of Health, Labour and Welfare of Japan, Roche, UCB Zsuzsanna Bata‐Csörgo: Research or institutional support ‐ NKFI, Orvostovábbképzo Szemle, Sanofi‐Genzyme Adrian Newland: Research or institutional support ‐ Amgen, Angle, argenx, Dova, Novartis, Ono, Rigel, Shionogi Jeffrey A. Allen: Research or institutional support ‐ Akcea, Alexion, Alnylam, Annexon, argenx, CSL Behring, Grifols, Immunovant, Immupharma, Johnson & Johnson, Pfizer, Takeda Yoshitaka Miyakawa: Research or institutional support ‐ Alexion, argenx, Asahi Kasei, BioMarin, Chugai, CSL Behring, Janssen, Novartis, Novo Nordisk, Pfizer, Sanofi, UCB Peter Ulrichts; Jana Podhorna; Sihui Zhao; Sophie Steeland; Jon Beauchamp: Employee ‐ argenx; Ownership interests ‐ argenx James F. Howard Jr: Research or institutional support ‐ Academic CME, Ad Scientiam, Alexion AstraZeneca Rare Disease, Amgen, argenx, Biohaven Ltd, Biologix, Cartesian Therapeutics, Centers for Disease Control and Prevention, CheckRare CME, CoreEvitas, Curie.bio, Hansa Biopharma, Medscape CME, Merck EMB Serono, MGFA, Muscular Dystrophy Association, NIH, NMD Pharma, Novartis, PCORI, PeerView CME, Physicians' Education Resource (PER) CME, PlatformQ CME, Regeneron Pharmaceuticals, Sanofi US, Toleranzia AB, UCB.

## EPO‐0145

### Italian real‐life experience with Zilucoplan for generalized myasthenia gravis: One year of ZILU25 study

#### G. Di Martino ^1^; N. Rini^1^; C. Antozzi^2^; P. Alboini^3^; L. Bevilacqua^4^; F. Biasini^5^; A. Bisecco^6^; S. Bonanno^7^; F. Brighina^1^; L. Codeluppi^8^; G. D'Alvano^6^; V. Damato^9^; C. Erra^10^; L. Fionda^11^; L. Florio^3^; M. Guida^12^; F. Habetswallner^10^; R. Iorio^13^; P. Luppino^1^; M. Maestri Tassoni^12^; L. Maggi^7^; M. Marini^13^; S. Marini^13^; S. Morino^11^; A. Pugliese^5^; E. Rossini^14^; F. Saccà^15^; A. Sarnataro^15^; F. Tuccillo^10^; F. Vanoli^7^; M. Verza^9^; C. Vinciguerra^4^; R. Frangiamore^7^; V. Di Stefano^1^

##### ^1^Department of Biomedicine, Neuroscience and Advanced Diagnostics (BiND), University of Palermo; 90127 Palermo, Italy; ^2^Neuroimmunology and Neuromuscular Diseases Unit, Fondazione IRCCS Istituto Neurologico Carlo Besta, Milan, Italy; Immunotherapy and Apheresis Unit, Fondazione IRCCS Istituto Neurologico Carlo Besta, Milan, Italy; ^3^Neurology Unit, Fondazione IRCCS Casa Sollievo della Sofferenza; 71013 San Giovanni Rotondo, Italy; ^4^Neurology Unit, Department of Medicine, Surgery and Dentistry “Scuola Medica Salernitana”, University of Salerno; 84131 Salerno, Italy; ^5^Department of Clinical and Experimental Medicine, University of Messina, Italy; ^6^Department of Advanced Medical and Surgical Sciences (DAMSS), University of Campania “Luigi Vanvitelli”, Naples, Italy; ^7^Neuroimmunology and Neuromuscular Diseases Unit, Fondazione IRCCS Istituto Neurologico Carlo Besta, Milan, Italy; ^8^Neurology Unit, Neuromotor & Rehabilitation Department, Azienda USL‐IRCCS di Reggio Emilia; 42122 Reggio Emilia, Italy; ^9^Department of Neurosciences, Psychology, Drug Research and Child Health (NEUROFARBA), University of Florence, Florence, Italy; ^10^UOC Neurophysiopathology, AORN Cardarelli, Via Antonio Cardarelli 9, Naples 80131, Italy; ^11^Neuromuscular and Rare Disease Centre, Neurology Unit, Sant'Andrea Hospital, Rome, Italy; ^12^Neurology Unit, Department of Neurosciences, Azienda Ospedaliero Universitaria Pisana, Pisa, Toscana, Italy; ^13^Department of Neuroscience, Università Cattolica del Sacro Cuore, Rome, Italy; Neurology Unit, Fondazione Policlinico Universitario A. Gemelli IRCCS, Rome, Italy; ^14^Department of Neuroscience, Mental Health and Sensory Organs (NESMOS), SAPIENZA University of Rome, Rome, Italy; ^15^Department of Neurological Sciences, Reproductive and Odontostomatological Sciences, University Federico II, Naples, Italy.

**Background and aims:** Generalized Myasthenia gravis (gMG) is a rare chronic autoimmune disease affecting the postsynaptic membrane of the neuromuscular junction. New treatment options, such as the C5 inhibitors, are growing with promising results. In this study we investigated the real‐world use of Zilucoplan for AChR‐positive gMG in Italy.

**Methods:** Zilucoplan was self‐administered by daily subcutaneous injections. Efficacy was assessed by Myasthenia Gravis Foundation of America Post‐intervention status (MGFA‐PIS), Myasthenia Gravis Activity of Daily Living (MG‐ADL), Myasthenia Gravis Composite (MGC), Myasthenia Gravis quantitative (QMG) scales and 15‐items Myasthenia Gravis Quality of Life (MG‐QOL‐15) questionnaire at baseline and after 1, 4, 12, 24 and 48 weeks.

**Results:** 54 patients (37 females, aged 57.3 years) were included in the follow‐up analysis. Clinical improvements observed in the initial study were sustained over time, with stable or further reduced MG‐ADL, QMG, MGC and MG‐QOL‐15r scored over weeks. Moreover, it was also observed a continued steroid‐sparing effect and a reduction of hospitalization due to MG worsening. Treatment adherence remained high, and no new safety report emerged during the extended follow‐up.

**Conclusion:** Zilucoplan has proven to be well‐tolerated and effective in most patients with AChR‐positive gMG. A clinical meaningful effect was reported since the first week and was sustained during the following weeks. Further studies are needed to confirm these preliminary real‐life data.

**Disclosure:** Nothing to disclose.

## EPO‐0146

### Quality of gravis patients achieving sustained versus transient clinical outcomes in the phase 3 vivacity‐MG3 trial

#### J. Vissing ^1^; C. Antozzi^2^; K. Gwathmey^3^; K. Gandhi^4^; I. Turkoz^5^; S. Pease^6^; N. Campbell^4^

##### ^1^University of Copenhagen, Copenhagen, Denmark; ^2^IRCCS Carlo Besta Neurological Institute Foundation, Milan, Italy; ^3^Virginia Commonwealth University, Richmond, USA; ^4^Johnson & Johnson, Horsham, USA; ^5^Johnson & Johnson, Titusville, USA; ^6^Johnson & Johnson, Raritan, USA

**Background and aims:** Minimal clinically important improvement in the Myasthenia‐Gravis‐Activities‐of‐Daily‐Living (MG‐ADL) score (MCI; ≥ 2‐point improvement) or a composite response (CR) of ≥ 2‐point improvement in MG‐ADL and ≥ 3‐point improvement in Quantitative‐Myasthenia‐Gravis scores are plausible treatment goals in generalized myasthenia gravis (gMG). This Vivacity‐MG3 posthoc analysis evaluated health‐related quality of life (HRQoL) in patients achieving sustained versus transient MCI or CR.

**Methods:** Nipocalimab‐ and placebo‐treated participants were stratified by MCI or CR status over the 24‐week double‐blind phase: never achieved MCI/CR (No‐MCI/CR), achieved MCI/CR sustained ≥8 weeks (sustained‐MCI/CR), or achieved MCI/CR ≥ 1 time but not sustained for ≥8 weeks (transient‐MCI/CR). Mean changes from baseline (CFB) to week 24 (W24) in MGQoL‐15r and Neuro‐QoL Fatigue scores were compared across groups using ANCOVA models.

**Results:** The MCI analysis included 129 patients (91 sustained‐MCI, 29 transient‐MCI, 9 no‐MCI) and the CR analysis included 125 patients (50 sustained‐CR, 37 transient‐CR, 38 no‐CR). Sustained‐ versus transient‐MCI patients demonstrated greater improvements in MGQoL‐15r (least‐square‐mean CFB, −6.2 versus −2.0, *p* < 0.001). Additionally, the sustained‐MCI group had greater improvements in Neuro‐QoL Fatigue (−19.5) versus transient‐MCI (−7.5, *p* < 0.001) and No‐MCI (−8.5, *p* = 0.04). Similarly, sustained‐CR patients demonstrated greater improvements in MGQoL‐15r (−9.6) versus transient‐CR (−5.4, *p* < 0.001) and No‐CR (−3.6, *p* < 0.001) and those with sustained‐CR had greater improvements in Neuro‐QoL Fatigue (−29.2) versus transient‐CR (−16.4, *p* < 0.001) and No‐CR (−12.2, *p* < 0.001).

**Conclusion:** Patients achieving sustained versus transient MCI or CR demonstrated greater improvements in HRQoL and fatigue, suggesting 8‐week sustained outcomes may be more impactful treatment goals than short‐term transient response for gMG patients receiving advanced treatments.

**Disclosure:** Funding This study was funded by Johnson & Johnson. Author Disclosures JV has received consultant fees for serving on advisory boards for Alexion Pharmaceuticals Inc, argenx BV, Dianthus Therapeutics, Horizon Therapeutics (now Amgen Inc.), Johnson & Johnson, Regeneron, Roche, and UCB Pharma SA. CA has received funding for travel, meeting attendance, and advisory board participation from Alexion, Momenta, Sanofi, argenx, UCB, and Johnson & Johnson. KGG has served as a paid consultant for Alexion Pharmaceuticals, argenx, and UCB; and she has served on the speakers' bureau for Alexion Pharmaceuticals. KG, IT, SP, and NC are employees of and may hold stock in Johnson & Johnson.

## EPO‐0147

### Myasthenia gravis Inebilizumab trial: 26‐week randomised‐placebo controlled and 26‐week open‐label results in anti‐muscle‐specific kinase subgroup

#### J. Vissing ^1^; M. Leite^2^; J. Howard; Jr.^3^; M. Benatar^4^; E. Ciafaloni^5^; K. Utsugisawa^6^; S. Bray^7^; A. Bhambri^8^; A. Kiran Kanchi^8^; S. Cheng^8^; R. Nowak^9^

##### ^1^University of Copenhagen, Copenhagen, Denmark; ^2^University of Oxford, Oxford, UK; ^3^University of North Carolina, Chapel Hill, USA; ^4^University of Miami Miller School of Medicine, Miami, USA; ^5^University of Rochester, Rochester, USA; ^6^Hanamaki General Hospital, Hanamaki, Japan; ^7^Amgen Ltd, Cambridge, UK; ^8^Amgen Inc, Thousand Oaks, USA; ^9^Yale University, New Haven, USA

**Background and aims:** Generalised myasthenia gravis (gMG) is caused by anti‐muscle‐specific kinase (MuSK+) and anti‐acetylcholine receptor (AChR+) antibodies targeting corresponding proteins in the synapse. The placebo (PBO)‐controlled MG Inebilizumab (INEB) Trial (MINT) assessed INEB, a monoclonal antibody targeting CD19+ B cells, in patients with MuSK+ or AChR+ gMG. INEB vs PBO yielded significantly greater improvements in MG Activities of Daily Living (MG‐ADL) and Quantitative MG (QMG) scores at week 26 in MINT. Here we report key outcomes for the MuSK+ subpopulation from the first 26 weeks of the open‐label period (OLP).

**Methods:** Participants with MuSK+ gMG in MINT (NCT04524273) were randomised 1:1 to 300 mg intravenous INEB or PBO for 26 weeks. Participants underwent a protocol‐specified steroid taper (≤5 mg/day by week 24). After the randomised, controlled period (RCP), participants could receive INEB in the OLP. This pre‐specified, interim, descriptive analysis included participants who received any dose of INEB during MINT. No statistical testing was done.

**Results:** Overall, 48 participants with MuSK+ were enrolled and randomised: 46 entered the OLP and 44 completed 26 weeks. Baseline MG‐ADL and QMG scores were similar between groups, and mean scores decreased from baseline to week 26 during the RCP (INEB –4.1, –5.0; PBO –3.1, –4.2, respectively). During the initial 26‐week OLP (week 52), the mean MG‐ADL and QMG scores continued to decrease from baseline (INEB/INEB: –5.6, –6.1; PBO/INEB: –5.1, –7.4, respectively; Figures 1–2; Table 1).

**Figure:**
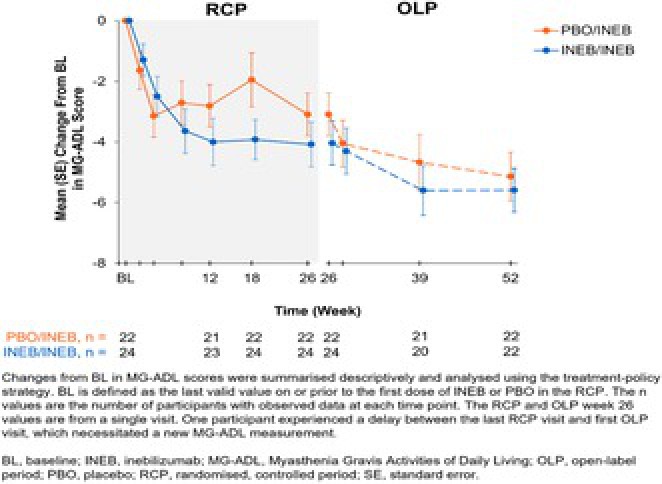
**FIGURE 1** Change from BL in MG‐ADL Score.

**Figure:**
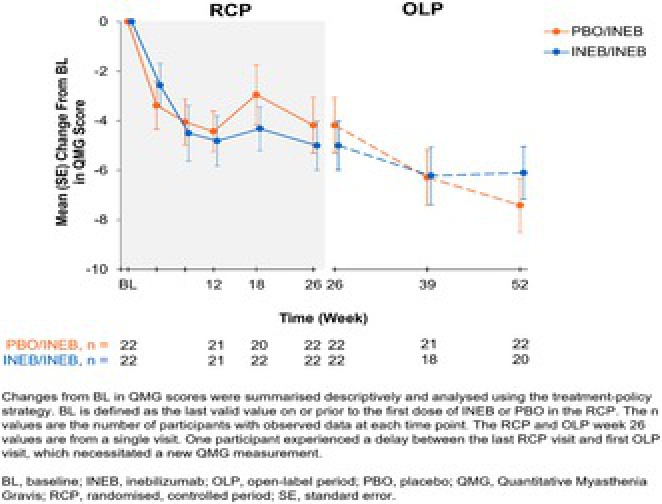
**FIGURE 2** Change from BL in QMG Score.

**Figure:**
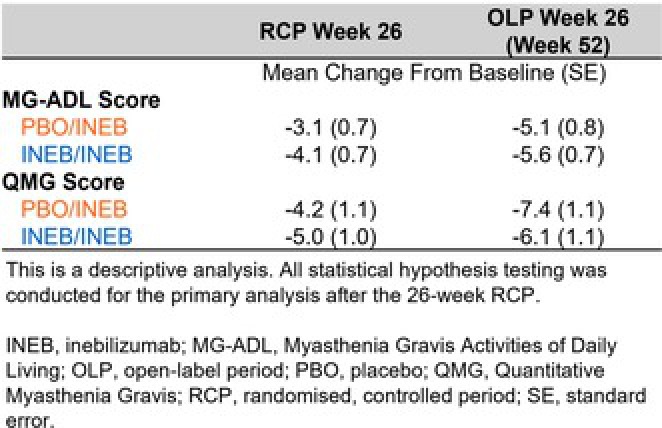
**TABLE 1** Change from BL in MG‐ADL and QMG Scores.

**Conclusion:** In this descriptive analysis, INEB demonstrated ongoing improvement in MG‐ADL and QMG scores compared to baseline irrespective of initial randomisation.

**Disclosure:** JV has been a consultant for and/or received speaker honoraria from Alexion Pharmaceuticals, Amgen Inc, argenx, Dianthus Therapeutics, Johnson & Johnson, NMD Pharma, Novartis, Regeneron Pharmaceuticals, Roche, Toleranzia AB, and UCB. MIL received funding from the National Health Service (MRDS and NSCG for NMOSD) and the University of Oxford. She was awarded research grants from the UK association for patients with myasthenia (myaware) and the University of Oxford. She received speaker honoraria/travel grants from the Guthy‐Jackson Charitable Foundation and UCB. She served on scientific/educational advisory boards for argenx, UCB, and Amgen Inc. JFH received research funding (paid to his institution) from Ad Scientiam, Alexion AstraZeneca Rare Disease, argenx, Cartesian Therapeutics, the Centers for Disease Control and Prevention, EMD Serono, Merck, the Muscular Dystrophy Association, Myasthenia Gravis Foundation of America, the National Institutes of Health, NMD Pharma, and UCB; honoraria/consulting fees from AcademicCME, Alexion AstraZeneca Rare Disease, Amgen Inc, Biohaven, CheckRare CME, CorEvitas, Curie.Bio, EMD Serono, Hansa Biopharma, Lundbeck, Merck, Novartis, PeerView CME, Physicians’ Education Resource CME, PlatformQ CME, Regeneron Pharmaceuticals, Sanofi, Seismic Therapeutics, TG Therapeutics, Toleranzia AB, and UCB; and nonfinancial support from Alexion AstraZeneca Rare Disease, argenx, Biohaven, Cartesian Therapeutics, Toleranzia AB, and UCB. MB received grant support from the ALS Association, the ALS Recovery Fund, Eli Lilly, the Kimmelman estate, the Muscular Dystrophy Association, the National Institutes of Health, and Target ALS. He reports research support from Alexion Pharmaceuticals and Immunovant and served as an investigator for clinical trials funded by Biogen, Novartis, and Orphazyme. He received personal consulting fees from Alaunos Therapeutics, Alector Therapeutics, Alexion Pharmaceuticals, Annexon Biosciences, Arrowhead Pharmaceuticals, Canopy Immunotherapeutics, CorEvitas, Denali Therapeutics, Eli Lilly, Evox Therapeutics, Amgen Inc, Immunovant, Janssen, Merck, Novartis, Roche, Sanofi, Takeda, Trace Neuroscience, UCB, uniQure, VectorY Therapeutics, Voyager Therapeutics, and Woolsey Pharmaceuticals. EC has no disclosures. KU served as a paid consultant for argenx, Chugai Pharmaceutical, HanAll BioPharma, Janssen, Merck, Mitsubishi Tanabe Pharma, UCB, and Amgen Inc. and received speaker honoraria from Alexion Pharmaceuticals, argenx, the Japan Blood Products Organization, and UCB. SB, AB, AKK, and SC are employees of Amgen Inc. and own stock. RJN received research support from Alexion Pharmaceuticals, Amgen Inc, argenx, Grifols,, Immunovant, Janssen, Myasthenia Gravis Foundation of America, the National Institutes of Health, and UCB and served as a consultant/adviser for Alexion Pharmaceuticals, Amgen Inc, argenx, Cabaletta Bio, Cour Pharmaceuticals, CSL Behring, Grifols, Immunovant, Janssen, and UCB.

## EPO‐0148

### Multiple responder analyses with claseprubart, an active C1s inhibitor, in patients with generalized myasthenia gravis

#### M. Dimachkie ^1^; F. Sacca^2^; M. Ait‐Tihyaty^3^; C. Briggs^3^; U. Siddiqui^3^; L. Hickey^3^; M. Lalla^3^; M. Smilowski^4^

##### ^1^University of Kansas Medical Center, Kansas City, USA; ^2^Azienda Ospedaliera Universitaria (AOU) Federico II, Naples, Italy; ^3^Dianthus Therapeutics, New York, USA; ^4^Neurologia Śląska Centrum Medyczne, Katowice, Poland

**Background and aims:** The classical complement pathway plays a significant role in acetylcholine receptor‐positive (AChR+) generalized myasthenia gravis (gMG) pathology. Claseprubart is an investigational, clinical‐stage, potent monoclonal antibody engineered to selectively target the classical complement pathway, by selectively inhibiting activated C1s protein. The clinical benefit of claseprubart in gMG may be best supported by improvement across multiple validated scales to capture multi‐dimensional improvement beyond single‐scale analyses.

**Methods:** AChR+ gMG patients treated with 300 mg claseprubart subcutaneously once every 2 weeks in the MaGic study (NCT06282159) – a global Phase 2, randomized, double‐blind, placebo‐controlled trial – were analyzed for responses based on multiple assessments compared to placebo. *p*‐values are one‐sided, with nominal significance assessed at an alpha of 0.1. A responder was defined as achieving an improvement in MG‐ADL ≥ 3 and QMG ≥ 4. Endpoints include proportion of dual MG‐ADL and QMG responders at Week 13, time to response, and proportion of responders sustaining response for at least 6 weeks.

**Results:** 63% of claseprubart patients had dual response improvement in MG‐ADL ≥ 3 and QMG ≥ 4, versus 14% for placebo (OR 29.00; *p* = 0.0006). Dual responders were seen as early as Week 1. The median time to response was Week 3. All claseprubart patients with dual response at Week 13 had maintained response for at least 6 weeks.

**Conclusion:** Claseprubart‐treated patients were more likely to achieve multi‐dimensional response than placebo. Responses emerged early and were sustained, confirming rapid, robust, and durable benefit from claseprubart across functional and quantitative measures from both patient and clinician perspectives.

**Disclosure:** MD grant or honoraria from Abcuro, Alexion/AstraZeneca, Alnylam, Amicus, Annexon, ArgenX, Astellas, AstraZeneca, Biosensics, Bristol‐Myers Squibb, Cabaletta, Candid, Catalyst, Covance, CSL‐Behring, Creyon, Dianthus, EMD Serono/Merck, GlaxoSmithKline, Genentech, Grifols, Horizon/Immunovant, Janssen, Kriya, Medlink, Nkarta, Novartis, Nuvig, Mitsubishi Tanabe, Octapharma, Orphazyme, Priovant, Sanofi Genzyme, Shire Takeda, TACT/Treat NMD, UCB Biopharma, and Vertex Pharmaceuticals. FS nothing to declare. MA employee of Dianthus Therapeutics CB employee of Dianthus Therapeutics US employee of Dianthus Therapeutics LH employee of Dianthus Therapeutics ML employee of Dianthus Therapeutics MS nothing to declare.

## EPO‐0149

### Treatment response in anti‐HMGCR and anti‐SRP immune‐mediated necrotizing myopathy: Impact of demographic and racioethnic factors

#### M. Seyam ^1^; N. Gonzalez Caldito^2^; J. Greenberg^3^; N. Goyal^2^; T. Mozaffar^2^; N. Goyal^3^; L. Chaitesipaseut^4^; C. Spahn^4^; S. Muppidi^3^

##### ^1^Graduate School of Life Sciences, Utrecht University, Netherlands & Department of Neurology and Neurological Sciences, Stanford University School of Medicine, Stanford, USA; ^2^Department of Neurology, University of California, Irvine, USA; ^3^Department of Neurology and Neurological Sciences, Stanford University School of Medicine, Stanford, USA; ^4^Department of Pharmacy, Stanford Health Care, Stanford, USA

**Background and aims:** Immune‐mediated necrotizing myopathy (IMNM) associated with anti‐HMGCR and anti‐SRP autoantibodies is a rare autoimmune neuromuscular condition characterized by significant phenotypic heterogeneity. Despite varying clinical presentations, the impact of demographic and racioethnic factors on treatment response remains poorly characterized.

**Methods:** This multi‐center retrospective study analyzed 56 patients with IMNM (48 anti‐HMGCR, 8 anti‐SRP). We assessed demographics, racioethnicity, number of immune therapies, and therapy escalations (defined as addition of therapy or increase in dose/frequency). Statistical analysis included Mann‐Whitney U, Kruskal‐Wallis, and Fisher's Exact tests. Significance was set at *p* < 0.05.

**Results:** The cohort (mean age 59.8 years, 54.6% female, 82.1% statin‐exposed) predominantly included anti‐HMGCR patients (85.7%). Anti‐HMGCR patients were significantly older (mean 62.3 vs. 44.8 years, *p* = 0.003) with higher statin exposure (91.7% vs. 25.0%, *p* < 0.001) than anti‐SRP patients. Mean time to diagnosis (12.3 months) was similar between groups. Peak CK levels varied significantly across racioethnic groups in the overall cohort, with the highest levels observed in Black (11,978 U/L) and Asian (10,332 U/L) patients compared to Hispanic (9,156 U/L) and White (6,751 U/L) patients. Younger patients (<59.8 years) required significantly more immune therapies (mean 3.4 vs. 2.8, *p* = 0.044) and treatment escalations (2.8 vs. 1.2, *p* = 0.025). Hispanic patients required significantly higher treatment escalations, nearly three times more than White patients (mean 2.9 vs. 0.9, *p* = 0.026). Subgroup analysis confirmed similar significant trends within the anti‐HMGCR cohort.

**Conclusion:** Demographic and racioethnic factors significantly influence IMNM disease severity and treatment intensity. This study underscores the need for larger studies to validate these findings and guide personalized therapeutic strategies.

**Disclosure:** Nothing to disclose.

## EPO‐0150

### Efficacy of long‐term zilucoplan treatment in subgroups of patients with generalised myasthenia gravis: A 120‐week analysis of RAISE‐XT

#### N. Savic^1^; M. Freimer^2^; C. Hewamadduma^3^; M. Leite ^4^; A. Maniaol^5^; K. Utsugisawa^6^; M. Weiss^7^; B. Boroojerdi^8^; P. Mahoney^9^; J. Howard^10^

##### ^1^UCB, Bulle, Switzerland; ^2^Department of Neurology, The Ohio State University Wexner Medical Center, Columbus, USA; ^3^Academic Neuromuscular Unit, Sheffield Teaching Hospitals NHS Foundation Trust & Sheffield Institute for Translational Neuroscience (SITraN), University of Sheffield, Sheffield, UK; ^4^Nuffield Department of Clinical Neurosciences, University of Oxford, Oxford, UK; ^5^Department of Neurology, Oslo University Hospital, Oslo, Norway; ^6^Department of Neurology, Hanamaki General Hospital, Hanamaki, Japan; ^7^Department of Neurology, University of Washington Medical Center, Seattle, USA; ^8^UCB, Monheim, Germany; ^9^UCB, Slough, UK; ^10^Department of Neurology, The University of North Carolina at Chapel Hill, Chapel Hill, USA

**Background and aims:** Clinically meaningful improvements in myasthenia gravis (MG)‐specific outcomes were observed with zilucoplan treatment up to Week 120 in patients with generalised MG (gMG) in the Phase 3, ongoing, open‐label extension study, RAISE‐XT (NCT04225871). Here, we evaluate the efficacy of long‐term zilucoplan treatment in patients with gMG, stratified by specific disease characteristics.

**Methods:** Adults with anti‐acetylcholine receptor antibody‐positive gMG who completed a qualifying double‐blind, placebo‐controlled study (NCT03315130 or NCT04115293 [RAISE]) could enter RAISE‐XT and self‐administer once‐daily subcutaneous zilucoplan 0.3 mg/kg injections. Change from baseline (CFB) to Week 120 in MG‐specific outcome measure scores was assessed (data cut‐off: 11 November 2023). Subgroups were pre‐specified according to the following baseline characteristics: age < 65 or ≥65 years, MG Activities of Daily Living (MG‐ADL) score ≤9 or ≥10, and disease duration < 5.2 or ≥5.2 years.

**Results:** Overall, 200 patients enrolled. At Week 120, mean (standard error) CFB in MG‐ADL scores across all subgroups were: age: < 65 years, *n* = 64: −7.3 (0.5) and ≥65 years, *n* = 22: −6.7 (0.8); MG‐ADL: ≤9, *n* = 68: −7.0 (0.4) and ≥10, *n* = 18: −7.6 (1.4); and disease duration: < 5.2 years, *n* = 42: −6.7 (0.7) and ≥5.2 years, *n* = 44: −7.5 (0.6). Improvements were also observed at Week 120 for Quantitative MG, MG Quality of Life 15‐item revised, and Quality of Life in Neurological Disorders Short Form Fatigue scores. Treatment‐emergent adverse events occurred in 97.0% (*n*/*N* = 194/200) of patients; most were mild or moderate and unrelated to the study drug.

**Conclusion:** Long‐term zilucoplan treatment demonstrated consistent improvements in MG‐specific outcomes up to Week 120, irrespective of disease severity, duration or patient age.

**Disclosure:** This study was funded by UCB. Natasa Savic, Babak Boroojerdi and Paul Mahoney are employees and shareholders of UCB. Full disclosure of all industry relationships will be made during congress presentation if accepted.

## EPO‐0151

### Health‐related quality of life in patients with generalised myasthenia gravis receiving gefurulimab in the PREVAIL trial

#### T. Ruck ^1^; F. Saccà^2^; T. Vu^3^; S. Rakhade^4^; A. Racine^4^; S. Pandeya^4^; A. Lizarraga^5^

##### ^1^Department of Neurology, Ruhr University Bochum, BG University Hospital Bergmannsheil, Bochum, Germany; ^2^NSRO Department, University of Naples Federico II, Napoli, Italy; ^3^Department of Neurology and Neuromuscular Medicine, University of South Florida Morsani College of Medicine, Tampa, USA; ^4^Alexion, AstraZeneca Rare Disease, Boston, MA, USA; ^5^Department of Neurology, University of Rochester, Rochester, USA

**Background and aims:** Gefurulimab is a novel dual‐binding nanobody complement component 5 inhibitor. Efficacy and safety of gefurulimab in adults with anti‐acetylcholine receptor antibody‐positive (AChR‐Ab+) generalised myasthenia gravis (gMG) were demonstrated in PREVAIL, a phase 3, randomised, placebo‐controlled trial (NCT05556096). To provide a holistic view of the treatment effect, we present health‐related (HR) quality of life (QoL) outcomes.

**Methods:** Patients with AChR‐Ab+ gMG were randomised to receive weekly subcutaneous self‐injection of gefurulimab or placebo. Exploratory endpoints assessed HRQoL changes from baseline through wk26 (randomised period completion) using: MG‐QOL 15‐Item Questionnaire‐Revised (MG‐QOL15r); QoL in Neurological Disorders (Neuro‐QoL) Fatigue Scale; EuroQol‐5 Dimensions‐5‐Level (EQ‐5D‐5L) Visual Analogue Scale (VAS) and Health State Index (HSI).

**Results:** Overall, 260 patients (female, 60.4%; mean ± SD age, 52.8 ± 15.7 yrs; mean ± SD baseline MG‐ADL total score, 9.0 ± 2.2) were randomised to gefurulimab (*n* = 131) or placebo (*n* = 129). Significantly greater improvements were observed with gefurulimab compared with placebo, starting at wk4 in MG‐QOL15r and Neuro‐QoL Fatigue and wk8 in EQ‐5D‐5L. These improvements were sustained throughout the randomised period. At wk26, the least squares mean (95% CI) changes from baseline among patients with available data receiving gefurulimab (*n* = 123) vs placebo (*n* = 117) were −4.9 (−6.0, −3.8) vs −2.3 (−3.2, −1.4) (*p* = 0.0005) in MG‐QOL15r; −4.8 (−6.2, −3.4) vs −2.5 (−3.8, −1.1) (*p* = 0.0164) in Neuro‐QoL Fatigue; 9.2 (6.2, 12.1) vs 4.6 (1.6, 7.6) (*p* = 0.0349) in EQ‐5D‐5L VAS; 0.12 (0.076, 0.155) vs 0.04 (−0.004, 0.079) (*p* = 0.0088) in EQ‐5D‐5L HSI.

**Conclusion:** In PREVAIL, patients with AChR‐Ab+ gMG receiving gefurulimab had significant improvements in HRQoL compared with those receiving placebo, demonstrated by MG‐specific and general HRQoL measures.

**Disclosure:** TR reports grants from the German Ministry of Education, Science, Research, and Technology; grants and personal fees from Alexion, AstraZeneca Rare Disease, argenx, Johnson & Johnson, Roche, Sanofi, and UCB; personal fees from Amgen, Merck, Novartis, and Roche outside of the submitted work. FS has received public speaking honoraria from Alexion, argenx, Biogen, Genpharm, Medpharma, Medison Pharma, Mylan, Neopharm Israel, Novartis, Roche, Sanofi, UCB, and Zai Lab; compensation for advisory boards from Alexion, Almirall, Amgen, argenx, AstraZeneca, Avexis, Biogen, Dianthus, Johnson & Johnson, Lexeo, Novartis GmBh, Reata, Sandoz, UCB, and Zai Lab; consultation fees from Alexion, argenx, Dianthus, EPG Communication Holdings Ltd, Johnson & Johnson, Medscape, PeerView, Vitaccess; clinical trial support from Almirall; funding from Agenzia Italiana del Farmaco (AIFA), Alexion, Associazione Italiana per la lotta alle Sindromi Atassiche (AISA), Friedreich Ataxia Research Alliance (FARA), Roche SpA, Tristan Allamby Research Fund (TARFfa); and was a clinical trial principal investigator from Alexion, argenx, Dianthus, Immunovant, Lediant, Lexeo, Novartis, Prilenia, RemeGen, and Sanofi. TV has served on advisory board and/or as consultant for Alexion, AstraZeneca Rare Disease, argenx, Amgen, Dianthus, Johnson & Johnson, ImmunAbs, NMD Pharma, and VOR; served as a clinical trial principal investigator for Alexion, AstraZeneca Rare Disease, Amgen, argenx, Cartesian, COUR, Dianthus, EMD Seono, ImmunAbs, Immunovant, Johnson & Johnson, NMD Pharma, Regeneron, VOR, and UCB; and participated in CME events sponsored by AcademicCME, CMEO, PER, and MedLive. SR, AMR, and SP are employees of Alexion, AstraZeneca Rare Disease, and hold stock or stock options in AstraZeneca. AAL has served as a consultant for argenx, Cartesian, and Johnson & Johnson.

## EPO‐0152

### Rapid, sustained improvements with efgartigimod in symptoms, daily life, and patient satisfaction in gMG: Initial results from the PREMIER study

#### T. Ruck ^1^; C. Qi^2^; M. Yang^3^; A. Anderson^4^; V. Bril^5^; C. Edmundson^6^; D. Gelinas^7^; T. Hughes^2^; G. Phillips^2^; L. Li^2^; B. Martins^3^; C. Xiang^3^; K. Heerlein^2^; C. Bergin^2^; B. Lin^3^; J. Liu^3^; G. Wolfe^8^

##### ^1^Department of Neurology, BG University‐Hospital Bergmannsheil, Ruhr‐University Bochum, Bochum, Germany; ^2^argenx US Inc, Boston, USA; ^3^Analysis Group, Inc. Boston, USA; ^4^Department of Neurology, Houston Methodist, Houston, USA; ^5^Ellen and Martin Prosserman Centre for Neuromuscular Diseases, University Health Network, Toronto General Hospital, University of Toronto, Toronto, Canada; ^6^Swedish Neuroscience Institute, Seattle, USA; ^7^Department of Neurology, UNC School of Medicine, Chapel Hill, USA; ^8^Department of Neurology, Jacobs School of Medicine and Biomedical Sciences, University at Buffalo, Buffalo, USA

**Background and aims:** Efgartigimod is a neonatal Fc receptor blocker indicated for the treatment of generalized myasthenia gravis (gMG) in adults. We report initial findings of the ongoing Prospective Real‐world study of EfgartigiMod for patIents with gEneralized myasthenia gRavis (PREMIER).

**Methods:** Adult patients prescribed efgartigimod were recruited from enrollees of the efgartigimod patient support program in the US. Patients completed surveys before (baseline) and at weeks (WKs) 4, 12, 20, and 32 after efgartigimod initiation. Surveys included questions on gMG symptoms and patients’ functional status using MG Activities of Daily Living (MG‐ADL), Patient‐Acceptable Symptom State (PASS), changes in ability to work, corticosteroid use, and overall treatment satisfaction.

**Results:** To date, 143 patients enrolled in PREMIER and 52 completed the WK32 survey, with 75.0% continuing on efgartigimod. Mean (standard deviation) age at baseline was 64.8 (14.1); 48.9% were female. Mean baseline MG‐ADL was 8.6, improving by 4.2 points by WK32 (*p* < 0.001), with larger improvements (4.7 points; *p* < 0.001) in those remaining on efgartigimod (Figure 1). Satisfaction with current disease (yes vs. no), assessed by PASS, increased from 25.2% at baseline to 51.9% at WK32 (*p* < 0.001) (Figure 2), and satisfaction with treatments improved from 38.5% to 71.2%. Ability to work improvements were reported by 42.9% (18 of 42 participants) by WK20. The proportion of patients without corticosteroids increased from 38.5% at baseline to 59.6% by WK32 (*p* < 0.05) (Figure 3).

**Figure:**
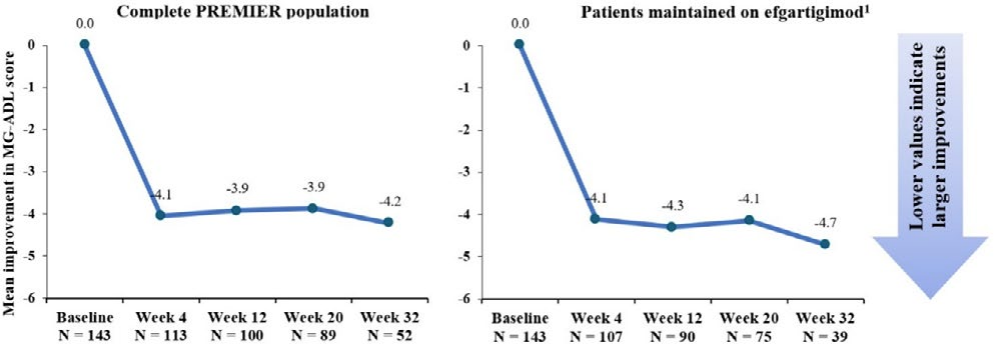
**FIGURE 1** Changes in MG‐ADL from baseline.

**Figure:**
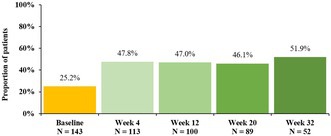
**FIGURE 2** Patient‐Acceptable Symptom State.

**Figure:**
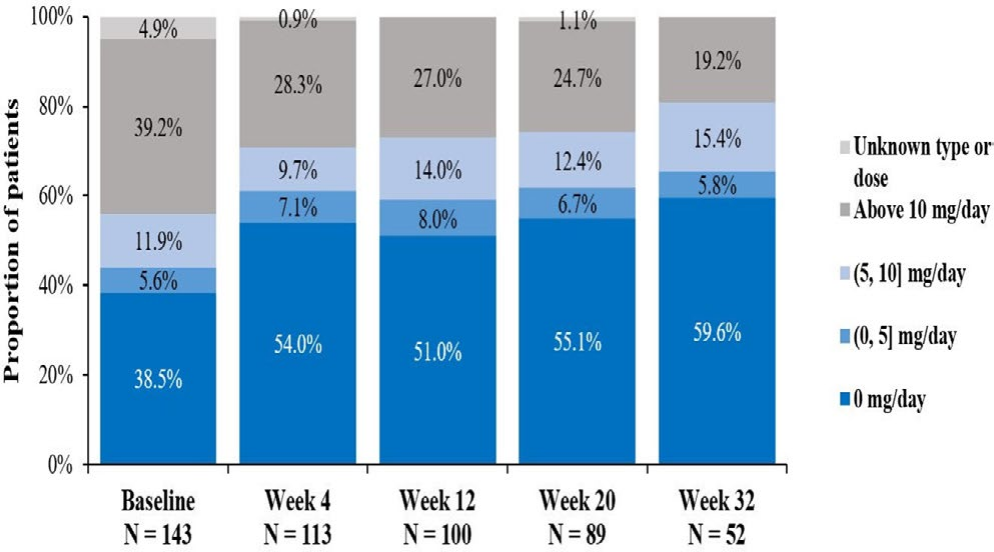
**FIGURE 3** Oral corticosteroids prednisone‐equivalent daily dose.

**Conclusion:** Initial results from PREMIER show rapid and sustained improvements in symptoms, daily life, ability to work, and patient satisfaction, along with reduction in corticosteroid use.

**Disclosure:** TR received honoraria and/or research support from Alexion, argenx, Biogen, Merck, Novartis, Sanofi, UCB, J&J, and Roche. CQ, TH, LL, GP, KH, CB are employees of argenx. AA is a Patient Education Speaker for argenx. VB is a consultant for Akcea Therapeutics, Inc.; Alexion Pharmaceuticals, Inc.; Alnylam Pharmaceuticals, Inc.; argenx; AstraZeneca; CSL; F. Hoffmann‐La Roche Ltd; Grifols; Immunovant, Inc.; Ionis Pharmaceuticals; Janssen Global Services, LLC; Japan Tobacco; Johnson & Johnson Services, Inc.; Momenta; Novo Nordisk A/S; Octapharma USA, Inc.; Pfizer Inc.; Powell Mansfield Inc.; Sanofi; Takeda Pharmaceutical Company Limited. V.B. has received research support from Akcea Therapeutics, Inc.; Alexion Pharmaceuticals, Inc.; argenx; AstraZeneca; CSL; Grifols; Immunovant, Inc.; Ionis Pharmaceuticals; Johnson & Johnson Services, Inc.; Momenta; Octapharma USA, Inc.; Takeda Pharmaceutical Company Limited; UCB S.A.CE reported receiving personal fees from Alexion, Alnylam, argenx, UCB, and Immunovant. DG was a former employee of argenx US, Inc, but no longer one during this study. MY, BM, CX, BL, and JL are employees of Analysis Group, Inc, which received funding from argenx US, Inc. to support this research. GW has served as member of advisory boards or provided paid consultations to Alexion, argenx, Cartesian, Janssen, and UCB; is on speaker bureaus for Alexion and UCB; and has received research support from Alexion, argenx, Immunovant, Roche, UCB, and the MG Foundation of America.

## Neurogenetics 1

## EPO‐0153

### Compound POMGNT1 variants associated with muscle‐eye‐brain disease: Necessity of comprehensive genetic testing

#### A. Frontistis ^1^; V. Poulidou^1^; E. Pityrigkas^1^; S. Kalampokini^1^; E. Liouta^1^; G. Pepe^2^; M. Spilioti^1^; V. Kimiskidis^1^

##### ^1^First Department of Neurology, AHEPA University Hospital, Aristotle University of Thessaloniki, Greece; ^2^Genekor Medical S.A, Athens, Greece

**Background and aims:** Congenital muscular dystrophies (CMD) are a heterogeneous group of rare autosomal recessive myopathies. Subtypes like dystroglycanopathies, collagen VI‐related dystrophies, and dystroglycan–sarcoglycan complex disorders pose significant diagnostic challenges due to overlapping ocular and neurological involvement.

**Methods:** We report a 32‐year‐old male patient presenting in a comatose and febrile state following status epilepticus. Neurological examination revealed spastic paraparesis with bilateral clonus and knee contractures, right‐arm hypotonia, and horizontal nystagmus. His history included global neurodevelopmental delay with subsequent loss of ambulation and speech, drug‐resistant epilepsy, and ophthalmological abnormalities including bilateral optic atrophy, severe refractive error, and cataract.

**Results:** Brain imaging demonstrated ventriculomegaly, lissencephaly and brainstem and cerebellum hypoplasia (Figure 1). Electroencephalography confirmed refractory epileptic encephalopathy, which ultimately responded to a four‐drug antiepileptic regimen (valproate sodium, brivaracetam, zonisamide, lacosamide). Muscle biopsy revealed myopathic changes with reduced α‐dystroglycan, laminin α2, and laminin β2 expression on immunohistochemistry. Next‐Generation‐Sequencing identified compound heterozygosity in POMGNT1, comprising a nonsense variant (c.1282C > T, p.Gln428*) and a missense variant (c.793C > T, p.Arg265Cys) (Figure 2). The missense variant is predicted to preserve partial enzymatic activity and likely contributes to the patient's phenotype.

**Figure:**
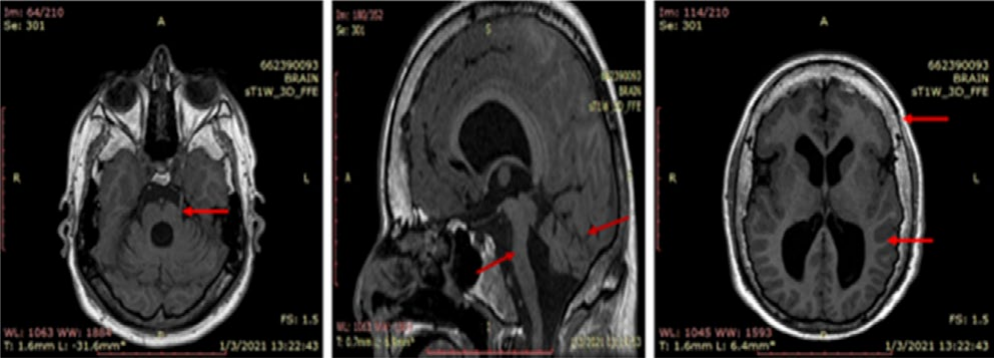
**FIGURE 1** T1‐weighted MRI imaging of the brain showing brainstem and cerebellar hypoplasia, ventricular enlargement and lissencephaly predominantly involving the frontal lobes (red arrows).

**Figure:**
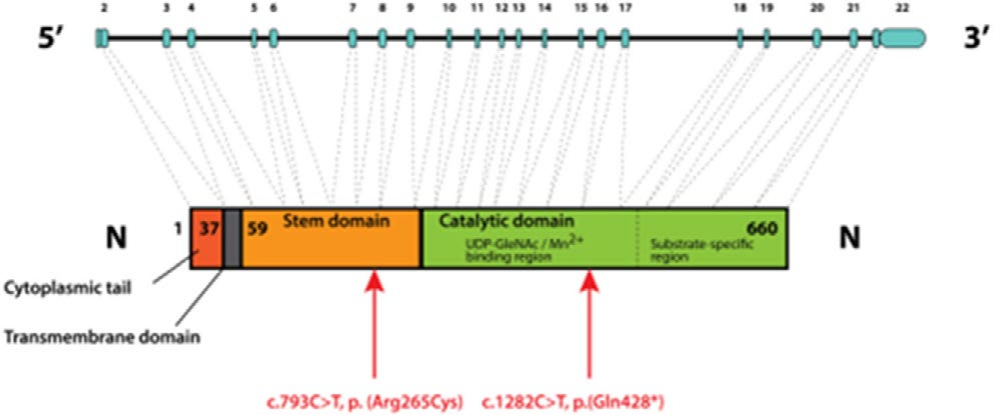
**FIGURE 2** POMGnT1 gene and the corresponding protein with its main domains; the variants found in the present case are shown with red arrows.

**Conclusion:** Combined genetic and histopathological results were consistent with Muscle‐Eye‐Brain disease (MEB), a dystroglycanopathy within the CMD spectrum. Over one‐third of MEB patients have epilepsy, approximately 20% of which suffer from refractory seizures. This case illustrates how compound heterozygosity with residual POMGNT1 activity permits prolonged survival, while predisposing to severe adult‐onset neurological complications, necessitating extensive genetic testing to resolve complex neuromuscular phenotypes.

**Disclosure:** The authors report no conflicts of interest or external funding.

## EPO‐0154

### Diagnostic clues: A multidisciplinary review of 600 cases with suspected inherited white matter disorder

#### C. Wade ^1^; D. Lynch^2^

##### ^1^Department of Neuroinflammation, UCL Queen Square Institute of Neurology, London, UCL Queen Square Institute of Neurology, London, UK; ^2^National Institute for Health Research, University College London Hospitals, Biomedical Research Centre, London, UK

**Background and aims:** Adult patients with suspected inherited white matter disorders (IWMDs present diagnostic challenges due to phenotypic overlap with acquired inflammatory, vascular, and toxic leukoencephalopathies. Delayed or incorrect classification may result in inappropriate treatment, missed therapeutic windows, and prolonged diagnostic odysseys. Multidisciplinary team (MDT) evaluation integrating clinical features, neuroimaging, cerebrospinal fluid (CSF) analysis, genetics, and longitudinal disease evolution is increasingly recognised as central to accurate diagnosis; however, real‐world data quantifying which features most reliably contribute to diagnostic confidence remain limited.

**Methods:** We performed a retrospective review of approximately 600 consecutive cases discussed at a tertiary Inherited White Matter Disorders MDT. Clinical, radiological, laboratory, and genetic data were extracted from our IWMD database supplemented with up to date electronic health records. Variables included presenting phenotype, age at onset, family history, MRI pattern and distribution, CSF findings, genetic testing results, and use of ancillary investigations (e.g. brain biopsy). Cases were classified as inherited, acquired, in work‐up, or unresolved following MDT consensus.

**Results:** Preliminary analysis demonstrates that no single clinical, radiological, or laboratory feature reliably distinguishes inherited from acquired white matter disease in isolation. Diagnostic confidence most commonly arose from the convergence of multiple domains, particularly characteristic MRI patterns, absence of inflammatory CSF markers, multisystem features, and longitudinal disease evolution. Brain biopsy was infrequently contributory and reserved for diagnostically ambiguous cases.

**Conclusion:** These findings highlight the value of integrated MDT assessment in complex adult white matter disorders and may inform more efficient diagnostic pathways, reducing unnecessary invasive investigations and supporting earlier recognition of genetic disease.

**Disclosure:** Nothing to disclose.

## EPO‐0155

### Miglustat in Alzheimer's disease associated with heterozygous NPC1 mutation: Exploratory case series and preliminary findings

#### D. Lopergolo ^1^; D. Gasparini^1^; S. Bianchi^1^; B. Pucci^1^; D. Tripodi^2^; V. Leoni^2^; A. Chincarini^3^; S. Sestini^4^; H. Zetterberg^5^; N. De Stefano^1^; A. Mignarri^1^

##### ^1^Department of Medicine, Surgery and Neurosciences, University of Siena, Siena, Italy; ^2^Laboratory of Clinical Pathology and Toxicology, Hospital Pio XI of Desio, ASST Brianza, School of Medicine and Surgery, University of Milano Bicocca, Milan, Italy; ^3^National Institute of Nuclear Physics (INFN), Genoa, Italy; ^4^Department of Diagnostic Imaging, Unit of Nuclear Medicine, PO‐S. Stefano, Azienda U.S.L. Toscana Centro, Prato, Italy; ^5^Department of Psychiatry and Neurochemistry, Institute of Neuroscience and Physiology, The Sahlgrenska Academy, University of Gothenburg, Mölndal, Sweden

**Background and aims:** Several studies have previously demonstrated an increased risk of dementia and brain amyloid deposition in individuals with heterozygous NPC1 mutations. Moreover, in a recent study, we identified the first family with autosomal dominant late‐onset Alzheimer's disease (AD) caused by a heterozygous NPC1 mutation. Unfortunately, there are currently no effective treatments available for this condition. Miglustat, which impacts the metabolism of oxysterols, has been shown to exert an anti‐amyloidogenic effect in a human cellular model of AD.

**Methods:** In our exploratory uncontrolled study, three patients from the previously published family were orally treated with miglustat for 12 months. They underwent monthly clinical evaluations and routine blood tests. Additionally, neuropsychological evaluations, brain amyloid‐PET imaging, and biochemical analyses on plasma and CSF were performed.

**Results:** All three patients achieved clinical stability, showed a sustained reduction in serum oxysterol levels, and experienced a marked decrease in brain amyloid burden.

**Conclusion:** Based on our preliminary observations and hypothesis‐generating findings, along with the growing evidence suggesting AD as a lipid disorder, miglustat should be further tested in a larger cohort of heterozygous NPC1 mutated patients and probably evaluated as a potential disease‐modifying treatment for AD.

**Disclosure:** H.Z. has served on scientific advisory boards and/or as a consultant for Abbvie, Acumen, Alector, Alzinova, ALZPath, Amylyx, Annexon, Apellis, Artery Therapeutics, AZTherapies, Cognito Therapeutics, CogRx, Denali, Eisai, LabCorp, Merry Life, Nervgen, Novo Nordisk, Optoceutics, Passage Bio, Pinteon Therapeutics, Prothena, Red Abbey Labs, reMYND, Roche, Samumed, Siemens Healthineers, Triplet Therapeutics, and Wave, has given lectures sponsored by Alzecure, BioArctic, Biogen, Cellectricon, Fujirebio, Lilly, Novo Nordisk, Roche, and WebMD, and is a co‐founder of Brain Biomarker Solutions in Gothenburg AB (BBS), which is a part of the GU Ventures Incubator Program (outside submitted work). None of the other authors declare conflicts of interest.

## EPO‐0156

### Predicting neurodevelopmental outcome in KCNQ2‐related disorders

#### E. Van Boxstael; C. Millevert; S. Weckhuysen

##### Department of Neurology, Antwerp University Hospital, Edegem, Belgium

**Background and aims:** KCNQ2‐related disorders (RD) are a major cause of neonatal‐onset epilepsy, presenting a spectrum from self‐limited (familial) neonatal epilepsy (SeL(F)NE) to severe developmental and epileptic encephalopathy (DEE). Haploinsufficient variants typically cause milder SeL(F)NE, whereas dominant‐negative variants often lead to DEE. Yet, clinical outcomes are variable, neonatal seizures occur across phenotypes, and functional data are often unavailable, complicating early prognostic assessment.

**Methods:** We performed a multicentric retrospective cohort study including patients with a (likely) pathogenic KCNQ2 variant and ≥ 3 years follow‐up. Mosaic variants were excluded, yielding 235 patients. Ten neonatal predictors were selected based on statistical significance (*p* ≤ 0.01), clinical relevance, and data availability. The cohort was randomly split into a training set (70%) and an independent validation set (30%). Three random forest models were trained using these predictors to forecast neurodevelopmental delay, age at first words, and age at walking.

**Results:** Six clinical predictors were retained: neonatal hypotonia, family history of neonatal seizures, EEG features, age at seizure onset, seizure type, and seizure frequency at onset. Four genetic predictors included exon location, truncating or de novo variant, and localization within KCNQ2‐DEE hotspot regions. Models predicted neurodevelopmental outcome (normal, mild, moderate to profound intellectual disability), age of language acquisition (<16 months, ≥16 months, never), and age of walking (<18 months, ≥18 months, never) with validation accuracies of 74%, 80%, and 81%, respectively.

**Conclusion:** These prediction models offer a practical tool for early, individualized prognostic counselling in KCNQ2‐RD, supporting patient stratification for early interventions, including sodium channel blockers, and guiding enrolment in precision medicine trials.

**Disclosure:** Nothing to disclose.

## EPO‐0157

### First reported case of SCA27b in Argentina

#### E. Gatto ^1^; M. Cesarini^1^; N. Gonzalez Rojas^1^; L. Schottlaender^3^; P. Iruzubieta Agudo^4^; S. Josefina^2^; M. Dicaire^2^; D. Pellerin^5^

##### ^1^Unidad de Trastornos del Movimiento, Instituto Neurociencias Buenos Aires (INEBA), Buenos Aires, Argentina; ^2^Servicio de Neurologia, Sanatorio de la Trinidad Mitre, Buenos Aires, Argentina; ^3^Instituto de Investigaciones en Medicina traslacional (IIMT), CONICET‐Universidad Austral, Pilar, Buenos Aires, Argentina; ^4^Dr. John T. Macdonald Foundation Department of Human Genetics and John P. Hussman Institute for Human Genomics, University of Miami Miller School of Medicine, Miami, USA; ^5^Montreal Neurological Hospital and Institute, McGill University, Montreal, Quebec, Canada

**Background and aims:** Deep intronic GAA repeat expansions in intron 1 of the FGF14 gene have recently been identified as a frequent cause of late‐onset cerebellar ataxia (LOCA), known as GAA‐FGF14–related ataxia (SCA27B). While SCA27B is increasingly recognized worldwide, data from South America remain scarce and limited to isolated reports.

**Methods:** We report the clinical, neuropsychiatric, neuroimaging, and genetic findings of a patient evaluated for progressive cerebellar ataxia. Molecular analysis of FGF14 was performed using PCR‐based techniques to identify GAA repeat expansions.

**Results:** We report a 62‐year‐old man with Spanish and Italian ancestry, carrying a pathogenic FGF14 GAA repeat expansion of 331 repeats. Originally referred due to paroxysmal ataxia and dysphonia, in the context of a long‐standing neuropsychiatric history and prolonged alcohol and substance abuse. Neurological examination revealed progressive cerebellar ataxia, multidirectional nystagmus, dysphonia, dysphagia, and autonomic dysfunction, including bladder urgency and orthostatic hypotension. Family history showed a maternal diagnosis of amyotrophic lateral sclerosis. Early clinical features and personal history led to an initial diagnosis of alcohol‐related cerebellar degeneration. Brain MRI showed nonspecific cerebellar atrophy. Subsequent progression to a pancerebellar ataxia‐plus syndrome and a marked symptomatic response to 4‐aminopyridine prompted genetic testing, confirming SCA27B.

**Conclusion:** This case represents, to our knowledge, the first genetically confirmed report of SCA27B in Argentina, expanding the phenotypic spectrum of SCA27B by highlighting concomitant neuropsychiatric manifestations and vulnerability to alcohol use as early disease features. In regions where alcohol‐related cerebellar ataxia is frequently diagnosed, careful phenotypic assessment may facilitate recognition of treatable late‐onset ataxias such as SCA27B

**Disclosure:** Nothing to disclose.

## EPO‐0158

### Developing a genetic risk card for sporadic Parkinson's disease: Evidence from the Italian population

#### G. Trastulli ^1^; G. Calvino^2^; D. Megalizzi^3^; M. Alborghetti^4^; F. Carrillo^5^; T. Esposito^6^; C. Pellicano^7^; C. Caltagirone^8^; R. Cascella^9^; E. Giardina^10^; C. Strafella^3^

##### ^1^UOC Diagnostica di Laboratorio e Genomica Medica, Santa Lucia Foundation IRCCS; Department of Systems Medicine, Tor Vergata University, Rome Italy; ^2^UOC Diagnostica di Laboratorio e Genomica Medica, Santa Lucia Foundation IRCCS; Department of Science, Roma Tre University, Rome, Italy; ^3^UOC Diagnostica di Laboratorio e Genomica Medica, Santa Lucia Foundation IRCCS, Rome, Italy; ^4^Department of Neuroscience, Mental Health and Sensory Organs, Sapienza University, Rome; Department of Neurology, IRCCS Neuromed, Pozzilli, Italy; ^5^Institute of Genetics and Biophysics “Adriano Buzzati‐Traverso”, National Research Council, Naples, Italy; ^6^Institute of Genetics and Biophysics “Adriano Buzzati‐Traverso”, National Research Council, Naples; IRCCS INM Neuromed, Pozzilli, IS, Italy; ^7^Laboratory of Neuropsychiatry, IRCCS Santa Lucia Foundation, Rome, Italy; ^8^Department of Clinical and Behavioral Neurology, IRCCS Santa Lucia Foundation, Rome, Italy; ^9^UOC Diagnostica di Laboratorio e Genomica Medica, Santa Lucia Foundation IRCCS, Rome, Italy; Department of Chemical‐Toxicological and Pharmacological Evaluation of Drugs, Catholic University Our Lady of Good Counsel; 1010 Tirana, Albania; ^10^UOC Diagnostica di Laboratorio e Genomica Medica, Santa Lucia Foundation IRCCS; Department of Biomedicine and Prevention, Tor Vergata University, Rome, Italy.

**Background and aims:** Genome‐wide association studies (GWAS) have substantially contributed to elucidating the genetic architecture of Parkinson's disease (PD). The present study investigated the burden of susceptibility variants associated with sporadic PD in the Italian population, with the goal of identifying specific genetic susceptibility profiles and candidate biomarkers.

**Methods:** A total of 180 Single Nucleotide Polymorphisms (SNPs) were analysed in 730 Italian patients with sporadic PD using OpenArray technology. Variants were selected from GWAS, meta‐analyses, public databases and PD‐related biological pathways. Case‐control association analyses with multiple testing correction and odds ratio estimation were integrated with machine‐learning and bioinformatic analyses.

**Results:** The study identified 72 SNPs significantly associated with PD susceptibility. Among the tested machine learning models, the neural network showed the best performance and identified five variants (rs2012070, ATP6V0A1; rs1800795, IL6; rs329652, MIR4697; rs2641116, PARK7; rs9275326, HLA‐DRB6/HLA‐DQA1) as the most predictive. eQTL analysis revealed predominant regulatory effects of six PD‐associated variants in the basal ganglia, cerebellum, cerebral cortex, hippocampus and hypothalamus regions of the brain. Pathway enrichment analysis indicated that genes harbouring the associated variants are involved in PD‐relevant biological processes, including immune and inflammatory response, neurodevelopment, synaptic function, protein degradation, cellular metabolism, membrane organization and intracellular trafficking, as well as regulation of neuronal survival and cell death.

**Conclusion:** These findings delineate a distinct genetic susceptibility profile for PD in the Italian population and provide a rationale for developing a population‐tailored genetic card as a tool to support risk stratification and identify individuals who may benefit from closer monitoring.

**Disclosure:** Nothing to disclose.

## EPO‐0159

### Landscape of dystrophin variants in Hungary

#### P. Balicza ^1^; S. Udvari^1^; A. Gal^1^; A. Palasti^1^; Z. Grosz^1^; S. Ando^2^; B. Bessenyei^2^; K. Koczok^2^; H. Piko^3^; L. Szabo^4^; S. Dobner^4^; Z. Liptai^4^; V. Karcagi^5^; I. Balogh^2^; M. Molnar^1^

##### ^1^Semmelweis University Institute of Genomic Medicine and Rare Disorders; ^2^University of Debrecen Clinical Genetics Centre; ^3^Semmelweis University Department of Internal Medicine and Oncology; ^4^Semmelweis University Pediatric Center; ^5^Istenhegyi Genetic Diagnostic Center

**Background and aims:** Dystrophinopathies are one of the most important causes of muscular dystrophies in boys. With the advent of targeted therapies, it became extremely important to know the exact molecular background of each patient.

**Methods:** We collected molecular results of patients across the country. We collected date of birth, date of diagnosis, and the genetic results from all patients. Cases, first tested by multiplex PCR, were retested with MLPA. For a subset of patients (cared at the Semmelweis University), we collected phenotypic data.

**Results:** Between 1992 and 2025, 558 DMD molecular tests were performed. Formerly, only copy number variant (CNV) analysis was easily available, but since 2020 single nucleotide variant (SNV) analysis is also more accessible. 169 in‐frame CNV (30.3%), 306 out‐of‐frame CNVs (54.8%), and 55 SNVs (9.9%) were detected. Among the out‐of‐frame CNVs, 115 different events occurred: in 9.3% duplication, and in 90.7% deletion. Most common events were del45‐50 (8% of cases), del45 (6.5%), and del48‐50 (6.5%). The number of theoretically amenable patients for exon skipping therapy (exon skipping 45/51/53) would be 122 (39.9%). We have available phenotype data from 109 patients in the whole cohort, with 43 Becker and 66 Duchenne phenotypes.

**Conclusion:** Routine availability of CNV and SNV testing, including NGS, allows reliable incidence estimates and reveals an increasing number of rare damaging variants and clinically affected females. Twelve common variants accounted for half of the cases, and a substantial proportion of patients are theoretically eligible for targeted therapies.

**Disclosure:** First author received support for congress attendance from the Medis, Medical Marketing Company.

## EPO‐0160

### Hereditary spastic paraparesis type 18: Shedding light upon a novel mutation in the ERLIN2 gene

#### R. Manso Calderón ^1^; B. Garcia^2^

##### ^1^Neurology Department. Complejo Asistencial Universitario de Salamanca, Salamanca, Spain; ^2^Genetics Department. Complejo Asistencial Universitario de Salamanca, Salamanca, Spain

**Background and aims:** Hereditary spastic paraparesis (HSP) type 18 is caused by mutations in the ERLIN2 gene, which encodes a protein involved in the endoplasmic reticulum‐associated degradation pathway. Autosomal recessive mutations are involved in complicated HSP, while autosomal dominant mutations present as pure HSP. We describe a case of a new mutation in the ERLIN2 gene.

**Methods:** A 60‐year‐old woman who developed progressive gait disturbance since the age of 16. At 50 years old, she also presented weakness in the lower limbs. Her maternal grandfather, a maternal great‐aunt, and two second‐degree cousins had gait abnormalities. However, her non‐consanguineous parents, two siblings, and one daughter had no neurological symptoms. On examination, muscle strength 4/5 in lower limbs with spasticity, hyperreflexia, Babinski sign, and bilateral clonus were observed. Modified Ashworth Scale: 3, and Timed 25‐Foot Walk Test (T25FW): 24 s.

**Results:** Brain and spinal MRI: normal. Whole exome sequencing (WES) identified the index case as a heterozygous carrier of the c.205G > C (p.D69H) mutation in the ERLIN2 gene. Previous treatments with oral baclofen, tizanidine, and botulinum toxin showed minimal response. Extended‐release fampridine (10 mg b.i.d.) was administered as a therapeutic alternative.

**Conclusion:** The proband case confirms the genotype‐phenotype correlation between monoallelic variants of the ERLIN2 gene and pure HSP. This finding suggests a potential dominant negative effect of the erlin2 protein on oligomerization, as the cause of a milder phenotype. Furthermore, this case introduces a new variant in the ERLIN2 gene, contributing to a better understanding of the genetic factors underlying HSP type 18.

**Disclosure:** Nothing to disclose.

## EPO‐0161

### Abstract withdrawn

## EPO‐0162

### Investigating natural history and genotype influence in ALS2‐related infantile‐onset ascending hereditary spastic paraplegia

#### S. Della Vecchia ^1^; E. NG'UMBI^2^; M. Rossi Sebastiano^3^; A. Vicidomini^2^; F. Cesca^4^; G. Caron^3^; G. Ermondi^3^; R. Battini^1^; F. Santorelli^1^

##### ^1^IRCCS Fondazione Stella Maris, Pisa; ^2^Università degli Studi di Trieste; ^3^Università di Torino; ^4^Università di Padova

**Background and aims:** Infantile‐onset ascending hereditary spastic paraplegia (IAHSP) is a rare motor neuron disorder caused by biallelic ALS2 mutations. Despite a distinctive phenotype, quantitative data on disease progression and genotype–phenotype correlations remain limited, hindering clinical trial design.

**Methods:** We conducted a natural history study of 83 individuals with biallelic‐ALS2 mutations, combining published cases with patients enrolled in a multicenter cohort. Collected variables included psychomotor development, age at onset of lower‐limb, upper‐limb and bulbar involvement, loss of independent ambulation, additional neurological features. Based on predicted residual protein function, patients were classified into three genotype groups: biallelic‐loss‐of‐function{00}, mixed{01}, non–loss‐of‐function{11}. Disease progression was analyzed using Kaplan–Meier curves and Cox proportional hazards models. Functional progression was assessed with the SPATAX‐disability score, allowing longitudinal modeling and preliminary sample size estimation. Unsupervised clustering was applied to identify phenotypic subgroups.

**Results:** Age at symptom onset was consistent across genotypes (median 1.4–1.6 years), identifying early lower‐limb involvement as a genotype‐independent hallmark of IAHSP. In contrast, progression rates differed by genotype: {00}‐patients showed faster and more homogeneous progression, {01}‐patients an early but more gradual course, and {11}‐patients the slowest progression. Longitudinal SPATAX analyses indicated a mean progression rate ~0.5 points/year. Based on this estimate, preliminary modeling suggested that approximately 10 patients may be sufficient to detect a 50% reduction in progression in an interventional trial. Unsupervised clustering identified three preliminary phenotypic groups that did not map one‐to‐one onto genotype classes, suggesting additional sources of clinical heterogeneity currently under further investigation.

**Conclusion:** These data support time‐based endpoints and data‐driven stratification for IAHSP trials.

**Disclosure:** Nothing to disclose.

## EPO‐0163

### Applicability of PPCS as diagnostic biomarker for Niemann Pick type C2 patients

#### S. Fischer; A. Bertoli‐Avella; L. Pardo; T. Böttcher; P. Bauer

##### Centogene GmbH, Rostock, Germany

**Background and aims:** N‐palmitoyl‐O‐phosphocholineserine (PPCS) formerly known as Lyso‐SM‐509 has been shown to be a diagnostic marker for Niemann Pick type C1 (NPC1) with high sensitivity and specificity (Griese 2015). It has been reported to correlate in NPC1 patients with the annual severity increment score (Sidhu R 2020) and with disease severity (clinical presentation) and progression (measured in dried blood spot ‐ DBS ‐ samples) (Guatibonza 2023). So far, this biomarker has only been investigated for NPC1 patients. For NPC2 patients however, biomarker data is not yet publicly available since pathogenic NPC2 variants are accountable for only 5 percent of all NPC cases which corresponds to an incidence rate of only 1 of 2,858,998 (Wassif 2016).

**Methods:** With this work, we aim to establish a PPCS reference range for NPC2 patients based on data that has been acquired in our laboratories over 10 years from DBS samples of 33 patients.

**Results:** All patients were characterized genetically with the predominant variants being p.Cys47Ter, p.Cys99Arg, and p.Ser63fs. The PPCS blood levels of all NPC2 patients were above the diagnostic cut‐off and hence above the 97th percentile of values measured in blood of healthy individuals. Furthermore, clinical data was dissected using HPO terms, and genotype‐phenotype analysis was performed.

**Conclusion:** This work should serve as evidence for the applicability of PPCS as diagnostic biomarker for NPC2 and should be the starting point for investigation of its use as monitoring biomarker in treatment efficacy studies.

**Disclosure:** Steffen Fischer, Luba Pardo, Aida Bertoli‐Avella and Peter Bauer are employees of Centogene GmbH.

## EPO‐0164

### Genetic, biochemical and clinical properties of GLA p.Asp313Tyr variant in a large global cohort – No indication of Fabry disease

#### T. Böttcher ^1^; C. Beetz^2^; D. Schulze^3^; E. Zonic^2^; S. Schroeder^2^; A. Kaune^2^; L. Bauer^4^; J. Pinto Basto^2^; P. Bauer^5^

##### ^1^Centogene GmbH, Rostock, Germany and Dept. Neurology, Medical Faculty, University of Greifswald, Germany; ^2^Centogene GmbH, Rostock, Germany; ^3^Institute of Biometry and Clinical Epidemiology, Charité‐University Medicine Berlin, Germany; ^4^Sigmund Freud Private University, Vienna, Austria; ^5^Centogene GmbH, Rostock, Germany and Pomeranian Medical University, Szczecin, Poland

**Background and aims:** Fabry disease (FD) is a multisystemic disorder caused by pathogenic variants in the GLA gene. Premature stroke and neuropathic pain are typical for neurological involvement. The disease‐causing properties of the GLA variant c.937G > T (p.Asp313Tyr), reclassified from “Variant of uncertain significance” to “Benign” by our lab, are still debated. Recently, we demonstrated no specific phenotype for this variant in a large German cohort. To confirm the data globally we extended our evaluation.

**Methods:** Leveraging Centogene's Biodatabank we analyzed all samples (*n* = 154,447) molecularly tested for Fabry disease during 2015 and 2020 from 72 countries for GLA genotypes, clinical presentations, and biochemical phenotypes.

**Results:** The allele frequency of GLA variant p.Asp313Tyr in this cohort was increased compared to gnomAD v4.1.0 data resulting in an odds ratio of 3.77 (95% CI: 3.61–3.94; *p* < 0.0001). Again, there was no specific clinical nor biochemical phenotype compared to GLA reference individuals, supporting a diagnosis of FD.

**Conclusion:** The GLA variant p.Asp313Tyr is classified as a benign variant and does not cause FD. Neither clinical symptoms nor biochemical characteristics evidenced a specific effect in our cohort. The fact that the allele frequency here is higher than expected from general population reference data and publications describing a higher prevalence of GLA p.Asp313Tyr in patients with peripheral nervous system involvement or cerebrovascular disease, demand further clarification of a potential role as risk factor for a microangiopathic/neurological dysfunction, possibly comparable to the role of GBA1 variants in Parkinson's disease, must be elucidated. Therefore, properly designed case‐control studies are needed.

**Disclosure:** No support has been received to conduct the study. T.B, C.B, E.Z, S.S, A.K, J.P.B. and P.B. are employees of Centogene GmbH.

Ageing and dementia 2

## EPO‐0165

### Mapping causal pathways from traumatic brain injury to dementia a systematic synthesis with computational inference

#### A. Shaheen ^1^; Dennstädt^2^; I. Filchenko^2^

##### ^1^Alexandria Faculty of Medicine, Egypt; ^2^Bern University Hospital and University of Bern, Switzerland

**Background and aims:** Traumatic brain injury (TBI) represents a critical global health burden, and serves as an environmental risk factor for neurodegenerative diseases, and increasing dementia risk. However, the mechanistic heterogeneity linking acute injury to chronic neurodegeneration remains poorly integrated across the vast clinical and preclinical literature.

**Methods:** 1,347 abstracts screened using a validated prompt engineering system, extracting structured biomedical triples from 823 relevant studies. Semantic path analysis was performed using SapBERT embeddings, dimensionality reduction (PCA‐UMAP), and hierarchical clustering (HDBSCAN) to construct directed acyclic graphs (DAGs) of causal evidence.

**Figure:**
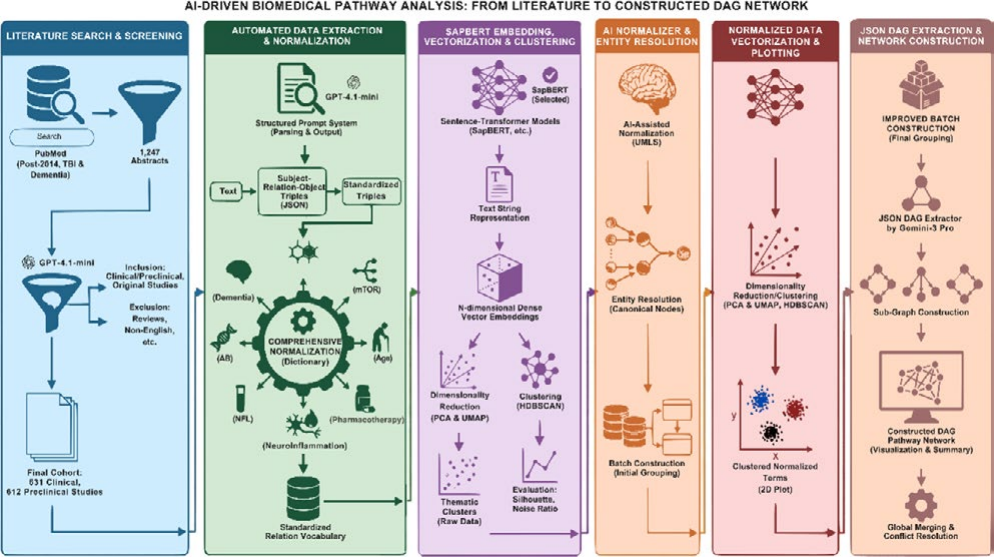
FIGURE 1

**Results:** Our analysis reconstructed the causal map of the post‐TBI events leading to dementia, delineating distinct phases of pathogenesis. Primary axonal degeneration is driven by SARM1‐mediated NAD+ depletion and calpain‐dependent spectrin proteolysis, distinct from classical apoptosis. This transitions into a chronic neuroinflammatory phase characterized by HMGB1‐TLR4 signaling, NF‐κB activation, and the assembly of the NLRP3 inflammasome, which drives gasdermin‐D mediated pyroptosis. We identified critical failures in clearance mechanisms, specifically AQP4 mislocalization leading to glymphatic dysfunction, which exacerbates the accumulation of hyperphosphorylated cis‐tau, amyloid‐beta, and TDP‐43. Furthermore, our synthesis highlights the integrated stress response (ISR) and mitochondrial fission (Drp1) as central metabolic drivers of synaptic failure. The constructed causal maps identify specific therapeutic targets, including SARM1 inhibitors, NLRP3 antagonists, and senolytics, and validate fluid biomarkers such as NfL and GFAP for prognostic stratification.

**Figure:**
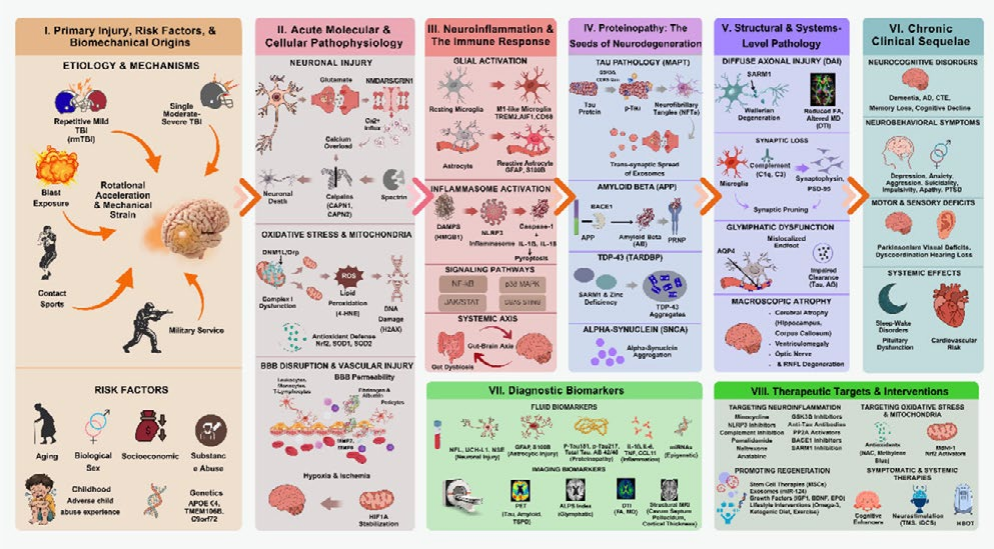
**FIGURE 2** Integrated evidence.

**Figure:**
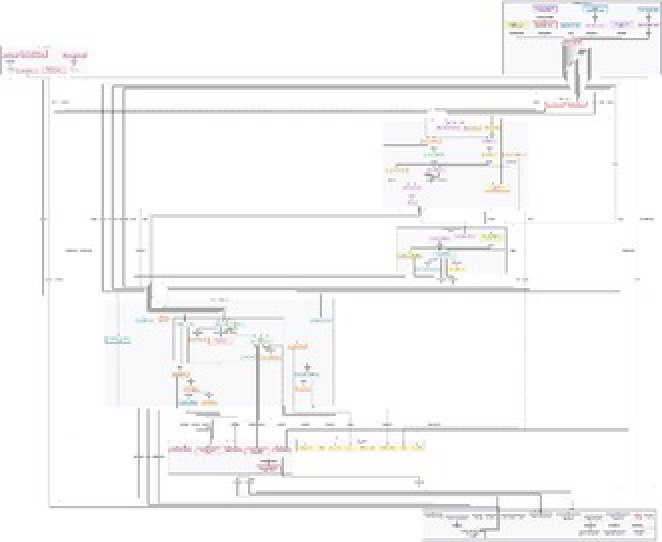
**FIGURE 3** Clinical evidence.

**Conclusion:** This computational synthesis bridges the gap between molecular reductionism and clinical phenomenology, offering a unified framework for precision intervention in TBI‐associated dementia.

**Disclosure:** Nothing to disclose.

## EPO‐0166

### Regional amyloid‐PET dynamics in patients treated with anti‐Aβ monoclonal antibody therapy

#### A. Ghirelli ^1^; E. Spinelli^1^; A. Samanes Gajate^2^; G. Cecchetti^3^; G. Rugarli^1^; S. Pisano^3^; E. Canu^3^; V. Castelnovo^3^; E. Sibilla^3^; A. Gilioli^3^; C. Tripodi^1^; F. Freri^3^; A. Panzacchi^2^; G. Pepe^2^; A. Chiti^2^; M. Filippi^4^; F. Agosta^5^

##### ^1^Center for Alzheimer's disease and related disorders (CARD), Neurology Unit, and Neuroimaging Research Unit, Division of Neuroscience, IRCCS San Raffaele Scientific Institute, Milan, Italy; and Vita‐Salute San Raffaele University, Milan, IT; ^2^Nuclear Medicine Service, IRCCS San Raffaele Scientific Institute, Milan, Italy; ^3^Center for Alzheimer's disease and related disorders (CARD), Neurology Unit, and Neuroimaging Research Unit, Division of Neuroscience, IRCCS San Raffaele Scientific Institute, Milan, Italy; ^4^CARD, Neurology Unit, Neurorehabilitation Unit, Neurophysiology Service, and Neuroimaging Research Unit, Division of Neuroscience, IRCCS San Raffaele Scientific Institute, Milan, Italy; and Neurotech Hub, Vita‐Salute San Raffaele University, Milan, Italy; ^5^Center for Alzheimer's disease and related disorders (CARD), Neurology Unit, and Neuroimaging Research Unit, Division of Neuroscience, IRCCS San Raffaele Scientific Institute, Milan, Italy; and Neurotech Hub, Vita‐Salute San Raffaele University, Milan, Italy

**Background and aims:** Anti–amyloid‐β monoclonal antibodies (AAA) have demonstrated substantial reductions in cerebral amyloid burden in early symptomatic Alzheimer's disease (AD). However, interpretation of amyloid PET changes largely relies on global metrics that may mask regional heterogeneity of amyloid clearance.

**Methods:** We analyzed real‐world amyloid PET data from 19 patients treated with Lecanemab or Donanemab who completed six months of therapy. Global Centiloid values and regional standardized uptake value ratios were quantified at baseline and follow‐up using [18F]flutemetamol or [18F]florbetaben. Regional amyloid changes were examined according to treatment, clinical diagnosis, and clinical disease stage using ANCOVA models adjusted for tracer and AAA.

**Results:** Amyloid reduction showed pronounced regional variability, with the largest decreases in anterior cingulate, caudate and precuneus, and smaller changes in mesial temporal and occipital regions. Patients with early‐onset AD showed higher baseline amyloid burden than those with late‐onset disease but similar longitudinal reductions. Despite comparable baseline amyloid burden, patients with mild cognitive impairment demonstrated greater cortical amyloid reduction than patients with mild dementia. Among patients reaching global amyloid positivity thresholds, residual regional uptake—most consistently in the occipital cortex—was frequently observed.

**Conclusion:** Early amyloid clearance following AAA therapy is regionally heterogeneous and incompletely captured by global amyloid PET measures.

**Disclosure:** A Ghirelli, EG Spinelli, AM Samanes Gajate, G Rugarli, S Pisano, V Castelnovo, E Sibilla, A Gilioli, C Tripodi F Freri, A Panzacchi, and G Pepe have nothing to disclose. Giordano Cecchetti received speaker honoraria from Neopharmed Gentili. Elisa Canu receives research support from the Italian Ministry of Health. A. Chiti reports consulting or advisory role for Blue Earth Diagnostics, Telix Pharmaceuticals, InnovaRadi Therapeutic, and General Electric Healthcare; and Speaker's Bureaus for Bracco Diagnostics, General Electric Healthcare, Novartis, Telix Pharmaceuticals, and United Imaging; he is Editor in Chief of The EANM Journal. M Filippi is Editor‐in‐Chief of the Journal of Neurology, Associate Editor of Human Brain Mapping, Neurological Sciences, and Radiology; received compensation for consulting services from Almirall, Biogen, Bristol‐Myers Squibb, Eli Lilly, Merck, Novartis, Roche, Sanofi; speaking activities from Amgen, Bayer, Biogen, Bristol‐Myers Squibb, Celgene, Chiesi Italia SpA, Eisai, Eli Lilly, Fujirebio, Genzyme, Janssen, Merck, Neopharmed Gentili, Neuraxpharm, Novartis, Novo Nordisk, Roche, Sanofi, Takeda; participation in Advisory Boards for Alexion, Biogen, Bristol‐Myers Squibb, Eli Lilly, GE Healthcare Ltd, Merck, Neuraxpharm, Novartis, Roche, Sandoz, Sanofi, Takeda; scientific direction of educational events for Biogen, Merck, Roche, Celgene, Bristol‐Myers Squibb, Lilly, Novartis, Sanofi‐Genzyme; he receives research support from Biogen Idec, Merck‐Serono, Novartis, Roche, the Italian Ministry of Health, the Italian Ministry of University and Research, and Fondazione Italiana Sclerosi Multipla. F Agosta is Associate Editor of NeuroImage: Clinical and the European Journal of Neurology; has received speaker honoraria from Biogen Idec, Bristol Myers Squibb, Eisai, Eli Lilly, GE Healthcare, Neuraxpharm, and Roche,; and receives or has received research supports from the Italian Ministry of Health, the Italian Ministry of University and Research, AriSLA (Fondazione Italiana di Ricerca per la SLA), the European Research Council (ERC), the EU Joint Programme – Neurodegenerative Disease Research (JPND), and Foundation Research on Alzheimer Disease (France).

## EPO‐0167

### Family history and associated risk factors in patients with dementia with Lewy bodies

#### C. Teles ^1^; J. Durães^1^; P. Faustino^1^; C. Bernardes^2^; M. Coelho^1^; C. Cunha^1^; M. Almeida^3^; D. Duro^1^; I. Baldeiras^4^; M. Tábuas Pereira^1^; I. Santana^1^

##### ^1^Neurology Department, Local Health Unit of Coimbra, Coimbra, Portugal; ^2^Neurology Department, Local Health Unit of Alto Ave, Guimarães, Portugal; ^3^Centre for Neuroscience and Cell Biology (CNC), University of Coimbra, Coimbra, Portugal; ^4^Centre for Innovative Biomedicine and Biotechnology (CIBB), University of Coimbra, Coimbra, Portugal

**Background and aims:** Dementia with Lewy bodies (DLB) is one of the most common neurodegenerative diseases. Its aetiology and possible risk factors remain largely unclear. Evidence on whether general family history of dementia confers higher risk of DLB remains scarce. We aim to assess whether family history of dementia is associated with increased risk of DLB. We explore inheritance patterns (maternal, paternal, and siblings), and possible link to consanguinity.

**Methods:** We conducted a tertiary hospital‐based cohort study with 1836 participants, including 152 patients with DLB, 843 patients with Alzheimer's disease (AD) and 841 sex‐ and age‐matched cognitively healthy controls over 75 years old. Participants were questioned regarding family history of dementia (maternal, paternal, siblings). Parental birthplace and history of consanguinity was also registered. Binary logistic regressions and comparative analyses were performed between DLB, AD, and controls.

**Results:** In the total 1836 participants, 152 had DLB, with mean current age 80.2 years (SD: 5.9), mean onset age 74.3 years (SD: 6.2), and 62.1% were women. We found that having a sister with dementia was associated with higher odds of DLB compared with controls (OR 51.33, 95% CI = 20.02–131.65) and AD (OR 2.61, 95% CI = 1.66–4.10). Higher odds of DLB were also associated with maternal dementia history (OR 1.89, 95% CI = 1.16–3.08) versus controls.

**Conclusion:** This study provides new evidence that maternal and sibling family history of dementia may be associated with an increased risk of DLB, refining our understanding of a possible heritable architecture, highlighting the importance of detailed family history in clinical assessment and risk stratification.

**Disclosure:** Nothing to disclose.

## EPO‐0168

### Comparison between Aβ42, Aβ40, and Aβ42/40 ratio as an in vivo biomarker of cerebral amyloid angiopathy

#### E. Sentieri ^1^; M. Losa^1^; M. Cotta Ramusino^2^; I. Gandoglia^3^; F. Massa^1^; L. Gualco^1^; F. Mazzacane^2^; G. Perini^2^; A. Costa^2^; M. Kozberg^4^; F. Piazza^5^; M. Del Sette^3^; L. Roccatagliata^3^; L. Farina^6^; M. Pardini^1^

##### ^1^Department of Neuroscience, Rehabilitation, Ophthalmology, Genetics, Maternal and Child Health (DINOGMI), University of Genoa, Genoa, Italy; ^2^Research Unit of Clinical Neuroscience of Dementia, IRCCS Mondino Foundation, Pavia, Italy; ^3^IRCCS Ospedale Policlinico San Martino, Genoa, Italy; ^4^J Philip Kistler Research Center, Department of Neurology, Massachusetts General Hospital and Harvard Medical School, Boston, USA; ^5^CAA and AD Translational Research and Biomarkers Laboratory, School of Medicine and Surgery, University of Milano‐Bicocca, Monza, Italy; ^6^Advanced Imaging and Radiomics Center, Neuroradiology Department, IRCCS Mondino Foundation, Pavia, Italy

**Background and aims:** The Boston Criteria v2.0 integrates clinical‐radiological features to diagnose Cerebral Amyloid Angiopathy (CAA), with deep hemorrhagic manifestations, the so‐called mixed small vessel disease (SVD), precluding a CAA diagnosis, given the high likelihood of underlying hypertensive SVD. This study aims to evaluate whether the Aβ42/40 ratio outperforms isolated biomarkers (Aβ42, Aβ40) as a proxy of neuropathology in a heterogeneous SVD cohort.

**Methods:** We analyzed a cohort of patients with probable CAA (Boston Criteria v2.0) or mixed SVD with available CSF biomarkers (Aβ42, Aβ40). We quantified MRI markers of SVD, including cerebral microbleeds (CMB), cortical superficial siderosis (cSS), enlarged perivascular spaces (EPVS), and the CAA‐SVD score. Associations between MRI scales and Z‐score normalized CSF biomarkers were evaluated using logistic regression models.

**Results:** We included 79 patients (58 probable CAA, 21 mixed SVD). Aβ40 showed a significant association with cSS (OR = 0.54; *p* = 0.005), Aβ42 with cSS (OR = 0.40; *p* < 0.001), CAA‐SVD score (OR = 0.55; *p* = 0.003), and deep EPVS (OR = 2.45; *p* < 0.001), while the Aβ42/40 ratio was associated with cSS (OR = 0.54; *p* = 0.010), CAA‐SVD score (OR = 0.63; *p* = 0.021), deep EPVS (OR = 2.42; *p* < 0.001), and deep CMBs (OR = 3.29; *p* < 0.001). The only CSF biomarker that showed a bimodal distribution was the Aβ42/40 ratio.

**Conclusion:** In our cohort, lower levels of Aβ42 and Aβ40 are closely linked to specific CAA features. However, the Aβ42/40 ratio gains more reliability in relation to deep hemorrhagic markers. This suggests, given also the binomial distribution, that the Aβ42/40 ratio may better differentiate the underlying SVD compared to isolated CSF biomarkers. This should be confirmed in further studies, verifying also the diagnostic accuracy of a biology‐integrated approach compared to the current radiological classification.

**Disclosure:** Nothing to disclose.

## EPO‐0169

### Epilepsy prevalence in memory clinics: Does amyloid matter?

#### G. Lepeu ^1^; A. Rossetti^1^; O. Rouaud^2^; A. Griffa^2^; G. Allali^2^

##### ^1^Service of Neurology, Department of Clinical Neurosciences, Lausanne University Hospital and University of Lausanne, Lausanne, Switzerland; ^2^Leenaards Memory Center, Department of Clinical Neurosciences, Lausanne University Hospital and University of Lausanne, Lausanne, Switzerland

**Background and aims:** Epilepsy is common in neurodegenerative diseases, particularly Alzheimer's disease (AD). However, the impact of amyloid‐β on epilepsy prevalence in clinical populations remains uncertain, as does the association between epilepsy and specific cognitive profiles or brain atrophy. This study aimed to compare cognitive characteristics, AD cerebrospinal fluid (CSF) biomarkers, and brain morphometry between patients with neurodegenerative diseases with and without epilepsy. Additionally, we examined whether epilepsy prevalence differs between patients with AD (as confirmed by CSF biomarkers) and patients with non‐Alzheimer's neurodegenerative diseases (Non‐AD).

**Methods:** In a cross‐sectional study, we retrospectively analyzed well‐characterized patients in a University Memory Clinic cohort (Leenaards Memory Center; Lausanne University Hospital, Switzerland). CSF biomarkers, global cognitive functioning, dominant impaired cognitive domain, brain morphometry, epilepsy diagnosis and EEG report were assessed. Patients were classified using the ATN system to differentiate AD from other neurodegenerative conditions (Non‐AD).

**Results:** In a Memory Clinic cohort (*n* = 479, age: 69.1 +/‐ 9.2, 44% female), no significant differences were found in either cognitive impairment, brain morphometry, CSF amyloid‐β and phosphorylated Tau levels or ATN classification between patients with or without epilepsy. In addition, epilepsy prevalence was comparable between AD and Non‐AD groups (5.2% and 5.4% respectively, *p* = 0.965).

**Conclusion:** Epilepsy was not associated with a distinct cognitive, morphometric, AD CSF biomarker or ATN profile. Importantly, Alzheimer's disease did not correlate with an increased prevalence of epilepsy. These findings point toward mechanisms beyond amyloid‐β ‐ potentially shared across neurodegenerative diseases – as contributors to epileptogenesis, emphasizing the need for further targeted research.

**Disclosure:** Gilles Allali was supported by a grant from the Swiss National Science Foundation (grant number # 214855), from the Leenaards Foundation, the Solis Foundation, the Empiris Foundation, the Marina Cuennet‐Mauvernay Foundation, the Faculty of Biology and Medicine of the University of Lausanne.

## EPO‐0170

### The utility of multimodal non‐invasive biomarkers for early and differential diagnosis of limbic‐predominant age‐related TDP‐43 encephalopathy

#### J. Laczó; K. Veverova; H. Horakova; O. Lerch; V. Matuskova; H. Hadzic; V. Matoska; M. Vyhnalek; J. Hort; M. Laczó

##### Department of Neurology, Motol and Homolka University Hospital, Prague, Czech Republic

**Background and aims:** Limbic‐predominant age‐related TDP‐43 encephalopathy (LATE) is a recently recognised neurodegenerative disease with clinical characteristics, cognitive deficits, and structural brain changes similar to those seen in Alzheimer's disease (AD). The diagnosis of LATE is challenging due to the absence of validated specific biomarkers. This study evaluated diagnostic and clinical usefulness of non‐invasive biomarkers in a memory clinic cohort, exploring the potential of these biomarkers as tools for the early diagnosis of LATE and for differentiating it from AD.

**Methods:** 83 participants from the Czech Brain Aging Study, including individuals with amnestic mild cognitive impairment (aMCI) and biomarker‐confirmed AD (*n* = 27), individuals with aMCI meeting criteria for probable LATE (*n* = 33), and cognitively normal older adults (CN, *n* = 23), underwent cognitive assessment, structural brain MRI, blood‐based biomarker assessment, and genetic testing. Additionally, the aMCI participants underwent amyloid PET imaging and cerebrospinal fluid biomarker assessment.

**Results:** Participants with LATE aMCI outperformed AD aMCI and were similar to CN on visuospatial tests (Figure 1), exhibited greater reductions in entorhinal cortical thickness and a lower ratio of entorhinal‐to‐parahippocampal cortical thickness compared to AD aMCI and CN (Figure 2), and had lower plasma glial fibrillary acidic protein levels and a lower frequency of the APOE4 allele than AD aMCI, and similar to CN (Figure 3). A combination of these markers differentiated between LATE and AD aMCI with 86% sensitivity and 74% specificity.

**Figure:**
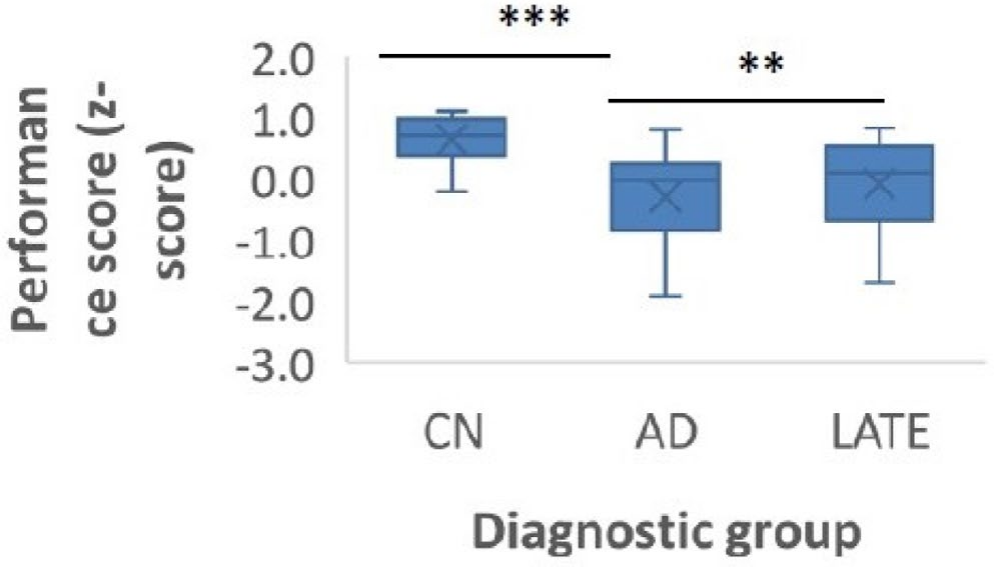
**FIGURE 1** Between‐group differences in visuospatial function.

**Figure:**
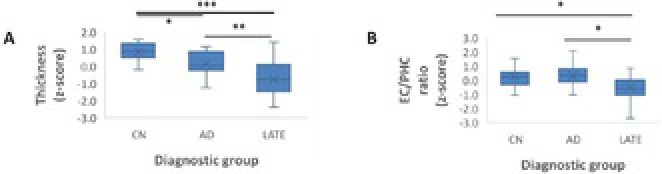
**FIGURE 2** Between‐group differences in A) entorhinal cortical thickness and B) entorhinal‐to‐parahippocampal cortical thickness ratio.

**Figure:**
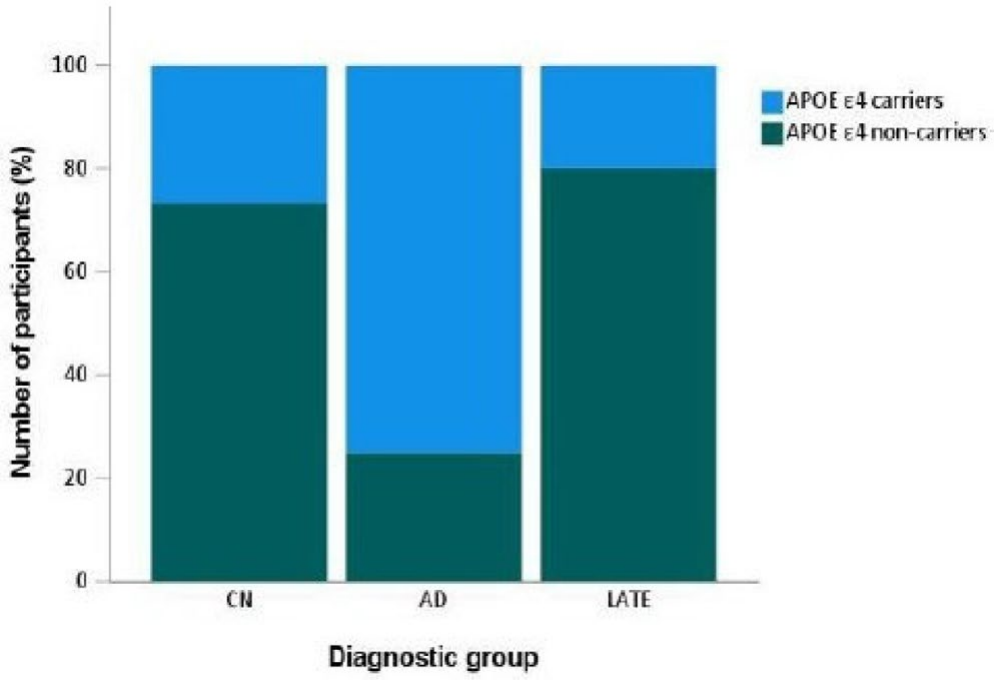
**FIGURE 3** Between‐group differences in the distribution of the APOE4 allele.

**Conclusion:** A combination of specific non‐invasive biomarkers may distinguish early LATE from early AD, providing moderate‐to‐high sensitivity and specificity. This makes them valuable tools for differential diagnosis in clinical settings.

**Disclosure:** This study was supported by the Ministry of Health of the Czech Republic, grant nr. NW25‐04‐00337, the Grant Agency of Charles University (Grant No. 40125), the Institutional Support of Excellence 3 2. LF UK (Grant No. 6980382), the Ministry of Health of the Czech Republic—conceptual development of research organisation, University Hospital Motol, Prague, Czech Republic grant nr. 00064203, and the Czech Science Foundation (GACR) registration number 26‐21480S.

## EPO‐0171

### GALECTIN‐3 as an emerging biomarker of neuroinflammation in Alzheimer's disease and frontotemporal dementia

#### L. Bonino ^1^; S. Boschi^1^; F. Roveta^1^; E. Piella^1^; G. Brodini^1^; A. Agostinelli^1^; G. Mengozzi^2^; I. Rainero^3^; E. Rubino^1^

##### ^1^Department of Neuroscience “Rita Levi‐Montalcini”, University of Turin, Turin, Italy; ^2^Department of Medical Sciences, University of Turin, Turin, Italy; ^3^Department of Neuroscience and Mental Health, AOU Città della Salute e della Scienza di Torino, Turin, Italy

**Background and aims:** Frontotemporal dementia (FTD) and Alzheimer's disease (AD) are the most common causes of dementia and neuroinflammation plays an important role in their pathogenesis. Elevated levels of Galectin‐3 (a galactoside‐binding lectin expressed by activated microglia, Gal‐3), were reported elevated in the cerebrospinal fluid (CSF) of AD and FTD patients. This study aims to evaluate CSF Gal‐3 levels in AD, FTD and patients without diagnosed neurodegenerative disorders (NND), and to investigate its relationship with established biomarkers of neurodegeneration.

**Methods:** CSF samples were collected from 118 patients recruited at Turin University Dementia Center: 51 with AD, 40 with FTD and 27 classified as NND. Gal‐3 concentrations were measured using an Enzyme‐Linked ImmunoSorbent Assay (ELISA) kit (Abcam, Cambridge, UK) while CSF Amyloid‐beta‐42 (Aβ42), Amyloid‐Beta‐42/40 ratio (Aβ40/42), total‐tau (t‐Tau), phosphorylated‐tau‐at‐threonine‐181 (p‐Tau181), and neurofilament‐light‐chain (NfL) using a Lumipulse platform (Fujirebio, Ghent, Belgium).

**Results:** Mean Gal‐3 levels were higher in the FTD group (562.1 ± 226.1 pg/mL) and in the AD group (473.1 ± 128.3 pg/mL) compared to the NND group (256.7 ± 164.6 pg/mL; *p* < 0.001). Gal‐3 levels did not differ between the FTD and AD groups (*p* = 0.064). In the FTD subgroup Gal‐3 showed a significant correlation with t‐Tau (*r* = +0.33, *p* = 0.045), while in the overall population Gal‐3 correlated with NfL (*r* = +0.28, *p* = 0.039).

**Conclusion:** CSF Gal‐3 levels were elevated in patients with AD and FTD, supporting the contribution of neuroinflammation to neurodegeneration and reinforcing Gal‐3 as a potential biomarker. The correlation between Gal‐3 and t‐Tau further suggests a relationship between microglial activation and tau‐related pathology.

**Disclosure:** Nothing to disclose.

## EPO‐0172

### Gut–brain communication via extracellular vesicles enables amyloid‐β transport to the central nervous system

#### M. Munir; T. Davis; I. Javed

##### Australian Institute for Bioengineering and Nanotechnology, The University of Queensland, St. Lucia, Queensland, Australia

**Background and aims:** Alzheimer's disease (AD) is defined by progressive neurodegeneration and cerebral deposition of amyloid‐β (Aβ). While central amyloid pathology has been extensively studied, emerging evidence suggests that peripheral tissues may contribute to disease initiation and progression. However, the cellular pathways enabling amyloid proteins to traverse the gut–brain axis remain poorly defined.

**Methods:** Gut‐to‐brain transport was examined using complementary in vitro and in vivo models. A co‐culture was established to model directional transport. Epithelial barrier integrity was confirmed using TEER measurements, Dextran‐FITC permeability assays, and ZO‐1 immunostaining. EV involvement in Aβ transport was assessed by tracking EV‐associated Aβ transfer. Neuronal oxidative stress and cytotoxicity were quantified following exposure to free or EV‐associated Aβ. In vivo validation was performed in zebrafish larvae.

**Results:** Intestinal barriers remained structurally and functionally intact across in vitro models. Aβ was preferentially packaged into EVs released by intestinal epithelial cells and subsequently internalized by neurons. Neurons exposed directly to free Aβ exhibited significantly higher oxidative stress and cytotoxicity compared to neurons receiving EV‐associated Aβ through epithelial–neuronal interfaces. In vivo imaging demonstrated that Aβ delivered into the zebrafish gut accumulated in the brain.

**Conclusion:** These findings establish extracellular vesicles as critical mediators of amyloid‐β transport from the gut to the brain via transcellular pathways, independent of intestinal barrier disruption.

**Disclosure:** Nothing to disclose.

## EPO‐0173

### Neurological perspectives in solid organ transplantation: Advancing ethical and equitable care for people with cognitive impairment

#### V. Lo Re ^1^; A. Grossi^2^

##### ^1^Neurology Service, IRCCS ISMETT (Istituto Mediterraneo per i Trapianti e Terapie ad Alta Specializzazione), Palermo, Italy; ^2^Department of Human Sciences, Innovation and Territory, University of Insubria, Varese‐Como, Italy

**Background and aims:** Cognitive impairment (CI) affects a significant proportion of liver (up to 80%), kidney (up to 55%), lung (45%), and heart transplant candidates (25–74%). As organ failure increasingly affects older adults, the number of solid organ transplant candidates with CI is expected to increase. However, the lack of standardized cognitive assessments leads to variability across centers, potentially resulting in inequities in transplant accessibility and outcomes. Incorporating CI assessments into pre‐transplant evaluations is therefore crucial to better predict post‐transplant outcomes and to support ethically sound decision‐making.

**Methods:** We reviewed the available literature on the debated issue of organ allocation for patients with CI, synthetizing arguments both in favor of and against transplantation, as well as proposed approaches to address these challenges.

**Results:** It has been recommended to establish independent regional boards to address allocation challenges and reduce the risk of discrimination against patients with CI. These boards should include transplant and non‐transplant professionals (e.g, social workers, psychologists, bioethicists) and other relevant stakeholders (e.g, patients and family members) to enhance transparency and consensus. Integrating ethical assessments and review into pre‐transplant evaluations may further support multidisciplinary discussions.

**Conclusion:** We propose that transplant eligibility for individuals with CI would be approached as a specialized clinical evaluation, conducted early in the transplant process by experts in cognitive disorders to assess factors relevant to perioperative and long‐term outcomes, including severity, etiology, and prognosis of the CI; this clinical assessment should be systematically integrated with structured ethical evaluation to support fair and transparent decision‐making.

**Disclosure:** The authors have nothing to disclose.

## EPO‐0174

### Plasma metabolic profiles predict future dementia and dementia subtypes: A prospective analysis of 274,160 participants

#### X. He

##### Department of Neurology and National Center for Neurological Disorders, Huashan Hospital, State Key Laboratory of Medical Neurobiology and MOE Frontiers Center for Brain Science, Shanghai Medical College, Fudan University, Shanghai, China.

**Background and aims:** Blood‐based biomarkers for dementia are gaining attention due to their non‐invasive nature and feasibility in regular healthcare settings. Here, we explored the associations between 249 metabolites with all‐cause dementia (ACD), Alzheimer's disease (AD), and vascular dementia (VaD) and assessed their predictive potential.

**Methods:** This study included 274,160 participants from the UK Biobank. Cox proportional hazard models were employed to investigate longitudinal associations between metabolites and dementia. The importance of these metabolites was quantified using machine learning algorithms, and a metabolic risk score (MetRS) was subsequently developed for each dementia type. We further investigated how MetRS stratified the risk of dementia onset and assessed its predictive performance, both alone and in combination with demographic and cognitive predictors.

**Results:** During a median follow‐up of 14.01 years, 5,274 participants developed dementia. Of the 249 metabolites examined, 143 were significantly associated with incident ACD, 130 with AD, and 140 with VaD. Among metabolites significantly associated with dementia, lipoprotein lipid concentrations, linoleic acid, sphingomyelin, glucose, and branched‐chain amino acids ranked top in importance. Individuals within the top tertile of MetRS faced a significantly greater risk of developing dementia than those in the lowest tertile. When MetRS was combined with demographic and cognitive predictors, the model yielded the area under the receiver operating characteristic curve (AUC) values of 0.857 for ACD, 0.861 for AD, and 0.873 for VaD.

**Figure:**
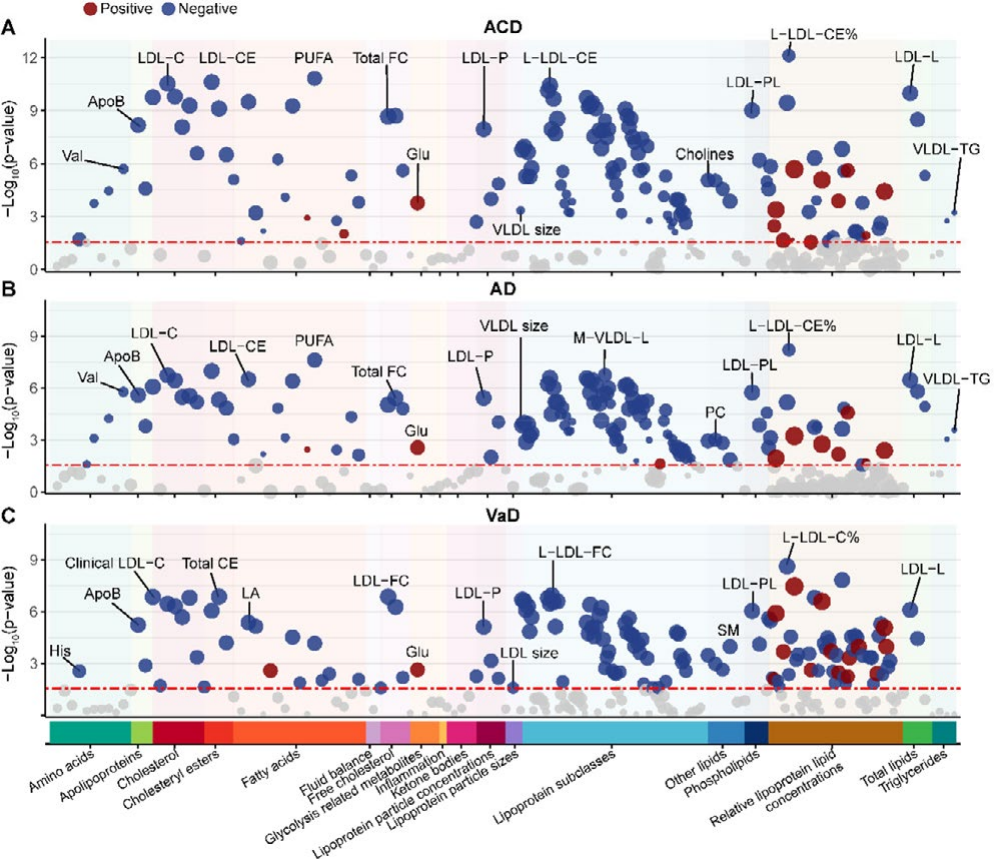
**FIGURE 1** Dementia‐associated metabolites in the association analysis.

**Figure:**
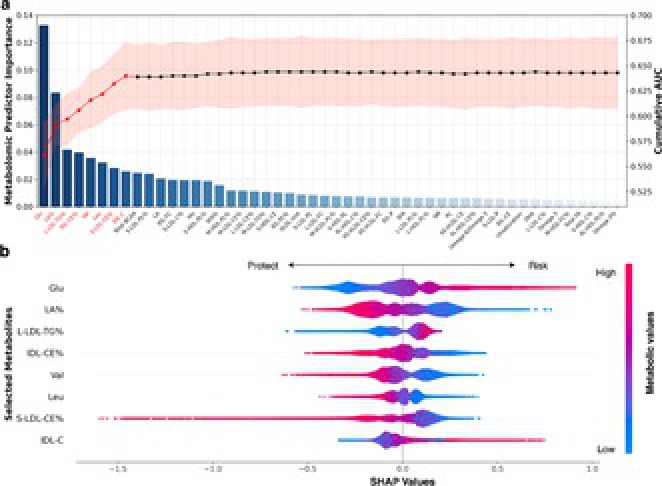
**FIGURE 2** Metabolites importance ranking and SHAP visualization of modeling based on incident ACD populations.

**Figure:**
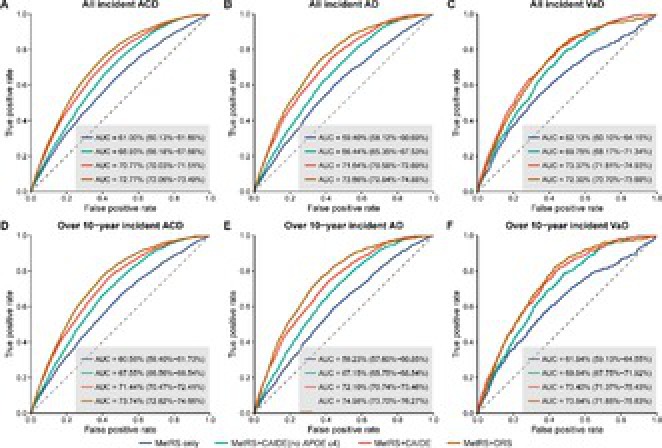
**FIGURE 3** Prediction of incident ACD, AD, and VaD.

**Conclusion:** We conducted the largest metabolome investigation of dementia to date, for the first time revealed the metabolite importance ranking, and highlighted the contribution of plasma metabolites for dementia prediction.

**Disclosure:** Nothing to disclose.

## EPO‐0175

### Serum clinical laboratory tests and risk of incident dementia: A prospective cohort study of 407,190 individuals

#### X. He

##### Department of Neurology and National Center for Neurological Disorders, Huashan Hospital, State Key Laboratory of Medical Neurobiology and MOE Frontiers Center for Brain Science, Shanghai Medical College, Fudan University, Shanghai, China.

**Background and aims:** Prevention of dementia is a public health priority, and the identification of potential biomarkers may provide benefits for early detection and prevention.

**Methods:** This study investigates the association of common serum laboratory tests with the risk of incident dementia. Among 407 190 participants from the UK Biobank (median follow‐up of 9.19 years), we investigated the linear and nonlinear effects of 30 laboratory measures on the risk of all‐cause dementia using Cox models and restricted cubic spline models.

**Results:** We found that dementia incidence was associated with low vitamin D concentration (hazard ratio 0.994, 95% confidence interval 0.993–0.996), indicators of endocrine disorders: IGF‐1 level (P for non‐linearity = 1.1E‐05), testosterone level (*p* for non‐linearity = 0.006); high sex‐hormone‐binding globulin level (HR 1.004, 95% CI 1.003–1.006); reduced liver function: lower alanine aminotransferase (HR 0.990, 95% CI 0.986–0.995); renal dysfunction: cystatin C level (*p* for non‐linearity = 0.028); oxidative stress: lower urate level (HR 0.998, 95% CI 0.998–0.999); lipids dysregulation: lower LDL (HR 0.918, 95% CI 0.872–0.965) and triglycerides (HR 0.924, 95% CI 0.882–0.967) concentrations; insulin resistance: high glucose (HR 1.093, 95% CI 1.045–1.143) and HbA1c (HR 1.017, 95% CI 1.009–1.025) levels; immune dysbiosis: C−reactive protein (*p* for non‐linearity = 5.5E‐09).

**Figure:**
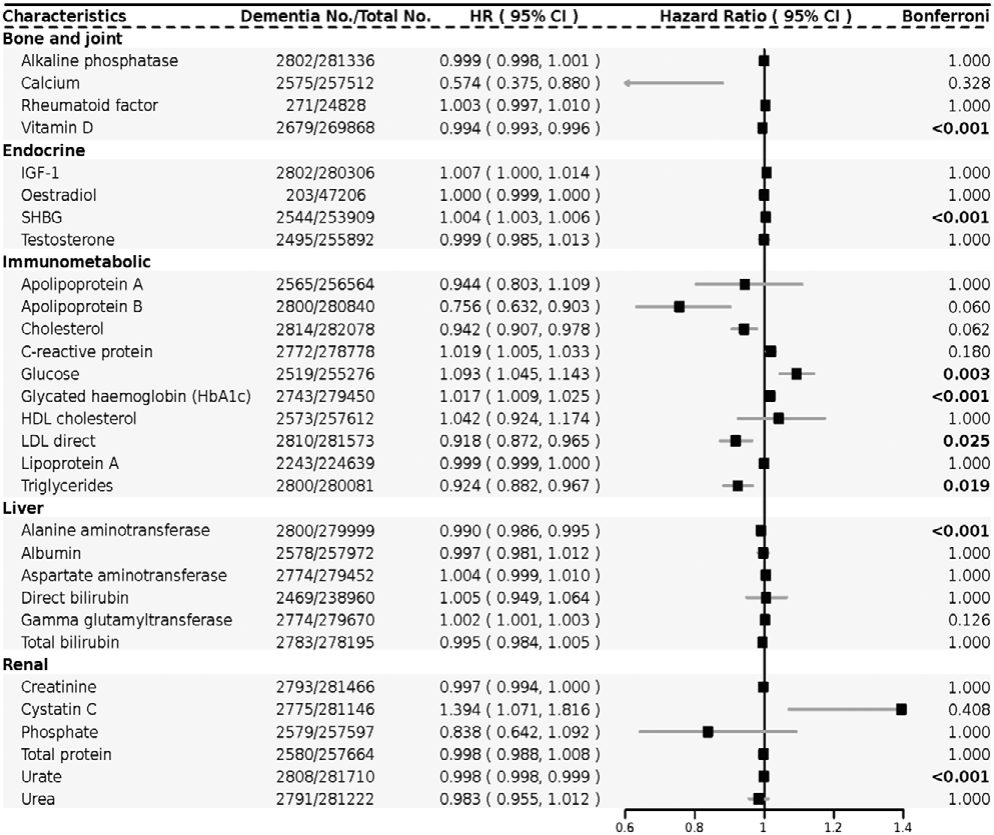
**FIGURE 1** Linear associations between serum laboratory tests and risk of incident dementia.

**Figure:**
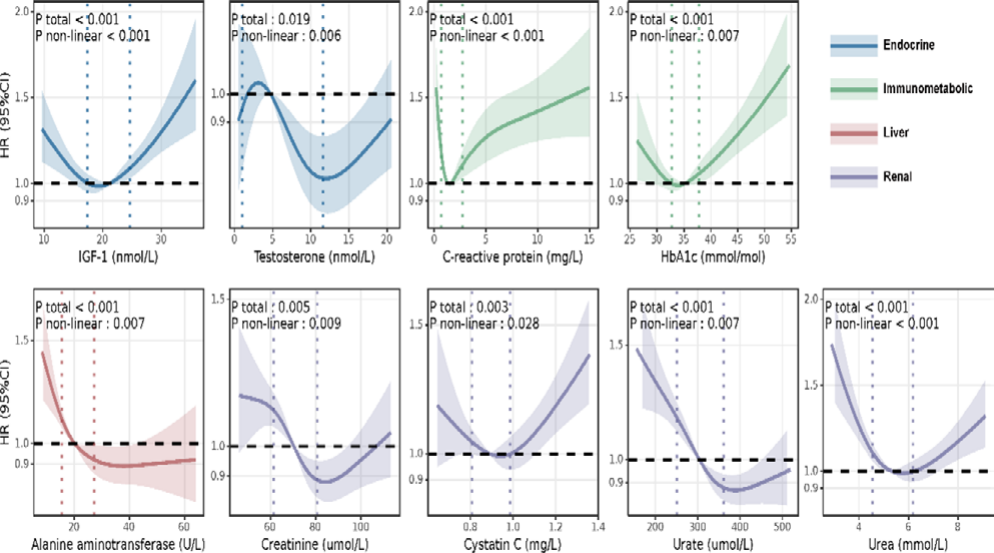
**FIGURE 2** Nonlinear associations between serum laboratory tests and risk of incident dementia.

**Figure:**
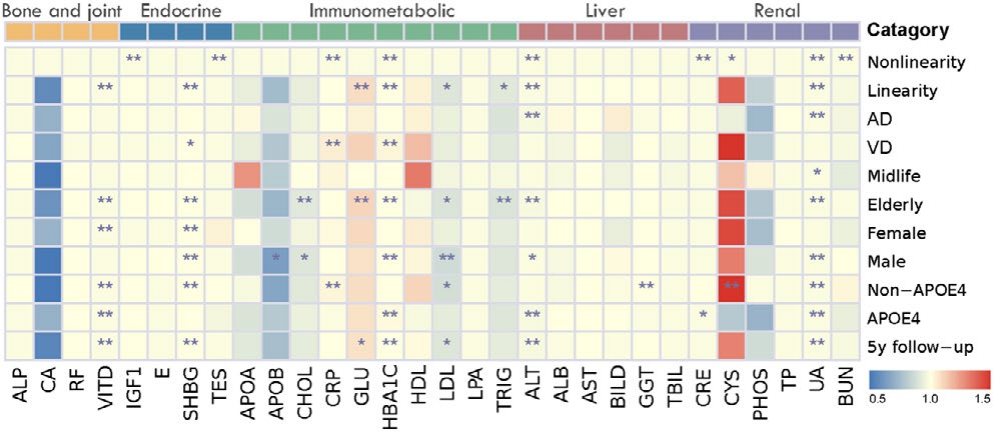
**FIGURE 3** Heatmap to overview the associations of serum laboratory tests with dementia risks.

**Conclusion:** In conclusion, markers of vitamin D deficiency, GH‐IGF‐1 axis disorders, bioactive sex hormone deficiency, reduced liver function, renal abnormalities, oxidation, insulin resistance, immune dysbiosis, and lipids dysregulation were associated with incident dementia. Our results support a contributory role of systemic disorders and diverse biological processes to onset of dementia.

**Disclosure:** Nothing to disclose.

## Neurotoxicology/Occupational Neurology

## EPO‐0176

### Cannabis‐induced PRES with acute cerebral infarctions involving the bilateral posterior territories and left thalamus

#### A. Jamal Eddine

##### Neurology Department, Klinik Hietzing, Vienna, Austria

**Background and aims:** Posterior Reversible Encephalopathy Syndrome (PRES) is an uncommon but clinically significant condition characterized by headache, visual disturbances, seizures, altered mental status, and posterior white‐matter changes. Cerebral infarction occurs in a minority of cases. Although many triggers have been described, cannabis‐associated PRES remains rare.

**Methods:** Case report.

**Results:** A 36 year old patient was admitted after mixed alcohol and cannabis intoxication. On admission, he reported several days of headache, vertigo, and visual perceptual disturbances. Blood pressure was 161/91 mmHg. Due to markedly elevated urinary tetrahydrocannabinol levels and recent heavy cannabis use, symptoms were initially attributed to intoxication. Because neurological symptoms persisted, MRI was performed and revealed subacute infarctions in the bilateral posterior territories and the left thalamus, with PRES‐compatible changes and minor bilateral haemorrhagic transformation. Transcranial and extracranial Doppler studies showed normal flow without vasospasm or stenosis. During monitored care, mildly elevated blood pressure stabilized under calcium channel blocker therapy. A follow up CT after three days confirmed stable haemorrhagic findings, allowing initiation of aspirin for secondary stroke prevention. With ongoing visuospatial rehabilitation, the patient showed gradual but incomplete improvement. Visual agnosia partially resolved, but deficits in colour discrimination and prosopagnosia persisted. He was discharged with a structured outpatient neurorehabilitation program.

**Figure:**
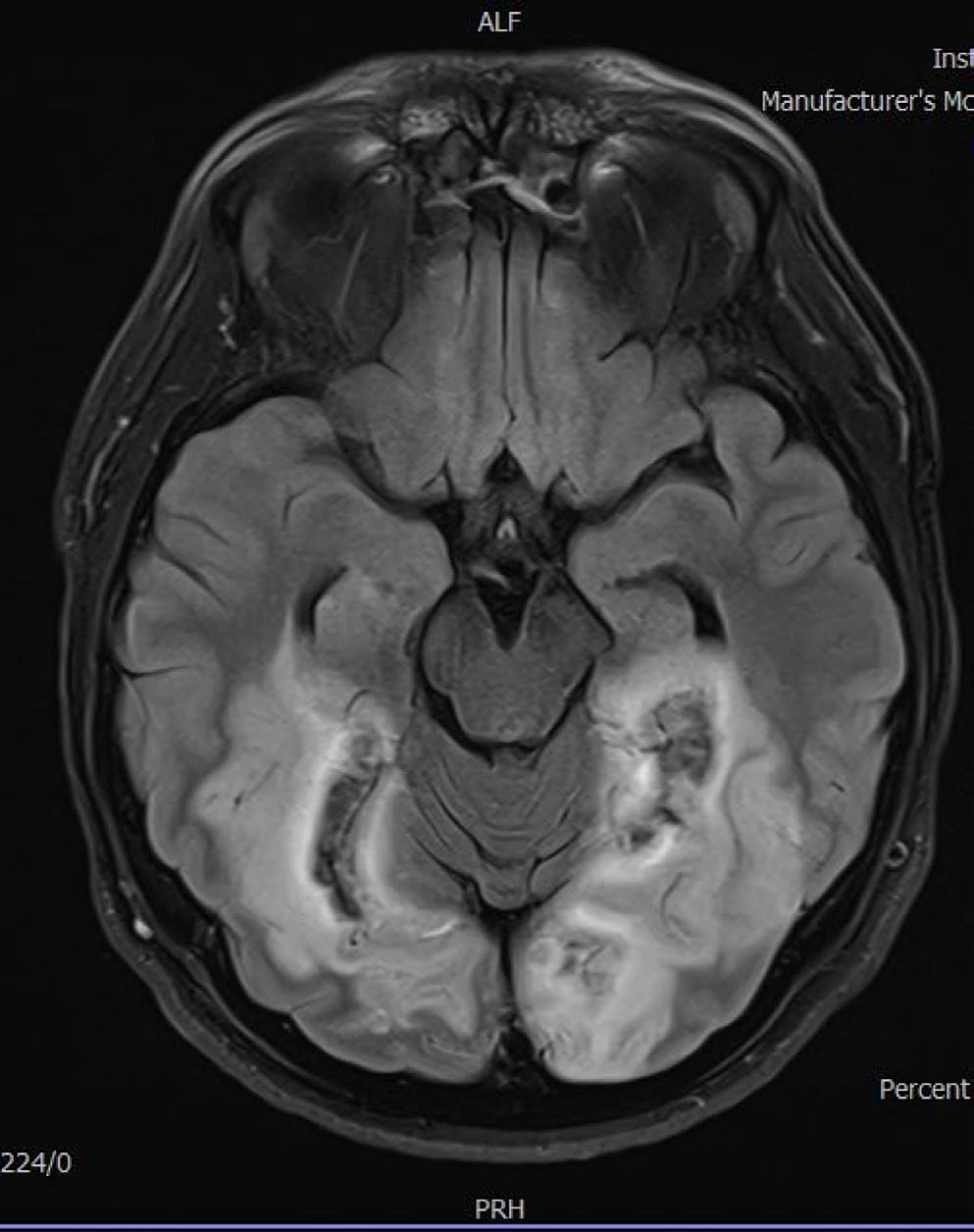
**FIGURE 1** MRI FLair.

**Figure:**
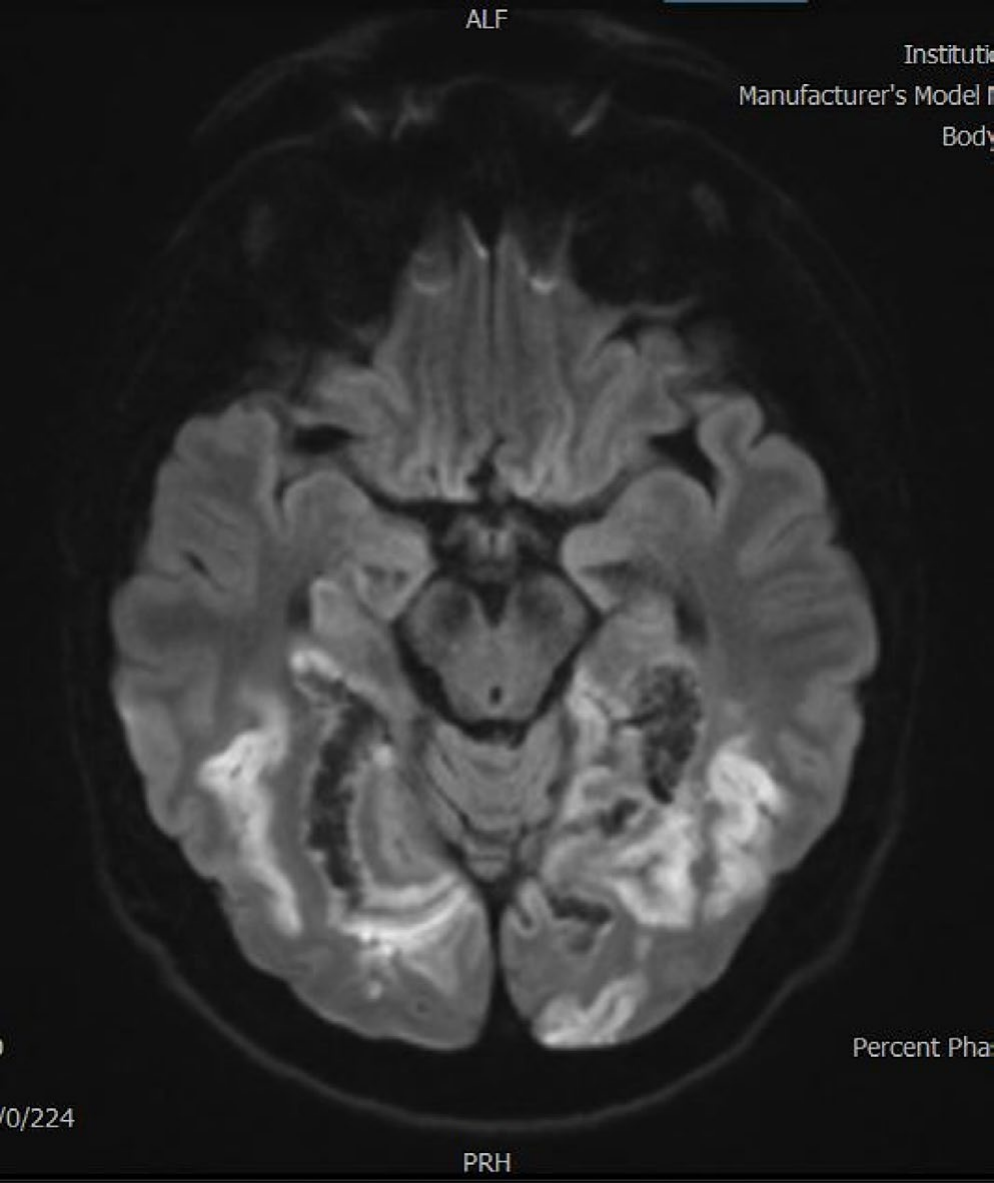
**FIGURE 2** MRI DWI.

**Figure:**
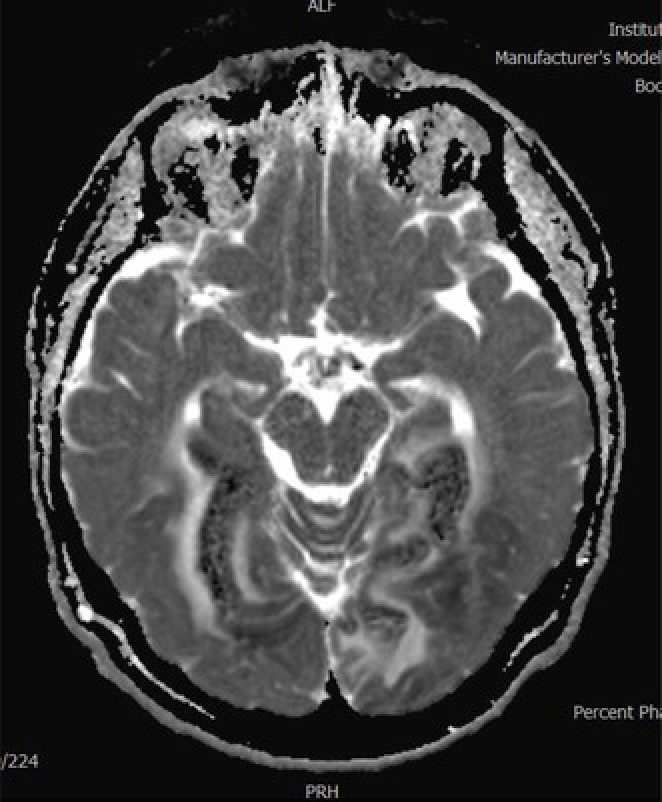
**FIGURE 3** MRI ADC.

**Conclusion:** This case illustrates an atypical form of PRES with concurrent posterior infarctions, reflecting severe autoregulatory dysfunction. Early recognition and blood pressure control supported partial recovery. The case highlights the importance of considering PRES in patients presenting with neuropsychiatric symptoms after substance use, where initial impressions may be misleading.

**Disclosure:** Nothing to disclose.

## EPO‐0177

### Abstract withdrawn

## EPO‐0178

### Greens are not always good for you!

#### G. Diniz de Pinho; S. Nunes Oliveira

##### Neurology Department, Hospital da Luz, Lisbon, Portugal

**Background and aims:** Hyperacute hour‐lasting confusional states frequently suggest exposure to intoxicating substances, but associations may be hard to establish outside the typical contexts.

**Methods:** We discuss a case of a healthy 69‐year‐old Portuguese female patient.

**Results:** She was brought to the emergency department after sudden confusion. Hypertension, sinus tachycardia and flushed skin were noted. Temperature was normal and she did not have headache or vomiting. Neurologic and mental exams were remarkable for disorientation, periods of delusional incoherent and disconnected ideas in between regular thought processing, slurred speech and extremity/truncal ataxia, with no other focal deficits. Laboratory tests were normal including: no detectable alcohol, amphetamines, cocaine, cannabinoids, opioid and barbiturates. Brain CT/MRI were unremarkable. She was kept under surveillance and all symptoms subsided within 12 hours. She recalled only parts of the episode, but remembered having eaten locally bought sprouts that day. Her son whom she had had lunch with described a dry mouth feeling afterwards. She was discharged upon clinical stability and photographed the greens, where different‐looking fragments with bristly stems could be seen among the other tuberous roots and leaves.

**Figure:**
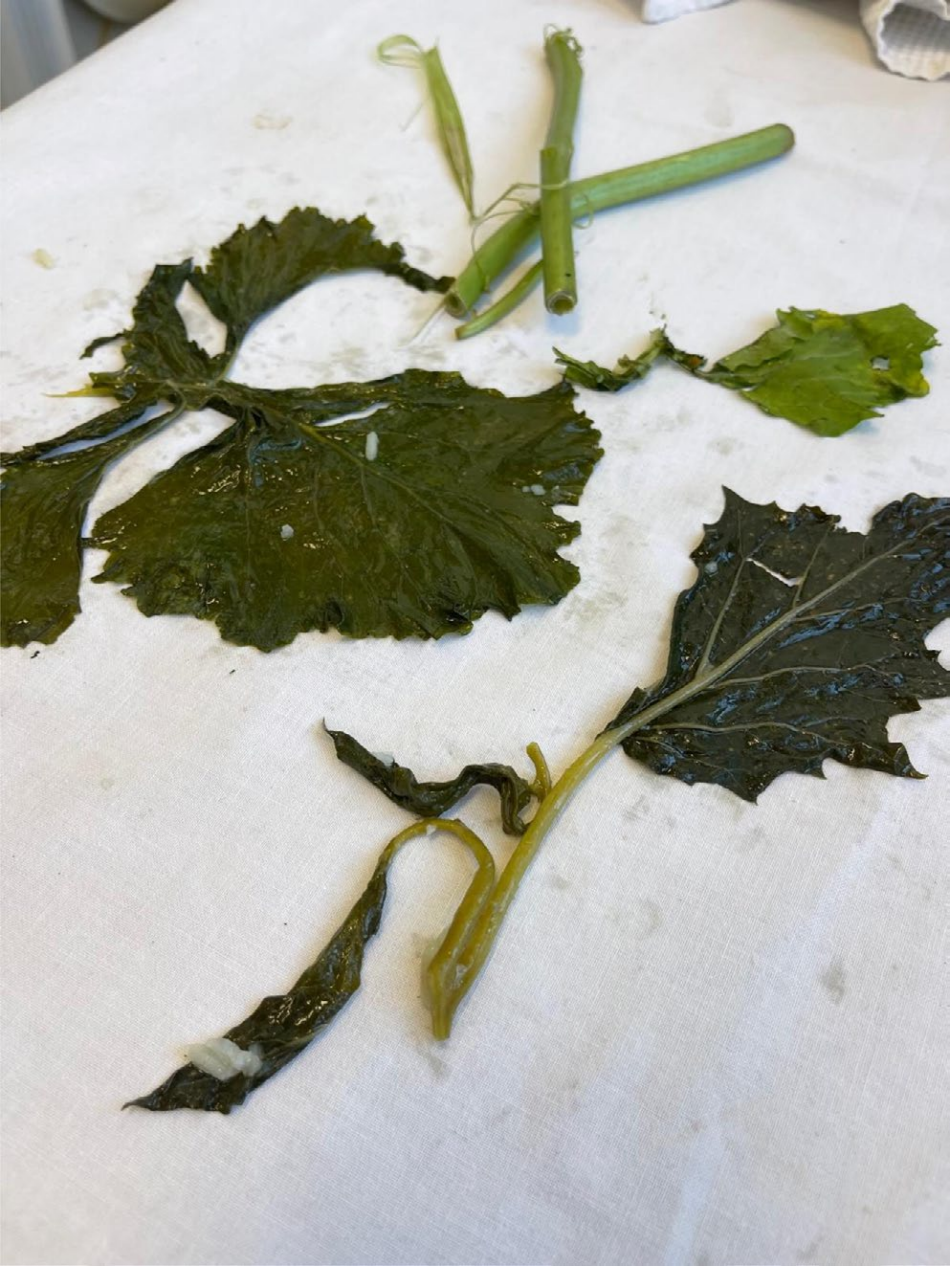
**FIGURE 1** Patient's own vegetable mix (the dark leaves are the sprouts, and the different‐looking tuberous root fragments are the lighter green, bulkier ones).

**Figure:**
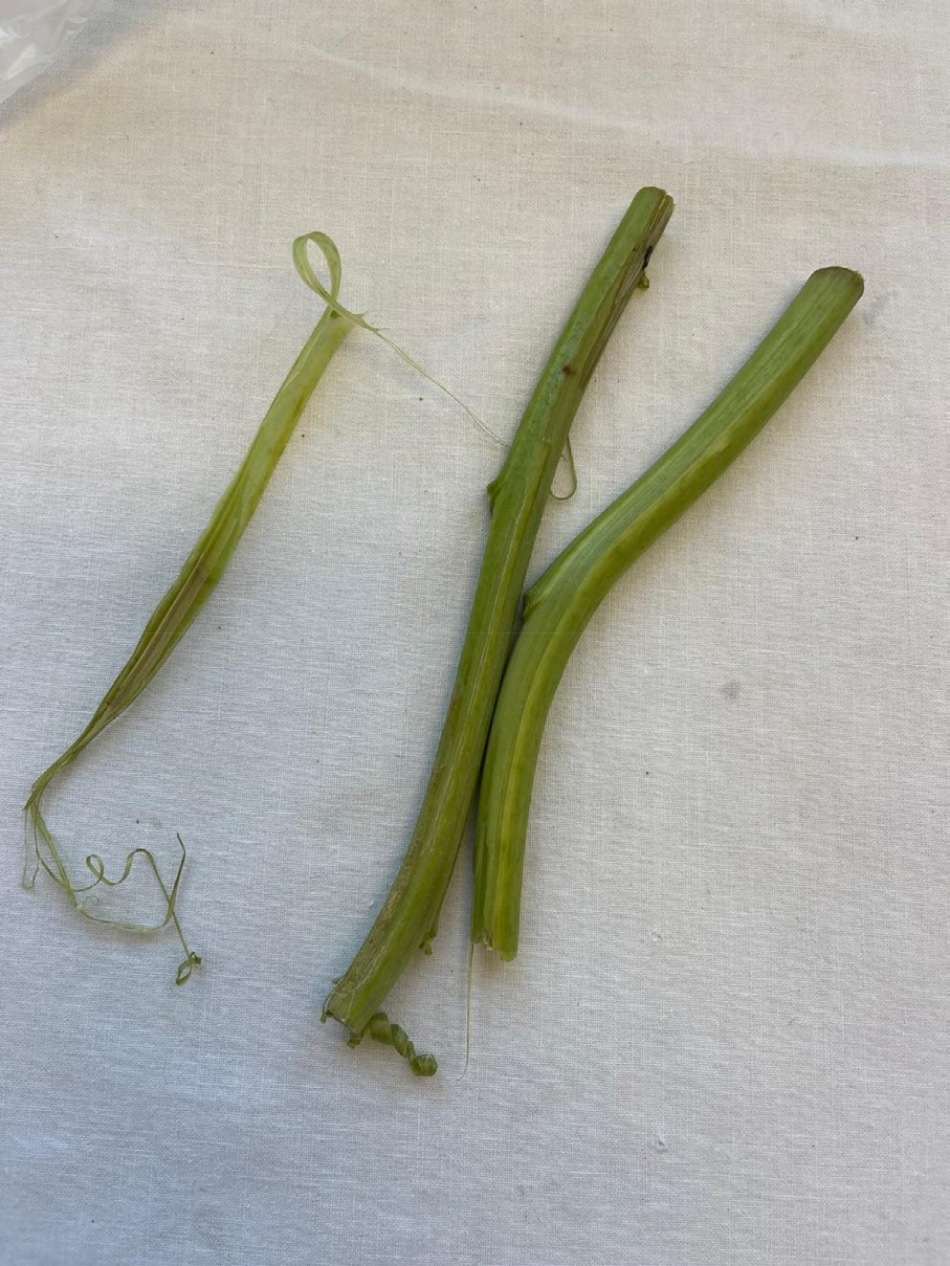
**FIGURE 2** Closer look at the fragments (1).

**Figure:**
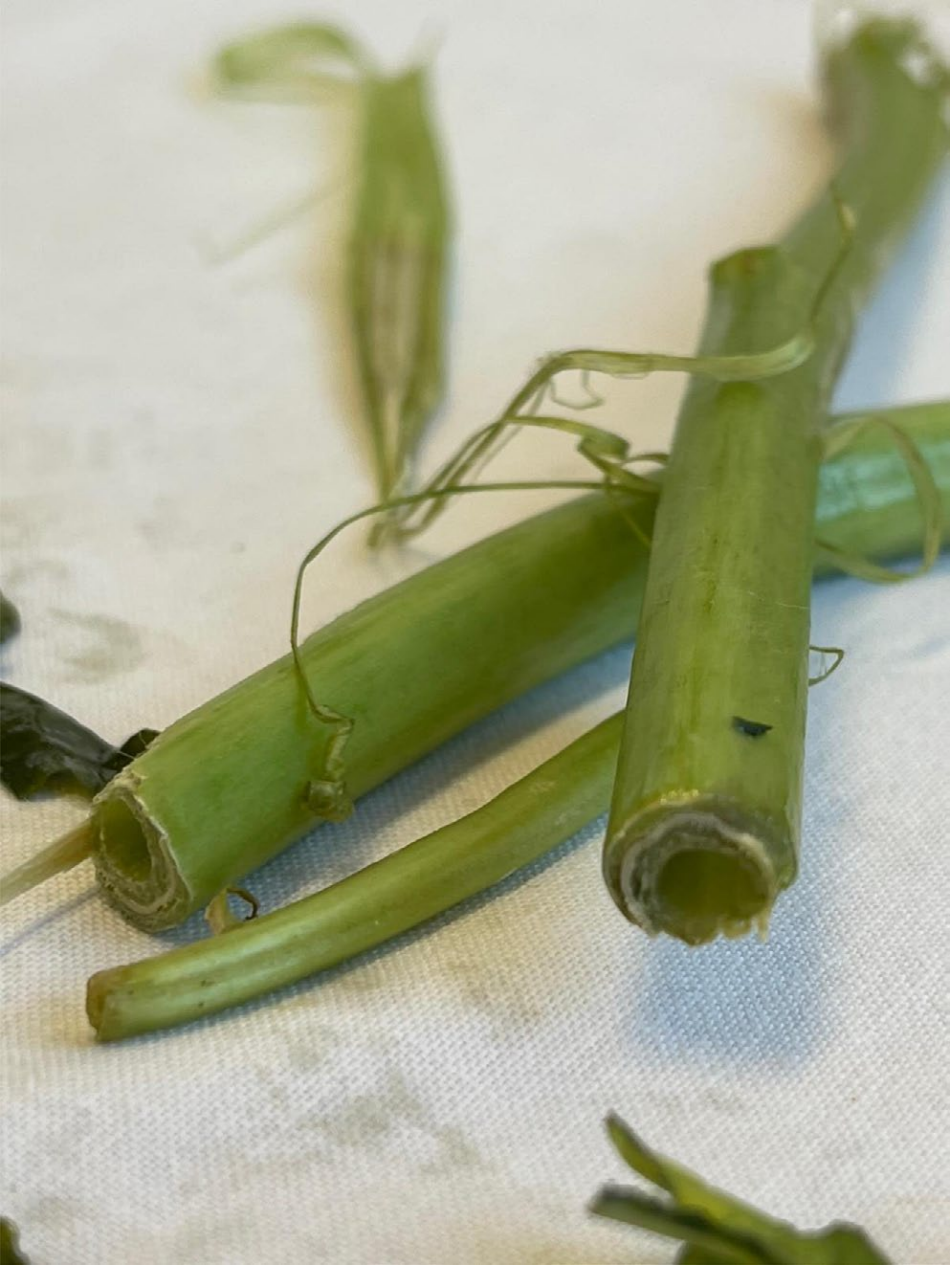
**FIGURE 3** Closer look at the fragments (2).

**Conclusion:** Fragments of Ecballium elaterium (squirting cucumber), a flowering plant found in some parts of southern Europe, including Portugal, and northwestern Africa, were probably inadvertently consumed by our patient. It is allergenic and a strong purgative but, unlike plants of the Solanaceae family, has not been implicated in anticholinergic or central nervous system toxicity. We emphasize reviewing the proliferation of these species in some rural areas for both health and cultivation control.

**Disclosure:** Nothing to disclose.

## EPO‐0179

### Neurological spectrum of recreational nitrous oxide use: A case series including monozygotic twins

#### L. Donneys Martán ^1^; M. De la Calle Cuevas^2^; L. Martín Gil^2^; D. Pérez Gil^2^; A. Gómez Domínguez^1^; B. Estrella León^1^; A. Díaz Negrillo^1^; T. Montalvo Moraleda^3^; L. López Trashorras^3^; I. Llera López^3^; N. Fontana García^4^; E. Escario Méndez^4^; L. Santos Sánchez de las Matas^2^

##### ^1^Department of Clinical Neurophysiology, Hospital Infanta Elena, Madrid, Spain; ^2^Department of Neurology, Hospital Infanta Elena, Madrid, Spain; ^3^Department of Neurology, Hospital Rey Juan Carlos, Madrid, Spain; ^4^Department of Clinical Neurophysiology, Hospital Rey Juan Carlos, Madrid, Spain

**Background and aims:** Nitrous oxide (N2O) is a gas widely used in medical and dental practice that has gained increasing popularity as a recreational substance due to its euphoric effects, earning the nickname “laughing gas.” Its abuse can induce a functional vitamin B12 deficiency through oxidation of the cobalt ion, reducing myelin synthesis and leading to neurological manifestations. The aim of this study is to describe the clinical presentations associated with recreational N2O use.

**Methods:** Case series of four patients with neurological impairment following prolonged N2O consumption.

**Results:** Four male patients aged 18 to 28 years were included, with an average N2O use of four months, presenting with muscle weakness and/or sensory symptoms. Neurological outcomes varied: one patient was asymptomatic, one presented with axonal polyneuropathy, another with cervical subacute combined degeneration (SCD), and another with both conditions. The series included monozygotic twins with identical exposure: one developed combined SCD and motor axonal polyneuropathy with elevated homocysteine and reduced B12 levels, whereas the twin who intermittently self‐supplemented with B‐group vitamins showed only mild hypoesthesia and had normal MRI, electromyography (EMG), nerve conduction studies (NCS) and B12 values. Clinical improvement was observed following N2O cessation and vitamin supplementation.

**Figure:**
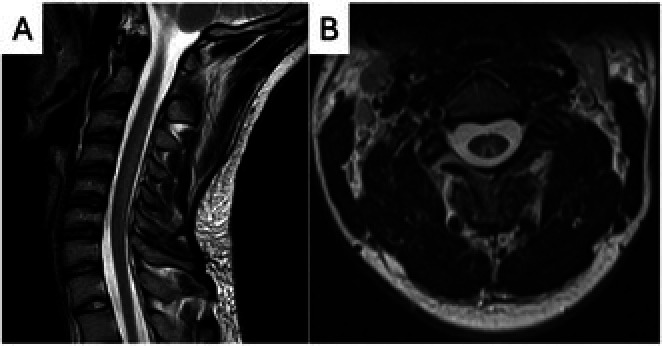
**FIGURE 1** Cervical MRI in SCD. (A) Sagittal T2‐weighted of Patient 1: longitudinal hyperintense signal in posterior columns. (B) Axial T2‐weighted of Patient 4: bilateral and symmetric posterior column hyperintensity forming the “inverted V sign”.

**Figure:**
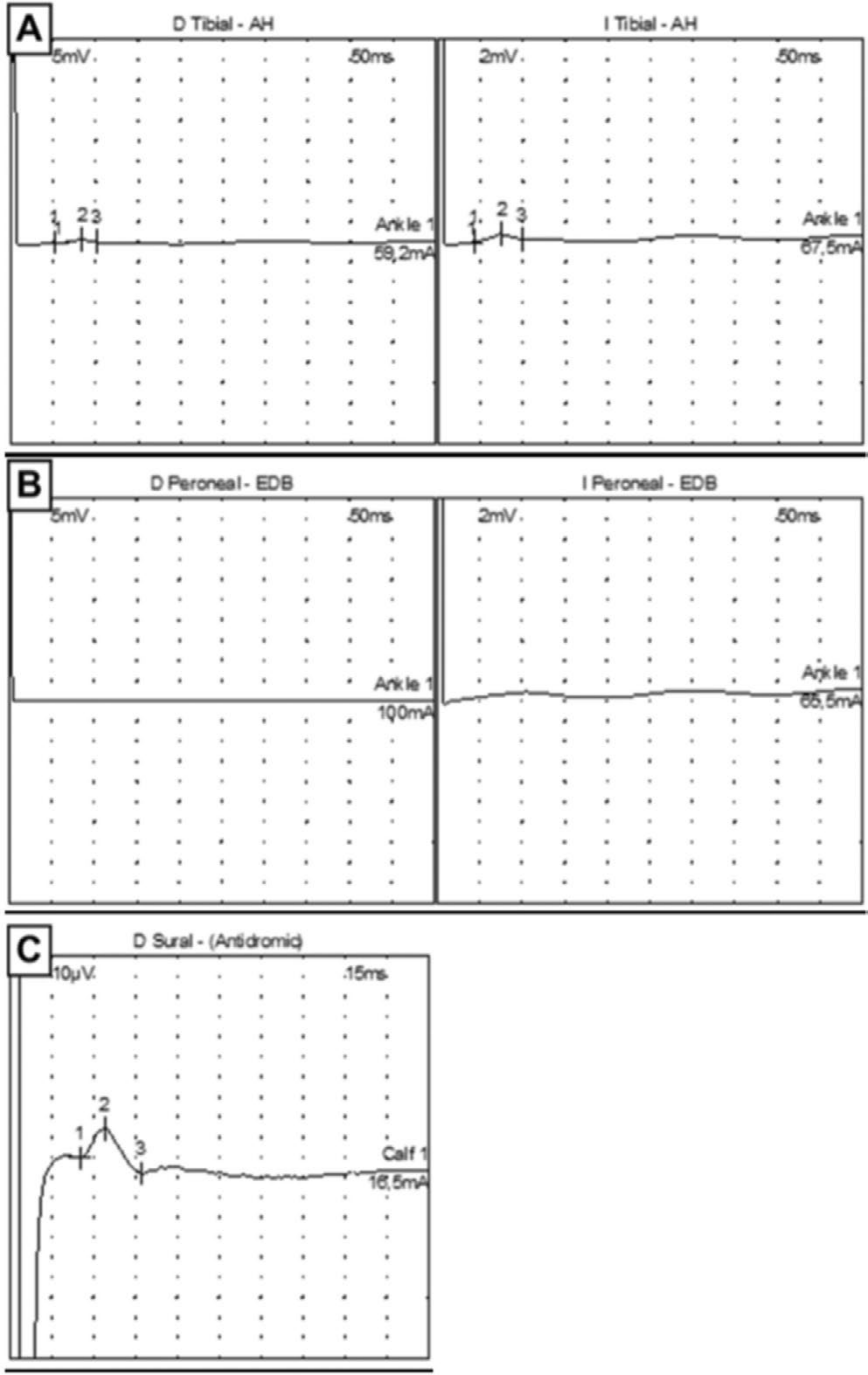
**FIGURE 2** NCS of Patient 1 showing a pure motor axonal polyneuropathy of the lower limbs. (A) Reduced compound muscle action potential amplitude in tibial nerves. (B) Absent peroneal nerves responses. (C) Preserved sural nerve sensory response.

**Figure:**
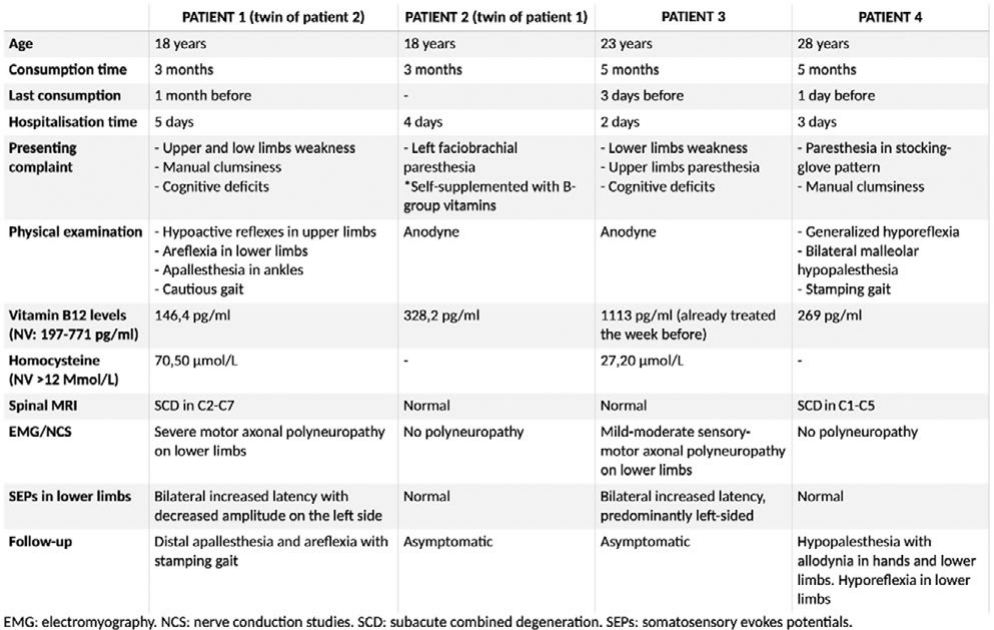
**TABLE 1** Main clinical characteristics of patients.

**Conclusion:** Inhalation of N2O may cause a spectrum of neurological injury ranging from axonal polyneuropathy to myelopathy. Normal B12 levels do not exclude neurotoxicity, limiting their usefulness as a sole diagnostic marker. Vitamin supplementation may delay symptom onset, but cessation of N2O use is essential to halt disease progression.

**Disclosure:** Nothing to declare.

## EPO‐0180

### Curcumin exhibits comparable effects to rapamycin in reducing neuronal soma size and intracellular calcium expression in SH‐SY5Y cells

#### M. Husna ^1^; R. Rakhmatiar^1^; A. Nur Rakhmani^2^; H. Anggu Maliga^1^

##### ^1^Department of Neurology, Faculty of Medicine, Brawijaya University/Saiful Anwar General Hospital, Malang, East Java, Indonesia; ^2^Department of Family Medicine, Faculty of Medicine, Brawijaya University, Malang, Indonesia

**Background and aims:** The mTORC2 complex is vital for neuronal function and structure. There are no specific inhibitors for mTORC2; however, chronic high‐dose rapamycin and curcumin may have indirect effects. This study compares their impact on mTORC2 inhibition in neuronal cells, focusing on calcium levels and soma size.

**Methods:** This in vitro study involved SH‐SY5Y neuroblastoma cell cultures divided into four groups: a control, one treated with 100 nM rapamycin, and two treated with curcumin at 5 μM and 10 μM for 48 hours to target mTORC2. Soma size was measured in μm^2^, and intracellular calcium levels were labeled with Fluo‐4 and quantified with confocal laser scanning microscopy (CLSM). Statistical analysis assessed data differences.

**Results:** Soma sizes were measured as: control (7892.07 μm^2^), 5 μM curcumin (6101.25 μm^2^), 10 μM curcumin (3413.44 μm^2^), and rapamycin (3341.95 μm^2^). Intracellular calcium intensities were: control (29.35 nPhoton), rapamycin (7.69 nPhoton), 10 μM curcumin (3.80 nPhoton), and 5 μM curcumin (2.19 nPhoton). Significant differences (*p* < 0.001) were found between all treatment groups and the control for both parameters. The 5 μM curcumin group differed significantly from rapamycin (*p* < 0.001), but there was no significant difference between the 10 μM curcumin and rapamycin groups (*p* = 0.84). The 5 μM curcumin had the lowest calcium intensity, indicating a non‐linear dose‐dependent effect

**Conclusion:** Rapamycin is comparable to 10 μM curcumin in inhibiting mTORC2, which reduces neuronal soma size and calcium levels. This suggests that curcumin may naturally modulate the mTOR pathway, highlighting its potential for treating neurological disorders related to mTOR.

**Disclosure:** Nothing to disclose.

## EPO‐0181

### Boldine and the nervous system: A case of reversible myoneuropathy

#### M. Verdeguer ^1^; M. García^1^; G. Esparza^1^; J. Cabello^2^; D. Górriz^1^; J. Jiménez^2^; E. Millet^3^; N. Muelas^2^; T. Sevilla^2^; R. Sivera^2^

##### ^1^Hospital Universitario y Politécnico la Fe, Neurology department, Valencia, Spain; ^2^Hospital Universitario y Politécnico la Fe, Neurology department. CIBER de enfermedades raras ‐ CIBERER, EURO‐NMD, Valencia, Spain; ^3^Hospital Universitario y Politécnico la Fe, Neurophysiology department, Valencia, Spain

**Background and aims:** Phytotherapeutic preparations are widely used and often perceived as safe; however, many contain bioactive compounds with potential neuromuscular toxicity that remains poorly characterized. Boldine is an aporphine alkaloid extracted from Peumus boldus and commonly used for its presumed hepatoprotective properties. We report a case of reversible myoneuropathy associated with rhabdomyolysis, likely related to boldine consumption.

**Methods:** A comprehensive clinical, laboratory, neurophysiological, and radiological evaluation was performed, including brain, spinal cord, and muscle magnetic resonance imaging (MRI), lumbar puncture, neurophysiological studies, serial measurements of creatine kinase (CK) and serum neurofilament light chain (sNfL).

**Results:** A 55‐year‐old man with alcoholic cirrhosis presented with a three‐week history of bilateral proximal lower limb weakness and ascending paresthesias. Neurological examination revealed weakness of hip and knee movements, thigh hypoesthesia, stocking‐type sensory loss in the feet, and absent lower limb reflexes. Symptom onset temporally coincided with the initiation of boldine for liver function improvement. Acute inflammatory polyradiculoneuropathy was initially suspected, and intravenous immunoglobulins were administered; however, this diagnosis was excluded due to normal electrophysiological findings and the absence of albuminocytological dissociation. Laboratory tests showed elevated CK levels, peaking at 4,176 U/L. Whole‐body MRI demonstrated focal muscle edema involving the right scalene, pectoral, vastus medialis, and left rectus femoris muscles, consistent with rhabdomyolysis. sNfL were elevated.

**Figure:**
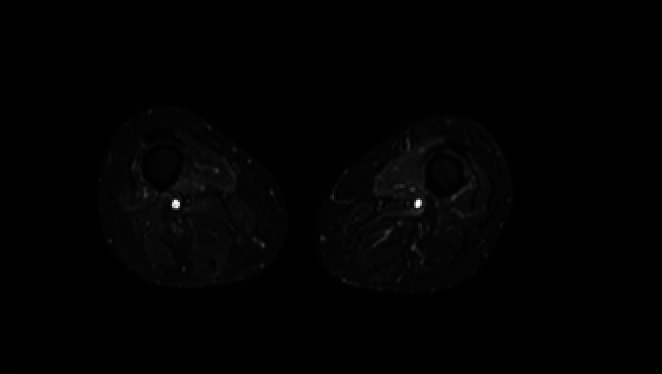
**FIGURE 1** Edema of the left quacriceps.

**Figure:**
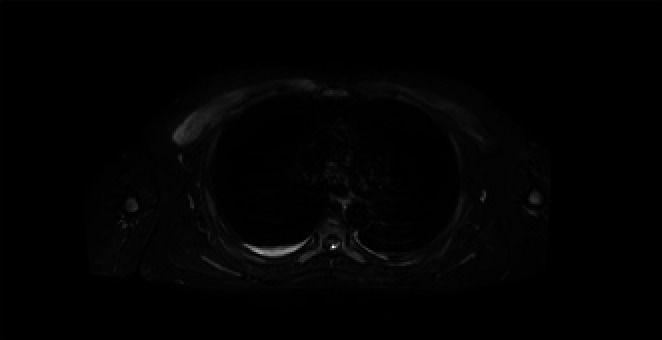
**FIGURE 2** Edema of the right pctoral muscle.

**Conclusion:** The clinical and laboratory course was favorable after discontinuation of boldine, with near‐complete recovery within one month, normalization of biomarkers, and electrophysiological improvement. The temporal relationship with boldine exposure, absence of alternative etiologies, and reversibility support a probable toxic myoneuropathic mechanism.

**Disclosure:** Nothing to disclose.

## EPO‐0182

### Wernicke encephalopathy: Learning from experience

#### N. Marginean; C. Herghelegiu; A. Niculae; A. Scurtu; L. Dumitrescu; E. Davidescu; B. Popescu

##### “Carol Davila” University of Medicine and Pharmacy, Faculty of Medicine, Clinical Neuroscience Department, Bucharest, Romania; Colentina Clinical Hospital, Neurology Department, Bucharest, Romania

**Background and aims:** Wernicke encephalopathy (WE) is a neurological emergency caused by thiamine deficiency, frequently associated with heavy alcohol drinking, malnutrition or consumptive diseases. Not all cases manifest with the complete classical triad (i.e, ophthalmoplegia, gait ataxia and encephalopathy), leading to underdiagnosis.

**Methods:** We conducted a descriptive study using the electronic medical record database of Colentina Clinical Hospital, Bucharest, Romania. We included data from patients admitted between January 2019 and December 2025 with a diagnosis of WE (ICD‐10 code E51.2). For statistical analysis we used Microsoft Excel and IBM SPSS.

**Results:** Datasets from 41 patients (90% male, 10% female) complied with the inclusion criteria. The median age was 66 years. Alcohol use disorder was the main identified risk factor for WE in 95% of cases. Gait ataxia was the most frequent sign upon evaluation. Only 21% of patients exhibited the full classical triad. All patients received high doses of intravenous thiamine for several days (mean total dose 5634 mg +/‐3607 mg). Brain MRI was performed in 12 patients (29%), of whom only 2 had typical signs of WE. Clinical improvement at discharge was observed in 82% of patients, only a third achieving previous neurological status. We found no significant correlation between the degree of clinical improvement and initial clinical presentation or dose of thiamine administered.

**Conclusion:** Our findings are in line with the published literature. We reinforce the importance of maintaining a high index of clinical suspicion and prompt initiation of parenteral thiamine treatment, particularly in patients with known risk factors for WE.

**Disclosure:** Nothing to disclose.

## EPO‐0183

### Chronic noise exposure induces cis‐P‐tau accumulation, an early Alzheimer's disease‐related pathological marker, and cognitive deficits in rats

#### S. Nabavi

##### Department of Regenerative Medicine, Cell Science Research Center, Royan Institute for Stem Cell Biology and Technology, ACECR, Tehran, Iran

**Background and aims:** Chronic noise exposure is a stressor linked to central nervous system dysfunction and cognitive decline, including Alzheimer's disease (AD). Hyperphosphorylation of tau protein, particularly Thr231, is a hallmark of AD. Cis p‐tau is highly neurotoxic and an early driver of tauopathy, causing microtubule disruption and neurodegeneration, a process termed Cistauosis.

**Methods:** Rats were exposed to 95 dB SPL white noise for 4 hours daily over 15 days. Cognitive and anxiety‐related behaviors were assessed using the elevated plus maze and Y‐maze. Western blotting and immunohistochemistry were performed to evaluate tau pathology.

**Results:** Noise‐exposed rats exhibited increased anxiety‐like behaviors and memory deficits. Molecular analyses demonstrated that chronic noise significantly induced cis p‐tau accumulation and Cistauosis in multiple brain regions.

**Conclusion:** Chronic noise exposure promotes Cistauosis, linking environmental stress to neurodegeneration and AD development. These findings highlight the potential role of noise‐induced tau pathology in the early stages of Alzheimer's disease.

**Disclosure:** The authors declare that they have no known competing financial interests or personal relationships that could have appeared to influence the work reported in this paper.

## EPO‐0184

### Neurological manifestations related to toxic exposure in children: A clinical study

#### S. Hachicha; M. Ben Hafsa; T. Ben Younes; Z. Miladi; A. Zyoudi; M. Jamoussi; H. Ben Rhouma; H. Klaa; I. Kraoua

##### Department of Pediatric Neurology, LR18SP04, National Institute Mongi Ben Hmida of Neurology, Tunis, Tunisia

**Background and aims:** Neurological manifestations due to toxic exposure in children represent a significant cause of pediatric morbidity. Their clinical heterogeneity often complicates diagnosis and delays appropriate management. This study aimed to describe the epidemiological characteristics, clinical presentation, management, and outcomes of toxic‐induced neurological manifestations in a pediatric population.

**Methods:** We conducted a retrospective study including patients aged under 18 years, hospitalized between 2017 and 2025 in the Pediatric Neurology Department of the National Institute of Neurology in Tunis, for neurological symptoms attributed to toxic exposure. Demographic, clinical, paraclinical, therapeutic, and outcome data were collected and analyzed.

**Results:** Eighteen patients were included, with a median age of 6 years. Toxic exposure was drug‐ related in 13 cases, including chemotherapy (5 cases), antiepileptic drugs (4 cases), haloperidol (1 case), and flunarizine (1 case). One case each was attributed to carbon monoxide and N‐hexane exposure. The circumstances were iatrogenic in nine cases, accidental in six cases, and intentional in five cases. Five patients presented with an encephalitic‐like syndrome. Peripheral neuropathy, movement disorders, and ataxia were each observed in four patients. The outcome was favorable in 13 cases, while two patients required admission to the intensive care unit.

**Conclusion:** In our series, neurological manifestations related to toxic exposure in children were predominantly iatrogenic. Early recognition and prompt discontinuation of the offending agent are crucial for a favorable prognosis. These conditions should be systematically considered in the differential diagnosis of acute or subacute neurological presentations in children.

**Disclosure:** The authors declare no conflicts of interest.

## Cerebrovascular Diseases 2

## EPO‐0185

### Asymptomatic hemorrhagic transformation after mechanical thrombectomy is not benign: Early and long‐term outcomes

#### A. Dębiec ^1^; K. Boniecka^1^; J. Winnicka^1^; P. Piasecki^2^; A. Stępień^1^

##### ^1^Clinic of Neurology, Military Institute of Medicine‐ National Research Institute, Warsaw, Poland; ^2^Department of Radiology, Military Institute of Medicine‐ National Research Institute, Warsaw, Poland

**Background and aims:** The prognostic relevance of asymptomatic hemorrhagic transformation (aHT) after mechanical thrombectomy (MT) for acute ischemic stroke (AIS) remains uncertain and is often considered clinically benign. We evaluated the impact of aHT on early and long‐term outcomes after MT.

**Methods:** We retrospectively analyzed consecutive patients with AIS treated with MT at a comprehensive stroke center. A follow‐up CT scan was performed after 24 hours and hemmorhagic transformation was assesed using Heidelberg Bleeding Classification. Patients were followed up for 12 months. Associations between aHT, functional outcomes, and mortality were analyzed using multivariable regression models.

**Results:** Among 363 patients, 237 (65.3%) had no HT, 84 (23.1%) developed aHT, and 35 (9.6%) developed symptomatic HT. Patients with aHT had worse functional outcomes than those without HT. Functional independence (mRS 0–2) at 90 days was achieved in 45.1% of patients without HT(23.8% with aHT). After adjustment, aHT was independently associated with reduced odds of functional independence at 90 days (OR 0.29, 95% CI 0.15–0.57), a shift toward worse functional outcome at 12 months (common OR 2.11, 95% CI 1.30–3.41), and increased mortality during follow‐up (HR 1.64, 95% CI 1.07–2.52). Patients developing aHT more frequently presented with higher baseline NIHSS, while no single baseline clinical or treatment‐related variable emerged as a strong independent predictor of aHT.

**Conclusion:** aHT after MT is associated with worse early and long‐term functional outcomes and increased mortality. Further validation of our findings in large cohort studies of MT‐treated patients is warranted.

**Disclosure:** Nothing to disclose.

## EPO‐0186

### Epidemiological trends in intracerebral haemorrhage: A decadal analysis of aetiology, prior blood pressure control, and direct oral anticoagulant use

#### A. Leung; Z. Ren; K. Teo

##### Division of Neurology, Department of Medicine, Queen Mary Hospital, LKS Faculty of Medicine, The University of Hong Kong, Hong Kong

**Background and aims:** The incidence of intracerebral haemorrhage (ICH) in Hong Kong remains high compared to developed countries globally. As the most severe form of stroke, where treatment offers only modest benefit, ICH prevention is crucial to reducing disease burden. We aim to study changes in ICH epidemiology, particularly focusing on pre‐ICH blood pressure (BP) control, direct oral anticoagulants (DOAC) use, and the cause of ICH. Our findings will help inform public health strategies to prevent ICH.

**Methods:** We conducted a single‐centre observational study of spontaneous ICH patients admitted from 2014 to 2022. Demographic and clinical data were collected, including pre‐ICH BP control, anticoagulant use and ICH aetiology using the modified Boston criteria. Temporal shifts in the aetiology of ICH and BP control were analysed.

**Results:** A total of 1,044 ICH patients were categorised into three temporal groups: 2014–16, 2017–19, and 2020–22. There was a numerical increase in the proportion of ICH attributed to cerebral amyloid angiopathy (CAA) from 19.8% (75/379; 2014–16) to 25.8% (86/333; 2020–22) (*p* = 0.110). The rate of undiagnosed/uncontrolled hypertension decreased from 67.3% (255/379; 2014–16) to 62.2% (207/333; 2020–22) (*p* = 0.023) but remained high. The prevalence of anticoagulant‐associated ICH roughly doubled from 2014–16 to 2020–22 (7.1% vs 13.0%, *p* = 0.028), driven by DOAC‐ICH. Among DOAC‐ICH, 54.3% had uncontrolled/ undiagnosed hypertension and 30.6% had CAA.

**Figure:**
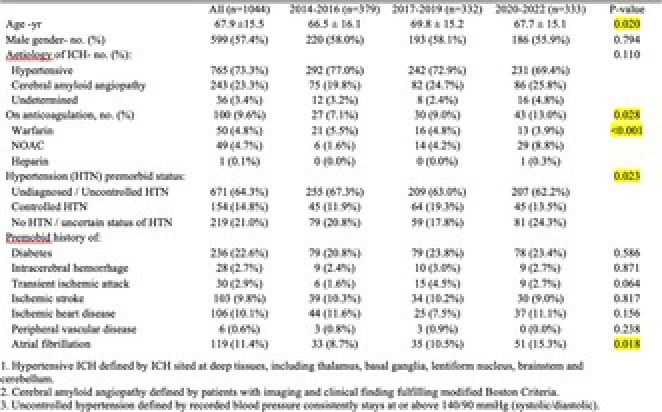
**TABLE 1** Baseline demographic and clinical characteristics of ICH patients over 3 temporal groups.

**Conclusion:** Pre‐ICH BP control had slightly improved over the decade, but the rate of undiagnosed/uncontrolled hypertension remains unacceptably high. Strengthening hypertension control in both the general population and, as importantly, DOAC users, is essential to reduce ICH burden.

**Disclosure:** Nothing to disclose.

## EPO‐0187

### Comparative cardiovascular and bleeding outcomes with PPIs versus H2‐receptor blockers: A nationwide cohort study in Korea

#### B. KIM ^1^; S. Cho^2^

##### ^1^Nowon Eulji Medical Center, Seoul, Republic of Korea; ^2^Uijeongbu Eulji Medical Center, Uijeongbu, Republic of Korea

**Background and aims:** Among stroke survivors on antithrombotic therapy, gastroprotection is common, yet the cardiovascular safety of proton pump inhibitors (PPIs) relative to H2‐receptor blockers (H2Bs) remains debated.

**Methods:** We performed a retrospective cohort study using linked Korean National Health Insurance claims and national stroke registry data. Adults with imaging‐confirmed index ischemic stroke (2016–2020) who received antithrombotics and initiated either a PPI or an H2B within 30 days after the index event were included. The primary outcome was major adverse cardiovascular events (MACE)—composite of myocardial infarction, recurrent stroke, or all‐cause death. Secondary outcomes were gastrointestinal (GI) bleeding, intracranial hemorrhage, and other bleeding. Propensity‐score matching balanced baseline covariates, and Cox models estimated hazard ratios (HRs).

**Results:** From 850,053 screened records, 4,569 eligible patients were identified and grouped as PPI (*n* = 2,515) and H2B (*n* = 2,054). After propensity‐score matching, there was no significant difference in MACE between PPI and H2B users across crude, PS‐matched, and fully adjusted analyses. In contrast, GI bleeding was significantly lower with PPIs than with H2Bs, while intracranial hemorrhage and other bleeding showed no excess risk with PPIs. Findings were consistent across sensitivity analyses.

**Conclusion:** In ischemic stroke patients treated with antithrombotics, PPIs did not increase MACE risk compared with H2Bs and were associated with lower GI‐bleeding risk. These results support the cardiovascular safety of PPIs and suggest they may be the preferred gastroprotective option in this setting, particularly for patients at heightened risk of GI bleeding.

**Disclosure:** Nothing to disclose.

## EPO‐0188

### Impact of tenecteplase implementation in a remote region without on‐site thrombectomy

#### C. Vera‐Cáceres; M. Regalado; D. Chi; A. López Traba; D. Vargas; Y. Aguilera; R. Mora; C. Marrero; D. Ramos; M. González Platas

##### Neurology Department, General Hospital of Fuerteventura, Canary Islands, Spain

**Background and aims:** Intravenous thrombolysis (IVT) remains the main treatment for acute ischemic stroke (AIS) in hospitals without endovascular treatment (EVT). Tenecteplase may simplify workflow and reduce treatment delays compared with alteplase. We aimed to evaluate the impact of switching from alteplase to tenecteplase on workflow metrics, safety, and early clinical outcomes in an insular hospital.

**Methods:** An observational study including consecutive patients with AIS treated with IVT at a hospital without on‐site EVT. Two periods were compared: 2024 (alteplase) and 2025 (tenecteplase). The primary outcome was DTN time. Secondary outcomes included the proportion treated < 60 minutes, intracranial hemorrhage, in‐hospital mortality, and functional outcomes at discharge.

**Results:** 14 patients in the alteplase group and 7 in the tenecteplase group. Mean age was 69 and 62 years, respectively (*p* 0.18), and mean baseline NIHSS was 12.5 vs 9 (*p* 0.17). Median DTN was significantly shorter with tenecteplase (45 vs 71 minutes), representing a reduction of 26 minutes (*p* < 0.01). The proportion treated within 60 minutes increased from 35.7% to 71.4% (*p* 0.04). Intracranial hemorrhage rates were similar (21.4% vs 14.3%; *p* 1). One in‐hospital death occurred in the alteplase group. NIHSS at discharge did not differ between groups (mean 5.4 vs 4.1; *p* 0.29). Functional independence at discharge (mRS 0–2) was observed in 61.5% of the alteplase group and 57.1% of the tenecteplase group.

**Conclusion:** In remote regions without onsite EVT, implementation of tenecteplase for IVT was associated with a significant improvement in workflow efficiency, particularly door‐to‐needle time, without compromising safety.

**Disclosure:** Nothing to disclose.

## EPO‐0189

### External validation of ASPECTS–NIHSS clinical–imaging equations for predicting 90‐day outcome after endovascular thrombectomy in acute ischemic stroke

#### H. Savsin ^1^; T. Homa^3^; A. Slowik^2^

##### ^1^Student Scientific Group of Cerebrovascular Diseases, Jagiellonian University Medical College. Krakow, Poland; ^2^Department of Neurology, Jagiellonian University Medical College, Krakow, Poland; ^3^Department of Neurology, University Hospital, Krakow, Poland

**Background and aims:** Despite the widespread adoption of endovascular thrombectomy (EVT) in acute ischemic stroke (AIS) patients with large vessel occlusions, rapid bedside tools for prognostication are limited. Novel simple clinical‐imaging equations based on Alberta Stroke Program Early CT Score (ASPECTS) and National Institutes of Health Stroke Scale (NIHSS) were recently developed (Nie et al, 2025) to predict post‐EVT outcomes. We aimed to externally validate the equations and the published logistic model for predicting 90‐day functional independence in an independent cohort.

**Methods:** Consecutive AIS patients undergoing anterior circulation EVT within 24 hours at the University Hospital Krakow (January‐December 2023) with available NIHSS, ASPECTS and 90‐day modified Rankin Scale (mRS) were included. Three predictive approaches were validated: (1) clinical–imaging equation (CIE = ASPECTS −0.5 × NIHSS), (2) age‐adjusted‐CIE (AdjCIE = ASPECTS−0.5 × NIHSS−0.2 × Age), and (3) published logistic model probability (p_FI). Primary outcome was functional independence (mRS 0–2). Discrimination was assessed using area under the ROC curve (AUC), calibration using intercept and slope and, overall accuracy using Brier score.

**Results:** 149 of 361 patients were included (mean age 72, 60.4% female, median NIHSS 15 [9]) and 67.8% achieved functional independence. Discriminative ability was good for p_FI (AUC 0.761; 95% CI 0.676–0.846) and AdjCIE (AUC 0.763; 95% CI 0.679–0.847), and moderate for CIE (AUC 0.705; 95% CI 0.616–0.793). Calibration of p_FI showed systematic underprediction (intercept 1.805, slope 1.404) and Brier score was 0.281.

**Figure:**
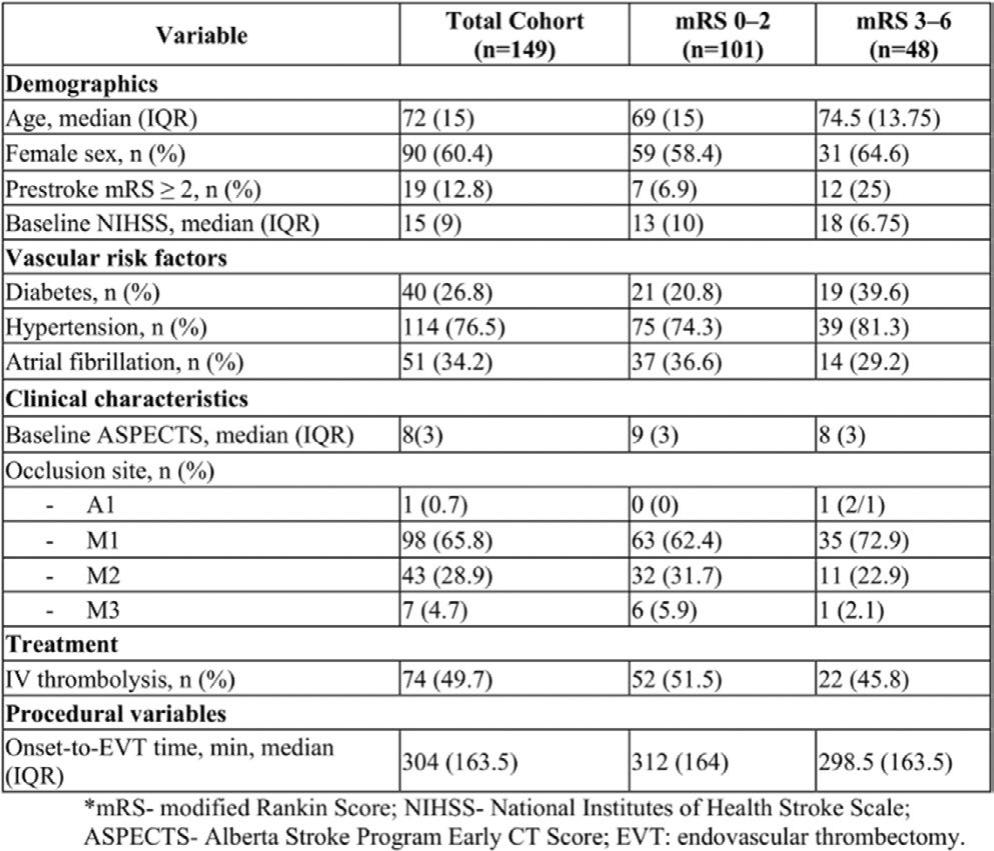
**TABLE 1** Baseline characteristics of the study cohort stratified by modified Rankin Score.

**Figure:**
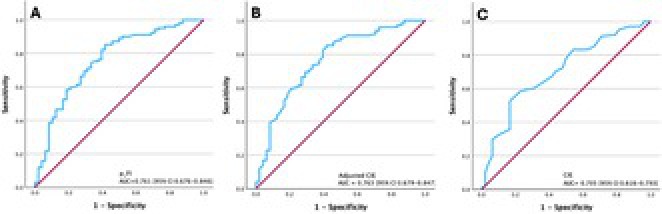
**FIGURE 1** ROC curves of (A) the published logistic regression model probability of functional independence (p_FI), (B) age‐adjusted clinical–imaging equation (AdjCIE) and (C) clinical–imaging equation (CIE) for 90‐day functional independence.

**Figure:**
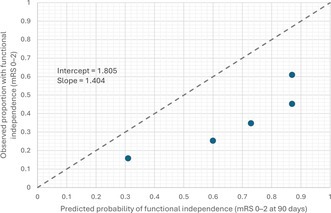
**FIGURE 2** Calibration plot for the published logistic model predicting functional independence after 90 days. Predicted probabilities grouped into quintiles of predicted risk in the validation cohort (*n* = 149). The dashed line is perfect calibration.

**Conclusion:** Proposed clinical‐imaging equations show good discrimination for 90‐day functional independence in our cohort but systematic underprediction in calibration, indicating the possible requirement for regional recalibration before clinical application.

**Disclosure:** Nothing to disclose.

## EPO‐0190

### Cancer‐related stroke in esus patients: A Tunisian cross‐sectional study

#### L. Bouafia ^1^; H. Slimene; K. Jemai; A. Mili; E. Jarrar; A. Rekik; A. Hssine; S. Naija; S. Benamor

##### Neurology department, Sahloul University Hospital, Sousse, Tunisia

**Background and aims:** Malignancy is identified in ~10% of patients with embolic stroke of undetermined source (ESUS). Ischemic stroke (IS) may precede or follow cancer diagnosis, yet no consensus exists on systematic malignancy screening in stroke patients. This study aimed to evaluate the clinical, biological, and radiological features of cancer‐related stroke (CRS) in a Tunisian cohort.

**Methods:** We conducted a 4‐year cross‐sectional study including patients with acute IS confirmed by CT or MRI and admitted to the Neurovascular Unit of Sahloul University Hospital, Tunisia. CRS was defined as ESUS with a negative etiological workup (brain MRI, CT angiography, 48‐hour Holter monitoring, transthoracic and transesophageal echocardiography) in patients with active cancer diagnosed before or during stroke evaluation.

**Results:** Twenty‐four patients were included (median age 66.2 ± 9.1 years; male‐to‐female ratio 3.8). Cancer was revealed during stroke workup in 16 patients (66.7%). Common malignancies were lung (41.7%), pancreatic (20.8%), colon (12.5%), and prostate (8.3%). Median NIHSS was 6. Five patients had prior venous thrombosis or pulmonary embolism, suggestive of Trousseau syndrome. Imaging showed multiple infarcts in ≥ 2 vascular territories in 50% and the Three Territory Sign in 37.5%. Median D‐dimer was 4.0 μg/mL [IQR 1.93–13.1], and mean fibrinogen was 3.18 ± 1.89 g/L. Eight patients (33.3%) died shortly after stroke; eleven received curative anticoagulation.

**Conclusion:** Elevated D‐dimer levels and embolic infarct patterns, particularly the three territory Sign, are key markers of CRS in ESUS patients. D‐dimer alone lacks specificity, but combined markers improve risk stratification and enable earlier detection in high‐risk patients.

**Disclosure:** Nothing to disclose.

## EPO‐0191

### Serum brain‐derived tau in moyamoya angiopathy: A case–control study of associations with cognitive impairment

#### J. Norsted Sømark ^1^; M. Skjelland^1^; B. Halvorsen^2^; T. Ueland^2^; V. Bjerkeli^2^; P. Aukrust^3^; T. Nordenmark^4^; M. Wiedmann^5^; A. Aamodt^1^

##### ^1^Dpt. of Neurology, Oslo University Hospital, Oslo, Norway; ^2^Research Institute for Internal Medicine, Oslo University Hospital, Rikshospitalet,, Faculty of Medicine, Institute of Clinical Medicine, University of Oslo, Oslo, Norway; ^3^Institute of Clinical Medicine, University of Oslo, Oslo, Norway, Section of Clinical Immunology and Infectious Diseases,, Oslo University Hospital Rikshospitalet, Oslo, Norway; ^4^Department of Psychology, Oslo University Hospital, Ullevål, Oslo, Norway; ^5^Department of Neurosurgery, Oslo University Hospital, Rikshospitalet, Oslo, Norway

**Background and aims:** Brain‐derived tau (BD‐tau) is a sensitive blood‐based biomarker of neuronal injury in acute ischemic stroke; but its relevance in conditions characterized by chronic cerebral hypoperfusion remains unclear. Moyamoya angiopathy (MMA) is a progressive steno‐occlusive disorder causing regional cerebral hypoperfusion, impaired vasomotor reserve, and heterogenous clinical phenotypes, including cognitive impairment, complicating risk stratification. Biomarkers reflecting hypoperfusion‐related neuronal injury may complement imaging and clinical assessment in neurosurgical decision‐ making. We investigated serum BD‐tau levels in MMA patients and healthy controls and explored relations with cognitive performance.

**Methods:** This cross‐sectional, register‐based case‐control study included MMA patients without recent stroke (*n* = 37) and healthy controls (*n* = 14). Serum total BD‐tau levels were compared using Mann‐Whitney U test. MMA patients underwent comprehensive neuropsychological assessment across nine cognitive domains. A Global Cognitive Impairment Index (GCII) was calculated as the proportion of domains with at least one test score ≥ 1 standard deviation below normative means. Associations between BD‐tau and GCII were assessed using Spearman´s rank correlation.

**Results:** Median serum BD‐tau levels were 12.38 [9.40–14.96] pg/mL in the MMA group and 9.97 [9.27–13.77] pg/mL in the control group, without significant between‐group difference (*p* = 0.263). Within the MMA group, BD‐tau levels showed no significant correlation with GCII (*p* = 0.061).

**Figure:**
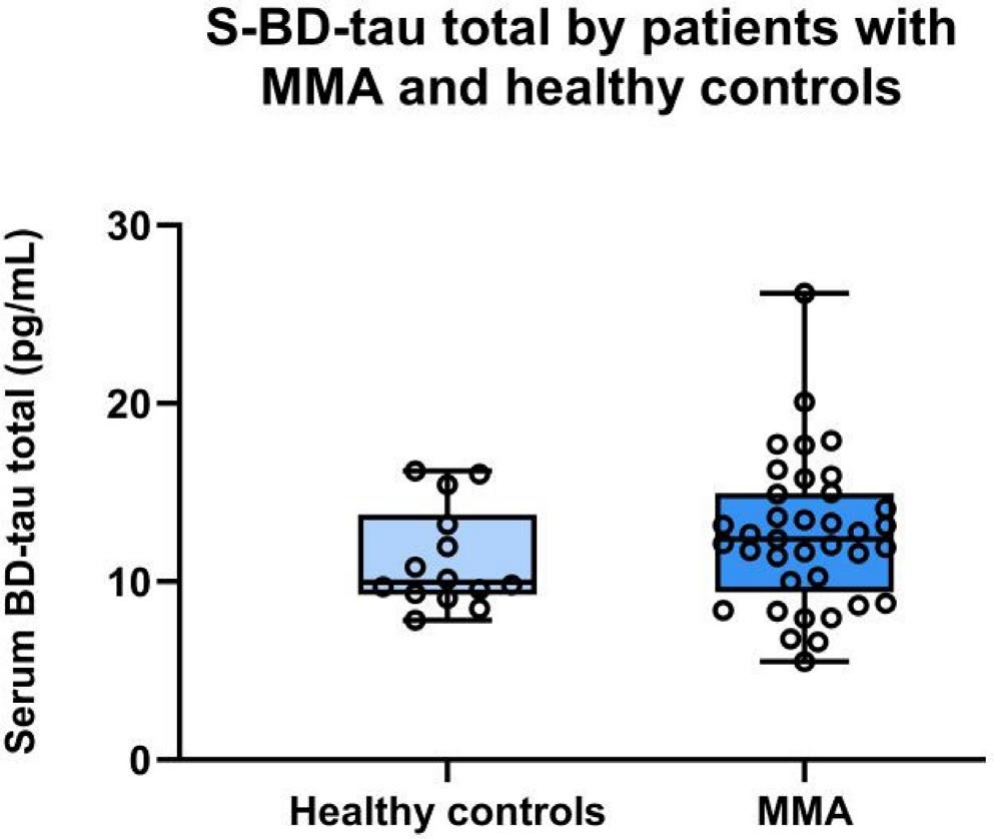
**FIGURE 1** Box‐plot comparing BD‐tau total levels between MMA‐patients (*n* = 37) and healthy controls (*n* = 14). Difference was non‐significant (*p* = 0.263, Mann‐whitney U test). S‐BD‐tau: serum brain‐derived tau total. MMA: moyamoya angiopathy.

**Figure:**
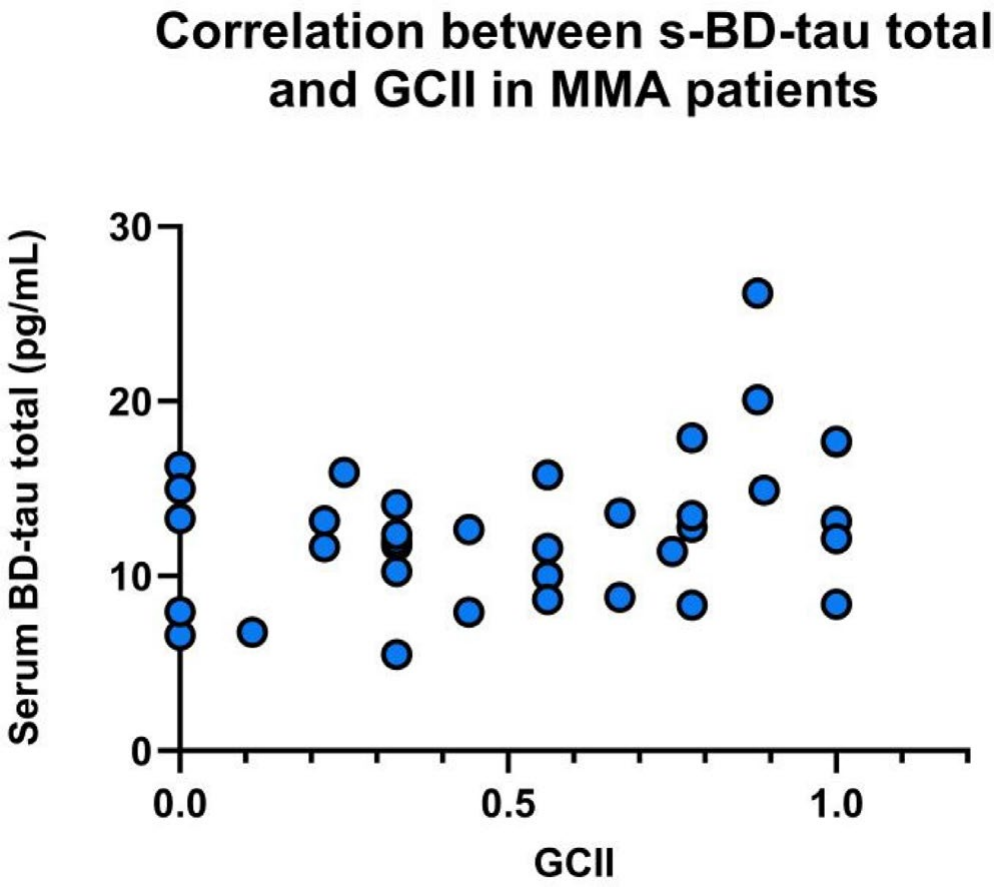
**FIGURE 2** Scatterplot demonstrating no statistical correlation between GCII and BD‐tau levels in MMA patients (*n* = 37, Spearman ρ = 0.311 *p* = 0.061). S‐BD‐tau: serum brain‐derived tau. MMA: moyamoya angiopathy. GCII: Global Cognitive Impairment Index.

**Conclusion:** Serum BD‐tau levels were not elevated in MMA patients and were not associated with global impairment. Despite small sample size, these findings suggest limited general utility of BD‐tau in MMA, though its potential in selected subgroups warrants further investigation in larger cohorts.

**Disclosure:** Anne Hege Aamodt has received personal fees for lectures/advisory boards; Novartis, Abbvis, TEVA, Roche, Lundbeck, Pfizer, Boehringer Ingelheim, non‐personal research grants from Norwegian National Association for Public Health, South‐Eastern Norway regional Health Authority, Odd Fellow, National Program for Clinical Treatment Research in the Specialist Health Service (KLINBEFORSK), EU, Boehringer Ingelheim, Medtronic, BMS. Bente Halvorsen is an SAB member in CircM, Linkøping, Sweden and is an evaluator in MH panel, Swedish Research council. The remaining authors have nothing to disclose.

## EPO‐0192

### Determinants of functional outcome in patients with intracranial atherosclerotic stenosis: Analysis of the bernese intracranial stenosis study

#### K. Antonenko ^1^; P. Radojewski^2^; A. Hoffmann^3^; S. Fenzel^3^; J. Kaesmacher^3^; M. Arnold^1^; R. Wiest^2^; P. Mordasini^4^; M. Heldner^1^

##### ^1^Department of Neurology, Inselspital, Bern University hospital and University of Bern, Bern, Switzerland; ^2^Institute of Diagnostic and Interventional Neuroradiology, Inselspital, University Hospital and University of Bern, Bern, Switzerland; Translational Imaging Center, sitem‐insel, Bern, Switzerland; ^3^Institute of Diagnostic and Interventional Neuroradiology, Inselspital, University Hospital and University of Bern, Bern, Switzerland; ^4^Institute of Diagnostic and Interventional Neuroradiology, Inselspital, University Hospital and University of Bern, Bern, Switzerland; Department of Diagnostic and Interventional Neuroradiology, Cantonal Hospital Aarau, Aarau, Switzerland

**Background and aims:** Functional outcome after intracranial atherosclerotic stenosis (ICAS) is highly variable, yet the prognostic determinants of outcome in these patients are still not well defined. Ultra–high‐field 7‐Tesla MRI enables detailed evaluation of ICAS.

**Methods:** We analyzed 131 ICAS patients (of which *n* = 58 symptomatic) under best medical management from the prospective Bernese Intracranial Stenosis Study, dichotomized by 1‐year functional outcome (favorable: mRS 0–1, *n* = 58; unfavorable: mRS 2–6, *n* = 73). Group comparisons were performed for demographics, vascular risk factors, laboratory values, and 3/7T MR imaging markers at baseline and follow‐up.

**Results:** At baseline, the unfavorable vs. favorable outcome group had a significantly higher prevalence of symptomatic ICAS (52.1% vs. 34.5%, *p* = 0.044), diabetes mellitus (39.7% vs. 20.7%, *p* < 0.02), physical inactivity (30.1% vs. 5.2%, *p* < 0.001), peripheral artery disease (15.1% vs. 3.4%, *p* = 0.027), higher median baseline NIHSS (1 vs. 0, *p* = 0.012), median baseline BDI (7 vs. 3, *p* < 0.001), alongside lower median baseline MoCA (26 vs. 27, *p* = 0.001) and median baseline EuroQoL VAS (70 vs. 83, *p* < 0.001). At 1‐year follow‐up, the unfavorable outcome group had significantly worse median NIHSS (2 vs. 1, *p* < 0.001), BDI‐II (6 vs. 3, *p* = 0.005), MoCA (26 vs. 28, *p* < 0.001), and EuroQoL VAS (75 vs. 87, *p* < 0.001) in univariable analysis. In multivariable analysis, symptomatic ICAS (OR 2.74, 95% CI 1.11–6.80, *p* = 0.029) remained to be the only independent predictor of unfavorable outcome.

**Conclusion:** In summary, symptomatic ICAS was independently associated with 2.74‐fold increased odds of an unfavorable functional outcome. This highlights the importance of early identification and close monitoring of symptomatic ICAS patients to optimize functional recovery.

**Disclosure:** Nothing to disclose.

## EPO‐0193

### Tenecteplase in the extended treatment window for acute ischemic stroke: Evidence from randomized trials

#### K. Moghib ^1^; M. Meshref^2^

##### ^1^Kasralainy medical school, Cairo University, Cairo, Egypt; ^2^Department of Neurology, Faculty of Medicine, Al‐Azhar University, Cairo, Egypt

**Background and aims:** The role of intravenous tenecteplase (TNK) for acute ischemic stroke treated between 4.5 and 24 hours remains debated, particularly across healthcare settings with variable access to endovascular thrombectomy (EVT).

**Methods:** Randomized controlled trials comparing TNK (0.25 mg/kg) with placebo or standard care in adults treated 4.5–24 hours after stroke onset were systematically reviewed. The primary outcome was excellent functional recovery (mRS 0–1) at 90 days. Secondary outcomes included recanalization, neurological improvement, and safety endpoints. Random‐effects meta‐analyses were performed overall and stratified by EVT availability. PROSPERO (CRD420251274200)

**Results:** Four multicenter RCTs (*n* = 1,278) were included. TNK significantly increased the likelihood of excellent functional outcome and vessel recanalization. Overall differences in good functional outcome and early neurological improvement were not significant. In settings without EVT access, TNK improved multiple functional and early recovery outcomes, whereas benefits in EVT‐capable settings were limited to recanalization. Rates of symptomatic intracerebral hemorrhage and 90‐day mortality were similar between groups.

**Conclusion:** TNK improves excellent functional recovery and recanalization in the 4.5–24‐hour window, particularly where EVT is unavailable, supporting its role as a pragmatic reperfusion option in resource‐limited contexts.

**Disclosure:** Nothing to disclose.

## EPO‐0194

### Greater occipital nerve block as a treatment for non‐traumatic subarachnoid haemorrhage related headache: Systematic review of the available evidence

#### M. Delgado‐Romeu ^1^; Á. Lambea‐Gil^1^; M. Guasch‐Jimenez^1^; P. Carbonell‐Fernández^1^; Ó. López‐Lombardía^1^; G. Ezcurra‐Díaz^1^; L. Prats‐Sánchez^1^; A. Martínez‐Domeño^1^; J. Fernández‐Vidal^1^; N. Morollón Sánchez‐Mateos^2^; N. Pérez de la Ossa^1^; P. Camps‐Renom^1^; A. Ramos‐Pachón^1^

##### ^1^Stroke Unit, Hospital de la Santa Creu i Sant Pau, Barcelona, Spain; ^2^Headache and Neuralgia Unit, Hospital de la Santa Creu i Sant Pau, Barcelona, Spain

**Background and aims:** Non‐traumatic subarachnoid haemorrhage (SAH) is characterized by severe headache refractory to conventional analgesia, leading to frequent use of opioids. Greater occipital nerve blocks (GONB) are commonly used in migraine and other headache disorders with great success. The goal of this study was to review available evidence on the use of GONB in the treatment of SAH‐related headache.

**Methods:** Systematic review of available evidence following the PRISMA‐2020 guidelines from inception to 01/2026 with a narrative synthesis of the results. We included studies with adult patients with non‐traumatic SAH‐related headache, which used GONB (with/without other nerve blocks) as adjunctive analgesia and monitored headache intensity with validated scores (VAS, NPRS [both 0–10]). The search was performed in electronic databases (PubMed, Embase, Cochrane CENTRAL, ClinicalTrials.gov) using controlled terms related to SAH, headache and GONB. Bias risk was assessed using validated tools (JBI/ROBINS‐I).

**Results:** 48 studies were identified, of which three met inclusion criteria (Figure 1). Two were case series (*n* = 2, *n* = 10) and one was a non‐randomized observational study with historical controls (*n* = 10). All studies reported a decrease in headache intensity scores after the procedure (ΔNPRS 2.67–6, timeframe 1 h–1 week), and a reduced need for rescue analgesia. No significant adverse events were reported. All studies had a moderate‐to‐high bias risk due to lack of proper controls and small sample sizes (Table 1).

**Figure:**
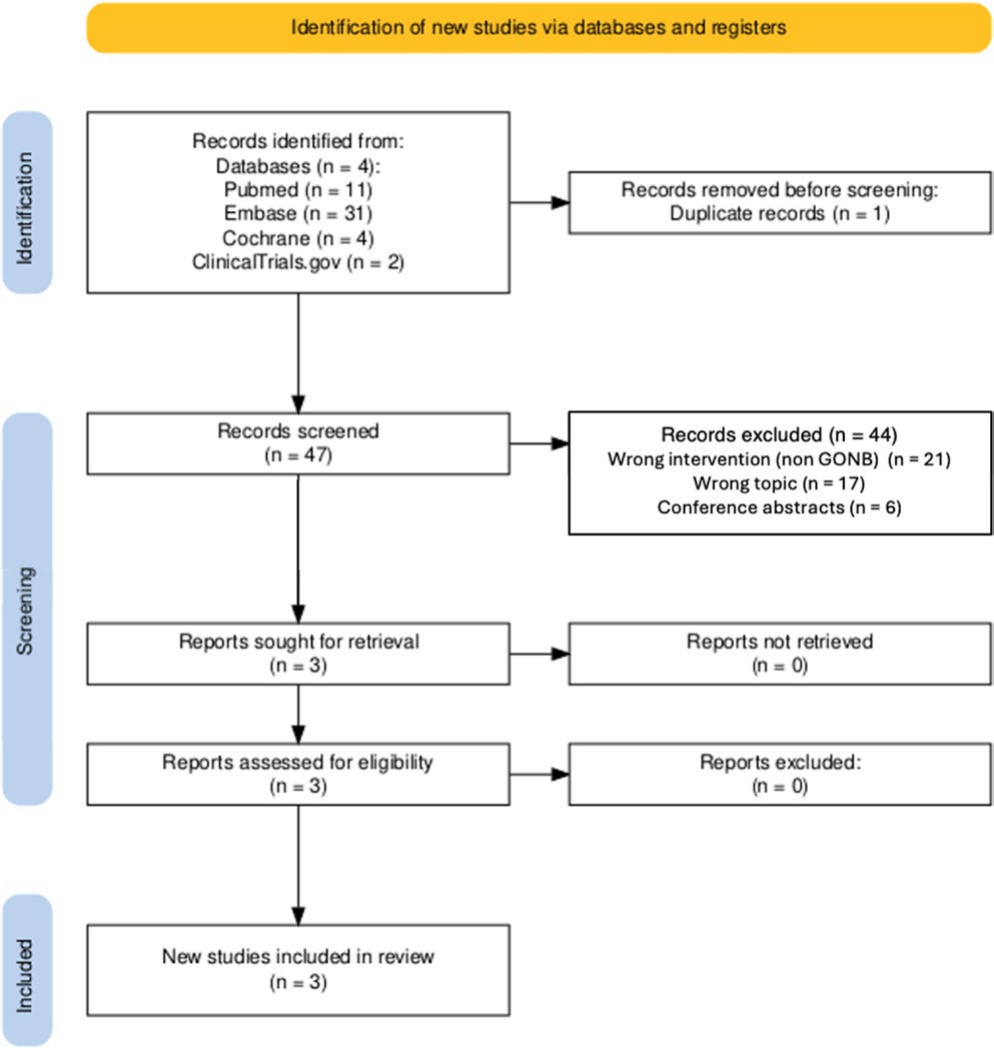
**FIGURE 1** PRISMA‐2020 flow diagram.

**Figure:**
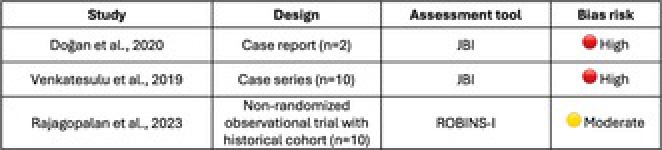
**TABLE 1** Risk of bias assessment Abbreviations: JBI = Joanna Briggs Institute; ROBINS‐I = Risk of Bias in Non‐randomized Studies – of Interventions.

**Conclusion:** Current evidence on GONB for treating non‐traumatic SAH‐related headache is limited due to small‐sample non‐controlled studies, but consistently suggests potential analgesic benefits. Randomized controlled data are needed to further examine its efficacy.

**Disclosure:** The authors declare no conflicts of interest.

## EPO‐0195

### Predicting neurological functional improvement using machine learning in acute minor stroke: A Vietnamese cohort study

#### M. Tran Nguyen Tuan ^1^; T. Duy Mai^2^; D. Tien Nguyen^2^; P. Viet Dao^2^; M. Cong Tran^3^; H. Xuan Nguyen^1^

##### ^1^London Digital Twin Research Center, Middlesex University, London, UK; ^2^Stroke Center, Bach Mai Hospital, Hanoi, Vietnam; ^3^Department of Clinical Neuroscience, University of Oxford, Oxford, UK

**Background and aims:** Although acute minor stroke is often considered benign, many patients develop persistent functional limitations. Early prediction of neurological functional improvement, assessed by the 90‐day modified Rankin Scale (mRS), may support treatment decisions and rehabilitation planning. This study aimed to develop machine learning models to predict 90‐day mRS improvement in patients with acute minor stroke using newly collected data from Vietnam.

**Methods:** We analyzed data from 736 patients with acute minor stroke admitted to Bach Mai Hospital, Vietnam, between 2023 and 2024. The dataset was split into a training set (70%, *n* = 515) and a test set (30%, *n* = 221). Predictor selection was performed in the training set using LASSO regression with 5‐fold cross‐validation. Four binary classifiers ‐ Extra Trees, Random Forest, Extreme Gradient Boosting, and Support Vector Machine with a Radial Basis Function kernel ‐ were trained using the selected predictors. Hyperparameters were optimized using GridSearch cross‐validation, and performance was evaluated in the test set.

**Figure:**
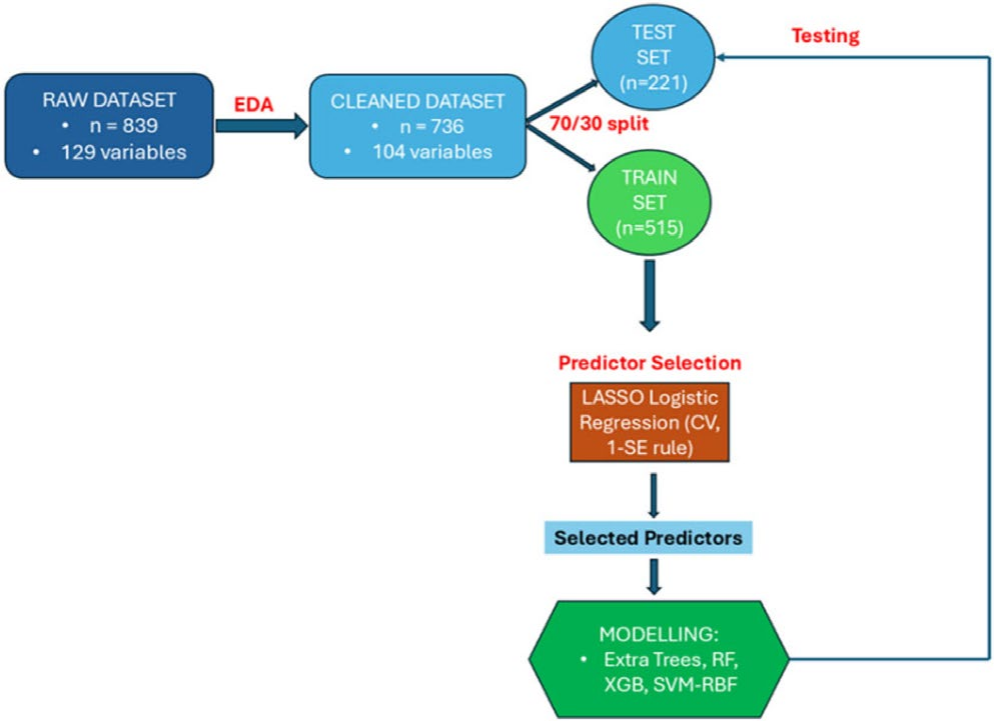
**FIGURE 1** The overall workflow diagram illustrating the main sequential steps

**Results:** Fourteen predictors were identified. All models achieved an area under the receiver operating characteristic curve above 0.75. The Extra Trees model showed the best performance, with an Area Under the Receiver Operating Characteristic Curve (AUC) of 0.780 (95% CI, 0.704–0.854), Precision‐Recall AUC of 0.890, accuracy of 0.810, and F1‐score of 0.878.

**Figure:**
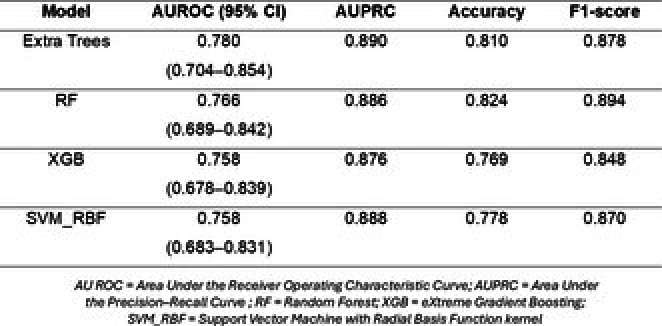
**FIGURE 2** Performance of the four models

**Figure:**
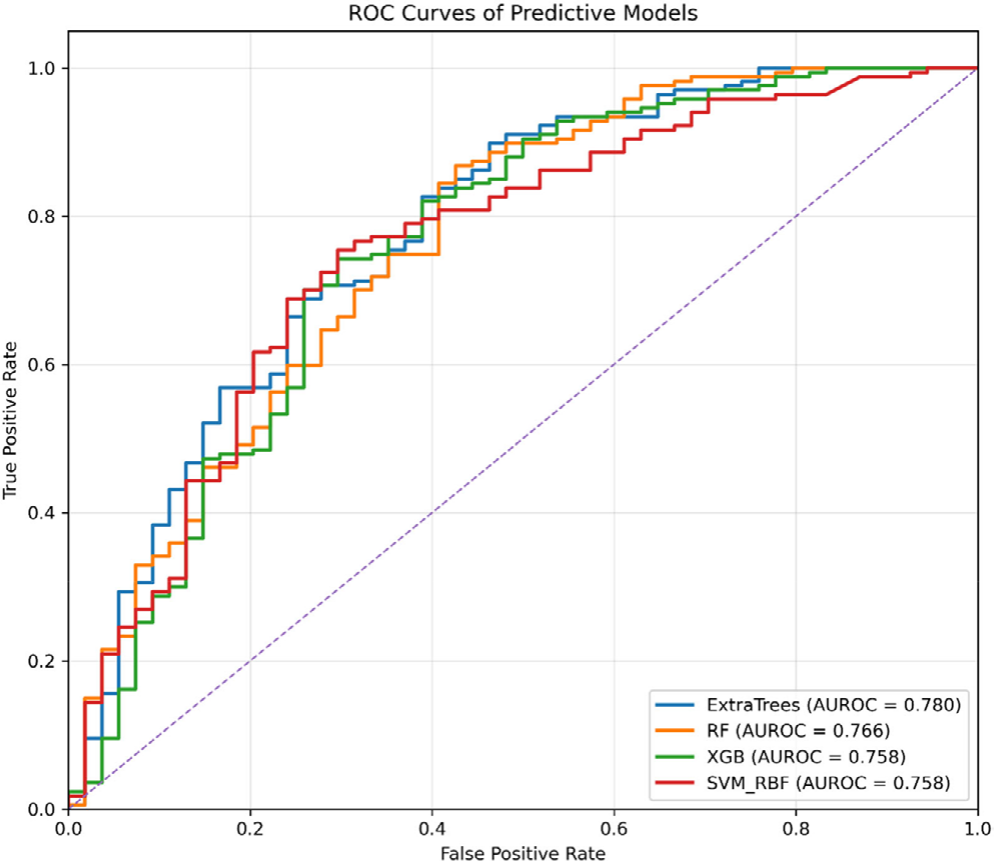
**FIGURE 3** ROC curves of the four models.

**Conclusion:** Machine learning models may help predict 90‐day functional improvement in patients with acute minor stroke using real‐world data from Vietnam. Further validation in larger and more diverse cohorts is required.

**Disclosure:** Nothing to disclose.

## EPO‐0196

### ATAD3A stabilizes Drp1 via lysosome‐dependent regulation to sustain pathological mitochondrial fission after cerebral ischemia/reperfusion

#### T. Xiong^1^; X. Wu^1^; Q. Wu^1^; H. Liang^1^; X. Luo^1^; M. Yang^2^; J. Wang^1^; N. Qin^1^; L. Tan^2^; L. Fu^1^; X. Qin^1^; R. Zhang ^1^

##### ^1^Department of Neurology, Chongqing Key Laboratory of Neurology, The First Affiliated Hospital of Chongqing Medical University, Chongqing, China; ^2^Department of Endocrinology, the Second Affiliated Hospital, Chongqing Medical University, Chongqing, China

**Background and aims:** Excessive mitochondrial fission is a key driver of neuronal death after cerebral ischemia/reperfusion (I/R), yet the mechanisms sustaining this process beyond the acute phase remain unclear. Dynamin‐related protein 1 (Drp1) is central to mitochondrial fission, but how its pathological activation is maintained following I/R is poorly understood. This study investigated whether ATPase family AAA domain‐containing protein 3A (ATAD3A) regulates mitochondrial fission and neuronal death through control of dynamin‐related protein 1 stability.

**Methods:** Mouse middle cerebral artery occlusion/reperfusion (MCAO/R) and neuronal oxygen–glucose deprivation/reoxygenation (OGD/R) models were established. ATPase family AAA domain‐containing protein 3A was suppressed genetically in vivo and in vitro. Protein interaction, degradation pathways, mitochondrial dynamics, and neuronal apoptosis were analyzed using biochemical, imaging, and functional approaches.

**Results:** Cerebral I/R induced sustained upregulation of ATAD3A in neurons. ATAD3A interacted with Drp1 and inhibited its lysosome‐dependent degradation, thereby preserving Drp1 protein stability. Persistent stabilization of Drp1 promoted excessive mitochondrial fission and disruption of mitochondrial dynamics, leading to increased neuronal apoptosis. Suppression of ATAD3A accelerated Drp1 degradation, attenuated pathological mitochondrial fission, and reduced neuronal apoptosis after ischemia/reperfusion.

**Figure:**
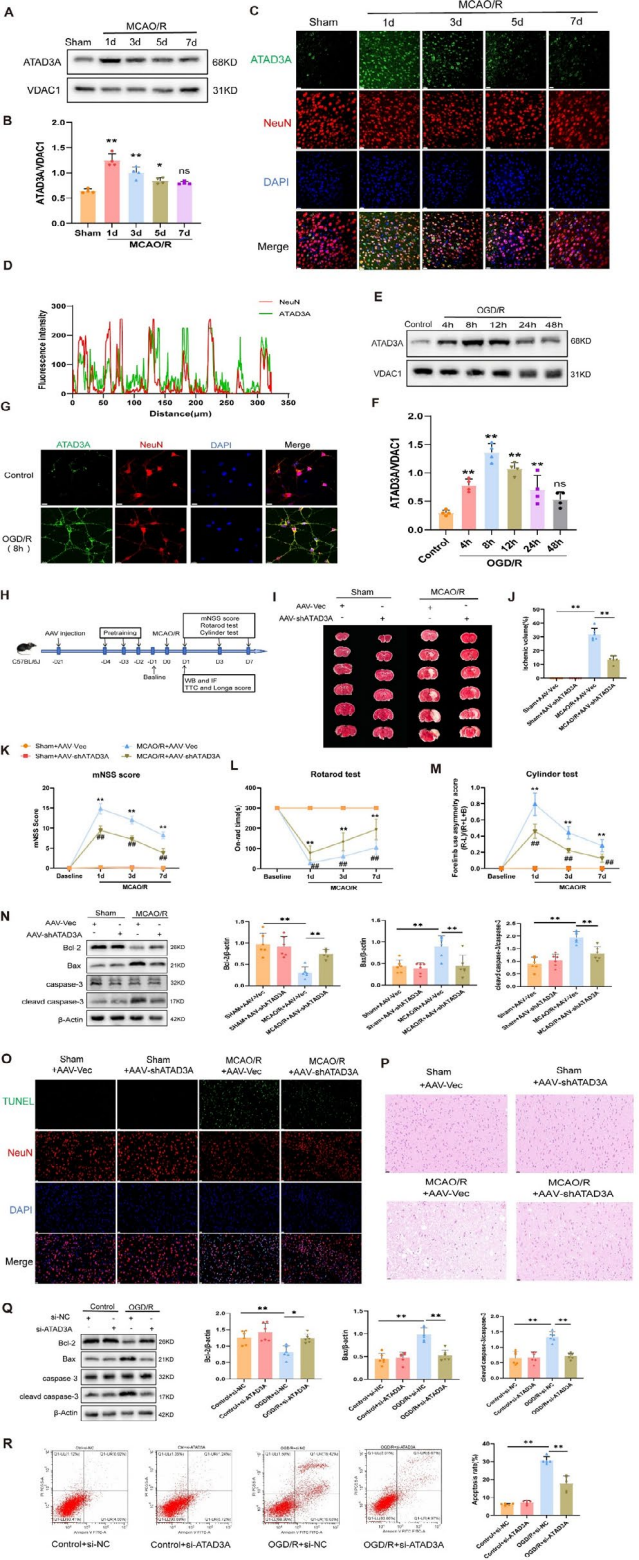
**FIGURE 1** ATAD3A is upregulated in neurons after and knockdown of ATAD3A improves neurological function and ameliorates neuronal apoptosis following cerebral ischemia/reperfusion injury.

**Figure:**
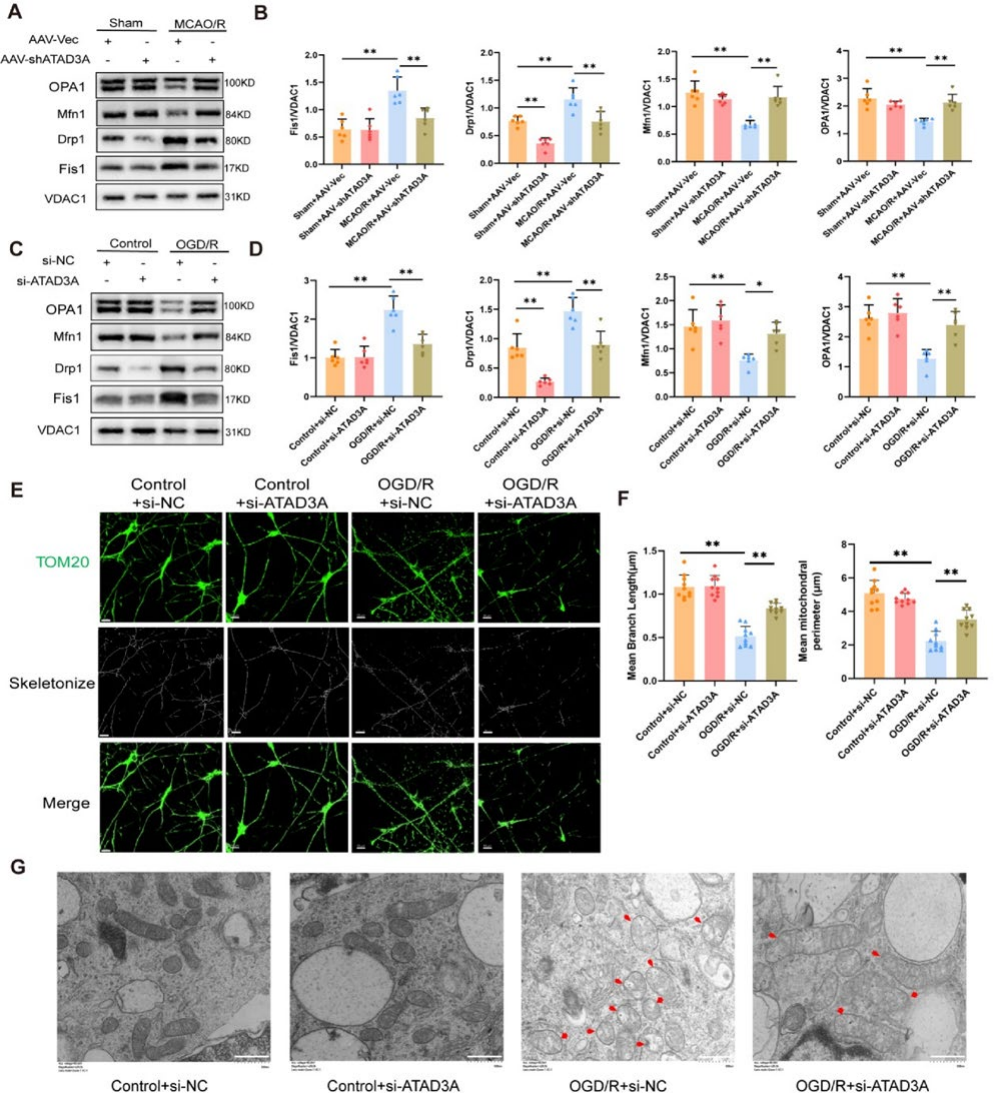
**FIGURE 2** ATAD3A promotes mitochondrial fission and suppresses mitochondrial fusion.

**Figure:**
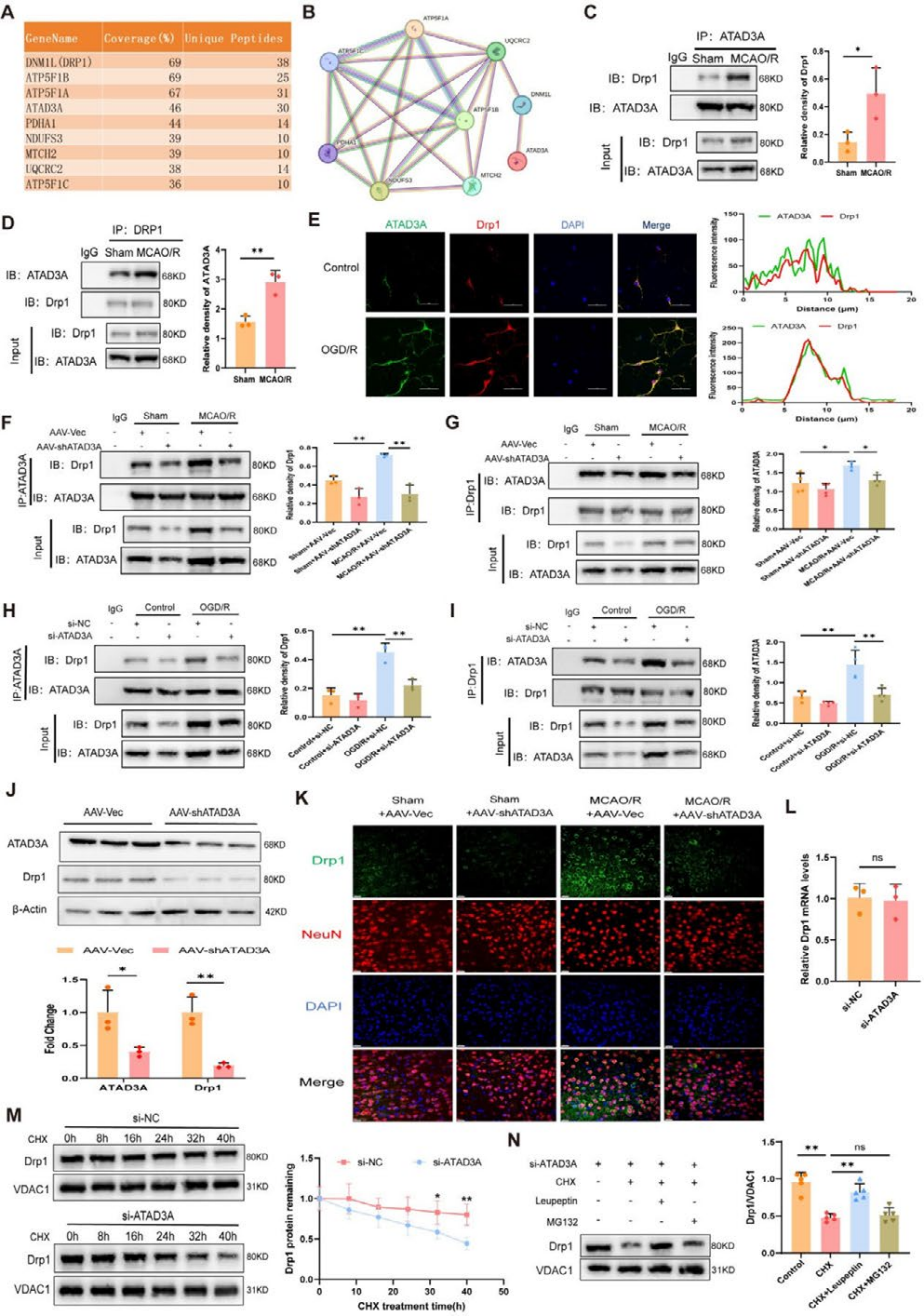
**FIGURE 3** ATAD3A interacts with and stabilizes Drp1 by inhibition of lysosome‐mediated degradation.

**Conclusion:** ATAD3A sustains pathological mitochondrial fission after cerebral I/R by preventing lysosome‐dependent degradation of Drp1. This proteostatic mechanism represents a key driver of persistent mitochondrial dynamics disruption and neuronal death following ischemic injury.

**Disclosure:** Nothing to disclose.

## Cerebrovascular Diseases 3

## EPO‐0197

### Recovery of post‐stroke dysphagia: A 16‐month follow‐up study in 455 post stroke patients using the Functional Oral Intake Scale (FOIS)

#### A. Dine; O. Çibuku; E. Ranxha

##### Neuro vascular Service, University Hospital “Mother Teresa” Tirane, Albania

**Background and aims:** Tracking swallowing function with validated tools is essential for understanding recovery patterns and guiding care. To evaluate the progression and predictors of dysphagia recovery in stroke patients over 16 months using the Functional Oral Intake Scale (FOIS).

**Methods:** This prospective cohort study included 455 patients with ischemic or hemorrhagic stroke and clinically confirmed dysphagia, admitted between June 2023 and January 2025. Swallowing ability was assessed at baseline, 1, 3, 6, 12, and 16 months post‐stroke using the FOIS score. Recovery was defined as reaching FOIS level ≥ 6. Multivariate logistic regression identified factors associated with persistent dysphagia (FOIS ≤ 5) at 16 months.

**Results:** At baseline, all patients scored between FOIS 1 and 3. At 3 months, 54.1% had reached FOIS ≥ 4. By 6 months, 68.3% had achieved FOIS ≥ 6, and by 16 months, 82.2% of patients reached FOIS levels 6–7, indicating functional oral intake. Persistent dysphagia (FOIS ≤ 5) at 16 months was independently associated with brainstem stroke (OR 3.5, *p* < 0.001), higher NIHSS scores on admission (*p* = 0.003), and age > 75 (*p* = 0.01). A total of 28 patients (6.2%) remained dependent on partial or full enteral feeding at the end of the follow‐up.

**Figure:**
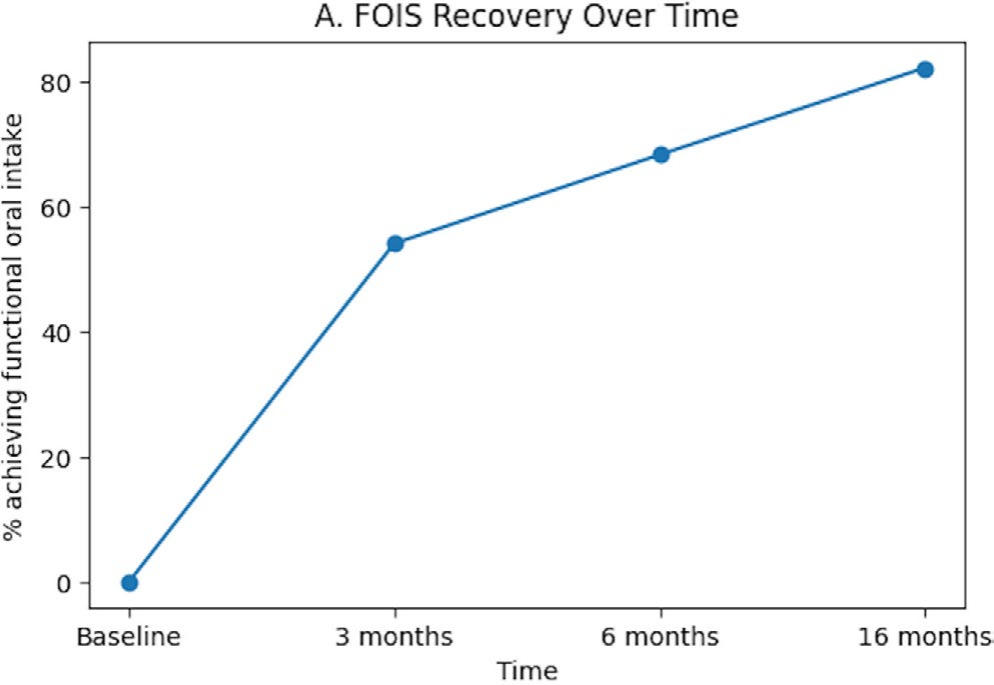
**FIGURE 1** FOIS in months.

**Figure:**
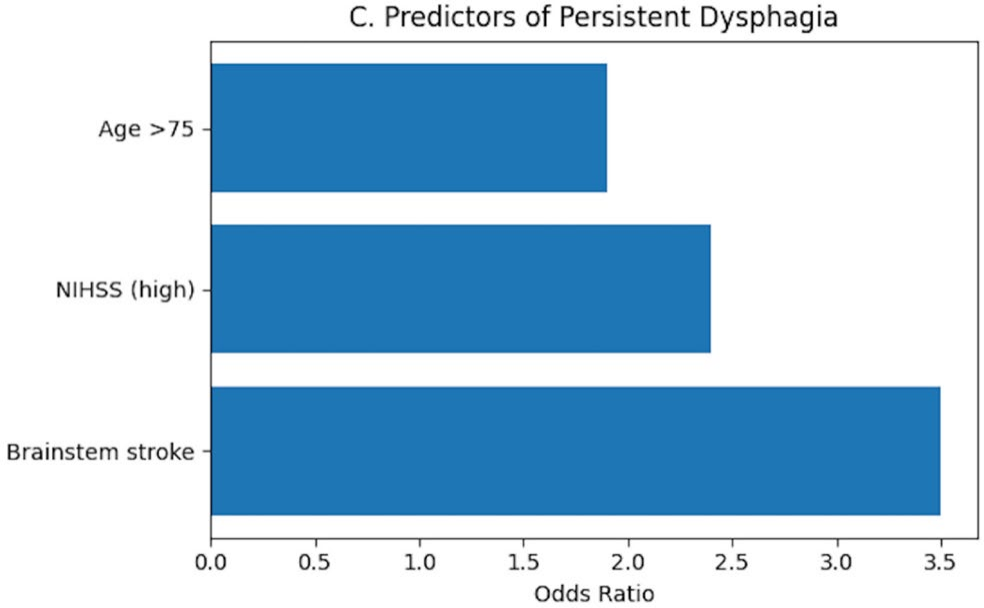
**FIGURE 2** Predictors of persistent dysfagia.

**Figure:**
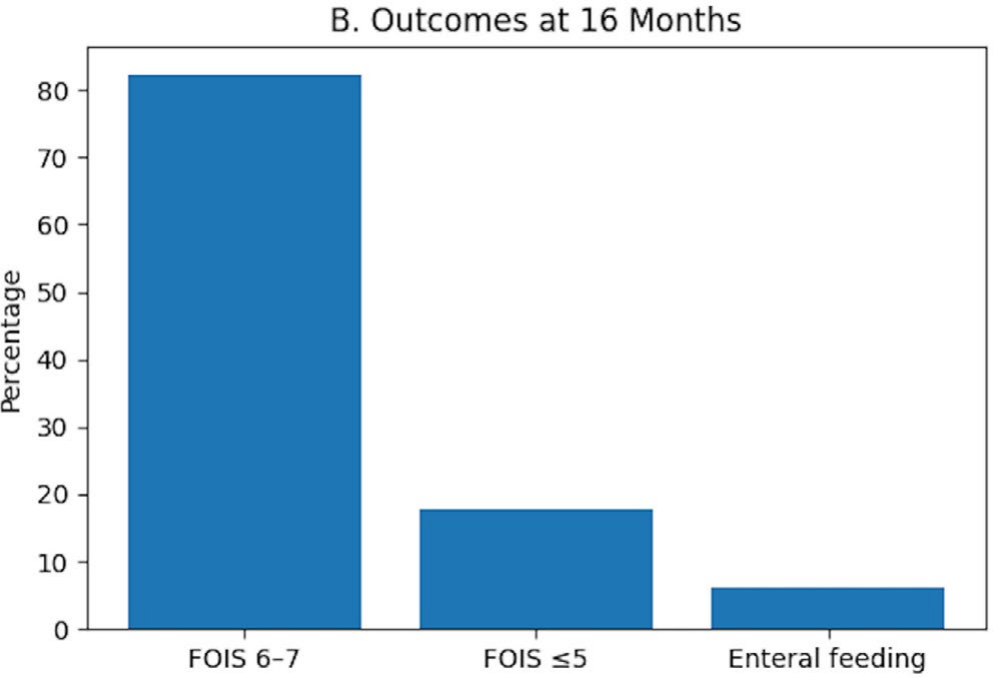
**FIGURE 3** Outcomes over time.

**Conclusion:** The majority of patients with post‐stroke dysphagia demonstrate significant improvement on the FOIS within the first 6 months, with gradual gains continuing through 16 months. Severe stroke, brainstem involvement, and advanced age are predictors of poor recovery.

**Disclosure:** Nothing to disclose.

## EPO‐0198

### Explainable achine learning–based prediction of poor functional outcome after ischemic stroke using routine clinical data

#### B. Özdemir

##### Ankara Univercity Medical Faculty, Neurology Department, Ankara, Türkiye

**Background and aims:** Early prediction of poor functional outcome after ischemic stroke remains challenging. Conventional prognostic models show limited performance in heterogeneous real‐world populations. We aimed to evaluate whether a machine learning model using routinely available clinical variables improves prediction of poor functional outcome compared with logistic regression.

**Methods:** We retrospectively analyzed 642 ischemic stroke patients with available baseline clinical, laboratory, electrocardiographic, and imaging data. Poor functional outcome was defined as modified Rankin Scale score greater than 3 at follow‐up. Data were split into training and test sets. A Random Forest classifier was developed and compared with logistic regression. Model performance was evaluated using receiver operating characteristic area under the curve. Model interpretability was assessed using SHapley Additive exPlanations.

**Results:** The Random Forest model showed good discriminative performance with a receiver operating characteristic area under the curve of 0.87 on the test set, outperforming logistic regression. Sensitivity and specificity varied according to probability thresholds. SHapley Additive exPlanations identified stroke severity, mortality, functional status, and selected laboratory markers as key contributors to model predictions.

**Figure:**
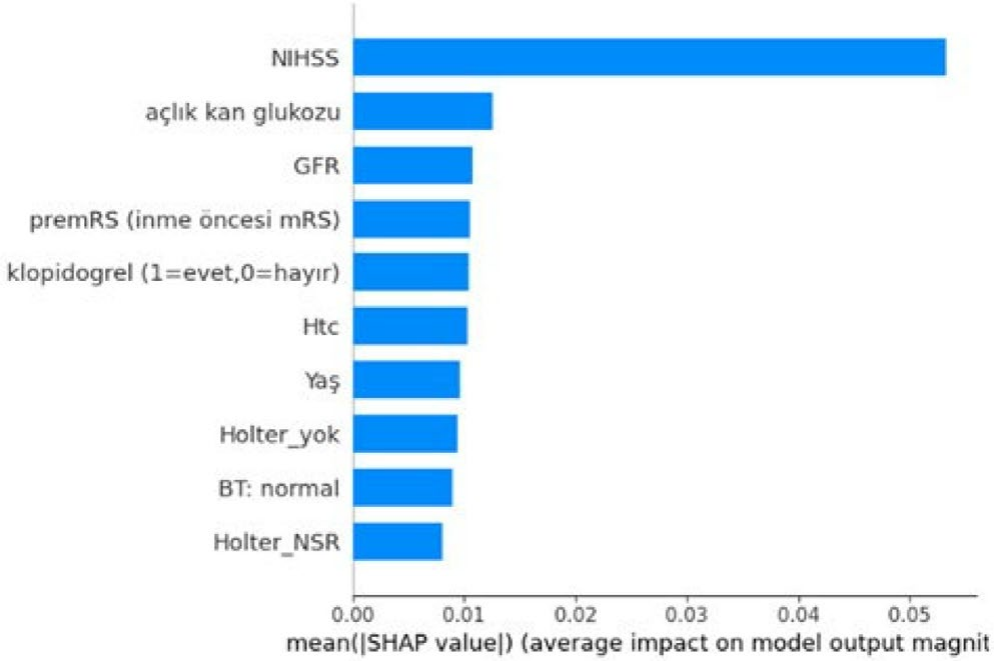
**FIGURE 1** Mean absolute SHAP values showing the relative contribution of each variable to poor outcome prediction. NIHSS had the strongest impact, followed by glucose, renal function, premorbid disability, and age.

**Figure:**
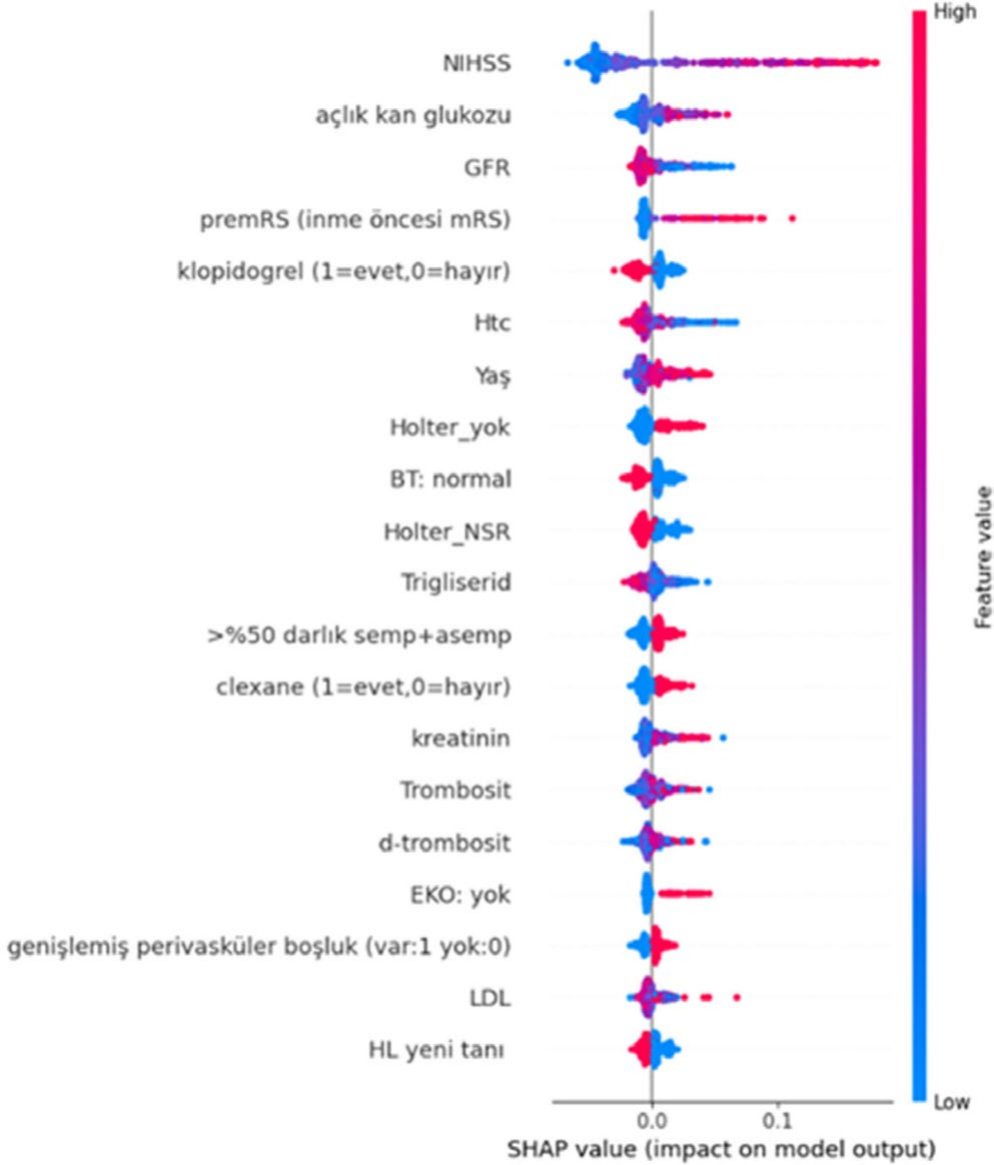
**FIGURE 2** SHAP summary plot illustrating direction and magnitude of feature effects. Higher NIHSS and glucose values increased poor outcome risk, while better renal function shifted predictions toward favorable outcome.

**Figure:**
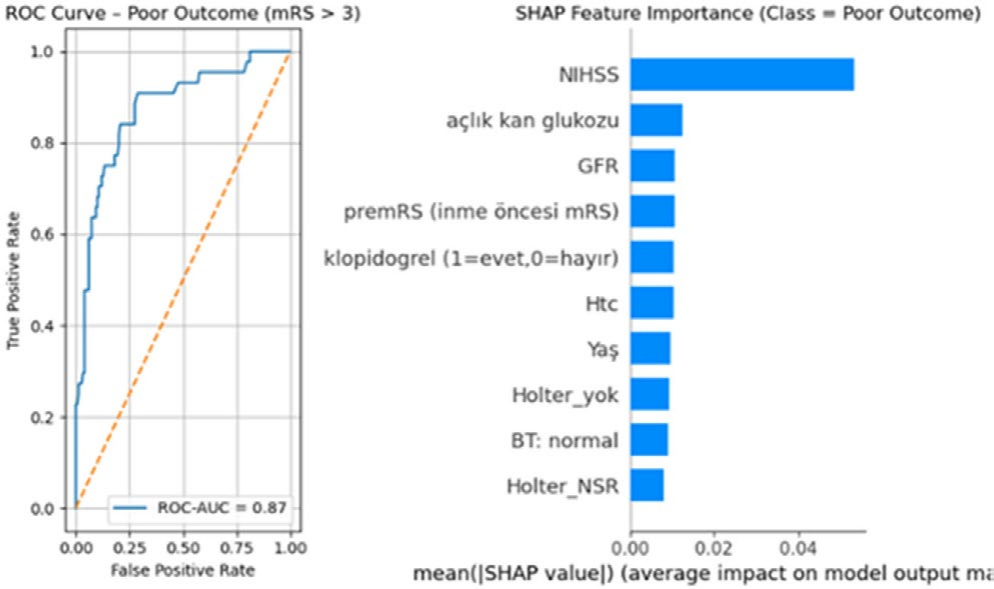
**FIGURE 3** Receiver operating characteristic curve demonstrating strong discrimination for poor outcome (area under the curve 0.87). SHAP importance confirms NIHSS as the dominant predictor.

**Conclusion:** Explainable machine learning using routine clinical data improved prediction of poor functional outcome after ischemic stroke. Explainable models may support individualized risk stratification in clinical practice.

**Disclosure:** Nothing to disclose.

## EPO‐0199

### The impact of advanced practice nurse‐led follow‐up model on medication adherence for secondary prevention in patients with acute ischaemic stroke

#### D. Hou

##### Department of Nursing, Beijing Tsinghua Changgung Hospital, School of Clinical Medicine, Tsinghua Medicine, Tsinghua University

**Background and aims:** This study explores the impact of a follow‐up model led by advanced practice nurses on medication adherence for secondary prevention in patients with acute ischaemic stroke, aiming to provide a reference for long‐term medication management in this patient group.

**Methods:** A follow‐up programme led by advanced practice nurses was developed, encompassing both in‐hospital and post‐discharge management. In‐hospital management involved advanced practice nurses providing structured health education and guidance during the patient's hospital stay. Upon discharge, patients were given personalised medication management plans. Post‐discharge management included follow‐ups conducted by advanced practice nurses at 1 month, 3 months, 6 months, and 1 year after discharge via telephone, WeChat, and online platforms. During these follow‐ups, individualised education and guidance were provided, and assistance with outpatient appointment bookings was offered when necessary.

**Results:** A total of 190 patients were included. After one year of intervention, the adherence rates to antithrombotic medication at 3 months, 6 months, and 1 year post‐discharge were 94.7% (180/190), 94.2% (179/190), and 95.3% (181/190), respectively; adherence rates to statins were 95.3% (181/190), 92.1% (175/190), and 89.5% (170/190), respectively; adherence rates to hypoglycaemic drugs were 100% (52/52), 96.2% (50/52), and 92.3% (48/52), respectively; adherence rates to antihypertensive drugs were 96.5% (110/114), 99.1% (113/114), and 96.5% (110/114), respectively.

**Conclusion:** The advanced practice nurse‐led follow‐up model has a positive impact on medication adherence for secondary prevention in patients with acute ischaemic stroke and merits further promotion and application.

**Disclosure:** Nothing to disclose.

## EPO‐0200

### Prevalence and predictors of impaired venous outflow in supratentorial intracerebral hemorrhage

#### F. Mazzacane ^1^; A. Dell'Orco^2^; B. Del Bello^3^; G. Busto^4^; E. Fainardi^5^; M. Wattjes^2^; A. Morotti^6^; J. Nawabi^2^

##### ^1^Department of Emergency Neurology and Stroke Unit, IRCCS Mondino Foundation, Pavia, Italy; ^2^Department of Neuroradiology, Charité ‐ Universitätsmedizin Berlin, Humboldt‐Universität zu Berlin, Freie Universität Berlin, Berlin Institute of Health, Germany; ^3^Department of Brain and Behavioral Sciences, University of Pavia, Pavia, Italy; ^4^Neuroradiology, Department of Radiology, AOU Careggi, Italy; ^5^Department of Biomedical Experimental and Clinical, Neuroradiology, University of Firenze, AOU Careggi, Firenze, Italy; ^6^Department of Clinical and Experimental Sciences, Neurology Unit, University of Brescia, Italy

**Background and aims:** Venous outflow (VO) is essential for maintaining cerebral hemodynamics. Impaired venous outflow (VO) has been associated with poor outcomes and hemorrhagic transformation in ischemic stroke; however, its prevalence and determinants in acute intracerebral hemorrhage (ICH) remain understudied.

**Methods:** We retrospectively included patients admitted to Charité University Hospital, Berlin (January 2019‐June 2025) with supratentorial ICH and available baseline CT angiography. VO assessment was performed according to the validated Comprehensive Venous Outflow (CVO) score of the ipsilateral hemisphere. The association between clinical and radiological variables and the CVO score was assessed using a multivariable Bayesian beta‐binomial model to account for overdispersion in the bounded count outcome. The variables included in the model were age, systolic blood pressure, ICH volume, intraventricular hemorrhage, and edema volume.

**Results:** A total of 533 patients were included in this analysis. Baseline characteristics revealed a mean age of 73 years (SD 13.8) and mean ICH volume of 41.41 ml (SD 42.9). A total of 300 (56.3%) patients had deep ICH. The median CVO was 8 (IQR 5–8), with a score < 6 in 149 (27.9%) patients, indicating significant impairment. ICH volume (aOR for 1 point reduction for 1 SD increase, 1.84, 95%CrI 1.34–2.49), IVH volume (aOR 1.41, 1.27–1.57), and edema volume (aOR 1.29, 0.95–1.77) along with deep location (aOR 1.54, 1.16–2.06) were associated with a decrease in the CVO score.

**Conclusion:** VO impairment is common in patients with ICH and is associated with ICH features such as ICH volume, edema, and location. Further research is warranted to evaluate its clinical significance.

**Disclosure:** F. Mazzacane was supported by EAN Research Fellowship grant. A. Dell’Orco: Nothing to disclose. B. Del Bello: Nothing to disclose. G. Busto: Nothing to disclose. E. Fainardi: Nothing to disclose. M. Wattjes: Nothing to disclose. A. Morotti: Nothing to disclose. J. Nawabi: Nothing to disclose.

## EPO‐0201

### Timing of initiating anticoagulants administration in stroke patient who developed atrial fibrillation: A systematic review and meta‐analysis

#### M. A. AlGhamdi^1^; R. A. Almalki^3^; M. M. Bukari^1^; M. S. Baselm^1^; H. A. AlShareef ^1^; R. A.Alharbi^4^; S. Makkawi^1^

##### ^1^College of Medicine, King Saud bin Abdulaziz University for Health Sciences, Jeddah, Saudi Arabia; ^3^College of Pharmacy, Umm Al‐Qura University, Makkah, Saudi Arabia; ^4^Faculty of Medicine, Jeddah University, Jeddah, Saudi Arabia

**Background and aims:** Atrial fibrillation (AF) is a major cause of ischemic stroke and is associated with higher rates of recurrence and worse outcomes. Oral anticoagulation is essential for secondary prevention; however, the optimal timing of initiation following acute ischemic stroke remains uncertain due to concerns regarding hemorrhagic complications. This study aims to evaluate the safety and efficacy of early compared with delayed initiation of oral anticoagulant therapy in patients with AF‐related ischemic stroke.

**Methods:** A systematic review and meta‐analysis were conducted following PRISMA guidelines. Randomized controlled trials and observational studies involving adult patients with ischemic stroke and non‐valvular AF were included. Studies comparing early and delayed initiation of oral anticoagulants were analyzed. Primary outcomes included recurrent ischemic events or transient ischemic attacks, intracranial hemorrhage, major bleeding, and all‐cause mortality. Random‐effects models were used for pooled analyses, with sensitivity analyses performed to assess result robustness.

**Figure:**
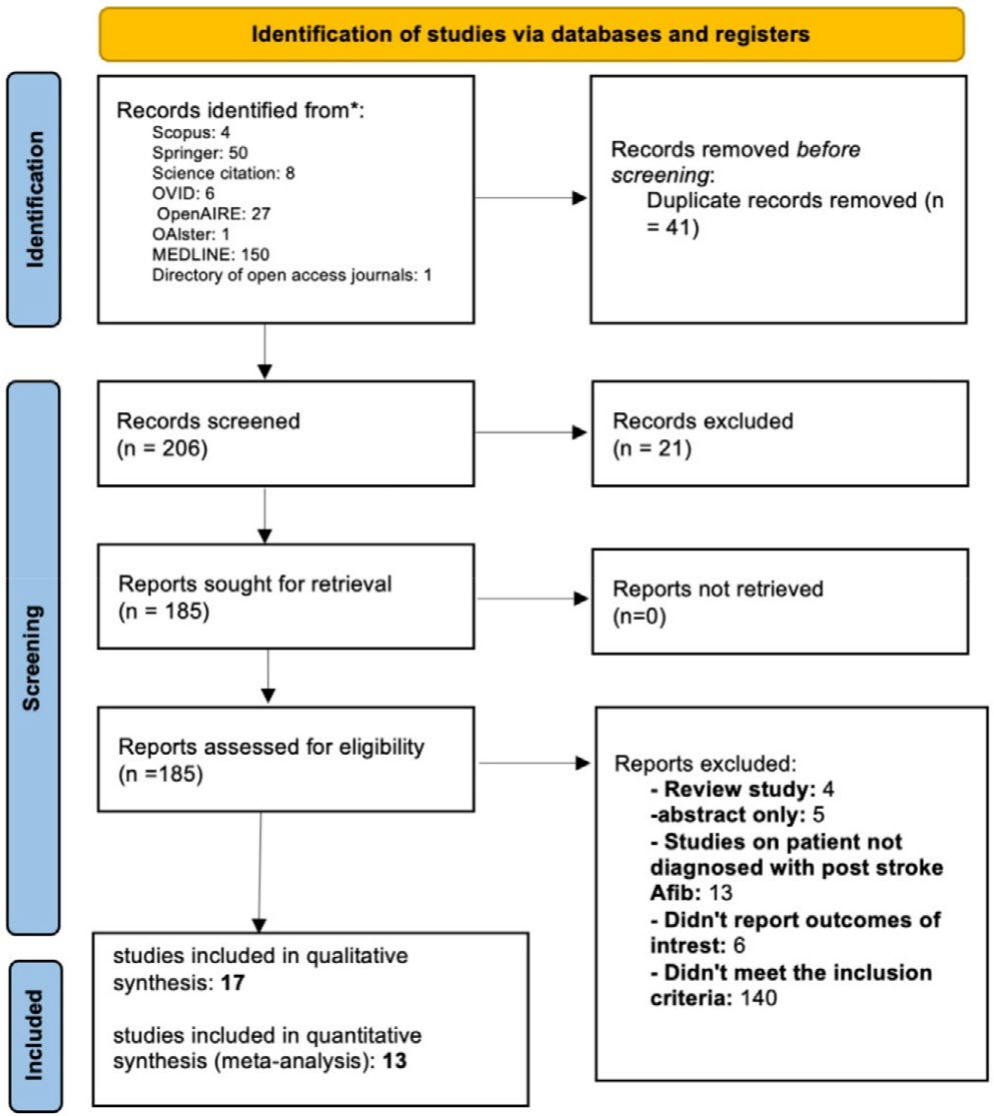
**FIGURE 1** PRISMA flow diagram illustrating the study selection process for included randomized controlled trials and observational studies.

**Results:** Seventeen studies met inclusion criteria, with thirteen eligible for quantitative synthesis, comprising more than 32,000 patients. Early initiation of oral anticoagulation was not associated with increased all‐cause mortality or major bleeding compared with delayed initiation. Sensitivity analyses demonstrated a lower risk of recurrent ischemic events without an increased risk of intracranial hemorrhage among patients receiving early therapy.

**Figure:**
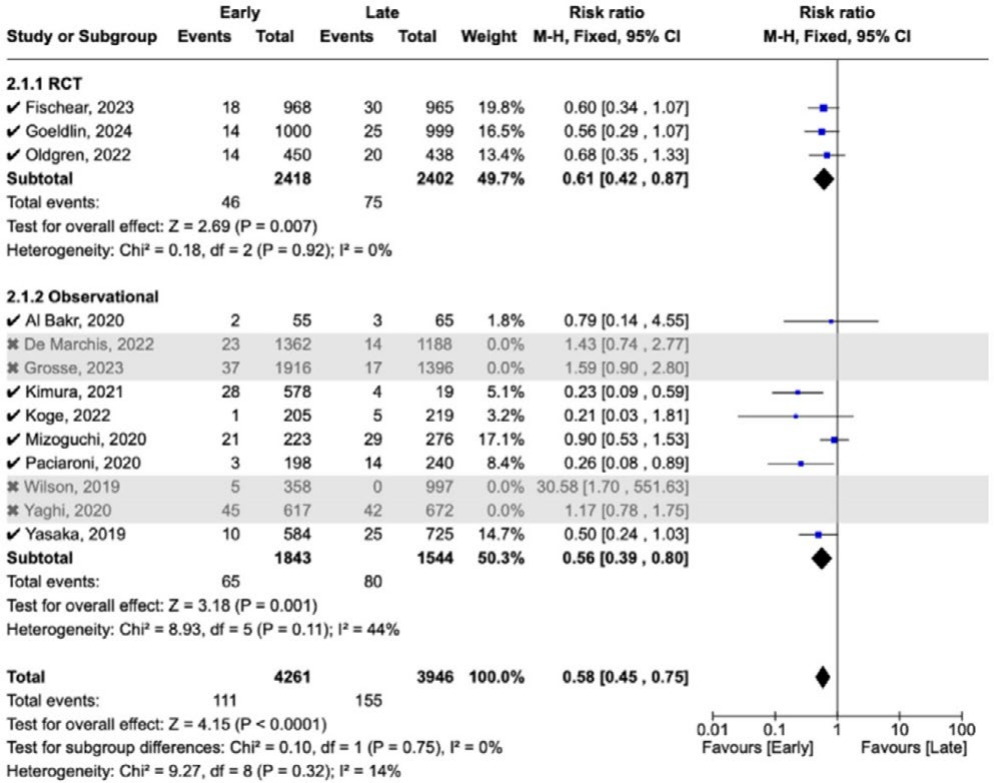
**FIGURE 2** Forest plot comparing early versus delayed initiation of oral anticoagulation for ischemic events or transient ischemic attack in patients with atrial fibrillation related ischemic stroke.

**Figure:**
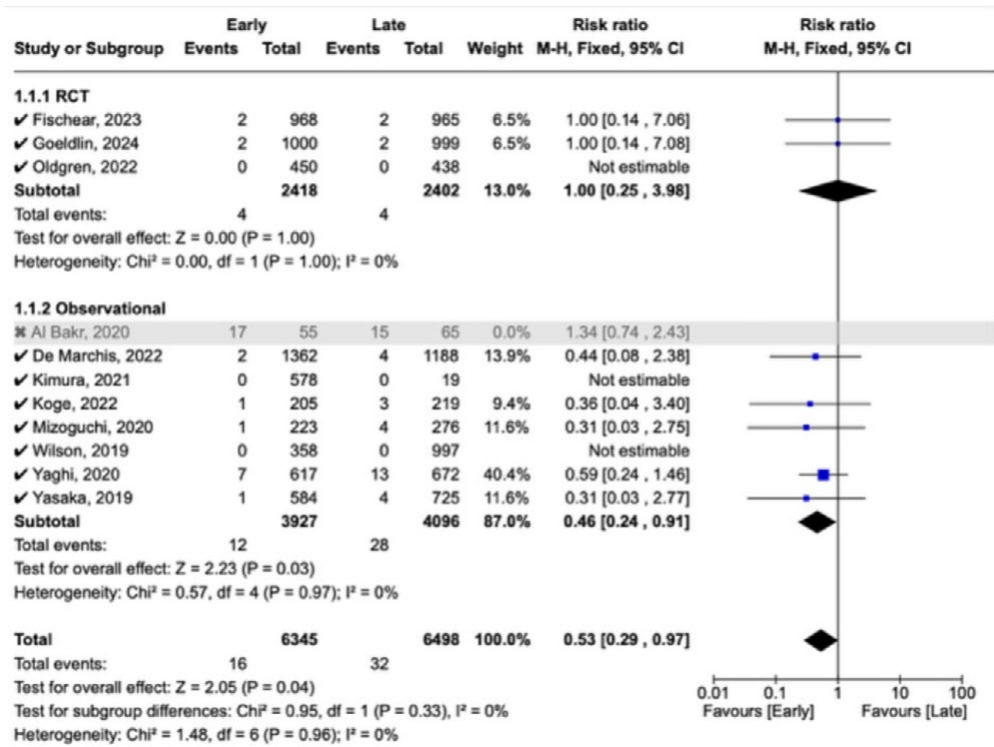
**FIGURE 3** Forest plot showing the risk of intracranial hemorrhage associated with early versus delayed oral anticoagulation initiation after atrial fibrillation related ischemic stroke.

**Conclusion:** Early initiation of oral anticoagulation following AF‐related ischemic stroke appears safe and may offer superior protection against recurrent ischemic events. These findings support early, individualized, imaging‐guided anticoagulation strategies and may inform future clinical guideline updates.

**Disclosure:** Nothing to disclose.

## EPO‐0202

### Endovascular treatment for anterior circulation strokes beyond 6 hours: Better late than never

#### I. Stoican ^1^; R. Stanciulescu^2^; R. Neamtu^2^; A. Cristache^2^; S. Dumitrescu^2^; A. Dimancea^1^; R. Naum^1^; S. Tuta^1^; M. Manea^1^

##### ^1^National Institute of Neurology and Neurovascular Diseases, Neurology Department, Bucharest, Romania; ^2^National Institute of Neurology and Neurovascular Diseases, Radiology Department, Bucharest, Romania

**Background and aims:** Endovascular treatment beyond 6 hours has been shown to improve outcomes in selected stroke patients, usually based on advanced imaging. This study aimed to evaluate the outcomes of patients undergoing thrombectomy beyond 6 hours without the use of advanced imaging.

**Methods:** We retrospectively included patients with anterior circulation strokes who underwent thrombectomy without prior thrombolysis and divided them into late window (LW) and non‐late window (NLW) groups. Baseline characteristics included age, sex, National Institutes of Health Stroke Scale (NIHSS), and Alberta Stroke Program Early CT Score (ASPECTS). Outcomes were assessed using parenchymal hemorrhagic transformation (PHT), ASPECTS at 24 hours, and modified Rankin Scale (mRS) at discharge. In the LW group, associations between age, baseline ASPECTS, number of thrombectomy passes and outcomes were analyzed.

**Results:** A total of 117 patients were included (47 LW, 70 NLW), with no significant baseline differences between groups. No statistically significant differences were observed in PHT rates (*p* = 0.3649) or mean ASPECTS at 24 hours (*p* = 0.12356). Mean mRS at discharge was 3.4043 (± 1.6896) in the LW group and 3.9857 (± 1.9523) in the NLW group (*p* = 0.05118). In the LW group, mRS at discharge was not associated with age but showed significant correlations with baseline ASPECTS (*r* = −0.30732, *p* = 0.03562) and number of thrombectomy passes (*r* = 0.41476, *p* = 0.00375).

**Conclusion:** Thrombectomy beyond 6 hours without advanced imaging resulted in outcomes comparable to those achieved within the conventional time window. Higher baseline ASPECTS and fewer thrombectomy passes were associated with better functional outcomes.

**Disclosure:** Nothing to disclose.

## EPO‐0203

### Cardiac ultrasound in stroke by neurologists: A comparative study of diagnostic accuracy

#### J. Thillen; Y. Winter; P. Mencke; L. Schwindling; G. Sergiu; P. Lochner

##### Saarland University Center, Saarbrücken, Germany

**Background and aims:** Cardioembolic stroke is one of the most prevalent causes of ischemic stroke, making it crucial to identify cardiac embolic sources accurately. Transthoracic echocardiography (TTE) is widely used for this purpose, but logistical barriers often delay its use when performed outside neurology departments, potentially, reducing the rate of cardiac imaging in stroke patients. Thus, the objective of the present study was to evaluate the diagnostic accuracy of neurologist‐performed TTEs following specialised training, using cardiologist readings as the reference standard.

**Methods:** Neurologists were trained using a simplified 10‐point echocardiographic protocol developed by the ESNCH Working Group. A total of 74 TTEs performed by neurologists in ischemic stroke patients were compared by cardiologists. Diagnostic sensitivity, specificity, interobserver agreement (Cohen's kappa), accuracy and predictive values were calculated.

**Results:** Perfect interobserver agreement was found for pericardial fluid (k = 1.0; accuracy 100%) and left atrial (LA) enlargement (k = 0.832; sensitivity 90.5%, specificity 93.9%). Moderate agreement was observed for significant valvular dysfunction (k = 0.572) and left ventricular (LV) function impairment (k = 0.489). Notably, the negative predictive value (NPV) was high (> / = 95%) for seven out of nine parameters, suggesting Neurologist‐performed TTE is a reliable tool for ruling out major structural pathologies.

**Conclusion:** Trained neurologists can accurately identify significant cardiac abnormalities on TTE relevant to stroke care, with strong specificity across parameters. Neurologist‐performed TTE may serve as a reliable diagnostic component in acute stroke settings, improving access and efficiency in interdisciplinary stroke care.

**Disclosure:** Nothing to disclose.

## EPO‐0204

### Adjunctive hypothermia after endovascular reperfusion therapy for acute ischemic stroke: Evidence from a meta‐analysis

#### K. Sarhan; R. Mohamed

##### Faculty of Medicine, Mansoura University, Mansoura, Egypt

**Background and aims:** Endovascular thrombectomy (EVT) is the standard of care for acute ischemic stroke due to large vessel occlusion; however, reperfusion injury limits neurological recovery. Selective or mild therapeutic hypothermia has emerged as a potential neuroprotective adjunct to EVT, aiming to mitigate ischemia‐reperfusion injury while preserving procedural safety.

**Methods:** We conducted a systematic review and meta‐analysis of clinical studies evaluating therapeutic hypothermia combined with EVT in patients with acute ischemic stroke. PubMed, Embase, Scopus, and Web of Science were searched from inception to December 2025. Primary outcomes were favorable functional outcome (modified Rankin Scale [mRS] 0–2 at 90 days). Secondary outcomes included excellent functional outcome (mRS 0–1), mortality, and symptomatic intracranial hemorrhage (sICH).

**Results:** Seven studies comprising 603 patients were included, of whom 294 received therapeutic hypothermia combined with EVT and 309 underwent thrombectomy alone. The addition of hypothermia was associated with a significantly higher rate of favorable functional outcome at 90 days (mRS 0–2: 59.2% vs 49.2%; RR = 1.23, 95% CI 1.06–1.42). Patients treated with hypothermia also demonstrated a greater likelihood of excellent functional recovery (mRS 0–1: 48.6% vs 33.1%; RR = 1.45, 95% CI 1.12–1.88). There were no significant differences between groups in mortality (RR = 0.97, 95% CI 0.75–1.26) or symptomatic intracranial hemorrhage (RR = 0.78, 95% CI 0.50–1.21). Statistical heterogeneity was low to moderate across outcomes.

**Conclusion:** Therapeutic hypothermia combined with EVT is associated with improved functional outcomes without increased mortality or hemorrhagic risk. Larger randomized trials are needed to confirm efficacy and optimize cooling strategies.

**Disclosure:** Nothing to disclose.

## EPO‐0205

### MicroRNA‐based point‐of‐care potential for stroke subtype differentiation

#### M. Nath; D. Vibha

##### Department of Neurology, Neurosciences Center, All India Institute of Medical Sciences, New Delhi, India

**Background and aims:** Timely differentiation between acute ischemic stroke and intracerebral hemorrhage is essential for safe treatment. However, neuroimaging may delay care in resource‐limited settings. Circulating microRNAs (miRNAs) are stable and linked to cerebrovascular injury, making them promising diagnostic markers.

**Methods:** Adults presenting within six hours of stroke onset were enrolled before neuroimaging, with STARD guidelines as reference. The diagnosis was confirmed with standard imaging. Blood samples were collected at presentation and analyzed via next‐generation sequencing to identify miRNA, then validated by qRT‐PCR. Participants included patients with acute ischemic stroke (*n* = 15), intracerebral hemorrhage (*n* = 15), and healthy controls (*n* = 30). miRNA cycle thresholds were normalized. Diagnostic performance was evaluated using nonparametric analysis, ROC curves, PCA, and machine‐learning classifiers. Pathway enrichment supported biological plausibility.

**Results:** Distinct circulating miRNA expression patterns were observed across stroke subtypes and controls. miR‐21 and miR‐146a were significantly downregulated, whereas miR‐155 was upregulated in stroke cohorts relative to healthy individuals. Diagnostic performance analyses demonstrated robust discriminatory capacity, with miR‐21 achieving the highest classification accuracy (AUC = 0.80). PCA revealed clear separation between ischemic, hemorrhagic, and control groups. Among machine‐learning approaches, Random Forest models achieved superior predictive performance, particularly in differentiating intracerebral hemorrhage from controls (AUC = 0.79). Pathway analyses linked these miRNAs to inflammatory signaling, immune modulation, and cell survival mechanisms relevant to acute cerebrovascular injury.

**Conclusion:** Circulating miRNA signatures enable early discrimination between ischemic and hemorrhagic stroke before imaging confirmation. These findings support further validation of miRNA‐based assays as adjunctive diagnostic tools to accelerate stroke triage and precision care.

**Disclosure:** Nothing to Disclose.

## EPO‐0206

### Combined impact of intracranial atherosclerotic stenosis and chronic kidney disease on functional outcome after ischemic stroke

#### M. Reichmuth ^1^; K. Mettler^1^; K. Antonenko^1^; P. Radojewski^2^; N. Slavova^1^; W. Almiri^3^; A. Hoffmann^3^; S. Fenzl^3^; J. Kaesmacher^3^; R. Wiest^3^; M. Heldner^1^

##### ^1^Department of Neurology, Inselspital, Bern University hospital and University of Bern, Bern, Switzerland; ^2^Institute of Diagnostic and Interventional Neuroradiology, Inselspital, University Hospital and University of Bern, Bern, Switzerland and Translational Imaging Center, sitem‐insel, Bern, Switzerland; ^3^Institute of Diagnostic and Interventional Neuroradiology, Inselspital, University Hospital and University of Bern, Bern, Switzerland

**Background and aims:** Intracranial atherosclerotic stenosis (ICAS) and chronic kidney disease (CKD) are both significant contributors to stroke risk and poor outcomes.

**Methods:** In this retrospective observational cohort study, 488 first‐ever ischemic stroke patients from the Bernese Stroke Registry (April 2021 – March 2023) were analyzed. Patients were stratified into four groups based on ICAS presence and eGFR: no ICAS/eGFR ≥ 60 (*n* = 226), no ICAS/eGFR < 60 (*n* = 73), ICAS/eGFR ≥ 60 (*n* = 107), and ICAS/eGFR < 60 (*n* = 82). Data on demographics, vascular risk factors, stroke aetiology, ICAS location, acute treatment, and 3‐month outcomes (mRS) were analyzed.

**Results:** Patients with ICAS and eGFR < 60 were the oldest (median 83.9 years, range 55.4–99.5) and had significantly worse renal function (median creatinine 117 μmol/L, median eGFR 44 ml/min/1.73 m^2^) compared to other groups (*p* < 0.001). Arterial hypertension was significantly more prevalent in the ICAS/eGFR < 60 group (85.4%) than in the no ICAS/eGFR ≥ 60 group (63.1%) (*p* < 0.001). Diabetes mellitus prevalence was highest in the ICAS/eGFR < 60 group (37.8%, *p* = 0.009). According to TOAST classification, large artery atherosclerosis was the dominant stroke mechanism in ICAS groups (44.9% in ICAS/eGFR ≥ 60 vs. 7.5% in no ICAS/eGFR ≥ 60, *p* < 0.001). At 3‐month follow‐up, patients with ICAS/eGFR < 60 had the poorest functional outcomes, with only 58.2% achieving a favourable mRS (0–2) compared to 81.3% in the no ICAS/eGFR ≥ 60 group (*p* < 0.001).

**Conclusion:** The co‐existence of ICAS and impaired renal function identifies a high‐risk stroke group characterized by a greater burden of vascular risk factors, large artery atherosclerosis as stroke aetiology, and worse short‐term functional recovery. These findings underscore the importance of integrated vascular risk assessment and tailored management strategies in this vulnerable patient group.

**Disclosure:** Nothing to disclose.

## EPO‐0207

### Symptomatic and asymptomatic hematoma growth: Definition, prevalence and association with outcomes in intracerebral hemorrhage

#### Q. Li ^1^; X. Lv^2^; A. Morotti^3^; A. Qureshi^4^; D. Dowlatshahi^5^; G. Falcone^6^; K. Sheth^7^; A. Shoamanesh^8^; S. Murthy^9^; A. Viswanathan^10^; J. Goldstein^11^

##### ^1^Department of Neurology, The Second Affiliated Hospital of Anhui Medical University, Hefei, China; ^2^Department of Neurology, Stroke Center, The First Hospital of Jilin University, Changchun, China; ^3^Neurology Unit, Department of Clinical and Experimental Sciences, University of Brescia, Brescia, Italy; ^4^Zeenat Qureshi Stroke Institute and Department of Neurology, University of Missouri, Columbia, USA; ^5^Department of Medicine, Division of Neurology, University of Ottawa and Ottawa Hospital Research Institute, Ottawa, Canada; ^6^Department of Neurology, Yale School of Medicine, New Haven, USA; ^7^Division of Neurocritical Care and Emergency Neurology, Departments of Neurology and Neurosurgery, and the Yale Center for Brain and Mind Health, Yale School of Medicine, New Haven, USA; ^8^Department of Medicine, Division of Neurology, McMaster University, Population Health Research Institute, Hamilton, Canada; ^9^Clinical and Translational Neuroscience Unit, Feil Family Brain and Mind Research Institute and Department of Neurology, Weill Cornell Medicine, New York, USA; ^10^J Philip Kistler Stroke Research Center, Department of Neurology, Massachusetts General Hospital, Harvard Medical School, Boston, USA; ^11^Department of Emergency Medicine, Massachusetts General Hospital, Harvard Medical School, Boston, USA

**Background and aims:** Hematoma growth occurs in approximately one‐third of patients with intracerebral hemorrhage (ICH) and is associated with a high rate of morbidity and mortality. However, radiological hematoma growth does not always coincide with neurological deterioration. This study aimed to differentiate symptomatic and asymptomatic hematoma growth and examine their respective clinical implications using data from the Antihypertensive Treatment of Acute Cerebral Hemorrhage 2 (ATACH‐2) trial.

**Methods:** Among 917 ICH patients (568 male patients) from ATACH‐2 with complete clinical and imaging data, hematoma growth was defined as a > 33% relative or > 6 mL absolute increase in volume on 24‐hour computed tomography. Symptomatic hematoma growth was defined by concurrent neurological deterioration (≥ 1‐point NIHSS increase), while asymptomatic growth occurred without NIHSS worsening. Associations between hematoma growth types and 90‐day functional outcomes (modified Rankin Scale [mRS]) were evaluated using multivariable logistic regression.

**Results:** Symptomatic hematoma growth occurred in 5.9% and asymptomatic growth in 16.7% of patients. Both were independently associated with poor functional outcomes (mRS 4–6), with the strongest effect observed for symptomatic growth (adjusted OR 6.891; 95% CI 3.411–13.921). Mortality was highest among symptomatic patients (25.9%), compared with asymptomatic (10.5%) and no‐growth groups (3.7%). Larger baseline hematoma volume, ultra‐early hematoma growth, and intraventricular hemorrhage growth were significantly more frequent in symptomatic hematoma growth.

**Figure:**
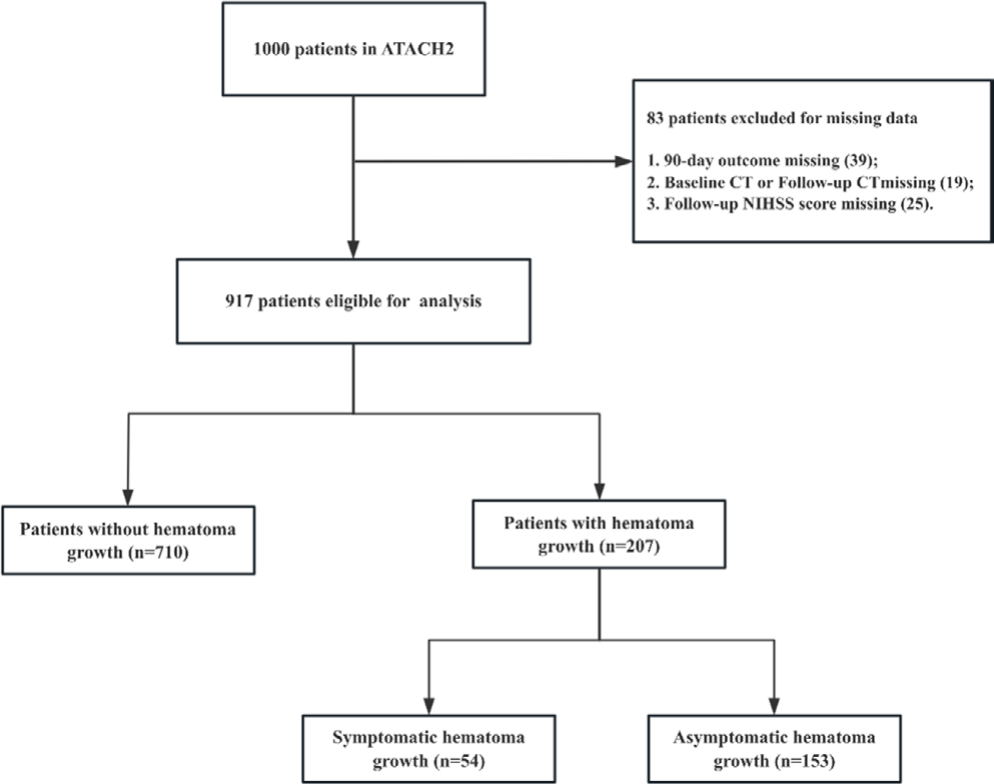
**FIGURE 1** Flow diagram of the study participants.

**Figure:**
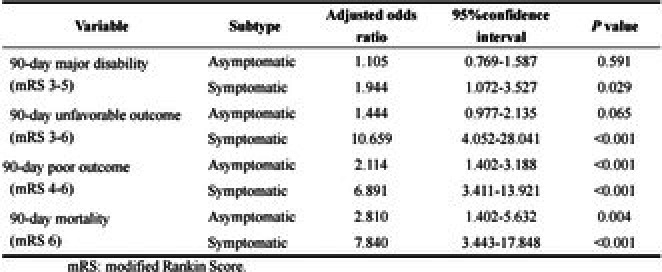
**TABLE 1** Relationship between different types of hematoma growth and prognosis.

**Conclusion:** Asymptomatic hematoma growth is not uncommon and predicts poor outcomes after ICH, although symptomatic growth carries a substantially higher risk of death and disability. Early identification through integrated clinical–radiological assessment and vigilant monitoring may improve prognostication and facilitate timely intervention.

**Disclosure:** JNG has received consulting from AstraZeneca, CSL Behring, Octapharma, Takeda, and Cayuga.

## EPO‐0208

### Morbidity and mortality of ruptured cerebral aneurysms in patients aged over 80 years: A systematic review and meta‐analysis

#### S. Soleimani ^1^; M. Dabbagh Ohadi^1^; R. Dashti^2^; M. Niemelä^1^; E. Winkler^3^; B. Rezai Jahromi^1^

##### ^1^1. Department of Neurosurgery, Helsinki University Hospital and University of Helsinki, Helsinki, Finland; ^2^2. Department of Neurosurgery, Stony Brook University Hospital, Stony Brook, USA; ^3^3. Department of Neurosurgery University of California–San Francisco San Francisco, USA

**Background and aims:** Aneurysmal subarachnoid hemorrhage (aSAH) in patients over 80 years is associated with high mortality and morbidity, yet evidence guiding management in this population is limited. As life expectancy increases, clarifying outcomes of different treatment strategies in very elderly patients has become increasingly important.

**Methods:** PubMed, Embase, Scopus, and Web of Science were systematically searched till October 2025 in accordance with PRISMA guidelines. Studies reporting outcomes of ruptured intracranial aneurysms in patients aged ≥80 years were included. Primary outcomes were favorable functional outcome and mortality and complications of ruptured cerebral aneurysms. Clinical severity was assessed using Hunt & Hess, or WFNS grading systems. Random‐effects meta‐analyses were performed, and study quality was evaluated using the ROBINS‐I tool.

**Results:** 26 retrospective studies including 14,469 patients were analyzed; approximately two‐thirds were female, and the mean age was 84.3 years. Surgical clipping was performed in 4,863 patients, endovascular treatment in 5,444, and conservative management in 4,135. Among 1,252 patients with reported admission grade, 577 had poor‐grade SAH. Interventional treatment was associated with a higher pooled rate of favorable outcomes than conservative management (28.6% vs 9.2%) and significantly improved odds of favorable outcome (OR 3.18) and reduced mortality (OR 0.16). Benefits were greatest in patients with good initial clinical grades. The pooled complication rate was 49%, with cerebral vasospasm and hydrocephalus each affecting about one‐quarter of patients.

**Figure:**
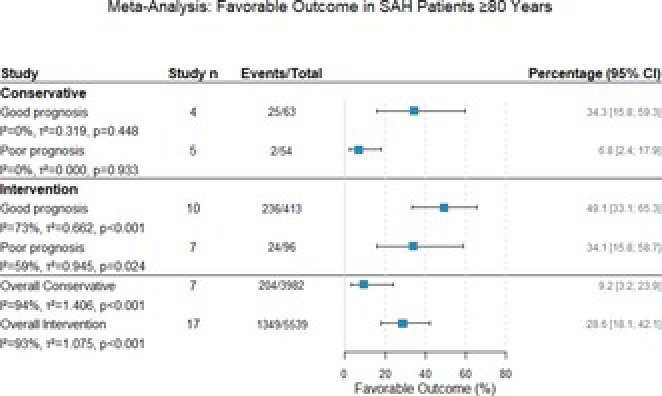
**TABLE 1** Favorable Outcome in SAH patients ≥80 years old.

**Figure:**
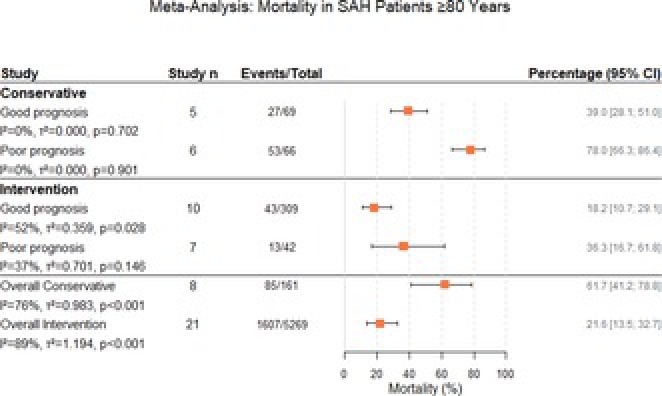
**TABLE 2** Mortality in SAH patients ≥80 years old.

**Figure:**
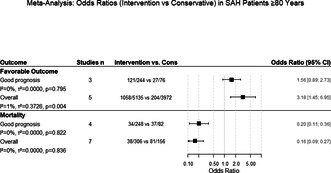
**TABLE 3** Odds Ratios between Intervention and Conservative management in SAH patient ≥80 years.

**Conclusion:** Despite high overall mortality, selected very elderly patients, particularly those with good clinical grades, may benefit from interventional treatment. Treatment decisions should be individualized rather than based on age alone.

**Disclosure:** Behnam Rezai Jahromi and Mika Niemelä have patents pending on aneurysm imaging.

Neuropathies 2

## EPO‐0209

### Management of disabling spasticity with functional neurosurgery (selective microsurgical neurotomy)

#### A. Afif

##### Department of Neurosurgery, Department of Anatomy, INSERM U 1028, Pain Center, Pierre Wertheimer Hospital, Lyon 1 University; European Hospital, Paris, France

**Background and aims:** Spasticity is one of the most common sequelae of central neurological disorders. It is defined as a motor disorder characterized by a velocity‐dependent increase in the tonic stretch reflex associated with an exaggeration of deep tendon reflexes. When disabling spasticity is focused on one or a few muscle groups, selective peripheral neurotomy may be considered.

**Methods:** Before undergoing these surgical programs, patients undergo a complete preoperative assessment using a block test of the targeted nerves using local anesthetics. After an approach tailored to the targeted nerve, the first step is microsurgical dissection of the nerve branches and the fascicles of each branch. The study of motor responses allows for the differentiation of motor fascicles from sensory fascicles, and then hyperexcited fascicles from others. After this identification, the fascicles are partially transected according to the preoperative plan.

**Results:** In our series, patients who underwent selective neurotomy of the upper and lower limbs experienced significant suppression or reduction of spasticity and a reduction or disappearance of spasticity‐induced pain. Functional improvement was significant (upper limb flexibility, hand and finger opening, limb reuse, improved gait and foot stability during stance and walking), with improvements in body image, comfort when dressing, and shoe fitting.

**Conclusion:** In the management of spasticity, selective microsurgical neurotomy has predictable effects based on preoperative testing. By eliminating excessive spasticity, correcting abnormal postures, and reducing associated pain, neurotomy allows for the resumption of active and effective rehabilitation and, in some cases, the reappearance or improvement of voluntary and useful motor skills.

**Disclosure:** Selective Microsurgical Neurotomy is a modular and adaptable technique for each patient. It allows for the long‐term reduction of focal spasticity at the cost of low morbidity.

## EPO‐0210

### Silent dysphagia in CANVAS: Videofluoroscopic abnormalities despite low EAT‐10

#### A. Alungulese; I. Catalina Alvarez; L. Del Pino Tejado

##### Department of Neurology, Gregorio Marañón University Hospital, Madrid, Spain

**Background and aims:** Dysphagia has been reported within the clinical spectrum of cerebellar ataxia, neuropathy and vestibular areflexia syndrome (CANVAS), but it is often underrecognized and not systematically assessed. The objective of this study was to characterize dysphagia in genetically confirmed CANVAS and evaluate concordance between symptom‐based screening and instrumental assessment.

**Methods:** We conducted a retrospective case series of 12 adult patients with CANVAS. All patients underwent dysphagia screening using the Eating Assessment Tool‐10 (EAT‐10) and videofluoroscopic swallowing study (VFSS). VFSS reports were reviewed for swallowing safety (laryngeal penetration, aspiration, cough reflex) and efficiency (pharyngeal residue), with analysis according to bolus consistency.

**Results:** The cohort comprised 12 patients (8 women), with a mean age of 66.5 years. Chronic cough was present in most patients (10/12). All patients exhibited sensory axonal polyneuropathy and bilateral vestibular hypofunction, consistent with the CANVAS phenotype. Cerebellar involvement, defined by neurological examination and/or brain MRI findings, was identified in 2/12 patients. EAT‐10 scores were below the pathological cut‐off (<3) in 11/12 patients, while one patient had advanced dysphagia requiring percutaneous endoscopic gastrostomy. In contrast, VFSS demonstrated objective abnormalities in 10/12 patients (83.3%) (Figure 1) Laryngeal penetration was predominantly observed with thin liquids. All patients with abnormal VFSS lacked an effective cough reflex (10/10, 100%). Aspiration occurred in 2/12 patients (16.7%). Minimal pharyngeal residue was reported in 2/12 patients (16.7%) (Figure 2).

**Figure:**
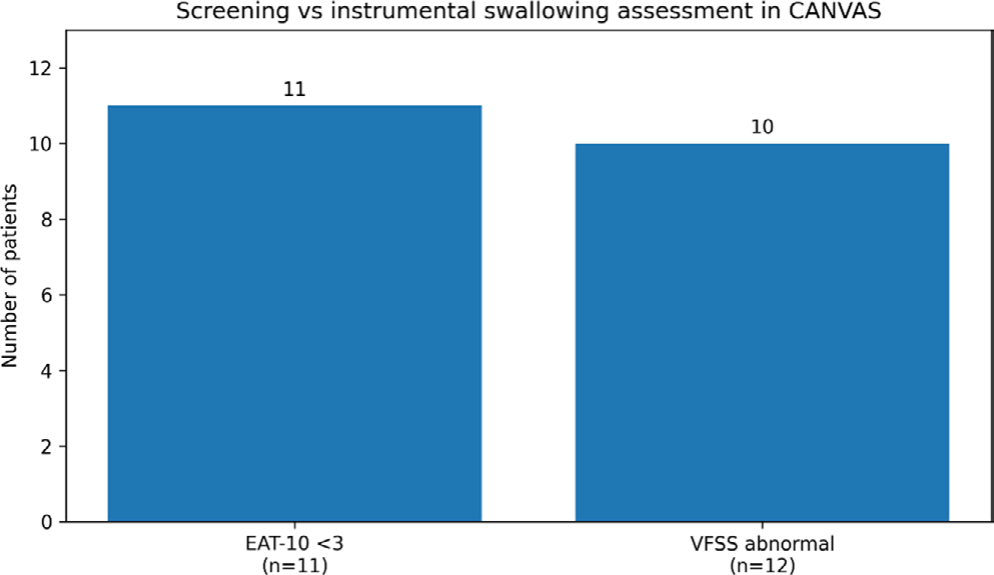
**FIGURE 1** Screening versus instrumental swallowing assessment in CANVAS.

**Figure:**
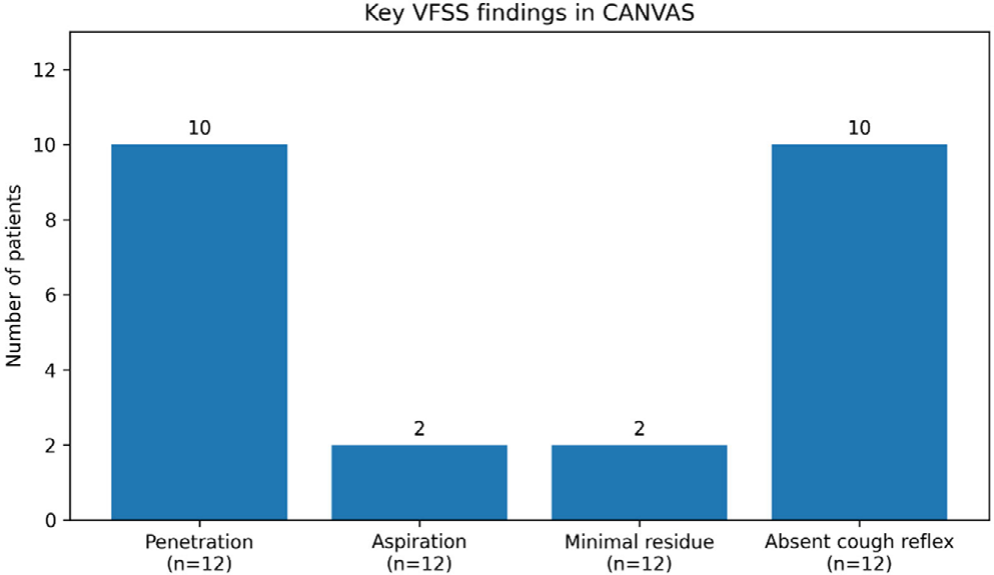
**FIGURE 2** Key videofluoroscopic swallowing findings in CANVAS.

**Conclusion:** In RFC1‐CANVAS, dysphagia appears to follow a silent progression, with normal EAT‐10 scores preceding objective videofluoroscopic abnormalities and aspiration risk.

**Disclosure:** Nothing to disclose.

## EPO‐0211

### Clinical characteristics and therapeutic response in patients with autoimmune nodopathies: A multi‐center study from the US

#### C. Koutsioumpa^1^; M. Weisman^2^; A. Morrison^3^; W. Khan^4^; S. Muppidi^5^; N. Chahin^6^; L. Davalos^7^; A. Deifes^2^; R. Sadjadi^4^; K. Gable^8^; M. Dimachkie^7^; C. Karam^2^; J. Allen^9^; B. Roy ^1^

##### ^1^Department of Neurology, Yale University, New Haven, USA; ^2^Department of Neurology, Perelman School of Medicine, Philadelphia, USA; ^3^Department of Neurology, The Ohio State University, Columbus, USA; ^4^Department of Neurology, Mass General Hospital, Boston, USA; ^5^Department of Neurology, Standford School of Medicine, Stanford, USA; ^6^Department of Neurology, Oregon Health and Science University, Oregon, USA; ^7^Department of Neurology, University of Kansas Medical Center, Kansas City, USA; ^8^Department of Neurology, Duke University Medical Center, Durham, USA; ^9^Department of Neurology, University of Minnesota Medical School, Minneapolis, USA

**Background and aims:** Autoimmune nodopathies (AINs) with circulating IgG4 autoantibodies targeting nodes of Ranvier antigens are rare. About 5–10% of patients with electrodiagnostic features of a demyelinating neuropathy have autoimmune nodopathies. AINs usually have distinct clinical features and often don’t respond to intravenous immunoglobulin (IVIg), but improve with B‐cell‐depleting therapy. Most of the current literature on AIN came from Europe and Japan, and data from the US is limited.

**Methods:** This multi‐center study, including over 12 centers in the US, is actively capturing the clinical features and therapeutic responses of patients with AINs.

**Results:** To date, we have included 33 patients, 25 (76%) with anti‐neurofascin 155 (NF155) and six (18%) with anti‐Contactin IgG4 autoantibodies, and two patients positive for both autoantibodies. The average age of disease onset was 50.3 ± 19.6 years, and 18 (54.5%) patients were male. About 55% of patients had both distal and proximal weakness, and 36% had predominant distal weakness. Distal loss of vibration sensation and ataxia were common. Areflexia was observed in > 80% patients. Over 90% of this cohort received IVIg, but only 33% had partial benefit. Steroids were used in 50% of cases, neonatal Fc receptor (FcRn) inhibitors in 13% of cases, and PLEX in 20% of cases. Over 70% of patients were treated with rituximab, and most showed objective benefit on clinical examination.

**Conclusion:** This important collaborative effort will provide new insights into the clinical characteristics of AIN and critical information on management strategies.

**Disclosure:** CK, MW, WK, DL, AD, have nothing to disclose. KG reports consulting or Adjudication committee for the following: Sanofi, Immunovant, Takeda, Argenx, CSL Behring, Grifols, Annexon, Dianthus C.Karam reports consultant and/or advisory board Acceleron: Alpine, Alexion, Alnylam, Amgen, Amicus, Annexon, Applied Therapeutics, Argenx, Astra Zeneca, Catalyst, Corino, Biogen, CSL Behring, Genentech, Immunovant, Intellia, Ionis, J&J, Medison, Neuroderm, Novo Nordisk, Nuvig, Octapharma, Pfizer, Sanofi, UCB, Takeda, Vertex, and Zai Lab. AM reports to be on the scientific advisory board for Candid Therapeutics MMD received consultancy fees, payment or honoraria for lectures, presentations, manuscript writing or educational events, or participated on a Data Safety Monitoring Board or Advisory Board for AAN, AANEM, Abcuro, Amicus, Annexon, ArgenX, Astellas, Astra Zaneca, Cabaletta Bio, Candid Therapeutics, Catalyst, Covance/Labcorp/Fortrea, CSL‐Behring, Creyon Bio, Inc, Dianthus, EMD Serono/Merck, Horizon, Ig Society, Inc, Janssen, Kriya Therapeutics, Inc, Medlink, Nuvig, Octapharma, Sanofi Genzyme, Shire Takeda, and TACT/Treat NMD, UCB and Vertex. MMD has received grants or from Abcuro, Alexion/ AstraZeneca, Alnylam Pharmaceuticals, Amicus, ArgenX, Biosensics, Bristol‐Myers Squibb, Cabaletta Bio, Catalyst, CSL‐Behring, EMD Serono, FDA/OOPD, GlaxoSmithKline, Genentech, Grifols, Horizon Immnunovant, Mitsubishi Tanabe Pharma, MDA, NIH, Nkarta, Novartis, Octapharma, Orphazyme, Priovant, Ra Pharma/UCB Biopharma, Sanofi Genzyme, Sarepta Therapeutics, Shire Takeda, Spark Therapeutics, and The Myositis Association. MMD received royalty for manuscript writing from Wolters Kluwer Health/UpToDate and from Elsevier and for discovery relating to health care from Zevra Therapeutics. NC reports to serve as a consultant for argenx, sanofi, amicus, and arcellx. JA has received honoraria/consultation fee or being in the advisory boards of advisory board: Akcea therapeutics, Amgen, Argenx, Alexion, Alnylam, Astra Zeneca, Annexon, BioCryst, CSL Behring, Grifols, Immunovant, Immunopharma, ImmunoAbs, Octapharma, Dianthus, Sanofi, Johnson & Johnson, Pfizer, Takeda. BR reports serving as a consultant/advisor for Alexion (now part of AstraZeneca), Takeda, Sanofi, and argenx. Additionally, BR has received research support from the Martin Shubik Fund for IBM at Yale University, NIH, Abcuro Pharmaceuticals, argenx, Immunovant, and Takeda. BR has small stocks in Cabaletta Bio, CAVA, and Pfizer.

## EPO‐0212

### Treating chronic inflammatory demyelinating polyneuropathy – analysis of specialized German centers

#### F. Konen ^1^; K. Kneer^2^; E. Bucak^1^; A. Fisse^3^; F. Stascheit^4^; K. Jendretzky^1^; S. Gingele^1^; T. Gasimli^2^; R. Klimas^3^; J. Motte^3^; A. Grimm^2^; K. Pitarokoili^3^; T. Skripuletz^1^

##### ^*1*^ *Department of Neurology, Hannover Medical School, Hannover, Germany;* ^*2*^ *Department of Neurology, Tuebingen University Hospital, Tuebingen, Germany;* ^*3*^ *Department of Neurology, St. Josef‐Hospital, Ruhr‐University Bochum, Bochum, Germany;* ^*4*^ *Neuroscience Clinical Research Center, Charité – Universitätsmedizin Berlin, Freie Universität Berlin and Humboldt Universität zu Berlin, Berlin, Germany*

**Background and aims:** Chronic inflammatory demyelinating polyneuropathy (CIDP) is a heterogeneous immune‐mediated neuropathy requiring long‐term immunomodulatory treatment. Although different effective therapies are available, real‐world data on treatment‐sequencing, dosing patterns, and escalation strategies remain limited.

**Methods:** This multicenter retrospective observational study included patients with CIDP fulfilling the 2021 EAN/PNS diagnostic criteria treated at specialized German centers (Neuritis Network). Individual treatment sequences and dosages across consecutive therapy lines were reconstructed. Disability and functional impairment were assessed using the Inflammatory Neuropathy Cause and Treatment (INCAT) score and the Inflammatory Rasch‐built Overall Disability Scale (RODS).

**Results:** 511 patients were included (median age at diagnosis 59 years, 29% female) with 79% diagnosed with typical CIDP and a median follow‐up of 80 months. Initial treatment consisted mostly of intravenous immunoglobulins (IVIg, 47%) or intravenous glucocorticoids (46%). While most patients starting on IVIg remained on immunoglobulin therapy, the majority of those initially treated with glucocorticoids later switched to IVIg. Over the disease course, IVIg was used in 90% of patients, intravenous glucocorticoids in 56%, subcutaneous immunoglobulins (SCIg) in 17%, and oral glucocorticoids in 8%. At data cut‐off, 62% of patients were treated with IVIg and 17% with SCIg. IVIg dosing was highly variable, ranging from 0.12 to 2.9 g/kg body weight per four weeks. Escalation therapies were applied in 24% of patients, most commonly rituximab.

**Conclusion:** These findings highlight immunoglobulin therapy as the cornerstone of CIDP management in real‐world practice, while heterogeneous dosing patterns and frequent escalation underscore ongoing disease activity and the need for more targeted therapeutic options.

**Disclosure:** The study received no external commercial or institutional funding. FFK received honoraria for lectures and travel compensation from Alexion, Argenx, Merck, Novartis, and Takeda. He received research support as fellow of the German Research Foundation (DFG)– and Hannover Medical School (MHH)‐funded Clinician Scientist Program (PRACTIS) at MHH and from Merck, Siemens and Erwin‐Röver‐Foundation. KK received honoraria for lectures and travel compensation from Argenx and AstraZeneca. ALF holds shares in Novartis, Novo Nordisk, Coloplast; received consulting fees by Takeda and travel expenses reimbursement by CSL Behring. FS received travel/accommodation/meeting expenses from Alexion Pharmaceuticals and argenx and received speaking honoria and honoria for attendance at advisory boards from Alexion Pharmaceuticals, argnx and UCB pharma. She receives financial research support (paid to her institution) from Alexion Pharmaceuticals and argenx. She serves as a member of the medical advisory of the German Myasthenia Gravis Society e.V. KFJ received research support from Else Kröner Fresenius Foundation and travel compensation and congress fee from Neuraxpharm, Merck and Novartis. SG reports research support from Alnylam Pharmaceuticals, CSL Behring, Else Kröner Fresenius Foundation, Deutsche Forschungsgemeinschaft and Hannover Biomedical Research School (HBRS) and consulting and/or speaker honoraria from Alexion, Alnylam Pharmaceuticals, AstraZeneca, CSL Behring, GSK, Pfizer, Merck and Takeda Pharmaceuticals. TG reports no conflict of interest. RK declares stock ownership from Novo Nordisk; research funding from LFB Group France and Ruhr‐University Bochum; travel funding from Grifols, Takeda, and Alnylam; and speaker honoraria from Argenx. JM declares stock ownership from Amgen, Bayer, and Sanofi; travel grants from Alnylam, Biogen Idec, Novartis, Teva, Eisai, Neuraxpharm, Bristol Myers Squibb, and Kyverna; consulting fees from Novartis and Alnylam; and research funding from Klaus Tschira Foundation, Ruhr‐University Bochum (FoRUM program), Deutsche Multiple Sklerose Gesellschaft, Hertie Foundation, Novartis, and Kyverna. AG reports research support from CSL Behring, Canon; honoraria for lectures, travel support for meeting attendance, and/or consultancy fees from Alexion, Alnylam, Amgen, argenx, CSL Behring, Grifols, Johnson and Johnson, Roche, Sanofi, Takeda and UCB. KP received research funding from Biogen, CSL Behring, Grünethal, Argenx, Ruhr University Bochum and speaker honoraria and travel support from Grifols, Argenx, Novartis and Sanofi. TS reports research support from Alnylam, CSL Behring, Merck, Novartis, Siemens; honoraria for lectures, travel support for meeting attendance, and/or consultancy fees from Alexion, Alnylam, argenx, Bayer, Biogen, Bristol Myers Squibb, Centogene, CSL Behring, Grifols, Hexal AG, Horizon, Janssen, Merck, Novartis, Pfizer, Purpose Pharma, Roche, Sanofi, Siemens, SOBI, Teva, Viatris.

## EPO‐0213

### Therapeutic effects of repeated immunoadsorption in CIDP: A long‐term, prospective, observational study

#### J. Dorst; Z. Elmas

##### Department of Neurology, University of Ulm, Ulm, Germany

**Background and aims:** Corticosteroids, intravenous immunoglobulins (IVIGs), and plasma exchange (PLEX) constitute the main therapeutic options in CIDP, but immunoadsorption (IA) represents a noteworthy alternative to PLEX due to its excellent tolerability. In this study, we sought to evaluate the short‐ and long‐term therapeutic effects of IA in CIDP compared to PLEX and preceding therapies (IVIGs and corticosteroids).

**Methods:** Between 02/12/2013 and 03/03/2025, we prospectively evaluated the course of disease of patients with CIDP who received at least one cycle of IA or PLEX. One cycle of IA or PLEX consisted of 5 treatments on 5 consecutive days. As the primary outcome parameter, we used a combined CIDP score of 3 validated scales comprising disability (Inflammatory Neuropathy Cause and Treatment (INCAT) score), motor score (Medical Research Council, MRC), and vibration sensitivity (tuning fork test).

**Results:** In total, 80 patients were included. We observed improvements of CIDP scores post‐treatment in the IA group (median improvement from 310 (224–374) to 321 (234–373) points; *p* < 0.0001), but not in the PLEX group (median 254 (214–358) vs. 254 (209–351), *p* = 0.12). Long‐term progression rates compared to preceding corticosteroid and IVIG therapies decreased from 3.8 (2.2–9.1) to 0.2 (−0.5–2.2) points/month in the IA group, and from 4.2 (2.6–17.2) to −1.1 (−1.6–0.5) points/month in the PLEX group, corresponding to a clinical stabilization of disease progression.

**Figure:**
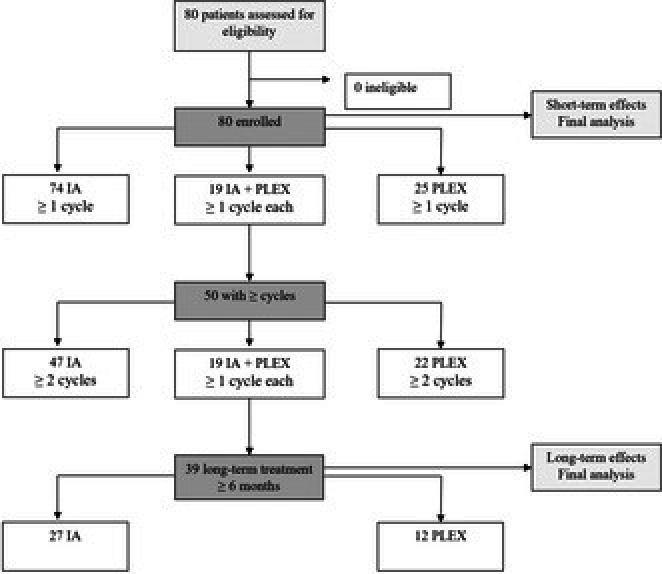
**FIGURE 1** Study flow diagram.

**Figure:**
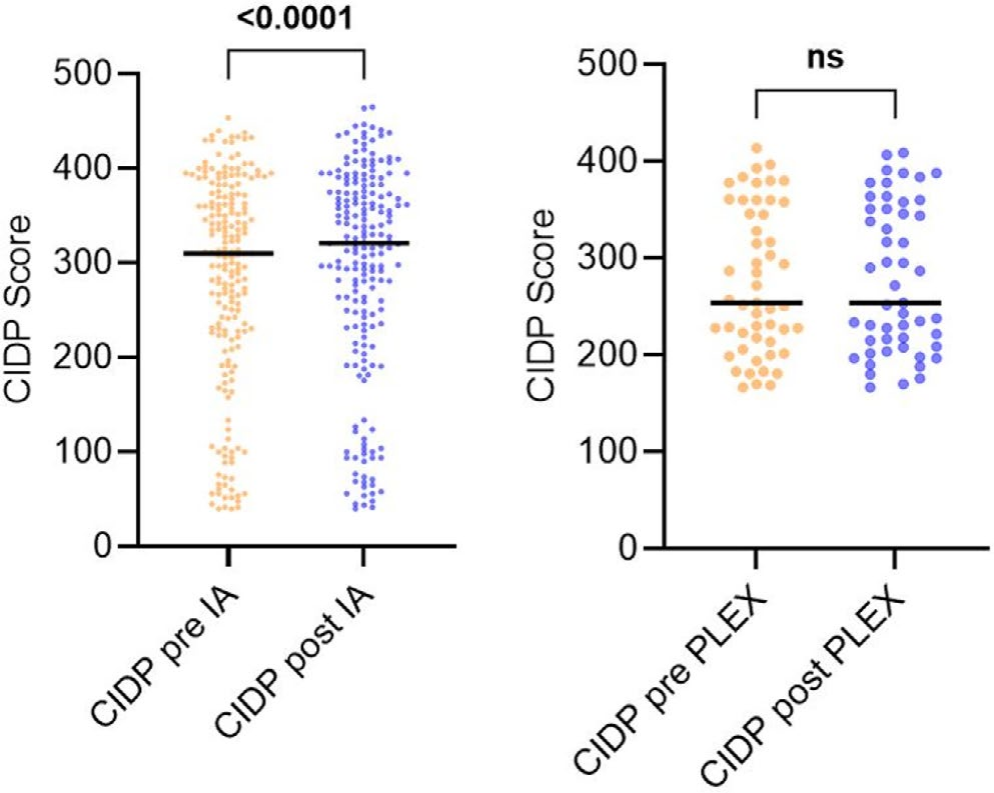
**FIGURE 2** Short‐term effects.

**Figure:**
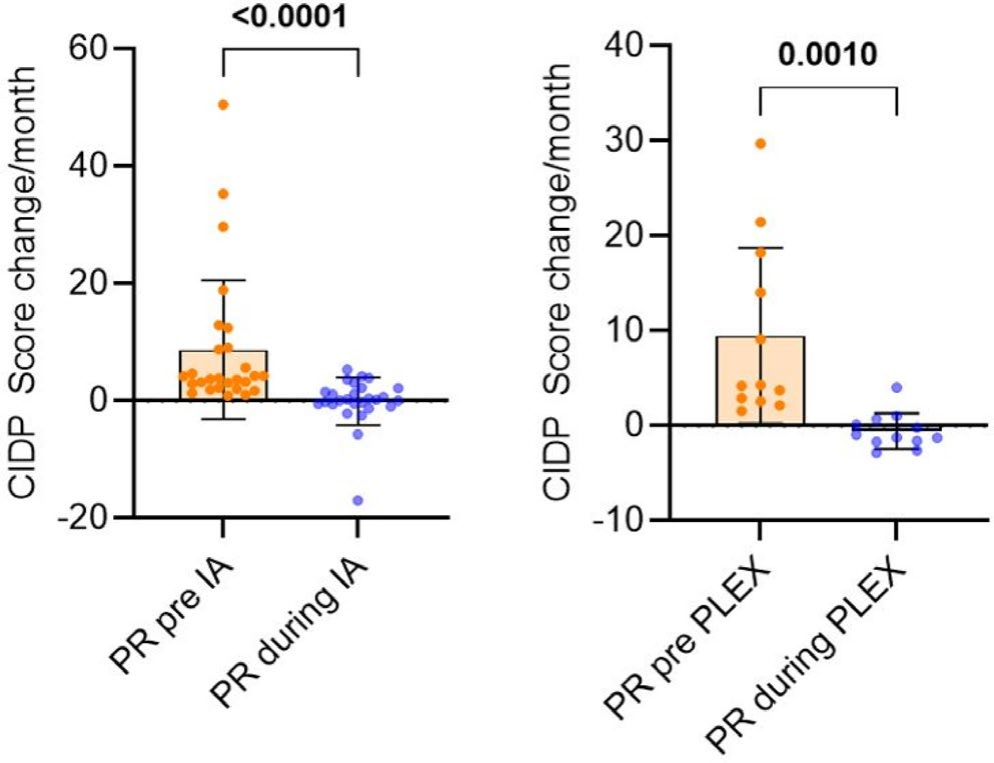
**FIGURE 3** Long‐term effects.

**Conclusion:** Our results suggest that repeated IA might constitute promising therapeutic options in patients with CIDP who do not sufficiently respond to corticosteroids and IVIGs, stabilizing the disease in the majority of patients.

**Disclosure:** JD reports research grants from Fresenius Medical Care GmbH, Fresenius Medical Care Deutschland GmbH, Miltenyi Biotec, and Diamed GmbH, as well as personal fees from Fresenius Medical Care GmbH, Fresenius Medical Care Deutschland GmbH, Miltenyi Biotec, Diamed GmbH, and argenx. ZE reports personal fees from Fresenius Medical Care GmbH. HT reports research and sponsoring grants from Fresenius Medical Care GmbH and Fresenius Medical Care Deutschland GmbH. MS has received honoraria for speaking and/or travel grants from Bayer, Biogen and TEVA and research funding from the Hertha‐Nathorff‐Program and University of Ulm, none related to this study. AR reports research grants from Deutsche Gesellschaft für Muskelkranke e. V. as well as personal fees from Amicus, Sanofi, Sun Pharmaceuticals, Argenx, Fulcrum, Alexion, outside the submitted work. CH, KK, OS, VR, LJ, LR, MW, TF, SJ, RG, JHW, and BM declare no competing interests.

## EPO‐0214

### Paediatric genetic neuropathies: Characterisation and molecular diagnosis in Central Portugal

#### J. Alves ^1^; J. Amaral^2^; A. Matos^1^; L. Almendra^1^; A. Geraldo^1^; I. Videira^1^; P. Almeida^3^; L. Ramos^3^; J. Ribeiro^2^

##### ^*1*^ *Neurology Department, Coimbra University Hospital, Coimbra Local Health Unit, Coimbra, Portugal;* ^*2*^ *Paediatric Neurology Department, Child Development Centre, Coimbra Paediatric Hospital, Coimbra Local Health Unit, Coimbra, Portugal;* ^*3*^ *Medical Genetics Department, Child Development Centre, Coimbra Paediatric Hospital, Coimbra Local Health Unit, Coimbra, Portugal*

**Background and aims:** Paediatric genetic neuropathies are a group of heterogeneous neuromuscular diseases that are often diagnosed late. This study aims to characterise patients with genetic neuropathy and evaluate the genetic tests that enabled diagnosis.

**Methods:** A retrospective study of cases since 2000 was conducted, focusing on those with genetic confirmation, with descriptive analyses of demographic, clinical data and diagnostic methods.

**Results:** We obtained a sample of 68 patients (53.5% male) with suspected genetic neuropathy, in which we identified pathogenic or likely pathogenic variants in 43 (median age of 20.9 years (IQR 16.0–29.5)). The median age at symptom onset was 4.5 years (1.7–8.0), and the median diagnostic delay was 6.7 years (IQR 2.3–11.8). A family history was present in 50% and consanguinity in 11.6%. Isolated neuropathies accounted for 81.4%, while 18.6% were associated with complex syndromes. Fifteen diagnoses were identified, most commonly CMT1A (48.8%), followed by HNPP (9.3%), and CMT4C, CMT‐SORD, CMT2A, Friedreich's ataxia and ARSACS (4.7% each); remaining diagnoses were single cases. Electrophysiology showed demyelinating neuropathy in 58.1%, axonal in 27.9% and intermediate in 2.3%, with sensory‐motor involvement in 76.7%. Genetic diagnosis was achieved by single‐gene testing in 65.1%, exome sequencing in 16.3%, gene panels in 11.6% and microarray in 4.7%. Prior genetic testing had been performed in 37.2%. Seven (10.8%) variants of uncertain significance were identified, for which we cannot reclassify their pathogenicity.

**Figure:**
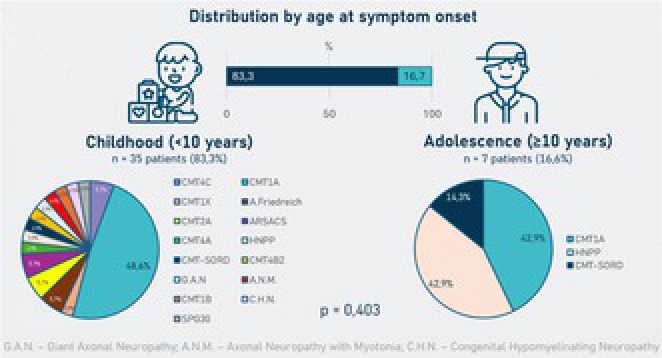
**FIGURE 1** Distribution of diagnoses by age at symptom onset.

**Figure:**
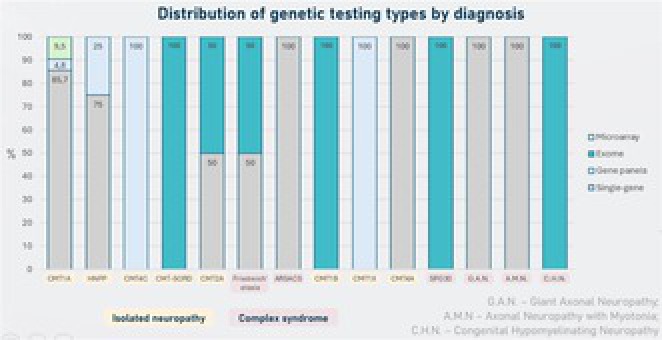
**FIGURE 2** Distribution of genetic testing types by diagnosis.

**Figure:**
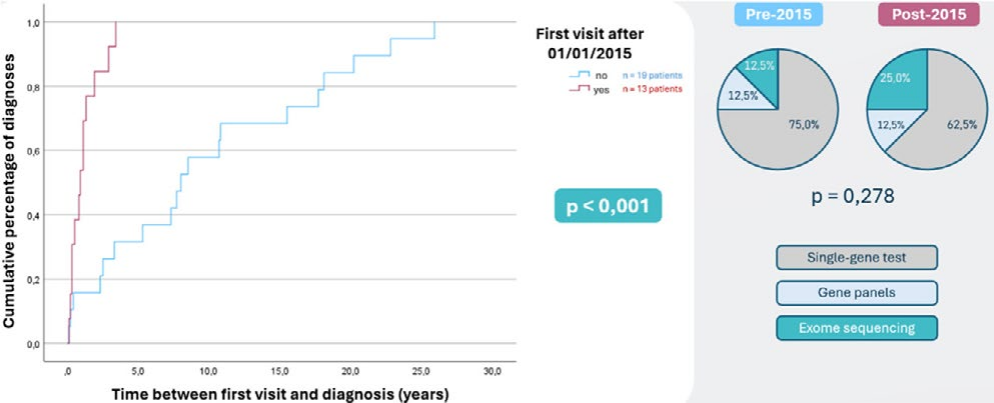
**FIGURE 3** Cumulative incidence of diagnosis: Pre‐ vs. Post‐2015 first visit cohorts.

**Conclusion:** Paediatric genetic neuropathies show marked heterogeneity and prolonged diagnostic delay. While targeted testing remains valuable in selected scenarios, advanced sequencing techniques are essential in complex cases, supporting individualised diagnostic strategies.

**Disclosure:** Nothing to disclose.

## EPO‐0215

### Long‐term real‐world use of intravenous immunoglobulin in CIDP and MMN

#### S. Pingel^1^; F. Klostermann^2^; M. Müngersdorf^3^; K. Pitarokoil^4^; A. Grimm^5^; F. Hoffmann^6^; M. Stettner ^7^

##### ^*1*^ *Grifols Deutschland GmbH, Frankfurt, Germany;* ^*2*^ *Department of Neurology and Experimental Neurology, Charité University Medicine Berli;* ^*3*^ *Private Practice of Neurology, Center for Movement Disorders and Diagnosis;* ^*4*^ *Neurological department, St. Josef Hospital, Ruhr University Bochum;* ^*5*^ *Department of Neurology, Department of Neurology, University Hospital Tuebingen, Hertie Center of Brain Research;* ^*6*^ *Department of Neurology, Martha‐Maria Hospital Halle‐Dölau;* ^*7*^ *Department of Neurology, University Medical Center Essen*

**Background and aims:** Intravenous immunoglobulin (IVIg) is recommended as a first‐line therapy for chronic inflammatory demyelinating polyradiculoneuropathy (CIDP) and multifocal motor neuropathy (MMN) according to EAN/PNS and EFNS/PNS guidelines. However, long‐term real‐world data on IVIg treatment and treatment decision‐making are limited and have thus been investigated during this study.

**Methods:** This non‐interventional, multicenter, longitudinal study evaluated treatment patterns in 84 patients (65 typical CIDP, 9 CIDP variants, 10 MMN) treated with IVIg (Gamunex® 10%) across 1,192 visits over up to 18 months. At each infusion cycle, physicians documented criteria for individual treatment decision making with regard of IVIg therapy.

**Results:** Patient and treatment characteristics are shown in Table 1 Treatment was continued without modification in 1,112 (93.3%) visits of which patients were evaluated clinically stable in 1,051 visits, supported by stable INCAT scores over time (Figure 1). Adaptations for medical reasons occurred in 22/84 (26.2%) patients, which were more frequent in patients with shorter time since diagnosis (≤ 1 year: adaptations in 36.4% of patients; > 1 year: 22.6%) and in patients with CIDP variants (adaptations in 55.6%) compared to typical CIDP (21.5%). Adverse drug reactions occurred in 8/1,176 Gamunex® 10% infusions (0.7%), were mild or moderate, and did not lead to any discontinuation.

**Figure:**
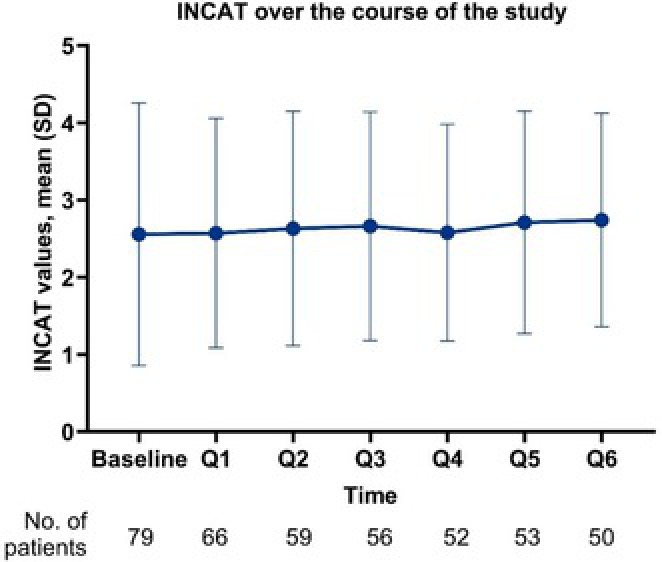
**FIGURE 1** INCAT measures remained stable over the course of the study (18 months).

**Figure:**
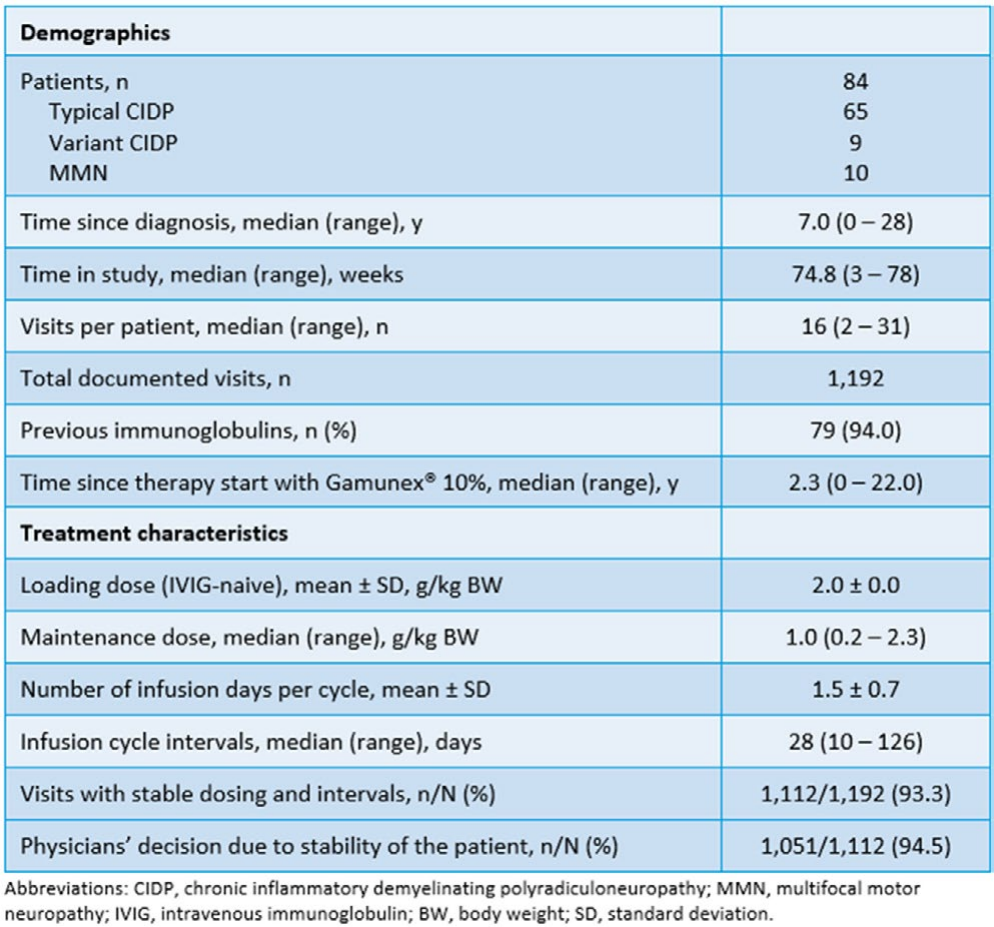
**TABLE 1** Patient demographics and treatment characteristics.

**Conclusion:** Therapeutic decisions were individualized, and therapy was adjusted during the observation period in a significant proportion of patients. Most patients remained clinically stable over 78 weeks. IVIg 10% demonstrated a favorable safety profile in real‐world practice.

**Disclosure:** The study was supported by Grifols, manufacturer of Gamunex®10%. SP: Employee at Grifols FK: Honoraria for lecturing and advisory activities from Abbvie, Stadapharm, Esteve, CSL Behring, Takeda, Zambon, Octapharma, and Argenx. MM: Served in advisory boards for Abbvie, Bial, Desitin, Zambon, UCB, Ipsen and Merz and received honoraria from Abbvie, Bial, UCB, Zambon, Merz, Ipsen, Sanofi‐Genyzme, Novartis, Merz and Takeda KP: Research funding from Biogen, CSL Behring, Grünenthal, GBS/CIDP Foundation International, LFB Group France and Ruhr University Bochum (FORUM) and has received speaker honoraria from ARGENX, CSL Behring, Pfizer, Grifols. AG: Received travel reimbursement and lecture fee by Grifols, CSL Behring, Argenx, Alexion, Alnylam, Akcea, AstraZeneca, Takeda, Pfizer, Johnson and Johnson and Amgen, not directly related to the research topic FH: Payment or honoraria for lectures, presentations, speakers bureaus, manuscript writing or educational events: Alexion, Bayer, Biogen, BMS, Grifols, Janssen, Merck, Novartis, Roche, Sanofi, TEVA. Support for attending meetings and/or travel: Merck, TEVA, Janssen MS: Served on the scientific advisory boards and/or received speaker honoraria, travel funding or honoraria for medical writing from Argenx; Bayer; Biogen Idec; Biotest; CSL Behring; Genzyme; Grifols; Immunovant; Kedrion; Merck; Novartis; Octapharma; PPTA; Roche; Sanofi‐Aventis; TEVA; UCB.

## EPO‐0216

### Neuropathic pain in RFC1‐related CANVAS: A systematic review and meta‐analysis

#### P. Neophytou ^1^; C. Avraam^3^; P. Zis^2^

##### ^1^ *Neurology Department, Nicosia General Hospital, Nicosia, Cyprus;* ^2^ *Medical School, University of Cyprus, Nicosia, Cyprus;* ^3^ *Neurosurgery Department, Nicosia General Hospital, Nicosia, Cyprus*

**Background and aims:** Cerebellar ataxia, neuropathy and vestibular areflexia syndrome (CANVAS), caused by biallelic intronic repeat expansions in RFC1, is a multisystem neurological disorder in which sensory neuronopathy is a core feature. Although neuropathic pain is increasingly reported in clinical cohorts and narrative reviews of RFC1‐related disease, its prevalence has not been systematically evaluated. We conducted a systematic review and meta‐analysis to determine the prevalence of neuropathic pain in RFC1‐related CANVAS.

**Methods:** A systematic literature search was performed on 13/01/26 using the terms (pain OR painful OR neuropathy OR ganglionopathy OR neuronopathy) AND (CANVAS OR RFC1). The search identified 243 records. After title and abstract screening, 146 articles underwent full‐text review. Eligible studies included genetically confirmed CANVAS reporting the presence neuropathic pain. Study selection followed PRISMA guidelines. Data were extracted independently by two reviewers. Statistically pooled proportion calculations were conducted in R language using the default settings of the “meta” package and the “metaprop” function with a random effects model, with heterogeneity assessed using the I^2^ statistic.

**Results:** The meta‐analysis included 433 patients with CANVAS. The pooled prevalence of sensory neuronopathy was 94.7% (95% CI 85.9%–98.2%). The pooled prevalence of neuropathic pain was 46.6% (95% CI 35.6%–57.9%). The heterogeneity was moderate (I^2^ = 73%).

**Figure:**
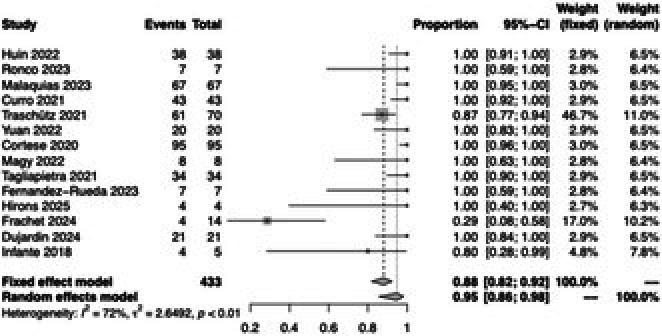
**FIGURE 1** Forest plot showing the pooled prevalence of sensory neuronopathy in patients with CANVAS across 14 studies. The pooled prevalence of sensory neuronopathy was 94.7% (95% CI 85.9%–98.2%).

**Figure:**
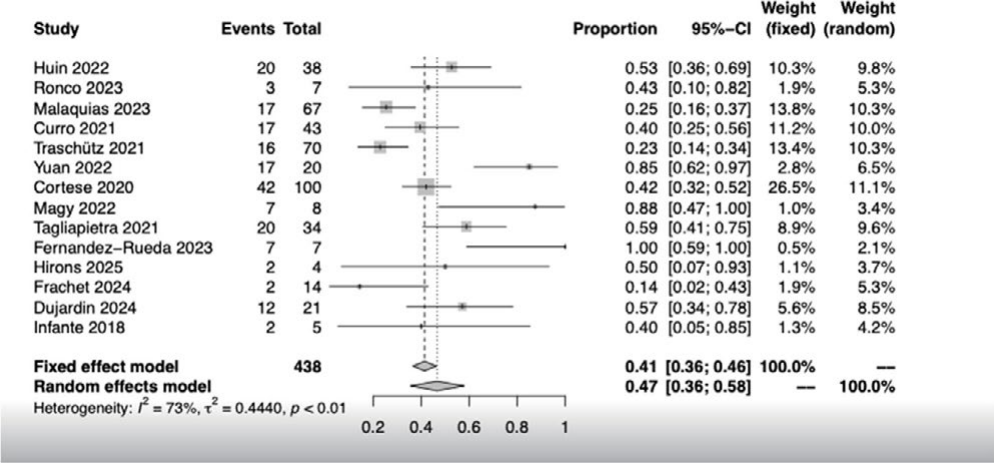
**FIGURE 2** Forest plot showing the pooled prevalence of pain in patients with CANVAS across 14 studies. The pooled prevalence of neuropathic pain was 46.6% (95% CI 35.6%–57.9%). The heterogeneity was moderate (I^2^ = 73%).

**Conclusion:** Neuropathic pain is common as it affects a substantial proportion of patients with RFC1‐related CANVAS. This systematic review and meta‐analysis provides the first pooled prevalence estimate of neuropathic pain in this disorder, highlighting the need for routine pain assessment and increased clinical awareness.

**Disclosure:** Nothing to disclose.

## EPO‐0217

### Electrical stimulation of spinal cord alleviates pain and restores sensory and autonomic functions in painful diabetic peripheral neuropathy

#### R. Martínez Marín ^1^; C. Rizea^1^; G. Gutiérrez^2^; B. Mansilla^3^; M. Román de Aragón^3^; H. Scholtes^4^; Q. Doan^4^; I. Huertas^4^; J. Paz Solís^3^

##### ^*1*^ *Servicio de Neurología. Hospital Universitario La Paz. Madrid. Spain;* ^*2*^ *Servicio de Neurología. Hospital Universitario Infanta Sofía. San Sebastian de los Reyes (Madrid). Spain;* ^*3*^ *Servicio de Neurocirugía. Hospital Universitario La Paz, Madrid, Spain;* ^*4*^ *Boston Scientific Neuromodulation, Valencia, USA*

**Background and aims:** Effective management of pain and other neurological symptoms remains challenging in painful Diabetic Peripheral Neuropathy (pDPN). We investigated the clinical and physiological effects of Spinal Cord Stimulation (SCS) in pDPN.

**Methods:** Twelve patients with “stocking‐and‐glove” pDPN were implanted with epidural electrodes at thoracic (T10‐T12) level. Assessments were performed pre‐ and post‐SCS implantation (3‐, 6‐ and 12‐months) and included questionnaires related to pain, neuropathic and autonomic symptoms and neurological tests examining large‐ and small‐fibers such as sensory and motor nerves conduction studies (NCS), sensorimotor physical exam, limbs skin conductance and thermography.

**Results:** Baseline assessments confirmed a severe neuropathy profile denoted by impaired pin sensation test and abundant NCS abnormalities: 40/96 of sensory and 15/72 of motor nerves with absent response. After implant, patients reported significant relief in pain (VRS from 8.2‐to‐2.0) neuropathic (NPSI from 50‐to‐15) and autonomic symptoms (BASQ from 28‐to‐14). We observed a progressive recovery in nerve function at both large‐ and small‐fibers. Specifically, to last visit, 29/40 (72%) of sensory and 3/15 of motor responses became detectable (∆SNAP~2μV). Also, in responding nerves, there were noticeable conduction improvements (e.g. ~∆SNCV at sural (+2.7 m/s), sup. peroneal (+4.1 m/s) and radial (+5.5 m/s)). This was congruent with improvements in large‐fiber‐related symptoms (e.g. dysesthesia from 7.2‐to‐2.5).

**Figure:**
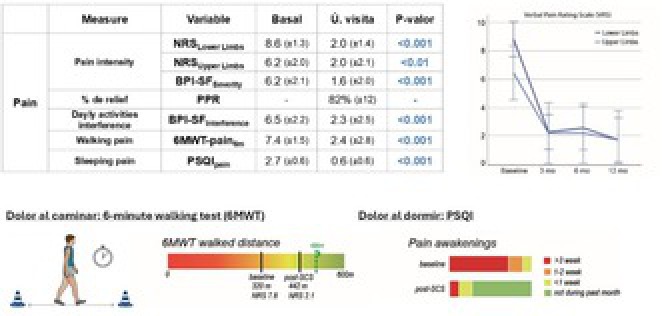
**FIGURE 1** Pain results.

**Figure:**
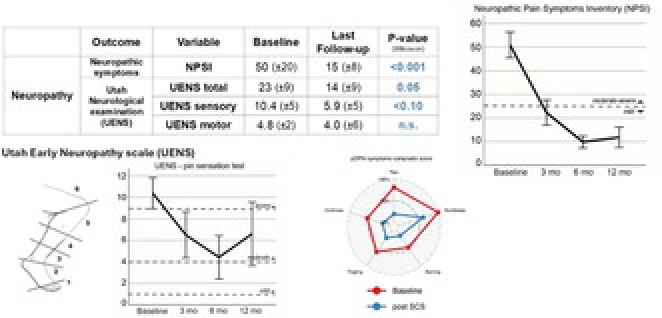
**FIGURE 2** Neuropathic symptoms and clinical examination.

**Figure:**
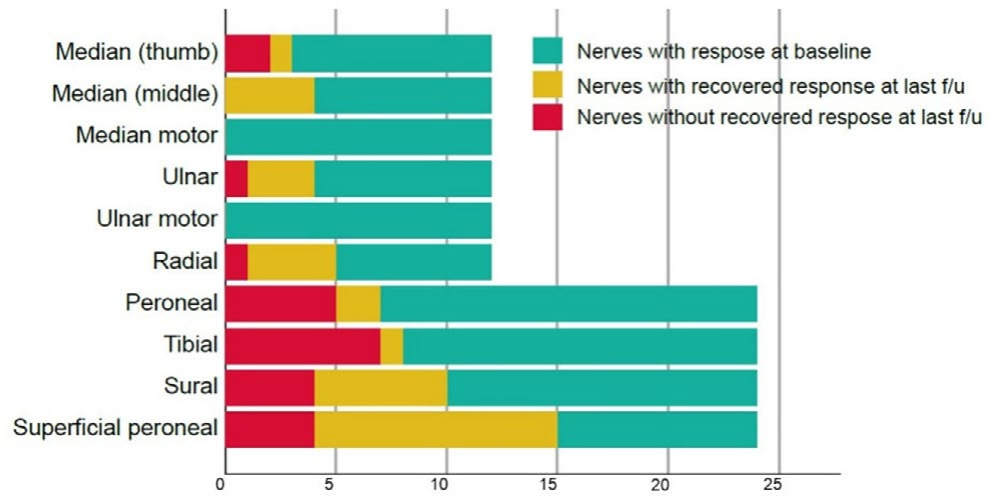
**FIGURE 3** Nerve conduction‐response recovery studies.

**Conclusion:** Our results indicate that SCS is able to not only effectively treat pain and other neurological symptoms but also to produce a quantifiable gain in nerve function in pDPN. This suggests that SCS might cause changes in excitability as well as, potentially, some degree of regeneration of nerve fibers.

**Disclosure:** This study was sponsored by Boston Scientific.

## EPO‐0218

### Empasiprubart versus placebo (EMNERGIZE) or immunoglobulin (EMVIGORATE) in chronic inflammatory demyelinating polyradiculoneuropathy: Study designs

#### S. Rinaldi ^1^; T. H. Brannagan^2^; P. Doneddu^3^; K. L. Gable^4^; L. Querol^5^; M. Stettner^6^; K. Budding^7^; S. Ellor^7^; H. Haddad^7^; M. Markov^7^; R. Patel^7^; O. Van de Steen^7^; I. Van de Walle^7^; J. A. Allen^8^

##### ^*1*^ *University of Oxford, Oxford, UK;* ^*2*^ *Vagelos College of Physicians & Surgeons, Columbia University Irving Medical Center, Columbia University, New York, USA;* ^*3*^ *Neuromuscular and Neuroimmunology Unit, IRCCS Humanitas Research Hospital, Milan, Italy; Humanitas University, Pieve Emanuele, Milan, Italy;* ^*4*^ *Duke University Medical Center, Duke University, Durham, USA;* ^*5*^ *Neuromuscular Diseases Unit, Hospital de La Santa Creu I Sant Pau, Universitat Autònoma de Barcelona, Barcelona, Spain;* ^*6*^ *University Medicine Essen, Essen, Germany;* ^*7*^ *argenx, Ghent, Belgium;* ^*8*^ *University of Minnesota, Minneapolis USA*

**Background and aims:** Chronic inflammatory demyelinating polyradiculoneuropathy (CIDP) is an immune‐mediated neuropathy involving complement‐driven macrophage‐mediated demyelination. Empasiprubart binds complement C2, blocking activation of classical and lectin complement pathways. Two phase 3, randomised, double‐blinded studies in participants with CIDP will compare intravenous (IV) empasiprubart versus placebo (EMNERGIZE, NCT07091630) and IV empasiprubart versus IV immunoglobulin (IVIg) (EMVIGORATE, NCT06920004).

**Methods:** EMNERGIZE (placebo‐controlled) will randomise ~160 adults with CIDP 2:1 to receive either empasiprubart or placebo on Days 1 and 8 then once every 4 weeks in a 24‐week double‐blind treatment period (part A) (Figure 1). EMVIGORATE (double‐dummy) will randomise ~218 adults with CIDP 1:1 on stable maintenance IVIg to receive either empasiprubart or continue the stable IVIg dose in a 24‐week double‐blind treatment period (part A) (Figure 2). Both studies are followed by a 4‐week double‐blind rollover, 23‐month open‐label period (part B) and 15‐month safety follow‐up.

**Results:** The primary endpoint for both studies is a reduction of > = 1 point versus baseline in adjusted Inflammatory Neuropathy Cause and Treatment (aINCAT) score at Week 24. Key secondary endpoints (Week 24) include changes from baseline in Inflammatory Rasch‐built Overall Disability Scale score, Medical Research Council sum score, dominant hand grip strength, Timed Up and Go and time to reduction of > = 1 point versus baseline in aINCAT score.

**Conclusion:** These two global phase 3 studies will evaluate the efficacy and safety of empasiprubart in participants with CIDP. Similar designs and endpoints with complementary study populations support the potential role of empasiprubart as a reference treatment across patients with CIDP, regardless of treatment status.

**Figure:**
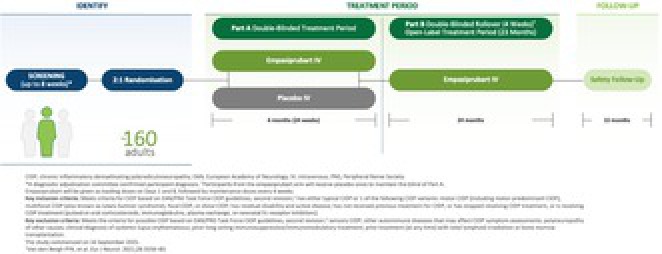
**FIGURE 1** EMNERGIZE study design.

**Figure:**
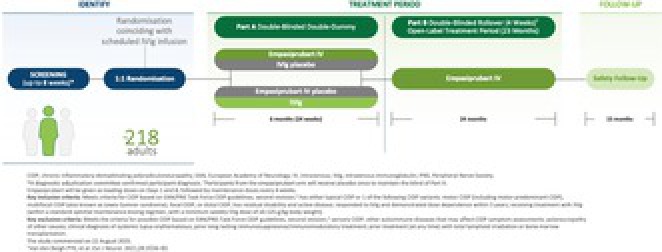
**FIGURE 2** EMVIGORATE study design.

**Disclosure:** Simon Rinaldi: Annexon, argenx, the Beijing Association of Holistic and Integrated Medicine, British Medical Association, CSL Behring, Dianthus, Excemed, Fresenius, GBS/CIDP Foundation International, Guillain‐Barré Syndrome and Related Inflammatory Neuropathies (GAIN) charity, Hansa Biopharma, the Irish Institute of Clinical Neuroscience, Medical Research Council (UK), National Institute of Health Research (NIHR), the Pathological Society of Great Britain & Ireland, Peripheral Nerve Society, Takeda, UCB, the University of Oxford's John Fell Fund, Wellcome Trust. Thomas H. Brannagan: argenx, Immunovant, Janssen, Sanofi. Pietro Emiliano Doneddu: argenx, CSL Behring, GBS‐CIDP Foundation International, Kedrion, Takeda. Karissa L. Gable: Annexon, argenx, CSL Behring, Dianthus, Grifols, Immunovant, Sanofi, Takeda. Luis Querol: Alnylam, Annexon, argenx, Avilar, Biogen, CIBERER, CSL Behring, Dianthus, Fundació La Marató, GBS‐CIDP Foundation International, Grifols, Instituto de Salud Carlos III – Ministry of Economy and Innovation (Spain), Janssen, LFB, Lundbeck, Merck, Novartis, Octapharma, Roche, Sanofi, UCB. Mark Stettner: argenx, Bayer, Biogen Idec, Biotest, CSL Behring, Genzyme, Grifols, Immunovant, Kedrion, Merck, Novartis, Octapharma, PPTA, Roche, Sanofi‐Aventis, TEVA, UCB. Kevin Budding, Susan Ellor, Hafedh Haddad, Martin Markov, Reena Patel, Olivier Van de Steen, Inge Van de Walle: Employees of argenx. Jeffrey A. Allen: Akcea, Alexion, Alnylam, Annexon, argenx, CSL Behring, Grifols, Immunovant, Immupharma, Johnson & Johnson, Pfizer, Takeda.

## EPO‐0219

### NF 140 and NF 186 positive nodpathy masquerading as GBS

#### S. Ratnasababathy

##### Department of General Medicine, Sundaram Medical Foundation, Chennai, India

**Background and aims:** Nodal and para‐nodal disorders are often misdiagnosed as GBS or CIDP. Diagnosis relies on clinical suspicion and re‐calibration of decisions in patients earlier diagnosed as GBS or CIDP not responding to treatment.

**Methods:** Nodal and para‐nodal disorders can have clinical features and nerve conduction abnormalities that mimic GBS or CIDP but there is no demyelination. The pathology is at the level of the node of Ranvier. These patients have poor responses to IVIG. This case report describes a 19 year old boy who presented after 10 days of difficulty walking and a preceding history of respiratory infection. He was diagnosed initially with GBS based on nerve conduction studies and CSF analysis. 1 year later, on further evaluation considering poor response to IVIG he was found to be positive for antibodies to Neurofascin 140 and 186 and was diagnosed with nodopathy.

**Results:** The patient was treated with 2 doses of Rituximab and he improved neurologically from mobility with walker to independent ambulation.

**Conclusion:** Nodal and para‐nodal disorders are now part of the spectrum of inflammatory neuropathies. This case reiterates the need to suspect nodopathies among other causes in patients earlier diagnosed with GBS/ CIDP with inadequate response to conventional IVIG or steroids. Whether antibody testing for nodopathy should be part of initial evaluation in patients with features of GBS/ CIDP needs further speculation.

**Disclosure:** Nothing to disclose.

## Cognitive Neurology/Neuropsychology 2

## EPO‐0220

### Cerebellar cognitive affective syndrome in hypertrophic olivary degeneration associated progressive ataxia and palatal tremor – A case series

#### B. Vlad ^1^; J. Hartung^1^; C. Sommer^2^; K. Schregel^2^; A. Günther^1^; P. Bublak^1^

##### ^*1*^ *Department of Neurology, Jena University Hospital, Jena, Germany;* ^*2*^ *Department of Neuroradiology, Institute of Diagnostic and Interventional Radiology, Jena University Hospital, Jena, Germany*

**Background and aims:** Hypertrophic olivary degeneration (HOD) is a rare trans‐synaptic degeneration of the inferior olivary nucleus caused by lesions within the Guillain–Mollaret triangle (GMT). This pathway, connecting inferior olivary nucleus, red nucleus, and dentate nucleus, plays a key role in cerebellar motor control. Lesions of the GMT may impair coordination, articulation, and precision movements. Previous studies have focused primarily on motor manifestations of HOD, particularly progressive ataxia and palatal tremor (PAPT), the characteristic clinical features. Potential cognitive consequences of cerebellar involvement remain largely unexplored. Only a single case report has described a cerebellar cognitive affective syndrome in HOD. We aimed to systematically assess cerebellar cognitive impairment in a series of three patients with HOD.

**Methods:** We conducted a monocentric retrospective case series of three patients with HOD who underwent multiparametric brain MRI including diffusion tensor imaging (DTI) and comprehensive neuropsychological assessment.

**Results:** All patients were male, aged 57–75 years. An autoimmune‐paraneoplastic etiology was suspected in two patients, and one had a post‐hemorrhagic structural lesion. Clinically, all presented with PAPT, with disease duration of 1.5–2 years. MRI revealed bilateral HOD in two patients. None of the patients reported subjective cognitive complaints, and global cognitive status was normal. However, all fulfilled criteria for definite cerebellar cognitive impairment on the CCAS‐Scale. Notably, despite normal semantic fluency and absence of language impairment, all showed deficits in switching between semantic categories.

**Conclusion:** This case series suggests that HOD‐associated PAPT may involve the GMT in cognitive functions, particularly executive aspects of language production, highlighting a broader cerebellar role beyond motor control.

**Disclosure:** The authors report nothing to disclose.

## EPO‐0221

### Emotion recognition deficits in frontotemporal degeneration: Exploring brain functional connectivity and emotional reserve

#### C. Tripodi^1^; E. Canu ^2^; A. Gilioli^3^; A. Bianchi^3^; S. Basaia^3^; V. Castelnovo^2^; E. Sibilla^2^; F. Freri^3^; E. Spinelli^4^; G. Cecchetti^2^; A. Ghirelli^4^; S. Pisano^2^; G. Rugarli^4^; G. Magnani^5^; F. Caso^5^; E. Aiello^6^; G. De Luca^6^; P. Caroppo^7^; S. Prioni^8^; C. Villa^7^; L. Tremolizzo^9^; I. Appollonio^9^; F. Verde^10^; N. Ticozzi^10^; V. Silani^10^; M. Gorno‐Tempini^11^; B. Poletti^12^; M. Filippi^13^; F. Agosta^4^

##### ^*1*^ *Neuroimaging Research Unit, Division of Neuroscience, IRCCS San Raffaele Scientific Institute, Milan, Italy; and Vita‐Salute San Raffaele University, Milan, Italy;* ^*2*^ *Neuroimaging Research Unit, Division of Neuroscience, and Neurology Unit, IRCCS San Raffaele Scientific Institute, Milan, Italy;* ^*3*^ *Neuroimaging Research Unit, Division of Neuroscience, IRCCS San Raffaele Scientific Institute, Milan, Italy;* ^*4*^ *Neuroimaging Research Unit, Division of Neuroscience, and Neurology Unit, IRCCS San Raffaele Scientific Institute, Milan, Italy; and Vita‐Salute San Raffaele University, Milan, Italy;* ^*5*^ *Neurology Unit, IRCCS San Raffaele Scientific Institute, Milan, Italy;* ^*6*^ *Department of Neurology and Laboratory of Neuroscience, IRCCS Istituto Auxologico Italiano, Milan, Italy;* ^*7*^ *Fondazione IRCCS Istituto Neurologico Carlo Besta, Unit of Neurology 5 – Neuropathology, Milan, Italy;* ^*8*^ *Fondazione IRCCS Istituto Neurologico Carlo Besta, Clinical Neuropsychology Unit, Milan, Italy;* ^*9*^ *Neurology Unit, IRCCS “Fondazione San Gerardo” and School of Medicine and Surgery, University of Milano‐Bicocca, Monza, Italy;* ^*10*^ *Department of Neurology and Laboratory of Neuroscience, IRCCS Istituto Auxologico Italiano, Milan, Italy; and Department of Pathophysiology and Transplantation, “Dino Ferrari” Center, Università degli Studi di Milano, Milan, Italy;* ^*11*^ *Memory and Aging Center, and Global Brain Health Institute, University of California San Francisco, San Francisco, USA;* ^*12*^ *Department of Neurology and Laboratory of Neuroscience, IRCCS Istituto Auxologico Italiano, Milan, Italy; and Department of Oncology and Hemato‐Oncology, Università degli Studi di Milano, Milano, Italy;* ^*13*^ *Neuroimaging Research Unit, Division of Neuroscience, Neurology Unit, Neurorehabilitation Unit, and Neurophysiology Service, IRCCS San Raffaele Scientific Institute, Milan, Italy; and Vita‐Salute San Raffaele University, Milan, Italy*

**Background and aims:** By examining emotion recognition (ER), direct functional connectivity (dFC) of ER‐related networks, and their relationship in frontotemporal degeneration (FTD), this study uncovers the neural underpinnings of socioemotional variability and explores emotional reserve, defined as better‐than‐predicted ER based on dFC.

**Methods:** 109 FTD patients [46 behavioral (bvFTD) and 10 semantic behavioral (sbvFTD); 13 nonfluent (nfvPPA) and 18 semantic (svPPA) primary progressive aphasia; 22 progressive supranuclear palsy (PSP)] and 109 HC completed the Comprehensive Affect Testing System‐abbreviated (CATS‐A), yielding discrete and composite ER scores. Resting‐state functional MRI was analyzed in 92 patients and 97 HC, by extracting dFC and nodal graph metrics across six ER‐related networks [salience (SN), semantic appraisal, anterior default mode (aDMN), visuo‐associative, sensorimotor (SMN), basal ganglia] reconstructed in 50 independent HC. Correlations between ER scores and network properties were examined. Emotional reserve was evaluated using a residual‐based approach.

**Results:** All FTD showed reduced positive and negative ER versus HC. SvPPA outperformed bvFTD and nfvPPA in happiness; sbvFTD performed worse than svPPA in disgust. FTDs exhibited altered dFC properties in SN, SMN, visuo‐associative and aDMN. In FTD, lower negative ER correlated with reduced aDMN integration. Emotional reserve analysis revealed lower residuals in FTD versus HC, for comparable SN connectivity, reflecting reduced emotional reserve.

**Conclusion:** FTD showed widespread ER deficits, particularly for negative emotions, with distinct temporal‐variants (svPPA vs sbvFTD) profiles. Findings highlight aDMN involvement in negative ER and identify reduced emotional reserve as a potential marker of socioemotional vulnerability in FTD.

**Disclosure:** Supported by European Research Council (StG‐2016_714388_NeuroTRACK); Foundation Research on Alzheimer Disease. Co‐funding Next Generation EU [DM 1557 11.10.2022]. C. Tripodi, A. Gilioli, A. Bianchi, V. Castelnovo, E. Sibilla, F. Freri, G. Magnani, F. Caso, E.N. Aiello, G. De Luca, P. Caroppo, S. Prioni, C. Villa, L. Tremolizzo, I. Apollonio, F. Verde, N. Ticozzi, V. Silani, M.L. Gorno‐Tempini, B. Poletti report no competing interests; E. Canu and S. Basaia received research support from the Italian Ministry of Health; E.G. Spinelli received honoraria from Eli Lilly; G. Cecchetti M. received honoraria from Eli Lilly and Neopharmed Gentili; M. Filippi is Editor‐in‐Chief of the Journal of Neurology, Associate Editor of Human Brain Mapping, Neurological Sciences, and Radiology; received compensation for consulting services from Almirall, Biogen, Bristol‐Myers Squibb, Eli Lilly, Merck, Novartis, Roche, Sanofi; speaking activities from Amgen, Bayer, Biogen, Bristol‐Myers Squibb, Celgene, Chiesi Italia SpA, Eisai, Eli Lilly, Fujirebio, Genzyme, Janssen, Merck, Neopharmed Gentili, Neuraxpharm, Novartis, Novo Nordisk, Roche, Sanofi, Takeda; participation in Advisory Boards for Alexion, Biogen, Bristol‐Myers Squibb, Eli Lilly, GE Healthcare Ltd, Merck, Neuraxpharm, Novartis, Roche, Sandoz, Sanofi, Takeda; scientific direction of educational events for Biogen, Merck, Roche, Celgene, Bristol‐Myers Squibb, Lilly, Novartis, Sanofi‐Genzyme; he receives research support from Biogen Idec, Merck‐Serono, Novartis, Roche, the Italian Ministry of Health, the Italian Ministry of University and Research, and Fondazione Italiana Sclerosi Multipla. F. Agosta is Associate Editor of NeuroImage: Clinical, has received speaker honoraria from Biogen Idec, Italfarmaco, Roche, Zambon and Eli Lilly, and receives or has received research supports from the Italian Ministry of Health, the Italian Ministry of University and Research, AriSLA (Fondazione Italiana di Ricerca per la SLA), the European Research Council, the EU Joint Programme – Neurodegenerative Disease Research (JPND), and Foundation Research on Alzheimer Disease (France).

## EPO‐0222

### **Identifying individuals at‐risk for Alzheimer**'**s Disease: The role of eye‐tracking in the NAP4A digital platform**

#### C. Tripodi^1^; E. Canu ^2^; A. Gilioli^3^; S. Musicco^3^; L. Flematti^4^; M. Nouri Janian^4^; M. Zullo^4^; A. Sanna^4^; F. Agosta^5^; M. Filippi^6^

##### ^*1*^ *Neuroimaging Research Unit, Division of Neuroscience, IRCCS San Raffaele Scientific Institute, Milan, Italy; and Vita‐Salute San Raffaele University, Milan, Italy;* ^*2*^ *Neuroimaging Research Unit, Division of Neuroscience, and Neurology Unit, IRCCS San Raffaele Scientific Institute, Milan, Italy;* ^*3*^ *Neuroimaging Research Unit, Division of Neuroscience, IRCCS San Raffaele Scientific Institute, Milan, Italy;* ^*4*^ *Center for Advanced Technology in Health and Wellbeing, IRCCS San Raffaele Scientific Institute, Milan, Italy;* ^*5*^ *Neuroimaging Research Unit, Division of Neuroscience, and Neurology Unit, IRCCS San Raffaele Scientific Institute, Milan, Italy; and Vita‐Salute San Raffaele University, Milan, Italy;* ^*6*^ *Neuroimaging Research Unit, Division of Neuroscience, Neurology Unit, Neurorehabilitation Unit, and Neurophysiology Service, IRCCS San Raffaele Scientific Institute, Milan, Italy; and Vita‐Salute San Raffaele University, Milan, Italy*

**Background and aims:** Early identification of individuals with amnestic mild cognitive impairment (aMCI) at risk of Alzheimer's disease (AD) requires sensitive digital biomarkers beyond standard neuropsychological testing. Eye‐tracking may detect subtle changes in attentional and mnemonic processing preceding overt cognitive decline. This study reports the first validation of the Neurocognitive Assessment Platform for Alzheimer's disease (NAP4A), integrating eye‐tracking and cognitive assessment.

**Methods:** Twenty‐one healthy controls were assessed at baseline, 6 months, and 12 months using the NAP4A platform, which collects physiological and behavioral data during cognitive tasks. Participants completed the CANTAB battery and a visual memory task involving personal/autobiographical and unfamiliar neuroaesthetic (“Neffie”) images. Eye movements were recorded with a TOBII™ eye tracker (90 Hz). Fixation number, fixation duration, saccade number, and saccade distance were analyzed. Wilcoxon signed‐rank tests compared encoding versus recall phases and stimulus types. Cognitive performance was examined longitudinally.

**Results:** CANTAB performance remained stable across timepoints, with no significant cognitive changes (*p* > 0.05). Eye‐tracking showed greater visual exploration during encoding than recall, with increased fixation number, fixation duration, saccade number, and saccade distance (*p* < 0.001). Neffie images elicited more fixations and shorter fixation durations than personal stimuli (*p* < 0.001), with no saccade differences.

**Conclusion:** Stable cognition over 12 months aligns with expected trajectories in healthy individuals. Eye‐tracking findings indicate higher attentional demands during encoding and greater exploration of unfamiliar stimuli, supporting eye‐tracking as a sensitive marker for early AD risk detection.

**Disclosure:** C. Tripodi, A. Gilioli, S Musicco, L Flematti, M Nouri Janian, M Zullo, A Sanna report no competing interests; E. Canu received research support from the Italian Ministry of Health; F. Agosta is Associate Editor of NeuroImage: Clinical, has received speaker honoraria from Biogen Idec, Italfarmaco, Roche, Zambon and Eli Lilly, and receives or has received research supports from the Italian Ministry of Health, the Italian Ministry of University and Research, AriSLA (Fondazione Italiana di Ricerca per la SLA), the European Research Council, the EU Joint Programme – Neurodegenerative Disease Research (JPND), and Foundation Research on Alzheimer Disease (France). M. Filippi is Editor‐in‐Chief of the Journal of Neurology, Associate Editor of Human Brain Mapping, Neurological Sciences, and Radiology; received compensation for consulting services from Almirall, Biogen, Bristol‐Myers Squibb, Eli Lilly, Merck, Novartis, Roche, Sanofi; speaking activities from Amgen, Bayer, Biogen, Bristol‐Myers Squibb, Celgene, Chiesi Italia SpA, Eisai, Eli Lilly, Fujirebio, Genzyme, Janssen, Merck, Neopharmed Gentili, Neuraxpharm, Novartis, Novo Nordisk, Roche, Sanofi, Takeda; participation in Advisory Boards for Alexion, Biogen, Bristol‐Myers Squibb, Eli Lilly, GE Healthcare Ltd, Merck, Neuraxpharm, Novartis, Roche, Sandoz, Sanofi, Takeda; scientific direction of educational events for Biogen, Merck, Roche, Celgene, Bristol‐Myers Squibb, Lilly, Novartis, Sanofi‐Genzyme; he receives research support from Biogen Idec, Merck‐Serono, Novartis, Roche, the Italian Ministry of Health, the Italian Ministry of University and Research, and Fondazione Italiana Sclerosi Multipla.

## EPO‐0223

### Feasibility study of the European inter‐societal recommendations for the biomarker‐based diagnosis of dementia: The multi‐center EuroValiCo project

#### I. Trescato^1^; M. Barbi^1^; C. Festari^1^; S. Orini^2^; M. Cotta Ramusino^3^; A. Kilpeläinen^4^; C. Manco^5^; F. Massa ^6^; K. Miettinen^4^; M. Pievani^7^; A. Sangiorgio^5^; A. Silva‐Spinola^8^; D. Plantone^5^; M. Riverol^9^; A. Solomon^4^; I. Santana^8^; E. Solje^4^; R. Gatta^10^; G. Frisoni^11^

##### ^*1*^ *Neuropsychology Unit, IRCCS Istituto Centro San Giovanni di Dio Fatebenefratelli, Brescia, Italy;* ^*2*^ *Alzheimer's Unit, Memory Clinic, IRCCS Istituto Centro San Giovanni di Dio Fatebenefratelli, Brescia, Italy;* ^*3*^ *Unit of Behavioral Neurology and Dementia Research Center, IRCCS Mondino Foundation, Pavia, Italy;* ^*4*^ *Institute of Clinical Medicine – Neurology, University of Eastern Finland, Kuopio, Finland/Neuro Center – Neurology, Kuopio University Hospital, Kuopio, Finland;* ^*5*^ *Department of Medicine, Surgery and Neuroscience, University of Siena, Siena, Italy;* ^*6*^ *Department of Neuroscience, Rehabilitation, Ophthalmology, Genetics, Maternal and Child Health (DINOGMI), University of Genoa, Genoa, Italy/IRCCS Policlinico San Martino, IRCCS Policlinico San Martino, Genoa, Italy;* ^*7*^ *Lab Alzheimer's Neuroimaging & Epidemiology, IRCCS Istituto Centro San Giovanni di Dio Fatebenefratelli, Brescia, Italy;* ^*8*^ *Faculty of Medicine/Neurology Service/Center for Innovative Biomedicine and Biotechnology, University of Coimbra/Centro Hospitalar e Universitário de Coimbra, Coimbra, Portugal;* ^*9*^ *Neurology Department, Clínica Universidad de Navarra, Pamplona, Spain;* ^*10*^ *Department of Clinical and Experimental Sciences, Università degli Studi di Brescia, Brescia, Italy;* ^*11*^ *Memory Centre, Division of Geriatrics and Rehabilitation, University Hospitals of Geneva, Geneva, Switzerland/Laboratory of Neuroimaging of Aging (LANVIE), University of Geneva, Geneva, Switzerland*

**Background and aims:** The increasing availability of disease‐modifying therapies for neurocognitive disorders has intensified the need for accurate and timely diagnosis supported by biomarker‐based approaches. Recent European intersocietal recommendations (Frisoni, 2023) proposed a structured, phenotype‐driven diagnostic workflow embedding biomarker use within a stepwise clinical pathway. This preliminary analysis from the EuroValiCo study aim to characterise real‐world diagnostic pathways in European memory clinics and to evaluate concordance between recommended and observed biomarker use.

**Methods:** EuroValiCo is an ongoing multicentre observational study, aiming at validating the European recommendations in clinical practice. Medical charts of consecutive patients at first clinical access for cognitive complaints in 2022 and 2024 are retrospectively reviewed, representing pre‐ and post‐recommendation cohorts. Demographic, clinical, neuropsychological, imaging, and biomarker data are collected. Process discovery techniques were applied to reconstruct real‐world diagnostic pathways and assess concordance between recommended and observed biomarker use.

**Results:** Recommendations indicate cerebrospinal fluid (CSF) analysis as the first‐line biomarker for suspected Alzheimer's disease (AD), FDG‐PET for frontotemporal lobar degeneration (FTLD) and tauopathies, and DAT‐SPECT for Lewy body disease (LBD). In a preliminary cohort of 268 patients (49% women; median age 72 years, range 48–85), concordance with recommendations was observed in 71% of suspected AD cases, 57% of FTLD cases, 0% of tauopathy cases, and 50% of LBD cases.

**Conclusion:** These findings provide insight into the alignment between guideline‐recommended and real‐world biomarker use, revealing substantial condition‐specific differences, with the highest concordance observed in suspected AD. Future analyses will assess adherence across the full diagnostic process, inter‐centre variability, and the impact of recommendation implementation.

**Disclosure:** We acknowledge unrestricted grants from EISAI Europe LTD, GE HealthCare LTD, and Novo Nordisk. The funders had no role in the conception, design, and implementation of the project nor in data collection, data analysis, interpretation, or discussion of the results. Funders had no privileged access to the project's outputs at any stage. CF reports institutional research funding from the Alzheimer's Association and the Italian Ministry of Health, and personal fees from Roche for speaker activities. MP reports institutional research funding from the Italian Ministry of Health (Ricerca Corrente), provided to her institution. ES has served on the advisory board of Novartis, Eisai, Eli Lilly and Roche, served as a consultant for Novo Nordisk, Eli Lilly, Eisai, BioArctic and Roche and received honoraria from lectures from Lundbeck, BioaArctic, TEVA, Eli Lilly and Roche and received travel support from Eli Lilly and Merck and received research funding from Roche and Eli Lilly. E Solje is editor of Alzheimer's Research & Therapy and associate editor of Journal of Alzheimer's disease. GBF reports institutional funding through the Private Foundation of Geneva University Hospitals from A.P.R.A. – Association Suisse pour la Recherche sur la Maladie d'Alzheimer, Fondation Segré, Ivan Pictet, Race Against Dementia Foundation, Fondation Child Care, Fondation Edmond J. Safra, Fondation Minkoff, Fondazione Agusta, McCall Macbain Foundation, Nicole et René Keller, and Fondation AETAS; institutional funding through the University of Geneva or Geneva University Hospitals for investigator‐initiated studies from Roche, OM Pharma, Eisai, Biogen, and Novo Nordisk, and for competitive research projects from H2020, the Innovative Medicines Initiative (IMI/IMI2), the Swiss National Science Foundation, and the VELUX Foundation; institutional consulting fees from Biogen, Diadem, Eisai, Lilly, Roche, and Synaptica; institutional honoraria for lectures and educational activities from Biogen, Roche, Novo Nordisk, and GE HealthCare; and participation on an advisory board for Acumen.

## EPO‐0224

### **Data‐efficient AI models for predicting three‐year cognitive decline in early Alzheimer**'**s disease**

#### M. Zeynali ^1^; X. Deng^2^; N. Kandiah^3^; S. Saffari^1^

##### ^*1*^ *Centre for Biomedical Data Science, Duke‐NUS Medical School, National University of Singapore, Singapore, Singapore;* ^*2*^ *Department of Neurology, National Neuroscience Institute, Singapore, Singapore;* ^*3*^ *Lee Kong Chian School of Medicine, Nanyang Technological University, Singapore, Singapore*

**Background and aims:** Alzheimer's disease has a prolonged preclinical phase in which pathological changes precede cognitive symptoms by many years. Blood‐based biomarkers offer a scalable and minimally invasive opportunity for early risk stratification; however, small sample sizes and cohort heterogeneity limit the clinical translation of advanced machine learning models.

**Methods:** Using the Alzheimer's Disease Neuroimaging Initiative (ADNI) dataset, Baseline blood biomarkers, including pTau217–amyloid beta interaction terms, along with demographics and cognitive assessments, were analysed. Cognitive decline was defined as a sustained decrease of 2 or more points on the Montreal Cognitive Assessment across two consecutive years. Artificial Neural Networks (ANNs) and Support Vector Machines (SVMs) were trained on original and synthetically augmented datasets using Conditional Tabular Generative Adversarial Networks (CTGAN), Tabular Variational Autoencoders (TVAE), and Synthetic Minority Over‐sampling Technique for Nominal and Continuous features (SMOTENC). Model performance was evaluated using area under the curve (AUC), sensitivity, and specificity, with Shapley value analysis used to assess feature importance.

**Results:** CTGAN augmentation achieved optimal performance with AUC improvements of 5.6% (ANN) and 3.2% (SVM) vs non‐augmented models. Models demonstrated the ability to predict cognitive decline outcomes over a three‐year period. Shapley analysis identified pTau217–amyloid beta interaction terms, baseline diagnosis, and age as primary predictors, with significant biomarker interaction effects.

**Figure:**
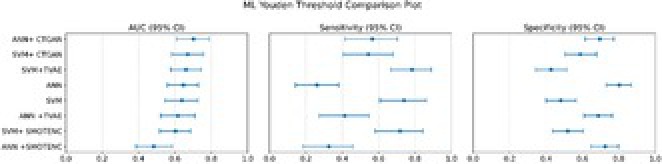
**FIGURE 1** Youden‐threshold performance comparison across models and augmentation strategies. ANN with CTGAN achieves the best balance of AUC, sensitivity, and specificity, while SMOTENC underperforms.

**Figure:**
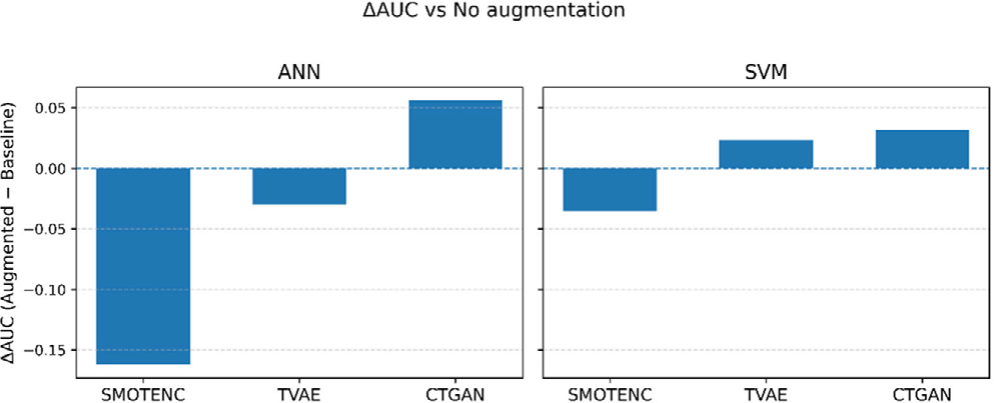
**FIGURE 2** ΔAUC relative to baseline across augmentation methods. CTGAN consistently improves performance in both ANN and SVM, whereas TVAE shows minor gains and SMOTENC reduces AUC.

**Figure:**
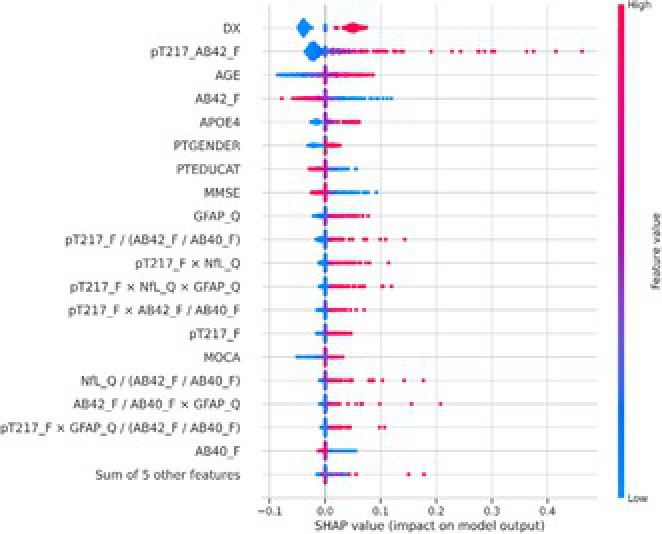
**FIGURE 3** SHAP summary plot for the ANN model predicting three‐year cognitive decline. Points represent individual participants; colors indicate feature values (blue = low, red = high).

**Conclusion:** Despite sample size limitations, synthetic data augmentation enables robust prediction of cognitive decline risk over three years using baseline blood biomarkers. This methodology supports translation of AI‐based screening tools into clinical practice, facilitating early identification of at‐risk patients and timely intervention strategies.

**Disclosure:** Nothing to disclose.

## EPO‐0225

### Associations between carotid atherosclerosis, dyslipidemia, activity of daily living and cognitive decline

#### M. Militaru ^1^; D. Lighezan^2^; A. Militaru^2^

##### ^*1*^ *Department of Neuroscience, Neurology II University Clinic, Victor Babes University of Medicine and Pharmacy Timisoara, Municipal Emergency Hospital Timisoara, Timisoara,Romania;* ^*2*^ *Department of Internal Medicine I, Medical Semiology I University Clinic, Victor Babes University of Medicine and Pharmacy Timisoara, Municipal Emergency Hospital Timisoara, Timisoara, Romania*

**Background and aims:** According to recent studies cardiovascular‐risk‐factors (CVRFs) and carotid‐atherosclerosis may contribute to cognitive‐decline (CD). The aim of the study is to evaluate the CD, instrumental‐activity‐of‐daily‐living (IADL), signs of depression, subclinical‐atherosclerosis (intima‐media‐thickness (IMT))/carotid‐atherosclerosis < 50% stenosis in carotid‐atherosclerosis/dyslipidemia patients versus patients without carotid‐atherosclerosis/dyslipidemia.

**Methods:** We included in the study 156CVRFs‐patients mean‐age 70.83 ± 9.35 (92(59.00%) female/64 (41.00%) male), with/without carotid‐atherosclerosis/dyslipidemia, without stroke/cardiovascular‐disease/carotid‐atherosclerosis > 50%stenosis. We compared the results from the CD, IADL and signs‐of‐depression‐screening of the 79 (50.60%) carotid‐atherosclerosis/dyslipidemia‐patients versus 77 (49.40%) patients‐without‐carotid‐atherosclerosis/dyslipidemia. They underwent clinical‐examination, BP, HR, lipid‐profile, Montreal‐Cognitive‐Assessment (MOCA), IADL and Geriatric‐Depression‐Scale (GDS‐15). We measured IMT (mm)/carotid‐plaque using a carotid‐duplex‐scan in both groups of patients.

**Results:** From 79 (50.60%) carotid‐atherosclerosis/dyslipidemia patients (34 (43.00%) male/45 (57.00%) female), there were 71 (89.90%) hypertension‐patients, 28 (35.40%) with obesity, 27 (34.20%) smokers, 43 (54.40%) with atrial‐fibrillation, 42 (53.20%) diabetes‐mellitus‐type‐II patients. MoCA, IADL, DBP (mmHg), LDL‐cholesterol (mg/dl) significant‐decrease and IMT (mm), GDS‐15 significant‐increase in carotid‐atherosclerosis/dyslipidemia patients versus patients without‐carotid‐atherosclerosis/dyslipidemia (*p* < 0.05). In carotid‐atherosclerosis/dyslipidemia patients we determined a significant‐weak‐negative‐correlation between age and MoCA (*p* = 0.027), a weak‐significant‐positive‐correlation with SBP (mmHg) (*p* = 0.002), IMT (mm) (*p* < 0.05) and moderate‐significant‐positive with GDS‐15 (*p* < 0.001).We determined a moderate‐positive‐statistically‐significant correlation between MoCA and IADL (*p* < 0.001) and weak‐positive with total‐cholesterol/LDL‐cholesterol (mg/dl) (*p* < 0.05) and a strong‐statistically‐significant‐negative correlation with GDS‐15 (*p* < 0.001), moderate‐negative with SBP (mmHg) (*p* < 0.05)/DBP (mmHg) (*p* < 0.001) and IMT (mm) (*p* < 0.001) and weak‐negative with HR (b/min.) (*p* < 0.05).We found a moderate‐statistically‐significant‐negative correlation between left/right IMT (mm) with MoCA, IADL (*p* < 0.05) and a moderate‐positive‐statistically‐significant correlation between left/right IMT with GDS‐15 (*p* < 0.001).

**Conclusion:** Early evaluation of CD, activities of daily‐living, and depressive symptoms is essential in patients with carotid‐atherosclerosis and dyslipidemia, as these patients are at increased risk of cognitive‐impairment. Correlating these findings with carotid‐atherosclerosis severity, age, BP, HR, lipid‐profile alterations and CVRFs may improve early identification and management of CD.

**Disclosure:** Nothing to disclose.

## EPO‐0226

### Neuropsychiatric symptoms and structural MRI correlates in major dementia subtypes: A retrospective cohort study

#### M. Canbuldu; S. Sahin; O. Totuk

##### University of Health Sciences, Hamidiye Faculty of Medicine, Prof. Dr. Ilhan Varank Training and Research Hospital, Department of Neurology, Istanbul, Türkiye

**Background and aims:** This study aimed to investigate the distribution of depression and anxiety symptoms among major dementia subtypes and to explore their associations with structural magnetic resonance imaging findings.

**Methods:** We retrospectively analyzed clinical and imaging data from 1,450 patients followed in a tertiary dementia clinic. Diagnoses included Alzheimer disease, vascular dementia, frontotemporal dementia, and dementia with Lewy bodies. Depression and anxiety were measured using the Geriatric Depression Scale and Geriatric Anxiety Scale. Structural brain changes were rated with the Medial Temporal Atrophy scale, Fazekas score, orbitofrontal sulcus score, collateral sulcus score, and Koedam posterior atrophy score. Group comparisons were conducted using the Kruskal–Wallis test; correlations were assessed with Spearman coefficients. A *p*‐value < 0.05 was considered statistically significant.

**Results:** Depression scores varied significantly across dementia types (*p* = 0.043), with the highest scores in vascular dementia. Anxiety scores also differed significantly (*p* = 0.007), being highest in dementia with Lewy bodies. Depression correlated positively with Fazekas score (*r* = 0.27, *p* = 0.01), while anxiety showed a weaker but significant association with Koedam posterior atrophy score (*r* = 0.18, *p* = 0.04).

**Figure:**
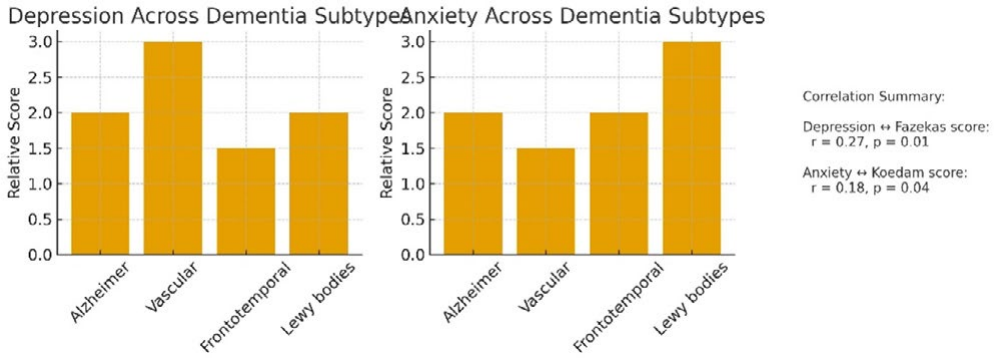
**FIGURE 1** Depression and anxiety scores across dementia subtypes. Depression peaked in vascular dementia, while anxiety was highest in dementia with Lewy bodies. Source: Created by the authors. Author's own analysis and visualization.

**Conclusion:** Distinct dementia subtypes exhibit heterogeneous neuropsychiatric profiles and imaging markers. The findings suggest different underlying mechanisms, such as white matter changes linked to depression and posterior atrophy linked to anxiety. Integrating neuroimaging and symptom data may aid personalized dementia care.

**Disclosure:** Nothing to disclose.

## EPO‐0227

### Probiotic *Lactiplantibacillus plantarum* KABP051 prevents depressive‐like symptoms in drosophila melanogaster and murine models of depression

#### P. Huedo ^1^; A. Castells‐Nobau^2^; R. Mariné‐Casadó^3^; J. Teichenné^3^; M. Martin^4^; J. del Bas^5^; A. Caimari^6^; J. Mayneris‐Perxachs^2^; J. Espadaler‐Mazo^1^

##### ^*1*^ *R&D Department, AB‐Biotics S.A. (Part of Kaneka Corporation), Barcelona, Spain; Basic Sciences Department, Universitat Internacional de Catalunya, Barcelona, Spain;* ^*2*^ *Integrative Systems Medicine and Biology Group, Girona Biomedical Research Institute (IDIBGI‐CERCA), Salt, Spain;* ^*3*^ *Eurecat‐Centre Tecnològic de Catalunya, Unitat de Nutrició i Salut, Reus, Spain;* ^*4*^ *Eurecat‐Centre Tecnològic de Catalunya, Unitat de Nutrició i Salut, Reus, Spain; Department of Morphological Sciences, Unit of Human Anatomy and Embryology, Faculty of Medicine, Universitat Autonoma de Barcelona, Bellaterra, Spain;* ^*5*^ *Biotechnology Area, Centre Tecnològic de Catalunya, Eurecat, Reus, Spain; Department of Biochemistry and Biotechnology, Center of Environmental, Food and Toxicological Technology, Universitat Rovira i Virgili, Tarragona, Spain;* ^*6*^ *Biotechnology Area, Centre Tecnològic de Catalunya, Eurecat, Reus, Spain*

**Background and aims:** Previous clinical evidence indicated that Lactiplantibacillus plantarum KABP051 may alleviate stress and anxiety in adults in association to modulation of serotonergic and kynurenine pathways. This study investigated its potential to prevent depressive‐like behaviors using two validated animal models.

**Methods:** Adult germ‐free flies and flies mono‐colonized with different probiotics (10^8^ CFU) were subjected to a 4‐day consecutive mechanical stress protocol to induce depression‐like states. Subsequently, voluntary behavioural responses were assessed using the gap climbing (GAP) and stop‐for‐sweet (SFS) test. Adult male C57BL/6 mice were administered with probiotic formulations (10^9^ CFU/day) for 43 days. From day 22, all groups except placebo‐unstressed underwent water avoidance stress (WAS) for seven days. Depressive‐like behaviors were evaluated using the open field (OFT) and forced swim (FST) tests on days 39 and 40.

**Results:** Mechanical stress and WAS induced depressive‐like phenotypes in flies and mice, respectively. Among the tested probiotics (including additional L. plantarum strains), strain KABP051 showed the strongest reduction of the susceptibility to depression index in both GAP and SFS tests (*p* < 0.0001 vs stressed germ‐free flies). In mice, all probiotics showed minor effects on OFT, but only KABP051 significantly reduced immobility time in FST (*p* < 0.05 vs stressed group), mirroring unstressed animals.

**Figure:**
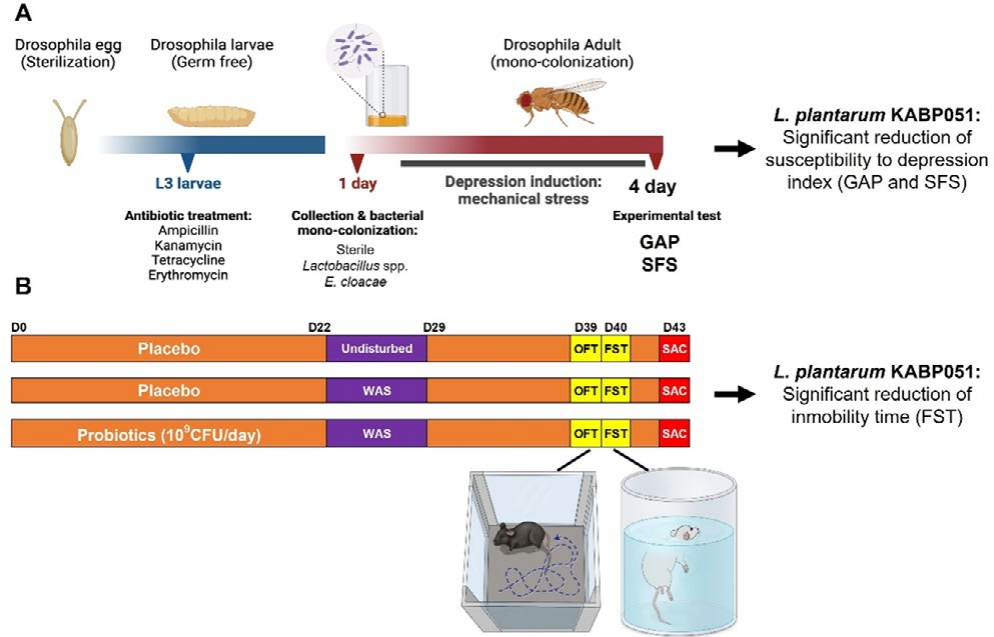
**FIGURE 1** Graphical abstract of the D. melanogaster (A) and mice (B) models of depression. L. plantarum KABP051 showed the greatest efficacy in preventing depressive‐like symptoms in both animal models.

**Conclusion:** L. plantarum KABP051 exhibits cross‐species depression‐preventive activity, likely via conserved pathways such as kynurenine modulation. Further research should elucidate underlying mechanisms and confirm translational benefits in humans.

**Disclosure:** PH and JE‐M are full time employees of AB‐BIOTICS S.A. (Kaneka Corporation).

## EPO‐0228

### Early identification of cognitive impairment in elderly patients with HFpEF: A simple clinical risk score

#### S. Arnautu ^1^; D. Jianu^2^; M. Andor^1^; D. Arnautu^1^

##### ^*1*^ *Department V Internal medicine, “Victor Babes” University of Medicine and Pharmacy, Timisoara, Romania;* ^*2*^ *Department of Neurosciences, “Victor Babes” University of Medicine and Pharmacy, Timisoara, Romania*

**Background and aims:** Cognitive impairment is a frequent yet under‐recognized comorbidity in elderly patients with heart failure with preserved ejection fraction (HFpEF), contributing to functional decline and poor outcomes. Simple and clinically applicable tools for early identification of patients at risk are currently lacking. We aimed to develop a practical clinical risk score for predicting cognitive impairment in elderly patients with HFpEF.

**Methods:** We conducted a cross‐sectional observational study including 326 HFpEF patients aged ≥ 65 years. Cognitive function was assessed using the Mini‐Mental State Examination‐2 (MMSE‐2), with cognitive impairment defined as a score < 24. Clinical, laboratory, vascular, and functional parameters were collected. Independent predictors were identified using multivariable logistic regression, and a point‐based clinical risk score was derived. Discriminative performance was evaluated using receiver operating characteristic (ROC) analysis.

**Results:** Cognitive impairment was identified in 9.8% of patients. Independent risk predictors included diabetes mellitus, prior stroke or transient ischemic attack, carotid artery disease, elevated NT‐proBNP levels, and reduced estimated glomerular filtration rate. Higher Kansas City Cardiomyopathy Questionnaire (12‐KCCQ) scores and anticoagulant therapy for atrial fibrillation were independently associated with lower cognitive risk. The clinical risk score demonstrated strong discriminative ability (AUC = 0.84). A threshold of ≥ 2 points identified cognitive impairment with 75% sensitivity and 83% specificity.

**Figure:**
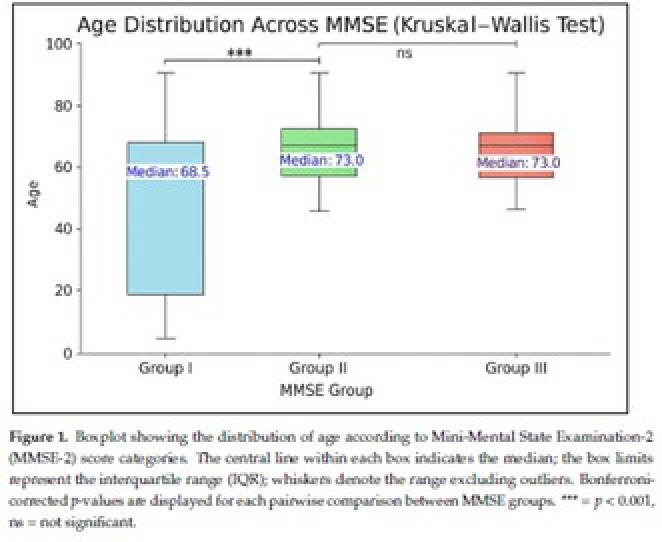
**FIGURE 1** Age distribution.

**Figure:**
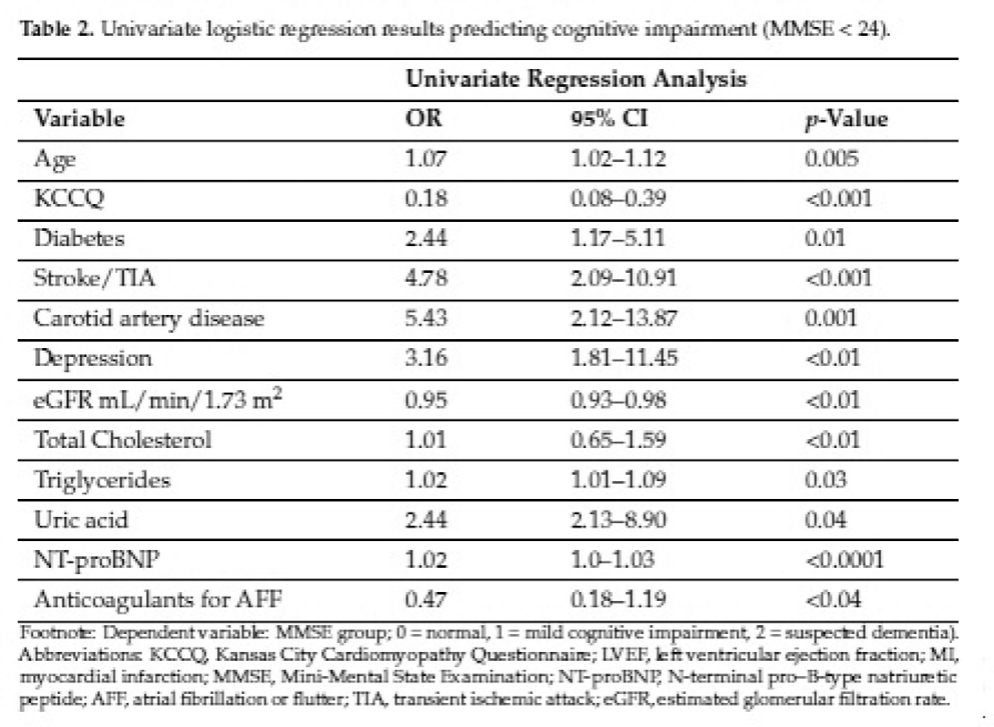
**FIGURE 2** Logistic regression.

**Figure:**
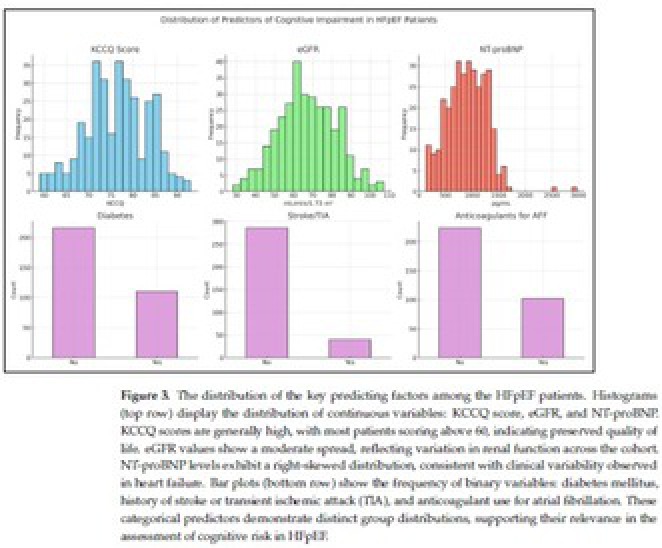
**FIGURE 3** Distribution of key predicting factors.

**Conclusion:** We propose a simple, clinically applicable risk score that enables early identification of cognitive impairment in elderly HFpEF patients and may support targeted cognitive screening within an integrated heart–brain care approach.

**Disclosure:** Nothing to disclose.

## EPO‐0229

### Clinical correlates of cognitive complaints in individuals with subjective cognitive decline

#### S. Buscarnera

##### Department of Human Neuroscience, La Sapienza University, Rome, Italy

**Background and aims:** Complains about cognitive performance in the absence of cognitive impairment represent the core feature of subjective cognitive decline (SCD), a common complaint in older adults and a potential risk factor for future cognitive impairment. This study examined clinical and psychological correlates of self‐ and partner‐reported cognitive complaints, as well as awareness of personal cognition, in SCD.

**Methods:** Data of 789 participants with SCD were obtained fromthe Alzheimer's Disease Neuroimaging Initiative 2 (ADNI2). Cognitive complaints were assessed using the Everyday Cognition scale, completed by both participants (ECog‐P) and study‐partners (ECog‐SP). Awareness of cognitive functioning was operationalized as the difference between ECog‐P and ECog‐SP scores (awareness). Additional measures included the Geriatric Depression Scale (GDS) and the Functional Activities Questionnaire (FAQ). Descriptive statistics summarized demographic and clinical characteristics. Linear regression models, adjusted for age, sex, and education, were used to examine associations between ECog‐P, ECog‐SP, awareness, and the clinical variables.

**Results:** The mean age was 71.0 years (SD = 6.3), with a mean education of 16.7 years (SD = 2.4). Higher FAQ scores were significantly associated with worse ECog‐P (*β* = 0.04, *p* < 0.001), ECog‐SP (*β* = 0.14, *p* < 0.001) (Figure 1), and greater awarenss (*β* = 0.10, *p* < 0.001) (Figure 3). Depressive symptoms were positively associated with both ECog‐P (*β* = 0.05, *p* < 0.001) and ECog‐SP (*β* = 0.03, *p* = 0.02) (Figure 2). No correlation was found with cognitive awareness (Figure 3).

**Figure:**
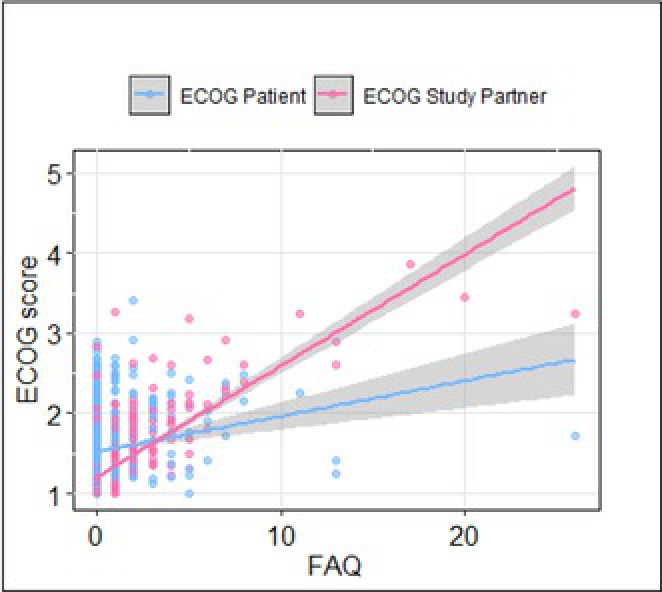
**FIGURE 1** Correlations of ECOG Patient (blue circles) and ECOG Study Partner (red triangles) scores with FAQ.

**Figure:**
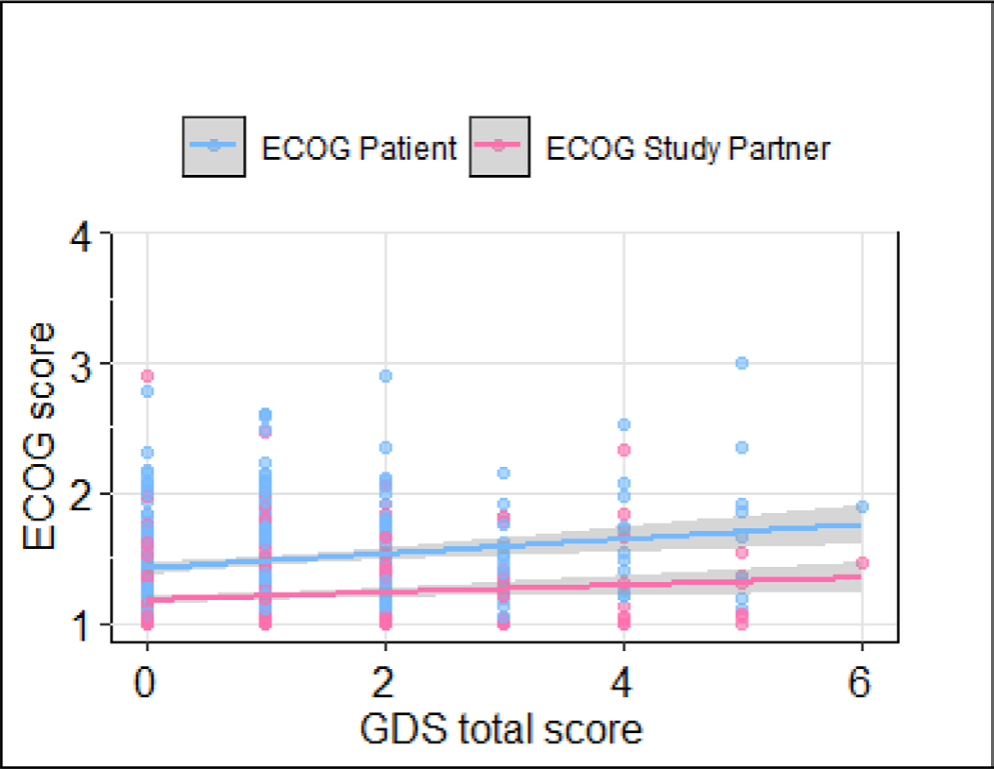
**FIGURE 2** Correlations of ECOG Patient (blue circles) and ECOG Study Partner (red triangles) scores with FAQ.

**Figure:**
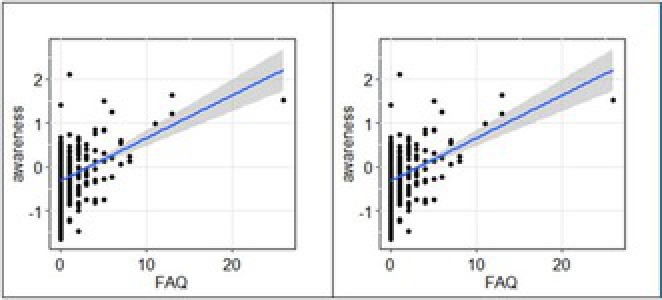
**FIGURE 3** Correlation of awareness with FAQ and GDS.

**Conclusion:** Cognitive complaints SCD are influenced by multiple factors. Understanding the clinical correlates of perceived cognitive decline can offer valuable insights into the complexity of SCD, supporting both prognosis and management.

**Disclosure:** Nothing to disclose.

## EPO‐0230

### Persistent cognitive, functional, and fatigue deficits in post‐COVID‐19 individuals: A comparative study

#### S. Hangün ^1^; Z. Kunduracilar^2^; E. Koca^3^; E. Yildirim^4^

##### ^*1*^ *Gulhane Faculty of Physiotherapy and Rehabilitation, University of Health Sciences, Ankara, Türkiye;* ^*2*^ *Faculty of Health Sciences,Istanbul Gelisim University, Istanbul, Türkiye;* ^*3*^ *Suleyman Yalcin City Hospital Psychiatry Clinic, Medeniyet University, Istanbul, Türkiye;* ^*4*^ *Süreyyapaşa Chest Dıseases And Thoracıc Surgery Traınıng and Research Hospıtal, Istanbul, Türkiye*

**Background and aims:** The long‐term impact of COVID‐19 extends beyond respiratory symptoms. This study aimed to compare cognitive function, functional capacity, fatigue, and healthy lifestyle behaviors between individuals with a history of COVID‐19 and healthy controls

**Methods:** We included 40 individuals with a history of COVID‐19 and 40 healthy controls. Cognitive status was assessed using the Montreal Cognitive Assessment (MoCA), and functional capacity was measured via the 2‐Minute Walk Test. Additional evaluations included the Fatigue Severity Scale, Pittsburgh Sleep Quality Index, Beck Depression Inventory, and Healthy Lifestyle Behaviors Scale II (HLBS‐II).

**Results:** The clinical characteristics of COVID‐19 infection in the study group are presented in Table 1 The study group demonstrated significantly lower scores on the MoCA and 2‐Minute Walk Test compared to controls (*p* < 0.05) (Table 2). Similarly, they reported higher fatigue levels and lower scores in the physical activity, stress management and interpersonal relations subdomains of the HLBS‐II (*p* < 0.05). Conversely, sleep quality and depression scores showed no significant differences between the groups (*p* > 0.05).

**Figure:**
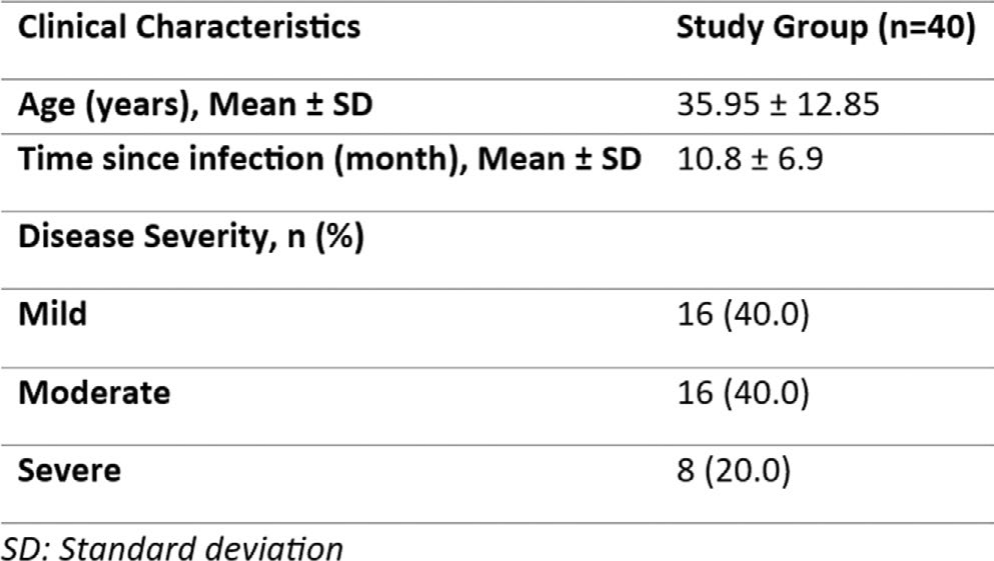
**TABLE 1** Clinical characteristics of COVID‐19 infection in the study group.

**Figure:**
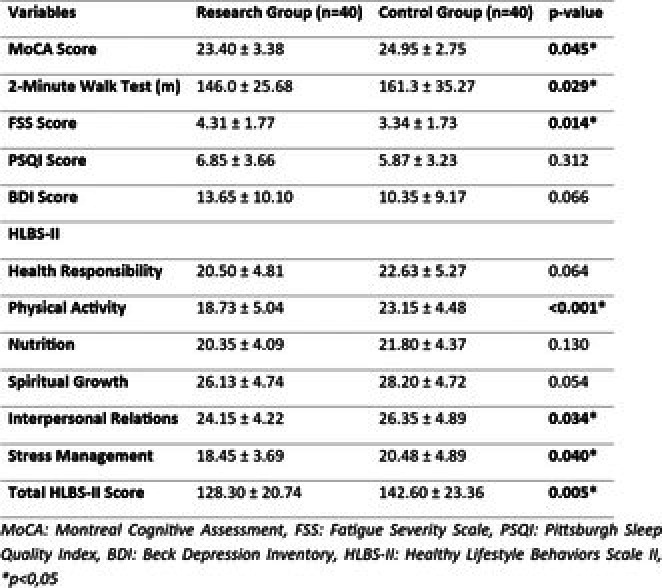
**TABLE 2** Comparison of variables between groups.

**Conclusion:** Individuals with a history of COVID‐19 exhibit persistent deficits in cognitive function and functional capacity, accompanied by increased fatigue. These impairments appear independent of sleep quality or depression. Clinicians should prioritize long‐term monitoring and implement targeted rehabilitation strategies focused on neuro‐cognitive recovery and lifestyle modification for this population.

**Disclosure:** Nothing to disclose.

## EPO‐0231

### The impact of molecular profile outweighs tumor location on preoperative cognitive‐emotional function in glioma patients

#### Y. Fang ^1^; Z. Gao^2^; R. Yang^3^; T. Ai^2^; L. Xu^1^; Q. Shi^1^; S. Lu^1^; X. Wang^2^; H. Yan^1^

##### ^*1*^ *Huanhu Hospital Affiliated to Tianjin Medical University, Tianjin, China;* ^*2*^ *Beijing Tiantan Hospital, Capital Medical University, Beijing, China;* ^*3*^ *School of Psychological and Cognitive sciences and Beijing Key Laboratory of Behavior and Mental Health, Peking University, Beijing, China*

**Background and aims:** Neuropsychological impairments are common in glioma patients, but their links to specific clinical and molecular features, beyond tumor location, remain unclear. This study examined whether molecular profiles influence preoperative neuropsychological function more than tumor location alone.

**Methods:** We prospectively assessed 99 preoperative glioma patients and 52 healthy controls with a comprehensive neuropsychological battery. After controlling for demographics, patients with insular (deep) and fronto‐temporo‐parietal (superficial) tumors were compared. Subgroup analyses were then conducted separately for insular and frontal gliomas to examine associations with molecular markers and clinical factors.

**Results:** Patients showed broad cognitive‐emotional impairments compared to controls. However, no significant difference was found between insular and superficial tumor groups. Subgroup analysis revealed: (1) Within insular gliomas, dominant hemisphere involvement correlated with poorer global cognition. Notably, IDH mutation here associated with worse attention and empathy, yet higher positive and lower negative affect. (2) In frontal gliomas, molecular and clinical features showed stronger associations: absence of epilepsy, lower grade, 1p19q codeletion, lack of TERT mutation, and presence of IDH mutation each linked to better performance across multiple cognitive and emotional domains.

**Figure:**
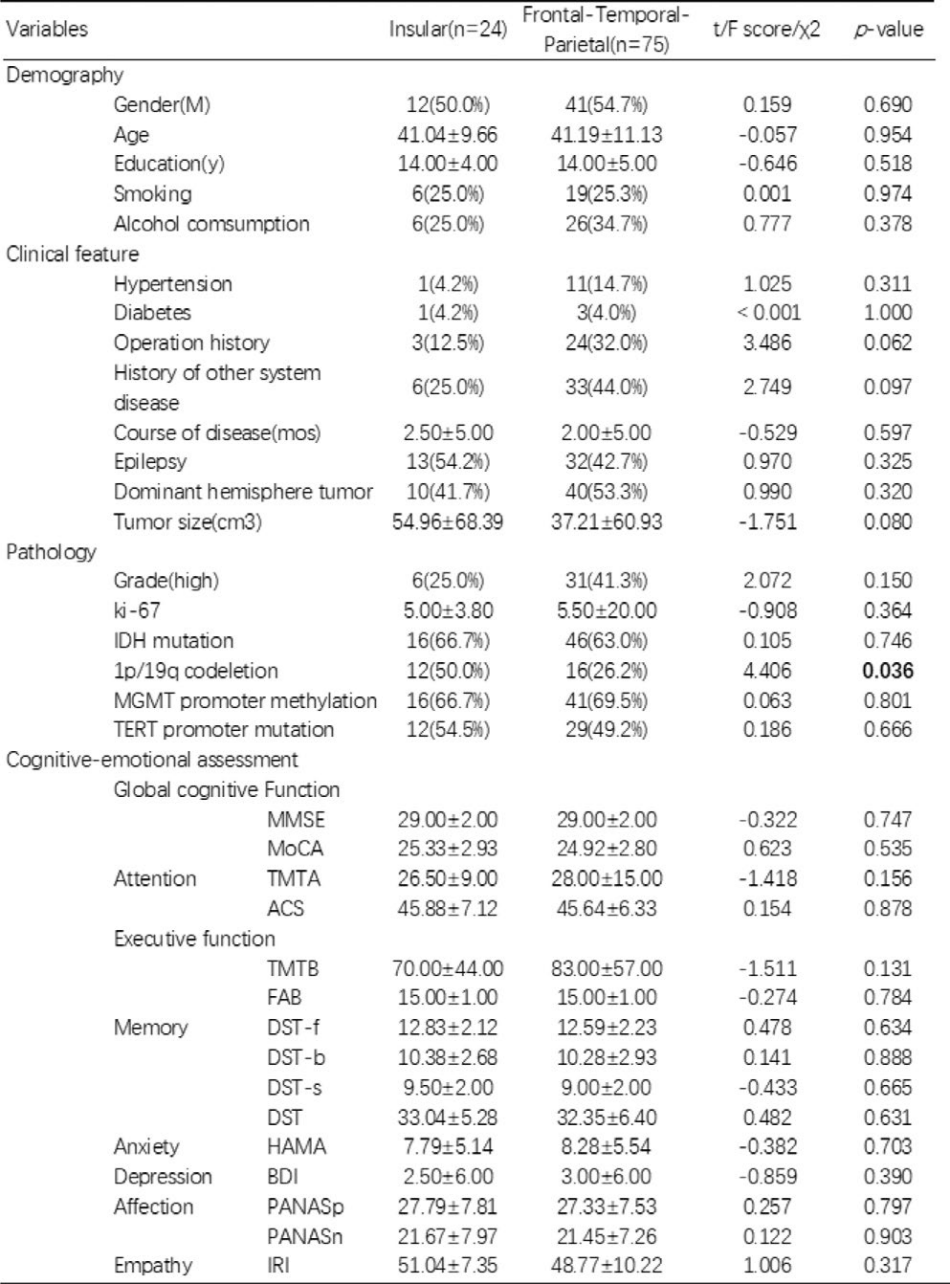
**TABLE 1** Demographic, Clinical, Molecular Characteristics and Cognitive and Emotional Measures of Glioma Patients by Lesion Location.

**Figure:**
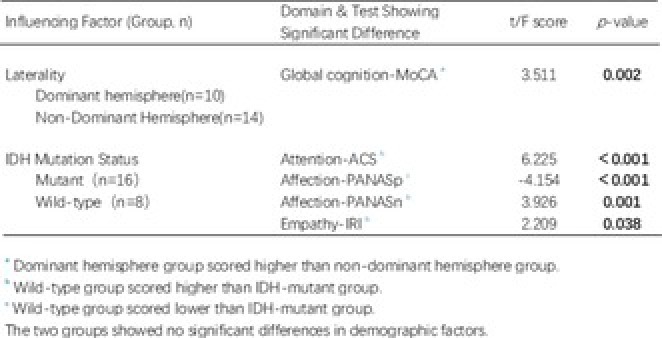
**TABLE 2** Comparisons of Neuropsychological Test Scores by Clinical and Molecular Features in the Insular Lobe Subgroup (*n* = 24).

**Figure:**
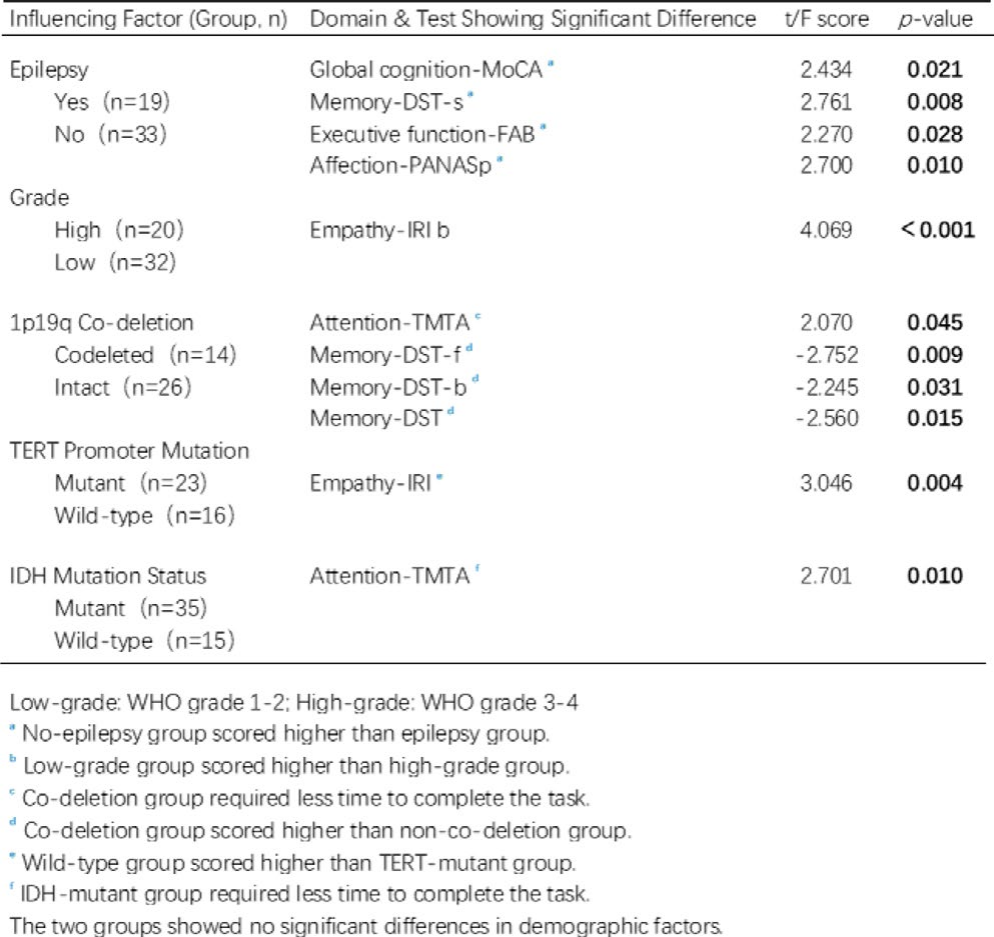
**TABLE 3** Comparisons of Neuropsychological Test Scores by Clinical and Molecular Features in the Frontal Lobe Subgroup (*n* = 52).

**Conclusion:** This study indicate that molecular profiles and key clinical factors outweigh tumor location in shaping preoperative neuropsychological function in glioma. This argues for a molecularly‐informed framework to better understand neurobehavioral manifestations and guide patient assessment and intervention.

**Disclosure:** Nothing to disclose.

## Epilepsy 2

## EPO‐0232

### Systematic review of Epilepsia partialis continua: Clinical profile, neurophysiological findings and etiological spectrum in a cohort of 287 patients

#### P. Miranda Cabral^1^; E. Reyes Almonte^1^; C. Peralta Sousa^1^; J. Pineda Amarilla^1^; E. Patricio^1^; A. Reyes ^1^; A. Tejada Henriquez^1^; C. Perez Lizardo^1^; K. Yorro^1^; M. Rodriguez^2^

##### ^*1*^ *Synaptic Research League;* ^*2*^ *Hospital Universitario Salvador Bienvenido Gautier, Santo Domingo, Dominican Republic*

**Background and aims:** Epilepsia partialis continua (EPC), also known as Kojevnikov epilepsy, is a rare form of focal status epilepticus with an incidence greater in males than females [1], characterized by continuous or near‐continuous focal motor seizures [2]. It affects both adults and children and is associated with diverse etiologies, including structural, inflammatory, and metabolic brain disorders [3].

**Methods:** We performed a systematic literature review following the PRISMA guidelines. S PubMed and BVS were searched using the keywords “Kojevnikov epilepsy”, “focal status epilepticus”, “physiopathology”, “continuous focal seizures”, “etiology”, and “neurophysiology”. Nonexperimental, nonanimal, English‐written studies were included.

**Results:** We included 287 patients. The mean age was 31 years. Sex was reported in a minority of cases (33 females, 17 males), and was not reported for the remaining cases. Continuous focal epileptiform activity (97.6%) and focal epileptic status (99.3%) were more often reported in most of the cases, commonly involving the cortical regions (96.9%). Structural brain lesions represented the most frequent etiologies (86.7%), preceded by autoimmune diseases (21.1%), of which Rasmussen syndrome is the most frequent (20.2%). Metabolic (0.70%) and mitochondrial (5.2%) causes were less commonly identified. Genetic involvement was uncommon and included POLG (0.7%), GFAP (0.3%), and DNM1L (0.3%), as well as ARF6 dysfunction. One case (0.3%)of anti‐GABA‐A receptor antibodies following alemtuzumab treatment was identified.

**Figure:**
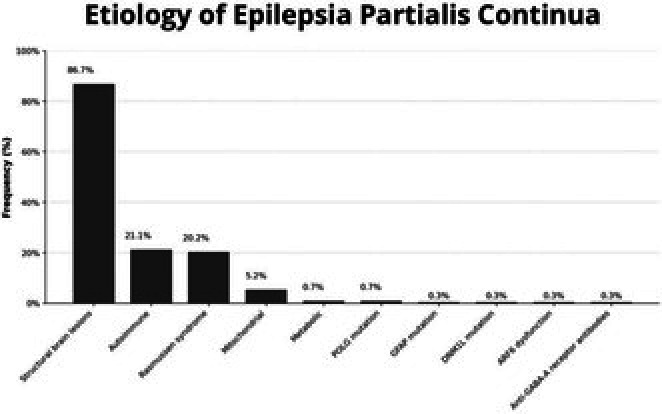
**FIGURE 1** Frequency of the Etiologies in Epilepsia Partialis Continua.

**Figure:**
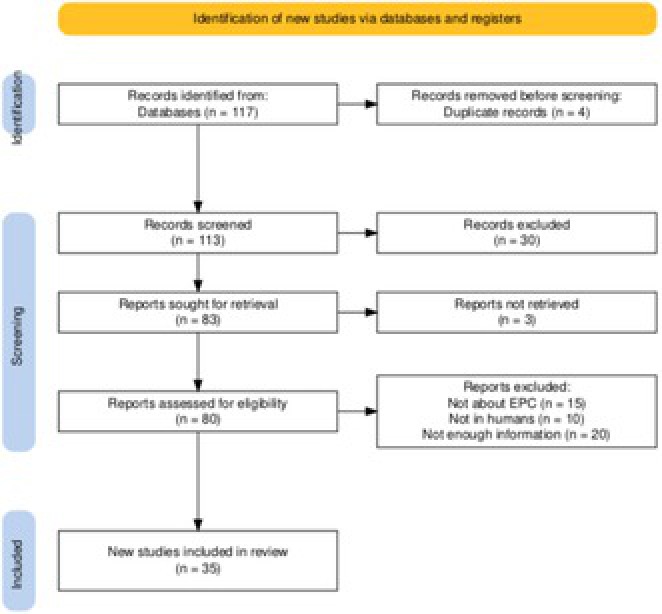
**FIGURE 2** Prisma‐P flowchart of the process of inclusion of studies in the systematic review.

**Conclusion:** EPC is predominantly a cortical focal status epilepticus with mainly structural etiologies, followed by autoimmune and inflammatory causes. These findings support a diagnostic approach and highlight the need for further research into less common immunological and genetic mechanisms.

**Disclosure:** Nothing to disclose.

## EPO‐0233

### Disconnection surgeries in refractory epilepsy: A retrospective study from a Tertiary Epilepsy Center

#### B. Turk ^1^; M. Iris^1^; H. Cerci^2^; E. Taskiran^1^; M. Delil^1^; C. İsler^2^; T. Tanriverdi^2^; S. Yeni^1^; C. Ozkara^1^; M. Uzan^2^

##### ^*1*^ *Department of Neurology, IUC‐Cerrahpasa Faculty of Medicine, Istanbul, Türkiye;* ^*2*^ *Department of Neurosurgery, IUC‐Cerrahpasa Faculty of Medicine, Istanbul, Türkiye*

**Background and aims:** Disconnection surgery is an established treatment option for selected patients with drug‐resistant epilepsy in whom resective surgery is not feasible. This study aimed to evaluate postoperative prognosis according to different disconnection surgery subtypes.

**Methods:** Patients followed between 2000 and 2023 at the Epilepsy Unit of Istanbul University–Cerrahpasa Faculty of Medicine were retrospectively reviewed. Fifty‐five patients who underwent disconnection surgery were included. Patients were classified into four subgroups based on surgical technique: Functional Hemispherectomy (FH), Posterior Quadrant Disconnection (PQD)—including temporo‐parietal (TP‐PQD) and parieto‐occipital disconnection (PO‐PQD)—and Subtotal Hemispherotomy (SH). Demographic and clinical characteristics were analyzed in relation to postoperative outcomes.

**Results:** The cohort consisted of 32 males and 23 females, with a mean age of 20.7 ± 12.5 years. Mean age at surgery was 14.2 ± 12.7 years, and the mean duration between seizure onset and surgery was 10.2 ± 10.8 years. FH was performed in 24 patients, PQD in 17 patients, and SH in 8 patients. At final follow‐up, Engel class I outcome was achieved in 38 patients, class II in 7, class III in 5, and class IV in 5. No statistically significant differences were observed in postoperative Engel outcomes or complication rates among the different surgical subgroups.

**Conclusion:** Disconnection surgery is an effective therapeutic option for refractory epilepsy, with favorable seizure outcomes across different techniques. Surgical strategy should be individualized based on patient characteristics and the anatomical localization of the epileptogenic zone.

**Disclosure:** Nothing to disclose.

## EPO‐0234

### A new non‐invasive approach to quantify pharmacological modulation of cortical excitability

#### C. Friedrichs‐Maeder ^1,2^; V. Santoro^2^; B. Clennell^3^; H. Clark^2^; L. Rocchi^2,4,5^; M. P. Richardson^2^

##### ^*1*^ *Sleep‐Wake‐Epilepsy Center, Center for Experimental Neurology, Department of Neurology, Inselspital Bern, University Hospital, University of Bern, Switzerland;* ^*2*^ *Department of Basic and Clinical Neuroscience, Institute of Psychiatry, Psychology and Neuroscience (IoPPN), King's College London, London, UK;* ^*3*^ *Centre for Human & Applied Physiological Sciences, King's College London, London, UK,* ^*4*^ *Department of Medical Sciences and Public Health, University of Cagliari, Cagliari, Italy,* ^*5*^ *Department of Clinical and Movement Neurosciences, UCL Queen Square Institute of Neurology, University College London, London, UK*

**Background and aims:** Cortical excitability (CE)—the cortex's responsiveness to perturbation—is fundamental and altered in neurological and psychiatric disorders, making it a pharmacological target. Yet a robust, scalable, drug‐sensitive marker is lacking. CE can be probed non‐invasively with transcranial magnetic stimulation combined with electroencephalography (TMS–EEG), but standard comparisons of evoked amplitudes in predefined time windows are variable and may miss medication effects. We introduce and validate an excitability index (ExI) derived from TMS–EEG stimulus–response curves (SRCs) across intensities to quantify pharmacological modulation of CE.

**Methods:** In a placebo‐controlled crossover study, 12 healthy men received levetiracetam (LEV; 3000 mg) and placebo (10 completed both). Single‐pulse TMS over left M1 at seven intensities (70–130% resting motor threshold) was delivered before and 2 h after intake while EEG was recorded. Responses were quantified using global mean field amplitude (GMFA) and ExI (area under the normalized SRC using EEG line‐length response magnitude). Wilcoxon signed‐rank tests assessed within‐session changes; precision–recall (PR) analyses compared ExI versus GMFA for discriminating LEV from placebo.

**Results:** LEV reduced GMFA in selected time windows with medium‐to‐large effect sizes, without consistent latency shifts. ExI significantly decreased following LEV (p < 0.01) but not placebo, and showed superior discrimination (AUC‐PR 0.79, baseline prevalence = 0.27) versus GMFA (AUC‐PR 0.27–0.43).

**Conclusion:** ExI is a scale‐free, medication‐sensitive marker of CE that outperforms standard TMS–EEG metrics, providing a non‐invasive readout of pharmacological effects on human cortical physiology. ExI could serve as a translational biomarker to monitor drug engagement and optimize dosing for epilepsy and other excitability disorders.

**Disclosure:** Nothing to disclose.

## EPO‐0235

### Early use of Cenobamate in a case of non‐convulsive status epilepticus in a woman with progressive multifocal leukoencephalopathy

#### E. Gentile ^1^; A. Alicino^2^; E. Colombo^3^; A. Campomori^1^; D. Lomonaco^1^; E. Mazzotta^1^; E. Rigoni^3^; E. Tartara^2^

##### ^*1*^ *Department of Brain and Behavioral Sciences, University of Pavia, Pavia, Italy;* ^*2*^ *Epilepsy Center, IRCCS Mondino Foundation, ERN Epicare Full Member, Pavia, Italy;* ^*3*^ *Multiple Sclerosis Center, IRCCS Mondino Foundation, Pavia Italy*

**Background and aims:** Non‐convulsive status epilepticus (NCSE) is a type of status epilepticus (SE) with no prominent motor signs. In NCSE without coma that is refractory to first and second line treatment, therapy with anaesthetics is less appropriate. ASMs are therefore used sequentially. Cenobamate (CNB) efficacy in SE is supported by limited data. We present a case of NCSE in which CNB was used early.

**Methods:** A 54‐year‐old woman with multiple sclerosis received natalizumab until she was diagnosed with progressive multifocal leukoencephalopathy (mRS4). One year later, she was admitted with acute aphasia, right head deviation, and impaired consciousness (GCS10). Brain MRI revealed hyperintensities and edema in the right hemisphere. EEG showed right focal SE with prominent contralateral motor signs, resistant to benzodiazepines, levetiracetam, and lacosamide.

**Results:** After a few days, the EEG evolved into NCSE with preserved consciousness, repetitive right and head deviation, and an inability to walk (mRS5). Despite the introduction of a third ASM (perampanel, 8 mg/day), there was no electroclinical improvement until CNB was added and titrated up to 200 mg/day within one month. The MRI scan showed resolution of the peri‐ictal alterations in the right hemisphere. A two‐month EEG follow‐up excluded NCSE relapse.

**Figure:**
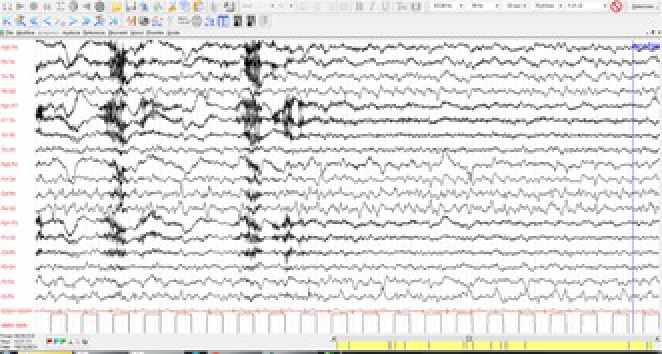
**FIGURE 1** EEG performed while taking levetiracetam and lacosamide.

**Figure:**
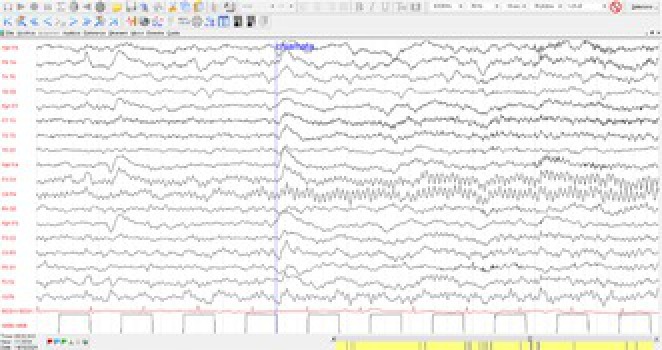
**FIGURE 2** Follow‐up EEG during levetiracetam and lacosamide treatment.

**Figure:**
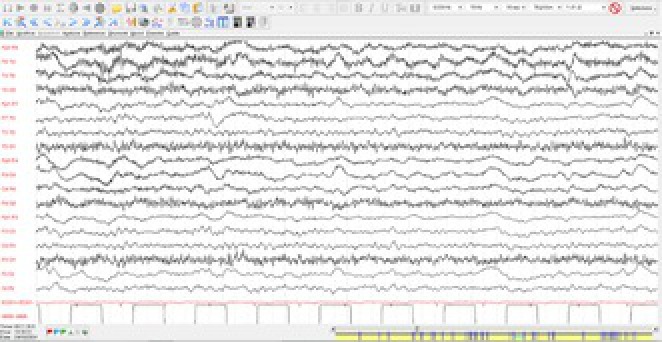
**FIGURE 3** Follow‐up EEG during treatment with levetiracetam, lacosamide, perampanel, and cenobamate.

**Conclusion:** Currently, it is not possible to recommend the use of cenobamate in NCSE, as only anecdotal cases have been reported to date. An important limitation is its slow titration, but it can be considered as a third‐line therapy in selected cases with subtle clinical manifestations. Furthermore, CNB may modulate the GABAergic dysfunction described in NCSE.

**Disclosure:** Nothing to disclose.

## EPO‐0236

### A retrospective cohort study of valproate and infertility in men with epilepsy or bipolar disorder using international health data

#### G. Mbizvo ^1^; S. Duncan^2^; L. Nancarrow^1^; T. Sagarino^3^; L. Watkins^4^; M. Mbizvo^5^; S. Lip^1^; T. Marson^1^

##### ^*1*^ *The University of Liverpool, Liverpool, UK;* ^*2*^ *The University of Edinburgh, Edinburgh, UK;* ^*3*^ *TriNetX LLC, Cambridge, USA;* ^*4*^ *University of South Wales, Pontypridd, UK;* ^*5*^ *Population Council, Lusaka, Zambia*

**Background and aims:** Against a background of limited animal data and existing restrictions, we assessed infertility in men with epilepsy or bipolar disorder exposed and unexposed to valproate.

**Methods:** We conducted a retrospective cohort study using data from TriNetX, an international network of healthcare organisations. Men aged < 55 years were identified in three cohorts: (A) epilepsy or bipolar disorder, (B) epilepsy, and (C) bipolar disorder. Validated diagnostic and prescription codes were used. Valproate‐exposed and unexposed men were compared for lifetime risks of infertility outcomes. Follow‐up began from the second valproate prescription (exposed) or second disease code (unexposed). Propensity‐score matching balanced > 140 baseline covariates. Survival analysis was undertaken using Cox proportional hazards models, generating hazard ratios (HRs) with 95% confidence intervals (CIs).

**Results:** Among 627,720 men with epilepsy or bipolar disorder, 91,917 were exposed and 535,803 unexposed to valproate. Serum valproate was detectable in 98% of tested exposed individuals. After matching, 78,971 men were included in each group. There was a < 1% difference in infertility outcomes between those exposed and unexposed to valproate (not significant): male infertility HR 1.057; CI 0.864–1.295, testicular hypofunction HR 0.916; CI 0.824–1.019, testicular atrophy HR 1.000; CI 0.682–1.465, and composite low sperm concentration, motility, vitality, normal forms, or semen volume HR 0.856; CI 0.613–1.196. Total testosterone was modestly lower in valproate‐exposed men but remained within the normal range; other hormones did not differ. Analyses stratified by diagnosis were similarly null.

**Conclusion:** Our findings do not support an association between valproate and infertility in men with epilepsy or bipolar disorder in real‐world settings.

**Disclosure:** A.G.M. declares (i) a UCB Pharma grant paid to University of Liverpool for the National Audit of Seizure Management in Hospitals (NASH) study, which is unrelated to the submitted work; (ii) an Angelini grant to be paid to University of Liverpool as co‐applicant for A multi‐method PRoject to maximise efficient and equitable pathways tO suPport from a rEgional epiLepsy centre (PROPEL), which is unrelated to the submitted work; (iii) Honoraria paid to University of Liverpool for lectures unrelated to the submitted work given at educational events sponsored by Sanofi, Eiasi, and GSK; (iii) Support from Angelini for attendance unrelated to the submitted work at the 2024 International League Against Epilepsy (ILAE) congress. G.K.M. declares an Angelini grant paid to the University of Liverpool as co‐applicant on the PROPEL study, which is unrelated to the submitted work; (ii) Honoraria paid to the University of Liverpool for delivering a lecture at an educational event sponsored by Angelini, which was unrelated to the submitted work. G.Y.H.L. is a consultant and speaker for BMS/Pfizer, Boehringer Ingelheim, Daiichi‐Sankyo, and Anthos, which is unrelated to the submitted work. No fees are received personally. G.Y.H.L. is a National Institute for Health and Care Research (NIHR) Senior Investigator and co‐principal investigator of the AFFIRMO project on multimorbidity in AF, which has received funding from the European Union's Horizon 2020 research and innovation programme under grant agreement No. 899871, which is unrelated to the submitted work. T.S. is an employee of TriNetX, which routinely provides data for research studies, including the data used in this study. The involvement of T.S. as a co‐author reflects her technical assistance as a data analyst and does not imply any compensation or influence from TriNetX on this study. The remaining authors declare no competing interests.

## EPO‐0237

### Beyond neuroprotection: A context‐dependent view of GLP‐1 receptor agonists and seizure susceptibility

#### J. Findeis; F. Kerling; M. Winterholler

##### Department of Neurology, Sana Hospital Rummelsberg, Schwarzenbruck, Germany

**Background and aims:** Glucagon‐like peptide‐1 receptor agonists (GLP‐1 RAs) are widely used in type 2 diabetes mellitus and obesity and are increasingly discussed for potential neuroprotective properties. Large‐scale clinical and real‐world studies from 2024/2025 report a predominantly neutral to protective association between GLP‐1 RA use and seizure‐ or epilepsy‐related endpoints. However, the effects of GLP‐1 RAs on neuronal excitability in patients with established epileptogenic circuitry remain insufficiently characterized.

**Methods:** We conducted a narrative review critically examining experimental and clinical evidence regarding the impact of GLP‐1 RAs on seizure propensity, with particular emphasis on discrepancies between population‐level outcomes and individual network‐level vulnerability.

**Results:** Preclinical studies demonstrate heterogeneous, network‐dependent actions of GLP‐1 RAs. Anticonvulsant properties are observed in chemically induced seizure models, whereas paradoxical pro‐seizure responses have been reported in genetic absence epilepsy models, suggesting circuit‐specific directionality. In contrast, population‐level analyses consistently report no increased risk of incident seizure‐related events under GLP‐1 RA therapy. However, these findings are largely derived from administrative diagnostic codes, lack epileptological phenotyping and are not designed to capture transient, exposure‐dependent alterations in ictogenic potential. Additional factors, including delayed gastric emptying affecting antiseizure medication absorption, rapid metabolic shifts during titration, blood‐brain barrier dysfunction and agent‐specific pharmacokinetics may further modulate individual susceptibility.

**Conclusion:** GLP‐1 RA effects on neuronal excitability appear highly context‐dependent, shaped by epilepsy subtype, network vulnerability, exposure dynamics and treatment phase. Prospective studies with higher temporal and clinical resolution, incorporating detailed phenotyping, therapeutic drug monitoring and longitudinal seizure assessment, are required to ensure neurological safety in patients with epilepsy.

**Disclosure:** Nothing to disclose.

## EPO‐0238

### A systematic review of Lamotrigine overdose: From uneventful to ICU admission and cardiac arrest

#### L. Taruffi ^1^; N. Orlandi^2^; M. Pugnaghi^2^; M. Burani^1^; L. Affronte^2^; A. Vaudano^3^; G. Giovannini^2^; S. Meletti^3^

##### ^*1*^ *Post‐graduate School of Neurology, Department of Biomedical Metabolic and Neural Sciences, University of Modena and Reggio Emilia, Italy;* ^*2*^ *Neurophysiology Unit, Neuroscience Dept, AOU Modena, Italy;* ^*3*^ *Department of Biomedical Metabolic and Neural Sciences, University of Modena and Reggio Emilia, Italy*

**Background and aims:** Lamotrigine (LTG) is a widely used drug to treat epilepsy and depression. We performed a systematic review of studies reporting LTG overdose. Additionally, we reported an original case.

**Methods:** PubMed was searched without date limits. 103 records were screened, of which 40 studies were finally included in the systematic review, and results reported according to PRISMA guideline, adding seven studies identified from included articles. We extracted demographics, LTG indication, overdose reason, complications, EEG findings, and outcomes.

**Results:** Forty‐seven patients (27 female; mean age 28.3 years) were included. Nineteen patients co‐ingested substances in addition to lamotrigine. Intent was suicidal in 33 cases. Lamotrigine was mainly prescribed for bipolar disorder (55%) and epilepsy (28%). Seizures occurred in 45% of overdoses (5 single, 10 cluster, 11 status epilepticus), including 6/13 patients with epilepsy. Median ingestion was 4.7 g (IQR 2.7 to 9.5) and median plasma concentration was 35.75 mg/L (IQR 25.3 to 52.8). There were 3 deaths and 5 cardiac arrests. Twenty‐five patients required ICU admission. Electroencephalography was reported in 23/47: 3 showed ongoing seizure, 12 with slowing pattern or epileptiform abnormalities, and the remainder unremarkable. Personal case: 75‐year‐old man with temporal lobe epilepsy admitted for tonic‐clonic status epilepticus. LTG concentration was 39.73 mg/L after erroneous intake. Patient required rehabilitation after prolonged ICU staying.

**Conclusion:** LTG overdose can result in severe neurotoxicity, coma, status epileptics and death (6%). Cardiac arrest was reported 11% of cases. Suicidal ideation was the most common reason for overdose but also erroneous intake in children and elders.

**Disclosure:** Nothing to disclose.

## EPO‐0239

### Does time since diagnosis or the number of failed ASMs matter more in people with DRE receiving VNS therapy?

#### M. Boffini ^1^; P. Kwan^2^; G. Giannicola^1^; X. Zhou^3^; S. Baeesa^4^; S. Shimada^5^; G. Morris^6^; G. Motamedi^7^; K. Myers^8^; F. Beraldi^9^; L. Acciari^9^; M. Dibue^1^; R. Verner^1^; C. Gordon^1^; K. Nichol^1^; A. Sen^10^

##### ^*1*^ *LivaNova PLC (or a subsidiary);* ^*2*^ *The Alfred Hospital, Monash University, and The Royal Melbourne Hospital, University of Melbourne, Melbourne, Victoria, Australia;* ^*3*^ *Upstate University Hospital and SUNY Upstate Medical University, Syracuse, New York;* ^*4*^ *Neurosciences Department, King Faisal Specialist Hospital & Research Centre, Jeddah, Saudi Arabia;* ^*5*^ *The University of Tokyo Hospital, Tokyo, Japan;* ^*6*^ *St. Mary's Hospital, Ascension, USA;* ^*7*^ *Department of Neurology, Medstar Georgetown University Hospital, Washington, DC;* ^*8*^ *Research Institute of the McGill University Medical Center, Montreal, Quebec, Canada;* ^*9*^ *Valos, LivaNova Partner CRO, Genoa, Italy;* ^*10*^ *Oxford Epilepsy Research Group, John Radcliffe Hospital, Oxford, UK*

**Background and aims:** Time since diagnosis and number of failed ASMs may have an impact of the outcomes of people who receive VNS Therapy for DRE.

**Methods:** Participants with DRE enrolled into the CORE‐VNS study and who were naïve to VNS Therapy were grouped into early (EI), < 5 years; intermediate (II), 5 to < 10 years; or late (LI), > 10 years. Participants were also grouped by number of failed ASMs: < 3, 4–6, 7–10. 11–16, or > 16. Clinical outcomes were assessed across seizure‐related (frequency) and non‐seizure (quality of life) domains at 3, 6, 12, 24, and 36 months.

**Results:** EI tended to have greater seizure frequency reduction than LI: median percentage change from baseline in seizure frequency at 36 months for the EI group was −88.2% (95% CI −100% to −76.5%) versus −71.4% (95% CI −81.6% to −55.0%) in the LI group. 40.2% of EI achieved a 100% reduction in seizure frequency at 36 months, compared to 22.6% in the LI group. Despite this, participants treated with fewer ASMs had a significant greater likelihood of response than those with more failed ASMs when adjusted for covariates including age and time since diagnosis (*p* < 0.05). At 36 months, participants implanted after 2–3 failed ASMs had a median change from baseline in seizure frequency of −94.4% compared with a −57.4% who failed 11–16 ASMs.

**Conclusion:** The benefits of VNS Therapy on seizure outcomes are better when initiated promptly. Continued trials of ASMs may limit the benefit of early implantation following DRE diagnosis.

**Disclosure:** GG, MD, RV, CG, and KN are employees of LivaNova PLC (or a subsidiary) and hold stock or stock options with the company, who is the manufacturer of the VNS Therapy System. All other authors are investigators associated with the CORE‐VNS Study, and in that capacity they or their institutions receive compensation from LivaNova for study‐related activities. No author received direct compensation from LivaNova.

## EPO‐0240

### VNS therapy is associated with a decrease in health care resource utilization over 36 months in people with drug‐resistant epilepsy

#### M. Dibue ^1^; J. Freeman^2^; K. Myers^3^; N. Kurwale^4^; G. Giannicola^1^; G. Motamedi^5^; P. Kwan^6^; Y. Al‐Said^7^; G. Delougne^1^; S. Shimada^8^; C. Gordon^1^; A. Sen^9^; K. Nichol^1^

##### ^*1*^ *LivaNova PLC (or a subsidiary);* ^*2*^ *The Royal Children's Hospital, Melbourne, Australia;* ^*3*^ *Research Institute of the McGill University Medical Center, Montreal, Quebec, Canada;* ^*4*^ *Deenanath Mangeshkar Hospital and Research Centre, Pune, India;* ^*5*^ *Department of Neurology, Georgetown University Hospital, Washington, DC;* ^*6*^ *The Alfred Hospital, Monash University, and The Royal Melbourne Hospital, University of Melbourne, Melbourne, Victoria, Australia;* ^*7*^ *Department of Neurosciences, King Faisal Specialist Hospital & Research Center, Jeddah, Saudi Arabia;* ^*8*^ *The University of Tokyo Hospital, Tokyo, Japan;* ^*9*^ *Oxford Epilepsy Research Group, John Radcliffe Hospital, Oxford, UK*

**Background and aims:** VNS Therapy is important for people resistant to antiseizure medications and for those in whom surgery is not feasible. People with DRE have increased HCRU due to seizures and seizure‐related health events. The impact of any therapy on HCRU is an important consideration when evaluating total benefit and costs of an intervention.

**Methods:** People with DRE were enrolled into a prospective, observational, multicenter, international study called the CORE‐VNS (NCT03529045). HCRU was evaluated for 12 months before baseline and at 12‐month intervals over the duration of the CORE‐VNS study. For this analysis, the HCRU related to seizure activity in people with DRE from baseline to 36 months was analyzed.

**Results:** 526 participants met the study criteria. Participants who reported seizure‐related ER visits and the number of events decreased from 32.3% at baseline (*n* = 599) to 14.8% at 36 months (*n* = 128). Participants with seizure‐related ER visits due to status epilepticus decreased from 12.2% at baseline (*n* = 195) to 3.6% at 36 months (*n* = 42), and those with seizure‐related ER visits leading to hospitalization decreased from 15.8% at baseline (*n* = 201) to 6.2% at 36 months (*n* = 32). Participants reporting seizure‐related hospitalizations decreased from 27.0% at baseline (*n* = 312) to 7.4% at 36 months (*n* = 45). Participants reporting hospitalizations due to status epilepticus decreased from 9.5% at baseline (*n* = 129) to 2.1% at 36 months (*n* = 11).

**Conclusion:** The use of VNS Therapy is associated with a decrease in HCRU in people with DRE.

**Disclosure:** PT, GG, MD, GD, CG, and KN are employees of LivaNova PLC (or a subsidiary) and hold stock or stock options with the company, who is the manufacturer of the VNS Therapy System. All other authors are investigators associated with the CORE‐VNS Study, and in that capacity they or their institutions receive compensation from LivaNova for study‐related activities. No author received direct compensation from LivaNova.

## EPO‐0241

### First Antiseizure medication choice in older adults with seizures: An observational cohort study based on a claims database

#### P. Vassallo ^1,2,3^; R. Thijs^1,2,3^; J. van der Palen^4,5^; J. Sander^1,2,6^

##### ^*1*^ *UCL Queen Square Institute of Neurology, London, and Chalfont Centre for Epilepsy, Chalfont St Peter, K;* ^*2*^ *Stichting Epilepsie Instellingen Nederland (SEIN), Heemstede, Netherlands;* ^*3*^ *Department of Neurology, Leiden University Medical Centre, Leiden, Netherlands;* ^*4*^ *Department of Epidemiology, Medisch Spectrum Twente, Enschede, The Netherlands;* ^*5*^ *Section Cognition, Data and Education, Faculty of Behavioural, Management, and Social Sciences, University of Twente, Enschede, The Netherlands;* ^*6*^ *Department of Neurology, West China Hospital, Sichuan University, Chengdu, Sichuan, China*

**Background and aims:** Data on first‐line antiseizure medication (ASM) use in older adults with new‐onset epilepsy are limited. Enzyme‐modulating ASMs may affect cholesterol levels, which is relevant in patients with cardiovascular comorbidity. We examined sex differences and cardiovascular risk profiles, focusing on enzyme‐modulating ASM use.

**Methods:** This nationwide, claims‐based study included adults aged ≥ 50 years with seizures diagnosed in 2014, using Diagnosis Treatment Combination codes in the Dutch health insurance database (Vektis). Pharmacy claims documented ASM prescriptions. Analyses were restricted to incident cases with ASM after diagnosis. Descriptive statistics explored prescribing patterns and sex differences. Cox regression assessed associations between ASM class and initiation of lipid‐lowering therapy. Consent was waived due to anonymization.

**Results:** Among 8728 adults with incident seizures, 3,583 received a single ASM after diagnosis. 42.5% (*n* = 1,524) were female; median age was 70 years (range 60–102). Levetiracetam (53%) and valproic acid (30%) were the most prescribed ASMs across sexes. Enzyme‐modulating ASMs were frequently used despite cardiovascular comedication. Carbamazepine was more often prescribed to women, while lamotrigine ranked fifth overall. Males had longer prescription intervals than females. Daily doses were similar between sexes, but lower in adults over 70. No significant association was found between enzyme‐modulating ASMs and time to lipid‐lowering agent initiation.

**Figure:**
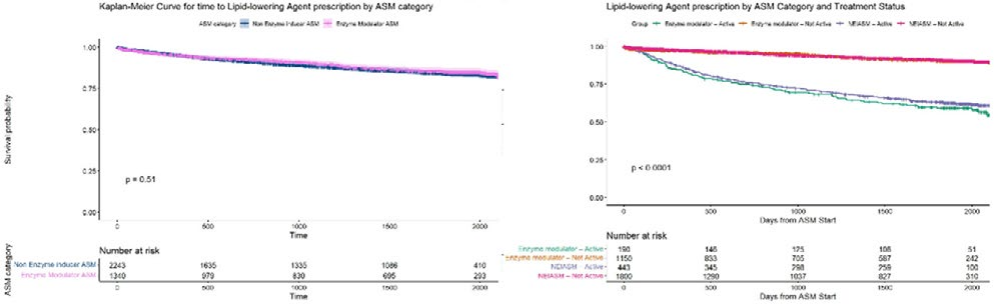
**FIGURE 1** 1A (left). Kaplan‐Meier curves for lipid‐lowering initiation by ASM class and 1B (right). active treatment in overall sample.

**Figure:**
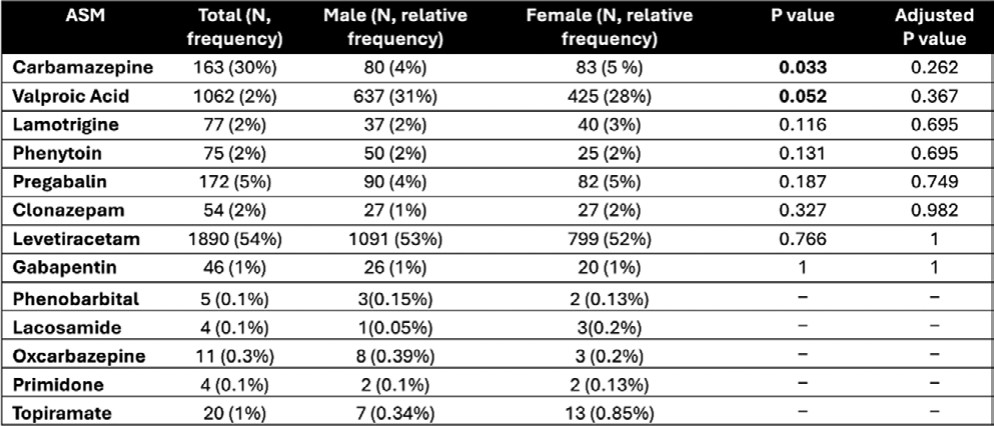
**TABLE 1** ASM prescription frequency (N and percentage of the total number of prescriptions) and relative frequencies comparison across sex groups. *p*‐values and adjusted *p*‐values after correction for multiple testing, indicate the level of significance.

**Figure:**
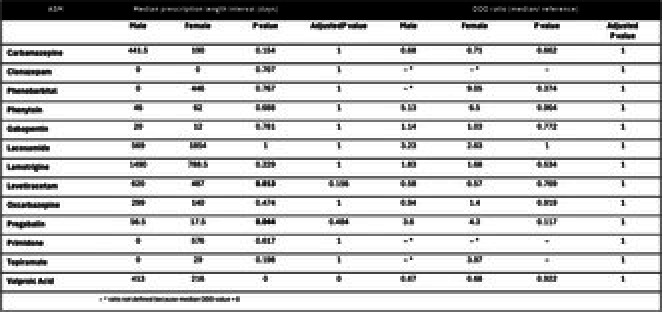
**TABLE 2** ASM prescriptions with the median prescribed daily doses ratio, and median prescription intervals length (in days). *p*‐values and adjusted *p*‐values for multiple testing indicate the level of significance.

**Conclusion:** Nationwide claims data offer insights into real‐world ASM prescribing. Treatment remains conservative, with older ASMs prescribed despite cardiovascular co‐medications. Women and the oldest adults may receive shorter treatment intervals and lower doses. Clinical linkage is lacking, but these findings may guide optimization of epilepsy management in specific subgroups.

**Disclosure:** PV is supported by Fondation Ancrage and Novartis Stiftung für medizinisch‐biologische Forschung. RDT receives research support from the Anna Teding van Berhout Stichting, Vriendenloterij, EpilepsieNL, ZonMW, EU Pathfinder, and the Michael J. Fox Foundation. JWS receives research support from the National Institute for Health Research (NIHR), the Marvin Weil Epilepsy Research Fund and the Academy of Medical Sciences. This study was partly based at the NIHR University College London Hospitals Biomedical Research Centre, which receives a proportion of funding from the UK Department of Health.

## EPO‐0242

### A ferroptosis‐based diagnostic index for severity stratification in symptomatic epilepsy

#### R. Azizova; F. Mallaev; A. Abboskhonov

##### Tashkent State Medical University, Uzbekistan

**Background and aims:** Reliable biomarkers for assessing disease severity in symptomatic epilepsy are limited. Given the emerging role of ferroptosis in neurological disorders, integrating ferroptosis‐related biomarkers into a composite diagnostic index may improve epilepsy severity stratification and support personalized management.

**Methods:** This prospective study included 103 patients with symptomatic epilepsy and 20 healthy individuals. Serum markers of iron metabolism (total iron, ferritin, transferrin), lipid peroxidation (MDA, 4‐HNE), and antioxidant defense (GSH, GPX4, SOD) were measured using standardized biochemical methods. A ferroptosis index (FI) was calculated using a composite formula incorporating pro‐oxidant and antioxidant parameters. Patients were stratified according to disease severity. Diagnostic performance was evaluated using ROC analysis.

**Results:** The ferroptosis index demonstrated progressive elevation with increasing epilepsy severity. Based on FI values, patients were classified into mild (FI < 2.5), moderate (FI 2.5–7.5), and severe epilepsy (FI > 7.5). ROC analysis revealed high diagnostic accuracy of the FI for identifying severe epilepsy, with an area under the curve of 0.89 (95% CI: 0.82–0.96), sensitivity of 87%, and specificity of 83%. Higher FI values were associated with increased seizure frequency and more pronounced cognitive impairment.

**Conclusion:** The proposed ferroptosis‐based diagnostic index is a reliable and clinically applicable tool for severity stratification in symptomatic epilepsy and may facilitate individualized therapeutic decision‐making.

**Disclosure:** Nothing to disclose.

## EPO‐0243

### Utility of the IsCHEMiA score in predicting post‐stroke epilepsy after stroke reperfusion therapy

#### W. Leung^1^; T. Tanaka^2^; I. Leung^1^; R. Ho^1^; F. Chu ^1^; A. Leung^1^; E. Yip^3^; M. Ihara^2^; K. Fukuma^2^; K. Teo^1^; G. Lau^1^

##### ^*1*^ *Division of Neurology, Department of Medicine, Queen Mary Hospital, University of Hong Kong, Hong Kong SAR, China;* ^*2*^ *Department of Neurology, National Cerebral and Cardiovascular Center, Osaka, Japan;* ^*3*^ *Department of Medicine and Geriatrics, Ruttonjee Hospital, Hong Kong SAR, China*

**Background and aims:** The IsCHEMiA score is a newly developed, internationally validated tool for predicting post‐stroke epilepsy (PSE) after acute ischemic stroke. We aimed to study its predictive performance in stroke patients after reperfusion therapy compared to existing risk scores.

**Methods:** We reviewed clinical and imaging features of patients with first‐ever acute ischemic stroke who received intravenous thrombolysis (IVT) and/or endovascular thrombectomy (EVT) at Queen Mary Hospital (Hong Kong), Ruttonjee Hospital (Hong Kong), and National Cerebral and Cardiovascular Center (Japan). We calculated the IsCHEMiA, SeLECT, and SeLECT 2.0 scores, and compared their performances using discrimination (c statistic).

**Results:** We included 627 patients with a mean age of 72.1 years, where 421 (67.1%) received IVT and 349 (55.7%) underwent EVT (Table 1). PSE, defined as the occurrence or recurrence of unprovoked seizure > 7 days after stroke, occurred in 42 (6.7%) patients. Haemorrhagic transformation (of any grade) occurred in 154 (24.6%) patients. The IsCHEMiA score outperformed the SeLECT score (c = 0.806 vs. 0.729, z = 2.772; *p* = 0.006) and SeLECT 2.0 score (c = 0.806 vs. 0.718, z = 2.784; *p* = 0.005) (Figure 1).

**Figure:**
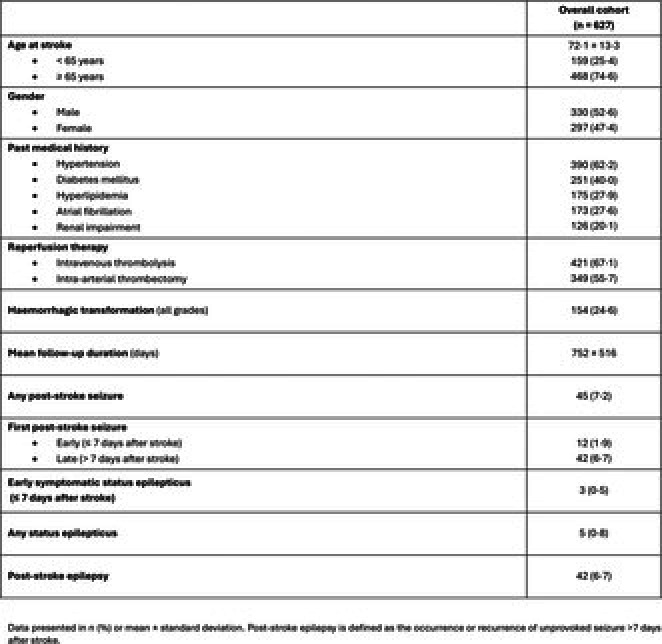
**TABLE 1** Baseline Characteristics of overall cohort.

**Figure:**
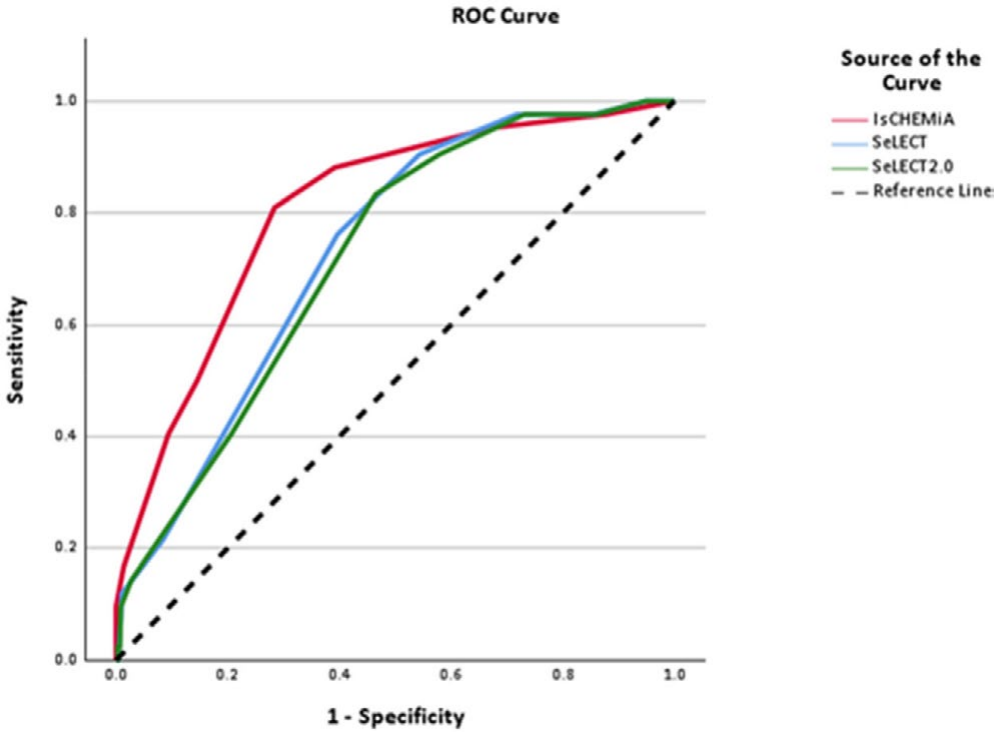
**FIGURE 1** Receiver Operating Characteristic (ROC) Curves of IsCHEMiA, SeLECT, and SeLECT2.0.

**Conclusion:** The IsCHEMiA score further improved the prediction of PSE in the stroke population who received acute reperfusion therapy. This reflects the strength of the IsCHEMiA score by accessing the characteristics of the final infarct instead of stroke severity. Our findings further suggest an important role haemorrhagic lesions in epileptogenesis. Future research is warranted to study the role of preventing post‐reperfusion haemorrhagic transformation in reducing the risk of PSE.

**Disclosure:** Nothing to disclose.

## Headache 2

## EPO‐0244

### Consistency of effect of Atogepant in the acute treatment of migraine across multiple attacks: Results from the ECLIPSE trial

#### A. Van Dycke ^1^; E. Brand‐Schieber^2^; R. Ornello^3^; M. Lee^4,5^; L. Luo^2^; T. Takeshima^6^; D. Holle‐Lee^7^; C. Tassorelli^8,9^; K. Carr^2^; K. Nagy^2^

##### ^*1*^ *Department of Neurology, General Hospital Sint‐Jan Bruges, Brugge, Belgium;* ^*2*^ *AbbVie Inc. North Chicago, IL, USA;* ^*3*^ *Department of Biotechnological and Applied Clinical Sciences, University of L'Aquila, L'Aquila, Italy;* ^*4*^ *Department of Neurology, Seoul National University Hospital, Seoul, South Korea;* ^*5*^ *Seoul National University College of Medicine, Seoul, South Korea;* ^*6*^ *Headache Center and Department of Neurology, Tominaga Hospital, Osaka, Japan;* ^*7*^ *Department of Neurology, West German Headache and Vertigo Center Essen, University of Essen, Essen, Germany;* ^*8*^ *Department of Brain and Behavioral Sciences, University of Pavia, Pavia, Italy;* ^*9*^ *Headache Science & Neurorehabilitation Centre, IRCCS C. Mondino Foundation, Pavia, Italy*

**Background and aims:** Atogepant is an FDA/EMA approved calcitonin gene‐related peptide receptor antagonist for migraine preventive treatment. This analysis evaluated within‐participant consistency of effect (CoE) of atogepant 60 mg in the acute treatment of migraine headache and its effect across multiple migraine attacks.

**Methods:** ECLIPSE was a double‐blind (DB), placebo‐controlled, multiple‐attack, crossover trial evaluating atogepant's efficacy, safety, and tolerability for acute treatment of migraine. Adults with 2–8 monthly moderate‐to‐severe attacks were randomised to receive atogepant for three qualifying attacks and placebo for one attack during the up to 16‐week DB period. CoE was assessed by six prespecified supportive secondary endpoints, defined as achievement of key outcomes in > = 2 out of 3 atogepant‐treated attacks, pain freedom at 2 hours postdose, pain relief at 2 hours, and sustained pain freedom/pain relief (from 2–24 to 2–48 hours postdose). Efficacy of atogepant versus placebo was evaluated for each of these endpoints across multiple attacks (Attacks 1–4).

**Results:** Atogepant demonstrated CoE for pain freedom and pain relief at 2 hours, as well as sustained pain freedom and relief from 2–24 to 2–48 hours (Table 1). Across all 4 treated attacks, atogepant demonstrated improvements versus placebo for pain freedom and pain relief at 2 hours, and sustained pain freedom and relief from 2–24 to 2–48 hours (Attack 1, adjusted *P* < 0.0001; Attacks 2–4, nominal *P* < 0.0001) (Figures 1 and 2).

**Figure:**
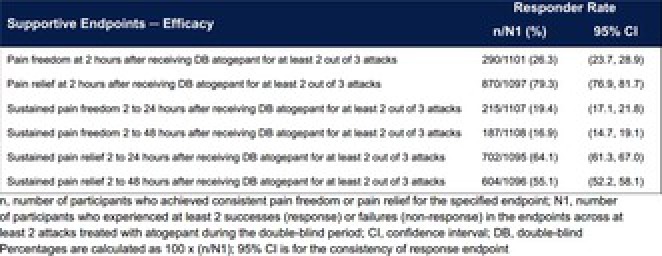
**TABLE 1** Supportive endpoints for consistency of effect: Responder rates across multiple attacks treated with double‐blind atogepant.

**Figure:**
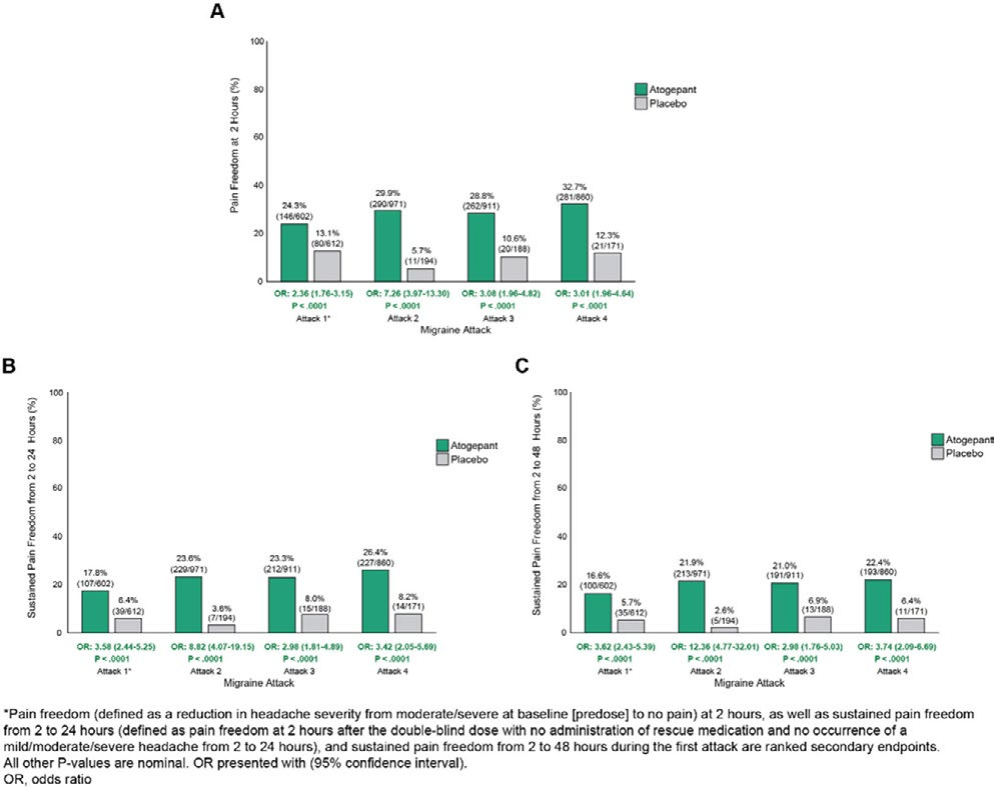
**FIGURE 1**: Proportion of participants achieving pain freedom (A) at 2 hours after double‐blind dose, (B) from 2 to 24 hours after double‐blind dose, and (C) from 2 to 48 hours after double‐blind dose.

**Figure:**
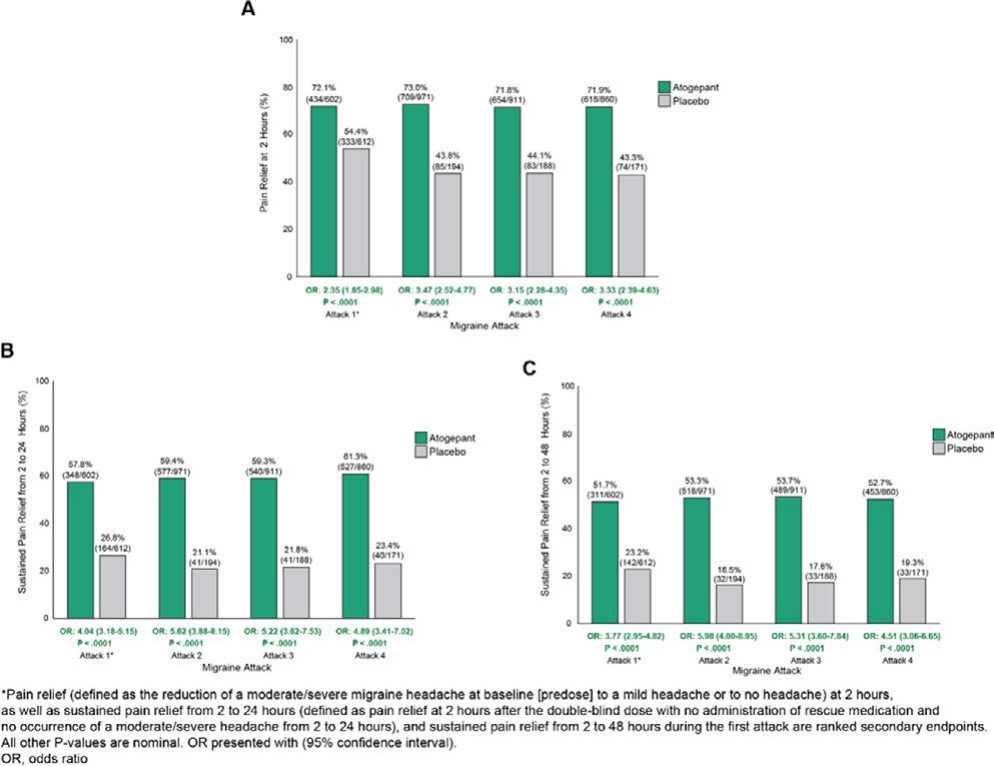
**FIGURE 2**: Proportion of participants achieving pain relief (A) at 2 hours after double‐blind dose; (B) from 2 to 24 hours after double‐blind dose, and (C) from 2 to 48 hours after double‐blind dose.

**Conclusion:** Among adults with migraine headache, atogepant demonstrated consistency of within‐participant effect and showed reliable efficacy compared with placebo across multiple migraine attacks.

**Disclosure:** AVD has served on advisory/scientific boards and/or given lectures for AbbVie/Allergan, Angelini Pharma, Eli Lilly, Lundbeck, Neuraxpharm, Novartis, Organon, Pfizer, TEVA, and UCB, for which she has received honoraria. RO reports personal fees from AbbVie, Bayer, Eli Lilly, Lundbeck, Novartis, Organon, Pfizer, and Teva. He is a member of the Editorial Board of The Journal of Headache and Pain, International Journal of Stroke, Arquivos de Neuropsiquiatria, and Confinia Cephalalgica. MJL has received honoraria as a consultant or speaker for Eli Lilly, Teva, AbbVie, Pfizer, Organon Korea, SK pharm, and YuYu Pharma; has been the principal investigator or co‐investigator in trials sponsored by Eli Lilly, Novartis, Teva (Otsuka), Allergan, Abbvie, Lundbeck, Biohaven, Yuhan Company, Ildong Pharm, Samjin Pharm, and DongAST; and has received research support from the National Research Foundation of Korea and Korea Medical Device Development Fund. TT has been on the speakers' bureau for Amgen K.K, Daiichi Sankyo Co, Ltd, Eli Lilly Japan K.K, and Otsuka Pharmaceutical Co, Ltd, and has received research funding/collaborative research expenses from AbbVie GK, Amgen K.K, Eli Lilly Japan K.K, Eisai Co, Ltd, Lundbeck Japan K.K, and Pfizer Japan Inc. TT has also acted as an advisor to Hedgehog MedTech, Inc, Sawai Pharmaceutical Co, Ltd, and TEIJIN Pharma Ltd. DHL received honoraria for consulting from AbbVie/Allergan, Amgen, Eli Lilly, Novartis, Teva, Lundbeck, Hormosan, and Zuellig Pharma. CT reports, over the last 36 months, personal fees for participating in advisory boards or for lecturing at sponsored symposia for AbbVie, Dompé, Eli Lilly, Ipsen, Lundbeck, Medscape, Organon Pfizer, and Teva. She is principal investigator or collaborator in clinical trials sponsored by AbbVie, Eli Lilly, Ipsen, Lundbeck, Pfizer and Teva. She has received research grants from the European Commission, the Italian Ministry of Health, the Italian Ministry of University, the Migraine Research Foundation, and the Italian Multiple Sclerosis Foundation. EB‐S, LL, KC, and KN are employees of AbbVie and may hold AbbVie stock.

## EPO‐0245

### Efficacy of acute treatment with Atogepant on migraine symptoms and rescue medication use across multiple attacks: Results from the ECLIPSE trial

#### A. Van Dycke ^1^; K. Carr^2^; R. Ornello^3^; M. Lee^4,5^; L. Luo^2^; T. Takeshima^6^; D. Holle‐Lee^7^; C. Tassorelli^8,9^; E. Brand‐Schieber^2^; K. Nagy^2^

##### ^*1*^ *Department of Neurology, General Hospital Sint‐Jan Bruges, Brugge, Belgium;* ^*2*^ *AbbVie Inc. North Chicago, IL, USA;* ^*3*^ *Department of Biotechnological and Applied Clinical Sciences, University of L'Aquila, L'Aquila, Italy;* ^*4*^ *Department of Neurology, Seoul National University Hospital, Seoul, South Korea;* ^*5*^ *Seoul National University College of Medicine, Seoul, South Korea;* ^*6*^ *Headache Center and Department of Neurology, Tominaga Hospital, Osaka, Japan;* ^*7*^ *Department of Neurology, West German Headache and Vertigo Center Essen, University of Essen, Essen, Germany;* ^*8*^ *Department of Brain and Behavioral Sciences, University of Pavia, Pavia, Italy;* ^*9*^ *Headache Science & Neurorehabilitation Centre, IRCCS C. Mondino Foundation, Pavia, Italy*

**Background and aims:** Atogepant, an FDA/EMA approved migraine preventive treatment, has recently demonstrated efficacy for the acute treatment of a single migraine attack. This analysis evaluated the efficacy of atogepant 60mg versus placebo for the acute treatment of migraine‐related symptoms and rescue medication use across multiple attacks in double‐blind (DB) trial.

**Methods:** ECLIPSE was a DB, placebo‐controlled, multiple‐attack, crossover trial evaluating atogepant's efficacy, safety, and tolerability for acute treatment of migraine. Adults with 2–8 monthly moderate‐to‐severe attacks were randomised to receive atogepant for three qualifying attacks and placebo for one attack during the up‐to‐16‐week DB period. This analysis evaluated the proportion of participants achieving absence of the most‐bothersome symptom (MBS: photophobia, phonophobia, or nausea) at 2‐hours postdose across attacks 1–4. Additionally, the absence of photophobia, phonophobia, and nausea at 2‐hours postdose, as well as rescue medication use within 24‐ and 48‐hours postdose, were examined across attacks.

**Results:** Atogepant resulted in a significantly (*P* < 0.0001, nominal or ranked secondary as indicated) higher proportion of participants achieving absence of the MBS (OR: 1.77–3.09), photophobia (OR: 1.74–2.77), and phonophobia (OR: 1.96–3.43) 2 hours postdose across attacks 1–4 compared to placebo (Figures 1, 2). Participants had lower rates of nausea with atogepant, but these results were not significant for the ranked attack (Figure 2). Rescue medication use was lower with atogepant versus placebo across both 24 and 48 hours postdose period amongst all attacks (OR: 0.13–0.16; all *P* < 0.0001) (Figure 3).

**Figure:**
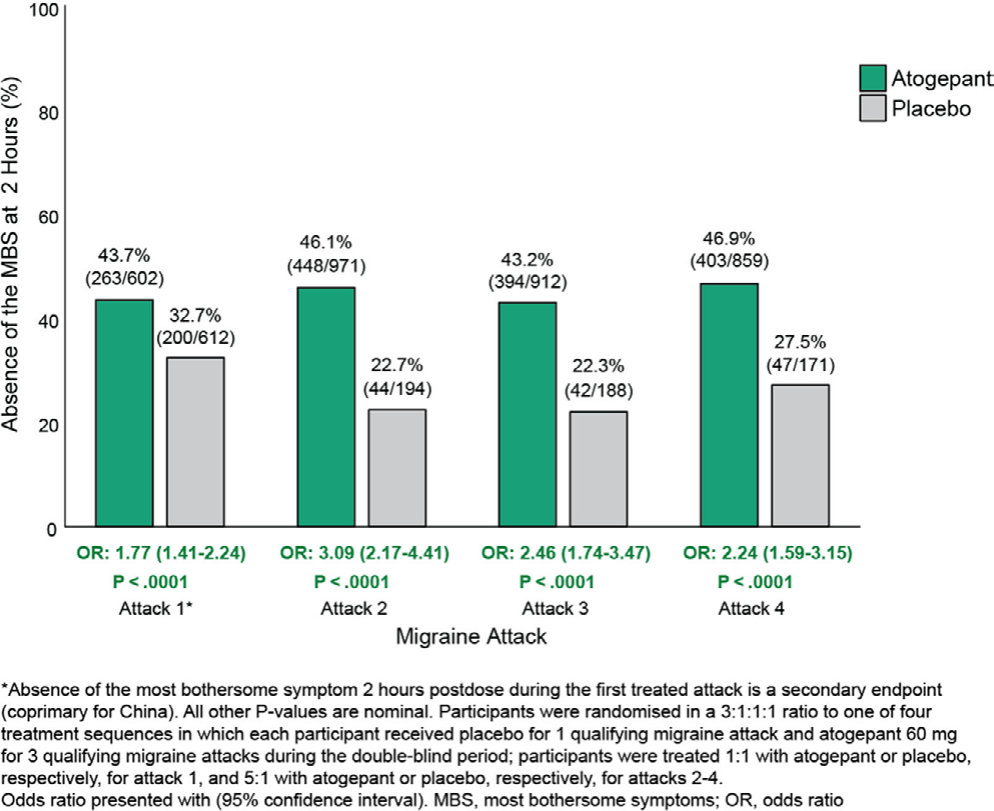
**FIGURE 1**: Proportion of participants free from the most bothersome symptom at 2 hours postdose during the double‐blind period across multiple attacks.

**Figure:**
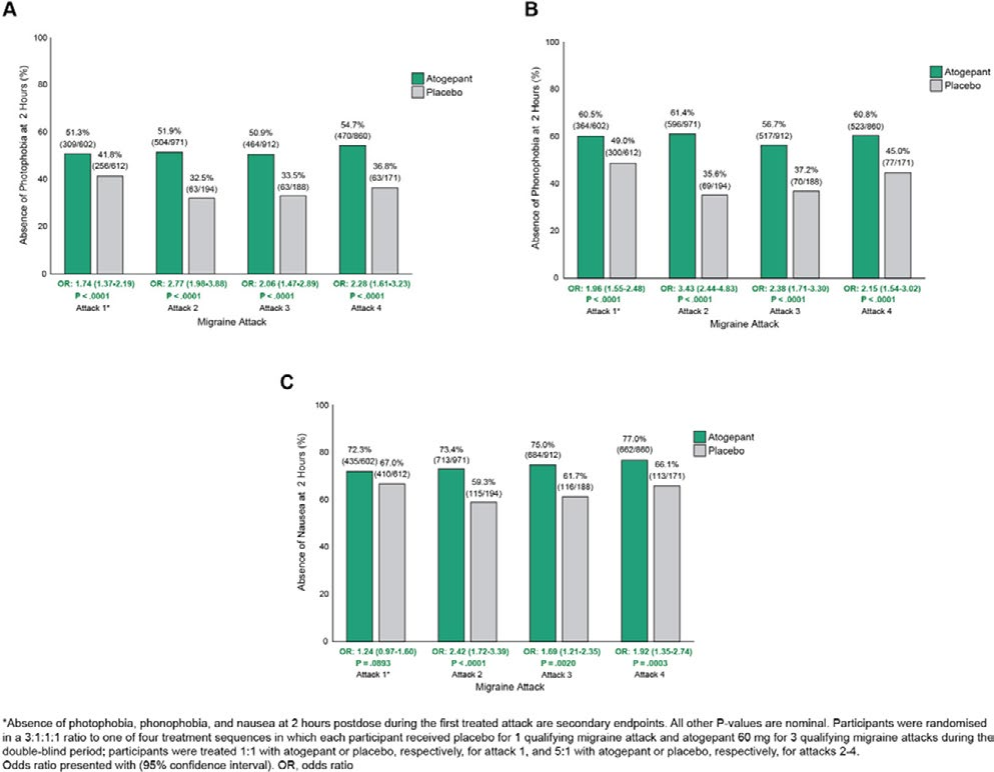
**FIGURE 2**: Proportion of participants with absence of photophobia, phonophobia, and nausea at 2 hours postdose during the double‐blind period across multiple attacks.

**Figure:**
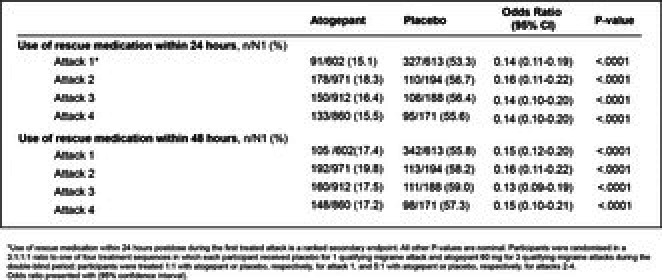
**FIGURE 3**: Proportion of participants using rescue medication within 24 and 48 hours postdose during the double‐blind period across multiple attacks.

**Conclusion:** Atogepant demonstrated reliable treatment effect compared to placebo by reducing migraine‐related symptoms and the need for rescue medication use across multiple migraine attacks.

**Disclosure:** AVD has served on advisory/scientific boards and/or given lectures for AbbVie/Allergan, Angelini Pharma, Eli Lilly, Lundbeck, Neuraxpharm, Novartis, Organon, Pfizer, TEVA, and UCB, for which she has received honoraria. RO reports personal fees from AbbVie, Bayer, Eli Lilly, Lundbeck, Novartis, Organon, Pfizer, and Teva. He is a member of the Editorial Board of The Journal of Headache and Pain, International Journal of Stroke, Arquivos de Neuropsiquiatria, and Confinia Cephalalgica. ML has received honoraria as a consultant or speaker for Eli Lilly, Teva, AbbVie, Pfizer, Organon Korea, SK pharm, and YuYu Pharma; has been the principal investigator or co‐investigator in trials sponsored by Eli Lilly, Novartis, Teva (Otsuka), Allergan, Abbvie, Lundbeck, Biohaven, Yuhan Company, Ildong Pharm, Samjin Pharm, and DongAST; and has received research support from the National Research Foundation of Korea and Korea Medical Device Development Fund. TT has been on the speakers' bureau for Amgen K.K, Daiichi Sankyo Co, Ltd, Eli Lilly Japan K.K, and Otsuka Pharmaceutical Co, Ltd, and has received research funding/collaborative research expenses from AbbVie GK, Amgen K.K, Eli Lilly Japan K.K, Eisai Co, Ltd, Lundbeck Japan K.K, and Pfizer Japan Inc. TT has also acted as an advisor to Hedgehog MedTech, Inc, Sawai Pharmaceutical Co, Ltd, and TEIJIN Pharma Ltd. DHL received honoraria for consulting from AbbVie/Allergan, Amgen, Eli Lilly, Novartis, Teva, Lundbeck, Hormosan, and Zuellig Pharma. CT reports, over the last 36 months, personal fees for participating in advisory boards or for lecturing at sponsored symposia for AbbVie, Dompé, Eli Lilly, Ipsen, Lundbeck, Medscape, Organon, Pfizer, and Teva. She is principal investigator or collaborator in clinical trials sponsored by AbbVie, Eli Lilly, Ipsen, Lundbeck, Pfizer and Teva. She has received research grants from the European Commission, the Italian Ministry of Health, the Italian Ministry of University, the Migraine Research Foundation, and the Italian Multiple Sclerosis Foundation. EB‐S, LL, KC, and KN are employees of AbbVie and may hold AbbVie stock.

## EPO‐0246

### Effect of Atogepant on cognitive function following acute treatment of migraine across multiple attacks: Results from the ECLIPSE trial

#### A. Van Dycke ^1^; G. Ahmadyar^2^; E. Brand‐Schieber^2^; C. Castro^2^; R. Ornello^3^; L. Luo^2^; P. Pozo‐Rosich^4^; U. Reuter^5^; K. Carr^2^; R. Gil‐Gouveia^6^

##### ^*1*^ *Department of Neurology, General Hospital Sint‐Jan Bruges, Brugge, Belgium;* ^*2*^ *AbbVie Inc. North Chicago, USA;* ^*3*^ *Department of Biotechnological and Applied Clinical Sciences, University of L'Aquila, L'Aquila, Italy;* ^*4*^ *Headache Unit, Neurology Department, Vall d'Hebron University Hospital, Barcelona, Spain; Headache and Neurological Pain Research Group, Vall d'Hebron Institute of Research, Universitat Autonoma de Barcelona, Barcelona, Spain;* ^*5*^ *University Hospital Bonn, Bonn, Germany; Charité Universitätsmedizin Berlin, Berlin, Germany;* ^*6*^ *Neurology Department, Hospital da Luz, Lisbon, Portugal; Center for Interdisciplinary Research in Health, Universidade Católica Portuguesa, Lisbon, Portugal*

**Background and aims:** Atogepant is approved for the preventive treatment of migraine in adults and has recently demonstrated its efficacy and safety for the acute treatment of migraine. This analysis evaluated the impact of atogepant 60mg administered as needed (PRN) for acute treatment of migraine on cognitive function assessed by the ability to think clearly.

**Methods:** ECLIPSE was a double‐blind (DB), placebo‐controlled, multiple‐attack, crossover trial evaluating atogepant's efficacy, safety, and tolerability for acute migraine treatment. Adults with 2–8 monthly moderate‐to‐severe attacks were randomised to receive atogepant for three qualifying attacks and placebo for one attack during the up to 16‐week DB period. Cognitive function scale is a single‐item, patient‐reported questionnaire measuring participants' difficulty in ability to think clearly, rated from 0 (not difficult at all) to 3 (very difficult). The proportion of participants achieving the ability to think clearly at 2‐, 4‐, and 8‐hours postdose across attacks 1–4 was an additional (exploratory) efficacy endpoint. Data for atogepant and placebo are presented with nominal *p*‐values.

**Results:** Atogepant treatment resulted in a nominally significant higher proportion of participants achieving the ability to think clearly compared with placebo as early as 2‐hours postdose in the first treated attack (OR: 1.78, Figure 1); this effect increased over time through 4‐ and 8‐hours postdose (OR: 3.39, 3.73, respectively; Figure 1). A similar reliable response was observed across attacks 2–4 (Figure 2).

**Figure:**
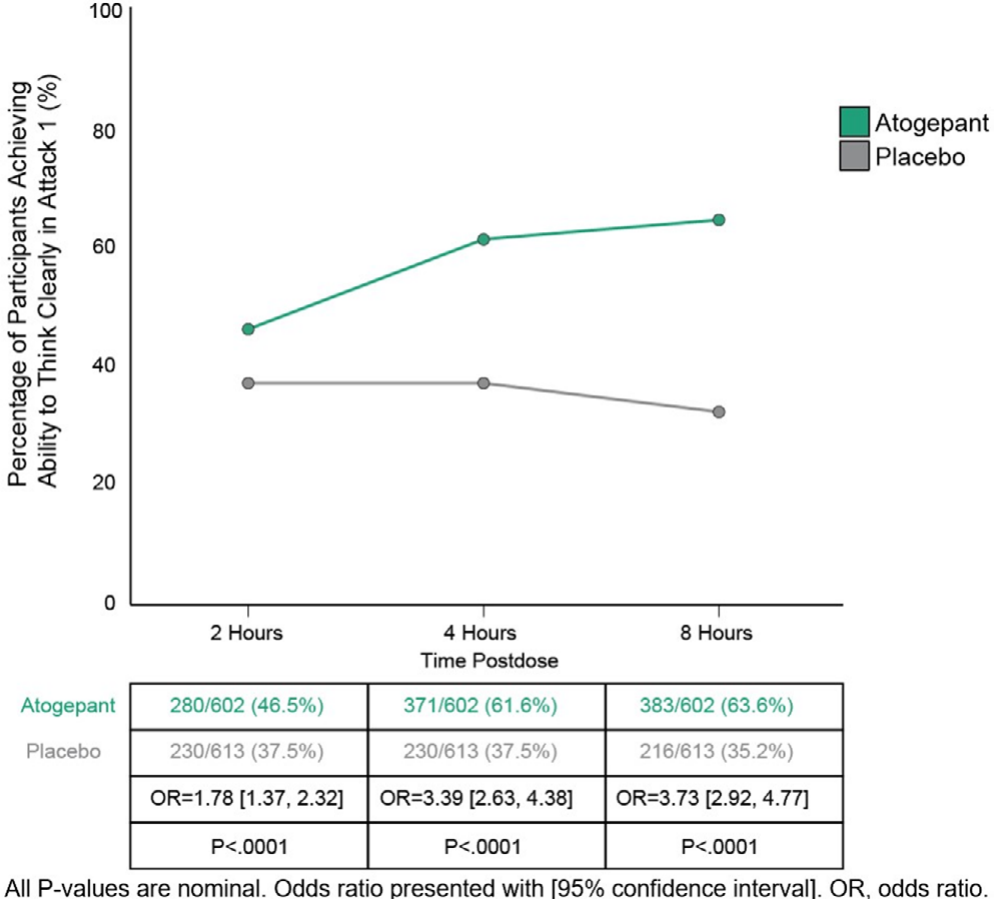
**FIGURE 1**: Proportion of participants who reported “not difficult at all” in ability to think clearly at 2‐, 4‐, and 8‐hours postdose in the first migraine attack.

**Figure:**
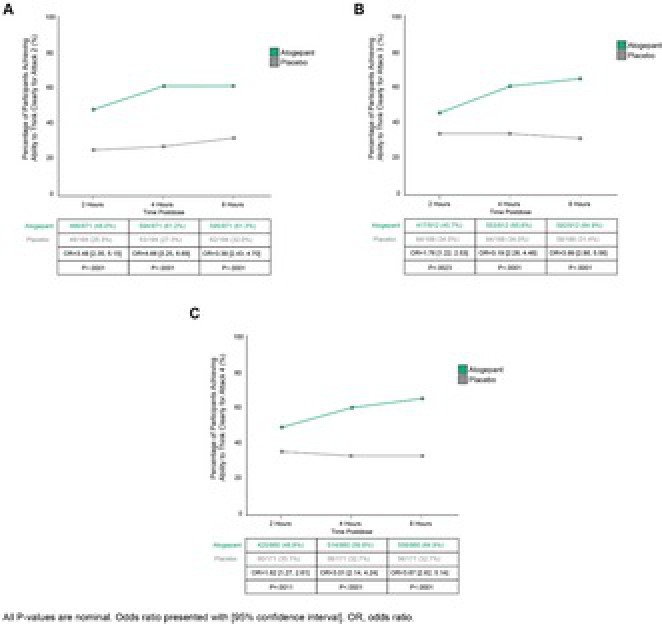
**FIGURE 2**: Proportion of participants who reported “not difficult at all” in ability to think clearly at 2‐, 4‐, and 8‐hours postdose across attacks 2, 3, and 4 (A, B, and C, respectively).

**Conclusion:** A greater proportion of participants treated with atogepant achieved the ability to think clearly compared to placebo as early as 2‐hours and up to 8‐hours postdose across multiple attacks.

**Disclosure:** AVD has served on advisory/scientific boards and/or given lectures for AbbVie/Allergan, Angelini Pharma, Eli Lilly, Lundbeck, Neuraxpharm, Novartis, Organon, Pfizer, TEVA, and UCB, for which she has received honoraria. RO reports personal fees from AbbVie, Bayer, Eli Lilly, Lundbeck, Novartis, Organon, Pfizer, and Teva. He is a member of the Editorial Board of The Journal of Headache and Pain, International Journal of Stroke, Arquivos de Neuropsiquiatria, and Confinia Cephalalgica. RG‐G has received honoraria for consultant and/or advisory boards from Allergan/Abbvie, Astra Zeneca, Almirall, Bial, Biogen, Bristol‐Myers Squibb, CMBE, FLOAT, Lilly, Lundbeck, Merk, Novartis, Novo Nordisk, Organon, Pfizer, Roche, Sanofi, Tecnifar and Teva; research funding from Fundação Ciência Tecnologia (Project 29675, MigN2Treat, 02/SAICT/2017); Learning‐Health, Luz Saúde (LiON, Luz Innovation on Neurosciences); Novartis‐Sociedade Portuguesa de Cefaleias; Bolsa –Projecto Centro de Investigação Interdisciplinar em Saúde and UCP. PPR reports personal fees for consulting from AbbVie, Amgen, Dr. Reddy's, Eli Lilly, Lundbeck, Medscape, Novartis, Organon, Pfizer, and Teva in the last three years. She has received personal fees for speaking from AbbVie, Amgen, Dr. Reddy's, Eli Lilly, Lundbeck, Medscape, Novartis, Organon, Pfizer and Teva, and has had grants paid to her research group from AbbVie, AGAUR, EraNet Neuron, FEDER RIS3CAT, Instituto Investigación Carlos III, Novartis, and Teva. UR has served on advisory boards for AbbVie, Eli Lilly, Lundbeck, Novartis, Pfizer, and Teva and has received institutional honoraria for lectures from AbbVie, Eli Lilly, Lundbeck, Novartis, StreaMedUp, Springer, and Teva, received institutional honoraria for consulting services from Lundbeck, Pfizer, and AbbVie; received research funding from Novartis (CHERUB01) and the German Federal Ministry of Education and Research; UR is an associate editor of the Journal of Headache and Pain, and is the president of the EHF. GA, EB‐S, LL, CC, and KC are employees of AbbVie and may hold AbbVie stock.

## EPO‐0247

### Longitudinal shifts in migraine frequency patterns: Transition from chronic to episodic migraine in a pooled analysis of the PEARL and FINESSE studies

#### M. Ashina^1^; G. Broessner^2^; C. Tassorelli ^3^; C. Gaul^4^; P. Pozo‐Rosich^5^; L. Neeb^6^; P. Kokturk^7^; X. Hamann^8^; H. Akcicek^7^; A. Straube^9^

##### ^*1*^ *Department of Neurology, Danish Headache Center, Copenhagen University Hospital–Rigshospitalet Glostrup, Copenhagen, Denmark; Department of Clinical Medicine, University of Copenhagen, Copenhagen, Denmark;* ^*2*^ *Department of Neurology, Medical University of Innsbruck, Innsbruck, Austria;* ^*3*^ *Department of Brain and Behavioral Sciences, University of Pavia, Pavia, Italy; IRCCS C. Mondino Foundation, Pavia, Italy;* ^*4*^ *Headache Center Frankfurt, Frankfurt am Main, Germany;* ^*5*^ *Headache Unit and Research Group, Vall d'Hebron Hospital and Research Institute, Universitat Autonoma de Barcelona, Barcelona, Spain;* ^*6*^ *Department of Neurology, Brandenburg Medical School Theodor Fontane, University Hospital Brandenburg/Havel, Brandenburg/Havel, Germany;* ^*7*^ *Teva Pharmaceuticals Europe B.V, Haarlem, The Netherlands;* ^*8*^ *Teva GmbH, Ulm, Germany;* ^*9*^ *Department of Neurology, Ludwig‐Maximilians‐University Munich, Munich, Germany*

**Background and aims:** Chronic migraine (CM) is associated with increased disability, greater reductions in health‐related quality of life and higher rates of medication overuse compared with episodic migraine (EM). Consequently, achieving reversion from CM to EM is an important therapeutic goal. This analysis assessed long‐term changes in migraine frequency among participants with CM in PEARL and FINESSE.

**Methods:** The 24‐month, prospective, observational, real‐world PEARL (pan‐European) and FINESSE (Germany and Austria) studies evaluated the effectiveness and tolerability of fremanezumab in routine clinical practice among adults with migraine. Mean monthly migraine days (MMD) were assessed using daily headache diaries completed by participants throughout the study. In this pooled analysis, the proportions of participants shifting from CM (diagnosed at baseline) to high‐frequency EM (HFEM; > = 8 and < 15 MMD), moderate‐frequency EM (MFEM; > = 4 and < 8 MMD) and low‐frequency EM (LFEM; < 4 MMD) were assessed over time.

**Results:** Of 2145 participants included in the pooled analysis, 1212 had CM at baseline (Table 1). By Month 6, 20.4%, 25.0% and 39.5% of participants with available data (*n* = 1070) had transitioned to HFEM, MFEM and LFEM, respectively (Table 2). At Month 24, 49.5% of participants with available data (*n* = 641) had shifted to LFEM and only 7.6% of participants still had > 15 MMD.

**Figure:**
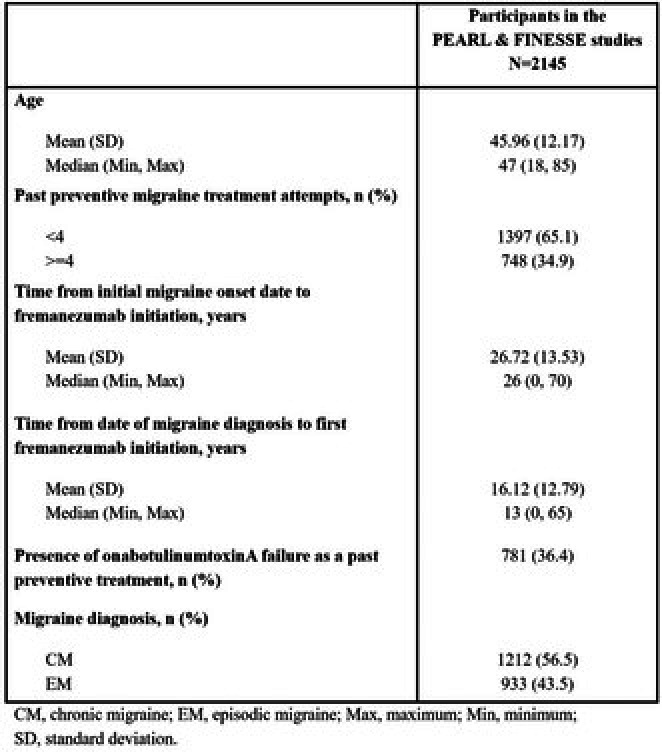
**TABLE 1** Baseline characteristics.

**Figure:**
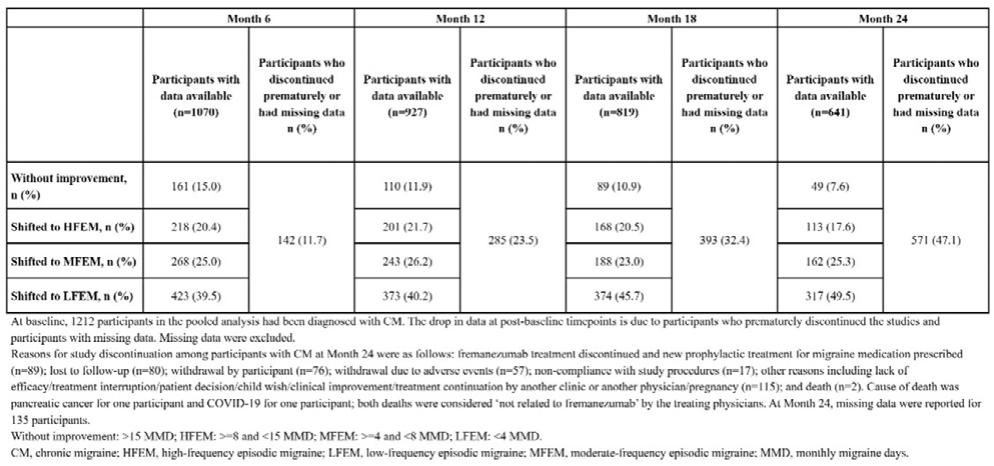
**TABLE 2** Proportion of participants transitioning from CM at baseline to HFEM, MFEM and LFEM at Months 6, 12, 18 and 24.

**Conclusion:** Fremanezumab facilitated meaningful reductions in migraine frequency over time, with nearly half of the participants with CM who persisted with treatment up to Month 24 transitioning to LFEM. These findings highlight the long‐term benefit of fremanezumab and its potential to restore low disease burden in a relevant subset of patients.

**Disclosure:** MA has received personal fees and declares PI/collaborator in clinical trials for AbbVie, AstraZeneca, Eli Lilly, GlaxoSmithKline, Lundbeck, Novartis, Pfizer and Teva Pharmaceuticals; and research grants from the Lundbeck Foundation, the Novo Nordisk Foundation, Lundbeck and Novartis. GB is, or was, a member of the following advisory boards: Grünenthal, Lilly, Lundbeck, Novartis, Pfizer and Teva Pharmaceuticals; is a speaker or member of the following speaker boards: Grünenthal, Lilly, Lundbeck, Novartis, Pfizer and Teva Pharmaceuticals; has acted as a consultant for Grünenthal, Lilly, Novartis and Teva Pharmaceuticals; and received grant support for research or education from Teva Pharmaceuticals. CT declares advisory boards, symposia and/or PI/collaboration in clinical trials for AbbVie, Chordate, Dompé, Eli Lilly, Ipsen, Lundbeck, Novartis, Pfizer and Teva Pharmaceuticals; and research grants from the European Commission, the Italian Ministry of Health, the Italian Multiple Sclerosis Foundation and the Migraine Research Foundation. CG is, or was, a member of the following advisory boards: AbbVie, Hormosan Pharma, Lilly, Lundbeck, Merz, Novartis Pharma, Organon, Sanofi‐Aventis, Teva Pharmaceuticals and Vectura Fertin Pharma; is a speaker or member of the following speaker boards: AbbVie, Hormosan Pharma, Lilly, Lundbeck, Novartis Pharma, Reckitt‐Benckiser, Sanofi‐Aventis and Teva Pharmaceuticals; has acted as a consultant for Chordate and Pfizer; has served the following editorial boards: Acta Neurologica Scandinavia, Frontiers in Neurology and Medizinischer Sachverständiger; and reports author royalties for Novartis Pharma. PP‐R has received honoraria as a consultant and speaker for AbbVie, Amgen, Eli Lilly, Lundbeck, Medscape, Novartis, Pfizer and Teva Pharmaceuticals; research grants from AbbVie, Novartis and Teva Pharmaceuticals; and clinical trial funding from AbbVie, Alder, Amgen, Biohaven, electroCore, Eli Lilly, Lundbeck, Novartis and Teva Pharmaceuticals. LN is, or was, a member of the following advisory boards: AbbVie, Lundbeck and Teva Pharmaceuticals; is a speaker or member of the following speaker boards: AbbVie, Lundbeck, Novartis Pharma and Teva Pharmaceuticals; has acted as a consultant for Perfood; has served the following editorial boards: Frontiers in Neurology. PK is an employee and/or shareholders of Teva Pharmaceuticals. XH is an employee of Teva GmbH. HA is an employee and/or shareholder of Teva Pharmaceuticals. AS is, or was, a member of the following advisory boards: Allergan, Lilly, Novartis, Sanofi and Teva Pharmaceuticals; and a speaker or member of the following speaker boards: Allergan, Lilly, Novartis, Sanofi and Teva Pharmaceuticals; has acted as a consultant for Sanofi and Teva Pharmaceuticals; received grant support for research or education from Novartis; and acted as an editorial board member for Lilly, Novartis and Sanofi.

## EPO‐0248

### Changes in IHS‐proposed migraine control categories over time with fremanezumab: A pooled longitudinal analysis of the PEARL and FINESSE studies

#### M. Ashina^1^; G. Broessner^2^; C. Tassorelli ^3^; C. Gaul^4^; P. Pozo‐Rosich^5^; L. Neeb^6^; P. Kokturk^7^; X. Hamann^8^; S. Zuppone^7^; A. Straube^9^

##### ^*1*^ *Department of Neurology, Danish Headache Center, Copenhagen University Hospital–Rigshospitalet Glostrup, Copenhagen, Denmark; Department of Clinical Medicine, University of Copenhagen, Copenhagen, Denmark;* ^*2*^ *Department of Neurology, Medical University of Innsbruck, Innsbruck, Austria;* ^*3*^ *Department of Brain and Behavioral Sciences, University of Pavia, Pavia, Italy; IRCCS C. Mondino Foundation, Pavia, Italy;* ^*4*^ *Headache Center Frankfurt, Frankfurt am Main, Germany;* ^*5*^ *Headache Unit and Research Group, Vall d'Hebron Hospital and Research Institute, Universitat Autonoma de Barcelona, Barcelona, Spain;* ^*6*^ *Department of Neurology, Brandenburg Medical School Theodor Fontane, University Hospital Brandenburg/Havel, Brandenburg/Havel, Germany;* ^*7*^ *Teva Pharmaceuticals Europe B.V, Haarlem, The Netherlands;* ^*8*^ *Teva GmbH, Ulm, Germany;* ^*9*^ *Department of Neurology, Ludwig‐Maximilians‐University Munich, Munich, Germany*

**Background and aims:** With advances in preventive therapy, treatment goals for migraine have evolved from reduced migraine frequency toward optimal disease control. To evaluate disease control among participants in the long‐term, real‐world PEARL and FINESSE studies, this pooled analysis assessed mean monthly migraine days (MMD) according to IHS‐defined migraine control categories in adults treated with fremanezumab.

**Methods:** The prospective, observational PEARL (pan‐European) and FINESSE (Germany and Austria) studies evaluated the effectiveness and tolerability of fremanezumab in routine clinical practice among adults with episodic or chronic migraine (EM; CM), over 24 months. MMD were assessed using daily headache diaries completed by participants throughout the study. In this pooled analysis, the proportions of participants achieving different treatment outcomes categories (migraine freedom [0 MMD], optimal [ < 4 MMD], modest [4–6 MMD] or insufficient [ > 6 MMD] control) were assessed over 24 months.

**Results:** A total of 2145 participants were included in the pooled analysis (Table 1). Optimal migraine control and migraine freedom was achieved by 28.0% and 11.2% of participants, respectively, as early as 1 month after starting fremanezumab and by 41.5% and 18.7% of participants at Month 24 (Figure 1). Among the subset of participants who had modest or insufficient control at Months 12 and 24 respectively, a substantive proportion achieved > = 50% reduction from baseline in MMD (Figure 2).

**Figure:**
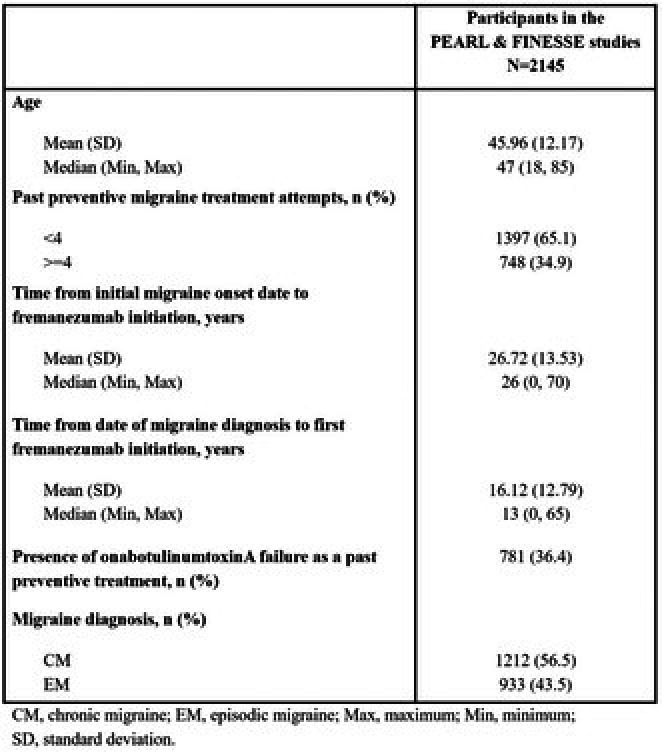
**TABLE 1** Baseline characteristics.

**Figure:**
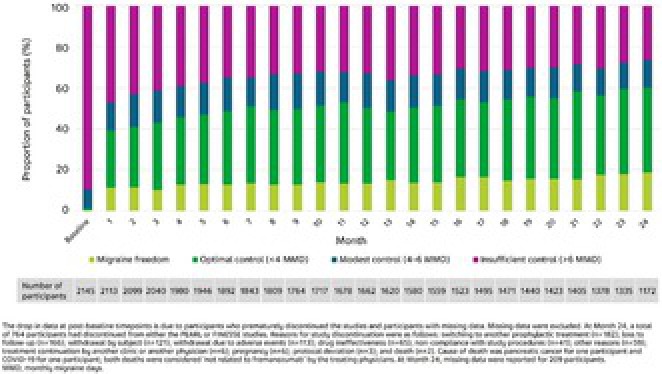
**FIGURE 1** MMD control over time.

**Figure:**
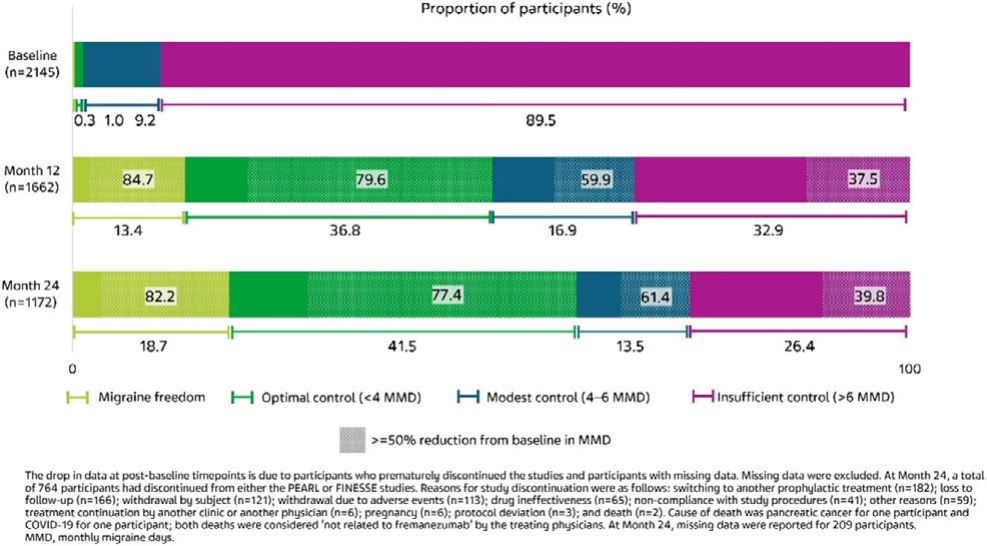
**FIGURE 2** Participants achieving >=50 reduction from baseline in MMD within each control category at Month 12 and Month 24

**Conclusion:** Using an outcome measure that aligns with individualised treatment goals beyond traditional responder rates, this pooled analysis of the PEARL and FINESSE studies demonstrated the early and sustained effectiveness of fremanezumab in substantially improving migraine control.

**Disclosure:** MA has received personal fees and declares PI/collaborator in clinical trials for AbbVie, AstraZeneca, Eli Lilly, GlaxoSmithKline, Lundbeck, Novartis, Pfizer and Teva Pharmaceuticals; and research grants from the Lundbeck Foundation, the Novo Nordisk Foundation, Lundbeck and Novartis. GB is, or was, a member of the following advisory boards: Grünenthal, Lilly, Lundbeck, Novartis, Pfizer and Teva Pharmaceuticals; is a speaker or member of the following speaker boards: Grünenthal, Lilly, Lundbeck, Novartis, Pfizer and Teva Pharmaceuticals; has acted as a consultant for Grünenthal, Lilly, Novartis and Teva Pharmaceuticals; and received grant support for research or education from Teva Pharmaceuticals. CT declares advisory boards, symposia and/or PI/collaboration in clinical trials for AbbVie, Chordate, Dompé, Eli Lilly, Ipsen, Lundbeck, Novartis, Pfizer and Teva Pharmaceuticals; and research grants from the European Commission, the Italian Ministry of Health, the Italian Multiple Sclerosis Foundation and the Migraine Research Foundation. CG is, or was, a member of the following advisory boards: AbbVie, Hormosan Pharma, Lilly, Lundbeck, Merz, Novartis Pharma, Organon, Sanofi‐Aventis, Teva Pharmaceuticals and Vectura Fertin Pharma; is a speaker or member of the following speaker boards: AbbVie, Hormosan Pharma, Lilly, Lundbeck, Novartis Pharma, Reckitt‐Benckiser, Sanofi‐Aventis and Teva Pharmaceuticals; has acted as a consultant for Chordate and Pfizer; has served the following editorial boards: Acta Neurologica Scandinavia, Frontiers in Neurology and Medizinischer Sachverständiger; and reports author royalties for Novartis Pharma. PP‐R has received honoraria as a consultant and speaker for AbbVie, Amgen, Eli Lilly, Lundbeck, Medscape, Novartis, Pfizer and Teva Pharmaceuticals; research grants from AbbVie, Novartis and Teva Pharmaceuticals; and clinical trial funding from AbbVie, Alder, Amgen, Biohaven, electroCore, Eli Lilly, Lundbeck, Novartis and Teva Pharmaceuticals. LN is, or was, a member of the following advisory boards: AbbVie, Lundbeck and Teva Pharmaceuticals; is a speaker or member of the following speaker boards: AbbVie, Lundbeck, Novartis Pharma and Teva Pharmaceuticals; has acted as a consultant for Perfood; has served the following editorial boards: Frontiers in Neurology. PK is an employee and/or shareholder of Teva Pharmaceuticals. XH is an employee of Teva GmbH. SZ is an employee and/or shareholder of Teva Pharmaceuticals. AS is, or was, a member of the following advisory boards: Allergan, Lilly, Novartis, Sanofi and Teva Pharmaceuticals; and a speaker or member of the following speaker boards: Allergan, Lilly, Novartis, Sanofi and Teva Pharmaceuticals; has acted as a consultant for Sanofi and Teva Pharmaceuticals; received grant support for research or education from Novartis; and acted as an editorial board member for Lilly, Novartis and Sanofi.

## EPO‐0249

### Eptinezumab reduced acute medication use in participants with chronic migraine and medication‐overuse headache: 24‐week data from the RESOLUTION trial

#### C. Lundqvist ^1^; R. Jensen^3^; H. Schytz^3^; C. Tassorelli^4^; F. Vernieri^6^; M. Lantéri‐Minet^8^; A. Blumenfeld^10^; S. Tepper^11^; R. Lipton^12^; M. Josiassen^13^; G. Jansson^13^; A. Ettrup^13^; A. Mittoux^13^; G. Terwindt^14^

##### ^*1*^ *Departments of Neurology and Health Services Research, Akershus University Hospital, Lørenskog, Norway;* ^*3*^ *Department of Neurology, Danish Headache Center, Rigshospitalet‐Glostrup, University of Copenhagen, Copenhagen, Denmark;* ^*4*^ *Department of Brain and Behavioral Sciences, University of Pavia, Pavia, Italy;* ^*6*^ *Unit of Headache and Neurosonology, Fondazione Policlinico Universitario Campus Bio‐Medico, Rome, Italy;* ^*8*^ *Pain Department and FHU InovPain, Centre Hospitalier Universitaire de Nice, Nice, France;* ^*10*^ *The San Diego Headache Center, San Diego, CA, United States;* ^*11*^ *The New England Institute for Neurology and Headache, Stamford, CT, United States;* ^*12*^ *Department of Neurology, Albert Einstein College of Medicine, New York, NY, United States;* ^*13*^ *H. Lundbeck A/S, Copenhagen, Denmark;* ^*14*^ *Department of Neurology, Leiden University Medical Centre, Leiden, Netherlands*

**Background and aims:** This phase 4 trial evaluated the 24‐week impact of eptinezumab on acute medication use in adults with chronic migraine (CM) and medication‐overuse headache (MOH) who also received patient education.

**Methods:** RESOLUTION (NCT05452239) included a 12‐week double‐blind placebo‐controlled period and 12‐week open‐label extension (OLE). At baseline, adults with CM and MOH were randomized 1:1 to IV eptinezumab 100mg or placebo; all participants received a brief educational intervention on MOH before infusion. At Week 12, all participants received eptinezumab 100mg. Acute medication use was captured daily via electronic diary. Analyses applied inferential statistics to the placebo‐controlled analyses and descriptive methods to the OLE.

**Results:** Participants randomized: *n* = 608; treated, baseline: *n* = 604; full‐analysis set, placebo‐controlled period: *n* = 602; treated/analyzed, OLE: *n* = 593; completed: *n* = 584. Baseline mean monthly acute medication days: 20.1 across arms. Eptinezumab was more favourable than placebo in reducing monthly acute medication use days over Weeks 1–4 (−11.3 vs. −7.7) and 1–12 (−11.2 vs. −7.8), particularly triptan days (*p* < 0.01 all comparisons). During the OLE, improvements were sustained for participants continuing eptinezumab; participants started on placebo and switched to eptinezumab achieved similar changes as those continuing eptinezumab. Eptinezumab provided greater decreases in mean Severity of Dependence Scale for Headache medication score than placebo at Week 12 (−2.2 vs. −1.2; *p* = 0.0002).

**Conclusion:** In participants with CM and MOH who also received patient education, eptinezumab resulted in greater reductions than placebo in acute medication use and dependence over Weeks 1–12. Similar reductions in acute medication use were seen during open‐label eptinezumab treatment, irrespective of treatment in the placebo‐controlled period.

**Disclosure:** CL has participated on an advisory board and received payment for lectures arranged by AbbVie, Lundbeck, Novartis, Pfizer, and Roche, and has received research sponsorship from AbbVie. RHJ has given lectures for Allergan, ATI, Eli Lilly, Lundbeck, Merck, Novartis, Pfizer, and Teva; served as investigator in clinical trials with ATI, Eli Lilly, Lundbeck, Novartis, and Novo Nordisk; is the director of the Danish Headache Center, Lifting The Global Burden of Headache, and Founder of Master of Headache Disorders at University of Copenhagen; and has received research funding from ATI, Lundbeck Foundation, Rigshospitalet, The Medical Society in Copenhagen, Novo Nordisk Foundation, Tryg Foundation, and University of Copenhagen. HWS has received personal fees from AbbVie, Eli Lilly, Lundbeck, Novartis, and Teva, and has received research grants from Novartis and Novo Nordisk Foundation. CT in the past 3 years has received support (financial or drugs) from AbbVie and Novartis for an investigator‐initiated trial; consulting fees for the participation in advisory boards for AbbVie, Dompé, Eli Lilly, Ipsen, Lundbeck, Organon, Pfizer, and Teva; honoraria for scientific lectures and presentations from AbbVie, Eli Lilly, Lundbeck, Pfizer, and Teva; support for attending meetings from AbbVie, Dompé, Eli Lilly, Ipsen, Lundbeck, Pfizer, and Teva; has been Principal Investigator in clinical trials sponsored by AbbVie, Biohaven, Chordate, Eli Lilly, Ipsen, Lundbeck, Pfizer, and Teva; and received grants from the European Commission, the Italian Ministry of Health, and the Italian Ministry of University. FV has received financial support from AbbVie, Angelini, and Lundbeck for investigator‐initiated trials; consulting fees for the participation in advisory boards from AbbVie, Angelini, Eli Lilly, Lundbeck, Organon, Pfizer, and Teva; honoraria for scientific lectures and presentations from AbbVie, Eli Lilly, Lundbeck, Pfizer, and Teva; support for attending meetings from AbbVie, Eli Lilly, Lundbeck, Pfizer, and Teva; and has been Principal Investigator in clinical trials sponsored by AbbVie, Eli Lilly, Lundbeck, Pfizer, and Teva. ML‐M reports personal fees for advisory boards, speaker panels, or investigation studies from Allergan, Amgen, Astellas, ATI, BMS, Boehringer, Boston Scientific, CoLucid, Convergence, Eli Lilly, GlaxoSmithKline, Grunenthal, Ipsen, Lundbeck, Medtronic, MSD, Novartis, Orion Pharma, Perfood, Pfizer, Reckitt Benckiser, St. Jude, Salvia BioElectronics, Sanofi‐Aventis, Teva, UCB, UPSA, and Zambon. AB has served on advisory boards for AbbVie, Aeon, Alder, Amgen, Biohaven, Eaglet, Eli Lilly, Impel, Lundbeck, Novartis, Promius, Revance, Supernus, and Teva; and has received funding for speaking from AbbVie, Amgen, Avanir, Biohaven, Depomed, Eli Lilly, Impel, Lundbeck, Pernix, Promius, Supernus, and Teva. [Disclosures for the remaining authors will be included in the presentation].

## EPO‐0250

### Real‐world effectiveness and adherence of atogepant in chronic and episodic migraine: Results from the ATOMUR study

#### D. López Segura ^1^; L. Calderón Soriano^2^; F. Salazar Hernández^1^; M. Ruiz Perelló^1^; J. Marín Marín^2^; S. Marín Balbuena^3^; I. Jiménez Díaz^1^; A. Torres Perales^4^; C. Lucas Ródenas^3^; M. Frutos Alegría^5^; M. Lozano Caballero^3^; H. Rodríguez Hilario^5^; J. García‐Carmona^1^

##### ^*1*^ *Department of Neurology, Santa Lucía University Hospital, Cartagena; Spain;* ^*2*^ *Department of Neurology, Reina Sofía University Hospital, Murcia; Spain;* ^*3*^ *Department of Neurology, Arrixaca University Hospital, Murcia; Spain;* ^*4*^ *Department of Neurology, Los Arcos‐Mar Menor University Hospital, San Javier; Spain;* ^*5*^ *Department of Neurology, Morales Meseguer University Hospital, Murcia; Spain*

**Background and aims:** Atogepant, an oral CGRP receptor antagonist, has shown efficacy in migraine prevention in clinical trials. Nonetheless, real‐world data on its long‐term effectiveness and treatment adherence in chronic (CM) and episodic migraine (EM) remain limited. We evaluated treatment compliance and changes in monthly migraine days (MMDs), MIDAS score and its side effects in patients with CM and EM treated with atogepant in clinical practice.

**Methods:** 101 patients diagnosed with chronic or episodic migraine who initiated preventive treatment with atogepant were included and followed at 3, 6, and 12 months.

**Results:** 57 (56.4%) patients had CM and 44 (43.6%) EM. Mean baseline MMDs were 18.6 ± 4.9 in CM and 9.8 ± 2.7 in EM patients. At 3 months, mean MMDs were reduced to 10.2 ± 4.6 in CM and 5.4 ± 2.9 in EM, corresponding to a reduction of 45% and 44%, respectively. These reductions were sustained at 6 months (CM: 9.6 ± 4.8; EM: 5.1 ± 2.6) and 12 months (CM: 9.1 ± 5.0; EM: 4.9 ± 2.8). At 12 months, 68% of patients achieved a ≥ 50% reduction in MMDs. Mean MIDAS scores decreased significantly from baseline (48.2 ± 19.6) to 3 months (28.5 ± 17.3; *p* < 0.001), with further reductions at 6 months (24.1 ± 16.8; *p* < 0.001) and 12 months (21.6 ± 17.1; *p* < 0.001). Treatment adherence remained high, with 80% of patients continuing therapy at 6 months and 66% at 12 months. Discontinuations were mainly due to lack of effectiveness or mild adverse events (nausea).

**Conclusion:** Atogepant showed sustained effectiveness in reducing monthly migraine days and high adherence over 12 months in patients with chronic and episodic migraine. These findings support its role as a long‐term preventive treatment.

**Disclosure:** Nothing to disclose.

## EPO‐0251

### Comparing AI with specialist ground truth on each headache attack using headache diary app

#### M. Katsuki ^1^; M. Okada^2^; T. Shimazu^3^; T. Takeshima^4^; H. Igarashi^5^; M. Tatsumoto^6^; Y. Ito^7^; T. Mitsufuji^2^; M. Hirayama^2^; K. Ohbayashi^7^; N. Imai^8^; S. Kikui^4^; D. Danno^4^; J. Miyahara^4^; K. Murakata^4^; Y. Matsumori^9^; Y. Nakazato^2^; K. Fujita^10^; E. Hoshino^3^; Y. Kato^11^; Y. Maruki^3^; R. Delyon^12^; I. Kizil^12^; U. Alexandre^12^; F. Cadiou^12^; G. Oyama^2^; S. O'Connor^13^; T. Ward^14^; K. Moran^15^; T. Yamamoto^2^

##### ^*1*^ *Physical Education and Health Center, Nagaoka University of Technology, Niigata 940‐2137, Japan 3 Insight Research Ireland Centre for Data Analytics and School of Health and Human Performance, Dublin City University, Dublin D09 V209, Ireland;* ^*2*^ *Department of Neurology, Saitama Medical University; 38 Morohongo, Moroyama‐machi, Iruma‐gun, Saitama 350‐0495, Japan;* ^*3*^ *Department of Neurology, Saitama Neuropsychiatric Institute, Saitama 338‐8577, Japan;* ^*4*^ *Headache Center and Department of Neurology, Tominaga Hospital, Osaka 556‐0017, Japan;* ^*5*^ *Headache Care Unit, Department of Internal Medicine, Fujitsu Clinic, Kanagawa 211‐8588, Japan;* ^*6*^ *Department of Neurology, Dokkyo Medical University, Tochigi 321‐0293, Japan. and Canon Marketing Japan;* ^*7*^ *Ohbayashi Clinic, Tochigi 321‐0933, Japan;* ^*8*^ *Department of Neurology, Japanese Red Cross Shizuoka Hospital, Shizuoka 420‐0853, Japan;* ^*9*^ *Sendai Headache and Neurology Clinic, Sendai 982‐0014, Japan;* ^*10*^ *Department of Neurology, Jichi Medical University Saitama Medical Center, Saitama 330‐8503, Japan;* ^*11*^ *Department of Neurology and Cerebrovascular Medicine, Saitama Medical University International Medical Center, Saitama 350‐1298, Japan;* ^*12*^ *Aptar Digital Health, Rueil‐Malmaison, France;* ^*13*^ *School of Health and Human Performance, Dublin City University, Dublin, Ireland;* ^*14*^ *Insight Research Ireland Centre for Data Analytics, Dublin City University, Dublin, Ireland;* ^*15*^ *School of Health and Human Performance, Dublin City University, Dublin, Ireland; Insight Research Ireland Centre for Data Analytics and Department of Sport Science and Nutrition, Maynooth University, Kildare, Ireland*

**Background and aims:** The International Classification of Headache Disorders, 3rd edition (ICHD‐3), defines migraine at the diagnostic level but is not suited for qualifying individual headache attacks, limiting accurate evaluation of acute treatments. This study aimed to construct an expert‐derived ground‐truth dataset of individual attacks and to compare three artificial intelligence (AI)‐based classifications of each attack with specialist judgment.

**Methods:** Patients from headache‐specialized clinics in Japan completed standardized clinical questionnaires and recorded attacks in a smartphone diary (Migraine Buddy) for 3 months. Three board‐certified headache specialists independently evaluated 10 attacks from each of 10 patients (100 attacks total), using full clinical context and diary data. Each attack was rated on a five‐level migraine‐probability scale. Majority voting classified attacks as migraine or non‐migraine and served as the ground truth. We evaluated three AI approaches against this reference: (1) a rule‐based ICHD‐3 decision tree, (2) large language models (LLMs), and (3) supervised machine‐learning models trained on expert labels.

**Results:** Ninety‐eight attacks were eligible for analysis; 82 were classified as migraine and 16 as non‐migraine. The ICHD‐3 rule‐based approach showed high specificity (0.81) but low sensitivity (0.43). LLMs demonstrated high sensitivity (0.95) and moderate specificity (0.69), with notable variability across repeated runs. XGBoost models achieved balanced performance, with sensitivity of 0.84 and specificity of 0.88.

**Conclusion:** Automated approaches can approximate specialist‐level classification of individual headache attacks, with XGBoost showing the most robust performance. These findings support the use of AI for per‐attack migraine qualification while reinforcing the continued role of expert clinical judgment.

**Disclosure:** RD, IK, UA, and FC are employees of Aptar Digital Health, Rueil‐Malmaison, France. Others do not have anything to disclose. Funding: This publication has emanated from research supported under the European Union's Horizon 2020 research and innovation programme under the Marie Skłodowska‐Curie grant agreement No 101034252 and the Insight Research Ireland Centre for Data Analytics under grant agreement 12/RC/2289_P2.

## EPO‐0252

### Magnetic resonance–guided focused ultrasound (MRgFUS) in pharmacoresistant trigeminal neuralgia: A single‐center pilot feasibility and safety study

#### P. Gutierrez Bedia ^1^; L. Franco Rubio^1^; M. Yus^2^; A. Lopez‐Frías^2^; C. Pérez García^2^; M. Valles^1^; J. Matías‐Guiu^1^; J. Porta‐Etessam^3^; N. González García^1^

##### ^*1*^ *Neurology, Hospital Clínico San Carlos, Madrid, Spain;* ^*2*^ *Radiology, Hospital Clinico San Carlos, Madrid, Spain;* ^*3*^ *Neurology, Hospital Fundación Jiménez Díaz, Madrid, Spain*

**Background and aims:** Pharmacoresistant trigeminal neuralgia (TN) represents a severe form of neuropathic pain and poses a major therapeutic challenge. Functional neurosurgery has identified central targets involved in pain perception, such as the central lateral nucleus (CLN) of the thalamus. Magnetic resonance–guided focused ultrasound (MRgFUS) enables precise thermal lesioning of deep brain structures, including the thalamus, in a non‐invasive manner. This technique is an innovative and expanding therapeutic option for refractory neuropathic pain.

**Methods:** This is a prospective, single‐center, uncontrolled pilot study designed to assess the feasibility and safety of MRgFUS targeting the CLN. Patients with pharmacoresistant TN evaluated at our Headache Unit are included. Clinical screening, neuroimaging, psychopathological and neuropsychological assessments are performed. The procedure is conducted under conscious sedation, with monitoring of adverse events and postoperative MRI. Validated scales are used for baseline and follow‐up assessments (CBD, DN4, PainDETECT, EQ‐5D‐5L, PDQ, PCS, PGIC, HADS).

**Results:** To date, one patient (male, 52 years) has been successfully treated. One week after the procedure, complete resolution of trigeminal pain and paroxysms was observed, with no clinically relevant adverse events. Additional patients will be included in the coming months.

**Conclusion:** MRgFUS targeting the CLN demonstrated good tolerability, absence of complications, and a relevant analgesic effect in this first case. This ongoing study aims to confirm the feasibility of MRgFUS as a non‐invasive therapeutic alternative for pharmacoresistant trigeminal neuralgia.

**Disclosure:** Nothing to disclose.

## EPO‐0253

### Biomolecular effects of ATOgepant in high‐frequency episodic Migraine: The ATOM Project

#### R. De Icco; F. Cammarota; V. Grillo; G. Vaghi; M. Corrado; B. Agostini; M. Giraudo; D. Martinelli; M. Allena; G. Sances; R. Greco; C. Demartini; M. Francavilla; S. Facchetti; C. Tassorelli

##### Department of Brain and Behavioral Sciences, University of Pavia & Headache Science and Neurorehabilitation Unit, IRCCS Mondino Foundation, Pavia, Italy

**Background and aims:** Atogepant is an oral CGRP receptor antagonist approved for migraine prevention. The aim of this study was to investigate the biomolecular changes induced by atogepant in individuals with high‐frequency episodic migraine (HFEM).

**Methods:** In this prospective study, 40 participants (age 35.5 ± 21.9 years, 30 females) underwent blood sampling in the interictal phase at baseline (T0) and after a 3‐month‐daily atogepant 60 mg treatment (T1). The biomolecular assessment included: (i) monoacylglycerol lipase (MAGL) and fatty acid amid hydrolase (FAAH) gene expression (mRNA) in peripheral blood mononuclear cells (PBMCs); (ii) microRNAs expression in PBMCs, namely miR‐382‐5p, miR‐34a, and miR‐155; and (iii) plasma levels of CGRP and PACAP (pg/mL).

**Results:** At T1, 70% of participants qualified as Responders (> 50% reduction in monthly migraine days). MAGL expression only decreased in Responders (Responders: T0: 9.8 ± 5.4 vs. T1: 13.2 ± 7.9 rela‐tive quantification – RQ, *p* = 0.021, Non‐Responders: T0: 7.4 ± 4.3 vs. T1: 8.3 ± 4.4 RQ, *p* = 0.552). At T0 MAGL was higher in Responders compared to Non‐Responders (*p* = 0.022). At T1, we did not find modifications in FAAH and microRNAs expression or CGRP and PACAP levels.

**Conclusion:** After a 3‐month atogepant treatment, we described a modulation of MAGL expression in Respond‐ers. Along with clinical benefit, atogepant treatment was associated with an improvement in the ho‐meostasis of our pain‐control system. It remains unclear whether the MAGL change reflects a direct pharmacological effect or, more plausibly, a downstream consequence of clinical improvement.

**Disclosure:** RDI received honoraria from AbbVie for advisory board participation and speaker invitation. The ATOM project was funded by a CORE – AMD Resident Research Grant (application ID#: 1273396).

## EPO‐0254

### Indomethacin for the symptomatic treatment of migraine: A clinical reappraisal

#### S. Braca; C. Russo; C. Giannini; G. Cretella; A. Stornaiuolo; R. De Simone

##### Department of Neuroscience, Reproductive Sciences and Odontostomatology, University of Naples “Federico II”, Naples, Italy

**Background and aims:** Indomethacin is a potent NSAID with distinctive neurovascular pharmacology, including interactions with nitric oxide pathways and significant effects on intracranial pressure regulation. Despite long‐standing clinical use in headache disorders, evidence supporting indomethacin for the symptomatic treatment of migraine remains limited. This study aims to provide new clinical data on its effectiveness and tolerability in the symptomatic treatment of migraine.

**Methods:** This is a pilot, observational, open‐label, head‐to‐head study. We enrolled adults with low‐frequency episodic migraine (2–8 attacks/month). Participants were sequentially assigned in alternating order to indomethacin 50 mg or ibuprofen 600 mg, taken at attack onset. The primary endpoint was 2‐hour pain freedom during the first treated attack. We also recorded the rate of adverse events.

**Results:** A total of 120 participants were included (indomethacin *n* = 60; ibuprofen *n* = 60). Two‐hour pain freedom occurred more frequently with indomethacin (43.3%) than with ibuprofen (21.7%) (*p* = 0.01). Adverse events were numerically more common with indomethacin (36.7%) than with ibuprofen (21.7%), although without a statistically significant difference (*p* = 0.07).

**Conclusion:** Indomethacin showed superior outcomes versus ibuprofen, supporting a clinical reappraisal of this drug as an acute migraine option. Its higher efficacy might relate not only to anti‐inflammatory activity but, importantly, also to modulation of nitric oxide–mediated neurovascular signaling and intracranial pressure dynamics. Although not statistically significant, indomethacin was associated with a higher number of adverse events, so its use should be preferably considered in more severe or refractory cases.

**Disclosure:** Nothing to disclose.

## EPO‐0255

### Erenumab versus OnabotulinumtoxinA for reducing triptan use: A target trial emulation study

#### M. Jayaraman ^1^; M. Bjørk^2^; J. Igland^3^; D. Daltveit^4^; s. Mathisen^5^; E. Tronvik^6^; N. Gilhus^7^

##### ^*1*^ *Department of Neurology,Stavanger University Hospital,Stavanger, University of Bergen(UiB), NorHead, Norway;* ^*2*^ *University of Bergen(UiB), Bergen, Deparatment of Neurology, Haukeland University Hospital, NorHead, Norway;* ^*3*^ *Department of Global Public Health and Primary Care, University of Bergen. Department of Health and Caring Sciences, Western Norway University of Applied Sciences, Department of Neurology, Haukeland University Hospital, Bergen, Norway;* ^*4*^ *Department of Global Public Health and Primary Care, University of Bergen. Norwegian Registry of Cleft Lip and Palate, Surgical Clinic, Haukeland University Hospital, Bergen, Norway;* ^*5*^ *Department of Neurology,Stavanger University Hospital, Stavanger, Norway;* ^*6*^ *NTNU, NorHead, Trondheim,Norway;* ^*7*^ *University of Bergen(UiB), Bergen, Deparatment of Neurology, Haukeland University Hospital, Bergen, Norway*

**Background and aims:** To compare the real‐world effectiveness of Erenumab and OnabotulinumtoxinA in reducing triptan use among patients with migraine

**Methods:** We used nationwide Norwegian health registry data to emulate a target trial comparing initiation of Erenumab with OnabotulinumtoxinA. Propensity score overlap weighting was applied to balance baseline characteristics. Outcomes were assessed over 12 months and included absolute change in triptan defined daily doses (DDDs) and achievement of ≥ 50% reduction in triptan use. Weighted linear mixed effects models were used for continuous outcomes, and weighted logistic mixed effects models for binary outcomes. Both intention‐to‐treat and per‐protocol analyses were conducted.

**Results:** A total of 3,062 Erenumab users and 2,431 OnabotulinumtoxinA users were included. After weighting, baseline characteristics were identical. As shown in Table 1, Erenumab produced greater reductions in triptan DDDs. In the intention‐to‐treat analysis, adjusted between‐group differences ranged from −8.35 (95% CI −10.69 to −6.01) at 0–3 months to −4.24 (−6.91 to −1.57) at 9–12 months; per‐protocol differences ranged from −9.59 to −6.01. For the secondary outcome (Table 2), Erenumab users had higher odds of achieving a ≥ 50% reduction in triptan use, with intention‐to‐treat ORs from 1.49 to 1.18 and per‐protocol ORs from 1.66 to 1.55. Triptan utilization trajectories are shown in Image 1.

**Figure:**
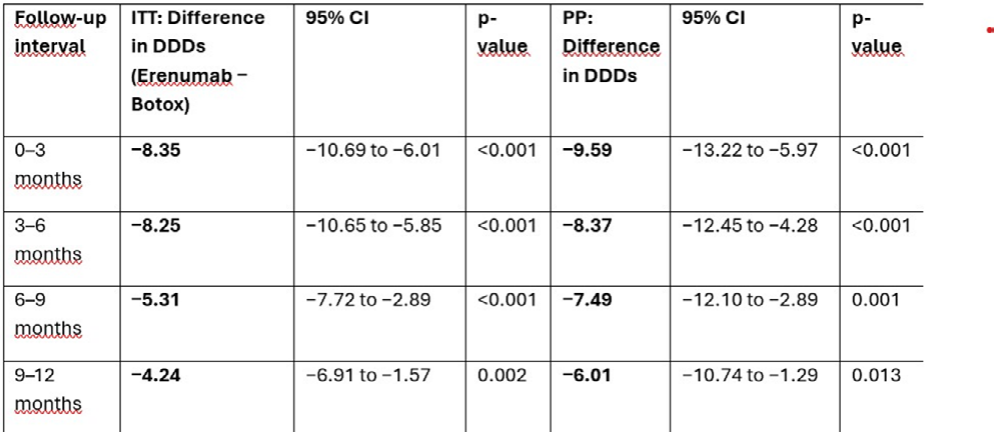
**TABLE 1** Primary outcome: Absolute change in triptan daily defined doses (DDD).

**Figure:**
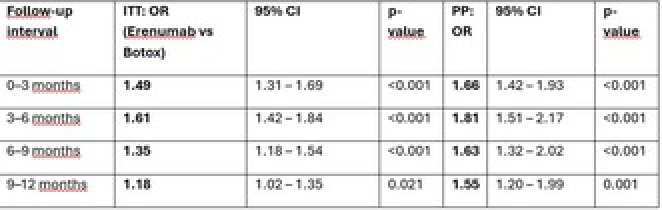
**TABLE 2** Secondary outcome: ≥ 50% reduction in triptan use.

**Figure:**
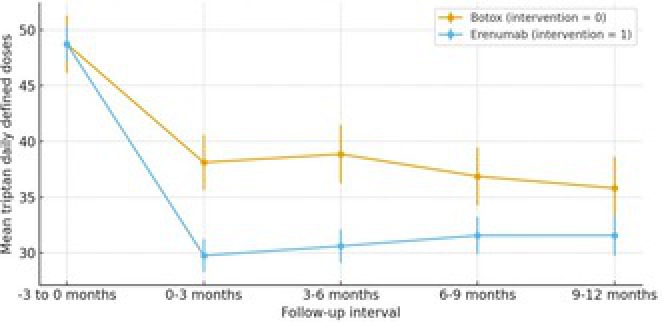
**FIGURE 1** Weighted mean triptan daily defined doses over time (ITT).

**Conclusion:** Erenumab showed a consistent advantage over OnabotulinumtoxinA in reducing triptan use, particularly among adherent patients. These findings support the superior real‐world effectiveness of Erenumab.

**Disclosure:** The author receives a PhD stipend from Helse Vest and this is a part of the NorHead project.

## Infectious Diseases 2

## EPO‐0256

### Overtreatment and delays in antiviral therapy for suspected herpesvirus CNS infections

#### B. Nieuwenhuijsen; M. Brouwer; S. Staal; D. van de Beek

##### Neuroinfection centre, Amsterdam UMC, Amsterdam, The Netherlands

**Background and aims:** Herpesviruses are a common cause of CNS infections. Guidelines recommend empirical antiviral therapy when herpesvirus encephalitis is suspected. This practice often results in treatment of patients without herpesvirus CNS infection.

**Methods:** We analysed adults with suspected CNS infection enrolled in two prospective cohorts (2012–2025). All patients receiving antiviral therapy for suspected CNS infection were included. All PCR‐confirmed herpesvirus CNS infections were considered as appropriately treated, and all others were classified as overtreatment. We quantified overtreatment and undertreatment, and examined the clinical context of delayed therapy and prolonged therapy. In addition, the use of dexamethasone was evaluated in patients with confirmed herpesvirus CNS infection.

**Results:** Among 1,785 patients, 506 (28%) received antiviral therapy. Of these, 436 (86% of treated; 24% of total) did not have herpesvirus CNS infection, while 70 (14% of treated; 4% of total) had PCR‐confirmed herpesvirus CNS infection. All patients with herpesvirus encephalitis received antiviral treatment. Delayed treatment (> 1 day after presentation) occurred in 9/70 (17%) herpesvirus CNS infection cases, mostly due to concurrent or alternative diagnoses. Continued treatment (≥ 7 days) in non‐herpesvirus cases occurred in 18/436 (4%), predominantly while awaiting repeat PCR results. Dexamethasone was administered in 20/70 (29%) patients with herpesvirus CNS infection and was not associated with differences in GOS at discharge.

**Figure:**
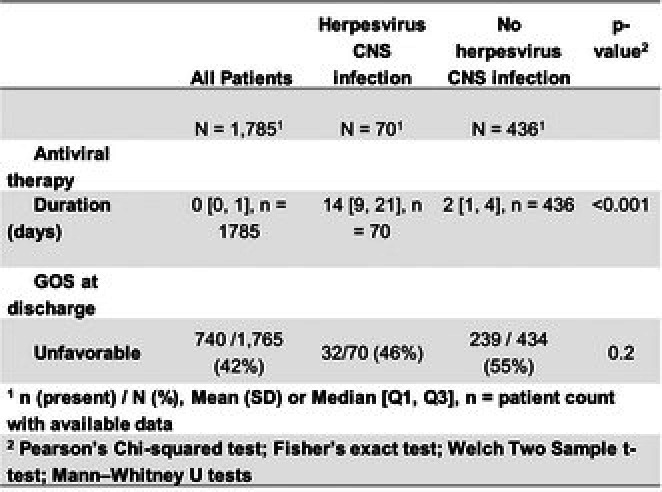
**TABLE 1** Antiviral therapy duration and Glasgow outcome scale in patients treated with antiviral therapy, comparing patients with herpesvirus CNS infection to those with other diagnoses.

**Figure:**
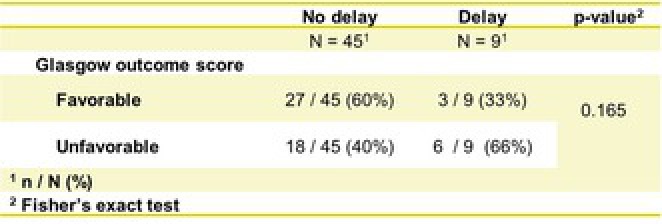
**TABLE 2** Glasgow outcome scale in patients who used antiviral therapy with PCR‐confirmed herpesvirus CNS infection (delayed start of antiviral therapy > 1 day vs no delay).

**Figure:**
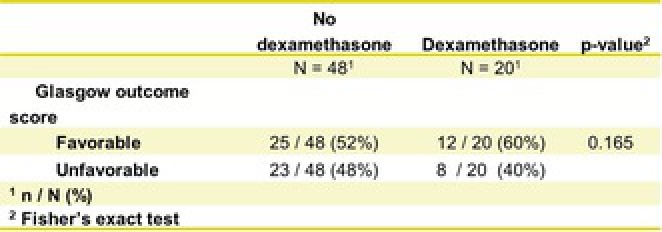
**TABLE 3** Glasgow outcome scale in patients who used antiviral therapy with PCR‐confirmed herpesvirus CNS infection (dexamethasone vs no dexamethasone).

**Conclusion:** In order to ensure all herpesvirus encephalitis cases were treated, nearly one‐quarter of patients with suspected CNS infection received antiviral therapy, exposing many to potential adverse effects. Nevertheless, delayed therapy remained common among herpesvirus CNS infections, highlighting the challenge of early identification.

**Disclosure:** Nothing to disclose.

## EPO‐0257

### Optimal timing of ARV initiation among patients with toxoplasma encephalitis: A meta‐analysis

#### C. Plan; K. San Diego; A. Barlin; A. Dimaano

##### Department of Neurology, The Medical City – Ortigas, Pasig City, Philippines

**Background and aims:** Toxoplasma encephalitis (TE) affects 10–40% of AIDS patients worldwide, with mortality rates of 10–50%. The optimal timing of antiretroviral therapy (ART) initiation remains controversial, balancing early immune recovery benefits against immune reconstitution inflammatory syndrome (IRIS) risks.

**Methods:** A meta‐analysis of six studies (2009–2025) encompassing 710 HIV patients with TE was conducted. Studies compared early (≤ 10 days) versus delayed (> 21 days) ART initiation after anti‐toxoplasma treatment. Primary outcomes included mortality and neurological sequelae. Random‐effects models were used with heterogeneity assessed by I^2^ statistics.

**Figure:**
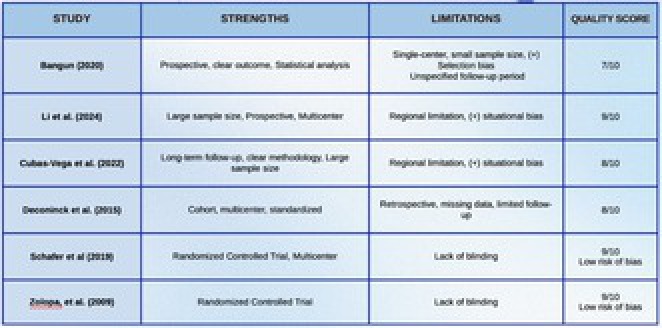
**TABLE 1** Critical appraisal of studies.

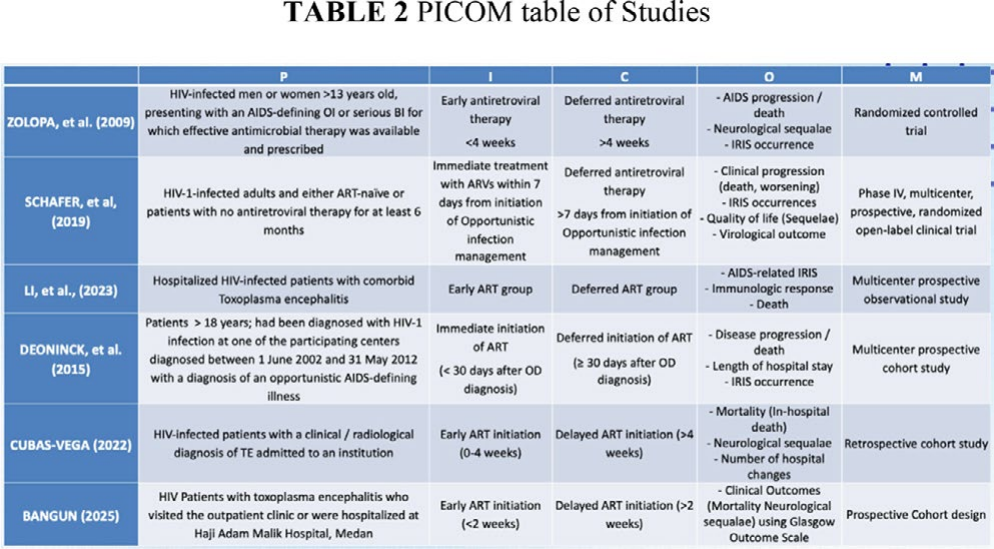

**Results:** Early ART initiation showed no significant overall mortality difference (pooled OR: 0.85, 95% CI: 0.42–1.72, *p* = 0.65) but demonstrated significant mortality reduction at 24‐week follow‐up (pooled OR: 0.65, 95% CI: 0.50–0.83, *p* < 0.001). Neurological sequelae increased non‐significantly with early initiation (pooled OR: 1.28, 95% CI: 0.97–1.67, *p* = 0.07). IRIS occurrence showed no significant difference between groups. Optimal outcomes occurred with ART initiation at 10–21 days post‐diagnosis, balancing mortality reduction with minimized complications. The mortality benefit of early ART supports immune reconstitution advantages, while increased neurological sequelae raise safety concerns. The absence of significant IRIS differences suggests concurrent anti‐toxoplasma therapy may reduce inflammatory responses. Study heterogeneity, timing definitions and geographic diversity affect interpretation.

**Figure:**
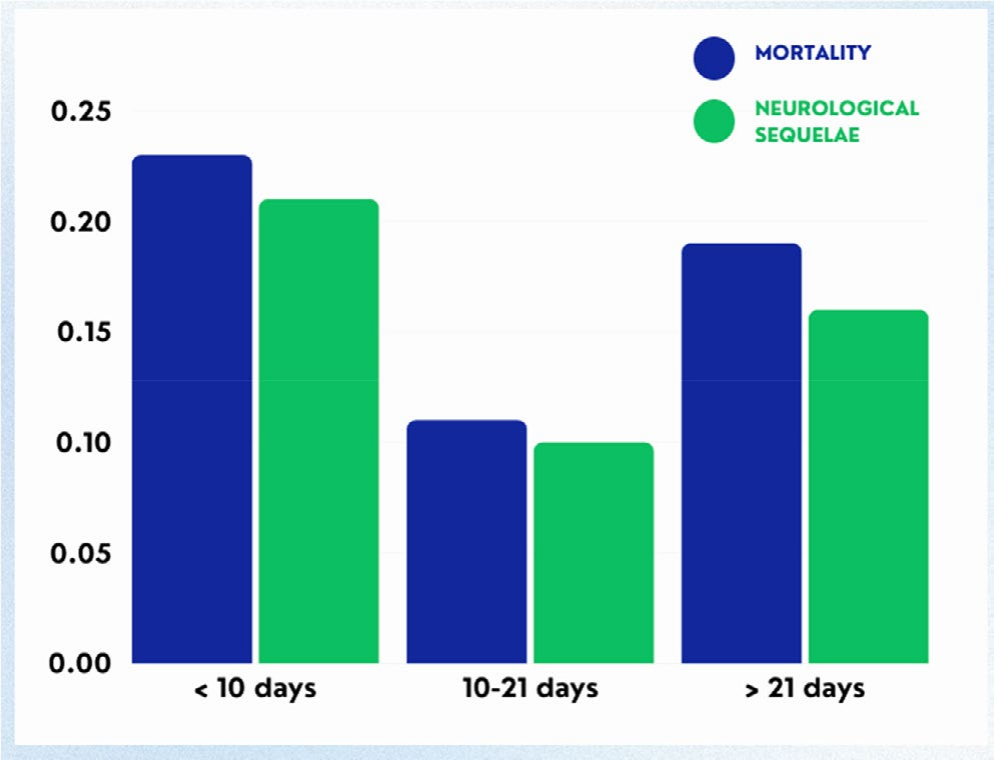
**FIGURE 1** Optimal timing of antiretroviral therapy initiation based on mortality and neurological sequelae.

**Conclusion:** ART initiation between 10–21 days after anti‐toxoplasma treatment provides optimal risk‐benefit balance for most patients. Clinical decisions should incorporate individual factors including CD4 count, neurological severity, and healthcare resources. Standardized timing definitions and long‐term outcome studies are needed.

**Disclosure:** The authors declare no conflicts of interest, external funding, and followed PRISMA guidelines. Ethical approval attained.

## EPO‐0258

### Asplenia and functional hyposplenia in community‐acquired bacterial meningitis

#### E. Schepers; V. Boon; E. Drost; M. Brouwer; D. van de Beek

##### Neurology, Amsterdam UMC, Amsterdam, The Netherlands

**Background and aims:** Bacterial meningitis is a serious disease characterized by high rates of complications and adverse outcomes. Disfunction of the spleen is a known risk factor for developing bacterial meningitis.

**Methods:** Patients with cerebrospinal fluid culture‐confirmed bacterial meningitis were enrolled in a nationwide prospective cohort study between March 1, 2006, and April 1, 2025. Splenectomy or conditions associated with functional hyposplenia were scored for each patient. Clinical characteristics and outcome of patients with a history of splenectomy or functional hyposplenia were assessed and compared with normosplenic patients.

**Results:** Hundred‐four of 3165 patients (3.3%) had an abnormal splenic function: 52 had a history of splenectomy and 52 had functional hyposplenia. Vaccination status was known for 76 patients – 26 of 45 splenectomized (58%) and 4 of 31 hyposplenic patients (13%) were vaccinated. The most common causative organisms were Streptococcus pneumoniae in splenectomized patients (51 of 52 patients, 98%), while 18 of 52 hyposplenic patients were infected with Listeria monocytogenes (35%). Asplenic patients presented with higher serum leukocytes (*P* < 0.01) and C‐reactive protein (*P* < 0.01), but lower levels of leukocytes in cerebrospinal fluid (*P* < 0.001) compared to normosplenic patients. Outcome was unfavorable in 47 patients (45%) and 17 patients died (16%).

**Conclusion:** Bacterial meningitis in splenectomized patients is caused by S. pneumoniae and is associated with a more severe systemic inflammatory response. In hyposplenic patients L. monocytogenes was a frequent cause of meningitis, likely due to a combination of immunocompromising factors and spleen dysfunction. Enhanced vaccination strategies should be employed to reduce the disease burden in these patients.

**Disclosure:** Nothing to disclose.

## EPO‐0259

### Influenza B–related mild encephalopathy with reversible splenial lesion Type II in an adult

#### F. Günel

##### Department of Neurology, Ondokuz Mayıs University, Samsun, Türkiye

**Background and aims:** Mild encephalopathy with a reversible splenial lesion (MERS) is a rare clinico‐radiological syndrome characterized by transient neurological symptoms and reversible lesions of the splenium of the corpus callosum. It is classified into Type I, limited to the splenium, and Type II, involving the entire corpus callosum and adjacent white matter. Adult cases associated with influenza infection are uncommon.

**Methods:** We report a case of influenza B–associated type II MERS in a 35‐year‐old woman.

**Results:** A previously healthy 35‐year‐old woman presented with acute upper respiratory tract symptoms and fever, followed by rapid onset of confusion on the same day. On examination, she was disoriented to time, place, and person, with a body temperature of 38°C. There were no signs of meningeal irritation. Cranial magnetic resonance imaging revealed hyperintense lesions on diffusion‐weighted imaging involving the splenium of the corpus callosum and bilateral frontoparietal deep white matter. Cerebrospinal fluid analysis, routine laboratory investigations, and electroencephalography were unremarkable. Polymerase chain reaction testing of a nasopharyngeal swab was positive for Influenza B virus. The patient was managed with supportive treatment alone. Her neurological symptoms resolved completely within two days. A follow‐up MRI demonstrated marked regression of the previously noted lesions.

**Figure:**
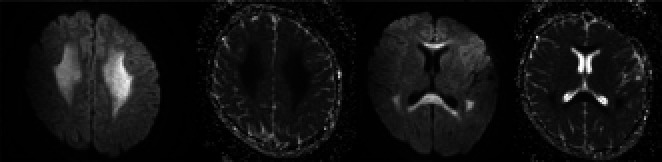
**FIGURE 1** Initial brain MRI demonstrating DWI hyperintense and ADC hypointense lesions in the splenium of the corpus callosum and bilateral frontoparietal deep white matter.

**Figure:**
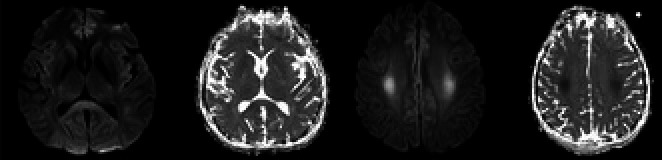
**FIGURE 2** Follow‐up brain MRI performed 4 days later demonstrating regression of the lesions observed on the initial MRI.

**Conclusion:** Influenza B–associated MERS Type II is exceedingly rare in adults. This case highlights the importance of diffusion‐weighted MRI in diagnosis and emphasizes that MERS typically follows a self‐limiting course with excellent clinical and radiological recovery under supportive management.

**Disclosure:** Nothing to disclose.

## EPO‐0260

### Can we predict meningitis before the tap? A machine learning approach to lumbar puncture decision‐making

#### G. Tsur ^1^; M. Schwartz Yoskovitz^1^; B. Yacobi^2^; S. Yust‐Katz^1^; E. Auriel^1^; K. Pardo^1^

##### ^*1*^ *Neurology, Rabin Medical Center, Petach Tikva, Israel;* ^*2*^ *Beilinson Medical Center Innovation, Artificial Intelligence Center, Rabin Medical Center, Petah Tikva, Israel*

**Background and aims:** Diagnosis of meningitis in the emergency department (ED) presents a challenge due to variable clinical presentations that overlap with other neurological and systemic conditions. The diagnosis today relies mostly on lumbar puncture (LP). Our aim was to develop clinical decision support tool for managing suspected meningitis patients in the ED.

**Methods:** Retrospective data of patients who underwent LP in the ER for suspicion of meningitis between 2019 and 2024 was collected. We used clinical and laboratory data to train machine learning (ML) model to predict meningitis prior to LP results. Model performance was evaluated using accuracy, precision, recall, and the area under the curve (AUC). Shapley value was used to identify important features in meningitis prediction.

**Results:** 1526 patients were included, among them 344 (22.54%) had meningitis. Most were aseptic meningitis (*n* = 270, 78.49%), followed by meningoencephalitis (*n* = 49, 14.24%), bacterial (*n* = 24, 6.97%) and brain abscess (*n* = 1, 0.29%). Meningitis patients were younger compared to non‐meningitis [median age of 36 (IQR30‐57) and 52 (IQR34‐71), respectively, *p* < 0.001] and had less cerebrovascular risk factors. Our ML model achieved AUC of 0.87 and accuracy of 0.85 for the diagnosis of meningitis. The most important features in our model were number of symptomatic days prior to ED visit, temperature, CRP, photophobia and absolute lymphocytes.

**Figure:**
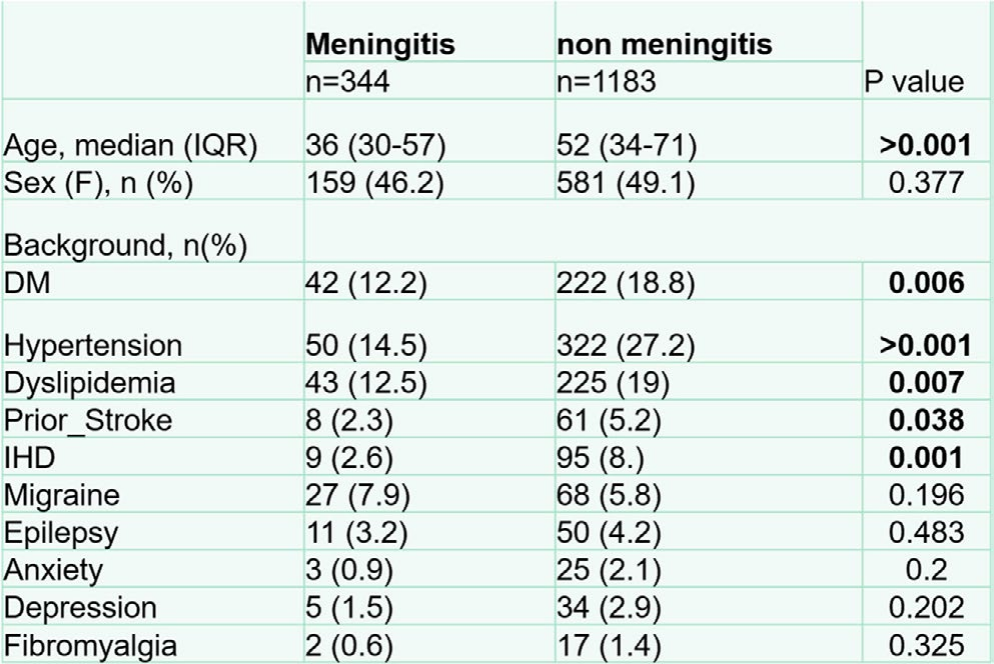
**TABLE 1** Demographic and characteristics.

**Figure:**
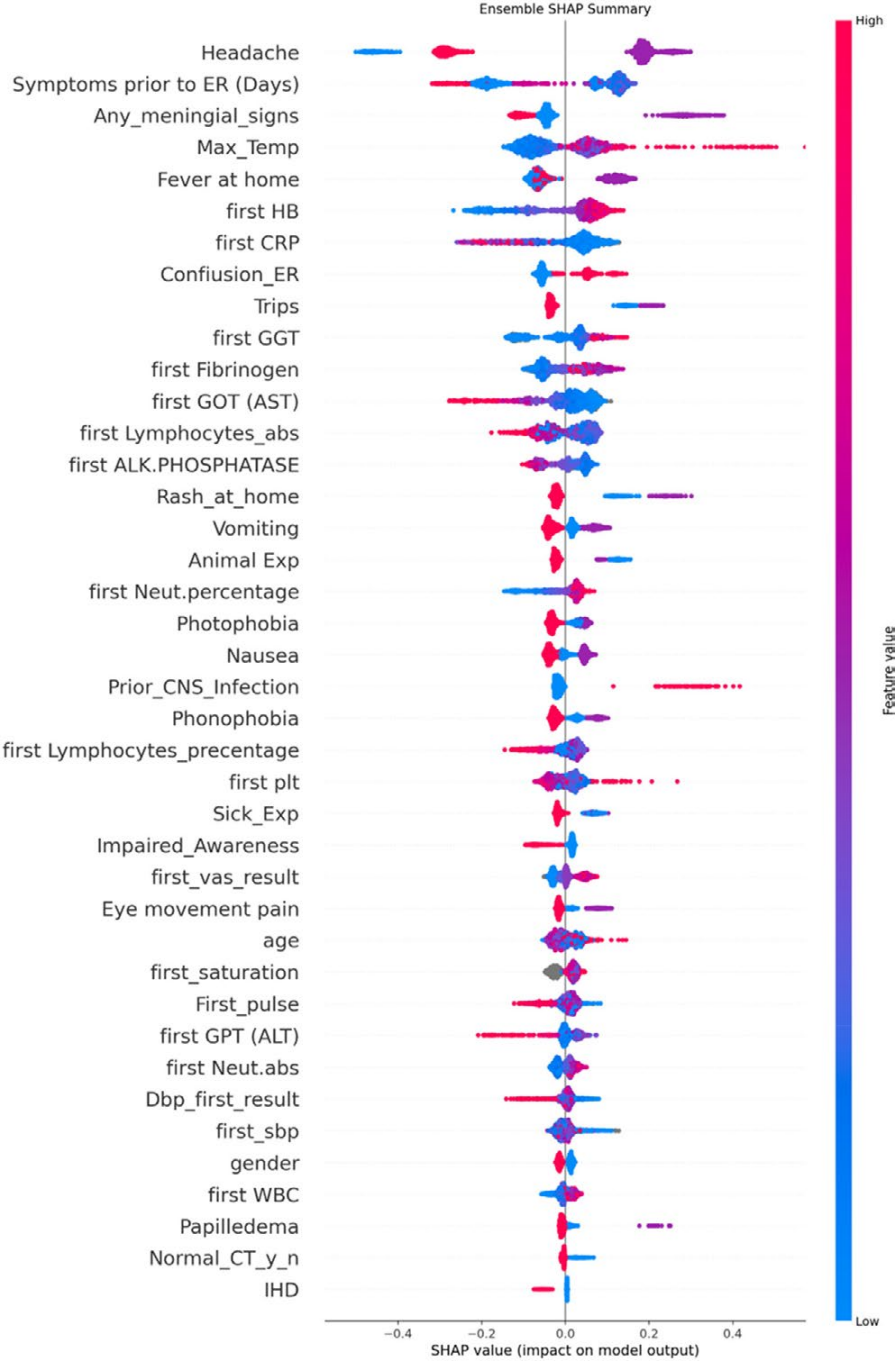
FIGURE 1

**Figure:**
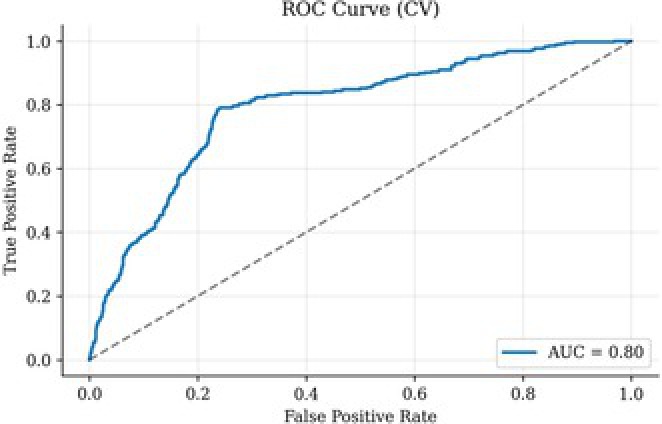
FIGURE 2

**Conclusion:** Our ML model was able to successfully predict patients with meningitis, providing a tool in clinical management of meningitis patients in the ED. Such tool can help determine whether a LP is indicated, avoiding unnecessary invasive tests.

**Disclosure:** Nothing to disclose.

## EPO‐0261

### Beta‐blockers in tuberculous meningitis associated cerebral salt wasting: An open label randomized controlled trial

#### J. Kalita; F. M Nizami; P. C Pandey; R. Mahajan; R. Kumar; U. Misra

##### Department of Neurology, Sanjay Gandhi Post Graduate Institute of Medical Sciences, Lucknow, Uttar Pradesh, India

**Background and aims:** More than 50% tuberculous meningitis (TBM) patients have hyponatremia. Cerebral salt wasting (CSW) is the commonest cause of hyponatremia, and has been attributed to higher catecholamine level as a result of stress response. Beta‐blockers may reduce stress response, and may be effective in TBM associated CSW. We report the efficacy and safety of propranolol in TBM associated CSW compared to placebo in a randomized placebo‐controlled trial.

**Methods:** Thirty‐eight TBM associated CSW patients were included, and were randomized to tablet propranolol 10mg twice daily on day 1, and 20mg twice daily from day 2 till one month or placebo. Both the arms received sodium chloride 10gm/day and normal saline till the normalization of serum sodium and fluid deficit. The primary outcome was day of normalization of serum sodium, and secondary outcomes were day of correction of fluid deficit and polyuria, adverse effects and a modified Rankin Scale (mRS) score at 1, 3 and 6 months. Both the groups received standard antitubercular treatment.

**Results:** Nineteen patients each received propranolol and placebo, and their baseline clinical, MRI, and laboratory findings were comparable. The propranolol group had an earlier normalization of serum sodium (median days 8vs20;*P* < 0.01), fluid deficit (median days 11vs42;*P* < 0.01) and polyuria (median days 4vs16;*P* < 0.01) compared to the placebo group. Death at 1 month and mRS score at 3 and 6 months were comparable between the two groups. Two patients required reduction of propranolol dose due to hallucinations and bradycardia.

**Conclusion:** Adjunctive propranolol results in earlier resolution of CSW in TBM patients

**Disclosure:** Nothing to disclose.

## EPO‐0262

### Plasma BD‐tau in post‐COVID neurological syndrome: A longitudinal study

#### M. Boldingh^1^; Pignatiello^2^; T. Ueland^3^; I. Gregersen^3^; m. argren^1^; S. Andersson^2^; M. Beyer^6^; V. Bjerkeli^3^; C. Brunborg^7^; B. Boye^2^; E. Bøen^2^; K. Devik^9^; A. Farmen^8^; H. Flemme^10^; K. Hestad^11^; H. Harbo^5^; T. Popperud^1^; E. Haugestøl^14^; Å. Morsund^12^; K. Otterdal^3^; B. Ratajczak‐Tretel^13^; Y. Schanke^3^; P. Selnes^14^; C. Samsonsen^15^; Ø. Torkildsen^16^; P. Aukrust^3^; T. Dahl^3^; B. Halvorsen^3^; A. Aamodt ^1^

##### ^*1*^ *Department of Neurology, Oslo University Hospital, Oslo, Norway;* ^*2*^ *Section of Psychosomatic Medicine, Oslo University Hospital, Oslo, Norway;* ^*3*^ *Research Institute of Internal Medicine, Oslo University Hospital, University of Oslo, Oslo, Norway;* ^*5*^ *Institute of Clinical Medicine, Faculty of Medicine, University of Oslo, Oslo, Norway;* ^*6*^ *Division of Radiology and Nuclear Medicine, Oslo University Hospital, Oslo, Norway;* ^*7*^ *Oslo Centre for Biostatistics and Epidemiology, Research Support Services, Oslo University Hospital, Oslo Norway;* ^*8*^ *Department of Neurology, Innlandet Hospital Trust, Lillehammer, Norway;* ^*9*^ *Department of Neurology, Namsos Hospital, Norway;* ^*10*^ *Department of Neurology, Telemark Hospital, Skien, Norway;* ^*11*^ *Department of Research, Innlandet Hospital Trust, Brumunddal, Norway;* ^*12*^ *Department of Neurology, Molde Hospital, Norway**;*** ^*13*^ *Department of Neurology, Østfold Hospital Trust, Norway;* ^*14*^ *Department of Neurology, Akershus University Hospital, Lørenskog, Norway;* ^*15*^ *Department of Neurology and Clinical Neurophysiology, St. Olavs Hospital, Trondheim, Norway;* ^*16*^ *Department of Neurology, Haukeland University Hospital & Section for Neurology, University of Bergen, Norway*

**Background and aims:** Post‐COVID‐19 condition (PCC) includes persistent neurological symptoms, raising concerns about long‐term neurodegeneration. Brain‐derived tau (BD‐tau) is a novel plasma biomarker with improved brain specificity compared to traditional tau assays. This study evaluated BD‐tau as a marker of cognitive impairment and symptom persistence in PCC.

**Methods:** We conducted a longitudinal analysis within the Norwegian NeuroCOVID cohort, including adults with SARS‐CoV‐2 infection and neurological symptoms (*n* = 112) and recovered controls without persistent symptoms (*n* = 33). Plasma BD‐tau, p‐tau217, p‐tau181, and total tau were measured at 6 and 12 months using ultrasensitive immunoassays. Clinical outcomes included modified Rankin Scale and neuropsychological testing. Associations were assessed using regression and correlation analyses.

**Results:** BD‐tau, p‐tau217, p‐tau181, and total tau remained stable between 6 and 12 months, regardless of symptom remission (43% at 12 months) or cognitive impairment (39% at 6 months; 25% at 12 months). None of the tau biomarkers correlated with cognitive domains. BD‐tau showed strong correlations with total tau (ρ = 0.66 at 6 months; ρ = 0.61 at 12 months, *p* < 0.001) and moderate associations with p‐tau217 and p‐tau181, but no significant relationship with cognitive scores or headache persistence. Higher BD‐tau was predicted by age, creatinine levels, comorbidities, and COVID‐19 severity, explaining up to 26% of variance at 6 months and 38% at 12 months.

**Figure:**
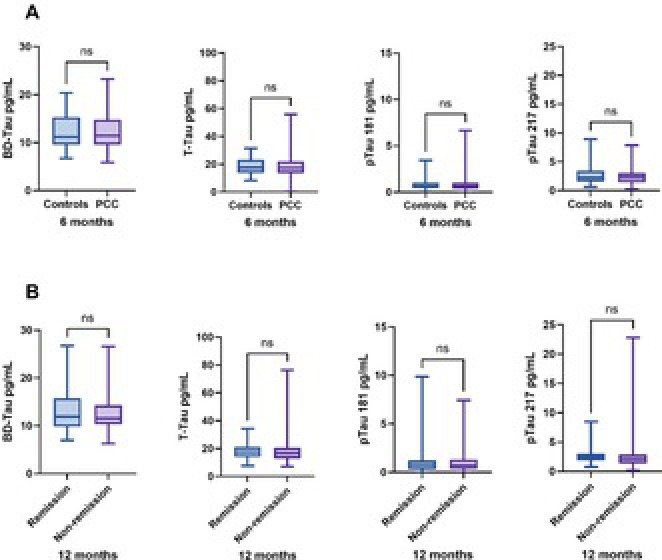
**FIGURE 1** (A) Plasma concentrations of BD‐tau, total tau (t‐tau), phosphorylated tau 181 (pTau181), and phosphorylated tau 217 (pTau217) in individuals with post‐COVID condition (PCC) compared to recovered controls at 6 months post‐infection. (B) Distribution of plasma BD‐tau, t‐tau, pTau181, and pTau217 in participants with and without symptom remission at 12 months post‐COVID‐19 infection. Biomarker concentrations remained stable over time, with no significant differences between groups, despite clinical remission in nearly half of the cohort (*n* = 48; 43.2%).

**Conclusion:** Plasma BD‐tau does not reflect remission or cognitive impairment in PCC, suggesting tau‐driven neurodegeneration is not a dominant mechanism. Larger studies with extended follow‐up are needed to clarify long‐term neurocognitive risk.

**Disclosure:** The authors report no disclosures relevant to this study. A.H. Aamodt has received honoraria for advice or lecturing from BMS‐Pfizer, Abbvie, Teva, Novartis, Roche and Lundbeck and research grant from Boehringer Ingelheim, EAH has received honoraria for advice or lecturing from Sanofi and research grant from MS union. ØT received honoraria for advice or lecturing from Merck, Sanofi and Novartis. MBA received honoraria for advice or lecturing from BMS‐Pfizer, Abbvie, Teva, Novartis and Lundbeck.

## EPO‐0263

### Neurological manifestations of dengue: Clinical spectrum, imaging diagnosis, and response to immunomodulatory treatment

#### P. Tejada^1^; J. Baez^1^; G. Fernández^1^; A. Tejada^1^; Y. Rodríguez^1^; L. Jiménez^1^; A. Reyes^1^; K. Yorro^1^; C. Pérez Lizardo^1^; M. Rodríguez ^2^

##### ^*1*^ *Synaptic Research League, Santo Domingo, Dominican Republic;* ^*2*^ *Neurology Department, Hospital Universitario Salvador Bienvenido Gautier, Santo Domingo, Dominican Republic*

**Background and aims:** Dengue virus infection is described as systemic febrile illness. Increasing evidence shows that the nervous system is a frequent and clinically relevant target. Neurological manifestations include encephalitis, encephalopathy, myelitis and demyelinating syndromes, cerebrovascular events, and immunes‐mediated neuropathies, occurring during acute infection or post‐infectious complications, often remaining underrecognized [1–3]. This study describes clinical profile and symptomatic treatment of cases of neurological dengue, aiming to better define its spectrum and outcomes.

**Methods:** We conducted a PRISMA systematic review. Pubmed was searched using the keywords “dengue fever” and “neurological complications”. Studies reporting clinical, radiological, or laboratory data on dengue‐associated neurological manifestations were included, excluding non‐human and literature reviews studies.

**Results:** We analyzed 142 patients with neurological complications of dengue infection. Mean age was 28 years. Neurological symptoms developed at a mean of 10.9 days after fever onset. The most frequent manifestations were encephalopathy (40%), seizures (25%), and focal neurological deficits (20%). The overall mortality rate was 5.6%. Diagnosis was confirmed by serology (NS1 and/or IgM) in 90% of cases and by cerebrospinal fluid PCR in 30%. MRI revealed abnormalities in 70% of patients, most commonly thalamic edema, myelitis, and corpus callosum lesions. Treatment with corticosteroids or intravenous immunoglobulins was administered in 50% of cases, with complete recovery observed in 60%.

**Figure:**
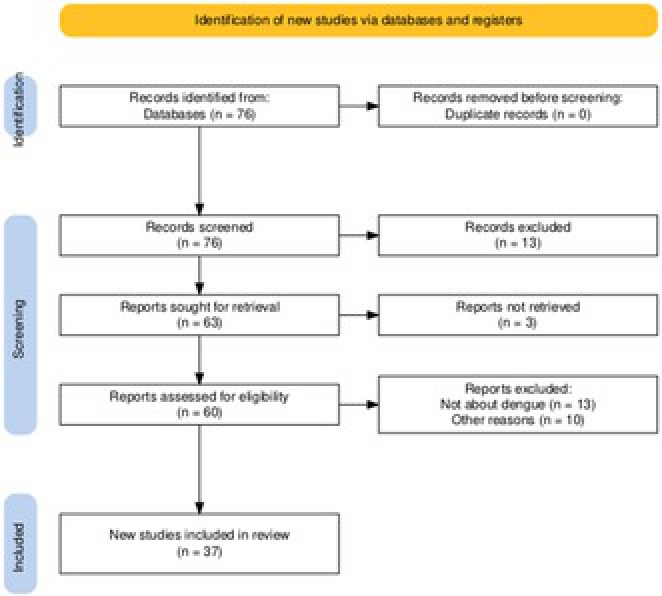
**FIGURE 1** Prisma‐P flowchart of the process of inclusion of studies in the systematic review.

**Figure:**
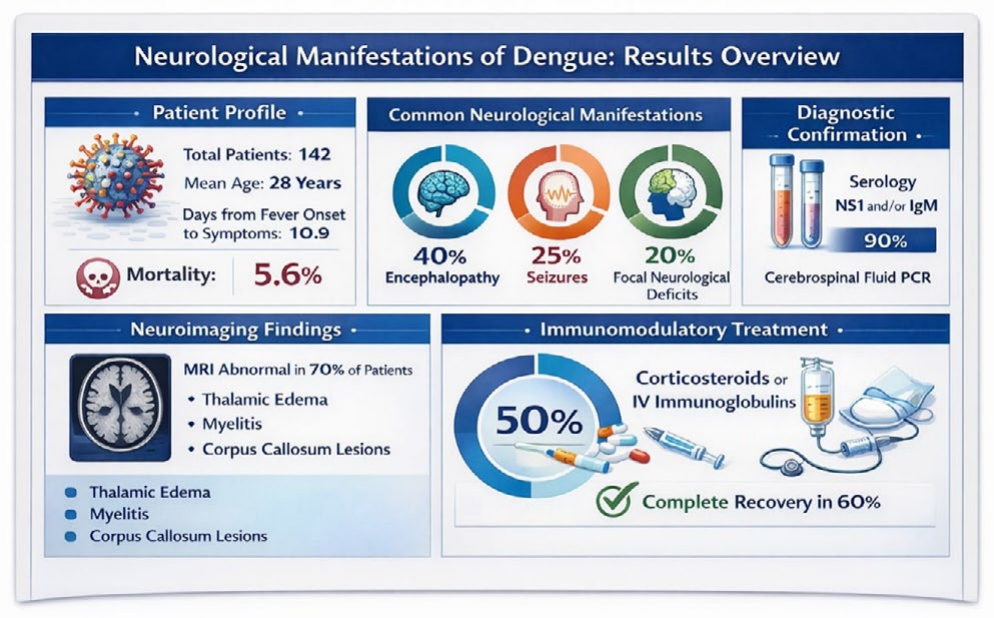
**FIGURE 2** Neurological manifestations of dengue, neuroimaging findings and immunomodulatory treatment.

**Conclusion:** Neurological complications of dengue are frequent and diverse, with significant mortality. Diagnosis is based on serology and neuroimaging, and immunomodulatory treatment is associated with high rates of complete recovery, highlighting the need for early recognition.

**Disclosure:** Nothing to disclose.

## EPO‐0264

### How long MRI abnormality persists in Tuberculous meningitis?

#### P. Chandra Pandey ^1^; F. M Nizami^2^; R. Mahajan^3^; J. Kalita^4^

##### ^*1*^ *Department Of Neurology, Sanjay Gandhi Postgraduate Institute of Medical Sciences, Lucknow, India;* ^*2*^ *Department Of Neurology, Sanjay Gandhi Postgraduate Institute of Medical Sciences, Lucknow, India;* ^*3*^ *Department Of Neurology, Sanjay Gandhi Postgraduate Institute of Medical Sciences, Lucknow, India;* ^*4*^ *Department Of Neurology, Sanjay Gandhi Postgraduate Institute of Medical Sciences, Lucknow, India*

**Background and aims:** There is paucity of information regarding resolution of MRI abnormalities, and its clinical relevance in patients with Tuberculous meningitis (TBM). We report the resolution of MRI abnormality at 6 and 12 months of treatment, and association with admission clinic‐laboratory findings.

**Methods:** 115 patients with TBM diagnosed on the basis of clinical, CSF and MRI findings were included during 2018–2024, and followed up clinically and radiologically at 3, 6 and 12 months. The baseline clinical severity, and MRI findings (exudates, Infarcts, hydrocephalous and granuloma) were noted. Patients were treated with four antitubercular drugs, prednisolone and aspirin. The outcome was assessed using modified Rankin Scale (mRS) and categorized into death, poor (3–5) and good (0–2). The predictors of complete resolution of MRI at one year was evaluated including baseline findings.

**Results:** Their median age was 25 years, and 45.2% were females. The median duration of illness was 60 days and majority were in stage II (*n* = 78 (67.8%) at admission). MRI revealed exudates in 103 (89.6%), Infarcts in 51 (44.3%), granuloma in 93 (80.9%) and hydrocephalous in 32 (27.8%) patients. The complete resolution occurred at 3 months in 10 (8.7%), 6 months in 25 (27.1%) and at 12months 25 (27.1%) patients. Enhancing granuloma was seen at 12months in 62 (53.9%), and exudates in 7 (6.1%) patients. On multivariate analysis, the predictors of abnormal MRI at one year included seizure (*p* = 0.026) and lymphopenia in blood (*p* = 0.005) at baseline.

**Conclusion:** Complete resolution of MRI abnormality at year occurs in 33.9% patients especially in nonlymphopenic and non‐seizure patients

**Disclosure:** Nothing to disclose.

## EPO‐0265

### Opportunistic central nervous system infections and transplant rejection in solid organ transplant recipients: A scoping review

#### P. Shekhawat ^2^; M. Kagan^1^; K. Thakur^3^

##### ^*1*^ *Columbia University Irving Medical Center, New York, NY;* ^*2*^ *Mass General Brigham, Dover, NH;* ^*3*^ *Columbia University Irving Medical Center, New York, NY*

**Background and aims:** Opportunistic central nervous system (CNS) infections are uncommon but devastating complications of solid organ transplantation (SOT), accounting for up to 10% of transplant‐associated complications and carrying disproportionate morbidity and mortality. While prior studies have emphasized short‐term neurologic outcomes, the impact of immunosuppressive modulation on graft rejection and longer‐term outcomes remains poorly characterized. To map and synthesize the existing literature describing transplant rejection in the setting of CNS infections among SOT recipients.

**Methods:** A scoping review was conducted following the Arksey and O'Malley framework, refined by the Joanna Briggs Institute, and reported according to PRISMA‐ScR guidelines (Figure 1). PubMed, EMBASE, and Scopus were searched from inception through October 2025. Eligible studies included human experimental and observational designs describing both opportunistic CNS infection and transplant rejection in SOT recipients of any age. Two reviewers independently screened studies and extracted data using a standardized form. Findings were synthesized narratively using the Population–Concept–Context framework.

**Figure:**
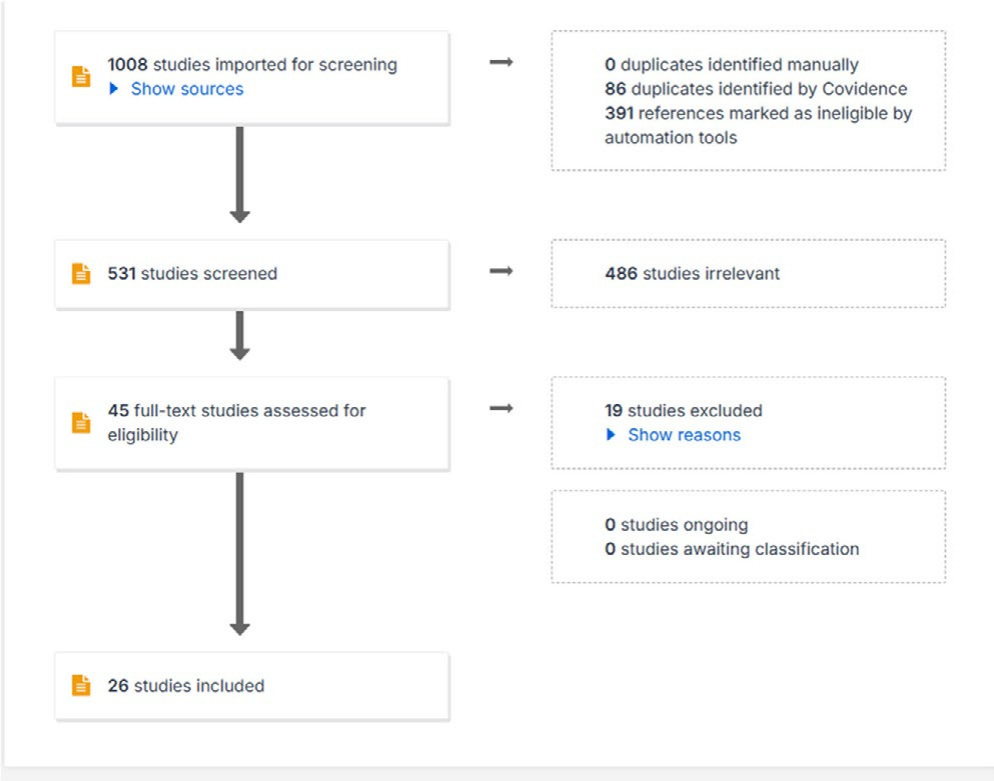
**FIGURE 1** PRISMA flow diagram illustrating study identification, screening, eligibility assessment, and inclusion for the scoping review of opportunistic CNS infections and transplant rejection in solid organ transplant recipients.

**Results:** CNS infections frequently occurred years after transplantation, often following intensified immunosuppression for rejection. Immunosuppressive regimens including calcineurin inhibitors and corticosteroids were consistently associated with pathogen‐specific CNS infections. Reduction of immunosuppression during infection management was followed by acute rejection in approximately 25–35% of patients within one year. Neurologic outcomes ranged from full recovery to permanent neurologic injury and graft dysfunction.

**Conclusion:** The literature addressing transplant rejection in the context of opportunistic CNS infections is limited and heterogeneous. Significant gaps remain regarding standardized immunosuppressive management and long‐term outcomes, highlighting the need for prospective studies and consensus‐based guidance.

**Disclosure:** Nothing to disclose.

## EPO‐0266

### A rare disease with a rarer manifestation: The neurobrucellosis therapeutic conundrum

#### J. Sowmya ^1^; P. Sripadma^1^; M. Shekar^2^

##### ^*1*^ *Department of Neurology, Kastruba Medical College, Manipal Academy of Higher Education Manipal, Karnataka, India;* ^*2*^ *Department of Intervention Radiology, Kastruba Medical College, Manipal Academy of Higher Education, Manipal, Karnataka, India*

**Background and aims:** Neurobrucellosis is a rare but severe complication of systemic brucellosis, accounting for 3–10% of cases. Clinical presentations range from meningitis to stroke, mimicking neuro‐tuberculosis in endemic countries, leading to delayed and often misdiagnosis.

**Methods:** A 58‐year‐old man presented with intermittent fever, polyarthralgia, and headache for three weeks. General and neurological examinations were unremarkable. Evaluation for pyrexia of unknown origin revealed lymphocytic pleocytosis, elevated protein, and hypoglycorrhachia on cerebrospinal fluid analysis, suggestive of infection. Blood cultures grew Brucella, and serum agglutination testing was positive. MRI of the spine demonstrated thoracic discitis. Treatment with ceftriaxone, rifampicin, and doxycycline was initiated.

**Figure:**
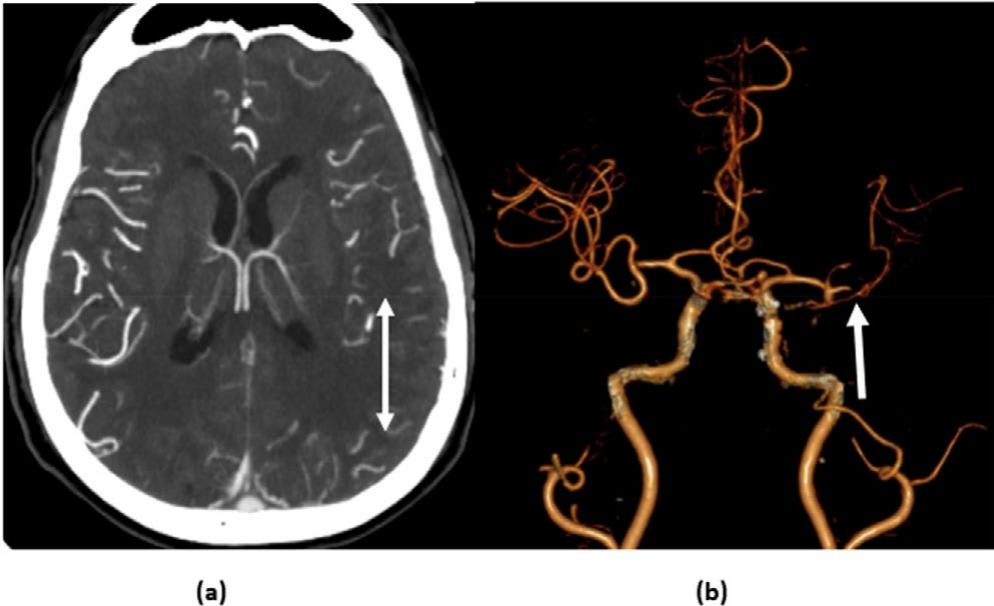
**FIGURE 1** Axial CT sections of CT angiogram study (a) and 3D reconstruction of intracranial vessels show paucity of left middle cerebral artery (MCA) territory M3 and M4 cortical branches (double headed arrow) and stenosis at distal left M1 MCA on 3D images (arrow).

**Figure:**
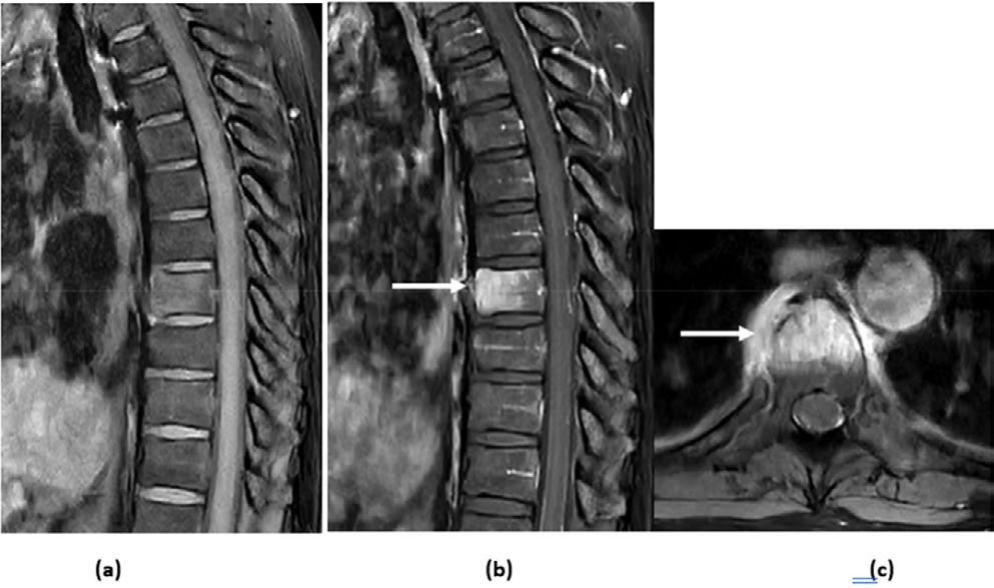
**FIGURE 2** T1 pre contrast (a) and T1 post contrast sagittal (b) and T1 post contrast axial (c) images of dorsal spine show abnormal contrast enhancement (arrow) at D8 vertebral body.

**Results:** Four weeks later, despite appropriate antimicrobial therapy, he developed recurrent transient neurological episodes followed by persistent dysarthria and right hemiparesis. Brain MRI revealed acute lentiform nucleus infarcts, and MR angiography showed focal stenosis of the left middle cerebral artery (M1 segment). Vessel wall imaging demonstrated concentric enhancement, consistent with cerebral vasculitis. High‐dose corticosteroids were added while continuing antibiotics. The patient stabilized without further neurological deterioration. Steroids were gradually tapered over six months, ceftriaxone was continued for six months, and doxycycline with rifampicin for one year. At one‐year follow‐up, he had minimal residual dysarthria without limb weakness.

**Figure:**
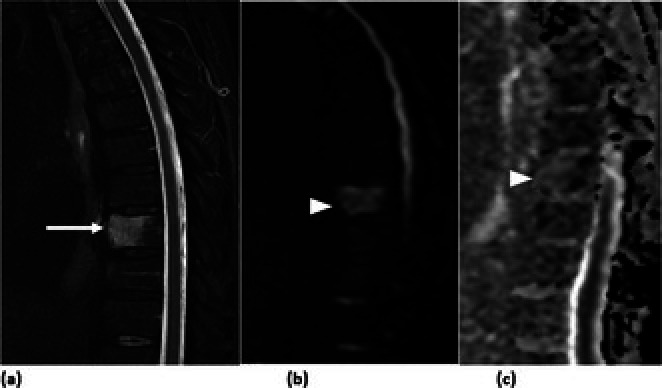
**FIGURE 3** T2 STIR (a) image of dorsal spine show abnormal signal intensity (arrow) along the D8 vertebral body with DWI (b) hyperintensity (arrowhead) and ADC (c) image showing partial signal drop suggesting partial diffusion restriction.

**Conclusion:** Vasculitis in neurobrucellosis is very rare, with no consensus on treatment protocol. Starting steroids is a cliffhanger with the risk of worsening an active systemic infection and the presumed benefit of neurological improvement. Our experience highlights that steroids, when judiciously used in selective complications like CNS vasculitis, along with antibiotics, can improve the outcome.

**Disclosure:** Nothing to disclose.

Motor neurone diseases 2

## EPO‐0267

### Functional status and prognostic predictors in the final six months of life in ALS: A retrospective analysis of 703 patients

#### B. Machado Antunes ^1^; M. Oliveira Santos^1^; V. Barão de Sá^2^; D. Lopes^2^; I. Alves^2^; M. de Carvalho^1^

##### ^*1*^ *Department of Neuroscience and Mental Health, Santa Maria Local Health Unit, Academic Center of Medicine of Lisbon, Lisbon, Portugal;* ^*2*^ *Institute of Physiology, Egas Moniz Research Center,Faculty of Medicine, University of Lisbon, Lisbon, Portugal*

**Background and aims:** In advanced Amyotrophic Lateral Sclerosis (ALS), transition to palliative care often leads to formal neurological follow‐up discontinuation. Consequently, revised ALS Functional Rating Scale (ALSFRS‐R) data near the end of life are scarce. This study aimed to characterize ALSFRS‐R total and subdomain scores during the final six months of life and evaluate their independent prognostic value.

**Methods:** We retrospectively analysed 703 patients with ALS (including progressive muscular atrophy) who underwent ALSFRS‐R assessment within six months of death. Clinical and functional data were analysed using descriptive statistics; scores were compared by onset site (spinal vs. bulbar) using the Mann–Whitney U test. Cox proportional hazards models were employed to evaluate the association between functional scores and mortality risk, adjusting for age at onset, sex, onset region, and the use of gastrostomy and non‐invasive ventilation.

**Results:** Median age at onset was 66.7 years and median survival was 29 months. Near death, median ALSFRS‐R total score was 20, with no significant differences between onset types. Notably, respiratory and bulbar subscores remained higher relative to limb and global domains. Multivariate analysis revealed that higher total ALSFRS‐R scores (HR 0.988, 95% CI 0.979–0.998) and higher bulbar subscores (HR 0.965, 95% CI 0.939–0.992) were independently associated with reduced risk of death. Other subscores did not significantly predict survival in this late stage.

**Conclusion:** Quantifying functional decline in the terminal phase of ALS is vital for informed end‐of‐life clinical decision‐making. In advanced disease, overall functional status and, specifically, bulbar dysfunction emerge as the most robust predictors of mortality.

**Disclosure:** The author has applied for a research grant related to this work and is currently awaiting the outcome.

## EPO‐0268

### Therapeutic comparison of edaravone and riluzole in amyotrophic lateral sclerosis: A systematic review and meta‐analysis

#### C. De Souza Pavao ^1^; Y. Bastos Faller^2^; N. Belen Toca^2^; J. Ferreira Jasmineiro Pitanga^1^; C. Rocha Dantas^2^; M. Sánchez espuelas^2^

##### ^*1*^ *Department of Microbiology, Parasitology and Immunology, University of Buenos Aires, Buenos Aires, Argentina;* ^*2*^ *Faculty of Medicine, University of Buenos Aires (UBA), Buenos Aires, Argentina*

**Background and aims:** Amyotrophic lateral sclerosis (ALS) is a rapidly progressive neurodegenerative disorder. Riluzole is the standard therapy, while edaravone is an approved adjunct treatment; however, the clinical benefit of combination therapy remains uncertain. This meta‐analysis evaluates the effect of riluzole plus edaravone versus riluzole alone on disease progression in ALS according to the Revised Amyotrophic Lateral Sclerosis Functional Rating Scale (ALSFRS‐R).

**Methods:** A systematic search was performed for studies published through October 2025 comparing combination therapy with riluzole monotherapy in patients with ALS. Extracted outcomes included ALSFRS‐R and all‐cause mortality. Standardized mean differences (SMDs) and odds ratios (ORs) with corresponding 95% confidence intervals (CIs) were calculated using random‐effects models. Statistical heterogeneity was assessed using the I^2^ statistic. All analyses were performed using RStudio.

**Results:** Five studies comprising 548 patients were included, of whom 39.4% received riluzole plus edaravone. For functional progression, pooled analysis showed no significant difference between combination therapy and riluzole monotherapy (SMD = 0.20, 95% CI −0.37 to 0.77; *p* = 0.49), with moderate heterogeneity (I^2^ = 63%). Similarly, no significant difference in all‐cause mortality was observed between treatment strategies (OR = 1.37, 95% CI 0.25 to 7.44; *p* = 0.71), also with moderate heterogeneity (I^2^ = 60%). Heterogeneity likely stemmed from differences in study design and disease stage at treatment initiation.

**Figure:**
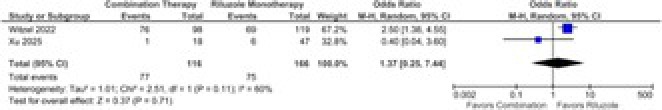
**FIGURE 1** Forest plot of all‐cause mortality comparing combination therapy (edaravone plus riluzole) versus riluzole monotherapy in patients with ALS.

**Figure:**
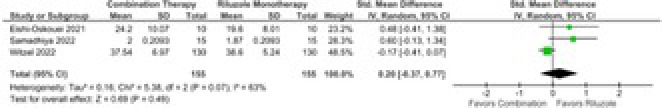
**FIGURE 2** Forest plot comparing functional progression (ALSFRS‐R scores) between edaravone plus riluzole combination therapy and riluzole monotherapy.

**Conclusion:** Riluzole plus edaravone did not demonstrate a significant advantage over riluzole alone in ALS. This quantitative synthesis clarifies the absence of a population‐level benefit of combination therapy. However, larger and standardized randomized trials are required to inform future treatment recommendations.

**Disclosure:** Nothing to disclose.

## EPO‐0269

### NLRP3 inflammasome and pyroptosis as contributors to neuroinflammation in amyotrophic lateral sclerosis

#### D. Shevchuk ^1^; I. Dolzhikova^2^; A. Kovyrshina^2^; M. Zakharova^1^

##### ^*1*^ *6th neurological department, Russian Center of Neurology and Neurosciences, Moscow, Russian Federation;* ^*2*^ *N. F. Gamaleya National Research Center for Epidemiology and Microbiology, Moscow, Russian Federation*

**Background and aims:** Amyotrophic lateral sclerosis is a fatal neurodegenerative disorder in which growing evidence points to a contributory role of innate immune mechanisms. Activation of inflammasome‐dependent inflammatory pathways and pyroptotic cell death has been demonstrated in experimental models; however, clinical evidence in patients remains scarce. This study aimed to evaluate circulating and cerebrospinal fluid markers related to NLRP3 inflammasome activation and pyroptosis in amyotrophic lateral sclerosis and to explore their clinical and genetic associations.

**Methods:** A total of 100 patients with amyotrophic lateral sclerosis and 36 neurologically healthy controls were enrolled. Serum and cerebrospinal fluid levels of interleukin‐1 beta, interleukin‐18, and gasdermin D were quantified using ELISA. Clinical phenotypes, disease staging, and progression characteristics were analyzed. Molecular genetic testing was performed to identify SOD1 mutations and c9orf72 repeat expansion. Group and subgroup comparisons were conducted using appropriate statistical methods.

**Results:** Compared with controls, patients with ALS exhibited higher serum concentrations of gasdermin D and increased cerebrospinal fluid levels of interleukin‐1 beta. Elevated cerebrospinal fluid interleukin‐1 beta was observed in carriers of c9orf72 repeat expansion. Serum gasdermin D concentrations varied across clinical phenotypes, with higher levels in patients with predominant lower motor neuron involvement. Interleukin‐18 levels did not significantly differ between groups. Associations between peripheral and central inflammatory markers were identified.

**Figure:**
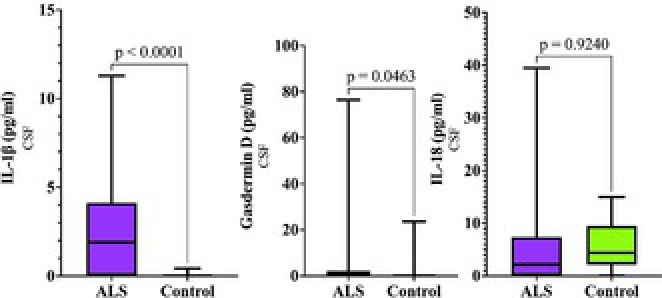
**FIGURE 1** Cerebrospinal fluid levels of interleukin‐1 beta, gasdermin D, and interleukin‐18 in patients with ALS and controls.

**Figure:**
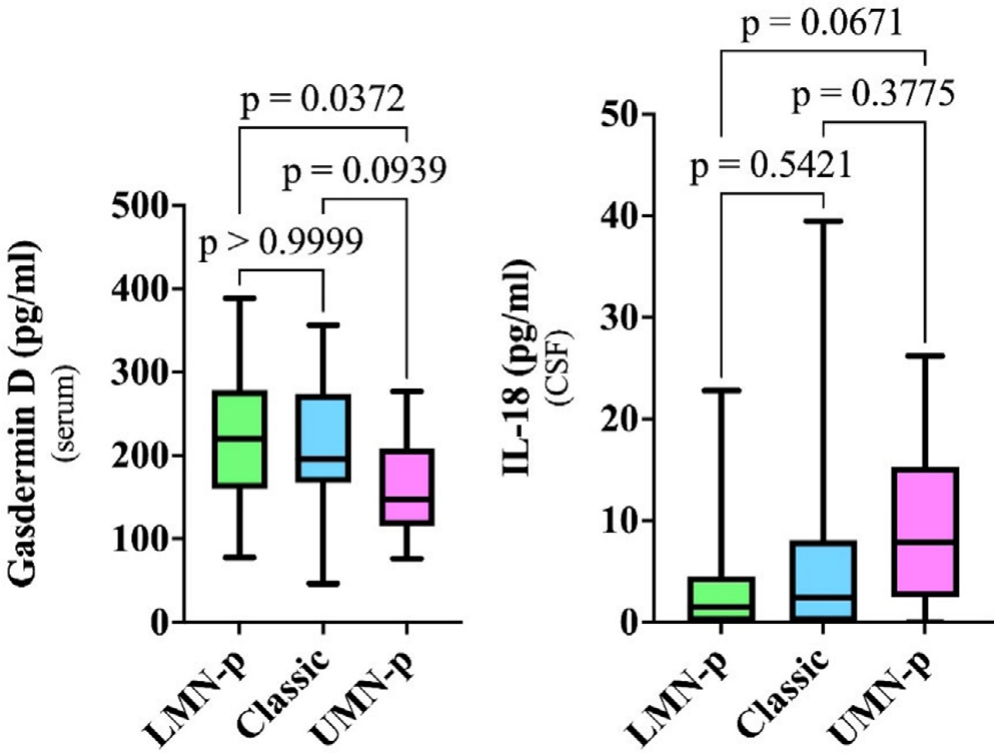
**FIGURE 2** Serum gasdermin D and CSF interleukin‐18 levels across ALS clinical phenotypes: lower motor neuron–predominant, classic, and upper motor neuron–predominant.

**Figure:**
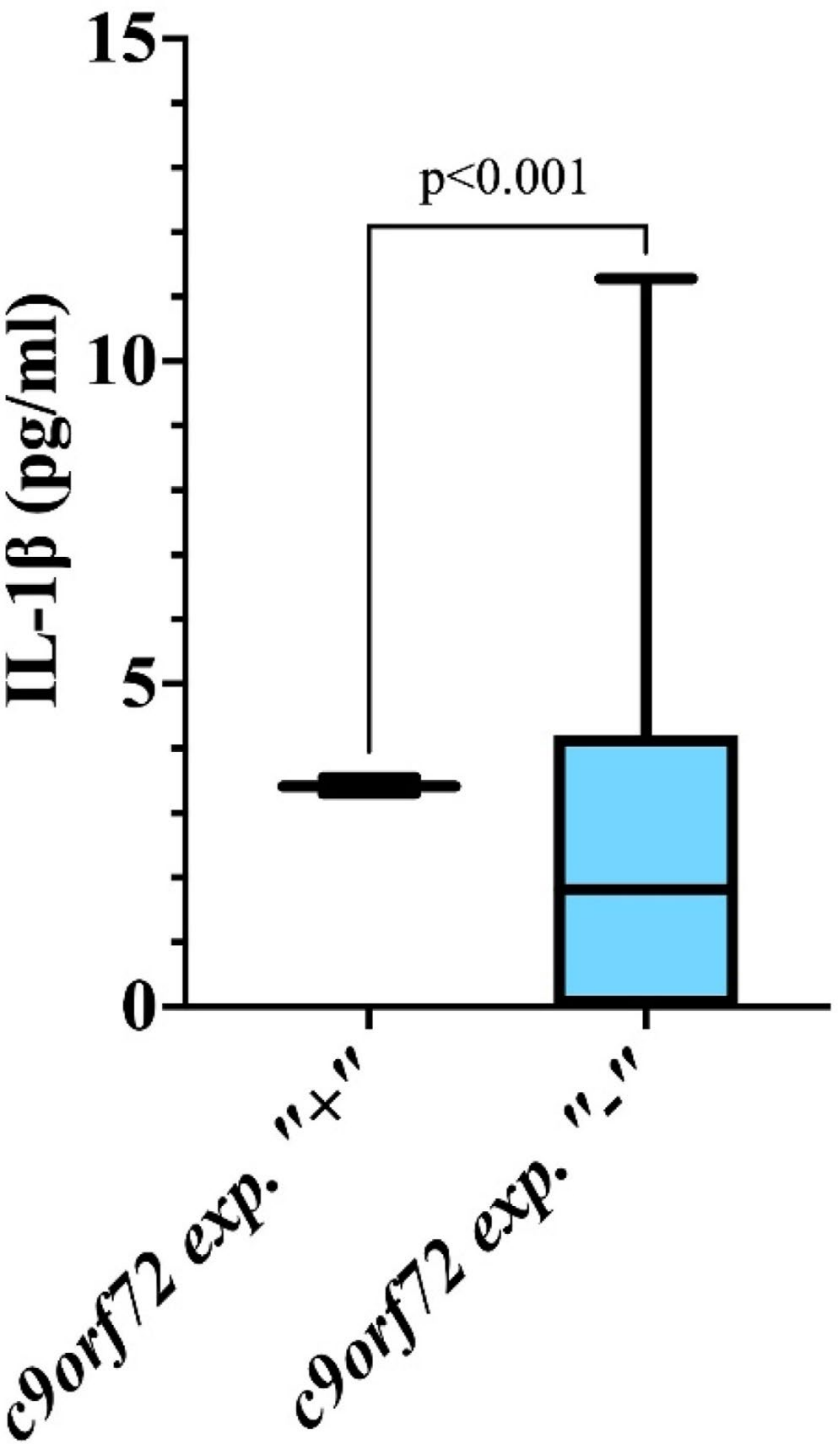
**FIGURE 3** CSF interleukin‐1 beta levels in ALS patients with and without c9orf72 repeat expansion.

**Conclusion:** The results indicate activation of inflammasome‐related inflammatory pathways and pyroptosis in amyotrophic lateral sclerosis. These findings support a role for innate immune dysregulation in disease pathogenesis and suggest that inflammasome‐associated molecules may represent promising biomarkers and therapeutic targets.

**Disclosure:** Nothing to disclose.

## EPO‐0270

### Efficacy of immunoglobulin in participants with post‐polio syndrome: Results from the multicenter, randomized, placebo‐controlled FORCE trial

#### F. Nollet ^1^; D. Trojan^2^; H. Andersen^3^; J. Jakobsen^4^; P. Sáiz^5^; J. Skopek^6^; S. Petri^7^; K. Piasecka–Stryczyńska^8^; L. Bertolasi^9^; K. Rejdak^10^; S. Camprubi^11^; B. Garcia^11^; D. Whyms^12^; E. Mondou^13^; M. Dalakas^14^

##### ^*1*^ *Department of Rehabilitation Medicine, Amsterdam UMC Location University of Amsterdam, Amsterdam, The Netherlands and Amsterdam Movement Sciences, Rehabilitation and Development, Amsterdam, The Netherlands;* ^*2*^ *Department of Neurology and Neurosurgery, Montreal Neurological Institute‐Hospital, McGill University Health Centre, McGill University, Montreal, Canada;* ^*3*^ *Århus Universitets Hospital, Aarhus, Denmark;* ^*4*^ *The Neuroscience Centre Rigshospitalet (National Hospital), Copenhagen, Denmark;* ^*5*^ *Hospital de Neurorehabilitación, Badalona, Spain;* ^*6*^ *Fakultní Thomayerova nemocnice, Praha, Czech Republic;* ^*7*^ *Hannover Medical School, Hannover, Germany;* ^*8*^ *Clinical Research Center Spółka z Ograniczoną Odpowiedzialnością MEDIC‐R Sp. K, Poznan, Poland;* ^*9*^ *Azienda Ospedaliera Universitaria Integrata Verona (Ospedale Borgo Roma), Verona, Italy;* ^*10*^ *Department of Neurology, Medical University of Lublin, Lublin, Poland;* ^*11*^ *Grifols, Sant Cugat, Spain;* ^*12*^ *Grifols Worldwide Operations, Dublin, Ireland;* ^*13*^ *Grifols, Research Triangle Park, NC, USA;* ^*14*^ *Thomas Jefferson University, Philadelphia, USA*

**Background and aims:** Post‐Polio Syndrome (PPS) is a chronic, slowly progressive neuromuscular condition with no specific medical treatments. This study evaluated intravenous immunoglobulin (IVIG, Flebogamma® 5% DIF) in participants with PPS, based on observations of immune/inflammatory markers activation.

**Methods:** This Phase II/III multicenter, randomized, double‐blind, placebo‐controlled trial included adult participants with PPS across Europe, Canada, and the US. Participants received monthly infusions of IVIG (1 g/kg) or placebo for 52 weeks, followed by a 24‐week observation. The primary endpoint was change from baseline in Two‐Minute Walking Distance (2MWD) at Week 52. Secondary and exploratory endpoints included Six‐Minute Walking Distance (6MWD), fatigue (Fatigue Severity Scale and Borg scale), muscle strength (Medical Research Council scale), and pain (Visual Analog Scale).

**Results:** 149 participants were randomized to IVIG 1 g/kg (*n* = 75) or placebo (*n* = 74). Mean age 63.2 years, 61.7% female. IVIG significantly improved 2MWD vs placebo at Week 52 (*p* = 0.0469), which was further confirmed in a sensitivity analysis (least squares mean difference [LSMD]: +6.07 m; *p* = 0.0147) (Figure 1). A clinically meaningful improvement in 6MWD was observed in the IVIG group vs placebo (+15.8 m; *p* = 0.1205); IVIG improvement from baseline was twice that of placebo (Figure 2). Muscle strength decreased in placebo group and increased in IVIG group (*p* = 0.0220). No notable differences were observed in fatigue or pain (Table 1). IVIG 1 g/kg was well tolerated.

**Figure:**
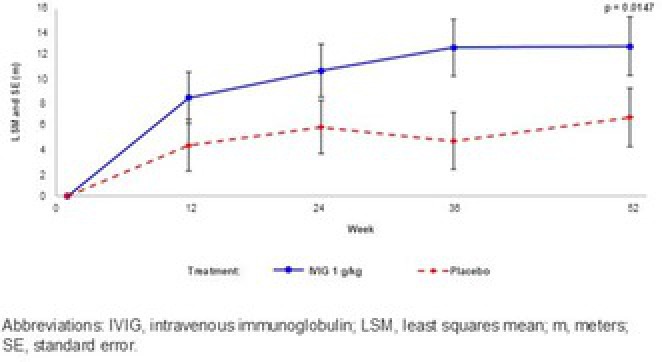
**FIGURE 1** Least squares mean change in 2MWD from baseline to Week 52.

**Figure:**
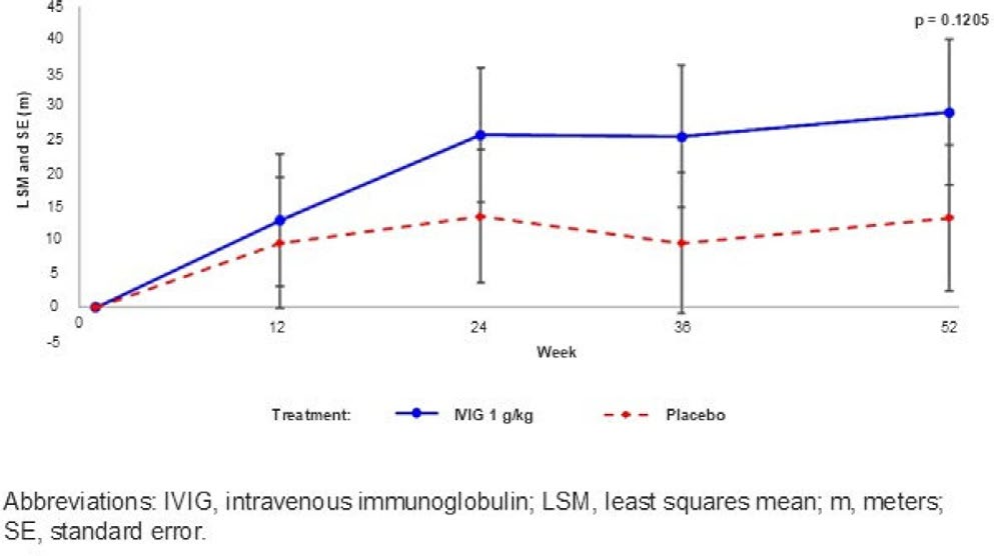
**FIGURE 2** Least squares mean change in 6MWD from baseline to Week 52.

**Figure:**
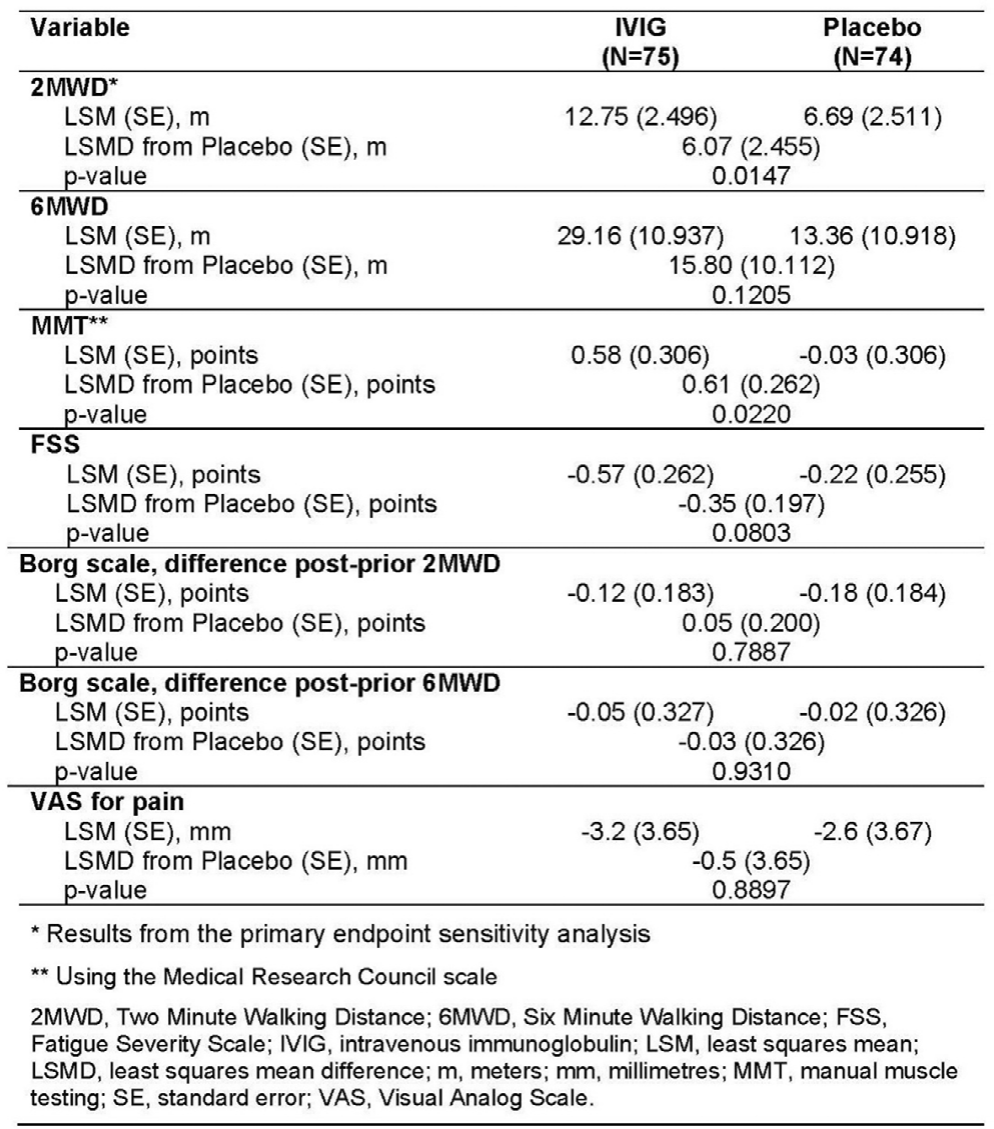
**TABLE 1** Summary of efficacy outcomes: Change from baseline to Week 52.

**Conclusion:** Monthly IVIG infusions demonstrated statistically and clinically meaningful improvements in walking capacity in participants with PPS, indicating IVIG 1 g/kg monthly for one year improves physical performance and endurance.

**Disclosure:** This study was funded by Grifols. S. Camprubi, B. Garcia, D. Whyms, and E. Mondou are full‐time employees of Grifols and have no other competing interests to declare. F. Nollet and D. Trojan received institutional grants for participation as study site in the FORCE trial, for providing the training of outcome assessors in the FORCE trial, and fees for consultation services and management of study participants. D. Trojan receives research support from the Polio Quebec Association and The Robert Lassner Research Fund. H. Andersen received research support, travel support, speaker honoraria and served as consultant on advisory boards from CSL Behring, NMD Pharma, Pfizer, Novo, Lundbeck, Alexion, Sanofi Genzyme, Biogen, Merck, UCB Pharma. J. Jakobsen received speaker honoraria from Takeda. The rest of authors declare no conflict of interest.

## EPO‐0271

### Neutrophil percentage‐to‐albumin ratio as a possible prognostic biomarker in amyotrophic lateral sclerosis: A PRO‐ACT study cohort

#### L. Ferullo ^1^; E. Olivieri^2^; B. Risi^1^; L. Poli^3^; A. Padovani^2^; M. Filosto^1^

##### ^*1*^ *NeMO‐Brescia Clinical Center for Neuromuscular Diseases, Brescia, Italy;* ^*2*^ *Department of Clinical and Experimental Sciences, University of Brescia, Brescia, Italy;* ^*3*^ *Unit of Neurology, ERN Euro‐NMD Center, ASST Spedali Civili, Brescia, Italy*

**Background and aims:** Increasing evidence suggests that immune dysregulation contributes to the pathophysiology and progression of amyotrophic lateral sclerosis (ALS), underscoring the need for inflammatory biomarkers to monitor disease progression. The neutrophil percentage‐to‐albumin ratio (NPAR) has been proposed as a marker of systemic inflammation and has shown prognostic relevance in ischemic stroke, intracerebral hemorrhage, and cognitive decline. However, its role in ALS remains unclear. This study aimed to investigate longitudinal changes in NPAR and to evaluate its prognostic value in ALS.

**Methods:** We conducted a retrospective longitudinal analysis of 917 ALS patients, comprising 2,168 NPAR measurements collected at baseline and at 6 and 12 months. NPAR values were log‐transformed to improve normality. Longitudinal trajectories of log‐NPAR were modeled using linear mixed‐effects models with random intercepts and slopes, adjusted for age, sex, site of onset, riluzole treatment, baseline ALSFRS‐R score, and body mass index. Survival was analyzed using Cox proportional hazards models with NPAR as a time‐dependent covariate, considering a composite endpoint of death or tracheostomy. Data were obtained from the PRO‐ACT database.

**Results:** Log‐NPAR increased significantly over time across the entire cohort (slope: 0.036/year, *p* < 0.001). Patients who reached the endpoint of death or tracheostomy exhibited a steeper increase in log‐NPAR (*p* = 0.0003). In survival analyses, higher time‐dependent NPAR was associated with an increased risk of death or tracheostomy (hazard ratio [HR] 12.75, 95% [CI] 3.09–52.57, *p* < 0.001), with attenuation of the effect over time.

**Conclusion:** NPAR demonstrates longitudinal changes in ALS, particularly among patients with poorer outcomes, supporting its potential role as a prognostic blood biomarker.

**Disclosure:** Nothing to disclose.

## EPO‐0272

### Tofersen in the treatment of SOD1 ALS – A two year experience from the Polish Early Access Program

#### M. Kuzma‐Kozakiewicz

##### Department of Neurology, Medical University of Warsaw, Warsaw, Poland

**Background and aims:** Tofersen, an ASO against SOD1, is now registered by FDA and EMA. Some patients obtained tofersen in the EAP and/or rescue therapy. The aim of the study was to follow up on clinical outcomes and NfL concentration in patients participating in the EAP in Poland for 24 months.

**Methods:** 20 SOD1‐ALS patients qualified to EAP (10 FALS and 10 SALS, a male:female ratio of 2.2:1) carried 11 SOD1 mutations (45% – L145S and 10% – K4E). Median age of onset was 49.1 years, age of introducing tofersen was 52.6 years. From the original cohort, 1 patient resign after the first dose of treatment, two withdrew (due to severe adverse event or respiratory insufficiency) and 2 passed away due to disease progression. The analysis included the demographic and genetic data, ALSFRS‐R, delta ALSFRS‐R, FVC and NfL CSF concentration.

**Results:** Mean treatment was 17 months (16–24 months), delta ALSFRS‐R ranged from 0.46 prior to treatment to 0.15 at the last assessment. Seven patients (37%) showed increase of ALSFRS‐R by 1–2 points in the treatment period, six patients (31%) stabilized, while 6 patients (31%) showed further progression. Mean ALSRFS‐R was stable after 6 and 24 months, and increased after 12 an 18‐months. NfL concentration decreased in 5/7 of analyzed patient. Treatment was well tolerated in 78% of patients. Three patients experienced SAE– two transverse myelitis, one exacerbation of colitis ulcerosa and one poliradiculopathy.

**Figure:**
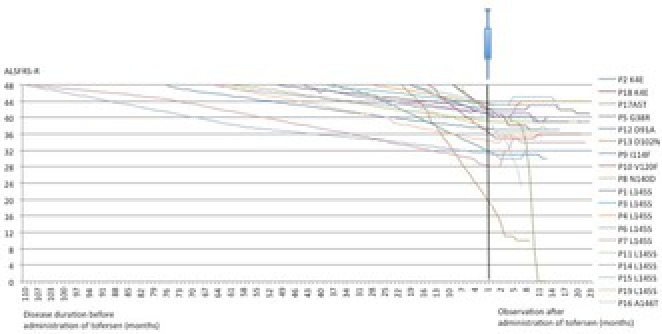
**FIGURE 1** Disease progression prior and after tofersen treatment.

**Conclusion:** The data from a long term follow‐up supports the efficiency of tofersen in SOD1‐ALS.

**Disclosure:** MKK obtained lecture grants from Biogen.

## EPO‐0273

### Sensor–based real‐world gait metrics as sensitive mobility outcomes in amyotrophic lateral sclerosis

#### M. Poleur ^1^; K. Burke^2^; C. Rasser^3^; C. Gonzalez Barral^3^; N. Calcagno^4^; K. Carney^2^; S. Hall^2^; S. Mandepudi^2^; O. Piquet^3^; C. Hermanne^1^; D. Eggenspieler^3^; S. Delstanche^1^; J. Berry^2^; L. Servais^5^

##### ^*1*^ *CRMN, University department of neurology, Citadelle Hospital of Liège, Belgium;* ^*2*^ *Sean M. Healey and AMG Center for ALS at Massachusetts General Hospital, USA;* ^*3*^ *Sysnav, Vernon, France;* ^*4*^ *Department of Neurology and Laboratory of Neuroscience, IRCCS Istituto Auxologico Italiano, Milan, Italy;* ^*5*^ *MDUK Oxford Neuromuscular Centre, John Radcliffe Hospital, Oxford, UK*

**Background and aims:** Amyotrophic lateral sclerosis (ALS) is a heterogeneous, rapidly progressive neurodegenerative disease. Clinical trials rely mainly on the Revised ALS Functional Rating Scale (ALSFRS‐R), which is subjective and often insensitive to subtle changes in ambulation. This study aimed to evaluate the analytical validity, feasibility, and clinical validity of real‐world gait outcomes derived from wearable digital health technology (wDHT) in ALS.

**Methods:** One analytical validation study and three longitudinal observational studies were conducted in Belgium and the United States. Ambulatory adults with ALS wore ankle‐mounted wDHTs daily for repeated 30‐day periods. Three digital mobility outcomes were derived: stride velocity 95th centile (SV95C), number of strides per hour, and peak walking distance. Analytical validation against optical motion capture assessed stride detection and gait parameter accuracy. Reliability, construct validity and sensitivity to change were evaluated.

**Results:** Analytical validation showed excellent precision and recall for stride detection (> 0.97), and mean error of 2.2cm and 1.7cm/s for stride length and velocity measurements, respectively (*N* = 10). In the longitudinal study (*N* = 46), adherence rate exceeded 80% over 9 months and SV95C demonstrated outstanding reliability (intraclass correlation coefficient of 0.99). SV95C correlated strongly with the ALSFRS‐R gross motor subscore (*R* = 0.67) and 6MWT (*R* = 0.89; *p* < 0.001), but weakly with ALSFRS‐R total score. SV95C declined significantly over 3, 6, and 9 months and showed greater sensitivity to change than ALSFRS‐R and 6MWT.

**Figure:**
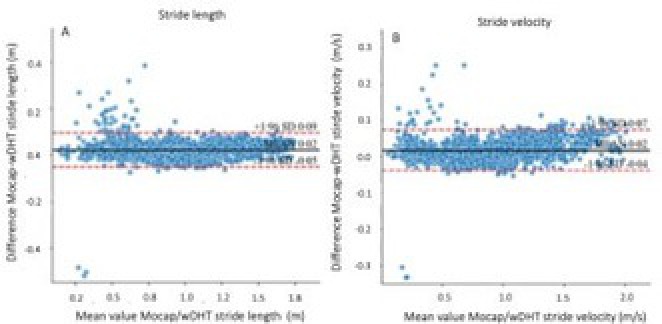
**FIGURE 1** Bland Altman plots for the error of the stride velocity and length.

**Figure:**
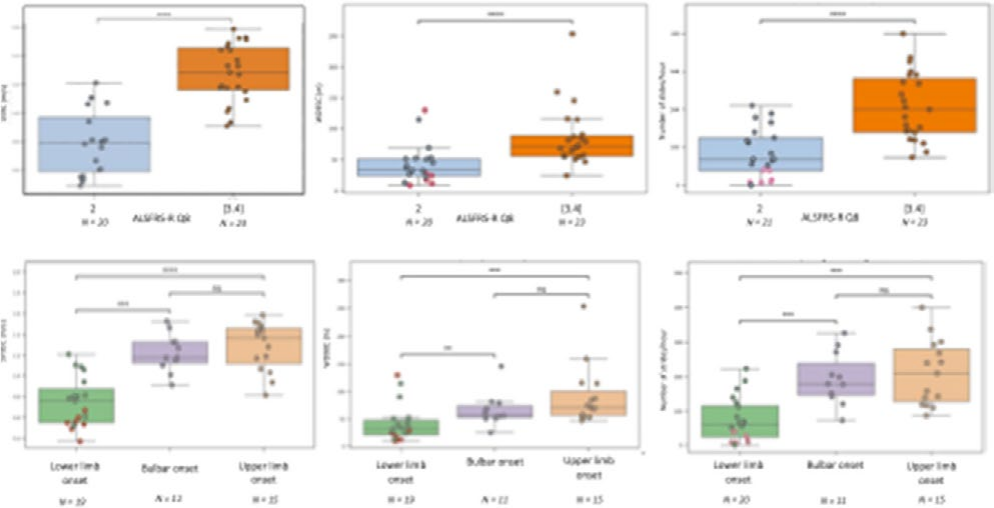
**FIGURE 2** Digital outcome measures by by ALSFRS‐r walking score and disease onset type (*p* < 0.05*, *p* < 0.01**, *p* < 0.001***, *p* < 0.0001****, ns = not significant). ALSFRS‐R Question 8 – Walking assessment: 4 = normal, 3 = early ambulation difficulties.

**Figure:**
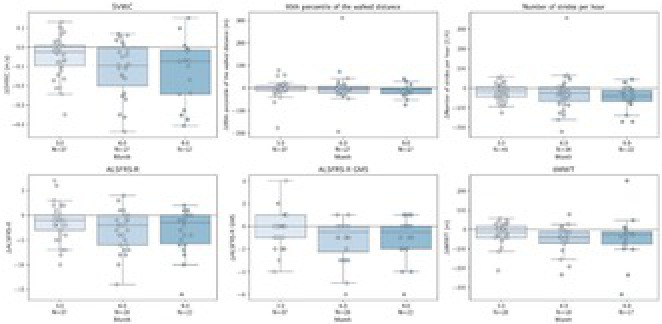
**FIGURE 3** Box plots with change at 3, 6, and 9 months for each digital and clinical outcome (*p* < 0.05*, *p* < 0.01**).

**Conclusion:** Continuous real‐world gait monitoring using wDHTs is feasible in ALS. SV95C provides a reliable and sensitive measure of ambulatory decline and represents a promising outcome for ALS clinical trials.

**Disclosure:** This project received funding from Belgian Association for Neuromuscular Disease (ABMM) and Muscular Dystrophy Association (MDA).

## EPO‐0274

### The role of hsa‐miR‐21‐5p, hsa‐miR‐210‐3p, hsa‐miR‐197‐3p, hsa‐miR‐125a‐5p and hsa‐miR‐206‐3p as potential biomarkers in amyotrophic lateral sclerosis

#### M. Sokratous ^1^; P. Stamati^1^; I. Liampas^1^; C. Marogianni^1^; D. Bogdanos^2^; E. Dardiotis^1^; V. Siokas^1^

##### ^*1*^ *Department of Neurology, University Hospital of Larissa, Faculty of Medicine, University of Thessaly, Larissa, Greece;* ^*2*^ *Department of Rheumatology and Clinical Immunology, Faculty of Medicine, University of Thessaly, Larissa, Greece*

**Background and aims:** Amyotrophic lateral sclerosis (ALS) is a fatal neurodegenerative disease characterized by progressive loss of motor neurons. MicroRNAs (miRNAs) are small, non‐coding RNA molecules that regulate gene expression. The purpose of this study is to identify selected miRNAs in the cerebrospinal fluid (CSF) and serum as biomarkers for ALS.

**Methods:** A total of 50 ALS patients and 50 age and sex‐matched non‐ALS controls were enrolled. MiRNAs were isolated from serum and CSF samples and quantified using quantitative reverse transcription polymerase chain reaction (qRT‐PCR) with specific primers for hsa‐miR‐21‐5p, hsa‐miR‐210‐3p, hsa‐miR‐197‐3p, hsa‐miR‐125a‐5p and hsa‐miR‐206‐3p. Internal and spike‐in controls were used for normalization. ΔCt values were compared between ALS and non‐ALS groups using non‐parametric tests with covariates for age, gender, amyotrophic lateral sclerosis functional rating scale – revised (ALSFRS‐R), disease duration and progression rate.

**Results:** Hsa‐miR‐210‐3p and has‐miR‐206‐3p were markedly up‐regulated in ALS CSF and serum and correlated with disease duration. Hsa‐miR‐210‐3p also correlated with progression rate in the CSF (*p* < 0.001). Hsa‐miR‐210‐3p exhibited the highest diagnostic performance, with an area under curve (AUC) of 0.925 in the serum and 0.902 in the CSF. Hsa‐miR‐21‐5p showed up‐regulation in ALS CSF and serum and strong association with poorer ALSFRS‐R. Hsa‐miR‐125a‐5p was significantly down‐regulated in ALS serum but remained stable in the CSF. Hsa‐miR‐197‐3p showed no diagnostic or prognostic value in either material.

**Figure:**
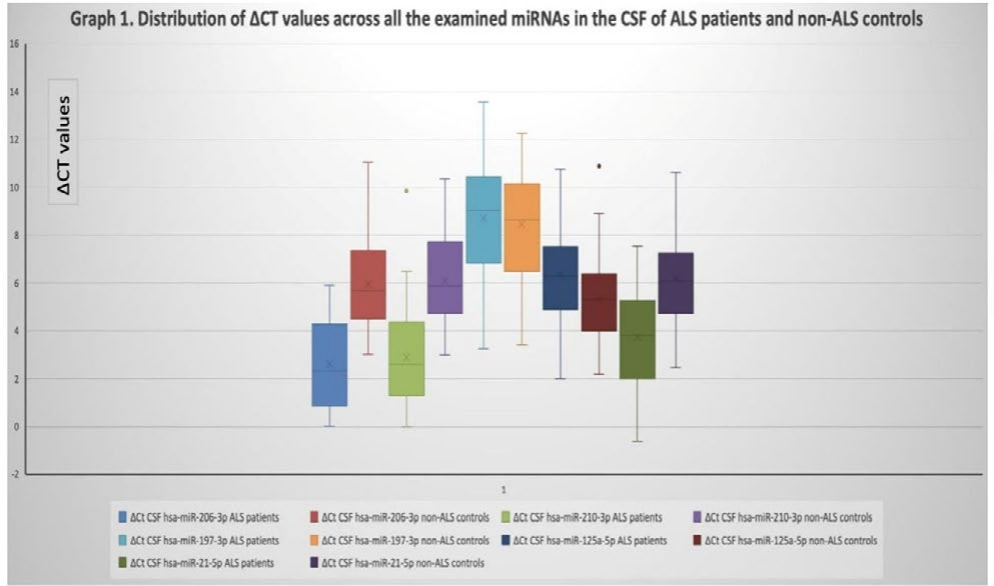
**FIGURE 1** Distribution of ΔCT values across all the examined miRNAs in the CSF of ALS patients and non‐ALS controls.

**Figure:**
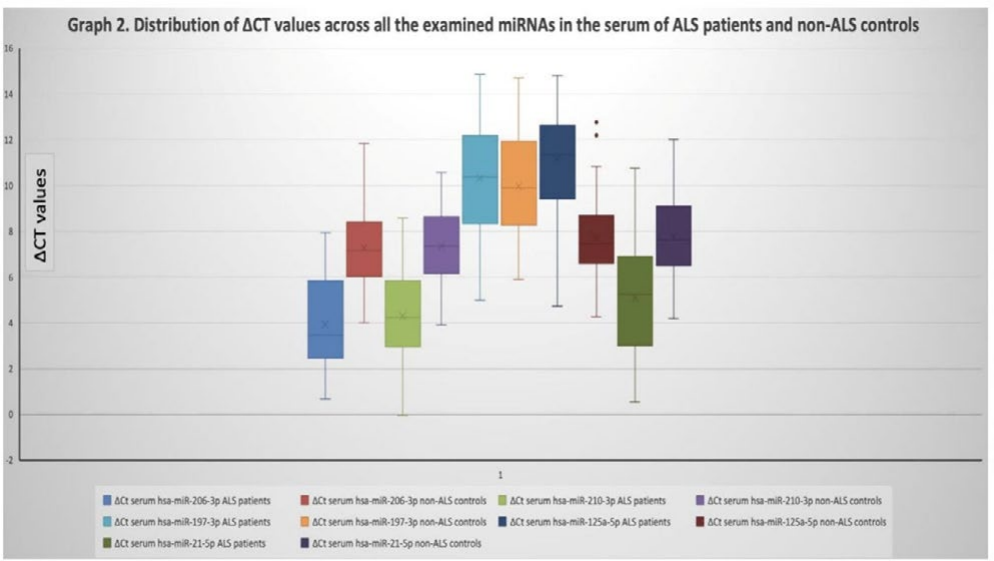
**FIGURE 2** Distribution of ΔCT values across all the examined miRNAs in the serum of ALS patients and non‐ALS controls.

**Conclusion:** Serum and CSF hsa‐miR‐210‐3p and hsa‐miR‐206‐3p could serve as strong diagnostic ALS biomarkers, while hsa‐miR‐21‐5p as a robust disease‐severity indicator. The combination of up‐regulated and down‐regulated miRNAs suggests a multimarker panel.

**Disclosure:** All authors declare that they have nothing to disclose and there is no conflict of interest.

## EPO‐0275

### Elevated CSF GFRa1 and NFL levels: A comparative study on ALS biomarkers

#### P. Bakkal ^1^; Ş. Aydın Türkoğlu^1^; T. Bakkal^2^; N. Yıldız^3^

##### ^*1*^ *Department of Neurology, Bolu Abant İzzet Baysal University Faculty of Medicine, Bolu, Türkiye;* ^*2*^ *Department of Neurology, Izzet Baysal State Hospital, Bolu, Türkiye;* ^*3*^ *Department of Neurology, Liv Hospital Ulus, Istanbul, Türkiye*

**Background and aims:** Amyotrophic lateral sclerosis (ALS) is a neurodegenerative disease characterized by complex axonal degeneration and impairments in neurotrophic support mechanisms, leading to the progressive loss of motor neurons.

**Methods:** In this study, cerebrospinal fluid (CSF) levels of GFAP, IL‐1B, IL‐22, IL‐6, GFRa1, and NFL were investigated using the ELISA method in 6 patients with ALS (2F, 4M) and 6 healthy controls (3F, 3M).

**Results:** The median age of the ALS group was 67 years (range: 38–73), while the median age of the healthy control group was 51 years (range: 23–72), no significant difference in age was found between the groups (*p* = 0.35). In the ALS group, cerebrospinal fluid (CSF) levels of GFRa1 and NFL (10.16ng/L ± 1.16 and 20.95 ± 6.29) were significantly higher compared to the control group (5.30 ng/L ± 3.23 and 13.37 ± 2.46) (*p* = 0.01, *p* = 0.02). No statistically significant differences were detected between the groups regarding CSF levels of GFAP, IL‐1B, IL‐22, IL‐6, or total protein (*p* = 0.85, *p* = 0.85, *p* = 0.68, *p* = 0.87). Furthermore, a statistically significant, moderate‐to‐strong positive correlation was identified between GFRa1 and NFL levels (*r* = 0.64; *p* = 0.03).

**Figure:**
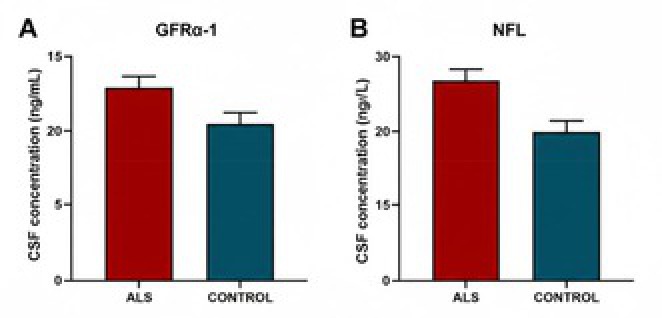
**FIGURE 1** Comparative analysis of CSF GFRa1 and NFL levels between ALS patients and healthy controls.

**Conclusion:** The significant elevation of GFRa1 accompanying the increase in NFL, coupled with the identified positive correlation, reflects a dynamic neurotrophic response that intensifies as motor neuron destruction deepens. This increase may represent a compensatory mechanism aimed at protecting motor neurons from apoptosis. The absence of inflammation suggests that the pathology is driven by an axis of neuronal integrity rather than glia‐mediated neuroinflammation. This pathogenic link holds significant potential for both understanding ALS pathogenesis and formulating novel therapeutic strategies.

**Disclosure:** Nothing to disclose.

## EPO‐0276

### Respiratory function improvement in spinal muscular atrophy patients after long‐term risdiplam treatment: The experience of two Italian centers

#### M. Sframeli^1^; N. Brolatti^2^; F. Trucco^4^; E. La Rosa^1^; R. Lauro^1^; R. Materia^1^; M. Maniaci^1^; N. Longoni^1^; I. Said^1^; C. Allegra^1^; M. Pedemonte^3^; C. Bruno^4^; S. Messina ^1^

##### ^*1*^ *Department of Clinical and Experimental Medicine, University of Messina, Messina, Italy;* ^*2*^ *Center of Translational and Experimental Myology and Maternal and Child Health, IRCCS Istituto Giannina Gaslini**;*** ^*3*^ *Pediatric Neurology and Muscular Diseases Unit, IRCCS Istituto Giannina Gaslini, Genoa, Italy;* ^*4*^ *Department of Neuroscience, Rehabilitation, Ophthalmology Genetics, University of Genoa, Genoa, Italy*

**Background and aims:** Risdiplam is an available treatment of all patients with spinal muscular atrophy (SMA). Although the respiratory impairment is inevitably progressive also in later‐onset SMA, no studies report the effect of this drug on respiratory function.

**Methods:** We report a case series of 14 patients, followed at two different Italian Centre, with long‐term disease duration, treated with risdiplam for more than 5 years. We describe changes in respiratory and motor function and the need for non‐invasive ventilation (NIV).

**Results:** Patients (mean age: 19.7 years, range: 14–28), 11 SMA2 (78.6%) and 3 SMA3 (21.4 %), were sitter (57.1%) or non‐sitter (43.9%). The Hammersmith Functional Motor Scale Expanded (HFMSE) score was ≤ 8 in 13/14 patients, and did not show significative changes after treatment (*p* = 0.9). Mean FVC at baseline was 1.27 l (SD: 1.02). After more than 5 years (mean follow‐up 5.3 years) the mean FVC improved significantly (1.41 l; SD: 1.06, *p* = 0.04). Overall 4 patients (28%) showed minimal FVC decrease (−0.12 l; *p* = 0.09), while the other 10 patients (72%) showed a significative improvement in FVC (+0.24; *p* = 0.009). None of the patients on NIV at baseline (*n* = 8) required changes in ventilation regime at follow‐up.

**Conclusion:** We report longer‐term respiratory follow‐up in treated patients and, for the first time, an improvement in commonly used pulmonary function tests, in contrast to motor functional outcome measures. Therefore, we propose to explore a high responsiveness of the FVC, mostly in severely affected SMA patients, in which commonly used motor functional outcome measures might not detect changes.

**Disclosure:** The study has been founded by Italian Health Ministry project PNRR‐MR1‐2022‐12376937. Nothing else to disclose.

## EPO‐0277

### Autologous SVF therapy modulates neuroinflammation in ALS: Phase I trial demonstrating safety and CSF biomarker dynamics

#### Z. Cai; R. Li; X. Li; J. Li

##### Department of Neurology, Suzhou Hospital, Affiliated Hospital of Medical School, Nanjing University, Suzhou, China

**Background and aims:** Amyotrophic lateral sclerosis (ALS) is a neurodegenerative disorder with limited treatments. Stromal vascular fraction (SVF), a cell population derived from autologous adipose tissue, exhibits multimodal immunomodulatory and neuroprotective properties, positioning it as a promising therapeutic candidate. This trial aimed to assess autologous stromal vascular fraction (SVF) safety and efficacy in patients with ALS.

**Methods:** This trial aimed to assess autologous stromal vascular fraction (SVF) safety and efficacy in patients with ALS. 26 patients received combined intravenous (0.5×10^6^cells/kg) and intrathecal (20×106 cells) autologous SVF.

**Figure:**
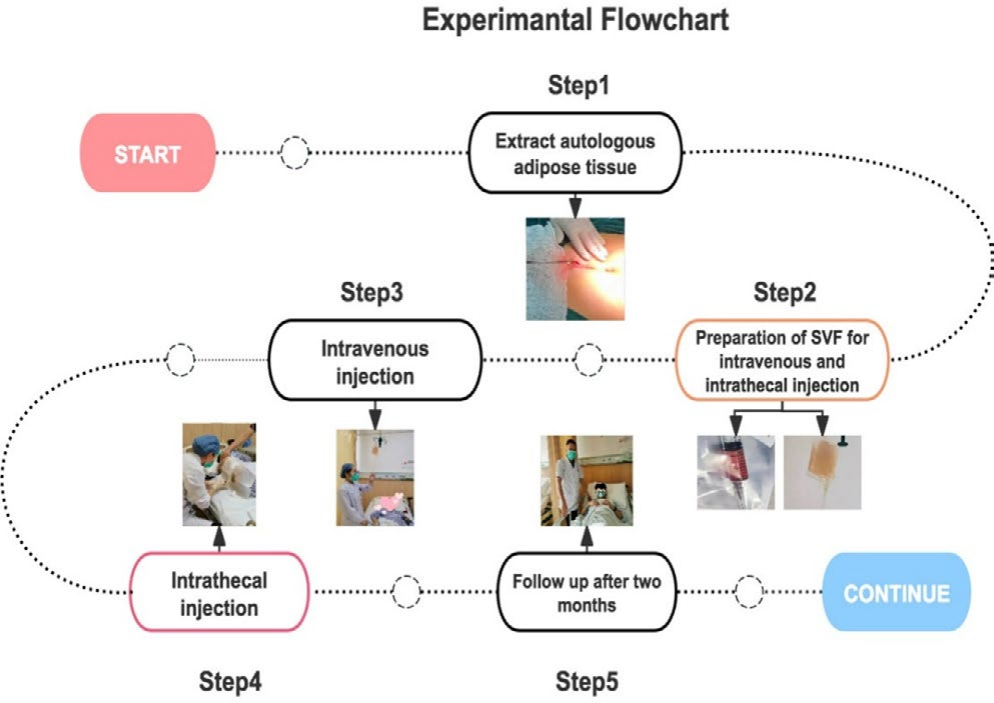
**FIGURE 2** The experimental process of this study.

**Results:** SVF administration was well‐tolerated. Five mild adverse events (AEs) (subcutaneous bleeding, headache, and low‐grade fever) occurred, with no serious AEs reported. Although ALSFRS‐R scores showed non‐significant improvement post‐treatment, 15/26 participants (57.7%) self‐reported symptomatic improvement after treatment. Critically, cerebrospinal fluid biomarker analysis revealed significant reductions in neurofilament light chain (NfL; Δ530.29 pg/mL, *P* = 0.039) and glial fibrillary acidic protein (GFAP; Δ622.23 pg/mL, *P* = 0.038), indicating attenuation of neuroaxonal degeneration and astroglial activation. While ALSFRS‐R scores showed no significant change (Δ−0.53, *P* = 0.384), prognostic modeling identified female sex (OR = 0.011, *p* = 0.008) and shorter disease duration (OR = 1.35/month, *P* = 0.005) as predictors of response.

**Figure:**
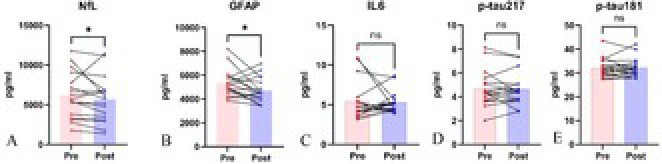
**FIGURE 1** CSF biomarker changes after SVF therapy.

**Conclusion:** These findings support Autologous SVF therapy demonstrates an acceptable safety profile in patients with ALS. The significant reduction in CSF NfL and GFAP levels provides objective evidence of their potential neuroprotective effects that modulates ALS‐relevant neuroinflammation pathways. Female participants and those with shorter disease duration may derive greater benefits.

**Disclosure:** Nothing to disclose.

## Movement Disorders 2

## EPO‐0278

### Real‐world outcomes of subcutaneous foslevodopa/foscarbidopa therapy in Parkinson's disease (DuoPark study)

#### C. Correia* ^1^; A. Costa*^3^; I. Pereira^1^; S. Lopes^3^; F. Ferreira^1^; A. Oliveira^1^; S. Varanda^3^; O. Costa^3^; A. Fernandes^1^; G. Carneiro^3^; M. Rosas^1^; M. Rodrigues^4^; C. Soares^1^

##### ^*1*^ *Serviço de Neurologia, Hospital Universitário de São João, Porto;* ^*3*^ *Serviço de Neurologia, ULS Braga, Braga;* ^*4*^ *CNS – Campus Neurológico, Braga*

**Background and aims:** Subcutaneous foslevodopa/foscarbidopa (fLD/fCD) is a continuous infusion therapy for Parkinson's disease (PD) with motor complications. Real‐world data are essential to assess its effectiveness and safety in clinical practice.

**Methods:** Interim analysis of a prospective, multicenter, observational study including people with PD treated with fLD/fCD between April/2024‐December/2025. Clinical and sociodemographic variables, “off” and “on” times, and MDS‐UPDRS scores (parts III–IV) at 3 and 6 months were analyzed. Infusion rates, levodopa equivalent daily dose, discontinuation of dopaminergic drugs, adverse events, and treatment discontinuation were also assessed. The effectiveness and safety of fLD/fCD for motor symptoms of PD were evaluated.

**Results:** Forty‐one patients were included (48.8% women; age 75 ± 15 years; MDS‐UPDRS‐III score: “off” 56.16 ± 15.65; “on” 34.5 ± 13.34). Mean daily “off” time was 6.02 ± 2.69 h/day, decreasing by 20.32% (−2.38 h/day, *p* = 0.017) and 14.10% (−2.08 h/day, *p* = 0.005) at 3 and 6 months, respectively. “On” time without troublesome dyskinesia increased by 35.26%(*p* = 0.003) and 33.08%(*p* = 0.002) at 3 and 6 months. MDS‐UPDRS‐IV scores improved significantly at both time points, with no significant differences in MDS‐UPDRS‐III “on” scores. Adverse events occurred in 26 patients (63.4%), mainly infusion‐site skin reactions, and neuropsychiatric symptoms. Seven patients (17.1%) discontinued treatment: 4 (9.8%) due to adverse events, 2 (4.9%) due to death, and 1 (2.4%) due to lack of family support.

**Conclusion:** fLD/fCD infusion significantly reduces “off” time and increases “on” time without troublesome dyskinesia during the first 6 months of treatment. Despite frequent but mostly mild to moderate adverse events. fLD/fCD demonstrated a favorable effectiveness and safety profile.

**Disclosure:** Nothing to disclose.

## EPO‐0279

### Neuroinflammatory marker MCP‐1 and its correlation with motor and non‐motor dysfunction in Parkinson's disease and atypical parkinsonism

#### D. Akramova; G. Rakhimbaeva; T. Bobomuratov; D. Bobamuratova; N. Vakhabova; U. Shamsieva; B. Ibodov

##### Tashkent State Medical University, Uzbekistan

**Background and aims:** Parkinson's disease (PD) and atypical parkinsonism (AP) represent a heterogeneous group of neurodegenerative disorders with overlapping motor manifestations but markedly different disease trajectories and prognoses. Increasing evidence highlights neuroinflammation as a central mechanism driving disease progression.

**Methods:** A total of 163 patients were enrolled, including 80 with PD and 83 with AP (MSA, PSP, CBD, DLB). Comprehensive neurological assessments were conducted using the Unified Parkinson's Disease Rating Scale (UPDRS) and standardized non‐motor symptom questionnaires. Serum MCP‐1 concentrations were measured by enzyme‐linked immunosorbent assay. Correlation and multivariate regression analyses were applied to explore associations between MCP‐1 levels and clinical parameters. Statistical significance was defined as *p* < 0.05.

**Results:** MCP‐1 levels were significantly higher in AP compared to PD patients (*p* < 0.01). Elevated MCP‐1 concentrations demonstrated a strong positive correlation with non‐motor symptom severity (*r* = 0.46, *p* < 0.001), particularly cognitive decline and autonomic dysfunction. Moderate correlations were observed with motor disability (*r* = 0.29, *p* < 0.05). Patients with high MCP‐1 levels exhibited accelerated clinical progression and reduced functional independence. Multivariate analysis confirmed MCP‐1 as an independent predictor of disease severity after adjustment for age, disease duration, and genetic status.

**Conclusion:** This study provides the first comprehensive evidence from a Central Asian cohort demonstrating that MCP‐1 is a robust biomarker of neuroinflammatory activity and neurological deterioration in parkinsonian disorders. Our findings support the integration of MCP‐1 into prognostic models for early risk stratification and personalized management. Presenting these results at an international congress will facilitate cross‐regional collaboration, enhance global data diversity, and promote precision medicine approaches in neurodegenerative diseases.

**Disclosure:** Nothing to disclose.

## EPO‐0280

### Mapping spatial and temporal evolution of dystonia in atypical parkinsonisms: A retrospective longitudinal cohort study

#### G. Vadi; J. Pasquini; E. Benevento; G. Palermo; E. Del Prete; E. Unti; R. Ceravolo

##### Center for Neurodegenerative diseases – Parkinson's disease and Movement disorders, Unit of Neurology, Department of Clinical and Experimental Medicine, University of Pisa, Pisa, Italy

**Background and aims:** Dystonia is a frequent yet inconsistently characterized feature of atypical parkinsonian disorders (APDs), including progressive supranuclear palsy (PSP), multiple system atrophy (MSA), and corticobasal syndrome (CBS). The objective of this study was to provide a first systematic, longitudinal comparison of dystonia across APDs focusing on its prevalence, topography, and timing of onset.

**Methods:** We retrospectively analysed longitudinal clinical data from a single‐center cohort of 182 patients diagnosed with PSP, MSA, or CBS. A five‐year subcohort with homogeneous follow‐up from symptom onset (*n* = 119) was examined to estimate dystonia prevalence and spatiotemporal trajectories. Complementary time‐to‐event analyses were used to model the evolution of dystonia across the entire disease course. Associations between dystonia and disease‐progression milestones, including dysphagia and loss of ambulation, were also explored.

**Results:** Within five years from symptom onset, dystonia occurred in 71% of PSP, 47% of MSA, and 86% of CBS patients. Cranial dystonia was most frequent in PSP (55.6%), whereas upper limb dystonia predominated in CBS (82.6%) (both *p* < 0.001). No significant differences were observed for cervical or truncal dystonia. Upper limb dystonia occurred earlier in CBS compared with PSP (*p* = 0.025). Dystonia‐related pain was significantly more frequent in CBS than in MSA and PSP (*p* < 0.001). Dysphagia and loss of ambulation were not significantly influenced by cervical and trunk dystonia, respectively.

**Conclusion:** Dystonia is a common and early feature of APDs, with distinct spatial and temporal trajectories across syndromes. Systematic longitudinal assessment of dystonia may improve clinical characterization and support diagnostic and therapeutic strategies.

**Disclosure:** Nothing to disclose.

## EPO‐0281

### Recruitment barriers for Parkinson's disease research at a tertiary centre in Kenya: A TraPCAf sub‐study

#### J. Nambafu ^1^; M. Pambo^1^; M. Parsemei^1^; J. Hooker^1^; D. Sokhi^1^; D. Sokhi^2^

##### ^*1*^ *Department of Medicine, Aga Khan University Medical College of East Africa, Nairobi, Kenya;* ^*2*^ *Brain and Mind Institute, Aga Khan University, Nairobi, Kenya*

**Background and aims:** Africa faces a rising burden of Parkinson's disease (PD), yet research is constrained by an unquantified failure to enrol hospital‐identified patients into clinical trials, limiting data on disease phenotypes and treatment needs. We have previously described these challenges in recruiting participants to a motor neurone disease (MND) study.

**Methods:** As part of the ongoing multinational Transforming Parkinson's Care in Africa (TraPCAf) study, we conducted a retrospective analysis of patient recruitment challenges at our study site in Nairobi, Kenya. Participants were chosen based on coding from our hospital's electronic health records.

**Figure:**
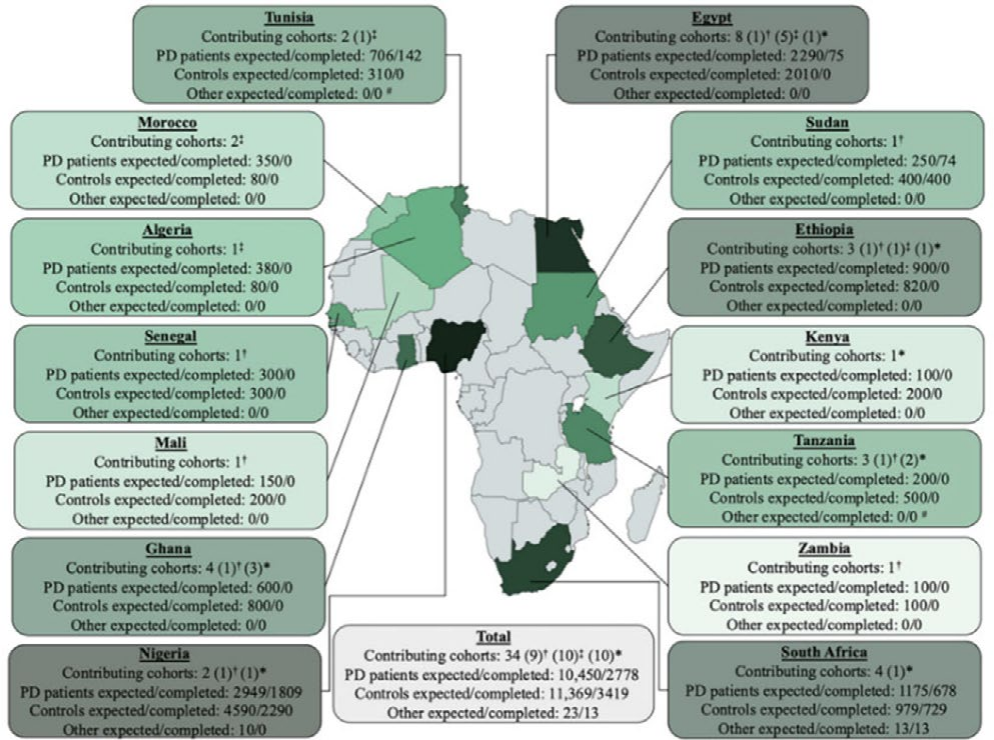
**FIGURE 1**: Study site map, adapted from Step et al, Mov Disord, 2024.

**Results:** Out of 432 potential participants, 20.8% (90/432) were recruited; of the remaining: 25.0% (108/432) had frailty including dementia; 13.7% (59/432) declined; 13.8% (60/432) were lost to follow‐up; 12% (52/432) lived too far, including outside Kenya; 8.3% (36/432) were misdiagnosed; and 6.3% (27/432) were deceased.

**Figure:**
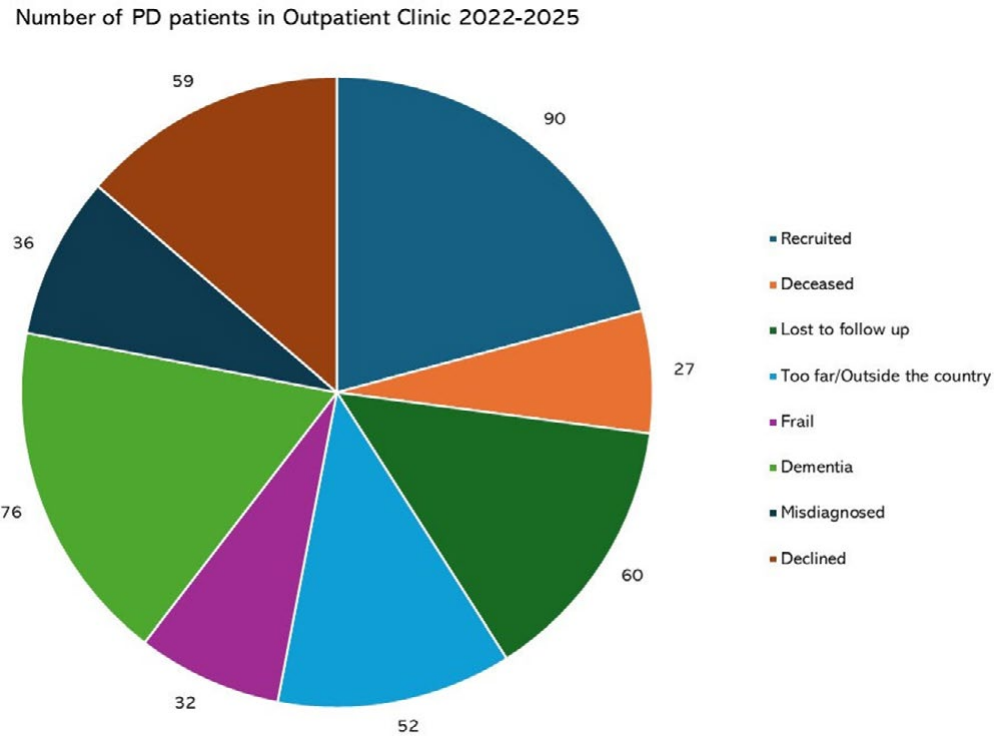
**FIGURE 2**: Patient final enrolment outcome.

**Conclusion:** Our findings underscore that patient identification from EHR does not translate to successful study enrolment. The spread of challenges is similar to those we encountered in our MND study. Our study suggests these factors should be consistently accounted for when establishing sample sizes for research studies in neurological disease in our part of the world.

**Disclosure:** This work was conducted as part of the TraPCAf study, funded by the UK National Institute for Health and Care Research (NIHR) via Newcastle University in partnership with Aga Khan University. The views expressed are those of the author(s) and not necessarily those of the NIHR, the UK Department of Health and Social Care, or Newcastle University. The authors have no other conflicts of interest or competing financial interests to declare relevant to this work.

## EPO‐0282

### Beyond motor symptoms of cervical dystonia – is cognitive deficit primary or secondary feature of the disease?

#### M. Dudzic ^1^; A. Pieczyńska^2^; A. Drużdż^1^; A. Rajewska^1^; K. Hojan^2^

##### ^*1*^ *Department of Neurology, Municipal Hospital in Poznań, Poland;* ^*2*^ *Department of Occupational Therapy, Poznań University of Medical Sciences, Poznań, Poland*

**Background and aims:** The study aimed to evaluate subtle cognitive impairment (CI) in patients with cervical dystonia (CD) and to determine whether it represents an independent non‐motor feature of the disease or arises secondary to interactions between motor manifestations and other non‐motor symptoms (NMS). Although CI is increasingly recognized in dystonia, its associations with motor severity and concomitant NMS remain insufficiently explored.

**Methods:** Patients with CD underwent comprehensive clinical assessment of motor symptoms (CD severity) and NMS of dystonia (cognition, depression, anxiety, sleep, pain). Matched controls underwent a single assessment (cognition, depression, anxiety, sleep).

**Results:** Patients with CD demonstrated significantly lower total MoCA scores compared with controls (*p* < 0.001). Relative to controls, CD patients showed poorer performance across multiple cognitive domains: executive function (*p* < 0.001), visuospatial abilities (*p* = 0.004), language (*p* < 0.001), memory (*p* = 0.002), and attention (*p* = 0.022). Patients also exhibited higher rates of depressive symptoms, anxiety, and sleep disturbances. To explore contributing factors, a multivariable model incorporating NMS and socioeconomic variables was constructed, revealing a combined influence of these factors on the severity of CI in CD.

**Figure:**
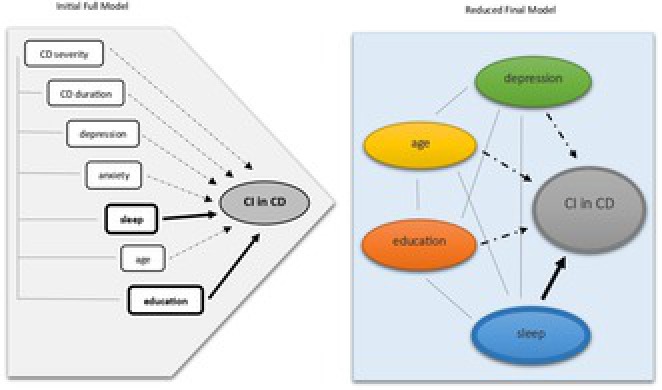
**FIGURE 1** Multivariable models of factors associated with cognitive impairment in cervical dystonia. The initial full model and the reduced final model are presented. CI denotes cognitive impairment; CD severity refers to the severity of cervical dystonia.

**Conclusion:** The study examined CI in CD in relation to motor severity and non‐motor domains, with multivariable analysis indicating a potential contribution of sleep disturbances. Although a definitive distinction between cognitive dysfunction as a core feature of dystonia or a secondary effect and overlap of other NMS cannot be made, the findings highlight interdependent mechanisms underlying cognitive deficit in CD. These results underscore the importance of comprehensive assessment of non‐motor domains in this population.

**Disclosure:** Nothing to disclose.

## EPO‐0283

### Investigation of SKY‐0515 in patients with Huntington's disease: Interim results of a phase 1 study

#### M. Miller ^2^; M. Brault^1^; J. Duffy^1^; S. Paushkin^1^; M. Mokhtarani^1^

##### ^*1*^ *Skyhawk Therapeutics, Inc., Waltham, USA;* ^*2*^ *Skyhawk Therapeutics Europe, Basel, Switzerland*

**Background and aims:** Huntington's disease (HD) is a neurodegenerative disorder caused by a trinucleotide (CAG) repeat expansion in the huntingtin (HTT) gene, resulting in a toxic mutant huntingtin (mHTT) protein. Disease progression correlates with the rate of somatic CAG expansion. SKY‐0515 has the potential to provide disease modifying benefits through reduction of toxic proteins, mHTT and PMS1, a key driver of expansion. The study aims to evaluate safety, PK/PD and efficacy of SKY‐0515, an orally available, brain penetrant small molecule RNA splicing modulator, in HD patients.

**Methods:** SKY‐0515‐001‐C is an ongoing 15‐month Phase 1 study in HD. Patients were randomized to 3mg (*N* = 10), 9mg (*N* = 10) or placebo (*N* = 6) for 84 days, after which patients rolled over onto active drug, receiving 4mg or 9mg (blinded) for an additional 12 months. Key enrollment criteria include genetic diagnosis of HD (CAG ≥ 40) and clinical baseline of TFC ≥ 10, IS ≥ 70, and TMS ≥ 6, reflecting an HD‐ISS disease stage 2 or mild stage 3.

**Results:** 9‐month interim results in patients who continuously received SKY‐0515 (excluding placebo) show positive trends in HD efficacy outcomes compared to expected natural history disease progression. In HD patients treated with SKY‐0515, early signs of disease stabilization are observed in components of the UHDRS, including function (TFC = +0.35 points), motor (TMS = −3.69 points), and cognition (Stroo*p* = +1.0 points). SKY‐0515 has been well tolerated up to 9 months.

**Conclusion:** These findings support further clinical evaluation of SKY‐0515 as a potential disease‐modifying therapy for HD. A confirmatory Phase 2/3 study (FALCON‐HD) is now enrolling.

**Disclosure:** The authors are full time employees of Skyhawk Therapeutics, a privately owned company, and the sponsor of the referenced study.

## EPO‐0284

### Objective assessment of long‐ and short‐term levodopa effects on hypomimia and head movement

#### M. Novotný ^1^; D. Škrabal^2^; T. Tykalová^1^; P. Dušek^2^; J. Rusz^1^

##### ^*1*^ *Department of Circuit Theory, Faculty of Electrical Engineering, Czech Technical University in Prague, Prague, Czech Republic;* ^*2*^ *Department of Neurology and Centre of Clinical Neuroscience, First Faculty of Medicine, Charles University, Prague, Czech Republic*

**Background and aims:** Hypomimia is a hallmark feature of Parkinson's disease (PD) that significantly impairs non‐verbal communication. Although levodopa is known to improve motor symptoms, its effect on facial and head movements, particularly in the early phase of treatment, has not been objectively and quantitatively assessed.

**Methods:** Video recordings were obtained using a single‐camera setup from 93 drug‐naïve PD patients at baseline and one year later after initiation of stable pharmacological treatment. At follow‐up, patients were examined in a defined OFF state (≥ 12 hours without dopaminergic medication) and in the ON state one hour after medication intake. Facial and head movements were quantified using a state‐of‐the‐art facial landmark tracking system and derived movement markers. A control group of 50 age‐ and sex‐matched healthy participants was also recorded.

**Results:** At baseline, PD patients showed significantly reduced facial and head movements compared with healthy controls (*p* < 0.001). After one year of treatment, a significant improvement was observed across all evaluated areas (*p* < 0.001) nearly matching performance of control group. However, no significant differences were found between the OFF and ON states during the short‐term levodopa challenge at follow‐up.

**Conclusion:** Levodopa treatment is associated with a significant long‐term improvement of hypomimia across facial and head movement domains in early PD. The absence of short‐term OFF–ON differences suggests that a 12‐hour washout period is insufficient to fully eliminate the sustained beneficial effects of stable dopaminergic treatment.

**Disclosure:** The authors report no disclosures. This study was supported by the Ministry of Health of the Czech Republic, grant No. NU23J‐04‐00042.

## EPO‐0285

### Unilateral MR‐guided laser interstitial thermal therapy thalamotomy for essential tremor: A 12‐month follow‐up study

#### M. Aubignat ^1^; M. Tir^1^; M. Ouendo^2^; J. Constans^3^; M. Lefranc^4^

##### ^*1*^ *Department of Neurology and Movement Disorders, Amiens Picardie University Hospital, Amiens, France;* ^*2*^ *Department of Anaesthesiology and Critical Care Medicine, Amiens Picardie University Hospital, Amiens, France;* ^*3*^ *Department of Radiology, Amiens Picardie University Hospital, Amiens, France;* ^*4*^ *Department of Neurosurgery, Amiens Picardie University Hospital, Amiens, France*

**Background and aims:** Essential tremor (ET) often resists medical therapy. Magnetic Resonance‐guided Laser Interstitial Thermal Therapy (MRgLITT) is a minimally invasive technique allowing real‐time MRI thermometry for precise thalamotomy. We evaluated the 12‐month efficacy and safety of unilateral MRgLITT thalamotomy in 21 pharmacoresistant ET patients.

**Methods:** Patients underwent unilateral MRgLITT thalamotomy (ROSA robot, Visualase system). Assessments at baseline, 3, and 12 months used the Fahn‐Tolosa‐Marin Tremor Rating Scale (FTM‐TRS). Primary outcome was the treated limb FTM‐TRS score (rest, posture, action, drawing). Secondary outcomes included Quality of Life in Essential Tremor Questionnaire (QUEST‐SI), Patient Global Impression of Change (P‐CGI), Montreal Cognitive Assessment (MoCA), Mini‐Mental State Examination (MMSE), and adverse events (AE). Linear mixed‐effects models were used for analysis.

**Results:** Twenty‐one patients (mean age 67.2 ± 12.8 years; 12 males; 17 right‐handed) underwent unilateral thalamotomy (17 left). Treated limb FTM‐TRS scores decreased by 75.1% (14.0 ± 3.1 baseline vs 3.5 ± 1.6 at 12 months; *p* < 0.001). All patients (100%) achieved > 50% improvement. QUEST‐SI improved from 41.2 ± 15.7% to 18.0 ± 14.6% (*p* < 0.001). Mean satisfaction (P‐CGI) was 82.0 ± 20.7%. Transient AEs (3 months: 71.4%) primarily included proprioceptive and balance disorders; only one patient (4.8%, gait ataxia) had persistent symptoms at 12 months. MoCA decreased slightly (25.4 ± 3.1 to 23.9 ± 3.5; *p* = 0.024) while MMSE remained stable (27.2 ± 2.3 vs 26.5 ± 2.6; *p* = 0.158).

**Figure:**
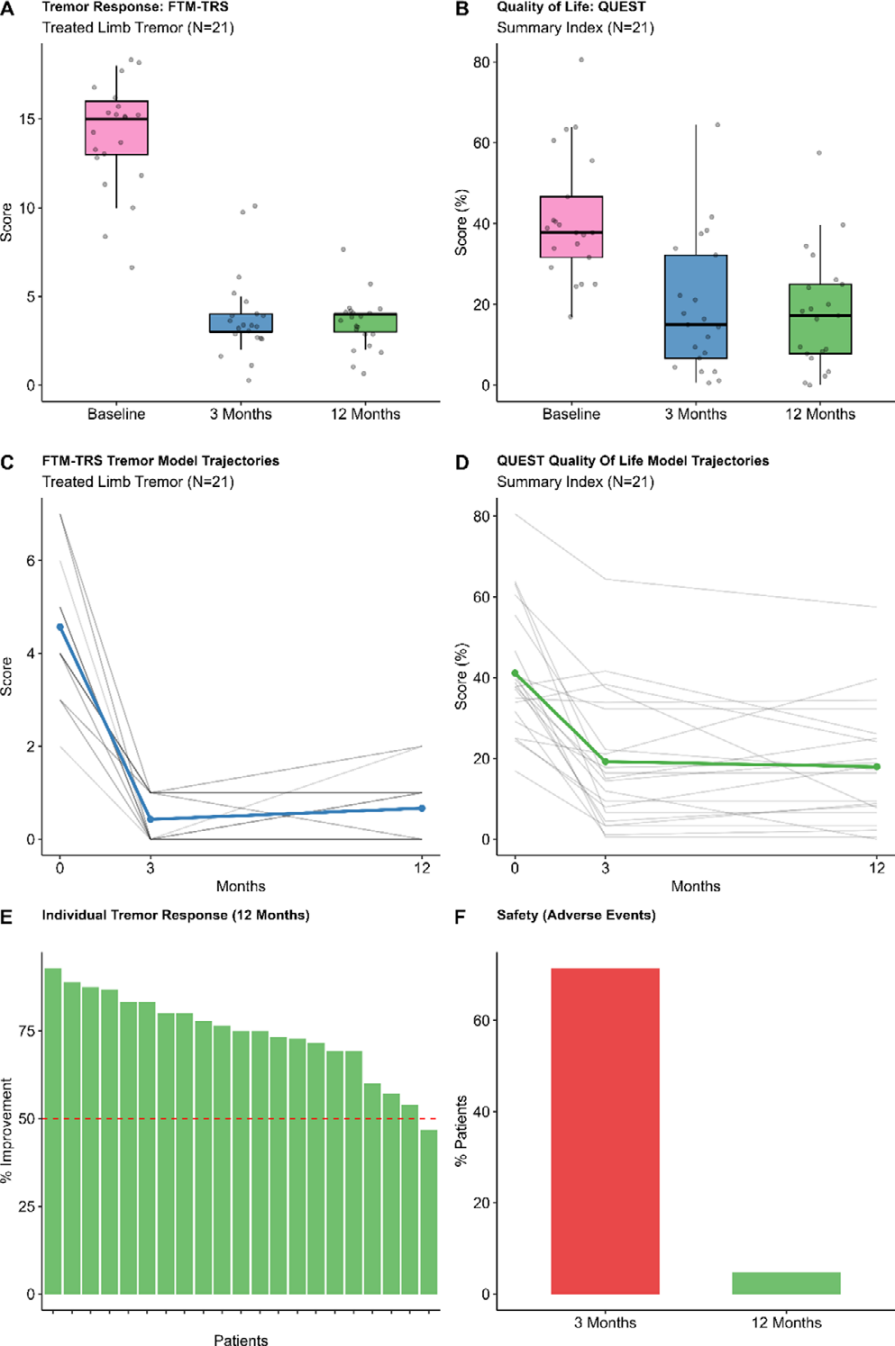
**FIGURE 1** Unilateral MRgLITT thalam outcomes. (A‐B) Group improvements in FTM‐TRS and QUEST‐SI. (C‐D) Longitudinal model trajectories. (E) Waterfall plot of 12‐month individual responders. (F) Transition from transient (M3) to resolved (M12) adverse events.

**Figure:**
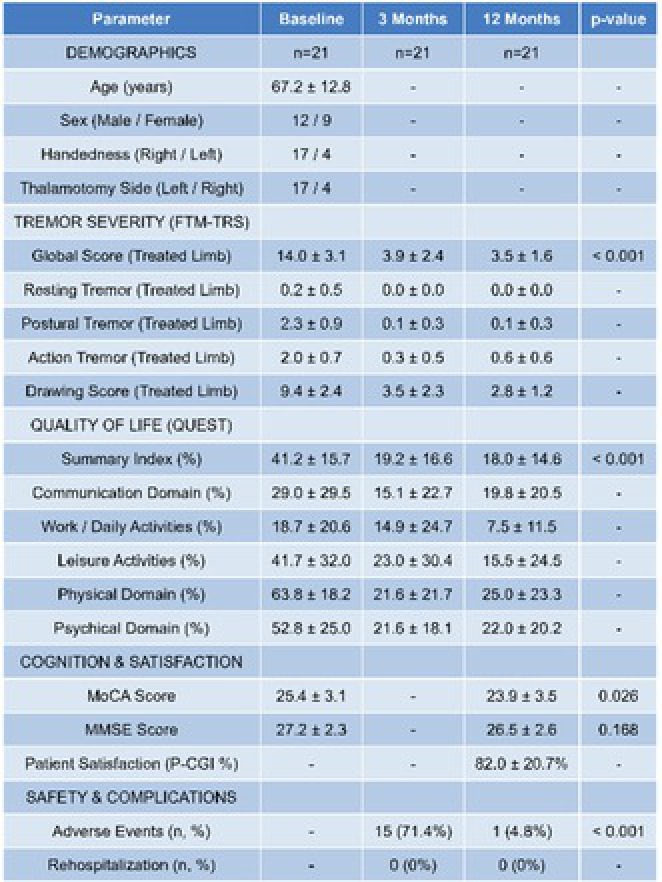
**TABLE 1** Clinical characteristics and longitudinal outcomes. Data presented as mean ± SD or n (%). *p*‐values compare baseline vs. 12 months for primary metrics (FTM‐TRS, QUEST‐SI, MoCA, MMSE). Adverse events decreased significantly by month 12.

**Conclusion:** Unilateral MRgLITT thalamotomy provides robust, sustained tremor relief and improved quality of life for pharmacoresistant ET. Despite the small cohort, it represents a safe, promising alternative to other surgical techniques. Larger longitudinal studies are warranted to establish its long‐term role.

**Disclosure:** Nothing to disclose.

## EPO‐0286

### SWITCH‐ON: Prospective, observational study of LECIG efficacy in Parkinson's disease patients previously treated with subcutaneous foslevodopa

#### S. Singh; N. Smith; B. Amlani;

##### Britannia Pharmaceuticals Limited, Green Park, Reading, UK

**Background and aims:** Levodopa–entacapone–carbidopa intestinal gel (LECIG) is a device‐aided therapy (DAT) for advanced Parkinson's disease (PD) patients who have severe motor fluctuations and hyperkinesia/dyskinesia that persists despite treatment with optimised oral therapy combinations (Figure 1). Subcutaneous (SC) infusion of foslevodopa/foscarbidopa (Fos) is an alternative DAT option in this setting. However, real‐world data suggest patient discontinuation from SC Fos is high, so other DAT options need to be considered. The SWITCH‐ON study (NCT07313176; Figure 2) will evaluate whether transition to intestinal infusion of LECIG is beneficial for those who discontinue SC Fos.

**Figure:**
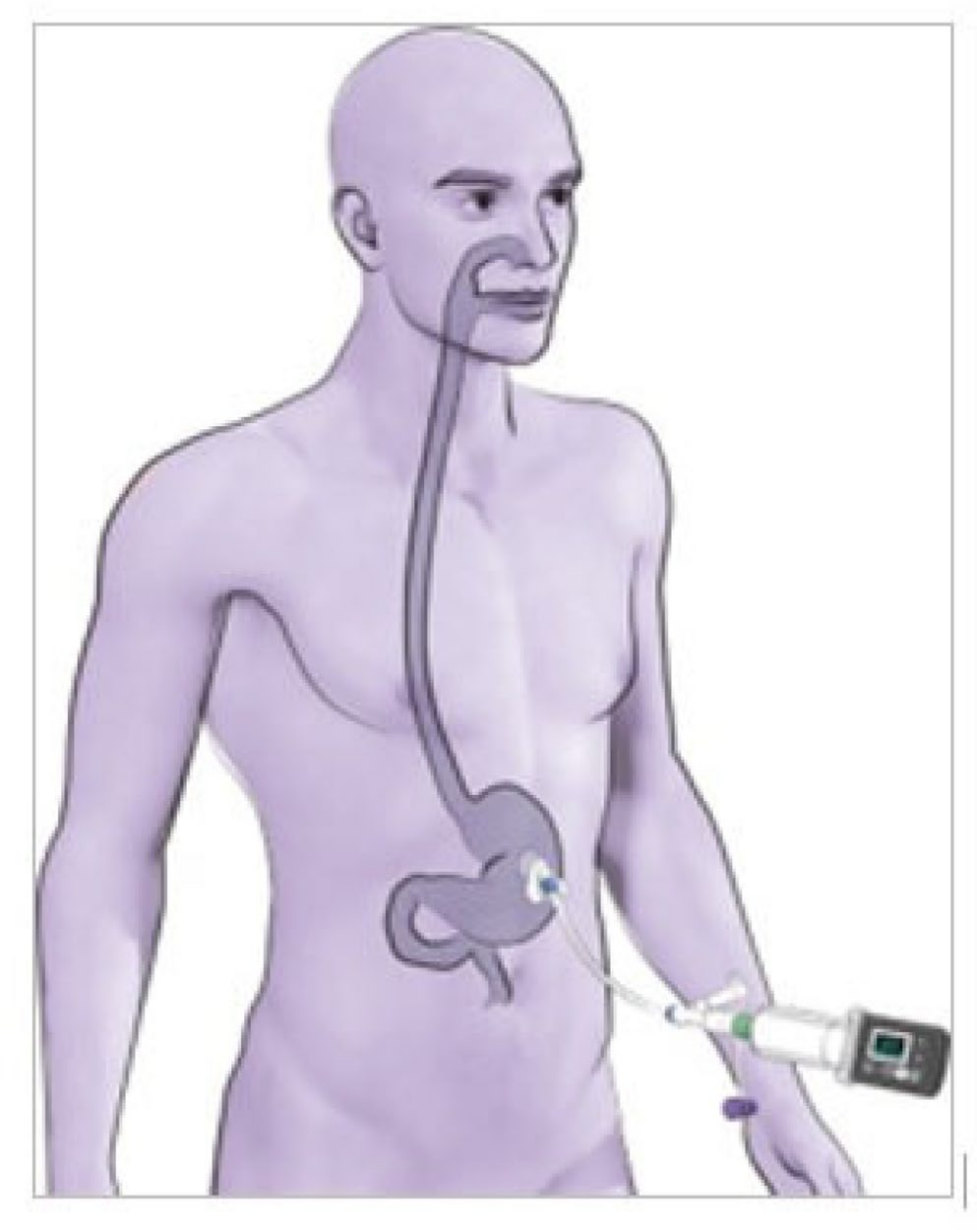
**FIGURE 1** Illustration of the placement of levodopa–entacapone–carbidopa intestinal gel (LECIG) infusion as a device‐aided therapy for advanced Parkinson's disease.

**Figure:**
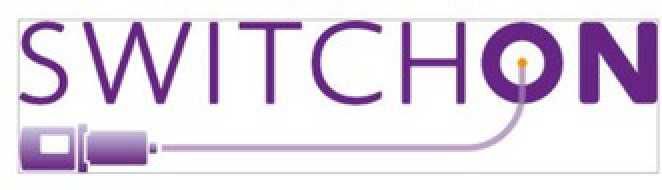
**FIGURE 2** SWITCH‐ON Study logo.

**Methods:** Target recruitment is ~215 adult PD patients from 30 centres in Spain, the UK, Germany, Romania and the Netherlands undergoing routine clinical care who will be followed‐up for 12‐months. Patients will have had prior SC Fos for ≥ 1 month. Data collection is planned from January 2026, with an interim analysis once 100 patients have completed ≥ 1 follow‐up visit. Data completion is planned for January 2028.

**Results:** The primary outcome is OFF time reduction with LECIG treatment at 12 months using the Movement Disorder Society Unified Parkinson's Disease Rating Scale part IV, with multiple secondary endpoints (Table 1). SWITCH‐ON aims to generate valuable real‐world evidence on the long‐term effectiveness, safety and tolerability of LECIG in routine clinical practice for advanced PD patients transitioning from SC Fos therapy.

**Figure:**
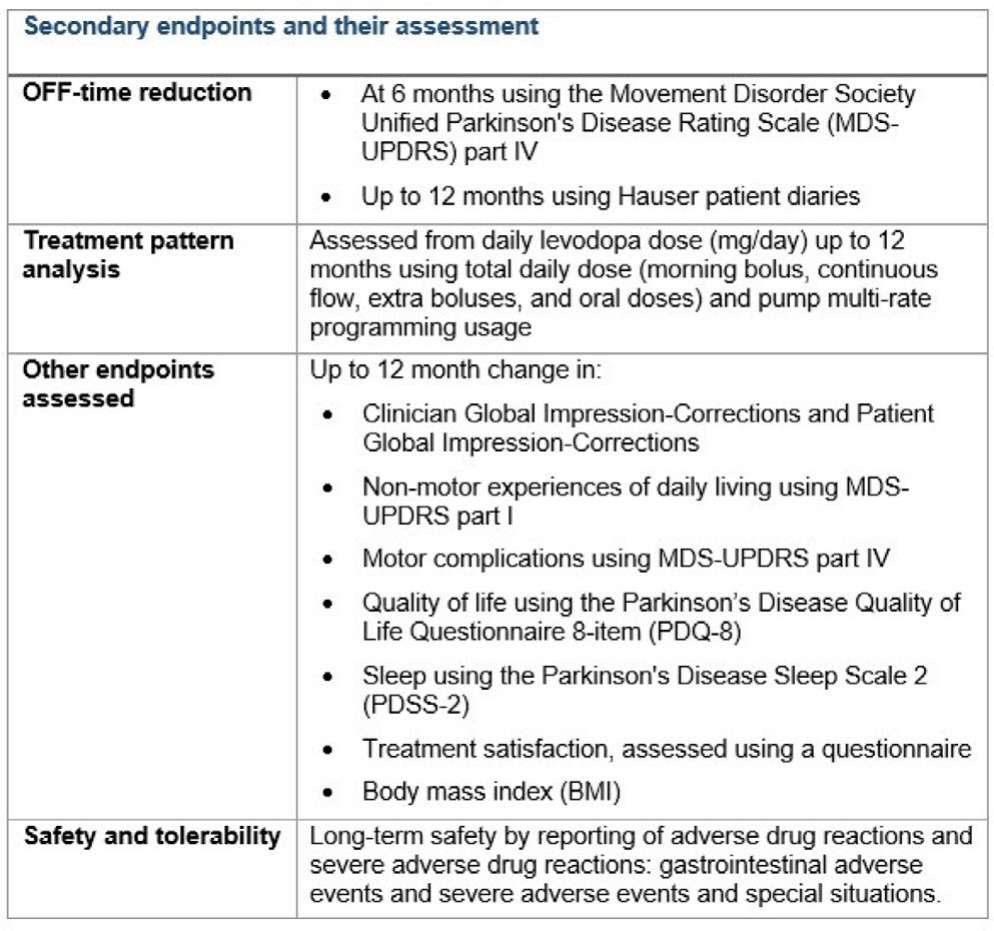
**TABLE 1** SWITCH‐ON Study secondary endpoints.

**Conclusion:** SWITCH‐ON hopes to demonstrate that intrajejunal therapy can be a feasible option after Fos, and that prior SC treatment failure is not the end of the road for patients.

**Disclosure:** The SWITCH‐ON study is sponsored by Britannia Pharmaceuticals Ltd. SS, NS and BA are employees of Britannia Pharmaceuticals Ltd.

## EPO‐0287

### Patient perceptions of the Crono® LECIG pump device in advanced Parkinson's disease treatment – data from the ELEGANCE observational study

#### S. Singh; N. Smith; B. Amlani

##### Britannia Pharmaceuticals Limited, Green Park, Reading, UK

**Background and aims:** Levodopa–entacapone–carbidopa intestinal gel (LECIG) infusion is a treatment option for advanced Parkinson's disease (PD). It is delivered using the specifically designed Crono® LECIG pump (Figure 1), which aims to provide convenience and usability for patients. ELEGANCE is an international, non‐interventional study (NCT05043103) capturing efficacy, safety and patient‐reported outcomes data from advanced PD patients treated with LECIG in routine clinical practice.

**Figure:**
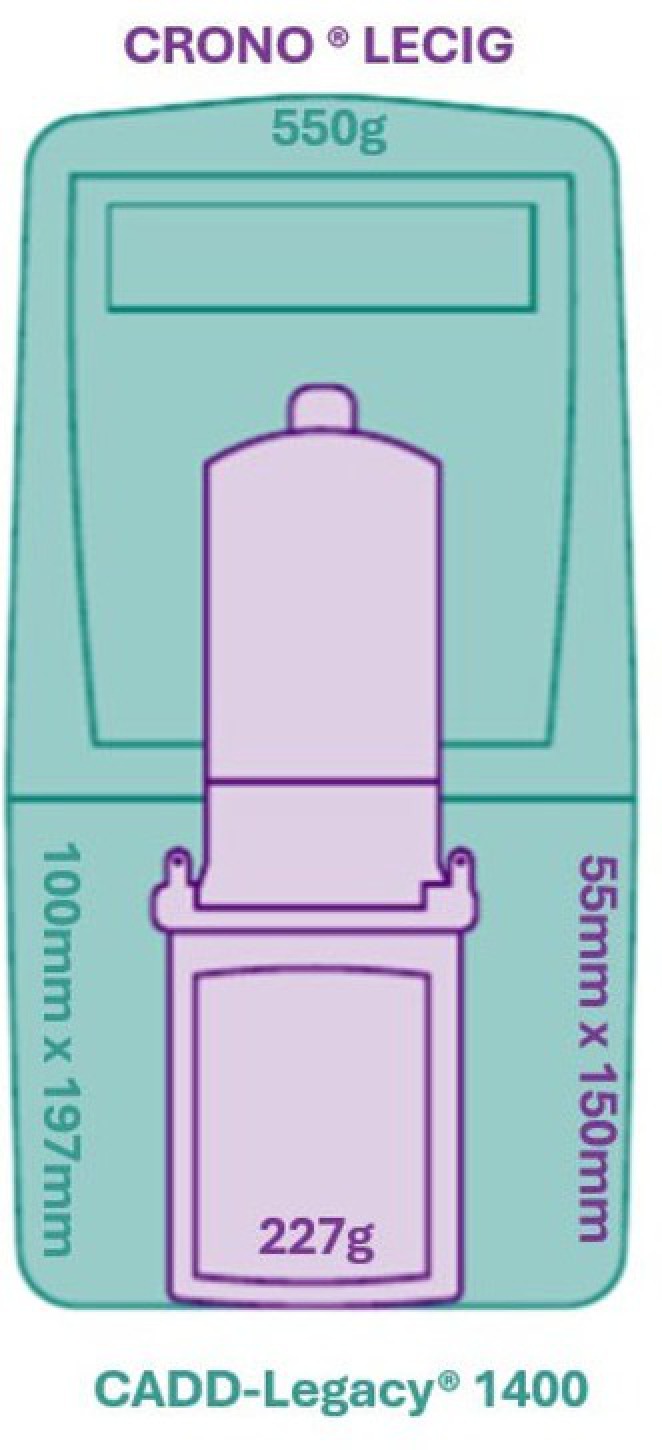
**FIGURE 1** Comparison of the Crono® LECIG pump and the Smith Medical CADD‐LEGACY® 1400 pump. Silhouettes show size and weight with full cassette (references: Crono® LECIG pump user manual and Smith Medical CADD‐LEGACY® 1400 pump user manual).

**Methods:** Here we report an analysis of interim data regarding patient perceptions of the Crono® LECIG pump for 161 patients treated with LECIG infusion for up to 24 months. Patient‐reported satisfaction with the device, as well as its handling, weight, size and noise, were assessed on scale of 1 = absolutely dissatisfied to 10 = absolutely satisfied, with a total score for all parameters (possible total score: 50).

**Results:** Mean satisfaction scores were high (≥ 8.2 for individual parameters), giving a mean total satisfaction score of 43.5. Scores stratified by previous treatment confirm high satisfaction, even in those who switched from another infusion therapy (Table 1; Figure 2). Levodopa infusion switch patients reported the highest satisfaction scores for most attributes, suggesting that the smaller, lighter design of the pump is meaningful. APO infusion switch patients had lower satisfaction scores which may reflect differences in device expectations.

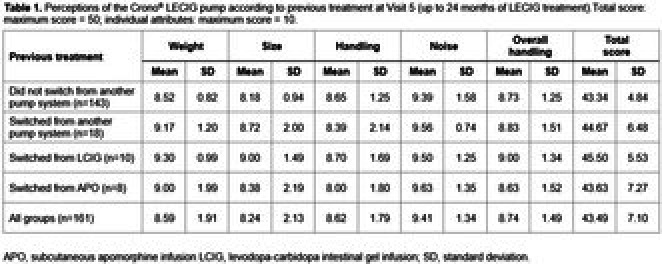

**Figure:**
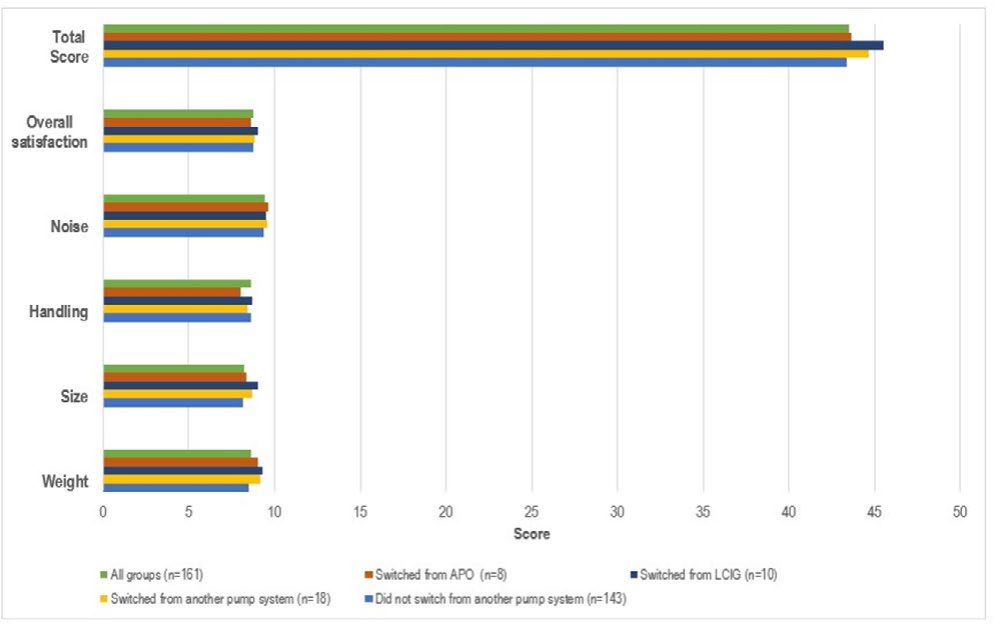
**FIGURE 2** Perceptions of the Crono® LECIG pump according to previous treatment at Visit 5. Total score: maximum score = 50; individual attributes: maximum score 10.

**Conclusion:** Higher device satisfaction scores in those who switched from another pump therapy, suggest the Crono® LECIG pump may address some limitations of other devices. These findings highlight the critical role of patient‐reported outcomes in evaluating drug‐delivery developments in PD.

**Disclosure:** The ELEGANCE study is sponsored by Britannia Pharmaceuticals Ltd. SS, NS and BA are employees of Britannia Pharmaceuticals Ltd.

## EPO‐0288

### Eslicarbazepine acetate shows anti‐tremorgenic properties in the mouse harmaline‐induced tremor model

#### A. Coelho^1^; A. Evrad^2^; C. Roucard^2^; V. di Foggia ^1^; P. Soares‐da‐Silva^3^; J. Holenz^1^; N. Pires^1^

##### ^*1*^ *BIAL – Portela & Cª, S.A, Coronado, Portugal;* ^*2*^ *SynapCell SAS, Grenoble, France;* ^*3*^ *Department of Biomedicine, Unit of Pharmacology and Therapeutics and MedInUp – Center for Drug Discovery and Innovative Medicines, University of Porto, Portugal*

**Background and aims:** Essential tremor (ET) is a common movement disorder, affecting around 5% of individuals over 65. Its hallmark feature is an 8–12 Hz kinetic tremor of upper limbs, often accompanied by head and voice tremor. Current treatment relies on pharmacological agents, yet almost 55% of patients respond inadequately. This study evaluated the anti‐tremorgenic effect of eslicarbazepine acetate (ESL), an antiseizure medication approved for treatment of focal‐onset epilepsy, in the harmaline‐induced tremor mouse model.

**Methods:** Quantitative electroencephalography (EEG) was recorded for 6 hours in freely moving mice (previously implanted with electrodes on motor cortex and cerebellum) under a cross‐over design. Tremor activity was assessed for 18 minutes in a tremor monitor chamber. Animals received ESL (75 and 150 mg/kg, gavage), propranolol (10–20 mg/kg, intraperitoneally) as reference, or vehicle‐control, 20–30 min before harmaline (20mg/kg, intraperitoneally).

**Results:** In vehicle‐treated (*n* = 10), harmaline induced sustained EEG low gamma oscillation increase in both brain regions. Similarly to propranolol, ESL treatment significantly, and dose‐dependently, prevented harmaline‐induced low gamma oscillation increase vs vehicle (ESL 75mg/kg *p* < 0.05 in motor cortex, ESL 150mg/kg *p* < 0.01 in both brain regions, at 2hours time‐point). In vehicle‐treated, harmaline induced a generalized tremor activity (*n* = 12) peaking at 12–14 Hz, whilst with ESL, similarly to propranolol, it was reduced (−18% by ESL 75mg/kg *p* = 0.002, −21% by ESL 150mg/kg *p* = 0.0002, vs vehicle).

**Conclusion:** ESL reversed harmaline‐induced EEG alterations and attenuated tremor in a mouse model of ET. This effect, possibly attributed to a T‐type calcium channel inhibition, suggests a therapeutic potential for this movement disorder.

**Disclosure:** Supported by BIAL – Portela & Cª, S.A. Ana Coelho, Valentina di Foggia, Joerg Holenz and Nuno Pires are current BIAL employees.

## EPO‐0289

### Functional bowel disorders in idiopathic Parkinson's disease: A Rome IV–based controlled study

#### Z. Yavuz

##### Neurology Clinic, Etlik City Hospital, Ankara, Türkiye

**Background and aims:** Functional bowel disorders (FBDs) are common non‐motor manifestations of idiopathic Parkinson's disease (iPD). Although constipation is well recognized, the prevalence and clinical relevance of broader Rome IV–defined FBDs in iPD remain insufficiently characterized. This study aimed to compare FBD frequency between iPD patients and controls and to examine their clinical associations within iPD.

**Methods:** This cross‐sectional controlled study included 64 patients with idiopathic Parkinson's disease and 64 age‐ and sex‐matched controls without neurological disease. Functional bowel disorders were assessed using the Rome IV Diagnostic Questionnaire. Non‐motor symptoms, mood, and quality of life were evaluated using the Non‐Motor Symptoms Questionnaire, Hamilton Depression Rating Scale, Hamilton Anxiety Rating Scale, Parkinson's Disease Questionnaire‐39, and the Gastrointestinal Quality of Life Index. Multivariable logistic regression was used to assess factors associated with FBD presence, and multivariable linear regression to identify predictors of gastrointestinal quality of life.

**Results:** The prevalence of functional bowel disorders did not differ significantly between iPD patients and controls. Within the iPD group, FBD positivity was associated with higher non‐motor symptom burden and poorer quality of life. Cognitive and sexual domains of the Parkinson's Disease Questionnaire‐39 were particularly affected. Anxiety‐related measures were significant predictors of reduced gastrointestinal quality of life.

**Figure:**
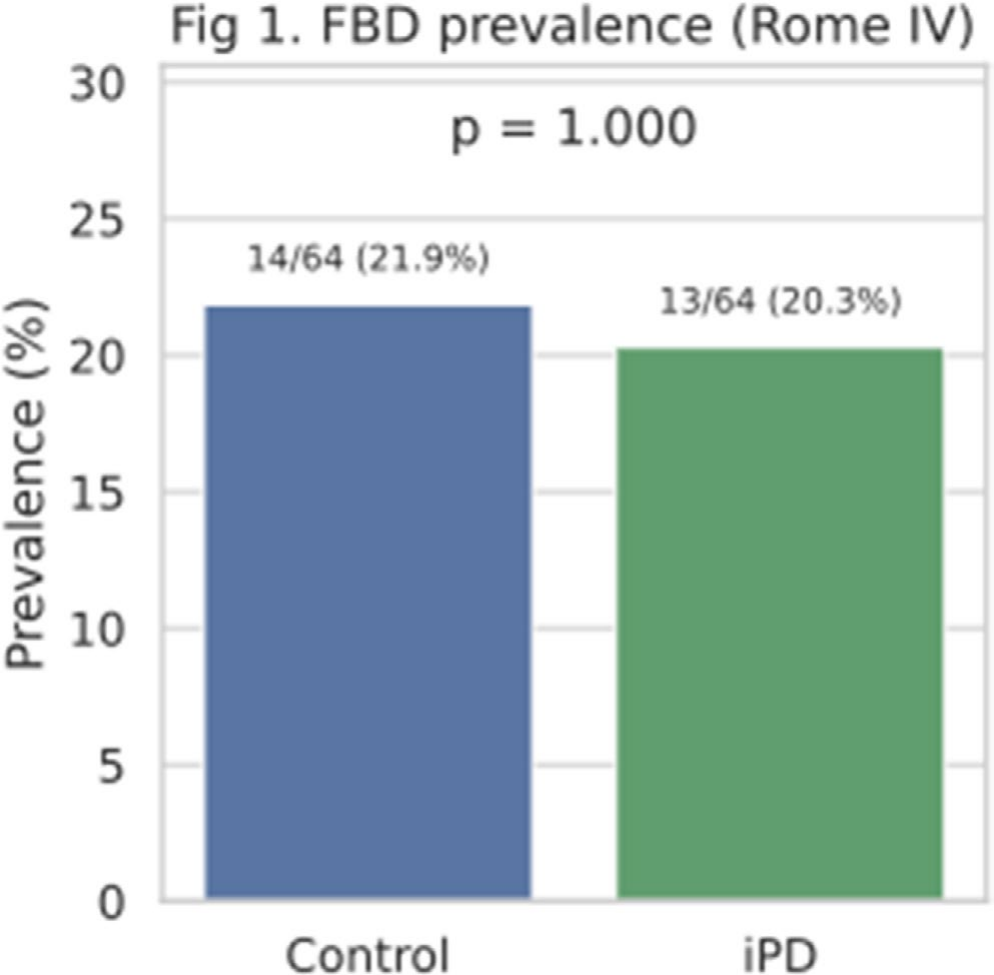
**FIGURE 1** Prevalence of Rome IV–defined functional bowel disorders in idiopathic Parkinson's disease patients and controls (*p* = 1.000).

**Figure:**
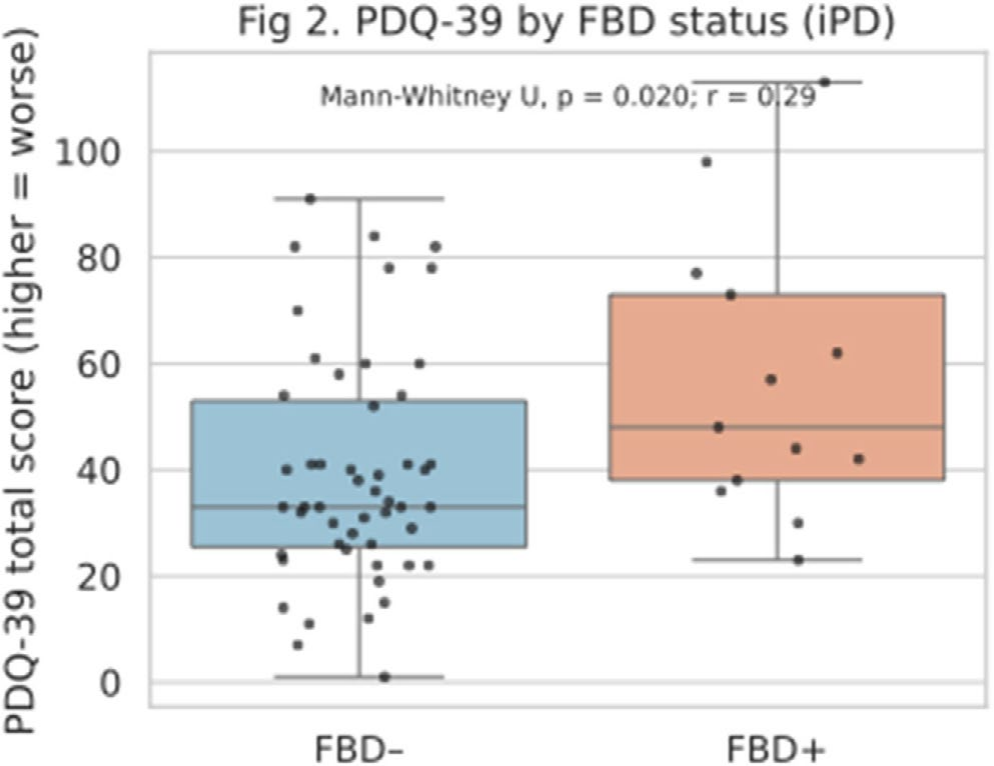
**FIGURE 2** Parkinson's Disease Questionnaire‐39 total scores according to functional bowel disorder status in idiopathic Parkinson's disease patients.

**Figure:**
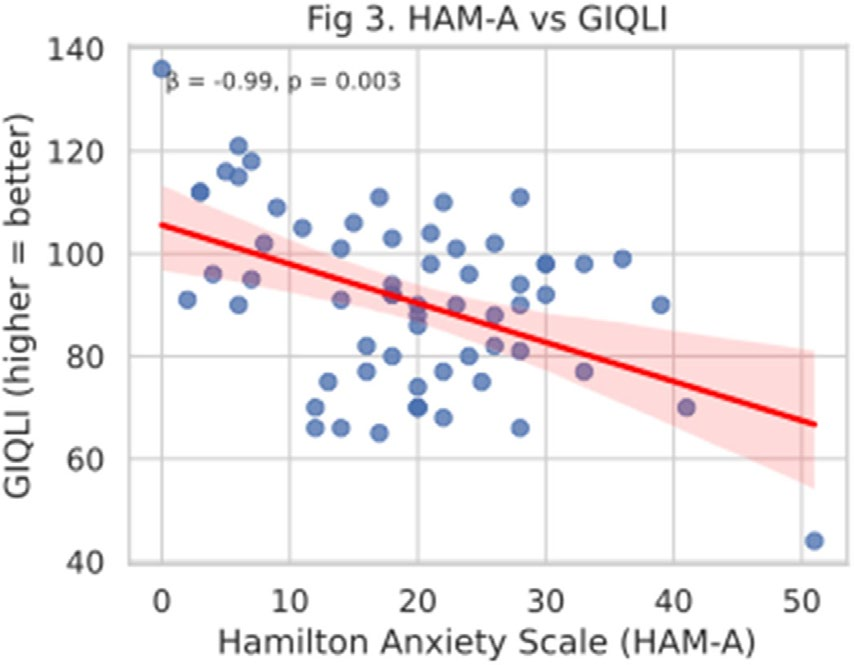
**FIGURE 3** Relationship between Hamilton Anxiety Scale scores and Gastrointestinal Quality of Life Index in idiopathic Parkinson's disease patients.

**Conclusion:** Functional bowel disorders do not appear to be disease‐specific markers in idiopathic Parkinson's disease but rather phenotype modifiers associated with increased non‐motor symptom burden. Targeting anxiety‐related factors may improve gastrointestinal well‐being in iPD.

**Disclosure:** Nothing to disclose.

## MS and Related Disorders 2

## EPO‐0290

### Microstructural damage and disability associations of paramagnetic rim and T1‐hypointense lesions in multiple sclerosis

#### A. Miscioscia ^1^; V. Mauceri^2^; C. Treaba^3^; E. Silvestri^4^; D. Landi^1^; D. Centonze^1^; G. Marfia^1^; F. Rinaldi^2^; P. Perini^2^; A. Bertoldo^4^; C. Mainero^3^; P. Gallo^2^; M. Puthenparampil^2^

##### ^*1*^ *Multiple Sclerosis Clinical and Research Unit, Department of Systems Medicine, University of Rome Tor Vergata, Rome, Italy;* ^*2*^ *Multiple Sclerosis Centre of the Veneto Region (CeSMuV), Padua University Hospital, Padua, Italy;* ^*3*^ *A. Martinos Center for Biomedical Imaging, Department of Radiology, Massachusetts General Hospital, Boston, USA**;*** ^*4*^ *Department of Information Engineering, University of Padua, Padua, Italy*

**Background and aims:** Recent microglia PET studies in MS show that most white matter (WM) T1‐hypointense lesions exhibit activated microglia within lesion core or at the border. Lesions with activated microglia, such as paramagnetic rim lesions (PRLs), are associated with smoldering inflammation and greater clinical disability. However, whether PRLs and non‐PRL‐T1‐hypointense lesions share similar tissue‐disrupting properties remains unclear. This diffusion MRI study investigated lesion‐level microstructural damage in PRLs, non‐PRL‐T1‐hypointense lesions, and T1‐isointense lesions, and assessed the relative contribution of their lesion burden to clinical disability.

**Methods:** In 34 newly diagnosed, treatment‐naïve patients with relapse‐onset MS (mean age 38.4 years; mean disease duration 0.65 years) WM lesions were classified using FLAIR, T1‐weighted and susceptibility‐weighted imaging as PRLs, non‐PRL‐T1‐hypointense lesions, or T1‐isointense lesions. Fractional anisotropy (FA), mean diffusivity (MD), and neurite density index (NDI) were extracted for each lesion class. Regression models compared diffusion metrics across lesion classes and evaluated associations between lesion burden and Expanded Disability Status Scale (EDSS) scores.

**Results:** Among 638 lesions, 23 were PRLs (4%), 586 non‐PRL‐T1‐hypointense (91%), and 29 T1‐isointense lesions (5%). PRLs showed lower FA than non‐PRL‐T1‐hypointense (*p* = 0.045) and T1‐isointense lesions (*p* = 0.005). Both PRLs and non‐PRL‐T1‐hypointense lesions had higher MD and lower NDI compared with T1‐isointense lesions (all *p* < 0.002). At patient level, PRL volume was more strongly associated with clinical disability (*p* < 0.001) than non‐PRL‐T1‐hypointense (*p* = 0.334) and T1‐isointense lesion volume (*p* = 0.737), independently of age and disease duration.

**Conclusion:** Non‐PRL‐T1‐hypointense lesions represent over 90% of MS WM lesions and exhibit intermediate tissue disruption. Despite their low prevalence, PRL burden most strongly affects clinical disability.

**Disclosure:** Nothing to disclose.

## EPO‐0291

### Identifying MS subtypes using MRI‐based machine learning

#### A. Bianchi^1^; R. Cortese^1^; M. Battaglini ^1^; A. Eshaghi^2^; G. Battaglia^1^; L. Luchetti^1^; F. Sforazzini^1^; G. Gentile^1^; F. Cacciante^1^; M. Leoncini^1^; F. Barkhof^2^; B. Bellenberg^4^; A. Bisecco^5^; B. Bodini^6^; A. Cagol^7^; M. Calabrese^8^; A. Calvi^9^; E. Colato^10^; S. Collorone^2^; M. Foster^2^; M. Filippi^11^; A. Gallo^5^; C. Gasperini^13^; C. Granziera^7^; M. Kudrna^14^; A. Lazzarotto^6^; S. Llufriu^9^; C. Lukas^4^; E. Martínez‐Heras^9^; M. Moccia^15^; D. van Nederpelt^3^; F. Prados^12^; G. Pontillo^2^; M. Rocca^11^; S. Ruggieri^13^; M. Schoonheim^10^; B. Stankoff^6^; E. Strijbis^16^; A. Tamanti^8^; C. Tortorella^13^; T. Uher^14^; P. Valsasina^11^; M. Vaneckova^14^; A. Toosy^2^; O. Ciccarelli^2^; N. De Stefano^1^

##### ^*1*^ *Department of Medicine, Surgery and Neuroscience, University of Siena, Siena, Italy;* ^*2*^ *Queen Square MS Centre, Department of Neuroinflammation, UCL Queen Square Institute of Neurology, Faculty of Brain Science, University College London, London, UK;* ^*3*^ *MS Center Amsterdam, Radiology and Nuclear Medicine, Vrije Universiteit Amsterdam, Amsterdam Neuroscience, Amsterdam UMC location VUmc, Amsterdam, the Netherlands;* ^*4*^ *Ruhr University Bochum, St. Josef Hospital, Institute of Neuroradiology, Bochum, Germany;* ^*5*^ *Department of Advanced Medical and Surgical Sciences, University of Campania “Luigi Vanvitelli”, Naples, Italy;* ^*6*^ *Neurology Department, Pitié‐Salpetrière Hospital, APHP, Paris, France;* ^*7*^ *Translational Imaging in Neurology (ThINk) Basel, Department of Biomedical Engineering, Faculty of Medicine, University Hospital Basel and University of Basel, Basel, Switzerland;* ^*8*^ *Department of Neurosciences and Biomedicine and Movement, The Multiple Sclerosis Center of University Hospital of Verona, Verona, Italy;* ^*9*^ *Neuroimmunology and Multiple Sclerosis Unit and Laboratory of Advanced Imaging in Neuroimmunological Diseases (ImaginEM), Hospital Clinic and Institut d'Investigacions Biomèdiques August Pi i Sunyer (IDIBAPS), University of Barcelona, Barcelona, Spain;* ^*10*^ *MS Center Amsterdam, Department of Anatomy and Neurosciences, Vrije Universiteit Amsterdam, Amsterdam Neuroscience, Amsterdam UMC, location VUmc, Amsterdam, the Netherlands;* ^*11*^ *Neuroimaging Research Unit, Division of Neuroscience, IRCCS San Raffaele Scientific Institute, Milan, Italy;* ^*12*^ *First Division of Neurology and Neurophysiopathology, AOU Luigi Vanvitelli, Naples, Italy;* ^*13*^ *Department of Neurosciences, San Camillo‐Forlanini Hospital, Rome, Italy;* ^*14*^ *Department of Radiology, First Faculty of Medicine, Charles University and General University Hospital, Prague, Czech Republic;* ^*15*^ *Department of Molecular Medicine and Medical Biotechnology, “Federico II” University of Naples, Naples Italy;* ^*16*^ *MS Center Amsterdam, Neurology, Vrije Universiteit Amsterdam, Amsterdam Neuroscience, Amsterdam UMC location VUmc, Amsterdam, the Netherlands*

**Background and aims:** Multiple sclerosis (MS) is a heterogeneous disorder whose clinical classification does not fully capture its pathological complexity. Machine learning (ML) approaches applied to research data have identified MRI‐based MS subtypes. The aim of this study is to validate this ML approach using real‐world data.

**Methods:** We analysed cross‐sectional MRI data from a multicentre MAGNIMS cohort of patients with MS (pwMS) and healthy controls (HC), including a training cohort (746 pwMS, 114 HC) and an independent validation cohort (199 pwMS). T1‐weighted and T2‐FLAIR MRI data were processed to extract measures of lesion burden, tissue integrity, brain and spinal cord volumes. MRI variables distinguishing pwMS from HC were normalised using Bayesian linear regression. The unsupervised ML algorithm SuStaIn was used to identify MRI‐driven subtypes and stages, and their associations with clinical phenotypes and disability were assessed using appropriate regression models.

**Results:** Three distinct MRI‐driven MS subtypes were identified and replicated across training and validation cohorts: (1) a lesion‐driven subtype, (2) a subtype characterised by early deep grey matter and brainstem damage, and (3) a cortical grey matter–driven subtype. Clinical phenotype distributions were consistent across cohorts, with no significant associations between MRI‐driven subtypes and standardised clinical phenotypes. ML‐derived stages correlated with disease duration (b = 0.072, *p* < 0.001) and were associated with clinical phenotypes (LR chi2 = 90.03, b = 0.545, *p* = 0.002) and disability (b = 0.263, *p* < 0.001).

**Figure:**
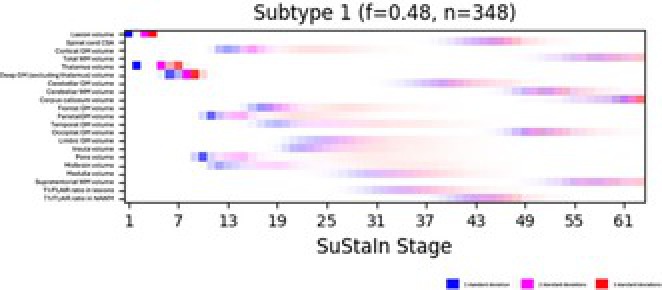
**FIGURE 1** Progression pattern of MRI abnormalities in MRI‐driven subtype 1 identified by SuStaIn. Subtype 1 was lesion‐driven, characterised by early lesion accumulation, followed by the involvement of deep grey matter structures.

**Figure:**
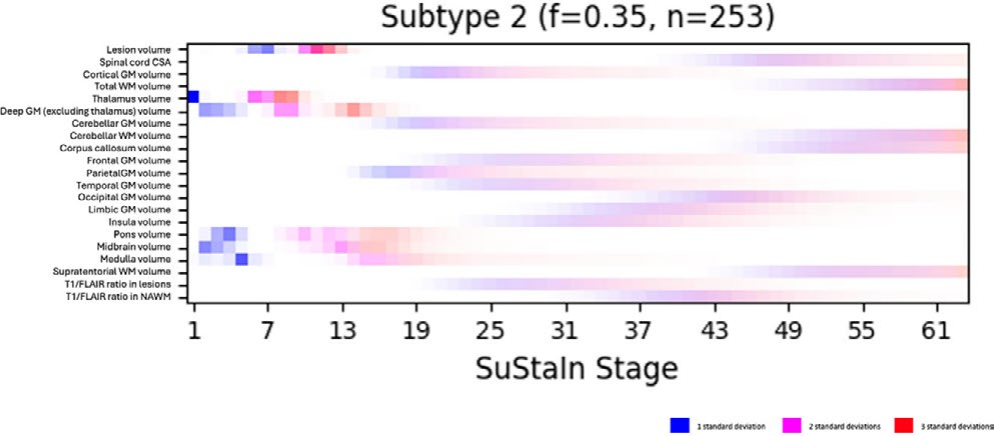
**FIGURE 2** Progression pattern of MRI abnormalities in MRI‐driven subtype 2 identified by SuStaIn. Subtype 2 showed diffuse and early deep grey matter and brainstem damage.

**Figure:**
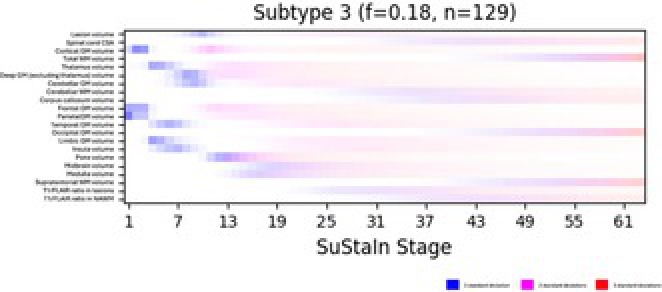
**FIGURE 3** Progression pattern of MRI abnormalities in MRI‐driven subtype 3 identified by SuStaIn. Subtype 3 was characterised by prominent cortical grey matter damage with early deep grey matter involvement.

**Conclusion:** These findings confirm that ML applied to MRI data can identify MS subtypes and stages of progression in real‐world multicentre cohorts, correlating with but extending beyond clinical phenotypes within a data‐driven classification framework.

**Disclosure:** ABia received research funding from the Italian Society of Neurology, a MAGNIMS–ECTRIMS fellowship (2023), and honoraria/travel support from Merck Serono and Biogen. RC received honoraria/travel support from Roche, Merck Serono, UCB, Sanofi‐Genzyme, Alexion, Novartis and Janssen, and research funding from the Italian Ministry of University and Research. FB serves/served on steering committees, DSMBs, advisory boards or as consultant for multiple pharmaceutical and research organizations, has research agreements with industry partners, and is co‐founder/shareholder of Queen Square Analytics Ltd. ABis, BB, ACag, MC, MF, CGa, SL, CL, MM, MAR, SR, BS, EMMS, CT, TU, MV, ATo, OC and NDS received honoraria, consulting fees, travel support and/or research funding from pharmaceutical companies active in MS research. Several authors (FB, MF, MMS, EMMS, ATo, MAR) hold editorial roles in scientific journals. CGr reports institutional research support and fees received by her employer. DRN, FP and ACal received fellowship or grant support from public or charitable bodies. MB, AE, GB, LL, FS, GG, FC, ML, BB, EC, SC, AG, MK, AL, EMH, GP, ATa and PV declare no disclosures. All the authors declare no disclosures for this work.

## EPO‐0292

### Brain lesion topography relates to cognitive functions in multiple sclerosis: Cross‐sectional and longitudinal analysis

#### C. Masciulli ^1^; M. Madsen^1^; T. Broeders^1^; I. Koubiyr^1^; E. Colato^1^; F. Barkhof^2^; E. Strijbis^3^; E. Portaccio^4^; M. Amato^4^; T. Fuchs^1^; M. Schoonheim^1^

##### ^*1*^ *Department of Anatomy and Neurosciences, MS Center Amsterdam, Amsterdam Neuroscience, Amsterdam UMC, Vrije Universiteit Amsterdam, Amsterdam, The Netherlands;* ^*2*^ *Department of Radiology and Nuclear Medicine, MS Center Amsterdam, Amsterdam Neuroscience, Amsterdam UMC, Vrije Universiteit Amsterdam, Amsterdam, The Netherlands;* ^*3*^ *Department of Neurology, Amsterdam University Medical Centers, Universiteit Amsterdam, Amsterdam, The Netherlands/MS Center Amsterdam, Amsterdam, The Netherlands/Amsterdam Neuroscience, Amsterdam, The Netherlands;* ^*4*^ *Department of NEUROFARBA, University of Florence, Florence, Italy*

**Background and aims:** Cognitive impairment (CI) is a common and disabling feature of multiple sclerosis (MS), affecting multiple domains. Understanding the biological mechanisms underlying CI in MS is key research goal. Conventional MRI measures have long been associated with cognitive deficits, reflecting the impact of white matter disconnection on large‐scale brain networks. This study investigated cross‐sectional and longitudinal associations between brain lesion distribution and domain‐specific cognitive performance in MS.

**Methods:** We analyzed longitudinal data from the Amsterdam MS cohort. Cognitive performance was assessed at baseline and 5‐year follow‐up using the Brief Repeatable Neuropsychological Battery. Longitudinal cognitive decline was defined using a regression‐based Reliable Change Index. Baseline T2‐weighted lesion masks were processed and analyzed using voxel‐based lesion–symptom mapping. All models were adjusted for demographic and clinical covariates, total lesion volume.

**Results:** Cross‐sectional analyses identified lesion–symptom associations across major white matter pathways. Processing speed and attention were associated with lesions in the corpus callosum, cingulum, and internal capsule, while memory and language domains involved temporo‐occipital and frontotemporal tracts. Baseline lesion distribution predicted cognitive performance at 5 years across several domains. Longitudinal decline occurred in a subset of patients and was specifically associated with lesions in the left superior temporal pole for executive function. These associations were visualized using atlas‐based representations of affected regions across cognitive domains.

**Figure:**
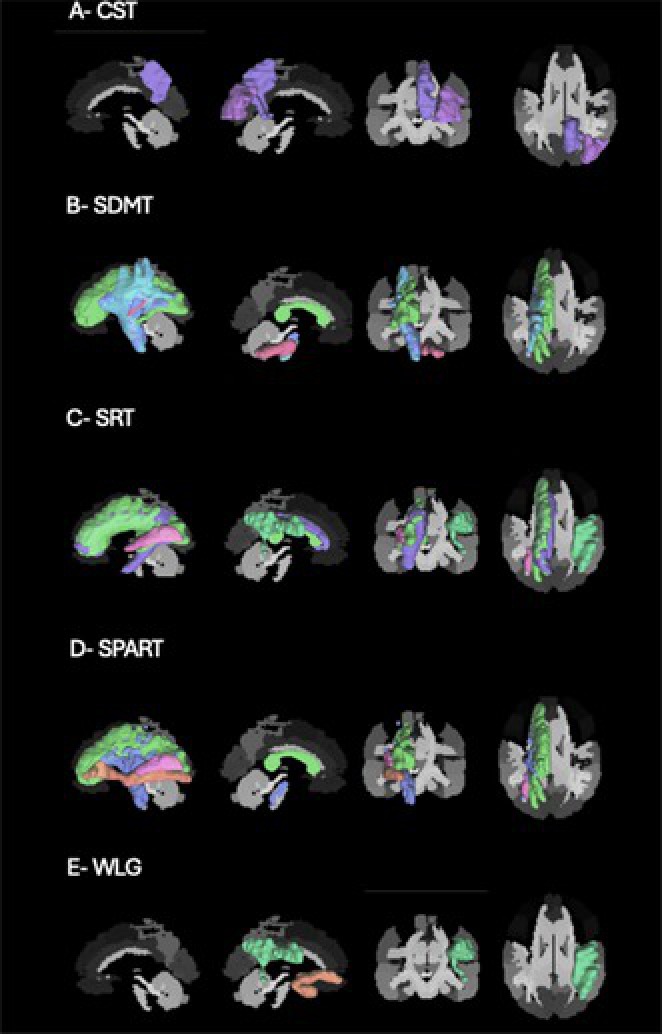
**FIGURE 1** Atlas‐based visualization of regions of interest across neuropsychological tests at year 0. Different colors indicate distinct anatomical regions across neuropsychological tests.

**Figure:**
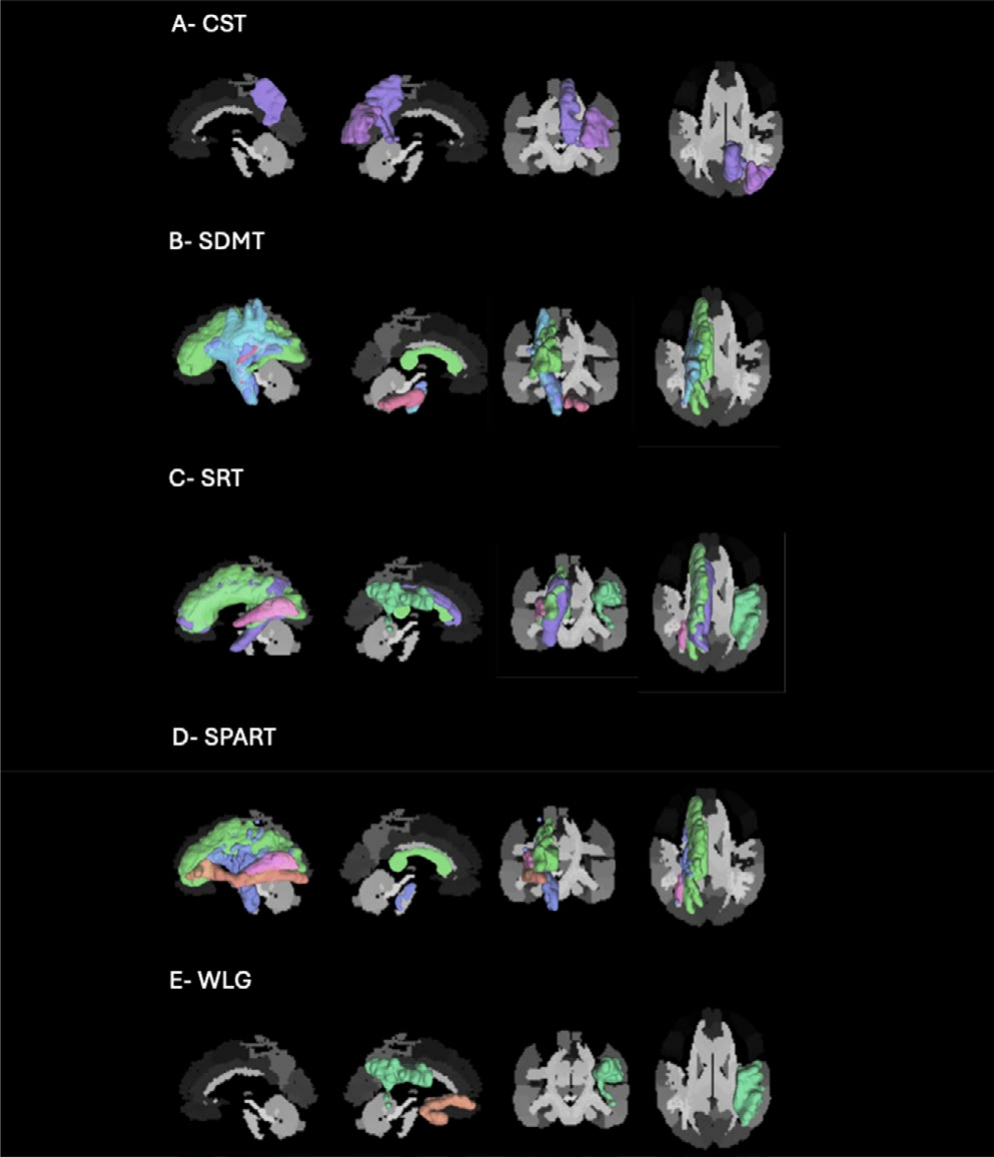
**FIGURE 2** Atlas‐based visualization of regions of interest across neuropsychological tests at year 5. Different colors indicate distinct anatomical regions across neuropsychological tests.

**Figure:**
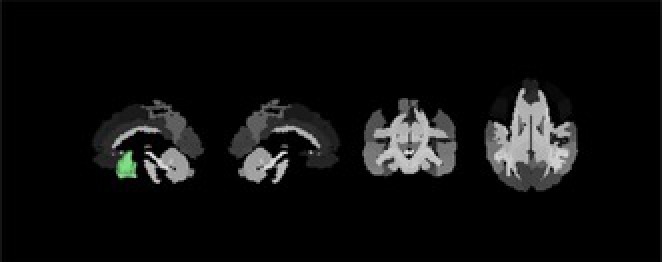
**FIGURE 3** Atlas‐based visualization of regions of interest associated with CST‐decliners.

**Conclusion:** Lesion location is a key determinant of current and future CI in MS. Shared involvement of major white matter tracts supports domain‐general disconnection mechanism, while selective regions contribute to domain‐specific vulnerability. Lesion mapping may provide prognostic information beyond global lesion measures.

**Disclosure:** Nothing to disclose.

## EPO‐0293

### Telomere shortening as a marker of biological aging in multiple sclerosis

#### D. Savasci ^1,2^; İ. Karacan^3^; S. Susgun^5^; B. Yigit^3^; D. Yozlu^3^; N. Korkmaz^5^; O. Duzenli^3^; A. Yabaci^6^; E. Yucesan^3^; A. Gursoy^1^

##### ^*1*^ *Department of Neurology, Basaksehir Cam and Sakura City Hospital, Istanbul, Türkiye,* ^*2*^ *Department of Immunology, Istanbul University, Aziz Sancar Institute of Experimental Medicine,* ^*3*^ *Department of Neurogenetics, Institute of Neurological Sciences, Istanbul University‐Cerrahpasa, Istanbul, Türkiye,* ^*4*^ *Department of Integrative Biology and Physiology, University of California, Los Angeles (UCLA), Los Angeles, USA,* ^*5*^ *Department of Biostatistics and Medical Informatics, Bezmialem Vakif University, Istanbul, Türkiye*

**Background and aims:** Multiple sclerosis (MS) is a chronic immune‐mediated disease of the central nervous system in which aging‐related mechanisms are thought to contribute to disease activity. Telomere length is a well‐established biomarker of biological aging and has been reported to be shortened in MS. However, the contribution of telomere‐regulating genes such as TERF1 and TERF2 to telomere dynamics in MS remains unclear.

**Methods:** This case–control study included 40 patients with relapsing–remitting MS (RRMS) and 39 age‐ and sex‐matched healthy controls aged 18–40 years. Relative leukocyte telomere length was measured by qPCR using the telomere‐to‐single copy gene ratio. The TERF1 polymorphisms rs10107605 and rs1545827, and the TERF2 polymorphism rs251796 were genotyped using high‐resolution melting analysis.

**Results:** Leukocyte telomere length was significantly shorter in patients with MS compared with healthy controls (*p* = 0.005). Telomere length was not associated with age, sex, smoking status, disease duration, or other clinical variables within the MS group. Receiver operating characteristic analysis demonstrated a moderate discriminatory capacity for telomere length between patients and controls (AUC = 0.70). All investigated single nucleotide polymorphisms were in Hardy–Weinberg equilibrium, and no significant associations were observed between TERF1 or TERF2 variants and telomere length across any inheritance model.

**Conclusion:** Accelerated leukocyte telomere shortening appears to reflect biological aging in multiple sclerosis, independent of common TERF1 and TERF2 genetic variants. These findings support the relevance of cellular aging mechanisms in early MS and highlight telomere length as a potential component of multimodal biomarker strategies. Longitudinal and multi‐omics studies are warranted to further clarify telomere dynamics in MS.

**Disclosure:** Nothing to disclose.

## EPO‐0294

### Call for unity: 348,000 patients, six registries, one common data model for MS research

#### J. Drahota^1^; L. Forsberg^2^; L. Pontieri^3^; E. Mouresan^2^; P. Iaffaldano^4^; G. Lucisano^5^; T. Spelman^2^; R. Casey^6^; H. Joensen^3^; M. Copetti^7^; F. Sandjo^6^; D. Stastna ^8^; H. Butzkueven^9^; J. Hilert^2^; D. Horakova^8^; M. Magyari^10^; M. Trojano^11^; S. Vukusic^6^; G. Fistravec^12^

##### ^*1*^ *ReMuS Registry, ReMuS Endowment Fund, Prague, Czech Republic; Department of Neurology and Centre of Clinical Neuroscience, First Faculty of Medicine, Charles University and General University Hospital in Prague, Prague, Czech Republic;* ^*2*^ *Department of Clinical Neuroscience, KarolinskaInstitutet, Stockholm, Sweden;* ^*3*^ *The Danish Multiple Sclerosis Registry, Danish Multiple Sclerosis Center, Department of Neurology, Copenhagen University Hospital – Rigshospitalet, Glostrup, Denmark;* ^*4*^ *Department of Translational Biomedicines and Neurosciences, University of Bari “AldoMoro”, Bari, Italy; DiBraiN Multiple Sclerosis Center, Policlinico of Bari, Bari, Italy;* ^*5*^ *Department of Translational Biomedicines and Neurosciences, University of Bari “AldoMoro”, Bari, Italy; Center for Outcomes Research and Clinical Epidemiology – CORESEARCH SRL, Pescara, Italy;* ^*6*^ *Université de Lyon, Lyon, France; Observatoire Français de la Sclérose en Plaques, Centre de Recherche en Neurosciences de Lyon, Lyon, France;* ^*7*^ *Unit of Biostatistics, Fondazione IRCCS Casa Sollievo della Sofferenza, San Giovanni Rotondo, Italy;* ^*8*^ *Department of Neurology andCentre of Clinical Neuroscience, First Faculty of Medicine, Charles University and General University Hospital in Prague, Prague, CzechRepublic;* ^*9*^ *Deptof Neuroscience, School of Translational Medicine, Monash University, Melbourne, Australia;* ^*10*^ *The Danish Multiple Sclerosis Registry, Danish Multiple Sclerosis Center, Department of Neurology, Copenhagen University Hospital – Rigshospitalet, Glostrup, Denmark; Institute for Clinical Medicine, University of Copenhagen, Copenhagen, Denmark;* ^*11*^ *Department of Translational Biomedicines and Neurosciences, University of Bari “AldoMoro”, Bari, Italy;* ^*12*^ *ReMuS Registry, ReMuS Endowment Fund, Prague, Czech Republic*

**Background and aims:** Heterogeneous registry structures limit cross‐border real‐world evidence generation in multiple sclerosis (MS). The European Medicines Agency calls for standardised, interoperable data approaches to support post‐authorisation safety studies and rare outcome detection. We developed and validated a common data model (CDM) within the Big MS Data (BMSD) Network to enable reproducible federated analyses while preserving data sovereignty across registries.

**Methods:** Six MS registries (Czech, Danish, French, Italian, Swedish, MSBase) collaboratively defined 120 standardised variables covering demographics, disease history, relapses, treatments, comorbidities, disability outcomes, and paraclinical data. Registry‐specific datasets were mapped to CDM format using automated transformation scripts with error logging. Validation included transformation success metrics, demographic plausibility checks, and pilot federated logistic regression analyses.

**Results:** The CDM successfully harmonised 348,371 patient records (ranging from 19,097 to 111,727 patients per registry). Transformation achieved 98–100% information retention across core domains (patients, visits, treatments, medical conditions). Demographic validation confirmed expected distributions: mean age at MS onset ranged 31.8–35.4 years across registries with approximately 70% female representation. Federated logistic regression demonstrated consistent effect estimates across registries without patient‐level data transfer. The framework now supports ongoing European research initiatives investigating treatment patterns and rare adverse events.

**Conclusion:** This CDM represents a scalable solution for privacy‐preserving multi‐registry MS research aligned with European regulatory requirements. By enabling federated analyses across six registries, the framework facilitates detection of rare outcomes and treatment patterns undetectable in single‐registry studies, with potential applicability to other neurological conditions.

**Disclosure:** Supported by Ministry of Health of the Czech Republic, grant nr. NW26J‐08‐00103.

## EPO‐0295

### High levels of p‐STAT1 are associated with lower brain volumes at baseline in patients with early Multiple Sclerosis

#### M. Manconi; R. Orlandi; S. Bartiromo; F. Gobbin; E. Butturini; A. Carcereri De Prati; S. Mariotto; A. Gajofatto

##### Department of Neurosciences, Biomedicine and Movement Sciences, University of Verona, Italy

**Background and aims:** The role of oxidative stress has been suggested in Multiple Sclerosis (MS) pathogenesis. Phosphorylated Signal Transducer and Activator of Transcription 1 (pSTAT1) is a key transcription factor that promotes proinflammatory processes in response to oxidative stress. This study investigates the association of blood levels of pSTAT1 with measures of disease severity in patients with early MS.

**Methods:** 29 patients aged 18–40 years, recently (≤ 2 years) diagnosed with MS, were consecutively enrolled in an observational study at Verona University Hospital; a brain MRI scan performed between 6 months before and 1 month after inclusion was mandatory. A clinical and MRI follow‐up was performed after 10–48 months in a subgroup of 20 of patients. We assessed the association of baseline values of pSTAT1 with clinical variables, global and regional brain volumes and their annualized variations.

**Results:** At inclusion, patients with medium‐to‐high compared to low levels of p‐STAT1 showed lower median volumes of global brain (1.13e+7 vs 1.033+7 mm3 *p* = 0.024 ), cerebellar cortex (101.7 vs 112.2 cm3, *p* = 0.028), thalamus (132.4 vs 150 cm3, *p* = 0.028), caudate (60.9 vs 69.5 cm3, *p* = 0.007) and putamen (87.56 vs 94.96 cm3, *p* = 0.041). In the longitudinal study, we didn't find any significant association between pSTAT1 and any of the imaging and clinical variables.

**Conclusion:** High pSTAT1 expression is associated with low brain volumes in early MS, to be confirmed in larger longitudinal studies.

**Disclosure:** Nothing to disclose.

## EPO‐0296

### Fatigue control: 24‐month real‐world MoOzaRt study Shows 88.8% stabilization and strong correlation with reduced MS burden

#### I. Penner ^1^; T. Wiemer^2^; H. Schreiber^3^

##### ^*1*^ *Department of Neurology, Inselspital, Bern University Hospital, University of Bern, Switzerland; COGITO Center for applied neurocognition and neuropsychological research, Düsseldorf, Germany;* ^*2*^ *Roche Pharma AG, Grenzach‐Wyhlen, Germany;* ^*3*^ *Neurological Practice Center & Neuropoint Academy, Ulm, Germany*

**Background and aims:** Fatigue is considered the most common and one of the most debilitating symptoms in multiple sclerosis (MS). MoOzaRt assesses the impact of ocrelizumab on patient‐reported persistent (trait) and transient (state) fatigue in patients with relapsing MS (RMS).

**Methods:** The ongoing non‐interventional MoOzaRt study (ISRCTN55332718) recruited 272 RMS patients initiating ocrelizumab. The primary combined endpoint is a clinically meaningful reduction (≥ 9 points) or stabilization (±8 points) from baseline in trait fatigue, measured by the Fatigue Scale for Motor and Cognitive Functions (FSMC) total score over 24 months. Secondary endpoints include state fatigue (Visual Analogue Scale (VAS)), Expanded Disability Status Scale (EDSS), Patient Reported Outcomes (PROs) and safety.

**Results:** The third interim analysis (cut‐off Dec 10, 2025, snapshot) included 261 patients (Table 1 67.4% female, mean age 38.3 years, mean EDSS 2.43). Over the 24‐month observation period, 88.8% of patients showed stable or reduced FSMC total scores, of which 30.1% achieved a clinically meaningful improvement. Treatment‐naive patients exhibited the highest proportion of fatigue stabilization or improvement (90.4%). Lower fatigue scores strongly correlated with reduced physical and psychological disease burden (MSIS‐29, WAPI:MS, PDQ‐20, HADS).

**Figure:**
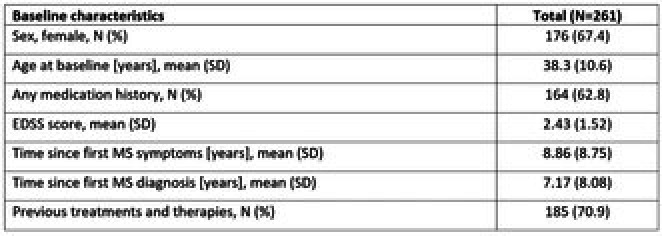
TABLE 1

**Conclusion:** This analysis confirms that ocrelizumab treatment is associated with stable or even improved fatigue in a very high proportion of RMS patients, particularly in treatment‐naive patients (90.4%). Given the strong correlation between reduced fatigue scores and reduced disease burden, these findings suggest that early high‐efficacy therapy (HET) is a valuable strategy for relieving and preventing disease burden.

**Disclosure:** IKP: Almirall, Biogen, BMS, Celgene, Genzyme, Janssen, Merck, Novartis, Roche, Teva//speakers bureau or advisory board, consulting fees; The German MS Society, Celgene, Novartis, Roche, Teva//research grants. TW: Roche//employee HS: Almirall, Biogen, BMS, Genzyme, Janssen, Merck, Novartis, Roche, Sanofi, Teva//speakers bureau or advisory board, consulting fees, travel reimbursement; Biogen, Novartis, Roche, Sanofi, Teva//research grants; Biogen, Novartis, Roche//data monitoring or steering committees.

## EPO‐0297

### Diagnostic guidelines for identifying and monitoring cognitive impairment in multiple sclerosis: An IMSCOGS and ECTRIMS Delphi consensus statement

#### I. Penner ^1^; S. Morrow^2^; L. Hancock^3^; F. Nonino^4^; D. Langdon^5^; M. Amato^6^; M. Schoonheim^7^

##### ^*1*^ *Department of Neurology, Inselspital, Bern University Hospital, University of Bern, Switzerland;* ^*2*^ *Department of Clinical Neurosciences, University of Calgary, Canada**;*** ^*3*^ *Department of Neurology, Cleveland University, USA**;*** ^*4*^ *Department of Epidemiology and Statistics, University of Bologna, Italy**;*** ^*5*^ *Department of Health and Medicine Royal Holloway University of London UK**;*** ^*6*^ *Department of Neurology, University of Florence Italy**;*** ^*7*^ *Department of Neuroscience University of Amsterdam, The Netherlands*

**Background and aims:** CI occurs frequently in MS, is a common cause of loss of employment and can have a significant impact on quality of life. The aim was to develop international guidelines to assist in the identification of cognitive impairment (CI) among adult persons with MS (PwMS) for both clinical and research applications. The three topics for these guidelines were: 1) cognitive screening, 2) cognitive measurement and diagnosis and 3) monitoring and diagnosing change over time.

**Methods:** The International Multiple Sclerosis Cognition Society (IMSCOGS) and European Committee for Treatment and Research in MS (ECTRIMS) partnered with Cochrane Multiple Sclerosis and Rare Diseases to conduct a scoping literature review to support the formation of guidelines. Based on this review, statements were formulated. Next, a modified Delphi approach was used to establish consensus via multiple virtual meetings and rounds of anonymous voting by a large group of clinical and research experts in this area with global representation. A minimum response and consensus of 80% was required for a statement to be accepted.

**Results:** Our scoping review provided an evidence gap map, identifying key inconsistencies in the assessment of CI in PwMS. Delphi voting resulted in a clear set of criteria and guidelines. Results on aims 1, 2 and 3: Cognitive screening; cognitive measurement and diagnosis; monitoring/diagnosing change over time will be presented.

**Conclusion:** These guidelines permit reliable diagnosis of CI and cognitive decline in MS, allowing generalizability of research findings in future studies of CI in MS and consistency across research and clinical settings.

**Disclosure:** IP, SM, LH, DL, MPA and MS are members of the board/steering committee of IMSCOGS. This project was financially supported by ECTRIMS.

## EPO‐0298

### Modifications of time‐varying thalamic connectivity underpin fatigue development in multiple sclerosis

#### M. Margoni^1^; P. Valsasina^2^; P. Preziosa^3^; D. Mistri ^2^; M. Rocca^3^; M. Filippi^4^

##### ^*1*^ *Neuroimaging Research Unit, Division of Neuroscience, Neurology Unit, and Neurorehabilitation Unit, IRCCS San Raffaele Scientific Institute, Milan, Italy;* ^*2*^ *Neuroimaging Research Unit, Division of Neuroscience, IRCCS San Raffaele Scientific Institute, Milan, Italy;* ^*3*^ *Neuroimaging Research Unit, Division of Neuroscience, and Neurology Unit, IRCCS San Raffaele Scientific Institute, Milan, Italy; and Vita‐Salute San Raffaele University, Milan, Italy;* ^*4*^ *Neuroimaging Research Unit, Division of Neuroscience, Neurology Unit, Neurorehabilitation Unit, and Neurophysiology Service, IRCCS San Raffaele Scientific Institute, Milan, Italy; and Vita‐Salute San Raffaele University, Milan, Italy*

**Background and aims:** Fatigue is common in multiple sclerosis (MS) and occurs independently from disease stages. Resting state (RS) functional connectivity (FC) studies showed an implication of thalamic circuits dysfunction in MS‐related fatigue pathogenesis. Here, we investigated changes over time of thalamic time‐varying (TV) FC and its association with fatigue development in MS.

**Methods:** Ninety‐two right‐handed MS patients and 55 healthy controls (HC) underwent clinical and 3.0T RS fMRI assessment at baseline and after 1.27‐year median follow‐up. The modified fatigue impact scale (MFIS) assessed fatigue at both visits; patients were considered as fatigued (F) if MFIS was > 38. TVFC of left/right thalamus was analyzed using sliding‐window seed‐voxel correlation.

**Results:** At baseline, 26 patients (28%) were F and 66 not fatigued (NF). Of these, 23 (35%) developed fatigue (devF) at follow‐up and 43 remained NF. At baseline, F‐MS patients exhibited increased TVFC (*p* < 0.001, conjunction analysis) between the left thalamus and hippocampus, and between the right thalamus, right insula, and bilateral frontal regions. Conversely, NF‐MS patients showed reduced baseline thalamic TVFC (*p* < 0.001, conjunction analysis) with temporal, occipital and cingulate regions. At follow‐up, NF‐MS patients showed increased thalamic TVFC with parietal, temporal and occipital regions. Conversely, devF‐MS patients showed increased TVFC over time (*p* < 0.001, time‐by‐group interaction) between the right thalamus and bilateral cingulate, left insular, left superior frontal and left inferior temporal regions. Finally, F‐MS patients presented decreased TVFC over time (*p* < 0.001, time‐by‐group interaction) between right thalamus and left insula.

**Conclusion:** Distinct patterns of thalamic TVFC changes characterized MS patients based on their fatigue status.

**Disclosure:** M. Margoni reports grants and personal fees from Sanofi Genzyme, Merck Serono, Roche, Biogen, Amgen and Novartis. P. Valsasina and D. Mistri have nothing to disclose. P. Preziosa received speaker honoraria from Roche, Biogen, Novartis, Merck, Bristol Myers Squibb, Genzyme, Horizon and Sanofi. M.A. Rocca received consulting fees from Biogen, Bristol Myers Squibb, Roche; and speaker honoraria from Alexion, Biogen, Bristol Myers Squibb, Celgene, Horizon Therapeutics Italy, Merck Serono SpA, Mitsubishi‐Tanabe Pharma, Neuraxpharm, Novartis, Roche, Sandoz, and Sanofi. She receives research support from the MS Society of Canada, the Italian Ministry of Health, the Italian Ministry of University and Research, and Fondazione Italiana Sclerosi Multipla. She is Associate Editor for Multiple Sclerosis and Related Disorders; and Associate Co‐Editor for Europe and Africa for Multiple Sclerosis Journal. M. Filippi is Editor‐in‐Chief of the Journal of Neurology, Associate Editor of Human Brain Mapping, Neurological Sciences, and Radiology; received compensation for consulting services from Almirall, Biogen, Bristol‐Myers Squibb, Eli Lilly, Merck, Novartis, Roche, Sanofi; speaking activities from Amgen, Bayer, Biogen, Bristol‐Myers Squibb, Celgene, Chiesi Italia SpA, Eisai, Eli Lilly, Fujirebio, Genzyme, Janssen, Merck, Neopharmed Gentili, Neuraxpharm, Novartis, Novo Nordisk, Roche, Sanofi, Takeda; participation in Advisory Boards for Alexion, Biogen, Bristol‐Myers Squibb, Eli Lilly, GE Healthcare Ltd, Merck, Neuraxpharm, Novartis, Roche, Sandoz, Sanofi, Takeda; scientific direction of educational events for Biogen, Merck, Roche, Celgene, Bristol‐Myers Squibb, Lilly, Novartis, Sanofi‐Genzyme; he receives research support from Biogen Idec, Merck‐Serono, Novartis, Roche, the Italian Ministry of Health, the Italian Ministry of University and Research, and Fondazione Italiana Sclerosi Multipla.

## EPO‐0299

### MRI‐derived subtypes identified by sustain capture distinct clinical, cognitive, and disability profiles in Multiple Sclerosis

#### P. Preziosa ^1^; L. Storelli^2^; E. Pagani^2^; N. Tedone^3^; M. Margoni^4^; F. Esposito^5^; M. Filippi^6^; M. Rocca^1^

##### ^*1*^ *Neuroimaging Research Unit, Division of Neuroscience, and Neurology Unit, IRCCS San Raffaele Scientific Institute, Milan, Italy; and Vita‐Salute San Raffaele University, Milan, Italy;* ^*2*^ *Neuroimaging Research Unit, Division of Neuroscience, IRCCS San Raffaele Scientific Institute, Milan, Italy;* ^*3*^ *Neuroimaging Research Unit, Division of Neuroscience, IRCCS San Raffaele Scientific Institute, Milan, Italy; and Vita‐Salute San Raffaele University, Milan, Italy;* ^*4*^ *Neuroimaging Research Unit, Division of Neuroscience, Neurology Unit, and Neurorehabilitation Unit, IRCCS San Raffaele Scientific Institute, Milan, Italy;* ^*5*^ *Neurology Unit; and Laboratory of Human Genetics of Neurological Disorders, IRCCS San Raffaele Scientific Institute, Milan, Italy;* ^*6*^ *Neuroimaging Research Unit, Division of Neuroscience, Neurology Unit, Neurorehabilitation Unit, and Neurophysiology Service, IRCCS San Raffaele Scientific Institute, Milan, Italy; and Vita‐Salute San Raffaele University, Milan, Italy*

**Background and aims:** Clinical multiple sclerosis (MS) classifications incompletely capture disease heterogeneity. Data‐driven subtyping may better reflect MS pathobiology and clinical variability. We applied SuStaIn to identify MRI‐based MS subtypes.

**Methods:** We retrospectively analyzed 1017 MS patients and 548 healthy controls with standardized clinical, neuropsychological, and brain 3T MRI assessments. Z‐scores of 21 MRI features, including white matter (WM) lesion volume major WM tract microstructural abnormalities, and global and regional normalized brain volumes, were input into SuStaIn to derive subtypes and stages. Associations with clinical phenotype (relapsing‐remitting [RR] vs progressive), age at onset (AAO) (pediatric [POMS], adult [AOMS], late [LOMS]), disability (Expanded Disability Status Scale [EDSS]), and cognition (Brief Repeatable Battery of Neuropsychological Tests) were explored.

**Results:** SuStaIn identified four MRI subtypes: lesion‐led (44%), cortex‐led (23%), tract‐led (23%), and deepGM‐led (10%). Subtypes differed by clinical phenotype and AAO (*p* < 0.001). Cortex‐led and deepGM‐led were enriched in progressive forms (47% and 56%); lesion‐led and tract‐led were mainly RRMS (66–71%). Tract‐led was overrepresented in POMS (38%) and underrepresented in AOMS (19%), lesion‐led predominated in AOMS (48%), whereas deepGM‐led was more frequent in LOMS (14%). Disability milestones were reached more often in cortex‐led and deepGM‐led patients (EDSS ≥ 4.0: 45% and 56%; EDSS ≥ 6.0: 28% and 29%) than in lesion‐led (34% and 20%) or tract‐led (32% and 19%) (*p* < 0.001). Cognitive performance did not differ across subtypes, but within each subtype higher SuStaIn stage correlated with worse global and domain‐specific performance (*p* ≤ 0.030).

**Conclusion:** SuStaIn‐derived MRI subtypes capture clinically relevant heterogeneity in MS and may support personalized therapeutic strategies.

**Disclosure:** P. Preziosa received speaker honoraria from Roche, Biogen, Novartis, Merck, Bristol Myers Squibb, Genzyme, Horizon and Sanofi. L. Storelli has nothing to disclose. E. Pagani has nothing to disclose. N. Tedone has nothing to disclose. M. Margoni reports grants and personal fees from Sanofi Genzyme, Merck Serono, Roche, Biogen, Amgen and Novartis. F. Esposito has nothing to disclose. M. Filippi is Editor‐in‐Chief of the Journal of Neurology, Associate Editor of Human Brain Mapping, Neurological Sciences, and Radiology; received compensation for consulting services from Almirall, Biogen, Bristol‐Myers Squibb, Eli Lilly, Merck, Novartis, Roche, Sanofi; speaking activities from Amgen, Bayer, Biogen, Bristol‐Myers Squibb, Celgene, Chiesi Italia SpA, Eisai, Eli Lilly, Fujirebio, Genzyme, Janssen, Merck, Neopharmed Gentili, Neuraxpharm, Novartis, Novo Nordisk, Roche, Sanofi, Takeda; participation in Advisory Boards for Alexion, Biogen, Bristol‐Myers Squibb, Eli Lilly, GE Healthcare Ltd, Merck, Neuraxpharm, Novartis, Roche, Sandoz, Sanofi, Takeda; scientific direction of educational events for Biogen, Merck, Roche, Celgene, Bristol‐Myers Squibb, Lilly, Novartis, Sanofi‐Genzyme; he receives research support from Biogen Idec, Merck‐Serono, Novartis, Roche, the Italian Ministry of Health, the Italian Ministry of University and Research, and Fondazione Italiana Sclerosi Multipla. M.A. Rocca received consulting fees from Biogen, Bristol Myers Squibb, Roche; and speaker honoraria from Alexion, Biogen, Bristol Myers Squibb, Celgene, Horizon Therapeutics Italy, Merck Serono SpA, Mitsubishi‐Tanabe Pharma, Neuraxpharm, Novartis, Roche, Sandoz, and Sanofi. She receives research support from the MS Society of Canada, the Italian Ministry of Health, the Italian Ministry of University and Research, and Fondazione Italiana Sclerosi Multipla. She is Associate Editor for Multiple Sclerosis and Related Disorders; and Associate Co‐Editor for Europe and Africa for Multiple Sclerosis Journal.

## EPO‐0300

### Gray matter damage and long‐term disability, cognitive outcome, and survival in patients with multiple sclerosis

#### P. Preziosa ^1^; A. Meani^2^; N. Tedone^3^; I. Gattuso^3^; M. Rovaris^5^; M. Rocca^1^; M. Filippi^6^

##### ^*1*^ *Neuroimaging Research Unit, Division of Neuroscience, and Neurology Unit, IRCCS San Raffaele Scientific Institute, Milan, Italy; and Vita‐Salute San Raffaele University, Milan, Italy;* ^*2*^ *Neuroimaging Research Unit, Division of Neuroscience, IRCCS San Raffaele Scientific Institute, Milan, Italy;* ^*3*^ *Neuroimaging Research Unit, Division of Neuroscience, IRCCS San Raffaele Scientific Institute, Milan, Italy; and Vita‐Salute San Raffaele University, Milan, Italy;* ^*5*^ *IRCCS Fondazione Don Carlo Gnocchi ONLUS, Milan;* ^*6*^ *Neuroimaging Research Unit, Division of Neuroscience, Neurology Unit, Neurorehabilitation Unit, and Neurophysiology Service, IRCCS San Raffaele Scientific Institute, Milan, Italy; and Vita‐Salute San Raffaele University, Milan, Italy*

**Background and aims:** The long‐term prognostic value of gray matter (GM) damage in multiple sclerosis (MS) is unclear; we examined MRI markers of GM and white matter (WM) damage associated with long‐term outcomes in MS.

**Methods:** Seventy‐three relapse‐onset MS patients underwent clinical and brain MRI at baseline and 12 months, including WM lesion volumes, brain, GM, WM, and thalamic volume fractions, and GM, thalamic, normal‐appearing (NA) WM, and WM lesions MTR. High‐efficacy disease‐modifying therapies (HE‐DMTs) escalation was recorded. Long‐term associations with Expanded Disability Status Scale (EDSS) worsening, evolution to a more severe disease stage, MS‐related death, and cognitive deterioration were analyzed using LASSO‐regularized logistic regression with bootstrap validation.

**Results:** Over a median 25.9‐year follow‐up, 79.5% patients experienced EDSS worsening, 72.6% evolved to a more severe disease stage, 24.7% died from MS, and 39.5% exhibited cognitive deterioration. EDSS worsening was associated with lower baseline GM and thalamic fractions, HE‐DMT escalation, lower baseline NAWM MTR peak height, and higher 12‐month decline in GM MTR histogram peak height (AUC = 0.843). Evolution to a more severe disease stage was associated with higher 12‐month EDSS score change and lower baseline GM fraction (AUC = 0.793). MS‐related death was associated with male sex, higher baseline EDSS score, lower baseline GMF, lower baseline GM MTR peak height, and greater 12‐month thalamic MTR decline (AUC = 0.887). Cognitive deterioration was associated with older age, lower baseline brain parenchymal fraction, and lower baseline mean GM MTR (AUC = 0.891).

**Conclusion:** GM damage is associated with worse 26‐year clinical outcomes in relapse‐onset MS, supporting the relevance of GM‐focused MRI markers.

**Disclosure:** P. Preziosa received speaker honoraria from Roche, Biogen, Novartis, Merck, Bristol Myers Squibb, Genzyme, Horizon and Sanofi. A. Meani has nothing to disclose. N. Tedone has nothing to disclose. I. Gattuso has nothing to disclose. M. Rovaris received speaker honoraria from Merck and Novartis, he has received research support from Italian Ministry of University and Research and Fondazione Italiana Sclerosi Multipla (FISM). M.A. Rocca received consulting fees from Biogen, Bristol Myers Squibb, Roche; and speaker honoraria from Alexion, Biogen, Bristol Myers Squibb, Celgene, Horizon Therapeutics Italy, Merck Serono SpA, Mitsubishi‐Tanabe Pharma, Neuraxpharm, Novartis, Roche, Sandoz, and Sanofi. She receives research support from the MS Society of Canada, the Italian Ministry of Health, the Italian Ministry of University and Research, and FISM. She is Associate Editor for Multiple Sclerosis and Related Disorders; and Associate Co‐Editor for Europe and Africa for Multiple Sclerosis Journal. M. Filippi is Editor‐in‐Chief of the Journal of Neurology, Associate Editor of Human Brain Mapping, Neurological Sciences, and Radiology; received compensation for consulting services from Almirall, Biogen, Bristol‐Myers Squibb, Eli Lilly, Merck, Novartis, Roche, Sanofi; speaking activities from Amgen, Bayer, Biogen, Bristol‐Myers Squibb, Celgene, Chiesi Italia SpA, Eisai, Eli Lilly, Fujirebio, Genzyme, Janssen, Merck, Neopharmed Gentili, Neuraxpharm, Novartis, Novo Nordisk, Roche, Sanofi, Takeda; participation in Advisory Boards for Alexion, Biogen, Bristol‐Myers Squibb, Eli Lilly, GE Healthcare Ltd, Merck, Neuraxpharm, Novartis, Roche, Sandoz, Sanofi, Takeda; scientific direction of educational events for Biogen, Merck, Roche, Celgene, Bristol‐Myers Squibb, Lilly, Novartis, Sanofi‐Genzyme; he receives research support from Biogen Idec, Merck‐Serono, Novartis, Roche, the Italian Ministry of Health, the Italian Ministry of University and Research, and FISM.

## Muscle and Neuromuscular Junction Disorder 2

## EPO‐0301

### Comparative efficacy of anti‐C5 vs. anti‐FcRn therapy in thymoma‐associated myasthenia gravis: A multicentre retrospective study

#### A. Cepele ^1^; M. Maestri Tassoni^1^; P. Alboini^2^; P. Businaro^3^; V. Damato^4^; C. Erra^5^; S. Falso^6^; L. Fionda^7^; L. Florio^2^; M. Gastaldi^3^; F. Habetswallner^5^; R. Iorio^6^; V. Iovino^1^; A. Lo Iacono^1^; S. Marini^6^; F. Matrone^5^; S. Natoli^8^; A. Pugliese^8^; E. Rossini^7^; C. Rodolico^8^; L. Tufano^9^; M. Verza^4^; M. Guida^1^

##### ^*1*^ *Department of Clinical and Experimental Medicine, Neurology Unit, University of Pisa, Pisa, Italy;* ^*2*^ *Neurology Unit, Fondazione IRCCS Casa Sollievo della Sofferenza, San Giovanni Rotondo, Italy;* ^*3*^ *Neuroimmunology Research Section, IRCCS Mondino Foundation, Pavia, Italy;* ^*4*^ *Department of Neurosciences, Psychology, Drug Research and Child Health (NEUROFARBA), University of Florence, Firenze, Italy;* ^*5*^ *UOC Neurophysiopathology, AORN Cardarelli, Via Antonio Cardarelli 9, Napoli, Italy;* ^*6*^ *Department of Neuroscience, Università Cattolica del Sacro Cuore, Roma, Italy;* ^*7*^ *Department of Neuroscience, Mental Health and Sensory Organs (NESMOS), SAPIENZA University of Rome, Roma, Italy;* ^*8*^ *Unit of Neurology and Neuromuscular Disorders, Department of Clinical and Experimental Medicine, University of Messina, Messina, Italy;* ^*9*^ *Department of Neuroscience, Mental Health and Sensory Organs (NESMOS), Faculty of Medicine and Psychology, SAPIENZA University of Rome, Sant'Andrea Hospital, Roma, Italy*

**Background and aims:** Thymoma‐associated Myasthenia Gravis (TAMG) represents a challenging clinical phenotype. While anti‐complement therapy (C5IT) and neonatal‐Fc‐receptor inhibitors (aFcRn) have expanded the therapeutic landscape, comparative data regarding their efficacy in TAMG are lacking. This study evaluated the comparative efficacy and corticosteroid (CS)‐sparing potential of C5IT versus aFcRn in TAMG patients.

**Methods:** We conducted a retrospective study of 93 AChR‐positive TAMG patients (58 C5IT maintenance; 35 aFcRn cycles) across eight Italian centers. The primary endopoint included longitudinal changes in MG‐ADL, QMG, daily CS dosage over 12 months (Table 1). LMM was performed to assess treatment‐by‐time interactions. aFcRn patients were stratified into “short responders” (mean cycles‐intervals < 8 weeks) and “long‐responders” (mean cycles‐intervals > = 8 weeks).

**Figure:**
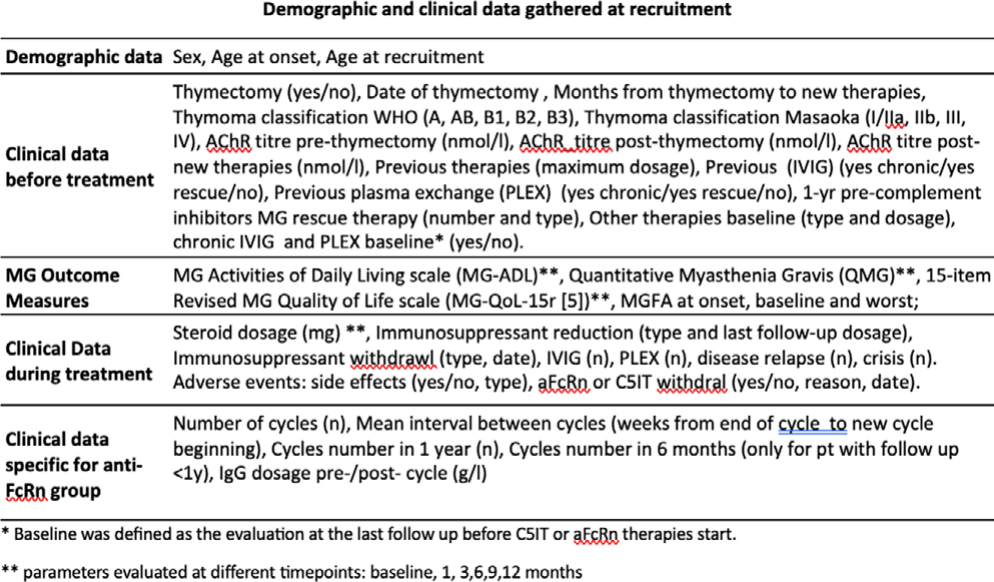
**TABLE 1** Demographic and clinical data gathered at recruitment.

**Results:** Baseline demographics and disease severity were comparable between cohorts. While both groups exhibited clinical improvement, LMM analysis demonstrated a significant treatment‐by‐time interaction favouring C5IT for MG‐ADL (F = 8.48, *p* = 0.004) and daily CS reduction (F = 6.284, *p* = 0.013). C5IT achieved greater mean reductions in MG‐ADL (−6.43 vs. −3.21) and daily CS dosage (−10.8 mg vs. −6.6 mg) compared to aFcRn. Subgroup analysis revealed that aFcRn “short responders” (43% of the cohort) had significantly inferior outcomes compared to both C5IT and aFcRn “long responders”.

**Conclusion:** In TAMG patients, chronic C5IT maintenance regimen provides a greater overall clinical improvement and daily corticosteroids compared to aFcRn cycle‐based treatment schedule in TAMG patients. aFcRn patients requiring frequent cycling (< 8 weeks) had suboptimal responses, whereas those with sustained durability mirrored C5IT efficacy. These founding highlight the necessity of predictive factors to identify potential “short responders” and optimize personalized treatment selection and administration schedules.

**Disclosure:** Carmelo Rodolico has received funding for meeting attendance and advisory boards partecipation from Argenx, Alexion Pharmaceuticals, Amgen, Johnson & Johnson and UCB Pharma. Valentina Damato has received public speaking honoraria and compensation for advisory boards and/or consultations fees from UCB, Alexion, Dianthus, Roche, Argenx, Amgen. Alboini Paolo Emilio has received compensation for advisory boards and/or consultations fees from Alexion, Amgen, Argnx, UCBA Pharma. Laura Fionda has received public speaking honoraria and/or compensation for advisory boards and/or consultations fees from Alexion, Argnx, UCB Pharma, Dianthus, J&J, Amgen. Alessia Pugliese has received public speaking honoraria and/or funding for meeting attendance from Alexion Pharmaceuticals, UCB Pharma and Sanofi Pharmaceuticals. Francesco Habetswallner has received honoraria as a speaker or for advisory boards from: Alfa Sigma, Angelini, Argenx, Amgen, Alnylam, Alexion, CLS Behring, Lusofarmaco, Johnson & Johnson, Merck, Roche, and Takeda. Raffaele Iorio has received consultancy fees and speaker honoraria from Alexion, Amgen, Argenx, Dianthus Therapeutics, Jonhson&Johnson, Merck, UCB. Silvia Falso has received consultancy fees and speaker honoraria from Alexion, Argenx, UCB. Sofia Marini has received consultancy fees and speaker honoraria from Alexion, Argenx, UCB. Michelangelo Maestri Tassoni has received personal fees from Alexion, UCB, Amgen and J&J. Matteo Gastaldi has received public speaking honoraria for Alexion, UCB, J&J. Alba Cepele has received personal fees from UCB. Melania Guida has received public speaking honoraria, advisory board and fees from Alexion, Argnx, UCB, Amgen, J&J.

## EPO‐0302

### Epigenetic profiling of cytokine gene promoters in MuSK myasthenia gravis

#### C. Elmas ^1^; A. Stoccoro^2^; M. Lari^2^; V. Iovino^3^; A. Cepele^3^; M. Guida^3^; M. Maestri^3^; F. Coppede^2^; I. Koneczny^1^

##### ^*1*^ *Division of Neuropathology and Neurochemistry, Department of Neurology, Medical University of Vienna, Vienna, Austria;* ^*2*^ *Department of Translational Research and of New Surgical and Medical Technologies, University of Pisa, Pisa, Italy;* ^*3*^ *Neurological Unit, Department of Neuroscience, University Hospital of Pisa, Pisa, Italy*

**Background and aims:** Myasthenia gravis associated with antibodies against muscle‐specific kinase (MuSK‐MG) is a well‐characterized IgG4‐autoimmune disease. This study investigated whether epigenetic modifications, specifically DNA methylation changes in cytokine gene promoters of interleukins implicated in IgG4 subclass switching, are associated with the IgG4‐predominant immune response in MuSK‐MG patients.

**Methods:** Peripheral blood mononuclear cells were isolated from MuSK‐MG patients (*n* = 36), acetylcholine receptor myasthenia gravis patients as disease controls (*n* = 7), and age‐ and sex‐matched healthy controls (*n* = 12). Genomic DNA was extracted and after sodium bisulfite conversion, DNA methylation of the promoter regions of interleukin (IL)‐4, IL‐10, and IL‐13 was assessed using methylation‐sensitive high‐resolution melting (MS‐HRM) analysis. Methylation levels were estimated by comparing the melting profiles of subjects' DNA samples with those obtained from DNA standards with known methylation percentages.

**Results:** MuSK‐MG patients showed 16% lower DNA methylation levels in the IL‐13 promoter compared with healthy controls (mean 14.51% vs 30.40%, *p* = 0.0038). IL‐4 promoter methylation was lower by 185 in MuSK‐MG patients compared with healthy controls (73.24% vs 91.18%, *p* = 0.0001), as well as disease control (98.08%, *p* < 0.0001). No significant differences were observed for IL‐10 promoter. These methylation patterns were independent of age, sex, and treatment status.

**Conclusion:** Selective hypomethylation of IL‐4 and IL‐13 promoters is observed in MuSK‐MG, while IL‐10 promoter methylation remains unaltered. This potentially facilitates increased cytokine expression, potentially favoring IgG4 subclass switching, although additional regulatory mechanisms are likely involved.

**Disclosure:** This project is conducted within the IgG4‐TREAT consortium, a Marie Skłodowska‐Curie Actions doctoral network funded by the European Union (Grant Agreement No: 101119457).

## EPO‐0303

### Clinical features and outcomes of very late‐onset myasthenia gravis: Insights from a greek center of reference

#### E. Strataki; D. Tzavella; K. Kournis; A. Vakrakou; V. Zouvelou

##### 1st Department of Neurology, National and Kapodistrian University of Athens, Athens, Greece, Eginition Hospital, ERN EURO‐NMD

**Background and aims:** The demographic profile of myasthenia gravis (MG) has shifted in recent decades, with increasing prevalence among older individuals. This study investigates the clinical characteristics and outcomes of patients with very late‐onset MG (VLOMG), defined as disease onset at ≥ 65 years.

**Methods:** We retrospectively reviewed patients with VLOMG registered in the Specialized Myasthenia Clinic database between Jan‐2008 and Dec‐2025.

**Results:** A total of 102 patients were included (72 males, 30 females), with a mean follow‐up of 47.86 months. Mean age at diagnosis was 73.88 ± 6.65 years (range: 65–91), with a mean diagnostic delay of 5.1 months. Thymoma was identified in 3.92%, autoimmune disease in 11.76%, malignancies in 17.65%, and other comorbidities in 91.18%. Overall, 99% were seropositive; 94.11% AChR‐Abs(+) and 4.9% MuSK‐Abs(+). AChR‐Abs titers ranged from 0.8 to 433nM/L (mean 49.07 ± 76.28nM/L). Generalized MG was present in 80.39%, most commonly with oculo‐bulbar involvement. Rescue therapies were required in 63.72%; 12.74% required intensive care admission and 5.88% mechanical ventilation. Long‐term immunosuppression was used in 93.13%. Advanced immunotherapies included rituximab (17.65%), FcRn antagonists (6.86%), ravulizumab (1.96%), and tocilizumab in one patient with concomitant thyroid eye disease. At last follow‐up, 68.62% achieved minimal manifestations or better and 19.6% showed clinical improvement, on a mean daily prednisolone dose of 6.75mg.

**Conclusion:** VLOMG represents a distinct MG subgroup characterized by male predominance, predominant bulbar phenotype, rare thymoma and high AChR positivity. Despite a significant comorbidity burden, early immunosuppression enables effective disease control with low maintenance corticosteroid doses. Novel and targeted immunotherapies may be safe and effective in elderly patients.

**Disclosure:** Nothing to disclose.

## EPO‐0304

### Electrophysiological assessment of motor unit loss in adult spinal muscular atrophy Types III/IV: A national study comparing MUNIX, CMAP, and MUSIX

#### E. Sole Cruz ^1^; E. Salort‐Campana^1^; T. Lenglet^2^; E. Fortanier^1^; Y. Pereon^3^; E. Berling^4^; S. Beloribi‐Djefaflia^1^; F. Bouhour^5^; P. Cintas^6^; E. Delmont^1^; M. Cavalli^7^; A. Ezaru^7^; E. De La Cruz^8^; S. Fernandes^1^; A. Kaminsky^9^; L. Kouton^1^; A. Nadaj‐Pakleza^10^; C. Tard^11^; A. Pegat^5^; G. Nicolas^4^; T. Stojkovic^2^; J. Hogrel^2^; S. Attarian^1^

##### ^*1*^ *Reference Center for Neuromuscular Diseases and ALS, CHU Timone, AP‐HM, Marseille, France;* ^*2*^ *Centre de référence de pathologie neuromusculaire Paris‐Est, groupe hospitalier Pitié‐Salpêtrière, institut de Myologie, AP‐HP, France;* ^*3*^ *Centre de Référence Maladies Neuromusculaires AOC, Filnemus, Euro‐NMD, Hôtel‐Dieu, CHU de Nantes, Nantes, France;* ^*4*^ *Service de Neurologie, Hôpital Raymond Poincaré, Centre de référence des maladies neuromusculaires Nord‐Est‐Ile de France, FHU Phenix, Garches, France;* ^*5*^ *ervice d'Electroneuromyographie et Pathologies Neuromusculaires, centre de référence des maladies neuromusculaires PACA‐Réunion‐Rhône Alpes, Hôpital Neurologique P. Wertheimer, Hospices Civils de Lyon, Lyon, France;* ^*6*^ *Service de Neurologie, Centre de référence des Maladies Neuromusculaires, CHU de Toulouse Purpan, Toulouse, France;* ^*7*^ *ervice de Neurologie: Système nerveux périphérique, Muscle et SLA, Hôpital Pasteur 2, CHU de Nice, Nice, France;* ^*8*^ *Service de Neurologie, CHU Gui de Chauliac, Montpellier, France;* ^*9*^ *Service de Neurologie, Centre Référent des Maladies Neuromusculaires Rares, CHU de Saint Etienne, Saint‐Etienne, France;* ^*10*^ *Service de Neurologie, Centre de référence des maladies neuromusculaires Nord/Est/Ile‐de‐France, CHU de Strasbourg, Strasbourg, France;* ^*11*^ *Service de Neurologie, U1172, CHU de Lille, Centre de Référence des Maladies Neuromusculaires Nord/Est/Ile‐de‐France, Lille, France*

**Background and aims:** Spinal muscular atrophy (SMA) types III–IV are the most common late‐onset forms and progress slowly, making sensitive biomarkers essential. The Motor Unit Number Index (MUNIX) estimates motor unit loss and may complement traditional electrophysiological measures such as Compound Muscle Action Potential amplitudes (CMAP). However, their respective performances have not been directly compared in adult SMA.

**Methods:** In a French multicentric study (NCT04690998), 71 adults with SMA and 24 healthy controls underwent clinical and electrophysiological assessment. MUNIX, CMAP, and Motor Unit Size Index (MUSIX) were recorded in four muscles, and sum scores (SumMUNIX, SumCMAP, SumMUSIX) were computed. Test–retest reliability was evaluated using intraclass correlation coefficients (ICCs). Associations with functional outcomes and strength measures were analysed, including multivariate models.

**Results:** MUNIX, MUSIX and CMAP effectively distinguished SMA patients from controls. MUNIX showed the highest discriminative performance (AUC = 0.92), while CMAP demonstrated the strongest and most consistent associations with clinical severity. In multivariate analyses, only CMAP remained independently associated with all functional and strength measures, whereas MUNIX and MUSIX lost significance. CMAP showed the strongest ability to discriminate between ambulant and non‐ambulant SMA patients.

**Figure:**
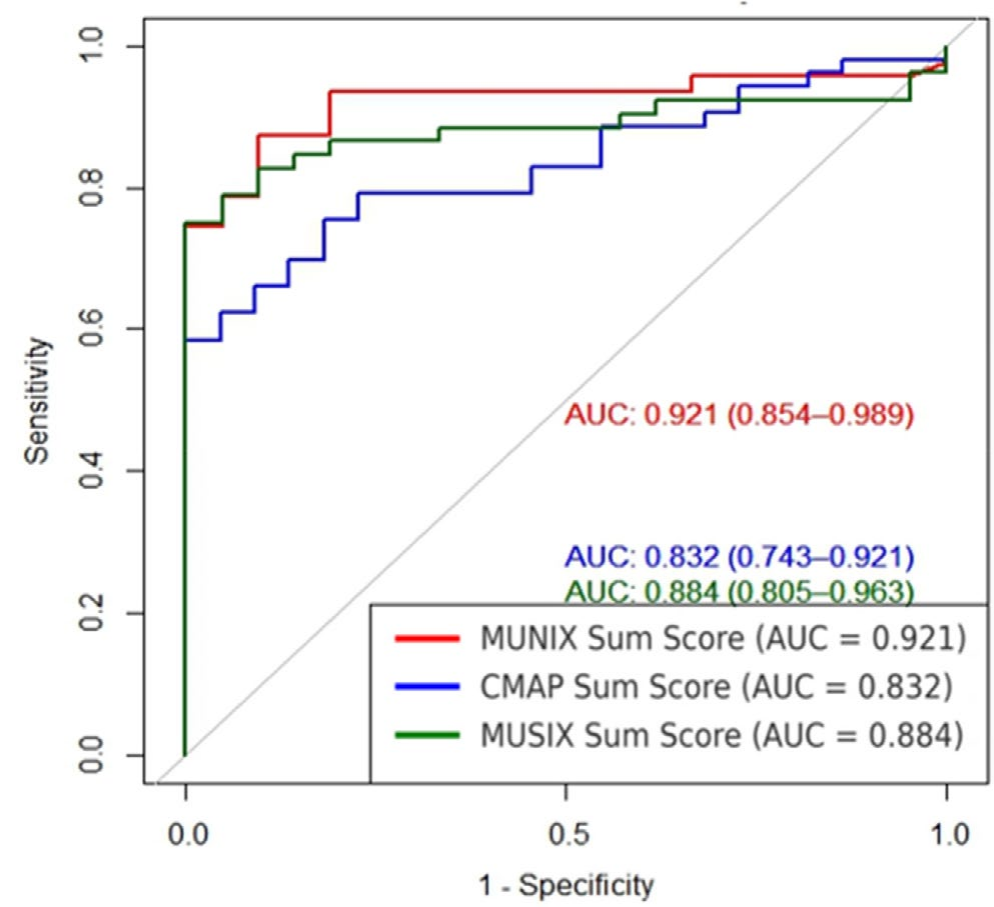
**FIGURE 1** ROC curves of Sum Scores (MUNIX, CMAP, MUSIX) discriminating patients with SMA from healthy controls.

**Figure:**
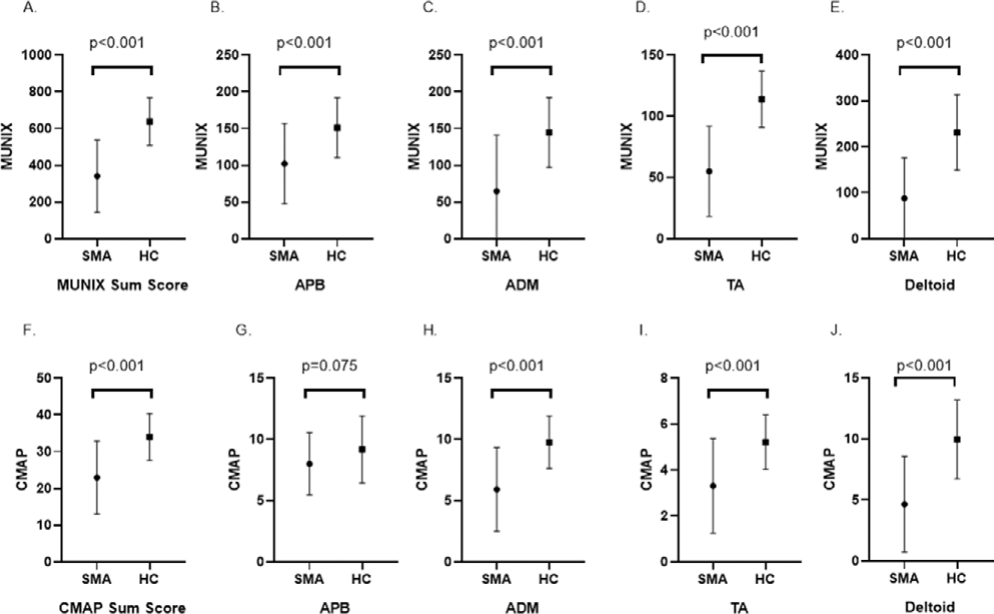
**FIGURE 2** MUNIX and CMAP differences between patients with SMA and healthy volunteers (HC). Upper row shows MUNIX differences and lower row CMAP differences; for MUNIX Sum Scores, CMAP Sum Scores, and MUNIX and CMAP individual muscle scores.

**Figure:**
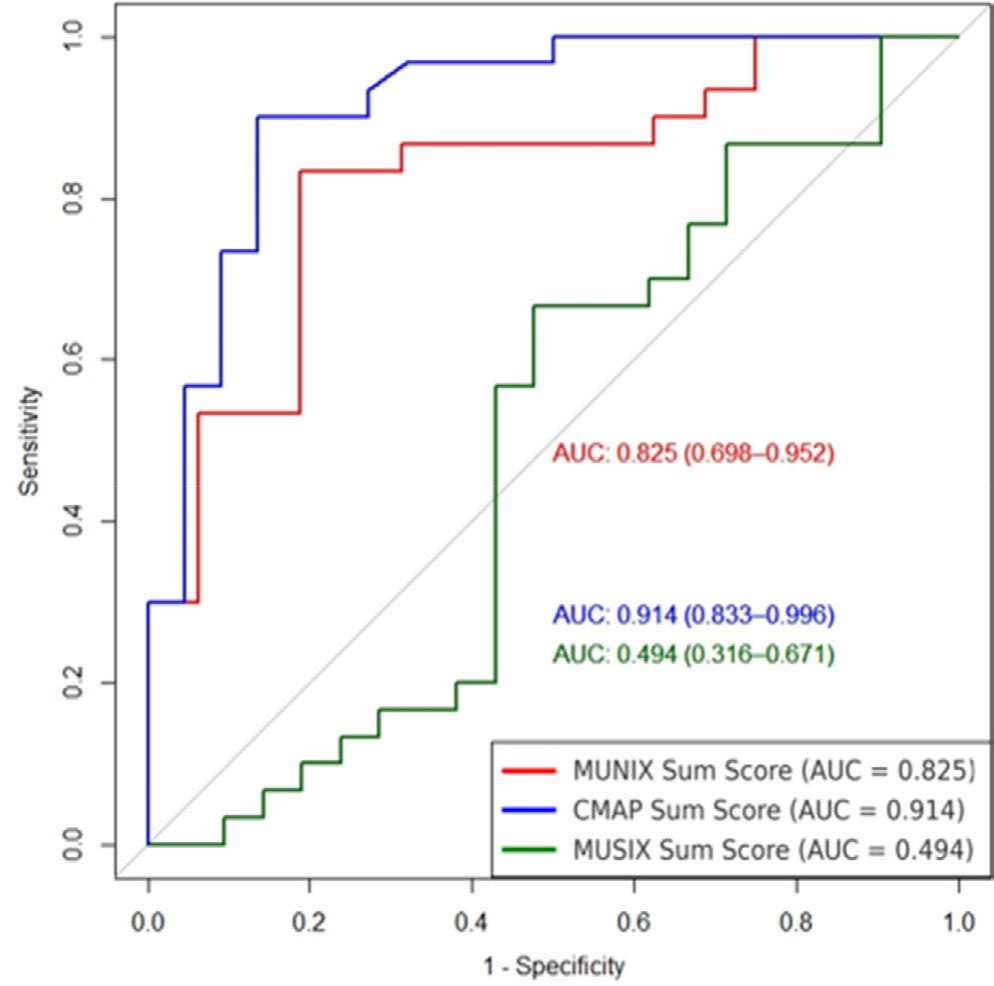
**FIGURE 3** ROC curves of MUNIX, CMAP, and MUSIX Sum Scores discriminative performances between ambulant and non‐ambulant patients with SMA.

**Conclusion:** MUNIX was the most discriminative biomarker for differentiating SMA from controls, detecting motor unit loss even when CMAP remained within normal limits. CMAP better reflected overall disease burden, likely because it integrates both motor unit loss and reinnervation. These complementary profiles support the concurrent use of MUNIX and CMAP in adult SMA. Longitudinal studies are needed to determine their responsiveness and suitability as endpoints in future clinical trials.

**Disclosure:** This study was supported by Biogen. The funder had no role in the design, data collection, analysis, interpretation of data, or in the writing of the manuscript.

## EPO‐0305

### Assessment of sustained health‐related quality of life in Phase 3 Vivacity‐MG3 Trial of Nipocalimab versus Placebo in Generalized Myasthenia Gravis

#### J. Vissing ^1^; K. Gandhi^2^; S. Pease^3^; I. Turkoz^4^; M. Fitzgibbon^3^; G. Coteur^5^; W. Karmous^6^; N. Campbell^2^; S. Ramchandren^4^; E. Cortés‐Vicente^7^

##### ^*1*^ *Department of Neurology, University of Copenhagen, Copenhagen, Denmark;* ^*2*^ *Johnson & Johnson, Horsham, USA;* ^*3*^ *Johnson & Johnson, Raritan, NJ, USA;* ^*4*^ *Johnson & Johnson, Titusville, NJ, USA;* ^*5*^ *Johnson & Johnson, Horsham, PA, USA & IPATH Solutions, Wemmel, Belgium;* ^*6*^ *Johnson & Johnson, Issy‐les‐Moulineaux, France;* ^*7*^ *Unitat Patologia Neuromuscular, Neurology Service, Hospital Santa Creu i Sant Pau, Barcelona, Spain*

**Background and aims:** Generalized myasthenia gravis (gMG), an autoantibody‐mediated disease causes fluctuating muscle symptoms and substantially impairs health‐related quality‐of‐life (HRQoL). In Vivacity‐MG3 (NCT04951622), nipocalimab+standard‐of‐care (SOC) demonstrated improved and sustained disease control versus placebo+SOC. HRQoL changes during the 24‐week (W) double blind phase were evaluated using patient reported MGQoL‐15r and EQ‐5D visual analogue scale (EQ‐5D‐VAS).

**Methods:** Mean change‐from‐baseline (CFB) between groups in MGQoL‐15r and EQ‐5D‐VAS over 24W were compared using ANCOVA models. Meaningful‐within‐person‐improvement (MWPI) response was a change of one‐half a standard deviation on MGQoL‐15r (> = 4‐points) and EQ‐5D‐VAS (> = 10‐points). Proportion of patients achieving MWPI and time‐to‐MWPI over 24W were examined using Chi‐square test and Kaplan‐Meier analyses. Logistic regression evaluated likelihood of achieving MWPI over 24W and sustaining MWPI for > = 8, 12, 16, and 20W between groups.

**Results:** LS‐mean (95% CI) difference in CFB on MGQoL‐15r and EQ‐5D‐VAS was greater with nipocalimab+SOC versus placebo+SOC (−1.38 [−3.14, 0.37], not significant [ns] versus 6.86 [2.07, 11.66], *p* < 0.05, respectively) at 4W and sustained (−1.61 [−3.55, 0.33], ns; 6.47 [1.05, 11.88], *p* < 0.05) at 24W. At 24W, 67.2% (45/67) versus 56.5% (35/62) achieved MWPI on MGQoL‐15r (absolute difference = 10.7%[ns]) and 55.2% (37/67) versus 37.1% (23/62) patients achieved MWPI on EQ‐5D‐VAS (absolute difference = 18.1%, [*p* < 0.05]) in nipocalimab+SOC versus placebo+SOC. For both measures, nipocalimab+SOC patients were twice more likely to achieve MWPI over 24W with median time‐to‐response approximately 4W versus 8W with placebo+SOC (*p* < 0.05). Similarly, nipocalimab+SOC patients were approximately twice more likely to sustain MWPI for > = 8, 12, 16, 20W (*p* < 0.05).

**Conclusion:** Nipocalimab+SOC‐treated patients achieved meaningful HRQoL improvements as early as 4W and were significantly more likely to achieve and sustain these improvements over 24W versus placebo+SOC.

**Disclosure:** This work was funded by Johnson & Johnson. John Vissing: Consultant on advisory boards or received speaker honoraria related to myasthenia gravis from Roche, Regeneron, Toleranzia, Hansa Biopharma, argenX BVBA, UCB Biopharma SPRL, Amgen, Dianthus Therapeutics, NMD Pharma, Alexion Pharmaceuticals, Novartis Pharma AG, Johnson & Johnson and is principal investigator on trials in myasthenia gravis for Roche, argenX BVBA, Toleranzia, Hansa Biopharma, Novartis Pharma AG, Alexion Pharmaceuticals, UCB Biopharma SPRL, Regeneron, Johnson & Johnson, Dianthus Therapeutics. Kavita Gandhi, Sheryl Pease, Ibrahim Turkoz, Marie Fitzgibbon, Wisam Karmous, Nolan Campbell, and Sindhu Ramchandren: Employees of Johnson & Johnson may hold stocks/stock options in Johnson & Johnson. Geoffroy Coteur: Contractor for Johnson and Johnson. Elena Cortés‐Vicente: Received consulting/advisory from argenX BV, Alexion Pharmaceuticals Inc, Janssen Pharmaceuticals Inc, UCB Pharma SA.

## EPO‐0306

### Safety and effectiveness of efgartigimod in Japanese patients with generalized myasthenia gravis by serostatus: Post‐marketing surveillance analysis

#### H. Teranishi ^1^; R. Aoyagi^1^; D. Harada^1^; M. Watanabe^2^

##### ^*1*^ *argenx Japan K.K, Tokyo, Japan;* ^*2*^ *Kyushu University Hospital,* Fukuoka, Japan

**Background and aims:** Efgartigimod for intravenous infusion (efgartigimod‐IV) is approved in Japan for generalized myasthenia gravis (gMG) regardless of auto‐antibody profiles. Post‐marketing surveillance for efgartigimod‐IV in gMG was performed to assess the safety and effectiveness in real‐world settings in Japan.

**Methods:** Patients with gMG who received efgartigimod‐IV between May 2022 and September 2023 were registered. Effectiveness was evaluated by Myasthenia Gravis‐Activities of Daily Living (MG‐ADL) total and subdomain (ocular, bulbar, limb/gross motor, respiratory) scores. The change of oral glucocorticoid dose was also evaluated.

**Results:** The safety analysis set consisted of 469 patients: 57.1% (*n* = 268) anti‐acetylcholine receptor antibody positive (AChR‐Ab+), 13.4% (*n* = 63) anti‐muscle‐specific receptor kinase antibody positive (MuSK‐Ab+), and 29.4% (*n* = 138) double‐seronegative. Adverse drug reaction and serious adverse drug reaction were reported in 22.6% and 4.2% of patients, respectively. No specific safety signals were observed in any of the serological profiles. The effectiveness analysis set consisted of 307 patients with a similar composition ratio of serological profile to the safety analysis set. After three weeks from the first administration, mean MG‐ADL total score improved from 7.2 to 4.2: −3.0 points (standard deviation: 2.98, *p* < 0.001). Substantial improvements were observed in all subdomain scores regardless of serological profiles. Mean dose of oral glucocorticoid (standard deviation) (mg/day) at baseline and one year later were 10.7 (7.42) and 7.7 (5.41), respectively.

**Conclusion:** In real‐world settings, efgartigimod‐IV was well tolerated and effective in patients with gMG regardless of serological profiles. Oral glucocorticoid dose reduction was observed after efgartigimod administration.

**Disclosure:** This surveillance was funded by argenx Japan K.K. The writing support was also funded by argenx Japan K.K. Hirofumi Teranishi, Ryohei Aoyagi, and Daisuke Harada are fulltime employees of argenx Japan K.K. Mitsuru Watanabe has received speakers' honoraria, manuscript fee, or research support from argenx Japan K.K, Alexion Pharma, Novartis Pharma, UCB Japan, Johnson&Johnson, Mitsubishi Tanabe Pharma, Chugai Pharmaceutical, Biogen Japan, Viatris, Daiichi Sankyo, and Takeda Pharmaceutical.

## EPO‐0307

### Efficacy and tolerability of nipocalimab in patients with generalized myasthenia gravis: Real‐world experience from an Academic Center

#### J. Greenberg; M. Seyam; S. Muppidi; N. Goyal

##### Department of Neurology and Neurological Sciences, Stanford University, Redwood City, USA

**Background and aims:** Nipocalimab, a neonatal Fc receptor (FcRn) inhibitor, was approved in April 2025 for treatment of antibody positive generalized myasthenia gravis (gMG). However, data from clinical practice is limited. Here we describe a single academic institution's experience with nipocalimab.

**Methods:** A retrospective review of 11 gMG patients who received nipocalimab between May–November 2025 was conducted. Data was collected regarding demographics, comorbidities, antibody status, treatment history, Myasthenia Gravis Activities of Daily Living (MG‐ADL) scores, side effects, and corticosteroid dose.

**Results:** Most patients were antibody positive (*n* = 9 acetylcholine receptor, *n* = 1 muscle‐specific tyrosine kinase, *n* = 1 seronegative). Mean baseline MG‐ADL was 8 (±3.98). In all patients, nipocalimab was started due to refractory symptoms and/or intolerable side effects with prior treatments. MG‐ADL scores improved by a mean of 1.9 points (*p* = 0.017), and 63.6% (7/11) of patients had a clinically significant improvement (reduction ≥ 2 in MG‐ADL score) at follow up 10.6 (±4.7) weeks after treatment initiation. Nine patients previously failed one or more FcRN inhibitors (*n* = 6 efgartigimod, *n* = 1 rozanolixizumab, *n* = 2 both) due to inefficacy (*n* = 2) or wearing off between cycles (*n* = 7). 77.8% (7/9) tolerated nipocalimab, and the reduction in MG‐ADL score remained statistically significant (*p* = 0.034). Only 1 patient reported wearing off. 100% (7/7) of patients on corticosteroids successfully lowered or discontinued dosage without symptom worsening.

**Conclusion:** We found nipocalimab to be effective, fast‐acting, and well tolerated in patients with refractory gMG, leading to modest clinical improvement and steroid tapering in most patients. Nipocalimab was effective in patients who had previously failed other FcRn inhibitors and wearing off between doses was uncommon.

**Disclosure:** Dr. Greenberg has no disclosures. Dr. Seyam has no disclosures. Dr. Muppidi has served on the advisory board for Alexion, UCB, Johnson & Johnson, Amgen, Kyverna, and Arcellex. Dr. Goyal has served as a consultant for Argenx, Alexion, UCB, Janssen, Amgen, Novartis, Immunovant, Dianthus, Annexon, Seismic, Cartesian, EMD Serono, and has grant funding from Argenx.

## EPO‐0308

### Progression and exacerbation timing in myasthenia gravis: Real‐world evidence from France (RELIEF study)

#### T. Gendre^1^; D. Theis^2^; E. Guyot^3^; M. Nolin^3^; M. Belhassen^3^; I. Rodriguez^4^; J. Meijer ^4^; L. Gautier^4^; J. Dupin^4^; J. Noury^5^

##### ^*1*^ *Département de Neurologie, APHP, CHU Henri Mondor, Créteil, France;* ^*2*^ *Département d'information médicale, CHU de Lille, Lille, France;* ^*3*^ *Epimentis (PELyon), Lyon, France**;*** ^*4*^ *J&J Innovative Medicine, Issy‐les‐Moulineaux, France;* ^*5*^ *Centre de Référence Des Maladies Neuromusculaires AOC, CHRU de Brest, Brest, France*

**Background and aims:** Understanding the timing of progression and acute events in myasthenia gravis (MG) is critical for optimizing care. This study aimed to estimate the time‐to‐progression from MG to its generalized (gMG) and refractory forms, and to assess the time‐to‐first exacerbation or crisis.

**Methods:** Adults with incident MG between 2014 and 2022 were identified in the French health claims database (SNDS) using algorithms based on ICD‐10 codes and treatment patterns, including first‐ and second‐line maintenance therapies and crisis treatments (IV immunoglobulins or plasma exchange). Patients were classified as MG, gMG, or refractory gMG accordingly. Exacerbations were defined as first acute immunoglobulin/plasma exchange, hospitalization for MG or MG crisis; crises included hospitalization with respiratory failure, intubation, ventilation, or enteral feeding. Kaplan‐Meier analyses estimated progression and first exacerbation/crisis event timing.

**Results:** Among 10,284 MG patients (49.3% males, mean age: 61.3 ± 18.2) the probability of gMG diagnosis was 49.0% within 12 months and 60.9% within 60 months (MST: 413 days [369; 459]) (see Figure 1). Of 6,905 gMG patients, the probability of refractory disease was 19.8% at 12 months and 34.1% at 60 months (see Figure 2). In MG patients, probability of exacerbation was 55.1% [54.1; 56.1] at 12 months and 63.5% [62.5;64.5] at 60 months (probability of crisis: 6.1% [5.6;6.6] and 10.0% [9.4;10.7], respectively).

**Figure:**
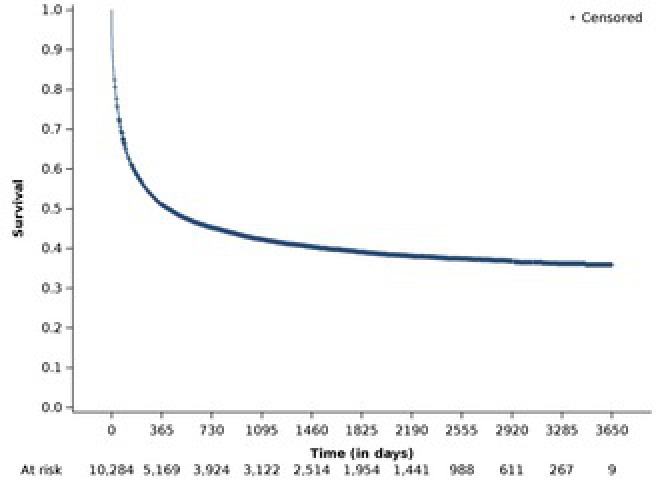
**FIGURE 1** Kaplan Meier curve for the occurrence of gMG in the incident population of MG adults with index date between 2014 and 2022 and at least one year of FU available (except in case of death).

**Figure:**
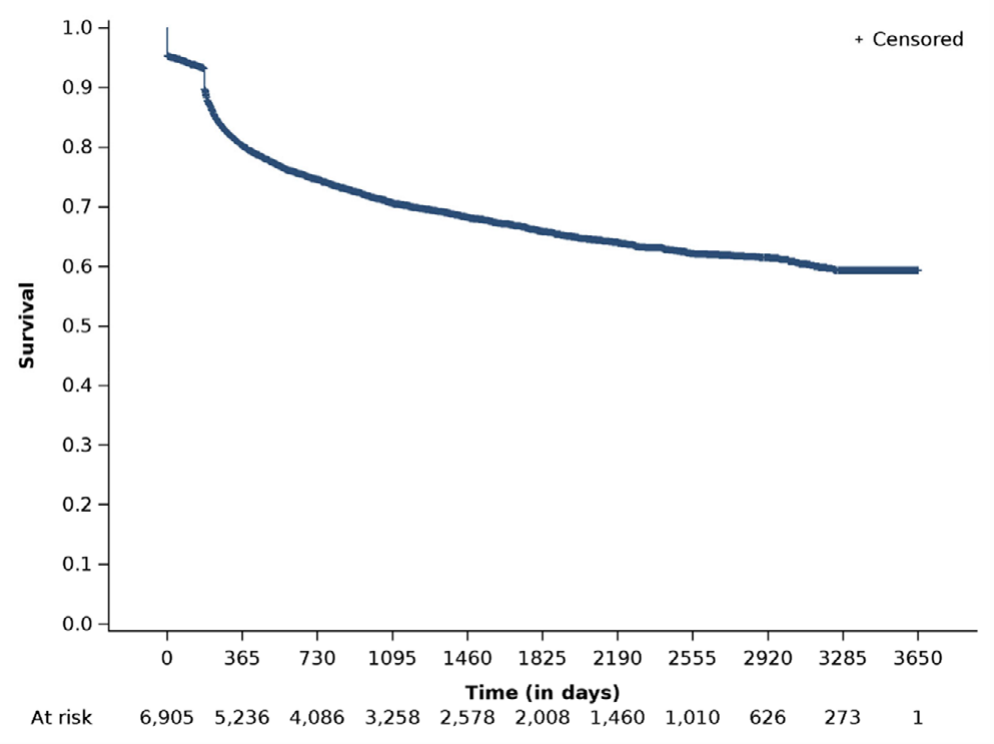
**FIGURE 2** Kaplan Meier curve for the occurrence of refractory gMG in the incident population of gMG adults with first diagnosis of gMG between 2014 and 2022 and at least one year of FU available (except in case of death).

**Conclusion:** These real‐world findings demonstrate rapid progression to gMG in majority of patients, with a significant proportion of these developing refractory disease. Most also experience exacerbation (including crisis) within one year of diagnosis, underscoring the need for early and sustained disease control.

**Disclosure:** Thierry Gendre has received consultancy fees from J&J, UCB, and Amgen; payments for oral communications from Argenx and CSL Behring; supports for congress from Alexion. Didier Theis, and Jean‐Baptiste Noury have received consultancy fees from J&J. Erika Guyot, Maeva Nolin, and Manon Belhassen are employees of Epimentis (PELyon). Ingrid Rodriguez, Julia Meijer, Laurène Gautier, and Julien Dupin are employees of J&J Innovative Medicine. This study was funded by J&J Innovative Medicine.

## EPO‐0309

### One‐year burden of crises and exacerbations in myasthenia gravis: Real‐world evidence from France (RELIEF Study)

#### J. Noury^1^; D. Theis^2^; E. Guyot^3^; M. Nolin^3^; M. Belhassen^3^; I. Rodriguez^4^; J. Meijer ^4^; L. Gautier^4^; J. Dupin^4^; T. Gendre^5^

##### ^*1*^ *Centre de Référence Des Maladies Neuromusculaires AOC, CHRU de Brest, Brest, France;* ^*2*^ *Département d'information médicale, CHU de Lille, Lille, France;* ^*3*^ *Epimentis (PELyon), Lyon, France**;*** ^*4*^ *J&J Innovative Medicine, Issy‐les‐Moulineaux, France;* ^*5*^ *Département de Neurologie, APHP, CHU Henri Mondor, Créteil, France*

**Background and aims:** Acute events drive the short‐term burden in myasthenia gravis (MG). This study evaluated the one‐year risk of MG crises (MGc) and exacerbations (MGe) after diagnosis of MG and its generalized form (gMG), and characterized the nature of these acute episodes.

**Methods:** This French health claims database (SNDS) study included patients diagnosed with MG or gMG in 2022 using algorithms based on ICD‐10 codes and treatment dispensing, including first‐ and second‐line maintenance therapies and crisis treatments (IVIg or plasma exchange). MGc were defined as hospitalization with respiratory failure, intubation, mechanical ventilation, or enteral feeding. MGe included MGc, acute IVIg/plasma exchange, or MG hospitalization. Analyses reported patients with ≥ 1 event within one year, incidence rates per 100 patient‐years (100PY) [95% CI], and event components.

**Results:** There were 1,197 patients with MG diagnosed in 2022 (50.7% males, age: 62.4 ± 18.0), and 859 patients with gMG. MGc occurred in 76 MG (6.3%) and 89 gMG (10.4%) patients. MGe affected 685 MG (57.2%) and 619 gMG (72.1%) patients. In MG, the incidence rates were 6.8/100PY [5.4;8.5] for MGc and 117.7/100PY [109.2;126.8] for MGe, while they were11.7/100PY [9.5;14.5] and 236.3/100PY [218.4;255.6], respectively in gMG. Of MG patients with MGc, 81.6% had mechanical ventilation, 46.1% intubation, and 44.7% respiratory failure.

**Conclusion:** MGe are frequent in the first year following the diagnosis, particularly in gMG patients. MGc is a life‐threatening acute event whose incidence is not negligible in the first year. These results highlight the importance of early prevention and prompt therapeutic intervention in gMG patients.

**Disclosure:** Jean‐Baptiste Noury and Didier Theis have received consultancy fees from J&J. Thierry Gendre has received consultancy fees from J&J, UCB, and Amgen; payments for oral communications from Argenx and CSL Behring; supports for congress from Alexion. Erika Guyot, Maeva Nolin, and Manon Belhassen are employees of Epimentis (PELyon). Ingrid Rodriguez, Julia Meijer, Laurène Gautier, and Julien Dupin are employees of J&J Innovative Medicine. This study was funded by J&J Innovative Medicine.

## EPO‐0310

### Efficacy of telitacicept in gMG: Domain‐specific and thymoma‐associated outcomes from a Phase 2 trial

#### M. Zhao ^1^; J. Yin^1^; X. Liu^1^; J. Zhang^1^; C. Liu^1^; M. Zhang^2^; Z. Xu^3^; Z. Li^4^; X. Qin^5^; Z. Li^6^

##### ^*1*^ *Department of Neurology, Beijing Hospital, National Center of Gerontology, Institute of Geriatric Medicine, Chinese Academy of Medical Sciences, China;* ^*2*^ *Department of Neurology, First Hospital of Shanxi Medical University, China;* ^*3*^ *Department of Neurology, Affiliated Hospital of Zunyi Medical University, China;* ^*4*^ *Department of Neurology, Xi'an Gaoxin Hospital, China;* ^*5*^ *Department of Neurology, First Affiliated Hospital of Chongqing Medical University, China;* ^*6*^ *Department of Neurology, Tangdu Hospital, Air Force Medical University of PLA (formerly known as the Fourth Military Medical University),* Shaanxi, China

**Background and aims:** This post hoc analysis aimed to evaluate the domain‐specific clinical efficacy of telitacicept and to explore its differential effect in the clinically relevant subgroup of patients with a history of thymoma.

**Methods:** Eligible patients from a phase 2 trial (NCT04302103) were randomized to receive weekly subcutaneous telitacicept at 160 mg (*n* = 14) or 240 mg (*n* = 15). Key changes from baseline to Week 24 in the total Quantitative Myasthenia Gravis (QMG) score and its bulbar, ocular, limb, and respiratory subdomain scores were analyzed. Outcomes were also assessed based on thymoma history.

**Results:** Both doses induced early (from Week 4) and sustained improvements across all QMG domains. The 240‐mg dose resulted in a significantly greater improvement in ocular scores at Weeks 8 and 12 compared to the 160‐mg dose (*p* = 0.037). Patients with a history of thymoma (*n* = 14) showed a numerically greater reduction in the total QMG score than those without (*n* = 15). The 240‐mg dose demonstrated superior efficacy regardless of thymoma status.

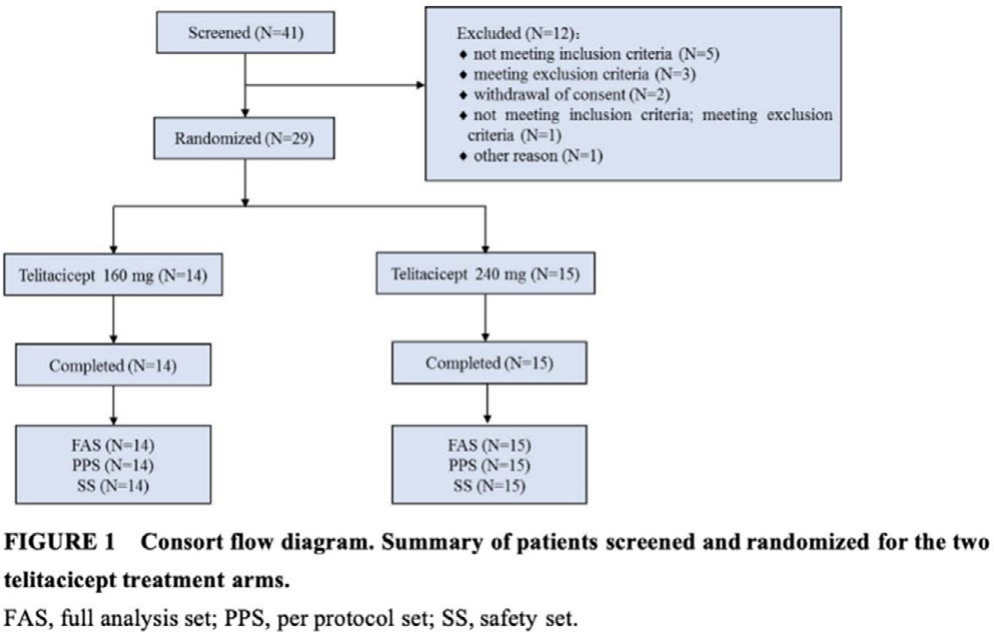

**Figure:**
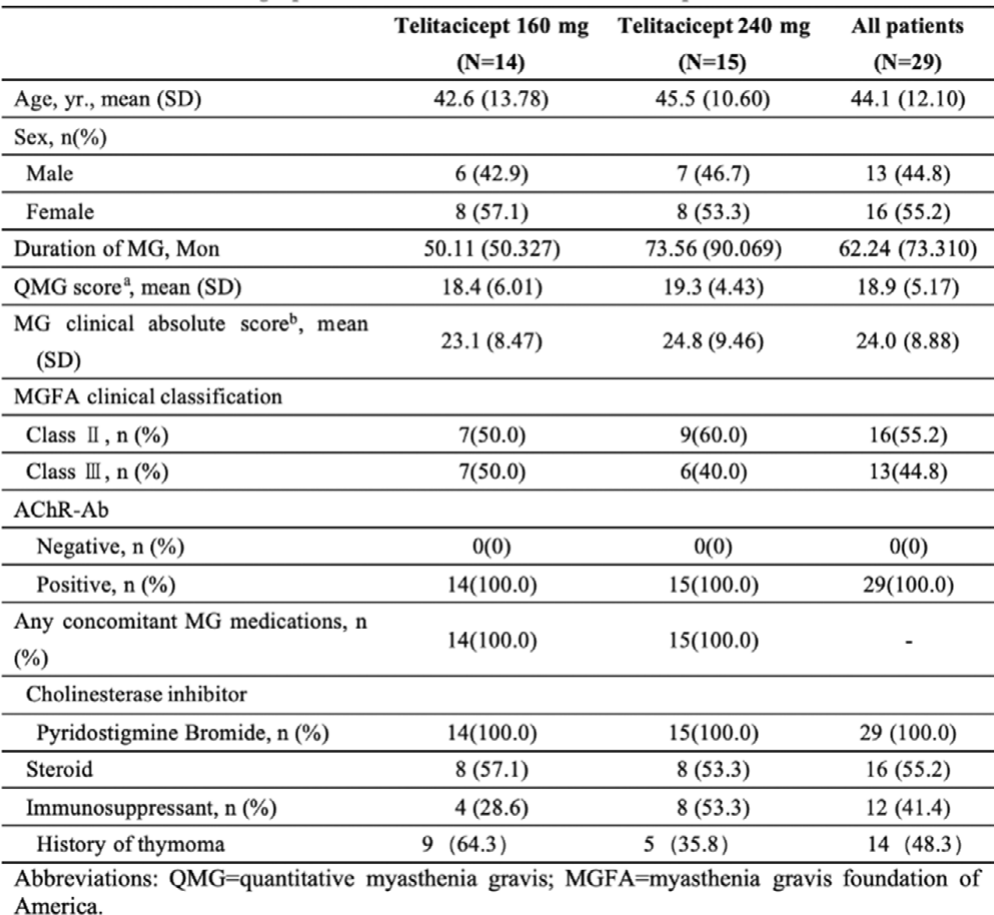
**TABLE 1** Demographics and clinical characteristics of the patients at baseline.

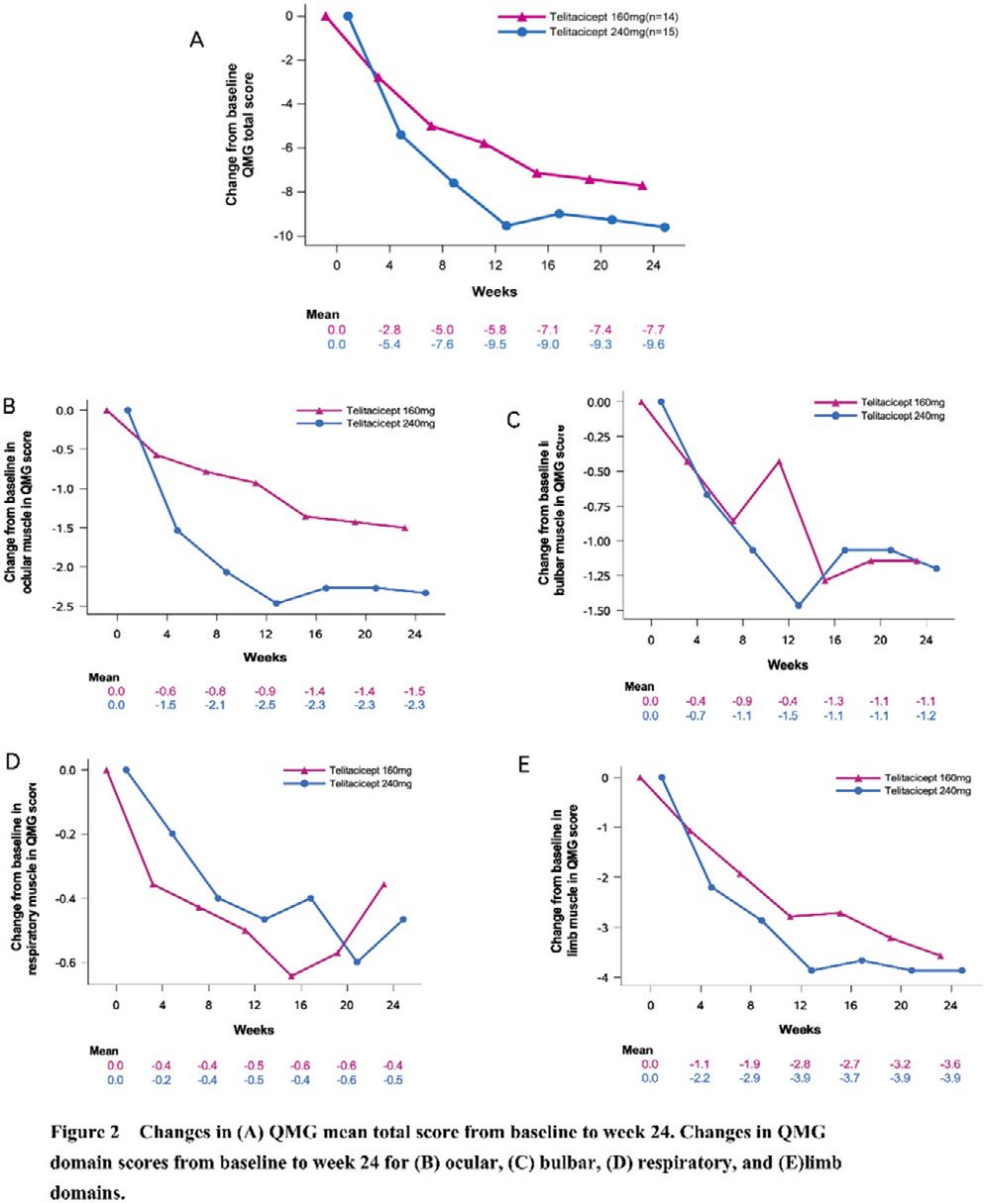

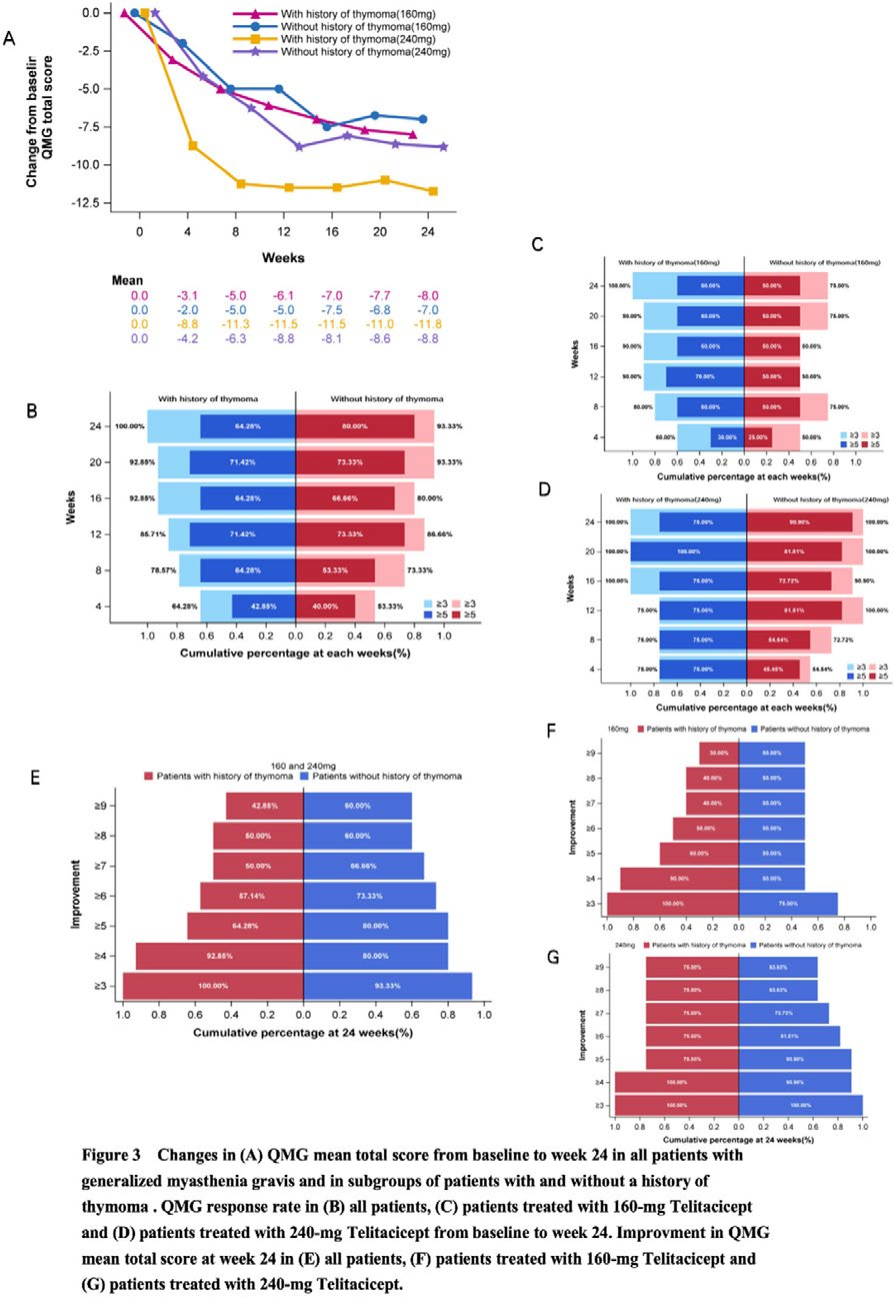

**Conclusion:** Telitacicept provides rapid and sustained improvement in muscle strength across all domains in generalized myasthenia gravis. The 240‐mg dose appears particularly beneficial for ocular involvement and in patients with a history of thymoma. These promising subgroup findings warrant further investigation in larger confirmatory studies.

**Disclosure:** Nothing to disclose.

## EPO‐0311

### Efficacy of long‐term rozanolixizumab treatment cycles in patients with generalised myasthenia gravis: A subgroup analysis of Phase 3 studies

#### S. Sacconi ^1^; G. Antonini^2^; E. Cortés Vicente^3^; A. Drużdż^4^; A. Habib^5^; Z. Mahuwala^6^; R. Mantegazza^7^; R. Pascuzzi^8^; K. Utsugisawa^9^; J. Vissing^10^; T. Vu^11^; P. Mahoney^12^; T. Tarancón^13^; V. Bril^14^

##### ^*1*^ *Université Côte d'Azur, Peripheral Nervous System & Muscle Department, Pasteur 2 Hospital, Centre Hospitalier Universitaire de Nice, Nice, France;* ^*2*^ *Department of Neuroscience, Mental Health and Sensory Organs (NESMOS), Sapienza University of Rome, Rome, Italy;* ^*3*^ *Neuromuscular Diseases Unit, Hospital de la Santa Creu i Sant Pau, Barcelona, Spain;* ^*4*^ *Department of Neurology, Municipal Hospital, Poznań, Poland;* ^*5*^ *MDA ALS & Neuromuscular Center, Department of Neurology, University of California, Irvine, USA;* ^*6*^ *Department of Neuromuscular Medicine, Epilepsy and Clinical Neurophysiology, University of Kentucky, Lexington, USA;* ^*7*^ *Emeritus and Past Director, Department of Neuroimmunology and Neuromuscular Diseases, Fondazione IRCCS, Istituto Nazionale Neurologico Carlo Besta, Milan, Italy;* ^*8*^ *Neurology Department, Indiana University School of Medicine, Indiana University Health, Indianapolis, USA;* ^*9*^ *Department of Neurology, Hanamaki General Hospital, Hanamaki, Japan;* ^*10*^ *Copenhagen Neuromuscular Center, Department of Neurology, Rigshospitalet, University of Copenhagen, Copenhagen, Denmark;* ^*11*^ *Department of Neurology, University of South Florida Morsani College of Medicine, Tampa, USA;* ^*12*^ *UCB, Slough, UK;* ^*13*^ *UCB, Madrid, Spain;* ^*14*^ *Ellen and Martin Prosserman Centre for Neuromuscular Diseases, Toronto General Hospital, University of Toronto, Toronto, Ontario, Canada*

**Background and aims:** In MycarinG (Phase 3; NCT03971422), one 6‐week cycle of rozanolixizumab (7 mg/kg or 10 mg/kg) improved myasthenia gravis (MG)‐specific outcomes versus placebo. Here, we evaluate the long‐term efficacy of repeated rozanolixizumab treatment cycles across MycarinG and its open‐label extension studies in subgroups of patients with generalised MG (gMG).

**Methods:** Following MycarinG, patients could enrol in MG0004 (NCT04124965) to receive once‐weekly rozanolixizumab for < = 52 weeks, or MG0007 (NCT04650854), which comprised an initial 6‐week rozanolixizumab cycle, with further cycles administered upon symptom worsening. Final efficacy data were pooled across MycarinG, MG0004 (first 6 weeks) and MG0007 for patients with > = 2 symptom‐driven rozanolixizumab cycles. Mean change from baseline (CFB) in MG Activities of Daily Living (MG‐ADL) score to Day 43 in Cycles 1–13 was assessed. Subgroups were prespecified according to baseline characteristics.

**Results:** Overall, 129 patients received > = 2 symptom‐driven cycles. Mean (standard deviation) CFB in MG‐ADL score at Day 43 across Cycles 1–13 for the overall population ranged from −3.2 (3.3) in Cycle 3 (*n* = 113) to −6.0 (3.9) in Cycle 12 (*n* = 24). Improvements from baseline in MG‐ADL scores at Day 43 were consistent in subgroups of autoantibody status (anti‐muscle‐specific tyrosine kinase and anti‐acetylcholine receptor antibody positive), disease duration (< 5.4 and > = 5.4 years), age (< 65 and > = 65 years), MG‐ADL score (< 5 and > = 5) and thymectomy status. Treatment‐emergent adverse events occurred in 93.1% (*n* = 175/188) of patients with > = 1 treatment cycle across MycarinG and MG0007; most were mild/moderate.

**Conclusion:** Repeated rozanolixizumab treatment cycles demonstrated long‐term efficacy in a broad range of patients with gMG.

**Disclosure:** This study was funded by UCB. Full disclosure of all industry relationships will be made during congress presentation if accepted.

## EPO‐0312

### Aspects of molecular genetic polymorphism of the dystrophin gene in Uzbekistan

#### U. Omonova; I. Akhmedov

##### Tashkent State Medical University, Tashkent, Uzbekistan

**Background and aims:** To investigate the genetic polymorphism of the dystrophin gene among patients with Duchenne muscular dystrophy and to determine the frequency of dystrophin gene mutation carriage among female relatives in families affected by DMD in the population of Uzbekistan.

**Methods:** Molecular genetic studies were conducted MLPA diagnostics to detect deletions and duplications in patients with DMD. MLPA diagnostics was performed in 91 patients with DMD from 81 families The analysis covered all 79 exons of the dystrophin gene.

**Results:** deletions were not detected in 33 patients (36.3%) from 32 families (39.5%), while deletions of the dystrophin gene of varying length—from one to nine exons—were identified in 58 patients (63.7%) from 49 families (60.5%). Extended deletions were verified in 65.3% of families, whereas single‐exon deletions were observed in 34.7% of families. The main deletion spectrum was located in the distal part of the dystrophin gene at the 3′ end (deletions of exons 40–60), accounting for 81.6% (40 families, 47 patients).

**Conclusion:** According to MLPA diagnostics in patients with Duchenne muscular dystrophy, mutations were identified in 16 of the 79 analyzed exons, which provides a basis for using the DMD‐del1 and DMD‐del2 kits for the diagnosis of “major” deletions in the dystrophin gene in the population of Uzbekistan.

**Disclosure:** Nothing to disclose.

## Neurogenetics 2

## EPO‐0313

### Binaural hearing in spinocerebellar ataxia type 3

#### A. Bazzoli ^1^; A. Rupp^1^; H. Jacobi^2^

##### ^*1*^ *Sektion für Biomagnetismus, Neurologische Klinik Heidelberg, Germany;* ^*2*^ *Neurologische Klinik, Heidelberg, Germany*

**Background and aims:** Brainstem degeneration in spinocerebellar ataxia type 3 (SCA3) affects binaural structures such as the medial superior olive (Faber et al, 2021), potentially impairing binaural processing. Huggins Pitch (HP), a psychoacoustic phenomenon dependent on binaural synchronization, may thus serve as a marker of early dysfunction. Previously, we demonstrated that the upper limit of Huggins Pitch (HP) differed significantly between SCA3 mutation carriers and age‐matched controls (Jacobi et al, 2024). Here, we investigated longitudinal HP processing in 18 SCA3 mutation carriers (pre‐ataxic and ataxic) to assess its potential as a biomarker for disease progression.

**Methods:** HP thresholds were determined via a 3‐alternative forced‐choice behavioral task, and cortical N100 responses were recorded with MEG. Ataxia severity was assessed using the SARA scale.

**Results:** HP thresholds declined significantly from baseline to follow‐up (1.8195 vs. 1.4375 kHz; *p* < 0.05), with ataxic carriers showing a stronger decline (1.6511 vs. 1.2976 kHz; *p* = 0.039) and a trend in pre‐ataxic carriers (2.1983 vs. 1.7522 kHz; *p* = 0.09). Thresholds correlated strongly with SARA sum score at follow‐up (*r* = −0.82, *p* < 0.001) and were predicted by time to onset in linear mixed‐effects models. Carriers–control Z‐scores declined significantly over time, and N100 amplitude was reduced compared to controls (*p* < 0.05).Linear mixed‐effects modeling showed progressive HP decline over time for all carriers (*β* = −0.173 kHz/year, *p* = 0.004).

**Figure:**
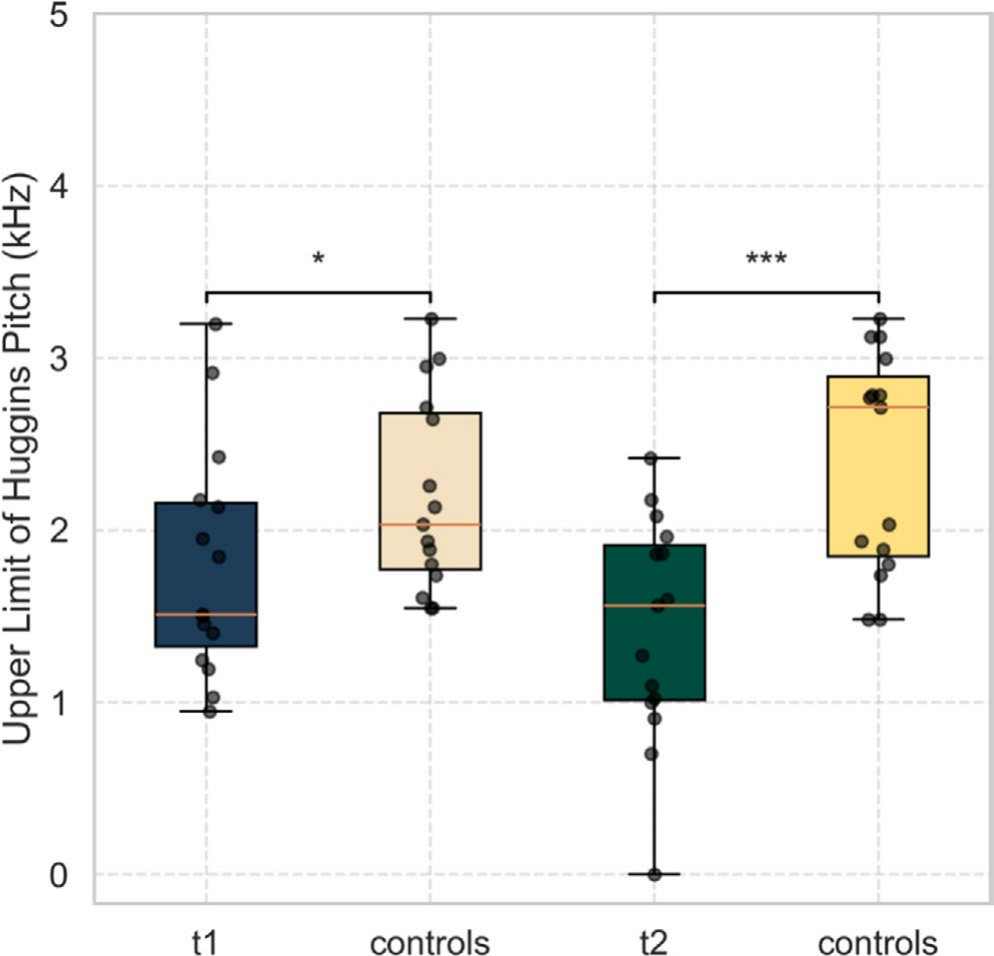
**FIGURE 1.1** Boxplots show Huggins Pitch Thresholds in SCA3 carriers (*N* = 15) vs healthy controls (*N* = 15) at baseline and at follow‐up. **FIGURE 1.2** Boxplot show HP Thresholds in SCA3 carriers at baseline vs follow‐up (1.80 ± 0.67 vs 1.49 ± 0.53 kHz, *p* = 0.019).

**Figure:**
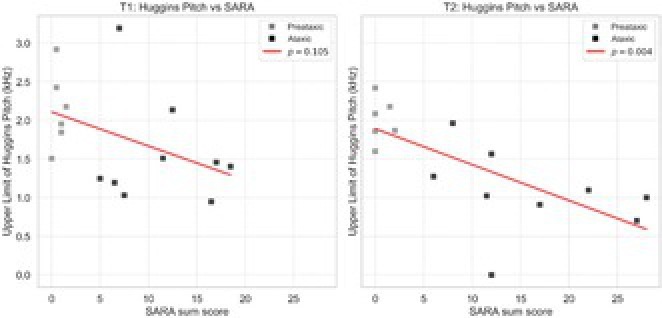
**FIGURE 2** Upper limit of Huggins Pitch frequency (kHz) vs SARA sum score in pre‐ataxic (*n* = 7) and ataxic (*n* = 9) SCA3 mutation carriers at baseline (T1, *r* = −0.44, *p* = 0.105) and at follow‐up (T2, *r* = −0.84, *p* < 0.001).

**Figure:**
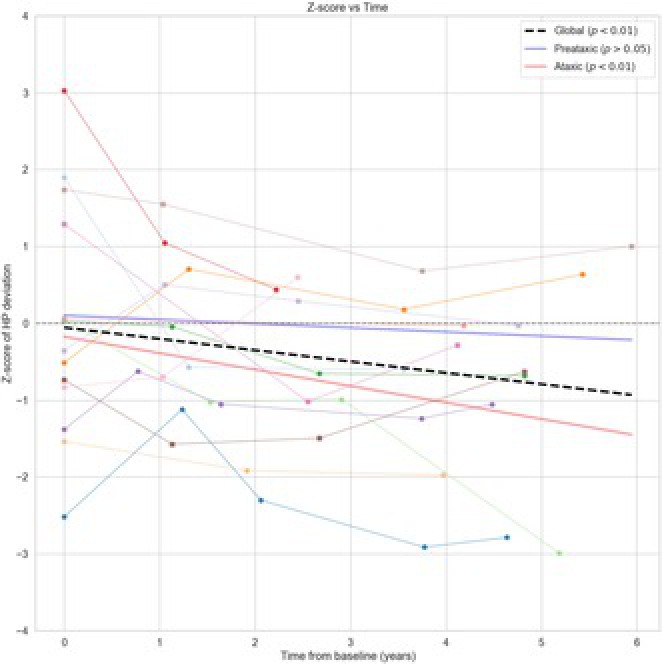
**FIGURE 3** LME of longitudinal development of Carriers–Controls Z‐scores shows a progressive decline over time for all carriers (*β*_1_ = −0.138 ± 0.055, *p* = 0.012), preataxic carriers (*β*_1_ = −0.054 ± 0.079, *p* = 0.499), and ataxic carriers (*β*_1_ = −0.195 ± 0.075, *p* = 0.009.

**Conclusion:** These findings indicate that HP thresholds and cortical responses reliably decline with SCA3 progression, suggesting HP as a sensitive marker for early detection and longitudinal tracking, including in pre‐ataxic mutation carriers.

**Disclosure:** Nothing to disclose.

## EPO‐0314

### KMT2B‐related disorders in Austria: Clinical features and long‐term outcome

#### E. Indelicato

##### Center for Rare Movement Disorders Innsbruck, Department of Neurology, Medical University Innsbruck, Innsbruck, Austria

**Background and aims:** Since its initial description in 2016, DYT‐KMT2B has emerged as one of the most common genetic causes of early‐onset dystonia. Subsequent reports have expanded its phenotypic spectrum, frequently including neurodevelopmental features. Deep brain stimulation of the globus pallidus internus (GPi DBS) has become a therapeutic mainstay; however, most published data are based on single cases or short‐term observations, and long‐term outcomes remain poorly characterized.

**Methods:** We report the clinical course and response to GPi DBS in nine patients with KMT2B variants prospectively followed at two Austrian national reference centers for rare movement disorders. Long‐term follow‐up data (range: 5–20 years) were available for six patients. Clinical features and treatment outcomes were compared with previously published cohorts.

**Results:** Non‐motor features such as developmental delay, intellectual disability, and epilepsy were more frequent in our cohort than in earlier reports. All patients developed generalized dystonia and laryngeal involvement over time, emphasizing the progressive nature of the disease. Despite secondary symptom worsening during long‐term follow‐up, GPi DBS preserved ambulation in three patients and enabled sustained recovery of walking ability in two, maintaining functional independence. Surgical correction of foot deformities further supported mobility. Notably, KMT2B variants were identified upon genetic re‐evaluation in two patients previously diagnosed with dyskinetic cerebral palsy.

**Conclusion:** Our long‐term data underscore the progressive but heterogeneous course of DYT‐KMT2B. GPi DBS offers durable clinical benefits, particularly when initiated before loss of ambulation. Early surgical intervention and multidisciplinary management are essential to optimize long‐term outcomes.

**Disclosure:** Nothing to disclose.

## EPO‐0315

### The first libyan patient diagnosed with POLR3B‐related leukodystrophy: A case study

#### E. Farag ^1^; M. Elshukri^2^

##### ^*1*^ *Ankara University Faculty of Medicine, Ankara, Türkiye;* ^*2*^ *Benghazi Medical Center, Benghazi, Libya*

**Background and aims:** Leukodystrophies are rare inherited white matter disorders with significant clinical and genetic heterogeneity. POLR3B‐related hypomyelinating leukodystrophy is typically diagnosed in childhood, while adult‐onset presentations are rare and often overlooked.

**Methods:** We report a 19‐year‐old Libyan male presenting with a two‐year history of progressive ataxia and tremor. Detailed neurological examination, laboratory testing, neurophysiological studies, brain magnetic resonance imaging (MRI), and genetic analysis were performed. Autoimmune, infectious, paraneoplastic, and post–COVID‐19 etiologies were systematically excluded.

**Results:** Neurological examination demonstrated ataxia, dysarthria, tremor, nystagmus, myoclonus, and impaired coordination. Brain MRI revealed diffuse hypomyelinating white matter abnormalities involving the parieto‐occipital regions, corpus callosum splenium, corticospinal tracts, and cerebellar peduncles without diffusion restriction or contrast enhancement. Genetic testing identified heterozygous variants in the POLR3B gene, confirming the diagnosis of POLR3B‐related hypomyelinating leukodystrophy. The progressive clinical course, characteristic imaging findings, negative immunological and oncological workup, and lack of response to immunotherapy unequivocally excluded post–COVID‐19 neurological involvement.

**Figure:**
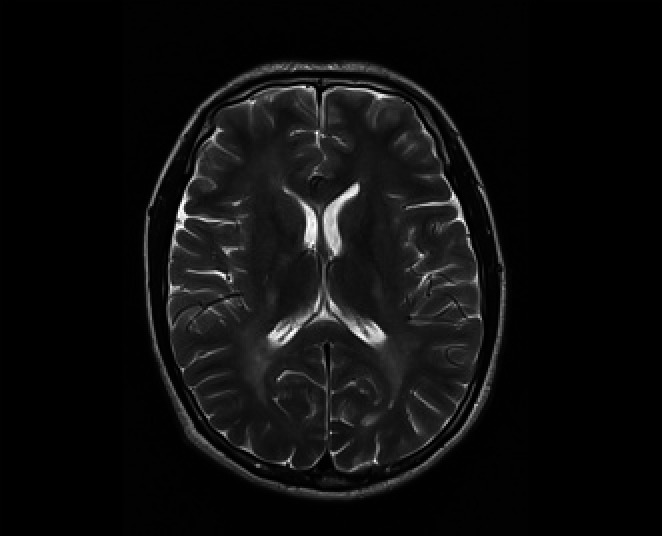
**FIGURE 1** Left/Axial T2‐weighted MRI of the brain showing confluent hyperintense lesions in the parieto‐occipital deep white matter, extending through the splenium of the corpus callosum.

**Figure:**
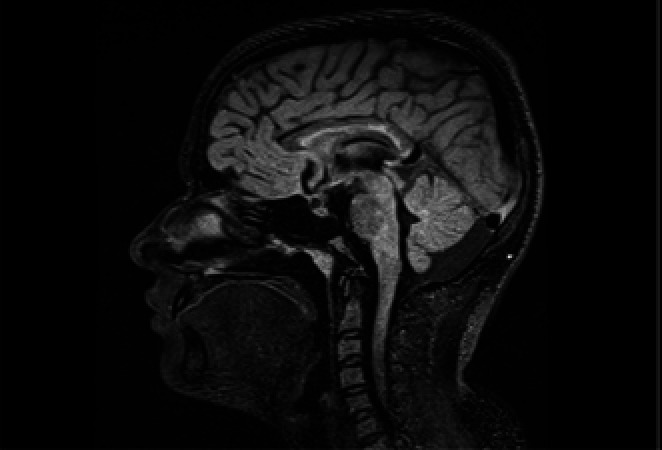
**FIGURE 2** Right/Sagittal T2‐weighted MRI of the brain demonstrating widespread hypomyelination with subtle involvement of the cerebellar peduncles, sparing the cerebellar tonsils.

**Figure:**
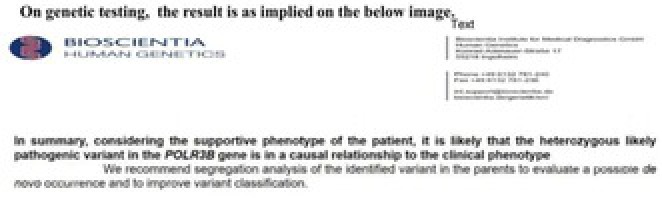
**FIGURE 3** POLR3B gene variant detected on genetic testing.

**Conclusion:** This case highlights the diagnostic challenges of adult‐onset POLR3B‐related leukodystrophy and emphasizes the importance of genetic testing in young adults with progressive ataxia. To our knowledge, this represents the first reported case from Libya.

**Disclosure:** AI Use Disclosure: Artificial intelligence tools were used solely for language editing, grammar correction, and improvement of writing clarity. All data, analyses, interpretations, and conclusions presented in this work are entirely original and were generated by the authors. The use of AI did not influence the study design, data collection, data analysis, or scientific content.

## EPO‐0316

### Genetic landscape of pediatric epilepsy associated with focal cortical dysplasia

#### F. Turdialiyeva

##### Center for the development of professional qualification of medical workers

**Background and aims:** Focal cortical dysplasia is a major structural cause of epilepsy in children and is frequently associated with drug‐resistant seizures. Increasing evidence indicates that genetic factors contribute significantly to the pathogenesis of focal cortical dysplasia–related epilepsy. The objective of this study was to characterize the genetic spectrum of pediatric epilepsy associated with focal cortical dysplasia and to describe associated clinical features.

**Methods:** We analyzed a cohort of 36 children with epilepsy associated with focal cortical dysplasia evaluated at a national tertiary pediatric center between 2021 and 2025. All patients underwent whole‐exome sequencing (WES). Available clinical data, electroencephalography (EEG), and magnetic resonance imaging (MRI) reports were reviewed. Genetic variants were interpreted according to current pathogenicity criteria. Demographic characteristics, including parental consanguinity, were assessed.

**Results:** Whole‐exome sequencing data were available for 100 percent of patients. Pathogenic and likely pathogenic variants were identified in genes associated with epilepsy and cortical development, including SCN1A, DEPDC5, PCDH19, SCN2A, SCN8A, and TSC2. Both ion channel genes and genes related to cortical development and mammalian target of rapamycin pathway regulation were represented. Parental consanguinity was documented in 5.6 percent of cases. MRI reports were available in 77 percent of patients, EEG reports in 58 percent, and detailed clinical discharge summaries in 97 percent.

**Conclusion:** This study demonstrates a heterogeneous genetic landscape underlying pediatric epilepsy associated with focal cortical dysplasia. Whole‐exome sequencing provides valuable diagnostic information even in settings with incomplete neuroimaging or electrophysiological data and supports its integration into routine clinical evaluation.

**Disclosure:** The author reports no conflicts of interest and no external funding related to this study.

## EPO‐0317

### Cerebellar cognitive affective syndrome in hereditary ataxias: A single‐center experience

#### G. Falcone ^1^; S. Drago^2^; I. Arena^1^; C. Rodolico^3^; O. Musumeci^3^

##### ^*1*^ *Department of Biomedical and Dental Sciences and Morphofunctional Images, University of Messina, Messina, Italy;* ^*2*^ *Department of Chemical, Biological Pharmaceutical and Environmental Sciences, University of Messina, Italy;* ^*3*^ *Unit of Neurological and Neuromuscular disorders, Department of Clinical and Experimental Medicine, University of Messina, Italy*

**Background and aims:** The Cerebellar Cognitive Affective Syndrome (CCAS) scale screens for cognitive and affective impairments in cerebellar disorders, but its prevalence in real‐world settings remains underexplored.

**Methods:** The Italian CCAS scale was administered to 68 patients from a single‐center (22 Friedreich ataxia (FA), 13 SCA2, 12 RFC1‐related disease, 4 SCA27B, 4 SPG7, 3 SCA8, 3 SCA42, 2 SCAR32, 2 HSD17B4, 1 each of GSS, PEX2, ATM, and SCA45) and 30 age‐, sex‐, and education‐matched healthy controls. CCAS was classified as definite (≥ 3 failed items), and a total sum score calculated. Motor impairment was evaluated with the SARA and 9‐Hole Peg Test (9HPT). Dysarthria was assessed with the PATA rate, and its correlation with verbal fluency tasks analyzed. Subtest comparisons among genotypes were adjusted for age, education, and SARA score. Neurodegenerative liquid biomarkers were also measured.

**Results:** Definite CCAS was present in 68% of patients. By genotype, prevalence was 100% in SPG7, 74% in FA, 62% in SCA2, 50% in SCA27B, and 20% in RFC1‐related disease. CCAS total score did not correlate with SARA (ρ = −0.075, *p* = 0.54) or 9HPT (ρ = −0.18, *p* = 0.14). Patients with FA, SCA2, and SPG7 performed worse in executive function, short‐term memory, and abstract reasoning than those with RFC1‐related disease and SCA27B. Word‐fluency subtest had the strongest discriminative ability between patients and controls.

**Conclusion:** Cognitive impairment is frequent in HCAs and varies by genotype. The lack of correlation with motor severity suggests cognitive deficits may occur independently of ataxia progression.

**Disclosure:** Nothing to disclose.

## EPO‐0318

### Cortical atrophy mediates the link between clinical severity and retinal neurodegeneration in spinocerebellar ataxia type three

#### L. Chuyi

##### The First Affliated Hospital of Fujian Medical University, Fuzhou, China

**Background and aims:** SCA3 pathology extends beyond the cerebellum, and OCT reveals retinal thinning. We tested whether cortical atrophy mediates the association between clinical severity and retinal neurodegeneration, consistent with a cortical–retinal axis.

**Methods:** Ninety genetically confirmed SCA3 patients and 32 age‐ and sex‐matched controls underwent OCT, structural MRI, and neurological assessment (SARA, ICARS). OCT metrics were compared between groups. In SCA3, correlations linked retinal thickness with disease duration, clinical scores, and cortical volumes. Mediation analyses evaluated 20 cortical regions.

**Results:** SCA3 patients showed significant retinal layer thinning versus controls (*p* < 0.05). Longer disease duration and higher SARA scores correlated with reduced perifoveal thickness. Six significant indirect pathways were detected. Left calcarine atrophy mediated the link between SARA and right‐eye inferior perifoveal thickness (*p* = 0.04) and mediated associations between disease duration and perifoveal thinning (*p* < 0.001). Right lingual and left precentral atrophy further mediated effects of disease duration on parafoveal thickness (*p* < 0.001).

**Conclusion:** Visual and motor cortical atrophy partially explains retinal thinning in SCA3, supporting OCT‐derived retinal measures as noninvasive indicators of cortical integrity and disease progression.

**Disclosure:** Nothing to disclose.

## EPO‐0319

### Broadening the clinical spectrum of TGM6‐related spinocerebellar ataxia: A pediatric case

#### S. Aloisio ^1^; L. Angelini^2^; G. Beligi^1^; M. De Riggi^1^; S. Grandolfo^1^; D. Birreci^1^; A. Spalice^3^; M. Bologna^1^

##### ^*1*^ *Department of Human Neurosciences, Sapienza University of Rome, Rome, Italy;* ^*2*^ *IRCCS Neuromed, Pozzilli, Italy;* ^*3*^ *Department of Maternal and Child Health and Urogynecological Sciences, Sapienza University of Rome, Rome, Italy*

**Background and aims:** Spinocerebellar ataxia type 35 (SCA35), caused by pathogenic variants in the TGM6 gene encoding transglutaminase 6, is classically a late‐onset cerebellar disorder characterized by progressive gait ataxia, tremor, and mild oculomotor abnormalities. Dystonia, myoclonus, and parkinsonism are less frequent, while neurodevelopmental impairment and epilepsy are rare. Although increasing evidence suggests broader phenotypic variability, early‐onset presentations remain poorly characterized. Here, we describe an early‐onset phenotype of SCA35 with objective kinematic characterization of associated movement disorders.

**Methods:** A 15‐year‐old boy with early developmental delay, focal epilepsy since infancy, and progressive gait impairment underwent neurological examination, longitudinal brain MRI review, and quantitative kinematic assessment of tremor and bradykinesia during repetitive finger tapping. Genetic testing was performed using a targeted next‐generation sequencing panel for ataxia and movement disorders.

**Results:** The patient carried a heterozygous pathogenic TGM6 c.980A > G (p.Asp327Gly) variant, also detected in his mildly affected mother, supporting intrafamilial variability. Serial brain MRI (2013–2021) showed no cerebellar or brainstem atrophy. Neurological examination revealed bilateral irregular tremor at rest, posture, and action, mild cerebellar dysdiadochokinesia, and upper limb bradykinesia with stable clinical course. Quantitative kinematic analysis demonstrated a right‐hand resting tremor peaking at ~8 Hz, a bilateral low‐amplitude irregular postural tremor, and bradykinesia with sequence effect (Figure 1).

**Figure:**
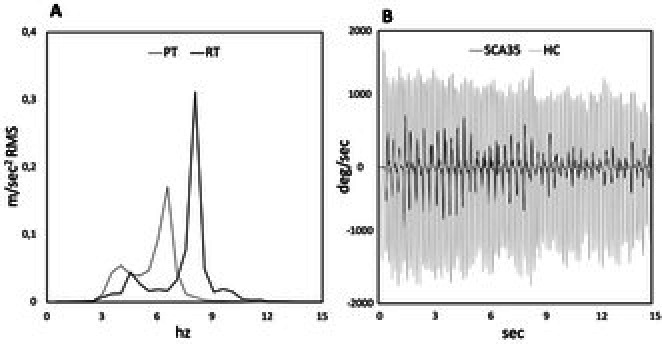
**FIGURE 1** Quantitative kinematic characterization of tremor and bradykinesia.

**Conclusion:** This case expands the phenotypic spectrum of TGM6‐related disease, showing that pathogenic variants may manifest in childhood with neurodevelopmental involvement, epilepsy, and complex movement disorders even in the absence of structural neuroimaging abnormalities. These findings support a network‐based pathophysiological mechanism potentially involving cerebellar–basal ganglia circuits.

**Disclosure:** Nothing to disclose.

## EPO‐0320

### Long‐term (30 Months) outcomes of N‐Acetyl‐L‐leucine in adults and children with niemann‐pick disease type C

#### T. Bremova‐Ertl ^1^; J. Kerthi^2^; B. Zanrucha^2^; J. Raymond^2^; A. Hatcher^2^; T. Fields^2^; I. Billington^2^; M. Strupp^3^; M. Patterson^2^

##### ^*1*^ *Department of Neurology and Center for Rare Diseases, University Hospital Inselspital Bern, Bern, Switzerland;* ^*2*^ *IntraBio Inc, Austin, USA;* ^*3*^ *Department of Neurology, LMU University Hospital, LMU Munich, Germany*

**Background and aims:** Niemann‐Pick disease type C (NPC) is a rare lysosomal storage disorder. IB1001‐301 is a Phase 3, placebo‐controlled, crossover trial encompassing a Parent Study and an ongoing Extension Phase (EP) evaluating the long‐term effects of N‐acetyl‐L‐leucine (NALL) in people with NPC.

**Methods:** IB1001‐301 enrolled participants with a genetically confirmed diagnosis of NPC. Participants received a total of 2–4 g/day orally administered NALL. The primary endpoint of the EP is the modified 5‐domain NPC Clinical Severity Scale (5‐D‐NPC‐CSS). Exploratory endpoints include the 15‐D‐NPC‐CSS (excluding hearing), 4‐D‐NPC‐CSS, and Scale for Assessment and Rating of Ataxia (SARA). Interim 30‐month results are reported here.

**Results:** Forty participants completed Visit 12 (30‐months) of the EP. The mean (±SD) baseline score on the 5‐D‐NPC‐CSS was 10.50 (±4.46). After 30 months, the mean (±SD) change from baseline in the 5‐D‐NPC‐CSS was −0.20 (±2.60) with NALL vs 3.75 (±7.9), i.e. worsening, in the historical cohort; mean difference between cohorts: −3.95; 95% CI −6.60 to −1.30; *P* = 0.0041. The mean (±SD) baseline score on the 15‐D‐NPC‐CSS was 17.48 (±7.15). After 30 months, the mean (±SD) change from baseline in the 15‐D‐NPC‐CSS was 0.38 (±4.00) with NALL vs +4.68 (±2.74) in the historical cohort. The mean (±SD) baseline scores on the 4‐D‐NPC‐CSS and SARA were 7.65 (±3.32) and 15.0 (±7.68), respectively. After 30 months, the mean change from baseline (±SD) on the 4‐D‐NPC‐CSS and SARA were −0.58 (±2.04) and −1.39 (±3.71), respectively.

**Conclusion:** Treatment with NALL demonstrated significant and meaningful long‐term improvement, i.e, disease‐modifying effects. NALL was well tolerated, and no serious adverse events occurred.

**Disclosure:** Tatiana Bremova‐Ertl received speaker's honoraria and consultancy fees from Actelion, Sanofi‐Genzyme and Zevra as well as blinded video‐rater fees from IntraBio. Jorgji Kerthi, Bethany Zanrucha, Janelle Raymond, Asante Hatcher, Taylor Fields, Ian Billington and Marc C Patterson are employees of and may have stock option ownership in IntraBio Inc. Michael Strupp received speaker's honoraria from Abbott, Actelion, Auris Medical, Biogen, Eisai, Grünenthal, GSK, Henning Pharma, Interacoustics, MSD, Otometrics, Pierre‐Fabre, TEVA, UCB, Viatris. Consultant for Abbott, Actelion, Auris Medical, Decibel, Heel, and Sensorion. Scientific founder and shareholder of IntraBio.

## EPO‐0321

### Expanding the phenotypic spectrum of FAT2‐associated disorders: An Italian case series

#### V. Gioiosa ^1^; E. Cioffi^1^; A. Tessa^2^; F. Santorelli^2^; C. Casali^1^

##### ^*1*^ *Department of Medico‐Surgical Sciences and Biotechnologies, University of Rome Sapienza; 04100 Latina, Italy;* ^*2*^ *IRCCS Stella Maris Foundation, Calambrone, via dei Giacinti 2; 56128 Pisa, Italy*

**Background and aims:** Spinocerebellar ataxias (SCAs) are a clinically and genetically heterogeneous group of autosomal dominant neurodegenerative disorders with more than 50 distinct subtypes described to date. Among them, SCA45, associated with FAT2 gene mutation, was first described in 2017. Reported cases are rare and typically exhibit a pure cerebellar phenotype with late onset. FAT2 is highly expressed in cerebellar granule cells and is thought to play a role in neuronal migration and cerebellar development. We describe four patients from three unrelated families, expanding the clinical spectrum of SCA45, with two cases showing additional pyramidal involvement.

**Methods:** Three patients were evaluated at the Neurogenetics Clinic of Polo Pontino/ICOT, Sapienza University of Rome, and one at the IRCCS Stella Maris Foundation. Genetic analyses were performed via targeted NGS panels.

**Results:** We identified four patients with adult‐onset gait disturbances and rare heterozygous FAT2 variants. P1: Ataxic gait after age 60; cerebellar atrophy on MRI; c.8218C > T (p.Phe2740Leu). P2: Spastic‐paretic gait after 50; cerebellar atrophy and axonal neuropathy; c.5240A > G (p.Asp1747Gly). P3: Sister of P2; ataxic gait after 60; same variant c.5240A > G. P4: Spastic‐paretic gait, hyperreflexia; SPRS 28/52, SARA 17/40, SPATAX stage 5, FARS 4.0, FARS‐ADL 15/36; c.5416G > A (p.Glu1806Lys).

**Conclusion:** These cases broaden the phenotypic spectrum of FAT2‐related ataxia, highlighting the possible presence of spasticity and pyramidal signs in addition to cerebellar features. FAT2 variants should be considered in patients with adult‐onset cerebellar ataxia, especially when common SCA repeat expansions are excluded.

**Disclosure:** Nothing to disclose.

## EPO‐0322

### Longitudinal experience with Omaveloxolone in Friedreich's ataxia: Insights from the french cohort at CHU Bordeaux

#### V. Gioiosa; C. Angelini; C. Goizet

##### 1. Bordeaux University, Reference Center for Neurogenetics Disorders (BRAIN‐TEAM network), Neurogenetics Unit, Bordeaux, France

**Background and aims:** Friedreich's Ataxia is a rare autosomal recessive neurodegenerative disease that typically presents in childhood or adolescence. It is caused by a GAA repeat expansion in the FXN gene, leading to frataxin deficiency, mitochondrial dysfunction, and progressive neurological decline. Impaired Nrf2 signaling exacerbates oxidative stress. Omaveloxolone is an oral Nrf2 activator. Following the MOXIe trials, it received FDA approval in 2023, and France initiated a national early access program in January 2024.

**Methods:** This study reports real life data from a French cohort treated with Omaveloxolone at CHU Bordeaux, with up to 18 months of follow up. Clinical evaluations included the Scale for the Assessment and Rating of Ataxia (SARA) and SF12. Blood samples for hepatic, lipid, and cardiac monitoring were collected at baseline and scheduled time points.

**Results:** The cohort initially included 32 patients, with a mean baseline SARA score of 22.8. After 18 months, 23 patients remained on treatment. The average SARA score at one year was 23. All patients received 150 mg/day of Omaveloxolone. Adverse events, particularly transient elevations in liver enzymes, were most frequent within the first 12 weeks and subsequently decreased or returned toward baseline, supporting good tolerability.

**Conclusion:** After 12 months, the mean change in SARA score from baseline was −0.02 SD 2.74, indicating overall stability. This finding is consistent with a slowing of disease progression and contrasts with natural history data from the EFACTS cohort, where ambulant patients show an average annual SARA increase of +0.82 SD 0.05, suggesting a potential attenuation of disease progression

**Disclosure:** Nothing to disclose.

## EPO‐0323

### Deep pheno‐genotyping of NDUFV1‐related mitochondrial disease

#### B. Pancaldi ^1^; R. Maroofian^2^; J. Raza Alvi^3^; I. Javed^3^; N. Lock‐Hock^4^; P. Najarzadehh Torbati^5^; S. Sedighzadeh^6^; M. Abdel‐Hamid^7^; G. Abdel‐Salam^7^; M. Zaki^7^; T. Sultan^8^; K. Bhatia^9^; H. Houlden^2^; F. Magrinelli^10^

##### ^*1*^ *Department of Medicine and Surgery – University of Parma – Parma;* ^*2*^ *Department of Neuromuscular Diseases – UCL Queen Square Institute of Neurology – London, UK;* ^*3*^ *Department of Paediatric Neurology – The Children's Hospital and the University of Child Health Sciences – Lahore, Pakistan;* ^*4*^ *Genetics Department – Hospital Kuala Lumpur – Kuala Lumpur, Malaysia;* ^*5*^ *Department of Medical Genetics – Next Generation Genetic Polyclinic – Mashhad, Iran;* ^*6*^ *Department of Biological Sciences, Faculty of Science, Shahid Chamran University of Ahvaz, Ahvaz, Iran;* ^*7*^ *Clinical Genetics Department – Human Genetics and Genome Research Division – Cairo, Egypt;* ^*8*^ *Department of Pediatric Neurology – Children's Hospital and Institute of Child Health – Lahore, Pakistan;* ^*9*^ *Sobell Department of Motor Neuroscience and Movement Disorders – UCL Queen Square Institute of Neurology – London, UK;* ^*10*^ *Department of Clinical and Movement Neurosciences – UCL Queen Square Institute of Neurology – London, UK*

**Background and aims:** NDUFV1 encodes a 51‐kDa core subunit of mitochondrial complex I. We aim to expand the phenotypic and genotypic spectrum of NDUFV1‐related mitochondrial disease.

**Methods:** We screened international next‐generation sequencing datasets for biallelic NDUFV1 variants. Detailed phenotypic data were collected for identified individuals. Haplotype analysis was performed for recurrent variants. A PubMed search (Dec 2025, keyword “NDUFV1”) was conducted to extract published genetic and clinical data.

**Results:** We identified 28 new affected individuals (16 males) from 22 families of Indian, Pakistani, Iranian, Malaysian, and Egyptian ancestry and 68 previously reported cases. In our cohort age at onset ranged from birth to 10 years, with gait impairment and seizures being the most common initial symptoms. Core clinical features are summarized in Figure 1. We also document orofacial dystonia and sleep disturbances as newly associated features. Oculomotor abnormalities, as well as respiratory insufficiency, frequently reported in the literature, were rare in our cohort. We identified 27 homozygotes and 1 compound heterozygote. Among six identified variants, two were novel:NM_007103.4:c.597delC p.(Ala201LeufsTer25) and NM_007103.4:c.466A > G p.(Ile156Val). The most frequent was NM_007103.4:c.1156C > T p.(Arg386His), found in 78% of patients (Fig. 2). Although it was found recurrently in individuals from neighboring Asian regions, haplotype analysis showed distinct genetic backgrounds, supporting the view that it is not a founder effect (Fig. 3).

**Figure:**
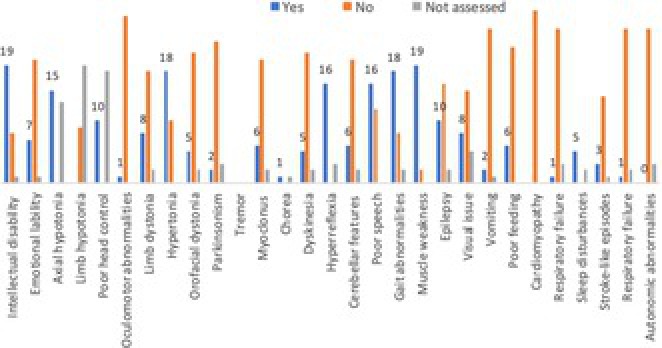
**FIGURE 1** Clinical features of the 28 patients of our cohort.

**Figure:**
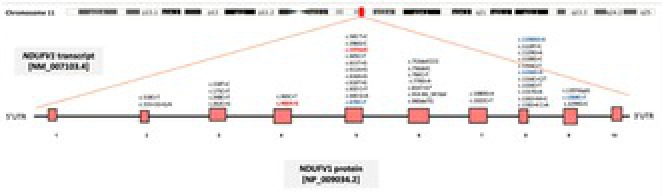
**FIGURE 2** NDUFV1 gene schematic with introns to scale; literature variants in black, known/cohort variants in blue, novel variants in red.

**Figure:**
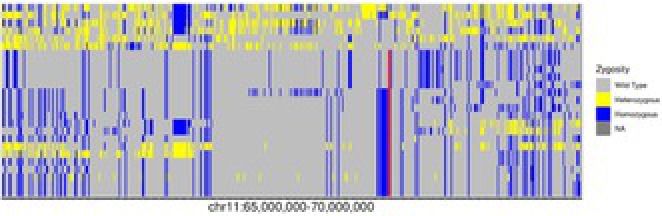
**FIGURE 3** Haplotype analysis on exome files performed in patients carrying the NM_007103.4:c.1156C > T variant.

**Conclusion:** This study broadens the phenotypic spectrum of NDUFV1‐related mitochondrial disease, highlighting orofacial dystonia and sleep disturbances as newly phenotypic features, and expands the genotypic landscape by identifying two novel variants.

**Disclosure:** Nothing to disclose.

## Sunday, June 28 2026

## Ageing and Dementia 3

## EPO‐0324

### Skin biopsy for detection of phosphorylated alpha‐synuclein by indirect immunofluorescence in a cohort of patients with cognitive impairment

#### A. Furia ^1^; A. Incensi^2^; C. Delprete^2^; R. Pantieri^3^; G. Rizzo^2^; S. Capellari^1^; M. Stanzani‐Maserati^2^; G. Plazzi^2^; E. Antelmi^4^; F. Pizza^1^; V. Vacchiano^2^; M. Giannoccaro^1^; R. Liguori^1^; W. Zou^5^; V. Donadio^1^

##### ^*1*^ *Dipartimento di Scienze Biomediche e Neuromotorie, Università di Bologna, Italy;* ^*2*^ *IRCCS Istituto delle Scienze Neurologiche di Bologna, UOC Clinica Neurologica, Ospedale Bellaria, Bologna, Italy,**;*** ^*3*^ *IRCCS Istituto delle Scienze Neurologiche di Bologna, UOC Neurologia, Ospedale Bellaria, Bologna, Italy,;* ^*4*^ *DIMI Dipartimento di Ingegneria per la Medicina di Innovazione, Università di Verona, Verona, Italy;* ^*5*^ *Institute of Neurology, Jiangxi Academy of Clinical Medical Instittue, The First Affiliated Hospital of Nanchang University, Nanchang, Jiangxi Province, China*

**Background and aims:** Techniques for detection of Dementia with Lewy Bodies (DLB) and distinction from other causes of cognitive impairment are in rapid establishment. Among these are Indirect Immunofluorescence (IF) for identification of phosphorylated α‐synuclein (p‐syn) in skin tissue and Seeding Amplification Assays (SAAs), including Real Time Quaking‐Induced Conversion (RT‐QuIC).

**Methods:** patients referred to our Clinic for major or minor cognitive impairment were prospectively recruited from 2015 to 2025, undergoing full neurological and neuropsychological examination. Subsequent examinations were guided by the clinical presentation and the final diagnosis, serving as gold standard, was established by experienced clinicians. Skin biopsy for detection of p‐syn by IF was performed in all patients at distal leg and cervical sites. Diagnostic accuracy (sensitivity, specificity) of skin IF was assessed, along with the correlation of quantitative p‐syn measures with clinical measures.

**Results:** 50 patients with DLB (syn group) diagnosis and 74 fulfilling different diagnoses (non‐syn cognitive impairment) were recruited. Skin biopsy detected p‐syn in 46 syn patients, resulting negative in all non‐syn cases. Quantitative measures did not correlate with clinical measures.

**Conclusion:** Skin biopsy is a promising technique for detection of p‐syn in patients with DLB diagnosis. The results suggest significant specificity. Current quantitative p‐syn measures do not correlate with disease parameters in DLB, warranting development of more specific indexes.

**Disclosure:** Nothing to disclose.

## EPO‐0325

### Speech biomarkers derived from explainable AI reveal therapeutic effects of transcranial pulse stimulation in Alzheimer's disease

#### A. Günes ^1^; D. Wiechmann^2^; E. Kerz^3^; Y. Qiao^3^; M. Köhne^1^; U. Sprick^4^

##### ^*1*^ *Department for Research and Development, Center for Neurostimulation, Alexius/ Josef Hospital, Neuss, Germany;* ^*2*^ *Institute for Logic, Language & Computation, University of Amsterdam, Amsterdam, Netherlands;* ^*3*^ *Exaia Technologies, Amsterdam, Netherlands;* ^*4*^ *Faculty of Medicine, Heinrich‐Heine University Düsseldorf, Düsseldorf, Germany*

**Background and aims:** We develop interpretable AI models using spontaneous‐speech digital biomarkers to support the differential diagnosis of Alzheimer's disease (AD) and to quantify individual therapeutic effects of transcranial pulse stimulation (TPS) on disease progression. TPS is a CE‐certified, non‐invasive, shockwave‐based neuromodulation technique. It enables neuronavigation‐guided targeting and modulation of cortical gray matter as well as deep brain structures.

**Methods:** Automated classification models were trained on spontaneous speech from 40 AD patients and 27 socio‐demographically matched healthy controls. Diagnosis followed German S3 guidelines and was supported by CERAD Plus (including MMSE), Stroop test and structural MRI. Interpretable AI identified the most discriminative speech features. Biomarkers were then used to quantify TPS‐associated modulation in 14 patients who received six sessions over 14 days, with 6000 pulses per session delivered bilaterally to the frontal and parietal lobes and the precuneus, using an energy level of 0.25 mJ/mm^2^ and a frequency of 4 Hz.

**Results:** Speech biomarkers correlated with cognitive performance and captured impairment better than the Stroop test. AD versus control classification achieved F1 = 0.81. Group‐level linguistic performance was stable or slightly improved during follow‐up, contrasting with the 3.3‐point annual MMSE decline expected in untreated AD. Individual t0/t1 biomarker trajectories varied markedly, indicating heterogeneous responsiveness. Discriminative speech features provided mechanistic insights into TPS‐related neurophysiological effects.

**Figure:**
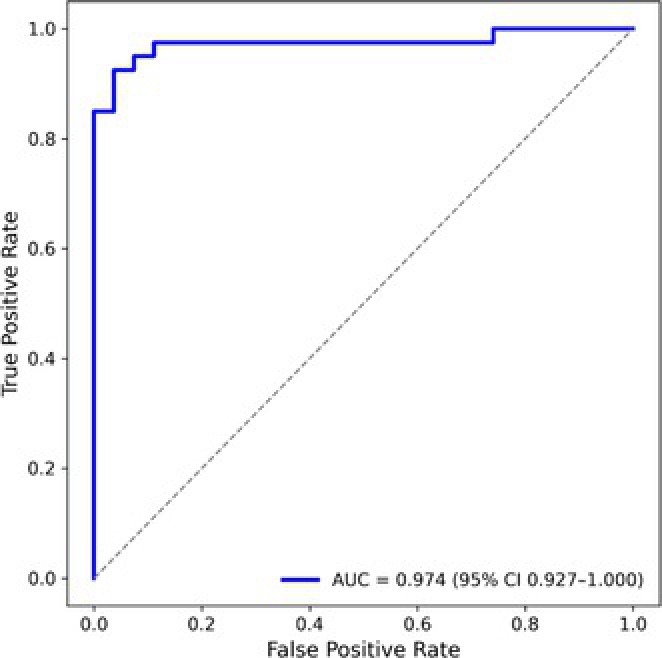
**FIGURE 1** Performance of the AD classification model. Receiver operating characteristic (ROC) curve for the final model trained on linguistic features using the full dataset. The model achieved an area under the curve (AUC) of 0.974 [95% CI: 0.927–1.000].

**Figure:**
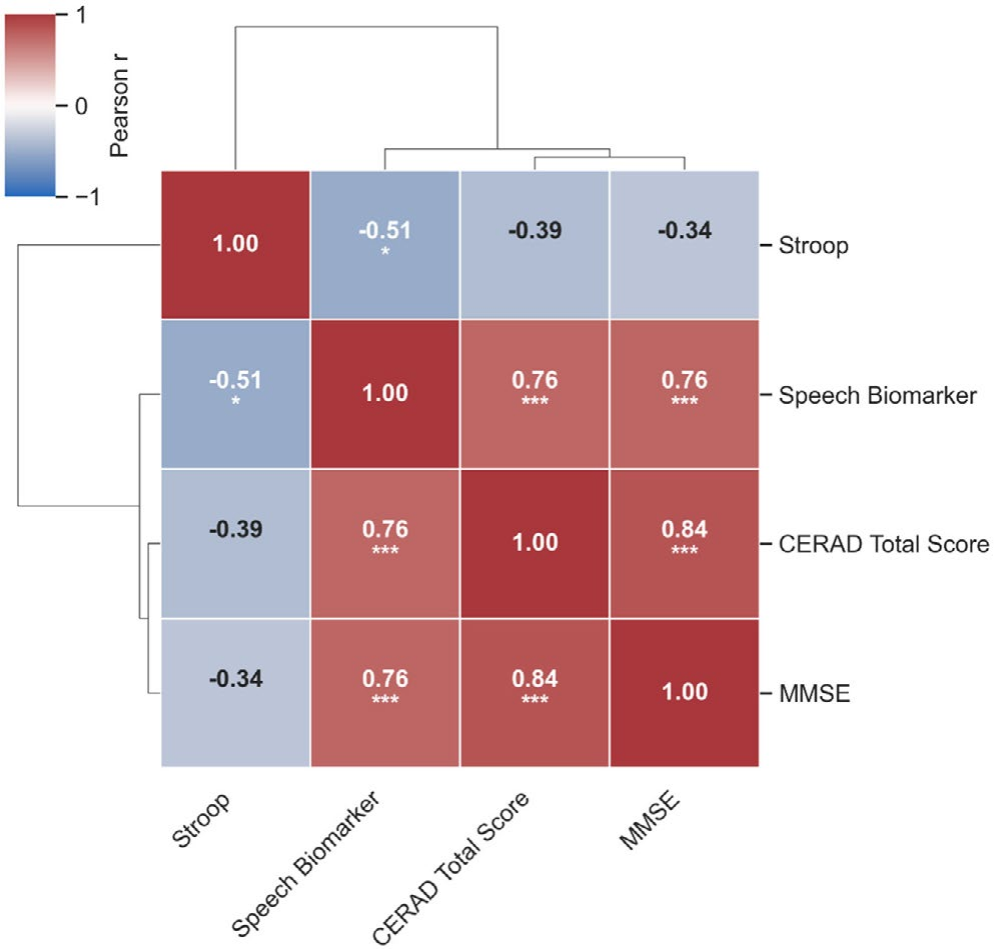
**FIGURE 2** Speech‐based biomarkers track global cognitive decline. Hierarchical clustering of Pearson correlation coefficients reveals a high‐fidelity association between speech‐biomarker and global cognitive measures (CERAD, *r* = 0.76; MMSE, *r* = 0.76).

**Figure:**
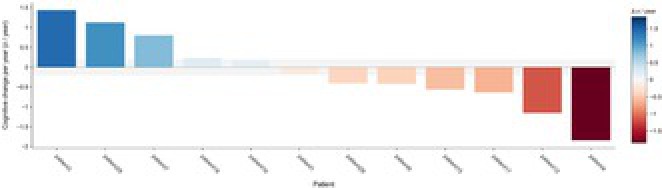
**FIGURE 3** Longitudinal trajectories of cognitive change captured by an AI‐derived speech biomarker. Waterfall plot showing annualized rates of change in speech biomarker indexing cognitive functioning for a representative sub‐cohort (AD group).

**Conclusion:** Interpretable AI with speech‐derived digital biomarkers provides a precision framework for AD diagnosis and treatment monitoring. TPS produces individualized, quantifiable effects on disease progression detectable with sensitive speech‐based measures that outperform conventional cognitive assessments.

**Disclosure:** Nothing to disclose.

## EPO‐0326

### Semiquantitative neuropathologic thresholds for cerebrospinal fluid biomarker alterations in Alzheimer's disease

#### A. Mastrangelo ^1^; S. Baiardi^1^; G. Bentivenga^1^; E. Ruggeri^2^; C. Vargiu^2^; A. Mammana^2^; S. Capellari^1^; P. Parchi^1^

##### ^*1*^ *Department of Biomedical and Neuromotor Sciences, University of Bologna, Bologna, Italy;* ^*2*^ *IRCCS Istituto delle Scienze Neurologiche di Bologna, Bologna, Italy*

**Background and aims:** Validation of Alzheimer's disease (AD) cerebrospinal fluid (CSF) biomarkers against pathology is limited by small sample cohorts and long CSF‐death intervals. Moreover, few studies assessed the burden of brain Aβ and tau pathologies using semiquantitative scoring. Using a large cohort, we defined the brain Aβ and tau burden threshold associated with significant alterations in CSF markers.

**Methods:** We studied 233 subjects with post‐mortem neuropathology (164 prion, 69 non‐prion) and antemortem CSF samples. Aβ and tau burden were semi‐quantitatively scored across nine and six brain regions, respectively. Thal phase, Braak stage and AD neuropathologic change (ADNC) category were assigned. CSF and plasma biomarkers were measured by the Lumipulse platform. Associations between pathology and log‐transformed biomarkers were tested using linear regression models. Age, sex, and CSF‐death interval were considered as covariates.

**Results:** Median sampling‐death interval was 1.5 months (IQR 0.5–5.1). CSF Aβ42/Aβ40 was already significantly reduced at Thal phase 2 (*p* = 0.019) and low‐ADNC (*p* < 0.001) when compared to non‐AD. The first significant reduction in CSF Aβ42/Aβ40 occurred at an Αβ density score > 15 (moderate neocortical and amygdala pathology; *p* = 0.047), with increasing pathological burden associated with greater biomarker changes (*p* < 0.001). Values of CSF Aβ42/Aβ40 ≤ 0.068 had 92% specificity and 90% sensitivity for distinguishing an Αβ density score > 25. In non‐prion cases, Aβ42/p‐tau181was earliest reduced at intermediate‐ADNC and Αβ density‐score > 35 (*p* < 0.001).

**Conclusion:** Group‐alterations of CSF Aβ42/Aβ40 appear at a relatively early stage of AD continuum, although initial Αβ deposits remain below detection. In contrast, a reduction in CSF Aβ42/p‐tau181 emerges only at higher levels of beta‐amyloid burden.

**Disclosure:** Nothing to disclose.

## EPO‐0327

### Validation of CogniPaz: A rapid, ecologically‐oriented digital biomarker for detection of mild cognitive impairment

#### C. Estebas Armas ^1^; A. Medina López^1^; D. Blanco de Miguel^1^; M. Hernández Barral^1^; E. Alonso López^1^; E. Alonso López^2^; S. Henche Rodriguez^1^; O. Ocampo Perez^1^; M. Alonso de Leciñana Cases^1^; A. Frank García^1^; A. Frank García^2^; Á. Martín Montes^1^

##### ^*1*^ *epartment of Neurology and Stroke Center. La Paz University Hospital. Universidad Autónoma de Madrid. La Paz University Hospital Research Institute (IdiPAZ). Madrid, Spain;* ^*2*^ *Networking Research Center on Neurodegenerative Diseases (CIBERNED), Instituto de Salud Carlos III, Madrid, Spain*

**Background and aims:** Differentiation between amnestic mild cognitive impairment (aMCI), subjective cognitive decline (SCD) and healthy controls (HC) is a diagnostic challenge. We developed CogniPaz, an ecologically oriented digital cognitive assessment composed of nine tasks evaluating memory and executive functions. We evaluated its diagnostic accuracy as a digital biomarker in discriminating MCI/aMCI from SCD and HC.

**Figure:**
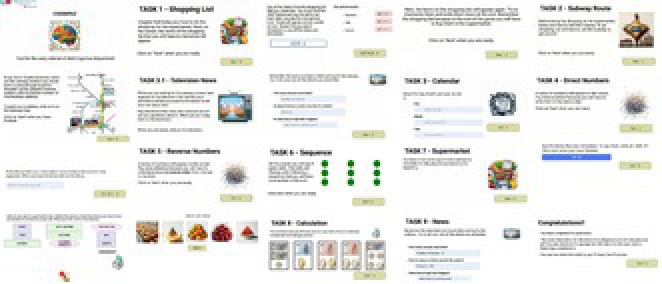
**FIGURE 1** 9‐task CogniPaz protocol and interface (translated from the original Spanish).

**Methods:** We conducted a prospective observational study including participants with subjective cognitive complaints or asymptomatic controls recruited between April 2025 and December 2025. Participants were classified based on clinical evaluation and Montreal Cognitive Assessment (MoCA) scores into HC (asymptomatic; MoCA > = 26), SCD (cognitive complaints; MoCA > = 26) and MCI/aMCI (cognitive complaints without functional impairment; MoCA < 26). CogniPaz was administered after MoCA by a blinded evaluator, following a standardized protocol. We assessed correlation with MoCA score (Pearson), diagnostic accuracy (ROC analysis) and usability (satisfaction questionnaire).

**Figure:**
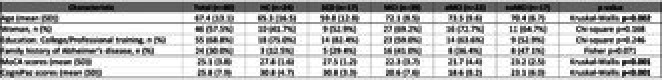
**TABLE 1** Demographic and clinical characteristics and mean scores of the study population.

**Results:** 80 participants were included: 39 MCI (22 aMCI), 17 SCD and 24 HC. Mean administration time was 9.65 minutes (SD 3.72). CogniPaz total scores correlated strongly with MoCA (*r* = 0.83) and memory tasks correlated with MoCA memory domains (*r* = 0.69). Diagnostic accuracy was excellent for distinguishing MCI from HC (AUC = 0.882), MCI from SCD (AUC = 0.916), aMCI from HC (AUC = 0.907) and aMCI from SCD (AUC = 0.937). CogniPaz also discriminated suspected neurodegenerative from non‐neurodegenerative cases (AUC = 0.835). Usability was rated highly (mean score 8.11 out of 9).

**Figure:**
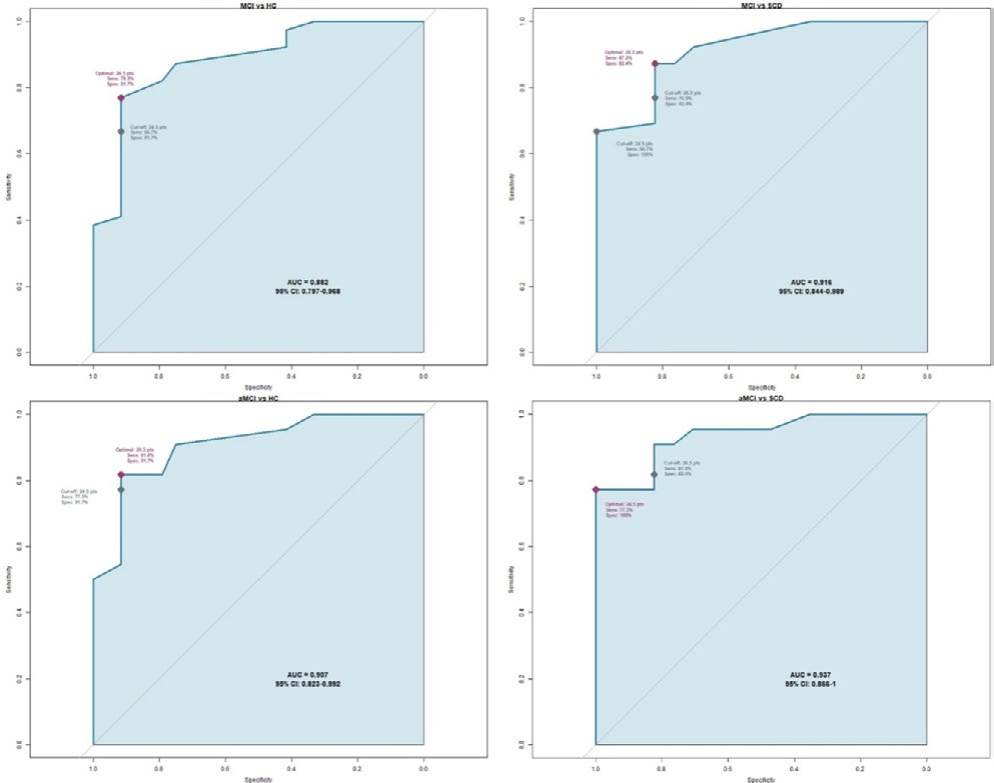
**FIGURE 2** Cognipaz ROC curves. MCI: mild cognitive impairment. aMCI: amnestic mild cognitive impairment. SCD: subjective cognitive decline. HC: healthy controls.

**Conclusion:** CogniPaz shows strong correlation with MoCA and excellent diagnostic accuracy. Its brevity and ease of use support its utility as an efficient digital screening tool for distinguishing MCI/aMCI from SCD and HC.

**Disclosure:** The authors declare that this study was conducted with institutional research support. No commercial sponsorship influenced the design or results of this study.

## EPO‐0328

### Lobar Cerebral microbleeds and white matter hyperintensities differentially associate with neuropsychiatric vulnerability in dementia with Lewy bodies

#### P. Stancu^1^; D. Janela ^1^; D. Botta^2^; F. Kurz^2^; F. Assal^1^; L. Sveikata^1^

##### ^*1*^ *Neurology Department, Geneva University Hospitals, Geneva, Switzerland;* ^*2*^ *Neuroradiology Department, Geneva University Hospitals, Geneva, Switzerland*

**Background and aims:** To explore the relationship between markers of small vessel disease (SVD)—lobar cerebral microbleeds (CMB) and white matter hyperintensities (WMH)—and neuropsychiatric symptoms (NPS) in dementia with Lewy bodies (DLB). Markers of SVD are associated with cognitive impairment in older adults, but their contribution to early neuropsychiatric manifestations in DLB remains insufficiently characterized.

**Methods:** Clinically diagnosed DLB cases (*n* = 70) were analyzed. CMB and WMH were assessed using standardized visual rating scales. NPS, including depression, anxiety, apathy, hallucinations and delirium, was assessed retrospectively. NPS other than delirium were evaluated at the first cognitive visit. Pre‐diagnostic delirium was defined via the Confusion Assessment Method scale within the year preceding diagnosis. Descriptive analyses were performed. Cross‐sectional associations between SVD imaging markers and NPS were examined using regression models adjusted for age, sex, and educational level.

**Results:** CMB were present in 17/54 (31.5%) cases, and 45/69 (65.2%) had at least one documented delirium episode in the year preceding diagnosis. CMB were selectively associated with depression (OR = 6.80, 95% CI 1.60–28.87, *p* = 0.009) and pre‐diagnostic delirium (OR = 5.11, 95% CI 1.02–25.71, *p* = 0.048). CMB were not associated with anxiety, apathy, or hallucinations. WMH showed no significant association with any NPS outcome.

**Conclusion:** In contrast to WMH, CMB were selectively associated with depression and pre‐diagnostic delirium in clinically diagnosed DLB. These findings suggest diverging pathophysiological mechanisms of SVD comorbidity in LBD and support a differential contribution of cerebrovascular markers to prodromal neurocognitive symptoms and clinical expression in DLB.

**Disclosure:** Funding: L.S. is supported by the Alzheimer's Association grant (AACSF‐22‐922907) and clinician‐scientist award at the University of Geneva.

## EPO‐0329

### Innovating brain health: An Italian experience with a community‐based service

#### E. Canu ^1^; B. Lamorgese^2^; E. Sibilla^1^; G. Cecchetti^3^; E. Spinelli^4^; V. Castelnovo^1^; A. Gilioli^1^; C. Tripodi^1^; F. Freri^1^; G. Rugarli^4^; A. Ghirelli^4^; S. Basaia^2^; R. Santangelo^5^; J. Lanzone^5^; M. Cursi^6^; L. Zanchi^2^; F. Agosta^4^; M. Filippi^7^

##### ^*1*^ *Neuroimaging Research Unit, Division of Neuroscience, and Neurology Unit, IRCCS San Raffaele Scientific Institute, Milan, Italy;* ^*2*^ *Neuroimaging Research Unit, Division of Neuroscience, IRCCS San Raffaele Scientific Institute, Milan, Italy;* ^*3*^ *Neuroimaging Research Unit, Division of Neuroscience, Neurology Unit, and Neurophysiology Service, IRCCS San Raffaele Scientific Institute, Milan, Italy;* ^*4*^ *Neuroimaging Research Unit, Division of Neuroscience, and Neurology Unit, IRCCS San Raffaele Scientific Institute, Milan, Italy; and Vita‐Salute San Raffaele University, Milan, Italy;* ^*5*^ *Neurology Unit, and Neurophysiology Service, IRCCS San Raffaele Scientific Institute, Milan, Italy;* ^*6*^ *Neurophysiology Service, IRCCS San Raffaele Scientific Institute, Milan, Italy;* ^*7*^ *Neuroimaging Research Unit, Division of Neuroscience, Neurology Unit, Neurorehabilitation Unit, and Neurophysiology Service, IRCCS San Raffaele Scientific Institute, Milan, Italy; and Vita‐Salute San Raffaele University, Milan, Italy*

**Background and aims:** To assess clinical/neuropsychological, neurophysiological, neuroimaging, genetic, and fluid biomarker profiles of individuals attending a Brain Health Service (BHS) designed for early identification of Alzheimer's disease (AD).

**Methods:** Fifty‐two participants were referred to the BHS and underwent clinical/neurological and neuropsychological evaluations, brain MRI, electroencephalography (EEG), optical coherence tomography (OCT), APOE genotyping, and, for research purposes, blood‐sampling for plasma AD‐biomarker‐analysis. Participants were classified as A+ (*n* = 22) or A‐ (*n* = 30) based on p‐tau217, and clinically stratified into cognitively normal (C*N* = 15; CN+ = 6; CN‐ = 9), subjective cognitive decline (SCD = 18; SCD+ = 3; SCD‐ = 15), and mild cognitive impairment (MCI = 19; MCI+ = 13; MCI‐ = 6). We analyzed: gray matter (GM) atrophy, white matter (WM) hyperintensities, EEG features and OCT data.

**Results:** A+ cases were older than A‐, reported higher alcohol consumption, showed poorer cognitive performances and engaged in less physical activity. CN+ showed reduced semantic fluency compared to SCD. Compared to A‐, A+ showed reduced GM volume in the left medial temporal lobe, left fusiform gyrus, right precuneus and anterior temporal gyrus, and increased WM burden in periventricular and deep regions. Compared to A‐, in A+ EEG analysis revealed lower frequencies in temporal and frontal regions, and OCT revealed increased global retinal nerve fiber layer thickness.

**Conclusion:** Our findings support the feasibility and clinical relevance of plasma biomarkers, offered within a research framework, for the early detection of AD in individuals accessing a BHS. Early biomarker identification enables timely lifestyle interventions that may delay symptoms onset. The BHS provides a promising framework to detect at‐risk individuals and guide preventive strategies.

**Disclosure:** E. Canu has received research support from the Italian Ministry of Health; B. Lamorgese, E. Sibilla, V. Castelnovo, G. Rugarli, A. Ghirelli, R. Santangelo, J. Lanzone, M. Cursi, L. Zanchi, report no competing interests; E.G. Spinelli received honoraria from Eli Lilly; G. Cecchetti received honoraria from Eli Lilly and Neopharmed Gentili; F. Agosta is Associate Editor of NeuroImage: Clinical and the European Journal of Neurology; has received speaker honoraria from Biogen Idec, Bristol Myers Squibb, Eisai, Eli Lilly, GE Healthcare, Neuraxpharm, and Roche,; and receives or has received research supports from the Italian Ministry of Health, the Italian Ministry of University and Research, AriSLA (Fondazione Italiana di Ricerca per la SLA), the European Research Council (ERC), the EU Joint Programme – Neurodegenerative Disease Research (JPND), and Foundation Research on Alzheimer Disease (France); M. Filippi is Editor‐in‐Chief of the Journal of Neurology, Associate Editor of Human Brain Mapping, Neurological Sciences, and Radiology; received compensation for consulting services from Almirall, Biogen, Bristol‐Myers Squibb, Eli Lilly, Merck, Novartis, Roche, Sanofi; speaking activities from Amgen, Bayer, Biogen, Bristol‐Myers Squibb, Celgene, Chiesi Italia SpA, Eisai, Eli Lilly, Fujirebio, Genzyme, Janssen, Merck, Neopharmed Gentili, Neuraxpharm, Novartis, Novo Nordisk, Roche, Sanofi, Takeda; participation in Advisory Boards for Alexion, Biogen, Bristol‐Myers Squibb, Eli Lilly, GE Healthcare Ltd, Merck, Neuraxpharm, Novartis, Roche, Sandoz, Sanofi, Takeda; scientific direction of educational events for Biogen, Merck, Roche, Celgene, Bristol‐Myers Squibb, Lilly, Novartis, Sanofi‐Genzyme; he receives research support from Biogen Idec, Merck‐Serono, Novartis, Roche, the Italian Ministry of Health, the Italian Ministry of University and Research, and Fondazione Italiana Sclerosi Multipla.

## EPO‐0330

### Greater burden of Alzheimer's co‐pathology in women with Parkinson's disease

#### E. Driver‐Dunckley

##### Neurology, Mayo Clinic Arizona, Scottsdale, USA

**Background and aims:** Alzheimer's disease (AD) and Parkinson's disease (PD) frequently co‐occur in older adults, yet the influence of sex on AD pathology in the context of PD remains unexplored. The objective of this study is to investigate sex differences in AD related pathology among autopsy‐confirmed PD cases.

**Methods:** All subjects were enrolled in the Arizona Study of Aging and Neurodegenerative Disorders (AZSAND) and Brain and Body Donation Program (BBDP) and had annual standardized research clinical assessments by neuropsychologists, subspecialty behavioral and movement disorders neurologists, as well as comprehensive neuropathological examinations after death

**Results:** Among 230 autopsy‐confirmed PD cases, females exhibited greater amyloid plaque pathology burden than males, regardless of a co‐occurring AD diagnosis. Specifically, females with PD had significantly higher mean cortical total plaque scores (mean 6.5/15 vs. 4.9/15, *p* = 0.045) and greater CERAD neuritic plaque density (mean 1.7/3 vs. 1.3/3, *p* = 0.035). Females were also more likely to have a higher cortical plaque burden (plaque total ≥ 5: 56.8% vs. 39.7%, *p* = 0.015). In multivariable logistic regression models, female subjects showed greater than twice the odds of having an amyloid plaque burden ≥ 5 compared to males (OR = 2.18; 95% CI = 1.17 – 4.06; *p* = 0.014), when controlling for ApoE ε4 status and age at death.

**Conclusion:** Female sex is associated with increased amyloid plaque pathology in PD, independent of ApoE ε4 status. These findings highlight a sex‐specific vulnerability to AD pathology in PD patients and support the need for sex‐informed approaches to diagnosis and treatment in mixed neurodegenerative disease.

**Disclosure:** Nothing to disclose.

## EPO‐0331

### Cognitive improvement following transcranial pulse stimulation (TPS) in patients with dementia: A randomized, sham‐controlled study

#### F. Pistoia ^1^; F. Vecchio^2^; C. Pappalettera^2^; P. Sucapane^3^; G. Saporito^3^; A. Cacciotti^2^; C. Carrarini^2^; L. Titti^2^; C. Decaro^2^; V. Socci^3^; N. Cimini^3^; P. Rossini^2^

##### ^*1*^ *Department of Biotechnological and Applied Clinical Sciences, University of L'Aquila, L'Aquila, Italy; San Salvatore Hospital L'Aquila, Italy;* ^*2*^ *Department of Neuroscience and Neurorehabilitation IRCCS San Raffaele Roma Rome Italy;* ^*3*^ *Department of Neurology, San Salvatore Hospital, L'Aquila, Italy*

**Background and aims:** The burden of dementia is increasing worldwide, resulting in a substantial social and healthcare burden. Hence, the increasing interest in both pharmacological and non‐pharmacological treatments, with potential to slow the clinical course of the disease. The objective of this study was to investigate whether Transcranial Pulse Stimulation (TPS) may induce cognitive improvement in patients with an early stage of dementia.

**Methods:** A double‐blind, sham‐controlled study was conducted, with patients randomly assigned to receive either TPS or sham TPS for four weeks in parallel groups. Patients received either TPS or sham treatment once daily, five days per week. TPS was applied to bilateral frontal cortex, bilateral parietal cortex and precuneus cortex via a neuronavigation system based on individual MRI spatial coordinates. Clinical assessments were performed through the Alzheimer's Disease Assessment Scale‐Cognitive Subscale (ADAS‐Cog) at baseline (t0), two weeks after the start of TPS (t1), one month after the start of TPS (t2), two months after the start of TPS (t3), and six months after the start of TPS (t4).

**Figure:**
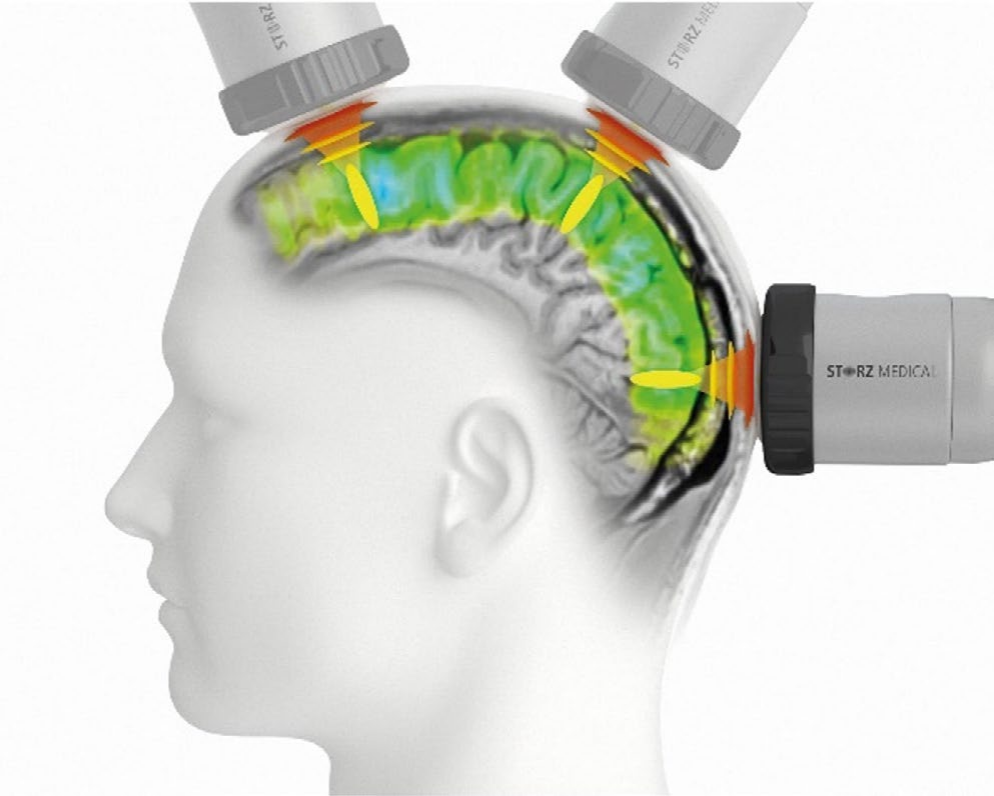
**FIGURE 2** TPS device (by courtesy of Storz Medical).

**Results:** 82 patients were enrolled. Sixty (mean age ± SD 74.3 ± 8; 20 males) completed assessments at baseline and after one month of stimulation. A significant improvement in ADAS‐Cog scores at one month was detected in patients receiving the active treatment (*n* = 34 mean age ± SD 73.2 ± 9; 11 males) compared with those receiving sham treatment (*n* = 26 mean age ± SD 75.8 ± 6.5; 9 males) (*p* < 0.009). No significant side‐effects were reported.

**Figure:**
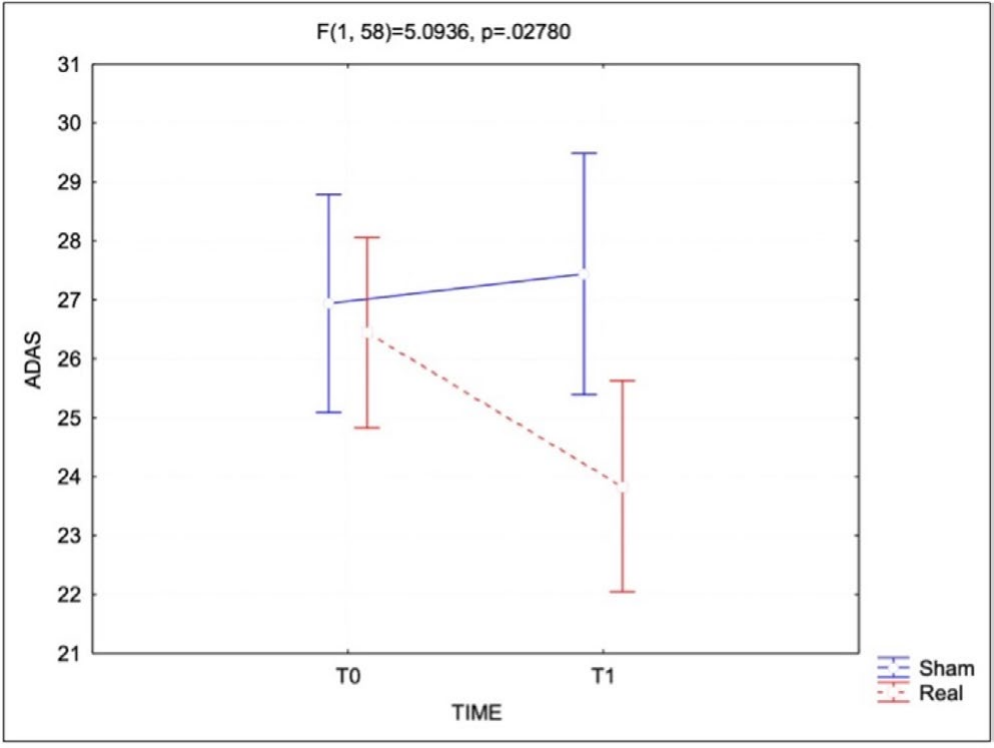
Pre‐ vs post‐stimulation ADAS‐Cog score comparison.

**Conclusion:** Preliminary findings suggest that TPS may be a promising tool for enhancing neuroplasticity‐related recovery in dementia.

**Disclosure:** This research was funded by the Italian Ministry of Health under the National Recovery and Resilience Plan (PNRR), Mission 6 – Component 2 – Investment 2.1 “Enhancement and strengthening of biomedical research within the National Health Service (SSN)”, Project code PNRR‐MCNT2‐2023‐12377235 (CUP: E13C24000930001), financed by the European Union – NextGenerationEU.

## EPO‐0332

### EEG dynamics in Lewy body diseases is related to clinical fluctuations and degeneration of locus coeruleus

#### E. Výtvarová^1^; K. Mitterová ^1^; M. Angiolelli^2^; A. Kovářová^1^; A. Šejnoha Minsterová^1^; L. Brabenec^1^; M. Lamoš^1^; P. Sorrentino^2^; I. Rektorová^1^; J. Fousek^1^

##### ^*1*^ *Central European Institute of Technology (CEITEC), Masaryk University, Brno, Czech Republic;* ^*2*^ *Institute de Neuroscience des Systems (INS), Institut National de la Santé et de la Recherche Médicale (INSERM), Aix Marseille Université, Marseille, France*

**Background and aims:** The complex relationships between the focal neurodegeneration, dynamics of the brain activity and cognitive decline are not well understood in the context of Lewy body diseases. Quantitative assessment of the transient cascades of increased brain activity (neuronal avalanches) has proven useful in capturing meaningful information across conditions. Here we examined whether the richness of brain dynamics was associated with clinical symptoms and with the microstructural integrity of catecholaminergic nuclei – the right caudal locus coeruleus (rcLC) and the substantia nigra pars compacta (SNpc).

**Methods:** The sample comprised 30 HC, 56 cognitively normal individuals with core clinical features of dementia with LB (CN‐CCF), 34 individuals with mild cognitive impairment with LB (MCI‐LB), and 30 advanced PD patients. Neuronal avalanches were extracted from source‐reconstructed resting‐state EEG and described by their count, the number of unique spatial patterns (flexibility), hemispheric symmetry of flexibility, and avalanche transition matrices. LC and SNpc integrity were assessed using neuromelanin‐sensitive MRI and free‐water diffusion imaging, respectively.

**Results:** Lewy body disease groups exhibited increased flexibility relative to HC, which was, in prodromal groups, associated with cognitive fluctuations and REM sleep behavior disorder symptoms, but not parkinsonism. Flexibility was negatively related to rcLC integrity, which was lowest in MCI‐LB.

**Figure:**
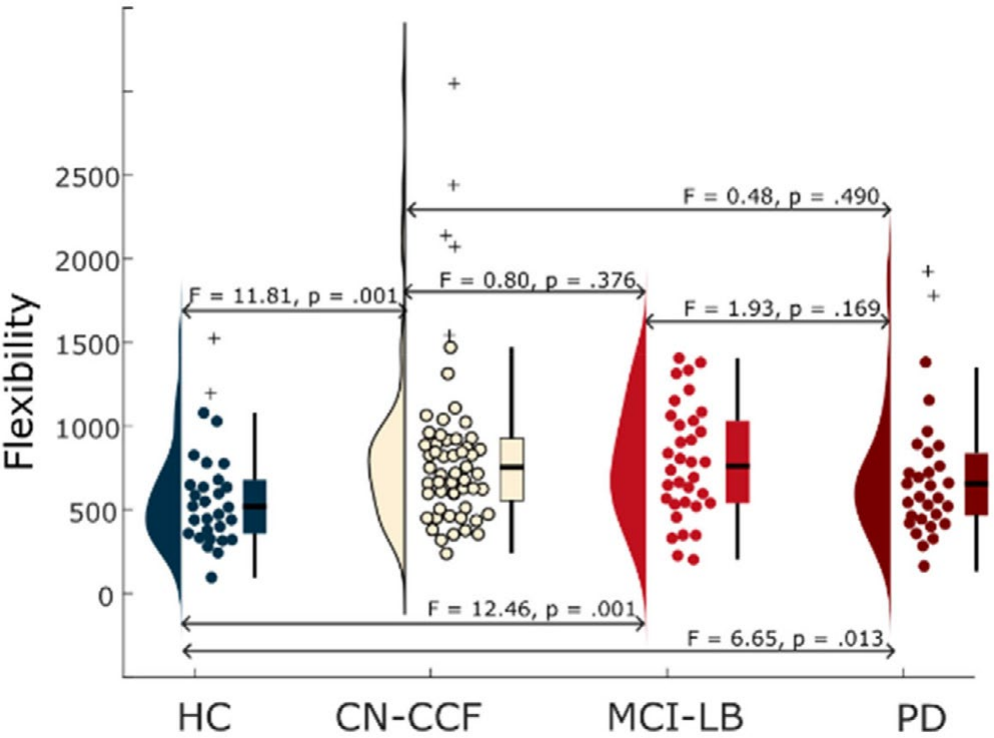
Group differences in flexibility. Note: Crosses (+) mark 9 outliers that were removed before statistical analysis; *n*(HC) = 28, *n*(CN‐CCF) = 51, *n*(MCI‐LB) = 34, *n*(PD) = 28.

**Conclusion:** These findings suggest that elevated flexibility reflects dysregulated brain dynamics linked to malignant non‐motor features of LB diseases and to the caudal LC involvemen.

**Disclosure:** This work has received funding from the project nr. LX22NPO5107 (MEYS): Financed by European Union – Next Generation EU; from the European Union's Horizon Europe research and innovation programme under grant agreement No 101147319 (EBRAINS 2.0); from the Ministry of Education, Youth and Sports of the CR (Joint Programme Neurodegenerative Disease), project nr. 9F25001 (TRACE‐PD): Tracking the mechanisms of disease progression and functional compensation in the early phase of Parkinson's disease; from EU Joint ProgramNeurodegenerative Disease (JPND) project entitled ‘TACKLing the Challenges of PREsymptomatic Sporadic Dementia (TACKL‐PRED),’ project nr. 8F22005. This work was supported by Ministry of Health of the Czech Republic, grant nr. NNW26J04‐00157; by the European Regional Development Fund project LangInLife, reg. no.: CZ.02.01.01/00/23_025/0008726.

## EPO‐0333

### A nested case‐control study of plasma extracellular vesicle biomarkers for Alzheimer's disease

#### L. Yang

##### Zhejiang Hospital, Hangzhou, China

**Background and aims:** Alzheimer's disease (AD) is a progressive neurodegenerative disorder posing global health challenges. Accessible, non‐invasive early biomarkers are urgently needed. Plasma‐derived extracellular vesicles (EVs) cross the blood‐brain barrier, carrying CNS cargo and offering biomarker potential. We aim to identify, validate, and determine the cellular origins of a novel plasma EV protein panel with high diagnostic accuracy for AD.

**Methods:** A two‐phase nested case‐control study was used. Discovery: untargeted 4D‐Label‐free proteomics (LC‐MS/MS) on plasma EVs from 25 probable AD patients and 25 matched healthy controls (HC). Bioinformatic analyses integrated public brain scRNA‐seq data to infer cellular origins. Validation: candidate panel quantified by targeted mass spectrometry (SRM) in an independent multi‐center cohort (30 AD, 25 MCI, 30 HC). Models adjusted for age and systolic blood pressure. Machine learning built and evaluated a multi‐marker diagnostic model.

**Results:** Several proteins were differentially expressed; genes encoding them were enriched in brain‐specific cells, notably endothelial cells and microglia. In validation, PRKCSH was upregulated in AD; ANGPT1, NCKAP1, and FLJ78516 were downregulated vs. controls (all *p* < 0.001). MCI showed intermediate levels, suggesting a disease continuum. A diagnostic model with these four biomarkers achieved AUC 0.95 (95% CI 0.91–0.99) using Random Forest, with 92% sensitivity and 91% specificity for AD vs. controls.

**Figure:**
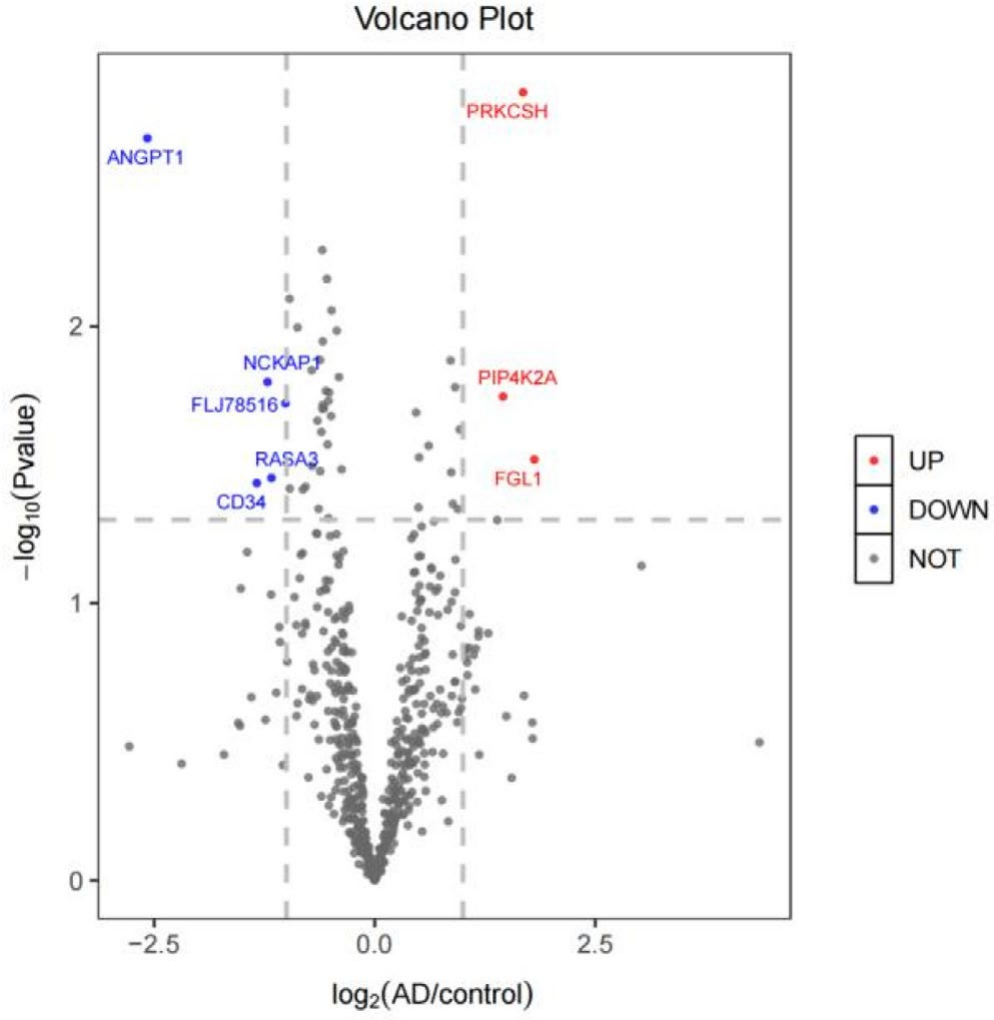
**FIGURE 1** Volcano plot of differentially expressed EV proteins in the discovery cohort (AD vs. HC). Proteins with FC > 2 and *p* < 0.05 are shown in red (upregulated), while those with FC < 0.5 and *p* < 0.05 are in blue (downregulated).

**Figure:**
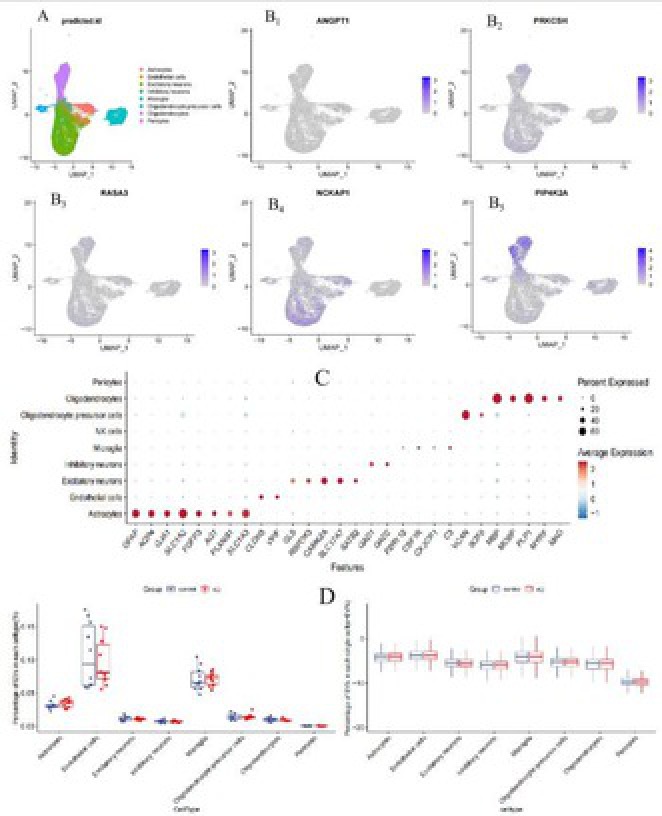
**FIGURE 2** Results of tissue origin tracing by cumulative calculation of AD exosome markers. *A: UMAP plot of cell clustering; B1‐5: UMAP plots of single gene expression distribution; C: Gene bubble plot; D: Box plot of cell proportion.

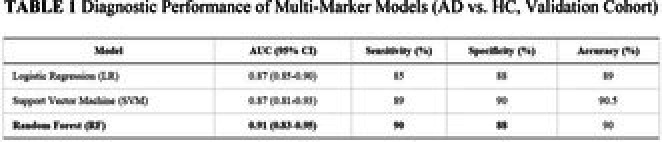

**Conclusion:** A four‐protein plasma EV panel (PRKCSH, ANGPT1, NCKAP1, FLJ78516) shows high diagnostic potential, likely originating from brain endothelial cells and microglia. This non‐invasive panel advances toward a clinically applicable AD blood test.

**Disclosure:** Nothing to disclose.

## EPO‐0334

### Alzheimer's disease, environment and genes – looking at the unexpected

#### M. Coelho ^1^; P. Faustino^1^; C. Bernardes^2^; J. Durães^1^; M. Lima^3^; D. Duro^1^; M. Almeida^3^; I. Baldeiras^3^; M. Tábuas‐Pereira^1^; I. Santana^1^

##### ^*1*^ *Neurology Department, Unidade Local de Saúde de Coimbra, Coimbra, Portugal;* ^*2*^ *Neurology Department, Unidade Local de Saúde do Alto Ave, Guimarães, Portugal;* ^*3*^ *Centre for Innovative Biomedicine and Biotechnology (CIBB), Universidade de Coimbra, Coimbra, Portugal*

**Background and aims:** Atypical forms of Alzheimer's disease (AD) are characterized by predominant symptoms in nonmemory cognitive domains. Apart from different clinical manifestations, these variants also have a younger age of onset, which may suggest a higher genetic contribution. With this work we aimed to study family history in AD subtypes, as a proxy of genetic contribution to their development.

**Methods:** Family history and APOE status in patients with typical and atypical AD diagnosis supported by biomarkers. Non‐parametric tests (χ2) were used to verify differences between groups. Logistic regression models were performed to investigate the association between family history and APOE status and the development of an atypical variant.

**Results:** The study included 392 patients: 293 with typical AD and 99 with atypical AD (53 frontal, 33 logopenic, and 14 posterior variants). A positive family history was more frequent in typical AD (48.5%) than in atypical AD (33.3%; χ^2^ = 6.775, *p* = 0.009). Family history prevalence did not differ significantly among atypical variants (χ^2^ = 1.107, *p* = 0.575). APOEε4 carriage was also more common in typical AD (50.5%) compared to atypical AD (38.4%; χ^2^ = 4.852, *p* = 0.028). In logistic regression analyses, the presence of an atypical AD variant was inversely associated with a positive family history (*β* = −0.548, 95% CI 0.347–0.963, *p* = 0.035) and APOEε4 status (*β* = −0.495, 95% CI 0.371–1.000, *p* = 0.050).

**Conclusion:** Our results suggest that genetic factors have a lower contribution to the development of atypical variants than typical AD, suggesting the contribution of other factors, namely environmental, warranting further studies.

**Disclosure:** Nothing to disclose.

## EPO‐0335

### Effects of different exercise modalities on cognitive function in older adults: A systematic review and network meta‐analysis

#### R. Lorandi ^1^; A. Rech^1^; R. Radaelli^2^; M. Petropoulou^3^; H. Almeida^1^; C. Silveira^1^; C. Muller^1^; L. Grolli^1^; P. Casara^1^; P. Lopez^1^

##### ^*1*^ *Grupo de Pesquisa Em Exercício Para Populações Clínicas (GPCLIN), Universidade de Caxias do Sul, Caxias do Sul, Brazil;* ^*2*^ *Egas Moniz Center for Interdisciplinary Research (CiiEM), Egas Moniz School of Health and Science, Almada, Portugal;* ^*3*^ *Institute of Medical Biometry and Statistics, Faculty of Medicine and Medical Center, University of Freiburg, Freiburg, Germany*

**Background and aims:** Globally, one in four older adults experiences cognitive impairment. Physical exercise has positive cognitive effects; however, the optimal exercise modality remains to be determined. Therefore, we aimed to systematically review and analyse the effects of different exercise modalities on cognitive function domains in older adults.

**Methods:** We included published randomised clinical trials from seven different databases on April 8, 2025, involving older adults (≥ 60 years), with or without cognitive impairment, randomised to supervised resistance (RE), aerobic (AE) and/or combined resistance and aerobic exercise (COMB) for at least 4 weeks. Outcomes included global cognition, attention, executive function and memory. Meta‐analysis was undertaken with a three‐level mixed effects model, and results were expressed as standardised mean difference (SMD) and 95% confidence intervals.

**Results:** We included 127 randomised controlled trials (*n* = 10,433) comprising 780 cognitive outcomes. Exercise significantly improved overall cognition (0.24 [0.17–0.30], *p* < 0.001). Similar benefits were observed following COMB (0.27 [0.09–0.45], *p* = 0.005) and RE (0.26 [0.14–0.37], *p* < 0.001), with smaller effects for AE (0.18 [0.11–0.26], *p* < 0.001). Exercise improvements were mainly for global cognition (0.42 [0.26–0.57], *p* < 0.001), followed by complex attention (0.18 [0.09–0.27], *p* < 0.001), executive function (0.14 [0.07–0.21], *p* < 0.001) and learning and memory (0.14 [0.05–0.24], *p* < 0.001). No significant effects were observed for language (0.09 [−0.03–0.21], *p* = 0.130) or perceptual‐motor function (0.05 [−0.11–0.22], *p* = 0.510).

**Conclusion:** Our findings indicate that resistance‐based exercise programs significantly improve specific cognitive domains in older adults, particularly attention, executive function, and learning/memory. These results underscore the importance of exercise to prevent cognitive decline and to support individuals presenting with cognitive impairment.

**Disclosure:** Nothing to disclose.

## Neuroimmunology 1

## EPO‐0336

### Cognitive and structural brain changes in ofatumumab‐treated multiple sclerosis: A longitudinal study

#### E. D'Amico; P. Di Filippo; C. Robusto; M. Moretti; C. Avolio; A. Zanghì

##### Brand Center, Department of Medical and Surgical Sciences, University of Foggia, Italy

**Background and aims:** Anti‐CD20 therapies control inflammatory activity in multiple sclerosis (MS), but their effects on neurodegeneration and cognition in real‐world settings remain uncertain. We aimed to characterize longitudinal cognitive and regional brain structural changes in ofatumumab‐treated MS patients. We also evaluated structure–cognition associations over time.

**Methods:** We conducted a longitudinal observational study of relapsing MS patients treated with ofatumumab. Cognitive performance was assessed using the Brief International Cognitive Assessment for Multiple Sclerosis (BICAMS), with processing speed as the primary outcome, and Magnetic Resonance Imaging (MRI) measures included thalamic volume, deep gray matter volume (DGM), and cortical thickness (Cth). Longitudinal changes and structure–cognition associations were analyzed using linear mixed‐effects models.

**Results:** Eighty‐five RMS patients treated with ofatumumab (mean age 37.9 ± 9.9 years; 69.4% female; 222 MRI scans) were included. Brain atrophy persisted but attenuated after 12 months: whole‐brain volume −0.15% vs −0.09%; DGM −1.01% to −0.36%; thalamus −0.94% −0.77%; CTh −1.67% −0.62% (all p for slope changes > 0.05). Cognitive performance improved (Symbol Digit Modalities Test, SDMT +3.79 points/year, 95% CI 1.35–6.23; *p* = 0.003), with improvements in verbal and visuospatial memory (all *p* < 0.05). No global longitudinal structure–cognition association was observed. In subgroups, thalamic atrophy was associated with SDMT decline in patients with Expanded Disability Status Scale ≥ 3 (*β* = −54.31; *p* = 0.013), and DGM atrophy with cognitive worsening in patients with baseline SDMT *z* ≤ −1 (*β* = −55.91; *p* = 0.012).

**Conclusion:** In ofatumumab‐treated relapsing MS, neurodegeneration persists but slows over time while cognition is preserved, and structural–functional coupling emerges only in vulnerable patients.

**Disclosure:** Nothing to disclose.

## EPO‐0337

### From intravenous to subcutaneous ocrelizumab in a high‐volume infusion center: A time‐and‐motion–informed capacity and service‐flow analysis

#### A. Zanghì; P. Di Filippo; C. Rutigliano; C. Avolio; E. D'amico

##### University of Foggia, Italy

**Background and aims:** Subcutaneous (SC) ocrelizumab offers a streamlined alternative to intravenous (IV) infusion in the management of multiple sclerosis (MS). Building on time‐and‐motion (T&M) methodologies validated in oncology, we conducted a singlecenter operational study to quantify procedure‐level time savings, estimate the resulting care‐time dividend, and assess changes in patient flow following SC implementation

**Methods:** We employed a cross‐sectional observational design in a five‐chair day hospital treating ~600 patients with MS treated with infusive therapies. T&M data were collected for 15 IV ocrelizumab administrations (April 2025) and 15 SC administrations (November 2025), capturing active clinical time across four phases: premedication, administration, observation, and checkout. Service‐flow metrics (monthly visit volume, mean waiting time) were extracted from the electronic queue system for May 2025 (early SC implementation) and November 2025 (mature SC adoption). A deterministic operational model projected capacity gains under different SC adoption scenarios.

**Results:** SC ocrelizumab reduced total active clinical time from 320 to 60 minutes (−81%), driven by administration (210→10 min) and observation (60→15 min) reductions. At 15 SC administrations/month, this translated into 65 clinical hours and 50 chair‐hours released—equivalent to 14 IV‐equivalent infusion slots. Despite unchanged ocrelizumab volumes, monthly visits increased (+7.9%) and mean waiting time decreased (−30%) from May to November 2025. Scenario modeling projected 100–133 chair‐hours released at 30–40 SC administrations/month.

**Conclusion:** Transitioning from IV to SC ocrelizumab yields substantial procedural efficiencies and frees infusion‐unit capacity. The care‐time dividend enables expanded access to complex therapies without additional staff or infrastructure, positioning SC ocrelizumab as a structural efficiency tool in MS care

**Disclosure:** Nothing to disclose.

## EPO‐0338

### Serum neurofilament light chain levels are associated with increased disability and impairment of specific cognitive domains in neuro‐Behçet disease

#### E. Tuzun ^1^; M. Tezel^1^; H. Karpuzoglu^2^; A. Emekli^3^; T. Gunduz^3^; V. Yılmaz^1^; M. Kurtuncu^3^; C. Küçükali^1^

##### ^*1*^ *Department of Neuroscience, Aziz Sancar Institute of Experimental Medicine, Istanbul University, Istanbul, Türkiye;* ^*2*^ *Department of Medical Biochemistry, Istanbul Faculty of Medicine, Istanbul University, Istanbul Türkiye;* ^*3*^ *Department of Neurology, Istanbul Faculty of Medicine, Istanbul University, Istanbul, Türkiye*

**Background and aims:** Neurological symptoms may emerge in around 10% of Behçet disease (BD) patients. Biomarkers for monitoring the clinical outcome of neuro‐BD have not been properly investigated.

**Methods:** Levels of neurofilament light chain (NFL), biomarker of neuroaxonal degeneration, were measured in sera of 27 patients with parenchymal neuro‐BD (16 men, 48 ± 9‐year‐old, 11 ± 5 years disease duration) and age/gender‐matched 30 patients with relapsing‐remitting multiple sclerosis (RRMS) and 25 healthy controls by the Siemens Healthineers Atellica Analyzer. Sera of all patients were obtained during remission. Potential correlations were sought between NFL levels and clinical‐cognitive aspects of neuro‐BD.

**Results:** NFL levels of neuro‐BD patients (13 ± 6 pg/mL; range, 6–31 pg/mL) were comparable with RRMS patients and significantly higher than healthy individuals (*p* < 0.0001). In neuro‐BD, NFL levels were correlated with EDSS (*p* = 0.019, *R* = 0.445), 9‐hole peg test (*p* = 0.002, *R* = 0.603), timed 25‐foot walk (*p* = 0.001, *R* = 0.612), visual‐spatial domain of Addenbrooke's cognitive examination revised (ACE‐R; *p* = 0.015, *R* = −0.490), Stroop interference test (*p* = 0.016, *R* = 0.506), complex figure test (*p* = 0.039, *R* = −0.422) and Wisconsin card sorting test (WCST) scores (*p* = 0.004, *R* = 0.592). No significant correlation was found between NFL levels versus disease duration, scores of MMSE and tests assessing verbal/visual memory, language and psychiatric symptoms.

**Conclusion:** Neuroaxonal degeneration is intimately involved in development of somatic disability, visual‐spatial and executive dysfunction in neuro‐BD, whereas memory impairment and psychiatric symptoms appear to occur via divergent disease mechanisms. ACE‐R, Stroop and WCST tests might potentially be used to monitor cognitive impairment in neuro‐BD.

**Disclosure:** Nothing to disclose.

## EPO‐0339

### Serum galectin‐3 as a biomarker in acute inflammatory polyradiculoneuropathies: A cohort study

#### G. Siconolfi ^1^; F. Vitali^1^; A. Sciarrone^1^; V. Guglielmino^1^; G. Ciasca^2^; U. Basile^3^; G. Primiano^4^; M. Luigetti^4^

##### ^*1*^ *Dipartimento di Neuroscienze, Università Cattolica del Sacro Cuore; 00168 Rome, Italy;* ^*2*^ *Sezione di Fisica, Dipartimento di Neuroscienze, Università Cattolica del Sacro Cuore; 00168 Rome, Italy;* ^*3*^ *Dipartimento di Patologia Clinica, Ospedale Santa Maria Goretti, A.U.S.L. Latina, Latina, Italy;* ^*4*^ *Fondazione Policlinico Universitario Agostino Gemelli IRCCS, Rome, Italy*

**Background and aims:** Guillain‐Barré Syndrome (GBS) refers to a group of acute inflammatory polyradiculopathies characterized by a diffuse inflammatory attack on the peripheral nervous system. In this study, we propose galectin‐3 (Gal‐3), a novel serum molecule identified expressed by Schwann cells and macrophages, as a potential biomarker in patients with GBS.

**Methods:** Serum levels of Gal‐3 and neurofilament light chain (NfL) were measured using the Simple Plex cartridge‐based immunoassay on the Ella platform in serum samples from a cohort of patients with GBS (*n* = 19) and in a validation cohort of healthy control subjects (*n* = 13). We also collected data on clinical history and patient outcomes.

**Results:** Gal‐3 levels were significantly higher in patients with GBS (median: 7041 pg/mL; IQR: 5576–8561) compared to healthy controls (median: 5030 pg/mL; IQR: 3877–5998). Within the GBS cohort, Gal‐3 levels were significantly elevated in patients with the axonal variant compared to those with classical AIDP variant (median: 8561 pg/mL vs. median: 5998 pg/mL, *p* < 0.05). In multivariate logistic regression, Gal‐3 remained significantly associated with the axonal variant of GBS after adjustment for age at sampling, diarrhea, GBS Disability Score at admission, and log‐transformed NfL. In ROC analysis, Gal‐3 demonstrated good discriminatory performance between axonal GBS and AIDP (AUC = 0.84), outperforming NfL (AUC = 0.76). Gal‐3 threshold of 6457.5 pg/mL distinguish axonal GBS with 100% sensitivity and 61.5% specificity

**Figure:**
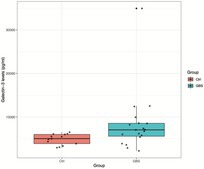
**FIGURE 1** Boxplot of serum levels of Gal‐3 in GBS patients and in control subjects.

**Figure:**
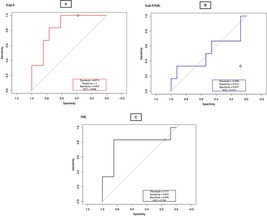
**FIGURE 2** Discriminatory performance of Galectin‐3 and Gal‐3/NfL Ratio Between Axonal and AIDP GBS Patients. ROC curves demonstrating the diagnostic performance of serum Gal‐3 levels (A), Gal3/NfL ratio(B), and serum NfL levels (C) in distinguishing the axonal variant.

**Figure:**
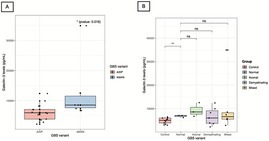
**FIGURE 3** Boxplot of serum levels of Gal‐3 in subgroup based on NCS findings firstly in axonal GBS variant (AMAN or AMSAN) and AIDP patient (A) and in relation to electrophysiological findings (B).

**Conclusion:** In our cohort, Gal‐3 was associated with both diagnostic and prognostic features in patients with GBS. These findings suggest that Gal‐3 may serve as a potential biomarker for differentiating axonal subtypes within the GBS spectrum.

**Disclosure:** Nothing to disclose.

## EPO‐0340

### Frequency of anti‐neural antibodies and autoimmune epilepsy in focal epilepsy of unknown etiology: An observational study in a singaporean cohort

#### J. Tan ^1^; J. Tan^2^; S. Park^2^; S. Saffari^2^; X. Peng^1^; P. Wong^1^; K. Tay^1^; A. Koe^1^; J. Tye^1^; J. Chai^1^; P. Koh^1^; Y. Tan^1^; Y. Tan^2^; S. Srinivasan^1^; N. Loh^1^; N. Loh^2^; N. Loh^4^; N. Tan^1^; N. Tan^3^; K. Tan^1^; K. Tan^2^; K. Tan^3^; T. Yeo^1^; T. Yeo^2^; T. Yeo^3^

##### ^*1*^ *Department of Neurology, National Neuroscience Institute, Singapore;* ^*2*^ *Duke‐NUS Medical School, Singapore;* ^*3*^ *Yong Loo Lin School of Medicine, National University of Singapore, Singapore;* ^*4*^ *Lee Kong Chian School of Medicine, Nanyang Technological University, Singapore*

**Background and aims:** Detection of anti‐neural autoantibodies for autoimmune epilepsy (AES) is crucial for early immunotherapy for improved outcomes. Conventional cell‐based assays (CBA) test for limited panel of known autoantibodies. Immunohistochemistry (IHC) using rodent brain tissue represents a more comprehensive repertoire. Prevalence of AES and anti‐neuronal antibodies in in this cohort is unknown in Singapore. We describe here 1. Establishment of IHC assay and comparison to CBA sensitivity and prevalence 2. Comparing clinical characteristics between seropositive and seronegative patients 3. Clinical utility of the ACES (Antibodies Contributing to Focal Epilepsy Signs and Symptoms)score.

**Methods:** This is a cross‐sectional, prospective, single‐center study performed at the National Neuroscience Institute (NNI), Singapore. Inclusion criteria were patients with focal epilepsy of unknown etiology; able to provide informed consent; ≥ 18 years old and had at least 1 seizure in the last 1 year prior to recruitment. Demographic data, medical history and epilepsy information were collected. ACES scores were calculated. Patients were stratified into definite/probable/possible/non‐AES using IHC and CBA. Clinical characteristics, MRI, EEG and ACES scores were analyzed.

**Results:** 120 patients were included. Demographics and clinical data are detailed in Table 1. Figure 1 shows selected staining patterns and table showing seropositive IHC and/or CBA. Clinical characteristics of seropositive patients are detailed in Table 2A. Comparative analysis of possible AES and non‐AES patients are shown in Table 2B. Diagnostic performance of ACES score is shown in Table 2C.

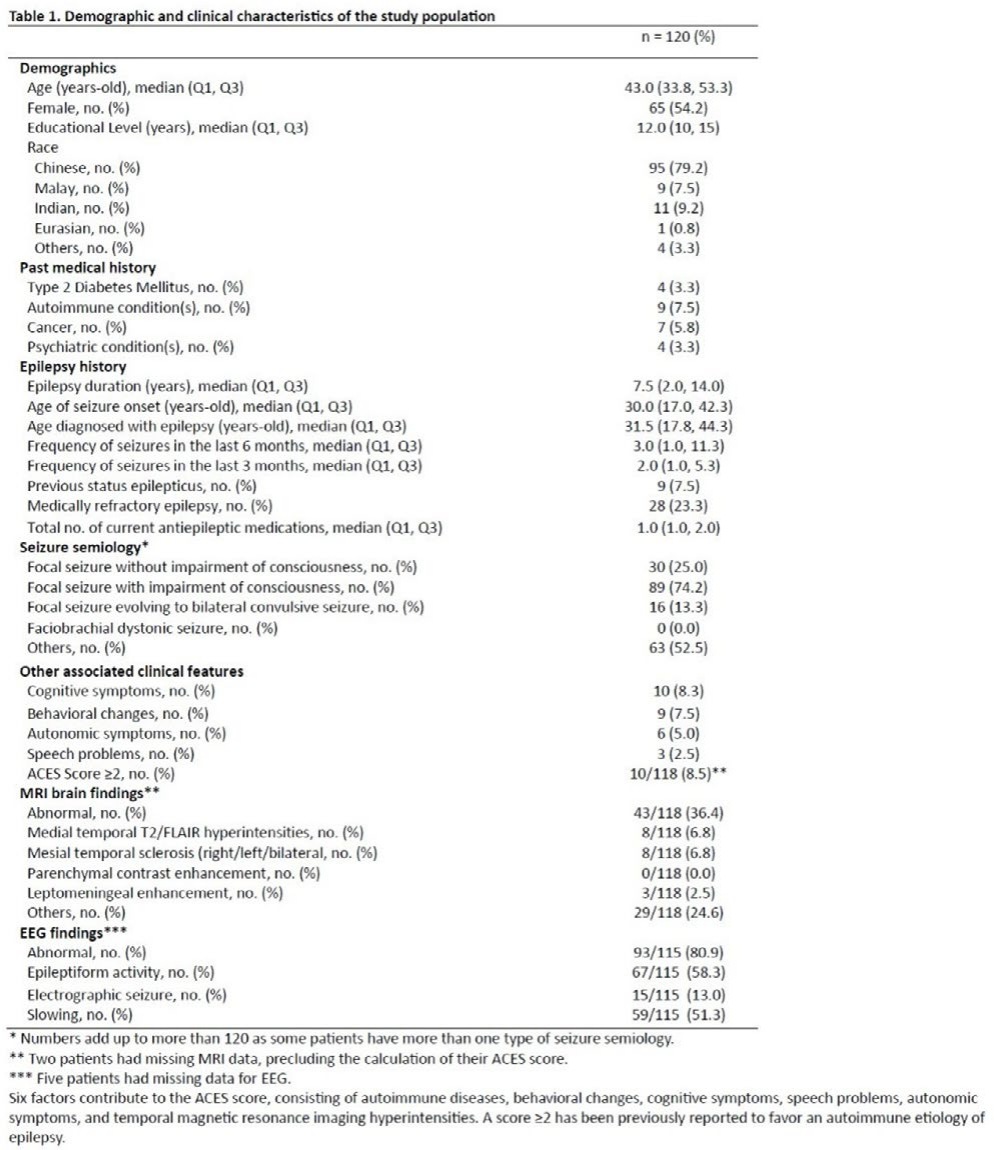

**Figure:**
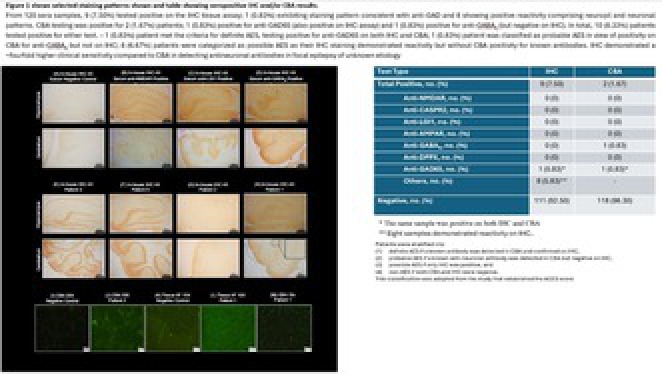
**FIGURE 1** shows selected staining patterns shown and table showing seropositive IHC and/or CBA results.

**Figure:**
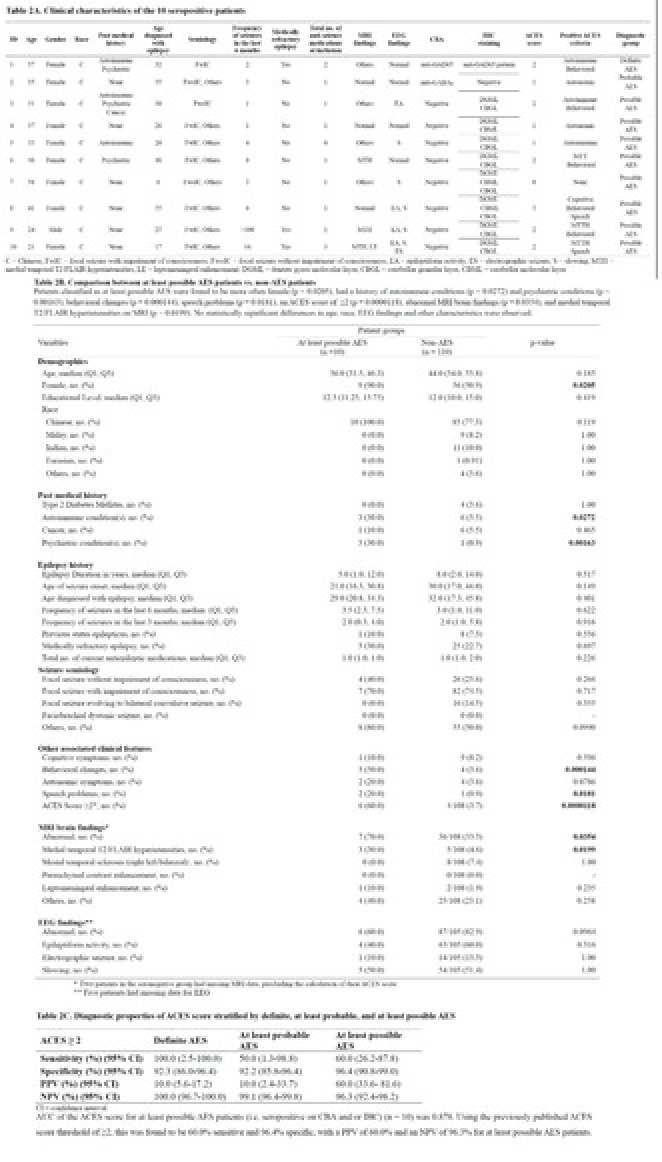
**TABLE 2** Clinical characteristics of seropositive patients are detailed in Table 2A. Comparative analysis of possible AES and non‐AES patients are shown in Table 2B. Diagnostic performance of ACES score is shown in Table 2C.

**Conclusion:** The combination of clinical characteristics, IHC screening, and ACES scoring can improve AES detection, enabling earlier diagnosis and immunotherapy even in patients with negative CBA results.

**Disclosure:** This study was funded by the NNI RIE2025 CG Pilot Grant award to Jeanne May May Tan, AM‐ETHOS grant awarded by Duke‐NUS Medical School to Seong Jin Park and National Medical Research Council (Singapore) Research Training Fellowship grant awarded to Tianrong Yeo. Jeanne May May Tan is funded by the Singhealth CIVA award (06/2024/CIVA/04‐A04). Tianrong Yeo is funded by the Singapore Ministry of Health's National Medical Research Council Clinician Scientist Award (CSAINV24jul‐0004). Jeanne May May Tan has received honoraria from AstraZeneca, Merck, and Roche for speaker's fees, and research grants from the National Medical Research Council (NMRC Singapore) and Singhealth Pte Ltd. She has also received travel grants from Roche and Merck. Tianrong Yeo has received honoraria from ASNA, AstraZeneca, Edanz Pharma, Euroimmun AG, Merck, Novartis, Roche, Terumo BCT for consulting services and speaker's fees, and research grants from the National Medical Research Council (NMRC Singapore), AstraZeneca and Roche. He has also received travel grants and awards from PACTRIMS, ACTRIMS, ECTRIMS, Orebro University, UCB, and Merck. The other co‐authors declare no competing interest. This study was conducted in Singapore under local ethics approval (CIRB 2016/2469). Written informed consent was obtained from every study participant.

## EPO‐0341

### GLP‐1/GLP‐1R‐mediated glycolytic reprogramming drives area postrema neuronal hyperexcitability in NMOSD‐associated area postrema syndrome

#### L. Yang; Q. Li; H. Qin; S. Sang; Y. Yao; Y. Li; Y. Jia

##### Department of Neurology, The First Affiliated Hospital of Zhengzhou University, China

**Background and aims:** Area postrema syndrome (APS) in neuromyelitis optica spectrum disorder (NMOSD) involves unclear metabolic mechanisms. This study investigates the unexplored role of glucagon‐like peptide‐1 receptor (GLP‐1R) signaling in driving AP neuronal hyperactivity via glycolytic reprogramming.

**Methods:** Mechanistic studies used a dual‐antigen (AQP4+MOG) EAE mouse model and SH‐SY5Y cells. Interventions included GLP‐1R antagonist Exendin‐(9–39), agonist Exendin‐4, and IL‐6 inhibitor Satralizumab. Analyses comprised metabolomics, patch‐clamp electrophysiology, and molecular assays.

**Figure:**
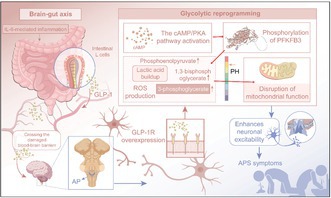
**FIGURE 1** Figure abstract.

**Results:** The EAEAQP4+MOG model developed APS‐like symptoms (reduced food intake, weight loss) with disrupted blood‐brain barrier integrity and AP region inflammation. AP neurons showed significant upregulation of GLP‐1/GLP‐1R and neuronal activity markers (c‐FOS, RAB5a). Metabolomic profiling revealed enhanced anaerobic glycolysis (elevated phosphoenolpyruvic acid, 1,3‐bisphosphoglycerate, 3‐phosphoglycerate) and lactate accumulation, alongside reduced TCA cycle intermediates (fumaric acid, succinic acid) in AP neurons. Patch‐clamp electrophysiology demonstrated decreased action potential threshold and increased neuronal hyperexcitability. Mechanistically, GLP‐1R activation triggered cAMP‐PKA pathway activation, PFKFB3 phosphorylation, and mitochondrial dysfunction (disorganized cristae, reduced ATP production, elevated ROS). In vitro, LPS‐induced inflammation or Exendin‐4 treatment replicated GLP‐1R overexpression, glycolytic reprogramming, and mitochondrial impairment in SH‐SY5Y cells. Administration of Exendin‐(9–39) or Satralizumab reversed these pathological changes, normalizing neuronal excitability, metabolic profiles, and mitochondrial function, and alleviating APS‐like symptoms in EAE mice.

**Figure:**
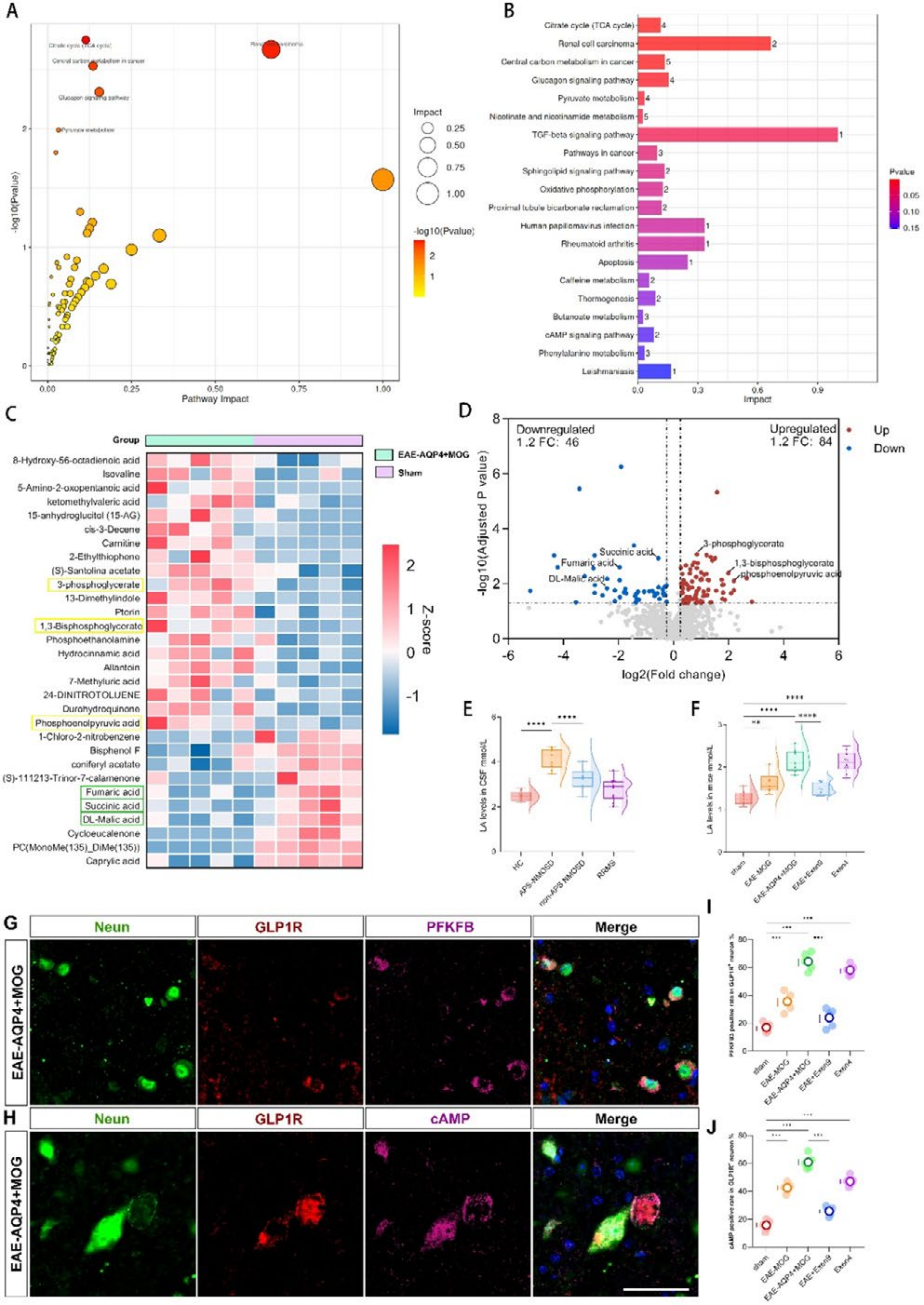
**FIGURE 2** EAE mice metabolomics.

**Figure:**
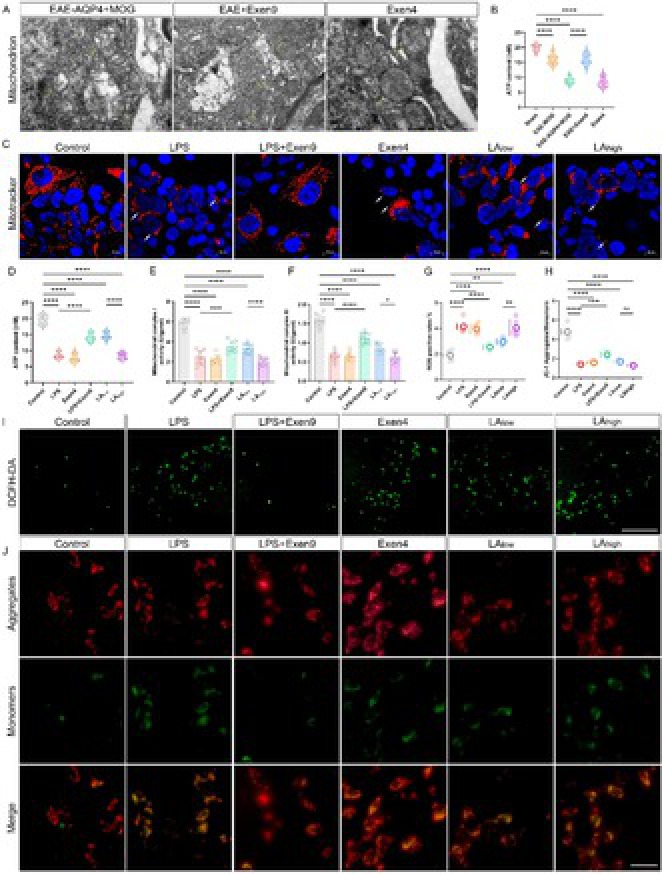
**FIGURE 3** EAE mice mitochondrial dysfunction.

**Conclusion:** GLP‐1/GLP‐1R‐mediated glycolytic reprogramming via cAMP‐PKA pathway drives AP neuronal hyperactivity in NMOSD‐APS. Targeting GLP‐1R signaling or IL‐6‐mediated inflammation offers a novel therapeutic strategy by correcting both metabolic dysregulation and neuronal dysfunction. This study highlights the importance of metabolic‐immune crosstalk in autoimmune neurological disorders and provides new insights into NMOSD pathophysiology.

**Disclosure:** Nothing to disclose.

## EPO‐0342

### Long‐term and patient‐reported outcomes (PROs) in patients with autoimmune encephalitis: A prospective cohort study

#### M. Borioni; A. Morano; A. Marocchi; E. Cerulli Irelli; A. Mazzeo; P. Moro; A. De Matteis; L. Fulignati; A. Giallonardo; C. Di Bonaventura

##### Department of Human Neurosciences, Policlinico Umberto I, Rome, Italy

**Background and aims:** Autoimmune encephalitis (AE) is a disorder presenting with acute neuropsychiatric manifestations and seizures. While AE may be associated with favorable recovery, prognosis can be variable. Outcomes in AE are traditionally assessed using the modified Rankin Scale (mRS) which fails to capture long‐term effects on mood, sleep, and quality of life (QoL). Here, we evaluated a cohort of patients with AE using patient‐reported outcomes (PROs) to provide a wider assessment of long‐term prognosis.

**Methods:** We prospectively enrolled adult patients previously diagnosed with AE according to Graus criteria, with a minimum follow‐up of two years. After collection of electroclinical data, the following validated PROs were administered: the Pittsburgh Sleep Quality Index (PSQI), Beck Depression Inventory‐II (BDI‐II), Hospital Anxiety and Depression Scale (HADS), and the Short Form Health Survey (SF‐36).

**Results:** Thirty‐two patients were enrolled (median age 67 years [IQR: 55–77]; 82% males), with a median follow‐up of 7.5 years. At last follow‐up, mRS scores ranged from 0 to 1 in 60% of patients. In contrast, the SF‐36 revealed impaired quality of life in up to 80% of patients. Clinically relevant anxiety and/or depressive symptoms were observed in 71% of patients based on BDI‐II and HADS scores, while 67% showed reduced sleep quality according to the PSQI. Diagnostic delay was associated with worse BDI‐II (*p* = 0.046) and PSQI scores (*p* = 0.022).

**Conclusion:** Although most patients with AE show improvement on traditional mRS assessment at follow‐up, a substantial proportion continues to experience mood disturbances and sleep impairment, which significantly affect their quality of life.

**Disclosure:** Nothing to disclose.

## EPO‐0343

### CSF lipidomics approach in CIDP: Sphingomyelin 18:1 as a potential biomarker?

#### N. Tjiang ^1^; F. Kohle^1^; C. Schroeter^2^; R. Klimas^3^; K. Pitarokoili^3^; A. Fisse^3^; J. Motte^3^; B. Schoser^4^; G. Fink^5^; M. Schroeter^1^

##### ^*1*^ *Department of Neurology, Faculty of Medicine, University of Cologne and University Hospital Cologne, Kerpener Strasse; 62; 50937 Cologne, Germany;* ^*2*^ *Department of Neurology, Medical Faculty, Heinrich Heine University Düsseldorf, Düsseldorf, Germany;* ^*3*^ *Department of Neurology, St. Josef‐Hospital, Ruhr‐University Bochum, Gudrunstrasse 56; 44791 Bochum, Germany;* ^*4*^ *Department of Neurology, Friedrich‐Baur‐Institute, Ludwig‐Maximilians‐University Clinic Munich, Ziemssenstr. 1; 80336 Munich, Germany;* ^*5*^ *University of Cologne, Faculty of Medicine and University Hospital, Department of Neurology, Cologne, Germany; Cognitive Neuroscience Section, Institute of Neuroscience and Medicine (INM‐3), Research Centre Juelich, Juelich, Germany*

**Background and aims:** Chronic Inflammatory Demyelinating Polyradiculoneuropathy (CIDP) is a heterogeneous immune‐mediated neuropathy marked by diagnostic complexity and variable treatment responses. Reliable biomarkers for diagnosis and disease stratification are lacking. Lipoproteins have emerged as regulators of neuroinflammation and potential autoimmune targets. However, their role in CIDP is unknown. We investigated the lipid profiles in serum and cerebrospinal fluid (CSF) treatment‐naïve CIDP patients.

**Methods:** Serum and CSF samples from CIDP patients at initial diagnosis and controls were analyzed using mass spectrometry–based lipidomics targeting glycerophospholipids and sphingomyelin species (*n* = 26). Patients with idiopathic intracranial hypertension and diabetic neuropathy served as control groups (*n* = 18, *n* = 22). Lipidomic findings were integrated with proteomic data and longitudinal clinical scores, including therapy response and disease severity, and confounders like age, gender, and total CSF protein.

**Results:** Preliminary proteomic data reveal a differential increase in proteins involved in lipid metabolism in the CSF of CIDP patients (including APOA2, APOA4, and APOL1). The preliminary lipidomic analysis shows a significant decrease of phosphatidylinositol 32:1 in CSF. In contrast, the sphingomyelin 18:1 CSF‐to‐serum‐ratio is markedly elevated, with significant increases in CSF and decreases in serum (*p* < 0.05).

**Conclusion:** Our findings provide evidence for lipid dysregulation in CIDP patients. Especially the altered CSF‐to‐serum ratio of 18:1 sphingomyelin species warrants further investigation. Further correlation with clinical measures and validation in independent cohorts are required to assess the potential of lipids as mechanistic biomarkers in CIDP.

**Disclosure:** The authors declare no conflicts of interest.

## EPO‐0344

### Cognitive teleREhabilitation in patients with AutoIMmune encephalitis, a pilot study to assess feasibility and potential efficacy: CoRE‐AIM study

#### P. Giglio ^1^; R. Barnabei^1^; A. Malvaso^1^; S. Bernini^2^; S. Bottiroli^2^; S. Gasverde^4^; F. Deleo^5^; S. Beretta^6^; A. Vogrig^7^; R. Iorio^8^; P. Businaro^3^; S. Masciocchi^3^; M. Gastaldi^3^

##### ^*1*^ *Department of Brain and Behavioral Sciences, University of Pavia; 27100 Pavia, Italy;* ^*2*^ *Cognitive Psychology Research Section, IRCCS Mondino Foundation, Pavia, Italy;* ^*3*^ *Neuroimmunology Research Unit, IRCCS Mondino Foundation‐National Neurological Institute,;* ^*4*^ *ASL TO4, Ciriè, Italy;* ^*5*^ *Istituto Neurologico Carlo Besta, Milan, Italy;* ^*6*^ *Department of Neuroscience, Università degli Studi di Milano Bicocca, Milano, Italy;* ^*7*^ *Clinical Neurology, Department of Head‐Neck and Neuroscience, Azienda Sanitaria Universitaria Friuli Centrale (ASUFC), Udine, Italy; Department of Medicine (DMED), University of Udine, Udine, Italy;* ^*8*^ *Department of Neuroscience, Università Cattolica del Sacro Cuore, Rome, Italy; Neurology Unit, Fondazione Policlinico Universitario A. Gemelli, IRCCS, Rome, Italy*

**Background and aims:** To assess feasibility of a cognitive telerehabilitation protocol (CTR) in patients with autoimmune encephalitis (AE).

**Methods:** Patients with confirmed diagnosis of AE and neuropsychological impairment were prospectively recruited from November 2022 to November 2025. Participants were evaluated through a battery of neuropsychological testing and a comprehensive neurological evaluation at baseline (T0) and after 3 months (T1). At T0, patients and caregivers received a portable touch screen computer installed with a tailored telerehabilitation program (3 weekly, 25 min‐sessions for a total of 12 weeks). User experience (UX) was assessed using the Italian‐validated User Experience Questionnaire (UEQ) and the System Usability Scale (SUS). Health status was evaluated with the 12‐Item Short Form Health Survey questionnaire (SF‐12).

**Results:** Twelve subjects were included (4 LGI1‐AE, 2 GAD‐AE, 3 CASPR2‐AE, 1 seronegative‐AE, 1 GABA‐B‐AE, 1 NMDAR‐AE). A median of 100% of the sessions and 94.57% of the tasks were completed with a median SUS of 78.75. UEQ scores were above the benchmark level for all the items included in the analysis. Median MoCA scores were similar between T0 (21.5, range 14.6–30) and T1 (23, range 18–28), but cognitive performances significantly improved in attention as assessed by faster TMT‐A (*p* < 0.05). In addition, patients showed an improved perception of physical health on the SF‐12 questionnaire (*p* < 0.05).

**Conclusion:** CTR is a feasible and potentially effective approach to treat cognitive dysfunction in AE. Further prospective studies are needed to define specific protocols and timing of administration.

**Disclosure:** Nothing to disclose.

## EPO‐0345

### Effectiveness of early efgartigimod use in adult gMG patients in Japan: A retrospective study

#### R. Aoyagi ^1^; H. Teranishi^1^; K. Tsuda^1^; Y. Zheng^2^; J. Luo^2^; S. Inagaki^2^; M. Takahashi^3^; C. Blein^4^

##### ^*1*^ *argenx Japan, Tokyo, Japan;* ^*2*^ *ZS Associates;* ^*3*^ *Osaka University, Japan;* ^*4*^ *argenx BVBA,* Ghent, Belgium

**Background and aims:** This study evaluated the impact of intravenous/subcutaneous efgartigimod (EFG) on treatment burden in Japanese patients with generalized myasthenia gravis (gMG) between heavily pre‐treated (HPT) and less heavily pre‐treated (LHPT) cohorts.

**Methods:** We conducted a retrospective cohort analysis using hospital‐based claims from Medical Data Vision of adult gMG patients initiating EFG between May 2022, and April 2025. Eligible patients had 12‐month baseline and follow‐up observation, oral glucocorticoids (GC) or non‐steroidal immunosuppressants within 90‐days pre‐EFG initiation, and no other biologics during follow‐up (Figure 1). HPT was defined as > = 2 baseline fast‐acting treatment (FT) cycles (intravenous immunoglobulin, plasma exchange, intravenous methylprednisolone) or > = 20 mg/day prednisolone‐equivalent oral GC dose within 90 days pre‐EFG initiation; all others were LHPT. Outcomes were oral GC average daily dose (ADD) and FT cycles counts.

**Figure:**
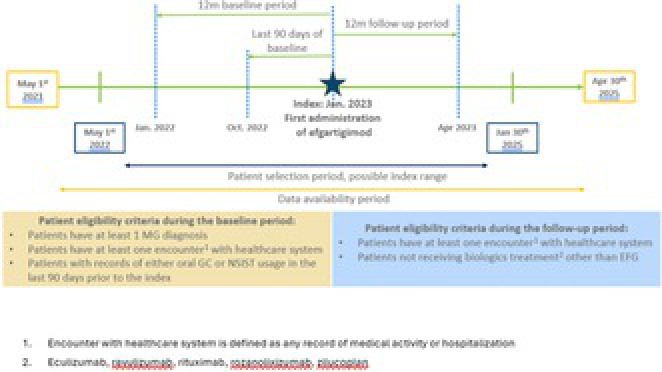
**FIGURE 1** Patient Selection Criteria and Study Design. Eligible patients in the study must meet all the criteria listed for both baseline and the follow‐up period.

**Results:** Among 260 patients identified, 58% were HPT and 42% were LHPT. Mean duration from first recorded gMG diagnosis to index, and EFG treatment duration did not differ between cohorts (HPT: 5.7 years, 455 days; LHPT: 5.9 years, 447 days; *p* = 0.635, *p* = 0.816 respectively). Mean oral GC ADD approximately halved after EFG initiation in both cohorts (HPT: 19.1 to 9.6 mg/day, LHPT: 14.2 to 7.1 mg/day, baseline to follow‐up). LHPT had higher proportion of patients achieving < = 5 mg/day throughout follow‐up (Figure 2) and one‐third as many FT cycles per patient versus HPT during follow‐up (0.45 vs. 1.46, Figure 3).

**Figure:**
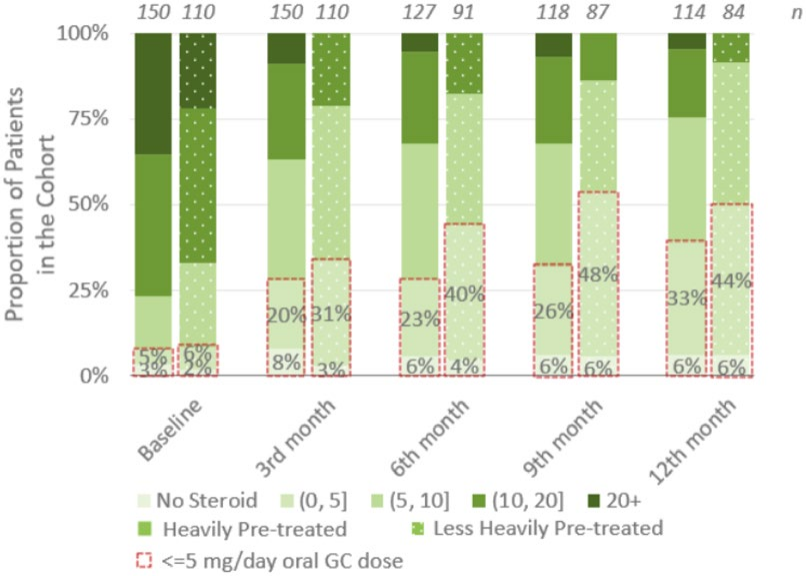
**FIGURE 2** Oral GC ADD in Patients with Continuous EFG Use. ADD within 3rd, 6th, 9th, and 12th month follow‐up was measured for the HPT (left) and LHPT cohorts (right), focusing on patients who continued EFG until the end of the months of measurement.

**Figure:**
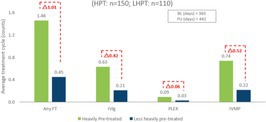
**FIGURE 3**: Average FT cycle count per patient in follow‐up. Average FT (IVIg, PLEX, IVMP, sum of all) during 12‐month follow‐up was measured for the HPT (left) and LHPT cohorts (right).

**Conclusion:** LHPT consistently showed lower treatment burden, while effective oral GC tapering was observed in both cohorts. EFG initiation in earlier disease management may improve real‐world treatment outcomes.

**Disclosure:** RA, HT, KT, and CB are employees of argenx. MPT reports unrestricted research grants from Japan Blood Products Organization and Astellas Pharma outside the submitted work, has served as a paid consultant for Alexion, Argenx, Hanall BioPharma, and UCB Pharma, and received honoraria for lectures from Argenx, Alexion Pharmaceuticals, and UCB Pharma. YZ, JL, and SI are employees of ZS Associates and serve as paid consultants for argenx.

## EPO‐0346

### Conformational antibodies to PLP1/DM20 define a clinical subgroup within immune mediated neuropathies

#### S. Masciocchi ^1^; M. Zuliani^2^; P. Businaro^1^; E. Vegezzi^3^; A. De Lorenzo^4^; L. Diamanti^3^; C. Morandi^1^; S. Scaranzin^1^; E. Zardini^1^; Schirinzi^5^; A. Salvalaggio^6^; G. Bruno^7^; L. Lorusso^8^; R. Milani^8^; V. Ziccone^9^; S. Bucello^9^; G. Macorig^10^; G. Cellante^11^; M. Fabris^12^; A. Vogrig^11^; S. Tozza^13^; F. Canale^14^; V. Todisco^14^; L. Benedetti^15^; G. Siciliano^5^; C. Briani^6^; E. Marchioni^3^; P. Doneddu^4^; D. Franciotta^16^; J. Devaux^17^; M. Gastaldi^1^

##### ^*1*^ *Neuroimmunology Laboratory and Neuroimmunology Research Section, IRCCS Mondino Foundation, Pavia, Italy;* ^*2*^ *Department of Brain and Behavioral Sciences, University of Pavia, Italy;* ^*3*^ *Neuroncology and Neuroinflammation Unit, IRCCS Mondino Foundation, Pavia, Italy;* ^*4*^ *Neuromuscular and Neuroimmunology Unit, IRCCS Humanitas Research Hospital, Milano, Italy;* ^*5*^ *Department of Clinical and Experimental Medicine, Neurological Clinic, University of Pisa, Italy;* ^*6*^ *Department of Neurosciences, Neurology Unit, University of Padova, Padova, Italy;* ^*7*^ *Pediatric Neurology Unit, Department of Neurosciences, Santobono‐Pausilipon Children's Hospital, Naples, Italy;* ^*8*^ *UOC Neurology and Stroke Unit, ASST Lecco, Merate, Italy;* ^*9*^ *Multiple Sclerosis Center, “E. Muscatello” Hospital – ASP8, Augusta, Italy;* ^*10*^ *San Giovanni di Dio Hospital, Neurology Unit, Gorizia, Italy;* ^*11*^ *Clinical Neurology, Department of Head‐Neck and Neuroscience, Azienda Sanitaria Universitaria Friuli Centrale, Udine, Italy;* ^*12*^ *Institute of Clinical Pathology, Santa Maria della Misericordia University Hospital, Udine,Italy;* ^*13*^ *University of Naples; Neurology Unit, Policlinico Federico II University Hospital, Italy;* ^*14*^ *Department of Advanced Medical and Surgical Sciences, University of Campania “Luigi Vanvitelli”, Naples, Italy;* ^*15*^ *IRCCS Ospedale Policlinico San Martino, Genoa, Italy;* ^*16*^ *UOM, Laboratory of Clinical Pathology, Department of Laboratories, APSS Santa Chiara Hospital, Trento, Italy;* ^*17*^ *Institut de Génomique Fonctionnelle, Université de Montpellier, CNRS, INSERM, Montpellier, France*

**Background and aims:** We have previously identified conformational antibodies to proteolipid protein‐1 (PLP1) and its peripheral isoform DM20 in patients with combined demyelinating disorders of the peripheral and central nervous systems. However, their presence in IMN has never been investigated.

**Methods:** We implemented a DM20 live cell‐based assay and used it to screen a retrospective exploratory cohort of defined IMN (*n* = 294), a prospective validation cohort of suspect IMN (*n* = 324), and controls (*n* = 81). Clinical features were compared with a cohort of DM20‐IgG‐negative chronic inflammatory demyelinating polyneuropathy (CIDP, *n* = 43).

**Results:** DM20‐IgG were identified in 20 of 537 cases (3.7%) of IMN and in none of the controls. DM20‐IgG–positive IMN fulfilled the criteria for CIDP (*n* = 12) or Guillain‐Barrè syndrome (GBS; *n* = 8). Most presented with acute onset (17/20) and had a preceding trigger (11/20, of which 5 SARS‐CoV‐2 vaccination). Compared with “typical CIDP”, DM20‐IgG positive patients showed frequent cranial nerve involvement (12/20 vs 10/43, *p* = 0.009), pain (12/20 vs 11/43, *p* = 0.012) and higher disability at acme (INCAT total score 4 [1–9] vs 2 [0–8], *p* = 0.06). A satisfactory response to intravenous immunoglobulin was obtained in 17/18 patients (94%). DM20‐IgG predominantly pertained to IgG1 (12/20) and bound myelin on rat teased nerve fibres, live oligodendrocytes and myelinated Schwann cell segments.

**Conclusion:** DM20‐IgG defines a subgroup of IMN characterized by acute onset, cranial nerve involvement, severe disability and a favorable response to immunotherapy. Their ability to bind their target on live cultures might suggest pathogenicity, that needs to be explored in future studies.

**Disclosure:** Nothing to disclose.

## EPO‐0347

### Abstract withdrawn

## Cerebrovascular Diseases 4

## EPO‐0348

### Is selective screening safe for stroke prevention? High rates of missed severe stenosis in Pre‐CABG: Validating ESVS 2023

#### A. Nusairat ^1^; M. Al‐Akhras^1^; A. Alanzi^1^; T. Bnayan^1^; Y. Al‐Hushki^1^; M. Abu‐Nukta^1^; T. Nusairat^2^; M. Al‐Mansi^1^

##### ^*1*^ *Jordan University of Science & Technology (JUST),* Irbid, Jordan*;* ^*2*^ *The Hashemite University,* Zarqa, Jordan

**Background and aims:** Perioperative stroke is a devastating complication of coronary artery bypass grafting (CABG), frequently driven by occult carotid atherosclerosis. The 2023 ESVS guidelines advise against routine carotid screening, favoring selective screening only for high‐risk subsets (Age > 70, Stroke/TIA, Left Main Disease). However, the safety of this restrictive strategy in populations with high metabolic burden is unproven. We validated whether following these guidelines leaves patients at risk of undetected carotid stenosis and subsequent cerebrovascular events.

**Methods:** We analyzed 203 consecutive patients scheduled for CABG at King Abdullah University Hospital (Jordan). All patients underwent comprehensive neurovascular imaging (Duplex/CTA). We stratified the cohort based on ESVS selective criteria to determine the “miss rate” for hemodynamically significant internal carotid artery (ICA) stenosis (≥ 50%). Multivariate logistic regression identified independent predictors of carotid disease relevant to neurological risk.

**Results:** he cohort had a high metabolic burden (Diabetes: 67%, Hypertension: 85%). Overall Carotid Artery Disease prevalence was 54.2%, with significant ICA stenosis (≥ 50%) in 17.7% (*n* = 36). Crucially, strict adherence to ESVS 2023 selective criteria would have failed to detect 11.1% (*n* = 4) of patients with significant stenosis. These “missed” patients were asymptomatic and younger (< 70 years) yet possessed critical plaque burden. In multivariate analysis, Left Main Coronary Disease (OR 1.66, 95% CI 1.05–2.8) and Hypertension (OR 2.47) were independent predictors of significant carotid stenosis.

**Conclusion:** In a high‐risk cohort, reliance on “selective screening” misses > 11% of patients with significant carotid stenosis, leaving them vulnerable to preventable perioperative stroke. To optimize neuroprotection, we propose that Left Main Disease should trigger mandatory carotid screening.

**Disclosure:** Nothing to disclose.

## EPO‐0349

### Early‐phase fluid diagnostic biomarkers in acute ischemic stroke: An umbrella metanalysis

#### A. Bombaci ^1^; F. Pozzi^1^; S. Mazzeo^1^; E. Bortolin^1^; G. Bruschi^1^; M. Corbari^1^; A. Astengo^4^; G. Stufano^4^; S. Rossi^4^; M. Perini^4^; G. Rotondo^4^; F. Novellino^2^; M. Filippi^3^; M. Salone^1^

##### ^*1*^ *Unità di Neurologia, IRCSS Policlinico San Donato, San Donato Milanese, Milano, Italy; Vita‐Salute San Raffaele University, Milan, Italy;* ^*2*^ *Neuroscience Research Center, Department of Medical and Surgical Science, University of “Magna Grecia” Catanzaro, Italy;* ^*3*^ *Vita‐Salute San Raffaele University, and Neurology Unit, Neurorehabilitation Unit, and Neurophysiology Service, IRCCS Ospedale San Raffaele, Milan, Italy;* ^*4*^ *Unità di Neurologia, IRCSS Policlinico San Donato, San Donato Milanese, Milano, Italy*

**Background and aims:** Acute ischemic stroke (AIS) is a time‐critical emergency in which rapid diagnosis within established therapeutic windows is essential to optimize outcomes. Fluid biomarkers offer a promising adjunct to clinical and neuroimaging assessment but their temporal dynamics in the acute phases remain incompletely characterized.

**Methods:** We performed an umbrella review of systematic reviews and meta‐analyses evaluating fluid biomarkers in AIS versus controls or stroke mimics. Quantitative synthesis of primary studies (random‐effects meta‐analysis of standardized mean differences) was stratified by clinically relevant time windows. Heterogeneity, small‐study effects, and excess significance bias were assessed.

**Figure:**
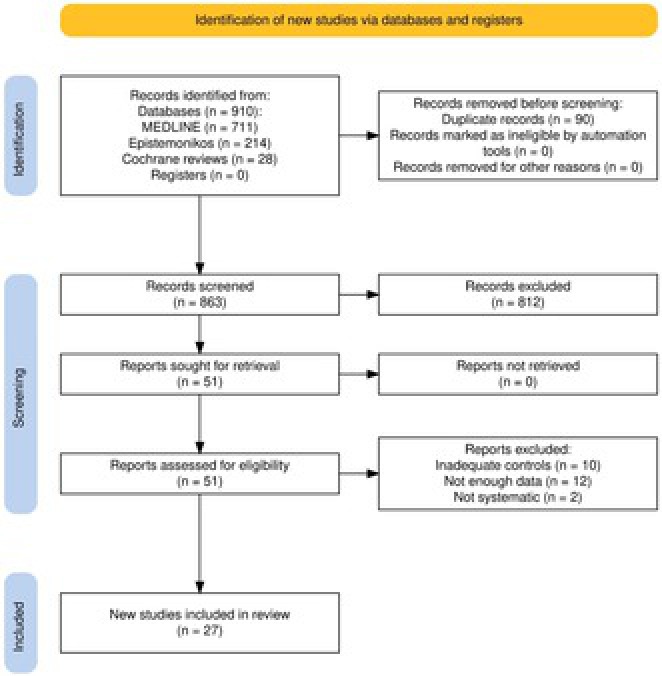
**FIGURE 1** Prisma flowchart of the selected studies.

**Results:** We included 27 publications (18 biochemistry, 1 metabolomic, 10 transcriptomic, 5 cell‐free‐DNA [cfDNA]). Across all time‐points the largest effect sizes were observed for neuron‐specific enolase (NSE), ischemia‐modified albumin (IMA), d‐Dimer, S100B, GFAP, and IL‐6. Looking at metabolites, studies revealed early accumulation of lactate, succinate, glutamate and lysophosphatidylcholines, alongside depletion of arginine, citrulline and citrate. A catalogue of 220 micro‐RNAs (132 upregulated; 108 downregulated) identified robust markers (miR‐16‐5p, let‐7e‐5p, miR‐107, miR‐451a, miR‐126‐3p). 46 circulating‐RNAs, and 55 long‐non‐coding‐RNAs were consistently dysregulated. Five studies reported elevated nuclear and mitochondrial cfDNA within 6h.

**Figure:**
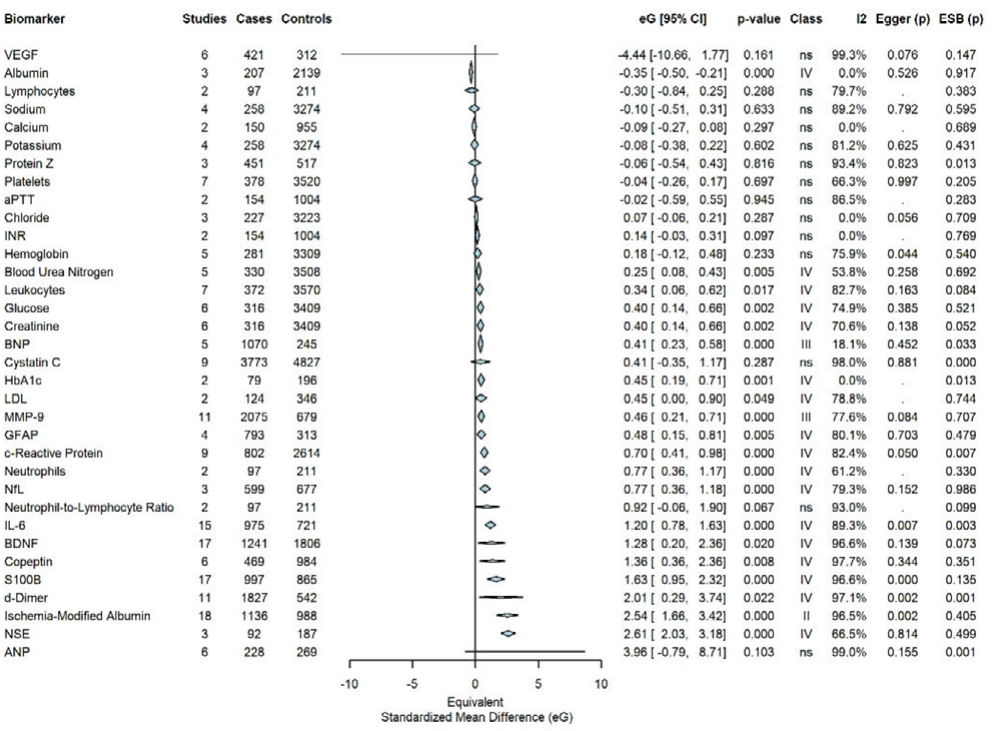
**FIGURE 2** Forest plot of the meta‐analyses for biochemistry biomarkers, comparing acute ischemic stroke (AIS) vs controls (HCs and mimics) across all time‐points.

**Figure:**
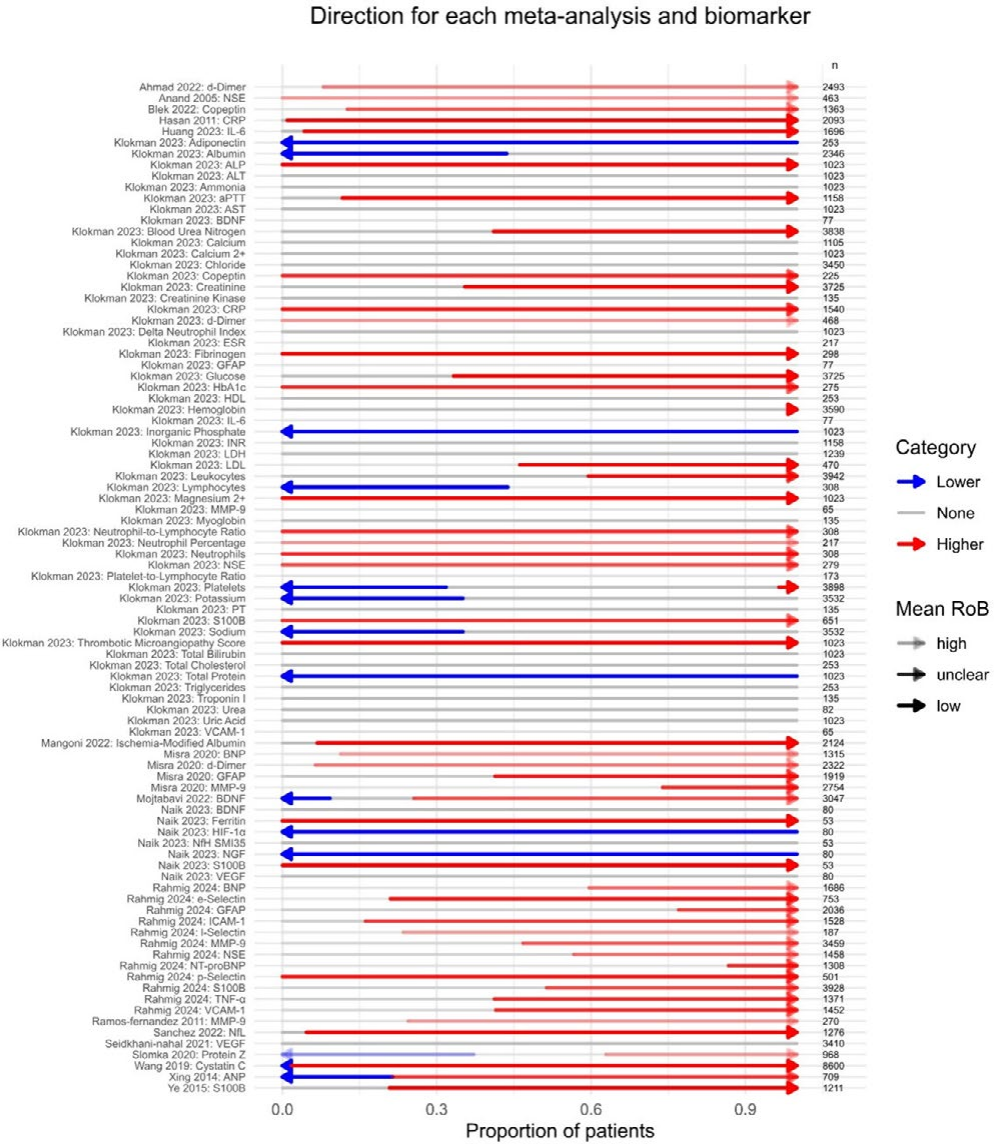
**FIGURE 3** Arrow plot of the biochemistry biomarkers, comparing acute ischemic stroke (AIS) vs controls across all time‐points. For each biomarker we reported the direction of the studies and the total number of patients across studies.

**Conclusion:** Fluid biomarkers exhibit a temporally evolving signature: early coagulopathy (D‐dimer), glial activation (GFAP, S100B) and inflammation (IL‐6), followed by neuronal necrosis (NSE) and oxidative stress (IMA) within 24 h. Multi‐omic integration, including metabolomics, transcriptomics and cfDNA, highlights convergent pathways (PI3K/Akt, NF‐κB, immunometabolism) and supports the development of rapid, point‐of‐care panels. Standardized sampling windows and harmonized assay protocols are essential for clinical translation and prospective validation in prehospital settings.

**Disclosure:** Nothing to disclose.

## EPO‐0350

### Why genetics can't be ignored in secondary stroke prevention: The CYP2C19 challenge in Asia

#### A. Raheja; D. De Silva; K. Narasimhalu

##### Department of Neurology, National Neuroscience Institute (Singapore General Hospital Campus), Singapore

**Background and aims:** Stroke is a leading cause of death and disability globally, with Asia disproportionately affected. A critical barrier to effective secondary prevention is the high prevalence of CYP2C19 loss‐of‐function alleles, present in almost 75% of South and East Asians, which reduce clopidogrel efficacy.

**Methods:** Evidence from trials including CHANCE‐2, shows that genotype‐guided alternatives, including ticagrelor and cilostazol, substantially lower recurrent stroke risk in loss‐of‐function carriers. Yet, clinical adoption remains limited by insufficient genetic testing infrastructure, cost, guideline gaps, and clinician training.

**Results:** Despite these challenges, genotype‐guided therapy is both feasible and cost‐effective, with potential to reduce recurrent strokes, disability, and healthcare burden.

**Figure:**
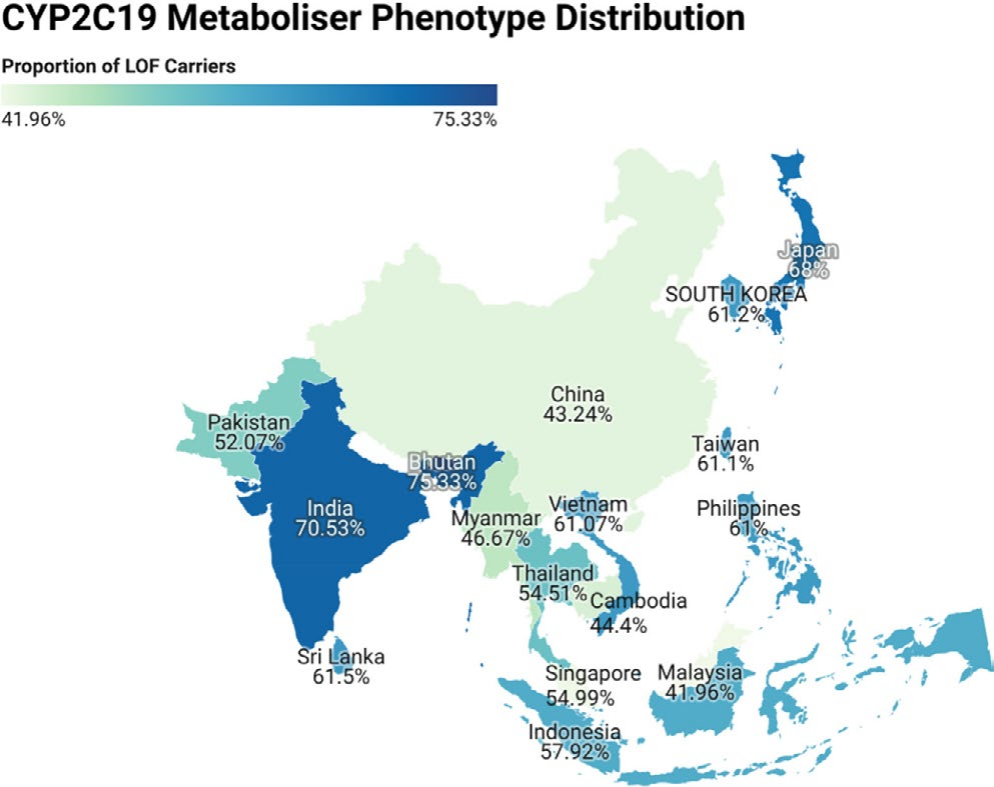
**FIGURE 1** Geographic distribution of CYP2C19 intermediate (IM) and poor metabolisers (PM) rates across selected Asian countries. 3, 9, 11 Shading intensity corresponds to the combined prevalence of these phenotypes.

**Conclusion:** Urgent action is required to implement precision antiplatelet strategies, update guidelines, and ensure equitable access, making pharmacogenomics a central component of stroke care in Asia.

**Disclosure:** Nothing to disclose.

## EPO‐0351

### Cerebral small vessel disease in early‐ vs late‐onset Alzheimer's disease in a memory clinic cohort

#### C. Tamba ^1^; A. Griffa^1^; P. Michel^2^; P. Salvioni Chiabotti^1^; S. Pistocchi^3^; Y. Aleman‐Gomez^3^; V. Dunet^3^; O. Rouaud^1^; P. Hagmann^3^; G. Allali^1^; G. Bommarito^1^

##### ^*1*^ *Leenaards Memory Center, Department of clinical neurosciences, Lausanne University Hospital and University of Lausanne (CHUV‐UNIL), Lausanne, Switzerland**;*** ^*2*^ *Stroke Center, Neurology Service, Department of clinical neurosciences, Lausanne University Hospital and University of Lausanne (CHUV‐UNIL), Lausanne, Switzerland**;*** ^*3*^ *Department of Diagnostic and Interventional Radiology, Lausanne University Hospital and University of Lausanne (CHUV‐UNIL), Lausanne, Switzerland*

**Background and aims:** Cerebral small vessel disease (CSVD) frequently co‐occurs with Alzheimer's disease and increases with age. Here, we evaluated CSVD burden in early onset and late‐onset Alzheimer's disease (EOAD and LOAD), distinguishing between hypertensive arteriopathy (HTNA) and cerebral amyloid angiopathy (CAA) co‐pathology, as they have different underlying mechanisms.

**Methods:** We retrospectively included 59 EOAD and 162 LOAD (clinically‐biologically diagnosed), retrieving clinical features including vascular risk factors. CSVD features were rated on MRI using established criteria, deriving HTNA and CAA prevalence. CSVD burden was compared between EOAD and LOAD, adjusting for sex and the difference between age at MRI and age at symptom onset.

**Results:** A probable CAA was present in 15.3% of EOAD and 29.6% of LOAD, while a moderate/severe HTNA was observed in 10.2% of EOAD and 30.2% of LOAD. LOAD present with higher rates of hypertension, diabetes, and atrial fibrillation (*p* < 0.05). LOAD showed a greater CSVD burden, driven primarily by more severe white matter hyperintensities, lacunes and perivascular spaces load (*p* < 0.05). HTNA severity and probable CAA prevalence were higher in LOAD (*p* < 0.001 and *p* = 0.035, respectively), despite the absence of significant differences in hemorrhagic features.

**Figure:**
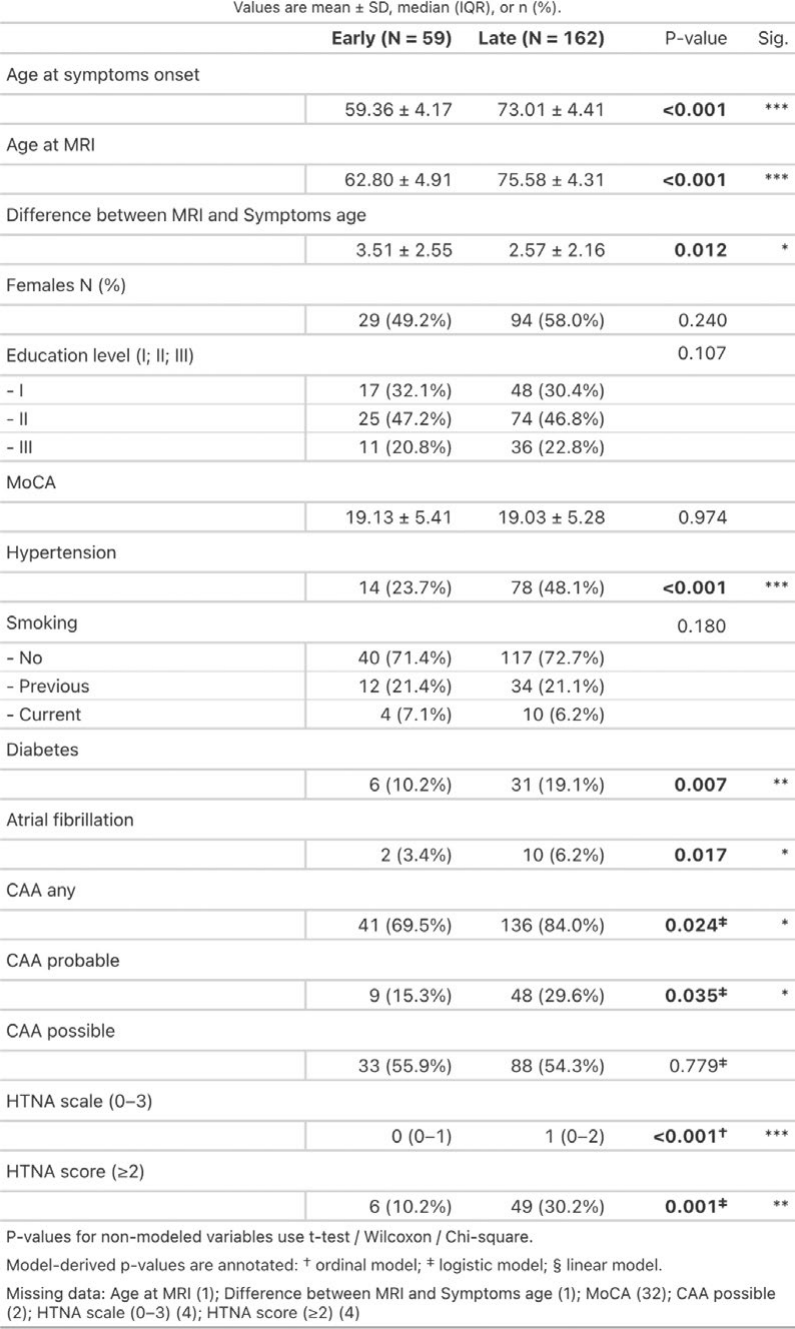
**TABLE 1** Demographics, vascular risk factors, and diagnosis markers in early‐onset (*n* = 59) and late‐onset (*n* = 162) Alzheimer's disease.

**Figure:**
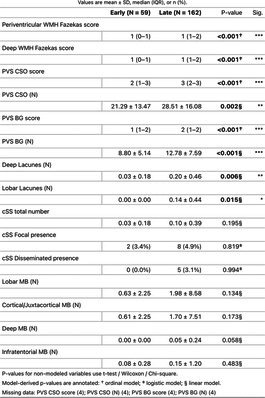
**TABLE 2** Magnetic resonance imaging (MRI) markers of cerebral small vessel disease in early‐onset (*n* = 59) and late‐onset (*n* = 162) Alzheimer's disease.

**Figure:**
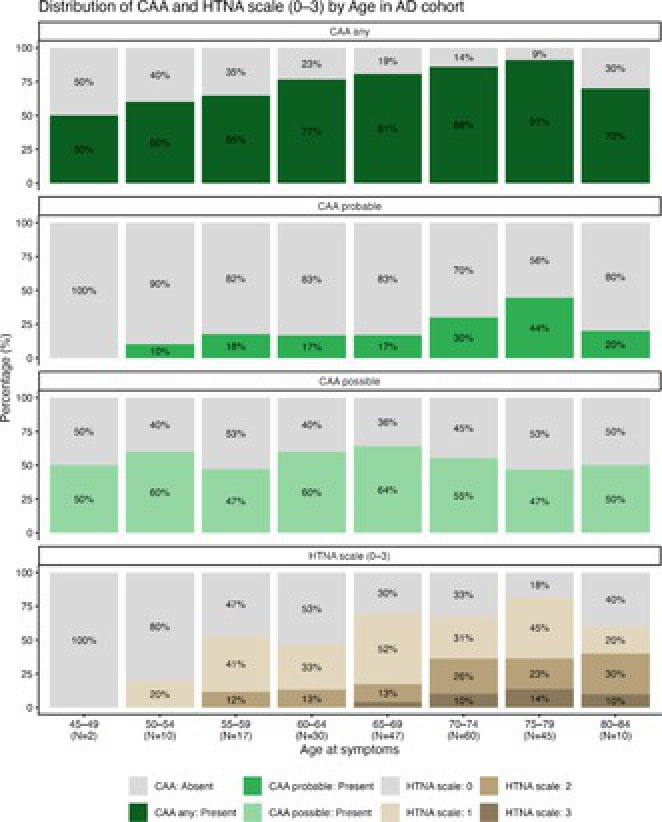
**FIGURE 1** Cerebral amyloid angiopathy and hypertensive arteriopathy across age at symptom onset. Stacked bars show percentages by age bin for any/possible/probable cerebral amyloid angiopathy (CAA) and hypertensive arteriopathy (HTNA) score.

**Conclusion:** LOAD is associated with heavier vascular risk and CSVD co‐pathology, in terms of both HTNA and CAA. The difference between groups seems to be mainly driven by CSVD features affecting the white matter, more severe in LOAD. However, CSVD burden in EOAD is not negligeable, supporting routine vascular co‐pathology assessment across age.

**Disclosure:** Nothing to disclose.

## EPO‐0352

### Medium‐vessel occlusion recanalisation after medical management, and clinical outcome

#### F. Fenter ^1^; V. Dunet^2^; P. Michel^1^; D. Strambo^1^

##### ^*1*^ *Stroke Center, Neurology Service, Department of Clinical Neurosciences, Lausanne, Switzerland;* ^*2*^ *Neuroradiology Unit, Service of Diagnostic and Interventional Radiology, Department of Medical Radiology, both at the Lausanne University Hospital and University of Lausanne, Lausanne, Switzerland*

**Background and aims:** In acute ischemic stroke (AIS) with medium‐vessel occlusion (MeVO), intravenous thrombolysis (IVT) is the currently recommended acute recanalisation therapy. We aimed to evaluate the association between imaging‐proven recanalisation (IPR) and clinical outcome in MeVO‐AIS treated with conservative treatment (CTr) or IVT.

**Methods:** From our local stroke registry we included all consecutive patients with MeVO‐AIS receiving CTr or IVT. IPR was defined as complete recanalisation on 12–24‐hour CTA/MRA. Clinical outcome was 24h‐NIHSS and 3‐month modified‐Rankin‐Scale (mRS). The association of treatment (CTr vs. IVT) and recanalisation (IPR vs. no IPR) with clinical outcomes was assessed using propensity‐score‐weighed regression models. Weighting accounted for pre‐stroke mRS, baseline NIHSS, age, sex, onset‐to‐door time, MeVO localisation, vascular risk factors.

**Results:** Among 229 patients with MeVO, 52 received CTr and 177 IVT. Overall, 114 had IPR. Patients with IPR had more M3 occlusions, fewer P1‐P3 occlusions, and shorter onset‐to‐door times. IVT was significantly associated with IPR (aOR = 16.8, 95% CI = 5.37–48.74) but not with 3‐month mRS. In the overall cohort, IPR was associated with greater 24‐hour NIHSS improvement (beta‐coeff = 1.93, 95% CI = 0.39–3.47), better 3‐month mRS (pswOR = 2.50, 95% CI = 1.56–4.00), and higher rates of excellent outcome (mRS 0–1, pswOR = 3.10, 95% CI = 1.72–5.59). The same associations between IPR and clinical outcomes were observed in the cohort of IVT‐treated patients

**Figure:**
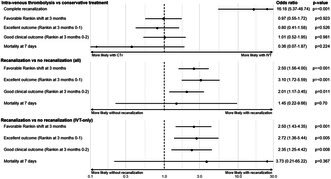
**FIGURE 1** Forest plot of binary/categorial outcomes.

**Conclusion:** In MeVO‐AIS treated with medical management, IPR is associated with improved functional outcome. While IVT increased the likelihood of IPR, persistent occlusion remained frequent, suggesting that more effective revascularisation strategies are needed to improve recanalisation rates and thus clinical outcomes.

**Disclosure:** François Fenter: nothing to disclose. Vincent Dunet: nothing to disclose. Patrik Michel: nothing to disclose. Davide Strambo: advisory board for Boehringer Ingelheim.

## EPO‐0353

### Neuroanatomical correlates in stroke‐related ipsilateral hemiparesis: Single center experience and review of the literature

#### F. Fenter ^1^; D. Strambo^1^; A. Vicino^1^; E. Dirren^3^; V. Dunet^2^; P. Michel^1^

##### ^*1*^ *Stroke Center, Neurology Service, Department of Clinical Neurosciences, Lausanne, Switzerland;* ^*2*^ *Neuroradiology Unit, Service of Diagnostic and Interventional Radiology, Department of Medical Radiology, Lausanne University Hospital and University of Lausanne, Lausanne, Switzerland;* ^*3*^ *Stroke Center, Neurology Service, Geneva University Hospital, Geneva, Switzerland*

**Background and aims:** Ipsilateral hemiparesis (ILH) in stroke is rare and challenges the conventional understanding of motor system organization. We aim to describe ILH strokes from our academic stroke center, complemented by a literature review.

**Methods:** All patients with hemiparesis and an acute ischemic lesion on neuroimaging were selected from 2003–2023 in our stroke registry. ILH cases were identified, and all their investigations were reviewed, including MRI‐tractography, functional MRI, and TMS (if performed. We also performed a review of the literature of ILH cases in ischemic and hemorrhagic strokes. We compared all identified cases with all patients with contralateral hemiparesis in our local registry.

**Figure:**
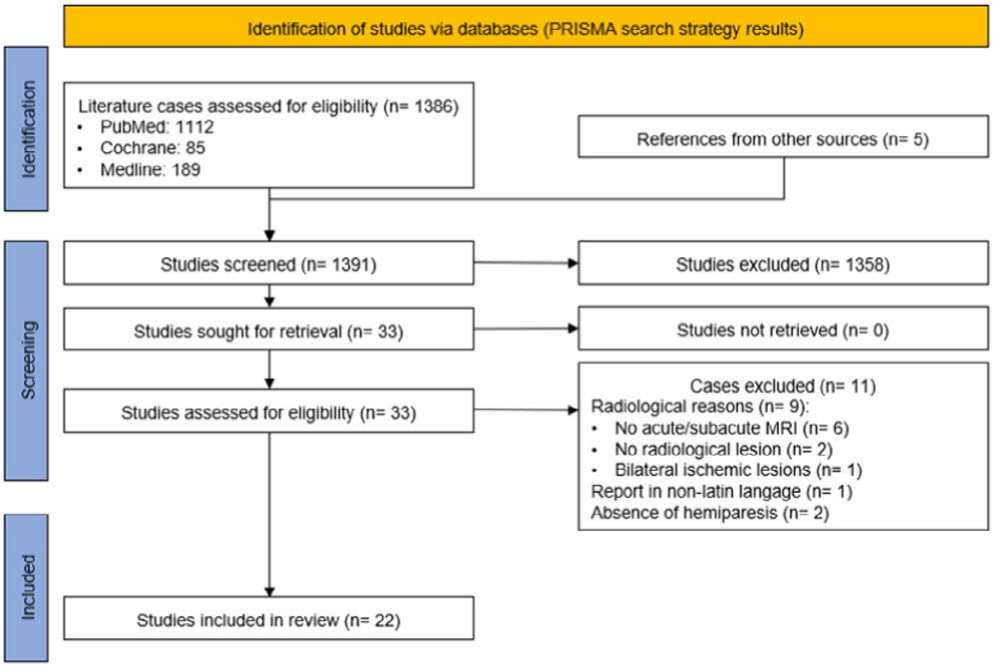
**FIGURE 1** PRISMA search strategy results realized in December 2024.

**Results:** We found 4 ILH cases among 2,426 consecutive hemiparetic stroke patients (0.16%). MRI‐tractography showed physiological corticospinal tract decussation and fMRI found bilateral motor activation in all 4. The systematic literature review yielded 61 additional cases of ILH related to stroke, resulting in a total of 14 TMS, 18 fMRI, and 14 MRI‐tractographic exams. In multivariable analysis, ILH was associated with younger age (beta = 0.94; *p* < 0.01) and history of previous stroke (OR = 7.99; *p* < 0.01). The additional investigations revealed evidence of corticospinal tract reorganization, aberrant interhemispheric connectivity, corticospinal tract anatomy variation, and involvement of secondary motor area as potential mechanisms.

**Figure:**
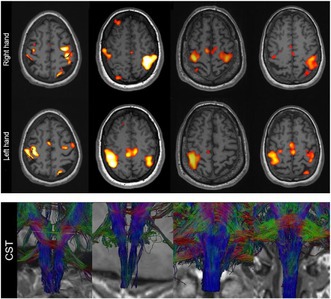
**FIGURE 2** fMRI (upper rows), and MR‐tractography (third row) of our 4 ILH patients who all had left MCA ischemic strokes.

**Figure:**
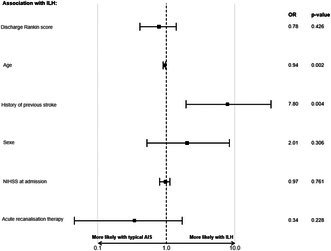
**FIGURE 3**: Multivariable binary regression model showing patients' ad stroke features independently associated with ILH. Results expressed as odds radio (OR) and *p*‐value. *X*‐axis on logarithmic scale.

**Conclusion:** ILH is a rare manifestation of acute stroke and seems to occur more frequently in younger patients with prior strokes. Our findings suggest that some ILH result from corticospinal reorganization and recruitment of secondary motor pathways, potentially highlighting the importance of neuroplasticity in post‐stroke motor control.

**Disclosure:** Nothing to disclose.

## EPO‐0354

### Clinical impact of systemic inflammatory response in ischaemic stroke due to atherosclerotic tandem lesions

#### J. Andrada Moreno ^1^; M. Medina Rodríguez^1^; P. Baena Palomino^1^; L. Ainz Gómez^1^; J. Cabezas Rodríguez^1^; A. Hermosín Gómez^1^; A. De Albóniga Chindurza^2^; M. Aguilar Pérez^2^; A. González García^2^; F. Moniche Álvarez^1^; E. Zapata Arriaza^2^

##### ^*1*^ *Neurology, Hospital Virgen del Rocío, Seville, Spain;* ^*2*^ *Interventional Neuroradiology, Hospital Virgen del Rocío, Seville, Spain*

**Background and aims:** Systemic inflammatory response (SIR) is frequently elevated in patients with atherosclerotic ischaemic stroke. This study aimed to assess the clinical impact of SIR in patients with tandem lesions treated with mechanical thrombectomy (MT) and emergent carotid artery stenting (eCAS).

**Methods:** We analysed data from the prospective ARTISTA registry, including patients with ischaemic stroke secondary to atherosclerotic tandem lesions treated with MT and eCAS between 2019 and 2023. Baseline SIR were assessed using the neutrophil‐to‐lymphocyte ratio (NLR) on admission. Associations between NLR and acute carotid stent thrombosis (< 24 hours), symptomatic intracranial haemorrhage (sICH), poor functional outcome (modified Rankin Scale [mRS] > 3), and 3‐month mortality were evaluated using multivariable binary logistic regression models adjusted for age, sex, and predefined covariates.

**Results:** 149 patients were included; 78.5% were male, with a median age of 67 years (IQR 60–77). The median NLR was 1.13 (IQR 0.67–2.16). Acute stent thrombosis occurred in 9 patients (6.7%), sICH in 14 (9.4%), poor functional outcome in 63 (42.3%), and 30 patients (20.1%) died within 3 months. After adjustment for age and sex, higher baseline NLR remained independently associated with acute carotid stent thrombosis (adjusted odds ratio [aOR] 1.08, 95% CI 1.01–1.16; *p* = 0.02), but not with sICH, poor functional outcome, or mortality. This association persisted after further adjustment for antiplatelet therapy.

**Figure:**
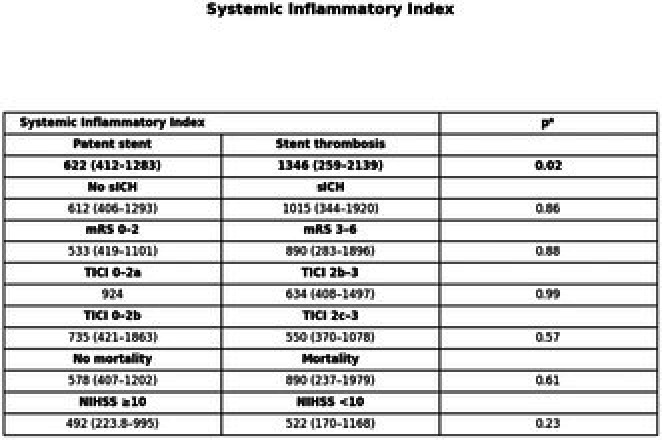
**TABLE 1** Association between baseline neutrophil‐to‐lymphocyte ratio and clinical outcomes (p value calculated using multivariable linear regression adjusted for age and sex).

**Figure:**
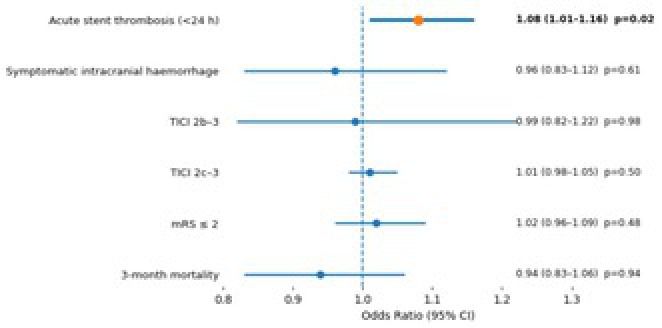
**FIGURE 1**: Association between baseline neutrophil‐to‐lymphocyte ratio and clinical outcomes (model adjusted for age, sex, and antiplatelet therapy).

**Conclusion:** Patients with tandem lesions and elevated baseline SIR are at increased risk of acute carotid stent thrombosis. These findings may guide future studies to identify patients who could benefit from intensified periprocedural antithrombotic strategies.

**Disclosure:** Nothing to disclose.

## EPO‐0355

### Impact of liver fibrosis on stroke outcomes in patients with intracranial atherosclerotic stenosis: A swiss cohort study

#### K. Mettler ^1^; L. Siegenthaler^1^; M. Reichmuth^1^; K. Antonenko^1^; N. Slavova^1^; W. Almiri^2^; A. Antonenko^3^; A. Hakim^2^; M. Heldner^1^

##### ^*1*^ *Department of Neurology, Inselspital, University Hospital and University of Bern, Bern, Switzerland;* ^*2*^ *Institute of Diagnostic and Interventional Neuroradiology, Inselspital, University Hospital and University of Bern, Bern, Switzerland;* ^*3*^ *Department of Visceral Surgery and Medicine, Inselspital, University Hospital and University of Bern, Bern, Switzerland*

**Background and aims:** Chronic liver disease (CLD) is increasingly recognized as a contributor to cardiovascular disease, but its relationship with intracranial atherosclerotic stenosis (ICAS) and stroke outcomes remains underexplored. This study aimed to investigate the association between liver fibrosis, assessed by the FIB‐4 score, and outcomes in acute ischemic stroke patients with‐/out ICAS.

**Methods:** We performed a retrospective observational cohort study using data from the Bernese Stroke Registry. A total of 500 patients with acute ischemic stroke (admitted 04/2022–12/2023) were categorized into four groups based on FIB‐4 score (≤ 3.25/> 3.25) and ICAS presence (yes/no). Clinical characteristics, vascular risk factors, ICAS location, treatment, and 3‐months outcomes were compared.

**Results:** Patients with high FIB‐4 scores were significantly older (median 82.5 vs. 71 years, *p* < 0.001) and had a higher burden of atrial fibrillation and renal insufficiency (both *p* < 0.001). Arterial hypertension was more prevalent in ICAS groups (83.6/86.7% vs. 67.3/68.2%, *p* < 0.001), while hypercholesterolemia was less frequent in high‐FIB‐4 groups (*p* = 0.028). No significant differences were found in acute revascularization therapies. Patients with FIB 4 > 3.25 and ICAS had significantly poorer 3‐months outcomes: only 10.3% achieved an excellent outcome (mRS ≤ 1) compared to 36.3% in the low FIB 4/no ICAS group (*p* = 0.030), recurrent ischemic stroke was more frequent (7.1% vs. 1.6%; *p* = 0.041) and mortality was significantly elevated (43.3% vs. 13.3%; *p* < 0.001).

**Conclusion:** High liver fibrosis (FIB‐4 > 3.25) is linked to worse outcomes in acute ischemic stroke, particularly with ICAS, including more recurrent stroke, and worse 3‐months functional outcome, highlighting the value of assessing liver fibrosis in stroke prognosis.

**Disclosure:** None.

## EPO‐0356

### Stroke in people living with HIV and 6‐month outcomes in northwestern Tanzania

#### L. Mwamba ^1^; E. Bukelebe^2^; I. Paul^2^; J. Ngimbwa^3^; M. Basinda^2^; B. Andrew^4^; A. Paul^2^; A. Mawazo^5^; D. Lucas^4^; L. Rudovick^4^; B. Wajanga^4^; S. Kalluvya^2^; P. Olowoyo^6^; R. Peck^7^; S. Matuja^2^

##### ^*1*^ *Department of Neurology, First Affiliated Hospital of Jinzhou Medical University, Liaoning, China;* ^*2*^ *Department of Internal Medicine, Catholic University of Health and Allied Sciences‐Weill Bugando School of Medicine, Mwanza, Tanzania;* ^*3*^ *Department of Internal Medicine, Aga Khan University, Dar‐es‐salaam, Tanzania**;*** ^*4*^ *Department of Internal Medicine, Bugando Medical Center, Mwanza, Tanzania;* ^*5*^ *Department of Microbiology and Immunology, Muhimbili University of Health and Allied Sciences, Dar es Salaam, Tanzania;* ^*6*^ *Department of Medicine, Federal Teaching Hospital Ido‐Ekiti, Afe Babalola University, Ado‐Ekiti, Nigeria;* ^*7*^ *Center for Global Health, Department of Internal Medicine, Weill Cornell Medicine, New York, USA*

**Background and aims:** HIV patients face a higher risk of stroke, either ischemic or hemorrhagic, which is often associated with traditional risk factors. We aimed to evaluate the current trends, characteristics, and 6‐month outcomes among people living with HIV (PLWH) compared to people without HIV (PWOH).

**Methods:** This prospective cohort study included 463 adults with stroke registered in the Lake Zone Stroke Registry at Bugando Medical Center between November 2023 and March 2025. Baseline characteristics, stroke severity, and outcomes were assessed using the National Institutes of Health Stroke Scale (NIHSS) and the modified Rankin Scale (mRS), respectively. The Kaplan–Meier analysis was used to describe survival, and the Cox regression model to determine predictors of mortality.

**Results:** We analyzed 295 adults, with an HIV proportion of 8.8% (95% CI: 5.9%–12.7%) (26/295). The mean age (SD) for the overall study cohort was 66.2 ± 37.4 years, and 57.2% (164/295) were males. Among PLWH, the mean age (SD) was 53.2 ± 14.5 years, and females were 61.5% (16/26). The median CD4 count was 518 IQR (151.5–571) cells/μl, and 84.6% (22/26) were naïve to antiretroviral therapy (ART). PLWH experienced longer hospital stay, 5 days (IQR 2–7.3), *p* = 0.001. Both groups experienced poor functional outcomes, mRS 3–6, 84.7% (250/295), and a mortality rate of 38.5% (114/295) at 6 months. In the multivariate analysis, difficulty in breathing, leukocytosis, and NIHSS scores of 21–42 were independent predictors of mortality.

**Figure:**
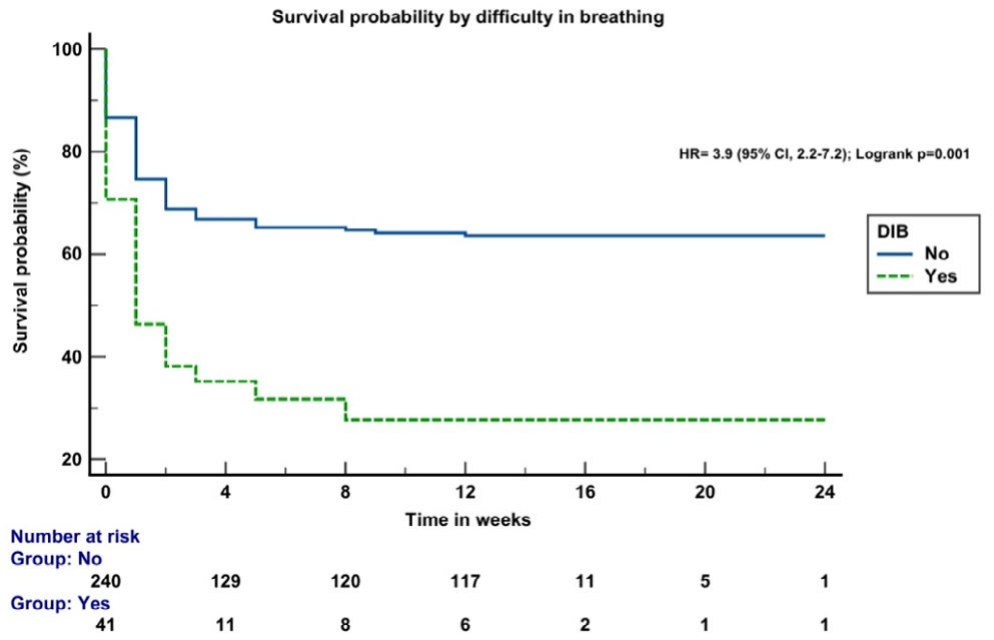
**FIGURE 1** Kaplan Meier survival probability by difficulty in breathing.

**Figure:**
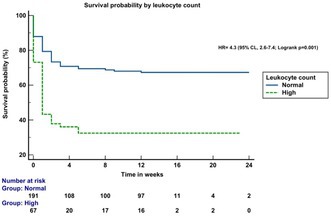
**FIGURE 2** Kaplan Meier survival probability by leukocyte count.

**Figure:**
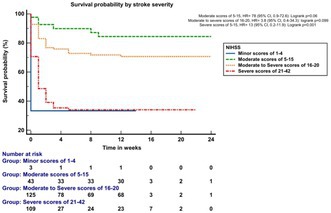
**FIGURE 3** Kaplan Meier survival probability by stroke severity based on NIHSS scores.

**Conclusion:** Although 6‐month outcomes were comparable between both groups, targeted strategies are needed to address healthcare disparities and reduce the additional burden of stroke in PLWH.

**Disclosure:** Nothing to disclose.

## EPO‐0357

### Validation of the DIAS^3^ score in cerebral venous thrombosis patients treated by decompressive surgery

#### M. Costa Taveira ^1^; M. Sanchez van Kammen^2^; S. Aaron^3^; J. M Ferreira^4^; J. M Coutinho^2^; P. Canhão^5^; A. B Conforto^6^; A. Arauz^7^; M. Carvalho^8^; J. Masjuan^9^; V. K Sharma^10^; J. Putaala^11^; M. Uyttenboogaart^12^; D. J Werring^13^; R. Bazan^14^; S. Mohindra^15^; J. Weber^16^; B. A Coert^17^; P. Kirubakaran^3^; P. Singh^3^; D. Aguiar de Sousa^18^; J. M Ferro^5^

##### ^*1*^ *Nuerology Service, Department of Neuroscience and Mental Health, Santa Maria Local Health Unit, Lisbon, Portugal;* ^*2*^ *Department of Neurology, Amsterdam University Medical Center, University of Amsterdam, Amsterdam, The Netherlands**;*** ^*3*^ *Neurology Unit, Department of Neurological Sciences, Christian Medical College & Hospital, Vellore, Tamil Nadu, India;* ^*4*^ *Neurology Service, São José* Local Health Unit*, Lisbon, Portugal;* ^*5*^ *Egas Moniz Study Center, Faculty of Medicine, University of Lisbon, Portugal**;*** ^*6*^ *Hospital das Clínicas, University of São Paulo, São Paulo, Brazil**;*** ^*7*^ *Stroke Clinic, Instituto Nacional de Neurología y Neurocirugía Manuel Velasco Suárez, Mexico City, Mexico**;*** ^*8*^ *Neurology Service, São João Local Health Unit; Department of Neuroscience and Mental Health, Faculty of Medicine, University of Porto, Porto, Portugal;* ^*9*^ *Neurology Service, Hospital Universitario Ramón y Cajal, IRYCIS, Department of Medicine, Universidad de Alcalá. Red RICORS, Madrid, Spain;* ^*10*^ *Department of Medicine, Yong Loo Lin School of Medicine, National University of Singapore, Singapore;* ^*11*^ *Department of Neurology, Helsinki University Hospital and University of Helsinki, Helsinki, Finland;* ^*12*^ *Department of Neurology and Medical Imaging Center University Medical Center Groningen, University of Groningen, Groningen, The Netherlands;* ^*13*^ *Stroke Research Centre, UCL Queen Square Institute of Neurology, London, UK;* ^*14*^ *Faculdade de Medicina Campus de Botucatu, Universidade Estadual Paulista Julio de Mesquita Filho, Botucatu, São Paulo, Brazil;* ^*15*^ *Department of Neurosurgery, Post Graduate Institute of Medical Education & Research (PGIMER), Chandigarh, India;* ^*16*^ *Department of Neurosurgery, Steinenberg Clinic, Reutlingen, Germany**;*** ^*17*^ *Department of Neurosurgery, Amsterdam University Medical Centers, University of Amsterdam, Amsterdam, The Netherlands**;*** ^*18*^ *Stroke Center, São José* Local Health Unit*, Lisbon, Portugal*

**Background and aims:** Cerebral venous thrombosis (CVT) is associated with a risk of late seizures, particularly in patients with severe disease requiring decompressive surgery. The DIAS^3^ score was developed to predict late seizures after CVT, but its performance in surgically treated patients is unknown. We aimed to validate the DIAS^3^ score in a high‐risk cohort of CVT patients treated by decompressive surgery.

**Methods:** This was a substudy of the DECOMPRESS2 – a prospective, multicentre cohort, including patients with CVT treated with decompressive surgery. Late seizures were defined as those occurring more than 7 days after CVT diagnosis, up to 1 year follow‐up. DIAS3 score was calculated for all included patients. Predictive performance was assessed only for the 1‐year risk of late seizures. Discrimination was evaluated using receiver operating characteristic analysis, and calibration by comparing predicted and observed seizure risks. Diagnostic performance was assessed across multiple probability thresholds.

**Results:** Eighty‐nine patients were included (mean age 37.3 years; 69.7% female). Late seizures occurred in 22 patients (24.7%). The mean predicted 1‐year seizure risk according to the DIAS^3^ score was 28.4%. The DIAS^3^ score showed poor discrimination (area under the curve 0.44, 95% confidence interval 0.30–0.59) and poor calibration. Across all tested thresholds, sensitivity and specificity showed an expected trade‐off, but overall predictive accuracy was limited.

**Figure:**
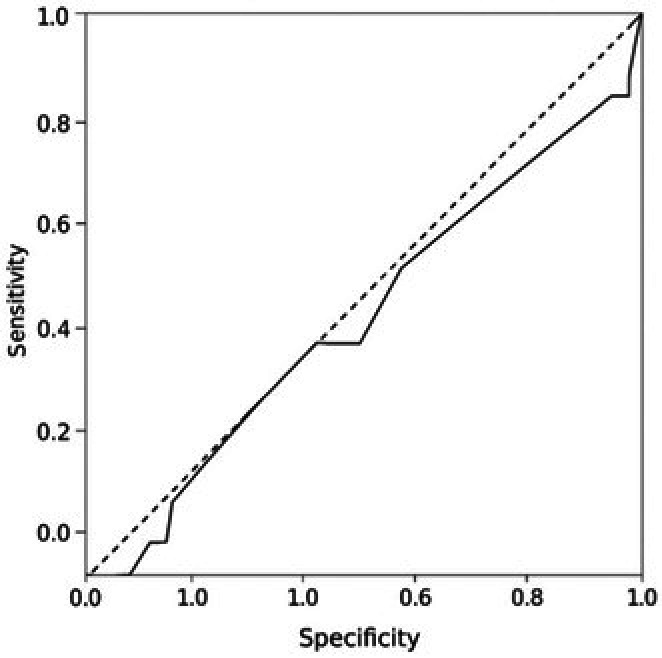
**FIGURE 1** ROC curve for late seizure prediction using the DIAS3 score in the DECOMPRESS2 cohort (AUC = 0.44, 95% CI 0.30–0.59). The dashed diagonal line represents the reference line of no discrimination.

**Figure:**
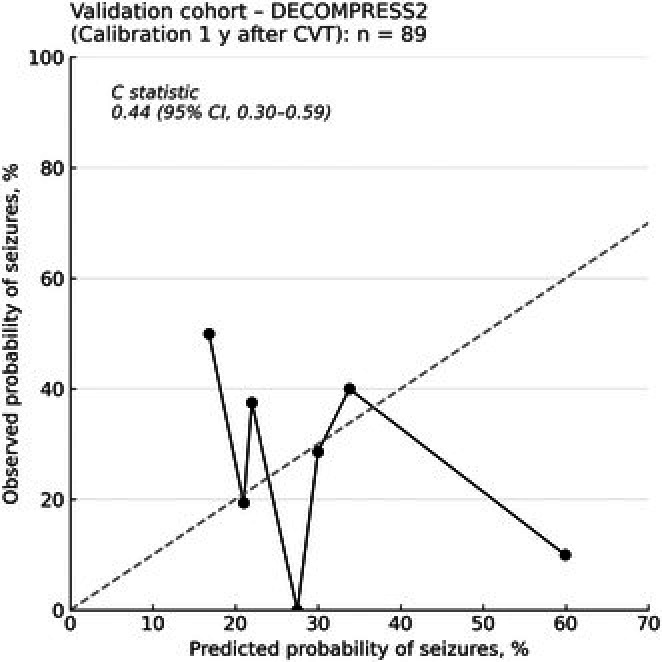
**FIGURE 2** Calibration plot for late seizure prediction using the DIAS3 score in the DECOMPRESS2 validation cohort (*n* = 89).

**Conclusion:** The DIAS^3^ score demonstrated poor discrimination and calibration in CVT patients treated by decompressive surgery. These findings suggest that the DIAS^3^ score should not be used to predict late seizures in this high‐risk surgical subgroup.

**Disclosure:** Nothing to disclose.

## EPO‐0358

### Impact of ambient temperatures on ischemic stroke characteristics and in‐hospital recurrence in a swiss cohort

#### I. Rizzo^1^; F. Huber^1^; C. Bouverat^1^; M. Costa^1^; L. Siegenthaler^1^; K. Antonenko^1^; T. Dobrocky^2^; E. Piechowiak^2^; T. Bremova‐Ertl^1^; M. Heldner ^1^

##### ^*1*^ *Department of Neurology, Inselspital, University Hospital and University of Bern, Bern, Switzerland;* ^*2*^ *Institute of Diagnostic and Interventional Neuroradiology, Inselspital, University Hospital and University of Bern, Bern, Switzerland*

**Background and aims:** The association between ambient temperatures and ischemic stroke remains poorly characterized in Europe. This study aimed to examine the influence of acute heat and cold exposure on stroke characteristics.

**Methods:** We conducted a retrospective cohort study of 500 patients experiencing a first ischemic stroke, using data from the Bernese Stroke Registry (09/2018–12/2020). Patients were categorized based on the temperature on the day of stroke: normal (0–30°C; *n* = 365), hot (> 30°C; *n* = 22), and cold (< 0°C; *n* = 113). Stroke etiology, vascular risk factors, and in‐hospital stroke recurrence were compared across temperature groups.

**Results:** Cardioembolism was the most prevalent stroke etiology across all temperature groups, with the highest proportion observed in the cold group on the day prior to stroke (43.9%). Coronary heart disease prevalence was significantly higher in the hot group (41.7%) compared with the normal temperature group (20.1%;*p* = 0.039). The incidence of new in‐hospital ischemic strokes was markedly elevated in the extreme temperature groups: 36.4% in the hot group and 18.0% in the cold group, versus 8.9% in the normal temperature group (*p* < 0.001). Temperature on the stroke day was also associated with significant differences in stroke etiology (*p* = 0.014), with cardioembolism observed in 38.9% of patients in the cold group. No significant differences were noted for other vascular risk factors, occlusion location, or acute interventions.

**Conclusion:** Extreme temperatures influence ischemic stroke profiles and in‐hospital recurrence. Heat is associated with increased cardiovascular comorbidity and recurrence risk, while cold exposure favors cardioembolic strokes, suggesting delayed cardiovascular effects. These findings underscore ambient temperature as a clinically relevant, modifiable stroke risk factor.

**Disclosure:** None.

## EPO‐0359

### Association of admission LDL cholesterol with clinical profile and 3 months outcomes after ischaemic stroke

#### M. Costa^1^; C. Berger^1^; K. Antoneko^1^; M. Peycheva^2^; M. Krasteva^3^; A. Scutelnic^1^; A. Hakim^4^; M. Kielkopf^1^; H. Sarikaya^1^; M. Heldner ^1^

##### ^*1*^ *Department of Neurology, Inselspital, University Hospital and University of Bern, Bern, Switzerland;* ^*2*^ *Department of Neurology, Medical University Plovdiv, Plovdiv, Bulgaria;* ^*3*^ *Department of Neurology, Inselspital, University Hospital and University of Bern, Bern, Switzerland and Department of Neurology, University Hospital Queen Giovanna, Sofia, Bulgaria**;*** ^*4*^ *Institute of Diagnostic and Interventional Neuroradiology, Inselspital, University Hospital and University of Bern, Bern, Switzerland*

**Background and aims:** While low‐density lipoprotein cholesterol (LDL‐C) is a well‐established modifiable risk factor for stroke, recent evidence suggests a “lipid paradox," where low LDL‐C levels may be associated with worse outcomes.

**Methods:** In this retrospective cohort study, we analyzed 1634 patients aged ≥ 18 years admitted to the Bernese Stroke Centre (02/2015–03/2023). We investigated the association between admission LDL‐C levels and clinical characteristics after first‐ever acute ischaemic stroke. Patients were stratified into five LDL‐C groups: > 3.0 mmol/L (*n* = 493), 2.6–3.0 mmol/L (*n* = 225), 1.8–2.6 mmol/L (*n* = 490), 1.4–1.8 mmol/L (*n* = 254), and < 1.4 mmol/L (*n* = 172). Functional outcome was assessed at 3 months.

**Results:** Patients with LDL‐C < 1.4 mmol/L were older (median 78 vs. 73 years;*p* < 0.001), more often male (59% vs. 44%;*p* < 0.001), and had a higher burden of comorbidities, including arterial hypertension, coronary heart disease, atrial fibrillation, heart failure, and renal impairment (all *p* < 0.001). Over 70% of patients in the lowest LDL‐C group were already receiving lipid‐lowering therapy at admission, versus < 10% in the highest group (*p* < 0.001). Laboratory markers associated with frailty showed significant differences: haemoglobin levels were lower (124 vs. 139 g/L;*p* < 0.001) and creatinine levels higher (92 vs. 79–83 μmol/L;*p* < 0.001) in the lowest versus highest LDL‐C group. Favourable (mRS 0–2) and excellent (mRS 0–1) functional outcomes at 3 months declined across groups (42% to 29% and 36% to 23%, respectively), as did survival (80% to 61%;all *p* < 0.05).

**Conclusion:** Low LDL‐C at stroke onset identifies older, multimorbid, and frail patients with poorer 3 months outcomes, likely reflecting underlying vulnerability and comorbidities rather than independently causing poor prognosis.

**Disclosure:** None.

## Treatment in MS and Related Disorders 1

## EPO‐0360

### CLadribine tablets level of respOnse preDIctors in cliNical practice: Interim analysis of CLODINA

#### R. Hupperts^1^; A. Kalinowska‐Lyszczarz^2^; T. Reynders^3^; J. Sequeira^4^; M. Vachová^5^; S. Deftereos^6^; A. Antonakopoulou ^6^; P. Vandenberk^7^; P. Rane^8^; N. Grigoriadis^9^

##### ^*1*^ *Department of Neurology, Zuyderland Medical Center Sittard, Maastricht, University Medical Centre, the Netherlands;* ^*2*^ *Poznan University of Medical Sciences, Department of Neurology, Division of Neurochemistry and Neuropathology;* ^*3*^ *Department of Neurology, Universitair Ziekenhuis Antwerpen, Edegem, Belgium; Research Group on Translational NeuroSciences, Faculty of Medicine and Health Sciences, University of Antwerp, Wilrijk, Belgium;* ^*4*^ *Multiple Sclerosis Centre of Integrated Responsability, Santo António dos Capuchos Hospital, São José* Local Health Unit*, Lisbon, Portugal; Lisbon Academic Clinical Center, Lisbon, Portugal;* ^*5*^ *Neurology Dpt with MS centre, Hospital Teplice‐Krajksa zdravotni a.s, Neurology DPT, General Hospital, Charles University Prague;* ^*6*^ *Merck S.A, Greece, an affiliate of Merck KGaA;* ^*7*^ *Merck N.V.‐S.A, Overijse, Belgium, an affiliate of Merck KGaA;* ^*8*^ *Merck Specialties, India, an affiliate of Merck KGaA;* ^*9*^ *Second Department of Neurology, Special Unit for Biomedical Research and Education (S.U.B.R.E.), School of Medicine, Aristotle University of Thessaloniki, Greece*

**Background and aims:** CLODINA is a multicenter, Phase IV ambispective, non‐interventional study in patients diagnosed with RMS who have received cladribine tablets, aiming to identify baseline patient characteristics predicting the time to treatment discontinuation in real‐world. Secondary objectives are to assess relapses, disability progression and improvement, MRI activity, and safety.

**Methods:** This interim analysis, conducted after 50% of patients completed the 2‐year follow‐up, reports Annualized Relapse Rate (ARR), disability progression and improvement, and MRI data descriptively. A predictive model for discontinuations was not created, as it would be superseded by the final analysis.

**Results:** The Full Analysis Set included 365 patients (33.7% prospective, 66.3% retrospective); median age was 38 years. 72.1% were female, and 73.4% (268) had received a DMT prior to cladribine tablets, with 79.4% (213) switching from platform therapies and 20.6% (55) from high‐efficacy therapies. The main reason for switching was inadequate response to prior DMT; psychiatric disorders were the most common comorbidities (15.3%). MRI assessment within 12 months before the index date was available for 98.9% of patients. At year 2, relapse data were available for 87.1% of patients, and ARR was 0.08 (0.03, 0.08). 298 patients (81.6%) were relapse‐free. 6‐month confirmed disability progression/improvement was seen in 4.1% and 3.3%. 233 patients had available MRI data at year 2, of whom 189 (81%) were free from MRI activity. 19.7% reported adverse events.

**Conclusion:** Cladribine tablets are highly effective, showing low ARR over a 2‐year follow‐up, with a generally safe and well‐tolerated profile.

**Disclosure:** RH received honoraria as consultant on scientific advisory boards from Merck, Biogen, Genzyme‐Sanofi and Teva, research funding from Merck and Biogen, and speaker honoraria from Sanofi‐Genzyme and Novartis. AKL received honoraria or consultation fees from: Bayer, Biogen, Merck, Novartis, Sanofi‐Genzyme, Bristol‐Meyers Squibb, Janssen, Teva, Sandoz. TR has received financial travel support, educational support, consultancy fees and/or speaker fees from Roche, Biogen, Sanofi, Merck, Teva, Novartis and Janssen Pharmaceuticals. She is a scientific board member of the Sanofi MS Academy in Flanders, Belgium. JS has received personal compensation for participation in advisory boards from Janssen, Merck, Novartis, Roche and Sanofi; consulting fees from Novartis; speaking honoraria from Almirall, Merck, Novartis, Roche and Sanofi; and travel expenses for scientific meetings from Bayer, Biogen, Janssen, Merck, Novartis, Sanofi and Teva. MV has received personal compensation for participation in advisory boards from Biogeen, BMS, Janssen, Merck, Novartis, Roche and Sanofi; consulting fees from Biogen, Roche and Novartis; speaking honoraria from Biogen, BMS, Merck, Janssen, Novartis, Roche and Sanofi; and travel expenses for scientific meetings from Bayer, Biogen, Janssen, Merck, Novartis, Sanofi and Teva. SD is an employee of Merck S.A, Greece, an affiliate of Merck KGaA AA is an employee of Merck S.A, Greece, an affiliate of Merck KGaA PV is an employee of Merck N.V.‐S.A, Overijse, Belgium, an affiliate of Merck KGaA PR is an employee of Merck Specialties, India, an affiliate of Merck KGaA NG has received honoraria and travel support from Biogen Idec, Biologix, Novartis, TEVA, Bayer, Merck, Genesis Pharma, Sanofi Genzyme, Roche, has received consultancy fees from Biogen Idec, Novartis, Teva, Bayer, Merck, Genesis Pharma, Sanofi Genzyme, Celgene, ELPEN, Roche, has received lecture fees from Biogen Idec, Novartis, Teva, Bayer, Merck S.A, Greece, an affiliate of Merck KGaA, Genesis Pharma, Sanofi Genzyme, ROCHE and research grants from Biogen Idec, Novartis, Teva, Merck S.A, Genesis Pharma, Sanofi Genzyme, Roche.

## EPO‐0361

### Post‐hoc analysis of the OPTIMUM long‐term extension study: Preservation of independent ambulation in patients treated with Ponesimod

#### M. Martínez Gines^1^; M. Heubl^2^; S. de Guitaut^2^; A. Sarfati^2^; A. Atallah ^2^

##### ^*1*^ *Neurology Department, Hospital Gregorio Marañón, Madrid, Spain;* ^*2*^ *Medical Affairs Department, Juvise Pharmaceuticals, Paris, France*

**Background and aims:** Disability accumulation in relapsing multiple sclerosis (RMS) is commonly quantified using the Expanded Disability Status Scale (EDSS). Reaching EDSS 6 marks the need for an ambulatory aid, while EDSS 7 reflects loss of independent ambulation and wheelchair dependence, representing major milestones in long‐term disease burden.

**Methods:** Following 108 weeks of double‐blind treatment with ponesimod 20 mg or teriflunomide 14 mg in the core OPTIMUM study, participants could enter the long‐term extension (LTE) and receive open‐label ponesimod 20 mg. This post hoc analysis confirmed EDSS 6 and EDSS 7 over a follow‐up of up to 7 years. Kaplan–Meier estimated the cumulative incidence of reaching these disability milestones.

**Results:** Overall, 1133 patients were randomized in the core study, and 877 entered the LTE. At core baseline, mean EDSS was 2.56 ± 1.20. Median cumulative exposure to ponesimod during the combined core plus LTE period was 6.3 years (IQR 2.8–6.7). Among patients treated continuously with ponesimod (*n* = 567), 86.8% had not reached EDSS 6 (Figure 1) and 98.7% had not reached EDSS 7 by week 360 (Figure 2). During the LTE period, 90.5% of patients remained free from EDSS 6 (Figure 3) (92.3% in those initially randomized to ponesimod and 88.8% in those initially randomized to teriflunomide), and 99.3% remained free from EDSS 7 (99.1% and 99.5%, respectively).

**Figure:**
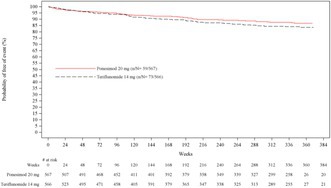
**FIGURE 1** EDSS superior or equal to 6 free probability, Kaplan‐Meier curve, combined analysis period.

**Figure:**
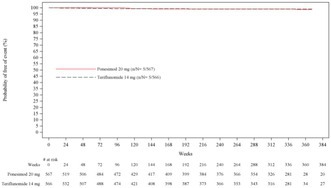
**FIGURE 2** EDSS superior or equal to 7 free probability, Kaplan‐Meier curve, combined analysis period.

**Figure:**
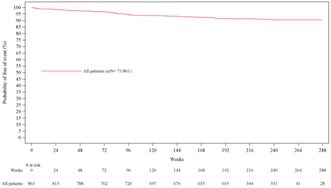
**FIGURE 3** EDSS superior or equal to 6 free probability, Kaplan‐Meier curve, extension period.

**Conclusion:** This post‐hoc analysis of OPTIMUM‐LTE shows a sustained preservation of mobility and functional independence with ponesimod over up to 7 years of follow‐up, supporting its durable efficacy in patients with RMS.

**Disclosure:** MLMG declare fees for lectures, consultations, travel support, assistance to congresses, advisory meetings, teaching or research from: Almirall, Amgen, AstraZeneca, Biogen, Bristol Myers Squibb, Janssen, Merck, Novartis, Roche, Sanofi, Teva, Neuraxpharm, Juvise. M.H, S.G, A.A and A.S are employees of Juvise Pharmaceuticals.

## EPO‐0362

### The effect of cladribine tablets on retinal microglial activity in relapsing multiple sclerosis: Optical coherence tomography findings from MAGNIFY‐MS

#### D. Jakimovski ^1^; J. Havla^2^; L. Leocani^3^; M. Danten^4^; L. Gardner^5^; N. De Stefano^6^

##### ^*1*^ *Buffalo Neuroimaging Analysis Center, Department of Neurology, Jacobs School of Medicine and Biomedical Sciences, University at Buffalo, State University of New York, Buffalo, USA;* ^*2*^ *Institute of Clinical Neuroimmunology, LMU University Hospital, LMU Munich, Munich, Germany;* ^*3*^ *University Vita‐Salute San Raffaele, Milan, Italy;* ^*4*^ *Global Biostatistics, Merck Santé S.A.S, Lyon, France, an affiliate of Merck KGaA;* ^*5*^ *EMD Serono Research & Development Institute Inc, Billerica, USA, an affiliate of Merck KGaA;* ^*6*^ *Department of Medicine, Surgery and Neuroscience, University of Siena, Siena, Italy*

**Background and aims:** Hyperreflective foci (HRF) are retinal punctate structures, observable with optical coherence tomography (OCT), and are potential neuroinflammatory biomarkers. Pathological correlates of HRF may be microglial accumulation. We evaluated the presence and change of HRF in cladribine tablets (CladT)‐treated people with highly‐active relapsing multiple sclerosis (pwRMS) over 24 months and determined relationships with clinical and magnetic resonance imaging (MRI) outcomes.

**Methods:** Overall, 36 pwRMS enrolled in the OCT sub‐study in MAGNIFY‐MS (NCT03364036). Clinical (progression independent of relapse activity [PIRA]) and MRI (lesions and brain volume) outcomes were analysed. Total HRF (sum of HRF in the ganglion cell inner plexiform layer [GCIPL] and inner nuclear layer [INL]), GCIPL‐HRF, and INL‐HRF were determined by OCT. All outcomes measured at baseline, month (M) 12, and M24. Linear regression models were used; data are presented as mean and standard deviation. HRF analysis was performed by an experienced rater (D.J.).

**Results:** From baseline to M24, CladT significantly decreased the total number of HRF (*p* = 0.007) and GCIPL‐HRF (*p* = 0.002; Table 1). Compared to MRI‐stable pwRMS, those with in‐trial MRI activity had higher baseline GCIPL‐HRF (5.6 vs. 3.0, *p* = 0.032), and a larger decrease in GCIPL‐HRF over 24 months (−2.6 vs. −0.6, *p* = 0.013; Table 2). PIRA activity was associated with baseline INL‐HRF (*p* = 0.034). Total baseline HRF was the only predictor of 12‐month brain volume loss (R2 = 0.323, *p* = 0.038).

**Figure:**
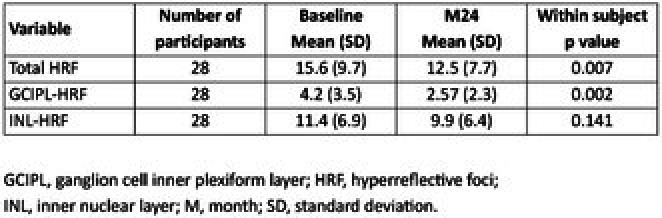
**TABLE 1** Results from a pairwise analysis of HRF in GCIPL and INL retinal layers.

**Figure:**
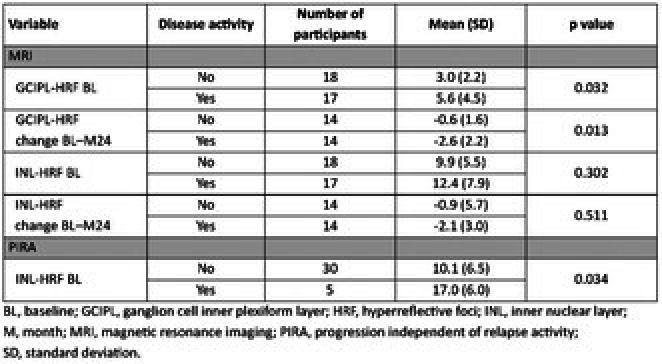
**TABLE 2** Differences in HRF between groups with and without disease activity over the 24‐month study period.

**Conclusion:** In our exploratory analysis, CladT treatment reduced overall HRF burden in pwRMS. Baseline GCIPL‐HRF was associated with early inflammatory activity, whereas INL‐HRF was associated with chronic neurodegenerative changes.

**Disclosure:** DJ has nothing to disclose JH reports a grant for OCT research from the Friedrich‐Baur‐Stiftung, Horizon/Amgen, Sanofi and Merck, personal fees and nonfinancial support from Alexion, Amgen, Bayer, Biogen, BMS, Merck, Novartis and Roche, and nonfinancial support of the Sumaira‐Foundation and Guthy‐Jackson Charitable Foundation, all outside the submitted work LL has received honoraria for consulting services or speaking activities from Merck and Roche. MD is an employee of Merck Santé S.A.S, Lyon, France, an affiliate of Merck KGaA. LG is an employee of EMD Serono Research & Development Institute, Inc, Billerica, MA, USA, an affiliate of Merck KGaA. NDS has received honoraria from Biogen‐Idec, Bristol Myers Squibb, Celgene, Genzyme, Immunic, Merck, Novartis, Roche, and Teva for consulting services, speaking, and travel support. He serves on advisory boards for Immunic, Merck, Novartis, Biogen‐Idec, Roche, and Sanofi. He has received research support from the Italian Ministry of University and Research, and Fondazione Italiana Sclerosi Multipla. He is Associate Editor of Neurological Sciences and co‐founder of Siena‐Imaging s.r.l.

## EPO‐0363

### Clinical and MRI outcomes of Siponimod versus ocrelizumab in secondary progressive multiple sclerosis: A real‐world study

#### E. Maida ^1^; G. Miele^1^; G. Abbadessa^1^; G. Maniscalco^2^; E. Prestipino^2^; E. D'Amico^3^; A. Zanghi^3^; E. Cocco^4^; G. Coghe^4^; L. Lavorgna^1^; G. Romano^1^; M. Sparaco^1^; L. Pasquali^5^; T. Guerra^6^; E. Signoriello^1^; G. Lus^1^; S. Bonavita^1^

##### ^*1*^ *Department of Advanced Medical and Surgical Sciences, University of Campania Luigi Vanvitelli, Naples, Italy;* ^*2*^ *Neurological Clinic and Stroke Unit and Multiple Sclerosis Center “A. Cardarelli” Hospital, Naples, Italy;* ^*3*^ *Department of Medical and Surgical Sciences, University of Foggia, Foggia, Italy;* ^*4*^ *Multiple Sclerosis Center, ATS Sardinia, Cagliari, Italy;* ^*5*^ *Department of Clinical and Experimental Medicine, Neurology Unit, University of Pisa, via Roma 67, Pisa 56126, Italy;* ^*6*^ *Department of Basic Medical Sciences, Neurosciences and Sense Organs, University of Bari “Aldo Moro,” Bari, Italy*

**Background and aims:** We compared the effectiveness and safety of siponimod (SIP) versus ocrelizumab (OCR) in real‐world cohort of patients with secondary progressive multiple sclerosis (SPMS).

**Methods:** We retrospectively included SPMS patients from five Italian centers initiating SIP or OCR and followed‐up for 18 months. Outcomes included time to first relapse, annualized relapse rate (ARR), confirmed disability progression (CDP), progression independent of relapse activity (PIRA), MRI activity at 18 months (new T2 lesion or Gd+ lesion), and safety. Baseline differences were addressed using stabilized inverse probability of treatment weighting and overlap weighting. Weighted Cox and negative binomial models were applied.

**Results:** Among 182 patients (98 OCR; 84 SIP;mean age 50.9 years), clinical outcomes were similar: time to first relapse SIP vs OCR (HR 1.41; 95% CI 0.58–3.45; *p* = 0.451); recurrent relapses (HR 1.20; 0.52–2.76; *p* = 0.667); CDP (HR 0.83; 0.38–1.82; *p* = 0.643); PIRA (HR 0.86; 0.38–1.94; *p* = 0.708); ARR (IRR 1.78; 0.83–3.81; *p* = 0.136). MRI activity at 18 months differed between groups, with new lesions occurring more frequently under SIP (OR 9.12; 1.26–66.34; *p* = 0.029), confirmed in overlap‐weighted analysis (OR 7.14; 1.09–46.63; *p* = 0.040). SIP was associated with lower white blood cells/lymphocytes/neutrophils (all *p* < = 0.0037) and higher liver enzymes (*p* < = 0.049); infection and adverse‐event rates were similar.

**Figure:**
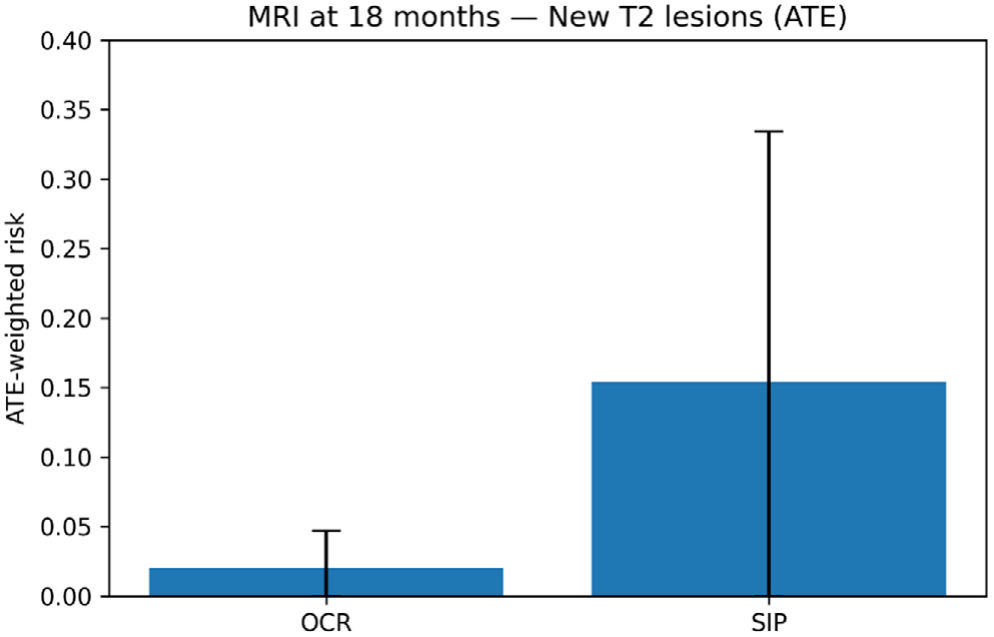
**FIGURE 1** ATE‐weighted risk of new T2 lesions at 18 months in patients treated with ocrelizumab (OCR) or siponimod (SIP). Error bars represent 95% confidence intervals derived from stabilized inverse probability weighting.

**Figure:**
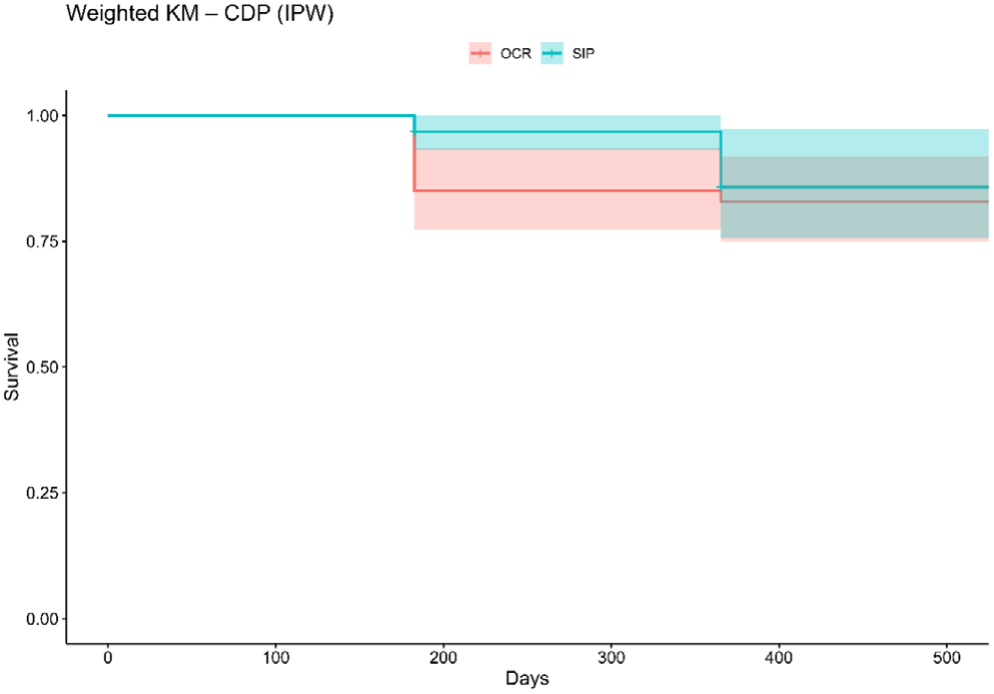
**FIGURE 2** IPW‐weighted Kaplan–Meier curves for confirmed disability progression over 18 months in patients treated with ocrelizumab or siponimod. Shaded areas indicate 95% confidence intervals.

**Conclusion:** In SPMS, short‐term clinical outcomes were comparable between SIP and OCR, whereas MRI activity at 18 months differed, with higher odds of new lesions under SIP. When disability endpoints fail to discriminate over short follow‐up, suppression of subclinical inflammatory MRI activity may represent the key differentiator guiding treatment choice, particularly in patients with residual inflammatory activity.

**Disclosure:** The authors report no relevant relationships/activities/interests related to this work.

## EPO‐0364

### Evaluating the value of serum neurofilament light chain after DMT initiation for monitoring early treatment response

#### F. Föttinger; J. Nolte; N. Krajnc; M. Ponleitner; F. Leutmezer; T. Monschein; P. Rommer; C. Schmied; B. Kornek; T. Zrzavy; G. Zulehner; T. Berger; G. Bsteh

##### Medical University of Vienna, Department of Neurology, Vienna, Austria

**Background and aims:** Serum neurofilament light chain (sNfL) is an established biomarker of axonal injury in multiple sclerosis (MS). While sNfL typically decreases with effective disease‐modifying therapy (DMT), it remains unclear whether early post‐initiation sNfL levels or their change predict treatment response. We evaluated sNfL as an early biomarker of DMT response in relapsing MS (RMS).

**Methods:** Within an ongoing prospective observational cohort at the Medical University of Vienna, we included patients with RMS newly initiating any DMT. Serum NfL was measured at treatment start (baseline) and at 6 months and expressed as age‐ and BMI‐adjusted z‐scores. Minimum follow‐up was 24 months. Treatment failure (EDA‐3) was defined by relapse, MRI activity, and/or confirmed disability progression.

**Results:** Ninety‐two patients were analyzed (mean age 35 ± 8 years; 71% on high‐efficacy DMT); 22 (24%) developed EDA‐3 during follow‐up. Baseline sNfL was not significantly associated with subsequent EDA‐3 (aOR 1.36, 95% CI 0.90–1.99; *p* = 0.20). At 6 months, median sNfL z‐scores were lower in those remaining relapse‐free (0.03 in NEDA‐3 vs 1.94 in EDA‐3; *p* < 0.001; change of Δz ‐0.94 vs 0.01; *p* = 0.014). Discrimination for future EDA‐3 was moderate for both absolute 6‐month z‐scores (AUC 0.752; optimal cut‐off 0.935) and Δz‐scores (AUC 0.733; cut‐off −0.811), with high negative predictive values (95.6% and 97.8%). In multivariable analysis, 6‐month absolute z‐scores predicted future EDA‐3 (aOR 3.40, 95% CI 1.91–7.07; *p* = 0.017).

**Conclusion:** Six‐month sNfL z‐scores, particularly their normalization after DMT initiation, may early identify patients with favorable treatment response in RMS.

**Disclosure:** No disclosures or relationships of interest relevant to the context of this study.

## EPO‐0365

### CAR T‐cell therapy in neuroimmunological diseases – The vienna model (Neuro CAR‐T Vienna)

#### G. Bsteh ^1^; P. Rommer^1^; T. Zrzavy^1^; L. Haider^2^; N. Worel^3^; E. Han^3^; K. Phan^3^; W. Rabitsch^4^; N. Dominik^4^; H. Lechner‐Radner^5^; J. Marschalek^6^; T. Berger^1^; A. Müller^3^

##### ^*1*^ *Department of Neurology, Medical University of Vienna, Vienna, Austria;* ^*2*^ *Department of Neuroradiology, Medical University of Vienna, Vienna, Austria;* ^*3*^ *Department of Transfusion Medicine and Cell Therapy, Medical University of Vienna, Vienna, Austria;* ^*4*^ *Department of Internal Medicine I, Bone Marrow Transplantation Unit, Medical University of Vienna, Vienna, Austria;* ^*5*^ *Division of Rheumatology, Department of Internal Medicine III, Medical University of Vienna, Vienna, Austria;* ^*6*^ *Department of Obstetrics and Gynaecology, Medical University of Vienna, Vienna, Austria*

**Background and aims:** Chimeric antigen receptor (CAR) T‐cell therapies have undergone rapid development in recent years, with increasing evidence supporting their efficacy and safety in severe, treatment‐refractory autoimmune diseases of the nervous system. In response, the Medical University of Vienna has established Austria's first center dedicated to the application of CAR T‐cell therapy in neuroimmunological disorders.

**Methods:** The Neuro CAR‐T Vienna model is based on a structured, interdisciplinary collaboration between Neurology, Transfusion Medicine & Cell Therapy, and Hemato‐Oncology, with additional involvement of Rheumatology, Neuroradiology, and Gynecology. Central to the model is an interdisciplinary CAR T‐cell board, where patients are systematically evaluated and indications reviewed. Referrals are accepted for patients with neuroimmunological diseases that are insufficiently controlled by conventional therapies and carry a high risk of a deleterious disease course. Evaluation requires comprehensive documentation of disease and treatment history, current imaging and laboratory findings, and confirmation of the patient's willingness and psychological suitability for an invasive therapy.

**Results:** CAR T‐cell therapy may be administered either as an individual compassionate‐use treatment using in‐house manufactured CAR T cells or within clinical trials. Patients receive comprehensive interdisciplinary counseling, including potential effects on fertility and family planning, with fertility preservation options discussed when appropriate. For pharmacovigilance purposes, treated patients commit to a structured long‐term follow‐up with at least annual assessments for a minimum of five years.

**Figure:**
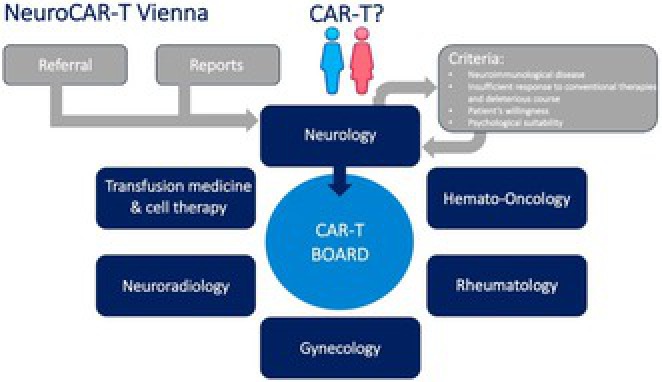
**FIGURE 1** Neuro CAR‐T Vienna.

**Conclusion:** The Neuro CAR‐T Vienna model represents Austria's first standardized interdisciplinary framework for CAR T‐cell therapy in neuroimmunological diseases, enabling safe, transparent, and patient‐centered implementation and serving as a reference model for future national expansion.

**Disclosure:** Nothing to disclose.

## EPO‐0366

### Efficacy and safety of fenebrutinib vs teriflunomide in relapsing multiple sclerosis: Results of the FENhance 1 and 2 studies

#### J. Oh ^1^; A. Bar‐Or^2^; G. Giovannoni^3^; M. Sormani^4^; M. Weber^5^; S. Stoll^6^; J. Nicholas^7^; H. von Büdingen^8^; M. Decker‐Palmer^9^; A. Loukitcheva^9^; M. Triyatni^8^; N. Hu^9^; J. Ratchford^9^; J. Napieralski^9^; A. Goodyear^9^; L. Kappos^10^; S. Hauser^11^

##### ^*1*^ *St. Michael's Hospital, University of Toronto, Toronto, ON, Canada;* ^*2*^ *Perelman School of Medicine, University of Pennsylvania, Philadelphia, USA;* ^*3*^ *Queen Mary University of London, London, UK;* ^*4*^ *University of Genoa, Genoa, Italy;* ^*5*^ *Institute of Neuropathology and Department of Neurology, University Medical Center, Göttingen, Germany and Fraunhofer Institute for Translational Medicine and Pharmacology, Göttingen, Germany;* ^*6*^ *Advocare Stoll Medical Group, Philadelphia, USA;* ^*7*^ *OhioHealth Multiple Sclerosis Centre, Riverside Methodist Hospital, Columbus, USA;* ^*8*^ *F. Hoffmann‐La Roche Ltd, Basel, Switzerland;* ^*9*^ *Genentech, Inc, South San Francisco, USA;* ^*10*^ *University Hospital and University Basel, Research Center for Clinical Neuroimmunology and Neuroscience (RC2NB), Basel, Switzerland;* ^*11*^ *University of California San Francisco, San Francisco, USA*

**Background and aims:** Bruton's tyrosine kinase inhibitors (BTKis) may address both relapsing and progressive disease biologies in multiple sclerosis (MS) via a dual mechanism of action. Phase III trials in patients with relapsing MS (RMS) have not met their primary endpoint in showing that covalent, irreversible BTKis reduce annualized relapse rate (ARR) vs teriflunomide. The objective of the FENhance Phase III studies was to evaluate the efficacy and safety of fenebrutinib—a noncovalent, reversible, highly selective BTKi—vs teriflunomide in adults with RMS.

**Methods:** FENhance 1 (NCT04586010) and FENhance 2 (NCT04586023) are two identical Phase III, multicenter, randomized, double‐blind, double‐dummy, parallel‐group studies enrolling adults aged 18–55 years with RMS (2017 revised McDonald criteria; Expanded Disability Status Scale [EDSS] score 0–5.5). Patients were randomized 1:1 to oral fenebrutinib 200 mg twice daily or teriflunomide 14 mg once daily for a minimum of 96 weeks. The primary endpoint was ARR.

**Results:** In total, 1497 patients were enrolled globally. The mean (SD) age at baseline was 35.8 (9.4) years, mean (SD) EDSS score was 2.5 (1.3) and 66.5% were female. The mean (SD) duration since MS symptom onset was 5.5 (5.9), and mean (SD) duration since diagnosis was 3.6 (5.1) years. Primary efficacy and safety data from both FENhance studies will be presented.

**Conclusion:** While other BTKis have not shown superiority to teriflunomide in suppressing disease activity, the FENhance trials will determine whether fenebrutinib's unique molecular design, optimized CNS exposure and promising Phase II results translate into significant benefits in RMS.

**Disclosure:** Sponsored by F. Hoffmann‐La Roche Ltd. Writing and editorial assistance were provided by Nucleus Global and funded by F. Hoffmann‐La Roche Ltd. J. Oh, A. Bar‐Or, G. Giovannoni, M.P. Sormani, M.S. Weber, S. Stoll, J.A. Nicholas, H.‐C. von Büdingen, M. Triyatni, M. Decker‐Palmer, A. Loukitcheva, N. Hu, J.N. Ratchford, J. Napieralski, A. Goodyear, L. Kappos and S.L. Hauser have conflicts of interest to disclosure. These disclosures will be included in full at the time of presentation.

## EPO‐0367

### Anti‐CD20 drugs and Natalizumab confer equal protection against progression independent of relapse activity (PIRA) in people with multiple sclerosis

#### L. Palazzo ^1^; F. Clarelli^2^; M. Surano^1^; A. Giordano^1^; L. Moiola^1^; M. Rocca^1^; M. Filippi^1^; F. Esposito^1^

##### ^*1*^ *Neurology Unit and MS Center, IRCCS Ospedale San Raffaele, Milan, Italy;* ^*2*^ *Laboratory of Human Genetics of Neurological Disorders, Division of Neuroscience, IRCCS San Raffaele Scientific Institute, Milan, Italy*

**Background and aims:** Highly effective (HE) disease‐modifying therapies (DMTs) reduce relapse‐associated worsening (RAW) and progression independent of relapse activity (PIRA). Natalizumab (NTZ) and anti‐CD20 agents show similar efficacy on relapse suppression, but long‐term comparative data on PIRA are limited. We aimed to compare PIRA accrual in relapsing‐remitting MS (RRMS) patients treated with NTZ and anti‐CD20 drugs over a long follow‐up.

**Methods:** We included RRMS patients followed at our centre and recorded in the Italian MS and Related Disorders Register with at least 5 years of continuous treatment with NTZ or anti‐CD20 agents (Ocrelizumab, Rituximab, Ofatumumab), at least 3 Expanded Disability Status Scale (EDSS) assessments, and complete demographic and clinical data. Patients were matched for sex, age at treatment initiation, and disease duration. Logistic regression and Cox proportional hazard models were applied.

**Results:** Of 267 eligible patients (178 NTZ, 89 anti‐CD20), optimal matching yielded 89 balanced pairs. A first PIRA event was observed in 37.1% of NTZ‐treated and 32.6% of anti‐CD20‐treated patients. Although the absolute number of PIRA events was higher in the NTZ group (47 vs 30; *p* = 0.015), adjusted analyses revealed no significant differences in cumulative risk (RR: 1.15; 95% CI: 0.57–2.37; *p* = 0.692) or time to first PIRA event (HR: 1.21; 95% CI 0.47–1.39; *p* = 0.446).

**Conclusion:** In this real‐world cohort with long‐term follow‐up, NTZ and anti‐CD20 therapies conferred comparable protection in controlling disability progression beyond relapse, leaving space for further survey, desiderabily in a larger cohort, to confirm and refine these observations.

**Disclosure:** Nothing to disclose.

## EPO‐0368

### Evolution of brain neuro‐inflammation following ocrelizumab therapy in multiple sclerosis: A 2‐years TSPO PET longitudinal study

#### M. Hamzaoui^1^; T. Soulier^2^; V. Ricigliano^3^; D. Hajage^4^; J. Dufour^2^; A. Lazzarotto^2^; I. Laurent^4^; J. Palmyre^5^; C. Louapre^2^; V. Lebon^8^; A. Kas^6^; M. Bottlaender^7^; B. Bodini^2^; B. Stankoff ^2^

##### ^*1*^ *Sorbonne Université, Paris Brain Institute, CNRS, Inserm, France;* ^*2*^ *AP‐HP, Hôpital Universitaire Pitié‐Salpêtrière, Sorbonne Université, Paris Brain Institute, CNRS, Inserm, Paris, France;* ^*3*^ *Neurology Unit, GHNE–Paris Saclay Hospital, Orsay, Université Paris‐Saclay, UNIACT, Neurospin CEA, Gif‐sur‐Yvette, France;* ^*4*^ *INSERM, Institut Pierre Louis d'Epidémiologie et de Santé Publique, AP‐HP, Hôpital Pitié‐Salpêtrière, Département de Santé Publique, Centre de Pharmacoépidémiologie, Sorbonne Université, Paris, France;* ^*5*^ *Unité de Recherche clinique, Hôpital Universitaire Pitié‐Salpêtrière, Paris, France;* ^*6*^ *Department of Nuclear Medicine, Pitié‐Salpêtrière Hospital, APHP Sorbonne Université, Paris, France; Sorbonne Université, INSERM, CNRS, Laboratoire d'Imagerie Biomédicale, LIB, Paris, France;* ^*7*^ *Université Paris‐Saclay, UNIACT, Neurospin CEA, CNRS, Inserm, BioMaps, Service Hospitalier Frédéric Joliot, Orsay, France;* ^*8*^ *Université Paris‐Saclay, CEA, CNRS, Inserm, BioMaps, Service Hospitalier Frédéric Joliot, Orsay, France*

**Background and aims:** Ocrelizumab has been approved for both relapsing and primary progressive multiple sclerosis (MS), but the impact of this treatment on brain innate immune cell neuro‐inflammation remains unknown.

**Methods:** People with MS (PwMS) aged 18–60 years with relapsing–remitting (RRMS), active secondary progressive (SPMS), or primary progressive MS (PPMS) underwent neurological evaluation and a 90‐min dynamic PET–MR using the [18F]DPA714 TSPO tracer at baseline and at months 6 and 24 after ocrelizumab initiation. Ocrelizumab was administered as 600‐mg infusions every 24 weeks. Age‐, sex‐, and TSPO‐genotype–matched healthy controls underwent a single PET–MR scan. The primary outcome was the change in active voxels in total white matter (TWM) between baseline and month 24, defined using [18F] DPA714 thresholds derived from comparisons with controls. Secondary analyses assessed longitudinal changes at months 6 and 24 and within MS subgroups.

**Results:** Thirty‐seven PwMS completed all evaluations. The proportion of DPA‐active voxels in TWM was not significantly reduced between month‐24 and baseline (median relative change: +3.58% [−43.32%,+23.98%]; *p* = 0.86), but decreased significantly between baseline and month 6 (−26.75% [−39.86%,−10.61%]; *p* = 0.017), with a subsequent increase trend between month‐6 and ‐24. Significant reductions between baseline and month 6 were observed in TWM and normal‐appearing WM in RRMS patients (*p* = 0.027, 0.037) and in lesions in RRMS and SPMS subjects (*p* = 0.005, 0.035). No significant longitudinal changes were detected in PPMS subjects.

**Conclusion:** Ocrelizumab initiation was associated with reduced white matter neuroinflammation at month 6, an effect that was no longer detectable at month 24.

**Disclosure:** M Hamzaoui, T Soulier: Nothing to disclose V AG Ricigliano reports fees for travel, consulting/advisory board fees and speaker's honoraria from Novartis, Merck, Roche, Sanofi, Sandoz, Biogen, Janssen, M3 Global Research and Atheneum Partners. D Hajage, J Dufour: Nothing to disclose A Lazzarotto reports fees for traveling and speaker's honoraria from Novartis, Roche, Merck and Sandoz. I Laurent, J Palmyre: Nothing to disclose C Louapre has received consulting or travel fees from Biogen, Merck, Novartis, Roche, Sanofi and Oculis, and research grant from Biogen. V Lebon, A Kas, M Bottlaender: Nothing to dislose B Bodini reports fees for traveling and speaker's honoraria from Novartis, Genzyme, Roche, and Merck Serono; B Stankoff has received research grants and personal fees for lectures from Roche, Novartis, and Merck‐Serono, personal fees for lectures from Alexion, and Janssen. The study was funded by Roche and sponsored by APHP.

## EPO‐0369

### Characterizing patient profiles linked to treatment persistence in multiple sclerosis: Federated analysis of clinical real‐world data in different healthcare systems

#### V. Illiano^1^; M. Elze^1^; G. Zilorri^1^; A. Morgan^2^; T. Görbing^3^; N. Orr^4^; E. Longbrake^2^; T. Ziemssen^3^; L. Steinman^4^; B. Tackenberg^1^; M. Antonin ^1^

##### ^*1*^ *F. Hoffmann‐La Roche AG, Basel, Switzerland;* ^*2*^ *Department of Neurology, Yale University, New Haven, Connecticut, USA;* ^*3*^ *Department of Neurology, Dresden University of Technology, Dresden, Germany;* ^*4*^ *Department of Neurology and Neurological Sciences, Stanford University, Stanford, California, USA*

**Background and aims:** The therapeutic landscape of Multiple Sclerosis (MS) has evolved significantly in recent years and the treatment strategy has become a contentious debate. It has been shown that persistence is critical to treatment effectiveness. However, more real‐world evidence is needed to understand correlates of persistence among people with MS (pwMS). In this study, we applied Federated Analysis (FA) to simplify data analysis across three sites in two countries, investigating covariates of treatment persistence and its effects on clinical and socio‐economic outcomes in MS across different healthcare systems.

**Methods:** The study cohorts included people with MS (pwMS) initiating a Disease Modifying Treatment (DMT) between 2018 and 2021 with at least 2 years of follow‐up data. Persisters were defined as pwMS having continuous records of a certain class of DMT within the 2‐year follow‐up period. Outcomes were collected retrospectively, harmonized, and made available for FA. This approach keeps data localized at the institutions and transmits only aggregated data.

**Results:** We analyzed data of 2528 pwMS across three clinical sites (Dresden University of Technology, Stanford University and Yale University). 76.5% of pwMS were persisters. The likelihood of persistence was positively associated with treatment efficacy, age, and conversely with a lower risk of hospitalization or need for walking aids or wheelchairs.

**Conclusion:** We explored covariates of persistence and their association with clinical outcomes in different healthcare systems. The results will foster an understanding of the influence of different treatment approaches in the real‐world and the benefits of leveraging FA spanning multiple healthcare systems.

**Disclosure:** Funding statement: Sponsored by F. Hoffmann‐La Roche Ltd Disclosures: V P Illiano, M C Elze, G Zilorri, B Tackenberg and M Antonin are employees of F. Hoffman‐La Roche Ltd. A Morgan has been a consultant for Genentech. T Görbing and N Orr have nothing to disclose. E Longbrake has received honoraria for consulting from Bristol Myers Squibb, Sanofi, Alexion, Novartis, TG Therapeutics, EMD Serono, Genentech. She has received research support from Biogen, Genentech. T Ziemssen reports scientific advisory board and/or consulting for Biogen, Roche, Novartis, Celgene, and Merck; compensation for speakers' bureaus for Roche, Novartis, Merck, Sanofi, Celgene, and Biogen; research support from Biogen, Novartis, Merck, and Sanofi. L Steinman has received research contracts from F.Hoffmann‐La Roche Ltd.

## EPO‐0370

### Real‐world short‐term outcomes of ofatumumab and ocrelizumab in RR‐MS

#### N. Cavalli ^1^; G. Boffa^1^; E. Cipriano^1^; S. Al Qudsi^1^; F. Baldisseri^1^; E. Capello^2^; A. Laroni^1^; M. Inglese^1^; C. Lapucci^2^

##### ^*1*^ *Department of Neurosciences, Rehabilitation, Ophthalmology, Genetics and Maternal and child sciences, University of Genoa, Genoa, Italy;* ^*2*^ *Neurology Unit, San Martino Hospital, Genoa, Italy*

**Background and aims:** Real‐world comparative data about ofatumumab (OFA) and ocrelizumab (OCR) efficacy and safety in multiple sclerosis (MS) are still scarce.

**Methods:** We compared efficacy and safety of our 103 relapsing–remitting (RR) MS OFA‐treated patients with those of the first consecutive 103 RR‐MS OCR‐treated patients. Only OFA‐treated patients with at least 6 months FU were included.

**Results:** OFA and OCR patients exhibited similar demographic and clinical features at baseline [OFA: 64.1% females, mean (SD) age 40.6 (10.4) years, disease duration (DD) mean 10.9 (9.7) years, median EDSS 1.5, 30.8% naïve; OCR: 59.2% females, mean age 39.5 (11) years, DD mean 11.5 (11.3) years, median EDSS 2.5, 15% naive]. No patients showed relapses at 1‐year and 2‐year FU. At 1‐year, comparing OFA and OCR no significant differences in terms of MRI activity [12/85 (14.1%) vs 19/103 (18.4%)] and PIRA events [3/85 (3.5%) vs 3/103 (2.9%)] were observed. 1‐year No Evidence of Disease Activity‐3 (NEDA‐3) was achieved by 70/85 (82.4%) and 84/103 (81.6%) respectively. At 2‐year, no significant differences in terms of MRI activity [8/38 (21.1%) vs 18/99 (18.2%)] and PIRA events [3/38 (7.9%) vs 9/99 (9.1%)] were observed. MRI activity occurred within the first 6 months of treatment (median 2.8 and 4 months respectively). At 2‐year FU only one new cortical lesion (CL) was observed in the OCR cohort. At 2‐year FU, severe infections were reported in 1 and 4 patients respectively (OFA: 1 urosepsis; OCR: 4 pneumonias, 2 during COVID‐19 pandemic).

**Conclusion:** OFA and OCR showed similar short‐term efficacy and safety. Our data demonstrated the efficacy of both therapies also in preventing CLs development.

**Disclosure:** GB received personal fees from Novartis and Roche. AL received fees for consultation, public speaking or both from Roche, Genzyme, Merck, Biogen, Novartis and Bristol‐Myers Squibb. MI received grants from NIH, NMSS, FISM, and Ministero dell'Università e ricerca (MUR) and received fees for consultation from BMS, Janssen, Roche, Genzyme, Merck, Biogen and Novartis. CL has received honoraria for speaking, travel grants and participating in advisory boards from Merck, Sanofi, Novartis, Roche and Alexion. NC, EC, SAQ, FB and EC have nothing to disclose.

## EPO‐0371

### Real‐world effectiveness of ocrelizumab: Altering the six‐year disease trajectory in multiple sclerosis

#### O. Mammadov ^1^; U. Samadzade^2^; S. Alizada^1^; C. Caliskan^2^; S. Ozakbas^2^

##### ^*1*^ *Department of Neurology, Dokuz Eylul University, Izmir, Türkiye;* ^*2*^ *Department of Neurology, Izmir University of Economics, Izmir, Türkiye*

**Background and aims:** Ocrelizumab is highly effective for relapsing and progressive multiple sclerosis (MS). We evaluated clinical trajectories during the three years preceding and six years following treatment initiation.

**Methods:** This retrospective study analyzed 268 patients (158 RRMS, 110 SPMS). Clinical outcomes were compared across three‐year intervals: pre‐treatment, Years 0–3, and Years 3–6. Effectiveness was assessed using annualized relapse rates (ARR), EDSS progression, and MRI activity. NEDA‐3 (no relapses, no disability progression, and no new MRI lesions) was the primary endpoint. Regression models identified predictors for long‐term NEDA‐3 achievement.

**Results:** Patients showed dramatic improvement, particularly in Years 3–6 versus Years 0–3. Mean ARR fell from 1.00 to 0.03, MRI activity dropped from 31.5% to 1.5%, and disability progression slowed from 42.9% to 13.4%. By the second period, 84% of patients achieved NEDA‐3. Notably, 76.5% of patients who lacked NEDA‐3 in the first three years reached stability during the second period. For SPMS, early NEDA‐3 achievement was the strongest predictor of long‐term success (OR = 4.30, *p* = 0.019).

**Conclusion:** Ocrelizumab almost completely stops MS activity over a six‐year period. The treatment's benefits increase over time, with the best results seen after the third year. Achieving stability early in treatment is key to long‐term success, particularly for those with progressive MS.

**Disclosure:** Nothing to disclose.

## Neuroimaging 1

## EPO‐0372

### Bayesian predictive validation of a multimodal Alzheimer disease modeling framework

#### B. Haji; Q. Zhang; A. Tahami Monfared

##### Eisai Inc., Tokyo, Japan

**Background and aims:** Disease‐progression models inform cost‐effectiveness analyses, payer decisions, and simulation studies of Alzheimer therapies. Multimodal latent models may improve biological fidelity, but their value for health‐economic modeling depends on predictive validation. We evaluated calibration, accuracy, and decision relevance of a Bayesian latent trajectory model.

**Methods:** We analyzed 932 β‐amyloid–positive participants from the Alzheimer's Disease Neuroimaging Initiative (ADNI) with repeated multimodal measures spanning neurodegeneration, amyloid/tau pathology, metabolism, neuroaxonal injury, and cognition. Probabilistic principal component analysis generated a harmonized latent severity score (PPC1). Hierarchical Bayesian regression estimated participant‐specific trajectories. Predictive validity was assessed using posterior predictive checks, cross‐validated predictive error, predictive‐interval coverage, widely applicable information criterion (WAIC), expected log predictive density, and Pareto‐smoothed importance sampling leave‐one‐out cross‐validation (PSIS‐LOO).

**Results:** Posterior predictive distributions closely matched observed latent trajectories. Adding apolipoprotein E (APOE) genotype improved predictive accuracy while preserving calibrated uncertainty. Predictive‐interval coverage was ~98%, supporting probabilistic sensitivity analysis. WAIC and expected log predictive density favored the genotype‐augmented model by 16–22 points. PSIS diagnostics were stable with fewer influential observations, and conclusions were consistent across sensitivity analyses.

**Conclusion:** This validated multimodal Bayesian framework provides accurate, well‐calibrated forward projections of Alzheimer progression with uncertainty suitable for decision modeling, supporting cost‐effectiveness analyses, payer simulations, and trial‐enrichment forecasting.

**Disclosure:** All authors are employees of Eisai Inc.

## EPO‐0373

### A multimodal latent severity axis for Alzheimer disease progression modeling

#### B. Haji; Q. Zhang; A. Tahami Monfared

##### *Eisai Inc.*, *Tokyo, Japan*

**Background and aims:** Reliable quantitative severity scales are essential for disease‐progression, value‐based modeling, and simulation studies. Single‐modality measures can misrepresent the interconnected biology of Alzheimer disease. We developed a multimodal latent disease‐severity metric using probabilistic principal component analysis (PPCA) to integrate structural, molecular, metabolic, and cognitive signals into a biologically anchored index related to—but distinct from—clinical and biological staging.

**Methods:** Thirteen biomarkers from an amyloid‐positive baseline cohort (*N* = 1,058)—MRI atrophy, CSF tau and Aβ species, plasma neurofilament light, amyloid PET, FDG‐PET, and cognitive scores—were harmonized, sign‐aligned, and z‐scaled after age and intracranial‐volume adjustment where appropriate. PPCA was fit to the baseline matrix to define a latent coordinate system. Follow‐up visits were projected using the PPCA conditional distribution to accommodate missing modalities, yielding a continuous severity axis (PPC1) robust to missingness. Structural stability was assessed by bootstrap resampling (100 replicates).

**Results:** PPC1 explained 38.7% of multimodal variance and was highly stable (loading correlations ≈0.997; score correlations ≈0.999). Loadings reflected coherent neurobiology spanning neurodegeneration, tau/amyloid dysregulation, metabolic decline, neuroaxonal injury, and cognitive impairment. Diagnostic groups aligned along an ordered continuum. PPC1 captures latent continuous severity inferred from multimodal patterns rather than a clinical stage, although its distribution tracked standard staging categories. Residual age effects were minimal after adjustment, and the multimodal score reduced noise relative to individual biomarkers.

**Conclusion:** PPCA yields a robust, biologically grounded severity index enabling cleaner longitudinal modeling, uncertainty propagation, and simulation inputs for value assessment.

**Disclosure:** All authors are employees of Eisai Inc.

## EPO‐0374

### Abstract withdrawn

## EPO‐0375

### Magnetic resonance neurography in neuralgic amyotrophy: Clinical and electrodiagnostic correlations

#### F. Greloni ^1^; J. Pastor Rueda^2^; E. Garat^3^; P. Totah^4^; F. Varela^5^; C. Cejas^6^; F. Barroso^7^

##### ^*1*^ *FLENI (Fundación para la Lucha contra las Enfermedades Neurológicas de la Infancia), Ciudad Autónoma de Buenos Aires, Argentina;* ^*2*^ *FLENI (Fundación para la Lucha contra las Enfermedades Neurológicas de la Infancia), Ciudad Autónoma de Buenos Aires, Argentina;* ^*3*^ *FLENI (Fundación para la Lucha contra las Enfermedades Neurológicas de la Infancia), Ciudad Autónoma de Buenos Aires, Argentina;* ^*4*^ *FLENI (Fundación para la Lucha contra las Enfermedades Neurológicas de la Infancia), Ciudad Autónoma de Buenos Aires, Argentina;* ^*5*^ *FLENI (Fundación para la Lucha contra las Enfermedades Neurológicas de la Infancia), Ciudad Autónoma de Buenos Aires, Argentina;* ^*6*^ *FLENI (Fundación para la Lucha contra las Enfermedades Neurológicas de la Infancia), Ciudad Autónoma de Buenos Aires, Argentina;* ^*7*^ *FLENI (Fundación para la Lucha contra las Enfermedades Neurológicas de la Infancia), Ciudad Autónoma de Buenos Aires, Argentina*

**Background and aims:** Neuralgic amyotrophy (NA) is a non‐traumatic peripheral nervous system disorder predominantly affecting the brachial plexus, typically presenting with unilateral involvement of the upper and middle trunks. Diagnosis is primarily clinical, while complementary studies mainly exclude alternative diagnoses. Advances in magnetic resonance neurography (MRN) allow detailed assessment of roots, trunks, and denervated muscles, revealing anatomical involvement in NA beyond clinical and electrodiagnostic evaluation. The objective is to describe MRN findings in patients with clinically diagnosed NA and assess concordance with clinical and electrodiagnostic features.

**Methods:** We retrospectively reviewed adult patients with NA undergoing MRN 2012–2025. Demographic and clinical variables, including symptom laterality and topography, were collected. Electrodiagnostic (EMG/NCS) and MRN findings were analyzed, focusing on root, trunk, and muscle involvement.

**Results:** Twenty‐four patients were included (79% male; mean age 45.7 years). Mean time from symptom onset to evaluation was 2.3 months. Pain and weakness were present in 95.8%, and muscle atrophy in 75%. Proximal upper limb involvement in 87.5%. Electrodiagnostic studies (*n* = 21) were abnormal in 85.7%, mainly involving the upper trunk (55.6%). MRN showed abnormal findings in all patients, predominantly affecting roots and trunks of the upper trunk territory (91.7%). Muscle denervation most commonly involved the supra‐ and infraspinatus muscles (41.7%). Concordance between clinical and electrodiagnostic topography was high, but MRN identified additional nerve and muscle involvement, including subtle or subclinical abnormalities.

**Figure:**
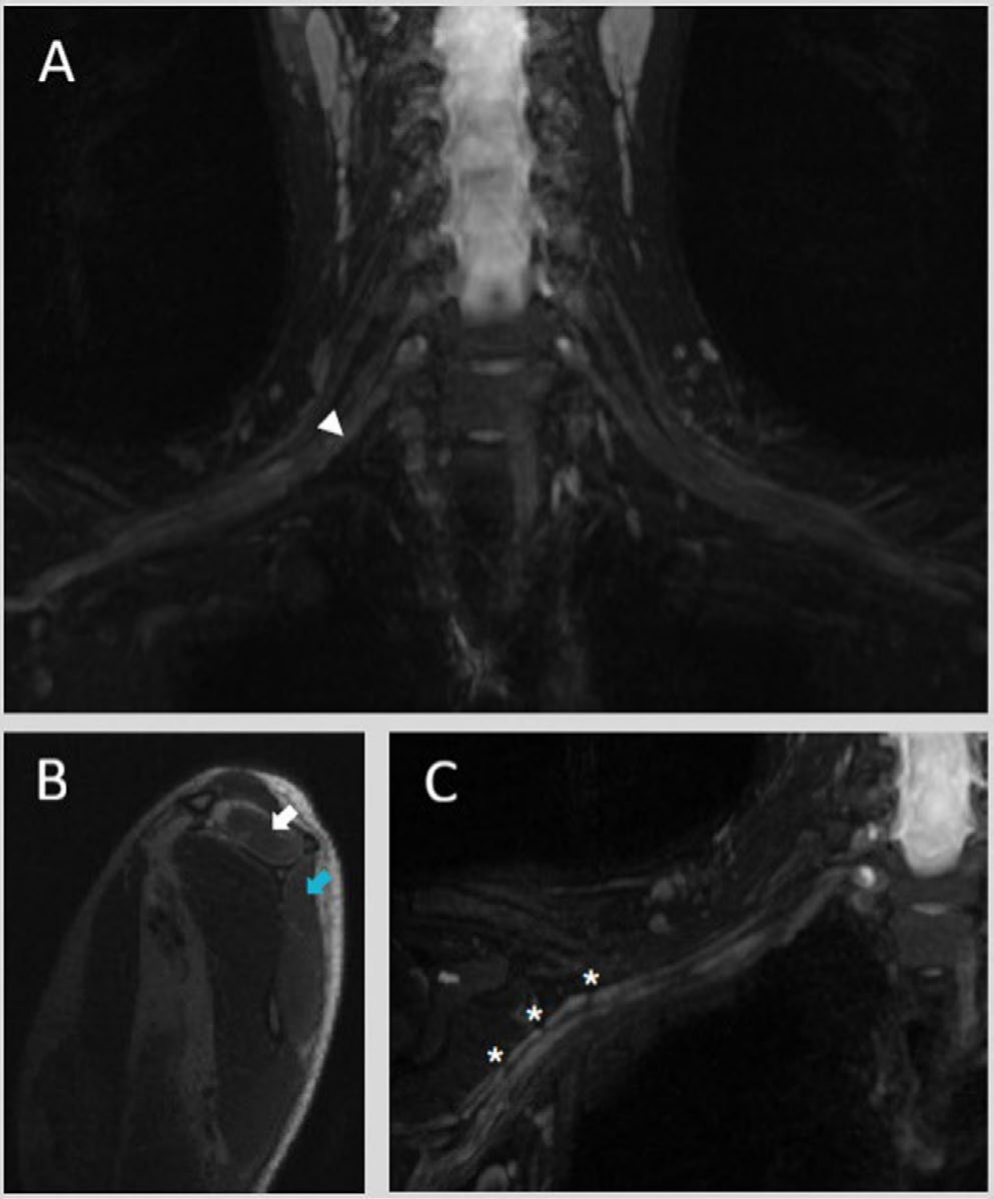
**FIGURE 1** (A) MR neurography showing C5–C6 nerve enlargement and hyperintensity (arrow). (B) Sagittal T2 images revealing hyperintensity in the supraspinatus (white arrow) and infraspinatus (blue arrow). (C) Focal constrictions of upper trunk nerves (ast).

**Figure:**
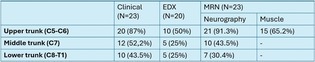
**TABLE 1** Concordance between clinical presentation, electrodiagnostic (EDX), and MR neurography (MRN) topography in patients with neuralgic amyotrophy.

**Conclusion:** MRN identified a broader extent of nerve and muscle involvement than clinical examination and electrodiagnostic studies. These findings support that NA may represent a more extensive, potentially bilateral and asymmetric radiculoplexopathy, usually unilateral clinically.

**Disclosure:** Nothing to disclose.

## EPO‐0376

### Spatial covariance of cortical metabolism with multiple metrics of nigrostriatal degeneration in Dementia with Lewy bodies (DLB)

#### G. Tomassini ^2^; L. Sofia^1^; L. Lombardo^2^; F. D'Amico^1^; T. Di Raimondo^1^; F. Massa^2^; D. Arnaldi^2^; B. Orso^2^; F. Lanfranchi^1^; S. Raffa^1^; N. Epis^2^; E. Sentieri^2^; G. Bozzo^2^; W. Kreshpa^2^; F. De Cesari^2^; M. Losa^2^; M. Pulze^2^; G. Sambuceti^1^; A. Uccelli^1^; M. Bauckneht^1^; S. Morbelli^3^; M. Pardini^2^

##### ^*1*^ *Department oh health sciences (DISSAL), University of Genoa, Genoa, Italy;* ^*2*^ *Department of Neuroscience, Rehabilitation, Ophthalmology, Genetics, Maternal and Child Health (DINOGMI), University of Genoa, Genoa, Italy;* ^*3*^ *Nuclear Medicine, University of Turin, Turin, Italy*

**Background and aims:** From a biomarker perspective, Dementia with Lewy bodies (DLB) is characterised by striatal dopamine transporter (DaT) deficits and by specific patterns of cortical hypometabolism, assessable through DaT‐SPECT and FDG‐PET, respectively. However, the relationship between dopaminergic and metabolic imaging features remains unclear. This study aimed to investigate the association between dopaminergic deafferentation in different striatal regions (caudate, anterior and posterior putamen) and cortical metabolic patterns in DLB.

**Methods:** 76 DLB patients (27 females, 49 males) who underwent both DaT‐SPECT and FDG‐PET were included. DaT‐SPECT scans were analysed using DaTQUANT 2.0 to obtain striatal binding ratios (SBR). FDG‐PET images were flipped to align the most affected hemisphere to the right. Correlations between SBR values and FDG‐PET metabolic patterns were assessed using SPM12, correcting for age, sex and MMSE. Family‐wise error–corrected p values < 0.05 at the cluster level were considered significant.

**Results:** Caudate SBR reduction correlated with hypometabolism in parietal, occipital and lateral temporal cortices, with the most significant cluster in the lateral temporal cortex (p[FWE] < 0.001). Anterior putamen SBR reduction was associated with a smaller hypometabolic cluster in similar regions, mainly in the angular gyrus (p[FWE] < 0.005). Posterior putamen SBR reduction correlated predominantly with occipital hypometabolism, particularly in the inferior occipital gyrus (p[FWE] < 0.001).

**Conclusion:** In DLB, progressive dopaminergic deafferentation from the posterior putamen to the caudate is associated with increasingly widespread cortical hypometabolism, with region‐specific striatal deficits linked to distinct cortical metabolic patterns.

**Disclosure:** Nothing to disclose.

## EPO‐0377

### Intranasal esketamine expands key frontal and hippocampal regions and reduces serum neurofilament levels in patients with major depressive disorder

#### J. Garcia‐Carmona ^1^; A. Rodriguez‐Lorente^2^; P. Campos‐Navarro^2^; P. Almela Rojo^3^; J. Navarro‐Zaragoza^3^; J. Martinez‐Riquelme^3^; D. López Segura^1^; F. Salazar Hernandez^1^; M. Ruiz Perello^1^; B. Gomez Gozalvez^1^; A. Lara Fernandez^4^; S. Benavente López^4^

##### ^*1*^ *Neurology, Santa Lucia University Hospital, Cartagena; Spain;* ^*2*^ *Psychiatry, Santa Lucia University Hospital, Cartagena; Spain;* ^*3*^ *Pharmacology, University of Murcia; Spain;* ^*4*^ *Psychiatry, Infanta Elena University Hospital, Madrid*

**Background and aims:** We investigated the effects of intranasal esketamine (ESK‐IN) on brain structural volumes and serum neurofilament light chain (sNfL) levels over a six‐month period in patients with treatment‐resistant depression (TRD).

**Methods:** 22 patients with TRD received ESK‐IN in addition to ongoing oral antidepressant therapy. Clinical assessments were performed at baseline and after 1, 3, and 6 months of treatment. Structural brain MRI and blood sampling were conducted at baseline and at the six‐month follow‐up. MRI data from 44 matched healthy controls were also analyzed to enable comparisons of basal brain volume measures. Brain volumetric analyses were performed using VolBrain2 and DSI Studio software.

**Results:** At baseline, TRD patients showed significantly reduced volumes compared to healthy controls in the insula, frontal cortex, superior parietal cortex, and both superior and inferior temporal cortices. Reduced volumes were also observed in subcortical structures, such as the nucleus accumbens, putamen, thalamus, and hippocampus. Following six months of ESK‐IN treatment, significant volumetric increases were detected in the frontal cortex, superior frontal gyrus, frontal operculum, and anterior cingulate cortex, as well as in the hippocampus. Additionally, increases were observed in the volume and integrity of white‐matter tracts involved in emotional regulation and cognitive control, including the frontoparahippocampal, frontoparietal, and frontal aslant tracts. Concomitantly, sNfL concentrations decreased after treatment, suggesting a reduction in ongoing neuronal damage.

**Conclusion:** Altogether, these findings indicate that ESK‐IN may induce neuroplastic structural changes in brain regions critical for mood regulation while exerting potential neuroprotective effects in patients with TRD.

**Disclosure:** Nothing to disclose.

## EPO‐0378

### Molecular changes induced by anti‐CGRP monoclonal antibodies for chronic migraine prophylaxis: A brain magnetic resonance spectroscopy study

#### M. Pengo ^1^; C. Manfredi^1^; C. Gambini^2^; J. Bottini^1^; M. Stroppi^3^; V. Patisso^4^; M. Olivero^5^; V. Marinato^6^; S. Sbaraini^7^; C. Uggetti^6^; S. Marceglia^6^; A. Priori^8^; M. Secchi^1^

##### ^*1*^ *Neurology Unit, ASST Santi Paolo e Carlo, San Paolo University Hospital, Milan, Italy;* ^*2*^ *Neurology and Stroke Unit, ASST Rhodense, Garbagnate M.se Hospital, Milan, Italy;* ^*3*^ *Medical Physics Unit, Fondazione IRCCS Ca' Granda Ospedale Maggiore Policlinico, Milan, Italy;* ^*4*^ *Department of Neurology, IRCCS Istituto Auxologico Italiano, Milan, Italy;* ^*5*^ *Department of Neurology, Maggiore della Carità Hospital, University of Piemonte Orientale, Novara, Italy;* ^*6*^ *Aldo Ravelli Center for Neurotechnology and Experimental Brain Therapeutics, Department of Health Sciences, University of Milan, Milan, Italy;* ^*7*^ *Neuroradiology Unit, Department of Radiology, San Carlo Borromeo Hospital, ASST Santi Paolo e Carlo, San Carlo Borromeo Hospital, Milan, Italy**;*** ^*8*^ *Aldo Ravelli Center for Neurotechnology and Experimental Brain Therapeutics, Department of Health Sciences, University of Milan, Milan, Italy. Neurology Unit, ASST Santi Paolo e Carlo, San Paolo University Hospital, Milan, Italy*

**Background and aims:** Calcitonin gene‐related peptide monoclonal antibodies (CGRP‐mAb) are effective treatments for preventing chronic migraine (CM). However, the molecular basis of indirect effects on the central nervous system (CNS) is still to be explored. We applied Brain Magnetic Resonance Spectroscopy (MRS) to investigate the neurochemical changes in the CNS induced by CGRP‐mAb in CM.

**Methods:** This longitudinal study included 9 patients with CM without aura, evaluated before and six months after CGRP‐mAb treatment initiation. At baseline, subjects underwent brain MRS (3T scanner, voxel 8 cm3, TE 135 ms) and comprehensive clinical evaluation including headache frequency, headache intensity (HIT‐6), disability (MIDAS), cognitive assessment (MOCA) and mood (BDI). At 6‐month follow‐up, patients were re‐evaluated with clinical assessment and MRS. All patients were scanned interictally. Linear mixed models were applied to evaluate changes in metabolite concentrations before and after treatment.

**Results:** After treatment neurochemical changes were observed: phosphocreatine (PCr) increased (*p* = 0.02) and glutathione (GSH) decreased (*p* = 0.02) in occipital cortex (OCC), whereas glycerophosphocholine (GPC) increased (*p* = 0.03) in anterior cingulate cortex (ACC). Trends suggested positive correlations between baseline migraine intensity and changes in PCr and GPC concentration in OCC and ACC, respectively.

**Figure:**
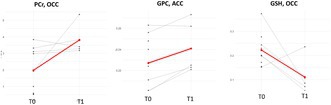
FIGURE 1

**Conclusion:** The main finding of this study is the increase of PCr concentration in OCC, in line with previous evidence of the key role of brain energetics in CM. Whether this represents a central correlate of treatment or an epiphenomenon related to the reduction in migraine frequency requires further investigation.

**Disclosure:** Nothing to disclose.

## EPO‐0379

### Multispiral computed tomographic angiography in diagnosing neurovascular conflict in classical trigeminal neuralgia: A 15‐year study

#### O. Zykova; B. Elena; A. Anastasia

##### Rostov State Medical University, Rostov‐on‐Don, Russian Federation

**Background and aims:** The aim of the study was to compare the accuracy of magnetic resonance imaging (MRI) and 3D multispiral computed tomographic angiography (MSCTA) in differentiating neurovascular conflict in patients with classical trigeminal neuralgia (CTN).

**Methods:** The study included 500 patients diagnosed with CTN based on the actual ICHD and the ICOP classification. Each patient underwent MRI scans and MSCTA in 3D mode with bolus contrast. The study focused on identifying neurovascular conflict (NVC) by examining the superior cerebellar artery loop's apex as it transitions from the anterior pontomesencephalic segment to the lateral segment, relative to the Meckel cave and the trigeminal nerve root. Microvascular decompression (MVD) was performed on patients who did not respond to conservative treatment. Intraoperative assessments were conducted to verify the MRI and MSCTA findings.

**Results:** The MRI performed on patients effectively excluded secondary causes of CTN, but it could not determine the type of NVC or distinguish between NVC and contact. MSCTA revealed NVC type 1 in 320 patients (64%), type 2 in 180 patients (36%). Intraoperative assessments in 412 patients indicated that MRI misdiagnosed neurovascular conflict in 64 cases (15.5%). Notably, 78 patients (18.9%) with MRI‐confirmed neurovascular conflict who underwent microvascular decompression did not experience pain relief.

**Figure:**
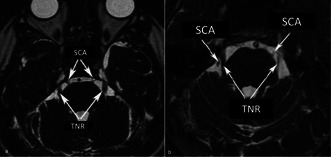
**FIGURE 1** Magnetic resonance imaging: a) there is no conflict between the trigeminal nerve root (TNR) and the superior cerebellar artery (SCA); b) neurovascular conflict between the trigeminal nerve root andthe superior cerebellar nerve on the left.

**Figure:**
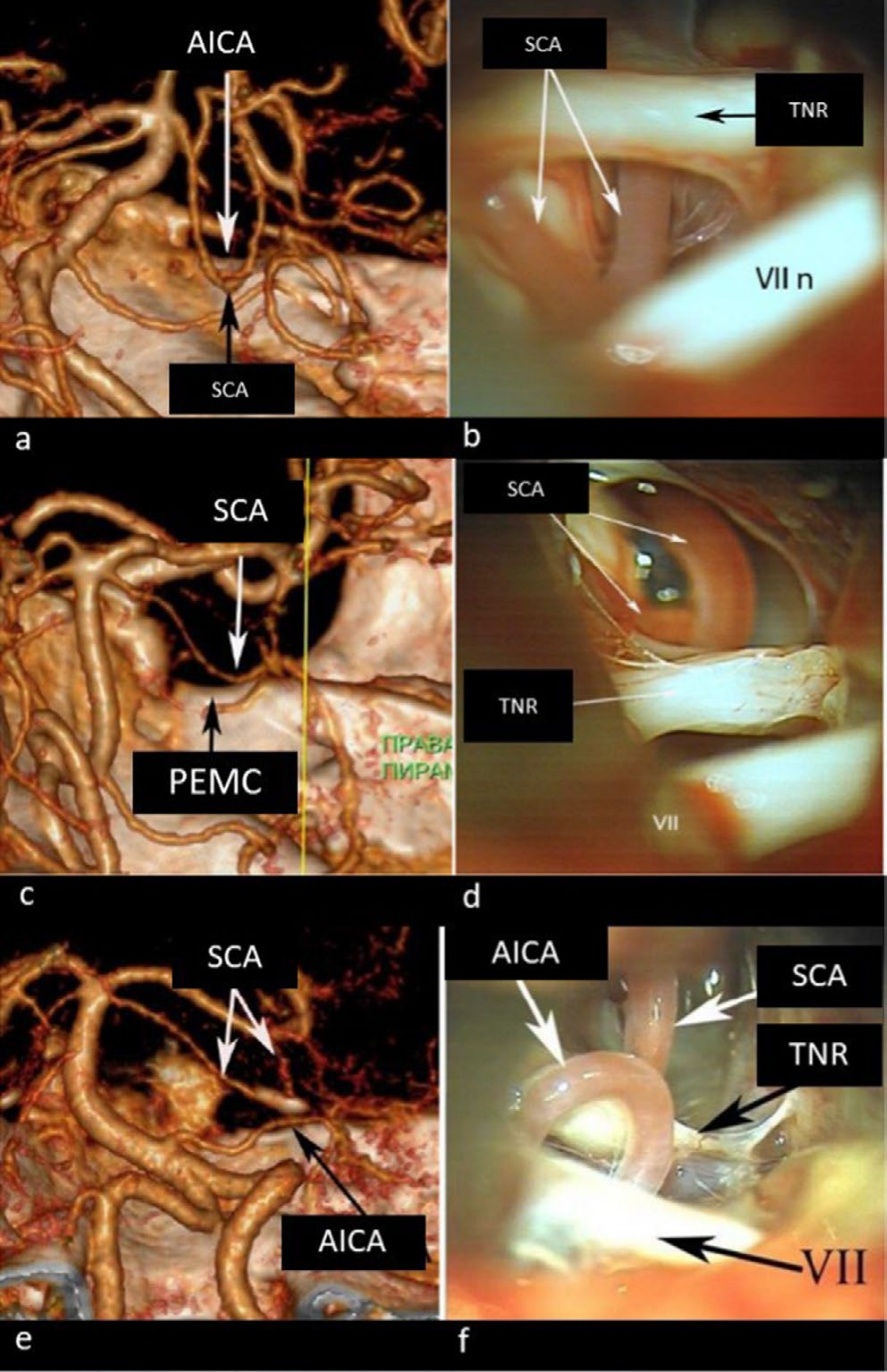
**FIGURE 2** Results of multispiral computed tomographic angiography (MSCTA) in 3D mode with bolus contrast and intraoperative pictures during microvascular decompression: a) Neurovascular conflict type 1 – MSCTA; b) Neurovascular conflict type 1.

**Figure:**
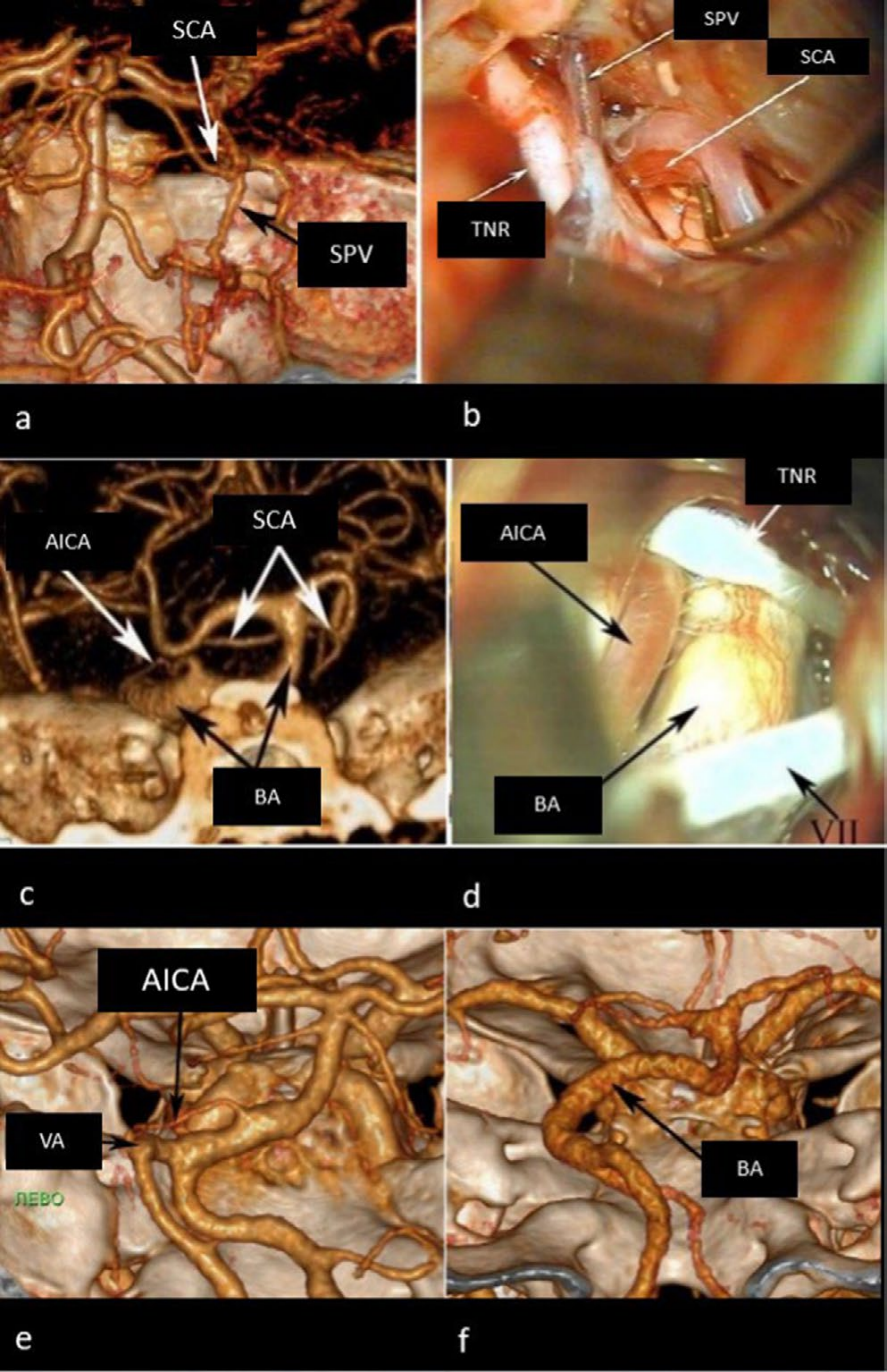
**FIGURE 3** Results of multispiral computed tomographic angiography (MSCTA) in 3D mode with bolus contrast and intraoperative pictures during microvascular decompression: a) Arterio‐venous sandwich (trigeminal nerve root between an artery and a vein).

**Conclusion:** MRI has limitations in differentiating between NVC and contact, leading to concerns about overdiagnosis and misinterpretation in CTN patients and healthy individuals. This reliance on MRI may reduce favorable surgical outcomes. MSCTA demonstrates higher accuracy in identifying the neurovascular relationship and indicating the need for MVD.

**Disclosure:** Nothing to disclose.

## EPO‐0380

### A progressive lesional mantle adjacent to the frontal cortices disclosing a CSF1R leukoencephalopathy in presymptomatic stage

#### E. Peczenik ^1^; P. Alegria^2^

##### ^*1*^ *Neurology, CHU Tivoli, La Louvière, Belgium;* ^*2*^ *Neurology, CHU Charleroi‐Chimay, Charleroi, Belgium*

**Background and aims:** Colony‐stimulating factor‐1 receptor (CSF1R) related leucoencephalopathy usually presents around the 4th decade of life and frequently progresses to death in a few years. Transmission is autosomal dominant but de novo mutations and incomplete penetrance may account for a negative family history. Allogenic bone marrow transplantation has been reported to stabilize patients and a therapeutic trial with a receptor agonist is ongoing. Early diagnosis will be essential to allow treatment before neurologic damage installs.

**Methods:** We report the evolution of a presymptomatic patient and describe a particular MRI pattern that could help to identify other patients.

**Results:** A 35‐year‐old woman with no relevant personal medical history, underwent a brain MRI for tension‐type headaches. Besides a few unspecific deep white matter point lesions, a few unusual small lesions adjacent to both prefrontal cortices (but sparing the U‐fibers) were present. A subsequent MRI at 39 years‐old showed a degree of coalescence of the frontal lesions adjacent to the cortex, starting to form a “mantle”. One year later this mantle started to appear also in the right temporal lobe and 2 small lesions showed restriction on diffusion. She remained asymptomatic but a first neurological examination by that time noted some mild attention deficits and a bilateral ankle clonus. She reported that her mother had died of an obscure neurological disease by the age of 44. Genetic testing disclosed a c.2625G > A mutation. At 41 years‐old she remains stable.

**Figure:**
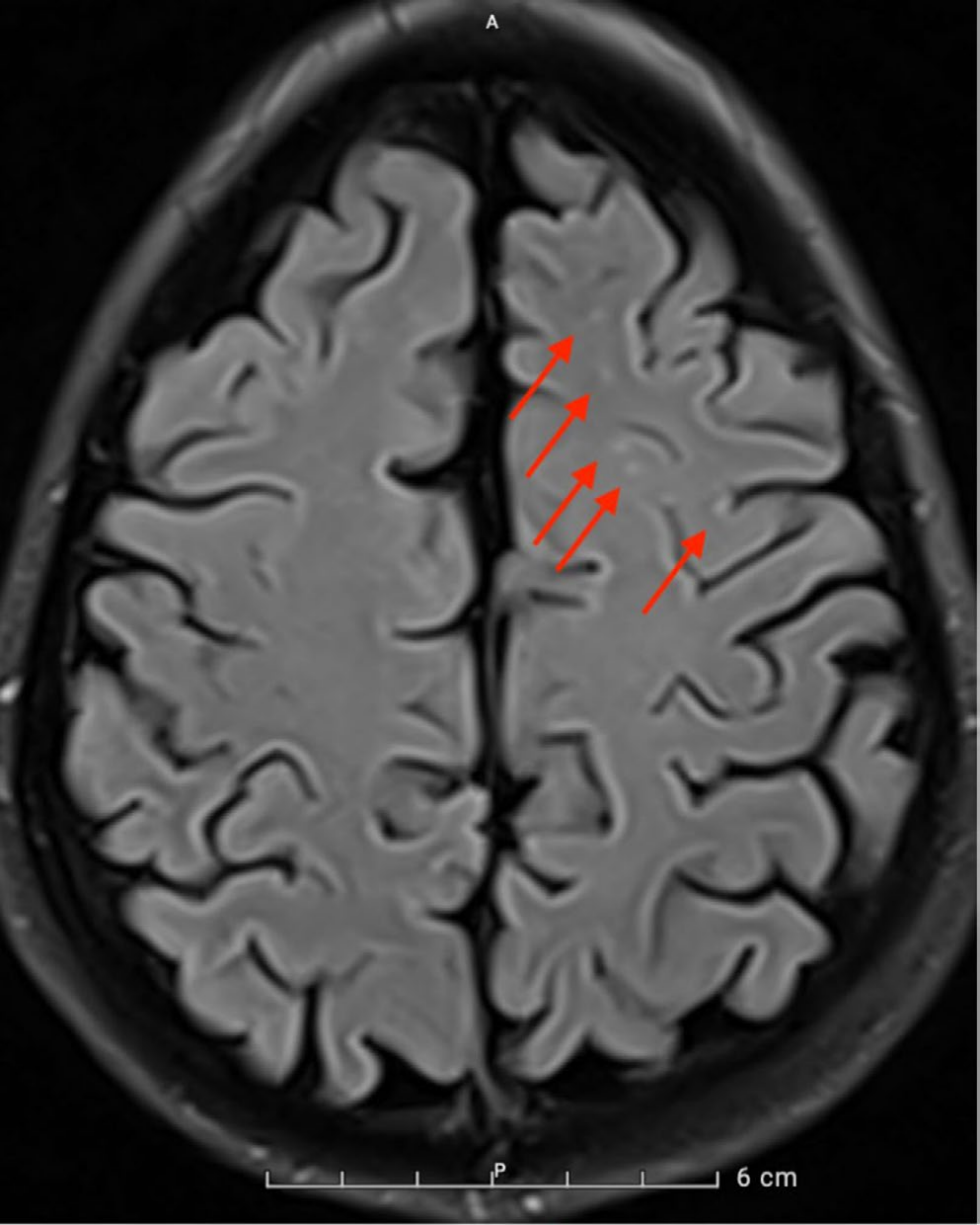
**FIGURE 1** 30 years‐old. FLAIR. Small lesions adjacent to the left frontal cortex.

**Figure:**
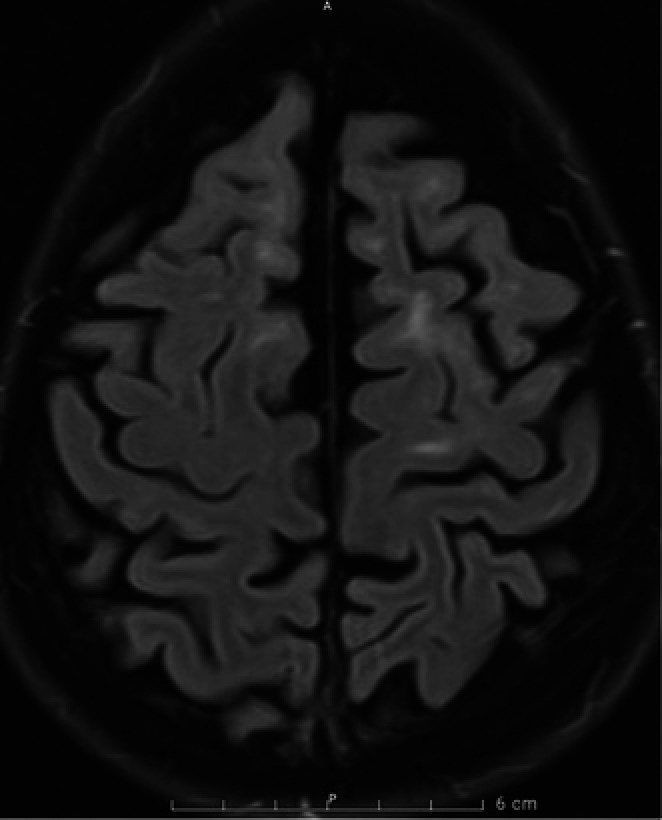
**FIGURE 2** 4O years‐old. FLAIR. This unusual pattern raised the hypothesis of a leukodystrophy.

**Figure:**
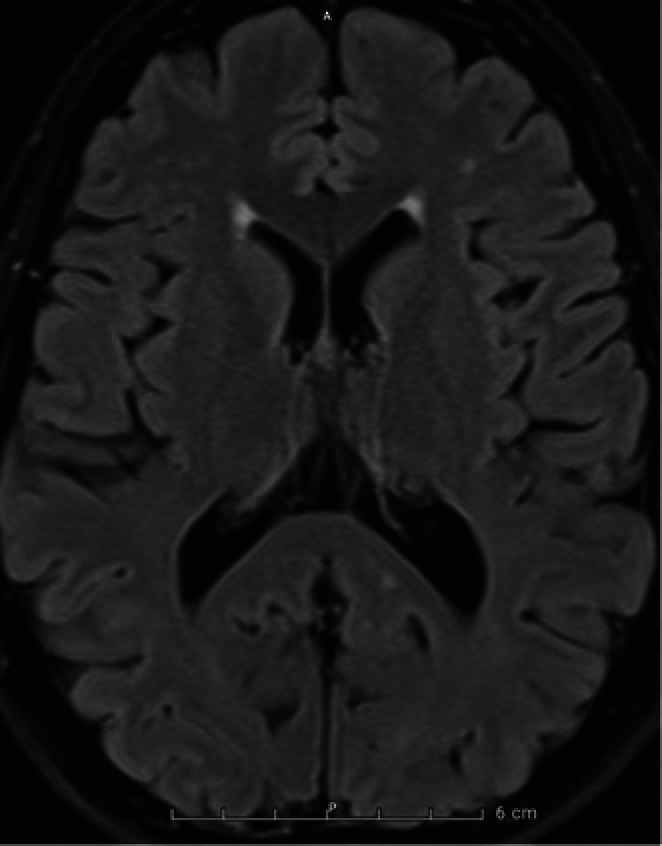
**FIGURE 3** 40 years‐old. Lesions elsewhere in the brain are sparse and unspecific and wouldn't disclose the diagnosis by themselves.

**Conclusion:** A patchy mantle of small frontal juxtacortical lesions can disclose this disease in a very early phase.

**Disclosure:** Nothing to disclose.

## EPO‐0381

### Substantia nigra neuromelanin magnetic resonance imaging in LRRK2 asymptomatic carriers

#### V. Carvalho ^1^; J. Freitas^2^; C. Guerreiro^2^; A. Travessa^3^; C. Correia Rodrigues^4^; D. Cruz^1^; R. Barreto^1^; A. Valadas^1^; P. Pita Lobo^1^; M. Grunho^4^; R. Simões^5^; M. Rosa^1^; M. Coelho^1^; A. Mendes^6^; J. Ferreira^7^; S. Reimão^2^; L. Correia Guedes^1^

##### ^*1*^ *Department of Neurosciences and Mental Health, Neurology, Santa Maria* Local Health Unit*, Lisbon, Portugal;* ^*2*^ *Department of Neurological Imaging, Santa Maria* Local Health Unit*, Lisboa, Portugal;* ^*3*^ *Department of Medical Genetics, Department of Pediatrics, Santa Maria Hospital, Santa Maria* Local Health Unit*, Lisbon, Portugal;* ^*4*^ *Department of Neurology, Garcia de Orta Hospital – Almada‐Seixal* Local Health Unit*, Almada, Portugal;* ^*5*^ *Neurology Department, Beatriz Ângelo Hospital, Loures, Portugal;* ^*6*^ *Department of Neurology, Santo António Hospital, Porto University Hospital Centre, Porto, PortugaL;* ^*7*^ *CNS – Campus Neurológico, Torres Vedras, Portugal*

**Background and aims:** Parkinson's disease (PD) is a progressive neurodegenerative disorder, with long pre‐clinical and prodromal stages. The development of non‐invasive biomarkers for pre‐diagnostic stages is of utmost importance. Neuromelanin (NM)‐sensitive magnetic resonance imaging (MRI) of the substantia nigra (SN) is an established biomarker of neurodegeneration in PD, which is expected to begin years before motor symptoms emerge, and for which LRRK2 mutation asymptomatic carriers are at higher risk. We aim to investigate NM MRI in asymptomatic LRRK2 carriers

**Methods:** Asymptomatic at‐risk first‐degree relatives of LRRK2‐PD patients were sequentially recruited. An extensive clinical evaluation, including motor and non‐motor symptoms, as well as a study of neuromelanin, using a specific T1‐weighted MRI sequence, was performed. Targeted genetic testing for LRRK2 variants was performed after clinical evaluation and imaging studies.

**Results:** Nineteen asymptomatic participants were included, of which 12 LRRK2 non‐carriers (NC) and 7 carriers (AC). Baseline demographics did not differ between groups (median age AC 46 years (IQR 35–53) vs NC 51.5 (39–59) *p* = 0.379). Motor scores did not differ between groups (MDS‐UPDRS III median 2 (2–4) in AC vs 2.5 (1.5–5.4) in NC, *p* = 0.863). NM area was significantly smaller in AC when compared to NC, (16 mm^2^ (14.9–19.1) vs 20.6 mm^2^ (19.3–24.0), *p* = 0.025).

**Conclusion:** NM‐MRI can be significantly decreased in LRRK2‐G2019S AC. This technique may be a promising biomarker of neurodegeneration in pre‐diagnostic stages of PD.

**Disclosure:** Nothing to disclose.

## EPO‐0382

### Association of glymphatic imaging markers with functional independence and white matter hyperintensity burden after intracerebral hemorrhage

#### X. Hu; Z. Wang; Q. Li

##### Department of Neurology, The Second Affiliated Hospital of Anhui Medical University, Hefei, China

**Background and aims:** Glymphatic system plays a critical role in cerebral metabolite clearance and white matter injury. However, its clinical relevance and underlying mechanisms in intracerebral hemorrhage (ICH) remain poorly understood. This study aimed to investigate imaging‐based glymphatic markers in relation to functional outcomes and white matter hyperintensity (WMH) burden in patients with ICH.

**Methods:** We included 70 patients with acute supratentorial ICH who underwent diffusion tensor imaging (DTI). Glymphatic‐related imaging parameters were derived using DTI and T1‐weighted MRI, including the contralateral DTI‐ALPS index, free water (FW) ratio in contralateral deep white matter (WM), and relative perivascular space (PVS) volume. Relative WMH volume was defined as absolute WMH normalized to intracranial volume and log‐transformed. Functional independence was defined as a 3‐month modified Rankin Scale score of 0–2. Associations were examined using multivariable logistic and linear regression models.

**Figure:**
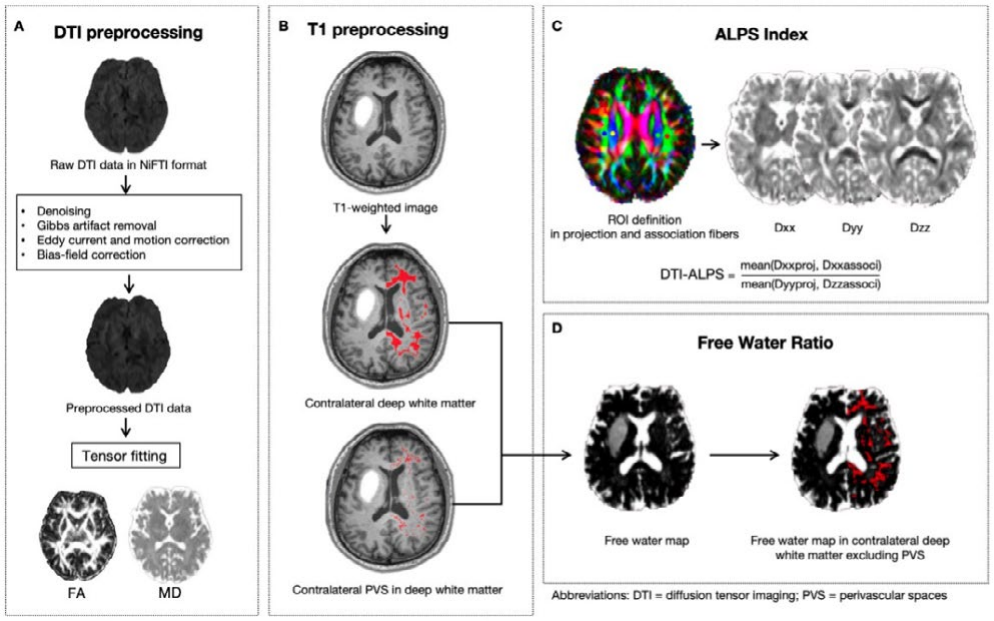
**FIGURE 1** Flowchart of imaging processing. (A) Diffusion tensor imaging preprocessing. (B) Generation of contralateral deep white matter (WM) and PVS masks. (C) Calculation of the DTI‐ALPS. (D) Free water ratio in contralateral deep WM excluding PVS.

**Results:** In fully adjusted logistic regression models, higher FW in deep WM was independently associated with a lower likelihood of functional independence at 3 months (OR = 0.59; 95% CI, 0.36–0.98; *P* = 0.041), with consistent findings across sequential models. Linear regression analyses further demonstrated that greater relative WMH burden was significantly associated with increased FW in deep WM (*β* = 0.143; 95% CI, 0.008–0.277; *P* = 0.038) and with smaller relative PVS volume in fully adjusted models.

**Figure:**
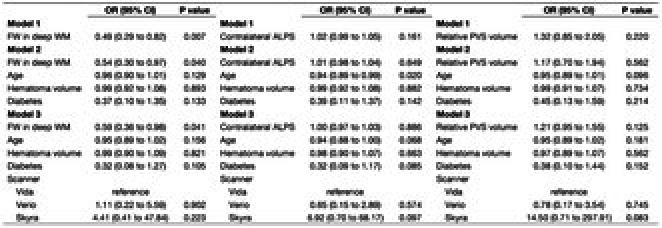
**TABLE 1** Associations between glymphatic imaging parameters and functional independence.

**Figure:**
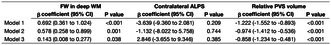
**TABLE 2** Linear regression analyses between relative WMH volume and glymphatic parameters. Model 1: Unadjusted. Model 2: Adjusted for age, NIHSS, and baseline hematoma volume. Model 3: Adjusted for variables in model 2, mean diffusivity and scanner.

**Conclusion:** These findings suggest that white matter injury may influence functional recovery after ICH through glymphatic system dysfunction, with FW emerging as the most sensitive imaging marker linking WMH burden to adverse functional outcomes.

**Disclosure:** We declare no conflict of interests.

## EPO‐0383

### Peri‐nidal hypoperfusion on multi‐delay ASL is associated with epilepsy in unruptured brain arteriovenous malformation

#### X. Li ^1^; C. Wu^1^; Y. Yin^1^; B. Xu^2^; T. Wu^2^; J. Lu^1^; Q. Qin^3^

##### ^*1*^ *Department of Radiology and Nuclear Medicine, Xuanwu Hospital, Capital Medical University, Beijing, China;* ^*2*^ *MR Research China, GE Healthcare, Beijing, China;* ^*3*^ *Diagnostic Imaging Sciences Center (DISC), University of Washington School of Medicine, Seallte, USA*

**Background and aims:** The hemodynamics of epilepsy in unruptured brain AVM remains unclear. This study aims to investigate the perfusion characteristics of the whole brain and peri‐nidal tissues and explore their relationships with AVM‐related epilepsy.

**Methods:** This study enrolled patients with unruptured brain AVMs. Time‐encoding multi‐delay ASL was performed to obtain corrected cerebral blood flow (cCBF), arterial transit time (ATT), and arterial cerebral blood volume (aCBV). After excluding draining veins, five concentric perinidal zones were delineated, each 2 mm thick. Within each zone, perfusion parameters were specifically quantified for the entire brain tissue, gray matter, and white matter separately. Independent samples t‐tests were applied to compare these parameters between AVM patients with and without epilepsy.

**Figure:**
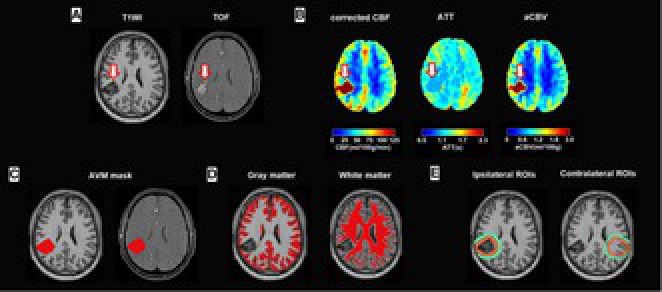
**FIGURE 1** Anatomical axial images, perfusion maps, and tissue segmentations.

**Results:** A total of 40 AVM patients (20 with epilepsy, 20 without) were included. While no global perfusion differences were found, patients with epilepsy showed significantly lower cCBF and aCBV in the tissue closest to the nidus (≤ 2 mm), particularly in gray matter, compared to non‐epilepsy patients. This difference diminished with increasing distance from the nidus, with the most severe impairment found in the peri‐nidal gray matter.

**Figure:**
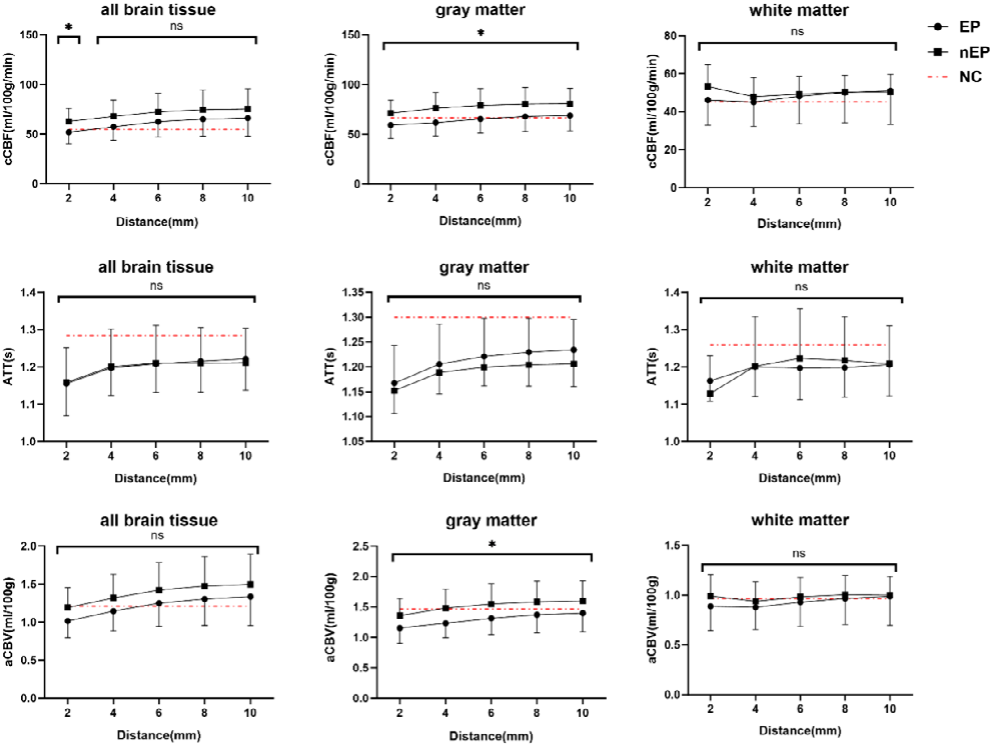
**FIGURE 2** Comparison of perinidal cCBF, ATT, and aCBV between AVM patients with and without epilepsy.

**Figure:**
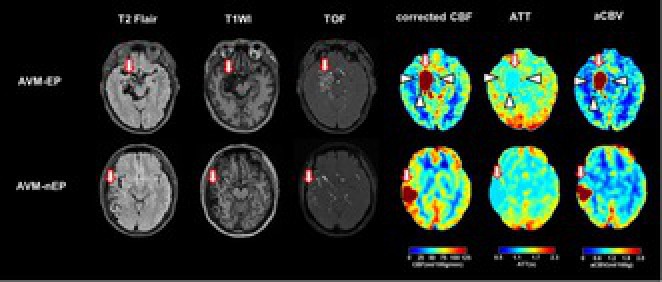
**FIGURE 3** Imaging comparison and the peri‐nidal steal phenomenon.

**Conclusion:** The findings of a pronounced and distance‐dependent steal effect in epilepsy patients support the hemodynamic steal hypothesis. This suggests that an AVM's high‐flow shunt critically diverts blood, causing chronic ischemia in the adjacent cortex. Such ischemia may contribute to neuronal dysfunction, selective loss, and gliosis, which collectively lower the seizure threshold and create an epileptogenic focus, revealing a potential hemodynamic mechanism for epilepsy.

**Disclosure:** Nothing to disclose.

## Cognitive Neurology/Neuropsychology 3

## EPO‐0384

### Augmenting clinical reasoning: Integrating artificial intelligence into neuropsychological and mental health assessment

#### A. Desmarais; A. Hudon

##### Département de psychiatrie et d'addictologie, Université de Montréal, Montréal, Canada

**Background and aims:** Artificial intelligence (AI) is increasingly entering neuropsychological and mental health practice, yet its adoption remains limited by ethical concerns, professional responsibility, and clinician acceptability. Beyond technical performance, effective integration of AI in neurology and neuropsychology depends on alignment with clinical reasoning, ethical standards, and professional identities. This presentation reflects on these challenges through the design of Flow, an AI‐enabled clinical platform developed in collaboration with mental health professionals.

**Methods:** This presentation adopts a conceptual, practice‐informed approach grounded in participatory and ethical‐by‐design principles. Flow was developed through iterative exchanges with neuropsychologists and clinicians, focusing on real‐world workflows. The platform functions as a supervisory tool that supports clinical reasoning without generating autonomous diagnoses. Transparency, clinician control, and clear boundaries of responsibility guide its design.

**Results:** Drawing on clinical feedback and field experience, this presentation synthesizes key themes related to clinician expectations, perceived risks, and conditions for acceptability of AI tools. Tensions between efficiency and clinical nuance, automation and professional judgment, and innovation and regulation are highlighted.

**Conclusion:** Overall, the future of AI in neuropsychology and mental health depends less on algorithmic sophistication than on ethical design, clinician trust, and alignment with professional values.

**Disclosure:** Annie Desmarais is the founder and CEO of Flow Factor, the company developing the Flow clinical AI platform. Her postdoctoral research is conducted on and in relation to the Flow platform.

## EPO‐0385

### What do we know about post‐stroke social cognition impairments? A systematic review of evidence across core sub‐domains

#### M. Favaro; A. Panzavolta; C. Cerami

##### IUSS Cognitive Neuroscience ICoN Center, Scuola Universitaria Superiore IUSS, Pavia, Italy

**Background and aims:** Growing evidence reported social cognition impairments in patients following stroke, with converging findings highlighting a higher prevalence in individuals with right‐hemisphere lesions. Despite increasing recognition of socio‐cognitive deficits after stroke, substantial variability remains across studies with respect to prevalence rates, affected sub‐domains, and underlying neural correlates. This systematic review therefore aimed to synthesize current evidence on post‐stroke social cognition impairments, with a specific focus on domain‐specific performance and hemispheric lesion patterns, to define the post‐stroke socio‐cognitive profile.

**Methods:** A systematic literature search was conducted in PubMed, Scopus, and Web of Science databases following PRISMA guidelines. The risk of bias assessment was assessed with the AXIS and the NIH Quality Assessment tools.

**Results:** Among the 1293 papers initially retrieved, 33 studies met eligibility criteria. Quality assessment showed a middle‐high overall quality of evidence. Emotion recognition is the most frequently investigated socio‐cognitive sub‐domain, with impairments reported in about 25–35% of post‐stroke patients without any consistent emotion‐specific pattern. Theory of Mind deficits are reported in both cognitive and affective components, especially following right hemisphere strokes. Findings on empathy are more heterogenous, with studies reporting reduced cognitive empathy alongside relatively preserved affective empathy.

**Conclusion:** Despite consistent evidence across studies, heterogeneity in assessment methods, timing and samples strongly prevents a clear profiling of social cognition impairments in post‐stroke patients. Standardization of cognitive assessment protocols and harmonization of neuropsychological procedures is needed to overcome current literature gaps and improve diagnostic precision, support targeted rehabilitation, and enhance functional and interpersonal outcomes in stroke survivors.

**Disclosure:** None.

## EPO‐0386

### Early‐life multidomain engagement relates to semantic fluency and episodic learning in aging and mild cognitive impairment

#### F. Cieri ^1^; J. Caldwell^2^; D. Cordes^1^; C. Cross^3^

##### ^*1*^ *Department of Neurology, Cleveland Clinic Lou Ruvo Center for Brain Health, Las Vegas, USA;* ^*2*^ *University of Wisconsin–Madison, Madison, USA;* ^*3*^ *School of Veterinary Medicine, Texas Tech University, Amarillo, USA*

**Background and aims:** Engagement in cognitively, physically, creatively, and socially stimulating activities is recognized as a modifiable protective factor against cognitive decline and dementia. However, whether early‐life periods represent sensitive windows for later‐life neuropsychological resilience remains insufficiently characterized, particularly across the continuum from cognitively unimpaired (CU) aging to mild cognitive impairment (MCI).

**Methods:** Fifty‐eight participants, including CU older adults and individuals with MCI, completed the dynamic Neurocognitive Adaptation (dNA) scale, assessing engagement across four domains (cognitive, physical, creative, social) over seven life‐course time windows. Neuropsychological outcomes included semantic fluency (vegetables, animals) and episodic learning (RAVLT). Participants also underwent amyloid PET assessment. Hierarchical regression analyses examined associations between domain‐ and time‐specific dNA scores and cognitive performance, controlling for age, sex, education, and amyloid status.

**Results:** Childhood emerged as a sensitive period for neuropsychological outcomes. Higher childhood cognitive engagement (COG1) was associated with better semantic fluency (vegetables: *β* = 0.410, *p* = 0.003; animals: *β* = 0.487, *p* = 0.005), while higher childhood physical engagement (PHY1) was associated with stronger episodic learning (*β* = 0.417, *p* = 0.006). No dNA domain predicted delayed recall. All associations survived false discovery rate correction and were not moderated by sex, education, or diagnosis.

**Figure:**
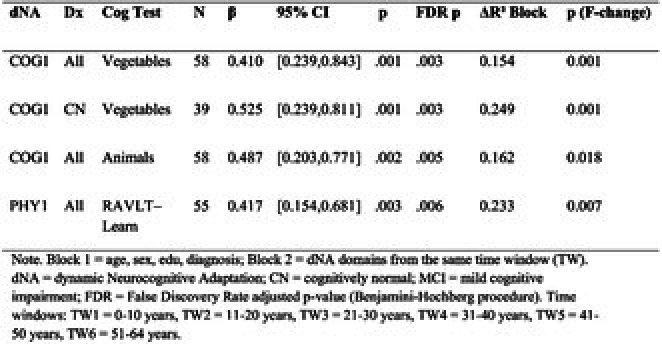
**TABLE 1** Significant HMR results — dNA → Cognitive.

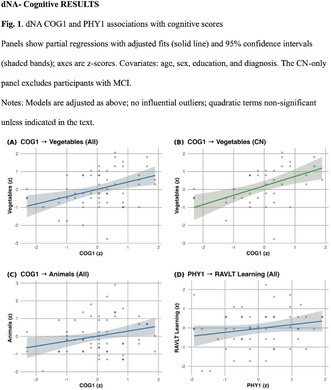

**Conclusion:** Early‐life multidomain engagement, particularly cognitive and physical activity during childhood, is associated with preserved semantic and episodic memory performance in CU and MCI. These findings support early‐life engagement as a protective factor for neuropsychological resilience and highlight the value of time‐resolved tools such as the dNA for prevention research.

**Disclosure:** Nothing to disclose.

## EPO‐0387

### Alzheimer's disease in stroke: Prevalence in 3 cohorts and links with clinical, imaging, and cognitive status

#### J. Neel ^1^; J. Morris^2^; V. Mok^3^; P. Cervellera^1^; M. Roussel^1^; T. Benzinger^4^; B. Lam^3^; C. Lamy^1^; B. Gordon^4^; S. Canaple^1^; B. Grant^5^; A. Courselle‐Arnoux^1^; A. Wong^3^; C. Leclercq^1^; M. Streitz^2^; O. Godefroy^1^

##### ^*1*^ *Department of Neurology Amiens University Hospital and Laboratory of Functional Neurosciences (UR 4559) University of Picardie Jules Verne, Amiens, France;* ^*2*^ *Department of Neurology, Washington University School of Medicine, St. Louis, USA;* ^*3*^ *Division of Neurology, Department of Medicine and Therapeutics, The Chinese University of Hong Kong, Shatin, Hong Kong**;*** ^*4*^ *Department of Radiology, Washington University School of Medicine, St. Louis, USA;* ^*5*^ *The Institute for Informatics, Data Science and Biostatistics, Washington University School of Medicine, St. Louis, USA*

**Background and aims:** Amyloidopathy is reported in stroke populations with varying prevalence and cognitive impact. To address this limitation, the present study pooled data from the 3 largest stroke cohorts assessing cognitive status and amyloid burden using PET (IDEA3, ref 1, St Louis, 2 and STRIDE, 3). Our primary objective was to estimate the prevalence of amyloidopathy in stroke. Secondary objectives included associations between amyloidopathy and clinical characteristics, Mini‐Mental State Examination (MMSE) score, cognitive status and brain imaging markers.

**Methods:** A cross‐sectional analysis was performed on 218 patients (age 70.8 ± 10.8 years; 59% male; education 9.5 ± 4.4 years), including infarct, TIA, and hemorrhage. Cognitive status was categorized as dementia, mild cognitive impairment, or normal. Data were analyzed using mixed models adjusted for age, education, stroke subtype, stroke severity, and prestroke dementia.

**Results:** Amyloidopathy prevalence was 19.7% and increased with age (*p* = 0.001), with no effect of stroke‐PET time interval (*p* = 0.1). Amyloidopathy was associated (*p* = 0.012) with lower MMSE score but not with cognitive status. Regarding imaging amyloidopathy was associated with lobar microbleeds (*p* = 0.04) but not with deep microbleeds, white matter hyperintensities and hippocampal volume.

**Conclusion:** Amyloidopathy affects about one fifth of stroke patients, increasing with age. It worsens cognitive performance as measured by MMSE but does not influence overall cognitive status, highlighting the role of vascular lesions in poststroke impairment. The presence of lobar microbleeds should prompt the search for amyloidopathy, even in ischemic stroke. References 1. Godefroy O et al. Stroke 2025;56:74–83 2. Koenig L.N et al. NeuroImage: Clinical 2021, 102553 3. Yang J et al. Alzheimers&dementia 2015, Vol: 11, Pages: 16–23.

**Disclosure:** Marissa Streitz and John C. Morris are funded by National Institutes of Health grants (P30AG066444, P01AG003991, P01AG026276). Tammie Benzinger and Brian Gordon report no conflicts of interest other than support from the main Alzheimer's Disease Research Center grants. Sandrine Canaple reports having received travel funding from Medtronic. Olivier Godefroy has served, within the last two years, on scientific advisory boards and as a speaker for GE Healthcare, ESAI SAS, and Novo Nordisk; he did not receive funding for travel or meetings from pharmaceutical companies. Jeremy Neel, Vincent C.T. Mok, Pierre‐A. Cervellera, Martine Roussel, Bonnie Y.K. Lam, Chantal Lamy, Betsy Grant, Audrey Courselle‐Arnoux, Adrian Wong, and Claire Leclercq declare no conflicts of interest.

## EPO‐0388

### Sex‐specific cognitive divergence in Parkinson's disease

#### J. Saltyte Benth ^1^; G. Sanchez Nido^2^; O. Tysnes^2^; G. Alves^3^; C. Tzoulis^2^; G. Skeie^2^

##### ^*1*^ *Department of Clinical Medicine, University of Bergen, Bergen, Norway;* ^*2*^ *Neuro‐SysMed, Department of Neurology, Haukeland University Hospital, Bergen, Norway;* ^*3*^ *Department of Neurology and Center for Movement Disorders, Stavanger University Hospital, Stavanger, Norway*

**Background and aims:** Sex differences in cognitive impairment in Parkinson's disease (PD) have been reported, with several studies suggesting greater cognitive decline in males. However, evidence on long‐term trajectories across specific cognitive domains is limited, as most prior work is cross‐sectional. Thus, we aim to investigate sex‐specific longitudinal changes across multiple cognitive domains over 9 years in idiopathic PD, compared to demographically matched controls.

**Methods:** We analyzed data from the prospective ParkWest study, including *n* = 190 persons with PD (PwPs) and *n* = 202 age‐ and sex‐matched controls, followed for 9 years. Regularly assessed cognitive domains included semantic fluency, processing speed, cognitive control, memory, and visuospatial ability. Linear mixed‐effect models were used, including a three‐way interaction between time, disease status and sex, to test whether sex‐specific cognitive change in PD differed from that observed in controls. Models were adjusted for age, years of education, baseline depression, and UPDRS part II.

**Results:** Male PwPs showed faster decline than female PwPs across most cognitive measures, resulting in gradually increasing sex‐differences over 9 years. However, compared with controls, the differential sex‐specific decline was statistically significant only for semantic fluency (*p* = 0.013) and processing speed (*p* = 0.027 and *p* = 0.035). These findings remained significant after covariate adjustment.

**Conclusion:** Over 9 years, PD was associated with increasing sex‐differences in semantic fluency and processing speed, driven by faster decline in males. These findings confirm the male susceptibility to cognitive decline in PD and suggest that sex‐specific vulnerabilities in cognitive trajectories in PD are domain‐specific.

**Disclosure:** Nothing to disclose.

## EPO‐0389

### Cognitive‐communicative profiles in mild cognitive impairment: Evidence from the bulgarian SCCAN

#### K. Chompalov ^1^; G. Spray^2^; D. Georgieva^1^; P. Atanasova^1^

##### ^*1*^ *Department of Neurology, St. George Hospital, Medical University of Plovdiv, Plovdiv, Bulgaria;* ^*2*^ *Department of Speech, Language, and Hearing Sciences, Auburn University, Auburn, USA*

**Background and aims:** Early identification of mild cognitive impairment due to Alzheimer's disease (MCI) is essential for timely intervention. Although episodic memory deficits are central to MCI, language performance may also be affected early. This study examined cognitive and language performance differences between individuals with MCI and cognitively healthy older adults using the Bulgarian version of the Scales of Cognitive and Communicative Ability for Neurorehabilitation (SCCAN).

**Methods:** Thirty individuals with MCI and 30 cognitively healthy controls were assessed with the Bulgarian SCCAN. Group differences were examined using independent samples t‐tests for the total SCCAN score and selected domains, including episodic memory, attention, problem solving, and oral expression.

**Results:** Participants with MCI showed significantly lower total SCCAN scores than healthy controls (mean 75.7, SD 8.0 vs. mean 85.2, SD 8.8; *p* < 0.001), with a large effect size (Cohen's d = 1.13). Episodic memory demonstrated the largest group difference (*p* < 0.001, Cohen's d = 1.75). Oral expression was also significantly reduced in the MCI group (*p* = 0.006, Cohen's d = 0.73). Differences in attention and problem solving were smaller and did not reach statistical significance, although moderate effect sizes were observed.

**Conclusion:** Beyond memory impairment, individuals with MCI exhibit clinically meaningful deficits in oral language performance. These findings highlight the importance of incorporating language performance measures into early cognitive assessment and support the clinical utility of the Bulgarian SCCAN for identifying cognitive‐communicative profiles in MCI.

**Disclosure:** The authors report no conflicts of interest.

## EPO‐0390

### Sustained cognitive and mood benefits of a prismatic adaptation–based digital therapy in stroke rehabilitation

#### M. Oliveri

##### Department of Biomedicine, Neurosciences and Advanced Diagnostics (BiND), University of Palermo, Palermo, Italy

**Background and aims:** Stroke survivors often experience persistent cognitive deficits and mood disturbances, which can hinder functional recovery. Non‐invasive neuromodulation combined with game‐based digital therapies has been proposed as a novel approach to enhance cognitive and emotional rehabilitation. This study investigated whether a prismatic adaptation–based digital therapy could provide sustained cognitive and mood benefits compared with standard pen‐and‐paper rehabilitation.

**Methods:** Patients with a first‐ever ischemic or hemorrhagic stroke affecting either hemisphere were recruited at the Santa Lucia Foundation IRCCS, Rome, Italy. Participants were randomly assigned to an experimental group (*n* = 28) receiving the digital therapy or a control group (*n* = 29) receiving standard cognitive rehabilitation. Cognitive outcomes were measured across five domains: visual perception, spatial attention/hemineglect, language, memory, and executive functions. Depressive symptoms were assessed using the Hamilton Depression Rating Scale (HAM‐D) at baseline, 3 months, and 6 months.

**Results:** Significant Group × Time interactions were observed for all cognitive domains. Participants in the experimental group demonstrated progressive improvements over time, whereas the control group showed no significant changes. Cognitive gains were most pronounced at the 3‐ and 6‐month follow‐ups. Additionally, depressive symptoms were significantly reduced in the experimental group at 3 months compared with baseline.

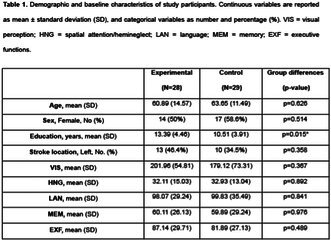

**Figure:**
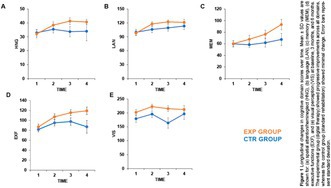
**FIGURE 1** Longitudinal changes in cognitive domain scores over time.

**Conclusion:** Prismatic adaptation–based digital therapy resulted in sustained cognitive improvements and reduced depressive symptoms post‐stroke. Combining non‐invasive neuromodulation with game‐based cognitive training may enhance rehabilitation outcomes by addressing both cognitive and emotional recovery in stroke survivors.

**Disclosure:** None.

## EPO‐0391

### Streamline‐based lesion‐symptom mapping reveals the anatomy for multimodal syntactic and semantic deficits after stroke

#### M. Rijntjes ^1^; A. Dressing^1^; M. Reisert^2^; E. Van den hoven^1^; V. Glauche^1^; H. Urbach^3^; K. Willmes^4^; C. Weiller^1^

##### ^*1*^ *Department of Neurology and Clinical Neuroscience, Medical Center – University of Freiburg, Faculty of Medicine, University of Freiburg, Freiburg, Germany;* ^*2*^ *Department of Radiology, Medical Physics, Medical Center, University of Freiburg, Faculty of Medicine, University of Freiburg, Germany**;*** ^*3*^ *Department of Neuroradiology, Medical Center – University of Freiburg, Faculty of Medicine, University of Freiburg, Freiburg, Germany;* ^*4*^ *Department of Neurology, RWTH Aachen University, Aachen, Germany*

**Background and aims:** The Dual Loop Model with hubs in inferior frontal and parieto‐temporal cortex, combining the dorsal pathway for processing sequences and the ventral pathway for hierarchical‐structural categorisation, was suggested as the anatomical substrate for syntax in all modalities. The caudal pathway, from multimodal associations in the angular gyrus to abstract concept formation in anterior temporal lobe (ATL), was suggested as essential for semantics. In stroke patients, we searched for the common anatomical substrate for syntactic and semantic deficits in apraxia, aphasia and neglect.

**Methods:** 165 patients with a single left medial cerebral artery infarction were tested for apraxia, the Token Test and spatial attention. We used the new method of streamline‐based lesion‐symptom mapping (SLSM, FDR 0.001) to detect white matter disconnections related to common behavioural deficits.

**Results:** Two Principal components (PC) explained 60% of variance across domains. PC1 (50% of variance) was associated with progressive loading for more complex apraxia tests, the Token Test and neglect. PC2 (an additional 10%) related to easier apraxia tests and extinction (Figure 1). Affected streamlines associated with PC1 included the dorsal and ventral pathway of the dual loop, additionally the Middle longitudinal Fascicle (MdlF) as the caudal pathway. PC2 was only associated with the dorsal pathway and MdlF. The interaction analysis showed that the ventral pathway, predominantly connecting inferior frontal cortex with ATL, was selectively involved in PC1 (Figure 2).

**Figure:**
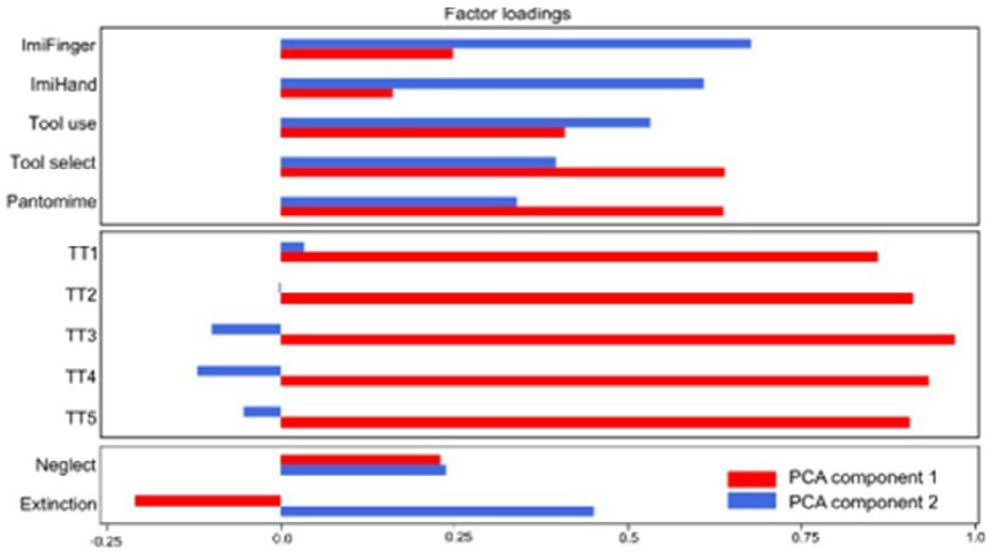
FIGURE 1

**Figure:**
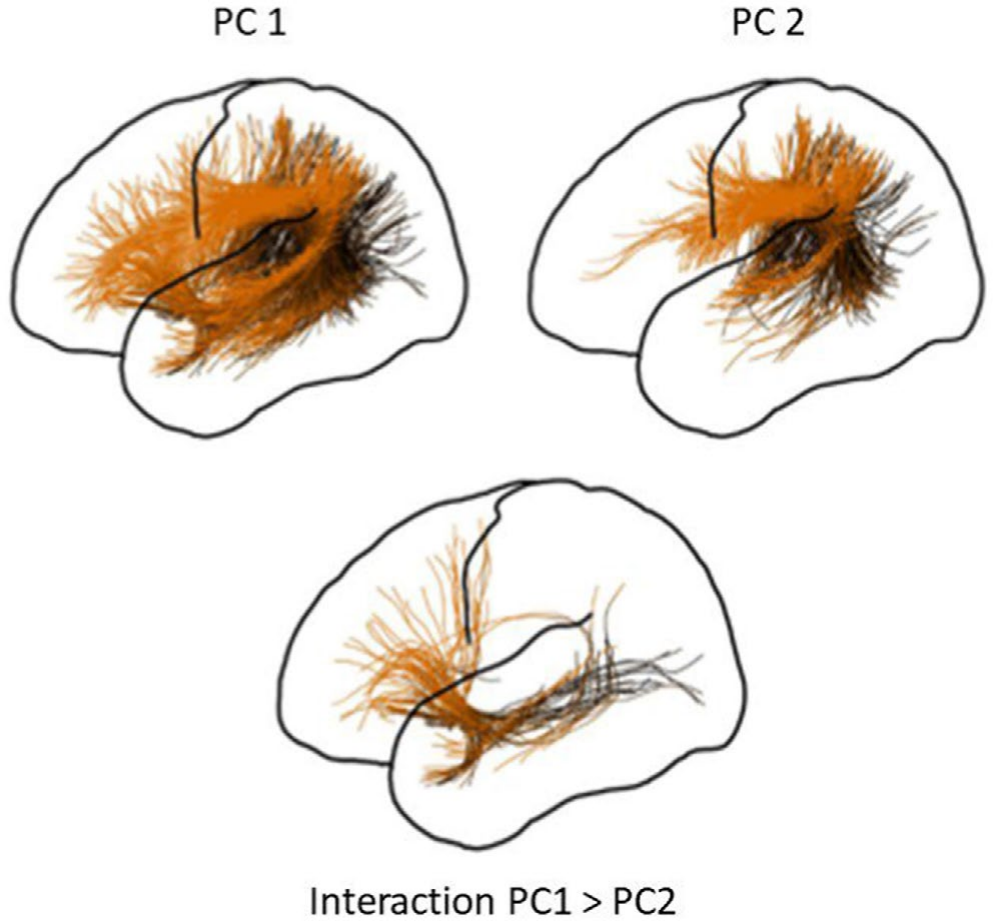
FIGURE 2

**Conclusion:** A lesion of the ventral pathway leads to multimodal deficits when syntax and semantics have to be combined.

**Disclosure:** MR, EvdH, and CW are supported by EBRAINS2.0 AD is supported by the Berta‐Ottenstein Advanced Clinical Scientists program.

## EPO‐0392

### From eye clinic to neurology: Diagnostic clues in Heidenhain‐variant Creutzfeldt–Jakob disease

#### N. Bocero Hanan; S. Sánchez Gamino; A. Sarmiento Pita; P. Dodu; J. Pérez Ceballos

##### Neurology, Hospital Regional Universitario Malaga, Malaga, Spain

**Background and aims:** Creutzfeldt–Jakob disease (CJD) is a rare and invariably fatal prion‐related neurodegenerative disorder, typically presenting with rapidly progressive dementia. The Heidenhain variant (HvCJD), however, manifests predominantly with isolated visual symptoms at onset, frequently leading to misdiagnosis and delayed neurological assessment. This report describes two cases of HvCJD initially attributed to ophthalmological pathology, highlighting key diagnostic challenges.

**Methods:** We retrospectively analyzed two patients who presented with isolated visual disturbances and underwent cataract surgery before a neurological diagnosis was established. Clinical course, neuroimaging findings, cerebrospinal fluid (CSF) biomarkers (14‐3‐3 protein and RT‐QUIC), and electroencephalographic (EEG) data were reviewed. Findings were contextualized through a focused review of the literature on HvCJD to identify common diagnostic pitfalls.

**Results:** An 80‐year‐old man presented with persistent visual impairment and hemianopia refractory to cataract surgery; brain MRI revealed posterior cortical hyperintensities consistent with prion disease. A second patient, a 60‐year‐old man, developed progressive visual symptoms followed by gait instability after cataract surgery, with MRI and EEG findings suggestive of CJD. CSF analysis was positive for both 14‐3‐3 protein and RT‐QUIC in both cases. Published series confirm that isolated visual symptoms frequently result in misdiagnosis, delayed referral to neurology, and exposure to unnecessary invasive procedures.

**Figure:**
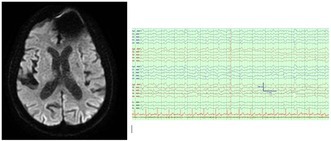
**FIGURE 1** MRI and EEG of the first pation explained.

**Figure:**
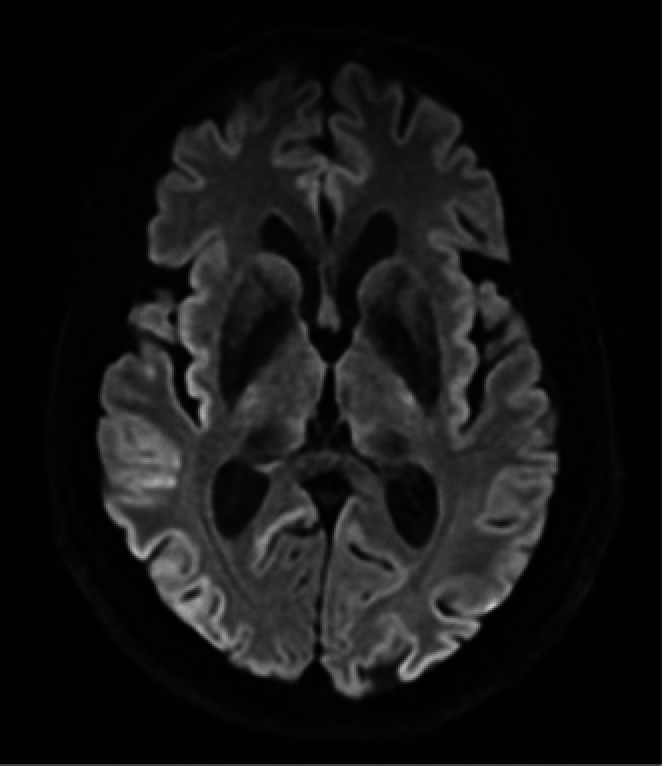
**FIGURE 2** MRI with restriction on diffusion (second patient).

**Figure:**
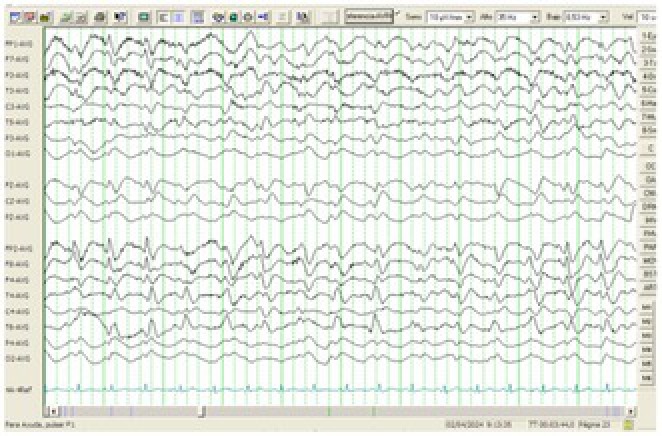
**FIGURE 3** EEG showing triphasic waves (second patient).

**Conclusion:** HvCJD should be considered in patients with unexplained or rapidly progressive visual disturbances, particularly when symptoms are inconsistent with primary ocular disease. Visual manifestations reflect cortical involvement rather than ophthalmological pathology. Increased awareness among ophthalmologists and neurologists, together with early neuroimaging and CSF biomarker assessment, is essential to reduce diagnostic delay and avoid iatrogenic risks.

**Disclosure:** Nothing to disclose.

## EPO‐0393

### Impact of a structured neurocognitive and psychosocial intervention on quality of life and psychosocial morbidity in drug‐resistant epilepsy: An RCT

#### N. Choudhary^1^; P. Kharbanda ^1^; D. Rana^2^; K. Chakravarty^1^; A. Sharma^2^; J. Baishya^1^

##### ^*1*^ *Department of Neurology, Post Graduate Institute of Medical Education and Research, Chandigarh, India;* ^*2*^ *Department of Psychiatry, Post Graduate Institute of Medical Education and Research, Chandigarh, India*

**Background and aims:** Patients with drug‐resistant epilepsy (DRE) experience substantial psychosocial morbidity, including depression, anxiety, stigma, and functional disability, which contribute significantly to reduced quality of life (QOL). Despite optimal medical management, these challenges remain under‐addressed in routine clinical practice. We assessed the effectiveness of a structured neuro‐cognitive and psychosocial intervention module in improving psychosocial outcomes and QOL in individuals with drug‐resistant epilepsy.

**Methods:** A randomized controlled trial was conducted on 60 adults with DRE, allocated to an intervention group (*n* = 30) and a control group (*n* = 30). The intervention consisted of psychoeducation, cognitive‐behavioral strategies, cognitive retraining, and family counseling, delivered over 8 weeks, in addition to standard medical treatment. Psychosocial outcomes were evaluated using QOLIE‐31, BDI, HARS, Stigma Scale for Epilepsy (SSE), WHODAS, and Dysfunctional Analysis Questionnaire (DAQ). Per‐protocol and intention‐to‐treat analyses were performed.

**Results:** Patients receiving the intervention showed a clinically meaningful improvement in quality of life, with QOLIE‐31 scores improving from 52.4 ± 8.5 at baseline to 67.8 ± 7.2 post‐intervention (*p* < 0.001). Significant reductions were observed in anxiety (HARS: 21.3 ± 5.8 to 12.1 ± 4.7, *p* < 0.001), depressive symptoms, perceived stigma (*p* = 0.03), psychosocial dysfunction, and disability scores. The control group showed no statistically significant changes over the same period. Seizure frequency remained stable, indicating that improvements were independent of seizure control.

**Conclusion:** The integrated neuro‐cognitive and psychosocial intervention is effective in reducing psychosocial morbidity and improving quality of life in patients with drug‐resistant epilepsy. Incorporating such structured interventions into routine epilepsy care may substantially enhance holistic patient outcomes.

**Disclosure:** Nothing to disclose.

## EPO‐0394

### Wernicke encephalopathy with normal imaging and labs in a severely malnourished elderly patient: A case report

#### S. Moudden; O. Cherkaoui Ghazouani

##### Neurology Department, Military Hospital of Avicenna, Marrakech, Morocco

**Background and aims:** Wernicke's encephalopathy is a neurological emergency secondary to thiamine deficiency, with a frequently atypical clinical presentation. The absence of the classic triad and normal complementary investigations may delay diagnosis. We report a case of probable Wernicke's encephalopathy revealed by a progressive neuropsychiatric syndrome.

**Methods:** We report the case of a 65‐year‐old female patient with a history of recent untreated hyperthyroidism, laryngeal carcinoma treated with radiotherapy, and hypertension. She was admitted for behavioral disturbances including psychomotor agitation, opposition to care, aggressiveness, and irritability, associated with memory impairment and visual hallucinations, with progression over 20 days. Investigations included blood tests, thyroid function assessment, brain imaging, electroencephalography, and lumbar puncture with cerebrospinal fluid analysis.

**Results:** On admission, the patient was fully conscious with a Glasgow Coma Scale score of 15/15, but severely malnourished with a low body mass index, dehydrated, and uncooperative, with no focal neurological deficits. Biological investigations were unremarkable. Brain computed tomography showed mild ventricular enlargement, while brain magnetic resonance imaging and electroencephalography were normal. Cerebrospinal fluid analysis revealed no abnormalities. Given the strong clinical suspicion of thiamine deficiency, vitamin B1 therapy was initiated, along with nutritional and hydration support. This resulted in a marked and rapid improvement in behavioral symptoms, cognitive functions, and general condition. Visual hallucinations partially persisted. The patient was discharged on chlorpromazine 25 mg nightly, with follow‐up scheduled one month later.

**Conclusion:** This case highlights the importance of considering Wernicke's encephalopathy in malnourished elderly patients with acute or progressive neuropsychiatric syndromes. Early thiamine administration should not be delayed when suspected.

**Disclosure:** Nothing to disclose.

## EPO‐0395

### When the past catches up: Iatrogenic Creutzfeldt–Jakob Disease with a 39‐year incubation period

#### S. Chowdhury ^1^; M. NG^2^; S. Mead^3^; S. Oghuvwu^4^; S. Lim^1^; S. Sedehizadeh^1^

##### ^*1*^ *Neurology, Nottingham University Hospitals NHS Foundation Trust, Nottingham, UK;* ^*2*^ *National Prion Clinic;* ^*3*^ *University College London Hospitals NHS Foundation Trust, London, UK;* ^*4*^ *Sherwood Forest Hospitals NHS Foundation Trust,* Sutton‐in‐Ashfield, UK

**Background and aims:** Creutzfeldt–Jakob disease (CJD) is a rare, fatal prion disease, most commonly occurring sporadically but occasionally transmitted through medical procedures, resulting in iatrogenic CJD (iCJD).

**Methods:** A 39‐year‐old man presented to Queen's Medical Centre, Nottingham, UK with a four‐week history of rapidly progressive cognitive and behavioural decline. His family reported impaired executive function, including leaving dirty cookware in cupboards, difficulty following instructions at work, misplacing personal items, and gait disturbance with leftward leaning. At six weeks of age, he developed meningitis complicated by seizures and bilateral subdural empyema. On 7 January 1986, he underwent craniotomy with dural repair using a cadaveric dura mater graft (Lyodura). He had lifelong learning difficulties, dyslexia, and communication and social difficulties.

**Results:** During admission, he deteriorated rapidly, becoming bedbound, non‐verbal, and doubly incontinent. He developed stimulus‐induced myoclonus, initially treated with levetiracetam but switched to clonazepam due to rash. MRI brain demonstrated diffusion restriction in the bilateral basal ganglia with multifocal cortical ribboning, more prominent on the left. EEG showed non‐specific encephalopathy. Cerebrospinal fluid was acellular with normal protein, and RT‐QuIC was positive. He was referred to palliative care and died in a hospice within two weeks of discharge.

**Figure:**
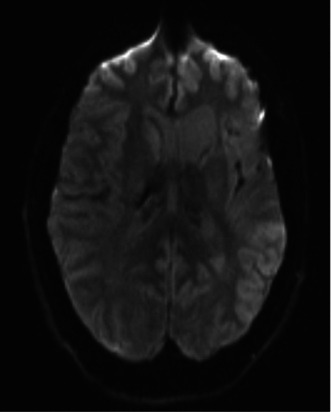
**FIGURE 1** MRI brain demonstrated diffusion restriction in the bilateral basal ganglia with multifocal cortical ribboning, more prominent on the left.

**Figure:**
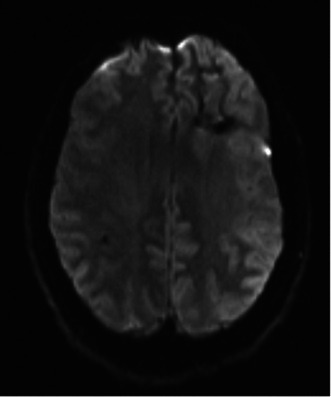
**FIGURE 2** MRI brain demonstrated diffusion restriction in the bilateral basal ganglia with multifocal cortical ribboning, more prominent on the left.

**Figure:**
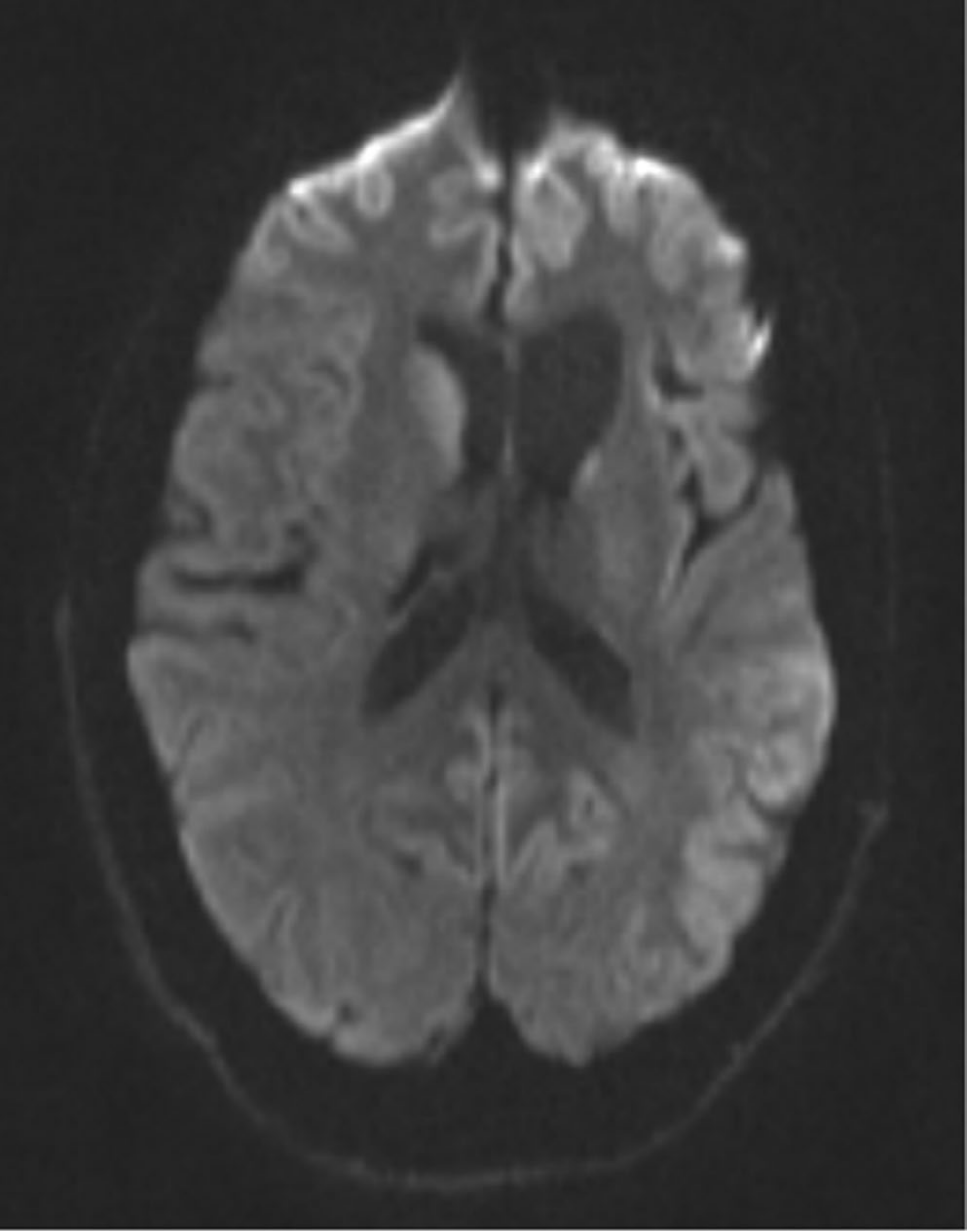
**FIGURE 3** MRI brain demonstrated diffusion restriction in the bilateral basal ganglia with multifocal cortical ribboning, more prominent on the left.

**Conclusion:** The case highlights the diagnostic value of a detailed lifetime surgical history, particularly childhood neurosurgical interventions that may otherwise be overlooked. Incubation periods in iCJD can exceed 30–40 years, making careful lifetime surgical history essential. Continued vigilance, prompt recognition, and systematic reporting are essential to support surveillance efforts and improve understanding of iatrogenic prion disease in neurological practice.

**Disclosure:** Nothing to disclose.

## Epilepsy 3

## EPO‐0396

### Transient epileptic amnesia as an underdiagnosed form of late‐onset temporal lobe epilepsy

#### A. Atrous^1^; O. Sakli^1^; A. Ghatbi^1^; I. Abdelkafi^2^; M. Kabbage^1^; Y. Abida^1^; A. Souissi^1^; I. Kacem ^1^; A. Gargouri^1^; R. Gouider^1^

##### ^*1*^ *Neurological department, Center of clinical investigation on neuroscience and mental health LR18 SP03, Razi Hospital, Manouba, Tunisia;* ^*2*^ *Hospital of interior security forces, Marsa, Tunisia*

**Background and aims:** Late‐onset epilepsy is defined as epilepsy with first seizure onset from the age of 50 years onward. Within this spectrum, temporal lobe epilepsy in older adults (TLE‐OA) is increasingly recognized. Among its clinical phenotypes, transient epileptic amnesia (TEA) represents a distinctive but frequently underdiagnosed form.

**Methods:** We conducted a retrospective analysis of patients followed for TLE‐OA at the Neurology Department of Razi Hospital. Clinical features, interictal electroencephalography (EEG) and brain magnetic resonance imaging (MRI) were reviewed. Diagnostic criteria included the presence of recurrent episodes of transient amnesia with preservation of other cognitive functions, associated with ictal features, EEG abnormalities, or a clear response to antiseizure medication.

**Results:** Ten out of fourteen patients fulfilled diagnostic criteria for TEA. The mean age was 65.2 years (range 51–74), with equal sex distribution. The median time to diagnosis was 16.5 months. Two patients reported persistent cognitive/behavioral symptoms. EEG revealed epileptiform abnormalities involving the frontal and/or temporal regions in 50% of cases. MRI revealed focal abnormalities in one case. Anti‐seizure therapy, most often with a single agent, resulted in improvement in all eight cases with available follow‐up.

**Conclusion:** TEA represents a frequent yet underrecognized presentation of TLE‐OA rather than a separate diagnostic entity. Because routine EEG and MRI findings may be normal, TEA is particularly prone to diagnostic delay and misclassification. Improved awareness of TEA as a form of epilepsy in older adults is essential, as early clinical recognition enables appropriate anti‐seizure treatment and may limit long‐term cognitive impact.

**Disclosure:** Nothing to disclose.

## EPO‐0397

### Long‐term outcomes and predictors of response to vagus nerve stimulation in drug‐resistant epilepsy

#### C. Dönmez ^1^; Y. Selek^1^; A. Akkan Suzan^1^; B. Türk^1^; M. Delil^1^; C. İşler^2^; T. Tanriverdi^2^; M. Uzan^2^; S. Yeni^1^; Ç. Özkara^1^

##### ^*1*^ *Department of Neurology, Istanbul University‐Cerrahpaşa, Cerrahpaşa Faculty of Medicine, Istanbul, Türkiye;* ^*2*^ *Department of Neurosurgery, Istanbul University‐Cerrahpaşa, Cerrahpaşa Faculty of Medicine, Istanbul, Türkiye*

**Background and aims:** Vagus nerve stimulation (VNS) is an established neuromodulatory treatment for drug‐resistant epilepsy. Long‐term studies suggest that its efficacy may increase over time. This study evaluate long‐term clinical outcomes and potential predictors of treatment response in patients who underwent VNS implantation at a tertiary epilepsy center.

**Methods:** We retrospectively reviewed patients who received VNS therapy between 2005 and 2025. Potential predictors of VNS response included epilepsy classification (focal vs. generalized), underlying etiology (structural vs. non‐structural), age at seizure onset, duration of epilepsy prior to VNS therapy, previous epilepsy surgery, maximum stimulation voltage, and duration of VNS therapy.

**Results:** A total of 122 patients were included. At the last follow‐up, 30% of patients achieved a ≥ 50% reduction in seizure frequency and were classified as responders. Patients with structural epilepsy (*n* = 64) demonstrated a significantly higher responder rate compared to those with non‐structural etiologies (*p* = 0.006). The duration of VNS therapy was significantly longer in responders compared to non‐responders (8.02 ± 4.73 years vs. 4.89 ± 4.02 years, *p* = 0.001), supporting a time‐dependent treatment effect. No significant associations were observed between treatment response and age at seizure onset (*p* = 0.373), age at VNS implantation (*p* = 0.195), duration of epilepsy prior to VNS therapy (*p* = 0.592), or maximum stimulation voltage (*p* = 0.202). Furthermore, maximum stimulation voltage was not associated with the occurrence of adverse effects.

**Conclusion:** VNS therapy demonstrates greater efficacy in patients with structural epilepsy and in those with longer treatment duration, supporting a cumulative and time‐dependent therapeutic effect.

**Disclosure:** Nothing to disclose.

## EPO‐0398

### Clinical and demographic correlates of multifocal epileptiform activity in hospitalized adults

#### D. Carapinha; J. Silva; P. Neves; A. Martins; J. Peres

##### Neurology Department, ULS Amadora/Sintra, Portugal

**Background and aims:** Multifocal epileptiform activity, defined as the presence of at least three independent epileptiform foci involving both cerebral hemispheres on EEG, is poorly characterized clinically. Clinical observations suggest an association with advanced age, comorbidities, and systemic illness; however, these relationships have not been studied. We aimed to evaluate the clinical and demographic correlates of multifocal epileptiform activity in hospitalized adults.

**Methods:** Methods: We conducted a retrospective observational study including 80 hospitalized patients selected in reverse chronological order from our institutional EEG database (accessed 30 November 2024): 40 with multifocal epileptiform activity and 40 with non‐multifocal activity. Demographic data, medical history, EEG indication, metabolic disturbances defined by the ILAE as causes of acute symptomatic seizures, and neuroimaging findings were collected. Statistical significance was set at *p* < 0.05.

**Results:** Patients with multifocal epileptiform activity were older than those with non‐multifocal activity (*p* = 0.003). EEG indications differed between groups (*p* = 0.002), with altered consciousness more frequent in the multifocal group and convulsive seizures in the non‐multifocal group. Cognitive decline (OR 2.9; 95% CI 1.1–7.2; *p* = 0.024) and sepsis (OR 9.8; 95% CI 1.2–82.1; *p* = 0.029) were more frequent in the multifocal group. In a multivariable logistic regression, explaining approximately 25% of the variability (*p* < 0.001), only age remained independently associated with multifocal activity (OR 1.04; 95% CI 1.01–1.08; *p* = 0.020).

**Conclusion:** Multifocal epileptiform activity in hospitalized adults occurs predominantly in older patients and is associated with altered consciousness and sepsis, suggesting that it reflects global disease severity rather than a specific epileptic etiology.

**Disclosure:** Nothing to disclose.

## EPO‐0399

### Efficacy and adverse event profile of pharmacological and non‐pharmacological therapies in Dravet Syndrome: A systematic review

#### C. Michel^1^; L. Pimentel ^1^; C. Quant^1^; P. Miranda^1^; M. Read^1^; D. Vásquez^1^; A. Tejada^1^; K. Yorro^1^; C. Pérez Lizardo^1^; A. Reyes^1^; M. Rodríguez^2^

##### ^*1*^ *Synaptic Research League, Santo Domingo, Dominican Republic;* ^*2*^ *Hospital Universitario Salvador Bienvenido Gautier, Santo Domingo, Dominican Republic*

**Background and aims:** Dravet Syndrome (DS) is a severe epileptic encephalopathy with onset in early infancy that requires long‐term pharmacological management [1]. Increasing evidence has highlighted significant treatment‐related adverse effects, including cognitive, behavioral, and systemic complications [2]. This study aims to analyze and summarize treatment‐associated complications in patients with Dravet Syndrome.

**Methods:** We conducted a systematic literature review following PRISMA guidelines. Of 74 studies, 7 were included. PubMed was searched using the keywords Dravet syndrome, treatment regimens, adverse effects, and treatment complications. Treatment regimens, outcomes, and adverse effects were evaluated.

**Results:** We analyzed 275 patients with Dravet syndrome. The mean age was 14.3 years. The most frequent treatments were fenfluramine, valproate, clobazam, and stiripentol, see Figure 1 Adjuvant therapies included cannabidiol/THC (19 patients) and a ketogenic diet (60 patients). A ≥ 50% reduction in seizures was achieved in 58–83% of patients, with seizure freedom in 7.6–25%. Adverse effects were common (up to 59.1% somnolence, 31.5% total events) but generally manageable, leading to discontinuation in < 21% of cases.

**Figure:**
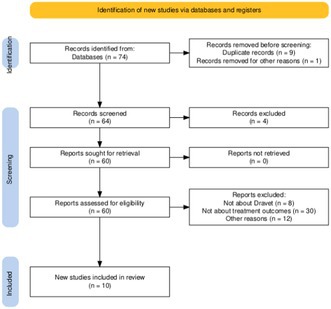
**FIGURE 1** Prisma‐P flowchart of the process of inclusion of studies in the systematic review.

**Figure:**
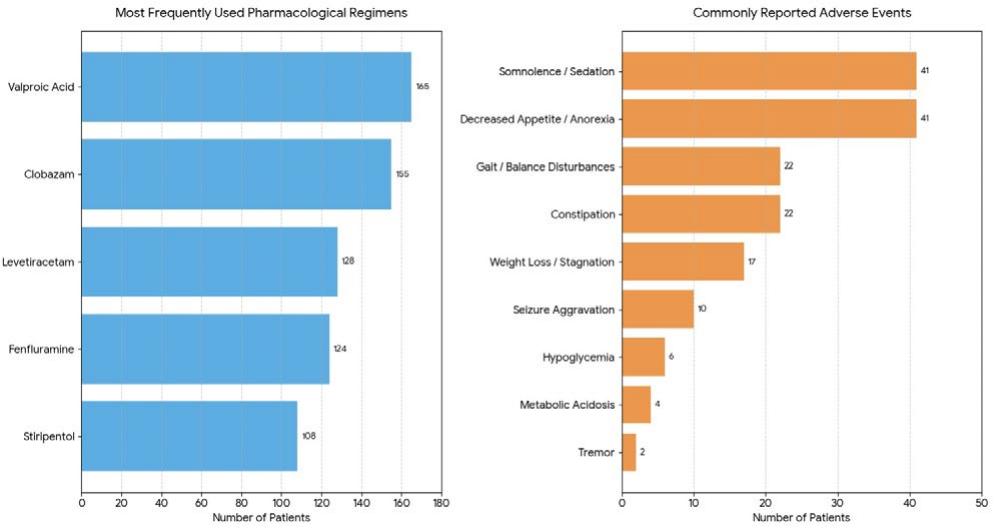
**FIGURE 2** Frequency of Pharmacological Regimens and Their Adverse Events of Dravet Syndrome.

**Conclusion:** Available evidence suggests that fenfluramine and ketogenic diet can reduce crisis in Dravet, with a minority achieving freedom from disease, while cannabinoids show variable answers. However, design heterogeneity, co‐interventions and incomplete security report limit comparisons and robust meta‐analysis. Studies with standardized structures are required and systematic follow‐up of adverse events to better define risk‐benefits.

**Disclosure:** Nothing to disclose.

## EPO‐0400

### Abstract withdrawn

## EPO‐0401

### Structures associated with ictal alpha and theta activity in temporal lobe epilepsy – A volumetric study

#### K. Stojić ^1^; M. Kovačević^3^; Z. Joković^2^; A. Pejović^2^; I. Berisavac^2^; A. Ristić^2^; N. Vojvodić^2^

##### ^*1*^ *Medical Faculty, University of Belgrade, Belgrade, Serbia;* ^*2*^ *Neurology Clinic, University Clinical Center of Serbia, Medical Faculty, University of Belgrade, Belgrade, Serbia;* ^*3*^ *Neurology Clinic, University Clinical Center of Serbia, Medical Faculty, University of Belgrade, Belgrade, Serbia, Department of Neurology, Medical University of Vienna, Vienna, Austria*

**Background and aims:** Ictal rhythms ≥ 5 Hz are well described as important predictors of favourable outcome after mesial temporal lobe epilepsy (MTLE) surgery. However, the precise anatomical structures involved in generation and propagation of these activities have not been fully understood. Aim of our study was to analyze structural brain alterations of anatomical regions typically involved in seizures in MTLE to determine associations with these rhythms.

**Methods:** This retrospective study included MRIs of 60 patients who underwent successful MTLE surgery and had at least one seizure documented during video‐EEG monitoring (Table 1). Patients were classified based on observed rhythmic ictal activity ≥ 5 Hz in alpha and fast theta frequency. Volumes of 25 brain structures were analyzed. Univariate and multivariate logistic regression models were used to determine structures associated with temporal alpha (TA) and fast temporal theta (FTT).

**Figure:**
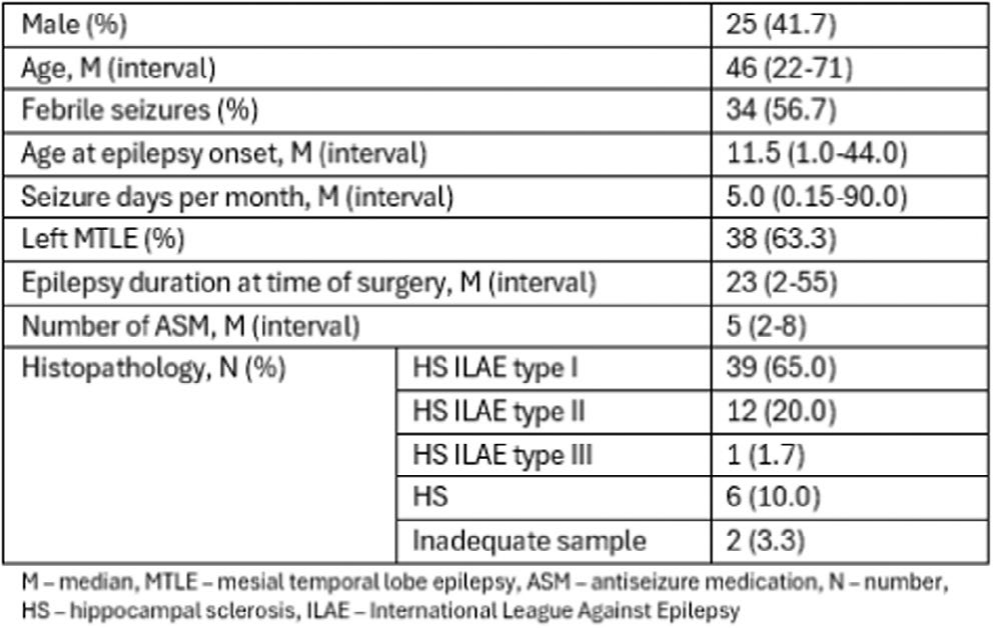
TABLE 1

**Results:** We observed TA in 20 seizures in seven patients and FTT in 219 seizures in 39 patients. Demographic and clinical variables (age, sex, and age at epilepsy onset) did not differ significantly between patients groups based on presence of TA and FTT. Univariate logistic regression analysis identified significant association between TT and three corpus callosum regions: mid‐posterior, central and mid‐anterior, while TA showed an association with ipsilateral thalamus and isthmus gyri cinguli. Multivariate analysis revealed that only the mid‐anterior corpus callosum was an independent predictor of TT, while ipsilateral thalamus and isthmus gyri cinguli were both independent predictors of TA.

**Conclusion:** Mid‐anterior corpus callosum, thalamus, and isthmus gyri cinguli are significantly associated with TT and TA in MTLE.

**Disclosure:** Nothing to disclose.

## EPO‐0402

### Cenobamate for refractory epilepsy secondary to primary CNS tumours: Real‐world outcomes across WHO classifications

#### M. Elbadri; S. Bose; S. Samarasekera

##### Department of Neurology, Queen Elizabeth Hospital, Mindelsohn Way, Edgbaston, Birmingham, UK

**Background and aims:** Epilepsy is the commonest chronic and life‐limiting neurological co‐morbidity associated with primary central nervous system (CNS) tumours. Cenobamate (CNB), a third generation antiseizure medication (ASM), has demonstrated high efficacy in focal epilepsy. Evidence for its efficacy in brain tumour‐related epilepsy (BTRE) remains limited. We evaluated the real‐world tolerability and effectiveness of CNB in BTRE.

**Methods:** Patients with BTRE attending the Queen Elizabeth hospital Birmingham who were prescribed CNB between 2022 and 2025 were observed. Tumours were classified according to the 2021 WHO CNS tumour classification. Data including tumour histology and grade, baseline seizure frequency and adverse events were recorded. The primary outcome was significant (≥ 50%) seizure reduction. Additional outcomes included ≥ 90% seizure reduction, change in ASM burden and tolerability.

**Results:** 25 Patients representing a broad spectrum of WHO tumour entities (grades I–IV) were included. Median duration of follow up was 19 months. Mean number of concomitant ASMs decreased from 2.75 at baseline to 1.79 following CNB initiation. 11/25 patients (44%) achieved a ≥ 50% reduction in seizure frequency. 6 patients (24%) achieved ≥ 90% seizure reduction. Response was observed across multiple tumour subtypes and WHO grades. CNB was generally well tolerated. Treatment discontinuation due to adverse effects (commonly somnolence and sedation) occurred in 3/25 patients (12%).

**Conclusion:** This is the largest real‐world cohort of patients with refractory BTRE treated with CNB. CNB was associated with significant seizure reduction in over 40%. These findings support Cenobamate as a valuable therapeutic option in BTRE, irrespective of tumour type.

**Disclosure:** Dr Shanika Samarasekera has received speaker honoraria from Angelini Pharma. QEHB has been supported by Angelini Pharma with a grant for an epilepsy pharmacist.

## EPO‐0403

### IPCEF1‐mediated NMDA receptor trafficking: A novel IL‐1β‐driven mechanism linking inflammation to epileptogenesis and NREM sleep desynchrony

#### P. Yi ^1^; S. Huang^2^; F. Chang^2^

##### ^*1*^ *Department of Sport Management, Aletheia University, New Taipei City, Taiwan;* ^*2*^ *Department of Veterinary Medicine, National Taiwan University, Taipei, Taiwan*

**Background and aims:** Epilepsy often co‐occurs with sleep disturbances, but molecular links between inflammation, excitatory plasticity, and these comorbidities are unclear. We hypothesised that IL‐1β signalling activates the PIP3–AKT–NF‐κB cascade to upregulate IPCEF1, promoting NMDA receptor (NMDAR) trafficking and hippocampal hyperexcitability, thereby driving seizures and non‐rapid eye movement (NREM) sleep desynchrony.

**Methods:** Pentylenetetrazol (PTZ) kindling (35 mg/kg i.p, every 48 h for 14 days) was induced in wild‐type and IL‐1R1 knockout mice. Interventions included IL‐1 receptor antagonist (IL‐1ra) and inhibitors of PIP3–AKT–NF‐κB signalling (PIT‐7, HS‐173, BMS‐345541). Continuous EEG/EMG monitored seizures and sleep architecture. Hippocampal inflammation (IL‐1β, microglia, astrocytes) and NMDAR subunit expression/trafficking were assessed by Western blotting and immunofluorescence. IPCEF1 was specifically silenced to isolate its role.

**Results:** PTZ kindling elevated hippocampal IL‐1β, glial activation, synaptic NMDAR enrichment, seizure frequency, and disrupted NREM slow‐wave synchrony. Blocking IL‐1R1 or downstream cascade prevented inflammation, reduced NMDAR trafficking, suppressed seizures, and normalised sleep. Notably, IPCEF1 silencing selectively reduced seizures and restored NREM synchrony without affecting total sleep–wake distribution.

**Conclusion:** IL‐1β‐driven IPCEF1‐mediated NMDAR trafficking links inflammation to epileptogenesis and cortical desynchrony in NREM sleep. The IL‐1‐IPCEF1‐NMDAR axis represents a precise therapeutic target for controlling seizures and sleep comorbidities in epilepsy.

**Figure:**
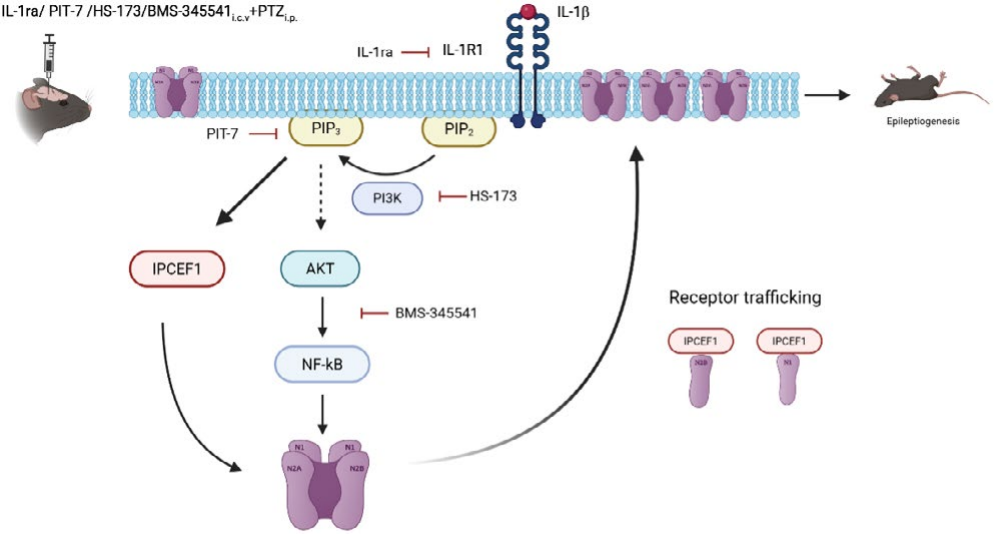
IL‐1β‐driven IPCEF1‐mediated NMDAR trafficking links inflammation to epileptogenesis and cortical desynchrony in NREM sleep.

**Disclosure:** Nothing to disclose.

## EPO‐0404

### From seizures to vascular risk: Carotid ultrasound markers in epilepsy

#### F. Dono; R. Agosto; M. Russo; M. Di Pietro; S. Sensi

##### Department of Neuroscience, Imaging and Clinical Science, “G. d'Annunzio” University of Chieti‐Pescara, Chieti, Italy

**Background and aims:** Cardiovascular risk may be increased in epilepsy, potentially influenced by chronic antiseizure medication (ASM) exposure. Carotid intima‐media thickness (IMT) is a validated surrogate marker of subclinical atherosclerosis. We investigated whether carotid IMT differs according to drug‐resistant epilepsy (DRE) and exposure to enzyme‐inducing ASMs.

**Methods:** We consecutively enrolled patients attending a tertiary epilepsy center between January 2025 and January 2026. Seventy‐one subjects underwent carotid ultrasound assessment. Right, left, mean, maximal IMT and side‐to‐side IMT difference were recorded, along with carotid stenosis. Participants were stratified by DRE status and by enzyme‐inducing ASM exposure.

**Results:** No demographic or lifestyle differences were observed between DRE and non‐DRE groups, nor between participants with vs without exposure to enzyme‐inducing ASMs. Carotid IMT measures were not significantly different between DRE vs non‐DRE (right IMT *p* = 0.673; left IMT *p* = 0.173; mean IMT *p* = 0.223; maximal IMT *p* = 0.143). A non‐significant trend toward higher left and maximal IMT was observed in DRE. No significant differences were found according to enzyme‐inducing ASM exposure (right IMT *p* = 0.813; left IMT *p* = 0.503; mean IMT *p* = 0.673; maximal IMT *p* = 0.513). Carotid stenosis prevalence was comparable between exposure groups (*p* = 0.972).

**Conclusion:** In this cohort, DRE and enzyme‐inducing ASM exposure were not associated with increased carotid IMT. Larger samples may clarify whether the observed DRE trend reflects a true biological effect.

**Disclosure:** Nothing to disclose.

## EPO‐0405

### Plasma NfL and phosphorylated tau as emerging biomarkers of neuronal injury in pediatric drug‐resistant epilepsy: A preliminary study

#### S. Drago ^1^; A. Butera^1^; F. Polito^2^; G. Irene^2^; L. Licitri^2^; V. Macaione^2^; G. Di Rosa^3^; M. Aguennouz^2^

##### ^*1*^ *Department of Chemical, Biological, Pharmaceutical and Environmental Sciences, University of Messina, Messina, Italy;* ^*2*^ *Department of Clinical and Experimental Medicine, University of Messina, Messina, Italy;* ^*3*^ *Dipartimento di Scienze biomediche, odontoiatriche e delle immagini morfologiche e funzionali, University of Messina, Messina, Italy*

**Background and aims:** Pediatric drug‐resistant epilepsies represent a significant clinical challenge, profoundly affecting neurocognitive development, quality of life, and long‐term prognosis. The identification of novel neurodegenerative biomarkers could improve patient stratification, clinical management, and support the development of therapies.

**Methods:** A prospective study was conducted analyzing blood samples from patients with drug‐resistant epilepsy followed at the Pediatric Neuropsychiatry Unit of Messina. Biomarker levels were measured. Descriptive analyses were performed, while Spearman's rank correlation analyses were conducted to evaluate the relationships between biomarker levels and clinical variables.

**Results:** The study included 14 pediatric patients with drug resistant epilepsy (mean age 8.5 years), and 8 (57.1%) were male. Plasma biomarker showed a mean neurofilament light chain (NfL) level of 31.997 pg/mL, pTau181 mean value of 1.172 pg/mL, pTau217 showed a mean of 0.179 pg/mL. Compared with age matched reference values, 10 patients (71.4%) exhibited elevated NfL levels, and 7 (50%) showed increased pTau217. The most frequent diagnosis was epileptic encephalopathy (42.9%), including Lennox–Gastaut (21.4%) and Dravet syndrome (7.1%). EEG abnormalities were mainly diffuse fronto‐centro‐temporal (28.6%), parieto‐temporal/post‐central (28.6%), or non‐localized (21.4%). Overall, patients with early onset complex encephalopathies showed the most severe biochemical profiles, whereas those with Lennox–Gastaut syndrome exhibited consistently lower NfL levels.

**Conclusion:** Correlation analyses showed NfL positively associated with pTau217 (ρ = 0.55, *p* = 0.041) and pTau181 with pTau217 (ρ = 0.70, *p* = 0.005). These results suggest NfL and pTau217 are sensitive, complementary biomarkers of neuronal injury in children with drug‐resistant epilepsy, with potential for disease stratification and clinical decision.

**Disclosure:** Nothing to disclose.

## EPO‐0406

### Evaluating gender related outcomes with adjunctive cenobamate in focal‐onset seizures

#### S. Lattanzi ^1^; B. Steinhoff^2^; E. Alvarez‐Baron^3^; J. Leach^3^; P. Perez‐Domper^3^; V. Villanueva^5^; Thangavelu^4^

##### ^*1*^ *Department of Experimental and Clinical Medicine, Neurological Clinic, Marche Polytechnic University, Ancona, Italy;* ^*2*^ *Department for Adults, Kork Epilepsy Center, Kehl‐Kork, Germany Clinic for Neurology and Neurophysiology, University of Freiburg, Germany;* ^*3*^ *Global Medical Department, Angelini Pharma S.p.A, Rome, Italy;* ^*4*^ *MeDaStats LLC, Tampa, USA;* ^*5*^ *Refractory Epilepsy Unit, Neurology Service, Hospital Universitari i Politècnic La Fe, Valencia, Spain, Member of ERN Epicare*

**Background and aims:** Sex differences are observed in neurological disorders and may be apparent in prevalence, clinical presentation, and treatment outcomes. In epilepsy, sex related factors can influence the selection and dosing of antiseizure medications (ASMs), yet little is known about variations in treatment response. This analysis explored potential differences in the efficacy and safety of adjunctive cenobamate (CNB).

**Methods:** This post‐hoc analysis of the C017 placebo‐controlled double‐blind trial evaluated efficacy and safety of CNB in adults with uncontrolled focal‐onset seizures despite 1–3 prior ASMs at stable doses. Outcomes were assessed by sex across CNB dose groups (100, 200, and 400 mg) and placebo.

**Results:** Females represented 49.5% of the randomized population. They had a median age of 40 years, 25 years since epilepsy diagnosis, and a baseline seizure frequency of 10 seizures/28 days. Males had a median age of 37 years, 21.4 years since diagnosis, and a baseline seizure frequency of 8.65 seizures/28 days. Seizure freedom rates during maintenance‐phase in males were 3.6%, 10.2%, and 25.0% for CNB 100, 200, and 400 mg, respectively, versus 1.8% for placebo. In females, rates were 4.3%, 12.2%, and 17.6% for the same CNB doses, with 0% for placebo. The incidence and nature of adverse events were similar between sexes across CNB groups.

**Figure:**
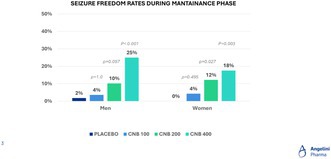
**FIGURE 1** Seizure Freedom Rates during the Maintenance Phase.

**Conclusion:** Cenobamate demonstrated consistent efficacy and a similar tolerability profile in both females and males. Although small numerical differences were observed, no clear meaningful sex related differences emerged. These findings suggest that treatment‐benefit from CNB is not sex dependent.

**Disclosure:** SL: Speaker/consultant: Angelini Pharma, Eisai, GW Pharmaceuticals, Medscape, NewBridge Pharmaceuticals, and UCB Pharma Advisor: Angelini Pharma, Arvelle Therapeutics, BIAL, EISAI, GW Pharmaceuticals, Rapport Therapeutics, and UCB Pharm VV: Consultant/advisor: Angelini Pharma, BIAL, Biocodex, Eisai, Esteve, GlaxoSmithKline, Jazz, Novartis, Sandoz, Takeda, UCB Pharma, Xenon; Speaker: Angelini Pharma, BIAL, Cevomed, Eisai, Esteve, Jazz, Newbridge, Paladin, UCB Pharma; Research support: Angelini Pharma, BIAL, Eisai, Jazz, UCB Pharma. BS: Consultant/advisor: Angelini, B. Braun Melsungen, Desitin,Eisai, GW Pharmaceuticals, UCB Pharma, Zogenix; Research support: Eisai, GW Pharmaceuticals, SK Life Science, UCB Pharma. JPL, PPD, EAB: Employees, Angelini Pharma. KT: Consultant: Angelini Pharma.

## EPO‐0407

### Time to pre‐randomization monthly seizure frequency for patients with uncontrolled focal onset seizures treated with cenobamate

#### S. Lattanzi ^1^; V. Villanueva^2^; E. Alvarez‐Baron^3^; J. Leach^3^; P. Perez‐Domper^3^; K. Thangavelu^4^; B. Steinhoff^5^

##### ^*1*^ *Department of Experimental and Clinical Medicine, Neurological Clinic, Marche Polytechnic University, Ancona, Italy;* ^*2*^ *Refractory Epilepsy Unit, Neurology Service, Hospital Universitari i Politècnic La Fe, Valencia, Spain, Member of ERN Epicar;* ^*3*^ *Global Medical Department, Angelini Pharma S.p.A, Rome, Italy;* ^*4*^ *MeDaStats LLC, Tampa, USA;* ^*5*^ *Department for Adults, Kork Epilepsy Center, Kehl‐Kork, Germany Clinic for Neurology and Neurophysiology, University of Freiburg, Germany*

**Background and aims:** The updated EMA guideline on clinical investigation in epileptic disorders (September 2025) recognizes time to event endpoints as a potential approach for primary analyses, with the aim of reducing exposure to placebo. This post hoc analysis evaluated the time required to reach pre randomization monthly seizure frequency as a measure of cenobamate (CNB) efficacy.

**Methods:** Data were derived from the C017 randomized, placebo‐controlled trial in adults with uncontrolled focal onset seizures receiving 1–3 concomitant ASMs at stable doses. Time to achieving the individual 28‐day baseline seizure rate was compared between placebo and CNB treatment groups (100, 200, and 400 mg/day). Analyses included the proportion of patients reaching baseline seizure frequency and the median time to event during the maintenance phase.

**Results:** The proportion of patients reaching baseline seizure rate was 89.1% with placebo versus 76.4% (*p* = 0.0161), 66.9% (*p* < 0.001), and 51.7% (*p* < 0.001) with CNB 100, 200, and 400 mg, respectively. Median (95% CI) time to baseline seizure frequency was 5.34 (4.71–6.29) weeks for placebo, compared with 6.00 (5.57–8.14) weeks for CNB 100 mg, 8.71 (7.43–11.57) weeks for CNB 200 mg, and 10.86 (8.43–NA) weeks for CNB 400 mg.

**Figure:**
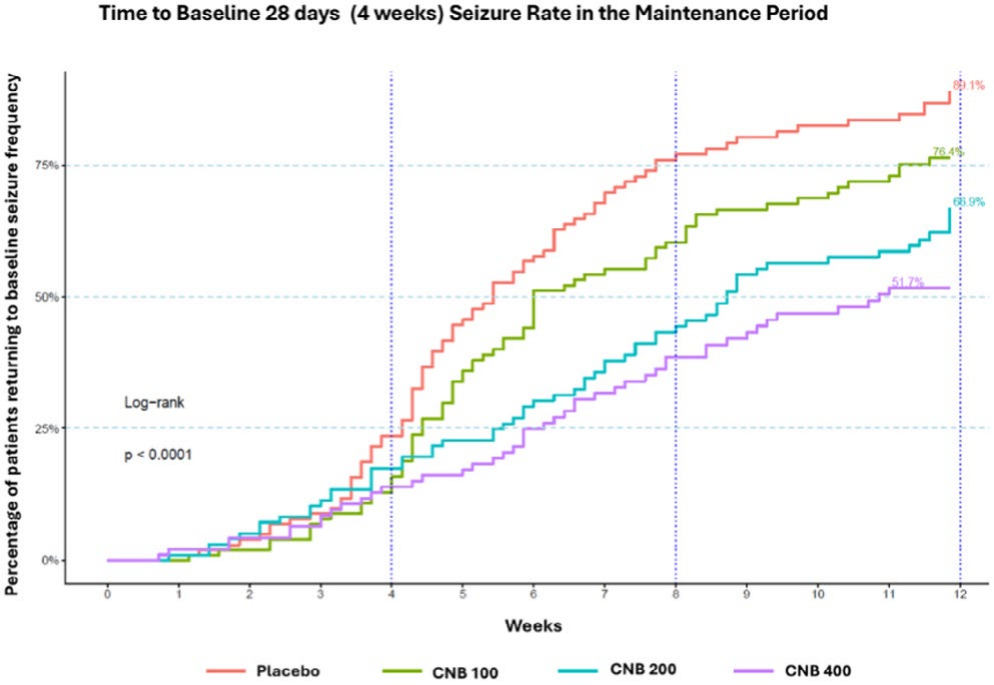
**FIGURE 1** Time to Baseline 28 days (4 weeks) Seizure Rate in the Maintenance Period.

**Conclusion:** Cenobamate significantly prolonged the time to pre randomization monthly seizure rate and reduced the proportion of patients reaching it compared with placebo in a dose‐dependent manner. Consistent with previously reported outcomes, higher CNB doses resulted in greater effects. These findings support time to baseline seizure rate as a promising endpoint for future epilepsy trials.

**Disclosure:** SL: Speaker/consultant: Angelini Pharma, Eisai, GW Pharmaceuticals, Medscape, NewBridge Pharmaceuticals, and UCB Pharma Advisor: Angelini Pharma, Arvelle Therapeutics, BIAL, EISAI, GW Pharmaceuticals, Rapport Therapeutics, and UCB Pharm VV: Consultant/advisor: Angelini Pharma, BIAL, Biocodex, Eisai, Esteve, GlaxoSmithKline, Jazz, Novartis, Sandoz, Takeda, UCB Pharma, Xenon; Speaker: Angelini Pharma, BIAL, Cevomed, Eisai, Esteve, Jazz, Newbridge, Paladin, UCB Pharma; Research support: Angelini Pharma, BIAL, Eisai, Jazz, UCB Pharma. BS: Consultant/advisor: Angelini, B. Braun Melsungen, Desitin,Eisai, GW Pharmaceuticals, UCB Pharma, Zogenix; Research support: Eisai, GW Pharmaceuticals, SK Life Science, UCB Pharma. JPL, PPD, EAB: Employees, Angelini Pharma. KT: Consultant: Angelini Pharma.

## Headache 3

## EPO‐0408

### Migraine without vascular risk factors versus controls: White matter hyperintensities align with systemic inflammation and atherogenic lipid ratios

#### A. Kale; N. Tepe; G. Ertürk Kale

##### Neurology, Balıkesir University Health Practice and Research Hospital, Balıkesir/Türkiye

**Background and aims:** To compare white matter hyperintensities (WMH) between a control group and migraine patients classified according to the Fazekas score, and to evaluate their relationships with disease burden, inflammatory and lipid indices.

**Methods:** Retrospective cross‐sectional analysis of 320 adults: controls (*n* = 90), migraine with Fazekas‐0 (*n* = 112), and migraine with Fazekas 1–3 (*n* = 118). Participants had no vascular comorbidities. WMH were graded on brain magnetic resonance imaging (MRI). We analyzed C‐reactive protein (CRP), systemic immune‐inflammation index (SII), total‐cholesterol to high‐density lipoprotein ratio (TC/HDL) and atherogenic index of plasma (AIP). Group comparisons used Welch or Kruskal‐Wallis tests with Holm correction. In migraine, Spearman correlations and logistic regression assessed associations with WMH.

**Results:** CRP increased across groups (median 1.22 in controls, 1.12 in Fazekas‐0, 2.19 in Fazekas 1–3; *p* = 1.1e−8). TC/HDL also increased (mean 3.00, 3.70, 5.15; *p* = 1.4e−30), and AIP was higher in Fazekas 1–3 (mean 0.14, 0.15, 0.17; *p* = 0.0011). SII did not differ (*p* = 0.256). In migraine, Fazekas score correlated with TC/HDL (Spearman *r* = 0.53, *p* = 5.6e−18) and CRP (*r* = 0.30, *p* = 4.3e−6). After adjustment for age, migraine duration, and monthly attack frequency, TC/HDL (odds ratio (OR) 3.84 per‐unit, *p* = 5.1e−8) and CRP (OR 1.47 per‐unit, *p* = 0.0019) remained associated with WMH. In the Fazekas 1–3 migraine group, disease duration and monthly attack frequency were significantly higher than in the Fazekas‐0 group (*p* < 0.001). Among aura patients, right‐to‐left shunt rates were higher in Fazekas 0 than Fazekas 1–3, but not statistically significant (38.7% vs 33.3%).

**Figure:**
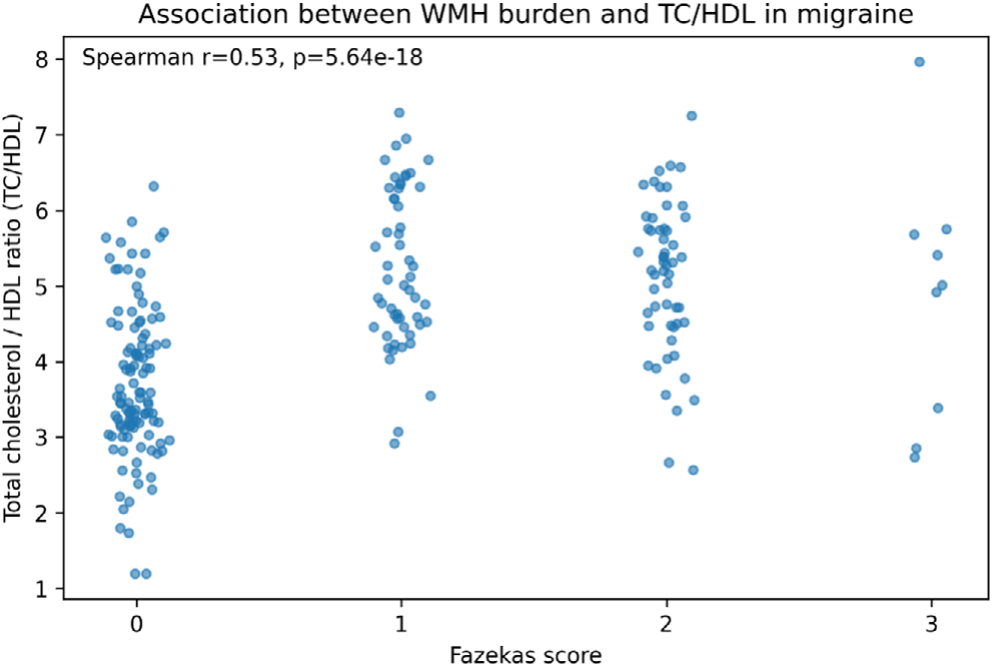
**FIGURE 1** Relationship between Fazekas score and TC/HDL in migraine group.

**Figure:**
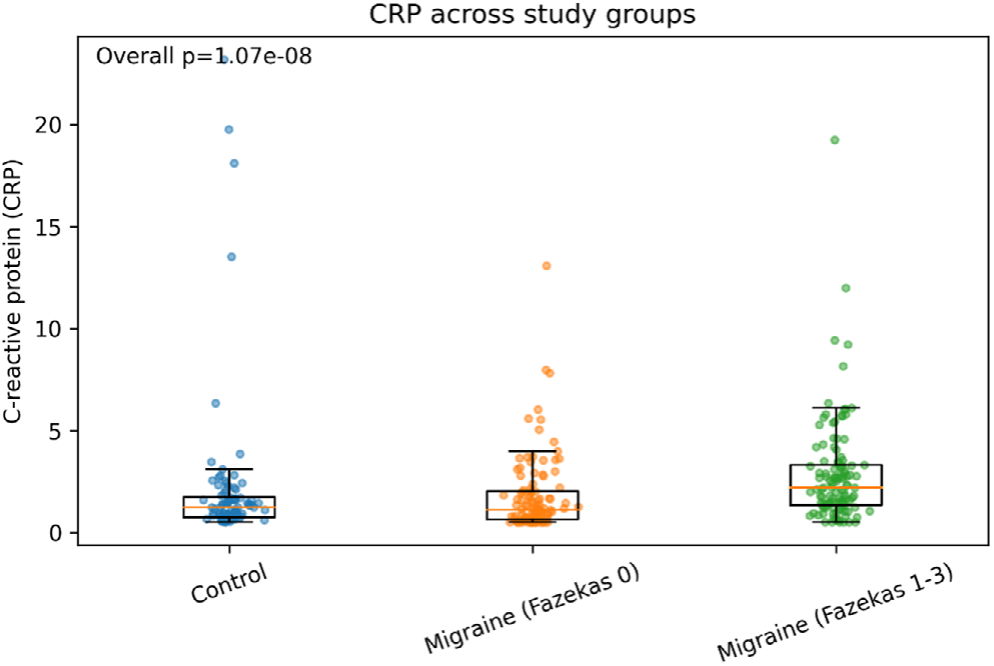
**FIGURE 2** CRP – Comparison of 3 groups; found to be statistically significant.

**Figure:**
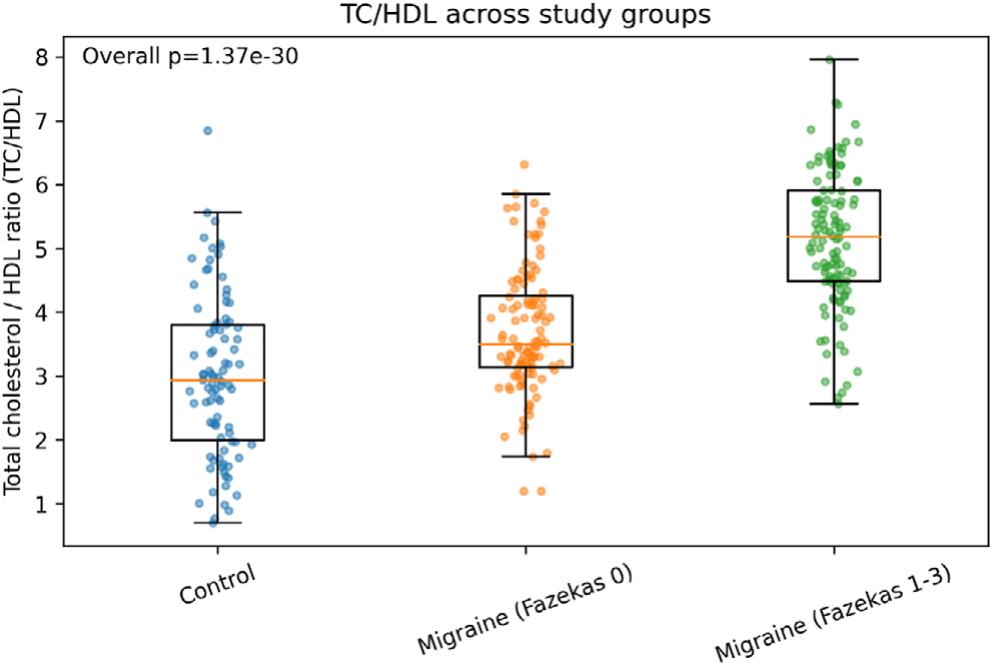
**FIGURE 3** TC/HDL – Comparison of 3 groups; found to be statistically significant.

**Conclusion:** In migraine without vascular comorbidities, higher WMH burden is linked to higher CRP and more atherogenic lipid profile (TC/HDL and AIP), while SII was not discriminative. Longitudinal studies are needed.

**Disclosure:** Nothing to disclose.

## EPO‐0409

### Most bothersome non‐pain symptoms in migraine and their response to CGRP monoclonal antibodies: A service evaluation

#### P. Amarasena; A. Gimson; F. Greenwood; S. Maniataki; U. Ashraf; M. Villar‐Martinez; N. Karsan; P. Goadsby

##### Headache Group, Wolfson Sensory, Pain and Regeneration Centre, King's College London, London, UK

**Background and aims:** Monoclonal antibodies targeting calcitonin gene‐related peptide (CGRP‐mAbs) have been in wide use as migraine preventives for five years. They have potential efficacy in improving the most bothersome non‐pain symptoms (MBS).

**Methods:** Patients currently on mAbs at King's College Hospital, London completed a questionnaire, and clinical and efficacy data were collected from patient records as part of a service evaluation. Associations between categorical variables were assessed using Pearson Chi‐Square tests.

**Results:** A total of 223 patients responded (median age 48 years; 82% female): chronic migraine (62%), episodic migraine (29%), and new daily persistent headache with migraine phenotype (9%). Fatigue was the most frequent MBS (*n* = 44, 20%), followed by brain fog (*n* = 38, 17%), neck stiffness (*n* = 31, 14%), nausea (*n* = 26, 12%) and photophobia (*n* = 21, 9%). Overall, 191 (88%) reported > 30% improvement in headache with their current CGRP‐mAb; however, only 107 (56%) of responders reported improvement in their MBS. Improvement of the MBS had a statistically significant positive effect on headache improvement (χ^2^ = 7.77, *P* = 0.005). Of those who reported headache improvement with mAbs, majority of those who identified fatigue (56%), brain fog (58%), photophobia (67%) and nausea (77%) as the MBS reported improvement and only 46% of those who identified neck stiffness as the MBS reported improvement.

**Conclusion:** Fatigue, brain fog and neck stiffness are the most common non‐pain symptoms among migraine patients. Despite headache improvement with CGRP‐mAbs, many patients continue to experience their most bothersome non‐pain symptoms, particularly neck stiffness.

**Disclosure:** PA, AG, FG, SM, UA, MV and NK have nothing to declare. PJG reports, over the last 36 months, personal fees from Aeon Biopharma, Abbvie, Aurene, CoolTech LLC, Dr Reddy's, Eli‐Lilly and Company, Kallyope, Linpharma, Lundbeck, Pfizer, PureTech Health LLC, Satsuma, Seaport Therapeutics, Shiratronics, and Teva Pharmaceuticals.

## EPO‐0410

### CGRP monoclonal antibodies in migraine: Persistence of premonitory symptoms in the absence of headache — A service evaluation

#### P. Amarasena; A. Gimson; F. Greenwood; G. Sebastianelli; S. Maniataki; U. Ashraf; M. Villar‐Martinez; D. Moreno‐Ajona; N. Karsan; P. Goadsby

##### Headache Group, Wolfson Sensory, Pain and Regeneration Centre, King's College London, London, UK

**Background and aims:** Monoclonal antibodies targeting calcitonin gene‐related peptide (CGRP‐mAbs) have been in wide use for migraine prevention for five years. They have efficacy on headache and potential effects on premonitory symptoms (PreS).

**Methods:** Patients currently receiving CGRP‐mAbs at King's College Hospital, London completed a questionnaire, including data on PreS as part of a service evaluation. Clinical data were extracted from patient records. Associations between categorical variables were assessed using McNemar's tests.

**Results:** A total of 223 patients responded (median age 48 years; 82% female): chronic migraine (62%), episodic migraine (29%), and new daily persistent headache with migraine phenotype (9%). Overall, 188 patients (84%) recognised PreS, with a median of 6 symptoms (IQR 2–9). Most common PreS were fatigue (67%), neck stiffness (58%) and brain fog (58%). Yawning (McNemar's χ^2^ = 4.985, *P* = 0.026) and euphoria (exact McNemar test, *P* < 0.001) were more likely to occur in the premonitory phase than the headache phase. Food cravings were more common in the premonitory phase without statistical significance. Build‐up of PreS without headache was reported by 114 (53%), which included 54% (*n* = 104) of patients with > 30% improvement in headache and 38% (*n* = 10) of those with < 30% improvement. Median number of PreS in this group was 5 (IQR 3–7). Brain fog (60%), fatigue (54%), and neck stiffness (51%) were most common and the duration of PreS was 1–24 hours in most (67%).

**Conclusion:** PreS are recognised by a significant majority of migraine patients. Over half of those responding to mAbs continue to experience PreS without headache.

**Disclosure:** PA, AG, FG, SM, UA, MV and NK have nothing to declare. PJG reports, over the last 36 months, personal fees from Aeon Biopharma, Abbvie, Aurene, CoolTech LLC, Dr Reddy's, Eli‐Lilly and Company, Kallyope, Linpharma, Lundbeck, Pfizer, PureTech Health LLC, Satsuma, Seaport Therapeutics, Shiratronics, and Teva Pharmaceuticals.

## EPO‐0411

### Persistent headache attributed to ischemic stroke: Impact on health‐related quality of life

#### H. Weber ^1^; M. Naumann^1^; D. Freuer^2^; C. Meisinger^2^; J. Linseisen^2^; M. Ertl^3^; L. Braadt^1^

##### ^*1*^ *Department of Neurology and Clinical Neurophysiology, University Hospital Augsburg, Augsburg, Germany;* ^*2*^ *Epidemiology, Faculty of Medicine, University of Augsburg, Augsburg, Germany;* ^*3*^ *Department of Neurology and Neurological Rehabilitation, BKH Günzburg, Günzburg, Germany*

**Background and aims:** Persistent headache attributed to ischemic stroke (PHAIS) is a chronic headache disorder developing after ischemic stroke. While demographic and clinical characteristics have already been described, impairment of the health‐related quality of life (QoL) in patients suffering from PHAIS has not yet been demonstrated. This study aims to investigate whether PHAIS impairs the QoL at 12 months after stroke using the Stroke Impact Scale (SIS).

**Methods:** Data was derived from the Prospective Stroke Cohort Augsburg (SCHANA I & II). After baseline assessment, patients completed a 3‐ and 12‐month follow‐up regarding headache characteristics (Rostock Headache Questionnaire) and QoL (SIS). PHAIS was defined as new onset headache since stroke persisting at 12 months. Statistical analyses comprised Kruskal‐Wallis‐Tests including post‐hoc tests and median regression adjusted for age, sex and history of migraine. Bonferroni‐correction was applied to account for multiple testing.

**Results:** Of 973 valid responses at 12‐month follow‐up, 35 (3.6%) met PHAIS criteria. Patients classified with PHAIS had significantly reduced scores in SIS Domain Memory compared to patients without headaches (*p* = 0.015). In multivariable median regression, SIS Domain Memory remained impaired (estimate: −5.03, 95% confidence interval: −7.02 to −3.05, *p* < 0.001). Phenotype of PHAIS was heterogenous with 15% of patients having migraine‐like, 12% tension‐like and 15% unspecific symptoms.

**Figure:**
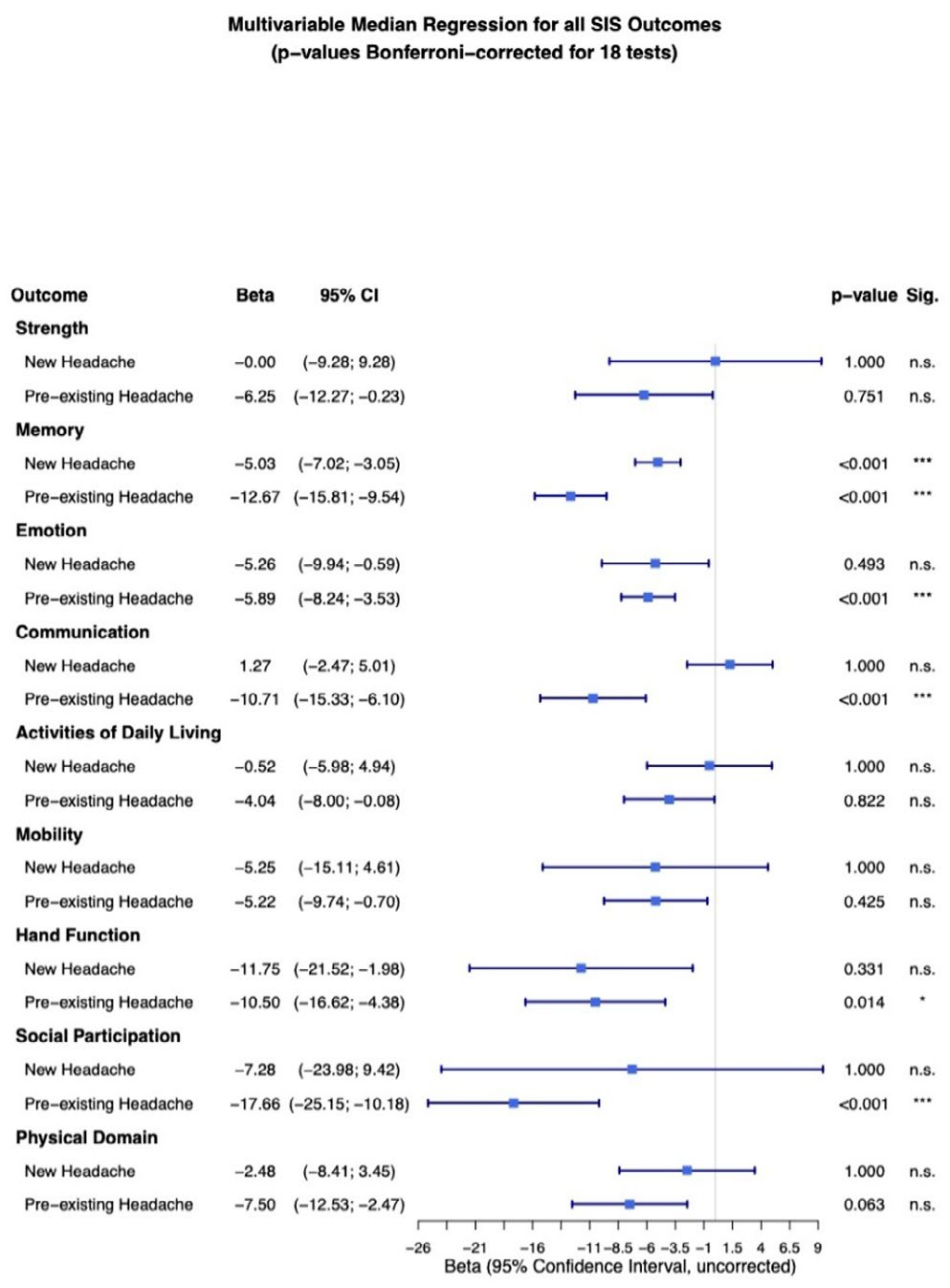
**FIGURE 1**: Multivariable median regression for all SIS outcomes.

**Conclusion:** PHAIS significantly impairs SIS Domain Memory at 12 months after stabilisation of stroke, independently of age, sex and history of migraine. Our findings support the consideration of routine headache assessment in poststroke follow‐ups and may indicate the necessity of future adaptations of PHAIS in poststroke guidelines.

**Disclosure:** Nothing to disclose.

## EPO‐0412

### Impact of trialing multiple triptans on work productivity and indirect costs in employed individuals with migraine: CaMEO‐I study findings

#### J. Versijpt^1^; K. Fanning^2^; P. Gandhi^3^; N. Chalermpalanupap^3^; E. Leroux^4^; P. Jacob^3^; K. Carr ^3^; D. Buse^5^; R. Gil‐Gouveia^6^

##### ^*1*^ *Department of Neurology, Universitair Ziekenhuis Brussel, Brussels, Belgium;* ^*2*^ *MIST Research, Wilmington, USA;* ^*3*^ *AbbVie, North Chicago, USA;* ^*4*^ *Montreal Neurological Clinic, Montreal, Canada;* ^*5*^ *Department of Neurology, Albert Einstein College of Medicine, Bronx, USA;* ^*6*^ *Neurology Department, Hospital da Luz, Lisbon, Portugal; Center for Interdisciplinary Research in Health, Universidade Católica Portuguesa, Lisbon, Portugal*

**Background and aims:** Individuals with migraine may experience suboptimal efficacy or adverse events with triptans, potentially contributing further to disease burden, particularly among working populations. CaMEO‐I, a multi‐country, cross‐sectional, observational survey study, assessed the burden of migraine internationally. This analysis evaluates the impact of the number of triptans trialed on work productivity loss and annual indirect costs, among employed individuals with migraine.

**Methods:** This post hoc analysis included employed adults with complete Work Productivity and Activity Impairment (WPAI) data from CaMEO‐I, stratified by number of triptans trialed: current user (0), 1, 2, and ≥ 3. Median work impairment and indirect costs were measured via WPAI. Indirect costs were estimated in respondents' local currency, scaled using country‐specific working weeks, and adjusted for inflation to October 2025 values.

**Results:** Here, data from US (*n* = 565), UK (*n* = 506), and Germany (*n* = 571) are reported; results for all six countries are in the complete analysis. Median WPAI: overall work productivity Impairment ranged from 50.0–85.8%, increasing with the number of triptans trialed (Figure 1). Parallel trends were observed in median annual indirect costs. Those who trialed ≥ 3 triptans generally demonstrated the highest indirect costs: $32,927 (US), £18,964 (UK), and €24,716 (Germany). Across all countries, indirect cost generally increased across response strata, with most countries showing the greatest cost burden among individuals who trialed ≥ 3 triptans (Figure 2).

**Figure:**
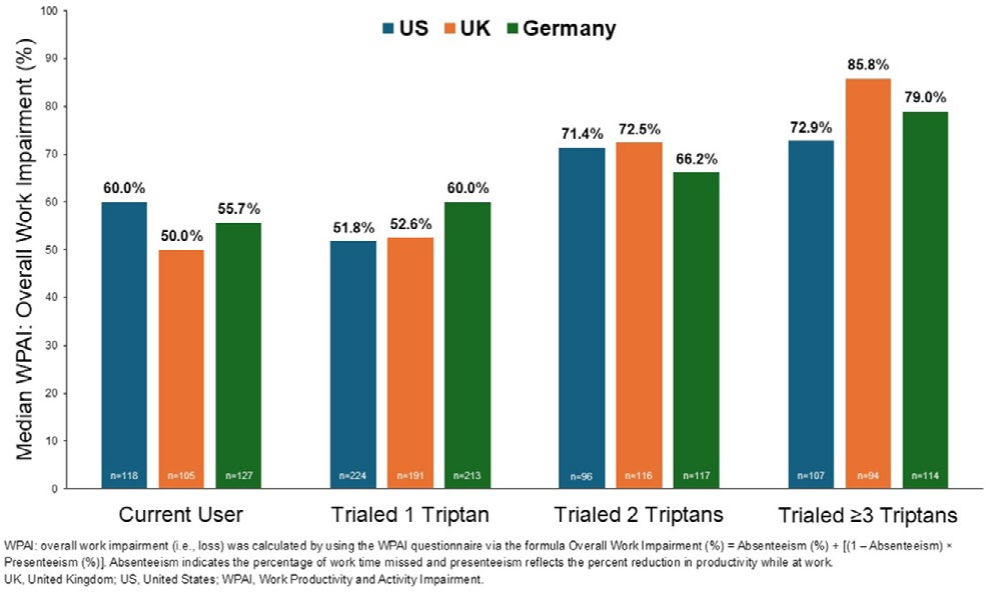
**FIGURE 1** Median WPAI: Overall Work Productivity Loss by Number of Triptans Trialed in the US, UK, and Germany.

**Figure:**
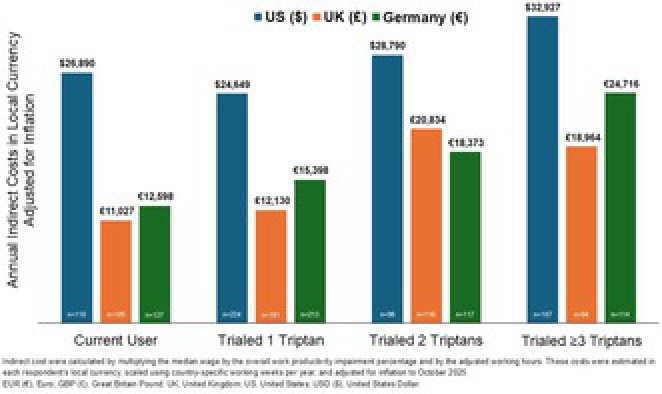
**FIGURE 2** Median Annual Indirect Costs by Number of Triptans Trialed in the US, UK, and Germany Adjusted for Inflation (USD/GBP/EUR).

**Conclusion:** Insights from the CaMEO‐I study suggest that among employed individuals with migraine, trialing multiple triptans is associated with increases in both work productivity loss and indirect costs, across multiple countries.

**Disclosure:** Jan Versijpt received personal fees and nonfinancial support from Teva, Pfizer and Medtronic, personal fees from Novartis and Lundbeck, and grants and nonfinancial support from Allergan/AbbVie. Kristina Fanning is the Managing Director of MIST Research, which has received research funding from AbbVie, AESARA, Aptar, CoolTech, Migraine Canada, NYC Langone Health, Pfizer, UC Irvine, Ipsen. Elizabeth Leroux has received consultancy fees and/or speaker fees from AbbVie, Eli Lilly, Miravo, Lin Pharmaceutical, Lundbeck, Novartis, Paladin, Pfizer, and Teva. Dawn Buse has received research funding from the US Food and Drug Administration, the National Headache Foundation and Amgen and received consulting fees from Amgen, Allergan/AbbVie, Eli Lilly, Ispen, Lundbeck, Pfizer, Theranica, and Teva. Raquel Gil‐Gouveia reports honoraria for consultant and/or advisory boards from Allergan/AbbVie, Astra Zeneca, Almirall, Bial, Biogen, Bristol‐Myers Squibb, CMBE, FLOAT, Lilly, Lundbeck, Merk, Novartis, Novo Nordisk, Organon, Pfizer, Roche, Sanofi, Tecnifar and Teva; research funding from Fundação Ciência Tecnologia (Project 29675, MigN2Treat, 02/SAICT/2017); Learning‐Health, Luz Saúde (LiON, Luz Innovation on Neurosciences); Novartis‐Sociedade Portuguesa de Cefaleias; Bolsa –Projecto Centro de Investigação Interdisciplinar em Saúde and UCP.

## EPO‐0413

### Familial hemiplegic migraine and treatment with CGRP‐mabs – A swiss cohort study

#### L. Krenger; F. Riederer; N. Slavova; A. Scutelnic; A. Maurer; L. Weichsel; C. Schankin

##### Neurology, Inselspital, Bern, Switzerland

**Background and aims:** Familial Hemiplegic Migraine (FHM) is a rare hereditary migraine subtype characterized by motor aura with potentially severe neurological manifestations. Treatment is mostly based on standard migraine therapies, as FHM is usually excluded from clinical trials and existing evidence mainly consists of case reports and series. We present a single‐center cohort study to evaluate disease course and treatment, focusing on calcitonin gene‐related peptide (CGRP)‐based therapies.

**Methods:** We retrospectively analyzed a cohort of 13 patients with FHM (*N* = 11) or probable FHM (*N* = 2) treated at Inselspital Bern, based on medical records and headache diaries. Two patients received the anti‐CGRP monoclonal antibody (mab) erenumab.

**Results:** Eight women and five men were followed for a median of 2, 35 years (range 0–11). Three patients had genetically confirmed FHM type 1 and two had type 2. Clinical course varied, ranging from infrequent mild attacks to severe migraines with prolonged neurological symptoms. Patients requiring prophylaxis, showed favorable responses to lamotrigine, flunarizine, or propranolol. Several patients used triptans acutely without reported adverse events. Two patients (one with FHM1, one with FHM2) with frequent, severe attacks refractory to multiple prophylactic therapies were treated with erenumab. In combination with other medications, antibody therapy reduced attack frequency and severity, although efficacy declined over time in one patient.

**Conclusion:** This case series highlights the heterogeneity of FHM and the need for individualized treatment strategies. While evidence remains limited, mabs, and combination therapies may represent effective and well‐tolerated prophylactic options in selected patients. Further data collection on CGRP mabs in FHM is warranted.

**Disclosure:** FR: Speaker and consultancy honoraria from Lundbeck, Teva, Abbvie, Merz The authors declare no potential conflicts of interest.

## EPO‐0414

### Lasmiditan efficacy and cardiovascular safety in high‐risk patients: A systematic review and meta‐analysis

#### S. Biswas^1^; S. Vasireddy ^2^; Y. Srivastava^1^; S. Arora^3^; J. Rajamani^4^; F. GOHIL^5^; K. Jindal^6^

##### ^*1*^ *Department of Internal Medicine, Ivano‐Frankivsk National Medical University, Ivano‐Frankivsk, Ukraine;* ^*2*^ *Department of Neurology, NMC Specialty Hospital, Abu Dhabi, United Arab Emirates;* ^*3*^ *Department of Internal Medicine, University of Debrecen, Hungary;* ^*4*^ *Department of Internal Medicine Medical College, Caucasus University, Tbilisi, Georgia;* ^*5*^ *Department of Internal Medicine, Università Cattolica del Sacro Cuore, Rome;* ^*6*^ *Department of Internal Medicine Government Medical College, Patiala*

**Background and aims:** Approximately 20% of migraine patients have cardiovascular contraindications to triptans. Lasmiditan, a selective 5‐HT₁F receptor agonist without vasoconstrictive properties, may offer a safer alternative, though systematic evidence in cardiovascular‐risk populations remains limited.

**Methods:** We systematically searched MEDLINE, Embase, Scopus, and Cochrane CENTRAL from inception to June 2025. Primary outcomes were pain freedom at 2 hours and major adverse cardiovascular events (MACE). We performed random‐effects meta‐analyses and assessed bias using Cochrane RoB 2 and ROBINS‐I tools.

**Results:** Sixteen studies were included (5 lasmiditan efficacy trials with 5,555 participants, 10 triptan safety studies). Lasmiditan significantly increased pain freedom versus placebo (OR 2.28, 95% CI 1.94–2.67, *p* < 0.001; NNT 4.2–15.4) with clear dose‐response relationships (50 mg: OR 1.29; 100 mg: OR 1.72; 200 mg: OR 2.10). Treatment effects remained consistent across cardiovascular risk categories (*p* = 0.56 for interaction). Cardiovascular adverse events occurred in < 1% of patients with no increase in highest‐risk patients (OR 1.48, *p* = 0.56). Zero vasoconstriction events occurred across > 19,000 treatment occasions. Central nervous system effects were common (dizziness 8.6–18.1% vs 2.5% placebo; NNH 6.4–16.4) but predominantly mild‐to‐moderate. Observational triptan data showed apparent MACE protection (HR 0.82, 95% CI 0.73–0.92), likely reflecting confounding.

**Figure:**
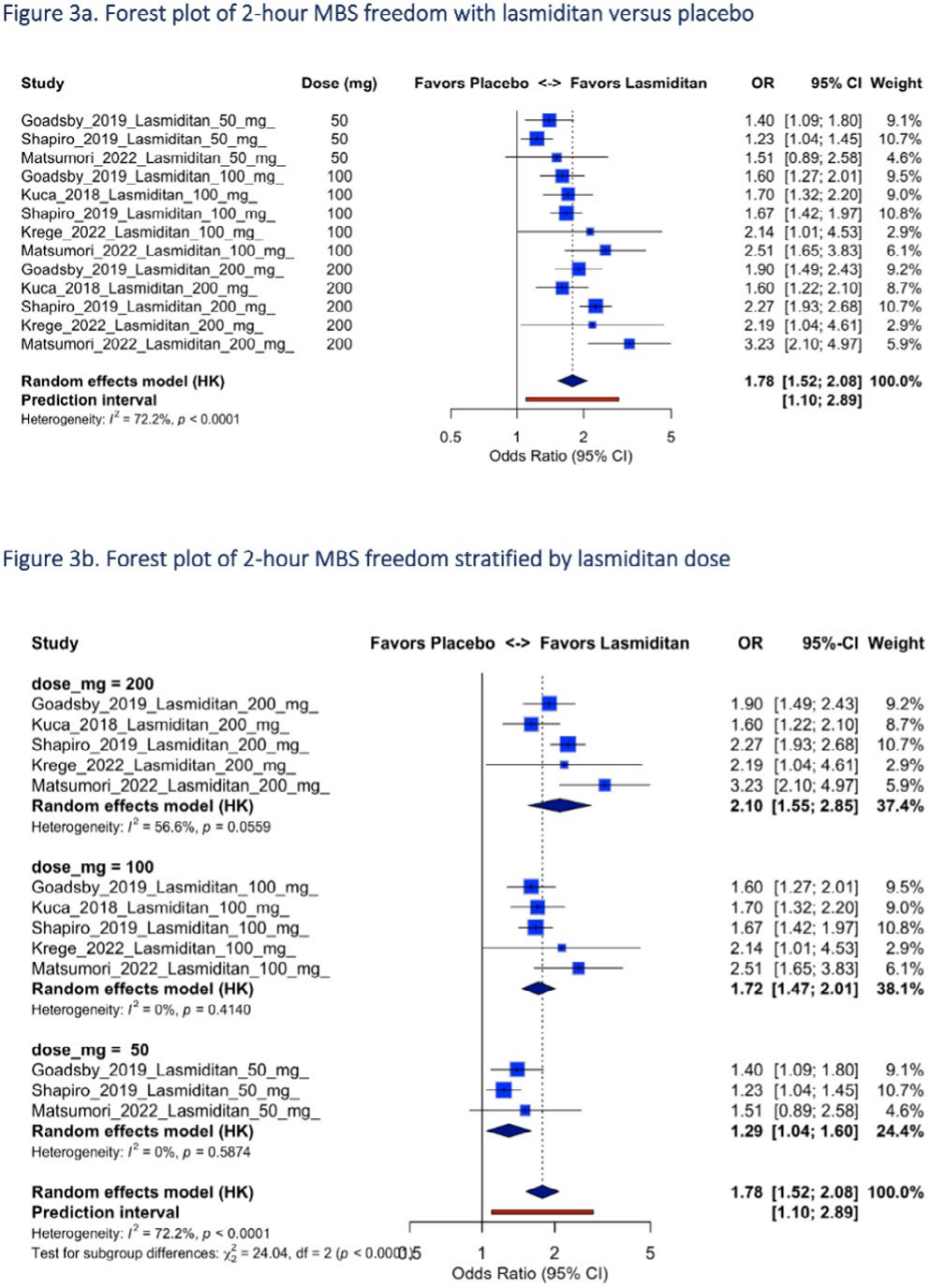
FIGURE 1

**Figure:**
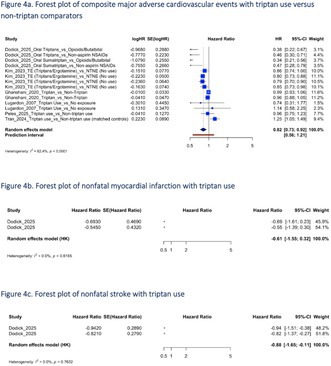
FIGURE 2

**Figure:**
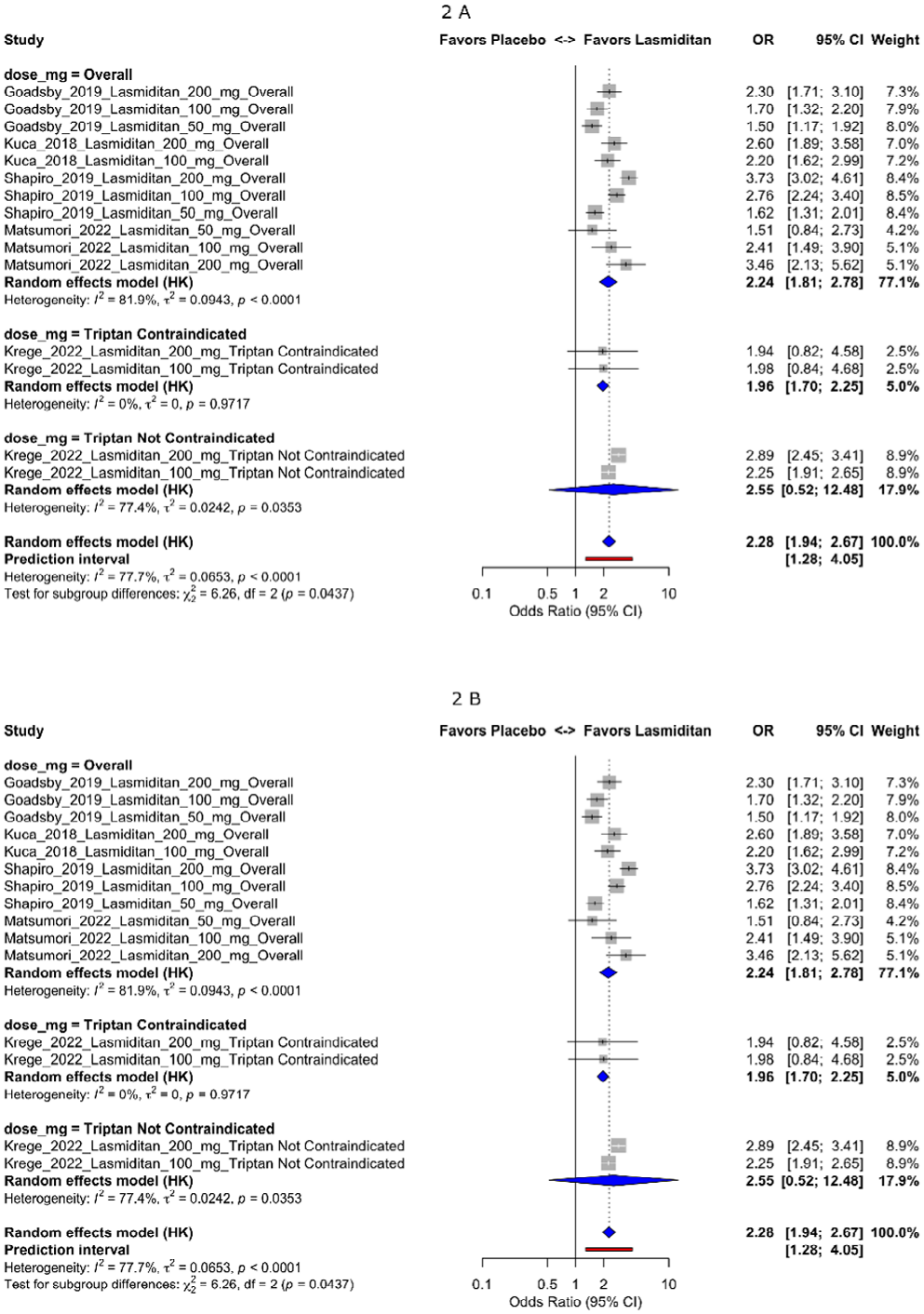
FIGURE 3

**Conclusion:** Lasmiditan demonstrates robust efficacy and favorable cardiovascular safety with complete absence of vasoconstriction events. Given tolerability profiles, gepants may represent preferred first‐line cardiovascular‐safe alternatives, with lasmiditan reserved for gepant non‐responders.

**Disclosure:** Nothing to disclose.

## EPO‐0415

### Impact of atogepant on functional outcomes for the preventive treatment of migraine: A Phase 3, open‐label, 156‐week, long‐term safety extension study

#### M. Ashina ^1^; P. Gandhi^2^; S. Ashina^3^; C. Tassorelli^4^; S. Cho^5^; R. Man^2^; J. Smith^2^; J. Stokes^2^; K. Nagy^2^; P. Goadsby^6^

##### ^*1*^ *Danish Headache Center, Department of Neurology, Copenhagen University Hospital – Rigshospitalet, Copenhagen, Denmark; University of Copenhagen, Copenhagen, Denmark;* ^*2*^ *AbbVie, North Chicago, USA;* ^*3*^ *Harvard Medical School, Boston, USA;* ^*4*^ *University of Pavia, Pavia, Italy; IRCCS Mondino Foundation, Pavia, Italy;* ^*5*^ *Hallym University College of Medicine, Hwaseong, Korea;* ^*6*^ *King Abdullah University of Science and Technology, Thuwal, Saudi Arabia; King's College London, UK*

**Background and aims:** Evaluate the long‐term impact of atogepant on functional outcomes for the preventive treatment of migraine.

**Methods:** An open‐label, 156‐week, long‐term safety extension study enrolled adult participants with chronic or episodic migraine who had an inadequate response to 2–4 classes of conventional oral preventive treatment, and completed the lead‐in studies PROGRESS or ELEVATE, respectively. The primary outcome was safety. Clinical efficacy and functional outcomes were prespecified exploratory analyses. Functional outcomes included change from baseline (CFB) in performance of daily activities (PDA) and physical impairment (PI) domain scores of the Activity Impairment in Migraine‐Diary (AIM‐D) and Migraine‐Specific Quality of Life Questionnaire v2.1 (MSQv2.1) Role‐Function Restrictive (RFR) domain score during 156 weeks.

**Results:** A total of 529 participants were included in the modified intent‐to‐treat population (PROGRESS, *n* = 285; ELEVATE, *n* = 244). Overall safety results were consistent with the known safety profile of atogepant. The mean (SD) change from lead‐in study baseline in PDA domain score at weeks 13–16 was −16.0 (14.1), and this reduction (improvement) was consistent at 153–156 weeks [CFB = −16.0 (14.1)] (Figure 1a). The mean (SD) change from lead‐in study baseline in PI domain score at weeks 13–16 was −13.2 (13.1), and this reduction was consistent through 153–156 weeks [CFB = −13.4 (13.6)] (Figure 1b). An improvement in MSQv2.1 RFR scores was demonstrated at week 12 and was consistent at week 156 (Figure 2).

**Figure:**
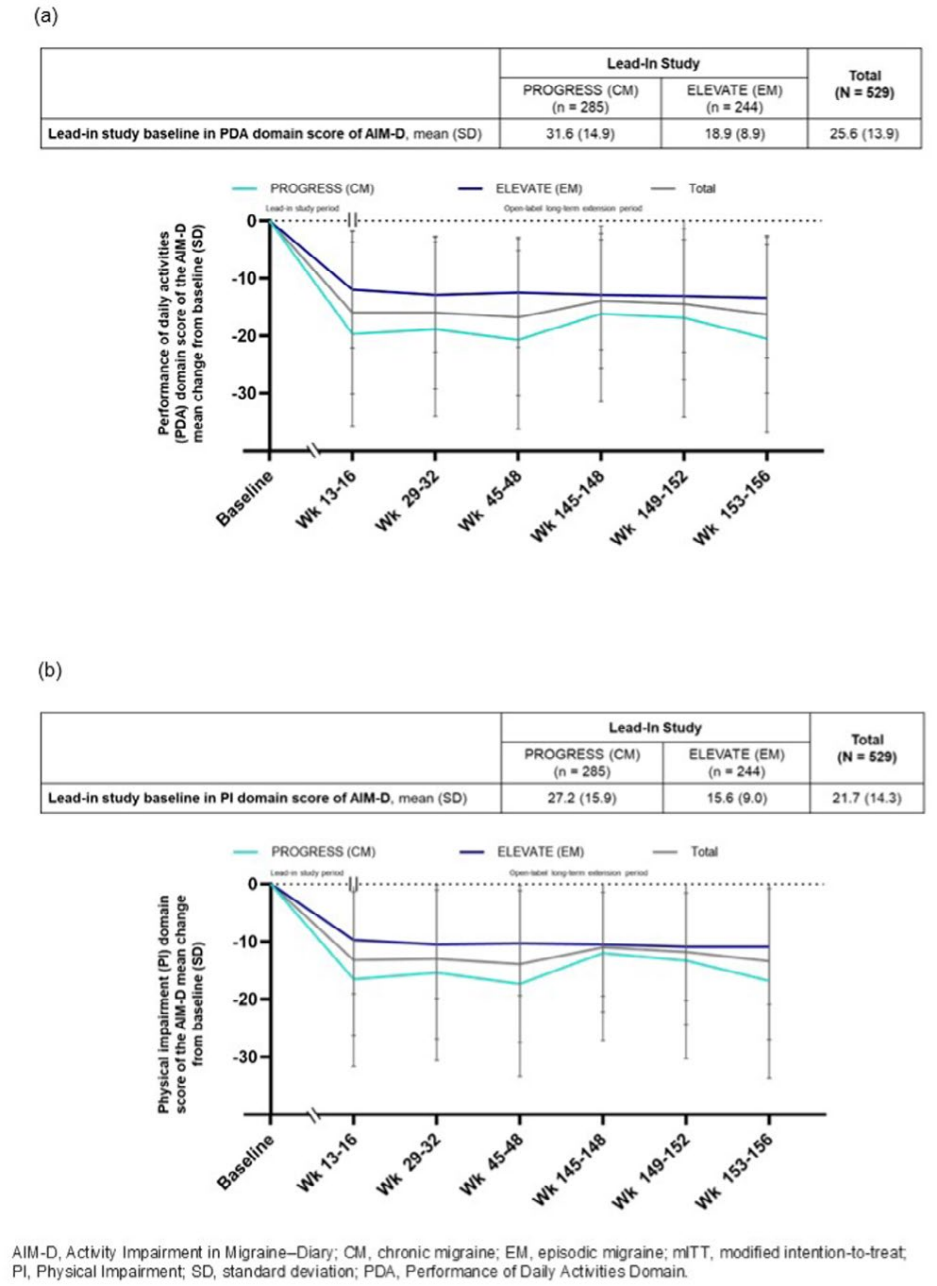
**FIGURE 1** Changes from lead‐in study baseline in mean Activity Impairment in Migraine‐Diary (AIM‐D) domains: (a) PDA and (b) PI at weeks 13–16, 29–32, 45–48, 145–148, 149–152, and 153–156 (mITT population).

**Figure:**
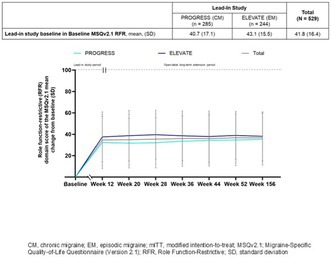
**FIGURE 2**: Migraine‐Specific Quality of Life Questionnaire v2.1 (MSQv2.1): Mean changes from lead‐in study baseline in MSQv2.1 (A) role function‐restrictive (RFR), at Weeks 12, 20, 28, 36, 44, 52, and 156 (in mITT population).

**Conclusion:** Atogepant demonstrated improvements in functional outcomes at Week 12 and was consistent at 156 (MSQv2.1 RFR), and at Weeks 13–16 and was consistent at 153–156 (PDA and PI).

**Disclosure:** Peter J. Goadsby over the last 36 months reports personal fees from CoolTech LLC, Dr Reddy's, Epalex, Ipsen, Kallyope, Linpharma, Lundbeck, Orion Pharma, Pfizer, PureTech Health LLC, Satsuma, Scilex Pharmaceuticals, Seaport Therapeutics, Septurna and Teva Pharmaceuticals, and personal fees for advice through Gerson Lehrman Group, Guidepoint, SAI Med Partners, and fees for educational materials from CME Outfitters and WebMD, and publishing royalties or fees from Massachusetts Medical Society, Oxford University Press, UptoDate and Wolters Kluwer Messoud Ashina has served as an advisory board member, consultant, and or speaker for AbbVie, AstraZeneca, Eli Lilly, GlaxoSmithKline, Incyte, Lundbeck, Pfizer, Teva, and Vedana Therapeutics Inc. He has received institutional research grants from the Danish National Research Foundation, Lundbeck Foundation, Novo Nordisk Foundation, and Novartis. He serves on the editorial board of Neurotorium and has received honoraria. Sait Ashina received honoraria for consulting from AbbVie/Allergan, Amgen, Biohaven, Eli Lilly, Impel NeuroPharma, Linpharma, Lundbeck, Novartis, Pfizer, Percept, Satsuma, Supernus, Teva, and Theranica. Cristina Tassorelli has participated in advisory boards for AbbVie, Dompé, Eli Lilly, Lundbeck, Pfizer and Teva, and has lectured at symposia sponsored by AbbVie, Eli Lilly, Lundbeck, Pfizer and Teva. She is principal investigator or collaborator in clinical trials sponsored by AbbVie, Eli Lilly, Lundbeck, Pfizer and Teva. She has received research grants from the European Commission, the Italian Ministry of Health, the Italian Ministry of University, the Migraine Research Foundation, and the Italian Multiple Sclerosis Foundation. Soo‐Jin Cho has received honoraria for speaking or consulting from Abbie, Allergan, Eli Lilly and Company, Lundbeck, Handok‐Teva, Pfizer, and SK chemicals. Pranav Gandhi, Jonathan Stokes, Jonathan H. Smith, Krisztian Nagy, and Rebeka Man are employees of AbbVie and may hold AbbVie stock.

## EPO‐0416

### Workplace productivity improvements following preventive treatment with eptinezumab: Region‐based analyses from the phase 3 SUNRISE trial

#### T. Takeshima^1^; P. Pozo‐Rosich ^2^; Y. Matsumori^3^; B. Kim^4^; D. Danno^1^; M. Chu^5^; K. Ranc^6^; A. Ettrup^6^; B. Sperling^6^; A. Mittoux^6^; S. Yu^7^

##### ^*1*^ *Tominaga Hospital, Osaka, Japan;* ^*2*^ *Headache Unit, Neurology Department, Vall d’Hebron University Hospital; Headache and Neurological Pain Research Group, VHIR, Barcelona, Spain;* ^*3*^ *Sendai Headache and Neurology Clinic, Sendai, Japan;* ^*4*^ *Eulji University School of Medicine, Seoul, Republic of Korea;* ^*5*^ *Neurology Department, Severance Hospital, Yonsei University, Seoul, Republic of Korea;* ^*6*^ *6. H. Lundbeck A/S, Copenhagen, Denmark;* ^*7*^ *Department of Neurology, The First Medical Center, Chinese PLA General Hospital, Beijing, China*

**Background and aims:** To evaluate migraine‐related impairments in productivity and impact of eptinezumab versus placebo in the SUNRISE trial in chronic migraine, and to explore regional differences.

**Methods:** SUNRISE (NCT04921384) confirmed the migraine‐preventive efficacy and safety of eptinezumab in participants with chronic migraine, predominantly from Asian countries. Adults (18–75 y) diagnosed with chronic migraine were randomized 1:1:1 to 12‐week double‐blind treatment with IV eptinezumab 100 mg, 300 mg, or placebo. The patient‐reported Migraine‐specific Work Productivity and Activity Impairment questionnaire (WPAI:M), captured at baseline and at Weeks 4, 8, and 12, includes sub‐scores for absenteeism, presenteeism, work productivity loss, and activity impairment. Region‐based subgroup analyses were conducted post hoc for Asia and Europe. *p*‐values are descriptive.

**Results:** Of 978 participants treated, 63%/37% were from Asia/Europe. Mean WPAI:M baseline sub‐scores (total population) were absenteeism: 7–10%; presenteeism: 53–57%; work productivity loss: 55–60%; and activity impairment: 58–60% (Europe > Asia across sub‐scores). In the full population, both eptinezumab doses were associated with greater improvements from baseline versus placebo (*p* < 0.05) in presenteeism, productivity loss, and activity impairment at Weeks 4, 8, and 12. Smaller changes from baseline in absenteeism may reflect lower baseline scores, which could suggest participants working despite migraine burden.

**Conclusion:** In a predominantly Asian population with chronic migraine, eptinezumab provided greater improvements than placebo in workplace productivity and activity from Week 4 through Week 12. Participants from Asia reported less absenteeism (at baseline and improvement) than participants from Europe, reflecting a more pronounced pattern of work attendance despite a similar high level of migraine burden.

**Disclosure:** SY declares no conflicts of interest. YM reports personal consultancy fees from Amgen Astellas BioPharma K.K, Daiichi Sankyo Company, Limited, Eli Lilly Japan K.K, and Otsuka Pharmaceutical Co, Ltd. B‐KK has received honoraria as a consultant and speaker from AbbVie, Teva Korea, Lundbeck Koreas, Organon Korea, Pfizer Korea, and SK Pharmaceuticals. His research group has received research grants from Pfizer Korea and funding for clinical trials from AbbVie, Lundbeck, Novartis, Pfizer, and Teva Pharmaceuticals. He is on editorial boards of Headache and Pain Research and Journal of Clinical Neurology. AG‐D has received fees from AbbVie, Amgen, Lundbeck, Pfizer, and Teva while serving as a principal investigator in clinical trials, as well as for speaking and training. GG received fees from Lundbeck while serving as a principal investigator in clinical trials. PP‐R has received honoraria, in the last 36 months, as a consultant and speaker from AbbVie, Almirall, Dr. Reddy's, Eli Lilly, Lundbeck, Medscape, Novartis, Organon Pfizer, and Teva Pharmaceuticals. Her research group has received research grants from AbbVie, AGAUR, EraNet Neuron, FEDER RIS3CAT, Instituto Investigación Carlos III, MICINN, Novartis, and Teva Pharmaceuticals, and has received funding for clinical trials from AbbVie, Amgen, Biohaven, Eli Lilly, Lundbeck, Novartis, Pfizer, and Teva Pharmaceuticals. She is the Honorary Secretary of the International Headache Society, is on the editorial board of Revista de Neurologia, and is an associate editor for Cephalalgia and Neurologia. She is a member of the Clinical Trials Guidelines Committee of the International Headache Society. She has edited the Guidelines for the Diagnosis and Treatment of Headache of the Spanish Neurological Society. She is the founder of www.midolordecabeza.org, a platform to give information and tools to physicians and people who suffer from migraine and other headaches. MKJ and BS are full‐time employees of H. Lundbeck A/S and own stock or stock options in H. Lundbeck A/S. KR, AE, and AM are all full‐time employees of H. Lundbeck A/S. TT is an advisor to Hedgehog MedTech, Inc. and Sawai Pharmaceutical Co, Ltd.

## EPO‐0417

### Hormonal migraine across the life course: European migraine in women survey results (EMHA), H2 2025

#### P. Pozo‐Rosich ^1^; E. Ruiz de la Torre^2^; Goadsby^3^; D. Watson^4^; E. Couturier^5^; A. MacGregor^6^; R. Nappi^7^; E. Merki^8^; D. Hurtado^9^; C. Fairley^10^

##### ^*1*^ *Headache Clinic, Neurology Department, Vall d’Hebron University Hospital, Barcelona, Spain;* ^*2*^ *European Migraine & Headache Alliance (EMHA);* ^*3*^ *Division of Biomedical Sciences, King Abdullah University of Science and Technology, Saudi Arabia; and King's College London, London, UK;* ^*4*^ *GPwER headache, Department of Neurology, Aberdeen Royal Infirmary, Aberdeen, Scotland;* ^*5*^ *MD, Consultant Neurologist, Head of Department of Neurology, Neurologie Centrum Amsterdam, Amsterdam, The Netherlands;* ^*6*^ *Centre for Neuroscience, Surgery and Trauma, Barts and the London School of Medicine and Dentistry, London, UK; and Centre for Reproductive Medicine, St Bartholomew's Hospital, London, UK;* ^*7*^ *Department of Clinical, Surgical, Diagnostic and Pediatric Sciences, University of Pavia, Pavia, Italy; and Research Center for Reproductive Medicine, Gynecological Endocrinology and Menopause, IRCCS S. Matteo Foundation, Pavia, Italy;* ^*8*^ *University of Zürich, Zürich, Switzerland;* ^*9*^ *Prescient Healthcare Group;* ^*10*^ *Prescient Healthcare Group.*

**Background and aims:** Migraine attack improvement or worsening has been attributed to hormonal life stages, yet prospective, longitudinal data is limited and tailored management remains inconsistent. EMHA, with oversight from an interdisciplinary Scientific Committee, launched the Migraine in Women Survey to generate patient‐reported evidence to inform education and care pathway improvements.

**Methods:** A cross‐sectional online survey was conducted across 13 European countries. Recruitment used GAD3 and dissemination via EMHA national patient associations. Women (18–70) were screened using the validated 3‐item ID Migraine tool (positive screen ≥2/3), yielding 5,410 screened‐positive respondents for analysis. Questions assessed headache patterns and care experiences across menstruation/perimenopause, pregnancy/postpartum, and menopause, with a separate path for transgender participants using hormone therapy.

**Results:** Overall, 42% reported no formal migraine diagnosis and 76% reported having discussed their attacks with a healthcare professional. Among menstruating/perimenopausal respondents (*n* = 3,783), 66% linked attacks to their period (38% most/every period; 28% some periods) and 47% reported attacks a few days before bleeding. Period‐related attacks were more painful (36%), longer‐lasting (28%), and more medication‐resistant (20%) than non‐menstrual attacks, yet only 23% reported being offered specific treatment options. 16% rated current treatments “very effective,” and 72% wanted more education on hormone–headache links.

**Conclusion:** Hormonal influences on migraine are commonly reported across Europe, but substantial gaps persist in diagnosis, healthcare engagement, and tailored management, supporting targeted clinician/patient education and improved hormonal migraine pathways.

**Disclosure:** ^1^ Nothing to declare*; ^2^ Nothing to declare*; ^3^ PJG reports, over the last 36 months, personal fees for consulting from Abbvie, CoolTech LLC, Dr Reddy's, Epalex, Ipsen, Kallyope, Linpharma, Lundbeck, Orion Pharma, Pfizer, PureTech Health LLC, Satsuma, Scilex Pharmaceuticals, Seaport Therapeutics, Septurna and Teva Pharmaceuticals.; ^4^ Nothing to declare; ^5^ Nothing to declare; ^6^ Nothing to declare; ^7^ REN has on‐going relationship with Abbott, Astellas, Bayer HealthCare AG, Besins Healthcare, Biocodex, Exeltis, Fidia, Gedeon Richter, Merck & Co, Novo Nordisk, Organon & Co, Shionogi Limited, Theramex, Viatris, and Vichy Laboratories. REN is the current president of the International Menopause Society (IMS). REN declares that none of these could be perceived as prejudicing the impartiality of the research reported; ^8^ reports fees for advisory board and presentations for/from Novartis, Eli Lilly, Almirall, and Lundbeck; ^9^ Nothing to declare; ^10^ Nothing to declare

## EPO‐0418

### Rimegepant for the prevention of migraine in adults with inadequate response to 3 or 4 categories of non‐migraine‐specific oral preventive medication

#### P. Mathew^1^; A. Van Dycke ^2^; L. Ramirez^3^; A. Thiry^4^; R. Fountaine^4^; K. de Schaetzen^5^

##### ^*1*^ *Harvard Medical School, Boston, USA;* ^*2*^ *AZ Sint‐Jan Brugge, Brugge, Belgium;* ^*3*^ *Pfizer Inc, Princeton, USA;* ^*4*^ *Pfizer Inc, Groton, USA;* ^*5*^ *Pfizer NV/SA, Brussels, Belgium*

**Background and aims:** A previous phase 4 trial (NCT05518123) conducted in 14 countries evaluated the efficacy and tolerability of rimegepant for the prevention of episodic migraine in participants with a documented history of inadequate response to 2 to 4 categories of non‐migraine‐specific oral preventive medications (OPMs). This exploratory analysis evaluated rimegepant efficacy in participants with inadequate response to 3 or 4 prior non‐migraine‐specific OPM categories.

**Methods:** Following a 28‐day observation phase (OP), adults not using preventive therapy with 4–14 monthly migraine days (MMDs) and <15 monthly headache days were stratified by OPMs with prior inadequate response and migraine days in the OP then randomized to double‐blind treatment (DBT) with rimegepant 75 mg orally disintegrating tablet (ODT) or placebo every other day (EOD) for 12 weeks.

**Results:** 114 participants with documented prior inadequate response to 3 or 4 OPM categories were enrolled into the rimegepant group and 109 into the placebo group. In both DBT groups, most participants had 3 prior OPMs (81.6% and 84.4%) and ≥8 migraine days in the OP (64.9% and 66.1%). The primary endpoint analysis favored rimegepant vs. placebo: mean change from the OP in MMDs over the entire DBT phase was −2.1 vs. −0.6 (difference = −1.5; 95% CI = −2.4, −0.6; nominal *p* = 0.0016; Figure). Key secondary endpoint analyses also favored rimegepant (all nominal *p* < 0.05; Figure).

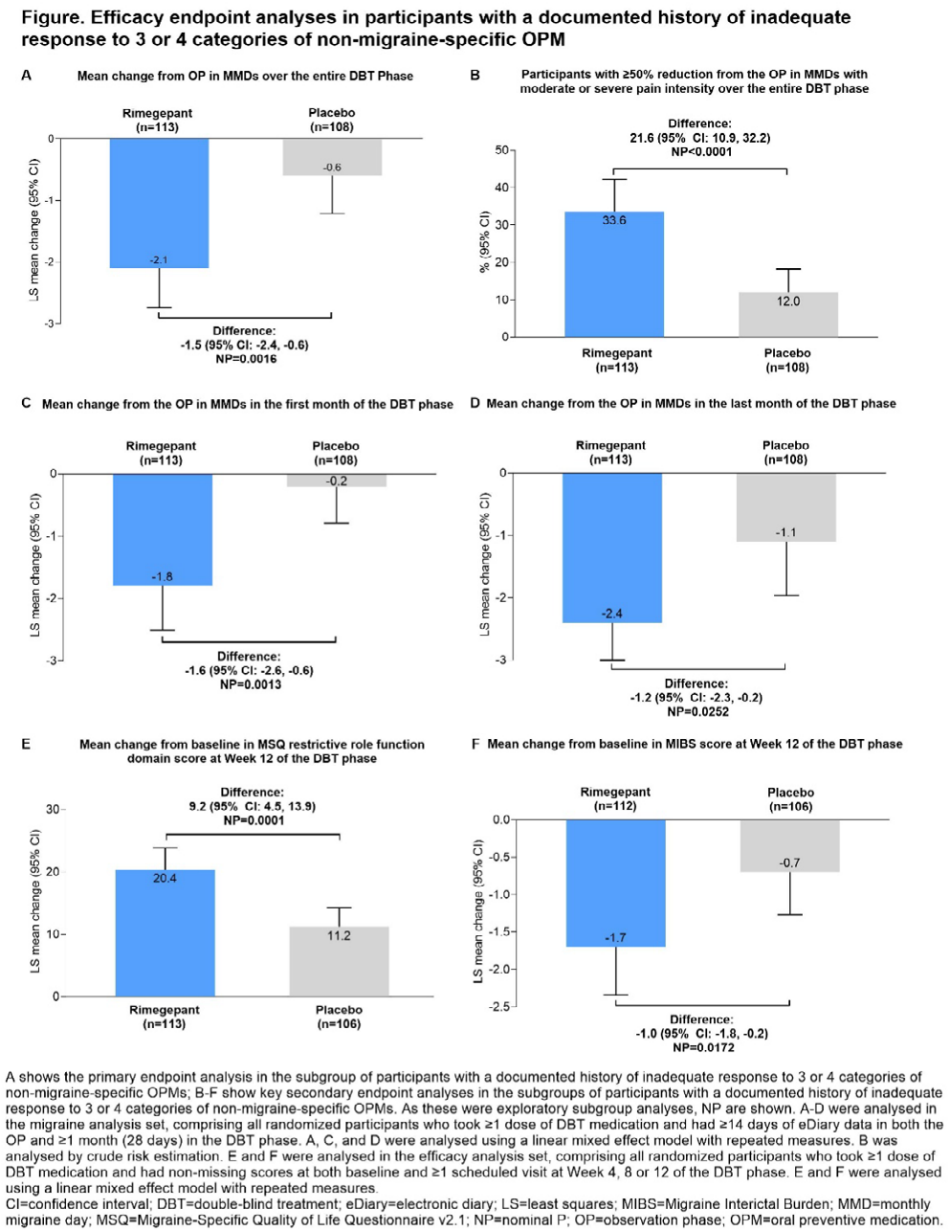

**Conclusion:** Rimegepant 75 mg ODT EOD demonstrated efficacy for the prevention of episodic migraine in participants with a documented history of inadequate response to 3 or 4 categories of non‐migraine‐specific OPM.

**Disclosure:** This study was sponsored by Pfizer. In the past 24 months, Paul G Mathew has served as a consultant for Pfizer, Abbvie, Axsome, Lilly, Theranica, and Tonix. For this abstract, he served as an uncompensated consultant and not in his capacity as a member of the HMS/MGB Faculty. Annelies Van Dycke has served on advisory/scientific boards and/or given lectures for AbbVie/Allergan, Angelini Pharma, Eli Lilly, Lundbeck, Neuraxpharm, Novartis, Organon, Pfizer and TEVA for which she has received honoraria. Luz M Ramirez, Alexandra Thiry, Robert J Fountaine, and Karine de Schaetzen are employees of Pfizer and hold stock/options.

## EPO‐0419

### Effectiveness And Safety of Monthly Versus Quarterly Fremanezumab for migraine prevention: A 12‐Month Italian multicenter real‐world study

#### R. Messina ^1^; A. Visentini^1^; E. Ratto^2^; I. Cetta^1^; F. Genovese^1^; L. Zanandrea^1^; C. Lovati^3^; S. Cevoli^4^; V. Favoni^4^; F. Vernieri^5^; C. Altamura^5^; B. Colombo^2^; M. Filippi^1^

##### ^*1*^ *Neuroimaging Research Unit and Neurology Unit, IRCCS San Raffaele Scientific Institute, and Vita‐Salute San Raffaele University, Milan, Italy;* ^*2*^ *Neurology Unit, IRCCS San Raffaele Scientific Institute, Milan, Italy;* ^*3*^ *L. Sacco Department of Biomedical and Clinical Sciences, University of Milan, Milan, Italy;* ^*4*^ *IRCCS Istituto delle Scienze Neurologiche di Bologna, Bologna, Italy;* ^*5*^ *Unit of Headache and Neurosonology, Department of Medicine and Surgery, University Campus Bio‐Medico di Roma, and Fondazione Policlinico Universitario Campus Bio‐Medico, Rome, Italy*

**Background and aims:** Fremanezumab, an anti–calcitonin gene‐related peptide (CGRP) monoclonal antibody approved for migraine prevention, can be administered monthly (225 mg) or quarterly (675 mg). In clinical practice, regimen selection is mainly based on patient preference, while real‐world evidence comparing the effectiveness of the two dosing strategies remains limited. We aimed to compare monthly versus quarterly fremanezumab in a real‐life setting over 12 months.

**Methods:** This prospective, multicenter, real‐world study involved four Italian headache centers. Patients received monthly or quarterly fremanezumab according to preference. Primary endpoints were changes in monthly migraine days (MMD) and monthly headache days (MHD) at 3, 6, and 12 months, and the occurrence of adverse events (AEs). Secondary endpoints included ≥30%, ≥50%, and ≥75% responder rates in migraine frequency, reductions in monthly acute medication days (AMD) and pills (AMP), changes in disability and clinical scores (MIDAS, HIT‐6, NRS, ASC‐12), and medication‐overuse headache (MOH) cessation.

**Results:** A total of 294 patients were enrolled (152 monthly; 142 quarterly). At 6 months, 138 and 133 patients, and at 12 months, 98 and 102 patients completed follow‐up in the monthly and quarterly groups, respectively. Baseline characteristics were comparable. Both regimens showed similar reductions in MMD and MHD across all time points, with no significant differences in AEs. Improvements in AMP, AMD, MIDAS, HIT‐6, NRS, and ASC‐12 scores were comparable, as were responder rates and MOH cessation.

**Conclusion:** Monthly and quarterly fremanezumab demonstrated comparable effectiveness and safety for migraine prevention over 12 months in a real‐world setting.

**Disclosure:** Nothing to disclose.

Movement disorders 3

## EPO‐0420

### [18F]‐ FDG‐PET metabolic patterns in idiopathic normal pressure hydrocephalus and neurodegenerative disorders: A voxel‐wise analysis

#### C. Espinoza‐Vinces ^1^; E. Prieto^2^; I. Avilés‐Olmos^1^; J. Núñez‐Córdoba^3^; G. Martí‐Andrés^4^; J. Arbizu^2^; M. Luquin^1^

##### ^*1*^ *Department of Nuclear Medicine, Clinica Universidad de Navarra, Pamplona, Spain;* ^*2*^ *Department of Nuclear Medicine, Clinica Universidad de Navarra, Pamplona, Spain;* ^*3*^ *Research Support Service‐Central Clinical Trials Unit, Clinica Universidad de Navarra, Pamplona, Spain;* ^*4*^ *Department of Neurology, University Hospital of Navarra, Pamplona, Spain*

**Background and aims:** Idiopathic normal pressure hydrocephalus (iNPH) is a potentially treatable cause of gait impairment that frequently overlaps clinically and radiologically with neurodegenerative disorders (ND). We aimed to identify [18F]‐FDG‐PET metabolic patterns associated with iNPH in comparison with ND in patients presenting with ventriculomegaly.

**Methods:** We retrospectively analysed 50 adults (mean age 72.0 ± 7.5 years) with gait impairment and ventriculomegaly, characterised by disproportionately enlarged subarachnoid‐space hydrocephalus, with supportive features including Evans Index >0.30 and callosal angle <90°. Diagnostic classification integrated neurological and cognitive assessments, brain MRI, CSF biomarkers when available, and dopaminergic imaging when required. Final diagnoses and key characteristics are summarised in Tables 1 and 2. FDG‐PET scans underwent blinded visual interpretation at baseline by an experienced nuclear medicine specialist. Voxel‐wise analyses were performed in SPM on spatially and intensity‐normalised images. Group comparisons used independent‐sample t‐tests and ANOVA at a voxel‐level threshold of *p* < 0.005. Statistical parametric maps were projected onto a T1‐weighted MRI template.

**Figure:**
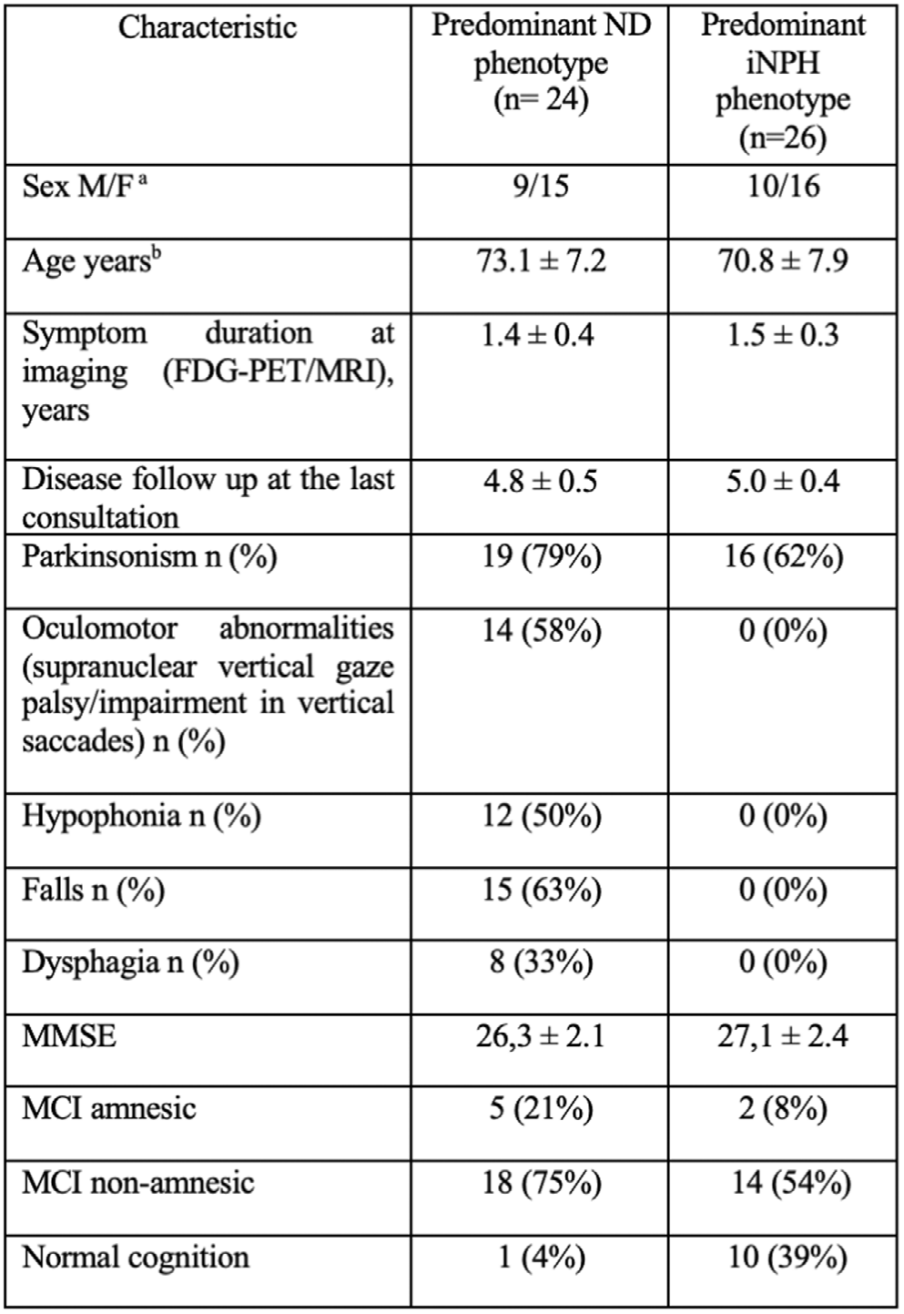
**TABLE 1** Demographic and clinical characteristics of patients with ventriculomegaly classified by predominant clinical phenotype.

**Figure:**
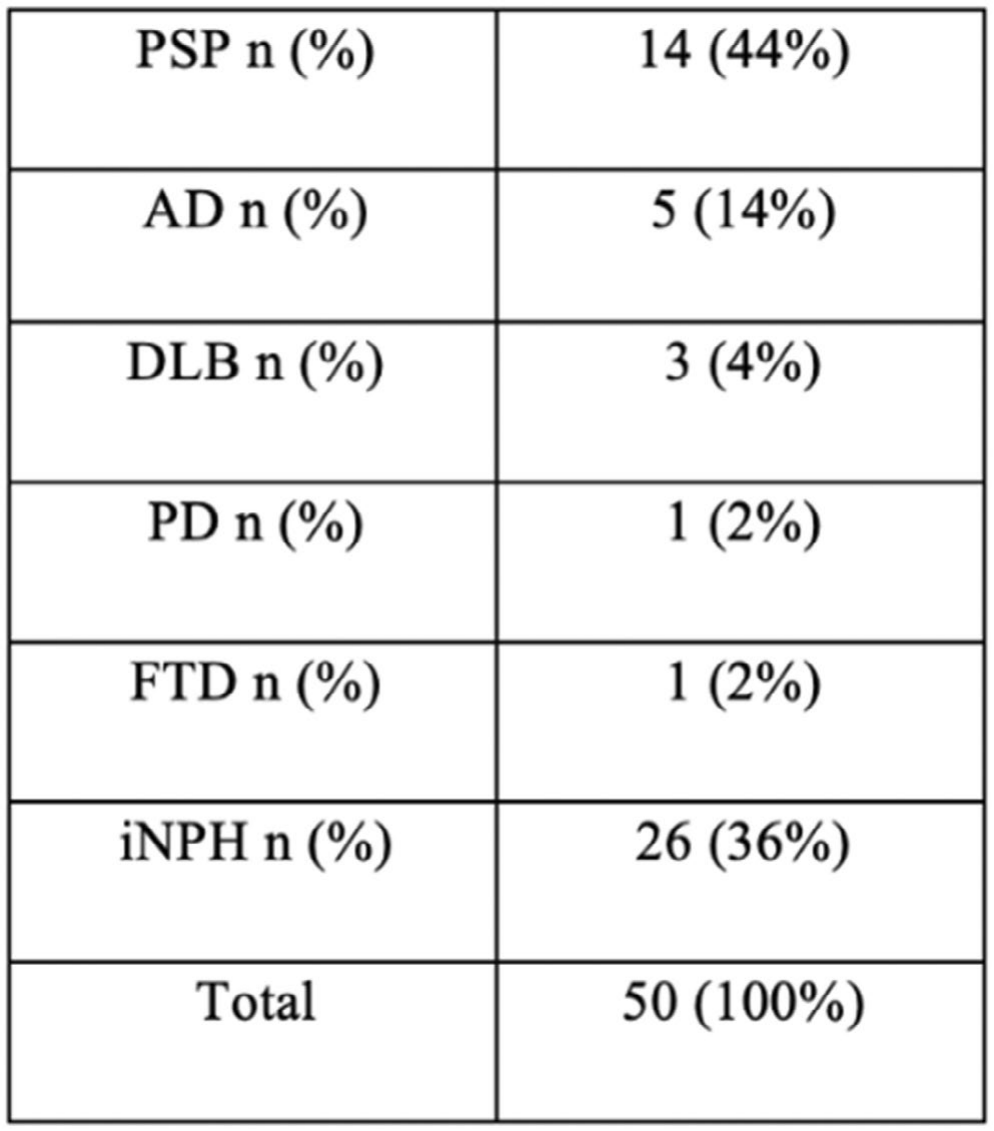
**TABLE 2** Final clinical diagnosis in patients with ventriculomegaly.

**Results:** Voxel‐wise comparisons demonstrated metabolic differences between iNPH and ND subgroups. Relative to progressive supranuclear palsy, iNPH showed higher metabolism in ventromedial frontal regions and basal ganglia. Compared with Alzheimer's disease, iNPH displayed increased metabolism in bilateral medial temporal cortices. Compared with Lewy body disease, iNPH demonstrated greater metabolism in bilateral posterior cortical regions. Relative to frontotemporal dementia and Parkinson's disease, iNPH showed higher metabolism in the cerebellar vermis (*p* < 0.005) (Figure 1).

**Figure:**
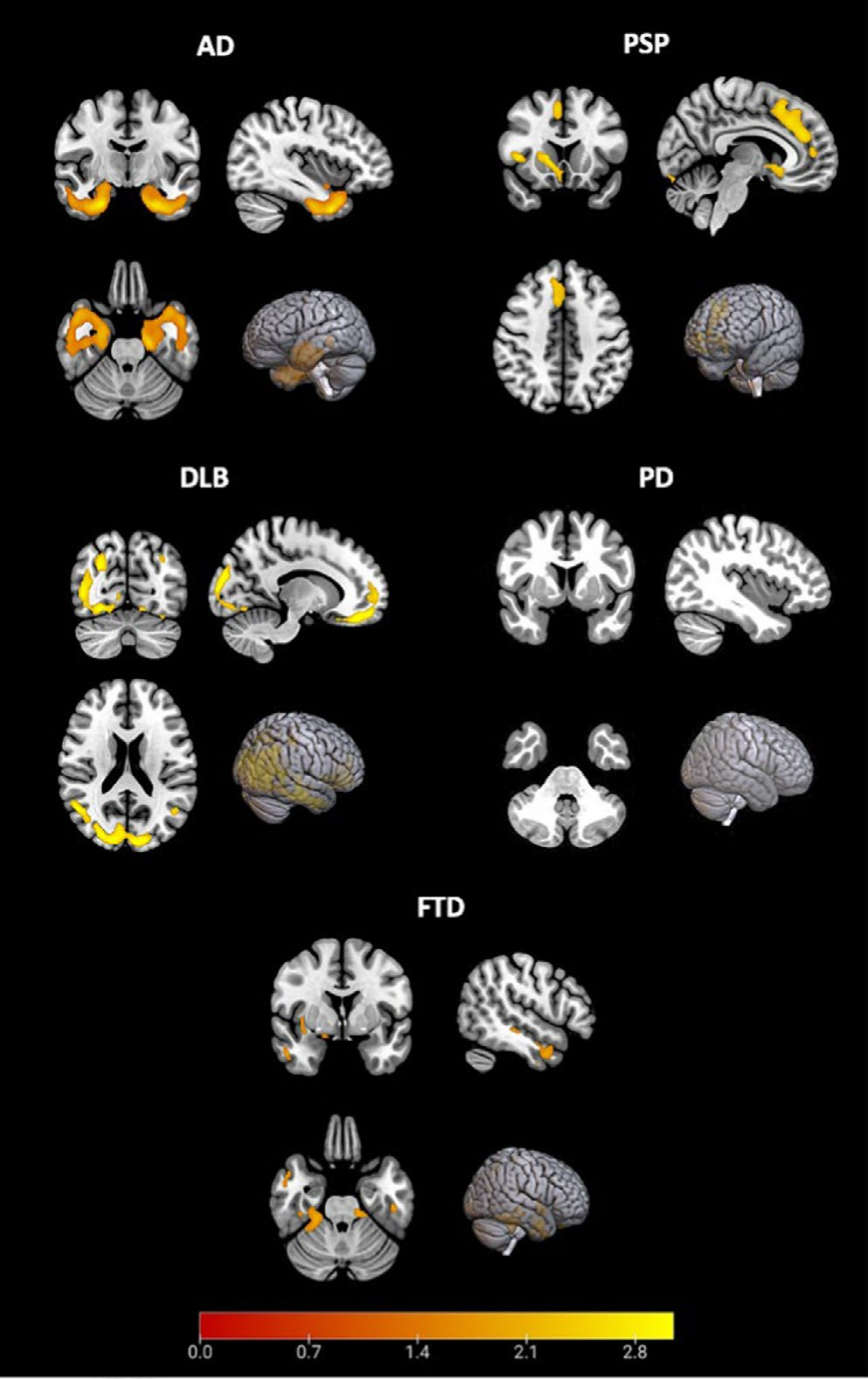
**FIGURE 1** Voxel‐wise FDG‐PET metabolic patterns across major neurodegenerative disorders in patients with ventriculomegaly.

**Conclusion:** Voxel‐wise FDG‐PET reveals regionally distributed metabolic differences in iNPH, supporting its role in early clinical characterisation within a spectrum of neurodegenerative disorders.

**Disclosure:** Nothing to disclose.

## EPO‐0421

### PRKRA‐related dystonia–parkinsonism presenting with acute encephalopathy and striatal necrosis: Expanding the genetic spectrum of rapid‐onset dystonia

#### C. Espinoza‐Vinces ^1^; M. Bradley^1^; S. Maher^2^; K. Gorman^3^; T. Lynch^1^; R. Walsh^1^; A. Espay^4^; C. Fearon^1^

##### ^*1*^ *Dublin Neurological Institute at the Mater Misercordiae University Hospital, Dublin, Ireland;* ^*2*^ *University Hospital Limerick, Limerick, Ireland**;*** ^*3*^ *Children's Health Ireland at Temple Street, Dublin, Ireland;* ^*4*^ *James J and Joan A Gardner Center for Parkinson's disease at the University of Cincinnati, Ohio, USA*

**Background and aims:** PRKRA‐related dystonia–parkinsonism (DYT‐PRKRA) is a rare autosomal recessive disorder typically presenting with childhood‐onset progressive generalised dystonia. We report a case with an acute encephalopathic presentation triggered by febrile illness, a phenotype that remains poorly characterised.

**Methods:** We describe the longitudinal clinical, neuroimaging, and genetic findings of a patient with an acute encephalopathic onset followed by progressive dystonia. Clinical data were obtained from serial neurological assessments. Brain MRI studies were reviewed longitudinally, with particular focus on basal ganglia involvement. Genetic investigations included metabolic testing, mitochondrial studies, and whole‐exome sequencing.

**Results:** A previously healthy child developed an acute encephalopathy at 3 years of age following a viral febrile illness, characterised by somnolence, loss of ambulation, axial hypotonia, ataxia, dysarthria, and pyramidal signs. Cerebrospinal fluid, metabolic, and infectious investigations were unremarkable. Brain MRI demonstrated bilateral putaminal T2 hyperintensities, subsequently evolving into progressive putaminal atrophy (Figure 1). Mitochondrial genetic testing and muscle biopsy were normal. Partial neurological recovery occurred, followed by progressive generalised dystonia with prominent oromandibular involvement during adolescence. Treatment with levodopa, biotin, and thiamine was ineffective. Whole‐exome sequencing identified a homozygous likely pathogenic variant in PRKRA (c.665C>T; p.Pro222Leu), confirming the diagnosis of DYT‐PRKRA.

**Figure:**
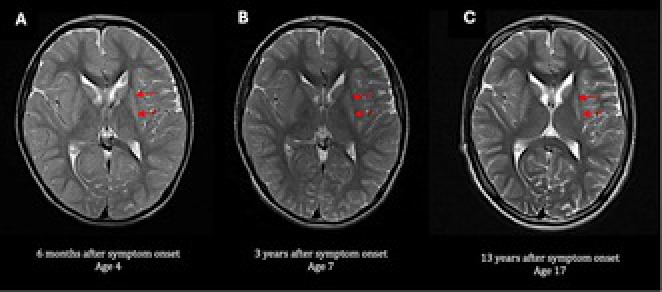
**FIGURE 1** Longitudinal MRI evolution of bilateral putaminal involvement in DYT‐PRKRA.

**Conclusion:** This case demonstrates that DYT‐PRKRA may present with an acute encephalopathic phenotype triggered by febrile illness, mimicking ATP1A3‐related rapid‐onset dystonia–parkinsonism, but distinguished by striatal necrosis on MRI. These findings support a shared clinicopathological spectrum among PRKRA‐, ATP1A3‐, and SLC19A3‐related disorders and highlight the importance of considering PRKRA in acute dystonia presentations with basal ganglia involvement.

**Disclosure:** Nothing to disclose.

## EPO‐0422

### Lower urinary tract symptoms in functional neurological disorder: A prospective cohort study

#### E. Louis ^1^; C. Hentzen^2^; M. Teng^2^; R. Assaraj^3^; S. Baltassis^3^; M. Houot^4^; B. Garcin^3^

##### ^*1*^ *Frontlab team, Paris Brain Institute (ICM), Paris, France;* ^*2*^ *Department of Neuro‐urology, AP‐HP, Pitié‐Salpêtrière Hospital, Paris, France;* ^*3*^ *Department of Neurology, AP‐HP, Avicenne Hospital, Bobigny, France**;*** ^*4*^ *Clinical Investigation Centre, Paris Brain Institute (ICM), Paris, France*

**Background and aims:** Lower urinary tract symptoms (LUTS) are common in neurological disorders but unexpected in functional neurological disorder (FND) in which neural pathways are structurally intact. Evidence on this association is scarce. The primary objective was to describe the prevalence and pattern of LUTS in FND, and the secondary objective was to identify associated factors.

**Methods:** In a two‐year prospective cohort study, we enrolled consecutive patients assessed for FND, with a diagnosis confirmed by positive clinical signs. Conditions causing LUTS were excluded. LUTS, pelvic symptoms, psychiatric comorbidity, pain, and quality of life were assessed using validated questionnaires; anticholinergic load was quantified by the Anticholinergic Drug Scale. The presence of LUTS was defined by cutoffs on the Urinary Symptom Profile or on a visual analog scale of pelvi‐perineal discomfort. Factors independently associated with LUTS were identified using stepwise multivariable logistic regression.

**Results:** Of 145 participants (mean [SD] age 43 [13.7] years; 81% women), 64 (44%) reported LUTS, predominantly storage‐type (30/64, 47%), frequently associated with coexisting bowel and sexual dysfunction (46/61, 72%). Independent factors associated with LUTS were (OR [95% CI] per 1‐SD increase): higher symptom count (1.98 [1.31–3.12]; *p* = 0.002), anticholinergic load (1.86 [1.23–2.93]; *p* = 0.005), pain intensity (1.81 [1.19–2.90]; *p* = 0.008), and childhood adversity (1.61 [1.09–2.43]; *p* = 0.019).

**Conclusion:** Prospective systematic assessment revealed frequent LUTS in FND (44%), mainly storage symptoms, exceeding previous prevalence estimates. The combination of multiple associated factors suggests multifactorial mechanisms. These findings support systematic screening and targeted management, including medication review and multimodal pain management.

**Disclosure:** Nothing to disclose.

## EPO‐0423

### Unique challenges and barriers to increasing equitable access to neuropalliative care in Parkinson's disease: A scoping review

#### F. Connolly ^1^; B. Kluger^2^; S. Seshadri^2^

##### ^*1*^ *Department of Neurology, University of Rochester, Rochester, USA;* ^*2*^ *Department of Medicine, Palliative Care, University of Rochester, Rochester, USA*

**Background and aims:** Persons with Parkinson's disease (PD) have significant palliative care needs that can be addressed through Neuropalliative care (NPC). NPC aims to improve quality of life through non‐motor symptom and pain management, caregiver support, emotional/spiritual support, advanced care planning and specialist/hospice referral. However, NPC is underutilized by individuals with PD from underserved communities and the reasons for this inequity are not fully understood.

**Methods:** We conducted a scoping review of the literature with the primary research question,”What are the barriers to equitable access to neuropalliative care in Parkinson's disease?” A literature search was completed on PubMed and Covidence was utilized to review the search results.

**Results:** Of the 383 articles identified and 27 were included in the final review. Four Themes were identified: a) Lack of knowledge of characteristics of normal aging as distinct from PD symptoms; b) Systemic barriers such as skewed geographic distribution of neurologists, lack of inclusive research, insurance coverage for specialist care etc. can impede access to NPC; c) Sociocultural misunderstanding and resistance to palliative care; d) Underutilization of innovative and culturally responsive models such as telehealth, and partnerships with community partners.

**Figure:**
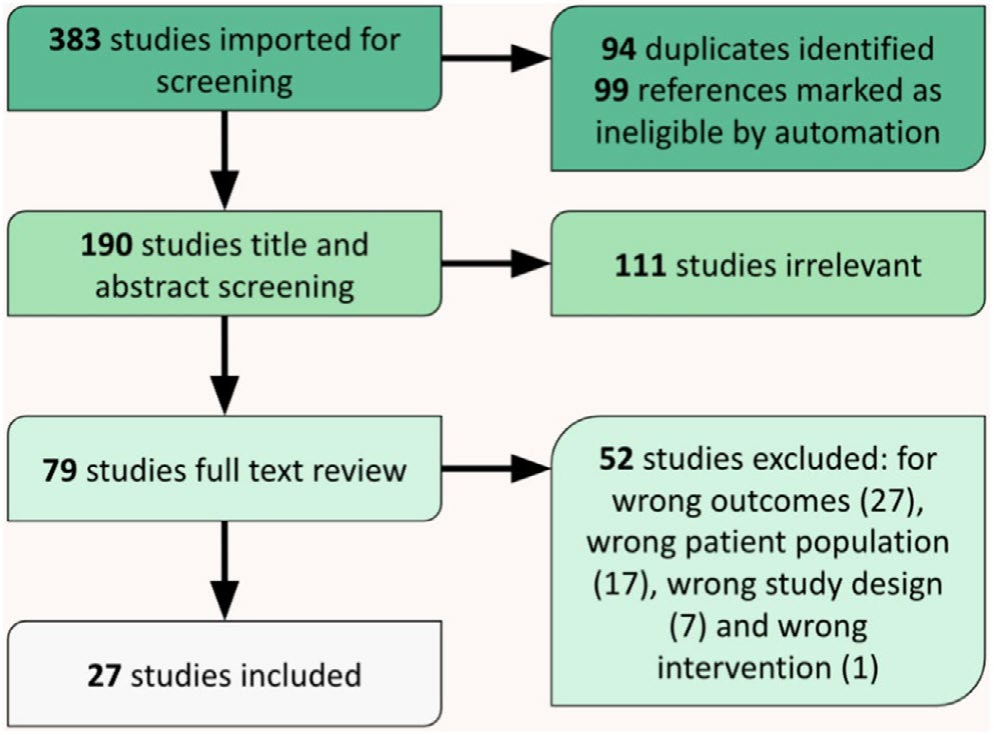
**FIGURE 1** Covidence Results.

**Conclusion:** Multifactorial barriers impede underserved persons with PD from accessing NPC. Future research is needed to further understand and address the barriers identified. Future directions for improving access to NPC among those from underserved communities could include increasing community outreach, improving awareness about resources provided by NPC, demystifying the term “palliative care” through community based programs, and increasing appropriate referrals from primary care to neurology.

**Disclosure:** Nothing to disclose.

## EPO‐0424

### Smoking and Huntington's Disease Symptom Onset ‐ Results from the Enroll‐HD Study

#### G. Witkowski ^1^; R. Rola^1^; D. Zielonka^2^; H. Sienkiewicz‐Jarosz^3^

##### ^*1*^ *Department of Neurology, Military Institute of Aviation Medicine, Warsaw, Poland;* ^*2*^ *Department of Public Health and Epidemiology, Poznan University of Medical Sciences, Poznan, Poland;* ^*3*^ *Maria Sklodowska‐Curie Medical Academy, Warsaw, Poland*

**Background and aims:** Huntington's disease (HD) is an incurable neurodegenerative disorder. Therefore, identifying lifestyle factors that influence its progression is of particular interest. This study aimed to evaluate the impact of smoking tobacco on the onset and progression of HD symptoms.

**Methods:** The study is based on the Enroll‐HD database. A cohort of 2,438 HD subjects (799 of whom were presymptomatic) who had four consecutive annual visits was extracted. Logistic regression models were used to evaluate the impact of lifetime smoking on progression to the symptomatic phase. For the premanifest group, a Cox proportional hazards model was applied to estimate the hazard ratio of HD diagnosis for smokers versus nonsmokers during the three‐year observation period. Multivariate linear regression models were used to investigate the relationship between smoking and the progression of HD clinical measures.

**Results:** Among presymptomatic HD females, but not males, we found that lifetime smoking was associated with a higher risk of transitioning to the symptomatic phase (odds ratio: 1.26, *p* = 0.026). Current female smokers were at a higher risk of progressing to the symptomatic phase than nonsmokers (hazard ratio: 1.35, *p* = 0.023). For the symptomatic HD cohort, regardless of sex, smoking was associated with faster progression of motor symptoms (Total Motor Score, *p* = 0.038; 95% confidence interval [CI], 0.03–0.9) and cognitive impairment (Stroop Word Reading Test, *p* = 0.04; 95% CI, 0.1–0.9).

**Conclusion:** Lifetime and current smoking may be associated with an earlier age of onset and faster progression of HD.

**Disclosure:** Nothing to disclose.

## EPO‐0425

### **Sex Differences in the Treatment of People with Parkinson´s Disease with a Device‐aided therapy. A Prospective Real‐world Study**.

#### D. Santos García ^1^; Á. Solleiro Vidal^1^; M. Blázquez Estrada^2^; P. Mir^3^; N. López Ariztegui^4^; D. Alonso Modino^5^; I. Legarda^6^; A. Peral^7^; R. García‐Ramos^8^; I. Cabo^9^; P. Sánchez Alonso^10^; J. Hernández Vara^11^; J. Ruíz Martínez^12^; M. Álvarez‐Sauco^13^; G. Fernández Pajarín^14^; L. Vela^15^; F. Escamilla^16^; Á. Sánchez Ferro^17^; D. Cerdán Santacruz^18^; G. González‐Ortega^19^

##### ^*1*^ *CHUAC (Complejo Hospitalario Universitario de A Coruña), A Coruña, Spain;* ^*2*^ *Hospital Universitario Central de Asturias (HUCA), Oviedo, Spain;* ^*3*^ *Hospital Universitario Virgen del Rocío, Sevilla, Spain;* ^*4*^ *Hospital Universitario de Toledo, Toledo, Spain;* ^*5*^ *Hospital Universitario de la Candelaria, Santa Cruz de Tenerife, Spain;* ^*6*^ *Hospital Universitario Son Espases, Palma de Mallorca, Spain;* ^*7*^ *Consorci Sanitari Integral, Hospital Moisés Broggi, Sant Joan Despí, Barcelona, Spain;* ^*8*^ *Hospital Universitario Clínico San Carlos, Madrid, Spain;* ^*9*^ *Complejo Hospitalario Universitario de Pontevedra (CHOP), Pontevedra, Spain;* ^*10*^ *Hospital Universitario Puerta de Hierro, Madrid, Spain;* ^*11*^ *Hospital Universitario Vall d´Hebron, Barcelona, Spain**;*** ^*12*^ *Hospital Universitario Donostia, San Sebastián, Spain**;*** ^*13*^ *Hospital General Universitario de Elche, Alicante, Spain;* ^*14*^ *Complejo Hospitalario Universitario de Santiago de Compostela (CHUS), Santiago de Compostela, Spain;* ^*15*^ *Hospital Universitario Fundación de Alcorcón, Madrid, Spain;* ^*16*^ *Hospital Universitario Virgen de las Nieves, Granada, Spain;* ^*17*^ *Hospital Universitario 12 de Octubre, Madrid, Spain;* ^*18*^ *Hospital General de Segovia, Segovia, Spain;* ^*19*^ *Hospital Universitario de Móstoles, Madrid, Spain*

**Background and aims:** Sex differences in the treatment of people with Parkinson´s disease (PwP) with a device‐aided therapy (DAT) have been poorly investigated. Our aim was to analyze sex differences in the management and response to a DAT in PwP in daily clinical practice (DCP).

**Methods:** Data collected in the DATs‐PD GETM Spanish Registry until October 30, 2025, were used. This is a descriptive, observational, prospective, multicenter clinical registry with progressive inclusion of PwP treated with a DAT in DCP conditions in more than 30 centers from Spain (Santos‐García. PLoS One 2025). Sex differences in the DAT received and changes in quality of life (QoL), motor symptoms (MS), non‐motor symptoms (NMS), and autonomy for activities of daily living (AADL) after 6 months of treatment were analyzed.

**Results:** A total of 618 PD patients (66.9 ± 9.5 years old; 57.6% males) were treated with a DAT. A significant difference was observed in the DAT type according to sex (*p* = 0.006), with 73.1% of PwP who were treated with deep brain stimulation being males (Figure‐1). At the time of DAT indication, females were older, received a higher levodopa equivalent daily dose, and had a worse health‐related QoL and AADL (Table‐1). OFF time decreased, whereas MS and NMS burden and health‐related QoL improved at 6 months follow‐up in both groups, males and females (Table‐2). AADL improved only in males during the OFF state (Table‐2).

**Figure:**
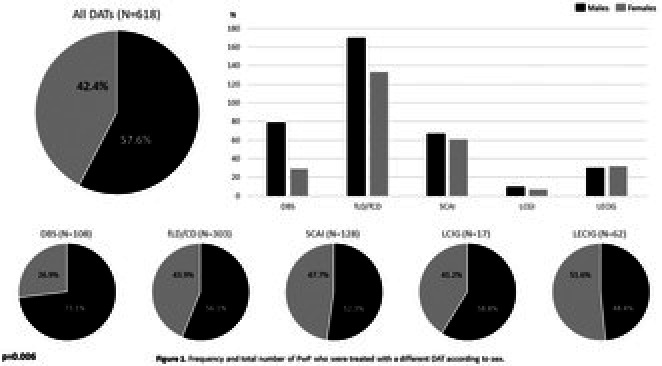
FIGURE 1

**Figure:**
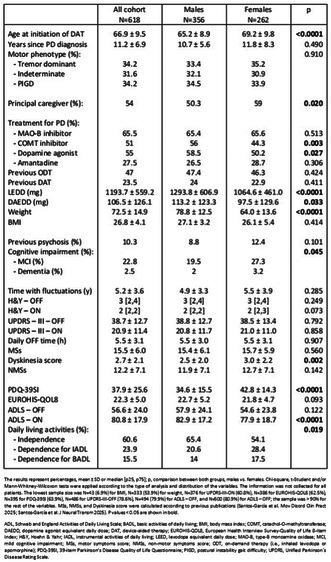
**TABLE 1** PD related variables at baseline (before initiation with a DAT) in males vs. females from the DATs‐PD GETM Spanish registry.

**Figure:**
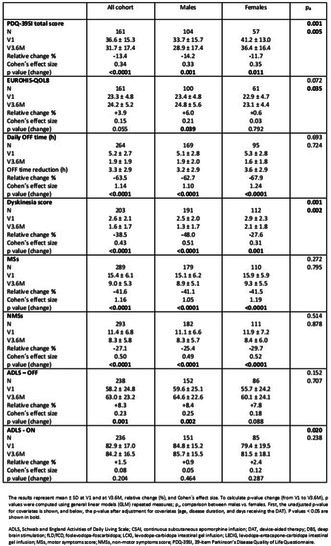
**TABLE 2** Covidence Results Comparison between males and females treated with a DAT in the change from the baseline to the final visit at 6 months in the main variables analyzed related to PD.

**Conclusion:** Sex differences were detected in the use of DATs in PwP. Improvement in OFF time, MS, NMS, and QoL were detected in both groups.

**Disclosure:** There are no financial disclosures.

## EPO‐0426

### Quantifying Upper Limb Coordination in Patients with Friedreich's Ataxia via Video Recordings and Unsupervised Anomaly Detection

#### L. Hourieh ^1^; C. Velasquez^1^; M. Praster^2^; M. Pishnamaz^2^; J. Schulz^1^; K. Reetz^1^; S. Lischewski^1^; R. Dadsena^1^; I. Dogan^1^; S. Sandro Romanzetti^1^; K. Konrad^3^; T. Clavel^4^; V. Jankowski^5^; J. Jankowski^5^; Pabst^6^; N. Marx^7^; J. Moellmann^7^; M. Jacobsen^7^; K. Marx‐Schütt^7^; J. Dukart^8^; S. Eickhoff^8^; R. Hilgers^9^

##### ^*1*^ *Department of Neurology, RWTH Aachen University Hospital, Aachen, Germany ;* ^*2*^ *Department for Orthopaedics, Trauma and Reconstructive Surgery, RWTH Aachen University Hospital, Aachen, Germany ;* ^*3*^ *Section Child Neuropsychology, Department of Child and Adolescent Psychiatry, Psychosomatics and Psychotherapy, RWTH Aachen University Hospital, Aachen, Germany ;* ^*4*^ *Functional Microbiome Research Group, Institute of Medical Microbiology, RWTH Aachen University Hospital, Aachen, Germany ;* ^*5*^ *Institute for Molecular Cardiovascular Research, University Hospital RWTH Aachen, Aachen, Germany ;* ^*6*^ *Institute of Molecular Medicine, RWTH Aachen University, Aachen, Germany ;* ^*7*^ *Department of Internal Medicine I, Cardiology, RWTH Aachen University, Aachen, Germany ;* ^*8*^ *Institute of Neuroscience and Medicine, Brain and Behaviour (INM‐7), Research Centre Jülich, Jülich, Germany;* ^*9*^ *Department of Medical Statistics, RWTH Aachen University, Aachen, Germany*

**Background and aims:** Friedreich's Ataxia (FRDA) is a rare neurodegenerative disorder characterized by progressive ataxia. Clinical assessment relies on observer‐based scales such as the Scale for the Assessment and Rating of Ataxia (SARA) and the modified Friedreich's Ataxia Rating Scale (mFARS), which may be insensitive to subtle motor changes. This study applies video‐based analysis and unsupervised anomaly detection to quantify upper‐limb coordination.

**Methods:** 778 video recordings of the finger‐nose (SARA, mFARS), finger‐tapping (mFARS) and pronation‐supination tasks (SARA, mFARS) of 64 individuals with FRDA (48% female), and 58 controls (47% female) acquired during routine clinical visits were analyzed. Features describing movement speed, frequency, smoothness, entropy, and variability were extracted. Group‐ and severity‐related differences were assessed using Mann‐Whitney U Test. To capture multivariate deviations in movement patterns not detectable by univariate testing, unsupervised anomaly detection models trained exclusively on controls were used. Here, subject‐specific anomaly scores and assess their relevance to disease status were extracted.

**Figure:**
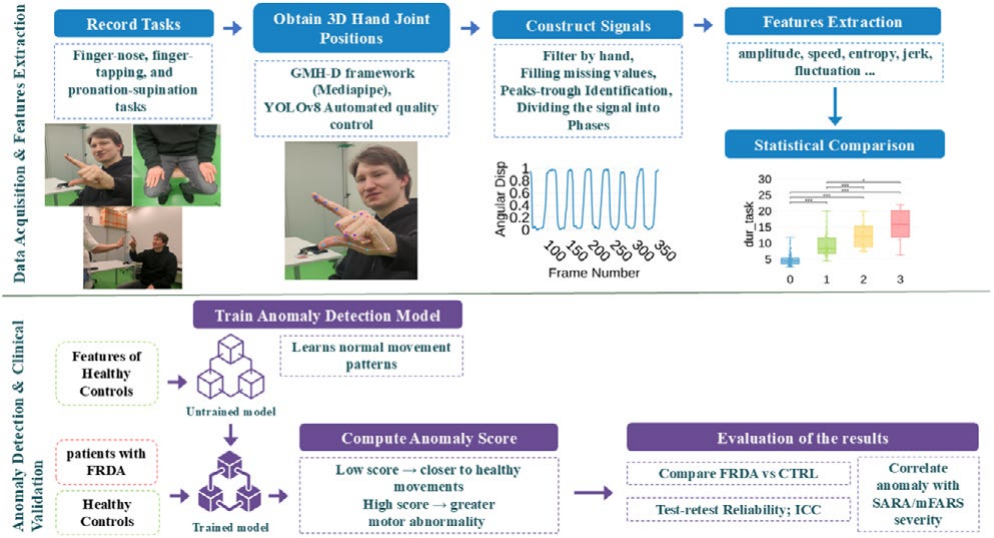
**FIGURE 1** Workflow for Feature Extraction and Anomaly Scoring in Upper‐Limb Task.

**Results:** Most features differed significantly between patients and controls and across increasing SARA and mFARS severity. Greater severity was associated with prolonged task execution, slower and less frequent movements, increased variability, and higher spectral entropy. Anomaly scores increased with severity, showing strong associations in finger fapping (Spearman's ρ ≈ 0.64–0.65) and pronation–supination (ρ ≈ 0.71–0.72), with moderate associations in nose–finger task (ρ ≈ 0.33–0.36).

**Figure:**
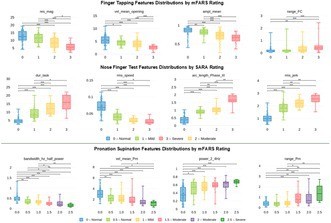
**FIGURE 2** Distributions of some features across clinical severity levels for all tasks.

**Figure:**
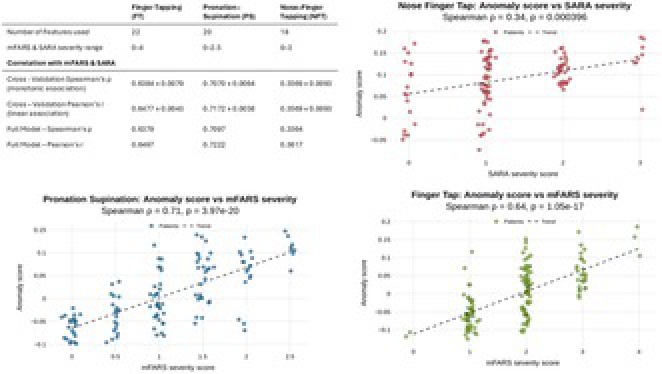
**FIGURE 3** Anomaly detection model performance across tasks and clinical severity levels.

**Conclusion:** Video‐based upper‐limb kinematic analysis combined with anomaly scoring provides an objective, feature‐driven measure of motor impairment with potential utility for quantifying disease severity and tracking progression in FRDA.

**Disclosure:** JBS received grants from the German Research Foundation, Interdisciplinary Center for Clinical Research within the faculty of Medicine at the RWTH Aachen University, Germany, Biogen and Eisai and for presentations or advisory boards from Biogen, Reata, Eisai, Lilly, Roche, Novo Nordisk. KR has received grants from the German Research Foundation, Friedreich's Ataxia Research Alliance (FARA), Interdisciplinary Center for Clinical Research within the faculty of Medicine at the RWTH Aachen University, Germany (OC2), German Federal Ministry of Education and Research (BMBF KP22‐ 106E) and honoraria for presentations or advisory boards from Biogen, Eisai, Lilly and Roche. SAL has received speaker and advisory honoraria from Biogen.

## EPO‐0427

### Cross‐over trial of four spinal cord stimulation paradigms for gait impairment in Parkinson's disease

#### M. Terkelsen ^1^; V. Hvingelby^2^; E. Johnsen^3^; M. Møller^3^; E. Danielsen^3^; T. Henriksen^4^; A. Glud^2^; Y. Tai^5^; A. Andersen^2^; R. Valdemarsen^6^; A. Knudsen^2^; K. Meier^2^; E. Moro^7^; J. Sørensen^2^; N. Pavese^8^

##### ^*1*^ *Department of Clinical Medicine, Aarhus University, Aarhus, Denmark;* ^*2*^ *Department of Neurosurgery, Aarhus University Hospital, Aarhus, Denmark;* ^*3*^ *Department of Neurology, Aarhus University Hospital, Aarhus, Denmark;* ^*4*^ *Department of Neurology, Bispebjerg Hospital, Copenhagen, Denmark;* ^*5*^ *Department of Neurosciences, Imperial College Healthcare NHS Trust, London, UK;* ^*6*^ *Department of Nuclear Medicine and PET Center, Aarhus University Hospital, Aarhus, Denmark;* ^*7*^ *Department of Psychiatry, Neurology, Neurological Rehabilitation and Forensic Medicine, Grenoble Alpes University Hospital, Grenoble, Auvergne‐Rhône‐Alpes, France;* ^*8*^ *Clinical Ageing Research Unit, Newcastle University, Newcastle Upon Tyne, UK*

**Background and aims:** Spinal cord stimulation (SCS) has recently been suggested to improve gait impairment in Parkinson's disease (PD). However, it remains unclear whether any specific stimulation paradigm is superior. Here, we aimed to compare different paradigms of SCS in PD patients with gait impairment.

**Methods:** Eight gait‐impaired PD patients who had previously received SCS were included. Four stimulation paradigms (Fast Acting Sub‐perception Therapy [FAST], MicroBurst, low frequency/high pulse width, and high frequency/low pulse width) were administered in a randomised order, each for two months. The Postural Instability and Gait Disorder (PIGD) score was assessed before initiation of SCS (approximately two years prior) and during each stimulation paradigm. A linear mixed model was used to compare the stimulation paradigms. In addition, the individually optimal paradigm was compared with a natural cohort of gait‐matched PD patients (*n* = 8) without SCS.

**Results:** Seven participants completed follow‐up for at least two settings (four completed all settings, one completed three, and two completed two). One participant was excluded due to non‐compliance. No stimulation paradigm was found to be overall superior to the others. After two years, participants receiving SCS demonstrated an improvement in PIGD when using their individually optimal paradigm, which was significantly better compared with the two‐year PIGD deterioration observed in a natural cohort of PD patients without SCS (*p* = 0.001).

**Conclusion:** Our findings suggest that the SCS paradigm should be individualised rather than based on a “one‐size‐fits‐all” approach. Furthermore, long‐term SCS may mitigate the natural progression of gait deterioration in PD.

**Disclosure:** This work was funded by the Independent Research Fund Denmark and the Danish Parkinson's Association. Funders had not impact on trial design, execution or analysis.

## EPO‐0428

### Impact of Pimavanserin on Longitudinal Trajectories of Parkinson's Disease Psychosis and Cognitive Symptoms

#### P. Oikonomou

##### Department of Neurosciences, UC San Diego Health System, University of California, San Diego, La Jolla, USA

**Background and aims:** Parkinson's disease psychosis (PDP) is a disabling non‐motor feature of PD. Treatment options are limited, with clozapine constrained by safety concerns, quetiapine demonstrating variable efficacy, and pimavanserin approved specifically for PDP but unavailable outside of the U.S. Long‐term real‐world data on treatment effects are scarce. This study examined whether initiation of PDP pharmacotherapy alters the longitudinal trajectory of psychosis and subjective cognitive symptoms.

**Methods:** We performed a retrospective longitudinal observational study using electronic medical records from University of California San Diego movement disorders clinics from 2015–2025. Eligible patients had PD and a Movement Disorder Society–Unified Parkinson's Disease Rating Scale Part I item 1.2 score of at least one. Patients were categorized by treatment (pimavanserin, quetiapine, combination (pimavanserin and quetiapine), or no treatment). Ordinal psychosis and cognitive symptom scores were analyzed using cumulative link mixed‐effects models. Treated groups were modeled with pre‐ and post‐treatment trajectories.

**Results:** 100 patients were included (pimavanserin *n* = 14; quetiapine *n* = 12; combination *n* = 16; untreated *n* = 58), followed for a mean of 4.3 years (SD 2.7; range 0–12.8). After adjustment for demographics and disease duration, only pimavanserin was associated with a statistically significant attenuation of psychosis progression after treatment initiation (Δ slope = −0.498; *p* = 0.045). Quetiapine and combination therapy showed non‐significant reductions. Cognitive symptoms worsened prior to treatment in all groups, with significant post‐treatment attenuation observed for pimavanserin and combination therapy, but not quetiapine.

**Figure:**
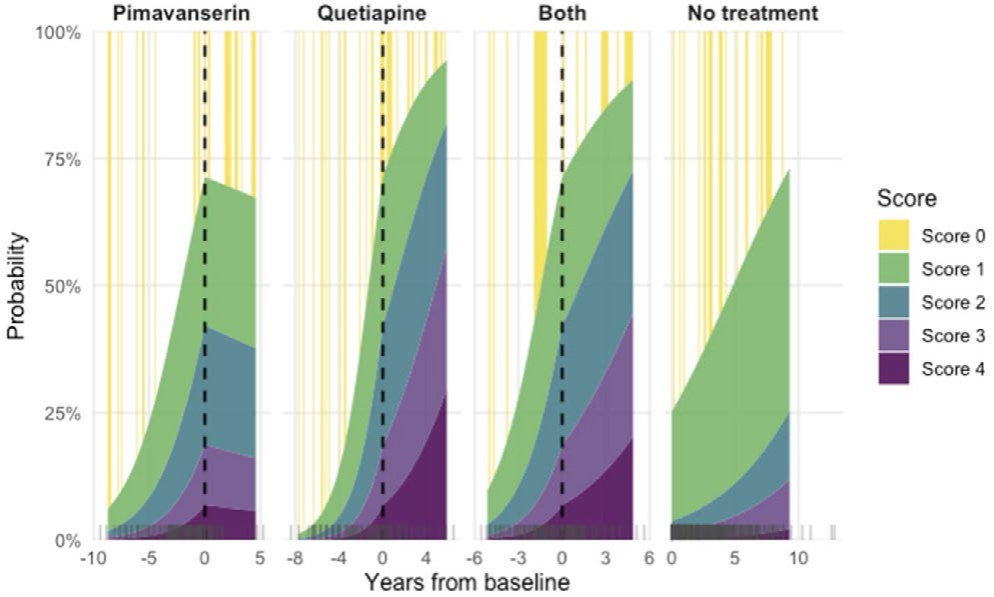
**FIGURE 1** Predicted distribution of Parkinson's disease psychosis symptoms (MDS‐UPDRS 1.2 score) by treatment group over time.

**Figure:**
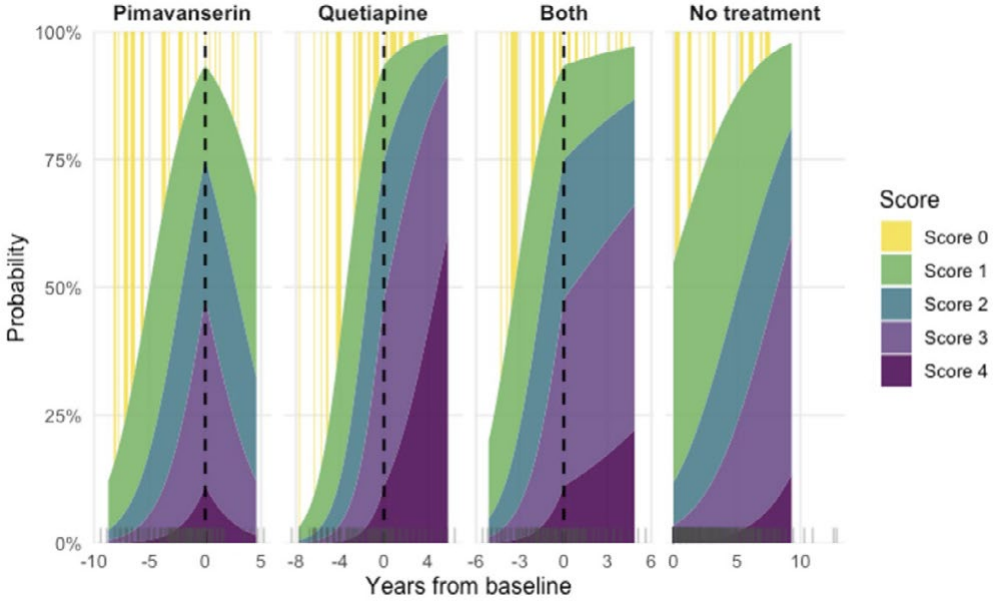
**FIGURE 2** Predicted distribution of Parkinson's disease cognitive symptoms (MDS‐UPDRS 1.1 score) by treatment group over time.

**Conclusion:** Pimavanserin was associated with long‐term attenuation of psychosis and cognitive symptom progression in this real‐world cohort, exceeding effects observed with other treatments.

**Disclosure:** This research was funded by Acadia Pharmaceuticals. The authors report no other disclosures.

## EPO‐0429

### Effects of proprioceptive–visual and vestibular–visual integration training on balance and gait in Parkinson's disease: A randomized controlled trial

#### Q. Zhao; X. Luo; L. Jin

##### Department of Movement Disorders, Neurorehabilitation Center, Shanghai Yangzhi Rehabilitation Hospital (Shanghai Sunshine Rehabilitation Center), Tongji University School of Medicine, Shangai, China

**Background and aims:** Postural instability and impaired sensory integration are major contributors to falls and disability in Parkinson's disease (PD), yet the optimal sensory integration training strategy remains unclear.

**Methods:** I84 individuals with PD were allocated randomly to the control group, proprioceptive–visual integration group A and vestibular–visual integration group B. All participants received 4 weeks of conventional rehabilitation, while group A and B additionally performed 30‐minute sensory integration training with center‐of‐mass visual feedback, with or without an unstable surface. The primary outcome was the Sensory Organization Test (SOT) composite score. Secondary outcomes included clinical balance, motor symptoms, sensory integration parameters, gait measures, and quality of life.

**Figure:**
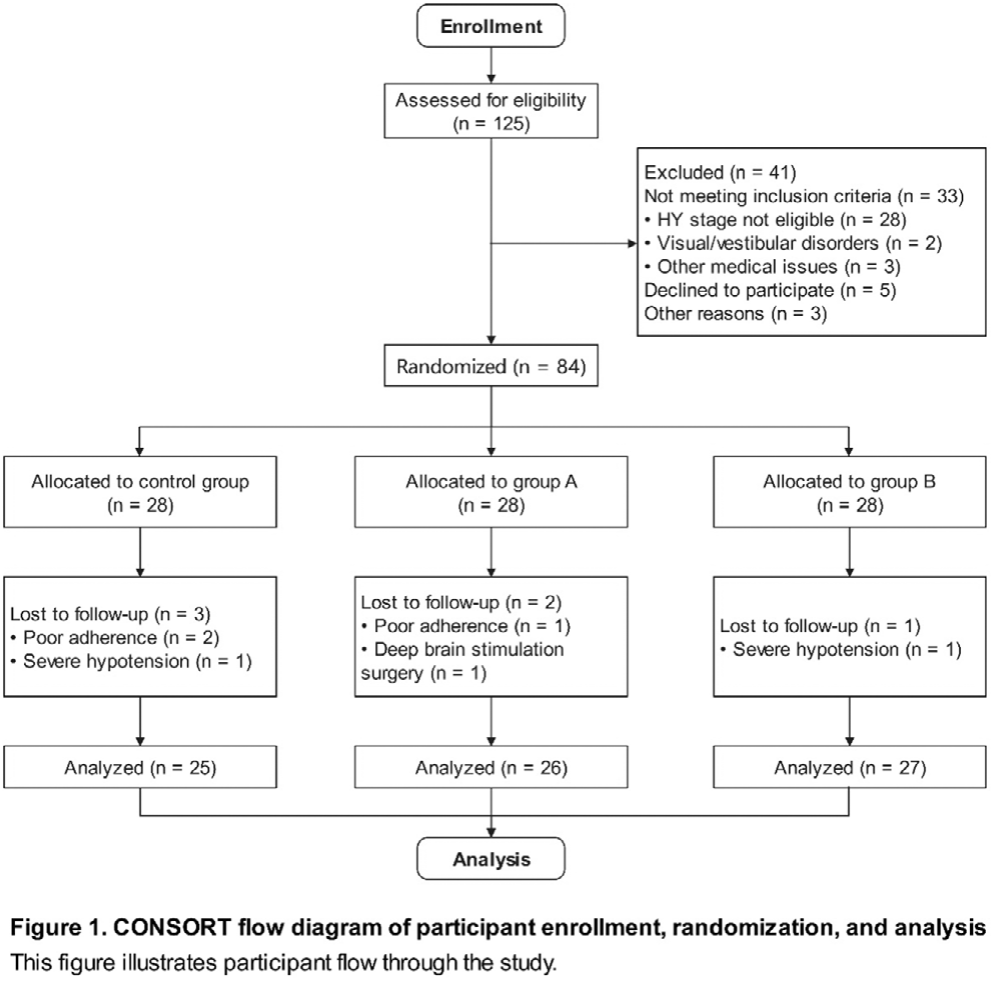
**FIGURE 1** CONSORT flow diagram.

**Results:** Vestibular–visual integration training combined with conventional rehabilitation can better improve balance control, motor symptoms and gait symmetry in patients with PD.

**Figure:**
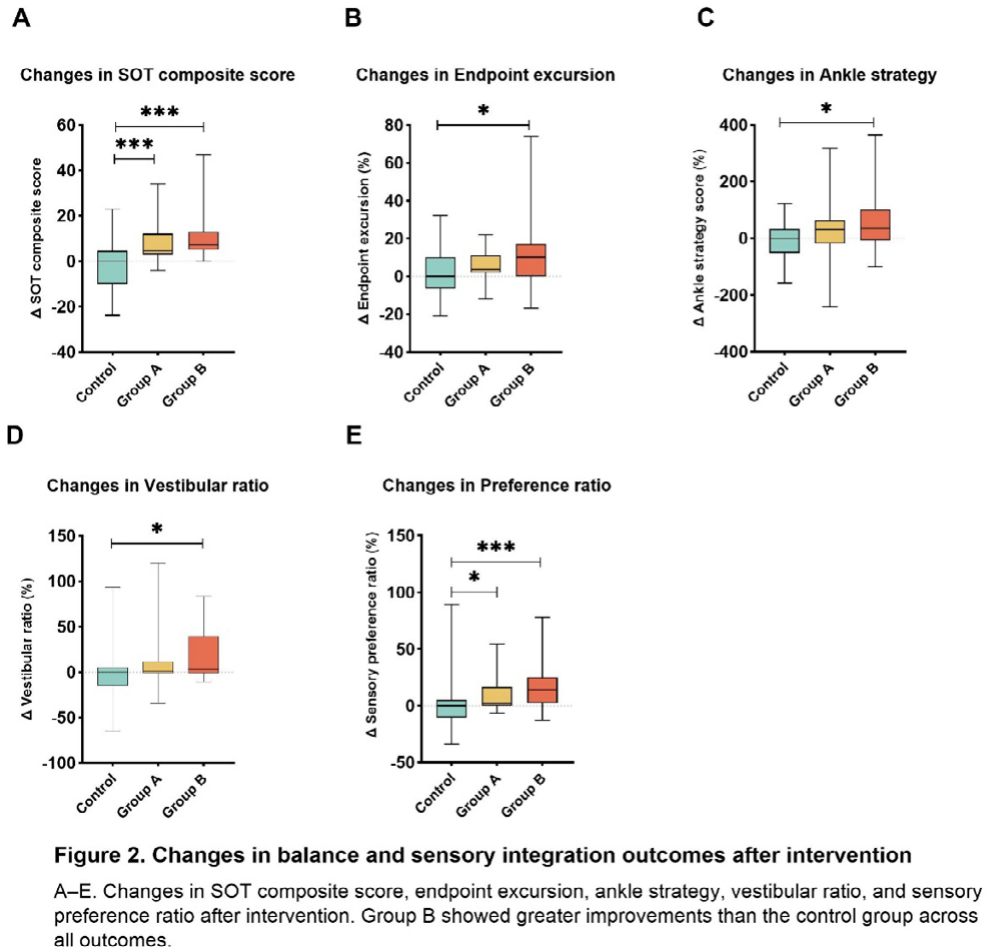
**FIGURE 2** Results.

**Conclusion:** Vestibular–visual integration training combined with conventional rehabilitation can better improve balance control, motor symptoms, and gait symmetry in patients with PD. This approach may be particularly beneficial for patients with prominent postural instability and elevated fall risk.

**Disclosure:** Nothing to disclose

## EPO‐0430

### Echinacoside Attenuates Pathogenic α‐Synuclein Aggregation by Inhibiting the PERK–eIF2α–ATF3/CHOP Pathway in the PFFs Models of Parkinson's Disease

#### Q. Zhao; Z. Wang; L. Jin

##### Department of Movement Disorders, Neurorehabilitation Center, Shanghai Yangzhi Rehabilitation Hospital (Shanghai Sunshine Rehabilitation Center), Tongji University School of Medicine

**Background and aims:** Abnormal aggregation of α‐synuclein (α‐Syn) is a core pathological hallmark of Parkinson's disease (PD), and the α‐Syn pre‐formed fibrils (PFFs) model closely mimics this process. Our previous work showed that echinacoside (ECH) protects against MPTP‐induced injury by suppressing endoplasmic reticulum (ER) stress. Here, we asked whether ECH ameliorates α‐Syn mediated dopaminergic damage in PFFs‐based models and whether this effect involves ER‐stress related ATF3 signaling.

**Methods:** In vivo, mice received unilateral striatal PFFs injections followed by intragastric ECH (40 or 20 mg/kg) for 1 month. Motor function was assessed by standard behavioral tests, and substantia nigra pathology was evaluated by tyrosine hydroxylase (TH) immunofluorescence and Western blot of phosphorylated α‐syn (p‐α‐syn), GRP78 and PERK–eIF2α–ATF3–CHOP proteins. In vitro, PFFs‐treated SH‐SY5Y cells were exposed to the ER‐stress inducer thapsigargin (Tg) or to ECH. Cell viability, TH, p‐α‐syn and the same ER‐stress markers were quantified.

**Results:** PFFs induced motor deficits, TH loss, p‐α‐syn accumulation and up‐regulation of GRP78 and PERK–eIF2α–ATF3–CHOP in mouse substantia nigra. ECH improved motor behavior, increased TH expression and significantly decreased p‐α‐syn and ER‐stress marker levels (*p* < 0.05; Figure 1). In SH‐SY5Y cells, Tg further enhanced PFFs‐induced GRP78, p‐PERK, p‐eIF2α, ATF3, CHOP and p‐α‐syn, whereas ECH reduced p‐α‐syn and down‐regulated these ER‐stress proteins (*p* < 0.05; Figure 2).

**Conclusion:** ECH protects nigrostriatal dopaminergic neurons and reduces pathological α‐Syn aggregation in PFFs‐induced cellular and mouse models of PD, an effect closely associated with inhibition of PERK–eIF2α–ATF3–CHOP‐mediated ER stress.

**Disclosure:** Nothing to disclose.

## EPO‐0431

### Significant elevation of serum pro‐inflammatory cytokines in GBA1‐associated Parkinson's disease: Implications for immune‐mediated pathogenesis

#### V. Busco ^1^; G. Di Lazzaro^2^; A. Cimmino^1^; J. Gambelli^1^; M. Petracca^2^; D. Genovese^2^; A. De Biase^2^; F. Musso^1^; P. Calabresi^2^; A. Bentivoglio^2^

##### ^*1*^ *Università Cattolica del Sacro Cuore, Rome, Italy;* ^*2*^ *Neurology Unit, Fondazione Policlinico Universitario Agostino Gemelli IRCCS, Rome, Italy*

**Background and aims:** Parkinson's disease (PD) associated with GBA1 mutations (GBA‐PD) is increasingly recognized as a clinically and biologically heterogeneous subtype[1]. This study aimed to investigate differences in serum inflammatory and neurodegeneration markers between Parkinson's disease (PD) patients carrying GBA1 variants (mPD) and patients with idiopathic sporadic PD (sPD), focusing on the potential role of interferon‐gamma (IFN‐γ), a key Th1 cytokine[2].

**Methods:** We enrolled 12 patients with GBA1‐associated PD and 12 with sporadic PD, matched for age, sex, and disease duration. Clinical data and serum samples were collected. PD diagnosis followed Movement Disorder Society criteria. Serum cytokines (IL‐1β, IL‐17A, IFN‐γ, IL‐4, TNF‐α, IL‐10, IL‐6) were measured by ELISA. Neurodegeneration markers included neurofilament light chain (NfL) and brain‐derived neurotrophic factor (BDNF). Group differences were analyzed with Mann–Whitney U test (*p* < 0.05).

**Figure:**
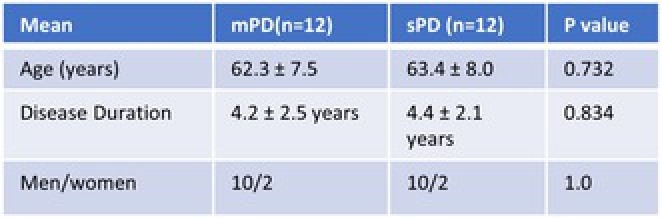
**TABLE 1** Demographics and clinical characteristics of mPD and sPD patients. Continuous variables are mean ± SD, categorical as counts. Groups were matched; no statistically significant differences were observed.

**Results:** IFN‐γ levels were significantly elevated in GBA‐PD compared to sPD (*p* = 0.0078), along with IL‐1β (*p* < 0.05), while other cytokines, NfL, and BDNF showed no differences. Comorbidities in GBA‐PD included depression (66%) and melanoma (25%). The selective IFN‐γ elevation suggests a Th1‐skewed systemic immune activation in GBA1 mutation carriers.

**Conclusion:** GBA‐PD patients exhibit a distinct peripheral immune‐inflammatory profile characterized by Th1‐associated IFN‐γ and IL‐1β elevation, supporting the concept of a biologically unique PD subgroup. This Th1‐skewed signature may contribute to both neurodegeneration and comorbid autoimmune or psychiatric manifestations[3]. IFN‐γ represents a potential biomarker and therapeutic target, and modulation of Th1 pathways could offer novel strategies to personalize treatment in this PD subgroup. Validation in larger cohorts is warranted.

**Disclosure:** Nothing to disclose

Neurorehabilitation 1

## EPO‐0432

### Temporal precision requirements for neuroplasticity‐based neurorehabilitation: A meta‐regression analysis

#### A. Oza

##### Science Department, Northern Highlands Regional High School, Allendale, USA

**Background and aims:** Brain–computer interface (BCI) systems combined with functional electrical stimulation (FES) demonstrate efficacy in research settings (pooled effect size d = 0.72), yet clinical adoption remains limited. This translation gap suggests that available systems fail to meet neurophysiological requirements, particularly temporal precision within the neuroplasticity window required for Hebbian learning.

**Methods:** Following PRISMA guidelines, a systematic review examined randomized controlled trials evaluating timing‐sensitive neurorehabilitation interventions for post‐stroke upper limb recovery. Interventions were classified as research prototypes or commercial systems. The primary outcome was change in the Fugl–Meyer Upper Extremity score. Meta‐regression assessed associations between neural‐to‐stimulation latency and efficacy while adjusting for intervention duration, stroke chronicity, and study quality. Segmented regression identified latency thresholds, and mediation analysis evaluated whether latency explained performance differences.

**Results:** Across 28 studies including 1,247 participants, a critical latency threshold of 300 ms (95% CI: 250–350 ms) was identified. Systems operating below this threshold achieved larger effects (d = 0.89) than those exceeding it (d = 0.38; *p* < 0.001). Research prototypes demonstrated lower latencies (median 220 ms) and greater efficacy (d = 0.85) than commercial systems (median 1,150 ms; d = 0.34). Mediation analysis indicated that 73% of the efficacy gap was attributable to latency, with each additional 100 ms associated with a 0.042 reduction in effect size (*p* < 0.001).

**Figure:**
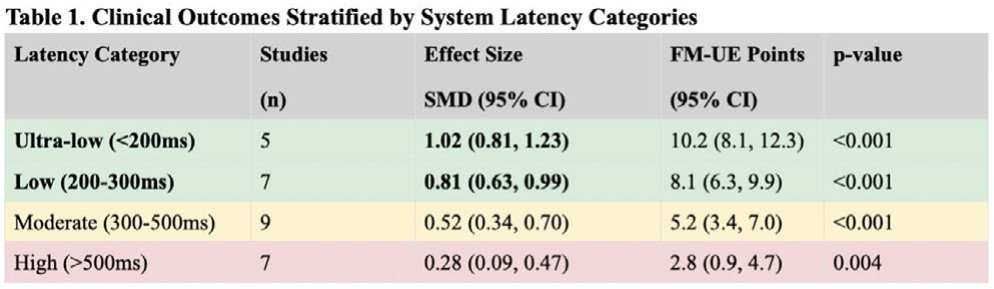
**TABLE 1** Systems with latency below 300ms achieve 3.6x better outcomes (d = 1.02 vs 0.28) than high‐latency systems, demonstrating temporal precision as the primary driver of neuroplasticity‐based recovery in stroke rehabilitation.

**Figure:**
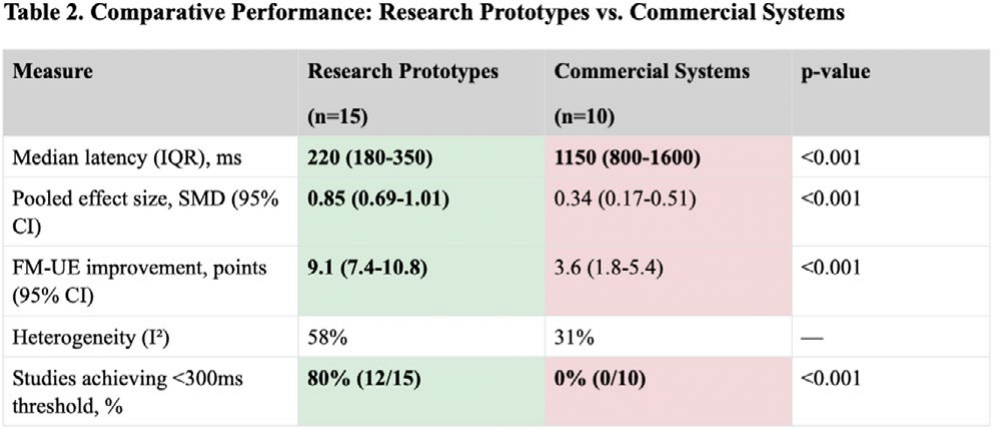
**TABLE 2** Commercial systems operate 5x slower than research prototypes (1150ms vs 220ms) which explains why marketed BCI devices fail clinically despite promising research trials.

**Figure:**
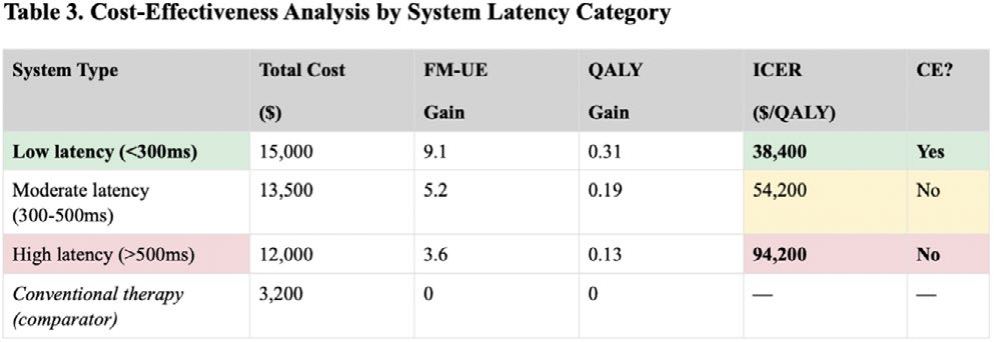
**TABLE 3** Low‐latency systems are cost‐effective ($38K/QALY) despite higher costs, while cheaper high‐latency systems exceed thresholds ($94K/QALY) due to poor outcomes.

**Conclusion:** Clinical translation failure is therefore attributable to latency‐mediated loss of efficacy operating beyond the identified 300 ms threshold. Enforcing engineering standards based on segmented and mediation analyses, including sub‐300 ms latency targets linked to superior cost‐effectiveness, is essential for improving therapeutic and economic outcomes.

**Disclosure:** Nothing to disclose.

## EPO‐0433

### Evaluation of reticulospinal plasticity using a novel wearable (PowerBead) for post‐stroke finger extension recovery

#### A. Das ^1^; Y. Wang^1^; L. Salisbury^1^; N. Ward^2^; S. Baker^3^

##### ^*1*^ *KnitRegen, London, UK;* ^*2*^ *Clinical Neuroscience Centre, Institue of Neurology, UCLH, Queens Square, London, UK;* ^*3*^ *Henry Welcome Building, Faculty of Medical Sciences, Newcastle University, Newcastle upon Tyne, UK*

**Background and aims:** Loss of finger extension excludes ~50% stroke survivors from rehabilitation. Strengthening the reticulospinal tract (RST) may aid recovery when the corticospinal tract (CST) is damaged. The RST, activated via muscle spindle afferents (MSA), can be stimulated by mechanical ‘taps’. Pairing these with timed auditory ‘clicks’ may induce neuroplastic changes through convergent input [Salisbury, Wang & Baker, n.d.].The objective of this study is to evaluate the feasibility and physiological efficacy of a wearable tap–click neuromodulation approach in inducing reticulospinal plasticity relevant to post‐stroke finger extension.

**Methods:** The PowerBead is a wearable delivering a ‘tap’ to the affected forearm and an earpiece providing a synchronised contralateral ‘click’. Co‐designed with clinicians and therapists, it provides 4‐hour treatment cycles to 20 healthy participants and 20 stroke survivors, guided by PPI feedback. Efficacy is assessed pre‐ and post‐cycle using TMS‐evoked EMG at the extensor digitorum, the Modified Ashworth Scale, grip/pinch strength, and Box & Block tests.

**Figure:**
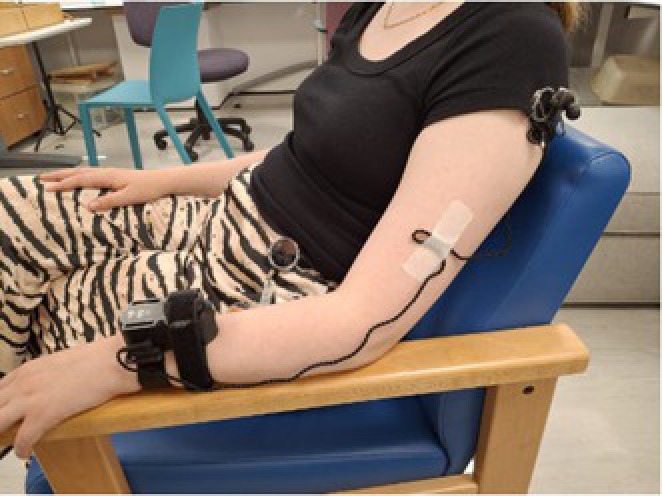
**FIGURE 1** Depicts the device 'The PowerBead'.

**Results:** Previous studies confirmed PowerBead induced MSA activation [Salisbury, Wang & Baker, n.d]. Building on work by Baker et al (2021), which showed MSA–auditory pairing supports recovery, our device integrates this ‘tap–click’ loop to promote passive finger extension. Early data demonstrated RST plasticity after 4 hours of treatment, with increased cortical excitability and suppressed brainstem activity.

**Figure:**
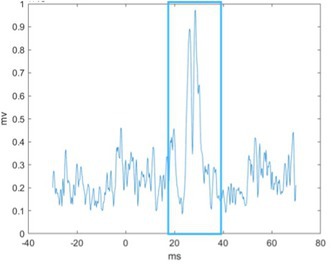
**FIGURE 2** The blue box in the graph denotes a sample MSA response generated by the PowerBead.

**Conclusion:** Early PowerBead findings indicate promising engagement of the RST through the ‘tap‐click’ loop, highlighting its novelty in translating lab‐based neuroplasticity into accessible home‐based therapy. Data collection is ongoing, with plans for clinical trials and public availability.

**Disclosure:** This study is funded by NIHR [National Institue for Health and Care Research] i4i award and led by Dr Laura Salisbury [CEO of KnitRegen] in collaboration with UCL [University College London].

## EPO‐0434

### Evolving use of botulinum toxin in neurological practice: A national audit from malta (2013–2025)

#### A. Ferriggi; J. Abela; M. Mallia

##### Department of Neurosciences ‐ Mater Dei Hospital, Msida, Malta

**Background and aims:** Botulinum toxin (BoNT) is an established treatment for neurological conditions associated with spasticity and dystonia. Since the establishment of a dedicated national BoNT service in Malta, clinical demand and service complexity have increased. This audit aimed to evaluate patient characteristics, indications, treatment patterns, clinical outcomes, and documentation practices in order to identify areas for service development and standardization.

**Methods:** A retrospective audit was conducted of electronic and paper records for patients attending the Neurology and Spasticity Clinic between 2013 and 2025. Data collected included demographics, neurological diagnosis, treatment status, BoNT dosing patterns, follow‐up intervals, documented symptomatic and functional outcomes, adjunct pharmacological therapy, and recording of multidisciplinary team (MDT) involvement and consent. Descriptive analyses were performed.

**Results:** A total of 187 patients were identified, of whom 146 received BoNT therapy and 134 remained in active follow‐up. Mean age was 56 years, with balanced sex distribution. Indications included post‐ischemic stroke spasticity, cerebral palsy, dystonia, and traumatic brain injury. Most patients were reviewed at clinically appropriate intervals, typically every 4–6 months. Mean BoNT dose per session was 79.4 units, with higher doses used for spasticity. Symptomatic and functional improvements were commonly documented.

**Figure:**
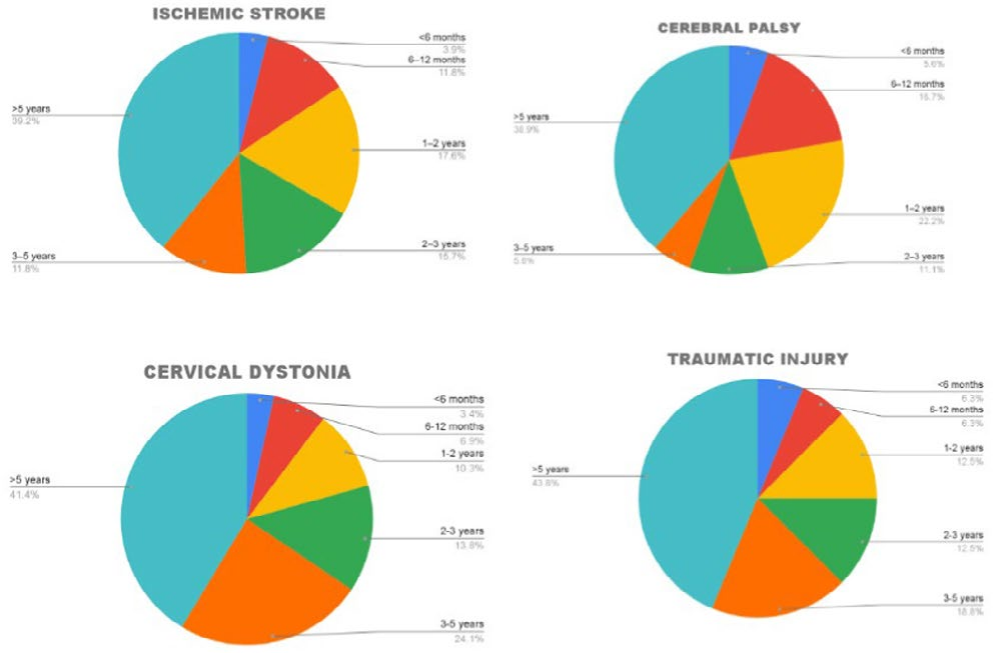
**FIGURE 1** Results ‐ Use of botulinum therapy in different neurological conditions.

**Figure:**
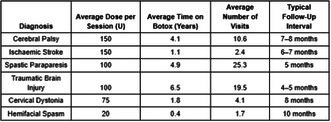
**FIGURE 2** Results ‐ Average does of botulinum therapy used in different neurological conditions with follow up periods.

**Figure:**
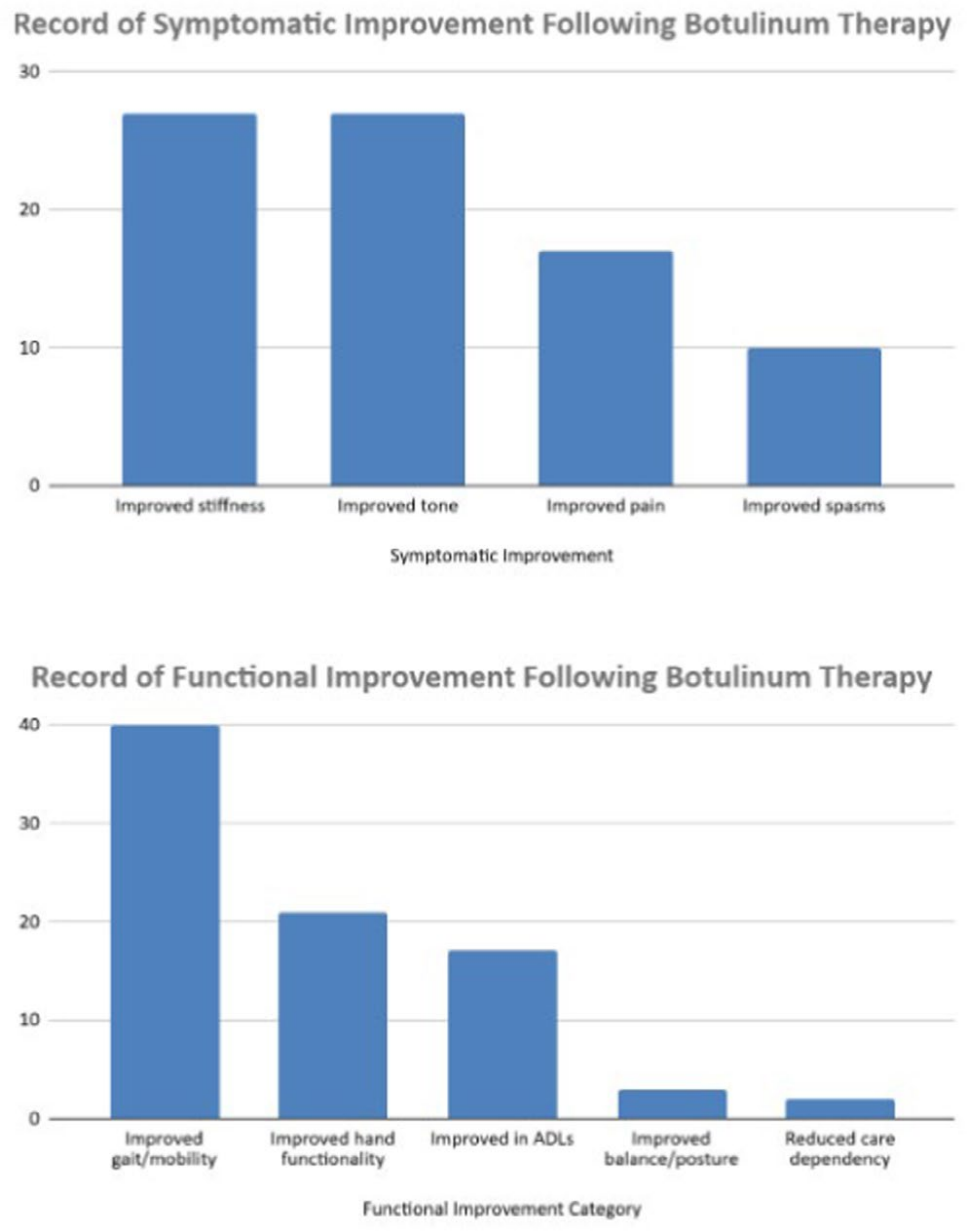
**FIGURE 3** Results – Symptomatic and functional improvement following botulinum therapy.

**Conclusion:** This national audit demonstrates effective real‐world use of BoNT across a heterogeneous neurological population. Variability in documentation of MDT input, dose titration, and follow‐up highlights opportunities for standardization. Findings will inform the development of structured documentation tools and a local protocol, with re‐audit planned to assess service improvement.

**Disclosure:** Nothing to disclose.

## EPO‐0435

### Long‐term follow‐up of patients with disorders of consciousness: Prognostic value of early CRS‐R Improvement

#### B. Hakiki ^1^; F. Draghi^2^; A. De Nisco^2^; A. Romoli^2^; D. Maccanti^2^; C. Macchi^2^

##### ^*1*^ *Dipartimento di Medicina Sperimentale e Clinica, Università di Firenze, Firenze, Italy;* ^*2*^ *IRCCS Fondazione Don Carlo Gnocchi ETS, Firenze, Italy*

**Background and aims:** The Coma Recovery Scale–Revised (CRS‐R) is the gold standard for assessing consciousness in prolunged Disorders of Consciousness (pDoC). We previously^1^˒^2^ showed that baseline CRS‐R and its 4‐week increase iCRS‐R predicted consciousness improvement at discharge from rehabilitazion setting. This longitudinal follow‐up evaluates the predictive value of baseline CRS‐R and its early change for long‐term survival and functional autonomy.

**Methods:** Using a cross‐sectional approach, all patients enrolled in the previous study^2^ were contacted. A structured telephone interview, including the Glasgow Outcome Scale–Extended (GOSE), was administered. Mortality rate, time to death were also recorded.

**Results:** Of 110 patients,96 (87.3%) completed the interview and 69 patients (71.8%) died [median time from event 15 months (IQR: 52.5; 3–124)]. Lower iCRS‐R (OR: 0.027; *p* < 0.001), non‐traumatic aetiology (OR: 0.125; *p* = 0.003), and older age (OR: 1.049; *p* = 0.020) were significantly associated with death in the multivariate analysis. Younger age (HR: 1.038; *p* = 0.001) and greater iCRS‐R (HR: 0.0284; *p* = 0.007) were associated with longer survival in the Cox‐regression analysis

**Conclusion:** The early improvement in CRS‐R during intensive rehabilitation represents a key prognostic marker of long‐term survival. Longitudinal CRS‐R assessment may help identify patients at higher risk of mortality and support clinical decision‐making. References: 1. Portaccio et al. doi:10.1080/02699052.2018.1440420 2. Portaccio et al. doi:10.1016/j.apmr.2018.01.015

**Disclosure:** Nothing to disclose.

## EPO‐0436

### Single‐Session Artificial Intelligence‐Assisted Gait Training Improves Spatiotemporal Parameters in Dementia and Parkinson's Disease

#### C. Hong

##### Taipei Medical University‐Shuang Ho Hospital, Taiwan

**Background and aims:** Gait impairment in dementia and Parkinson's disease (PD) drives falls and loss of independence, yet therapist‐intensive rehabilitation limits scalability. Artificial intelligence (AI)–assisted gait training may standardize delivery, reduce manpower, and provide real‐time feedback. The present study aims to test whether a single session of AI‐assisted gait training improves spatiotemporal gait parameters in dementia and PD.

**Methods:** Adults aged 60–85 years with dementia or PD who could ambulate completed one training session (~23.2 ± 9.6 min) comprising standardized walking tasks with real‐time feedback, delivered under “Support” or “Escort” mode walking with the AI‐assisted gait training device as needed. Primary outcomes were pre–post changes in gait speed (m/s) and the secondary outcomes included step length (cm), step count, and gait variability obtained by the device. Two‐sided paired t tests were performed within four strata: Dementia–Support (*n* = 67), Dementia–Escort (*n* = 43), PD–Support (*n* = 64), and PD–Escort (*n* = 43).

**Results:** Gait speed increased within session across all strata. Step length increased and step count decreased in Support walking for both diagnoses, whereas Escort walking showed non‐significant trends for these spatial parameters. Gait variability showed no significant changes in any stratum. Cognitive status did not affect the changing of gait parameter, supporting feasibility for targeting elders.

**Figure:**
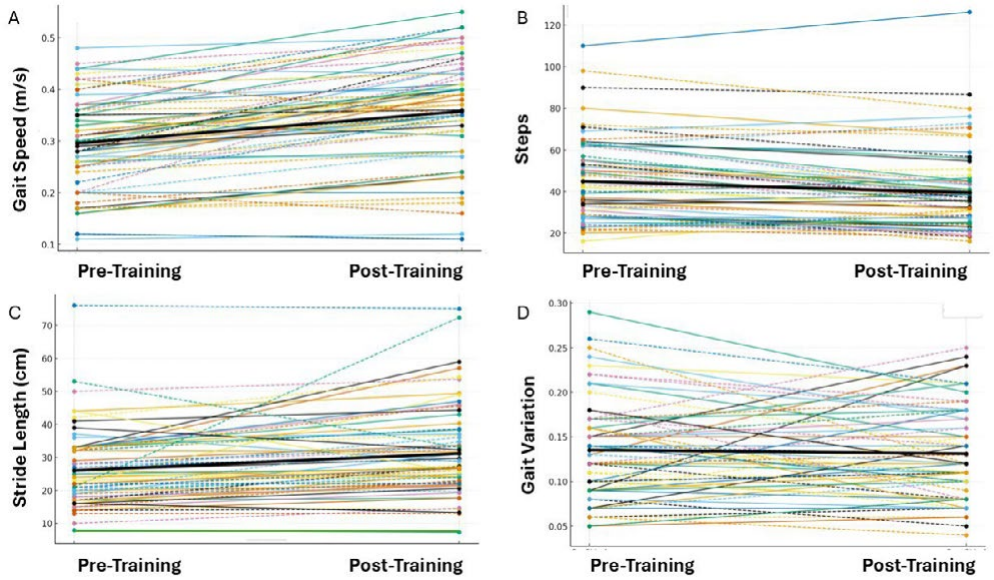
**FIGURE 1** Individual changes in gait parameters following the training intervention in dementia subjects.

**Figure:**
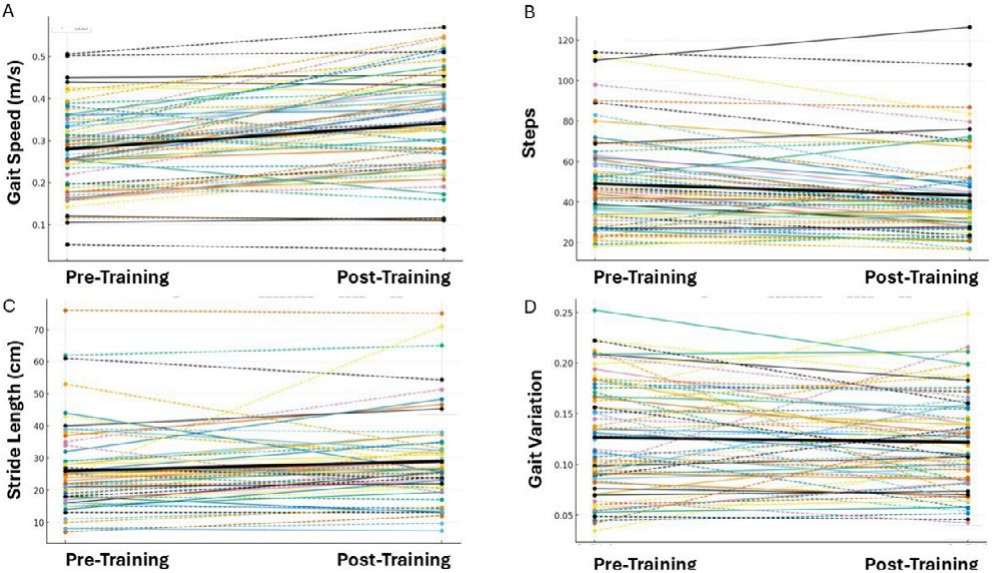
**FIGURE 2** Individual changes in gait parameters following the training intervention in Parkinson's disease subjects.

**Conclusion:** A single AI‐assisted gait training produced short‐term functional gains in spatiotemporal gait parameters in dementia and PD, demonstrating a therapist‐sparing, scalable training model. Longer‐term trials should evaluate durability, dose–response, and transfer to everyday gait and falls, and refine automation for home or remote deployment.

**Disclosure:** Nothing to disclose.

## EPO‐0437

### Enhancing post‐stroke extubation swallowing recovery with pharyngeal electrical stimulation: A meta‐analysis of randomized controlled trials

#### K. Sarhan; R. Mohamed

##### Faculty of Medicine, Mansoura University, Mansoura, Egypt

**Background and aims:** Post‐stroke dysphagia is associated with aspiration, prolonged hospitalization, and poor recovery. Pharyngeal electrical stimulation (PES) is an emerging neurostimulation technique designed to enhance swallowing function through modulation of pharyngeal sensory pathways. This study aimed to systematically review and meta‐analyze the effects of PES on swallowing outcomes and safety in patients with acute stroke.

**Methods:** We performed a systematic review and meta‐analysis of randomized controlled trials evaluating PES in patients with post‐stroke dysphagia. PubMed, Scopus, and Web of Science were searched from inception to December 2025. Outcomes included dysphagia severity, swallowing function, functional oral intake, feeding tube withdrawal, length of stay, and safety.

**Results:** Nine randomized controlled trials involving 448 patients were included. PES significantly improved overall swallowing function compared with control (SMD = −0.30, 95% CI: −0.50 to −0.10), with dysphagia severity scores also showing greater improvement (SMD = −0.35, 95% CI: −0.55 to −0.15). Patients receiving PES had higher rates of nasogastric tube withdrawal (RR = 2.5, 95% CI: 1.8–3.5) and improved functional oral intake. The analysis demonstrated a significant reduction in hospital length of stay, driven largely by post‐extubation stroke populations (SMD = −0.5, 95% CI: −0.80 to −0.20). PES was well tolerated, with no increase in serious adverse events.

**Conclusion:** PES improves swallowing function and reduces dysphagia severity in patients with acute stroke. It increases the likelihood of nasogastric tube removal, improves oral intake, and shortens hospital stay, while remaining safe and well tolerated, supporting its use as an effective adjunct in stroke rehabilitation.

**Disclosure:** Nothing to disclose.

## EPO‐0438

### Abstract withdrawn

## EPO‐0439

### Upper‐extremity functional outcomes following bilateral rTMS after stroke: Systematic review and meta‐analysis

#### M. Kodounis ^1^; D. Onan^2^; R. Ornello^3^; S. Sacco^3^; D. Mitsikostas^1^; E. Dardiotis^4^; V. Siokas^4^

##### ^*1*^ *First Neurology Department, Eginitio Hospital, School of Medicine, National & Kapodistrian University of Athens, Athens, Greece;* ^*2*^ *Department of Physiotherapy and Rehabilitation, Faculty of Health Sciences, Yozgat Bozok University, Yozgat Türkiye;* ^*3*^ *Department of Biotechnological and Applied Clinical Sciences – University of L’Aquila, L’Aquila, Italy;* ^*4*^ *Department of Neurology, University Hospital of Larissa, School of Medicine, University of Thessaly, Larissa, Greece*

**Background and aims:** The literature largely examines unilateral high‐frequency stimulation of the lesioned hemisphere or low‐frequency inhibition of intact hemisphere using repetitive Transcranial Magnetic Stimulation (rTMS) on stroke patients. In contrast, bilateral protocols combining facilitative and inhibitory effects remain underexplored. This review evaluates the effects of bilateral rTMS on upper extremity function.

**Methods:** A systematic search was conducted in PubMed, Web of Science, and Scopus up to November 22, 2025 (Figure‐1). Randomized controlled trials (RCTs) were included if stroke patients were assessed with Fugl‐Meyer Assessment (FMA) and received bilateral rTMS. Bilateral rTMS and sham groups were compared. The meta‐analysis was conducted in RStudio (PROSPERO: CRD420251230168).

**Figure:**
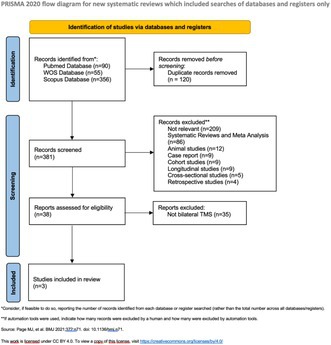
**FIGURE 1** Flow Diagram.

**Results:** Three RCTs were included in the study and demonstrated low risk and good methodological quality (Figure‐2a,2b). The bilateral rTMS intervention demonstrated a significant advantage over sham in improving upper‐extremity functional outcomes. Based on the common‐effect model, the post‐treatment effect was substantial (SMD = 1.54, 95% CI:1.10–1.99), while the random‐effects model yielded a wider and more uncertain estimate (SMD = 3.30, 95% CI:–1.06–7.66) due to heterogeneity (I^2^ = 96.6%, *p* < 0.0001). At the 3‐month follow‐up, the improvement was sustained and even greater under the common‐effect model (SMD = 2.09, 95% CI: 1.60–2.58), whereas the random‐effects model again showed uncertainty (SMD = 4.23, 95% CI:–0.67–9.13) due to heterogeneity (I^2^ = 96.8%, *p* < 0.0001). Although the overall direction of effect consistently favors active rTMS, the heterogeneity warrants cautious interpretation of the pooled estimates (Figures‐3a,3b).

**Figure:**
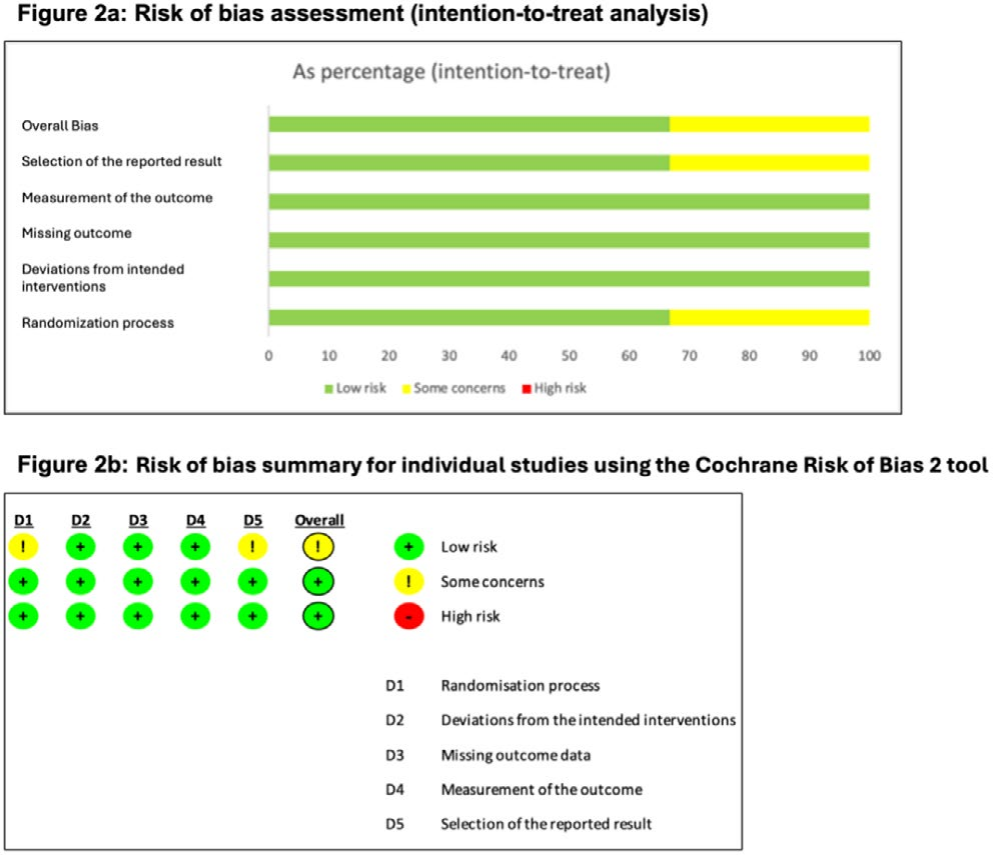
**FIGURE 2**a, 2b. RoB2 Assessment.

**Figure:**
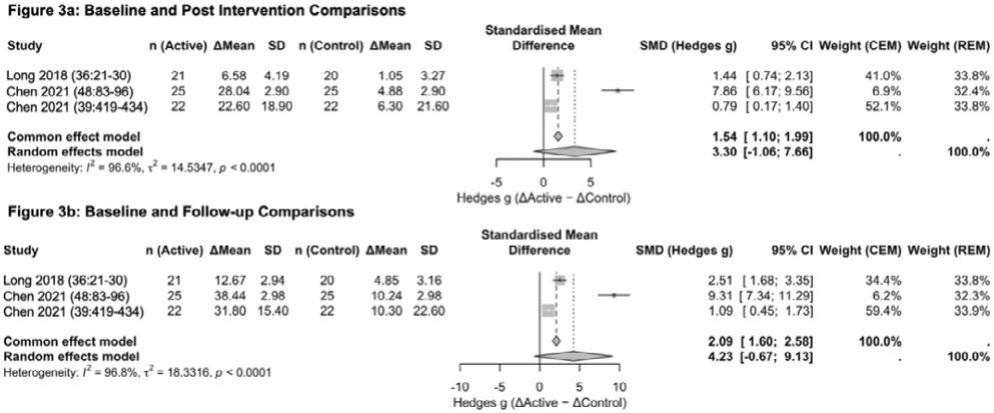
**FIGURE 3**a, 3b. Baseline and Post Intervention Comparisons.

**Conclusion:** Bilateral rTMS may provide benefits for upper‐extremity functional recovery after stroke, although the limited number of included studies contributes to substantial heterogeneity. These findings indicate the need for more well‐designed RCTs.

**Disclosure:** Nothing to disclose.

## EPO‐0440

### Observational gait analysis tools for neurological populations: A COSMIN meta‐analysis

#### M. Etoom

##### Department of Rehabilitation Sciences, Faculty of Applied Medical Sciences, Jordan University of Science and Technology, Jordan

**Background and aims:** Observational gait analysis (OGA) tools are widely used due to their simplicity, low cost, and feasibility. However, the psychometric properties of OGA tools remain unclear. The aim was to conduct a systematic review and meta‐analysis investigating psychometric properties of OGA tools among neurological populations.

**Methods:** COnsensus‐based Standards for the selection of health Measurement Instruments (COSMIN) guidelines were followed. COSMIN psychometric properties involve Validity (Content, Structural, Criterion, cross‐cultural and Construct), Reliability (Internal Consistency, reliability and Measurement Error), and Responsiveness. The measurement Property for the will be rated as sufficient (+), insufficient (‐), or indeterminate (?) using COSMIN criteria for good measurement properties. The recommendations for scales were mainly based on content validity and internal consistency.

**Results:** There were 26 included articles involving 8 OGA tools; Wisconsin Gait Scale (WGS), Gait Assessment and Intervention tool, Tinetti Gait Scale, Rivermead Visual Gait Assessment, Gait Abnormality Rating Scale, Malta Gait scale, Hemiplegic Gait Analysis Form, and New York Medical School Orthotic Gait Analysis. Included articles assessed through 64 studies all psychometric proprieties except structural validity. The 64 results were synthesized into 48 results in stroke, multiple sclerosis and Parkinson's disease. Thirty‐one results rated as (+), 10 (‐), and 8 (?). No OGA tool is currently recommended for use. All included OGA have potential for recommendation. WGS has the best potential.

**Conclusion:** No OGA tool is currently recommended due to absence of evidence for unidimensionality. Structural validity studies are highly required and should be prioritized to confirm unidimensionality and level of recommendations for OGA tools.

**Disclosure:** Nothing to disclose.

## EPO‐0441

### Putting family before patient: Physicians' reasons for prolonging life‐sustaining treatment in chronic disorders of consciousness

#### W. van Erp^1^; C. Span^1^; J. van Gurp^2^; H. van Delden^3^; J. Lavrijsen^1^; N. Kok ^2^

##### ^*1*^ *Primary Care, Radboudumc, Nijmegen, the Netherlands;* ^*2*^ *IQ Health, Radboudumc, Nijmegen, the Netherlands**;*** ^*3*^ *Julius Center, UMC Utrecht, the Netherlands*

**Background and aims:** The Netherlands report the lowest prevalence of prolonged disorders of consciousness (PDOC), often related to an ethical–legal context allowing the discontinuation of life‐sustaining treatment (LST) when recovery is unlikely or presumed patient consent is absent. Despite this, some PDOC patients receive LST for decades. This study explores physicians' reasons for doing this.

**Methods:** Semi‐structured interviews were conducted with physicians responsible for patients diagnosed with unresponsive wakefulness syndrome, based on repeated Coma Recovery Scale–Revised assessments. Interviews, conducted at a mean of 14 years post‐injury, began with the question: "Which factors influenced your treatment decision?" Transcripts were analysed using qualitative thematic analysis supported by ATLAS.ti software.

**Results:** Ten elderly care physicians in nursing homes, and responsible for nine patients were interviewed. In all cases, family requests to continue treatment overruled diagnosis, prognosis, and the patient's presumed perspective. Physicians prioritized maintaining family relationships and avoided conflict, without consulting external experts. Advance care planning was often delayed, even though physicians rated the patients' quality of life as very poor (mean 1.6/10). None of the physicians would want similar treatment for themselves. When asked about their preferred treatment for the patient if they had no family, all stated they would discontinue life‐sustaining treatment.

**Conclusion:** Even in the unique Dutch medico‐ethical context, life‐sustaining treatment in PDOC can continue for decades. Family preferences largely dictate these decisions, while patient interests are often overlooked. This highlights an ethical tension in PDOC care, emphasizing the need for clear role definitions, advance care planning, and better support for physicians facing moral distress.

**Disclosure:** Nothing to disclose.

## EPO‐0442

### Introducing Disorders of Consciousness Program Accreditation Standards

#### P. Lucesoli de Valyi; T. Carolan

##### *CARF International,* Tucson, USA

**Background and aims:** Despite evidence and published guidelines demonstrating the need for specialized DoC rehabilitation, barriers to accessing these skilled programs remain. Recent evidence has provided guidance on the care for those with DoC, minimal competency recommendations for interdisciplinary teams providing care for persons with DoC, timing of withdrawal of life saving treatment, recovery and functional outcomes, and presence of consciousness in those with covert awareness. CARF International has worked with the field to create standards for transdisciplinary DoC rehabilitation programs which will focus on quality and performance improvement, advocate for individuals with DoC their family members and demonstrate the value of rehabilitation to all stakeholders including external payers and referrers.

**Methods:** referrers. CARF International conducted interviews with individuals with lived experience, family members, researchers, providers and payers to determine the need to create DoC Program standards. An International Standards Advisory Committee used a consensus‐driven process to create draft DoC program standards, which were shared in a public field review where 133 individuals and organizations provided feedback which was then incorporated into the final DoC progam standards.

**Results:** In July, 2026, these 24 DoC program standards will be used to provide the first DoC program accreditation, using peer‐surveyors and an on‐site survey process.

**Conclusion:** DoC Program accreditation standards will be used to establish a minimum standard for DoC programs which will allow easier identification of DoC programs for family members seeking DoC rehabilitation and create an enduring process to assess the quality of DoC rehabilitation programs.

**Disclosure:** I am a consultant with CARF International.

## EPO‐0443

### Overground gait training with body‐weight support in individuals with neurological disorders: A scoping review

#### A. Luís; R. Brandão; I. Baleia

##### *Alcoitão School of Health Sciences,* Alcabideche, Portugal

**Background and aims:** Neurological disorders are a major cause of disability and are frequently associated with gait impairments that reduce functional independence and quality of life. Overground body‐weight supported gait training has emerged as a potential alternative to treadmill‐based approaches, but its clinical application remains insufficiently explored.

**Methods:** A scoping review was conducted following Joanna Briggs Institute methodology and PRISMA‐ScR guidelines. Searches were performed in PubMed, PEDro, CENTRAL, EBSCO, and Web of Science. Studies investigating overground gait training with body‐weight support in neurological populations were included and critically appraised using JBI tools.

**Results:** Seven studies were included, involving individuals with stroke, cerebral palsy, spinal cord injury, and Parkinson's disease. Overground body‐weight supported gait training was associated with improvements in gait independence, walking speed, endurance, gait symmetry, motor function, fall risk, and freezing of gait. Outcomes were comparable or superior to treadmill‐based or conventional gait training.

**Conclusion:** Evidence on overground body‐weight supported gait training in neurological populations is limited and heterogeneous. Although promising gait‐related outcomes have been reported, methodological limitations prevent strong conclusions, highlighting the need for high‐quality randomised controlled trials with standardised protocols.

**Disclosure:** Nothing to disclose

Movement disorders 4

## EPO‐0444

### Abstract withdrawn

## EPO‐0445

### Greater clinical benefit with tiomolibdate choline versus standard‐of‐care in neurologic WD patients in the Phase 3 FoCus Trial

#### A. Poujois ^1^; J. Bronstein^2^; M. Lorincz^3^; P. Hedera^4^; D. Bega^5^; C. Robinson^6^; A. Cittadine^6^; D. Tuffy^6^; P. Dusek^7^; I. Mohr^8^; T. Litwin^9^

##### ^*1*^ *Department of Neurology, Adolphe de Rothschild Foundation Hospital, Paris, France;* ^*2*^ *Department of Neurology, David Geffen School of Medicine at UCLA, Los Angeles, United States;* ^*3*^ *Department of Neurology, University of Michigan Health Systems, Ann Arbor, United States;* ^*4*^ *Department of Neurology, School of Medicine, University of Louisville, Louisville, United States;* ^*5*^ *Department of Neurology, Feinberg School of Medicine, Chicago, United States;* ^*6*^ *Monopar Therapeutics, Wilmette, United States;* ^*7*^ *Department of Neurology and Centre of Clinical Neuroscience, Charles University and General University Hospital, Prague, Czech Republic;* ^*8*^ *Department of Gastroenterology and Hepatology, Heidelberg University Hospital, Heidelberg, Germany;* ^*9*^ *Second Department of Neurology, Institute of Psychiatry and Neurology, Warsaw, Poland*

**Background and aims:** Wilson disease (WD) is a rare genetic disorder where excess copper (Cu) accumulation causes progressive neurologic disability. Tiomolibdate choline (TMC; ALXN1840) is an oral, once‐daily, high affinity Cu‐binding agent. This analysis evaluates outcomes in neurologic WD patients, a population at greater risk of symptomatic worsening on current WD therapy than asymptomatic patients.

**Methods:** In the open‐label Phase 3 FoCus trial (NCT03403205), 207 WD patients were randomized 2:1 to TMC or SoC for 48 weeks (W), with an optional TMC extension phase. Neurologic and clinical outcomes were analysed at 48W (primary) and 96W (extension) for patients with neurologic symptoms at baseline, defined as Unified WD Rating Scale [UWDRS] Part III score exceeding the minimum clinically important difference [MCID] of 4.668.

**Results:** Neurologic WD patients treated with TMC (*n* = 77) demonstrated increasing clinical benefit over time, with statistically significant improvements in UWDRS Part III observed at 48W and continuing through 96W (mean change: −7.2; *p* < 0.001). Benefit of TMC over SoC was evident by 48W, with significant UWDRS Part III improvement in the TMC arm (*p* = 0.002) but not the SoC arm (*p* = 0.235). Additionally, fewer TMC patients worsened by ≥MCID at 48W vs. SoC (9% vs 25%). Disease improvement per Clinical Global Impression‐Improvement (CGI‐I) scale was statistically significantly greater for TMC vs. SoC at 48W (*p* = 0.003).

**Conclusion:** In neurologic WD patients, 48 weeks of TMC treatment led to greater clinical benefit than SoC, with continued improvement through 96 weeks. These findings support the potential for TMC to improve neurologic outcomes in this at‐risk population.

**Disclosure:** ML has received a travel grant from Monopar Therapeutics. CR, AC, and DT are employees of Monopar Therapeutics. AP, JB, DB, PD, IM, and TL have nothing to disclose.

## EPO‐0446

### Abstract withdrawn

## EPO‐0447

### Clinical and kinematic assessment of the “Head snap” sign in patients with essential and dystonic tremor

#### D. Birreci ^1^; L. Angelini^2^; A. Grandolfo^1^; M. De Riggi^1^; S. Aloisio^1^; F. Santachiara^1^; S. Cirinei^1^; M. Bologna^1^

##### ^*1*^ *Department of Human Neurosciences, Sapienza, University of Rome, Rome, Italy;* ^*2*^ *IRCCS Neuromed, Pozzilli, IS, Italy*

**Background and aims:** The head snap sign (HSS) is a brief, involuntary jerk of the head observed during the finger–to–nose maneuver in patients with tremor [1], [2]. Originally described in essential tremor (ET), its prevalence and clinical significance across different tremor syndromes, particularly dystonic tremor (DT), remain unclear. We aimed to assess the prevalence and reliability of HSS in ET and DT and to explore its clinical and kinematic correlates.

**Methods:** Seventy‐five patients (55 ET, 20 DT) underwent standardized video examination and optoelectronic kinematic recordings. Five blinded raters evaluated the presence of HSS and dystonic features. Inter‐rater reliability was assessed using Krippendorff's alpha. Tremor severity and cognition were assessed with standardized scales. Associations with diagnosis and dystonia were tested using Fisher's exact test. Clinical and kinematic variables were then compared between HSS‐positive (HSS+) and HSS‐negative (HSS‐) patients.

**Results:** HSS was identified in 20/75 (20.0% ET, 45.0% DT). HSS was more frequent in DT than in ET (OR = 3.21, *p* = 0.04) and was significantly associated with head dystonia (OR = 3.91, *p* = 0.03). HSS+ patients exhibited lower cognitive performance (*p* = 0.01). Kinematic analysis revealed a more severe tremor phenotype in HSS+ patients, with higher postural, kinetic, and head tremor amplitude (all *p* < 0.02).

**Conclusion:** HSS is more prevalent in dystonic tremor and strongly associated with head dystonia, supporting a dystonia‐related origin. Although also observed in ET, HSS emerges mainly in more advanced and severe tremor phenotypes, representing a potential clinical marker of dystonic involvement within the tremor spectrum.

**Disclosure:** Nothing to disclose.

## EPO‐0448

### Exploring the phenotypic features of a large hereditary ataxia cohort: Insights from a single‐center experience

#### E. Sahin ^1^; B. Samanci^1^; B. Atasu^2^; E. Lohmann^2^; Z. Tufekcioglu^3^; E. Erzurumluoglu Gokalp^4^; S. Artan^4^; B. Bilgic^1^; H. Hanagasi^1^

##### ^*1*^ *Behavioral Neurology and Movement Disorders Unit, Department of Neurology, Istanbul Faculty of Medicine, Istanbul University, Istanbul, Türkiye;* ^*2*^ *Department of Neurology, University of Tübingen, Tübingen, Germany;* ^*3*^ *Department of Neurology, Faculty of Medicine, Istanbul Atlas University;* ^*4*^ *Department of Medical Genetics, Faculty of Medicine, Eskisehir Osmangazi University, Eskisehir, Türkiye*

**Background and aims:** Hereditary cerebellar ataxias (HCAs) are rare, genetically heterogeneous neurodegenerative disorders causing progressive cerebellar and multisystem involvement. Their diagnosis is challenging, particularly in populations with high consanguinity, such as Türkiye, where autosomal recessive (AR) forms are more prevalent. The aim of this study was to investigate the phenotypic features of hereditary ataxias in a large Turkish cohort.

**Methods:** A total of 481 individuals (207 index cases and 274 unaffected family members) were recruited from a tertiary referral center over 15 years. Clinical evaluation, pedigree analysis, and targeted genetic testing or whole‐exome sequencing were performed. Variants were classified according to ACMG guidelines, and segregation analyses were completed when possible.

**Results:** Pathogenic variants were identified in 50 patients (24%). Of these, 83% displayed AR inheritance, reflecting the high prevalence of consanguinity. The most frequent disorder was autosomal recessive spastic ataxia of Charlevoix‐Saguenay (ARSACS; 14%), followed by Friedreich's ataxia (6%), ANO10‐related SCAR10 (8%), and CANVAS due to RFC1 expansions (8%). Less frequent variants included mutations in ATM, APTX, SETX, SYNE1, NPC1, and others. Overall, 14% of patients had late‐onset disease, and several novel variants were documented.

**Figure:**
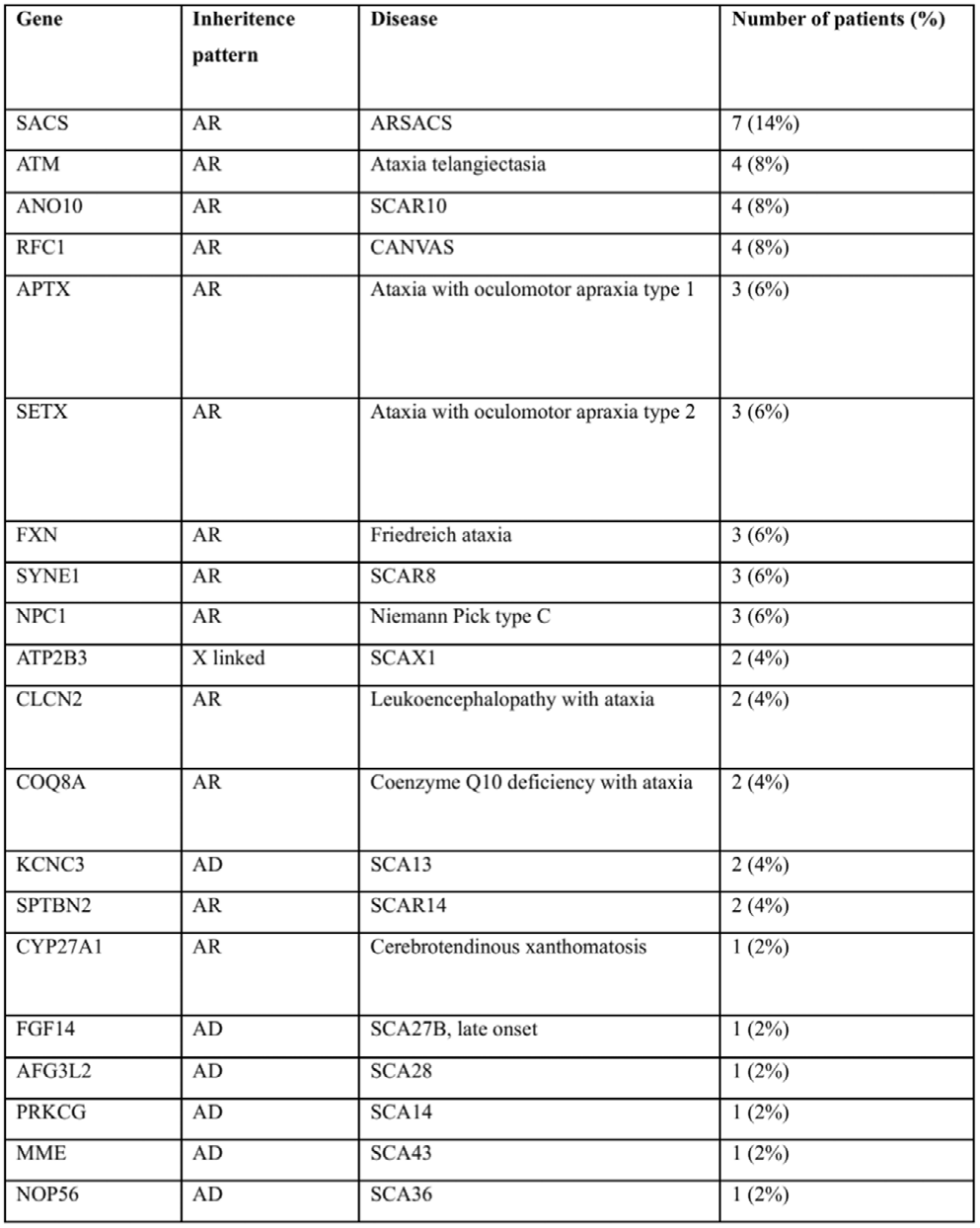
**TABLE 1** Molecular analysis results of the patients.

**Conclusion:** This single‐center study represents one of the largest hereditary ataxia cohorts reported in Türkiye. The predominance of AR inheritance highlights the impact of consanguinity on the genetic landscape. Recognition of specific phenotypic patterns, particularly treatable forms like CTX, is critical for timely diagnosis and therapeutic intervention. Advances in sequencing and emerging gene‐targeted therapies emphasize the importance of accurate molecular diagnosis to improve patient outcomes.

**Disclosure:** Nothing to disclose.

## EPO‐0449

### Sex differences in non‐motor symptoms and quality of life in Parkinson's disease: A systematic review and meta‐analysis

#### S. Reis Pimenta ^1^; S. Moura de Sousa Brasil^2^; M. Carolina Soares^3^; M. de Medeiros Fernandes^4^; D. Leonel Boone^5^

##### ^*1*^ *Heinrich‐Heine‐Universität Düsseldorf, Düsseldorf, Nordrhein‐Westfalen, Germany;* ^*2*^ *State University of Ceará, Fortaleza, Brazil;* ^*3*^ *Federal University of São Paulo, Department of Neurology and Neurosurgery, São Paulo, Brazil;* ^*4*^ *State University of Rio Grande do Norte, Medicine Department, Mossoró, Brazil;* ^*5*^ *Federal University of São Paulo, Department of Neurology and Neurosurgery, São Paulo, Brazil*

**Background and aims:** Observational studies suggests that there are sex‐specific symptom profiles in Parkinson's Disease (PD), with potential differences in perceived quality of life (QoL).

**Methods:** PubMed, Embase, and Cochrane were systematically searched to identify sex‐related differences in the Non‐Motor Symptoms Scale (NMSS) and Parkinson's Disease Questionnaire–39 (PDQ‐39). Statistical heterogeneity was evaluated with I2statistics. A random‐effects model was employed for outcomes with substantial heterogeneity.

**Results:** Seven reports were included (*n* = 2,968; 1,733 men, 1,235 women). The total NMSS Mean Difference (MD) 5.00 (95% CI 2.26–7.73), *p* = 0.0003, I^2^ = 19% shows a greater overall non‐motor symptom burden in women. Similarly, the PDQ‐39 MD 4.68 (95% CI 0.43 to 8.93), *p* = 0.03, I^2^ = 88% shows a worse QoL in women. Regarding the NMSS domains, women have worse scores in the urinary domain MD 0.55 (95% CI 0.17–0.92), *p* = 0.004, I^2^ = 65%, attention MD 0.27 (95% CI 0.02–0.51), *p* = 0.03, I^2^ = 15%, sleep/fatigue MD 1.38 (95% CI 0.70–2.07), *p* < 0.00001, I^2^ = 93%.

**Figure:**
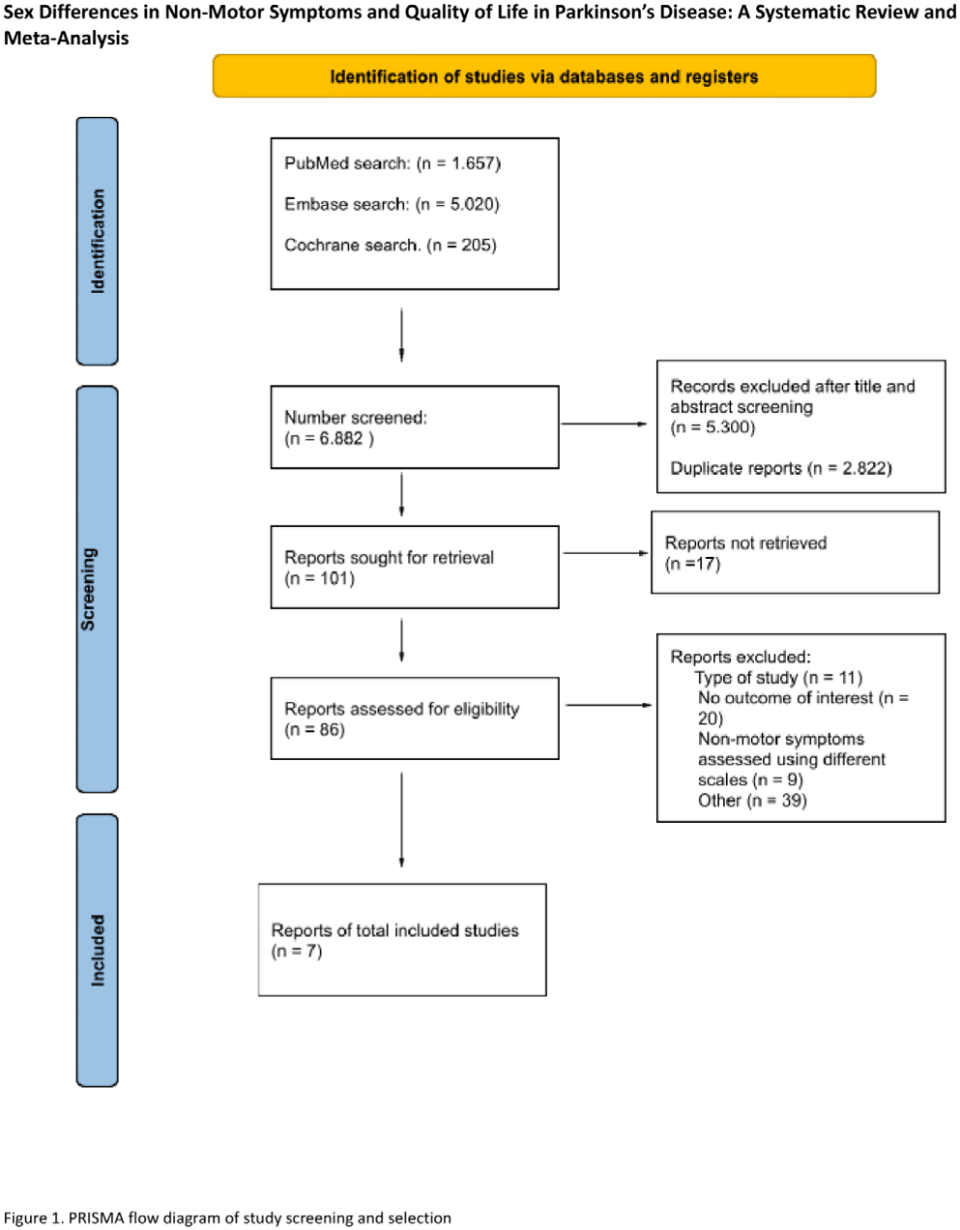
**FIGURE 1** PRISMA ‐ "Sex Differences in Non‐Motor Symptoms and Quality of Life in Parkinson's Disease: A Systematic Review and Meta‐Analysis".

**Figure:**
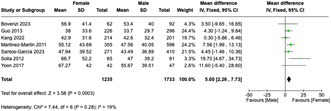
**TABLE 1** NMSS total forest plot.

**Figure:**
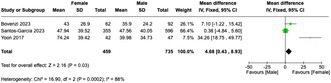
**TABLE 2** PDQ 39 forest plot.

**Conclusion:** Women with PD exhibit a higher non‐motor symptom burden and worse QoL, particularly in sleep/fatigue and mood/cognition domains. In contrast, men tend to have higher scores in the cardiovascular domain. However, substantial heterogeneity was observed in some analyses, indicating that the strength of these findings varies across domains.

**Disclosure:** Nothing to disclose.

## EPO‐0450

### Dynamic STN beta oscillations and fronto–subcortical connectivity track state anxiety in Parkinson's disease

#### L. Ricciardi

##### Neuromodulation and Motor Control Section, Department of Psychology and Neuroscience, School of Health and Medical Sciences, City St George's, University of London, London, UK

**Background and aims:** Anxiety in Parkinson's disease (PD) involves altered autonomic regulation and cortico–subcortical network dynamics. Mechanisms underlying state anxiety remain unclear.

**Methods:** We examined the effects of experimentally induced state anxiety and recovery via mindfulness/deep breathing on autonomic function, STN and cortical oscillations, and STN–cortical connectivity in PD, and their interaction with DBS. Thirteen PD patients with STN‐DBS completed a protocol including stress induction and deep breathing/mindfulness. STN local field potentials, EEG, and ECG were recorded with DBS ON and OFF (ON medication). Oscillatory power (theta, alpha, beta), STN–cortical connectivity, and heart rate variability (HRV) were analysed using repeated‐measures ANOVAs.

**Results:** Stress increased state anxiety (*F*(1.22) = 22.24, *p* < 0.001), which decreased after deep breathing (*p* < 0.001), with no stimulation effect. State anxiety correlated with trait anxiety ON (rho = 0.678, *p* = 0.016) and OFF DBS (rho = 0.820, *p* = 0.001). STN beta power showed a stimulation×condition interaction (*F*(2) = 5.0, *p* = 0.04): reduced beta during deep breathing vs rest (*p* = 0.02) and stress (*p* = 0.02) with DBS ON, and DBS‐related beta suppression during rest (*p* = 0.03) and deep breathing (*p* = 0.01). No theta or alpha effects were found. Root Mean Square of Successive Differences (RMSSD), a key HRV metric, showed a condition effect (*F*(2) = 5.5, *p* = 0.01). Stress‐related anxiety changes correlated negatively with RMSSD (rho = −0.69, *p* = 0.02) and STN beta power (rho = −0.91, *p* < 0.001) with DBS ON. Recovery was associated with increased right STN–frontal beta connectivity (rho = −0.681, *p* = 0.03).

**Conclusion:** State anxiety in PD is associated with dynamic modulation of STN beta oscillations and STN–frontal connectivity. DBS enhances coupling between subjective anxiety, autonomic regulation, and STN beta activity.

**Disclosure:** Nothing to disclose.

## EPO‐0451

### Multimodal mixed‐reality screening for Parkinson's disease: Accuracy and key features

#### M. Wójcik‐Pędziwiatr ^1^; D. Hemmerling^2^; B. Krawczyk^3^; W. Szecowka^2^; M. Dudek^2^; M. Rudzinska‐Bar^1^

##### ^*1*^ *Department of Neurology, Andrzej Frycz Modrzewski Cracow University, Cracow, Poland;* ^*2*^ *AGH University of Cracow, Cracow, Poland;* ^*3*^ *Samsung R&D Institute, Poland*

**Background and aims:** Clinical evaluation of PD is often time‐consuming and partially subjective. Objective, sensor‐based screening tools may enable scalable triage and longitudinal monitoring. The objective was to benchmark multimodal machine‐learning models for Parkinson's disease (PD) detection during a mixed‐reality assessment and to identify the tasks and sensor modalities most critical to diagnostic performance.

**Methods:** We analyzed 137 participants (66 with PD, 71 healthy controls) who performed 17 guided tasks with synchronized speech and motion capture. A total of 4,850 engineered features per participant were extracted. Data were split into training (60%) and testing (40%) sets. Missingness was substantial and group‐dependent (51.8% controls; 27.6% PD). Imputation methods were compared using masked‐value reconstruction RMSE; AttentionVAE achieved the lowest error (0.9481), outperforming MICE (0.9540) and mean/median imputation (1.0172/1.0424).

**Results:** Representation‐learning approaches achieved strong classification performance. CrossModalAVAE combined with random forest reached a mean accuracy of 0.915 ± 0.060 (AUC 0.985 ± 0.019; F1 0.917 ± 0.059), while CrossTaskAVAE with gradient boosting achieved 0.901 ± 0.084 (AUC 0.982 ± 0.018; F1 0.904 ± 0.074). Speech features dominated (87.2% of informative variables). Removing head‐movement features reduced accuracy from 0.873 to 0.691 and excluding both acoustic and head‐movement modalities reduced accuracy to ~58%. Task‐level ablations identified picture/story description and sustained vowel/a/ as most informative.

**Conclusion:** Mixed‐reality multimodal assessment enables accurate PD screening and informs task prioritization to reduce patient burden. Larger external validation is required to establish generalizability and clinical utility.

**Disclosure:** Supported by the National Centre for Research and Development, Poland (LIDER/6/0049/L‐12/20/NCBIR/2021).

## EPO‐0452

### When personality and polygenic risk scores interact to predict impulse control disorders in Parkinson's disease

#### M. Boussac ^1^; P. André Dias Bastos^2^; T. Wirth^3^; A. Lanore^4^; E. Harroch^2^; L. Mariani^4^; M. Anheim^3^; J. Corvol^4^; C. Brefel‐Courbon^2^

##### ^*1*^ *Toulouse NeuroImaging Center (ToNIC), Inserm UMR1214, University of Toulouse III, UPS, France;* ^*2*^ *Department of Clinical Pharmacology and Neurosciences, Parkinson Expert Center, Clinical Investigation Center, University Hospital of Toulouse, NeuroToul COEN (Center of Excellence in Neurodegeneration), Toulouse, NS‐PARK/FCRIN Network, France;* ^*3*^ *Service de Neurologie, Hôpitaux Universitaires de Strasbourg, Strasbourg, France;* ^*4*^ *Sorbonne Université, Institut du Cerveau ‐ Paris Brain Institute ‐ ICM, Inserm, CNRS, Paris, France*

**Background and aims:** In Parkinson's disease (PD), patients may develop impulse control disorders (ICDs), as adverse effect of dopamine agonists. Some factors have already been associated with ICDs such as genetic polymorphisms (Cormier‐Dequaire et al. 2018), as well as personality (Novelty Seeking/Self‐Directedness dimensions) related to impulsivity in PD (Boussac et al. 2022; Meira et al. 2022). We aim to evaluate interactions effects between personality and polygenetic risk scores (PRS) on the risk of ICDs.

**Methods:** PD patients with (*n* = 139) and without (*n* = 111) ICDs were included in the BADGE‐PD study (NCT02319395). Personality dimensions were assessed using the Temperament and Character Inventory‐Revised (TCI‐R) (Cloninger et al. 1994), while 18 PRS associated with psychiatric or behavioral phenotypes were calculated. Logistic regression models were done to evaluate main and interaction effects between TCI‐R dimensions and PRS on ICD risk by calculating odds ratios (OR).

**Results:** Main significant effects were found for the Self‐Directedness personality dimension, for the attention deficit hyperactivity disorder (ADHD, PGS003753) and the major depressive disorder (MDD, PGS000907) PRS: higher scores being associated with higher risk to develop ICDs (ORs between 1.31 and 1.54). Moreover, some personality*PRS interactions significantly modulated this risk either amplifying or attenuating it. Highest ICD risk was found from the interaction between Self‐Directedness and MDD PRS (global OR of 2.78) corresponding to a 278% increased risk of ICD for each increase of one standard deviation in both variables.

**Conclusion:** This study highlights some important interactions between genetic and personality to consider in personalized risk assessment for ICDs in PD.

**Disclosure:** I declare no conflict of interest for this study. This study was funded by a grant of the French Ministry of Health (PHRC AOM 1008), with the support of the Mutuelles AXA health sponsorship program for the ancillary part of this study.

## EPO‐0453

### A Bayesian reappraisal of disease‐modifying drug candidates for Parkinson's disease

#### M. Russo ^1^; T. Costa^2^; G. Polito^1^; D. Calisi^1^; N. Spano^1^; E. Cammarano^1^; L. Miccoli^1^; S. Sensi^1^

##### ^*1*^ *Department of Neuroscience, Imaging and Clinical Sciences, G. d'Annunzio University of Chieti‐Pescara, Chieti, Italy;* ^*2*^ *GCS‐fMRI, Koelliker Hospital and Department of Psychology, University of Turin, Turin, Italy*

**Background and aims:** Parkinson's disease (PD) remains without a confirmed disease‐modifying therapy (DMT). Numerous clinical trials have investigated candidate compounds, yet conclusions often rely on frequentist analyses that may obscure the strength or weakness of the evidence.

**Methods:** We performed a Bayesian reappraisal of major DMT trials—covering cinpanemab, deferiprone, CoQ10, ambroxol, NLY01, liraglutide, lixisenatide, and exenatide—focusing on motor and non‐motor outcomes, with standardized effect sizes, Bayes Factors (BF₁₀), and posterior probabilities.

**Results:** Deferiprone (FAIRPARK‐II) showed extreme evidence for a detrimental effect on 36‐week MDS‐UPDRS total scores (BF₁₀ = 5.03 × 10^6^; P(H₁) = 0.993). Liraglutide demonstrated strong evidence for improvement in Non‐Motor Symptoms Scale scores (BF₁₀ = 41.7; P(H₁) = 0.977). Lixisenatide showed moderate evidence for a small motor improvement on MDS‐UPDRS‐III at 12 months (BF₁₀ = 4.75; P(H₁) = 0.826). CoQ10, cinpanemab, ambroxol, NLY01, and exenatide (2025) all showed BF₁₀ < 1, indicating no support for efficacy.

**Conclusion:** Bayesian analysis aligns with the strongest frequentist signals while clarifying ambiguous or inconsistent results, underscoring that the magnitude of the effect and evidential strength vary substantially across compounds. GLP‐1R agonists show promising efficacy as DMT candidates for PD treatment. Pharmacokinetic and pharmacodynamic differences among the compounds could underlie the different efficacy profiles concerning the disease stage and different symptoms (i.e, motor or non‐motor).

**Disclosure:** Nothing to disclose.

## EPO‐0454

### The longitudinal Progression of Gait Symptoms in Early Parkinson's Disease: A 7‐year study from the PPMI dataset

#### R. Iredale ^1^; V. Foster^1^; S. Sahana Sathyanarayana^1^; D. Galley^1^; C. Stewart^1^; J. Pasquini^2^; D. Ledingham^1^; N. Pavese^1^

##### ^*1*^ *Clinical Ageing Research Unit, Newcastle University, Campus for Ageing and Vitality, Newcastle upon Tyne, UK;* ^*2*^ *Department of Clinical and Experimental Medicine, University of Pisa, Pisa, Italy, Clinical Ageing Research Unit, Newcastle University, Campus for Ageing and Vitality, Newcastle upon Tyne, UK*

**Background and aims:** Gait disturbances are cardinal symptoms of Parkinson's disease (PD). Existing studies in early PD focused on aggregate scores, lacking sensitivity to sub‐item level changes. Correlations between gait and cognitive impairment were demonstrated but findings remain inconsistent. This study longitudinally tracked the progression of early gait disturbances along with cognitive impairment to understand their impact on Activities of Daily Living (ADL) over 7‐years.

**Methods:** Data was obtained from 150 PD participants in the Parkinson's Progression Markers Initiative (PPMI) database. Motor symptoms, cognition and ADL were measured using the Movement Disorder Society‐ Unified Parkinson's Disease Rating Scale (MDS‐UPDRS), the Montreal Cognitive Assessment (MoCA) and the Modified‐Schwab England (ADL‐ SE) scale respectively from PPMI annual visits.

**Results:** Patient reported outcomes (PROs) identified walking and balance dysfunction in 32% of the cohort at baseline, 53% by year 3 and 69% at year 7. Whereas, clinicians identified gait disturbances in 55% at baseline, 60% at year 3 and 74% at year 7 with a significant impact on ADL's. Freezing of gait (FOG) presented between year 4 (3%) and 5 (5%) in clinician scores, but 5% of patients already reported FOG at baseline that increased to 17% by year 5 and 35% at year 7 on PROs. At year 7, FOG, gait, and postural stability scores correlated negatively with MoCA scores (*p* < 0.012).

**Conclusion:** This study shows that gait disturbances are widely prevalent in early PD with a fast rate of progression, highlighting the importance of appropriate physiotherapy and management early in the disease course.

**Disclosure:** Nothing to disclose.

## EPO‐0455

### Expanding the differential diagnosis of MSA: A systematic review on dysautonomia in genetic movement disorders

#### U. Lazic ^1^; A. Milovanovic^1^; R. Damjanovic^2^; M. Svetel^1^; V. Kostic^1^; N. Dragasevic Miskovic^1^; I. Stankovic^1^

##### ^*1*^ *Neurology Clinic, University Clinical Center of Serbia,* Belgrade, Serbia; ^*2*^ *University Clinical Center Nis, Neurology Clinic, Nis, Serbia*

**Background and aims:** Adult‐onset genetic movement disorders can present with autonomic failure alongside parkinsonism, ataxia, and other movement disorders. Autonomic dysfunction, a frequent cause of misdiagnosis of multiple system atrophy (MSA), has not been systematically evaluated across these disorders. This study aimed to comprehensively characterize dysautonomia in adult‐onset genetic movement disorders.

**Methods:** A systematic PubMed search using predefined terms identified original English‐language research articles. Of 313 papers retrieved on September 8, 2025, 82 were included (61 original studies, 5 case series, and 16 case reports). Exclusions comprised 7 review articles, 12 chapters, 206 unrelated publications, and 6 non‐English papers. Extracted data included study population, age at onset, inheritance pattern and genetic mutation, presence of control groups, clinical phenotype, autonomic assessment methodology, and key findings related to autonomic dysfunction. Results were summarized in a table of evidence.

**Results:** Thirty‐four adult‐onset genetic movement disorders caused by mutations in 52 genes were identified as manifesting autonomic failure in association with ataxia, parkinsonism, dystonia, spastic paraplegia, chorea, or complex phenotypes. Several conditions—including spinocerebellar ataxias, hereditary spastic paraplegias, certain genetic parkinsonian syndromes, fragile X tremor–ataxia syndrome, LMNB1‐related autosomal dominant leukodystrophy, and RFC1‐related disease—emerge as MSA mimics. The phenotype and severity of dysautonomia varied considerably across disorders.

**Conclusion:** Many adult‐onset genetic movement disorders can closely resemble MSA, creating significant diagnostic challenges. Autonomic dysfunction is an important but often overlooked feature in these conditions and should be considered in the differential diagnosis of MSA. Comprehensive clinical history, autonomic testing, and targeted genetic testing in selected cases may improve diagnostic accuracy and reduce misdiagnosis.

**Disclosure:** Nothing to disclose.

MS and related disorders 3

## EPO‐0456

### Epigenetic modifications of cerebrospinal fluid cell‐free DNA as a marker of neuronal damage

#### C. Starvaggi Cucuzza^1,2,3^; C. Rao Prakash^1,2^; C. Nolan^4^; A. Simeone ^4^; L. Sarre^4^; T. Charlesworth^4^; F. Puddu^4^; Y. Han^1,2^; M. Khademi^1,2^; B. Evertsson^1,5^; K. Fink^1,2,3,5^; P. Svenningsson^1,3^; C. Ingre^1,5^; C. Seitz^6^; F. Fang^6^; E. Thelin^1,5^; L. Kular^1,2^; F. Piehl^1,2,3,5^; M. Needhamsen^1,2^; M. Jagodic^1,2,3^

##### ^*1*^ *Department of Clinical Neuroscience, Karolinska Institutet, Stockholm, Sweden;* ^*2*^ *Center for Molecular Medicine, Karolinska University Hospital, Stockholm, Sweden;* ^*3*^ *Center for Neurology, Academic Specialist Center, Stockholm, Sweden;* ^*4*^ *biomodal Limited, Chesterford Research Park, Cambridge, UK;* ^*5*^ *Department of Neurology, Karolinska University Hospital, Stockholm, Sweden;* ^*6*^ *Institute of Environmental Medicine, Karolinska Institutet, Stockholm, Sweden*

**Background and aims:** Degenerative processes during Multiple Sclerosis (MS) progression are scarcely captured by currently available biomarkers. Cell‐free DNA (cfDNA) are fragments of DNA shed into biological fluids by dying cells and retain the cell‐of‐origin DNA methylation. Our aim is to utilize DNA methylation signatures in cfDNA from cerebrospinal fluid (CSF) to detect ongoing neuronal death in MS and other neurological diseases.

**Methods:** We profiled bulk methylation (mC) using enzymatic methylation sequencing (EMSeq) and/or true methylation (5mC) and hydroxymethylation (5hmC) using 6‐base sequencing in cfDNA extracted from CSF of healthy controls and people with MS, amyotrophic lateral sclerosis (ALS), narcolepsy type 1, movement disorders, or traumatic brain injury (TBI).

**Results:** While cfDNA extraction from CSF yielded a low amount of DNA for all but TBI samples, EMSeq and 6‐base sequencing successfully generated sequencing libraries, although with low genome coverage. To overcome the latter, we aggregated EMSeq reads and calculated methylation scores for neuronal or glial damage. TBI samples showed significantly higher scores than HC, followed by increased scores in ALS, suggesting more extensive neural cell death in these conditions. To increase sensitivity for neuronal damage, we quantified the neuron‐enriched hmC with 6‐base sequencing, and we confirmed higher neuronal DNA content in CSF cfDNA from TBI. Clustering of samples based on regions enriched for hmC successfully separated HC from neurological conditions, including MS.

**Conclusion:** Analysis of DNA methylation modifications of cfDNA has the capacity of capturing a neuronal signature in cell‐free CSF. Further studies are needed to implement cfDNA epigenetic analysis as real‐time biomarker of neurodegeneration.

**Disclosure:** CN, AS, LS, TC and FPuddu are current employees of biomodal Limited and hold share options. KF has received research grants from UCB, Merck and AMICUS, all outside the submitted work. PS has received lecture honoraria grants from AbbVie, MERZ, AstraZeneca, and fees for serving on DMC in clinical trials with Lundbeck. CI has consulted for Cytokinetics, Pfizer, BioArctic, Novartis, Tikomed, Ferrer, Amylyx, Prilenia, Mitsubishi and Abbvie. She is also a board member of Tobii Dynavox and of the Stiching TRICALS Foundation; all outside the submitted work. FPiehl has received research grants from Denka, Janssen, Merck KGaA, Novartis, Pfizer and UCB, and fees for serving on DMC in clinical trials with Lundbeck and Roche, and preparation of expert witness statement for Novartis. CSC, CRP, YH, MK, BE, CS, FF, EPT, LK, MN and MJ have nothing to disclose.

## EPO‐0457

### Smelling and tasting neurodegeneration: Chemosensory dysfunction and retinal changes in multiple sclerosis: A pilot study

#### E. Idini ^1^; G. Sollai^3^; M. Melis^2^; G. Coghe^3^; J. Frau^3^; I. Tomassini Barbarossa^2^; E. Cocco^3^

##### ^*1*^ *Department of Medical Sciences and Public Health, University of Cagliari, Cagliari, Italy**;*** ^*2*^ *Department of Biomedical Sciences, University of Cagliari, Cagliari, Italy;* ^*3*^ *Multiple Sclerosis Center, Binaghi Hospital, Cagliari, Italy*

**Background and aims:** To characterize gustatory and olfactory function in relapsing‐remitting multiple sclerosis (RRMS) and explore associations with clinical variables and optical coherence tomography (OCT) retinal measures.

**Methods:** Single‐centre cross‐sectional pilot study (Cagliari, Italy; February to April 2025), 78 adults with RRMS completed Taste Strips (Total Taste score 0–16; hypogeusia defined as less than 9). A subgroup (*n* = 60) completed Sniffin Sticks battery (Threshold, Discrimination, Identification; TDI score 1–48). Spectral‐domain OCT measured peripapillary retinal nerve fibre layer (pRNFL) and ganglion cell‐inner plexiform layer (GCIPL) thickness in eyes without prior optic neuritis. Correlations analyses and multivariable linear regression assessed associations between chemosensory measures, clinical variables and pRNFL thickness.

**Results:** Mean Total Taste score was 11.5 (standard deviation 2.85) and 13 (16.7%) had hypogeusia; sour required higher concentrations for correct identification than sweet, salty and bitter (*p* < 0.05). Higher body mass index was associated with lower taste scores (*r* = −0.30; *p* = 0.033) and smoking with hypogeusia (*p* = 0.023); taste performance was not associated with Expanded Disability Status Scale (EDSS), disease duration, pRNFL or GCIPL. Mean TDI was 29.5 (3.38) and 23 (38%) were hyposmic. TDI correlated with disease duration (*r* = −0.56; *p* < 0.001) and pRNFL thickness (*r* = 0.28; *p* = 0.038). In multivariable models for pRNFL thickness, only disease duration remained independently associated (*p* = 0.029).

**Conclusion:** Chemosensory dysfunction is common in RRMS and should be recognized clinically. Taste impairment appears mainly linked to modifiable factors (BMI, smoking), whereas olfactory performance reflects cumulative disease burden; the olfaction‐pRNFL association was not independent of disease duration. Larger longitudinal, multimodal studies including magnetic resonance imaging and cognition are needed.

**Disclosure:** Nothing to disclose.

## EPO‐0458

### Enhancing clinical assessment in multiple sclerosis: The role of patient‐reported outcomes and the PDDS scale

#### E. Idini ^1^; I. Schiavetti^2^; G. Mura^3^; G. Coghe^3^; E. Carta^3^; E. Cocco^3^; J. Frau^3^

##### ^*1*^ *Department of Medical Sciences and Public Health, University of Cagliari, Cagliari, Italy**;*** ^*2*^ *University of Genoa, Department of Health Sciences, Genoa, Italy;* ^*3*^ *Multiple Sclerosis Center, Binaghi Hospital, ASL Cagliari*

**Background and aims:** The multiple sclerosis (MS) management increasingly integrates traditional clinical measures with Patient‐Reported Outcomes (PROs) to capture dimensions like fatigue and psychological well‐being often missed by physician‐led scales. Aim of the Study: To evaluate the efficacy of the “Patient Determined Disease Steps” PDDS as a simplified self‐assessment tool, equivalent to the Expanded Disability Status Scale (EDSS), for monitoring functional status.

**Methods:** MS patients were assessed using PDDS and various PROs. Clinical and demographic data have been collected for each participant. Agreement between PDDS and EDSS was evaluated via quadratically weighted Cohen's kappa (κ) and Spearman's rho. Potential effect modifiers (demographics, clinical data, and PROs) were analyzed to assess stability of the agreement.

**Results:** 221 participants (71.9% female; mean age 48.4) were enrolled, predominantly RRMS (94.6%). Median EDSS and PDDS were 2.0 and 1.0, respectively. High prevalence of depression (38.5%), fatigue (37.6%), and anxiety (26.7%) was observed. A substantial agreement was found between EDSS and PDDS (κ = 0.84, 95% CI:0.783–0.879; rho = 0.73; *p* < 0.001). Subgroup analysis revealed higher concordance in RRMS (κ = 0.831) than in progressive form (κ 0.600; Δκ = 0.231, *p* < 0.05). No other factors significantly influenced the agreement.

**Conclusion:** The robust agreement between EDSS and PDDS confirms its validity as an accessible tool for disability monitoring. Its stability across most variables supports PDDS use in remote monitoring and research. The lower concordance in progressive forms suggests differing disability perceptions in advanced stages. Integrating PDDS into clinical follow‐up promotes a more personalized, patient‐centered approach to MS care.

**Disclosure:** Nothing to disclose.

## EPO‐0459

### Sensor‐based gait and balance testing can detect subclinical sensorimotor dysfunction in radiologically isolated syndrome

#### K. Akar ^1^; S. Bünül^2^; F. Akkoç^2^; H. Efendi^2^; A. Vural^3^

##### ^*1*^ *Graduate School of Health Sciences, Koç University, Istanbul, Türkiye;* ^*2*^ *Faculty of Neurology, Kocaeli University, Kocaeli/Izmit, Türkiye;* ^*3*^ *School of Medicine, Koç University, Istanbul, Türkiye*

**Background and aims:** Radiologically isolated syndrome (RIS) presents with MRI features suggestive of multiple sclerosis (MS) without overt clinical signs or symptoms. We hypothesized that highly sensitive sensor‐based gait and balance assessments can reveal subclinical sensorimotor impairment in individuals with RIS.

**Methods:** Ninety‐two participants (RIS *n* = 28, MS *n* = 34, healthy controls [HC] *n* = 30) underwent inertial measurement unit sensor‐based gait and postural assessment alongside clinical measures. Group comparisons used Kruskal‐Wallis tests with post‐hoc analyses. Binary logistic regression (adjusted for age and sex) identified RIS discriminators. Spearman's correlations assessed clinical validity using EDSS in MS patients.

**Results:** Groups were age‐and sex matched (MS median EDSS = 1.5). RIS showed longer Timed 25‐Foot Walk duration (*p* = 0.003) and reduced Symbol Digit Modalities Test scores (*p* = 0.007) versus HC. Sensor‐derived feet apart‐eyes open‐foam stance (sway area, jerk, path length, root mean square [RMS] sway and range) and gait parameters (gait speed, stride length, heel strike angle, turn angle and speed) significantly discriminated RIS from HC (*p* < 0.05, all). Sensor‐based balance parameters demonstrated significant correlations with EDSS in pwMS: range (*r* = 0.53, *p* < 0.001), RMS sway (*r* = 0.48, *p* < 0.001), range sagittal (*r* = 0.50, *p* < 0.001), RMS sway coronal (*r* = 0.46, *p* = 0.01), RMS sway sagittal (*r* = 0.45, *p* = 0.01), sway area (*r* = 0.43, *p* = 0.01), jerk sagittal (*r* = 0.38, *p* = 0.03), path length (*r* = 0.37, *p* = 0.03).

**Conclusion:** Sensor‐based gait and balance analysis detects subclinical sensorimotor dysfunction in RIS and demonstrates clinical validity through EDSS correlations in pwMS. Sensor‐based gait and balance parameters hold significant potential as biomarkers of subtle clinical impairment in early MS and even RIS.

**Disclosure:** Nothing to disclose.

## EPO‐0460

### Updated pregnancy and infant outcomes from an ongoing worldwide enhanced pharmacovigilance program of Cladribine tablets: 8‐year results of MAPLE‐MS

#### K. Hellwig ^1^; H. Tilson^2^; S. Thiel^1^; Y. Khunte^3^; M. Danten^4^; N. Dubois^5^; M. Sabidó^6^

##### ^*1*^ *Department of Neurology, St. Josef Hospital, Katholisches Klinikum Bochum, Ruhr University Bochum, Bochum, Germany;* ^*2*^ *School of Global Public Health, University of North Carolina, USA;* ^*3*^ *Merck Specialties Pvt. Ltd, Bangalore, India, an affiliate of Merck KGaA;* ^*4*^ *Global Biostatistics, Merck Santé S.A.S, Lyon, France, an affiliate of Merck KGaA;* ^*5*^ *EMD Serono Research & Development Institute, Inc, Billerica, USA, an affiliate of Merck KGaA;* ^*6*^ *Merck Healthcare KGaA, Darmstadt, Germany*

**Background and aims:** Data concerning pregnancy and infant outcomes following exposure to cladribine tablets (CladT) before or during pregnancy are limited. MAPLE‐MS assesses pregnancy and foetal outcomes for women with multiple sclerosis (MS) exposed to CladT ≤6 months before pregnancy or during pregnancy (maternal cohort), and pregnancies fathered by men with MS exposed to CladT ≤6 months before their partner's pregnancy (paternal cohort).

**Methods:** MAPLE‐MS uses a global patient safety database to assess pregnancy outcomes after CladT exposure. Prospectively and retrospectively reported cases for this 8‐year interim analysis were captured between August 2017 and April 2025. The primary outcome is the prevalence of major congenital anomalies (MCAs) among live births excluding genetic anomalies. Secondary outcomes include live birth, elective termination, spontaneous abortion, ectopic pregnancy, and stillbirth. Outcome prevalence including 95% confidence interval (CI) were calculated.

**Results:** There were 441 pregnancies in the maternal cohort and 53 pregnancies in the paternal cohort. Pregnancies with known outcomes were 214 (48.5%) and 19 (35.8%), respectively. In the maternal cohort, the prevalence of MCA was 0.8% of live births (1/132; 95% CI: 0.0, 4.6; an atrial septal defect). There remain no cases of MCA in the paternal cohort. Prevalence of known pregnancy outcomes are shown in Table 1.

**Figure:**
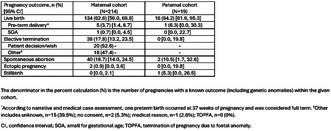
**TABLE 1** Prevalence of Pregnancy Outcomes in Pregnancies with Known Outcomes, by Maternal and Paternal Cohorts.

**Conclusion:** Among pregnancies resulting in live birth, one case of MCA has been observed since the launch of CladT. Pregnancy outcomes are in line with published estimates from the general and MS populations. To date, no safety signals have been identified.

**Disclosure:** KH has received honoraria and research support from Bayer, Biogen, Novartis, Sanofi, Teva, and Merck. HHT provides services as an epidemiologist on multiple pregnancy and drug‐exposure registries, for which he receives honoraria. ST received speakers’ honoraria from Bayer Healthcare and Biogen GmbH, payment for manuscript writing from HEXAL AG as well as sponsorship for congress participation from Biogen GmbH. YKK is an employee of Merck Specialities Pvt. Ltd, Bangalore, India, an affiliate of Merck KGaA. MD is an employee of Merck Santé S.A.S, Lyon, France, an affiliate of Merck KGaA. ND is an employee of EMD Serono Research & Development Institute, Inc, Billerica, MA, USA, an affiliate of Merck KGaA. MS is an employee of Merck Healthcare KGaA, Darmstadt, Germany.

## EPO‐0461

### Targeting store‐operated CA2+ entry with CIC‐39: A novel non‐immunosuppressive therapeutic approach for multiple sclerosis

#### L. Azzarone ^1^; H. Abreu^2^; C. Muschitiello^1^; M. Cerina^3^; E. Gallo^3^; D. Raineri^2^; R. Di Martino^3^; T. Pirali^3^; A. Chiocchetti^2^; D. Vecchio^4^; G. Cappellano^2^; B. Riva^1^

##### ^*1*^ *ChemiCare srl, Novara, Italy;* ^*2*^ *Department of Health Sciences, Interdisciplinary Research Center of Autoimmune Diseases‐IRCAD, University of Eastern Piedmont, Novara, Italy;* ^*3*^ *Department of Pharmaceutical Sciences, University of Eastern Piedmont, Novara;* ^*4*^ *Department of Translational Medicine, Neurology Unit, Maggiore Della Carità Hospital, University of Eastern Piedmont, Novara, Italy*

**Background and aims:** Multiple Sclerosis (MS) is an autoimmune demyelinating disease of the CNS for which current high‐efficacy disease‐modifying therapies often cause severe immunosuppression. Dysregulated Ca^2+^–calcineurin–NFAT signalling, driven by overactivated Store‐Operated Ca^2+^ entry (SOCE), promotes immune activation and pro‐inflammatory cytokine release (Fig1). This study evaluates SOCE as a therapeutic target using CIC‐39, a novel SOCE inhibitor in preclinical development, to restore immune homeostasis. Targeting SOCE may provide a safer, immune‐preserving strategy for MS, reducing immunosuppressive side effects (Fig2).

**Figure:**
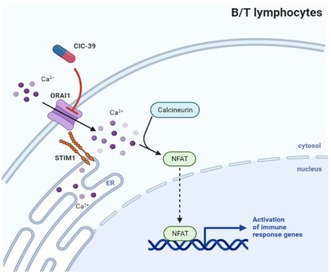
**FIGURE 1** SOCE‐Calcineurin‐NFAT pathway in B and T lymphocytes.

**Figure:**
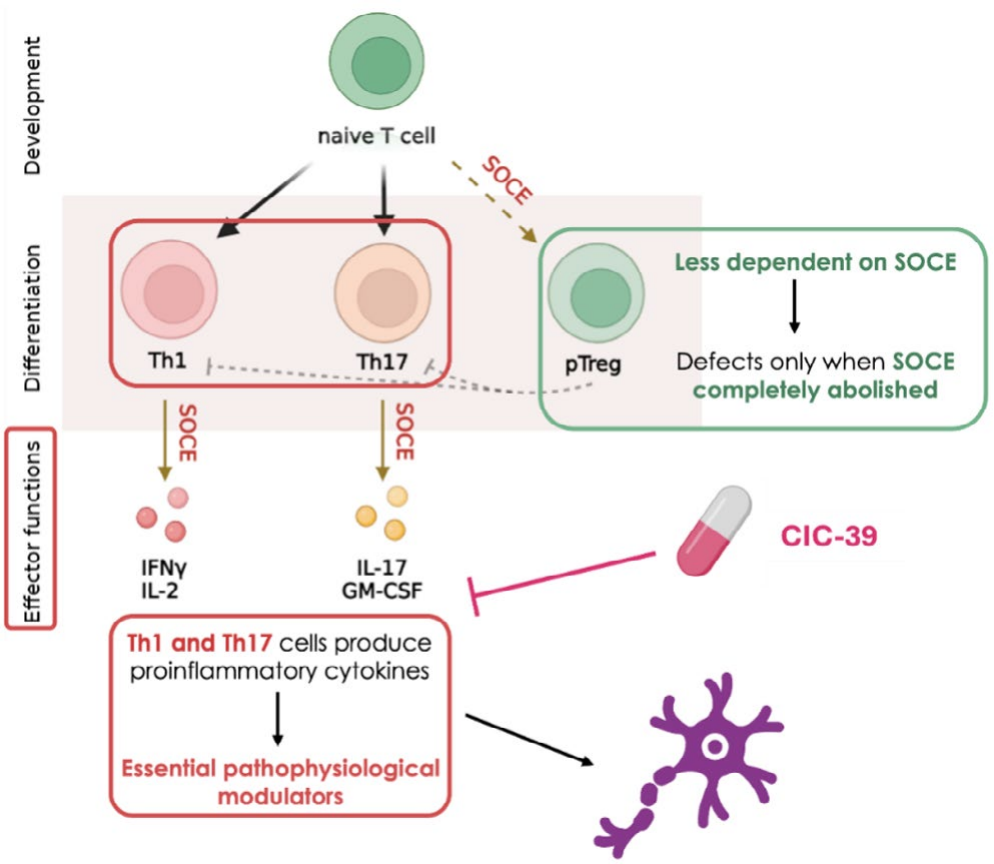
**FIGURE 2** Role of SOCE in Th1 and Th17 pro‐inflammatory cytokine release.

**Methods:** Peripheral blood mononuclear cells from MS patients and healthy controls were collected (Ethical Committee CE152/2024). CD4^+^ T cells were isolated, activated, and differentiated. CIC‐39 (1–3 μM) was tested in vitro for SOCE, proliferation, and cytokine release. In vivo, EAE mice were treated with CIC‐39 60 mg/kg via minipumps for 15 days (prot. 503/2024‐PR).

**Results:** SOCE was overactivated in PBMCs from MS patients, and CIC‐39 effectively restored Ca^2+^ homeostasis. Unlike the immunosuppressant teriflunomide, CIC‐39 did not impair CD4^+^ T‐cell proliferation but significantly reduced Th1/Th17‐associated cytokines (IL‐17A, TNF‐α, IFN‐γ, GM‐CSF) while preserving iTreg differentiation and regulatory function. In vitro and preliminary EAE studies demonstrated that CIC‐39 attenuated pro‐inflammatory responses, decreased circulating cytokines, and effectively arrested disease progression, highlighting its potential as an immune‐preserving therapeutic strategy for MS.

**Conclusion:** Our findings demonstrate that CIC‐39 selectively modulates overactive SOCE in MS, restoring physiological Ca^2+^ levels and preserving lymphocyte proliferation. It reduces Th1/Th17 cytokines, and preliminary studies in EAE mice confirmed rapid disease arrest with decreased IFN‐γ and IL‐17A, supporting CIC‐39 as a promising safer and immune‐preserving disease‐modifying therapy for MS.

**Disclosure:** Beatrice Riva owns stocks of ChemiCare srl. Luigi Azzarone and Carlotta Muschitiello are at present employees of ChemiCare srl.

## EPO‐0462

### Preliminary results from the brain‐age MS study: NAWM CEST and T1 show no association with paramagnetic rim lesion count

#### L. Pirpamer ^1^; S. Navarrete^2^; M. Capiglioni^3^; S. Marti^4^; R. McKinley^1^; R. Wiest^1^; R. Hoepner^4^; A. Betancourt^4^; P. Radojewski^5^

##### ^*1*^ *Institute for Diagnostic and Interventional Neuroradiology, Support Center for Advanced Neuroimaging, University of Bern, Bern, Switzerland;* ^*2*^ *Faculty of Engineering, Universidad de Concepción, Chile;* ^*3*^ *High Field Magnetic Resonance Center, Max Planck Institute for Biological Cybernetics, Tübingen, Germany;* ^*4*^ *Department of Neurology, Inselspital, Bern University Hospital and University of Bern, Switzerland;* ^*5*^ *Translational Imaging Center (TIC), Sitem‐insel, Bern, Switzerland*

**Background and aims:** Paramagnetic rim lesions (PRLs) are established imaging markers of chronic active multiple sclerosis (MS), reflecting focal inflammatory pathology. While PRLs are well characterised at the lesion level, it remains unclear whether PRL‐count is associated with diffuse alterations in normal‐appearing white matter (NAWM). Chemical exchange saturation transfer (CEST) imaging is sensitive to metabolic tissue properties beyond conventional MRI. In this preliminary analysis of the brain‐age MS study (NCT06221631), we investigated whether PRL‐count is associated with CEST‐metrics and T1 relaxation in clinically stable MS.

**Methods:** Clinically stable MS patients underwent 7T MRI including CEST imaging (amide, amine, rNOE, MT) and MP2RAGE‐based T1 mapping. PRLs were identified on susceptibility‐weighted imaging, and PRL‐count was determined per subject. NAWM regions of interest were conservatively defined by excluding T2‐FLAIR–visible lesions. Linear associations between PRL‐count and median NAWM quantitative metrics were assessed.

**Results:** No significant linear associations were observed between PRL‐count and NAWM CEST or T1. Substantial inter‐individual variability was present, but no consistent monotonic trends were observed with increasing PRL‐count.

**Figure:**
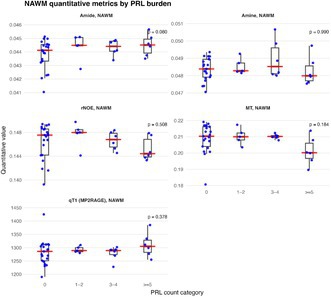
**FIGURE 1** NAWM CEST metrics and quantitative T1 stratified by PRL count. Boxplots show distributions across PRL categories with overlaid individual values; red lines indicate medians. *p*‐values derive from linear regression trend tests between PRL count and NAWM met.

**Conclusion:** In this preliminary analysis of clinically stable MS patients from the brain‐age MS study, PRL count was not associated with metabolically sensitive NAWM CEST metrics or quantitative T1. These findings suggest that PRL‐related pathology remains spatially focal and is not reflected by diffuse NAWM alterations at this stage. Larger longitudinal studies are needed to determine whether NAWM metabolic changes emerge with disease progression.

**Disclosure:** Nothing to disclose.

## EPO‐0463

### OCT and OCT angiography in differential diagnosis of MS: The retina as a window on the CNS

#### M. Di Cristinzi ^1^; L. Alonzo^1^; C. Lenzetti^2^; F. Azzolini^3^; A. Mariottini^1^; A. Poggesi^1^; L. Massacesi^1^; G. Virgili^1^; A. Repice^4^

##### ^*1*^ *Department of Neurofarba, University of Florence,* Florence, Italy; ^*2*^ *Department of Ophtalmology, AOU Careggi;* ^*3*^ *Department of Neurology, IRCCS Neuromed;* ^*4*^ *Department of Neurology, AOU Careggi,* Florence, Italy

**Background and aims:** MS is an inflammatory demyelinating disease of the CNS characterized by immune‐mediated perivenular inflammation, reflected by the perivenular distribution of white matter lesions (PVL) on MRI by the central vein sign (CVS). In contrast, several MS‐like and atypical MS disorders share overlapping clinical and radiological features but show a lower prevalence of PVL, suggesting alternative pathogenic mechanisms in which vascular dysfunction may play a prominent role. Optical coherence tomography (OCT) and OCTangiography (OCTA) enable non‐invasive assessment of retinal neuroaxonal integrity and microvascular perfusion, providing insights into CNS vascular involvement.

**Methods:** 56 patients underwent OCT‐OCTA and 19 received 3T MRI with CVS assessment (CVS+ if PVL>50%). OCT measured retinal nerve fiber layer (RNFL) and ganglion cell complex (GCC) thickness, while OCTA assessed optic nerve head vessel density and superficial (SCP) and deep (DCP) capillary plexus density.

**Results:** Patients were classified as MS (*n* = 34), atypical‐MS (*n* = 8), and not‐MS (nMS;*n* = 15), including other inflammatory, vascular, and neoplastic CNS disorders. Previous optic neuritis was associated with RNFL and GCC thinning, without significant changes in vessel density. MS showed significant temporal RNFL thinning compared with atypical‐MS and nMS (*p* < 0.001). Conversely, atypical‐MS and not‐MS exhibited a marked reduction in SCP and DCP density, in the absence of major structural retinal damage. In the CVS subgroup, CVS‐eyes demonstrated significantly lower vessel density than CVS+eyes, with high concordance between CVS+ and SCP status.

**Figure:**
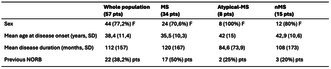
**TABLE 1** Basal characteristics of the whole population and subgroups.

**Figure:**
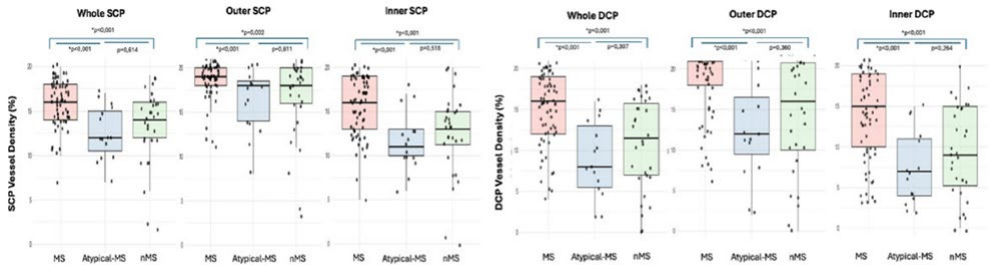
**FIGURE 1** Superficial (SCP) and Deep (DCP) capillary plexus vessel density of different zones in subgroups MS, atypical‐MS and not‐MS.

**Figure:**
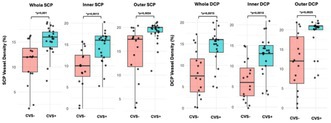
**FIGURE 2** Superficial (SCP) and Deep (DCP) capillary plexus vessel density of different zones in CVS+ and CVS‐.

**Conclusion:** Atypical‐MS and not‐MS are characterized by predominant retinal microvascular impairment with minimal neuroaxonal loss, supporting a primary vascular pathogenic contribution. OCT‐OCTA may assist early differential diagnosis of MS‐like syndromes and help identify non‐demyelinating disease mechanisms.

**Disclosure:** Nothing to disclose.

## EPO‐0464

### Exploring the impact of multiple sclerosis on social determinants of health: Results from the SocialMS study

#### M. Ponzano ^1^; A. Signori^2^; D. Landi^3^; A. Bellavia^4^; I. Schiavetti^2^; L. Lavorgna^5^; A. Di Sapio^6^; R. Fantozzi^7^; F. Marinelli^8^; M. Sormani^2^

##### ^*1*^ *Department of Life Sciences, Health and Health Professions, Link Campus University, Rome, Italy;* ^*2*^ *Department of Health Sciences, University of Genoa, Genoa, Italy;* ^*3*^ *Department of Systems Medicine, University of Rome Tor Vergata, Rome, Italy;* ^*4*^ *Department of Environmental Health, Harvard T.H. Chan School of Public Health, Boston, USA;* ^*5*^ *Department of Advanced Medical and Surgical Sciences, University of Campania Luigi Vanvitelli, Naples, Italy;* ^*6*^ *Department of Neurology & Regional referral Multiple Sclerosis Centre (CRESM), University Hospital San Luigi Gonzaga, Orbassano, Italy;* ^*7*^ *Department of Neurology, IRCCS Neuromed, Pozzilli (IS), Italy;* ^*8*^ *MS Center, Neurology Unit, F. Spaziani Hospital, Frosinone, Italy*

**Background and aims:** Multiple Sclerosis (MS) can affect several social determinants of health (SDH), but research remains limited and fragmented, with no studies exploring multiple SDH simultaneously. We examined four SDH (education, work, financial resources and social life) to assess the impact of MS and interrelationships, to identify risk factors, and to study associations with social support.

**Methods:** SocialMS is a cross‐sectional Italian questionnaire‐based study including adults with MS. The number of SDH affected was the primary endpoint, while secondary outcomes included impact on at least one domain and on individual domains. Risk factors were identified using Poisson and logistic regression.

**Results:** We included 1,039 participants followed at 68 MS centers (mean age: 43.82 years (standard deviation: 11.53); 68.82% females; 86.53% relapsing remitting, median Patient Determined Disease Steps (PDDS): 1.00 (interquartile range (IQR): 0.00–3.00)). Median number of SDH affected was 1 (IQR: 0–3), with social life (51%) and work (48%) most impacted; high interrelationships among domains were observed (Figure 1). Relevant risk factors included socioeconomic factors (education incomplete at diagnosis: incidence‐rate ratio (IRR) = 1.19, *p* = 0.004; non‐working or early retired: IRR = 1.18, *p* = 0.007; hardly making ends meet: IRR = 1.47, *p* < 0.001); comorbidities (IRR = 1.23, *p* < 0.001) and PDDS (IRR = 1.17, *p* < 0.001) (Table 1). Economic strain and disability were associated with all secondary outcomes (*p* < 0.05). Almost 90% received tangible (64.29%) or intangible (85.18%) social support, and number of impacted SDH was positively associated with both (odds ratios = 1.88 and 1.25, *p* < 0.05, Figure 2).

**Figure:**
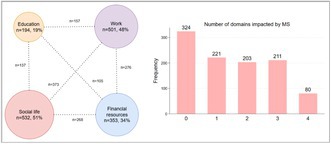
**FIGURE 1** Impact of multiple sclerosis (MS) on social determinants of health (SDH). Left: Frequency of participants reporting impact of MS on the four domains. Right: Distribution of participants according to the number of domains impacted by MS (0–4).

**Figure:**
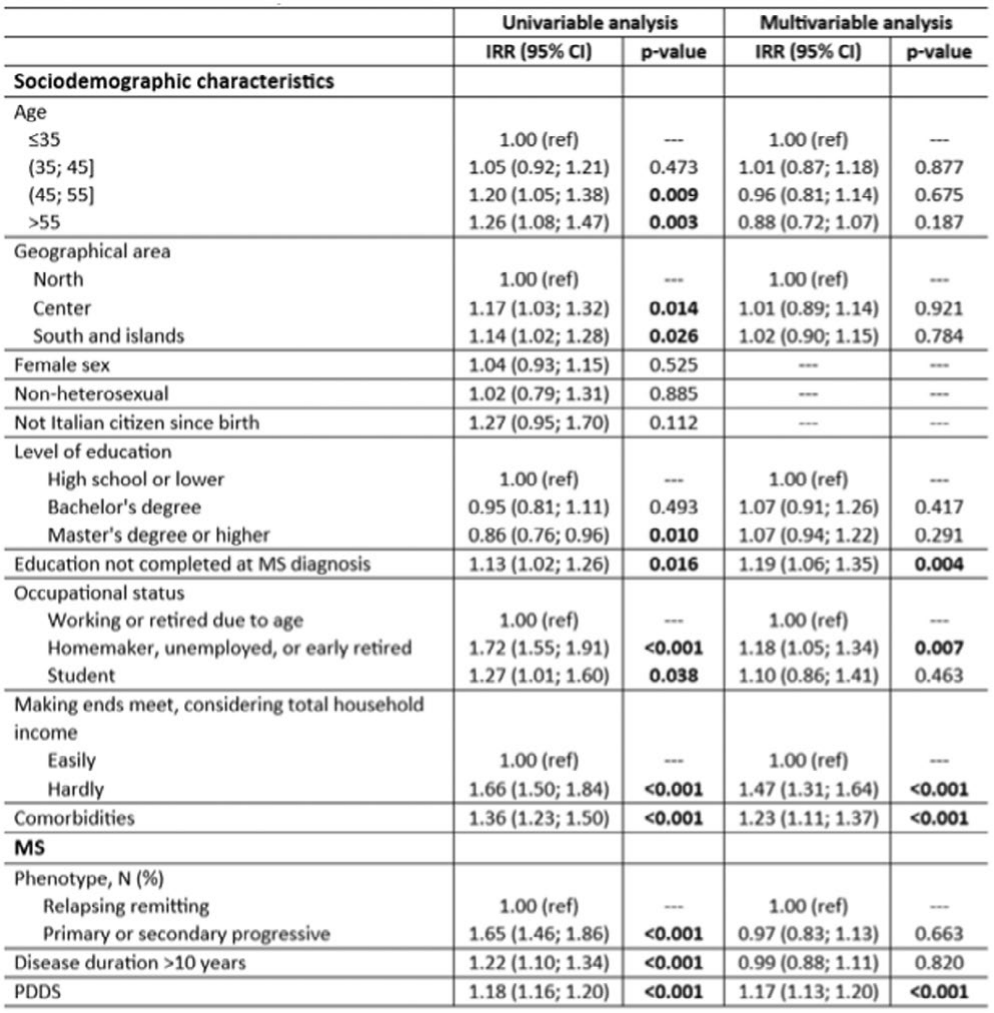
**TABLE 1** Poisson regression models examining the association of sociodemographic and multiple sclerosis (MS) characteristics with the number of SDH impacted by MS. Variables with *p* < 0.10 in univariable models were included in the multivariable analysis.

**Figure:**
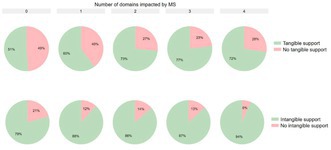
**FIGURE 2** Frequency of tangible and intangible social support according to the number of domains impacted by MS (0–4).

**Conclusion:** MS substantially affects multiple SDH, with greater impact in individuals with socioeconomic and MS‐related vulnerabilities.

**Disclosure:** MP has nothing to disclose, AS received speaker's honoraria from Chiesi, Novartis, and Horizon and grant from MSBase outside from this work, DL received travel funding from Biogen, Merck Serono, Sanofi, Teva, Bristol Myers Squibb, Mylan, Neuraxapharm, speaking or consultation fees from Sanofi, Merck Serono, Teva, Biogen, Roche, Novartis, Bristol Myers Squibb, Janssen, Alexion, Amgen, AB reports consulting fees (Arboretum LifeSciences, Inc), payment or honoraria for lectures, presentations, speakers bureaus, manuscript writing or educational events (Columbia University and BiostatEpi) and participation on a data safety monitoring board or advisory board (MarvelBiome, Inc), IS has received personal compensation for speaking and consulting from Roche, Biogen, Hoya, Hippocrates Research, D.M.G. Italia, Eyepharma, and DreamsLab. None of these relationships are related to the submitted work, LL received consultancy and speaker fees from Biogen, Novartis, Roche, Idorsia, Bristol, Lilly, Roche, Amgen, Alexion, Merck, and Sanofi for advisory services and participation in conferences, ADS reports grants or contracts from any entity (Roche, Sanofi), payment or honoraria for lectures, presentations, speakers bureaus, manuscript writing or educational events (Novartis, Alnylam, Roche, Merck, Janssen, Bristol Myers Squibb, Horizon, Alexion), support for attending meetings and/or travel (Novartis, Merck, Roche, Sanofi, Biogen) and participation on a data safety monitoring board or advisory board (Sanofi, Merck, Roche, Alexion, Sandoz), RF received travel support, consultations fees and honoraria for advisory boards from Novartis, Merk‐Serono, Biogen, Roche, Alexion, Neuraxpharm, FM has received personal compensation for consulting services and speaker activities from Merck Serono, Novartis, Roche, Biogen, BMS, MPS received consulting fees from Roche, Biogen, Merck, Novartis, Sanofi, Celgene, Immunic, Geneuro, GSK, Medday; received payment or honoraria for lectures, presentations, speakers’ bureaus, manuscript writing or educational events from Roche, Biogen Merck, Novartis, Sanofi, Celgene; participated on a Data Safety Monitoring Board or Advisory Board for Roche, Sanofi, Novartis, Merck.

## EPO‐0465

### Microglial activation in MS brain contributes significantly to brain age gap between MS patients and controls

#### O. Hartiala; J. Tuomaala; M. Saraste; M. Matilainen; L. Airas

##### Neurocenter, Turku PET Centre, Clinical Neurosciences and InFLAMES Research Flagship, Turku University Hospital, and University of Turku, Finland

**Background and aims:** Disease‐ and aging‐related factors impair brain health in people with MS. Aging MS patients typically experience gradual disease progression without relapses due to glial activation causing neuroaxonal damage. Aging‐, MS‐CNS pathology‐ and autoimmunity‐related factors may exacerbate detrimental glial activation and neuroaxonal damage in MS brain. We sought to investigate how aging associates with microglial activation and white matter damage and evaluate differences in brain age gap among healthy controls (HCs) and people with MS.

**Methods:** 128 MS patients and 69 age‐ and sex‐matched HCs underwent 18‐kDa translocator protein (TSPO)‐binding radioligand 11C‐PK11195 positron emission tomography for assessment of glial activation using distribution volume ratio (DVR) and concomitant magnetic resonance imaging (MRI) including diffusion tensor imaging (DTI). Brain age gap was calculated as difference between chronological and biological age estimated using data on 1) MRI‐measured atrophy, 2) atrophy and DTI and 3) the machine‐learning tool BraineageR.

**Results:** Normal‐appearing white matter (NAWM) DVR values increased (*p* = 0.001) and DTI metrics worsened (*p* < 0.01 for all measures) with increasing age among MS patients but not HCs. Brain age gap was significantly elevated in MS patients vs. HCs and this gap increased further when NAWM DVR was included in the model.

**Figure:**
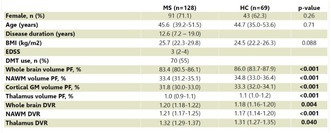
**TABLE 1** Clinical characteristics, MRI volumes, and TSPO Distribution Volume Ratios (DVR) for MS patients and healthy controls. Expressed as median (interquartile range) unless otherwise stated.

**Figure:**
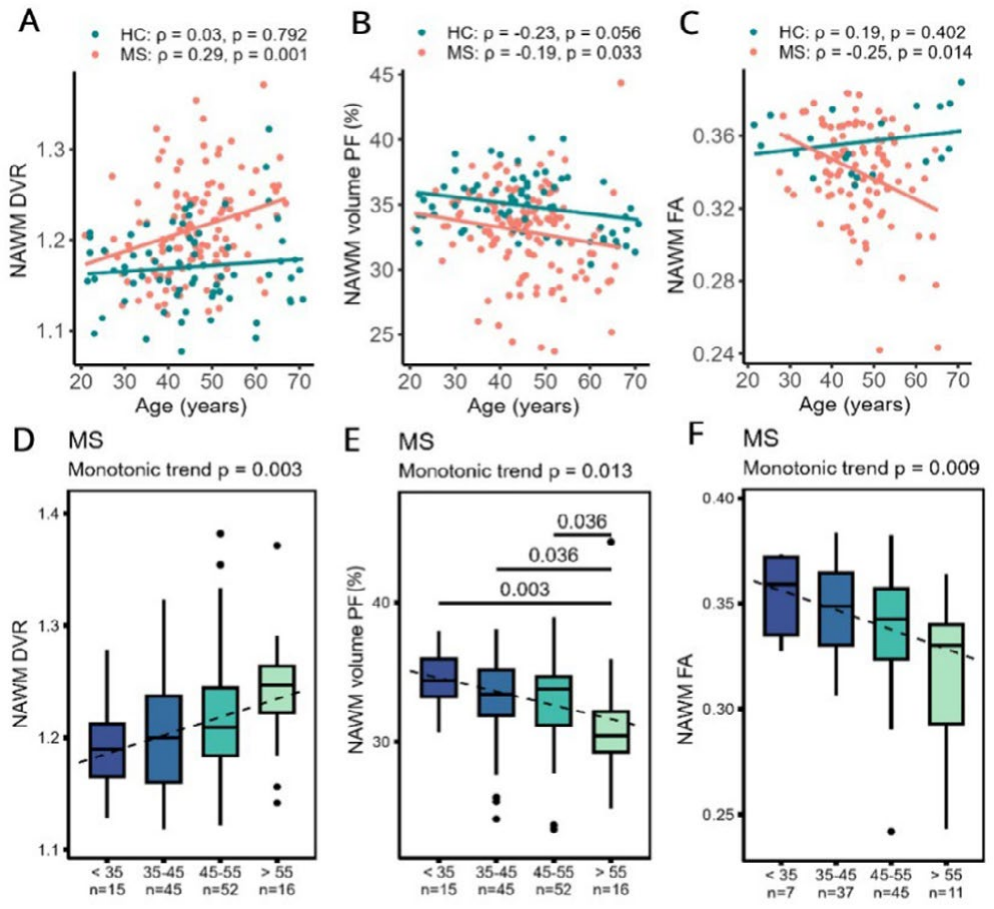
**FIGURE 1** Age and NAWM DVR (A) correlate positively, age and volume PF% (B) and FA (C) negatively in MS but not in HCs. NAWM DVR (D), volume PF% (E), and FA (F) by age group in MS. Correlation coefficients, trend lines, *p*‐values reported above panels

**Figure:**
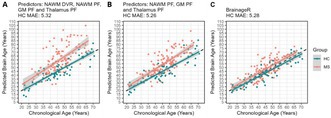
**FIGURE 2** Biological brain age is highest in MS patients compared to controls in model using brain parenchymal fractions and DVR (A) compared to models using PFs alone (B) or BraineageR (C). Mean absolute error displayed.

**Conclusion:** Higher age associated with increased glial activation among MS patients but not among HCs and co‐occurred with loss of white matter structural integrity in the NAWM and increases in biological brain age in MS. Increased TSPO signal in aging MS patients likely represents increasing compartmentalized inflammation, which can be accelerated by general inflammaging leading to premature brain neurodegeneration.

**Disclosure:** Olli Hartiala has received support for congress participation and travel from Novartis, Merck and Roche. Maija Saraste has received support for attending meetings and/or travel from Turku University Foundation, the InFLAMES Flagship Programme of the Academy of Finland and Merck. Joel Tuomaala: nothing to disclose. Markus Matilainen: nothing to disclose. Laura Airas has received honoraria and institutional research grant support from Sanofi and Merck Serono. This work was funded by the Research Council of Finland (decision number: 330902), the InFLAMES Flagship Programme of the Research Council of Finland (decision numbers: 337530, 357910 and 358823), and the State Research Funding (SRF) for university‐level health research in Turku University Hospital, Wellbeing Services County of Southwest Finland.

## EPO‐0466

### Serum neurofilament light chain predicts early subsequent disability progression in progressive MS

#### R. Klein Kranenbarg ^1^; K. Blok^1^; A. Marques^2^; J. van Rooij^3^; N. Asahaad^4^; C. de Brabander^5^; F. Claes^6^; I. Hoppenbrouwers^7^; N. Jafari^8^; L. van Rooij^9^; J. Samijn^9^; M. Ciaccio^10^; L. Agnello^10^; J. Kuhle^11^; A. Tintu^12^; J. de Beukelaar^13^; B. Wokke^14^; J. Smolders^15^

##### ^*1*^ *Department of Neurology, MS Centre ErasMS, Erasmus MC, University Medical Centre Rotterdam, Rotterdam, the Netherlands | Department of Neurology, MS Centre, Albert Schweitzer Hospital, Dordrecht, the Netherlands;* ^*2*^ *Department of Immunology, MS Centre ErasMS, Erasmus MC, University Medical Centre Rotterdam, Rotterdam, the Netherlands;* ^*3*^ *Department of Internal Medicine, Erasmus MC, University Medical Centre, Rotterdam, the Netherlands;* ^*4*^ *Department of Neurology, IJsselland Hospital, Capelle aan den IJssel, the Netherlands;* ^*5*^ *Department of Neurology, Admiraal de Ruijter Hospital, Goes, the Netherlands;* ^*6*^ *Department of Neurology, Fransiscus Gasthuis and Vlietland Hospital, Rotterdam, the Netherlands;* ^*7*^ *Department of Neurology, Van Weel‐Bethesda Hospital, Dirksland, the Netherlands;* ^*8*^ *Department of Neurology, Amphia Hospital, the Netherlands;* ^*9*^ *Department of Neurology, Maasstad Hospital, the Netherlands;* ^*10*^ *Department of Biomedicine, Neurosciences and Advanced Diagnostics, Institute of Clinical Biochemistry, Clinical Molecular Medicine and Clinical Laboratory Medicine, University of Palermo, Palermo, Italy;* ^*11*^ *Department of Neurology, Research Centre for Clinical Neuroimmunology and Neuroscience, University of Basel and University Hospital Basel, Basel, Switzerland;* ^*12*^ *Department of Clinical Chemistry, Erasmus MC, University Medical Centre, Rotterdam, the Netherlands;* ^*13*^ *Department of Neurology, MS Centre, Albert Schweitzer Hospital, Dordrecht, the Netherlands;* ^*14*^ *Department of Neurology, MS Centre ErasMS, Erasmus MC, University Medical Centre Rotterdam, Rotterdam, the Netherlands;* ^*15*^ *Department of Neurology and Immunology, MS Centre ErasMS, Erasmus MC, University Medical Centre Rotterdam, Rotterdam, the Netherlands | Neuroimmunology Research Group, Netherlands Institute for Neuroscience, Amsterdam, the Netherlands*

**Background and aims:** Disability progression in multiple sclerosis (MS) is dominantly driven by chronic compartmentalized rather than acute focal inflammation, emphasizing the need for biomarkers to capture this biology. Evidence supporting the prognostic value of common genetic variants, serum neurofilament light chain (sNfL) and glial fibrillary acidic protein (sGFAP) in progressive MS is limited. We assessed whether sNfL and sGFAP predict disability progression in a cohort of people diagnosed with primary progressive MS (pwPPMS) and evaluated the interaction with relevant common genetic variants.

**Methods:** Baseline sNfL and sGFAP Z‐scores were acquired. Risk‐carriership of rs10191329 and HLA‐DRB1*15:01 were collected from SNP‐array data and MS severity and susceptibility weighted genomic risk scores were calculated. Logistic regression models including age and immunomodulatory treatment (IMT) assessed prediction of disability progression at 1, 2, and 3 years.

**Results:** 173 pwPPMS (median age 60 years, 51% female) were included. sNfL Z‐scores correlated positively with disability severity, as measured by Expanded Disability Status Scale (EDSS) (ρ = 0.19, *p* = 0.014) and Age‐Related Multiple Sclerosis Severity Score (ARMSS) (ρ = 0.25, *p* < 0.001). Higher sNfL Z‐scores predicted EDSS progression at 1 year (OR 1.83 [95% CI 1.03–3.26], *p* = 0.04) and 2 years (OR 1.67 [95% CI 1.02–2.67], *p* = 0.04), but not at 3 years. Results of GFAP and genetic analyses will be presented.

**Conclusion:** Elevated sNfL predicted early disability progression. These findings support sNfL as a clinically relevant biomarker in progressive MS, with potential implications for prognostication and personalised treatment.

**Disclosure:** This work was funded by Stichting BeterKeten, the Dutch National MS Foundation (OZ2021‐019 and 2019‐019), and Siemens Healthineers. Joost S received research support from Biogen, Roche, Siemens Healthineers and Hansa Biopharma, and lecture and/or consultancy fee from Biogen, Merck, Novartis, Roche, and Sanofi. The remaining authors have nothing to disclose.

Muscle and neuromuscular junction disorder 3

## EPO‐0467

### Risk of myasthenia gravis exacerbation during pregnancy and postpartum: A nationwide cohort study

#### L. O´Connor ^1^; K. Gabrysch^2^; A. Wikström^3^; A. Rostedt Punga^1^

##### ^*1*^ *Department of Medical Sciences, Clinical Neurophysiology, Uppsala University, Uppsala, Sweden**;*** ^*2*^ *Uppsala Clinical Research Center, Uppsala University, Uppsala, Sweden;* ^*3*^ *Department of Women´s and Children's Health, Uppsala University, Uppsala, Sweden*

**Background and aims:** Pregnancy and postpartum represent unique physiological states that may influence autoimmune diseases such as myasthenia gravis (MG). Evidence on MG exacerbations during these periods is scarce and often based on small case series. We aimed to assess the risk of MG exacerbation during pregnancy and up to one year postpartum using nationwide longitudinal register data.

**Methods:** We conducted a population‐based cohort study including women with MG and their pregnancies in Sweden between 1987 and 2019. Data were obtained through linkage of the Swedish MG Register with national health and sociodemographic registers. The primary outcome was hospital admissions for MG during pregnancy and up to one year postpartum, compared with the year preceding pregnancy. Cox regression models estimated hazard ratios (HR), adjusting for disease duration and thymectomy. Secondary outcomes included changes in immunosuppressive MG medications and whether women conceived during periods of relative disease stability.

**Results:** Our findings indicate that pregnancy is generally associated with clinical stability, whereas the postpartum period may carry an increased risk of exacerbation in a subset of women. Patterns of immunosuppressive therapy appear to reflect these trends. These results provide population‐level evidence on MG risk dynamics surrounding pregnancy.

**Conclusion:** This nationwide cohort study offers novel insights into MG risk dynamics during pregnancy and postpartum. These insights will inform clinical counselling and management strategies for women with MG planning pregnancy.

**Disclosure:** Nothing to disclose.

## EPO‐0468

### Effect of exercise on functional decline in Inclusion body myositis based on the Yale IBM registry

#### A. Ozonoff^1^; G. Cutter^2^; E. Chakraborty^1^; K. Petschke^3^; A. Paltiel^3^; B. Roy ^1^

##### ^*1*^ *Department of Neurology, Yale University, New Haven, USA;* ^*2*^ *Department of Biostatistics, UAB School of Public Health, Birmingham, USA;* ^*3*^ *Health Policy and Management, Yale School of Public Health, New Haven, USA*

**Background and aims:** Inclusion body myositis (IBM) is a rare, progressive muscle disease with preferential involvement of quadriceps and long finger flexors, leading to muscle weakness and atrophy, gait impairment, and loss of hand function. The potential beneficial role of exercise has been implicated, but the impact of exercise in IBM, including the safety of exercise in IBM, is not well established.

**Methods:** In this study, we leveraged IBM functional rating scale (IBM‐FRS) scores, longitudinally collected by the Yale IBM registry (IBMR). A linear mixed‐effects model was used to examine the role of exercise on functional decline in IBM.

**Results:** We included 401 patients (age 68.4 ± 8.4 years), of whom 139 were women (34.7%). The baseline mean IBM‐FRS score was 26.9 ± 6.8. All patients showed a decline in IBM‐FRS over time. We did not notice any major differences in disease progression by gender or age. The habit of exercise was associated with a slower rate of decline (*p*‐value < 0.01), with a graded benefit for more active patients (*p*‐value < 0.001). Patients actively engaged in physical therapy and walking showed slower deterioration than those with no exercise (*p*‐values of 0.003 and 0.04, respectively). Analyses of within‐person changes emphasized the role of exercise continuity.

**Conclusion:** These findings, based on patients’ self‐reports, suggest safety and the potential benefit of remaining physically active in IBM. Large and long‐duration clinical trial with a structured exercise regimen to delineate the true impact of exercise on IBM disease progression is warranted.

**Disclosure:** AO, GC, EC, KP have nothing to disclose. GC reports to be in the Data and Safety Monitoring Boards of Applied Therapeutics, AI therapeutics, Amgen‐NMO peds, AMO Pharma, Argenx, Astra‐Zeneca, Bristol Meyers Squibb, CSL Behring,, DiamedicaTherapeutics, Horizon Pharmaceuticals, Immunic, Inhrbx‐sanfofi, Karuna Therapeutics, Kezar Life Sciences, Medtronic, Merck, Meiji Seika Pharma, Mitsubishi Tanabe Pharma Holdings, Prothena Biosciences, Novartis, Pipeline Therapeutics (Contineum), Regeneron, Sanofi‐Aventis, Teva Pharmaceuticals, United BioSource LLC, University of Texas Southwestern, Zenas Biopharmaceuticals. He is also in the Consulting or Advisory Boards: Alexion, Antisense Therapeutics/Percheron, Avotres, Biogen, Clene Nanomedicine, Clinical Trial Solutions LLC, Endra Life Sciences, Genzyme, Genentech, Immunic, Klein‐Buendel Incorporated, Kyverna Therapeutics, Inc, Linical, Merck/Serono, Noema, Neurogenesis, Perception Neurosciences, Protalix Biotherapeutics, Regeneron, Revelstone Consulting, Roche, Sapience Therapeutics, Tenmile. He is employed by the University of Alabama at Birmingham and President of Pythagoras, Inc. a private consulting company located in Birmingham AL. BR has served as a consultant/advisor for Alexion (now part of AstraZeneca), Takeda, Sanofi, and argenx. Additionally, BR has received research support from the Martin Shubik Fund for IBM at Yale University, NIH, MDA, Abcuro Pharmaceuticals, argenx, Immunovant, and Takeda. BR has small stocks in Cabaletta Bio, CAVA, and Pfizer.

## EPO‐0469

### Continuing medical education improves competence in implementing new therapies for myasthenia gravis

#### S. Rohani‐Montez^1^; M. Calle^1^; F. David ^1^; E. Santos^2^; N. Gilhus^3^

##### ^*1*^ *Medscape Education Global, London, UK;* ^*2*^ *Biomedical Sciences School of University of Porto, Santo Antonio University Hospital, Porto, Portugal;* ^*3*^ *University of Bergen, Haukeland University Hospital, Bergen, Norway*

**Background and aims:** Treatments for myasthenia gravis (MG) are evolving, requiring physicians to consider new mechanisms including FcRN or complement inhibition. We sought to improve knowledge of these new therapies and their application in today's clinical practice.

**Methods:** Physicians participated in a 3‐part video‐based, online CME activity (available at www.medscape.org/viewarticle/1002773). The education effects were assessed using a 3‐question, repeated pairs, pre‐assessment/post‐assessment study design. One question assessed confidence. Pre‐ to post‐assessment differences were evaluated using the McNemar's test. *p* ≤ 05 is the significance level. The activity launched in 08‐2025 and data were collected through 10‐2025.

**Results:** 53 neurologists and 80 primary care physicians (PCPs) completed all pre‐ and post‐assessment questions. Overall significant improvements were seen after participation for neurologists (correct pre‐assessment response rate of 47% vs 73% at post‐assessment; *p* < 0.001, Cohen's d = 0.9), and PCPs (correct pre‐assessment response rate of 31% vs 54% at post‐assessment; *p* < 0.001, Cohen's d = 0.98). Improvements were observed in clinicians’ understanding of the rationale for FcRn inhibition in MG, and how best to tailor new therapies in different clinical scenarios (Figure 1). After participating, 38% of neurologists and 26% of PCPs had measurable improved confidence in determining which patients with MG may benefit from a targeted therapy. Post‐education, 52% of neurologists and 42% of PCPs said they intend to consider targeted therapies earlier for patients with severe or rapidly progressive MG (other responses in Figure 2).

**Figure:**
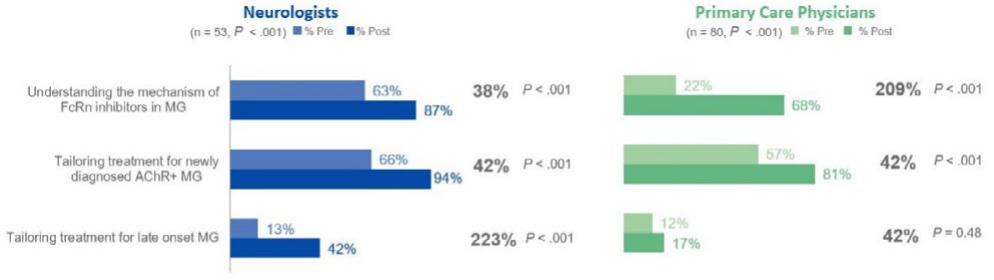
**FIGURE 1** Changes in Knowledge and Competence Pre‐ vs Post‐Education.

**Figure:**
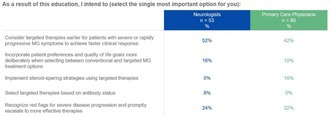
**FIGURE 2** Intended practice changes post‐education.

**Conclusion:** This study demonstrates the success of online, multi‐component, video‐based education in improving competence in implementing new targeted therapies for MG in practice.

**Disclosure:** Nothing to disclose.

## EPO‐0470

### Effect of subcutaneous self‐administered Gefurulimab on ocular muscle function in myasthenia gravis in the PREVAIL Trial

#### F. Saccà ^1^; D. Annane^2^; M. Mäurer^3^; M. Takahashi^4^; S. Rakhade^5^; J. Scholz^5^; S. Shang^5^; T. Vu^6^

##### ^*1*^ *NSRO Department, University of Naples Federico II, Napoli, Italy;* ^*2*^ *General ICU, Hôpital Raymond Poincaré, Garches, France;* ^*3*^ *Clinic for Neurology and Neurological Early Rehabilitation, Würzburg‐Mitte Hospital, Würzburg, Germany;* ^*4*^ *Department of Clinical Laboratory and Biomedical, The University of Osaka Graduate School of Medicine Sciences, Osaka, Japan;* ^*5*^ *Alexion, AstraZeneca Rare Disease, Boston, USA;* ^*6*^ *Department of Neurology and Neuromuscular Medicine, University of South Florida Morsani College of Medicine, Tampa, FL*

**Background and aims:** Gefurulimab, a novel dual‐binding nanobody, blocks complement component 5 activation. The phase 3 PREVAIL trial, assessing the efficacy and safety of gefurulimab in adults with anti‐acetylcholine receptor antibody‐positive (AChR‐Ab+) generalised myasthenia gravis (gMG) (NCT05556096), met all primary and secondary endpoints and demonstrated clinically meaningful improvement from baseline in Myasthenia Gravis Activities of Daily Living (MG‐ADL) total score at week 26 compared with placebo. Here, we present the effect of gefurulimab on ocular symptoms.

**Methods:** Adults with AChR‐Ab+ gMG were randomised 1:1 to weekly subcutaneous self‐administration of gefurulimab or placebo. The primary endpoint was change from baseline in MG‐ADL total score at wk26. MG‐ADL and Quantitative Myasthenia Gravis (QMG) subdomain scores were also assessed.

**Results:** 260 patients were randomized (gefurulimab: *n* = 131; placebo: *n* = 129). Mean±SD baseline MG‐ADL and QMG total scores were 9.0±2.16 and 14.8±4.37, respectively. Improvements in ocular subdomain scores were observed at first assessment after treatment initiation (MG‐ADL, wk1; QMG, wk4) and sustained through wk26. Least squares mean±SEM change from baseline at wk26 in MG‐ADL and QMG ocular subdomain scores for gefurulimab versus placebo were −1.2 ± 0.14 versus −0.6±0.13 (treatment difference, −0.6±0.19; 95% CI: −1.0, −0.2; *p* = 0.0016) and −1.7±0.14 versus −0.9±0.14 (treatment difference, −0.7±0.19; 95% CI: −1.1, −0.4, *p* = 0.0002), respectively. Improvements were also observed in other MG‐ADL and QMG subdomains. Adverse events were similar between groups; no meningococcal infections were reported.

**Conclusion:** Gefurulimab, self‐administered once weekly via subcutaneous injection, showed early and sustained improvements in MG‐ADL and QMG ocular subdomain scores, demonstrating benefits for patients with gMG experiencing ocular symptoms.

**Disclosure:** FS has received public speaking honoraria from Alexion, argenx, Biogen, Genpharm, Medpharma, Medison Pharma, Mylan, Neopharm Israel, Novartis, Roche, Sanofi, UCB, and Zai Lab; compensation for advisory boards from Alexion, Almirall, Amgen, argenx, AstraZeneca, Avexis, Biogen, Dianthus, Johnson & Johnson, Lexeo, Novartis GmBh, Reata, Sandoz, UCB, and Zai Lab; consultation fees from Alexion, argenx, Dianthus, EPG Communication Holdings Ltd, Johnson&Johnson, Medscape, PeerView, Vitaccess; clinical trial support from Almirall; funding from Agenzia Italiana del Farmaco (AIFA), Alexion, Associazione Italiana per la lotta alle Sindromi Atassiche (AISA), Freidreich Ataxia Research Alliance (FARA), Roche SpA, Tristan Allamby Research Fund (TARFfa); and was a clinical trial principal investigator from Alexion, argenx, Dianthus, Immunovant, Lediant, Lexeo, Novartis, Prilenia, Remgen, and Sanofi. DA has received study funding from Alexion, AstraZeneca Rare Disease; and serves on the CHAMPION‐MG study steering committee. MM has received compensation for lectures, consulting and travel expenses from Alexion, AstraZeneca Rare Disease, Almirall, argenx, Biogen, BMS, CSL Behring, Hexal, Genzyme, GSK, Merck, Novartis, Roche, Sanofi, Teva, and UCB. MT has received consulting fees from Alexion Pharma GK, Alexion AstraZeneca Rare Disease, argenx, Hanall Biopharma, and UCB Pharma; research support from the Japan Blood Products Organization; and speaker honoraria from Alexion Pharma GK, AstraZeneca Rare Disease, argenx, and UCB. SR, JS, and SS are employees of Alexion, AstraZeneca Rare Disease, and hold stock or stock options in AstraZeneca. TV has received research support from Alexion/AstraZeneca Rare Disease, Amgen, Amylyx, argenx, Cartesian, CSL Behring, Dianthus, Healy Platform Trials, Immunovant, Ipsen, Johnson & Johnson, PTC Therapeutics, Regeneron, Sanofi, UCB, and Woolsey Pharma; served on speakers bureaus for Alexion, AstraZeneca Rare Disease, argenx, and CSL Behring; and served on ad boards or as consultant for Alexion/AstraZeneca Rare Disease, Amgen, argenx, ImmunAbs, Johnson & Johnson, and UCB.

## EPO‐0471

### Expanded phenotypic and ultrasound characterization of the p.Ser55Phe myotilinopathy variant: The MYOT MUR study

#### G. Zmork Martínez ^1^; L. Pulido Fraiz^1^; Ó. Camejo Mas^1^; M. Aledo Serrano^1^; A. Mena Bravo^1^; M. Lorenzo Diéguez^1^; A. García Leal^1^; J. Pérez Lucas^2^; A. Terry Sanz‐Pastor^3^; E. Pérez García^3^; R. Martínez Marín^1^

##### ^*1*^ *Neurology, La Paz University Hospital and Stroke Center, Madrid, Spain;* ^*2*^ *Neurology, El Tajo University Hospital, Madrid, Spain;* ^*3*^ *Neurosurgery, La Paz University Hospital and Stroke Center, Madrid, Spain*

**Background and aims:** Myotilinopathies are the most frequent myofibrillar myopathy in Spain, mainly the p.Ser55Phe MYOT variant. We characterized the phenotype and disease progression in the largest known cohort of this variant from Murcia, Spain.

**Methods:** We conducted an ambispective observational study (2022–2025) on 46 p.Ser55Phe carriers. Annual evaluations included history, pedigree analysis, neuromuscular ultrasound (NMUS) and neurological examination including North Star Ambulatory Assessment (NSAA), Medical Research Council (MRC) scale, and 6‐minute walk test (6mWT). Sex differences were analyzed using non‐parametric tests.

**Results:** The cohort (*n* = 46; 44% female) had a mean onset age of 47.4 years. 71.7% were symptomatic. Weakness was asymmetric (89.7%). Lower limb weakness (67.4%) typically had distal onset (*n* = 15) with frequent foot drop (60.6%), progressing proximally. Upper limb weakness (54.3%) was mostly proximal at onset (68.8%), progressing distally. Pain (60.9%) and dyspnea (37%) were frequent. Males were more symptomatic (84% vs 55%, *p* = 0.049) with earlier onset than females (45.3 vs 51.5 years, *p* = 0.013). Males exhibited lower limb predominance (*p* = 0.006), more foot drop (*p* < 0.001), atrophy (*p* < 0.001), and lower NSAA, MRC, and 6mWT scores across most visits. Females showed axial predominance. NMUS revealed increased echogenicity initially in the tibialis anterior and extensor digitorum brevis, progressing to the vastus intermedius and brachialis.

**Conclusion:** We describe the largest *p*.Ser55Phe myotilinopathy cohort to date, an expanded clinical phenotype, and a characteristic ultrasound pattern. Weakness is asymmetric and distal‐predominant. We highlight a significant sex difference, with males experiencing a more severe course and worse functional prognosis.

**Disclosure:** Nothing to disclose.

## EPO‐0472

### Immediate FcRn inhibitor initiation following fast‐acting treatment in generalised myasthenia gravis: A single‐centre retrospective cohort study

#### K. Yanagisawa; T. Ishii; T. Saito; M. Zenko; T. Aoki; A. Sugie; H. Myojin; T. Saito; Y. Kondo; K. Sato; T. Uchiyama

##### Department of Neurology, Seirei Hamamatsu General Hospital, Shizuoka, Japan

**Background and aims:** Early initiation of fast‐acting treatment (FT; intravenous immunoglobulin, plasma exchange, or intravenous methylprednisolone) improves long‐term outcomes in patients with generalised myasthenia gravis (gMG). However, the optimal approach to achieve durable disease control after FT remains unclear. We hypothesised that the timely initiation of an FcRn inhibitor (FcRn‐i) as a "consolidation therapy" following FT may promote sustained benefit, particularly in patients with shorter disease duration.

**Methods:** This retrospective single‐centre cohort study included adults with gMG treated with FcRn‐i between January 2022 and September 2025. Immediate initiation was defined as FcRn‐i administration within 60 days after the last FT and disease duration ≤2 years; all others were classified as deferred. The primary outcome was the time to treatment escalation to additional FT or C5 inhibitor therapy. Group differences were analysed using Kaplan–Meier curves with log‐rank tests and Firth's penalised Cox models adjusted for age and MGFA classification.

**Results:** Thirty‐eight patients were included (immediate, *n* = 8; deferred, *n* = 30). Median follow‐up was 355 days (IQR 190–572). Treatment escalation occurred in 0/8 immediate patients and 15/30 deferred patients. Kaplan–Meier analysis showed a significant difference (log‐rank *p* = 0.02). In Firth's Cox model, immediate initiation was associated with a markedly lower hazard of treatment escalation (HR 0.09, 95% CI 0.001–0.70).

**Figure:**
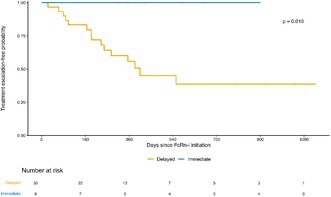
**FIGURE 1** Kaplan–Meier curves of treatment escalation–free probability since FcRn‐i initiation in gMG (immediate *n* = 8 vs delayed *n* = 30). Tick marks indicate censoring; numbers at risk are shown below. Log‐rank *p* = 0.019.

**Conclusion:** Immediate post‐FT initiation of FcRn‐i was associated with a reduced hazard of treatment escalation to additional FT or C5 inhibitor therapy. This consolidation‐type strategy may support more stable disease control in patients early in the disease course and will be further evaluated in larger cohorts.

**Disclosure:** Nothing to disclose.

## EPO‐0473

### Measuring daily living ambulation of patients with high accuracy to derive meaningful clinical conclusions

#### M. Poleur^1^; N. Noblot^2^; O. Piquet^2^; N. Benmhammed^1^; M. Duclos^1^; L. Médard^1^; A. Tricot^2^; D. Eggenspieler^2^; L. Servais ^3^

##### ^*1*^ *University department of neurology, CHR Citadelle, Liège, Belgium;* ^*2*^ *Sysnav, Vernon, France;* ^*3*^ *University of Oxford, Oxford, UK & Department of Paediatrics, University and University Hospital of Liège, Liège, Belgium*

**Background and aims:** Wearable devices are increasingly used in clinical trials in neurology. However, their actual capacity to precisely identify and measure movements in different conditions is never reported. The qualification of a digital measure, stride velocity 95th centile, as a primary endpoint in Duchenne muscular dystrophy (DMD) showed that suitable wearable digital health technologies (wDHT) can be reliable tools to measure disease progression and efficacy of therapeutic interventions in diseases impacting ambulation. It is crucial to establish in various populations the wDHT accuracy to detect strides and to precisely measure gait features.

**Methods:** This study enrolled 100 participants, including patients with amyotrophic lateral sclerosis, Charcot‐Marie‐Tooth disease, myotonic dystrophy type 1, limb girdle muscular dystrophy, Pompe disease, myasthenia gravis, spinal muscular atrophy, Huntington disease, Parkinson's disease, chronic inflammatory demyelinating polyneuropathy, DMD and healthy volunteers. Participants wore Syde ankle sensors and walked at different paces on path with turns, in a room equipped with a motion capture system (reference).

**Results:** Individual strides were correctly detected with an average precision of 99.5% and average recall of 97.8% (Table 1). The mean difference between the wDHT and the reference on stride length and stride velocity were −0.8 to 4.8 cm and −0.9 to 4.3 cm/s, respectively. Standard deviations of the absolute errors were between 1.5–4.1 cm for stride length and 1.5–3.5 cm/s for stride velocity.

**Figure:**
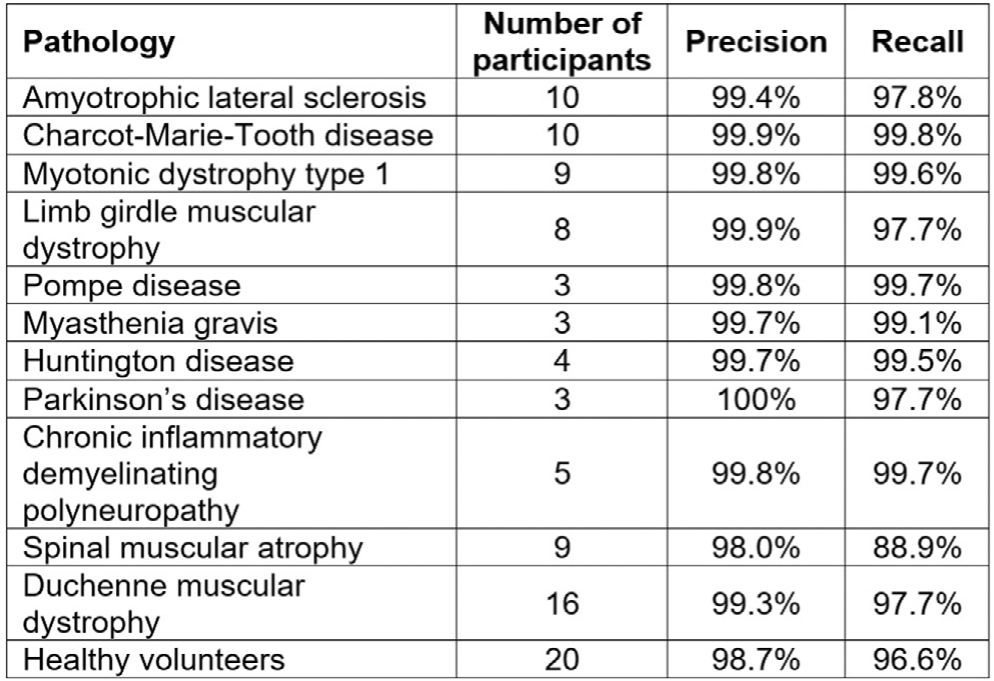
**TABLE 1** Stride detection results by pathology. Precision is defined as the ratio of well‐detected strides among all strides detected by the wDHT. Recall is defined as the ratio of well‐detected strides among all existing strides.

**Conclusion:** We demonstrated Syde excellent precision to detect individual strides and similar performance in measuring gait parameters in several pathologies affecting ambulation, supporting its application to develop digital endpoints.

**Disclosure:** This study was funded by Sysnav. NN, OP, AT and DE are employees of Sysnav. LS has given consultancy for Sysnav.

## EPO‐0474

### Anti‐Titin antibodies and thymoma in myasthenia gravis: A 10‐year age‐adjusted portuguese cohort study

#### M. Almeida ^1^; I. Carvalho^1^; A. Geraldo^1^; R. Cunha^2^; L. Negrão^1^; A. Matos^1^; L. Almendra^1^

##### ^*1*^ *Neuromuscular and Neurophysiology Unit, Department of Neurology – Unidade Local de Saúde de Coimbra, Coimbra, Portugal;* ^*2*^ *Clinical Pathology Laboratory – Unidade Local de Saúde de Coimbra, Coimbra, Portugal*

**Background and aims:** Anti‐titin antibodies are detected in approximately one‐third of patients with myasthenia gravis (MG) and are considered a sensitive biomarker for thymoma in patients aged ≤50 years. However, their high prevalence in late‐onset MG reduces specificity, limiting their clinical utility in older patients. We aimed to assess the predictive value of anti‐titin antibodies for thymoma in a Portuguese MG cohort.

**Methods:** We conducted a retrospective study of all patients newly diagnosed with MG between January 1, 2015, and August 1, 2025. Anti‐titin antibody status was assessed by immunoblotting, and thymoma was evaluated using chest CT, PET‐CT, or histopathology. Associations between anti‐titin positivity, age, and thymoma were analyzed.

**Results:** Among 114 MG patients (mean age 66.6 years), 34 had thymoma and 81 were anti‐titin–positive. In patients <50 years, anti‐titin positivity showed a non‐significant trend toward association with thymoma (OR 4.44, *p* = 0.181). In patients ≥50 years, anti‐titin positivity was not predictive of thymoma (OR 0.44, *p* = 0.171) and was inversely associated with thymoma in those aged 50–75 years (OR 0.23, *p* = 0.042). Age significantly modified the predictive value of anti‐titin antibodies (*p* = 0.039). Anti‐titin positivity was markedly more frequent in older non‐thymoma patients (≥50: 81.2% vs <50: 27.3%, *p*<0.001), while sensitivity among thymoma patients was similar across age groups.

**Conclusion:** In MG patients over 50 years, anti‐titin antibodies show reduced specificity for thymoma due to high positivity among non‐thymoma cases. In patients aged 50–75 years, anti‐titin positivity may favor the absence of thymoma. These findings highlight the age‐dependent limitations of anti‐titin antibodies in thymoma prediction.

**Disclosure:** The authors have nothing to disclosure.

## EPO‐0475

### Clinical and immunological features of thymoma patients with and without myasthenia gravis: A pilot study

#### M. Corbo ^1^; F. Beretta^1^; R. Taliani^1^; S. Bongiolatti^2^; L. Voltolini^2^; L. Massacesi^1^; V. Damato^1^; G. Spagni^1^

##### ^*1*^ *Department of Neurosciences, Psychology, Drug Research and Child Health (NEUROFARBA), University of Florence, Italy;* ^*2*^ *Thoracic Surgery Unit, Careggi University Hospital, Florence, Italy*

**Background and aims:** Thymoma‐associated myasthenia gravis (TAMG) is nearly invariably associated with acetylcholine receptor antibodies (AChRAb). However, approximately 15% of thymoma patients have AChRAb in the absence of MG and rarely may develop post‐thymectomy MG (PTMG). This study evaluates the clinical and immunological features of thymoma patients with and without MG to identify predictive biomarkers of PTMG.

**Methods:** Patients with thymoma treated in our Centre between 2011 and 2025 were included in the study. Demographic and clinical data were collected, and available serum samples were evaluated for in vitro complement activation using a flow cytometry live cell‐based assay.

**Results:** Fifty thymoma patients were included in the study: 17/50 (34%) TAMG, 27/50 (54%) thymoma patients with no MG and AChRAb negative (AChRAb‐MG‐), 6/50 (12%) AChRAb positive thymoma patients without MG (AChRAb+MG‐), of whom 2/6 (33%) developed PTMG. Types AB/B thymomas predominated across groups (*n* = 40/50,80%) without statistically significant difference between MG and non‐MG patients (with/without AChRAb). Most had a Masaoka stage I–II (*n* = 43/50, 86%). The majority of patients had maximum MGFA>II (13/19, 68%). Sixteen serum samples were available for further analyses (10 TAMG, 2 PTMG, 4 AChRAb+MG‐). Complement activation was significantly lower in AChRAb+MG‐ thymoma patients compared to TAMG and PTMG patients (*p* = 0.0332, Mann‐Whitney U test). In one PTMG patient with available serial serum samples, level of complement activation increased significantly from thymoma diagnosis to MG onset.

**Conclusion:** In vitro assessment of complement activation could represent a novel biomarker of PTMG. Future studies are warranted to investigate in‐depth antibody as well as thymic and B‐cells properties in PTMG patients.

**Disclosure:** The authors report no disclosures or conflicts of interest relevant to this work.

## EPO‐0476

### Omaveloxolone for Friedreich ataxia: What does 'Cochrane' have to say about it?

#### S. lyons

##### Department of Neurology, University Hospital Waterford, Waterford, Ireland

**Background and aims:** Omaveloxolone (RTA 408) is thought to activate the nuclear factor erythroid‐derived 2‐related factor (Nrf2) signalling which is grossly impaired in Friedreich ataxia (FRDA) which is a rare autosomal recessive, progressive disease, typically presents around puberty. Approximately one third of people with FRDA are diagnosed after the age of 20. It usually presents with unsteadiness, followed by progressive limb and gait ataxia. It can be associated with scoliosis, pes cavus, cardiomyopathy and diabetes mellitus. Cochrane Reviews are systematic reviews and preform meta‐analyses, where possible. They are a key resource in evidence‐based medicine.

**Methods:** The aim of the review was to assess the effects of pharmacological treatments in Friedreich ataxia after one year's treatment. We found 7 randomised controlled trials of 1 year duration. We synthesised results for each outcome using meta‐analysis, used GRADE to assess the certainty of evidence.

**Figure:**
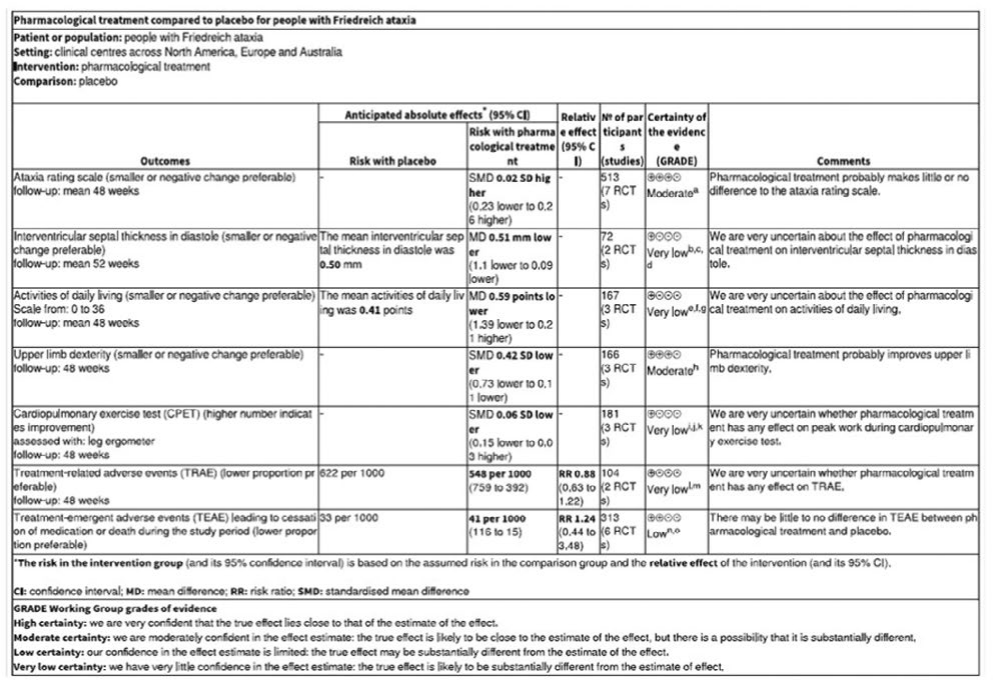
**FIGURE 3** Summary of findings table.

**Results:** Pharmacological treatments probably make little or no difference to change in ataxia rating scale (ARS) but improved upper limb dexterity (ULD) There was variation in that omaveloxolone helped ARS and leriglitazone helped ULD. The evidence for both outcomes was moderate and was an unexpected finding given that both outcomes share similar competencies.

**Figure:**
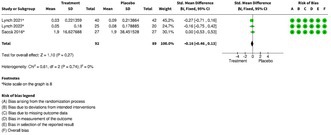
**FIGURE 1** Forest plot‐ change in ataxia rating score after 12 months treatment.

**Figure:**
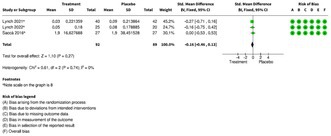
**FIGURE 2** Forest plot ‐ change in upper limb dexterity after 12 months treatment.

**Conclusion:** The seven RCTs' outcomes were downgraded due to imprecision to small number of participants or wide confidence intervals. This would suggest that studies with approximately 200 or more events are required to help capture the subtle changes that occur over time in this slowly progressive disease.

**Disclosure:** Shane Lyons, Prashant Janjal, Patricia Patton, Michael Fahey: None known. Mary Kearney attended a Biogen Advisory Board meeting; Massimo Pandolfo was involved in two studies in this review. This abstract is based on a pre‐peer review draft of a Cochrane review submitted to the Cochrane Database of Systematic Reviews (www.cochranelibrary.com).

## EPO‐0477

### Safety and efficacy of claseprubart, an active C1s inhibitor, in patients with generalized myasthenia gravis

#### S. Peric ^1^; P. Narayanaswami^2^; M. Smilowski^3^; A. Slowik^4^; S. Gokhale^5^; C. Briggs^5^; U. Siddiqui^5^; S. Randhawa^5^; M. Truman^5^; L. Hickey^5^; S. Shelly^6^; T. Vu^7^; J. Vissing^8^

##### ^*1*^ *University of Belgrade, Belgrade, Serbia;* ^*2*^ *Beth Israel Deaconess Medical Center/Harvard Medical School, Boston, USA;* ^*3*^ *Neurologia Śląska Centrum Medyczne, Katowice, Poland;* ^*4*^ *Jagiellonian University, Kraków, Poland;* ^*5*^ *Dianthus Therapeutics, USA;* ^*6*^ *Rambam Medical Center, Haifa, Israel;* ^*7*^ *University of South Florida, Tampa, USA;* ^*8*^ *University of Copenhagen, Copenhagen, Denmark*

**Background and aims:** The classical complement pathway plays a significant role in the pathogenesis of generalized myasthenia gravis (gMG). Claseprubart is a monoclonal antibody that targets the classical pathway by inhibiting active C1s. The MaGic trial assessed the safety and efficacy of claseprubart in adults with acetylcholine receptor antibody positive (AChR+) gMG.

**Methods:** MaGic (NCT06282159) is a randomized, double‐blind, placebo‐controlled Phase 2 study. 65 participants with AChR+ gMG were randomized 1:1:1 to receive: claseprubart 300 mg (Q2W), claseprubart 600 mg (Q2W), or placebo for 13 weeks, followed by a 52‐week open‐label extension and 40‐week safety follow‐up. Endpoints at 13 weeks included safety, tolerability, efficacy (MG activities of daily living (MG‐ADL), Quantitative MG score (QMG), Minimal Symptom Expression (MSE) and MG Composite scale (MGC).

**Results:** Claseprubart was well tolerated with no serious adverse events, no serious bacterial infections, and no autoimmune activation. Injection site reactions were infrequent and mild to moderate. At Week 13, claseprubart improved MG‐ADL by 4.6 (*p* = 0.0113) for 300 mg, 5.4 (*p* = 0.0006) for 600 mg, and 2.8 for placebo (no significant difference between active treatment arms). Significant improvement in MG‐ADL was observed as early as Week 1. At Week 13, claseprubart 300 mg improved QMG by 4.4 (*p* = 0.0144), claseprubart 600 mg by 4.5 (*p* = 0.0111), and 2.0 for placebo. MSE was achieved by 37% (300 mg) and 27% (600 mg) versus 14% for placebo. MGC for 300 mg was 8.7 (*p* = 0.0008), 8.6 (*p* = 0.0008) for 600 mg versus 3.1 for placebo.

**Conclusion:** With a favorable safety profile, claseprubart demonstrated rapid, statistically significant, and clinically meaningful improvements in MG‐ADL, QMG, MGC.

**Disclosure:** SP grants or honoraria from Dianthus, Amgen, Avidity, Amicus Swixx, Argenx, AstraZeneca, Immunabs, Octapharma, Roche, Viatris, Zentiva. PN grants or honoraria from AHRQ, PCORI, Argenx, Cabaletta Bio, AstraZeneca, Amgen, Cartesian, CVS, Dianthus, Serono, Immuneabs, Immunovant, J&J, UCB, Viridian, Vor‐Biol; Sanofi, Argenx, NMD. MS nothing to declare. AS nothing to declare. SG nothing to declare. CB employee of Dianthus Therapeutics US employee of Dianthus Therapeutics SR employee of Dianthus Therapeutics MT employee of Dianthus Therapeutics LH employee of Dianthus Therapeutics SS grants or honoraria from Dianthus therapeutics, J&J, Argenex, Astrazenica, Alexion, Mediaon, Neopharm, Novartis and Sanofi. TV grants or honoraria from Alexion/AstraZeneca, Amgen, Argenx, Cartesian, COUR, Dianthus, EMD Serono, ImmunAbs, Immunovant, J&J, NMD, Regeneron, VOR, UCB, CSL Behring, and VOR. JV grants or honoraria from Roche, Regeneron, Argenx, UCB, Amgen, Novartis, Dianthus, Lundbeck A/S, Merck Toleranzia, NMD, AstraZeneca, J&J, Hansa.

## EPO‐0478

### Interim analysis of an observational, prospective, multicenter study of efgartigimod as consolidation therapy after acute exacerbation in MG

#### Y. Da ^1^; H. Yang^2^; Y. Guan^3^; C. Zhao^4^; H. Xiao^5^; Q. Jiang^6^; G. Qi^7^; T. Chang^8^; C. Yang^9^; Z. Li^10^; Y. Xu^11^; Y. Zhang^12^; H. Zhou^13^; Q. Wang^14^; R. Duan^15^; M. Ding^16^; R. Zhang^17^; H. Feng^18^; Y. Wang^19^; H. Zhang^20^

##### ^*1*^ *Department of Neurology, Xuanwu Hospital Capital Medical University, Beijing, China;* ^*2*^ *Department of Neurology, Xiangya Hospital of Central South University, Changsha, China;* ^*3*^ *Department of Neurology, Peking Union Medical College Hospital, Chinese Academy of Medical Sciences, Beijing, China;* ^*4*^ *Department of Neurology, Huashan Hospital, Fudan University, Shanghai, China;* ^*5*^ *Department of Neurology, Neuromedicine Center, The University of Hong Kong – Shenzhen Hospital, Shenzhen, Guangdong Province, China; Shenzhen**;*** ^*6*^ *Department of Neurology, The First Affiliated Hospital of Guangzhou University of Chinese Medicine, Guangzhou, China;* ^*7*^ *Center of Treatment of Myasthenia Gravis, People's Hospital of Shijiazhuang, Shijiazhuang, China;* ^*8*^ *Department of Neurology, Tangdu Hospital, The Fourth Military Medical University, Xi'an, China;* ^*9*^ *Department of Neurology, Tianjin Medical University General Hospital, Tianjin, China;* ^*10*^ *Department of Neurology, Tongji Hospital, Tongji Medical College, Huazhong University of Science and Technology, Wuhan, Hubei, China;* ^*11*^ *Department of Neurology, The Second Affiliated Hospital of Zhejiang University School of Medicine, Hangzhou, China;* ^*12*^ *Department of Neurology, The Affiliated Hospital of Xuzhou Medical University, Xuzhou, China;* ^*13*^ *Department of Neurology, West China Hospital, Sichuan University, Chengdu, China;* ^*14*^ *Department of Neurology, Qilu Hospital of Shandong University, Jinan, China;* ^*15*^ *Department of Neurology, The First Affiliated Hospital of Shandong First Medical University & Shandong Provincial Qianfoshan Hospital, Jinan, China**;*** ^*16*^ *Department of Neurology, Renmin Hospital of Wuhan University, Wuhan, China;* ^*17*^ *Department of Neurology, First Affiliated Hospital of Zhengzhou University, Zhengzhou, China;* ^*18*^ *Department of Neurology, The First Affiliated Hospital of Sun Yat‐sen University, Guangdong Provincial Key Laboratory of Diagnosis and Treatment of Major Neurological Diseases, Guangzhou, China;* ^*19*^ *Department of Neurology, The Third Affiliated Hospital of Sun Yat‐sen University, Guangzhou, China;* ^*20*^ *Department of Neurology, Beijing Hospital, Beijing, China*

**Background and aims:** Patients with myasthenia gravis (MG) still require consolidation therapy after acute exacerbation to control symptoms and prevent relapse. This interim analysis aims to explore whether efgartigimod in addition to standard of care (SOC) provides added benefit as consolidation therapy.

**Methods:** Adult patients with Acetylcholine Receptor (AChR) antibody‐positive generalized MG who achieved clinically meaningful improvement after one treatment cycle of efgartigimod for acute exacerbation were enrolled in the study (NCT07079020). Patients were allocated to either efgartigimod plus SOC (EFG) group or SOC alone (SOCA) group based on real‐world clinical decision‐making, with a 6‐month follow‐up period. The primary endpoint was achievement of minimal symptom expression (MSE) within the second month of the consolidation phase and sustained MSE for more than four consecutive months.

**Results:** A total of 112 patients with ≥2 months of follow‐up by October 2025 were included (48 in SOCA group, 64 in EFG group). MSE was achieved in 64.58% of patients in the SOCA group and in 75% of patients in the EFG group (*p* = 0.010) (Figure 1). MG worsening was less frequently observed in the EFG group compared with the SOCA group, either relative to the previous visit (37.50% vs 50.00%, *p* = 0.013) or to the baseline (18.75% vs 35.42%, *p* = 0.016) (Figures 2 and 3).

**Figure:**
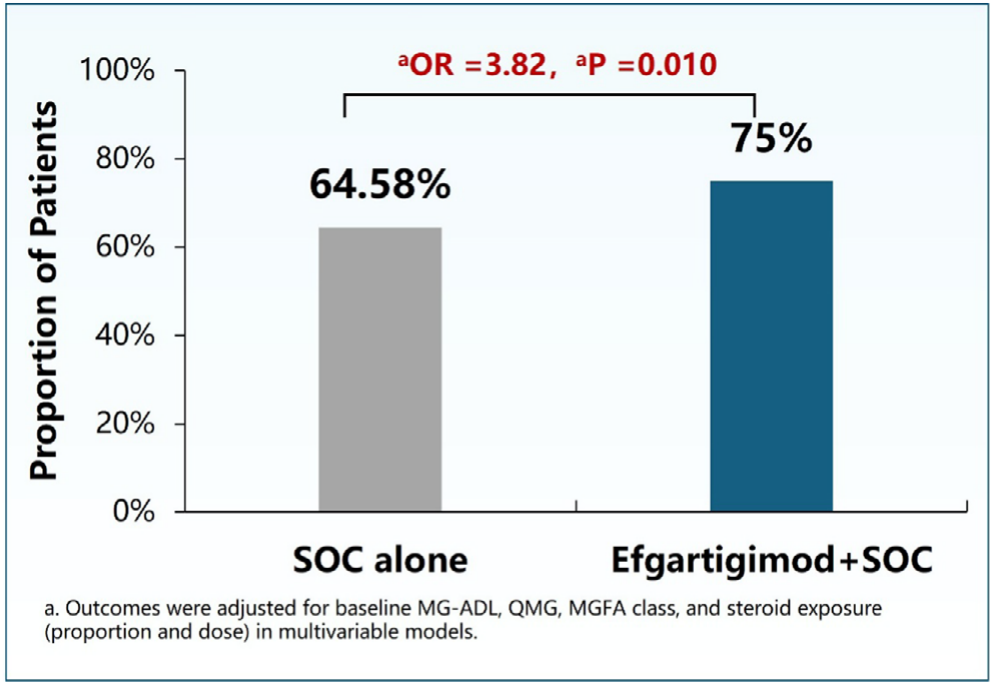
**FIGURE 1** Proportion of patients achieving MSE at the interim analysis.

**Figure:**
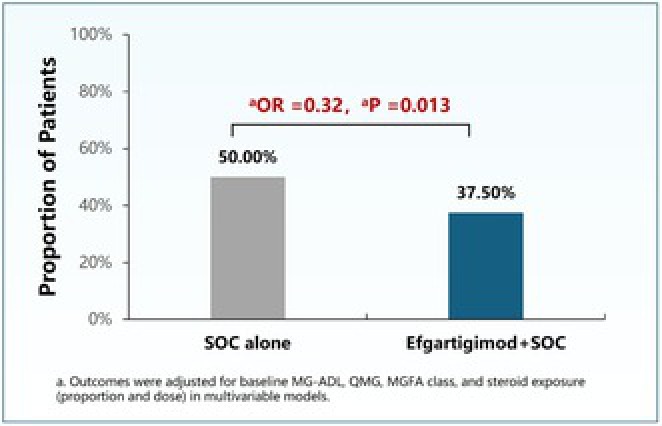
**FIGURE 2** Proportion of patients with MG worsening compared with the previous visit.

**Figure:**
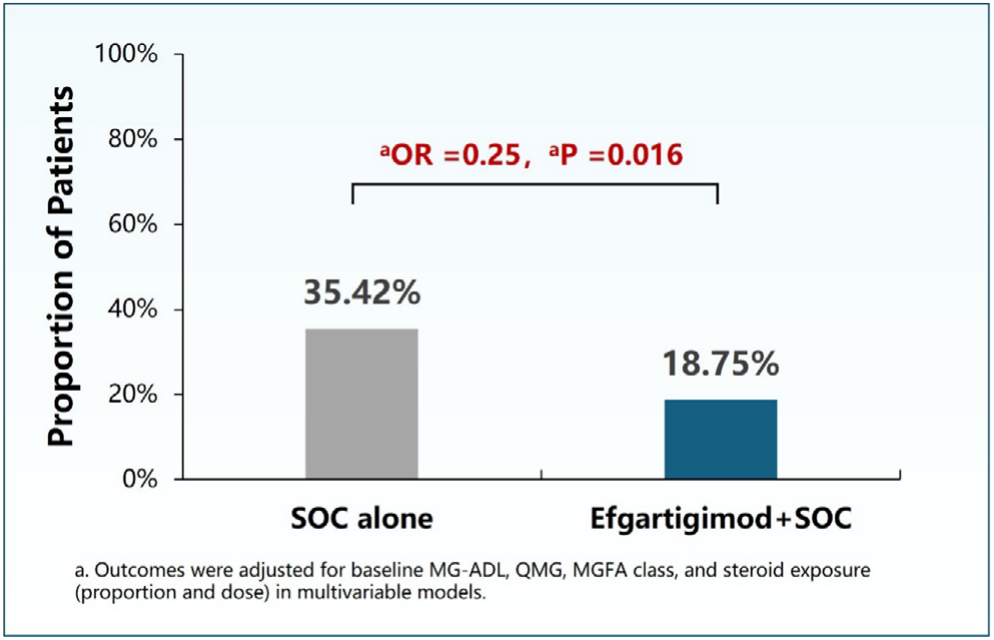
**FIGURE 3** Proportion of patients with MG worsening compared with the baseline.

**Conclusion:** In this interim analysis, efgartigimod was associated with higher rates of achieving MSE, and reduced risk of MG worsening in consolidation therapy. These findings support the role of efgartigimod as a therapeutic strategy in post‐exacerbation MG management.

**Disclosure:** This study was supported by the China Alliance for Rare Diseases (non‐profit organization).

Neurogenetics 3

## EPO‐0479

### Intrathecal AAV‐based gene therapy for metachromatic leukodystrophy: Preclinical efficacy and safety in a large animal model

#### A. Ayupova; A. Ibrahim; A. Fattakhova; A. Sharshakova; V. Solovyeva; Y. Mukhamedshina; A. Yakubova; A. Rizvanov

##### Institute of Fundamental Medicine and Biology, Kazan Federal University, Kazan, Russian Federation

**Background and aims:** Metachromatic leukodystrophy (MLD) is an autosomal recessive neurodegenerative lysosomal storage disorder caused by deficiency of arylsulfatase A (ARSA), leading to progressive motor dysfunction, spastic tetraparesis, ataxia, seizures, optic atrophy, and cognitive decline. The aim of this study was to develop and evaluate a gene therapy product based on recombinant adeno‐associated virus serotype Olig001 (AAVOlig001) carrying a codon‐optimized human ARSA gene.

**Methods:** AAVOlig001‐ARSA containing a unique codon‐optimized ARSA sequence was generated. Following intravenous administration of mannitol (1 g/kg), pigs received an intrathecal injection of AAVOlig001‐ARSA at a dose of 2 × 10^13^ gc/kg. ARSA enzymatic activity was dynamically assessed in cerebrospinal fluid (CSF) and plasma using pNCS. One month after administration, central nervous system (CNS) tissues were collected for analysis of ARSA activity, gene expression (qPCR), and protein expression by immunohistochemistry (IHC). Safety was evaluated using biochemical and hematological blood analyses.

**Results:** No significant differences in plasma ARSA activity were observed between experimental and control animals, while ARSA activity in CSF was detectable on day 28. In contrast, CNS tissue homogenates showed a marked increase in ARSA activity, most prominently in the cerebellum, cervical, thoracic, and lumbar spinal cord, and brainstem. Overexpression of the codon‐optimized ARSA gene and ARSA protein expression in the CNS were confirmed by qPCR and IHC. Blood biochemical parameters remained within physiological ranges; however, hematological changes observed on day 28 suggested systemic exposure to the vector and a potential immune response (Figure 1).

**Figure:**
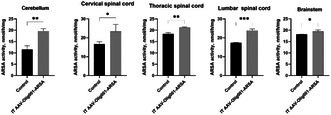
**FIGURE 1** ARSA enzymatic activity analysis in homogenates of various CNS regions on day 28 after AAV‐Olig001‐ARSA administration. administration. Data represent the mean of five biological replicates +‐ SD. * — *p* < 0.001.

**Conclusion:** In summary, intrathecal administration of AAVOlig001‐ARSA enabled efficient CNS delivery and induced ARSA overexpression.

**Disclosure:** This work was supported by the Kazan Federal University Strategic Academic Leadership Program (PRIORITY‐2030).

## EPO‐0480

### Giant axonal neuropathy: A homogeneous phenotype, a diverse genetic landscape, and the emergence of gene therapy

#### P. Tejada^1^; G. Fernández^1^; L. Jiménez^1^; S. Campusano^1^; Y. Rodríguez^1^; C. Pérez Lizardo^1^; K. Yorro^1^; A. Tejada^1^; A. Reyes ^1^; M. Rodríguez^2^

##### ^*1*^ *Synaptic Research League, Santo Domingo, Dominican Republic;* ^*2*^ *Neurology Department, Hospital Universitario Salvador Bienvenido Gautier, Santo Domingo, Dominican Republic*

**Background and aims:** Giant Axonal Neuropathy (GAN) is a genetic autosomal disorder caused by mutations in the GAN gene, patients usually develop a progressive degeneration of the central and peripheral nervous systems [1]. This study aims to investigate the correlation between specific genetic variants and clinical profiles to better define the disease spectrum.

**Methods:** We conducted a systematic literature review following PRISMA protocols, emphasizing abnormal presentations. PubMed and Google Scholar databases were searched using keywords "Giant Axonal Neuropathy". We evaluated age, genotype, CNS signs, cranial nerve involvement and physical signs.

**Results:** We included 29 studies, reporting 128 patients with Giant Axonal Neuropathy (GAN) revealed a uniform and severe clinical presentation. The mean age of onset was 2.8 years, with diagnosis at 9.3 years. Motor (100%) and sensory (99%) neuropathy were nearly universal, accompanied by cerebellar ataxia (93%) and curly hair (93%). Scoliosis was present in (52%) of patients. Autonomic dysfunction was less common (<10%). More than 20 pathogenic variants were identified in the GAN gene, see Figure 1 Intrathecal gene therapy (NCT02362438) in 14 patients showed stabilization of motor function (MFM‐32) and neurophysiological improvements, offering the first disease‐modifying intervention.

**Figure:**
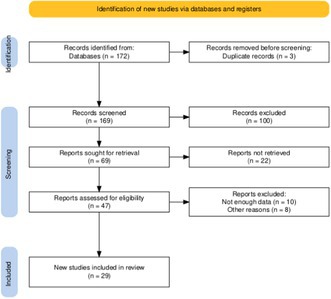
**FIGURE 1** Prisma‐P flowchart of the process of inclusion of studies in the systematic review.

**Figure:**
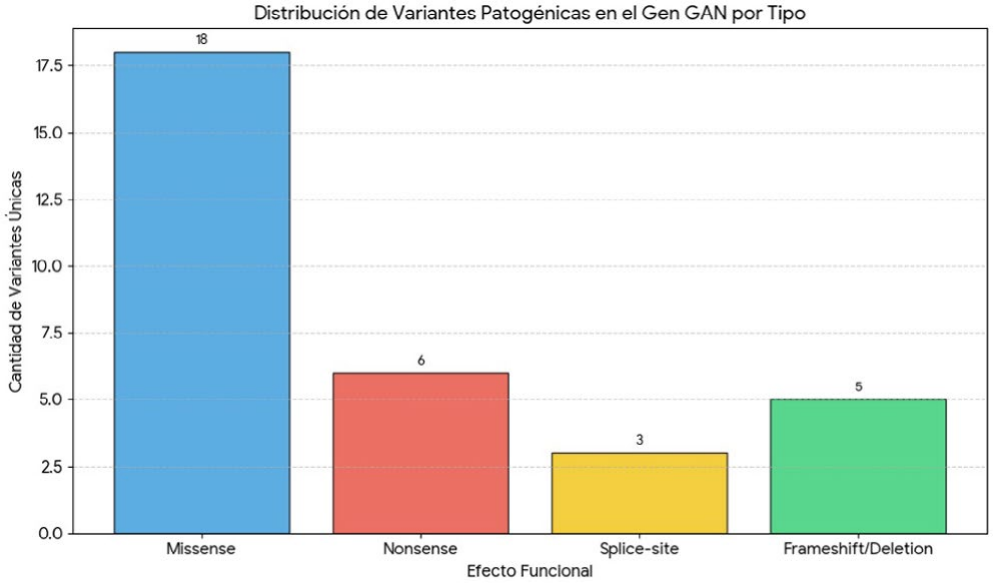
**FIGURE 2** Distribution of Pathogenic Giant Axonal Neuropathy Gene Variants by Type.

**Conclusion:** Despite the various genetic variants, GAN presents a remarkably uniform clinical profile, dominated by early‐onset neuropathy, ataxia, and characteristic capillary changes. This phenotypic consistency, along with new gene therapy data, provides a clear framework for diagnosis and highlights a promising intervention for disease modification.

**Disclosure:** Nothing to disclose

## EPO‐0481

### Multiple systemic lipomatosis associated with the 8344A>G tRNALys mtDNA variant in a large Italian family: Searching for a threshold effect

#### V. Gioiosa ^1^; E. Cioffi^1^; A. Tessa^2^; F. Santorelli^2^; C. Casali^1^

##### ^*1*^ *Department of Medico‐Surgical Sciences and Biotechnologies, University of Rome Sapienza, 04100 Latina, Italy;* ^*2*^ *IRCCS Stella Maris Foundation, Calambrone, via dei Giacinti 2, 56128 Pisa, Italy*

**Background and aims:** Myoclonic epilepsy with ragged red fibers (MERRF) is a rare mitochondrial DNA (mtDNA) disorder commonly caused by the 8344A>G mutation in the MT‐TK gene encoding tRNALys. Clinical expression is highly variable due to tissue‐specific heteroplasmy and threshold effects. In addition to the classic neurological and systemic features, multiple systemic lipomatosis (MSL) has been described. The aim of this study was to investigate a large Italian family carrying the 8344A>G variant and to explore a possible threshold effect for the development of MSL.

**Methods:** The proband was a 28‐year‐old woman presenting with myoclonic epilepsy, cerebellar ataxia, peripheral neuropathy, and a large cervical lipoma. Following molecular confirmation of the 8344A>G variant, 14 maternally related family members were evaluated. Clinical assessment focused on neurological features and the presence of MSL. Molecular analysis was performed on peripheral blood leukocytes (PBLs) and urinary epithelial cells to determine heteroplasmy levels.

**Results:** Ten out of 14 family members presented isolated MSL with variable extension. All affected individuals carried the 8344A>G variant. Mean mutation load was higher in affected subjects. In the proband, heteroplasmy exceeded 84% in both tissues. Individuals without MSL showed lower mutation loads. Overall, variant levels were higher in urinary epithelial cells than in PBLs.

**Conclusion:** This study describes a large family in which MSL was often an isolated manifestation. Differences in heteroplasmy between tissues and between affected and unaffected individuals suggest an apparent threshold effect for the development of MSL. These findings support considering MERRF in families with maternally inherited MSL and warrant further investigation.

**Disclosure:** Nothing to disclose.

## EPO‐0482

### Breaking the age barrier: Adult‐onset GM2A gangliosidosis with reclassification of two likely pathogenic variants

#### C. Guerreiro ^1^; F. Godinho^2^

##### ^*1*^ *Neurology department, Unidade Local de Saúde São José, Lisbon, Portugal;* ^*2*^ *Centro Neurológico Sénior, Torres Vedras, Portugal*

**Background and aims:** GM2 gangliosidoses are rare autosomal recessive lysosomal storage disorders, with a classically fatal infantile onset. GM2 activator protein deficiency (GM2, AB variant), caused by variants in GM2A, is the rarest subtype, with adult‐onset cases being exceptionally rare. To date, only one adult‐onset case has been reported. We describe two adult siblings with late‐onset disease and provide genetic and functional evidence supporting reclassification of two GM2A variants as likely pathogenic.

**Methods:** Two affected siblings underwent clinical, neurophysiological, imaging, enzymatic, genetic, and functional evaluation, including plasma ganglioside quantification.

**Results:** A 57‐year‐old man developed progressive lower limb weakness from age 30. His 53‐year‐old brother had presented with symmetric areflexic paraparesis, thigh muscle atrophy, gait impairment and Gowers’ sign, from age 20. distal sensory involvement was present in one sibling. Electromyography revealed a chronic, long‐standing motor neuronopathy associated with a milder sensory neuronopathy. Muscle MRI was significant for quadriceps atrophy, and muscle biopsy showed angular fiber atrophy. β‐hexosaminidase activity was normal. Genetic testing identified compound heterozygosity for GM2A variants c.428G>A and c.496G>A, both initially classified as variants of uncertain significance. Plasma ganglioside analysis showed increased GM2 levels compared to controls in both siblings, with a profile comparable to patients with confirmed GM2A pathogenic variants and Tay–Sachs disease, supporting reclassification of both variants as likely pathogenic.

**Conclusion:** These cases represent an exceptionally rare adult‐onset GM2 AB variant, expanding the phenotypic and molecular spectrum of GM2A‐related disease and highlighting the diagnostic value of functional studies for variant reclassification in late‐onset neuromuscular phenotypes.

**Disclosure:** Nothing to report.

## EPO‐0483

### Genotype–phenotype patterns in genetic Parkinson's disease in a tertiary center from Türkiye

#### F. Bayır; D. Tezen; H. Apaydın; A. Gündüz

##### Department of Neurology, Istanbul University‐Cerrahpasa, Cerrahpasa Medical Faculty, Istanbul, Türkiye

**Background and aims:** Genetic forms of Parkinson's disease (PD) are clinically heterogeneous and increasingly recognized, particularly in regions with elevated consanguinity such as Türkiye. Despite growing international data, national evidence remains limited. This study evaluates the clinical and genetic characteristics of patients with confirmed pathogenic variants to explore genotype–phenotype relationships.

**Methods:** In this retrospective cohort, medical records of 1812 patients diagnosed with PD between 2010 and 2024 were reviewed. Thirty‐one individuals with confirmed pathogenic or likely pathogenic variants were included and stratified into three groups: PRKN, GBA, and other genetic causes. Demographic, motor, and non‐motor features were compared. Logistic regression with correction was used to mitigate small‐sample bias, with cognitive involvement as the outcome.

**Results:** Homozygous or compound heterozygous PRKN variants were the most frequent variations and were associated with earlier onset and a relatively homogeneous, motor‐predominant phenotype. Heterozygous GBA variants demonstrated a more heterogeneous profile, with higher frequencies of cognitive impairment and postural instability; exploratory regression indicated increased odds of cognitive impairment (OR≈5.7). Rare variants—including ATP13A2, FBXO7, HTRA1, and GCH1—occurred as single cases and were linked to atypical or complex presentations.

**Conclusion:** This cohort highlights the substantial genotypic and phenotypic diversity of PD in Türkiye. PRKN and GBA predominate, while rare variants contribute to atypical disease profiles.

**Disclosure:** Nothing to disclose.

## EPO‐0484

### Genetic contributions in Parkinson's disease and Parkinsonism from the PADUA‐CESNE cohort

#### G. Bonato ^1^; M. Carecchio^2^; A. Guerra^2^; M. Campagnolo^2^; V. Misenti^2^; R. Biundo^2^; M. Ginevrino^2^; L. Salviati^3^; A. Antonini^1^

##### ^*1*^ *Neurodegenerative Disease Unit, Department of Neuroscience, University of Padova, Padova, Italy; Foundation IRCCS Ca' Granda Ospedale Maggiore Policlinico, Neurology Unit, University of Milan, Milan, Italy;* ^*2*^ *Neurodegenerative Disease Unit, Department of Neuroscience, University of Padova, Padova, Italy;* ^*3*^ *Clinical Genetics Unit, Department of Women and Children's Health, Azienda Ospedale Università Padova, University of Padova, Padova, Italy*

**Background and aims:** In Parkinson's disease (PD), monogenic causes account for 5–10% of cases and are rarer in atypical parkinsonism. In our previous study of 218 selected patients, genetic causes were identified in 20%. The PADUA‐CESNE cohort as a biocollection program has now doubled these data, so we aim at further investigating genetic contributions to parkinsonism in our Italian population.

**Methods:** NGS multigene panel analysis, MLPA, 409 patients with PD/parkinsonism (377 PD, 32 atypical parkinsonism or DLB).

**Results:** Overall, 266 genetic variants in genes associated with movement disorders and/or cognitive decline were identified in 204/409 patients; 40 carried multiple variants. According to ACMG‐AMP criteria, 98/266 variants were classified as likely pathogenic. Most variants were missense (212/266), followed by frameshift/nonsense variants (23/266); splice‐site and copy number variants were less frequent. A diagnosis of monogenic parkinsonism was established in 67 patients (25% of the cohort), all but four with PD. GBA1 mutations were the most frequent finding (39 patients, 58.2% of positive cases), with N370S accounting for 41% of GBA1‐PD cases, followed by LRRK2 pathogenic variants (13 patients, 19.4%, half G2019S) and biallelic PRKN variants (9 patients). Rare genetic etiologies (GRN, OPTN) were also identified. Variants of uncertain significance were found in 137 patients, mainly in recessive PD‐related genes or other movement disorder genes.

**Figure:**
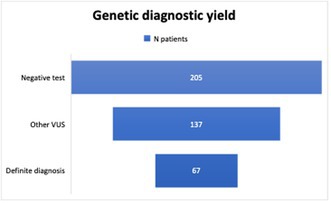
**FIGURE 1** Gene panel diagnostic yield in Padua‐CESNE cohort.

**Figure:**
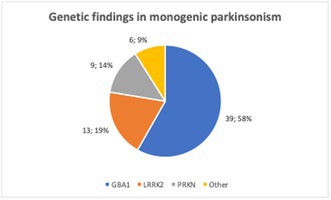
**FIGURE 2** Main genetic findings in positive cases.

**Conclusion:** NGS analysis revealed a substantial proportion of monogenic parkinsonism, confirming the central role of GBA1 and LRRK2 and supporting the clinical utility of genetics in movement disorders.

**Disclosure:** Data partially submitted for 2026 Italian movement disorders congress as abstract/poster Nothing to disclose

## EPO‐0485

### Clinical characterization of a neurodegeneration with brain iron accumulation 4 cohort from Estonia

#### K. Kukumägi ^1^; E. Õiglane‐Šlik^2^; K. Õunap^3^

##### ^*1*^ *Department of Neurology, Tartu University Hospital, Tartu, Estonia;* ^*2*^ *Department of General Paediatrics and Neurology, Tartu University Hospital, Tartu, Estonia;* ^*3*^ *Department of Genetics and Personalized Medicine, Institute of Clinical Medicine, University of Tartu, Tartu, Estonia*

**Background and aims:** Biallelic pathogenic variants in the C19orf12 gene have been shown to cause neurodegeneration with brain iron accumulation 4 (NBIA4), and the same variants have now been identified in Estonia. This study aimed to characterize the phenotype of Estonian NBIA4 patients.

**Methods:** This retrospective study included eight patients from Tartu University Hospital, born between 1981 and 2010. All patients had radiological and clinical features consistent with NBIA and a homozygous 11‐base‐pair C19orf12 deletion c.171_181del (p.Gly58Argfs*10). Clinical data were collected from medical records.

**Results:** In a cohort of eight patients, six were male and two female. At the time of the study, two patients were alive and six were deceased. All patients presented with their first symptoms in early childhood; the mean (±SD) age at symptom onset was 4.6 ± 2.1 years. Initial symptoms included vision loss (50%), spasticity of the lower limbs (50%) and gait impairment (37.5%). Optic nerve atrophy was diagnosed in seven of eight patients at a mean age of 9.1 ± 3.1 years. All patients attended school up to the 9th grade and developed movement disorders and neuropsychiatric abnormalities during the disease course. The mean life expectancy of the six deceased patients was 30 ± 4.7 years (range 23–35 years).

**Figure:**
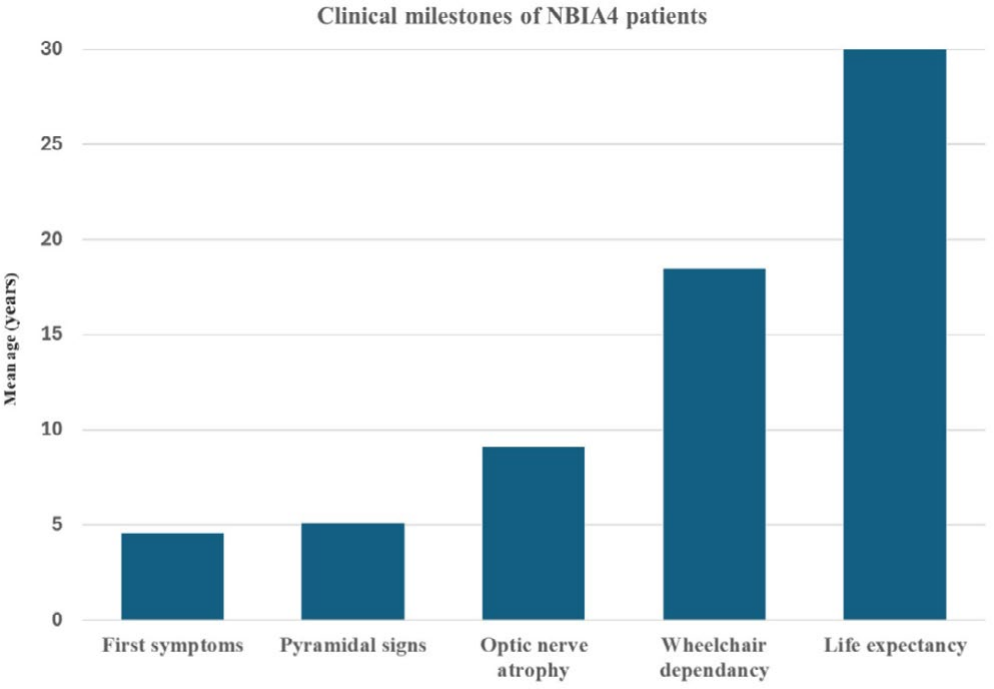
**FIGURE 1** Clinical milestones of NBIA4 patients..

**Conclusion:** The Estonian NBIA4 patients with homozygous C19orf12 deletion c.171_181del exhibited similar clinical features, including pyramidal signs and optic nerve atrophy from early childhood, with survival extending into early adulthood.

**Disclosure:** An author reports research funding from Estonian Research Council (grant PRG2040).

## EPO‐0486

### NFκB1 associations with Parkinson's disease risk: Systematic literature review and cross‐ancestry analysis of > 50,000 individuals

#### L. Furnica ^3^; S. Bandrivska^1^; W. Sun^4^; Popescu^6^; A. Zirra^5^; M. Ostrožovičová^1^

##### ^*1*^ *Department of Neuromuscular Disorders, UCL Queen Square Institute of Neurology, University College London, London, UK;* ^*3*^ *Department of Neurology, Colentina Clinical Hospital, Bucharest, Romania;* ^*4*^ *Department of Neurodegenerative Diseases, Hertie Institute for Clinical Brain Research, University of Tuebingen and German Center for Neurodegenerative Diseases (DZNE), Tuebingen, Germany;* ^*5*^ *Centre for Preventive Neurology, Wolfson Institute of Population Health, Queen Mary University of London, UK;* ^*6*^ *"Carol Davila" University of Medicine and Pharmacy,, Bucharest, Romania*

**Background and aims:** Nuclear factor kappa B1 (NFκB1), a key transcription factor in inflammatory signalling, has been linked to Parkinson's disease (PD) and neurodegeneration. This study intends to clarify the effect of NFκB1 variants in the pathogenesis of PD through a systematic review, and by investigating variants in this gene in the Global Parkinson's Disease (GP2) genotyping data.

**Methods:** For the systematic review, out of 51 identified records, 11 studies met the inclusion criteria, encompassing a wide methodological spectrum. We also conducted an analysis on genotyping imputed data from GP2 release 10 (33,813 PD cases and 18,714 controls across 11 different ancestries).The NFkB1 gene region was extracted with PLINK2, variants were annotated using ANNOVAR. Fisher's exact test was applied to examine differences between allele frequencies for common variants. Single‐nucleotide polymorphism (SNP) associations were also evaluated using logistic regression, with Bonferroni correction applied within each ancestry. To assess the cumulative effect of low‐frequency variants we ran burden analyses using RV tests.

**Results:** Evidence across all studies indicates that NF‐κB1 is consistently upregulated in Parkinson's disease and plays a key role in neuroinflammation and dopaminergic neuronal injury. In the large GP2 genotyping data 167 nominal associations with PD risk were observed across multiple ancestries. None remained significant after Bonferroni correction. Burden analyses showed no statistical significance.

**Conclusion:** While existing literature supports a pathogenic role for NF‐κB1 pathway in PD, our large‐scale multi‐ancestry genetic analysis finds no significant association between NFκB1 variants and PD risk, highlighting the need for future whole‐genome sequencing studies to further clarify this relationship.

**Disclosure:** Nothing to disclose

## EPO‐0487

### A new pathogenic variant of AARS2 gene causing leukoencephalopathy, progressive, with ovarian failure

#### M. Marconi ^1^; S. Diamant^2^; S. Petrucci^3^; L. Travaglini^4^; M. Piane^3^; M. Salvetti^2^; S. Romano^2^; G. Ristori^2^

##### ^*1*^ *Department of Neurology, Sant'Andrea University Hospital, Rome, Italy;* ^*2*^ *Department of Neurosciences, Mental Health and Sensory Organs, Sapienza University of Rome, Rome, Italy;* ^*3*^ *Department of Clinical and Molecular Medicine, Sapienza University of Rome, Rome, Italy;* ^*4*^ *Laboratory of Molecular Genetics, Bambino Gesu' Children`s Hospital, Rome, Italy*

**Background and aims:** The nuclear gene AARS2 encodes the mitochondrial alanyl‐tRNA synthetase. Pathogenic biallelic AARS2 variants cause mitochondrial disease, presenting as early‐onset cardiomyopathy or leukoencephalopathy, progressive, with ovarian failure (LKENP). LKENP is an autosomal recessive neurodegenerative disorder characterized by gait impairment, ataxia, spasticity, cognitive decline, and premature ovarian failure, usually beginning in young adulthood. Muscle biopsy may show ragged‐red fibers and brain MRI typically reveals progressive white‐matter abnormalities. We report a patient with LKENP carrying a previously unreported pathogenic AARS2 variant.

**Methods:** Clinical and neurological data were collected at Sant’Andrea Hospital (Rome). Brain MRI and genetic testing were performed as part of routine diagnostic evaluation.

**Results:** The patient is a 34‐year‐old woman with a history of cardiac arrest at birth, early menopause, clumsiness, and corrected bilateral strabismus, and a family history of cerebrovascular disease and autism. Karyotype and FMR1 testing at age 24 were normal. At 28, she had a transient loss of consciousness; brain MRI showed small bilateral frontal subcortical lesions, progressing to diffuse white‐matter abnormalities five years later. She subsequently developed cognitive decline, dysmetria, and gait impairment. Next‐generation sequencing identified two AARS2 variants: a previously unreported frameshift variant c.2821_2832dupTG, p.(Ala945fs*33), classified as pathogenic by ACMG criteria, and the known pathogenic variant c.595C>T, p.(Arg199Cys). Parental testing confirmed compound heterozygosity.

**Figure:**
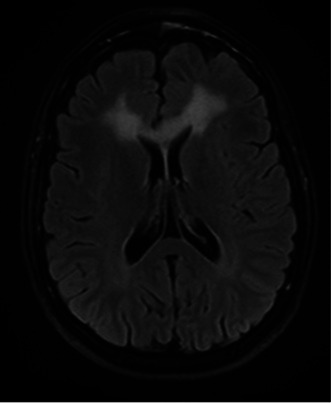
**FIGURE 1** Brain MRI (axial FLAIR) obtained at age 34 showing diffuse white matter hyperintensity involving both cerebral hemispheres, mainly in periventricular regions, corpus callosum, and posterior hippocampal areas.

**Figure:**
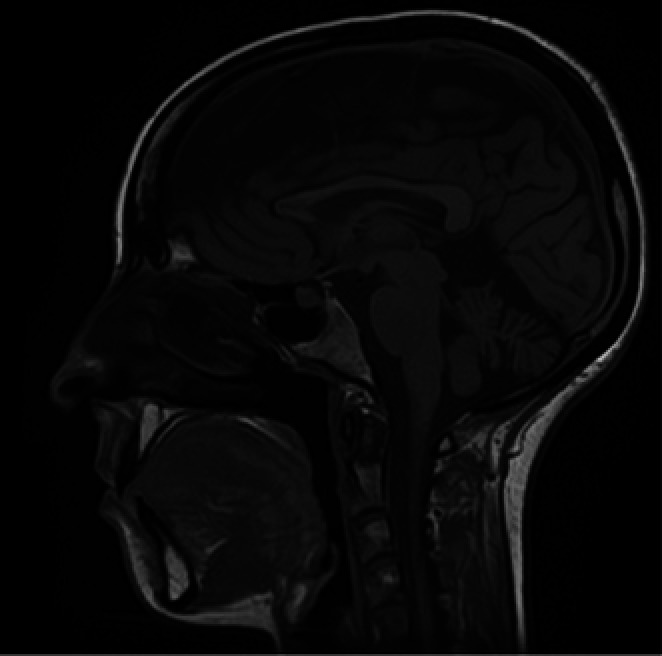
**FIGURE 2** Brain MRI (sagittal T1) obtained at age 34 demonstrating thinning of the corpus callosum, bilateral enlargement of frontal pericortical spaces, and mild widening of pericerebellar cisterns.

**Conclusion:** Genetic findings and clinical presentation confirm LKENP diagnosis. No effective therapies are currently available; the patient is receiving symptomatic management, including oral coenzyme Q10 supplementation, which has shown potential benefit in recent reports.

**Disclosure:** Nothing to disclose

## EPO‐0488

### Leukoencephalopathy and cerebral venous thrombosis secondary to autosomal recessive MTHFR deficiency: A case report

#### N. MacAlevey ^1^; K. Lau^1^; K. Teo Soon Hwee^1^; H. Chin^2^; Y. Chan^1^

##### ^*1*^ *Division of Neurology, Department of Medicine, National University Hospital, Singapore;* ^*2*^ *Department of Paediatrics, National University of Singapore, Singapore, Singapore*

**Background and aims:** Methylenetetrahydrofolate reductase (MTHFR) is an enzyme catalysing the conversion of homocysteine to methionine. Genetic variants that result in MTHFR deficiency may cause hyperhomocysteinemia and its associated complications.

**Methods:** A middle‐aged female presented with progressive unsteady gait and cognitive decline occurring over several years. Clinically, the patient had spastic paraparesis with brisk lower limb reflexes and a scissoring gait. She had a significant background of dietary folate and B12 deficiency which was treated with maintenance B12 and folate supplementation. MRI brain showed confluent periventricular T2‐weighted hyperintensities suggestive of an underlying leukodystrophy and identified an incidental venous sinus thrombosis in the junction between the left sigmoid sinus and left internal jugular vein. MRI Whole Spine showed no abnormal cord signal or enhancement, and cerebrospinal fluid analysis was unremarkable. A workup for her venous sinus thrombosis identified a high serum homocysteine (>50.0umol/L) with normal B12, folate levels and low‐normal serum methionine levels.

**Results:** Expanded Exome Proband Analysis confirmed homozygosity for a pathogenic variant c.971A>G in the MTHFR gene. She was diagnosed with autosomal recessive MTHFR deficiency and managed with betaine and B12/folate replacement. Betaine contributes an alternative methyl donor for remethylation of homocysteine to methionine, treating her hyperhomocysteinemia. Apixaban was started to treat her venous sinus thrombosis in the presence of a prothrombotic state.

**Conclusion:** MTHFR deficiency is a rare but treatable genetic metabolic disorder that may predispose patients to leukoencephalopathy, cognitive decline, spastic paraparesis, spinal cord atrophy, or thrombosis due to hyperhomocysteinemia. It should be suspected in patients with severe hyperhomocysteinemia and hypomethioninemia without folate/B12 deficiency.

**Disclosure:** Nothing to disclose.

## EPO‐0489

### Late‐onset RANBP2‐associated acute necrotizing encephalopathy (ANE1) in two siblings from an Italian family

#### V. Ciampana ^1^; L. Paciolla^1^; G. Filisetti^1^; S. Asaro^1^; F. Caushi^2^; L. Corrado^2^; C. Comi^1^; S. D'Alfonso^2^; D. Vecchio^1^

##### ^*1*^ *Neurology Unit, Department of Translational Medicine, University of Piemonte Orientale, University‐Hospital Maggiore della Carita', Novara, Italy;* ^*2*^ *Laboratory of Human Genetics, Department of Health Sciences, University of Piemonte Orientale, Novara, Italy*

**Background and aims:** Acute Necrotizing Encephalopathy (ANE) is a rare para‐infectious encephalopathy of childhood, characterized by symmetric bilateral thalamic brain magnetic resonance imaging (MRI) lesions. Adolescent or adult onset is exceptional. Familial or recurrent forms are linked to heterozygous pathogenic RANBP2 variants (ANE1). We report two siblings with late‐onset ANE1, diagnosed after the second presentation.

**Methods:** Both underwent neurological evaluation, MRI, cerebrospinal fluid analysis, and infectious, autoimmune, and metabolic testing. Genetic investigations included whole‐exome sequencing in the second sibling and targeted Sanger sequencing in the first.

**Results:** The male developed fulminant post‐febrile encephalopathy at age 15, rapidly progressing to coma. MRI showed bilateral thalamic, pontine, and external capsule involvement. He recovered with motor and cognitive sequelae. He remained undiagnosed for over 20 years. His sister presented at age 20 with milder post‐infectious encephalopathy and visual‐sensory symptoms. MRI revealed bilateral thalamic lesions involving the external capsules, optic tracts, and mammillary bodies. She received high‐dose corticosteroids followed by intravenous immunoglobulin, achieving recovery. Extensive investigations were non‐diagnostic in both. In the second sibling, whole‐exome sequencing revealed a heterozygous missense RANBP2 variant (c.1754C>T; p.Thr585Met) confirmed by Sanger sequencing in her brother.

**Conclusion:** A concordant phenotype with variable severity supports a familial ANE1. These cases confirm RANBP2‐associated ANE1 as a cause of late‐onset encephalopathy and expand the phenotypic spectrum, highlighting intrafamilial variability. Our family is the third reported in Italy and the first with delayed onset and relatively favorable prognosis. Early recognition and immunomodulatory therapy may improve outcomes. Awareness of late‐onset ANE1 and RANBP2 testing in adults is warranted.

**Disclosure:** Nothing to disclose.

## EPO‐0490

### Genetic insights into somatic symptoms and related disorders versus psychiatric phenotypes

#### V. Fominykh; P. Jaholkowski; A. Shadrin; O. Andreassen

##### Institute of Clinical Medicine, University of Oslo, Oslo, Norway

**Background and aims:** The genetic mechanisms underlying somatic symptoms and related disorders (SSRD) are poorly known, even though they have a significant impact on healthcare. We aimed to identify additional significant loci using statistical genetics methods and first large‐scale SSRD GWAS meta‐analysis including functional neurological (FND) and somatoform disorders (SomD), and to examine genetic overlap with psychiatric disorders.

**Methods:** We performed the cross‐sectional analysis of SSRD GWAS [Fominykh et al, 2025, 22,203 and 1,831,107 controls] and three psychiatric disorders: anxiety [ANX, N 175,163], major depression [MDD, N 250215] and post‐traumatic symptoms disorder [PTSD, N 146,660] from Million Veterans Projects. Genetic correlations were assessed using LDSC. The conditional/conjunctional false discovery rate framework (cond/conjFDR) was used to identify new and shared loci for SSRD. FUMA and Open Targets were used for functional annotations.

**Results:** LDSC analysis shows significant genetic correlation between SSRD and MDD (rg = 0.8), ANX (rg = 0.75) and PTSD (rg = 0.7) in range with previous publication. We revealed 9 new loci for SSRD using condFDR. ConjFDR analysis identified 2 loci jointly associated with SSRD and MDD, 2 loci – with SSRD and PTSD. The lead SNP common for all three phenotypes was mapped to EXD3 gene. The remaining lead SNPs mapped to BPTF and GPM6A, which is linked to psychiatric and neurological conditions.

**Conclusion:** This study explores the genetic aspects of the SSRD. We reveal new SNPs associated with SSRD, as well as common SNPs involved in SSRD development and most co‐morbid psychiatric disorders.

**Disclosure:** No special disclosures. This work was supported by an RCN grant 324252. MVP dbGaP accession number phs001672.

Ageing and dementia 4

## EPO‐0491

### Abstract withdrawn

## EPO‐0492

### Investigating synaptic and neuroinflammation biomarkers in the Alzheimer's disease continuum

#### F. Roveta ^1^; L. Bonino^1^; S. Boschi^1^; A. Chiarandon^1^; G. Brodini^1^; E. Piella^1^; A. Cermelli^1^; M. Zotta^2^; G. Rovera^2^; S. Morbelli^2^; E. Rubino^1^; I. Rainero^1^

##### ^*1*^ *Department of Neuroscience “Rita Levi‐Montalcini”, University of Turin, Turin, Italy;* ^*2*^ *Nuclear Medicine Unit, University Hospital “Città della Salute e della Scienza di Torino”, Turin, Italy*

**Background and aims:** Synaptic dysfunction and neuroinflammation are key mechanisms in Alzheimer's disease (AD), potentially preceding neurodegeneration. Synaptosome‐associated protein 25 (SNAP‐25) reflects synaptic damage, whereas glial fibrillary acidic protein (GFAP) and soluble TREM2 (sTREM2) index glial activation. Human data on their interplay across the AD continuum remain limited.

**Methods:** We prospectively enrolled 69 patients with AD (age 70.9±7.0; 59% female). Based on Clinical Dementia Rating (CDR), participants were classified as mild cognitive impairment (MCI; CDR = 0.5; *n* = 34) or dementia (CDR≥1; *n* = 35). All underwent standardized neuropsychological assessment including MMSE. Cerebrospinal fluid (CSF) levels of GFAP, sTREM2, SNAP‐25, and neurofilament light chain (NfL) were measured using Simoa. Associations with AD biomarkers were assessed using Spearman correlations. Longitudinal cognition over two years was analyzed with linear mixed‐effects models. Voxel‐wise analyses explored relationships with FDG‐PET metabolism.

**Results:** CSF NfL and GFAP were higher in dementia than MCI (*p* = 0.029 and *p* = 0.030), while sTREM2 and SNAP‐25 did not differ. GFAP correlated with p‐Tau181 (rho = 0.41, *p* = 0.001). sTREM2 correlated positively with p‐Tau181 (rho = 0.32, *p* = 0.012) and negatively with the Aβ42/40 ratio (rho = −0.38, *p* = 0.003). SNAP‐25 correlated with t‐Tau (rho = 0.30, *p* = 0.019). Baseline biomarkers were not associated with cognition. Higher NfL predicted faster MMSE decline (β = −0.16, *p* < 0.001). Higher GFAP was associated with reduced FDG uptake in the right medial and orbitofrontal prefrontal cortex (*p* < 0.001).

**Conclusion:** GFAP differentiated clinical stages and was linked to frontal hypometabolism, highlighting the contribution of astrocytic activation to AD‐related neurodegeneration. These findings support the relevance of neuroinflammatory biomarkers for staging and monitoring disease progression across the AD continuum.

**Disclosure:** Nothing to disclose.

## EPO‐0493

### Plasma GFAP improves Alzheimer's disease classification within the pTAU217 gray zone: A real‐world multi‐biomarker analysis

#### G. Rugarli ^1^; G. Cecchetti^2^; F. Coraglia^1^; E. Spinelli^1^; A. Ghirelli^1^; S. Pisano^3^; E. Canu^3^; F. Caso^4^; G. Magnani^4^; F. Agosta^1^; M. Filippi^5^

##### ^*1*^ *Neuroimaging Research Unit, Division of Neuroscience, and Neurology Unit, IRCCS San Raffaele Scientific Institute, Milan, Italy; and Vita‐Salute San Raffaele University, Milan, Italy;* ^*2*^ *Neuroimaging Research Unit, Division of Neuroscience, Neurology Unit, and Neurophysiology Service, IRCCS San Raffaele Scientific Institute, Milan, Italy;* ^*3*^ *Neuroimaging Research Unit, Division of Neuroscience, and Neurology Unit, IRCCS San Raffaele Scientific Institute, Milan, Italy;* ^*4*^ *Neurology Unit, IRCCS San Raffaele Scientific Institute, Milan, Italy;* ^*5*^ *Neuroimaging Research Unit, Division of Neuroscience, Neurology Unit, Neurorehabilitation Unit, and Neurophysiology Service, IRCCS San Raffaele Scientific Institute, Milan, Italy; and Vita‐Salute San Raffaele University, Milan, Italy*

**Background and aims:** To refine the diagnostic performance of plasma pTau217 for Alzheimer's disease (AD) in a real‐world setting, we evaluated double‐cutoff strategies and the added discriminative value of GFAP within the pTau217 “gray zone”.

**Methods:** We analyzed 441 consecutive patients from the San Raffaele Memory Center, including 181 with cerebrospinal fluid (CSF) A/T classification. Plasma pTau217, pTau181, GFAP, NfL, and Aβ42/40 were measured using automated immunoassays (Lumipulse G600II). The CSF‐defined subgroup was used to derive optimal pTau217 double cutoffs across all sensitivity–specificity pairs ≥0.90, quantifying the proportion of indeterminate (“gray”) cases. GFAP discriminative ability (AUC) was assessed within each gray zone. Associations between GFAP, age, APOE‐ε4, sex, and cognition were explored using generalized additive and linear models.

**Results:** No pTau217 cutoff combination achieved ≥95% sensitivity/specificity while limiting gray cases to ≤20%. The optimal balanced pair (90% sensitivity, 91% specificity) resulted in 28% indeterminate subjects. Within this gray zone, GFAP discriminated A+ from A− individuals (AUC = 0.83), reducing indeterminate cases by 37% when used as a triage marker. GFAP increased with age, particularly in A+ individuals, and remained independently associated with A‐positivity after adjustment for APOE and sex (*p* < 0.001). Adding GFAP to pTau217 modestly improved explained MMSE variance (ΔR^2^ = 0.04).

**Conclusion:** Systematic sensitivity–specificity mapping shows that plasma GFAP provides robust complementary value in resolving intermediate pTau217 results. Integrating GFAP into multimarker workflows may enhance diagnostic certainty and support the clinical implementation of blood‐based biomarkers for AD.

**Disclosure:** Giulia Rugarli has nothing to disclose; Giordano Cecchetti received speaker honoraria from Neopharmed Gentili; F Coraglia has nothing to disclose; Edoardo Gioele Spinelli has nothing to disclose; Alma Ghirelli has nothing to disclose; Stefano Pisano has nothing to disclose; Elisa Canu receives research support from the Italian Ministry of Health; Francesca Caso has nothing to disclose; Giuseppe Magnani has nothing to disclose; F Agosta is Associate Editor of NeuroImage: Clinical and the European Journal of Neurology; has received speaker honoraria from Biogen Idec, Bristol Myers Squibb, Eisai, Eli Lilly, GE Healthcare, Neuraxpharm, and Roche,; and receives or has received research supports from the Italian Ministry of Health, the Italian Ministry of University and Research, AriSLA (Fondazione Italiana di Ricerca per la SLA), the European Research Council (ERC), the EU Joint Programme – Neurodegenerative Disease Research (JPND), and Foundation Research on Alzheimer Disease (France). M Filippi is Editor‐in‐Chief of the Journal of Neurology, Associate Editor of Human Brain Mapping, Neurological Sciences, and Radiology; received compensation for consulting services from Almirall, Biogen, Bristol‐Myers Squibb, Eli Lilly, Merck, Novartis, Roche, Sanofi; speaking activities from Amgen, Bayer, Biogen, Bristol‐Myers Squibb, Celgene, Chiesi Italia SpA, Eisai, Eli Lilly, Fujirebio, Genzyme, Janssen, Merck, Neopharmed Gentili, Neuraxpharm, Novartis, Novo Nordisk, Roche, Sanofi, Takeda; participation in Advisory Boards for Alexion, Biogen, Bristol‐Myers Squibb, Eli Lilly, GE Healthcare Ltd, Merck, Neuraxpharm, Novartis, Roche, Sandoz, Sanofi, Takeda; scientific direction of educational events for Biogen, Merck, Roche, Celgene, Bristol‐Myers Squibb, Lilly, Novartis, Sanofi‐Genzyme; he receives research support from Biogen Idec, Merck‐Serono, Novartis, Roche, the Italian Ministry of Health, the Italian Ministry of University and Research, and Fondazione Italiana Sclerosi Multipla.

## EPO‐0494

### Integrating retinal OCT and blood biomarkers to characterize cognitive decline across the Alzheimer's disease spectrum

#### G. Rugarli ^1^; G. Cecchetti^2^; R. Santangelo^3^; F. Coraglia^1^; E. Spinelli^1^; A. Ghirelli^1^; S. Pisano^4^; E. Canu^4^; F. Caso^5^; G. Magnani^5^; F. Agosta^1^; M. Filippi^6^

##### ^*1*^ *Neuroimaging Research Unit, Division of Neuroscience, and Neurology Unit, IRCCS San Raffaele Scientific Institute, Milan, Italy; and Vita‐Salute San Raffaele University, Milan, Italy;* ^*2*^ *Neuroimaging Research Unit, Division of Neuroscience, Neurology Unit, and Neurophysiology Service, IRCCS San Raffaele Scientific Institute, Milan, Italy;* ^*3*^ *Neurology Unit, and Neurophysiology Service, IRCCS San Raffaele Scientific Institute, Milan, Italy;* ^*4*^ *Neuroimaging Research Unit, Division of Neuroscience, and Neurology Unit, IRCCS San Raffaele Scientific Institute, Milan, Italy;* ^*5*^ *Neurology Unit, IRCCS San Raffaele Scientific Institute, Milan, Italy;* ^*6*^ *Neuroimaging Research Unit, Division of Neuroscience, Neurology Unit, Neurorehabilitation Unit, and Neurophysiology Service, IRCCS San Raffaele Scientific Institute, Milan, Italy; and Vita‐Salute San Raffaele University, Milan, Italy*

**Background and aims:** To investigate the relationship between retinal structural alterations, assessed by optical coherence tomography (OCT), and plasma biomarkers of neurodegeneration across the Alzheimer's disease (AD) continuum, from cognitively unimpaired individuals to patients with dementia.

**Methods:** A total of 205 participants were recruited at the Neurology Unit, IRCCS San Raffaele Scientific Institute, including subjects with subjective cognitive decline (SCD), mild cognitive impairment (MCI), dementia (DEM), and healthy controls (HC). Plasma levels of Aβ42, Aβ40, pTau‐181, pTau‐217, and neurofilament light chain (NfL) were quantified using automated CLEIA technology. OCT was used to measure peripapillary retinal nerve fiber layer (RNFL), ganglion cell layer (GCL), and inner plexiform layer (IPL) thickness. Correlations between retinal parameters, plasma biomarkers, cognitive performance (MMSE), and carotid ultrasound findings were analyzed, with a one‐year longitudinal follow‐up in a subsample.

**Results:** Inner retinal layer thinning—particularly in the GCL and IPL—showed significant associations with plasma NfL and pTau‐217 levels, suggesting that retinal structural loss parallels systemic markers of neuroaxonal damage. These correlations were strongest in early disease stages (SCD and SCD_AD) and diminished with advanced cognitive decline. Longitudinal data demonstrated a significant reduction in GCL thickness over one year, reinforcing its sensitivity as an early indicator of neurodegeneration.

**Conclusion:** Retinal OCT may serve as a non‐invasive biomarker for early detection and monitoring of AD‐related neurodegeneration. Combining retinal imaging with plasma biomarkers could improve diagnostic accuracy, disease staging, and patient stratification in clinical trials, ultimately advancing personalized therapeutic approaches in Alzheimer's disease.

**Disclosure:** Giulia Rugarli has nothing to disclose; Giordano Cecchetti received speaker honoraria from Neopharmed Gentili; R Santangelo has nothing to disclose; F Coraglia has nothing to disclose; Edoardo Gioele Spinelli has nothing to disclose; Alma Ghirelli has nothing to disclose; Stefano Pisano has nothing to disclose; Elisa Canu receives research support from the Italian Ministry of Health; Francesca Caso has nothing to disclose; Giuseppe Magnani has nothing to disclose; F Agosta is Associate Editor of NeuroImage: Clinical and the European Journal of Neurology; has received speaker honoraria from Biogen Idec, Bristol Myers Squibb, Eisai, Eli Lilly, GE Healthcare, Neuraxpharm, and Roche,; and receives or has received research supports from the Italian Ministry of Health, the Italian Ministry of University and Research, AriSLA (Fondazione Italiana di Ricerca per la SLA), the European Research Council (ERC), the EU Joint Programme – Neurodegenerative Disease Research (JPND), and Foundation Research on Alzheimer Disease (France). M Filippi is Editor‐in‐Chief of the Journal of Neurology, Associate Editor of Human Brain Mapping, Neurological Sciences, and Radiology; received compensation for consulting services from Almirall, Biogen, Bristol‐Myers Squibb, Eli Lilly, Merck, Novartis, Roche, Sanofi; speaking activities from Amgen, Bayer, Biogen, Bristol‐Myers Squibb, Celgene, Chiesi Italia SpA, Eisai, Eli Lilly, Fujirebio, Genzyme, Janssen, Merck, Neopharmed Gentili, Neuraxpharm, Novartis, Novo Nordisk, Roche, Sanofi, Takeda; participation in Advisory Boards for Alexion, Biogen, Bristol‐Myers Squibb, Eli Lilly, GE Healthcare Ltd, Merck, Neuraxpharm, Novartis, Roche, Sandoz, Sanofi, Takeda; scientific direction of educational events for Biogen, Merck, Roche, Celgene, Bristol‐Myers Squibb, Lilly, Novartis, Sanofi‐Genzyme; he receives research support from Biogen Idec, Merck‐Serono, Novartis, Roche, the Italian Ministry of Health, the Italian Ministry of University and Research, and Fondazione Italiana Sclerosi Multipla.

## EPO‐0495

### Early‐onset dementia: Etiological and clinical insights from a 10‐year cohort

#### G. Yılmaz Çakan ^1^; B. Samancı^1^; M. Alaylıoğlu^2^; E. Şahin^1^; H. Gürvit^1^; E. Dursun^2^; H. Hanağası^1^; D. Gezen Ak^2^; B. Bilgiç^1^

##### ^*1*^ *Istanbul University, Istanbul Faculty of Medicine, Behavioral Neurology and Movement Disorders Unit, Istanbul, Türkiye;* ^*2*^ *Brain and Neurodegenerative Disorders Research Laboratories, Department of Neuroscience, Institute of Neurological Sciences, Istanbul University‐Cerrahpasa, Istanbul, Türkiye*

**Background and aims:** Early‐onset dementia (EOD) represents 5–9% of all dementia cases and differs clinically from late‐onset dementia (LOD), often delaying recognition and complicating management. Despite its clinical relevance, large cohort data remain limited, particularly in Türkiye. This study presents a decade‐long EOD cohort from a tertiary referral center.

**Methods:** A total of 1,656 patients were evaluated between 2015–2025. After excluding LODs and secondary dementias, 656 patients with complete CSF assessments were included. Clinical and demographic features were analyzed.

**Results:** The cohort included 52.1% female. Mean age at onset was similar across groups except for atypical Alzheimer's disease (AD) versus mixed dementia (*p* = 0.017). The most common diagnoses were typical AD (24.5%), atypical AD (18.1%), and frontotemporal dementia (FTD) (15.6%). Posterior cortical atrophy (PCA) was the leading atypical AD subtype (30.3%), while behavioral‐variant FTD (bvFTD) predominated within FTD (47.1%). When staged at the time of biomarker assessment, dementia was more frequent in typical AD than atypical AD, and in atypical AD than FTD (*p* = 0.001), whereas no difference between FTD and typical AD (*p* = 0.759).

**Conclusion:** This cohort resembles previous EOD populations but differs in etiological distribution and clinical stage at diagnosis. Findings underscore the importance of early symptom recognition and timely diagnostic evaluation, given that EOD follows a distinct clinical course compared to LOD. As a decade‐long cohort from a high‐volume tertiary center, this study may reflect the broader population, addressing gaps in epidemiological and etiological data on EOD globally and within Türkiye.

**Disclosure:** Nothing to disclose.

## EPO‐0496

### GFPaint: A novel technique for visualizing alpha‐synuclein strains across synucleinopathies

#### N. Graves; E. Sierecki

##### Department of Molecular Medicine, School of Biomedical Sciences, University of New South Wales, Sydney, Australia

**Background and aims:** Misfolded alpha‐synuclein aggregates are a defining feature of Parkinson's disease, dementia with Lewy bodies, and multiple system atrophy. However, distinguishing disease‐relevant alpha‐synuclein strains remains a major challenge. The objective of this study was to develop a single‐molecule approach to discriminate alpha‐synuclein strain polymorphs derived from different synucleinopathies and defined synthetic conditions based on selective protein interactions.

**Figure:**
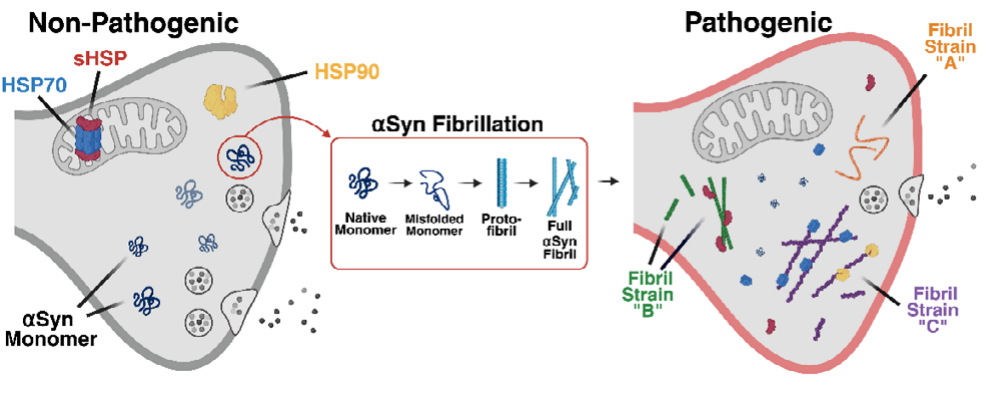
**FIGURE 1** Conceptual schematic of alpha‐synuclein fibrillation and strain diversity. Chaperone‐mediated proteostasis maintains soluble alpha‐synuclein, whereas pathogenic conditions promote formation of distinct fibril strains with differential chaperone engagement.

**Methods:** We developed GFPaint, a single‐molecule fluorescence assay combining cell‐free expression of green fluorescent protein tagged heat shock proteins with dual‐channel coincidence spectroscopy. A panel of chaperones spanning HSP40, HSP70, HSP90, and small heat shock protein families was incubated with alpha‐synuclein fibrils generated under traditional, metal ion, and saccharide conditions, as well as with brain‐derived samples from Parkinson's disease, dementia with Lewy bodies, and multiple system atrophy. Binding interactions were quantified using coincidence, binding affinity, and binding specificity metrics, including peak‐ and integral‐based analyses.

**Figure:**
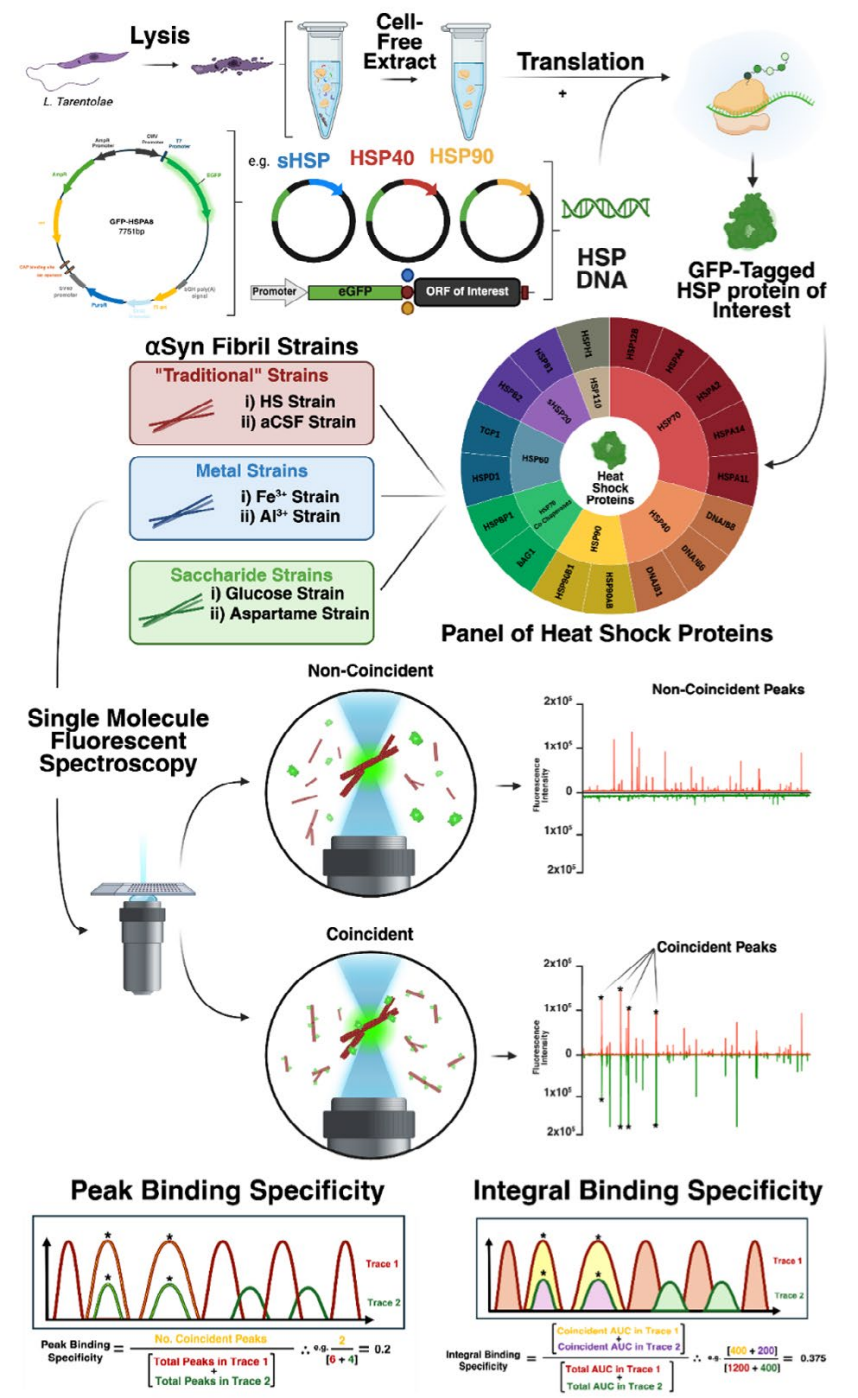
**FIGURE 2** Overview of the GFPaint workflow. Green fluorescent protein tagged heat shock proteins are expressed in a cell‐free system and incubated with defined alpha‐synuclein fibril strains. Dual‐channel single‐molecule data produced.

**Results:** GFPaint revealed pronounced strain‐dependent differences in chaperone engagement. HSP40 family members, particularly DNAJB1 and DNAJB6, showed strong and selective binding to specific alpha‐synuclein polymorphs, whereas HSP70 and HSP90 family members displayed weak or nonspecific interactions. Distinct binding fingerprints differentiated Parkinson's disease, dementia with Lewy bodies, and multiple system atrophy brain samples and aligned with defined synthetic strain profiles.

**Figure:**
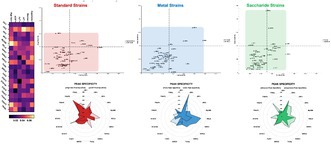
**FIGURE 3** Strain‐dependent protein interaction profiles across alpha‐synuclein fibril classes. Heat maps, quadrant analyses, and radar plots reveal distinct peak and integral binding specificity patterns for standard, metal ion, and saccharide induced aSyn strains.

**Conclusion:** GFPaint enables single‐molecule strainotyping of alpha‐synuclein based on selective protein interactions, providing a scalable platform to distinguish disease‐specific alpha‐synuclein conformations across synucleinopathies.

**Disclosure:** Nothing to disclose.

## EPO‐0497

### Relationship between cortical neurotransmitter target impairment and core features of behavioural variant frontotemporal dementia

#### N. Epis; M. Losa; E. Sentieri; S. Garbarino; F. Massa; L. Lorenzini; S. Raffa; L. Sofia; S. Morbelli; L. Roccatagliata; D. Arnaldi; B. Orso; M. Pardini

##### Department of Neuroscience, Rehabilitation, Ophthalmology, Genetics, Maternal and Child 3 IRCCS Ospedale Policlinico San Martino, Genoa, Italy

**Background and aims:** Behavioural variant frontotemporal dementia (bvFTD) is characterized by distinct patterns of neurodegeneration and neurotransmitter deficits, but the relationship between specific neurotransmitter vulnerability and core symptoms of bvFTD is poorly explored. We aimed to investigate the spatial colocalization between hypometabolism and normative distribution of neurotransmitter receptors, assessing their association with the main neuropsychiatric and cognitive symptoms of bvFTD.

**Methods:** We analysed [18F]FDG‐PET scans from bvFTD patients compared to healthy controls to derive relative hypometabolism maps. Using the JuSpace toolbox, we computed spatial correlations between hypometabolism and normative density maps of the neurotransmitter systems target known to be impaired in bvFTD, such as serotonergic (5HT), dopaminergic (D), GABAergic (GABA), and glutamatergic (mGluR) receptors. Multivariate logistic or linear regression models were applied to evaluate the association between the receptor‐specific spatial colocalization coefficients, bvFTD‐related subitems of the Neuropsychiatric Inventory (NPI), and executive performance.

**Results:** In bvFTD patients (*n* = 86; age 73±7 years), the hypometabolism distribution significantly colocalized with the distribution of 5HT1a (*r* = −0.24), 5HT2a (*r* = −0.15), 5HT4 (*r* = −0.21), D1 (*r* = −0.29), D2 (*r* = −0.21), and mGluR5 (*r* = −0.21) receptors (all *p* < 0.001). In multivariate models, apathy presence was associated with 5HT2a vulnerability (OR = 0.029; *p* = 0.040), while disinhibition with D1 (OR = 0.058; *p* = 0.038) and D2 (OR = 0.098; *p* = 0.049) vulnerability. mGLUr5 impairment was associated with Trail Making Test A (TMT‐A; B = −0.060; *p* = 0.033), TMT‐B (B = −0.075; *p* = 0.023), Corsi test (OR:3.56; *p* = 0.03) and phonemic fluency (B = 0.76; *p* = 0.042), while D1 was associated with TMT‐B (B = −0.06; *p* = 0.040).

**Conclusion:** Our findings highlighted a distinct receptor vulnerability in bvTFD patients, which correlated with disease‐specific symptoms, suggesting possible pharmacological targets. Further studies should evaluate the implications at the individual‐patient level, potentially guiding therapeutic strategies.

**Disclosure:** The authors declare no conflicts of interest.

## EPO‐0498

### 3D convolutional neural network predicts MCI‐to‐AD conversion: Systematic review and meta‐analysis

#### R. Mohamed ^1^; Z. Hasan^2^; A. Kertam^3^; *N*. A. Mohammed^4^; E. M.Soliman^5^; A. Wael Moftah^6^; A. Essam El‐dain Said^7^; S. Ali^8^; A. Ahmed Radi^9^; B. Ayoub^10^

##### ^*1*^ *Faculty of Medicine, Alexandria University, Egypt;* ^*2*^ *Jordan University of Science and Technology, Irbid, Jordan;* ^*3*^ *Tu Lab for Diagnostic Research, Yale School of Medicine, New Haven, USA; Faculty of Medicine, Ain‐Shams University, Cairo, Egypt;* ^*4*^ *Biochemistry Division, Cairo University, Cairo, Egypt;* ^*5*^ *Faculty of Medicine Zagazig University, Zagazig, Egypt;* ^*6*^ *Faculty of Medicine, King Salman International university, South Sinai, Egypt;* ^*7*^ *Faculty of Science Cairo University, Cairo, Egypt;* ^*8*^ *Biochemistry Department Hazara University Mansehra Kpk, Pakistan;* ^*9*^ *Faculty of Medicine, Minia University, Minia, Egypt;* ^*10*^ *Faculty of Medicine, Cairo University, Egypt*

**Background and aims:** Early identification of patients with Mild Cognitive Impairment (MCI) who are likely to progress to Alzheimer disease (AD) remains a major clinical challenge. In recent years, three‐dimensional (3D) deep learning approaches have been increasingly applied to neuroimaging data to improve risk stratification. This meta‐analysis examines the performance of 3D deep learning models in predicting MCI‐to‐AD conversion and compares their results with two‐dimensional (2D) models, traditional machine learning techniques, and physician‐based assessment.

**Methods:** A total of 1,987 patients diagnosed with MCI were included. The intervention consisted of 3D deep learning architectures, primarily 3D Convolutional Neural Networks, applied to volumetric neuroimaging modalities, including resting‐state functional Magnetic Resonance Imaging and Positron Emission Tomography. Comparator methods included 2D Convolutional Neural Networks, conventional machine learning models, and physician consensus diagnoses. The primary outcomes were the pooled values of classification accuracy and Relative Risk for the conversion to Alzheimer's disease.

**Results:** Across the five studies, the overall accuracy of the 3D CNN models stood at 92.6% with a relative risk of 1.85 for the conversion to AD, clearly outperforming the consensus of doctors (69.5% accuracy). The AUC values for the discrimination of the FDG‐PET 3D CNN were excellent, with values between 0.93 and 0.99. The attention models SwinNet‐CATNet had an accuracy of 98.2% for the staging of AD, and the hippocampus‐centered PCANet‐BLS models surpassed the 95% threshold.

**Conclusion:** 3D deep learning has a substantial advantage over 2D models, as well as clinical assessment, in predicting MCI‐to‐AD transition (*p* < 0.01). Models including PET + hippocampal data have high discrimination.

**Figure:**
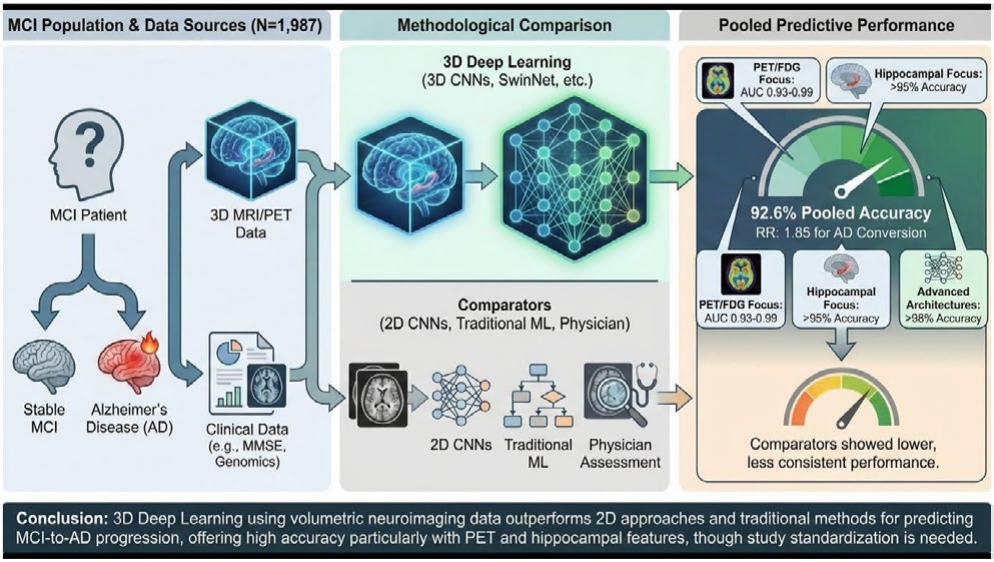
**FIGURE 1** Graphical abstract

**Disclosure:** Nothing to disclose.

## EPO‐0499

### Impact of the 2024 NIA‐AA revised criteria on biological AD classification in a clinical Memory cohort from Central Italy

#### S. Corsi; M. Del Chicca; E. Bergamin; L. Petrozzi; R. Ceravolo; G. Tognoni

##### Department of Clinical and Experimental Medicine, Neurology Unit, University of Pisa, Italy

**Background and aims:** Updates in Alzheimer's disease (AD) diagnostic criteria have significantly influenced clinical practice over the past decade. The AT (N) research framework introduced by NIA‐AA in 2018 stratifies individuals based on biomarkers of amyloid deposition (A), tau pathology (T), and neurodegeneration (N). The 2024 revision introduced two new core biomarkers—p‐tau/Aβ42 and t‐tau/Aβ42 ratios—and defined a hierarchy of Core 1 and Core 2 markers, establishing that the presence of just one Core 1 biomarker is sufficient to define biological AD.

**Methods:** To assess the impact of the updated NIA‐AA 2024 criteria, we retrospectively re‐evaluated cerebrospinal fluid (CSF) profiles of 81 patients (mean age 68 years; 46 male; mean education 10 years) at our cognitive neurology clinic, including patients with mild cognitive impairment and established dementia.

**Results:** Of the 81 patients, 27 (33%) didn’t meet the 2018 criteria for biological AD. Among these, 10 patients (37%) were reclassified as having biological AD according to the 2024 criteria. All reclassified patients showed pathological values in p‐tau/Aβ42, t‐tau/Aβ42, and Aβ42 or Aβ42/40 ratios, except for one case, who showed an isolated elevation of the t‐tau/Aβ42 ratio. This patient was later diagnosed with prion disease, highlighting the need for careful interpretation of neurodegeneration markers in non‐AD conditions. P‐tau alone was the least informative marker in triggering diagnostic reclassification. In contrast, p‐tau/Aβ42, Aβ42, and Aβ42/40 ratio performed consistently and equivalently in identifying amyloid pathology.

**Conclusion:** The 2024 NIA‐AA criteria substantially increase the number of individuals meeting the definition of biological AD.

**Disclosure:** Nothing to disclose.

## EPO‐0500

### Fluid Neurofilament Light captures antero/posterior cortex metabolic gradient in behavioral variant of frontotemporal dementia

#### S. Anderl‐Straub ^1^; F. Massa^2,3^; A. Cirone^4^; S. Garbarino^4,5^; S. Malagò^5^; M. Pardini^2,3^; L. Werner^1^; P. Steinacker^6^; I. Uttner^1^; J. Weishaupt^1^; J. Strobel^7^; A. Beer^7^; M. Otto^6^

##### ^*1*^ *1University of Ulm, Department of Neurology, Ulm, Deutschland;* ^*2*^ *University of Genoa, Department of Neuroscience, Rehabilitation, Ophthalmology, Genetics, Maternal and Child Health (DINOGMI), Genoa, Italy;* ^*3*^ *IRCCS Ospedale Policlinico San Martino, Genoa, Italy;* ^*4*^ *IRCCS Ospedale Policlinico San Martino, Life Science Computational Laboratory, Genoa, Italy;* ^*5*^ *University of Genoa, Department of Mathematics, Genoa, Italy;* ^*6*^ *Martin‐Luther‐University Halle‐Wittenberg, Department of Neurology, Halle (Saale), Deutschland;* ^*7*^ *University of Ulm, Department of Nuclear Medicine, Ulm, Deutschland*

**Background and aims:** Neurofilament light (NfL) is a biofluid marker of axonal injury in frontotemporal lobar degeneration (FTLD), but its regional metabolic correlates on [^18^F]FDG PET remain poorly defined. We aimed to characterize cortical [^18^F]FDG correlates of NfL in a well‐defined bvFTD cohort and to explore associated cognitive effects.

**Methods:** We studied 48 bvFTD patients who underwent resting‐state [18F]FDG‐PET and serum and/or CSF NfL sampling. CSF NfL values were converted to serum‐equivalent concentrations using a fixed factor (serum = 0.43 × CSF) and log‐transformed. [18F]FDG uptake was parcellated with the Desikan–Killiany atlas (68 cortical ROIs), normalized to the pons, and related to log‐transformed NfL using age‐adjusted linear models. ROIs showing significant or trend‐level NfL effects were grouped into anterior and posterior composites. From these we derived an A/P gradient and a posterior‐residual index (posterior variance unexplained by anterior uptake and age, indexing relative posterior sparing), which were related to logNfL. Associations with neuropsychological scores were tested in separate age‐adjusted models.

**Results:** Higher NfL was significantly associated with lower [18F]FDG uptake in frontal regions and higher uptake in posterior cortices. Anterior–posterior correlations were weak, and frontal uptake did not predict posterior uptake. The posterior‐residual index correlated positively with logNfL (*r* = 0.43, *p* = 0.003), indicating that increasing NfL values are linked to a steeper A/P metabolic gradient—greater frontal hypometabolism with relative posterior sparing. At the uncorrected level, the A/P gradient showed small, directionally consistent associations with executive dysfunction.

**Conclusion:** Integrating NfL with [18F]FDG PET may help quantify biologically meaningful fronto–posterior imbalance in FTLD and support patient stratification.

**Disclosure:** This work was supported by the Josef and Luise Kraft Foundation. The funding was provided to author Sarah Anderl‐Straub. The Co‐authors have nothing to disclose.

## EPO‐0501

### Plasma microglia related biomarker predicting faster cognitive decline

#### S. Kang^1^; M. Noh^1^; Y. Moon^3^; D. Chang^2^; S. Kim ^1^

##### ^*1*^ *Department of Neurology, Hanyang University College of Medicine, Seoul, Republic of Korea;* ^*2*^ *Department of Neurology, Gil Medical Center, Gachon College of Medicine;* ^*3*^ *Department of Radiology, Konkuk University Medical Center, Seoul, Republic of Korea*

**Background and aims:** Stratifying subgroups with rapid cognitive decline is a critical challenge in the management and research of Alzheimer's disease (AD) and frontotemporal lobar degeneration (FTLD). Previously identified as a marker of microglial dysfunction in amyotrophic lateral sclerosis, we investigated whether plasma microRNA‐214‐3p could serve as a predictor for rapid progression across the AD–FTLD spectrum.

**Methods:** We analyzed a multicenter prospective cohort of 169 participants, including AD, FTLD, and control groups. We evaluated plasma microRNA‐214‐3p, cerebrospinal fluid (CSF) amyloid and inflammatory markers, APOE4 status, and longitudinal Mini‐Mental State Examination (MMSE) scores. Fast progression was defined as an annual MMSE decline of >3 points. Additionally, we assessed phagocytic function and pathology in induced microglial‐like cells from participants.

**Results:** Plasma microRNA‐214‐3p was the strongest predictor of cognitive decline among all biomarkers. Baseline levels demonstrated excellent accuracy in identifying fast progressors (AUC = 0.92), significantly outperforming CSF neurofilament light chain (AUC = 0.73). High baseline levels correlated with rapid MMSE decline, exhibiting a synergistic interaction with APOE4. Longitudinal increases in microRNA‐214‐3p independently predicted decline, particularly in individuals with lower amyloid burden. Mechanistically, fast progressors displayed elevated microRNA‐214‐3p alongside reduced microglial phagocytosis and increased phosphorylated TDP‐43 levels.

**Conclusion:** Plasma microRNA‐214‐3p is a sensitive blood‐based surrogate for microglial phagocytic dysfunction. It effectively identifies individuals at risk of rapid cognitive decline across the AD–FTLD spectrum, offering significant prognostic value, especially for APOE4 carriers or those with non‐amyloid pathologies such as TDP‐43.

**Disclosure:** Funding: This research was supported by a grant of the Korea Dementia Research Project through the Korea Dementia Research Center (KDRC), funded by the Ministry of Health & Welfare and Ministry of Science and ICT, Republic of Korea (grant number: RS‐2024‐00348451). The authors have no interests to disclosure.

## EPO‐0502

### Digital cognitive training via mobile applications in Lewy body dementia: A systematic review and meta‐analysis

#### C. Pace

##### Federal University of Rio de Janeiro, Rio de Janeiro, Brazil

**Background and aims:** Lewy body dementia (LBD) is associated with rapid cognitive and functional decline, and pharmacological treatment options remain limited. Digital cognitive training (CT) has emerged as a potential non‐pharmacological strategy to improve cognitive outcomes in neurodegenerative disorders.

**Methods:** This systematic review and meta‐analysis followed PRISMA guidelines. PubMed, Embase, PsycINFO, Cochrane Library, and ClinicalTrials.gov were searched (January 2010‐December 2024) for randomized controlled trials investigating app‐based cognitive training in Lewy body dementia. The primary outcome was change in global cognition (MMSE or MoCA). Data were pooled using random‐effects models, with standardized mean differences (SMD), 95% confidence intervals (CI), and I^2^ for heterogeneity assessment.

**Results:** From 845 screened records, 8 RCTs (*n* = 412) were included. Digital cognitive training significantly improved global cognition compared with control (SMD = 0.45, 95% CI 0.18–0.72; I^2^ = 35%). Greater effects were observed in attention and executive function (SMD = 0.58, 95% CI 0.30–0.86). Effects on quality of life were positive but not statistically significant (SMD = 0.22, 95% CI −0.05–0.49). Adherence to app‐based training was high (>80%), and no serious adverse events were reported.

**Figure:**
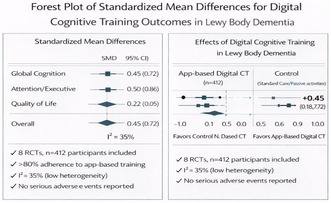
Forest plot showing positive effects of app‐based digital cognitive training on cognitive outcomes in Lewy body dementia.

**Conclusion:** Structured digital cognitive training is a safe and effective non‐pharmacological intervention for improving cognitive function in Lewy body dementia. These findings support the integration of validated digital applications as adjunct therapy in LBD management.

**Disclosure:** Nothing to disclose.

Neuroimmunology 2

## EPO‐0503

### Making the invisible audible: A real‐world connected speech study in myasthenia gravis

#### A. Zanghì; P. Di Filippo; C. Rutigliano; C. Avolio; E. D'Amico

##### University of Foggia, Italy

**Background and aims:** Bulbar involvement in myasthenia gravis (MG) impairs voice and speech, yet subtle changes may be overlooked.

**Methods:** We conducted a cross‐sectional study of consecutively enrolled adults with confirmed MG at a single center. Participants completed standardized voice recordings and contemporaneous clinical assessments (Myasthenia Gravis–Activities of Daily Living [MG‐ADL] and revised Myasthenia Gravis Quality of Life [rMG‐QOL15]). Tasks included sustained vowels, standardized reading, and spontaneous speech. Acoustic metrics comprised root‐mean‐square intensity (RMS, dB) and fundamental frequency (F0, Hz).

**Results:** Seventeen patients were enrolled (median age 58 years; 52.9% female). Median MG‐ADL was 6.0 and mean rMG‐QOL15 was 16.6, with no significant correlation (? = 0.09, *p* = 0.74). Male participants exhibited significant intensity reductions in connected speech: reading (‐3.22 dB, IQR ‐5.98 to ‐2.09; pBH = 0.031; *r* = 0.84) and spontaneous speech (‐5.46 dB, IQR ‐6.11 to ‐4.93; pBH = 0.031; *r* = 0.84). Female participants showed smaller, non‐significant changes ( = ‐2.2 dB; all pBH > 0.7). F0 shifts were modest and non‐significant after correction. Across the cohort, MG‐ADL correlated with both RMS and F0 drops in unadjusted models but not after adjusting for sex. rMG‐QOL15 showed no overall association, although females demonstrated a correlation between F0 drop and QOL burden (*r* = 0.77, *p* = 0.014).

**Conclusion:** Connected‐speech intensity decrements, particularly in males, represent a robust acoustic marker of bulbar involvement in MG not captured by patient‐reported outcomes.

**Disclosure:** Nothing to disclose related to the submitted manuscript

## EPO‐0504

### Demyelinating diseases and Sjögren's Syndrome: Clinical intersections and pathogenetic links

#### M. Akuç^1^; E. Koc ^2^; F. Saridas^2^; B. Yagız^1^; B. Coskun^1^; H. Dalkilic^1^; Y. Pehlivan^1^

##### ^*1*^ *Rheumatology, Faculty of Medicine, Uludag University, Bursa, Türkiye;* ^*2*^ *Neurology, Faculty of Medicine, Uludag University, Bursa, Türkiye*

**Background and aims:** The pathological relationship between demyelinating diseases and Sjögren's syndrome (SS) remains incompletely understood. Autoimmune mechanisms play a central role in the pathogenesis of both conditions, leading to overlapping clinical manifestations and responses to similar immunomodulatory therapies. We aimed to evaluate the frequency of coexisting SS in patients with suspected demyelinating diseases and to assess their clinical, serological, and histological features.

**Methods:** Over a one‐year period, 52 patients admitted to the neurology ward with suspected demyelinating disease and consulted by rheumatology were included. All patients underwent systematic rheumatological evaluation, including assessment of oral and ocular dryness. Antinuclear antibodies (ANA) and ANA profiles were analyzed in all cases. Salivary gland biopsy was performed in patients with sicca symptoms and/or relevant autoantibody positivity. Patients without clinical or serological evidence suggestive of SS were excluded from further evaluation.

**Results:** Among the 52 patients, 14 (26.9%) reported oral dryness and 7 (13.4%) ocular dryness. ANA positivity was detected in 11 patients (21.1%), and SS‐related autoantibodies were identified in 5 patients (10.5%). Salivary gland biopsy was performed in 27 patients and yielded positive results in 6 cases. Additionally, 3 patients with strong clinical and serological findings but negative biopsy results were diagnosed with SS. Overall, SS was diagnosed in 9 patients (17.3%). Concomitant diagnoses included MS, NMO, and myositis.

**Figure:**
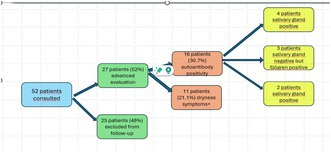
**FIGURE 1**: Study population and results.

**Figure:**
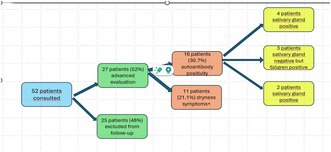
**FIGURE 1**: Study population and results.

**Figure:**
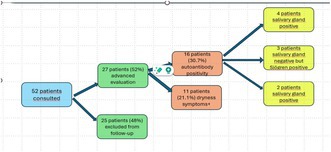
**FIGURE 1**: Study population and results.

**Conclusion:** Sicca symptoms and autoantibody positivity are more frequent in patients with demyelinating diseases than expected in the general population. Even in the absence of autoantibody positivity, SS should be considered when suggestive clinical features are present.

**Disclosure:** Nothing to disclose.

## EPO‐0505

### Autoimmune comorbidities affect thymoma‐associated Myasthenia Gravis outcome

#### F. Habetswallner; S. Falso; S. Marini; M. Marini; E. Aulitto; C. Bressi; M. Larossi; A. Evoli; R. Iorio

##### Catholic University of the Sacred Heart, Rome, Italy

**Background and aims:** Thymoma‐associated Myasthenia Gravis (TAMG) represents a clinical entity with poorer prognosis when compared to other MG subtypes. Outcome predictors are lacking. We conducted a retrospective study to identify clinical variables associated with favorable MG outcome as minimal manifestation or better according to MGFA post‐intervention status (MGFA‐PIS).

**Methods:** We retrieved information from clinical records of consecutive TAMG patients seen at our Institution between January 2005 and May 2025. Outcome predictors as patient demographics, MG severity (at onset and at maximum severity) and related treatment, pathologic variables, thymoma stage (according to Masaoka classification) and histotypes (according to WHO classification were assessed by univariate and multivariate analyses performed in R 4.3.1 Software Version.A favorable outcome was defined as achieving MM‐or‐better.

**Results:** A total of 118 patients [59 (50%) females, median age (IQR, years)= 63 (54–78)] with acetylcholine receptor antibody positive MG (AChR‐MG), and histologically confirmed thymoma were included. The outcome was assessed by MGFA‐PIS at the last follow‐up. Fourteen cases were excluded either due to missing data or to follow‐up period < 1 year. The univariate logistic regression revealed that the presence of autoimmune comorbidities (AIC) known or unknown to be linked to thymoma, and occurring at any time of patient history, was significantly associated with a lower chance to achieve ‘MM‐or‐better’, OR 0.25 (95% CI, 0.08–0.72; *p* = 0.006). There were no significant differences in follow‐up duration between patients with and without AICs.

**Figure:**
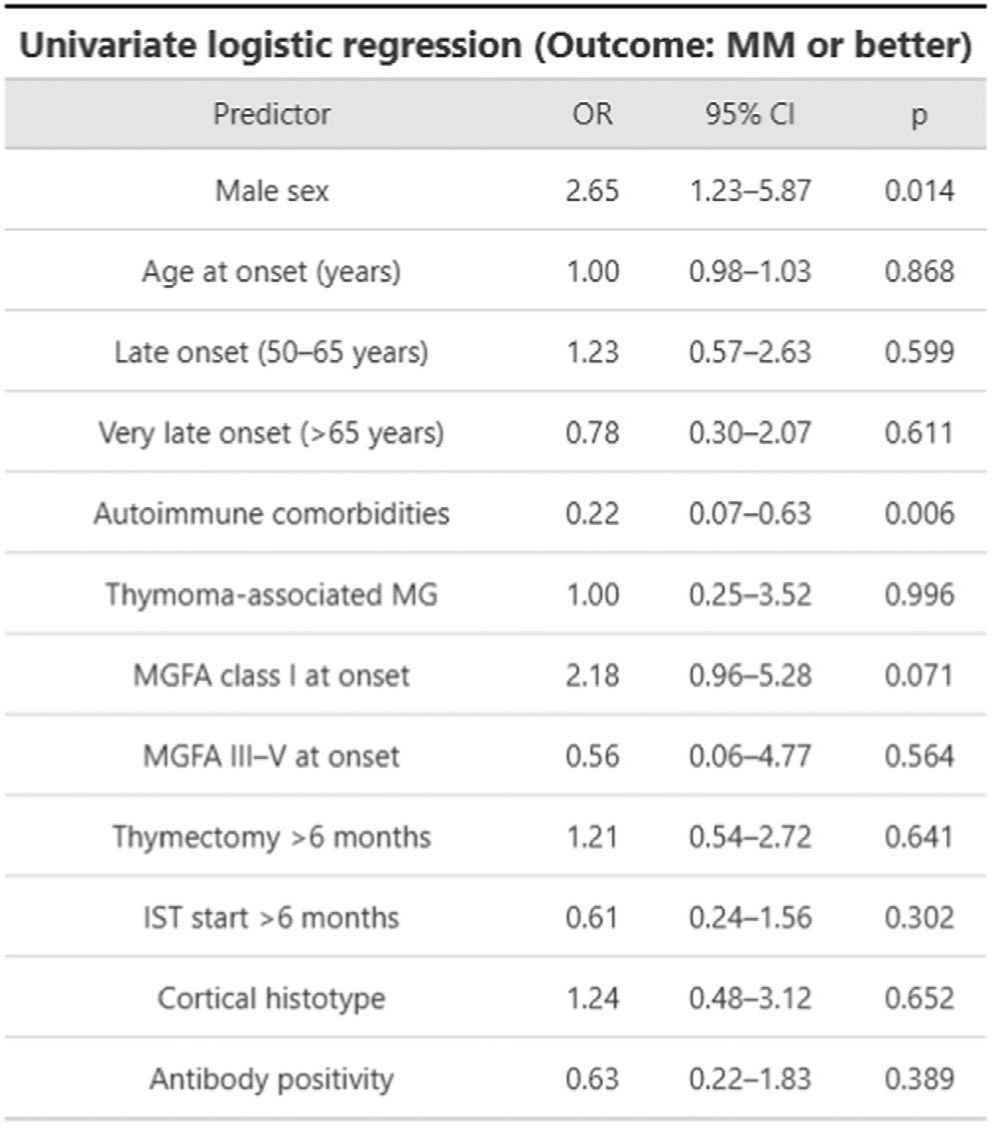
Univariate Analysis for predictors included across the TAMG cohort followed at out institution

**Figure:**
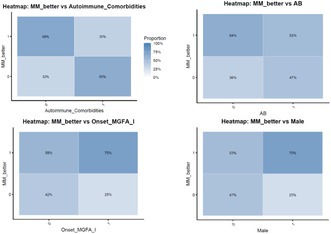
Heatmap by single predictor prevalence acroos the cohort of the study.

**Conclusion:** AICs can influence clinical outcome of patients with TAMG and should be carefully considered in therapeutic decision‐making

**Disclosure:**

## EPO‐0506

### Serum Nfl and GFAP as differential biomarkers of response to dimethyl fumarate and ocrelizumab in multiple sclerosis

#### A. Mingione^1^; A. Corona^1^; C. Monzani^1^; A. Zollo^1^; C. Cirocco^2^; T. Zaccone^3^; F. Massaro^1^; M. Scavone^4^; G. Podda^4^; P. Signorelli^5^; M. Miozzo^6^; A. Priori^7^; F. Martinelli Boneschi ^8^

##### ^*1*^ *Laboratory of Precision Medicine of Neurological Diseases, Aldo Ravelli Center for Neurotechnology and Experimental Brain Therapeutics, Department of Health Sciences, University of Milan, Milan, Italy;* ^*2*^ *Laboratory of Precision Medicine of Neurological Diseases, Department of Health Sciences, University of Milan, Milan, Italy;* ^*3*^ *Clinical Neurology Unit, ASST Santi Paolo e Carlo, Milan, Italy;* ^*4*^ *Division of General Medicine SP, ASST Santi Paolo e Carlo, Laboratory of Haemostasis and Thrombosis, Department of Health Sciences, University of Milan, Milan, Italy;* ^*5*^ *Aldo Ravelli Center for Neurotechnology and Experimental Brain Therapeutics, Department of Health Sciences, University of Milan, Biochemistry Laboratory, IRCCS Policlinico San Donato, Milan, Italy;* ^*6*^ *Medical Genetics, Department of Health Sciences, University of Milan, Medical Genetics Unit, ASST Santi Paolo e Carlo, Milan, Italy;* ^*7*^ *Aldo Ravelli Center for Neurotechnology and Experimental Brain Therapeutics, Department of Health Sciences, University of Milan, Clinical Neurology Unit, ASST Santi Paolo e Carlo, Milan, Italy;* ^*8*^ *Laboratory of Precision Medicine of Neurological Diseases, Aldo Ravelli Center for Neurotechnology and Experimental Brain Therapeutics, Department of Health Sciences, University of Milan, Clinical Neurology Unit, ASST Santi Paolo e Carlo, Milan, Italy*

**Background and aims:** Multiple Sclerosis (MS) is a chronic inflammatory and neurodegenerative disease. Quantifying neuronal damage is a critical step for patient care and treatment selection. Neurofilament light chain (sNfL) and glial fibrillary acidic protein (sGFAP) are the two most promising serum biomarkers reflecting neuronal damage and astroglial activation, respectively.

**Methods:** This study analyzed sNfL and sGFAP in 177 consecutive MS patients and 71 healthy controls (HCs) enrolled at Ospedale San Paolo in Milan using SIMOA technology, classifying patients as Responders (R) or Non‐Responders (NR) based on "No Evidence of Disease Activity 3" (NEDA‐3) status after two years of treatment. Longitudinal analyses were performed for Dimethyl fumarate (DMF) and Ocrelizumab (OCRE) treatment.

**Results:** Biomarker‐age correlation analysis in HCs confirmed correlation between either NfL and GFAP with age and cut‐off values specific for age decades were calculated. Both biomarkers were higher in MS patients compared to HCs. sNfL showed a significant increase in NR patients overall. In contrast, sGFAP was elevated in the low‐efficacy treatment agents (LETAs) NR group and also in the DMF NR subgroup, suggesting that it monitors persistent astrogliosis. Longitudinal analysis showed that either biomarkers decreased during DMF treatment after one year. During OCRE treatment sNfL rapidly reduced to HC levels within one year, while sGFAP decreased only after two years.

**Conclusion:** This study highlights the differential temporal dynamics of sNfL and sGFAP under ocrelizumab and the potential role of sGFAP as treatment‐response biomarker in DMF‐treated patients.

**Disclosure:** AM nothing to disclose AC nothing to disclose CM nothing to disclose AZ nothing to disclose CC nothing to disclose TZ received travel support from Merck, Biogen and Novartis FM nothing to disclose MS nothing to disclose GP nothing to disclose PS nothing to disclose MM nothing to disclose AP nothing to disclose FMB received travel support from Roche, Biogen and Novartis and speaking honoraria from Roche

## EPO‐0507

### Uncovering novel antigenic targets of CNS‐reactive autoantibodies in autoimmune neurological syndromes

#### I. Senyuz ^1^; S. Jones^1^; M. Canales^1^; A. Meringa^1^; R. Bernard‐Valnet^1^; R. Genolet^2^; M. Theaudin^1^; A. Harari^2^; C. Pot^1^; R. Du Pasquier^1^; A. Mathias^1^

##### ^*1*^ *Laboratory of Neuroimmunology and Service of Neurology, Department of Clinical Neurosciences, Lausanne University Hospital and University of Lausanne, Lausanne, Switzerland;* ^*2*^ *Ludwig Institute for Cancer Research, Lausanne Branch, Department of Oncology, Lausanne University Hospital and University of Lausanne, Agora Cancer Research Center, Lausanne, Switzerland*

**Background and aims:** The identification of auto‐antibodies (auto‐Abs) and their antigens has improved the diagnosis and stratification of autoimmune neurological syndromes such as autoimmune encephalitis (AIE) and neuromyelitis optica spectrum disorders (NMOSD). Yet, 7–30% of AIE/NMOSD patients remain seronegative. Auto‐Abs are also reported in multiple sclerosis, but their targets and pathogenic relevance remain unclear. Our objective is to identify novel antigenic targets to advance our understanding of disease immunopathology and improve diagnosis/prognosis.

**Methods:** We established a human induced pluripotent stem cell (hiPSC)‐derived astrocyte or neuron cell‐based assay (CBA) to identify patients with astrocyte‐ and/or neuron‐reactive IgG in serum or cerebrospinal fluid. Memory B cells from selected patients were immortalized to generate B lymphoblastoid cell lines (BLCLs) secreting monoclonal antibodies (mAbs). These mAbs were screened using the CBA, and B‐cell receptor variable regions of autoreactive clones were sequenced for recombinant mAb production, enabling antigen discovery. Enrolled patients presented with inflammatory neurological diseases (IND) including people with MS (pwMS), as well as non‐IND control patients.

**Results:** We identified astrocyte‐ and neuron‐reactive auto‐Abs in 10 seronegative AIE/NMOSD patients; 15 astrocyte‐ and 14 neuron‐reactive auto‐Abs among 97 pwMS; and astrocyte‐reactive auto‐Abs in one other‐IND (OIND) patient seronegative for known auto‐Ags. We successfully produced astrocyte‐reactive mAbs from selected patients (AQP4‐positive NMO, pwMS and OIND), which are being studied for downstream antigen discovery.

**Conclusion:** We present a robust pipeline combining hiPSC‐derived CBA with patient‐derived mAbs for Ag discovery. This approach enables identification of novel autoreactive targets in pwMS and seronegative autoimmune neurological patients, enhancing immunopathological understanding and diagnostic accuracy.

**Disclosure:** Caroline Pot is supported by the Panacée Foundation, the Swiss MS Society and the Swiss National Science Foundation.

## EPO‐0508

### Progression independent of relapse and MRI activity in disability accumulation throughout the course of multiple sclerosis

#### K. van Tulder ^1^; C. Corsten^1^; R. Klein Kranenbarg^2^; I. Smets^1^; J. de Beukelaar^3^; M. van Luijn^4^; B. Wokke^1^; J. Smolders^5^

##### ^*1*^ *Department of Neurology, MS center ErasMS, Erasmus MC, University Medical Center, Rotterdam, the Netherlands;* ^*2*^ *Department of Neurology, MS center ErasMS, Erasmus MC, University Medical Center, Rotterdam, the Netherlands; Department of Neurology, Albert Schweitzer Hospital, Dordrecht, the Netherlands;* ^*3*^ *Department of Neurology, Albert Schweitzer Hospital, Dordrecht, the Netherlands;* ^*4*^ *Department of Immunology, MS Center ErasMS, Erasmus MC, University Medical Center, Rotterdam, the Netherlands;* ^*5*^ *Department of Neurology, Department of Immunology, MS center ErasMS, Erasmus MC, University Medical Center, Rotterdam, the Netherlands; Neuroimmunology research group, Netherlands Institute for Neuroscience, Amsterdam, the Netherlands*

**Background and aims:** Progression independent of relapses and MRI activity (PIRMA) is an important driving factor of disability accumulation in relapsing multiple sclerosis (MS) trial‐cohorts treated with high‐efficacy therapies (HET). It is unclear how PIRMA during HET relates to PIRMA as observed in (untreated) early‐stage and primary progressive MS. In this study we assessed PIRMA in 3 independent cohorts to explore applicability of definitions and overlap in clinical characteristics associated with PIRMA.

**Methods:** Repetitive data on EDSS, PDDS, T25FW, SDMT and walking distance were collected from a retrospective HET‐cohort (*N* = 369), a prospective first attack of demyelination cohort (PROUD‐cohort, *N* = 458) and a prospective PPMS cohort (SPIN‐P‐cohort, *N* = 188). Worsening of these outcomes was defined as potentially attributable to relapses or MRI‐activity if any of these events were reported between measurements or <30 days after new measurement. Otherwise, worsening was attributed to PIRMA.

**Results:** Median age at respective baseline was 34 years (HET‐cohort), 33 (PROUD‐cohort) and 60 (SPIN‐P‐cohort). Both in HET‐cohort and PROUD‐cohort around 70% was female, while in SPIN‐P‐cohort 52% was female. EDSS and PDDS were the most used measurements in all cohorts (HET‐cohort *N* = 630, *N* = 261; PROUD‐cohort *N* = 854, *N* = 478; SPIN‐P‐cohort *N* = 463, *N* = 467, respectively). Worsening in outcome measures during follow‐up was low in all cohorts (20.5% (268/1311), 11.6% (179/1537), 18.5% (320/1729), respectively). The proportion of worsening attributed to PIRMA was 63.1 (HET‐cohort), 58.7 (PROUD‐cohort) and 92.5% (SPIN‐P‐cohort) respectively.

**Conclusion:** PIRMA is proportionally the most contributing factor of disability accumulation throughout the whole disease course of MS. Participant characteristics associated with worsening of EDSS and PDDS will be presented.

**Disclosure:** Conflicts of interest K. van Tulder: Nothing to disclose. C. Corsten: Nothing to disclose R. Klein Kranenbarg: Nothing to disclose I. Smets: has received lecture fees from Merck, Biogen Idec and Sanofi. J. de Beukelaar: Nothing to disclose. M. M. van Luijn: has received research support from EMD Serono, Merck, Novartis, GSK and Idorsia Pharmaceutical Ltd. B. Wokke: Nothing to disclose J. Smolders: reports grant for scientific research from Roche and Siemens Healthineers and received speaker and/or consultancy fee of Biogen, Merck, Novartis, Roche and Sanofi.

## EPO‐0509

### Population‐normalised smartwatch Z‐scores quantify real‐world disability in multiple sclerosis

#### L. Masanneck ^1^; P. Kirschner^1^; N. Werner^1^; T. Koelsche^1^; B. Danajka^1^; U. Ceylan^3^; B. Vittrant^4^; J. Voth^1^; R. Hagler^1^; P. Epping^1^; N. Huntermann^1^; B. Lechat^5^; A. Stern^2^; P. Benkert^6^; J. Kuhle^6^; R. Gold^3^; M. Schuette^1^; M. Heibel^7^; S. Faissner^3^; S. Meuth^1^; M. Pawlitzki^1^

##### ^*1*^ *Department of Neurology, Medical Faculty and University Hospital Düsseldorf, Heinrich‐Heine‐Universität Düsseldorf, Düsseldorf, Germany;* ^*2*^ *Hasso Plattner Institute, University of Potsdam, Potsdam, Germany;* ^*3*^ *Department of Neurology, Ruhr‐University Bochum, St. Josef‐Hospital, Bochum, Germany;* ^*4*^ *Withings, Issy Les Moulineaux, France;* ^*5*^ *Flinders Health and Medical Research Institute: Sleep Health, College of Medicine and Public Health, Flinders University, Adelaide, SA, Australia;* ^*6*^ *Multiple Sclerosis Centre and Research Center for Clinical Neuroimmunology and Neuroscience (RC2NB), Departments of Biomedicine and Clinical Research, University Hospital and University of Basel, Basel, Switzerland;* ^*7*^ *Sauerlandklinik, Hachen, Germany*

**Background and aims:** Wearables quantify real‐world function, but missing reference values limit clinical interpretation. Objective: to test whether population‐normalised smartwatch Z‐scores and a composite digital deviation from norm track disability in multiple sclerosis.

**Methods:** We pooled Withings ScanWatch data from prospective observational studies. Participants with > = 30 valid days and complete age, sex, body mass index (BMI), and Expanded Disability Status Scale (EDSS) data were included. Steps, moderate‐intensity activity, total sleep time, and nightly resting heart rate were converted to matched Z‐scores using an aggregated reference dataset of 2,655,433 users (2023–2024). Digital Deviation Composite (DDC2‐4) averaged only abnormal deviations across 2–4 domains.

**Results:** 143/180 participants met criteria. Step and activity Z‐scores declined with disability, becoming significantly below norms from EDSS 4.0–4.5 (median step Z‐score −0.92) and reaching −1.66 at EDSS > = 7.0 (Figure 1). EDSS correlated with steps (Spearman *r* −0.71) and activity (*r* −0.63), weakly with resting heart rate (*r* 0.27), and not with sleep (*r* 0.05). DDC2, DDC3, and DDC4 correlated with EDSS (*r* 0.72, 0.68, 0.66) (Figure 2). Serum glial fibrillary acidic protein (GFAP) showed weak positive associations with DDC3 and DDC4, whereas serum neurofilament light chain (NfL) showed none; in migraine controls (*n* = 45), activity Z‐scores were near norms.

**Figure:**
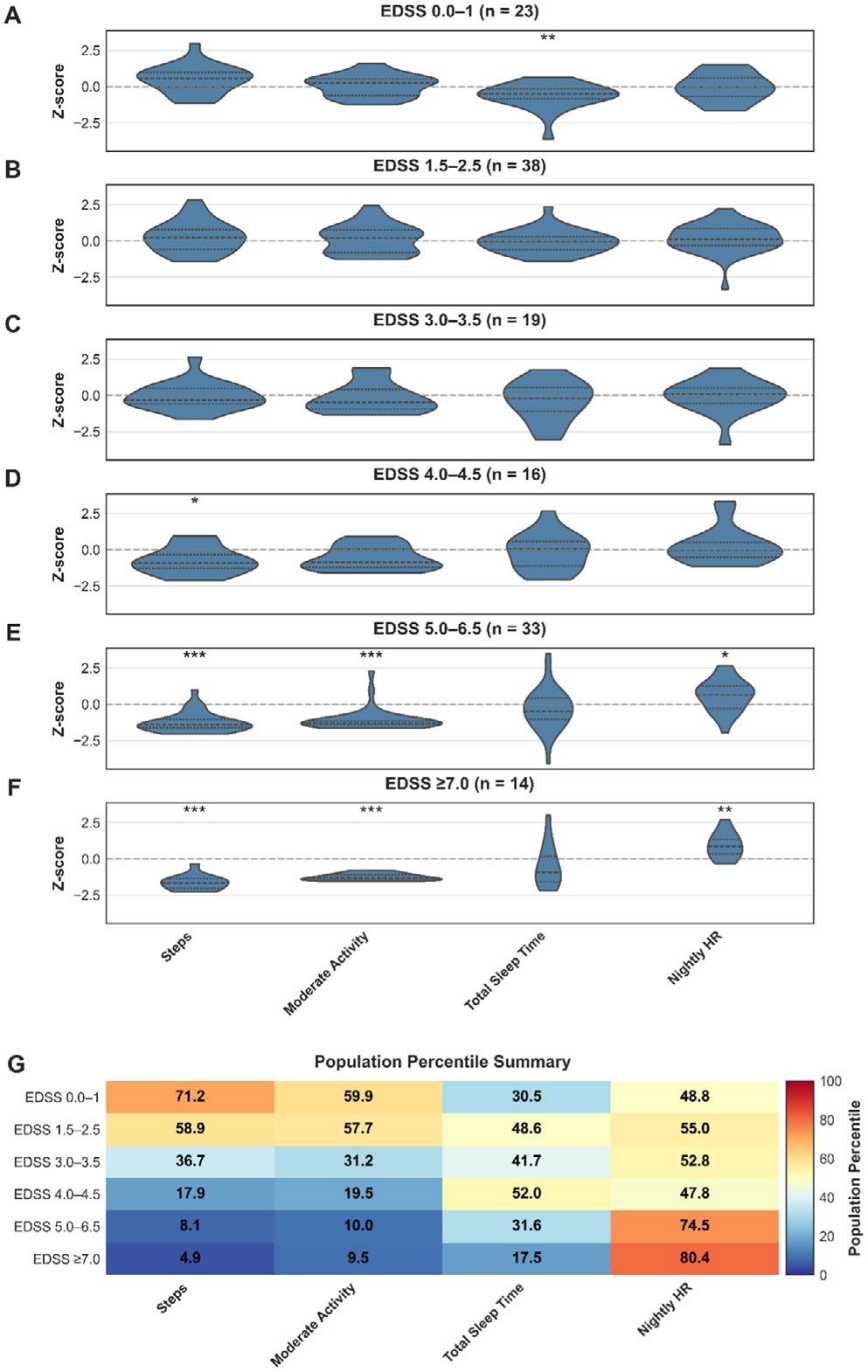
**FIGURE 1** Age‐, sex‐, and BMI‐matched smartwatch Z‐scores (steps, moderate activity, sleep time, resting heart rate) across Expanded Disability Status Scale (EDSS) groups; heatmap shows median population percentiles. *n* = 143.

**Figure:**
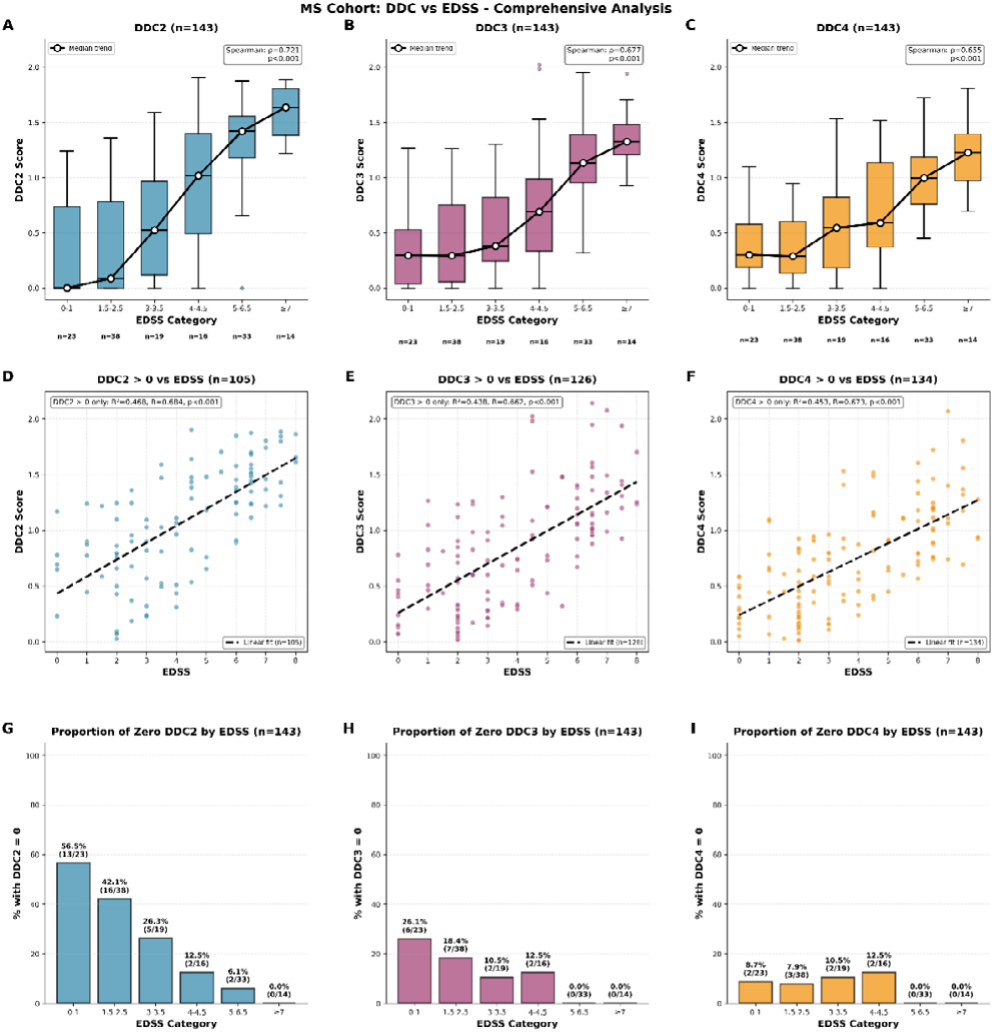
**FIGURE 2** Digital Deviation Composite (DDC2‐4) versus Expanded Disability Status Scale (EDSS): boxplots by EDSS group, scatter among participants with DDC>0 (linear fit), and bars showing proportion with DDC = 0. *n* = 143.

**Conclusion:** Population‐normalised wearable Z‐scores and DDCs provide an interpretable distance‐from‐norm framework for multiple sclerosis disability and may complement episodic clinic‐based scales; a public web app supports Z‐score calculation. Longitudinal studies should test responsiveness and prognostic value.

**Disclosure:** Supported by Deutsche Forschungsgemeinschaft (DFG) 493659010; Withings provided aggregated reference data; Benjamin Vittrant is employed by Withings and Bastien Lechat has received Withings research support outside the submitted work; all other authors report no relevant relationships for this abstract.

## EPO‐0510

### Neurofilament light chain as a biomarker in chronic inflammatory demyelinating polyradiculoneuropathy: Insights from ADHERE

#### R. Collet Vidiella ^1^; M. Caballero‐Avila^1^; T. Casneuf^2^; E. Hofman^2^; G. Istas^2^; P. Llarch^1^; L. Martín‐Aguilar^1^; E. Pascual‐Goñi^1^; A. De Roeck^2^; B. Balbino^2^; L. Querol^1^

##### ^*1*^ *Neuromuscular Diseases Unit, Hospital de La Santa Creu I Sant Pau, Universitat Autònoma de Barcelona, Barcelona, Spain,* ^*2*^ *argenx, Ghent, Belgium*

**Background and aims:** Chronic inflammatory demyelinating polyradiculoneuropathy (CIDP) is a debilitating, progressive autoimmune neuropathic disease. Neurofilament light chain (NfL) levels indicate axonal damage and may serve as a biomarker in CIDP. NfL levels were analysed in ADHERE (NCT04281472), the largest CIDP cohort to date.

**Methods:** Participants with probable/definite CIDP received weekly subcutaneous (SC) efgartigimod PH20 1000 mg (stage A) for ≤12 weeks. Responders entered stage B and were randomised (1:1) to weekly efgartigimod PH20 SC 1000 mg or placebo for ≤48 weeks. Serum NfL (sNfL) was measured longitudinally in 214 participants, from which NfL z‐score (zNfL) was determined.

**Results:** During stage A, sNfL levels remained stable in responders with baseline levels within healthy reference range (≤20 pg/mL, *n* = 114); sNfL reduced by 18% over time in responders with elevated baseline levels (>20 pg/mL, *n* = 36). In participants receiving continuous efgartigimod through stage B, sNfL reduced by 35% by week 24 (*n* = 13). Mean (SD) sNfL levels at stage A baseline were 18.9 (22.6) pg/mL (*n* = 214), corresponding to a mean (SD) zNfL of 0.64 (1.57). Elevated baseline zNfL levels were associated with higher CIDP disease activity status (CDAS 5). zNfL scores at stage A baseline were highest in samples from CIDP‐treatment‐naïve participants versus those who had previously received CIDP treatment.

**Conclusion:** At stage A, higher baseline NfL levels correlated with higher (worse) CDAS scores. Among efgartigimod‐treated responders with elevated baseline sNfL, we observed a reduction in sNfL in both stages A and B. Further analysis will determine whether NfL can serve as a contextual biomarker and inform on disease monitoring.

**Disclosure:** Roger Collet Vidiella, Marta Caballero‐Avila, Paula Llarch, Lorena Martín‐Aguilar: Nothing to disclose. Tineke Casneuf, Erik Hofman, Geoffrey Istas, Arne De Roeck, Bianca Balbino: Employees of argenx. Elba Pascual‐Goñi: Speaker honoraria from argenx. Luis Querol: Alnylam, Annexon, argenx, Avilar, Biogen, CIBERER, CSL Behring, Dianthus, Fundació La Marató, GBS‐CIDP Foundation International, Grifols, Instituto de Salud Carlos III – Ministry of Economy and Innovation (Spain), Janssen, LFB, Lundbeck, Merck, Novartis, Octapharma, Roche, Sanofi, UCB.

## EPO‐0511

### Autoimmune brainstem encephalitis: A cohort study showing wide variety in phenotype and outcomes

#### M. Jin ^1^; J. Kerstens^1^; R. van Steenhoven^1^; E. Erdag Turgeon^1^; M. Nagtzaam^1^; M. van Duijn^1^; S. Veenbergen^2^; J. de Vries^1^; M. Titulaer^1^

##### ^*1*^ *Department of Neurology, Erasmus Medical Center, Rotterdam, the Netherlands;* ^*2*^ *Department of Immunology, Laboratory Medical Immunology, Erasmus Medical Center, Rotterdam, the Netherlands*

**Background and aims:** Autoimmune brainstem encephalitis (ABE) manifests diversely due to the complexity of brainstem nuclei. We aimed to comprehensively characterize the phenotype and paraclinical features to improve diagnostic approaches for ABE, and describe treatment and outcomes.

**Methods:** Patients suspected of or diagnosed with ABE at Erasmus Medical Center between January 2017 and April 2025 were retrospectively reviewed.

**Results:** Initial search returned 122 patients. After excluding 25 patients, 97 patients were included. The median age was 61 (range 1–86) years, and 56 (58%) individuals were male. Common brainstem symptoms were: ataxia (47%), eye movement disorders (45%),, pyramidal tract signs (44%), and dysarthria (42%), while neuropsychiatric (45%) and movement disorders (25%) were the most common additional symptoms. Cancer was identified in 35 patients (36%): hematologic (12), breast carcinoma (6), colorectal (5), lung cancers (small cell lung cancer 4, adenocarcinoma 1), germ cell (2), neuroendocrine carcinoma (1), urogenital (1), and others (3). Identified antibodies (20%) included: anti‐KLHL11 (3), anti‐Ma2 (3), anti‐Hu (2), anti‐NMDAR (2), anti‐glycine receptor (2), anti‐IgLON5, anti‐amphiphysin, anti‐GAD65, anti‐AQP4, anti‐MOG, anti‐GFAP, anti‐GQ1b (all *n* = 1). Inflammatory changes in cerebrospinal fluid were observed in 61/88 (69%), and MRI brain abnormalities were observed in 64/95 patients (67%). Outcomes were available in 59 patients: improvement or stabilization was achieved in 44 patients (31 and 13 respectively, 77%). Patients with extracellular or non‐neuronal antibodies generally respond better than those with intracellular antibodies, while antibody‐negative group tends to be in between.

**Figure:**
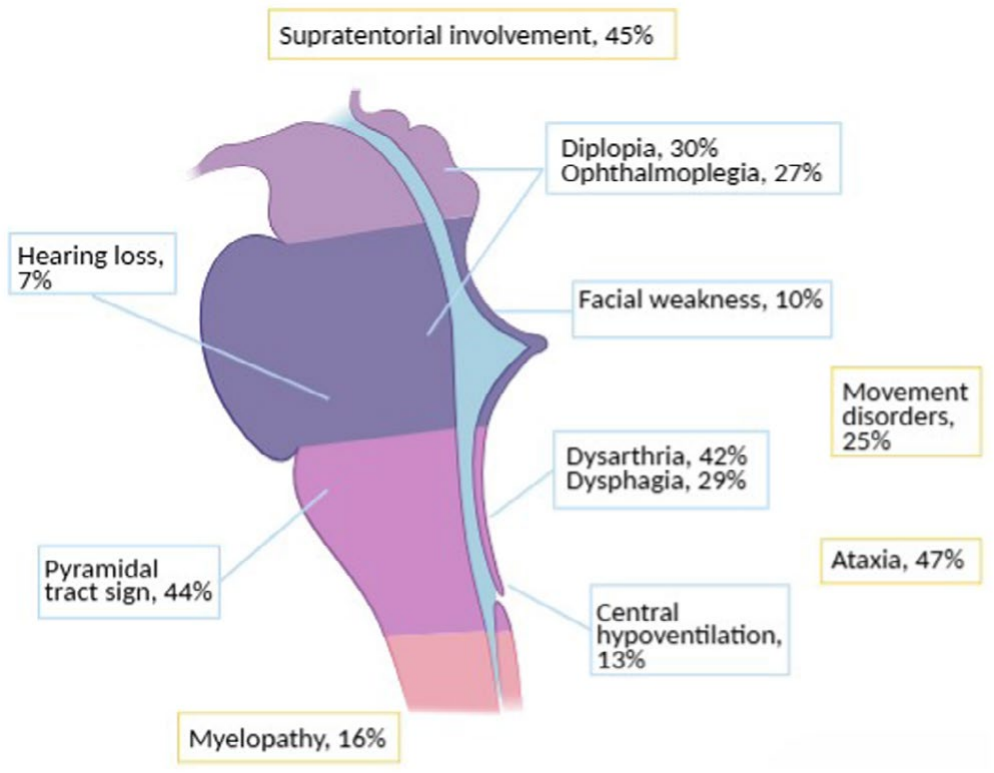
**FIGURE 1** Neurological symptom spectrum in autoimmune brainstem encephalitis.

**Figure:**
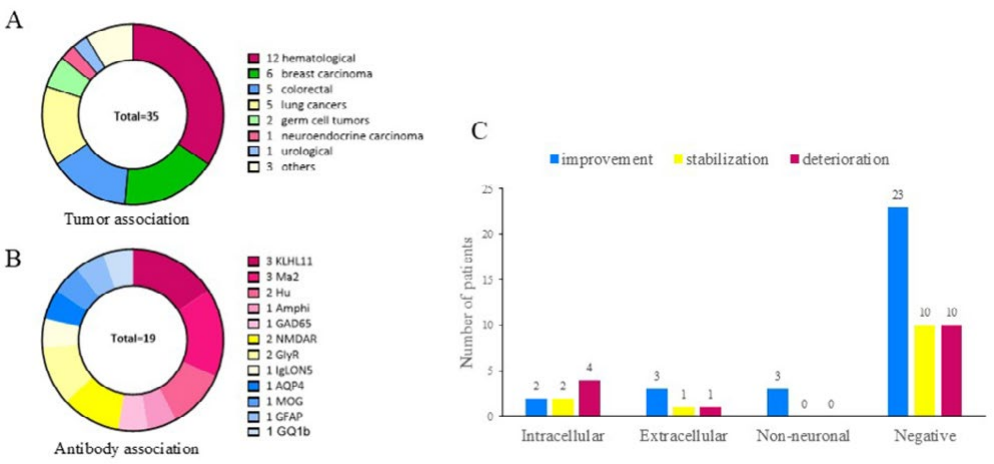
**FIGURE 2** A. The tumor associations of autoimmune brainstem encephalitis (ABE). B. The antibody associations of ABE. C. The prognosis of patients with intracellular, extracellular, non‐neuronal antibodies or patients without known antibodies.

**Conclusion:** ABE has complicate aetiologies, broad tumor and antibody associations. Most treated patients respond to immunotherapy.

**Disclosure:** M.J. is supported by the Chinese scholarship council program (Project ID 202308430038).

## EPO‐0512

### Adapted physical exercise can promote microglia–neuron interactions to enhance recovery in EAE

#### V. Pantazou ^1,2^; G. Dorcet^1,3^; M. S Aigrot^1^; B. Stankoff^1,3^; C. Lubetzki^1,3^; A. Desmazières^1^

##### ^*1*^ *Paris Brain Institute, Sorbonne Université, CNRS, Inserm, GH Pitié‐Salpêtrière, Paris, France;* ^*2*^ *2Service of neurology, Department of clinical neurosciences, Lausanne University Hospital and University of Lausanne, Lausanne, Switzerland;* ^*3*^ *3Neurology Department, Assistance Publique‐Hôpitaux de Paris, Pitié Salpêtrière Hospital Paris, France*

**Background and aims:** In multiple sclerosis (MS), remyelination is frequently inefficient and highly heterogeneous between individuals. Physical exercise has been shown to enhance oligodendrogenesis and new myelin sheath formation in demyelinating models, and to attenuate disease severity in experimental autoimmune encephalomyelitis (EAE) when performed prior to disease onset. Whether physical activity can promote repair in vivo after disease onset remains unknown.

**Methods:** We compared two exercise paradigms in EAE induced mice: an earlier‐onset, higher‐intensity protocol (initiated 36 h after peak disease; 2x15‐min treadmill sessions/day, at 5 cm/s, for four consecutive days), with a later‐onset, low‐intensity protocol (initiated 48 h after peak; 2x10‐min sessions/day, at 5 cm/s, for 4 days) and sedentary controls.

**Results:** Early‐onset, higher‐intensity exercise failed to improve clinical outcome compared with sedentary mice and was associated with a transient worsening of motor deficits. In contrast, mice subjected to later‐onset, low‐intensity exercise displayed significantly improved motor performance during remission. At the tissue level, this improvement was associated with enhanced lesion resolution, increased microglial‐neuron interactions and higher expression of IGF‐1 and P2Y12R in microglia/macrophages, consistent with a pro‐repair phenotype and a partial return toward homeostasis.

**Conclusion:** Together, these findings identify a critical temporal window after demyelination during which, appropriately timed, low‐intensity exercise promotes microglial phenotypic changes and improves clinical recovery in mice with EAE. Defining exercise paradigms that are both biologically effective and translatable to patients with MS remains a key challenge.

**Disclosure:** Nothing to disclose.

## EPO‐0513

### The Comprehensive Live Cell‐Based Cytotoxicity Assay for Predicting Rescue Therapy in Acute Attacks of NMOSD

#### X. Xu^1^; X. Zhao^2^; J. Ding^1^; Y. Du^1^; G. Luo^1^; J. Li^1^; R. Liu^1^; X. Xu^1^; X. Li^1^; C. Yang ^1^; W. Jiang^1^

##### ^*1*^ *Department of Neurology, Tianjin Medical University General Hospital, Tianjin, China;* ^*2*^ *Department of Radiology, The Third Affiliated Hospital of Zhengzhou University, Zhengzhou, China*

**Background and aims:** Neuromyelitis optica spectrum disorder (NMOSD) lacks serological biomarkers that accurately reflect disease activity and treatment response. We established a novel live cell‐based complement‐dependent cytotoxicity assay (NMO‐CBA‐CDC) using endogenous complement to quantify serum cytotoxicity. This assay was developed to offer a novel tool for monitoring disease activity, assessing therapeutic efficacy, and determining the necessity of rescue therapy during acute attacks.

**Methods:** 111 samples from 65 aquaporin‐4 immunoglobulin G seropositive (AQP4‐IgG+) NMOSD patients were collected. We compared serum complement levels (C1q, C5a, soluble membrane attack complex [sC5b‐9]), 50% hemolytic complement (CH50), AQP4‐IgG titers, and the cytotoxic index (CI) determined by NMO‐CBA‐CDC between the remission and acute phases, including patients receiving rescue therapies.

**Results:** Serum C5a and sC5b‐9 levels were elevated during acute attacks (*p* < 0.05). Intravenous methylprednisolone (IVMP) lowered complement components but not AQP4‐IgG titers or CI. Notably, the CI was significantly higher in the acute phase than in remission (*p* = 0.0011) and effectively distinguished IVMP responders from non‐responders. Among rescue therapies, plasma exchange/immunoadsorption (PE/IA) most consistently reduced the CI, while intravenous immunoglobulin (IVIG), neonatal Fc receptor (FcRn) antagonists, and C5 inhibitors showed variable effects.

**Figure:**
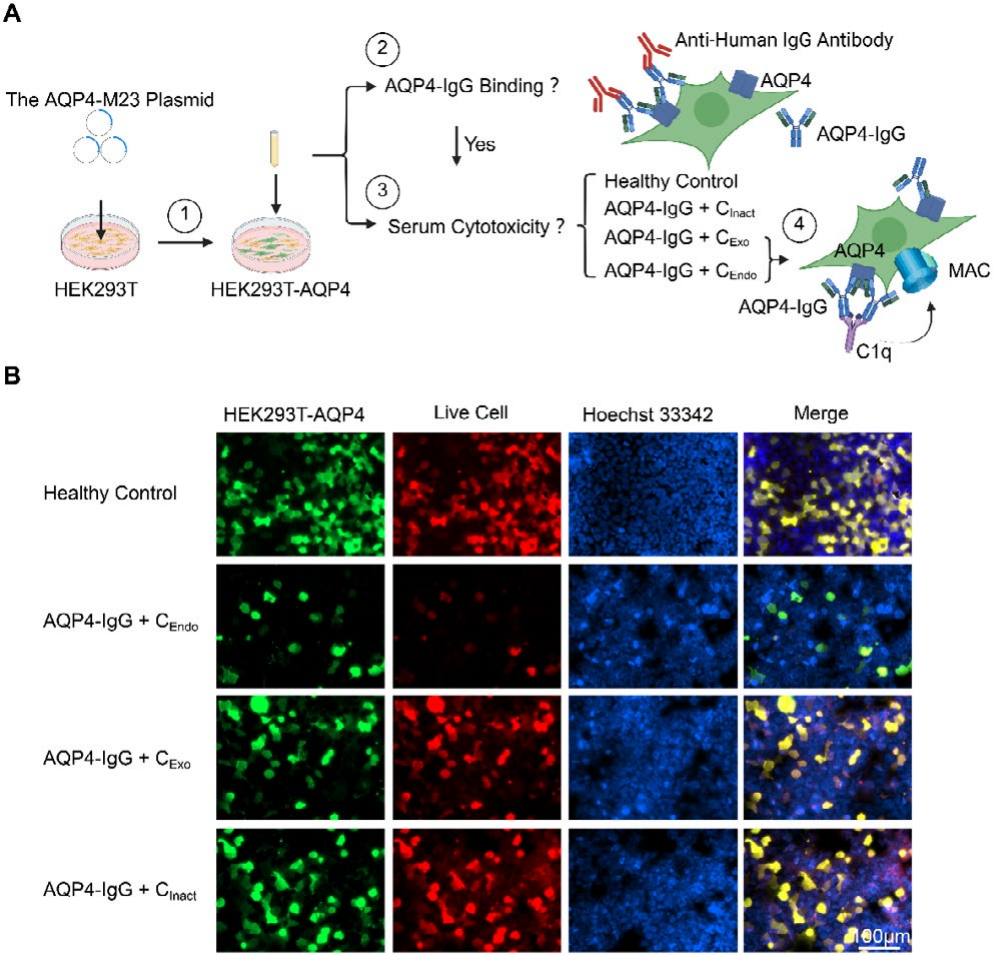
**FIGURE 1** Development a comprehensive NMO‐CBA‐CDC assay to assess serum cytotoxicity in HEK293T‐AQP4 cells.

**Figure:**
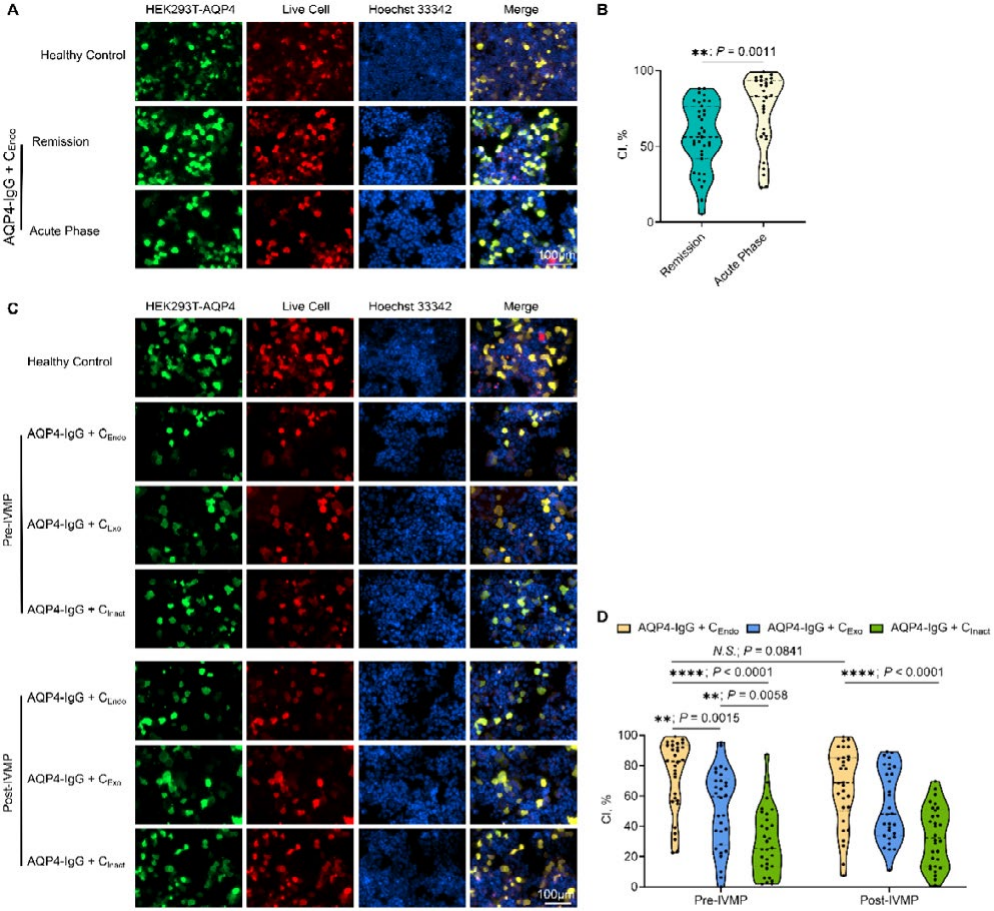
**FIGURE 2** The cytotoxic index increased during the acute attack of NMOSD, which failed to be suppressed following intravenous methylprednisolone puls therapy.

**Figure:**
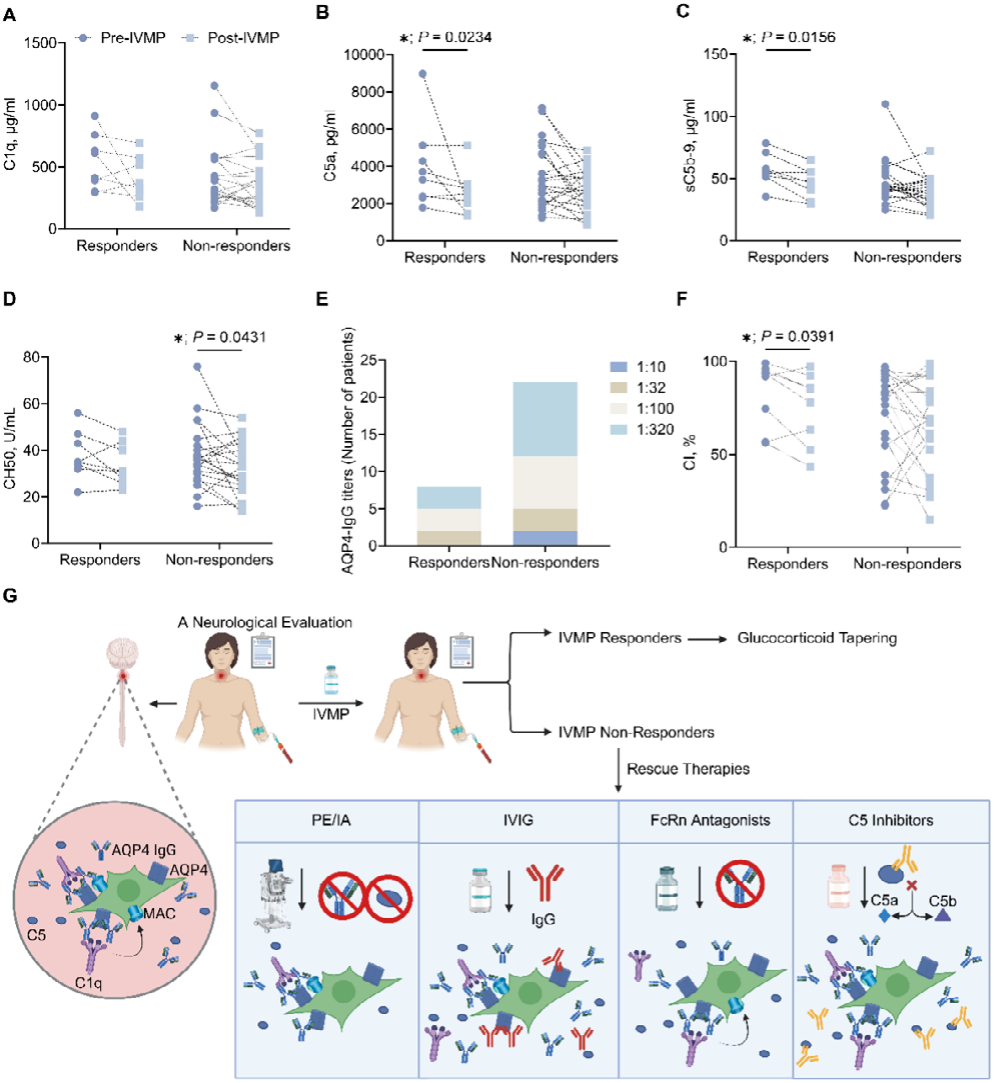
**FIGURE 3** The serological indicators for the responsiveness to IVMP during acute attack of NMOSD and the implication for further rescue therapy.

**Conclusion:** The NMO‐CBA‐CDC assay combined endogenous AQP4‐IgG and complement activity to comprehensively assess the serum cytotoxicity in AQP4‐IgG+ NMOSD patients. The CI served as an efficient tool for monitoring and predicting disease activity, evaluating therapeutic efficacy, and guiding the necessity of rescue therapy. Among rescue therapies, PE/IA demonstrated superior efficacy as reflected by NMO‐CBA‐CDC assay.

**Disclosure:** The authors have no conflicts of interest to declare. This study was supported by the National Natural Science Foundation of China (82171277, 82571535, 82171338), and Young Scientific and Technological Talents (Level Three) in Tianjin.

## EPO‐0514

### Eculizumab in Myasthenia Gravis—A Multicenter Retrospective Real‐World Study in China

#### Y. Li^1^; T. Chang^2^; H. Yang^3^; S. Tan^4^; L. Luo^5^; X. Cao^6^; J. Hu^7^; B. Bu^1^; Z. Li ^1^

##### ^*1*^ *Department of Neurology, Tongji Hospital, Tongji Medical College, Huazhong University of Science and Technology, Wuhan, China;* ^*2*^ *Department of Neurology, Tangdu Hospital, Air Force Medical University, Xi'an, China;* ^*3*^ *Department of Neurology, Xiangya Hospital, Central South University, Changsha, China;* ^*4*^ *epartment of Rare Diseases, Sichuan Provincial People's Hospital, School of Medicine, University of Electronic Science and Technology of China, Chengdu, China;* ^*5*^ *Department of Neurology, Wuhan No.1 Hospital, Wuhan, China;* ^*6*^ *Department of Neurology, Union Hospital, Tongji Medical College, Huazhong University of Science and Technology, Wuhan, China;* ^*7*^ *Department of Neurology, Taihe Hospital, Hubei University of Medicine, Shiyan, China*

**Background and aims:** Myasthenia gravis (MG) is a chronic autoimmune neuromuscular disorder needing long‐term immunosuppressive therapy. Eculizumab is a promising treatment, but real‐world evidence on its efficacy and safety is limited.

**Methods:** A retrospective cohort study was done across six medical centers, enrolling 60 MG patients on eculizumab. Clinical data, including MG‐Activities of Daily Living (ADL) scores, quantitative MG scores (QMG), and oral corticosteroid dosage at different time points (baseline, Weeks 1, 4, 8, and final follow‐up before November 2024), were extracted from medical records Study outcomes included efficacy measures (changes in MG‐ADL and QMG scores, Myasthenia Gravis Foundation of America [MGFA] Post‐Intervention Status [PIS], glucocorticoid dosage), and safety profile of eculizumab.

**Results:** In this cohort, 30.95% of patients achieved minimal manifestation status or better by Week 8, increasing to 32.69% at final follow‐up. Meanwhile, mean ADL scores significantly dropped from 8.13±3.99 at baseline to 5.59±3.72 by Week 1 and to 3.33±3.56 by the final follow‐up (*p* < 0.0001). Clinically meaningful improvement up to 76.27% at final follow‐up was also observed. In the thymoma‐associated MG (TAMG) subgroup, ADL decreased rapidly compared to baseline (*p* < 0.001), and the non‐TAMG cohort also improved significantly at each time point (*p* ≤ 0.026). Additionally, no treatment‐related adverse events were observed. The quick drop in MG‐ADL scores indicates its efficacy, and the steroid‐sparing effect is crucial for long‐term management. In TAMG, improvement was early, reaching maximum benefit in the first week.

**Figure:**
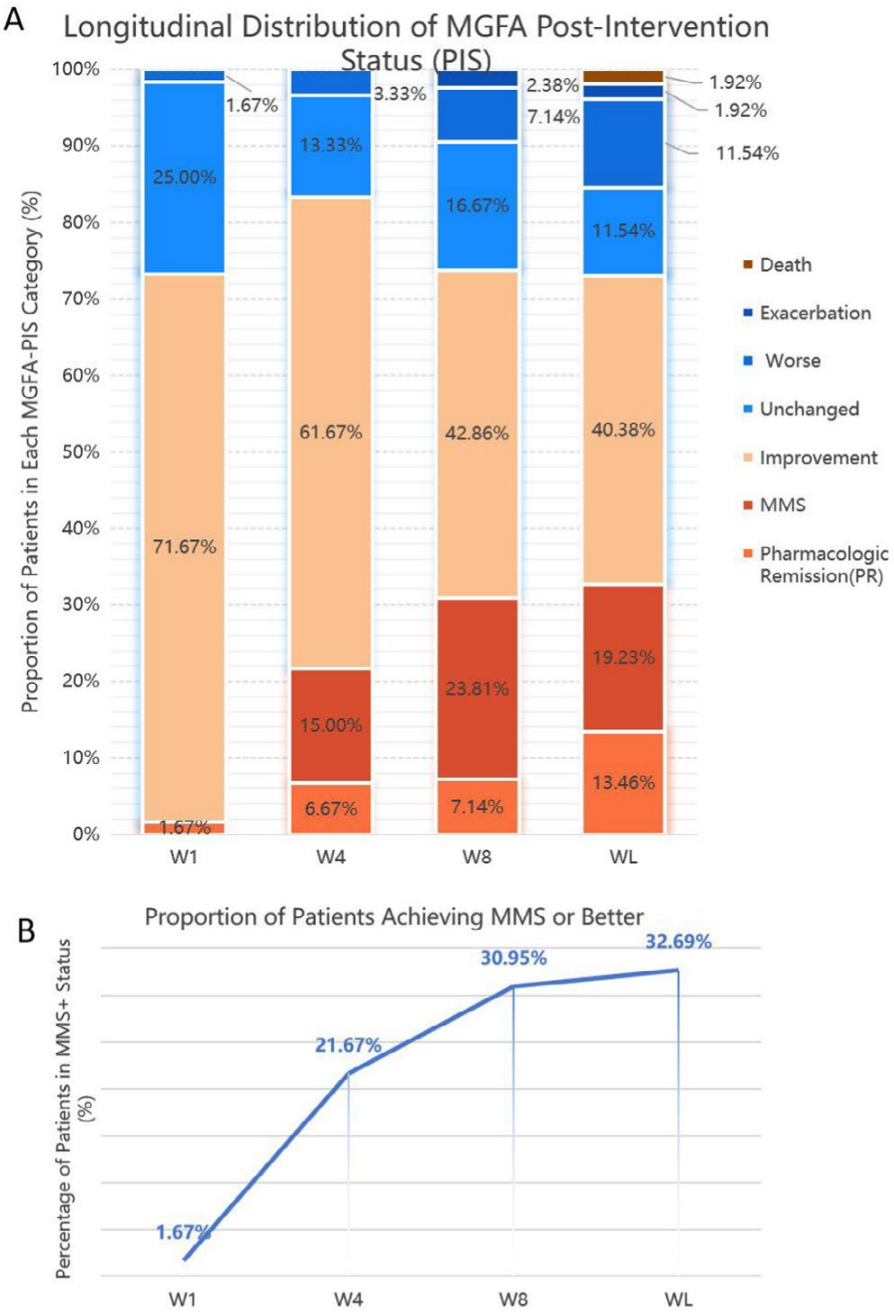
**FIGURE 1** Longitudinal Distribution of MGFA Post‐Intervention Status (PIS) (A) Percentage of patients classified as MMS+, improved, unchanged, worse, or deceased at baseline, Weeks 1, 4, 8, and the last follow‐up. (B) Percentage of patients achieving MMS.

**Figure:**
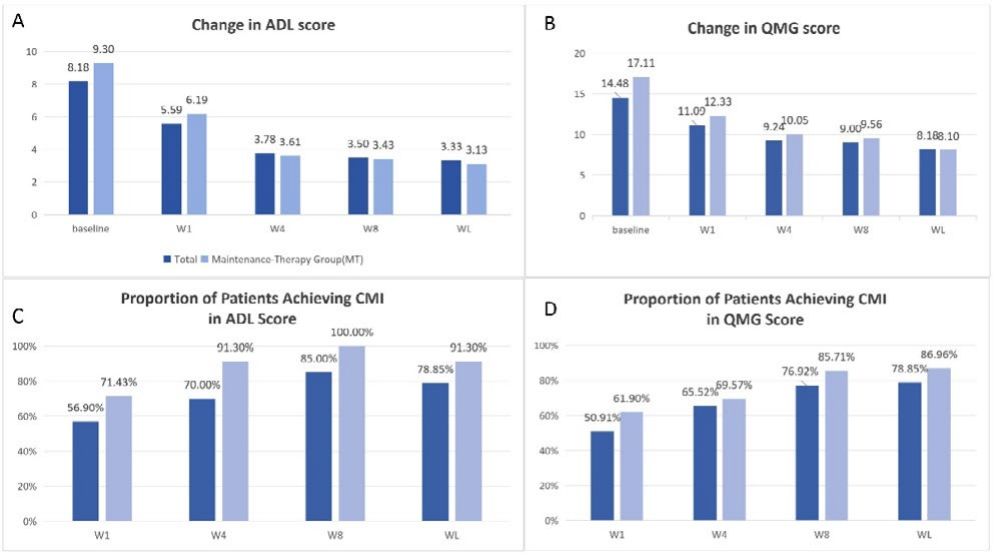
**FIGURE 2** ADL and QMG score changes following initiation of eculizumab.

**Figure:**
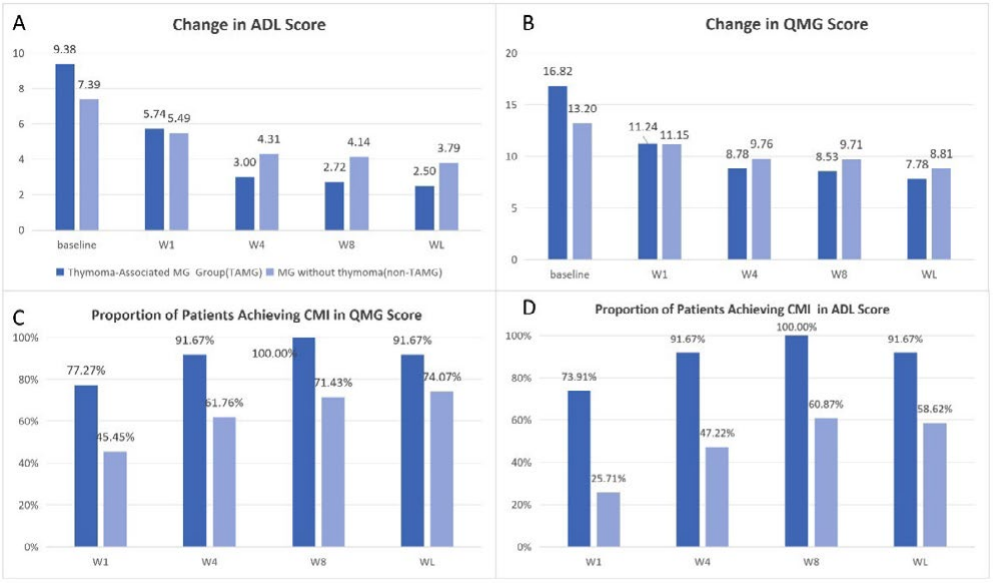
**FIGURE 3** ADL and QMG score changes after initiation of eculizumab in the TAMG and non‐TAMG groups.

**Conclusion:** This multicenter analysis shows eculizumab improves clinical outcomes in MG patients, reducing disease activity and corticosteroid use.

**Disclosure:** Nothing to disclose

Cerebrovascular diseases 5

## EPO‐0515

### MRI lesion patterns and embolic sources in patients with embolic stroke of undetermined source

#### B. Del Bello ^1^; F. Mazzacane^2^; S. Marcheselli^3^; F. Vodret^3^; G. Mazza^4^; A. Cascio Rizzo^5^; S. Beretta^6^; A. Cavallini^2^

##### ^*1*^ *Department of Brain and Behavioural Sciences, University of Pavia, Pavia, Italy;* ^*2*^ *Department of Cerebrovascular Disease/Stroke Unit, IRCCS Mondino Foundation, Pavia, Italy;* ^*3*^ *Neurology Unit and Stroke Unit, Istituto di Ricovero e Cura a Carattere Scientifico, Humanitas Research Hospital, Milan, Italy;* ^*4*^ *Neurology Unit, Maggiore Hospital, Lodi, Italy;* ^*5*^ *Department of Neurology and Stroke Unit, ASST Grande Ospedale Metropolitano Niguarda, Milan, Italy;* ^*6*^ *Department of Neurology and Stroke Unit, Fondazione IRCCS San Gerardo dei Tintori, Monza, Italy*

**Background and aims:** Embolic stroke of undetermined source (ESUS) represents a significant proportion of ischemic stroke cases. Advanced diagnostic techniques (e.g. transesophageal echocardiography (TEE), implantable loop recorder, and vessel wall MRI (VWMRI)) improve diagnosis but are not widely available. The distribution of lesions on DWI‐MRI may provide clues to the underlying embolic source and help guide the diagnostic workup.

**Methods:** We prospectively included patients with ESUS admitted to five Italian hospitals. At three months, they were classified as “cardio‐ESUS” if atrial fibrillation or a probable PFO was detected and “athero‐ESUS” if complicated aortic arch plaques on TEE or vulnerable non‐stenosing plaques on VWMRI were identified. Patients with no identifiable etiology remained classified as having ESUS. Lesion patterns on DWI‐MRI were classified according to a validated classification 1.

**Results:** A total of 128 patients were included in this study. At three months, 36 patients were diagnosed as athero‐ESUS, 19 as cardio‐ESUS, and 73 remained ESUS. The posterior cerebral artery pattern was significantly more prevalent in cardio‐ESUS group vs ESUS (36,8% vs 8,2%, *p* = 0.0014), and a trend was observed for multiple small scattered lesions (present in ESUS but absent in cardio‐ESUS, 16,4% vs 0%, *p* = 0.058). No significant differences were observed between patients with ESUS and athero‐ESUS.

**Conclusion:** The lesion pattern in ESUS patients may suggest the potential embolic source. Patients with no potential embolic sources detected at 3 months resembled those with probable artery‐to‐artery embolism. The integration of clinical and additional radiological data may further help identify patients who would benefit from targeted diagnostic testing.

**Disclosure:** Source of funding: Italian Ministry of Health Ricerca Corrente 2022–2024

## EPO‐0516

### Safety of antiplatelet medication discontinuation more than 12 months after stent‐assisted coil embolization

#### C. Kim; S. Lee; J. Yu

##### Department of Neurosurgery, Pusan National University Yangsan Hospital, Pusan National University School of Medicine, Yangsan, Korea

**Background and aims:** Antiplatelet maintenance is essential to avoid ischemia following stent‐assisted coiling (SAC) procedures. However, indications for antiplatelet medication discontinuation (AMD) remain controversial, and optimal timing of cessation has yet to be determined. Our goal, which we achieved through a multicenter, prospectively enrolled, non‐interventional study, was to investigate the safety of AMD conducted more than 12 months after SAC.

**Methods:** Data were retrieved from the records of 495 consecutive patients prospectively enrolled at 10 institutions during a 3‐year period (between January 2021 and December 2023). Each subject had discontinued antiplatelet therapy >12 months after SAC. Maintenance duration and cessation were both at physician discretion, based on patient clinical status. We investigated clinical outcomes for at least 6 months after AMD.

**Results:** A majority of patients engaged in AMD (292/495, 59.0%) were not at high risk for ischemia. Mean±SD time to AMD was 20.0±12.9 months after SAC. Treated aneurysms were largely confined to the internal carotid artery (332/495, 67.1%), followed by the anterior (95/495, 19.2%) and middle (43/495, 8.7%) cerebral arteries. A laser‐cut open‐cell stent was most often applied (60.5%); laser‐cut closed‐cell (22.2%) and braided closed‐cell (17.3%) stents were used to a lesser extent. Four patients underwent double stenting. Despite sizeable (41.0%) high‐risk group representation, there were no ischemic events in relation to AMD.

**Conclusion:** Our results suggest that AMD >12 months after SAC procedures is safe in patients who are not at high risk for ischemia. Randomized controlled trials are warranted to confirm these results.

**Disclosure:** Nothing to disclose

## EPO‐0517

### Fibrinolytic therapy in late‐window stroke: A Bayesian network meta‐analysis of randomized trials

#### M. Lenzi^1^; G. Damaceno^2^; A. Santana^3^; T. Oliveira^4^; E. Assis ^5^; B. Souza^6^; J. Cardoso^7^; P. Lajczak^8^; J. Andrade^9^; T. Fagundes^10^

##### ^*1*^ *State University of Rio Grande do Norte;* ^*2*^ *University of Western Paulista;* ^*3*^ *Faculty of Medicine of the University of Sao Paulo;* ^*4*^ *Federal University of São João del Rei;* ^*5*^ *Baltic Federal University**;*** ^*6*^ *Mass General Brigham;* ^*7*^ *University of Pernambuco;* ^*8*^ *Medical University of Silesia;* ^*9*^ *Federal University of São Paulo;* ^*10*^ *Barretos Cancer Hospital*

**Background and aims:** Time from symptom onset to hospital arrival remains the primary determinant for intravenous thrombolysis (IVT) eligibility in acute ischemic stroke (AIS). Although traditional windows were capped at 4.5 hours, recent evidence supports IVT in extended windows. However, the comparative efficacy and safety of different thrombolytic agents remain unclear.

**Methods:** We searched PubMed, Embase, and Cochrane Central for randomized controlled trials (RCTs) comparing alteplase, tenecteplase, and best medical therapy (BMT) in AIS patients beyond 4.5 hours or with unknown onset. A Bayesian network meta‐analysis was performed using JAGS and BUGSnet to estimate odds ratios (OR) with 95% credible intervals (CrI). Ranking was performed via the surface under the cumulative ranking curve (SUCRA).

**Results:** Ten RCTs (*n* = 2,862) were included. Alteplase significantly improved functional independence (mRS 0–1) compared to BMT (OR 1.53; 95% CrI 1.18–1.96). Tenecteplase showed a numerical trend toward benefit (OR 1.33; 95% CrI 0.98–1.78) but did not reach statistical significance. Alteplase was associated with a higher risk of symptomatic intracranial hemorrhage (sICH) vs BMT (OR 3.82; 95% CrI 1.55–13.39), while tenecteplase demonstrated a more favorable safety ranking (SUCRA 49.1% vs 6.6% for alteplase).

**Conclusion:** In late‐window ischemic stroke, intravenous alteplase confers the highest probability of functional independence but at the cost of increased hemorrhagic risk. Tenecteplase demonstrated a more favorable safety profile with comparable efficacy estimates, suggesting it may represent a balanced alternative for patients at higher bleeding risk. These findings clarify the trade‐off between efficacy and safety among fibrinolytic strategies beyond 4.5 hours.

**Disclosure:** Nothing to disclose.

## EPO‐0518

### Progressive lacunar syndrome and 12‐months stroke recurrence in acute ischemic lacunar stroke: Associated factors, long‐term outcomes and mortality

#### G. Kurian; P. Michel; D. Strambo

##### Stroke Center, Service of Neurology, Department of Clinical Neurosciences, University Hospital of Lausanne and University of Lausanne, Lausanne, Switzerland

**Background and aims:** Acute ischemic strokes (AIS) of microangiopathic etiology usually result in mild‐to‐moderate neurological deficits and favorable short‐term outcomes. However, some patients develop early neurological deterioration, called progressive lacunar syndromes (PLS), or ischemic stroke recurrence (ISR). We aimed to identify predictors of PLS and ISR and assess their long‐term prognostic relevance.

**Methods:** We included consecutive AIS of microangiopathic etiology from a single‐center prospective stroke registry. The first outcome was PLS, defined as early (≤24 hours) neurological deterioration (≥2 point NIHSS) or fluctuation (≥4 point rise and drop), or progressive worsening (≥4 point) within one week. The second outcome was 1‐year ISR. Multivariable logistic regression models were developed for each outcome.

**Results:** Among 843 consecutive lacunar strokes (median age 71, IQR 62–81, 43% female), 74 (8.8%) developed PLS. Among 651 patients with 12‐month follow‐up, 36 (5.5%) experienced ISR. Only two patients had both PLS and ISR. Higher admission NIHSS (adjusted‐OR = 1.09, 95% CI = 0.99–1.8) and diabetes (aOR = 2.04, 95% CI = 1.11–3.67) were independently associated with PLS, while no acute variable predicted ISR. PLS was independently associated with more unfavorable modified‐Rankin‐scale‐shift (mRS) at 3 (acOR = 4.47, 95% CI = 2.56–7.79) and 12 months (acOR = 2.97, 95% CI = 1.55–5.69), as well as higher 12‐month mortality (18.4% versus 5.7%, OR = 3.75, *p* = 0.003). ISR occurred in 36 patients (5.5%). After adjustment using the IPSW method among survivors, ISR was not independently associated with worse 12‐month functional outcome.

**Figure:**
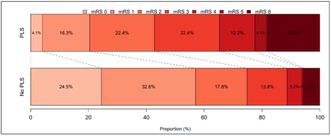
Distribution of functional outcome distribution at 12 months according to presence of progressive lacunar syndrome (PLS) with Grotta bars.

**Figure:**
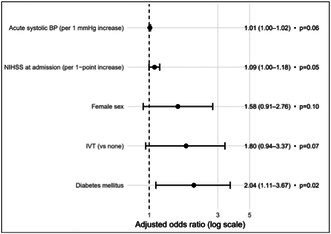
Multivariable predictors of progressive lacunar syndrome (PLS).

**Figure:**
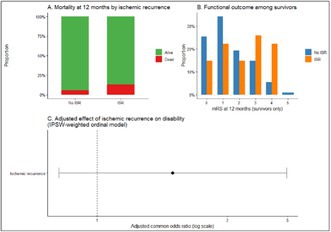
Impact of ISR on mortality and functional outcome. Panel (A) Proportion of patients alive and dead at 1 year according to ISR. Panel (B) Distribution of 1‐year functional outcome (mRS) scores among survivors, according to ISR. Panel (C) Adjusted associati.

**Conclusion:** In acute lacunar stroke, PLS is associated with greater baseline stroke severity and diabetes mellitus, as well as worse functional outcome. Distinct mechanisms likely underlie PLS and ISR.

**Disclosure:** Nothing to disclose

## EPO‐0519

### Impact of the Absence of Standard Modifiable Cardiovascular Risk Factors on Short‐ or Long‐Term Vascular Outcomes in Patients with Ischemic Stroke

#### J. Kim ^1^; S. Ryu^2^

##### ^*1*^ *Department of Neurology, Chonnam National University Hospital, Gwangju, Korea;* ^*2*^ *Department of Rehabilitation, Cheomdan Wooam Hospital, Gwangju, Korea*

**Background and aims:** This study aimed to investigate whether the absence of standard modifiable risk factors (SMuRFs; hypertension, diabetes, dyslipidemia, and current smoking) influences short‐ and long‐term outcomes in patients with ischemic stroke and to explore the prognostic relevance of SMuRF burden.

**Methods:** This study was an analysis of a prospective, multicenter, stroke registry between January 2011 and December 2023. Patients without any of SMuRFs were classified as no SMuRF. SMuRF burden was defined as the number of risk factors present (0–4). Each risk factor was weighted equally in the analysis. The primary outcome was a composite of stroke, myocardial infarction (MI), and all‐cause mortality within 3‐month and 1‐year.

**Results:** A total of 67,214 patients (age 68.3±13.4years, 58.8% male) were finally analyzed. No‐SMuRF patients were 9.4%, and those with 1–4 SMuRFs were 27.0%, 34.8%, 22.7%, and 6.2%, respectively. The composite outcome did not differ between patients without and with SMuRFs at 3‐months (7.9% vs 7.6%, respectively) or 1‐year (12.2% vs 12.3%, respectively). After adjustment, patients with SMuRFs had higher rates of primary composite outcomes at both 3‐months and 1‐year than those without SMuRFs. SMuRFs were independently associated with increased risk of primary composite outcomes at 3‐months (aHR, 1.30[1.19–1.44]) and 1‐year (aHR, 1.25[1.06–1.35]), with a graded increase in risk according to the number of SMuRFs.

**Conclusion:** In patients with ischemic stroke, SMuRF burden—but not the absence of SMuRFs—was independently associated with a higher risk of composite of stroke, MI and all‐cause mortality at both 3‐months and 1‐year, suggesting the prognostic relevance of cumulative vascular risk and the importance of comprehensive risk factor management.

**Disclosure:** Nothing to disclose

## EPO‐0520

### Comprehensive prestroke risk factor control and functional outcomes after acute ischemic stroke

#### J. Kim ^1^; S. Ryu^2^; H. Bae^3^

##### ^*1*^ *Chonnam National University Hospital, Gwangju, Korea;* ^*2*^ *Cheomdan Wooam Hospital, Gwangju, Korea;* ^*3*^ *Seoul National University Bundang Hospital, Seongnam, Korea*

**Background and aims:** This study evaluated whether comprehensive prestroke risk factor (RF) control—by degree of RF control or comprehensive RF control—is associated with 3‐month functional outcomes after ischemic stroke.

**Methods:** We analyzed ischemic stroke patients from the CRCS‐K‐NIH registry (2011–2022), a multicenter stroke registry in Korea. Hypertension, diabetes, dyslipidemia, and atrial fibrillation were considered as major RFs for ischemic stroke. The comprehensive RF control was categorized as well‐controlled (all present RFs well‐controlled), partially‐controlled (some RFs well‐controlled), or poorly‐controlled (none RFs well‐controlled). The primary outcome was 3‐month mRS of 0–2, indicating functional independence (good outcome).

**Results:** Of the 63,570 patients with one or more major RFs, 28.6% had poorly‐controlled, 35.6% had partially‐controlled, and 35.8% had well‐controlled RF control. The rate of 3‐month good outcome was 66.0% among those with one RF, 60.8% in two, and 55.0% in three or more. After adjusting for covariates, well‐controlled groups had higher odds of good outcomes (aOR[95% CI], 1.16[1.08–1.25] for one RF, 1.34[1.21–1.48] for two, and 1.23[1.03–1.48] for ≥3), as did partially‐controlled groups (aOR 1.16[1.06–1.26] for two; 1.22[1.05–1.42] for ≥3), versus poorly‐controlled. Across all RF levels, well‐controlled (aOR 1.22[1.15–1.29]) and partially‐controlled (aOR 1.14[1.07–1.22]) statuses were significantly linked to higher odds of good 3‐month outcomes.

**Conclusion:** Comprehensive prestroke RF control was independently associated with a higher likelihood of favorable 3‐month outcomes, both within and across levels of RF burden. These findings highlight the importance of not only identifying but also optimal comprehensive prestroke RF control to improve post‐stroke functional outcomes, regardless of the number of RF present.

**Disclosure:** Nothing to disclose.

## EPO‐0521

### Clinical outcomes of endovascular thrombectomy beyond 24 hours in large vessel occlusion stroke: A meta‐analysis

#### K. Moghib ^1^; M. Meshref^2^

##### ^*1*^ *Kasralainy midical school, Cairo University, Cairo, Egypt;* ^*2*^ *Department of Neurology, Faculty of Medicine, Al‐Azhar University, Cairo, Egypt*

**Background and aims:** The effectiveness of endovascular thrombectomy (EVT) for acute ischemic stroke due to large vessel occlusion beyond 24 hours from last known well remains unclear, with existing evidence largely derived from observational studies.

**Methods:** We systematically searched major databases through February 2025 for studies comparing EVT with best medical therapy in patients treated more than 24 hours after symptom onset. Outcomes included 90‐day functional independence (mRS 0–2), excellent outcome (mRS 0–1), symptomatic intracranial hemorrhage (sICH), and mortality. Random‐effects models were used to pool adjusted and unadjusted odds ratios.PROSPERO (CRD420251274201)

**Results:** Ten observational studies (*n* = 1,871) were included. EVT was associated with higher odds of functional independence (adjusted OR 4.62) and excellent outcome (adjusted OR 5.68). EVT was linked to an increased risk of sICH, while 90‐day mortality was not significantly different between groups. Subgroup analyses across imaging strategies, stroke severity, and occlusion territory were consistent with primary findings. Certainty of evidence ranged from low to very low.

**Conclusion:** EVT beyond 24 hours may improve functional outcomes without increasing mortality, despite a higher hemorrhagic risk. Randomized controlled trials are needed to validate these findings and refine patient selection in the ultra‐late window.

**Disclosure:** Nothing to disclose.

## EPO‐0522

### Sex‐based inequities in prehospital stroke identification: A systematic review and meta‐analysis

#### K. Moghib ^1^; M. Meshref^2^

##### ^*1*^ *Faculty of Medicine, Cairo University, Cairo, Egypt;* ^*2*^ *Department of Neurology, Faculty of Medicine, Al‐Azhar University, Cairo, Egypt*

**Background and aims:** Evidence suggests that sex‐related disparities may influence early stroke recognition and management before hospital arrival. This systematic review and meta‐analysis evaluated differences between female and male patients across dispatch and emergency medical service (EMS) pathways and identified gaps requiring further investigation.

**Methods:** A systematic search of PubMed, EMBASE, CINAHL, and EMCARE identified studies reporting sex differences in prehospital stroke care. Random‐effects meta‐analyses using inverse variance weighting were conducted. The primary outcome was the likelihood of correct prehospital stroke identification. Secondary outcomes included dispatch coding, EMS prenotification, transport destination, and time metrics. PROSPERO (CRD420251274202)

**Results:** Sixteen studies including 1,193,788 patients (622,764 women; 571,024 men) were analyzed. Women were less likely to receive a correct prehospital stroke diagnosis compared with men (RR 0.92, 95% CI 0.89–0.96), with substantial heterogeneity. No significant sex differences were observed in dispatch stroke coding, EMS prenotification, transport to stroke centers, or call‐to‐door times. Data were insufficient to draw conclusions regarding on‐scene clinical management and interprofessional communication.

**Conclusion:** Female patients experience lower rates of accurate prehospital stroke recognition. Considerable heterogeneity and limited reporting across care stages highlight the need for standardized, high‐quality studies to address sex‐based inequities in prehospital stroke systems.

**Disclosure:** Nothing to disclose.

## EPO‐0523

### Cerebrovascular risk factors impact on brain atrophy and glymphatic dysfunction in neuromyelitis optica spectrum disorder

#### M. Rubin ^1^; P. Preziosa^1^; M. Margoni^2^; L. Storelli^3^; E. Pagani^3^; P. Valsasina^3^; M. Albergoni^3^; L. Moiola^4^; M. Rocca^1^; M. Filippi^5^

##### ^*1*^ *Neuroimaging Research Unit, Division of Neuroscience, and Neurology Unit, IRCCS San Raffaele Scientific Institute, Milan, Italy; and Vita‐Salute San Raffaele University, Milan, Italy;* ^*2*^ *Neuroimaging Research Unit, Division of Neuroscience, Neurology Unit, and Neurorehabilitation Unit, IRCCS San Raffaele Scientific Institute, Milan, Italy;* ^*3*^ *Neuroimaging Research Unit, Division of Neuroscience, IRCCS San Raffaele Scientific Institute, Milan, Italy;* ^*4*^ *Neurology Unit, IRCCS San Raffaele Scientific Institute, Milan, Italy;* ^*5*^ *Neuroimaging Research Unit, Division of Neuroscience, Neurology Unit, Neurorehabilitation Unit, and Neurophysiology Service, IRCCS San Raffaele Scientific Institute, Milan, Italy; and Vita‐Salute San Raffaele University, Milan, Italy*

**Background and aims:** Neuromyelitis optica spectrum disorder (NMOSD) is an autoimmune astrocytopathy where disability is largely relapse‐driven. Cerebrovascular risk factors (cVRFs) may exacerbate astrocytic and small‐vessel injury, therefore we investigated their impact on MRI damage and disability in NMOSD.

**Methods:** Twenty‐eight aquaporin 4‐positive NMOSD patients and 56 age‐ and sex‐matched healthy controls (HC) underwent 3T brain and cervical MRI, cVRF and neurological assessment, including Expanded Disability Status Scale (EDSS). Brain T2‐hyperintense white matter lesion volume (WMLV), normalized global and regional volumes, normal‐appearing WM fractional anisotropy and mean diffusivity, diffusion tensor imaging analysis along the perivascular spaces (DTI‐ALPS) index, spinal cord (SC) LV and normalized cross‐sectional area (nCSA) were quantified.

**Results:** cVRFs were present in 57% of NMOSD and 39% of HC, with smoking being the most frequent (FDR‐p = 0.734). Compared to HC, NMOSD showed higher brain WMLV and SCLV, lower normalized brain, deep gray matter volumes (nDGMV), and nCSA (FDR‐*p* ≤ 0.030). Significant diagnosis×cVRF interactions were found for nDGMV and DTI‐ALPS (FDR‐p≤0.038). In NMOSD, higher EDSS correlated with lower DTI‐ALPS (r = –0.46/FDR‐*p* = 0.048) and higher SCLV (*r* = 0.53, FDR‐p = 030). At moderation analysis, cVRFs amplified the association between DTI‐ALPS and disability (FDR‐p = 0.045).

**Conclusion:** cVRFs amplify CNS damage and the clinical impact of glymphatic dysfunction in NMOSD, suggesting convergent vascular and astrocytic injury.

**Disclosure:** M. Rubin has nothing to disclose. P. Preziosa received speaker honoraria from Roche, Biogen, Novartis, Merck, Bristol Myers Squibb, Genzyme, Horizon and Sanofi. M. Margoni reports grants and personal fees from Sanofi Genzyme, Merck Serono, Roche, Biogen, Amgen and Novartis. L. Storelli, E. Pagani, P. Valsasina, and M. Albergoni have nothing to disclose. L. Moiola received speaker honoraria from Roche. M. Filippi is Editor‐in‐Chief of the Journal of Neurology, Associate Editor of Human Brain Mapping, Neurological Sciences, and Radiology; received compensation for consulting services from Almirall, Biogen, Bristol‐Myers Squibb, Eli Lilly, Merck, Novartis, Roche, Sanofi; speaking activities from Amgen, Bayer, Biogen, Bristol‐Myers Squibb, Celgene, Chiesi Italia SpA, Eisai, Eli Lilly, Fujirebio, Genzyme, Janssen, Merck, Neopharmed Gentili, Neuraxpharm, Novartis, Novo Nordisk, Roche, Sanofi, Takeda; participation in Advisory Boards for Alexion, Biogen, Bristol‐Myers Squibb, Eli Lilly, GE Healthcare Ltd, Merck, Neuraxpharm, Novartis, Roche, Sandoz, Sanofi, Takeda; scientific direction of educational events for Biogen, Merck, Roche, Celgene, Bristol‐Myers Squibb, Lilly, Novartis, Sanofi‐Genzyme; he receives research support from Biogen Idec, Merck‐Serono, Novartis, Roche, the Italian Ministry of Health, the Italian Ministry of University and Research, and Fondazione Italiana Sclerosi Multipla. M.A. Rocca received consulting fees from Biogen, Bristol Myers Squibb, Roche; and speaker honoraria from Alexion, Biogen, Bristol Myers Squibb, Celgene, Horizon Therapeutics Italy, Merck Serono SpA, Mitsubishi‐Tanabe Pharma, Neuraxpharm, Novartis, Roche, Sandoz, and Sanofi. She receives research support from the MS Society of Canada, the Italian Ministry of Health, the Italian Ministry of University and Research, and Fondazione Italiana Sclerosi Multipla. She is Associate Editor for Multiple Sclerosis and Related Disorders; and Associate Co‐Editor for Europe and Africa for Multiple Sclerosis Journal.

## EPO‐0524

### Clinical safety and efficacy of stent‐assisted coil embolization with ACCERO stent in cerebral aneurysm: Short‐term follow‐up and precaution for use

#### S. Lee; C. Kim; J. Yu

##### Department of Neurosurgery, Pusan National University Yangsan Hospital, Pusan National University School of Medicine, Yangsan, Korea

**Background and aims:** Stent‐assisted coil embolization (SAC) is an effective method of treating intracranial aneurysms. The aim of the study was to assess the safety and efficacy of the new ACCERO stent for the treatment of cerebral aneurysms.

**Methods:** Nine ruptured and 41 unruptured cerebral aneurysms were treated using the ACCERO stent between February 2021 and December 2023. Patient demographics, aneurysm characteristics, procedural parameters, grade of occlusion, complications, and clinical outcomes were analyzed. Follow‐up was conducted with magnetic resonance angiography (MRA) or Digital subtraction angiography (DSA) was performed 6 to 12 months after the procedure.

**Results:** The ACCERO stent deployment was attempted in 51 cases, with replacement by the Neuroform Atlas stent in 1 case. Successful stent deployment was achieved in 50 cases, and appropriate wall apposition to the parent artery. Intimal hyperplasia was observed in 1 case, but no other clinical complications related to the stent occurred. Favorable clinical outcomes were observed in 92% of patients (46/50), including those with subarachnoid hemorrhage. Immediate favorable angiographic outcomes and complete occlusion were achieved in 90% (45/50) and 74% (37/50) of cases, respectively. Among the 45 patients who had imaging follow‐up, favorable angiographic outcomes and complete occlusion were observed in 93.3% (43/45) and 82.2% (37/45) of cases, respectively.

**Conclusion:** The ACCERO stent is a braided‐type stent that requires more attention than stents, such as the Neuroform Atlas or Enterprise stents. However, since the struts of the stent are fully visible, it can be more useful in treating challenging aneurysms once the user becomes familiar with its use.

**Disclosure:** Nothing to disclose

## EPO‐0525

### Neuroanatomic correlates of conjugate eye deviation in left hemispheric stroke

#### O. Saprikis ^1^; T. van der Bij^1^; M. Reisert^2^; C. Weiller^1^; M. Rijntjes^1^

##### ^*1*^ *Department of Neurology and Clinical Neuroscience, Faculty of Medicine, University of Freiburg, Freiburg i.Br, Germany;* ^*2*^ *Department of Medical Physics, Faculty of Medicine, University of Freiburg, Freiburg i.Br, Germany*

**Background and aims:** Conjugate eye deviation (ED) is a frequent clinical sign in acute stroke, a marker of large vessel occlusion and a predictor for the occurrence of neglect. While its occurrence has been extensively studied in right hemispheric stroke, data for left hemispheric lesions remain limited.

**Methods:** We retrospectively analysed patients with first time left hemispheric middle cerebral artery stroke from the Freiburg Large Scale Project registry. ED was quantitatively measured on acute CT or MRI. Neglect was assessed using item 11 of the NIH Stroke Scale (NIH‐SS). Lesion–symptom relationships were examined with the novel Streamline‐based Lesion‐symptom Mapping (SLSM).

**Results:** Seventy patients met inclusion criteria. Larger ED was associated with the presence of neglect but not with age or lesion volume. SLSM revealed that ED severity was associated with lesions along a number of tracts: superior longitudinal and arcuate fasciculi (dorsal pathway), the extreme capsule complex (ventral pathway), and the middle and inferior longitudinal fasciculi (caudal pathway) (Figure 1). Subgroup analysis (Figure 2) showed that larger ED (in purple) was linked to dorsal and caudal disconnections. Smaller ED (in green) was associated with ventral pathway involvement, because in CT/MRI, no detection of stimuli or reorienting was required.

**Figure:**
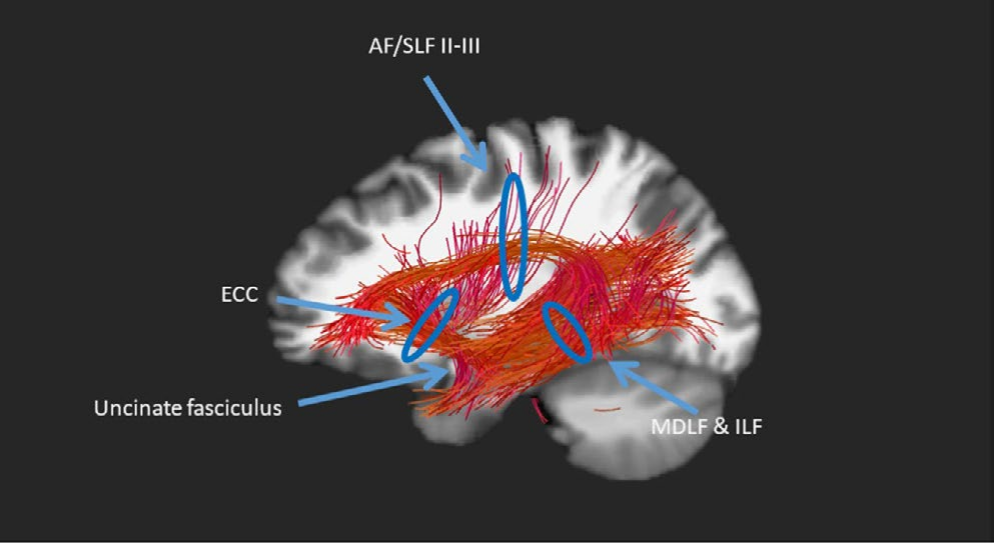
FIGURE 1

**Figure:**
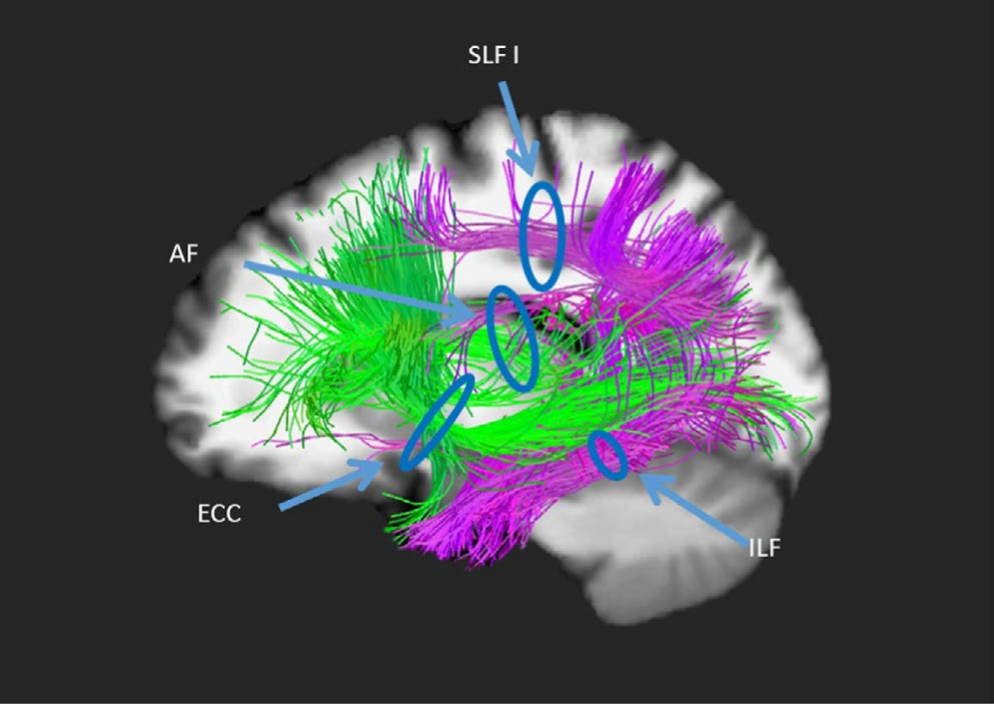
FIGURE 2

**Conclusion:** In left hemispheric stroke, ED at rest without external stimuli reveals disconnection of three distinct networks: dorsal, ventral and caudal, associated with different aspects of neglect: control of eye movements and gaze orientation, attention to novel and behaviourally relevant stimuli, and spatial awareness.

**Disclosure:** Nothing to disclose.

MS and related disorders 4

## EPO‐0526

### Efficacy of Neurofeedback in Cognitive Impairment in Multiple Sclerosis

#### A. Tursunov; K. Olga; Y. Madjidova

##### Department of Neurology, Pediatric Neurology and Medical Genetics, Tashkent State Medical University, Tashkent, Uzbekistan

**Background and aims:** Cognitive impairment is a common manifestation of multiple sclerosis (MS), affecting approximately 43–72% of patients and significantly reducing quality of life and social functioning. Pharmacological treatment options for MS‐related cognitive deficits remain limited. Therefore, there is a growing interest in non‐invasive and non‐pharmacological neurorehabilitation approaches, including neurofeedback, aimed at improving cognitive function through modulation of brain activity.

**Methods:** This study included 40 patients with multiple sclerosis who were divided into two comparable groups. The neurofeedback group (*n* = 20) received standard therapy combined with neurofeedback training, while the control group (*n* = 20) received standard therapy alone. Cognitive function was assessed using the Montreal Cognitive Assessment (MoCA), Mini‐Mental State Examination (MMSE), and Mini‐Cog tests. Cortical evoked potentials (P300) were recorded before and after treatment. The neurofeedback protocol consisted of 10–15 training sessions.

**Results:** At baseline, no significant differences in cognitive performance were observed between the two groups (*p* > 0.05). After treatment, the neurofeedback group demonstrated significant improvements in cognitive scores: MoCA increased from 22.6±1.9 to 25.3±1.6, MMSE from 25.5±1.7 to 28.0±1.3, and Mini‐Cog scores also improved (*p* < 0.05). In contrast, cognitive improvements in the control group were less pronounced. A reduction in P300 latency by 18–22 ms was observed only in the neurofeedback group.

**Conclusion:** Neurofeedback significantly enhances cognitive outcomes in patients with multiple sclerosis when used as an adjunct to standard therapy. This method may be considered an effective and non‐invasive approach for cognitive rehabilitation in MS.

**Disclosure:** Nothing to disclose

## EPO‐0527

### Adding OCT‐Based Retinal Thinning into NEDA‐3 for Improved Clinical Monitoring in Multiple Sclerosis

#### F. Föttinger ^1^; J. Nolte^1^; N. Krajnc^1^; M. Ponleitner^1^; F. Di Pauli^2^; H. Hegen^2^; F. Leutmezer^1^; T. Monschein^1^; P. Rommer^1^; C. Schmied^1^; B. Kornek^1^; G. Zulehner^1^; T. Zrzavy^1^; T. Berger^1^; G. Bsteh^1^

##### ^*1*^ *Medical University of Vienna, Department of Neurology, Vienna, Austria;* ^*2*^ *Medical University of Innsbruck, Department of Neurology, Innsbruck, Austria*

**Background and aims:** No evidence of disease activity 3 (NEDA‐3) is a proposed composite treatment target in multiple sclerosis (MS). Optical coherence tomography (OCT) derived retinal layer thinning is a promising paraclinical marker of future disability progression. We investigated whether integrating OCT into NEDA‐3 (NEDA‐3+OCT) improves monitoring of response to disease‐modifying therapy (DMT).

**Methods:** We screened two observational cohorts at the Medical Universities of Vienna and Innsbruck for patients with relapsing MS who (i) newly initiated DMT, (ii) underwent MRI and OCT at baseline and 12 months, and (iii) had ≥24 months of clinical follow‐up. Retinal thinning was defined as ≥0.5% reduction in the ganglion cell and inner plexiform layer and/or ≥1.0% reduction in peripapillary retinal nerve fibre layer at 12 months. NEDA‐3/NEDA‐3+OCT status was determined 12 months after DMT initiation. The primary endpoint was confirmed disability progression occurring after month 12.

**Results:** Overall, 124 individuals were included (72% female; mean age 33.1±7.7 years; median baseline EDSS 2.0 [IQR 0.0–2.5]). Over a median follow‐up of 3.4 years, 28 (23%) experienced disability progression. Time to progression did not significantly differ between EDA‐3 and NEDA‐3 at month 12 (*p* = 0.067), and EDA‐3 was not significantly associated with progression in multivariable Cox regression (aHR 1.52; 95% LL‐CI 0.64; *p* = 0.173). In contrast, EDA‐3+OCT was associated with increased progression risk (aHR 4.47; 95% LL‐CI 1.87; *p* = 0.002) and shorter time to progression (RMST 42.5±1.9 vs 51.6±1.1 months; *p* < 0.001).

**Conclusion:** Adding OCT to NEDA‐3 may better identify individuals at risk of future disability progression than NEDA‐3 alone, particularly among those receiving high‐efficacy DMT.

**Disclosure:** No disclosures or relationships of interest relevant to the context of this study.

## EPO‐0528

### Factors associated with progression independent of relapse and magnetic resonance imaging activity in relapsing multiple sclerosis

#### F. Romeo ^1^; E. Portaccio^1^; M. Betti^1^; L. Pastò^2^; A. Lugaresi^3^; E. Cocco^4^; G. De Luca^5^; F. Patti^6^; V. Torri Clerici^7^; D. Ferraro^8^; V. Brescia Morra^9^; G. Salemi^10^; M. Vianello^11^; R. Cerqua^12^; M. Inglese^13^; M. Rovaris^14^; P. Perini^15^; S. Romano^16^; C. Pozzilli^17^; C. Tortorella^18^; A. Di Sapio^19^; P. Annovazzi^20^; M. Simone^21^; P. Iaffaldano^22^; M. Filippi^23^; M. Trojano^22^; E. De Meo^1^; M. Amato^1^

##### ^*1*^ *Department of NEUROFARBA, University of Florence, Florence, Italy;* ^*2*^ *Department of Neurology, Careggi University Hospital, Florence, Italy;* ^*3*^ *UOSI Riabilitazione Sclerosi Multipla, IRCCS Istituto delle Scienze Neurologiche di Bologna;* ^*4*^ *Department Medical Science and Public health, Centro Sclerosi Multipla, University of Cagliari, Italy;* ^*5*^ *Neuroscience, Imaging and Clinical Sciences, University G. D'Annunzio di Chieti‐Pescara, Chieti, Italy;* ^*6*^ *Department of Medical and Surgical Sciences and Advanced Technologies "G.F. Ingrassia", University of Catania, Catania, Italy;* ^*7*^ *Neuroimmunology Unit, Fondazione IRCCS Istituto Neurologico C. Besta, Milan, Italy;* ^*8*^ *Dipartimento di Neuroscienze ‐ Ospedale Civile di Baggiovara ‐ Azienda Ospedaliero‐Universitaria, Modena, Italy;* ^*9*^ *Multiple Sclerosis Clinical Care and Research Center, Department of Neuroscience (NSRO), Federico II University, Naples, Italy;* ^*10*^ *Department of Biomedicine, Neuroscience and Advanced Diagnostics, University of Palermo, Palermo, Italy;* ^*11*^ *Neurology, Ca' Foncello Hospital, AULSS2, Treviso, Italy;* ^*12*^ *Clinica Neurologica, AOU delle Marche, Ancona, Italy;* ^*13*^ *Centro per lo Studio e la Cura della Sclerosi Multipla e Malattie Demielinizzanti ‐ Dipartimento di Neuroscienze, Riabilitazione, Oftalmologia, Genetica e Scienze Materno‐Infantili ‐ Clinica Neurologica ‐ Ospedale Policlinico San Martino (DiNOGMI), Genova;* ^*14*^ *Centro SM ‐ Fondazione Don Carlo Gnocchi IRCCS, Milan, Italy;* ^*15*^ *Centro Specializzato Regionale per la Sclerosi Multipla (CeSMuV), Regione Veneto, Dipartimento di Neuroscienze DNS, Azienda Ospedaliera, Università degli Studi di Padova, Padova, Italy**;*** ^*16*^ *Centro Neurologico Terapie Sperimentali, Università La Sapienza di Roma, AO S. Andrea, Rome, Italy;* ^*17*^ *Centro SM ‐ Policlinico S. Andrea, Università La Sapienza di Roma, Rome, Italy;* ^*18*^ *Centro Sclerosi Multipla, AO S. Camillo Forlanini, Rome, Italy;* ^*19*^ *Centro di riferimento Regionale SM (CRESM) ‐ SCDO Neurologia ‐ AOU San Luigi Gonzaga, Orbassano, Torino, Italy;* ^*20*^ *Neurologia ad Indirizzo Neuroimmunologico ‐ Centro Sclerosi Multipla, ASST della Valle Olona, Ospedale di Gallarate, Gallarate (VA), Italy;* ^*21*^ *Child Neuropsychiatric Unit, Department of Biomedical Sciences and Human Oncology, University ‘Aldo Moro’ of Bari, Bari, Italy;* ^*22*^ *Department of Basic Medical Sciences, Neurosciences and Sense Organs, University of Bari Aldo Moro, Bari, Italy;* ^*23*^ *Neurology Unit and MS Center, IRCCS San Raffaele Scientific Institute; Neuroimaging Research Unit, Division of Neuroscience; Neurorehabilitation Unit and Neurophysiology Service, IRCCS San Raffaele Scientific Institute, Milan, Italy*

**Background and aims:** The proportion of Progression Independent of Relapse Activity (PIRA) events is significantly reduced when subclinical inflammatory activity detected by magnetic resonance imaging (MRI) is considered. This study aims to identify factors associated with Progression Independent of Relapse and MRI Activity (PIRMA) in patients with relapsing multiple sclerosis (MS).

**Methods:** This multicenter, retrospective, cohort study used prospectively collected data from the Italian MS Register. Inclusion criteria were clinically isolated syndrome or relapsing–remitting MS, first visit on/after January 1, 2000, > = 3 Expanded Disability Status Scale (EDSS) assessments, > = 5 years of follow‐up. Confirmed Disability Accrual (CDA) was defined as a EDSS progression confirmed at 24 weeks, using roving baseline. PIRA was defined in absence of relapses <90 days before and <30 days after CDA onset. In patients undergoing annual brain and/or spinal cord MRI, PIRA events temporally associated with MRI activity were reclassified as PIRMA. Cox proportional hazards models were applied to identify associated factors.

**Results:** Overall, 30203 patients met inclusion criteria and were followed for a mean of 11.3 years; 18187 (60.2%) experienced at least one CDA, of which 12501 (68.7%) were classified as PIRA. Among 605 patients with annual MRI, 357 (59%) CDA events occurred. The proportion of PIRA decreased from 63.6% to 45.1% after accounting for MRI activity. PIRA was associated with older age (HR = 1.04,95% CI:1.02–1.05, *p* < 0.001) and visit density (HR = 1.59,95% CI:1.20–2.10, *p* = 0.001). PIRMA was associated with older age (HR = 1.04,95% CI:1.02–1.06, *p* < 0.001), higher EDSS (HR = 1.12,95% CI:1.01–1.24, *p* < 0.047) and visit density (HR = 1.65,95% CI:1.20–2.28, *p* = 0.002).

**Conclusion:** Approximately one third of PIRA events were associated with MRI inflammatory activity. PIRA and PIRMA showed largely overlapping factors, suggesting similar underlying pathological mechanisms.

**Disclosure:** E. Portaccio has received compensation for travel, advisory boards and/or speaking activities from Biogen, Merck Serono, Sanofi, Teva, Roche, BMS Celgene, Janssen, and Novartis, and serves on the editorial board of Frontiers in Neurology and Brain Sciences. L. Pastò received research support from Novartis and Biogen and speaker honoraria from Teva. A. Lugaresi has served on advisory boards and received honoraria or travel support from multiple companies including Biogen, Merck, Novartis, Roche, Sanofi/Genzyme, and others; her institutions received research grants from Novartis, Roche, and Sanofi/Genzyme. E. Cocco, F. Patti, V.L.A.M. Torri Clerici, D. Ferraro, V. Brescia Morra, G. Salemi, M. Vianello, R. Cerqua, P. Perini, S. Romano, C. Pozzilli, C. Tortorella, A. Di Sapio, P. Annovazzi, P. Iaffaldano, and M. Trojano received grants, honoraria, travel support, and/or served on advisory boards for pharmaceutical companies including Almirall, Alexion, Bayer, Biogen, BMS/Celgene, Janssen, Merck (Serono), Novartis, Roche, Sanofi/Genzyme, Teva, and others. M. Inglese received research grants from NIH, NMSS, and FISM and consultation fees from Roche, Genzyme, Merck, Biogen, and Novartis. M. Filippi is Editor‐in‐Chief of the Journal of Neurology and received consulting fees, speaker honoraria, and research support from multiple pharmaceutical companies and public institutions. M.P. Amato served on advisory boards and received speaker honoraria and research support from Biogen Idec, Merck Serono, Bayer Schering Pharma, and Sanofi Aventis and serves on editorial boards of Multiple Sclerosis Journal and BMC Neurology. All other authors report no relevant disclosures.

## EPO‐0529

### Kappa free light chains and oligoclonal bands in the diagnosis of multiple sclerosis: Preliminary results of the PRO‐KFLC study

#### F. Konen ^1^; S. Tauber^2^; A. Bayas^3^; C. Otto^4^; R. Gold^5^; C. Seliger‐Behme^6^; A. Pröbstel^7^; A. Dressel^8^; T. Ziemssen^9^; S. Meuth^10^; V. Rothhammer^11^; Y. Yalachkov^12^; H. Wiendl^13^; C. Warnke^14^; S. Pfeuffer^15^; M. Süße^16^; M. Otto^17^; M. Friese^18^; B. Wildemann^19^; S. Groppa^20^; C. Geis^21^; S. Vay^22^; S. Schreiber^23^; T. Kümpfel^24^; B. Hemmer^25^; C. Gross^26^; R. Linker^27^; U. Zettl^28^; F. Leypoldt^29^; M. Kowarik^30^; H. Tumani^31^; A. Haarmann^32^; A. Harrer^33^; J. Kuhle^34^; M. Khalil^35^; Ø. Torkildsen^36^; D. Zeman^37^; K. Rejdak^38^; X. Montalban^39^; A. Gary Ciceron^40^; M. Moccia^41^; I. Rosenstein^42^; S. Gnanapavan^43^; I. Galea^44^; C. Teunissen^45^; V. Van Pesch^46^; S. Laakso^47^; U. Rot^48^; M. Willrich^49^; T. Skripuletz^1^

##### ^*1*^ *Department of Neurology, Hannover Medical School, Hannover, Germany;* ^*2*^ *Department of Neurology, University Hospital Aachen, Aachen, Germany;* ^*3*^ *Department of Neurology, University of Augsburg, Augsburg, Germany;* ^*4*^ *Department of Neurology, Charité University Medicine Berlin, Berlin, Germany;* ^*5*^ *Department of Neurology, St. Josef‐Hospital, Ruhr‐Universität Bochum, Bochum, Germany;* ^*6*^ *Department of Neurology, University Hospital Knappschaftskrankenhaus Bochum, Germany;* ^*7*^ *Department of Neuroimmunology, University Hospital and University Bonn, Bonn, Germany;* ^*8*^ *Department of Neurology, Medical University Lausitz‐ Carl Thiem, Cottbus, Germany,;* ^*9*^ *Center of Clinical Neuroscience, Carl Gustav Carus University Clinic, University Hospital of Dresden, Dresden, Germany;* ^*10*^ *Department of Neurology, University Hospital Düsseldorf, Düsseldorf, Germany;* ^*11*^ *Department of Neurology, University Hospital Erlangen, Erlangen, Germany;* ^*12*^ *Department of Neurology, Goethe University Frankfurt, Frankfurt am Main, Germany;* ^*13*^ *Department of Neurology, Medical Center – University of Freiburg, Freiburg, Germany;* ^*14*^ *Deparment of Neurology Marburg, University Hospital Giessen and Marburg, Marburg, Germany;* ^*15*^ *Department of Neurology Giessen, University Hospital Giessen and Marburg, Justus‐Liebig‐University Giessen, Giessen, Germany;* ^*16*^ *Department of Neurology, University Medicine Greifswald, Greifswald, Germany;* ^*17*^ *Department of Neurology, University Hospital Halle/Wittenberg, Halle, Germany;* ^*18*^ *Institute of Neuroimmunology and Multiple Sclerosis, Hamburg, Germany;* ^*19*^ *Department of Neurology, University Hospital Heidelberg, Heidelberg, Germany;* ^*20*^ *Department of Neurology, University of Saarland, Homburg, Germany;* ^*21*^ *Department of Neurology, University Hospital Jena, Jena, Germany;* ^*22*^ *Department of Neurology, University Hospital Köln, Köln, Germany;* ^*23*^ *Department of Neurology, Otto‐von‐Guericke University, Magdeburg, Germany;* ^*24*^ *Institute of Clinical Neuroimmunology, LMU University Hospital, LMU Munich, Germany;* ^*25*^ *Deparment of Neurology, TU München, München, Germany;* ^*26*^ *Department of Neurology, University Hospital Münster, Münster, Germany;* ^*27*^ *Department of Neurology, University Hospital Regensburg, Regensburg, Germany;* ^*28*^ *Department of Neurology, University Hospital Rostock, Rostock, Germany;* ^*29*^ *Department of Neurology, University Hospital Kiel, Kiel, Germany;* ^*30*^ *Department of Neurology, UKT Tübingen, Tübingen, Germany;* ^*31*^ *Department of Neurology, University Hospital Ulm, Ulm, Germany;* ^*32*^ *Department of Neurology, University of Würzburg, Würzburg, Germany;* ^*33*^ *Department of Neurology, University of Salzburg, Paracelsus Medical University, Salzburg, Austria;* ^*34*^ *Department of Neurology, Universitätsspital Basel, Basel, Switzerland;* ^*35*^ *Department of Neurology, Medical University of Graz, Graz, Austria;* ^*36*^ *Neuro‐SysMed, Department of Neurology, Haukeland University Hospital, Bergen, Norway;* ^*37*^ *Department of Neurology, University Hospital Ostrava, Ostrava, Czech Republic;* ^*38*^ *Department of Neurology, Medical University of Lublin, Lublin, Poland;* ^*39*^ *Multiple Sclerosis Centre of Catalonia (Cemcat), Barcelona, Spain;* ^*40*^ *Department of Neurology, University of Girona, Girona, Spain;* ^*41*^ *Department of Neuroscience, University of Naples Federico II, Naples, Italy;* ^*42*^ *Department of Neurology, University of Gothenburg, Göteborg, Sweden;* ^*43*^ *Department of Neurology, Barts Health NHS Trust & Princess Alexandra Hospital NHS Trust, London, Great Brittain;* ^*44*^ *Department of Neurology, University of Southampton, Great Brittain;* ^*45*^ *Department of Neurology, Amsterdam UMC, Amsterdem, The Netherlands;* ^*46*^ *Department of Neurology, Cliniques Universitaires Saint‐Luc, Université Catholique de Louvain, Louvain, Belgium;* ^*47*^ *Department of Neurology, University of Helsinki, Helsinki, Finland;* ^*48*^ *Department of Neurology, University Medical Centre Ljubljana, Slovenia;* ^*49*^ *Department of laboratory medicine and pathology, Mayo Clinic, Rochester, USA*

**Background and aims:** The 2024‐revised McDonald criteria for diagnosing multiple sclerosis (MS) include kappa free light chains (KFLC) alongside oligoclonal bands (OCB) as markers as diagnostic features. However, the role of OCB and KFLC in the diagnosis of MS requires further clarification.

**Methods:** The PRO‐KFLC study (prospective, multicenter, real‐world analysis of KFLC in the diagnosis of MS; NCT07183020) is coordinated by the German Society for Cerebrospinal Fluid (CSF) Diagnostics and Clinical Neurochemistry. The study recruits CSF/serum sample pairs from patients with MS, and clinically or radiologically isolated syndromes (CIS, RIS) across international MS‐centers. OCB are detected by different externally performed site‐specific methods and by the centrally performed silver‐staining and immunofixation (Sebia) method. KFLC concentrations are determined using nephelometric and turbidimetric assays on different analytical platforms (Atellica NEPH 630, BN ProSpec II, Optilite).

**Results:** To date, 764 CSF/serum sample pairs have been analyzed from over 50 centers across Europe. The female‐to‐male ratio was 1.9, and the median age at diagnosis was 34 years. Among the cohort, 63 patients were diagnosed with CIS or RIS. CSF‐specific OCB were detected by center‐specific methods in 87% of the patients. An intrathecal KFLC synthesis was identified in 91% using the KFLC index (6.1) and in 96% using Reiber's hyperbolic function (Figure 1).

**Figure:**
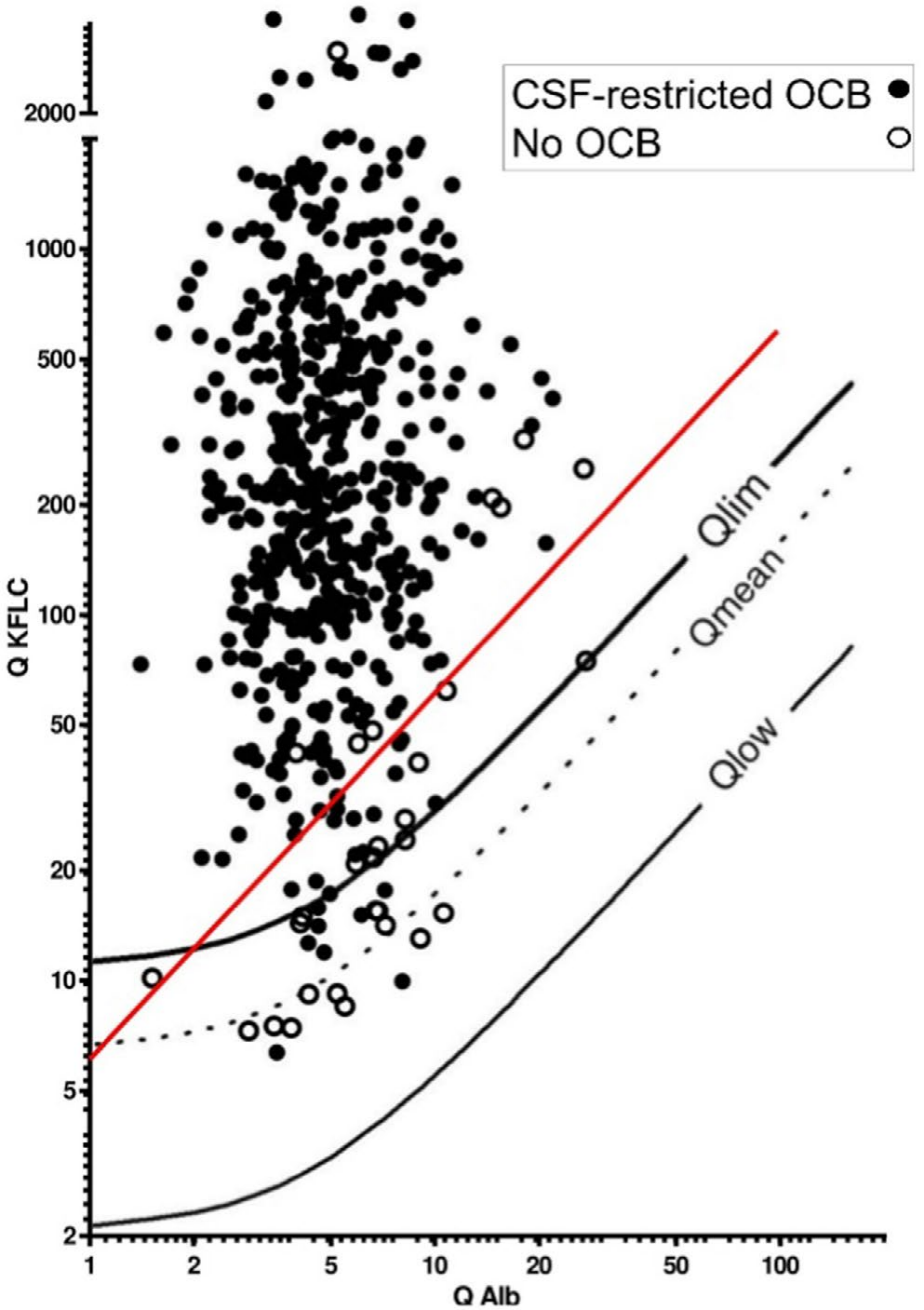
**FIGURE 1** The black line “QLim” of the Reiber quotient diagram (kappa free light chain quotient, Q KFLC; albumin quotient, Q Alb) represents the upper reference limit for intrathecal KFLC synthesis. The red line represents the linear KFLC index of 6.1. Black dots i.

**Conclusion:** The preliminary results indicate that KFLC are a valuable biomarker for detecting intrathecal inflammation in patients with MS, CIS and RIS. Ongoing recruitment and analysis are expected to further elucidate the diagnostic performance and clinical utility of KFLC assays and the role of OCB.

**Disclosure:** This is a prospective, diagnostic biomarker study and none of the authors and centers have to report a conflict of interest. Outside the submitted work: Franz Felix Konen received honoraria from Alexion, Argenx, Merck, Novartis, and Takeda. He received research support from Merck, Siemens and Erwin‐Röver‐Foundation. Thomas Skripuletz reports research support from Alnylam, CSL Behring, Merck, Novartis, Siemens; honoraria for lectures, travel support for meeting attendance, and/or consultancy fees from Alexion, Alnylam, Amgen, argenx, Bayer, Biogen, Bristol Myers Squibb, Centogene, CSL Behring, Grifols, Hexal AG, Horizon, Janssen, Merck, Novartis, Pfizer, Roche, Sanofi, Siemens, SOBI, Teva, Viatris. For this study, institutional research support was granted from Erwin‐Röver‐Foundation. Complete author list: Konen, Franz Felix; Albers, Lina Marie; Bucak, Erda; Streichert, Anna Lena; Schwenkenbecher, Philipp; Jendretzky, Konstantin Fritz; Nay, Sandra; Grote‐Levi, Lea Martha; Tauber, Simone; Bohn, Amelie; Bayas, Antonios; Braun, Marie; Ruprecht, Klemens; Höpner, Lena Katharina; Otto, Carolin; Schaebitz, Wolf; Bytyqi, Adonis; Gold, Ralf; Motte, Jeremias; Horstkemper, Lea; Ruck, Tobias; Boeckers, Joshua; Seliger‐Behme, Corinna; Lazaridis, Lazaros; Pröbstel, Anne‐Katrin; Möhn, Nora; Dressel, Alexander; Bock, Henry; Ziemssen, Tjalf; Goerbing, Tina; Meuth, Sven; Pawlitzki, Marc; Rothhammer, Veit; Tsaktanis, Thanos; Yalachkov, Yavor; Hauck, Lilith; Koerbel, Kimberley; Wiendl, Heinz; Dersch, Rick; Warnke, Clemens; Svacina, Martin; Pfeuffer, Steffen; Süße, Marie; Budde, Kathrin; Otto, Markus; Bentrup, Jana; Friese, Manuel A.; Schubert, Charlotte; Wildemann, Brigitte; Groppa, Sergiu; Decker, Yann; Fousse, Mathias; Geis, Christian; Schwab, Matthias; Vay, Sabine; Konitsioti, Agni; Schreiber, Stefanie; Vielhaber, Stefan; Al‐Dubai, Marwa; Stephanik, Heike; Kuempfel, Tania; Engels, Daniel; Hemmer, Bernhard; Busch, Theresa; Gross, Catharina; Schulte‐Mecklenbeck, Andreas; Witt, Karsten; Linker, Ralf; Angstwurm, Klemens; Winkelmann, Alexander; Zettl, Uwe; Leypoldt, Frank; Ehmke, Lena; Kowarik, Markus; Tieck‐Fernandez, Maria; Tumani, Hayrettin; Bachhuber, Franziska; Haarmann, Axel; Schuhmann, Michael; Harrer, Andrea; Kliushnikova, Dariia; Kuhle, Jens; Oechtering, Johanna; Khalil, Michael; Torkildsen, Oivind Fredvik Grytten; Zeman, David; Kusnierova, Pavlina; Rejdak, Konrad; Montalban, Xavier; Arrambide, Georgina; Espejo, Carmen; Alvarez, Gary; Quiroga, Ana; Moccia, Marcello; Rosenstein, Igal; Axelsson, Markus; Novakova, Lenka; Schmierer, Klaus; Gnanapavan, Sharmilee; Galea, Ian; Teunissen, Charlotte; Wessels, Mark; Van Pesch, Vincent; Benyahia, Zohra; Soares, Mafalda; Ladeira, Ana; Laakso, Sini; Tienari, Pentti; Emersic, Andreja; Uros, Rot; Willrich, Maria Alice; Manin, Analisa; Villa, Andres Maria; Tuzun, Erdem; Yilmaz, Vuslat; Skripuletz, Thomas

## EPO‐0530

### c‐Met: A new signature of pro‐inflammatory CD4+ T cells in multiple sclerosis

#### G. Breville ^1^; M. Benkhoucha^2^; A. Rezk^3^; N. Lan Tran^2^; I. Senoner^2^; A. Bar‐Or^3^; P. Lalive^1^

##### ^*1*^ *Department of Clinical Neurosciences, Division of Neurology, University Hospital of Geneva, Geneva, Switzerland;* ^*2*^ *Department of Pathology and Immunology, Faculty of Medicine, University of Geneva, Geneva, Switzerland;* ^*3*^ *Center for Neuroinflammation and Experimental Therapeutics, Department of Neurology, Perelman School of Medicine, University of Pennsylvania, Philadelphia, USA*

**Background and aims:** Tyrosine kinase receptor c‐Met is the unique receptor for hepatocyte growth factor (HGF). The HGF/c‐Met axis is reported to modulate cell migration, cytokine production, and antigen presentation. Here, we analyzed c‐Met expression on human CD4+ T cells from multiple sclerosis (MS) patients versus patients with non‐inflammatory neurological disease (NIND).

**Methods:** Cells were isolated from blood and CSF samples of MS and NIND patients from December 2020 to December 2022 at Geneva University Hospital. Phenotypic characterization were determined by RNA sequencing (bulk) and flow cytometry. Adhesion and transmigration capacity were studied to further characterize the function of activated c‐Met+CD4+ T cells.

**Results:** c‐Met+CD4+ T cells are detected at higher levels in both blood and CSF of MS patients compared to NIND with a blood to CSF gradient. The phenotype of CD4+c‐Met+ T cells is oriented toward Th17.1 polarization with a predominance of central memory and effector memory activity profiles. c‐Met+ CD4+ T cells have increased proinflammatory activity, showing higher intracellular levels of IL‐17+ and IL‐17+IFNγ+ compared with c‐Met‐CD4+ T cells. c‐Met+CD4+ T cells showed increased transmigration capacity correlated with increased expression of integrins (Itgα4, Itgβ1, Itgβ7, Itgα4β1, Itgα4β7, ItgαLβ2) in transcriptomic (bulk mRNA sequencing) and surface protein (flow cytometry) analyses compared with c‐Met‐CD4+ T cells. Anti‐Itgα4 (natalizumab) and anti‐ItgαLβ2 (odulimomab) inhibit CD4+ T cell transmigration with predominant inhibition of c‐Met+CD4+ T cells compared with c‐Met‐CD4+ T cells.

**Figure:**
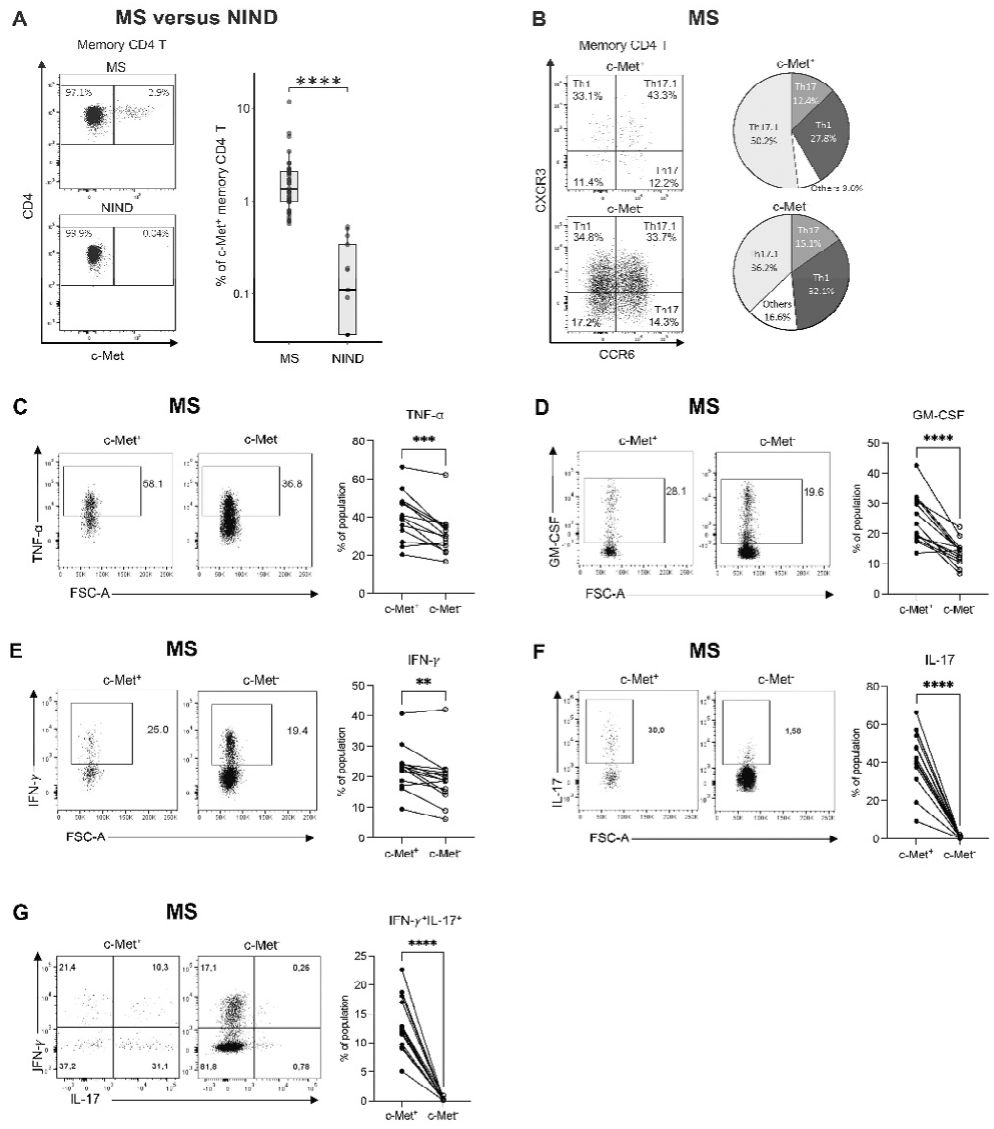
Increased frequency of blood c‐Met+ CD4 T cells displaying enhanced inflammatory propensity in MS patients.

**Figure:**
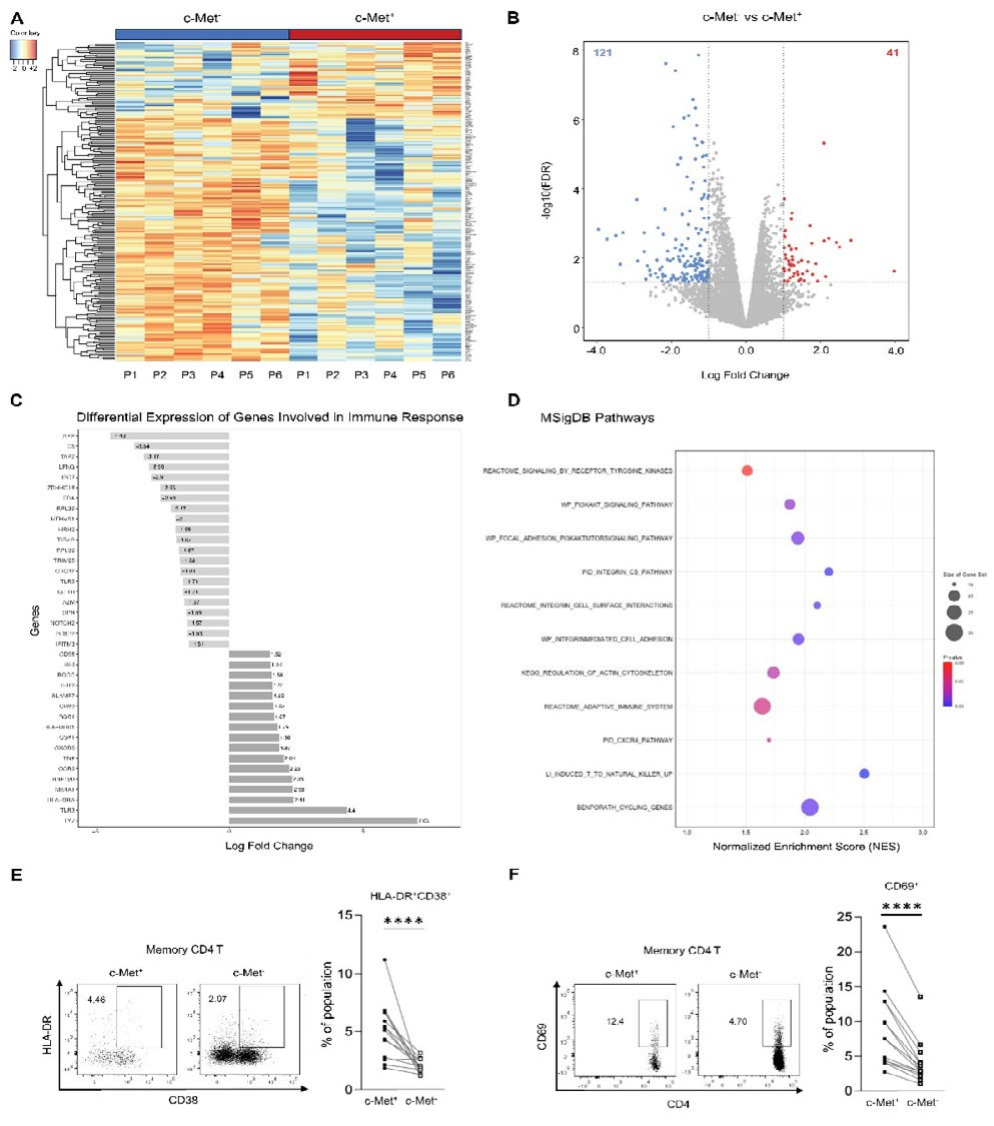
Bulk RNA‐seq analysis of c‐Met+ and c‐Met‐ CD4 T cells isolated from the blood of MS patients.

**Figure:**
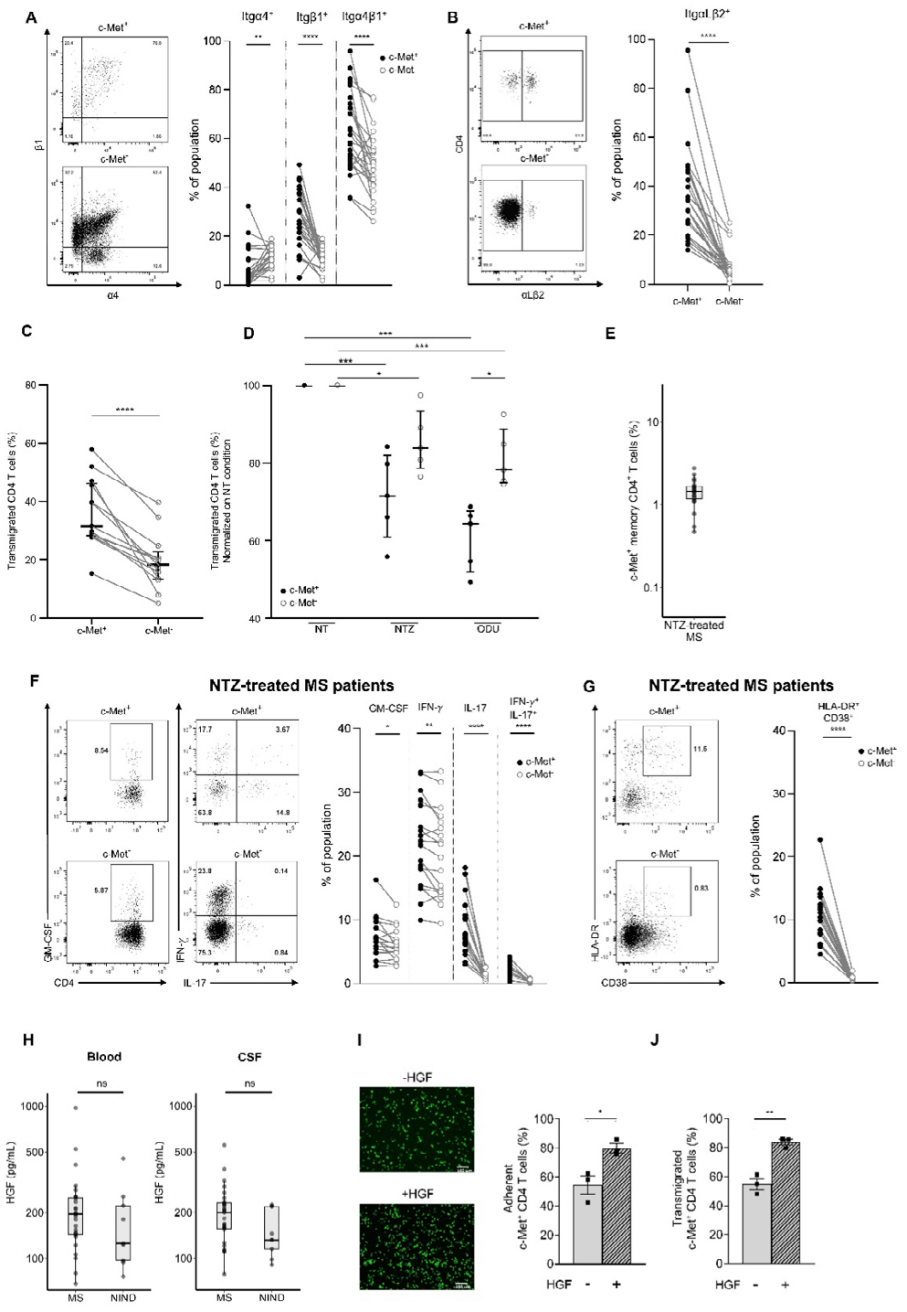
c‐Met+ CD4 T cells show increased expression of VLA‐4 and LFA‐1, and are associated with enhanced migratory capacities in the blood of MS patients.

**Conclusion:** These results emphasize c‐Met as an immune marker of highly pro‐inflammatory and pro‐migratory CD4+ T lymphocytes in MS.

**Disclosure:** None

## EPO‐0531

### Stride‐level gait monitoring as a sensitive marker of relapse‐associated and relapse‐independent disability progression in multiple sclerosis

#### M. Poleur ^1^; O. Piquet^2^; B. Willekens^3^; B. Degos^4^; D. Ricard^5^; V. Van Pesch^6^; A. Mélin^7^; E. Lommers^8^; A. Tricot^2^; A. De Keersmaecker^3^; I. Coman^4^; M. Michaud^5^; L. Médard^1^; C. Cluzeau^2^; D. Eggenspieler^2^; L. Servais^9^

##### ^*1*^ *University department of neurology, Citadelle Hospital of Liège, Belgium**;*** ^*2*^ *Sysnav, Vernon, France**;*** ^*3*^ *Department of Neurology, Antwerp University Hospital, Antwerp, Belgium;* ^*4*^ *Neurology Department, Avicenne Hospital, APHP, Hôpitaux Universitaires de Paris‐Seine Saint Denis (HUPSSD), Sorbonne Paris Nord;* ^*5*^ *Service de Neurologie, Service de Santé des Armées, Hôpital d’Instruction des Armées de Percy, France**;*** ^*6*^ *Cliniques Universitaires Saint‐Luc, UCLouvain, Belgium;* ^*7*^ *Department of Neurology, CHC Mont‐Legia, Belgium;* ^*8*^ *Department of neurology, Centre Hospitalier Universitaire de Liège, Belgium**;*** ^*9*^ *MDUK Oxford Neuromuscular Centre, John Radcliffe Hospital, UK*

**Background and aims:** In multiple sclerosis (MS), high‐efficacy disease‐modifying therapies substantially reduce inflammatory activity, yet long‐term disability progression persists. Both progression independent of relapse activity (PIRA) and relapse‐associated worsening (RAW) contribute to disability accumulation. Sensitive methods to capture these processes are needed.

**Methods:** We conducted a multicenter, 3‐year observational study using an ankle‐worn wearable device enabling stride‐level gait reconstruction and the computation of the fastest 5% of strides recorded (SV95C). Participants completed one‐month periods of daily monitoring at baseline, one year, and every six months over the subsequent two years. The Expanded Disability Status Scale (EDSS) and Timed 25‐Foot Walk Test (T25FWT) were performed before each monitoring period. Reliability, clinical validity and sensitivity to change were evaluated.

**Results:** Seventy‐eight participants (median age 48.5 years; EDSS range 0–5.5), including 61 with relapsing‐remitting MS (RRMS) and 17 with progressive MS (PMS), were enrolled. SV95C showed excellent reliability (intra‐class correlation coefficient of 0.99) and strong correlations with EDSS (*R* = −0.65) and T25FWT (*R* = −0.71; all *p* < 0.001) (Figure 1). SV95C differentiated MS subtypes and EDSS subgroups (Figure 2). Significant SV95C decline was detected in relapse‐free PMS (standardized response mean, SRM −0.89 at 1Y; −1.06 at 2Y; both *p* < 0.05) and RRMS (SRM −0.84 at 1.5Y, *p* < 0.01; −0.87 at 2Y, *p* < 0.001) (Figure 3). EDSS identified change only in PMS (SRM 0.67, *p* < 0.05 at 1Y). Relapses were associated with marked SV95C declines.

**Conclusion:** Passive, stride‐level gait monitoring is a valid and sensitive marker of ambulatory decline in MS, capturing both RAW and PIRA. Full three‐year results will be presented at the congress.

**Disclosure:** This study was funded by F. Hoffmann‐La Roche Ltd.

## EPO‐0532

### Temporal Dynamics of Proteomic Profiling in Multiple Sclerosis: Insights from a retrospective longitudinal study

#### M. Martinez‐Serrat ^1^; G. Datta^2^; C. Tafrali^1^; R. Demjaha^1^; M. Haindl^1^; B. Helmlinger^3^; T. Kaiser^1^; D. Pinter^3^; B. Heschl^4^; A. Damulina^1^; E. Hofer^5^; S. Ropele^4^; C. Enzinger^4^; D. Brazel^2^; F. Qureshi^2^; M. Khalil^1^

##### ^*1*^ *Neurology Biomarker Research Unit and Department of Neurology, Medical University of Graz, Graz, Austria;* ^*2*^ *Octave Bioscience, Inc, Menlo Park, USA;* ^*3*^ *Research Unit for Neuronal Plasticity and Repair and Department of Neurology, Medical University of Graz, Austria;* ^*4*^ *Department of Neurology, Medical University of Graz, Graz, Austria;* ^*5*^ *Institute for Medical Informatics, Statistics and Documentation, Medical University of Graz, Austria*

**Background and aims:** Identifying blood‐based biomarkers to enhance clinical decision‐making in multiple sclerosis (MS) is essential, yet no single protein captures the disease's complex pathophysiology. The MS Disease Activity (MSDA) panel, comprising 18 proteins reflecting neuroinflammation, immunomodulation, myelin biology, and neuroaxonal integrity, has been associated with radiological and clinical outcomes. However, its relationship with evidence of disease activity (EDA) and temporal patterns remains underexplored. This study investigates temporal variations in the MSDA score in relation to MS disease activity and its potential utility in clinical evaluations.

**Methods:** Retrospective longitudinal data from 102 MS patients (mean±SD age 31.7±7.7 years, 61.8% female, median disease duration 1.3 years [IQR:0.7–6.8]) were analyzed, with a median of 7 samples [IQR:6–9] per patient over a mean±SD follow‐up of 10.4±3.4 years. Protein levels were measured using the MSDA panel on the Olink™ platform. Radiological activity was defined as Gadolinium‐enhanced or new/enlarging T2 lesions on 3T MRI scans. EDA included clinical relapse, confirmed disability worsening (assessed by Expanded Disability Status Scale scores), or radiological activity within six months of sampling.

**Results:** Temporal analysis showed a significant MSDA‐score increase at relapse (*p* < 0.001), remaining elevated for six months (*p* < 0.01). Inflammation‐related pathways peaked at relapse, while myelin and neuroaxonal scores peaked three months later. The MSDA score was higher in patients who developed future EDA within 1.5 years, but only when samples were collected during stable periods (*p* < 0.05).

**Conclusion:** These findings highlight the value of dynamic proteomic profiling. The MSDA score may help identify patients at risk of future disease activity, potentially improving routine MS care.

**Disclosure:** Maria Martinez‐Serrat has received travel funding and speaker honoraria from Novartis. Gargi Datta is an employee of Octave Bioscience Cansu Tafrali has received travel funding and speaker honoraria from Merck and Novartis. Rina Demjaha has received travel funding from Janssen, Novartis and Sanofi. Michaela Tanja Haindl has nothing to disclose. Birgit Helmlinger has received speaking honoraria from Roche, Sanofi, and Bristol‐Myers Squibb, and travel funding from Janssen. Timothy Kaiser has nothing to disclose. Daniela Pinter is a member of the advisory board for “Cognition and MS” for Novartis and has received speaking honoraria from Biogen, Novartis, MedAhead and Bristol‐Myers Squibb. Bettina Heschl has nothing to disclose. Anna Damulina has participated in meetings sponsored by, received speaker honoraria or travel funding from Sanofi‐Aventis, Novartis and Janssen. Edith Hofer has nothing to disclose. Stefan Ropele has nothing to disclose. Christian Enzinger has received funding for traveling and speaker honoraria from Biogen Idec, Bayer Schering Pharma, Merck Serono, Novartis, Genzyme and Teva Pharmaceutical Industries Ltd./Sanofi‐Aventis, Shire; received research support from Merck Serono, Biogen Idec, and Teva Pharmaceutical Industries Ltd./Sanofi‐Aventis; and serves on scientific advisory boards for Bayer Schering Pharma, Biogen Idec, Merck Serono, Novartis, Genzyme, Roche, and Teva Pharmaceutical Industries Ltd./Sanofi‐ Aventis. David Brazel is an employee of Octave Bioscience Ferhan Qureshi is an employee of Octave Bioscience Michael Khalil has received travel funding and speaker honoraria from Bayer, Biogen, Novartis, Merck, Sanofi and Teva and serves on scientific advisory boards for Biogen, Bristol‐Myers Squibb, Gilead, Merck, Novartis, Alexion, Amgen and Roche. He received research grants from Biogen, Novartis and Teva.

## EPO‐0533

### Change in excess and relative mortality in multiple sclerosis over time and by sex

#### M. Magyari ^1^; R. Holm^2^; N. Koch‐Henriksen^3^

##### ^*1*^ *The Danish Multiple Sclerosis Registry, Copenhagen University Hospital – Rigshospitalet, Glostrup, Denmark, and Institute for Clinical Medicine, University of Copenhagen, Denmark;* ^*2*^ *The Danish Multiple Sclerosis Registry, Copenhagen University Hospital – Rigshospitalet, Glostrup, Denmark;* ^*3*^ *Department of Clinical Epidemiology, Aarhus University Hospital, Aarhus, Denmark, and The Danish Multiple Sclerosis Registry, Copenhagen University Hospital – Rigshospitalet, Glostrup, Denmark*

**Background and aims:** People with multiple sclerosis (PWMS) die at a lower age than matched background populations, but how much the disease shortens lifetime, differ between published studies, older as well as more recent.

**Methods:** The nationwide Danish Multiple Sclerosis Registry has followed people with multiple sclerosis (MS) for up to 70 years after onset with complete registration of vital status. In this study we describe the trend over time of excess mortality in PWMS and focus on the difference between the sexes.

**Results:** 28,118 cases had onset of MS between 1950 and 2022. Of them 9875 had died before follow‐up at the end of year 2022, whereas only 3579 deaths were expected in matched Danish background population, matched by sex, current age and current calendar year. The relative mortality in terms of standardized mortality rate (SMR) was 2.84 (95% CI 2.71–2.81). The median survival time from onset was 31.4 years (95% CI 30.6–32.4) as opposed to 49.8 years in the matched population within the same time‐ and age‐frame. The median age of death was 74.2 years in PWMS (95% CI 73.8 – 84.6) and 83.4 years in the matched population. 20‐years SMR changed from 5.21 in the 1950–1964 onset cohort to 2.9 in the 1980–1994 onset cohort. The ratio of relative mortalities between women and men was 1.04 (*p* = 0.085).

**Conclusion:** Excess mortality in MS has decreased over the last several decades, but it does not differ between the sexes.

**Disclosure:** Melinda Magyari has served on the scientific advisory board for Sanofi, Novartis, Merck, and Moderna and has received honoraria for lecturing from Biogen, Merck, Novartis, Roche, Sanofi, Moderna, and Neuroxpharm. Rolf Pringler Holm has received speaker honoraria from Sanofi and Novartis, has served on an advisory board for Novartis, and has received a travel grant from The Danish MS Society. Nils Koch‐Henriksen has nothing to disclose.

## EPO‐0534

### Ponesimod efficacy and safety in relapsing multiple sclerosis patients with mild to moderate disability: Results from OPTIMUM and long‐term extension

#### O. Heinzlef ^1^; M. Heubl^2^; S. de Guitaut^2^; A. Atallah^2^; A. Sarfati^2^

##### ^*1*^ *Department of Neurology, Centre Hospitalier Intercommunal de Poissy‐Saint‐Germain‐en‐Laye, Poissy, France;* ^*2*^ *Medical Affairs Department, Juvise Pharmaceuticals, Paris, France*

**Background and aims:** OPTIMUM was a phase 3 trial comparing ponesimod with teriflunomide in relapsing multiple sclerosis (RMS). In the long‐term extension (LTE), all participants received ponesimod 20 mg for up to 5 years. This post hoc analysis assessed efficacy and safety in patients with baseline Expanded Disability Status Scale (EDSS) ≤3.5 at core study entry.

**Methods:** Annualized relapse rate (ARR) was estimated using a negative binomial model. Disability outcomes included 24‐week confirmed disability accumulation (24wCDA). Magnetic resonance imaging activity was assessed using cumulative combined unique active lesions (CUAL), defined as T1 gadolinium‐enhancing lesions and new or enlarging T2 lesions. Safety assessments included serious and fatal adverse events (AE).

**Results:** Overall, 946 patients were included (core study: 472 ponesimod; 474 teriflunomide). During the core period, ARR was lower with ponesimod versus teriflunomide (0.17 vs 0.29), rate ratio 0.59 (95% confidence interval [CI] [0.47‐ 0.74]). Over the combined period, ARR remained lower in patients receiving continuous ponesimod versus those switching from teriflunomide (0.13 vs 0.21), rate ratio 0.63 (95% CI [0.51‐ 0.79]). 24wCDA estimates at 360 weeks were 20.5% (95% CI [16.7 ‐ 25.0]) with continuous ponesimod versus 24.6% (95% CI [20.5 ‐ 29.3]) in switchers. No CUALs were observed in 69.7% and 75.9% of patients receiving continuous ponesimod at end of core and extension studies visits respectively. 12.3% of patients experienced severe AE in the ponesimod group.

**Conclusion:** In patients with baseline EDSS ≤3.5, ponesimod showed sustained clinical and magnetic resonance imaging efficacy across OPTIMUM and the LTE with a favorable safety profile.

**Disclosure:** O.H. reports consulting and lecturing fees from Bayer Schering, Merck, Teva, Genzyme, Novartis, Almirall and BiogenIdec, travel grants from Novartis, Teva, Genzyme, Merck Serono and Biogen Idec and research support from Roche, Merck and Novartis, all of the above unrelated to this work. M.H, S.G, A.A and A.S are employees of Juvise Pharmaceuticals.

## EPO‐0535

### Defining Progression Independent of Relapse Activity in Early Multiple Sclerosis

#### T. Ayaz; A. Emekli; T. Gündüz; M. Kürtüncü

##### Department of Neurology, Istanbul University Faculty of Medicine, Istanbul University, Istanbul, Türkiye

**Background and aims:** Progression independent of relapse activity (PIRA) and relapse‐associated worsening (RAW) are primary mechanisms of disability accumulation in multiple sclerosis (MS). Various PIRA definitions in literature limit cross‐study comparability, and most studies rely on clinical trials rather than real‐world data. We aimed to determine a clinically applicable PIRA definition in early MS by comparing 54 detection models.

**Methods:** We included 392 relapsing‐remitting MS patients meeting 2024 McDonald criteria, evaluated within one year of symptom onset, with ≥2 years follow‐up. Missing EDSS data were imputed by employing a 90‐day interval linear interpolation‐based imputation method, in which missing values were estimated along the trajectory defined by the preceding and subsequent EDSS assessments. Fifty‐four PIRA detection models were compared across four parameters: (1) baseline relapse‐free criteria (3 months, 6 months, 12 months), (2) confirmation timeframe (6 months, 12 months, end‐of‐follow‐up), (3) confirmation type (threshold‐based vs. absolute), and (4) worsening relapse‐free criteria (90 days before worsening, no relapses baseline‐to‐worsening, no relapses baseline‐to‐confirmation).

**Results:** Among 392 patients (257 female), median follow‐up was 6.4 years (IQR: 3.2–10.2) and median age at disease onset was 26.4 years (IQR: 21.9–33.3). During up to 5 years of follow‐up (1,592.5 patient‐years), PIRA prevalence ranged from 1.5% to 7.4% across definitions. We identified the 3‐month relapse‐free roving baseline, 12‐month absolute confirmation with baseline‐to‐worsening relapse‐free criteria as optimal for clinical use. This model yielded 3.3% PIRA and 7.9% RAW prevalence.

**Conclusion:** We propose 3‐month relapse‐free roving baseline, 12‐month absolute confirmation with baseline‐to‐worsening relapse‐free criteria as the most clinically applicable PIRA definition in early MS.

**Disclosure:** This study was funded by the Turkish Neuroimmunology Association.

## EPO‐0536

### Can Immune Cell Dynamics After Short Course Cladribine Tablets Predict MRI Lesion Activity in MS? Insights From The 4 year MAGNIFY MS Study

#### T. Derfuss^1^; F. Sellebjerg^2^; D. Piani Meier^3^; D. Guala^4^; K. Thangavelu^5^; L. Gardner^6^; H. Wiendl^7^; N. De Stefano ^8^; K. Schmierer^9^

##### ^*1*^ *Department of Neurology, University Hospital Basel, Basel, Switzerland**;*** ^*2*^ *Danish MS Center, Department of Neurology, Copenhagen University Hospital ‐ Rigshospitalet, Glostrup, Denmark;* ^*3*^ *Neurology & Immunology Medical Unit, Ares Trading SA, Eysins 1262, Switzerland, an affiliate of Merck KGaA;* ^*4*^ *Data Sciences, Merck AB, Solna, Sweden, an affiliate of Merck KGaA;* ^*5*^ *Statistics Department, MeDaStats, LLC, Tampa, USA;* ^*6*^ *EMD Serono Research & Development Institute, Inc, Billerica, USA, an affiliate of Merck KGaA;* ^*7*^ *Department of Neurology and Neurophysiology, Medical Center ‐ University of Freiburg, Freiburg, Germany;* ^*8*^ *Department of Medicine, Surgery and Neuroscience, University of Siena, Siena, Italy;* ^*9*^ *The Blizard Institute, Centre for Neuroscience, Surgery & Trauma, Faculty of Medicine & Dentistry, Queen Mary University of London, London, UK*

**Background and aims:** It is unknown whether immune reconstitution patterns during years (Y)3–4 (treatment‐free period) after cladribine tablets (CladT) treatment relate to MRI‐lesion activity. We investigated lymphocyte reconstitution dynamics and MRI‐lesion activity during Y3‐4 in people with MS (PwMS) after treatment with CladT.

**Methods:** Absolute and relative changes from baseline (Month [M] 0) in 14 predefined T‐cell and 9 B‐cell subsets were compared in PwMS with/without Y3‐4 MRI‐lesion activity. 215 B‐ and T‐cells ratios were also compared. Data were evaluated using Wilcoxon rank tests for univariate comparative analyses, and adjusted, multivariate Cox regression analyses using M15‐48 data (post‐lymphocyte‐count‐nadir). *p* < 0.05, and *p* < 0.1 for sensitivity analysis with adjustment for multiple testing, considered nominally significant.

**Results:** 159/182 PwMS without lesion‐activity after treatment completion (M18‐24) were evaluated in the comparative analysis; 59 experienced Y3‐4 MRI‐lesion activity and 100 remained lesion free. Patients with new/active lesions had increased B‐memory, B‐activated and B‐transitional cells, proportionally decreased B‐naïve cells, and increased central memory (CM) and Th1 T‐cells (Figure 1). Multivariate Cox regression confirmed increased risk for MRI‐lesion activity in patients with higher levels of these cells in addition to plasma B‐cells, and decreased risk with higher levels of CD8‐naïve cells (Figure 2). Adjustment for multiple testing supported the relevance of B‐activated and B‐plasma cell ratios to CD4 naïve and/or Th2 T‐cells (Figure 3).

**Figure:**
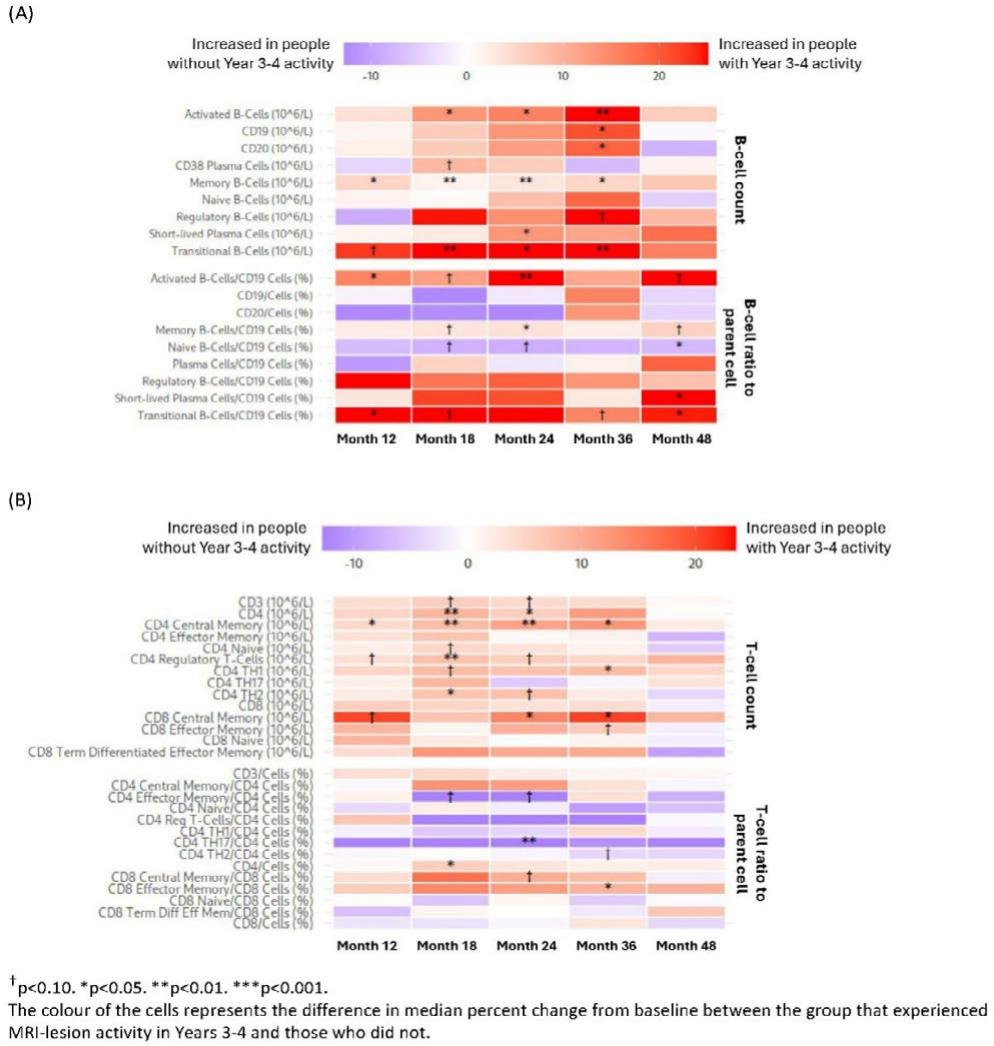
**FIGURE 1** Difference in median percent change from baseline in (A) B‐cells and (B) T‐cells comparing patients resuming MRI lesion activity during Years 3–4 with those remaining lesion‐free.

**Figure:**
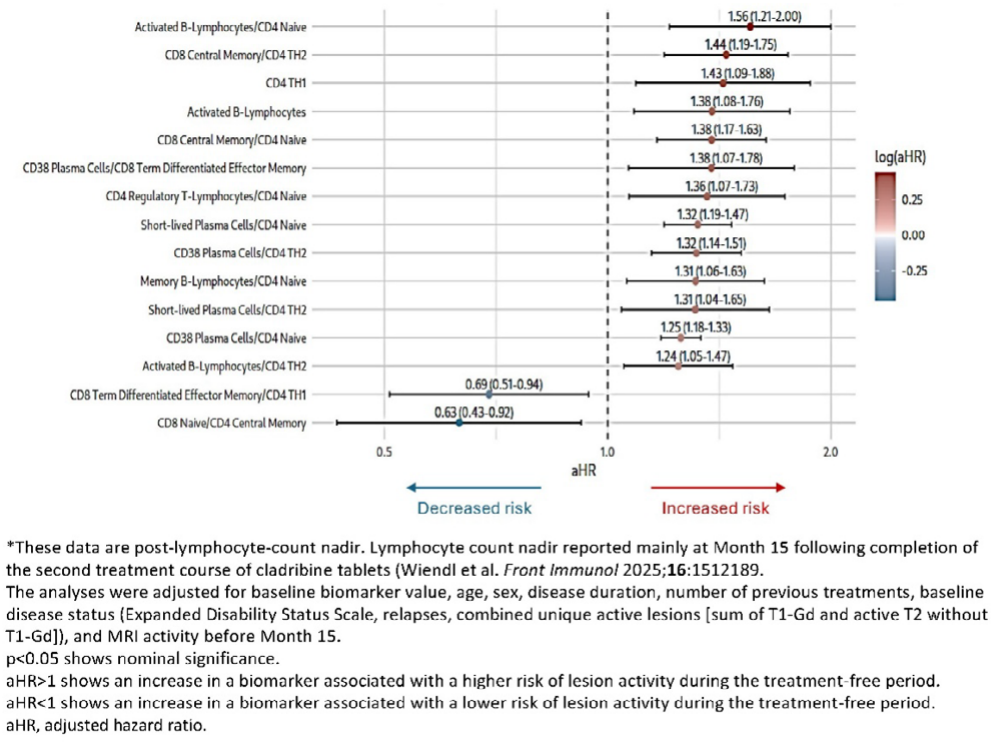
**FIGURE 2** Multivariate Cox‐regression analyses of MRI activity according to immune cell markers during Months 15–48* (absolute values and ratios; *n* = 197): top 15 associations (*p* < 0.05).

**Figure:**
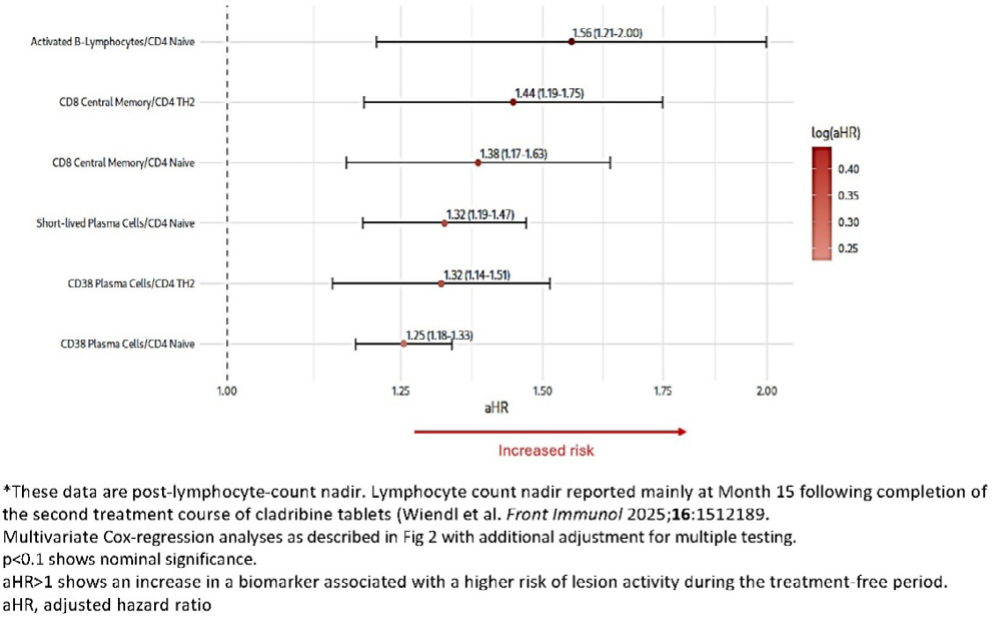
**FIGURE 3** Multivariate Cox‐regression analyses of MRI activity according to immune cell markers during Months 15–48 (absolute values and ratios; *n* = 197): adjusted for multiple testing (*p* < 0.1).

**Conclusion:** CM and Th1 T‐cells, along with plasma (CD38+ and short‐lived), B‐memory, and B‐activated cells were associated with MRI‐lesion activity. Assessing reconstitution dynamics, especially B‐ vs T‐cell ratios, during the treatment‐free period may help predict disease activity and guide long‐term disease management.

**Disclosure:** TD: Scientific ad boards: Actelion (Janssen/J&J), Alexion, Amgen, Biogen, Celgene (Bristol Myers Squibb), GeNeuro, Merck, Novartis, Roche, Sanofi. Funding for travel and/or speaker honoraria: Biogen, Merck, Novartis, Roche, Sanofi. Research support: Alexion, the EU, Novartis, Roche, the Swiss MS Society, the Swiss National Foundation HW: Research support: DFG, Deutsche Myasthenie Gesellschaft e.V, the EU, Alexion, Amicus Therapeutics Inc, Argenx, Biogen, CSL Behring, F. Hoffmann‐La Roche, Genzyme, Merck, Novartis, Roche, UCB Biopharma. Speaker and/or consultancy fee: Alexion, Argenx, Argobio, AOCN, AstraZeneca, BGP Products Operations GmbH, Biogen, Bristol Myers Squibb, CEMCAT, Dianthus, EMD Serono Research & Development Institute Inc, Billerica, USA, an affiliate of Merck KGaA, EPG Health/Medthority, Fondazione Cariplo, Genzyme, Idorsia, Immunic, Immunovant, INmune Bio_Syneos Health, Kohlhammer, LTS, Lundbeck, Merck, MS at the Limits, Muna Therapeutics, Myrobalan Therapeutics, Neurodiem, NMSS, Novartis, Ology, PSL Group, Red Nucleus, Roche, Samsung, Sangamo, Sanofi, Springer, StreamedUp, Swiss MS Society, Teladochealth, Toleranzia, Teva, UCB, Uvet, Viatris, WebMD global. Scientific Ad Boards: Alexion, Argenx, Biocryst, Bristol Myers Squibb, Cellerys, Galapagos, Janssen, Merck, Novartis, Sandoz‐Hexal, uniQure biopharma B.V FS: Scientific ad boards, clinical trial steering committees, consultancy, support for congress participation, speaker honoraria, or research support for his laboratory: Biogen, Celgene (Bristol Myers Squibb), EMD Serono, Merck, Novartis, Roche, Sanofi, Teva NdS: Honoraria: Biogen, Celgene (Bristol Myers Squibb), Genzyme, Immunic, Novartis, Roche, Teva. Advisory boards: Biogen, Immunic, Merck, Novartis, Roche, Sanofi. Research grant support: Italian Ministry of University and Research, Fondazione Italiana Sclerosi Multipla. Associate Editor of Neurological Sciences and co‐founder of Siena‐Imaging s.r.l DPM: Employee of Ares Trading SA, Eysins, Switzerland, an affiliate of Merck KGaA DG: Employee of Merck AB, Solna, Sweden, an affiliate of Merck KGaA KT: Employee of MeDaStats, LLC, Tampa, USA, funded by Merck KGaA to perform statistical analyses LG: Employee of EMD Serono Research & Development Institute, Inc, Billerica, USA, an affiliate of Merck KGaA KS: Chief investigator of ChariotMS (NCT04695080) supported by NIHR, MS Societies, Barts Charity, Merck, and AttackMS (NCT05418010) supported by Biogen & Sandoz. Co‐chief investigator of AssistMS supported by UK Department of Health and Social Care and icometrix. Advisory committee: Merck. Research and NHS service support: Merck, Neuraxpharm, Novartis, Roche, Sanofi. Speaking honoraria and/or advisory role: Biogen, icometrix, Merck, Neuraxpharm, Novartis, Roche, Sanofi; remuneration for teaching activities from AcadeMe & Medscape

Neuroimaging 2

## EPO‐0537

### Brain parenchyma displacement and deformation in patients with Spontaneous Sntracranial Hypotension (SIH)

#### A. El Rahal ^1^; P. Scheffler^1^; K. Wolf^1^; F. Volz^1^; M. Overstijns^1^; H. Urbach^2^; N. Lützen^2^; C. Zander^2^; J. Beck^1^

##### ^*1*^ *Department of Neurosurgery, Medical Center, University of Freiburg, Freiburg, Germany;* ^*2*^ *Department of Diagnostic and Interventional Neuroradiology, Medical Center University of Freiburg, Freiburg, Germany*

**Background and aims:** Spontaneous intracranial hypotension (SIH) is mainly associated with orthostatic headache, but can also cause neurological symptoms, with symptoms mimicking behavioral‐type frontotemporal dementia in severe cases. SIH is known to lead to brain sagging. The aim of this study was to investigate whether brain MRI imaging in SIH patients shows quantifiable displacement, strain, and compression of brain structures.

**Methods:** We designed an AI‐driven image analysis pipeline for automated segmentation, volumetry and diffeomorphic registration of pre‐ and postoperative MRI imaging (AssemblyNet, Synthmorph CNNs). We calculated voxel‐wise perioperative spatial displacements for all brain areas in 96 patients with SIH. We calculated the Jacobian determinant as a measure of local brain expansion and compression, and the von Mises equivalent strain as a measure of local distortion.

**Results:** Brain structures demonstrated consistent predominantly upward–outward postoperative shift. Most major brain regions exhibited statistically significant movement, with the highest magnitude seen in the parietal lobes and the left lateral ventricle (> 0.7 mm, *p* < 0.0001). Ventricles and transverse sinuses showed relevant perioperative expansion and contraction, respectively. Apart from the ventricles, high strain was observed in temporomesial structures and the ventromedial prefrontal cortex, indicating structural strain.

**Figure:**
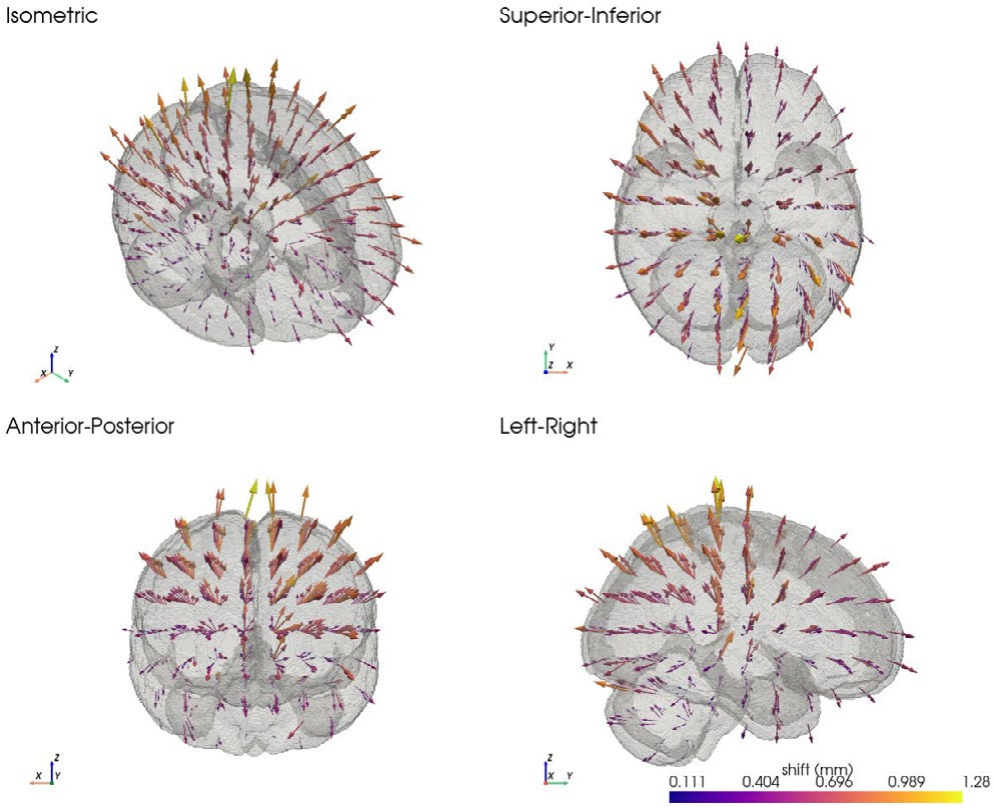
Three‐dimensional illustration of perioperative brain parenchyma shifts.

**Figure:**
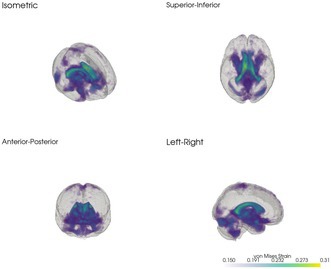
Three‐dimensional illustration of perioperative deviatoric strain in brain parenchyma.

**Figure:**
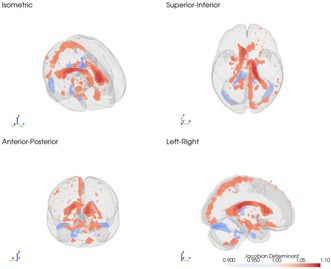
Three‐dimensional illustration of Jacobian determinant as a measure of local brain expansion.

**Conclusion:** Surgical CSF leak closure in SIH is associated with consistent, quantifiable postoperative brain shifts on MRI. We demonstrate strain in brain regions of SIH patients usually affected by frontotemporal dementia, which could explain a similar clinical presentation in severe cases of SIH. AI‐assisted image analysis in SIH may provide valuable prognostic information in clinical practice, potentially aiding the assessment of surgical outcome.

**Disclosure:** Nothing to disclose

## EPO‐0538

### Altered functional connectivity in sensorimotor, emotional, and cognitive networks and changes of cerebellar volumes in functional movement disorders

#### A. Gardoni ^1^; E. Sarasso^2^; M. Gandolfi^3^; S. Basaia^1^; E. Canu^4^; E. Sibilla^1^; C. Tripodi^1^; A. Sandri^5^; I. Di Vico^5^; M. Fiorio^3^; G. Pedrotti^3^; A. Paolicelli^5^; P. Barone^6^; M. Pellecchia^6^; R. Erro^6^; S. Cuoco^7^; I. Carotenuto^7^; C. Vinciguerra^7^; A. Botto^8^; M. Amboni^7^; M. Russo^9^; G. Mansueto^3^; F. Pizzini^3^; M. Barillari^3^; M. Lauriola^5^; M. Tozzi^5^; F. Rusciano^5^; C. Geroin^3^; M. Fasoli^3^; A. Marotta^3^; E. Pizzolla^3^; F. Salaorni^3^; I. Lozzi^3^; F. Bombieri^3^; G. Squintani^5^; S. Mariotto^5^; S. Tamburin^3^; F. Paio^3^; G. De Biasi^7^; G. Piscosquito^7^; M. Tinazzi^3^; M. Filippi^10^; F. Agosta^4^

##### ^*1*^ *Neuroimaging Research Unit, Division of Neuroscience, IRCCS San Raffaele Scientific Institute, Milan, Italy; and Neurotech Hub, Vita‐Salute San Raffaele University, Milan, Italy;* ^*2*^ *Neuroimaging Research Unit, Division of Neuroscience, IRCCS San Raffaele Scientific Institute, Milan, Italy; and Neurotech Hub, Vita‐Salute San Raffaele University, Milan, Italy; and DINOGMI, University of Genoa, Genoa, Italy;* ^*3*^ *University of Verona, Verona, Italy;* ^*4*^ *Neuroimaging Research Unit, Division of Neuroscience, and Neurology Unit, IRCCS San Raffaele Scientific Institute, Milan, Italy; and Neurotech Hub, Vita‐Salute San Raffaele University, Milan, Italy;* ^*5*^ *Azienda Ospedaliera Universitaria Integrata Verona, Verona, Italy;* ^*6*^ *University of Salerno, Salerno, Italy;* ^*7*^ *Neurological Clinic, AOU San Giovanni di Dio e Ruggi d’Aragona, Salerno, Italy;* ^*8*^ *Department of Neuroradiology, AOU San Giovanni di Dio e Ruggi d’Aragona, Salerno, Italy;* ^*9*^ *Neurological Clinic, AOU San Giovanni di Dio e Ruggi d’Aragona, Salerno, Italy; and Dipartimento di Ingegneria Elettrica e delle Tecnologie dell’Informazione, Università degli Studi di Napoli Federico II, Naples, Italy;* ^*10*^ *Neuroimaging Research Unit, Division of Neuroscience, Neurology Unit, Neurorehabilitation Unit, and Neurophysiology Service, IRCCS San Raffaele Scientific Institute, Milan, Italy; and Neurotech Hub, Vita‐Salute San Raffaele University, Milan, Italy*

**Background and aims:** Preliminary evidence in functional movement disorders (FMD) suggested that symptoms might result from faulty neural processing. This study aimed at investigating alterations in resting‐state brain functional connectivity (FC) and deep gray matter volume in patients with FMD.

**Methods:** Sixty patients with FMD and 65 age‐ and sex‐matched healthy controls underwent brain MRI. We assessed the FC within the main resting‐state networks using independent component analysis (MELODIC), cerebellar volumes using SUIT, and deep gray matter (GM) volumes using FMRIB's Integrated Registration and Segmentation Tool and FreeSurfer.

**Results:** FMD patients relative to healthy subjects exhibited an increased FC of bilateral caudate, and right putamen/pallidum, insula, inferior/superior frontal gyrus and olfactory gyrus within the basal ganglia network; an increased FC of bilateral precuneus and left cuneus within the ventral default‐mode network (DMN); an increased FC of right middle cingulum within the precuneus network; and an increased FC of left cerebellum crus I‐II within the primary visual network. Moreover, FMD patients showed increased volume of vermis crus II, VIIIa/b, and left lobules VIIIa/b. The altered FC within the basal ganglia and ventral DMN correlated with worse alexithymia, depression and fatigue in FMD patients.

**Conclusion:** The observed alterations of cerebellar volumes and resting‐state FC may represent a mismatch between sensorimotor, emotional, and cognitive systems, leading to impaired self‐awareness and motor intention, heightened attention to bodily signals, and compromised voluntary movement control mechanisms. These results support the neural basis of FMD, confirming that distinct mechanisms underlie the symptoms of this complex disorder.

**Disclosure:** Funded by the European Union ‐ Next Generation EU ‐ NRRP M6C2 ‐ Investment 2.1 Enhancement and strengthening of biomedical research in the NHS (PNRR‐MAD‐2022‐12376826) A Gardoni, E Sibilla, C Tripodi, A Sandri, I Di Vico, M Fiorio, G Pedrotti, A Paolicelli, S Cuoco, I Carotenuto, A Botto, M Amboni, M Russo, G Mansueto, FB Pizzini, M Barillari, MF Lauriola, M Tozzi, F Rusciano, C Geroin, M Fasoli, A Marotta, E Pizzolla, F Salaorni, I Lozzi, F Bombieri, GM Squintani, S Tamburin, F Paio, P Barone, R Erro, G De Biasi, and G Piscosquito declare no financial competing interests.. E Sarasso, S Basaia, E Canu, S Mariotto, and M Tinazzi received research support from the Italian Ministry of Health. M Gandolfi received research support from Fondazione Italiana Sclerosi Multipla. MT Pellecchia received research support from the Italian Ministry of Health and the Italian Ministry of University. C Vinciguerra received honoraria for speakers, manuscript writing, and educational events from Alexion, UCB, ArgenX. M Filippi is Editor‐in‐Chief of the Journal of Neurology, Associate Editor of Human Brain Mapping, Neurological Sciences, and Radiology; received compensation for consulting services from Almirall, Biogen, Bristol‐Myers Squibb, Eli Lilly, Merck, Novartis, Roche, Sanofi; speaking activities from Amgen, Bayer, Biogen, Bristol‐Myers Squibb, Celgene, Chiesi Italia SpA, Eisai, Eli Lilly, Fujirebio, Genzyme, Janssen, Merck, Neopharmed Gentili, Neuraxpharm, Novartis, Novo Nordisk, Roche, Sanofi, Takeda; participation in Advisory Boards for Alexion, Biogen, Bristol‐Myers Squibb, Eli Lilly, GE Healthcare Ltd, Merck, Neuraxpharm, Novartis, Roche, Sandoz, Sanofi, Takeda; scientific direction of educational events for Biogen, Merck, Roche, Celgene, Bristol‐Myers Squibb, Lilly, Novartis, Sanofi‐Genzyme; he receives research support from Biogen Idec, Merck‐Serono, Novartis, Roche, the Italian Ministry of Health, the Italian Ministry of University and Research, and Fondazione Italiana Sclerosi Multipla. F Agosta is Associate Editor of NeuroImage: Clinical, has received speaker honoraria from Biogen Idec, Italfarmaco, Roche, Zambon, Eli Lilly, GE Healthcare and Bristol Myers Squibb, and receives or has received research supports from the Italian Ministry of Health, the Italian Ministry of University and Research, AriSLA (Fondazione Italiana di Ricerca per la SLA), the European Research Council, the EU Joint Programme—Neurodegenerative Disease Research (JPND) and Foundation Research on Alzheimer Disease (France).

## EPO‐0539

### Improvements in ultra‐low field portable MRI and applications for Multiple Sclerosis and Stroke

#### A. Traboulsee ^1^; S. Balaji^2^; A. Rosati^2^; N. Wiley^3^; M. Sekhon^4^; L. Mechtler^5^; W. Kimberly^6^; A. Sorby‐Adams^7^; E. Knopp^8^; S. Williams^9^; S. Deoni^10^; S. Kolind^1^

##### ^*1*^ *Neurology, University of British Columbia, Vancouver, Canada;* ^*2*^ *Physics, University of British Columbia, Vancouver, Canada;* ^*3*^ *Radiology, University of British Columbia, Vancouver, Canada;* ^*4*^ *Critical Care Medicine, University of British Columbia, Vancouver, Canada;* ^*5*^ *Neurology, Dent Neurologic Institute, Amherst, USA;* ^*6*^ *Neurocritical Care, Harvard Medical School, Cambridge, USA;* ^*7*^ *Neurology, Harvard Medical School, Cambridge, USA;* ^*8*^ *Hyperfine Inc, Guilford, USA;* ^*9*^ *Neuroimaging, Institute of Psychiatry, Psychology & Neuroscience, King's College, London, UK;* ^*10*^ *GE Division, Gates Foundation, Seattle, USA*

**Background and aims:** Ultra‐low field MRI (0.064T) is a point of care, portable imaging tool that has a small physical foot print, runs off of a standard electric outlet, does not require specialized shielding, and a low technician operating burden. It could improve access to brain imaging in resource limited or remote locations and is ideal for patients who can’t tolerate a conventional MRI study. Technical advances in image reconstruction have improved image quality and user satisfaction. AIM: demonstrate improvements in scan‐rescan reproducibility and illustrative case examples of lesion detection in MS and Stroke.

**Methods:** Healthy volunteers were scanned multiple times on Hyperfine Inc's portable Swoop 64mT MRI scanner before and after software upgrade. Image acquisition included Fluid Attenuated Inversion Recovery (FLAIR, thickness = 5mm; before: 7.5min, in‐plane resolution = 1.7mm; after: 7min, in‐plane resolution = 0.85mm) and T2‐weighted (thickness = 5mm; before: 5.5min, in‐plane resolution = 1.5mm; after: 6min, in‐plane resolution = 0.65mm) sequences. The Structural Similarity Index Measure (SSIM), measuring image contrast, luminance and structure, was used to assess reproducibility of scans before and after this software upgrade.

**Figure:**
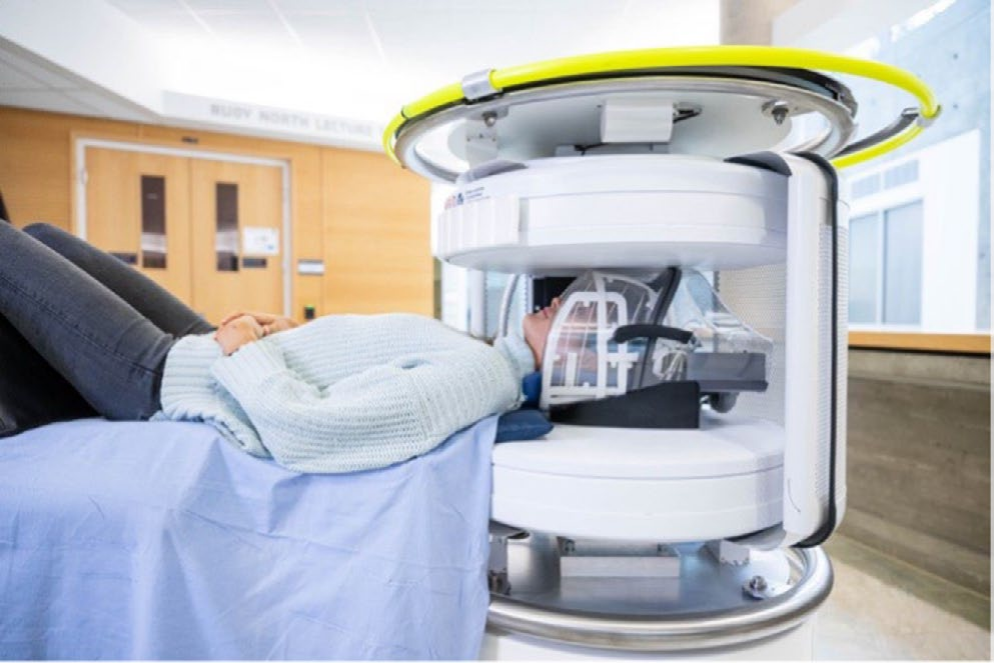
Hyperfine Swoop Ultra Low Field 0.064T MRI.

**Results:** The average SSIM scores before and after upgrade increased (T2w: 0.958 to 0.962, FLAIR: 0.931 to 0.943), showing improved reproducibility. Qualitatively, both periventricular and juxtacortical demyelinating white matter lesions could be identified, with further improvements seen in identifying infratentorial lesions.

**Figure:**
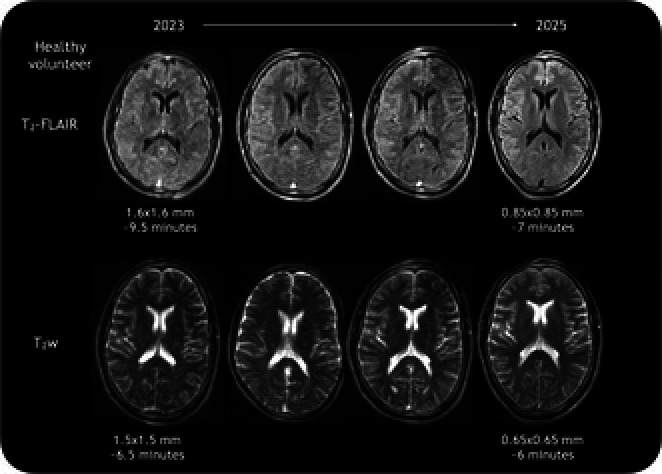
Sequential improvements in image quality with subsequent software versions.

**Figure:**
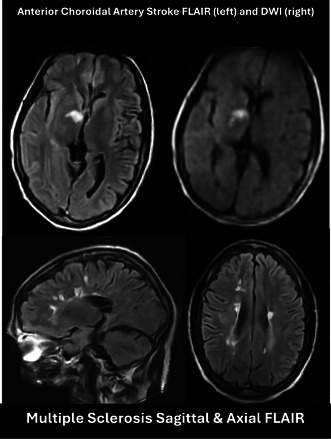
Illustrative example of stroke and MS lesions visible at 0.064T Ultra Low Field MRI.

**Conclusion:** Image quality acquired on an ultra‐low field portable MRI scanner has improved with subsequent iterations of scanner software. Potential applications include monitoring of multiple sclerosis patients on treatment, rapid relapse assessment, assessing MS patients with poor mobility, rapid assessment of stroke, and neurocritical care.

**Disclosure:** E Knopp is an employee of Hyperfine Inc.

## EPO‐0540

### Olfactory Identification and Structural MRI Correlates Across cognitively unimpaired, Mild Cognitive Impairment, and Parkinson's Disease

#### P. Giriprakash^1^; J. Caldwell^2^; R. Doty^3^; D. Cordes^1^; F. Cieri ^1^

##### ^*1*^ *Department of Neurology, Cleveland Clinic Lou Ruvo Center for Brain Health, Las Vegas, USA;* ^*2*^ *University of Wisconsin–Madison, Madison, WI, USA;* ^*3*^ *Smell and Taste Center, Department of Otorhinolaryngology, Head and Neck Surgery, Perelman School of Medicine, University of Pennsylvania, Philadelphia, USA*

**Background and aims:** Olfactory dysfunction is among the earliest and most prevalent clinical features of several neurodegenerative disorders, including Alzheimer's disease and Parkinson's disease. Despite its diagnostic and prognostic relevance, the neuroanatomical substrates linking hyposmia to cognitive syndromes across aging and neurodegeneration remain undefined.

**Methods:** Seventy‐four participants divided in cognitively unimpaired (CU), mild cognitive impairment (MCI), and Parkinson's disease‐cognitive impairment (PD‐CogImp), completed the Brief Smell Identification Test (B‐SIT) and underwent high‐resolution structural MRI. Cortical thickness (CT) measure was obtained using FreeSurfer. Partial correlation analyses assessed associations between olfactory identification and CT across 34 bilateral cortical regions, controlling for age, sex, education, and APOE ε4 carrier status. Analyses were conducted within diagnostic groups to characterize syndrome‐specific structure–olfaction relationships.

**Results:** Olfactory identification differed across groups, with lowest B‐SIT scores in PD‐CogImp, followed by MCI and CU. In CU individuals, higher performance was associated with greater CT, predominantly right‐hemispheric, including the lateral orbitofrontal cortex (*p* = 0.04), cuneus (*p* = 0.04), pericalcarine cortex (*p* = 0.01), and insula (*p* = 0.04). Trend‐level associations were observed in left cuneus (ρ = 0.54), pericalcarine cortex (ρ = 0.53), and rostral middle frontal cortex (ρ = 0.53). MCI showed no significant associations (ρ range = −0.29 to 0.27; all *p* ≥ 0.35). PD‐CogImp showed absent or inverse structure–olfaction relationships in posterior and associative cortices, consistent with syndrome‐specific neuroanatomical disruption.

**Conclusion:** Olfactory identification shows syndrome‐specific associations with cortical thickness across CU aging, MCI, and PD‐CogImp, supporting its utility for differential diagnosis and neurobiological characterization of neurodegenerative disease.

**Disclosure:** Richard Doty is president and major shareholder of Sensonics International, the manufacturer of the olfactory test used in this study. All the other authors have no competing interests and nothing to disclose.

## EPO‐0541

### Intra‐ and Inter‐Hemispheric Connectivity and Clinical Worsening in Multiple Sclerosis

#### F. Romanò^1^; S. Zucchelli^1^; E. Pagani^1^; P. Valsasina^1^; P. Preziosa ^2^; M. Rocca^2^; M. Filippi^3^

##### ^*1*^ *Neuroimaging Research Unit, Division of Neuroscience, IRCCS San Raffaele Scientific Institute, Milan, Italy;* ^*2*^ *Neuroimaging Research Unit, Division of Neuroscience, and Neurology Unit, IRCCS San Raffaele Scientific Institute, Milan, Italy; and Vita‐Salute San Raffaele University, Milan, Italy;* ^*3*^ *Neuroimaging Research Unit, Division of Neuroscience, Neurology Unit, Neurorehabilitation Unit, and Neurophysiology Service, IRCCS San Raffaele Scientific Institute, Milan, Italy; and Vita‐Salute San Raffaele University, Milan, Italy*

**Background and aims:** Brain connectivity is an important determinant of disability in multiple sclerosis (MS). It varies across the disease course and can lead to “network collapse”, causing clinical deficits. We investigated how changes in short‐ and long‐range functional and structural connectivities within the sensorimotor network (SMN) contribute to disability and physical performance worsening over one year in MS patients.

**Methods:** We studied 100 MS patients and 89 healthy controls (HC), who underwent 3.0‐Tesla MRI and clinical evaluation, including Nine‐Hole Peg Test (9‐HPT), Timed 25‐Foot Walk Test (T25FWT), and Expanded Disability Status Scale (EDSS). MS patients were reassessed clinically after one year. Clinical worsening was defined using the EDSS‐Plus (EDSS progression or ≥20% deterioration in 9‐HPT or T25FWT). Group comparisons between HC and MS, and between stable/worsened MS patients, evaluated functional connectivity (FC) and structural connectivity (SC) of the SMN at intra‐ and inter‐hemispheric levels.

**Results:** Compared with HC, MS patients had worse baseline motor performance (*p* ≤ 0.001) and presented low disability levels (median EDSS = 1.5). They showed reduced inter‐hemispheric SMN SC (*p* ≤ 0.001), while other connectivity measures did not differ. After one year, 36 patients were classified as worsened by EDSS‐Plus. No baseline demographic or clinical differences emerged between stable and worsened patients. Worsened patients showed higher intra‐hemispheric SMN SC at baseline (*p* = 0.013), which significantly decreased at follow‐up (*p* = 0.006). No differences were observed in other SMN connectivity measures.

**Conclusion:** SMN connectivity evolves nonlinearly in MS. Inter‐hemispheric SC disruption appears early, while increased intra‐hemispheric connectivity may reflect compensatory reorganization and is associated with subsequent clinical worsening.

**Disclosure:** F Romanò, S Zucchelli, E Pagani, P Valsasina have nothing to disclose. P. Preziosa received speaker honoraria from Roche, Biogen, Novartis, Merck, Bristol Myers Squibb, Genzyme, Horizon and Sanofi. M.A. Rocca received consulting fees from Biogen, Bristol Myers Squibb, Roche; and speaker honoraria from Alexion, Biogen, Bristol Myers Squibb, Celgene, Horizon Therapeutics Italy, Merck Serono SpA, Mitsubishi‐Tanabe Pharma, Neuraxpharm, Novartis, Roche, Sandoz, and Sanofi. She receives research support from the MS Society of Canada, the Italian Ministry of Health, the Italian Ministry of University and Research, and FISM. She is Associate Editor for Multiple Sclerosis and Related Disorders; and Associate Co‐Editor for Europe and Africa for Multiple Sclerosis Journal. M. Filippi is Editor‐in‐Chief of the Journal of Neurology, Associate Editor of Human Brain Mapping, Neurological Sciences, and Radiology; received compensation for consulting services from Almirall, Biogen, Bristol‐Myers Squibb, Eli Lilly, Merck, Novartis, Roche, Sanofi; speaking activities from Amgen, Bayer, Biogen, Bristol‐Myers Squibb, Celgene, Chiesi Italia SpA, Eisai, Eli Lilly, Fujirebio, Genzyme, Janssen, Merck, Neopharmed Gentili, Neuraxpharm, Novartis, Novo Nordisk, Roche, Sanofi, Takeda; participation in Advisory Boards for Alexion, Biogen, Bristol‐Myers Squibb, Eli Lilly, GE Healthcare Ltd, Merck, Neuraxpharm, Novartis, Roche, Sandoz, Sanofi, Takeda; scientific direction of educational events for Biogen, Merck, Roche, Celgene, Bristol‐Myers Squibb, Lilly, Novartis, Sanofi‐Genzyme; he receives research support from Biogen Idec, Merck‐Serono, Novartis, Roche, the Italian Ministry of Health, the Italian Ministry of University and Research, and FISM.

## EPO‐0542

### Clinical relevance of baseline brain MRI‐derived risk groups for accelerated disability progression in Multiple Sclerosis

#### A. Liseune^1^; D. Sima^1^; T. Billiet^1^; T. Phan^1^; V. Anania^1^; R. Khan^1^; C. Lukas^2^; R. Schneider^2^; B. Bellenberg^2^; A. Roenneke^2^; F. Paul^3^; L. Anderhalten^3^; I. Meňkyová^4^; J. Krásenský^5^; D. Horáková^4^; M. Vaněčková ^5^

##### ^*1*^ *icometrix, Leuven, Belgium;* ^*2*^ *Ruhr University Bochum, St. Josef Hospital, Institute of Neuroradiology, Bochum, Germany;* ^*3*^ *Ruhr University Bochum, St. Josef Hospital, Department of Neurology, Bochum, Germany;* ^*4*^ *Department of Neurology, Charité ‐ Universitätsmedizin Berlin, Berlin, Germany;* ^*5*^ *Department of Neurology and Centre of Clinical Neuroscience, First Faculty of Medicine, Charles University in Prague and General University Hospital, Prague, Czech Republic*

**Background and aims:** MRI metrics offer potential for assessing disease progression in patients with Multiple Sclerosis (pwMS). We aim to validate whether baseline MRI metrics can estimate the risk of faster progression.

**Methods:** The study utilized a subset of the RECLAIM dataset. A parametric Weibull survival model was trained to predict time‐to‐event for reaching Expanded Disability Status Scale (EDSS) ≥ 3 on 1002 pwMS with baseline EDSS < 3. Features included 29 MRI‐based metrics computed by icobrain, age, and sex. Model's risk predictions were used to stratify an independent test set (329 patients) into low‐risk group (LRG; *n* = 294) and high‐risk group (HRG; *n* = 35). We assessed the risk group characteristics in terms of EDSS, brain and lesion volumes.

**Results:** The MRI‐based risk clustering showed statistically significant faster disease progression of HRG compared with LRG (*p* = 0.0006) over a 15 year follow‐up (Figures 1 and 2). HRG showed significantly lower brain tissue volumes (1467.06 ± 65.98 ml vs. 1555.71 ± 51.45 ml in LRG) and higher lesion volumes (19.55 ± 10.94 ml vs. 3.41 ± 3.36 ml in LRG) at baseline, suggesting a link between brain atrophy, lesion burden, and a higher risk for conversion to EDSS ≥ 3 (Figure 3).

**Figure:**
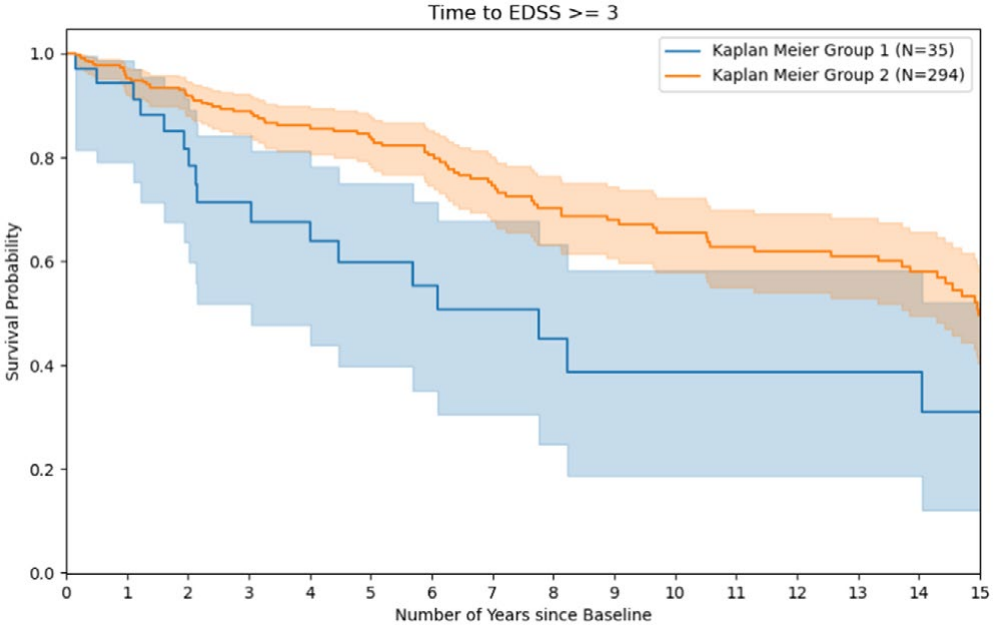
**FIGURE 1** Faster progression represented by an accelerated decrease of the survival probability (Kaplan‐Meier curve) over a 15‐year follow‐up in the high‐risk group (Group 1 in blue) in comparison with the low‐risk group (Group 2 in orange).

**Figure:**
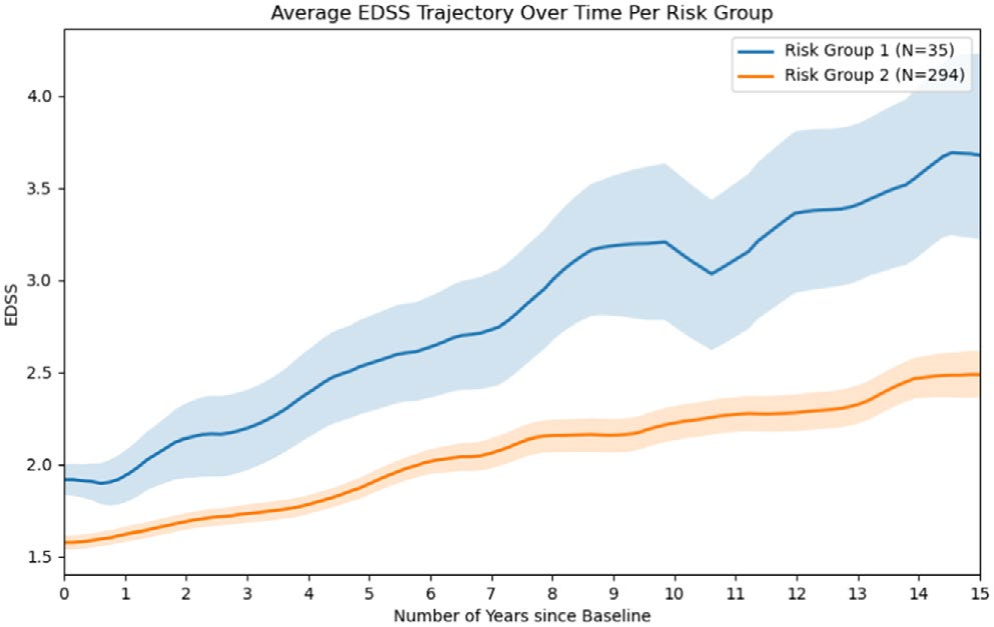
**FIGURE 2** Faster progression represented by accelerated increase of disability (EDSS trajectory) over a 15‐year follow‐up in the high‐risk group (Group 1 in blue) in comparison with the low‐risk group (Group 2 in orange).

**Figure:**
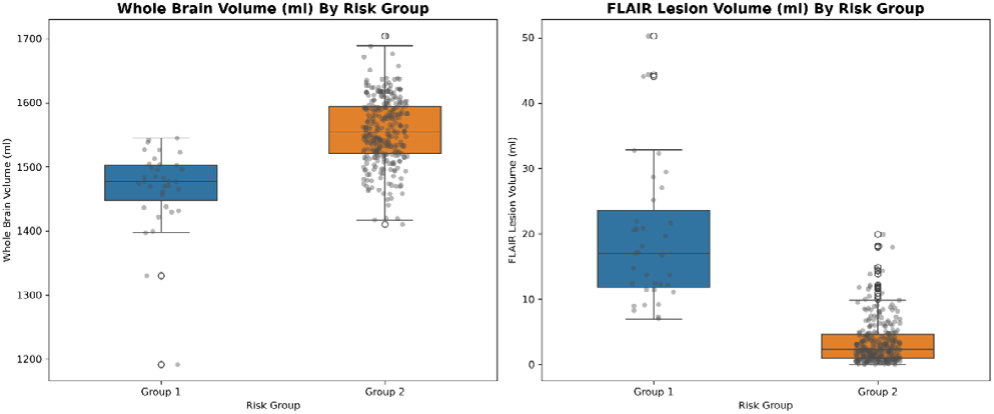
**FIGURE 3** Boxplot showing the distribution of the whole brain volumes normalized for head size (left) and FLAIR lesion volumes (right) of the high risk group (Group 1 in blue) and the low‐risk group (Group 2 in orange).

**Conclusion:** These findings support the hypothesis that brain tissue loss and lesion accumulation contribute to an increased risk of disease progression. With further validation, this approach could help clinicians better interpret brain MRI volumetric measurements for making clinical decisions.

**Disclosure:** 1 icometrix, Leuven, Belgium 2 Ruhr University Bochum, St. Josef Hospital, Institute of Neuroradiology, Bochum, Germany 3 Ruhr University Bochum, St. Josef Hospital, Department of Neurology, Bochum, Germany 4 Department of Neurology, Charité ‐ Universitätsmedizin Berlin, Berlin, Germany 5 Department of Neurology and Centre of Clinical Neuroscience, First Faculty of Medicine, Charles University in Prague and General University Hospital, Prague, Czech Republic 6 MR unit, Department of Radiology, First Faculty of Medicine, Charles University and General University Hospital, Prague, Czech Republic

## EPO‐0543

### Superior anatomical fidelity of multi‐shell (MS) vs single‐shell (SS) tractography for deep brain stimulation (DBS) targeting in tremor disorders

#### M. Buhlan ^1^; A. Divanbeighi Zand^2^; R. Mills^1^; A. Dmytriw^1^; J. Eraifej^2^; A. Green^2^; J. Grist^1^

##### ^*1*^ *Oxford Centre for Clinical Magnetic Resonance Research, Medical Sciences Division, University of Oxford, Oxford, UK;* ^*2*^ *Department of Neurosurgery, Oxford University Hospitals NHS Foundation Trust, Oxford, UK*

**Background and aims:** Tremor improvement following DBS is increasingly linked to modulation of the dentatorubrothalamic tract (DRTT). Targeting the DRTT, surgical planning often relies on SS diffusion tractography, which may limit anatomical fidelity in areas with complex fibre architecture. This study compared the reliability and anatomical precision of MS vs SS tractography for DRTT reconstruction in patients with Parkinson's disease, essential tremor and dystonic tremor.

**Methods:** We conducted a unicentral, retrospective analysis of 41 movement disorder patients undergoing DBS using 3‐Tesla SS and MS diffusion MRI. DRTT reconstruction was performed on individual patient data without template registration. Evaluation focused on reliability of DRTT visualisation within the posterior subthalamic area (PSA, **Figure 1**.i) and detection of decussating fibres in the brainstem (**Figure 1**.ii) across streamline thresholds from >150 to >500 streamlines per voxel (spv).

**Figure:**
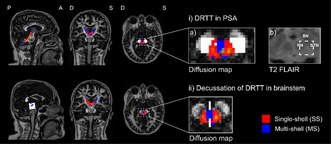
**FIGURE 1**i, SS and MS DRTT projections within the PSA (white box), shown on diffusion image (a) and T2‐FLAIR (b), highlighting the subthalamic nucleus (STN), substantia nigra (SN) and red nucleus (RN). ii, Decussating fibres crossing the brainstem midline.

**Results:** MS tractography consistently demonstrated higher reliability than SS. Bilateral DRTT reconstruction was achieved in all patients using MS data, whereas SS imaging failed to detect the DRTT in 27–29% of cases at >150 and >200 spv (*p* < 0.001). Decussating fibres were identified in 98% of cases using MS compared to 70–73% using SS (*p* < 0.006). These findings were consistent across higher streamline thresholds.

**Conclusion:** Patient‐specific MS diffusion tractography provides superior reliability and anatomical fidelity for DRTT reconstruction compared to SS imaging. Our results support the use of MS reconstructions at lower streamline thresholds to refine DBS targeting which may translate into improved tremor outcomes.

**Disclosure:** Nothing to disclose.

## EPO‐0544

### Lesion Histogram Features for Distinguishing Multiple Sclerosis And Systemic Lupus Erythematosus

#### M. Pawlak ^1^; J. Nikadon^3^; A. Wypych^4^; E. Więsik‐Szewczyk^7^; K. Pawlak‐Buś^6^; P. Leszczynski^6^; S. Michalak^1^; Z. Serafin^8^; W. Kozubski^1^; A. Kalinowska^1^

##### ^*1*^ *Institute of Neurological Disorders, Poznan University of Medical Sciences, Poznan, Poland;* ^*3*^ *Faculty of Philosophy and Social Sciences, Department of Cognitive Science, Faculty of Philosophy and Social Sciences, Interdisciplinary Centre for Modern Technologies, Torun, Poland;* ^*4*^ *Interdisciplinary Centre for Modern Technologies, Torun, Poland;* ^*6*^ *Struś Municipal Hospital, Department of Rheumatology, Systemic Connective Tissue Diseases and Immunotherapy of Rheumatic Diseases, Poznan, Poland;* ^*7*^ *Central Clinical Hospital of the Ministry of National Defense, Military Institute of Medicine, National Research Institute, Department of Internal Medicine, Pneumonology, Allergology and Clinical Immunology, Warsaw, Poland;* ^*8*^ *Bydgoszcz University of Science and Technology, Faculty of Medical Sciences, Bydgoszcz, Poland*

**Background and aims:** Evolution of white matter lesions in the course of autoimmune inflammatory disorders differs depending on severity of demyelination and recovery dynamic. Multiple Sclerosis (MS) and Systemic Lupus Erythematosus (SLE) with neuropsychiatric presentation may be difficult to distinguish in clinical practice. We aimed identify the features of the signal distribution of T1‐weighted and FLAIR sequences in the lesions and the cortex in the MS and SLE populations.

**Methods:** We investigated distributions of regions of interest signal using skewness and kurtosis and creating 2D histograms to differentiate data from 42 MS patients and 35 SLE patients acquired at 3T MR 750 GE scanner. The FLAIR and T1‐weighted images were coregistered, bias field corrected and segmented using SAMSEG pipeline (Puonti 2021). Then extracted skewness and kurtosis using python script and computed diagnostic performance of the receiver operator characteristic curves.

**Results:** The most effective features discriminating systemic lupus erythematosus patients from multiple sclerosis were lesion 2D PCA receiver operator characteristic area under the curve Skewness 0.77 and Kurtosis 0.69. For cortex 2D PCA receiver operator characteristic area under the curve skewness 0.59 and kurtosis 0.51. For each mode separately FLAIR lesion kurtosis AUC is 0.76 and for skewness 0.64; for cortex kurtosis 0.48 and skewness 0.52. For T1‐weighted lesion signal both kurtosis and skewness were 0.62; for the cortex skewness was 0.43 and kurtosis was 0.60.

**Conclusion:** The multimodal 3D MRI acquisitions enables generation of reliable 2D signal histograms characterized by signal features that help distinguish populations of patients with multiple sclerosis and systemic lupus erythematosus.

**Disclosure:** Mikolaj A. Pawlak: Speakers fee from Merck range $500–4900 two years ago. J. Nikadon: nothing to disclose. A.Wypych: nothing to disclose. A. Kalinowska received honoraria and/or consultation fees and/or support for attending meetings and/or travel from: Bayer, Biogen, Bristol Myers Squibb, GSK, Janssen‐Cilag, Merck, Novartis, Roche, Sandoz, Sanofi‐Genzyme.

## EPO‐0545

### Amide Proton Transfer Imaging Detects Hippocampal Proteostasis Disturbance Induced by Simulated Microgravity at 7.0 T MRI

#### S. Liu; R. Wu

##### Department of Radiology, Second Affiliated Hospital, Shantou University Medical College, Shantou, China

**Background and aims:** Many studies have confirmed that spacflight and simulated microgravity can affect learning and memory, resulting in cognitive decline; however, the effects of simulated microgravity on cognitive function remain unclear. The relationship between cognitive decline and proteostasis disorders in the brain under microgravity has not been elucidated. While amide proton transfer (APT) imaging is a chemical exchange saturation transfer technique that utilizes amide protons in proteins and peptides, it can serve as a non‐invasive imaging biomarker for identifying brain proteostasis disorders.

**Methods:** We employ a 14‐day hindlimb unloading (HU) rat model to simulate microgravity conditions and utilize APT imaging on HU Rats and control rats. To explore the mechanisms of microgravity‐induced cognitive impairment in spaceflight through mass spectrometry‐based proteomics analysis combined with hematoxylin and eosin (HE) staining and Nissl staining.

**Results:** Our results show that 14 days of tail suspension can cause disturbed proteostasis, and the brain APT signal increases with increasing tail suspension time, which is significantly higher in HU rats than in normal rats, and this signal showed a negative correlation with the number of viable neurons counted in Nissl staining. Through mass spectrometry‐based proteomics analysis, differentially expressed proteins within the protein homeostasis network pathway were identified in the hippocampi of both normal and HU rats, providing biological evidence for the altered APT signal.

**Conclusion:** This study shows that APT imaging can monitor the disturbance of protein homeostasis in the brain of simulated microgravity rats in vivo and provides more references for the study of cognitive decline in simulated microgravity rats.

**Disclosure:** Nothing to disclose

## EPO‐0546

### Double Dat: Clinical impact of repeated brain dopaminergic neurotransmission nuclear imaging ‐ A retrospective cohort

#### V. Shkatova ^1^; J. Alves^1^; M. Rangel^1^; I. Casimiro^2^; A. Moreira^2^; M. Cunha^2^; C. Machado^1^; D. Reis Carneiro^1^

##### ^*1*^ *Neurology Service, Local Health Unit of Coimbra, Coimbra, Portugal;* ^*2*^ *Nuclear Medicine Service, Local Health Unit of Coimbra, Portugal*

**Background and aims:** The clinical role of repeating dopamine transporter studies remains unclear, particularly in the diagnostic workflow for parkinsonian syndromes. Objectives: To characterize patients undergoing repeated dopaminergic imaging and evaluate its repetition on clinical management.

**Methods:** Retrospective descriptive analysis of patients who underwent multiple dopamine transporter imaging studies between 2007 and 2025 at a Portuguese center.

**Results:** 2930 studies were performed (90.0% DATScan, 10.0% PET‐BCIT) in 2828 patients. Ninety‐seven patients (50.5% male; 49.5% female) underwent repeated scanning (94.8% twice; 5.2% thrice). Median age at first examination was 66.0 years (57.0–72.0). Tremor was the most frequent motor symptom (44.2%), followed by akinesia (27.9%) and gait disturbances (16.3%). Median symptom duration was 4.0 years (1.0–7.5) and median MDS‐UPDRS‐III score was 12.0 (8.0–25.0). DATScan was the first requested exam in 92.8% of cases; 62.0% were initially normal. Median inter‐scan interval was 43.0 months (22.0–72.0). While 65.6% of patients initially evaluated with DATScan repeated it, all PET‐BCIT patients later had DATScan. Imaging results changed in 28.3% of cases (16.3% from “abnormal” to “normal”; 12.0% from “normal” to “abnormal”). Normalization occurred in 78.6% of patients who underwent PET‐BCIT after an initially abnormal DATScan. The second scan led to diagnostic changes in 20.5% and therapeutic modifications in 39.2% of patients.

**Conclusion:** Most patients did not benefit from repeat imaging. High rates of normalization were found in the transition from DATScan to PET‐BCIT.

**Disclosure:** Nothing to disclose.

## EPO‐0547

### Noninvasive Prediction of Ki‐67 expression in gliomas using MRI‐based artificial intelligence: A systematic review and meta‐analysis

#### Y. Faller ^1^; F. Lara‐Freire^2^; G. Itaya^3^; G. de Oliveira^4^

##### ^*1*^ *Faculty of Medicine, University of Buenos Aires (UBA), Buenos Aires, Argentina;* ^*2*^ *Faculty of Medicine, Pontificia Universidad Católica del Ecuador, Quito, Ecuador;* ^*3*^ *ECPE–PPCR Program, Harvard T. H. Chan School of Public Health, Boston, MA, United States;* ^*4*^ *Department of Pathology, Brigham and Women's Hospital, Boston, MA, United States*

**Background and aims:** Gliomas are aggressive central nervous system tumors, and the Ki‐67 proliferation index is a key prognostic marker. However, assessment via immunohistochemistry (IHC) is invasive and subject to sampling bias. Magnetic resonance imaging (MRI)‐based artificial intelligence (AI) has emerged as a promising noninvasive alternative, but its diagnostic performance remains uncertain. This meta‐analysis evaluates the diagnostic accuracy of MRI‐based AI in predicting Ki‐67 status in gliomas.

**Methods:** A systematic search was performed for studies through August 2025. Eligible studies compared MRI‐based AI models with IHC as the reference standard. Pooled sensitivity, specificity, and area under the summary receiver operating characteristic (SROC) curve were estimated using a univariate random‐effects model. Heterogeneity was assessed using the I^2^ statistic. Statistical analyses were performed using RStudio (R version 4.2.3)

**Results:** From 1,704 identified records, eleven studies comprising 893 patients were included. MRI‐based AI models achieved a pooled sensitivity of 0.849 (95% confidence interval (CI): 0.730–0.921) and a pooled specificity of 0.744 (95% CI: 0.636–0.829) for Ki‐67 prediction. The SROC curve yielded an area under the curve (AUC) of 0.82 (95% CI: 0.676–0.872), reflecting good diagnostic accuracy. Heterogeneity was substantial for sensitivity (I^2^ = 77%) and moderate for specificity (I^2^ = 40%). Variability likely stemmed from differences in MRI protocols, AI architectures, and Ki‐67 thresholds across studies.

**Figure:**
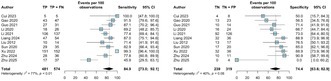
**FIGURE 1** Forest plots of sensitivity (left) and specificity (right) across included studies.

**Figure:**
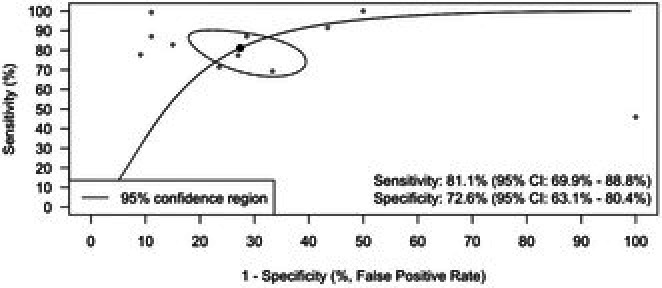
**FIGURE 2** SROC curve derived from bivariate analysis for MRI‐based AI models predicting Ki‐67 expression in gliomas.

**Conclusion:** MRI‐based AI demonstrates good diagnostic accuracy (AUC = 0.82) for noninvasive prediction of Ki‐67 status in gliomas. These findings highlight its potential to support preoperative decision‐making. However, standardized imaging protocols, consistent thresholds, and robust prospective multicenter studies are crucial for clinical implementation

**Disclosure:** Nothing to disclose

## EPO‐0548

### The value of quantitative susceptibility mapping in differential diagnosis of Parkinson's disease and atypical parkinsonian syndrome

#### Y. Li; Q. Ye

##### The Union Hospital Affiliated to Fujian Medical University, Fuzhou, China

**Background and aims:** To assess brain iron deposition features in Parkinson's disease (PD) and atypical parkinsonian syndromes (APS) using Quantitative Susceptibility Mapping (QSM) and evaluate its differential diagnostic value.

**Methods:** This study included 80 patients (with 2‐year follow‐up for diagnosis confirmation) and 33 matched controls. Regional susceptibility values were obtained via manual ROI sketching. Statistical analyses (including logistic regression and ROC curve analysis) identified differential features and evaluated diagnostic performance.

**Figure:**
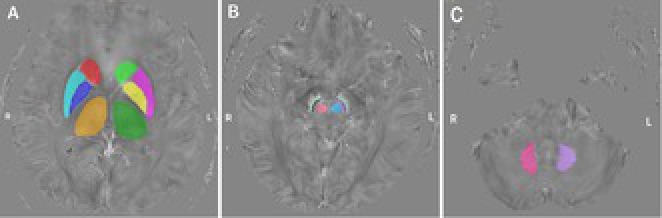
QSM Region of Interest (ROI) Mapping Diagram

**Results:** 1) Specific brain regions showed higher susceptibility in each group versus controls. 2) MSA‐P and PSP groups had higher UPDRS‐III scores and H‐Y stages than PD. 3) Significant inter‐group differences were found in susceptibility of the putamen, globus pallidus, and red nucleus. 4) In PD only, disease severity scores correlated with susceptibility in the putamen and red nucleus. 5) Combined putamen/globus pallidus susceptibility effectively differentiated MSA‐P from PD (AUC = 0.838) and from PSP (AUC = 0.873). Red nucleus susceptibility differentiated PD from PSP (AUC = 0.760).

**Figure:**
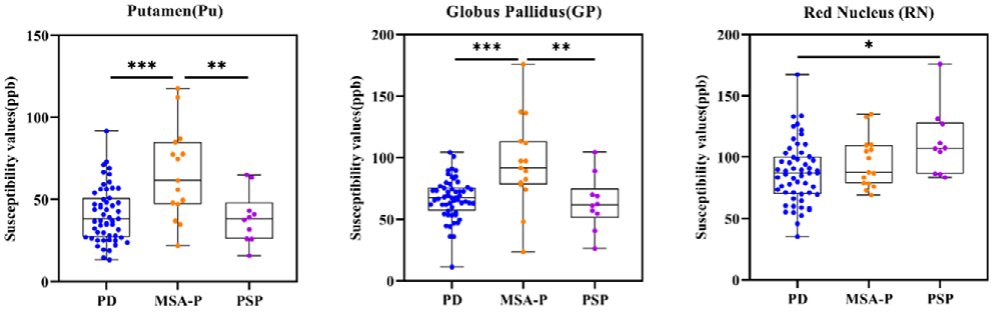
Intergroup Comparison of QSM Susceptibility Values for Different Nuclei.

**Figure:**
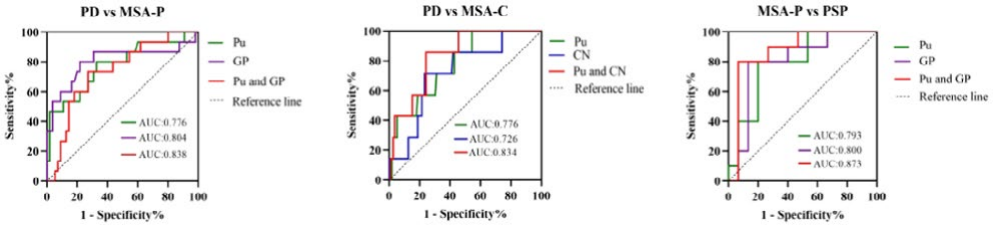
ROC Curve of QSM in the Differential Diagnosis of PD.

**Conclusion:** QSM reveals distinct iron deposition patterns (PD: substantia nigra; MSA‐P: putamen/globus pallidus; PSP: red nucleus). These patterns, along with clinical correlations in PD, demonstrate the utility of QSMfor differential diagnosis among Parkinsonian disorders.

**Disclosure:** Nothing to disclose.

Cognitive neurology/neuropsychology 4

## EPO‐0549

### Neurological Manifestations of Preeclampsia

#### S. Khudayarova; G. Rakhmatullayeva; A. Kadirova

##### Tashkent State Medical University, Tashkent, Uzbekistan

**Background and aims:** Preeclampsia is one of the leading causes of maternal morbidity and is frequently associated with central nervous system involvement, increasing the risk of neurological and cognitive complications.

**Methods:** A total of 120 pregnant women in the third trimester were examined: 80 patients with preeclampsia and 40 with normal pregnancy. All participants underwent clinical and neurological examination, blood pressure assessment, evaluation of proteinuria, and cognitive testing using the Montreal Cognitive Assessment (MoCA). Statistical analysis included Student's t‐test, chi‐square test, and Pearson correlation analysis. Differences were considered statistically significant at *p* < 0.05.

**Results:** Neurological symptoms were observed in 67.5% of patients with preeclampsia compared to 12.5% in the control group (*p* < 0.001). The most common manifestations were headache (58.7%), visual disturbances (32.5%), and dizziness (28.7%). The mean MoCA score was significantly lower in the preeclampsia group (24.1 ± 2.3) than in controls (27.6 ± 1.8, *p* < 0.01). A positive correlation was found between systolic blood pressure and severity of neurological symptoms (*r* = 0.62; *p* < 0.01), while proteinuria showed a negative correlation with cognitive performance (*r* = −0.54; *p* < 0.05).

**Conclusion:** Neurological and cognitive impairments are common in preeclampsia and are significantly associated with disease severity, highlighting the importance of early neurological monitoring in affected patients.

**Disclosure:** Nothing to disclose.

## EPO‐0550

### Long‐term cognitive, emotional and quality‐of‐life outcomes after recovery from prolonged disorders of consciousness

#### D. Driessen^1^; C. Utens ^2^; G. Ribbers^1^; W. van Erp^2^; M. Heijenbrok ‐ Kal^1^

##### ^*1*^ *Rehabilitation Medicine, Erasmus MC, Rotterdam, the Netherlands;* ^*2*^ *Primary Care, Radboudumc, Nijmegen, the Netherlands*

**Background and aims:** Epidemiological studies on prolonged disorders of consciousness (PDOC) usually focus on recovery of consciousness and functional independence. However, whether recovery is meaningful to patients depends on cognitive, emotional and behavioural functioning and health‐related quality of life (HRQoL) as well. We studied these factors in a nationwide PDOC cohort.

**Methods:** Prospective cohort study including PDOC patients ≥16 years admitted to the nationwide Dutch PDOC chain of care (2019–2023) with follow‐up until 2 years post‐admission. Outcomes included cognition (Montreal Cognitive Assessment, MoCA), emotional functioning (Hospital Anxiety and Depression Scale, HADS), neuropsychiatric symptoms (Neuropsychiatric Inventory, NPI), and HRQoL (Quality of Life after Brain Injury questionnaire, QOLIBRI). Longitudinal changes were analysed using generalized estimating equations.

**Results:** For 61 patients, data were available at two‐year follow‐up (mean age 37 years; 68% traumatic aetiology). Cognitive functioning improved significantly over time, but remained impaired in most patients: only 11% achieved a normal MoCA score at two years. Anxiety and depression decreased over time, yet persisted in a substantial proportion of patients who could complete questionnaires (15% and 30%, respectively). Neuropsychiatric symptoms were absent in 67% of patients at two years. Condition‐specific HRQoL was within the normal range in 32% of patients able to complete the QOLIBRI.

**Conclusion:** Patients recovering from PDOC constitute a highly heterogeneous group with wide variability in cognitive, emotional and quality‐of‐life outcomes. While a meaningful recovery is achievable for a subset of patients, persistent cognitive and emotional sequelae are common and should be central in long‐term outcome evaluation and shared decision‐making.

**Disclosure:** Nothing to disclose

## EPO‐0551

### Neuropsychological assessment in obesity: Cognitive vulnerability in patients with Type 2 diabetes mellitus

#### D. Righi; C. Manco; A. Salvemini; G. Iraci Sareri1;; A. Bufano; N. Beneati; A. Sagnella; F. Maino; A. Cartocci; A. Tirone; C. Voglino; S. Pisirinu; N. De Stefano; M. Castagna; D. Plantone

##### Department of Medical, Surgical and Neurological Sciences, University of Siena, Siena, Italy

**Background and aims:** This study aims to assess cognitive functions, mood, and sleep quality in obese patients and evaluate their association with comorbidities, given the elevated risk of neurodegenerative diseases linked to obesity and the limited understanding of these associations

**Methods:** One hundred and one obese patients (80 females) were recruited and underwent a neuropsychological assessment including Montreal Cognitive Assessment (MoCA), a Rey Auditory Verbal Learning Test (RAVLT), the Beck Depression Inventory (BDI‐II), and the Pittsburgh Sleep Quality Index (PSQI).

**Results:** Comorbidities were carefully recorded. A non‐parametric statistical analysis was performed with age and BMI as covariates. The median age of our cohort was 48 years [25th–75th percentile 34–55]; the median body weight was 116 kg (25th–75th percentile 105–129); the median BMI was 43.1 (25th–75th percentile 40–45.7), and 27 patients had type 2 diabetes. The median education level was 12 years, with no difference between diabetic and non‐diabetic obese individuals. Comparing the subcohort of diabetic obese patients with those without diabetes, we found significantly lower scores for MoCA (*p* = 0.012; median value 24 vs 26; 25th–75th percentile 23–25.5 vs 25th–75th percentile 24–28), immediate RAVLT (*p* = 0.005; median value 38 vs 46.5; 25th–75th percentile 33–50 vs 25th–75th percentile 41–53) and delayed recall (*p* < 0.001; median value 7 vs 10; 25th–75th percentile 4.5–9.5 vs 25th–75th percentile 7.25–12). BDI‐II scores correlated with PSQI scores (*r* = 0.54; *p* < 0.001).

**Figure:**
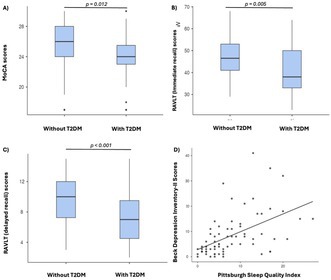
Cognitive and psychological measures in participants with and without type 2 diabetes mellitus (T2DM). (A–C) Boxplots comparing performance on the MoCA (A), RAVLT immediate recall (B), and RAVLT delayed recall (C). Participants with T2DM scored significan.

**Conclusion:** Our ongoing study indicates a potential link between type 2 diabetes mellitus, obesity, and reduced cognitive performance, as evidenced by borderline/impaired MoCA scores.

**Disclosure:** No disclosure to declare.

## EPO‐0552

### Core components of apraxia differentially predict disability after stroke: Evidence from principal component analysis

#### E. Rounis

##### MRC Cognition and Brain Sciences Unit, University of Cambridge

**Background and aims:** Apraxia is a common but heterogeneous higher‐order motor disorder after stroke, affecting 30–50% of survivors and strongly associated with disability. However, the relationship between specific apraxic deficits and functional independence remains unclear. Traditional subtypes (ideational, ideomotor, constructional) may not capture functionally relevant dimensions, and prior studies were limited by small samples and multicollinearity. Here, we identify core apraxia components that independently predict disability in the largest UK post‐stroke cohort comprehensively assessed for apraxia.

**Methods:** Eighty‐five stroke patients (mean age 60.5±11 years; 42% female; median 3.6 years post‐stroke) completed a comprehensive apraxia battery assessing gesture production, object use, imitation, visuospatial processing, biological motion, and executive sequencing. Eleven composite z‐scores underwent principal component analysis with varimax rotation following KNN imputation. Factorability was assessed using KMO and Bartlett's test. Multiple regression examined independent predictors of instrumental activities of daily living (Frenchay Activities Index), comparing rotated and unrotated solutions.

**Results:** Data showed excellent factorability (KMO = 0.807, Bartlett's *p* < 0.001). Varimax rotation revealed three components explaining 62% of variance: (1) Gesture/Praxis (23.2%); (2) Visuospatial/Imitation (21.4%); and (3) Executive/Sequential (17.4%). All three independently predicted iADL performance (Adj‐R^2^ = 0.338, *p* < 0.001), with Gesture/Praxis showing the strongest effect (β = 0.82, *p* < 0. 0001), followed by Visuospatial/Imitation (β = 0.70, *p* < 0.0001) and Executive/Sequential abilities (β = 0.36, *p* = 0.038).

**Conclusion:** Apraxia fractionates into three dissociable components with differential functional impact, challenging unitary deficit models. Gesture production is the strongest determinant of independence, explaining divergent functional outcomes among patients with similar overall severity. These components likely reflect distinct neural mechanisms and argue for targeted, component‐specific rehabilitation strategies.

**Disclosure:** Nothing to disclose

## EPO‐0553

### Metabolic and bioimpedance changes associated with dietary ketosis in early cognitive decline: A pilot study

#### F. Filippi ^1^; S. Tartaglia^2^; I. Cancelli^3^; A. Pittino^1^; C. Del Regno^2^; C. Prezza^1^; N. Ditta^2^; V. Miele^2^; M. Milia^2^; G. Ermanis^1^; G. Gigli^2^; M. Valente^2^

##### ^*1*^ *Department of Medicine (DMED), University of Udine, Udine, Italy;* ^*2*^ *Clinical Neuroloy Unit, Department of Head‐Neck and Neurosciences, Azienda Sanitaria Universitaria Friuli Centrale (ASUFC), Udine, Italy;* ^*3*^ *Neurology Unit, Udine University Hospital, Udine, Italy*

**Background and aims:** Systemic metabolic alterations and chronic low‐grade inflammation are increasingly recognized as contributors to early neurodegenerative processes. Bioelectrical impedance vector analysis (BIVA), particularly phase angle, has emerged as a potential marker of cellular integrity and inflammatory status in neurological disorders.

**Methods:** In this single‐center pilot study, patients aged 60–85 years with Mild Cognitive Impairment (MCI) or Subjective Cognitive Decline (SCD) followed one of three dietary interventions for three months: a modified ketogenic diet, a Mediterranean diet supplemented with medium‐chain triglycerides (MCT), or a standard Mediterranean diet. Anthropometric measures, ketonemia, and BIVA‐derived parameters (fat mass, fat‐free mass, phase angle) were assessed at baseline and follow‐up, alongside clinical neurological evaluation.

**Results:** The ketogenic diet was associated with a significant reduction in body weight and body mass index compared to the Mediterranean diet. Although changes in fat mass, lean mass, and phase angle did not reach statistical significance, patients following the ketogenic intervention showed a consistent favorable trend toward increased phase angle, suggestive of improved cellular integrity. These changes occurred in parallel with modest increases in circulating ketone levels. No adverse effects on lean mass were observed.

**Figure:**
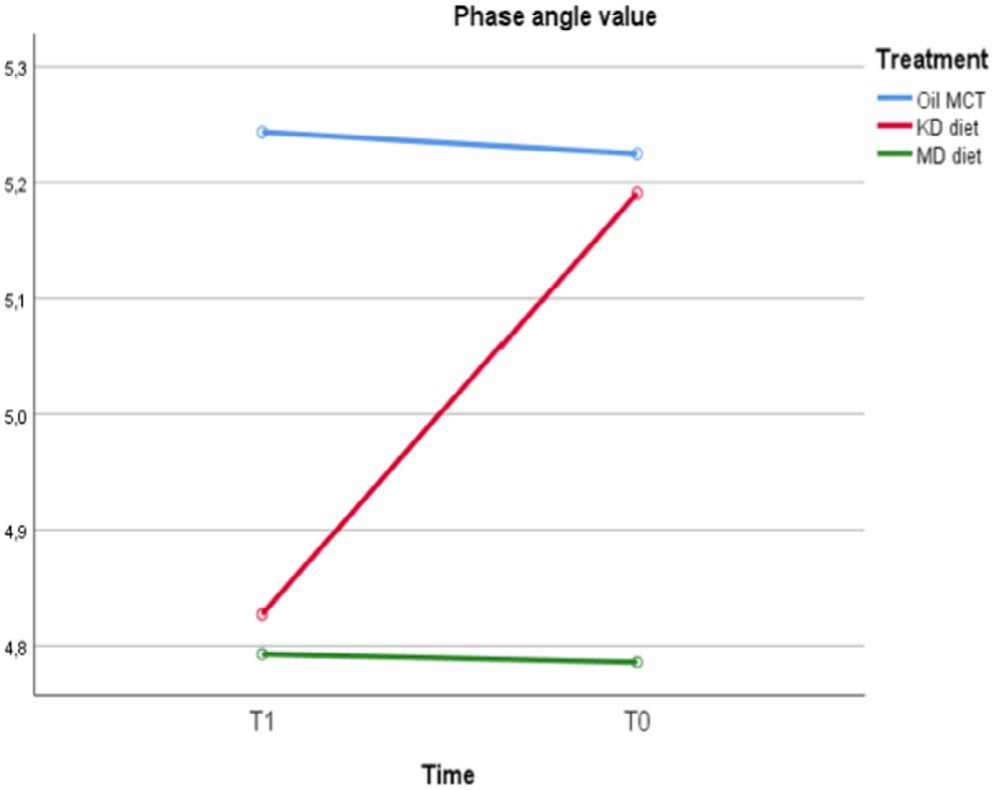
**FIGURE 1**: The graph highlights a metabolic improvement in patients on the ketogenic diet, evidenced by an increase in phase angle, which is indicative of a reduction in systemic inflammatory status.

**Conclusion:** Diet‐induced ketosis was associated with favorable metabolic and bioimpedance trends in patients with early cognitive decline. BIVA‐derived parameters, particularly phase angle, may represent useful adjunctive markers to monitor systemic and potentially neuroinflammatory responses to non‐pharmacological interventions in prodromal cognitive disorders. Larger studies integrating biochemical and neuroinflammatory biomarkers are warranted.

**Disclosure:** Nothing to disclose.

## EPO‐0554

### Assessment of posterior and temporal functions in diverse populations: Systematic review and development of a neuropsychological screening battery

#### G. Sarti ^1^; J. Palisson^1^; K. Benrahmoune Idrissi^1^; S. Franzen^2^; M. Canevelli^3^; L. Merlay^4^; B. Garcin^1^; P. Narme^4^

##### ^*1*^ *Service de Neurologie, Hôpital Avicenne, AP–HP, Bobigny, France**;*** ^*2*^ *Erasmus MC University Medical Center, Rotterdam, Netherlands;* ^*3*^ *Department of Human Neuroscience, Sapienza University, Rome, Italy;* ^*4*^ *Laboratoire Mémoire Cerveau et Cognition, Université Paris Cité, Boulogne‐Billancourt, France*

**Background and aims:** Ongoing demographic changes require a culturally sensitive approach to neuropsychological assessment for diverse populations with cognitive disorders. This review examines tools developed, adapted, or validated to assess posterior and temporal cognitive functions across culturally and educationally diverse groups, as these variables significantly influence outcomes. Based on these findings, we aim to develop an adapted tool targeting these domains.

**Methods:** An electronic search was conducted in PubMed and APA PsycINFO for articles published between 1972 and July 2025, following PRISMA guidelines. A total of 503 studies were screened by title and abstract, and 62 full texts were assessed for eligibility. Each study's characteristics related to tools, populations, sociocultural effects, normative and validation data were documented. Articles focused on the adaptation of cognitive screening instruments were excluded.

**Figure:**
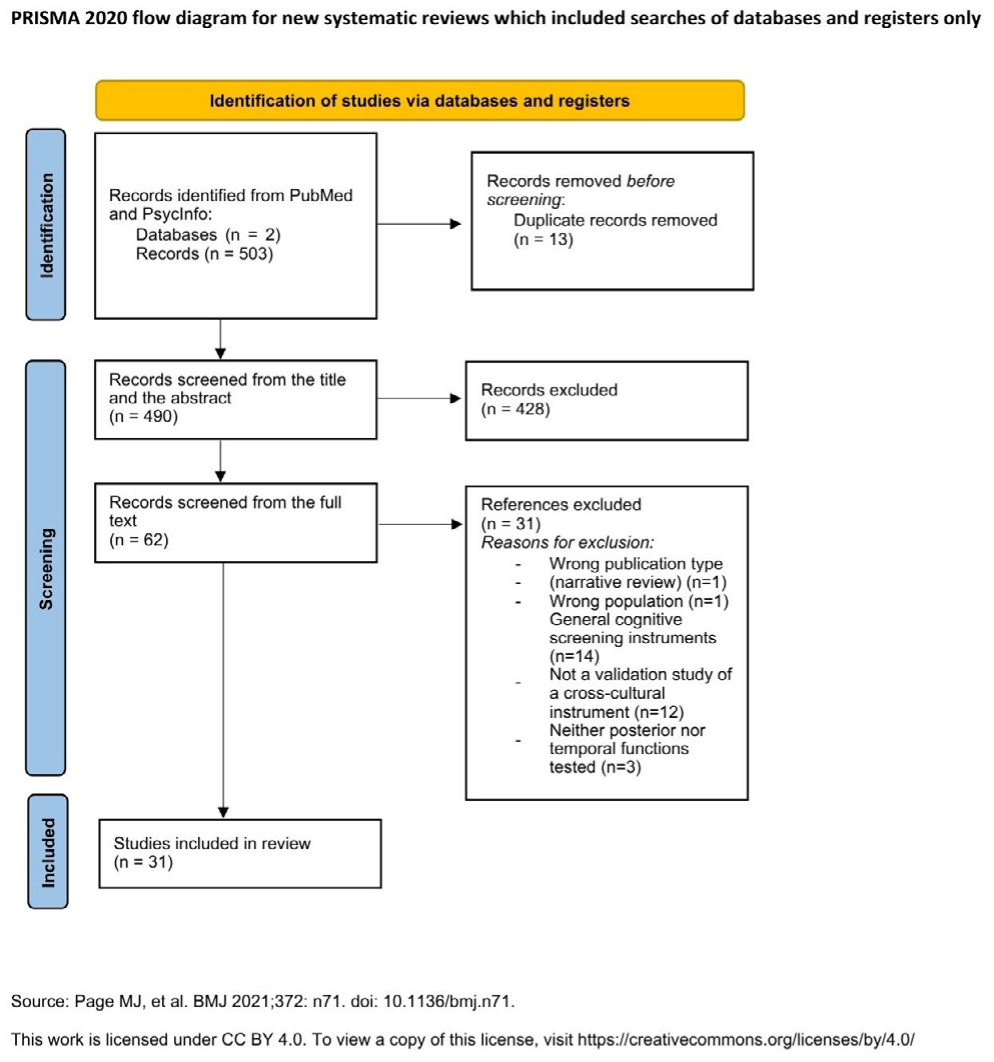
PRISMA 2020 flow diagram.

**Results:** A total of 31 studies were included, featuring 22 assessment tools, mainly adaptations of multidomain cognitive batteries. Only four tools evaluated language functions (FAST, LCT, MINT, C‐CLNT), while two assessed visuospatial functions (Simplified Taylor Complex Figure Test and Visuoconstructional Copy Stimuli). Education influenced performance in 61% of cases, while cultural/linguistic effects were explored in only 29% of studies. Although 97% assessed psychometric properties, only 77% reported satisfactory performance.

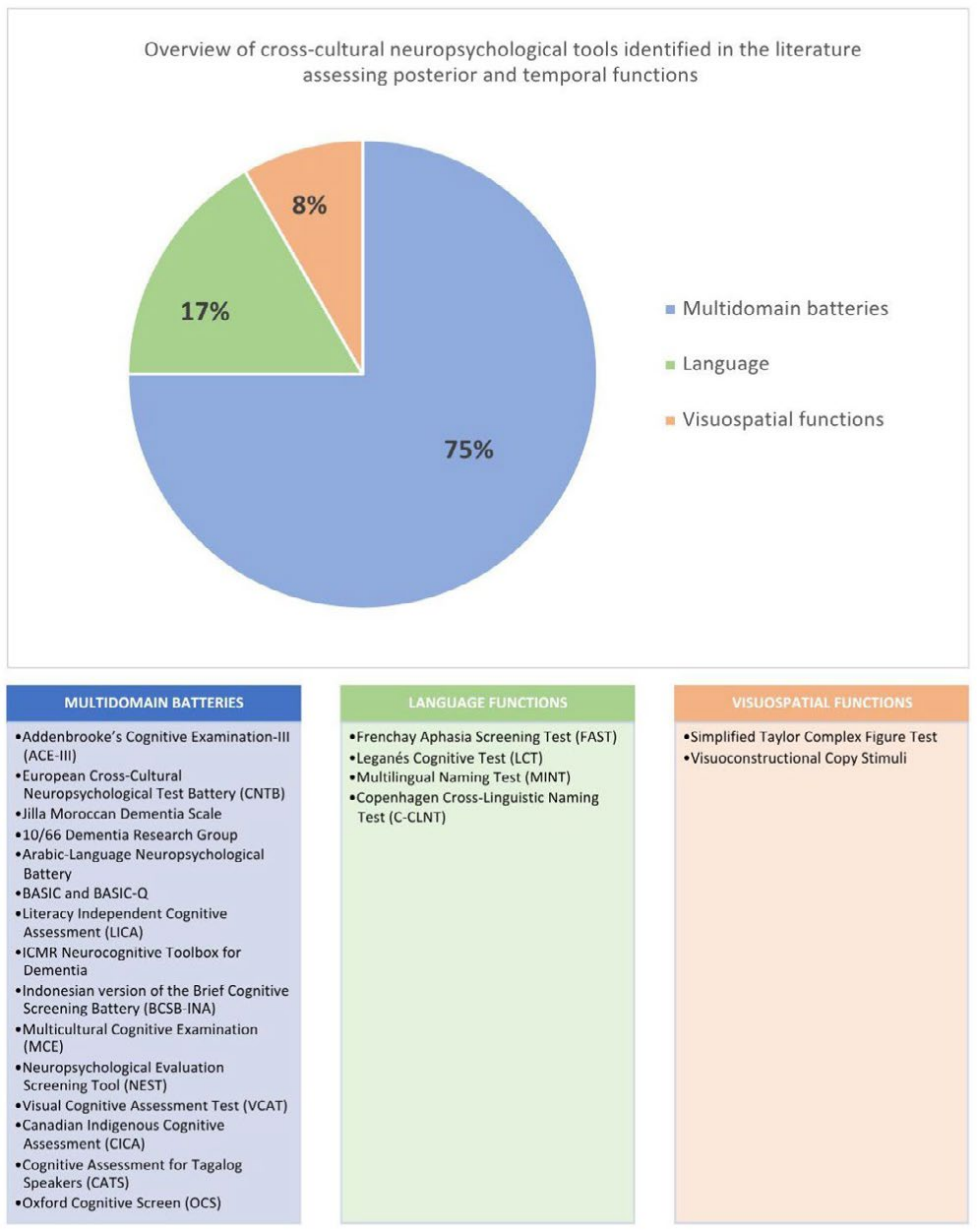

**Conclusion:** This review highlights the impact of education on neuropsychological tools and the lack of culturally validated assessments for posterior and temporal functions. To address these gaps, a new cross‐cultural tool, the TFP‐93, is being developed at Avicenne Hospital (Bobigny, France) to evaluate these functions while reducing biases related to education and culture.

**Disclosure:** Nothing to disclose.

## EPO‐0555

### Insular stroke: Neuropsychological correlates and Functional Connectivity in the subacute and chronic phases

#### G. Patanè ^1^; A. Digiovanni^1^; M. De Angelis^2^; G. Lovero^1^; E. Grande^1^; G. Committeri^1^; M. Caulo^1^; A. Baldassarre^1^; S. Sensi^1^

##### ^*1*^ *Department of Neuroscience, Imaging and Clinical Science, University “G. d’Annunzio” Chieti‐Pescara, Chieti, Italy;* ^*2*^ *Emergency Neurology and Stroke Unit, “SantoSpirito” Hospital, Pescara, Italy*

**Background and aims:** The insular cortex is considered a cerebral hub involved in multiple cognitive domains. Ischemic strokes involving the insular cortex are often clinically underrated, as routine assessments across acute/subacute and chronic phases often fail to capture the multifaceted complexity of this subtype of stroke. This study aims to characterize the neuropsychological and resting‐state Functional Connectivity (rs‐FC) profiles of patients with ischemic stroke involving the insular cortex, evaluated during the subacute and chronic stages.

**Methods:** Fifteen patients with subacute ischemic stroke were enrolled: seven with insular involvement and eight with strokes in other cerebral regions. All participants underwent a comprehensive neuropsychological assessment and resting‐state functional MRI (rs‐fMRI). Six patients with insular stroke completed a follow‐up evaluation at 3–3.5 months.

**Results:** Compared with patients with non‐insular strokes, patients with insular involvement showed significant impairments on the Trail Making Test and phonemic fluency, along with trend‐level reductions on the Reading the Mind in the Eyes Test, the Ekman 36‐item Faces Test, and the Rey–Osterrieth Complex Figure recall. At follow‐up, emotion recognition and verbal fluency improved, whereas attentional/executive functions and visuospatial memory did not show significant recovery. Rs‐FC analysis revealed reduced intra‐network connectivity within the Salience Network and decreased anticorrelation between the Default Mode Network and the Frontoparietal and Dorsal Attention Networks. At follow‐up, inter‐network connectivity involving the Salience and Ventral Attention Networks showed a partial tendency toward normalization.

**Figure:**
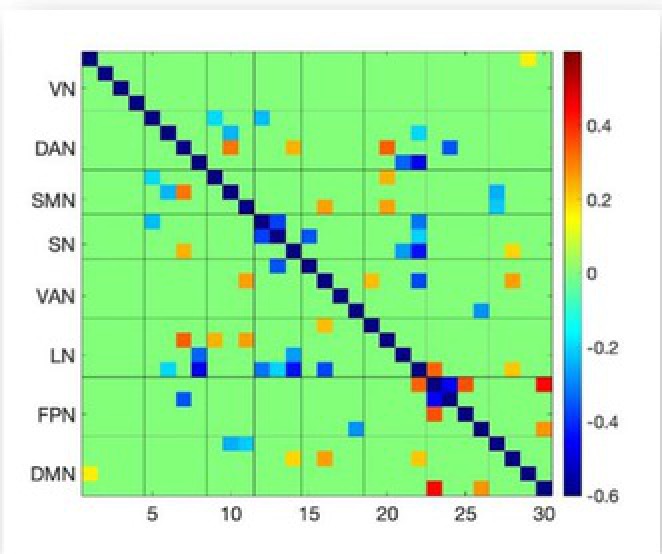
Functional connectivity matrix showing between‐group differences (two‐sample t‐test) between patients with insular stroke and those with non‐insular stroke during the acute phase.

**Conclusion:** Insular stroke is associated with crucial Salience Network dysfunction and related executive–attentional deficits, with only partial recovery during the first months after the acute event.

**Disclosure:** Nothing to disclose.

## EPO‐0556

### Perfusion Abnormality is Associated with Cognitive Impairment in Patients with Chronic Carotid or MCA Artery Occlusion

#### H. Park^1^; J. Cha^2^; J. Park^3^; D. Kim ^4^

##### ^*1*^ *Inha University Hospital, Incheon, Korea, Republic of;* ^*2*^ *Dong‐A University Hospital, Busan, Republic of Korea;* ^*3*^ *Catholic University of Korea Eunpyeong St. Mary's Hospital, Seoul, Republic of Korea;* ^*4*^ *Department of Neurology, Konkuk University Hospital, Seoul, Republic of Korea*

**Background and aims:** Cognitive impairment is well known to be associated with Chronic occlusion of large vessel occlusion like carotid or middle cerebral artery. It is still unclear what factors influence the occurrence of cognitive impairment in these patients. Here, we investiage the radiological and clinical parameters associated with cognitive dysfunction in the patients with chronic occlusion of carotid artery.

**Methods:** From unicentric, prospectively collected stroke registry, we enrolled the patients who had chronic carotid or MCA occlusion and who took MMSE at admission and 2 years later simultaneously. We excluded patients with severe neurologic deficiet or aphasia or the patients with stroke recurrence. We analyzed the results of MMSE, the clinical history, and radiological findings in these patients.

**Results:** Among the patients with chronic carotid or MCA artery occlusion, a total of 105 patients were enrolled. The mean age was 76 ± 15 years and 62 patients were men. During the median follow‐up of 26.8 months, 75 patients showed aggravation of MMSE score. The multivariate analysis demonstrated the old age (75 or older), diabetes mellitus, atrial fibrillation and perfusion abnormality in lesion area. The site of occlusion, Hba1c level, or type of antithrombotic agent was not associated with cognitive deterioration.

**Conclusion:** Cognitive aggravation is common in the patients with chronic occlusion of carotid or MCA artery occlusion. Perfusion status at baseline appears to be a good predictor of cognitive impairment, suggesting that interventions aimed at improving perfusion deficits may have beneficial effects.

**Disclosure:** Nothing to disclose.

## EPO‐0557

### Selective Executive and Attentional Dysfunction in Chronic Obstructive Pulmonary Disease

#### H. Dikbas ^1^; R. Cörüt^2^

##### ^*1*^ *Department of Neurology, Giresun University/Giresun, Turkiye**;*** ^*2*^ *Department of Pulmonary Diseases, Giresun University/Giresun, Turkiye*

**Background and aims:** Chronic obstructive pulmonary disease (COPD) is associated with chronic hypoxia, systemic inflammation, and oxidative stress, mechanisms also implicated in neurodegenerative disorders. While an increased prevalence of dementia has been reported in COPD, data regarding mild cognitive impairment (MCI) and Alzheimer's disease (AD) remain limited.

**Methods:** This cross‐sectional case–control study included 147 participants: 86 patients with COPD and 61 age‐ and sex‐matched controls. Cognitive assessment comprised the Mini‐Mental State Examination (MMSE), Montreal Cognitive Assessment (MoCA), semantic and phonemic fluency tests, clock drawing test, and Stroop test. Clinical characteristics, pulmonary function parameters, oxygen saturation (SpO₂), inflammatory markers, and COPD severity indices were recorded. Group comparisons, correlation analyses, and multivariable path analysis were performed.

**Results:** The prevalence of MCI was higher in patients with COPD compared to controls (30.2% vs. 18.0%), whereas AD prevalence was similar between groups (Figure 1). MMSE and MoCA did not differ significantly. COPD patients demonstrated significantly prolonged Stroop word reading times, indicating impaired executive function and attention (Table‐1). Lower SpO₂ levels and higher dyspnea severity were significantly associated with worse cognitive performance. In multivariable path analysis adjusting for age, sex, smoking status, body mass index, and C‐reactive protein, COPD presence remained independently associated with prolonged Stroop word reading time (β = 0.22, *p* = 0.004), explaining 14.7% of the variance.

**Figure:**
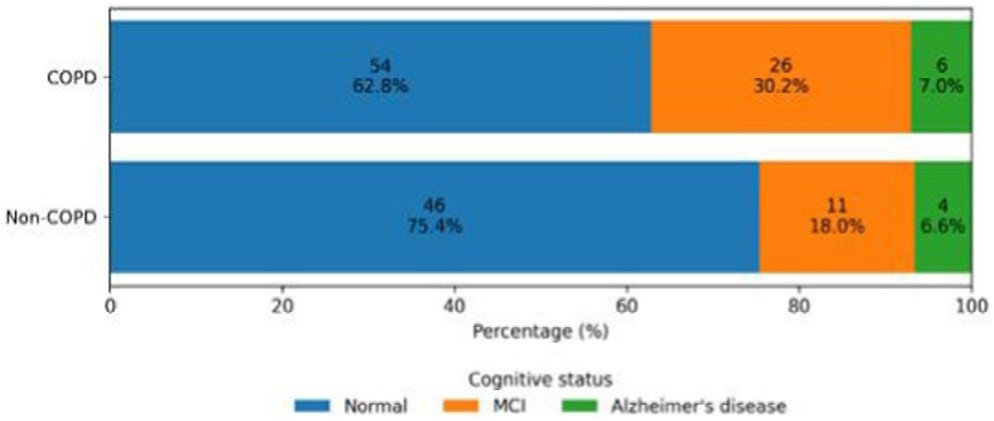
**FIGURE 1** Distribution of cognitive status in individuals with and without COPD.

**Figure:**
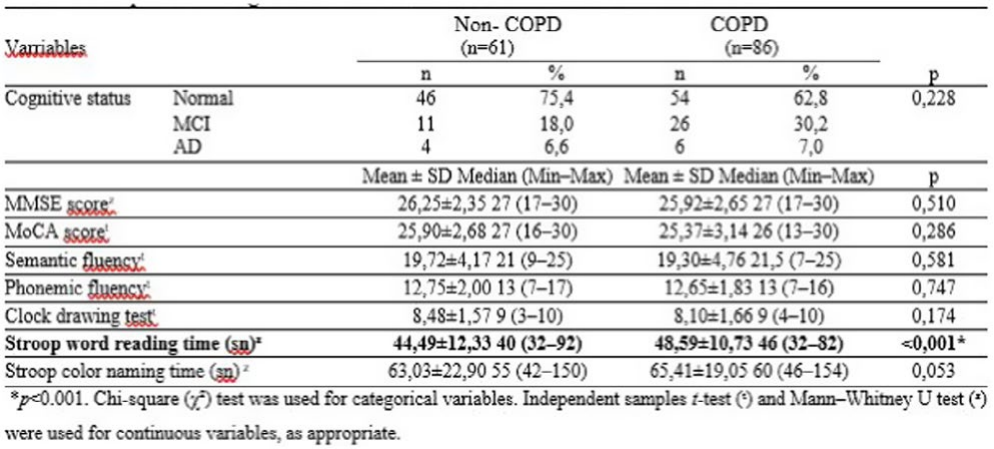
**TABLE 1** comparison of cognitive test results in individuals with and without COPD.

**Conclusion:** COPD is associated with selective cognitive impairment, predominantly affecting executive functions and attention, independent of systemic inflammation. Hypoxemia and dyspnea may play an important role in this association. Patients with COPD may benefit from cognitive screening targeting executive functions.

**Disclosure:** Nothing to disclose.

## EPO‐0558

### Mild cognitive impairment detection using digital speech biomarkers: A systematic review and meta‐analysis

#### M. Rodrigues dos Santos ^1^; G. Portela Macedo^1^; P. Rodrigues Queiroz^1^; J. Alves Santana^1^; C. Aires Brasil^1^; B. Storti^2^; A. Cronemberger Andrade^3^

##### ^*1*^ *Bahiana's School of Medicine and Public Health, Salvador, Brazil;* ^*2*^ *Carlo Besta Neurological Institute, Milan, Italy;* ^*3*^ *Federal University of Bahia, Salvador, Brazil*

**Background and aims:** Alzheimer's disease can begin as mild cognitive impairment (MCI), a prodromal stage that requires sensitive detection. Traditional screening may show insufficient sensitivity and access to neuropsychological testing is limited. As speech problems could precede other clinical symptoms, this study reviews digital/automated speech‐based methods and evaluates their diagnostic performance.

**Methods:** A systematic search was conducted in PubMed, EMBASE, WoS, SCOPUS, IEEE‐Xplore, ACM Digital Library, and Cochrane Central in studies published between 2015 and 2025. Observational studies involving adults with MCI and cognitively unimpaired (CU) controls using automated speech biomarkers and reporting diagnostic accuracy were included. A diagnostic accuracy meta‐analysis was performed using hierarchical models to pool sensitivity, specificity, and summary ROC estimates. Quality and risk of bias were assessed using QUADAS‐2.

**Results:** After duplicate removal, 3,735 records were screened, of which 34 studies met the predefined inclusion criteria; 68% were observational, cross‐sectional studies. Overall, 9,957 participants were analyzed, including 1,852 individuals with MCI and 3375 CU control. Most of the studies employed automated analysis (67.7%) with supervised machine learning and hybrid acoustic‐linguistic biomarkers (41.2%). Diagnostic accuracy demonstrated considerable heterogeneity, with pooled analysis sensitivity and specificity of 0.78 (95% CI = 0.74–0.82) and 0.85 (95% CI = 0.81–0.89), respectively. There was moderate between‐study heterogeneity for sensitivity (σ^2^ = 0.23) and substantial heterogeneity for specificity (σ^2^ = 1.13).

**Figure:**
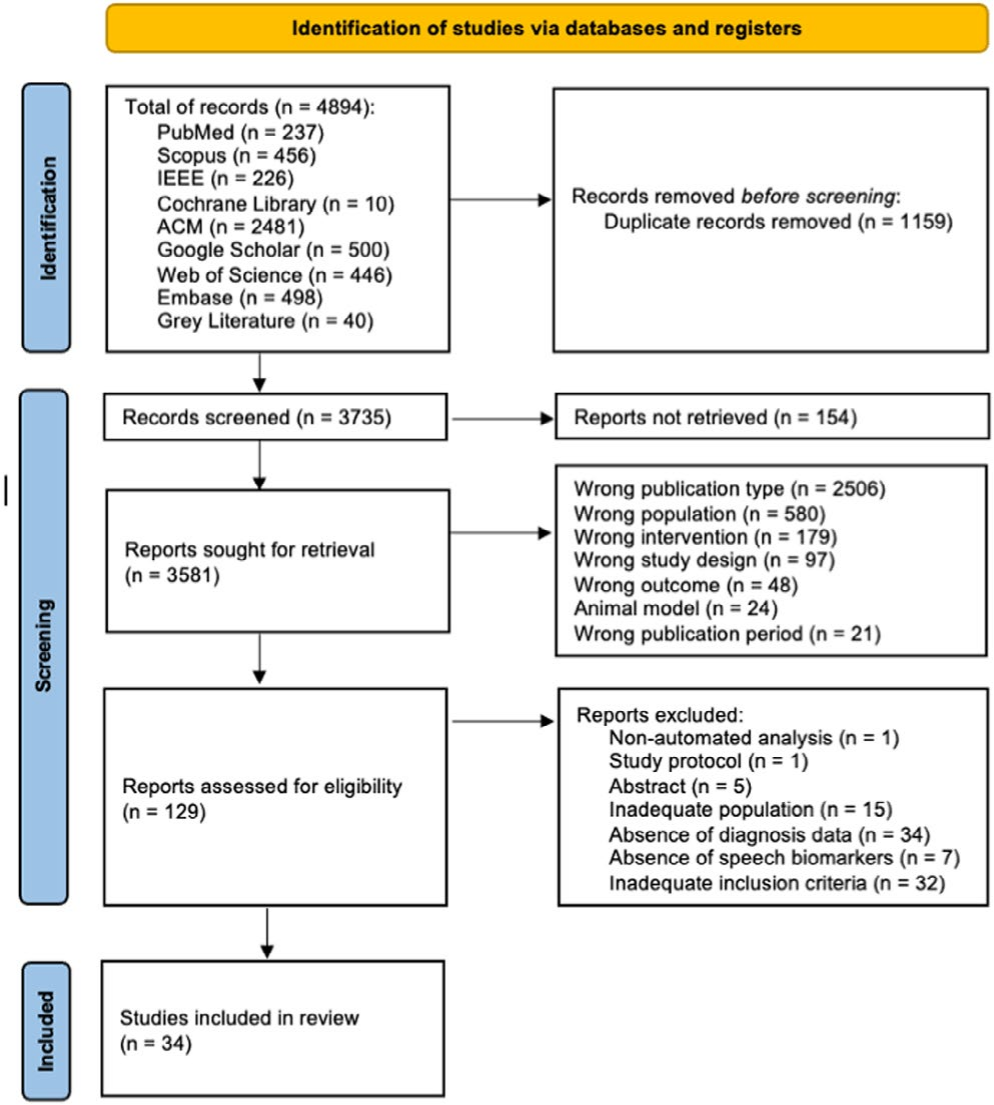
**FIGURE 1** PRISMA 2020 flow diagram.

**Figure:**
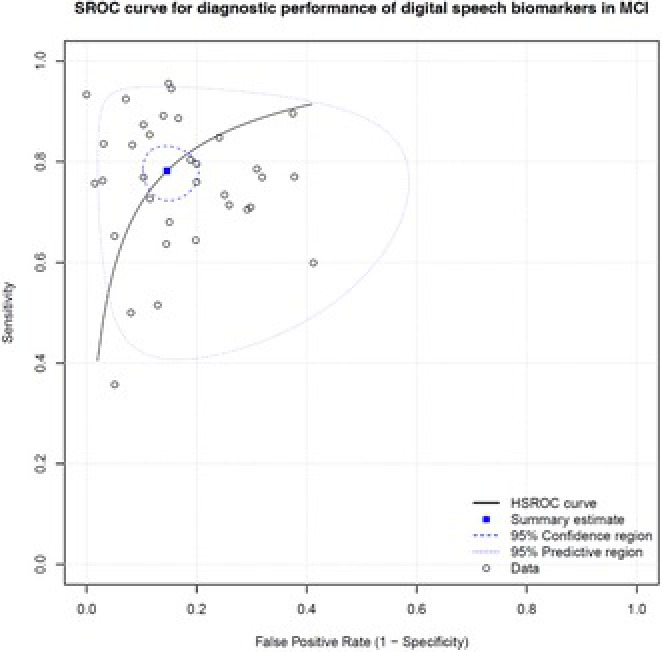
**FIGURE 2** SROC curve for diagnostic performance of digital speech biomarkers in MCI. The positive likelihood ratio was 5.34 (95% CI 4.06–7.02) and the negative likelihood ratio was 0.26 (95% CI 0.21–0.31).

**Conclusion:** Automated speech‐based biomarkers show promising diagnostic performance for MCI, supporting potential integration into dementia screening pathways.

**Disclosure:** Nothing to disclose.

## EPO‐0559

### Cognitive, neuropsychiatric and electrophysiological changes in post COVID‐19 patients ‐ A 6 months trajectory

#### S. Panda ^1^; R. Naik^1^; S. Tiwari^2^; G. Bohra^3^

##### ^*1*^ *Department of Neurology, All India Institute of Medical Sciences, Jodhpur, India;* ^*2*^ *Department of Diagnostic and Interventional Radiology All India Institute of Medical Sciences, Jodhpur, India;* ^*3*^ *Department of Medicine All India Institute of Medical Sciences, Jodhpur, India*

**Background and aims:** Post–Coronavirus disease 2019 (COVID‐19) condition is frequently associated with persistent cognitive decline, neuropsychiatric symptoms, and fatigue. This study aimed to evaluate the trajectory of cognitive function, neuropsychiatric manifestations, and electrophysiological changes in post‐COVID‐19 patients over a 6‐month follow‐up period.

**Methods:** This prospective, hospital‐based observational study included patients with a confirmed history of COVID‐19 infection at least 6 months prior to enrolment. Cognitive function, anxiety, depression, and fatigue were assessed using standardized clinical scales, and P300 event‐related potentials were recorded at baseline and after 6 months.

**Results:** Among 102 patients, cognitive impairment was observed in 38.2% at baseline, predominantly mild in severity. At 6‐month follow‐up, cognitive dysfunction was present in 43.8% of patients. Anxiety (80.4%), depression (69.6%), and fatigue (46.1%) were highly prevalent **Figure 1** Electrophysiologically, prolonged P300 latency was noted in 48% of patients at baseline, increasing to 61.6% at follow‐up (Table 1). Patients with baseline cognitive impairment demonstrated significantly worse frontal lobe function, anxiety, depression, and fatigue scores compared to cognitively preserved individuals (*p* 0.0001, 0.002, 0.003 and 0.043 respectively) (Table 2). Notably, P300 abnormalities were also observed in patients without clinically evident cognitive decline.

**Figure:**
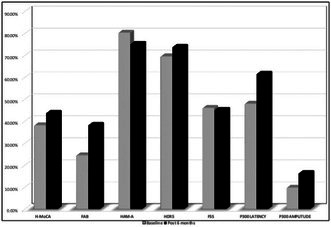
**FIGURE 1**: Bar graph illustrating the proportion of patients with abnormal scores on neuropsychiatric scales at baseline and at 6‐month follow‐up. Anxiety, depression, fatigue and P300 abnormalities remained highly prevalent with an observed increase over.

**Figure:**
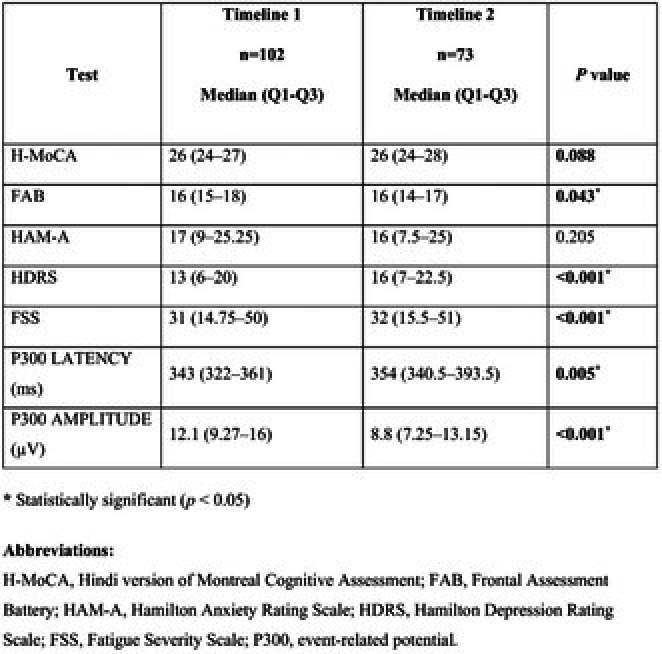
**TABLE 1** Comparison of neuropsychiatric and electrophysiological parameters at baseline and 6‐month follow‐up.

**Figure:**
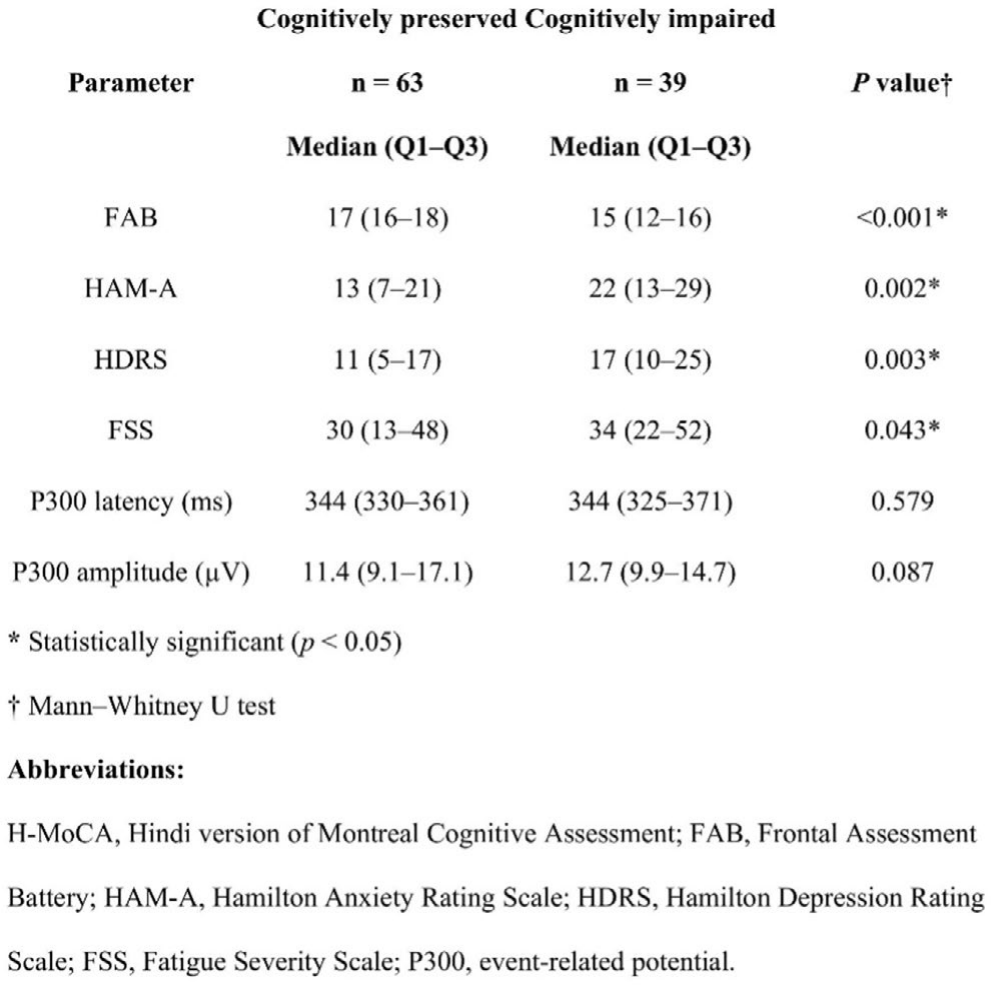
**TABLE 2** Baseline comparison of neuropsychiatric and electrophysiological parameters between cognitively preserved and cognitively impaired groups.

**Conclusion:** Persistent and progressive cognitive, neuropsychiatric, and electrophysiological abnormalities were observed in post‐COVID‐19 patients over a 6‐month period. Cognitive dysfunction, depression, and fatigue may worsen over time, even long after acute infection. P300 abnormalities may precede clinically apparent cognitive decline, highlighting their potential role as an objective marker for subclinical cognitive dysfunction in post‐COVID‐19 condition.

**Disclosure:** Nothing to disclose.

## EPO‐0560

### Multidimensional fatigue relates to disability severity and personal well‐being in multiple sclerosis

#### J. Duranović^1^; S. Pavelin ^2^; M. Rogić Vidaković^1^

##### ^*1*^ *Laboratory for Human and Experimental Neurophysiology (LAHEN), Department of Neuroscience, School of Medicine, University of Split, Split, Croatia**;*** ^*2*^ *Department of Neurology, University Hospital of Split, Split, Croatia*

**Background and aims:** Fatigue is one of the most common symptoms of multiple sclerosis (MS), negatively affecting daily functioning and well‐being. Understanding the relationships between different fatigue domains, disability status, and personal well‐being is necessary for practical interventions. The aim of this study was to examine the relationships between multidimensional fatigue, disability status, and personal well‐being in people with MS.

**Methods:** A total of 141 MS subjects participated in the study, with an average age of 47 years, of whom 104 (74%) were female. The median EDSS was 3, and the average disease duration was 9 years. All subjects completed the Modified Fatigue Impact Scale (MFIS) and the Personal Wellbeing Index – Adult (PWI‐A), while an expert neurologist completed the Expanded Disability Status Scale (EDSS).

**Results:** Pearson correlation analyses showed moderate to strong positive associations between all fatigue domains (physical, cognitive, psychosocial, and total) and disability (*r* = 0.43–.56, *p* < 0.001), and strong negative associations with personal well‐being (*r* = –.71 to –.74, *p* < 0.001).

**Conclusion:** Higher multidimensional fatigue is strongly associated with higher disability status and lower personal well‐being.

**Disclosure:** This study was conducted as part of the Croatian Science Foundation project [HRZZ‐IP‐2022‐10‐6203] at the Department of Neurology, University Hospital Split, and the Department of Neuroscience, Laboratory for Human and Experimental Neurophysiology (LAHEN), University of Split School of Medicine.

Epilepsy 4

## EPO‐0561

### IP3R2 deletion alters glutamate dynamics and increases mortality in a mouse model of temporal lobe epilepsy

#### C. G. Nome ^1^; C. B. Bakketu^2^; T. Berger^2^; V. Jensen^1^; L. Roth^1^; K. Heuser^2^; R. Enger^1^

##### ^*1*^ *GliaLab and Letten Centre, Division of Anatomy, Department of Molecular Medicine, Institute of Basic Medical Sciences, University of Oslo, Oslo, Norway;* ^*2*^ *Department of Neurology, Oslo University Hospital, Oslo, Norway*

**Background and aims:** Inositol 1,4,5‐trisphosphate receptor 2 (IP3R2)–mediated Ca2+ signaling has been implicated in epilepsy pathogenesis, potentially through Ca2+‐dependent astrocytic release of glutamate.

**Methods:** In this study, we examined the contribution of IP3R2 to activity‐evoked astrocytic Ca2+ signals and extracellular glutamate dynamics during epileptogenesis in a mouse model of temporal lobe epilepsy with hippocampal sclerosis. To do this, we used adeno‐associated viral vectors to genetically express fluorescent reporters for intracellular Ca2+ and extracellular glutamate in hippocampal slices from mice

**Results:** We found that IP3R2 knockout mice showed markedly increased mortality in the days following kainate‐induced status epilepticus. Among survivors, activity‐evoked glutamate responses were reduced 10 days after status epilepticus, which may reflect enhanced neuronal loss.

**Conclusion:** Overall, our findings indicate that IP3R2‐mediated Ca2+ signaling, despite its proposed proconvulsant role in acute seizures, may be neuroprotective during epileptogenesis by supporting survival and preserving activity‐evoked glutamate signaling.

**Disclosure:** Nothing to disclose

## EPO‐0562

### Continuous versus routine EEG impact on the management of critically ill adults: Analysis of the CERTA study

#### C. Robitaille ^1^; J. Novy^1^; V. Alvarez^2^; K. Schindler^3^; S. Rüegg^4^; A. Rossetti^1^

##### ^*1*^ *1. Department of Neurology, Lausanne University Hospital, University of Lausanne, Lausanne, Switzerland;* ^*2*^ *2. Department of Neurology, Hôpital du Valais, Sion, Switzerland;* ^*3*^ *3. Sleep‐Wake‐Epilepsy‐Center, Department of Neurology, Inselspital, Bern University Hospital and University of Bern, Bern, Switzerland;* ^*4*^ *4. Department of Neurology, University Hospital Basel and University of Basel, Basle, Switzerland*

**Background and aims:** Continuous electroencephalogram (cEEG) is increasingly utilized in the intensive care unit for consciousness‐impaired patients with various conditions. It is however costly in terms of material and human resources, and its impact on prognosis compared with routine EEG (rEEG) remains debated. We aimed to characterize the effect of cEEG vs rEEG on clinical management modifications, and to determine if these modifications were linked to a better outcome.

**Methods:** This is an ancillary analysis using data of the patients enrolled in the CERTA trial (NCT03129438), a randomized control trial comparing cEEG versus repeated rEEG in critically ill adults with altered consciousness. Clinical management modifications due to the EEG intervention, defined as performed within 60 hours of EEG start, were prospectively recorded (anti‐seizure medication ‐ASM, sedation, new radiological examination, surgical intervention, intensive care withdrawal). Correlation with the EEG type were analyzed by 2x2 tables; Mantel‐Haenszel tests explored the impact on mortality and functional outcome at 6 months.

**Results:** We included 182 patients each in the cEEG and rEEG groups. ASM were modified in 22.0% (cEEG) and 12.1% (rEEG) (*p* = 0.012), while 58.2% of cEEG and 48.4% of rEEG patients had any change of management (*p* = 0.018) (ASM, sedation, new radiological examination, new surgical intervention and care withdrawal). Adjusting for the impact of ASM modifications, mortality (*p* = 0.845) and functional outcome (*p* = 0.729) remained similar across EEG intervention groups.

**Conclusion:** In critically ill adults, cEEG is statistically associated with modifications of clinical management, particularly regarding ASM, compared to repeated rEEG, but does not translate into improved patients’ outcomes.

**Disclosure:** Nothing to disclose.

## EPO‐0563

### The many faces of Lafora progressive myoclonus epilepsy – the Swiss cohort

#### E. Boudriot ^1^; O. Kohnen^1^; L. Imbach^2^

##### ^*1*^ *Swiss Epilepsy Center, Klinik Lengg, Zurich, Switzerland;* ^*2*^ *Institute for Neuroscience, Department of Health Sciences and Technology, ETH Zurich, Zurich, Switzerland*

**Background and aims:** Lafora progressive myoclonus epilepsy (PME) is an ultra‐rare autosomal‐recessive neurodegenerative disorder with onset in adolescence. The prevalence is estimated around four cases per million individuals with higher numbers in Mediterranean countries. It is caused by loss‐of‐function mutations in EPM2A or EPM2B/NHLRC1 genes. With currently no directed therapy available and increasing pharmacoresistance in the disease course, most patients die within a decade after symptom onset. Comprehensive data on the electroclinical disease course are rare due to scarcity of the disease, delayed diagnosis, and rapid disease progression.

**Methods:** We retrospectively analysed clinical and electroencephalographic data from the medical records of three patients with a genetic diagnosis of Lafora PME at the Swiss epilepsy center between 2019 and 2025. To our knowledge, these are currently the only Lafora patients in Switzerland.

**Results:** Patient 1 presented with discrete bilateral myoclonia, followed by focal visual seizures, clustering of atypical absences, and progressing to super‐refractory non‐convulsive status epilepticus within 8 months, being rescued only by the implantation of deep brain stimulation into the anterior nucleus of the thalamus (ANT‐DBS). Patient 2 was diagnosed after an initial bilateral tonic‐clonic seizure and later developed prominent myoclonic‐tonic‐clonic seizures. Patient 3 showed frequent eyelid myoclonia and eye closure sensitivity on EEG, leading to the initial diagnosis of epilepsy with eyelid myoclonia (formerly Jeavons syndrome). All patients showed progressive drug‐resistant myoclonus, progressive cognitive deterioration and electrographic status epilepticus with background slowing over time.

**Conclusion:** Lafora disease can present with many different initial symptoms, clinically prominent features, and dynamics of disease course.

**Disclosure:** Nothing to disclose.

## EPO‐0564

### Duration matters: Prognostic cut‐offs of status epilepticus beyond established predictors

#### F. Iannaccone; C. Scarpitta; E. Bonanni; R. Ceravolo; C. Pizzanelli

##### Department of Clinical and Experimental Medicine, Neurology Unit, University of Pisa, Pisa, Italy

**Background and aims:** Status epilepticus (SE) is a time‐dependent neurological emergency. While baseline clinical predictors are well established, the prognostic role of SE duration remains incompletely defined. We aimed to identify clinically relevant duration cut‐offs for major outcomes and to assess the impact of diagnostic delay on SE duration in nonconvulsive SE (NCSE).

**Methods:** We retrospectively analyzed 73 consecutive adult patients admitted for SE between January 2024 and August 2025. Outcomes were SE resolution, 30‐day mortality, and functional worsening at discharge. Multivariable models included established prognostic predictors and SE duration. Receiver operating characteristic analysis identified prognostic duration cut‐offs. In NCSE, the association between onset‐to‐electroencephalography time and total SE duration was evaluated.

**Results:** SE resolved in 82.2% of patients; 30‐day mortality was 39.7%, and functional worsening occurred in 57.5%. When compared with established predictors, SE duration showed an independent prognostic value for non‐resolution and functional worsening. A duration > = 108 hours was associated with an approximately eleven‐fold increased risk of non‐resolution, while a duration > = 52 hours increased the risk of functional worsening nearly twelve‐fold. SE duration was not independently associated with 30‐day mortality. In NCSE, longer onset‐to‐electroencephalography time was significantly associated with longer SE duration.

**Conclusion:** SE duration provides prognostic information beyond established predictors, particularly for refractoriness and functional outcome. In NCSE, diagnostic delay contributes to prolonged SE, highlighting electroencephalography timing as a modifiable determinant of outcome.

**Disclosure:** Nothing to disclose.

## EPO‐0565

### Real‐world effectiveness and safety of fenfluramine in adults with Dravet syndrome and Lennox–Gastaut syndrome: A retrospective single‐centre study

#### I. Dolezalova; O. Strycek; J. Kocvarova; M. Pail; M. Brazdil

##### Brno Epilepsy Center, member of ERN‐EpiCare, St. Anne's University Hospital and Masaryk University, Brno, Czech Republic

**Background and aims:** Evidence for fenfluramine in Dravet syndrome (DS) and Lennox–Gastaut syndrome (LGS) is largely derived from paediatric cohorts, while real‐world data in adults are scarce. We evaluated the effectiveness and safety of fenfluramine in adults with long‐standing, drug‐resistant epilepsy.

**Methods:** We retrospectively identified adults with DS or LGS who initiated fenfluramine between January 2024 and August 2025. Fifteen patients were included (DS *n* = 9; LGS *n* = 6). All DS cases had confirmed SCN1A variants; LGS etiologies were structural (*n* = 2), genetic (*n* = 1), or unknown (*n* = 3). Patients were highly pharmacoresistant (9–17 lifetime antiseizure medications; 2–5 at initiation). Median disease duration was 31 years. Effectiveness was assessed as percentage seizure reduction at 6 months (*n* = 15) and 12 months (*n* = 9). Safety and discontinuations were recorded retrospectively.

**Results:** At 6 months, 86.7% achieved a clinical response and 13.3% were non‐responders, most commonly with ≥50% seizure reduction. At 12 months, all evaluable patients (100%) showed sustained benefit. Treatment was discontinued in 13.3% due to inefficacy with or without adverse events. Adverse events occurred in 53.3%, mainly gastrointestinal symptoms, fatigue/somnolence, reduced appetite with weight loss, and behavioural changes; 20% reported improved attention.

**Conclusion:** In this real‐world adult cohort with long‐standing, highly refractory DS and LGS, fenfluramine was associated with substantial and sustained seizure reduction and an acceptable safety profile. These findings support the use of fenfluramine as an effective add‐on therapy beyond childhood and highlight its potential benefit even in chronically treated adult patients.

**Disclosure:** Nothing to disclose.

## EPO‐0566

### Fast‐ripples are an emergent property of neural networks

#### L. Sheybani ^1^; Y. Qiu^1^; P. Singh^4^; U. Vivekananda^5^; N. Burgess^5^; B. Diehl^1^; A. McEvoy^1^; A. Miserocchi^1^; J. Bisby^6^; T. Shekh‐Ahmad^4^; G. Lignani^1^; D. Bush^7^; M. Walker^1^

##### ^*1*^ *Research Department of Epilepsy, UCL Queen Square Institute of Neurology, University College London, London, UK;* ^*4*^ *The Institute of Drug Research, The School of Pharmacy, Faculty of Medicine, The Hebrew University of Jerusalem, Israel;* ^*5*^ *Institute of Cognitive Neuroscience, University College London, London, UK;* ^*6*^ *Division of Psychiatry, University College London, London, UK;* ^*7*^ *Department of Neuroscience, Physiology and Pharmacology, University College London, London, UK*

**Background and aims:** Fast‐ripples have been proposed as a promising biomarker in epilepsy, but their specificity remains unclear. In particular, it is unclear whether they reflect chance coincident neural activity or distinctly generated pathological entities.

**Methods:** Here, we used a multi‐modal approach combining in silico simulations, neuronal cultures, a rodent model of temporal lobe epilepsy and microwire recordings in humans with epilepsy to test, using a within‐subjects design, whether fast‐ripples occur more frequently than predicted by the random firing of neurons. For chance level, we used signal shuffling to disrupt any distinct pathological entities and to generate only fast‐ripples arising from stochastic aggregation of action potentials. Furthermore, since evidence suggests that fast‐ripples incidence varies across the sleep‐wake cycle, we assessed whether the relative proportion of “chance” fast‐ripples is modulated across vigilance states.

**Results:** We found that, both in vitro and in sleeping rodents, the incidence of fast‐ripples did not exceed chance levels. Our simulations predicted an increased rate of fast‐ripples during wakefulness, when firing rates are higher and less synchronized across neurons. This was confirmed in a rodent model of epilepsy, where fast‐ripples were also more frequent than expected by chance but still represented a minority of all detected events. This was also the case in awake human recordings.

**Conclusion:** Our findings suggest that most fast‐ripples reflect general network activity rather than distinctly generated pathological entities, challenging prevailing assumptions about their pathogenesis and biomarker specificity.

**Disclosure:** M.W. has acted as a consultant for Seer and EpilepsyGtx. He is a founder shareholder in EpilepsyGtx. He has received honoraria from Eisai, Angelini, and UCB pharma.

## EPO‐0567

### Can statins influence the risk of remote seizure after a first new onset status epilepticus? Data from the adult population of Modena, Northern Italy

#### N. Orlandi ^1^; G. Giovannini^2^; J. Ngnintedem Dontsop^3^; L. Taruffi^1^; M. Burani^1^; M. Malerba^2^; S. Scolastico^1^; L. Madrassi^1^; L. Affronte^2^; A. Vaudano^2^; M. Pugnaghi^2^; S. Lattanzi^4^; S. Meletti^2^

##### ^*1*^ *Department of Biomedical, Metabolic and Neural Science, University of Modena and Reggio Emilia, Modena, Italy**;*** ^*2*^ *Neurophisiology Unit, Neuroscience Department, Azienda Ospedaliera‐Universitaria, Modena, Italy;* ^*3*^ *School of Medicine, University of Modena and Reggio Emilia, Modena, Italy**;*** ^*4*^ *Neurological Clinic, Department of Experimental and Clinical Medicine, Marche Polytechnic University, Ancona, Italy*

**Background and aims:** Preclinical evidence supports the role of statins as antiepileptogenic agents. In this study, we investigated the risk of remote unprovoked seizures (RS) according to the use of statin therapy in a cohort of first ever status epilepticus (SE) survivors.

**Methods:** Observational, single‐center, retrospective study in adult patients with a first SE who were consecutively admitted to Modena Academic Hospital, Italy, from September 2015 to December 2023. Kaplan–Meier survival analyses were used to calculate the probability of RS following the index event, while Cox proportional hazard regression models were used to assess predictors of RS occurrence. Competing‐risk regression model was used as sensitivity analysis to assess the impact of mortality as a competing event with the occurrence of RS.

**Results:** 314 patients were included (mean age 70 y/o; 63% females), 86 (27.4%) of whom were taking statins. Overall, 67 patients (21.3%) developed RS (mean follow‐up: 31.5 months). Cumulative probability of RS occurrence was 16% and 32% at 12 months and 5 years after SE, respectively. Cumulative probability of RS was significantly lower in statin users compared to non‐users (log rank *p* = 0.038). After adjusting for potential confounders, the risk of RS was lower in patients who assumed statins after the index event (HR 0.48 95% CI 0.26 – 0.90; *p* = 0.023).

**Conclusion:** In our cohort, statin exposure was associated to a lower incidence of RS. Our findings expand the knowledge regarding a possible neuroprotective and antiepileptogenic role of statins after SE and support further studies on this field.

**Disclosure:** Nothing to disclose

## EPO‐0568

### Real‐world evidence on the use of Perampanel in the management of brain tumor‐related epilepsy

#### R. Cutellè ^1^; A. Pascarella^1^; D. Abelardo^1^; A. Santoro^1^; C. Mummolo^1^; D. Filippelli^1^; V. Cianci^2^; S. Gasparini^1^; A. Iudice^3^; F. Bisulli^4^; P. Bonanni^5^; E. Caggia^6^; A. D'Aniello^7^; C. Di Bonaventura^8^; S. Beretta^9^; E. Domina^10^; F. Dono^11^; A. Gambardella^1^; A. Marrelli^12^; S. Matricardi^13^; A. Morano^8^; F. Paladin^14^; R. Renna^15^; M. Piccioli^16^; P. Striano^17^; E. Le Piane^18^; A. Orsini^19^; C. Torino^20^; U. Aguglia^1^; P. Study Group^21^; E. Ferlazzo^1^

##### ^*1*^ *Department of Medical and Surgical Sciences, Magna Græcia University of Catanzaro, Catanzaro, Italy;* ^*2*^ *Neurology Unit, "Great Metropolitan "Bianchi‐Melacrino‐Morelli Hospital", Reggio Calabria, Italy;* ^*3*^ *Department of Neurosciences, Section of Neurology, University of Pisa, Pisa, Italy;* ^*4*^ *Department of Biomedical and Neuromotor Sciences, University of Bologna, Bologna, Italy;* ^*5*^ *Epilepsy and Clinical Neurophysiology Unit, Scientific Institute, IRCCS Eugenio Medea, Conegliano, Treviso, Italy;* ^*6*^ *Neurology Unit, Ospedale Giovanni Paolo II, Ragusa, Italy;* ^*7*^ *IRCCS Neuromed, Pozzilli, Italy;* ^*8*^ *Epilepsy Unit, Department of Human Neurosciences, "Sapienza" University of Rome, Rome, Italy;* ^*9*^ *Department of Neurology, Fondazione IRCCS San Gerardo dei Tintori, Monza, Italy;* ^*10*^ *U.C. Neurology, Ospedale Maggiore di Lodi ASST, Lodi, Italy;* ^*11*^ *Department of Neuroscience, Imaging and Clinical Science, "G. D'Annunzio" University of Chieti‐Pescara, Chieti, Italy;* ^*12*^ *Neurophysiopathology Unit, Epilepsy Center, San Salvatore Hospital, L'Aquila, Italy;* ^*13*^ *Department of Pediatrics, University of Chieti, Chieti, Italy;* ^*14*^ *Neurology Unit, Epilepsy Center, Venice, Italy;* ^*15*^ *Neurological Clinic and Stroke Unit, AORN San Pio, Benevento, Italy;* ^*16*^ *UOC Neurology, PO San Filippo Neri, ASL Roma 1, Rome, Italy;* ^*17*^ *Department of Neurosciences, Rehabilitation, Ophthalmology, Genetics, Maternal and Child Health, University of Genova, Genoa, Italy;* ^*18*^ *Dipartimento di Neurologia, Ospedale Pugliese‐Ciaccio, Catanzaro, Italy;* ^*19*^ *Pediatric Neurology, Pediatric Department, AOUP Santa Chiara Univeristy Hospital, Pisa, Italy;* ^*20*^ *Clinical Epidemiology and Physiopathology of Renal Diseases and Hypertension of Reggio Calabria, National Research Council, Institute of Clinical Physiology, Reggio Calabria, Italy;* ^*21*^ *PEROC Study Group*

**Background and aims:** Seizures are a frequent complication in patients with brain tumors, with more than 50% developing epilepsy that is often pharmacoresistant [1]. This study aimed to assess the efficacy and tolerability of perampanel (PER) as the sole additional antiseizure medication (ASM) in brain tumor‐related epilepsy (BRTE).

**Methods:** we performed a subgroup analysis of patients with BRTE included in the PEROC study cohort [2], a retrospective, longitudinal, multicentre observational study enrolling patients with focal or generalized epilepsy receiving PER added to a single concomitant ASM. Retention, responder rate, seizure‐freedom and adverse event (AE) frequency at 3, 6 and 12 months after PER introduction were evaluated, also comparing early (≤1 prior ASM) vs late PER add‐on.

**Results:** forty‐six patients were included (23 female, median age: 51 years). The median PER dose at 12 months was 4 mg. Retention rates were 90%, 82.9%, and 70.8% at 3, 6, and 12 months. Responder rates were 60% at 3, 80.6% at 6, and 76.5% at the same time points. Seizure freedom was achieved in 36.7%, 45.2% and 47.1% of patients after 3, 6 and 12 months. AEs were reported in 15.2% of patients, leading to treatment discontinuation in 6.5%. No significant differences were observed between early and late add‐on groups for any outcome.

**Figure:**
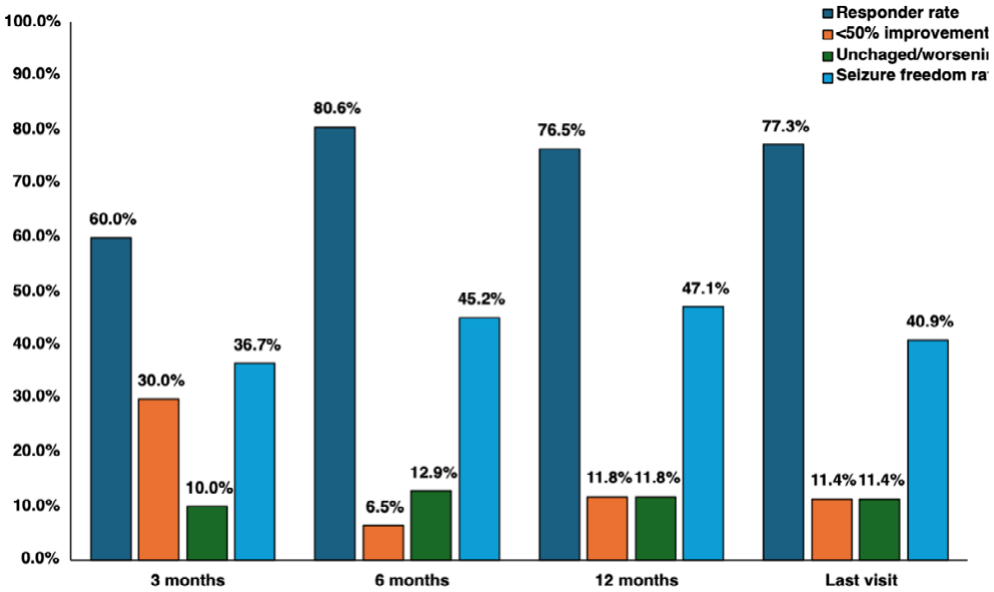
**FIGURE 1** Clinical response to add‐on Perampanel treatment in the overall population. The figure shows the proportion of responder patients, patients achieving seizure freedom, patients 3with

**Figure:**
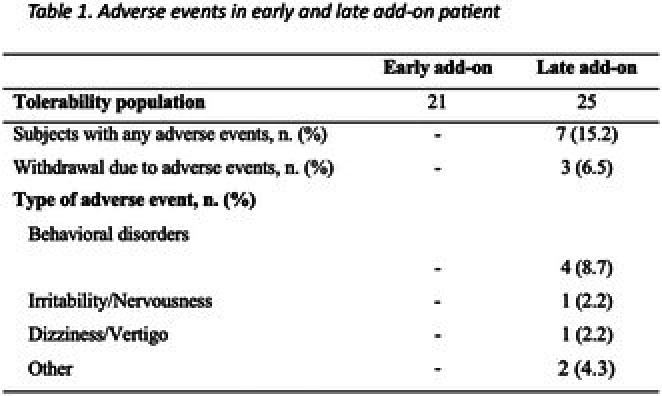
Adverse events in early and late add‐on patient.

**Conclusion:** PER proved to be an effective and well‐tolerated treatment option for BTRE, providing satisfactory seizure control and good treatment retention. Although confirmation from larger prospective studies is needed, PER may represent a valuable strategy to improve seizure management and quality of life in this population

**Disclosure:** Nothing to disclose.

## EPO‐0569

### Clinical characteristics, comorbidities, and healthcare utilization in patients with psychogenic nonepileptic seizures: A retrospective study

#### S. Bashayreh ^1^; A. Momani^1^; K. Kheirallah^1^; A. Yassin^1^; M. Alhazaimeh^2^

##### ^*1*^ *Neurology, Jordan University of Science and Technology, Irbid, Jordan;* ^*2*^ *Neurosurgery, Yarmouk University, Irbid, Jordan*

**Background and aims:** Psychogenic nonepileptic seizures (PNES) are paroxysmal events that resemble epileptic seizures but occur without ictal epileptiform activity. Diagnosis is frequently delayed, and many patients are initially misdiagnosed with epilepsy, leading to unnecessary anti‐seizure medication (ASM) use and increased healthcare utilization. Regional data describing the clinical characteristics and burden of PNES remain limited.

**Methods:** We retrospectively reviewed patients evaluated for suspected psychogenic nonepileptic seizures (PNES) using outpatient video‐EEG (1–6 hours) at King Abdullah University Hospital between 2018 and 2022. PNES was defined as a typical clinical event captured without ictal EEG changes. Patients with exclusively subjective symptoms were excluded. Demographic, clinical, diagnostic, treatment, and healthcare utilization data were analyzed descriptively.

**Results:** Among 59 patients with PNES, 57.6% were female and psychiatric comorbidities were common. Events were captured on video‐EEG in 83.1%, with 74.6% being typical habitual events, and epileptiform discharges were uncommon (13.6%). Semiology was heterogeneous using Pooya and Wadwekar classifications. MRI was mostly normal or non‐specific, ≈68% had received ASMs, often polytherapy, and healthcare utilization prior to diagnosis was high.

**Conclusion:** Psychogenic nonepileptic seizures are associated with heterogeneous clinical presentations, high psychiatric comorbidity, substantial healthcare utilization, and frequent exposure to anti‐seizure medications prior to diagnosis. This study adds regional, real‐world data on the clinical characteristics, diagnostic evaluation, and healthcare utilization associated with PNES, reinforcing existing evidence while highlighting the burden of delayed diagnosis in routine practice.

**Disclosure:** Nothing to disclose

## EPO‐0570

### An optimized clinical algorithm for selecting DEE‐SWAS patients for whole exome sequencing

#### S. Shokhimardonov; N. Tuychibaeva

##### Tashkent State Medical University, Tashkent, Uzbekistan

**Background and aims:** Developmental and epileptic encephalopathy with spike‐wave activation in sleep (DEE‐SWAS) is a clinically heterogeneous disorder with a substantial genetic contribution. Whole exome sequencing (WES) is not routinely accessible for all patients, highlighting the need for rational selection strategies.

**Methods:** A retrospective cohort of 125 pediatric patients with DEE‐SWAS was analyzed. Clinical characteristics, cognitive outcomes, treatment response, family history, and brain magnetic resonance imaging (MRI) findings were collected. Electroencephalography (EEG) recordings during non‐rapid eye movement sleep were reviewed, and spike‐wave index (SWI) was calculated. A scoring algorithm was developed assigning one point to each of seven criteria: SWI >85% or pronounced SWAS pattern, full‐scale intelligence quotient (FSIQ) <70, pharmacoresistant epilepsy or need for intensified therapy, absence of a convincing structural MRI cause, epilepsy onset ≤4 years, positive family history of epilepsy or febrile seizures, and parental consanguinity. Patients were stratified into low (0–2), moderate (3–4), and high (≥5) genetic risk groups. Multivariable logistic regression was performed to identify independent predictors of monogenic etiology.

**Results:** High SWI, severe cognitive impairment, pharmacoresistance, absence of structural MRI abnormalities, positive family history, and parental consanguinity were independently associated with monogenic disease (odds ratios 1.8–4.9; all *p* < 0.05).

**Conclusion:** A weighted clinical, EEG, and MRI‐based algorithm effectively stratifies DEE‐SWAS patients according to genetic risk and supports targeted referral for WES. This approach improves diagnostic efficiency and may facilitate earlier etiological clarification in routine clinical practice.

**Disclosure:** Nothing to disclose.

## EPO‐0571

### Vagus Nerve Stimulation in Failed Epilepsy Surgery: 36 Month Outcomes From the CORE‐VNS Study

#### S. Fetzer ^1^; E. Nicolai^1^; K. Sierazdan^2^; M. Fry^3^; O. Schijns^4^; K. Rijkers^4^; R. Verner^1^; S. Baeesa^5^; N. Kurwale^6^; G. Giannicola^1^; C. Gordon^1^; A. Moon^1^; F. Beraldi^7^; A. Sen^8^; K. Nichol^1^

##### ^*1*^ *LivaNova PLC (or a subsidiary);* ^*2*^ *Southmead Hospital (North Bristol NHS Trust), Bristol, England, UK;* ^*3*^ *Department of Neurosurgery, Cleveland Clinic, Ohio;* ^*4*^ *Department of Neurosurgery, Academic Center for Epileptology, Maastricht University Hospital, The Netherlands;* ^*5*^ *Neurosciences Department, King Faisal Specialist Hospital and Research Centre, Jeddah, Saudi Arabia;* ^*6*^ *Deenanath Mangeshkar Hospital and Research Centre, Pune, India;* ^*7*^ *Valos S.r.L, Genova Italy;* ^*8*^ *8Oxford Epilepsy Research Group, John Radcliffe Hospital, Oxford, UK*

**Background and aims:** The safety and effectiveness of VNS Therapy are evaluated in individuals who have (+Hx) and have not (‐Hx) previously undergone brain surgical procedures for epilepsy.

**Methods:** The impact of +Hx on the safety profile and change in seizure frequency of severe seizure types (Focal Impaired Awareness Motor [FIA‐M], Focal Impaired Awareness Non‐Motor [FIA‐NM], Focal‐to‐Bilateral Tonic‐Clonic [FBTC], and Generalized Tonic‐Clonic [GTC]) is evaluated.

**Results:** Of the 464 participants with ‐Hx 446 were included in the modified safety population (mSAF). Lesionectomy, callosotomy, and lobar resection were the 3 most frequently reported therapeutic brain surgeries in the 84 participants with +Hx in the mSAF. At 36 months, participants with +Hx experienced a median percentage change in frequency of FIA‐M by ‐36.1% (*n* = 28), FIA‐NM by ‐83.3% (*n* = 18), FBTC by ‐91.7% (*n* = 19), and GTC by ‐84% (*n* = 5). In the ‐Hx participants this was ‐86.1% (*n* = 131), ‐100% (*n* = 72), ‐96.7% (*n* = 73), and ‐83.5% (*n* = 78) respectively. In those with a +Hx and ‐Hx, complete seizure freedom (zero seizures over the previous 3 months) was reported at the first 3 month visit in 6.9% (5/72) and 8.8% (29/329) respectively. At the 36‐month visit, 8.8% (6/68) and 18% (58/323) reported complete seizure freedom, respectively. The overall incidence of adverse events (AEs) was similar between groups, 36.9% +Hx and 41.0% ‐Hx. Serious AEs were observed in 3.6% and 7.2% respectively.

**Conclusion:** Adjunctive VNS Therapy was found to have consistent safety and effectiveness in participants with +Hx and ‐Hx epilepsy brain surgery. In addition, SF was reported in 8.8% of participants.

**Disclosure:** EN, KN, GG, CG, MD, and AM are employees of LivaNova PLC (or a subsidiary) and hold stock or stock options with the company, who is the manufacturer of the VNS Therapy System. All other authors are investigators associated with the CORE‐VNS Study, and in that capacity they or their institutions receive compensation from LivaNova for study‐related activities. No author received direct compensation from LivaNova related to this abstract.

## EPO‐0572

### VNS Therapy is Associated with Reduced Disabling Seizure Burden in Children Early in Treatment (3 M) of DRE with Continued Improvement Over 36 Months

#### S. Fetzer ^1^; J. Wheless^2^; M. Zafar^3^; G. Giannicola^1^; F. Babtain^5^; G. Motamedi^4^; S. Baeesa^5^; M. Fry^6^; R. El Tahry^7^; G. Groeppel^8^; T. O'Brien^9^; K. Liiow^11^; P. Lyons^10^; R. Verner^1^; F. Beraldi^1^; C. Gordon^1^; D. Urry^1^; A. Sen^12^; K. Nichol^1^; M. Dibue^1^

##### ^*1*^ *LivaNova PLC (or a subsidiary);* ^*2*^ *University of Tennessee Health Science Center & Le Bonheur Children's Hospital, Memphis, USA;* ^*3*^ *Duke University Hospital, Durham, USA;* ^*4*^ *Department of Neurology, Medstar Georgetown University Hospital, Washington, USA;* ^*5*^ *Neurosciences Department, King Faisal Specialist Hospital and Research Centre, Jeddah, Saudi Arabia;* ^*6*^ *Cleveland Clinic, Cleveland, USA;* ^*7*^ *Centre for Refractory Epilepsy, Department of Neurology, Cliniques Universitaires Saint‐Luc, and Institute of Neuroscience (IONS), Université Catholique de Louvain, Brussels, Belgium;* ^*8*^ *Department of Pediatrics and Adolescent Medicine, Johannes Kepler University, Linz, Austria;* ^*9*^ *Department of Neuroscience, School of Translational Medicine, Monash University, and Department of Neurology, Alfred Hospital, Melbourne, Victoria, Australia;* ^*10*^ *Virginia Commonwealth Epilepsy Program, Winchester, USA;* ^*11*^ *Comprehensive Epilepsy Center, Hawaii Pacific Neuroscience, University of Hawaii at Mānoa, John A. Burns School of Medicine, Honolulu, USA;* ^*12*^ *Oxford Epilepsy Research Group, John Radcliffe Hospital, Oxford, UK*

**Background and aims:** The CORE‐VNS represents the first large cohort of prospectively gathered pediatric data on the effectiveness of VNS Therapy for DRE.

**Methods:** The CORE‐VNS (NCT03529045) study collected data on seizure and non‐seizure outcomes following treatment with VNS Therapy. Participants 4‐<18 (children) who received their first VNS Therapy were selected for this analysis. Seizure frequency reduction of defined most severe seizures and patient‐described outcome measures (quality of life and quality of sleep) were collected at baseline and at 3, 6, 12, 24, and 36 months.

**Results:** 213 met the criteria for this analysis. Certain seizure types were more commonly described in this analysis as most disabling (focal impaired aware motor (FIA‐M), focal impaired aware non‐motor (FIA‐NM), focal to bilateral tonic‐clonic (FBTC), and generalized tonic‐clonic seizures (GTC)). Seizure frequency reduction was reported in all seizure types across age groups early (3‐month visit) in the study. At the 36‐month follow‐up, children had a median seizure reduction of 86.7% FIA‐M, 100% FIA‐NM, 100% FBTC, and 75.2% GTC. At 36 months, 80.3% reported their quality of life as very good/pretty good/good and bad. The most reported (~5%) adverse events were cough and dysphonia.

**Conclusion:** Adjunctive VNS Therapy reduced the most severe disabling seizures in children early after implantation with sustained effectiveness and improved quality of life at 36 months.

**Disclosure:** GG, RV, KN, FB, CG, MD, and DU are employees of LivaNova PLC (or a subsidiary) and may hold stock or stock options with the company, who is the manufacturer of the VNS Therapy System. All other authors are investigators associated with the CORE‐VNS Study, and in that capacity they or their institutions receive compensation from LivaNova for study‐related activities. No author received direct compensation from LivaNova.

Headache 4

## EPO‐0573

### Venous sinus stenting in idiopathic intracranial hypertension: A prospective interventional study of safety and efficacy

#### A. Mohamed Abd El‐Moneam ^1^; H. Rashad El‐habashy^2^; A. Abdelkader Nemr^3^; M. Atallah^4^

##### ^*1*^ *MSc, Armed Forces College of Medicine, Neurology department, Maadi Armed Forces Medical Complex, Cairo, Egypt;* ^*2*^ *MD, Armed Forces College of Medicine, Cairo, Egypt;* ^*3*^ *MD, Neurology department, Maadi Armed Forces Medical Complex, Cairo, Egypt;* ^*4*^ *MD, Armed Forces College of Medicine, Cairo, Egypt"*

**Background and aims:** Idiopathic Intracranial Hypertension (IIH) with bilateral transverse–sigmoid venous sinus stenosis is a debilitating condition with limited effective treatments. This study aimed to evaluate the safety and effectiveness of venous sinus stenting (VSS) in medically refractory IIH patients, focusing on clinical outcomes and objective physiological parameters.

**Methods:** A prospective, single‐arm interventional study was conducted across two tertiary centers in Cairo, Egypt, over nine months. Twenty‐four patients (median age 35.5 years; 95.8% female) with IIH and bilateral venous sinus stenosis confirmed by magnetic resonance venography underwent VSS using self‐expanding stents across stenotic segments. Inclusion criteria included failure of medical therapy, papilledema grade ≥II, and trans‐stenotic pressure gradient >8–10 mmHg. Pre‐ and post‐intervention assessments comprised intracranial pressure (CSF opening pressure), venous pressure gradients, optical coherence tomography, visual evoked potentials, and visual field perimetry. Follow‐up was performed at 1 and 6 months.

**Figure:**
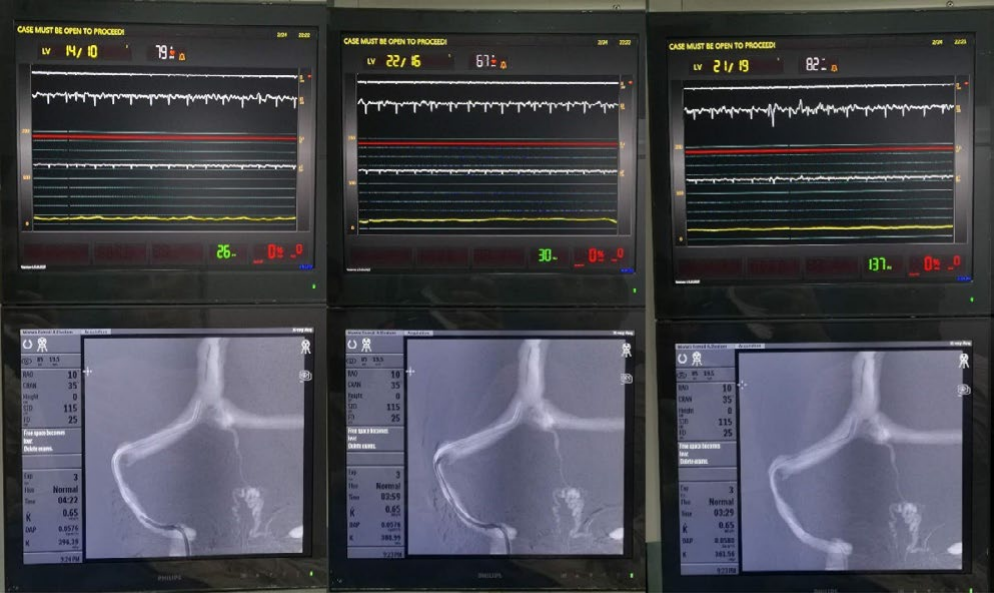
Pre venous sinus stenting pressure gradient.

**Figure:**
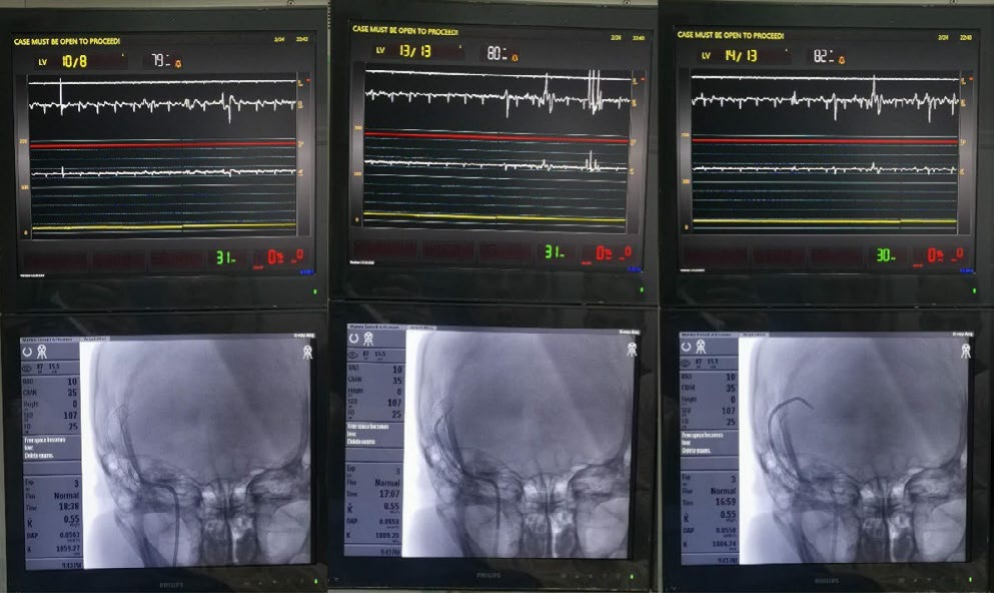
Post venous sinus stenting pressure gradient.

**Results:** All patients demonstrated significant improvement in symptoms and objective measures. Headache severity reduced markedly (*p* < 0.001); papilledema resolved in 100% of affected cases. Median venous pressure gradient decreased from 12.5 to 2 mmHg, and cerebrospinal fluid opening pressure reduced from 36.45±5.83 to 19.54±2.6 Cm H₂O (*p* < 0.001). Visual evoked potential parameters improved significantly, reflecting enhanced optic nerve function. No complications, stent migration, or symptom recurrence occurred during follow‐up.

**Figure:**
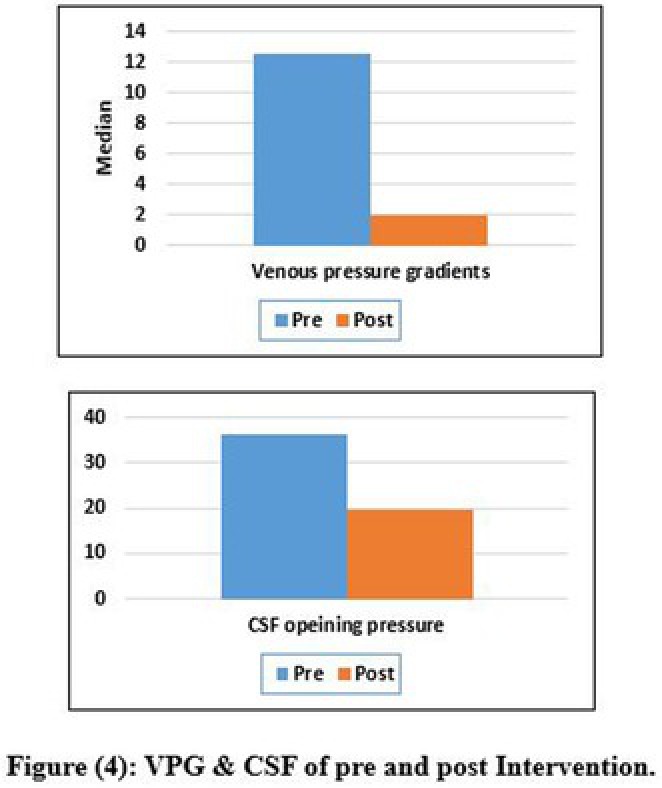
VPG & CSF of pre and post Intervention.

**Conclusion:** Venous sinus stenting is a safe and effective therapeutic option for medically refractory IIH with venous sinus stenosis. It significantly lowers intracranial pressure, resolves papilledema, and improves visual function, supporting its role as a minimally invasive alternative in selected patients.

**Disclosure:** The authors declare no conflicts of interest or financial support related to this study.

## EPO‐0574

### Increased neuroimmune and neurotrophic responses to a migraine trigger after prenatal immune activation

#### A. Yakubova ^1^; S. Svitko^2^; K. Gilizhdinova^2^; A. Rizvanov^1^; G. Sitdikova^2^

##### ^*1*^ *Openlab “Gene and Cell Technologies”, Kazan Federal University, Kazan, Russian Federation;* ^*2*^ *Department of Human and Animal Physiology, Kazan Federal University, Kazan, Russian Federation*

**Background and aims:** Prenatal immune activation (PIA) induced by maternal lipopolysaccharide (LPS) exposure is considered as a factor capable of altering neuroimmune reactivity in adulthood. In this study, we analyzed how prenatal LPS exposure modifies migraine responses by assessing biochemical markers in a model of migraine induced by a single nitroglycerin (NTG) injection.

**Methods:** This study included 16 adult male Wistar rats. Levels of CGRP, TNF‐alpha, IL‐1beta, IL‐6 and BDNF were measured in homogenates of isolated brain regions (hippocampus, cerebral cortex and brainstem) using ELISA.

**Results:** At baseline, the animals exposed to PIA demonstrated significantly decreased IL‐1beta levels in all examined brain regions (*p* < 0.05) and reduced BDNF levels in the cortex (*p* = 0.04) compared to the control group. Levels of TNF‐alpha, CGRP, IL‐6 were nearly equal or slightly decreased, indicating the absence of active neuroinflammation and the development of a compensated state. After administration of NTG, a region‐dependent increase in the central inflammatory response was observed in the PIA group. CGRP concentrations increased significantly in the hippocampus and brainstem compared to control animals with migraine (*p* < 0.05), with a similar trend in the cortex. TNF‐alpha levels were significantly elevated in all examined brain regions (*p* < 0.05), IL‐6 level increased only in the brainstem, while in the cortex remained equal. IL‐1beta levels tended to be lower in the PIA group and BDNF levels did not change significantly.

**Figure:**
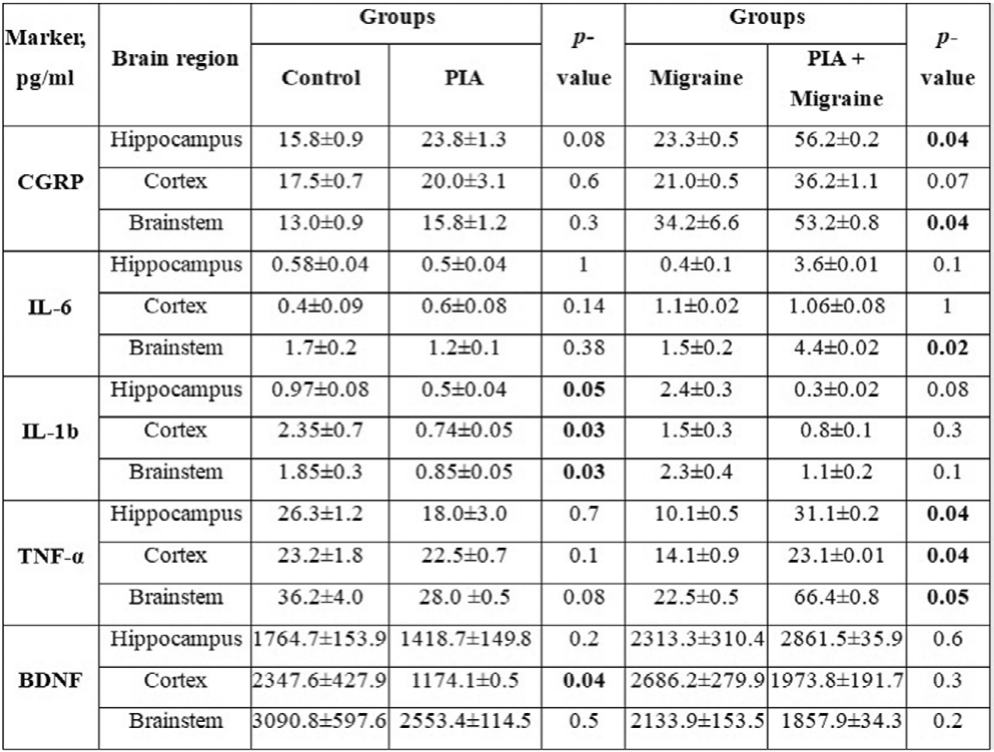
**TABLE 1** Levels of CGRP, IL‐6, IL‐1beta, TNF‐alpha, BDNF in hippocampus, cortex, brainstem in experimental groups (mean ± SD); Mann‐Whitney U test; significant *p* < 0.05 in bold.

**Figure:**
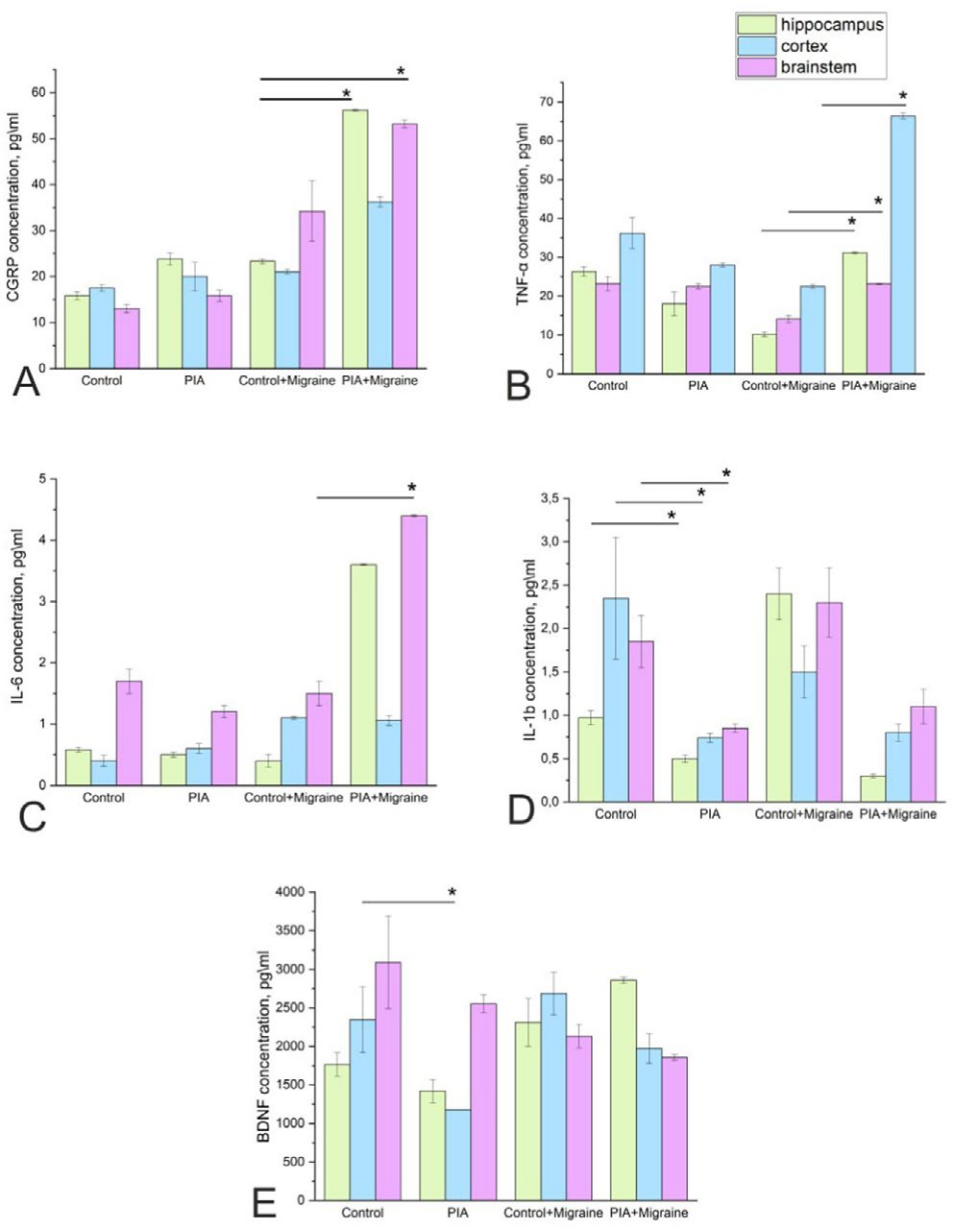
**FIGURE 1** Comparison of CGRP, TNF‐alpha, IL‐6, IL‐1beta, and BDNF levels in the hippocampus, cerebral cortex and brainstem between control and prenatal immune activation (PIA) groups under baseline conditions and after migraine induction. * *p* < 0.05.

**Conclusion:** Thus, PIA does not lead to a sustained increase of inflammatory markers, but induces heightened sensitivity to the migraine trigger, manifested by an enhanced and heterogeneous biochemical response from central brain structures.

**Disclosure:** This study was supported by the Kazan Federal University Strategic Academic Leadership Program (PRIORITY‐2030).

## EPO‐0575

### Genetic associations of TRPM8 with migraine chronification

#### A. Yakubova ^1^; T. Nasyrov^1^; P. Elboeva^1^; A. Belopasova^2^; Y. Davidyuk^1^; A. Rizvanov^1^

##### ^*1*^ *Openlab “Gene and Cell Technologies”, Kazan Federal University, Kazan, Russian Federation;* ^*2*^ *Research Centar of Neurology, Moscow, Russian Federation*

**Background and aims:** TRPM8 receptors are highly expressed in migraine pathophysiology. Genome‐wide association studies have previously identified the strong association of single nucleotide polymorphism rs10166942 with migraine. However, its specific role in disease progression remains unstudied. Here was evaluated the genotype frequency distribution of rs10166942 in the TRPM8 gene in healthy individuals and patients with episodic (EM) and chronic migraine (CM) to test its influence on susceptibility to these forms of migraine.

**Methods:** The study included 238 participants ‐ patients with migraine (47 EM and 42 CM) and 149 healthy controls. DNA from peripheral blood was used to test rs10166942 using allele‐specific PCR.

**Results:** The genotype and allele frequency distributions in EM were comparable with control (*p* = 0.77 and *p* = 0.7). In the CM group distribution of genotypes differed significantly from both control and EM groups (both *p* = 0.03), (Table 1). Notably, in the CM group, in contrast to control and EM groups, the TT genotype frequency was nearly halved, while the TC and CC genotypes frequencies increased nearly twofold (Figure 1). Allele analysis confirmed this trend and showed a significant accumulation of the C allele in CM patients (*p* = 0.03).

**Figure:**
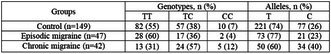
**TABLE 1** Genotypes and alleles distributions of rs10166942 in TRPM8 gene in the control group, episodic and chronic migraine patients.

**Figure:**
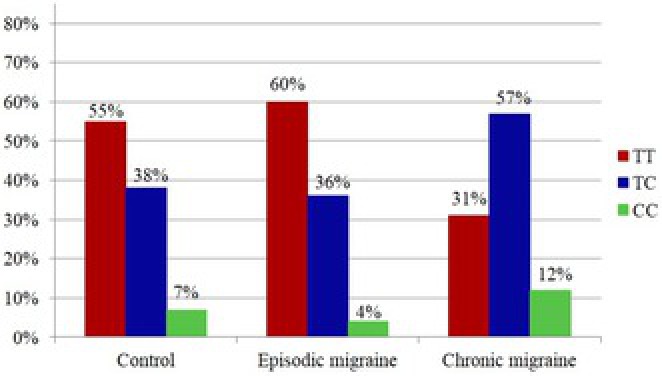
**FIGURE 1** TRPM8 rs10166942 genotypes distribution in the control group, episodic and chronic migraine patients.

**Conclusion:** These are the first indications of distinctive involvement of rs10166942 TRPM8 genotypes in EM and CM. Our data reveal a different predisposition to chronic pain in migraine and propose that this SNP play a potential role in migraine chronification.

**Disclosure:** This study was supported by the Kazan Federal University Strategic Academic Leadership Program (PRIORITY‐2030).

## EPO‐0576

### Migraine prevalence and Burden in Africa: A systematic review and meta‐analysis

#### H. Atwan ^1^; M. Atef^1^; O. Rageh^2^; M. Nada^3^

##### ^*1*^ *Faculty of Medicine, Assiut University, Assiut, Egypt;* ^*2*^ *Faculty of Medicine, Tanta University, Tanta, Egypt;* ^*3*^ *Department of Neurology, Cairo University, Giza, Egypt*

**Background and aims:** Migraine is a leading cause of years lived with disability worldwide, yet data from Africa are limited. We aimed to estimate migraine prevalence, patterns, and associated burden across African populations.

**Methods:** We systematically searched PubMed, Scopus, Web of Science, African Journals Online, and Google Scholar (inception–August 2025) for community‐based studies reporting migraine prevalence or burden. Two reviewers independently screened studies, extracted data, and assessed quality using the Joanna Briggs Institute checklist. Random‐effects meta‐analyses estimated pooled prevalence, with subgroup analyses by sex, migraine subtype, and setting. Heterogeneity and publication bias were assessed.

**Results:** Sixty‐one studies (*n* = 201,437) from 18 African countries were included. Overall migraine prevalence was 18% (95% CI: 12–26%), with high heterogeneity (I^2^ = 99.7%). Standardized prevalence was 140 per 1,000 population (14%; 95% CI: 11–17%). Females were more affected than males (63% vs. 37%; female‐to‐male ratio ≈1.7:1). Migraine without aura predominated, with 84% lower odds of aura. Urban–rural differences were inconsistent. Migraines accounted for 31% of all headaches. Burden was substantial, with moderate‐to‐severe disability, reduced quality of life, productivity loss, and low healthcare utilization. Evidence of small‐study effects and potential publication bias was detected.

**Figure:**
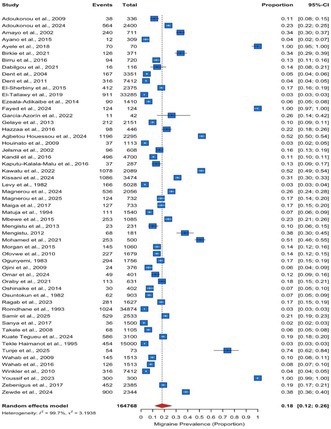
**FIGURE 1** Forest plot showing the proportion of migraine cases across included studies. Each study is represented by its effect estimate (proportion) and 95% confidence interval.

**Figure:**
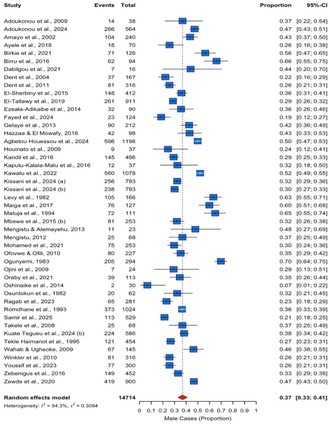
**FIGURE 2** Forest plot showing the proportion of male migraine cases across included studies.

**Figure:**
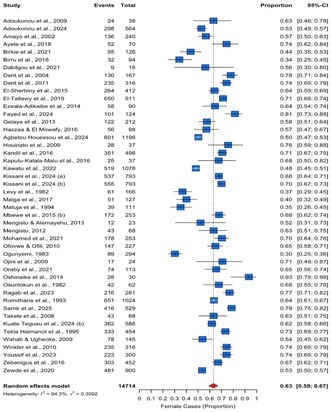
**FIGURE 3** Forest plot showing the proportion of female migraine cases across included studies.

**Conclusion:** Migraine affects a considerable proportion of Africans, disproportionately females, and are associated with significant disability. Heterogeneity across regions and populations highlights the need for improved recognition, standardized diagnostics, and better resource allocation for headache disorders in Africa.

**Disclosure:** Nothing to disclose.

## EPO‐0577

### Temporal burden synchronization and machine learning‐identified dietary nuances in migraine‐anxiety comorbidity

#### H. Hu; F. Yang

##### The First Medical Center, Chinese PLA General Hospital, Beijing, China

**Background and aims:** Migraine and anxiety disorders are highly prevalent and frequently comorbid, leading to substantial functional impairment. However, the long‐term temporal evolution of this comorbidity and the role of modifiable dietary factors remain poorly understood. We aimed to characterize the evolving disease burden in the USA and identify specific dietary micronutrients and whole‐food patterns associated with anxiety risk in individuals with migraine.

**Methods:** We utilized data from the Global Burden of Disease (GBD) Study 2023 to analyze US trends (1990–2023) and generate projections to 2030 using ARIMA models. We further integrated NHANES (2001–2004) data and the MyPyramid Equivalents Database to assess dietary correlates in migraineurs (*n* = 984). Six machine‐learning algorithms were benchmarked, with Random Forest selected for interpretation via SHAP and LIME frameworks to identify key dietary predictors.

**Figure:**
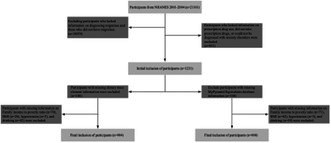
Flowchart of participant selection.

**Results:** GBD analysis revealed a stable migraine burden but a sharp increase in anxiety disorders after 2018, peaking during the COVID‐19 pandemic. A "burden‐synchronization window" was identified between ages 20 and 49, where peak disability‐adjusted life years (DALYs) for both conditions overlapped. Random Forest demonstrated superior predictive performance. SHAP analysis identified caffeine, theobromine, and protein as protective factors, while higher intakes of vitamin E, dietary fiber, and polyunsaturated fatty acids were unexpectedly associated with increased risk. Red/orange vegetables and whole grains were inversely associated with anxiety risk.

**Figure:**
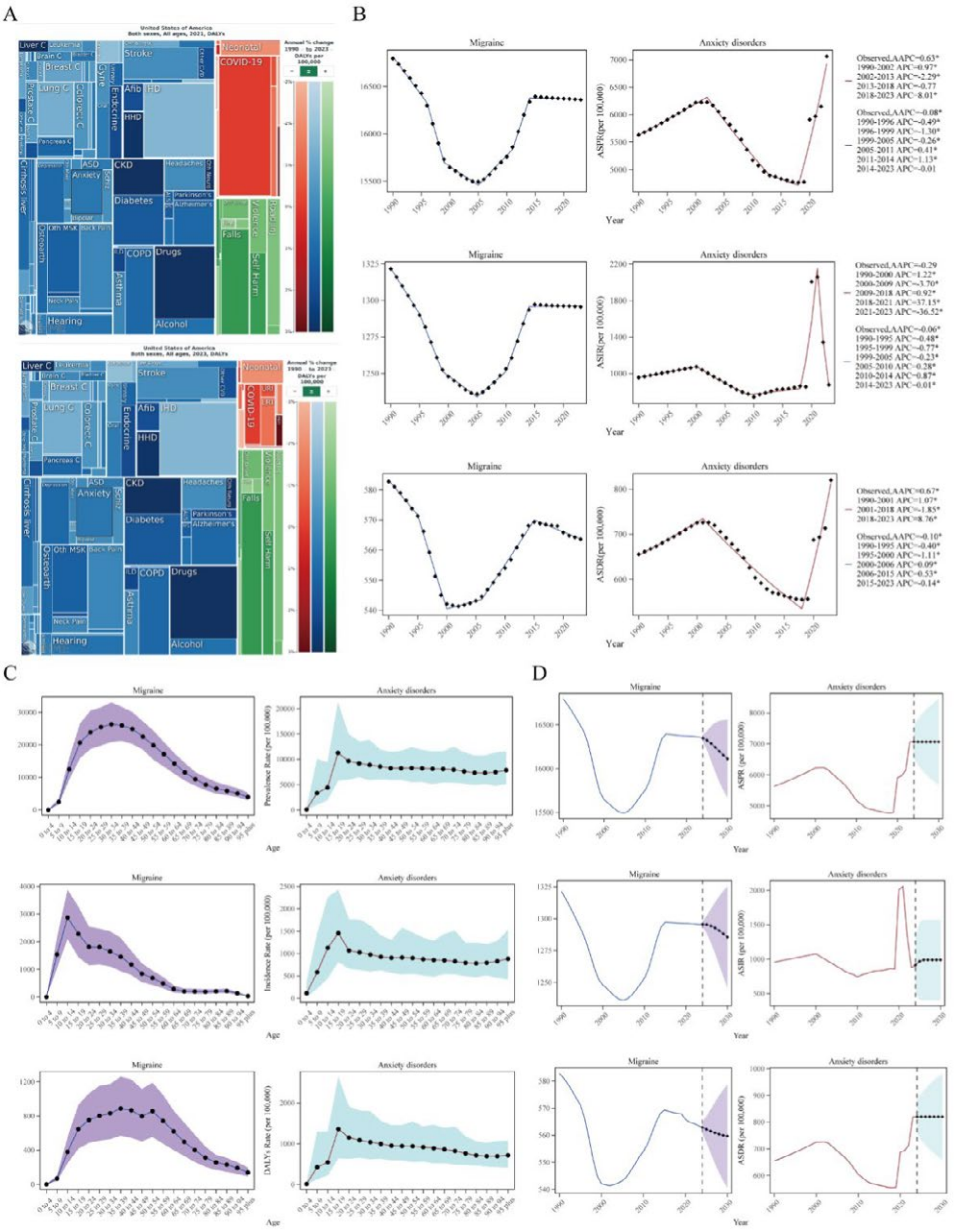
Trends in the burden of migraine and anxiety disorders in the United States (1990–2023) and projections based on GBD 2023. (A) Heatmap comparison of age standardized DALY rates for leading diseases in the United States between GBD 2021 and GBD 20

**Figure:**
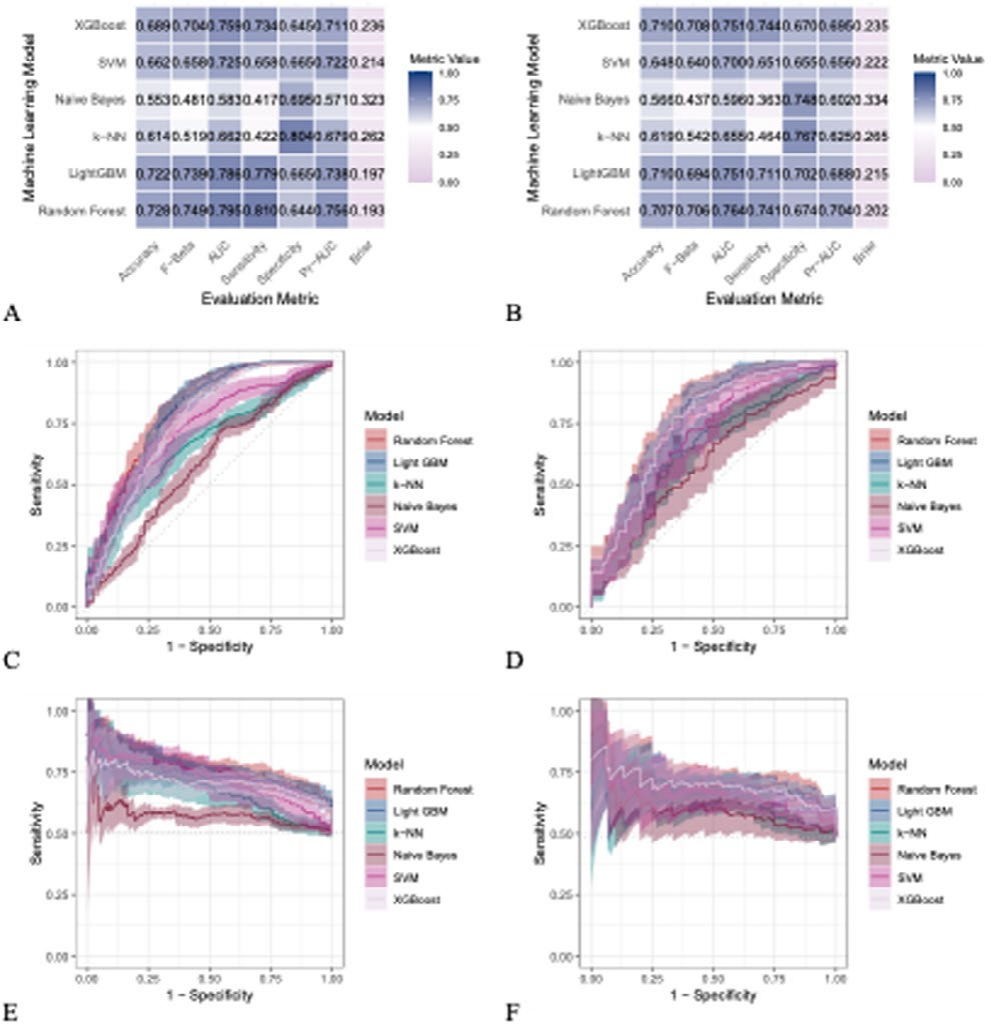
Time‐series analysis validating the machine‐learning results based on the MyPyramid Equivalents Database.

**Conclusion:** The rising burden of anxiety among migraineurs during mid‐adulthood necessitates integrated clinical management. Specific dietary interventions may modulate anxiety risk, challenging simplified antioxidant theories and emphasizing personalized nutritional strategies.

**Disclosure:** Nothing to disclose

## EPO‐0578

### Development and Assessment of a Headache Medicine Fellowship for Advanced Practice Providers (APPs)

#### J. Rothrock; A. Koutsandreas; K. Hoang

##### Inova Health Neurology/University of Virginia

**Background and aims:** There exists a mismatch between the volumes of individuals with headache disorders and providers possessing the skills and inclination to manage those individuals effectively. To help redress this imbalance we developed and assessed a 1 year headache medicine fellowship program intended for APPs.

**Methods:** One of us (JR) developed a 1 year fellowship curriculum blending didactic instruction, self‐learning, in‐clinic observation and subsequent clinical work involving a progressive increase in independence. In the last quarter of the fellowship we conducted a prospective study to assess clinical concordance and patient satisfaction for the APP fellow vs an MD neurologist with subspecialty training in headache (JR). All patients initially were evaluated by the fellow and then subsequently by the neurologist blinded to the results of the fellow's evaluation.

**Results:** Two nurse practitioners (NPs:AK and KH) successfully completed the 1 year fellowship, including the last quarter's prospective study. In 98 and 96 of the 100 consecutive new headache patients evaluated by each NP, diagnoses matched those of the neurologist. There were no major differences in the management recommended by the NPs versus the neurologist. Patient satisfaction with the NPs was slightly higher than with the neurologist.

**Conclusion:** Assessing the clinical effectiveness of a fellowship‐trained APP vs that of a subspecialist physician, we found patient satisfaction to be essentially equal and both diagnostic and management concordance to be high. Utilization of appropriately trained APPs may represent one means of coping more effectively with the existing imbalance between headache patients seeking expert care and the volume of providers available.

**Disclosure:** This research was funded by AbbVie, Inc and the Inova Health Care system of Northern Virginia. All 3 authors are full‐time employees of Inova Health and the University of Virginia School of Medicine, and Dr. Rothrock has served as a paid consultant and speaker for AbbVie. His parent institutions have received funding for research he has conducted on behalf of AbbVie.

## EPO‐0579

### Early wearing‐off of onabotulinumtoxin A in chronic migraine: A retrospective study

#### K. Dos Santos Ferreira ^1^; A. Velly^2^

##### ^*1*^ *Neurologist, Headache Clinic, Montreal Neurological Clinic, Montreal, Quebec, Canada;* ^*2*^ *Associate Professor, Faculty of Dentistry, Jewish General Hospital/ McGill University, Montreal, Quebec, Canada*

**Background and aims:** OnabotulinumtoxinA is a well‐established prophylactic therapy for chronic migraine, administered every 12 weeks. However, many patients report a wearing‐off of efficacy prior to the end of the dosing cycle. This retrospective review investigates the frequency of early wearing‐off in patients receiving onabotulinumtoxin A and explores associated clinical factors.

**Methods:** Medical records of chronic migraine patients treated with onabotulinumtoxinA were reviewed. Patient demographics, headache frequency, use of rescue therapies, dosing regimens, and adjunctive procedures were recorded. Patients were divided into two groups based on whether they experienced wearing‐off before 12 weeks.

**Results:** Among the 89 patients (91% female), 24 (26.9%) reported wearing‐off prior to 12 weeks. It was significantly associated with higher baseline headache frequency (79% vs. 47.7%, *p* = 0.007), earlier injection requirements (87.5% vs. 1.5%, *p* = 0.000), and Greater Occipital Nerve blocks (GON blocks) for recurrent pain (41.6% vs. 9.2%, *p* = 0.000). Patients with wearing‐off prior to 12 weeks received the full 195 U dose of onabotulinumtoxin A more frequently (58.3% vs. 20%, *p* = 0.000). Ubrogepant was also more commonly used in the wearing‐off group (33.4% vs. 6.2%, *p* = 0.000). No significant differences were noted for Rimegepant (16.7% vs 6.2%, *p* = 0.123), Atogepant (8.3% vs 3.0%, *p* = 0.288) or Monoclonal antibodies against CGRP (12.5% vs 10.8%, *p* = 0.818) use.

**Figure:**
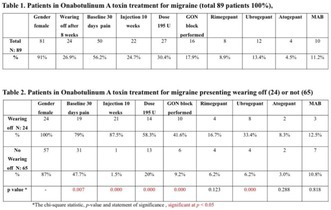
Patients using Onabotulinumtoxin A.

**Conclusion:** Wearing‐off was associated with higher baseline migraine frequency, earlier reinjection intervals, and increased use of acute therapies, suggesting that this subgroup may require tailored management strategies, including reassessing injection technique, dose optimization, shorter injection intervals (10 weeks), GON blocks inter procedures, optimizing other preventive treatment or using Gepants to improve outcomes.

**Disclosure:** Nothing to disclose.

## EPO‐0580

### Biomolecular Effects of Atogepant in High‐Frequency Episodic Migraine

#### M. Giraudo ^1^; R. De Icco^1^; F. Cammarota^1^; G. Vaghi^1^; V. Grillo^1^; M. Corrado^2^; B. Agostini^1^; M. Allena^2^; E. Guaschino^2^; N. Ghiotto^2^; R. Greco^2^; C. Demartini^2^; M. Francavilla^2^; S. Facchetti^2^; G. Sances^2^; C. Tassorelli^1^

##### ^*1*^ *1) Department of Brain and Behavioral Sciences, University of Pavia, Pavia, Italy**;*** ^*2*^ *2) Headache Science and Neurorehabilitation Unit, IRCCS Mondino Foundation, Pavia, Italy*

**Background and aims:** Atogepant is a novel migraine preventive drug acting as calcitonin gene‐related peptide (CGRP) receptor antagonist. We conducted a prospective, open label study aimed to evaluate whether clinical response to atogepant is associated with biomolecular changes in subjects with high‐frequency episodic migraine (HFEM ‐ at least 8 migraine days/month ‐ MMDs). As a secondary aim, we assessed differences between Responders, those achieving at least a 50% reduction in MMDs after atogepant treatment, and Non‐Responders.

**Methods:** We enrolled 40 subjects (75% women, age 35.5 ± 21.9, MMDs 9.9 ± 2.3). All participants underwent a biomolecular profiling at baseline (T0) and after a 3‐month daily atogepant 60 mg treatment (T1). We analyzed the expression of endocannabinoid enzymes, monoacylglycerol lipase (MAGL) and fatty‐acid amide hydrolase‐(FAAH), and microRNAs (miR‐155, miR‐34a, miR‐382‐5p) expression in peripheral blood mononuclear cells, and neuropeptides (CGRP, PACAP) plasma levels.

**Results:** At T1 70% of participants classified as Responders (28/40). At T1, MAGL gene expression decreased in Responders (T0: 9.8±5.4 relative quantification – RQ vs. T1: 13.2±7.9 RQ, *p* = 0.021), without changes inNon‐Responders (T0: 7.4 ± 4.3 RQ vs. T1: 8.3 ± 4.4 RQ, *p* = 0.552). At T0, MAGL was higher in future Responders compared to Non‐Responders (*p* = 0.022). No baseline differences or after‐treatment modifications were found for FAAH expression, microRNAs expression, or neuropeptides levels.

**Conclusion:** We confirmed atogepant effectiveness in HFEM. We found a modulation of MAGL expression in Responders participants. Our data suggests an indirect modulation of the endocannabinoid system homeostasis during atogepant treatment.

**Disclosure:** Nothing to disclose

## EPO‐0581

### Migraine‐free days following acute rimegepant treatment in the real‐world: Findings from CONFIDENCE

#### P. Goadsby^1^; R. Lipton^2^; L. Abraham^3^; A. Urani^4^; G. Lambru^5^; P. Pozo‐Rosich^6^; T. Schwedt^7^; K. Fanning^8^; K. Hygge Blakeman ^9^

##### ^*1*^ *King Abdullah University of Science and Technology, Thuwal, Saudi Arabia and King's College London, London, UK;* ^*2*^ *Montefiore Medical Center and Albert Einstein College of Medicine, Bronx, USA;* ^*3*^ *Pfizer R&D UK Ltd, Tadworth, Surrey, UK;* ^*4*^ *Aptar Digital Health, Paris, France;* ^*5*^ *The Headache and Facial Pain Service, Guy's and St. Thomas' NHS Foundation Trust, London, UK;* ^*6*^ *Headache Unit, Neurology Department, Vall d'Hebron Hospital, and Headache Research Group, Vall d’Hebron Research Institute (VHIR), Universitat Autònoma de Barcelona, Barcelona, Spain;* ^*7*^ *Department of Neurology, Mayo Clinic, Scottsdale, USA;* ^*8*^ *MIST Research, Wilmington, USA;* ^*9*^ *Pfizer AB, Stockholm, Sweden*

**Background and aims:** CONFIDENCE (NCT06467370) was a prospective observational study evaluating the effectiveness of acute rimegepant treatment over multiple migraine attacks. As rimegepant is also a preventive migraine treatment, this analysis evaluated duration of migraine freedom after acute use.

**Methods:** Participants with 3‐14 headache days in the past 30 completed a 28‐day migraine diary via the Migraine Buddy® app. Preventive therapy was permitted. This analysis included all acutely treated (prescription/over‐the‐counter) migraine attacks recorded >3 days before the end of study and without missing diary data before the next attack. Generalized linear mixed models with a logit link were used to calculate the odds ratio for ≥1, ≥2, ≥3, or ≥4 consecutive migraine free days following the day of acute treatment with rimegepant vs other medications.

**Results:** 429 participants recorded 2563 eligible migraine attacks. Independent of which acute treatment was used, 64.9% of attacks were followed by ≥1, 47.6% by ≥2, 35.4% by ≥3, and 25.0% by ≥4 migraine‐free days. In models evaluating treatment alone, rimegepant was associated with a significantly higher chance of migraine‐freedom in the following ≥1 (77%), ≥2 (71%), ≥3 (58%), and ≥4 days (44%) vs other acute medications (Table 1). In models controlling for migraine days during the study, pain level, and functional ability at the time of treatment, rimegepant was associated with a significantly higher chance of migraine‐freedom in the following ≥1 (54%), ≥2 (48%), ≥3 days (30%) vs other acute medications.

**Figure:**
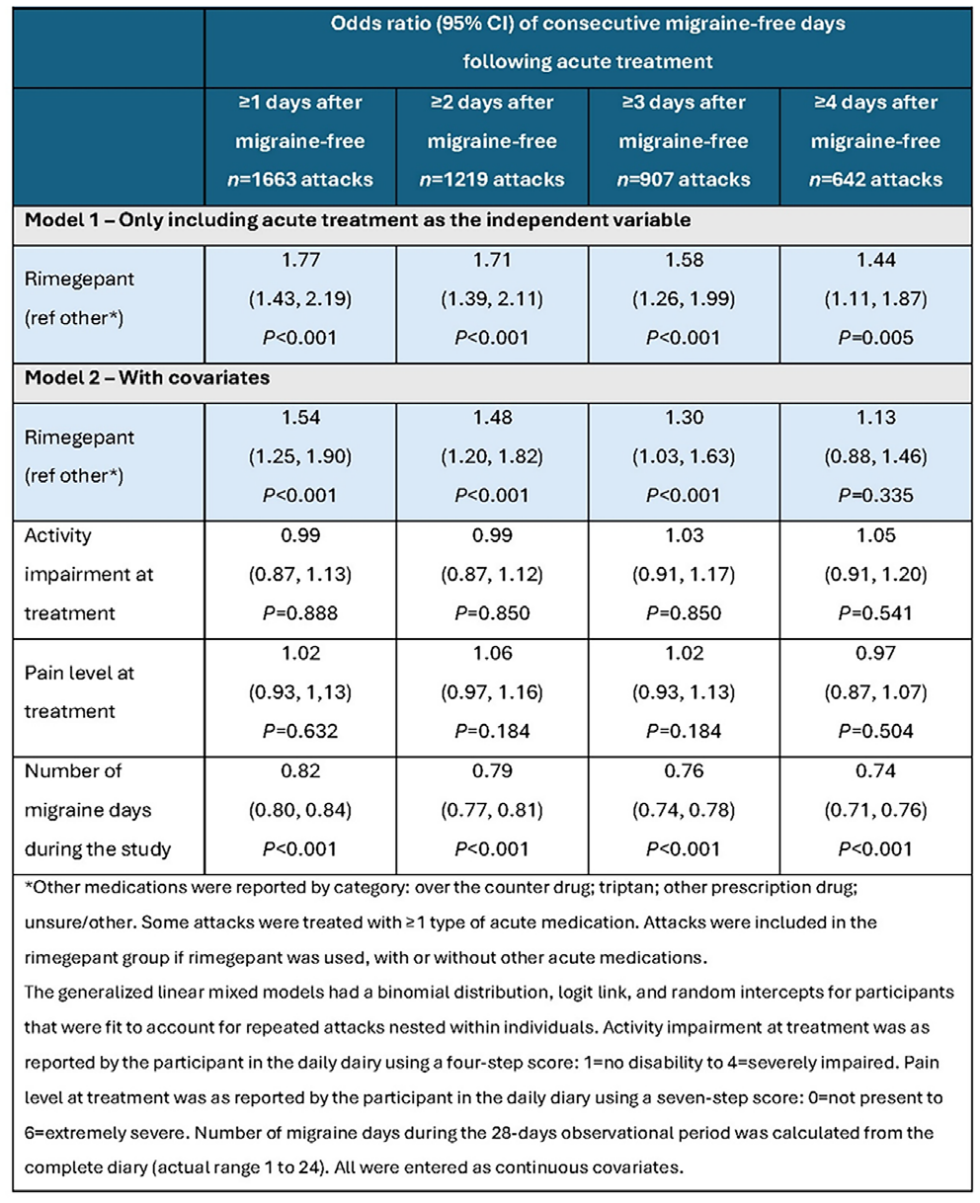
**TABLE 1** Odds ratios for consecutive migraine‐free days following rimegepant vs other acute medication treatment of a migraine attack.

**Conclusion:** Higher odds of migraine‐free days following treatment was observed with rimegepant vs other acute migraine medications.

**Disclosure:** This study was sponsored by Pfizer. PJG reports consulting fees from Aeon, AbbVie, CoolTech, Dr Reddy's Laboratories, Eli Lilly, Epalex, Ipsen, Kallyope, Linpharma, Lundbeck, Orion Pharma, Pfizer, PureTech Health, Satsuma, Seaport Therapeutics, Shiratronics, and Teva; personal fees for advice from Gerson Lehrman Group, Guidepoint, SAI Med Partners, and Vector Psychometric; fees for educational materials from CME Outfitters and WebMD; publishing royalties or fees from Massachusetts Medical Society, Oxford University Press, UptoDate, and Wolters Kluwer. RBL reports research support from NIH, FDA, and the National Headache Foundation; research grants, consultant, advisory board member or honoraria from Aeon, Allergan/AbbVie, Amgen, Axsome, Dr Reddy's Laboratories, Eli Lilly, GSK, Ipsen, Lundbeck, Pfizer, Merck, Teva, and Vedanta; royalties from Informa and Wolff's Headache (8th edition, Oxford University Press, 2009); and stock options in Biohaven Pharmaceuticals, CoolTech, and NuVieBio. AU is an employee of Aptar Digital Health, which was a paid consultant to Pfizer for the conduct of this study. GL reports consulting fees from AbbVie, Dr Reddy's Laboratories, Eli Lilly, Lundbeck, Novartis, Pfizer, and Teva. PP‐R reports in the last 36 months consultant or speaker fees/support from AbbVie, Almirall, Dr. Reddy's, Eli Lilly, Lundbeck, Medscape, Novartis, Organon, Pfizer, and Teva; research grants from AbbVie, AGAUR, EraNet Neuron, FEDER RIS3CAT, Instituto Investigación Carlos III, MICINN, Teva; funding for clinical trials to her research group from AbbVie, Amgen, Eli Lilly, Ipsen, Lundbeck, Novartis, Pfizer and Teva. TJS reports in the last 24 months consulting fees from AbbVie, Lundbeck, and Salvia; royalties from UpToDate; stock options/ownership interest in Allevalux and Nocira; research grants from AbbVie, American Heart Association, Flinn Foundation, Henry Jackson Foundation, National Headache Foundation, United States National Institutes of Health, Patient Centered Outcomes Research Institute, Pfizer, and United States Department of Defense. KMF is the Managing Director of MIST Research, which has received research funding from AbbVie, AESARA, Aptar, CoolTech, Migraine Canada, NYC Langone Health, Pfizer, UC Irvine, Ipsen. LA and KHB are employees of and own stock/options in Pfizer.

## EPO‐0582

### Rimegepant for the Acute Treatment of Migraine in People With ≥1 Lapsed Triptans, With and Without Current Triptan Use, in the CONFIDENCE study

#### R. Lipton^1^; L. Abraham^2^; A. Urani^3^; G. Lambru^4^; P. Goadsby^5^; P. Pozo‐Rosich^6^; T. Schwedt^7^; K. Fanning^8^; K. Hygge Blakeman ^9^

##### ^*1*^ *Montefiore Medical Center and Albert Einstein College of Medicine, Bronx, USA;* ^*2*^ *Pfizer R&D UK Ltd, Tadworth, Surrey, UK;* ^*3*^ *Aptar Digital Health, Paris, France;* ^*4*^ *The Headache and Facial Pain Service, Guy's and St. Thomas' NHS Foundation Trust, London, UK;* ^*5*^ *King Abdullah University of Science and Technology, Thuwal, Saudi Arabia and King's College London, London, UK;* ^*6*^ *Headache Unit, Neurology Department, Vall d'Hebron Hospital, and Headache Research Group, Vall d’Hebron Research Institute (VHIR), Universitat Autònoma de Barcelona, Barcelona, Spain;* ^*7*^ *Department of Neurology, Mayo Clinic, Scottsdale, USA;* ^*8*^ *MIST Research, Wilmington, USA;* ^*9*^ *Pfizer AB, Stockholm, Sweden*

**Background and aims:** The prospective, observational CONFIDENCE study (NCT06467370) evaluated the effectiveness of rimegepant for the acute treatment of migraine over multiple attacks. This analysis evaluated rimegepant treatment outcomes in participants with ≥1 previously discontinued (lapsed) triptans, both with/without current triptan use.

**Methods:** CONFIDENCE recruited participants using rimegepant for the acute treatment of migraine from the Migraine Buddy® app. Participants completed a baseline survey and 28‐day migraine diary. On days with a migraine attack, treatment characteristics, time to meaningful pain relief (MPR), and meaningful functional improvement (MFI) were collected. Generalized linear mixed models with a logit link were used to control for within person correlation and evaluate predictors of MPR and MFI within 2 and 4 h of rimegepant intake.

**Results:** Participants (*n* = 370) with ≥1 lapsed triptan reported 1948 rimegepant‐treated migraine attacks. Of these, 662 (34%) attacks were in participants with 1 lapsed triptan, 719 (37%) with 2, and 567 (29%) with ≥3; 538 (28%) were in participants who currently used a triptan to treat some attacks. Models identified significantly lower odds of MPR within 2 (58%) and 4 h (50%), and MFI within 2 (54%) and 4 h (57%) of rimegepant treatment in participants with ≥3 lapsed triptans (vs 1; Table 1). Having 2 lapsed triptans (vs 1) and current triptan use were associated with numerically lower odds of MPR and MFI but were not consistently statistically significant.

**Figure:**
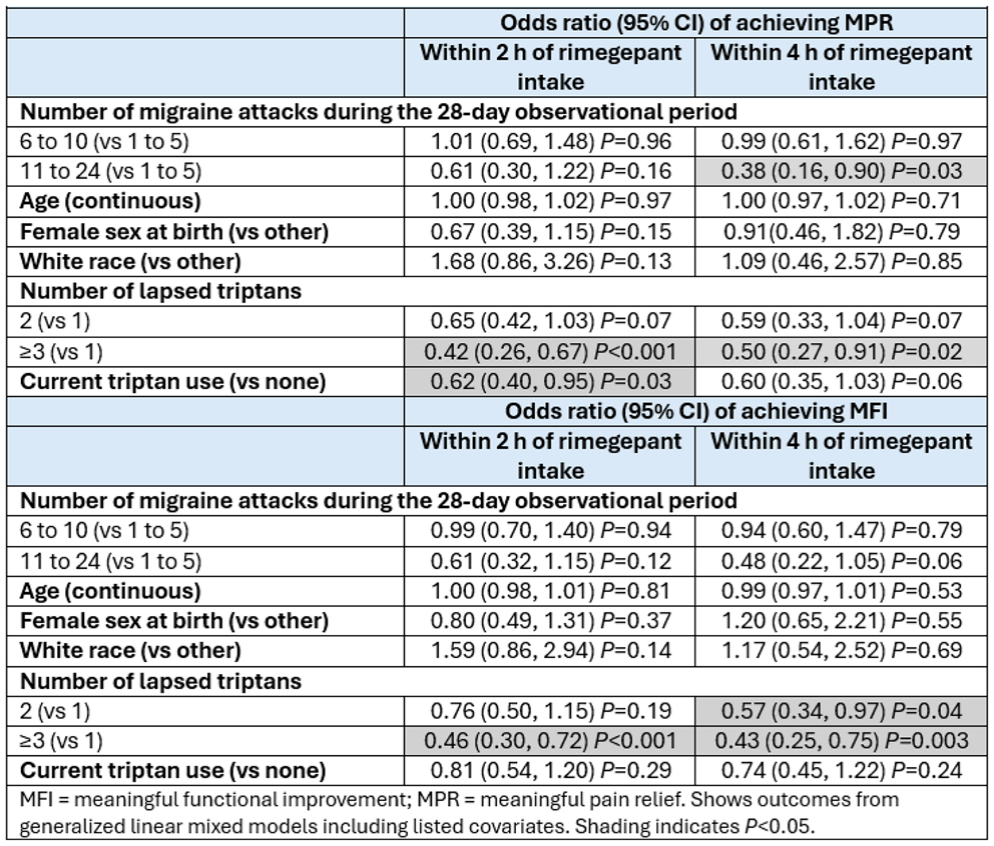
**TABLE 1** Predictors of meaningful pain relief and meaningful functional improvement following acute rimegepant treatment.

**Conclusion:** Rimegepant efficacy was higher in participants with lower numbers of lapsed triptans and without current triptan use, possibly reflecting clinical differences between subgroups.

**Disclosure:** This study was sponsored by Pfizer. RBL reports research support from NIH, FDA, and the National Headache Foundation; research grants, consultant, advisory board member or honoraria from Aeon, Allergan/AbbVie, Amgen, Axsome, Dr Reddy's Laboratories, Eli Lilly, GSK, Ipsen, Lundbeck, Pfizer, Merck, Teva, and Vedanta; royalties from Informa and Wolff's Headache (8th edition, Oxford University Press, 2009); and stock options in Biohaven Pharmaceuticals, CoolTech, and NuVieBio. AU is an employee of Aptar Digital Health, which was a paid consultant to Pfizer for the conduct of this study. GL reports consulting fees from AbbVie, Dr Reddy's Laboratories, Eli Lilly, Lundbeck, Novartis, Pfizer, and Teva. PJG reports consulting fees from Aeon, AbbVie, CoolTech, Dr Reddy's Laboratories, Eli Lilly, Epalex, Ipsen, Kallyope, Linpharma, Lundbeck, Orion Pharma, Pfizer, PureTech Health, Satsuma, Seaport Therapeutics, Shiratronics, and Teva; personal fees for advice from Gerson Lehrman Group, Guidepoint, SAI Med Partners, and Vector Psychometric; fees for educational materials from CME Outfitters and WebMD; publishing royalties or fees from Massachusetts Medical Society, Oxford University Press, UptoDate, and Wolters Kluwer. PP‐R reports in the last 36 months consultant or speaker fees/support from AbbVie, Almirall, Dr. Reddy's, Eli Lilly, Lundbeck, Medscape, Novartis, Organon, Pfizer, and Teva; research grants from AbbVie, AGAUR, EraNet Neuron, FEDER RIS3CAT, Instituto Investigación Carlos III, MICINN, Teva; funding for clinical trials to her research group from AbbVie, Amgen, Eli Lilly, Ipsen, Lundbeck, Novartis, Pfizer and Teva. TJS reports in the last 24 months consulting fees from AbbVie, Lundbeck, and Salvia; royalties from UpToDate; stock options/ownership interest in Allevalux and Nocira; research grants from AbbVie, American Heart Association, Flinn Foundation, Henry Jackson Foundation, National Headache Foundation, United States National Institutes of Health, Patient Centered Outcomes Research Institute, Pfizer, and United States Department of Defense. KMF is the Managing Director of MIST Research, which has received research funding from AbbVie, AESARA, Aptar, CoolTech, Migraine Canada, NYC Langone Health, Pfizer, UC Irvine, Ipsen. LA and KHB are employees of and own stock/options in Pfizer.

## EPO‐0583

### Efficacy of eptinezumab in adults with CM and MOH who also received patient education: Country‐level results from the RESOLUTION trial

#### G. Terwindt^1^; S. Tepper^2^; R. Jensen ^3^; C. Lundqvist^4^; H. Schytz^3^; F. Vernieri^6^; M. Lantéri‐Minet^8^; A. Blumenfeld^10^; R. Lipton^11^; K. Ranc^12^; G. Jansson^12^; A. Ettrup^12^; A. Mittoux^12^; C. Tassorelli^13^

##### ^*1*^ *Department of Neurology, Leiden University Medical Centre, Leiden, Netherlands;* ^*2*^ *The New England Institute for Neurology and Headache, Stamford, CT, United States;* ^*3*^ *Department of Neurology, Danish Headache Center, Rigshospitalet‐Glostrup, University of Copenhagen, Copenhagen, Denmark;* ^*4*^ *Departments of Neurology and Health Services Research, Akershus University Hospital, Lørenskog, Norway;* ^*6*^ *Unit of Headache and Neurosonology, Fondazione Policlinico Universitario Campus Bio‐Medico, Rome, Italy;* ^*8*^ *Department of Neurology, Albert Einstein College of Medicine, New York, NY, United States;* ^*10*^ *INSERM U1107 Migraine and Trigeminal Pain, Auvergne University, Clermont‐Ferrand, France;* ^*11*^ *The San Diego Headache Center, San Diego, CA, United States;* ^*12*^ *H. Lundbeck A/S, Copenhagen, Denmark;* ^*13*^ *Department of Brain and Behavioral Sciences, University of Pavia, Pavia, Italy*

**Background and aims:** These country‐level analyses evaluated the migraine‐preventive efficacy of eptinezumab versus placebo in adults with chronic migraine (CM) and medication‐overuse headache (MOH), together with a brief educational intervention (BEI).

**Methods:** The multinational phase 4 RESOLUTION (NCT05452239) trial included a 12‐week double‐blind, placebo‐controlled period. Adults (18–75yrs) with CM and MOH were randomized 1:1 to IV eptinezumab 100 mg or placebo at baseline; all participants received a BEI on MOH before infusion. Post hoc subgroup analyses were conducted by country (those with > = 20 participants per arm). Endpoints included change from baseline in monthly migraine days (MMDs) and monthly days with acute medication use (Weeks 1–12; full‐analysis set).

**Results:** The full‐analysis set included 602 of 608 randomized participants. Countries with > = 20 participants per arm included Denmark, France, Georgia, Italy, the Netherlands, and Spain (Table 1). In comparison with placebo with BEI, eptinezumab with BEI improved treatment outcomes, with directionally consistent results seen across countries. Participants from the Netherlands had large reductions in acute medication use in both treatment groups, but concurrently the largest difference between both treatment groups in change from baseline in MMDs.

**Figure:**
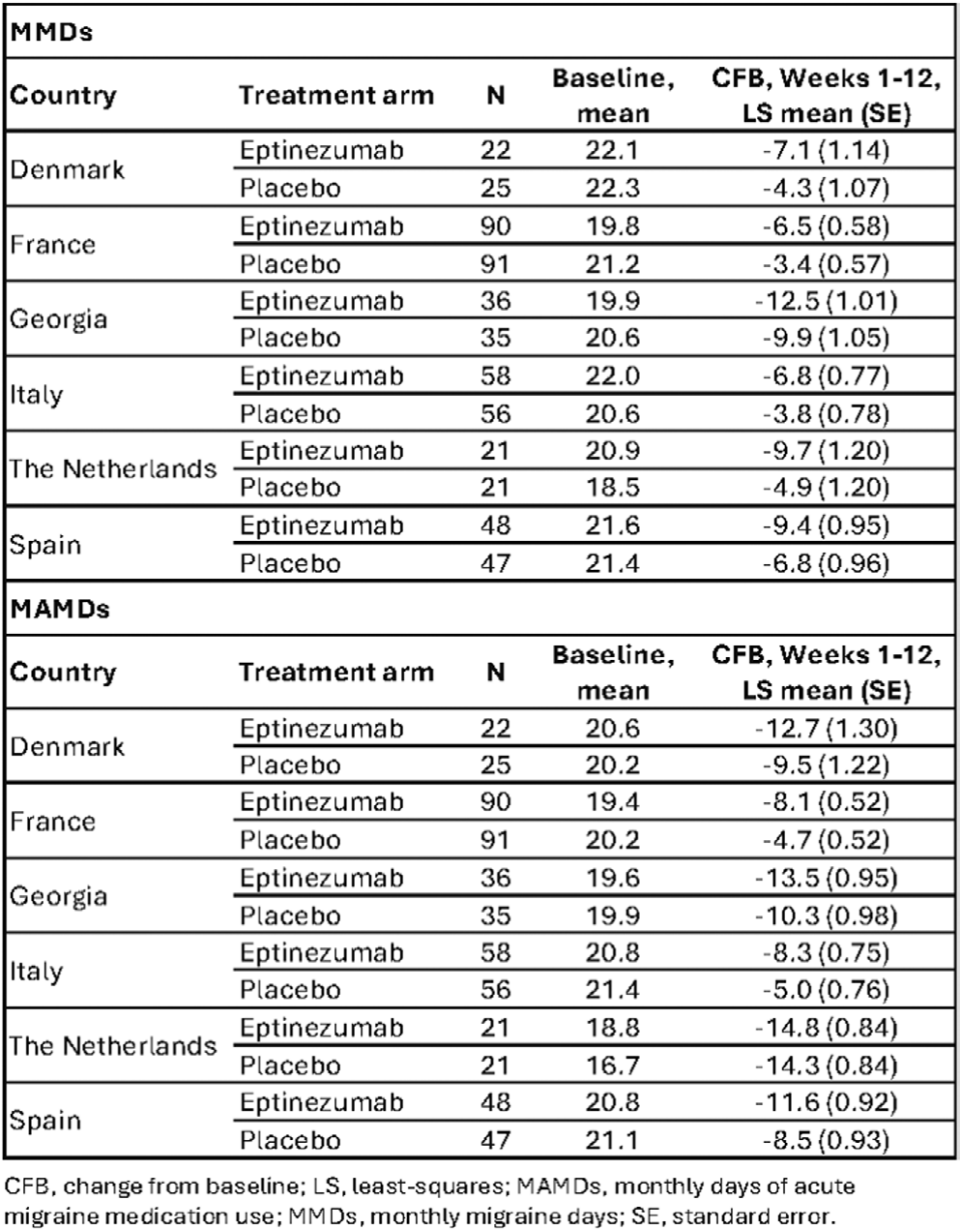
**TABLE 1** Change from baseline over Weeks 1–12 in MMDs and MAMDs.

**Conclusion:** In this post hoc analysis of the RESOLUTION trial, the treatment effect of eptinezumab among participants with CM and MOH who received patient education appeared to be homogenous across countries, supporting the robustness of the intervention and its relative independence of country effects. Results from the Netherlands suggest that, while patient education can help reduce acute medication use, concurrent initiation of preventive treatment with eptinezumab has additional benefit for reversing migraine chronification.

**Disclosure:** GMT reports grants or consultancy support from AbbVie, Eli Lilly, Lundbeck, Novartis, Organon, Pfizer, and Teva, and independent support from the Clayco Foundation, Dioraphte Foundation, Dutch Research Council, Dutch Heart Foundation, Dutch Brain Foundation, IRRF, and the European Community. SJT reports grants for research from AbbVie, Aeon, Amgen, Annovis, Axsome, Cassava, Cognition, Eli Lilly, Inhibikase, Ipsen, Lundbeck, Merz, Neurolief, Pfizer, PrecisionMed, Revance, Scilex, Suven, and UCB; has served as a consultant and/or on advisory boards (honoraria) for AbbVie, Aeon, Alphasights, Amgen, Aruene, Atheneum, Axsome Therapeutics, Becker Pharmaceutical Consulting, BioDelivery Sciences International, Biohaven, Catch Therapeutics, ClearView Healthcare Partners, Click Therapeutics, CoolTech, CRG, Decision Resources, Defined Health, DRG, Dr. Reddy's, Eli Lilly, ExpertConnect, FCB Health, Fenix, Gilmartin Capital, GLG, Guidepoint Global, Health Advances, Health Science Communications, HMP Communications, Impel, Initiator Pharma, Interactive Forums, IQVIA, Keyquest, Ki Health Partners, Krog and Partners, Lundbeck, M3 Global Research, Magellan Health, Magnolia Innovation, Miravo Healthcare, MJH Holdings, Neurofront Therapeutics, Neurolief, Nocira, Novartis, *p* Value Communications, Pain Insights, Palion Medical, Perfood, Pfizer, Pulmatrix, Putnam Associates, Rehaler, SAI MedPartners, Satsuma, Scilex, Slingshot Insights, Spherix Global Insights, Strategy Inc, Synapse Medical Communication, System Analytic, Taylor and Francis, Tegus, Teva, Theranica, Third Bridge, Tonix, Trinity Partners, Unity HA, Vial, and Xoc; receives salary from Dartmouth‐Hitchcock Medical Center, Thomas Jefferson University, and Ki Health Partners; serves as a speaker for AbbVie, Dr. Reddy's, Eli Lilly, Lundbeck, Pfizer, Scilex, Teva, and Tonix; and received continuing medical education honoraria from the American Academy of Neurology, American Headache Society, Annenberg Center for Health Sciences, Catamount Medical Education, Diamond Headache Clinic, Forefront Collaborative, Haymarket Medical Education, HMP Global, Medical Education Speakers Network, Medical Learning Institute, Migraine Association of Ireland, Miller Medical Education, National Association for Continuing Education, North American Center for CME, The Ohio State University, PeerView, Physicians’ Education Resource, PlatformQ Education, Primed, Vindico Medical Education, and WebMD/Medscape. [Disclosures for the remaining authors will be included in the presentation.]

## EPO‐0584

### Migraine Prevention with Eptinezumab in Real Life – A Retrospective Swiss Cohort Study

#### F. Riederer^1^; V. Fankhauser ^2^; R. Agosti^3^, B. Anders^4^, P. Balcerak^5^, L. Granata^6^, J. Held^7^, C. Kamm^8^, A. Palla^4^, A. Papadopoulou^9^, A. Pfister^10^, H. Pohl^11^, J. Rossel^12^, P. Ryvlin^10^, L. Schmid^1^, K. Schönholzer^9^, A. Scutelnic^1^, N. Slavova^1^, A. Ungureanu^3^, S. Wegener^11^, C. Zecca^13^, A. Gantenbein^14^, C. Schankin^15^

##### ^*1*^ *Department of Neurology, Inselspital, University Hospital Bern, Bern, Switzerland;* ^*2*^ *Department of Neurology, Inselspital, University Hospital Bern, Bern, Switzerland. Faculty of Medicine, University of Bern, Bern, Switzerland;* ^*3*^ *Kopfwehzentrum Hirslanden, Zurich, Switzerland;* ^*4*^ *Bellevue Medical Group, Zurich, Switzerland;* ^*5*^ *Department of Neurology, Kantonsspital St.Gallen, St.Gallen, Switzerland;* ^*6*^ *Schmerzzentrum Granata, Zurich, Switzerland;* ^*7*^ *Bellevue Medical Group, Zurich, Switzerland. Department of Neurology, University Hospital Zurich, Zurich, Switzerland;* ^*8*^ *Department of Neurology, Luzerner Kantonsspital, Lucerne, Switzerland;* ^*9*^ *Department of Neurology, University Hospital Basel, Basel, Switzerland. Department of Clinical Research, University of Basel, Basel, Switzerland;* ^*10*^ *Department of Neurology, CHUV, Lausanne, Switzerland;* ^*11*^ *Department of Neurology, University Hospital Zurich, Zurich, Switzerland;* ^*12*^ *Department of Clinical Research, University of Bern, Bern, Switzerland;* ^*13*^ *Neurocenter of Southern Switzerland, EOC, Lugano, Switzerland. Faculty of Biomedical Sciences, Università della Svizzera Italiana, Lugano, Switzerland;* ^*14*^ *Neurologische Praxis Bülach, Bülach, Switzerland;* ^*15*^ *Bellevue Medical Group, Zurich, Switzerland. Department of Neurology, Inselspital, University Hospital Bern, Bern, Switzerland*

**Background and aims:** To evaluate real‐world data to establish safety and efficacy of eptinezumab for the prevention of episodic and chronic migraine in clinical practice in Switzerland.

**Methods:** This multicenter retrospective cohort study included patients treated with eptinezumab at eleven Swiss headache centers. Over 12‐months, patients received up to four infusions of eptinezumab 100 mg or 300 mg administered every three months. Data on efficacy and tolerability were extracted from patients’ charts, including prospective headache diaries. The primary efficacy endpoint was the proportion of patients achieving a > = 50% reduction in monthly migraine days (MMDs) three months after the first infusion.

**Results:** A total of 201 patients were included. Mean baseline MMDs were 17.5. After eptinezumab administration, 56% (95% CI [48.7, 63.1]) of patients reached the primary endpoint of > = 50% response. Mean MMDs decreased significantly from baseline by 7.3 days (95% CI [6.19, 8.33], *p* < 0.001). This was observed for both, the 100 mg and 300 mg doses (100 mg, −7.2, *p* < 0.001; 300 mg, −7.4, *p* < 0.001). Prior treatment failure with CGRP ligand‐targeting antibodies was associated with a significantly lower > = 50% response rate (OR = 0.42, 95% CI 0.2–0.89, *p* = 0.024). Efficacy was not related to comorbid anxiety or depression. Adverse events within three months after infusion were reported by 15.1% of patients, most commonly rhinosinusitis (8%), constipation (6%), and fatigue (4%).

**Conclusion:** In a real‐world clinical setting, eptinezumab for the prevention of episodic and chronic migraine was effective and well tolerated. Prior treatment failure with CGRP ligand‐targeting antibodies was associated with reduced treatment response.

**Disclosure:** This work was funded by a research grant from Lundbeck The following authors have potential conflicting interests to disclose: Franz Riederer: Inselspital Bern, Bern University Hospital received compensation for F.R.'s speaking activities, consulting fees from Teva, Lundbeck, Abbvie. Chiara Zecca: Ente Ospedaliero Cantonale (employer) or C.Z. received compensation for C.Z.'s speaking activities, consulting fees, or grants from Abbvie, Alexion, Almirall, Biogen, Bristol Meyer Squibb, Eisai, Lilly, Lundbeck, Merck, Merz, Neuraxapharm, Novartis, Organon, Pfizer, Pharmaticino, Roche, Sandoz, Sanofi, Spirig, Takeda, Teva Pharma. C.Z. is recipient of a grant for senior researchers provided by AFRI (Area Formazione Accademica, Ricerca e Innovazione), EOC. Athina Papadopoulou: A.P.'s institution (University Hospital of Basel) received speaker fees/fees for advisory boards/for consulting from Sanofi‐Genzyme, Eli Lilly, AbbVie, Lundbeck, Pfizer and Teva. She received travel support from Bayer AG, Abbvie, Teva and Hoffmann‐La Roche. Her research was/is being supported by the University and University Hospital of Basel, the Swiss Multiple Sclerosis Society, the "Stiftung zur Förderung der gastroenterologischen und allgemeinen klinischen Forschung sowie der medizinischen Bildauswertung", the "Freie Akademische Gesellschaft Basel" and the Swiss National Science Foundation (Project numbers: P300PB_174480 and currently: PZ00P3_216468). Philipp Balcerak: Advisory Board/Speaker for Novartis, AbbVie and Alexion.

Movement disorders 5

## EPO‐0585

### Limb‐specific cervical spinal cord microstructure in Parkinson's disease: A clinically focused re‐analysis of an open quantitative MRI dataset

#### M. Elkasaby ^1^; A. Hassan^1^; M. Ahmed^2^

##### ^*1*^ *Faculty of Medicine, Al‐Azhar University, Cairo, Egypt;* ^*2*^ *Faculty of Medicine, Mansoura University, Mansoura, Egypt*

**Background and aims:** Gait disturbance and postural instability are major contributors to disability in Parkinson's disease (PD), yet conventional brain MRI and global clinical scores only partially account for these deficits. The cervical spinal cord—particularly the dorsal columns—may contribute to limb‐specific motor impairment but remains understudied.

**Methods:** We performed a cross‐sectional re‐analysis of an open NeuroLibre dataset including quantitative cervical spinal cord MRI (C2–C5) from 68 individuals with PD and 38 healthy controls. Metrics included cord cross‐sectional area, diffusion tensor imaging, and NODDI measures in the dorsal columns, magnetisation transfer ratio (MTR) in white and grey matter, and grey‐matter T2. Motor impairment was assessed using UPDRS III total and limb‐specific subscores.

**Results:** Cord cross‐sectional area did not differ between groups, but PD showed altered dorsal column microstructure, with higher orientation dispersion and lower axial diffusivity. Within PD, dorsal column fractional anisotropy, orientation dispersion, and intracellular volume fraction were independently associated with worse lower‐limb/gait impairment, explaining ~23–25% of variance. Grey‐matter MTR preferentially correlated with upper‐limb impairment. Lower‐limb‐dominant patients exhibited greater dorsal column microstructural disruption, with lateralisation to the clinically more affected side and progression across disease stages. An exploratory model combining dorsal column metrics with age achieved modest discrimination between PD and controls (AUC = 0.64).

**Conclusion:** Cervical dorsal column microstructure is selectively altered in PD and closely linked to lower‐limb and gait impairment, motor phenotype, and disease stage, even in the absence of cord atrophy. Grey‐matter metrics show a complementary relationship with upper‐limb dysfunction.

**Disclosure:** Nothing to disclose.

## EPO‐0586

### Knowledge, perceptions, and attitudes toward influenza vaccination in people with Parkinson's disease

#### S. Diaconu ^1^; I. Murasan^2^; D. Batrinu^2^; C. Falup‐Pecurariu^1^

##### ^*1*^ *Department of Neurology, County Clinic Hospital, Transilvania University Brasov, Romania;* ^*2*^ *Department of Neurology, County Clinic Hospital, Brasov, Romania*

**Background and aims:** Influenza infection represents a significant health risk for people with Parkinson's disease (PD), being associated with increased morbidity, functional decline, and hospitalization. Although annual influenza vaccination is recommended for older adults and individuals with chronic neurological disorders, reported vaccination rates in PD remain low. The aim of this study was to evaluate knowledge, perceptions, and attitudes toward influenza vaccination in patients with Parkinson's disease and to determine influenza vaccine acceptance rates in this population.

**Methods:** We conducted a cross‐sectional observational study including 123 people with PD. Participants completed a structured questionnaire assessing socio‐demographic variables, disease duration, comorbidities, influenza vaccination history, knowledge of influenza and vaccination, perceived risks and benefits, sources of information, and attitudes toward vaccination.

**Results:** The mean age of participants was 68.4 ± 8.9 years, and 56% were male. Only 11.4% (*n* = 14) reported receiving the influenza vaccine during the most recent season. Although 74% of patients considered influenza a potentially serious disease, only 23% correctly identified vaccination as the most effective preventive measure. Fear of adverse effects was reported by 61% of unvaccinated patients, concerns regarding worsening of Parkinson's symptoms by 56%, and doubts about vaccine efficacy by 42%. A lack of explicit recommendation from a healthcare professional was reported by 53% of participants.

**Conclusion:** Influenza vaccination acceptance among patients with Parkinson's disease was extremely low. Despite moderate awareness of influenza‐related risks, misconceptions, safety concerns, and insufficient physician recommendations remain major barriers.

**Disclosure:** Nothing to disclose.

## EPO‐0587

### Local and global microstructural and functional thalamomotor connectivity alterations in Parkinson's disease following motor learning

#### A. Amedi ^1^; T. Tamir^2^; E. Sasson^3^; N. Saporta^2^; S. Shelly^4^; R. Sivan Hoffmann^5^; M. Catalogna^1^

##### ^*1*^ *The Baruch Ivcher Institute for Brain, Cognition, and Technology, Baruch Ivcher School of Psychology, Reichman University, Herzliya, Israel;* ^*2*^ *Remepy Health Ltd, Ramat Gan, Israel;* ^*3*^ *WiseImage, Utrecht, Netherlands;* ^*4*^ *Department of Neurology, Rambam Medical Center, Haifa, Israel;* ^*5*^ *Radiology Department, Meir Medical Center, Kfar Sava, Israel*

**Background and aims:** Diffusion tensor imaging (DTI) provides a sensitive approach to detect microstructural decline in Parkinson's disease (PD) and is emerging as a method to identify rapid learning induced white‐matter changes, yet such evidence in PD remains limited.

**Methods:** In this prospective, double‐blind, randomized, placebo‐controlled study, 32 levodopa‐treated patients were randomized to a 3‐week digital intervention (DopApp) or placebo app. The DopApp involved a digital spatial navigation protocol combining egocentric and allocentric strategies with multisensory‐deprivation principles, alongside psychological and rehabilitation modules. Patients were assessed pre‐ and post‐treatment using clinical and psychological scales and MRI scans, including resting‐state fMRI (rsFC) and DTI, to investigate motor learning neuroplasticity.

**Results:** DopApp treatment significantly improved the total MDS‐UPDRS score compared to placebo‐app, driven by gains in motor function (Part III) and activities of daily living (Part II). Neuroimaging revealed increased fractional anisotropy in the right ventrolateral posterior (VLp) thalamus and strengthened functional connectivity between VLp and precentral gyrus (PreCG), both correlated with clinical motor improvements. Graph‐theory analysis indicated increased local efficiency in PreCG and adjacent motor network, suggesting enhanced structural integration.

**Figure:**
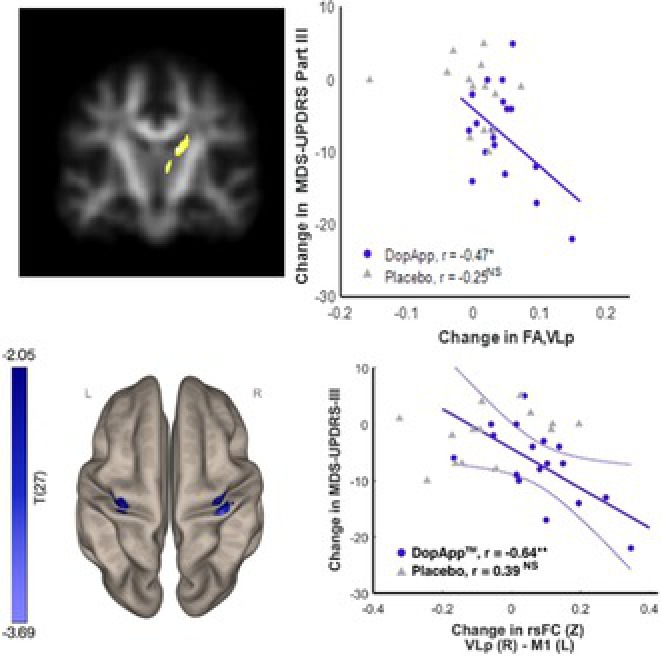
Neuroimaging analysis results and correlation with clinical measures

**Conclusion:** These findings demonstrate regionally specific, short‐term plasticity in PD, and support DTI's sensitivity to capture clinically relevant motor training‐related white‐matter adaptations beyond dopaminergic medication effects.

**Disclosure:** Amir Amedi is a co‐founder and employee of Remepy Health Ltd. and holds shares in Remepy Inc, the company that developed the DopApp.

## EPO‐0588

### Sublingual Apomorphine Tolerability in Clinical Practice: 18‐months Post launch experience in Europe

#### J. Kassubek^1^; R. García‐Ramos^2^; M. F. Gago^3^; H. C. Brigas^4^; C. Denecke‐Muhr ^4^; D. Lopes^4^; C. Meneses^4^; J. Holenz^4^

##### ^*1*^ *Department of Neurology, University of Ulm, Ulm, Germany;* ^*2*^ *Department of Neurology, Hospital Clínico San Carlos, 28040 Madrid, Spain;* ^*3*^ *Department of Neurology, ULS Alto Ave, Hospital da Senhora da Oliveira, Guimarães, Portugal; CNS ‐ Campus Neurológico, Braga, Portugal;* ^*4*^ *Research & Development Division, BIAL‐Portela & Cª, S.A, S. Mamede do Coronado, Portugal*

**Background and aims:** Sublingual apomorphine (SL‐APO) is an on‐demand therapy for the management of OFF episodes in Parkinson's disease. This analysis describes the post‐marketing safety profile of SL‐APO in Europe during the first 18 months after launch.

**Methods:** The drug global safety database was searched for European valid post‐marketing reports (spontaneous reports, health authorities, literature reports, non‐interventional studies, excluding marketing studies) from launch (April 2025) to October end 2025. Expectedness was assessed based on the current European Summary of Product Characteristics (SmPC) for SL‐APO.

**Results:** A total of 346 events were collected from 159 valid cases: 94 male (59%), mean age 65 years, from Germany (90, 57%), Portugal (44, 28%) and Spain (25, 16%). The 3 most common reported events were “nausea” (48, 14%), “dizziness” (22, 6%) and “vomiting” (15, 4%), consistent with known safety profile of SL‐APO. Oropharyngeal events accounted for 47 (14%) of all events, most commonly “aphthous ulcer”(7) and “oral pain”(4) (Table1). Overall, 326 (94%) events were considered non‐serious, and “syncope”(4) was the only serious event reported more than once. SL‐APO was withdrawn, temporarily or permanently, in 72 (45%) cases, including 18 (11%) due to oropharyngeal events.

**Figure:**
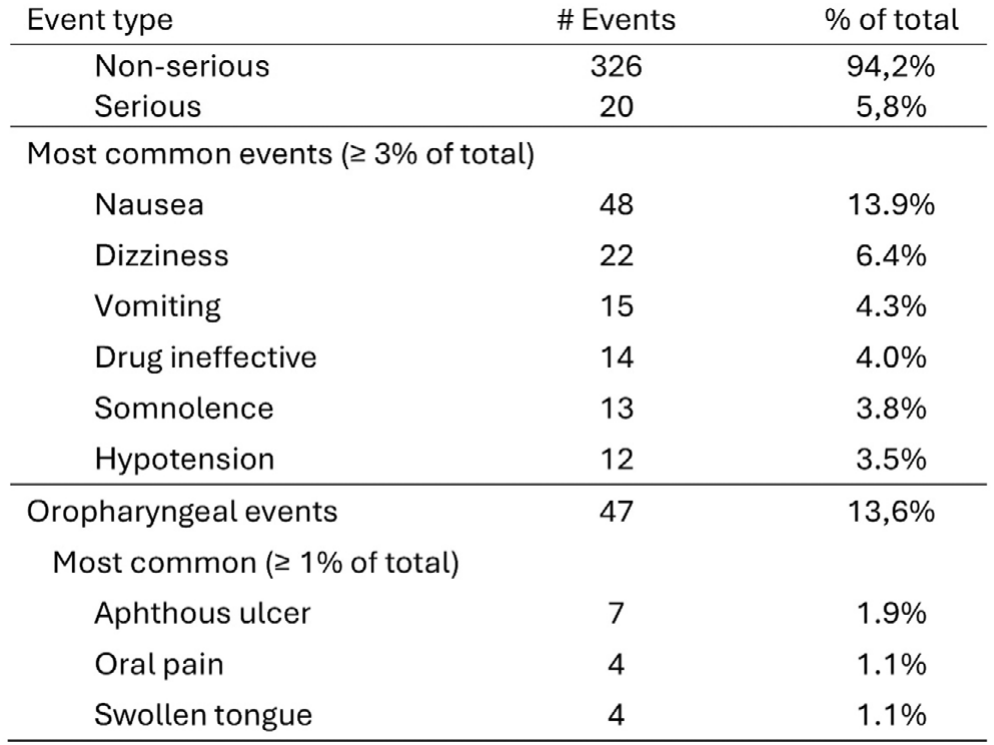
**TABLE 1** Summary of 346 reported events from 159 cases with sublingual apomorphine (SL‐APO) in European post‐marketing surveillance.

**Conclusion:** Reported events were predominantly non‐serious and consistent with SL‐APO known safety profile, supporting its favorable tolerability in real‐world practice. The apparent discontinuation rate should be interpreted cautiously, given the reporting bias inherent to spontaneous post‐marketing data and increased vigilance during the early post‐launch period. These observations underscore the importance of ongoing pharmacovigilance, proactive management of tolerability issues, and patient education to optimize adherence.

**Disclosure:** Supported by Bial‐Portela & Cª, S.A.

## EPO‐0589

### Advancing Deep Brain Stimulation management in Parkinson's Disease through wearable technologies: A systematic review

#### I. D'Ascanio ^1^; L. Baldelli^2^; I. Cani^2^; S. Belfiori^1^; M. Paolillo^1^; G. Lopane^3^; L. Chiari^1^; G. Giannini^2^; L. Palmerini^2^

##### ^*1*^ *Department of Electrical, Electronic and Information Engineering, Alma Mater Studiorum – University of Bologna, Bologna, Italy;* ^*2*^ *UOC Clinica Neurologica Rete Metropolitana NEUROMET, IRCCS Istituto delle Scienze Neurologiche di Bologna, Bologna, Italy;* ^*3*^ *Unit of Rehabilitation Medicine, IRCCS Istituto delle Scienze Neurologiche di Bologna, Bologna, Italy*

**Background and aims:** Deep Brain Stimulation (DBS) is a well‐established advanced treatment in Parkinson's disease (PD) [1]. The effects of DBS have been investigated using laboratory‐based systems [2], which are restricted to specialized clinical settings and therefore limit broader clinical integration. In contrast, wearable sensors have emerged as a complementary approach to objectively assess PD symptoms in both clinical and real‐world settings [3], thereby extending traditional assessments. This study aims to conduct a systematic review to examine the application of wearable technology in supporting DBS management.

**Methods:** The literature review was conducted analyzing Pubmed and Scopus databases. Studies were eligible for inclusion if they involved individuals with PD treated with DBS and used wearable devices. Studies were excluded if they involved non‐commercial prototype devices, did not specify the device used, and were not peer‐reviewed.

**Results:** After full‐text screening, 71 articles were included and categorized into two main research topics: 49 studies (69%) focused on digital monitoring, aiming to evaluate DBS efficacy; 22 studies (31%) investigated wearable‐based approaches to support DBS programming, exploring brain‐activity patterns during disease‐related symptoms. The majority of studies were conducted in clinical settings (53, 74%) and used a single device (42, 58%). Considerable heterogeneity was observed across study protocols in terms of tasks, treatment conditions, and time measurements.

**Conclusion:** Current literature, while heterogeneous and largely exploratory, highlights the promising potential of wearables to assess treatment efficacy, characterize motor impairments alongside associated brain activity patterns, and support personalized approaches to DBS management. Large‐scale studies employing standardized protocols are required to improve their generalizability.

**Disclosure:** Nothing to disclose.

## EPO‐0590

### Alpha–fetoprotein level in dystonia patients: Follow ‐ up

#### J. Rajmonová ^1^; P. Havránková^1^; L. Kunc^1^; J. Roth^1^; D. Springer^2^; M. Zech^3^; R. Jech^1^

##### ^*1*^ *Department of Neurology and Center of Clinical Neuroscience, First Faculty of Medicine, Charles University and General University Hospital in Prague**;*** ^*2*^ *Department of Clinical Biochemistry and Laboratory Medicine, First Faculty of Medicine Charles University and General University Hospital in Prague;* ^*3*^ *Institute of Neurogenomics, Helmholtz Zentrum München, Munich, Germany*

**Background and aims:** In our previous study, we observed elevated alpha‐fetoprotein (AFP) levels in patients with dystonia. The aim of this project was to monitor AFP levels over one‐year period in dystonic patients with elevated AFP, to exclude other causes of increased AFP (tumor, hepatopathy, or gene‐related persistence), and to compare clinical characteristics between patients with normal and elevated AFP levels.

**Methods:** Patients with primary idiopathic dystonia (including negative WES) and healthy controls were enrolled. AFP levels were measured using an immunoassay.

**Results:** Of 171 patients with primary idiopathic dystonia, 156 (47 men) had normal AFP levels (3 ± 1.7 ug/L), whereas 15 (2 men) had elevated AFP levels (17.7 ± 22 ug/L). At one‐year follow‐up, AFP levels normalized in only two patients; in the remaining 13 patients, AFP levels either remained stable or increased. Oncological screening was negative in all patients and mild hepatopathy was observed in two cases, HNF–1 mutations, (causing AFP persistence) were excluded. All 57 age‐and sex‐matched healthy controls had normal AFP levels (3.3 ± 1.8 μg/L) and no significant differences between groups were detected.

**Conclusion:** These findings suggest that AFP elevation in dystonia was predominantly long‐term and occured without an identifiable triggering cause. This unexpected persistence raises the possibility of an association between AFP elevation and neurodevelopmental pathology. Increased AFP levels have been observed in neural tube defects, spina bifida, and in autosomal recessive cerebellar ataxias. We conclude that long‐term AFP elevation may be present in patients with dystonia; however, its clinical relevance and relationship to disease pathophysiology remain unclear.

**Disclosure:** Nothing to disclose.

## EPO‐0591

### Concomitant recording of streamed local field potentials from the STN and GPI to investigate Parkinson's disease neurophysiology: A feasibility study

#### M. Mancuso ^1^; C. Gonzalez^1^; S. Xu^1^; J. Candelario‐Mckeown^1^; J. Esperida^1^; C. Duraffourd^3^; L. Mooney^3^; H. Akram^1^; M. Krueger^1^; L. Zrinzo^1^; T. Foltynie^1^; V. Litvak^2^; P. Limousin^1^

##### ^*1*^ *Unit of Functional Neurosurgery, Department of Clinical and Movement Neurosciences, UCL Queen Square Institute of Neurology, University College London, London, UK;* ^*2*^ *Department of Imaging Neuroscience, UCL Queen Square Institute of Neurology, London, UK;* ^*3*^ *Medtronic, Watford, UK*

**Background and aims:** Globus pallidus internus (GPi) and subthalamic nucleus (STN) deep brain stimulation (DBS) are both effective treatments for Parkinson's disease (PD). The development of stimulators able to stream local field potentials (LFP) such as Medtronic Percept® allows to study the effect of DBS in chronic implants, and to elucidate the role of STN and GPi neurophysiology in the parkinsonian system. We recorded simultaneously from two patients with dual STN and GPi implant, to assess the feasibility of concomitant recording, and to investigate the effect of DBS on the interaction between nuclei.

**Methods:** 2 patients with dual STN‐GPi Medtronic Percept® implants underwent resting‐state simultaneous LFP recording with increasing levels of stimulation (0 to 4 mAmp, 0.5 mAmp steps). After synchronization, we computed time‐frequency resolved power, coherence, and granger causality at each step.

**Results:** We successfully synchronized the STN and GPi signal in both patients. Stimulation of either nucleus resulted in suppression of high‐beta peak power in both the stimulated and non‐stimulated nucleus, with accompanying coherence loss at the same frequency. Granger analysis showed an STN to GPi directionality of high‐beta connectivity, which was abolished with increasing stimulation to either nucleus.

**Conclusion:** In this preliminary study we demonstrate the feasibility of simultaneous LFP recording from the STN and GPi. We identify an indirect modulatory effect of stimulation on the high beta peak of the non‐stimulated nucleus and show that high beta‐band connectivity within the subthalamo‐pallidal system is directed from the STN to the GPi, in line with previously proposed models.

**Disclosure:** Nothing to disclose.

## EPO‐0592

### Non‐motor symptom burden does not reliably discriminate between PSP subtypes

#### N. Shakarishvili ^1^; S. Lyons^1^; N. O'Shea^2^; M. Finnegan^3^; S. Fullam^1^; A. O'Connor^1^; D. Bradley^4^; S. Hutchinson^1^; O. Hardiman^5^; E. McGovern^1^; S. O'Riordan^6^; L. Williams^6^; T. Lynch^7^; C. Fearon^7^; R. Walsh^7^; S. O'Dowd^1^

##### ^*1*^ *Department of Neurology, Tallaght University Hospital, Dublin, Ireland;* ^*2*^ *Department of Palliative Medicine, University Hospital Kerry, Tralee, Ireland;* ^*3*^ *Department of Old Age Psychiatry, Tallaght University Hospital, Dublin, Ireland;* ^*4*^ *Department of Neurology, St James's Hospital, Dublin, Ireland;* ^*5*^ *Department of Neurology, Beaumont Hospital, Dublin, Ireland;* ^*6*^ *Department of Neurology, St Vincent's University Hospital, Dublin, Ireland;* ^*7*^ *Department of Neurology, Mater Misericordiae University Hospital, Dublin, Ireland*

**Background and aims:** Progressive supranuclear palsy (PSP) can be sub‐classified into Richardson's syndrome (RS), cortical, and subcortical subtypes. We aimed to characterise symptom severity across PSP variants using validated scales, adjusting for disease duration, to determine whether phenotypes exhibit differential patterns of non‐motor burden.

**Methods:** 92 patients with PSP were classified into cortical (*n* = 17), subcortical (*n* = 28) and RS subtypes (*n* = 47). Each was assessed with PSPRS, ACE‐III, SCOPA‐AUT, BDI, CBI, NPI, and SF‐36. Descriptive statistics summarised total‐cohort and subtype distributions. Group differences were examined using multivariate general linear modelling.

**Results:** PSP subtypes demonstrated a significant overall multivariate effect on PSPRS domain scores (*p* < 0.001). Univariate tests showed differences in Mentation (*p* = 0.029), Bulbar (*p* < 0.001), Ocular (*p* = 0.008), Limb (*p* < 0.001) and Gait (*p* = 0.014) with PSP‐RS exhibiting greatest impairment and PSP‐Subcortical least. No significant subtype differences were detected across autonomic symptom domains by SCOPA‐AUT. While there was no overall multivariate subtype effect on cognitive profile, higher fluency scores characterised PSP‐Subcortical versus PSP‐RS (*p* = 0.047). In the neuropsychiatric domain, neither BDI scores nor CBI caregiver‐informed behavioural scores differed significantly between subtypes. Multivariate analysis did not display an overall subtype effect on total NPI scores. Quality‐of‐life did not differ significantly between subtypes across any SF‐36 domain.

**Conclusion:** Our results suggest that motor manifestations remain the principal differentiator between PSP phenotypes, with PSP‐RS demonstrating most severe bulbar, ocular, limb and gait impairment. In contrast, non‐motor domains—including cognition, autonomic function, mood and behaviour—did not reliably discriminate between subtypes, suggesting a shared non‐motor burden across the PSP spectrum.

**Disclosure:** No conflicts of interest to disclose. Acknowledgment given to the PSP Association of Ireland for providing funding to support this research.

## EPO‐0593

### Motor, nonmotor and cognitive correlates of iron deposition within subcortical nuclei in early drug‐naïve Parkinson's disease patients

#### N. Piramide; R. De Micco; M. Pirozzi; M. Siciliano; G. Caiazzo; V. Sant'Elia; F. Esposito; A. Tessitore; O. Ciaramaglia

##### Department of Advanced Medical and Surgical Sciences, University of Campania “Luigi Vanvitelli”, Naples, Italy

**Background and aims:** Iron deposition using Quantitative Susceptibility Mapping (QSM) has been reported in several cortical/subcortical areas within the dopaminergic pathways in patients with Parkinson's disease (PD). This study was aimed at exploring 3T MRI‐derived iron deposition content within bilateral subcortical nuclei in early drug‐naive PD patients compared to healthy controls (HC), and as well as potential associations with motor, non‐motor and cognitive/behavioral symptoms.

**Methods:** 3T MRI images of 59 drug‐naïve PD patients were analyzed and compared with 18 age‐, sex‐‐matched HC. QSM values were extracted from several subcortical deep gray matter nuclei and 16 thalamic subregions using the HybraPD atlas. Partial Spearman correlation analysis was performed between QSM metrics significantly different in PD compared to HC, and motor, non‐motor and cognitive/behavioral variables. Finally, a ROC curve analysis was performed to test the ability to discriminate PD patients from HC using QSM values.

**Results:** Compared to HC, PD patients showed significantly higher iron deposition in the bilateral substantia nigra pars compacta, left substantia nigra pars reticulata, left subthalamic nucleus, and in the right red and intralaminar thalamic nuclei. Correlation analyses revealed greater motor and non‐motor severity associated with increased iron within the right intralaminar thalamic and red nuclei, respectively. ROC analysis demonstrated that QSM values may accurately discriminate PD patients from HC.

**Conclusion:** Our findings demonstrated that using a disease‐specific HybraPD atlas, a specific iron accumulation within subcortical nuclei can be found in PD patients even at early stages, and may be proposed as a potential biomarker of dopaminergic neurodegeneration.

**Disclosure:** Disclosures: no disclosures to declare

## EPO‐0594

### MRgFUS thalamotomy may reduce long‐term motor complications in tremor‐dominant Parkinson's disease: A five‐year prospective case‐control study

#### R. Cancilla ^1,2^; N. Golfrè Andreasi^1^; A. Braccia^1^; F. Colucci^1^; G. Gaudiano^1^; L. Romito^1^; A. Elia^1^; R. Cilia^1^; V. Leta^1,3^; V. Levi^4^; N. Castelli^4^; G. Devigili^1^; S. Rinaldo^1^; M. Stanziano^5^; V. Caldiera^6^; M. Grisoli^5^; E. Ciceri^6^; R. Eleopra^1^

##### ^*1*^ *Fondazione IRCCS Istituto Neurologico Carlo Besta, Department of Clinical Neurosciences, Parkinson and Movement Disorders Unit, Milan, Italy;* ^*2*^ *Neurology Department of Medicine and Surgery, University of Parma, Parma, Italy;* ^*3*^ *Parkinson's Centre of Excellence at King's College Hospital and King's College London, London, UK;* ^*4*^ *Fondazione IRCCS Istituto Neurologico Carlo Besta, Neurosurgery Department, Functional Neurosurgery Unit, Milan, Italy;* ^*5*^ *Fondazione IRCCS Istituto Neurologico Carlo Besta, Neuroradiology Unit, Milan, Italy;* ^*6*^ *Fondazione IRCCS Istituto Neurologico Carlo Besta, Diagnostic Radiology and Interventional Neuroradiology, Milan, Italy*

**Background and aims:** Magnetic resonance–guided focused ultrasound (MRgFUS) thalamotomy targeting the ventral intermediate nucleus (VIM) is an established treatment for medication‐refractory tremor in tremor‐dominant Parkinson's disease (TDPD). Long‐term data on tremor improvement, dopaminergic therapy management and development of motor complications after MRgFUS are limited. This study evaluated the long‐term impact of VIM MRgFUS thalamotomy compared with optimized medical therapy in TDPD.

**Methods:** We conducted a prospective case–control study including patients with TDPD without baseline motor fluctuations or dyskinesias. Patients treated with unilateral VIM MRgFUS thalamotomy (PD‐FUS) were compared with patients receiving optimized drug therapy alone (PD‐ODT). Groups were matched 1:2 for sex, age, disease duration and baseline levodopa equivalent daily dose (LEDD). Assessments were performed in On‐medication state at baseline, one and five years. The primary outcome was motor complications severity assessed by the Movement Disorder Society–Unified Parkinson's Disease Rating Scale (MDS‐UPDRS) Part IV.

**Results:** 21 PD‐FUS and 38 PD‐ODT patients were included. At five years, median MDS‐UPDRS‐IV scores were lower in PD‐FUS than PD‐ODT (3 [0;4] vs 5 [4;7] respectively; *p* = 0.016) (Figure 1). Tremor improved in PD‐FUS, with a greater median reduction in total tremor score than PD‐ODT (−60%[−75; −27] vs −13% [−43; 25]; *p* = 0.049). Over five years, total LEDD increased in both groups but remained lower in PD‐FUS (median increase 185 mg [83–463] vs 419 mg [303–690]; *p* = 0.014) (Figure 2). Adverse events were mild and transient.

**Figure:**
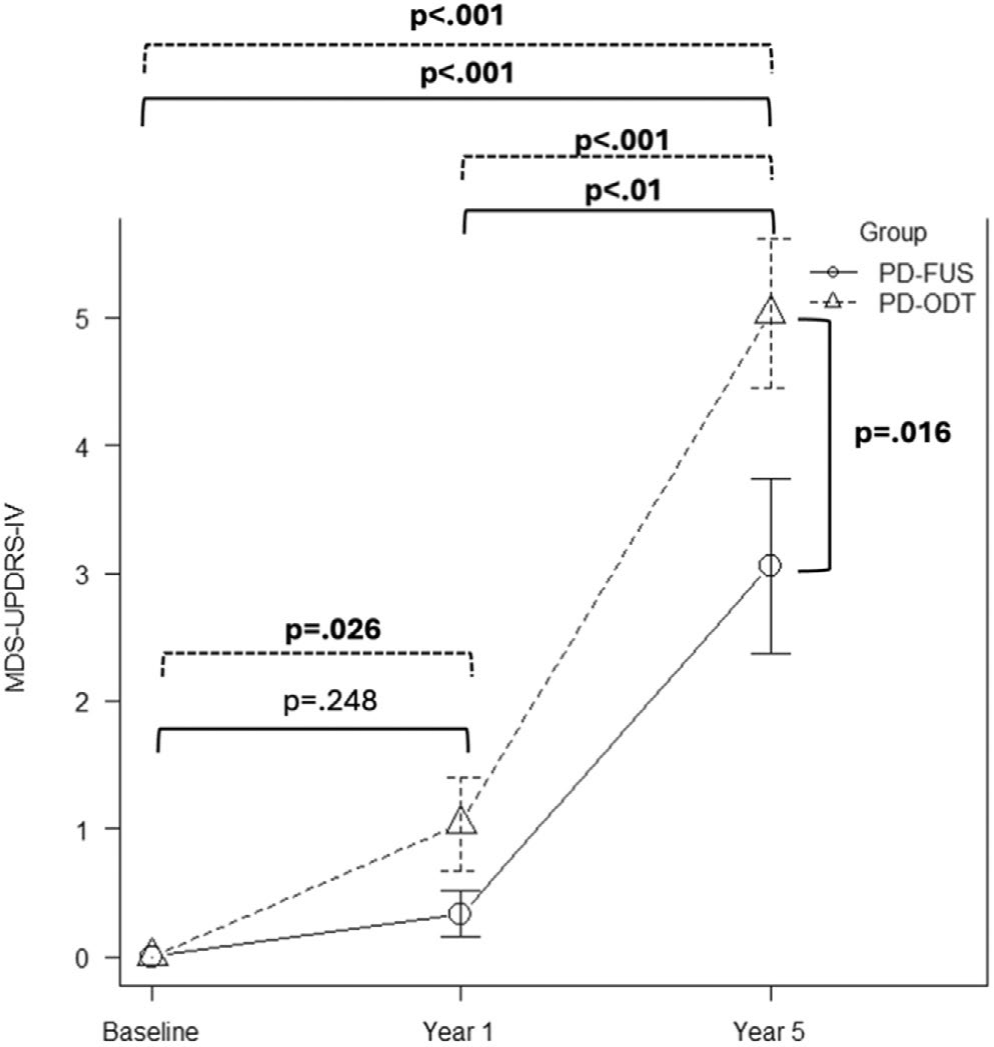
**FIGURE 1** Changes in MDS‐UPDRS Part IV scores over follow‐up in patients treated with magnetic resonance–guided focused ultrasound (PD‐FUS) and optimized drug therapy (PD‐ODT). *p* values indicate between‐group comparisons.

**Figure:**
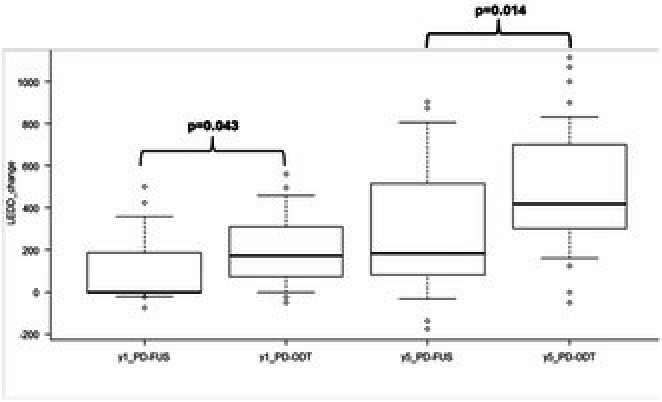
**FIGURE 2** Changes in total levodopa equivalent daily dose (LEDD) from baseline to 1 and 5 years in PD‐FUS and PD‐ODT patients. *p* values indicate between‐group comparisons.

**Conclusion:** VIM MRgFUS thalamotomy provides durable tremor control in TDPD and is associated with reduced long‐term severity of motor complications and lower increase in LEDD.

**Disclosure:** Nothing to disclose.

## EPO‐0595

### Olfactory impairment severity and cognitive performance in Parkinson's disease: A 5‐year longitudinal study with baseline functional connectivity

#### S. Sathyanarayana ^1^; M. Firbank^2^; D. Ledingham^3^; V. Foster^1^; R. Iredale^1^; D. Galley^1^; B. Mollenhauer^5^; N. Pavese^4^

##### ^*1*^ *Clinical Ageing Research Unit, Newcastle University, Newcastle Upon Tyne, UK**;*** ^*2*^ *Translational and Clinical Research Institute and NIHR Newcastle Biomedical Research Centre, Newcastle University, Newcastle Upon Tyne, UK;* ^*3*^ *Clinical Ageing Research Unit, Newcastle University, and The Royal Victoria Infirmary, The Newcastle Upon Tyne Hospitals NHS Foundation Trust, Newcastle Upon Tyne, UK;* ^*4*^ *Clinical Ageing Research Unit, Newcastle University, Newcastle Upon Tyne, UK and Department of Nuclear Medicine and PET Centre, Aarhus University Hospital, Aarhus, Denmark;* ^*5*^ *University Medical Center, Clinic for Neurology, Göttingen, Germany*

**Background and aims:** Olfactory impairment is a recognised early marker of Parkinson's disease (PD). However, the extent to which graded hyposmia severity relates to longitudinal cognitive performance remains unclear. This study examined longitudinal cognitive outcomes and baseline resting‐state functional connectivity (rs‐fMRI) across five olfactory categories: normosmia, mild, moderate and severe hyposmia, and anosmia.

**Methods:** Data were obtained from the Parkinson's Progression Markers Initiative (PPMI) database. Participants were classified into five olfactory groups based on age‐adjusted percentile scores from the University of Pennsylvania Smell Identification Test (UPSIT). Longitudinal Montreal Cognitive Assessment (MoCA) scores were analysed using linear mixed‐effects models in SPSS. Baseline rs‐fMRI data were processed using CONN. ROI‐to‐ROI and seed‐to‐voxel analyses focused on regions involved in olfactory and cognitive networks, including the insula, orbitofrontal cortex, anterior cingulate cortex, amygdala, hippocampus and thalamus.

**Results:** A total of 254 PD patients and 133 healthy controls showed comparable demographic characteristics. Greater olfactory impairment severity significantly predicted lower MoCA scores (*p* = 0.004), independent of time. Each incremental increase in olfactory severity category was associated with a 0.14‐point reduction in MoCA score. Baseline rs‐fMRI analyses (PD = 91; controls = 17) revealed no significant functional connectivity differences across olfactory categories within predefined regions of interest.

**Conclusion:** Greater olfactory impairment severity is associated with reduced cognitive performance in PD, even without detectable baseline functional connectivity differences. These findings suggest olfactory testing may be a sensitive marker of early cognitive vulnerability, while connectivity alterations may emerge later or require alternative imaging approaches to detect subtle network changes.

**Disclosure:** Nothing to disclose.

## EPO‐0596

### Neuromodulation Specialist Nurse‐Led support system for caregivers of patients with PD post DBS based on Omaha system: A randomized controlled trial

#### Y. Zhao ^1^; Q. Zhao^1^; Y. Ma^1^; B. Shen^1^; J. Wu^2^; Y. Xu^1^

##### ^*1*^ *Department of Nursing, Huashan Hospital, Fudan University, Shanghai, China;* ^*2*^ *Department of Neurology, Huashan Hospital, Fudan University, Shanghai, China*

**Background and aims:** Deep Brain Stimulation (DBS) is an effective treatment for the motor symptom of Parkinson's disease (PD), but it seems to have a limit effect on caregiver burden. Caregivers continually confront the dual challenges of navigating symptom fluctuations and handling the DBS device. Moreover, there are limit intervention were used to decrease caregiver burden of PD patients post DBS. Therefore, we have constructed an caregiver intervention program based on Omaha system and to verify it's effectiveness on caregiver burden.

**Figure:**
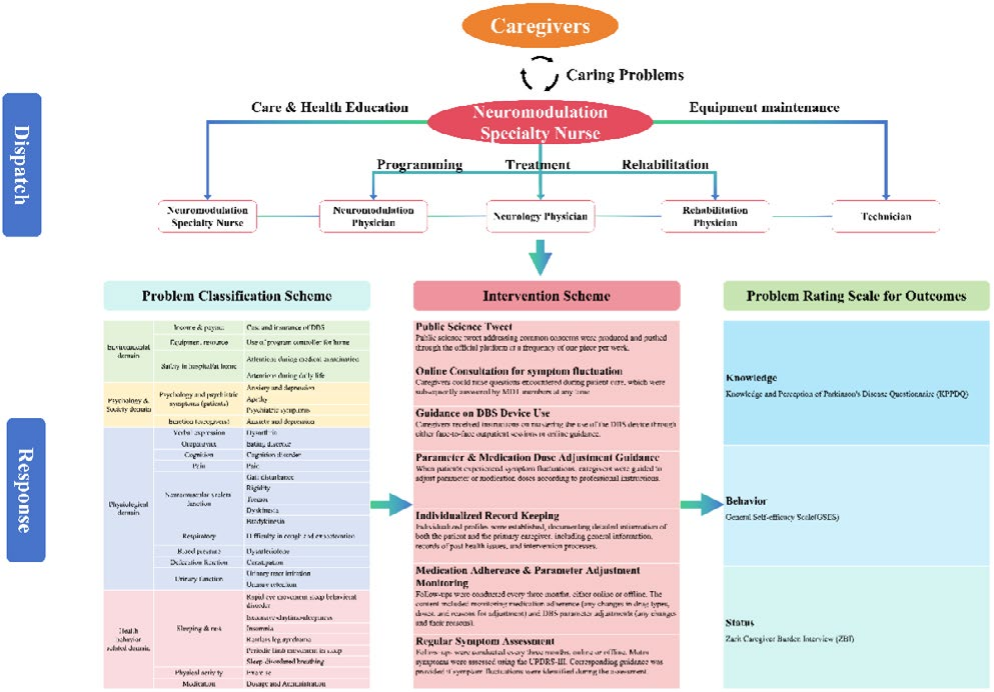
**FIGURE 1**Neuromodulation Specialist Nurse‐Led support system for caregivers of patients with PD post DBS based on Omaha system.

**Methods:** A randomized controlled trial was designed. The stratified block randomization was used. 120 caregivers were randomly separated in either intervention (Nursing care based on Omaha system) or control (General nursing care) group. The demographic information, score of ZBI, GSES and KPPDQ were collected at baseline (T0), 3‐ and 9‐month follow.

**Results:** Using repeated‐measures analysis of variance to compare difference between two groups. And Caregiving knowledge only revealed a significant main effect of time (F(1.83, 215.83) = 17.66, *p* < 0.001, η^2^ = 0.13). A significant main effect of group (F(1, 118) = 9.19, *p* = 0.003, η^2^ = 0.072) and a significant time‐by‐group interaction (F(2, 236) = 5.23, *p* = 0.006, η^2^ = 0.042) for caregiver self‐efficacy. Caregiver burden revealed a significant main effects of time (F(2, 118) = 4.13, *p* = 0.017, η^2^ = 0.034) and group (F(1, 118) = 8.08, *p* = 0.005, η^2^ = 0.064), as well as the time‐by‐group interaction (F(2, 118) = 5.20, *p* = 0.006, η^2^ = 0.042)

**Figure:**
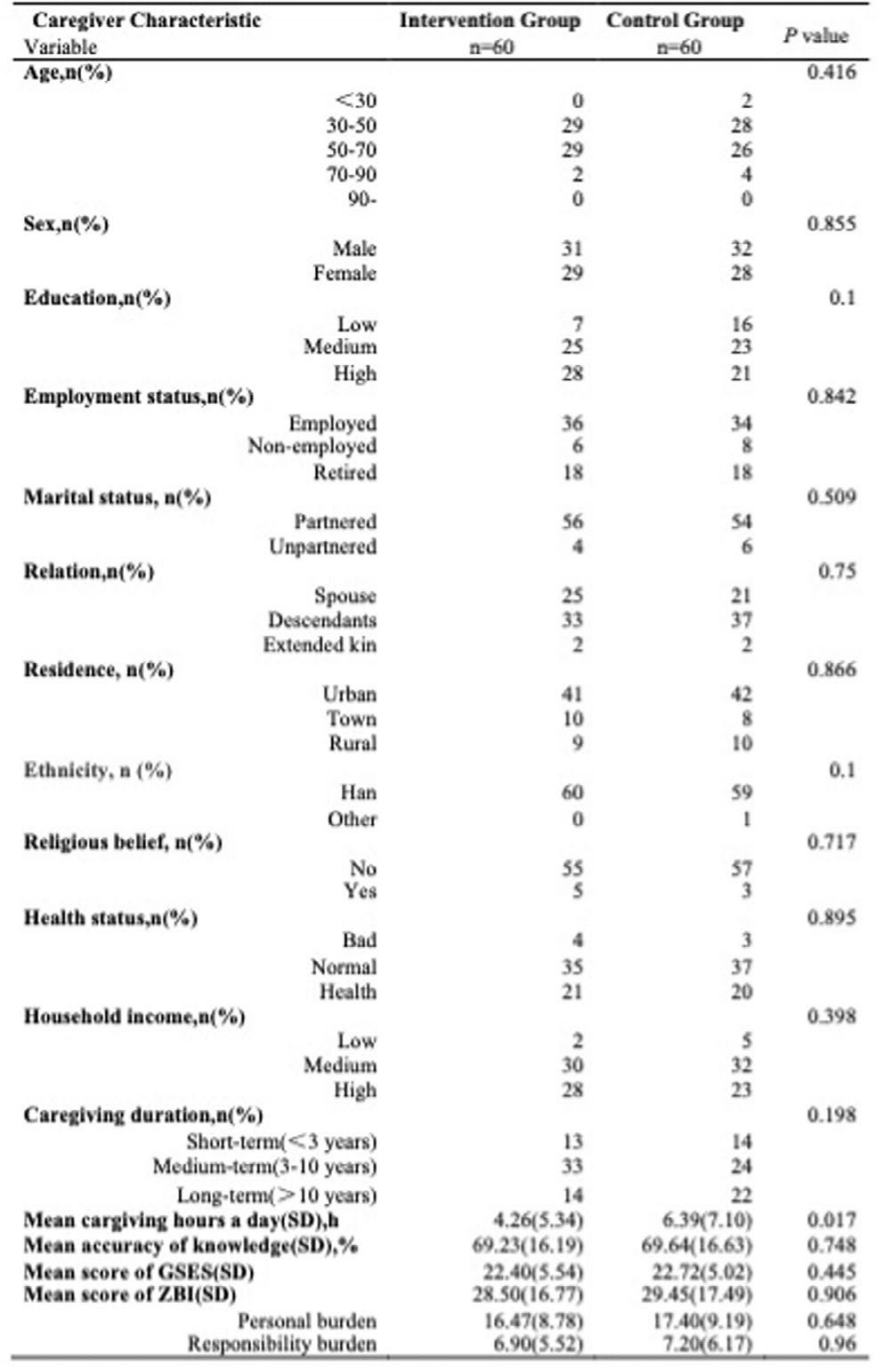
**TABLE 1** Baseline characteristics.

**Figure:**
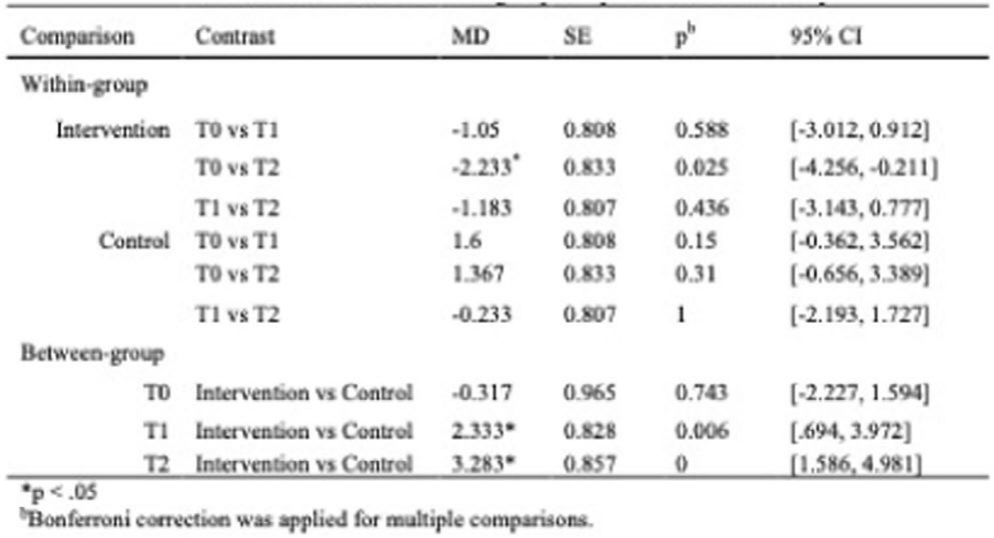
**TABLE 2** Within‐ and between‐group comparison for self efficacy.

**Figure:**
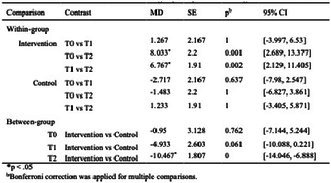
**TABLE 3** Within‐ and between‐group comparison for caregiver burden.

**Conclusion:** Neuromodulation Specialist Nurse‐Led support system for caregivers of PD patients post DBS based on Omaha system provide a long‐term, multi‐dimensional and multi‐form to improve the knowledge, self‐efficiency and burden of caregivers.

**Disclosure:** The work described in this paper was supported in full by grants from the Fu Xing Nursing Scientific Research Fund of Fudan University, Shanghai, China [grant number FNF202324], and Shanghai Zhou Liangfu Medical Development Foundation (Nursing Research), Shanghai, China [grant number XM00036‐2022‐1]

Neurorehabilitation 2

## EPO‐0597

### Biological motion recognition in multiple sclerosis: Toward behavioral biomarkers for personalized neurorehabilitation

#### A. Antonioni ^1^; A. Casile^2^; M. Govoni^1^; M. Buongiorno^1^; F. Bonzagni^1^; G. Palma^1^; G. Fregna^3^; G. Perachiotti^1^; L. Mencarelli^4^; L. Micillo^5^; G. Mioni^5^; A. Baroni^1^; S. Straudi^1^

##### ^*1*^ *Department of Neuroscience and Rehabilitation, University of Ferrara, Ferrara, Italy;* ^*2*^ *Department of Biomedical and Dental Sciences and Morphofunctional Imaging, University of Messina, Messina, Italy;* ^*3*^ *Department of Neuroscience, Ferrara University Hospital, Ferrara, Italy;* ^*4*^ *Experimental Neuropsychophysiology Laboratory, Santa Lucia Foundation IRCCS, Rome, Italy;* ^*5*^ *Department of General Psychology, University of Padua, Padua, Italy*

**Background and aims:** Multiple sclerosis (MS) is a chronic disease associated with complex neurorehabilitation needs. The Action Observation Network (AON) supports motor learning and the prediction of biological motion (BM). However, evidence on AON functioning and BM recognition in MS remains limited. We aimed to (i) characterize BM recognition performance in people with MS (pwMS), (ii) compare their performance with that of young and age‐matched healthy controls (HCs), and (iii) investigate the role of clinical, neuroimaging, and neuropsychological covariates.

**Methods:** In this cross‐sectional study, 37 pwMS, 30 age‐matched HCs, and 22 young HCs were enrolled. Participants completed a computerized BM task in which they observed arm movements displayed as either photorealistic avatars or point‐light stimuli to estimate perceptual temporal extrapolation at the point of subjective equality (PSE). Clinical, neuropsychological, and subjective fatigue measures were collected; structural neuroimaging was available for pwMS.

**Results:** Two response patterns emerged: a “good” psychometric curve that allows PSE estimation and an inverted (“bad”) pattern. Among “good” participants, pwMS showed significantly altered temporal extrapolation compared with both control groups, independent of stimulus type. Age‐matched HCs exhibited a bimodal distribution for impoverished stimuli, and performance was associated with fatigue: increased fatigue was associated with greater temporal extrapolation (similar to young HCs), whereas reduced fatigue was associated with diminished extrapolation.

**Figure:**
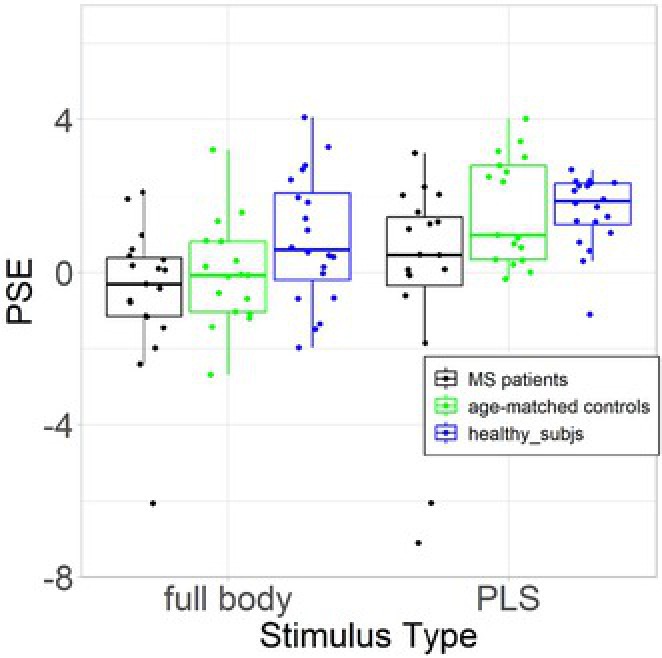
Performance on the BM at the two levels of detail (i.e, full‐body and point‐light stimuli) in pwMS, age‐matched HCs, and young HCs.

**Conclusion:** Preliminary findings indicate altered BM recognition in pwMS, possibly reflecting AON dysfunction. Fatigue appears to modulate BM perception not only in pwMS but also in physiological aging. These results support the potential of BM recognition as a biomarker for personalizing action‐observation–based rehabilitation.

**Disclosure:** Nothing to disclose.

## EPO‐0598

### Multimodal integrative interventions in korean medicine for persistent postural‐perceptual dizziness: A systematic review and network meta‐analysis

#### H. Yu^1^; E. Lee ^1^; S. Ahn^1^; J. Yoo^2^

##### ^*1*^ *College of Korean Medicine, Kyung Hee University, Seoul, Republic of Korea;* ^*2*^ *College of Nursing Science, Kyung Hee University, Seoul, Republic of Korea*

**Background and aims:** Persistent Postural‐Perceptual Dizziness (PPPD) is a chronic functional vestibular disorder with complex pathophysiology. Evidence regarding multimodal integrative strategies remains limited. This study compared the relative effectiveness of Korean medicine–based single and multimodal interventions for PPPD using Bayesian network meta‐analysis.

**Methods:** We analyzed RCTs published between 2000 and September 2024 involving patients with PPPD or chronic functional dizziness. Interventions included herbal medicine, acupuncture, physical/psychological therapy, and combinations. Primary outcomes were Dizziness Handicap Inventory (DHI) and emotional well‐being, with treatment hierarchies estimated via SUCRA values.

**Results:** Ten RCTs (956 participants) were analyzed across three subnetworks (**Figure 1**, Figure 2). In the control–monotherapy subnetwork, psychological therapy ranked highest (SUCRA = 99%). Within the VRT‐based subnetwork, CBT‐informed VRT was most effective (SUCRA = 86.1%). In the pharmacologic–integrative subnetwork, multimodal interventions consistently outperformed monotherapy, with combined psychological, physical, and pharmacological therapy ranking highest (SUCRA = 99.71%) (Figure 3).

**Figure:**
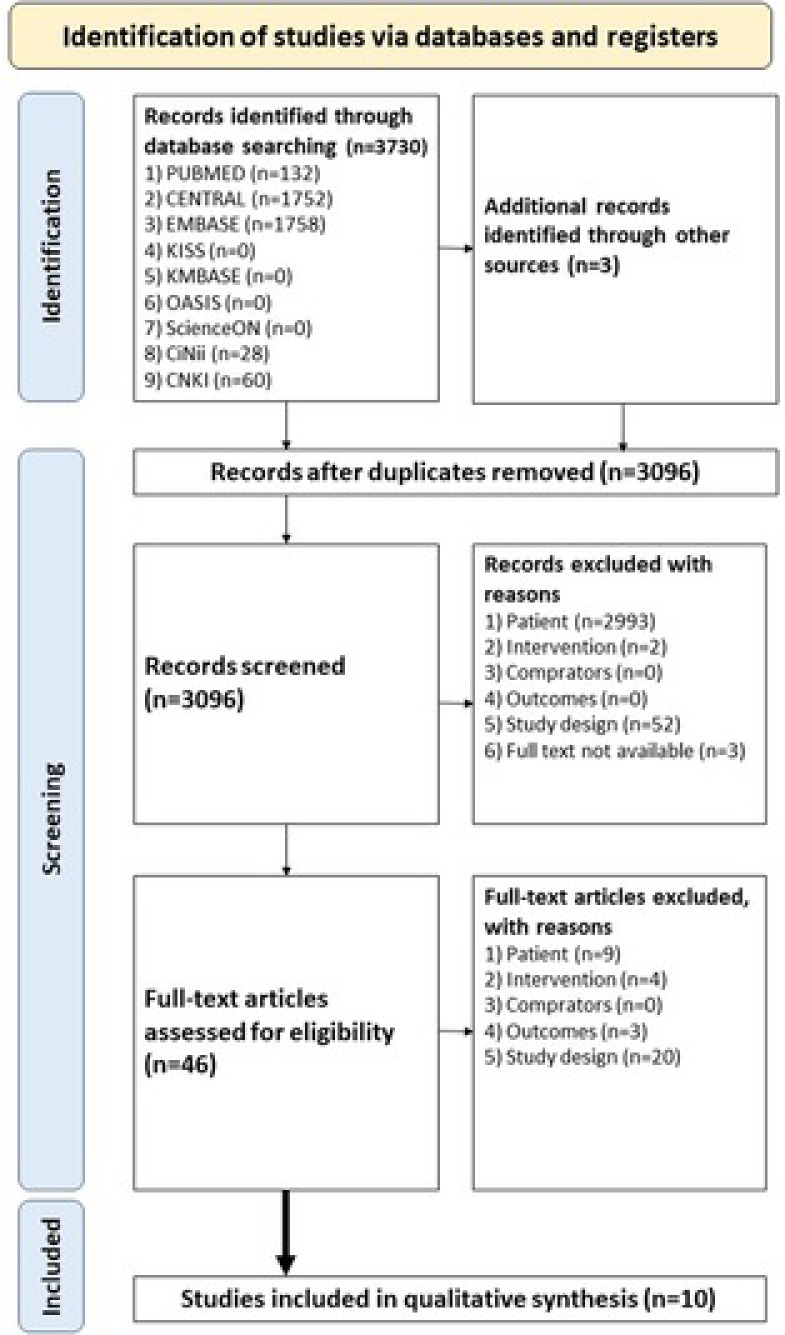
**FIGURE 1** PRISMA Flow Diagram of Study Selection. From an initial 3,733 records identified across nine databases, 10 RCTs involving 956 participants were finally included for the network meta‐analysis after a rigorous screening based on PICOS criteria.

**Figure:**
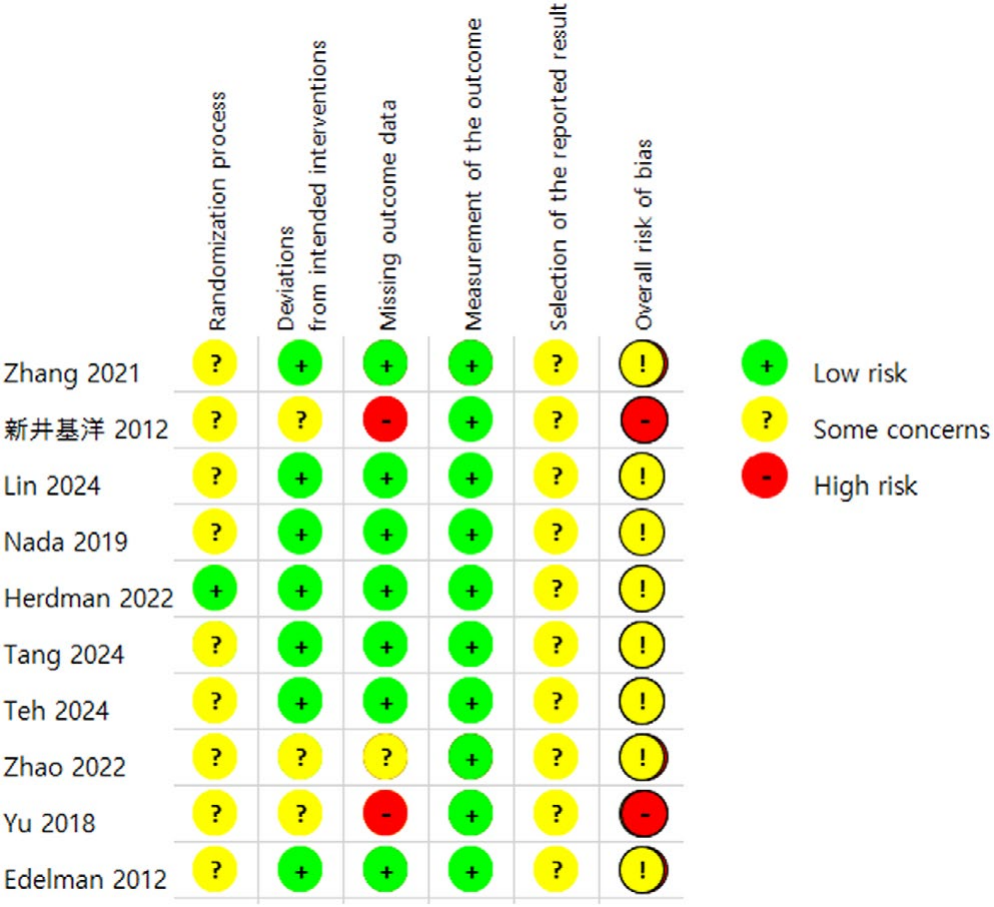
**FIGURE 2** RoB of the included studies. Most included studies (8/10) were classified as having "some concerns," while two studies were judged as "high risk" primarily due to issues with missing outcome data.

**Figure:**
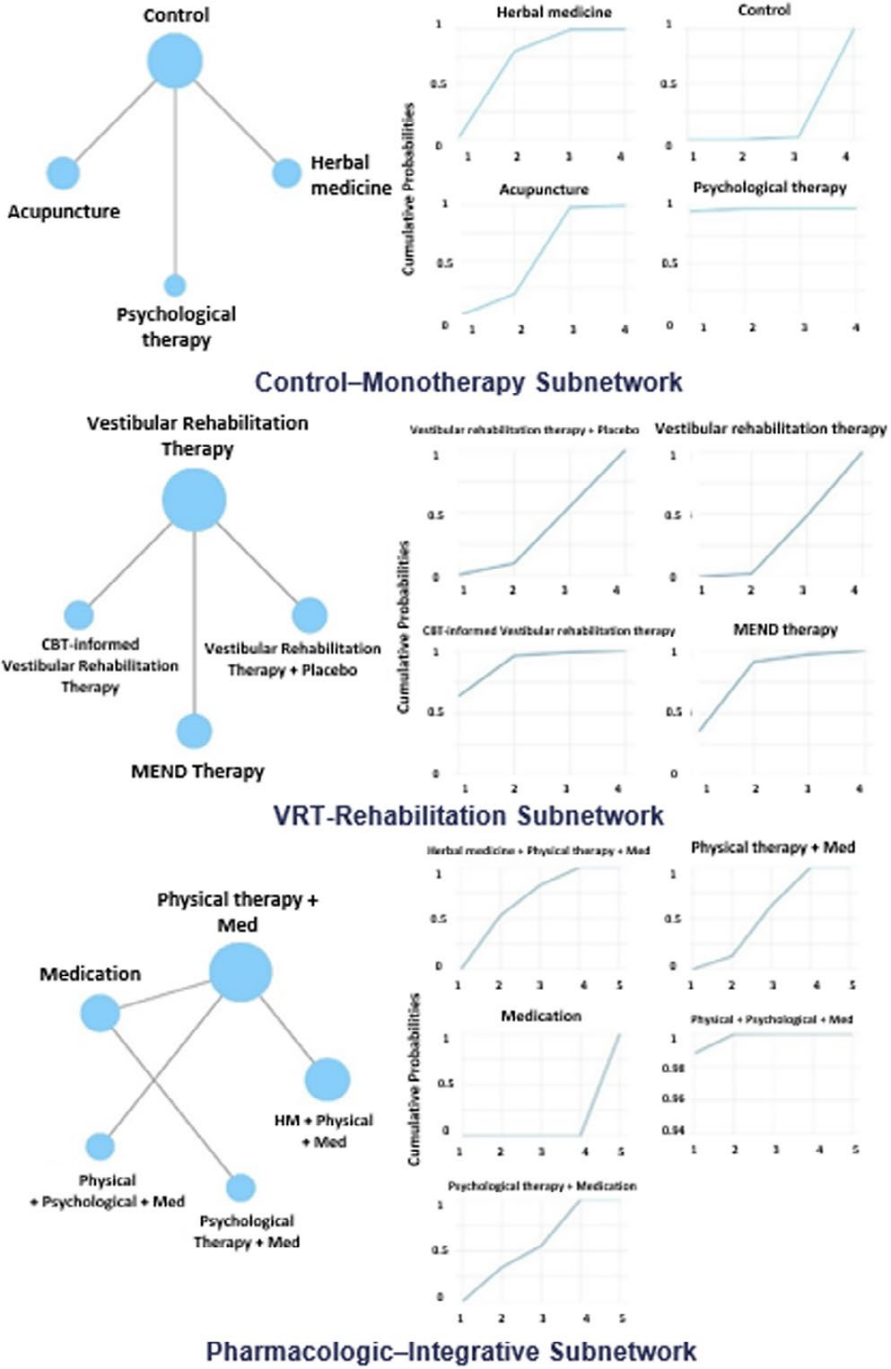
**FIGURE 3** Multimodal strategies (SUCRA 99.71%) outperformed monotherapy. Psychological therapy (99%) and CBT‐informed VRT (86.1%) ranked highest in their subnetworks, demonstrating that integrated physical and psychological care is most effective.

**Conclusion:** This first network meta‐analysis on PPPD highlights the clinical relevance of multimodal strategies addressing both physical and psychological dimensions. Although findings are promising, evidence certainty is low due to small sample sizes and fragmented network structure. Future large‐scale randomized trials are needed to strengthen the evidence base.

**Disclosure:** Nothing to disclose.

## EPO‐0599

### Abstract withdrawn

## EPO‐0600

### Polypharmacy Deprescribing Does Not Affect Muscle Mass, Strength, and Physical Function in Post‐Stroke Geriatric Patients with Sarcopenia

#### K. Siregar ^1^; P. Prawiroharjo^2^; S. Tanumihardja^3^

##### ^*1*^ *School of Medicine Maranatha Christian University, Bandung, Indonesia;* ^*2*^ *Department of Neurology, Faculty of Medicine, Universitas Indonesia, Jakarta, 10430/ RSUPN dr. Cipto Mangunkusumo, Jakarta, 10320/ Rumah Sakit Universitas Indonesia, Depok, 16424, Indonesia;* ^*3*^ *Department of Neurology, School of Medicine Maranatha Christian University/Immanuel Hospital, Bandung, Indonesia*

**Background and aims:** One‐third of geriatric patients with multimorbidity take five or more prescribed medications (polypharmacy). We evaluated whether polypharmacy deprescribing affect muscle mass, strength, and physical function in post‐stroke geriatric patients with sarcopenia.

**Methods:** Relevant studies were identified from PubMed, Scopus, and Web of Science until December 2025. Observational studies involving post‐stroke geriatric patients with sarcopenia and polypharmacy were included, comparing deprescribing and non‐deprescribing groups. Two reviewers independently screened studies and assessed risk of bias. Forest plots were generated, with SMI (Skeletal Muscle mass Index), HG (Hand Grip strength), FIM (Functional Independence Measure), BRS (Brunnstrom Recovery Stage) as the outcome.

**Results:** Four studies comprising 432 participants in the deprescribing group and 534 in the non‐deprescribing group were included. There were no significant differences between groups in SMI (MD −0.04 kg/m^2^; 95% CI −0.20 to 0.11; I^2^ = 0%), handgrip strength (MD −0.17 kg; 95% CI −1.93 to 1.59; I^2^ = 37%), FIM motor scores (MD 0.25; 95% CI −7.21 to 7.71; I^2^ = 72%), BRS of the upper limb (MD −0.07; 95% CI −0.46 to 0.31; I^2^ = 0%), and lower limb (MD −0.25; 95% CI −0.59 to 0.08; I^2^ = 0%).

**Figure:**
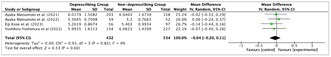
**FIGURE 1** Forest plot for SMI (Skeletal Muscle mass Index).

**Figure:**
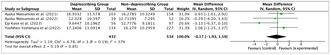
**FIGURE 2** Forest plot for HG (Hand grip Strength).

**Figure:**
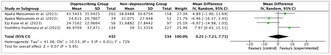
**FIGURE 3** Forest plot for FIM (Functional Independence Measure) Motor.

**Conclusion:** This study showed that polypharmacy deprescribing was not associated with difference in muscle mass, muscle strength, or physical function. Limited study numbers and heterogeneity highlight the need for future prospective studies focusing on the effects of specific drug classes on sarcopenia.

**Disclosure:** Nothing to disclose.

## EPO‐0601

### Combined high‐frequency rTMS over the non‐lesional hemisphere and rPMS for severe upper limb paralysis: Functional compensation via ipsilateral fibers

#### K. Yoshida; M. Abo

##### The Jikei University School of Medicine, Tokyo, Japan

**Background and aims:** Standard repetitive transcranial magnetic stimulation (rTMS) for stroke‐induced upper limb paralysis often shows limited efficacy in severe cases. This study investigates a novel therapeutic approach combining high‐frequency (10Hz) rTMS over the non‐lesional hemisphere and repetitive peripheral magnetic stimulation (rPMS) of paretic proximal muscles. The objective was to activate ipsilateral descending pathways, such as the anterior corticospinal and reticulospinal tracts, to promote functional compensation in severe paralysis.

**Methods:** We included 16 patients (mean age 61.4 +/‐ 10.0 years) with severe paralysis, defined as a Fugl‐Meyer Assessment for Upper Extremity (FMA‐UE) score of 22 or less. Participants received 10Hz rTMS over the non‐lesional motor cortex for two weeks and rPMS on paralyzed shoulder muscles (e.g, deltoid) for two to four weeks. Clinical outcomes were evaluated using FMA‐UE scores, comparing pre‐ and post‐treatment results via the Wilcoxon signed‐rank test.

**Results:** Total FMA‐UE scores significantly improved from 12.5 ± 4.4 to 14.8 ± 4.6 (*p* = 0.0051). Sub‐score analysis revealed a significant increase in Part A (proximal function) from 10.8 ± 3.8 to 12.6 ± 3.8 (*p* = 0.0074). Conversely, no significant differences were observed in Parts B, C, or D. No serious adverse events were reported during the intervention.

**Figure:**
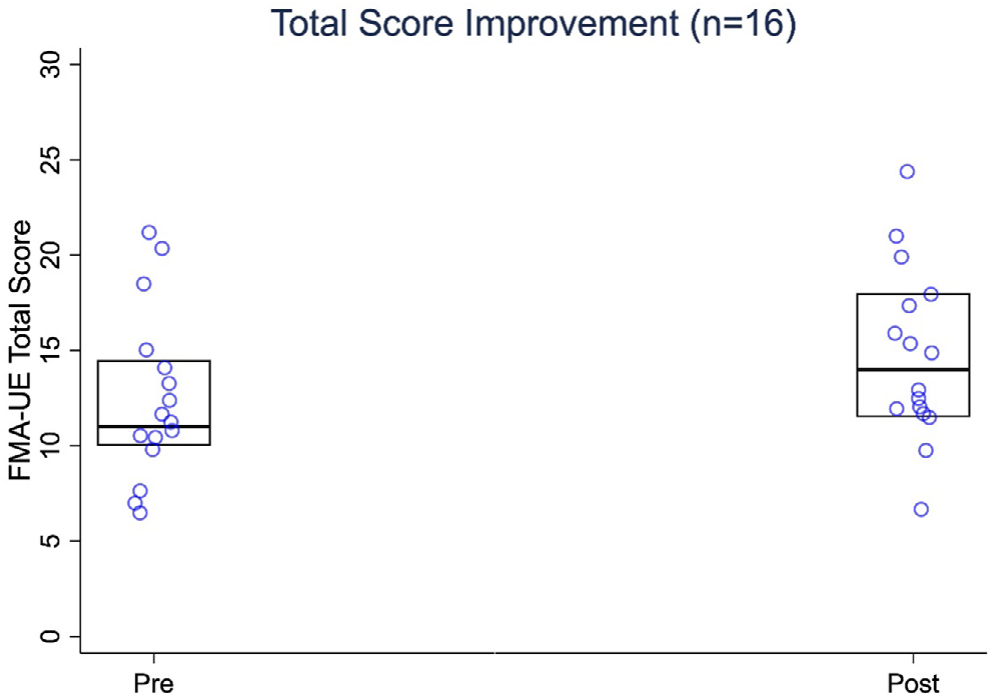
**FIGURE 1** Changes in total FMA‐UE scores before and after the combined stimulation protocol.

**Figure:**
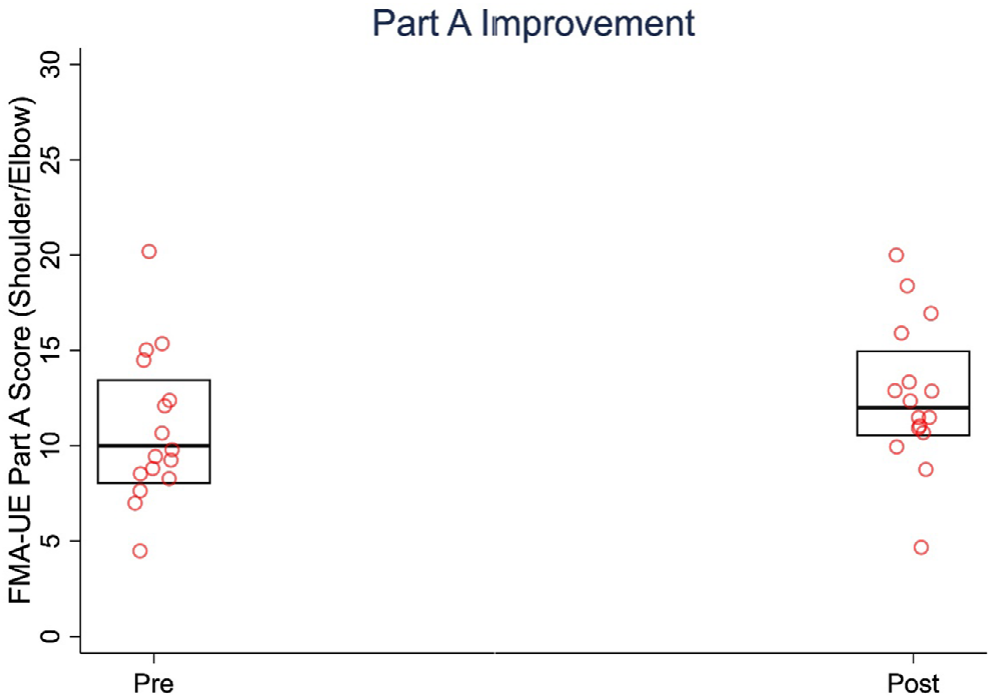
**FIGURE 2** Changes in FMA‐UE Part A scores before and after the combined stimulation protocol.

**Conclusion:** Simultaneous central and peripheral stimulation significantly improved proximal upper limb function in severe stroke cases. This combined protocol likely promotes functional activation of ipsilateral descending pathways, offering a valuable new strategy for functional recovery in patients with severe paralysis where conventional treatment options are extremely limited.

**Disclosure:** This work was supported by JSPS KAKENHI Grant Number 24K14390. The authors declare no other conflicts of interest associated with this manuscript.

## EPO‐0602

### Exploring the relationship between balance and quality of life in individuals with Parkinson's disease: Preliminary findings

#### B. Özcan^1^; B. Farhoomand^1^; N. Cetisli‐Korkmaz^1^; L. Bi̇ ^2^

##### ^*1*^ *Pamukkale University, Faculty of Physiotherapy and Rehabilitation, Denizli, Türkiye;* ^*2*^ *Pamukkale University, Faculty of Medicine, Department of Neurology, Denizli, Türkiye*

**Background and aims:** The aim of this study was to investigate the relationship between health‐related quality of life (QoL) and commonly used clinical balance and functional performance tests in individuals with PD.

**Methods:** Nineteen individuals with PD at Modified Hoehn and Yahr stages ≤2.5 were included in the study. Health‐related quality of life was assessed using the Parkinson's Disease Questionnaire‐39 (PDQ‐39). Balance and lower extremity function were evaluated using the 30‐Second Sit‐to‐Stand Test (30STS), Six‐Spot Step Test (SSST), Four Square Step Test (FSST), 3‐Meter Backward Walk Test (3MBWT), Timed Up and Go Test (TUG), and the Berg Balance Scale (BBS). The Modified Falls Efficacy Scale (MFES) was used to assess the perceived fear of falling.

**Results:** No significant relationships were found between the PDQ‐39 total score and the performance‐based balance tests (30STS, SSST, FSST, 3MBWT, and TUG) (*p* > 0.05). In contrast, statistically significant associations were observed between the total PDQ‐39 scores and both the BBS and MFES scores (*p* < 0.05). The simple linear regression analyses indicated that BBS and MFES explained 20% and 32% of the variance in QoL, respectively.

**Conclusion:** The findings of this study suggest that the reduced QoL in individuals with PD is more strongly associated with balance‐related perceptual factors, such as fear of falling, and balance confidence, than with objective, performance‐based balance and mobility measures alone. These results highlight the importance of incorporating interventions that not only aimed physical performance but also address fear of falling, and enhance balance confidence within physiotherapy and rehabilitation programs.

**Disclosure:** Nothing to disclose.

## EPO‐0603

### Longitudinal Effects of Two Courses of HF‐rTMS and Intensive Speech Therapy for Aphasia

#### M. Abo

##### Department of Rehabilitation Medicine, The Jikei University School of Medicine, Tokyo, Japan

**Background and aims:** The longitudinal effects of combining intensive speech therapy (ST) with two separate courses of high‐frequency repetitive transcranial magnetic stimulation (HF‐rTMS) for aphasia remain unclear. This study retrospectively investigated longitudinal changes in language function in patients who underwent this combined protocol.

**Methods:** Sixteen patients with chronic aphasia underwent two courses of 2‐week inpatient treatment combining fMRI‐guided HF‐rTMS and intensive ST between April 2018 and February 2025. The Standard Language Test of Aphasia (SLTA) total and modality‐specific raw scores were assessed at four time points: at the 1st admission, 3 months after the 1st discharge, at the 2nd admission, and 3 months after the 2nd discharge.

**Results:** The mean SLTA total raw score significantly improved over time: 147 +/‐ 51.6 at the 1st admission, 157 +/‐ 49.9 at 3 months after the 1st discharge, 159 +/‐ 48.8 at the 2nd admission, and 163 +/‐ 50.2 at 3 months after the 2nd discharge. Significant improvements in speech function were observed in the Overall, Fluent Aphasia, and Non‐fluent Aphasia groups. Notably, therapeutic effects were maintained without decline between the two admissions.

**Conclusion:** Two courses of HF‐rTMS combined with intensive ST may be effective for the long‐term improvement of language function, particularly speech function, in patients with chronic aphasia.

**Disclosure:** This work was supported by JSPS KAKENHI Grant Number JP 23K10567, and the authors declare no conflicts of interest.

## EPO‐0604

### Quantifying phenotype: A task‐standardised, multimodal protocol for next‐generation hyperkinetic assessment

#### M. Coll i Omaña ^1^; I. Varela^2^; K. Sampson^1^; I. Platt^3^; U. Hernandez^1^; V. Tahtis^4^

##### ^*1*^ *Department of Clinical and Movement Neuroscience, Institute of Neurology, University College London, UK;* ^*2*^ *Gatsby Computational Neuroscience Unit, University College London, London, UK;* ^*3*^ *National Hospital for Neurology and Neurosurgery, London, UK**;*** ^*4*^ *Faculty AI, King's College Hospital NHS Foundation Trust, London, UK*

**Background and aims:** Hyperkinetic movement disorders are defined by behavioural phenotypes that guide diagnosis and treatment, yet these phenotypes remain imprecisely specified, inconsistently sampled, and lack objective biomarkers. This limits mechanistic inference, complicates cross‐disorder comparison, and challenges clinical decision‐making. Clinicians nonetheless elicit rich motor behaviour using shared bedside tasks, without a clear framework for identifying which examination items provide the strongest discriminative signal or how variability should be interpreted across syndromes

**Methods:** We developed a unified assessment framework with two components: (i) a patient‐reported questionnaire capturing common symptom modifiers and contextual factors influencing movement (ii) a cross‐disorder motor assessment created by deconstructing existing rating scales and applying explicit inclusion criteria (Figure 1). Tasks were supplemented with naturalistic paradigms from motor control and functional rehabilitation contexts (Figure 2). The framework is currently being tested in 100 patients with isolated, archetypal hyperkinetic phenotypes and 100 healthy controls. Data acquisition is multimodal and time‐synchronised, combining marker‐based motion capture, multi‐view video for marker‐less pose estimation, wireless surface electromyography with inertial measurement units and auxiliary data streams including facial video, audio, tablet‐based tasks, and human‐computer interaction (Figure 3).

**Figure:**
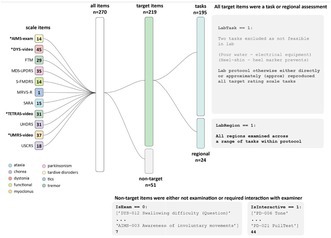
**FIGURE 1**: Items from validated movement disorder scales were systematically characterised and algorithmically selected, with most content reproduced to ensure cross‐disorder comparability while maintaining syndrome‐specific constructs.

**Figure:**
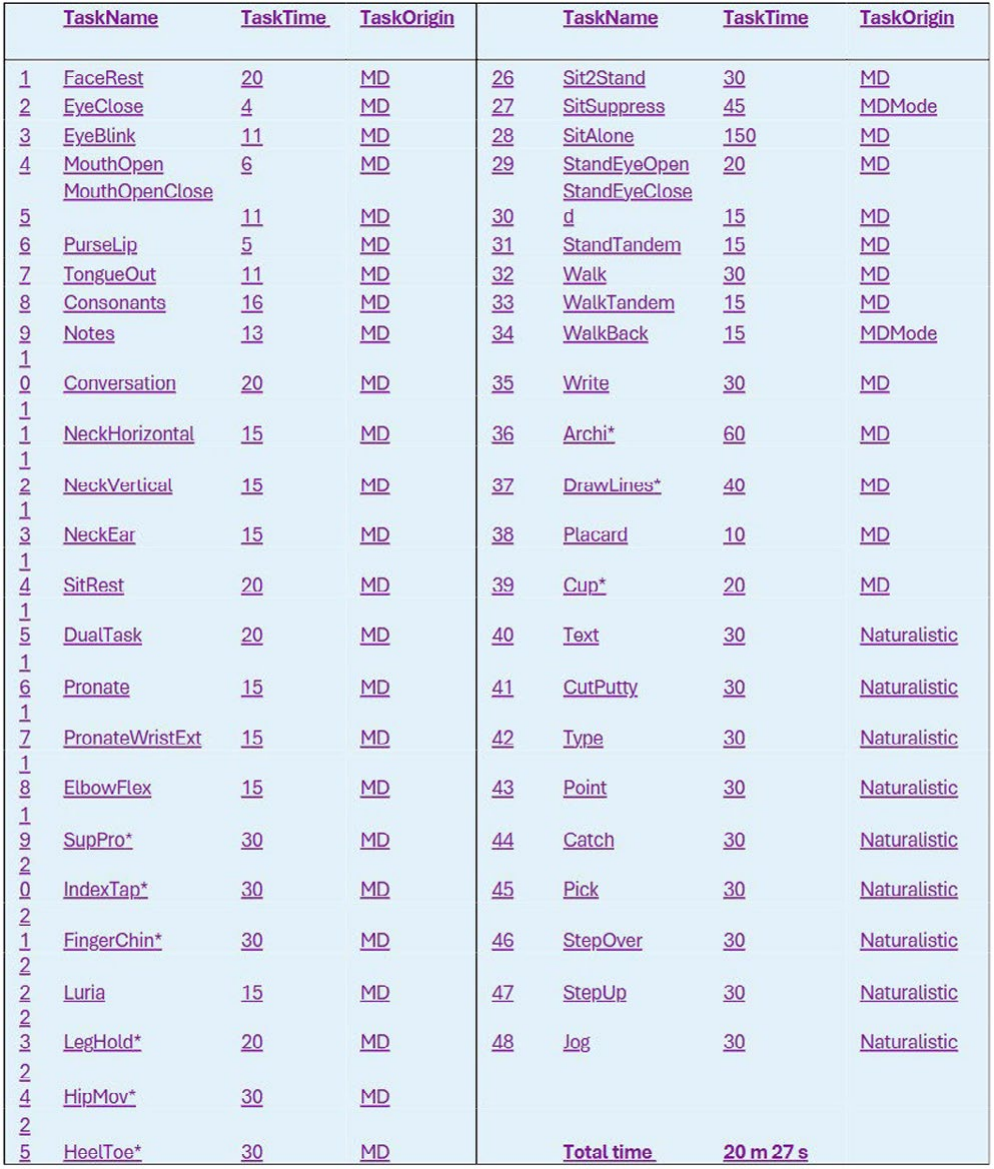
**FIGURE 2** Table listing protocol tasks indicating whether each was derived from movement disorder scales (MD), targeted motor control modes (MDMode), or adapted from ecological tasks. Asterisked tasks were performed bilaterally.

**Figure:**
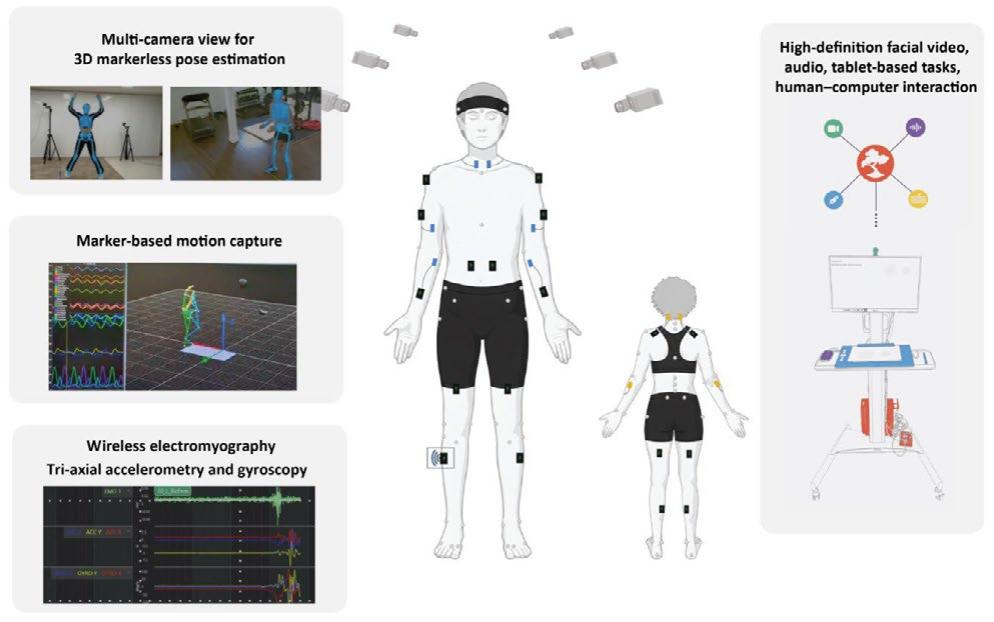
**FIGURE 3** Multimodal data acquisition from anterior and posterior views, integrating synchronised multi‐view video, motion capture, surface EMG with IMU's, and additional streams including facial video, audio, tablet tasks, and human–computer interaction.

**Results:** The resulting standardised questionnaire and 20‐minute, 48‐task motor assessment sample core bedside constructs and naturalistic behaviours, enabling harmonised acquisition across sites and future remote or ambulatory extension.

**Conclusion:** We consolidate fragmented, syndrome‐specific assessments into a reproducible cross‐disorder protocol, enabling principled characterisation of variability and invariance, longitudinal tracking, and data‐driven inference. This strengthens behavioural examination as a quantitative foundation for biomarker discovery and treatment optimisation.

**Disclosure:** Nothing to disclose.

## EPO‐0605

### Treatment effects on spasticity and motor outcomes in pediatric cerebral palsy: A systematic review and meta‐analysis

#### M. Alghadier ^1^; A. Alsuwayegh^2^

##### ^*1*^ *Department of Health and Rehabilitation Sciences, Prince Sattam bin Abdulaziz University, Alkharj, Saudi Arabia;* ^*2*^ *Corporate Department of Pharmacy, King Saud University Medical City, Riyadh, Saudi Arabia.*

**Background and aims:** Botulinum toxin type A (BoNT‐A) is a cornerstone intervention for managing focal spasticity in children with Cerebral Palsy (CP). Despite its widespread use, variability in trial designs has complicated the assessment of its magnitude, durability, and impact on functional gains. This study aimed to quantify the short‐term therapeutic effects of BoNT‐A and synthesize evidence regarding dosing and rehabilitation contexts.

**Methods:** We conducted a systematic review of Randomized Controlled Trials (RCTs) published between January 2015 and November 2025 across four major medical databases (PubMed, Cochrane, Web of Science, Google Scholar). Inclusion was restricted to pediatric CP populations receiving pharmacological spasticity management. Primary outcomes included spasticity severity (Modified Ashworth Scale, MAS) and gross motor function (GMFM). Data were synthesized to calculate pooled mean differences and evaluate heterogeneity across limb targets and dosing strategies.

**Results:** Eighteen RCTs involving 892 participants met the inclusion criteria. Meta‐analysis of placebo‐controlled lower‐limb studies demonstrated a statistically significant reduction in spasticity at 4–6 weeks (pooled mean difference on MAS: −0.31). While tone reduction was consistent across licensed doses, functional improvements (GMFM) were modest and primarily observed when injections were paired with structured rehabilitation (1–3 months post‐intervention). The therapeutic effect typically attenuated by week 12. Adverse events were predominantly mild and transient.

**Conclusion:** BoNT‐A is effective for short‐term focal spasticity reduction in pediatric CP. However, translation into functional motor gains is inconsistent and strongly dependent on concurrent rehabilitation. Clinicians should view BoNT‐A as an adjunct to therapy rather than a standalone solution, with re‐administration required to sustain benefits.

**Disclosure:** Nothing to disclose.

## EPO‐0606

### Comparison of Respiratory Functions and Physical Fitness Levels in Individuals Diagnosed with Multiple Sclerosis and Healthy Individuals

#### N. Duruturk ^1^; I. Gözlekci^1^; M. Kılınc^2^

##### ^*1*^ *Baskent Univeristy, Physiotherapy and Rehabilitation Department, Ankara, Türkiye;* ^*2*^ *Baskent University, Neurology Department, Ankara, Türkiye*

**Background and aims:** Multiple sclerosis (MS) is defined as a chronic, inflammatory and autoimmune disease affecting the white matter of the central nervous system. Depending on the location of the lesions, symptoms may vary and can significantly affect the daily life of patients. The study aimed to investigate the comparison of pulmonary function and physical fitness levels in individuals with Multiple Sclerosis and healthy individuals.

**Methods:** The study included 34 individuals with MS with EDSS scores of ≤ 4.5 ‐ 6 and 34 healthy individuals of similar age and gender. Respiratory function was measured by spirometer, inspiratory muscle strength by respiratory muscle strength meter, inspiratory muscle endurance by ascending threshold loading test, biceps and quadriceps muscle strength by digital hand dynamometer, cardiorespiratory endurance by Six Minutes Walking Test, flexibility with sit and reach and back scratch test and balance by interactive balance system.

**Results:** There were significant differences between the MS group's respiratory function test and inspiratory muscle strength and biceps, quadriceps muscle strength, 6MWT walking distance, expected percentage, stability test parameters and the values of the healthy group (*p* < 0.05).

**Figure:**
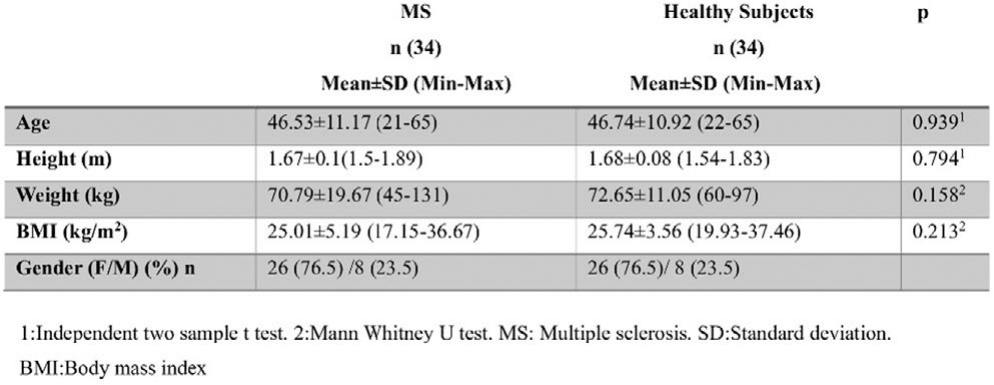
**TABLE 1** Demographic characteristics of participants.

**Figure:**
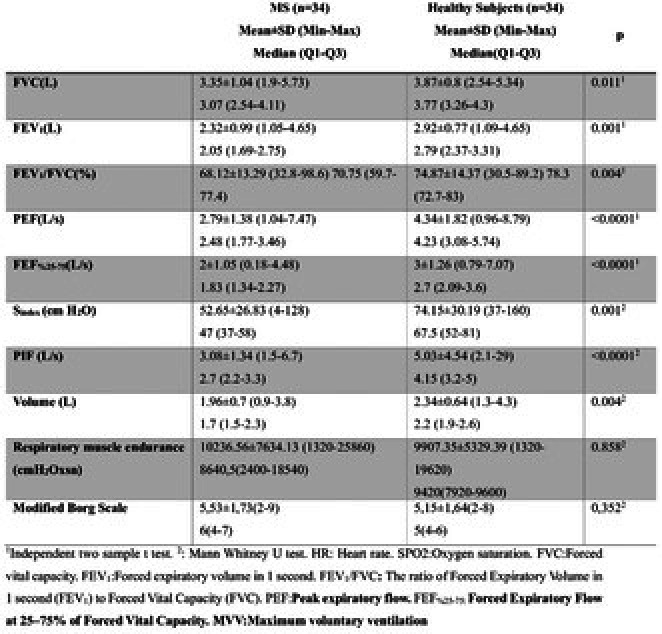
**TABLE 2** Comparison of pulmonary function test results of MS and healthy group.

**Figure:**
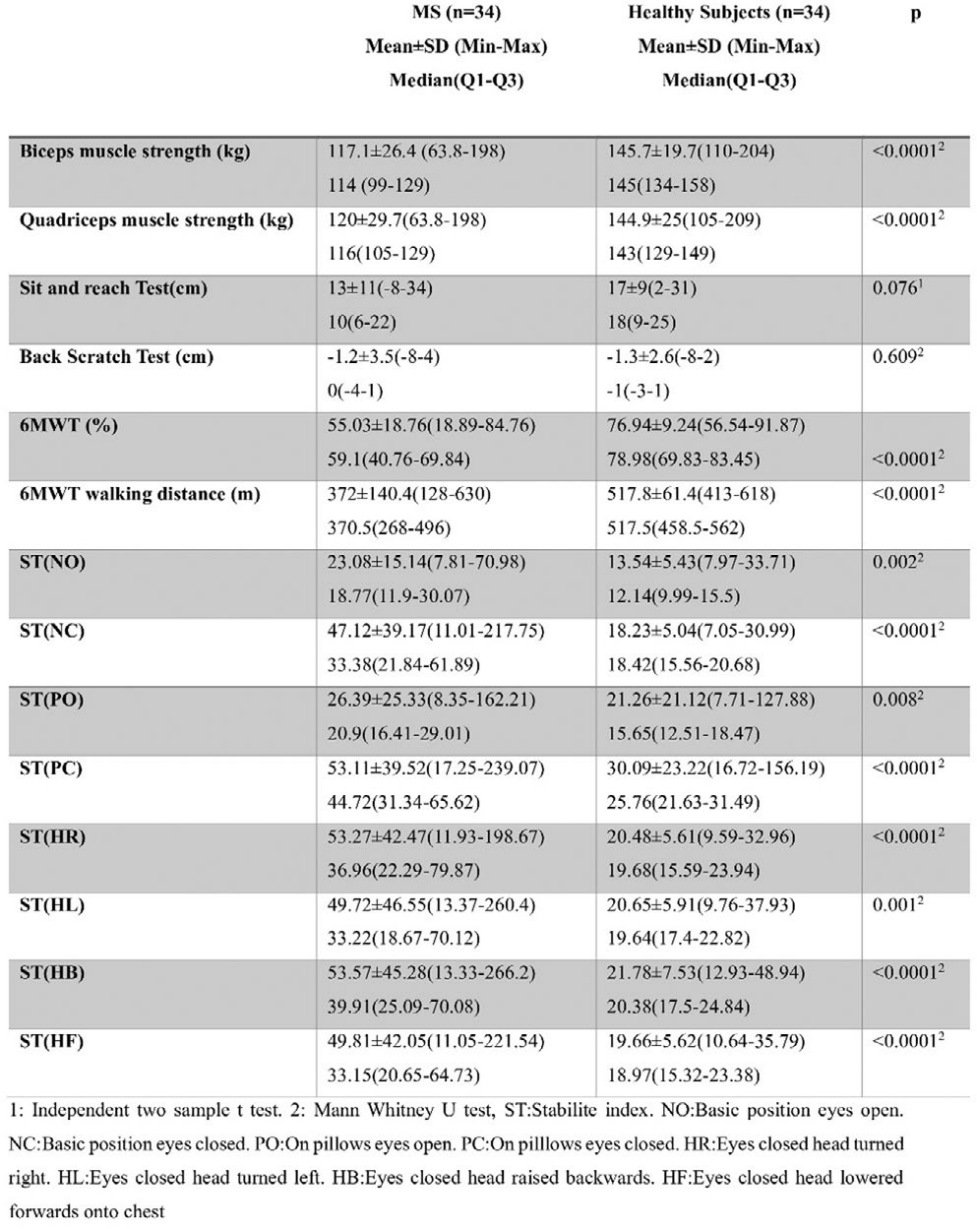
**TABLE 3** Comparison of physical fitness test results of MS and healthy group.

**Conclusion:** As a result of our study we determined that respiratory functions and physical fitness parameters were lower in MS individuals than in healthy individuals. We think that appropriate evaluation and treatment should be made by considering the effects of these factors in physiotherapy and rehabilitation programs specific to MS individuals and developing them.

**Disclosure:** Nothing to disclose

## EPO‐0607

### Stride width can be a descriptor of reduced ability of gait modulation in people with Multiple Sclerosis

#### R. Rossanigo ^1^; M. Caruso^1^; G. Cicalò^1^; N. Leo^1^; A. Audisio^1^; P. Tasca^1^; V. Agostini^1^; G. Olmo^1^; M. Ghislieri^1^; R. Chirico^2^; I. Bertoni^2^; E. Favano^3^; D. Vecchio^3^; C. Comi^3^; E. Virgilio^4^; S. Rolla^4^; M. Morra^4^; A. Dal Molin^3^; M. Clerico^4^; A. Cereatti^1^

##### ^*1*^ *Department of Electronics and Telecommunications, Politecnico di Torino, Turin, Italy;* ^*2*^ *University Hospital Maggiore della Carità, Novara, Italy;* ^*3*^ *Department of Translational Medicine, Neurology Unit, University of Piemonte Orientale, Novara, Italy;* ^*4*^ *Department of Clinical and Biological Sciences, University of Turin, Turin, Italy*

**Background and aims:** Gait modulation—the ability to adapt gait to maintain stability while minimizing energetic cost across different contexts—can be impaired in people with Multiple Sclerosis (pwMS) due to proprioceptive deficits and motor rigidity, increasing fall risk. Traditional gait measures such as stride velocity describe overall performance but limited insight into dynamic balance. Stride width (SW), the mediolateral distance between the feet, is a sensitive marker of lateral stability but is typically restricted to lab‐based assessment. We monitored SW and stride velocity in healthy subjects (HS) and pwMS during in‐lab and ecological walking, to examine how different contexts shape gait modulation.

**Methods:** Ten healthy subjects (HS) and ten pwMS (EDSS 1–6) performed a 2‐min walking test (2MWT), an in‐lab simulation of daily activities (SDA), and a 2.5‐h free‐living session while wearing a validated wearable system estimating stride velocity and SW (Figure 1). Linear mixed‐effects models evaluated the impact of walking condition and group on these parameters.

**Figure:**
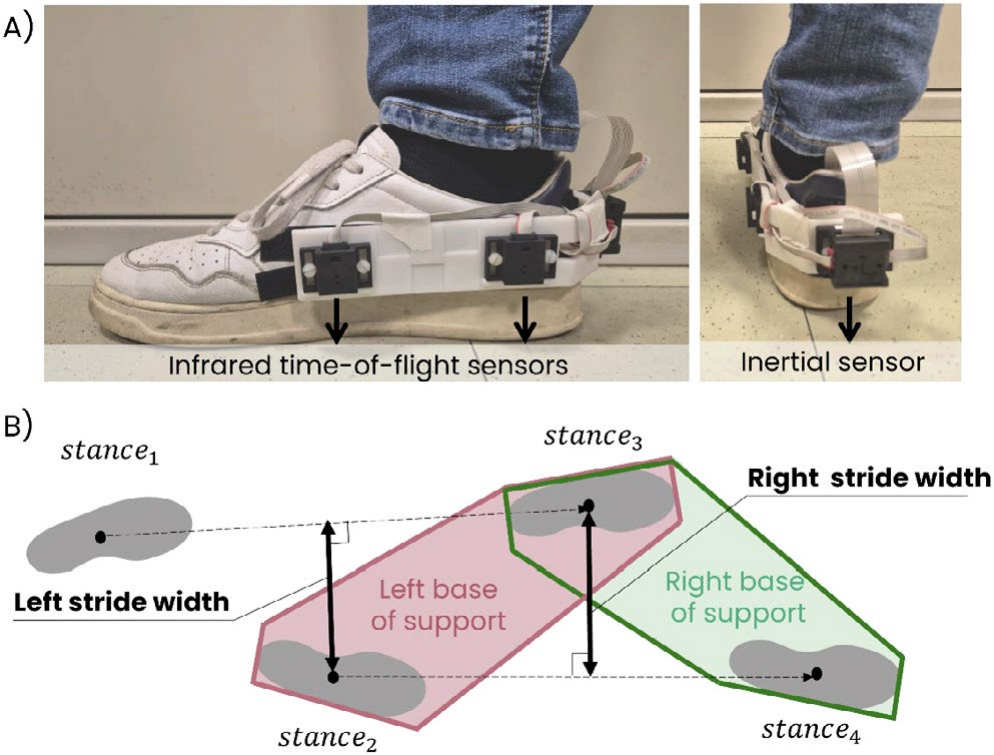
**FIGURE 1** Participant's right foot instrumented with a novel miniaturized wearable system (A) which enables to estimate bilateral stride width (B).

**Results:** Stride velocity was consistently lower in pwMS than HS, while SW revealed context‐specific patterns (Figure 2).

**Figure:**
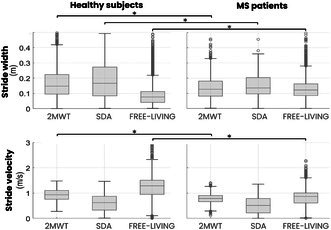
**FIGURE 2** Distributions of stride width (above) and stride velocity (bottom) of healthy subjects (left) and people with Multiple Sclerosis (right) across different walking conditions. Over 45000 steps were analyzed. *p < 0.05.

**Conclusion:** Wearable‐based SW captures context‐dependent gait adaptation in pwMS that stride velocity alone cannot. SW variability was reduced in pwMS during 2MWT and SDA, indicating limited modulation of the base of support, whereas free‐living walking showed wider SW, suggesting a compensatory strategy prioritizing postural safety over gait economy. Impaired SW modulation may represent a novel biomarker of lateral instability, proprioceptive deficits, and motor rigidity. Future work will investigate its longitudinal relationship with clinical disability and fall incidence.

**Disclosure:** Part of the project NODES, MUR – M4C2 1.5 of PNRR funded by the European Union ‐ NextGenerationEU (ECS00000036 – CUP E13B22000020001).

## EPO‐0608

### Validation of a Vision Screening Tool for Detecting Visual Abnormalities in Acute Stroke Care

#### S. Ryan ^1^; A. Litzen Jørstad^2^; M. Moe^2^; M. Skjelland^1^; F. Rowe^3^; Ø. Jørstad^2^; A. Aamodt^1^

##### ^*1*^ *Department of Neurology, Oslo University Hospital, Oslo, Norway;* ^*2*^ *Department of Ophthalmology, Oslo University Hospital, Oslo, Norway**;*** ^*3*^ *Institute of Population Health, Faculty of Health and Life Sciences, University of Liverpool, Liverpool, UK*

**Background and aims:** Visual abnormalities are common after stroke but are under‐recognised in acute care. The Vision Impairment Screening Assessment (VISA) was developed to enable structured bedside screening by non‐specialist stroke clinicians. We aimed to validate the Norwegian translated version of VISA against comprehensive neuro‐ophthalmic examination and describe the prevalence of post‐stroke visual abnormalities in a hyperacute stroke population.

**Methods:** StrokeVIS was a prospective diagnostic accuracy study including consecutive patients admitted for acute stroke management at a tertiary thrombectomy centre. Participants underwent VISA screening and neuro‐ophthalmic examination within 72 hours of admission. VISA was administered by trained stroke staff blinded to the reference standard. Diagnostic performance was assessed using sensitivity, specificity, predictive values, and overall accuracy. Secondary analyses examined prevalence across visual domains and evaluated the performance of VISA and the visual items of the National Institutes of Health Stroke Scale (NIHSS).

**Results:** A total of 127 patients were included, of whom 81 completed both assessments. Neuro‐ophthalmic examination identified visual abnormalities in 70.1% of patients, most frequently ocular motility disturbances (46.5%), visual inattention (38.3%), reduced distance visual acuity (25.5%), and visual field loss (15.7%). VISA demonstrated high sensitivity (94.5%) but low specificity (34.6%), with an overall accuracy of 75.3%. The NIHSS visual items showed lower sensitivity (60.7%) and accuracy (62.0%).

**Figure:**
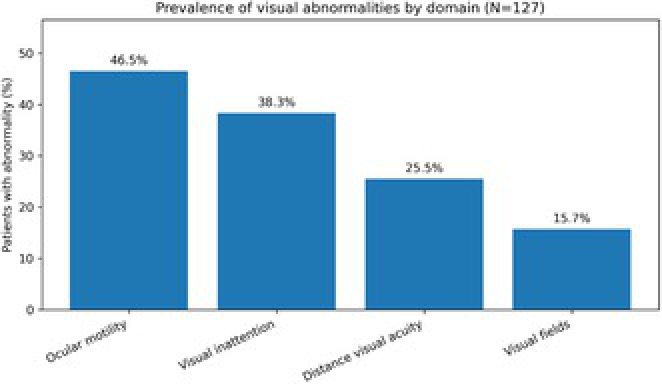
**FIGURE 1** Prevalence of visual abnormalities detected on neuro‐ophthalmic examination in the full study cohort (*N* = 127). Percentages represent patients with at least one abnormality per domain. Multiple abnormalities per patient were allowed.

**Figure:**
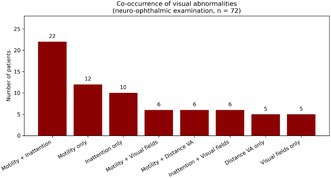
**FIGURE 2** Co‐occurrence of visual abnormalities identified on neuro‐ophthalmic examination among patients with complete data across all domains (*n* = 72). Bars show single‐domain abnormalities and the most frequent multi‐domain combinations.

**Figure:**
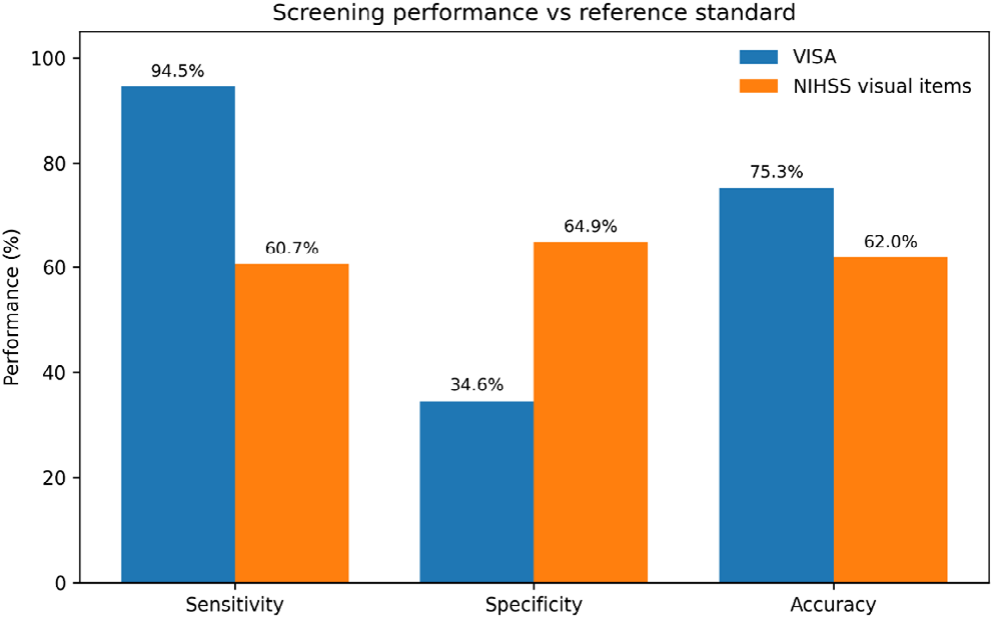
**FIGURE 3** Screening performance of the Vision Impairment Screening Assessment (VISA) and visual items of the National Institutes of Health Stroke Scale (NIHSS) compared with neuro‐ophthalmic examination as the reference standard.

**Conclusion:** The Norwegian version of VISA demonstrates high sensitivity for detecting early post‐stroke visual abnormalities in acute care. Given the high prevalence of visual abnormalities and the limited sensitivity of routine neurological assessment, structured visual screening may improve early identification of vision‐related deficits in stroke pathways.

**Disclosure:** Nothing to disclose.

Movement disorders 6

## EPO‐0609

### Disease course in primary brain calcification: A monocentric longitudinal study from an Italian Cohort

#### G. Bonato ^1^; D. Gasparini^3^; F. Pistonesi^2^; C. Bertolin^4^; L. Salviati^5^; A. Antonini^2^; M. Carecchio^2^

##### ^*1*^ *Neurodegenerative Disorders Unit, Neuroscience Department, University of Padova, Padova, Italy; Foundation IRCCS Ca' Granda Ospedale Maggiore Policlinico, Neurology Unit, University of Milan, Milan, Italy;* ^*2*^ *Neurodegenerative Disorders Unit, Neuroscience Department, University of Padova, Padova, Italy;* ^*3*^ *Biostatistics, Epidemiology and Public Health Unit, University of Padua, Padova, Italy;* ^*4*^ *Clinical Genetics Unit, University Hospital of Padova, Padova, Italy**;*** ^*5*^ *Clinical Genetic and Epidemiology Unit, Department of Women and Children's Health, University of Padova, Padova, Italy*

**Background and aims:** Primary Brain Calcification (PBC) is a rare neurodegenerative disorder characterized by brain calcium deposition, featuring movement disorders and psychiatric/cognitive disturbances[1–3]. Quantitative longitudinal data describing disease progression are limited[2–4]. We here investigate motor‐symptoms evolution and clinical predictors of functional outcome.

**Methods:** 83 PBC patients from Padova University, diagnosed by CT‐scan and metabolic screening; NGS genetic test; neurological assessment (MDS‐UPDRS‐III, H&Y, SARA, UHDRS, BFMDRS, TETRAS, MoCA); mean prospective follow‐up of 3.5±2 years, combined with retrospective data collection covering 9±4 years; linear‐mixed‐models/hinge‐LMM, Kaplan‐Meier‐survival‐curves, multivariate analysis.

**Results:** Neurological symptoms were documented in 64 subjects; movement disorders were the most frequent manifestation (*n* = 52, predominantly parkinsonism); over time, combined motor and cognitive/psychiatric involvement occurred in 65% of patients. Mean age‐at‐onset was 53.3 years but neuropsychiatric symptoms presented earlier (mean 42 years, *p* = 0.002). Genetic diagnosis was achieved in 46%, mostly involving MYORG and SLC20A2 mutations. Parkinsonism, cerebellar signs and cognitive decline predicted worse functional outcome (*p* = 0.01), older age was a risk‐factor of symptom development (*p* = 0.004), genetics influenced specific symptoms penetrance. Clinical progression occurred in 63% of the cohort, with significant worsening in UPDRS‐III, SARA and MoCA (*p* < 0.001) scales. Hinge‐LMM, aligning to symptom onset, demonstrated progression rates of +1.7/year at UPDRS‐III, +0.7/year at SARA (*p* < 0.001). Isolated dystonia/tremor showed minimal progression. MYORG mutation carriers showed faster progression compared to other genetic subgroups (UPDRS‐III:+3.6/y, *p* = 0.01; SARA:+1.7/y, *p* = 0.01).

**Figure:**
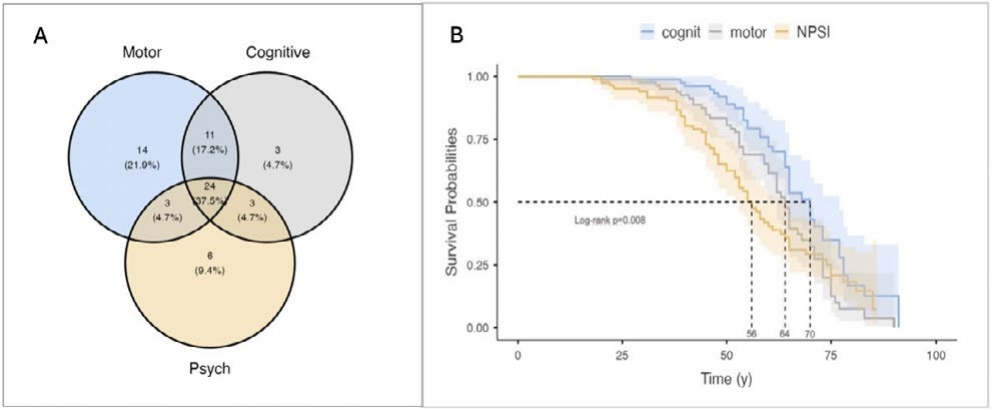
**FIGURE 1** A) Combination of clinical symptoms at last evaluation (Venn diagram). B) Kaplan‐Meier survival curves for core symptoms over time.

**Figure:**
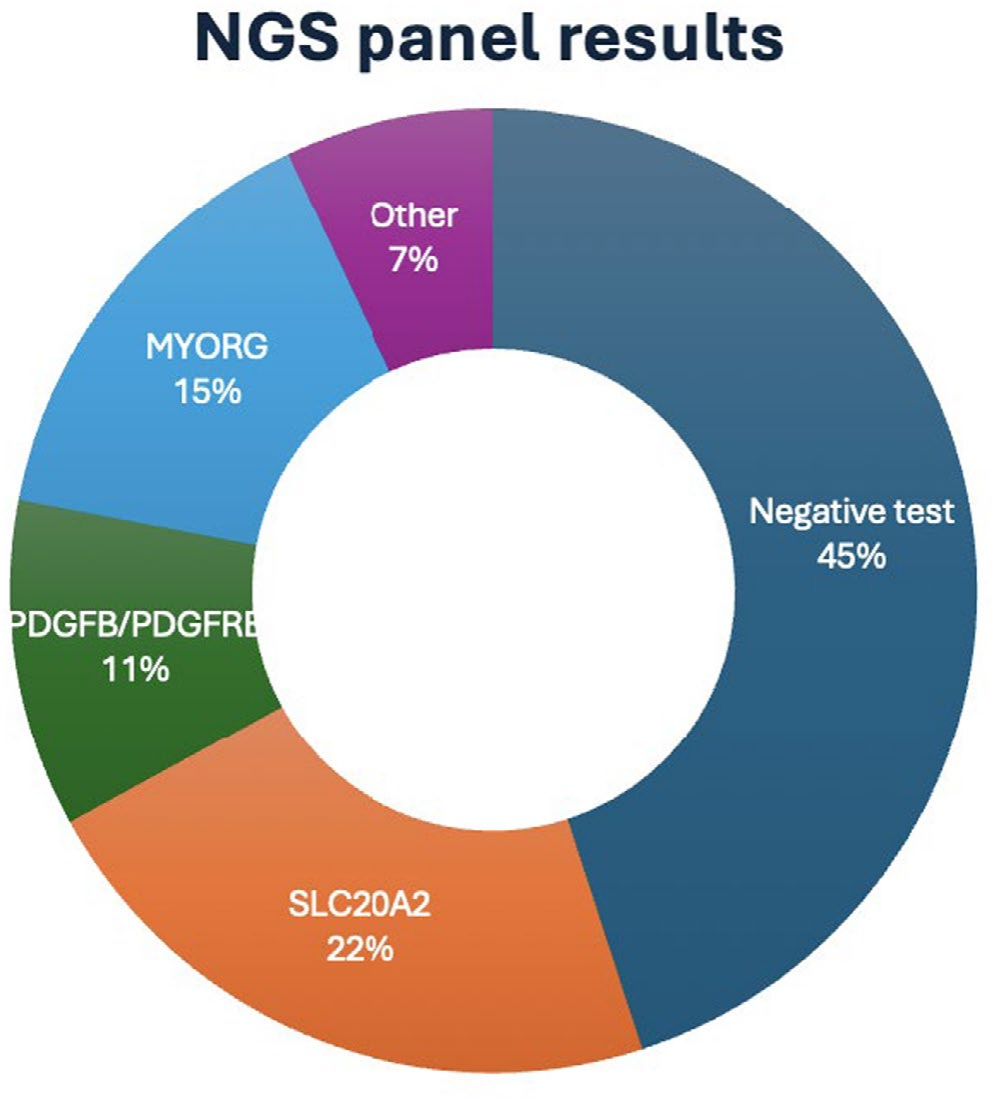
**FIGURE 2** Genetic findings in the cohort.

**Figure:**
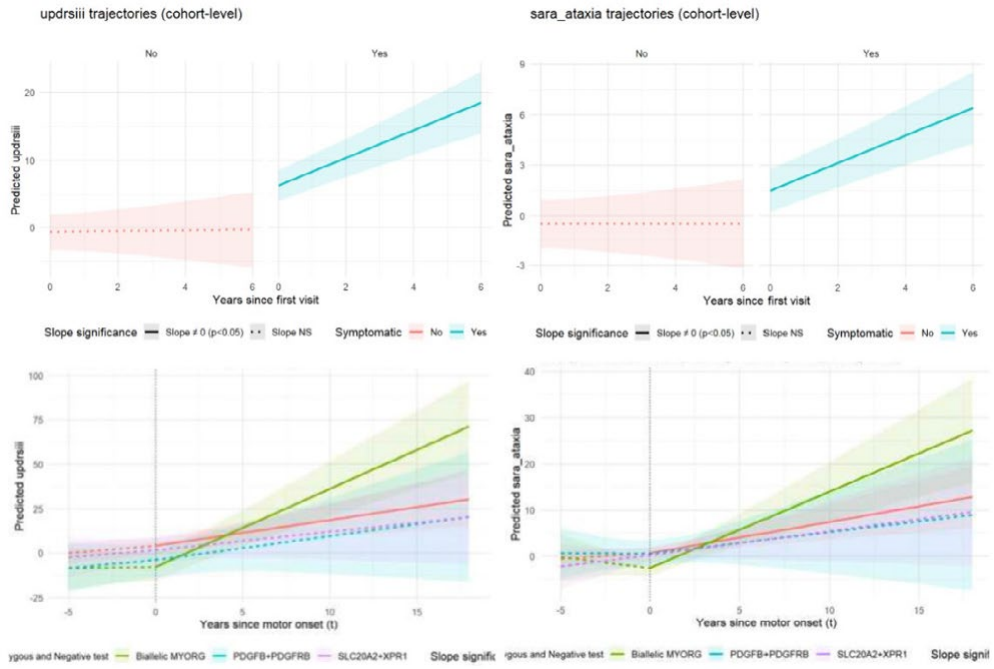
**FIGURE 3** Disease trajectories for significant motor scales (UPDRS‐III and SARA).

**Conclusion:** PBC is a progressive disorder with distinct disease trajectories influenced by symptom profile and genetic background. Comprehensive assessment and longitudinal monitoring are essential to define individual disease prognosis and support future therapeutic trials.

**Disclosure:** Data partially submitted to 2026 Italian Movement Disorders Congress as abstract Nothing to disclose

## EPO‐0610

### Baseline variables associated with sublingual apomorphine film maintenance dose

#### J. Kassubek^1^; J. Schwarz^2^; L. López Manzanares^3^; J. Reis^4^; H. C. Brigas ^4^; C. Denecke‐Muhr4^4^

##### ^*1*^ *Department of Neurology, University Hospital Ulm, Ulm, Germany;* ^*2*^ *Department of Geriatrics, Kreisklinik Ebersberg, Ebersberg, Germany;* ^*3*^ *La Princesa University Hospital, Madrid, Spain;* ^*4*^ *Bial – Portela & Ca, S.A, Coronado, Portugal*

**Background and aims:** This analysis aimed to identify baseline demographic and clinical characteristics associated with the final titrated dose of sublingual apomorphine (SL‐APO) in patients with Parkinson's disease experiencing OFF episodes, to support more individualized titration strategies and optimized treatment.

**Methods:** Baseline characteristics from the CTH‐301 dose‐optimisation phase were examined across five SL‐APO doses (10–30 mg) from de novo patients. Associations between variables and SL‐APO dose were evaluated using univariate ordinal logistic regression. Odds ratios with 95% confidence intervals were calculated for continuous and categorical variables, and statistical significance was set at *p* < 0.05.

**Results:** Several baseline factors showed significant associations with higher SL‐APO doses. Patients with greater motor severity, reflected in higher MDS‐UPDRS Part I, II, III, and Total scores (all *p* < 0.001), were more likely to titrate to higher doses. A higher total daily levodopa dose was also significantly associated with higher SL‐APO doses (*p* < 0.001). In addition, the presence of delayed ON (*p* = 0.018) and a greater number of daily OFF episodes (*p* = 0.005) were associated with requiring higher doses. No significant associations were observed for age, sex, BMI, Hoehn&Yahr stage, cognitive status, PD duration, motor fluctuations duration, tremor, wearing‐off, morning akinesia, sudden OFF, or duration of OFF episodes.

**Figure:**
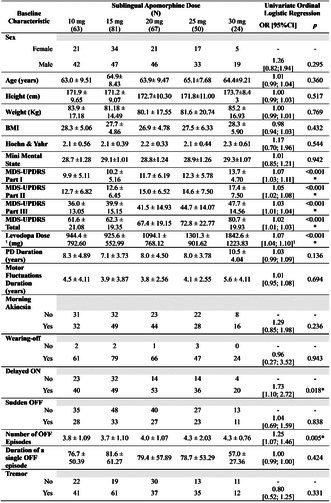
**TABLE 1** Baseline demographic and clinical characteristics associated with the final titrated dose of sublingual apomorphine.

**Figure:**
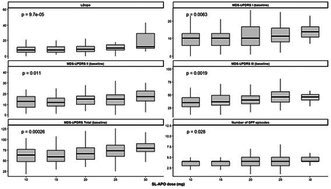
**FIGURE 1** Baseline demographic and clinical characteristics associated with the final titrated dose of sublingual apomorphine.

**Conclusion:** Patients who required higher SL‐APO doses presented with more severe baseline motor symptoms, higher levodopa requirements, delayed ON, and more frequent OFF episodes. These findings indicate that individuals with a greater motor burden and more complex fluctuation profiles may require higher SL‐APO doses, providing useful guidance for clinicians during dose titration to achieve an optimal therapeutic effect.

**Disclosure:** Supported by Bial‐Portela & Cª, S.A.

## EPO‐0611

### Long‐term Safety of Opicapone in Parkinson's Disease Patients with First Signs of Wearing‐off

#### J. Ferreira^1^; F. Stocchi^2^; A. Antonini^3^; O. Rascol^4^; G. Ebersbach^5^; J. Kulisevsky^6^; N. Pavese^7^; G. Hoglinger^8^; D. Ramos^9^; J. Rocha^9^; H. Brigas ^9^; J. Holenz^9^; W. Poewe^10^

##### ^*1*^ *Laboratory of Clinical Pharmacology and Therapeutics, Faculty of Medicine, University of Lisbon, Lisbon, Portugal;* ^*2*^ *Department of Neurology, IRCCS San Raffaele Pisana, Rome, Italy;* ^*3*^ *Department of Neurosciences, University of Padova, Padova, Italy;* ^*4*^ *University of Toulouse, University Hospital of Toulouse, INSERM, Clinical Investigation Center CIC1436 Departments of Neurosciences and Clinical Pharmacology and NS‐Park/FCRIN network, Toulouse, France;* ^*5*^ *Movement Disorder Clinic Beelitz‐Heilstaetten, Beelitz‐Heilstätten, Germany;* ^*6*^ *Movement Disorders Unit, Sant Pau Hospital, Barcelona, Spain;* ^*7*^ *Clinical Ageing Research Unit, Newcastle University, Newcastle, UK;* ^*8*^ *Ludwig‐Maximilians‐University Hospital, Munich, Germany;* ^*9*^ *BIAL – Portela & Ca S.A, Coronado, Portugal;* ^*10*^ *Department of Neurology, Medical University of Innsbruck, Innsbruck, Austria*

**Background and aims:** Opicapone is used to manage motor fluctuations in Parkinson's patients, and its earlier initiation at wearing‐off onset demonstrated benefits in OFF‐ and Good‐ON‐time without increasing dyskinesia over 1 year. This post‐hoc analysis evaluated opicapone’ s long‐term safety and tolerability in Parkinson's patients with early fluctuations.

**Methods:** Data were pooled from double‐blind, placebo‐controlled BIPARK I and II studies (3.5 months) and their 1‐year open‐label extensions. Analyses included participants randomized to placebo or opicapone 50 mg who continued opicapone during the open‐label phase and had developed wearing‐off within ≤2 years prior to screening. Treatment‐emergent adverse events (TEAEs), TEAEs considered related to treatment, and TEAEs leading to discontinuation were evaluated.

**Results:** Safety Set included 218 patients with recent‐onset wearing‐off (placebo, *n* = 110; opicapone, *n* = 118). During the double‐blind phase, TEAEs occurred in 55.9% of opicapone‐treated and 51.8% of placebo‐treated patients, with treatment‐related TEAEs being reported in 30.5% and 20.0%, respectively (Table 1). Most frequent treatment‐related TEAEs with opicapone were dyskinesia (9.3%) and insomnia (3.4%). In the 1‐year open‐label extension, treatment‐related TEAEs occurred in 39.8% of patients continuing opicapone and 35.5% of those switching from placebo, most commonly dyskinesia (8.5% and 13.6%, respectively) (Table 2). Treatment‐related TEAEs leading to discontinuation were infrequent (2.5% and 0.0%, respectively); no deaths were reported.

**Figure:**
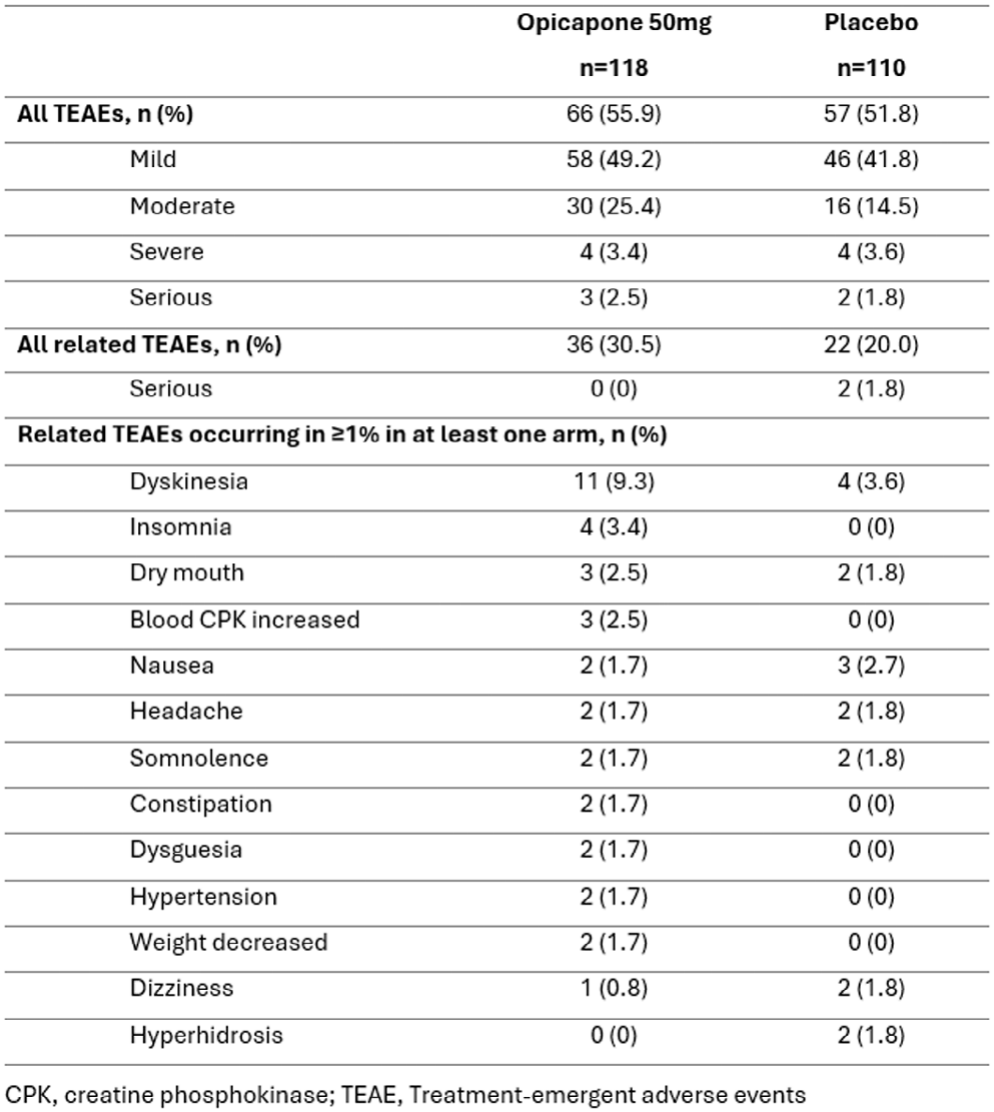
**TABLE 1** Safety and tolerability during the double‐blind phase (14–15 weeks).

**Figure:**
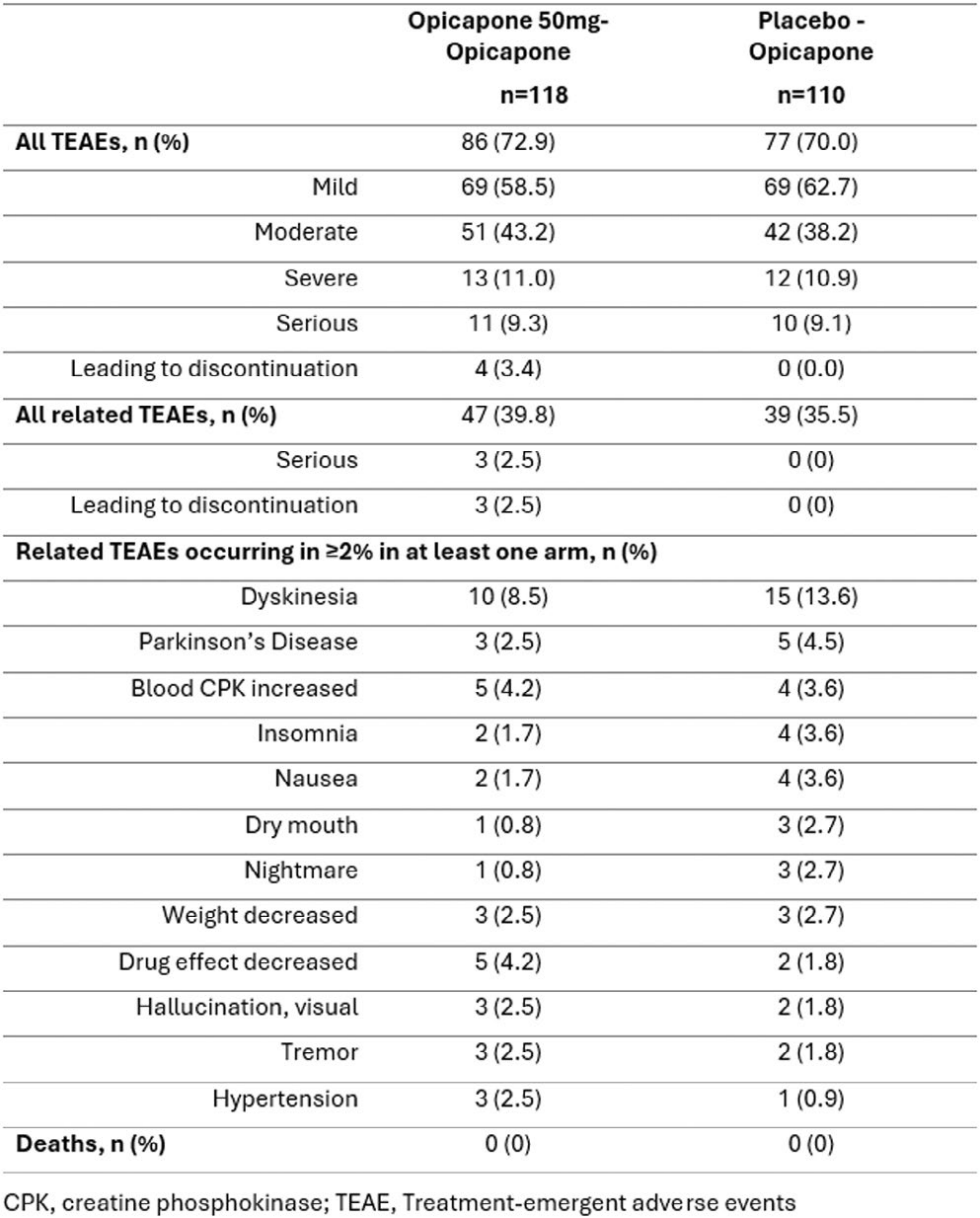
**TABLE 2** Safety and tolerability during the open‐label extension phase (1 year).

**Conclusion:** Long‐term opicapone exposure was generally safe and well‐tolerated in patients with recent‐onset fluctuations. Most TEAEs were mild/moderate and consistent with dopaminergic therapy. Dyskinesia occurred less frequently in early versus later starters during the open‐label phase, supporting opicapone’ s favorable safety profile when initiated at wearing‐off onset.

**Disclosure:** Supported by Bial.

## EPO‐0612

### Perceptions of Neuropalliative Care in Parkinsonism: Implications from a Multicenter Survey in Korea

#### C. Lee ^1^; S. You^2^; J. Yun^3^; H. Kim^3^; M. Kim^4^; Y. Hwang^5^; J. Lee^6^; K. Park^7^; H. Chang^8^; S. Lee^9^; I. Kwak^10^

##### ^*1*^ *Department of Neurology, Ewha Womans University Mokdong Hospital, Ewha Womans University College of Medicine, Seoul, Korea;* ^*2*^ *Department of Neurology, Seoul medical center, Seoul, Korea;* ^*3*^ *Department of Neurology, Ewha Womans University Seoul Hospital, Ewha Womans University College of Medicine, Seoul, Korea;* ^*4*^ *Department of Neurology, Bobath Memorial Hospital, Seongnam, Korea;* ^*5*^ *Department of Neurology, Jeonbuk National University Medical School and Hospital, Jeonju, Korea;* ^*6*^ *Department of Neurology, Dongsan Hospital, Keimyung University School of Medicine, Daegu, Republic of Korea;* ^*7*^ *Department of Neurology, Gangneung Asan Hospital, University of Ulsan College of Medicine, Gangneung, Republic of Korea;* ^*8*^ *Department of Neurology, Chungnam National University College of Medicine, Chungnam National University Hospital, Daejeon, Republic of Korea;* ^*9*^ *Department of Neurology, Konyang University Hospital, Konyang University College of Medicine, Daejeon, Korea;* ^*10*^ *Department of Neurology, Hallym University Sacred Heart Hospital*

**Background and aims:** Parkinsonism, particularly Parkinson's disease (PD), is a progressive neurodegenerative disorder with motor and non‐motor symptoms that negatively affect quality of life and increase caregiver burden. Neuropalliative care has recently emerged for chronic neurological disorders, including PD. This study evaluated awareness and perceptions of palliative care among patients with parkinsonism, their caregivers, and physicians in Korea, and identified factors associated with caregiver burden.

**Methods:** A multicenter, cross‐sectional study was conducted with 112 patients, 110 caregivers, and 61 physicians. Structured questionnaires assessed awareness and attitudes toward neuropalliative care. Caregiver burden was measured using validated Korean version of the Parkinson's Disease Questionnaire‐Carer (PDQ‐Carer).

**Results:** Awareness of palliative care differed markedly: 31.3% of patients and 27.5% of caregivers versus 90.2% of physicians. Caregivers were stratified by PDQ‐Carer score into low‐ (≤34, *n* = 63) and high‐burden (>34, *n* = 47) groups. High‐burden caregivers supported patients with longer disease duration, poorer cognition, higher Hoehn and Yahr stage, and greater levodopa equivalent daily doses (LEDD). Regression analysis showed that caregiver demographics were not associated with burden, whereas caregiving duration, patient cognitive decline, Hoehn and Yahr stage, LEDD, and disease duration were correlated. In the multivariable model, only patient disease duration remained significant (B = 2.048, 95% CI 0.083–4.012, *p* = 0.041).

**Conclusion:** There is a significant awareness gap in palliative care between physicians and patients/caregivers. The caregiver burden was mainly linked to disease duration as the strongest predictor. Education and structured neuropalliative strategies are needed to support patients with PD and their families

**Disclosure:** The authors declare no conflicts of interest.

## EPO‐0613

### Dopaminergic modulation of DBS‐evoked cortical responses in STN‐DBS for Parkinson’ disease

#### J. Marae; B. Bahners; R. Spooner; A. Schnitzler; E. Florin

##### Institute of Clinical Neuroscience and Medical Psychology, Medical Faculty, Heinrich‐Heine University, Duesseldorf, Germany

**Background and aims:** Subthalamic deep brain stimulation elicits short‐ and long‐latency cortical responses reflecting activation of hyperdirect and polysynaptic basal ganglia–thalamo–cortical pathways (Miocinovic et al, 2018). While dopaminergic medication alters network synchrony and basal ganglia coupling (Kühn et al, 2008; Tinkhauser et al, 2017), its influence on DBS‐evoked cortical responses remains unclear. Human EEG and primate studies suggest a medication‐related dampening of late cortical components (Eusebio et al, 2009; Campbell et al, 2022). However, systematic MEG studies examining the influence of dopaminergic state on DBS‐evoked cortical activity in Parkinson's disease are lacking. The present study aimed to determine whether dopaminergic state modulates the amplitude, latency, or spatial distribution of STN‐DBS‐evoked cortical responses.

**Methods:** Nineteen patients with Parkinson's disease and STN‐DBS underwent MEG recordings during low‐frequency (6 Hz) stimulation of the left STN. Stimulation was applied omnidirectionally at the clinically effective contact using the individual therapeutic amplitude and a pulse width of 60 μs. Each patient was recorded in medication ON and OFF states. Source activity was reconstructed using linearly constrained minimum variance beamforming, focusing on the primary motor cortex and supplementary motor area. Peak amplitudes and latencies were compared between medication states using paired two‐tailed t‐tests.

**Results:** DBS evoked consistent early (~6 ms), mid (~13 ms) and long‐latency (~21 ms) cortical responses in both conditions. No significant differences in response amplitude, latency, or cortical topography were observed between medication states.

**Conclusion:** DBS‐evoked cortical responses were robust and reproducible in both medication states, suggesting that DBS programming based on evoked cortical responses is feasible without medication withdrawal.

**Disclosure:** Nothing to disclose.

## EPO‐0614

### Opicapone therapy during continuous foslevodopa/foscarbidopa infusion: A good choice?

#### F. Galbiati ^1^; A. Anzani^1^; S. Cascino^1^; G. Pezzoli^2^; I. Isaias^1^; G. Lazzeri^1^

##### ^*1*^ *Parkinson Institute, ASST Gaetano Pini‐CTO, Milan, Italy;* ^*2*^ *Fondazione Pezzoli Per La Malattia Di Parkinson, Milan, Italy*

**Background and aims:** Continuous subcutaneous infusion of foslevodopa/foscarbidopa (CSFI) is an effective therapy for advanced Parkinson's disease (PD). However, the role of concomitant opicapone (OPC) during CSFI has not been systematically assessed. This study evaluated the impact of OPC co‐administration on clinical outcomes, dopaminergic requirements, and safety.

**Methods:** We performed a single‐center, retrospective study including 59 patients with advanced PD treated with CSFI. Motor outcomes (MDS‐UPDRS‐III and IV), infusion parameters, levodopa equivalent daily dose (LEDD), and adverse events were compared between patients treated with and without concomitant OPC.

**Results:** The cohort comprised 36 men and 23 women (mean age 66.7 years; mean disease duration 14.5 years). Twenty‐nine patients received OPC (24 continued from baseline, 5 initiated de novo). Treatment retention was high; only two patients discontinued OPC for non‐safety reasons, with one requiring prompt reintroduction due to severe OFF worsening. Pre‐infusion LEDD was significantly higher in the OPC group. At 8 months, both groups showed comparable motor improvement with identical infusion flow rates (0.28 mL/h). Mean improvement in MDS‐UPDRS‐III was similar between groups, while a trend toward greater improvement in UPDRS‐IV was observed in OPC‐treated patients. Total LEDD remained higher in the OPC group at follow‐up. Neuropsychiatric adverse events were more frequent in OPC‐treated patients, though differences were not statistically significant.

**Conclusion:** OPC appears to be a feasible adjunct during CSFI, supporting higher dopaminergic requirements without increasing infusion flow rates. While generally well tolerated, careful monitoring for neuropsychiatric adverse events is recommended.

**Disclosure:** G Lazzeri has received speaker honoraria by Zambon, abbvie, Everpharma IU Isaias is a Newronika S.p.A. consultant and shareholder and received funding for research activities from Newronika S.p.A. IUI received lecture honoraria and research funding from Medtronic Inc. IUI is Adjunct Professor at the Department of Neurology, NYU Grossman School of Medicine. The other authors declare no conflict of interest.

## EPO‐0615

### Wearable sensors may detect subtle gait alterations associated with autonomic dysfunction in drug‐naïve Parkinson's disease patients

#### F. Ambrosio ^1^; N. Piramide^1^; R. De Micco^1^; L. Donisi^1^; M. Siciliano^2^; C. Cabato^1^; O. Ciaramaglia^1^; F. Esposito^1^; A. Tessitore^1^

##### ^*1*^ *Department of Advanced Medical and Surgical Sciences, University of Campania “Luigi Vanvitelli”, Naples, Italy;* ^*2*^ *Neuropsychology Laboratory, Department of Psychology, University of Campania “Luigi Vanvitelli”, Caserta, Italy*

**Background and aims:** Gait and balance disorders significantly affect functional activities of daily life in patients with Parkinson's disease (PD). Autonomic dysfunction has been associated with increased risk of falls in patients with advanced PD. Recent studies suggest that earlier development of dysautonomia is associated with more rapid disease progression and shorter survival time. We aimed at investigating potential correlations between gait and autonomic dysfunction in early drug‐naive PD patients using wearable sensors.

**Methods:** Fifty drug‐naive PD patients (disease duration ≤ 3 years, Hoehn & Yahr ≤2.5) and 20 age and sex‐matched healthy controls were consecutively enrolled. Gait and balance parameters were acquired using six Opal V2R wearable sensors, when performing the Timed up and go, 7 and 10 Meters walking tests in single and dual task (ST/DT) conditions. Motor and autonomic symptoms were evaluated using the Unified Parkinson's disease Rating scale part III (UPDRS‐III) and the Scales for Outcomes in Parkinson's Disease ‐ Autonomic Dysfunction (SCOPA‐AUT). Bivariate correlation analysis between clinical and wearable sensors metrics were performed.

**Results:** Higher SCOPA‐AUT scores significantly correlated with worse gait and postural performances as detected by wearable sensors in both ST and DT conditions. Interestingly, no correlations have been found between SCOPA‐AUT and UPDRS III, including axial and gait subscores.

**Conclusion:** Our findings revealed that application of wearable sensors could provide useful information for the characterization of drug‐naive PD patients, even in the absence of clinically detectable gait and postural disturbances. These subtle changes are already associated with autonomic dysfunctions, suggesting a potential role to predict worse clinical outcome.

**Disclosure:** Nothing to disclose.

## EPO‐0616

### **Short‐ and long‐term effects of continuous dopaminergic infusion on cortical excitability and plasticity in Parkinson's disease**.

#### F. Marchet ^1^; M. De Bartolo^1^; F. Aiello^2^; M. Costanzo^2^; N. Modugno^1^; S. Pietracupa^1^; G. Leodori^1^; A. Conte^2^; G. Fabbrini^2^; A. Berardelli^1^; D. Belvisi^2^

##### ^*1*^ *IRCSS Istituto Neurologico Mediterraneo Neuromed, Pozzilli (IS), Italy;* ^*2*^ *Department of Human Neuroscieces, Sapienza University of Rome, Rome (RM), Italy*

**Background and aims:** Continuous subcutaneous infusion of foslevodopa/foscarbidopa has been recently developed for patients with Parkinson's disease (PD) experiencing motor complications inadequately controlled by oral dopaminergic therapy. The pathophysiology of motor complications involves abnormally reduced plasticity of the primary motor cortex (M1) in PD. Once motor complications emerge, intermittent dopaminergic stimulation fails to adequately influence motor cortical plasticity. The aim of the present study was to evaluate whether continuous dopaminergic stimulation can modulate cortical plasticity in patients with PD.

**Methods:** Fifteen patients (7 female, age 67 ± 6.41) with complicated PD receiving oral L‐dopa were consecutively enrolled. Clinical evaluation included standardized scales assessing motor and non‐motor manifestations. Neurophysiological evaluation of M1 plasticity was performed using intermittent theta‐burst stimulation (iTBS), transcranial magnetic stimulation paradigm. The after‐effects of iTBS were evaluated by measuring motor evoked potential amplitudes recorded before and at 5, 15, and 30 minutes after stimulation. Patients were assessed while still receiving oral therapy (T0), immediately after initiation of foslevodopa/foscarbidopa infusion (T1), and after 24 weeks of continuous treatment (T2).

**Results:** Foslevodopa/foscarbidopa treatment was associated with a significant improvement in motor and non‐motor manifestations, including a reduction in the severity of motor complications. At T2, foslevodopa/foscarbidopa treatment induced an improvement in abnormally reduced M1 plasticity in patients with PD, as evidenced by an increased response to the iTBS protocol compared with baseline (T0).

**Conclusion:** In contrast to intermittent dopaminergic stimulation, continuous dopaminergic stimulation induced by foslevodopa/foscarbidopa can modulate M1 plasticity in patients with PD. This mechanism may contribute to the clinical effects of this therapeutic approach.

**Disclosure:** Nothing to disclose.

## EPO‐0617

### Plasma NfL, GFAp and P‐Tau181 as stratifyers of alpha‐synucleinopthies, from prodromal to early stages

#### K. Eshja ^1^; A. Pilotto^1^; I. Girotto^2^; C. Tolassi^2^; A. Lupini^1^; C. Zatti^1^; A. Rizzardi^1^; L. Purin^1^; M. Pasolini^3^; C. Liguori^4^; N. Mercuri^5^; A. Chiaravallotti^5^; M. Fernandes^5^; K. Kulcsárová^6^; M. Skovarnek^6^; E. Schaeffer^7^; D. Berg^7^; A. Padovani^1^

##### ^*1*^ *Neurology Unit, Department of Clinical and Experimental Sciences, University of Brescia, Italy;* ^*2*^ *Laboratory of Digital Neurology and Biosensors, University of Brescia, Italy;* ^*3*^ *Sleep disorder Center, ASST Spedali Civili of Brescia, Italy**;*** ^*4*^ *Sleep Medicine Centre, Neurology Unit, University Hospital of Rome Tor Vergata, Rome, Italy;* ^*5*^ *Department of Systems Medicine, University of Rome Tor Vergata, Rome, Italy;* ^*6*^ *Department of Neurology, Faculty of Medicine, Pavol Jozef Šafárik University, 04001 Košice, Slovakia;* ^*7*^ *Department of Neurology, Christian‐Albrechts‐University of Kiel, Kiel, Germany*

**Background and aims:** plasma markers have recently been implemented in neurodegenerative disorders as they might contribute to stratify subjects at different risk for Parkinson Disease (PD) or Lewy Body Disease (DLB). Rem Sleep Behaviour disorders (RBD) patients seem to be biologically closer to DLB rather than PD. Aim of the study was to evaluate the distribution of NfL, GFAp and pTau 181 in PD, DLB and subjects with different prodromal risk factors for alpha‐synucleinopathies

**Methods:** early PD, DLB, as well as subjects with idiopathic Sleep behavioral disorders or isolated hyposmia and matched controls RBD and 136 Healthy Controls (HC), underwent an extensive PD prodromal marker evaluation and plasma markers assessment using Single molecule array (Simoa) technology. Cut‐off values were calculated based on 1 standard deviation of healthy controls.

**Results:** The study included 559 individuals, 108 DLB, 206 PD, 91 idiopathic RBD and 136 Healthy Controls (HC) of which 18 hyposmic, DLB globally showed higher levels of GFAp and a significant increase of pTau181 when compared to PD. iRBD exhibited higher NfL and GFAp levels compared to HC and PD, and a lower score at sniffing test suggesting that hyposmia can be detected together with RBD. GFAp levels seemed to be higher in subjects at risk for alpha synucleinopathies.

**Conclusion:** Plasma neurodegenerative markers stratify people at risk for alpha‐synucleinopathies and are higher in DLB patients. In prodromal cases, the increase of GFAp correlates with clinical risk of conversion. Future goal is identifying which prodromic symptoms correlate the more with a biomarker increase.

**Disclosure:** no disclosures

## EPO‐0618

### Results from the Global Phase 3 Trial (IB1001‐303) Evaluating Levacetylleucine in Ataxia‐Telangiectasia

#### K. Martakis^1^; M. Patterson^2^; M. Strupp ^3^; B. Zanrucha^2^; J. Raymond^2^; J. Kerthi^2^; A. Hatcher^2^; T. Fields^2^; I. Billington^2^; T. Bremova‐Ertl^4^

##### ^*1*^ *Department of Neuropediatrics, Justus Liebig University, Giessen, Germany,* ^*2*^ *IntraBio Inc, Austin, USA,* ^*3*^ *Department of Neurology, LMU University Hospital, LMU Munich, Germany,* ^*4*^ *Department of Neurology and Center for Rare Diseases, University Hospital Inselspital Bern, Bern, Switzerland*

**Background and aims:** Ataxia‐Telangiectasia (A‐T) is a rare, inherited cerebellar ataxia. Levacetylleucine (also known as *N*‐acetyl‐L‐leucine) is a modified amino acid that enters enzyme‐controlled pathways to correct metabolic dysfunction and restore cellular communication. In this Phase 3 randomised, double‐blind, placebo‐controlled trial (IB1001‐303), we evaluated the safety and efficacy of levacetylleucine for pediatric and adult individuals with A‐T.

**Methods:** IB1001‐303 enrolled A‐T participants aged 4 years or older across 10 multinational trial sites. Primary efficacy endpoint was the Scale for the Assessment and Rating of Ataxia (SARA). Secondary endpoints included Spinocerebellar Ataxia Function Index (SCAFI), International Cooperative Ataxia Rating Scale (ICARS), Neurology Quality of Life‐Upper Extremity Function (NeuroQOL‐UEF), Quality of Life (EQ‐5D), and investigator, caregiver, and patient Clinical Global Impression of Improvement (CGI‐I). Safety assessment included adverse event incidence and severity.

**Results:** Seventy‐three participants aged 5 to 50 were enrolled. The primary endpoint was met; mean change in the SARA total score with levacetylleucine was ‐1.92 (SD = 2.81) and −0.14 (SD = 2.38) with placebo; Linear Mixed Model (LMM) treatment effect −1.88 (SD = 0.41) [95% CI −2.70, −1.06; *p* < 0.0001]). The trial met secondary endpoints, including ICARS and CGI‐I. NALL was observed to be safe and well‐tolerated, consistent with its established safety profile.

**Conclusion:** Levacetylleucine demonstrated a significant benefit versus placebo, and clinically meaningful improvements in neurological manifestations of A‐T, functioning, and quality of life, and a favorable benefit‐risk profile for the treatment of A‐T.

**Disclosure:** MCP, BZ, JR, JK, AH, TF and IB are employees of and may have stock option ownership in IntraBio, Inc. MS received speaker's honoraria from Abbott, Actelion, Auris Medical, Biogen, Eisai, Grünenthal, GSK, Henning Pharma, Interacoustics, MSD, Otometrics, Pierre‐Fabre, TEVA, UCB, Viatris. Consultant for Abbott, Actelion, Auris Medical, Decibel, Heel, and Sensorion. Scientific founder and shareholder of IntraBio. TB received speaker's honoraria and consultancy fees from Actelion, Sanofi‐Genzyme and Zevra as well as blinded video‐rater fees from IntraBio.

## EPO‐0619

### Cerebrospinal fluid proteomics in progressive supranuclear palsy: Single center study

#### N. Madetko‐Alster ^1^; A. Malinowska^2^; D. Otto‐Ślusarczyk^3^; M. Struga^3^; A. Wiercińska‐Drapało^4^; P. Alster^1^

##### ^*1*^ *Department of Neurology, Medical University of Warsaw, Warsaw, Poland;* ^*2*^ *Mass Spectrometry Laboratory, Institute of Biochemistry and Biophysics, Polish Academy of Sciences, Warsaw, Poland;* ^*3*^ *Department of Biochemistry, Medical University of Warsaw, Warsaw, Poland;* ^*4*^ *Department of Infectious and Tropical Diseases and Hepatology, Medical University of Warsaw, Warsaw, Poland.*

**Background and aims:** Progressive supranuclear palsy (PSP) is a rare neurodegenerative disorder characterized by tau pathology and rapid clinical progression. Reliable cerebrospinal fluid (CSF) biomarkers reflecting disease‐specific molecular mechanisms are still lacking. Proteomic analysis of CSF may provide insight into pathophysiological processes and identify potential biomarkers for diagnosis and disease monitoring.

**Methods:** In this single‐center study, CSF samples from 24 PSP patients and 12 neurologically healthy age‐matched controls were analyzed. Samples were collected using standardized procedures. High‐resolution mass spectrometry–based proteomics was performed, enabling the identification and quantification of approximately 2,500 CSF proteins. Differential expression analyses were conducted to compare PSP subgroup with controls, followed by functional and pathway enrichment analyses.

**Results:** Proteomic profiling revealed distinct CSF protein signatures in PSP patients compared with controls. Differentially expressed proteins were mainly associated with neurodegeneration‐related pathways, including synaptic dysfunction, neuroinflammation, cytoskeletal organization, and protein aggregation. Several proteins involved in tau‐related processes and glial activation showed altered abundance in PSP. Exploratory analyses suggested potential protein panels that may discriminate PSP from controls.

**Conclusion:** CSF proteomics identified disease‐related molecular alterations in PSP and highlighted biological pathways relevant to its pathogenesis. These findings support the utility of CSF proteomic profiling as a tool for biomarker discovery in PSP. Further studies in larger, multicenter cohorts are warranted to validate candidate biomarkers and assess their clinical applicability.

**Disclosure:** Nothing to disclose.

## EPO‐0620

### Individualisation of LECIG therapy in advanced Parkinson's disease: Adapting treatment to suit patients’ lives

#### R. García‐Ramos ^1^; A. Fernández Revuelta^1^; C. Ribacoba^1^; A. Aldaz^1^; E. López Valdés^1^; N. Smith^2^

##### ^*1*^ *Movement Disorders Unit, Hospital Clínico San Carlos, Madrid, Spain;* ^*2*^ *Britannia Pharmaceuticals Limited, Green Park, Reading, UK*

**Background and aims:** Advanced Parkinson's disease (PD) is a heterogeneous condition requiring an individualised treatment approach. Levodopa–entacapone–carbidopa intestinal gel (LECIG) is a device‐aided therapy for advanced PD designed to allow flexible dosing, enabling adaptation to patients’ specific and fluctuating motor needs throughout the day.

**Methods:** We describe clinical cases from our centre illustrating how LECIG therapy, administered via the Crono® LECIG pump, can be adjusted to achieve optimal motor control over a 24‐hour period and across different levels of daily activity (Table 1).

**Results:** (1) A 51‐year‐old woman with PD from age 35, treated with levodopa–carbidopa intestinal gel (LCIG) infusion since 2015; switched to LECIG in 2023 due to disabling dyskinesias and evening motor worsening. She now receives 24‐hour LECIG infusion with three adjustable flow rates tailored to daily demands. (2) A 76‐year‐old woman with PD since age 44, treated with LCIG since 2011; switched to LECIG in 2025 due to evening motor deterioration and technical difficulties managing two pumps required for 24‐hour LCIG infusion. She currently uses LECIG at three flow rates, with marked motor improvement and better caregiver quality of life. (3) A 75‐year‐old man with PD since 2011, treated with high‐dose 24‐hour LCIG infusion since 2020; switched to LECIG due to unpredictable and uncontrollable OFF periods and now shows marked clinical improvement using three flow rates.

**Figure:**
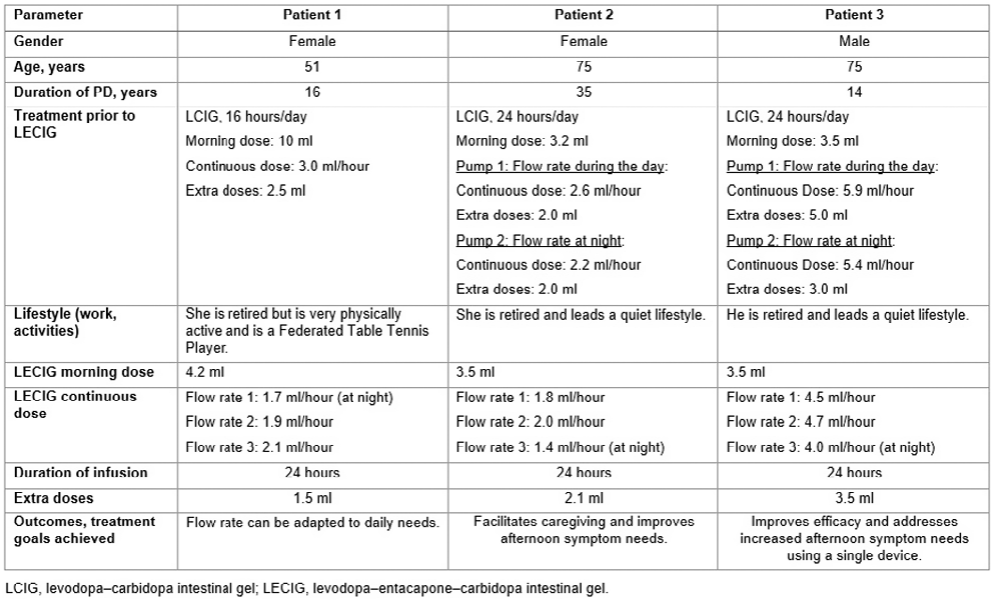
**TABLE 1** Characteristics and outcomes of patients treated with LECIG at our centre.

**Conclusion:** The use of multiple flow rates with the Crono® LECIG pump allows individualised medication delivery aligned with daily activities and symptom fluctuations, supporting functional independence and an active lifestyle.

**Disclosure:** NS is an employees of Britannia Pharmaceuticals Ltd. Other authors have nothing to disclose.

MS and related disorders 5

## EPO‐0621

### Shifting paradigms in multiple sclerosis: Long‐term outcomes across diagnostic eras

#### U. Samadzade; S. Ozakbas; C. Caliskan

##### Izmir University of Economics, Medical Point Hospital, Izmir, Türkiye

**Background and aims:** Advances in diagnostic criteria, neuroimaging, and disease‐modifying therapies have substantially changed the clinical course of multiple sclerosis (MS). This study compared long‐term outcomes of patients diagnosed with MS in two different diagnostic eras to assess the real‐world impact of evolving clinical practice.

**Methods:** In this retrospective cohort study, patients diagnosed with MS in two eras were analyzed: Group 1 (1990–1996, *n* = 133) and Group 2 (2008–2014, *n* = 579). Demographic characteristics, time from symptom onset to diagnosis, Expanded Disability Status Scale (EDSS) scores at diagnosis and at 10‐year follow‐up, annualized relapse rates (ARR), and conversion to secondary progressive MS (SPMS) were recorded.

**Results:** A total of 712 patients were included (mean age: 50.7 ± 12.2 years; mean disease duration: 20.7 ± 8.8 years; 70.4% female). Time from symptom onset to diagnosis was significantly shorter in Group 2 compared with Group 1 (3.2 ± 4.6 vs 4.2 ± 4.7 years; *p* < 0.005). After 10 years, disability outcomes remained significantly better in Group 2 (mean EDSS: 1.85 ± 2.02) compared with Group 1 (3.97 ± 2.23; *p* < 0.001). ARR was also lower in Group 2 (0.18 ± 0.20 vs 0.23 ± 0.20; *p* < 0.05). Conversion to SPMS occurred in 52.6% of Group 1 patients versus only 5.4% of Group 2.

**Conclusion:** Patients diagnosed in the more recent era demonstrated markedly improved long‐term disability outcomes, lower relapse activity, and substantially reduced progression to SPMS, underscoring the positive impact of advances in MS diagnosis and treatment.

**Disclosure:** Nothing to disclose.

## EPO‐0622

### Serum IgM Index as a Biomarker of Motor and Cognitive Performance in Multiple Sclerosis

#### U. Samadzade; C. Calıskan; S. Ozakbas

##### Izmir University of Economics, Medical Point Hospital, Izmir, Türkiye

**Background and aims:** The immunoglobulin M (IgM) index, reflecting intrathecal IgM synthesis, has been proposed as a biomarker of disease activity in multiple sclerosis (MS). This study examined functional differences between MS patients with persistently elevated IgM index values and those with normal IgM index.

**Methods:** This retrospective observational study included MS patients with elevated IgM index values and long‐term clinical follow‐up. A control group with normal IgM index was selected from a larger cohort of 4,191 MS patients and matched for disease duration and baseline Expanded Disability Status Scale (EDSS). Motor performance was assessed using the Timed 25‐Foot Walk (T25FW) and Nine‐Hole Peg Test (9HPT). Cognitive function was evaluated with the Symbol Digit Modalities Test (SDMT), Brief Visuospatial Memory Test–Revised (BVMT‐R), and California Verbal Learning Test–II (CVLT‐II).

**Results:** A total of 268 patients with high IgM index and 150 patients with normal IgM index were analyzed. The high IgM index group demonstrated significantly worse motor performance on the T25FW (*p* = 0.040) and 9HPT (*p* = 0.001). In cognitive assessments, patients with normal IgM index achieved higher scores on the SDMT (*p* = 0.022) and BVMT‐R (*p* = 0.011), indicating better processing speed and visuospatial memory. No significant difference was observed in CVLT‐II scores (*p* = 0.173).

**Conclusion:** MS patients with elevated IgM index showed poorer motor performance and selective cognitive impairment compared with those with normal IgM index. These findings support the IgM index as a potential prognostic biomarker and emphasize the value of integrating laboratory markers into long‐term functional evaluation in MS.

**Disclosure:** Nothing to disclose.

## EPO‐0623

### Spinal Cord Lesions and Atrophy Predict Disability Progression and PIRA in Multiple Sclerosis

#### E. DAROL^1^; V. Sezer^1^; S. Aksu^1^; O. Turhan^1^; Ş. Dalkılıç^1^; A. Tunç ^2^

##### ^*1*^ *Sakarya Training and Research Hospital, Clinic of Neurology, Sakarya, Türkiye;* ^*2*^ *Department of Neurology, Faculty of Medicine, Sakarya University, Türkiye*

**Background and aims:** Spinal cord pathology is a major contributor to long‐term disability in multiple sclerosis (MS). This study aimed to evaluate spinal cord lesion patterns and spinal cord atrophy and to determine their association with long‐term disability progression, progression independent of relapse activity (PIRA), and conversion to SPMS.

**Methods:** We retrospectively analyzed 223 patients with MS followed for at least three years. Spinal cord MRI was assessed for lesion presence, level, axial distribution, number, length, gadolinium enhancement, and spinal cord atrophy. Outcomes included confirmed disability accumulation, attainment of EDSS ≥3, PIRA, and SPMS conversion. Kaplan–Meier analyses and multivariate Cox regression models were applied.

**Results:** Spinal cord lesions were present in 172 patients (77.1%) and were associated with higher disability, increased risk of EDSS ≥3, PIRA, and conversion to SPMS. Lesion‐specific characteristics did not independently predict long‐term outcomes. In contrast, spinal cord atrophy independently predicted EDSS ≥3 (HR 1.98) and PIRA (HR 2.29). Despite similar baseline disability, patients with spinal cord lesions showed progressive divergence in disability over time.

**Figure:**
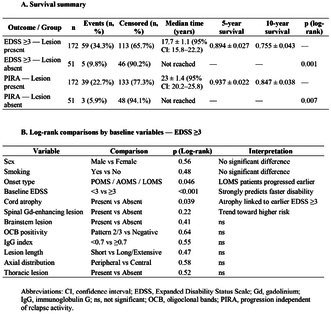
**TABLE 1** Kaplan‐Meier survival and log‐rank analyses for time to EDSS ≥3 and PIRA according to spinal cord lesion status.

**Figure:**
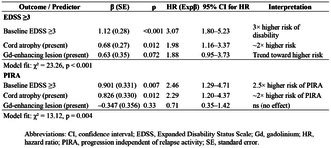
**TABLE 2** Cox proportional hazards models for predictors of EDSS ≥ 3 and PIRA among patients with spinal cord lesions (*n* = 172).

**Conclusion:** Spinal cord atrophy is a strong, lesion‐independent prognostic marker of disability progression and PIRA in multiple sclerosis. While lesion presence identifies patients at risk, lesion morphology has limited prognostic value, highlighting the importance of quantitative spinal cord assessment in longitudinal follow‐up.

**Figure:**
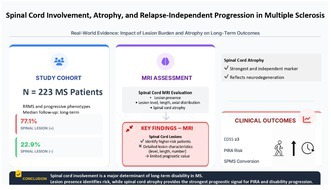
**FIGURE 1** Graphical abstract.

**Disclosure:** Nothing to disclose.

## EPO‐0624

### Prevalence and clinical correlates of sexual dysfunction in multiple sclerosis: A single‐center cross‐sectional study

#### B. Alberti‐Vall ^1^; A. Hernando‐Andres^1^; T. Alba‐Isasi^1^; V. Guerra‐Fernandez^1^; Y. Blanco‐Morgado^1^; S. Llufriu‐Duran^1^; A. Calvi^1^; C. Castelo‐Branco^2^; M. Sepulveda‐Gazquez^2^

##### ^*1*^ *Neuroimmunology Unit, Hospital Clinic of Barcelona, Barcelona, Spain;* ^*2*^ *Clinical Sexology Working Group, Hospital Clinic of Barcelona, Barcelona, Spain*

**Background and aims:** Sexual dysfunction (SD) is a common but under‐recognized complication of multiple sclerosis (MS) that substantially affects patients´quality of life. Although the etiology of SD in MS is multifactorial, reported prevalence and associated clinical factors vary widelyacross studies.

**Methods:** We conducted a single‐center cross‐sectional observational study including MS patients followed at our Neuroimmunology‐MS Unit between June and Octuber 2025. Sexual function and satisfaction were assessed using self‐administered questionnaires: the Multiple Sclerosis Intimacy and Sexuality Questionnaire (MSISQ‐15), the New Sexual Satisfaction Scale (NSSS), the Female Sexual Function Index (FSFI), and the International Index of Erectile Function (IIEF). Demographic and clinical variables were collected. Bivariate analyses and multivariable logistic regression were performed. Correlations were assessed using Spearman's rank correlation coefficient.

**Results:** 115 patients were included of whom 69 (60.5%) were female, with a mean age of 46, and with 90 (78.9%) being relapsing‐remitting forms. A total of 100 (87.0%) patients showed SDbased on MSISQ‐15 score. Patients with SD had greater neurological disability than those without SD (median EDSS 2.0 vs 1.5, *p* = 0.018). In multivariable analysis, EDSS remained independently associated with SD (OR 2.29, 95% CI 1.23–5.24). SD severity was strongly and inversely correlated with sexual satisfaction measured by the NSSS (ρ = −0.57, *p* < 0.001). In men, MSISQ‐15 scores were strongly and negatively correlated with IIEF scores, whereas in women the correlation with FSFI scores did not reach statistical significance.

**Figure:**
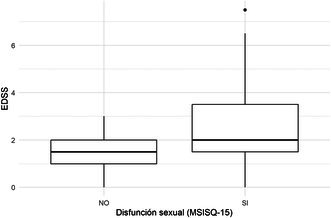
**FIGURE 1** Neurological disability according to sexual dysfunction status.

**Figure:**
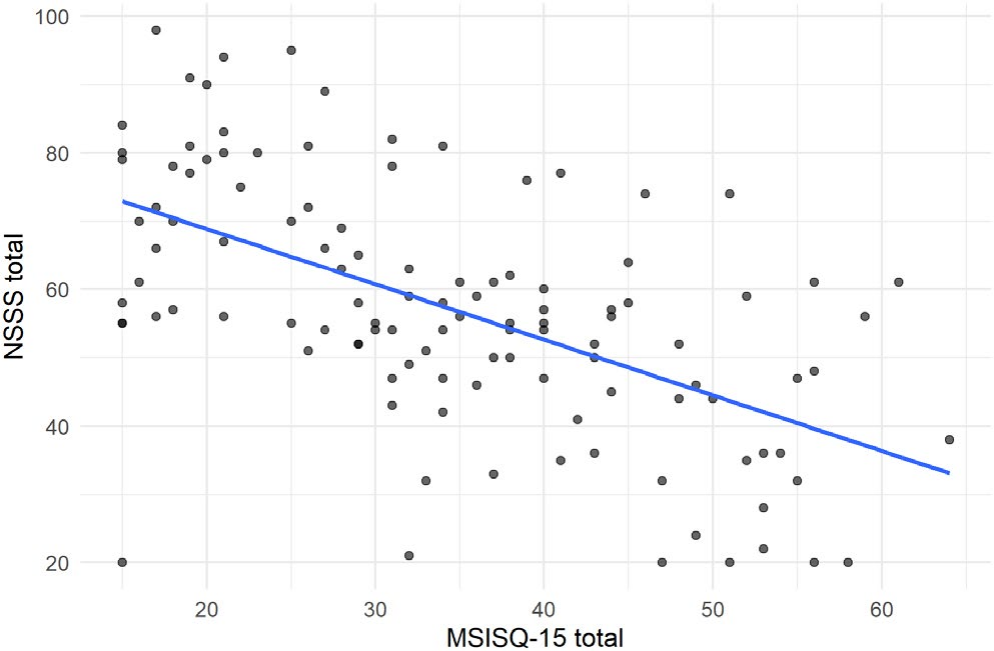
**FIGURE 2** Correlation between sexual dysfunction severity and sexual satisfaction.

**Conclusion:** SD is highly prevalent in patients with MS and is strongly associated with neurological disability and reduced sexual satisfaction.

**Disclosure:** Nothing to disclose.

## EPO‐0625

### Disease activity across pregnancy in NMOSD and MOGAD: Data from the Oxford National Service

#### C. Dal Bo ^1^; P. Faustino^4^; F. Chan^3^; M. Miron^2^; Y. Sharawakanda^2^; A. Francis^5^; M. Leite^1^; J. Palace^1^

##### ^*1*^ *Nuffield Department of Clinical Neurosciences, University of Oxford, Oxford, UK;* ^*2*^ *Department of Clinical Neurology, John Radcliffe Hospital, Oxford University Hospitals Trust, Oxford, UK;* ^*3*^ *Translational Neuroimmunology Group, Anzac Research Institute and Kids Neuroscience Centre, Sydney Medical School, Faculty of Medicine and Health, University of Sydney, Sydney, Australia;* ^*4*^ *Multiple Sclerosis Centre, ULS São José, Lisbon Clinical Academic Centre, Lisbon, Portugal;* ^*5*^ *Neurology Department, Musgrove Park Hospital, Taunton, UK*

**Background and aims:** Neuromyelitis optica spectrum disorder (NMOSD) and myelin oligodendrocyte glycoprotein antibody‐associated disease (MOGAD) predominantly affect women of reproductive age. This study aimed to assess pregnancy impact on disease activity

**Methods:** This retrospective study included informative pregnancies in women with NMOSD or MOGAD followed in the Oxford national service. Annualized relapse rates (ARR) were calculated in patients who had a pregnancy after disease onset.

**Results:** In MOGAD, 72 of 105 informative pregnancies occurred after disease onset. Mean ARR in the two years before pregnancy was 0.23 ± 0.30 and decreased significantly during pregnancy. Post‐pregnancy ARR showed a numerical increase in the early months (0.39 ± 1.19 at 0‐3 months), followed by a decline by 12 months, without significant differences compared with the pre‐pregnancy period. Spontaneous miscarriage occurred in 5/72 pregnancies (6.9%). In NMOSD, 43 of 51 informative pregnancies occurred after disease onset. Mean ARR before pregnancy was 0.36 ± 0.47. During pregnancy, ARR was significantly lower in the first and third trimesters, and similar to pre‐pregnancy in the second trimester (0.37 ± 1.18). Post‐pregnancy ARR showed a numerical increase at 0–3 months (0.74 ± 1.80) and declined thereafter, without significant differences compared with pre‐pregnancy. Spontaneous miscarriage occurred in 10/43 pregnancies (23.2%). Post‐pregnancy disease onset occurred in 31 MOGAD (4.62 ± 3.10 months) and 7 NMOSD (3.97 ± 3.43 months) patients.

**Figure:**
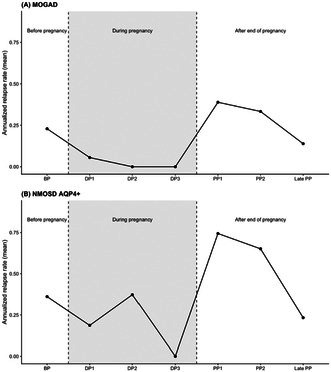
**FIGURE 1** Annualized relapse rate across time periods in relation to pregnancy in (A) MOGAD and (B) NMOSD: before pregnancy (BP), during pregnancy by trimester (DP1, DP2, DP3), post‐pregnancy 0–3 months (PP1), 3–6 months (PP2), and 6–12 months (late PP).

**Figure:**
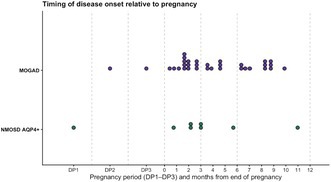
**FIGURE 2** Timing of disease onset relative to pregnancy in NMOSD and MOGAD. Each dot represents one patient. Disease onset is shown during pregnancy by trimester (DP1–DP3) and after the end of pregnancy (in months).

**Figure:**
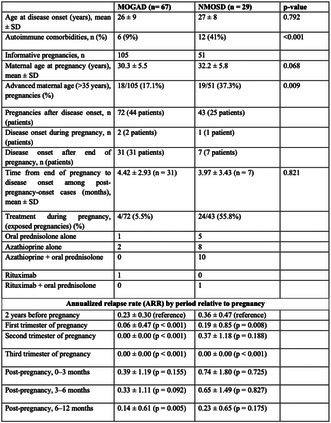
**TABLE 1** Clinical and pregnancy characteristics of the study cohort: interim analysis. Values are presented as mean ± SD or *n* (%). Continuous variables were compared using the Wilcoxon rank‐sum test, and categorical variables using Fisher's exact test.

**Conclusion:** Disease activity was significantly reduced during pregnancy in MOGAD. In both NMOSD and MOGAD, ARR showed a numerical increase in the early post‐pregnancy period (0–3 months), followed by a decline over the first year.

**Disclosure:** This project was supported by a scholarship grant from the ECTRIMS Clinical Training Fellowship awarded to Carolina Rodrigues Dal Bo. Disclosures Jacqueline Palace has received support for scientific meetings and honoraria for advisory work from Merck Serono, Novartis, Chugai, Alexion, Roche, MedImmune, argenx, Amgen, Vitaccess, UCB, Mitsubishi, Amplo, and Janssen. She has received research grants from Alexion, argenx, Clene, Roche, MedImmune, and Amplo Biotechnology. She holds a patent (ref. P37347WO) and a licence agreement with Numares for multimarker MS diagnostics, and holds shares in AstraZeneca. She acknowledges partial funding to the Trust by NHS England Highly Specialised Services. She serves on the medical advisory boards of the Sumaira Foundation and The MOG Project charities, is a member of the Guthy‐Jackson Foundation, a board member of the European Charcot Foundation, a member of MAGNIMS, the UK NHSE IVIG Committee, and has been an ECTRIMS Council member since June 2023. She currently serves on ABN advisory groups for MS and neuroinflammation and is a medical advisor to The Lancet Neurology editorial board. Maria Isabel Leite is funded by the NHS (Myasthenia & Related Disorders Service; National Specialised Commissioning Group for Neuromyelitis Optica, UK) and the University of Oxford. She has received research grants from Myaware, MDUK, and the University of Oxford. She has served on advisory boards for UCB Pharma, argenx, and Amgen, and has received speaker honoraria and travel grants from UCB Pharma, argenx, and Amgen. She has provided consultancy services for UCB Pharma. Patricia Faustino has received conference and travel support for scientific meetings and courses from Roche, Novartis, Biogen, BMS, and Merck. Anna Francis received a travel grant from Alexion in 2024. Fiona Chan, Yvonne Sharawakanda, and Madalina Miron: No disclosures to declare.

## EPO‐0626

### Language and voice analysis as multidimensional biomarkers of silent progression in relapsing–remitting multiple sclerosis

#### G. Carlucci^1^; M. Di Cristinzi ^1^; S. Astore^3^; A. Poggesi^1^; A. Repice^2^

##### ^*1*^ *Department of NEUROFARBA, University of Florence, Florence, Italy;* ^*2*^ *Department of Neurology, Careggi University Hospital, Florence, Italy;* ^*3*^ *University of Florence, Florence, Italy*

**Background and aims:** Progression independent of relapse activity (PIRA) is a major contributor to disability accumulation in MS but is difficult to detect in patients with low EDSS scores. Language and speech impairments are common yet underrecognized in MS and may reflect early cognitive and motor network dysfunction not captured by conventional disability measures. This study investigated whether multidimensional language and voice assessments could serve as sensitive functional biomarkers of subclinical progression in relapsing–remitting MS (RRMS).

**Methods:** Twenty‐eight patients with RRMS and mild disability and eight age‐ and sex‐matched healthy controls underwent comprehensive cognitive, linguistic, and speech evaluations. Cognitive assessment included MoCA and domain‐specific neuropsychological tests. Language was examined using verbal fluency and comprehension tasks. Speech and voice were evaluated with a standardized dysarthria profile, the Voice Handicap Index, and automated acoustic analysis using VoxPlot software. Acoustic parameters included Cepstral Peak Prominence Smoothed (CPPS), Acoustic Voice Quality Index (AVQI), and Acoustic Breathiness Index (ABI).

**Results:** Compared with controls, patients with RRMS showed reduced phonemic and semantic verbal fluency with preserved language comprehension. Mild but significant dysarthria was observed, mainly affecting respiration, diadochokinesis, and reflexes. Acoustic analysis revealed reduced CPPS and increased AVQI and ABI, indicating impaired voice quality. While several measures correlated with EDSS and disease duration, multivariate analyses showed that acoustic and dysarthric parameters were largely independent of global disability, age, and disease duration.

**Figure:**
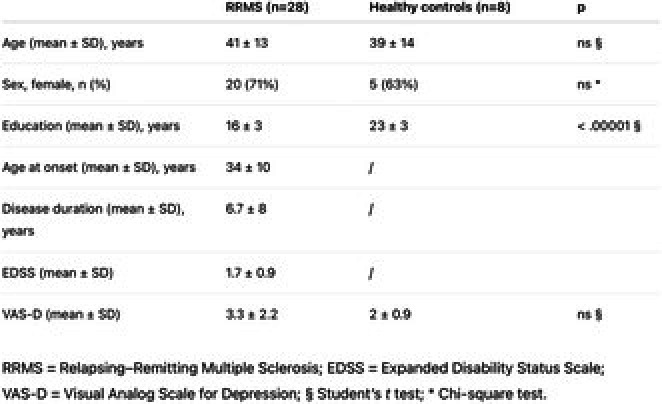
**TABLE 1** Demographic and clinical characteristics of the study population.

**Figure:**
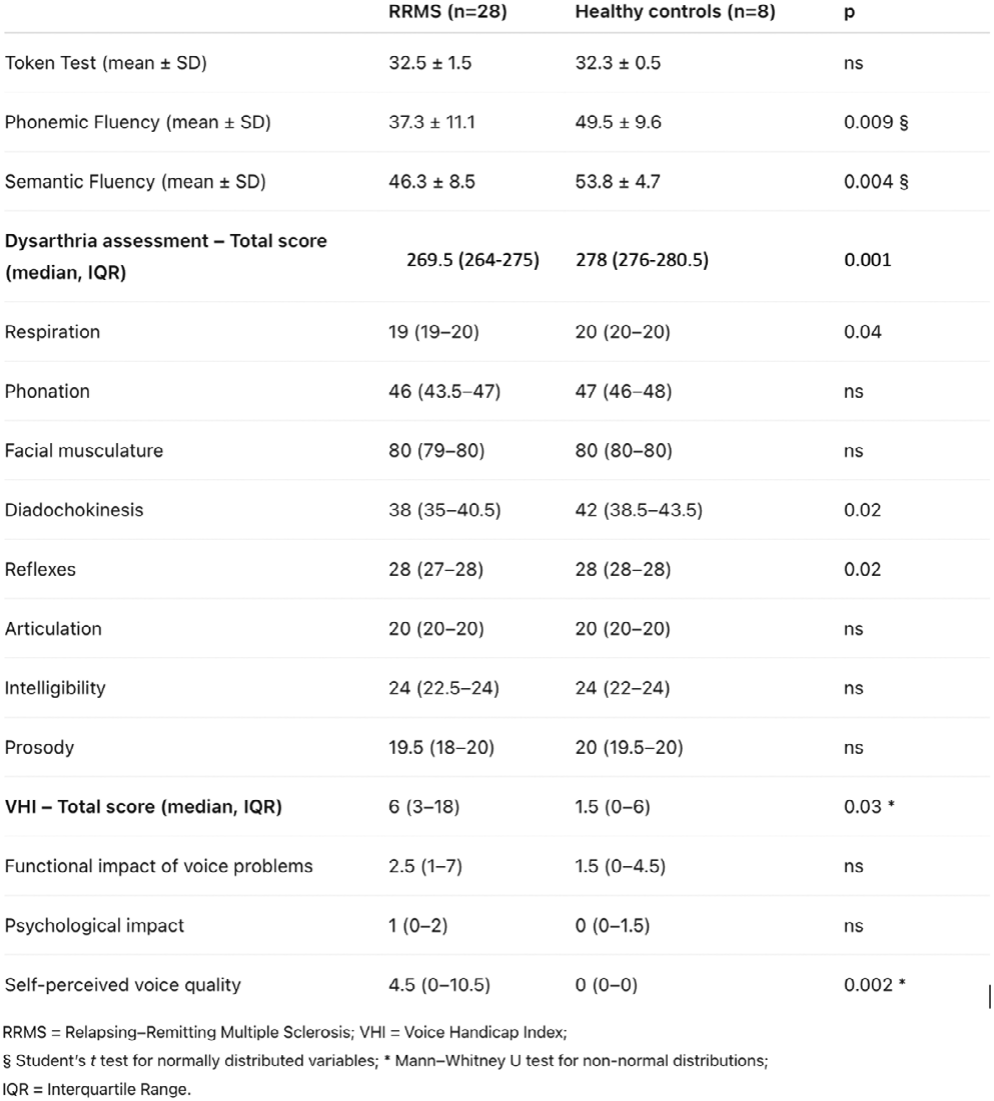
**TABLE 2** Language assessment of the study population (adjusted for age and education).

**Conclusion:** Language and voice assessments detect subtle multidimensional dysfunction in RRMS not captured by conventional disability scales, supporting their potential role as biomarkers of silent progression and PIRA particularly in patients with low physical disability.

**Disclosure:** Nothing to disclose.

## EPO‐0627

### Clinical relevance of evoked potentials for disease progression in early multiple sclerosis and clinically isolated syndrome in the ProVal‐MS study

#### M. Braun ^1^; J. Havla^3^; M. Kowarik^4^; H. Tumani^5^; L. Behrens^6^; M. Naumann^1^; H. Zimmermann^7^; J. Kirschke^8^; M. Senel^5^; M. Kreschensteiner^3^; L. Braadt^1^; V. Steuerwald^1^; K. Holzapfel^1^; A. Muzalyova^9^; H. Spengler^10^; M. Mühlau^11^; E. Oswald^3^; M. Hagedorn^12^; B. Sailer^13^; B. Bender^14^; I. Vardakas^5^; N. Sollmann^15^; O. Kohlbacher^13^; H. Kestler^19^; F. Kohlmayer^20^; U. Ziemann^4^; F. Albashiti^12^; F. Kramer^21^; M. Boeker^22^; P. Uibel^11^; S. Buchka^23^; I. Soto‐Rey^9^; S. Rohr^9^; U. Mansmann^23^; B. Hemmer^11^; A. Bayas^1^

##### ^*1*^ *Department of Neurology and Clinical Neurophysiology, Medical Faculty, University of Augsburg, Augsburg, Germany;* ^*3*^ *Institute of Clinical Neuroimmunology, LMU Hospital, LMU Munich, Munich, Germany;* ^*4*^ *Department of Neurology and Stroke, and Hertie‐Institute for Clinical Brain Research, Eberhard‐Karls University of Tübingen, Tübingen, Germany;* ^*5*^ *Department of Neurology, University Hospital Ulm, Ulm, Germany;* ^*6*^ *Diagnostic and Interventional Neuroradiology, Faculty of Medicine, University of Augsburg, Augsburg, Germany;* ^*7*^ *Institute of Neuroradiology, LMU Hospital, Ludwig‐Maximilians‐Universität Munich, Munich, Germany;* ^*8*^ *Department of Diagnostic and Interventional Neuroradiology, School of Medicine, Klinikum rechts der Isar, Technical University of Munich, Munich, Germany;* ^*9*^ *Institute for Digital Medicine, Faculty of Medicine, University Hospital of Augsburg, Augsburg, Germany;* ^*10*^ *Data Integration Center, Medical Center rechts der Isar, School of Medicine, Technical University of Munich, Munich, Germany;* ^*11*^ *Department of Neurology, School of Medicine, Technical University of Munich, Klinikum rechts der Isar, Munich, Germany;* ^*12*^ *Medical Data Integration Center, University Hospital, LMU Munich, Munich, Germany;* ^*13*^ *Medical Data Integration Center (meDIC), University Hospital Tübingen, Tübingen, Germany;* ^*14*^ *Department of Diagnostic and Interventional Neuroradiology, University Hospital Tübingen, Tübingen, Germany;* ^*15*^ *Department of Diagnostic and Interventional Radiology, University Hospital Ulm, Ulm, Germany;* ^*19*^ *Institute of Medical Systems Biology, Ulm University, Ulm, Germany;* ^*20*^ *Bitcare GmbH, Munich, Germany;* ^*21*^ *IT‐Infrastructure for Translational Medical Research, University of Augsburg, Augsburg, Germany;* ^*22*^ *Institute for Artificial Intelligence and Informatics in Medicine, Medical Center rechts der Isar, School of Medicine, Technical University of Munich, Munich, Germany;* ^*23*^ *Institute for Medical Information Processing, Biometry and Epidemiology (IBE), Faculty of Medicine, LMU Munich, Munich, Germany*

**Background and aims:** The individual clinical course of multiple sclerosis (MS) is highly variable. Prospective studies evaluating the clinical relevance of evoked potentials (EP) in correlation with clinical and paraclinical parameters are scarce. This study investigates the association of somatosensory (SEP), visual (VEP), and motor evoked potentials (MEP) at diagnosis for clinical and paraclinical endpoints after 24 months in the ProVal‐MS study (DRKS00014034), a multicentre prospective study at five German universities.

**Methods:** Assessments included VEP, tibial nerve SEP, and abductor hallucis MEP, clinical assessments (Expanded Disability Status Scale [EDSS], Symbol Digit Modalities Test [SDMT]), cerebral and spinal MRI, serum neurofilament light chain [sNfL], and serum GFAP [sGFAP]. Statistical analyses included Spearman correlations and multilevel linear regression.

**Results:** 367 patients (CIS, *n* = 27, RRMS, *n* = 340; median age 30.6 years; 64.9% female) were included. Spearman analyses revealed significant correlations between all baseline EPs and both EDSS and SDMT at baseline and after 24 months. Multilevel linear regression showed higher ( = worse) VEP scores associated with poorer cognition (beta = ‐4.44, 95% CI ‐7.68, ‐1.21, *p* = 0.007), while even mildly abnormal VEP scores were already associated with higher disability (VEP score 1: beta = 0.18, 95% CI 0.02, 0.33, *p* = 0.023). However, SEP showed no significant associations with cognition or disability. Further results, including associations with blood biomarkers, neuroimaging findings, and clinical scores will be presented at the meeting.

**Conclusion:** Baseline EPs correlate with disability and cognitive function over 24 months. Multilevel regression highlights VEP as associated with both cognitive impairment and disability, supporting it as a complementary tool for MS monitoring.

**Disclosure:** M. Braun: nothing to disclose. J. Havla: reports a grant for OCT research from the Friedrich‐Baur‐ Stiftung, Horizon, Sanofi and Merck, personal fees and nonfinancial support from Alexion, Amgen, Bayer, Biogen, BMS, Merck, Novartis and Roche, and nonfinancial support of the Sumaira‐Foundation and Guthy‐Jackson Charitable Foundation, all outside the submitted work. M. Kowarik: has served on advisory boards and received speaker fees/travel grants from Merck, Sanofi‐Genzyme, Novartis, Biogen, Janssen, Alexion, Celgene/Bristol‐Myers Squibb and Roche. He has received research grants from Merck, Roche, Novartis, Janssen, Sanofi‐Genzyme and Celgene/Bristol‐Myers Squibb. H. Tumani: received institutional research support and/or consulting/speaker honoraria from Alexion, Bayer, Biogen, Bristol‐Myers Squibb, Celgene, Diamed, Fresenius, Fujirebio, GlaxoSmithKline, Horizon, Janssen‐Cilag, Merck, Novartis, Roche, Sanofi Genzyme, TEVA. His research is also funded by Ministry of Education and Research (BMBF), Ministry of Science, Research and Arts Baden Württemberg (MWK‐BW), German Society of Multiple Sclerosis (DMSG), DMS‐Stiftung, AMSEL‐Stiftung, Bayern‐DMSG and Chemische Fabrik Karl Bucher GmbH. J.S. Kirschke: has received speaker honoraria from Novartis. He is shareholder from Bonescreen GmbH. M. Senel: has received consulting and/or speaker honoraria from Alexion, Amgen/Horizon, Bayer, Biogen, Biotest, Bristol‐Myers‐Squibb/Celgene, Janssen, Merck, Roche, Sanofi Genzyme, and UCB. B. Bender: Co‐Founder and CMO AIRAmed GmbH, received speaker honoraria and/or travel support from Eisai GmbH and Roche. I. Vardakas: has received consulting and/or speaker honoraria and/or travel support from Alexion, Novartis, Sanofi and UCB. F. Kohlmayer: funding for providing and maintaining of the study software, DIS from Bitcare GmbH. B. Hemmer: served on advisory boards for Novartis, Polpharma und Hoffmann LaRoche, as well as DMSC boards for AllergyCare, Polpharma, Sandoz, Biocom, and TG Therapeutics. He received honoraries for counseling clients of the Gerson Lehrman Group and educational activities by neuro.today andpatients.today. BH received funding for research projects by Regeneron, Polpharma and Hoffmann LaRoche. BH also received research funding from the EC as part of the Multiple MS and WISDOM Consortia, the Clinspect‐M Consortium funded by the Bundesministerium für Bildung und Forschung and by the Deutsche Forschungsgemeinschaft as a member of the Munich Cluster for Systems Neurology (EXC 2145 SyNergy – ID 390857198). A. Bayas: received personal compensation from Merck, Biogen, Novartis, TEVA, Roche, Sanofi/Genzyme, Celgene/Bristol Myers Squibb, Janssen, Sandoz/HEXAL, Alexion, Horizon, Argenx, UCB, grants for congress travel and participation from Biogen, TEVA, Novartis, Sanofi/Genzyme, Merck, Celgene, and Janssen, and research support from Novartis. All other authors: nothing to disclose.

## EPO‐0628

### Automated analysis of longitudinal language changes in multiple sclerosis

#### M. Šubert ^1^; M. Novotný^1^; T. Tykalová^1^; B. Srpová^2^; L. Friedová^2^; D. Horáková^2^; T. Uher^2^; J. Rusz^1^

##### ^*1*^ *Czech Technical University in Prague, Faculty of Electrical Engineering, Department of Circuit Theory, Prague, Czech Republic;* ^*2*^ *Charles University, First Faculty of Medicine, Department of Neurology and Centre of Clinical Neuroscience, Prague, Czech Republic*

**Background and aims:** Sensitive and objective biomarkers for monitoring cognitive decline in multiple sclerosis (MS) are critically needed. Language is often impaired in MS and may reflect underlying cognitive changes. Automated analysis of spontaneous speech offers a promising, non‐invasive approach to capture longitudinal disease‐related alterations. This study investigates whether longitudinal speech analysis can detect language changes in MS.

**Methods:** We analyzed 213 participants, including 97 patients with MS and 116 age‐, sex‐, and education‐matched healthy controls. All participants completed a story‐telling task, which was automatically transcribed using automatic speech recognition system. MS patients underwent follow‐up assessment after two years. Six state‐of‐the‐art linguistic features, three lexical and three syntactic, were extracted using natural language processing methods to quantify language performance. MS severity was ranked using the Expanded Disability Status Scale (EDSS).

**Results:** At two‐year follow‐up, MS patients demonstrated overall lexical deterioration, including reduced vocabulary diversity (*p* = 0.031) and increased phrase repetition (*p* = 0.037) (Figure 1). Among 97 MS patients, 24 exhibited clinical progression (EDSS change ≥0.5). This MS subgroup was associated with pronounced syntactic decline, including shorter sentences (*p* = 0.044) and reduced sentence development (*p* = 0.038). MS patients without clinical progression primarily showed lexical impairments, such as restricted vocabulary (*p* = 0.028) and increased phrase repetition (*p* = 0.049).

**Figure:**
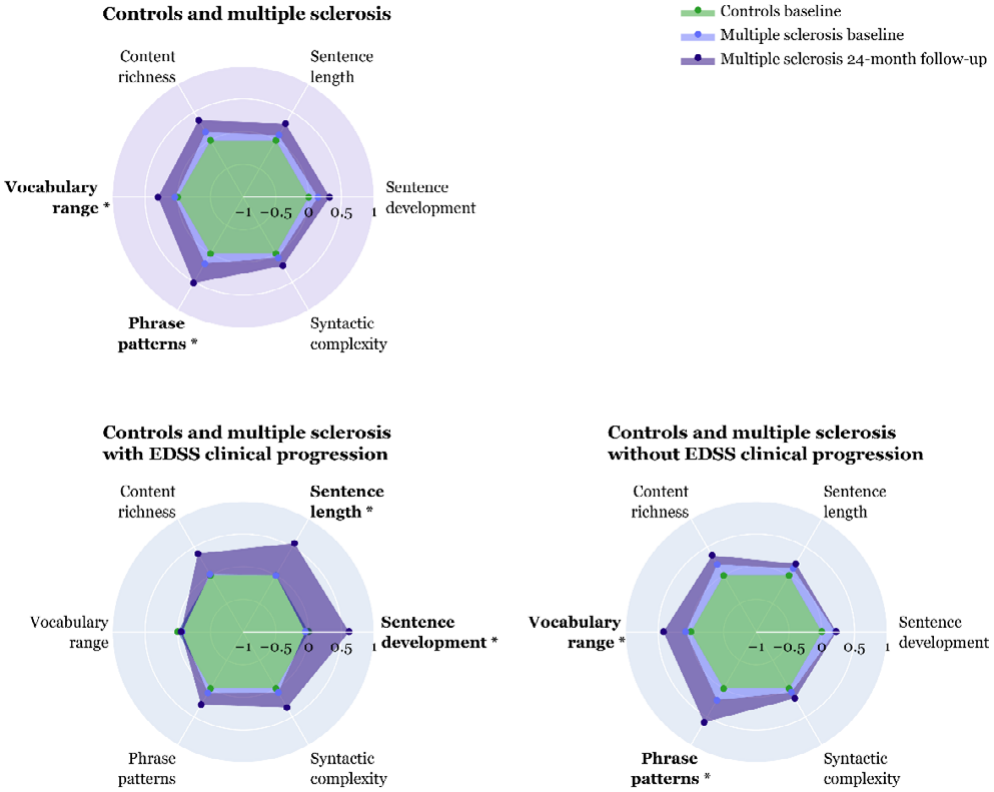
**FIGURE 1** Longitudinal changes in language dimensions over 24 months. Spider plots show z‐scored baseline and follow‐up values; higher scores indicate greater severity. *p.

**Conclusion:** Automated speech analysis detects longitudinal language changes in MS, even in subgroup of patients without progression detected on gold‐standard clinical scales. These findings highlight the potential of automated linguistic metrics as non‐invasive biomarkers for disease progression, complementing traditional cognitive and clinical assessments.

**Disclosure:** This project was supported by the ERC–CZ program from the Ministry of Education Youth and Sports (project no. LL2504).

## EPO‐0629

### Stage‐dependent relationship between retinal structure and visual function in radiologically isolated syndrome and early multiple sclerosis

#### R. Borgo ^1^; A. d'Ambrosio^1^; R. Capuano^2^; M. Risi^1^; G. Rummo^1^; M. Altieri^1^; V. Rippa^1^; M. Favale^1^; R. Docimo^3^; A. Bisecco^1^; A. Tessitore^1^; A. Gallo^1^

##### ^*1*^ *Department of Advanced Medical and Surgical Sciences, University of Campania Luigi Vanvitelli, Naples, Italy;* ^*2*^ *Neurology Unit, University Hospital "San Giovanni di Dio e Ruggi d'Aragona, Salerno, Italy;* ^*3*^ *First Division of Neurology and Neurophysiopathology, AOU Luigi Vanvitelli, Naples, Italy*

**Background and aims:** Radiologically isolated syndrome (RIS) represents a preclinical stage of multiple sclerosis (MS). In this context, structural and functional alterations of the anterior visual pathway remain poorly characterized. We aimed to evaluate retinal integrity and visual performance across the RIS‐MS spectrum.

**Methods:** We conducted a cross‐sectional study including healthy controls (HC), people with RIS (pwRIS) and people with early relapsing‐remitting MS (pwMS), excluding subjects with prior optic neuritis or relevant ophthalmological comorbidities. Optical coherence tomography (OCT) was used to measure mean peripapillary retinal nerve fiber layer (pRNFL), ganglion cell‐inner plexiform layer (GCIPL) and inner nuclear layer (INL) thickness, with age‐/sex‐adjusted z‐scores from published normative data. An exploratory retinal neurodegeneration score (RNS) was calculated as the mean of pRNFL and GCIPL z‐scores. Visual function was evaluated using low‐contrast letter acuity (LCLA) Sloan charts. Statistics included non‐parametric tests and Spearman correlations.

**Results:** We included 123 subjects (50 HC, 23 pwRIS, 50 pwMS). pRNFL thickness, pRNFL z‐scores and RNS differed significantly across groups (all *p* < 0.05), driven by significant reductions in pwMS versus HC, while pwRIS did not differ from either HC or pwMS. No significant differences were observed for GCIPL, INL and LCLA measures across all groups. While no structure‐function correlations emerged in HC or pwRIS, pwMS showed significant positive correlations between LCLA and both pRNFL metrics and RNS (rho range = 0.38–0.53, *p* < 0.05).

**Conclusion:** Our findings support the concept of a pathological continuum; while pwRIS maintain a near‐normal retinal profile, the transition to MS appears to be characterized by significant axonal loss and the emergence of structure‐function coupling.

**Disclosure:** RMB, AdA, RC, MR, GR, MA, VR, MLF: nothing to disclose. RD: received honoraria as a speaker and member of advisory boards by: Merck‐Serono, Roche, Novartis, BMS and travel funding from Almirall, Biogen, Novartis, Sanofi‐Genzyme, Roche and Merck‐Serono. AB: received speaker's honoraria and/or compensation for consulting services and for speaking activities from Biogen, Roche, Merck, Celgene, and Genzyme. AT: received honoraria as speaker or consultant from Lundbeck, AbbVie, UCB Pharma, Allergan, Almirall, and Bial. He is an editorial board member of European Journal of Neurology. AG: received speaker, consulting fees and travel support from Biogen, Genzyme, Merck Serono, Mylan, Novartis, Roche, and Teva, and receives research support from Fondazione Italiana Sclerosi Multipla and Italian Ministry of Health.

## EPO‐0630

### Patient‐reported outcomes after autologous haematopoietic stem cell transplantation compared with rituximab in multiple sclerosis

#### Y. Noui ^1^; A. Mitrache Desaga^1^; E. Freyhult^2^; K. Kultima^1^; J. Burman^1^

##### ^*1*^ *Department of Medical Sciences, Uppsala University, Uppsala, Sweden;* ^*2*^ *Department of Cell and Molecular Biology, Uppsala University, Uppsala, Sweden*

**Background and aims:** Autologous haematopoietic stem cell transplantation (AHSCT) has emerged as a highly‐effective treatment for relapsing‐remitting multiple sclerosis (RRMS). Patient‐reported outcomes have been shown to improve following AHSCT, but how this compares to other established disease‐modifying treatments is unclear. We aimed to describe the Multiple Sclerosis Impact Scale‐29 (MSIS‐29) questionnaire scores in comparable patients treated with AHSCT and rituximab.

**Methods:** This was a retrospective observational study using the Swedish MS registry. Eligbility for inclusion was on the basis of: a diagnosis of RRMS; treatment in Sweden with either AHSCT or rituximab between 2008 and 2020; a minimum clinical, radiological and MSIS‐29 dataset. Propensity scores were derived from prespecified confounders and used for 1:4 matching. Linear mixed‐effect models assessed differences in scaled scores between the matched groups.

**Figure:**
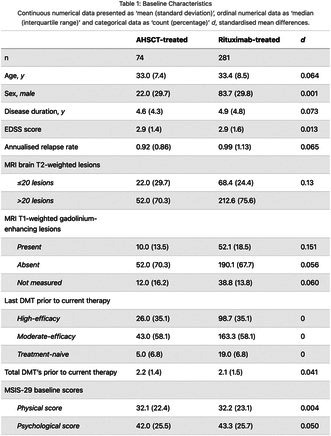
**TABLE 1** Baseline Characteristics of included participants. Participants matched on propensity score between rituximab‐ and AHSCT‐treated groups.

**Results:** Of the eligible 95 AHSCT and 2196 rituximab patients, 74 AHSCT were matched to 281 rituximab patients with good covariate balance (Table 1, standardised mean differences <0.1). Changes from baseline MSIS‐29 scores are shown in Figure 1 Both treatments were associated with a decrease in physical MSIS‐29 scores over time, with AHSCT associated with a greater decrease over time (−0.90 points‐per‐year, *p* = 0.019, Table 2). With regards to psychological MSIS scores, both treatments were again associated with a decrease in scores over time, with AHSCT again associated with greater decreases over time (−0.99 points‐per‐year, *p* = 0.022, Table 2).

**Figure:**
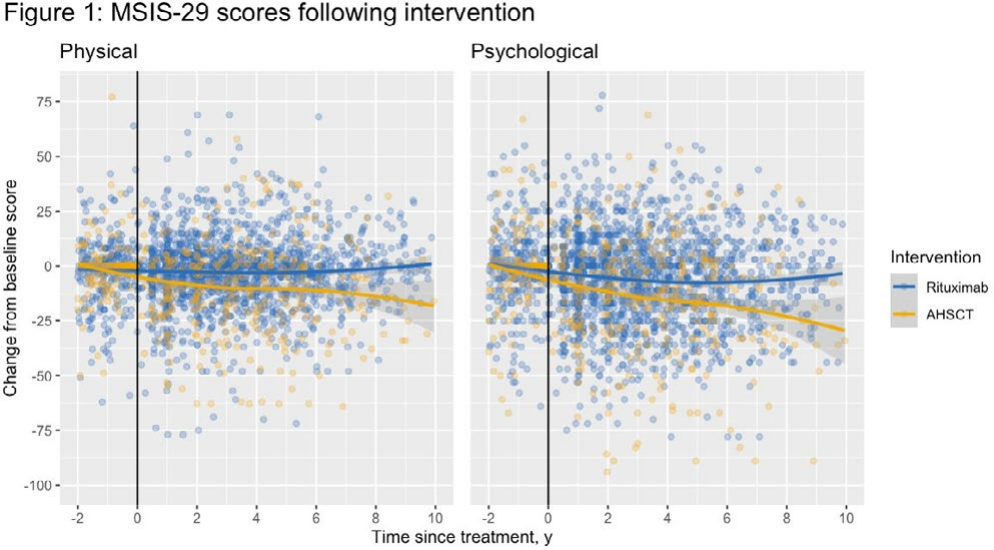
**FIGURE 1** Trends in MSIS‐29 scores in participants following intervention with either rituximab or AHSCT. Scores are presented relative to each individuals pre‐treatment baseline.

**Figure:**
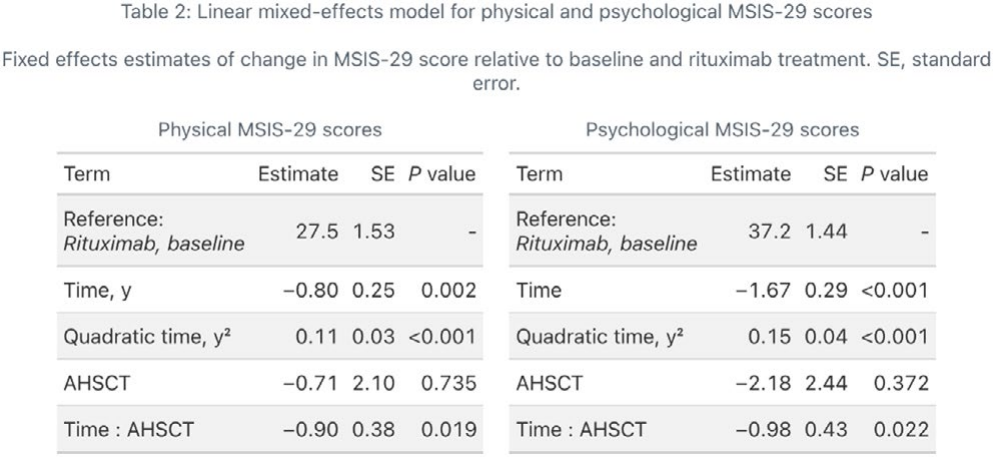
**TABLE 2** Linear mixed‐effects model estimates for change in physical and psychological MSIS‐29 scores with respect to time and intervention.

**Conclusion:** In propensity‐matched RRMS patients, AHSCT was associated with greater improvement in both physical and psychological MSIS‐29 scores than rituximab, supporting benefits on symp‐toms and wellbeing.

**Disclosure:** Yassine Noui: nothing to disclose. Ann‐Christine Mitrache‐Desaga: nothing to disclose. Eva Freyhult: nothing to disclose. Kim Kultima: nothing to disclose. Joachim Burman: nothing to disclose.

## EPO‐0631

### Dysphagia in neuromuscular disorders: A histopathological study of the cricopharyngeal muscle

#### B. Labella ^1^; G. Brochier^1^; M. Beuvin^2^; A. Chanut^2^; A. Madelaine^1^; C. Labasse^2^; E. Lacene^2^; T. Stojkovic^3^; B. Eymard^3^; J. Lacau Saint Guily^4^; T. Evangelista^2^

##### ^1^Neuromuscular functional Unit, Department of Neuropathology, Groupe Hospitalier Universitaire La Pitié‐Salpêtrière, Paris, France; ^2^Neuromuscular Morphology Unit, Myology Institute, Paris, France; ^3^Reference Center for Neuromuscular Disorders, Myology Institute, Groupe Hospitalier Universitaire La Pitié‐Salpêtrière, Paris, France; ^4^Department of Otolaryngology‐Head and Neck Surgery, Assistance Publique‐Hôpitaux de Paris (AP‐HP), Rothschild Foundation Hospital, Paris, France

**Background and aims:** Dysphagia is a debilitating condition with complex etiology. Potential therapeutic interventions include cricopharyngeal myotomy. This study aims to investigate the histopathological characteristics of the cricopharyngeal muscle (CM) in patients with neuromuscular disorders (NMDs), to evaluate its diagnostic utility.

**Methods:** CM tissue was collected from 10 patients that underwent cricopharyngeal myotomy, including 4 patients with Oculopharyngeal Muscular Dystrophy, 1 with Oculopharyngeal Distal Myopathy, 3 with Inclusion Body Myositis (IBM) and 1 with idiopathic achalasia. The specimens were processed for routine histoenzymology and immunohistochemistry (antibodies against PABPN1, MHC class 1/2, p62, LC3). Seven samples were also analyzed using ultrastructural analysis.

**Results:** Common histological findings in CM include horizontal and transverse fiber orientation, the presence of nemaline rods and mitochondrial abnormalities, type I fiber predominance, and increased connective tissue (Figure 1). Rimmed or non‐rimmed vacuoles were identified in four patients. Scattered necrosis was observed in five. Inflammatory infiltrates were found in two patients within the IBM cohort. Immunohistochemical staining is the most reliable method for detecting PABPN1 aggregates in CM and is also simpler to perform than ultrastructural studies. No distinctive histological pattern was identified in idiopathic achalasia. Ultrastructural analyses revealed also abundant autophagic structures.

**Figure:**
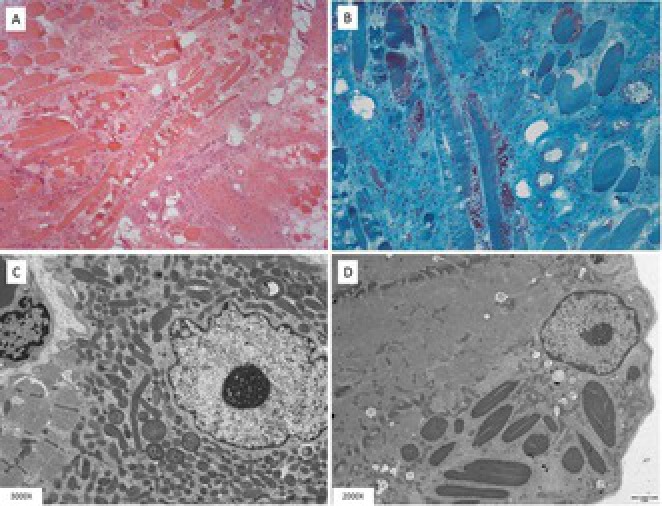
**FIGURE 1** Histological and ultrastructural findings of the CM.

**Conclusion:** Histological examination of CM is rarely performed but still can provide critical insights for diagnosis. Our study emphasizes its potential value in the diagnostic process, particularly in patients whose clinical presentation is exclusively limited to dysphagia, thereby reinforce its role as complementary tool.

**Disclosure:** Nothing to disclosure.

## EPO‐0632

### MG patients facing heat waves. Heat waves exposure differentially worsens MG‐ADL Scores in anti–acetylcholine receptor but not MuSK myasthenia gravis

#### E. Lagrange ^1^; S. Sacconi^2^; J. Reis^3^

##### ^1^Grenoble University Hospital Department of Neurology, Reference Center of Neuromuscular Disease and ALS consultations, Grenoble University Hospital; Grenoble, France; ^2^Université Côte d'Azur, Peripheral Nervous System and Muscle Department, Pasteur 2 Hospital, Centre Hospitalier Universitaire de Nice, Nice, France; ^3^INSERM‐UNISTRA UMR 1260, Centre de Recherche en Biomédecine de Strasbourg (CRBS); 1 rue Eugene Boeckel; Strasbourg, France

**Background and aims:** To assess the impact of heat wave exposure on symptom severity, as measured by the Myasthenia Gravis Activities of Daily Living (MG‐ADL) scores in patients with autoimmune myasthenia gravis (MG), comparing anti–acetylcholine receptor (anti‐AChR) and anti‐MuSK subtypes.

**Methods:** We conducted a cross‐sectional questionnaire‐based study including 206 adult MG patients. Heat‐related symptom worsening during heat waves, hot bath sensitivity, cooling strategies, and MG‐ADL–derived heat‐aggravation scores were analyzed according to antibody status.

**Figure:**
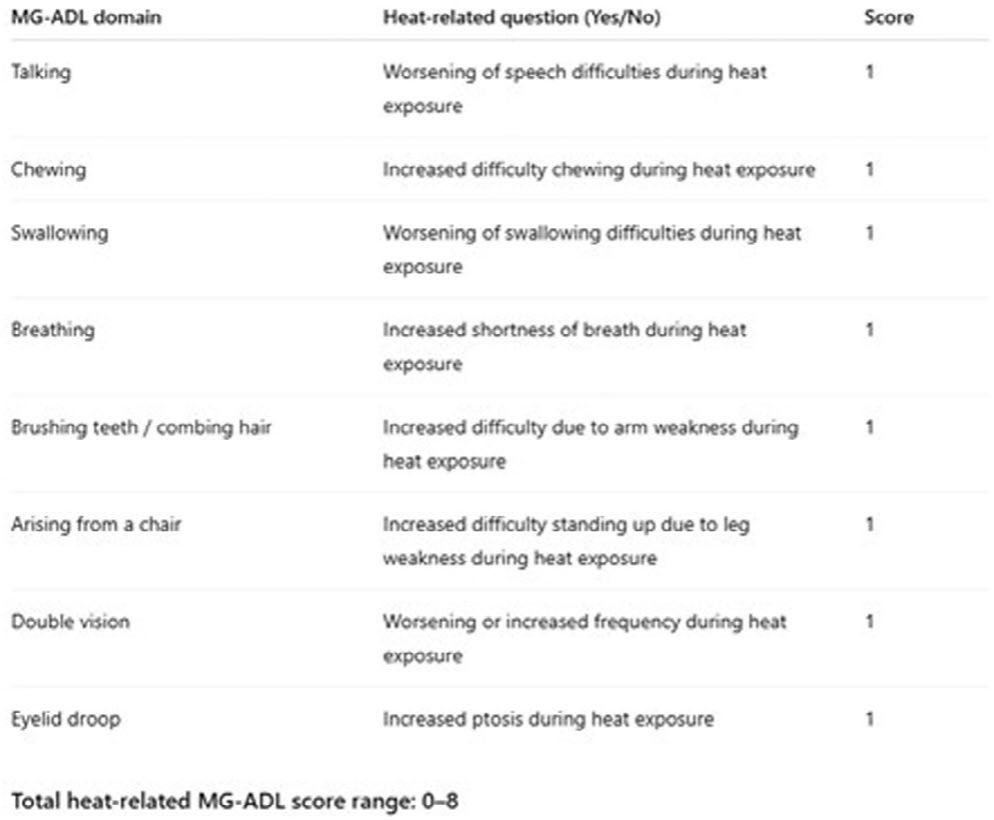
**TABLE 1** Composition of the heat related MG‐ADL score.

**Results:** Heat wave exposure was associated with a significant worsening of MG‐ADL scores in anti–AChR patients, whereas no significant effect was observed in MuSK patients Anti‐AChR patients frequently reported symptom exacerbation during days of heatwaves, after hot showers or baths and commonly required air conditioning. Cold exposure was associated with symptom improvement predominantly in the anti‐AChR group.

**Figure:**
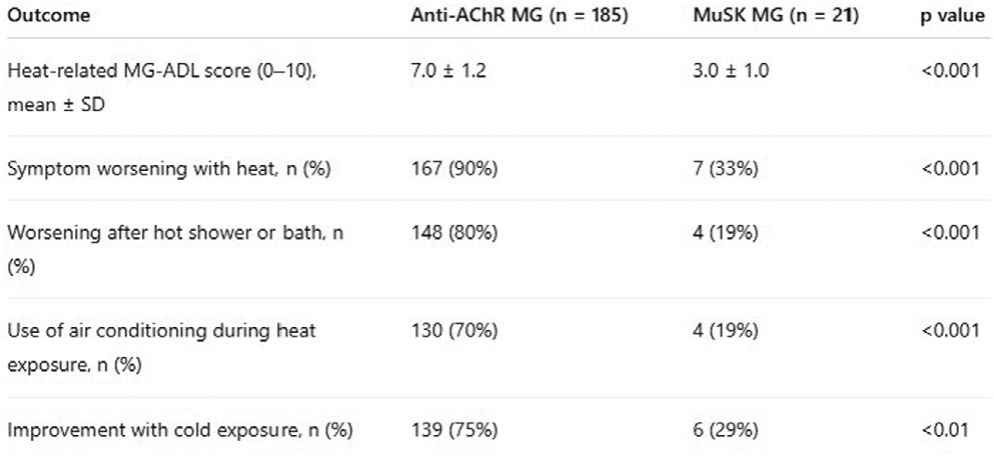
**TABLE 2** Baseline demographic and heat‐related clinical characteristics of patients with myasthenia gravis according to antibody subtype Values are presented as mean ± SD, median (interquartile range), or number (percentage), as appropriate. Heat‐related MG‐ADL sc.

**Figure:**
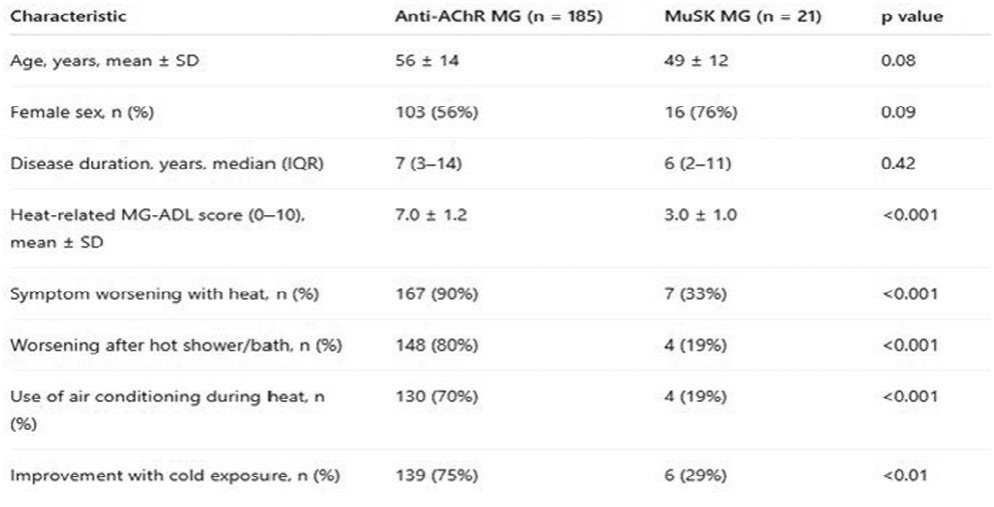
**TABLE 3** Heat waves ‐related clinical outcomes in myasthenia gravis according to antibody subtype. Values are presented as mean ± SD or number (percentage). Group comparisons were performed using Welch's t‐test for continuous variables and χ^2^ tests for cat.

**Conclusion:** Heat wave represents a clinically relevant aggravating factor in anti‐AChR MG, supporting targeted environmental management strategies in the perspectives of climate change, in such a vulnerable population.

**Disclosure:** Disclosure interests Dr Lagrange.

## EPO‐0633

### Tanruprubart rapidly improves health‐related quality of life in patients with Guillain–Barré syndrome: A randomised, placebo‐controlled Ph3 study

#### G. Morrison^1^; J. Du^1^; Q. Deen Mohammad^2^; *N*. Papri^3^; M. Kim ^1^; H. Kroon^1^; K. Gorson^4^

##### ^1^Annexon Biosciences, Brisbane, USA*;* ^2^National Institute of Neuroscience (NINS), Dhaka, Bangladesh*;* ^3^icddr,b, Dhaka, Bangladesh*;* ^4^Tufts University School of Medicine, Boston, USA

**Background and aims:** Guillain‐Barré syndrome (GBS) is a rare, life‐altering, neuromuscular emergency, causing long‐lasting disabilities severely impacting quality of life. Tanruprubart (ANX005) inhibits complement component C1q. In a Ph3, double‐blind, placebo‐controlled study (GBS‐02; NCT04701164), a single infusion of tanruprubart 30 mg/kg improved muscle strength and disability in patients with GBS at Day 8 and Week 8. This analysis presents patient‐reported outcomes (PROs) with tanruprubart 30 mg/kg versus placebo.

**Methods:** Patients aged ≥16 years with a recent GBS diagnosis were randomised to tanruprubart 30 mg/kg (*n* = 79), 75 mg/kg (*n* = 81) or placebo (*n* = 81). PROs included the EuroQol 5‐Dimenstion 5‐Level (EQ‐5D‐5L), Rasch‐built Modified Fatigue Severity Scale (rFSS) and Patient Global Impression of Change (PGIC). Impact of treatment on HRQoL was evaluated using proportional odds logistic regression adjusted for baseline covariates.

**Results:** By Day 8 significant improvements in EQ‐5D‐5L occurred in mobility and self‐care with tanruprubart 30 mg/kg versus placebo (Table 1). Rapid improvements were observed with tanruprubart versus placebo in all domains of the rFSS except for pain (Table 2). In the PGIC, eight (10.1%) patients reported being ‘very much improved’ with tanruprubart at Day 8, compared with three (3.7%) patients with placebo (aOR 2.5 [95% confidence interval (CI): 1.4, 4.6] *p* = 0.0028). Tanruprubart‐associated improvements in EQ‐5D‐5L mobility, self‐care and usual activities domains continued to Week 8 (Tables 1, 2). PGIC improvements at Week 8 still favoured tanruprubart (aOR 1.4 [95% CI 0.8, 2.6, *p* = 0.2917).

**Figure:**
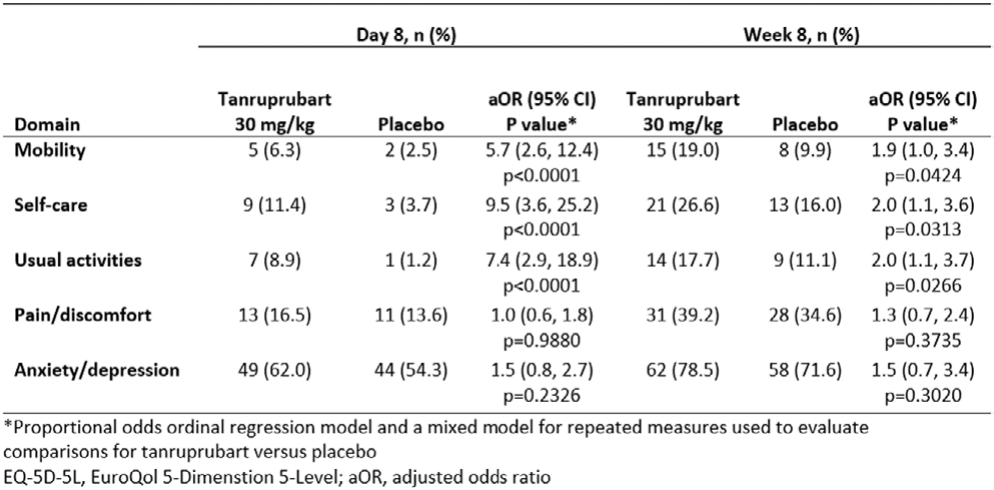
**TABLE 1** Patients reporting no problems across EQ‐5D‐5L domains on Day 8 and Week 8.

**Figure:**
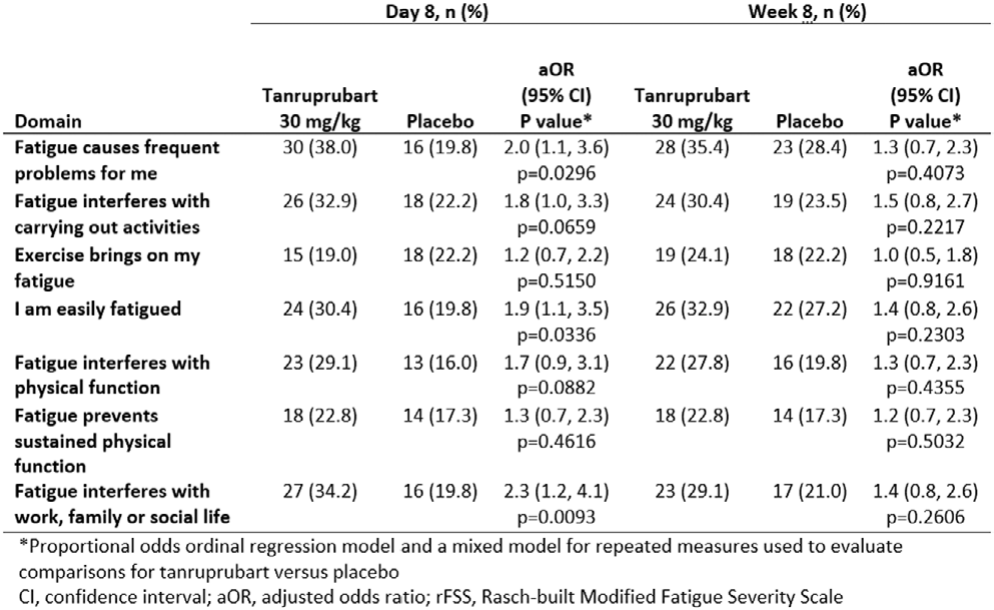
**TABLE 2** Patients who answered “disagree” to statements across rFSS domains at day 8 and week 8.

**Conclusion:** Tanruprubart demonstrated rapid and sustained improvements in HRQoL, consistent with observed objective clinical benefits, supporting its potential as a targeted immunotherapy for GBS.

**Disclosure:** The study was sponsored by Annexon Biosciences (Brisbane, CA, USA). MK, GM, JD, H‐AK: Employment with and shareholder of Annexon Biosciences QDM: Consultancy/advisory role with Annexon Biosciences NP: Nothing to disclose KCG: Consultancy/advisory role with Annexon Biosciences, argenx, Janssen, and Sanofi.

## EPO‐0634

### Late‐onset **TK2** deficiency in adults: **Long**‐term clinical outcomes of deoxynucleoside therapy

#### H. Durmus ^1^; A. Gedikbaşı^2^; A. Çakar^1^; E. Kıyan^3^

##### ^1^Department of Neurology, Istanbul Faculty of Medicine, Istanbul University, Istanbul, Türkiye; ^2^Institute of Child Health, Department of Pediatric Basic Sciences, Division of Medical Genetics, Istanbul University, Türkiye; ^3^Department of Pulmonology, Istanbul Faculty of Medicine, Istanbul University, Istanbul, Türkiye

**Background and aims:** Thymidine kinase 2 deficiency is a mitochondrial myopathy causing progressive weakness, respiratory insufficiency, and early mortality. While nucleoside supplementation benefits pediatric cases, evidence in adults remains limited.

**Methods:** Six genetically confirmed adults with TK2d were treated under compassionate‐use protocols in Türkiye, receiving escalating doses of oral or PEG‐administered Doxecitine and Doxribtimine up to 800 mg/kg/day. Functional and clinical outcomes included the 6‐minute walk test (6MWT), HFMSE, forced vital capacity (FVC), Fatigue Severity Scale (FSS), and BMI.

**Results:** Over a median follow‐up of 24 (range 6–36) months, therapy was associated with sustained functional and respiratory improvements. Mean 6MWT increased from 152 m at baseline to 468 m at 24 months and exceeded 600 m in patients with 36‐month data. The mean HFMSE increased from 20.5 at baseline to 24.5 at 3 months, 29 at 6 months. Patients with the longest follow‐up reached a mean HFMSE of 58 at 36 months. FVC, which had declined before treatment, stabilized or improved in all patients (mean increase from 36% to 55.6% at 36 months). The youngest patient achieved near‐normal respiratory function. Fatigue severity decreased by ~30%, and BMI improved notably in underweight individuals. Treatment was generally well tolerated; transient liver enzyme elevations and mild gastrointestinal side effects resolved spontaneously.

**Figure:**
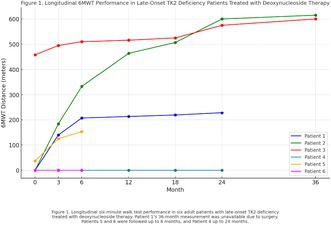
**FIGURE 1** Longitudinal six‐minute walk test performance in six adult patients with late‐onset TK2 deficiency treated with deoxynucleoside therapy. Patient 1's 36‐month measurement was unavailable due to surgery. Patients 5 and 6 were followed up to 6 mo.

**Figure:**
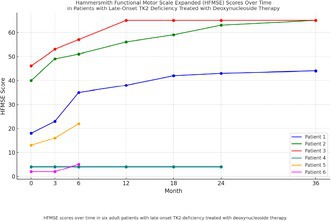
**FIGURE 2** Hammersmith Functional Motor Scale scores over time in six adult patients with late‐onset TK2 deficiency treated with deoxynucleoside therapy.

**Figure:**
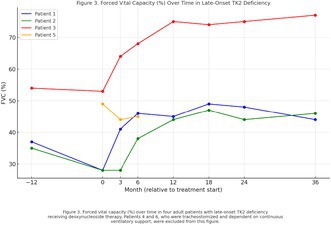
**FIGURE 3** Forced vital capacity (%) over time in four adult patients with late‐onset TK2 deficiency receiving deoxynucleoside therapy. Patients 4 and 6, who were tracheostomized and dependent on continuous ventilatory support, were excluded from this figure.

**Conclusion:** Deoxynucleoside therapy appears effective and well tolerated in adults with TK2d, producing sustained functional and respiratory gains. Early treatment initiation is crucial to maximize benefit; though partial improvements may occur even in advanced disease.

**Disclosure:** The authors have no conflicts of interest to declare.

## EPO‐0635

### A novel Guillain–Barré syndrome clinical progression scale to characterise the patient's care journey: Utilisation in a phase 3 trial of **t**anruprubart

#### H. Kroon ^1^; K. Gorson^2^; Q. Deen Mohammad^3^; T. Harbo^4^

##### ^1^Annexon Biosciences, Brisbane, USA; ^2^Tufts University School of Medicine, Boston, USA; ^3^National Institute of Neuroscience (NINS), Dhaka, Bangladesh; ^4^Aarhus University, Aarhus, Denmark

**Background and aims:** Guillain‐Barré syndrome (GBS) is a rare, acute, life‐altering autoimmune polyneuropathy with rapidly progressive weakness and prolonged recovery. In GBS‐02, a Ph3, randomised, double‐blind, placebo‐controlled trial, tanruprubart (ANX005) 30 mg/kg led to rapid complement inhibition and clinical improvement with sustained clinical benefit over 6 months in patients with GBS (NCT04701164). We characterised the patient's care journey using a novel GBS Clinical Progression Scale (CLIPS).

**Methods:** Patients aged ≥16 years with a recent GBS diagnosis (*n* = 241) were randomised to a single infusion of tanruprubart 30 mg/kg, 75 mg/kg or placebo. CLIPS was adapted from the WHO Covid‐19 Clinical Progression Scale by the study sponsor and GBS experts. This ordinal measure is based on 8 levels of care (Table 1). During GBS‐02, clinical status on the scale was physician recorded weekly for 4 weeks and at 2, 3, 4, and 6 months in a subset of patients (tanruprubart 30 mg/kg *n* = 41, 75 mg/kg *n* = 45, placebo *n* = 45). Treatment impact on level of care was evaluated using a proportional odds logistic regression adjusted for baseline covariates.

**Figure:**
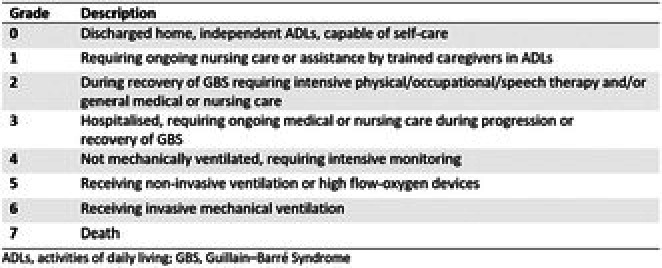
**TABLE 1** Eight grades of the ordinal GBS Clinical Progression Scale.

**Results:** All patients were hospitalised and most required medical intervention (Grade ≥3; Figure 1). By Day 8, care requirements decreased. At Week 4 the odds of requiring less care favoured tanruprubart 30 mg/kg (aOR 2.3, 95% CI (0.99–5.39; *p* = 0.054) over placebo, consistent with overall clinical outcome.

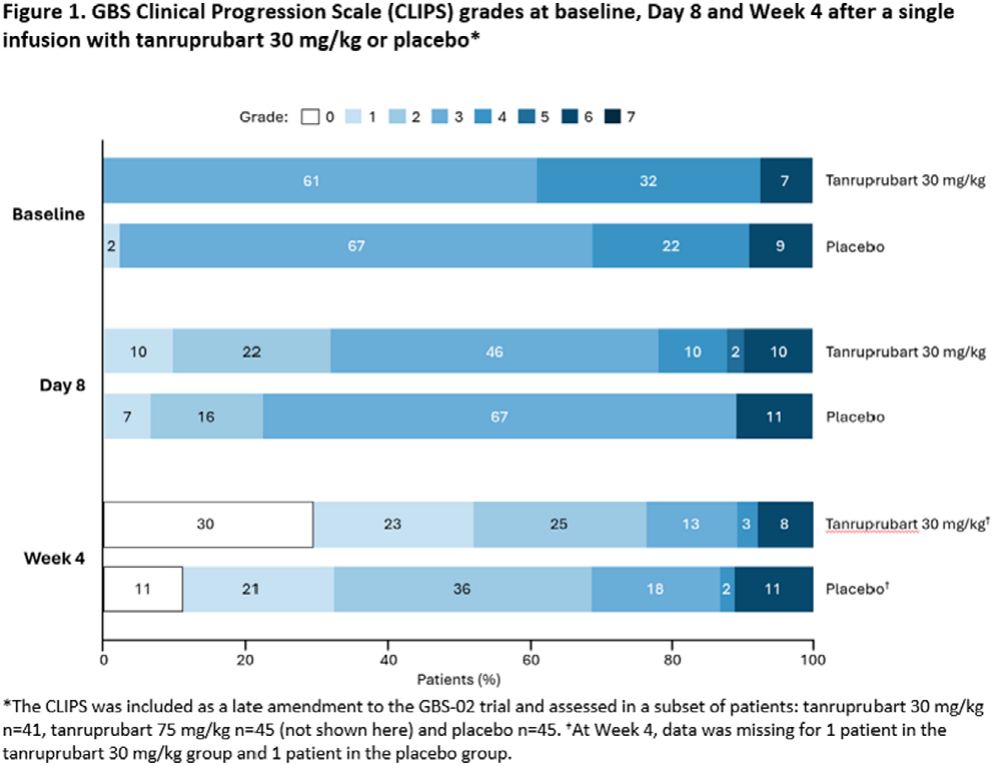

**Conclusion:** CLIPS captured patients’ transitions through levels of care, providing a pragmatic lens to interpret treatment benefit. Early separation at Week 4 favouring tanruprubart supports a clinically meaningful shift toward lower resource intensity during early GBS.

**Disclosure:** The study was sponsored by Annexon Biosciences (Brisbane, CA, USA). H‐AK: Employment with and shareholder of Annexon Biosciences KCG: Consultancy/advisory role with Annexon Biosciences, argenx, Janssen, and Sanofi QDM: Consultancy/advisory role with Annexon Biosciences TH: Received grant and honoraria from Dianthus Therapeutics, Sanofi, Janssen, Nuvig, Biocryst, Argenx, Abvie and Annexon.

## EPO‐0636

### Validating the 2024 ENMC inclusion body myositis criteria: Diagnostic utility in early “Polymyositis‐Mitochondrial” stages

#### I. Lopera‐Rodríguez; J. Ciriero‐Macías; J. Andrada‐Moreno; L. García‐Granados; J. Román‐Rueda; C. Villar‐Rodríguez; A. Luque‐Ambrosiani; E. Rivas‐Infante; C. Paradas; F. Gómez‐Fernández

##### Hospital Universitario Virgen del Rocío, Sevilla, Spain

**Background and aims:** The 2024 European Neuromuscular Centre (ENMC) criteria for Inclusion Body Myositis (IBM) integrates mitochondrial pathology as a key histological supportive feature. We evaluated their diagnostic capacity in a single‐centre cohort, specifically focusing on the “Polymyositis‐Mitochondrial” (PM‐Mito) phenotype, an early recognised stage of IBM.

**Methods:** We retrospectively reassessed 15 IBM patients following the 2024 ENMC consensus and evaluated the clinical phenotype, anti‐cN1A serology, muscle imaging and histological features.

**Results:** Mean onset age was 60.6 ± 9.5 years with a diagnostic delay of 3.7 ± 2.4 years. Mean Creatine kinase level was 711 ± 651 U/L. The “common” clinical phenotype (finger flexor/knee extensor weakness) was observed in 93% of patients and dysphagia in 67%. An inflammatory myopathic electromyogram pattern was found in 75% of analysed cases. Muscle magnetic resonance imaging revealed the classic distal/anterior thigh pattern in 70% (Figure 1). Anti‐cN1A antibodies were positive in 62.5% of tested patients. Histologically, all biopsies showed endomysial lymphocyte infiltration (Figure 2), p62 aggregates were identified in 77%, whereas TDP‐43 was rarely assessed. Two patients presented the PM‐Mito pattern: endomysial inflammation with cyclooxygenase (COX)‐negative fibres (Figure 3) exceeding the expected level for the patient's age, without additional supportive findings. Under previous criteria, this finding mimicked a polymyositis pattern. However, using the new criteria, the combination of “common” phenotype and mitochondrial pathology confirmed the IBM diagnosis.

**Figure:**
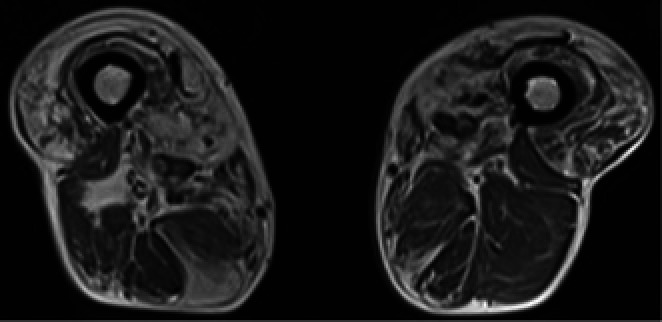
T1‐weigthed MRI image of both thighs showing anterior distal fatty infiltration.

**Figure:**
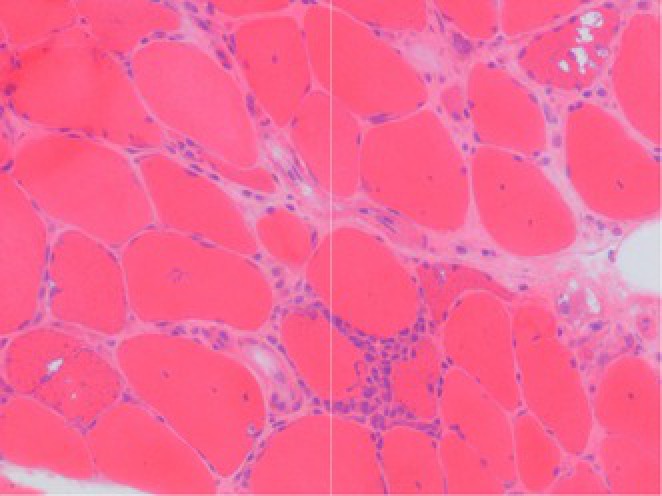
H&E muscle biopsy with endomysial lymphocyte infiltration.

**Figure:**
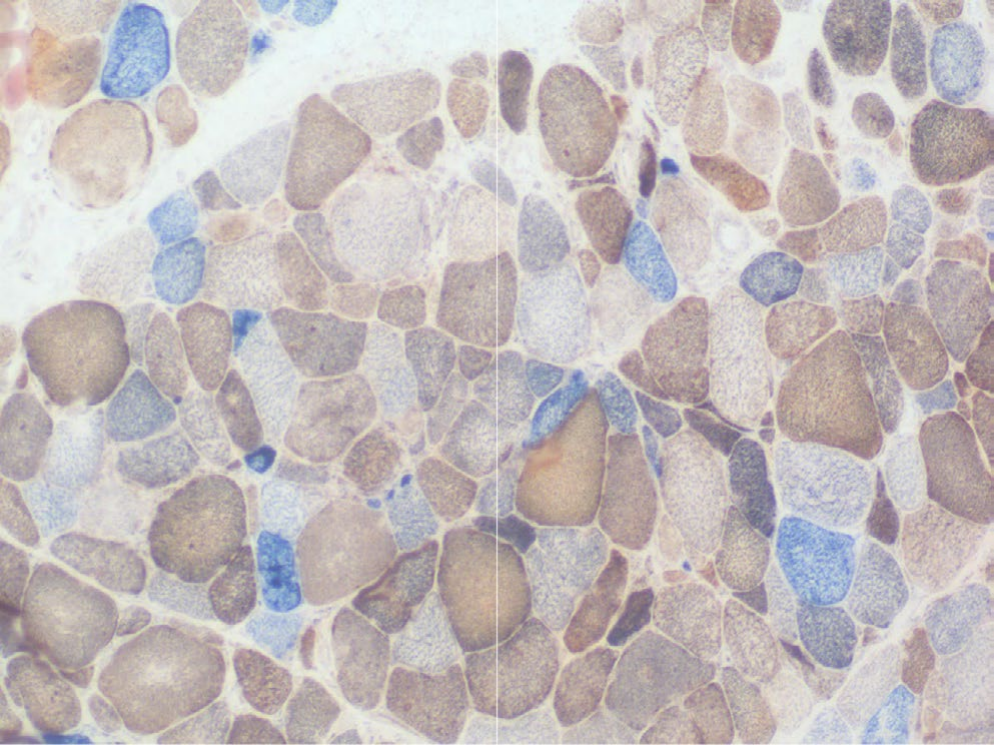
Pathological increase of COX negative muscle fibers.

**Conclusion:** The 2024 ENMC criteria enhance diagnostic sensitivity for early IBM. Elevating mitochondrial abnormalities to “supportive criterion” enables the definitive diagnosis of PM‐Mito cases, preventing misdiagnosis and avoiding ineffective immunosuppressive therapies.

**Disclosure:** Nothing to disclose.

## EPO‐0637

### The immune modulatory effect of eculizumab on B cells in myasthenia gravis beyond the complement inhibition

#### J. Li; G. Luo; W. Jiang; C. Yang

##### Department of Neurology, Tianjin Neurological Institute, Tianjin Medical University General Hospital, Tianjin, China

**Background and aims:** Eculizumab has demonstrated high efficacy in patients with anti‐acetylcholine receptor (AChR) antibody–positive myasthenia gravis (MG). It prevents the cleavage of C5 into C5a and C5b, thereby blocking the formation of the membrane attack complex (MAC) in neuromuscular junction. However, the potential off‐target effects of eculizumab on immune cell modulation—mediated via C5a‐C5aR1 axis during long‐term treatment—remain incompletely understood.

**Methods:** Patients with AChR Ab+ gMG receiving regular eculizumab or prednisolone treatment were enrolled and divided into exploratory and validation cohorts. Bulk RNA‐seq and flow cytometry were performed on peripheral immune cells. C5aR1 antagonist was administered to experimental autoimmune myasthenia gravis (EAMG) rats, and clinical scores, electromyograms, immunofluorescence staining of nicotinic AChR (nAChR), and flow cytometric analysis of immune cells were performed. B cells were co‐cultured with monocytes for in vitro analysis.

**Results:** Bulk RNA sequencing revealed significant transcriptomic alterations in MG patients following eculizumab treatment, enriched in B‐cell‐related pathways. Long‐term eculizumab markedly reduced peripheral CD19^+^ B cells and their subsets (including memory B cells and antibody‐secreting cells). C5aR1 is predominantly expressed on myeloid cells and is upregulated during acute exacerbations of MG. In vivo and in vitro co‐culture studies indicated that blocking C5a‐C5aR1 signaling pathway in monocytes inhibited B‐cell differentiation, promoted B‐cell apoptosis, and reduced circulating B‐cell numbers, likely through decreased BAFF secretion.

**Figure:**
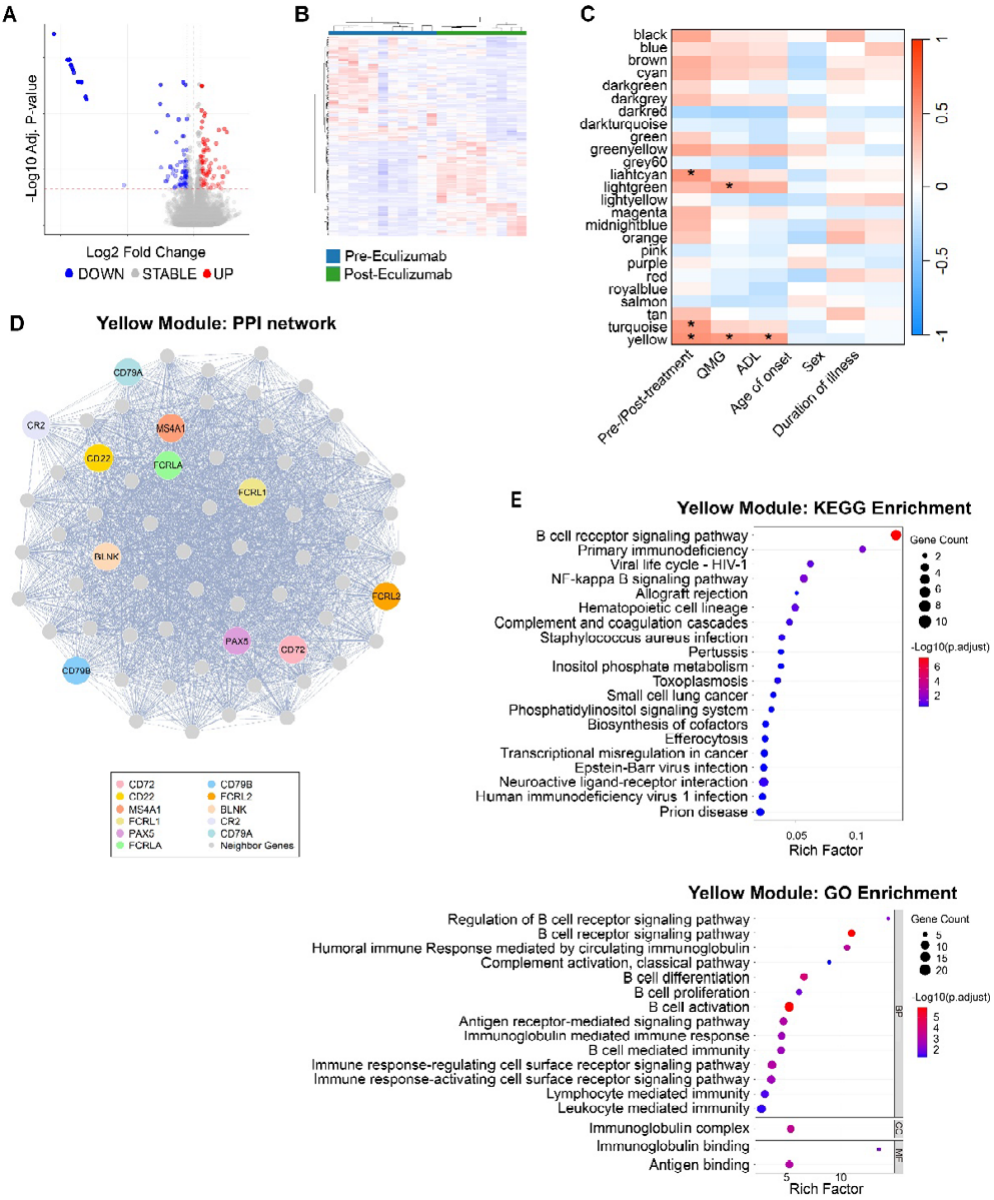
**FIGURE 1** Bulk RNA‐Seq analysis of differential gene expression and functional associations in AChR antibody‐positive generalized myasthenia gravis patients before and after eculizumab treatment.

**Figure:**
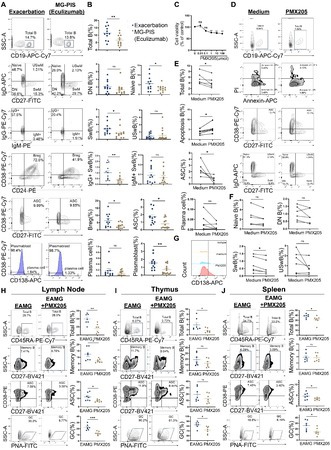
**FIGURE 2** Modulation of B‐cell subsets and viability by a C5aR1 antagonist in myasthenia gravis as shown by flow cytometry.

**Conclusion:** In addition to blocking the terminal complement pathway, eculizumab modulates peripheral B‐cell differentiation and survival by inhibiting the monocyte C5a‐C5aR1‐mediated BAFF secretion. This reveals its off‐target immunomodulatory effects and the mechanism of systemic immunoregulation during long‐term therapy.

**Disclosure:** This study was supported by the National Natural Science Foundation of China (82571535, 82171338, 82171277), and Young Scientific and Technological Talents (Level Three) in Tianjin.

## EPO‐0638

### Significant correlations between ME&MGTM digital biomarkers and equivalent MG‐ADL and MG‐QoL15r sub‐items scores

#### C. Barnett‐Tapia^1^; S. Lehnerer^2^; S. Bieuvelet^3^; L. Carment ^3^; C. Gorin^3^; N. Pesic‐Heuvrard^3^; D. Ravindra^3^; N. Sellami^3^; B. Dutta^4^; B. Yungher^4^; S. Zinaï^3^; J. Howard Jr^5^

##### ^1^Ellen and Martin Prosserman Centre for Neuromuscular Diseases, Division of Neurology, Department of Medicine, University Health Network, University of Toronto, Toronto, Ontario, Canada; ^2^Charité – Universitätsmedizin Berlin, corporate member of Freie Universität Berlin and Humboldt‐Universität zu Berlin, Department of Neurology with Experimental Neurology, Charitéplatz 1; 10117 Berlin, Germany; ^3^Ad Scientiam, Paris, France; ^4^Alexion, AstraZeneca Rare Disease, Boston, USA; ^5^University of North Carolina, Department of Neurology, Chapel Hill, USA

**Background and aims:** Symptoms of generalized Myasthenia Gravis (gMG) are routinely captured via patient‐reported outcomes (PROs) questionnaires. MG‐ADL and MG‐QoL15r assess daily activities and quality of life; high scores indicate higher severity and worse quality of life. Digital biomarkers (dBMKs) provide objective measures for remote symptoms’ self‐assessment through smartphone‐based active tests. ME&MGTM evaluates five gMG symptoms: ptosis, dysarthria, breathing, upper and lower limb weakness mirrored in 5 MG‐ADL and 3 MG‐QOL15r items. In the decentralized ME&MGopen study (NCT05566964), patients performed ME&MGTM tests for one year. Here, we describe the correlation between dBMKs and corresponding MG‐ADL and MG‐QoL15r sub‐items.

**Methods:** Completed ME&MGTM tests were reviewed for quality: inaccurate dBMKs values and agreement with human measurements. The relationship between dBMKs and sub‐items was determined using mixed models analyses. We expected negative associations with MyBreathing, MyEyelids, Mylegs and MyArms and positive correlations with MyVoice.

**Results:** 156 participants completed digital tests and questionnaires monthly (Figure 1). A total of 9106 evaluations were collected, of which 90.4% were of good quality. DBMKs scores for ptosis, breathing, and limb weakness decreased with increasing PRO assessed symptom severity and scores for dysarthria decreased with severity (Figure 2). Ptosis, dysarthria and limb weakness scores showed significant associations (all *p <* 0.019) with aforementioned sub‐items of the MG‐ADL and MG‐QoL‐15r questionnaires.

**Figure:**
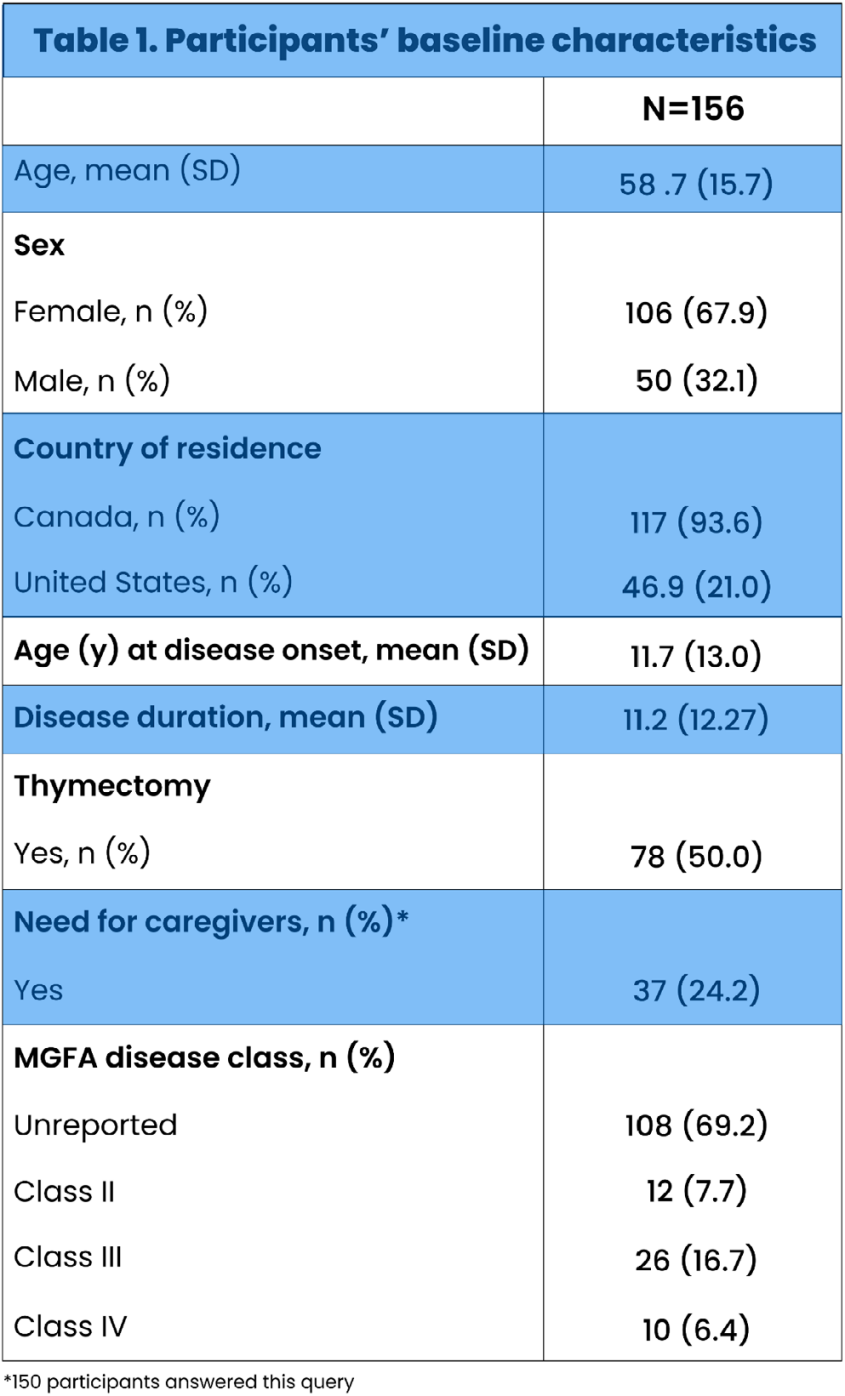
**FIGURE 1** Baseline characteristics of patients who completed all ME&MGTM during the ME&MGopen study.

**Figure:**
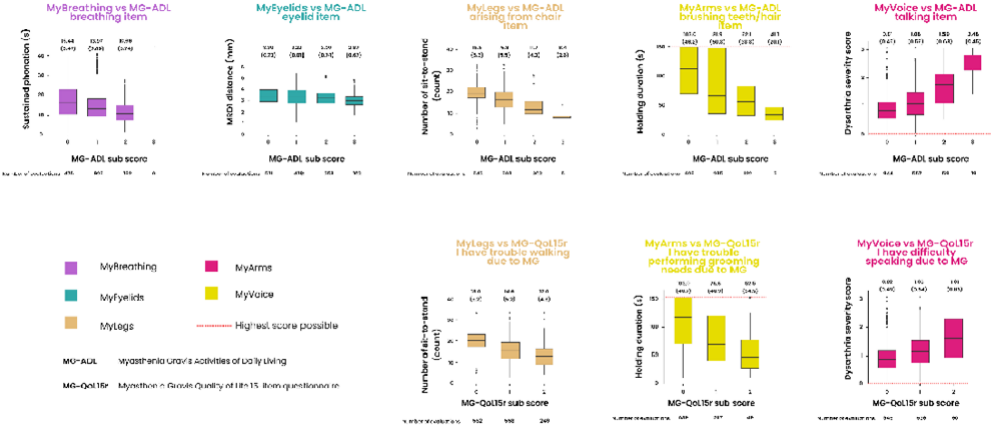
**FIGURE 2** Correlations between ME&MGTM scores and MG‐ADL or MG‐QoL15r sub‐items.

**Conclusion:** Significant associations between clinical ME&MGTM dBMKs and clinical assessments support their potential use in clinical settings to track gMG manifestations. These findings highlight the importance of the ongoing DOMYA validation study (NCT05564936).

**Disclosure:** C. Barnett‐Tapia: Ad Scientiam, Alexion, AstraZeneca rare Disease, Cartesian Therapeutics, US Department of Defence, Muscular Dystrophy Canada, MGNet, Grifols and Octapharma—research grants. Argenx, Alexion, AstraZeneca Rare Disease, Janssen and UCB—advisory board member. Argenx, Alexion, AstraZeneca Rare Disease, UCB, Novartis and Janssen—consulting fees. S. Lehnerer: Ad Scientiam, Alexion, AstraZeneca Rare Disease, argenx, Biogen, Hormosan, HUMA, Johnson & Johnson, Merck, UCB and Roche—speaker and consulting fees, research grants RareLink digital health GmbH—shareholder. S. Bieuvelet, L. Carment, C. Gorin, N. Pesic‐Heuvrard, D. Ravindra, N. Sellami and S. Zinaï: employees of Ad Scientiam; may hold stock and/or stock options. B.Dutta, B. Yungher: employees of Alexion, AstraZeneca Rare Disease; may hold stock and/or stock options. J.F.Howard Jr : Ad Scientiam, Alexion AstraZeneca Rare Disease, argenx, Cartesian Therapeutics, Centers for Disease Control and Prevention, Merck EMD Serono, MGFA, Muscular Dystrophy Association, NIH, NMD Pharma, and UCB Pharma—research grants. AcademicCME, Alexion AstraZeneca Rare Disease, Amgen, argenx, Biohaven Ltd, Cartesian Therapeutics, CheckRare CME, CoreEvitas, Curie.bio, H. Lundbeck A/S, Japan Tobacco Company, Kyverna Therapeutics, Merck EMD Serono, NMD Pharma, Novartis Pharma, PeerView CME, Physicians' Education Resource (PER) CME, PlatformQ CME, Regeneron Pharmaceuticals, Seismic Therapeutics, TG Therapeutics, Toleranzia AB, and UCB Pharma—consulting fees. Alexion AstraZeneca Rare Disease, argenx, Biohaven Ltd, Cartesian Therapeutics, Toleranzia AB, and UCB Pharma━ non‐financial support.

## EPO‐0639

### Minimal symptom expression with claseprubart, an active C1S inhibitor, in patients with generalized myasthenia gravis

#### M. Smilowski ^1^; N. Gilhus^2^; M. Ait‐Tihyaty^3^; C. Briggs^3^; U. Siddiqui^3^; L. Hickey^3^; M. Lalla^3^; S. Beydoun^4^

##### ^1^Neurologia Śląska Centrum Medyczne, Katowice, Poland; ^2^Haukeland University Hospital, Bergen, Norway; ^3^Dianthus Therapeutics, New York, USA; ^4^University of Southern California, Los Angeles, USA

**Background and aims:** The classical complement pathway plays a significant role in generalized myasthenia gravis (gMG) pathology. Claseprubart is a potent monoclonal antibody that selectively targets the classical pathway by inhibiting active C1s (aC1s). Remission is a key goal in gMG and achieving minimal symptom expression (MSE) with claseprubart is a key treatment objective.

**Methods:** MaGic (NCT06282159), is a global Phase 2, randomized, double‐blind, placebo‐controlled trial. Patients treated with claseprubart (300 mg, Q2W) were evaluated for MSE (MG‐ADL ≤1 or QMG ≤3) compared with placebo. *p*‐Values were one‐sided and nominal significance was assessed at an alpha = 0.1. Outcomes include proportion of patients achieving MSE at Week 13 and anytime during the study, earliest timepoint of MSE achievement, and durability defined as sustained MSE for at least 6 weeks.

**Results:** At Week 13, 37% of claseprubart treated patients achieved MG‐ADL‐MSE versus 14% with placebo (OR 3.81; *p* = 0.0550). 43% achieved MG‐ADL‐MSE at least once during the study versus 14% of placebo (OR: 4.72; *p* = 0.0231). This effect was observed as early as Week 1 (median time to response was Week 3). Sustained remission‐like states (for at least 6 weeks) were seen in all patients who achieved MSE. Similar results were observed using QMG minimum symptom expression threshold.

**Conclusion:** Claseprubart patients were more than four times more likely to achieve MSE than placebo patients and MSE was observed as early as week 1. Patients who achieved MSE maintained responses for at least 6 weeks. These results highlight the therapeutic potential of aC1s inhibition in AChR+ gMG.

**Disclosure:** MS nothing to declare. NG grants or honoraria from Dianthus, Amgen, Alexion, Argenx, UCB, Novartis, J&J, Medison, NMD Pharma, Lundbeck, and Merck. MA employee of Dianthus Therapeutics CB employee of Dianthus Therapeutics US employee of Dianthus Therapeutics LH employee of Dianthus Therapeutics ML employee of Dianthus Therapeutics SB nothing to declare.

## EPO‐0640

### Myasthenia Gravis Inebilizumab Trial (MINT): Adverse events of special interest during the randomized controlled period (RCP)

#### R. Nowak ^1^; K. Utsugisawa^2^; M. Benatar^3^; E. Ciafaloni^4^; M. Leite^5^; J. Vissing^6^; F. Tang^7^; B. Canales^7^; Q. Li^7^; S. Cheng^7^; J. Howard Jr.^8^

##### ^1^Yale University, New Haven, USA; ^2^Hanamaki General Hospital, Hanamaki, Japan; ^3^University of Miami Miller School of Medicine, Miami, USA; ^4^University of Rochester, Rochester, USA; ^5^University of Oxford, Oxford, UK; ^6^University of Copenhagen, Copenhagen, Denmark; ^7^Amgen Inc, Thousand Oaks, USA; ^8^University of North Carolina, Chapel Hill, USA

**Background and aims:** B cells play a key upstream mechanistic role in autoimmune generalized myasthenia gravis (gMG) pathogenesis through the production of antibodies targeting the acetylcholine receptor (AChR+), muscle‐specific kinase (MuSK+), or other related proteins. Inebilizumab is a monoclonal antibody targeting CD19+ B cells. B‐cell‐depleting therapies may impair humoral responses, increase the risk of infections and reduced response to vaccinations. Here we examine whether inebilizumab is associated with an increased risk of adverse events of special interest (AESIs) in gMG participants.

**Methods:** MINT (NCT04524273) was a phase 3 trial in adults with gMG. During the RCP (52‐weeks for AChR+; 26‐weeks for MuSK+), participants were randomized (1:1) to receive 300mg of inebilizumab or placebo on Day‐1, Day‐15, and Day‐183 (AChR+ only). Subsets of adverse events were identified as AESIs due to their relevance to inebilizumab's mechanism of action.

**Results:** 238 patients were randomized: inebilizumab 119 (95 AChR+, 24 MuSK+), placebo 119 (95 AChR+, 24 MuSK+). Baseline demographics and disease characteristics were generally balanced between treatment groups. AESIs were infrequent, occurring in 10.1% (*n* = 12) of the inebilizumab‐ and 16% (*n* = 19) of the placebo‐treated groups. Cytopenias occurred in 2.5% (*n* = 3) of inebilzumab‐treated and 9.2% (*n* = 11) of placebo‐treated participants. Serious and/or opportunistic infections were infrequent (inebilizumab: 3.4% [*n* = 4]; placebo: 5.0% [*n* = 6]), with COVID‐19 being the most common (inebilizumab: 2/4; placebo: 3/6) and no instances of progressive multifocal leukoencephalopathy. Infusion related reactions occurred in 5.0% (*n* = 6) of inebilizumab‐ and 2.5% (*n* = 3) of placebo‐treated participants (Table 1).

**Figure:**
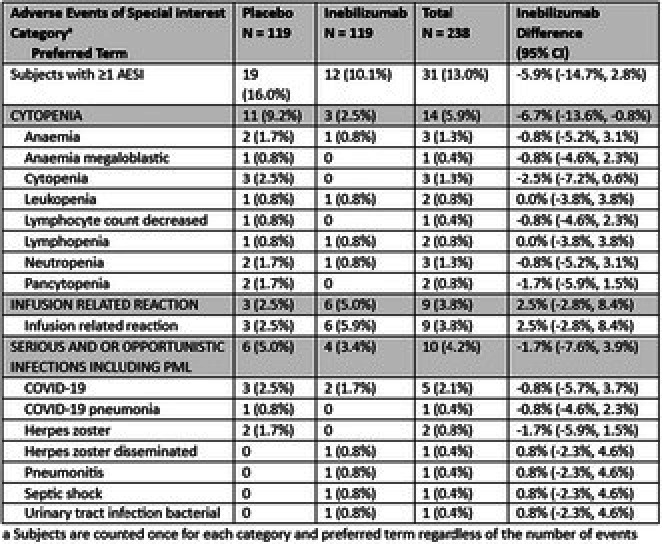
**TABLE 1** Treatment‐emergent adverse events of special interest.

**Conclusion:** These results support a favorable safety profile of inebilizumab over a 26–52‐week period.

**Disclosure:** RJN received research support from Alexion Pharmaceuticals, Amgen Inc, argenx, Grifols, Immunovant, Janssen, Myasthenia Gravis Foundation of America, the National Institutes of Health, and UCB and served as a consultant/adviser for Alexion Pharmaceuticals, Amgen Inc, argenx, Cabaletta Bio, Cour Pharmaceuticals, CSL Behring, Grifols, Immunovant, Janssen, and UCB. KU served as a paid consultant for argenx, Chugai Pharmaceutical, HanAll BioPharma, Janssen, Merck, Mitsubishi Tanabe Pharma, UCB, and Amgen Inc. and received speaker honoraria from Alexion Pharmaceuticals, argenx, the Japan Blood Products Organization, and UCB. MB received grant support from the ALS Association, the ALS Recovery Fund, Eli Lilly, the Kimmelman estate, the Muscular Dystrophy Association, the National Institutes of Health, and Target ALS. He reports research support from Alexion Pharmaceuticals and Immunovant and served as an investigator for clinical trials funded by Biogen, Novartis, and Orphazyme. He received personal consulting fees from Alaunos Therapeutics, Alector Therapeutics, Alexion Pharmaceuticals, Annexon Biosciences, Arrowhead Pharmaceuticals, Canopy Immunotherapeutics, CorEvitas, Denali Therapeutics, Eli Lilly, Evox Therapeutics, Amgen Inc, Immunovant, Janssen, Merck, Novartis, Roche, Sanofi, Takeda, Trace Neuroscience, UCB, uniQure, VectorY Therapeutics, Voyager Therapeutics, and Woolsey Pharmaceuticals. EC has no disclosures. MIL received funding from the National Health Service (MRDS and NSCG for NMOSD) and the University of Oxford. She was awarded research grants from the UK association for patients with myasthenia (myaware) and the University of Oxford. She received speaker honoraria/travel grants from the Guthy‐Jackson Charitable Foundation and UCB. She served on scientific/educational advisory boards for argenx, UCB, and Amgen Inc. JV has been a consultant for and/or received speaker honoraria from Alexion Pharmaceuticals, Amgen Inc, argenx, Dianthus Therapeutics, Johnson & Johnson, NMD Pharma, Novartis, Regeneron Pharmaceuticals, Roche, Toleranzia AB, and UCB. FT, BC, QL, and SC are employees of Amgen Inc. and own stock. JFH received research funding (paid to his institution) from Ad Scientiam, Alexion AstraZeneca Rare Disease, argenx, Cartesian Therapeutics, the Centers for Disease Control and Prevention, EMD Serono, Merck, the Muscular Dystrophy Association, Myasthenia Gravis Foundation of America, the National Institutes of Health, NMD Pharma, and UCB; honoraria/consulting fees from AcademicCME, Alexion AstraZeneca Rare Disease, Amgen Inc, Biohaven, CheckRare CME, CorEvitas, Curie.Bio, EMD Serono, Hansa Biopharma, Lundbeck, Merck, Novartis, PeerView CME, Physicians’ Education Resource CME, PlatformQ CME, Regeneron Pharmaceuticals, Sanofi, Seismic Therapeutics, TG Therapeutics, Toleranzia AB, and UCB; and nonfinancial support from Alexion AstraZeneca Rare Disease, argenx, Biohaven, Cartesian Therapeutics, Toleranzia AB, and UCB.

## EPO‐0641

### Efgartigimod in acetylcholine receptor antibody seronegative generalized myasthenia gravis: ADAPT SERON initial results

#### S. Hoffmann ^1^; J. F. Howard^2^; T. Vu^3^; C. Zhao^4^; S. Luo^4^; K. G. Claeys^5^; R. H. Jimenez^6^; I. Seghers^6^; D. Masschaele^6^; J. T. Guptill^6^; W. Huang^6^; Ł. Rzepiński^7^; E. Chroni^8^; A. Alshehri^9^; A. Breiner^10^

##### ^1^Department of Neurology and Neuroscience Clinical Research Center, Charité – Universitätsmedizin Berlin, Berlin, Germany; ^2^Department of Neurology, The University of North Carolina, Chapel Hill, North Carolina; ^3^Department of Neurology, University of South Florida Morsani College of Medicine, Tampa, Florida; ^4^Department of Neurology, Huashan Hospital, Fudan University, Shanghai, China; ^5^Department of Neurology, University Hospitals Leuven, Leuven, Belgium; Laboratory for Muscle Diseases and Neuropathies, KU Leuven, Leuven, Belgium; ^6^argenx, Ghent, Belgium; ^7^Department of Neurology; 10th Military Research Hospital and Polyclinic, Bydgoszcz, Poland; Department of Clinical Medicine, Faculty of Medicine, University of Science and Technology, Bydgoszcz, Poland; ^8^Department of Neurology, University Hospital of Patras, Rion, Greece; ^9^Department of Neurology, King Faisal Specialist Hospital and Research Centre, Riyadh, Saudi Arabia; ^10^Division of Neurology, The Ottawa Hospital/The University of Ottawa, Ottawa, Ontario, Canada

**Background and aims:** Approximately 15%–20% of patients with generalized myasthenia gravis (gMG) are acetylcholine receptor antibody (AChR‐Ab) seronegative. Efgartigimod is a human immunoglobulin G1 (IgG1) antibody Fc fragment that reduces IgG levels (including pathogenic autoantibodies) via neonatal Fc receptor blockade. ADAPT SERON is a Phase 3 trial investigating the efficacy and safety of intravenous (IV) efgartigimod in adults with AChR‐Ab seronegative gMG.

**Methods:** Diagnosis of gMG was confirmed by an MG diagnostic adjudication committee. In the double‐blinded, placebo‐controlled Part A, adult participants were randomized 1:1 to receive 4 once‐weekly infusions of 10 mg/kg efgartigimod IV or placebo followed by a 5‐week follow‐up period. Part B includes an ongoing open‐label extension (≤2 years).

**Results:** Results included 119 participants (*n* = 40, MuSK‐Ab+; *n* = 6, LRP4‐Ab+; *n* = 73, triple seronegative), 58 received efgartigimod IV, and 61 received placebo. Change in Myasthenia Gravis Activities of Daily Living (MG‐ADL) total score from baseline to week 4 (primary endpoint) was significantly (*p* = 0.007) different between efgartigimod IV and placebo groups (overall population), with least squares mean change from baseline (90% CI) of −3.35 (−3.98 to −2.72) and −1.90 (−2.51 to −1.28), respectively. Clinically meaningful improvement in mean MG‐ADL total score with efgartigimod IV (overall population) was observed in Part A and continued over subsequent cycles in Part B. Efgartigimod IV was well tolerated, with no new safety signals observed. Results from additional analyses will be presented at EAN 2026.

**Figure:**
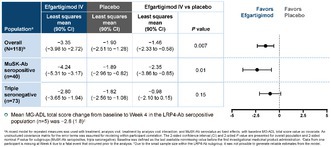
**FIGURE 1** MG‐ADL total score changes from baseline to Week 4.

**Figure:**
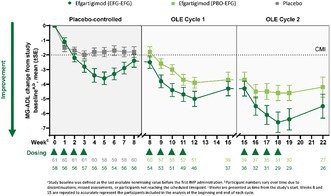
**FIGURE 2** MG‐ADL total score changes from study baseline over time‐overall population.

**Figure:**
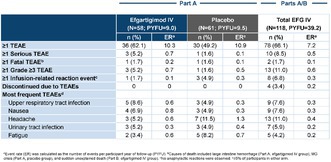
**TABLE 1** Summary of treatment emergent adverse events (TEAEs)‐overall population.

**Conclusion:** Efgartigimod IV demonstrated statistically significant improvement in MG‐ADL total score compared with placebo and was well tolerated in AChR‐Ab seronegative gMG.

**Disclosure:** This study was sponsored by argenx. RHJ, IS, DM, JTG, and W‐YH are employees of argenx. SH, JFH, TV, CZ, SL, KGC, LR, EC, AA, and AB have reported financial/nonfinancial relationships with argenx at the time of submission.

## EPO‐0642

### Effect of nipocalimab on pathogenic IgG and complement markers in patients with seropositive gMG: Exploratory analysis in Vivacity‐MG3 study

#### S. Miller^1^; J. Xi^2^; Y. Zhang^2^; R. Faelens^3^; M. Fitzgibbon^4^; S. Ramchandren^5^; S. Hoffmann ^6^

##### ^1^Johnson & Johnson, San Diego, USA**;** ^2^Johnson & Johnson, Spring House, USA; ^3^Johnson & Johnson, Beerse, Belgium; ^4^Johnson & Johnson, Raritan, USA; ^5^Johnson & Johnson, Titusville, USA; ^6^Neurology, Charité ‐ Universitätsmedizin Berlin, Berlin, Germany

**Background and aims:** Immunoglobulin (Ig)G class autoantibodies block and internalize the acetylcholine receptor (AChR) and activate complement at the neuromuscular junction, leading to symptoms of myasthenia gravis. Nipocalimab, a fully human neonatal‐Fc‐receptor blocker, substantially reduced circulating IgG, including pathogenic subclasses, and demonstrated sustained efficacy versus placebo during 24‐week Vivacity‐MG3 study (NCT04951622). This post‐hoc analysis evaluated whether nipocalimab reduces circulating complement components and activity indicative of complement activation, in Vivacity‐MG3.

**Methods:** Available serum samples from seropositive (positive for AChR/muscle‐specific receptor tyrosine kinase [MuSK]/Low‐density Lipoprotein Receptor‐related Protein‐4 [LRP4]) participants were analysed at baseline and Week‐24 and least square means were compared between placebo and nipocalimab using linear mixed model. Complement markers measured were: C3d circulating immune complexes (C3d‐CIC), C1q immune complex binding (C1q‐ICB), and total complement activity (CH50). Parallel assays quantified total IgG, IgG1/2/3, and anti AChR titers, which informed a nonlinear mixed‐model previously presented.

**Results:** Baseline levels of complement markers were comparable across nipocalimab and placebo groups (Table‐1). At Week‐24, nipocalimab was associated with substantial and sustained reductions in IgG1/2/3, and anti AChR titers (Table‐2). Concomitant decreases were observed in selected immune complex–associated complement markers (C3d‐CIC and C1q‐ICB, *p <* 0.05); no significant change in CH50 was detected (Table 1).

**Figure:**
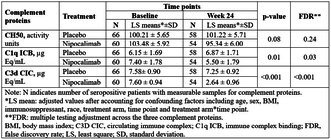
**TABLE 1** Characteristics of complement protein measurements among seropositive patients.

**Figure:**
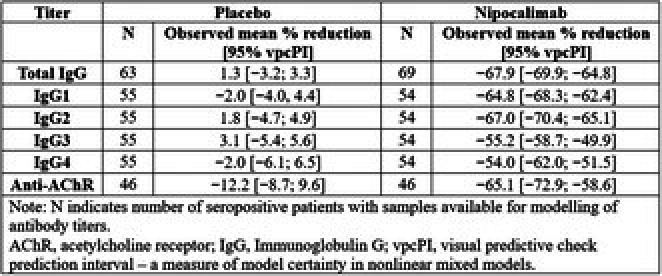
**TABLE 2** Relative change from baseline of IgG and anti‐AChR antibodies in seropositive patients with gMG at week 24.

**Conclusion:** Nipocalimab led to sustained reductions in IgG subclasses and anti‐AChR titers, accompanied by significant decreases in selected circulating immune complex–associated complement markers, while no significant change in total complement activity was observed. These data indicate changes in selected complement‐related markers following nipocalimab treatment; further exploration is needed to better understand the clinical relevance.

**Disclosure:** Sarah Hoffmann: Speakers´ honoraria: Alexion, argenx, Grifols, Johnson & Johnson, Kyverna, Roche, UCB; honoraria/attendance at advisory boards: Alexion, argenx, Johnson & Johnson, Kyverna, Novartis, Roche; member of the medical advisory board: the German Myasthenia Society, DMG. Silke Miller, Yanfei Zhang, Jingyue Xi, Ruben Faelens, Marie Fitzgibbon, and Sindhu Ramchandren: Employees of Johnson & Johnson and may hold stock or stock options in the company.

## Neurogenetics 4

## EPO‐0643

### Phenotypic and genotypic characterization of STUB1 variants in Portuguese pedigrees and analysis of TBP repeat expansions

#### C. Guedes Vaz ^1^; M. Seco^2^; G. Madureira^3^; R. Castro^4^; S. Costa^1^; J. Freixo^5^; J. Oliveira^5^; P. Salgado^2^; M. Calejo^2^; M. Santos^5^; J. Damásio^1^

##### ^1^Neurology Service, Santo Antonio Local Health Unit, Porto, Portugal; ^2^Neurology Service, Matosinhos Local Health Unit, Porto, Portugal; ^3^Neuroradiology Service, Santo Antonio Local Health Unit, Porto, Portugal; ^4^Neurology Service, Local Health Unit of Entre Douro e Vouga, Santa Maria da Feira, Portugal; ^5^Institute of Molecular and Cellular Biology, University of Porto, Porto, Portugal

**Background and aims:** SCA48 (ATX‐STUB1) is an adult‐onset ataxia caused by heterozygous pathogenic variants in STUB1. Recently, families carrying pathogenic STUB1 variants and intermediate alleles in TBP gene have been described, raising the hypothesis of STUB1/TBP digenic inheritance or phenotypic modulation by TBP intermediate alleles. This study aimed to characterize the clinical, genetic and neuroimaging features of Portuguese families with SCA48 and analyze TBP repeat expansions.

**Methods:** SCA48 patients were identified through two sources: (a) Portuguese population‐based survey on hereditary ataxias and (b) hospital‐based cohorts from Northern Portugal. TBP repeat length was analyzed in all patients except one (deceased). Genetic, clinical and neuroimaging data were collected according to a standardized protocol.

**Results:** Seven pedigrees comprising 18 patients were identified, four from Northern Portugal and three from South. Five distinct STUB1 variants were detected: one in‐frame deletion, one frameshift, two missense, and one nonsense variant. TBP intermediate alleles were present in 2 families. Mean age at onset was 41.5 years (range 28–64). Initial manifestations included gait ataxia (*n* = 12), chorea (*n* = 1), and diplopia (*n* = 1). All patients developed cerebellar syndrome. Additional features included cognitive decline (*n* = 7), depressive symptoms, chorea, and pyramidal signs (*n* = 6, each). Neuroimaging revealed cerebellar vermian and hemispheric atrophy (*n* = 8) and caudate nucleus atrophy (*n* = 3). Twelve patients died after a mean disease duration of 17 ± 10.2 years.

**Conclusion:** Most Portuguese SCA48 pedigrees lacked TBP intermediate alleles, supporting a primary pathogenic role of heterozygous STUB1 variants. SCA48 presented as a complex adult‐onset ataxia with prominent neuropsychiatric features and chorea.

**Disclosure:** Nothing to disclose.

## EPO‐0644

### A new loss of function mutation in KCNMA1 is associated with an hyperkinetic disorder, psychiatric disturbances and a distinct metabolic profile

#### D. Fontanesi ^1^; G. Saccardin^2^; E. Monfrini^3^; V. Yayha^3^; M. Bisquoli^1^; S. Coniglio^2^; A. Di Fonzo^3^; V. Rispoli^2^

##### ^1^Department of Biomedical, Metabolic and Neural Science, University of Modena and Reggio Emilia, Modena, Italy; ^2^Neurology Unit, Neuroscience Head Neck Department, Ospedale Civile Baggiovara, Azienda Ospedaliero‐Universitaria di Modena, Modena, Italy; ^3^Foundation IRCCS Ca' Granda Ospedale Maggiore Policlinico, Neurology Unit, Milan, Italy

**Background and aims:** KCNMA1 encodes one subunit of the BK large conductance voltage and Ca2+‐dependent K+ channel. Mutations in KCNMA1 are associated with paroxysmal non‐kinesigenic dyskinesias (PNKD) and generalized epilepsy due to a gain of function (GOF) mechanism. Otherwise, loss of function (LOF) mutations have been described in Liang Wang syndrome (LIWAS), a neurological and systemic disorder characterized by a large phenotypic variability and severity. The phenotypic spectrum may vary from severe developmental delay, craniofacial dysmorphism and visceral tissue abnormalities to milder signs such as ataxia, nystagmus or strabismus. Defining non‐canonical LOF phenotypes is critical to distinguish emerging multisystemic presentations from established LIWAS or epilepsy/PNKD spectrums.

**Methods:** An index case and two relatives were evaluated due to presence of an hyperkinetic movement disorder of upper limb and psychiatric symptoms. A subsequent WES‐based interrogation utilizing a virtual movement disorder panel revealed a shared mutation in KCNMA1. The mutation was analysed through online tools and classified as likely pathogenic.

**Figure:**
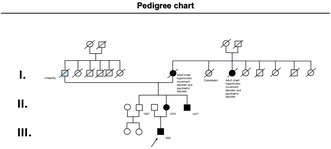
**FIGURE 1** Pedigree chart.

**Figure:**
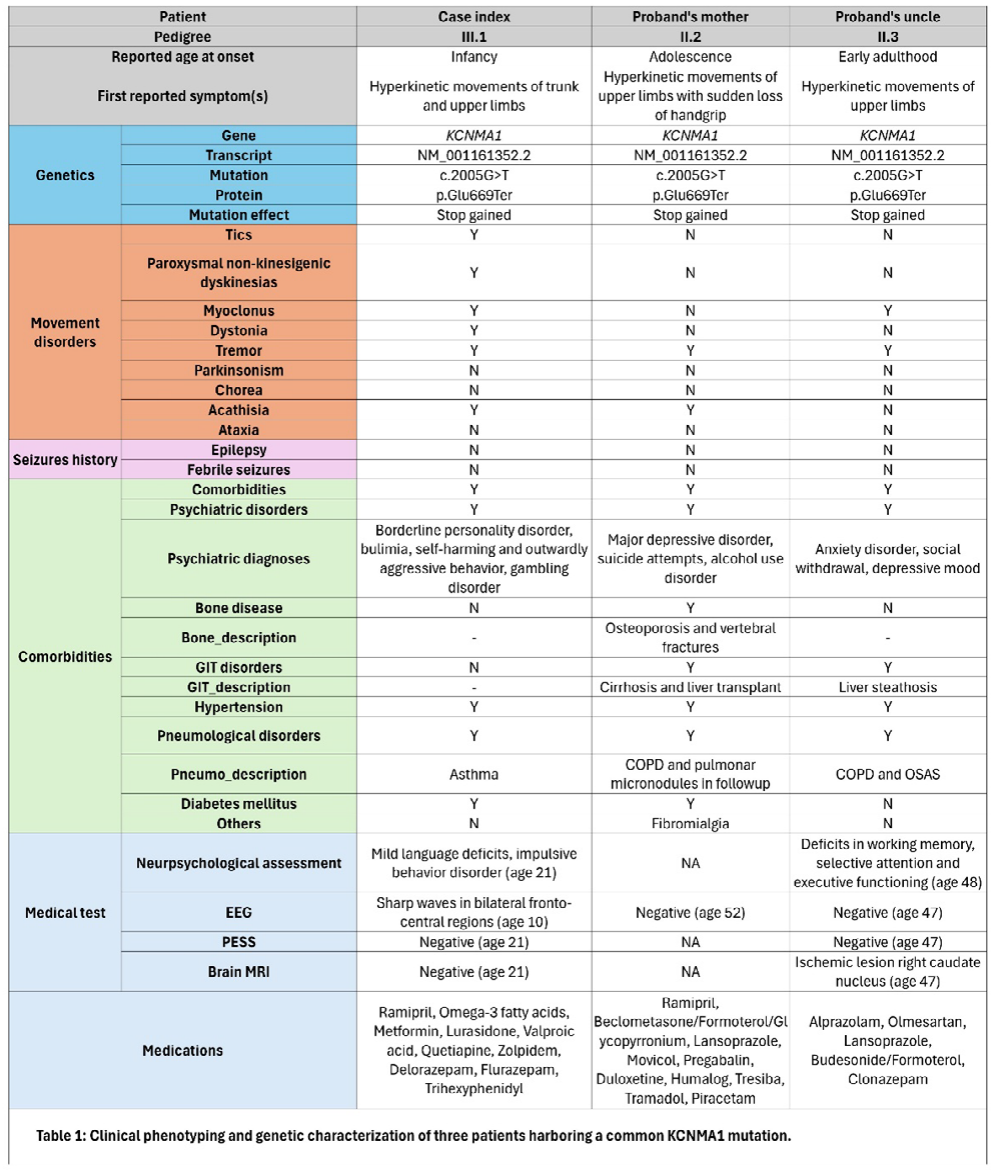
**TABLE 1** Clinical and genetic characterisation of three patients harbouring a KCNMA1 mutation.

**Results:** We identified a novel heterozygous nonsense variant in KCNMA1 (NM_001161352.2) [c.2005G>T p.Glu669Ter]. The phenotype manifests as upper limb hyperkinetic movements, psychiatric disturbances, and a shared metabolic phenotype characterized by chronic bronchial involvement (asthma/COPD), diabetes mellitus and liver disease, expanding the previous phenotype of KCNMA1 LOF‐related disorders.

**Conclusion:** We present a pedigree with a LOF likely pathogenic mutation in KCNMA1, manifesting with the coexistence of movement disorders, psychiatric disturbances and a distinct metabolic profile.

**Disclosure:** Nothing to disclose.

## EPO‐0645

### Clinical experience with omaveloxolone in Friedreich's ataxia in Portugal (OMAF‐PT): Establishment of a prospective national registry

#### J. Damásio ^1^; V. Carvalho^2^; M. Malaquias^3^; T. Morais^4^; A. Fernandes^5^; C. Marques‐Matos^6^; D. Fitas^7^; F. Godinho^8^; J. Lourenço^8^; C. Vaz^1^; S. Costa^1^; R. Barreto^2^; E. Lourenço^9^; M. Gago^10^; J. Martins^11^; A. Velon^12^; R. Lobato^13^; A. Sousa^14^; J. Massano^5^; J. Durães^4^; L. Correia Guedes^2^

##### ^1^Neurology Service, Santo António Hospital, Santo António University Hospital Centre, ULS Santo António, Porto, Portugal; ^2^Neurology Service, Santa Maria Hospital, Lisboa Norte University Hospital Centre, ULS Santa Maria, Lisboa, Portugal; ^3^Neurology Service, Vila Nova de Gaia University Hospital Centre, ULS VNGaia, VNGaia, Portugal; ^4^Neurology Service, Coimbra University Hospital Centre, ULS de Coimbra, Coimbra, Portugal; ^5^Neurology Service, São João e RISE‐HEALTH University Hospital Centre – Department of Neuroscience and Mental Health, Faculty of Medicine, University of Porto; ^6^Serviço de Neurologia Pediátrica, Hospital D Estefânia, ULS S. José, Lisbon, Portugal; ^7^Serviço de Neurologia, Hospital de Pedro Hispano, ULS Matosinhos, Matosinhos, Portugal; ^8^Neurology Service, Hospital de S. José, Centro Hospitalar Lisboa Central, ULS S. José, Lisbon, Portugal; ^9^Neurology Service, Hospitalar de Braga, ULS Braga, Braga, Portugal; ^10^Neurology Service, Hospitalar da Senhora da Oliveira, ULS Alto Ave, Guimarães, Portugal; ^11^Serviço de Neurologia Pediátrica, Centro Materno Infantil Norte, Centro Hospitalar Universitário de Santo António, ULS Santo António, Porto, Portugal; ^12^Neurology Service, Centro Hospitalar de Tras‐os‐Montes e Alto Douro, ULS de Tras‐os‐Montes e Alto Douro, Vila Real, Portugal; ^13^Neurology Service, ULS de Alto Minho, Viana do Castelo, Portugal; ^14^Neurology Service, Centro Hospitalar Entre Douro e Vouga, ULS Entre Douro e Vouga, Santa Maria da Feira, Portugal

**Background and aims:** Omaveloxolone, an activator of Nrf2, has been approved by EMA for the treatment of Friedreich ataxia (FA) in patients aged ≥16 years. In Portugal, it was initially provided through a limited early access program from July 2024 to January 2025 (pending central approval), while reimbursement negotiations were ongoing. This study aimed to establish a national, prospective registry to systematically evaluate real‐world effectiveness and safety in Portuguese FA patients receiving omaveloxolone.

**Methods:** Neurologists from 31 Portuguese hospitals were invited to participate. Study documentation was submitted for approval by ethics committees. Prospective assessments included mFARS, SARA, and FA‐ADL at baseline, every 3 months during the first year, followed by 6‐monthly assessments. Laboratory parameters and adverse events were monitored monthly during the first 3 months, quarterly until the end of the first year, and semiannually thereafter. Data were collected using REDCap.

**Results:** Fifteen hospitals (48.4%) agreed to participate, of which 13 actively followed FA patients. In Portugal, all genetically confirmed FA patients aged ≥16 years were eligible for treatment, irrespective of functional staging, provided no contraindications were present. A total of 67 patients were included: 39 (58.2%) receiving omaveloxolone, 27 (40.3%) waiting treatment initiation pending reimbursement negotiations, and 1 (1.5%) with cardiac contraindications to treatment. Two patients discontinued omaveloxolone during follow‐up.

**Conclusion:** This registry demonstrates the feasibility of nationwide, prospective real‐world monitoring of omaveloxolone in FA. While early access enabled treatment initiation in over half of eligible patients, a substantial proportion remained untreated. Continued follow‐up will be essential to evaluate long‐term safety and clinical outcomes as treatment availability expands.

**Disclosure:** Nothing to disclose.

## EPO‐0646

### Cortical metabolic signatures of visuospatial and gait dysfunction in GBA1‐related Parkinson's disease

#### L. Gallo ^1^; S. Caminiti^1^; P. Mitrotti^2^; R. Calabrese^2^; M. Picascia^2^; R. Malito^3^; T. Filidei^1^; A. Panzacchi^3^; A. Samanes Gajate^3^; G. Pepe^3^; F. Cavallieri^4^; G. Di Rauso^5^; V. Fioravanti^4^; D. Perani^3^; A. Chiti^3^; E. Valente^6^; M. Avenali^1^

##### ^1^Department of Brain and Behavioural Sciences, University of Pavia, Pavia, Italy; ^2^IRCCS Mondino Foundation, Pavia, Italy; ^3^IRCCS San Raffaele Scientific Institute, Milan, Italy; ^4^Neurology Unit, Neuromotor and Rehabilitation Department, Azienda USL‐IRCCS di Reggio Emilia, Reggio Emilia, Italy; ^5^Department of Biomedical, Metabolic and Neural Sciences, University of Modena and Reggio Emilia, Modena, Italy; ^6^Department of Molecular Medicine, University of Pavia, Pavia, Italy

**Background and aims:** GBA1 variants are the most common genetic risk factor for Parkinson's disease and are associated with an increased risk of cognitive decline. However, their impact on brain metabolism and related cognitive and motor features remains poorly understood: how visuospatial deficits reflect posterior cortical dysfunction and how these changes relate to other clinical manifestations? This study investigates cerebral metabolism and its association with clinical features in PD patients with and without GBA1 variants.

**Methods:** 38 GBA‐PD and 46 non‐GBA‐PD underwent clinical, neuropsychological, and FDG‐PET evaluation. Posterior cortical hypometabolism was quantified by single‐subject SPM‐analysis, and its association with visuospatial, motor, and non‐motor features was assessed using Spearman correlations.Visuospatial function was assessed using a standardized posterior visuospatial composite score (PAROVIS).

**Results:** Compared with non‐GBA‐PD, GBA‐PD patients were younger, had earlier disease onset, greater motor disability (including fluctuations and freezing of gait), higher depression, and worse quality of life, despite similar disease duration and education. FDG‐PET revealed greater posterior cortical hypometabolism in GBA‐PD. Cognitively, GBA‐PD showed poorer visuospatial and executive performance, while other cognitive domains were comparable to non‐GBA‐PD. Within GBA‐PD, the PAROVIS score correlated with posterior hypometabolism and freezing of gait severity, an association absent in non‐GBA‐PD (α = 0.77).

**Conclusion:** Visuospatial dysfunction is a core cognitive feature of GBA‐PD, associated with posterior cortical hypometabolism and gait impairment, even without global cognitive decline. These findings support a distinct posterior cortical–cognitive phenotype in GBA‐PD, while longitudinal validation is needed to establish FDG‐PET and visuospatial measures as reliable tools for monitoring disease progression and patient stratification.

**Disclosure:** Nothing to disclose.

## EPO‐0647

### Dystonia in Mowat‐Wilson syndrome: A yet underrecognized neurological feature

#### L. Kunc ^1^; P. Havránková^1^; M. Škorvánek^2^; J. Necpál^3^; J. Kothaj^3^; M. Zech^4^; R. Jech^1^

##### ^1^Department of Neurology and Center of Clinical Neuroscience, First Faculty of Medicine, Charles University and General University Hospital in Prague, Czech Republic; ^2^Department of Neurology, University Hospital L. Pasteur, Kosice, Slovakia; ^3^Department of Neurology, Zvolen Hospital, Zvolen, Slovakia; ^4^Institute of Human Genetics, Technical University of Munich, Munich, Germany

**Background and aims:** Mowat–Wilson syndrome (MOWS) is a rare neurodevelopmental disorder caused by heterozygous pathogenic variants in the ZEB2 gene. The neurological phenotype is characterized by intellectual disability, epilepsy, and characteristic craniofacial features. Based on the current literature, more than 300 individuals with MOWS have been reported. To date, only a single case report has documented dystonia in MOWS. The true frequency and clinical relevance of dystonia in MOWS therefore remain unclear.

**Methods:** We retrospectively identified three unrelated patients with genetically confirmed MOWS through a whole‐exome sequencing (WES) project focused on individuals with dystonia. All patients were evaluated by a movement disorder specialist. Detailed clinical, genetic, and neuroimaging data were reviewed and compared with available literature.

**Results:** The case series included two females and one male, aged 5–21 years, all of whom exhibited dystonia. The onset of dystonia occurred between 3 and 18 years of age. Two patients developed generalized dystonia while one presented with a segmental type. WES identified heterozygous de novo ZEB2 variants in all cases: two missense variants classified as likely pathogenic and one frameshift variant classified as pathogenic. All patients showed a typical MOWS phenotype, including intellectual disability and distinctive craniofacial features. Epilepsy was present in one patient.

**Conclusion:** Our findings demonstrate that dystonia can occur in Mowat–Wilson syndrome and may represent an underrecognized feature of its neurological phenotype. Awareness of movement disorders in patients with ZEB2 mutations is important for accurate phenotyping and may facilitate earlier diagnosis, particularly in individuals with developmental delay and dysmorphic features.

**Disclosure:** The study was supported by the Czech Ministry of Health under grant AZV ČR No. NW24‐04‐00067. LK, PH, and RJ were supported by the institutional research: General University Hospital in Prague project MH CZ‐DRO‐VFN64165. MS and JN received support from the EU Renewal and Resilience Plan “Large projects for excellent researchers” under grant No. 09I03‐03‐V03‐0000.

## EPO‐0648

### Therapeutic impact of iron chelation in PKAN and related NBIA subtypes: A systematic review of 552 cases

#### J. Pineda^1^; P. Miranda^1^; C. Peralta^1^; E. Reyes^1^; E. Patricio^1^; A. Reyes^1^; A. Tejada^1^; K. Yorro^1^; C. Pérez^1^; M. Rodríguez ^2^

##### ^1^Synaptic Research League, Santo Domingo, Dominican Republic; ^2^Neurology Department, Hospital Universitario Salvador Bienvenido Gautier, Santo Domingo, Dominican Republic

**Background and aims:** Neurodegeneration with Brain Iron Accumulation type 1 (NBIA1), or Pantothenate Kinase‐Associated Neurodegeneration, is a rare inherited neurodegenerative disorder characterized by iron deposition in the basal ganglia [1]. It affects fewer than one per million individuals. Iron chelation therapy has shown heterogeneous radiological and clinical outcomes [1].

**Methods:** A systematic review of the literature was performed in accordance with PRISMA guidelines. PubMed, BVS and Google Scholar were searched using the keywords “Neurodegeneration with Brain Iron”, “Accumulation”, “NBIA1”, “Chelation Therapy”, “Deferiprone”.

**Results:** A total of 552 patients with NBIA with a mean age of 22.1 years were included. Pantothenate kinase‐associated neurodegeneration (PKAN) was the predominant subtype accounting for 535 of the patients (96.9%); followed by other subtypes including MPAN (*n* = 2), BPAN (*n* = 1), neuroferritinopathy (*n* = 4), VAC14‐related NBIA (*n* = 1) and idiopathic NBIA cases (*n* = 9). 195 of the cases corresponded to males (53.6%) and 169 to females (46.4%). Defiprone was the most frequently used chelator, used at doses of 30 mg/kg/day varying in treatment duration from 17 weeks to 2 years. Reduction in brain iron was demonstrated radiologically in most cohorts leaning to stabilization and improvement with minimal reports of reversal. Hematologic events were reported as the main adverse effects accounting to 21% in the randomized data, the main cause of treatment discontinuation.

**Figure:**
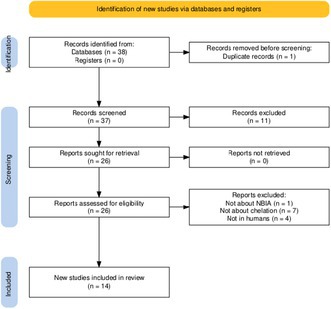
Prisma‐P flowchart of the process of inclusion of studies in the systematic review.

**Figure:**
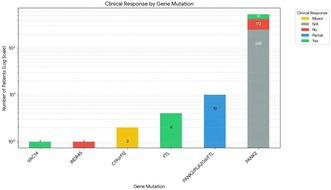
Clinical response by gene mutation in iron chelation in PKAN and related NBIA subtypes.

**Conclusion:** Iron chelation therapy with Deferipone is associated with reduced brain iron and clinical stabilization in most NBIA1 patients, although neurological reversal is uncommon. Hematologic adverse effects limit treatment, emphasizing the need for safer long‐term therapies and standardized outcome measures.

**Disclosure:** Nothing to disclose.

## EPO‐0649

### Clinical profile and treatment options in Nasu‐Hakola syndrome: Systematic literature review

#### J. Baez^1^; K. Pérez^1^; S. Campusano^1^; A. Rosario^1^; *P*. Tejada^1^; C. Michel^1^; P. Miranda^1^; A. Tejada^1^; K. Yorro^1^; C. Pérez Lizardo^1^; A. Reyes^1^; M. Rodríguez ^2^

##### ^1^Synaptic Research League, Santo Domingo, Dominican Republic; ^2^Neurology Department, Hospital Universitario Salvador Bienvenido Gautier, Santo Domingo, Dominican Republic

**Background and aims:** Nasu‐Hakola disease, or polycystic lipomembranous osteodysplasia with sclerosing leukoencephalopathy (PLOSL), is a rare, autosomal‐recessive, and fatal disorder caused by mutations in the TREM2 or TYROBP genes. It is uniquely characterized by the co‐occurrence of presenile dementia and cystic bone lesions, leading to death typically by the fifth decade [1–2]. This descriptive study aims to review its clinical manifestations, diagnostic findings and treatment.

**Methods:** A systematic review was performed following PRISMA guidelines. PubMed and BVS were searched using the keywords “Nasu‐Hakola,” “PLOSL,” and “brain‐bone‐fat disease”. Eligible studies were included and data were extracted into a standardized database for comparative analysis of clinical and treatment variables.

**Results:** Analysis of 12 patients (mean age 35.87 years; 33% familial) showed predominantly neurological onset (58%). Key findings included diffuse cerebral atrophy on MRI (91%), periventricular lesions (73%), basal ganglia abnormalities (64%), and bone cysts/lytic lesions (82%, 18% asymptomatic). Mutations were in TREM2 (58%) or TYROBP. Supportive treatments included antipsychotics/antiepileptics and fracture surgery; one case benefited from virtual reality neurorehabilitation.

**Figure:**
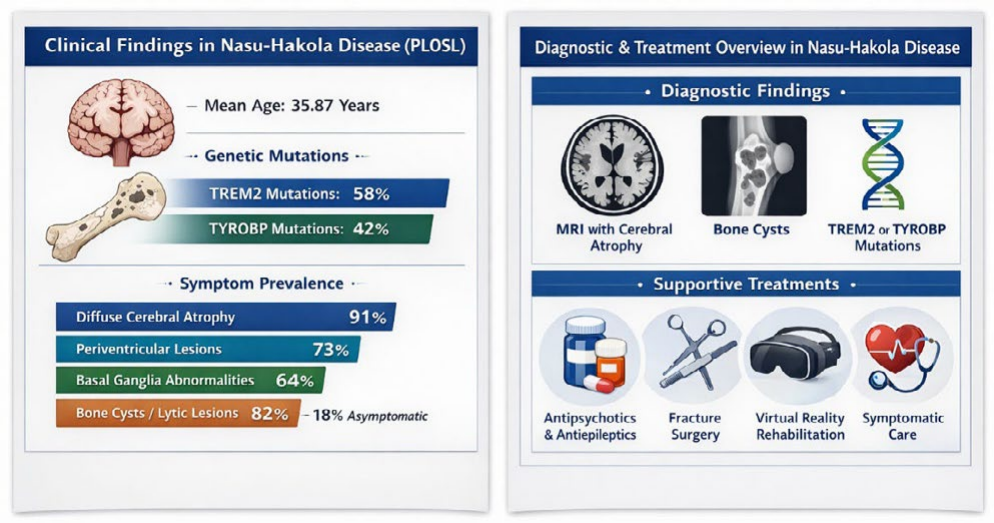
Clinical findings, diagnostic and treatment overview in Nasu‐Hakola disease.

**Conclusion:** This review confirms early neuropsychiatric presentation despite radiographic bone cysts. The diagnostic triad of frontal‐predominant cerebral atrophy, characteristic bone lesions, and TREM2/TYROBP mutation is consistent. The universally fatal progression underscores the absence of disease‐modifying therapies. Supportive care, including tailored neurorehabilitation, may ameliorate specific symptoms, but early genetic diagnosis remains crucial for definitive management and counseling.

**Disclosure:** Nothing to disclose.

## EPO‐0650

### Cerebrotendinous xanthomatosis mimicking or coexisting with juvenile ALS: A case with CYP27A1 and SETX mutations

#### N. Kahveci Erdemir; A. Çolakoğlu; A. Aksoy Gündoğdu

##### Department of Neurology, Tekirdag Namik Kemal University, Tekirdag, Türkiye

**Background and aims:** Cerebrotendinous xanthomatosis (CTX) is a rare autosomal recessive lipid storage disorder caused by CYP27A1 mutations, resulting in sterol 27‐hydroxylase deficiency and systemic cholestanol accumulation. Amyotrophic lateral sclerosis (ALS) is the most common motor neuron disease; its juvenile form begins before age 25, and autosomal dominant ALS type 4 is associated with SETX mutations. Coexistence of CTX and ALS type 4 has not been previously described.

**Methods:** A 34‐year‐old male presented with progressive difficulty walking. Symptoms began 6 years earlier with gait imbalance, worsening weakness, and diffuse limb pain. His history included chronic diarrhea since infancy, febrile seizures in early childhood, bilateral cataract surgery at age 10, and hypophonia and dysarthria developing in his mid‐20s. Neurological examination revealed severe tetraparesis with muscle atrophy, fasciculations, global hyperreflexia, bilateral clonus, and positive Babinski and Hoffman signs.

**Results:** Brain Magnetic Resonance Imaging showed bilateral cerebellar T2/FLAIR hyperintensities and periventricular white matter changes. Electromyography demonstrated widespread anterior horn cell involvement. Genetic testing identified a heterozygous SETX c.6625A>T (p.Lys2209Ter) mutation, confirming ALS type 4. Considering long‐standing gastrointestinal, ophthalmologic, and neurological symptoms, CYP27A1 sequencing was performed and demonstrated a homozygous pathogenic variant (c.1263+1G>A), establishing CTX diagnosis. Treatment with riluzole and chenodeoxycholic acid was initiated.

**Conclusion:** This rare coexistence of CTX and ALS type 4 highlights the importance of recognizing metabolic warning signs such as chronic diarrhea and childhood cataract. Early metabolic and genetic evaluation is essential, as timely chenodeoxycholic acid therapy may slow neurological progression and improve long‐term outcomes.

**Disclosure:** Nothing to disclose.

## EPO‐0651

### Beyond the classic phenotype: Optic neuropathy in a patient with Charcot‐Marie‐Tooth type 2Q

#### P. Dodu; G. García‐Marín

##### Regional Universitary Hospital of Málaga, Málaga, Spain

**Background and aims:** Charcot‐Marie‐Tooth disease type 2 (CMT2) is a genetic disorder characterized by axonal involvement of the peripheral nerves, with a normally autosomal dominant inheritance pattern. There are multiple subtypes of CMT2, each defined by a different affected gene.

**Methods:** We present the case of a patient with optic neuropathy and axonal polyneuropathy caused by a mutation in the DHTKD1 gene.

**Results:** We have a 53‐year‐old male patient with no family history and a personal history of alcoholism who presents with sequential loss of visual acuity, as well as chronic paresthesia in all four limbs. Ophthalmological examination revealed bilateral papillary atrophy, and neurological examination revealed glove‐and‐stocking paresthesia, decreased vibratory sensitivity in all four extremities, and difficulty walking on heels and tiptoes. A blood test was performed with negative autoimmunity, normal cranial MRI, normal cerebrospinal fluid study, and neurophysiological study with data of bilateral axonal optic neuropathy and chronic axonal peripheral neuropathy. Genetic testing showed the c.1897‐1G>A variant classified as pathogenic in heterozygosity in the DHTKD1 gene, associated with CMT2Q.

**Conclusion:** CMT2Q is a rare condition caused by mutations in the DHTKD1 gene, which usually presents with muscle weakness and progressive distal atrophy. The presence of optic neuropathy in our patient is an atypical finding, more commonly described in the CMT2A subtype. We believe that the mitochondrial metabolism disorder caused by the DHTKD1 mutation, together with alcohol abuse, may have acted synergistically, amplifying the phenotype of this disorder.

**Disclosure:** Nothing to disclose.

## EPO‐0652

### NOTCH3 rs201118034 is not an independent risk factor of vascular Parkinsonism among Taiwanese population

#### Y. Liaw ^1^; Y. Liaw^2^; S. Hsu^2^; Y. Liaw^2^

##### ^1^Division of Neurology, Department of Internal Medicine, National Yang Ming Chiao Tung University Hospital, Yi‐Lan, Taiwan; ^2^Department of Public Health and Institute of Public Health, Chung Shan Medical University, Tai‐Chung, Taiwan

**Background and aims:** NOTCH3 *p*.R544C mutation is a common hotspot for developing monogenetic leukoencephalopathy among Taiwanese population. Clinically, this mutation can cause a variable clinical presentations, including vascular Parkinsonism (VaP). This study aimed to investigate the risk of VaP in the generalized Taiwanese population utilizing single nucleotide polymorphisms (SNPs).

**Methods:** Data were retrieved from the Taiwan Biobank Database (2008–2020) and the National Health Insurance Research Database (2000–2020), and they were linked using encrypted personal identification numbers. After excluding participants with a history of stroke, dementia, or Parkinson's disease other than VaP, a total of 147 patients with VaP and 98,699 control subjects were included in the analysis. In this study, VaP was defined specifically with ICD‐10 of G21.4 and G21.8. rs201118034 was used to represented the NOTCH3 p.R544C mutation.

**Results:** Among the multivariable logistic regression analysis, the AG+AA genotype on rs201118034 has a higher, but not significant, odds ratio of developing VaP (OR = 2.71, *p* = 0.090). However, in the regression model, a higher age group between 61 and 70 years‐old was a significantly independent risk factor of VaP (OR = 6.56, *p* < 0.001). Lastly, diabetes (OR = 1.90, *p* = 0.001), hypertension (OR = 2.15, *p* < 0.001), and hyperlipidemia (OR = 1.73, *p* = 0.012) still remained independent predicting factors of developing VaP in our population.

**Figure:**
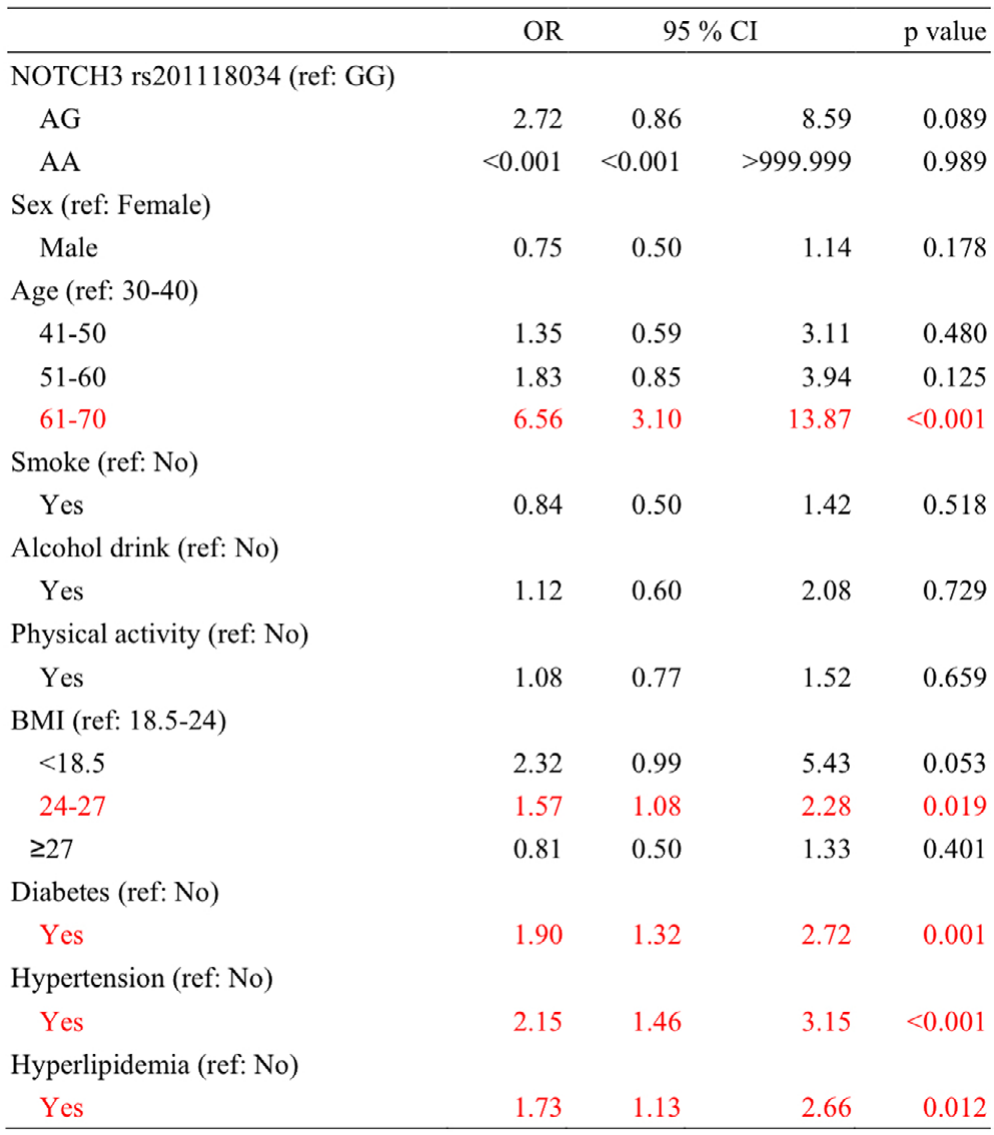
**TABLE 1** Multivariable logistic regression analysis of vascular Parkinsonism focusing on NOTCH3 rs201118034.

**Figure:**
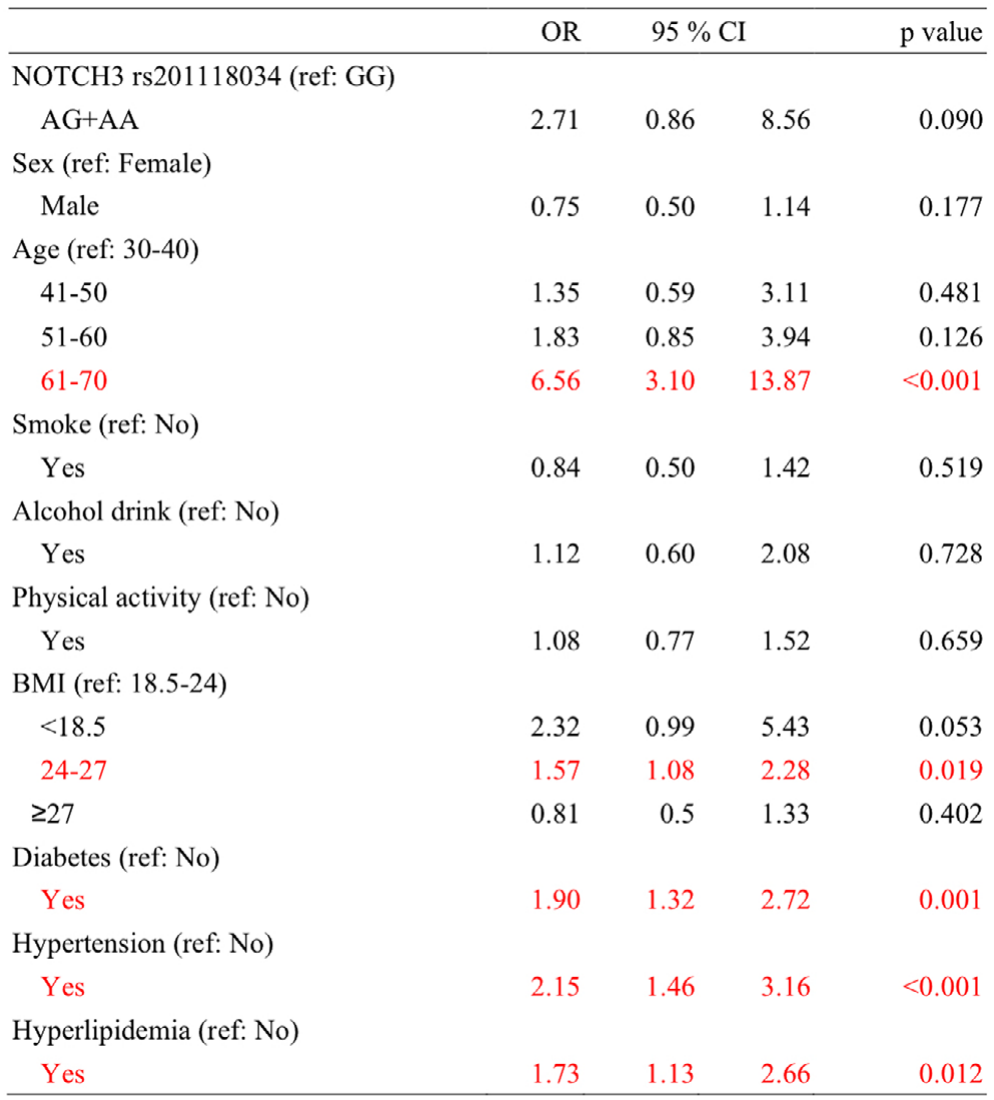
**TABLE 2** Multivariable logistic regression analysis of vascular Parkinsonism focusing on NOTCH3 rs201118034 (dominant model).

**Conclusion:** Our findings suggested that the risk of VaP of NOTCH3 *p*.R544C carrier is not determined independently via this mutation. An elder age, hypertension, diabetes and hyperlipidemia remained independent and critical factors in developing VaP.

**Disclosure:** Disclosure: The authors no conflicts of interest. Fundings: This work was funded by the Ministry of Science and Technology (MOST 111‐2121‐M‐040‐002; NSTC 112‐2121‐M‐040‐002; 113‐2121‐M‐040‐001; 114‐2121‐M‐040‐002) IRB: CS1‐23101.

## Monday, June 29 2026

## Movement Disorders 7

## EPO‐0653

### Depression and mood changes in Parkinson**’**s disease over time. A 5‐year follow‐up study

#### Á. Solleiro Vidal^1^; D. Santos García ^1^; A. Puy Núnez^2^; T. De Deus Fonticoba^2^; P. Mir^3^; G. Pons Pons^4^; J. García Caldentey^5^; N. Caballol^6^; J. Hernández Vara^7^; L. López Manzanares^8^; B. Vives Pastor^9^; M. Ávila Rivera^10^; I. González Aramburu^11^; R. García‐Ramos^12^; C. Borrué^13^; J. Dotor García‐Soto^14^; M. Álvarez Sauco^15^; I. Cabo López^16^; G. González Ortega^17^; COPPADIS Study Group^1^

##### ^1^Complejo Hospitalario Universitario de A Coruña (CHUAC), A Coruña, Spain*;* ^2^Complejo Hospitalario Universitario de Ferrol (CHUF), Ferrol, Spain*;* ^3^Hospital Universitario Virgen del Rocío, Sevilla, Spain*;* ^4^Hospital Universitari Mutua de Terrassa, Terrassa, Barcelona, Spain*;* ^5^Centro Neurológico Oms 42, Palma de Mallorca, Spain*;* ^6^Complex Hospitalari Universitari Moisés Broggi, Sant Joan Despí, Barcelona, Spain*;* ^7^Hospital Universitario Vall d´Hebron, Barcelona, Spain*;* ^8^Hospital Universitario La Princesa, Madrid, Spain*;* ^9^Hospital Universitario Son Espases, Palma de Mallorca, Spain*;* ^10^Consorci Sanitari Integral, Hospital General de L*'*Hospitalet, L´Hospitalet de Llobregat, Barcelona, Spain*;* ^11^Hospital Universitario Marqués de Valdecilla, Santander, Spain*;* ^12^Hospital Clínico San Carlos, Madrid, Spain*;* ^13^Hospital Infanta Sofía, Madrid, Spain*;* ^14^Hospital Universitario Virgen Macarena, Sevilla, Spain*;* ^15^Hospital General Universitario de Elche, Elche, Spain*;* ^16^Complejo Hospitalario Universitario de Pontevedra (CHOP), Pontevedra, Spain*;* ^17^Hospital Universitario de Móstoles, Madrid, Spain

**Background and aims:** Depression is frequent in Parkinson's disease (PD), but it is unclear how mood changes and impacts patient's quality of life (QoL) over time. Our objective was to analyze the frequency of depression and mood changes in people with PD (PwP) over 5 years of follow‐up, comparing it with a control group, as well as its relationship with the patients' QoL.

**Methods:** PwP and healthy controls (HC) recruited from the COPPADIS cohort from January/2016 to November/2017 were included in this 5‐year follow‐up study. Mood was assessed by the Beck Depression Inventory II (BDI‐II), and participants were classified as having major depression, minor depression, subthreshold depression, or non‐depression at baseline and at 2, 4, and 5 years of follow‐up. Correlation analysis and linear regression models were conducted to determine how different factors contribute to mood changes.

**Results:** The BDI‐II total score increased (*p <* 0.0001) from 8.1 ± 6.2 at baseline to 10.3 ± 8.1 at the 5‐year follow‐up visit in PwP but not in HC (from 4.1 ± 5.2 to 4.0 ± 5.7 [*p* = 0.896]). The prevalence of depression remained around 50% in the PwP group and 25% in the HC group throughout the follow‐up period, but the mood state showed variability for each patient between visits (Figure 1). Patients´ QoL was associated with the depressive state throughout the entire follow‐up period (Figure 2). Worsening QoL, sleep, longer disease duration, and an increase in neuropsychiatric symptoms were identified as independent factors associated with a worsening of mood over time in PwP (*N* = 348) (Table 1).

**Figure:**
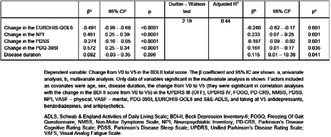
**TABLE 1** Factors associated with mood (BDI‐II score) change from baseline (V0) to the 5‐year follow‐up visit (V5) (BDI‐IIV5 – BDI‐IIV0) in PD patients (*N* = 346).

**Figure:**
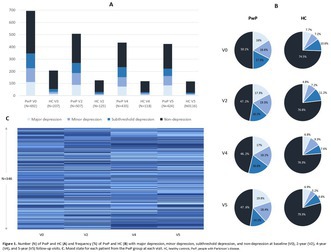
**FIGURE 1** Number (*N*) (A) and frequency (%) (B) of PwP and HC with major, minor, subthreshold and no depression at baseline (V0), 2‐year (V2), 4‐year (V4) and 5‐year (V5) follow‐up. (C) Mood state for each patient from the PwP group at each visit.

**Figure:**
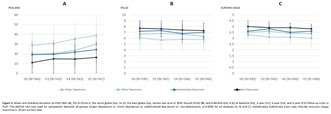
**FIGURE 2** Mean and standard deviation of PDQ‐39SI (A), PQ‐10 (B) and EUROHIS‐QOL8 (C) at baseline (V0), 2‐year (V2), 4‐year (V4) and 5‐year (V5) follow‐up in PwP. ANOVA comparisons between depression groups showed *p* < 0.0001.

**Conclusion:** Mood changes over time in PwP and are associated with QoL.

**Disclosure:** Nothing to disclose.

## EPO‐0654

### Inhaled levodopa improves quality of life and non‐motor symptoms associated with OFF episodes in people with Parkinson**'**s disease

#### D. Santos García ^1^; M. Cerdán Sánchez^2^; R. Yáñez Baña^3^; I. Cabo^4^; M. Cimas Hernando^5^

##### ^1^Neurology Department, CHUAC (Complejo Hospitalario Universitario de A Coruña), A Coruña, Spain; ^2^Neurology Department, Hospital Clínico Universitario Virgen de la Arrixaca, Murcia, Spain; ^3^Neurology Department, Complejo Hospitalario Universitario de Ourense (CHUO), Ourense, Spain; ^4^Neurology Department, Complejo Hospitalario Universitario de Pontevedra (CHOP), Pontevedra, Spain; ^5^Neurology Department, Complejo Hospitalario Universitario de Vigo (CHUVI), Vigo, Spain

**Background and aims:** CVT‐301 (Inbrija®) has demonstrated its effectiveness in treating OFF periods and improving motor symptoms in people with Parkinson's disease (PwP). However, its effect on non‐motor symptoms (NMS) and quality of life has not been researched yet. Our aim was to analyze the effect of CVT‐301 on PwP's health‐related quality of life (HRQoL) and NMS associated with OFF episodes.

**Methods:** INLEVO‐LIFE PD (an open‐label study of the effect of INhaled LEVOdopa on quality of LIFE and non‐motor symptoms in Parkinson**'**s Disease) is a prospective open‐label study conducted in five centers in Spain. The change from baseline (Vb) to the end of the observational period (12 weeks ± 2 weeks) (V12w) in the 39‐item Parkinson's disease Quality of Life Questionnaire (PDQ‐39) total score was the primary efficacy measure.

**Results:** Forty PD patients (age 62.3 ± 9.2 years; 55% males) were included between March 2024 and November 2025. At 12 weeks, 34 patients were using CVT‐301 and completed the follow‐up (85%). The PDQ39 total score decreased from 43.6 ± 24.2 at Vb to 31.4 ± 20.6 at V12w (*p <* 0.0001). A significant decrease was observed in the mean score of 5 domains of the PDQ39 (Figure 1). NMS burden was reduced at v12w from the OFF state to 30, 60, and 90 minutes after using CVT‐301 (Figure 2). Mean daily OFF time decreased by 1.6 ± 1.9 hours (*p <* 0.0001) (Table 1). Adverse events related to CVT‐301 were reported in 35% of the patients.

**Figure:**
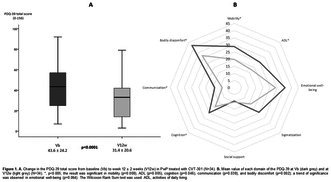
FIGURE 1

**Figure:**
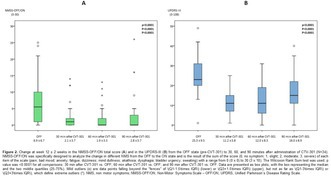
FIGURE 2

**Figure:**
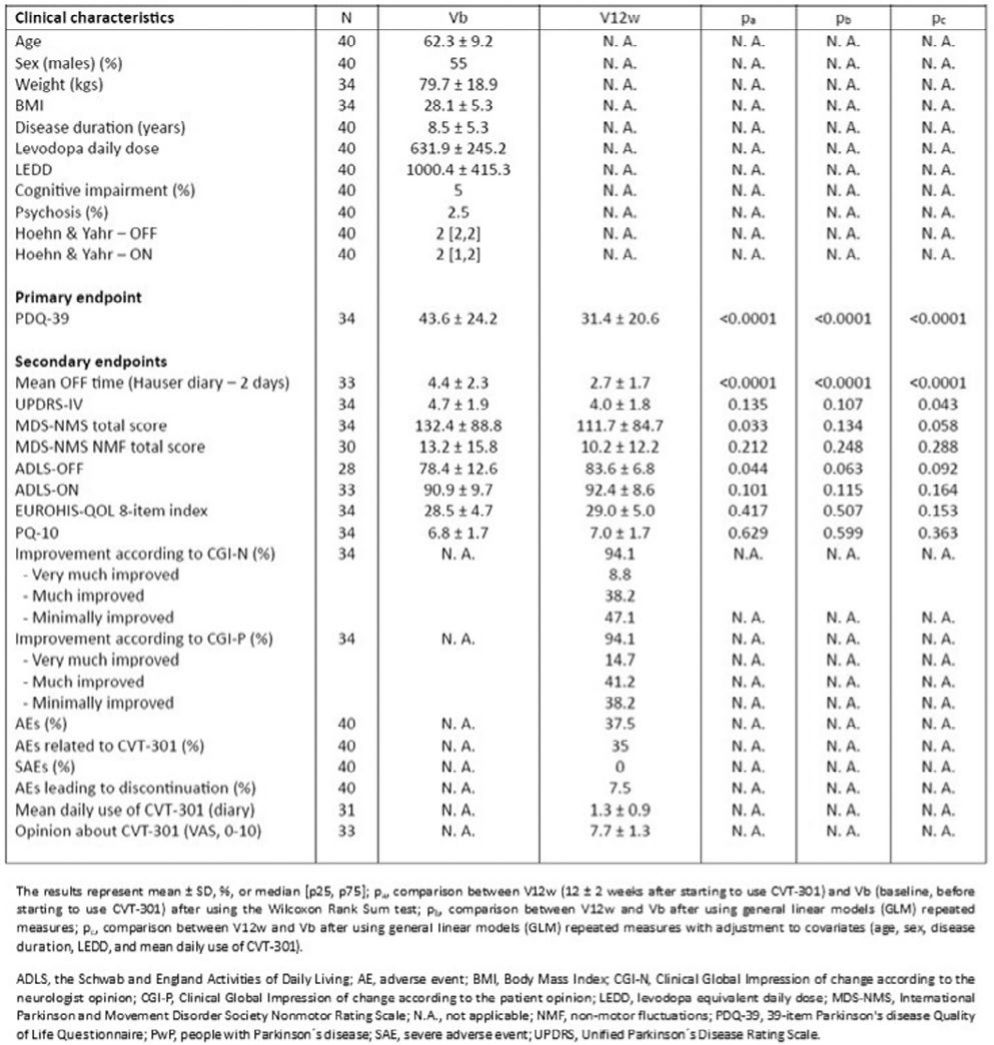
**TABLE 1** Baseline characteristics of PwP included in the INLEVO‐LIFE study (*N* = 40) and comparison of some clinical data between the final visit (12 weeks ± 2 weeks after starting to use CVT‐301) and the baseline visit (before starting to use CVT‐301).

**Conclusion:** HRQoL and NMS associated with OFF episodes improved in PwP after 12 weeks using CVT‐301.

**Disclosure:** This study received funding from Esteve. Moreover, María Sánchez, the data manager hired for this purpose, participated in the role of data entry.

## EPO‐0655

### Alterations of amygdala volume and functional connectivity in Parkinson's disease subjects with freezing of gait

#### E. Sarasso^1^; A. Gardoni ^2^; A. Grassi^2^; S. Basaia^2^; G. Bonardi^3^; S. Carta^3^; S. Mariotto^3^; I. Di Vico^3^; E. Mantovani^3^; E. Canu^4^; M. Bressan^3^; V. Castelnovo^2^; E. Sibilla^2^; C. Tripodi^2^; F. Freri^2^; R. Balestrino^5^; M. Trentinaglia^3^; F. Maffei^3^; V. Chiodega^3^; S. Tamburin^3^; M. Volontè^6^; M. Tinazzi^3^; F. Agosta^4^; M. Filippi^7^

##### ^1^Neuroimaging Research Unit, Division of Neuroscience, IRCCS San Raffaele Scientific Institute, Milan, Italy; Neurotech Hub, Vita‐Salute San Raffaele University, Milan, Italy; and DINOGMI, University of Genoa, Genoa, Italy; ^2^Neuroimaging Research Unit, Division of Neuroscience, IRCCS San Raffaele Scientific Institute, Milan, Italy; and Neurotech Hub, Vita‐Salute San Raffaele University, Milan, Italy; ^3^University of Verona, Verona, Italy; ^4^Neuroimaging Research Unit, Division of Neuroscience, and Neurology Unit, IRCCS San Raffaele Scientific Institute, Milan, Italy; and Neurotech Hub, Vita‐Salute San Raffaele University, Milan, Italy; ^5^Neurology Unit, and Neurorehabilitation Unit, IRCCS San Raffaele Scientific Institute, Milan, Italy; and Neurotech Hub, Vita‐Salute San Raffaele University, Milan, Italy; ^6^Neurology Unit, IRCCS San Raffaele Scientific Institute, Milan, Italy; ^7^Neuroimaging Research Unit, Division of Neuroscience, Neurology Unit, Neurorehabilitation Unit, and Neurophysiology Service, IRCCS San Raffaele Scientific Institute, Milan, Italy; and Neurotech Hub, Vita‐Salute San Raffaele University, Milan, Italy

**Background and aims:** Amygdala is crucial in emotional regulation and integrating interoceptive information, autonomic control, and motor behavior. The study aimed to assess amygdala volume and functional connectivity (FC) alterations in patients with Parkinson's disease with and without freezing of gait (PD‐FoG, PD‐NO‐FoG).

**Methods:** Thirty PD‐FoG patients, 30 PD‐NO‐FoG patients (with postural instability and gait disorders), and 30 matched healthy controls underwent neuropsychological, structural, and functional magnetic resonance imaging (MRI) assessments. Amygdala was segmented using FSL‐FIRST. Based on volumetric findings, right amygdala was used as region of interest for seed‐based FC analyses.

**Results:** PD‐FoG and PD‐NO‐FoG patients showed comparable cognitive profiles, with impairments in memory, visuospatial, and executive‐attentional domains, as well as increased depressive symptoms. MRI analyses revealed increased amygdala volume in PD‐FoG compared with healthy controls. Resting‐state fMRI analyses showed that, relative to controls, PD‐FoG patients exhibited increased FC between the right amygdala and limbic–striatal regions, including the left thalamus, putamen, insula, and inferior frontal orbital cortex, along with reduced connectivity with the middle temporal gyrus and cerebellar regions (Crus I and lobules VI–VIII). Compared with PD‐NO‐FoG patients, PD‐FoG patients showed reduced FC between the right amygdala and middle/superior temporal, inferior parietal, postcentral gyri, insula, and fusiform cortex.

**Conclusion:** Increased amygdala–limbic‐striatal connectivity in PD‐FoG may reflect stronger emotional influence on motor circuits, contributing to impaired automatic gait control. Reduced connectivity with temporal, parietal, and cerebellar regions suggests disrupted integration across emotional, cognitive, and motor networks, supporting the hypothesis that FoG arises from impaired network communication underlying gait automaticity.

**Disclosure:** Supported by Italian Ministry of Health grant number GR‐2021‐12374005 Elisabetta Sarasso, Silvia Basaia, Sara Mariotto, Elisa Canu, Roberta Belestrino and Michele Tinazzi are recipient of a grant from the Italian Ministry of Health. Andrea Gardoni, Andrea Grassi, Giulia Bonardi, Sara Carta, Ilaria Di Vico, Elisa Mantovani, Miriana Bressan, Veronica Castelnvo, Elisa Sibilla, Chiara Tripodi, Fabiola Freri, Milena Trentinaglia, Francesco Maffei, Vanessa Chiodega, Stefano Tamburin, and Maria Antonietta Volontè declare no financial competing interests. Federica Agosta is Associate Editor of NeuroImage: Clinical, has received speaker honoraria from Biogen Idec, Italfarmaco, Roche, Zambon, Eli Lilly, GE Healthcare and Bristol Myers Squibb, and receives or has received research supports from the Italian Ministry of Health, the Italian Ministry of University and Research, AriSLA (Fondazione Italiana di Ricerca per la SLA), the European Research Council, the EU Joint Programme—Neurodegenerative Disease Research (JPND) and Foundation Research on Alzheimer Disease (France). Massimo Filippi is Editor‐in‐Chief of the Journal of Neurology, Associate Editor of Human Brain Mapping, Neurological Sciences, and Radiology; received compensation for consulting services from Almirall, Biogen, Bristol‐Myers Squibb, Eli Lilly, Merck, Novartis, Roche, Sanofi; speaking activities from Amgen, Bayer, Biogen, Bristol‐Myers Squibb, Celgene, Chiesi Italia SpA, Eisai, Eli Lilly, Fujirebio, Genzyme, Janssen, Merck, Neopharmed Gentili, Neuraxpharm, Novartis, Novo Nordisk, Roche, Sanofi, Takeda; participation in Advisory Boards for Alexion, Biogen, Bristol‐Myers Squibb, Eli Lilly, GE Healthcare Ltd, Merck, Neuraxpharm, Novartis, Roche, Sandoz, Sanofi, Takeda; scientific direction of educational events for Biogen, Merck, Roche, Celgene, Bristol‐Myers Squibb, Lilly, Novartis, Sanofi‐Genzyme; he receives research support from Biogen Idec, Merck‐Serono, Novartis, Roche, the Italian Ministry of Health, the Italian Ministry of University and Research, and Fondazione Italiana Sclerosi Multipla.

## EPO‐0656

### Emotion in motion: Gait pattern and neural circuits modulation in Parkinson's disease

#### E. Sarasso^1^; M. Putzolu^2^; E. Canu^3^; M. Forghieri^3^; S. Basaia^3^; A. Gardoni ^3^; A. Grassi^3^; A. Botta^4^; S. Terranova^5^; L. Avanzino^6^; R. Balestrino^7^; M. Filippi^8^; E. Pelosin^2^; F. Agosta^9^

##### ^1^Neuroimaging Research Unit, Division of Neuroscience, IRCCS San Raffaele Scientific Institute, Milan, Italy; Neurotech Hub, Vita‐Salute San Raffaele University, Milan, Italy; and Department of Experimental Medicine, University of Genoa, Genoa, Italy; ^2^Department of Neuroscience, Rehabilitation, Ophthalmology, Genetics and Maternal Child Health, University of Genoa, Genoa, Italy; and IRCCS Ospedale Policlinico San Martino, Genoa, Italy; ^3^Neuroimaging Research Unit, Division of Neuroscience, IRCCS San Raffaele Scientific Institute, Milan, Italy; and Neurotech Hub, Vita‐Salute San Raffaele University, Milan, Italy; ^4^IRCCS Ospedale Policlinico San Martino, Genoa, Italy; ^5^Department of Experimental Medicine, Section of Human Physiology, University of Genoa, Genoa, Italy; ^6^Department of Experimental Medicine, Section of Human Physiology, University of Genoa, Genoa, Italy; and IRCCS Ospedale Policlinico San Martino, Genoa, Italy; ^7^Neurology Unit, and Neurorehabilitation Unit, IRCCS San Raffaele Scientific Institute, Milan, Italy; ^8^Neuroimaging Research Unit, Division of Neuroscience, Neurology Unit, Neurorehabilitation Unit, and Neurophysiology Service, IRCCS San Raffaele Scientific Institute, Milan, Italy; and Neurotech Hub, Vita‐Salute San Raffaele University, Milan, Italy; ^9^Neuroimaging Research Unit, Division of Neuroscience, and Neurology Unit, IRCCS San Raffaele Scientific Institute, Milan, Italy; and Neurotech Hub, Vita‐Salute San Raffaele University, Milan, Italy

**Background and aims:** Gait disturbances are a core feature of people with Parkinson's disease (pwPD) and are influenced by motor, cognitive, and emotional factors, reflecting the reciprocal link between emotion and movement described by embodied emotion theories. This study examined whether observing emotional gait modulates gait performance and locomotor network activity in pwPD and healthy controls, focusing on the effects of positive and negative emotions on gait parameters and sensorimotor activation.

**Methods:** Thirty‐one pwPD and twenty‐six age‐/sex‐matched HC underwent gait analysis and task‐based functional MRI (fMRI). Gait was assessed during spontaneous walking, top‐down embodiment (self‐evoked emotions), and bottom‐up embodiment (imitation after observing emotional gait videos depicting happiness, sadness, anxiety). fMRI data were collected during emotional gait observation and analyzed using permutation‐based nonparametric statistics focusing on sensorimotor regions.

**Results:** Both groups accurately recognized emotional gait and reported similar valence and intensity. Happiness improved gait in pwPD and HC, especially during bottom‐up embodiment, enhancing stride length, arm kinematics, and turning velocity, whereas sadness and anxiety worsened gait performance, increasing variability and reducing pace. fMRI showed emotion‐specific sensorimotor modulation, with happiness enhancing basal ganglia and sensorimotor activation and negative emotions reducing it. In pwPD, these neural patterns correlated with emotion‐specific changes in gait during bottom‐up embodiment.

**Conclusion:** Emotions modulate gait and its neural substrates in pwPD and HC. Positive emotional cues enhance motor system engagement even in pwPD, whereas negative emotions exert detrimental effects. These findings support the integration of emotionally enriched action observation into gait rehabilitation and highlight the relevance of emotional states in clinical evaluation.

**Disclosure:** Work supported by #NEXTGENERATIONEU (NGEU) and funded by the Ministry of University and Research (MUR), National Recovery and Resilience Plan (NRRP), project MNESYS (PE0000006) – A Multiscale integrated approach to the study of the nervous system in health and disease (DN. 1553 11.10.2022); and Italian Ministry of Health grant GR‐2021‐12374005 E Sarasso, E Canu, S Basaia and R Balestrino received research support from the Italian Ministry of Health M Putzolu was supported by #NEXTGENERATIONEU (NGEU) and funded by MUR, NRRP, project MNESYS (PE0000006) – A Multiscale integrated approach to the study of the nervous system in health and disease (DN. 1553 11.10.2022). M Forghieri, A Gardoni, A Grassi, A Botta, S Terranova have nothing to disclose. L Avanzino received speaker honoraria from Zambon and Bial and received research support from the EU Joint Programme for Neurodegenerative Disease Research (JPND) and Michael J. Fox Foundation. She received research support from FRESCO foundation and the Italian Ministry of Health. M Filippi is Editor‐in‐Chief of the Journal of Neurology, Associate Editor of Human Brain Mapping, Neurological Sciences, and Radiology; received compensation for consulting services from Almirall, Biogen, Bristol‐Myers Squibb, Eli Lilly, Merck, Novartis, Roche, Sanofi; speaking activities from Amgen, Bayer, Biogen, Bristol‐Myers Squibb, Celgene, Chiesi Italia SpA, Eisai, Eli Lilly, Fujirebio, Genzyme, Janssen, Merck, Neopharmed Gentili, Neuraxpharm, Novartis, Novo Nordisk, Roche, Sanofi, Takeda; participation in Advisory Boards for Alexion, Biogen, Bristol‐Myers Squibb, Eli Lilly, GE Healthcare Ltd, Merck, Neuraxpharm, Novartis, Roche, Sandoz, Sanofi, Takeda; scientific direction of educational events for Biogen, Merck, Roche, Celgene, Bristol‐Myers Squibb, Lilly, Novartis, Sanofi‐Genzyme; he receives research support from Biogen Idec, Merck‐Serono, Novartis, Roche, the Italian Ministry of Health, the Italian Ministry of University and Research, and Fondazione Italiana Sclerosi Multipla. E Pelosin is part of an Advisory Board for the M.J. Fox Foundation. She received grants from the Italian Ministry of University and Research and Michael J. Fox Foundation, and research supports from the Italian Ministry of Health. F Agosta is Associate Editor of NeuroImage: Clinical, has received speaker honoraria from Biogen Idec, Italfarmaco, Roche, Zambon and Eli Lilly, and receives or has received research supports from the Italian Ministry of Health, the Italian Ministry of University and Research, AriSLA (Fondazione Italiana di Ricerca per la SLA), the European Research Council, the EU Joint Programme – Neurodegenerative Disease Research (JPND), and Foundation Research on Alzheimer Disease (France).

## EPO‐0657

### Reduced falling with ND0612: Supportive analyses from the BouNDless phase 3 trial and an integrated safety database

#### N. Schröter^1^; S. Isaacson^2^; F. Stocchi^3^; A. Ellenbogen^4^; N. Lopes ^5^; I. Tacken^6^; N. Vostokova^5^; A. Salah^7^

##### ^1^Department of Neurology, Saarland University Hospital, Homburg, Germany; ^2^Parkinson's Disease and Movement Disorders Center of Boca Raton, Boca Raton, USA; ^3^Institute for Research and Medical Care IRCCS San Raffaele, Roma, Italy; Department of Neurology, San Raffaele University, Rome, Italy; ^4^Quest Research Institute, Farmington Hills, USA; ^5^NeuroDerm Ltd, Rehovot, Israel; ^6^Tanabe Pharma, Dusseldorf, Germany; ^7^Tanabe Pharma, Jersey City, USA

**Background and aims:** We previously reported that patients receiving an optimized ND0612 regimen experienced a lower incidence of falls during the double‐blind phase of the BouNDless trial, vs those treated with levodopa/carbidopa (IR‐LD/CD) therapy (7% vs 12%, respectively).

**Methods:** We (a) examined the baseline characteristics of patients who experienced recurrent (>1) falls using an integrated safety database (ISD), and (b) explored potential factors contributing to the reduced fall rates. Postural instability and gait disorder (PIGD) and freezing of gait (FOG) were measured using MDS‐UPDRS items; cognitive function via PDQ‐39 cognitive scores; and dyskinesia using Hauser diaries.

**Results:** Patients with recurrent falls were older (62% aged ≥65 years vs 49% in the overall cohort), had lived with their PD for longer (time since diagnosis of 10.7 vs 9.4 years), and had higher (worse) motor severity (MDS‐UPDRS Part III scores of 37.6 vs 34.5). In the BouNDless trial, ND0612 improved PIGD scores vs IR‐LD/CD (improvement of +0.26 vs –0.02), reduced FOG (improvement of +0.33 vs +0.18) and enhanced cognition (improvement of +0.90 vs +0.31). Mean ON‐ time with troublesome dyskinesia decreased with ND0612 from 0.71 to 0.45 h.

**Conclusion:** Treatment with ND0612 has a positive effect on the various factors associated with falling in PD, and support the finding of reduced falls with optimized ND0612 therapy.

**Disclosure:** Nothing to disclose.

## EPO‐0658

### Long‐term efficacy of ND0612 for motor fluctuations in Parkinson's disease

#### W. Jost^1^; A. Espay^2^; R. Pahwa^3^; A. Ellenbogen^4^; F. Stocchi^5^; *N*. Lopes ^6^; A. Salah^7^; O. Rascol^8^

##### ^1^Parkinson‐Klinik Ortenau, Wolfach, Germany; ^2^James J. and Joan A. Gardner Center for Parkinson's Disease and Movement Disorders, University of Cincinnati, Cincinnati, USA; ^3^University of Kansas Medical Center, Kansas City, KS, USA; ^4^Quest Research Institute, Farmington Hills, USA; ^5^Institute for Research and Medical Care IRCCS San Raffaele, Roma, Italy; Department of Neurology, San Raffaele University, Rome, Italy; ^6^NeuroDerm Ltd, Rehovot, Israel; ^7^Tanabe Pharma America, Inc, Jersey City, USA; ^8^University Hospital of Toulouse, INSERM, Clinical Investigation Center CIC1436 Toulouse, France

**Background and aims:** Primary efficacy analyses from the double‐blind phase of the BouNDless study demonstrated that treatment with ND0612 provided patients with Parkinson's disease (PD) an additional 1.72 hours (h) of Good ON‐time compared with levodopa/carbidopa (IR‐LD/CD) (*p <* 0.0001). We describe here one‐year efficacy outcomes from the ongoing open‐label extension (OLE) phase.

**Methods:** Patients who completed the double‐blind phase of the BouNDless study were eligible to enter the ongoing OLE phase (up to 54 months). All anti‐PD medications were adjusted according to individual response. Efficay was assessed with the Hauser diary at Months 6 and 12 of the OLE phase. Changes in ON‐time with troublesome dyskinesia were analyzed for the subgroup of patients who had ≥1 h troublesome dyskinesia at treatment onset.

**Results:** Of 232 participants (*n* = 113 previously randomized to ND0612, *n* = 119 previously randomized to IR‐LD/CD), 167 (72%) completed 1 year of ND0612 treatment in the OLE phase. By Month 6, patients had a mean ± SE change of −2.2 ± 0.2 h in OFF‐time and +2.4 ± 0.2 h in Good ON‐time. Efficacy benefits were sustained at Month 12 with a change of −2.1 ± 0.2 h in OFF‐time and +2.1 ± 0.2 h in Good ON‐time. The proportion of patients with ≥12 hours of Good ON‐time increased from 16.4% before initiating ND0612 to 57.9% at Month 6 and 54.6% at Month 12. Patients who had ≥1h of ON‐time with troublesome dyskinesia at ND0612 initiation (*n* = 45) showed a change of −1.2 ± 0.3 h at Month 6 and −1.5 ± 0.3 h at Month 12.

**Conclusion:** These data support the long‐term efficacy of ND0612 for PD patients experiencing motor fluctuations.

**Disclosure:** Nothing to disclose.

## EPO‐0659

### Efficacy of inhaled levodopa by age‐based subgroups in patients with Parkinson disease: Post hoc analysis of pooled data from three phase III trials

#### A. Scheschonka; M. Ngure; C. Walter; S. Freisens; G. Comes

##### Merz Therapeutics GmbH, Frankfurt, Germany

**Background and aims:** Many people with Parkinson disease (PwP) develop motor response fluctuations (OFF episodes) within 5 years of starting oral levodopa. CVT‐301 is an orally inhaled formulation of levodopa developed for the treatment of episodic OFF periods. This analysis aimed to assess the efficacy of CVT‐301 (84 mg) in reducing motor symptoms and improving patient‐reported outcomes in PwP across age‐based subgroups.

**Methods:** This descriptive post hoc analysis pooled data from three phase III clinical trials (CVT‐301‐004, CVT‐301‐004e, and CVT‐301‐005). Analyses were conducted across subgroups based on age at time of diagnosis, age at start of first OFF episode, and age at initiation of study treatment, defined as <55, 55–64, 65–74, and ≥75 years. Efficacy endpoints included Patient Global Impression of Change (PGIC) at Week 12 and change from baseline in Unified Parkinson's Disease Rating Scale Part III (UPDRS III) score from pre‐dose to 30 minutes post‐dose at Week 12.

**Results:** A total of 437 PwP were pooled. The mean age (range) was 63.5 (37–82) years; 64.3% were male and 96.3% were White. The mean (standard deviation [SD]) time since diagnosis was 8.7 (4.54) years. The mean (SD) time since first OFF episode was 3.8 (3.07) years. The mean change in UPDRS‐III score from pre‐dose to 30 minutes post‐dose at Week 12 showed improvements across all age subgroups. Similarly, the PGIC consistently showed improvements at Week 12.

**Figure:**
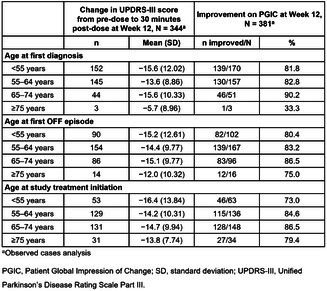
**TABLE 1** Change in UPDRS‐III score from pre‐dose to 30 minutes post‐dose and improvement on PGIC at Week 12 across age subgroups.

**Conclusion:** Use of CVT‐301 is an effective option for managing OFF episodes in PwP, with consistent motor symptom relief and patient‐reported improvement across all ages.

**Disclosure:** This study was funded by Merz Therapeutics GmbH.

## EPO‐0660

### Familial tremor linked to a heterozygous variant in the ceruloplasmin gene

#### G. De Vita^1^; G. Blasio^2^; G. Riccio ^3^; R. Coppola^3^; D. Dell'Aversana^3^; S. Cocozza^4^; E. Gragnano^4^; M. Esposito^5^; G. De Michele^3^; A. De Rosa^3^

##### ^1^Department of Molecular Medicine and Medical Biotechnology, University of Naples Federico II, Naples, Italy; ^2^CEINGE‐Biotecnologie Avanzate Franco Salvatore, Naples, Italy; ^3^Department of Neurosciences and Reproductive and Odontostomatological Sciences, Federico II University, Naples, Italy; ^4^Departments of Advanced Biomedical Sciences and Electrical Engineering and Information Technology, University of Naples Federico II, Naples, Italy; ^5^Clinical Neurophysiology Unit, Cardarelli Hospital, Naples, Italy

**Background and aims:** Biallelic mutations in the ceruloplasmin (CP) gene cause aceruloplasminemia, a rare autosomal recessive disorder characterized by systemic and cerebral iron overload. Here we describe the clinical, genetic, and radiologic features of a family carrying a heterozygous variant in the CP gene.

**Methods:** The proband was an 18‐year‐old girl presenting action tremor in the upper limbs, which was also observed in her father and older sister. Serum ceruloplasmin levels and brain Magnetic Resonance Imaging was assessed in all three subjects. The proband was tested by New Generation Sequencing for a panel of genes associated with movement disorders. The results were validated and extended to the parents and the older sister by Sanger sequencing.

**Results:** The analysis revealed a heterozygous variant c.188T>C in the CP gene, introducing a missense change p.(Ile63Thr) in the proband. The variant was inherited from the father and shared with the affected sister.

**Figure:**
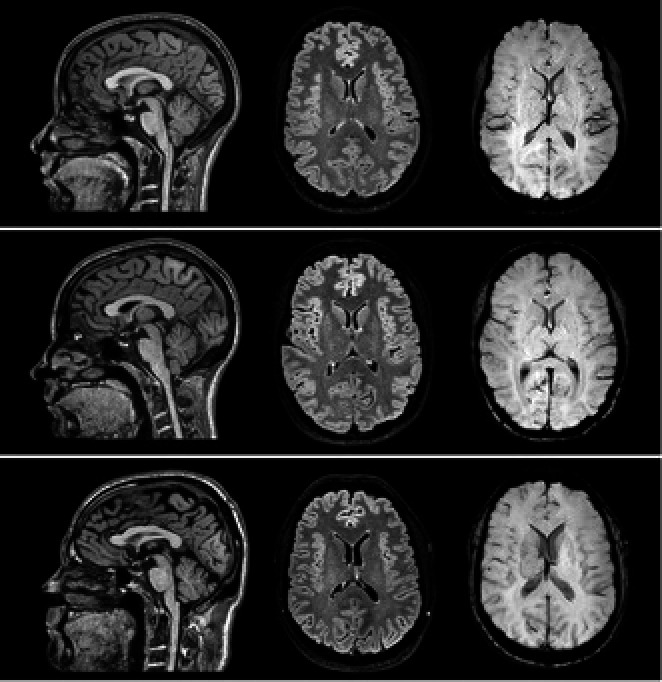
**FIGURE 1** Brain MRI scans of the three subjects harboring the heterozygous CP gene variant: the proband, the older sister, and the father, from top to bottom. From left to right are shown a volumetric T1‐weighted, a T2‐weighted FLAIR and an SWI sequences.

**Figure:**
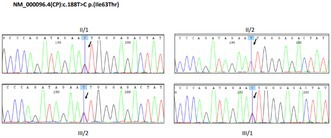
**FIGURE 2** Sanger sequencing showing the CP mutation (c.188T>C, arrows) in the proband (III/2), her older sister (III/1) and her father (II/1), but not in the mother (II/2).

**Figure:**
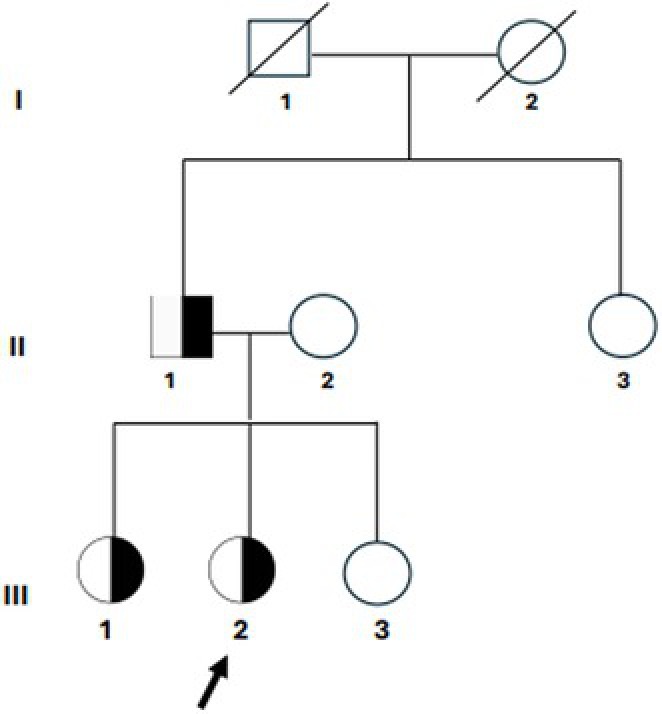
**FIGURE 3** Family pedigree showing that the heterozygous variant, carried by the proband (arrow), her father and older sister, segregates with the phenotype. The younger sister of the proband has not been tested due to the minor age and absence of symptoms.

**Conclusion:** In our family the CP variant segregated with the clinical phenotype, characterized by action tremor, suggesting that this mild neurological manifestation may be likely attributable to the variant rather than representing an incidental finding. Neurological symptoms may occur in heterozygous carriers of CP gene variants, although they are usually believed to be asymptomatic.

**Disclosure:** Nothing to disclose.

## EPO‐0661

### fMRI activity during working memory manipulation is associated with caudate integrity in early Parkinson's disease

#### H. Theis ^1^; K. Giehl^2^; A. Asendorf^3^; M. Banwinkler^3^; V. Dzialas^3^; R. Lubomierski^3^; M. Hönig^2^; T. van Eimeren^1^

##### ^1^University of Cologne, Faculty of Medicine and University Hospital Cologne, Department of Neurology, Cologne, Germany. University of Cologne, Faculty of Medicine and University Hospital Cologne, Department of Nuclear Medicine, Cologne, Germany; ^2^University of Cologne, Faculty of Medicine and University Hospital Cologne, Department of Nuclear Medicine, Cologne, Germany. Research Center Juelich, Institute for Neuroscience and Medicine (INM‐II), Juelich, Germany; ^3^University of Cologne, Faculty of Medicine and University Hospital Cologne, Department of Nuclear Medicine, Cologne, Germany

**Background and aims:** Cognitive difficulties are common in Parkinson's disease (PD). Specifically, manipulation in working memory (WM) seems particularly vulnerable as it relies on frontostriatal networks. However, it is still relatively unclear how early alterations in nigrostriatal integrity – quantified as dopamine transporter (DaT) availability and iron depositions via Quantitative Susceptibility Mapping (QSM) – may shape cognitive neural responses. Thus, we aimed to investigate the relationship between caudate integrity and neural activity during WM manipulation in early PD.

**Methods:** 25 patients and 35 matched healthy controls (HC) performed a validated WM paradigm to identify neural activity specifically associated with WM manipulation. A two‐sample t‐test was used to test for group differences. Mean specific binding ratios (SBRs) from DaT‐SPECT and susceptibility values from QSM MRI were computed for the caudate. Mean BOLD activity was partially correlated with caudate SBR and QSM values.

**Results:** Both groups showed robust widespread activation in the frontoparietal, cerebellar and anterior striatal regions (Figure 1). There were no group differences in activation and task performance. Within the PD group, higher caudate DaT SBRs were trend associated with stronger BOLD responses (partial *r* = 0.342, one‐sided *p* = 0.055), while higher caudate iron load as indexed via QSM correlated with reduced BOLD activity (partial *r* = –0.681, one‐sided *p* = 0.003) during WM manipulation (Figure 2).

**Figure:**
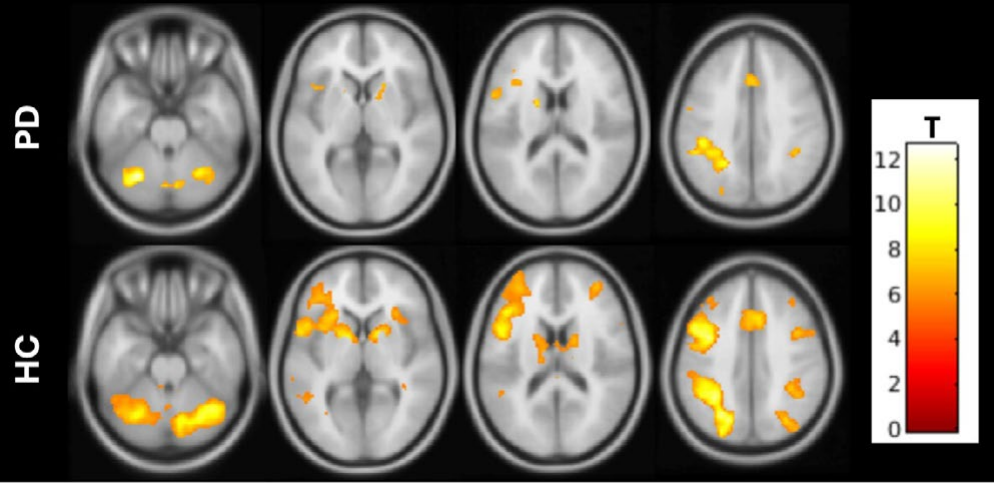
T‐maps for the WM manipulation contrast (*p* < 0.05, FWE‐corrected). Upper row: PD, lower row: HC.

**Figure:**
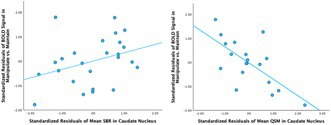
Scatterplots of the partial correlations between individual BOLD signal during WM manipulation and mean caudate DaT SBR (left) or mean caudate QSM (right) corrected for age and sex. For illustration, standardized residuals of BOLD activity and DaT SBR or.

**Conclusion:** Patients show preserved large‐scale WM activation patterns. However, task‐related BOLD modulation during WM manipulation relates to nigrostriatal integrity within the caudate nucleus. These results highlight caudate‐dependent modulation of executive processes within WM in early PD.

**Disclosure:** This work was supported by the Deutsche Forschungsgemeinschaft (DFG, German Research Foundation)—Project‐ID 431549029—SFB 1451/C03. H.T. was supported by the Cologne Clinician Scientist Program (CCSP)/ Faculty of Medicine/University of Cologne, funded by the DFG—Project ID 413543196. 1 Giehl K, Ophey A, Hammes J, Rehberg S, Lichtenstein T, Reker P, Eggers C, Kalbe E, van Eimeren T. Working memory training increases neural efficiency in Parkinson's disease: a randomized controlled trial. Brain communications. 2020;2(2):fcaa115.

## EPO‐0662

### Multisite ultrasound investigation of vagus nerve echostructure, regional substantia nigra, extraneural markers in Parkinsons disease: MULTIVERSE‐PD.

#### E. Bianchini^1^; G. Sergi^1^; G. Anzini^1^; S. Toro^1^; N. Casasoli^1^; M. Frizzi^2^; F. Spagnolo^2^; G. Vivacqua^3^; M. Marano ^1^

##### ^1^Fondazione Policlinico Universitario Campus Bio‐Medico, Rome, Italy; ^2^Department of Neurology, A. Perrino Hospital, Brindisi, Italy; ^3^Department of Microscopic and Ultrastructural Anatomy, Campus Biomedico University of Roma, Rome, Italy.

**Background and aims:** Ultrasound sonography (US) is a reliable cost‐effective tool to investigate brain and nerve tissue features. Substantia Nigra (SN) US has been extensively evaluated in Parkinson's Disease (PD) [1]. Along with SN, recent studies also examined vagus nerve (VN) US features, with alternate results [2]. The MULTIVERSE‐PD study aims at implementing the use of US biomarkers in PD. In this preliminary phase we aimed at implementing the use of SN and VN sonography in a well characterized sample of PD with mobility and fluid biomarkers.

**Methods:** VN cross sectional area (VN‐CSA) and SN hyperechogenic area (SN‐HA) were consecutively collected in mild‐to‐moderate PD patients. Participants were clinically assessed through MDS‐UPDRS, SCOPA, PDSS2, ESS, PDQ8 and other scales. All subjects underwent real‐world mobility evaluations through IMUs (McRoberts MoveMonitor) and plasmatic inflammatory and neurodegeneration biomarker dosing.

**Results:** Twenty patients were recruited (10 male, 10 female; age 66 ± 9; disease duration 7.3 ± 4; MDS UPDRS III 26 ± 12). Mean SN‐HA was 0.4 ± 0.1 cm2, VN‐CSA was 1.4 ± 0.5 cm^2^. SN‐HA was significantly related (*p <* 0.01) to total scores of MDS‐UPDRS part I and II, SCOPA, NMSS (*r* = 0.5–0.8), PDSS2 (*r* = 0.7) and PDQ8 (*r* = 0.5). VN‐CSA inversely related (*p <* 0.01) to MoCA (*r* = 0.7) and directly to ESS (*r* = 0.5).

**Conclusion:** SN and VN echographic features are promising biomarkers in PD. The MULTIVERSE‐PD study will collect more echographic biomarkers from neural and extraneural targets that will be harmonized with mobility and biochemical parameters through artificial intelligence. A multicenter effort is required.

**Disclosure:** Nothing to disclose.

## EPO‐0663

### Adult Tourette syndrome: Patient experience and clinical insights from a UK Regional Neurology Centre

#### R. McGinty ^1^; Jessica Lythgoe^1^; Z. Htet^1^; A. Arun^1^; A. Macerollo^1,2^

##### ^1^The Walton Centre NHS Foundation Trust, Liverpool, UK*;* ^2^Institute of Systems, Molecular and Integrative Biology, The University of Liverpool, UK

**Background and aims:** This study investigated tic phenomenology, psychiatric comorbidity and clinical pathways in adults with Tourette Syndrome (TS). Variation in access to appropriate services can prolong the diagnostic journey and pose challenges for management.

**Methods:** Retrospective analysis of electronic clinical records for adults with TS attending a regional neurology Centre, The Walton Centre NHS Foundation Trust (Liverpool, UK), between November 2020 and November 2025. Data collected included demographics, tic characteristics and medication use.

**Results:** Of 128 patients, 56 were women and the mean age was 30.6 years. The mean age of tic onset was 8.9 years and at TS diagnosis was 24 years. The estimated mean time from symptom‐onset to diagnosis was 184 months. In childhood, 14 patients had contact with paediatric neurology services. The clinical course of TS worsened for 46.9%. Tic‐related pain was experienced 26.6%. For tic management, 20.3% tried behavioural interventions and 66.4% tried medications (most commonly aripiprazole, clonidine and risperidone). Two patients were treated with deep brain stimulation at University College of London. Coexisting psychiatric disorders affected 118 (92.2%) individuals and, of these, 62 (52.5%) had three or more psychiatric diagnoses.

**Conclusion:** Most patients did not have access to appropriate TS services in childhood and faced a prolonged diagnostic delay. Only one‐fifth of patients had access to gold standard tic treatment with behavioural interventions, as this is not funded by the National Health Service. The high rate of psychiatric comorbidity underlines the importance of multidisciplinary management.

**Figure:**
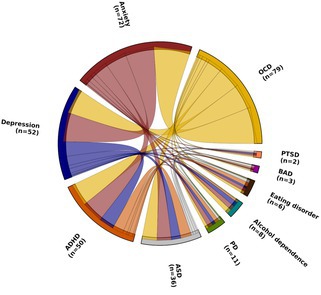
**FIGURE 1** Co‐existing mental health and neurodevelopmental disorders in adults with TS. Data shown for 118 patients (ten others did not have any mental health or neurodevelopmental diagnoses).

**Disclosure:** Nothing to disclose.

## Neuroimmunology 3

## EPO‐0664

### The invisible enemy : Shedding light on antibody‐negative autoimmune encephalitis

#### A. Dupic ^1^; B. Grall^1^; G. Picard^2^; C. Bost^3^; M. Benaiteau^2^; M. Rafiq^4^; D. Psimaras^1^; B. Stankoff^5^; J. Pariente^4^; J. Honnorat^2^; G. Dorcet^5^

##### ^1^Pitié‐Salpêtrière Hospital, AP‐HP, Paris, France; ^2^French Reference Centre on Paraneoplastic Neurological Syndromes, HCL, Lyon, France; ^3^Toulouse University Hospital, Toulouse, France; ^4^Toulouse Neuroimaging Centre, Paul Sabatier Toulouse‐3 University, Inserm, CNRS, Toulouse, France; ^5^Paris Brain Institute, Sorbonne University, Inserm, CNRS, Paris, France

**Background and aims:** Despite diagnostic criteria proposed in 2016, antibody‐negative autoimmune encephalitis (AE−) remain diagnostically elusive, often delaying access to effective treatments. While antibody‐positive AE (AE+) has been extensively characterized, AE‐ remains underexplored.

**Methods:** We built a multicenter database from the three French AE reference centers (Lyon, Paris, Toulouse), including patients diagnosed between 2016 and 2023, and followed for 2 years. This preliminary analysis includes 139 patients from Paris and Toulouse.

**Results:** We identified 110 AE+ (79%) and 29 AE‐ (21%). Mean age (56 years old) and sex‐ratio (1:1) were similar. AE‐ occurred more frequently in a post‐infectious context (17% vs 3%, *p* = 0.01) and showed less epilepsy (21% vs 43%, *p* = 0.03), and a trend to more neuro‐ophthalmologic involvement (31% vs 15%, *p* = 0.07). Non‐limbic presentations were also more frequent in AE− (45% vs 20%, *p* = 0.01). Despite these differences, the clinical severity was comparable between AE− and AE+, with similar rates of intensive care unit admissions (30% and 28%, respectively) and clinical scores (median modified Rankin score = 3 and CASE = 5 in both groups). Nevertheless, the prognostic RAPID score was higher in AE− (3 vs 1, *p* = 0.0001). On complementary investigations, AE− more frequently exhibited basal ganglia hypermetabolism on brain positron‐emission tomography (100% vs 39%, *p* = 0.0169), and diffuse or focal slowing on electroencephalography (63% vs 36%, *p* = 0.02). CSF cell count and proteins were similar, but interleukin‐6 levels were higher in AE− (21.5 pg/mL vs 3.4 pg/mL, *p* = 0.0265).

**Conclusion:** These preliminary findings suggest that AE‐ follows a distinct clinico‐radiological profile. Early identification may help guide more timely and targeted treatment strategies.

**Disclosure:** Nothing to disclose.

## EPO‐0665

### Real‐world experience of transition from intravenous to subcutaneous Ocrelizumab in multiple sclerosis

#### C. Guedes Vaz ^1^; M. Seco^2^; S. Costa^1^; J. Lopes^1^; D. Costa^1^; E. Santos^1^; R. Samões^1^

##### ^1^Serviço de Neurologia, Unidade Local de Saúde de Santo António, Porto, Portugal; ^2^Serviço de Neurologia, Unidade Local de Saúde de Matosinhos, Porto, Portugal

**Background and aims:** Subcutaneous (SC) Ocrelizumab has demonstrated non‐inferiority to the intravenous (IV) formulation in Multiple Sclerosis (MS). However, real‐world data on the transition from IV to SC remain limited. We aimed to evaluate patient‐reported outcomes (PROM), safety and logistical impact associated with its transition in clinical practice.

**Methods:** We conducted a retrospective study including all patients treated with Ocrelizumab in 2025, at a tertiary MS center. Demographic and clinical data were collected. Adverse events (AEs) were graded according to CTCAE v5.0. Patient satisfaction and experience with SC administration were assessed using a modified version of Treatment Administration Satisfaction Questionnaire.

**Results:** Of 115 patients treated with Ocrelizumab, 61 (53%) received the SC formulation, and 51 of these completed PROMs. Thirty (58.8%) were men, with a mean age of 45.5 ± 13.0 years and a median disease duration of 9 (IQR 1–35) years. Forty‐four (86.3%) patients previously received IV Ocrelizumab. The median number of SC administrations was 1 (IQR 2). AEs occurred in 70.6% (36/51), predominantly mild (grade 1) local injection‐site reactions. Overall, 82.3% of patients (42/51) were satisfied or very satisfied with the administration, 86.3% found it convenient, 81.8% (36/44) perceived time savings compared with IV infusion. Forty‐eight percent preferred SC formulation, 40.9% were neutral. No clinical relapses were observed, CD19+ B‐cell counts remained ≤5 cells/μL in 96% (24/25). The first SC administration reduced hospital time by 3h/patient (183h total); subsequent administration will reduce it 290h/session.

**Conclusion:** Transitioning from IV to SC Ocrelizumab was safe, well‐tolerated, and associated with high patient satisfaction and substantial logistical benefits in real‐world clinical practice.

**Disclosure:** Nothing to disclose.

## EPO‐0666

### Plasma neurofilament light chain, glial fibrillary acidic and Tau proteins dynamic in patients receiving CAR‐T treatment

#### C. Lapucci ^1^; M. Gambella^2^; S. Mariotto^3^; V. Chiodega^3^; R. Varaldo^2^; A. Ghiso^2^; M. Centanaro^4^; E. Angelucci^2^; A. Raiola^2^; M. Inglese^5^

##### ^1^IRCCS Ospedale Policlinico San Martino, Genoa, Italy; ^2^UO Ematologia e Terapie cellulari. IRCCS Ospedale Policlinico San martino. Genova, Italy; ^3^Neurology Unit, Department of Neurosciences, Biomedicine, and Movement Sciences, University of Verona, Verona, Italy; ^4^Anesthesiology and Intensive Care Unit, IRCCS Ospedale Policlinico San Martino, Genoa, Italy; ^5^Department of Neuroscience, Rehabilitation, Ophthalmology, Genetics, Maternal and Child Health (DINOGMI), University of Genoa, Genoa, Italy

**Background and aims:** ICANS is a severe adverse event of CAR‐T cell therapy with limited predictive factors. Neuroaxonal and astroglial biomarkers may reflect pre‐existing vulnerability and coexistent damage. This study evaluates the dynamic of neurofilament light chain (NfL), glial fibrillary acidic protein (GFAP), and Tau levels as predictors of ICANS.

**Methods:** Plasma NfL, GFAP, and Tau levels were measured at baseline (pre), day 7 (T1), and day 30 (T2) post‐infusion. Their levels were analysed to assess associations with ICANS occurrence and severity.

**Results:** Among 20 patients (median age 56.6 years), moderate‐severe ICANS occurred in 6/20 (30%), while 14/20 patients (70%) developed no or mild ICANS. Pre and T1 NfL, GFAP, and Tau levels did not differ between patients with moderate‐severe ICANS and no or mild ICANS. NfL increased at T1 in moderate–severe ICANS (*p* = 0.021). ΔNfL (T1–pre) predicted moderate‐severe ICANS (*p* = 0.04). NfL increased at T2 versus pre (*p* = 0.026) and T1 (*p* = 0.027) timepoints in moderate‐severe ICANS. A trend toward higher GFAP levels was observed at T1 in moderate‐severe ICANS (*p* = 0.054) with a decrease at T2 in both groups. Tau showed no significant associations at any timepoint.

**Figure:**
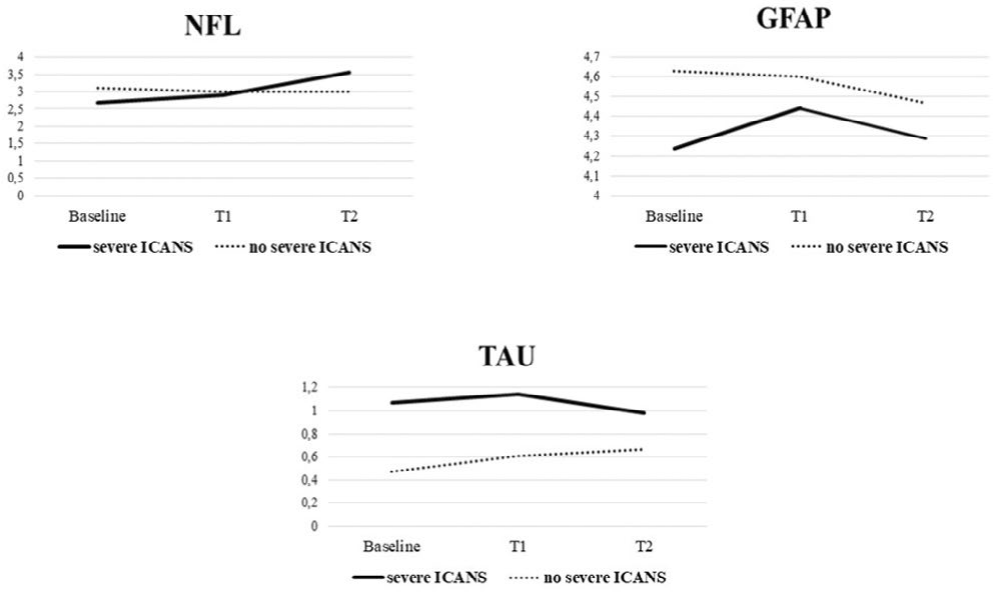
NFLs, GFAP and Tau dynamics in CAR‐T treated patients.

**Conclusion:** NfL, GFAP and Tau display distinct dynamics after CAR‐T. An early increase of NfL levels after baseline predicts moderate–severe ICANS. Patients with moderate–severe ICANS also exhibit a higher late rise of NfL compared with baseline and early measurements. GFAP increase may play a role in the early phase but does not appear to be involved in established ICANS.

**Disclosure:** Nothing to disclose.

## EPO‐0667

### Persistent synapse‐engulfing phagocytes shape the aftermath of encephalitis

#### G. Di Liberto; A. Mariotte; I. Vincenti; N. Page; G. Shammas; M. Piccinno; K. Egervari; M. Kreutzfeldt; I. Wagner; S. Lemeille; D. Merkler

##### Department of Pathology and Immunology, University of Geneva, Geneva, Switzerland

**Background and aims:** T cell–mediated encephalitides often cause neurological deficits that persist after resolution of overt inflammation, suggesting that long‐lived innate immune responses may sustain pathology beyond T cell contraction. While IFN‐γ–producing T cells initiate disease, how IFN‐γ enables phagocytes to acquire persistent pathogenic phenotypes remains unclear. As IFN‐γ induces durable epigenetic remodeling of synaptic genes in neurons, we hypothesized that IFN‐γ is required for acquisition of a persistent disease‐associated phagocyte state driving ongoing synaptic pathology independent of continued T cell activity.

**Methods:** We used a murine viral déjà vu model of T cell–mediated encephalitis, combining CSF1R‐mediated phagocyte depletion with genetic ablation of IFNGR2 or TREM2/APOE signaling. Lineage tracing tracked microglia and macrophages across disease stages. Sorted phagocytes were analyzed by single‐cell RNA sequencing, bulk ATAC‐seq, and SCENIC. Motor function and synaptic integrity were assessed behaviorally and histologically.

**Results:** CSF1R‐mediated phagocyte depletion ameliorated neurological deficits. Microglia and macrophages persisted in the CNS during late disease stages, when T cell responses declined. Bulk ATAC‐seq revealed sustained chromatin remodeling at phagocytosis‐, antigen presentation‐, and interferon‐responsive loci shared by both cell types. During acute disease, phagocytes acquired a synapse‐engulfing phenotype that persisted at lower levels later. Single‐cell RNA sequencing showed early convergence toward a disease‐associated state regulated by interferon‐responsive transcription factors. Disruption of TREM2/APOE signaling was not protective, whereas IFNGR2 ablation significantly improved motor performance and synaptic pathology. Similar phagocyte signatures were observed in Rasmussen encephalitis.

**Conclusion:** Persistent IFN‐γ–dependent phagocyte reprogramming sustains synaptic pathology after encephalitis, identifying CNS phagocytes as key drivers of long‐term neurological dysfunction.

**Figure:**
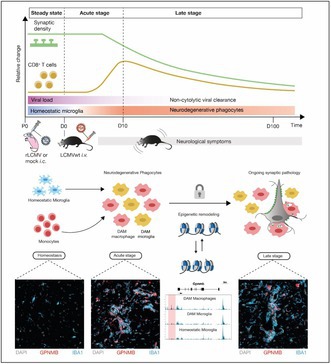
Synapse‐engulfing phagocytes persist after encephalitis. Acute CD8^+^ T‐cell responses coincide with synaptic loss and phagocyte activation. Microglia and macrophages adopt durable DAM‐like states with sustained chromatin remodeling and ongoing synaptic pat.

**Disclosure:** Nothing to disclose.

## EPO‐0668

### The role of the C5a–C5aR1 axis in mediating peripheral immune activation and central nervous system demyelination in MOGAD

#### H. Xue; C. Yang; W. Jiang

##### Department of Neurology, Tianjin Medical University General Hospital, Tianjin, China

**Background and aims:** Complement activation is involved in the pathogenesis of myelin oligodendrocyte glycoprotein antibody–associated disease (MOGAD), but whether it acts mainly through complement‐dependent cytotoxic effects or through bystander effects on the immune system—and to what extent it contributes—remains unclear.

**Methods:** Twenty‐two patients with MOGAD (10 acute, 12 remission) were enrolled. Peripheral blood was collected for serum complement profiling, immune‐cell analysis by flow cytometry, and peripheral blood mononuclear cell (PBMC) bulk RNA sequencing. The C5aR1 antagonist PMX205 was administered in a MOG‐IgG–augmented MOG35–55 experimental autoimmune encephalomyelitis (EAE) model. Histopathological and immunophenotypic analyses were performed on the brain and spinal cord; additional co‐culture experiments used naïve CD4^+^ T cells and myeloid cells.

**Results:** Acute MOGAD showed enrichment of inflammatory and chemotactic pathways, along with immune‐cell redistribution. The complement axis was upregulated; C5aR1 correlated most strongly with the inflammatory module and was also associated with chemotaxis and antigen‐presentation modules. Flow cytometry and enzyme‐linked immunosorbent assay (ELISA) indicated increased circulating C5a with increased cellular C5aR1 expression. In augmented EAE, PMX205 improved neurological outcomes, reduced central nervous system (CNS) myeloid infiltration, shifted T‐cell polarization (↓Th1, ↑Treg), and mitigated demyelination; co‐culture supported myeloid‐driven T‐cell differentiation.

**Figure:**
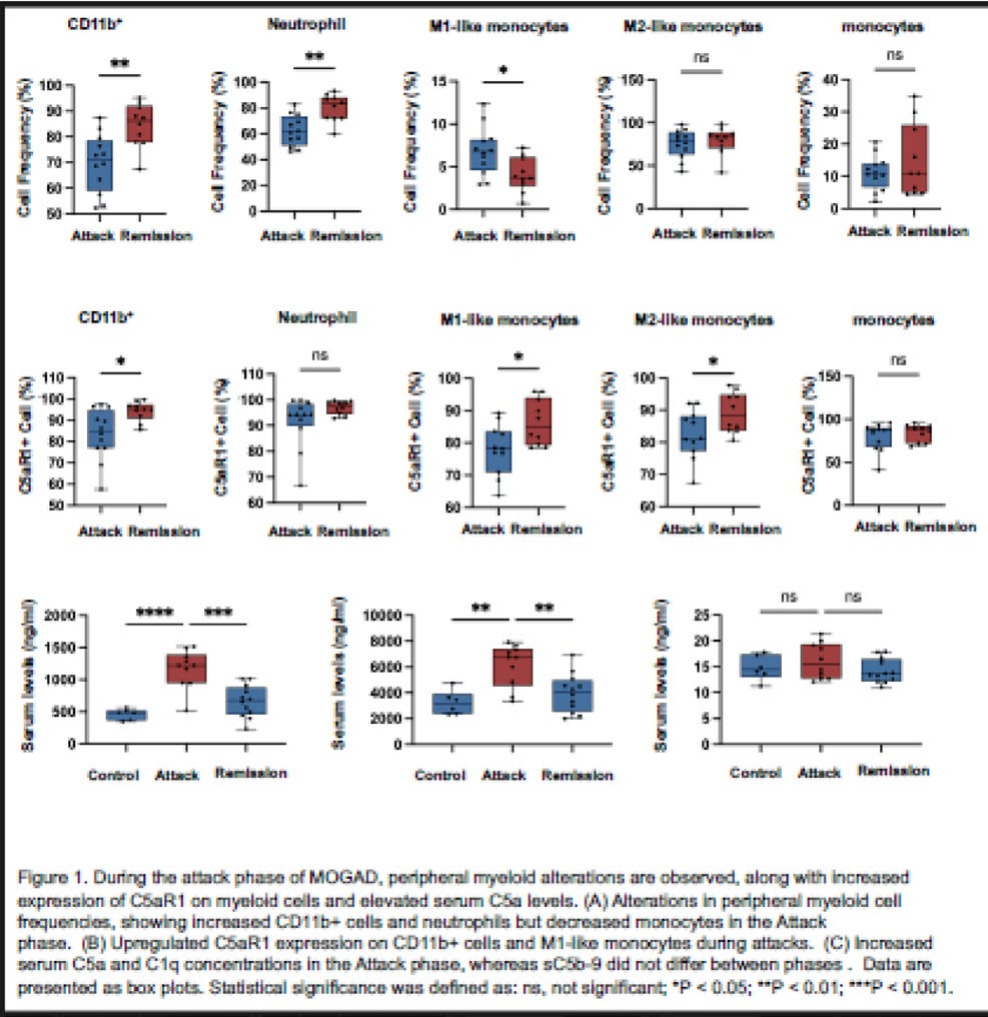
**FIGURE 1** Acute‐phase MOGAD exhibits a “low C5a–high C5aR1” imbalance.

**Figure:**
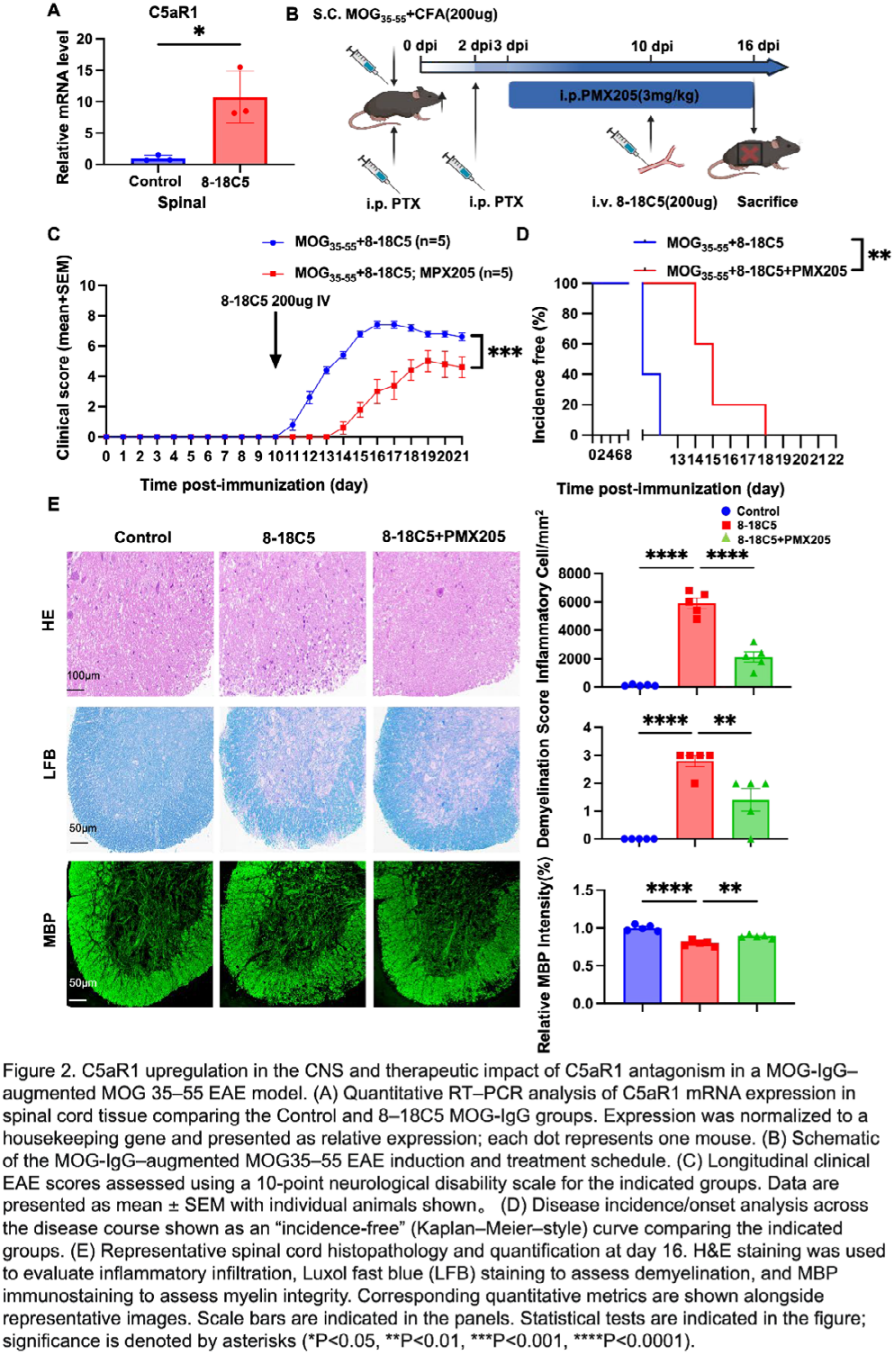
**FIGURE 2** C5aR1 upregulation in the CNS and therapeutic impact of C5aR1 antagonism in a MOG‐IgG–augmented MOG 35–55 EAE model.

**Figure:**
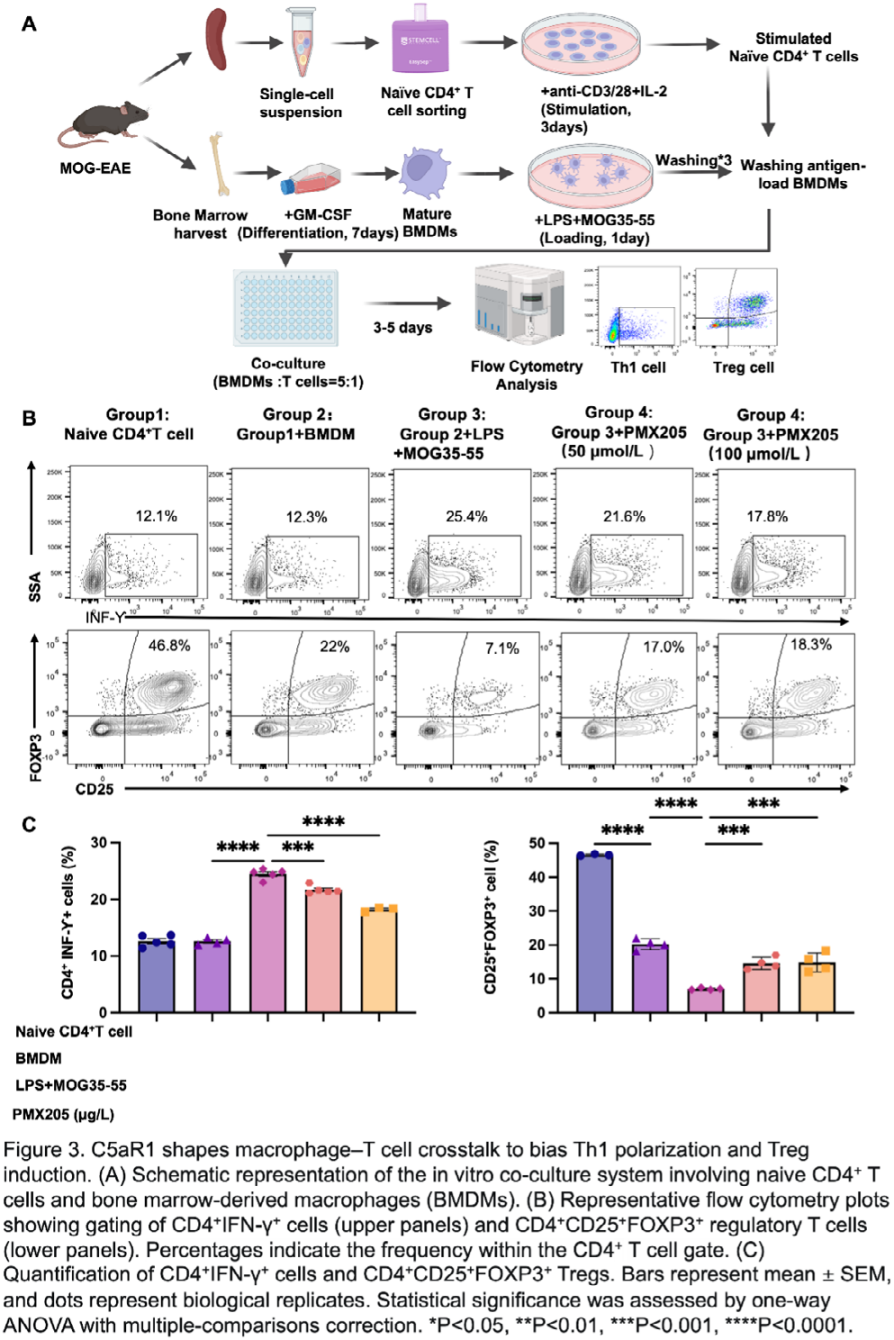
**FIGURE 3** C5aR1 shapes macrophage–T cell crosstalk to bias Th1 polarization and Treg induction.

**Conclusion:** The C5a–C5aR1 axis may contribute to peripheral immune activation in active MOGAD and, via myeloid recruitment and myeloid–T cell crosstalk, relate to CNS inflammation and demyelination.

**Disclosure:** This study was supported by the National Natural Science Foundation of China (82171277, 82571535, 82171338), and Young Scientific and Technological Talents (Level Three) in Tianjin.

## EPO‐0669

### Real‐world effectiveness and use of Efgartigimod in chronic inflammatory demyelinating polyradiculoneuropathy: ADHERE REAL study design

#### J. Zschüntzsch ^1^; J. Aldridge^2^; J. A. Allen^3^; A. De Roeck^2^; F. Eftimov^4^; D. Gneißl;^2^; S. Haghighi^2^; G. Istas^2^; B. Knier^5^; C. Pashos^2^; R. Traub^6^; C. Karam^7^

##### ^1^University Medical Center Göttingen, Göttingen, Germany; ^2^argenx, Ghent, Belgium; ^3^University of Minnesota, Minneapolis, USA; ^4^Amsterdam UMC, University of Amsterdam, Amsterdam, the Netherlands; ^5^Diak‐Klinikum Landkreis Schwäbisch Hall, Schwäbisch Hall, Germany and TUM Klinikum rechts der Isar, TUM School of Medicine and Health, Technical University of Munich, Munich, Germany; ^6^University of North Carolina, Chapel Hill, USA; ^7^University of Pennsylvania, Philadelphia, USA

**Background and aims:** Chronic inflammatory demyelinating polyradiculoneuropathy (CIDP), an immune‐mediated neuropathy, causes progressive muscle weakness and sensory disturbance. Efgartigimod, a human IgG1 antibody Fc fragment, reduces IgG recycling by blocking FcRn, reducing impact on CIDP pathophysiology. The efficacy and safety of subcutaneous efgartigimod PH20 in patients with CIDP was demonstrated in the registrational ADHERE trial. ADHERE REAL will assess the real‐world effectiveness of subcutaneous efgartigimod PH20 in patients with CIDP and describe their treatment journey and healthcare resource utilisation.

**Methods:** This real‐world, prospective, multicentre study will assess adults with CIDP across the US and Germany. Patients will be efgartigimod PH20 naïve at screening and initiate treatment in routine clinical practice. Clinician‐assessed and patient‐reported outcomes will be recorded regularly, and treatment/dosage changes or relapses will be recorded.

**Results:** Change over time in scores, including adjusted Inflammatory Neuropathy Cause and Treatment and Inflammatory Rasch‐built Overall Disability Score, will assess real‐world effectiveness of efgartigimod PH20 and will be monitored via patient questionnaires, nurse assessments and medical record review. Proportions of participants with CIDP relapse and with evidence of clinical improvement will be assessed. Assessment of patient‐treatment journeys will include disease course, comorbidities, CIDP treatment, modifications, and timing/rationale for these changes. Healthcare resource utilisation will be assessed by frequency, duration, and reasons for healthcare provider follow‐ups, emergency room visits and hospitalisations. As of December 2025, 12 participants have been enrolled in Germany; enrolment has begun in the US.

**Conclusion:** This study will expand on the ADHERE trial and provide insights into the real‐world usage of efgartigimod in patients with CIDP.

**Disclosure:** Jana Zschüntzsch: Alexion, Amicus, argenx, Biogen, CSL Behring, German Society for Muscle Diseases (DGM e.V.), German Society for Myasthenia Gravis (DMG), Hormosan, Janssen, Kedrion, Roche, Sanofi and UCB. Jeffrey A. Allen: Consultant fees: Akcea, Alexion, Alnylam, Annexon, argenx, CSL Behring, Grifols, Immunovant, ImmuPharma, Johnson & Johnson, Pfizer and Takeda. Jamie Aldridge, Arne De Roeck, Désirée Gneißl, Saman Haghighi, Geoffrey Istas, Chris Pashos: Employees of argenx. Filip Eftimov: Received investigator‐initiated research grants for CIDP from ZonMw, Prinses Beatrix Spierfonds, the GBS‐CIDP Foundation and Dianthus. As principal investigator of INCbase, reports investigator‐initiated grants from Kedrion, Terumo BCT, CSL Behring, Grifols, argenx and Takeda. The institution has received fees from Hansa, Sanofi, Dianthus, CSL Behring, Grifols and Takeda for advisory board participation and/or lectures. All grants and fees were paid to the institution. Benjamin Knier: Alexion, argenx, CSL Behring, Heidelberg Engineering, Hexal, Merck, Novartis, Novo Nordisk, Roche, Teva, Deutsche Forschungsgemeinschaft (DFG). Rebecca Traub: Consultant: argenx, Intellia, Sanofi. Clinical trials/research funding: Alnylam, argenx, Immunovant, Ionis, Takeda. Chafic Karam: Alexion, Alnylam, Annexon, Alpine, argenx, AstraZeneca, Biogen, Corino, CSL Behring, Genentech, Ionis, Neuroderm, Novo Nordisk, Pfizer, Sanofi, UCB, Takeda and Zai Lab.

## EPO‐0670

### Clinical outcomes with exploratory biomarker analyses from phase 2 of KYSA‐6, an open‐label, single arm, multicenter study of miv‐cel in gMG

#### J. Motte ^1^; S. Muppidi^2^; M. Hunter^3^; S. Hoffmann^4^; T. Hegelmaier^5^; R. Gold^1^; C. Schubert^6^; B. Hunter^7^; E. Meyer^8^; M. Hütter‐Krönke^9^; C. Schultze‐Florey^10^; R. Schroers^11^; F. Ayuk^12^; B. Xing^13^; J. Chou^13^; T. Van Blarcom^13^; N. Gehchan^13^; A. Haghikia^5^

##### ^1^Department of Neurology, Ruhr University Bochum, Bochum, Germany; ^2^Department of Neurology, Stanford Healthcare, Palo Alto, USA; ^3^Department of Neuromuscular Medicine, Intermountain Medical Center, Murray, Utah, USA; ^4^Department of Neurology with Experimental Neurology and Neuroscience Clinical Research Center (NCRC), Charité‐Universitätsmedizin Berlin, Berlin, Germany; ^5^Department of Neurology with Clinical Neurophysiology, Hannover Medical School, Hannover, Germany; ^6^Institute of Neuroimmunology and Multiple Sclerosis and Department of Neurology, University Medical Center Hamburg‐Eppendorf, Hamburg, Germany; ^7^Department of Hematology Oncology, Blood and Marrow Transplantation, Intermountain LDS Hospital, Salt Lake City, USA; ^8^Department of Medicine, Blood and Marrow Transplantation, Stanford Medicine, Palo Alto, USA; ^9^Department of Hematology, Oncology and Cancer Immunology, Charité Universitätsmedizin Berlin, Berlin, Germany; ^10^Department of Hematology, Hemostaseology, Oncology and Stem Cell Transplantation, Hannover Medical School, Hannover, Germany; ^11^Department of Hematology and Oncology, Ruhr University Bochum, Bochum, Germany; ^12^Department for Stem Cell Transplantation, University Medical Center Hamburg‐Eppendorf, Hamburg, Germany; ^13^Kyverna Therapeutics, Emeryville, USA

**Background and aims:** Generalized myasthenia gravis (gMG) is a B‐cell and antibody‐mediated neuromuscular autoimmune disease that can result in substantial disability. Novel therapies are needed to improve disease symptoms. Miv‐cel (mivocabtagene autoleucel; KYV‐101) is a fully‐human, autologous CD19 CAR T‐cell therapy with CD28 costimulation, designed for potency and tolerability. Updated data for miv‐cel in gMG from KYSA‐6 phase 2 (NCT06193889) are presented.

**Methods:** Adults (18–75 y) with gMG (class IIb–IV), history of autoantibodies (AChR, MuSK, LRP4), MG activities of daily living (MG‐ADL) score ≥6, and ≥2 immunosuppressant/immunomodulator failures, received lymphodepletion followed by 1×10^8^ CAR T cells.

**Results:** As of October 3, 2025, 6 patients were dosed (Table). Follow‐up was 28 days‐9 months (≥24 weeks, *n* = 3). Miv‐cel rapidly expanded with CAR T‐cell activation evidenced by deep B‐cell depletion and transient immunomodulatory cytokine elevation. Of 3 patients with ≥24‐weeks follow‐up, mean reductions from baseline were 8.0 and 7.7 points for MG‐ADL and QMG, respectively, at 24 weeks and 67% achieved minimal symptom expression (Figures 1 and 2). All 6 patients remained immunosuppressant therapy‐free up to 24 weeks. No ICANS or high‐grade CRS occurred. After miv‐cel, alterations in T‐cell subsets were observed in the 3 patients with ≥24‐weeks follow‐up and reconstituted B cells in 2 patients 3–4 months post‐infusion were predominantly naïve, supporting immune reset.

**Figure:**
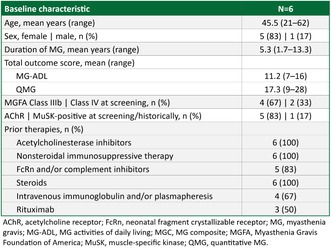
Patient demographics.

**Figure:**
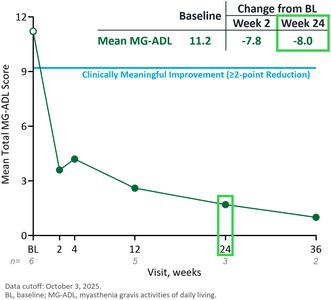
Miv‐cel showed rapid, robust, and sustained reduction in MG‐ADL (primary endpoint).

**Figure:**
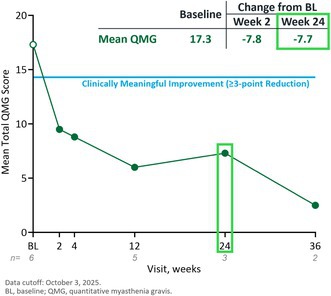
Miv‐cel showed rapid, robust, and sustained reduction in QMG scores.

**Conclusion:** With a single dose, miv‐cel induced deep B‐cell depletion with evidence of immune reset. Patients experienced rapid, robust, and sustained improvements in disease severity with acceptable safety. Miv‐cel demonstrates the potential to deliver durable, drug‐free, disease‐free remission in patients with gMG. Phase 3 is ongoing.

**Disclosure:** JM reports research support from Novartis and Kyverna, consulting fees from Novartis, payment or honoraria from Grifols and Novartis, support for meetings and/or travel from Sanofi, Candit, Novartis, and Johnson & Johnson, stock/stock options for Sanofi, Bayer, and Amgen, and receipt of equipment, materials, drugs, medical writing, gifts or other services from Candit. SM has served on advisory boards for AstraZeneca, Argenx, J&J, UCB, Amgen, Kyverna, Arcellx, and Seismic Pharma, and DSMB for Dianthus Therapeutics. SH has received speaker honoraria from Alexion, Argenx BV, Grifols, Johnson&Johnson, Merck, Roche, and UCB, honoraria for attendance at advisory boards from Alexion, Argenx BV, Johnson&Johnson, Novartis and Roche, and participated in trials from Alexion, Argenx, Immunovant, Johnson&Johnson, Kyverna and Regeneron. RG has received research support and speaker‘s honoraria from Bayer Schering, Biogen‐Idec, BMS, Chugai, Eisai, ELAN, Janssen, Kyverna, Merck Serono, Nikkiso Pharma, Novartis, Roche, Sanofi‐Genzyme, and TEVA, and consulting honoraria from ZLB Behring, Baxter, Kyverna, and Talecris, and has private stock from Merck, Kyverna, Novartis, Sanofi and Roche. CS received honoraria from Alexion and Argenx. BDH reports a research grant from Johnson&Johnson, honoraria from Kite Pharma, Novartis, BMS, ADC Therapeutics, Johnson&Johnson, Genmab, Notable Labs, AbbVie, Genentech, Astellas, In8 Bio, Regeneron, Poseida Therapeutics, Caribou Biosciences, and stock holdings for Actinium Pharmaceuticals. EM has received research grant support from Kyverna and Orca Biosystems, has done consulting for AstraZeneca and has equity as consultant in TRACT therapeutics, Indee, Jura and GigaMune. MLH‐K has received speakers' honoraria, and honoraria at advisory boards from Amgen, Grifols, Jazz, Sanofi, and Kyverna. CS‐F has received honoraria from BMS, Johnson&Johnson, AbbVie, Kite Pharma, Mikrogen, Pierre Fabre and Roche. RS has served on an advisory board for Kyverna. FA has received honoraria from AbbVie, BMS, Kite/Gilead, Janssen, Therakos, Miltenyi Biomedicine, Novartis, Takeda, Medac, Neovii; and research funding from Therakos and Neovii. BX, JC, TVB, NG are employees of Kyverna Therapeutics and may have stock/stock options. AH reports honoraria from Merck Serono, Kyverna, BMS, Amgen, Neuraxpharm, Sanofi, and Galapagos. MCH and TH have nothing to disclose.

## EPO‐0671

### Cortical excitability changes in neuromyelitis optica spectrum disorder: Insights from transcranial magnetic stimulation and clinical correlations

#### L. Luise; C. Siniscalchi; L. Mollo; L. Ruggiero; R. Dubbioso; R. Russo; R. Iodice

##### Department of Neurosciences, Reproductive Sciences and Odontostomatology, University of Naples *“*Federico II*”*, Via S. Pansini, Naples, Italy

**Background and aims:** NMOSD is a severe inflammatory demyelinating disease of the central nervous system, primarily affecting the optic nerves and spinal cord. Although NMOSD is classically considered to spare the brain, emerging evidence suggests functional cortical involvement. Altered cortical excitability may provide insights into the pathophysiology of NMOSD and its clinical manifestations.

**Methods:** A cross‐sectional study was conducted on 30 NMOSD patients and 30 age‐ and sex‐matched healthy controls. Transcranial magnetic stimulation (TMS) was used to assess key cortical excitability parameters, including resting motor threshold (RMT), short‐interval intracortical inhibition (SICI), intracortical facilitation (ICF), and cortical silent period (CSP). Correlations between TMS measures and clinical parameters, including disease duration, EDSS scores, relapse history, and presence of brain lesions on MRI, were analyzed.

**Results:** NMOSD patients exhibited significant cortical excitability alterations compared to controls, including increased RMT (*p* < 0.05), reduced SICI (*p* < 0.01), prolonged CSP (*p* < 0.05), and enhanced ICF (*p* < 0.05). Reduced SICI correlated with higher EDSS scores (*r* = −0.45, *p* < 0.01) and greater relapse frequency (*r* = −0.42, *p* < 0.05), suggesting impaired GABAergic inhibition in patients with more severe disease. Patients with supratentorial MRI lesions showed greater cortical excitability changes than those without.

**Conclusion:** Our findings suggest that NMOSD is associated with significant alterations in cortical excitability. These changes correlate with clinical severity and may reflect underlying neurophysiological dysfunction beyond the spinal cord. TMS could serve as a useful biomarker for cortical involvement in NMOSD and a potential tool for monitoring disease progression and therapeutic response.

**Disclosure:** Nothing to disclose.

## EPO‐0672

### Phenotype‐driven identification of autoimmune central vitamin B12 deficiency in idiopathic neuroinflammatory myelopathy

#### A. García Sarreón ^1^; A. Gifreu‐Fraixinó^1^; J. Gutierrez Naranjo^1^; A. Boix Lago^1^; J. Pluvinage^2^; G. Álvarez‐Bravo^1^

##### ^1^Multiple Sclerosis and Neuroimmunology Unit, Neurology Department, Dr. Josep Trueta University Hospital and Santa Caterina Hospital, Girona, Spain; ^*2*^Department of Neurology, University of California, San Francisco (UCSF), San Francisco, USA

**Background and aims:** The recent description of autoimmune vitamin B12 central deficiency mediated by antibodies against the transcobalamin receptor (CD320) provides a novel pathophysiological framework for selected idiopathic neuroinflammatory phenotypes. We applied this mechanistic concept to idiopathic neuroimmunological disease with myelopathy resembling subacute combined degeneration.

**Methods:** Patients were identified from an institutional registry of idiopathic neuroimmunological disorders and screened for predominant spinal cord involvement. Based on the proposed pathophysiological model of autoimmune central vitamin B12 deficiency, patients with clinical and magnetic resonance imaging features suggestive of subacute combined degeneration were selected for targeted anti‐CD320 testing in serum and cerebrospinal fluid at reference laboratories.

**Results:** Eighty‐four patients were included in the registry, of whom 23 showed predominant spinal cord involvement (double‐seronegative neuromyelitis optica spectrum disorder, *n* = 8; systemic autoimmune disease–associated neurological disease, *n* = 5; other idiopathic inflammatory phenotypes, *n* = 10). Six patients with posterior column–predominant myelopathy and compatible imaging features were prioritised; samples were available in all six (Table 1). Anti‐CD320 antibodies were detected in four cases, including one with double‐seronegative neuromyelitis optica spectrum disorder and three with other idiopathic inflammatory phenotypes. Seropositive patients presented with progressive myelopathy, posterior column dysfunction and non‐inflammatory cerebrospinal fluid profiles. Following antibody identification, two patients initiated rituximab and high‐dose intramuscular vitamin B12; follow‐up is ongoing.

**Figure:**
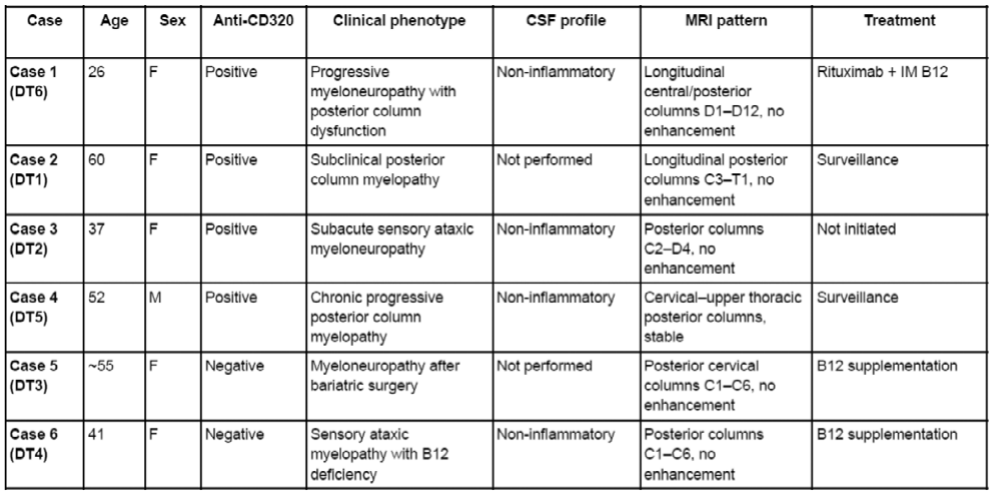
**TABLE 1** Clinical, cerebrospinal fluid, and MRI features of patients prioritised for anti‐CD320 antibody testing.

**Conclusion:** Anti‐CD320 antibodies were identified in a subset of patients with idiopathic neuroinflammatory myelopathy characterised by posterior column–predominant, subacute combined degeneration–like phenotypes, supporting a phenotype‐driven approach to targeted autoantibody screening with potential diagnostic and therapeutic relevance.

**Disclosure:** Garcia‐Sarreón, A.: has served as a speaker at events funded by Amgen and Sanofi; received travel grants from Roche, Novartis, and UCB Pharma; and completed an ECTRIMS Clinical Training Fellowship (2023–2024). Gifreu‐Fraixinó, A.: has received academic support from UCB Pharma, Bial Pharmaceutical, Angelini Pharma, Merck, Bristol‐Myers‐Squibb, Biogen, Janssen, Novartis, Roche, Neuraxpharm, Sanofi, Amgen. Boix‐Lago, A.: has received academic support from Merck, Bristol‐Myers‐Squibb, Biogen, Janssen. Gutiérrez Naranjo, J.: has received academic support from Merck, Lilly, Teva, Novartis, Abbvie, and speaking fees from Teva, Lilly, and Abbvie. Pluvinage, J. V.: has nothing to disclose. Álvarez‐Bravo, G.: has received compensation for consulting services and speaking fees from Biogen, Novartis, Merck, Sanofi, Amgen, Roche, Bristol‐Myers‐Squibb, Teva.

## EPO‐0673

### Serum GFAP versus NfL for predicting disability progression in multiple sclerosis: **A** systematic review and meta‐analysis

#### S. Biswas^1^; S. Vasireddy^1^; Y. Srivastava^1^; S. Arora ^2^; J. Rajamani^3^; P. Kulkarni^4^; R. Falodia^5^; K. Jindal^6^

##### ^1^Department of Internal Medicine, Ivano‐Frankivsk National Medical University, Ivano‐Frankivsk, Ukraine; ^*2*^Department of Internal Medicine, University of Debrecen, Hungary; ^*3*^Department of Internal Medicine Medical College, Caucasus University, Tbilisi, Georgia; ^*4*^Department of Internal Medicine, Ballari medical college and research centre, Ballari‐583104; ^*5*^Department of Internal Medicine, All India Institute of Medical Sciences, Jodhpur; ^*6*^Department of Internal Medicine Government Medical College, Patiala

**Background and aims:** Progression independent of relapse activity (PIRA) is increasingly recognised as a major driver of disability in multiple sclerosis (MS). Serum neurofilament light chain (NfL) is established for monitoring inflammatory activity, whilst glial fibrillary acidic protein (GFAP) has emerged as a potential marker of astrocytic damage and smouldering neurodegeneration. However, their comparative prognostic value for disability progression remains unclear. We aimed to compare serum GFAP and NfL as predictors of disability progression across MS subtypes.

**Methods:** We searched PubMed, Embase, Scopus, and Cochrane Library through December 2025. We included cohort studies and post‐hoc trial analyses reporting hazard ratios (HRs) for PIRA or confirmed disability worsening (CDW). Random‐effects meta‐analyses with Hartung‐Knapp adjustment were performed. Risk of bias was assessed using Newcastle‐Ottawa Scale.

**Results:** Eighteen studies (*n* = 15,944) were included. For PIRA, pooled HRs were 1.85 (95% CI 1.50–2.29; I‐squared=0%) for GFAP and 1.51 (95% CI 1.23–1.85; I‐squared=0%) for NfL. Overall pooled effects were HR 1.53 (1.28–1.82) for GFAP and HR 1.54 (1.41–1.67) for NfL. The formal GFAP/NfL ratio was 0.91 (0.80–1.04; *p* = 0.17), indicating no significant difference between biomarkers. In mutual adjustment analyses, GFAP remained significant after NfL adjustment (HR 1.80; *p* = 0.008), whilst NfL became non‐significant after GFAP adjustment (HR 1.45; *p* = 0.09).

**Figure:**
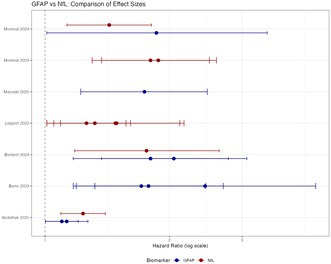
FIGURE 1

**Figure:**
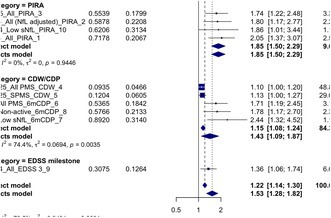
FIGURE 2

**Figure:**
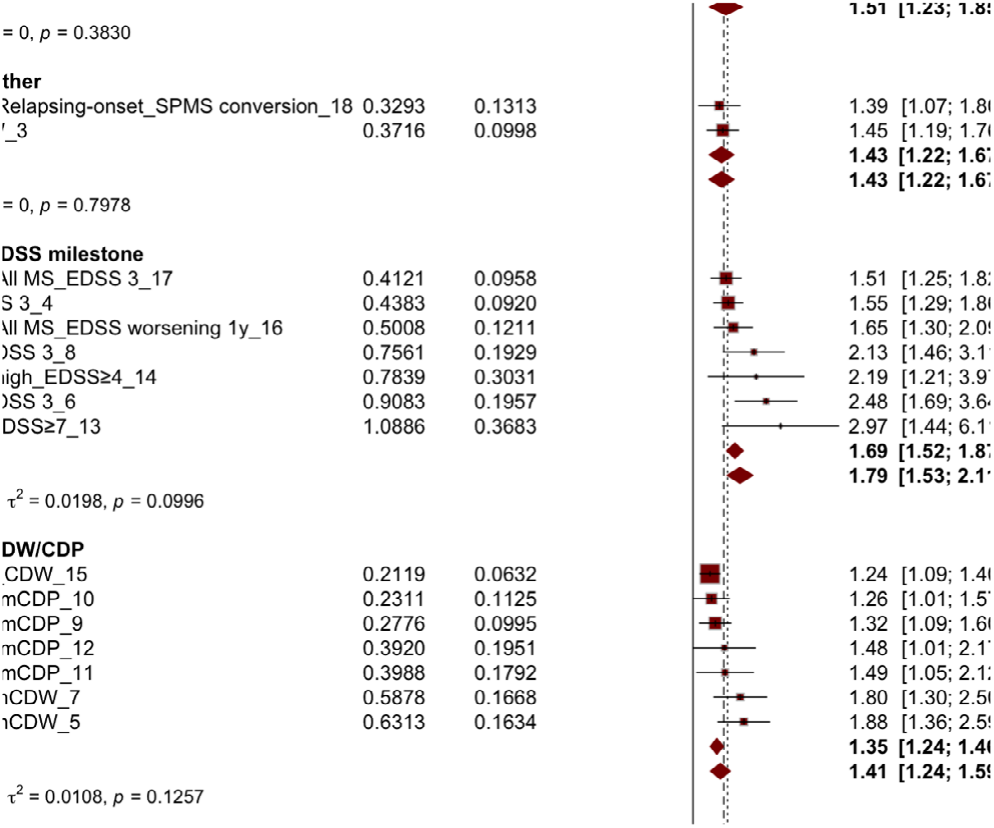
FIGURE 3

**Conclusion:** Both serum GFAP and NfL predict disability progression with similar overall effect sizes. GFAP shows stronger association with PIRA and provides independent prognostic information beyond NfL, supporting its complementary role in MS monitoring.

**Disclosure:** Nothing to disclose.

## EPO‐0674

### Early escalation of acute immunotherapy with plasma exchange and IVIG is associated with reduced relapse risk in pediatric MOGAD

#### S. Bartolomeo ^1^; R. Nistri^1^; N. Kim^1^; O. Ciccarelli^2^; C. Hemingway^1^; Y. Hacohen^1^

##### ^1^Department of Neurology, Great Ormond Street Hospital for Children, University College London, UK; ^2^Queen Square MS Center, Department of Neuroinflammation, UCL Queen Square Institute of Neurology, Faculty of Brain Sciences, University College London, UK

**Background and aims:** Acute treatment of myelin oligodendrocyte glycoprotein antibody–associated disease (MOGAD) includes high‐dose intravenous corticosteroids (IVCS), intravenous immunoglobulin (IVIG), and plasma exchange (PLEX), but the impact of different treatment strategies on long‐term outcome remains unclear. We evaluated whether early escalation of acute immunotherapy at the first pediatric MOGAD attack reduces relapse risk.

**Methods:** We conducted a retrospective single‐center cohort study of pediatric‐onset MOGAD (<18 years) evaluated between 2015 and 2016 and followed from first attack to last visit (median 3.1years). Of 114 screened patients, 98 were included. Patients received IV methylprednisolone (IVMP) alone or IVMP followed by escalation with IVIG, PLEX, or both based on disease severity or steroid response. Outcomes were early (≤1 year) and overall relapse. Cox regression assessed relapse risk.

**Figure:**
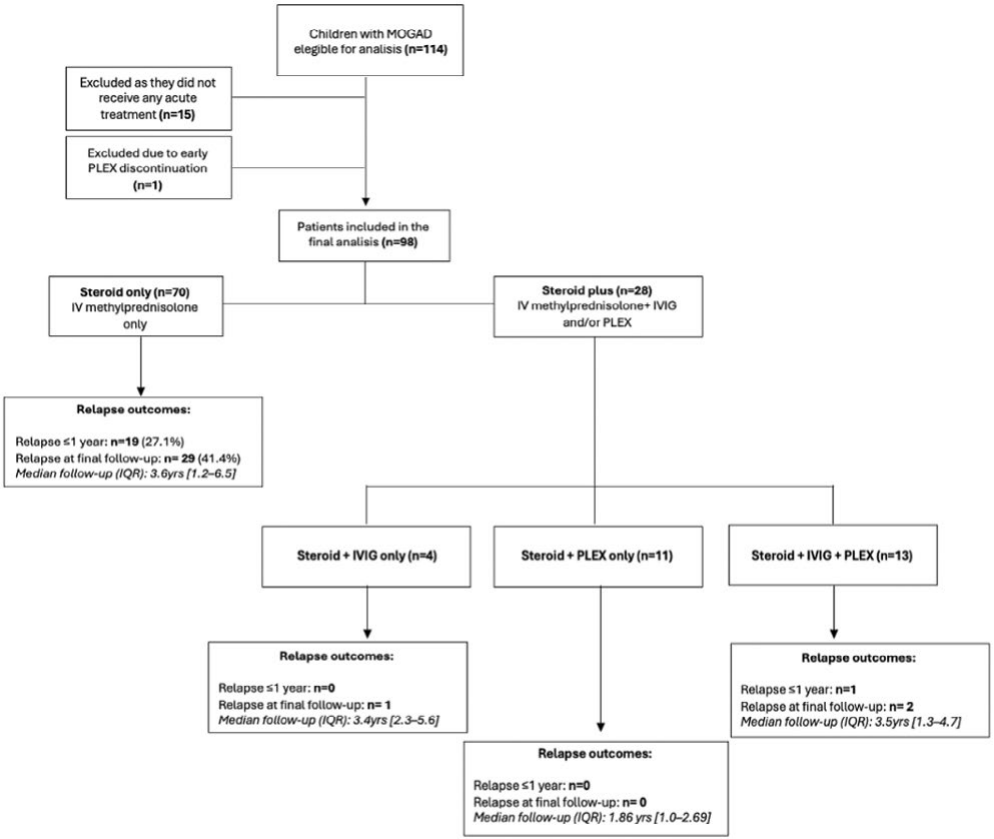
Study flow‐chart and relapse outcomes. Summarises the study flow‐chart and reports relapse outcomes in paediatric MOGAD patients grouped according to acute treatment strategy.

**Results:** Among 98 patients (57.1% female; median age 6.3years), onset phenotypes included optic neuritis (42.9%), acute disseminated encephalomyelitis (42.9%), cortical encephalitis (9.2%), and myelopathy (5.1%). Seventy received steroids alone and 28 received escalation therapy. Early relapse occurred in 27.1% of steroid‐only patients versus 3.6% with escalation (*p* = 0.011); overall relapse rates were 41.4% versus 10.7% (*p* = 0.004). Escalation was associated with reduced relapse risk (adjusted HR0.24, 95% CI 0.07–0.82).

**Figure:**
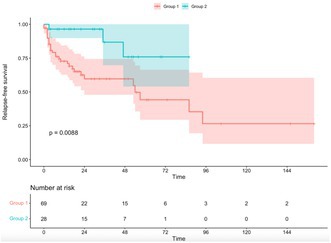
Kaplan–Meier survival curves illustrating time to first relapse (months) by treatment group. Patients treated with intravenous steroids alone experienced earlier relapses, with a median time to relapse of less than one year, whereas those who underwent t.

**Figure:**
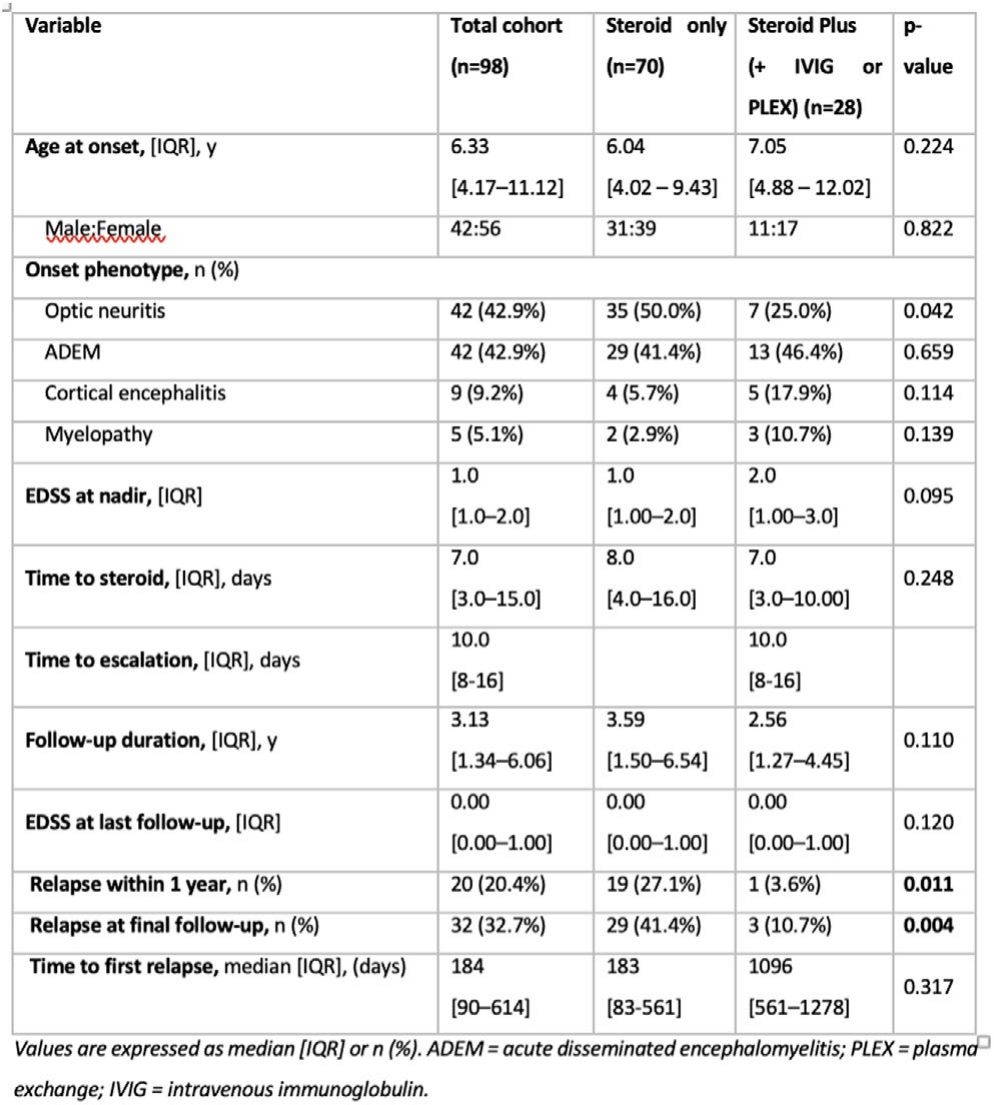
Demographic and clinical characteristics of the cohort (*N* = 98).

**Conclusion:** In pediatric MOGAD, early escalation with IVIG and/or PLEX following corticosteroids was associated with a substantially lower relapse risk compared with corticosteroids alone. These findings support the first clinical attack as a critical therapeutic window. Early aggressive therapy may reduce immune‐mediated relapses and improve long‐term disease trajectory. Prospective studies are needed to determine whether routine treatment escalation should be recommended to minimize relapse risk.

**Disclosure:** Bartolomeo S. received travel fundings from Biogen, Novartis, Sanofi, Bristol‐Myers Squibb. O. Ciccarelli is an NIHR Research Professor (RP‐2017‐08‐ST2‐004); acts as a consultant for Biogen, Merck, Novartis, Roche, and Teva; and has received research grant support from the MS Society of Great Britain and Northern Ireland, the NIHR UCLH Biomedical Research Centre, the Rosetree Trust, the National MS Society, and the NIHR‐HTA. Y. Hacohen received research grant support from the MS Society of Great Britain and Northern Ireland. Other authors have nothing to disclose.

## EPO‐0675

### Neuromyelitis optica spectrum disorder in Finland: **A** population‐based study

#### T. Aronen ^1^; S. Laakso^2^; M. Viitala^3^

##### ^1^Translational Immunology Research Program, University of Helsinki, Helsinki, Finland; ^2^Translational Immunology Research Program, University of Helsinki, and Neurocenter, Helsinki University Hospital, Helsinki, Finland; ^3^Aastat Ltd, Turku, Finland

**Background and aims:** Neuromyelitis optica spectrum disorder (NMOSD) is a demyelinating autoimmune disease of the central nervous system. Epidemiology of NMOSD in Finland has not been previously investigated on a national level. We set out to determine the incidence, prevalence and mortality for patients with NMOSD (pwNMOSD) in Finland during the last decades.

**Methods:** National care registries were searched for all diagnosed pwNMOSD (ICD‐10 code 36.0, ICD‐9 341.0) during 1994–2023. To evaluate confounders, the Helsinki University Hospital cohort was collected in detail using patient medical records during 2005–2023 and using the 2015 International Panel for NMO Diagnosis (IPND) criteria. Standardized mortality ratio (SMR) and causes of death were assessed using national care registries for 2015–2023.

**Results:** We identified 128 pwNMOSD in Finland, of which 78% were female. During 2015‐2023 standardized national incidence of NMOSD was 0.13/100,000 person‐years (95% CI 0.10–0.17). National prevalence increased from 1.10/100,000 in 2014 to 2.05/100,000 in 2023, with no clear regional differences. Peak in incidence was seen for the ages 30–49. SMR was 2.36 (95% CI 1.23–4.53) for pwNMOSD and NMOSD was the underlying or contributing cause of death for 46.7% of all the deaths recorded (*n* = 15).

**Conclusion:** NMOSD incidence in Finland is somewhat higher compared to other Nordic countries, and unlike reported for multiple slerosis incidence in Finland, shows no clear East‐West gradient. NMOSD has a clear impact on mortality in this population.

**Disclosure:** TA: Nothing to disclose. MV: Nothing to disclose. SML: Lecture fees Alexion, Argenx, Jansen, Lundbeck, Merck, Novartis, Sanofi, Teva; congress and travel expenses Merck, Novartis, UCB Pharma; advisory fees Argenx, Johnson & Johnson, Novartis, Sanofi, UCB Pharma. Funding: Maire Taponen Foundation (to SML).

## Cerebrovascular Diseases 6

## EPO‐0676

### Insight from Helsinki aneurysm database: Functional recovery in severely disabled survivors of aneurysmal subarachnoid hemorrhage

#### M. Dabbagh Ohadi; R. Tulamo; M. Niemelä; B. Rezai Jahromi

##### Department of Neurosurgery, Helsinki University Hospital, Helsinki, Finland

**Background and aims:** Aneurysmal subarachnoid hemorrhage (aSAH) frequently results in severe neurological disability. With advances in neurocritical and neurosurgical care, prognostication and assessment of functional recovery in patients discharged with severe disability have become increasingly important.

**Methods:** We retrospectively reviewed our aneurysm database from 1995 to 2015. Patients admitted within 72 hours of aSAH onset who survived hospitalization and were discharged with a mRS score of 4 or 5 were included. The primary outcome was good functional recovery at 3 months (mRS 0–3), with additional assessment at 1 year.

**Results:** A total of 1253 patients were included; 315 were discharged with mRS 4. Mean age was 57.4 ± 12.7 years, and 65% were women. Good functional recovery occurred in 730 patients (58.3%) at 3 months and in 266 (63.2%) at 1 year. In patients discharged with mRS 4, lower likelihood of recovery was associated with older age (OR 0.92), diabetes (OR 0.03), larger ICH volume (OR 0.96), and postoperative meningitis (OR 0.34). In patients discharged with mRS 5, reduced recovery was associated with older age (OR 0.95), Hunt and Hess grade 5 (OR 0.46), larger ICH volume (OR 0.98), cerebrospinal fluid diversion for hydrocephalus (OR 0.51), postoperative symptomatic brain infarction (OR 0.50), postoperative meningitis (OR 0.36), and pneumonia (OR 0.64).

**Figure:**
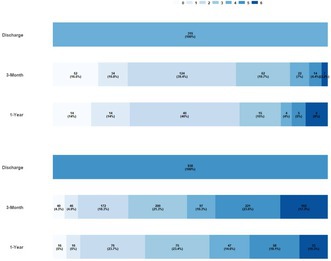
FIGURE 1

**Conclusion:** More than half of patients discharged with severe disability after aSAH achieved good functional recovery within 3 months. Increasing age, sever hemorrhage, and postoperative complications were consistently associated with reduced recovery.

**Disclosure:** Behnam Rezai Jahromi and Mika Niemelä have patents pending on aneurysm imaging.

## EPO‐0677

### Endovascular treatment versus best medical treatment in ischemic stroke due to distal to middle vessel occlusions: A meta‐analysis

#### B. Dell'Acqua ^1^; C. Costa^1^; V. Caso^2^; S. Vidale^1^

##### ^1^Department of Neurology & Stroke Unit ASST Sette Laghi, Varese, Italy; ^2^Department of Neurology, ASST Valle Olona Saronno, Italy

**Background and aims:** While mechanical thrombectomy (MT) is indicated in the treatment of large vessel occlusions, the consensus is still lacking regarding its superiority over the best medical treatment (BMT) in distal to middle vessel occlusions (DMVO). Aim of this pooled analysis was the investigation of the efficacy and safety of MT in DMVO compared to the BMT, including endovenous thrombolysis.

**Methods:** The study followed PRISMA guidelines. PubMed, EMBASE, and Cochrane Central were searched for studies comparing MT and BMT in adults with acute DMVO. The primary endpoint was good functional outcome at 180 days (mRS <3); secondary endpoints included excellent outcome at 3 months (mRS <2), sICH, and 180‐day mortality. Outcomes were pooled using meta‐analysis.

**Results:** A total of 43 studies including 13,049 patients were analyzed. Good functional outcome at 180 days was similar between MT and BMT (OR 1.00, 95% CI 0.84–1.19). The two treatment strategies had no differences in excellent outcome at 3 months (OR 1.09; 95% CI: 0.95–1.26; pheterogeneity < 0.0001), as well as mortality (OR 1.09; 95% CI: 0.95–1.26; pheterogeneity < 0.0001), while sICH rates were significantly higher in the MT group (OR=1.57; 95% CI: 1.20–2.05; pheterogeneity = 0.06).

**Figure:**
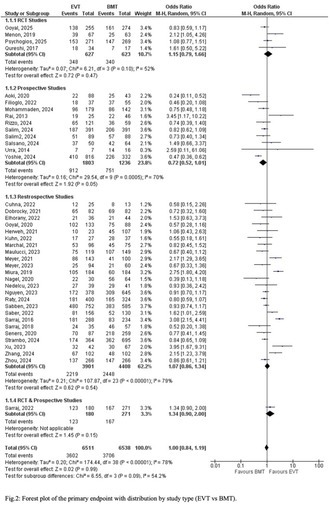
**FIGURE 1** Forest plot of the primary endpoint with distribution by study type (EVT vs BMT).

**Figure:**
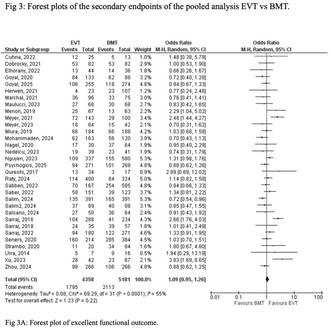
**FIGURE 2A** Forest plot of excellent functional outcome.

**Figure:**
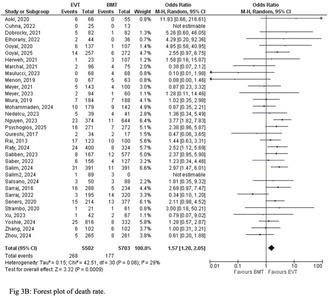
**FIGURE 2B** Forest plot of death rate.

**Conclusion:** This meta‐analysis shows similar functional outcomes between endovascular treatment and BMT in DMVO, with a higher rate of sICH in the MT group. These findings highlight the importance of patient selection based on occlusion site, collateral status, and clinical presentation, and support the need for further studies using stricter selection criteria.

**Disclosure:** Nothing to disclose.

## EPO‐0678

### Multiple fusiform cerebral aneurysms and inflammatory vasculopathy associated with cardiac myxoma

#### C. Ragucci ^1^; E. Matteo^2^; F. Naldi^3^; L. Piccolo^3^; M. Gentile^3^; F. Palombo^4^; L. Simonetti^5^; A. Zini^3^

##### ^1^Dipartimento di Scienze Biomediche e Neuromotorie, Università di Bologna, Bologna, Italy; ^2^IRCCS Istituto delle Scienze Neurologiche di Bologna, Ospedale Maggiore, Neurologia Rete Stroke Metropolitana, Bologna, Italy; Dipartimento di Scienze Biomediche e Neuromotorie, Università di Bologna, Bologna, Italy; ^3^IRCCS Istituto delle Scienze Neurologiche di Bologna, Ospedale Maggiore, Neurologia Rete Stroke Metropolitana, Bologna, Italy; ^4^IRCCS Istituto delle Scienze Neurologiche di Bologna, Programma di Neurogenetica, Ospedale Bellaria, Bologna, Italy; ^5^IRCCS Istituto delle Scienze Neurologiche di Bologna, Ospedale Maggiore, Neuroradiologia Rete Stroke Metropolitana, Bologna, Italy

**Background and aims:** Fusiform cerebral aneurysms are a rare complication of cardiac myxomas, possibly due to myxomatous cell embolization to the vasa vasorum. This phenomenon may lead to diagnostic uncertainty in acute cerebrovascular presentations.

**Methods:** We report a comprehensive diagnostic workup in a patient with recurrent ischemic events, including multimodal computed tomography (CT), digital subtraction angiography (DSA), brain MRI with vessel wall (VW) imaging, cerebrospinal fluid (CSF) analysis, autoimmune and infectious screening, and whole exome sequencing (WES).

**Results:** A 41‐year‐old woman with a previous left atrial myxoma resection and multiple cerebral aneurysms presented with acute headache and visual disturbances. Brain CT revealed a right occipital hypodensity of uncertain nature. DSA showed in situ thrombosis of a fusiform aneurysm of the right posterior cerebral artery. Acetylsalicylic acid was started. VW MRI demonstrated concentric wall thickening and enhancement of multiple aneurysms, with leptomeningeal enhancement in cerebellar and temporo‐occipital regions. CSF analysis showed pleocytosis, elevated proteins, blood–brain barrier dysfunction, and oligoclonal band mirror pattern. Autoimmune, infectious, and genetic investigations were unremarkable. Whole‐body CT angiography revealed an additional small splenic artery aneurysm. During follow‐up, she experienced a recurrent ischemic event due to thrombosis of a fusiform aneurysm in the right middle cerebral artery territory. Six‐month VW MRI showed persistent enhancement without size change.

**Figure:**
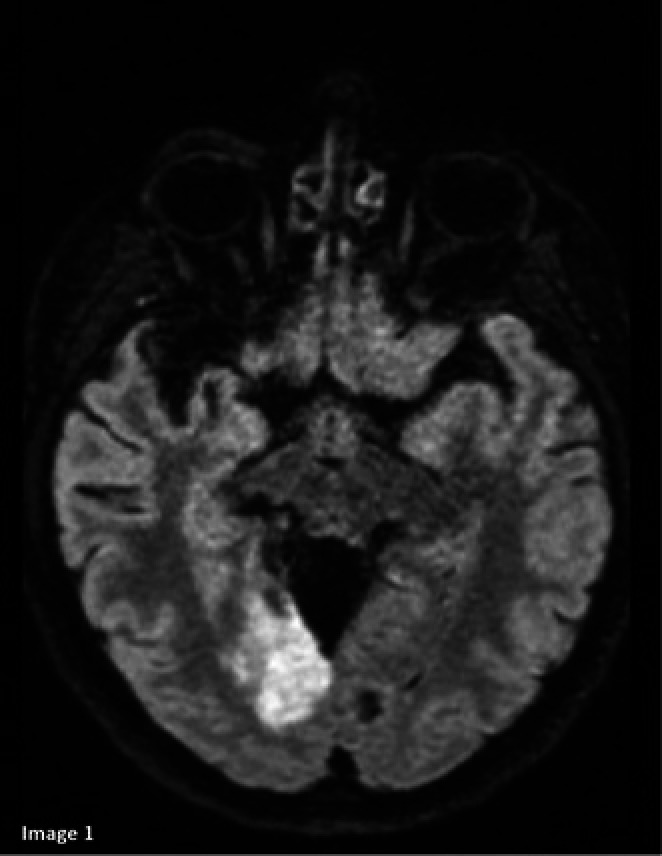
**IMAGE 1** Axial FLAIR MRI showing hyperintense signal in the right occipital lobe.

**Figure:**
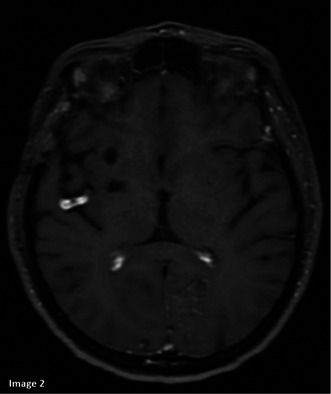
**IMAGE 2** Axial T1‐weighted VW MRI after contrast injection showing concentric thickening of one of the aneurysms located in the M2 segment of the right middle cerebral artery, with strong contrast enhancement.

**Figure:**
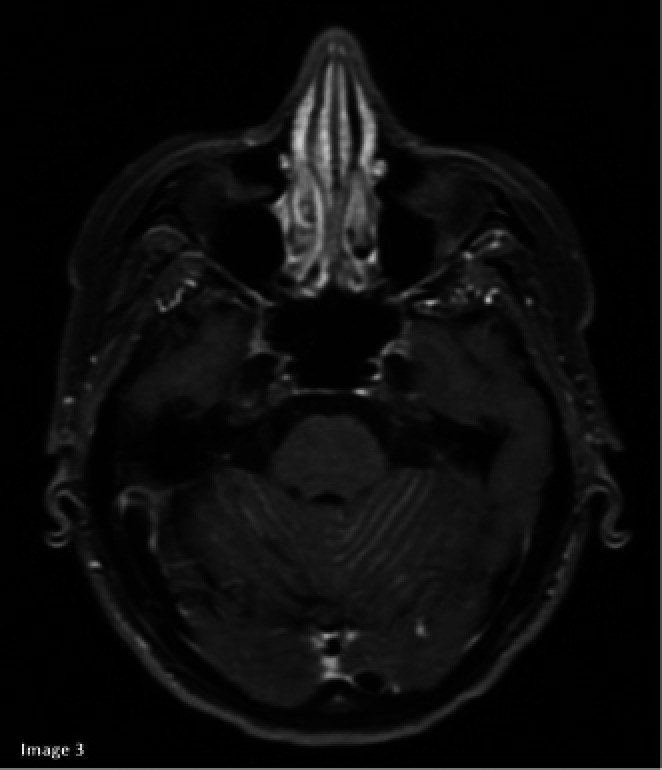
**IMAGE 3** Axial T1‐weighted MRI showing showing leptomeningeal contrast enhancement of the cerebellar folia.

**Conclusion:** This case highlights a dynamic inflammatory cerebral vasculopathy related to cardiac myxoma. Detailed vascular characterization is crucial to guide management and avoid misdiagnosis in the acute stroke setting.

**Disclosure:** Nothing to disclose.

## EPO‐0679

### Determinants of acute symptomatic seizures and post‐stroke epilepsy after reperfusion therapy: an 18‐month Dual Center Cohort Study

#### E. Ranxha; O. Çibuku

##### Neuro *Vascular* Service, University Hospital “Mother Teresa”, Tirane, Albania

**Background and aims:** Seizures are a frequent complication of stroke and are associated with worse functional outcomes. With the widespread use of reperfusion therapies (RTs), including intravenous thrombolysis and mechanical thrombectomy, their potential impact on acute symptomatic seizures (ASS) and post‐stroke epilepsy (PSE) requires clarification.

**Methods:** We conducted a retrospective cohort study over 18 months at the Neuro vascular Department of the University Hospital Centre “Mother Teresa” and Stroke Center American Hospital, Tirana. Consecutive patients with acute ischemic stroke were grouped as intravenous thrombolysis (IV tPA, *n* = 88), endovascular thrombectomy with or without thrombolysis (EVT, *n* = 55), and non‐revascularized controls (*n* = 66). ASS was defined as seizures within 7 days and PSE as unprovoked seizures beyond 7 days. Hemorrhagic transformation (HT) was assessed on follow‐up imaging.

**Results:** ASS occurred in 8/88 (9.1%) IV tPA patients, 6/55 (10.9%) EVT patients, and 4/66 (6.1%) controls (*p*>0.05). PSE developed in 6/88 (6.8%), 2/55 (3.6%), and 3/66 (4.5%) patients, respectively (*p*>0.05). When reperfusion‐treated patients were pooled, ASS occurred in 14/143 (9.8%) versus 4/66 (6.1%) in controls. HT was present in 4/18 (22.2%) patients with ASS and in 7/11 (63.6%) patients with PSE, indicating a strong association between HT and late epilepsy. Cortical involvement was frequent among seizure patients across all groups. Median NIHSS scores among patients with ASS were similar between groups.

**Figure:**
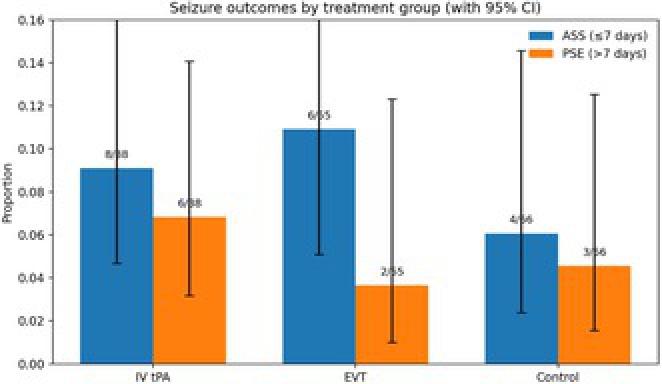
Seizure outcomes by treatment group with 95% confidence intervals.

**Figure:**
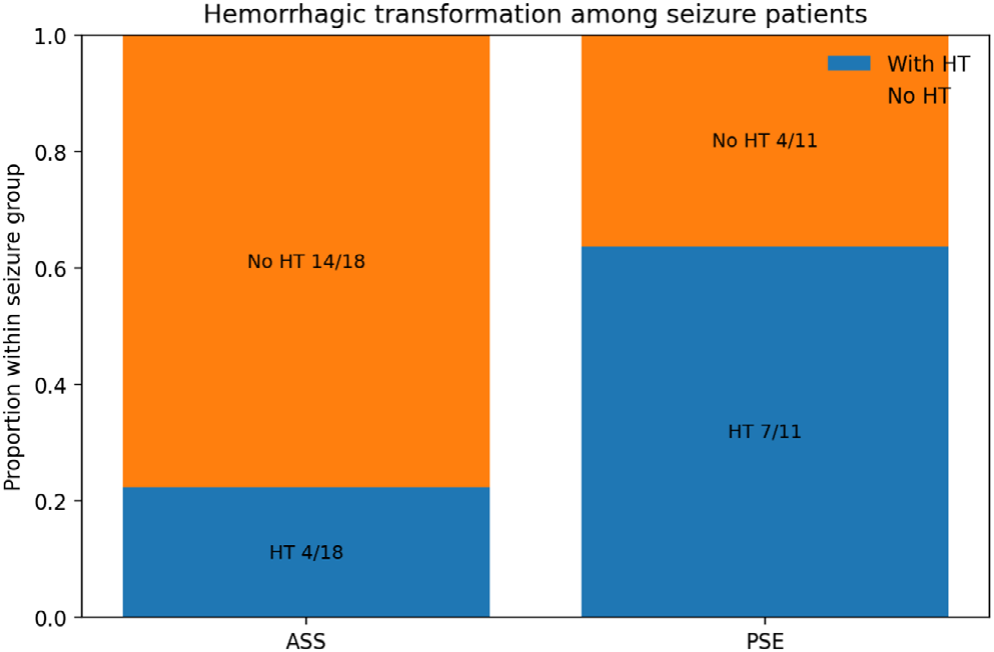
Proportion of hemorrhagic transformation (HT) in patients with ASS and PSE.

**Figure:**
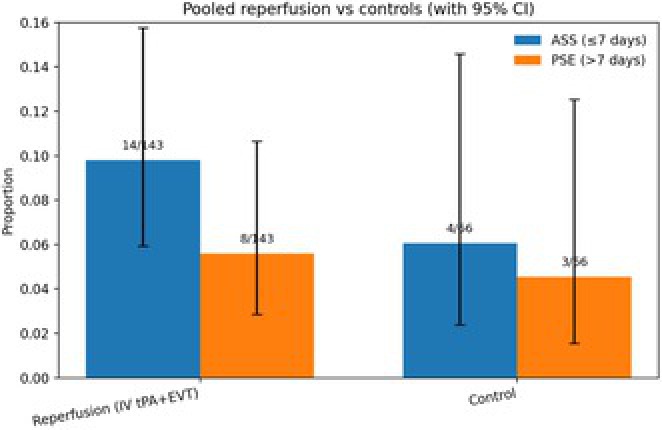
Pooled reperfusion versus controls with 95% confidence intervals.

**Conclusion:** Reperfusion therapies were not associated with an increased risk of ASS or PSE. Hemorrhagic transformation and cortical involvement appear to be the principal determinants of post‐stroke epileptogenesis, particularly for late epilepsy.

**Disclosure:** Nothing to disclose.

## EPO‐0680

### Elevated cerebral venous pulsatility as an independent predictor of hemodynamic compromise and functional outcomes in atherosclerotic ischemic stroke

#### F. Mallaev; G. Rakhimbaeva; S. Shokirov

##### Tashkent *State Medical University*, Tashkent, Uzbekistan

**Background and aims:** Venous pulsatility measurements provide insights into distal vascular resistance and intracranial hemodynamic burden, yet remain underinvestigated in cerebrovascular disease. We examined cerebral venous pulsatility as a potential biomarker for stroke severity and three‐month functional recovery in patients with major artery atherosclerosis.

**Methods:** This observational cohort investigation enrolled 120 consecutive patients presenting with acute ischemic stroke attributable to ≥50% stenosis of major cerebral vessels. Venous pulsatility index measurements were obtained from deep cerebral veins and principal venous sinuses using transcranial Doppler ultrasonography within 48 hours of symptom onset. Initial neurological impairment was quantified using the National Institutes of Health Stroke Scale, while 90‐day functional status was determined through modified Rankin Scale assessment.

**Results:** Elevated venous pulsatility demonstrated strong associations with greater baseline neurological compromise (NIHSS ≥ 10: venous pulsatility index 1.46 ± 0.18 versus 1.29 ± 0.16, *p <* 0.001). Unfavorable three‐month outcomes (mRS 3–6) occurred in 52 patients (43.3%) and showed significant correlation with increased baseline venous pulsatility (*p <* 0.001). Multivariable logistic regression analysis, adjusting for patient age, lesion volume, stenosis severity, and collateral status, confirmed elevated venous pulsatility as an independent predictor of poor functional recovery (OR 2.7, 95% CI 1.5–5.0).

**Conclusion:** Cerebral venous pulsatility effectively identifies hemodynamic stress and independently forecasts functional outcomes following atherosclerotic ischemic stroke. Assessment of venous pulsatility may offer a valuable non‐invasive prognostic instrument for stroke management.

**Disclosure:** Nothing to disclose.

## EPO‐0681

### Analysis of the racial differences in the prevalence of risk factors at stroke onset in Italy: a prospective, multicentric study on 2402 patients

#### I. Scala ^1^; P. Rizzo^2^; M. Monforte^3^; A. Zini^4^; A. Falcou^5^; M. Del Sette^6^; M. Guarino^7^; A. Cavallini^8^; L. Chiapparini^9^; M. Bagnato^10^; V. Andreone^11^; C. Rapillo^12^; L. Caputi^13^; M. Diomedi^14^; D. Centonze^15^; D. Toni^5^; A. Morotti^16^; S. Sacco^17^; A. Bersano^1^; P. Calabresi^3^; G. Frisullo^3^

##### ^1^Cerebrovascular Unit, IRCCS Fondazione Istitutito Neurologico C. Besta, Milan; ^2^Catholic University of the Sacred Heart, Rome; ^3^Department of Neuroscience, Fondazione Policlinico Universitario A. Gemelli IRCCS, Rome; ^4^IRCCS Istituto delle Scienze Neurologiche di Bologna, Department of Neurology and Stroke Center, Maggiore Hospital, Bologna, Italy; ^5^Emergency Department, Stroke Unit, Policlinico Umberto I, Rome, Italy; ^6^Neurology Unit, IRCCS San Martino Hospital, Largo R. Benzi 10, Genoa, Italy; ^7^IRCCS Istituto delle Scienze Neurologiche di Bologna, Neurology Unit, Sant’Orsola‐Malpighi University Hospital, Bologna, Italy; ^8^Department of Emergency Neurology and Stroke Unit, IRCCS C. Mondino, Pavia, Italy; ^9^Fondazione IRCCS Policlinico San Matteo, Pavia; ^10^Ospedale F. Spaziani, Frosinone; ^11^AORN Antonio Cardarelli, Napoli; ^12^IRCCS Humanitas Research Hospital, Milan; ^13^Ospedale Maggiore di Crema, Crema; ^14^Policlinico Tor Vergata, Roma; ^15^Istituto Neurologico Mediterraneo Neuromed IRCCS, Pozzilli; ^16^Spedali Civili di Brescia, Brescia; ^17^Ospedale SS Filippo e Nicola, Avezzano

**Background and aims:** Risk factors for Ischemic Stroke (IS) and adherence to primary prevention strategies may differ substantially across racial and ethnic groups. However, available evidence derives from the USA, where the racial/ethnic composition differs markedly from that of Europe. The primary aim of our study was to investigate racial/ethnic differences in the prevalence of stroke risk factors and primary prevention therapies in Italy.

**Methods:** Consecutive adult patients with IS presenting to 14 Italian stroke centers between October 2024 and November 2025 were prospectively enrolled. Based on self‐reported race/ethnicity, patients were classified as White or non‐White. Outcomes were the prevalence of stroke risk factors and preventive therapies in use at time of stroke onset. The Mann‐Whitney *U*‐test and χ^2^‐test were used for statistical comparisons. To identify independent associations between race/ethnicity and stroke risk factors, multivariable logistic regression models adjusted for sex and age were performed.

**Results:** A total of 2402 patients were enrolled in the study, of whom 2257 were Whites (94.0%). Compared with White patients, non‐White patients were younger (62.9 ± 16.7 vs 74.1 ± 13.9 years; *p <* 0.001) and less frequently women (33.8% vs 47.2%, *p* = 0.002). Cardiopathy (*p* = 0.037), dyslipidaemia (*p* = 0.016), and atrial fibrillation (*p* = 0.001) were more prevalent among White patients, whereas non‐White patients more frequently reported no ongoing therapy at admission (*p <* 0.001). However, after adjustment for age and sex, White race was not independently associated with any of these factors.

**Conclusion:** Non‐White patients experience ischemic stroke at a younger age than White patients, potentially reflecting earlier exposure to cardiovascular risk factors and lower awareness of them and their management.

**Disclosure:** Nothing to disclose.

## EPO‐0682

### Longer onset‐to‐ door times in non‐white patients in Italy: A prospective, multicentric study on 2402 patients

#### I. Scala ^1^; P. Rizzo^2^; M. Monforte^3^; A. Zini^4^; A. Falcou^5^; M. Del Sette^6^; M. Guarino^7^; A. Cavallini^8^; L. Chiapparini^9^; M. Bagnato^10^; V. Andreone^11^; C. Rapillo^12^; L. Caputi^13^; M. Diomedi^14^; D. Centonze^15^; A. Morotti^16^; S. Sacco^17^; A. Bersano^1^; P. Calabresi^3^; G. Frisullo^3^

##### ^1^Cerebrovascular Unit, IRCCS Fondazione Istitutito Neurologico C. Besta, Milan, Italy; ^2^Catholic University of the Sacred Heart, Rome, Italy; ^3^Department of Neuroscience, Fondazione Policlinico Universitario A. Gemelli IRCCS, Rome, Italy; ^4^IRCCS Istituto delle Scienze Neurologiche di Bologna, Department of Neurology and Stroke Center, Maggiore Hospital, Bologna, Italy; ^5^Emergency Department, Stroke Unit, Policlinico Umberto I, Rome, Italy; ^6^Neurology Unit, IRCCS San Martino Hospital, Genoa, Italy; ^7^IRCCS Istituto delle Scienze Neurologiche di Bologna, Neurology Unit, Sant’Orsola‐Malpighi University Hospital, Bologna, Italy; ^8^Department of Emergency Neurology and Stroke Unit, IRCCS C. Mondino, Pavia, Italy; ^9^Fondazione IRCCS Policlinico San Matteo, Pavia, Italy; ^10^Ospedale F. Spaziani, Frosinone, Italy; ^11^AORN Antonio Cardarelli, Napoli, Italy; ^12^IRCCS Humanitas Research Hospital, Milan, Italy; ^13^Ospedale Maggiore di Crema, Crema, Italy; ^14^Policlinico Tor Vergata, Rome, Italy; ^15^Istituto Neurologico Mediterraneo Neuromed IRCCS, Pozzilli, Italy; ^16^Spedali Civili di Brescia, Brescia, Italy; ^17^Ospedale SS Filippo e Nicola, Avezzano, Italy

**Background and aims:** Studies from the USA have reported lower access to reperfusion treatments (RTs) for Ischemic Stroke (IS) among racial/ethnic minorities; however, evidence from Italy is lacking. We aimed to assess the impact of race/ethnicity on RTS administration and onset‐to‐door time (ODT).

**Methods:** Consecutive adult patients with IS presenting to 14 Italian stroke centers between October 2024 and November 2025 were prospectively enrolled. Based on self‐reported race/ethnicity, patients were classified as White or non‐White. Outcomes were Intravenous Thrombolysis (IVT), Endovascular Treatment (EVT), and ODT. Associations between ODT and race/ethnicity were assessed using linear regression adjusted for demographics, comorbidities, baseline NIHSS, premorbid mRS, education level, mode of arrival, occupational status, language barriers, and cohabitation status. Logistic regressions adjusted for the same variables, plus large vessel occlusion, anticoagulant use, and ASPECT score were used to identify predictors of IVT and EVT.

**Results:** In total 2402 patients were enrolled in the study, of whom 2257 were Whites (94.0%). Overall, 830 patients (35.7%) received IVT and 648 (27.9%) EVT. Compared with White patients, non‐White patients were younger (62.9 ± 16.7 vs 74.1 ± 13.9 years; *p <* 0.001) and less frequently women (33.8% vs 47.2%, *p* = 0.002). White category was not associated with IVT and EVT administration (aOR 0.70 95% CI (0.34–1.67), *p* = 0.497 and aOR 2.50 95% CI (0.84–7.40), *p* = 0.098, respectively) but was independently associated with shorter ODT (aβ −631.69 95% CI (−1100.34 to −163.03); *p* = 0.008).

**Conclusion:** Our study suggests that racial/ethnic disparities in timely access to stroke care exist even in countries with universal healthcare systems, highlighting the need for public health interventions.

**Disclosure:** Nothing to disclose.

## EPO‐0683

### Abstract withdrawn

## EPO‐0684

### Beyond objective measures: Perceived environment associates with post‐stroke quality of life

#### L. Braadt ^1^; M. Naumann^1^; C. Meisinger^2^; J. Linseisen^2^; M. Ertl^3^; E. Hertig^4^

##### ^1^Department of Neurology and Clinical Neurophysiology, University Hospital Augsburg, Augsburg, Germany; ^2^Epidemiology, Faculty of Medicine, University of Augsburg, Augsburg, Germany; ^3^Department of Neurology and Neurological Rehabilitation, BKH Günzburg, Günzburg, Germany, Department of Neurology and Clinical Neurophysiology, University Hospital Augsburg, Augsburg, Germany; ^4^Regional Climate Change and Health, Faculty of Medicine, University of Augsburg, Augsburg, Germany

**Background and aims:** Stroke causes substantial disability and reduced health‐related quality of life (HRQoL). Environmental and atmospheric factors are increasingly recognized as health predictors, yet current research predominantly relies on heterogeneous objective measurements (e.g, satellite‐based green space indices, air quality monitors), while subjective environmental perception remains underexplored. We investigated associations between perceived environment quality and HRQoL after stroke.

**Methods:** In a prospective cohort study, stroke patients were surveyed during hospitalization and at 3‐ and 12‐month follow‐ups. HRQoL was measured using the Stroke Impact Scale (SIS). Subjective environmental ratings (air quality/noise, green spaces, thermal comfort) were assessed using the Perceived Residential Environment Quality questionnaire (PREQ) and Building Environmental Quality Questionnaire (BEQQ). Multivariable regression models adjusted for age, sex, baseline mRS, baseline NIHSS, and educational background were employed to analyse associations between subjective environmental perception and long‐term HRQoL.

**Results:** Of 531 baseline participants (mean age 68.8 years, 39.4% female), 456 (85.9%) completed 12‐month follow‐up. First analyses revealed that thermal comfort at 3 months is significantly associated with multiple SIS domains at 12 months. Green spaces and air quality showed no significant associations after correction for multiple testing.

**Conclusion:** While prior research emphasizes objective environmental measures, our findings highlight subjective perception, particularly thermal comfort, as a potentially relevant factor for post‐stroke HRQoL. Thus, a rather easily adaptable factor might already improve long‐term outcomes of stroke survivors, if considered in rehabilitation and post‐stroke care.

**Disclosure:** Nothing to disclose.

## EPO‐0685

### Association between cortical microinfarcts and cortical superficial siderosis in cerebral amyloid angiopathy: Evidence for a double‐hit mechanism?

#### M. Losa ^1^; M. Zanon Zotin^2^; T. van Harten^1^; H. van den Brink^1^; R. Carr^1^; L. Roccatagliata^3^; M. Pardini^4^; S. Greenberg^1^; A. Viswanathan^1^; S. van Veluw^5^; M. Kozberg^1^

##### ^1^J Philip Kistler Research Center, Department of Neurology, Massachusetts General Hospital and Harvard Medical School, Boston, USA; ^*2*^Center for Imaging Sciences and Medical Physics, Department of Medical Imaging, Hematology and Clinical Oncology, Ribeirão Preto Medical School University of São Paulo Ribeirão Preto SP Brazil; ^*3*^Neuroradiology, Department of Health Sciences, University of Genoa, Genoa, Italy; ^*4*^Department of Neuroscience, Rehabilitation, Ophthalmology, Genetics, Maternal and Child Health (DINOGMI), University of Genoa, Genoa, Italy; ^5^BHF‐UK DRI Centre for Vascular Dementia Research, University of Edinburgh, Scotland, UK

**Background and aims:** Cerebral amyloid angiopathy (CAA) is characterized by hemorrhagic and non‐hemorrhagic injuries. Cortical cerebral microinfarcts (CMI) have been associated with cortical superficial siderosis (cSS), although data are inconclusive. This study aims to determine the spatiotemporal relationship between CMI and cSS in CAA, with the hypothesis that cSS can promote local ischemic injury.

**Methods:** Probable CAA (Boston Criteria v2.0) were recruited from a prospective Massachusetts General Hospital cohort. CAA‐related radiological manifestations—including CMI and cSS—were quantified at baseline and at 24 months. Multivariate logistic regression and generalized linear models were applied to assess associations between baseline radiological features and their predictive value on radiological progression. We compared the local density of CMIs—defined as CMI Density Index, CDI—in regions with and without cSS.

**Results:** Among 74 probable CAA (age: 73.0 [67.9–78.2] years), 26 (35%) had CMIs (*n* = 135, 39% underlying cSS). At baseline, CMI count demonstrated an exponential relationship with cSS volume (Exp(B) = 1.12 [95% CI 1.07–1.18]; *p <* 0.001) and with lobar lacunes count (Exp(B) = 1.65 [95% CI 1.52–1.80]; *p <* 0.001). The CDI was markedly higher in cSS‐covered vs. non‐cSS regions (median: 20.84 [0.00–56.95] vs. 0.49 [0.11–0.89]; *p* = 0.010). Follow‐up scans (*n* = 36) revealed 23 incident CMIs (43% underlying cSS). Baseline cSS volume predicted CMI occurrence (OR: 1.41 [95% CI 1.02–1.95]; *p* = 0.036), whereas baseline CMI burden did not predict cSS progression.

**Conclusion:** Our findings demonstrate a CMI/cSS interplay, with cSS predicting incident CMI but not vice versa. Higher CDI in cSS‐covered regions suggests local ischemia synergistically worsened by global ischemic burden. Further studies should identify the driving pathophysiology, a possible therapeutic target for preventing cSS‐induced injury.

**Disclosure:** Nothing to disclose.

## EPO‐0686

### Stress hyperglycemia ratio and risk of cerebral edema in acute ischemic stroke: A systematic review and meta‐analysis

#### M. Serageldin; A. Hegazi; H. O. Hussien; A. Hafez; M. Elfadali; M. Abd EL‐Hamied; S. Abouelenein

##### Faculty of Medicine, Mansoura University, Egypt

**Background and aims:** Stress hyperglycemia is common following acute ischemic stroke (AIS) and correlates with adverse outcomes. While absolute hyperglycemia is a known risk factor, the Stress Hyperglycemia Ratio (SHR) offers a more physiological metric of relative hyperglycemic stress. However, its specific relationship with life‐threatening complications like cerebral edema remains incompletely defined. We aimed to evaluate the association between high SHR and the development of cerebral edema in patients with AIS.

**Methods:** A systematic review and meta‐analysis were conducted in accordance with PRISMA guidelines. We searched major electronic databases including PubMed, Scopus, Web of Science and Cochrane Library up to December 2025 for observational studies that evaluate the predictive impact of SHR on cerebral edema formation in adult AIS patients. The primary objective was to quantify the risk magnitude associated with elevated SHR levels. Pooled odds ratios (ORs) with 95% confidence intervals (CIs) were calculated using a random‐effects model.

**Results:** Five studies comprising a total of 2634 patients were included in the meta‐analysis. The pooled analysis demonstrated that high SHR was significantly associated with an increased risk of cerebral edema following ischemic stroke (OR 2.56, 95% CI 1.32–4.96, *p* = 0.006). This indicates more than a twofold higher odds of cerebral edema among patients with elevated SHR.

**Conclusion:** Stress hyperglycemia ratio is a significant predictor of cerebral edema after ischemic stroke. Early calculation of SHR may facilitate the rapid identification of high‐risk patients, informing closer neurological monitoring and targeted therapeutic strategies to mitigate edema progression.

**Disclosure:** Mohamed Serageldin, Amir Hegazi, Hussein o. Hussein, Ahmed Hafez, Mohamed Elfadali, Mahmoud Abd EL‐Hamied and Sherif Abouelenein: Nothing to disclose.

## EPO‐0687

### A decade of audio‐vestibular presentations to TIA service: A high‐risk cerebrovascular presentation

#### S. Mariathasan ^1^; R. Alijam^1^; C. Tierney^1^; T. Serafimova^1^; A. Hammam^2^; N. Koohi^3^; A. Chandratheva^1^; D. Kaski^4^; M. Mahmud^4,^ R. Periyasami^1^

##### ^1^Department of Stroke Medicine, National Hospital for Neurology and Neurosurgery, London, United Kindom*;* ^2^Department of Neuroradiology, National Hospital for Neurology and Neurosurgery, London, UK*;* ^3^Department of Clinical and Movement Neurosciences, University College London, London, UK*;* ^4^Department of Neurology, National Hospital for Neurology and Neurosurgery, London, UK

**Background and aims:** Vestibular symptoms are common in emergency settings but are perceived as low risk for cerebrovascular disease. Sudden‐onset hearing loss and pulsatile tinnitus, whilst recognised as necessitating urgent input, are commonly seen through ENT pathways. We aimed to quantify the burden of respectively vestibular and auditory presentations to a large tertiary transient ischaemic attack (TIA) service and determine associated stroke and TIA diagnoses.

**Methods:** Prospectively collated consecutive referrals to a central London TIA service between June 2015 and November 2025 were analysed. Referral reasons were categorised into neurological symptom clusters. Vestibular presentations were defined using Bárány Society‐informed descriptors, including vertigo, dizziness, imbalance, unsteadiness, and giddiness. Auditory symptoms were defined as hearing loss and tinnitus. We calculated the proportion of referrals for these symptom clusters and subsequent stroke/TIA diagnoses, comparing outcomes using logistic regression.

**Results:** Vestibular symptoms accounted for 14% (*n* = 1783) of 12,760 new referrals. Patients referred with isolated vestibular symptoms had a definite or probable stroke/ TIA diagnosis rate of 24% (*n* = 241/994), which significantly increased to 39% (*n* = 306/789) for vestibular presentations referred with additional neurological symptoms (OR 1.98, 95% CI 1.62–2.43; *p <* 0.001). By contrast, just 46 patients (<0.5%) presented with auditory symptoms, but 5/46 were diagnosed with definite or probable stroke/TIA and 2/46 with cervical artery dissection. One dissection presented with isolated sudden‐onset pulsatile tinnitus and headache without other neurological features.

**Conclusion:** Auditory and vestibular presentations to a specialist TIA clinic have high cerebrovascular diagnostic rates. Findings support low thresholds for specialist assessment to reduce missed neurovascular events.

**Disclosure:** Nothing to disclose.

## Neuro‐Ophthalmology/Neuro‐Otology 1

## EPO‐0688

### Comparative evaluation of large language models in differentiating grades of papilledema using optical coherence tomography

#### A. Mishra; R. Shukla; A. Shukla

##### All India Institute of Medical Sciences, Raebareli, Uttar Pradesh, India

**Background and aims:** Accurate grading of papilledema is essential for monitoring disease progression and guiding management in patients with intracranial hypertension. Large language models (LLMs) have emerged as potential clinical decision‐support tools; however, their performance in differentiating papilledema grades using Optical Coherence Tomography (OCT) ‐derived features remains unclear.

**Methods:** This observational comparative study included 15 de‐identified OCT datasets from patients with idiopathic intracranial hypertension, stratified by papilledema severity: mild (Frisén grades I–II, *n* = 5), moderate (grade III, *n* = 5), and severe (Frisén grades IV–V, *n* = 5). Structured OCT descriptions, including peripapillary retinal nerve fiber layer (RNFL) thickness, optic nerve head contour, and macular changes, were independently analyzed by ChatGPT, Google Gemini, and Microsoft Bing. Model‐assigned severity grades were compared with clinician‐determined Frisén grades.

**Results:** Overall accuracy for correct papilledema severity classification was highest for ChatGPT (77%), followed by Google Gemini (69%) and Microsoft Bing (55%). For mild papilledema (grades I–II), ChatGPT correctly classified 80% of cases, Google Gemini 72%, and Microsoft Bing 60%. In moderate papilledema (grade III), accuracy declined across all models, with correct classification rates of 68% for ChatGPT, 70% for Google Gemini, and 48% for Microsoft Bing. For severe papilledema (grades IV–V), ChatGPT demonstrated the highest accuracy (88%), followed by Google Gemini (75%) and Microsoft Bing (58%). Misclassification most frequently occurred between adjacent grades, particularly between mild and moderate papilledema, and moderate and severe papilledema.

**Conclusion:** Large language models demonstrate moderate accuracy in differentiating papilledema severity using OCT‐derived features, with more reliable performance at the extremes of disease severity and reduced accuracy for intermediate grades.

**Disclosure:** Nothing to disclose.

## EPO‐0689

### Vestibular agnosia in elderly subjects with small vessel disease and imbalance – preliminary results from an ongoing prospective study

#### F. Honegger^1^; E. Capodaglio^2^; M. Dapprich^2^; C. Nickel^3^; H. Gensicke^4^; S. Engelter^4^; B. Seemungal^5^; C. Stieger^6^; H. Rust ^2^

##### ^1^Division of Neurootology, Department of ORL, University of Basel Hospital, Basel, Switzerland; ^2^Department of Neurology, University of Basel Hospital, Basel, Switzerland; ^3^Department of Emergency Medicine, University of Basel Hospital, Basel, Switzerland; ^4^Neurology and Neurorehabilitation, University Department of Geriatric Medicine Felix Platter, University of Basel, Basel, Switzerland; ^5^Centre for Vestibular Neurology, Department of Brain Sciences, Imperial College London, London, UK; ^6^Division of Audiology, Department of ORL, University of Basel Hospital, Basel, Switzerland

**Background and aims:** Vestibular agnosia (VA) is a brain disconnection syndrome that manifests with imbalance and an altered sensation of self‐motion perception despite an intact peripheral vestibular system. VA can be measured and quantified employing vestibular psychophysics. Only recently VA was described for the first time in acute traumatic brain injury. VA has been reported to occur in elderly patients in whom there is disconnection within the brain due to small vessel disease (SVD). We assess vestibular perception and its relation to balance control in elderly patients with SVD and imbalance, dizziness and falls.

**Methods:** Ongoing exploratory prospective two‐centre study. 9 patients with SVD (Fazekas 2–3) and imbalance/ falls were assessed, 2 males, 7 females (age 78–93, average 82.8 years). 4 elderly controls were assessed, 1 male, 3 females (age 78, 72, 74, 89 years). Vestibular perceptual thresholds (VPTs) were determined for yaw‐plane rotations with randomly presented half cosine stimuli (0.1 Hz). Peripheral vestibular function, balance control, reaction times, cognition and self‐reported symptoms of dizziness, anxiety and depression were assessed.

**Results:** In those subjects with SVD elevated VPTs (overall range 1.1–11.4 deg/sec, mean 6.1 deg/sec) were correlated with impaired balance control and lower subjective symptom scores of dizziness. VPTs in elderly control subjects were 2.4 deg/sec (1.7–3.1 deg/sec) which is within limits of normal subject´s performances aged < 65 years (cut‐off 5.0 deg/sec).

**Conclusion:** VPTs in elderly subjects >70 years might be within a normal range. VPTs might serve as a biomarker for imbalance and risk of falls in elderly people.

**Disclosure:** Flurin Honegger has worked as a consultant for the company which made the equipment used in this study to assess balance control. This work was supported by Freiwillige Akademische Gesellschaft (FAG) Basel.

## EPO‐0690

### Square wave jerk in de novo idiopathic Parkinson's disease: Insights into the dynamic overshoot subtype

#### H. Kim ^1^; J. Choi^1^; S. Choi^2^; K. Choi^2^; J. Lee^1^; S. Lee^3^

##### ^1^Neurology, Pusan National University School of Medicine, Research Institute for Convergence of Biomedical Science and Technology, Pusan National University Yangsan Hospital, Yangsan, Republic of Korea; ^2^Neurology, Pusan National University Hospital, Pusan National University School of Medicine and Biomedical Research Institute, Busan, Republic of Korea; ^3^Neurology, Chonnam National University Hospital, Chonnam National University Medical School, Gwangju, Republic of Korea

**Background and aims:** Square wave jerks (SWJs) are pairs of small saccades that takes the gaze away from the target and then return it, commonly observed in neurodegenerative disorders. Although prominent in progressive supranuclear palsy, their clinical significance in Parkinson's disease (PD) is not well defined.

**Methods:** We retrospectively reviewed 145 de novo PD patients who underwent video‐oculography, UPDRS part III, Hoehn and Yahr staging, cognitive testing, and assessments for orthostatic hypotension (OH) and probable REM sleep behavior disorder (RBD). SWJs were categorized as macro (≥5°), staircase, or dynamic overshoot. Associations with motor severity, cognition, non‐motor features, and PD motor subtypes (tremor‐dominant [TD], postural instability/gait difficulty [PIGD], indeterminate) were analyzed.

**Results:** Among 67 TD, 41 PIGD, and 37 indeterminate patients, RBD (47.6%) and OH (29.7%) were most frequent in PIGD, though without statical significance. Overall SWJ frequency did not differ across motor subtypes. Macro SWJs were rare (3.4%). Staircase SWJs (49.7%) correlated with higher SWJ counts (*p <* 0.001) but no with clinical parameters. Dynamic overshoot SWJs (42.1%) were associated with higher SWJ frequency (*p <* 0.001), worse motor severity (UPDRS part III, *p <* 0.001), and higher PIGD scores (*p* = 0.032). No associations were observed with cognition, OH, or RBD.

**Conclusion:** Dynamic overshoot SWJs, unlike other subtypes, were linked to more severe motor impairment in de novo PD. These findings suggest that dynamic SWJs may reflect cortical inhibitory dysfunction, in which diminished inhibitory control from the frontal eye field results in disinhibition and subsequent hyperactivation of the superior colliculus.

**Disclosure:** Nothing to disclose.

## EPO‐0691

### Magnetic resonance imaging of the visual pathway in Lebers hereditary optic neuropathy: A systematic review

#### M. Almeida ^1^; R. Pires^2^; A. Jorge^1^; J. Sargento^1^; M. Jóia^3^; D. Pereira^2^; J. Lemos^1^

##### ^1^Department of Neurology, ULS Coimbra, Portugal; ^2^Department of Neuroradiology, ULS Coimbra; ^3^CTU‐CACC, Clinical Trial Unit – Coimbra Academic and Clinical Center, ULS Coimbra, Portugal

**Background and aims:** Leber's hereditary optic neuropathy (LHON) is a rare but important cause of blindness in young adults. Similarly to what is observed in other acquired optic neuropathies, paraclinical tests such as MRI could aid in LHON diagnosis; however, the typical MRI features of the visual pathway in LHON remain largely unknown. We conducted a systematic review on brain and/or orbit MRI findings in LHON.

**Methods:** We performed a search of published articles between 2008 and 2024 using databases from PubMed, EMBASE, SCOPUS and Web of Science Core Collection in accordance with PRISMA guidelines.

**Results:** We included 128 studies with 423 patients, plus 5 from our own series. The mean age at presentation was 34.3 years (SD 17.0). MRI abnormalities in patients presenting with simultaneous or unilateral vision loss, were observed in 48% of eyes. The most frequent pattern was a combination of intraorbital and intracranial/prechiasmal involvement of the optic nerve (19.5%), followed by isolated intraorbital involvement (18.2%), intraorbital, intracanalicular, and intracranial/prechiasmal involvement (19.5%), and intraorbital with intracanalicular involvement (6.1%). MRI abnormalities included T2 hyperintensity (77.4%), atrophy (7.5%), T1 gadolinium enhancement (3%), and combined T2 hypersignal with T1 gadolinium enhancement (11.3%).

**Conclusion:** Optic nerve MRI abnormalities are present in half of LHON patients and become less frequent by the time of second eye involvement. Importantly, these may mimic MRI patterns commonly seen in non‐genetic acquired optic neuropathies such as aquaporin‐4 and MOG‐related diseases and multiple sclerosis. Awareness of MRI patterns in LHON is crucial, prompting early diagnosis and better treatment outcomes.

**Disclosure:** Nothing to disclose.

## EPO‐0692

### Effects of noisy galvanic vestibular stimulation on gait and balance in bilateral vestibulopathy: A randomised sham‐controlled crossover pilot study

#### M. Bancroft; B. Jones; H. O'Neill; D. Kaski; M. Mahmud

##### Queen Square Institute of Neurology, Faculty of Brain Sciences, University College London (UCL), UK

**Background and aims:** Noisy galvanic vestibular stimulation (nGVS) has been suggested as a tool to improve gait and balance function in bilateral vestibulopathy (BVP). We conducted a pilot study to assess the effect of nGVS on gait and balance in BVP.

**Methods:** Subjects performed gait and balance tasks under stimulation and sham conditions: (1) standing on firm ground/foam with/without vision; (2) the timed‐up‐and‐go (TUG) test; (3) 6m walk at a self‐selected comfortable speed and slow speed (50% of self‐selected comfortable speed). The order of stimulation and sham was randomised and counterbalanced. A consistent nGVS stimulus (white noise, 0–100 Hz, peak‐to‐peak 0.4 mA, ramped up and down in 5 seconds) was used in all conditions. Gait and balance behaviour was quantified using a force plate, optical and video‐based motion capture systems. Primary outcome measures were: postural sway during standing; TUG completion time and turn speed; gait pace, variability, rhythm, asymmetry, and postural control metrics during walking.

**Results:** Ten subjects (72 yr median) with BVP participated in the study. No subject was able to distinguish stimulation from sham, confirming double‐blinding efficacy and that nGVS stimulation was sub‐perceptual. We did not find evidence of a difference between stimulation and sham conditions in gait or balance function during the standing, TUG, normal or slow speed walking tasks.

**Conclusion:** At the group level, we did not find evidence that nGVS improved gait and balance in patients with BVP. Individual improvements across some parameters were observed with nGVS and further studies may seek to characterise these further to develop more tailored treatment approaches.

**Disclosure:** Cochlear Technology Centre Belgium financially supported this study.

## EPO‐0693

### Bilateral vestibulopathy: Strong association with the ZNF91/LINC01224 locus

#### M. Strupp ^1^; F. Heindl^1^; A. Hartmann^2^; A. Zwergal^1^; I. Giegling^2^; B. Konte^2^; D. Rujescu^2^

##### ^1^Department of Neurology and German Center for Vertigo and Balance Disorders, Ludwig Maximilians University, Munich, Germany; ^2^Department of Psychiatry and Psychotherapy, Comprehensive Center for Clinical Neurosciences and Mental Health, Medical University of Vienna, Vienna, Austria

**Background and aims:** Bilateral vestibulopathy (BVP) is a chronic disabling vestibular disorder. Known etiologies are, e.g, ototoxic exposure or genetic syndromes including RFC1‐related disorders and FGF14‐GAA‐Ataxia (SCA27b). They explain only a minority of cases, leaving a substantial proportion idiopathic. To address this gap, we conducted a genome‐wide association study (GWAS) in patients with idiopathic BVP.

**Methods:** We analyzed a European cohort of 132 individuals diagnosed with idiopathic BVP and 3410 unaffected controls. Diagnosis was based on the current diagnostic criteria of the Bárány Society. Following quality control and removal of outliers, GWA was performed on approximately 7.5 million variants, complemented by gene‐based analysis with MAGMA.

**Results:** GWAS identified two loci reaching genome‐wide significance. The strongest signal mapped to chromosome 19 (ZNF91/LINC01224), with rs12185481 (*p* = 3.60E‐16) as the lead variant. A second, more tentative signal emerged on chromosome 2 represented by rs73006457 (*p* = 4.46E‐08) with GPC1 and OTOS as the nearest candidate genes. Gene‐based analysis revealed six significant loci, including ZNF91, highlighting novel candidate genes with potential relevance to BVP pathogenesis.

**Conclusion:** GWAS points to ZNF91/LINC01224 as a major contributor with a functional link between transcriptional regulation, retrotransposon silencing, and vestibular pathology with age‐related decline in ZNF91 expression potentially amplifying genetic risk. The second locus near OTOS on chromosome 2 represents a less robust plausible candidate, as OTOS is expressed in the inner ear, previously associated with susceptibility to ototoxic‐induced hearing loss. Together, these findings highlight novel pathways in BVP pathogenesis and merit replication in larger, independent cohorts.

**Disclosure:** Nothing to disclose.

## EPO‐0694

### Head circumduction paradigm as a model to stimulate the vestibulo‐spinal activity

#### M. Corrado ^1^; L. Martinis^2^; V. Grillo^2^; M. Gjini^2^; I. Campese^2^; B. Al‐Saheli^2^; B. Guerra^3^; S. Sozzi^3^; M. Schmid^3^; S. Ramat^3^; C. Tassorelli^2^; R. De Icco^2^; T. Ellmers^4^; A. Bronstein^4^

##### ^1^Movement Analysis Research Section, IRCCS Mondino Foundation, Pavia, Italy; ^2^Department of Brain and Behavioural Sciences, University of Pavia, Pavia, Italy; ^3^Bioengineering Lab, University of Pavia, Pavia, Italy; ^4^Department of Brain Sciences, Imperial College, London, UK

**Background and aims:** Vestibulospinal reflex (VSR) mechanisms are difficult to isolate. Here we applied the head circumduction paradigm (Corrado et al., 2025), which induces a transient vertical semicircular canal asymmetry, to assess the VSR and compare responses with those of the vestibulo‐ocular reflex (VOR)

**Methods:** We asked nine healthy participants to perform circumduction (circular) head rotations (0.75 Hz, i.e. a full head rotation every 1.33s) while standing on a force plate. We recorded baseline and post‐rotational (“stopping”) posturography [centre of pressure (CoP) deviation and trunk kinematics with infrared motion markers, focusing primarily on post‐rotational CoP shift. 3D video‐oculography was simultaneously obtained. We assessed whether head reorientation in pitch could influence the post‐rotational sway.

**Results:** On stopping head rotation 6 (66.7%) subjects’ trunk and CoP deviated towards the less active vestibular side (see Figure 1) by a mean of 13.4 ± 10.1 mm (CoP). The time constant of this direction‐specific sway response was 12.9 ± 13.8 s. The “stopping” eye nystagmus was prominently torsional as in (Corrado et al., 2025). Head reorientation in pitch did not influence the time constant of the postural response.

**Figure:**
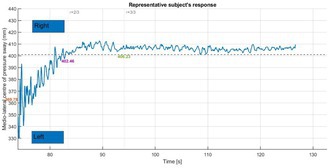
Representative subject's centre of pressure shift immediately on stopping after a right circumduction. The stopping response elicitates a right vertical canals hyperactivity, whereas the subject's COP shifts towards the left (less active) side.

**Conclusion:** The head circumduction paradigm can isolate the vertical canal vestibulospinal response. The direction‐specificity of the postural response makes it potentially useful for clinical purposes and not necessarily requiring complex or expensive equipment.

**Disclosure:** Nothing to disclose.

## EPO‐0695

### Vestibular migraine – Characterization of the long‐term clinical course

#### L. Fabritius^1^; F. Wullenweber^2^; R. Strobl^3^; E. Grill^1^; M. Dieterich^2^; S. Becker‐Bense ^2^

##### ^1^Department of Neurology, University Hospital, LMU Munich, Germany; ^2^German Center for Vertigo and Balance Disorders (DSGZ), University Hospital, LMU Munich, Germany; ^3^Institute for Medical Information Processing, Biometry, and Epidemiology, LMU Munich, Germany

**Background and aims:** Vestibular migraine (VM) is the most common cause of episodic vertigo attacks with considerable clinical variability. Data on its natural course are sparse, as on effectiveness of treatment. Aim of the study was to evaluate its clinical presentation in the long‐term course.

**Methods:** Data of 459 adult patients (~70% females, mean 42.5 years) with newly diagnosed VM according to Bárány Society's diagnostic criteria at a tertiary vertigo centre (2010–2020) were retrospectively analyzed. All patients underwent comprehensive clinical and instrumental neuro‐ophthalmological/otological examination. Long‐term course of VM symptoms including therapeutic interventions was systematically explored using standardized questionnaires.

**Results:** At first presentation 51% of VM patients presented at least one ocular motor impairment, while extended vestibular testing was usually unremarkable. The questionnaires were completed by 111 patients after a mean of 9.7 years: 63.1% reported continued episodic vertigo, mostly improved in frequency (91%), duration (89%) and/or intensity (90%). Vertigo‐related headaches decreased from 73% to 56%. Conservative migraine prophylaxis was carried out in 62% of patients, rated as helpful more for headaches (38%) than vestibular symptoms (34%). Pharmacological prophylaxis was used, at least temporarily, by 43%, rated as similarly effective for headaches and dizziness (34% vs. 37%).

**Conclusion:** After 10 years, approximately one third of VM patients became symptom‐free, while the other two‐thirds experienced less frequent and/or milder vertigo attacks. Conservative migraine prophylactic treatments were preferred, however, both conservative and pharmacological approaches were only partially effective. These results highlight the critical need for thorough patient education and further research regarding the effectiveness of various treatment strategies.

**Disclosure:** The study was supported by the Deutsche Stiftung Neurologie (project 80766125 and 80721017) and German Research Foundation (DFG) under Germany‘s Excellence Strategy (Munich Cluster for Systems Neurology: EXC 2145 SyNergy) to MD. All other authors reported no disclosures.

## EPO‐0696

### Strategic postural control for differential diagnosis of persistent postural‐perceptual dizziness (PPPD) and vestibular migraine (VM)

#### S. Becker‐Bense ^1^; L. Fabritius^2^; V. Flanagin^1^; M. Dieterich^1^

##### ^1^German Center for Vertigo and Balance Disorders (DSGZ), University Hospital, LMU Munich, Germany; ^2^Department of Neurology, University Hospital, LMU Munich, Germany

**Background and aims:** Persistent postural‐perceptual dizziness (PPPD) is a frequent cause of chronic dizziness/vertigo with subjective unsteadiness, whereas Vestibular Migraine (VM) is characterized by episodic vertigo attacks. However, some VM patients report persistent symptoms resembling PPPD. Clinical balance examination usually reveals no abnormalities in both. Aim of the current study was to determine whether PPPD and VM can be differentiated using posturographic stance parameters.

**Methods:** A retrospective analysis of a well‐characterised cohort recruited at a tertiary vertigo centre included 51 PPPD and 86 VM patients. Forty‐one of the 86 VM patients also presented with secondary persistent symptoms. Beside comprehensive clinical and instrumental neuro‐ophthalmological/otological examination all patients underwent testing of postural control under simple and challenging sensory conditions on a stabilometer platform. Sixty‐one age‐matched healthy subjects served as controls.

**Results:** Posturographic analysis revealed distinct patterns of postural regulation in VM and PPPD: The sway variability was significantly higher in VM compared to PPPD and controls in all conditions with eyes closed. On the other hand, PPPD patients showed a stereotypical sway pattern restricted almost exclusively to the fore‐aft axis. VM patients with secondary persistent symptoms presented the same postural regulation as those without PPPD‐like symptoms.

**Conclusion:** VM and PPPD showed different postural control mechanisms providing quantitative parameters to enrich differential diagnosis. PPPD patients developed highly regulated postural control, whereas VM patients with secondary persisting symptoms maintained stance pattern of episodic VM patients with higher variability. Further analysis is still needed to understand the nature of the chronic component of this VM phenotype.

**Disclosure:** The study was supported by the Deutsche Stiftung Neurologie (project 80766125 and 80721017) and German Research Foundation (DFG) under Germany‘s Excellence Strategy (Munich Cluster for Systems Neurology: EXC 2145 SyNergy) to MD. All other authors reported no disclosures.

## EPO‐0697

### High‐frequency vestibulo‐ocular reflex in spinocerebellar ataxias with slow saccades: SHIMP to unmask saccadic dysfunction

#### S. Lee; J. Kim; M. Park; S. Choi

##### Department of Neurology, Chonnam National University Hospital and Chonnam National University Medical School, Gwangju, Republic of Korea

**Background and aims:** The vestibulo‐cerebellum modulates the vestibulo‐ocular reflex (VOR) and saccadic eye movements. High‐frequency VOR can be quantified with video head impulse testing (vHIT) using the head impulse paradigm (HIMP) and the suppression head impulse paradigm (SHIMP). Spinocerebellar ataxias (SCAs), particularly SCA2 and SCA7, often show slow saccades, but the value of SHIMP in this setting is unclear. We aimed to determine whether SHIMP can reveal saccadic dysfunction in SCA patients with slow saccades but normal high‐frequency VOR gains.

**Methods:** We retrospectively analyzed five patients with genetically confirmed SCA (SCA2, *n* = 3; SCA7, *n* = 2). All showed horizontal saccades slowing on neurological and video‐oculographic examination. VOR was assessed with an ICS Impulse vHIT system using horizontal HIMP and SHIMP, and we measured horizontal canal gains and the presence and pattern of compensatory (HIMP) and anti‐compensatory (SHIMP) saccades.

**Results:** *n* all patients, horizontal canal VOR gains on HIMP were within the normal range (mean right 0.97, left 0.97). SHIMP gains were similarly normal (mean right 0.98, left 1.02), but the expected anti‐compensatory saccades were absent in two patients and small‐amplitude and scattered in three. Thus, despite preserved gains, SHIMP unmasked a failure to generate robust, time‐locked anti‐compensatory saccades in all cases, consistent with saccadic dysfunction.

**Conclusion:** In SCAs, SHIMP is a useful alternative VOR paradigm to unmask saccadic dysfunction that may be missed by conventional head impulse testing alone. The dissociation between normal gains and abnormal or absent SHIMP saccades suggests impaired VOR–saccade coupling and may help refine phenotyping in cerebellar ataxias.

**Disclosure:** Nothing to disclose.

## Neuroimaging and Neurosonology

## EPO‐0698

### Towards metabolic biomarkers of aging

#### A. León Betancourt ^1^; R. Hoepner^1^; S. Marti^1^; M. Capiglioni^2^; P. Radojewski^2^

##### ^1^Department of Neurology, Inselspital, Bern University Hospital and University of Bern, Bern, Switzerland; ^2^Translational Imaging Center, Swiss Institute for Translational and Entrepreneurial Medicine, Bern, Switzerland

**Background and aims:** Ageing is influenced by lifestyle and diseases. Multiple Sclerosis (MS) contributes to chronic low‐grade inflammation (“inflammaging”) and neurodegeneration, thereby affecting ageing processes. To date, brain ageing in MS has predominantly been studied using volumetric MRI approaches.

**Figure:**
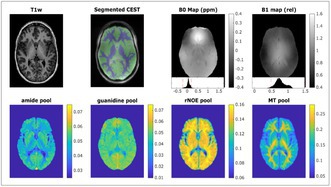
**FIGURE 1** Representative MS patient included in the analysis (female, 23 years old). High‐resolution anatomical image, CEST, B0 and B1 maps, and CEST contrast maps are displayed.

**Methods:** This interim analysis of an ongoing investigator‐initiated trial (NCT06221631). We evaluated age‐related variations in CEST pools in 7T MRI of healthy individuals (*n* = 47, aged 18–65 years) as potential biomarkers of ageing, and compared them to MS (*n* = 22, 18–63 years). CEST contrast was quantified using Lorentzian fitting of denoised, normalised, and B0‐ and B1‐corrected z‐spectra (55 offsets). Associations between age and CEST pool amplitudes were analysed for white matter (WM) and grey matter (GM), using Pearson correlations and a physics‐informed deep learning model (PICAE). Due to the interim nature, no covariate adjustment was applied. CEST pool maps (amide, amine, magnetisation transfer [MT], and relayed Nuclear Overhauser Effect [rNOE]) of MS were compared to age‐matched controls.

**Figure:**
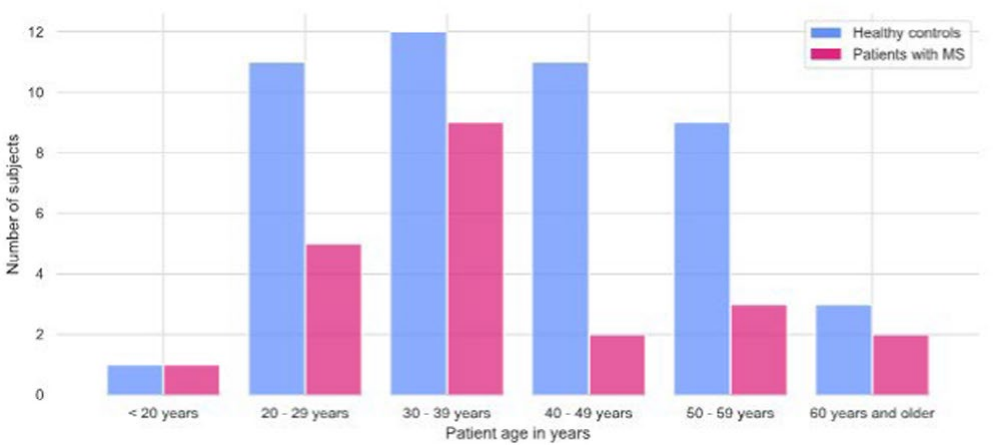
**FIGURE 2** Age group distribution of the data set.

**Results:** We observed a consistently negative correlation of different pools with age, post pronounced in GM. A statistically significant effect of age and an MS‐associated age dependency was detected especially on amides in GM (Holm corrected *p* = 0.013 and *p* = 0.014, respectively).

**Figure:**
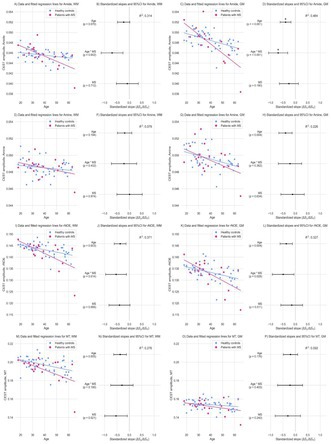
**FIGURE 3** Linear regression results for amide, amine, rNOE, and MT in white matter and grey matter. Panels (A), (C), (E), (G), (I), (K), M), O): Measured CEST amplitudes and fitted CEST amplitude for healthy controls and patients with MS.

**Conclusion:** This preliminary analysis shows a decrease of most CEST pools with age, making them potential metabolic biomarkers of biological brain aging. After recruitment finalization (foreseen 100 HC and 100 MS) we will look more in detail into these age‐associated changes overall. Aditionally we will look at metabolic markers in MS lesions.

**Disclosure:** Betancourt A has no conflict of interests related to this work. Hoepner R rhas no conflict of interests related to this work. Marti S has no conflict of interests Capiglioni M has no conflict of interests Radojewski P has no conflict of interests.

## EPO‐0699

### Evaluation of cerebral circulatory regulation using blood flow velocity spectrum analysis

#### A. Gaál ^1^; H. Pál^1^; B. Csányi^2^; L. Ajtai^3^; Á. Kovács^3^; K. Nagy^2^; T. Horváth^4^; Á. Koller^4^; R. Debreczeni^1^

##### ^1^Department of Neurology, Semmelweis University, Budapest, Hungary; ^2^Semmelweis University, Doctoral College, Budapest, Hungary; ^3^Faculty of Medicine, Semmelweis University, Budapest, Hungary; ^4^University of Physical Education, Research Center for Sport Physiology, Budapest, Hungary

**Background and aims:** Near‐constant global cerebral blood flow (CBF) is ensured by the simultaneous regulation of perfusion pressure and cerebral flow resistance (CVR). The CVR of a cerebral proximal vascular segment can be estimated from arterial blood pressure CBF velocity relationships during the cardiac cycle, particularly in diastole. We hypothesized that mathematical analysis of the diastolic phase of the flow velocity spectrum provides a novel and reliable method for assessing CVR. Our objective was to define a novel diastolic parameter that characterizes changes in CVR to a vasoactive stimulus and to compare its sensitivity with that of established indices.

**Methods:** 35 healthy volunteers’ CBF velocity was measured in the middle cerebral artery using transcranial Doppler ultrasound, with noninvasive arterial blood pressure monitoring. Orthostatic stress induced by a tilt table was applied, with measurements performed at 0°, 30°, 60°, and 90°. In addition to the pulsatility index (PI) calculated from velocity ratios and the cerebral arterial resistance (CAR) expressed as the blood pressure‐velocity ratio, the diastolic time constant (Tau) was introduced. Tau represents the time required for the initial diastolic flow velocity to decrease to 1/e of its value, determined by the decay constant (λ).

**Results:** Tau was significantly more sensitive than PI in detecting CVR changes (0–30°: *p* = 0.044, *r* = 0.75; 0–60°: *p* = 0.025, *r* = 0.76; 0–90°: *p* = 0.043, *r* = 0.81). Tau also showed greater sensitivity than CAR (0–30°: *p* = 0.047, *r* = 0.754; 0–60°: *p* = 0.017, *r* = 0.907; 0–90°: *p* = 0.043, *r* = 0.504).

**Conclusion:** The diastolic flow time constant (Tau) is a more sensitive marker of CVR changes than the PI or CAR.

**Disclosure:** Nothing to disclose.

## EPO‐0700

### Neurophysiological processes in speech production and speech perception

#### D. Grevenstein; A. Gutschalk

##### Department of Neurology, University Hospital Heidelberg, Heidelberg, Germany

**Background and aims:** Neuronal processes underlying perception of self‐produced naturalistic continuous speech remain unclear. While prior research has used temporal response function (TRF) analyses to investigate passive listening, this study established a novel MEG‐based paradigm comparing passive speech perception with self‐produced speech in an ecologically valid experimental setting.

**Methods:** Participants completed an experimental study alternating between 10‐second passive listening and active speaking sections across 100 trials. They heard audiobook segments and repeated what they had just heard as closely as possible. Audio was recorded during active speaking trials. TRF analyses were computed using the first derivative of the speech envelope, marking syllable onset, as predictor of MEG measurements. Source localization extracted neuronal activation within the ROI of the early auditory cortex.

**Results:** An N1 component centered at 90–100 ms was visually recovered in both conditions across both hemispheres. P1 and P2 components emerged with much smaller amplitudes. Statistical analyses of defined time frames (10–50 ms for P1; 70–110 ms for N1; 140–180 ms for P2) revealed no overall differences between conditions or hemispheres. A significant N1 was found in both hemispheres of the passive condition and the right hemisphere of the active condition. P2 reached significance only in the left hemisphere of the passive condition. A glossokinetic artifact synchronous with syllable onset emerged specifically in the active speaking condition. Signal Subspace Projection (SSP) successfully corrected for this artifact.

**Figure:**
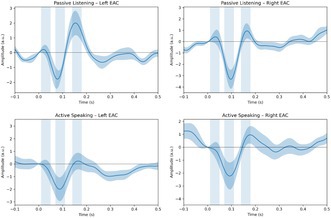
**FIGURE 1** TRF wave forms extracted from the Early Auditory Cortex ROI in the passive (top row) and active condition (bottom row) in the left and right hemispheres.

**Conclusion:** This research successfully established a paradigm to measure neuronal responses to self‐produced speech, demonstrating that measuring active speech in MEG using TRF requires specific artifact correction techniques.

**Disclosure:** Nothing to disclose.

## EPO‐0701

### Cerebral venous outflow impairment in acute ischemic stroke associated with large artery atherosclerosis: Impact on **severity and early deterioration**

#### F. Mallaev; G. Rakhimbaeve

##### Tashkent State Medical University, Tashkent, Uzbekistan

**Background and aims:** Although large artery atherosclerosis represents a leading cause of ischemic stroke, the contribution of venous circulation abnormalities to stroke pathophysiology has received limited attention. We investigated cerebral venous hemodynamic alterations in acute stroke patients with atherosclerotic arterial disease and examined their relationship with initial stroke severity and early clinical worsening.

**Methods:** This prospective investigation enrolled 120 consecutive acute ischemic stroke patients presenting with ≥50% stenosis affecting extracranial or intracranial major arteries. Clinical assessment utilized the National Institutes of Health Stroke Scale at hospital admission and on day seven. Neuroimaging evaluation within 72 hours comprised brain MRI incorporating diffusion‐weighted sequences and MR venography. Hemodynamic measurements included superior sagittal sinus flow velocity, venous pulsatility index, and venous sinus caliber, obtained through transcranial Doppler ultrasonography and MR venography.

**Results:** Study participants exhibited substantially diminished superior sagittal sinus flow velocity (21.9 ± 4.6 cm/s) alongside elevated venous pulsatility index values (1.38±0.17). Statistical analysis revealed significant correlations between impaired venous flow and admission NIHSS scores (*r* = −0.42, *p <* 0.001) as well as ischemic lesion volume (*r* = −0.39, *p* = 0.002). Twenty‐six patients (21.7%) experienced early neurological deterioration, which occurred predominantly among those demonstrating marked venous drainage impairment (*p* = 0.004). Multivariable regression modeling identified venous flow velocity as an independent determinant of early clinical worsening.

**Conclusion:** Disturbed cerebral venous drainage patterns occur frequently in atherosclerotic stroke and independently correlate with initial neurological severity and subsequent early deterioration. Integration of venous hemodynamic evaluation into acute stroke assessment could strengthen early prognostic stratification.

**Disclosure:** Nothing to disclose.

## EPO‐0702

### Cerebrovascular reactivity in Parkinson's disease: Acute effects of Levodopa assessed by transcranial Doppler

#### F. Garramone; I. Madonia; M. Sforza; V. Teresi; E. Bianchini; L. De Carolis; S. Galli; P. Pacilio; P. Lombardo; M. Salvetti; D. Rinaldi; G. Sette

##### Department of Neuroscience, Mental Health and Sensory Organs (NESMOS), Sapienza University of Rome, Via di Grottarossa; Rome, Italy

**Background and aims:** Parkinson's disease (PD) is associated with neurovascular unit dysfunction and reduced cerebrovascular reactivity (CVR). Levodopa may affect cerebral hemodynamics, but its acute effect on CVR remains unclear.

**Methods:** Twenty people with PD with Hoehn and Yahr stage below 4 were enrolled. CVR was assessed using transcranial Doppler Breath Holding Index (BHI) at the middle cerebral arteries in OFF state after medication withdrawal and in ON state one hour after Levodopa intake. Sixteen patients completed Doppler assessment. Clinical evaluation included MDS UPDRS part III. Autonomic evaluation included SCOPA AUT and orthostatic blood pressure testing. Cognitive evaluation included MoCA, Stroop test and verbal fluency. Patients with cerebrovascular stenosis, MMSE below 18 or incomplete data were excluded.

**Results:** Mean BHI was reduced compared with normative values (0.757 vs 1.29). BHI showed a trend toward reduction in ON compared with OFF (*p* = 0.054). In ON state, BHI was lower in the hemisphere contralateral to the more affected motor side (*p* = 0.035), while within the more affected hemisphere BHI was higher in OFF than ON (*p* = 0.030). Basal flow velocity and pulsatility index did not differ between states. Change in BHI correlated inversely with OFF BHI (*r* = −0.668, *p* = 0.003). Four patients had neurogenic orthostatic hypotension without BHI differences. Levodopa improved UPDRS III scores (*p* < 0.001) and Stroop interference performance (*p* = 0.016) without increasing CVR.

**Figure:**
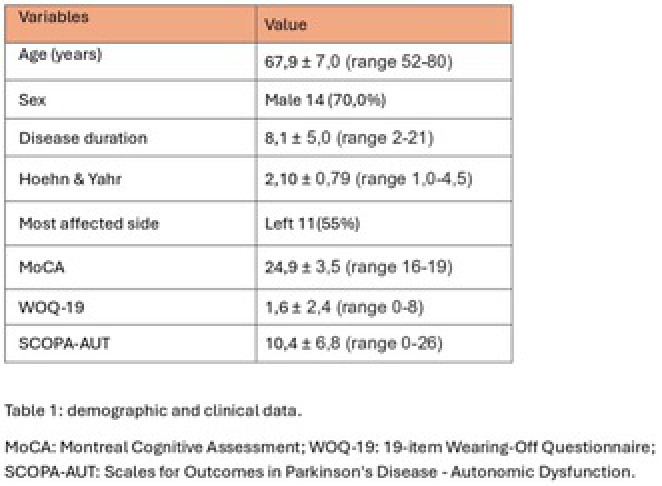
**TABLE 1** Demographic and clinical data.

**Conclusion:** CVR is reduced in PD. Acute Levodopa administration does not improve vasodilatory reserve and may further compress CVR, supporting a vascular ceiling effect.

**Disclosure:** Nothing to disclose.

## EPO‐0703

### Non‐**invasive quantification of motor unit recruitment using ultrasound optical flow**

#### J. Scott; W. Hay Mar; R. Whittaker; K. Suetterlin; S. Maitland

##### Translational and Clinical Research Institute, Newcastle University, Newcastle upon Tyne, UK

**Background and aims:** Motor unit assessment is commonly performed using electromyography (EMG), which records muscle fibre electrical activity but cannot directly measure muscle contraction or force generation—the processes most relevant to clinical weakness. EMG therefore provides limited insight into how neuronal activation translates into movement, including excitation–contraction coupling. As a result, surface and needle EMG incompletely reflect motor unit output. This study assesses whether clinical ultrasound with optical flow analysis can non‐invasively quantify motor unit recruitment and contractile behaviour during peripheral nerve stimulation.

**Methods:** Twenty‐two healthy participants underwent peripheral nerve stimulation of the abductor digiti minimi, abductor pollicis brevis, and tibialis anterior muscles with simultaneous surface EMG and high‐frame‐rate ultrasound (100–150 frames/s). Stimulation current was reduced from supramaximal levels to generate recruitment curves. Stimulus–response relationships were modelled using four‐parameter logistic regression, with EC50 defined as the current producing 50% of the maximal response.

**Results:** 11 male and 11 female participants took part; mean age 28 ± 9.16. Forty‐six paired recordings were analysed. Ultrasound detected motor units at lower stimulation thresholds (0.71 ± 0.82 mA earlier, *p* < 0.0001) and reached saturation earlier (2.6 ± 5.3 mA lower, *p* < 0.0001) than surface EMG. EC50 occurred at lower currents for ultrasound than EMG (7.89 ± 3.37 mA vs 8.94 ± 3.90 mA, *p* = 2.35 × 10^−11^).

**Conclusion:** Ultrasound with optical flow analysis enables non‐invasive measurement of motor unit recruitment by directly quantifying muscle contraction, including deep motor units not detected by surface EMG, with potential application in neuromuscular disease assessment.

**Disclosure:** Nothing to disclose.

## EPO‐0704

### Cortical damage and tracts disconnection as predictors of 3‐month aphasia severity after left ischemic stroke

#### S. Magno ^1^; T. Jacquemont‐Fulneau^2^; C. Rosso^2^; F. Mazzacane^1^; G. Perini^3^; A. Costa^1^; A. Cavallini^1^

##### ^1^Department of Emergency Neurology and Stroke Unit, IRCCS Mondino Foundation, Pavia, Italy; ^*2*^Institut du Cerveau, ICM, Inserm U1127, CNRS UMR 7225, Sorbonne Université, Paris, France; ^*3*^Unit of Behavioral Neurology and Center for Cognitive Disorders and Dementia (CDCD), IRCCS Mondino Foundation, Pavia, Italy

**Background and aims:** Tract‐based disconnection metrics are increasingly used to predict aphasia, but their value beyond lesion burden remains debated. We compared atlas‐based cortical lesion overlap and tract disconnection as predictors of aphasia severity at 3 months.

**Methods:** Fifty patients with left ischemic stroke (mean age 73.2 ± 12.8 years; 44% female) were included. Aphasia severity was assessed using NIHSS language subitem 9 at 3 months. Lesion masks were registered to MNI space. Using Tractotron, we computed the mean and maximum proportion of disconnection over the dorsal (gathering the arcuate fasciculus, the SLF and the frontal aslant tracts), ventral pathways (gathering the IFOF, ILF, uncinate tracts) and combination of both. Language cortical regions and lesion overlap percentages were computed using the Harvard–Oxford atlas. Correlations between cortical overlaps, tracts disconnection and aphasia severity were computed using the Spearman correlation test. Each feature was volume corrected by linearly regressing it out. Ordinal regression models were adjusted for lesion volume, baseline total NIHSS, age, education, and reperfusion status.

**Results:** Composite language‐network disconnection was associated with aphasia severity at 3 months (Spearman ρ ≈ 0.55, *p <* 0.001). Single‐tract analyses showed strongest associations for dorsal pathways (arcuate long segment: ρ = 0.51, *p* = 0.00018; SLF III: ρ = 0.50, *p* = 0.00026). After lesion‐volume adjustment, composite and dorsal residualized indices were not associated with outcome (ρ = 0.17, *p* = 0.24; ρ = 0.16, *p* = 0.26). Central Opercular Cortex overlap independently predicted aphasia severity (β = 0.077, *p* = 0.003; OR ≈ 2.16 per 10% overlap).

**Conclusion:** In this cohort, in this cohort, cortical involvement showed the most robust volume‐independent association with 3‐month outcome.

**Disclosure:** Nothing to disclose.

## EPO‐0705

### Carbon dots conjugated with procyanidin B2 function as diamagnetic CEST MRI theranostic agents for Alzheimers disease

#### S. Wu ^1^; f. Liu^2^; X. Li^1^; H. Gong^2^; C. Zhuang^1^; D. Shi^1^; X. Tan^2^; R. Wu^1^

##### ^1^Department of Medical Imaging, The Second Affiliated Hospital, Medical College of Shantou University, China; ^2^Center for Molecular Imaging Probe, Cancer Research Institute & Institute of *Pharmacy* and Pharmacology, School of Pharmaceutical Science, Hengyang Medical School, University of South China, China

**Background and aims:** Oxidative stress and neuroinflammation are recognized as central pathological mechanisms underlying Alzheimer's disease (AD). While natural procyanidins exhibit therapeutic potential by reducing reactive oxygen species (ROS) accumulation and neuroinflammation, their efficacy is limited by poor blood‐brain barrier (BBB) penetration. Recent advances highlight arginine‐derived carbon dots (C‐dots) as biocompatible nanocarriers for brain‐targeted delivery, owing to their small size, modifiable surface, and multifunctional properties, including optoelectronic behavior and size‐dependent fluorescence.

**Methods:** We synthesized a novel MRI contrast agent C‐dots@ PB2. The agent has chemical exchange saturation transfer (CEST), which enabled the use of CEST imaging for monitoring c‐dots distribution and provided the information for ROS and neuroinflammation accumulation.

**Results:** Based on in vitro and in vivo studies, we demonstrate that C‐dots@PB2 exhibits CEST effects. Moreover, C‐dots@PB2 efficiently mitigates ROS levels. Furthermore, the agent rapidly accumulates in the brains of AD mice and alleviate pathological features including Aβ plaque deposition, neuronal loss, and neuroinflammation. Additionally, they significantly enhance learning ability and memory function in AD mice.

**Figure:**
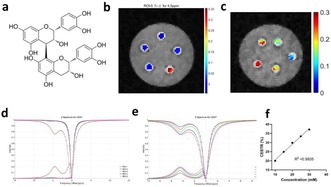
The chemical exchange saturation transfer (CEST) of PB2.

**Figure:**
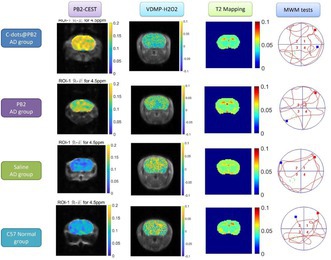
In 8‐month‐old APP/PS1 mice and C57 mice, therapeutic efficacy was assessed by dividing subjects into C‐dots@PB2 AD, PB2 AD, saline AD, and C57 normal control groups.

**Figure:**
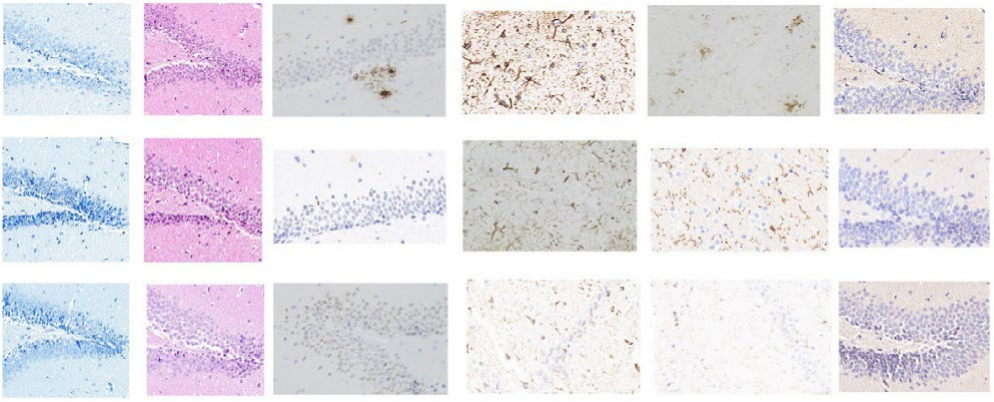
Histology and molecular analysis confirmed that PB2 treatment improved neuronal health, reduced amyloid plaques, and lowered neuroinflammation.

**Conclusion:** This study demonstrates that C‐dots@PB2 can attenuate the progression of AD by reducing levels of ROS and neuroinflammation accumulation. Additionally, its CEST effects offer a novel approach for developing theranostic agents targeting AD.

**Disclosure:** Nothing to disclose.

## EPO‐0706

### Abstract withdrawn

## EPO‐0707

### The importance of neuroimaging methods in assessing the aggressiveness of glioblastomas of cerebral hemispheres

#### S. Ahmedov ^1^; R. Egamberdiev^1^; B. Muminov^2^

##### ^1^Republican Specialized Scientific and Practical Medical Center of Neurosurgery; ^2^Tashkent State Medical University, Tashkent, Uzbekistan

**Background and aims:** This study evaluates the clinical value of a newly developed MRI‐based phenotypic aggressiveness scale for supratentorial glioblastomas and examines how phenotype‐guided surgical strategy, including selective internal decompression, influences resectability, neurological outcomes, and early postoperative radiological dynamics.

**Methods:** A total of 155 patients with newly diagnosed supratentorial glioblastoma were retrospectively analyzed. Preoperative MRI findings (tumor volume, peritumoral edema, midline shift, and infiltration pattern) were used to classify tumors into three phenotypes: low‐risk (A), intermediate‐risk (B), and high‐risk (C). Surgical outcomes, extent of resection, postoperative NANO scores, and early MRI changes were compared among groups. Selective internal decompression techniques were used selectively in B and C phenotypes.

**Results:** Phenotypic groups demonstrated distinct preoperative clinical and radiographic characteristics. Postoperative improvements were significant across all groups but varied by phenotype. Group A showed the most rapid recovery, with substantial reduction in edema and near‐complete normalization of midline shift. Group B demonstrated moderate but significant improvement. Group C—initially the most compromised—achieved markedly better resectability (up to 80–89%) and neurological recovery when internal decompression was applied, compared with standard resection alone (*p* < 0.01). All key radiological and clinical parameters showed statistically significant differences between phenotypes (*p* < 0.05).

**Conclusion:** The MRI‐based phenotypic aggressiveness scale reliably characterizes glioblastoma behavior and predicts surgical complexity. Phenotype‐guided decision‐making, particularly the use of selective internal decompression in high‐risk tumors, improves extent of resection, reduces mass effect, and enhances early postoperative neurological outcomes. This combined radiologic–surgical approach represents a practical framework for optimizing treatment in supratentorial glioblastoma.

**Disclosure:** Nothing to disclose.

## EPO‐0708

### BioSerenity‐E1: A unique EEG foundation model for rapid AI development in neurological diagnostics, including Alzheimer's, sleep, or EEG abnormality

#### U. Gimenez; M. Rahmouni; A. Honoré; R. Bettinardi; A. Bussalb; F. Le Gac; H. Launay; P. Di Piazza; C. Flipo; G. Jubien; P. Emerich

##### BIOSerenity, Paris, France

**Background and aims:** The development of AI algorithms for EEG‐based diagnostics is hindered by limited annotated data, and the complexity of neurophysiological signals. BioSerenity‐E1 is a unique general‐purpose EEG foundation model that addresses these challenges by learning universal representations, enabling rapid fine‐tuning for diverse clinical applications.

**Methods:** BioSerenity‐E1 is a transformer‐based foundation model pretrained on unlabeled EEG. The model employs intra‐ and inter‐attention mechanisms to capture spatiotemporal dependencies. We showcase its adaptability through three key applications: Normal/abnormal EEG classification on internal multicentric (*n* = 4480) and public (TUAB, *n* = 276) datasets. Alzheimer's disease detection using an independent external cohort (*n* = 65) and an internal test set (*n* = 779). Pediatric sleep stage classification on internal (*n* = 262) and external datasets (*n* = 52).

**Results:** Normal/abnormal classification: BioSerenity‐E1 achieves a balanced accuracy of 93.1 [90.2 – 95.3] on a rigorously annotated test set (*n* = 198), outperforming the commercial autoSCORE algorithm. On the unseen public TUAB benchmark, it reaches 82.25 [78.27–87.48] accuracy. Alzheimer's disease detection: The model demonstrates balanced accuracies of 0.74–0.78 on an independent external dataset and 0.69–0.70 on the internal test set, confirming its ability to generalize. Pediatric sleep stage classification: BioSerenity‐E1 achieves a macro‐F1 score of 0.746 on the internal test set and 0.65 on an external dataset, proving generalist viability without sleep pretraining.

**Figure:**
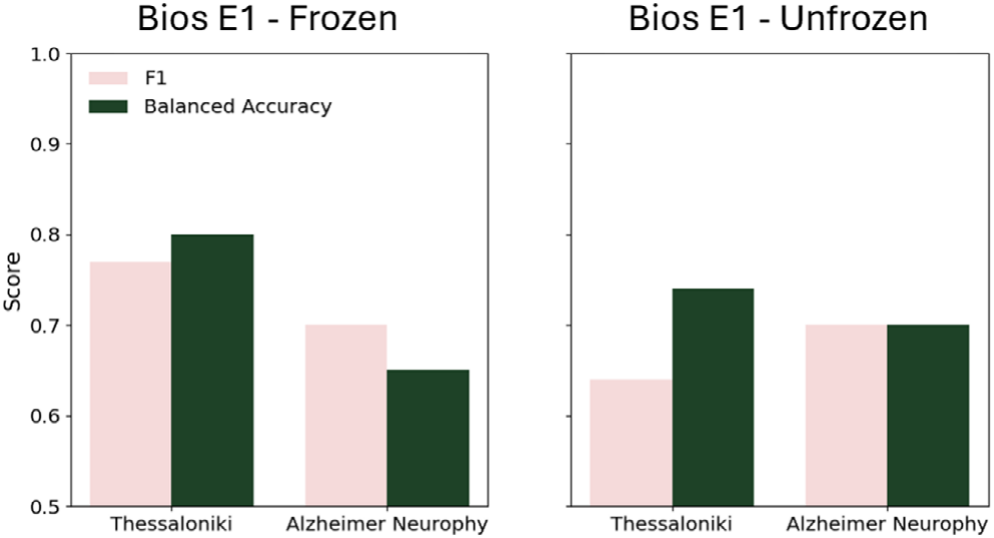
**FIGURE 1** Balanced accuracy and F1‐score for Bioserenity‐E1 Unfrozen and Bioserenity‐E1 Frozen models evaluated on internal (Neurophy) anx external (Thessaloniki) test datasets.

**Figure:**
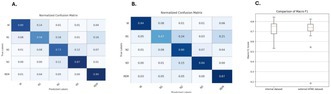
**FIGURE 2** Pediatrics sleep stages confusion matrices for internal (A, *n* = 53) and external (B, *n* = 49) datasets and macro F1 score boxplots for both datasets (C).

**Figure:**
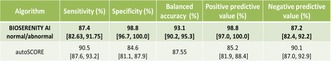
**TABLE 1** Normal/abnormal classification performances with 95% CI of Bioserenity‐E1 derived algorithm VS autoSCORE algorithm.

**Conclusion:** BioSerenity‐E1 accelerates the development of AI‐driven EEG diagnostics, delivering state‐of‐the‐art performance across multiple tasks. Its adaptability to heterogeneous EEG setups, and high specificity make it a powerful tool for developing additional specialized algorithms, addressing rare conditions and expanding the scope of EEG‐based diagnostics.

**Disclosure:** Nothing to disclose.

## EPO‐0709

### Improvement and validation of deep learning models in detection of superficial siderosis and microhemorrhages from T2* GRE images

#### S. Van Eyndhoven; R. Magalhaes; R. Khan; T. Phan; A. Liseune; A. Brys; D. Sima; D. Smeets; W. Van Hecke

##### icometrix, Leuven, Belgium

**Background and aims:** Eligibility for amyloid‐targeting monoclonal antibody treatments in Alzheimer's disease requires fewer than four microhemorrhages (MH) and no superficial siderosis (SS) on baseline T2*GRE MRI. icobrain is a deep learning–based system designed to detect MH and SS. The latest version, icobrain 5.17, was trained on additional clinical trial and real‐world data. This study compares its performance with the prior version, icobrain 5.16.

**Methods:** icobrain 5.16 was trained on 247 clinical trial cases. icobrain 5.17 incorporated an additional 356 cases (85 clinical trial, 271 real‐world), totaling 603 datasets. Evaluation was performed using subsets of the Alzheimer's disease neuroimaging initiative dataset across four experiments: MH detection in MH/SS‐positive cases (*n* = 300); MH detection in MH‐negative cases (*n* = 300); SS detection in MH/SS‐positive cases (*n* = 54); and SS detection error in MH/SS‐negative cases (*n* = 54). Performance was assessed using the 90th percentile of lesion quantification error (p90) of MH and SS across cases.

**Results:** p90 for SS detection in SS‐positive cases decreased from 3 (icobrain 5.16) to 2 (icobrain 5.17). p90 for SS in SS‐negative cases decreased from 1 to 0. p90 for MH detection in MH‐positive cases was 3 for both versions; and in MH‐negative cases was 1 for both versions.

**Figure:**
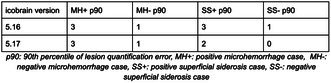
**TABLE 1** 90th percentile of lesion quantification error (p90) of MH and SS for icobrain 5.16 and 5.17.

**Conclusion:** icobrain 5.17 demonstrated reduced SS quantification error compared with icobrain 5.16. Given that any SS finding may render patients ineligible for amyloid‐targeting therapy, this improvement supports more accurate treatment eligibility assessment. MH error rates remained stable and below clinically relevant ineligibility thresholds.

**Disclosure:** All authors are employees of icometrix.

## Pain 1

## EPO‐0710

### How brain morphology predicts pain quality in trigeminal neuralgia patients: **A** neuroradiological regularized multivariate approach

#### D. Litewczuk; G. De Stefano; D. Chiffi; C. Leone; E. Galosi; G. Di Pietro; P. Falco; E. Evangelisti; G. Di Stefano; F. Caramia; A. Truini

##### Department of Human Neuroscience, Sapienza University of Rome, Italy

**Background and aims:** Trigeminal neuralgia (TN) is a facial pain condition characterized by unilateral, very short‐lasting pain paroxysms. In up to 50% of patients, it can be associated with a distinct, more prolonged type of pain, which is commonly referred to as concomitant continuous pain. We aimed to elucidate the contribution of brain morphology into the development of this condition.

**Methods:** We collected 66 subjects with a definite diagnosis of classical or idiopathic right TN and acquired their brain magnetic resonance imaging (MRI) scans. We constructed a predictive model of concomitant continuous pain based on MRI‐based data of cortical thickness and gray matter volume of 74 cerebral regions of interest (ROIs).

**Results:** According to our final model, we estimated that the most robust predictors of concomitant continuous pain in TN patients are contralateral orbital, precentral, superior frontal, superior and inferior temporal gyri, and temporal pole.

**Conclusion:** Our clinical and neuroradiological study can be considered a proof‐of‐concept for the capacity of brain morphology to predict concomitant continuous pain in TN patients. In particular, it could provide an innovative insight into the role of brain plasticity in the pathophysiology of TN.

**Disclosure:** The authors have no potential conflict of interest to disclose.

## EPO‐0711

### Small fiber dysfunction in fibromyalgia: A multimodal study

#### D. Dell'Aversana ^1^; F. Masciarelli^1^; F. Vitale^1^; G. Ciccarelli^1^; G. Caporaso^2^; S. Tozza^1^; V. Provitera^2^; L. Santoro^1^; F. Manganelli^1^; M. Nolano^1^; M. Nolano^2^

##### ^1^Department of Neurosciences, Reproductive Sciences and Odontostomatology, University of Naples Federico II, Naples 80131, Italy; ^2^Skin Biopsy Laboratory, Department of Neurology, ICS, Istituti Clinici Scientifici Maugeri, Telese Terme, Italy

**Background and aims:** Fibromyalgia (FMS) is characterized by widespread musculoskeletal pain associated with symptoms like depression, migraine and fatigue. A subset of FMS patients presents small fiber pathology. This study compares small fiber dysfunction in FMS patients with neuropathic pain and autonomic symptoms (FMS‐SFN) versus pure small fiber neuropathy (SFN) patients.

**Methods:** 30 FMS‐SFN patients and 67 SFN patients underwent sensory and autonomic evaluation trough the “Small‐Fiber‐Neuropathy‐Symptoms‐Inventory‐Questionnaire” (SFN‐SiQ), “Composite‐Autonomic‐Symptoms‐Score”(COMPASS‐31), Quantitative‐Sensory‐Testing (QST), cardiovascular reflexes, Sympathetic‐Skin‐Response (SSR) and Dynamic Sweat Test (DST). Cutaneous innervation was analysed on punch biopsies from three body sites applying indirect Immunofluerescence procedures.

**Results:** Compared to SFN patients (46 female, age 46 ± 16), FMS‐SFN (27female, age 41 ± 13) more frequently reported endometriosis (13% vs 1%), headaches (77% vs 16%) and depression (47% vs 7%). Dysimmune causes affected 47% of FMS‐SFN and 36% of SFN patients, who had more glucose metabolism disorders (3% vs. 15%). FMS‐SFN patients complained diffuse neuropathic pain, alongside more severe sensory and autonomic symptoms. Abnormal sensory thresholds were present in both groups with higher frequency of heat hyperalgesia in FMS‐SFN (17% vs 1%) and higher thermal hypoesthesia at distal sites in SFN. FMS‐SFN patients presented a lower RR variability at rest and non‐length‐dependent hypohidrosis with lower sweat volume per gland at proximal sites. Cutaneous sensory and autonomic innervation did not differ between the two groups.

**Conclusion:** FMS‐SFN patients had a non‐length‐dependent SFN with a more diffuse neuropathic pain and more severe autonomic symptoms. They, also, presented a mild cardiovascular parasympathetic impairment and a proximal lower sweat production per gland. Skin biopsies did not differentiate the two groups.

**Disclosure:** Nothing to disclose.

## EPO‐0712

### Electrophysiological evidence of systemic neural hyperexcitability in trigeminal neuralgia: **A** nerve excitability study

#### E. Evangelisti; C. Leone; D. Litewczuk; G. De Stefano; G. Di Pietro; P. Falco; E. Galosi; G. Di Stefano; A. Truini

##### Department of Human Neuroscience, Sapienza University of Rome, Rome, Italy

**Background and aims:** Primary trigeminal neuralgia (TN) is a representative neuropathic facial pain disorder and is classified into classical TN, which is associated with neurovascular compression, and idiopathic TN, in which no clear etiology is identified. Increasing evidence, including the identification of pathogenic genetic variants within the sensory electrogenisome, supports a multifactorial pathophysiology that converges on neuronal hyperexcitability. This hyperexcitability may extend beyond the trigeminal nerve, reflecting a more generalized axonal predisposition. Aim: to determine whether patients with TN exhibit generalized axonal hyperexcitability detectable in peripheral motor nerves and to assess the effects of sodium channel–blocking therapy on axonal excitability.

**Methods:** Motor nerve excitability testing of the median nerve was performed in 35 patients with classical or idiopathic TN and compared with healthy controls. The primary outcome measure was the strength–duration time constant (SDTC), an index of persistent sodium conductance. Secondary measures included recovery cycle and threshold electrotonus parameters. Subgroup analyses compared treated and untreated patients and classical versus idiopathic TN.

**Results:** TN patients showed a significant prolongation of SDTC compared with controls, independent of treatment status. Superexcitability was significantly reduced in patients receiving Na‐blockers compared with untreated patients. No differences in excitability parameters were observed between classical and idiopathic TN.

**Conclusion:** These findings demonstrate generalized axonal hyperexcitability in TN, characterized by increased persistent sodium conductance and detectable in peripheral motor nerves. This supports the hypothesis of an intrinsic predisposition to neuronal hyperexcitability in TN. Na–blocking therapy modulates superexcitability but does not normalize SDTC, indicating distinct pharmacodynamic effects and a shared pathophysiological substrate across TN subtypes

**Disclosure:** Nothing to disclose.

## EPO‐0713

### Real‐world evaluation of botulinum toxin A in focal peripheral neuropathic pain: Longitudinal outcomes

#### E. Sole Cruz ^1^; E. Van Obberghen^2^; C. Hirtz^3^; I. Bennour^2^; M. Lanteri‐Minet^2^; A. Donnet^3^

##### ^1^Reference Center for Neuromuscular Diseases and ALS, CHU Timone, Assistance Publique des Hopitaux de Marseille, Marseille, France; ^2^Pain treatment and evaluation Department, CHU de Nice, Nice, France; ^3^Pain treatment and evaluation center, CHU Timone, Assistance Publique des Hôpitaux de Marseille, Marseille, France

**Background and aims:** Neuropathic pain (NP) is frequently resistant to conventional treatments. Botulinum toxin type A (BT‐A) is an authorized option for refractory focal peripheral NP, but the dynamics of its effect outside clinical trials in real‐life conditions remain poorly characterized. The objective was to assess BT‐A efficacy in a real‐world study of patients with focal peripheral NP, over a one‐year follow‐up period.

**Methods:** In this prospective, observational study, adult patients with chronic focal peripheral NP refractory to standard therapies were treated with BT‐A in two French pain centers. Injections were individualized and administered at ~3‐month intervals. The primary outcome was the patient‐reported percentage of improvement since the first injection, assessed at each follow‐up visit (Cycles 2 to 5). Secondary outcomes included the Patient Global Impression of Change (PGIC), average pain intensity over the previous 8 days, and exploratory analysis of response trajectories distinguishing early (≥30% improvement at Cycle 3) from late responders.

**Results:** 82 patients received ≥2 BT‐A cycles and where included in the analysis. Mean self‐reported improvement increased from 30.7% at Cycle 2 to 51.0% at Cycle 5 (*p* < 0.001). PGIC and pain intensity also showed significant improvements. Among 59 patients evaluable at Cycle 3, 66% were early responders; late responders showed significant benefit after the third injection. BT‐A was well tolerated: 63.6% reported end‐of‐dose effects, and 25.8% experienced only mild, transient adverse events.

**Figure:**
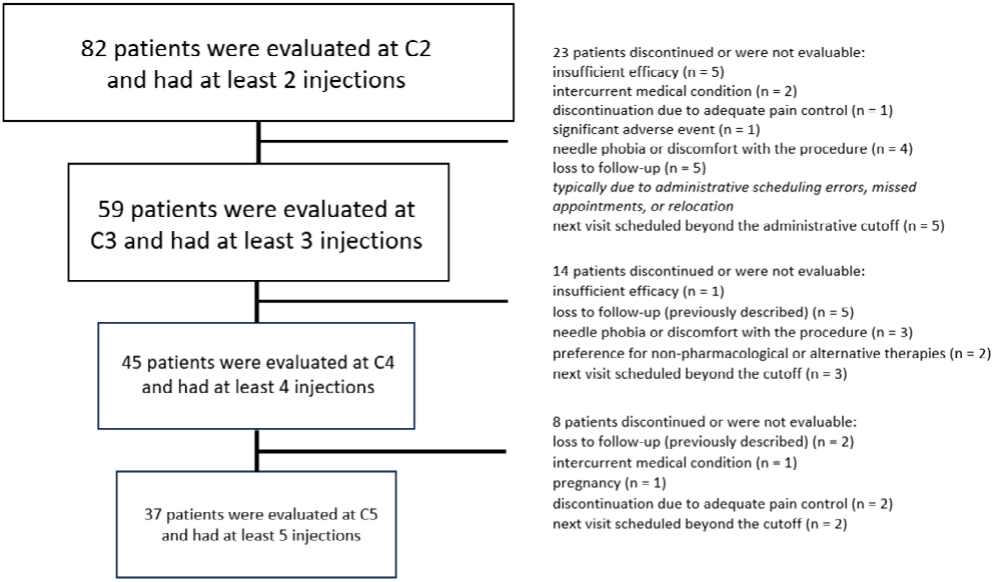
Patient flow from C2 to C5. Reasons for discontinuation include insufficient efficacy, sufficient pain relief, needle phobia, loss to follow‐up, and scheduling beyond study cutoff.

**Figure:**
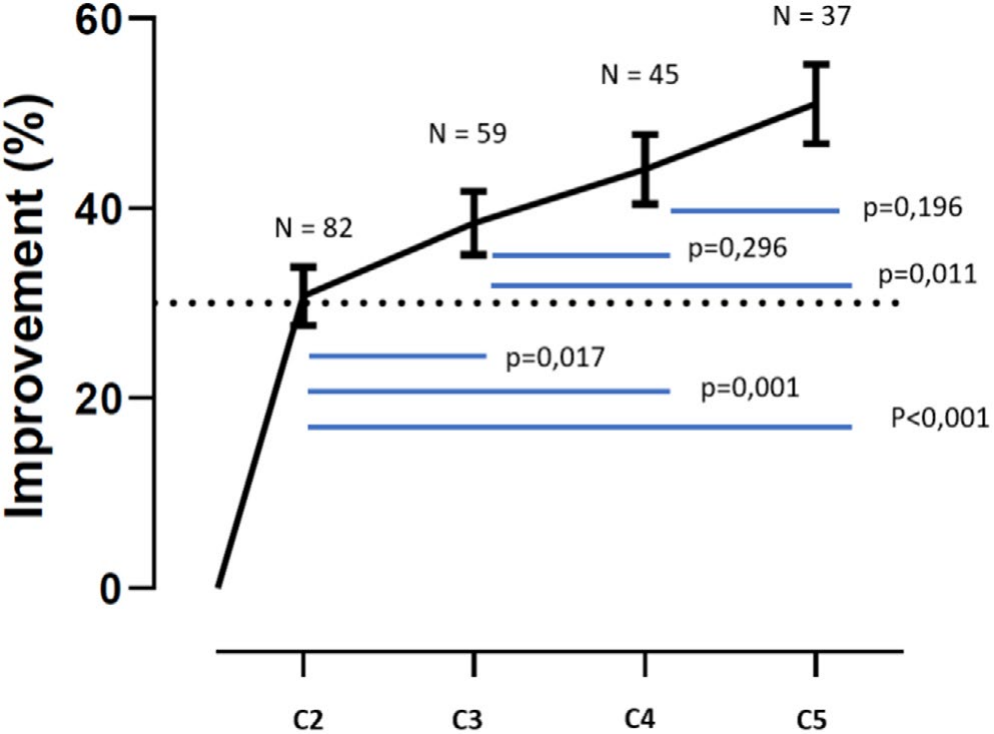
Mean patient‐reported percentage of improvement from baseline across treatment cycles C2 to C5 in the overall study population. The dotted horizontal line represents the threshold for clinically meaningful improvement (30%). (Bonferroni‐adjusted *p*‐values).

**Figure:**
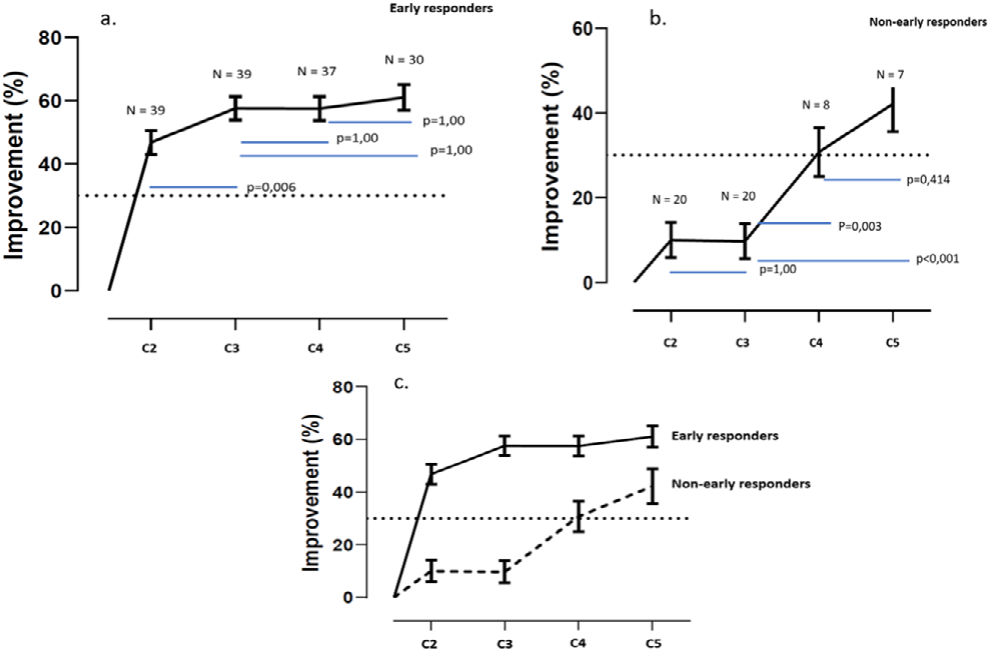
Improvement (%) from C2 to C5 in early responders (a), non‐early responders (b), and direct comparison (c). Early responders improved rapidly then plateaued, while non‐early responders showed delayed but progressive gains, crossing the 30% threshold by C4.

**Conclusion:** This real‐life study over one year suggests that BT‐A provides progressive and sustained benefit in focal peripheral NP, supporting continued treatment beyond two cycles.

**Disclosure:** Nothing to disclose.

## EPO‐0714

### Repeated capsaicin patch therapy in elderly neuropathic pain: Progressive real‐world improvements

#### M. Überall ^1^; P. Müller‐Schwefe^2^; M. Küster^3^

##### ^1^IFNAP *–* Institute of Neurological Sciences, Nürnberg, Germany; ^2^Interdisciplinary Center for Pain & Palliative Care Medicine, Göppingen, Germany; ^3^Interdisciplinary Center for Pain & Palliative Care Medicine, Bonn, Germany

**Background and aims:** Localized peripheral neuropathic pain (PNP) is frequent in older adults. Systemic treatments often show limited effectiveness and are associated with relevant safety risks. Long‐term real‐world data on topical high‐concentration capsaicin patch (HCCP) therapy in geriatric patients are limited. To assess real‐world effectiveness, treatment progression, and safety of repeated HCCP applications in patients aged ≥75 years with localized PNP.

**Methods:** This retrospective cohort analysis used depersonalized 12‐month routine‐care data from the German Pain e‐Registry (CASPAGE subgroup of CASPAR). Elderly patients receiving one to four consecutive HCCP applications were evaluated. Patient‐reported outcomes included pain intensities, neuropathic pain phenotype, pain‐related disability, sleep disturbance, quality of life, mood, and systemic analgesic use. The primary endpoint was a composite response defined by clinically meaningful improvement in highest pain intensity, pain‐related disability, and neuropathic pain symptoms.

**Results:** Data from 576 patients (mean age 81.4 years) and 1576 HCCP applications were analyzed. Pain and functional outcomes improved after the first treatment and showed progressive benefits with additional applications. The composite response increased from 0.9% after one treatment to 27.5% after four. Patients receiving ≥3 treatments demonstrated large reductions in pain, disability, and systemic analgesic use. Adverse drug reactions were local, transient, and decreased with repeated use.

**Conclusion:** Repeated HCCP therapy is associated with progressive, clinically meaningful improvements and good tolerability in very elderly patients with localized PNP, supporting its role as a long‐term topical treatment option.

**Disclosure:** The parent CASPAR registry study was supported by Grünenthal GmbH. The present CASPAGE subgroup analysis was conducted independently, without external financial support. Grünenthal had no influence on data selection, analysis, interpretation, or manuscript preparation. M.A. Überall holds leadership roles at IFNAP and O.Meany MDPM, which operate the German Pain e‐Registry. Other authors declare no conflicts of interest.

## EPO‐0715

### Efficacy & safety of IncobotulinumtoxinA in patients with postherpetic neuralgia: Results from the PaiNT proof‐of‐ concept phase 2 trial

#### N. Attal ^1^; I. Bicker^2^; I. Pulte^2^; R. Baron^3^; G. Klein^2^; N. Finnerup^4^; T. Geister^2^; U. Munzel^2^; E. Viel^5^

##### ^1^Paris Saclay University, Hôpital Ambroise Paré, Paris, France; ^2^Merz Therapeutics GmbH, Frankfurt am Main, Germany; ^3^University Hospital Schleswig‐Holstein, Kiel, Germany; ^4^Aarhus University, Aarhus, Denmark; ^5^Nîmes University Hospital and Montpellier‐Nîmes Faculty of Medicine, Montpellier, France

**Background and aims:** Postherpetic neuralgia (PHN) is a common peripheral neuropathic pain (PNP) and remains a major clinical challenge. Therapies often provide insufficient relief or cause systemic side effects. IncobotulinumtoxinA (incoBoNT‐A, Xeomin®) may offer a promising local treatment. The PaiNT Phase 2 proof‐of‐concept trial evaluated incoBoNT‐A in PHN and post‐surgical/post‐traumatic neuropathic pain (PS/PTNP).

**Methods:** The trial enrolled adults with moderate to severe PNP. The present results focus on PHN. Patients received subcutaneous injections of incoBoNT‐A (≤300 U in ≤60 sites into painful area) or placebo and were followed for 20 weeks. The primary endpoint was the change from baseline in average daily pain (ADP) intensity (0–10 NRS) at weeks 2–12. Secondary endpoints included the change in Neuropathic Pain Symptom Inventory (NPSI, 0–100 points) total score and dimensions at weeks 2–12 and safety.

**Results:** 123 PNP patients including 63 with PHN were randomised. The endpoints did not significantly separate from placebo in the combined set. However, the PHN group showed a numerically greater reduction in ADP intensity from baseline throughout weeks 2–12 compared to placebo (LSM difference: ‐0.56 points) and statistically significantly greater reduction at week 6 (−1.14 points; *p* = 0.0488). LSM difference to placebo in NPSI (total) change was −3.14. 50% of PHN patients received the maximum dose of 300 U. No new safety concerns were detected for incoBoNT‐A.

**Conclusion:** The PaiNT proof‐of‐concept study demonstrated clinically meaningful results with a favourable tolerability profile in treatment of PHN with IncoBoNT‐A, supporting further research potentially with higher dosages in this PNP indication.

**Disclosure:** This study was funded by Merz Therapeutics GmbH. IB, IP, GK, TLG, and UM are employees of Merz Therapeutics NA has served on the advisory boards or speakers panels of Grünenthal, Merz therapeutics, Medtronic, Viatris, Noema pharma and Pfizer. RB has received grant/research support from Pfizer, Genzyme, Grünenthal, German Federal Ministry of Education and Research (BMBF): German Research Network on Neuropathic Pain, NoPain system biology and German Research Foundation (DFG). He has received speaker honorarium from Pfizer, Genzyme, Grünenthal, Mundipharma, Sanofi Pasteur, Medtronic, Eisai, Lilly, Boehringer Ingelheim, Astellas, Desitin, Teva Pharma, Bayer‐Schering, MSD and served as consultant for Pfizer, Genzyme, Grünenthal, Mundipharma, Allergan, Sanofi Pasteur, Medtronic, Eisai, Lilly, Boehringer Ingelheim, Astellas, Novartis, Bristol‐Myers Squibb, Biogenidec, AstraZeneca, Merck, Abbvie. NBF has been a consultant for Mitsubishi Tanabe Pharma, Merck, Almirall, and NeuroPN. EV declares no competing interests RB has received grant/research support from EU Projects: „Europain“ (115007). DOLORisk (633491). IMI Paincare (777500). BMBF Projects: NoChro (13GW0338C), DFNS (01EM0903), NoPain, ERA‐NET (IM‐PAIN) Industry: Pfizer, Grünenthal, Mundipharma, Alnylam, Zambon, Sanofi Aventis, Viatris Trademarks: „fiberASSESS®" EU No. 018527050, „FabryScan®" EU No. 012094959, „AmyloScan®" EU No. 018188607, US No. 6168141. He has received Consultant /speaker honorarium from: Pfizer, Sanofi Aventis, Grünenthal, Eli Lilly, Desitin, Bayer, AbbVie, Janssen, Amicus, Novo Nordisk, Chiesi, Takeda, Stada, Ever, Unilab, Hexal, Viatris, AstraZeneca, Aristo, Esanum, Docflix, Vertanical, Denk, Teva, Glenmark, Novartis, Biotest, Desitin, Lateral, Alnylam, Confo, EVA, Merz, Neumentum, Algo, Nanobiotix, Stada, Heat2Move, Hoba, BioNTech, Esteve, SIMR, Desitin, SelectION, Max‐Delbrück‐C NBF has been a consultant for Merz, NeuroPN, Saniona, PharmNovo, Tris Pharma, AKIGAI, and Zyneyro. EV has served on the advisory boards or speakers panels of Grünenthal, Merz therapeutics, Ehyparm and Viatris; and received research grants from Grünenthal, Merz therapeutics and Vertex.

## EPO‐0716

### UUnderstanding patient diversity & placebo response in post‐surgical/traumatic neuropathic pain: Results from the PaiNT proof‐of‐concept phase 2 trial

#### N. Attal ^1^; I. Bicker^2^; I. Pulte^2^; R. Baron^3^; G. Klein^2^; N. Finnerup^4^; T. Geister^2^; U. Munzel^2^; E. Viel^5^

##### ^1^Paris Saclay University, Hôpital Ambroise Paré, Paris, France; ^2^Merz Therapeutics GmbH, Frankfurt am Main, Germany; ^3^University Hospital Schleswig‐Holstein, Kiel, Germany; ^4^Aarhus University, Aarhus, Denmark; ^5^Nîmes University Hospital and Montpellier‐Nîmes Faculty of Medicine, Montpellier, France

**Background and aims:** Post‐surgical/post‐traumatic neuropathic pain (PS/PTNP) is characterised by diverse etiologies and pain therapies are often insufficient and cause systemic side effects. IncobotulinumtoxinA (incoBoNT‐A, Xeomin®) may provide a promising local treatment of peripheral NP (PNP). The proof‐of‐concept PaiNT trial evaluated incoBoNT‐A in postherpetic neuralgia (PHN) and PS/PTNP.

**Methods:** These results focus on the PS/PTNP subgroup. Patients received subcutaneous injections of incoBoNT‐A (≤300 U in ≤60 sites into painful area) or placebo and followed for 20 weeks. The primary endpoint was the change from baseline in average daily pain (ADP) intensity (0–10 NRS) at weeks 2–12 (combined PHN & PS/PTNP). Secondary endpoints included the change in neuropathic pain symptom inventory (NPSI, 0–100 points) total score at weeks 2–12 and safety.

**Results:** The 60 PS/PTNP patients (28 incoBoNT‐A, 31 placebo) were highly heterogeneous, with 48 distinct diagnoses (many potentially indicating mixed pain). Groups differed in diagnoses, PNP duration, pre‐treatment history, and baseline characteristics. The endpoints did not significantly separate from placebo in the combined set. For PS/PTNP, LSM changes from baseline of ADP intensity and NPSI at weeks 2–12 for incoBoNT‐A were −0.13 and −22.15. Placebo response was high (ADP intensity: −0.64; NPSI: −22.84), exceeding the changes for placebo observed in the PHN group (ADP intensity: −0.16; NPSI: −5.63). No new safety concerns were detected.

**Conclusion:** Improvements in ADP/NPSI in the PS/PTNP population were masked by very high placebo response. Treatment with incoBoNT‐A is safe, but group heterogeneities make the comparison to placebo inconclusive. Careful patient selection is essential for future research.

**Disclosure:** This study was funded by Merz Therapeutics GmbH. IB, IP, GK, TLG, and UM are employees of Merz Therapeutics NA has served on the advisory boards or speakers panels of Grünenthal, Merz therapeutics, Medtronic, Viatris, Noema pharma and Pfizer. RB has received grant/research support from Pfizer, Genzyme, Grünenthal, German Federal Ministry of Education and Research (BMBF): German Research Network on Neuropathic Pain, NoPain system biology and German Research Foundation (DFG). He has received speaker honorarium from Pfizer, Genzyme, Grünenthal, Mundipharma, Sanofi Pasteur, Medtronic, Eisai, Lilly, Boehringer Ingelheim, Astellas, Desitin, Teva Pharma, Bayer‐Schering, MSD and served as consultant for Pfizer, Genzyme, Grünenthal, Mundipharma, Allergan, Sanofi Pasteur, Medtronic, Eisai, Lilly, Boehringer Ingelheim, Astellas, Novartis, Bristol‐Myers Squibb, Biogenidec, AstraZeneca, Merck, Abbvie. NBF has been a consultant for Mitsubishi Tanabe Pharma, Merck, Almirall, and NeuroPN. EV declares no competing interests RB has received grant/research support from EU Projects: “Europain” (115007). DOLORisk (633491). IMI Paincare (777500). BMBF Projects: NoChro (13GW0338C), DFNS (01EM0903), NoPain, ERA‐NET (IM‐PAIN) Industry: Pfizer, Grünenthal, Mundipharma, Alnylam, Zambon, Sanofi Aventis, Viatris Trademarks: “fiberASSESS®” EU No. 018527050, “FabryScan®” EU No. 012094959, “AmyloScan®” EU No. 018188607, US No. 6168141. He has received Consultant /speaker honorarium from: Pfizer, Sanofi Aventis, Grünenthal, Eli Lilly, Desitin, Bayer, AbbVie, Janssen, Amicus, Novo Nordisk, Chiesi, Takeda, Stada, Ever, Unilab, Hexal, Viatris, AstraZeneca, Aristo, Esanum, Docflix, Vertanical, Denk, Teva, Glenmark, Novartis, Biotest, Desitin, Lateral, Alnylam, Confo, EVA, Merz, Neumentum, Algo, Nanobiotix, Stada, Heat2Move, Hoba, BioNTech, Esteve, SIMR, Desitin, SelectION, Max‐Delbrück‐C NBF has been a consultant for Merz, NeuroPN, Saniona, PharmNovo, Tris Pharma, AKIGAI, and Zyneyro. EV has served on the advisory boards or speakers panels of Grünenthal, Merz therapeutics, Ehyparm and Viatris; and received research grants from Grünenthal, Merz therapeutics and Vertex.

## EPO‐0717

### Neuropathic pain in multiple sclerosis: Discrepancies between DN4 screening and neurophysiological measures

#### N. Drobinska; E. Sukockiene; M. Uginet; P. Lalive; A. Lascano

##### Department of Clinical Neurosciences, Division of Neurology, Geneva University Hospitals and University of Geneva, Switzerland

**Background and aims:** Pain is a common and disabling symptom in multiple sclerosis (MS), yet its pathophysiological mechanisms remain poorly understood. Clinical assessment of neuropathic pain relies largely on subjective screening tools such as the DN4 questionnaire. This study aimed to characterize pain phenotype in MS using the DN4 score and to determine its relationship with objective neurophysiological markers of nociceptive and non‐nociceptive pathway function assessed by laser‐evoked (LEPs) and somatosensory evoked potentials (SEPs).

**Methods:** Fifty MS patients reporting pain underwent standardized clinical evaluation, including DN4, pain intensity (VAS), fatigue (FSMC), anxiety and depression (HADS), and disability (EDSS). LEPs were recorded after laser stimulation of the hand and foot dorsum, and SEPs following median and tibial nerve stimulation. Neurophysiological parameters were compared between patients with DN4 <4 and ≥4. Logistic regression, non‐parametric group comparisons, and correlation analyses were performed.

**Results:** Neuropathic pain phenotype (DN4 ≥4) was identified in 56% of patients. DN4 showed moderate correlation with pain intensity and fatigue, and weakly with anxiety, but showed no significant association with LEPs or SEPs. Logistic regression confirmed that no neurophysiological measure independently predicted DN4 ≥4 status. LEP and SEP abnormalities were not significantly correlated, suggesting dissociated involvement of nociceptive and large‐fiber pathways.

**Conclusion:** In MS, DN4‐defined pain phenotype reflects subjective symptom burden rather than objective sensory pathway dysfunction. Its limited correlation with neurophysiological measures highlights pain's multidimensional nature in MS. LEPs and SEPs provide complementary mechanistic insights, supporting a multimodal, mechanism‐oriented approach to pain assessment and management.

**Disclosure:** Natalia Drobinska, Eglé Sukockiene, Marjolaine Uginet, Patrice H. Lalive have nothing to disclose. Agustina M. Lascano received honoraria for advisory from ATC Therapeutics, Biogen, Johnson and Johnson, Roche, Argenx, Amgen and Alexion, and speakers’ fees from Sanofi, CSL‐Behring, UCB, Takeda non‐related to the current study.

## EPO‐0718

### Sexually dimorphic neuroimmune pathways in chronic pain: A comprehensive systematic review of cellular and molecular mechanisms

#### N. Brezic ^1^; S. Gligorevic^2^; A. Sic^3^; V. Tseriotis^4^; N. Knezevic^3^

##### ^1^Department of Medicine, NYCHH/Lincoln, Bronx, USA; ^2^Department of Medicine, NYCHH/Jacobi Medical Center – North Central Bronx Hospital, Bronx, USA; ^3^Department of Anesthesiology, Advocate Illinois Masonic Medical Center, Chicago, USA; ^4^Department of Neurology, Agios Pavlos General Hospital of Thessaloniki, Leoforos Ethnikis Antistaseos 161, Kalamaria, Thessaloniki, Greece

**Background and aims:** Chronic pain shows marked sex differences in prevalence, severity, and persistence, suggesting underlying biological mechanisms beyond psychosocial factors. Increasing evidence indicates that sexually dimorphic neuroimmune pathways play a central role in chronic pain pathophysiology.

**Methods:** This systematic review was conducted according to PRISMA 2020 guidelines and synthesized human clinical and translational studies examining sex‐specific neuroimmune and glial mechanisms in adult chronic pain. Multiple biomedical databases were searched from inception to December 2025, and studies reporting sex‐stratified neuroimmune outcomes were included.

**Results:** Across pain conditions, males predominantly exhibited microglia‐driven mechanisms, particularly P2X4 receptor–mediated signaling and BDNF‐dependent neuronal disinhibition. In contrast, females showed greater involvement of adaptive immune pathways, including CD4^+^ and CD8^+^ T cell signaling, chemokine cascades, and astrocyte‐mediated neuroimmune interactions. Cytokine profiles, immune cell phenotypes, and neuroimaging markers further distinguished sex‐specific pain mechanisms. Heterogeneity and limited sex‐stratified power constrained quantitative synthesis.

**Conclusion:** Chronic pain comprises biologically distinct, sex‐dependent neuroimmune mechanisms, supporting the need for sex‐informed biomarkers and precision medicine approaches in pain management.

**Disclosure:** Nothing to disclose

## EPO‐0719

### What predicts pain in cognitively impaired patients?

#### M. Sinkovic; S. Rakusa; M. Rakusa

##### Division of Neurology, University Medical Centre Maribor, Slovenia

**Background and aims:** Pain is highly prevalent in older adults. However, it may be overlooked when cognitive deficits limit self‐report. Our study aimed to determine whether demographic factors and cognitive status are associated with pain.

**Methods:** We recruited individuals who completed two pain questionnaires (Doloplus‐2 and NOPPAIN) and a cognitive evaluation. Descriptive statistics were performed, and group comparisons between cognitively impaired and cognitively preserved individuals were conducted using t‐tests. Pain presence was defined as any non‐zero subjective pain rating (VAS ≥1, range 0–6). Factors associated with pain presence were examined using multivariable logistic regression.

**Results:** A total of 153 patients were included; 48 (31%) had cognitive impairment. The prevalence of pain was similar in cognitively impaired and unimpaired patients (86.0% vs 83.5%). Patients with cognitive impairment were significantly older (76.8 SD 9.5 vs 61.5 SD 15.4 years, *p* < 0.001), with higher subjective pain intensity (VAS 2.54 SD 1.47 vs 1.94 SD 1.24, *p* = 0.010), higher NOPPAIN total scores (2.54 SD 1.47 vs 1.95 SD 1.23, *p* = 0.011) and substantially higher Doloplus‐2 total scores (13.6 SD 6.5 vs 7.8 SD 5.1, *p <* 0.001) compared with cognitively preserved patients. In adjusted logistic regression analysis, higher age was independently associated with pain presence (OR 1.04 per year, 95% CI 1.00–1.07, *p* = 0.026), while cognitive impairment and sex were not.

**Conclusion:** Pain is common across cognitive groups and increases with age. Cognitive impairment doesn’t independently influence pain presence and shouldn't reduce clinical awareness of pain detection.

**Disclosure:** Nothing to disclose.

## EPO‐0720

### Restoring circadian rhythms with a citrus flavonoid to improve sleep and reduce neuropathic pain

#### W. Dai; V. Palada

##### Department of Physiology, SleepWell Research Program, Faculty of Medicine, University of Helsinki, Helsinki, Finland

**Background and aims:** Neuropathic pain (NP) disrupts sleep architecture and circadian regulation, impairing quality of life. Nobiletin, a polymethoxylated citrus flavonoid, amplifies molecular clock oscillations and may stabilise sleep–wake dynamics. We hypothesised that nobiletin alleviates NP by restoring circadian rhythmicity and improving sleep in mice after spared nerve injury (SNI).

**Methods:** Adult male and female PER2::LUC C57BL/6 mice underwent SNI. Nobiletin (10 mg/kg/day) or vehicle was continuously delivered via osmotic mini‐pumps. Telemetric EEG/EMG, body temperature and locomotor activity were recorded at baseline and post‐injury. Mechanical allodynia was assessed with von Frey filaments and dynamic brush testing. Ex vivo bioluminescence from DRG, spinal cord and prefrontal cortex explants was monitored to quantify Per2‐driven rhythmicity; circadian metrics were analysed using cosinor and CircaCompare. Procedures complied with institutional animal‐care guidelines.

**Results:** SNI induced robust ipsilateral mechanical hypersensitivity. Continuous nobiletin significantly attenuated dynamic brush allodynia (*p <* 0.01) but did not alter von Frey thresholds. At day 7 post‐SNI, REM sleep was reduced in both sexes, while NREM exhibited phase‐dependent changes (increase during dark phase; reduction in males during light phase). In female DRG, Per2::Luc bioluminescence rhythms showed altered mesor, reduced amplitude and shifted acrophase versus sham (all *p <* 0.001). Ongoing analyses test whether nobiletin rescues REM sleep and tissue‐clock amplitude.

**Conclusion:** Preliminary data support nobiletin as a chronotherapeutic candidate that selectively mitigates NP‐related behavioural hypersensitivity and stabilises sleep and circadian oscillations. If confirmed, this non‐opioid, clock‐targeting approach could inform timed interventions to improve sleep and reduce NP.

**Disclosure:** Nothing to disclose.

## EPO‐0721

### Headache during IVF treatment: A comparative evaluation of GnRH agonist and antagonist protocols

#### Z. Kalı^1^; F. Algül ^2^; Y. Kablan^3^

##### ^1^Obstetrics and Gynecology Department, Gozde Hospital, Malatya, Türkiye*;* ^2^Department of Neurology, Inonu University, Malatya, Türkiye*;* ^3^Department of Neurology, Inonu University, Malatya, Türkiye

**Background and aims:** Controlled ovarian hyperstimulation during in vitro fertilization (IVF) involves marked hormonal changes. GnRH agonist and antagonist protocols differ in the timing and dynamics of hypothalamic–pituitary axis suppression, yet headache during IVF treatment remains poorly investigated. This study aimed to compare headache frequency between GnRH agonist and antagonist protocols and to assess the temporal association between headache occurrence and antagonist administration.

**Methods:** Infertile women undergoing IVF/ICSI were retrospectively evaluated by protocol, with recombinant FSH stimulation and recorded headache reports, including post‐cetrorelix events

**Results:** A total of 46 women undergoing IVF were included in the analysis (GnRH agonist: *n* = 18; GnRH antagonist: *n* = 28). Headache was reported more frequently in patients treated with the GnRH antagonist protocol compared with those receiving the agonist protocol (53.6% vs. 22.2%). In the GnRH antagonist group, headache symptoms were most commonly reported within the first 24 hours following cetrorelix administration, with 9 of 15 patients (60.0%) experiencing headache during this period. The temporal distribution of headache episodes was evaluated descriptively. Headache frequency appeared to increase across rising estradiol categories, being reported in 25.0% of patients at 250–1000 pg/mL, 43.8% at 1001–2000 pg/mL, 64.3% at 2001–3000 pg/mL, and 87.5% at levels above 3000 pg/mL; this pattern may reflect a descriptive trend rather than a causal association.

**Figure:**
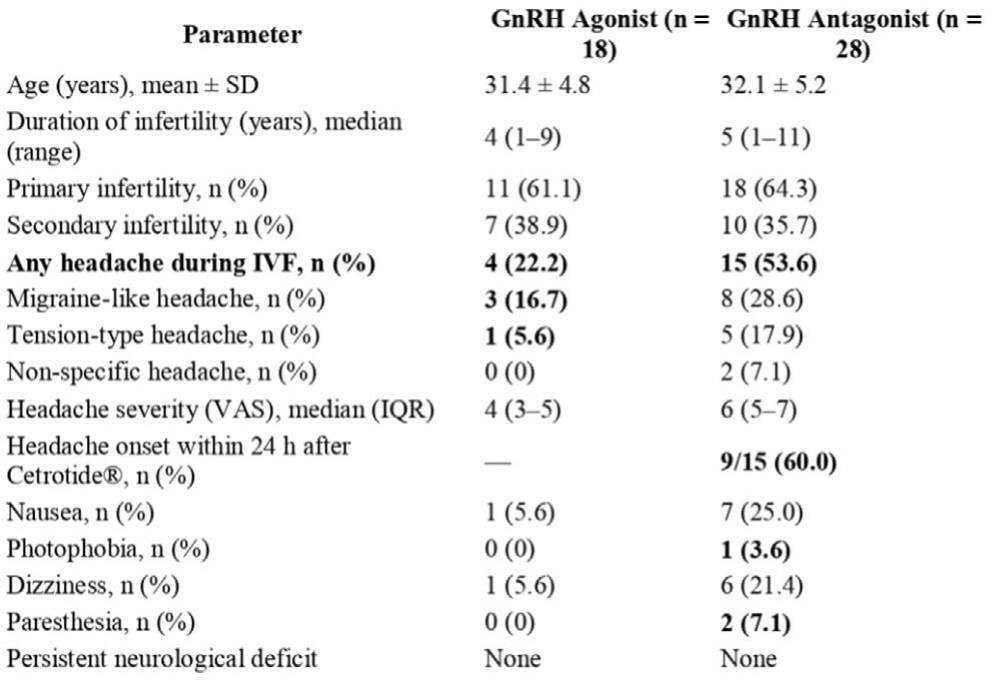
**TABLE 1** Clinical characteristics and headache features according to ovarian stimulation protocol.

**Figure:**
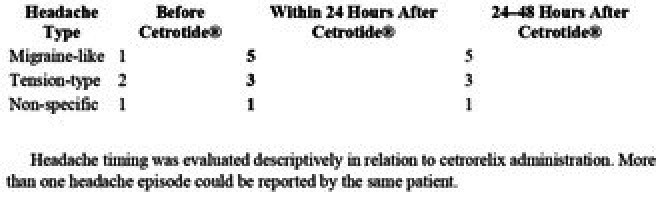
**TABLE 2** Temporal distribution of headache occurrence in the GnRH antagonist protocol.

**Figure:**
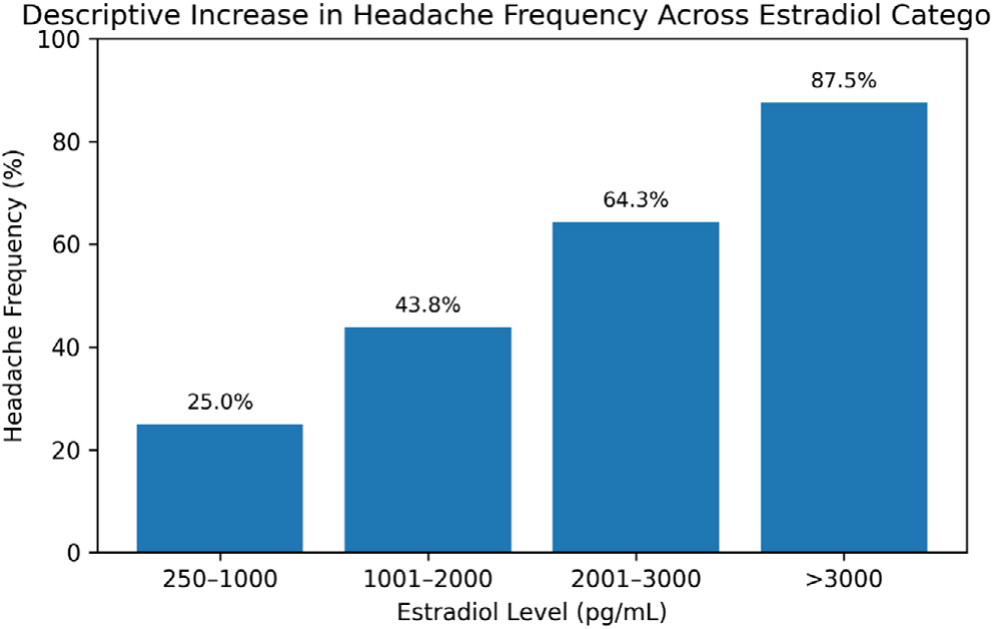
**FIGURE 1** Descriptive distribution of headache frequency across estradiol level categories.

**Conclusion:** Headache is more frequently reported during IVF cycles using GnRH antagonist protocols compared with agonist protocols. The early onset of symptoms following antagonist administration may be related to acute hormonal changes, rather than the ovarian stimulation process itself.

**Disclosure:** The authors declare no financial or non‐financial conflicts of interest related to this study.

## Peripheral Nerve Disorders 1

## EPO‐0722

### Reliability of predicted outcomes by modified Erasmus Guillain Barre outcome score mEGOS versus followup outcomes in Guillain Barré syndrome patients

#### A. Pai ^1^; N. Mukhi^1^; D. Varma^2^

##### ^1^Department of Neurology, Kasturba Medical College Manipal Academy Of Higher Education, Manipal, India; ^2^Department of Infectious Diseases, Kasturba Medical College, Manipal Academy of Higher Education, Manipal*, India*

**Background and aims:** Neuroprognostication in GBS relies on clinical assessments and neurophysiological evaluations. The mEGOS score, determined at hospital admission, predicts the likelihood of GBS patients achieving independent ambulation during follow‐up.

**Methods:** This study aimed to evaluate the reliability of predicted outcomes based on the mEGOS score at hospital admission by comparing them to actual follow‐up outcomes assessed using the Hughes Disability Scale at three months. A Hughes Disability Scale score of 0–2 was stratified as a good outcome and 3–6 as a poor outcome. A mEGOS score of 0–3 indicated a good outcome and 4–9 indicated a poor outcome.

**Results:** Among the 113 patients analysed from records of a GBS cohort admitted to our tertiary hospital, 64% were male, and 72% presented within one week of symptom onset. Nineteen per cent required mechanical ventilation. Nerve conduction studies at admission were demyelinating in 21%, axonal in 14%, equivocal in 44%, and normal in 13%. Among the 88 patients with all parameters, the mEGOS score at admission had a sensitivity of 45.5% and a specificity of 50.6% for predicting a poor outcome at three months. The positive predictive value was 11.6% while the negative predictive value was 86.7%.

**Conclusion:** The mEGOS score, therefore, is primarily an early triage and reassurance tool rather than a robust predictor of disability. Its high negative predictive value emphasises its clinical utility in identifying patients who are unlikely to experience poor outcomes, but since it tends to overestimate the risk of poor outcome it has a low positive predictive value.

**Disclosure:** Nothing to disclose.

## EPO‐0723

### Safety profile of tanruprubart (ANX005) in patients with Guillain–Barré syndrome: A randomised, placebo‐controlled phase 3 study

#### E. McCracken; H. Kroon; G. Morrison; E. Pulkstenis; A. Johnson

##### Annexon Biosciences, Brisbane, USA

**Background and aims:** Guillain‐Barré syndrome (GBS) is a rare, life‐altering, neuromuscular emergency, where classical complement activation contributes to progressive muscle weakness. Tanruprubart (ANX005), a humanised monoclonal IgG4 antibody, selectively inhibits complement component C1q. Infections with encapsulated bacteria and systemic autoimmunity were events of interest.

**Methods:** In GBS‐02 (Phase 3), participants ≥16 years with a recent GBS diagnosis without planned plasma exchange or intravenous immunoglobulins were randomised to receive a single intravenous dose of tanruprubart 30 or 75 mg/kg or placebo. Antihistamine and acetaminophen were administered pre‐infusion to mitigate for infusion related reactions (IRRs). Vaccinations and prophylactic antibiotics were not mandated. Safety over 6 months included treatment‐emergent adverse events (TEAEs), serious adverse events (SAEs), investigator‐assessed treatment‐related TEAEs, and events of special interest (IRRs; encapsulated‐bacteria infections; systemic immune disorders other than GBS).

**Results:** The safety set comprised 241 participants; 95% did not receive prophylactic antibiotics. 98.8% reported ≥1 TEAE (Table 1). Treatment‐related TEAEs were mostly IRRs (rash), which were generally mild/moderate and transient without infusion interruption. One participant per group discontinued due to an IRR; none withdrew due to TEAEs. Encapsulated‐bacteria infection rates were similar across groups. TEAEs and SAEs were consistent with GBS morbidity. No systemic autoimmune diseases other than GBS occurred. Three non‐treatment related deaths occurred in each group.

**Figure:**
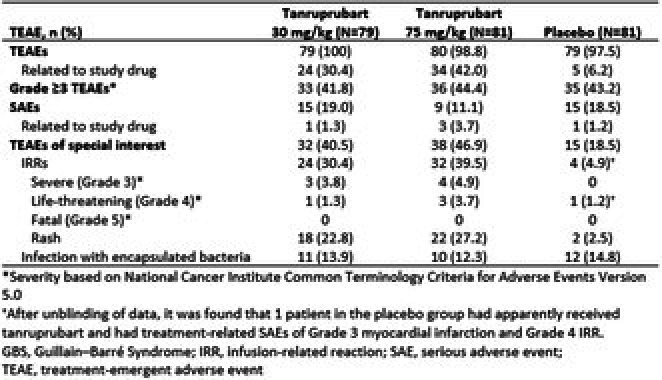
**TABLE 1** Summary of TEAEs.

**Conclusion:** Single‐dose tanruprubart was generally well‐tolerated. Common TEAEs were associated with disease course. Tanruprubart did not increase risk of infections with encapsulated bacteria, despite most participants not receiving prophylactic antibiotics. Findings support the promising potential of tanruprubart in GBS.

**Disclosure:** The study was sponsored by Annexon Biosciences (Brisbane, CA, USA). EM, H‐AK, GM, EP, AJ: Employment with and shareholder of Annexon Biosciences.

## EPO‐0724

### Tanruprubart, anti‐C1q therapy, accelerates recovery and reduces ventilation in severe Guillain‐Barré Syndrome: Results from the phase 3 GBS‐02 trial

#### H. Kroon ^1^; G. Morrison^1^; E. Pulkstenis^1^; Q. Deen Mohammad^2^; K. Gorson^3^

##### ^1^Annexon Biosciences, Brisbane, USA; ^2^National Institute of Neuroscience (NINS), Dhaka, Bangladesh; ^3^Tufts University School of Medicine, Boston, USA

**Background and aims:** Guillain‐Barré syndrome (GBS) is a severe, autoimmune, life‐altering disorder mediated by classical complement affecting peripheral nerves, resulting in muscle weakness and often prolonged recovery. The safety and efficacy of tanruprubart, a C1q inhibitor designed to treat patients with GBS, was evaluated in the randomised placebo‐controlled Phase 3 trial GBS‐02 (NCT04701164). Here, we report on prespecified measures of clinical benefit associated with patient recovery time and resource utilisation.

**Methods:** Patients with GBS aged ≥16 years and a baseline GBS disability score (GBS‐DS) of 3, 4, or 5 were randomised 1:1:1 to a single intravenous (IV) infusion of tanruprubart (30 or 75 mg/kg) or placebo. Neither IV immunoglobulin or plasma exchange was given. Time to walking independently (GBS disability score ≤2) was analysed using time‐to‐event methods. Duration of mechanical ventilation and intensive care unit (ICU) stay were assessed using the zero‐inflated negative binomial (ZINB) model (imputed for death).

**Results:** A total of 241 patients were randomised. Patients who received tanruprubart 30 mg/kg (*n* = 79) could walk independently a median 31 days earlier (*p* = 0.0211) compared with placebo (*n* = 81). The duration of mechanical ventilation was a median 28 days less (*p* = 0.0356) compared with placebo. The median ICU stay for the tanruprubart group was 7 days less than placebo (*p* = 0.5306).

**Conclusion:** In this Phase 3 study of severe GBS, tanruprubart improved recovery‐related outcomes, including earlier independent walking and shorter ventilation duration, with the possibility of shorter time in the ICU, consistent with clinically meaningful benefit.

**Disclosure:** The study was sponsored by Annexon Biosciences (Brisbane, CA, USA). HAK, GM, EP: Employment with and shareholder of Annexon Biosciences QDM: Consultancy/advisory role with Annexon Biosciences KCG: Consultancy/advisory role with Annexon Biosciences, argenx, Janssen, and Sanofi.

## EPO‐0725

### Targeted immunotherapy with Tanruprubart (ANX005) reduces ventilation requirements in Guillain–Barré syndrome: A phase 3 subgroup analysis

#### H. Kroon ^1^; Q. Deen Mohammad^2^; J. Navarro^3^; G. Morrison^1^; J. Du^1^; R. Gerwien^1^; K. Abul Kalam Azad^4^; Z. Islam^5^; K. Gorson^6^

##### ^1^Annexon Biosciences, Brisbane, USA; ^2^National Institute of Neuroscience (NINS), Dhaka, Bangladesh; ^3^Cotabato Regional Medical Center, Cotabato City, Philippines; ^4^Dhaka Medical College and Hospital, Dhaka, Bangladesh; ^5^Gut–Brain Axis Laboratory, Infectious Diseases Division, icddr,b, Dhaka, Bangladesh; ^6^Tufts University School of Medicine, Boston, USA

**Background and aims:** Guillain‐Barré syndrome (GBS) is a rare, life‐altering neuromuscular emergency requiring hospitalisation, and mechanical ventilation in severe cases. Tanruprubart (ANX005) is a targeted, humanised monoclonal IgG4 antibody that rapidly and selectively inhibits classical complement component C1q. In GBS‐02, a Phase 3, randomised, double‐blind, placebo‐controlled trial of tanruprubart 30 and 75 mg/kg (NCT04701164), a single infusion of tanruprubart 30 mg/kg led to early and sustained clinical benefit, and was well tolerated. This analysis evaluated the effect of tanruprubart on the duration of mechanical ventilation in GBS‐02.

**Methods:** Individuals aged ≥16 years with a recent GBS diagnosis and a GBS‐disability score (GBS‐DS) of 3 (can walk 10 metres with help) to 5 (requiring ventilation) were enrolled. Ventilation duration was analysed using a zero‐inflated negative binomial model; participants who died were imputed to 26 weeks (trial end). Ventilation duration was assessed overall and by baseline disease characteristics assessing muscle weakness (Medical Research Council sumscore [MRCss], 0 = tetraplegic, 60 = normal), a stratification factor (GBS‐DS) and axonal damage (neurofilament‐light [NfL] levels).

**Results:** Mechanical ventilation requirements were similar across treatment groups (Table 1). Among ventilated patients, approximately 75% were already ventilated at randomisation. Duration of ventilation was median 27 days shorter with tanruprubart 30 mg/kg versus placebo (Table 1). This difference was particularly notable in participants with greater muscle weakness (lowest MRCss tertile 0–20) and worse axonal damage (median NfL ≥352.64) at baseline compared with their healthier counterparts (Table 1).

**Figure:**
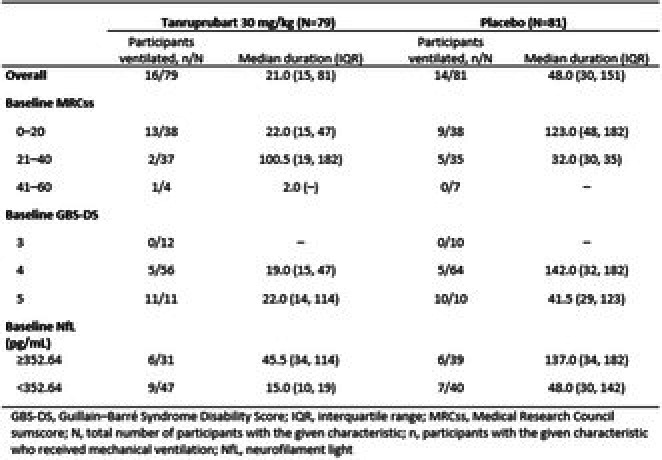
**TABLE 1** Duration of ventilation by baseline disease characteristics.

**Conclusion:** Tanruprubart reduced the duration of mechanical ventilation in individuals with GBS, especially among those with more severe disease.

**Disclosure:** The study was sponsored by Annexon Biosciences (Brisbane, CA, USA). H‐AK, GM, JD, RG: Employment with and shareholder of Annexon Biosciences QDM: Consultancy/advisory role with Annexon Biosciences JN: Consultancy role with Annexon Biosciences KAKA: No relevant disclosures ZI: Research funding from Fogarty International Center, National Institute of Neurological Disorders and Stroke of the National Institutes of Health, USA, and Annexon Biosciences KCG: Consultancy/advisory role with Annexon Biosciences, argenx, Janssen, and Sanofi.

## EPO‐0726

### Comparison of anti‐MAG antibody–associated neuropathy and distal or predominantly sensory CIDP: An analysis of the Portuguese CIDP registry

#### M. Henriques; S. Bernardo; D. Carapinha; S. Cruz; P. Study Consortium

##### Department of Neurology, Hospital Prof. Dr. Fernando Fonseca, Lisbon, Portugal

**Background and aims:** Distal or predominantly sensory chronic inflammatory demyelinating polyneuropathy (CIDP) and anti–myelin‐associated glycoprotein (anti‐MAG) neuropathy share overlapping clinical features but differ in prognosis and treatment response. Early differentiation is important, particularly when anti‐MAG antibody testing is unavailable.

**Methods:** Patients from the Portuguese CIDP registry were retrospectively analyzed. Diagnoses were reviewed according to the 2021 EAN/PNS criteria. Anti‐MAG neuropathy was defined by antibody titers >7000 BTU. Demographic data, disease duration, initial symptoms and signs, cerebrospinal fluid (CSF) findings, nerve conduction studies, and response to therapy were evaluated.

**Results:** Sixty patients were included: 48 with distal/sensory CIDP and 12 with anti‐MAG neuropathy. Time to diagnosis was significantly longer in the anti‐MAG group (*p* = 0.013), while age at onset was similar. Albuminocytologic dissociation and higher CSF protein levels were more frequent in CIDP (*p <* 0.001 and *p* = 0.014, respectively). Muscle atrophy at presentation was more common in CIDP (*p* = 0.016). Neurophysiological studies showed abnormalities predominantly restricted to the lower limbs in anti‐MAG neuropathy (*p <* 0.001), whereas sural sparing was exclusive to CIDP (*p* = 0.003). CIDP patients also exhibited higher peroneal and tibial CMAP amplitudes and shorter distal peroneal latency. Improvement with intravenous immunoglobulin (IVIg) was observed in 45.8% of CIDP patients compared with 8.3% of anti‐MAG patients (*p* = 0.044). In multivariate analysis, sural sparing and response to IVIg remained independent predictors of CIDP.

**Conclusion:** Differentiating distal/sensory CIDP from anti‐MAG neuropathy remains challenging. However, sural sparing, albuminocytologic dissociation, and particularly response to IVIg support a diagnosis of CIDP and may guide earlier therapeutic decisions.

**Disclosure:** Nothing to disclose.

## EPO‐0727

### Sensory recovery following digital nerve repair using conduits versus autologous nerve grafts: A systematic review and meta‐analysis

#### M. Elkasaby ^1^; A. Omran^2^; Hamdeno^1^; A. Hassan^1^

##### ^1^Al‐Azhar University, Cairo, Egypt; ^2^Plastic Surgery Department, Al‐Azhar University, Damietta, Egypt

**Background and aims:** Digital nerve injuries can impair sensory function and hand performance. Autologous nerve grafting (ANG) remains the standard for repairing nerve gaps but is associated with donor site morbidity. Nerve conduits—such as collagen‐based, chitosan‐based, and muscle‐in‐vein types—are emerging alternatives that avoid these complications. This systematic review and meta‐analysis compared the effectiveness and safety of nerve conduits versus ANG for sensory recovery after digital nerve repair.

**Methods:** A systematic search of PubMed, Embase, Scopus, and Web of Science was conducted through July 2025 following PRISMA guidelines. Included studies were randomized controlled trials and observational studies comparing conduits with ANG. Primary outcomes were static and moving two‐point discrimination (S2PD and M2PD); complication rates were secondary. Data were pooled using a random‐effects model with mean differences (MD) and risk ratios (RR) and 95% confidence intervals (CI).

**Results:** Five studies with 214 patients were included. Sensory recovery was similar between conduits and ANG for S2PD (MD = −0.21; 95% CI: −2.25 to 1.83; *p* = 0.84) and M2PD (MD = −0.67; 95% CI: −2.00 to 0.66; *p* = 0.84). Chitosan‐based conduits showed significantly better S2PD outcomes than ANG (MD = −2.57; 95% CI: −3.40 to −1.74; *p* < 0.0001). Complication rates were comparable overall (RR = 2.36; *p* = 0.08), but RCTs showed fewer complications with conduits (RR = 3.32; *p* = 0.03).

**Conclusion:** Nerve conduits provide similar sensory outcomes to ANG and may reduce complications, especially with chitosan‐based designs. They represent a promising alternative for digital nerve repair.

**Disclosure:** Nothing to disclose.

## EPO‐0728

### Tanruprubart in GBS: Targeting early complement‐associated inflammation conserved across the clinical spectrum and regions

#### P. Paliwal ^1^; H. Kroon^1^; Z. Islam^2^; D. Lorenz^3^; E. Chang^1^; Y. Ru^4^; T. Yednock^1^; R. Artis^1^; K. Sheikh^5^; C. Sommer^6^

##### ^1^Annexon Biosciences, Brisbane, USA; ^2^Gut‐Brain Axis Laboratory, icddr,b, Dhaka, Bangladesh; ^3^Excelra Inc, Boston, USA; ^4^Soley Therapeutics, South San Francisco, CA, USA; ^5^McGovern Medical School, UTHealth, Houston, USA; ^6^University Hospital Würzburg, Würzburg, Germany

**Background and aims:** Guillain‐Barré syndrome (GBS) is an acute, immune‐mediated polyradiculoneuropathy resulting in mild weakness to severe paralysis. Converging evidence supports an early pathogenic window where classical complement activation and innate immune effector mechanisms drive neuroinflammation and early nerve injury.

**Methods:** We conducted serum proteome‐wide longitudinal profiling of acute inflammatory effectors using the Olink® Explore HT platform in GBS patients from a Ph3, randomised, placebo‐controlled study of anti‐C1q therapy tanruprubart in Asia (*n* = 139), an IVIg/PE‐treated US cohort (*n* = 14) and healthy controls (*n* = 10). Prespecified prognostic and clinical strata were defined.

**Results:** Across the tanruprubart Ph3 and IVIg/PE US cohorts, 352 proteins were elevated at baseline, including 81 proteins mapping to the top 3 Reactome inflammatory pathways, with ~2–4‐fold higher levels versus healthy controls. In placebo‐treated subjects, inflammatory signature rose/remained high, reaching a consistent peak at ~Day 8, then declining below baseline by Day 29, indicating a brief early inflammatory phase. In contrast, tanruprubart produced rapid, broad suppression of these pathway‐linked proteins by Day 8, confirmed in the top 12 marker panel spanning cytokine signaling, interferon signaling, innate immune pathways, and neutrophil associated markers. The IVIg/PE US cohort exhibited no early reduction in these markers.

**Conclusion:** Across geographically and clinically diverse cohorts, GBS demonstrated a conserved, complement‐linked early inflammatory signature peaking at Day 8, defining a narrow window where short‐duration upstream complement inhibition is most impactful. Tanruprubart rapidly and durably suppressed this inflammatory cascade across strata and regions, highlighting C1q inhibition as a mechanism‐targeted strategy for GBS and supporting extrapolation of these biomarker effects to US patients.

**Disclosure:** The study was sponsored by Annexon Biosciences (Brisbane, CA, USA). PP, HAK, EC, TY, RA: Employees and shareholders of Annexon Biosciences ZI: Research funding from Fogarty International Center, National Institute of Neurological Disorders and Stroke of the National Institutes of Health, USA, and Annexon Biosciences DL: External contractor of Annexon Biosciences to perform data analysis YR: Nothing to disclose KS: Research funding from NINDS/NIH and DoD. Consultant Annexon Biosciences CS: Nothing to disclose.

## EPO‐0729

### Role of magnetic resonance neurography in CIDP: Experience from a single center

#### S. Palma ^1^; D. Pinto^2^; V. Teixeira^2^; C. Santos Silva^1,3^; D. Tribovane^2^; P. Pereira^1^

##### ^1^Department of Neurology, Garcia de Orta Hospital, Almada‐Seixal Local Health Unit, Almada, Portugal; ^2^Department of Neuroradiology, Garcia de Orta Hospital, Almada‐Seixal Local Health Unit, Almada, Portugal; ^3^Faculty of Medicine, Institute of Molecular Medicine, University of Lisbon, Lisbon, Portugal

**Background and aims:** According to the European Academy of Neurology/Peripheral Nerve Society guidelines, magnetic resonance (MR) imaging is a supportive diagnostic criterion for chronic inflammatory demyelinating polyneuropathy (CIDP). The aim of this study was to evaluate MR neurography findings in patients with suspected or confirmed CIDP and to compare them with patients with alternative diagnoses.

**Methods:** A longitudinal retrospective analysis included all patients who underwent MR neurography at Hospital Garcia de Orta between July 1, 2024, and June 31, 2025, excluding examinations for traumatic indications. Patients were stratified into two groups: those with suspected or confirmed CIDP and those with other diagnoses.

**Results:** Thirteen patients were included: eight with suspected or diagnosed CIDP and seven controls (83.3% vs. 57.1% male; median ages 64.5 and 56.7 years, respectively). Two patients with newly diagnosed CIDP/possible CIDP showed MR neurography abnormalities: a 65‐year‐old woman with suspected classic CIDP demonstrated a diffuse thickening of the bilateral cervical plexus; and a 79‐year‐old man with suspected right lower limb focal CIDP showed nerve thickening and post‐gadolinium enhancement of the right lumbosacral plexus. No MR neurography abnormalities were observed in clinically stable CIDP patients receiving immunoglobulin therapy (*n* = 2), patients with possible CIDP without electrophysiological criteria (*n* = 2), or in controls.

**Figure:**
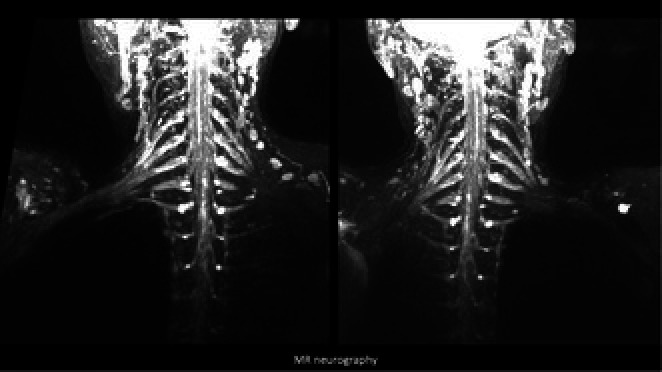
**FIGURE 1** MR neurography.

**Figure:**
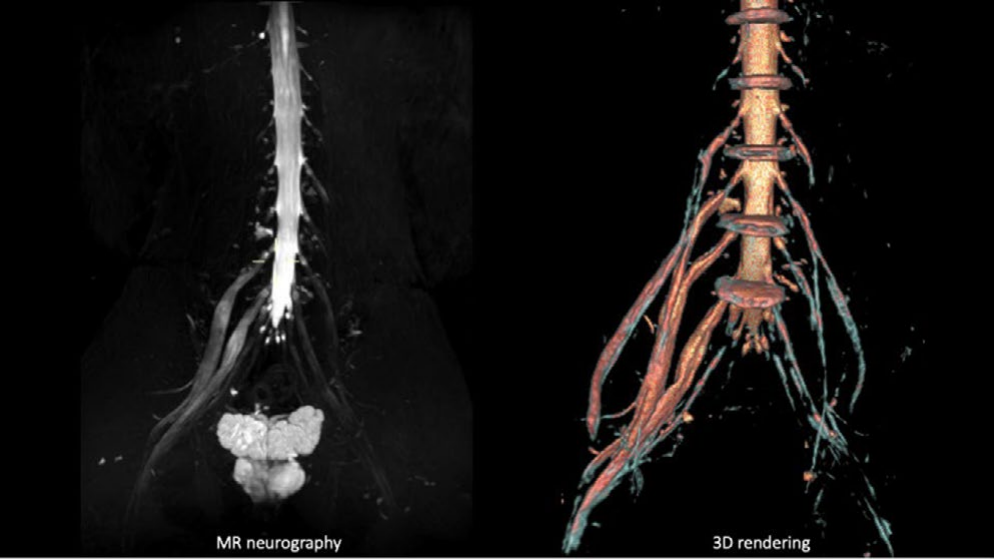
**FIGURE 2** MR neurography.

**Conclusion:** MR neurography revealed structural abnormalities exclusively in patients with a newly CIDP diagnosis, supporting its utility as a diagnostic tool. The absence of abnormalities in clinically stable, treated CIDP patients, suggests that MR neurography may also reflect treatment response and disease activity, underlying its potential value as a prognostic biomarker.

**Disclosure:** Nothing to disclose.

## EPO‐0730

### Early ultrasonographic swelling ratio of the facial nerve as a prognostic threshold for functional recovery in acute facial palsy

#### S. Park; S. Lee; S. Kim; Y. Kim

##### Department of Acupuncture and Moxibustion Medicine, Kyung Hee University Medical Center, Seoul, Republic of Korea

**Background and aims:** Early prognostic evaluation in acute facial palsy is difficult because early clinical grading is subjective and electroneurography (ENoG) is unreliable during the first 1–2 weeks after onset. An objective marker applicable in the ealry stage is needed. We investigated whether early ultrasonographic measurement of facial nerve swelling predicts outcomes at 3 months.

**Methods:** A retrospective review was conducted in 22 patients with early peripheral facial palsy within 14 days of onset. Facial nerve ultrasonography was performed using a standardized protocol, with nerve diameter measured below the earlobe. The swelling ratio was defined as the affected‐to‐unaffected side diameter ratio. Outcomes at 3 months were assessed using the House–Brackmann (HB) grade and Sunnybrook Facial Grading System (SFGS). Poor outcome was defined as HB grade >1 or SFGS <90. Prognostic performance was evaluated using receiver operating characteristic (ROC) analysis.

**Results:** ROC analysis showed the facial nerve swelling ratio predicted 3‐month outcomes. A cut‐off value of 1.27 discriminated incomplete recovery (HB grade >1; AUC 0.750, sensitivity 75.0%, specificity 100.0%). Functional impairment (SFGS <90) was predicted with an AUC of 0.886, sensitivity 85.7%, and specificity 100.0%. Patients with ratios ≤1.27 showed near‐complete recovery (mean SFGS 93.2), whereas those with ratios >1.27 had poorer outcomes (mean SFGS 70.2; *p* = 0.0182). In contrast, ENoG thresholds (≥85% and ≥90%) showed low discriminative ability (AUC 0.21–0.33).

**Figure:**
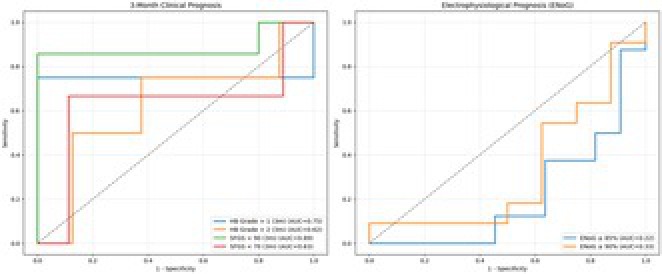
**FIGURE 1** Receiver operating characteristic curves comparing early ultrasonographic facial nerve swelling ratio and electroneurography (ENoG) for prediction of 3‐month outcomes in acute facial palsy.

**Conclusion:** An early facial nerve diameter ratio greater than 1.27 was associated with incomplete recovery and functional impairment at 3 months. Ultrasonography may provide a objective tool for early prognosis in facial palsy.

**Disclosure:** This research was supported by a grant of the Korea Health Technology R&D Project through the Korea Health Industry Development Institute (KHIDI), funded by the Ministry of Health & Welfare, Republic of Korea (RS‐2022‐KH127692).

## EPO‐0731

### Defining a standardized facial movement protocol for AI‐based video analysis in facial nerve palsy: A Delphi study

#### S. Lee ^1^; S. Bae^2^; I. Jang^2^; S. Lee^1^

##### ^1^Department of Acupuncture and Moxibustion, Kyung Hee University College of Korean Medicine, Kyung Hee University Medical Center, Seoul, Republic of Korea; ^2^Department of Computer Engineering, Hankuk University of Foreign Studies, Seoul, Republic of Korea

**Background and aims:** Reliable facial nerve palsy assessment is hindered by rater dependency and the limited precision of gross grading scales like House–Brackmann. Although our static image‐based artificial intelligence (AI) model achieved high accuracy (Quadratic Weighted Kappa: $0.7594$), static analysis cannot evaluate essential dynamic features such as movement trajectories and velocity. This study aimed to define a standardized dynamic assessment framework via expert consensus, serving as a reference foundation for next‐generation video‐based AI applications.

**Methods:** A Delphi study was conducted with 10 specialists ($> 3$ years of clinical experience). Twenty‐eight video‐based assessment items—encompassing basic movements, contracture, synkinesis, and functional metrics—were derived from clinical practice and literature. Items were rated on a 9‐point Likert scale. Consensus was predefined as a Median (Mdn) ≥7.0, Interquartile Range (IQR) ≤2.0, Content Validity Ratio (CVR) ≥0.62, and an agreement rate ≥70%.

**Results:** In the first round, consensus was achieved for 20 of 28 proposed items by meeting all predefined statistical thresholds. Core facial movements (eye closure, smiling, and lip puckering) and primary metrics (symmetry and range of motion) demonstrated the highest consensus (Mdn: 9.0, IQR: 0.0, CVR ≥0.80). In contrast, items involving natural speech tasks and speech‐rate variability showed lower agreement and were identified for further refinement in subsequent validation steps.

**Figure:**
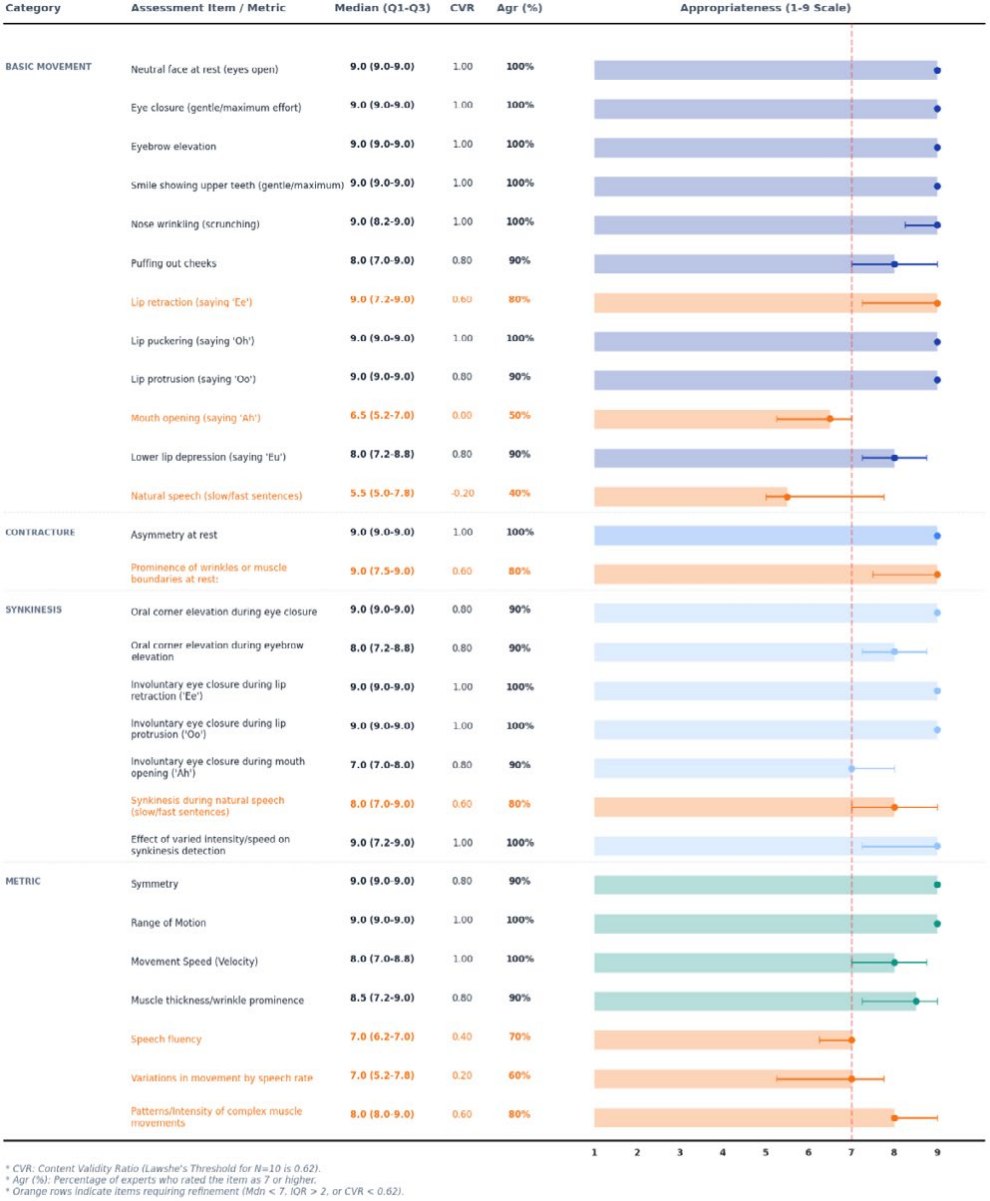
**FIGURE 1** Main results of the first Delphi round for video‐based facial nerve palsy assessment. Median scores and interquartile ranges are shown. Items in orange did not meet consensus criteria and require further refinement.

**Conclusion:** This study identifies expert‐validated components for video‐based assessment, addressing the functional gap between static image evidence and dynamic clinical requirements. The ongoing second Delphi round will finalize this framework, providing a critical foundation for high‐precision, objective digital monitoring of facial nerve palsy in clinical and research settings.

**Disclosure:** This research was supported by a grant of the Korea Health Technology R&D Project through the Korea Health Industry Development Institute (KHIDI), funded by the Ministry of Health & Welfare, Republic of Korea (RS‐2025‐02220534). The authors declare no other conflicts of interest.

## Headache 5

## EPO‐0732

### Epidemiology and clinical characteristics of nummular headache in observational studies: A systematic review and meta‐analysis

#### A. Osiowski; M. Osiowski; D. Taterra

##### Jagiellonian University Medical College, Kraków, Poland

**Background and aims:** Nummular headache (NH) is a rare primary headache disorder with unknown epidemiology and prevalence of the most common clinical characteristics ‐ this study aimed to establish all of the crucial aspects of NH.

**Methods:** Major databases were thoroughly searched for observational studies reporting the relevant data regarding NH.

**Results:** Out of initial 2441 records, 17 studies met all of the inclusion criteria. The pooled mean age of onset of NH was 46.91 (95% CI: 43.85–49.96). The PPE of NH in adult patients evaluated for a headache in a clinic‐based setting was 0.7% (95% CI: 0.2–2.4), with slight female predominance (females = 0.5%, 95% CI: 0.2–1.4 vs males = 0.3%, 95% CI: 0.1–0.8). The majority of patients (69.4%, 95% CI: 58.1–78.8) experience chronic course of NH. The shape of the headache was round/circular in 78.4% (95% CI: 71.9–83.7) and oval/elliptical in 21.6% (95% CI: 16.3–28.1) of patients. In 7.5% (95% CI: 2.7–19.0) of individuals pain had multifocal location and 59.1% (95% CI: 49.7–68.0) of NH patients experienced pain exacerbations. The pain was most prevalent in the strictly parietal region (43.0%, 95% CI: 37.4–48.7) of the cranium and had pressing quality (51.4%, 95% CI: 41.6–61.1).

**Figure:**
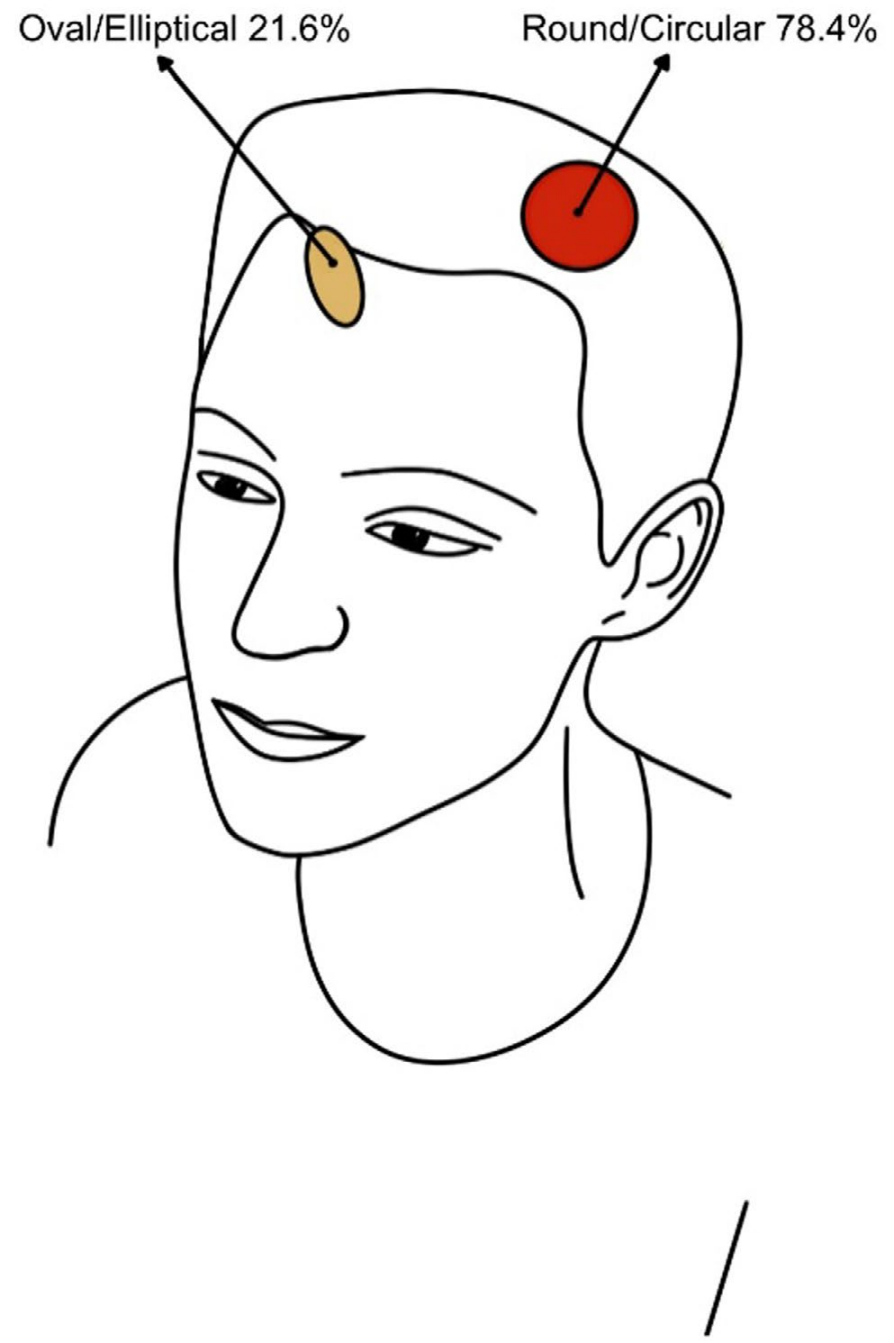
**FIGURE 1** Round/eliptical.

**Figure:**
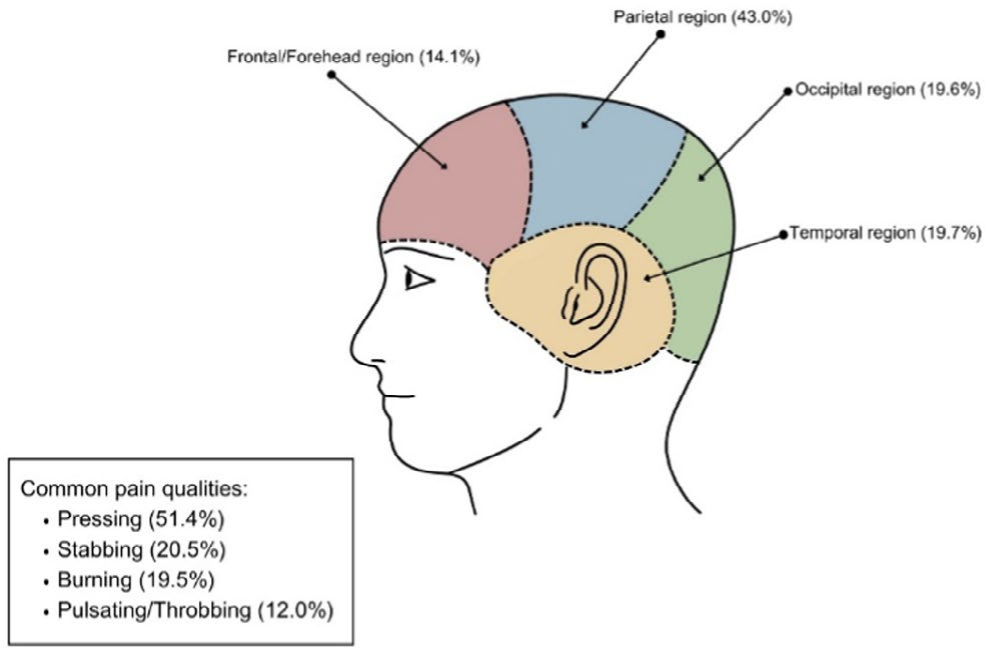
**FIGURE 2** Pain qualities and location.

**Conclusion:** The results of our study showed that NH is a very distinct and relatively rare to encounter headache disorder. Due to its unique clinical phenotype, physicians need to be aware when a patient presents with a small, well‐localized round/oval headache in the cranium region.

**Disclosure:** Nothing to disclose.

## EPO‐0733

### Headache as the most common manifestation of giant cell arteritis? A systematic review with meta‐analysis

#### A. Osiowski; M. Osiowski; D. Taterra

##### Jagiellonian University Medical College, Kraków, Poland

**Background and aims:** This study aimed to assess the relative frequency of clinical features of giant cell arteritis (GCA) and to investigate the predictors of temporal artery biopsy (TAB) outcomes.

**Methods:** A literature search of major electronic databases was conducted for observational studies that reported original data on clinical features of patients with GCA. A random‐effects meta‐analysis was performed to determine the pooled prevalence estimates. The study's design adhered closely to the MOOSE standards. The JBI appraisal tool was used to evaluate the risk of bias. The study's protocol was pre‐registered on PROSPERO (ID: CRD42024584763).

**Results:** Out of initial 12,628 records, 62 articles (9971 patients) met all of the eligibility criteria. Mean patients’ age upon diagnosis was 74.33 years (95% CI: 74.12–74.54 years). The most prevalent clinical feature of GCA was new‐onset headache (75.7%; 95% CI: 72.2–79.0; 95% PI: 0.47–0.92). Other common symptoms of GCA were temporal artery abnormalities (51.5%; 95% CI: 45.2–57.7; 95% PI: 0.25–0.77), weakness/malaise (46.7%; 95% CI: 35.4–58.4; 95% PI: 0.09–0.88), and scalp tenderness (39.1; 95% CI: 35.3–43.1; 95% PI: 0.22–0.59). Positive TAB results were present in 73.8% of patients (95% CI: 68.1–78.8%; 95% PI: 0.35–0.94). The presence of headache (LogOR= −1.11; 95% CI: −1.92 to −0.29) or PMR (−0.71; 95% CI: −1.09 to −0.32) significantly decreases the chance of receiving positive TAB results.

**Figure:**
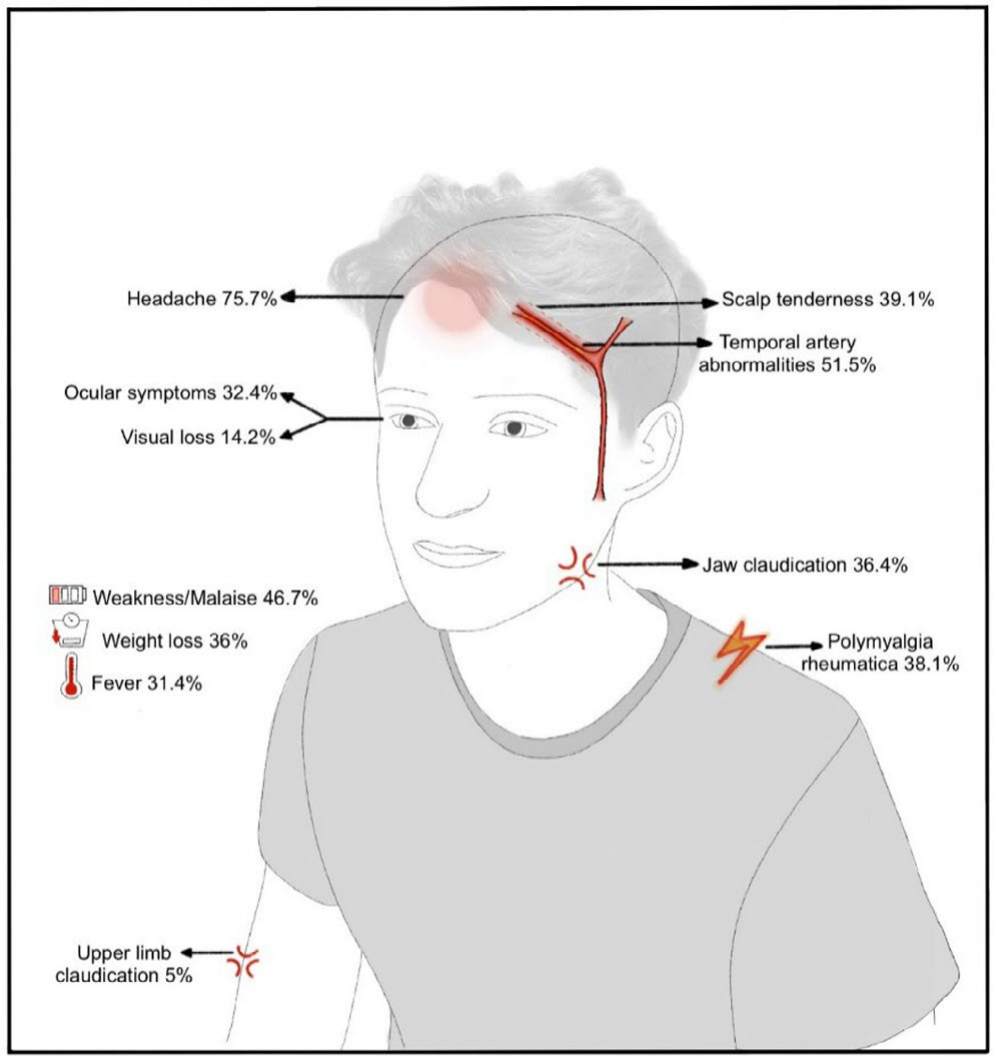
**FIGURE 1** Presentation of headache as the most common manifestation of GCA with comparison to other symptoms.

**Figure:**
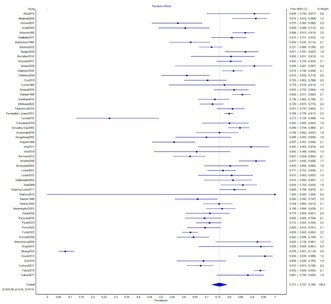
**FIGURE 2** Forest plot presenting pooled prevalence estimate of headache in GCA.

**Conclusion:** Since there is a greater likelihood of obtaining negative biopsy results, the TAB may not be required when a patient exhibits a headache along with other clinical symptoms that enable them to be diagnosed with GCA.

**Disclosure:** Nothing to disclose.

## EPO‐0734

### Glial neurovascular unit protein alterations and risk of idiopathic intracranial hypertension: A systematic review and meta‐analysis

#### B. Arora ^1^; A. Pandit^1^; M. Nath^1^; S. Shree^1^; P. Tangri^1^; P. Gupta^2^

##### ^1^Department of Neurology, AIIMS, New Delhi, India; ^2^Clinical Research Unit (CRU), AIIMS, New Delhi, India

**Background and aims:** Idiopathic intracranial hypertension (IIH) is characterized by raised intracranial pressure with incompletely understood pathophysiology. Increasing evidence implicates dysfunction of the glial neurovascular unit, including astrocytic, vascular, and neuroaxonal components, in disease development. This systematic review and meta‐analysis evaluated the association between glial neurovascular unit–related protein alterations and the risk of IIH.

**Figure:**
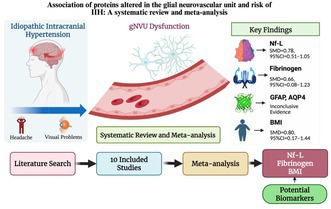
**FIGURE 1** This systematic review and meta‐analysis demonstrate that elevated cerebrospinal fluid neurofilament light chain and plasma fibrinogen levels are significantly associated with idiopathic intracranial hypertension, indicating underlying neuroaxonal injury.

**Methods:** A systematic search of PubMed, EMBASE, Cochrane Library, Scopus, and Web of Science was conducted up to August 2025. Studies comparing IIH patients with non‐IIH controls and reporting levels of aquaporin‐4, glial fibrillary acidic protein, fibrinogen, or neurofilament light chain were included. Data extraction and quality assessment using the Newcastle–Ottawa Scale were performed independently. Random‐effects models were used to calculate pooled standardized mean differences (SMDs) with 95% confidence intervals (CIs). BMI was analyzed as a secondary outcome.

**Results:** Ten studies comprising 327 IIH cases and 216 controls were identified, of which seven were included in quantitative synthesis. Cerebrospinal fluid NfL levels were significantly higher in IIH patients compared with controls (SMD 0.78, 95% CI 0.51–1.05), indicating neuroaxonal injury. Plasma fibrinogen levels were also elevated in IIH (SMD 0.66, 95% CI 0.08–1.23), suggesting a hypercoagulable and inflammatory state. Due to methodological heterogeneity, data on AQP4 and GFAP could not be pooled. Higher BMI was significantly associated with increased IIH risk (SMD 0.80, 95% CI 0.17–1.44).

**Figure:**
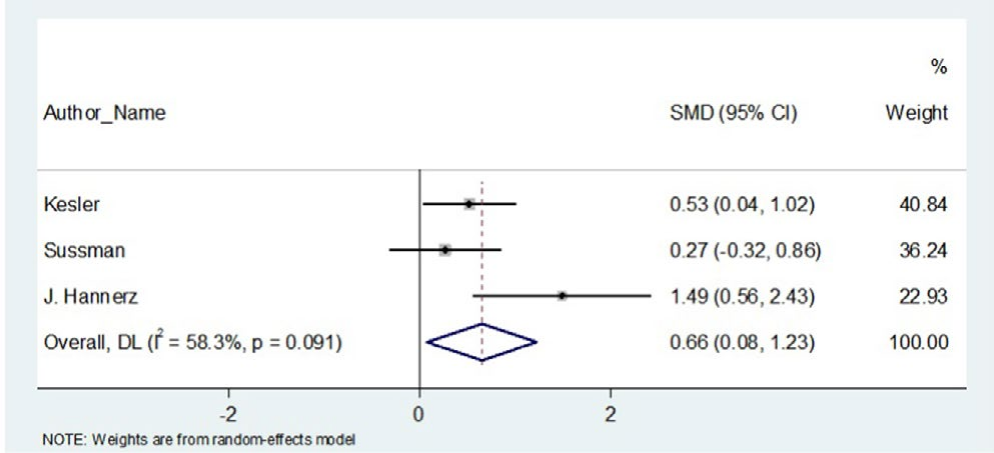
**FIGURE 2** Forest plot showing the association between Fibrinogen and the risk of IIH.

**Figure:**
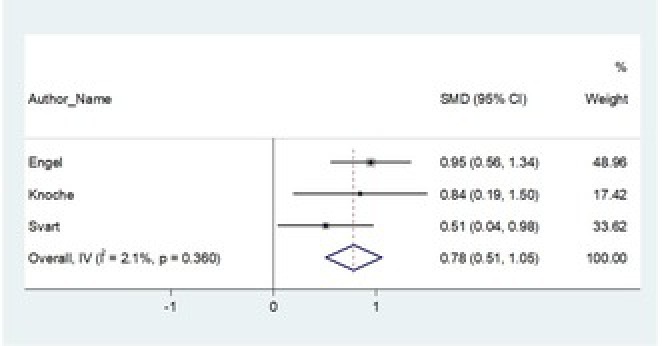
**FIGURE 3** Forest plot showing the association between Neurofilament – Light and risk of IIH.

**Conclusion:** Elevated NfL and fibrinogen levels are associated with IIH, supporting roles for neuroaxonal damage and vascular–inflammatory mechanisms in disease pathophysiology. Well‐designed multicentre studies are needed to validate glial neurovascular unit biomarkers and improve risk stratification.

**Disclosure:** Nothing to disclose.

## EPO‐0735

### People with migraine and two years treatment with anti‐CGRP therapies. Second report from the prospective national registry “Hercules”

#### D. Mitsikostas ^1^; C. Deligianni1^2^; E. Giannouli^3^; E. Kouremenos^4^; I. Spanou^4^; T. Constantinidis^5^; T. Sagona^6^; E. Chroni^6^; Z. Tsouris^7^; E. Dardiotis^7^; C. Tsironis^8^; S. Konitsiotis^8^

##### ^1^1st Neurology Department, Aeginition Hospital, National and Kapodistrian University of Athens, Athens, Greece; ^2^Neurology Department, Athens Naval Hospital, Athens, Greece; ^3^Headache Clinic, Athens Medical Center, Athens, Greece; ^4^Neurology Department; 251 General Hospital of Air Forces; ^5^Neurology Clinic, Corinth, Greece; ^6^Department of Neurology, Patras University Hospital, Patras, Greece; ^7^Neurology Department, Medical School, University Hospital of Larissa, University of Thessaly, Larissa, Greece; ^8^Neurology Department, Medical School, University Hospital of Ioannina, University of Ioannina, Ioannina, Greece

**Background and aims:** To record the long‐term efficacy and safety of anti‐CGRP treatments in people with migraine in Hellas.

**Methods:** HERCULES is a 2‐year, patient‐centered, national, multicenter, longitudinal (5 total assessments every 6 months), prospective, observational study enrolling individuals with migraine treated with anti‐CGRP agents. The primary endpoints are discontinuation of treatment due to adverse events and reduction in monthly migraine days (MDM).

**Results:** 425 subjects with migraine and prior failure to at least 3 repurposed anti‐migraine prophylactic treatments attending 8 headache centers in Greece have been recorded so far [erenumab 41 (9.6%), galganezumab 57 (13.4%), fremnezumab 287 (67.4%), eptinezumab 39 (9.2%) and rimegepant 2 (0.5%)], of whom 180 (42.3%) completed the 2‐year treatment with anti‐CGRP agents [29/180 (16.1%) subjects with erenumab; 22/180 (12.2%) with galcanezumab; and 129/180 (71.7%) with fremanezumab]. 57/425 (13.4%) participants discontinued treatment [fremanezumab 28/287 (9.7%); galcanezumab 17/57 (29.8%); erenumab 10/41 (24.4%); eptinezumab 2/39 (5.1%)], due to patient's decision [29/57 (50.9%)], failure [14/57 (24.6%)], adverse events [2/57 (3.5%)], complete remission of migraine [6/57 (10.5%)], or pregnancy [1/57 (1.7%)]. The most common AE recorded were constipation [47/425 (11%)] and injection site reactions [57/332 (13.4%)]. Only one severe AE has been recorderd [1/425 (0.2%)] during infusion of eptinezumab (allergic reaction). After 24 doses a ≥50%, ≥75%, or 100% reduction in MDM compared to pretreatment was recorded in 147/180 (81.6%), 81/425 (19%), and 13/180 (7.2%) subjects, respectively.

**Conclusion:** Anti‐CGRP mAbs are effective, safe, and well tolerated after long‐term administration and should be considered as a first‐line prophylactic treatment for migraine.

**Disclosure:** Hercules is supported by grants from Pharmaserve Lilly SACI, Pfizer Hellas AE and Teva Hellas SA. D.D.M. has received fees for participation in advisory committees and honoraria for lectures from AbbVie, Amgen, AstraZeneca, Bennett, Bristol‐Meyers Sqibb, Eli Lilly, Cross, ELPEN, Haleon, Intermed, Genesis Pharma, Lundbeck, Merck, Mylan, Orion Pharma, Novartis, Pfizer, Roche, and Teva Pharmaceuticals; has participated in clinical trials for Amgen, Eli Lily, Lundbeck, Novartis, Pfizer, Swixx, and Teva Pharmaceuticals as principal investigator; is the president of the Hellenic Headache Society and a member of the Management Group of the Coordinating Panel for the Functional Neurological Disorders at the European Academy of Neurology. C.D. has received personal fees/ honoraria/ travel expenses from Pfizer, Merck, Astra‐Zeneca, Teva, Orion Pharma, Haleon, Bennett. C.D. serves as member at large of European Headaceh Federation. E.G. has received travel grants, speaking fees and participated in advisory boards for AbbVie, Eli Lilly, Lundbeck, Novartis, Pfizer, and Teva/Specifar. E.K. has received travel grants and honoraria from Bristol‐Meyers Sqibb, Eli Lilly and Company, Innovis, Merck, Novartis, Lundbeck, Roche and Teva. I.S. has received travel grants from Lundbeck and Teva. T.S.C. received honoraria for participation in advisory boards, preparation and addressing of lectures in satellite symposia, as well as funding for congress participation from Abbvie, Teva, Sanofi, Cross, Elpen, Pharmathen‐Innovis, Pharmaserv‐Lilly, Novartis, Braintherapeutics Neuraxpharm, Merck, C.A. Papaellinas, Bayer, Swixx, Vianex, UCB and Genesis Pharma‐Biogen. T.S. received honoraria from UCB. E. C. received travel funds and honoraria for participation in Advisory Meetings and Satellite Symposia from Merck, Sanofi Genzyme, Genesis Pharma, Teva Pharmaceuticals, UCB, ITF, Medison Pharma, Alexion, AstraZeneca, CSL Behring, and Takeda. Z. T. has received compensation for lecture honoraria and travel expenses, participation in scientific meetings, clinical trial membership, or clinical advisory board participation from Teva, Αrgenx, Sanofi, Novartis, Janssen, Immunovant, Roche, Lundbeck, Bayer, Shionogi, Merck, Genesis Pharma, Bristol‐Myers Squibb, and UCB. E.D. has received compensation for lecture honoraria and travel expenses, participation in scientific meetings and advisory boards, clinical trial membership, from Abbvie, Astra‐Zeneca, Bayer, Bristol‐Myers Squibb, Elpen, Lundbeck, Merck, Mylan, Novartis, Sanofi, UCB, Teva, Tikun and Genesis. C.T. received honoraria for lecturing, travel grants for travel and participation in advisory meetings from Cross, Lundbeck and Teva. S.S.K. has received grants and honoraria from Abbvie, AstraZeneca, Bristol‐Myers Squibb, Sanofi Aventis, Innovis, Ipsen, ITF Hellas, Genesis Pharma, ERKIM Pharma, Medison Pharma, Medtronic Hellas, Merck, Novartis, Pharmaserv, Rosche, Sandoz, Teva, Viatris, and UCB.

## EPO‐0736

### Do European Headache Federation criteria reflect response to acute treatments in resistant and refractory migraine? The REFINE study

#### I. Gragnaniello

##### Università degli Studi dell'Aquila, L'Aquila, Italy

**Background and aims:** The European Headache Federation (EHF) defines resistant and refractory migraine by failure of ≥3 (resistant) or all (refractory) pharmacological preventive treatment classes, without considering acute treatments. This study aimed to evaluate whether these definitions also correlate with response to acute treatment.

**Methods:** We conducted a multicenter, prospective, international study (REFINE) to test the EHF definitions in a real‐life setting in 15 European headache centers. The Headache Under Response to Treatment (HURT) Questionnaire assessed the impact of migraine on daily activities and effectiveness of acute medications in patients with resistant, refractory, and non‐resistant non‐refractory (NRNR) migraine.

**Results:** We included 689 patients, of whom 261 (37.9%) had resistant, 73 (10.6%) refractory, and 355 (51.5%) NRNR migraine. Patients with refractory migraine experienced more significant impairment in daily activities (HURT‐2) and social activities (HURT‐3) versus those with resistant and NRNR migraine (46 [63.0%] vs 110 [42.2%] vs 51 [14.4%], *p <* 0.001; 37 [50.7%] vs 67 [25.7%] vs 25 [7.0%], *p <* 0.001). Regarding acute medication efficacy (HURT‐5), more patients with refractory migraine stated that one dose “never” relieved their headache, and delayed or avoided taking acute medication due to concerns about adverse events (HURT‐7), versus those with resistant and NRNR migraine (29 [39.7%] vs 28 [10.7%] vs 25 [7.0%], *p <* 0.001; 16 [21.9%] vs 7 [2.7%] vs 11 [3.1%], *p <* 0.001).

**Conclusion:** Patients with refractory and resistant migraine report poorer acute treatment response compared with those with NRNR migraine, which significantly affect daily lives. Management of difficult‐to‐treat migraine should focus on optimizing acute treatments, alongside preventive therapies.

**Disclosure:** Nothing to disclose.

## EPO‐0737

### Longitudinal effects of CGRP monoclonal antibodies for migraine prophylaxis: Impact of treatment type and age group

#### I. Mattioli ^1^; A. Rondina^2^; A. Lanfranchi^3^; G. Ceccardi^4^; M. Di Pasquale^2^; E. Cresta^3^; A. Pilotto^5^; A. Padovani^5^; R. Rao^3^

##### ^1^Department of Frailty and Continuity of Care, ASST Spedali Civili, Brescia, Italy; NDM Experimental Medicine, University of Oxford, Oxford, UK; ^2^Department of Clinical and Experimental Sciences, University of Brescia, Brescia, Italy; ^3^Department of Frailty and Continuity of Care, ASST Spedali Civili, Brescia, Italy; ^4^Neurology Unit, ASST Cremona, Cremona, Italy; ^5^Department of Clinical and Experimental Sciences, University of Brescia, Brescia, Italy; Department of Frailty and Continuity of Care, ASST Spedali Civili, Brescia, Italy

**Background and aims:** Calcitonin gene‐related peptide (CGRP) monoclonal antibodies (mAbs) are established preventive treatments for episodic and chronic migraine, but comparative longitudinal data across agents and age groups remain limited. This study aimed to evaluate long‐term clinical responses to different CGRP mAbs, assess whether age or treatment type influences response trajectories, and characterize multidimensional patterns of treatment response.

**Methods:** We conducted a retrospective, single‐centre observational study including 443 adults with high‐frequency episodic or chronic migraine treated with erenumab, galcanezumab, fremanezumab, or eptinezumab between 2018 and 2025. Patients were stratified by age (<50 vs ≥50 years). Longitudinal mixed‐effects models assessed changes in monthly migraine days, acute medication use, pain intensity, disability, quality of life, and mood over 12 months. Generalized linear mixed models evaluated predictors of ≥50% response. Principal component analysis (PCA) was used to identify multidomain response patterns.

**Results:** All CGRP mAbs produced rapid and sustained improvements across migraine frequency, disability, and patient‐reported outcomes over 12 months. Age did not significantly influence baseline severity, longitudinal trajectories, or responder status. Treatment differences were modest overall; however, at 12 months, fremanezumab and eptinezumab were associated with higher odds of response compared with erenumab, while galcanezumab showed consistently higher response odds independent of time. PCA identified a “General improvement” component capturing multidomain benefit, which was more pronounced in patients treated with eptinezumab.

**Figure:**
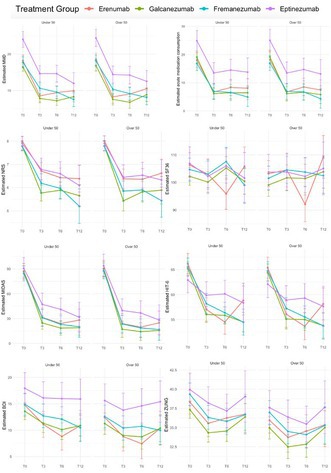
**FIGURE 1** Clinical and migraine‐related variables change over time across age and treatment groups.

**Figure:**
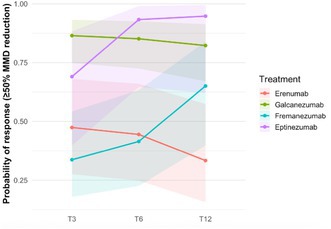
**FIGURE 2** probability of response over time across treatment groups (MMD=monthly migraine days).

**Figure:**
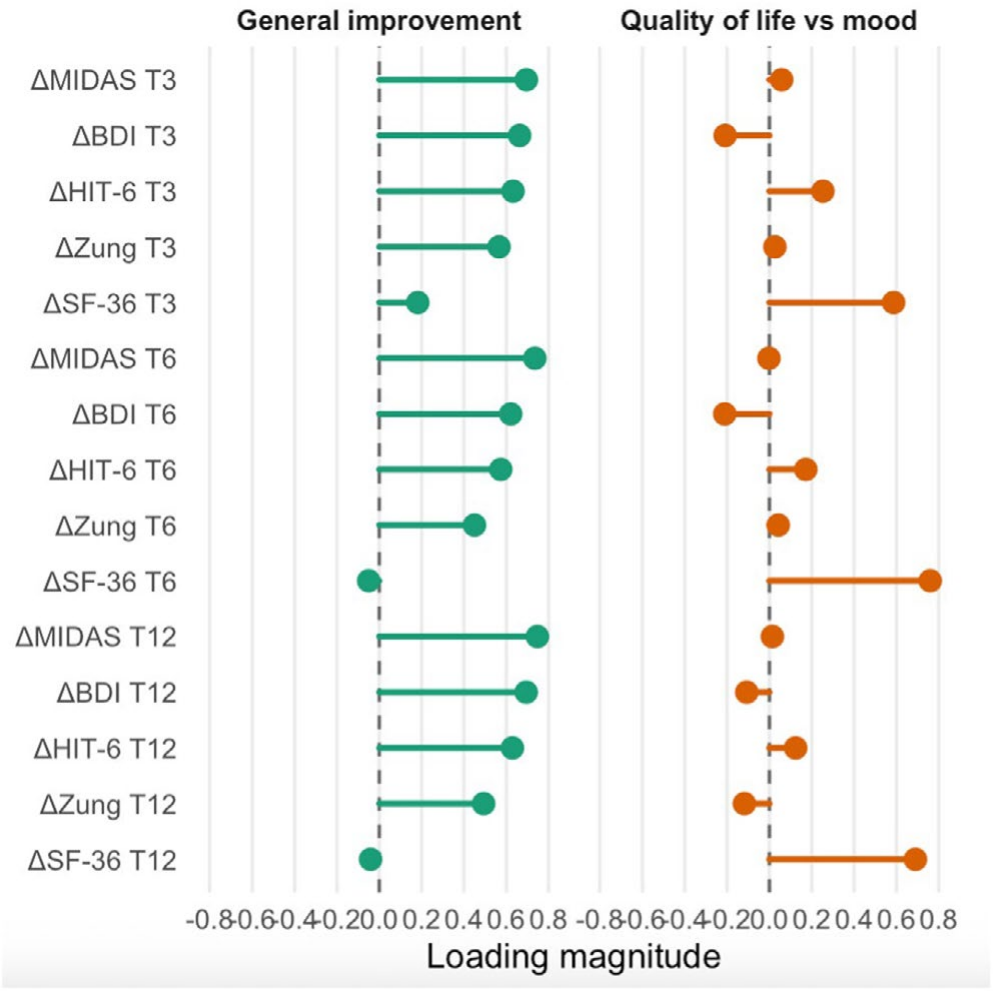
**FIGURE 3** Component loadings from PCA.

**Conclusion:** In real‐world clinical practice, CGRP mAbs demonstrate broadly comparable and durable effectiveness across age groups. Subtle long‐term differences between agents may emerge, particularly for multidimensional clinical improvement, supporting individualized treatment selection.

**Disclosure:** IM has received support for conference participation from Allergan/AbbVie, Pfizer, and Lundbeck; has received funding for an advanced training course from Teva; and is currently enrolled in a DPhil program funded by GlaxoSmithKline (Oxford–GSK). GC and MDP has received support for conference participation from Allergan/AbbVie, Pfizer, Teva and Lundbeck. APa has served on the scientific advisory board of GE Healthcare, Eli‐Lilly, and Actelion Ltd. Pharmaceuticals, and has received speaker honoraria from Nutricia, PIAM, Langstone Technology, GE Healthcare, Lilly, Lundbeck, UCB Pharma, and Chiesi Pharmaceuticals. RR has received speaker honoraria from Eli‐Lilly, Novartis, Lundbeck, and Teva. All other authors declare no conflicts of interest.

## EPO‐0738

### Work productivity and indirect cost savings of atogepant versus topiramate: Results from the TEMPLE trial

#### J. Díaz de Terán ^1^; P. Gandhi^2^; H. Guo^2^; F. Haile^2^; K. Nagy^2^; K. Carr^2^; J. Versijpt^3^; R. Gil‐Gouveia^4^; D. Buse^5^; R. Lipton^5^

##### ^1^La Paz University Hospital, Madrid, Spain; Hospital La Paz Institute for Health Research; ^2^AbbVie Inc, North Chicago, USA; ^3^Department of Neurology, Universitair Ziekenhuis Brussel, Brussels, Belgium; ^4^Neurology Department, Hospital da Luz, Lisbon, Portugal; Center for Interdisciplinary Research in Health, Universidade Católica Portuguesa, Lisbon, Portugal; ^5^Department of Neurology and Headache Center, Albert Einstein College of Medicine, Bronx, USA

**Background and aims:** Using the Work Productivity and Activity Impairment (WPAI): Migraine, we contrasted presenteeism, absenteeism, overall work productivity, and indirect cost savings of participants who received atogepant versus topiramate for the preventive treatment of migraine.

**Methods:** TEMPLE was a phase 3b, randomized, multicenter, double‐blind (DB), double‐dummy, active‐controlled trial to evaluate tolerability, safety, and efficacy of atogepant compared with topiramate for the preventive treatment of migraine. WPAI:Migraine was evaluated at each 4‐week time point during the 24‐week DB period. An excel‐based cost calculator used WPAI data to calculate the per‐patient‐per‐year (PPPY) work productivity cost by multiplying the percent overall work productivity loss, weeks per year, standard weekly full‐time work hours (40), and the average hourly wage in Canada, Portugal, United Kingdom, and United States. Productivity benefit from using atogepant was calculated as the difference between atogepant and topiramate productivity loss PPPY.

**Results:** Based on WPAI data from TEMPLE, atogepant demonstrated greater improvements in presenteeism and overall work productivity (Figures 1 and 2), and lower absenteeism and activity impairment versus topiramate. The cost calculator using overall work productivity data demonstrated atogepant provided greater productivity benefits and indirect cost savings compared with topiramate across all 4 countries (Table 1).

**Figure:**
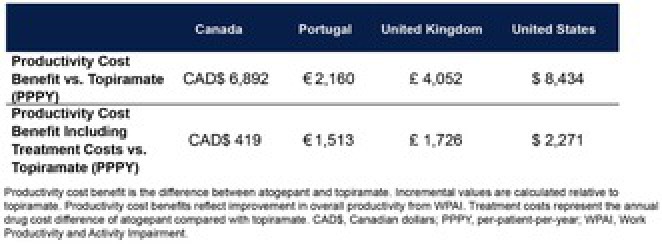
**TABLE 1** Work productivity benefit for Atogepant vs. Topiramate per patient per year.

**Figure:**
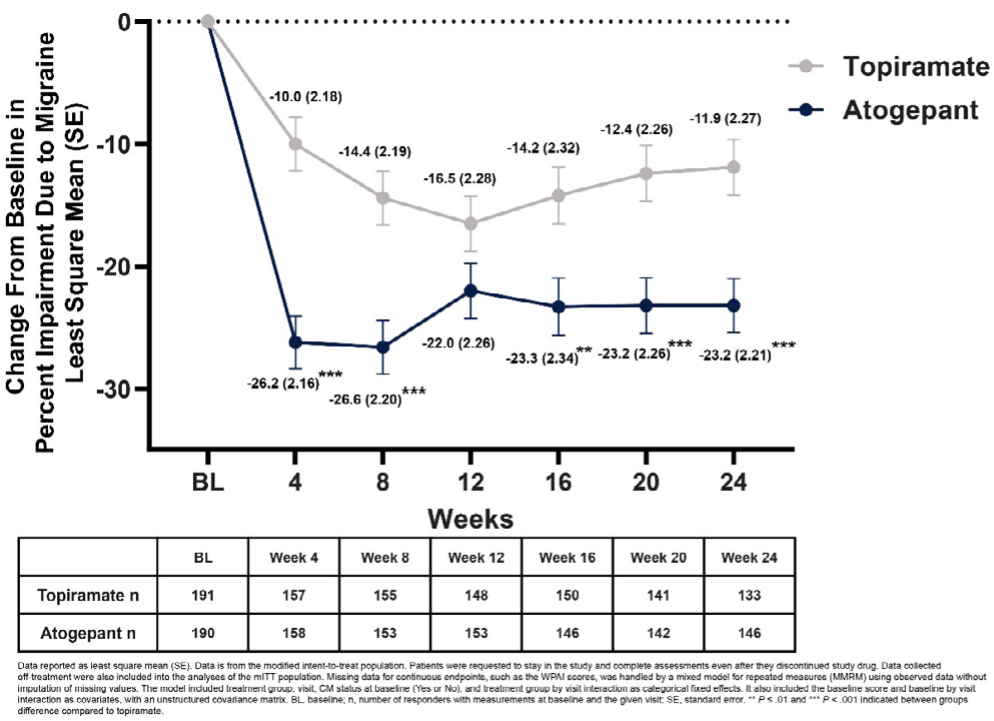
**FIGURE 1** Change from baseline in overall work productivity loss.

**Figure:**
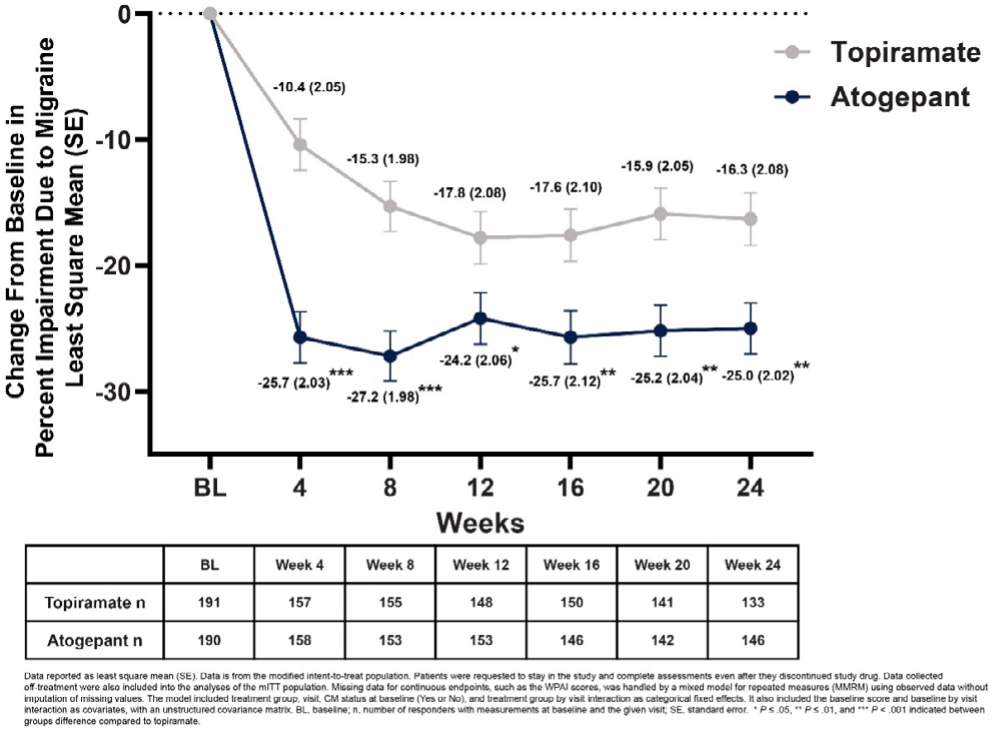
**FIGURE 2** Change from baseline in presenteeism.

**Conclusion:** Atogepant consistently demonstrated greater improvement compared to topiramate across all four WPAI domains. Using TEMPLE WPAI data, atogepant demonstrated work productivity benefit compared to topiramate in Canada, Portugal, UK, and United States.

**Disclosure:** Disclosures Javier Díaz de Terán declares public research funding from the Instituto de Salud Carlos III (Ministry of Health, Spain), project PI24/01254, outside the submitted work; and reports honoraria for consultant and/or advisory boards from AbbVie, Almirall, Lilly, Lundbeck, Novartis, Organon, Pfizer, Boston Scientific, and Teva. Jan Versijpt received personal fees and nonfinancial support from Teva, Pfizer and Medtronic, personal fees from Novartis and Lundbeck, and grants and nonfinancial support from Allergan/AbbVie. Raquel Gil‐Gouveia reports honoraria for consultant and/or advisory boards from Allergan/Abbvie, Astra Zeneca, Almirall, Bial, Biogen, Bristol‐Myers Squibb, CMBE, FLOAT, Lilly, Lundbeck, Merk, Novartis, Novo Nordisk, Organon, Pfizer, Roche, Sanofi, Tecnifar and Teva; research funding from Fundação Ciência Tecnologia (Project 29675, MigN2Treat, 02/SAICT/2017); Learning‐Health, Luz Saúde (LiON, Luz Innovation on Neurosciences); Novartis‐Sociedade Portuguesa de Cefaleias; Bolsa–Projecto Centro de Investigação Interdisciplinar em Saúde and UCP. Dawn Buse has received research funding from the US Food and Drug Administration, the National Headache Foundation and Amgen and received consulting fees from Amgen, Allergan/AbbVie, Eli Lilly, Lundbeck and Teva. Richard B. Lipton has received research support from the National Institutes of Health, the FDA, and the National Headache Foundation. He serves as consultant for, advisory board member of, or has received honoraria or research support from AbbVie/Allergan, Aeon, Amgen, Axsome, Biohaven, Dr. Reddy's Laboratories, electroCore, Eli Lilly, GlaxoSmithKline, Linpharma, Lundbeck, Pfizer, Merck, Novartis, Teva, and Vector. He receives royalties from Wolff's Headache, 8th edition (Oxford University Press, 2009), and Informa. He holds stock/options in Biohaven, CoolTech, and NuVieBio. PG, HG, FH, KN, and KC are employees of AbbVie and may hold AbbVie stock.

## EPO‐0739

### Antidepressant‐associated reversible cerebral vasoconstriction syndrome: Neuropsychiatric insights from a cohort study

#### J. Mirtil ^1^; D. Doukhi^1^; C. Tesnieres^2^; G. Tuloup^1^; C. Roos^1^; M. Mazighi^1^; A. Ducros^3^; J. Mawet^1^

##### ^1^Emergency Headache Center (Centre d’Urgences Céphalées), Department of Neurology, Lariboisière Hospital, Assistance Publique–Hôpitaux de Paris (AP‐HP), Paris, France; ^2^Department of Psychiatry, University Hospital of Caen, Caen, France; ^3^Department of Neurology, Montpellier University Hospital (CHU), Gui de Chauliac Hospital, Montpellier, France

**Background and aims:** Reversible cerebral vasoconstriction syndrome (RCVS) is a cerebrovascular disorder characterized by acute headaches and reversible cerebral arterial narrowing. While case series suggest an association between serotonergic antidepressant use and RCVS onset, clinical and long‐term outcomes data in this population remain scarce. We aimed to characterize the presentation and prognosis of antidepressant‐associated RCVS.

**Methods:** We conducted a retrospective analysis of adult patients with RCVS and concurrent antidepressant use from the Lariboisière Hospital RCVS cohort (2004–2023). Clinical and neuroimaging features were compared with those of non‐exposed patients. Long‐term outcomes were assessed in the exposed group through structured telephone interviews.

**Results:** Among 501 RCVS patients, 56 (11.2%) were using antidepressant at RCVS onset. Exposed patients were more often female (82.1% vs. 60.7%, *p* = 0.002) and older (median age: 48 vs. 41 years, *p* = 0.001), with acute emotional stress more frequently reported at onset (41.1% vs. 20.2%, *p* = 0.001). Focal deficits (25.0% vs. 13.0%; adjusted OR = 2.62, 95% CI [1.26–5.46]) and hemorrhagic complications (32.1% vs. 16.4%; OR = 2.28, 95% CI [1.15–4.52]) were more frequent in antidepressant users. Antidepressants were reintroduced in 53.6% of cases. Among the 39 patients with long‐term follow‐up, RCVS recurrence was rare (5.1%). Persistent psychiatric symptoms, particularly stress‐related disorders, anxiety and depressive features, remained highly prevalent.

**Figure:**
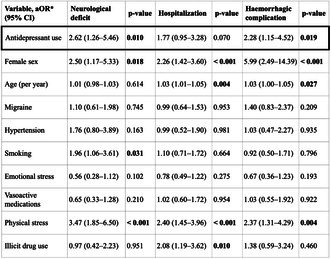
**TABLE 1** Multivariate analysis of RCVS severity outcomes associated with antidepressant use. Adjusted odds ratios (OR) with 95% confidence intervals are reported for neurological deficits, hospitalization, and hemorrhagic complications.

**Figure:**
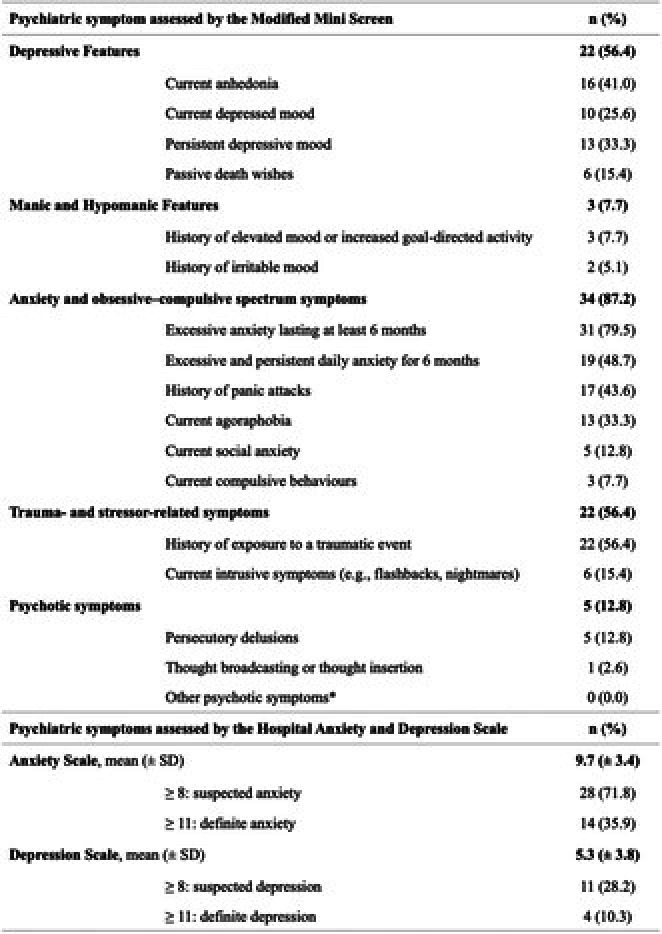
**TABLE 2** Psychiatric outcomes at long‐term follow‐up in 39 antidepressant‐associated RCVS. Values are no./total no. (%) or mean (25th–75th percentiles). Percentages are based on the subgroup with complete follow‐up (*n* = 39).

**Figure:**
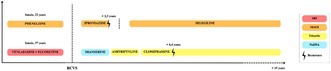
**FIGURE 1** Two cases of long‐term recurrence in antidepressant‐associated RCVS.

**Conclusion:** Antidepressant‐associated RCVS is associated with increased neurological severity and hemorrhagic risk. Recent serotonergic exposure and emotional stress may act synergistically. Antidepressant delayed reintroduction appears feasible with individualized risk assessment and close neurological and psychiatric monitoring.

**Disclosure:** Nothing to disclose.

## EPO‐0740

### Epidemiology of primary exercise headache: A systematic review and meta‐analysis with comparison between clinic‐ and population‐based settings

#### A. Osiowski; M. Osiowski; D. Taterra; K. Majka

##### Jagiellonian University Medical College, Kraków, Poland

**Background and aims:** Primary exercise headache (PEH) is a rare primary headache disorder that occurs specifically during or right after physical exertion and lasts up to 48 hours. This study aimed to evaluate the epidemiology of PEH in clinic‐ and population‐based settings.

**Methods:** Major databases were thoroughly searched for articles that presented original data on the epidemiology of PEH. Random‐effects meta‐analysis was performed in order to calculate the pooled prevalence estimates (PPE). The study's structure adhered closely to the MOOSE and PRISMA requirements. The protocol of the study was pre‐registered on PROSPERO (CRD42024612283).

**Results:** Out of the original 2083 records, 12 articles satisfied all inclusion requirements. The majority of the studies showed a low risk of bias. The headache is a rare finding in clinical‐based settings (PPE = 0.46%, 95% CI: 0.18–1.20, 95% PI: 0.01%–14.29%, I2 = 94.86), more common in population‐based surveys (PPE = 4.92%, 95% CI: 1.84–12.49, I2 = 98.73), shows a female predominance, and, moreover, typically has onset in the late twenties (27.47 years).

**Figure:**
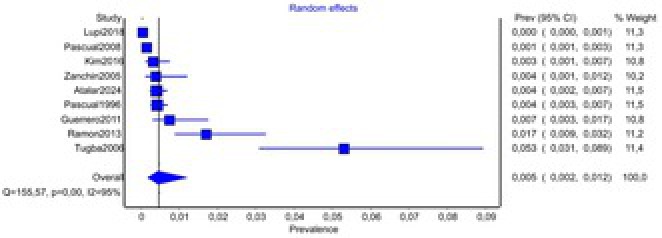
**FIGURE 1** Forest plot presenting the prevalence of PEH in general adult population.

**Conclusion:** Our study showed that PEH is a rare primary headache disorder among adults evaluated for a headache in a clinic‐based setting and is significantly more common in the general population. Furthermore, PEH is diagnosed within similar frequency among both sexes in headache‐oriented clinics and moreover, predominantly late twenties. However, in the general population, there is a notable female preponderance.

**Disclosure:** Nothing to disclose.

## EPO‐0741

### Assessing physician and patient alignment in migraine severity: Evidence from real‐world clinical practice

#### W. Whitton; L. Hargreaves; S. Cotton; S. Barlow

##### Adelphi Real World, Cheshire, UK

**Background and aims:** Migraine is characterised by unilateral headache pain. The burden of migraine is well established, yet potentially equivocal across different stakeholders, such as patients with migraine (PwM) and their physicians. This study aimed to assess the alignment between physician and patient reported migraine severity in real world clinical practice.

**Methods:** Data were drawn from the 2022/23 Adelphi Migraine Disease Specific Programme™, a cross‐sectional survey of physicians and their patients in France, Germany, Italy, Spain, the United Kingdom, China, Japan, and the United States. Physicians and their PwM reported average migraine severity over the past three months. The alignment between physician and patient reported severity was assessed by calculating Cohen's weighted kappa (κ) statistic. Interpretation of the κ value was based on Landis and Koch (1977).

**Results:** Overall, 938 physicians (391 PCPs, 527 neurologists, and 20 neurosurgeons) reported data for 7357 PwM, of which patient‐reported data were available for 3125. Matched severity data were available for 3078 PwM. Physicians reported 55% of patients (*n* = 1688) experienced very mild/mild migraine, 37% (*n* = 1137) experienced moderate migraine, and 8% (*n* = 253) experienced severe/very severe migraine. Conversely, 47% (*n* = 1443) of PwM reported experiencing very mild/mild migraine, 38% (*n* = 1171) reported moderate migraine, and 15% (*n* = 464) reported severe/very severe migraine. A kappa value of 0.57 was observed, suggesting only moderate alignment between physicians and their patients (Figure 1).

**Figure:**
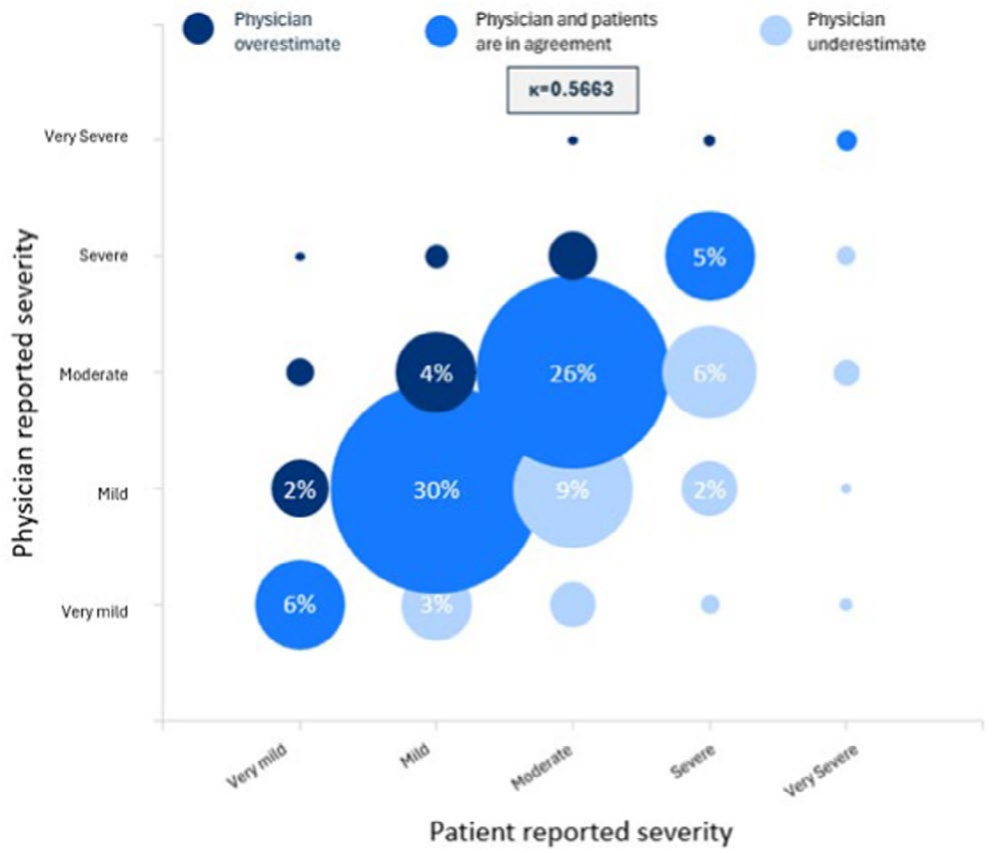
**FIGURE 1** Physician and patient‐reported migraine severity over the past 3 months (relative to time of data collection). Data are total percentages.

**Conclusion:** This study indicates physicians may under‐recognise the severity of migraine, highlighting the importance of open dialogue to support a shared understanding of migraine severity and guide optimal management decisions.

**Disclosure:** William Whitton, Lucy Hargreaves, Sarah Cotton, and Sophie Barlow are all employees of Adelphi Real World.

## EPO‐0742

### Migraine care gaps: A tale of two clinics in Ontario, Canada

#### P. Kim Chiaw^1^; S. Keshwani ^1^; L. Bou Khzam^1^; A. Grajales^1^; N. Nightingale^2^; I. Wang^2^; C. Neish^2^; J. Murray^2^

##### ^1^Pfizer Canada, Kirkland, Quebec, Canada; ^2^IQVIA Solutions Canada Inc, Mississauga, Ontario, Canada

**Background and aims:** Migraine is a leading cause of disability, yet substantial gaps in diagnosis, documentation, and treatment continue to impact care quality. Applying natural language processing (NLP) to real‐world electronic medical record (EMR) data provides an opportunity to reveal these systemic challenges and pinpoint critical patient care gaps.

**Methods:** We performed a descriptive retrospective analysis of Accuro EMR data from one general practice (GP) (2012–2023) and one neurology clinic (2022–2023) in Ontario using the DARWEN NLP engine. Extracted variables included demographics, monthly migraine days (MMDs), migraine classification, comorbidities, acute and preventive therapies, and medication overuse headache (MOH).

**Results:** The cohort comprised 967 GP and 149 neurology patients, predominantly female, with mean ages 41–42 years. Psychiatric and sleep comorbidities were common in both groups ‐ anxiety (28.9%; 27.5%), depression (11.7%; 4.0%), and insomnia (40.1%; 26.8%), respectively—underscoring migraine complexity. Migraine classification was rarely documented in GP, with 72.7% of patients having an unknown classification compared with 22.1% in neurology; MMDs were likewise underreported. Treatment patterns diverged, GP relying heavily on NSAIDs and opioids, while neurology commonly used triptans and a broader, though still limited, use of preventive therapies. MOH was rarely documented in GP (0.6%) but frequently identified in neurology (42.3%).

**Figure:**
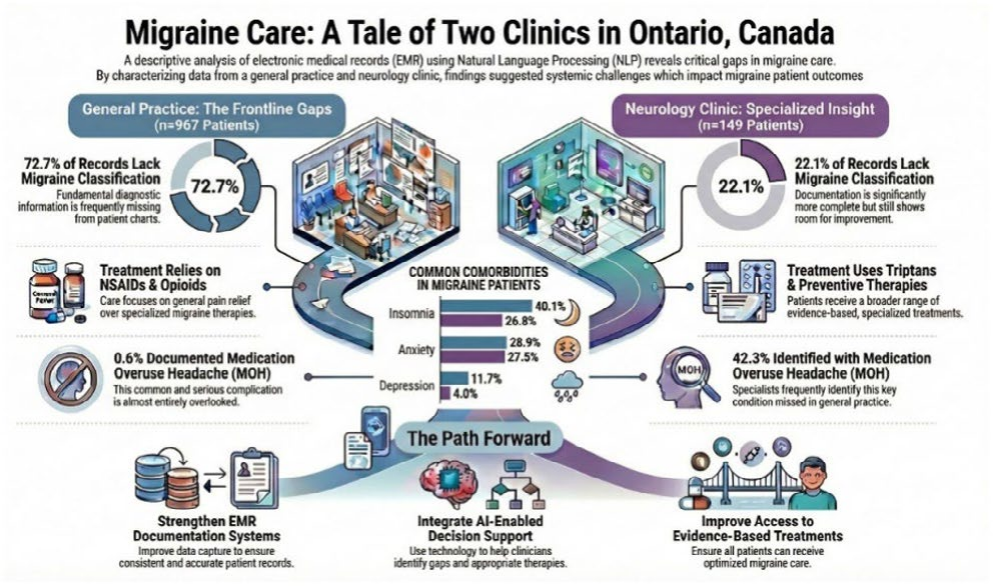
**FIGURE 1** Abstract infographic.

**Conclusion:** Findings highlight system level barriers—poor documentation, underdiagnosis, and inconsistent access to migraine‐specific therapy—which fragment migraine care. Enhancing EMR systems, integrating AI‐enabled decision support, and improving equitable access to evidence‐based treatments are essential to advancing migraine care in Canada.

**Disclosure:** This study was sponsored by Pfizer.

## EPO‐0743

### Real‐world utilization of acute treatments for cluster headache: Insights from the French national open health data

#### V. Arcani ^1^; A. Bruyat^2^; G. Hache^1^; A. Donnet^3^; S. Redon^3^

##### ^1^Aix‐Marseille Université, AP‐HM, Hôpital de La Timone, Service Pharmacie, Marseille, France; ^2^Aix‐Marseille Université, INSERM 1263, Marseille, France; ^3^Aix‐Marseille Université, AP‐HM, Hôpital de La Timone, Centre d’Evaluation et de Traitement de la Douleur, Marseille, France

**Background and aims:** Cluster headache (CH) is a primary headache disorder historically considered more prevalent in men. As a chronobiological condition, it has been hypothesised CH may be influenced by seasonality or sunlight‐related factors. The French open healthcare databases offer an opportunity to analyse real‐world dispensing patterns of CH treatments. The aim of this study was to provide an innovative interpretation of the clinical expression of this condition though healthcare data, and to highlight the evolving economic burden of its treatments.

**Methods:** We extracted consumption data for the two validated attacks treatments for CH: 6 mg subcutaneous sumatriptan (SS) and oxygen therapy (OT). Treatments patterns were analysed by sex, age group, and French administrative region, and compared with climatic, demographic and economic variables.

**Results:** Between 2014 and 2024, the total amount for OT in CH tripled, while SS costs increased by only 1,6‐fold. A consistent decrease in the male‐to‐female sex ratio was observed across all age group for both treatments. A distinct peak in SS dispensing was observed in July. However, no statistically significant associations were found between SS dispensing and sunshine or rainfall. Younger men exhibited significantly higher consumption than older men, and overall consumption was higher in men than in women.

**Conclusion:** Our findings highlight evolving treatment patterns and shifting sex ratio in the delivery of CH therapies in France. While no clear environmental drivers of treatment used were identified, the study underscores the value of open health data in monitoring prescribing trends and evaluation the economic impact of CH therapies.

**Disclosure:** Nothing to disclose.

## Movement Disorders 8

## EPO‐0744

### Movement disorders in multisystem proteinopathies: A systematic review

#### C. Terravecchia ^1^; G. Fazzina^1^; A. Bondarieva^2^; H. Houlden^2^; A. Nicoletti^1^; K. Bhatia^3^; F. Magrinelli^3^

##### ^1^Department *“*G.F. Ingrassia*”*, Section of Neurosciences, University of Catania, Catania, Italy; ^2^Department of Neuromuscular Diseases, UCL Queen Square Institute of Neurology, University College London, London, UK; ^3^Department of Clinical and Movement Neurosciences, UCL Queen Square Institute of Neurology, University College London, London, UK

**Background and aims:** Multisystem proteinopathies (MSP) represent a group of genetic disorders classically manifesting with a combination of inclusion body myopathy, Paget disease of bone (PDB) and motor neuron disorders (MND)/frontotemporal dementia. In the last years, a growing body of evidences expanded this phenotypical spectrum, including a variety of movement disorders (MSP‐MD). We aimed to systematically review MSP‐MD phenomenology.

**Methods:** The search was conducted in the Medline and Scopus databases by the following terms: “Multisystem proteinopathy”; “MSP”; “valosin containing protein”; “VCP”; “MSP1”; “heterogeneous nuclear ribonucleoprotein A2B1”; “hnRNPA2B1”; “MSP2”; “heterogeneous nuclear ribonucleoprotein A1”; “hnRNPA1”; “MSP3”; “Sequestosome 1”; “SQSTM1”; “MSP4”; “Matrin 3”; “MATR3”; “MSP5”; “T‐cell restricted intracellular antigen 1”; “TIA1”; “optineurin”; “OPTN”; “annexin A11”; “ANXA11”. All kind of studies reporting single cases or case series of MSP‐MD published up to 02/01/2026 were included.

**Results:** A total of *N* = 49979 studies were assessed, of whom *N* = 52 were included. A total of *N* = 132 MSP‐MD cases were identified [*N* = 68 VCP (51.1%), *N* = 46 SQSTM1 (34.6%), *N* = 9 OPTN (6.8%), *N* = 9 ANXA11 (6.8%), *N* = 1 hnRNPA2B1 (0.7%)]. A family history for MSP‐MD was most frequently reported, followed by dementia, myopathy, MND and PDB. Atypical parkinsonism was the most frequent manifestation (32.3%), followed by cerebellar syndrome (29.3%), dystonia (28.6%), Parkinson's disease (27.1%), choreo‐athetosis (9.8%), myoclonus (6.8%) and spastic paraplegia (4.5%). The most common concomitant MSP phenotype was dementia, followed by myopathy, PDB and MND. Heterogeneous MD phenomenology was reported across different involved genes (Table 1).

**Figure:**
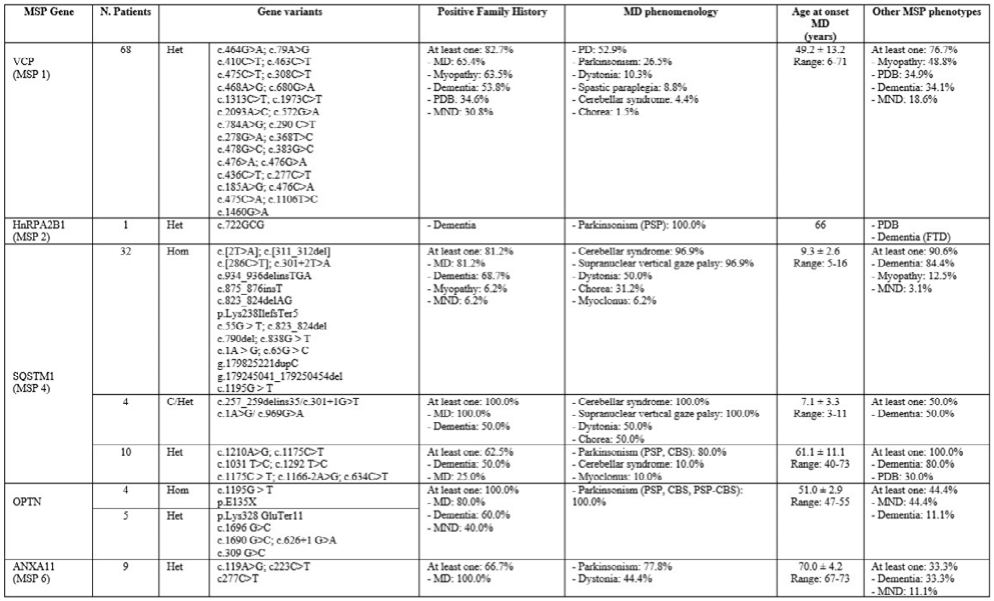
**TABLE 1** MSP‐MD related gene variants and MD phenomenology.

**Conclusion:** The spectrum of MSP‐MD was summarized suggesting possible clinical scenarios for a proper MSP syndromic diagnosis.

**Disclosure:** Nothing to disclose.

## EPO‐0745

### Incidence of calvarial bone infarction after focused ultrasound thalamotomy for refractory tremor: Associated factors

#### P. Gutierrez Bedia ^1^; A. Fernández Revuelta^1^; A. Aldaz Burgoa^1^; C. Ribacoba^1^; C. Perez Garcia^2^; M. Yus^2^; A. López‐Frías^2^; A. Trondin^3^; E. Lopez Valdes^1^; R. García Ramos^1^

##### ^1^Neurology, Hospital Clinico San Carlos, Madrid, Spain; ^2^Radiology, Hospital Clinico San Carlos, Madrid, Spain; ^3^Neurosurgery. Hospital Clínico San Carlos, Madrid, Spain

**Background and aims:** Magnetic resonance‐guided focused ultrasound (MRgFUS) thalamotomy has emerged as a non‐invasive and effective treatment for medication‐refractory essential tremor (ET) and Parkinsonian tremor. As its clinical use expands, rare adverse events have been increasingly observed, including calvarial bone infarction, but remain poorly characterized.

**Methods:** We conducted a retrospective observational study including 66 patients (median age 71 years, 33 females) who underwent unilateral MRgFUS thalamotomy between January 2022 and December 2024. All patients received clinical and MRI follow‐up at least one month after treatment. Demographic and procedural variables were compared between patients with calvarial bone infarction (*n* = 6) and those without (*n* = 60) using independent‐samples t‐tests.

**Results:** Calvarial bone infarction was an incidental MRI finding in six patients (incidence 9.09%; IC 95% 2.3–16.1%). These patients had a lower skull density ratio (0.455 vs 0.55, *p* = 0.004), a higher number of sonications (10.5 vs 8, *p* = 0.047), longer added sonication duration (22.5 s vs 12 s, *p <* 0.001), and greater maximum delivered energy (22,592 J vs 8572 J, *p <* 0.001). On multivariate analysis, no statistical significance was achieved. No significant differences were found in age, maximum temperature, or the number of ablative sonications.

**Conclusion:** Calvarial bone infarction is an infrequent complication after MRgFUS thalamotomy, potentially linked to lower skull density ratio warranting higher energy procedures, although small sample size warrants cautious interpretation. Its clinical relevance remains unknown. These findings may guide future optimization of MRgFUS safety protocols.

**Disclosure:** Nothing to disclose.

## EPO‐0746

### The effect of Botulinum neurotoxin injection on non‐motor symptoms of cervical dystonia: A prospective cohort

#### S. Kassaye ^1^; F. Dijkstra^2^; J. Maes^3^; K. Paredis^3^; C. Taelman^3^; E. Gudina^4^; W. Hertogh^1^; J. De Pauw^1^; D. Crosiers^3^

##### ^1^Department of Rehabilitation Sciences and Physiotherapy/Movant, Faculty of Medicine and Health Science, University of Antwerp, Antwerp, Belgium; ^2^Department of Neurology, Antwerp University Hospital, Antwerp, Belgium; ^3^Translational Neurosciences, Faculty of Medicine and Health Sciences, University of Antwerp, Belgium; ^4^Institute of Health, Jimma University, Jimma, Ethiopia

**Background and aims:** Evidence on the effect of intramuscular injections of botulinum neurotoxin (BoNT) treatment on non‐motor symptoms (NMS) and quality of life (QoL) in Cervical Dystonia (CD) remains scarce and inconsistent. Thus, this study aimed to determine the effect of intramuscular BoNT injections on NMS and QoL.

**Methods:** A prospective cohort was conducted with adult CD patients attending treatment at Antwerp University Hospital, Belgium (UZA) and a specialized private physiotherapy practice. Depression and Anxiety were measured using the Beck Depression inventory (BDI‐II) and the Beck Anxiety Inventory (BAI‐II), respectively. Sleep quality was measured using Pittsburgh Sleep Quality Index (PSQI); and QoL was measured using EuroQol‐5 dimensions (EQ‐5D) at baseline and 6‐weeks post treatment. Paired T‐test and Wilcoxon signed rank was used for comparison.

**Results:** A total of 29 CD patients were included. The mean age was 61.6 + 9.25 years. The mean + standard deviation (SD) score of the global PSQI score decreased from 10.1 + 4.6 at baseline to 7.41 + 4.7 post treatment. The mean (SD) BAI‐II score decreased slightly from 13.5 + 9.43 at baseline to 12.52 + 10.2 post treatment. The median (IQR) of BDI‐II score changed from 7 (5–19) to 8 (2.5–20). There was significant improvement of PSQI following BoNT treatment (MD = 2.65, *p* < 0.001).

**Conclusion:** BoNT injection significantly improved sleep quality. However, no statistical difference was observed in depressive and anxiety symptoms. The persistence of psychiatric symptoms underscores the necessity of comprehensive bio‐psychosocial interventions. Considering this, future studies targeting development of intervention modality is needed.

**Disclosure:** Nothing to disclose.

## EPO‐0747

### The immediate effect of peripheral stimulation on cervical dystonia disease outcomes: Single‐case experimental design

#### S. Kassaye ^1^; D. Crosiers^2^; E. Kebede Gudina^3^; N. Nadjmi^4^; E. Van de Casteele^5^; J. De Pauw^1^; W. De Hertogh^1^

##### ^1^Department of Rehabilitation Sciences and Physiotherapy/Movant, Faculty of Medicine and Health Science, University of Antwerp, Antwerp, Belgium; ^2^Department of Neurology, Antwerp University Hospital, Antwerp, Belgium; ^3^Institute of Health, Jimma University, Jimma, Ethiopia; ^4^Department of Cranio‐Maxillofacial Surgery, Antwerp University Hospital, Antwerp, Belgium; ^5^ZMACK/Associate MKA, AZ, Antwerp, Belgium

**Background and aims:** Limited Cervical Range of Motion (CROM) and pain are among the most common symptoms of Cervical Dystonia (CD) which are treated with Botulinum NeuroToxin injections. However, adjuvant interventions may offer additional benefit. Therefore, this study evaluated the immediate effects of peripheral stimulation comprising conventional transcutaneous electrical nerve stimulation (TENS), muscle stimulation (NMES), and muscle vibration on CROM and pain in patients with CD.

**Methods:** A Single‐Case Experiment Design (SCED), alternating treatment design was conducted with 19 CD patients, recruited at the Antwerp University Hospital (UZA). Participants randomly received conventional TENS, NMES and muscle vibration on three different days. Main outcome measures were CROM, measured using a 3D‐stereophotogrammetric scanning system (3dMD Inc, GA, USA) and pain, quantified by Visual Analogue Scale (VAS).

**Results:** Mean age of participants was 59.87 + 8.9 years. Baseline CROM for rotation ranged from 87o to 137.6o and from 79o to 132.4o for flexion/extension. Improvements in neck rotation were observed in 5 (33.3%) participants after conventional TENS, 8 (42.1%) after NMES, and 8 (42.1%) after muscle vibration. Similarly, improvement in flexion and extension was observed in 10 (55.6%) after conventional TENS, 8 (53.3%) after NMES, and 7 (46.7%) after muscle vibration. Significant reductions in pain shortly after the three interventions were observed in patients experiencing pain.

**Figure:**
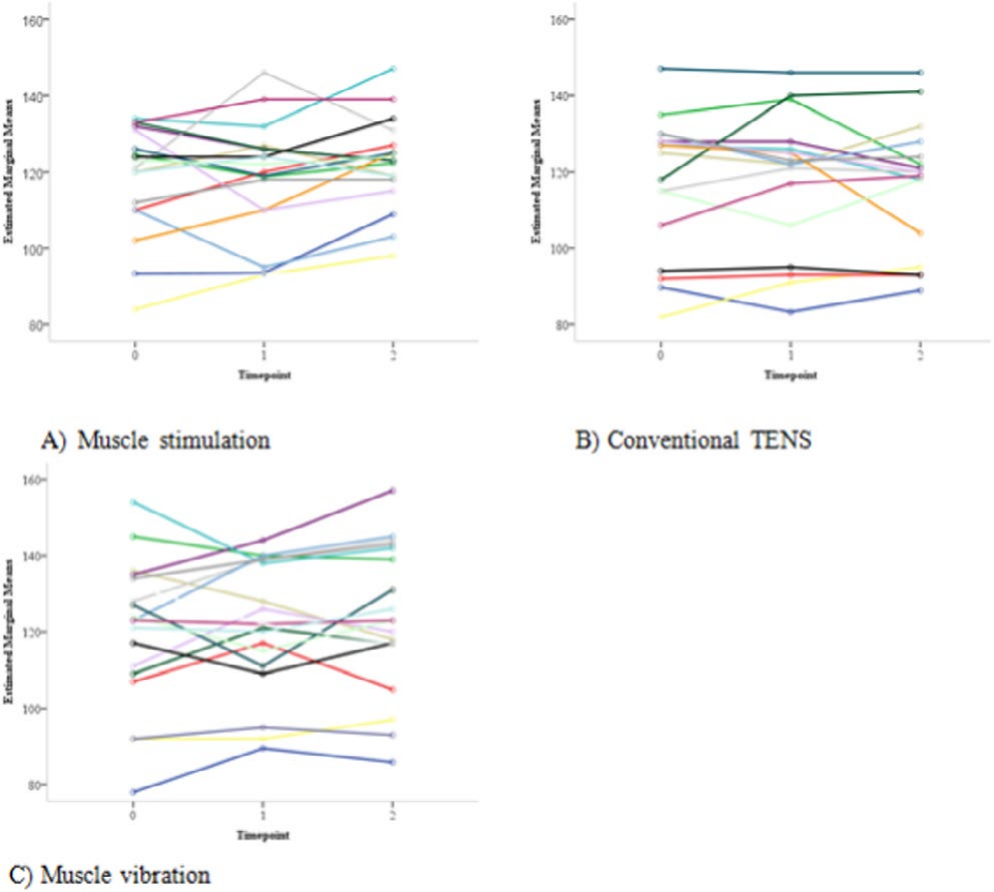
**FIGURE 1** Individual mean score of full range of neck rotation at baseline (0), immediately post stimulation (1), 10 min. post stimulation (2) for the different interventions.

**Conclusion:** These findings suggest that peripheral stimulation may provide immediate symptomatic relief in CD, particularly for improving cervical range of motion and reducing pain. While promising, these results should be considered as exploratory and warrant further investigation in larger, controlled studies.

**Disclosure:** Nothing to disclose.

## EPO‐0748

### Divergent clinical phenotypes in siblings with a shared GBA1 mutation and posterior cortical hypometabolism

#### T. Filidei ^1^; R. Malito^2^; L. Gallo^1^; R. Calabrese^3^; P. Mitrotti^3^; L. Bandirali^3^; A. Samanes Gajate^2^; D. Perani^2^; C. Tassorelli^1^; E. Valente^1^; S. Caminiti^3^; M. Avenali^1^

##### ^1^Department of Brain and Behavioral Sciences, University of Pavia, Pavia, Italy; ^2^IRCCS San Raffaele Scientific Institute, Milan, Italy; ^3^IRCCS Mondino Foundation, Pavia, Italy

**Background and aims:** Variants in the glucocerebrosidase gene (GBA1) are among the strongest genetic risk factors for Parkinson's disease (PD), yet disease penetrance is incomplete and marked intrafamilial heterogeneity is observed. Neuroimaging biomarkers may capture early neurobiological changes that are not reflected by clinical severity. The objective of this study was to describe the dissociation between clinical phenotype and brain metabolic patterns in siblings carrying a GBA1 mutation.

**Figure:**
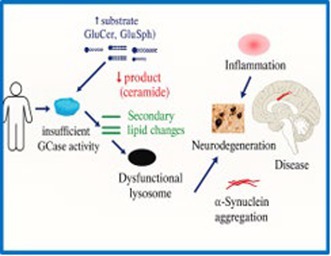
**FIGURE 1** Schematic representation of the proposed pathogenic pathway linking GBA1 mutations to reduced glucocerebrosidase activity, lysosomal dysfunction, lipid accumulation, and alpha‐synuclein aggregation in Parkinson's disease.

**Methods:** Two siblings carrying the same severe GBA1 mutation (*p*.H255Q) underwent a comprehensive cross‐sectional evaluation. Assessments included motor and non‐motor examination using the Movement Disorder Society Unified Parkinson's Disease Rating Scale, standardized cognitive testing, and brain metabolic imaging with fluorodeoxyglucose positron emission tomography (FDG‐PET). Peripheral biomarkers included glucocerebrosidase enzymatic activity and monomeric alpha‐synuclein levels measured in peripheral blood mononuclear cells.

**Figure:**
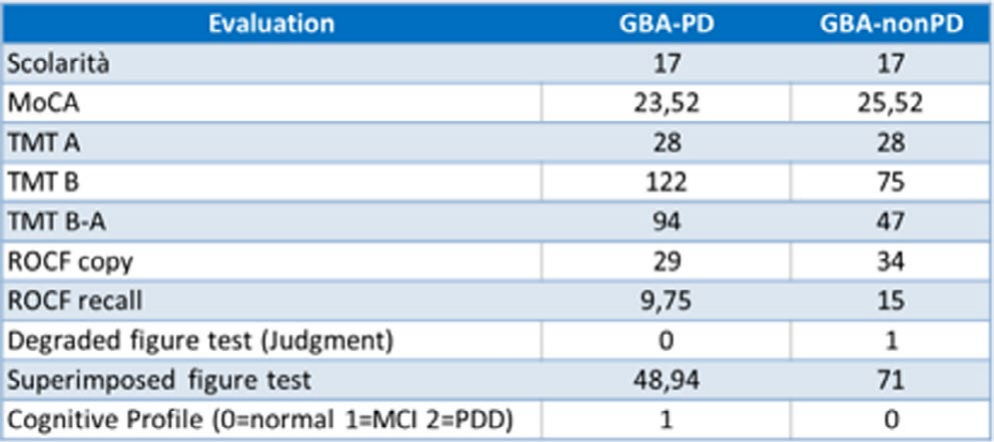
**TABLE 1** Comparison of cognitive test performance between the sibling with GBA1‐associated Parkinson's disease (GBA‐PD) and the non‐affected GBA1 carrier (GBA‐nonPD) across global cognition, memory, and visuospatial domains.

**Results:** The proband, a 59‐year‐old man with Parkinson's disease, showed motor impairment, multidomain cognitive deficits consistent with Parkinson's disease–mild cognitive impairment, and a high non‐motor symptom burden. His 56‐year‐old sister exhibited only minimal motor signs, preserved cognition, and negligible non‐motor symptoms. Despite the marked clinical divergence, FDG‐PET demonstrated a similar pattern of bilateral posterior cortical hypometabolism in both siblings. Glucocerebrosidase activity was comparable, while monomeric alpha‐synuclein levels were higher in the affected sibling.

**Figure:**
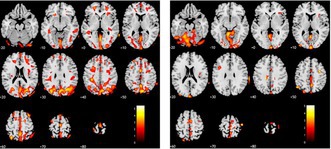
**FIGURE 2** FDG‐PET hypometabolism patterns in a GBA1‐associated Parkinson's disease patient (59‐year‐old male) and an unaffected GBA1 carrier (56‐year‐old female), obtained using single‐subject SPM analysis versus 125 age‐corrected healthy controls (*p* < 0.05).

**Conclusion:** This intrafamilial observation highlights a dissociation between cortical hypometabolism and clinical expression in carriers of a severe GBA1 mutation. These findings suggest that metabolic brain changes may precede or evolve independently of overt clinical manifestations, supporting the potential role of FDG‐PET in the phenotypic characterization of genetic Parkinson's disease.

**Disclosure:** Nothing to disclose.

## EPO‐0749

### Intrafamilial biochemical correlates of disease expression in GBA1 mutation carriers discordant for Parkinson's disease

#### T. Filidei ^1^; L. Gallo^1^; R. Calabrese^2^; P. Mitrotti^2^; L. Bandirali^1^; R. Stiuso^2^; C. Tassorelli^1^; E. Valente^1^; S. Caminiti^1^; M. Avenali^1^

##### ^1^Department of Brain and Behavioral Sciences, University of Pavia, Pavia, Italy; ^2^IRCCS Mondino Foundation, Pavia, Italy

**Background and aims:** Pathogenic variants of the glucocerebrosidase gene (GBA1) are a major genetic risk factor for Parkinson's disease, yet disease penetrance is incomplete. Discordant sibling designs allow intrafamilial comparisons that control for shared genetic background and early‐life environment. The objective of this study was to investigate intrafamilial differences in peripheral glucocerebrosidase activity and total alpha‐synuclein levels in siblings carrying the same GBA1 variant but discordant for Parkinson's disease.

**Methods:** We conducted a family‐based cross‐sectional study including sibling pairs carrying identical GBA1 variants and discordant for Parkinson's disease. The most recent peripheral blood mononuclear cell sample with available biochemical data was selected for each subject. Glucocerebrosidase activity and total alpha‐synuclein levels were quantified and normalized to total protein content. Intrafamilial paired differences were calculated as values in the Parkinson's disease–affected sibling minus those in the non‐manifesting carrier. Analyses focused on paired descriptive statistics and resampling approaches.

**Figure:**
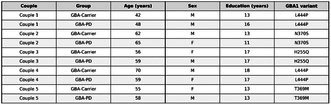
**TABLE 1** Demographic characteristics of discordant sibling pairs carrying the same GBA1 variant. Each couple includes one Parkinson's disease–affected sibling (GBA‐PD) and one non‐manifesting GBA1 carrier.

**Results:** Five discordant sibling pairs were available for analysis. Glucocerebrosidase activity showed heterogeneous intrafamilial differences without a consistent directional pattern. In contrast, total alpha‐synuclein levels were higher in the Parkinson's disease–affected sibling in most pairs. Resampling analyses supported a positive intrafamilial difference for alpha‐synuclein despite limited statistical power. Larger intrafamilial differences in alpha‐synuclein tended to associate with greater motor and non‐motor burden.

**Figure:**
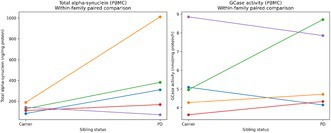
**FIGURE 1** Within‐family paired comparison of total alpha‐synuclein and glucocerebrosidase activity in peripheral blood mononuclear cells from GBA1 discordant sibling pairs. Each line represents one family, comparing non‐manifesting carriers and Parkinson.

**Conclusion:** In this discordant sibling design, the Parkinson's disease–carrier difference captures biological processes associated with disease expression beyond shared genetic risk. While peripheral glucocerebrosidase activity appears variable within families, total alpha‐synuclein emerges as a more consistent downstream readout associated with Parkinson's disease manifestation in GBA1 mutation carriers.

**Figure:**
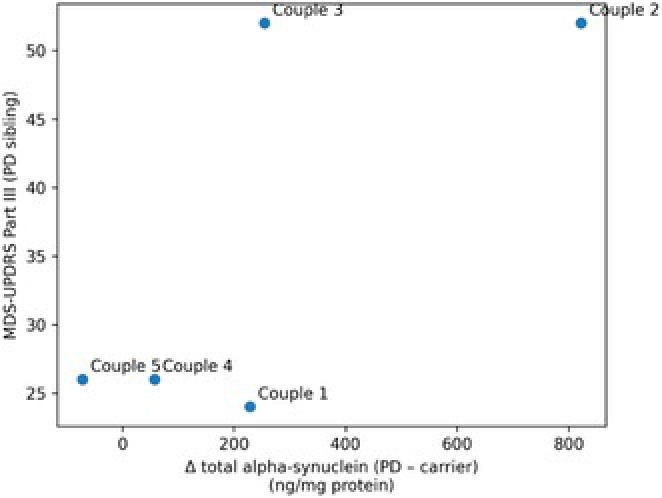
**FIGURE 2** Exploratory association between intrafamilial differences in total alpha‐synuclein (Parkinson's disease minus carrier) in peripheral blood mononuclear cells and motor severity, assessed by MDS‐UPDRS Part III, in GBA1 discordant sibling pairs.

**Disclosure:** Nothing to disclose.

## EPO‐0750

### RTA‐408 enhances Nrf2 activation and mitochondrial function to improve SCA3 cell and drosophila models

#### C. Liu ^1^; W. Lin^2^; F. Leng^2^; W. Cheng^2^; W. Lin^3^; S. Pan^2^; J. Chang^4^

##### ^1^Department of Neurology, Changhua Christian Hospital, Changhua, Taiwan; ^2^Department of Vascular and Genomic Center, Institute of ATP, Changhua Christian Hospital, Changhua, Taiwan; ^3^Graduate Institute of Integrated Medicine, College of Chinese Medicine, China Medical University, Taichung, Taiwan; ^4^Center of Regenerative Medicine and Tissue Repair, Institute of ATP, Changhua Christian Hospital, Changhua, Taiwan

**Background and aims:** Spinocerebellar ataxia type 3 (SCA3) is a polyglutamine neurodegenerative disorder characterized by mutant ataxin‐3 aggregation, oxidative stress, mitochondrial dysfunction, and progressive neuronal degeneration. Dysregulation of the Nrf2 antioxidant pathway contributes to SCA3 pathogenesis. RTA‐408 (omaveloxolone), a potent Nrf2 activator, has therapeutic potential in Friedreich's Ataxia. This study evaluated the effects of RTA‐408 on Nrf2 signaling, mitochondrial integrity, and disease‐related phenotypes in SCA3 cellular and Drosophila models.

**Methods:** Human SK‐*N*‐SH neuroblastoma cells expressing normal (MJD26) or expanded polyglutamine (MJD78) ataxin‐3 were treated with RTA‐408 (0.1–0.5 μM) for 24 hours. Cell viability, apoptosis, Nrf2 nuclear translocation, antioxidant enzyme expression, mutant ataxin‐3 protein levels, mitochondrial morphology, and mitochondrial copy number were assessed. In vivo efficacy was examined in ELAV‐ and GMR‐driven SCA3 transgenic Drosophila treated with RTA‐408 (1–4 μM), assessing survival, locomotor performance, and retinal degeneration.

**Figure:**
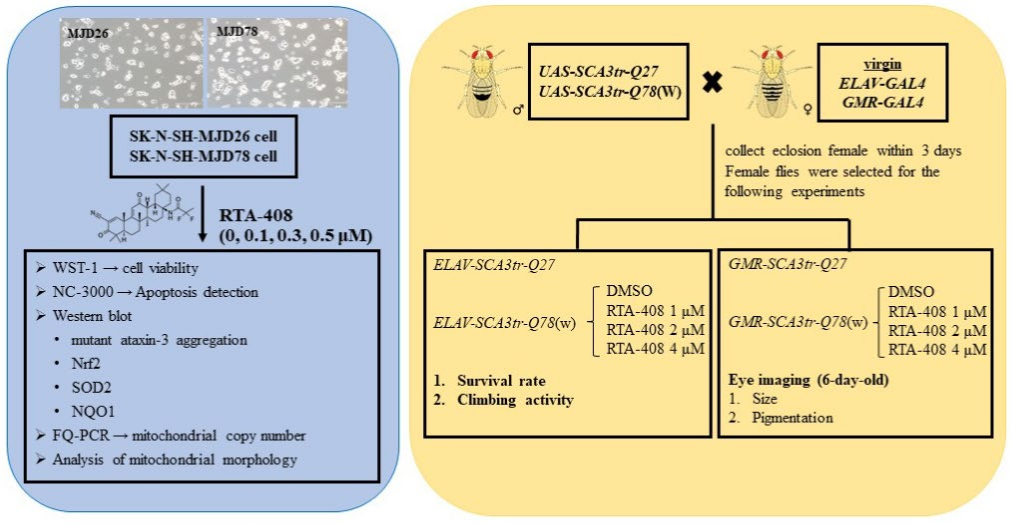
**FIGURE 1** Material and methods.

**Results:** MJD78 cells showed reduced viability, increased apoptosis, impaired Nrf2 nuclear localization, elevated mutant ataxin‐3 levels, and fragmented mitochondria. RTA‐408 significantly improved cell viability, enhanced Nrf2 nuclear translocation, increased antioxidant enzyme expression, reduced mutant ataxin‐3 protein levels, and partially restored mitochondrial morphology and copy number. In SCA3 transgenic Drosophila, RTA‐408 significantly prolonged survival, improved climbing activity, and ameliorated retinal degeneration.

**Figure:**
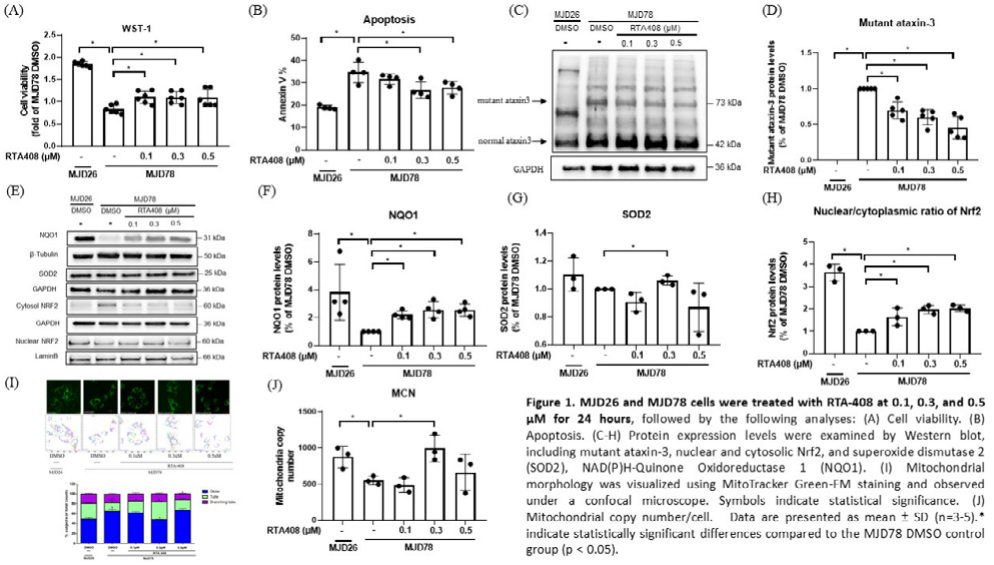
**FIGURE 2** MJD26 and MJD78 cells were treated with RTA‐408 at 0.1, 0.3, and 0.5 μM for 24 hours.

**Figure:**
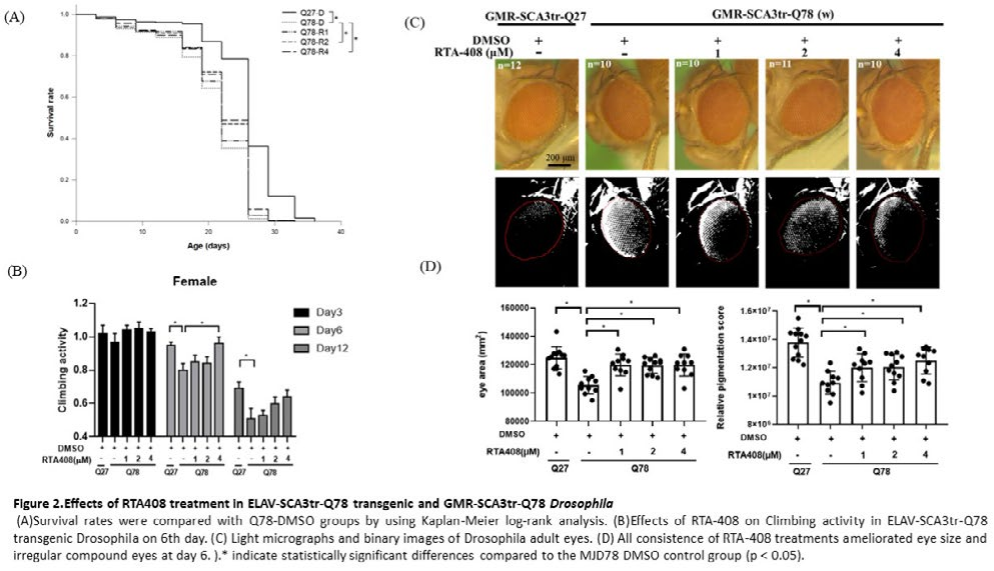
**FIGURE 3** Effects of RTA408 treatment in ELAV‐SCA3tr‐Q78 and GMR‐SCA3tr‐Q78 Drosophila.

**Conclusion:** RTA‐408 confers neuroprotection in SCA3 models by activating Nrf2 signaling and improving mitochondrial function. The reduction of mutant ataxin‐3 likely reflects decreased protein accumulation or enhanced clearance secondary to improved cellular redox homeostasis, supporting Nrf2‐targeted mitochondrial modulation as a potential disease‐modifying strategy for SCA3.

**Disclosure:** Nothing to disclose.

## EPO‐0751

### Sensor‐based extended timed up and go parameters and cognitive performance in idiopathic Parkinson's disease

#### D. Almonti ^1^; R. Zangari^2^; L. Brighina^3^; A. Pilotto^4^; G. Cairoli^2^; V. D'Agostino^3^; A. Oppo^3^; A. Rizzardi^4^; A. Padovani^5^; F. Biroli^2^

##### ^1^Department of Neurology, Papa Giovanni XXIII Hospital, Bergamo, Italy; ^2^FROM Research Foundation, Papa Giovanni XXIII Hospital, Bergamo, Italy; ^3^Department of Neurology, Fondazione IRCCS San Gerardo dei Tintori, Monza, Italy; ^4^Neurology Unit, Department of Clinical and Experimental Sciences, University of Brescia, Italy; ^5^Brain Health Center, University of Brescia, Italy

**Background and aims:** This study investigated whether Extended Timed Up and Go (eTUG) parameters capture global and composite cognitive impairment in Parkinson's disease (PD).

**Methods:** Sixty‐two PD patients (Hoehn‐Yahr 2–3, age 55–74, MMSE >24) underwent sensor‐based eTUG assessment using a single lumbar inertial measurement unit. Temporal (duration, turning time) and kinematic (velocity, trunk acceleration) parameters were extracted. Cognitive assessment included Montreal Cognitive Assessment (MoCA), MMSE, Frontal Assessment Battery (FAB) and composite scores reflecting attentive‐executive functions. Motor severity was evaluated using Unified Parkinson's Disease Rating Scale Part III (UPDRS‐III) and Postural Instability and Gait Difficulty (PIGD scores), and functional endurance was assessed with the six‐minute walk test (6MWT). Spearman's correlations examined associations among eTUG, cognitive, motor, and 6MWT measures, considering |r| ≥ 0.3 and *p* < 0.05 as significant.

**Results:** Turning time showed moderate correlations with global cognition (MoCA: r = ‐0.39) and attentive‐executive function (*r* = −0.38), *p* <0.01 (Figure 1). eTUG duration correlated with motor severity (UPDRS‐III: *r* = 0.51, and PIGD: *r* = 0.37, *p <* 0.01) and 6MWT parameters (distance: *r* = −0.76; gait speed: *r* = −0.40, *p <* 0.01). Kinematic parameters of eTUG showed weaker cognitive associations but correlated moderately‐to‐strongly with motor impairment and sustained walking performance (6MWT).

**Figure:**
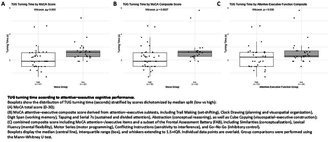
**FIGURE 1** TUG turning time according to attentive‐executive cognitive performance.

**Conclusion:** Sensor‐based eTUG turning metrics showed greater sensitivity to global and attentive‐executive cognitive performance compared to straight‐walking measures, highlighting the added value of turning analysis. Total eTUG duration captured both motor impairment and functional endurance. Overall, these findings support eTUG as a feasible motor‐cognitive marker for clinical stratification and research applications in PD.

**Disclosure:** Nothing to disclose.

## EPO‐0752

### Switching to CREXONT/IPX203 improves good on time and reduces motor fluctuations: Interim real‐world results from the first 100 patients in ELEVATE‐PD

#### R. Hauser^1^; S. Isaacson^2^; S. Bette^2^; A. Richmond^3^; M. Jay^4^; G. Banisadr ^4^; S. Fisher^4^; H. Visser^4^

##### ^1^University of South Florida Parkinson's Disease and Movement Disorders Center, USA; ^2^Parkinson's Disease and Movement Disorders Center of Boca Raton, Boca Raton, USA; ^3^University of Kansas Medical Center, Kansas, USA**;** ^4^Amneal Pharmaceuticals, Evanston, USA

**Background and aims:** CREXONT (IPX203), a novel extended‐release carbidopa‐levodopa formulation containing a mucoadhesive polymer, is designed to improve levodopa delivery and absorption. Here, we evaluate interim outcomes from the real‐world study of CREXONT, assessing its impact to extend “Good On” time and reduce “Off” time in PD patients after switching from other levodopa‐based therapies.

**Methods:** ELEVATE‐PD is an ongoing, open‐label phase 4 study where PD patients are converted from prior levodopa‐based therapies (IR CD/LD, IR+COMTinhibitors, Rytary) to CREXONT. Following a 5‐week dose‐optimization period and a 1‐week stable dosing, patients are followed for 12 months. Eligible participants had ≥2.5 hours of daily “Off” time at baseline. The primary endpoint is change in daily “Good On” time from baseline/Visit 1 to Day 42/Visit 4. This interim analysis includes 111 patients who completed Day 42.

**Results:** At baseline, mean daily “Good On” time was 9.39±2.53 hours, “Off” time was 6.21±2.31 hours: • CREXONT treatment increased daily “Good On” time:+3.40 hours for patients switching from IR CD/LD (*n* = 74), +2.91 hours from IR+COMTinhibitors (*n* = 9), +3.07 hours from Rytary (*n* = 22). Corresponding reductions in “Off” time were:–3.18, –3.09, –2.90 hours, respectively. CREXONT increased “Good On” time per dose by:+2.09, +0.91, +0.98 hours, respectively. CREXONT also improved MDS‐UPDRS total scores:–15.3, –10.0, –10.3 points, respectively. Majority of TEAEs were mild‐to‐moderate; most common (≥3%) were dizziness (9.0%), nausea (7.2%), fall (7.2%), dyskinesia (4.5%), hallucination (3.6%), headache (3.6%).

**Conclusion:** Switching from other levodopa‐based therapies to CREXONT significantly improved daily symptom control by increasing “Good On” time, decreasing “Off” time, and enhancing motor function across all treatment groups.

**Disclosure:** Supported by Amneal Pharmaceuticals.

## EPO‐0753

### Effect of cognitive and motor dual‐task activities on balance in patients with PPPD, FGD, and healthy controls

#### I. Klopotovska ^1^; E. Finegan^2^; M. Bancroft^3^; D. Shivji^1^; Z. Gunes^4^; M. Bautista^5^; L. Flix Diez^6^; D. Kaski^3^

##### ^1^University College London Hospitals NHS Foundation Trust, UK; ^2^Dublin Neurological Institute, The Mater Misericordiae University Hospital, Dublin, Ireland; ^3^UCL Queen Square Institute of Neurology, London, UK; ^4^Bezmialem Vakif University, Istanbul, Türkiye; ^5^Hospital Universitario Royo Villanova, Zaragoza, Spain; ^6^Clínica Universidad de Navarra, Madrid, Spain

**Background and aims:** Balance control relies on integrating sensory inputs and motor responses and can be challenged by simultaneous cognitive or motor tasks, known as the dual‐task effect. This study investigated balance under cognitive and motor dual‐task conditions in individuals with FGD, PPPD, and healthy controls.

**Methods:** In this observational cross‐sectional study, 15 participants with PPPD (61.7 ± 12.5 years [mean ± SD]), 16 with FGD (56.1 ± 15.5 years), and 15 healthy controls (55.3 ± 12.5 years) were assessed. Diagnoses followed Bárány Society criteria (PPPD) and Gupta and Lang criteria (FGD). Postural sway was measured using force plate during three 20‐second conditions: eyes closed, eyes closed with a motor dual task (dominant‐hand prono‐supination), and eyes closed with a cognitive dual task (listing months backward) (Figure 1).

**Figure:**
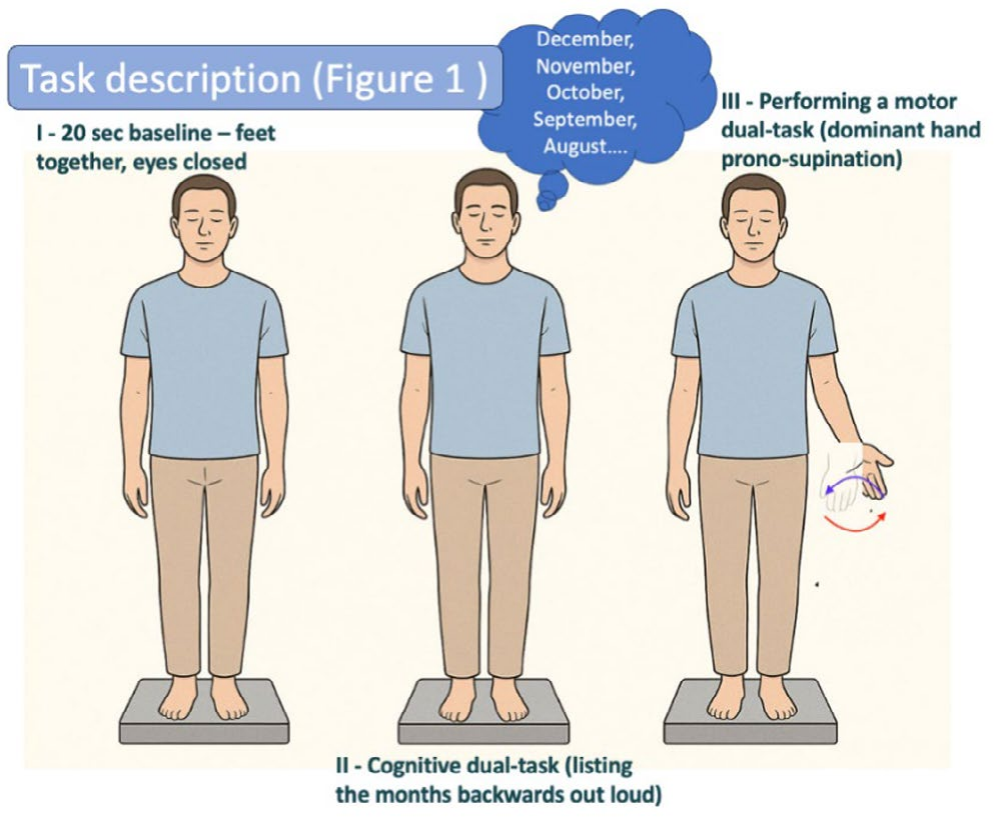
**FIGURE 1** Task description.

**Results:** FGD participants showed significantly higher sway than PPPD (*p* = 0.035) and healthy controls (*p* = <0.001) across all conditions. Healthy controls and PPPD did not show changes in sway during the dual‐task. A small, non‐significant reduction in sway was observed in the FGD group during the cognitive dual‐task. (Figure2) Dual‐task performance, measured by task completion time (*p* = 0.996) and months‐backward errors (*p* = 0.491), did not differ between groups. No learning or adaptation effects were detected.

**Figure:**
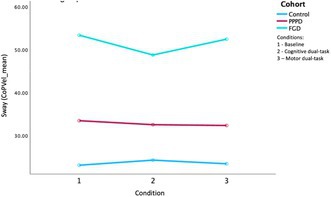
**FIGURE 2** Sway across conditions in FGD, PPPD, and healthy control groups.

**Conclusion:** FGD and PPPD demonstrate distinct postural control profiles, with higher sway in FGD under all conditions. Dual‐tasking did not significantly influence performance, although a trend toward reduced sway during cognitive dual‐tasking was noted in FGD. Additional analyses will examine balance changes across monthly intervals during the cognitive task.

**Disclosure:** Nothing to disclose.

## EPO‐0754

### Features of the cerebellar fMRI connectivity in Parkinson's disease patients with cognitive decline, REM sleep behavior disorder and freezing of gait

#### O. Alenikova ^1^; A. Chumak^1^; L. Parhach^1^; E. Mikitchuk^2^

##### ^1^Republican Research and Clinical Center of Neurology and Neurosurgery, Minsk, Belarus; ^2^Belarusian State University, Minsk, Belarus

**Background and aims:** Sleep disturbances like RBD, postural instability (PI), freezing of gait (FOG) and cognitive decline in Parkinson's disease (PD) are closely linked, and often untreatable. These symptoms are caused by disturbances in brain networks involving the cerebellum, which determines the nature and severity of the motor and non‐motor PD manifestations. We investigated the altered cerebellar functional connections (FC) in relation to RBD, PI, FOG severity and cognitive impairment in PD.

**Methods:** We used questionnaires and tests for assessment sleep problems, gait disturbances and cognition. Rs‐fMRI data were analyzed using the SPM12 with the CONN toolbox. The PD patients were divided into 2 groups according to REM Sleep Behavior Disorder scoring (Table 1).

**Figure:**
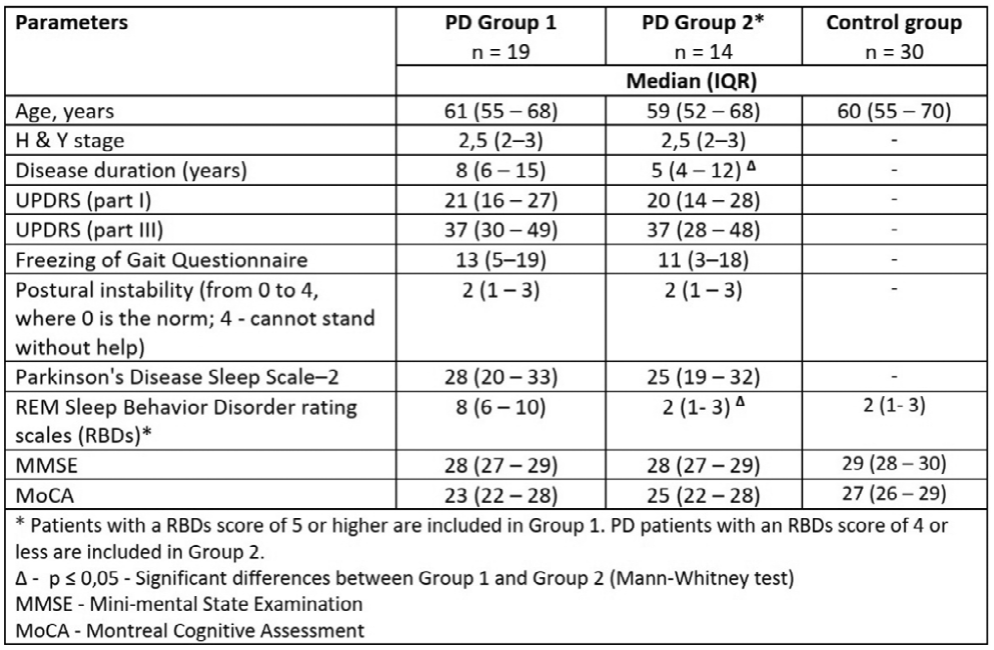
**TABLE 1** Comparative assessment of the PD patient's groups and the control group.

**Results:** PD patients both groups demonstrated altered FC between cerebellum (lobule VI, Crus I‐II, vermis) and Somatomotor, Default Mode (DMN), Central Executive Network (CEN) nodes. The 1stgroup had a longer PD duration and increased FC within the cerebellar network and between the cerebellum and the Salience Network (SN) compared to 2ndgroup. A significant negative correlation was found between the RBDs score and pedunculopontine nucleus (PPN)‐cerebellum FC. In addition to reduced PPN‐cerebellum FC, FOG and PI severity negatively correlated with cognitive impairment and reduced PPN – CEN FC.

**Conclusion:** The cerebellum is the key structures involved in the large‐scale functional network, which is compromised by neurodegeneration, contributing to sleep problems, gait disturbances, and cognitive decline in PD. These clinical manifestations have their own specific patterns of the «brainstem (PPN)‐cerebellum‐cerebrum» FC alteration, what can be considered as a target for neuromodulation.

**Disclosure:** Nothing to disclose.

## EPO‐0755

### Clinical decision‐making for patients with Friedreich ataxia: Real‐world insights from a survey of health care providers in four European countries

#### A. Adarmes‐Gomez^1^; M. Graf^2^; I. Brewer^2^; N. Land^2^; J. Chou^2^; R. Lawson^3^; M. Urbich ^4^; P. Guaraldi^5^

##### ^1^Nerology, Unidad de Trastornos del Movimiento, Neurology Service, Instituto de Biomedicina de Sevilla, IBiS/Hospital Universitario Virgen del Rocío/CSIC/Universidad de Sevilla, Seville, Spain; ^2^HEOR, Precision AQ, Bethesda, USA**;** ^3^Biogen, Inc, Cambridge, USA; ^4^Biogen International GmbH, Baar, Zug, Switzerland; ^5^Nerology, IRCCS Istituto delle Scienze Neurologiche di Bologna, Bologna, Italy

**Background and aims:** Friedreich ataxia (FA) is a rare genetic neurological disease. This study assessed knowledge of FA disease characteristics and clinical decision‐making practices of European health care providers (HCPs) in real‐world clinical settings.

**Methods:** Between May‐October 2025, we conducted a cross‐sectional survey of HCPs in France, Italy, Poland, and Spain to assess disease knowledge and decision‐making practices in the management of patients with FA. HCPs were asked about prior experience with diagnosing and managing patients with FA.

**Results:** The sample included 549 HCPs comprising 135 pediatricians, 155 primary care physicians, 216 adult neurologists, and 43 pediatric neurologists. 75% of HCPs reported experience managing ≥1 patient with FA and 36% of all HCPs indicated being confident or very confident with identifying the symptoms of FA, while 36% and 25% were somewhat or not at all confident, respectively. 48% of all HCPs estimated confirming an FA diagnosis takes a minimum of 7 months, and a majority of HCPs indicated delayed FA diagnosis has an above average or very high impact on worse quality of life (QoL, 69%), delays in treatment initiation (63%), worse survival outcomes (56%), and disease progression (53%). Improving QoL was the most important treatment objective (mean rank = 2.9 out of 6), followed closely by delaying loss of ambulation (3.2) and improving function (3.4).

**Conclusion:** In our sample of HCPs, confidence with FA disease characteristics and management was limited, potentially contributing to delayed diagnoses and treatment. Across all four European countries and HCP specialties, improving QoL was seen as the most important treatment objective.

**Disclosure:** Dr Pietro Guaraldi received speaker fees and honoraria from Alnylam, AstraZeneca, Akcea Therapeutic, Biogen, Chiesi, and Theravance Biopharma and received congress and travel accommodation expense compensations from AbbVie, Alnylam, Bial, and Zambon.

## Movement Disorders 9

## EPO‐0756

### Long‐term global survival analysis from the Ferrara registry of device‐aided therapies for Parkinson's disease (ReFerDAT study)

#### P. Antenucci ^1^; F. Colucci^2^; A. Gozzi^3^; A. Mechelli^3^; D. Gragnaniello^3^; M. Pugliatti^1^; M. Paciaroni^1^; M. Sensi^3^

##### ^1^Unit of Clinical Neurology, Department of Neurosciences and Rehabilitation, University of Ferrara, Ferrara, Italy; ^2^Fondazione IRCCS Istituto Neurologico Carlo Besta, Department of Clinical Neurosciences, Parkinson and Movement Disorders Unit, Milan, Italy; ^3^Unit of Neurology, Department of Neuroscience, Sant'Anna University‐Hospital, Ferrara, Italy

**Background and aims:** Long‐term follow‐up data in advanced Parkinson's patients receiving Device‐Aided Therapy (DAT) remain limited, our objective is to assess survival and determinants over up to 15 years of therapy.

**Methods:** Clinical and demographic data of all patients who underwent DAT implantation (Deep Brain Stimulation (DBS) or Levodopa–Carbidopa Intestinal Gel (LCIG)) at the Advanced Therapies for Movement Disorders Clinic, Neurology Department of Ferrara, from 2000 to 2023, were retrospectively analysed and compared.

**Results:** A total of 207 patients underwent DAT implantation during the observation period, 141 with DBS and 66 with LCIG. LCIG patients were older at implantation and had more advanced PD, with higher dropout at follow‐up, whereas the DBS group showed a higher proportion of mortality from cardiopulmonary and infection‐related complications. Fifteen‐year Kaplan–Meier analysis showed similar mean and median survival (DBS: mean 10.99 years, median 12.0 years; Duodopa: mean 10.62 years, median 13.17 years, χ^2^ = 0.021, *p* = 0.884). Age at implantation was the only independent predictor of mortality at 10‐ and 15‐year follow‐up (10‐year: HR 1.09 per year, 95% CI 1.03–1.15, *p* = 0.003; 15‐year: HR 1.08 per year, 95% CI 1.02–1.14, *p* = 0.004) by Cox models, while DAT type, sex, preoperative Hoehn–Yahr, and time from disease onset to implantation were not significantly associated with mortality.

**Figure:**
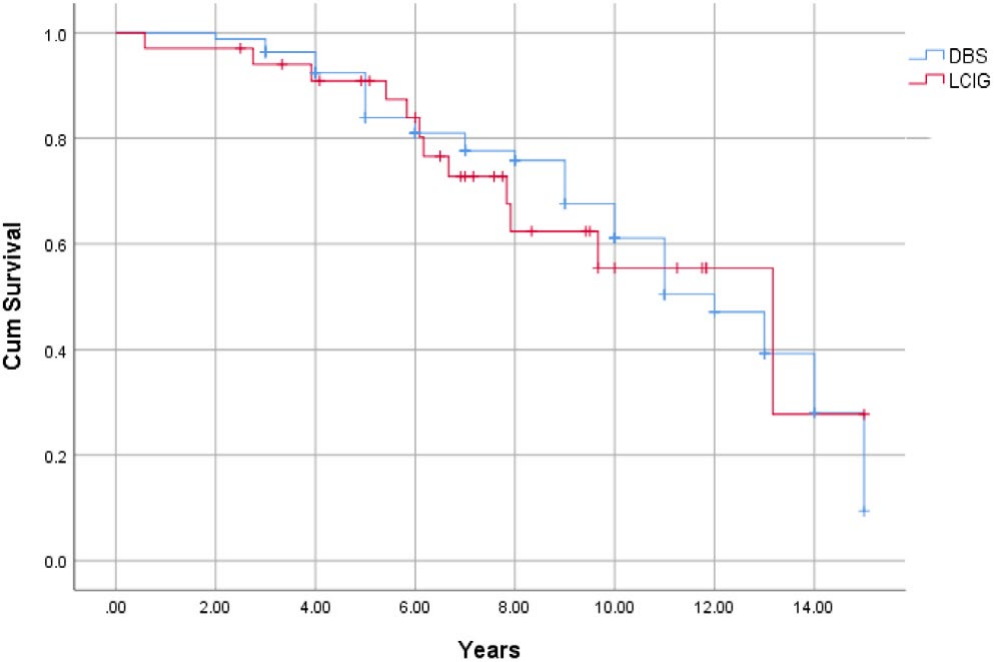
**FIGURE 1** Kaplan–Meier survival analysis over a 15‐year follow‐up comparing patients receiving Deep Brain Stimulation (DBS) and Levodopa–Carbidopa Intestinal Gel (LCIG/Duodopa). Censored observations are indicated on the curves.

**Figure:**
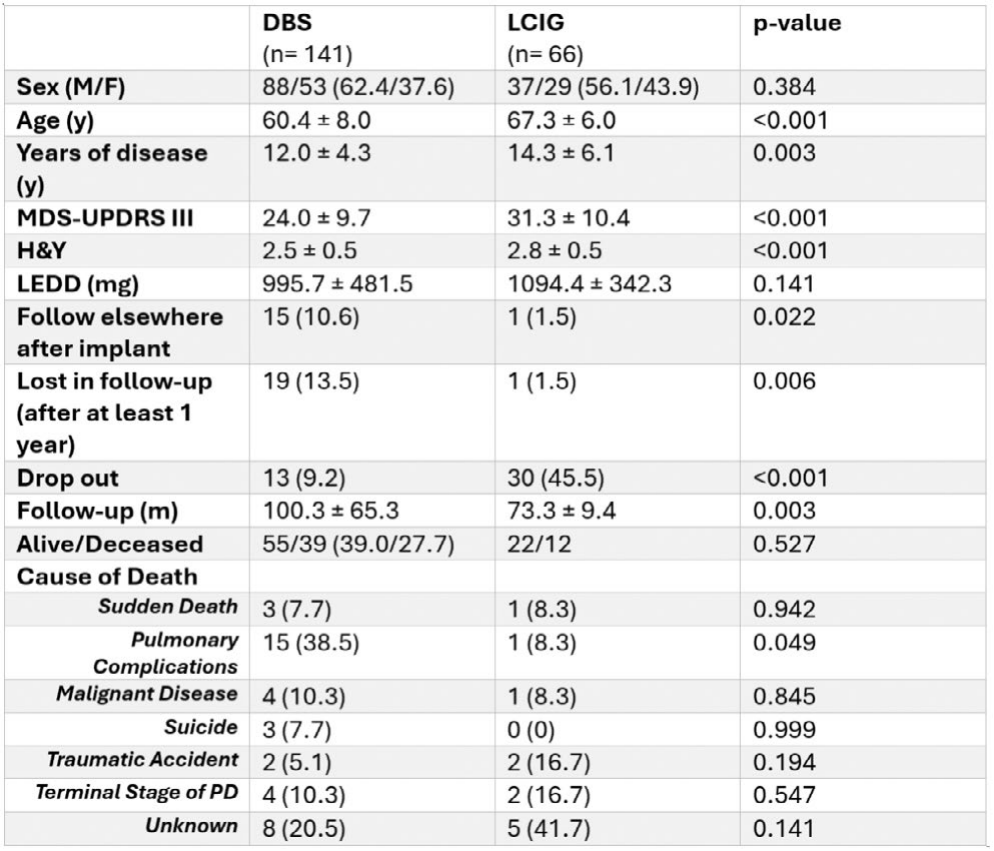
**TABLE 1** Patients characteristics: deep brain stimulation (DBS) vs Levodopa‐Carbidopa Intestinal Gel (LCIG). Data are reported as mean ± standard deviation or count (percentage). Patient characteristics and age refer to the time of implantation.

**Figure:**
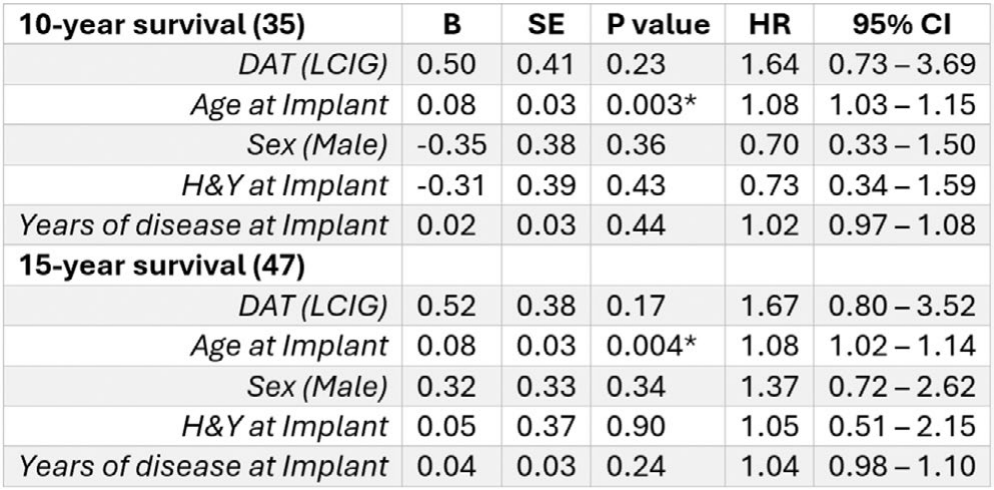
**TABLE 2** Results of Cox proportional hazards models at 10‐ and 15‐year follow‐up, number of events (deaths) shown in parentheses.

**Conclusion:** Although causes of death differ between groups, overall long‐term survival is comparable across DAT types. Age at implantation is the only factor exerting a stable long‐term effect on survival, independent of device type.

**Disclosure:** Nothing to disclose.

## EPO‐0757

### Sensor‐based kinematic markers of subthalamic deep brain stimulation efficacy in Parkinsonian tremor

#### A. Babakhani ^1^; A. Papp^1^; L. Halász^2^; L. Erőss^2^; A. Szilágyi^1^; G. Miklós^2^; G. Fekete^3^; L. Bognár^3^; M. Muthuraman^4^; G. Tamás^1^

##### ^1^Department of Neurology, Semmelweis University, Budapest, Hungary; ^2^Department of Neurosurgery and Neurointervention, Semmelweis University, Budapest, Hungary; ^3^Department of Neurosurgery, University of Debrecen, Debrecen, Hungary; ^4^Department of Neurology, Julius‐Maximilians‐Universität of Würzburg, Würzburg, Germany

**Background and aims:** Subthalamic nucleus deep brain stimulation (STN‐DBS) improves tremor in Parkinson's disease, but objective kinematic outcome markers remain limited.We quantified STN‐DBS–related changes in tremor frequency, amplitude, and rhythmicity in tremor‐dominant Parkinson's disease as objective outcome measures.

**Methods:** Thirty‐one patients with tremor‐dominant Parkinson's disease underwent bilateral STN‐DBS.Resting and postural tremor of the more affected hand were recorded with a three‐dimensional gyroscope preoperatively and at follow‐up (≥6 months). Fourier‐based analysis extracted peak tremor frequency, peak amplitude, rhythm (coefficient of variation), and tremor frequency range. Motor symptoms were assessed with the Movement Disorder Society Unified Parkinson's Disease Rating Scale (MDS‐UPDRS) part III.Active contact locations were measured relative to the subthalamic nucleus center.

**Results:** Mean age was 63.2 years (standard deviation, SD 7.67).Disease duration at surgery was 8.1 years (SD 4.37). MDS‐UPDRS part III scores improved from 15.8 (SD 10.85) to 8.3 (SD 7.07) with stimulation ON.Resting tremor scores decreased from 1.9 (SD 1.16) to 0.26 (SD 0.51).Postural tremor scores decreased from 1.3 (SD 1.14) to 0.2 (SD 0.42).Peak tremor frequency increased for resting (4.8–7.2 Hz) and postural tremor (5.3–8.0 Hz).Peak amplitude decreased by 56.2% for resting and 87.4% for postural tremor.Rhythmicity became less regular, with a 55% reduction for resting and 42% for postural tremor.

**Conclusion:** STN‐DBS in Parkinson's disease shifts tremor toward higher‐frequency, less rhythmic oscillations and improves tremor scores, especially for postural tremor.Quantitative kinematic measures provide objective markers of STN‐DBS efficacy and may support sensor‐based outcome assessment and adaptive DBS programming.

**Disclosure:** Nothing to disclose.

## EPO‐0758

### Effect of Opicapone on non‐motor burden in fluctuating PD patients: A NoMoFA prospective study

#### S. Galli^1^; E. Bianchini^1^; L. De Carolis^1^; P. pacilio^1^; L. Soccini^2^; C. Artusi^2^; D. Rinaldi ^1^

##### ^1^SAPIENZA, University of Rome, Italy; ^2^University of Verona, Verona, Italy

**Background and aims:** Non‐motor symptoms (NMS) in Parkinson's disease (PD), particularly those fluctuating with motor states (non‐motor fluctuations, NMF), significantly impact patient's quality of life but are often underrecognized and undertreated. The aim of the study is to evaluate the effect of adjunctive therapy with the COMT inhibitor Opicapone (OPC) on static and fluctuating NMS in PD patients with motor fluctuations.

**Methods:** In this prospective observational study we enrolled twenty‐one PD patients with motor fluctuations who initiated OPC 50 mg/day as add‐on therapy to levodopa. Clinical evaluations at baseline (T0) and after 8–10 weeks (T1) included the MSD‐UPDRS part III‐IV and the Non‐Motor Fluctuation Assessment (NoMoFA) Questionnaire, including the subscores pertaining to fluctuating symptoms (NoMoFA NMF), non‐fluctuating symptoms (NoMoFA NoDif). Pre/post‐treatment comparisons were analyzed using the Wilcoxon signed‐rank test.

**Results:** We observed a significant reduction in the severity of nonmotor symptoms reported in OFF and in the severity of nonmotor symptom fluctuations (NoMoFA NMF) at T1 (*p* < 0.001). No significant changes were found in the ‘static’ nonmotor symptoms (NoMoFA NoDif subscore; *p* = 0.936). The total NoMoFA score showed a trend toward improvement (median 18 to 14; *p* = 0.083).

**Figure:**
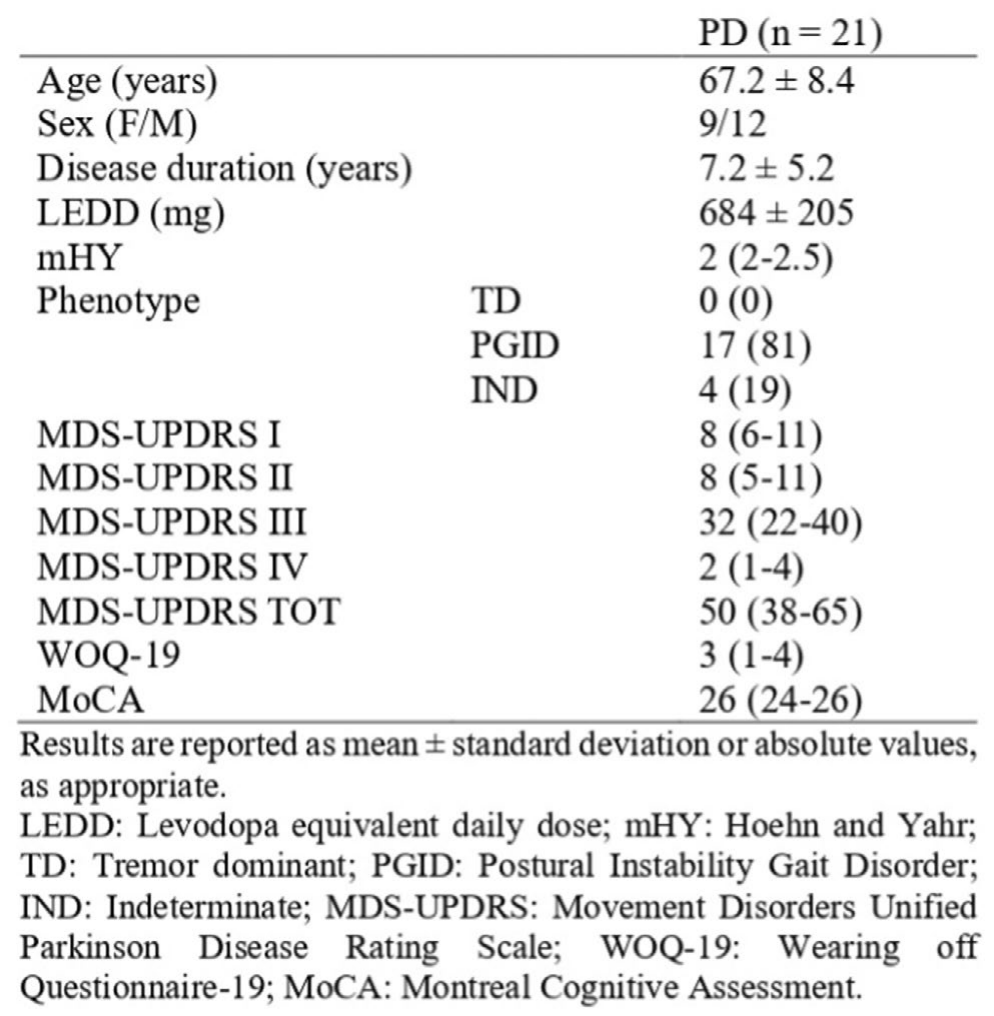
**TABLE 1** Demographic and clinical characteristics of included patients.

**Figure:**
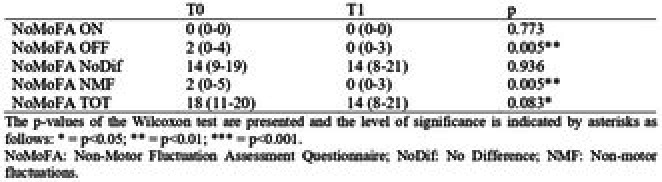
**TABLE 2** NoMoFa scores at T0 and T1.

**Figure:**
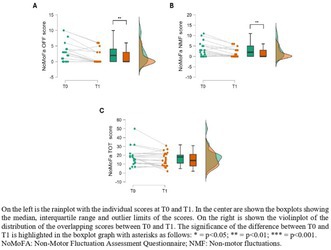
**FIGURE 1** NoMoFA scores change between T0 and T1 in OFF (A), NMF (B) and Total (C) section.

**Conclusion:** Adjunctive OPC therapy was associated with a significant reduction in fluctuating NMS, particularly during OFF periods, in PD patients with motor fluctuations. These real‐world findings support the hypothesis that improved dopaminergic stability may benefit the non‐motor domain, reducing the burden of non‐motor symptom fluctuations

**Disclosure:** Nothing to disclose.

## EPO‐0759

### Somatosensory timing captures early disease heterogeneity and predicts disease trajectory in Parkinson's disease

#### F. Aiello ^1^; M. De Bartolo^2^; M. Costanzo^3^; C. Cutrona^4^; G. Leodori^1^; F. Marchet^1^; G. Vivacqua^5^; G. Ferrazzano^1^; G. Fabbrini^1^; A. Berardelli^6^; A. Conte^1^; D. Belvisi^1^

##### ^1^Department of Human Neurosciences, Sapienza University of Rome, Rome, Italy; ^2^Department of Radiological Sciences, Oncology and Anatomical Pathology, Sapienza University of Rome, Rome, Italy; ^3^Department of Neuroscience, Istituto Superiore di Sanità, Rome, Italy; ^4^Department of Neurology and Stroke Unit, San Giovanni Addolorata Hospital, Rome, Italy; ^5^Department of Experimental Morphology and Microscopy Integrated Research Center (PRAAB), Campus Biomedico University of Rome, Italy; ^6^IRCCS Neuromed, Pozzilli (IS), Italy

**Background and aims:** The somatosensory temporal discrimination threshold (STDT) is a neurophysiological parameter reflecting the shortest time interval between two tactile stimuli required to perceive them as temporally separate. STDT is a stage‐, severity‐ and disease duration‐dependent parameter, possibly reflecting dopaminergic degeneration. However, its long‐term evolution and predictive value remain underexplored. We aimed to investigate longitudinal STDT changes and its potential to predict clinical progression in PD and discriminate divergent disease trajectories.

**Methods:** We conducted a 4‐year longitudinal study involving 60 early‐stage PD patients and 50 healthy controls (HC). In PD patients, clinical and STDT assessments were performed at baseline and follow‐up. STDT was measured at the distal phalanx of the index finger using an ascending stepwise paradigm. Longitudinal changes, correlations between STDT and clinical measures, regression analyses for progression prediction, and k‐means clustering based on baseline STDT were performed.

**Results:** At baseline, STDT values were similar between patients and HC, but significantly worsened over time in PD. Initially associated with REM sleep behaviour disorder (RBD) symptoms, STDT later correlated with cognitive decline and predicted motor progression. STDT‐based clustering identified two subtypes: cluster 1, defined by higher baseline STDT and more severe RBD, exhibited greater motor and non‐motor burden and faster progression at 4‐year follow‐up than cluster 2.

**Figure:**
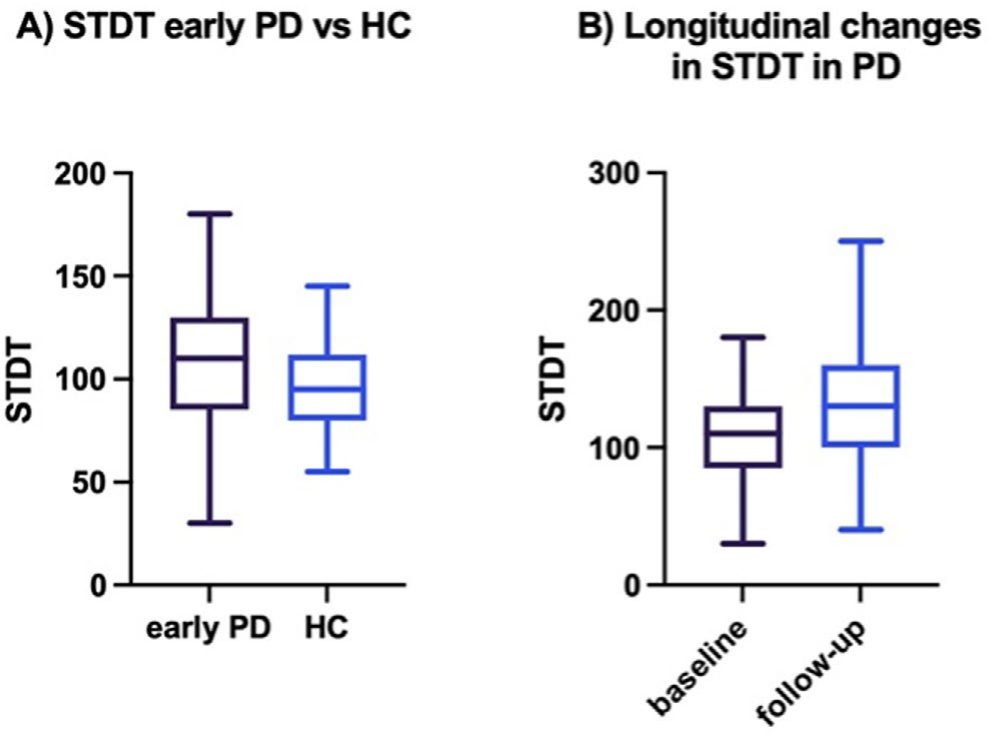
**FIGURE 1** Differences in Somatosensory Discrimination Threshold among groups: (A) Comparison of STDT values between early PD patients and HC; (B) Longitudinal changes of STDT in PD patients.

**Figure:**
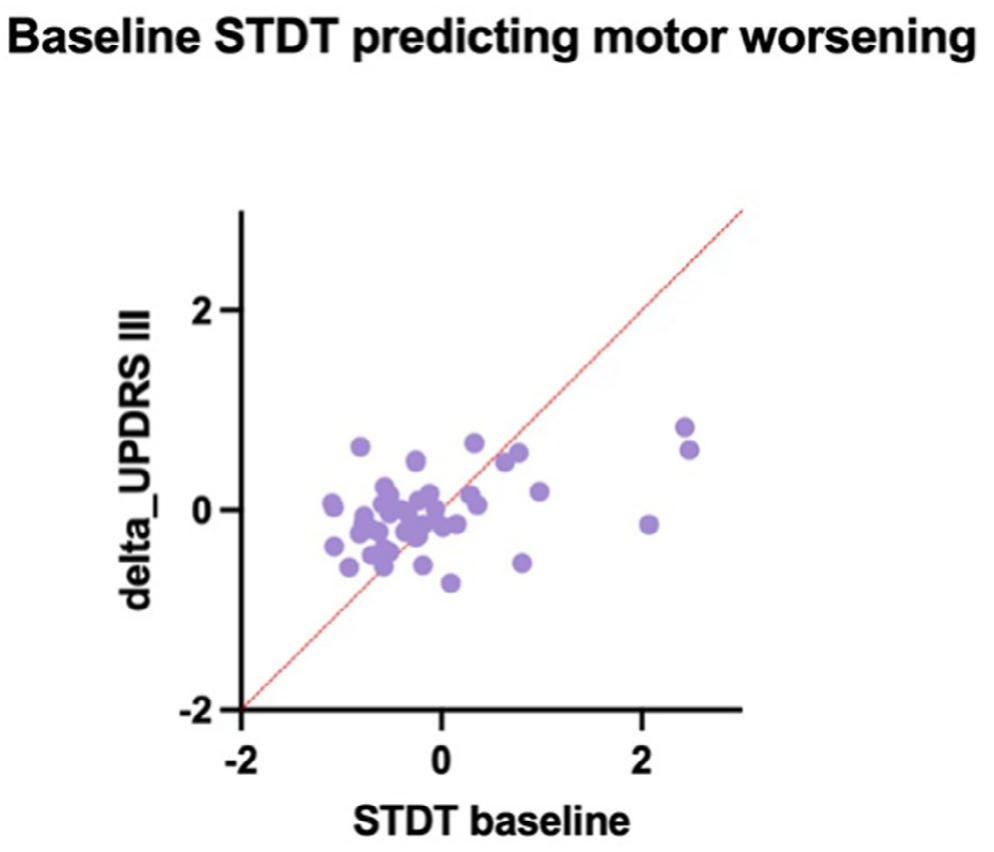
**FIGURE 2** Baseline STDT values predict motor symptom progression in Parkinson's disease. Scatter plot showing the relationship between STDT z‐scores at baseline and changes in UPDRS III scores (delta_UPDRS III) over a 4‐year follow‐up in PD patients.

**Figure:**
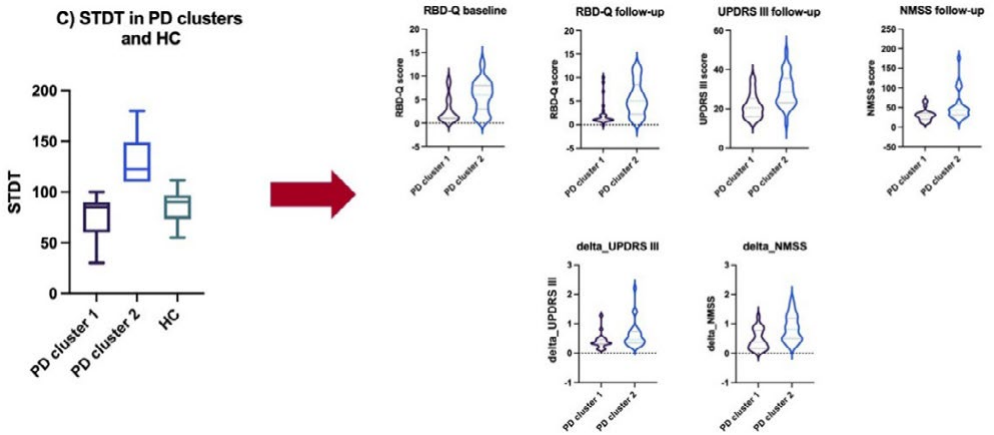
**FIGURE 3** Significant clinical differences between PD clusters. Box and violin box illustrate significant differences between PD cluster 1 and cluster 2 patients in clinical scores at baseline, follow‐up and in the progression rate.

**Conclusion:** Early STDT differentiates patients with distinct clinical profiles and divergent disease trajectories, capturing early inter‐individual variability and predicting long‐term motor outcomes in PD. STDT‐based clustering aligns with brain‐first vs body‐first models, supporting its value in early PD stratification.

**Disclosure:** The authors declare no financial disclosures relevant to the present manuscript. Over the past twelve months, A.C. has received speaker honoraria from Roche, Biogen, Novartis, Almirall and Merck, all unrelated to the content of this manuscript.

## EPO‐0760

### Longitudinal assessment of smartphone‐based motor and speech biomarkers in isolated REM sleep behaviour and Parkinson's disease

#### V. Illner^1^; M. Novotny^1^; T. Kouba^1^; T. Tykalova^1^; M. Simek^1^; P. Sovka^1^; J. Svihlik^1^; E. Ruzicka^2^; K. Sonka^2^; P. Dusek^2^; J. Rusz ^1^

##### ^1^Department of Circuit Theory, Faculty of Electrical Engineering, Czech Technical University in Prague, Prague, Czech Republic; ^2^Department of Neurology and Centre of Clinical Neuroscience, First Faculty of Medicine, Charles University and General University Hospital, Prague, Czech Republic

**Background and aims:** The sensitivity of smartphone assessments to disease progression in isolated rapid eye movement sleep behaviour disorder (iRBD) and Parkinson's disease (PD) remains unexplored.

**Methods:** Participants completed a set of active smartphone tasks every 14 days over a two‐year period in their home environments. The cohorts included 23 patients with iRBD, 24 patients with early PD, and 26 healthy controls. The tasks comprised (i) speech assessments (sustained vowel phonation, rapid syllable repetition, and reading a passage), (ii) psychomotor assessments (tapping speed, alternating tapping, and word typing), (iii) a tremor assessment (holding the phone with an outstretched arm), and (iv) a walking turnaround task.

**Results:** Most tasks, including sustained vowel phonation, rapid syllable repetition, tapping speed, alternating tapping, and the tremor task, demonstrated strong learning effects, limiting their ability to differentiate between groups or detect progression. Word typing with the dominant hand showed group differentiation (iRBD and PD vs. controls; *p* < 0.05). Measures of monopitch during reading and turnaround walking performance exhibited significant longitudinal worsening in the iRBD group (*p* < 0.05).

**Conclusion:** Measures capturing monotone speech and walking turnaround performance show potential for inclusion in future clinical trials in iRBD. Most simple active tasks demonstrated limited sensitivity to disease progression due to pronounced learning effects.

**Disclosure:** This project was supported by the Czech Ministry of Health (grant no. NW24‐04‐00211) and the ERC–CZ program from the Ministry of Education Youth and Sports (project no. LL2504).

## EPO‐0761

### Assessing dysarthria severity via long‐term average spectrum in neurological diseases

#### M. Simek^1^; T. Tykalova^1^; J. Klempir^2^; J. Rusz^1^; J. Svihlik ^3^

##### ^1^Department of Circuit Theory, Faculty of Electrical Engineering, Czech Technical University in Prague, Prague, Czech Republic; ^2^Department of Neurology and Centre of Clinical Neuroscience, First Faculty of Medicine, Charles University and General University Hospital, Prague, Czech Republic; ^3^Faculty of Electrical Engineering, CTU Prague, Faculty of Chemical Engineering, UCT Prague, Czech Republic

**Background and aims:** Speech is one of the most complex quantitative markers of motor function and is often affected already in most progressive neurological diseases. This study aimed to evaluate sensitivity of long‐term averaged spectrum (LTAS) descriptors computed for reading passage in a broad range of neurological diseases and various types and severities of dysarthria.

**Methods:** Four spectral moments (spectral mean, spectral standard deviation, spectral skewness and spectral kurtosis) based on LTAS were computed for reading passage collected from 462 speakers, including 306 healthy controls and 156 neurological patients secondary to multiple sclerosis, Parkinson's disease, supranuclear palsy, multiple‐system atrophy, Huntington's disease, essential tremor, cerebellar ataxia, and amyotrophic lateral sclerosis. One‐way analysis of covariance with post hoc Fisher's least significant difference test was applied.

**Results:** Compared to controls, Parkinson's disease showed lower mean (*p* = 0.015) and higher skewness (*p* = 0.014) and kurtosis (*p* = 0.01). Among parkinsonian groups, multiple system atrophy also had lower mean (*p* = 0.042). Conversely, mean was higher (*p* = 0.006) and skewness lower (*p* = 0.036) for cerebellar ataxia. Multiple sclerosis demonstrated only a lower mean (*p* = 0.023). Among diseases, mean (*p <* 0.001), skewness (*p <* 0.001) and kurtosis (*p* = 0.008) were able to separate cerebellar ataxia and multiple system atrophy.

**Conclusion:** Our results confirmed LTAS as a suitable approach to objectively describe speech changes due to neurological diseases. Furthermore, spectral moments were able to significantly separate diseases with similar clinical manifestations, which could have important implications for differential diagnosis.

**Disclosure:** The project was supported by the Czech Ministry of Health, project number NW24‐04‐00211 and by the ERC–CZ program from the Ministry of Education Youth and Sports, project number LL2504.

## EPO‐0762

### Artificial intelligence–driven identification of Parkinson's disease using oculometrics

#### E. Han^1^; A. Coito^2^; H. Fadavi^3^; B. Hauser^2^; P. Massatsch^2^; V. Stozitzky^4^; D. Li^4^; B. Balint^1^; H. Dawes^3^; K. Weber ^1^

##### ^1^University Hospital of Zurich, Switzerland; ^2^machineMD, Switzerland; ^3^University of Exeter, UK; ^4^gaitQ, UK

**Background and aims:** Parkinson's disease (PD) diagnosis remains largely clinical, motivating the search for objective biomarkers to support diagnosis, early detection, and disease monitoring. Eye movement and pupillary abnormalities in PD can be quantified as oculometrics and provide objective features for data‐driven analysis. We aimed to develop and evaluate artificial intelligence (AI) models for PD detection using quantitative ocular motor and pupillary measurements acquired with a virtual reality (VR)–based medical eye‐tracker.

**Methods:** Eighty‐one individuals with idiopathic PD were recruited at clinical centres in Zurich (Switzerland) and Exeter (UK). Participants completed two study visits including standardized VR‐based ocular motor assessments and routine clinical examinations. The analysis included 132 PD examinations and 148 examinations from an independent healthy control cohort. Each PD examination was matched to an age‐ and sex‐matched control using a globally optimal matching algorithm. Group differences were assessed using standardized mean differences (SMDs) and Welch's t‐tests. A supervised machine learning classifier was trained using the most discriminative oculometric features, with site‐specific performance evaluation.

**Results:** The largest PD–control differences were observed in saccadic parameters (accuracy, peak velocity, and main sequence), vergence measures, ocular alignment, and pupillary dynamics (constriction and dilation velocity and latency). Classification performance was high across sites: Zurich—AUC 0.96 (sensitivity 0.96; specificity 0.89); Exeter—AUC 0.91 (F1‐score 0.84; sensitivity 0.89; specificity 0.79).

**Conclusion:** VR‐derived oculometrics can help differentiate individuals with PD from healthy controls, supporting the potential of an oculomics‐based framework as an objective biomarker approach for PD. Further work focuses on cohort expansion, cross‐site data integration, and correlation with clinical disease measures.

**Disclosure:** Ana Coito Employee of machineMD, Switzerland. No other disclosures reported. Erika Han No disclosures to report. Hassan Fadavi No disclosures to report. Bruno Hauser Employee of machineMD, Switzerland. No other disclosures reported. Pia Massatsch Employee of machineMD, Switzerland. No other disclosures reported. Valentina Stozitzky Employee of gaitQ, UK. No other disclosures reported. Dongli Li Employee of gaitQ, UK. No other disclosures reported. Bettina Balint No disclosures to report. Helen Dawes No disclosures to report. Konrad *P*. Weber No disclosures to report.

## EPO‐0763

### Morbidities and estimated prodromal Parkinson's disease risk in the healthy brain aging (HeBA) study

#### M. De la Cruz‐Puebla ^1^; D. Pilco‐Janeta^1^; A. Garrido^1^; F. Farfan^1^; P. Mahlknecht^2^; C. Horlings^2^; C. Theyer^2^; S. Leiter^2^; I. Egner^2^; S. Ghosh^3^; K. Rege^3^; V. Satagopam^3^; K. Seppi^3^; W. Poewe^3^; R. Krüger^3^; B. Mollenhauer^3^; E. Tolosa^1^; M. Martí^1^

##### ^1^Parkinson's Disease and Movement Disorders Unit, Neurology Service, Hospital Clínic de Barcelona, Barcelona, Spain; ^2^Medical University of Innsbruck, Department of Neurology, Innsbruck, Innsbruck, Austria; ^3^University of Luxembourg, Luxembourg Centre for Systems Biomedicine, Esch‐sur‐Alzette, Luxembourg

**Background and aims:** Parkinson's disease (PD) has a prodromal phase characterized by non‐motor symptoms and clinical features that can be integrated into probabilistic risk estimates. Data on medical and neurological comorbidities across levels of estimated prodromal PD risk remain limited.

**Methods:** We conducted a cross‐sectional analysis within the Healthy Brain Aging (HeBA) study, an online population‐based cohort. Prodromal PD risk was estimated using the Movement Disorder Society probabilistic research criteria. Participants were classified as high (MDS score >30%) or low (MDS score <1%) risk and matched 1:1 by age, sex, and recruitment site (*n* = 326 total; 163 high‐risk and 163 low‐risk participants). Type II Diabetes mellitus and depression/anxiety were excluded from the risk calculation and analyzed separately. Medical and neurological conditions and clinical symptom scores were compared between high and low risk groups.

**Results:** High‐risk participants had more medical conditions, including hypertension (58.9% vs. 30.1%), depression/anxiety (55.8% vs. 14.1%), hypercholesterolemia (52.1% vs. 30.1%), diabetes mellitus (20.2% vs. 8.0%), and peripheral vascular disease (14.1% vs. 3.1%) (all *p* < 0.01). Restless legs syndrome (17.8% vs. 0.6%) and essential tremor (8.6% vs. 1.2%) were also more frequent. Multimorbidity increased the odds of high‐risk classification (odds ratios: 3.47 for two conditions, 12.38 for three, and 19.50 for > 4). Clinical symptom scores including NMS total, GDS, UPDRS‐II, and Quality of Life (VAS), differed significantly between groups (all *p* < 0.001).

**Figure:**
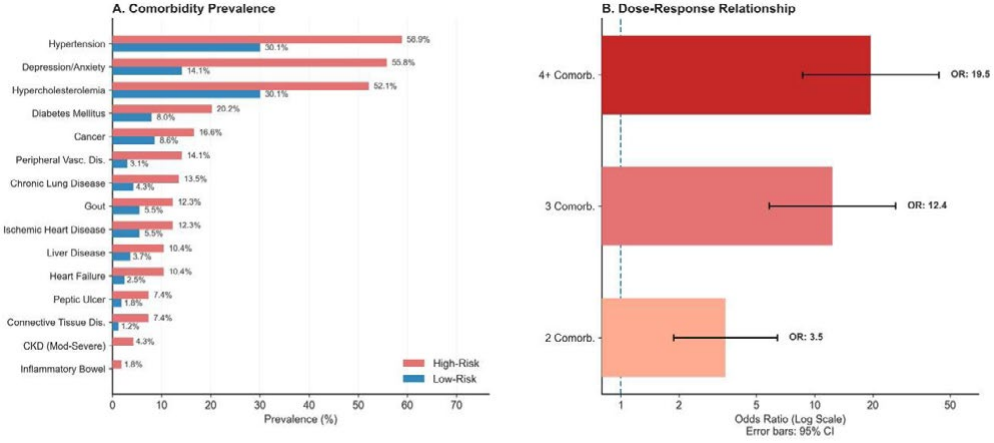
**FIGURE 1** Morbidity phenotype associated with prodromal Parkinson's disease risk. (A) Prevalence of selected medical conditions by risk group. (B) Dose–response association between morbidity burden and High‐Risk status, expressed as odds ratios.

**Conclusion:** Higher estimated prodromal PD risk was associated with increased multimorbidity and worse clinical symptom profiles, according to the scales used, in individuals without a PD diagnosis.

**Disclosure:** The HeBA study is supported by a grant from The Michael J. Fox Foundation for Parkinson's Research (grant MJFF‐PI047794). All authors report no disclosures related to this work.

## EPO‐0764

### Language as an early indicator of cognitive impairment in Parkinson's disease: Development and validation of an AI‐based application

#### L. Justi; L. Uhlemann; S. Brodoehl

##### Biomagnetic Center, Department of Neurology, Jena University Hospital, Jena, Germany

**Background and aims:** Parkinson's disease (PD) is not only defined by motor symptoms but also by early changes in speech that affect linguistic, semantic and executive functions. Because speech production engages widespread neural networks, subtle alterations can signal early cognitive decline. With recent advances in artificial intelligence (AI), these patterns can now be captured and analyzed automatically, making speech a promising non‐invasive marker of cognitive impairment in PD.

**Methods:** We developed an automated pipeline that records speech, transcribes it using Whisper AI, and analyzes linguistic and semantic features with spaCy and an OpenAI large language model. Extracted markers included word count, sentence structure, verb–noun ratios, semantic coherence and content complexity. These features were used to train three machine‐learning models—Support Vector Classifier, Random Forest Classifier and Logistic Regression—to differentiate patients with cognitive impairment (MoCA < 24) from those without impairment (MoCA ≥ 24). The entire workflow was implemented in a web‐based application for standardized and scalable data processing.

**Results:** Automated linguistic measures showed a high degree of concordance with manually obtained reference values, confirming the validity of the pipeline. All machine‐learning models demonstrated good discriminative performance. Logistic Regression performed best, achieving an AUC‐ROC of 0.804 and showing the most robust classification across repeated evaluations. The integrated web application processed recordings reliably and consistently.

**Figure:**
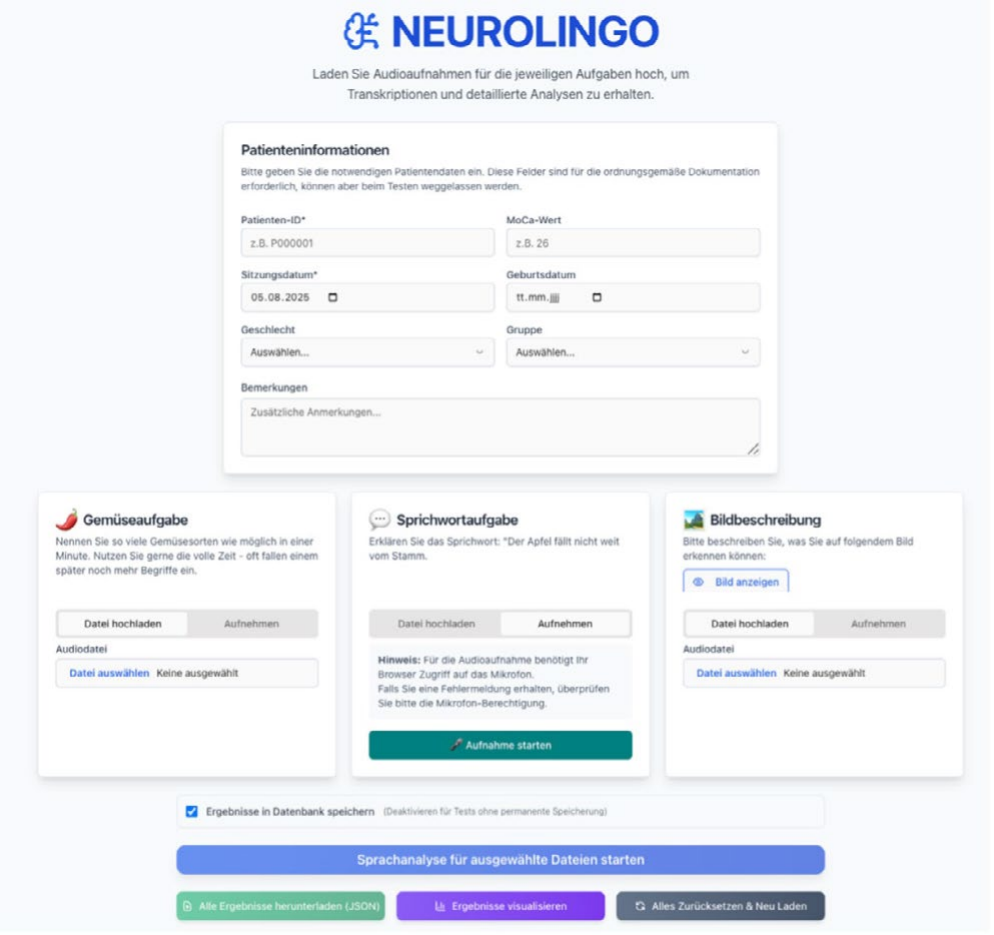
**FIGURE 1** Screenshot of the web‐based application prototype showing audio input, automated transcription, feature extraction, and model output.

**Figure:**
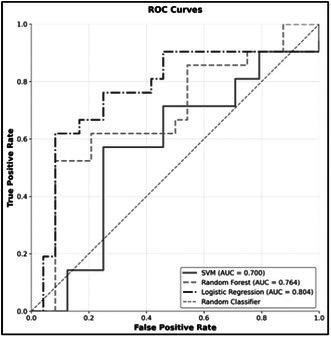
**FIGURE 2** ROC curves for all ML models, with Logistic Regression achieving the highest AUC (0.804).

**Figure:**
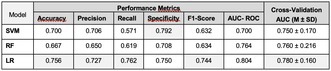
**TABLE 1** Performance metrics of the trained Support Vector Machine, Random Forest, and Logistic Regression models. Shown are accuracy, precision, recall, specificity, F1‐score, AUC‐ROC, and the mean AUC values from the stratified 5‐fold cross‐validation.

**Conclusion:** Our study suggests that AI‐based speech analysis can reliably detect speech alterations that indicate cognitive impairment in Parkinson's disease. The validated pipeline and its integration into a web application support its potential as a scalable, non‐invasive screening tool.

**Disclosure:** Nothing to disclose.

## EPO‐0765

### Abstract withdrawn

## EPO‐0766

### Cortical oscillatory and connectivity profiles of stimulation‐induced bradykinesia in dystonia

#### A. Mak^1^; B. Tenge‐Bahners^2^; D. Aravitska^2^; H. Voss^1^; L. Feldmann^1^; L. Goede^1^; A. de Almeida Marcellino^1^; W. Neumann^1^; T. Sander^3^; E. Florin^2^; A. Kühn^1^; R. Lofredi ^1^

##### ^1^Charité – Universitätsmedizin, Berlin, Germany; ^2^Heinrich‐Heine‐Univeristät, Düsseldorf, Germany; ^3^Physikalisch Technische Bundesanstalt, Berlin, Germany

**Background and aims:** Pallidal neuromodulation is an established treatment for dystonia, likely acting through modulation of distributed brain networks and their oscillatory dynamics. However, in some patients, pallidal deep brain stimulation (DBS) induces bradykinesia. While this adverse effect has been linked to local stimulation sites and oscillatory changes, its network‐level correlates remain unclear.

**Methods:** We recorded whole‐head magnetoencephalography (MEG) in 16 dystonia patients with pallidal DBS at rest with stimulation ON and after a ≥30‐minute washout (DBS OFF). In both conditions, patients performed a video‐recorded finger‐tapping task. Kinematics were extracted using computer vision–based pose estimation. DBS electrodes were localized using Lead‐DBS, and functional connectivity from active contacts was estimated using a normative fMRI connectome (*n* = 1000). Connectivity profiles were correlated with DBS‐induced changes in tapping frequency. To assess oscillatory network effects, we computed cortical spectral power (theta 4–8 Hz, alpha 8–12 Hz, beta 13–30 Hz, gamma 40–90 Hz) and related whole‐cortex power changes to movement slowing.

**Results:** At the group level, DBS did not significantly alter tapping frequency, indicating marked interindividual variability. Across patients, however, the connectivity profile of the active contact predicted DBS‐induced slowing (*R* = 0.45, *p* = 0.04): stronger connectivity to sensorimotor and supplementary motor areas was associated with greater bradykinesia. Cortical modulation in the alpha and gamma bands explained 50% (*R* = 0.77, *p* = 0.001) and 31% (*R* = 0.56, *p* = 0.01) of the variance, respectively. These signatures were spatially and spectrally distinct from those associated with therapeutic DBS effects, suggesting separable network mechanisms.

**Conclusion:** Integrating connectivity and oscillatory profiling may support individualized DBS programming to minimize motor side effects.

**Disclosure:** Nothing to disclose.

## EPO‐0767

### Abstract withdrawn

## Movement Disorders 10

## EPO‐0768

### Efficacy and safety of magnetic resonance‐guided focused ultrasound thalamotomy in essential tremor: A systematic review and meta‐analysis

#### A. Shiramba ^1^; S. Lane^2^; N. Ray^3^; T. Gilbertson^4^; R. Srinivasaiah^5^; J. Panicker^5^; M. Radon^5^; J. Osman‐Farah^5^; A. Macerollo^6^

##### ^1^School of Medicine, University of Liverpool, Liverpool, UK; ^*2*^Institute of Data Health Sciences, University of Liverpool, Liverpool, UK; ^*3*^Department of Psychology, Manchester Metropolitan University, Manchester, UK; ^*4*^Department of Neurology, Ninewells Hospital & Medical School, Dundee, UK; ^*5*^The Walton Centre NHS Foundation Trust for Neurology and Neurosurgery, Liverpool, UK; ^*6*^Institute of Systems, Molecular and Integrative Biology, The University of Liverpool, Liverpool, UK

**Background and aims:** Magnetic resonance‐guided focused ultrasound is a surgical intervention for medication‐refractory essential tremor, however, meta‐analyses have been limited in their exploration of the durability of the treatment effect. This study aimed to assess the treatment effect and safety of this procedure over time. Different to other meta‐analyses, this study assessed treatment effect durability over time from 1 month to 5 years follow‐up, as well as tremor recurrence.

**Methods:** A systematic search of the literature using set criteria was conducted via PubMed, Scopus, Web of Science, and Cochrane library databases, in accordance with the Preferred Reporting Items for Systematic Reviews and Meta‐Analyses (PRISMA) guidelines. Data analysis was conducted in R, utilising a random‐effects model for meta‐analysis and a mixed‐effects model for meta‐regression.

**Results:** 45 studies met the inclusion criteria, with 42 included in analyses. Significant changes in hand tremor, total tremor, disability scores, and quality of life scores were demonstrated across the time points investigated, the pooled standardized mean differences were −2.36 (*p* < 0.0001), −2.08 (*p* < 0.0001), −2.85 (*p* < 0.0001), and −1.41 (*p* < 0.0001) 1 year post‐operation. Sensory symptoms and unsteadiness adverse events were frequently observed, with pooled proportions of 22% (95% CI 15%; 31%) and 23% (95% CI 16%; 31%) 1 month post‐operation.

**Conclusion:** Although the procedure demonstrated efficacy and safety across the studies evaluated, meta‐regression analysis suggests a decrease in treatment effect over time that requires further investigation.

**Disclosure:** Nothing to disclose.

## EPO‐0769

### Multi‐target deep brain stimulation for dystonia and complex tremor syndromes: A single‐centre case series and literature review

#### A. Shiramba ^1^; J. Panicker^2^; D. Damodaran^2^; D. Bhargava^2^; J. Osman Farah^2^; A. Macerollo^3^

##### ^1^School of Medicine, University of Liverpool, Liverpool UK; ^2^The Walton Centre NHS Foundation Trust for Neurology and Neurosurgery, Liverpool, UK; ^3^Institute of Systems, Molecular and Integrative Biology, The University of Liverpool, UK

**Background and aims:** Deep Brain Stimulation (DBS) is an established neurosurgical procedure for pharmacologically refractory dystonia and complex tremor syndromes. Multiple target stimulation, such as internal globus pallidus (GPi) and ventral intermediate nucleus (VIM) stimulation, can be used to achieve the control of dystonic features as well as the severe tremor at the same time. This clinical study aims to highlight 10 cases treated at the Walton Centre NHS Foundation Trust (Liverpool, UK) wherein DBS has been implemented beyond unilateral and singular target procedures. It describes the immediate and long‐term response and effects of multiple‐target DBS in patients with dystonia and complex tremor syndromes (including Holmes tremor).

**Methods:** Case notes were reviewed retrospectively for the included patients. Data were collected from baseline and subsequent appointments, including treatment details, quantitative and qualitative findings, and side effects. Metrics included the cervical dystonia scale TWSTRS, Fahn‐Marsden dystonia scale, FTM scale for tremor, EQ‐5D for quality of life, and HADS Anxiety and HADS Depression scores.

**Results:** Our cohort experienced symptom relief following multi‐target DBS, with varying levels of symptom suppression and stimulation‐related side effects (i.e. slurred speech, gait disturbance); stimulation adjustments were made accordingly. Importantly, there were discrepancies in objective clinical improvement and perceived symptom relief by the patients.

**Conclusion:** Multi‐target DBS can be useful in managing dystonia and complex tremor cases; it requires balancing symptoms improvement with side effects through continued follow‐up and setting adjustments. Tremor suppression and side effect profiles are patient dependent, with varying levels of success demonstrated in our case series.

**Disclosure:** Nothing to disclose.

## EPO‐0770

### Early COMT inhibition improves motor stability and real‐world mobility in Parkinson's disease: A randomized single‐blind study with digital biomarkers

#### A. Zampogna ^1^; E. Fusco^2^; M. Patera^2^; M. Falletti^2^; V. Bellia^2^; L. Longo^2^; C. Pauletti^2^; A. Suppa^1^

##### ^1^Department of Human Neurosciences, Sapienza University of Rome, Italy; ^2^Department of Human Neurosciences, Sapienza University of Rome, Italy

**Background and aims:** Whether early COMT inhibition provides superior real‐world mobility compared with levodopa dose escalation in patients with Parkinson's disease (PD) and emerging motor fluctuations remains unclear. This study compared the efficacy of early add‐on therapy with Opicapone versus an additional levodopa dose, using clinician‐rated outcomes (CROs), patient‐reported outcome measures (PROMs), and digital biomarkers.

**Methods:** Forty‐six PD patients were randomized into two clinically‐matched groups (*n* = 23 each) receiving either add‐on Opicapone 50 mg or an additional levodopa/carbidopa dose (100/25 mg). Single‐blind clinical evaluations were performed at baseline and after one month using CROs (MDS‐UPDRS III, CGI‐C) and PROMs (WOQ‐19, PDQ‐9, PGI‐C). Also, home monitoring with a validated waist‐worn wearable sensor provided digital biomarkers of daily steps, step length, gait velocity, cadence, their coefficients of variation (CV), and estimated ON/OFF time.

**Results:** While CROs did not show between‐group differences after treatment, PROMs and digital biomarkers consistently indicated that patients receiving Opicapone had reduced fluctuation severity (WOQ‐19), greater perceived improvement (PGI‐C), longer objective daily ON time, and more stable gait performance (step length, gait velocity, and cadence CVs) than those taking an additional levodopa dose (Figure 1). MDS‐UPDRS III scores correlated with daily ON/OFF time and objective gait measures, indicating worse real‐world mobility with increasing motor severity (Figure 2).

**Figure:**
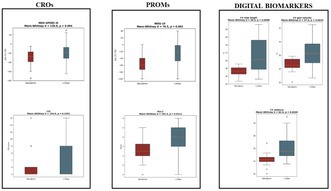
**FIGURE 1** Main findings from clinician‐rated outcomes (CROs), patient‐reported outcome measures (PROMs), and digital biomarkers.

**Figure:**
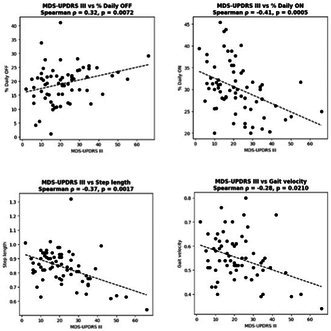
**FIGURE 2** Main clinical–behavioural correlations.

**Conclusion:** Early add‐on therapy with Opicapone appears more effective than an additional levodopa dose in improving motor stability and patient‐perceived well‐being in PD patients with emerging motor fluctuations. While CROs may fail to capture changes in long‐term motor phenomena, digital biomarkers provide an ecologically valid assessment of real‐world motor behaviours.

**Disclosure:** Nothing to disclose.

## EPO‐0771

### Cognitive performances are associated with subclinical gait alterations in drug‐naive cognitively unimpaired patients with Parkinson's disease

#### C. Cabato ^1^; V. Sant’Elia^1^; N. Piramide^1^; R. De Micco^1^; O. Ciaramaglia^1^; M. Siciliano^1^; M. Siciliano^2^; F. Ambrosio^1^; L. Donisi^1^; F. Esposito^1^; A. Tessitore^1^

##### ^1^Department of Advanced Medical and Surgical Sciences, University of Campania “Luigi Vanvitelli”, Naples, Italy; ^2^Neuropsychology Laboratory, Department of Psychology, University of Campania “Luigi Vanvitelli”, Caserta, Italy

**Background and aims:** Gait impairment is the most disabling symptom in Parkinson's disease (PD) patients and is associated with high falling risk, worse disease progression and reduced survival. Cognitive impairment has been consistently associated with impaired walking ability and higher fall risk in PD. Detection of subclinical gait alterations could identify early PD patients at risk to develop cognitive impairment.

**Methods:** Thirty‐four drug‐naive cognitively unimpaired PD patients (disease duration ≤3 years, Hoehn & Yahr ≤2.5) and 20 age‐ and sex‐matched healthy controls were consecutively enrolled. All patients underwent a II‐level neuropsychological assessment to exclude cognitive impairment. Motor symptoms were evaluated using the Unified Parkinson's Disease Rating Scale part III (UPDRS‐III). Gait and balance parameters were acquired using six Opal V2R wearable sensors when performing the Timed up and go test in single and dual task (ST/DT) conditions. Bivariate correlation analysis between clinical and wearable sensors metrics was performed.

**Results:** Worse gait and postural parameters detected by wearable sensors in ST/DT conditions significantly correlated with cognitive tests exploring visuo‐spatial processing, psychomotor coordination and executive functions involved in retrieving words and information recalling. No correlations have been found between cognitive tests and UPDRS‐III scores.

**Conclusion:** Subclinical gait and postural changes are already associated with cognitive performances in PD patients with normal cognition. Wearable sensors could provide useful information to characterize early PD patients, even when gait and postural disturbances are not clinically detectable. Integrating clinical, neuropsychological and digital data may identify patients more prone to develop clinical overt cognitive impairment useful for prevention strategies.

**Disclosure:** Nothing to disclose.

## EPO‐0772

### Neurophysiological markers of motor compensatory mechanisms in early Parkinson's disease: **A** 2‐year longitudinal study

#### F. Pirone ^1^; R. Cilia^1^; M. Passaretti^2^; M. Bologna^2^; A. Braccia^1^; F. Colucci^1^; G. Gaudiano^1^; N. Golfrè Andreasi^1^; M. Tosi^1^; S. Rinaldo^1^; L. Scolari^1^; E. Orunesu^3^; V. Leta^1^; G. Devigili^1^; A. Elia^1^; L. Romito^1^; A. Berardelli^2^; R. Eleopra^1^

##### ^1^Parkinson and Movement Disorders Unit, Department of Clinical Neurosciences, Fondazione IRCCS Istituto Neurologico Carlo Besta, Milan, Italy; ^2^Department of Human Neurosciences, Sapienza University of Rome, Rome, Italy; ^3^Nuclear Medicine Unit, Fondazione IRCCS Cà Granda, Ospedale Maggiore Policlinico, Milan, Italy

**Background and aims:** Parkinson's disease (PD) often shows asymmetric motor onset despite bilateral nigrostriatal degeneration, suggesting cortical compensation. To investigate 24‐month longitudinal clinical and TMS changes in early PD with unilateral motor signs, comparing stable versus progressive patients.

**Methods:** Following our previous study [1], 16 PD patients with unilateral motor signs and bilateral putaminal DAT‐SPECT loss, and 28 healthy controls (HC), were assessed at baseline; 13 completed follow‐up. Clinical scales and bilateral TMS measures of corticospinal excitability, intracortical excitability, interhemispheric inhibition, and paired associative stimulation were collected.

**Figure:**
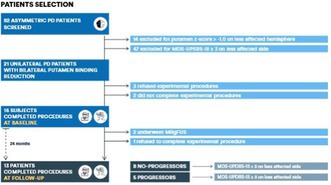
**FIGURE 1** Screening and selection of Parkinson's disease patients. The initial cohort of 82 subjects had already been screened for other clinical exclusion criteria.

**Results:** Motor disability increased with reduced asymmetry [2]; 5 out of the original 13 patients with unilateral motor signs converted to bilateral involvement (defined as ‘progressors’; presymptomatic‐side MDS‐UPDRS‐III ≥3), while others remained unilateral (‘non‐progressors’). Compared with HC, PD showed increased corticospinal excitability in both hemispheres and reduced intracortical facilitation. In the total PD cohort, presymptomatic hemispheres showed longitudinal motor worsening, accompanied by increased corticospinal excitability (RMT reduction *p* = 0.003) and reduced intracortical facilitation (ICF *p* = 0.013; slope *p* = 0.039). In progressors, presymptomatic hemispheres converting to symptomatic showed increased RMT (*p* = 0.042), reduced ICF (*p* = 0.043), flattening of the intracortical slope (*p* = 0.043), and loss of long‐interval IHI (*p* = 0.043). In non‐progressors, the presymptomatic hemisphere showed increased excitability (RMT reduction *p* = 0.018) with SICI enhancement (*p* = 0.050), while ICF, IHI, and motor scores remained stable.

**Figure:**
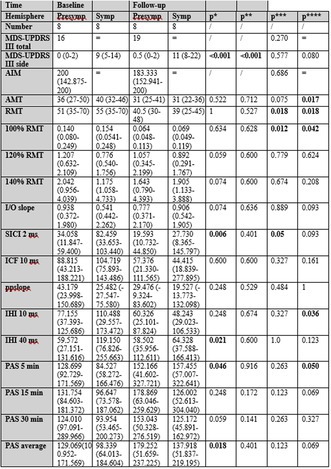
**TABLE 1** Neurophysiological data in patients with Parkinson's disease at baseline and follow‐up of the no‐progressors group.

**Figure:**
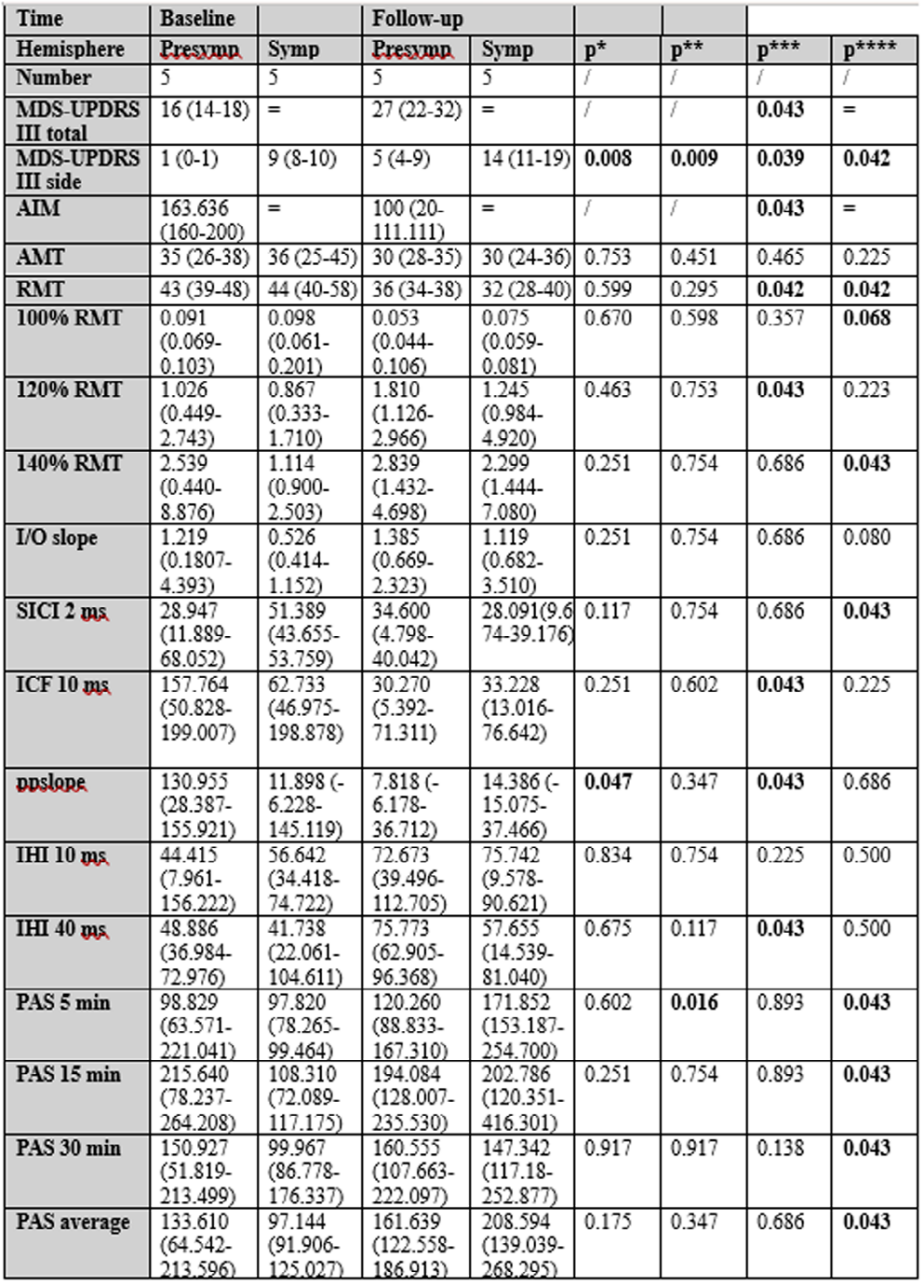
**TABLE 2** Neurophysiological data in patients with Parkinson's disease at baseline and follow‐up of the progressors group.

**Conclusion:** Corticospinal hyperexcitability reflects PD state and progression, whereas preserved intracortical and interhemispheric inhibition supports compensation. Loss of facilitation and interhemispheric control marks maladaptive changes linked to clinical conversion. TMS measures appear promising biomarkers of compensatory capacity and motor progression in early PD.

**Disclosure:** Nothing to disclose.

## EPO‐0773

### Real‐world post‐market outcomes of magnetic resonance–guided focused ultrasound in movement disorders

#### G. Schiff ^1^; A. Sanchez Fraga^2^; A. Sokolov^1^; K. Gant^3^; C. Ferrer^2^; A. Grinspan^3^

##### ^1^Insightec *Ltd*, Tirat Carmel, Israel; ^2^Insightec Europe GmBH, Munich, Germany; ^3^Insightec Inc, Miami, USA

**Background and aims:** MR–guided focused ultrasound (MRgFUS) is an established, CE‐approved therapy for unilateral Parkinson's disease (PD), unilateral and staged‐bilateral essential tremor (ET) and neuropathic pain (NP). Over 30,000 procedures, including 8000 in Europe, have been performed. This analysis reports updated safety outcomes based on accumulated clinical experience.

**Methods:** Long‐term safety was evaluated using published studies and manufacturer safety reports of MRgFUS in ET and PD.

**Results:** In the original unilateral pivotal ET trials, adverse events (AEs) were predominantly mild (74%), with paresthesia (38%) and gait disturbance (36%) most reported, generally resolving within six months. In the staged‐bilateral ET study (*N* = 51), no severe or life‐threatening device‐related events or unanticipated adverse device effects occurred. Within the first month posttreatment, common AEs included paresthesia or numbness (33%), dysarthria (29%), ataxia (23%), imbalance (18%), and dysgeusia (14%). By six months, ongoing AEs decreased in frequency, with a similar profile at 12 months. All ongoing AEs were mild. For unilateral PD treatment, reported AEs were predominantly mild, including paresthesia, gait disturbance, and dysarthria (0–35%, 3–26%, and 3–19%, respectively), with resolution typically observed within six months and no deterioration seen with long term follow‐up. Post‐marketing surveillance of 30,798 procedures reported 252 AEs (>1%), primarily paresthesia and gait disturbances. Additional data and updated analyses will be available at the time of presentation.

**Conclusion:** Long‐term safety data demonstrate a consistent, well‐characterized safety profile for unilateral and staged‐bilateral MRgFUS, supporting its recognized role as a safe treatment. Continued evaluation of lessons learned and real‐world improvements is warranted.

**Disclosure:** All authors are Insightec employees.

## EPO‐0774

### Outcome of people with Parkinson**'**s disease treated with levodopa‐entacapone‐carbidopa Intestinal gel who failed previous foslevodopa/foscarbidopa

#### D. Santos García ^1^; I. Legarda^2^; T. González Fernández^3^; A. Rodríguez Sanz^4^; M. Morales^5^; A. Peral^6^; N. Caballol^6^; M. Álvarez Sauco^7^; I. Campos Rodríguez^8^; D. Alonso Modino^9^; L. López Manzanares^10^; J. Olivares Romero^11^; A. Blanco Ollero^12^

##### ^1^Neurology Department, CHUAC (Complejo Hospitalario Universitario de A Coruña), A Coruña, Spain; ^2^Hospital Son Espases, Palma de Mallorca, Spain; ^3^Hospital del Bierzo, Ponferrada, Spain; ^4^Hospital Universitario La Paz, Madrid, Spain; ^5^Complejo Hospitalario Universitario de Toledo, Spain; ^6^Hospital de Sant Joan Despí Moises Broggi, Barcelona, Spain; ^7^Hospital Universitario de Elche, Elche, Spain; ^8^Hospital de Galdakao Usansolo, Vizcaya, Spain; ^9^Hospital de la Candelaria, Santa Cruz de Tenerife, Spain; ^10^Hospital La Princesa, Madrid, Spain; ^11^Hospital Universitario de Torrecárdenas, Almería, Spain; ^12^Hospital Universitario Juan Ramón Jiménez, Huelva, Spain

**Background and aims:** The clinical outcome of switching to levodopa‐entacapone‐carbidopa intestinal gel (LECIG) after failure of subcutaneous foslevodopa/foscarbidopa (fLD/fCD) is unknown. We analyze it in people with Parkinson's disease (PwP) treated in Spain.

**Methods:** Retrospective analysis of PwP who had previously received FLD/fCD but dropped out for different reasons and started before this LECIG in Spain up to November 30, 2025. Non‐parametric tests were applied to evaluate the changes between the pre‐ (Vpre) and post‐treatment (Vpost) (LECIG) periods.

**Results:** Data about 14 patients (57.1% males; 66.6 ± 8.6 years old) from 12 hospitals out of a total of 15 who were treated with LECIG, were included. The mean time with fLD/fCD was 98.6 ± 92.3 days, with 92.9% and 57.1% experiencing side effects and lack of response, respectively (Table 1). Specifically, significant subcutaneous nodules were reported in up to 64.3% of patients. LECIG was a direct switch from fLD/fCD in 35.7% of the patients. LECIG was well tolerated, with only 1 dropout due to complications related to dementia. Adverse events were reported in 28.6% and 35.7% of the patients in the optimization and final follow‐up evaluation (mean follow‐up of 233.7 ± 157.4 days) phases, respectively (Table 2). Daily OFF time was reduced from Vpre to Vpost in 3.2 ± 1.9 hours (*p* = 0.002). PwP improved significantly from Vpre to Vpost in motor symptoms, whereas a trend of significance was found for non‐motor symptoms burden and quality of life (Figure 1).

**Figure:**
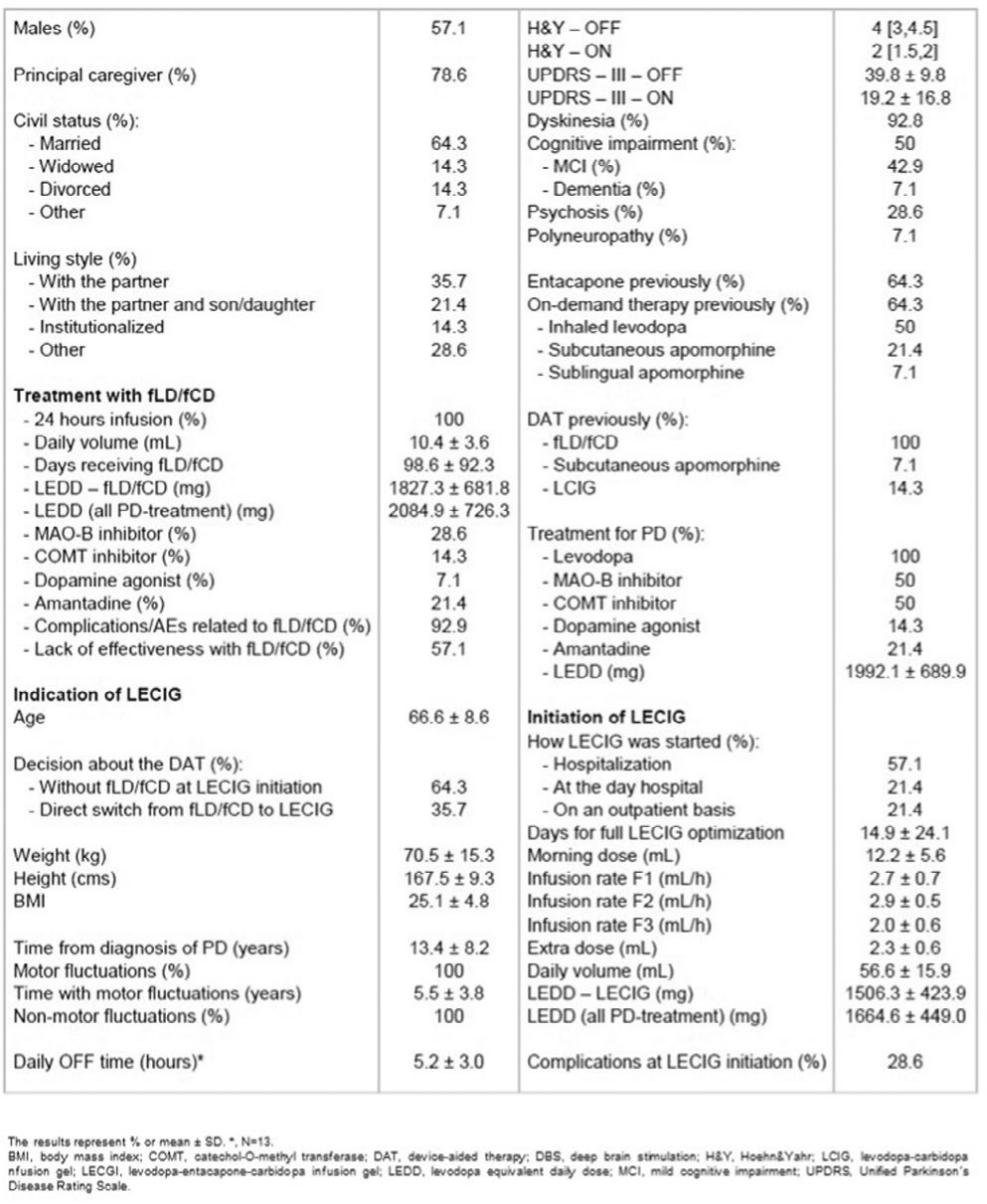
**TABLE 1** Data about sociodemographic aspects, comorbidities, antiparkinsonian drugs and other therapies regarding treatment with fLD/fCD, indication of LECIG, and initiation of LCIG (*N* = 14).

**Figure:**
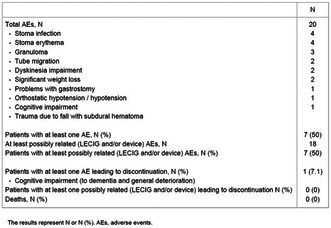
**TABLE 2** Adverse events collected by the neurologist in patients receiving LECIG (*N* = 14).

**Figure:**
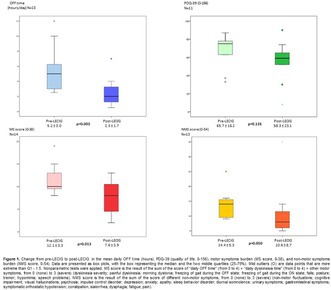
FIGURE 1

**Conclusion:** LECIG could be a good therapeutic option in PwP who failed fLD/fCD.

**Disclosure:** Nothing to disclose.

## EPO‐0775

### Low‐income settings, low dyskinesia? A treatment paradox in Parkinson's disease care

#### I. Teacă ^1^; M. Gavriliuc^1^; A. Arabadji^1^; E. Costru‐Tașnic^2^; M. Cebuc^1^; N. Rîbac^2^; O. Gavriliuc^2^

##### ^1^Neurology Department no.1, Nicolae Testemițanu University, Chișinău, Republic of Moldova; ^2^Diomid Gherman Institute of Neurology and Neurosurgery, Chisinau, Republic of Moldova

**Background and aims:** Levodopa‐induced dyskinesia (LID) is a common complication of long‐term dopaminergic therapy in Parkinson's disease (PD) in high‐income countries. In contrast, low‐resource settings report a paradoxically lower prevalence. This study evaluated whether limited access to optimized dopaminergic treatment and a conservative approach is linked to a distinct motor complication profile in PD patients from a low‐income country.

**Methods:** We conducted an observational study of 113 adults with idiopathic PD at a tertiary neurological center in Moldova during 2025. Data collected included disease duration, diagnostic delay, Hoehn and Yahr stage, current dopaminergic therapy (dose, frequency, duration), use of adjunctive and anticholinergic medications, and dyskinesia presence. Access to advanced and dyskinesia‐specific therapies was described.

**Results:** Despite a median disease duration of 3 years (IQR 1.55–6) and advanced stages at presentation (Hoehn & Yahr stage 2: 50%; stage 3: 36%; stage 4: 9%; stage 5: 1%), dyskinesia was rare (8%). Levodopa was often started late (median 1.775 years, IQR 0.6–3.5) and given at relatively low doses (median LEDD 600 mg, IQR 375–750), reflecting limited availability and concern over complications. Individual dose adjustment was restricted by the absence of alternative formulations and adjunct therapies. Effective dyskinesia treatments, such as amantadine (2 patients) or device‐aided therapies, were seldom accessible. Anticholinergics were commonly used (17%), indicating compensatory strategies in a resource‐limited setting.

**Conclusion:** In conclusion, the low prevalence of dyskinesia in this low‐income setting likely reflects chronic underexposure to dopaminergic therapy rather than disease‐related protection, highlighting the need for earlier diagnosis and improved access to diversified PD treatments.

**Disclosure:** This study was funded by the National Agency for Research and Development (NARD) of the Republic of Moldova, research project no. 25.80012.8007.04TC.

## EPO‐0776

### Brain**–**heart i**n**teraction in patients with Parkinson's disease treated by STN‐DBS

#### M. Lamoš ^1^; A. Žáčková^2^; J. Halámek^3^; P. Jurák^3^; V. Kincl^4^; M. Bočková^5^

##### ^1^Brain and Mind Research Program, Central European Institute of Technology, Masaryk University, Brno, Czech Republic**;** ^2^Faculty of Medicine, Masaryk University, Brno, Czech Republic**;** ^3^Institute of Scientific Instruments, Czech Academy of Sciences, Brno, Czech Republic**;** ^4^Department of Internal Medicine/Cardiology, Masaryk University School of Medicine, St. Anne's Hospital, Brno, Czech Republic**;** ^5^First Department of Neurology, Masaryk University School of Medicine, St. Anne's Hospital, Brno, Czech Republic

**Background and aims:** Parkinson's disease (PD) is recognized as a multisystem disorder with a variety of symptoms including autonomic dysfunctions. There is increasing evidence of cardiovascular symptoms including altered heart rate variability (HRV) compared to healthy controls. Deep brain stimulation of the subthalamic nucleus (STN‐DBS) is an established therapy for motor symptoms. This study used simultaneous EEG and ECG recordings to examine the effects of STN‐DBS on cortical–autonomic interactions.

**Methods:** EEG and ECG were recorded in 24 PD patients treated with STN‐DBS at rest in DBS OFF and ON condition. For ECG, HRV metrics, SD1 and SD2 were computed from Poincaré plots. In EEG, spectral parameterization (FOOOF) was performed to extract oscillatory power (delta, theta, alpha, beta, low/high gamma) and aperiodic features (offset, exponent). DBS‐induced change was defined as Δ = ON – OFF. Associations between ΔEEG and ΔHRV were assessed using Spearman correlations.

**Results:** Short‐term HRV (ΔSD1) was positively correlated with Δalpha power, strongest at F4 (*r* = 0.54, *p* = 0.011). Overall autonomic flexibility, reflected by ΔSD2, was negatively associated with Δexponent. This coupling was most prominent in central and temporoparietal areas, with significant effects in C4 (*r* = −0.59, *p* = 0.005), T8 (*r* = −0.46, *p* = 0.038), and Cz (*r* = −0.44, *p* = 0.048).

**Conclusion:** DBS exerts no coherent autonomic effect. However, our main findings reveal a structured pattern underlying this heterogeneity. Increases in SD2, reflecting increased autonomic flexibility considered beneficial, were associated with shifts toward cortical excitation, while reductions in SD2 corresponded to increased cortical inhibition.

**Disclosure:** Nothing to disclose.

## EPO‐0777

### Interpretable digital gait biomarkers for multiclass screening of Parkinson's disease, Alzheimer's disease, and healthy controls

#### S. Kim ^1^; J. Jung^1^; M. Son^2^

##### ^1^Department of Neurology, Busan Paik Hospital, Busan, Republic of Korea; ^2^Research Institute, JEIOS Inc, Busan, Republic of Korea

**Background and aims:** Parkinson's disease (PD) and Alzheimer's disease (AD) are prevalent neurodegenerative disorders that present with overlapping gait impairments, limiting the utility of clinical observation for differential screening.

**Methods:** We investigated whether shoe‐embedded inertial measurement units (IMUs) can support multiclass classification of PD, AD, and healthy controls while revealing interpretable, disease‐specific digital gait biomarkers. Ninety‐four participants (41 PD, 26 AD, 27 controls) performed 10‐meter walking tasks (196 trials total). From bilateral tri‐axial acceleration and angular velocity signals (100 Hz), 373 gait features spanning spatiotemporal, time‐domain, frequency‐domain, and entropy measures were extracted. To prevent information leakage from repeated trials, we applied nested cross‐validation with GroupKFold at the participant level.

**Results:** Among 11 classifiers, Linear Discriminant Analysis achieved optimal performance (test macro‐F1 0.840, 95% CI: 0.77–0.92; AUC 0.946; balanced accuracy 0.843) using only 20 features with the smallest generalization gap (0.040). Class‐wise sensitivity was 0.99 for controls, 0.82 for PD, and 0.73 for AD. SHAP analysis revealed distinct pathophysiological signatures: PD classification was driven by increased variability in inversion‐eversion angular velocity and double‐support phase duration, consistent with basal ganglia‐mediated motor control deficits; AD classification relied on entropy measures, gait velocity reduction, and timing alterations, reflecting impaired cognitive–motor integration.

**Conclusion:** These findings demonstrate that shoe‐embedded IMU gait analysis combined with explainable machine learning can effectively differentiate neurodegenerative conditions while providing clinically interpretable biomarkers aligned with established disease mechanisms, supporting scalable screening and hypothesis‐driven biomarker discovery.

**Disclosure:** Nothing to disclose.

## EPO‐0778

### Prevalence of nocturnal hypertension and pathological nocturnal dipping profile in patients with synucleinopathies in a tertiary care center

#### S. Loueslati ^1^; A. Gandhi^2^; B. Thompson^3^; N. Hankov^4^; M. Castro‐Jimenez^5^; C. Hubsch^6^; G. Courtine^7^; J. Bloch^8^; G. Wuerzner^9^; J. Bally^10^

##### ^1^Service of Neurology, Lausanne University Hospital and University of Lausanne, Lausanne, Switzerland**;** ^2^NeuroX Institute, School of Life Sciences, Swiss Federal Institute of Technology, Lausanne, Switzerland; ^3^Service of Nephrology and Hypertension and University of Lausanne, Lausanne University Hospital, Lausanne Switzerland; ^4^NeuroX Institute, School of Life Sciences, Swiss Federal Institute of Technology, Lausanne, Switzerland**;** ^5^Service of Neurology, Lausanne University Hospital and University of Lausanne, Lausanne Switzerland; ^6^Service of Neurology, Lausanne University Hospital and University of Lausanne, Lausanne Switzerland; ^7^NeuroX Institute, School of Life Sciences, Swiss Federal Institute of Technology, Lausanne, Switzerland; ^8^Department of Neurosurgery, Lausanne University Hospital (CHUV) and University of Lausanne (UNIL), Lausanne, Switzerland; ^9^Service of Nephrology and Hypertension and University of Lausanne, Lausanne University Hospital, Lausanne Switzerland; ^10^Service of Neurology, Lausanne University Hospital and University of Lausanne, Lausanne Switzerland

**Background and aims:** Nocturnal hypertension and pathological nocturnal dipping profiles are adverse prognostic factors in α synucleinopathies (Parkinson's Disease and Multiple System Atrophy), yet they remain under diagnosed because they are usually asymptomatic. This study evaluated their prevalence in a cohort of synucleinopathy patients followed at a tertiary centre.

**Methods:** Forty adults (26 M/14 F) with synucleinopathies and history of orthostatic hypotension were recruited from the CHUV‐Movement Disorders Unit. All underwent 24 hour Ambulatory Blood Pressure Monitoring (ABPM). Nocturnal hypertension was defined as mean nighttime SBP ≥ 120 mmHg or DBP ≥ 70 mmHg. Dipping status was classified as dippers (10–20% night time fall), non dippers (< 10%) and risers (night time BP higher than daytime). Isolated nocturnal hypertension meant elevated night time BP with normal daytime BP (< 135/85 mmHg). Association between variability indices and mean nocturnal BP were assessed using Pearson correlation coefficients and linear regression.

**Results:** 80.0 % percent of participants had nocturnal hypertension, and 81.3% of those met criteria for isolated nocturnal hypertension. Pathological nocturnal BP profiles were present in 82.5% of the cohort (Figure 1), more severe in male than in female. Higher daytime BP variability was moderately associated with higher nocturnal mean SBP (*r* = 0.44) and DBP (*r* = 0.47) (Figure 2).

**Figure:**
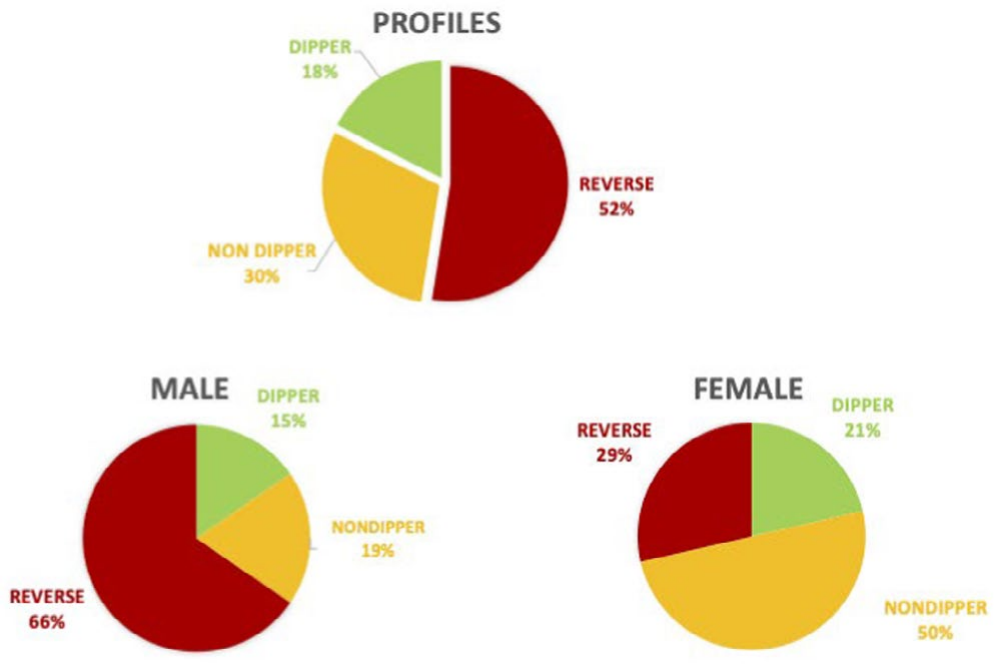
**FIGURE 1** Dipping status classification and their prevalence.

**Figure:**
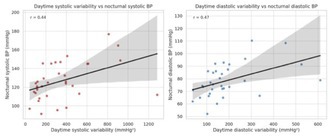
**FIGURE 2** Association between daytime variability and nocturnal blood pressure levels.

**Conclusion:** Nocturnal hypertension and abnormal dipping are highly prevalent in synucleinopathy patients, supporting routine 24 hour ABPM—especially in those with orthostatic hypotension complaints —to enable early cardiovascular management and improve quality of life. Larger prospective studies are needed to confirm these findings and assess their impact on disease morbidity and mortality.

**Disclosure:** Nothing to disclose.

## Treatment in MS and Related Disorders 2

## EPO‐0779

### Efficacy and safety of fenebrutinib vs ocrelizumab in primary progressive multiple sclerosis: Primary results of the phase III FENtrepid study

#### A. Bar‐Or ^1^; J. Oh^2^; G. Giovannoni^3^; M. Sormani^4^; M. Weber^5^; S. Stoll^6^; J. Nicholas^7^; H. von Büdingen^8^; J. Lopez^9^; L. Roberts^9^; M. Triyatni^8^; Q. Qi^9^; J. Ratchford^9^; J. Napieralski^9^; A. Goodyear^9^; S. Hauser^10^; L. Kappos^11^

##### ^1^Perelman School of Medicine, University of Pennsylvania, Philadelphia, USA; ^2^St. Michael's Hospital, University of Toronto, Toronto, Canada; ^3^Queen Mary University of London, London, UK; ^4^University of Genoa, Genoa, Italy; ^5^Institute of Neuropathology and Department of Neurology, University Medical Center, Göttingen, Germany and Fraunhofer Institute for Translational Medicine and Pharmacology, Göttingen, Germany; ^6^Advocare Stoll Medical Group, Philadelphia, USA; ^7^OhioHealth Multiple Sclerosis Centre, Riverside Methodist Hospital, Columbus, USA; ^8^F. Hoffmann‐La Roche Ltd, Basel, Switzerland; ^9^Genentech, Inc, South San Francisco, USA; ^10^University of California San Francisco, San Francisco, USA; ^11^University Hospital and University Basel, Research Center for Clinical Neuroimmunology and Neuroscience (RC2NB), Basel, Switzerland

**Background and aims:** Bruton's tyrosine kinase inhibitors (BTKi) have the potential to meet the need for multiple sclerosis (MS) treatments targeting both relapsing and progressive disease biologies. The Phase II FENopta study (NCT05119569) showed rapid reduction of acute inflammatory disease activity in relapsing MS with fenebrutinib, an oral, highly selective, noncovalent, reversible, central nervous system–penetrant BTKi. The effect of fenebrutinib on progressive MS subtypes has not been demonstrated. FENtrepid (NCT04544449) is the first clinical trial of fenebrutinib in primary progressive MS (PPMS), using ocrelizumab (the only approved treatment for PPMS) as an active comparator.

**Methods:** FENtrepid is a Phase III, multicentre, randomised, double‐blind, double‐dummy, parallel‐group study evaluating the noninferiority of fenebrutinib vs ocrelizumab in people with PPMS (2017 McDonald criteria), age 18–65 years and Expanded Disability Status Scale [EDSS] score 3.0–6.5. Participants (randomised 1:1) received 200‐mg oral fenebrutinib twice daily or 600‐mg intravenous ocrelizumab every 24 weeks. The primary endpoint was time to onset of 12‐week composite confirmed disability progression via worsening on EDSS, Timed 25‐Foot Walk Test and 9‐Hole Peg Test.

**Results:** PwPPMS (*N* = 985) had a mean (SD) age of 48.9 (10.3) years and median baseline EDSS score of 5.0. Half were female (49.5%) and most (84%) had no recent exposure to prior disease‐modifying therapies. Mean (SD) durations since MS symptom onset and diagnosis were 9.0 (6.7) and 4.7 (5.4) years, respectively. Primary efficacy and safety results will be presented.

**Conclusion:** FENtrepid will provide evidence on the benefits and risks of fenebrutinib vs ocrelizumab on disability progression in PPMS.

**Disclosure:** Sponsored by F. Hoffmann‐La Roche Ltd. Writing and editorial assistance were provided by Nucleus Global and funded by F. Hoffmann‐La Roche Ltd. A. Bar‐Or, J. Oh, G. Giovannoni, M.*P*. Sormani, M.S. Weber, S. Stoll, J.A. Nicholas, H.‐C. von Büdingen, J. Lopez, L. Roberts, M. Triyatni, Q. Qi, J.*N*. Ratchford, J. Napieralski, A. Goodyear, S.L. Hauser and L. Kappos have conflicts of interest to disclosure. These disclosures will be included in full at the time of presentation.

## EPO‐0780

### Long‐term frexalimab treatment modulates the activity of immune networks

#### B. Djukic ^1^; R. Gandhi^1^; G. Boldina^2^; V. Truong^3^; N. Ternes^4^; B. Zhang^1^; M. Levit^1^; P. Krishnaswami^1^; S. S. Geertsen^1^; S. Saubadu^5^; P. Truffinet^5^; H. Wiendl^6^; T. Chitnis^7^; A. Bar‐Or^8^

##### ^1^Sanofi, Cambridge, USA; ^2^Sanofi, Vitry‐sur‐Seine, France; ^3^Ividata Life Sciences, Paris, France; ^4^Sanofi, Montpellier, France; ^5^Sanofi, Gentilly, France; ^6^Department of Neurology and Neurophysiology, University of Freiburg, Freiburg, Germany; Brain & Mind Institute, University of Sydney, Sydney, Australia; ^7^Department of Neurology, Mass General Brigham, Harvard Medical School, Boston, USA; ^8^Department of Neurology, University of Pennsylvania, Philadelphia, USA

**Background and aims:** Frexalimab, a second‐generation anti‐CD40L monoclonal antibody, inhibits the costimulatory CD40/CD40L pathway, and may recalibrate immune networks involving adaptive and innate immunity. During the double‐blind‐period of the phase‐2 trial (NCT04879628) in participants with relapsing multiple sclerosis (pwRMS), frexalimab‐1200mg/intravenous (IV) reduced new gadolinium‐enhancing T1‐lesions by 89% vs placebo at Week (W) 12. Proteomic and transcriptomic analyses revealed modulation of B‐cell activation and antibody production, and reduced plasma levels of innate immune cell‐secreted chemokines and cytokines implicated in MS. Here, we explore frexalimab's long‐term effects through W120 of the open‐label‐extension (OLE) using plasma proteomics.

**Figure:**
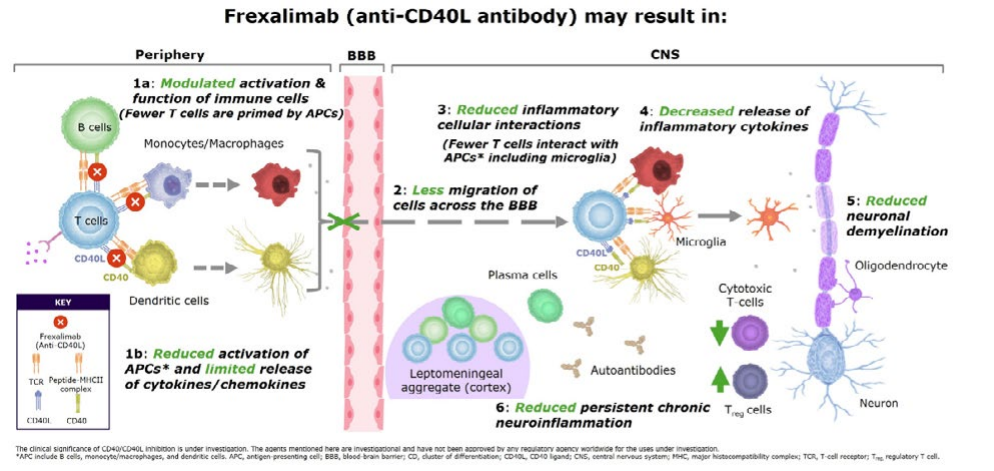
**FIGURE 1** Proposed mechanism of action of frexalimab (anti‐CD40L antibody) in multiple sclerosis.

**Methods:** 129 pwRMS were randomised (4:4:1:1) to frexalimab‐1200mg/intravenous (IV) every‐4‐weeks (q4w), frexalimab‐300mg/subcutaneous (SC)‐q2w, or matching placebo. After W12, placebo recipients switched to frexalimab and entered the OLE. All SC participants switched to 1800mg‐q4w for pharmacokinetic comparability with 1200mg/IV‐q4w. Plasma proteomic analysis from frexalimab‐1800mg/SC (*n* = 26) and 1200mg/IV (*n* = 30) arms was performed using NULISAseq with 249‐plex‐Inflammation panel, and analysed by a paired t‐test and Benjamini‐Hochberg correction.

**Results:** At W120, frexalimab treatment yielded 79 differentially abundant soluble proteins (76 downregulated, 3 upregulated) vs baseline, primarily involved in cytokine‐receptor interactions, immune‐cell communication, and chemotaxis. Frexalimab significantly downregulated adaptive and innate immune cell receptors/co‐receptors, cytokines, chemokines, antigen‐presentation and adhesion‐molecules (CXCL13, CD27, BCMA, GZMA, CSF1R) associated with MS pathophysiology, including progressive disease.

**Conclusion:** Long‐term frexalimab treatment exerts comprehensive immunomodulatory effects by downregulating inflammatory mediators associated with disease progression. These findings support understanding of frexalimab's mechanism of action, which targets immune networks involving adaptive and innate immunity, and holds the potential to mitigate acute and chronic neuroinflammation in MS.

**Disclosure:** Biljana Djukic, Roopali Gandhi, Galina Boldina, Nils Ternes, Bailin Zhang, Mikhail Levit, Pavithra Krishnaswami, Svend Geertsen, Stephane Saubadu, Philippe Truffinet: Employees of Sanofi (may hold shares and/or stock options in the company). Vinh Truong: an employee of Ividata Life Sciences (contracted by Sanofi), and may hold shares and/or stock options in the company. Heinz Wiendl: Honoraria for acting as a member of Scientific Advisory Boards for Biogen, Genzyme, Merck Serono, Novartis, Roche Pharma AG and Sanofi and UCB; speaker honoraria and travel support from Alexion, Biogen, Biologix, Cognomed, F. Hoffmann‐La Roche Ltd, Gemeinnützige Hertie‐Stiftung, Merck, Novartis, Roche Pharma AG, Genzyme, Teva and WebMD Global; paid consultant for Actelion, Argenx, Biogen, Bristol Myers Squibb, EMD Serono, Idorsia, IGES, Immunic, Immunovant, Janssen, Johnson & Johnson, Novartis, Roche, Sanofi, the Swiss Multiple Sclerosis Society and UCB; research funding by the German Ministry for Education and Research (BMBF), Deutsche Forschungsgemeinschaft (DFG), Else Kroner Fresenius Foundation, Fresenius Foundation, the European Union, Hertie Foundation, NRW Ministry of Education and Research, Interdisciplinary Center for Clinical Studies (IZKF) Muenster and Biogen, GlaxoSmithKline, Roche Pharma AG and Sanofi. Tanuja Chitnis: Received consulting fees from Genentech‐Roche and Novartis; member of advisory boards of Novartis, Roche‐Genentech, and Sanofi; research support Novartis, Sanofi, Serono, and Verily; and speaking fees from Medscape. Amit Bar‐Or: Received grant support from the University of Pennsylvania from Biogen Idec, EMD Serono, Novartis and Roche Genentech. Participated as a speaker in meetings sponsored by and received consulting fees from Accure, Atara Biotherapeutics, Biogen, Bristol‐Myers Squibb, GlaxoSmithKline, Gossamer, Janssen, Medimmune, EMD Serono, Novartis, Roche Genentech and Sanofi.

## EPO‐0781

### Baseline data of Austrian RMS patients treated earlier vs. later with Ofatumumab: The KRONOS study population

#### C. Vater ^1^; F. Leutmezer^2^; H. Assar^3^; F. Deisenhammer^4^; J. Diez^5^; C. Enzinger^6^; C. Gradl^7^; M. Guger^8^; T. Hofer^9^; P. Kapeller^10^; D. Oel^11^; K. Scigulinsky^12^; G. Traxler^13^; J. Weber^14^; P. Wipfler^15^; B. Mraz^16^; A. Holly^1^

##### ^1^Novartis Pharma GmbH, Vienna, Austria*;* ^*2*^Department of Neurology, Medical University of Vienna, Vienna, Austria; ^3^Department of Neurology, NeuroMed Campus, Kepler Universitätsklinikum GmbH, Linz, Austria; ^4^Department of Neurology, Medical University of Innsbruck, Innsbruck, Austria; ^5^Department of Neurology, Elisabethinen Hospital Graz, Graz, Austria; ^6^Department of Neurology, Medical University of Graz, Graz, Austria; ^7^Department of Neurology, Medical University of St. Pölten, St. Pölten, Austria; ^*8*^Department of Neurology, Pyhrn‐Eisenwurzen Hospital Steyr, Steyr, Austria; ^*9*^Department of Neurology, Barmherzige Brüder Hospital Linz, Linz, Austria; ^1*0*^Department for Neurology, Landeskrankenhaus Villach, Villach, Austria; ^1*1*^Department of Neurology, Klinikum Wels‐Grieskirchen, Wels, Austria; ^1*2*^Department of Neurology, Landesklinikum Mistelbach‐Gänserndorf, Mistelbach, Austria; ^1*3*^Department of Neurology, Med Campus III, Kepler Universitätsklinikum GmbH, Linz, Austria; ^1*4*^Department for Neurology, Klinikum Klagenfurt, Klagenfurt, Austria**;** ^1*5*^Department of Neurology, Christian Doppler Medical Center, Salzburg, Austria

**Background and aims:** The non‐interventional KRONOS study investigates effects of early versus late treatment with Ofatumumab in RMS patients in real‐world Austrian clinical practice. Current evidence suggests patients benefit from early highly effective therapy. KRONOS aims to support this evidence for Ofatumumab.

**Methods:** KRONOS (NCT05776888) is an ongoing 24‐month, non‐interventional study collecting retrospective and prospective data. Eligible RMS patients received Ofatumumab for ≥3 and ≤12 months at baseline. The primary endpoint is the proportion of patients with NEDA‐3 in the second year after starting Ofatumumab, comparing early (Cohort1) versus late use (Cohort 2). Secondary endpoints include demographics, individual NEDA‐3 components, and safety.

**Results:** A total of 106 patients were enrolled at 15 centers. Cohort 1 includes treatment‐naive patients or those on disease‐modifying therapy (DMT) <3 years; Cohort 2 includes patients with ≥3 years of prior DMT. Mean age was 34.3 years in Cohort 1 and 41.0 years in Cohort 2; overall, 37.5 years. Gender distribution was 40% male/60% female in Cohort 1 and 23.5%/76.5% in Cohort 2. Since disease onset, 79.5% in Cohort 1 and 40.5% in Cohort 2 had <2 relapses. Since starting Ofatumumab, 94.5% in Cohort 1 and 92.2% in Cohort 2 had no relapses. Most patients had EDSS 0.0–1.0. Overall, 13.2% showed a change in EDSS, mainly reductions (64.3% −1.0 point; 7.1% −2.0 points).

**Figure:**
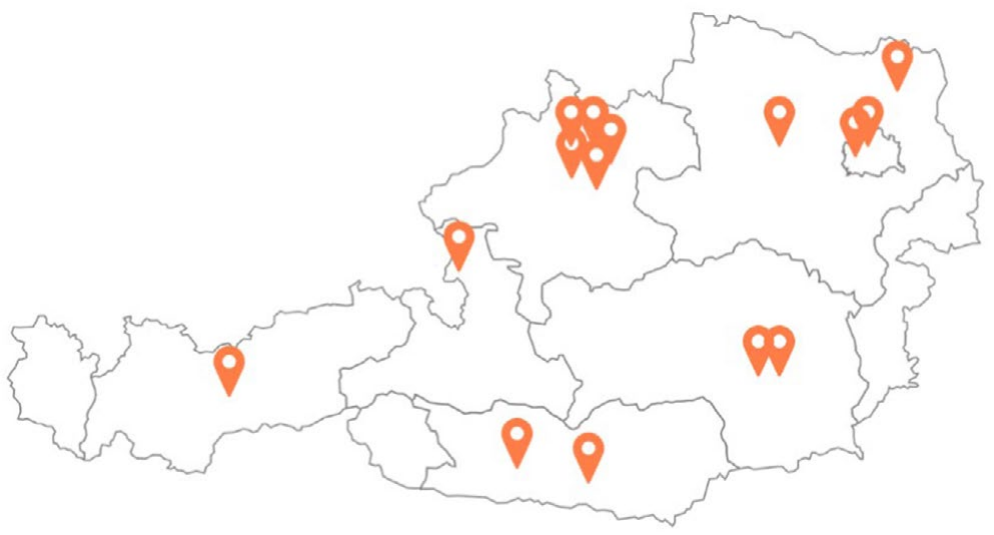
**FIGURE 1** KRONOS study centers.

**Figure:**
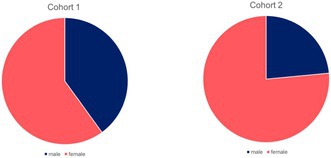
**FIGURE 2** KRONOS sex distribution.

**Figure:**
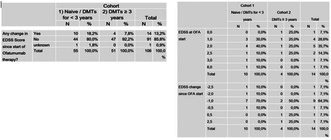
**TABLE 1** KRONOS EDSS change.

**Conclusion:** KRONOS patients were predominantely female with low baseline disability. Most remained relapse‐free after starting Ofatumumab, and a minority showed improved EDSS. Longitudinal data will clarify longer‐term outcomes and effects of early versus late Ofatumumab use.

**Disclosure:** This study is fully funded by Novartis Pharma GmbH, Austria.

## EPO‐0782

### Comparative efficacy analysis of ocrelizumab and natalizumab in a single‐centre study

#### E. De Matteis ^1^; M. Andolina^2^; A. Gokgul^3^; V. Singh‐Curry^3^; A. Nandoskar^3^; R. Dorsey^3^; D. Delacruz^3^; P. Muraro^1^; R. Nicholas^3^; A. Scalfari^3^

##### ^1^Department of Brain Sciences, Imperial College London, London, UK; ^2^Department of Biomedicine, Neurosciences and Advanced Diagnostics, University of Palermo, Palermo, Italy; ^3^Imperial College Healthcare NHS Trust, UK

**Background and aims:** Real‐world data directly comparing efficacy outcomes among RRMS patients treated with high‐efficacy disease modifying treatments remain limited. In this retrospective single centre study, we compared the efficacy of natalizumab (NTZ) and ocrelizumab (OCR).

**Methods:** We analysed relapse‐free survival (RFS), MRI activity‐free survival (MFS), progression‐free survival (PFS – 6‐month confirmed increase EDSS score) and evidence of disease activity (NEDA‐3), among RRMS patients treated with OCR (*n* = 677) and NTZ (*n* = 368) at Imperial College Healthcare Trust between 2008 and 2024. After propensity score matching (1:1), outcomes were compared by using Log‐rank tests. Cox model regressions were performed across pre‐specified subgroups.

**Results:** After matching (*n* = 276 per group), 394 (71.3%) patients were females, at treatment start median age was 38 years (IQR 30–47), disease duration 4 years (IQR 1–9), and median EDSS score 2.5 (IQR 2.0–5.0). At 4 years follow up, RFS (NTZ: 89% vs OCR; 85%, *p* = 0.064) and MFS (NTZ: 86% vs OCR: 82%, *p* = 0.120) were similar between the groups, while higher proportions of NTZ‐treated patients were free of progression (89% vs 85%, *p* = 0.050) and maintained NEDA‐3 (74% vs 62%, *p* = 0.002) status. In subgroup analyses, NTZ was associated with a lower risk of losing NEDA 3 than OCR in patients≤45 years (HR 0.54, 95% CI 0.37–0.78), with EDSS <4 (HR 0.60, 95% CI 0.42–0.87), and with disease duration ≤5 years (HR 0.52, 95% CI 0.35–0.76).

**Conclusion:** In our cohort, NTZ was superior to OCR in achieving sustained disease remission, driven by more effective prevention of disability progression

**Disclosure:** Nothing to disclose.

## EPO‐0783

### FCGR3A V158F affects ocrelizumab binding to natural killer cells and B cell repopulation during extended dosing

#### G. Abbadessa ^1^; U. Chianese^1^; C. Russo^2^; G. Miele^1^; E. Maida^1^; G. Esposito^3^; G. Lus^1^; G. Romano^1^; F. Bile^1^; E. Signoriello^1^; R. Benedetti^1^; C. Procaccini^3^; G. Matarese^4^; L. Altucci^1^; S. Bonavitq^1^

##### ^1^University of Campania Luigi Vanvitelli, Naples, Italy; ^2^AOU Federico II, Naples, Italy; ^3^Istituto degli Endotipi in Oncologia, Metabolismo e Immunologia (IEOMI) *–* CNR, Naples Italy; ^4^University of Naples Federico II, Naples, Italy

**Background and aims:** Ocrelizumab is highly effective in multiple sclerosis (MS). We tested whether FCGR3A (CD16) V158F variant (rs396991) modifies B cell kinetics and outcomes during ocrelizumab and whether ocrelizumab binding to natural killer (NK) cells differs by genotype.

**Methods:** We enrolled 101 people with MS receiving ocrelizumab (at least two cycles). B cell repopulation (BR) was defined as CD19 positive B cells at least 10 cells/ul. Genotype was modelled dominantly (F carriers vs VV) and as a genotype by interval interaction, adjusted for age, sex, baseline disability, disease duration, and prior therapy class. Flow cytometry binding assays tested whether rs396991 affects ocrelizumab binding to NK cells using fluorescein labelled ocrelizumab and rituximab in eight donors (four FF, four VV).

**Results:** While the polymorphism did not affect B cell kinetics under standard dosing, interval extension increased early B cell repopulation in F carriers (odds ratio 2.17; *p* equals 0.04), and no effects were observed on disease activity or progression outcomes; per month, the odds were 3.08 in F carriers (*p* equals 0.00026) and 1.42 in VV (*p* equals 0.195). Ocrelizumab binding to NK cells was lower in FF than VV (*p* less than 0.001), with the same pattern for rituximab (positive control). No genotype effect was seen in monocytes or B cells, supporting a CD16 specific effect.

**Figure:**
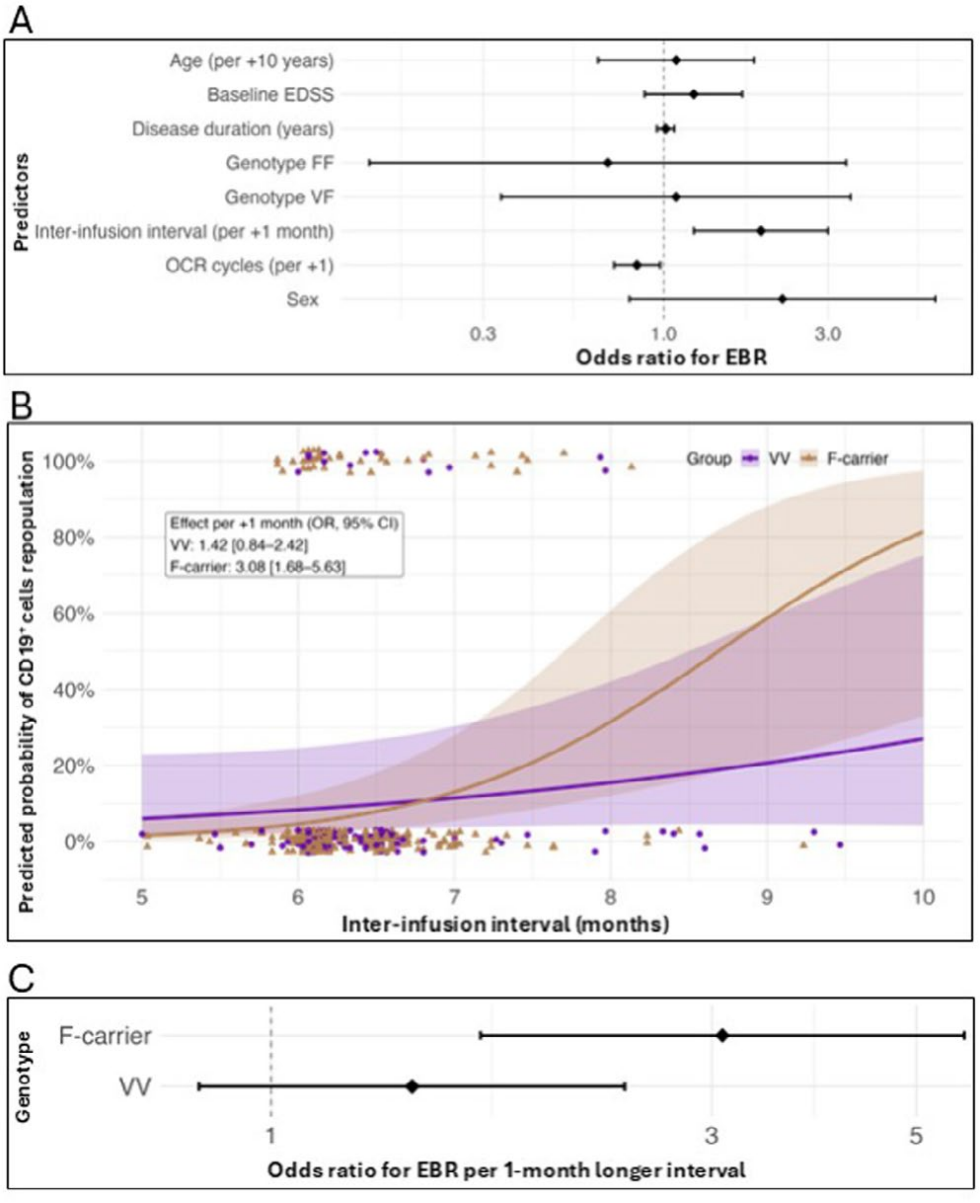
**FIGURE 1** FCGR3A polymorphism effects on B cell kinetics and clinical outcumes under ocrelizumab treatment in MS subjects.

**Figure:**
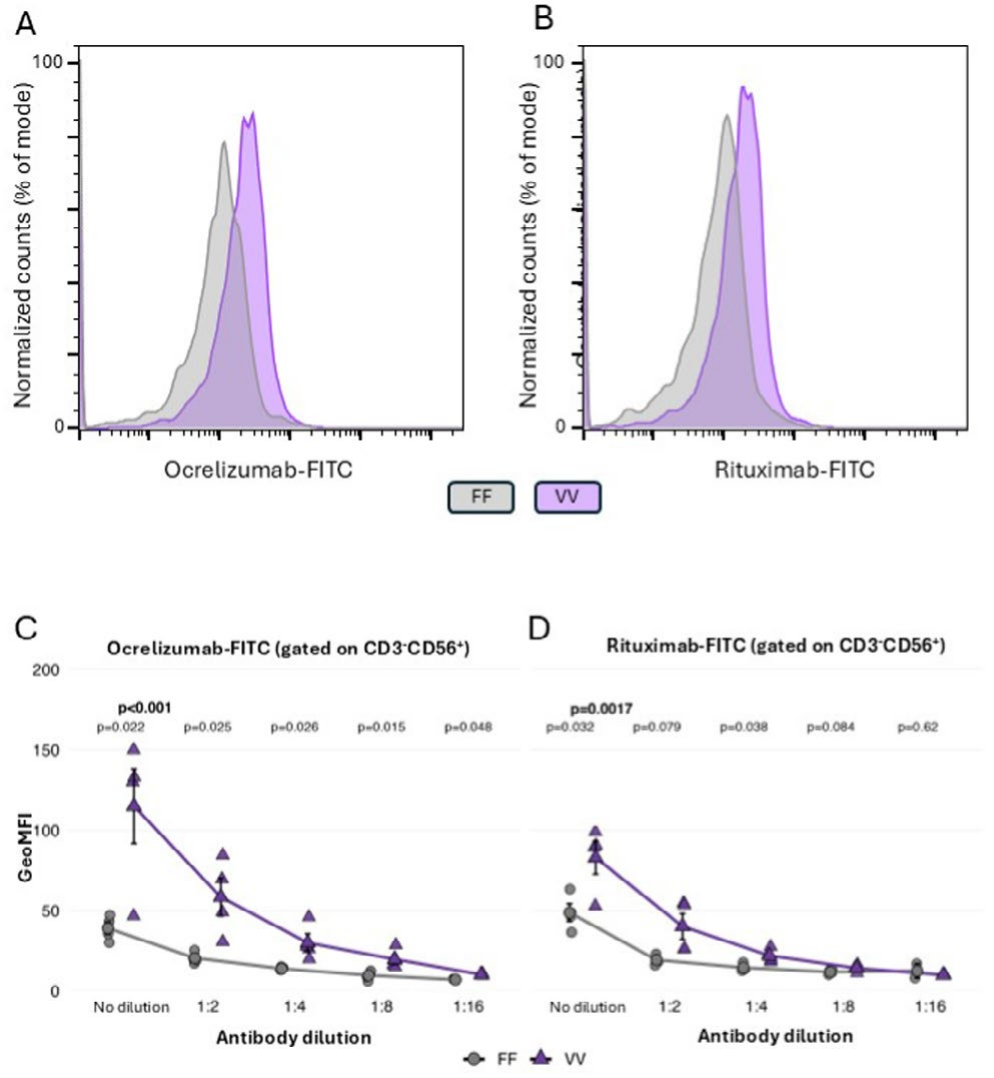
**FIGURE 2** FCGR3A polymorphism effects on ocrelizumab and rituximab binding to NK cells.

**Conclusion:** FCGR3A V158F identifies patients with earlier B cell repopulation when ocrelizumab intervals are extended, consistent with genotype dependent ocrelizumab binding on NK cells.

**Disclosure:** Nothing to disclose.

## EPO‐0784

### Switching natalizumab to cladribine: A monocentric observational trial in multiple sclerosis

#### G. Borriello ^1^; M. Peresson^1^; F. Gurreri^1^; V. Panetta^2^

##### ^1^Multiple Sclerosis Center, San Pietro Fatebenefratelli Hospital, Rome, Italy; ^2^Altrastatistica SRL ‐Consultancy & Training‐ Biostatistics office, Rome, Italy

**Background and aims:** To test the feasibility of transitioning patients from Natalizumab to Cladribine tablets, an open‐label, single‐arm, one year, phase IV, research study has been performed to obtain data on safety and efficacy in Multiple Sclerosis (MS).

**Methods:** patients with Relapsing Remitting (RR) or Secondary Progressive MS were included. All patients were discontinued because of JCV high positivity at both STRATIFY JCV™ and IMMUNOWELL™ Anti‐JCV assay. Concomitant administration of corticosteroid therapy was administered orally at the dosage of prednisone 50mg/day, tapered over 30 days or Betametasone 4 mg/day intramuscular for two weeks. Cladribine tablets were administered with the first dose given between days 3 to day 7 after last Natalizumab infusion.

**Results:** Sixty‐five patients mean age of 42.0 years (min–max: 23.0–64.0), 80.0% female were included and all completed the study at 1 year. Most patients had RRMS (66.2%). The mean time from diagnosis was 15.0 years (SD 7.22). At baseline, none of the patients reported relapse in the previous 12 months with the mean EDSS of 3.0. All patient tested for JCV Index resulted positive above 1.5 cut‐off. Three patients (4.6%) experienced a relapse at days 110, 180 and 240 after the switch. The median EDSS score was 3.0 at both 6 and 12 months. During Natalizumab treatment in the previous year, infections occurred in 30.8% of patients and 10.8% experienced an infection during Cladribine treatment.

**Conclusion:** Cladribine maintains disease stability and is well tolerated in patients discontinuing Natalizumab. The study provides real‐world evidence supporting Cladribine as a successor therapy.

**Disclosure:** Nothing to disclose.

## EPO‐0785

### Efficacy and safety of frexalimab in participants with relapsing multiple sclerosis: 3‐year results from the phase 2 open‐label extension

#### P. Vermersch^1^; S. Krieger^2^; H. Wiendl ^3^; C. Granziera^4^; Y. Mao‐Draayer^5^; G. Cutter^6^; O. Kalbus^7^; I. Staikov^8^; M. Dufek^9^; X. Montalban^10^; S. Saubadu^11^; X. Luo^12^; S. Patel^12^; B. Djukic^13^; P. Truffinet^11^; E. Wallstroem^13^; G. Giovannoni^14^; on behalf of Frexalimab Phase 2 Trial Group

##### ^1^University of Lille, Inserm U1172 LilNCog, CHU Lille, FHU Precise, Lille, France; ^2^Corinne Goldsmith Dickinson Center for Multiple Sclerosis, Icahn School of Medicine at Mount Sinai, New York, NY, United States; ^3^Department of Neurology and Neurophysiology, University of Freiburg, Freiburg, Germany; Brain & Mind Institute, University of Sydney, Sydney, Australia; ^4^Translational Imaging in Neurology (ThINk) Basel, Department of Biomedical Engineering, Faculty of Medicine; Neurologic Clinic and Policlinic, MS Center and Research Center for Clinical Neuroimmunology and Neuroscience Basel (RC2NB), University Hospital Basel and University of Basel, Basel, Switzerland; ^5^Autoimmunity Center of Excellence, Oklahoma Medical Research Foundation, Oklahoma City, OK, United States; ^6^Department of Biostatistics, UAB School of Public Health, Birmingham, AL, United States; ^7^Department of Neurology, Dnipro State Medical University, Dnipro, Ukraine; ^8^Clinic of Neurology and Sleep Medicine, Acibadem City Clinic University Hospital Tokuda, Sofia, Bulgaria; ^9^First Department of Neurology, St. Anne's University Hospital, Faculty of Medicine, Masaryk University, Brno, Czech Republic; ^10^Multiple Sclerosis Centre of Catalonia, Department of Neurology, Vall d'Hebron University Hospital, Barcelona, Spain; ^11^Sanofi, Gentilly, France; ^12^Sanofi, Morristown, NJ, United States; ^13^Sanofi, Cambridge, MA, United States; ^14^Queen Mary University of London, London, UK

**Background and aims:** Frexalimab is a second‐generation anti‐CD40L monoclonal antibody that inhibits the costimulatory CD40/CD40L pathway and may recalibrate immune networks involving adaptive and innate immunity. During the double‐blind‐period of the phase‐2 trial (NCT04879628) in participants with relapsing multiple sclerosis (pwRMS), frexalimab‐1200mg/intravenous (IV) reduced new gadolinium‐enhancing (Gd+) T1‐lesions by 89% vs placebo and was well‐tolerated at Week (W) 12. Here, we report W144 (3‐year) results of the open‐label‐extension (OLE).

**Figure:**
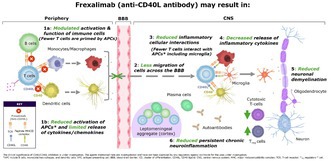
**FIGURE 1** Proposed mechanism of action of frexalimab (anti‐CD40L antibody) in multiple sclerosis.

**Methods:** 129 participants were randomised (4:4:1:1) to frexalimab‐1200 mg/IV every‐4‐weeks (q4w) or frexalimab‐300 mg/subcutaneous (SC)‐q2w or matching placebo. After W12, placebo recipients switched to frexalimab and 125 participants entered the OLE. All SC participants switched to 1800 mg‐q4w for pharmacokinetic comparability with 1200 mg/IV‐q4w. By W112, all SC participants switched to 1800 mg‐q4w, with W144 imaging data available for 55/57 participants. Key endpoints included efficacy (Gd+ T1‐lesions, new/enlarging T2‐lesions, annualised relapse rate [ARR]) and safety.

**Results:** 100 participants (78%) remained on treatment at the W144 cut‐off. The total number of Gd+ T1‐lesions (mean [SD]) remained low in all treatment groups (IV: 0.1 [0.3], placebo»IV: 0.3 [1.0]; SC: 0.0 [0.0], placebo»SC: 0.1 [0.3]). New/enlarging T2‐lesions monthly count also remained low through W144. Further, ARR (baseline–W144) was low in the frexalimab‐1200 mg/IV arm; 86% of participants were relapse‐free. Frexalimab was well‐tolerated through W144, with stable lymphocyte counts, immunoglobulin levels and no new safety signals.

**Conclusion:** Frexalimab continues to exert sustained reduction in disease activity with favourable safety in pwRMS over 3 years, supporting its further development in phase‐3 trials for RMS (FREXALT) and non‐relapsing secondary progressive MS (FREVIVA) as a potential high‐efficacy, non‐lymphocyte‐depleting therapy.

**Disclosure:** P.V: Janssen, Biogen, Sanofi, Novartis (NVS), Teva, Merck, Roche, Imcyse, AB Science, BMS‐Celgene, Ad Scientiam. C.G: Actelion, NVS, Sanofi, GeNeuro, Hoffmann La Roche, Siemens, Biogen, Teva, Merck, Janssen. Y.M‐D: Acorda, Bayer, Biogen, BMS/Celgene, Chugai, EMD Serono, Genentech‐Roche, Horizon/Amgen, Janssen, NVS, Sanofi, Teva), NIAID Autoimmune Center of Excellence, NIH NINDS, PCORI. G.C: Applied Therapeutics, AI therapeutics, AMO Pharma, Astra‐Zeneca, Avexis, Biolinerx, Brainstorm Cell Therapeutics, BMS/Celgene, CSL Behring, Galmed, Green Valley Pharma, Horizon, Immunic, Karuna Therapeutics, Mapi, Merck, Mitsubishi Tanabe Pharma Holdings, Opko Biologics, Prothena Biosciences, NVS, Regeneron, Sanofi, Reata, Teva, NHLBI, UT Southwestern, UPenn, Visioneering Technologies, Alexion, Antisense Therapeutics, Biogen, Clinical Trial Solutions LLC, Entelexo Biotherapeutics, Genentech, GW Pharma, Immunic, Immunosis, Klein‐Buendel, Merck/Serono, Perception Neurosciences, Protalix Biotherapeutics, Regeneron, Roche, SAB Biotherapeutics, University of Alabama, Pythagoras. O.K: Sanofi, Roche, Genentech, Merck, NVS, GeNeuro, BMS/Celgene, Mapi, VielaBio, Teva. I.S: Sanofi, Ewopharma‐Biogen, Shire, Gedeon‐Richter, Teva, Boehringer Ingelheim, Pfizer, Bayer, F. Roche, Mylan, Polpharma, Penumbra, Adapt, Merck, Gerot Lannach, Medochemie, NVS, Viatris, Nobel Pharma. M.D: Sanofi. X.M: Abbvie, Actelion, Alexion, AstraZeneca, Autolus, Bial PD, Biogen, Bristol‐Myers Squibb/Celgene, EMD Serono, Genzyme, Hoffmann‐La Roche, Immunic Therapeutics, Indivi, Janssen Pharmaceuticals, Juvisé Pharmaceutical, Lilly, MedDay, Medscape, Merck, Merz Therapeutics, Mylan‐Viatris, Nervgen, Neuraxpharm, NVS, PeerVoice, Rewind Therapeutics, Samsung‐Biosys, Sandoz, Sanofi‐Genzyme, Teva Pharmaceutical, TG Therapeutics, Zenas Biopharma, Excemed, ECTRIMS, MSIF, and NMSS or any of their affiliates. S.K: Consulting: Biogen, Cycle, EMD Serono, Genentech, MedRX, NVS and TG Therapeutics, and non‐promotional speaking with Biogen, EMD Serono, Genentech, and TG. Grant and research support from Biogen, BMS, NVS and Sanofi. H.W: Biogen, Genzyme, Merck Serono, NVS, Roche Pharma AG, Sanofi, UCB, Alexion, Biologix, Cognomed, F. Hoffmann‐La Roche Ltd, Gemeinnützige Hertie‐Stiftung, Genzyme, Teva, WebMD Global, Actelion, Argenx, Bristol Myers Squibb, EMD Serono, Idorsia, IGES, Immunic, Immunovant, Janssen, Johnson & Johnson, Sanofi, the Swiss Multiple Sclerosis Society, German Ministry for Education and Research (BMBF), Deutsche Forschungsgemeinschaft (DFG), Else Kroner Fresenius Foundation, Fresenius Foundation, the European Union, Hertie Foundation, NRW Ministry of Education and Research, Interdisciplinary Center for Clinical Studies (IZKF) Muenster, GlaxoSmithKline. S.S, X.L, B.S, B.D, *P*.T, and E.W: Sanofi Employee. G.G: AbbVie, Actelion, Atara Bio, Biogen, Canbex, Celgene, EMD Serono, Japanese Tobacco, Sanofi, Genentech, GSK, GW Pharma, Merck, NVS, Roche, Synthon BV, Teva.

## EPO‐0786

### Efficacy and safety of ofatumumab in clinical practice: A multicenter study

#### I. Gómez Estévez ^1^; M. Martínez Ginez^2^; J. Chico Garcia^3^; I. Moreno Torres^4^; S. Pastor‐Yvorra^5^; L. Rodríguez de Antonio^6^; I. Esain González^7^; A. Garcia Leal^8^; Y. Aladro Benito^9^; L. Borrega Canelo^10^; F. Valenzuela Rojas^11^; N. Juárez Torrejón^12^; M. Gómez‐Moreno^13^; A. Paz Tamayo^1^; J. l García Domínguez^2^; L. Costa‐Frossard França^3^; A. Orviz Gárcia^4^; C. González‐Ávila^5^; I. García Castañon^6^; R. Blasco Quilez^7^; I. Puertas Muñoz^8^; J. Pablo Cuello^2^; S. Sainz de la Maza Cantero^3^; S. De la Fuente Batista^4^; J. Sabin Muñoz^7^; L. Garcia‐Vasco^1^; C. Oreja_Guevara^1^

##### ^1^Hospital Clínico San Carlos, IdISSC, Madrid, Spain; ^2^Hospital Gregorio Marañon, Madrid, spain**;** ^3^Hospital Universitario Ramón y Cajal, IRYCIS, Universidad de Alcalá, Madrid, Spain; ^4^Hospital Universitario Fundación Jiménez Díaz, Madrid, Spain; ^5^Hospital General de Villalba, Madrid, spain Madrid, Spain; ^6^Hospital Universitario de Fuenlabrada,, Madrid, spain Madrid, Spain; ^7^Hospital Puerta de Hierro, Madrid, Spain**;** ^8^Hospital Universitario La Paz, Madrid, Spain; ^9^Hospital Universitario de Getafe, Madrid, Spain; ^10^Hospital universitario Fundación Alcorcón, Madrid, Spain; ^1*1*^Hospital Central de La Defensa Gómez Ulla, Madrid, Spain*;* ^1*2*^Hospital Universitario Severo Ochoa, Madrid, Spain; ^1*3*^Hospital Universitario Infanta Leonor, Madrid, Spain

**Background and aims:** The main goal was to analyze the efficacy and safety in MS patients treated with ofatumumab.

**Methods:** Retrospective, multicentre cohort study including patients treated with ofatumumab from the EMCAM cohort (Multiple Sclerosis Study Group of Madrid). Demographic and clinical characteristics were collected, and effectiveness and safety outcomes were evaluated during the first two years of treatment.

**Results:** A total of 473 patients were included. The clinical and demographic characteristics are shown in Table 1. 20% of patients have been undergoing treatment for more than a year, and 13%for more than two years. 19.4%of patients switched to ofatumumab due to relapse, 31%due to radiological activity and 13.5%for both reasons. 36.1% switched for other reasons such as safety concerns or adverse events. Two pregnancies have been reported, one full‐term and one that ended in miscarriage. 4.8% Patients have discontinued treatment. After one year of treatment with ofatumumab, the ARR decreased significantly to 0.02 and gadolinium‐enhancing lesions decreased to 0.04. EDSS remained stable at one year 2.2 (0–8). Adverse events were reported in 46% of patients after the first dose, and all were mild. One patient experienced a pulmonary thromboembolism shortly after treatment initiation; this event was deemed unrelated to therapy and did not require discontinuation. One patient suffered an ischemic stroke secondary to vertebral artery dissection three months after starting ofatumumab and has remained on treatment to date.

**Figure:**
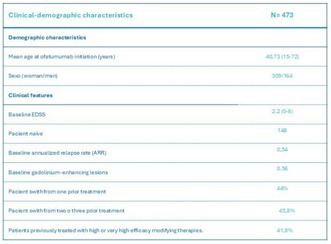
**TABLE 1** Clinical and demographic characteristics.

**Conclusion:** Ofatumumab was well‐tolerated, with no moderate or severe adverse events. two years Significant reductions in ARR and Gd+ lesions were observed at 1 and 2 years supporting its effectiveness and safety in relapsing multiple sclerosis.

**Disclosure:** Nothing to disclose.

## EPO‐0787

### Effectiveness and safety of ponesimod in relapsing multiple sclerosis: Results from an Italian multicenter real‐world observational study

#### M. Risi ^1^; A. d'Ambrosio^1^; F. Patti^2^; A. Alecci^2^; C. Chisari^2^; G. Marfia^3^; C. Gabri Nicoletti^3^; C. Guaschino^4^; P. Annovazzi^4^; A. Carotenuto^5^; V. Brescia Morra^5^; J. Frau^6^; E. Cocco^6^; A. Zanghì^7^; P. Di Filippo^7^; E. Signoriello^8^; M. Bruno^8^; V. Tomassini^9^; G. Sibilia^10^; R. Marziolo^11^; F. Matta^12^; R. Docimo^13^; A. Bisecco^1^; A. Gallo^1^

##### ^1^Department of Advanced Medical and Surgical Sciences, University of Campania Luigi Vanvitelli, Napoli, Italy; ^2^Department GF Ingrassia, Scienze Mediche e Chirurgiche e Tecnologie Avanzate, University of Catania, Italy; ^3^Multiple Sclerosis Clinical and Research Unit, Department of Systems Medicine, Tor Vergata University; 00133 Rome, Italy; ^4^Neuroimmunology Unit ‐ MS Centre, ASST Valle Olona, Gallarate Hospital, Italy; ^5^Department of Neuroscience, Reproductive Science and Odontostomatology, Federico II University of Naples, Naples, Italy; ^6^Multiple Sclerosis Center, Department of Medical Sciences and Public Health, University of Cagliari, Italy; ^7^Department of Medical and Surgical Sciences, University of Foggia, Foggia, Italy; ^8^Multiple Sclerosis Centre, Second Division of Neurology, University of Campania Luigi Vanvitelli, Naples, Italy; ^9^Department of Neurosciences, Imaging and Clinical Sciences, University G. d’Annunzio, Chieti‐Pescara, Italy; ^10^Dipartimento di Neurologia e Centro Sclerosi Multipla *P*.O. San Paolo, Napoli, Italy; ^11^Centro Sclerosi Multipla, SOC Neurologia e Stroke Unit, Azienda Ospedaliera Cannizzaro, Catania, Italy; ^12^Centro Sclerosi Multipla, UOC Neurologia, Azienda Ospedaliera Garibaldi, Catania, Italy; ^13^Multiple Sclerosis Center, First Division of Neurology, AOU "Luigi Vanvitelli," Naples, Italy

**Background and aims:** In 2021, the phase III OPTIMUM trial demonstrated the efficacy of Ponesimod (PON) ‐ a sphingosine‐1‐phosphate receptor modulator ‐ over teriflunomide in treating Relapsing Multiple Sclerosis (RMS). However, real‐world evidence on its effectiveness and safety is still limited.

**Methods:** We conducted a retrospective, multicenter observational study across 12 Italian MS centers, including adult patients with RMS treated with PON. Effectiveness was evaluated in patients with at least 12 months of follow‐up (FU), assessing relapse rate, MRI activity, and disability progression. Safety and tolerability were investigated by reviewing patients’ clinical records, focusing on adverse events (AEs), lymphocyte counts and treatment discontinuation.

**Results:** We included 106 patients treated with PON. Twenty‐two patients did not reach the minimum 12‐month FU period: 16 for a shorter FU, 6 for treatment discontinuation (4 for AEs and 2 for breakthrough disease activity). Eighty‐four (79.2%) patients completed ≥12 months follow‐up, and 40 (37.7%) reached 24 months. During the first 12‐months of FU, disease activity/PIRA occurred in 5 patients (5.8%), while 81 (94.2%) achieved NEDA‐3 status. Lymphopenia developed within the first 3‐months in most (90%) patients: 23% grade 1, 42% grade 2, 25% grade 3, 0% grade 4. No severe opportunistic infections were observed. Over the entire FU period, treatment discontinuation occurred in 10 patients (9.4%), most commonly due to AEs (*n* = 7), with no unexpected safety signals.

**Conclusion:** In this Italian real‐world study, PON showed high effectiveness in controlling clinical and radiological disease activity, with a favourable safety and tolerability profile.

**Disclosure:** A.d.A. received speaker's honoraria and/or compensation for consulting service and/or travel grants from Bristol Myers Squibb, Janssen, Juvisè, and Novartis. F.*P*. reports participation in conferences, advisory boards, speaking fees, and research grants with Alexion, Almirall, Amgen, Biogen, Bristol Myers Squibb, Merck, Neuraxpharm, Novartis, Roche, Sandoz, Sanofi, Reload, FISM, the Italian Ministry of Universities and Research, and the Italian Ministry of Health. A.A. reports no disclosures. C.C. received sponsorship for conferences and speaking fees from Alexion, Amgen, Biogen, Novartis, and Roche. G.A.M. served on advisory boards for Biogen Idec, Genzyme, Merck Serono, Novartis, and Roche; received honoraria from Almirall, Amgen, Alexion, Biogen Idec, Merck Serono, Neuraxpharm, Novartis, Sanofi‐Genzyme, Roche, Juvisè, and BMS; and was principal investigator in clinical trials for Merck Serono, Novartis, Roche, Sanofi‐Genzyme, and BMS. C.G. received advisory board fees, speaking honoraria, and travel grants from Biogen, Merck Serono, Sanofi‐Genzyme, Roche, Teva, Novartis, Bristol Myers Squibb, Juvisè, Almirall, and Horizon. *P*.A. received honoraria, advisory board fees, and/or travel support from Alexion, Almirall, Amgen, Biogen, BMS‐Celgene, Janssen, Juvisè, Lundbeck, Merck, Neuraxpharm, Novartis, Roche, Sanofi‐Genzyme, and Viatris. C.G. served on scientific advisory boards and received travel support from Biogen, Roche, Novartis, Almirall, Sanofi‐Genzyme, Merck Serono, Janssen, Alexion, Juvisè, and Neuraxpharm. A.C. received research grants from ECTRIMS‐MAGNIMS and Almirall; travel support from Novartis, Janssen, Roche, and Merck; and speaking honoraria from Merck, BMS, Biogen, Novartis, Roche, and Almirall. V.B.M. received research grants from the Italian Multiple Sclerosis Federation and Roche, and honoraria from Almirall, Biogen, BMS Celgene, Janssen, Merck, Novartis, Roche, Sanofi‐Genzyme, and Viatris. E.C. and J.F. served on scientific advisory boards and as speakers or consultants for Alexion, Amgen, Biogen, Bristol, Janssen, Merck, Neuraxpharm, Novartis, Roche, Sandoz, Sanofi, and Teva. E.S. received compensation from Almirall, Biogen, Sanofi, Roche, Alexion, Amgen, Novartis, and Teva for travel and advisory board activities. A.G. received honoraria from Biogen, Merck Serono, Mylan, Novartis, Roche, Sanofi‐Genzyme, and Teva for consulting, speaking, and/or travel support. V.T. received honoraria, research funding, and travel grants from Alexion, Viatris, Amgen, Merck, Sanofi, Roche, Novartis, Janssen, Bristol Myers Squibb, Horizon, and Biogen. R.D. received honoraria from Merck Serono, Roche, and Novartis, and travel funding from Almirall, Biogen, Novartis, Sanofi‐Genzyme, Roche, and Merck Serono. A.B. received honoraria for consulting and/or speaking from Biogen, Roche, Merck, Celgene, and Genzyme. M.R, A.Z, *P*.D.F, M.B, G.S, R.M, and F.M. declare no conflicts of interest.

## EPO‐0788

### Long‐term efficacy and safety of ponesimod in females and males: Post‐hoc analysis of the ponesimod long‐term extension trial

#### K. Hellwig^1^; K. Dost‐Kovalsky^1^; S. Vignali^2^; S. Sliepen^2^; A. Sarfati^2^; S. de Guitaut^2^; A. Atallah^2^; M. Heubl ^2^

##### ^1^Department of Neurology, St. Josef‐Hospital, Bochum, Germany; ^2^Medical Affairs Department, Juvise Pharmaceuticals, Paris, France

**Background and aims:** Potential sex‐related differences in response to disease‐modifying therapies in relapsing‐remitting multiple sclerosis (RRMS) are not well characterized. We assessed long‐term efficacy and safety of ponesimod in females versus males in the OPTIMUM trial and its long‐term extension (LTE).

**Methods:** After 108 weeks of double‐blind treatment with ponesimod 20 mg (P) or teriflunomide 14 mg (T) in the core OPTIMUM study, participants were offered ponesimod 20 mg in the OLE for up to 5 years. Outcomes were analyzed by sex subgroups.

**Results:** 735 females and 398 males were included. Baseline characteristics were comparable. In the core study, mean annualized relapse rate (ARR) was 0.21 (95% CI 0.18–0.26) vs 0.31 (95% CI 0.26–0.37) in females and 0.23 (95% CI 0.18–0.29) vs 0.31 (95% CI 0.25–0.38) in males for *P* vs T, respectively. ARR remained low in the extension period (0.11 females; 0.12 males) and the 24‐week Confirmed Disability Accumulation at Week 360 was similar for female and male patients (22.0%, 21.2%) in patients continuously treated with Ponesimod. Safety patterns across the combined (core + OLE) period were broadly similar between sexes and consistent with previously reported OPTIMUM findings. Sex‐related differences were observed, with urinary tract infection more common in females (14% vs <5% males), while alanine aminotransferase elevation occurred more often in males (32% vs 22%). Headache, fatigue and nausea were more frequent in females.

**Conclusion:** Ponesimod demonstrated sustained long‐term efficacy and safety with comparable outcomes in both sexes. These results support consistent use of ponesimod across both sub‐populations.

**Disclosure:** K.DK has nothing to disclose K.H. has received speaker honoraria and research support from Bayer, Biogen, Merck, Novartis, Sanofi‐Genzyme, Roche and Teva, has received support for congress participation from Bayer, Biogen, Merck, Roche, Sanofi Genzyme and Teva, and has served on scientific advisory boards for Bayer, Biogen, Sanofi, Teva, Roche, Novartis and Merck M.H, S.G, A.A and A.S are employees of Juvise Pharmaceuticals.

## EPO‐0789

### Efficacy and safety of ponesimod in patients aged 50 years or older in OPTIMUM and its long‐term extension

#### P. Vermersch ^1^; A. Atallah^2^; M. Heubl^2^; S. de Guitaut^2^; A. Sarfati^2^

##### ^1^Univ. Lille, Inserm U1172 LilNCog, CHU Lille, FHU Precise, Lille, France; ^2^Medical Affairs Department, Juvise Pharmaceuticals, Paris, France

**Background and aims:** OPTIMUM was a 2‐year phase 3 trial comparing ponesimod with teriflunomide in relapsing multiple sclerosis. In the 5 years extension period, all participants received ponesimod 20 mg. This post‐hoc analysis evaluated the efficacy and safety of ponesimod in patients aged 50 years or older.

**Methods:** This post‐hoc analysis included participants aged 50 years or older at core study entry enrolled in OPTIMUM. Clinical efficacy outcomes include Annualized relapse rate (ARR) and disability accumulation. Imaging efficacy was assessed using cumulative combined unique active lesions (CUAL), defined as T1 gadolinium‐enhancing lesions plus new or enlarging T2 lesions. Safety assessments included adverse events (AE).

**Results:** 68 patients were aged 50 years or older (34 ponesimod and 34 teriflunomide in the core study). During the core period, ARR was numerically lower with ponesimod (0.11 vs 0.23) compared with teriflunomide [rate ratio 0.47 (95% CI 0.16 to 1.35)] (Table 1). 96.4% of ponesimod patients remained CUAL free at the end of OPTIMUM compared to 86.2% of teriflunomide patients. During the combined period, ARR remained lower in patients receiving continuous ponesimod compared with those switching from teriflunomide (0.09 versus 0.14). At extension week 240 visit, none of the patients treated continuously with ponesimod presented CUALs. 9.1% of patients treated continuously with ponesimod during the combined period experienced severe AE and no fatal AE were reported. (Table 2).

**Figure:**
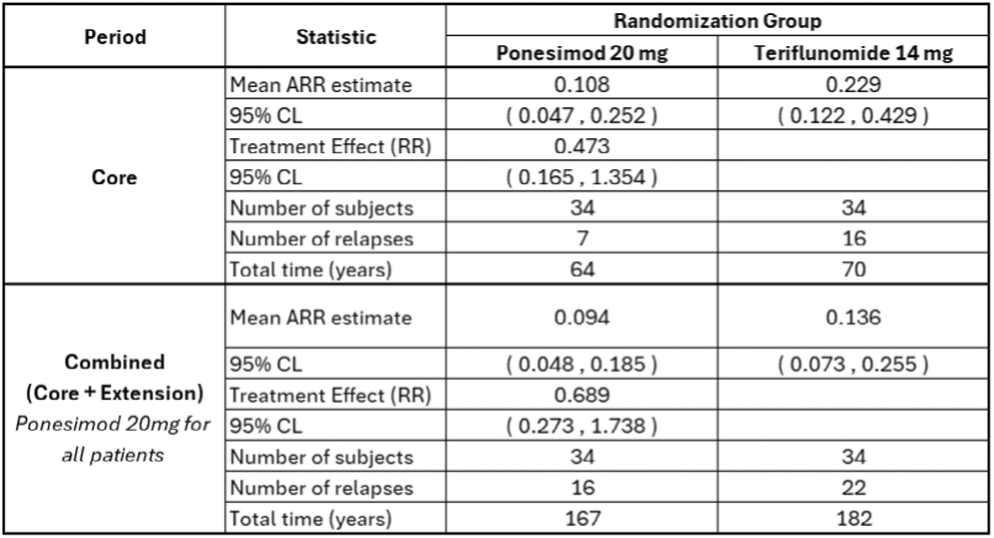
**TABLE 1** Annualized relapse rate (ARR) estimates by randomization group and study period in patients aged over 50 years; CL, confidence level; RR, rate ratio.

**Figure:**
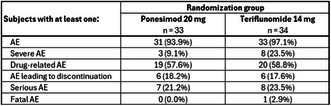
**TABLE 2** Adverse events by randomization group during combined period (core + extension) in patients aged over 50 years; AE: adverse events.

**Conclusion:** In this post‐hoc analysis of patients aged 50 years or older, ponesimod demonstrated sustained clinical and imaging efficacy with a favorable safety profile. These findings require confirmation in larger real‐world cohorts.

**Disclosure:** P.V. received honorarium, contributions to meetings from Biogen, Sanofi‐Genzyme, Novartis, Merck, Roche, AB Science, Janssen, Ad Scientiam and Juvise Pharmaceuticals. Research supports from Novartis, Sanofi‐Genzyme and Merck. M.H, S.G, A.A and A.S are employees of Juvise Pharmaceuticals.

## EPO‐0790

### Long‐term treatment effects of frexalimab on NfL, CXCL13, and brain volume loss in relapsing multiple sclerosis

#### C. Granziera^1^; P. Vermersch ^2^; B. Djukic^3^; S. S. Geertsen^3^; A. T. Shafer^3^; P. Truffinet^4^; G. Giovannoni^5^

##### ^1^Translational Imaging in Neurology (ThINk) Basel, Department of Biomedical Engineering, Faculty of Medicine; Neurologic Clinic and Policlinic, MS Center and Research Center for RC2NB, University Hospital Basel and University of Basel, Basel, Switzerland; ^2^University of Lille, Inserm U1172, Lille Neuroscience and Cognition, CHU Lille, FHU Precise, Lille, France; ^3^Sanofi, Cambridge, MA, United States; ^4^Sanofi, Gentilly, France; ^5^Queen Mary University of London, London, UK

**Background and aims:** Frexalimab, a second‐generation anti‐CD40L monoclonal antibody, inhibits the costimulatory CD40/CD40L pathway, and may recalibrate immune networks involving adaptive and innate immunity. During the double‐blind‐period of the phase‐2 trial (NCT04879628) in participants with relapsing multiple sclerosis (pwRMS), frexalimab‐1200mg/intravenous (IV) reduced new gadolinium‐enhancing T1‐lesions by 89% vs placebo at Week (W) 12, with 24% and 21% reductions in plasma neurofilament light chain (NfL) and chemokine (C‐X‐C motif) ligand‐13 (CXCL13) levels, respectively. Here, we report W144 (3‐year) results of NfL, CXCL13, and brain volume loss (BVL) during the open‐label‐extension (OLE).

**Figure:**
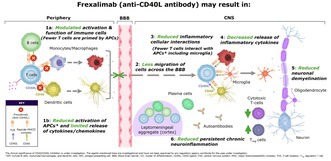
**FIGURE 1** Proposed mechanism of action of frexalimab (anti‐CD40L antibody) in multiple sclerosis.

**Methods:** 129 pwRMS were randomised (4:4:1:1) to frexalimab‐1200 mg/IV every‐4‐weeks (q4w), frexalimab‐300 mg/subcutaneous (SC)‐q2w, or matching placebo. After W12, placebo recipients switched to frexalimab and entered the OLE. The SC dose was increased to 1800 mg‐q4w for pharmacokinetic comparability with 1200 mg/IV‐q4W. Plasma NfL and CXCL13 were measured by Quanterix Simoa® and Meso Scale Discovery assays. BVL was estimated using Jacobian integration. Results are reported as: % change from baseline to W144 (NfL/CXCL13 [geometric mean]; BVL [median]).

**Results:** From baseline to W144, frexalimab reduced plasma NfL by 47% (IV), 49% (SC), 35% (placebo»IV), 45% (placebo»SC). CXCL13 decreased by 48%, 61%, 34%, 28%, respectively. In the IV arm (Phase 3 dose), BVL from baseline to W144 was −0.84% (IQR: −1.14, −0.50) for whole brain, −1.18% (−1.85, −0.13) for thalamus, and −0.85% (−1.67, −0.39) for cerebral cortex.

**Conclusion:** Three years of frexalimab treatment markedly reduced NfL and CXCL13, and limited BVL in pwRMS. These data demonstrate its effects on neuroinflammation and neurodegeneration, supporting further investigation in RMS and non‐relapsing secondary progressive MS.

**Disclosure:** C. Granziera: The University Hospital Basel, as the employer of C.G, has received the following fees for research support: advisory boards/consulting fees (Actelion, GeNeuro, Hoffmann La Roche, Novartis, Sanofi, Siemens); speaker fees (Biogen, Hoffmann La Roche, Janssen, Merck, Novartis, Sanofi, Teva); research grants (Biogen, GeNeuro, Hoffmann La Roche, Sanofi). *P*. Vermersch: Honoraria and/or consulting fees (AB Science, Ad Scientiam, Biogen, BMS, Celgene, Imcyse, Janssen, Merck, Novartis, Roche, Sanofi, Teva); research support (Novartis, Roche, Sanofi). B. Djukic, S. S. Geertsen, A.T. Shafer, Philippe Truffinet: Employees of Sanofi (may hold shares and/or stock options in the company). G. Giovannoni: Research support/consulting/speaker fees (AbbVie, Actelion, Ad Scientiam, Atara Bio, Biogen, Canbex, Celgene, EMD Serono, Genentech, GSK, GW Pharma, Japanese Tobacco, Merck, Novartis, Roche, Sanofi, Synthon BV, Teva).

## Sleep‐Wake Disorders 1

## EPO‐0791

### Human ponto‐geniculo‐occipital waves: Local field potentials analysis in people with Parkinson's disease during deep brain stimulation surgery

#### D. Sandri ^1^; S. Marceglia^2^; A. Priori^2^; T. Bocci^2^

##### ^1^Neurology Unit, ASST Santi Paolo e Carlo, University of Milan, Milan, Italy; ^2^Aldo Ravelli Research Center for Experimental Brain Therapeutics, Department of Health Sciences, University of Milan, Milan, Italy

**Background and aims:** Ponto‐geniculo‐occipital (PGO) waves are considered the hallmark of REM sleep and their presence is correlated to oneiric activity. Due to ethical concerns, PGO waves were studied only in animals, owing to their registration as local field potentials (LFP) by means of deep cerebral and cortical surface electrodes. PGO waves appear as singlets and clusters of biphasic sharp potentials recorded in meso‐diencephalic area and occipital cortex. With deep brain stimulation (DBS), in particular subthalamus's DBS for advanced Parkinson's disease (PD) treatment, this study aims to record the presence of PGO waves in humans.

**Methods:** 10 PD patients were enrolled. LFP signal was decoded from subthalamic electrodes during DBS surgery anaesthesia: 2 patients with ketamine (REM sleep model) and 8 with propofol (NREM sleep model). They also had an extracephalic electromyography canal. LFPs were visually scored in double‐blind by two neurologists, then checked by an AI algorithm trained on recognizing waves with characteristics similar to PGO.

**Results:** In the propofol group no graphoelements resembling PGO waves were seen. In the ketamine group, with concordance between neurologists and AI, lateralized biphasic sharp potentials were seen, with 200–400 μV amplitude range, 200–350 msec duration, 3–5 Hz frequency. The majority of graphoelements were organized in clusters occurring at 3–50 seconds range containing 3–9 waves each.

**Conclusion:** Waves seen during ketamine anaesthesia have similar morphology, frequency, amplitude and cluster organization of PGO waves recorded in animals. Despite the small sample, it is reasonable to think that PGO waves are present in humans and could be related to dream‐like activity.

**Disclosure:** Nothing to disclose.

## EPO‐0792

### Short‐form video consumption as a trigger for de novo tension‐type headache and sleep disorganization: A prospective cohort study

#### D. Satanovsky

##### Ukrainian Society of Lifestyle Medicine, Kyiv, Ukraine

**Background and aims:** The rapid shift to short‐form video content (TikTok and Reels) has introduced a high density of sensory stimulation into the medium. The aim of this study was to assess the neurophysiological impact of evening grazing on the development of primary headache and sleep disorder in clinically normal subjects.

**Methods:** The prospective cohort study involved 260 patients (15–45 years of age) who had no history of chronic pain or insomnia. Participants participated in two hours of evening video‐based short‐form for 60 days. Data were collected through headache diaries and sleep records. Statistical analysis has been carried out with SPSS 26.0.

**Results:** 27 percent (*n* = 70) developed de novo tension headache (TTH) after 60 days (*p <* 0.01). The mean total amount of sleep has decreased by 52 minutes a night. The sleep latency ranged from 15 to 60 minutes (*p <* 0.001). A specific behavioural marker, night‐time digital cravings (compulsive use of a smartphone when waking up in the night) was identified in 68 percent of sleep‐deprivation patients. A comparative analysis showed that the effect of digital stimulation on sleep latency was greater than that of caffeine and nicotine.

**Figure:**
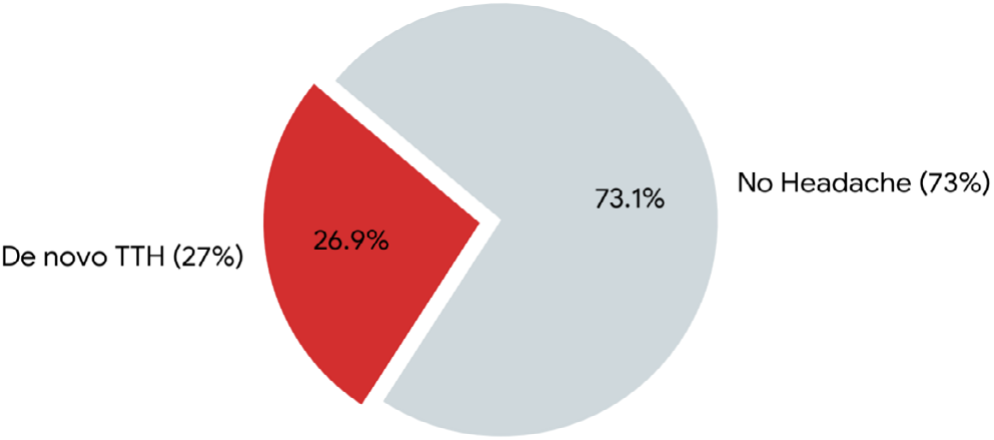
**FIGURE 1** Incidence of de novo tension‐type headache (*n* = 260 participants).

**Figure:**
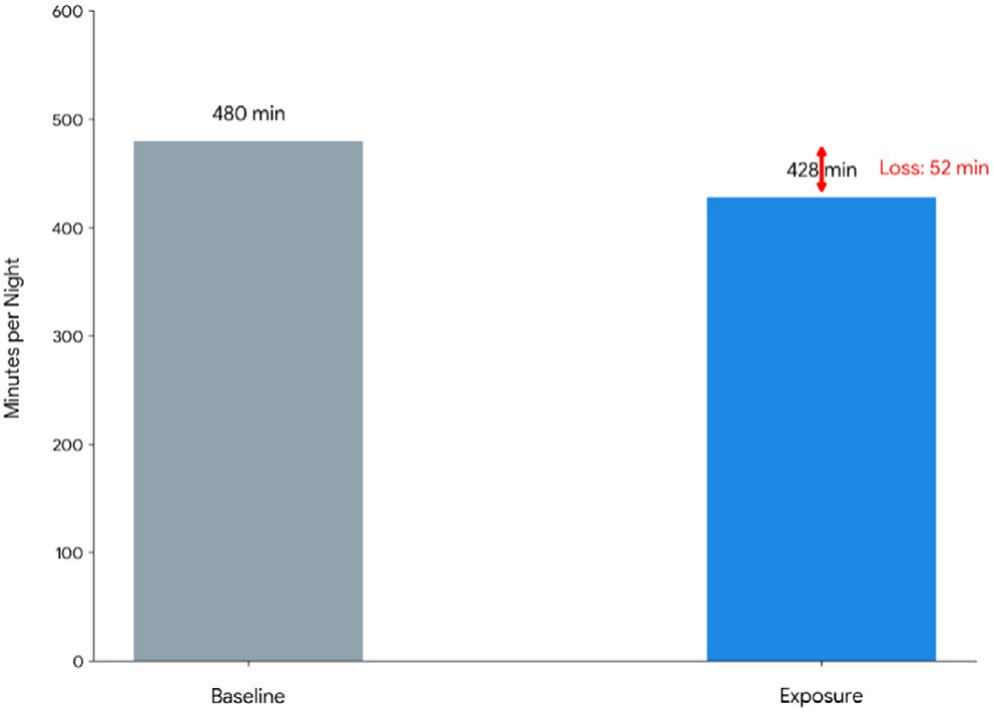
**FIGURE 2** Average reduction in sleep duration (study sample: *n* = 260).

**Figure:**
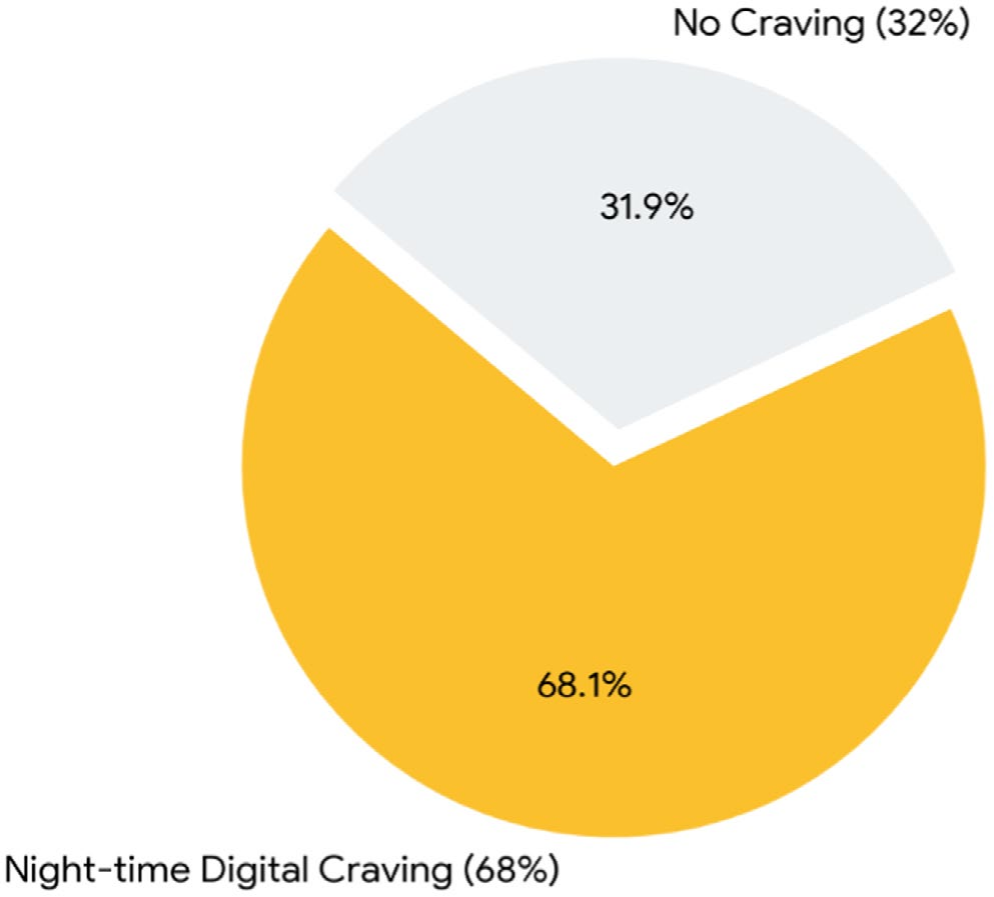
**FIGURE 3** The prevalence of night time digital cravings (68 percent) as a behavioral marker identified in sleep architecture disorder participants.

**Conclusion:** Results Regular consumption of short‐form video content is a self‐reinforcing trigger of TTH and severe sleep architecture disorder. These findings support the inclusion of digital silence protocols (at least 60 minutes before bedtime) in the clinical guidelines for the management of headache and insomnia. The night time digital cravings serve as a key indicator of the behavioural addiction of this population.

**Disclosure:** Nothing to disclose.

## EPO‐0793

### Psychiatric and affective: Behavioural profile in patients with isolated REM sleep behaviour disorder

#### F. Di Laudo ^1^; L. Baldelli^1^; L. Sambati^2^; D. Cimatti^2^; M. Binda^1^; P. Guaraldi^2^; A. Cerere^1^; G. Loddo^3^; G. Mainieri^2^; F. Mignani^1^; G. Calandra‐Buonaura^1^; F. Provini^1^

##### ^1^Department of Biomedical and NeuroMotor Sciences (DiBiNeM), University of Bologna, Italy; ^2^IRCCS ‐ Istituto delle Scienze Neurologiche di Bologna, Italy; ^3^Department of Primary Care, Azienda AUSL di Bologna, Italy

**Background and aims:** Isolated REM sleep behaviour disorder (iRBD) is considered a prodromal stage of overt synucleinopathies. Increasing interest has focused on identifying biomarkers of iRBD and predictors of phenoconversion. Psychiatric comorbidities in this population remain poorly investigated. This study aimed to describe the prevalence of psychiatric comorbidities in isolated iRBD and to evaluate differences in clinical features.

**Methods:** Eighty‐nine patients with polysomnography‐confirmed isolated RBD were enrolled. Clinical history was collected; patients underwent neuropsychological testing, cardiovascular reflex tests and olfactory function assessment. Affective‐behavioural features were assessed using Beck Depression Inventory‐II (BDI‐II), State–Trait Anxiety Inventory (STAI‐S, STAI‐T), Apathy Evaluation Scale (AES) and Neuropsychiatric Inventory (NPI).

**Results:** Among 89 patients (66 males, 23 females), 29 (32.6%) had a psychiatric diagnosis, mainly depressive and anxiety disorder; in 31% psychiatric diagnosis preceded iRBD. In the whole cohort, depressive symptoms were present in 39.3% and anxiety symptoms in 14.6%; 18.3% of patients without psychiatric diagnosis reported at least one psychiatric symptom. Patients with psychiatric comorbidity showed higher affective symptom burden for depression, anxiety and apathy (all except AES, *p* = 0.061). NPI data showed significant differences in anxiety and irritability domains (*p <* 0.05). No differences were observed in hyposmia, neurogenic orthostatic hypotension or mild cognitive impairment. Seventeen patients converted to a synucleinopathy, 31.1% of patients with psychiatric diagnosis vs 13.3% without (*p <* 0.05).

**Conclusion:** Psychiatric comorbidities are common in iRBD, and psychiatric symptoms may occur even in the absence of a formal diagnosis. The presence of a psychiatric diagnosis seems associated with phenoconversion, but larger studies are needed to further characterize this clinical phenotype.

**Disclosure:** Nothing to disclose.

## EPO‐0794

### Skin thermal stimuli and hypothalamic thermosensitivity dynamically dissociate REM sleep and cataplexy expression in narcolepsy

#### F. Colò ^1^; B. Viberti^1^; S. Bellini^1^; A. Chancel^2^; P. Luppi^2^; P. Fort^2^; M. Schmidt^1^

##### ^1^Center for Experimental Neurology, Department of Neurology, Bern University Hospital (Inselspital) and University Bern; Bern, Switzerland; ^2^Université Claude Bernard Lyon 1, CNRS, INSERM, Lyon Neuroscience Research Center CRNL UMR 5292 U1028, SLEEP, Bron, France

**Background and aims:** REM sleep is preferentially expressed under thermoneutral conditions, likely due to reduced thermoregulatory demands. Recent evidence suggests that skin temperature (Tskin) and hypothalamic circuits critically modulate REM sleep. Here, we investigated how Tskin and melanin‐concentrating hormone (MCH) neurons regulate REM sleep and cataplexy in a mouse model of narcolepsy, considering sex, thermal dynamics, and afferent thermoregulatory inputs.

**Methods:** Using hypocretin‐knockout mice, we selectively manipulated Tskin during the active phase while monitoring sleep–wake states and cataplexy. To assess causality, we optogenetically inhibited or activated MCH neurons in MCH‐Cre; Hcrt‐KO mice. Fiber photometry was used to assess MCH neuronal activity during thermal manipulation. Monosynaptic rabies tracing combined with cFos mapping was employed to identify thermoregulatory afferent inputs to MCH neurons.

**Results:** Thermoneutral skin warming robustly increased REM sleep and reduced cataplexy, whereas skin cooling produced opposite effects; these dissociating effects were observed in both sexes. Non–state‐specific MCH inhibition reduced REM sleep and increased cataplexy, while state‐specific inhibition during cataplexy prolonged episode duration. Thermoneutral warming overrode the pro‐cataplectic effect of MCH inhibition, suggesting parallel temperature‐dependent mechanisms partially independent of MCH signaling. Conversely, optogenetic activation of MCH neurons increased REM sleep and decreased cataplexy. Monosynaptic rabies tracing combined with cFos mapping identified thermoregulatory inputs to MCH neurons, with the median preoptic nucleus (MnPO) showing the strongest warm‐activated connectivity.

**Conclusion:** These findings identify skin temperature as a key physiological variable that dissociates REM sleep and cataplexy and highlight MnPO–MCH circuits as a critical interface between thermoregulation and behavioral state control.

**Disclosure:** Nothing to disclose.

## EPO‐0795

### The burden of insomnia and excessive daytime sleepiness: Switzerland**'**s pilot study preliminary results

#### M. Tüzün ^1^; U. Kallweit^2^; S. Seidel^3^; O. Endrich^4^; M. Leone^5^; O. Bruni^6^; R. Dodel^7^; A. Fiorillo^8^; I. Holmerová^9^; Jaarsma^10^; M. Lolich^11^; M. Konti^11^; D. Ramakulov^11^; D. Pevernagie^12^; E. Pupillo^5^; W. Randerath^13^; L. Vignatelli^14^; C. Meyer‐Massetti2^15^; M. Schmidt^1^; C. Bassetti^16^

##### ^1^Interdisciplinary Sleep‐Wake‐Epilepsy‐Center, Bern University Hospital (Inselspital) and University of Bern, Bern, Switzerland; ^2^University Witten/Herdecke, Faculty of Medicine, Professorship for Narcolepsy and Hypersomnolence Research, Witten, German; ^3^Rehabilitation Clinic Pirawarth, Bad Pirawarth, Austria; ^4^University Institute of Clinical Chemistry, Inselspital, Bern University Hospital, University of Bern, Switzerland; ^5^Department of Neurosciences, Istituto di Ricerche Farmacologiche “Mario Negri” IRCCS, Milano, Italy; ^6^Department of Developmental and Social Psychology, Sapienza University, Rome, Italy; ^7^Department of Geriatric Medicine, University Duisburg‐Essen, Essen, Germany; ^8^Department of Psychiatry, University of Campania “L. Vanvitelli”, Naples, Italy, European Psychiatric Association; ^9^Centre of Expertise in Longevity and Long‐term Care, Charles University, Prague, Czech Republic, Alzheimer Europe; ^10^A European Alliance for Restless Legs Syndrome, Brussels, Belgium; ^11^European Academy of Neurology, Vienna, Austria; ^12^Department of Internal Medicine and Paediatrics, Faculty of Medicine and Health Sciences, Ghent University, Ghent, Belgium; ^13^Institute of Pneumology, University of Cologne, Cologne, Germany; ^14^IRCCS Istituto delle Scienze Neurologiche di Bologna, Bologna, Italy; ^15^Institute for Pharmacology PKI, University of Bern, Bern, Switzerland; ^16^Department of Neurology, Bern University Hospital (Inselspital) and University of Bern, Bern, Switzerland

**Background and aims:** Excessive daytime sleepiness (EDS) and insomnia (IN) are common sleep–wake complaints with substantial personal and societal burden, yet they are often underrecognized in primary care. This pilot study evaluated the feasibility of 12‐month recruitment and follow‐up and estimated the prevalence and correlates of EDS/IN with health‐related quality of life (HRQoL), health status, and economic burden using standardized definitions and validated tools.

**Methods:** Adult patients were recruited from five general practices and five community pharmacies in Switzerland. A two‐step approach was used: consecutive identification of self‐reported sleep complaints, followed by screening with validated questionnaires. Participants exceeding clinical cutoffs (ESS >10 and/or ISI >7) were enrolled. Baseline measures included PSQI, HRQoL (SF‐12, EQ‐5D‐5L), and a validated cost‐of‐illness questionnaire. Follow‐up lasted 12 months.

**Results:** Of 821 pre‐screened patients, 757 had complete data. Overall, 38% reported EDS/IN (IN 32%, EDS 19%, both 13%), and 82% had symptoms >3 months. Self‐medication was reported by 22% and prescribed therapy by 28% (benzodiazepines, Z‐drugs, antidepressants). Among 92 screened for follow‐up, 15% did not meet questionnaire cutoffs despite subjective complaints. HRQoL was lowest in those with both EDS and IN, with mental domains more affected than physical. Health‐related costs were reported by 74% (median CHF 5340). After 12 months, symptoms largely persisted, with only modest improvements in PSQI and ISI.

**Conclusion:** This study demonstrates feasibility and highlights the chronicity, clinical impact, and economic burden of EDS/IN in primary care, underscoring the need for improved guideline‐based management.

**Disclosure:** Funded by the European Academy of Neurology.

## EPO‐0796

### Effect of oveporexton (TAK‐861), an oral orexin receptor 2 agonist, on narcolepsy type 1 symptoms: A pooled analysis of two phase 3 studies

#### M. Schmidt ^1^; Y. Dauvilliers^2^; R. Del Rio Villegas^3^; G. Lammers^4^; E. Mignot^5^; G. Plazzi^6^; A. Cai^7^; Y. Du^7^; M. Etherton^7^; T. Olsson^7^

##### ^1^University Clinic for Neurology, Inselspital, Bern, Switzerland; ^2^Sleep‐Wake Disorders Center, Department of Neurology, Gui‐de‐Chauliac Hospital, CHU, Institute of Neurosciences of Montpellier, INSERM, University of Montpellier and National Reference Network for Narcolepsy, Montpellier, France; ^3^Neurophysiology and Sleep Disorders Unit, Vithas Hospitals and Department of Clinical Medical Sciences, Universidad Ceu San Pablo, CEU Universities, Madrid, Spain; ^4^Sleep Wake Centre, Stichting Epilepsie Instellingen Nederland, Heemstede and Department of Neurology, Leiden University Medical Centre, Leiden, Netherlands; ^5^Stanford Center for Sleep Sciences and Medicine, Palo Alto, USA; ^6^Department of Biomedical, Metabolic and Neural Sciences, University of Modena and Reggio‐Emilia, Modena and IRCCS, Istituto delle Scienze Neurologiche, Bologna, Italy; ^7^Takeda Development Center Americas, Inc, Cambridge, USA

**Background and aims:** Narcolepsy type 1 (NT1) is caused by loss of orexin producing neurons. We report a pooled analysis of data from two randomized, double‐blind, phase 3 studies (The First Light: NCT06470828; The Radiant Light: NCT06505031) that evaluated the effect of oveporexton (TAK‐861) on NT1 symptoms and their severity.

**Methods:** Participants were aged 16–70 years, had an established diagnosis of NT1, and were randomized to twice daily oral oveporexton 1 mg (The First Light only), 2 mg, or placebo, ≥3 hours apart for 12 weeks. The primary endpoint was change from baseline to week 12 in mean sleep latency on the Maintenance of Wakefulness Test (MWT). Key secondary endpoints included changes from baseline in Epworth Sleepiness Scale (ESS) and Narcolepsy Severity Scale for Clinical Trials scores to week 12, weekly cataplexy rate at week 12, and Patient Global Impression of Change (PGI‐C) in overall narcolepsy symptoms at week 12.

**Results:** Overall, 273 participants were randomized to oveporexton 1 mg/1 mg (*n* = 61), 2 mg/2 mg (*n* = 136), or placebo (*n* = 76). At week 12, the least square mean differences from placebo (95% CI) for change from baseline in MWT mean sleep latencies were 15.90 (12.65, 19.15) minutes (1 mg/1 mg oveporexton) and 19.16 (16.65, 21.68) minutes (2 mg/2 mg; both unadjusted *P <* 0.0001). Results for the other endpoints are shown in Table 1.

**Figure:**
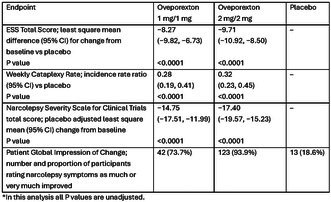
**TABLE 1** Secondary endpoint data at week 12.*

**Conclusion:** In this pooled analysis, oveporexton treatment improved measures of wakefulness, sleepiness, and cataplexy frequency and reduced the severity of narcolepsy symptoms over 12 weeks vs placebo.

**Disclosure:** This research was funded by Takeda Development Center Americas, Inc, who also provided funding to Envision Catalyst, an Envision Medical Communications agency, a part of Envision Pharma Group, to provide writing assistance under the direction of the authors. Markus Schmidt participated in an advisory board for Takeda. Yves Dauvilliers received funds for seminars, board engagements, and travel to conferences from Avadel, Bioprojet, Idorsia, Jazz Pharmaceuticals, Centessa, Alkermes, Pharmanovia, and Takeda. Rafael Del Rio Villegas received consultancy fees from Alkermes, Bioprojet, and Takeda, and travel funds from Bioprojet, Jazz Pharmaceuticals, and Takeda. Gert J. Lammers has received consulting fees, grants, and travel funds from Alkermes, Bioprojet, Daiichi Sankyo, Eisai, Jazz Pharmaceuticals, and Takeda. Emmanuel Mignot received consulting fees from Alkermes, Jazz Pharmaceuticals, and Takeda; research grant or trials to Stanford from Apple, Avadel, Eisei, Jazz Pharmaceuticals, and Takeda; travel funding from Harmony Biosciences, Paladin Labs, and Takeda; and stock options from Centessa. Giuseppe Plazzi received consultancy fees from Bioprojet, Jazz Pharmaceuticals, Orexia, and Takeda. Alice Cai, Ye‐Ting Du, Mark Etherton, and Tina Olsson are employees of Takeda Development Center Americas, Inc, and stockholders in Takeda Pharmaceuticals Company Limited.

## EPO‐0797

### A hospital‐based, retrospective analysis of the characteristics of periodic limb movements in sleep in children

#### M. İriş ^1^; D. Onar^1^; D. Karadeniz^1^; L. DelRosso^2^; G. Benbir Şenel^1^

##### ^1^Neurology, Istanbul University‐Cerrahpasa, Cerrahpasa Medical Faculty, Istanbul, Turkiye; ^2^Sleep Medicine, University of California, San Francisco, Fresno, USA

**Background and aims:** Periodic limb movements in sleep (PLMS) plays a growing role in screening patients for sleep disorders, as it is commonly associated with other sleep‐related diagnoses, deteriorates nighttime sleep quality and sleep architecture. While well‐studied in adults, pediatric data remains scarce. We investigated PLMS characteristics in children in our hospital‐based retrospective study.

**Methods:** We reviewed medical records and polysomnography (PSG) of 286 patients (<18 years). 109 (38.1%) children had a PLMS index ≥5/hour. Among these, 22 (20.2%) had PLMS only, and were diagnosed with Periodic Leg Movement Disorder (PLMD) due to associated symptoms. Additionally, 48 (44.0%) had pediatric obstructive sleep apnea (pOSA), and 19 (17.4%) had NREM parasomnias (NP). Overlapping diagnoses were excluded. Controls had normal PSG and no clinical sleep disorders.

**Results:** Demographics showed 52.8% of controls were boys, compared to 40.9% in PLMD (*p* = 0.272), 72.3% in pOSA and PLMS (*p* = 0.030), and 63.2% in NP and PLMS (*p* = 0.093). Mean age was significantly lower in pOSA+PLMS group (6.6+4.3 years, *p* = 0.010) versus others (11.1 + 4.7 in controls; 7.3 + 3.6 in PLMD; 9.6 + 3.0 in NP+PLMS groups). Regression analysis adjusted for age and sex. Aside from PLMS and apnea‐hypopnea indexes, PSG parameters (latency, efficiency, stages) showed no significant differences between groups. Mean ferritin levels were below 50 μg/L in all groups except pOSA, with the lowest levels in NP+PLMS (26.8 + 16.1 μg/L, *p* = 0.059).

**Figure:**
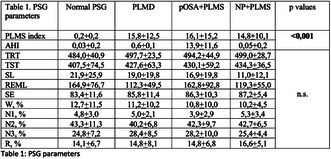
**TABLE 1** PSG parameters.

**Figure:**
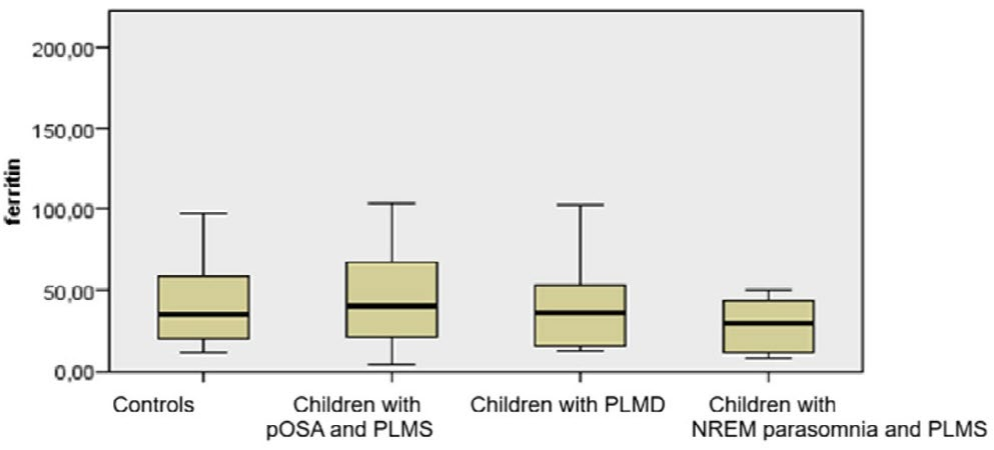
**FIGURE 1** Ferritin levels across groups.

**Conclusion:** PLMS is quite common in pediatric sleep center populations (~33%), frequently co‐existing with pOSA and NREM parasomnias. Low ferritin levels in groups except pOSA emphasize the importance of iron deficiency. Furthermore, a 20% hospital‐based PLMD prevalence is striking, suggesting this group is underestimated and warrants larger prospective studies.

**Disclosure:** Nothing to disclose.

## EPO‐0798

### Effect of the oral orexin receptor 2 agonist oveporexton (TAK‐861) on functional impacts of narcolepsy type 1: Pooled data from two phase 3 studies

#### R. Khatami ^1^; E. Mignot^2^; F. Pizza^3^; G. Plazzi^4^; S. Crawford^5^; M. Etherton^5^; T. Olsson^5^; H. Romero^5^; Y. Savva^5^; B. Yao^5^; Y. Dauvilliers^6^

##### ^1^Center of Sleep Medicine and Sleep Research, Klinik Barmelweid, Barmelweid and Department of Neurology, University Hospital of Bern, Bern, Switzerland; ^2^Stanford Center for Sleep Sciences and Medicine, Palo Alto, USA; ^3^Department of Biomedical and Neuromotor Sciences, University of Bologna and IRCCS Istituto delle Scienze Neurologiche di Bologna, Bologna, Italy; ^4^Department of Biomedical, Metabolic and Neural Sciences, University of Modena and Reggio‐Emilia, Modena and IRCCS, Istituto delle Scienze Neurologiche, Bologna, Italy; ^5^Takeda Development Center Americas, Inc, Cambridge, USA; ^6^Sleep‐Wake Disorders Center, Department of Neurology, Gui‐de‐Chauliac Hospital, CHU, Institute of Neurosciences of Montpellier, INSERM, University of Montpellier and National Reference Network for Narcolepsy, Montpellier, France

**Background and aims:** Narcolepsy type 1 (NT1), a central hypersomnolence disorder with deficiency of hypothalamic orexin signaling, affects functioning and quality of life. We conducted a pooled analysis of data from two randomized, double‐blind, oveporexton (TAK‐861) phase 3 studies (The First Light: NCT06470828; The Radiant Light: NCT06505031) to investigate its effect on functional impacts using the Functional Impacts of Narcolepsy Instrument (FINI).

**Methods:** Eligible participants were aged 16–70 years and had a confirmed NT1 diagnosis. Participants were randomized to twice daily oral oveporexton 1 mg (The First Light only), 2 mg, or placebo, ≥3 hours apart for 12 weeks. Change from baseline to week 12 in FINI domain scores was a secondary endpoint in both trials. The FINI comprises 6 independent domains scored from 0 (best functioning) to 100 (worst functioning): Tiredness, Cognitive Functioning, Cataplexy, Social Activities, Everyday Activities, and Everyday Responsibilities.

**Results:** Improvements with oveporexton were observed across all FINI domains. Mean (SD) change from baseline at week 12 with oveporexton (overall) was −44.6 (23.7) for the Tiredness domain, −33.6 (25.0) for Cognitive Functioning, −24.5 (22.6) for Cataplexy, −33.8 (27.1) for Social Activities, −45.9 (23.0) for Everyday Activities, and −40.0 (28.5) for Everyday Responsibilities; corresponding changes with placebo were −5.1 (22.2), −2.4 (24.4), −5.5 (18.5), −2.6 (23.8), −6.6 (21.5), and −7.7 (28.5). Comparisons versus placebo showed improvements with oveporexton across all domains and doses (Table 1).

**Figure:**
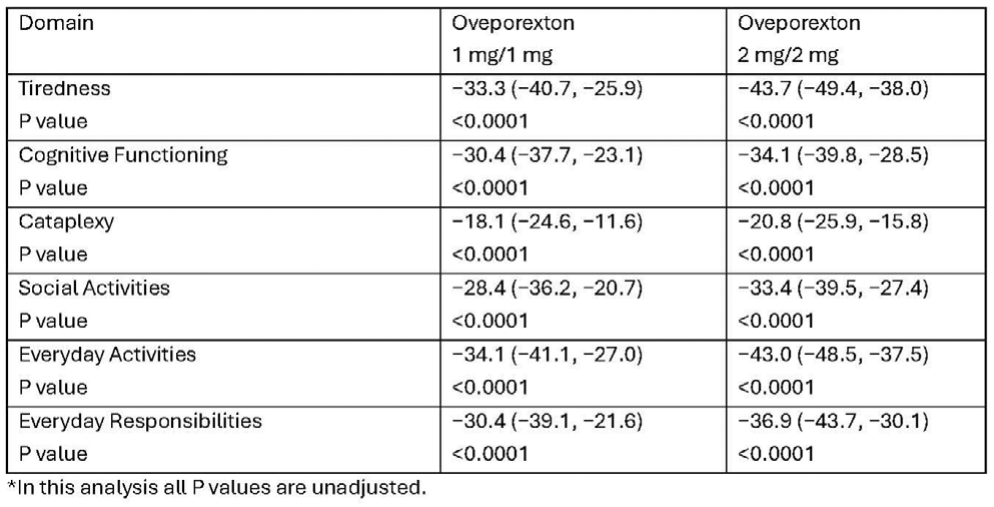
**TABLE 1** Least square mean change (95% CI) from baseline to week 12 versus placebo for the 6 FINI domains.*

**Conclusion:** In this pooled analysis of data from two phase 3 trials, oveporexton showed improvements in narcolepsy‐specific functioning versus placebo.

**Disclosure:** This research was funded by Takeda Development Center Americas, Inc, who also provided funding to Envision Catalyst, an Envision Medical Communications agency, a part of Envision Pharma Group, to provide writing assistance under the direction of the authors. Ramin Khatami received consulting fees, travel support, or board engagement from Bioprojet, Idorsia, Jazz Pharmaceuticals, and Takeda. Emmanuel Mignot received consulting fees from Alkermes, Jazz Pharmaceuticals, and Takeda; research grant or trials to Stanford from Apple, Avadel, Eisei, Jazz Pharmaceuticals, and Takeda; travel funding from Harmony Biosciences, Paladin Labs, and Takeda; and stock options from Centessa. Fabio Pizza participated in an advisory board for Takeda and received support for congress participation and funding from Bioprojet. Giuseppe Plazzi received consultancy fees from Bioprojet, Jazz Pharmaceuticals, Orexia, and Takeda. Stephen Crawford, Mark Etherton, Tina Olsson, Heather Romero, Yulia Savva, and Baiyun Yao are employees of Takeda Development Center Americas, Inc, and stockholders in Takeda Pharmaceuticals Company Limited. Yves Dauvilliers received funds for seminars, board engagements, and travel to conferences from Avadel, Bioprojet, Idorsia, Jazz Pharmaceuticals, Centessa, Alkermes, Pharmanovia, and Takeda.

## EPO‐0799

### Differential effects of digital self‐monitoring and multimodal training on sleep and psychological outcomes in adults with sleep difficulties

#### S. Shelly^1^; T. Tamir^2^; M. Catalogna^3^; N. Saporta^2^; A. Amedi ^3^

##### ^1^Department of Neurology, Rambam Medical Center, Haifa, Israel; ^2^Remepy Health Ltd, Ramat Gan, Israel; ^3^The Baruch Ivcher Institute for Brain, Cognition, and Technology, Reichman University, Herzliya, Israel

**Background and aims:** Sleep difficulties and insomnia are widespread conditions that impair emotional well‐being, daily functioning, and overall quality of life. Although digital tools are increasingly used to address sleep difficulties, the specific contribution of individual intervention components remains unclear.

**Methods:** We conducted a 4‐week randomized study in 63 adults with sleep complaints, comparing a digital sleep diary (DSD) to a multimodal app combining self‐monitoring, psychological training, and a cognitive multisensory task. Sleep and psychological outcomes were assessed pre‐ and post‐intervention. A subsample completed resting‐state fMRI to explore neural mechanisms.

**Results:** Both groups showed improvements in PSQI, indicating better subjective sleep quality. However, only the multimodal group showed significant ISI reductions and broader gains in depression, anxiety, stress, and attention scores. Neuroimaging revealed shared increases in nucleus accumbens (NAc) connectivity with default mode regions across groups. Unique to the multimodal group were reductions in NAc‐insula and NAc‐anterior cingulate connectivity, key salience network regions involved in both emotion regulation and attentional processing. Improvements in insomnia severity correlated with psychological improvements only in the multimodal group.

**Figure:**
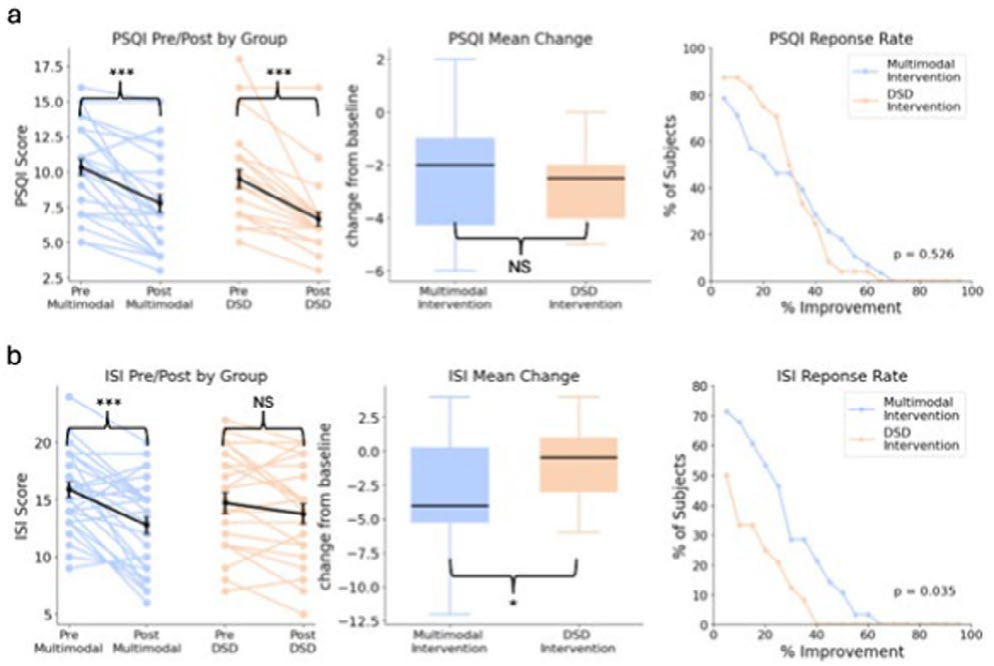
**FIGURE 1** Effects of the digital interventions on sleep outcomes across groups.

**Figure:**
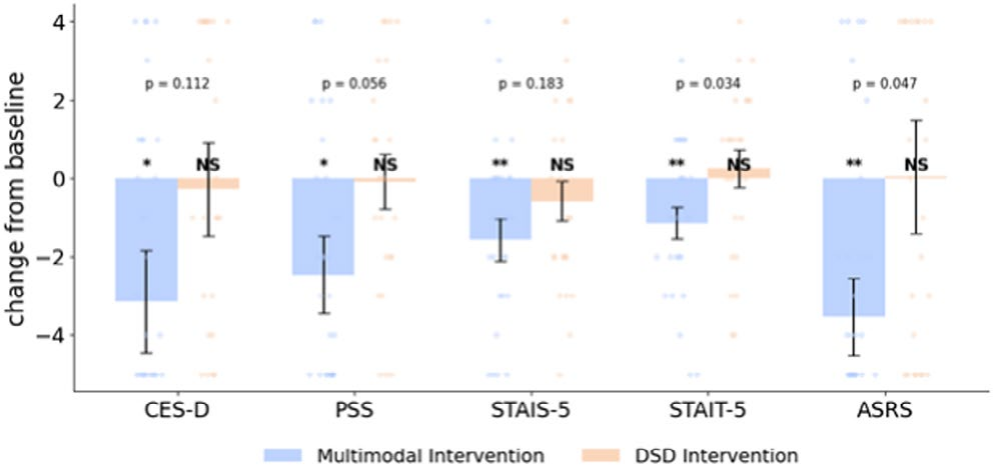
**FIGURE 2** Significant improvements in affective symptoms following the Multimodal intervention.

**Conclusion:** While self‐monitoring improved sleep quality, only the multimodal intervention significantly reduced insomnia severity and emotional symptoms. These findings highlight the value of incorporating targeted psychological and multisensory tools within digital sleep platforms and suggest that such multimodal interventions may offer a scalable and effective solution for addressing both sleep disturbance and its associated emotional symptoms in real‐world settings.

**Disclosure:** The authors of this abstract are employees or consultants of Remepy Health Ltd, the company who developed the app and the sponsor of the study.

## EPO‐0800

### A multimodal neurophysiological signature of REM sleep behavior disorder: A pilot study

#### A. Quercia ^1^; G. Lanza^2^; A. Galeano^1^; A. Garifoli^1^; B. Lanuzza^1^; M. Manuela^1^; M. Tripodi^1^; A. Palmigiano^2^; M. Figorilli^3^; F. Ginatempo^4^; C. Liguori^5^; R. Ferri^1^

##### ^1^Department of Neurology and Sleep Research Centre, Oasi Research Institute‐IRCCS, Troina, Italy; ^2^Department of Surgery and Medical‐Surgical Specialties, University of Catania, Catania, Italy; ^3^Sleep Disorder Research Center, Department of Medical Sciences and Public Health, University of Cagliari, Cagliari, Italy; ^4^Department of Biomedical Sciences, University of Sassari, Sassari, Italy; ^5^Department of Systems Medicine, University of Rome Tor Vergata, Rome, Italy

**Background and aims:** REM Sleep Behavior Disorder (RBD) is a strong prodrome of alpha‐synucleinopathies. Although brainstem dysfunction explains the loss of REM atonia, the interaction between sleep architecture, cortical activity, and motor excitability remains poorly understood. We aimed to map these multimodal interactions to clarify how cortical source signals from wake EEG relate to sleep macrostructure and daytime motor control.

**Methods:** Seven patients with RBD (6 males, 72.86 ± 4.01 years) underwent clinical evaluation (MMSE, MoCA, UPDRS‐I, UPDRS‐III), 5 minutes of resting‐state High‐Density EEG, full‐night Polysomnography (PSG), and Transcranial Magnetic Stimulation (TMS). Cortical current density was reconstructed via eLORETA. To mitigate statistical bias from the limited sample size, we applied a Domain‐Specific PCA to extract the main variance factors: EEG‐Source Activity, PSG indices, TMS inhibitory metrics (Silent period, contralateral and ipsilateral), and Clinical Outcomes. Interactions were mapped using Graph Theory and Hierarchical Clustering.

**Results:** Source analysis revealed significant cortical hypo‐activity within the Salience and the Default Mode networks (Figure 1). The PCA identified an Insular‐Source Factor as a central hub. This cortical node showed a strong coupling with Sleep Efficiency (rho = 0.83) and a secondary association with inhibition Motor Latencies (rho = −0.60) (Figure 2). In contrast, the network revealed a striking dissociation of both REM atonia and clinical motor symptoms (UPDRS‐III), which exhibited the lowest centrality.

**Figure:**
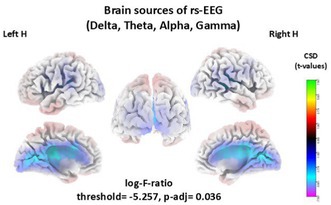
**FIGURE 1** eLORETA statistical maps of source activity differences in RBD. Three‐dimensional maps showing the log‐F ratio distribution for reconstructed sources averaged across frequency bands (Delta, Theta, Alpha, Gamma).

**Figure:**
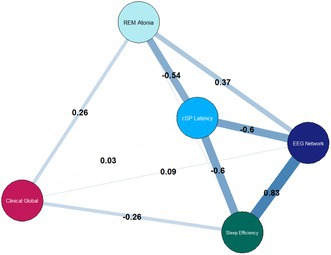
**FIGURE 2** Integration of multimodal biomarkers via Graph Theory. Correlation network representing the interactions between the main variance factors extracted through Domain‐Specific PCA. The nodes include the EEG‐Source Activity (EEG Network), PSG indice.

**Conclusion:** RBD may involve a systemic failure of cortical integration between the Salience and Default mode networks, a neurophysiological substrate for both sleep instability and slowed motor inhibitory processing. This multimodal approach provides a preliminary but promising window into the cortical network reorganization underlying RBD pathophysiology.

**Disclosure:** This research was supported by the following institutional grants: Italian Ministry of University and Research (MUR), BANDO PRIN 20222, project title “Caratteristiche neurofisiologiche multimodali e rischio di fenoconversione nell’iRBD” (grant number: MUR 20228XKKCM_001) to Claudio Liguori, Giuseppe Lanza, and Aurora Palmigiano; Italian Ministry of Health (MinSal), BANDO RF 2021, project title “Translational correlates of multimodal electrophysiological markers of disease process in REM Sleep Behavior Disorder (RBD) and its progression into neurodegenerative disorders: diagnostic hints and therapeutic implications” (grant number: GR‐2021‐12372863) to Giuseppe Lanza, Michela Figorlli, and Francesca Ginatempo.

## EPO‐0801

### Abstract withdrawn

## Neuroepidemiology

## EPO‐0802

### Leveraging a unified electronic health records system through community outreach centres to deliver tele‐neurology services in Kenya: A pilot study

#### C. Chirchir; J. Shah; D. Sokhi

##### Department of Medicine, Aga Khan University Medical College of East Africa (Nairobi Campus), Nairobi, Kenya

**Background and aims:** Access to neurologists in Sub‐Saharan Africa is significantly below global recommendations; Kenya has ~20 neurologists for 55 million people. While our 2021 study demonstrated tele‐neurology consultations (TNCs) were feasible, critical limitations included the inability to use private insurance or access onsite investigations and/or prescriptions. We aimed to address these gaps by leveraging our tertiary hospital's (TH) network of 55 community outreach centres (COCs)—the country's largest, with half spread across the capital—all interlinked by a unified electronic health record (EHR) implemented in 2023.

**Methods:** Prospective patients booked in the TH neurology clinic were offered TNC at their nearest COC. The new integrated workflow permitted insurance processing and direct ordering of investigations/prescriptions via EHR after the TNC. We undertook a post‐TNC survey after consenting participants and present our interim results.

**Results:** We surveyed 30 participants [median (IQR) age 45 (37.0–51.0) years, 73.3% (22/30) female]: the majority of conditions for TNC were epilepsy and migraine [53.3% (16/30), equal weighting). 90% (27/30) reported “high satisfaction” including for accessing investigations/prescriptions at COCs. Median (IQR) savings in travel, time and overall cost were 17 (11.5–216.0) km, 3 (2.0–48.0) hours and €16.5 (IQR 7–70) per encounter respectively, and total carbon emissions saved were 2.36 tCO2e.

**Figure:**
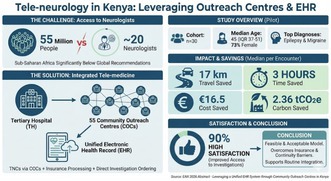
**FIGURE 1** Visual Abstract. Summary of the pilot study demonstrating the feasibility, economic impact, and high acceptability of tele‐neurology delivered via Community Outreach Centres (COCs) in Kenya.

**Conclusion:** Our enhanced workflow, utilizing the TH's vast COC network and EHR, proved highly acceptable and successfully overcame previous insurance and investigation barriers. Interim data confirm significant reductions in travel burden, costs, and carbon emissions, supporting the integration of this model into routine specialist care.

**Disclosure:** Nothing to disclose.

## EPO‐0803

### The International Hospital Federation Geneva Sustainability Centre Toolkit to improve the sustainability of neurology services

#### C. Gericke

##### School of Medicine, Australian National University, Canberra, Australia & International Hospital Federation (IHF) Geneva Sustainability Centre, Geneva, Switzerland

**Background and aims:** Climate change is the biggest threat to human health in the 21st Century. As a systemic issue that impacts all aspects of the healthcare sector, a common understanding of key sustainability terms and concepts is essential. Many hospitals and doctors, including neurologists, are interested in reducing their department or hospital's carbon footprint.

**Methods:** Presentation of the GSC Toolbox to improve the sustainability of healthcare services and practical case studies on how this can be used in clinical services.

**Results:** Many hospitals and health organisations around the world have now implemented the GSC Toolbox and other GSC tools. After a brief overview of all GSC tools to improve sustainability (sustainability accelerator tool, accreditation and certification, action planning, capacity strengthening), several international case studies of hospitals that have successfully implemented the GSC Toolbox will be presented.

**Conclusion:** Participants will learn practical tools to reduce their department or hospital's carbon footprint through case studies presented by a practising neurologist and public health physician.

**Disclosure:** CG is a member of the Steering Committee of the IHF Geneva Sustainability Centre.

## EPO‐0804

### Effectiveness of mobile stroke (MSUs) in acute ischemic stroke: An updated systematic review and meta‐analysis of the five **“**T‐Letter**”**

#### I. Kamal^1^; M. Hemida^2^; D. Abouda^2^; H. Elshiekh^3^; Z. Rezk^4^; M. El‐mezayen^2^; E. Radwan^5^; N. Kebir^6^; R. Hamdy^7^; K. Zinhom ^8^; M. Mamdouh^9^; . Fathallah^10^; A. Zakarya Nourelden^1^

##### ^1^Faculty of Medicine, Al‐Azhar University, Cairo, Egypt; ^2^Faculty of Medicine, Alexandria University, Alexandria, Egypt; ^3^Faculty of Medicine, Zagazig University, Zagazig, Egypt; ^4^Faculty of Medicine, Port Said University; ^5^Faculty of Medicine, Kafer Elshiekh University, Kafer Elshiekh, Egypt; ^6^Faculty of Medicine, University of Oran 1, Oran, Algeria**;** ^7^Faculty of Medicine, Tanta University, Egypt; ^8^Faculty of Medicine, October 6 University, Giza, Egypt; ^9^Faculty of Medicine, Beni‐Suef University, Beni‐Suef, Egypt; ^10^Faculty of Medicine, Minya University, Minya, Egypt

**Background and aims:** Mobile stroke units may accelerate acute ischemic stroke management, but updated evidence comparing their effectiveness and safety with standard emergency medical services (EMS) remains limited, necessitating a contemporary, comprehensive meta‐analysis.

**Figure:**
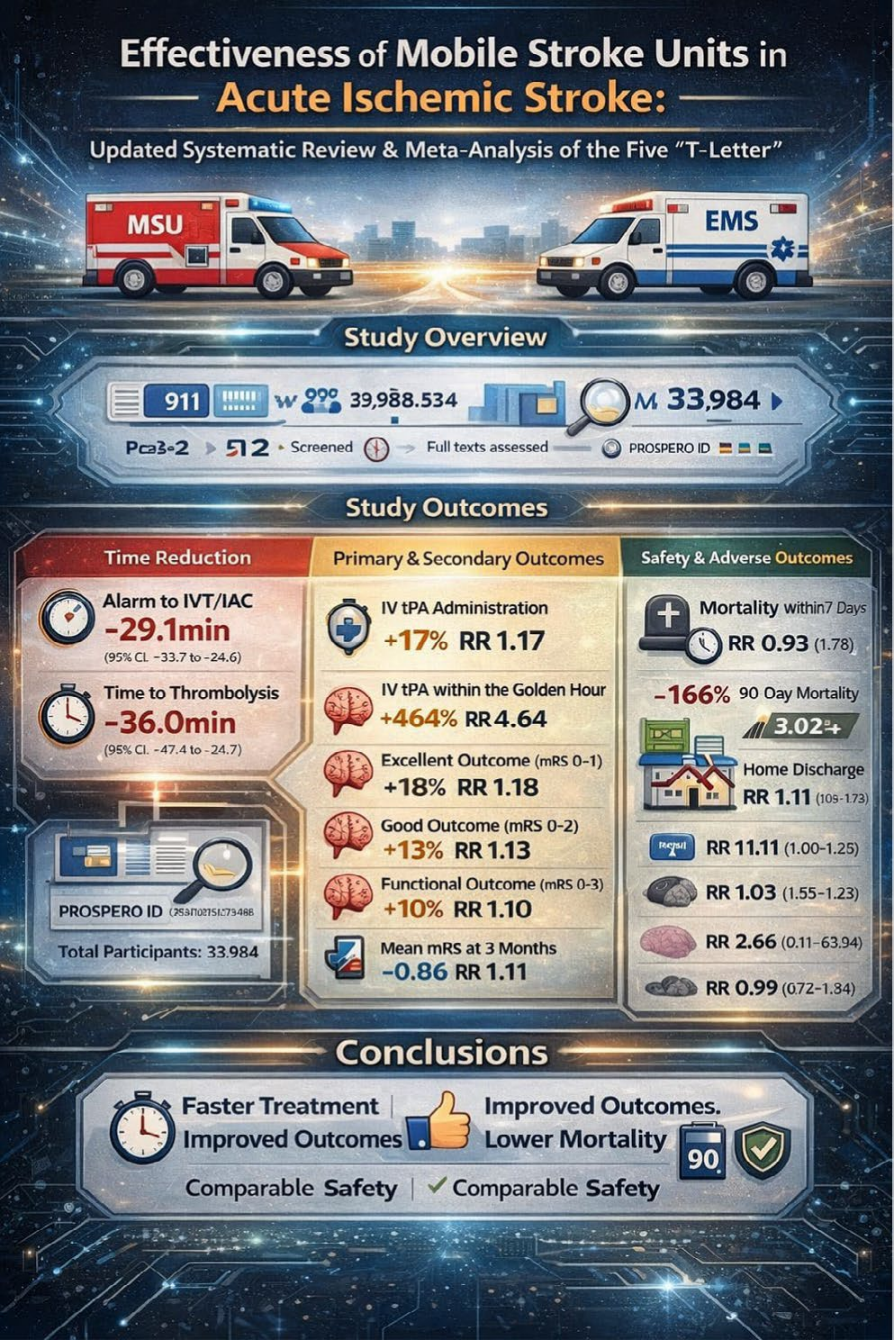
**FIGURE 1** Graphical abstract.

**Methods:** This PRISMA‐compliant systematic review (PROSPERO CRD420251273498) searched 5 databases for comparative studies of MSUs versus standard EMS. Random‐effects models pooled treatment time, functional, mortality, and safety outcomes, with risk of bias assessed using RoB‐2, ROBINS‐I, and NOS tools.

**Results:** Across thirty three eligible studies identified, pooled analyses showed substantial reductions in treatment delays, with alarm‐to‐IVT/IAC time shortened by 29.1 minutes (95% CI −33.7 to −24.6; *I* ^2^ = 97.6%) and time to thrombolysis reduced by 36.0 minutes (95% CI −47.4 to −24.7; *I* ^2^ = 89.9%). Functional outcomes favored MSUs care across multiple thresholds: the likelihood of excellent outcome (mRS 0–1) increased by 18% (RR 1.18), good outcome (mRS 0–2) by 13% (RR 1.13), and mRS 0–3 by 10% (RR 1.10). MSUs management increased IV tPA use by 17% (RR 1.17) and markedly improved golden‐hour thrombolysis (RR 4.64). While 7‐day mortality did not differ (RR 0.93), 90‐day mortality was reduced by 16% (RR 0.84, 95% CI 0.74–0.94). Safety outcomes, including intracranial hemorrhage (RR 1.01), seizures (RR 0.99), stroke mimics (RR 1.03), and TIA (RR 0.84), showed no significant differences, supporting the numerical efficacy and safety profile of MSU‐based stroke care.

**Figure:**
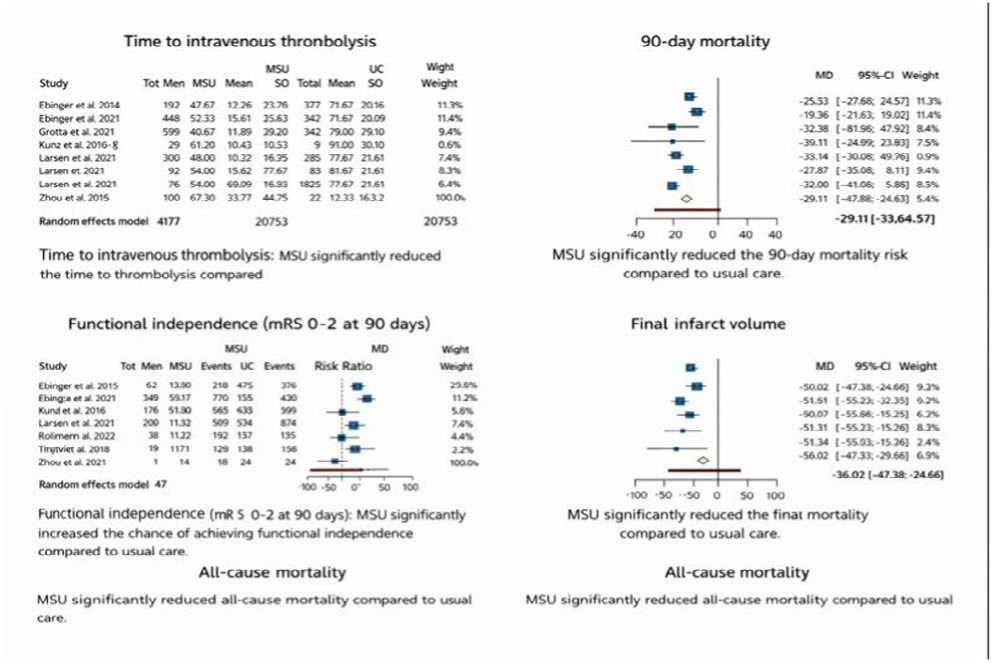
**FIGURE 2** Outcome analysis.

**Figure:**
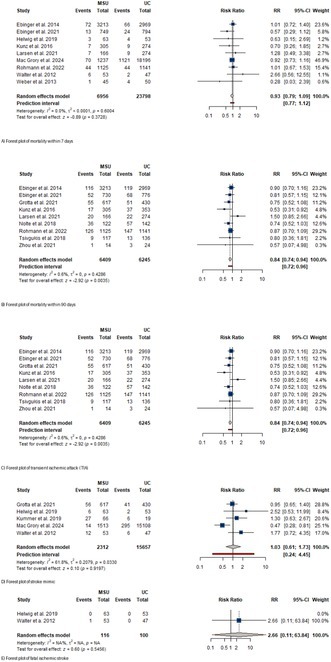
**FIGURE 3** Comparison between MSU and ESU.

**Conclusion:** MSUs care significantly reduced treatment times, increased thrombolysis rates, improved functional outcomes, and lowered 90‐day mortality without increasing adverse events, demonstrating superior effectiveness and comparable safety versus standard EMS.

**Disclosure:** Nothing to disclose.

## EPO‐0805

### Dementia burden in Europe, 1990–2023: Evidence from the global burden of disease study

#### D. Urso^1^; E. Nichols^2^; S. Giannoni‐Luza ^3^; C. Brayne^4^; N. Ray^1^; G. Logroscino^3^

##### ^1^GeoHealth group, Institute of Global Health, Faculty of Medicine, University of Geneva, Geneva, Switzerland; ^2^Leonard Davis School of Gerontology, University of Southern California, Los Angeles, USA; ^3^Department of Translational Biomedicine and Neuroscience (DiBraiN), University of Bari ‘Aldo Moro’, Bari, Italy; ^4^Cambridge Public Health, University of Cambridge, Cambridge, UK

**Background and aims:** Dementia is a leading cause of disability and mortality in Europe, yet no recent harmonised assessment has described its burden across the European Union (EU‐27) and the WHO European Region.

**Methods:** We used data from the Global Burden of Disease Study 2023 (GBD 2023) to estimate prevalence, mortality, and disability‐adjusted life years (DALYs) for Alzheimer's disease and other dementias from 1990 to 2023. Estimates were produced for the EU‐27 and the WHO European Region, by age and sex. Non‐fatal outcomes were modelled using DisMod‐MR 2.1, and dementia‐attributable mortality was estimated using an excess‐mortality framework. We also quantified DALYs attributable to six modifiable risk factors.

**Results:** In 2023, 7.76 million people were living with dementia in the EU‐27 and 12.28 million in the WHO European Region, representing ~90% increases since 1990 despite modest declines in age‐standardised prevalence. Prevalence was nearly twice as high in women as in men. Dementia rose from the eighth to the third leading cause of death in the EU‐27. Dementia accounted for 5.49 million DALYs in the EU‐27. An estimated 41% of DALYs in the EU‐27 were attributable to modifiable risk factors, particularly ambient particulate matter pollution, high fasting plasma glucose, and high body‐mass index, which are disproportionately concentrated in socioeconomically disadvantaged populations.

**Conclusion:** Despite modest declines in age‐standardised rates, the absolute burden of dementia in Europe continues to rise, driven by population ageing. The substantial contribution of modifiable risk factors highlights major opportunities for prevention. Robust, country‐specific estimates are essential to guide integrated strategies combining prevention and care planning.

**Disclosure:** Nothing to disclose.

## EPO‐0806

### Bern sleep–wake Registry: Extraction of longitudinal health trajectories from real‐world clinical data with large language models

#### I. Filchenko ^1^; K. Zub^1^; F. Pompizii^1^; J. van der Meer^1^; N. Cihoric^2^; M. Schmidt^1^; F. Dennstädt^2^; C. Bassetti^3^

##### ^1^Department of Neurology, Bern University Hospital (Inselspital) and University of Bern, Bern, Switzerland; ^2^Department of Radiation Oncology, Bern University Hospital (Inselspital) and University of Bern, Bern, Switzerland; ^3^Medical Faculty, University of Bern, Bern, Switzerland

**Background and aims:** Large registries with real‐world clinical data are essential for understanding long‐term health trajectories. Large language models (LLM) reliably extract binary‐coded diagnoses from clinical text (Dennstadt et al, 2024). Little is known about their ability to capture the temporal structure of diagnoses, including repeated instances. Classification of diagnostic trajectories is essential for prognostic modeling, with particular relevance for preventive medicine.

**Methods:** This interim analysis is part of the ongoing “Sleep and Longevity” project within the Bern Sleep–Wake Registry (Figure 1). Six ICD‐11 comorbidities with 25 subcategories were selected. They were manually scored in 1000 randomly selected plain‐text diagnostic records by two independent medical doctors. Disagreements were resolved by a third medical doctor to define the ground truth. For each diagnosis, scorers recorded its presence, type, and date, allowing up to five instances per diagnosis. LLM‐based extraction was performed using the data‐element‐extractor Python package (v0.2.0) with GPT‐OSS‐20B. Only basic prompting was applied. LLM performance was evaluated descriptively against the ground truth for exact matches.

**Figure:**
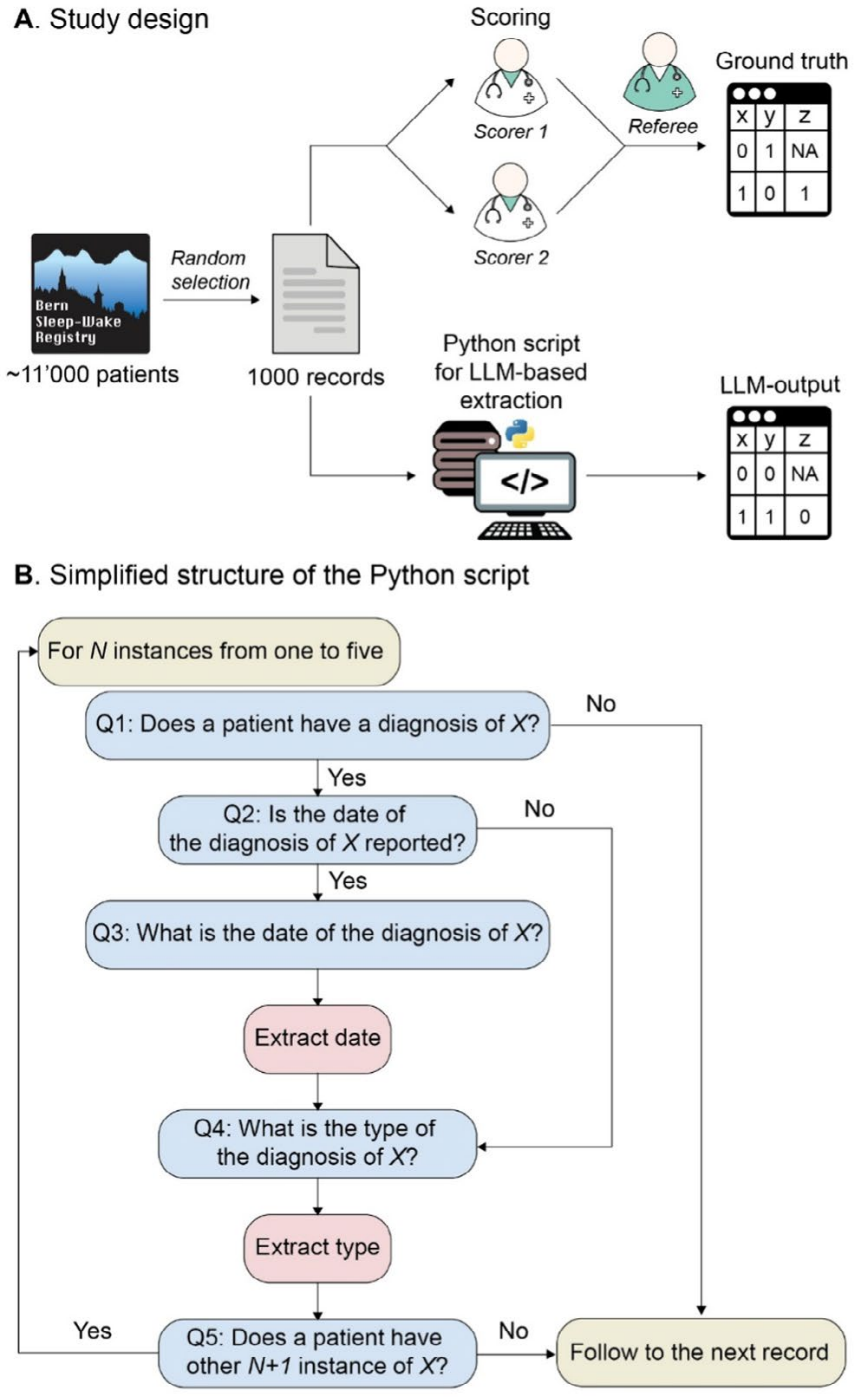
**FIGURE 1** (A) Study design. (B) Simplified structure of Python script.

**Results:** Interrater agreement between scorers was high (Cohen's kappa: median 0.96, IQR [0.79–0.98]). The LLM reliably identified diagnoses but struggled to differentiate repeated instances and their dates, occasionally returning no date (Table 1). For instances with an identified date, agreement with the ground truth was moderate to high.

**Figure:**
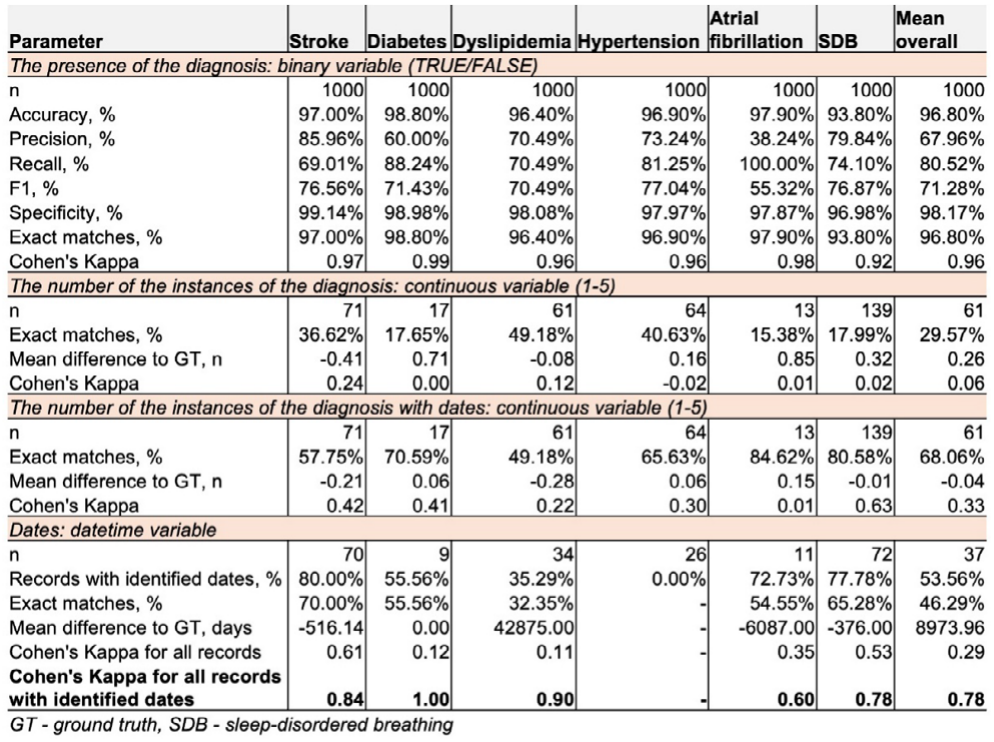
**TABLE 1** LLM performance evaluated descriptively against the ground truth for exact matches.

**Conclusion:** This is the first study to extract diagnostic trajectories from clinical text in patients with neurologic disorders using LLM. We will improve the extraction through optimized prompting, constrained outputs, and computational strategies (e.g, use of a larger model).

**Disclosure:** Nothing to disclose.

## EPO‐0807

### Risk and causes of hospitalization in persons with Parkinson's disease or atypical parkinsonism: The ParkLink Bologna cohort

#### L. Vignatelli ^1^; C. Zenesini^1^; M. Marinelli^1^; F. Baccari^1^; L. Apollonio^2^; L. Baldelli^3^; C. Calabrò^2^; I. Cani^3^; P. Guaraldi^1^; G. Giannini^3^; G. Loddo^1^; R. Pantieri^1^; E. Sancisi^2^; C. Scaglione^1^; C. Stipa^2^; V. Tontini^2^; E. Baldin^1^; G. Calandra‐Buonaura^3^; F. Nonino^1^

##### ^1^IRCCS Istituto delle Scienze Neurologiche di Bologna, Bologna, Italy; ^2^Azienda USL di Bologna, Bologna, Italy; ^3^Department of Biomedical and NeuroMotor Sciences (DiBiNeM), Alma Mater Studiorum *–* University of Bologna, ItalyDepartment of Biomedical and NeuroMotor Sciences (DiBiNeM), Alma Mater Studiorum *–* University of Bologna, Italy

**Background and aims:** Parkinson's disease is among the leading neurological causes of global burden in the elderly. Risk of hospitalization in people with Parkinson's disease (PPD) is an essential aspect to consider for improving healthcare strategies. We aimed to investigate the risk and causes of hospitalization admission in 2‐year observation in PPD or people with atypical parkinsonism (PAPS) compared to a matched general population cohort.

**Methods:** Prospective cohort of PPD or APS compared to a general population sample, matched for sex, age, residence, and comorbidity index within the ParkLink Bologna record linkage system, an ongoing project started in February 2015 in the Local Health Care Trust of Bologna, Northern Italy. PPD/PAPS were diagnosed and recruited by neurologists during routine outpatient clinical practice. Clinical data were linked to administrative health data. Hospitalization at 2‐year after recruitment was reported as Incidence Rate Ratio (IRR) between PPD/PAPS and controls.

**Results:** 816 PPD, 148 PAPS and 9447 controls were included. A higher risk of hospitalization was observed among PPD (IRR 1.60, 95% CI 1.45–1.76) and PAPS (IRR 1.61, 95% CI 1.28–2.02). The acute leading causes of hospitalisation were infections and metabolic imbalances for both groups, while severe trauma specifically affected PPD and thromboembolism specifically affected PAPS (Table).

**Figure:**
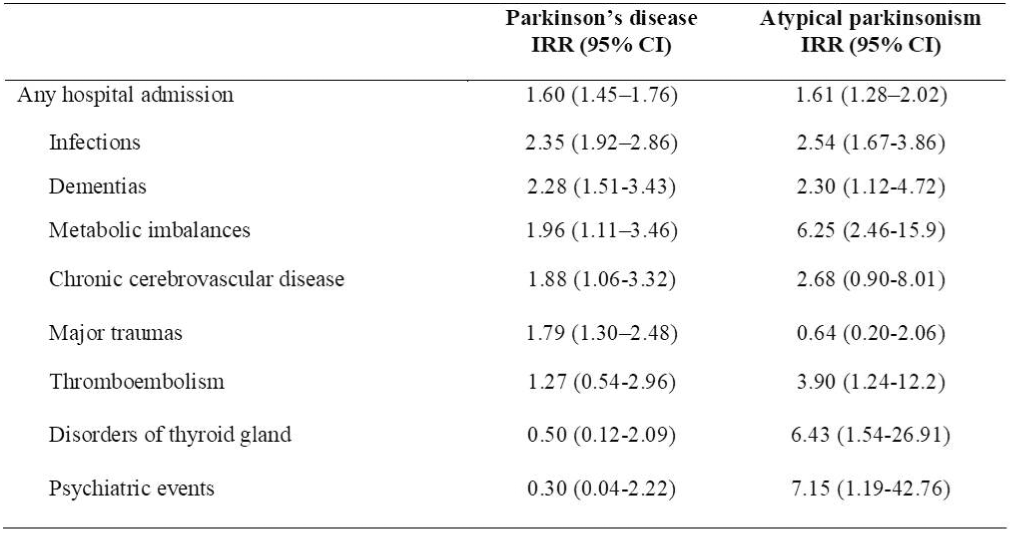
**TABLE 1** Incidence Rate Ratios (IRR) with 95% Confidence Intervals (95% CI) for the significant hospital clinical features, comparing the Parkinson's disease and atypical Parkinsonism cohorts with their respective control cohorts.

**Conclusion:** Linking administrative data with reliable clinical data allow exploring relevant outcomes of the clinical course of PPD. Over a 2‐year period, PPD and PAPS had an approximately 60% higher risk of hospitalization, respectively, mainly due to a doubled risk of infections and metabolic imbalances, compared to a matched general population cohort.

**Disclosure:** The study is funded by IRCCS ISNB (Bando Ricerca Corrente 2025).

## EPO‐0808

### 5q‐associated spinal muscular atrophy in Austria: Epidemiology and co‐morbidities

#### O. Keritam ^1^; G. Zulehner^1^; R. Wurm^1^; F. Gruber^2^; J. Rath^1^; M. Krenn^1^; D. Bormann^1^; M. Erdler^3^; S. Mahal^4^; A. Wiesenhofer^4^; M. Gosk‐Tomek^4^; M. Rappold^5^; A. Hüpper^4^; K. Vettori^4^; T. Griedl^6^; C. Kiss^6^; V. Gold^6^; J. Wanschitz^7^; A. Hotter^7^; V. Kleinveld^7^; C. Horlings^7^; M. Gräßl^8^; A. Schwerin‐Nagl^8^; J. Troger^9^; S. Grinzinger^10^; E. Stögmann^11^; P. Müller^12^; D. Langenscheidt^13^; B. Plecko^8^; F. Zimprich^1^; R. Topakian^12^; C. Eggers^14^; S. Quasthoff^6^; F. Knipp^4^; G. Bernert^15^; M. Baumann^16^; W. Löscher^7^; H. Cetin^1^

##### ^1^Department of Neurology, Medical University of Vienna, Vienna General Hospital, Vienna, Austria; ^2^Austrian Federation of Social Insurances, Vienna, Austria; ^3^Department of Neurology, Klinik Donaustadt, Vienna, Austria; ^4^Department of Paediatrics, Klinik Favoriten, Vienna, Austria; ^5^Primary Care Centre Nepomuk, Vienna, Austria; ^6^Department of Neurology, Medical University of Graz, Graz, Austria; ^7^Department of Neurology, Medical University of Innsbruck, Innsbruck, Austria; ^8^Department of Paediatrics and Adolescent Medicine, Medical University of Graz, Graz, Austria; ^9^Department of Neurology, Klinikum Klagenfurt, Klagenfurt, Austria; ^10^Department of Neurology, Paracelsus Medical University, Salzburg, Austria; ^11^Department of Paediatrics, Landesklinikum Mödling, Mödling, Austria; ^12^Department of Neurology, Academic Teaching Hospital Wels‐Grieskirchen, Wels, Austria; ^13^Department of Neurology, Landeskrankenhaus Rankweil, Rankweil, Austria; ^14^Department of Neurology, Johannes Kepler University, Linz, Austria; ^15^Department of Paediatrics, Medical University of Vienna, Vienna, Austria**;** ^16^Division of Paediatric Neurology, Department of Paediatrics I, Medical University Innsbruck, Innsbruck, Austria

**Background and aims:** Spinal muscular atrophy (SMA) is a genetic motor neuron disease caused by homozygous deletions or other pathogenic variants in the SMN1 gene, resulting in the degeneration of lower motor neurons associated with progressive weakness and muscle wasting. The development and approval of disease‐modifying therapies (DMT) in recent years has fundamentally transformed the management, as well as the clinical and epidemiological landscape of patients with SMA.

**Methods:** In this study, hospitalisation and prescription databases together with the capture‐recapture method are utilised to estimate the prevalence of SMA in Austria and to identify co‐morbidities associated with SMA. Patients were matched to healthy controls (1:10) by sex, age and district of residence.

**Results:** We estimated a total population of 236 patients (95% CI 231–241) with SMA in Austria and a corrected prevalence of 2.5/100,000 persons in the year 2023. Patients with SMA showed significantly higher use of several medication classes, particularly therapies related to respiratory and infectious conditions. Healthy controls, by contrast, exhibited higher prescribing rates of cardiovascular medications, including beta‐blockers and ACE inhibitors, as well as lipid‐lowering agents and muscle relaxants.

**Conclusion:** Our findings provide a robust nationwide estimate of SMA prevalence in Austria and highlight a distinct pattern of co‐morbidities in the era of disease‐modifying therapies, supporting informed healthcare planning and long‐term management of patients with SMA.

**Disclosure:** This study was financially supported by Biogen, Novartis and Roche.

## EPO‐0809

### Increasing prevalence of amyotrophic lateral sclerosis in Austria correlates with reduced mortality

#### O. Keritam ^1^; R. Wurm^1^; D. Bormann^1^; F. Gruber^2^; M. Krenn^1^; G. Zulehner^1^; J. Rath^1^; F. Zimprich^1^; H. Cetin^1^

##### ^1^Department of Neurology, Medical University of Vienna, Vienna General Hospital, Vienna, Austria; ^2^Austrian Federation of Social Insurances, Vienna, Austria

**Background and aims:** Amyotrophic lateral sclerosis (ALS) is a neurodegenerative disorder characterised by the progressive loss of motor neurons, ultimately leading to death within a few years of disease onset. Although recent studies suggest that the global burden of the disease has increased over past decades, the trajectory of epidemiological metrics in Austria remain unknown.

**Methods:** Hospital discharge records and riluzole prescription databases were used to identify ALS cases between 2016 and 2023. The capture–recapture method was applied to estimate the incidence and prevalence of ALS in Austria. The relationship between disease prevalence and the ratio of deaths to prevalent cases (death‐to‐case ratio) in a given year was analysed using Pearson's correlation.

**Results:** A total of 3344 patients were identified during the study period, of whom 44.3% were female. While the corrected annual incidence remained stable (5.2/100,000 person‐years in 2016 and 4.8/100,000 person‐years in 2023), the prevalence increased steadily over the same period, rising from 8.6 to 14.5/100,000 individuals. Median survival was 605 days (95% CI 569–638). The death‐to‐case ratio decreased during the study period, from 0.32 in 2016 to 0.22 in 2023, which correlated negatively with the corresponding prevalence numbers (*r* = −0.85, *p* = 0.0019).

**Conclusion:** In this study, we report on the epidemiology of ALS in Austria between 2016 and 2023. Notably, while the annual incidence remained stable over the study period, our findings indicate an increase of prevalence, most likely due to reduced mortality rates. Our results underscore the need for national healthcare policies to address the challenges posed by the growing disease burden.

**Disclosure:** This study was not financially supported.

## EPO‐0810

### Lower BMI thresholds for cardiometabolic risk in Hungary: Implications for stroke prevention

#### T. Jarecsny ^1^; D. Janoska^1^; F. Fazekas^1^; R. Schwab^2^; C. Árváné Egri^3^; R. Kosik^3^; G. Jozsef Szollosi^4^; M. Fekete^5^

##### ^1^Department of Neurology and Stroke, Saint John's Central Hospital of North Buda, Budapest, Hungary; ^2^BiomTeam@ MiND Brain‐Gut Center, Budapest, Hungary; ^3^National Public Health and Pharmaceutical Center, Budapest, Hungary; ^4^Faculty of Economics and Business, University of Debrecen, Debrecen, Hungary; ^5^Institute of Preventive Medicine and Public Health Faculty of Medicine, Semmelweis University, Budapest Hungary

**Background and aims:** Hungary has one of Europe's highest obesity rates (72.5% of adults). Excess weight is a major risk factor for stroke, contributing to substantial health burden (~€1 billion annual stroke care costs). We aimed to examine BMI associations with cardiometabolic risk factors in Hungarian adults and identify optimal thresholds for stroke prevention.

**Methods:** Cross‐sectional study of 868 Hungarian adults assessing BMI associations with hypertension, type 2 diabetes, dyslipidemia, and atherosclerosis. BMI was calculated from measured height and weight; cardiometabolic conditions determined via clinical examination and laboratory‐based point‐of‐care tests. Sex‐stratified logistic regression identified BMI cut‐offs for elevated cardiometabolic risk, compared to WHO standards.

**Results:** Higher BMI significantly associated with hypertension, diabetes, hypertriglyceridemia, and atherosclerosis (*p <* 0.05), but not hypercholesterolemia. Hungarian adults exhibited lower BMI thresholds than international standards. Women showed elevated risk at BMI ~21.8–22.3 kg/m^2^ (normal range) and further elevation at ~25–27.8 kg/m^2^. Men demonstrated risk inflection points at BMI ~23.8–24.3 and ~28.8–29.9 kg/m^2^.

**Figure:**
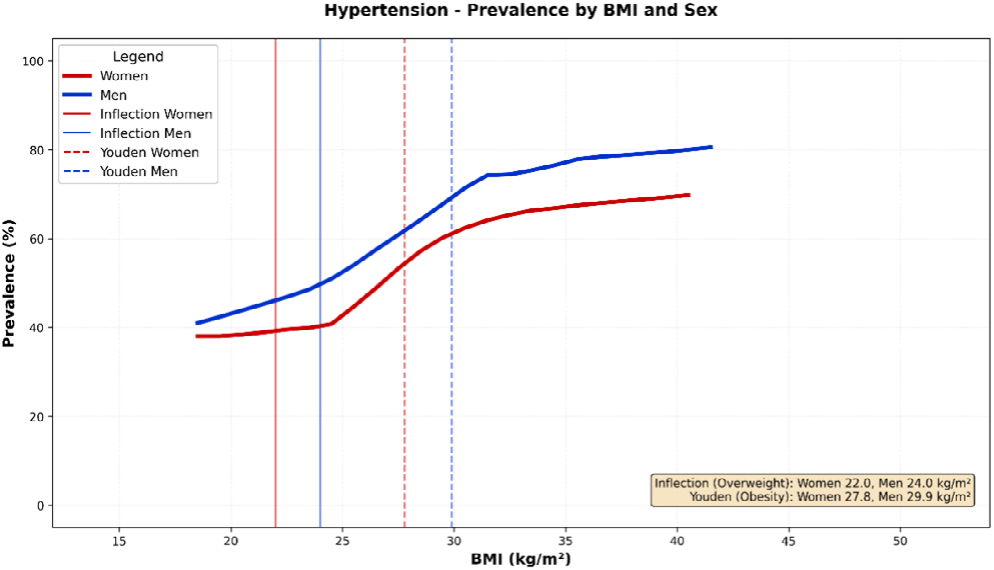
**FIGURE 1** Sex‐specific prevalence of hypertension across BMI.

**Figure:**
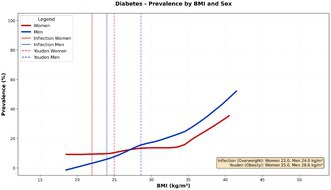
**FIGURE 2** BMI‐related prevalence of diabetes by sex.

**Figure:**
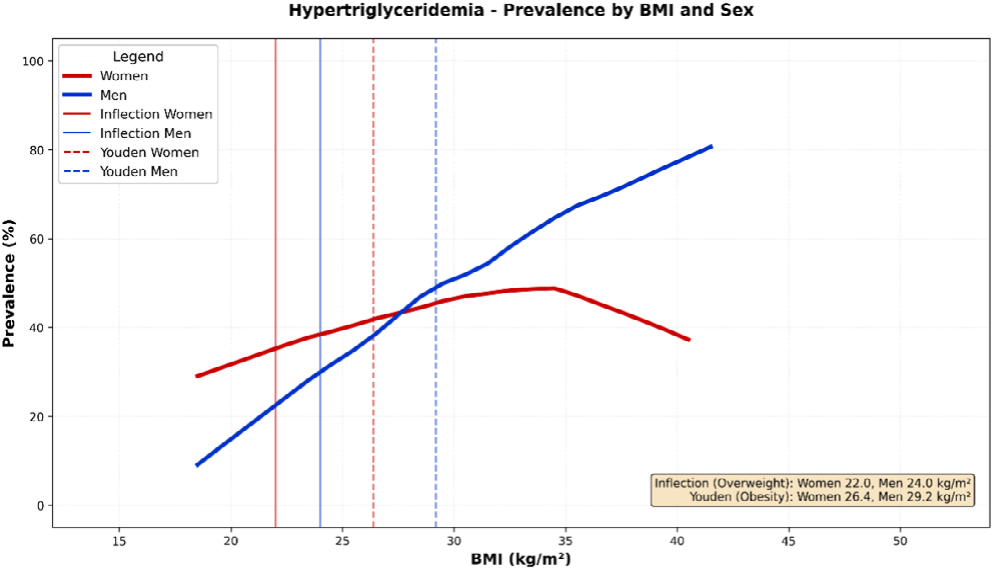
**FIGURE 3** Prevalence of hypertriglyceridemia by BMI and sex.

**Conclusion:** BMI thresholds for cardiometabolic risk are lower in Hungarians than WHO definitions. Hungary‐specific cut‐offs improve identification of high‐risk individuals and enable timely intervention to prevent stroke, substantially reducing incidence and burden.

**Disclosure:** Nothing to disclose.

## EPO‐0811

### A nationwide analysis: Memantine in the treatment of dementia in Hungary

#### P. Halmos^1^; D. Bereczki^2^; B. Dobi^3^; P. Vinnai^3^; A. Ajtay^2^; T. Kovács ^2^

##### ^1^Doctoral College János Szentágothai Neurosciences Division, Semmelweis University, Budapest, Hungary; ^2^Department of Neurology, Semmelweis University, Budapest, Hungary; ^3^HUN‐REN SE Neuroepidemiological Research Group, Semmelweis University, Budapest, Hungary

**Background and aims:** Dementia is a leading cause of dependency and disability, placing a substantial burden on patients, caregivers and healthcare. The aim of this study was to provide a comprehensive overview of the use of anti‐dementia drugs (ADD) – especially memantine –and their association with survival outcomes.

**Methods:** Using the NEUROHUN database, we analyzed health data covering the entire population of Hungary between 2010 and 2020. Descriptive statistics were calculated to assess disease incidence and prevalence, followed by Kaplan–Meier survival analyses and Cox proportional hazard regression to evaluate survival outcomes.

**Results:** We identified 398,584 patients with a diagnosis of dementia between 2010 and 2020. Of the total cohort, only 13.3 % of the patients were treated with at least one of the ADDs examined (memantine, donepezil or rivastigmine). Among the medication‐fillers, 24.04 % patients were treated with memantine only. Kaplan–Meier analysis demonstrated that the untreated group had the shortest median survival (3.105 years, 95% CI: 3.083–3.129), whereas among patients on medication, those treated with memantine monotherapy had the shortest median survival (3.704 years, 95% CI: 3.606–3.784). In the Multivariable Cox proportional hazards model, patients receiving a combination of memantine and donepezil had the lowest mortality risk (HR: 0.581, 95% CI: [0.566–0.597]) compared to the untreated group.

**Conclusion:** These findings suggest a high proportion of delayed diagnoses, as memantine is typically prescribed only in advanced dementia stages. Timely diagnosis—particularly in the era of emerging disease‐modifying therapies—along with prevention programs and evidence‐based healthcare planning is therefore essential.

**Disclosure:** Nothing to disclose.

## Neuroimmunology 4

## EPO‐0812

### Efficacy of eculizumab in the severe acute attacks of aquaporin‐4 IgG‐positive neuromyelitis optica spectrum disorder

#### K. Zeng; W. Chen; A. Lin

##### Department of Neurology, the First Affiliated Hospital, Fujian Medical University, Fuzhou, China

**Background and aims:** To compare the therapeutic efficacy of intravenous methylprednisolone (IVMP) versus IVMP combined with eculizumab in patients with neuromyelitis optica spectrum disorder (NMOSD) experiencing severe acute attacks.

**Methods:** The study performed a retrospective analysis of 25 severe acute attacks occurring in 17 patients with NMOSD during the acute phase. Treatment outcomes were evaluated using the Expanded Disability Status Scale (EDSS) and changes in visual acuity.

**Results:** Thirteen of the 17 patients received adjunctive eculizumab during the acute phase. The IVMP plus eculizumab group exhibited substantial clinical improvement at discharge and follow‐up, with a mean ΔEDSS (defined as discharge EDSS minus attack EDSS) of ‐1.5, in contrast to a mean ΔEDSS of ‐0.1 in the IVMP monotherapy group.

**Conclusion:** The combination of IVMP and eculizumab provides superior clinical benefit over IVMP for patients with NMOSD presenting with severe acute attacks.

**Disclosure:** Nothing to disclose.

## EPO‐0813

### Impact of blood‐brain barrier integrity on the clinical interpretation of serum neurofilament light as a biomarker of neuroaxonal damage

#### A. Imeri‐Merseli ^1^; M. Haindl^2^; R. Demjaha^2^; C. Tafrali^2^; M. Martinez Serrat^2^; D. Pinter^2^; R. Bichler^2^; S. Wurth^2^; S. Hochmeister^2^; C. Enzinger^2^; A. Lass^3^; E. Hofer^4^; M. Khalil^2^

##### ^1^Diagnostic & Research Institute of Human Genetics, Medical University of Graz, Austria; ^2^Department of Neurology, Medical University of Graz, Austria; ^3^Institute of Molecular Biosciences, University of Graz, Austria; ^4^Department of Neurology, Medical University of Graz, Austria; Institute for Medical Informatics, Statistics and Documentation, Medical University of Graz, Austria

**Background and aims:** Neurofilament light chain (NfL) is an established biomarker of neuroaxonal damage. Despite its relevance in disease monitoring and treatment follow‐up, the influence of BBB integrity on serum NfL levels, however, remains insufficiently investigated. This study aimed to assess the impact of BBB integrity on serum NfL concentrations and its clinical relevance.

**Methods:** NfL concentrations were analyzed in paired cerebrospinal fluid (CSF) and serum samples. BBB integrity was assessed using the CSF/serum albumin quotient (QAlb). 109 patients were included in correlation and regression analyses, 79 patients were classified by BBB integrity (intact BBB: *n* = 35; disrupted BBB: *n* = 44). NfL was measured using HD‐X (single molecule array assay) and Lumipulse G600 II (chemiluminescent enzyme immunoassay).

**Results:** Patients with a disrupted BBB showed higher NfL concentrations compared to patients with an intact BBB: HD‐X CSF 453 [276–1268] pg/mL vs. 1252 [569–4622] pg/mL (*p <* 0.001); HD‐X serum 9.98 [6.26–28.58] pg/mL vs. 25.46 [11.51–53.56] pg/mL (*p* = 0.005). Lumipulse CSF 334 [213–1191] pg/mL vs. 1060 [544–4260] pg/mL (*p* = 0.001); Lumipulse serum 12.33 [9.24–40.79] pg/mL vs. 35.60 [15.26–64.96] pg/mL (*p* = 0.006). CSF/serum correlations were stronger in patients with an intact BBB (HD‐X: rₛ = 0.84; Lumipulse: rₛ = 0.87; both *p <* 0.001) than with a disrupted BBB (HD‐X: rₛ = 0.73; Lumipulse: rₛ = 0.71; both *p <* 0.001). In the overall cohort (*N* = 109), strong CSF/serum correlations were observed (HD‐X: rₛ = 0.80, *p <* 0.001; Lumipulse: rₛ = 0.83, *p <* 0.001). Linear regression showed no significant influence of QAlb on this relationship (interaction *p* > 0.9).

**Conclusion:** BBB integrity influences absolute serum NfL concentrations but not the CSF/serum NfL relationship, supporting serum NfL as a valid biomarker of neuroaxonal damage, even in BBB dysfunction.

**Disclosure:** Arijeta Imeri‐Merseli has nothing to disclose Michaela Tanja Haindl has nothing to disclose Rina Demjaha has received travel funding from Janssen, Novartis and Sanofi. Cansu Tafrali has nothing to disclose Maria Martinez‐Serrat has nothing to disclose Roberta Bichler has nothing to disclose Sebastian Wurth has nothing to disclose Sonja Hochmeister has nothing to disclose Daniela Pinter is a member of the advisory board for “Cognition and MS” for Novartis and has received speaking honoraria from Biogen, Novartis, MedAhead and Bristol‐Myers Squibb. Christian Enzinger has received travel funding and speaker honoraria from Biogen Idec, Bayer Schering Pharma, Merck Serono, Novartis, Genzyme and Teva Pharmaceutical Industries Ltd./sanofi‐aventis, Shire; received research support from Merck Serono, Biogen Idec, and Teva Pharmaceutical Industries Ltd./sanofi‐aventis; and serves on scientific advisory boards for Bayer Schering Pharma, Biogen Idec, Merck Serono, Novartis, Genzyme, Roche, and Teva Pharmaceutical Industries Ltd./sanofi‐ Aventis. Achim Lass has nothing to disclose Edith Hofer has nothing to disclose Michael Khalil has received travel funding and speaker honoraria from Bayer, Biogen, Novartis, Merck, Sanofi and Teva and serves on scientific advisory boards for Biogen, Bristol‐Myers Squibb, Gilead, Merck, Neuraxpharm, Novartis, Alexion, Amgen and Roche. He received research grants from Biogen, Novartis and Teva.

## EPO‐0814

### Diagnostic performance and pitfalls of a commercial assay for CASPR2 autoantibodies

#### C. Milano ^1^; L. Naranjo^2^; R. Ruiz Garcia^2^; L. Arlettaz^3^; C. Papi^1^; L. Marmolejo^1^; T. Armangué^4^; M. Guasp^1^; J. Dalmau^1^; F. Graus^1^; M. Spatola^5^

##### ^1^Neuroimmunology Program, Fundació de Recerca Clínic Barcelona‐Institut d'Investigacions Biomédiques August Pi i Sunyer (FRCB‐IDIBAPS), University of Barcelona, Spain; La Caixa Research Institute, Barcelona, Spain**;** ^2^Neuroimmunology Program, Fundació de Recerca Clínic Barcelona‐Institut d'Investigacions Biomédiques August Pi i Sunyer (FRCB‐IDIBAPS), University of Barcelona, Spain; Immunology Service, Biomedical Diagnostic Center, Hospital Clínic, Barcelona, Spain; ^3^Service d’Immuno‐Allergologie, Institut Central des Hôpitaux, Sion, Switzerland; ^4^Neuroimmunology Program, FRCB‐IDIBAPS, University of Barcelona, Spain; La Caixa Research Institute, Barcelona, Spain; Pediatric Neuroimmunology Unit, Neurology Department, Sant Joan de Déu Children's Hospital, University of Barcelona, Barcelona, Spain; ^5^Neuroimmunology Program, FRCB‐IDIBAPS, University of Barcelona, Spain; La Caixa Research Institute, Barcelona, Spain; Service d’Immuno‐Allergologie, Institut Central des Hôpitaux, Sion, Switzerland

**Background and aims:** Widespread use of commercial cell‐based assays (cCBA) has enhanced detection of contactin‐associated protein‐like 2 antibodies (CASPR2‐abs) but has also raised concerns about false positive results, particularly when testing is limited to serum or antibody titres are low. However, their diagnostic performance has not been systematically validated.

**Methods:** We analysed serum and CSF samples from patients referred to our Neuroimmunology laboratory (Barcelona) for suspected autoimmune neurological disorders who tested positive for CASPR2‐abs by cCBA (Euroimmun). Samples were re‐tested using in‐house rat brain immunohistochemistry and CBA.

**Results:** Of 105 patients with CASPR2‐abs detected by cCBA, 74 (71%) were confirmed by in‐house techniques (true‐positive) and 31 (29%) were not (false‐positive). Compared to true‐positive, false‐positive results occurred more frequent in females (4% vs. 52%, *p <* 0.0001) and younger patients (median age 68 vs. 58 years, *p* = 0.02). Among patients with false positive results, 23 (74%) presented with symptoms initially compatible with CASPR2 autoimmunity (although most had a final alternative diagnosis). However, only 1/20 (5%) had clinico‐radiological evidence of limbic encephalitis, compared with 19/64 (30%) patients with confirmed CASPR2‐abs (*p* = 0.03). False positive results occurred exclusively in serum (31 samples available). All paired CSF (21 available) from these patients were negative by both CBAs and in‐house testing. In contrast, all the available CSF (37/37) from patients with true‐positive serum results were also positive by cCBA.

**Conclusion:** CASPR2‐abs detected exclusively in serum by cCBA, in the absence of CSF positivity, should be interpreted with caution, particularly in younger patients and females. CSF antibody testing is essential to minimize false‐positive diagnoses.

**Disclosure:** Nothing to disclose.

## EPO‐0815

### Neurosarcoidosis mimics: Diagnostic revision following referral to a UK national neurosarcoidosis multidisciplinary team meeting

#### D. Omar ^1^; T. Arun^1^; C. Rice^2^; H. Mehta^1^; B. Quirk^3^; P. Fernandes^4^; P. Brex^5^; A. Gontsarova^6^; J. Mathews^7^; V. Singh‐Curry^8^

##### ^1^University Hospitals Coventry Warwickshire NHS Trust, UK; ^2^North Bristol NHS Trust, UK; ^3^Royal Free London NHS Foundation Trust, UK; ^4^University Hospital Southampton NHS Foundation Trust, UK; ^5^King's College Hospital NHS Foundation Trust, UK; ^6^Imperial College Healthcare NHS Trust, UK; ^7^Barts Health NHS Trust, UK; ^8^Chelsea And Westminster Hospital NHS Foundation Trust, UK

**Background and aims:** Neurosarcoidosis (NS) is a diagnosis of exclusion, with significant clinical and radiological overlap with infectious, neoplastic, and neuroinflammatory conditions. We report outcomes from the UK NS multidisciplinary team (MDT) meeting, focusing on cases where the diagnosis of NS was not confirmed.

**Methods:** Twenty‐eight cases of suspected NS were reviewed since the MDT launch (May 2025). Clinical presentation, imaging features, diagnostic consensus, recommended investigations, onward referrals, and treatment decisions were reviewed.

**Results:** The primary reason for referral was diagnostic uncertainty (19/28), and, in 6/28 (21%), the MDT found insufficient evidence to confirm a diagnosis of NS. Differential diagnoses included vasculopathy and tuberculosis (6/28 each), IgG4‐related disease (4/28), neoplastic and demyelinating conditions (3/28 each) and Progressive multifocal leukoencephalopathy and fungal infection (2/28 each). Of those, MDT suggested unlikely to be NS (4/6) had radiological evidence of recurrent infarcts. The most frequent clinical manifestations were brainstem symptoms (7/28), seizures, and headache (5/28 each). Frequently recommended investigations included cardiac assessment (MRI/PET), additional CSF analysis, vascular imaging, ophthalmological evaluation and full‐thickness skin biopsy. Suggested management changes included discontinuation of immunosuppressive treatment (5/28), initiation of tuberculosis therapy (2/28), escalation to infliximab (7/28), cyclophosphamide (3/28), tocilizumab or adalimumab.

**Conclusion:** National MDT review frequently identified opportunities for additional work‐up with the aim of increasing diagnostic confidence and treatment outcomes, including the rationalisation of immunosuppression. These early data highlight the importance of the national MDT in supporting neurologists managing neurosarcoidosis and other complex neuroinflammatory conditions.

**Disclosure:** Nothing to disclose.

## EPO‐0816

### Acute leukoencephalo‐myelo‐radiculopathy after anti‐CD19 CAR T‐cell therapy for refractory large B‐cell lymphoma: A case report

#### D. Lomonaco ^1^; F. Oggiano^1^; M. Modesto^1^; M. Caccamo^1^; A. Tedesco^1^; P. Colella^2^; V. De Marco^1^; D. Pastore^3^; A. Rini^1^

##### ^1^Neurological Department A. Perrino's Hospital Brindisi Italy; ^2^Department of Radiology, A. Perrino's Hospital, Brindisi, Italy; ^3^Hematological Department, A. Perrino's Hospital, Brindisi, Italy

**Background and aims:** Chimeric antigen receptor (CAR) T‐cell therapy has profoundly changed the therapeutic landscape of relapsed/refractory B‐cell lymphoma. Cytokine release syndrome (CRS) and immune effector cell‐associated neurotoxicity syndrome (ICANS) represent relevant adverse events. Rare manifestations, including severe leukoencephalopathy, and non‐ICANS complications such as myelopathy and peripheral neuropathy, have been reported.

**Methods:** We report the clinical course, neuroimaging findings, and treatment of a patient who developed acute leukoencephalo‐myelo‐radiculopathy after anti‐CD19 CAR T‐cell therapy.

**Results:** A 28‐year‐old woman with stage IVB diffuse large B‐cell lymphoma resulted refractory to first‐line chemotherapy. She underwent cyclophosphamide/fludarabine lymphodepletion and axicabtagene ciloleucel infusion. On day 1, she developed grade 2 CRS, treated with tocilizumab and corticosteroids. On day 4, she developed grade 2 ICANS, progressing to grade 4 on day 5. High‐dose intravenous methylprednisolone was initiated. Brain magnetic resonance imaging (MRI) revealed diffuse leukoencephalopathy extending to the upper cervical spinal cord. On day 7, the patient was awake, but severe tetraparesis was evident. Spinal MRI demonstrated longitudinally extensive acute myelitis and thickening of the cauda equina nerve root with faint contrast enhancement. Lumbar puncture was not performed due to severe thrombocytopenia. Polymerase chain reaction testing for neurotropic viruses on peripheral blood was negative. High‐dose intravenous methylprednisolone was continued, and intravenous immunoglobulin was administered, with mild neurological improvement. By day 23, clinical and radiological evidence of abdominal mass progression emerged. The patient died on day 45.

**Figure:**
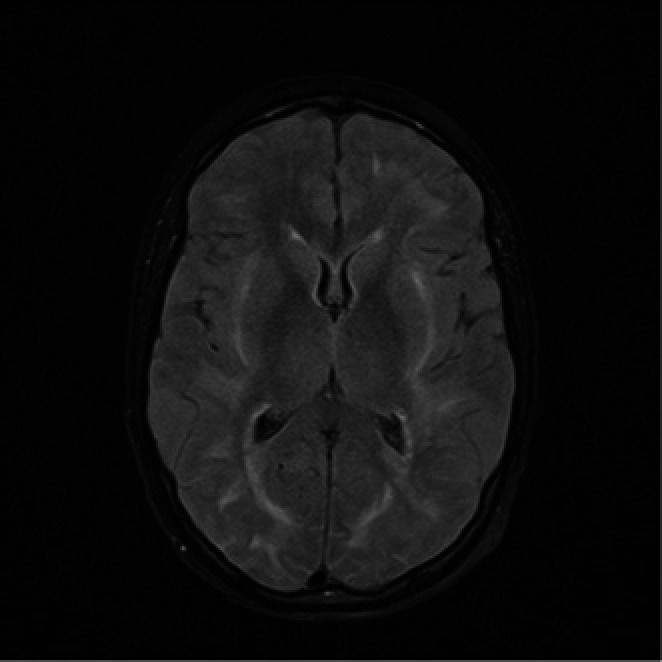
**FIGURE 1** Leukoencephalopathy changes on magnetic resonance imaging: T2‐weighted fluid attenuation inversion recovery axial image of the brain demonstrate symmetrical T2 hyperintensity of the deep white matter, most pronounced in the centrum semiovale.

**Figure:**
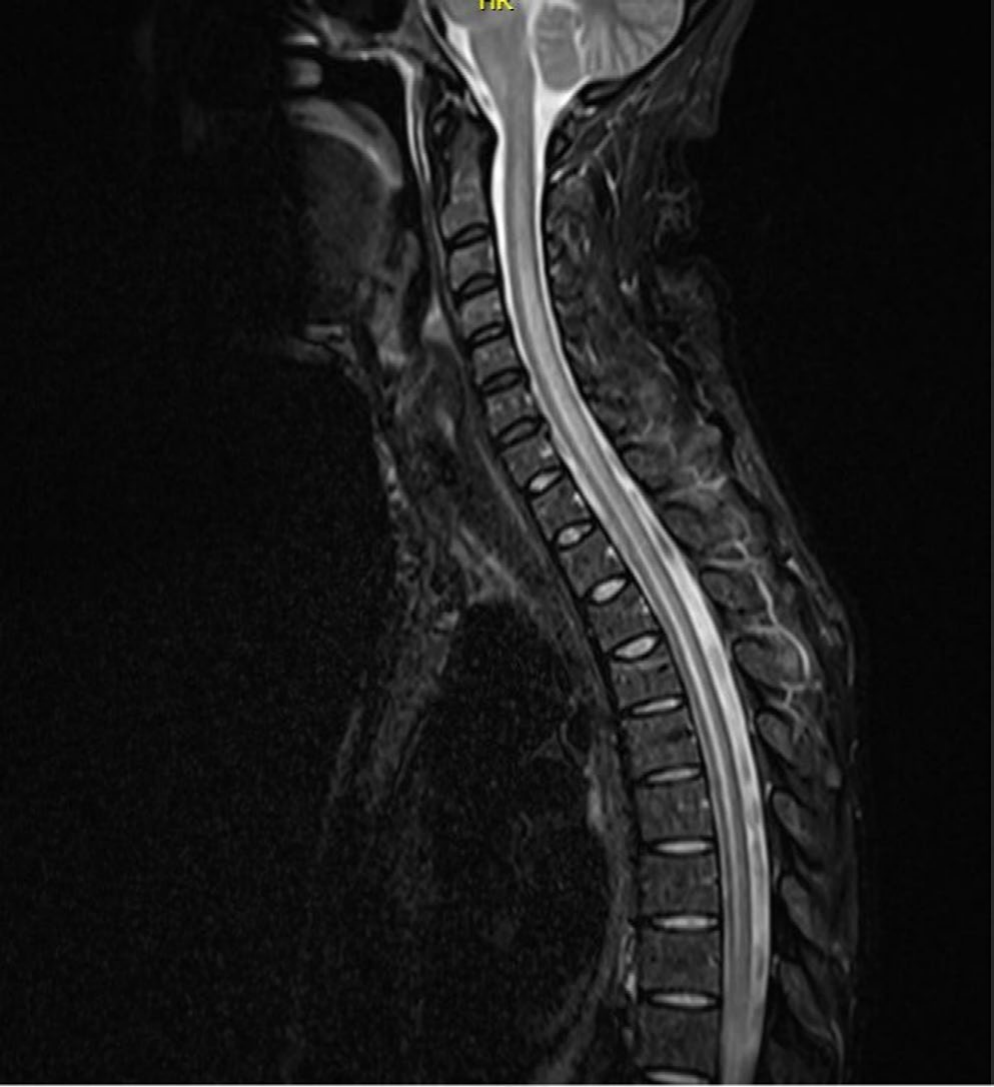
**FIGURE 2** Acute inflammatory myelopathy: Sagittal T2‐weighted fat‐suppressed MRI spinal magnetic resonance imaging demonstrates a longitudinally extensive intramedullary hyperintense signal involving the cervical and thoracic spinal cord, associated with mild cord.

**Figure:**
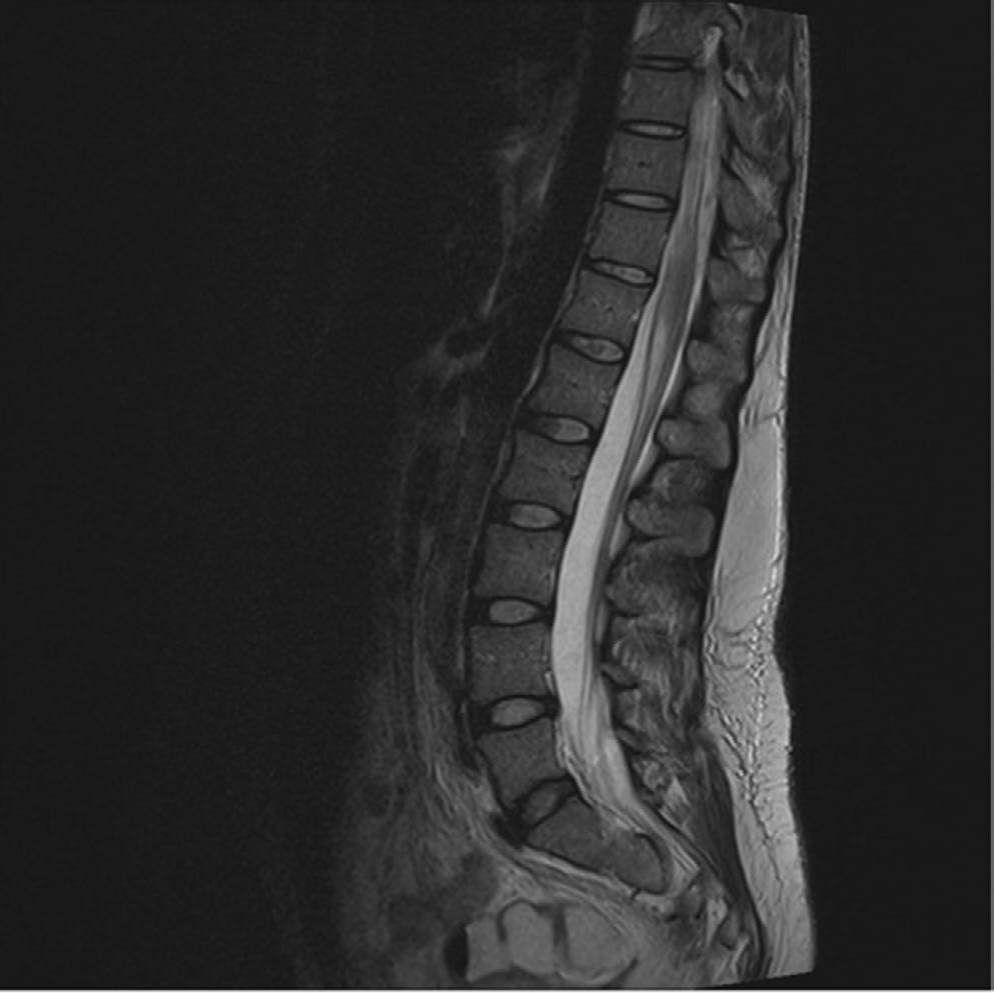
**FIGURE 3** Conus medullaris involvement in acute inflammatory myelopathy. Sagittal T2‐weighted lumbar spine magnetic resonance imaging demonstrates hyperintense signal changes and mild enlargement of the conus medullaris, with associated thickening of the cauda equi.

**Conclusion:** CAR T‐cell therapy may rarely cause severe neurotoxicity affecting both the central and peripheral nervous systems. Systematic reporting is needed to improve risk stratification and management.

**Disclosure:** Nothing to disclose.

## EPO‐0817

### From childhood to adulthood: Myelin autoantibody profile in acute demyelinating encephalomyelitis (ADEM)

#### E. Acquafredda ^1^; G. Romano^2^; E. Signoriello^2^; G. Lus^2^; M. Risi^3^; E. Maida^3^; A. Varone^3^; E. Spreafico^4^; T. Foiadelli^4^; D. Abruzzese^4^; M. Mancardi^5^; A. Santagostino Barbone^5^; R. Biddeci^5^; S. Bova^6^; A. Gadda^6^; C. Giovannini^6^; S. Masciocchi^7^; M. Gastaldi^7^

##### ^1^Department of Brain and Behavioral Sciences, University of Pavia, Pavia, Italy; ^*2*^Department of Advanced Medical and Surgical Sciences, University of Campania Luigi Vanvitelli, Naples, Italy; ^*3*^Pediatric Neurology Unit, Department of Neurosciences, Santobono Pausilipon Children Hospital, Naples, Italy; ^*4*^Paediatric Department, IRCCS Fondazione Policlinico S. Matteo, University of Pavia, Pavia, Italy; ^*5*^Unit of Child Neuropsychiatry, EpiCARE Network, IRCCS Istituto Giannina Gaslini, Genova, Italy; ^*6*^Child Neurology Unit, Buzzi Children's Hospital, Milan, Italy*;* ^*7*^Neuroimmunology Laboratory and Neuroimmunology Research Section, IRCCS Mondino Foundation, Pavia, Italy

**Background and aims:** Acute disseminated encephalomyelitis (ADEM) is a rare CNS demyelinating disease, in some cases associated with antibodies to the myelin oligodendrocyte glycoprotein (MOG‐IgG). Recently, antibodies to the proteolipid protein‐1 (PLP1‐IgG) has recently been identified in a subset of ADEM patients, though its frequency remains unknown. We aim to define the ADEM serological and clinical/paraclincal features in children versus adults.

**Methods:** We retrospectively screened patients with suspected ADEM referred to our laboratory and included those meeting the International Pediatric Multiple Sclerosis Study Group criteria. We collected clinical and radiological information from the clinical charts. In patients with available samples, we performed serological screening including MOG‐IgG and PLP1‐IgG.

**Results:** Ninety‐eight ADEM patients were included in the study. The median age at onset was 7 years (0.03–76.27), and 71% were pediatric (≤16 years). Adults presented more frequently myelitis (*p* = 0.002), PNS involvement (*p <* 0.001), and sensory/sphincteric symptoms (*p <* 0.001). Among 78 of 98 patients tested for anti‐myelin autoantibodies, 28/78 were MOG‐IgG+ (mostly children; *p* = 0.054); 10/78 were PLP1‐IgG+ (mostly adults; *p* < 0.001); 34/78 were double‐negative; and 6/78 were double‐positive. PLP1‐IgG+ patients showed more frequently PNS involvement (*p <* 0.001) and higher mRS at follow‐up (*p* = 0.002). Over a median follow‐up of 1.35 years, 30/98 patients relapsed without correlation with auto‐antibody profile. Good recovery (mRS < 3) was associated with younger age (*p <* 0.001), myelitis (*p* = 0.006), MOG‐IgG+ (*p* = 0.030) and PLP1‐IgG‐ (*p <* 0.001).

**Conclusion:** Adult‐onset ADEM exhibits distinct clinical/paraclinical and serological profile compared to pediatric cases. Further studies are needed to better define the relevance of PLP1‐IgG in this subgroup.

**Disclosure:** Nothing to disclose.

## EPO‐0818

### Thalamic subnuclei atrophy in MOG antibody–associated disease following optic neuritis

#### I. Elosua‐Bayés ^1^; M. Alberich^2^; L. Bollo^1^; G. Arrambide^1^; N. Mongay‐Ochoa^1^; M. Tintoré^1^; À. Rovira^2^; X. Montalban^1^; J. Sastre‐Garriga^1^; Á. Cobo‐Calvo^1^; D. Pareto^2^

##### ^1^Neurology‐Neuroimmunology Department, Centre d’Esclerosis Múltiple de Catalunya and Neurology Department, University Hospital Vall d’Hebron, Barcelona, Spain; ^2^Section of Neurorradiology. Department of Radiology, University Hospital Vall d’Hebron, Barcelona, Spain

**Background and aims:** MOG antibody‐associated disease (MOGAD) is a rare autoimmune demyelinating central nervous system condition, typically presenting optic neuritis (ON). The thalamus is a key structure for processing and relaying visual information. This study aims to assess volumetric differences in intra‐thalamic nuclei between MOGAD, multiple sclerosis (MS), and healthy controls (HC), following ON (MOGAD‐ON; MS‐ON).

**Methods:** Retrospective, single‐center observational study including MOGAD‐ON (*n* = 25), MS‐ON (*n* = 37) and (HC) (*n* = 38), matched for age, sex, and onset topography. Demographic and clinical variables were collected. Brain MRIs were acquired in a 3.0 T magnet, and the 3D T1‐weighted was segmented with FreeSurfer. Volume fractions were calculated with the total estimated brain volume. Between‐group differences were assessed using Wilcoxon rank‐sum test, *p <* 0.05 considered statistically significant.

**Results:** The median disease duration was 5 years for MOGAD and MS, with MOGAD presenting a more severe onset and a higher number of ON episodes. Brain parenchymal fraction was reduced in MOGAD and MS, compared to HC (Table 1). Three of 25 thalamic nuclei were significantly reduced in MOGAD compared to HC: lateral geniculate (LGN) (relative difference (RD): 7.1%), lateral posterior (LP) (RD: 9.2%) and pulvinar lateral (PL) (RD: 7.6%) (Figure 1). In MS, 5 nuclei were significantly reduced compared to HC, two overlapping with MOGAD (LGN RD: 3.4%; LP RD: 10.3%).

**Figure:**
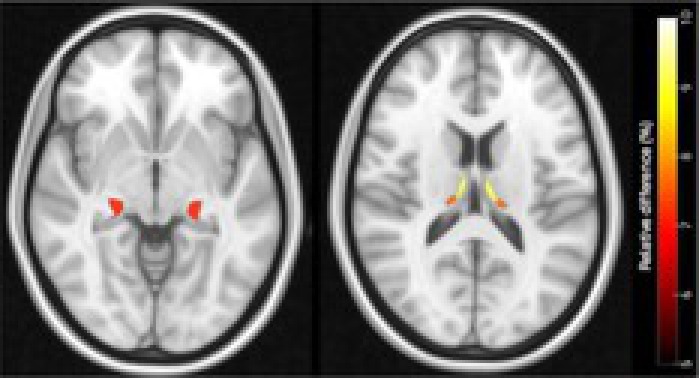
**FIGURE 1** Map showing thalamic nuclei significantly decreased in MOGAD compared to healthy controls.

**Figure:**
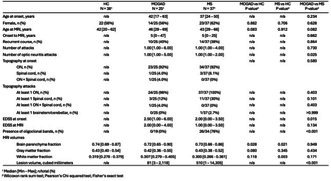
**TABLE 1** Clinical and demographic variables of patients with MOGAD, multiple sclerosis and healthy controls.

**Conclusion:** MOGAD‐ON presents a distinct pattern of thalamic atrophy. The marked reduction in LGN volume and selective atrophy of the PL in MOGAD suggest distinctive pathophysiological mechanisms. The PL may represent a radiologically specific signature of MOGAD, requiring confirmation in further studies.

**Disclosure:** Nothing to disclose.

## EPO‐0819

### Comparative analysis of three serum neurofilament light chain platforms in healthy controls and multiple sclerosis: Data and literature review

#### J. Dargvainiene ^1^; A. Torge^1^; K. Wandinger^1^; R. Junker^1^; R. Markewitz^1^; P. Benkert^2^; E. Willemse^2^; A. Maleska^2^; S. Schuster^3^; M. Ziemann^3^; C. Tafrali^4^; M. Martinez‐Serrat^4^; M. Khalil^4^; J. Kuhle^2^; F. Leypoldt^1^

##### ^1^Institute of Clinical Chemistry, University Hospital Schleswig‐Holstein, Campus Kiel and Lübeck, Germany; ^2^Multiple Sclerosis Centre and Research Center for Clinical Neuroimmunology and Neuroscience (RC2NB), Neurology, Departments of Biomedicine and Clinical Research, University Hospital and University of Basel, Basel, Switzerland; ^3^Institute of Transfusion Medicine, University Hospital Schleswig‐Holstein, Campus Kiel and Lübeck, Germany; ^4^Department of Neurology, Medical University of Graz, Graz, Austria

**Background and aims:** As multiple analytical platforms for serum NfL (sNfL), an established biomarker of neuroaxonal damage, are now available, assay comparability and harmonization are critical for clinical and research use. This study compared sNfL measurements across three assays—NF‐light Advantage V2/Plus (Quanterix), Lumipulse G NfL blood (FujiRebio), and Elecsys NfL (Roche Diagnostics) and suggested conversion formulas to support standardization.

**Methods:** Serum samples from healthy donors (*n* = 303) and patients with multiple sclerosis (MS; *n* = 181) were analyzed. Inter‐assay agreement was assessed using Passing–Bablok regression and correlation analyses. Assays were further evaluated for their ability to detect MS disease activity. In addition to original data, this poster will integrate and compare published conversion formulas and provide data on NfL levels across neurological diseases.

**Results:** All assays demonstrated strong correlations (Pearson *r* > 0.98; Spearman ρ > 0.90; *p <* 0.001). Fourteen of 484 samples (2.9%) showed stable, platform‐specific deviations (>4 SD). After excluding outliers, conversion equations to the NF‐light Advantage scale were derived: NfL = 6.31 × [Elecsys] + 2.33 ng/L; NfL = 0.94 × [Lumipulse] + 1.30 ng/L. All platforms detected significant differences between MS relapse and remission (*p <* 0.001). Age‐adjusted Z scores values yielded the largest effect sizes.

**Conclusion:** The three platforms show high concordance and comparable clinical performance. However, rare but marked assay‐specific deviations underscore the need for continued harmonization. Age‐adjusted Z scores may further enhance interpretability. The consistency and generalizability of published conversion formulas across broader NfL ranges warrant further investigation.

**Disclosure:** Assay kits were provided by Roche Diagnostics and FujiRebio; all analyses were conducted independently.

## EPO‐0820

### Baseline cortical excitability as a predictor of 5‐year cognitive outcomes in neuromyelitis optica spectrum disorders: A prospective study

#### L. Mollo; C. Siniscalchi; L. Luise; L. Ruggiero; R. Dubbioso; R. Russo; R. Iodice

##### Department of Neurosciences, Reproductive Sciences and Odontostomatology, University of Naples *“*Federico II*”*, Naples, Italy

**Background and aims:** Cognitive impairment is increasingly recognized as a relevant clinical manifestation of neuromyelitis optica spectrum disorders (NMOSD), but its underlying mechanisms and early predictors remain poorly understood. Alterations in cortical excitability, measurable through transcranial magnetic stimulation (TMS), may reflect subclinical cortical dysfunction and represent a potential biomarker of future cognitive decline.

**Methods:** Forty‐eight patients with newly diagnosed NMOSD and twenty‐five matched healthy controls underwent baseline neurophysiological assessment using TMS, including measures of resting motor threshold, short‐interval intracortical inhibition (SICI), and intracortical facilitation (ICF). Comprehensive neuropsychological testing was performed at baseline and at 5 years. Cognitive impairment was defined as performance ≥1.5 SD below controls on ≥2 cognitive domains. Multivariate regression analyses were used to identify baseline predictors of cognitive decline, adjusting for age, education, disease duration, and relapse rate.

**Results:** At diagnosis, NMOSD patients showed significantly reduced SICI and increased ICF compared with controls (*p* < 0.01), indicating early cortical disinhibition. After 5 years, 37.5% of patients developed cognitive impairment, primarily affecting attention, executive function, and processing speed. Baseline SICI reduction independently predicted cognitive decline (β = −0.42, *p* = 0.004), even after controlling for demographic and clinical covariates. No association was found between relapse frequency or EDSS progression and cognitive outcomes.

**Conclusion:** Early alterations in cortical excitability, reflecting GABAergic dysfunction, are strong predictors of long‐term cognitive deterioration in NMOSD. TMS‐derived measures may serve as valuable biomarkers to identify patients at higher risk for cognitive decline and guide early neuroprotective interventions.

**Disclosure:** Nothing to disclose.

## EPO‐0821

### International evaluation of the **a**quaporin‐4 antibody rapid idot test

#### M. Gastaldi ^1^; M. Vacca^1^; R. Li^2^; Y. Fu^2^; Y. Yan^3^; K. Li^3^; W. Qiu^4^; X. Sun^4^; M. Reindl^5^; K. Schanda^5^; N. Baumgartner^5^; A. Bornes^6^; D. Sato^6^; R. Marignier^7^; A. Ruiz^7^; E. Hirlimann^7^; L. Malaise^7^; A. Ballal^8^; L. Pandit^8^; S. Mariotto^9^; H. Kim^10^; J. Chung^10^; S. Han^10^; U. Amarasekara^11^; T. Chang^11^; N. Malavige^11^; Á. Cobo‐Calvo^12^; G. Arrambide^12^; J. Bennett^13^; S. Pittock^14^; E. Vieillard^15^; M. Woodhall^15^; R. Cakla^15^; M. Leite^15^; J. Palace^15^; R. Geraldes^15^; G. De Luca^15^; S. Rinaldi^15^; P. Waters^15^

##### ^1^IRCCS Fondazione Istituto Neurologico Nazionale Casimiro Mondino, Pavia, Italy; ^2^Department of Neurology and Institute of Neurology, The First Affiliated Hospital of Fujian Medical University, Fuzhou, China; ^3^Key Laboratory of the Ministry of Education for Medicinal Resources and Natural Pharmaceutical Chemistry, College of Life Sciences, Shaanxi Normal University, Xi’an, China; ^4^Department of Neurology of The Third Affiliated Hospital of Sun Yat‐sen University, Guangzhou, China; ^5^Medical University of Innsbruck, Innsbruck, Austria; ^6^Instituto do Cerebro do Rio Grande do Sul/PUCRS ‐ Centro de Pesquisa Pre‐Clínica (CPPC), Rio Grande do Sul, Brazil; ^7^Center for Research in Neuroscience of Lyon, Lyon, France; Centre de référence des maladies inflammatoires rares du cerveau et de la moelle, Lyon, France; ^8^Center for Advanced Neurological Research, KS Hegde Medical Academy, Nitte (deemed to be university), Mangalore, India; ^9^Neurology Unit, Department of Neurosciences, Biomedicine, and Movement Sciences, University of Verona, Verona, Italy; ^10^Research Institute and Hospital of Korea, National Cancer Center Research Building Gyeonggi‐do, Korea; ^11^Department of Immunology and Molecular Medicine, Faculty of Medical Sciences, University of Sri Jayawardenepura, Sri Lanka; ^12^Neurology‐Neuroimmunology Department, Multiple Sclerosis Centre of Catalonia (Cemcat), Vall d’Hebron Barcelona Hospital Campus, Barcelona, Spain; ^13^Department of Neurology, University of Colorado Anschutz, Aurora, USA; ^14^Department of Neurology, Center for Multiple Sclerosis and Autoimmune Neurology, Laboratory Medicine and Pathology, Mayo Clinic, Rochester, MN; ^15^Nuffield Department of Clinical Neurosciences, University of Oxford, UK

**Background and aims:** International evaluation of rapid iDOT aquaporin‐4 assay

**Methods:** Sera from centres in China, France, Italy, Korea, Spain, UK were sent coded to the coordinating centre in Italy from four patient groups (1. AQP4 antibody positive NMOSD patients 2. MOG‐IgG clear positive MOGAD patients 3. Clinically definite pwMS 4. Sera difficult on any assay). iDOT kits from China were sent to Italy. All samples were recoded and shipped with kits to 8 testing centres (Austria, Brazil, India, Sri Lanka, France, China, Korea, UK). iDOTs were performed at all centres; lCBAs in Korea and UK; fCBA in China. Test sensitivity was based on positive results in group 1; specificity was based on negative results in groups 2–4.

**Results:** Unblinding revealed 381 sera were tested: Group 1: 126, Group 2: 106, Group 3: 93, Group 4: 56. Kits were employed close to/after expiry date in seven centres. The median sensitivity was 84.9% (range 39.7–92.9%); median specificity was 99.2% (range 98.8–99.6%). 72% of group 1 samples were positive on all iDOTs across 7 centres with only 7 (6%) samples positive in fewer than 4/7 centres. The China team performed the assay on iDOT kits 2 months old with a sensitivity of 100% and specificity of 98.8%. The fCBA was 91.2% sensitive. lCBAs were 83.7% and 100% sensitive. All three were 100% specific.

**Conclusion:** The iDOT kit performed well despite being near the end of their shelf‐life. A relatively new kit performed in‐house had equal sensitivity to the best lCBA and highlights great promise for this system.

**Disclosure:** Nothing to disclose.

## EPO‐0822

### Efficacy and safety analysis of efgartigimod in patients with very‐late‐onset myasthenia gravis

#### Y. Zhao; M. Qu; X. Rong; X. Sun; L. Wang; M. Liu

##### Department of Neurology, the Affiliated Hospital of Qingdao University, Qingdao, China

**Background and aims:** Myasthenia gravis (MG) is an autoimmune disorder caused by pathogenic IgG autoantibodies disrupting the neuromuscular junction. Very‐late‐onset MG (VLOMG, onset ≥65 years) poses greater management challenges, with scarce data on efgartigimod (a novel FcRn antagonist) in this population.

**Methods:** Retrospective analysis of 62 AChR antibody‐positive VLOMG patients treated with efgartigimod (10 mg/kg IV weekly for 4 weeks, repeated after 4‐week intervals) at Affiliated Hospital of Qingdao University (Nov 2023–Sep 2025), assessing MSE rate, corticosteroid reduction, MG‐ADL/MG‐QoL15r scores, muscle function, and adverse events (AEs).

**Results:** A total of 62 eligible VLOMG patients were enrolled, of these, 15 completed 3 or more treatment cycles, 10 completed 2 cycles, and 37 completed 1 cycle. At the study endpoint, 41.9% (26/62) of patients had achieved MSE. MG‐ADL/MG‐QoL15r scores and corticosteroid doses significantly decreased across subgroups (1, 2, ≥3 cycles, all *P <* 0.05). MG‐ADL/MG‐QoL15r scores with progressive improvement in ≥3‐cycle patients. No serious AEs/deaths occurred; only 1 allergic rash.

**Figure:**
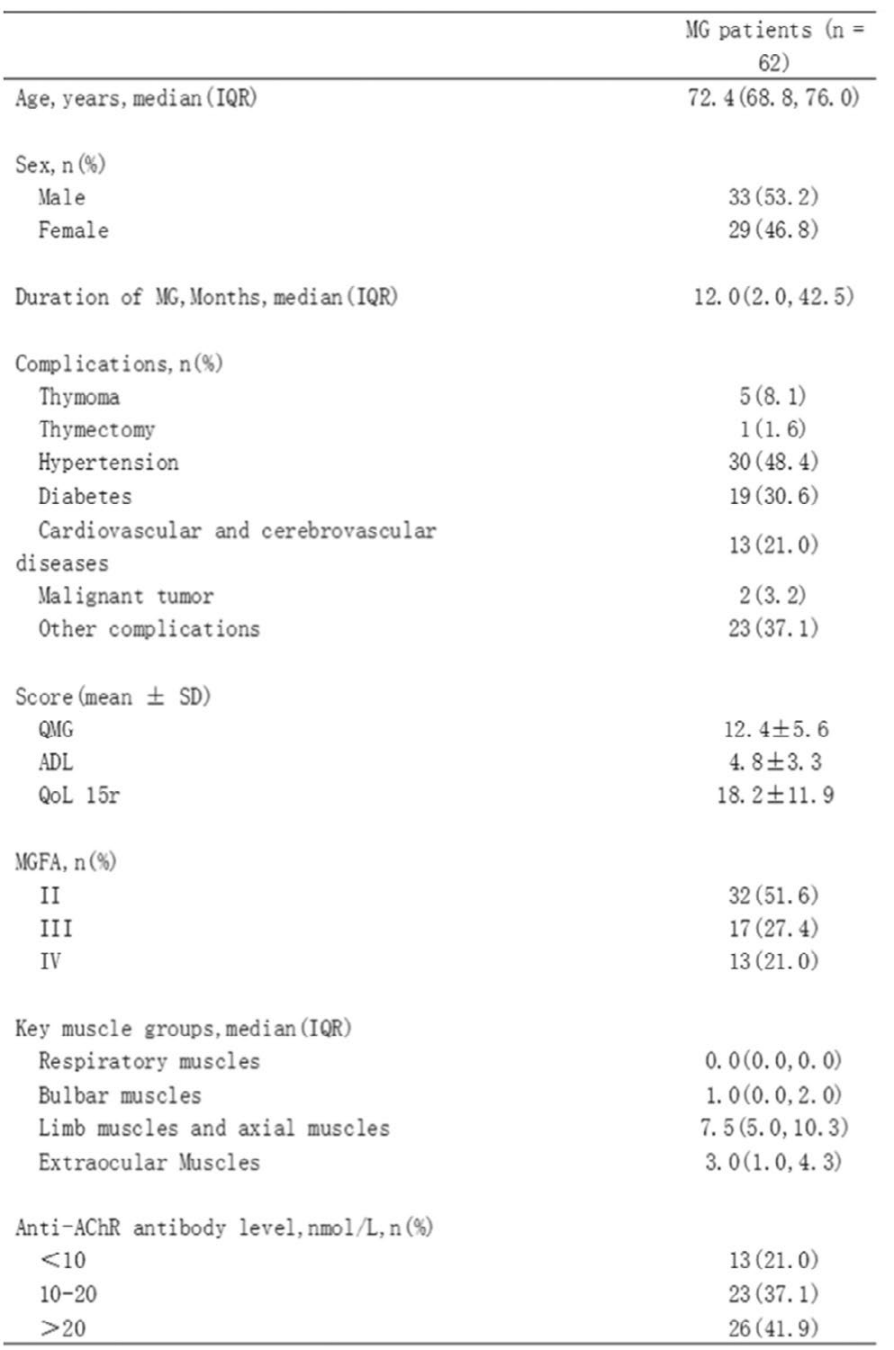
**TABLE 1** Patient characteristics at efgartigimod initiation.

**Figure:**
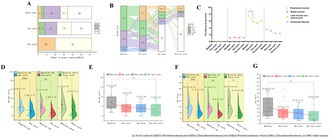
**FIGURE 1** (A) Dynamics of MSE criteria‐meeting patients (*n* = 62). (B) MG‐ADL score changes (*n* = 62). (C) Key muscle group changes (≥3cyc, *n* = 15). (D) MG‐ADL scores by treatment cycle. (E) MG‐ADL scores (≥3cyc). (F) QoL scores by treatment cycle. (G) QoL scores (≥3 cyc).

**Figure:**
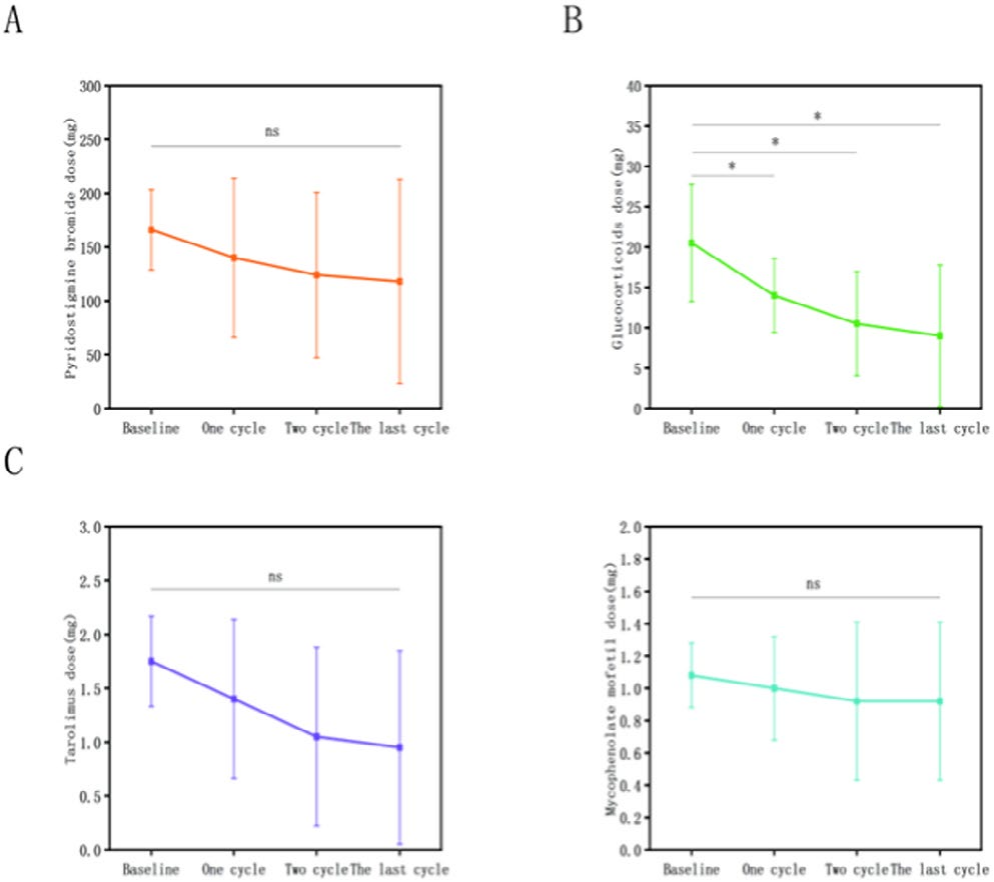
**FIGURE 2** Conventional immunosuppressant usage changes in MG patients with ≥3 efgartigimod cycles.

**Conclusion:** Efgartigimod is safe and effective for VLOMG, ameliorating symptoms, improving quality of life, and facilitating corticosteroid reduction.

**Disclosure:** Nothing to disclose.

## EPO‐0823

### Steroids with or without intravenous immunoglobulin for immune checkpoint inhibitor‐related neuropathies: A French multicenter cohort study

#### S. Cuzzubbo ^1^; E. Berling^2^; P. Lozeron^3^; F. D'Oria^4^; R. Ursu^4^; A. Echaniz Laguna^5^; C. Guémy^6^; G. Nicolas^2^; E. Fortanier^7^; S. Attarian^7^; G. Corazza^8^; S. Frachet^9^; C. Tard^10^; E. Meppiel^11^; S. Ng Wing Tin^12^; C. Denier^5^; A. Carpentier^1^; C. Cauquil^5^

##### ^1^Service de Neurologie, Université Paris Cité, AP‐HP, Hôpital Saint‐Louis, F‐75010 Paris, France; ^2^Service de Neurologie, AP‐HP, Hôpital Raymond Poincaré, Centre de référence Nord‐Est‐Ile‐de‐France, FHU PHENIX, Garches. Université de Versailles Saint‐Quentin‐En‐Yvelines, U 1179 INSERM, Paris‐Saclay, France; ^3^Service de Physiologie Clinique‐Explorations Fonctionnelles, DMU DREAM, AP‐HP, Hôpital Lariboisière, Paris, France. Université Paris Cité, INSERM U1148, Laboratory for Vascular Translational Science, Paris, France; ^4^Service de Neurologie, AP‐HP, Hôpital Saint‐Louis, Paris, France; ^5^Service de Neurologie, AP‐HP, Hôpital Bicêtre, Paris Saclay University, Centre de référence national des neuropathies amyloïdes familiales et autres neuropathies périphériques rares, CERAMIC, FILNEMUS Network, Le Kremlin‐Bicêtre, France; ^6^Service de Neurologie, AP‐HP, Hôpital Raymond Poincaré, Centre de référence Nord‐Est‐Ile‐de‐France, FHU PHENIX, Garches, France; ^7^Neurology Department, Reference Center for Neuromuscular Diseases and ALS, Aix‐Marseille University, AP‐HM, La Timone Hospital, Marseille, France. FILNEMUS Network, European Reference Network of Rare Diseases (ERN), France; ^8^Neurology Department, Reference Center for Neuromuscular Diseases and ALS, Aix‐Marseille University, La Timone Hospital. FILNEMUS Network, European Reference Network of Rare Diseases. Oncology Department, Paoli‐Calmettes Institute, Marseille, France; ^9^Department of Neurology, UR20218 NEURIT, University Hospital of Limoges, Dupuytren Hospital, Limoges, France; ^10^Service de Neurologie, U1172, Centre de référence des Maladies Neuromusculaires Nord/Est/Ile de France, CHU de Lille, Lille, France; ^11^Service de Neurologie, Centre Hospitalier de Saint‐Denis, F93200 Saint‐Denis, France; ^12^Service de Physiologie et Explorations Fonctionnelles, Hôpital Avicenne, Hôpitaux Universitaires de Paris Seine‐Saint‐Denis, AP‐HP, INSERM UMR 1272, UFR SMBH, Université Sorbonne Paris Nord, Bobigny, France

**Background and aims:** Current guidelines for immune‐related neurotoxicities recommend discontinuation of immune checkpoint inhibitors (ICIs) and steroid therapy in severe cases. However, the management of ICI‐related neuropathies remains less standardised, with intravenous immunoglobulin (IVIg) frequently combined with steroids despite limited evidence.

**Methods:** We conducted a retrospective analysis of patients with ICI‐related neuropathies from six French centers. Clinical, biological and electrophysiological data were collected at baseline, 3 months, and 6 months. The outcome was defined by changes in the modified Rankin Scale (mRS) and the Inflammatory Neuropathy Cause and Treatment (INCAT) disability scores between baseline and follow‐up visits.

**Results:** Forty‐eight patients with ICI‐related neuropathies were identified including 24 demyelinating neuropathies, 18 axonal neuropathies, and 6 multifocal radiculopathies. Most presented with subacute onset and predominant sensory symptoms. Immunomodulatory therapy was initiated for severe cases, primarily steroids alone (*n* = 25) or steroids combined with IVIg (*n* = 14). 90% of patients achieved a favourable and rapid outcome: 11 experienced complete recovery, 32 partial or subtotal improvement. No significant advantage was observed in adding IVIg to steroids. In the analysis of other variables, a higher baseline disability score (INCAT) was the only factor significantly associated with poorer outcomes. ICIs were reintroduced in ten patients, with good tolerance observed.

**Conclusion:** Our study does not support the routine use of IVIg in addition to steroids as first‐line treatment for ICI‐related neuropathies, even in demyelinating cases. Reintroduction of ICIs after resolution of neurological impairment appears safe and feasible.

**Disclosure:** AFC is a consultor for BMS. No disclosures for the other authors.

## Cerebrovascular Diseases 7

## EPO‐0824

### Imaging‐informed cerebral hemodynamic insights into Moyamoya disease and stroke risk: A systematic review

#### A. Shrivastava ^1^; R. Singh^2^; A. Garg^3^; S. Sanghi^1^; A. Suri^2^; S. Roy^1^

##### ^1^Department of Applied Mechanics, Indian Institute of Technology Delhi, New Delhi, India; ^2^Department of Neurosurgery, All India Institute of Medical Sciences New Delhi, Delhi, India; ^3^Department of Neuroimaging and Interventional Neuroradiology, All India Institute of Medical Sciences New Delhi, Delhi, India

**Background and aims:** Moyamoya disease (MMD) is a progressive cerebrovascular disorder characterized by intracranial arterial stenosis, leading to impaired cerebral perfusion and increased risk of ischemic and hemorrhagic stroke. Although advanced imaging is routinely used for diagnosis and follow‐up, the hemodynamic mechanisms underlying disease progression, stroke risk, and post‐revascularization outcomes remain incompletely understood. This systematic review synthesizes imaging‐informed hemodynamic evidence to support pathophysiological understanding and clinical decision‐making in MMD.

**Methods:** This systematic review followed PRISMA guidelines. A comprehensive literature search of PubMed, Scopus, Web of Science, and IEEE Xplore was conducted up to March 2024 using the keywords “Moyamoya disease” AND “computational fluid dynamics.” Original computational studies investigating cerebral hemodynamics, cerebrovascular biomechanics, patient‐specific blood‐flow modeling, stroke prediction, or relevant idealized models were included. Eighteen studies were selected for qualitative synthesis.

**Figure:**
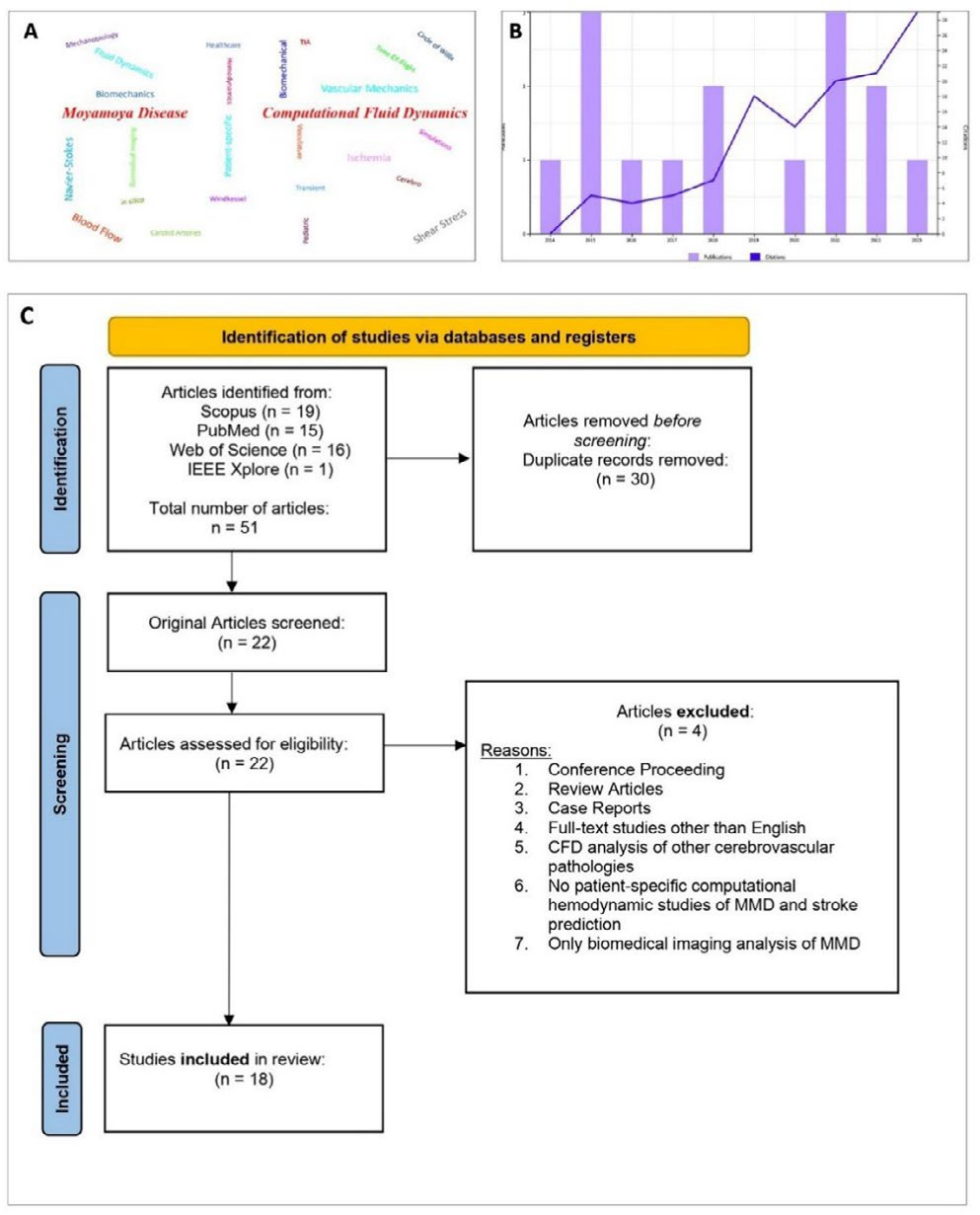
**FIGURE 1** Overview of the systematic review methodology for computational bio‐fluid mechanics studies in Moyamoya disease: (A) keyword cloud illustrating the literature search strategy, (B) temporal trend of publications and citations.

**Results:** Eighteen imaging‐informed computational studies were analyzed. Neuroimaging modalities, including digital subtraction angiography, CT perfusion, ASL‐MRI, and phase‐contrast MRI, were frequently integrated with computational fluid dynamics to quantify flow redistribution, perfusion deficits, and vascular remodeling. Consistent findings revealed abnormal flow patterns, elevated wall shear stress, pressure drops, and impaired distal perfusion in stenotic internal carotid and middle cerebral arteries. Pressure‐ and shear‐based metrics emerged as potential biomarkers for disease progression, surgical outcome assessment, and stroke risk stratification.

**Figure:**
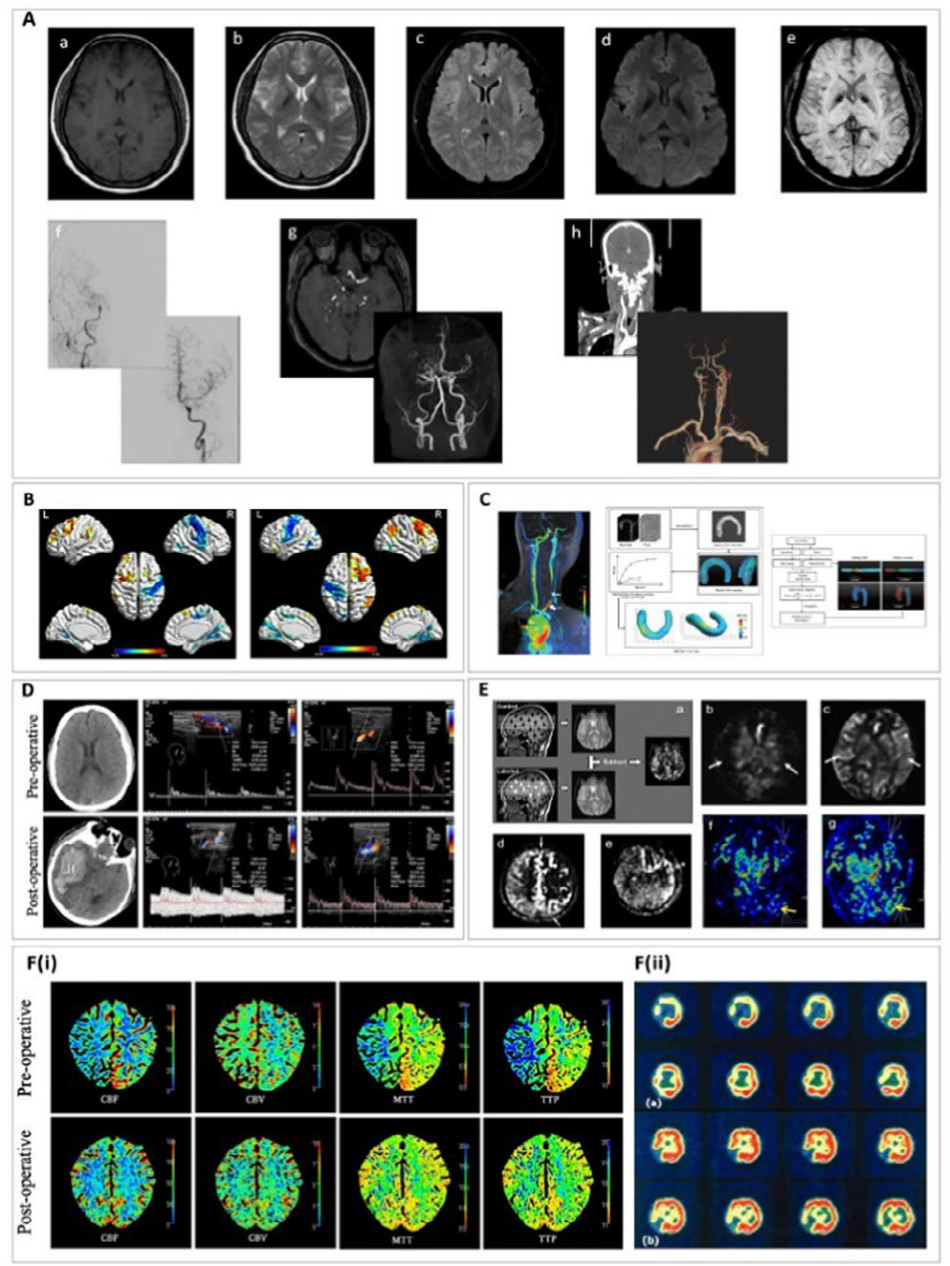
**FIGURE 2** Multimodal clinical imaging approaches for evaluating cerebrovascular structure, perfusion, and hemodynamics in Moyamoya disease. Shown are representative MRI sequences and angiographic techniques (DSA, TOF‐MRA, CTA), functional MRI, 4D flow MRI.

**Figure:**
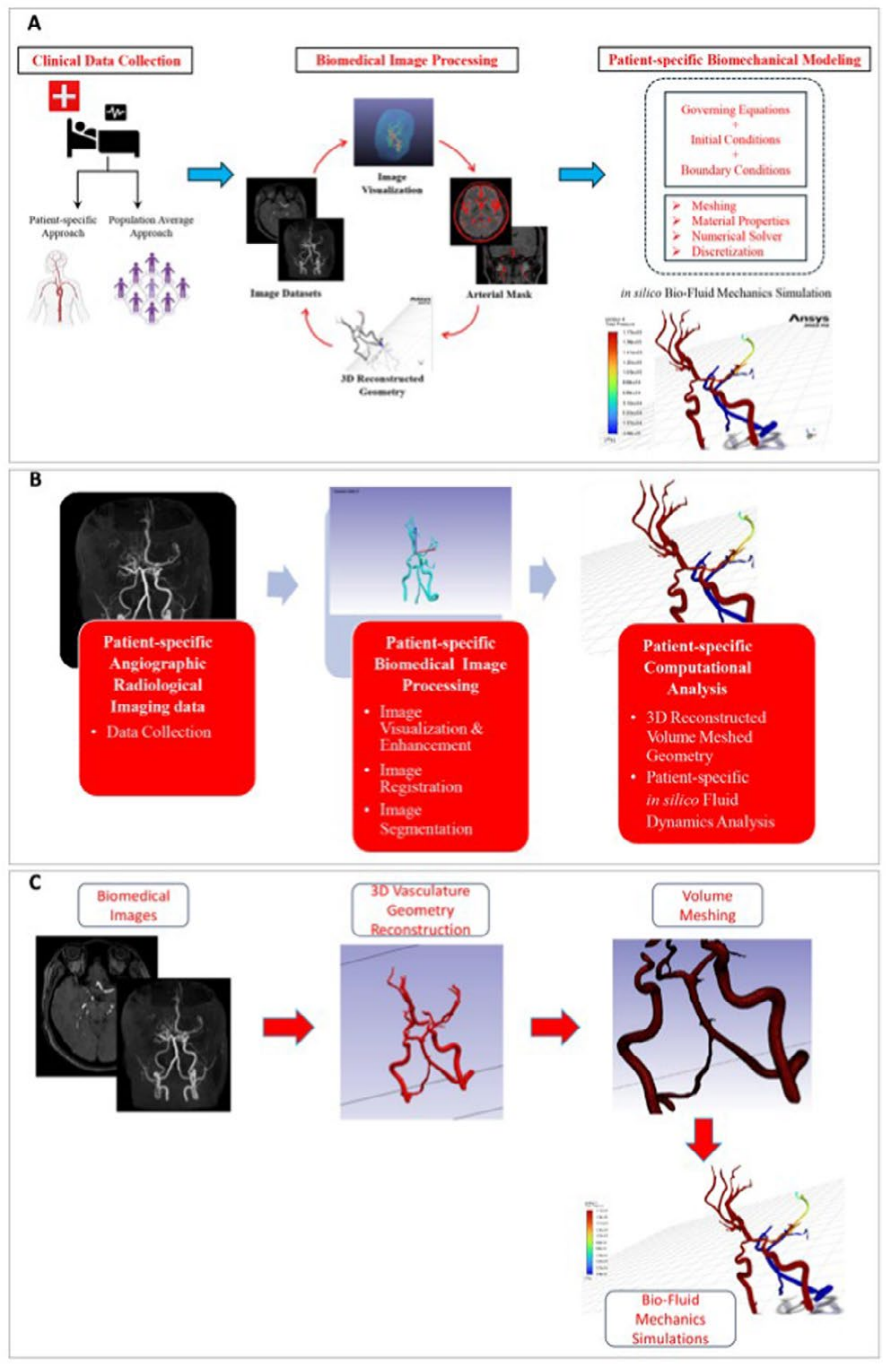
**FIGURE 3** Schematic overview of the in silico biomechanical analysis pipeline for Moyamoya disease and cerebral stroke risk assessment, illustrating biomedical image processing, patient‐specific vascular reconstruction, and computational fluid dynamics–ba.

**Conclusion:** Patient‐specific modeling integrated with clinical imaging shows promise for disease assessment, surgical planning, and postoperative evaluation. Future work should emphasize physiologically realistic fluid–structure interaction frameworks, standardized boundary condition calibration, and integration with advanced imaging and physics‐guided AI to enable robust, non‐invasive, personalized clinical decision‐making.

**Disclosure:** Nothing to disclose.

## EPO‐0825

### Impact of endovascular treatment in patients aged ≥85 years with large vessel occlusion ischemic stroke

#### A. Sánchez Asensio ^1^; C. Moreno López^1^; L. Gil Martínez^1^; J. Cortina García^1^; J. Romero Ferro^1^; P. González‐Barquero^2^; A. Iglesias Mohedano^3^; M. Vales Montero^3^; A. García Pastor^3^; C. Gómez Escalonilla^4^; P. Simal Hernández^4^; M. Moreu Gamazo^5^; L. Vidal Guerrero^6^; J. Rodríguez Pardo De Donlebun^6^; E. Mariño Trillo^6^; J. Alonso Maroto^7^; S. Trillo Senin^7^; C. Ramos Martín^7^; M. Gutiérrez Sánchez De La Fuente^8^; P. Calleja Castaño^8^; F. Ostos Moliz^8^; L. Fernández Gil^9^; R. Velasco Calvo^9^; J. Carneado‐Ruiz^9^; A. Cruz Culebras^1^; A. De Felipe Mimbrera^1^; C. Matute Lozano^1^; S. García Madrona^1^; J. Masjuan Vallejo^1^; R. Vera Lechuga^1^

##### ^1^Neurology, Hospital Universitario Ramón y Cajal, Madrid, Spain; ^2^Biostatistics, Instituto UC3M‐Santander de Big Data, Universidad Carlos III de Madrid, Madrid, Spain; ^3^Neurology, Hospital General Gregorio Marañón, Madrid, Spain; ^4^Neurology, Hospital Clínico San Carlos, Madrid, Spain; ^5^Radiology, Hospital Clínico San Carlos, Madrid, Spain; ^6^Neurology, Hospital Universitario La Paz, Madrid, Spain; ^7^Neurology, Hospital Universitario de La Princesa, Madrid, Spain**;** ^8^Neurology, Hospital Universitario 12 de Octubre, Madrid, Spain; ^9^Neurology, Hospital Universitario Puerta de Hierro Majadahonda, Madrid, Spain

**Background and aims:** To evaluate functional outcomes and mortality in patients aged ≥85 years with large vessel occlusion (LVO) ischemic stroke managed with endovascular therapy (EVT).

**Methods:** We conducted a prospective, multicenter observational study including patients aged ≥85 years with LVO treated in seven Stroke Centres in Madrid over one year (from February 2024). Patients treated with EVT were compared with those managed without EVT. Inverse probability of treatment weighting (IPTW) based on propensity scores was applied to adjust for baseline differences between groups.

**Results:** A total of 277 patients were included (71% women), with a mean age of 89.2 ± 3.11 years and a median baseline mRS of 1 (IQR 0–4). EVT was performed in 188 patients (67.8%). Patients treated with EVT had less pre‐stroke cognitive impairment and higher NIHSS scores. After IPTW adjustment, there were no significant differences in functional independence at 3 months (mRS 0–2) between EVT and non‐EVT patients (31.1% vs 24.7%; *p* = 0.31; OR = 1.41, 95% CI 0.72–2.72). However, EVT was associated with a higher proportion of favorable outcomes when considering the mRS 0–3 (50.0% vs 31.9%; *p* = 0.035; OR = 1.92, 95% CI 1.05–3.52) and with lower 3‐month mortality (29.3% vs 50.9%; *p* = 0.038; OR = 0.51, 95% CI 0.27–0.96).

**Figure:**
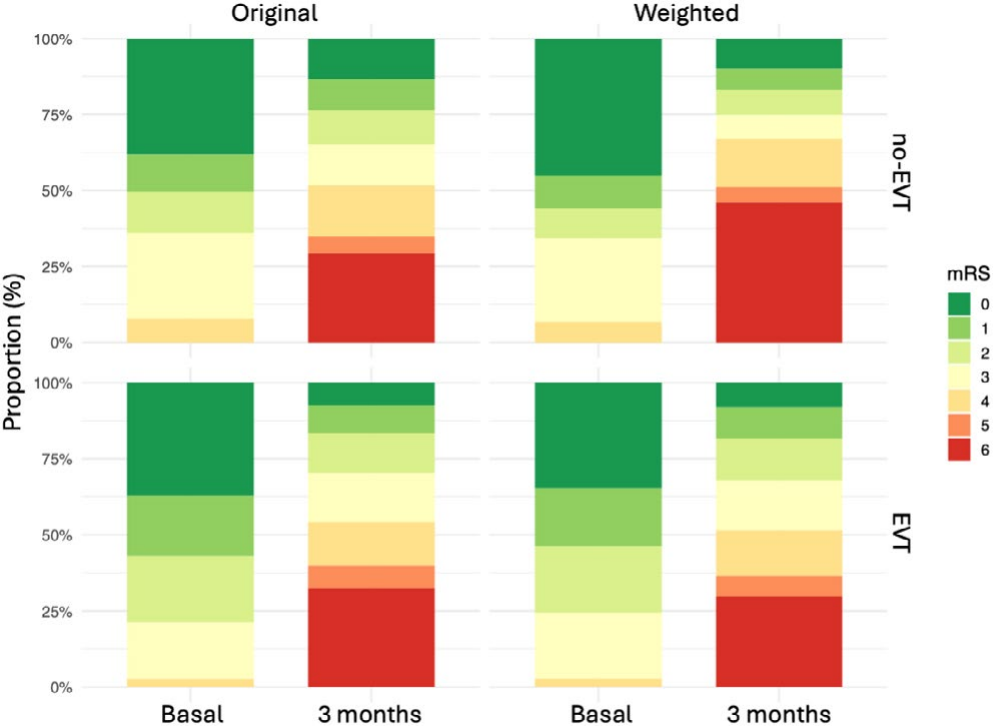
**FIGURE 1** Grotta bar charts comparing baseline and 3‐month modified Rankin Scale (mRS) distributions in the basal and weighted cohorts.

**Conclusion:** In our cohort, EVT did not significantly increase the likelihood of full independence or mild disability (mRS 0–2) at 3 months. However, EVT was associated with reduced mortality and a lower degree of disability among dependent patients.

**Disclosure:** Nothing to disclose.

## EPO‐0826

### Two circulations, different outcomes? Clinical and paraclinical results of mechanical thrombectomy in anterior and posterior circulation strokes

#### B. Moutinho ^1^; A. Fernandes^1^; S. Vedor^2^; M. Silva^2^; D. Ferro^1^

##### ^1^Neurology Department, Unidade Local de Saúde São João, Porto, Portugal; ^2^Neuroradiology Department, Unidade Local de Saúde São João, Porto, Portugal

**Background and aims:** Mechanical thrombectomy (MT) is the standard of care for acute ischemic stroke due to large vessel occlusion (LVO). The outcomes of thrombectomy for anterior circulation infarction (ACI) and posterior circulation infarction (POCI) may differ due to inherent clinical and paraclinical particularities of the respective vascular territories. Objective: To compare technical and clinical outcomes of MT in patients with POCI versus ACI.

**Methods:** Retrospective analysis of consecutive patients who underwent MT for POCI between July/2023‐July/2025, and for ACI between January‐July/2025. Demographic, clinical, and procedural variables were systematically collected.

**Results:** A total of 148 patients were included (49 POCI, 99 ACI), 78 (52.7%) female. No significant differences were found in age (*p* = 0.321) or interhospital transfer rates (*p* = 0.666). POCI patients more frequently presented with Glasgow Coma Sclae (GCS) score ≤ 8 at admission than ACI patients (17/49 vs. 4/99, *p <* 0.001). Compared with ACI, POCI was associated with lower neurological improvement at 24 hours (38.8% vs. 69.7%, *p <* 0.001), longer median onset‐to‐puncture time (627 vs. 416 minutes, *p* = 0.001), and higher two‐month mortality (16/49 vs. 9/99, *p <* 0.001). Puncture‐to‐recanalization time (31.9 ± 24.6 vs. 36.4 ± 22.1 minutes, *p* = 0.324) and successful reperfusion rates (TICI ≥ 2C; 63.3% vs. 59.6%, *p* = 0.720) were similar between groups. However, procedure‐related complications occured more frequently in POCI (8/49 vs. 2/99, *p* = 0.001).

**Conclusion:** Despite similar technical performance regarding time to recanalization and rate of successful reperfusion, POCI patients exhibited distinct clinical trajectories, which may be associated with lower admission GCS and delayed symptom‐to‐puncture times.

**Disclosure:** Nothing to disclose.

## EPO‐0827

### Early evolocumab reduces early neurological deterioration in acute non‐cardioembolic stroke: A real‐world retrospective cohort study

#### D. Jeong

##### Department of Neurology, Veterans Health Service Medical Center, Seoul, Republic of Korea

**Background and aims:** Early neurological deterioration (END) complicates 15–20% of acute ischemic strokes despite standard care. The acute neuroprotective potential of PCSK9 inhibition, mediated by rapid lipid‐lowering and anti‐inflammatory mechanisms, remains unexplored.

**Methods:** This retrospective cohort study at Central Veterans Hospital, Republic of Korea (2022–2025) enrolled consecutive patients with acute non‐cardioembolic ischemic stroke (large artery atherosclerosis or small vessel occlusion) presenting within 48 hours. The evolocumab group (*n* = 96, 100% Korean Asian, mean age 72 ± 10 years, 58% male) received subcutaneous evolocumab 140mg within 24 hours plus moderate‐to‐high‐intensity statins (atorvastatin 40–80 mg or rosuvastatin 20–40 mg). Historical controls (*n* = 120) received high‐intensity statins alone. matched 1:1.25 for age (±5 years), sex, and baseline NIHSS (±2 points). The primary endpoint was END (NIHSS increase ≥2 points) within 7 days, assessed daily by certified neurologists blinded to treatment allocation.

**Results:** Baseline characteristics were balanced (baseline NIHSS 5.2±3.1 vs 5.4 ± 3.3, *p* = 0.63). Evolocumab achieved superior LDL‐C reduction versus controls (149 ± 32→61 ± 18 mg/dL [59% reduction] vs 145 ± 35→89 ± 24 mg/dL [39% reduction]; between‐group difference ‐32 mg/dL, 95% CI −40 to −24, *p <* 0.001) and greater hs‐CRP reduction (5.2 ± 2.1→2.8 ± 1.5 mg/L [46% reduction] vs 5.0 ± 2.3→4.1 ± 1.9 mg/L [18% reduction], *p <* 0.001). END occurred in 8/96 (8.3%) versus 22/120 (18.3%) patients (unadjusted OR 0.41, 95% CI 0.17–0.95, *p* = 0.04; adjusted OR 0.38, 95% CI 0.15–0.91, *p* = 0.03), representing 55% relative risk reduction and a number‐needed‐to‐treat of 10. Functional independence at 90 days was 71% versus 63% (*p* = 0.31). No symptomatic intracranial hemorrhage or serious adverse events occurred.

**Figure:**
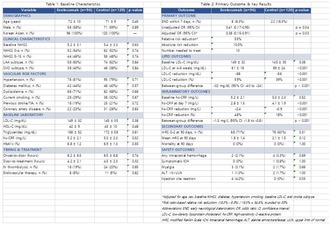
**FIGURE 1** Baseline characteristics, primary outcome.

**Conclusion:** Early evolocumab demonstrated potent dual lipid‐lowering and anti‐inflammatory effects, independently associated with reduced END.

**Disclosure:** Nothing to disclose.

## EPO‐0828

### Predictors and clinical impact of incomplete recanalization from endovascular treatment in acute ischemic stroke: A single‐centre cohort study

#### E. Maslias ^1^; D. Strambo^1^; S. Hajdu^2^; F. Puccinelli^2^; B. Bartolini^2^; G. Saliou^2^; P. Michel^1^

##### ^1^Stroke Center, Neurology Service, Department of Clinical Neurosciences, Lausanne University Hospital and University of Lausanne, Lausanne, Switzerland; ^2^Service of Diagnostic and Interventional Radiology, Interventional Neuroradiological Unit, Lausanne University Hospital and University of Lausanne, Switzerland

**Background and aims:** Poor recanalization at the end of endovascular treatment (EVT) despite successful catheter access to the target occlusion is associated with unfavorable outcome in acute ischemic stroke (AIS), but little is known about their predictors and clinical impact in the real world.

**Methods:** We retrospectively analyzed consecutive AIS patients from the Acute STroke Registry and Analysis of Lausanne (ASTRAL) who underwent EVT between 2015 and 2024 within 24 hours of symptom onset. Poor recanalization was defined as a final modified Thrombolysis In Cerebral Infarction (mTICI) score <2b. We assessed frequency and predictors of poor recanalization using multivariable logistic regression. Clinical impact was evaluated with adjusted models for 24‐hour neurological change (delta‐NIHSS) and 3‐month disability (ordinal mRS).

**Results:** Among 1448 EVT‐treated AIS patients with target‐lesion access, 9.9% had poor recanalization (mTICI<2b). In multivariable analysis, the number of device passes was the only independent predictor of poor recanalization (OR 1.73 per additional pass; *p <* 0.001). Poor recanalization was associated with significantly worse early and medium‐term outcomes: adjusted 24‐hour delta‐NIHSS worsened (+3.25) versus improved with complete recanalization (−4.39; *p <* 0.001), and 3‐month mRS was higher (4.1 vs 2.7; *p* = 0.001). Patients with poor recanalization were less often discharged home and had higher mortality.

**Conclusion:** In routine clinical practice, poor recanalization despite reaching the target lesion occurs in ~10% and has a major adverse impact on early recovery, 3‐month disability, and mortality. The number of device passes is the key predictor, supporting the clinical relevance of the first‐pass effect as a primary EVT goal.

**Disclosure:** Nothing to disclose.

## EPO‐0829

### Clinically relevant thrombophilia testing and atheroinflammatory indices in young cryptogenic ischemic stroke

#### G. Ertürk Kale; A. Kale; Ü. Sarı

##### Neurology, Balıkesir University Health Practice and Research Hospital, Balıkesir, Türkiye

**Background and aims:** Thrombophilia screening is common in young adults with cryptogenic ischemic stroke, but its clinically relevant yield and links with the systemic immune‐inflammation index (SII) and the triglyceride‐to‐high‐density lipoprotein cholesterol ratio (TG/HDL‐C) are unclear. We evaluated genetic and serologic testing for clinically relevant thrombophilias and associations with SII, TG/HDL‐C, and stroke severity.

**Methods:** We analyzed 112 patients (age range: 18–55) with Trial of Org 10172 in Acute Stroke Treatment (TOAST)‐defined cryptogenic ischemic stroke. Clinically relevant thrombophilia positivity was defined as any abnormal serologic or genetic test excluding methylenetetrahydrofolate reductase (MTHFR) variants, hyperhomocysteinemia, and Factor V Leiden (sensitivity definition). Outcomes included positive testing and management change after testing. Group comparisons used Fisher exact and Mann‐Whitney tests; correlations used Spearman.

**Results:** Clinically relevant thrombophilia testing was positive in 57/112 (50.9%). Management changed in 27/112 (24.1%) and was more frequent in thrombophilia‐positive than thrombophilia‐negative patients (46% vs 2%, *p <* 0.001). Thrombophilia‐positive patients were younger (42.4 (4.9) vs 45.9 (6.1) years, *p* = 0.001) and had higher National Institutes of Health Stroke Scale (NIHSS) scores (7.8 (7.9) vs 1.8 (3.7), *p <* 0.001), more frequent SII‐high status (40% vs 7%, *p <* 0.001), and higher TG/HDL‐C (11.7 (11.0) vs 4.2 (4.9), *p <* 0.001). Very high TG/HDL‐C risk level was more common in the thrombophilia‐positive group (53% vs 15%, *p <* 0.001). TG/HDL‐C correlated strongly with NIHSS (Spearman correlation coefficient 0.85, *p <* 0.001).

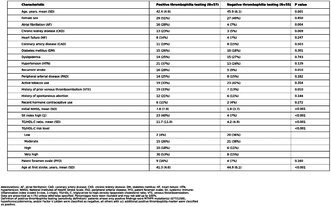

**Figure:**
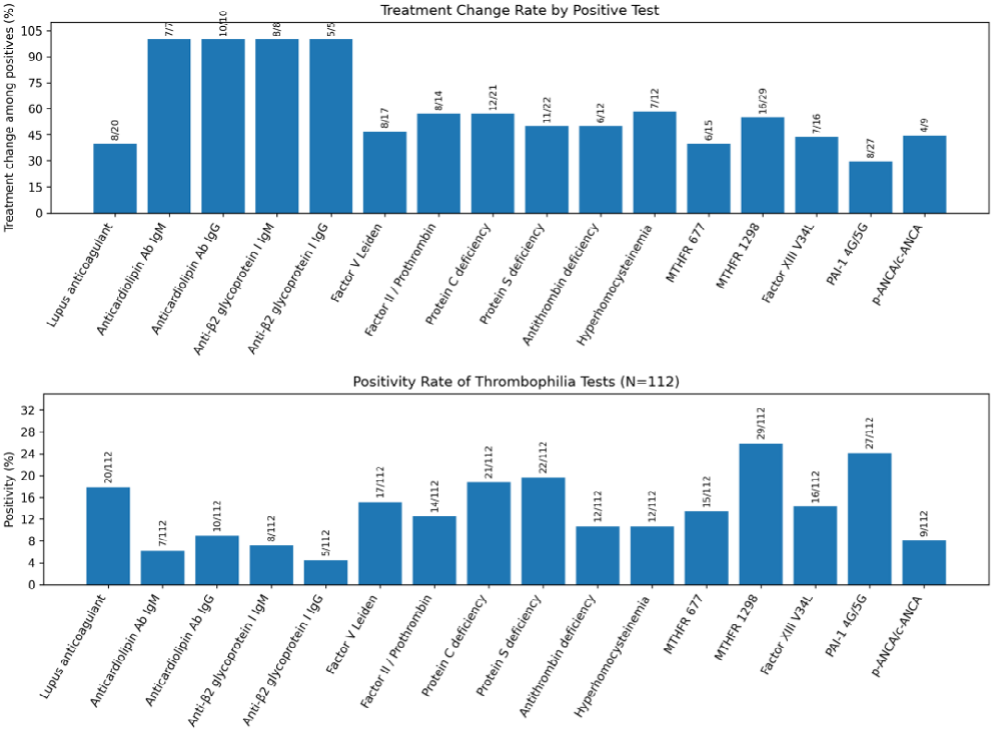
**FIGURE 1** Yield of thrombophilia testing in young adults with ischemic stroke and positivity rate of thrombophilia tests.

**Figure:**
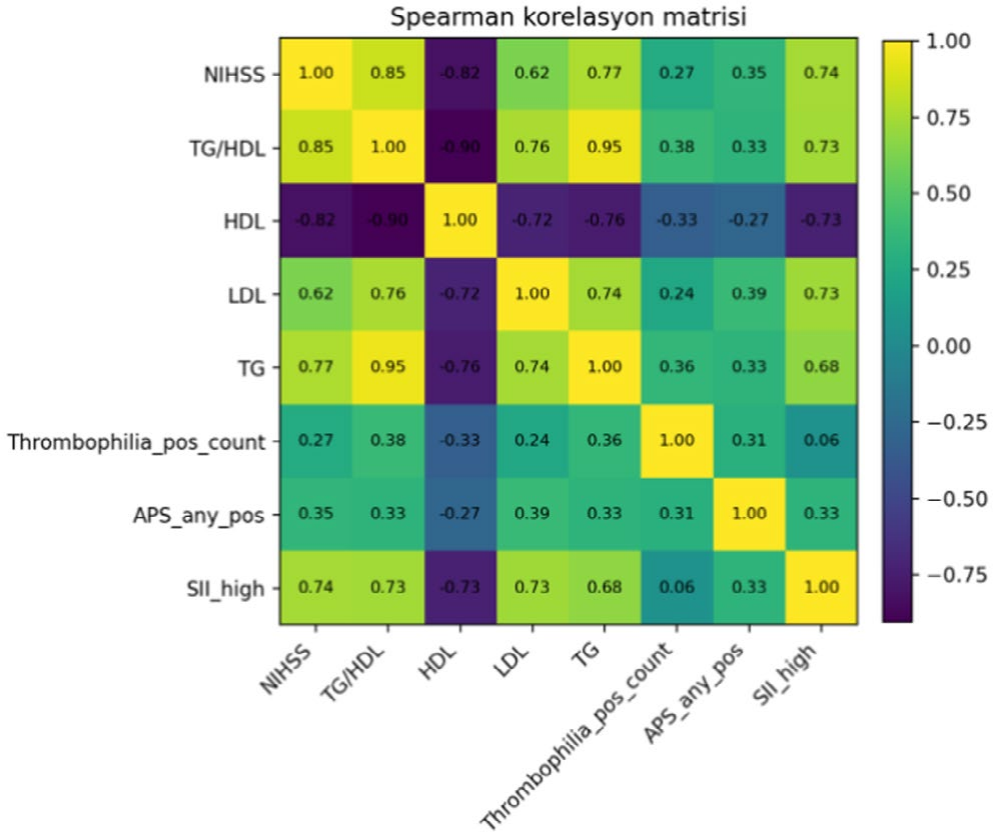
**FIGURE 2** Higher atherogenic lipid burden (higher TG/HDL, TG, LDL) and a higher inflammatory status (SII_high) tend to co‐occur with higher NIHSS, while higher HDL tends to co‐occur with lower NIHSS.

**Conclusion:** In young cryptogenic ischemic stroke, clinically relevant thrombophilia findings were frequent and were associated with higher NIHSS and adverse atheroinflammatory profiles (SII and TG/HDL‐C). The management impact of thrombophilia testing was higher than prior reports, suggesting a more favorable benefit‐harm balance when testing is focused on selected young cryptogenic patients.

**Disclosure:** Nothing to disclose.

## EPO‐0830

### Efficacy and safety of bridging intravenous thrombolysis prior to endovascular treatment in patients over 80 years old with acute ischemic stroke

#### I. Siow ^1^; Y. Chen^2^; K. Lee^3^; Y. Dai^1^; X. Xiao^4^; A. Garg^5^; A. Gopinathan^6^; C. Yang^7^; B. Tan^8^; M. Jing^8^; M. Zin^8^; H. Teoh^8^; C. Sia^9^; M. Feng^10^; J. Ma^11^; L. Chen^12^; S. Zhou^13^; Z. Xu^14^; Y. Zhou^15^; Y. Lai^16^; W. Peng^17^; Y. He^18^; M. Mofatteh^19^; T. Nguyen^20^; S. Pan^2^; L. Yeo^8^

##### ^1^Department of Medicine, Ministry of Health Holdings, Singapore; ^2^Department of Neurology, Nanfang Hospital, Southern Medical University, Guangzhou, China; ^3^Department of Neurosurgery, National Neuroscience Institute, Singapore; ^4^Department of Medicine, National University Health System, Singapore; ^5^Department of Medicine, Yong Loo Lin School of Medicine, National University of Singapore, Singapore; ^6^Department of Diagnostic Imaging, National University Health System, Singapore; ^7^Department of Radiology, Woodlands health; ^8^Division of Neurology, Department of Medicine, National University Health System, Singapore; ^9^National University Heart Centre, Singapore; ^10^Department of Neurology and Advanced National Stroke Center, Foshan Sanshui District People's Hospital, Foshan City, Guangdong Province, China; ^11^Department of Neurology, The Affiliated Yuebei People's Hospital of Shantou University Medical College, Shaoguan, China; ^12^Department of Neurology, The Eighth Affiliated Hospital of Southern Medical University, Foshan City, Guangdong Province, China; ^13^Department of Surgery of Cerebrovascular Diseases, First People's Hospital of Foshan, Foshan City, Guangdong Province, China; ^14^Neuromedical Center Ward 2, The Affiliated Panyu Central Hospital, Guangzhou Medical University, Guangzhou, China**;** ^15^Department of Neurology, Huadu District People's Hospital of Guangzhou, Guangzhou, China; ^16^Department of Neurology, Guangdong Provincial Hospital of Integrated Traditional Chinese and Western Medicine, Foshan City, Guangdong Province, China; ^17^Department of Neurology and Advanced National Stroke, Shaoguan First People's Hospital, Shaoguan, Guangdong Province, China; ^18^Department of Neurology, Nanfang Hospital, Southern Medical University, Guangzhou, China; ^19^School of Medicine, Dentistry and Biomedical Sciences, Queen's University Belfast, UK; ^20^Department of Neurology, Radiology, Boston University Chobanian & Avedisian School of Medicine, Boston, MA, United States

**Background and aims:** Endovascular treatment (EVT) is an effective treatment for patients with acute ischemic stroke (AIS). However, it is unclear if treatment with intravenous thrombolysis (IVT) prior to EVT confers any benefit in octogenarians and older. This study aimed to address if bridging tPA has improved functional outcomes or complications in patients 80 years

**Methods:** This multicentre retrospective cohort study included patients 80 years old and above who underwent endovascular therapy for large vessel occlusion acute ischaemic stroke in 10 compressive stroke centres across China and Singapore between 2018 and 2024. Clinical and procedural factors of patients in Singapore and China were compared using multivariate binary logistic regression. The primary outcome measured was achieving successful recanalization. Secondary outcomes included 3‐month functional independence defined as modified rankin scale (mRS) 0–2, 3‐month independent ambulation as defined as mRS 0–3, and 3‐month mortality rates. Data on complications such as parenchymal haematoma and any haemorrhage was also collected.

**Results:** Bridging IVT was not associated with increased rates of successful recanalisation (89.36% vs 87.63%; *p* = 0.672), improvement in 3‐month functional independence, improvement in 3‐month independent ambulation or 3‐month mortality rates. Instead, patients who underwent bridging IVT had higher rates of haemorrhage compared to patients who did not undergo bridging IVT even after adjusting for confounding factors (OR = 1.921; 95% CI 1.026–3.596; *p* = 0.041).

**Figure:**
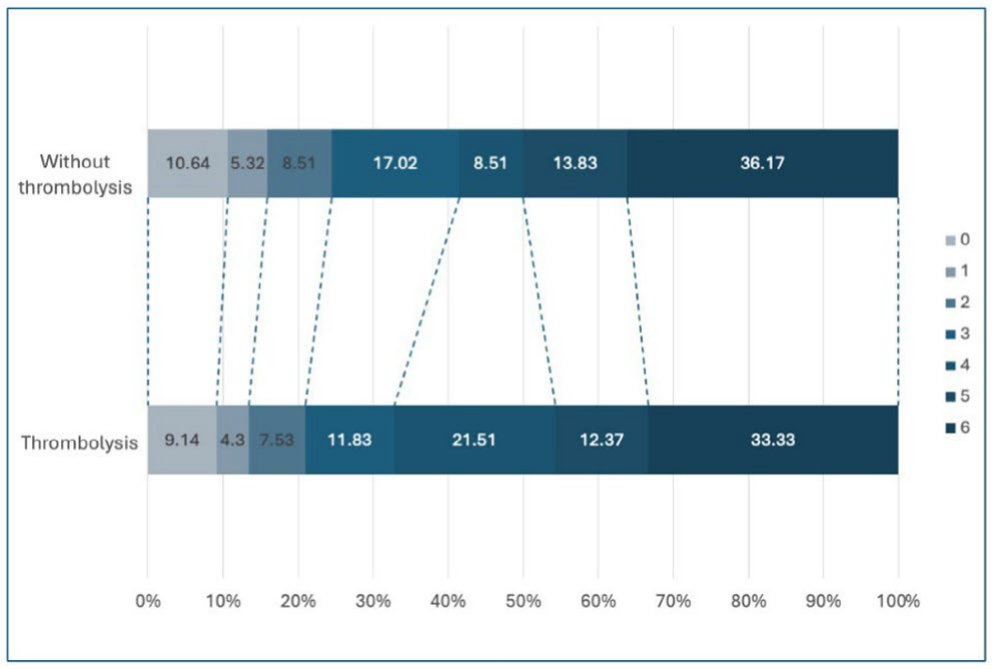
**FIGURE 1** Comparison of modified rankin scale scores between patients with bridiging intravenous thrombolysis prior to endovascular therapy and those without.

**Figure:**
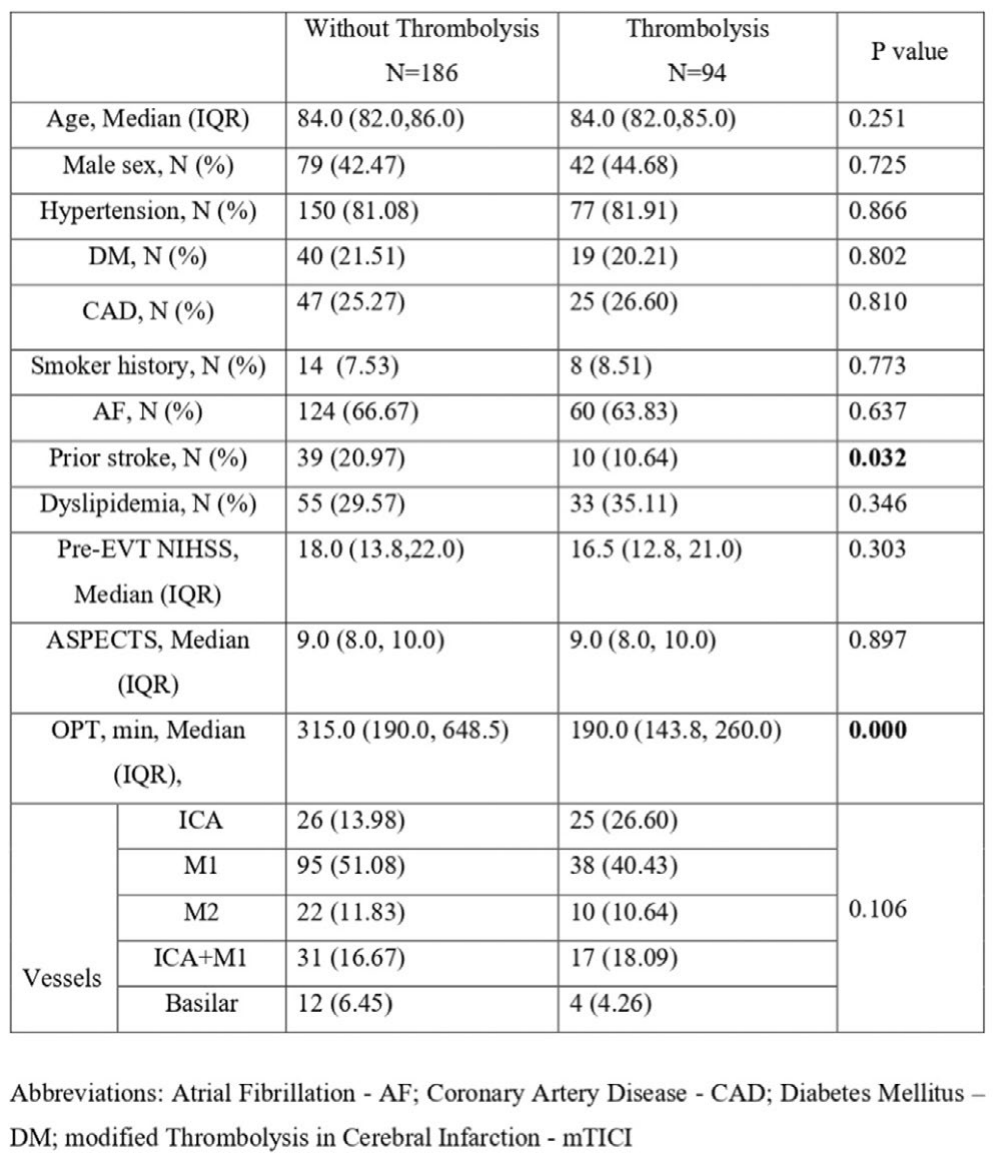
**TABLE 1** Baseline characteristics of patients.

**Figure:**
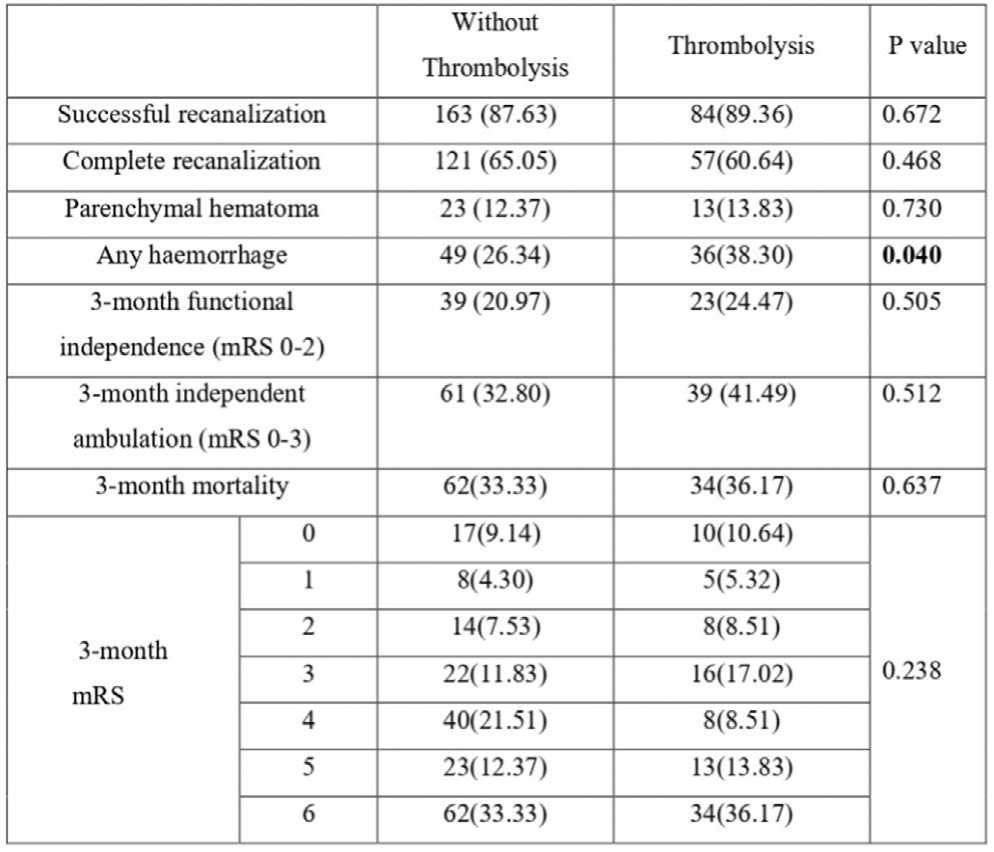
**TABLE 2** Comparing recanalization, safety and outcomes between elderly patients who underwent bridging thrombolysis and those who did not.

**Conclusion:** In patients over 80 years old with AIS, bridging IVT is not associated with improved functional independence or reduced mortality at three months. However, it is associated with increased risk of hemorrhage.

**Disclosure:** Nothing to disclose.

## EPO‐0831

### Anticoagulation and stroke risk in dialysis patients with atrial fibrillation: A single‐centre study in Malta

#### J. Darmanin; M. Schembri

##### Mater Dei Hospital, Malta

**Background and aims:** Anticoagulation is central to stroke prevention in atrial fibrillation (AF), but its use in end‐stage renal failure (ESRF) patients on dialysis is challenging. Warfarin has traditionally been used, though it is associated with labile INR, drug interactions, vascular calcification, and increased bleeding risk. Direct oral anticoagulants (DOACs), particularly apixaban, are increasingly prescribed off‐label due to predictable pharmacokinetics and no need for routine monitoring, but evidence of their safety and efficacy in ESRF is limited and conflicting.

**Methods:** This single‐centre retrospective study included all ESRF patients on chronic dialysis in Malta with documented AF. Data collected included demographics, comorbidities, anticoagulation type (warfarin, DOAC, or none), and major bleeding events. Patients were followed for one year to assess cerebrovascular outcomes, including ischaemic stroke or transient ischemic attack (TIA).

**Results:** Of 358 ESRF patients, 43 (12%) had AF: 7 (16.3%) received apixaban, 30 (69.8%) warfarin, and 5 (11.6%) no anticoagulation. Preliminary findings show 3 patients (60%) without anticoagulation experienced a cerebrovascular event, while no events occurred in warfarin or apixaban groups. Labile INR was noted in 11 warfarin patients (36.7%), and 4 experienced major bleeding; no major bleeding occurred with apixaban.

**Conclusion:** These results suggest warfarin and apixaban may be similarly effective for stroke prevention in ESRF patients with AF, but warfarin carries a higher bleeding risk. Although limited by small sample size, this audit included the entire ESRF population in Malta, providing valuable insight into real‐world anticoagulation practices in this high‐risk group.

**Disclosure:** Nothing to disclose.

## EPO‐0832

### Diagnostic value of microbiological analysis of cerebral thrombi in infective endocarditis–related stroke: A tertiary‐center descriptive study

#### L. García‐Granados ^1^; C. Villar Rodríguez^1^; D. Villagrán Sancho^1^; M. Medina Rodríguez^1^; J. Cabezas Rodríguez^1^; A. de Albóniga‐Chindurza Barroeta^2^; M. Aguilar Pérez^2^; M. Poyato Borrego^3^; A. González García^2^; E. Zapata Arriaza^2^; F. Moniche Álvarez^1^

##### ^1^Department of Neurology, Hospital Universitario Virgen del Rocío, Sevilla, Spain; ^2^Department of Radiology, Hospital Universitario Virgen del Rocío, Sevilla, Spain; ^3^Infectious Diseases Unit, Department of Internal Medicine, Hospital Universitario Virgen del Rocío, Sevilla, Spain

**Background and aims:** Infective endocarditis (IE) is an uncommon but severe cause of cardioembolic ischemic stroke. Since 2023, a positive microbiological analysis of the retrieved thrombus has been recognized as a pathological criterion for definite IE, enabling diagnosis to be established based on a single microbiological isolation.

**Methods:** Patients with ischemic stroke secondary to IE were included and divided into two groups according to the clinical sequence of diagnosis. Clinical, radiological, microbiological and prognostic characteristics were compared between groups (Table 1). The primary objective was to assess the diagnostic value of microbiological analysis of thrombi retrieved during mechanical thrombectomy.

**Figure:**
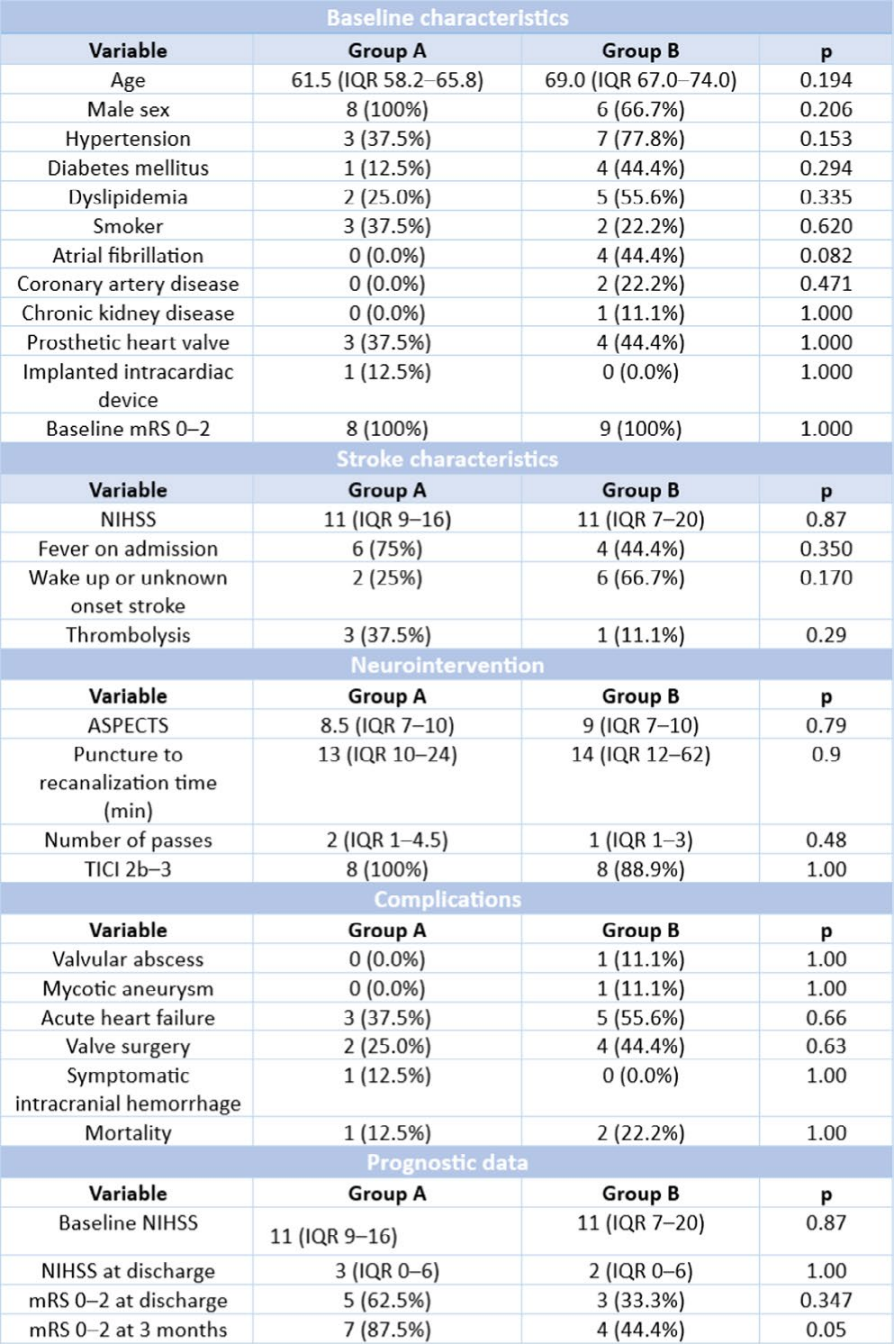
**TABLE 1** Characteristics of patients with ischemic stroke associated with infective endocarditis, stratified by study group: Group A (stroke as first clinical manifestation) and Group B (infective endocarditis diagnosed before stroke).

**Results:** Seventeen patients were included (8 presenting with stroke as the first manifestation and 9 with a previous diagnosis of IE). The median age was 67 years and 82.4% were male. Thrombus microbiological analysis was performed in 13 cases. In patients presenting with stroke as the first manifestation, thrombus cultures were positive in 100% of cases compared with 33.3% in patients with prior infective endocarditis (*p* = 0.021), with 100% concordance with blood culture isolates (Table 2). Patients with stroke as the initial manifestation showed a higher proportion of favorable functional outcome at 3 months (mRS 0–2: 87.5% versus 44.4%), showing a trend toward statistical significance (*p* = 0.05).

**Figure:**
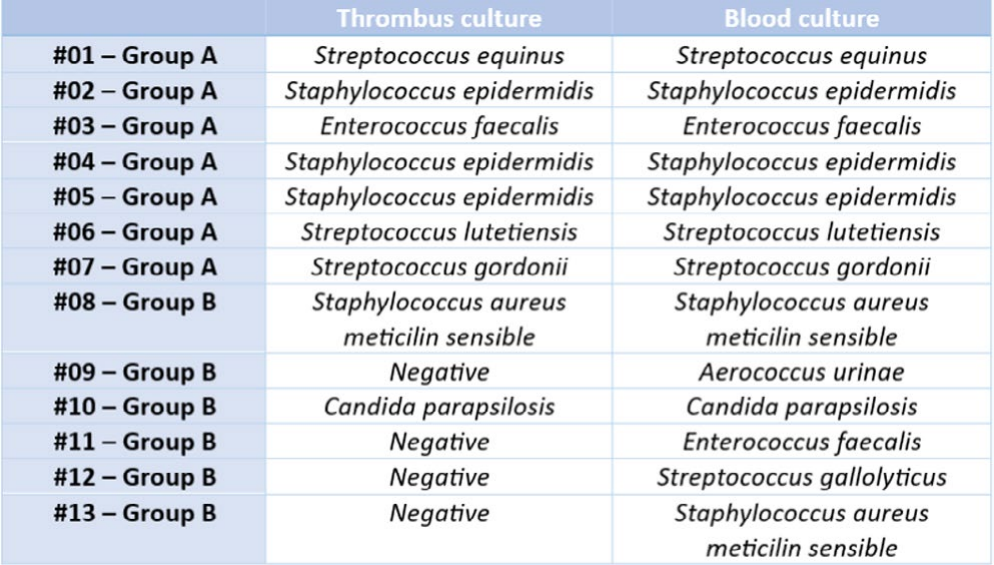
**TABLE 2** Comparison of thrombus and blood culture microorganisms in endocarditis‐related stroke by group (A: stroke first, B: endocarditis first). In Group A, thrombus culture showed 100% concordance with blood cultures.

**Conclusion:** Microbiological analysis of cerebral thrombi may provide relevant diagnostic value in IE, particularly when stroke is the first clinical manifestation. Early diagnosis of IE supported by thrombus analysis may enable earlier targeted treatment and be associated with improved long term functional outcomes.

**Disclosure:** Nothing to disclose.

## EPO‐0833

### Development of the GLaSSES score for predicting neurological deterioration after acute ischaemic stroke

#### M. De Sampaio; M. Grecco; A. Carrá; G. Di Fonzo; P. Guillermo; J. Pozo

##### Neurology Department, Hospital Churruca Visca, Buenos Aires, Argentina

**Background and aims:** Patients with acute ischaemic stroke (AIS) may experience neurological deterioration (ND) within the first seven days, defined as an increase in the National Institutes of Health Stroke Scale (NIHSS) score. ND incidence ranges from 5% to 40% and is linked to increased morbidity and mortality. Despite ongoing research, no practical predictive tool is currently available.

**Methods:** We conducted a retrospective observational study of patients aged >18 years hospitalised for AIS (excluding reperfusion therapies). ND occurrence, causes, and associated mortality were recorded. A multivariable logistic regression model with forward stepwise selection identified independent predictors. Model performance was evaluated using receiver operating characteristic (ROC) curve analysis.

**Results:** Of 444 patients (mean age 71.3 years), ND occurred in 20.3%, mainly due to ischaemic core progression (46%). Five independent risk factors were identified: hyperglycaemia (OR 15.9, 95% CI 8.1–33.2), leukocytosis (OR 10.1, 95% CI 4.8–23.1), baseline NIHSS severity (OR 3.3, 95% CI 1.3–8.9), symptomatic intracranial stenosis (OR 3.8, 95% CI 2.5–5.5), and extracranial stenosis (OR 6.0, 95% CI 1.6–8.9). The GLaSSES score stratified patients into four risk categories: ND incidence 1.9% (very low), 17.7% (low), 47.5% (moderate), 85.7% (high). The model showed good discrimination (AUC 0.85, 95% CI 0.82–0.88). Mortality was 47.8% in ND patients.

**Figure:**
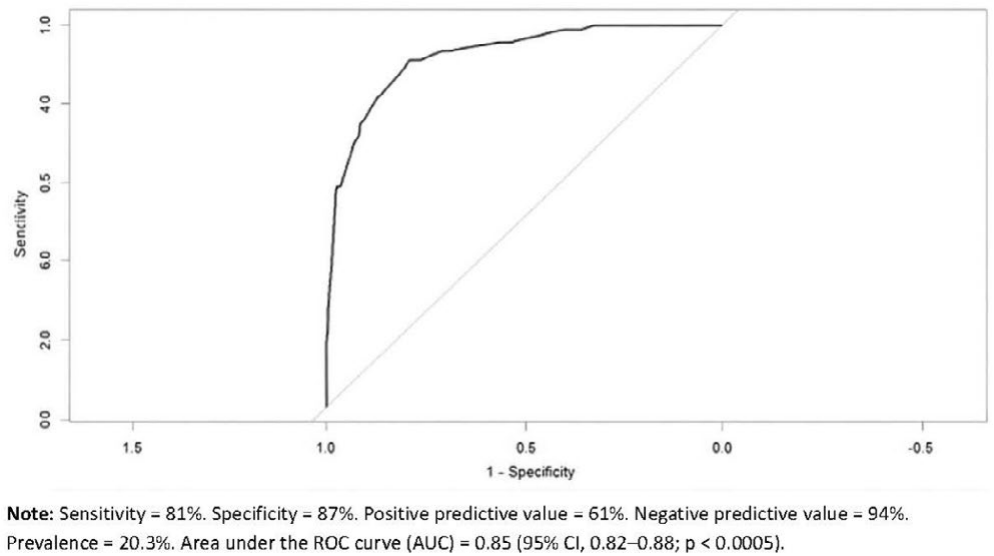
**FIGURE 1** Receiver operating characteristic (ROC) curve of the predictive model.

**Figure:**
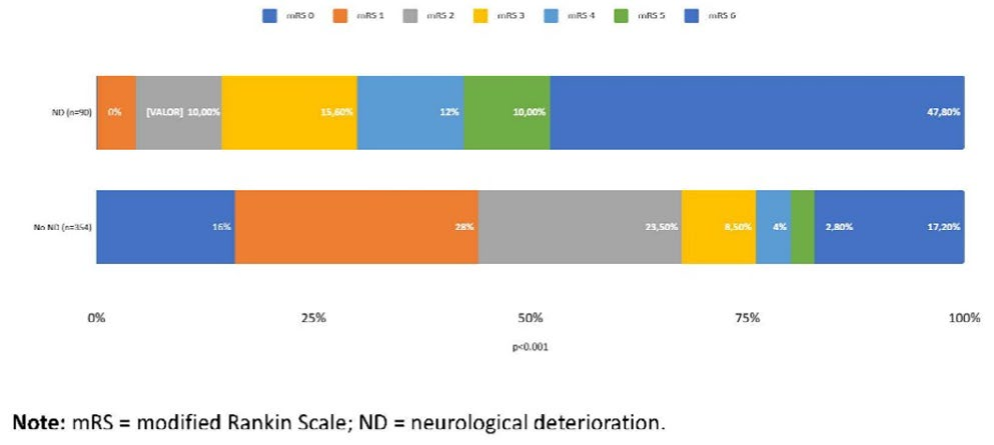
**FIGURE 2** Distribution of the modified Rankin Scale score at 7 days in patients with neurological deterioration.

**Conclusion:** The GLaSSES score is a promising, simple tool for predicting ND risk in AIS patients. External validation in larger, prospective cohorts is warranted to confirm its clinical utility and improve outcomes.

**Disclosure:** Nothing to disclose.

## EPO‐0834

### Mechanical thrombectomy in patients with low ASPECTS: Real‐world outcomes compared with SELECT‐2 and LASTE

#### P. Diaz‐Corta; B. Lara‐Rodríguez; A. Paipa‐Merchan; H. Quesada‐García; A. Núñez‐Guillén; P. Cardona‐Portela

##### Neurology, Hospital Universitario Bellvitge, Barcelona, Spain

**Background and aims:** Randomised trials (SELECT‐2, LASTE) support mechanical thrombectomy (MT) in patients with large ischaemic core infarction defined by low ASPECTS (≤5). Real‐world safety and functional outcomes remain less well described.

**Methods:** We conducted a retrospective observational study including consecutive patients with acute ischemic stroke and low ASPECTS treated with MT at a tertiary hospital.

**Results:** Sixty‐six patients were included (mean age 67 years; 56.1% women). Median baseline NIHSS was 20 and median ASPECTS was 4. Intravenous fibrinolysis was given in 22.7%. Successful reperfusion (mTICI 2b‐3) was achieved in 83.3%. sICH occurred in 10.6%. Median 90‐day mRS was 4 and 18.8% achieved mRS 0–2. Baseline characteristics and functional outcomes were broadly comparable to SELECT‐2 and LASTE, with similar reperfusion rates and functional status (both, median 90‐day mRS of 4).

**Conclusion:** In routine practice, MT in patients with ASPECTS ≤5 achieved high reperfusion rates with functional outcomes consistent with trial populations, supporting external validity and ongoing implementation in carefully selected patients.

**Disclosure:** Nothing to disclose.

## EPO‐0835

### Cerebral hemodynamic signatures linking mild cognitive impairment and late‐life depression: A multimodal transcranial doppler and MRI Study

#### S. Arnautu ^1^; D. Jianu^2^; D. Arnautu^1^

##### ^1^Department V Internal Medicine, “Victor Babes” University of Medicine and Pharmacy, Timisoara, Romania; ^2^Department of Neurosciences, “Victor Babes” University of Medicine and Pharmacy, Timisoara, Romania

**Background and aims:** Late‐life depression (LLD) and mild cognitive impairment (MCI) frequently coexist in older adults and share common neurovascular mechanisms. Cerebral small vessel dysfunction has been implicated in both cognitive and affective decline, yet clinically accessible markers linking these conditions remain limited. We investigated whether cerebral hemodynamic parameters assessed by transcranial Doppler (TCD), combined with MRI markers, identify shared neurovascular signatures between MCI and LLD.

**Methods:** In this cross‐sectional study, 96 older adults with depressive symptoms underwent standardized cognitive assessment (MMSE‐2, MoCA), affective evaluation (GDS‐15), brain MRI with Fazekas scoring of white matter lesions (WMLs), and TCD examination of the middle cerebral artery. Hemodynamic parameters included mean blood flow velocity (MBFV), end‐diastolic velocity (EDV), pulsatility index (PI), and resistive index (RI). Logistic regression and receiver operating characteristic (ROC) analyses identified independent predictors of MCI.

**Results:** Compared with cognitively unimpaired participants, individuals with MCI showed significantly lower MBFV and EDV and higher PI and RI (all *p* < 0.05). In multivariable analysis, altered MBFV, EDV, PI, and RI remained independently associated with MCI. RI demonstrated the highest discriminative accuracy (AUC = 0.919), followed by MBFV (AUC = 0.879). PI correlated with depressive symptom severity, while RI was associated with greater WML burden.

**Figure:**
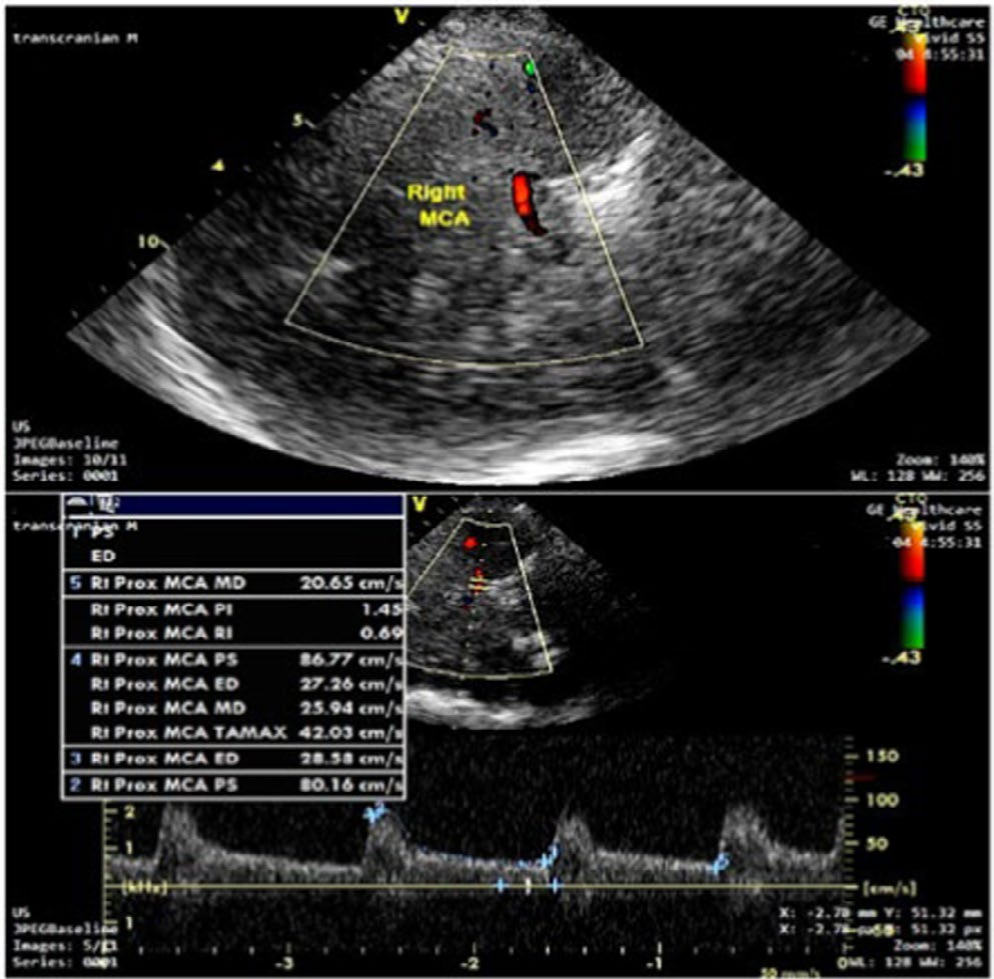
**FIGURE 1** Transcranial Doppler ultrasound recording of the middle right cerebral artery (MCA). The spectrum shows peak systolic velocity (PSV), end‐diastolic velocity (EDV), and mean blood flow velocity (MBFV) used for the calculation of the pulsatility index (PI) and resistivity index (RI).

**Figure:**
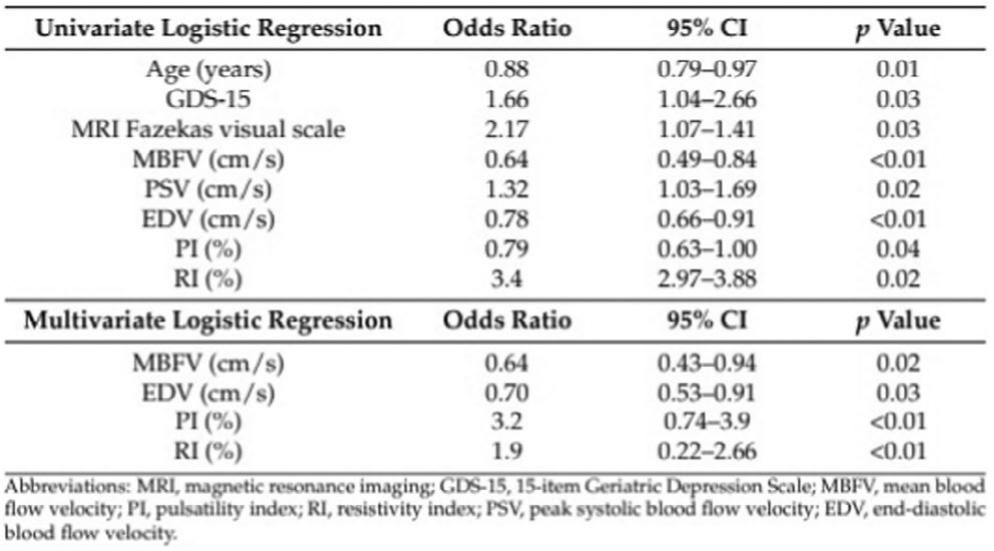
**TABLE 1** Predictors for MCI.

**Figure:**
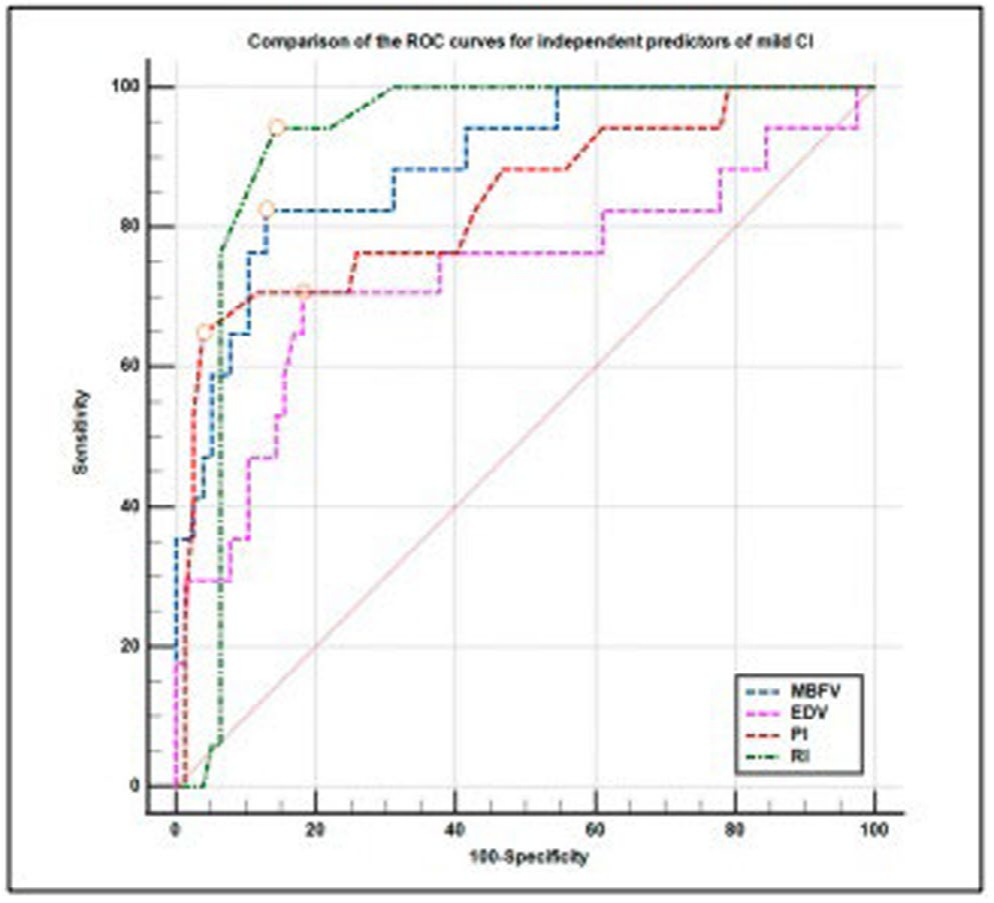
**FIGURE 2** A comparative ROC curve of all four predictors.

**Conclusion:** Altered cerebral hemodynamics represent a shared neurovascular substrate linking MCI and late‐life depression. Integrating TCD‐derived indices with MRI markers offers a clinically feasible multimodal approach for early identification of vascular cognitive and affective vulnerability in older adults.

**Disclosure:** Nothing to disclose.

## Neuro‐Ophthalmology/Neuro‐Otology 2

## EPO‐0836

### The vertigo challenge: Diagnostic variability across acute referral pathways

#### A. Heatley; I. Zia; E. Cortese; J. Tierney; M. Mahmud; S. Haider; A. Chandratheva; R. Simister; D. Kaski; N. Koohi

##### NHNN and NeuroSDEC at UCLH, London, UK

**Background and aims:** Acute vertigo, a common emergency presentation, places considerable strain on acute care resources. Stroke‐focused pathways drive extensive investigations, despite a low likelihood of a central cause. We evaluated diagnostic agreement between the emergency, stroke referral services, and specialist neuro‐otology in acute vertigo patients to identify gaps and inform targeted strategies.

**Methods:** This single‐centre retrospective cohort included new referrals with acute vertigo between August 2023 and July 2025. Of approximately 300 adult patients, 151 were analyzed. Data included referral source (ED, Acute Medicine Unit (AMU), Stroke Unit, TIA clinic, Neurology SDEC), vascular risk factors, imaging, and initial versus specialist diagnosis based on clinical history and imaging as a gold standard.

**Results:** Referrals originated from the ED/AMU (42.4%) and neuro‐SDEC/stroke/TIA services (57.6%). Overall diagnostic agreement was 32% higher for neurology‐led services (39.8%) than emergency services (23.5%). Overall, agreement was highest for stroke/TIA (75%) and lowest for chronic cardiovascular causes (6.7%). Additionally, 27.8% had no referral diagnosis, and most (65.56%) underwent neuroimaging.

**Conclusion:** Neurology‐led teams showed higher diagnostic accuracy than emergency services, which have limited neurology expertise, and this may explain the variability. Structured triage systems are essential to reduce misclassification, avoid unnecessary imaging, and support frontline teams.

**Disclosure:** Nothing to disclose.

## EPO‐0837

### Tolosa‐Hunt syndrome versus its mimics: Comparative analysis of clinical and radiological characteristics for accurate diagnosis

#### E. Patricio^1^; C. Peralta^1^; P. Miranda^1^; J. Pineda^1^; E. Reyes^1^; K. Yorro^1^; A. Tejada^1^; C. Pérez Lizardo^1^; A. Reyes ^1^; M. Rodríguez^2^

##### ^1^Synaptic Research League, Santo Domingo, Dominican Republic; ^2^Neurology Department, Hospital Universitario Salvador Bienvenido Gautier, Santo Domingo, Dominican Republic

**Background and aims:** Tolosa‐Hunt syndrome (THS) is a rare cause of painful ophthalmoplegia, diagnosed by its steroid response [1]. However, its presentation is mimicked by serious pathologies, often leading to initial misdiagnosis. This study analyzes the key differences between true THS and its clinical mimics to improve diagnosis accuracy.

**Methods:** A systematic review was performed following PRISMA guidelines. Out of 22 studies, 15 were included. PubMed and BVS were searched using the keywords “Tolosa‐Hunt syndrome,” “painful ophthalmoplegia,” and “cavernous sinus syndrome.” Data was extracted for comparative analysis of clinical and diagnostic variables.

**Results:** 222 patients, 125 females and 97 males, were included, with a mean age of 35.87 years. True THS accounted for 168 patients (75%). The other 58 cases were diagnosed with conditions including Rosai‐Dorfman Disease and neoplastic conditions involving cavernous sinus and orbit. True THS has slight predominance in females (86, 52.4%) than in male (78, 47.6%). True THS was involved unilaterally (>90%), more frequent on the left side in majority of patients. MRI abnormalities identified in 90% of cases with true TSH predominant to isolated cavernous sinus inflammation; >95% of false TSH showed mass‐like infiltrative lesions extending beyond the cavernous sinus. 85–90% of false cases demonstrated partial improvement after corticosteroid therapy, true cases showed >95% response and pain relief.

**Figure:**
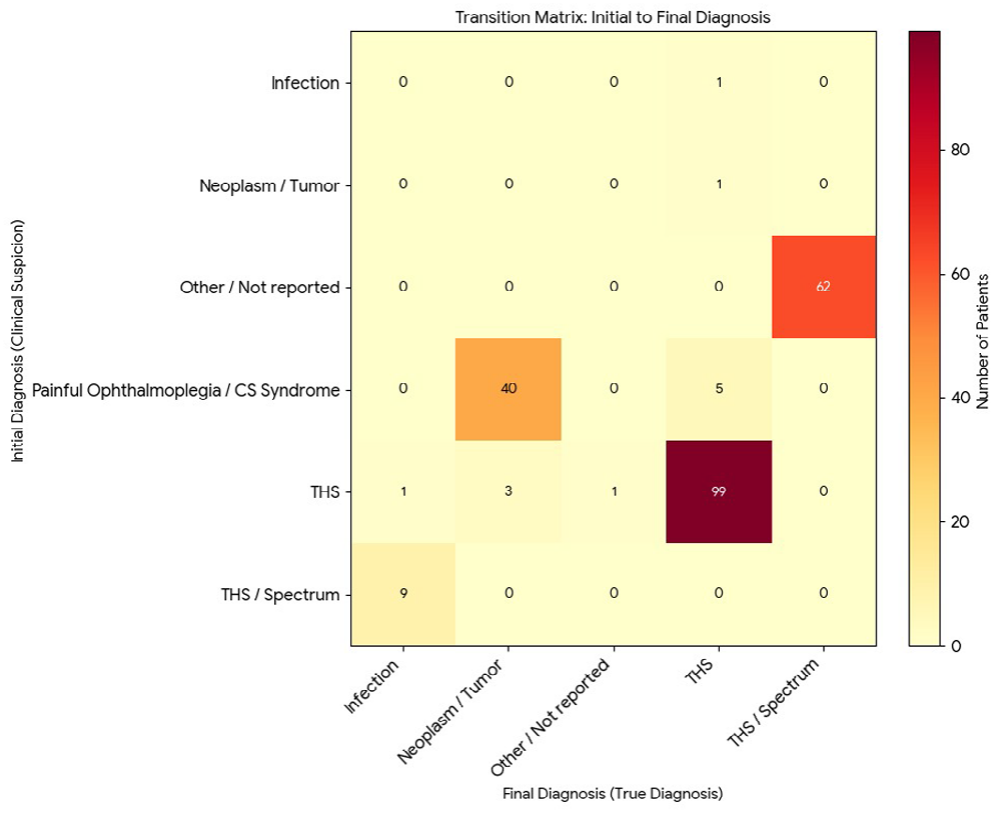
**FIGURE 1** Initial diagnosis versus final diagnosis in clinical suspicion of Tolosa‐Hunt Syndrome.

**Figure:**
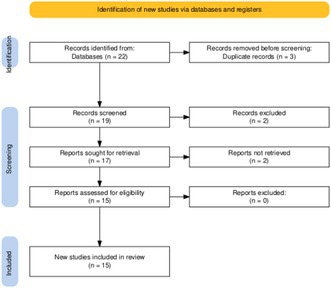
**FIGURE 2** Prisma‐P flowchart of the process of inclusion of studies in the systematic review.

**Conclusion:** Most cases labeled as THS are true THS; but some represent neoplastic disorders. Persistent or bilateral symptoms, mass‐like MRI lesions beyond the cavernous sinus, and incomplete steroid response suggest false‐positive THS, supporting early reassessment and targeted testing.

**Disclosure:** Nothing to disclose.

## EPO‐0838

### Brain MRI abnormalities along the visual pathways in subacute leber hereditary optic neuropathy

#### C. La Morgia ^1^; G. Amore^2^; M. Carbonelli^1^; M. Romagnoli^2^; C. Tonon^1^; R. Lodi^1^; R. Liguori^1^; V. Carelli^1^

##### ^1^Department of Biomedical and Neuromotor Sciences, University of Bologna, Bologna, Italy; ^2^IRCCS Institute of Neurological Sciences of Bologna, Bologna, Italy

**Background and aims:** Brain MRI abnormalities along the visual pathways are historically considered an uncommon feature of LHON at difference with inflammatory optic neuritis. We aimed at describing the prevalence of such MRI abnormalities in subacute LHON patients, correlating with clinical and genetic characteristics.

**Methods:** We retrospectively analysed clinical, genetic and ophthalmological data from 40 genetically‐confirmed LHON patients. MRI data performed within 6 months from onset were collected. Best corrected visual acuity (BCVA), retinal nerve fiber layer (RNFL) thickness and visual fields parameters were compared between patients with and without MRI abnormalities at time of MRI execution and at follow‐up (≥24 months). Linear mixed models were applied to account for inter‐eye correlation, with clinical and genetic predictors as covariates.

**Results:** MRI abnormalities along the visual pathways were found in 20/40 (50%) patients. Optic nerve hyperintensity was present in 45%, chiasm and optic tract involvement in 20%, and contrast enhancement of the optic nerve in 22%. At time of MRI execution, optic nerve hyperintensity resulted significantly associated with worse BCVA and visual field parameters, while optic nerve contrast enhancement was selectively associated with thinner temporal RNFL. At follow‐up (25 patients), long‐term BCVA was significantly influenced by mutation type, with rare mutations showing better outcomes, while MRI abnormalities were not prognostic. As exploratory analysis, high‐dose steroid therapy at onset showed mild influence on functional parameters at follow‐up.

**Conclusion:** MRI abnormalities are frequent in LHON and likely reflect early axonal damage and possibly an ongoing inflammatory component, but their independent prognostic value is still under evaluation.

**Disclosure:** Nothing to disclose.

## EPO‐0839

### Saccadic velocity dynamics abnormalities and levodopa‐induced dyskinesia in Parkinson's **disease**: An exploratory study

#### C. Terravecchia ^1^; A. Rufa^2^; C. Cicero^1^; G. Donzuso^1^; G. Mostile^1^; A. Nicoletti^1^

##### ^1^University of Catania, Department *“*G.F. Ingrassia*”*, Section of Neurosciences, Catania, Italy; ^2^Eye tracking and Visual Application Lab (EVA Lab), Department of Medicine, Surgery and Neurosciences, University of Siena, Siena, Italy

**Background and aims:** Oculomotor abnormalities were described in Parkinson's disease (PD) as an expression of disease‐related basal ganglia dysfunction, showing coherent relations with the disease's clinical features. Nevertheless, the possible relation between saccadic performances and levodopa‐induced dyskinesia (LID) has never been addressed to date. This exploratory study aimed to investigate possible associations between saccadic eye movements features and LID in PD.

**Methods:** Horizontal and vertical visually‐guided saccades (VGS) were analysed through Eyelink 1000 Plus in healthy controls (HCs), PD without LID (PD‐LID−) and PD with LID (PD‐LID+) in their OFF motor state. The following VGS metrics were assessed: latency, amplitude, duration, mean and peak velocity, gain. VGS dynamics were also assessed by: peak velocity/amplitude ratio, reflecting main sequence linear relation, and Q‐ratio (peak/mean velocity ratio), used to quantify and detect abnormalities of VGS velocity profile (i.e. higher Q‐ratio).

**Results:** Thirty‐two HCs, *N* = 16 PD‐LID‐ and *N* = 26 PD‐LID+ were enrolled. After adjusting by possible confounders, lower horizontal and vertical VGS amplitude, mean, peak velocity and gain were found in PD‐LID‐ as compared to HCs while no significant differences on VGS dynamics were found. On the other hand, reduced amplitude and gain as well as increased peak velocity/amplitude and Q‐ratios were shown in PD‐LID+ as compared to HCs. Significantly higher peak velocities, peak velocity/amplitude and Q‐ratios were demonstrated in PD‐LID+ as compared to PD‐LID−.

**Conclusion:** This exploratory study firstly highlights the association between saccadic velocity dynamics abnormalities as assessed in the OFF motor state and LID in PD. Clinical applications should be further investigated.

**Disclosure:** Nothing to disclose.

## EPO‐0840

### Vestibular impairment in Alzheimer's disease and amnestic mild cognitive impairment: Associations with domain‐specific cognitive decline

#### K. Koçoğlu^1^; H. Eraslan Boz^1^; P. Özçelik^1^; M. Akkoyun^1^; I. Tüfekci^1^; G. Halmágyi^2^; G. Akdal ^3^

##### ^1^Department of Neurosciences, Institute of Health Sciences, Dokuz Eylül University, Izmir, Türkiye; ^2^Department of Neurology, Royal Prince Alfred Hospital, Sydney, NSW Australia; ^3^Department of Neurology, Faculty of Medicine, Dokuz Eylül University, Izmir, Türkiye

**Background and aims:** This study investigated vestibular function in Alzheimer's disease (AD) and amnestic mild cognitive impairment (aMCI), to examine the prevalence of vestibular impairment and its association with domain‐specific cognitive decline.

**Methods:** The study included 91 participants: 30 AD, 30 aMCI, and 31 controls. All participants underwent neuropsychological testing and vestibular assessments using video Head Impulse Test (vHIT) for semicircular canals (SCCs) and vestibular‐evoked myogenic potentials (VEMPs) for otolith function. Overall vestibular scores were defined as abnormal results on ≥ 2 of 10 subtests. Cognitive domains assessed included memory, executive function, attention, visuospatial, and language (Table 1).

**Figure:**
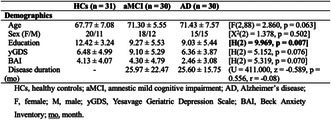
**TABLE 1** The descriptive statistics of the demographics and neuropsychological tests.

**Results:** (1) Abnormal vHIT results were significantly higher in AD and aMCI (50% each) versus controls (12.9%; *p* = 0.002) while VEMPs and overall vestibular scores showed no group differences; (2) Specific vestibular impairments were associated with distinct cognitive domains; (2a) in AD with vestibular impairment (ADwVi), memory linked to posterior SCC impairment and visuospatial to overall vestibular scores (*p* = 0.029). In aMCI with vestibular impairment (aMCIwVi), visuospatial associated with abnormal ocular VEMP (oVEMP; *p* = 0.046); (2b) oVEMP showed contrasting associations: language in aMCIwVi (*p* = 0.009) versus, Mini‐Mental State Examination (MMSE) and executive function in AD without vestibular impairment (*p* = 0.033 and *p* = 0.023, respectively); and (3) the overall vestibular scores, though not differing between groups (*p* = 0.063), correlated with memory, visuospatial, language performance, and age (Table 2).

**Figure:**
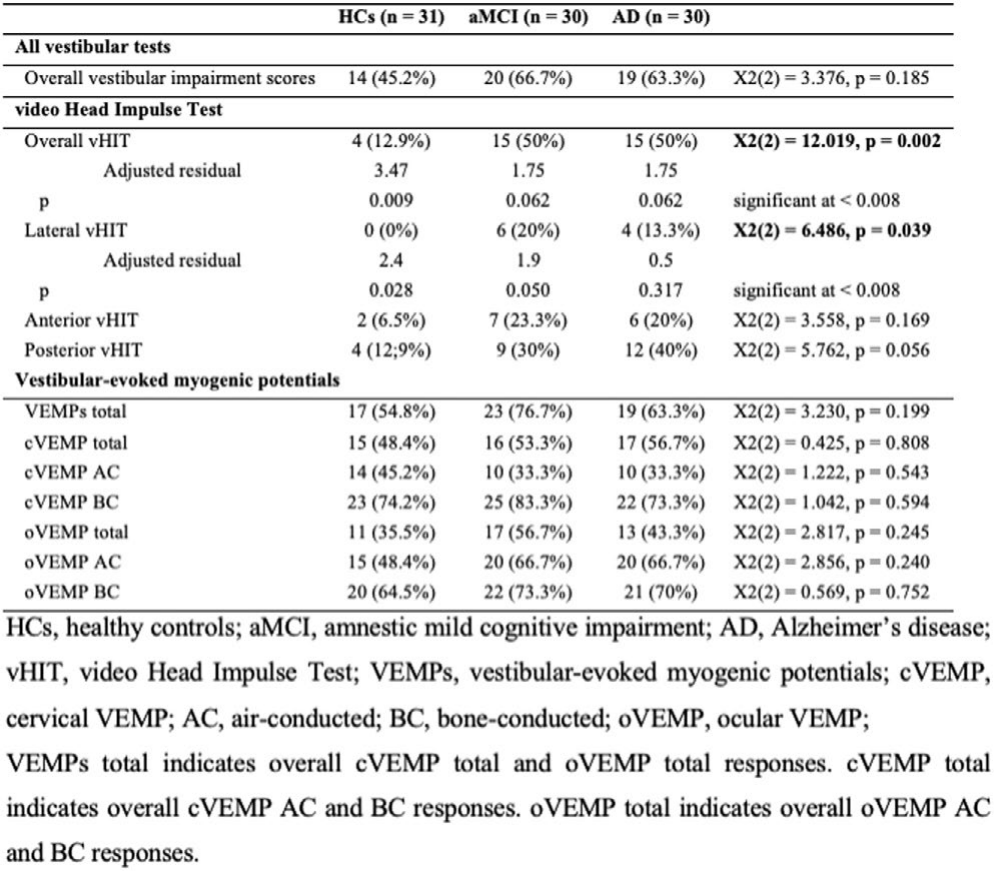
**TABLE 2** The proportions of subjects with abnormal responses in vestibular tests.

**Conclusion:** These findings indicate subclinical vestibular impairment, particularly involving SCCs, is prominent in AD and aMCI. Specific vestibular‐cognitive associations suggest vestibular assessment could serve as a valuable supplementary tool for identifying and monitoring cognitive decline in at‐risk populations.

**Disclosure:** Nothing to disclose.

## EPO‐0841

### Can perfusion‐weighted imaging improve diagnostic accuracy in transient vascular vertigo?

#### H. Lee; H. Park

##### Department of Neurology and Institute of Wonkwang Medical Science, Wonkwang University School of Medicine, Wonkwang University Hospital, Regional Cardiocerebrovascular Center, Iksan, Republic of Korea

**Background and aims:** Transient vestibular syndrome (TVS) is a frequent manifestation of transient ischemic attacks (TIA) in the vertebrobasilar territory. However, the underlying etiology often remains unidentified in more than half of these patients, even with detailed neuro‐otological examinations and standard MRI, including diffusion‐weighted imaging (DWI). The aim of this study is to evaluate the clinical utility of perfusion‐weighted imaging (PWI) in diagnosing acute transient vascular vertigo (TVV).

**Methods:** We retrospectively reviewed patients with recurrent transient vertigo/dizziness between October 2023 and November 2025. Inclusion criteria were: no alternative vestibular diagnosis, negative findings on initial brain MRI/DWI, and evaluation within 7 days of onset. Comprehensive neuro‐otological assessments (VOG, vHIT, cVEMP, SVV/SVH), lipid profiles, and PWI were analyzed.

**Results:** Thirty‐one patients were included (mean age 67 ± 13 years; 14 males). While all patients were DWI‐negative, PWI revealed perfusion deficits in 18 patients (58%). Posterior circulation vascular stenosis or hypoplasia was identified in 24 patients (77%). Regarding neuro‐otological findings, none showed spontaneous nystagmus or skew deviation, though apogeotropic nystagmus was observed in 12 patients. Abnormal cVEMP and vHIT were found in 13% and 22.5% of patients, respectively. All patients possessed at least three vascular risk factors, most commonly diabetes and dyslipidemia. Notably, 7 patients (22.6%) progressed to cerebral infarction within one week.

**Conclusion:** PWI is a valuable diagnostic tool for identifying transient vascular vertigo, especially when DWI is negative. The presence of posterior circulation stenosis and multiple vascular risk factors should raise clinical suspicion of TVV to prevent subsequent stroke.

**Disclosure:** Nothing to disclose.

## EPO‐0842

### Interpretation of HINTS findings in idiopathic sudden sensorineural hearing loss with vertigo

#### J. Choi ^1^; H. Kim^1^; S. Choi^1^; Y. Lee^1^; S. Choi^2^; K. Choi^2^; M. Kim^2^; W. Hwang^2^; S. Lee^3^

##### ^1^Department of Neurology, Pusan National University School of Medicine, Research Institute for Convergence of Biomedical Science and Technology, Pusan National University Yangsan Hospital, Yangsan, Republic of Korea; ^2^Department of Neurology, Pusan National University Hospital, Pusan National University School of Medicine and Biomedical Research Institute, Busan, Republic of Korea; ^3^Department of Neurology, Chonnam National University Hospital, Chonnam National University Medical School, Gwangju, Republic of Korea

**Background and aims:** The head impulse‐nystagmus‐test of skew (HINTS) examination is widely used to distinguish peripheral from central causes of acute vestibular syndrome, but its diagnostic performance in idiopathic sudden sensorineural hearing loss (SSNHL) with vertigo remains uncertain. This study aimed to assess the accuracy and clinical utility of the HINTS examination in patients with idiopathic SSNHL accompanied by vertigo.

**Methods:** We prospectively evaluated 121 patients with idiopathic SSNHL and vertigo at two university hospitals. The patients underwent detailed neuro‐otological examinations, including nystagmus assessment, caloric testing, head impulse tests (HITs), and, as well as brain MRI to exclude central lesions. HINTS findings were classified as “peripheral” or “central” according to established criteria, and false‐positive and true‐negative rates were calculated.

**Results:** Spontaneous nystagmus (SN) was present in 59 patients (49%), with no cases of direction‐changing nystagmus and only one case of skew deviation. Abnormal caloric responses were observed in 34/71 (48%), whereas abnormal horizontal HIT findings occurred in 35/119 (29%). Concordance between the two tests was noted in 80% of patients, while 17% showed abnormal caloric responses despite normal horizontal HIT. Among 59 patients with SN, 36 (61%) demonstrated “central” HINTS patterns despite normal brain MRI findings, indicating a high false‐positive rate. Misclassification was significantly more frequent in patients with ipsilesional SN than in those with contralesional SN (89% vs 31%, *p <* 0.001).

**Conclusion:** In idiopathic SSNHL with vertigo, the HINTS examination frequently yields central patterns despite peripheral pathology, reflecting limited specificity. Thus, careful interpretation with complementary audiovestibular testing is required for accurate diagnosis.

**Disclosure:** Nothing to disclose.

## EPO‐0843

### When LHON mimics demyelination: Area postrema syndrome in Biallelic DNAJC30 variant

#### K. Dzwilewski; M. Krygier; J. Szymarek; M. Zawadzka; M. Mazurkiewicz‐Bełdzińska

##### Department od Developmental Neurology, Medical University of Gdańsk, Gdańsk, Poland

**Background and aims:** Biallelic pathogenic variants in a nuclear‐encoded gene DNAJC30 cause autosomal recessive form of Leber hereditary optic neuropathy (arLHON), commonly considered to be mitochondrially inherited. Although most affected individuals present with isolated optic neuropathy, further research expanded phenotypic spectrum of diseases linked to DNAJC30. We report a patient with LHON and area postrema syndrome (APS).

**Methods:** 13‐year‐old patient was referred due to blurred vision with central scotoma of the left eye, recurrent vertigo, and vomiting for 2 months. He presented horizontal gaze‐evoked nystagmus and convergence insufficiency of the left eye. Ophthalmological evaluation showed bilateral papilledema with telangiectatic vessels and significant vision impairment of the left eye. Laboratory tests did not detect any specific antibodies. MRI identified brain stem T2 high‐signal lesions of suspected inflammatory etiology. Thus, intravenous steroids were commenced, followed by immunoglobulin therapy, without significant vision improvement. Follow‐up MRI showed T2 high‐signal lesions in AP and optic nerves enhancement, indicative for NMOSD. Despite further immunosuppressive treatment visual function did not improve. On the contrary, progressive vision impairment was observed. Hence, whole exome sequencing was performed, identifying homozygous pathogenic DNAJC30 variant c.152A>G, p.(Tyr51Cys).

**Results:** The patient was diagnosed with arLHON with symptoms of APS and provided with idebenone, resulting in significant vision improvement.

**Conclusion:** DNAJC30‐associated disease may mimic neuromyelitis optica spectrum disorder (NMOSD). We highlight the need of early genetic diagnosis in patients with optic neuropathy and APS who do not respond to immunotherapy, as targeted treatment with idebenone may lead to significant clinical and radiological improvement.

**Disclosure:** Nothing to disclose.

## EPO‐0844

### When atypical is common: Rethinking optic neuritis in multiple sclerosis

#### L. Silva; B. Silva; A. Martins; I. Monteiro; R. Machado; I. Correia; C. Nunes; C. Macário; A. Jorge; S. Batista; J. Lemos

##### Neurology Department, Coimbra University Hospital Centre, Coimbra, Portugal

**Background and aims:** Optic neuritis (ON) is a classic manifestation of multiple sclerosis (MS). If atypical features are present (AON), these generally suggest a distinct underlying etiology from MS. We aimed to ascertain the prevalence of AON in MS and to further investigate it as a subgroup.

**Methods:** A retrospective analysis of clinical/paraclinical data from MS patients who presented with acute optic neuritis in our department was conducted. All patients underwent neuro‐ophthalmic observation, retinography, optical coherence tomography, and perimetry at baseline, and 1‐ and 6‐month follow‐ups. These were further separated into typical (TON) and AON groups.

**Results:** We included 62 patients (47 females [75.8%]; mean age 38.9 ± 11.9). AON was present in 62.9% patients. If not considering lack of pain as an atypical feature, AON was still present in 40.3%. Visual acuity was ≤20/400 at baseline, ≤20/200 at 1‐month and ≤20/100 at 6‐months, in 21%, 4.8% and 4.8% patients, respectively. Ten (16.1%) patients were ≥50 years old at the time of optic neuritis. There was no pain in 35.5% patients. There was severe optic disc edema in 3.2%, and peripapillary hemorrhages in 1 (1.6%) patient. Four (6.5%) patients had bilateral involvement. In 3.2% patients, chiasmal involvement was documented. EDSS score, disease duration and number of relapses in the last two years did not significantly differ between groups.

**Conclusion:** Approximately 40% of MS patients show ≥1 atypical feature at the time of their optic neuritis. While AON still requires the active exclusion of an alternative diagnosis, atypical optic neuritis is not uncommon in MS.

**Disclosure:** Nothing to disclose.

## EPO‐0845

### Pupillodynamics: A potential indicator of autonomic dysfunction in Parkinson disease

#### L. Lehner; G. Laudes; L. Schwach; A. Jäck; S. Christoph; L. Marth; F. Schöberl; F. Hopfner; G. Höglinger; M. Strupp

##### Department of Neurology, LMU University Hospital, LMU Munich, Germany

**Background and aims:** The pupillary light reflex is an important metric of autonomic function. In Parkinson's disease (PD) autonomic function is often impaired, even in early stages of the disease. Therefore, we aimed to identify disease specific alterations by dynamic pupillometry.

**Methods:** Dynamic pupillometric parameters were assessed in 116 patients with PD and 188 healthy controls (HC) using BulbiCamTM. Group differences were analyzed with univariate general linear models for each pupillometric parameter, including diagnosis as the between‐subject factor and age and sex as covariates. Additional age‐stratified analyses were performed within the 51–60, 61–70, and 71–80 years subgroups.

**Results:** Patients with PD showed larger baseline pupil size after dark adaptation and after light exposure, as well as significantly larger minimal pupil size at 10 and 15 s, indicating reduced and less sustained pupillary constriction (medium to large effect sizes, ηp^2^ up to 0.137). In PD pupillary dynamics were selectively altered, with markedly reduced late phase constriction (8–10 and 13–15 s). Peak velocity of dilatation was modestly reduced in PD, whereas peak acceleration and pupillary latencies did not differ significantly between groups (Table). Age‐stratified analyses demonstrated that group differences in tonic pupil size and minimal pupil diameter were preserved or enhanced in older age groups (Figure 1), while impaired late phase pupillary constriction were consistently present across all age strata (Fig. 2).

**Figure:**
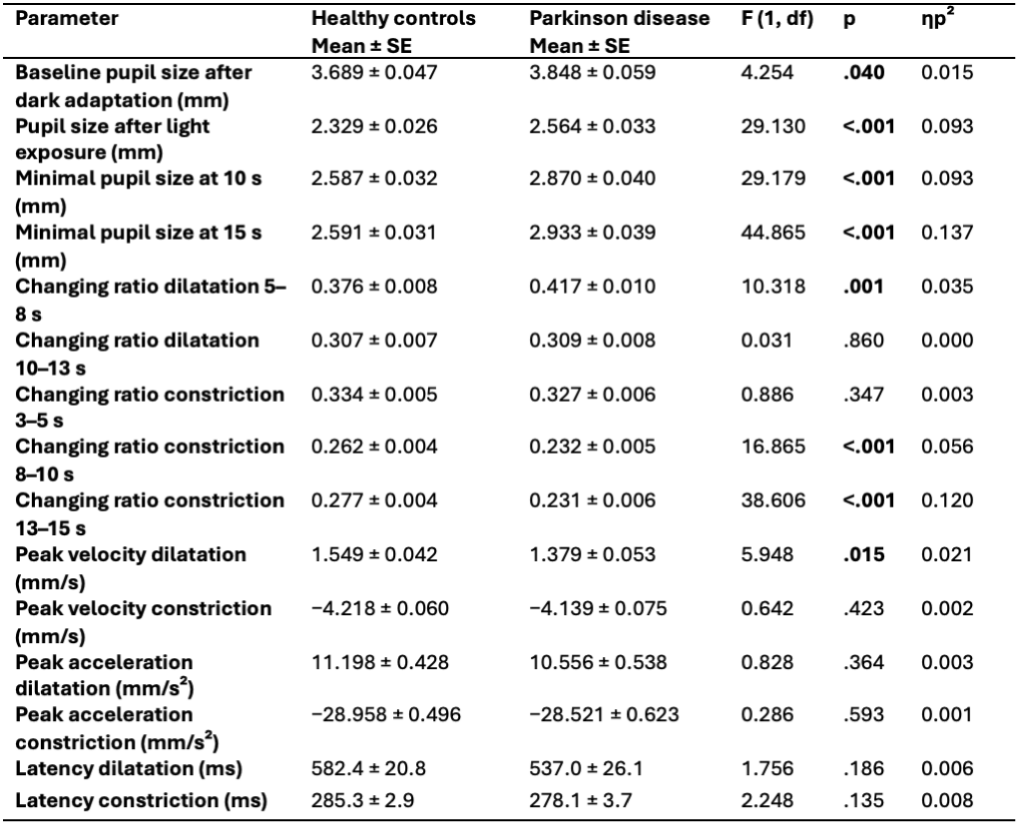
**TABLE 1** Group differences in pupillometric parameters between Parkinson's disease and healthy controls adjusted for age and sex. *F*, *F* statistic from the general linear model; ηp^2^, partial eta squared (effect size); SE, standard error.

**Figure:**
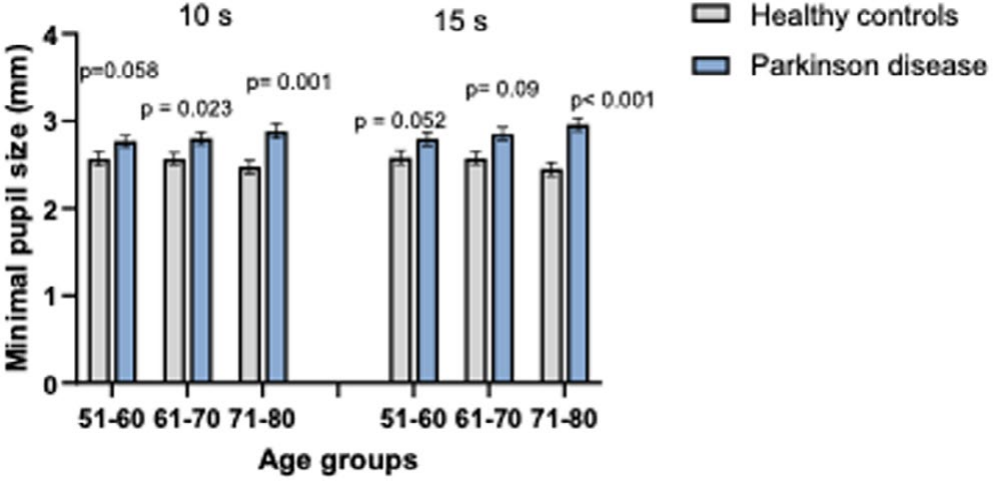
**FIGURE 1** Age‐stratified comparison of minimal pupil size at 10 and 15 s in HC and PD. Bars represent estimated marginal means ± SE. Group sizes: 51–60 years: HC *n* = 27, PD *n* = 29; 61–70 years: HC *n* = 37, PD *n* = 37; 71–80 years: HC *n* = 29, PD *n* = 31.

**Figure:**
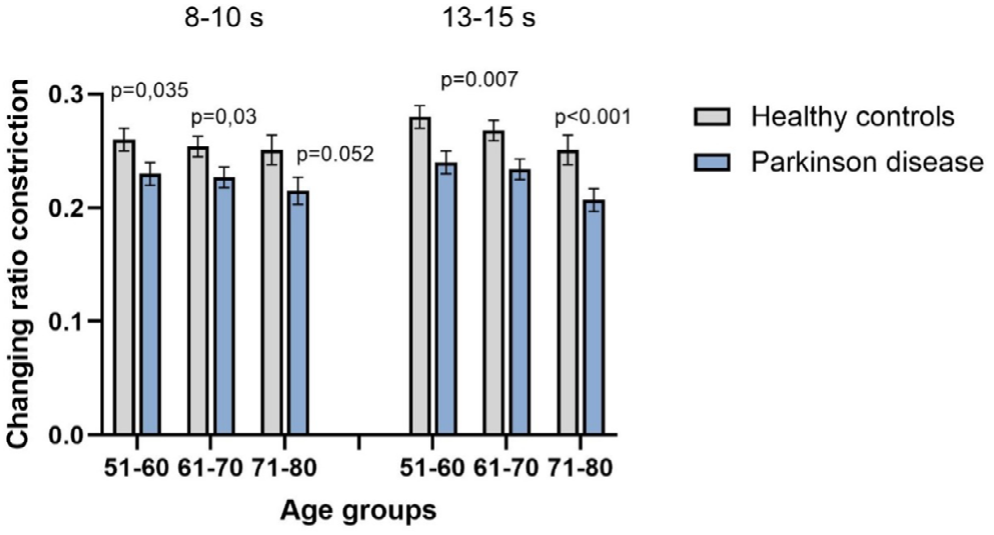
**FIGURE 2** Age‐stratified comparison of constriction dynamics (8–10 s and 13–15 s) in HC and PD. Bars represent estimated marginal means ± SEM. Group sizes: 51–60 years: HC *n* = 27, PD *n* = 29; 61–70 years: HC *n* = 37, PD *n* = 37; 71–80 years: HC *n* = 29, PD *n* = 31.

**Conclusion:** PD is associated with selective alterations of pupillary control, predominantly affecting pupil size regulation and late‐phase pupillary constriction. These findings support disease‐specific autonomic pupillary dysfunction in PD.

**Disclosure:** M. Strupp is Joint Chief Editor of the Journal of Neurology, Editor in Chief of Frontiers of Neuro‐otology and Section Editor of F1000. He has received speaker's honoraria from Abbott, Auris Medical, Biogen, Eisai, Grünenthal, GSK, Henning Pharma, Interacoustics, J&J, MSD, NeuroUpdate, Otometrics, Pierre‐Fabre, TEVA, UCB, and Viatris. He received support for clinical studies from Decibel, U.S.A, Cure within Reach, U.S.A. and Heel, Germany. He distributes “M‐glasses” and “Positional vertigo App”. He acts as a consultant for Abbott, AurisMedical, Bulbitec, Heel, Sensorion, Vifor and Vertify. He is a scientific founder, investor and share‐holder of IntraBio.

## EPO‐0846

### Translating RNFL thickness into papilledema grades in idiopathic intracranial hypertension

#### Y. Huang‐Link ^1^; S. Eriksson^1^; J. Schmiauke^1^; G. Yang^2^

##### ^1^Department of Neurology, Linköping University Hospital, Linköping, Sweden; ^2^Huizhou Aier Eye Hospital, Huizhou, China

**Background and aims:** The Frisén scale is widely used to grade papilledema, but it remains subjective with notable inter‐observer variability. Quantitative OCT provides objective and reproducible assessment of papilledema with micrometer‐level precision. This study aimed to define retinal nerve fiber layer (RNFL) thickness thresholds that correspond to modified Frisén papilledema grades.

**Methods:** A total of 49 patients with newly diagnosed idiopathic intracranial hypertension (98 eyes) were followed for up to two years. 64 healthy controls were included. RNFL thickness was compared across papilledema grades, and ROC was applied to identify cut‐off values.

**Results:** RNFL thickness correlated strongly with papilledema levels according Frisén grading and moderately with intracranial pressure. RNFL thickness increased progressively with higher papilledema grades and differed significantly between all grade groups (*p* < 0.001). Threshold values of approximately 105, 140, 232, and 400 μm distinguished Grade 0 from I, I from II, II from III, and III from IV, respectively, with AUCs ranging from 0.77 to 0.95.

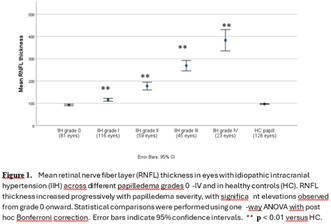

**Conclusion:** The results demonstrate that RNFL measurements can be translated into clinically meaningful papilledema severity levels. Defining RNFL‐based cut‐offs offers a more objective, sensitive, precise and reproducible approach to grading papilledema, supporting improved monitoring and management in IIH.

**Disclosure:** Nothing to disclose.

## Spinal Cord and Root Disorders

## EPO‐0847

### Spinal cord compression due to plexiform neurofibromas in NF1

#### A. Elsaddig

##### Neurology Department, Sheikh Tahnoon Bin Mohammed Medical City, Al Ain, United Arab Emirates

**Background and aims:** Neurofibromatosis type 1 (NF1) is an autosomal dominant neurocutaneous disorder caused by mutations in the NF1 gene. It is characterised by Schwann cell proliferation, leading to cutaneous neurofibromas and plexiform neurofibromas, which may cause neurological complications due to mass effect.

**Methods:** We present the case of a 26‐year‐old male with a known diagnosis of NF1 who presented with a six‐month history of intermittent, sudden‐onset jerking sensations affecting the chest and upper limbs, associated with pins and needles and tingling. Clinical examination and neuroimaging findings were reviewed.

**Results:** Neurological examination revealed multiple cutaneous neurofibromas, particularly over both wrists, with bilateral thenar eminence wasting. Muscle tone was normal, with preserved power except for weakness of thumb abduction. Upper limb reflexes were brisk. In the lower limbs, there was pyramidal‐pattern weakness with an absent left ankle reflex. MRI of the brain and spine demonstrated extensive cervical plexiform neurofibromas causing compression of the cervical spinal cord at the C2–3 level, with no radiological evidence of malignant transformation.

**Figure:**
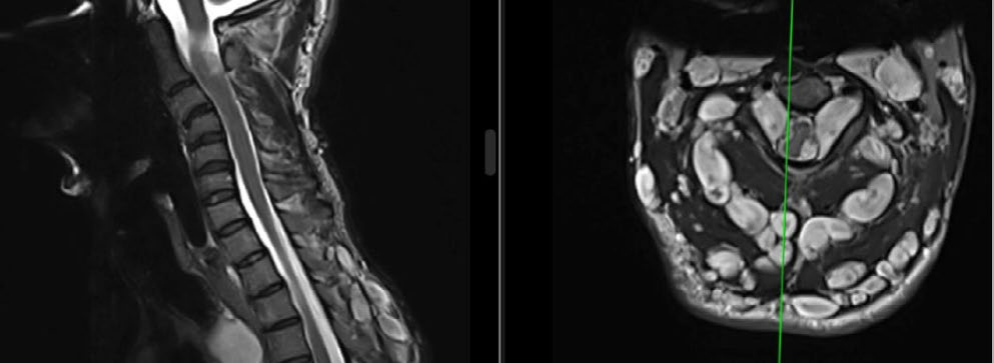
**FIGURE 1** Sagittal T2 and axial T2 showing distortion and compression of cervical cord.

**Figure:**
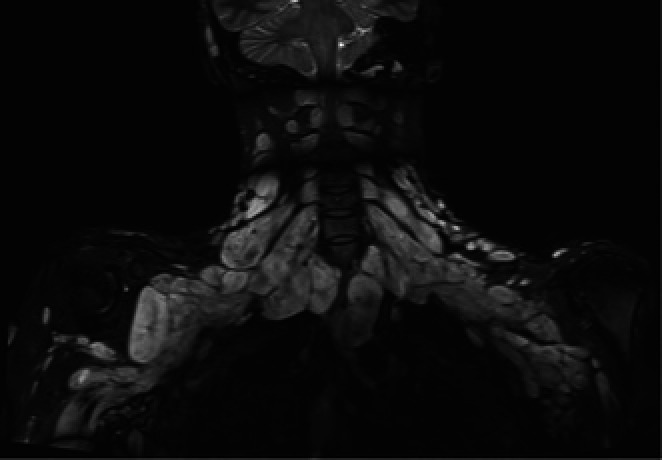
**FIGURE 2** Brachial plexus STIR view showing huge large plexiform neurofibromas.

**Conclusion:** NF1 is an important cause of nerve root hypertrophy and spinal cord compression. Plexiform neurofibromas can result in significant cervical cord compromise, highlighting the need for early recognition and surveillance imaging in symptomatic patients.

**Disclosure:** Nothing to disclose.

## EPO‐0848

### A systematic review of the natural history of degenerative thoracic myelopathy

#### E. Koubeh; S. Möbus; T. Rujeedawa; O. Mowforth; M. Kotter

##### Department of Clinical Neurosciences, University of Cambridge, Cambridge, UK

**Background and aims:** Degenerative thoracic myelopathy (DTM), a recently proposed term, encompasses a spectrum of degenerative spine conditions that cause thoracic spinal cord compression and progressive neurological dysfunction. The natural history of DTM is poorly characterised, hindering prompt diagnosis and management. This systematic review aimed to assess the initial symptoms, progression, and prognostic factors. Studying DTM as a unified condition provides a larger body of evidence to draw conclusions from, analogous to Degenerative Cervical Myelopathy.

**Methods:** A systematic literature search was performed in Embase and MEDLINE, identifying papers assessing DTM. Relevant outcomes included initial symptoms, symptom progression, duration of disease and prognostic factors. This review followed PRISMA guidelines.

**Results:** Sixteen studies met the inclusion criteria. Sensory disturbances were more common than motor symptoms in the early stages of DTM, with all 8 studies reporting initial symptoms finding that numbness, tingling or pain were most common. Gait disturbances and motor symptoms such as weakness were commonly reported later in disease progression. The prevalence of symptoms increased over time. Symptom progression was highly variable, with a mean duration from symptom onset to surgery of 25.5 months. Risk factors for progression include the underlying aetiology, age and the presence of T2 hyperintensity on spinal MRI.

**Conclusion:** Despite a paucity of literature on the natural history of DTM, this review identifies several patterns which provide actionable guidance for clinicians. Recognising early symptoms may reduce diagnostic delay, prognostic features can inform risk‐stratified surveillance and understanding progression may inform treatment thresholds. Importantly, diagnostic delay is associated with poorer post‐operative outcomes in DTM.

**Disclosure:** Nothing to disclose.

## EPO‐0849

### Abstract withdrawn

## EPO‐0850

## Clinical characteristics of toxocara canis myelitis in Lebanon: Largest single center cohort study

### K. Eid; N. El Ayoubi; T. El Halabi; S. F. Atweh

#### Neurology Department, American University of Beirut Medical Center, Beirut, Lebanon

**Background and aims:** Neurotoxocariasis is a rare manifestation of human toxocariasis presenting as myelitis and literature is limited to small case series. This study describes the clinical characteristics, diagnostic findings, treatment results, and prognostic factors of Toxocara‐associated myelitis.

**Methods:** This is a retrospective cohort study conducted at a tertiary center, including 44 patients diagnosed with Toxocara myelitis between 1998 and 2025. Diagnosis was based on clinical presentation, magnetic resonance imaging (MRI), serology, cerebrospinal fluid (CSF), eosinophilia, and treatment response. Clinical improvement was defined as ≥50% or <50% from baseline. Predictors of outcome were obtained by bivariate and multivariate analyses.

**Results:** Majority of patients were males (84%) with median age 41 years. All patients were ambulatory at baseline with sensory symptoms, however autonomic dysfunction and motor weakness were present in 59% and 57% of cases, respectively. Peripheral eosinophilia was in 46%, CSF pleocytosis in 21%, CSF eosinophilia in 5% and CSF oligoclonal bands in 44%. 95% had positive serology in blood and/or CSF. All patients were treated with Albendazole, 82 % also received corticosteroids. Of the 41 reevaluated patients: 71% improved, and 51% attained ≥50% recovery. Early Albendazole initiation was the only predictor of a favorable outcome (median 10 vs. 32 weeks from symptom onset; *p* = 0.012), confirmed controlling for age, sex, and treatment with corticosteroids. Importantly, demographic characteristics, clinical presentation, laboratory data, and corticosteroid use were not associated with recovery.

**Conclusion:** Toxocara myelitis is considered a potentially reversible subacute myelitis. The most important factor for neurological recovery is the early diagnosis and administration of albendazole.

**Disclosure:** Nothing to disclose.

## EPO‐0851

## Paraneoplastic sensory ganglionopathy as the initial manifestation of a small pancreatic neuroendocrine tumor

### M. Henriques; J. Almada Silva; D. Carapinha; M. Saraiva; R. Pinheiro; A. Rêgo

#### Department of Neurology, Hospital Prof. Dr. Fernando Fonseca, Lisbon, Portugal

**Background and aims:** Paraneoplastic neurological syndromes (PNS) are rare immune‐mediated disorders, affecting fewer than 1% of patients with cancer, and may precede the diagnosis of an underlying malignancy. Sensory ganglionopathy is a severe and disabling manifestation, classically associated with small‐cell lung carcinoma but also described in other tumors, including neuroendocrine neoplasms. The frequent absence of detectable onconeural antibodies may delay diagnosis, emphasizing the importance of systematic malignancy screening in unexplained sensory neuronopathies.

**Methods:** We report a 56‐year‐old woman presenting with subacute progressive sensory ataxia, marked proprioceptive loss, generalized areflexia, postural instability, and functional dependence. Motor weakness was predominantly secondary to sensory deafferentation. Cranial nerve examination were normal.

**Results:** Brain and spinal MRI showed no structural abnormalities. Cerebrospinal fluid analysis revealed mild protein elevation without pleocytosis, oligoclonal bands, or infectious markers. Extensive metabolic, infectious, toxic, and autoimmune investigations, including neuronal surface antibodies, were negative. Neurophysiological studies were consistent with moderate sensory ganglionopathy. Given the unexplained progressive course, systemic malignancy screening was pursued. Abdominal imaging identified a small lesion in the pancreatic head. Functional imaging with PET‐DOTANOC confirmed a somatostatin receptor–positive pancreatic neuroendocrine tumor. The patient underwent pancreaticoduodenectomy following multidisciplinary discussion. While awaiting surgery, recurrent hypoglycaemic episodes occurred, consistent with hormonally active disease, requiring glucagon therapy. Histopathological examination confirmed a well‐differentiated, low‐grade pancreatic neuroendocrine tumor (WHO grade 1). Despite tumor resection and immunomodulatory therapy, neurological deficits persisted, consistent with irreversible dorsal root ganglion damage.

**Conclusion:** Sensory ganglionopathy may be the initial manifestation of pancreatic neuroendocrine tumors, supporting early comprehensive oncological screening in unexplained subacute sensory neuronopathy.

**Disclosure:** Nothing to disclose.

## EPO‐0852

### Spontaneous spinal epidural hematoma secondary to a dural arteriovenous fistula as a cause of acute myelopathy

#### M. Serrano Alarcón ^1^; A. Trondin^3^; E. Huertas Muñoz^1^; L. Guirao Guillen^1^; L. Centeno Pons^1^; C. Gómez‐Escalonilla Escobar^1^; L. Franco Rubio^1^; A. López‐Frías López‐Jurado^2^

##### ^1^Neurology Department, Hospital Clínico San Carlos, Madrid, Spain; ^2^Neurointerventional Radiology Department, Hospital Clínico San Carlos, Madrid, Spain; ^3^Neurosurgery Department, Hospital Clínico San Carlos, Madrid, Spain

**Background and aims:** Spinal dural arteriovenous fistulas are vascular malformations that typically cause slowly progressive myelopathy. However, a rare but severe complication in this setting is spontaneous spinal epidural hematoma, which may present as acute spinal cord compression.

**Methods:** We report the case of an 81‐year‐old man who presented to the emergency department with acute lumbar pain followed by sudden bilateral lower‐limb weakness. Neurological examination revealed paraparesis with muscle strength graded as follows: hip flexion/extension 3−/2, knee flexion/extension 1/1, ankle dorsiflexion/plantarflexion 3+/0. Patellar and Achilles reflexes were absent, with bilateral indifferent plantar responses. Sensory examination showed bilateral hypoesthesia to pain and touch below the knees. Acute myelopathy was suspected, and urgent spinal MRI demonstrated an extensive extradural collection compatible with spinal epidural hematoma from T7 to L3, with maximal compression and myelopathy associated at T11–T12. Tortuous perimedullary vessels of uncertain significance were also observed. Emergency D11–D12 laminoplasty with hematoma evacuation was performed by the Neurosurgery ward.

**Figure:**
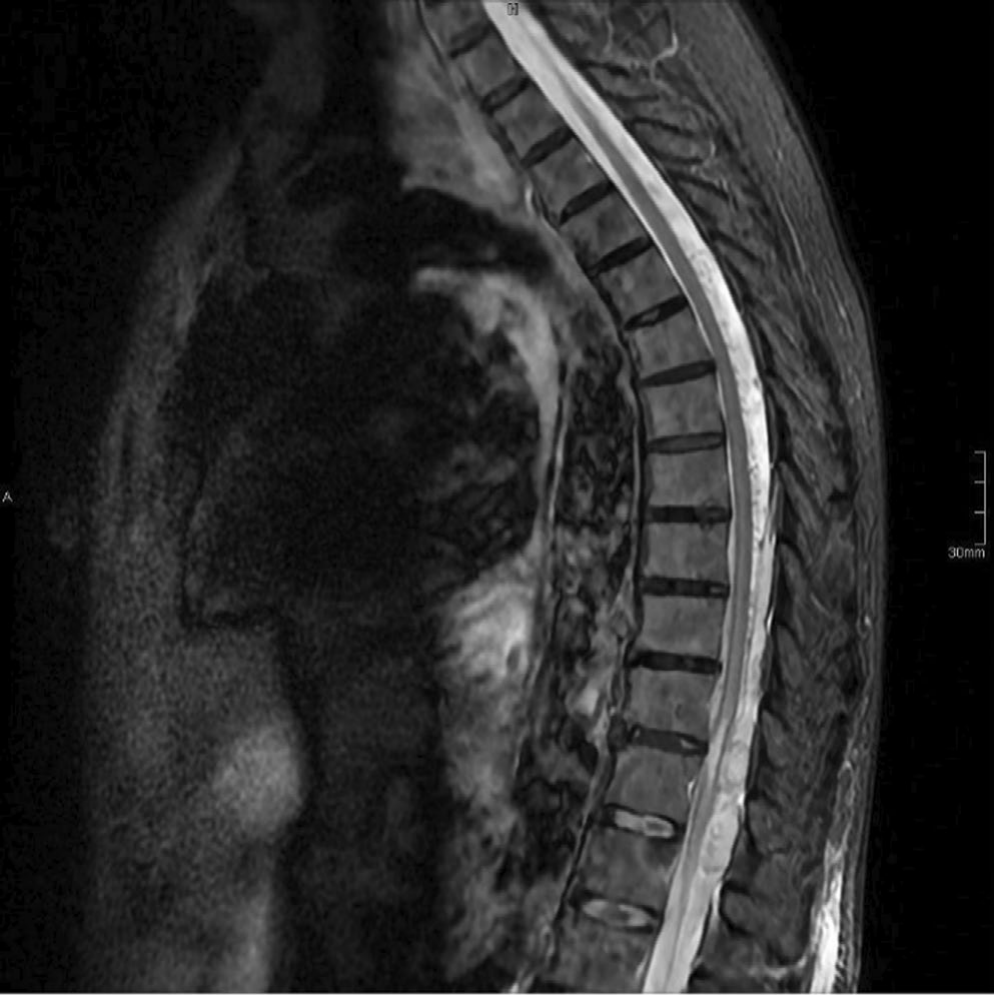
**FIGURE 1** Sagittal STIR spinal MRI demonstrating an extensive hyperintense extradural collection consistent with an acute spinal epidural hematoma extending from T7 to L3, with maximal spinal cord compression and associated myelopathy at T11–T12.

**Figure:**
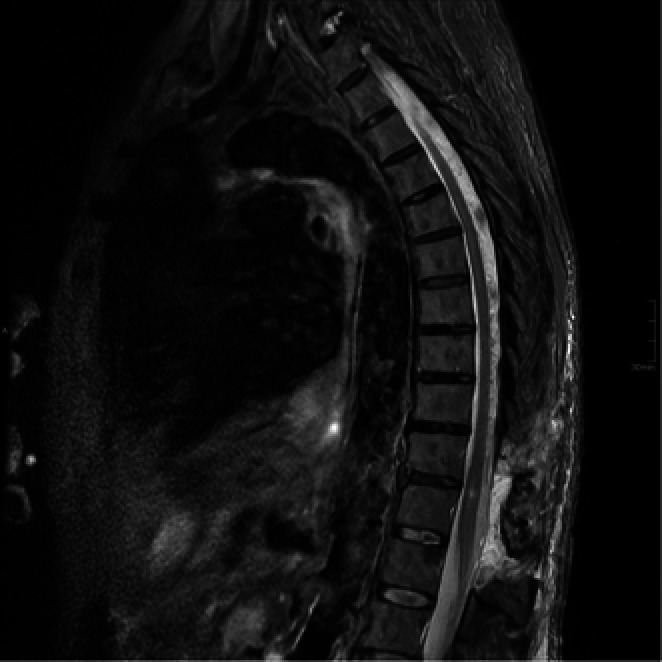
**FIGURE 2** Sagittal STIR spinal MRI showing postoperative changes at the level of the previous hematoma, with persistent but reduced extent of myelopathy. Engorged perimedullary vessels surrounding the spinal cord are observed, raising suspicion of an underlying vas.

**Results:** Given the suspicion of an underlying vascular malformation, the case was discussed with the Neurointerventional Radiology service, and spinal angiography was performed 48 hours later, identifying a dural arteriovenous fistula at the left D6 neural foramen. Neurological outcome was favorable, with muscle strength improving to 5/5 in the right lower limb and 2/5 in the left lower limb within 24 hours. Assisted ambulation was achieved after two weeks.

**Figure:**
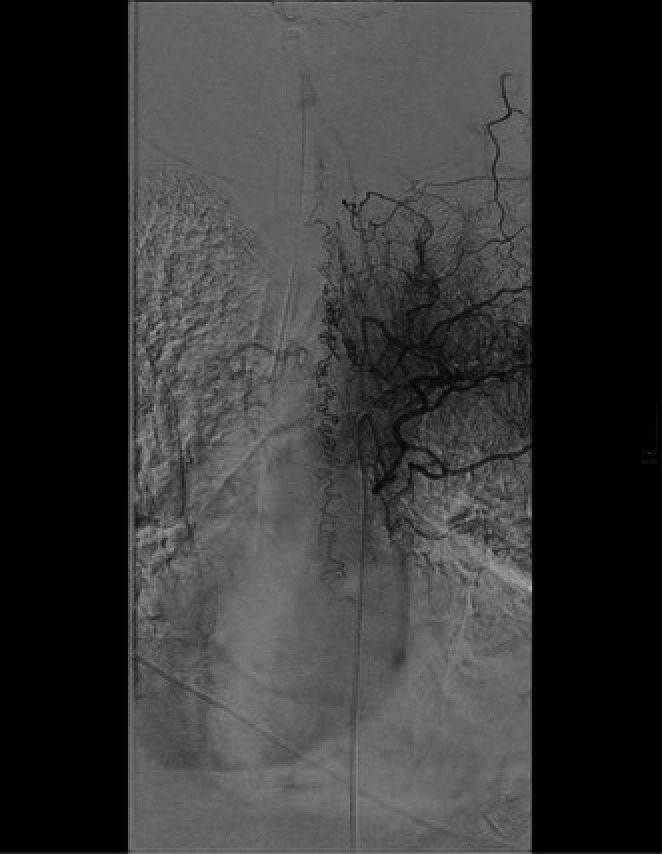
**FIGURE 3** Spinal angiography demonstrating an arteriovenous shunt between radiculomedullary arteries and perimedullary veins, consistent with a dural arteriovenous fistula at the left D6 neural foramen.

**Conclusion:** Dural arteriovenous fistula should be considered in cases of acute myelopathy secondary to spontaneous spinal epidural hematoma. Spinal angiography is essential for diagnosis and enables definitive treatment.

**Disclosure:** Nothing to disclose.

## EPO‐0853

### Isolated seronegative longitudinally extensive transverse myelitis in children: A Tunisian case series

#### M. Elloumi; A. Zioudi; Z. Miladi; M. Jamoussi; T. Ben Younes; M. Ben Hafsa; H. Ben Rhouma; H. Klaa; I. Kraoua

##### LR18SP04 and Department of Pediatric Neurology, National Institute Mongi Ben Hmida of Neurology, Tunis, Tunisia

**Background and aims:** Pediatric longitudinally extensive transverse myelitis (LETM) classification has evolved with antibody discovery. These cases are heterogeneous with variable clinical features and outcomes.

**Methods:** We retrospectively reviewed eight Tunisian children diagnosed with isolated seronegative inflammatory LETM from 2020 to 2025. Demographics, clinical presentation, neuroimaging, cerebrospinal fluid (CSF) findings, treatments, and outcomes were collected. Functional status and follow‐up MRI findings were analyzed. All patients were tested for serum and CSF myelin oligodendrocyte glycoprotein antibodies and serum anti‐aquaporin 4.

**Results:** Eight patients (five females, three males) had a median age of 13.5 years (range 5–17). A history of a preceding infection was noted in three patients. All presented with paraparesis. Six had sensory symptoms. Four had urinary dysfunction. CSF pleocytosis was present in six patients. CSF oligoclonal bands were positive in three. MRI showed cervical cord involvement in seven and conus medullaris involvement in two. Two patients had tumefied lesions. Two showed multifocal contrast enhancement. All received intravenous methylprednisolone. Six continued oral corticosteroids for 3–8 months. One received intravenous immunoglobulin. Five patients improved clinically. Two patients relapsed. One patient worsened. At a median follow‐up of two years, five had improved motor function. Follow‐up MRI showed spinal cord atrophy in two, stable myelitis in two, and lesion enlargement in one.

**Conclusion:** Seronegative pediatric LETM presents with variable clinical and radiological features. Despite early corticosteroid therapy, many patients have incomplete recovery. Careful long‐ term follow‐up is required to better understand this entity. Further studies are needed to identify prognostic factors and optimize treatment strategies.

**Disclosure:** Nothing to disclose.

## EPO‐0854

### Spinal dural arteriovenous fistula as a diagnostic pitfall: A case report and literature

#### O. Cibuku ^1^; V. Leka^2^; E. Xhukellari^1^; V. Thimjo^1^; E. Enesi^3^; A. Rroji^3^

##### ^1^Neuro vascular Service, University Hospital “Mother Teresa” Tirane, Albania; ^2^Stroke Center, American Hospital, Tirane, Albania; ^3^Neuro radiology Department, Univerisity Hospital “Mother Teresa” Tirane, Albania

**Background and aims:** Spinal dural arteriovenous fistulas (sDAVFs) are rare but potentially reversible vascular causes of progressive myelopathy resulting from venous hypertensive congestion of the spinal cord. Because of their insidious onset and nonspecific clinical and radiological features, they are frequently misclassified in acute and pre‐hospital settings, leading to delayed neurology referral and preventable neurological disability.

**Methods:** We report a 61‐year‐old man with several months of progressive gait impairment, lower limb numbness, and urinary retention. Examination showed spastic paraparesis (Aminoff–Logue G4 U3). Spinal MRI demonstrated longitudinal T2 hyperintensity from T7 to L1 with dilated perimedullary flow voids. Cerebrospinal fluid showed elevated protein, initially suggesting inflammatory or degenerative etiologies. However, progressive symptoms with sphincter dysfunction and characteristic imaging raised suspicion for a vascular cause. Digital subtraction angiography confirmed a right‐sided dural arteriovenous fistula at L2. Endovascular embolization resulted in clinical improvement with recovery of motor function and resolution of urinary symptoms (Aminoff–Logue G3 U1).

**Figure:**
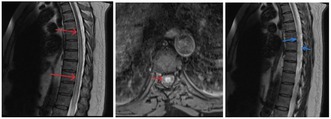
**FIGURE 1** MRI shows medullary edema (red arrow) from T7 to L1 with the presence of perimedullary vessels (blue arrow).

**Figure:**
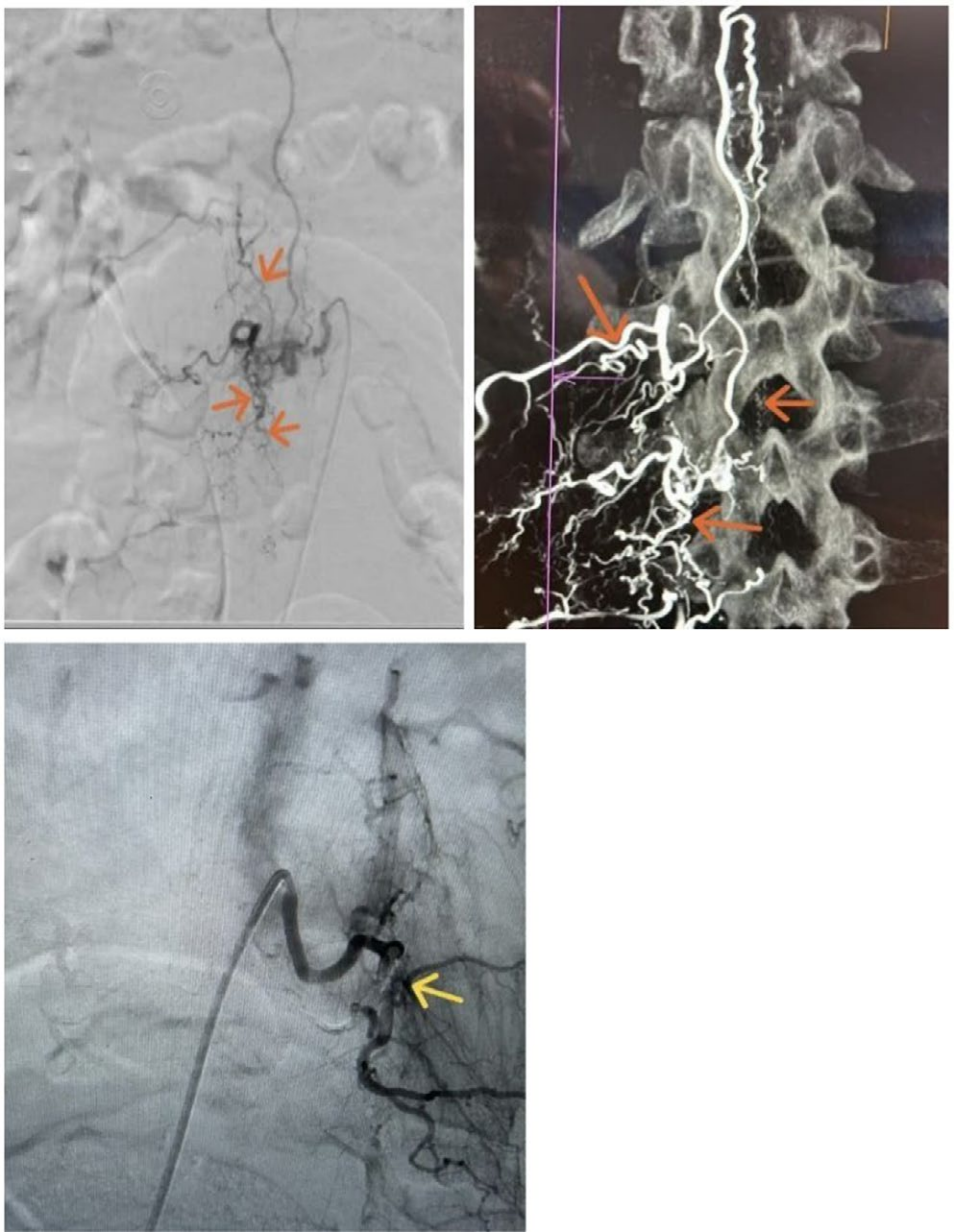
**FIGURE 2** Medullary angiography reveals aright lateral dural arteriovenous fistula at the L2 level (yellow arrow), with hypertrophic perimedullary vessels (orange arrow).

**Results:** A brief literature review indicates that median diagnostic delays range from 12 to 44 months, primarily due to misinterpretation of MRI, attribution of symptoms to nonvascular spinal disorders, and low awareness during stroke‐mimic triage and early neurological assessment. Prolonged delays are consistently associated with poorer neurological outcomes.

**Figure:**
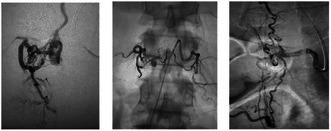
**FIGURE 3** Post‐embolization arteriography demonstrates reduced hypertrophic plexuses.

**Conclusion:** This case emphasizes the importance of including sDAVF in stroke‐mimic algorithms and neurology referral pathways for patients with unexplained progressive myelopathy, to enable timely angiographic confirmation and definitive treatment.

**Disclosure:** Nothing to disclose.

## EPO‐0855

### Impact of diagnostic delay on disability and early recovery in spinal dural AV fistulas: A 3‐year endovascular cohort

#### O. Cibuku ^1^; V. Leka^2^; E. Ranxha^1^; E. Enesi^3^; A. Rroji^3^

##### ^1^Neuro Vascular Service, University Hospital “Mother Teresa” Tirane, Albania; ^2^Stroke Center, American Hospital, Tirane, Albania; ^3^Neuro Radiology Department, University Hospital “Mother Teresa”, Tirane, Albania

**Background and aims:** Spinal dural arteriovenous fistulas (sDAVF) are the most common intradural spinal vascular malformations, yet they remain frequently underdiagnosed. Incidious clinical onset and misinterpretation of initial MRI can lead to delayed recognition and progressive venous hypertensive myelopathy. Using statistical techniques, this research analyzes the clinical features, diagnostic delay, and short‐term outcomes following endovascular therapy to investigate disability predictors.

**Methods:** A retrospective observational study including 9 patients diagnosed with sDAVF from 2022–2025. All patients underwent complete spinal MRI and selective spinal angiography. Functional status was assessed using the Aminoff–Logue (AL) gait and sphincter scores and the modified Rankin Scale (mRS). Associations between diagnostic delay and disability scores were explored with Pearson or Spearman correlation, as appropriate. A simple linear regression model evaluated diagnostic delay as a predictor of AL scores (*β*‐coefficient, 95% CI, *p*‐value). Statistical significance was set at *p* < 0. 05, outcomes before and after treatment compared using paired t‐tests.

**Results:** Mean age was 54.5 years; 7/9 were male. Median diagnostic delay was 7.11 months. Common comorbidities included degenerative spine disease (56.4%), hypertension (35.6%), and type 2 diabetes (23.4%). Post‐treatment analysis demonstrated significant improvement in AL gait and sphincter scores (Wilcoxonp < 0.05). Linear regression showed a positive β‐trend between longer diagnostic delay and worse baseline AL scores, although not statistically significant (*p* > 0.05), likely due to the small sample size.

**Figure:**
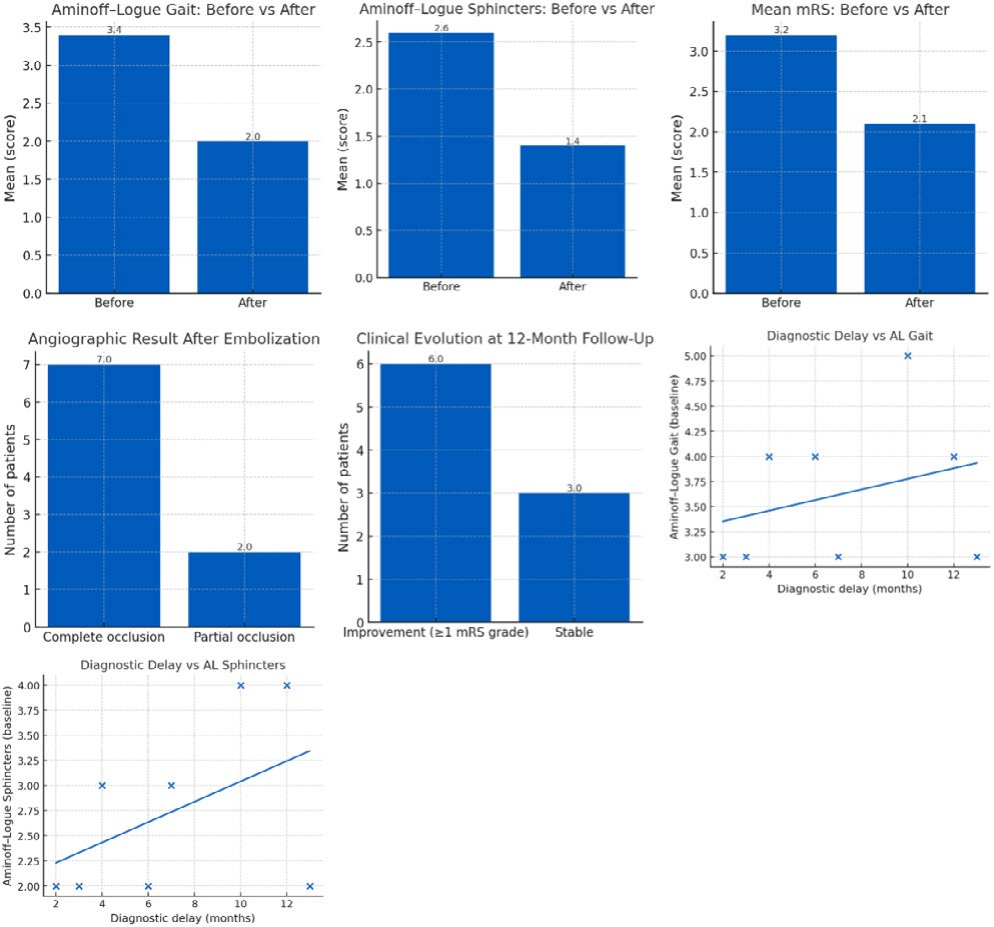
**FIGURE 1** Statistical results of the study.

**Conclusion:** Diagnostic delays in sDAVF remain common and correlate with greater neurological disability. Endovascular treatment achieved marked functional improvement. Incorporating structured MRI protocols and multidisciplinary evaluation is essential to reduce delays and improve outcomes.

**Disclosure:** Nothing to disclose.

## EPO‐0856

### Peripheral arteriovenous fistula and non‐traumatic myelopathy: Diagnostic challenges in a case of acute tetraparesis

#### P. Tavares de Almeida ^1^; L. Carvalho Rosas^2^; M. Rodrigues^3^; P. Pires^3^; T. Lima^2^; J. Novo^1^

##### ^1^Neurology Department, Local Health Unit of Gaia and Espinho, Vila Nova de Gaia, Portugal; ^2^Neurosurgery Department, Local Health Unit of Gaia and Espinho, Vila Nova de Gaia, Portugal; ^3^Cerebrovascular Intervention Neuroradiology Unit, Radiology Department, Local Health Unit of Gaia and Espinho, Vila Nova de Gaia, Portugal

**Background and aims:** Acute tetraparesis is a neurological emergency requiring prompt diagnostic evaluation. While traumatic or intrinsic spinal cord vascular causes are more frequent, rarer conditions such as hemodynamic changes induced by peripheral arteriovenous fistulas (AVFs) should be considered in selected patients.

**Methods:** Retrospective review of a patient's clinical file.

**Results:** A 57‐year‐old man presented with severe back pain followed by sudden tetraparesis evolving over 1 hour. He had no history of trauma. Past medical history included chronic kidney disease and prior hemodialysis via a right elbow AVF. Neurological examination revealed tetraparesis with lower limb plegia and MRC 3/5 upper limbs weakness, sensory level at C4, and absent reflexes. Cervical MRI revealed a posterior epidural collection from C3‐D10, suggestive of acute hemorrhage, and spinal cord hyperintensity from C5–C7 with diffusion restriction, compatible with ischemic injury. Emergent C5–C7 laminectomy and drainage of the epidural collection were performed, however follow‐up MRI revealed progression of spinal cord hyperintensity from C4–D1. Dural AVF was suspected and the patient underwent diagnostic angiography, excluding this hypothesis, but revealing an AVF of the elbow with a broad thrill and exuberant development of the right brachial territory and cervical collaterals, associated with subclavian‐steal syndrome. The elbow AVF was considered the likely cause of cervical vascular ectasia and consequent spinal ischemia. The patient underwent elbow AVF ligation and was discharged after 40 days, referred for rehabilitation.

**Conclusion:** This rare case highlights the importance of considering peripheral vascular causes as a potential etiology of non‐traumatic myelopathies, especially in patients with a history of hemodialysis.

**Disclosure:** Nothing to disclose.

## Pain 2

## EPO‐0857

### From peripheral hemodynamics to central connectivity: A multi‐modal framework towards precision medicine in trigeminal neuralgia

#### C. Cao ^1^; A. Oswal^2^; H. Chen^3^; X. Jiang^1^; Y. Wang^1^

##### ^1^Department of Neurosurgery, The First Affiliated Hospital of USTC, Division of Life Sciences and Medicine, University of Science and Technology of China, China; ^2^MRC Brain Networks Dynamics Unit, University of Oxford, Oxford, UK; ^3^Department of Radiology, The First Affiliated Hospital of USTC, Division of Life Sciences and Medicine, University of Science and Technology of China, China

**Background and aims:** Trigeminal neuralgia (TN) management is evolving towards precision medicine, necessitating biomarkers that delineate pathological neurovascular conflict (NVC) and its central consequences. We propose a multi‐modal framework integrating peripheral hemodynamics and central connectivity to guide treatment.

**Methods:** In Study 1 (CFD‐MVD), In 56 classical TN patients, computational fluid dynamics (CFD) quantified hemodynamic parameters (e.g, peak systolic flow‐PSF, max wall shear stress) at neurovascular conflict (NVC) sites to predict microvascular decompression (MVD) efficacy. In Study 2 (TGS‐fMRI), In 29 TN patients, longitudinal resting‐state fMRI assessed thalamo‐somatosensory functional connectivity (FC) changes before and after trigeminal ganglion stimulation (TGS). Machine learning evaluated FC as a treatment‐response biomarker.

**Results:** Study 1 revealed that “effective” NVC for MVD was characterised by significantly lower PSF (0.202 ± 0.136 vs. 0.306 ± 0.142 mL/s, *p* = 0.007) and higher Max WSS (3.231 vs. 2.197 Pa, *p* = 0.024). A prediction model incorporating these parameters achieved an AUC of 0.920 for MVD efficacy. Study 2 demonstrated that TN patients exhibited pathological hyperconnectivity within the thalamo‐somatosensory circuit compared to healthy controls. Successful TGS significantly normalised this aberrant FC in a region‐specific manner. The SVM classifier reliably distinguished pre‐ from post‐TGS states (AUC = 0.74), validating these FC patterns as stable neuromodulation biomarkers.

**Figure:**
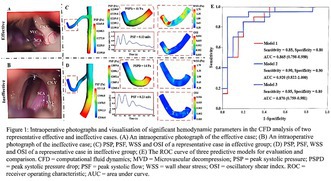
**FIGURE 1** Effective vs. ineffective surgical outcomes in CFD analysis. (A, B) Intraoperative views. (C, D) Key hemodynamic parameters (PSP, PSF, WSS, OSI). (E) ROC curve comparison of predictive models.

**Figure:**
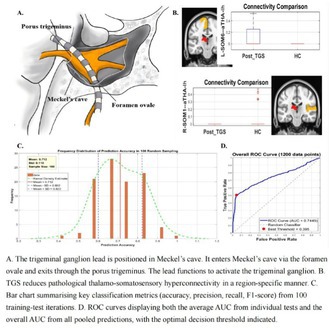
**FIGURE 2** (A) Trigeminal ganglion lead placement. (B) TGS reduces thalamo‐somatosensory hyperconnectivity. (C) Classification performance metrics. (D) ROC curves with AUC and optimal threshold.

**Conclusion:** This framework links peripheral CFD biomarkers (guiding MVD selection) with central fMRI biomarkers (assessing TGS response), enabling personalised TN therapy based on distinct pathophysiological profiles.

**Disclosure:** Nothing to disclose.

## EPO‐0858

### Clinical features distinguishing fibromyalgia from small fiber neuropathy

#### F. Masciarelli ^1^; D. Dell'Aversana^1^; F. Vitale^1^; G. Ciccarelli^1^; S. Tozza^1^; G. Caporaso^2^; V. Provitera^2^; L. Santoro^1^; F. Manganelli^1^; M. Nolano^1^

##### ^1^University of Naples Federico II, Department of Neurosciences, Reproductive Sciences, and Odontostomatology, Naples, Italy; ^2^Neurology Department – Skin Biopsy, Lab Istituti Clinici Scientifici Maugeri, Spa SB Institute of Telese Terme – IRCCS, Italy

**Background and aims:** Fibromyalgia syndrome (FMS) has been described associated with small fiber pathology and it manifests with a distinct clinical picture compared with pure small fiber neuropathy (SFN). However, some severe fibromyalgic patients may present with overlap phenotypes. We compared sensory and autonomic symptoms and signs across FMS, FMS associated with SFN and pure SFN.

**Methods:** We retrospectively analyzed 115 patients with FMS and 67 with biopsy‐confirmed SFN without FMS. Within FMS, 91 patients were classified as FMS + SFN, when associated with SFN symptoms and signs, otherwise as FMS (*n* = 24). We collected pain descriptors and distribution, sensory signs (allodynia, hyperalgesia, hypoesthesia to pinprick/touch), autonomic symptoms and comorbidities.

**Results:** Our population (mean age 46.1 ± 14.3 years) was meanly composed of women (FMS + SFN 90.1%; FMS 79, 2%; SFN 68,7%). Compared with FMS, FMS + SFN more often reported burning pain (91.2% vs 58.3%), hyperalgesia (23.1% vs 0%) and allodynia (47.3% vs 0%) and less frequently was associated with dysmetabolic comorbidities (15.4% vs 37.5%). Compared with SFN, FMS+SFN more frequently reported excruciating pain (91.2% vs 67.2%), painful cold (19.8% 7.5%), pins and needles (19.8% vs 7.5%), tingling (54.9% vs 32.8%), pinprick hyperalgesia (23.1% vs 3.0%), mechanical allodynia (47.3% vs 29.9%), itch (65.9% vs 38.8%), hypoesthesia to pinprick (82.4% vs 54.0%) and widespread pain distribution (95.6% vs 40.2 %). Overall, FMS+SFN presented more frequently autonomic symptoms (mostly urinary) and autoimmune comorbidities.

**Conclusion:** Patients with FMS/SFN phenotype overlap show a more severe sensory/autonomic burden than FMS and should prompt evaluation for the search of potentially treatable causes.

**Disclosure:** Nothing to disclose.

## EPO‐0859

### Sympathetic skin response in fibromyalgia patients with small fibers pathology

#### G. Paparella ^1^; E. Ammendola^1^; E. Ladisa^1^; G. Paparella^1^; G. Devigili^2^; M. Delussi^3^; K. Ricci^1^; L. Clemente^1^; M. de Tommaso^1^

##### ^1^Neurophysiopathology Unit, Department of Translational Biomedicine and Neuroscience (DiBraiN), University of Bari Aldo Moro, Bari, Italy; ^2^Movement Disorders Unit, Clinical Neurophysiology Unit Fondazione IRCCS Istituto Neurologico Carlo Besta, Milan, Italy; ^3^Department of Education, Psychology, Communication (For.Psi.Com.), University of Bari Aldo Moro, Bari, Italy

**Background and aims:** Fibromyalgia (FM) is a chronic disease characterised by small fibre pathology and autonomic dysfunction. Sympathetic Skin Response (SSR) is easily obtained in clinical practice as a marker of autonomic dysfunction. The present study aims to assess SSR abnormalities in small fibre pathology associated with the disease.

**Methods:** This was a retrospective, observational, monocentric, cross‐sectional study including 393 patients with FM, who underwent skin biopsy from the thigh and ankle, SSR obtained with a standard electromyographic active electrode attached to the right palm and sole, and clinical evaluation.

**Results:** SSR at the hand and foot was similarly absent in the three FM groups: those with normal skin biopsy, proximal denervation, and proximal and distal denervation. Latency at the foot was significantly prolonged in patients with proximal denervation (*z* = 2.33, *p* = 0.019). Amplitude was reduced in patients with distal denervation compared with the normal skin biopsy group (*z* = 2.30, *p* = 0.020). SSR had low predictive performance with respect to skin biopsy results (*r* ^2^ = 0.04). Latency of SSR at the foot and intraepidermal nerve fibre density (IENFD) correlated with the score for associated symptoms. Amplitude of SSR at the foot also negatively correlated with anxiety, depression, and fatigue scores.

**Conclusion:** SSR abnormalities did not correspond with small fibre impairment as shown by skin biopsy in FM patients, but instead appear to reflect different aspects of the complex clinical picture, including psychopathological features and fatigue, as distinct biomarkers of the symptom constellation.

**Disclosure:** Nothing to disclose.

## EPO‐0860

### MRI spectroscopy in the differential diagnosis of somatoform pain disorder and chronic neuropathic pain

#### K. Basiakova ^1^; O. Alenikova^1^; L. Parchach^1^; S. Tolkach^2^; A. Zelenko^2^

##### ^1^Republican Research and Clinical Center of Neurology and Neurosurgery, Minsk, Belarus; ^2^Republican Center of Hygiene, Epidemiology and Public Health, Minsk, Belarus

**Background and aims:** Differential diagnosis of somatoform pain disorder (SPD) from other chronic pain (CP) conditions presents significant diagnostic challenges due to the overlap in symptoms. It is known that CP leads not only to anxiety, depression and sleep disturbances, but also to a specific neurochemical imbalance in the brain. In addition, sleep disorders and CP negatively affect brain homeostasis by reducing glymphatic clearance. To identify differences in neurochemical imbalance and glymphatic clearance using MRI spectroscopy (MRS) and MRI tractography in patients with SPD and CP.

**Methods:** 27 patients were diagnosed with SPD according to DSM‐5 criteria (1st group), and 32 patients with chronic secondary musculoskeletal pain according to ICD‐11 criteria (2nd group). We used MRS and MRI with DTI‐ALPS index analysis, questionnaires and scales to assess pain characteristics, anxiety levels, depression and quality of life (table).

**Figure:**
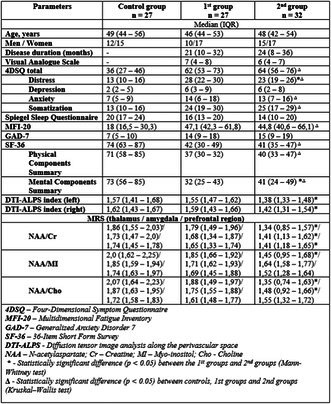
**TABLE 1** Comparative assessment of patients with somatoform pain disorder, chronic neuropathic pain and a control group.

**Results:** No differences were found in anxiety, depression, VAS, or quality of life. 1st group had the worst scores in the mental health component and the distress subscale. The 2nd group showed a decrease in the DTI‐ALPS index and NAA with an increase of the glial metabolites, which was expressed in NAA/Cr, NAA/MI and NAA/Cho ratio reduced in the thalamus, amygdala, prefrontal region compared to the 1st group and the controls (Table).

**Conclusion:** Despite the fact that both types of pain disorders are characterized by disturbances in the functional relationship between the affective and cognitive systems, the present study shows that SPD does not lead to significantly metabolic changes and glymphatic dysfunction characteristic of CP, which can be used to differentiate these conditions.

**Disclosure:** Nothing to disclose.

## EPO‐0861

### Immune activity in skull bone marrow and the periphery in chronic pain: A TSPO PET/MRI and LPS‐stimulated cytokines study

#### M. Menduni De Rossi ^1^; M. Mohammadian^3^; L. Brusaferri^6^; J. Cooper‐Hohn^3^; M. Kim^3^; J. P. Murphy^3^; Z. Alshelh^3^; G. Grmek^3^; J. Schnieders^3^; C. Chane^3^; T. Carmichael^3^; D. Yang^3^; V. Napadow^4^; M. Loggia^3^

##### ^1^Department of Translational Research on New Technologies in Medicine and Surgery, University of Pisa, Pisa, Italy; ^3^Department of Radiology, Athinoula A. Martinos Center for Biomedical Imaging, Massachusetts General Hospital, Harvard Medical School, Boston, USA; ^4^Spaulding Rehabilitation Hospital, Boston, MA, United States; ^6^Department of Computer Science and Digital Technology, College of Technology and Environment, London South Bank University, London, UK

**Background and aims:** Chronic pain is increasingly recognized as a neuroinflammatory condition involving interactions between peripheral immune processes and the central nervous system. The skull bone marrow (SBM) is a critical interface in this peripheral‐central immune axis, yet cytokine signatures and SBM activation patterns in distinct chronic pain conditions remain unclear.

**Methods:** We assessed lipopolysaccharide (LPS)‐induced cytokine reactivity in peripheral blood cells from patients with chronic lower back pain (cLBP; *n* = 13) and knee osteoarthritis (KOA; *n* = 17), alongside healthy controls (*n* = 10). Cytokine fold changes were analyzed in relation to pain intensity, pain interference, depression, and anxiety using partial linear regression adjusting for baseline cytokine levels. SBM immune cell density was quantified via [^11^C] PBR28 PET/MRI, indexing 18 kDa translocator protein (TSPO) expression.

**Results:** Compared to controls, only cLBP patients exhibited significantly reduced LPS‐induced IL‐4 and IL‐10 reactivity (*p* < 0.05). In cLBP, pain intensity showed associations with IL‐1β, TNF‐α, GM‐CSF, and IFN‐γ reactivity (*p* < 0.05), with a trend for IL‐6. In contrast, KOA demonstrated broader clinical associations, with both pain intensity and pain interference correlating with IL‐1β, IL‐6, MCP‐1, and MIP‐1α (*p* < 0.05). Notably, depressive and anxiety symptoms correlated with IL‐1 receptor antagonist (IL‐1RA) reactivity exclusively in cLBP (*p* < 0.001). Voxelwise analyses revealed significant associations between SBM TSPO signal and IL‐1RA, IL‐6, IL‐8, and GM‐CSF reactivity (*p* < 0.01).

**Figure:**
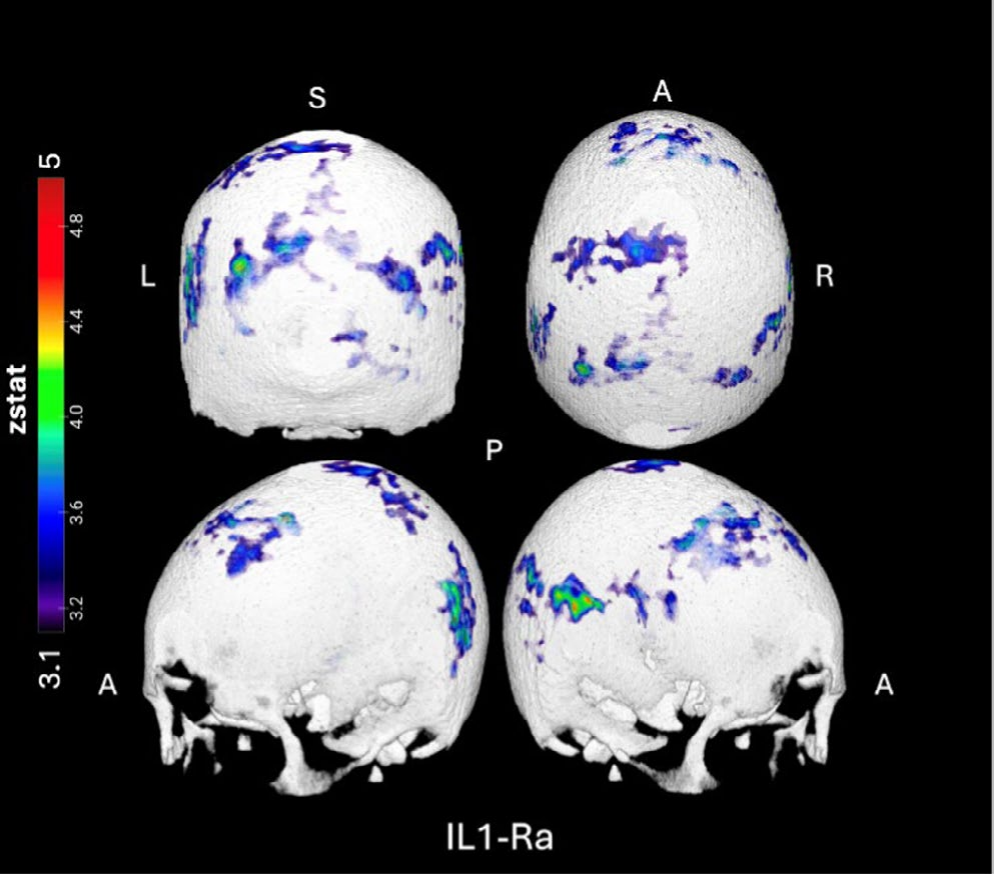
**FIGURE 1** 3D mapping of voxels in which patients with chronic pain showed a voxel‐wise positive correlation between skull bone marrow [11C]PBR28 TSPO signal and IL‐1Ra fold change (LPS‐stimulated/baseline), overlaid onto a 3D skull model in standard MNI152 space.

**Figure:**
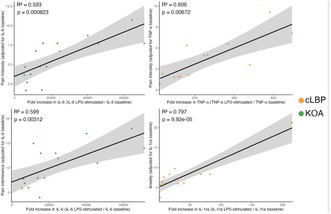
**FIGURE 2** Scatter plots of the partial linear regression and 95% confidence interval showing associations between PROMIS‐29 scores and cytokine fold change (LPS‐stimulated/baseline) in cLBP and KOA patients. All analyses were adjusted for baseline cytokine levels.

**Figure:**
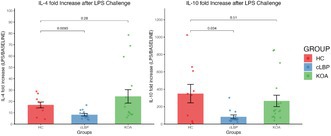
**FIGURE 3** Bar plots showing group differences in IL‐10 and IL‐4 fold change (LPS‐stimulated/baseline) between healthy controls, KOA, and cLBP. Bars represent group means ± SEM. Group comparisons were assessed using *t*‐tests

**Conclusion:** These findings reveal condition‐specific peripheral immune signatures in chronic pain and provide in vivo evidence linking SBM activation with peripheral cytokine reactivity, supporting a functional skull‐immune‐brain axis in human pain pathophysiology.

**Disclosure:** Nothing to disclose.

## EPO‐0862

### Characteristics of migraine patients who successfully discontinued anti‐CGRP monoclonal antibodies: A single‐center retrospective study

#### N. Imai ^1^; R. Narita^1^; A. Moriya^1^; R. Suzuki^1^; E. Kitamura^2^

##### ^1^Department of Neurology and Headache Center, Japanese Red Cross Shizuoka Hospital, Japan; ^2^Department of Neurology, Kitasato University School of Medicine, Japan

**Background and aims:** Randomized controlled trials have established anti‐CGRP monoclonal antibodies (mAbs) as effective for migraine prevention. While some patients discontinue treatment following substantial improvement—a process termed "graduation"—the clinical characteristics associated with success remain poorly understood. This study investigated factors associated with successful graduation from anti‐CGRP mAbs in a real‐world setting.

**Methods:** We retrospectively evaluated 257 migraine patients (ICHD‐3 criteria) treated with anti‐CGRP mAbs at our institution. Clinical characteristics, including medication overuse headache (MOH), baseline monthly migraine days (MMDs), and MIDAS/HIT‐6 scores, were compared between “graduates” and “non‐graduates.” Multivariable logistic regression analysis was performed to identify independent factors associated with graduation.

**Results:** The anti‐CGRP mAbs administered included galcanezumab (*n* = 96), erenumab (*n* = 66), and fremanezumab (*n* = 95). Thirty patients (11.7%) successfully graduated. Univariate analyses showed graduates had a significantly lower prevalence of MOH (20% vs. 66%, *p* = 0.015) and fewer baseline MMDs (median 11 vs. 16, *p* = 0.042). In multivariable logistic regression, absence of MOH (adjusted OR: 0.22; 95% CI: 0.05–0.89; *p* = 0.034) and lower baseline MMDs (adjusted OR: 0.93; 95% CI: 0.87–0.99; *p* = 0.048) were independently associated with successful graduation. No significant interaction was observed between MOH and MMDs.

**Figure:**
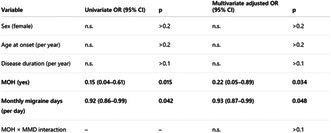
**TABLE 1** Logistic regression analysis of factors associated with “graduation.”

**Conclusion:** Successful discontinuation of anti‐CGRP mAbs was independently associated with fewer baseline MMDs and absence of MOH. Early initiation of preventive therapy—before high frequency and medication overuse develop—may enhance the likelihood of successful treatment withdrawal, supporting a treat‐to‐target strategy aimed at eventual treatment graduation.

**Disclosure:** Noboru Imai reports being an advisor for Sawai and received speaker fees from Daiichi Sankyo, Eli Lilly, Otsuka, and Amgen; however, these companies had no relation to the study.

## EPO‐0863

### Pain phenotypes in multiple sclerosis: Neuropathic pain is linked to higher disability and worse quality of life in a large real world cohort

#### S. Alizada ^1^; A. Ozdagar^2^; I. Kara^3^; T. Kahraman^4^; S. Ozakbas^3^

##### ^1^Department of Neurology, Dokuz Eylul University, Izmir, Türkiye; ^2^Health Sciences, Van Yuzuncu Yıl University, Van, Türkiye; ^3^Izmir University of Economics, Neurology Clinic, Izmir, Türkiye; ^4^Izmir Katip Celebi University, Izmir, Türkiye

**Background and aims:** Pain is a common and disabling symptom in multiple sclerosis (MS), but the clinical and functional impact of neuropathic pain (NP) versus musculoskeletal pain (MSP) is not well defined in large real‐world cohorts.

**Methods:** In this cross‐sectional study, 1,503 people with MS attending a tertiary MS center were assessed. Pain subtypes were classified using PainDETECT and the Nordic Musculoskeletal Questionnaire into no pain, MSP, or NP. Disability was measured with the Expanded Disability Status Scale (EDSS). Health‐related quality of life (HRQoL) was evaluated with EQ‐5D‐3L and EQ‐VAS.

**Results:** Pain was reported by 68.2% of participants; 50.5% had MSP and 17.7% had NP. The NP group had higher disability (median EDSS 2.0) than MSP or no‐pain groups (*p* < 0.001). HRQoL was poorest in NP, with significantly worse EQ‐5D domain scores and EQ‐VAS compared with MSP and no pain (all *p* < 0.001). MSP most frequently involved the lower back (33.4%), neck (31.3%), knees (31.0%), and ankles/feet (29.4%).

**Figure:**
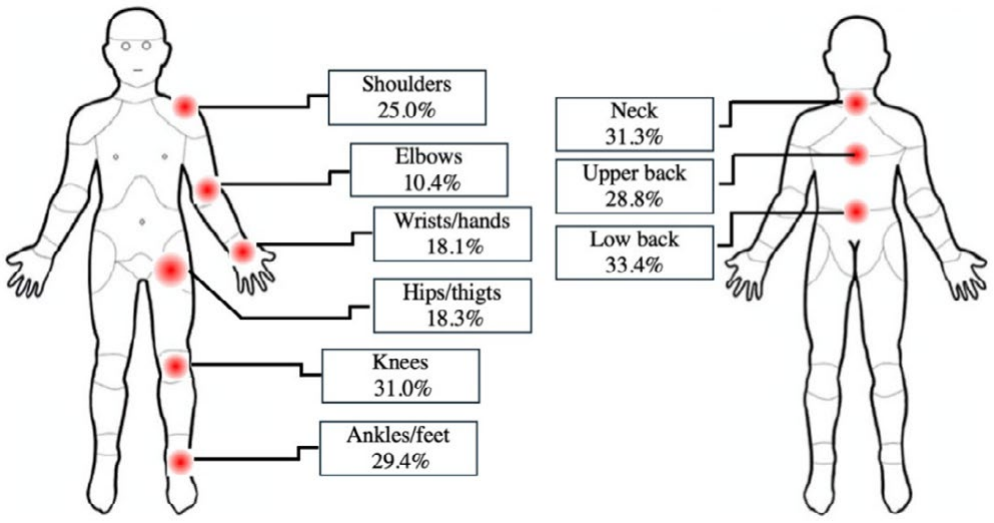
**FIGURE 1** Anatomical distribution of musculoskeletal pain in people with multiple sclerosis (pwMS).

**Figure:**
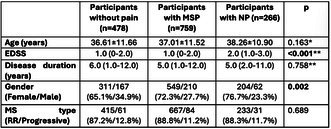
**TABLE 1** Comparison of demographic and clinical features of the participants (*n* = 1503) Data are presented as mean ± SD or median (IQR) unless otherwise indicated. Significant *p*‐values (<0.05) are shown in bold. EDSS, Expanded Disability Status Scale.

**Figure:**
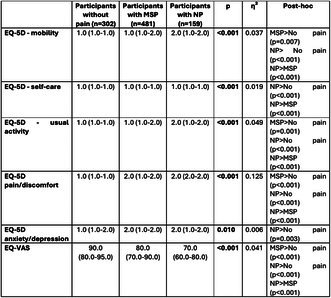
**TABLE 2** Comparison of HRQoL between groups.

**Conclusion:** In MS, NP is associated with greater disability and markedly worse HRQoL than MSP or no pain, supporting routine screening for pain subtypes and early, targeted management to reduce functional and psychosocial burden.

**Disclosure:** Nothing to disclose.

## EPO‐0864

### Microstructural changes at the trigeminal root entry zone and their role in classical trigeminal neuralgia: A systematic review

#### S. Nelson

##### Barts London Health, Whitechapel, UK

**Background and aims:** Classical trigeminal neuralgia is linked to neurovascular compression at the root entry zone. This review aimed to summarise evidence on structural and physiological mechanisms contributing to pain generation.

**Figure:**
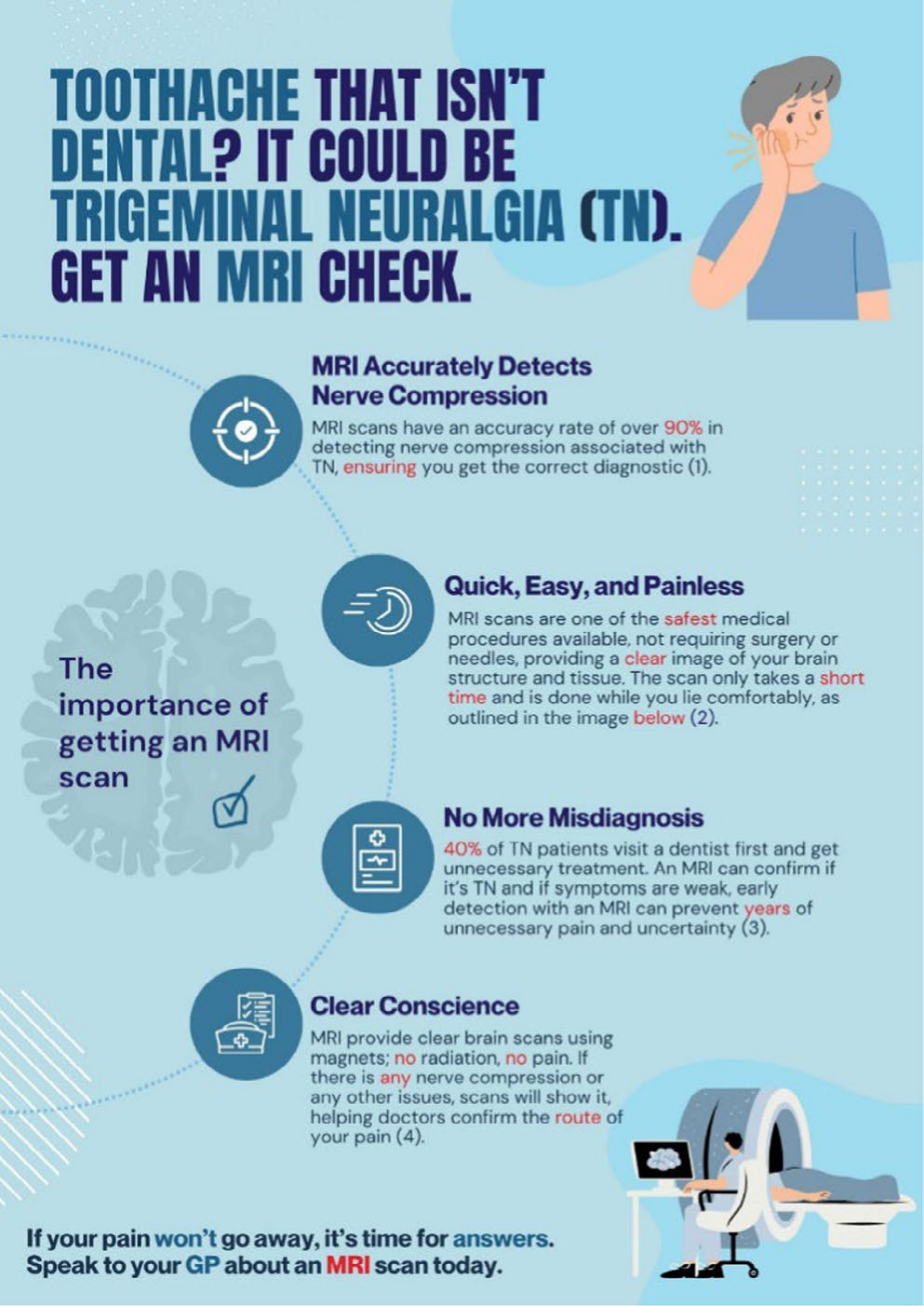
**FIGURE 1** Educational infographic illustrating the role of MRI in identifying neurovascular compression in trigeminal neuralgia. Included to contextualise the diagnostic relevance of imaging referenced in this review.

**Methods:** A systematic search of PubMed, Scopus, and Web of Science was conducted (2000–2024). Eligible studies included human clinical, neuroimaging, neurophysiological, or histopathological investigations examining the REZ in classical TN. Data were extracted on REZ morphology, diffusion tensor imaging (DTI) findings, electrophysiological abnormalities, and correlations with pain severity or treatment outcomes. Screening and qualitative synthesis followed PRISMA guidelines.

**Results:** A total of 47 studies met inclusion criteria. Neurovascular compression was consistently associated with focal demyelination at the REZ, with 78% of imaging studies reporting reduced fractional anisotropy in the affected nerve. Electrophysiological data demonstrated abnormal trigeminal reflex latencies consistent with impaired axonal conduction. Across clinical studies, greater structural or electrophysiological disruption was associated with higher pain intensity, increased attack frequency, and reduced responsiveness to sodium‐channel blockers. Evidence also showed that patients with severe REZ distortion were more likely to benefit from microvascular decompression compared with medical therapy alone.

**Conclusion:** Across diverse methodologies, current evidence supports a shared mechanism in TN centred on REZ demyelination, abnormal impulse generation, and ectopic cross‐talk between trigeminal fibres. These mechanistic insights reinforce the value of integrating neuroimaging and neurophysiology into routine evaluation to improve treatment selection. A unified mechanistic framework may also guide future biomarker development for personalised TN management.

**Disclosure:** Nothing to disclose.

## EPO‐0865

### An artificial intelligence‐based decision‐support tool to predict outcomes in low back pain

#### S. Singh; P. Kumar

##### Department of Neurosurgery, AIIMS, Raebareli, India

**Background and aims:** The study aimed to create a predictive tool (an artificial intelligence‐based decision‐support tool), which merges Clinical Intelligence and Radiomics to generate customized therapy plans for LBP patients.

**Methods:** The tool uses deep learning models to perform automated segmentation which enables the extraction of geometrical parameters including disc height and width, vertebrae height and width, canal diameter, disc height index, signal intensity, and disc volume. A total of 402 patient's mid‐sagittal T2‐weighted MRIs have been utilized for the segmentation model while 4260‐disc slice with radiological geometric parameters for spondylosis grading. The creation of a labeled dataset utilized expert‐verified Pfirrmann and spondylosis severity gradings (11 grades, shown in fig. 2) to address the clinical issues stemming from manual grading variability and subjectivity. The machine learning algorithms use this combined dataset to predict results and combined with clinical scores (Oswestry Disability Index and Numerical Rating Scale) to recommend personalized treatment plans.

**Figure:**
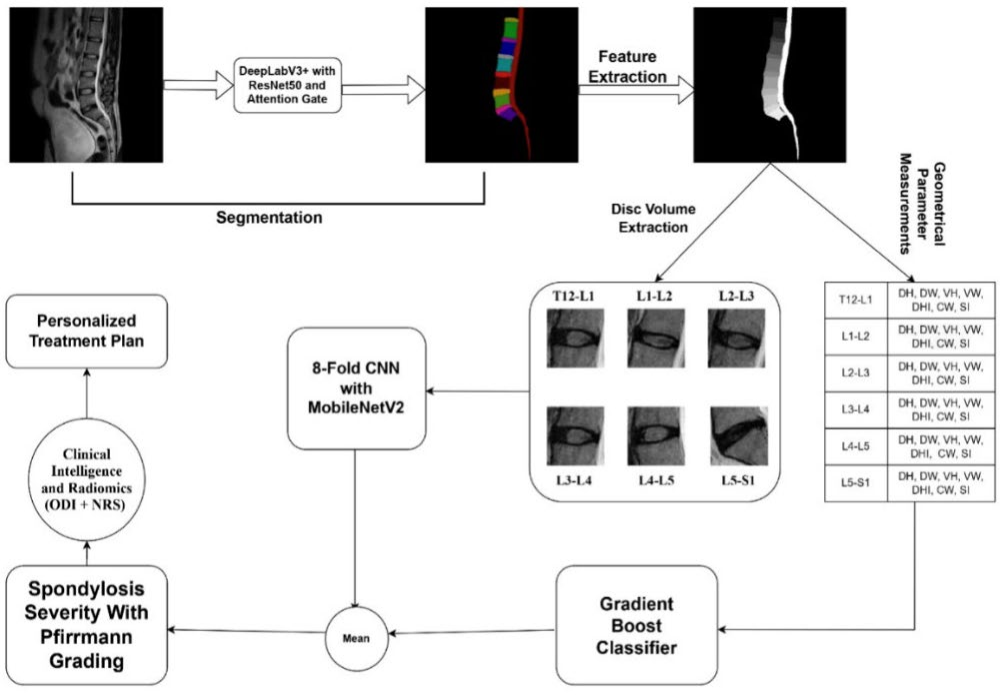
**FIGURE 1** The workflow of our study.

**Results:** The DeepLabV3+ segmentation model with ResNet50 encoder reached 95.5% accuracy, which increased to 98.7% after 8‐fold cross‐validation and simultaneously improved precision (96.95%), recall (97.1%), Dice coefficient (96.9%), and IoU (94.8%). The CNN with MobileNetV2 achieved 97.84% accuracy and 96.76% IoU for spondylosis severity prediction after cross‐validation. The Gradient Boost classifier demonstrated the best results with geometrical data by achieving 91.65% accuracy and 84.59% IoU.

**Conclusion:** The tool introduces an innovative method to customize LBP treatment through the combination of AI technology with radiological data and clinical expertise.

**Disclosure:** The study was sponsored by DHR‐MRU grant. Nothing to disclose Part of work is published (doi:10.1007/s10278‐025‐01517‐3).

## EPO‐0866

### A role for glycine transporter 2‐inhibitors in modulating levels of dopamine in the nucleus Accumbens

#### Y. Olsson ^1^; K. Danielsson^2^; A. Domi^2^; H. Lidö^2^; L. Engström Ruud^3^; M. Ericson^2^; B. Söderpalm^2^

##### ^1^Department of Neurology, Sahlgrenska University Hospital, Gothenburg, Sweden; ^2^Addiction Biology Unit, Department of Psychiatry and Neurochemistry, Institute of Neuroscience and Physiology, Sahlgrenska Academy, University of Gothenburg, Sweden; ^3^Department of Physiology, Institute of Neuroscience and Physiology, Sahlgrenska Academy, University of Gothenburg, Sweden

**Background and aims:** Impaired glycinergic neurotransmission in the spinal cord which disinhibits nociceptive signaling to higher cortical centers is implicated in states of pathological pain. Glycine transporter 2 (GlyT2)‐inhibition, which augments glycine receptor (GlyR) signaling has emerged as a novel pharmacological treatment concept for pain, devoid of adverse effects and effective in achieving allodynia in a phase III‐trial. In rats, GlyRs also regulate dopamine (DA) levels in the nucleus Accumbens (nAc), a pivotal region for motivational processes. Recently, the presence of GlyT2‐expressing glycinergic axons in the mouse nAc has been established.

**Figure:**
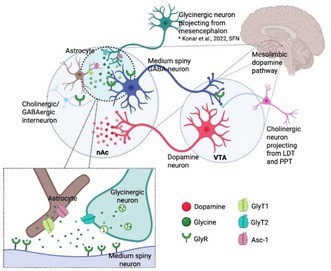
**FIGURE 1** Overview of hypothesized glycinergic signaling in the nAc. Enhanced glycinergic tone in the nAc, produced by e.g. GlyT1‐ or GlyT2‐inhibition disinhibits VTA‐nAc dopaminergic projections, thereby slightly elevating accumbal dopamine output.

**Methods:** This study in rats examined if GlyT2‐inhibition alters accumbal DA and glycine content as measured by in vivo microdialysis. The presence of GlyT2‐expressing axons and GlyRs in the nAc was examined by immunohistochemistry (IHC).

**Results:** The partial GlyT2‐inhibitors NAGly (10 mg/kg i.p.) and Opiranserin (20 mg/kg i.p.), but not the full GlyT2‐inhibitor Org 25543, slightly elevated accumbal DA levels, while no effects on extrasynaptic glycine levels were observed. The reversible GlyT1‐inhibitor Bitopertin (10 mg/kg p.o.) produced a pronounced glycine and DA elevation but combined GlyT1‐ and GlyT2 inhibition did not yield any statistically significant additive effects. The IHC study confirmed the presence of GlyT2 and GlyRs in the nAc.

**Figure:**
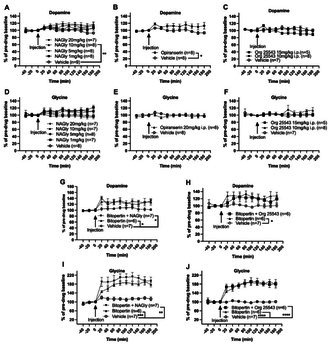
**FIGURE 2** Systemic treatment with partial but not full GlyT2‐inhibitors slightly elevate extracellular dopamine but not glycine levels in vivo in the nAc. Combined GlyT1‐ and GlyT2‐inhibition did not produce any statistically significant additive effects.

**Figure:**
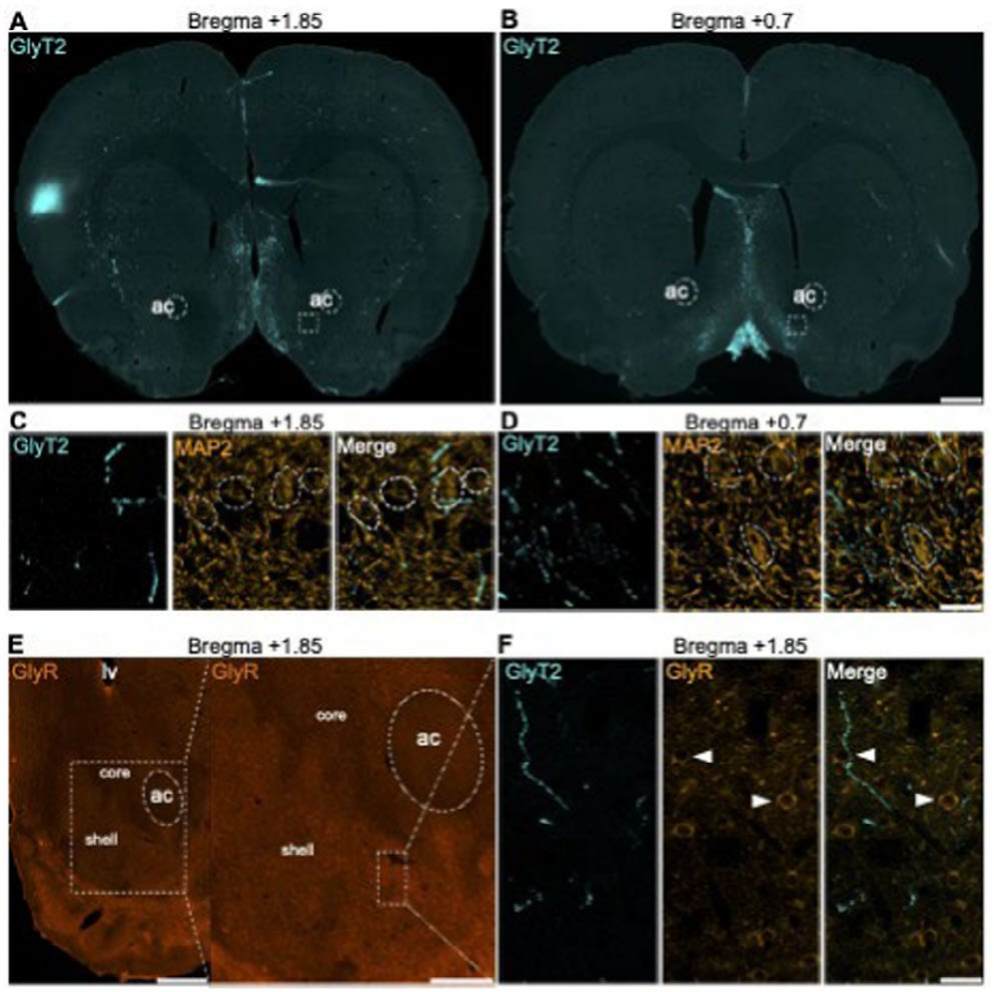
**FIGURE 3** Immunoreactivity for GlyT2 and GlyR was confirmed in the nAc at the A/P level of probe insertion for microdialysis (bregma +1.85 mm) and more caudally (+0.7), although with a higher signal for GlyT2 in e.g. septal regions.

**Conclusion:** To conclude, this study indicates that GlyT2‐expressing glycinergic neurons are involved in regulating nAc DA levels, albeit to a lesser extent than extrasynaptic GlyRs situated nearby GlyT1s. The extraspinal effects mediated by systemic treatment with GlyT2‐inhibitors, i.e. involvement of the mesolimbic DA system, should be further explored.

**Disclosure:** Nothing to disclose.

## EPO‐0867

### Central mechanisms and therapeutic interventions in trigeminal neuralgia: A systematic neuroimaging and neuromodulation study

#### Y. Wang; C. Cao; H. Chen; X. Jiang

##### The First Affiliated Hospital of USTC, Hefei, China

**Background and aims:** Microvascular decompression (MVD) targets peripheral neurovascular conflict in trigeminal neuralgia (TN), yet its variable efficacy highlights the critical role of central nervous system adaptations. Vascular compression pathophysiology, central sensitization neuroimaging correlates, and their treatment response predictive value remain unclear. This study combined multimodal neuroimaging with MVD, rTMS and TGS interventions to elucidate TN central mechanisms and explore personalized therapeutic strategies.

**Methods:** Three complementary studies: ① 60 TN patients (30 MVD responders/non‐responders) and 30 HCs underwent preoperative rs‐fMRI and DTI, with thalamo‐S1 FC analyzed (pipeline: Fig. 1a) and an SVM prediction model constructed; ② 34 early TN patients were randomized to active M1‐rTMS, sham stimulation, active DLPFC‐rTMS (Fig. 2a), with clinical scales and rs‐fMRI assessed pre‐/post‐intervention for network reorganization; ③ 29 TN patients received longitudinal rs‐fMRI before/after TGS (30 HCs as controls, electrode placement: Fig. 3a), with FC dynamics analyzed and feature stability verified via SVM.

**Results:** ① MVD non‐responders showed enhanced thalamo‐somatosensory FC (Fig. 1b), milder vascular compression, and reduced subcortical FA with structural‐functional decoupling (Fig. 1c); ② SVM predicted MVD outcomes with 67.33% accuracy (Fig. 1d); ③ All rTMS groups achieved pain relief (Fig. 2b), with active M1‐rTMS suppressing insula activation and sham/DLPFC groups enhancing prefrontal activity (Fig. 2c, targeted vs placebo‐mediated); ④ TGS normalized aberrant thalamo‐somatosensory hyperconnectivity (Fig. 3b, with regional heterogeneity), and SVM distinguished pre‐/post‐TGS states with 71.17% accuracy (Fig. 3c).

**Figure:**
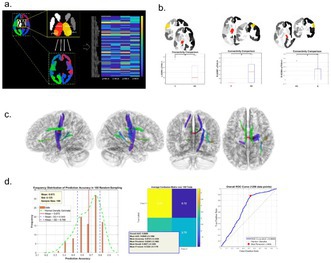
**FIGURE 1** (a) rs‐fMRI workflow: acquisition→preprocessing→ROI extraction→FC matrix construction; (b) NE vs E: enhanced thalamo‐somatosensory FC (p < 0.05); (c) Subcortical somatomotor FA ↓ with longer disease duration (FDR ≤0.05); (d) SVM MVD prediction performance.

**Figure:**
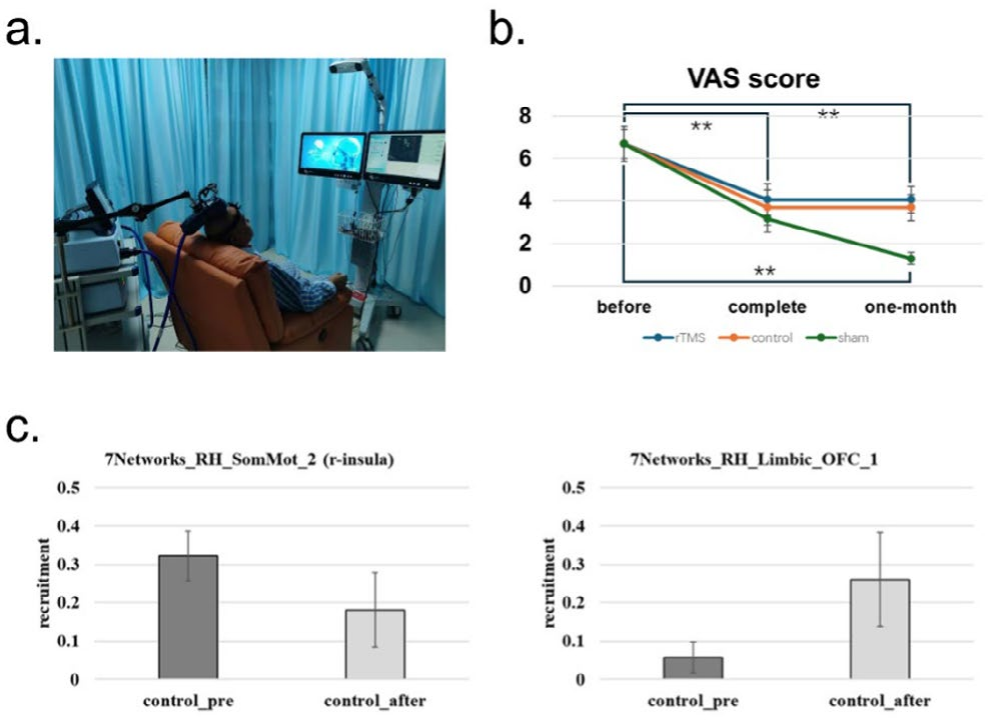
**FIGURE 2** rTMS neural mechanisms. (a) Study design: randomization to active M1‐rTMS, sham, active DLPFC‐rTMS; (b) Post‐intervention VAS pain reduction in all groups; (c) Divergent activation: M1‐rTMS ↓ insula recruitment, sham/DLPFC ↑ prefrontal activity.

**Figure:**
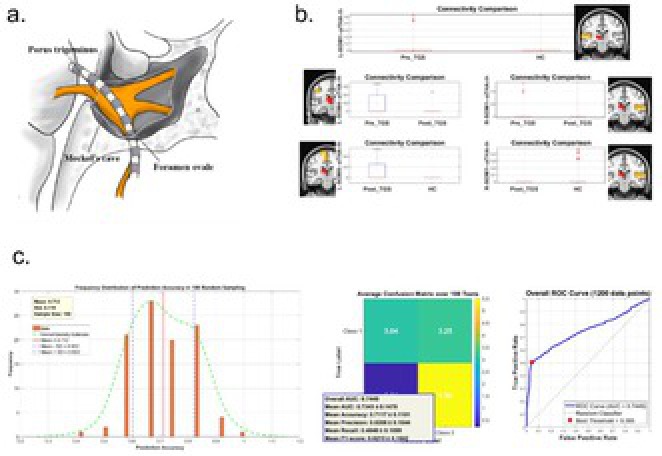
**FIGURE 3** TGS study: (a) Electrode placement; (b) Pre‐/post‐TGS connectivity normalization; (c) SVM validation of treatment response (71.17% accuracy).

**Conclusion:** This study confirms TN as a spectrum disorder from peripheral compression to central sensitization, with thalamo‐somatosensory FC as a core biomarker. It enables preoperative MVD patient stratification, guides neuromodulation target optimization, and advances TN management from empirical surgery to circuit biomarker‐informed personalized therapy.

**Disclosure:** Nothing to disclose.

Peripheral Nerve Disorders 2

## EPO‐0868

### Atherogenic lipid indices and electrophysiological severity in Carpal Tunnel Syndrome

#### A. Aksoy Gündoğdu^1^; D. Kotan ^2^; E. Çiçekli^2^

##### ^1^Department of Neurology, Tekirdağ Namık Kemal University Faculty of Medicine, Tekirdağ, Türkiye; ^2^Department of Neurology, Sakarya University Faculty of Medicine, Sakarya, Türkiye

**Background and aims:** Carpal tunnel syndrome (CTS) is the most common entrapment neuropathy, yet the role of metabolic and vascular factors in disease severity is not fully understood. Composite lipid indices may better reflect microvascular and metabolic alterations than conventional lipid fractions. This study compared atherogenic lipid indices between CTS patients and healthy controls and evaluated their association with electrophysiological severity.

**Methods:** In this cross‐sectional case‐control study, 124 CTS patients and 113 age‐ and sex‐matched controls were enrolled. Electrophysiological grading followed the Padua classification. Fasting total cholesterol, high‐density lipoprotein cholesterol (HDL‐C), low‐density lipoprotein cholesterol (LDL‐C), triglycerides (TG), and remnant cholesterol (RC) were measured. Composite indices included the TG/HDL, non–HDL/HDL, and atherogenic index of plasma (AIP). Between‐group comparisons and correlations with severity were analyzed using non‐parametric tests and Spearman correlation.

**Results:** Compared with controls, CTS patients had higher fasting glucose, HbA1c, total cholesterol, LDL‐C, HDL‐C, and remnant cholesterol (all *p* < 0.05). Composite indices (TG/HDL, non‐HDL/HDL, AIP) were similar between groups. Electrophysiological severity showed significant associations with multiple lipid measures, with total cholesterol, LDL‐C, TG, TG/HDL, non‐HDL/HDL, AIP, and remnant cholesterol increasing progressively from mild to moderate and severe CTS (all *p* < 0.05). No significant sex‐related differences were observed.

**Figure:**
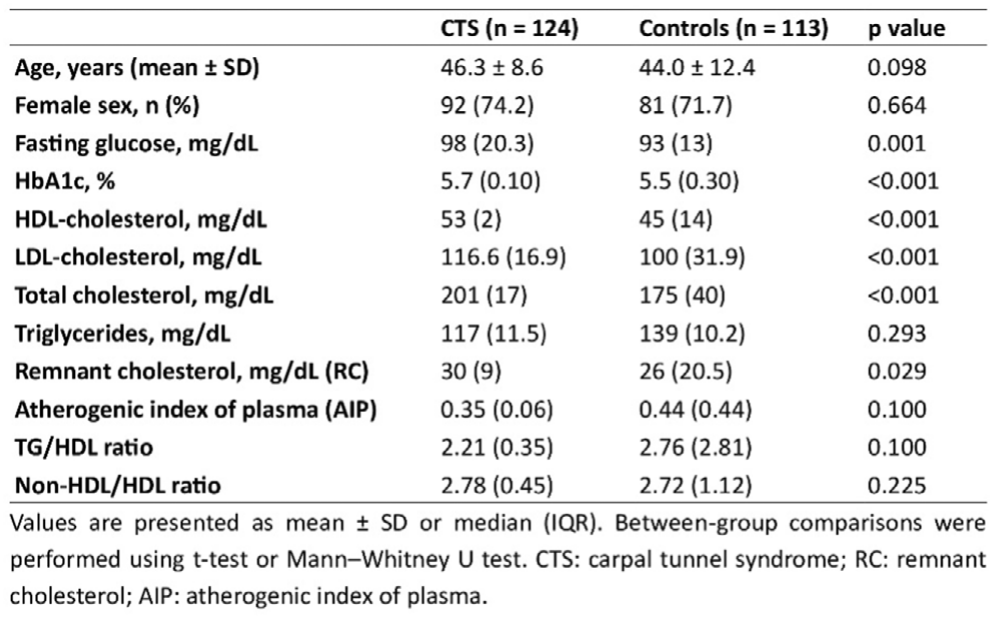
**TABLE 1** Demographic and baseline biochemical characteristics of patients with carpal tunnel syndrome and healthy controls.

**Figure:**
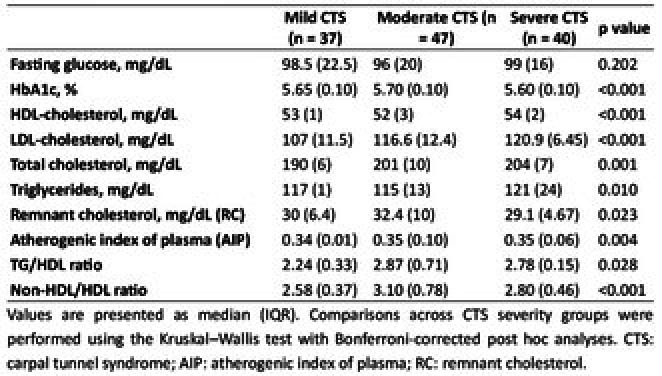
**TABLE 2** Biochemical parameters according to carpal tunnel syndrome severity.

**Figure:**
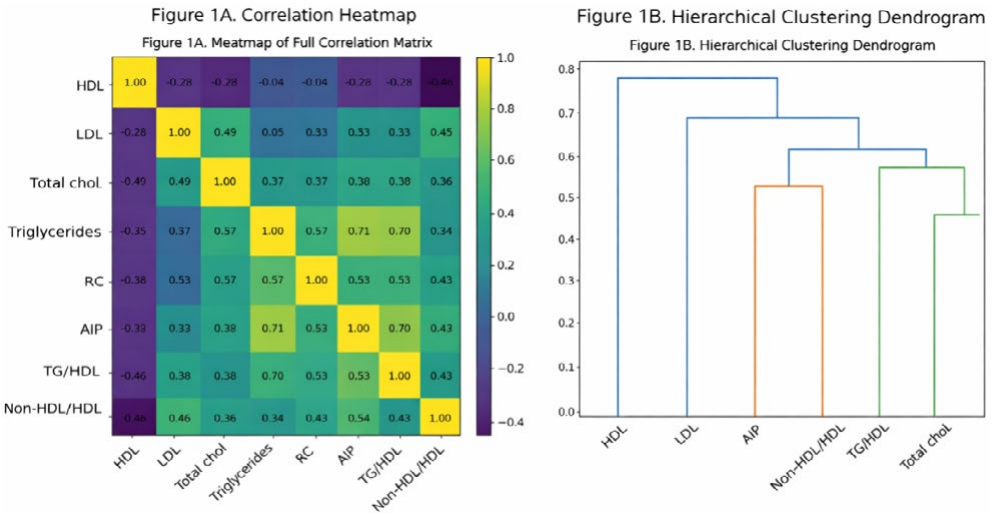
**FIGURE 1** Correlation heatmap and hierarchical clustering of lipid parameters in CTS.

**Conclusion:** Composite atherogenic indices and RC were associated with electrophysiological severity rather than CTS presence, supporting a severity‐focused metabolic framework. Atherogenic burden may contribute to progressive median nerve dysfunction beyond mechanical compression. Routine lipid‐derived indices may aid risk stratification and enhance understanding of CTS pathophysiology.

**Disclosure:** Nothing to disclose.

## EPO‐0869

### Autonomic dysfunction in hereditary transthyretin amyloidosis: Real‐world experience of stabilization under vutrisiran therapy

#### C. Fasano ^1^; M. Gianoli^1^; S. Longhi^2^; I. Ruotolo^3^; R. D'Angelo^1^; R. Rinaldi^1^; F. Provini^1^; G. Calandra Buonaura^1^; P. Guaraldi^1^

##### ^1^IRCCS Istituto delle Scienze Neurologiche di Bologna, University of Bologna, Bologna, Italy; ^2^Cardiology Unit, Cardiac Thoracic and Vascular Department, IRCCS Azienda Ospedaliero‐Universitaria di Bologna, Bologna, Italy; ^3^University of Bologna, Department of Medical and Surgical Sciences (DIMEC), Bologna, Italy

**Background and aims:** Hereditary transthyretin amyloidosis (ATTRv) is a systemic disease caused by the deposition of misfolded transthyretin (TTR), frequently affecting both peripheral nerves and the heart. Amyloid deposition prematurely involves autonomic ganglia, leading to early and often progressive autonomic dysfunction. Vutrisiran (Amvuttra), an RNA interference therapeutic targeting TTR, is approved for the treatment of hATTR and has demonstrated benefits on both neurological and cardiac outcomes.

**Methods:** We retrospectively analysed 57 patients with ATTRv treated with vutrisiran and followed within the Emilia‐Romagna regional network. Demographic and clinical data were collected from 9 months before treatment initiation through 18 months of follow‐up. Autonomic symptoms were assessed using the Composite Autonomic Symptom Score‐31 (COMPASS‐31), and disability was evaluated with the Norfolk Quality of Life–Diabetic Neuropathy (Norfolk QOL‐DN) questionnaire.

**Results:** Repeated measures analyses of COMPASS‐31 domains demonstrated overall longitudinal stability across follow‐up, with particular regard to the gastrointestinal domain, which exhibited a directional trend toward improvement. Norfolk QOL‐DN scores displayed a descriptive reduction from baseline to 18 months. Baseline COMPASS‐31 domains were not independently associated with Norfolk QOL‐DN at 9 months after adjustment for baseline disability; however, orthostatic intolerance showed a positive directional trend, suggesting that higher autonomic symptom burden at baseline may contribute to worse outcome.

**Figure:**
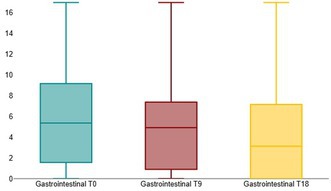
Longitudinal distribution of COMPASS‐31 gastrointestinal domain scores at baseline (T0), 9 months (T9), and 18 months (T18). Box plots show median, interquartile range and overall distribution of scores over time.

**Figure:**
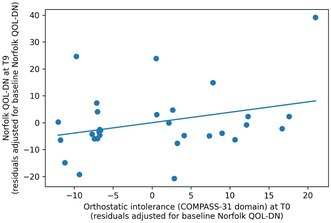
The figure shows the association between baseline orthostatic intolerance (COMPASS‐31 domain) at T0 and Norfolk Quality of Life–Diabetic Neuropathy (Norfolk QOL‐DN) at T9, with both variables adjusted for baseline Norfolk QOL‐DN.

**Conclusion:** Our real‐world findings highlight the role of autonomic dysfunction in determining clinical disability in ATTRv and suggest that treatment with vutrisiran may be effective in stabilizing autonomic symptom progression. These results support the relevance of targeting dysautonomia as a key component of disease management in ATTRv.

**Disclosure:** Alnylam Pharmaceuticals.

## EPO‐0870

### Sensory neuronopathy in chronic alcohol users: A retrospective bicentric cohort study

#### E. Louis ^1^; G. Beaudonnet^2^; B. Bardel^3^; C. Cauquil^4^; C. Labeyrie^4^; G. Fargeot^4^; I. Iliescu^2^; J. Lefaucheur^3^; A. Creange^1^; T. Gendre^1^

##### ^1^AP‐HP, Henri Mondor University Hospital, Department of Neurology, Créteil, France; ^2^AP‐HP, Bicêtre University Hospital, Unit of Clinical Neurophysiology, Le Kremlin‐Bicêtre, France; ^3^AP‐HP, Henri Mondor University Hospital, Unit of Clinical Neurophysiology, Créteil, France; ^4^AP‐HP, Bicêtre University Hospital, Department of Neurology, Le Kremlin‐Bicêtre, France

**Background and aims:** Chronic alcohol misuse typically causes length‐dependent axonal polyneuropathy. Purely sensory ataxic presentations have been reported, but data are limited. We aimed to characterise sensory neuronopathy (SNN) associated with chronic alcohol misuse.

**Methods:** In a retrospective two‐centre cohort (2008–2024), we analysed adults meeting clinical and electrophysiological criteria for SNN, reporting chronic alcohol misuse (≥100 g ethanol/week for ≥3 months), without any alternative aetiology. Severity was assessed using the modified Rankin Scale and a neurophysiological sensory score based on ten sensory nerves. Patients with and without a favourable outcome (mRS improvement ≥ 1) were compared.

**Results:** Twenty‐five patients were included (median age 50 years; 64% female). They presented pure sensory non‐length‐dependent ataxic neuropathy (100%), of acute/subacute onset (88%), highly disabling (mRS ≥ 3: 76%), painful (68%), asymmetrical (44%), with central nervous system involvement (32%). Precipitating events included unintentional weight loss (64%) and alcohol intake escalation (32%). Reported weekly consumption did not correlate with baseline severity. Markers of malnutrition (11/23, 48%) and vitamin deficiencies (18/23, 78%) were frequently observed, mostly involving B1, B9, and B12 pathways. Outcome was favourable in 16/21 (71%) and was more frequent among patients receiving B1 supplementation (*p* = 0.047).

**Conclusion:** Our findings are consistent with an association between chronic alcohol misuse and a distinct SNN phenotype marked by rapid onset, pain, and favourable outcomes despite high baseline disability. Findings support a predominantly nutritional contribution, without ruling out direct alcohol neurotoxicity. Rapid‐onset SNN should prompt assessment of alcohol exposure and nutritional status, with early vitamin supplementation when misuse is identified.

**Disclosure:** Nothing to disclose.

## EPO‐0871

### The electrophysiology of disease remission in chronic inflammatory demyelinating polyneuropathy

#### J. Freiha ^1^; Y. Rajabally^2^; R. Iqbal^2^; D. Allen^1^; C. Osman^1^

##### ^1^Wessex Neurological Centre, University Hospital Southampton, Southampton, UK; ^2^Inflammatory Neuropathy Service, Department of Neurology, University Hospitals Birmingham, Birmingham, UK

**Background and aims:** Whether serial electrophysiology may be useful in chronic inflammatory demyelinating polyneuropathy (CIDP), is unknown.

**Methods:** We retrospectively studied electrophysiological findings during active disease and after remission off‐treatment, in subjects with CIDP, at 2 UK neuropathy centres.

**Results:** We included 24 consecutive subjects. Eighteen subjects (75%) had typical CIDP, 3 (12.5%) had motor CIDP and 3 (12.5%) had distal CIDP. Mean age was 61.3 years. Mean interval between electrophysiological study during active disease and post‐remission, was 63.25 months (SD: 52.0). Mean distal motor latency improved for median (*p* < 0.001), ulnar (*p* < 0.001), fibular (*p* = 0.01) and tibial (*p* = 0.002) nerves. Mean motor conduction velocity improved for median (*p* < 0.001), ulnar (*p* < 0.001) and fibular (*p* = 0.012) nerves. Mean minimum F‐wave latency improved for median (*p* = 0.003), ulnar (*p* = 0.002), and tibial (*p* = 0.027) nerves. The proportion of nerves showing conduction block was reduced post‐remission compared to during active disease (10/67 vs. 26/66; *p* = 0.002). Demyelinating criteria were fulfilled by all subjects during active disease vs. 14/24 (58.3%) after remission (*p* = 0.006) . Electrophysiological quasi‐normalization correlated with shorter pre‐treatment disease duration (*p* = 0.002). Amplitude of functional improvement correlated with fibular nerve CMAP amplitude at the first study (*p* = 0.024).

**Conclusion:** Electrophysiological quasi‐normalisation may occur in a third of subjects with CIDP in remission off‐treatment and may be more likely with prompt diagnosis/treatment. Amelioration of most commonly studied parameters may be observed in all tested nerves. Pre‐treatment fibular nerve axonal loss may predict clinical improvement amplitude.

**Disclosure:** YAR has received consultancy honoraria from Sanofi, Argenx, Janssen, LFB, Polyneuron, Grifols, Takeda, Dianthus, Vitaccess; has received educational sponsorships from LFB and CSL Behring; has obtained research grants from LFB. CO has received speaker/consultancy honoraria from Takeda, Grifols and Terumo BCT. JF, RI and DA have no disclosures.

## EPO‐0872

### Comparative efficacy of treatments for chronic inflammatory demyelinating polyneuropathy: A network meta‐analysis

#### O. Uwishema

##### Department of Research and Education, Oli Health Magazine Organization, Kigali, Rwanda

**Background and aims:** Chronic inflammatory demyelinating polyneuropathy is an immune‐mediated neuropathy with multiple therapeutic options but limited head‐to‐head randomized comparisons. This study aimed to systematically compare and rank the efficacy and safety of available treatments for chronic inflammatory demyelinating polyneuropathy using a network meta‐analysis of randomized controlled trials.

**Methods:** A systematic literature search was conducted across PubMed, EMBASE, Scopus, CENTRAL, and ClinicalTrials.gov from inception to January 14, 2025. Randomized controlled trials evaluating pharmacological treatments for chronic inflammatory demyelinating polyneuropathy were included. Outcomes assessed were disability measured by the Inflammatory Neuropathy Cause and Treatment score, functional independence measured by the Inflammatory Rasch‐built Overall Disability Scale, muscle strength measured by the Medical Research Council sum score, grip strength, and safety outcomes. A frequentist network meta‐analysis with random‐effects modeling was performed, with Bayesian sensitivity analyses conducted to assess ranking consistency.

**Results:** Seventeen randomized controlled trials involving 1,079 participants were included. Subcutaneous immunoglobulin at 0.4 g per kilogram demonstrated the greatest improvement in disability. Intravenous immunoglobulin at 1.0 g per kilogram showed the strongest improvement in muscle strength. Efgartigimod significantly improved functional independence and ranked consistently across analyses. Plasma exchange showed the largest improvement in grip strength but was supported by a single trial. Corticosteroids ranked lowest across efficacy outcomes. Treatment rankings were consistent between frequentist and Bayesian analyses.

**Conclusion:** Intravenous immunoglobulin, subcutaneous immunoglobulin, and efgartigimod are the most effective therapies for improving disability, function, and strength in chronic inflammatory demyelinating polyneuropathy. These findings support individualized treatment selection based on patient‐specific clinical goals.

**Disclosure:** Nothing to disclose.

## EPO‐0873

### Burden of Guillain‐Barré syndrome in the United States in 2025

#### S. Rinaldi ^1^; M. Kim^2^; S. Vaghela^3^; E. Brouwer^4^; L. Bloudek^4^; B. Turner^5^; J. Duchen^5^; J. Allen^6^

##### ^1^Oxford University Hospitals NHS Foundation Trust, Oxford, UKLon; ^2^Annexon Biosciences, Brisbane, USA; ^3^Annexon Biosciences, Brisbane, USA, and HealthEcon Consulting, Inc, Ancaster, Canada; ^4^Curta Inc, Seattle, USA; ^5^Magnolia Market Access, Bridgewater, USA; ^6^University of Minnesota, Minneapolis, USA

**Background and aims:** Guillain‐Barre Syndrome (GBS) imposes substantial disability and economic burden, extending well beyond the initial hospitalisation. Real‐world data quantifying this burden remain limited. This study characterised real‐world GBS incidence, healthcare resource utilisation (HCRU), and costs in the US.

**Methods:** We analysed 2017–2023 data from a large US commercial claims database and Medicare Fee‐for‐Service (FFS) 5% sample to identify patients with GBS (Figure 1). Annual incidence rates were projected to the 2025 US population, and costs were adjusted for age and sex. Direct medical costs were estimated with paid amounts during the index hospitalisation and subsequent year, adjusted to 2024 USD. HCRU was measured during the 12 months following discharge from index hospitalisation.

**Figure:**
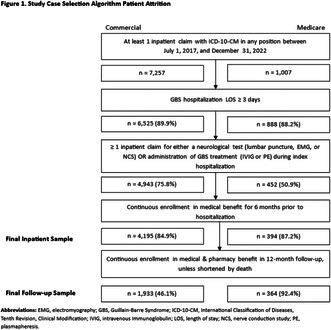
**FIGURE 1** Study case selection algorithm patient attrition.

**Results:** An estimated 8,039 GBS cases [95% CI: 7,734–8,364] occurred in the US in 2025, corresponding to an incidence of 2.39 per 100,000. During the index hospitalisation, 28.0% of commercial and 34.9% of Medicare patients required ICU care [mean (SD) ICU stay of 7.8 (14.2) and 9.1 (13.3) days, respectively] (Table 1), and age/sex‐adjusted GBS‐related medical costs averaged $77,096 and $50,429 for commercial and Medicare patients, respectively (Table 2). During the 12‐month follow‐up, GBS‐related and all‐cause medical costs averaged $32,786 and $67,822 for commercial and $62,322 and $104,919 for Medicare patients. Total GBS‐related and all‐cause medical costs averaged $109,883 and $144,918 for commercial and $112,751 and $155,347 in Medicare patients.

**Figure:**
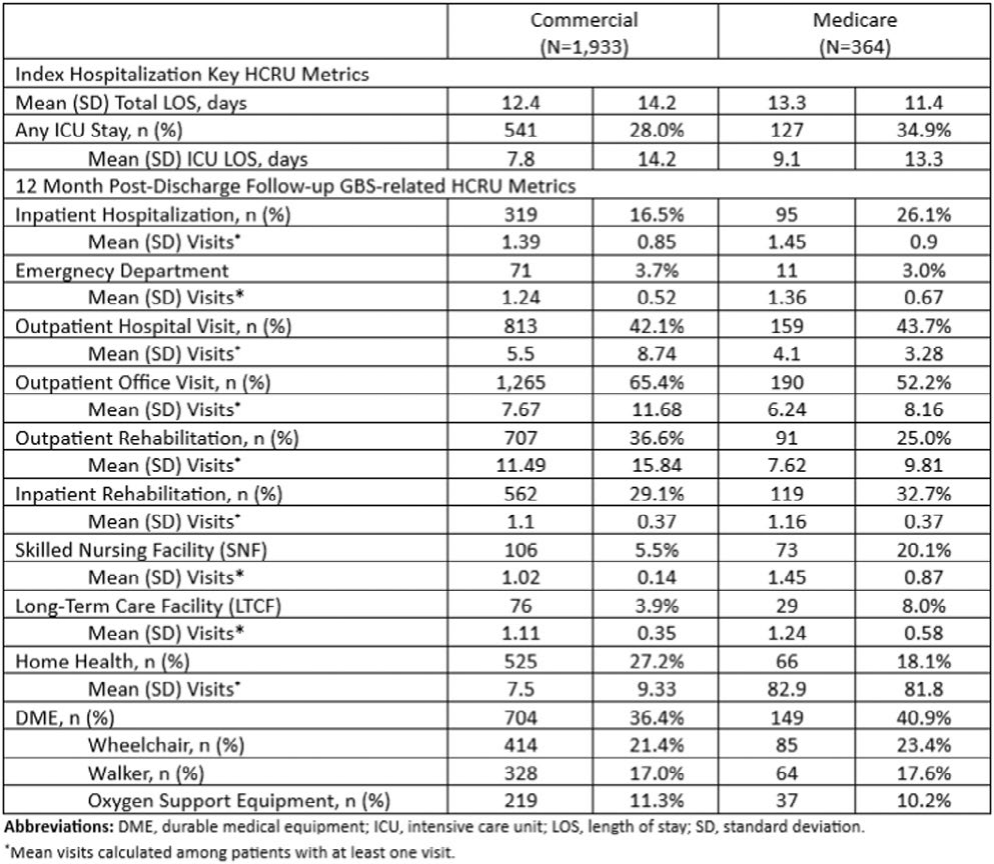
**TABLE 1** Post‐discharge GBS‐related healthcare resource utilization in 12‐month follow‐up.

**Figure:**
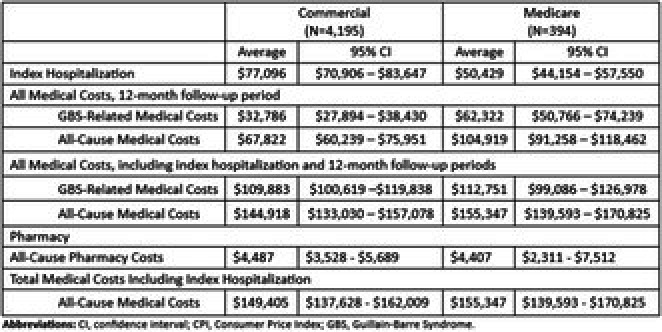
**TABLE 2** Weighted medical direct cost components of resource utilization for hospitalization and 12‐month follow‐up, adjusted to 2024 dollars using the US medical CPI.

**Conclusion:** GBS imposes a substantial and persistent burden on patients and the US healthcare system, highlighting the need for more effective treatments.

**Disclosure:** This study was sponsored by Annexon. SR: Medical Advisory Board member of the Guillain‐Barré syndrome and Associated Inflammatory Neuropathies (GAIN) patient charity. His department receives payment for the provision of diagnostic antibody testing, including of nodal/paranodal antibodies. He has received payment from Argenx for preparation and delivery of conference presentation on clinical trial, payment for serving on Argenx, Annexon, Dianthus and Hansa clinical trial advisory boards, and payment from CSL for delivering a conference symposium lecture. MK: Employment with and shareholder of Annexon Biosciences SV: Employee of HealthEcon Consulting and employee and shareholder of Annexon EB, LB: Employee of Magnolia Market Access BT, JD: Employee of Curta Inc JAA: Consultant fees: Akcea therapeutics, Alexion, Alnylam Pharmaceuticals, Annexon Biosciences, Argenx, CSL Behring, Grifols, Immunovant, Immupharma, Johnson & Johnson, Pfizer, and Takeda

## EPO‐0874

### Premature death as a major driver of total societal cost of Guillain‐Barré syndrome in the United States

#### S. Rinaldi ^1^; M. Kim^2^; S. Vaghela^3^; S. Katsandres^4^; L. Bloudek^4^; B. Bloudek^4^; E. Brouwer^4^; B. Turner^5^; J. Duchen^5^; J. Allen^6^

##### ^1^Oxford University Hospitals NHS Foundation Trust, London, UK; ^2^Annexon Biosciences, Brisbane, USA; ^3^Annexon Biosciences, Brisbane, USA and HealthEcon Consulting, Inc, Ancaster, Canada; ^4^Curta Inc, Seattle, USA; ^5^Magnolia Market Access, Bridgewater, USA; ^6^University of Minnesota, Minneapolis, USA

**Background and aims:** Guillain‐Barre Syndrome (GBS) is associated with substantial economic burden, including direct costs (hospitalisation and rehabilitation) and indirect costs (lost productivity and increased risk of premature death). We present the indirect cost of premature deaths among US patients hospitalised with GBS (USD 2025).

**Methods:** Incidence and mortality rates for patients hospitalised with GBS, derived from a claims analysis of commercially insured and Medicare Fee‐For‐Service 5% standard analytical files from 2017–2023, were projected to the 2025 US populations aged <65 and ≥65 years (average age: 45 and 70, respectively). Excess mortality was estimated using age‐adjusted background mortality (Centers for Disease Control and Prevention Life Tables). A value of statistical life (VSL) was applied to excess deaths ≤12 months post‐hospitalisation to estimate total premature mortality cost.

**Results:** 8,039 US GBS cases (95% CI: 7,734–8,364) were projected for 2025 (<65 years: 67%, ≥65: 33%). Excess mortality rates ≤12 months from hospitalisation were 4.98% (<65) and 22.1% (≥65) (including inpatient mortality: 1.8% vs 7.7%). Premature mortality costs (using the US Department of Health and Human Services age‐agnostic 2025 central VSL estimate; $13.2M) were $3.446B (<65) and $7.669B (≥65). The total annual estimate was $11.114B for deaths ≤12 months from hospitalisation. Applying age‐specific central estimates for VSL (age‐specific US EQ‐5D scores, Hamner 2026. 45 yr: $10.8M; 70 yr: $5.6M), resulted in premature mortality costs of $6.086B (<65: $2.812B, ≥65: $3.274B). Results were robust regardless of statistical method.

**Conclusion:** Premature death in patients hospitalised with GBS is a major driver of GBS total costs from a US societal perspective.

**Disclosure:** This study was funded by Annexon Biosciences. SR: Medical Advisory Board member of the Guillain‐Barré syndrome and Associated Inflammatory Neuropathies (GAIN) patient charity. His department receives payment for the provision of diagnostic antibody testing, including of nodal/paranodal antibodies. He has received payment from Argenx for preparation and delivery of conference presentation on clinical trial, payment for serving on argenx, Annexon, Dianthus and Hansa clinical trial advisory boards, and payment from CSL for delivering a conference symposium lecture. JAA: Consultant fees: Akcea therapeutics, Alexion, Alnylam Pharmaceuticals, Annexon Biosciences, argenx, CSL Behring, Grifols, Immunovant, Immupharma, Johnson & Johnson, Pfizer, and Takeda MK: Employee and shareholder of Annexon Biosciences SV: Employee of HealthEcon Consulting and employee and shareholder of Annexon Biosciences SCK, LB, BB, EB: Employees of Curta Inc. BT, JD: Employees of Magnolia Market Access.

## EPO‐0875

### De novo mutations in charcot‐marie‐tooth disease of Korean origin

#### S. Nam ^1^; A. Lee^2^; K. Chung^2^; B. Choi^3^

##### ^1^Cell and Gene Therapy Institute, Samsung Medical Center, Seoul, Republic of Korea; ^2^Department of Biological Sciences, Kongju National University, Gongju, Republic of Korea; ^3^Department of Neurology, Samsung Medical Center, Sungkyunkwan University School of Medicine, Seoul, Republic of Korea

**Background and aims:** Charcot–Marie–Tooth disease (CMT), a genetically and clinically heterogeneous group of peripheral neuropathies, is characterized by progressive muscle weakness and significant limb deformities. De novo mutations are an important source of pathogenic variation in autosomal dominant CMT, yet their overall frequency, parental origin, and clinical implications beyond recurrent copy number variants remain poorly defined. The aim of this study was to systematically identify de novo mutations in autosomal dominant CMT and to investigate their genetic features, parental origin, and associated clinical phenotypes.

**Methods:** We examined 151 genetically diagnosed Korean parent–offspring trio families with autosomal dominant CMT, excluding cases with CMT1A duplication. Pathogenic variants were identified using Sanger sequencing, whole‐exome sequencing, or targeted gene panels, and de novo status was confirmed by parental testing and paternity verification. Parental origin, CpG methylation status, mutation spectra, and clinical characteristics were analyzed.

**Results:** We identified 49 de novo point or small insertion/deletion mutations in 61 patients, corresponding to a de novo frequency of 40.4%. De novo mutations showed marked gene‐specific differences, with high frequencies in INF2, MORC2, PMP22, and KIF5A. Most mutations were paternally derived and preferentially occurred at highly methylated CpG sites. Clinically, affected children carrying de novo mutations demonstrated significantly earlier disease onset compared with their parental founders, consistent with phenotypic anticipation.

**Conclusion:** De novo mutations contribute substantially to autosomal dominant CMT and exhibit distinct molecular and clinical patterns. These findings emphasize the value of trio‐based analyses for genetic diagnosis and provide insights into mutational mechanisms and disease progression in inherited peripheral neuropathies.

**Disclosure:** Nothing to disclose.

## EPO‐0876

### Phenotypic severity in patients with hereditary motor and sensory neuropathy with somatic mosaic mutations

#### S. Nam ^1^; A. Lee^2^; K. Chung^2^; B. Choi^3^

##### ^1^Cell and Gene Therapy Institute, Samsung Medical Center, Seoul, Republic of Korea; ^2^Department of Biological Sciences, Kongju National University, Gongju, Republic of Korea; ^3^Department of Neurology, Samsung Medical Center, Sungkyunkwan University School of Medicine, Seoul, Republic of Korea

**Background and aims:** Hereditary motor and sensory neuropathies (HMSN) are inherited peripheral neuropathies characterized by progressive muscle weakness and sensory dysfunction. Although somatic mosaicism in HMSN‐associated genes has been reported, most cases show asymptomatic or mild phenotypes. However, some mosaic cases resemble patients with germline mutations. This study aimed to characterize somatic mosaicism and its clinical impact in a Korean HMSN family.

**Methods:** Whole exome sequencing (WES) followed by variant filtering was used to identify genetic causes. The degree of somatic mosaicism was quantified by analyzing approximately 100 cloned sequences per mutation site and calculating variant ratios. Clinical phenotypes were compared with those of patients carrying typical germline mutations.

**Results:** Two cases of somatic mosaicism were identified: p.Cys104Tyr in INF2 and p.Ser729Arg in TRPV4. The estimated mosaicism levels in blood were approximately 24% and 30%, respectively. The individual with the INF2 mutation exhibited mild peripheral neuropathy, whereas the individual with the TRPV4 mutation showed severe clinical manifestations. The INF2 case was considered gonadal mosaicism. Notably, the p.Cys104Tyr mutation in INF2 was associated with demyelinating peripheral neuropathy without focal segmental glomerulosclerosis (FSGS), expanding the known phenotype of INF2‐related disease.

**Conclusion:** This study represents one of the first reports describing somatic mosaicism involving INF2 and TRPV4 in HMSN. The severity of clinical phenotypes in mosaic cases appears to depend on both the mutation site and the proportion of mutant alleles in affected tissues. These findings suggest that somatic mosaicism may play an important role in inter‐ and intra‐familial phenotypic heterogeneity in HMSN.

**Disclosure:** Nothing to disclose.

## EPO‐0877

### Patient‐reported outcomes and safety from the phase 2 ARDA study of Empasiprubart in multifocal motor neuropathy

#### M. Pasnoor^1^; K. Beadon^2^; K. Gable^3^; T. Harbo ^4^; C. Hewamadduma^5^; C. Karam^6^; S. Cadour^7^; E. Persson^8^; M. Vujcic^8^; L. Querol^9^; J. Allen^10^; S. Peric^11^

##### ^1^University of Kansas Medical Center, Kansas City, USA; ^2^University of British Columbia, Vancouver, BC, Canada; ^3^Duke University Medical Center, Durham, USA; ^4^Aarhus University, Aarhus, Denmark; ^5^Sheffield Teaching Hospitals NHS Foundation Trust, Sheffield, UK; Sheffield Institute of Translational Neuroscience, University of Sheffield, Sheffield, UK; ^6^University of Pennsylvania, Philadelphia, USA; ^7^argenx (via PPD, part of Thermo Fisher Scientific), Ghent, Belgium; ^8^Argenx, Ghent, Belgium; ^9^Universitat Autònoma de Barcelona, Barcelona, Spain; Centro De Investigación Biomédica en Red en Enfermedades Raras (CIBERER), Madrid, Spain; ^10^University of Minnesota, Minneapolis, USA; ^11^University of Belgrade, Belgrade, Serbia

**Background and aims:** Multifocal motor neuropathy (MMN) is a rare, chronic, predominantly complement‐driven neuropathy, causing axonal degeneration and progressive, disabling, asymmetric limb weakness. Empasiprubart binds C2, blocking the classical and lectin complement pathways while leaving the alternative pathway intact. The phase 2 ARDA study (NCT05225675) assessed safety and efficacy of empasiprubart in adults with MMN.

**Methods:** Enrolled participants had probable/definite MMN (2010 European Federation of Neurological Societies/Peripheral Nerve Society guidelines) and proven intravenous immunoglobulin (IVIg) dependency and were on a stable IVIg regimen before randomisation. Participants were assigned to 1 of 2 dosing cohorts, each randomised 2:1 to empasiprubart or placebo. Efficacy endpoints included patient‐reported outcomes (PROs) measured by the Chronic Acquired Polyneuropathy Patient‐Reported Index (CAP‐PRI), the Fatigue Severity Scale (FSS), the Patient Global Impression of Change (PGI‐C), the Treatment Satisfaction Questionnaire for Medication (TSQM‐14), and the Health‐Related Productivity Questionnaire (HRPQ). Safety and tolerability were evaluated throughout the study.

**Results:** Overall, 27 participants (empasiprubart, *n* = 18; placebo, *n* = 9) were randomised per cohort (total *N* = 54). Participants in cohort 2 were older, with longer duration of disease and time since first IVIg treatment versus cohort 1. Overall, empasiprubart was generally well tolerated. Most adverse events were mild/moderate. Improvements in PROs were generally observed with empasiprubart compared with placebo in both cohorts, including CAP‐PRI (Figure 1A), FSS (Figure 1B), PGI‐C (improvement: 94% vs 11% in cohort 1; 83% vs 44% in cohort 2), TSQM‐14 (Figure 2A–C), and HRPQ (Table 1).

**Figure:**
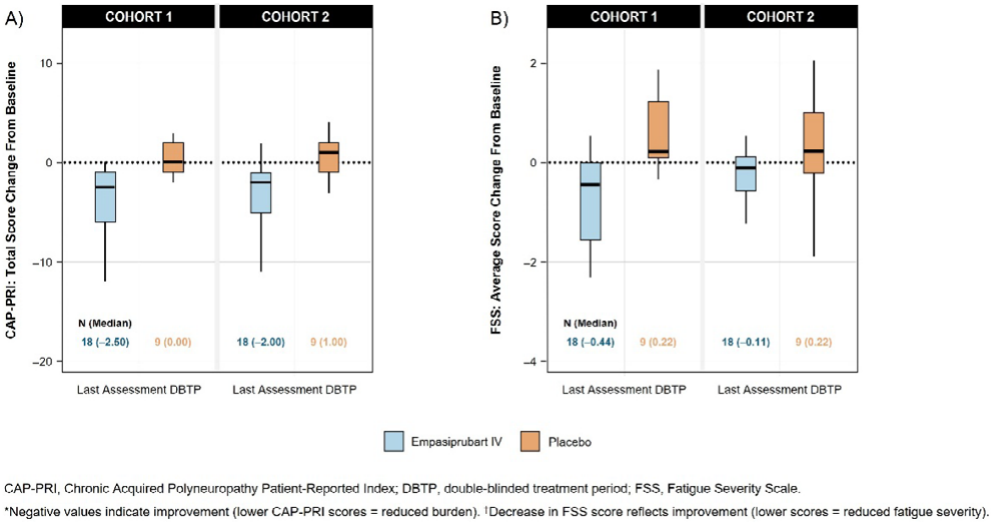
**FIGURE 1** Box plots of (A) CAP‐PRI* total score and (B) FSS† average score of change from baseline at last assessment during the DBTP.

**Figure:**
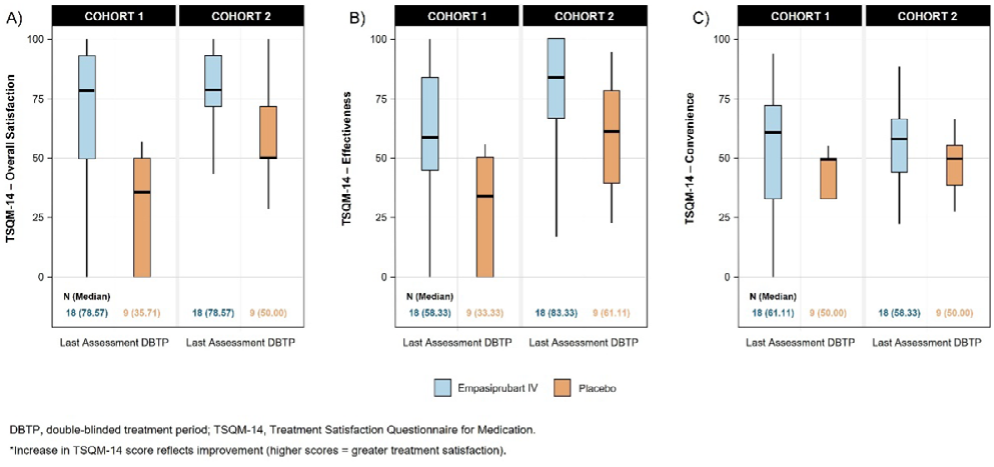
**FIGURE 2** Box Plots of values of (A) TSQM‐14* (overall satisfaction), (B) TSQM‐14* (Effectiveness), and (C) TSQM‐14* (Convenience).

**Figure:**
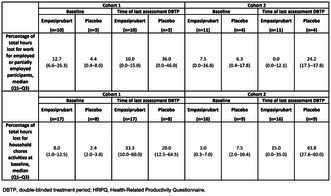
**TABLE 1** HRPQ results for work activities and for household chores activities.

**Conclusion:** Empasiprubart improved PROs versus placebo, with patients consistently reporting less fatigue and better quality of life.

**Disclosure:** MP: advisory board for Alexion, Amgen, Annexon, argenx, Catalyst, CSL Behring, Dianthus, EMD Serono/Merck, Immunovant, Janssen, Johnson & Johnson, Medlink, Momenta, Octapharma, Sanofi Genzyme, Takeda, TerumoBCT, UCB; KB: advisory board and research support for argenx, CSL Behring, Johnson & Johnson, Takeda, Sanofi, Octapharma; KLG: nothing to disclose; TH: research or institutional support for Annexon, argenx, Dianthus, Immunovant, Janssen, Nuvig, Sanofi, Takeda; CH: research or institutional support, advisory board, speaker honoraria for argenx, Alexion, Avidity, Biogen, Dyne, Immunovant, Johnson & Johnson, Lupin, Remgen, Roche, UCB, Vertex; CK: consulting agreement, honoraria, advisory board for Acceleron, Alpine, Alexion, Alnylam, Amgen, Amicus, Annexon, argenx, AstraZeneca, Biogen, Corino, CSL Behring, Genentech, Ionis, Johnson & Johnson, Neuroderm, Novo Nordisk, Octapharma, Pfizer, Sanofi, Takeda, UCB, Vertex, Zai lab; SC: consulting agreement for argenx, via PPD, part of Thermo Fisher Scientific; EKP and MV: employees of argenx; LQ: research or institutional support for Alnylam, Annexon, argenx, Avilar, Biogen, CIBERER, CSL Behring, Dianthus, Fundació La Marató, Grifols, Janssen, LFB, Lundbeck, Merck, Novartis, Octapharma, Roche, Sanofi, UCB; JAA: consulting agreement for argenx, Alexion, Alnylam, Annexon, CSL Behring, Grifols, Immunovant, Immupharma, Pfizer, Takeda; SP: research or institutional support for ADOC, argenx, Berlin‐Chemie Menarini, Kedrion, Mylan, Octapharma, Pfizer, Roche, Salveo, Sanofi Genzyme, Teva Actavis, Wörwag.

## Neuro‐Oncology 1

## EPO‐0878

### Early onset radiation‐induced leukoencephalopathy in patients treated for a glioblastoma by STUPP protocol and risk factors evaluation

#### A. Balcerac ^1^; S. Cuzzubbo^2^; F. Bompaire^1^; F. Ducray^3^; S. Kremer^4^; M. Sanson^5^; D. Psimaras^6^; E. Vicaut^7^; A. Carpentier^2^; D. Ricard^1^

##### ^1^Department of Neurology, Hôpital d'Instruction des Armées Percy, Service de Santé des Armées, Clamart; 92140, France; ^2^Neurology Department, AP‐HP, Saint Louis Hospital, Université Paris Cité, Paris, France; ^3^Department of Neuro‐Oncology, East Group Hospital, Hospices Civils de Lyon, Lyon, France; ^4^Department of Neuro‐Radiology, Hôpitaux Universitaires de Strasbourg, Strasbourg, France; ^5^The Paris Brain Institute, Sorbonne University, Paris, France; ^6^Sorbonne University ‐ AP‐HP, Pitié‐Salpêtrière Hospital, Department of Neurology, INSERM U974, Paris, France; ^7^Clinical Research Unit, Saint Louis‐Lariboisière‐Fernand Widal Hospital, and Paris Cité Université, Paris, France

**Background and aims:** Radiation‐induced leukoencephalopathy (RIL) is a known late complication of brain radiotherapy in glioblastoma (GBM) survivors, whereas early‐onset RIL remains poorly described. This ancillary study of the phase III ASTER trial evaluated the incidence of early‐onset RIL and explored associated risk factors, including exposure to losartan, a peroxisome proliferator‐activated receptor gamma (PPARγ) agonist.

**Methods:** We retrospectively analyzed ASTER trial participants treated with standard radiotherapy plus temozolomide (STUPP protocol). Brain MRI performed before radiotherapy and at six months post‐treatment was centrally reviewed. Early‐onset RIL was defined by new or progressive confluent T2/FLAIR white‐matter hyperintensities with associated cortico‐subcortical atrophy and no contrast enhancement, quantified using a simplified Scheltens scale. Cognitive function and quality of life were assessed using the Montreal Cognitive Assessment (MoCA) and EORTC QLQ‐C30/BN20 questionnaires. Clinical, treatment‐related, and genetic factors (PPARγ) were analyzed.

**Results:** Thirty‐one patients were evaluable. Early‐onset RIL was identified in 29% (*n* = 9), with high inter‐rater diagnostic agreement. No significant associations were observed with age, MGMT promoter methylation, IDH1 mutation, or PPARγ polymorphism. Losartan exposure was not associated with reduced RIL incidence. At six months, patients with RIL exhibited significantly lower MoCA scores and impaired physical and emotional functioning compared with patients without RIL.

**Conclusion:** Early‐onset RIL affects nearly one‐third of GBM patients treated with the STUPP protocol and is associated with early cognitive and functional decline. No specific clinical, genetic, or treatment‐related risk factors, including losartan exposure, were identified. Early monitoring and larger prospective studies are warranted.

**Disclosure:** No disclosure

## EPO‐0879

### Application of the PNS‐care score in real world clinical practice: Insights from a neuro‐immuno‐oncology perspective

#### M. García‐Castillo^1^; S. Jorquera‐Ortega^1^; A. Alfranca^2^; A. Barbosa^3^; F. Gilo^1^; S. Sánchez^4^; M. Conversa^3^; J. Rogado^5^; B. Hernández^5^; A. Gonzalez‐Martinez ^1^

##### ^1^Facultad de Medicina, Universidad Autónoma de Madrid, Madrid, Spain; Neurology Service, Hospital Universitario de la Princesa, Madrid, Spain; Instituto de Investigación Sanitaria Princesa (IIS‐Princesa), Madrid, Spain; ^2^Immunology Service, Hospital Universitario de la Princesa, Madrid, Spain; ^3^Neuroradiology Service, Hospital Universitario de la Princesa, Madrid, Spain; ^4^Laboratory, Hospital Universitario de la Princesa, Madrid, Spain; ^5^Oncology Service, Hospital Universitario de la Princesa, Madrid, Spain

**Background and aims:** To describe the diagnostic criteria compliance of patients with suspicion of paraneoplastic neurologic syndromes (PNSs) who have positive antibody testing.

**Methods:** We performed an observational retrospective study of a prospectively collected cohort of patients under PNSs suspicion with positive paraneoplastic antibodies between January‐2024 and December‐2025. Patients were classified using the PNS‐Care Score.

**Results:** We included 14 patients, 8 (57%) were male, median of age of 66 (SD: 19) years. The PNSs‐Care Score was definite in 3 (21%), probable in 1 (7%) and possible in 4 (29%) with 6 (43%) patients classified eventually as non‐PNSs. In the definite PNS group, 1/3 ( 33%) patient presented with Morvan syndrome with anti‐titin, anti‐LGI1 and anti‐CASPR2 positive antibodies, and was diagnosed with metastatic thymoma; 1/3 (33%) patient had anti‐Hu and Anti‐SOX1 positive antibodies presenting with Eaton‐Lambert phenotype and small‐cell lung cancer (SCLC); and 1/3 (33%) patient had also anti‐Hu positive antibody and SCLC presenting with sensory neuronopathy. The patient with probable PNS showed a positive anti‐SOX1 antibody, but did not present with a defined phenotype nor and a tumor was not found after diagnosis workup. In patients with possible PNSs 2 (50%) had anti‐NMDAR encephalitis, 1 (25%) had epileptic crisis with anti‐recoverin, anti‐Ma2 and anti‐CV2 antibodies, and 1 (25%) had anti‐Yo positivity with atypical parkinsonism.

**Conclusion:** These findings offer an insight of the application of the PNS‐Care Score in real world clinical practice, supporting the association between “high‐risk antibodies” and “high or intermediate risk neurologic phenotypes” with cancer. Further multicenter studies focusing on PNS‐Care Score applications are granted.

**Disclosure:** ISCIII, EU and FSE funding through JR23/0005; PI24/01085.

## EPO‐0880

### Drug sensitivity of patient‐derived organoids from recurrent GBM correlates with patients' response to second‐line chemotherapy

#### M. Morelli^1^; O. Santonocito^2^; F. Lessi^1^; S. Franceschi^1^; F. Pieri^2^; C. Gambacciani^2^; F. Aquila^2^; S. Cupini^3^; I. Stasi^3^; V. Zucchi^4^; A. Giusti^5^; L. Testaverde^6^; E. Franceschi^7^; G. Berzero^8^; C. Mazzanti^1^; A. Di Stefano ^2^

##### ^1^Section of Genomics and Transcriptomics, Fondazione Pisana per la Scienza, San Giuliano Terme, Italy; ^2^Neurosurgery Division, Azienda USL Toscana Nordovest, Hospital of Livorno, Italy; ^3^Department of Oncology, Azienda USL Toscana Nordovest, Hospital of Livorno, Italy; ^4^Unit of Pathology, Hospital of Livorno, Italy; ^5^ASL Toscana Nord Ovest, UO Anatomia Patologica Carrara, Italy; ^6^Unit of Neuroradiology, Hospital of Livorno, Italy; ^7^Nervous System Medical Oncology Department, IRCCS Istituto Delle Scienze Neurologiche di Bologna, Bologna; ^8^8Neurology Unit, IRCCS Ospedale San Raffaele Scientific Institute, Milan, Italy

**Background and aims:** This study aimed to explore the correlation between the effectiveness of second‐line chemotherapy in patients with recurrent glioblastoma (rGBM) who underwent second surgery and drug treatment tests on patient‐derived explants.

**Methods:** Sixteen adult patients diagnosed with IDH wild‐type glioblastoma underwent surgery for recurrent GBM, with 9 cases studied retrospectively and 7 prospectively. Patient‐derived 3D glioblastoma organoids (GBM‐EXPs) were established from resected tumors and treated with chemotherapy. NADH FLIM method was used to assess drug‐sensitivity in vitro.

**Results:** Among patients progressing during first‐line temozolomide (TMZ) therapy or within three months after discontinuation, most exhibited TMZ resistance by NADH‐FLIM (*p* = 0.02), with a longer PFS1 in TMZ‐sensitive compared with TMZ‐resistant rGBM‐EXPs (15.9 vs 10.9 months; *p* = 0.07), independent of MGMT promoter methylation. In the second‐line setting, patients treated with regorafenib whose rGBM‐EXPs were regorafenib‐sensitive experienced a significantly longer PFS2 than those with resistant organoids (7.5 vs 3.6 months; *p* = 0.04). In the prospective cohort, ex‐vivo testing prior to second‐line therapy was associated with a trend toward longer PFS2 (7.4 vs 4.6 months; *p* = 0.08) and a significantly improved OS2 (not reached vs 6.3 months; *p* = 0.03) compared with the retrospective cohort, with no difference in MGMT promoter methylation rates. Paired longitudinal analyses demonstrated acquisition of TMZ resistance in rGBM‐EXPs compared with untreated GBM‐EXPs.

**Conclusion:** This study shows that the sensitivity of rGBM‐EXP to TMZ mirrors the timing of recurrence observed in vivo. NADH‐FLIM‐based drug response assessment in rGBM‐EXP may play a role in predicting the efficacy of second‐line therapies.

**Disclosure:** Nothing to disclose.

## EPO‐0881

### Current pathways of rehabilitation for patients with high grade gliomas: Insights from AINO, SINch and SIRN

#### A. Di Stefano ^1^; A. Pace^2^; A. Silvani^3^; T. Ius^4^; D. Aiudi^5^; O. Santonocito^1^; M. Zampolini^6^; R. Rudà^7^; M. Bartolo^8^

##### ^1^Unit of Neurosurgery, Spedali Riuniti di Livorno, Italy; ^2^Neuro‐Oncology Unit, Regina Elena National Cancer Institute, Rome, Italy; ^3^Fondazione IRCCS Istituto Neurologico Carlo Besta, Milano, Italy; ^4^Neurosurgery Unit, Head‐Neck and NeuroScience Department, University Hospital of Udine, Udine, Italy; ^5^Department of Neurosurgery, Università Politecnica delle Marche ‐ Azienda Ospedaliero Universitaria delle Marche – Ancona; ^6^Physical and Rehabilitation Medicine Section of UEMS, Department of Rehabilitation, Hospital of Foligno, Foligno, Italy; ^7^Division of Neuro‐Oncology, Department of Neuroscience "Rita Levi Montalcini", University and City of Health and Science University Hospital, Turin, Italy; ^8^Department of Rehabilitation, Neurorehabilitation Unit, HABILITA Zingonia, Ciserano, Bergamo, Italy

**Background and aims:** In the management of patients diagnosed with high‐grade glioma (HGG), practices and rehabilitation programs display significant variability in real‐world settings. The HGG Rehab survey aimed to glean insights from physicians specialized in neuro‐oncology and affiliated with scientific societies in Italy: AINO, SINch and SIRN.

**Methods:** The questionnaire was released in June 2023, and responses were recorded until February 2024. One hundred eleven responses were collated.

**Results:** A median value of 40% of HGG patients from Neurosurgical and Neuro‐oncologic/Oncologic Divisions were evaluated for rehabilitation, and a median value of 20% were deemed eligible for a rehabilitation. The rate of requests for physiatric evaluation was progressively higher as KPS increased, especially patients with KPS 60–70 (80%). Over 50% of responders reported viable outpatient rehabilitation programs during adjuvant radio‐chemotherapy, with good tolerance (>55%). Seventy percent of practitioners recommended rehabilitation evaluation for newly diagnosed GBM patients and over 50% for those with recurrent GBM. Clinical benefits were observed in a median of 80% of patients, ranging from 30% to 100%. Recognized benefits of rehabilitation encompassed facilitating home discharges (82%), psychological benefits (65%), aiding the progress of adjuvant oncologic therapies (58%), and enhancing quality of life (40%). Key rehabilitation time points for newly diagnosed HGG patients included 5 days post‐surgery, after histo‐molecular diagnosis/treatment plan definition, and at recurrence.

**Conclusion:** The HGG Rehab Survey offers invaluable insights shared by three scientific societies into rehabilitation pathways for HGG patients in Italy. It highlights the usual criteria employed for the selection of patients and time points for outcome evaluation.

**Disclosure:** Nothing to disclose.

## EPO‐0882

### From single‐cell emergent behaviors to clinical outcome: Migratory strategy as a potential vulnerability in glioblastoma

#### C. Mazzanti^1^; M. Morelli^1^; F. Lessi^1^; S. Franceschi^1^; G. Ferri^1^; A. Pastore^1^; F. Marchetto^1^; F. Di Lorenzo^1^; F. Geraci^2^; P. Grigolini^3^; O. Santonocito^4^; L. Palatella^5^; A. Di Stefano^4^; G. Tancreda ^1^

##### ^1^Fondazione Pisana per la Scienza ONLUS, Italy; ^2^Institute for Informatics and Telematics, National Research Council; ^3^Department of Physics, North Texas University, Denton, USA; ^4^Neurosurgery Department Spedali Riuniti di Livorno Azienda Toscana Nord‐Ovest, Livorno, Italy ^5^Liceo Scientifico Statale “C. De Giorgi” viale De Pietro, Lecce, Italy

**Background and aims:** Glioblastoma (GBM) lethality stems from its aggressive infiltration of healthy brain tissue. Traditional metrics often fail to capture the complexity of this movement. This study aimed to characterize GBM cell motility using advanced modeling to identify new therapeutic vulnerabilities and stratification markers.

**Methods:** We developed Single‐Cell Behavior Live Imaging (ScBLI), integrating live imaging with Diffusion Entropy Analysis (DEA) across 28 primary patient‐derived cultures. By analyzing 3,842 trajectories, we classified cells into Low (L), Medium (M), and High (H) groups based on their $\delta$ scaling parameter.

**Figure:**
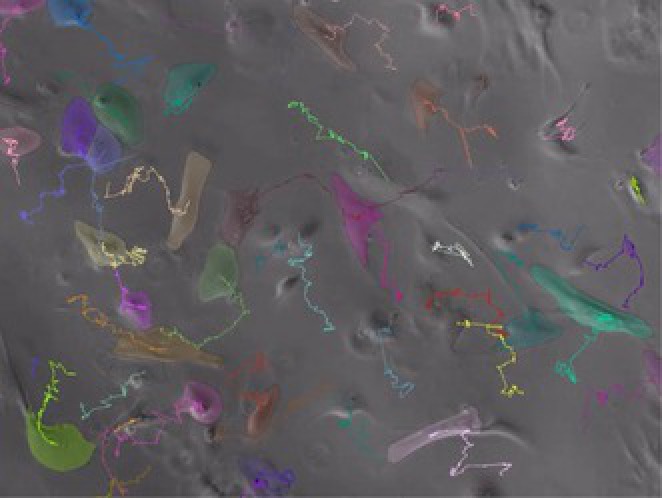
**FIGURE 1** Automated segmentation and tracking of patient‐derived glioblastoma (GBM) cells. Representative single frame from a 72‐hour live‐cell microscopy video showing patient‐derived GBM cells. The cells were automatically segmented using the Cellpose software.

**Results:** High $\delta$ scaling cells exhibited Lévy‐like, persistent migration. Paradoxically, while these cells were slower and covered less total distance, they demonstrated the highest migratory efficiency and superior chemotactic performance. Clinical correlation revealed a clear linear progression:High $\delta$ Scaling: Shortest patient survival (poorer prognosis).Low $\delta$ Scaling: Longest patient survival.Transcriptomic analysis confirmed distinct gene expression signatures corresponding to these behavioral clusters, suggesting a biological basis for these movement strategies.

**Figure:**
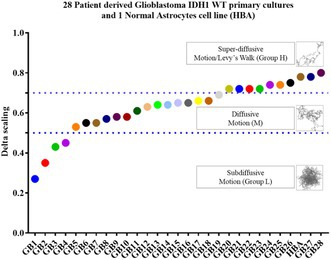
**FIGURE 2** Delta Scaling (δ) Analysis of Glioblastoma Cell Lines via Diffusion Entropy Analysis This figure illustrates the distribution of the Delta scaling parameter values, derived from the Diffusion Entropy Analysis (DEA), for 28 patient‐derived GB IDH1 Wild Type.

**Figure:**
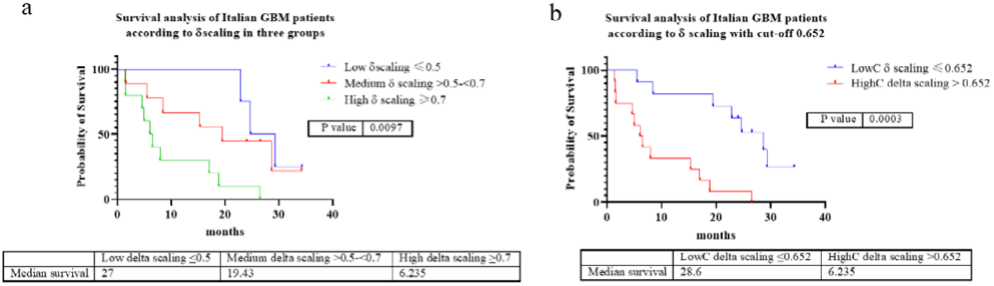
**FIGURE 3** Survival stratification of GBM IDH‐wildtype patients based on migration efficiency. (a) Kaplan‐Meier survival curves for the initial three‐tier stratification (low, medium, and high δ scaling).

**Conclusion:** his study introduces migratory efficiency as a superior predictor of GBM malignancy over traditional metrics like speed or distance. We demonstrate that a highly efficient spatial exploration strategy—rather than the sheer magnitude of movement—is a critical driver of tumor invasiveness and poor clinical outcomes. These findings redefine GBM motility, establishing $\delta$ scaling as a novel biomarker for aggressive disease.

**Disclosure:** Nothing to disclose.

## EPO‐0883

### Clinical, molecular, and transcriptomic factors associated with seizures at onset in patients with glioblastoma

#### J. Rossi ^1^; B. Donati^2^; E. Vitale^2^; E. Froio^3^; S. Serra^3^; A. Picca^4^; C. Ascione^2^; P. Pugliese^5^; F. Cavallieri^1^; G. Toschi^1^; M. Russo^1^; R. Rizzi^1^; M. Napoli^6^; C. Iaccarino^7^; G. Pavesi^7^; A. Pisanello^1^; M. Sanson^4^; G. Biagini^8^; A. Ciarrocchi^2^; S. Montepietra^1^; F. Valzania^1^

##### ^1^Neurology Unit, Neuromotor & Rehabilitation Department, Azienda USL‐IRCCS of Reggio Emilia, Reggio Emilia, Italy; ^2^Laboratory of Translational Research, Azienda USL‐IRCCS of Reggio Emilia, Reggio Emilia, Italy; ^3^Pathological Anatomy Service, Oncology Department and Advanced Technologies, Azienda USL‐IRCCS of Reggio Emilia, Reggio Emilia, Italy; ^4^Service de Neuro‐oncologie, Institut de Neurologie, Hôpital Pitié‐Salpêtrière, AP‐HP, Paris, France; ^5^Paris Brain Institute (ICM), Sorbonne Université, Inserm, CNRS, UMR S 1127, Paris, France; ^6^Reparto di Diagnostica per immagini, Salus Hospital, GVM Care & Research, Reggio Emilia, Italy; ^7^Neurosurgery Unit, Neuromotor and Rehabilitation Department, Azienda USL‐IRCCS of Reggio Emilia, Reggio Emilia, Italy; ^8^Department of Biomedical, Metabolic and Neural Sciences, University of Modena and Reggio Emilia, Modena, Italy

**Background and aims:** Epileptogenesis in glioblastoma (GBM) primarily arises within the peritumoral cortex, yet the contribution of this region to tumor progression and seizure generation remains poorly understood. This study investigated the interactions between the tumor core and the peritumoral microenvironment by comparing gene expression profiles between GBM patients presenting with seizures at onset (GBM‐EP+) and those with other symptoms (GBM‐EP−). We also evaluated differences in clinical and molecular characteristics, as well as overall survival (OS) and progression‐free survival (PFS).

**Methods:** We analyzed a retrospective cohort of 32 GBM patients using spatial transcriptomics. All patients had confirmed GBM, and complete clinical and molecular data. Molecular profiling, including IDH mutation and MGMT promoter methylation status, was performed by next‐generation sequencing. Spatial transcriptomic analyses were conducted using the GeoMx DSP® platform.

**Results:** Seizures at presentation were not associated with OS or PFS, and no clinical or molecular variables were significantly linked to seizure occurrence. Spatial analyses revealed limited differences between tumor and peritumoral compartments. However, distinct transcriptional programs characterized the two groups. EP^‐^ tumors exhibited broader gene deregulation with a mesenchymal‐like invasive phenotype, whereas EP^+^ GBMs showed a more proliferative profile marked by enhanced protein synthesis and mitochondrial activity, along with a relatively more inflamed immune microenvironment. Transcription factor analysis identified BRD4 as a key regulator of the mesenchymal program in EP^‐^ GBMs.

**Conclusion:** These results highlight the peritumoral microenvironment's role in shaping GBM phenotype and demonstrate the value of spatial transcriptomics in linking tumor biology with epilepsy.

**Disclosure:** Nothing to disclose.

## EPO‐0884

### A new diagnostic and therapeutic care pathway for early palliative care integration in patients with glioblastoma: A single centre experience

#### L. Panicari ^1^; B. Muoio^2^; G. Pesce^3^; V. Espeli^2^; A. Cassarino^3^; E. Brumana^1^; M. Zanon^2^; T. Fusi‐Schmidhauser^1^

##### ^1^Palliative and Supportive Care Clinic, Ente Ospedaliero Cantonale, Bellinzona, Switzerland; ^2^Oncology Service, Oncology Institute of Southern Switzerland, Bellinzona, Switzerland; ^3^Radiation Oncology Service, Oncology Institute of Southern Switzerland, Bellinzona, Switzerland

**Background and aims:** Glioblastoma is the most aggressive primary brain tumour in adults characterised by major clinical challenge due to multidimensional symptoms, rapid progression and poor prognosis. Early integration of palliative care (PC) from diagnosis can improve quality of life, symptom control and psychosocial well‐being. We developed and implemented an integrated care pathway for early PC in patients with glioblastoma.

**Methods:** The diagnostic and therapeutic care pathway (PDTA) was designed between February 15th 2024 and December 13th 2024 in a tertiary oncological institute involving oncologists, radiotherapists, PC specialists, nurses, and quality department. Implementation began on January 1st 2025 as standard care. Demographic and clinical data were collected from patient charts and analysed descriptively.

**Results:** The PDTA allows systematic identification of PC needs in patients with glioblastoma hospitalised for diagnosis or surgery. A neuro‐oncology nurse specialist assesses PC needs and arranges either an in‐hospital PC consultation or an outpatient PC visit with oncologist/radiotherapist within four to seven weeks after diagnosis. Between January 1st to December 31st 2025, 24 patients underwent in‐hospital or or outpatient PC consultations. The PDTA significantly shortened the time to the first PC consultation—from 80 days in 2024 to 37 days in 2025. Main clinical issues addressed included symptom management, advance care planning, and respite care.

**Conclusion:** Integrating early PC as a standard of care for glioblastoma enables systematic needs assessment, enhances quality of life, and fosters communication on treatment goals and preferred care settings. These findings encourage broader adoption of coordinated and patient‐centred models to optimize outcomes.

**Disclosure:** Nothing to disclose.

## EPO‐0885

### Machine learning to predict distant recurrence spread in Glioblastoma: A radiomic signature from standard T1‐CE tumor core and peritumoral margins

#### L. Conte ^1^; E. Lo Turco^2^; R. Abbritti^3^; C. Accettura^4^; G. Raso^1^; E. Iaboni^5^; U. De Giorgi^4^; G. De Nunzio^6^; D. Cascio^1^; M. Caffo^5^

##### ^1^Department of Physics and Chemistry, University of Palermo, Palermo, Italy; ^2^Azienda Ospedaliero Universitaria “R. Dulbecco”, Catanzaro, Italy; ^3^Service de Neurochirurgie Hôpital “Lariboisière”, Paris, France; ^4^University Oncology Unit, University of Salento, Lecce, Italy; ^5^Unit of Neurosurgery, Department of Biomedical and Dental Sciences and Morphofunctional Imaging, University of Messina, Messina, Italy; ^6^Department of Mathematics and Physics, University of Salento, Lecce, Italy

**Background and aims:** Predicting tumor spread in Glioblastoma (GB) is essential for prognosis and guiding treatment decisions. The clinical challenge lies in distinguishing whether recurrence will remain confined to the original tumor bed (local) or manifest as distant spread. While perfusion MRI is often used for this purpose, it requires complex protocols. This study evaluated whether Radiomic features derived solely from standard contrast‐enhanced T1‐weighted (T1‐CE) images could effectively predict this dissemination pattern.

**Methods:** We analyzed 109 patients with confirmed recurrence. Tumor was manually segmented by two experienced radiologists. Peritumoral margins were generated via a 5mm isotropic dilation of the core mask. Radiomic features were extracted separately from both regions using PyRadiomics. The pipeline included *Z*‐score normalization and Mutual Information feature selection. Eight classifiers were benchmarked: Multi‐Layer Perceptron, Support Vector Machine, XGBoost, K‐Nearest Neighbors, Naive Bayes, Logistic Regression, Random Forest, and Linear Discriminant Analysis. Class weighting was applied where applicable, and all models were validated via Leave‐One‐Out Cross‐Validation (LOOCV).

**Results:** The MLP model achieved the highest performance (AUC 0.80, accuracy 82.6%, Figure 1). Feature selection analysis revealed a robust "Radiomic signature" of 8 features consistently selected across different classifiers. Notably, 3 of these predictors were derived from the geometric dilated peritumoral margins.

**Figure:**
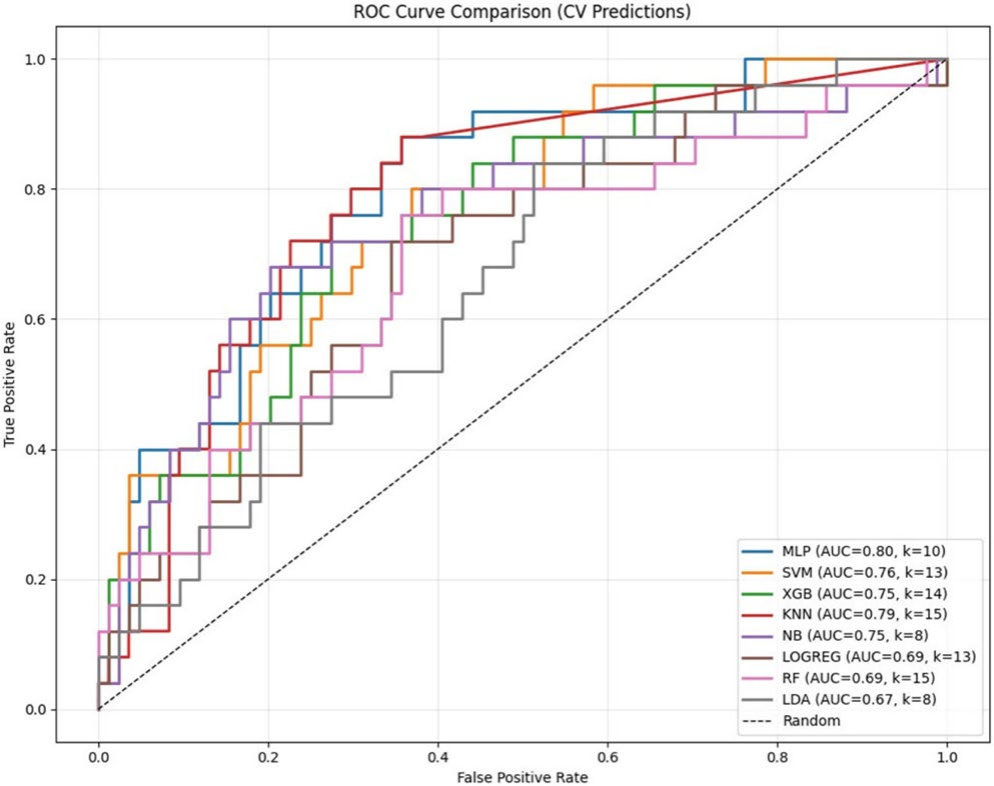
**FIGURE 1** Receiver operating characteristic (ROC) curve comparison. The plot displays the performance of the eight machine learning classifiers benchmarked for distinguishing local from distant Glioblastoma recurrence.

**Conclusion:** This analysis demonstrates that T1‐CE Radiomics provides a robust method for predicting GB recurrence topography. The significant contribution of marginal features confirms that infiltration signals are detectable in structural images. While these preliminary findings warrant prospective validation, this approach transforms widely available standard baseline imaging into robust non‐invasive prognostic biomarkers.

**Disclosure:** Nothing to disclose.

## EPO‐0886

### Neurological immune related adverse events of checkpoint inhibitors: A prospective study

#### M. Havlíček ^1^; D. Krýsl^1^; H. Boček^1^; M. Elišák^1^; L. Casas Mendez^2^; A. Bulová^3^; T. Buchler^3^; P. Marusič^1^

##### ^1^Department of Neurology, Second Faculty of Medicine, Charles University, Motol and Homolka University Hospital; ^2^Department of Pneumology, Second Faculty of Medicine, Charles University, Motol and Homolka University Hospital; ^3^Department of Oncology, Second Faculty of Medicine, Charles University, Motol and Homolka University Hospital

**Background and aims:** Neurological immune related adverse events (nirAEs) of checkpoint inhibitors (ICIs) are increasingly recognized in clinical practice. Retrospective studies may underreport nirAEs. The aim of this study was to prospectively determine the incidence of nirAEs, including grade 1 events.

**Methods:** Electronic PRO‐CTCAE based questionnaire was administered to immunotherapy‐naïve ICIs treatment candidates at baseline and at 3, 6 and 12 months to assess neurological and psychiatric symptoms including: insomnia, fatigue, symptoms of depression, poor concentration, memory problems, dizziness, problems with vision, swallowing, urination, numbness/weakness/clumsiness in extremities, nausea, vomiting, headache, muscle pain, joint pain. Reported symptoms were grouped into putative clinical syndromes and analyzed in relation to other clinical characteristics (age, sex, type of tumor and treatment).

**Results:** Seventy‐nine of 89 patients responded at baseline. Fifty‐nine patients responded to at least one follow‐up questionnaire (age median 66 [36–79], twenty‐three [39.0%] females). The most common tumor was non‐small cell lung carcinoma (22; 37.3%). Nineteen (32.2%) patients reported new or worsened symptoms indicative of polyneuropathy (10), myositis/myopathy (5) and/or encephalopathy (4). 7/10 patients with polyneuropathy symptoms had prior or ongoing chemotherapy. Thus, possible nirAEs occurred in 12/59 (20.3%) patients. Age, gender, tumor type, treatment (PD1/PDL1 ICIs alone or in combination with CTLA4 ICIs) and a history of autoimmune disease were not found to be statistically significant risk factors.

**Conclusion:** The incidence of adverse reactions, especially those of a lower grade, may be underestimated. Dedicated questionnaires can improve the detection of these events.

**Disclosure:** This work was supported by the Charles University project GA UK No 171324. PRO‐CTCAE questionnaire (https://healthcaredelivery.cancer.gov/pro‐ctcae/) was used in this study.

## EPO‐0887

### Neurological vulnerability to ICANS in CAR T‐cell therapy: Impact of comorbidity and EEG–cognitive monitoring

#### P. Dumez; H. Marquez; P. Devic; C. Seurret; S. Souci; B. Joubert

##### Neurology Unit, University Hospital of Lyon Sud, Hospices Civils de Lyon, France

**Background and aims:** Immune effector cell–associated neurotoxicity syndrome (ICANS) frequently complicates chimeric antigen receptor (CAR) T‐cell therapy, yet neurological vulnerability factors remain poorly defined. This study aimed to identify neurological predictors of ICANS and assess combined EEG–MoCA monitoring.

**Methods:** We conducted a retrospective monocentric study (Hospices Civils de Lyon), including adults treated with CAR T‐cells for B‐cell lymphoma between 2019 and 2024. Neurological comorbidity was defined as any pre‐existing central nervous system disorder, including lymphoma involvement, cerebrovascular, neuroinflammatory, traumatic, neoplastic, or neurodegenerative disorders, or prior seizure history. EEG and MoCA were obtained at baseline and day 7. ICANS risk factors were assessed using logistic regression, and longitudinal MoCA changes using linear mixed‐effects models.

**Results:** Among 356 patients, 152 (43%) developed ICANS, including 29 (18%) with severe forms. Neurological comorbidity was independently associated with ICANS (adjusted odds ratio [aOR] 2.07, 95% confidence interval [CI] 1.22–3.55; *p* = 0.007), along with older age (per 10‐year increase: aOR 1.42, 95% CI 1.19–1.70; *p* < 0.001) and treatment with Yescarta versus other CAR T‐cell products (aOR 2.44, 95% CI 1.53–3.94; *p* < 0.001). EEG abnormalities during days 4–10 affected up to 68% of patients with ICANS. A MoCA decline of at least 2 points at day 7 was more frequent in ICANS (31.2% vs 12.8%; *p* < 0.001), with greater longitudinal decline (beta −0.09 points per day; *p* = 0.038).

**Figure:**
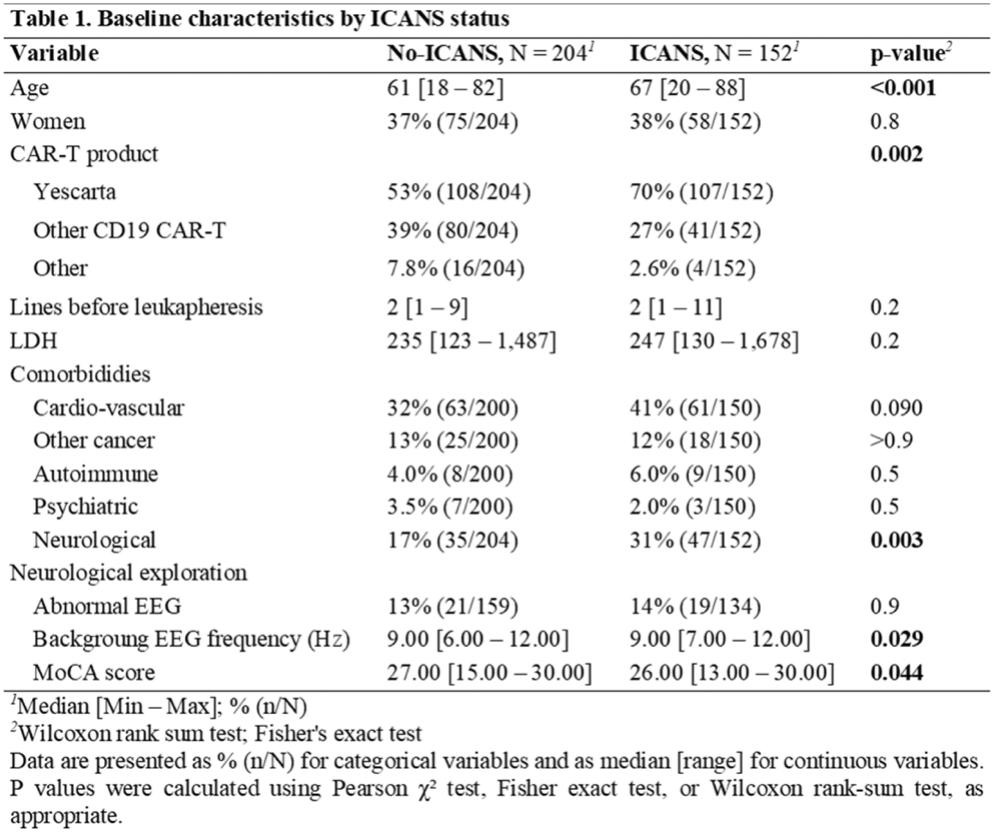
**TABLE 1** Baseline characteristics according to immune effector cell–associated neurotoxicity syndrome status.

**Figure:**
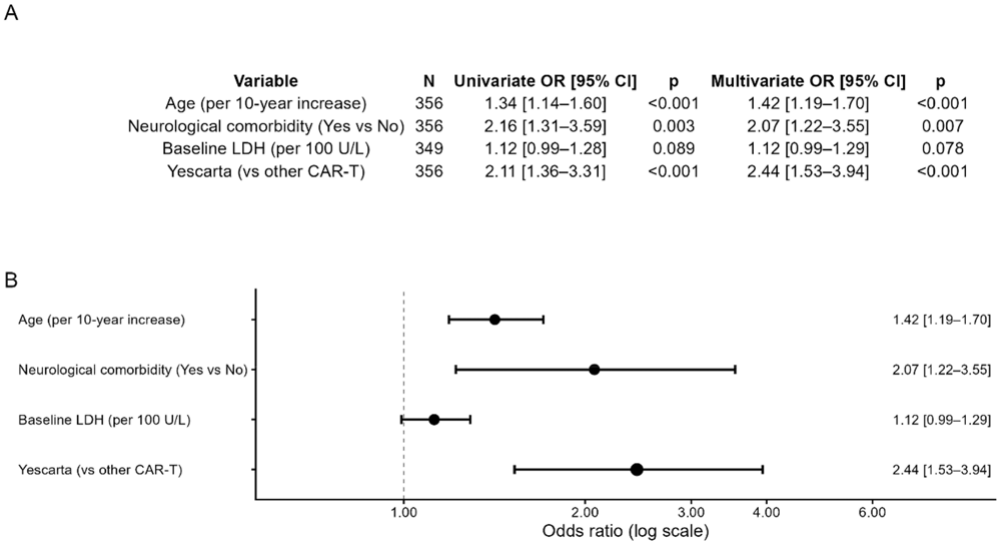
**FIGURE 1** Neurological predictors of immune effector cell–associated neurotoxicity syndrome (ICANS). (A) Univariable and multivariable logistic regression analyses. (B) Forest plot of adjusted odds ratios with 95% confidence intervals.

**Figure:**
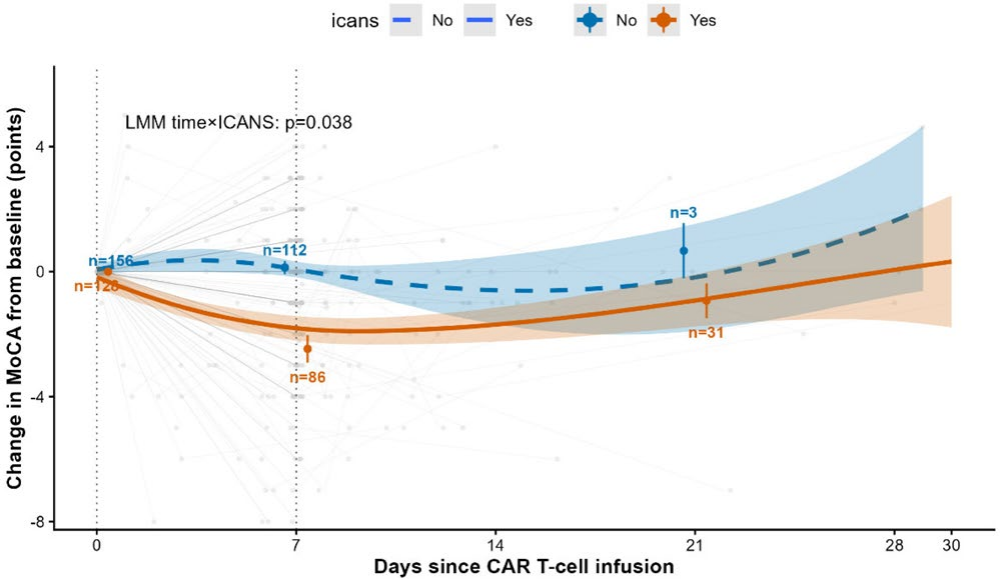
**FIGURE 2** Change in Montreal Cognitive Assessment score after CAR T‐cell infusion according to ICANS status. Individual trajectories, smoothed group trends with 95% confidence intervals, and group means at days 0, 7, and 21 are shown.

**Conclusion:** Pre‐existing neurological comorbidities are independently associated with an increased risk of ICANS. Combined EEG and MoCA monitoring provides a pragmatic framework to characterize neurological vulnerability and follow ICANS‐related cognitive trajectories.

**Disclosure:** Nothing to disclose.

## EPO‐0888

### Peripheral nerve toxicity of immune checkpoint inhibitors and CAR‐T therapy: A multicentric cohort study

#### V. Guerra‐Fernandez ^1^; E. Fonseca^1^; I. Martin^1^; M. Alaña^2^; G. Lafuente^3^; L. del Pino^3^; G. Velilla^4^; D. Perez‐Rancangel^4^; S. Garcia‐Bellido Ruiz^4^; A. Llaurado^5^; H. Ariño^5^; E. Muñoz^6^; A. Ramos‐Fransi^1^; J. Padrosa^7^; N. Martinez^8^; L. Naranjo^9^; R. Ruiz^9^; Y. Blanco^1^; J. Cabrera‐Maqueda^1^; E. Martinez‐Hernandez^1^

##### ^1^Neuroimmunology and Multiple Sclerosis Unit, Neurology Department, Hospital Clinic Barcelona, Fundació de Recerca Clínic Barcelona IDIBAPS, Barcelona, Spain; ^2^Neurology Department, Hospital Clinico Universitario de Salamanca, Salamanca, Spain; ^3^Neurology Department, Hospital General Universitario Gregorio Marañon, Madrid, Spain; ^4^Neurology Department, Hospital Universitario 12 de Octubre, Madrid, Spain; ^5^Neurology Department and Centre d’Esclerosi Múltiple de Catalunya (Cemcat), Hospital Universitari de Vall d’Hebron, Barcelona, Spain; ^6^Neurology Department, Hospital Clinico Universitario Lozano Blesa, Zaragoza, Spain; ^7^Medical Oncology Department, Hospital Clinic Barcelona, Fundació de Recerca Clínic Barcelona IDIBAPS, Barcelona, Spain; ^8^Hematology Department, Hospital Clinic Barcelona, Fundació de Recerca Clínic Barcelona IDIBAPS, Barcelona, Spain; ^9^Immunology Department, Hospital Clinic Barcelona, Fundació de Recerca Clinic Barcelona IDIBAPS, Barcelona, Spain

**Background and aims:** Among neurological complications of cancer immunotherapy peripheral neuropathies are recognized immune‐related adverse events (NP‐irAE) of checkpoint inhibitors (ICI), whereas CART‐related neuropathies (NP‐CART) are rarely reported. We aim to describe clinical features, treatment, and outcomes of this complication.

**Methods:** Retrospective multicenter study of NP‐irAE and NP‐CART across 20 Spanish hospitals (2018–2025). Neuropathies were classified as acute (<4 weeks), subacute (4–8w) or chronic (>8w). We assessed severity with INCAT scale and outcomes at one month and last follow‐up (good outcome: complete recovery or ≥2‐point mRS decrease).

**Results:** NP‐irAE occurred in 13/144 (9%) neurological irAE; 3 male (23%), median age 72 years (IQR: 68–74). Median onset was 44 days (24–113) post‐ICI initiation, median INCAT 0+3.5. Twelve (92%) had polyradiculopathy, and 1 (8%) sensory neuronopathy. Three (23%) had CNS involvement with high‐risk antibodies (2 anti‐Hu, 1 anti‐Ma2). Twelve (92%) received steroids and 8 (62%) IVIg. At 1 month, 11 (85%) improved; at last follow‐up (median 33 months, 15–54), 7 (54%) had good outcomes. Among 600 anti‐CD19 CAR‐T recipients, 4 (0.6%) developed NP‐CART; 3 male (75%), median age 54.5 years (45–57). One patient (25%) had prior severe ICANS. Median onset was 26 days (16–35) post‐infusion. Three (75%) had polyradiculopathy with median INCAT 2+5; 1 (25%) cranial neuropathy. Two received steroids and 2 IVIg. All improved at 1 month; at last follow‐up (median 40 months, 9–86), 2 (50%) had good outcomes.

**Conclusion:** NP‐CART occurred earlier than NP‐NiRAE. Although both are severe, high therapy responsiveness highlights the importance of prompt recognition and treatment.

**Disclosure:** Nothing to disclose.

## Movement Disorders 11

## EPO‐0889

### Characteristics of the intestinal microbiome in Parkinson's disease patients

#### N. Mansurova ^1^; D. Rakhmatova^2^; S. Akhrorova^2^

##### ^1^Tashkent State Medical University Tashkent, Uzbekistan; ^2^Bukhara State Medical Institute, Bukhara, Uzbekistan

**Background and aims:** Growing evidence implicates the gut–brain axis and gut microbiome dysbiosis in PD pathogenesis via immune mediators, the enteric nervous system and the vagus nerve, leading to microglial activation and chronic inflammation that may promote α‐synuclein aggregation.

**Methods:** The cohort included 73 PD patients followed at a municipal outpatient clinic in Tashkent. Gastrointestinal autonomic dysfunction was quantified using the gastrointestinal domain of SCOPA‐AUT, with a working severity grading (0–3 minimal; 4–7 mild; 8–12 moderate; ≥13 severe).Gut microbiota composition was analyzed using Osipov's gas chromatography–mass spectrometry (GC–MS) method, based on detection of microbial markers (specific fatty acids and related compounds) without culture.

**Results:** Signs of dysbiosis were identified in 90% of patients. Total bacterial load ranged from 16,848 to 38,784 units (mean 27,488 ± 8,012), exceeding reference values for healthy individuals; 44.4% showed hypercolonization (>35,000), potentially linked to slowed motility and constipation. The “microbiota core” (Firmicutes + Actinobacteria) varied between 28–48% (mean 39.2 ± 6.9%), and was reduced in 20% of patients, indicating depletion of obligate anaerobes. Fungal flora (mainly Candida) exceeded the accepted threshold in 55.5%. The viral component averaged 7.0 ± 2.3% (reference ~6%), with elevated levels in ~20% of patients, accompanied by higher endotoxin and a more pronounced anaerobic deficit; a positive correlation between viral load and Clostridium spp. was reported (*r* ≈ 0.58).

**Conclusion:** Overall, the findings indicate a persistent dysbiotic profile in PD, with depletion of obligate flora and increased opportunistic and viral components, potentially contributing to chronic inflammation and disease progression.

**Disclosure:** Nothing to disclose.

## EPO‐0890

### Inflammatory mechanisms in Parkinson's disease and vascular Parkinsonism: IL‐6, fecal calprotectin, and *Helicobacter pylori*

#### N. Mansurova ^1^; Y. Madjidova^2^; D. Khodjieva^3^; D. Rakhmatova^3^

##### ^1^Central Asian University*,* Tashkent, Uzbekistan; ^2^Tashkent State Medical University, Tashkent, Uzbekistan; ^3^Bukhara State Medical Institute, Uzbekistan

**Background and aims:** This study aimed to evaluate local and systemic inflammatory markers (interleukin‐6, fecal calprotectin) and Helicobacter pylori infection in patients with Parkinson's disease (PD) and vascular parkinsonism, and to determine their clinical and laboratory associations.

**Methods:** A total of 52 patients were consecutively enrolled (32 with PD and 20 with vascular parkinsonism) and followed in an outpatient setting in Tashkent during 2020–2025. Clinical and demographic parameters were recorded; disease stage was assessed using the Hoehn and Yahr scale, and motor severity was evaluated with UPDRS. Fecal calprotectin was measured by ELISA, serum IL‐6 by ELISA, and *H. pylori* infection by the urease breath test. Statistical analysis included parametric and non‐parametric comparisons, chi‐square/Fisher's exact tests, and Spearman correlations, with *p* < 0.05 considered significant.

**Results:** Elevated fecal calprotectin was detected in 34.4% of PD patients versus 9.3% in vascular parkinsonism (*p* < 0.05). Calprotectin levels correlated with Hoehn and Yahr stage (*r* = 0.42; *p* < 0.05) and UPDRS III (*r* = 0.38; *p* < 0.05), showing a tendency toward higher values at later disease stages. Increased IL‐6 was more frequent in PD (85%; mean 10.28 ± 4.30 pg/mL) than in vascular parkinsonism (59.4%; 8.13 ± 3.13 pg/mL; *p* < 0.05). In addition, *H. pylori* prevalence was higher in PD (37.5% vs 8.3%; *p* < 0.05) and was associated with longer disease duration and greater motor impairment. Positive correlations were also observed between *H. pylori* parameters and both calprotectin and IL‐6.

**Conclusion:** The findings support the contribution of intestinal and systemic inflammation to parkinsonism mechanisms and suggest that these markers may be useful for differential diagnosis, risk stratification, and more personalized clinical.

**Disclosure:** Nothing to disclose.

## EPO‐0891

### Rapidly progressive posterior cortical syndrome with alien limb: Diagnostic clues to sporadic Creutzfeldt–Jakob disease

#### C. Santoro; G. Guglielmini; N. Pilolli; G. Boero

##### Complex Structure of Neurology, SS Annunziata Hospital, Taranto, Italy

**Background and aims:** Corticobasal syndrome (CBS) is classically attributed to corticobasal degeneration (CBD), yet sporadic Creutzfeldt–Jakob disease (sCJD) can present with a CBS phenotype, often driven by posterior network dysfunction.

**Methods:** We reviewed the “CJD‐CBS/alien limb” literature to extract specific discriminators—particularly patterns of cognitive involvement and structural involvement that can meaningfully shift pre‐test probability early.

**Results:** Posterior alien limb phenomenon with non‐dominant parietal dysfunction and visual‐parietal signs (optic ataxia/neglect) supported a reproducible clinico‐anatomical motif in CJD‐CBS. While most CJD‐CBS reports include striatal/basal ganglia abnormalities, a small subset shows complete structural and metabolic sparing of basal ganglia with dominant posterior cortical DWI changes and posterior hypometabolism on FDG‐PET. This “striatum‐sparing, posterior‐cortical” phenotype is unusual for typical sCJD and can misdirect clinicians toward CBD unless specifically recognized. Contrary to the heuristic that early dementia is obligatory in CJD, several CJD‐CBS cases show minimal cognitive impairment at onset, with later emergence of visuospatial deficits consistent with posterior network primacy and helping explain early diagnostic anchoring on CBD. A parallel biomarker–imaging dissociation with markedly elevated total tau and normal p‐tau plus RT‐QuIC positivity supports prion disease even when amyloid markers are reduced, as low Aβ42 in CJD may not imply concomitant Alzheimer pathology.

**Conclusion:** The combination of posterior ALP + visual‐parietal signs should trigger a topographic differential: assess posterior cortical DWI ribboning, actively interrogate basal ganglia involvement (or sparing) on MRI/FDG‐PET, and integrate RT‐QuIC with tau patterns—especially when cognition is initially preserved. Recognizing the “posterior, striatum‐sparing CJD‐CBS” subgroup may prevent systematic misclassification as CBD and accelerate appropriate prion‐focused work‐up.

**Disclosure:** Nothing to disclose.

## EPO‐0892

### Essential tremor five years follow‐up study of MRgFUS in a real‐world setting

#### C. Ferrer ^1^; A. Sanchez Fraga^1^; R. Martuscello^2^; L. Knight^2^; I. Pyle^2^; K. Gant^2^; A. Grinspan^2^

##### ^1^Insightec Europe GmbH, Munich, Germany; ^2^Insightec, Inc, Miami, USA

**Background and aims:** This study evaluated the long‐term outcomes of magnetic resonance‐guided focused ultrasound (MRgFUS) thalamotomy in patients with medication‐refractory essential tremor (ET). ET is a common neurological condition. Unilateral and staged‐bilateral (≥ 9 months apart) MRgFUS thalamotomy are CE‐approved. This global, open‐label FDA Mandated extension study enrolled participants after completion of the pivotal trial as the first cohort in a real‐world setting. It further investigates long‐term follow‐up outcomes after MRgFUS thalamotomy.

**Methods:** Treatment effectiveness was assessed by changes in tremor severity and motor function using the Clinical Rating Scale for Tremor (CRST) and quality of life with the Quality of Life in Essential Tremor (QUEST) questionnaire. Safety was monitored through documentation of adverse events (AEs).

**Results:** Among 61 subjects (mean age 69.5 ± 14.0 years; 67.2% male), 26 (42.6%) completed 5‐year follow‐up (Figure 1). MRgFUS thalamotomy produced sustained improvements in tremor, motor function, and disability. From baseline to 1 and 5 years, percentage improvements were: CRST A+B 62.2% and 51.9%, tremor severity 75.6% and 67.4%, and CRST‐C 65.4% and 35.4%, respectively. Quality‐of‐life scores improved 53.6% at 1 year and 43.7% at 5 years (Figure 2). Most treatment‐related AEs were mild (85%) or moderate (12%), 3% were severe; over half resolved within six months (Table 1).

**Figure:**
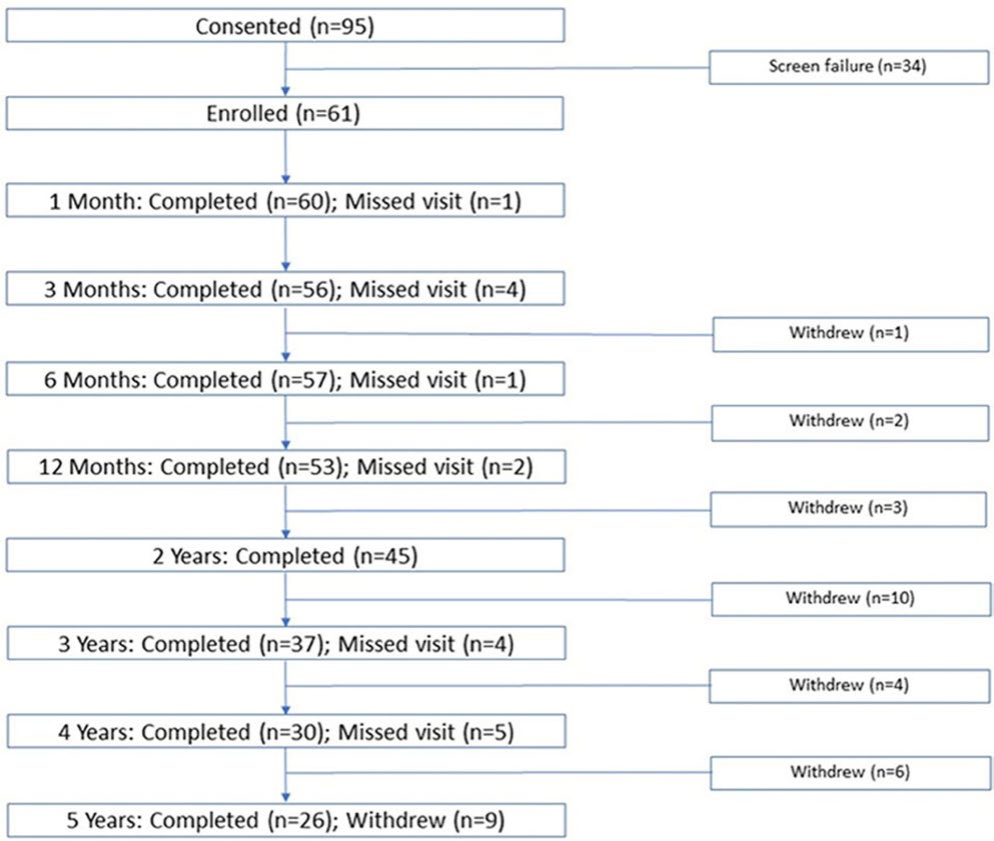
**FIGURE 1** Patient disposition.

**Figure:**
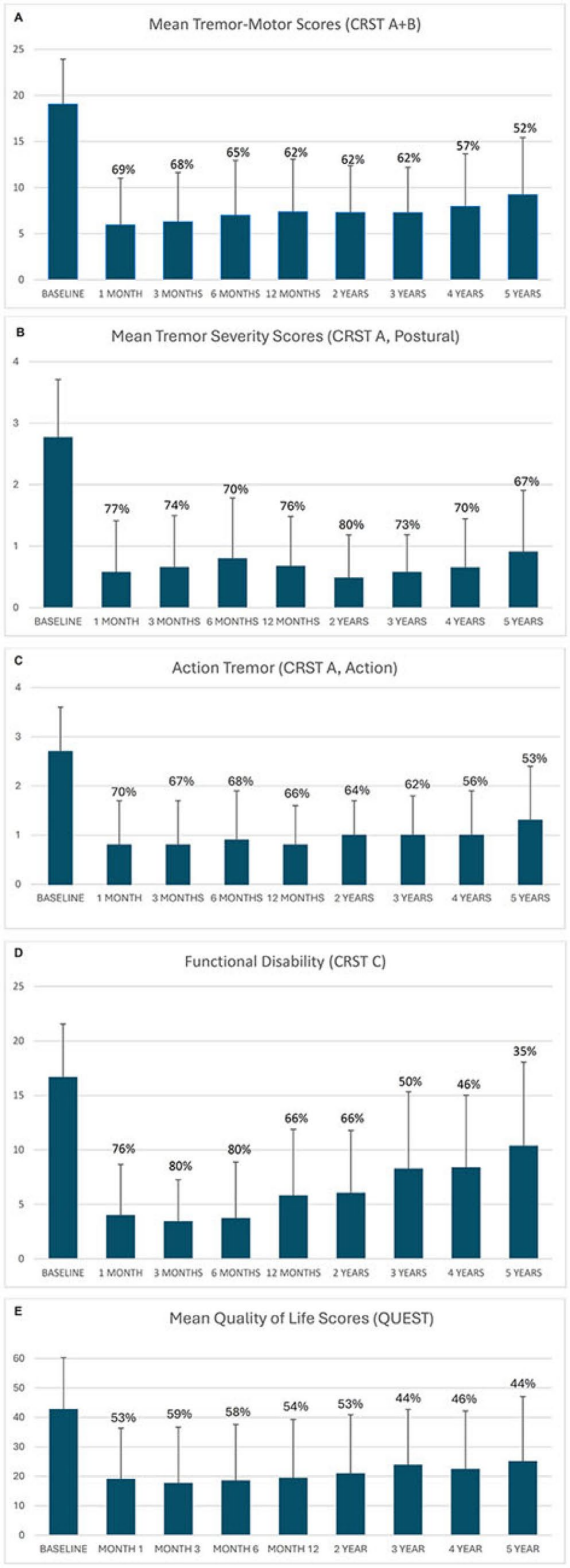
**FIGURE 2** CRST scores and QUEST.

**Figure:**
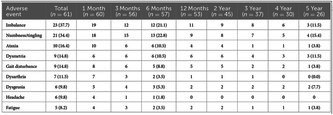
**TABLE 1** Number of subjects reporting adverse events.

**Conclusion:** These findings complement the original pivotal data outcome supporting long‐term safety and effectiveness of MRgFUS thalamotomy in 1st side medication‐refractory ET patients. Further studies should assess 2nd‐side treatment requirement and long‐term outcomes. Meanwhile, MRgFUS thalamotomy offers a safe, durable, and meaningful option for improving quality of life in ET.

**Disclosure:** All authors are Insightec employees.

## EPO‐0893

### Homer 3 antibodies: Emerging biomarkers in cerebellar dysfunction

#### E. Karatzioula; V. Poulidou; S. Kalampokini; V. Kimiskidis; M. Arnaoutoglou

##### 1st Department of Neurology, AHEPA University Hospital, Aristotle University of Thessaloniki, Thessaloniki, Greece

**Background and aims:** Homer‐3 is a postsynaptic scaffolding protein expressed in Purkinje cells and plays a crucial role in calcium signaling mediated by metabotropic glutamate receptors. Cerebellitis associated with anti‐Homer‐3 antibodies is rare. Most reported patients present with vertigo and gait disturbance, while neurological examination almost invariably reveals ataxia and nystagmus. Cerebrospinal fluid (CSF) analysis typically shows pleocytosis and type 2 oligoclonal bands, whereas brain MRI findings are usually non‐specific. Anti‐Homer antibodies may be detected in serum or CSF. Various immunotherapeutic strategies have been used, including corticosteroids, intravenous immunoglobulin (IVIG), plasmapheresis, mycophenolate mofetil, and rituximab, with variable clinical responses

**Methods:** A 51‐year‐old woman presented with subacute cerebellar ataxia, choreiform movements, and inability to walk, since five months. Corticosteroid therapy worsened choreiform movements. Neurological examination revealed gaze‐evoked nystagmus, dysmetria, choreiform movements in all four limbs, impaired deep sensation, and inability to stand unassisted. Deep tendon reflexes were absent. CSF analysis showed a normal cell count and type 1 oligoclonal bands. Brain MRI and EEG were unremarkable. Extensive immunological, virological, and screening for malignancy, revealed no pathological findings. Serum testing was positive for anti‐Homer‐3 antibodies, while CSF was negative.

**Results:** Treatment with IVIG resulted in significant clinical improvement, allowing ambulation with bilateral support.

**Conclusion:** Anti–Homer‐3 cerebellitis should be considered in the differential diagnosis of subacute‐onset ataxia, as early identification and timely immunotherapy may improve outcome if initiated before irreversible neuronal damage occurs.

**Disclosure:** Nothing to disclose.

## EPO‐0894

### Phase angle as a robust indicator of clinical severity in patients with Huntington's disease

#### G. Foresti ^1^; A. Rizzardi^1^; C. Agosti^2^; N. Agostini^1^; N. Martinazzi^1^; A. Pilotto^1^; A. Padovani^1^

##### ^1^Department of Clinical and Experimental Sciences, Neurology Unit, University of Brescia, Italy; ^2^Neurology Unit, Department of continuity of care and frailty, ASST Spedali Civili Brescia Hospital, Italy

**Background and aims:** Huntington's disease (HD) is a hereditary neurodegenerative disorder involving progressive motor, cognitive, and neuropsychiatric impairment, alongside systemic alterations in body composition. Bioelectrical impedance analysis (BIA)–derived parameters, specifically phase angle (PA), are potential markers of cellular integrity but remain underexplored in HD.

**Methods:** We enrolled 23 patients with genetically confirmed HD and 25 age‐ and sex‐matched healthy controls (HC). Body composition and hydration were assessed via BIA. Within the HD group, we analysed clinical severity (UHDRS motor score ≤25 vs >25) and genetic burden (CAG repeats <42 vs ≥42). Multivariable regression models adjusted for age, sex, and height were used to control for anthropometric confounding. Partial correlations controlling for age were also performed.

**Results:** Compared to HC, HD patients showed significantly lower PA (*p* = 0.008) and body cell mass index (BCMI) (*p* = 0.013), with higher extracellular and lower intracellular water percentages (*p* < 0.02). Within the HD cohort, higher UHDRS scores were independently associated with lower PA (*p* < 0.03), while differences in muscle mass did not survive adjustment. Higher CAG repeats were associated with increased extracellular water volume (*p* < 0.02) but not with PA. In multivariable models, the UHDRS score was the primary predictor of PA.

**Conclusion:** HD is associated with reduced cellular integrity and altered fluid distribution. Phase angle appears to be a robust, severity‐sensitive marker independent of body size and genetic load. BIA‐derived PA may represent a clinically meaningful biomarker of disease severity in HD, warranting further longitudinal investigation.

**Disclosure:** Nothing to disclose.

## EPO‐0895

### Eight‐year outcome of MRI focused ultrasound thalamotomy in essential tremor patients

#### I. Schlesinger ^1^; A. Sinai^2^; M. Nassar^1^; I. Senderova^1^; M. Katson^1^; S. Sobol^1^; I. Erikh^1^; L. Lev Tov^2^

##### ^1^Movement Disorders Institute, Rambam Health Care Campus, Haifa, Israel; ^2^Department of Neurosurgery, Rambam Health Care Campus, Haifa, Israel

**Background and aims:** MRI Focused Ultrasound Thalamotomy is a technology that enables non invasive intracranial focal thermal ablation. It has been effective in ameloriating tremor in medication resistant patients. The effect of treatment beyond 5 years has not been reported.

**Methods:** Six ET patients who completed 8 years follow‐up were included. Tremor was assessed with Clinical Rating Scale for Tremor (CRST) and in the treated hemibody (hemi‐CRST).

**Results:** Median age before MRgFUS was 74.5 years (range 67–78 years). Median disease duration of 15 years (range 4–30). Tremor was significantly improved immediately following MRgFUS in all patients. At 8‐year following MRgFUS, Median hemi‐CRST score was significantly improved as compared with baseline (baseline median score 23, range 16–32 vs. median score at 8 years 11.5, range 5–18, *p* = 0.001). At 8‐year following MRgFUS, Median total CRST score was improved as compared with baseline (baseline median score 58, range 42–71 vs. median score at 8 years 38.5, range 30–61) but this did not reach statistical significance. Adverse events following the procedure were mils and transient

**Conclusion:** This study found that improvement in tremor following unilateral MRgFUS thalamotomy in ET was sustained at 8 years with no long standing adverse effects.

**Disclosure:** Nothing to disclose.

## EPO‐0896

### Clinical features and brain structural correlates of blood lipid profile in idiopathic normal‐pressure hydrocephalus

#### J. Bissacco ^1^; R. Bovenzi^1^; M. Conti^1^; C. Simonetta^1^; D. Mascioli^1^; M. Mancini^1^; V. Buttarazzi^1^; F. Veltri^1^; V. Nesci^1^; S. Bagetta^1^; M. Pierantozzi^1^; A. Stefani^1^; F. Buttari^2^; D. Centonze^2^; T. Schirinzi^1^

##### ^1^Department of Systems Medicine, University of Rome “Tor Vergata”, Rome, Italy; ^2^Neurology Unit ‐ IRCCS Neuromed, Pozzilli (IS), Italy

**Background and aims:** Idiopathic normal pressure hydrocephalus (iNPH) has multifactorial pathogenesis. Blood lipids may contribute to the pathogenic cascade; however, their role in iNPH has not been assessed. Here, we outline the blood lipid profile, including index of metabolic dyshomeostasis, in a longitudinal iNPH cohort, investigating relationships with neuroimaging markers of glymphatic dysfunction and clinical outcomes.

**Methods:** We conducted a dual‐center retrospective‐longitudinal study involving 67 iNPH patients and 38 controls. At baseline we collected “iNPH Grading Scale”, “Modified Ranking Scale” (mRS), lipid profile and blood cell count, and APOE genotype. Enlarged perivascular spaces (EPVS) were quantified on baseline 3T‐MRI using Potter scale in centrum semiovale (CSO), basal ganglia, and midbrain dividing subjects with “high‐grade” (Potter scale > 1) from “low‐grade” EPVS (Potter scale ≤ 1). Depending on five‐year outcome, iNPH patients were grouped into “poor outcome” (PO; mRS ≥ 5) and “favorable outcome” (FO; mRS < 5). Biomarkers were compared with ANCOVA, correlations tested, and ROC analysis performed.

**Figure:**
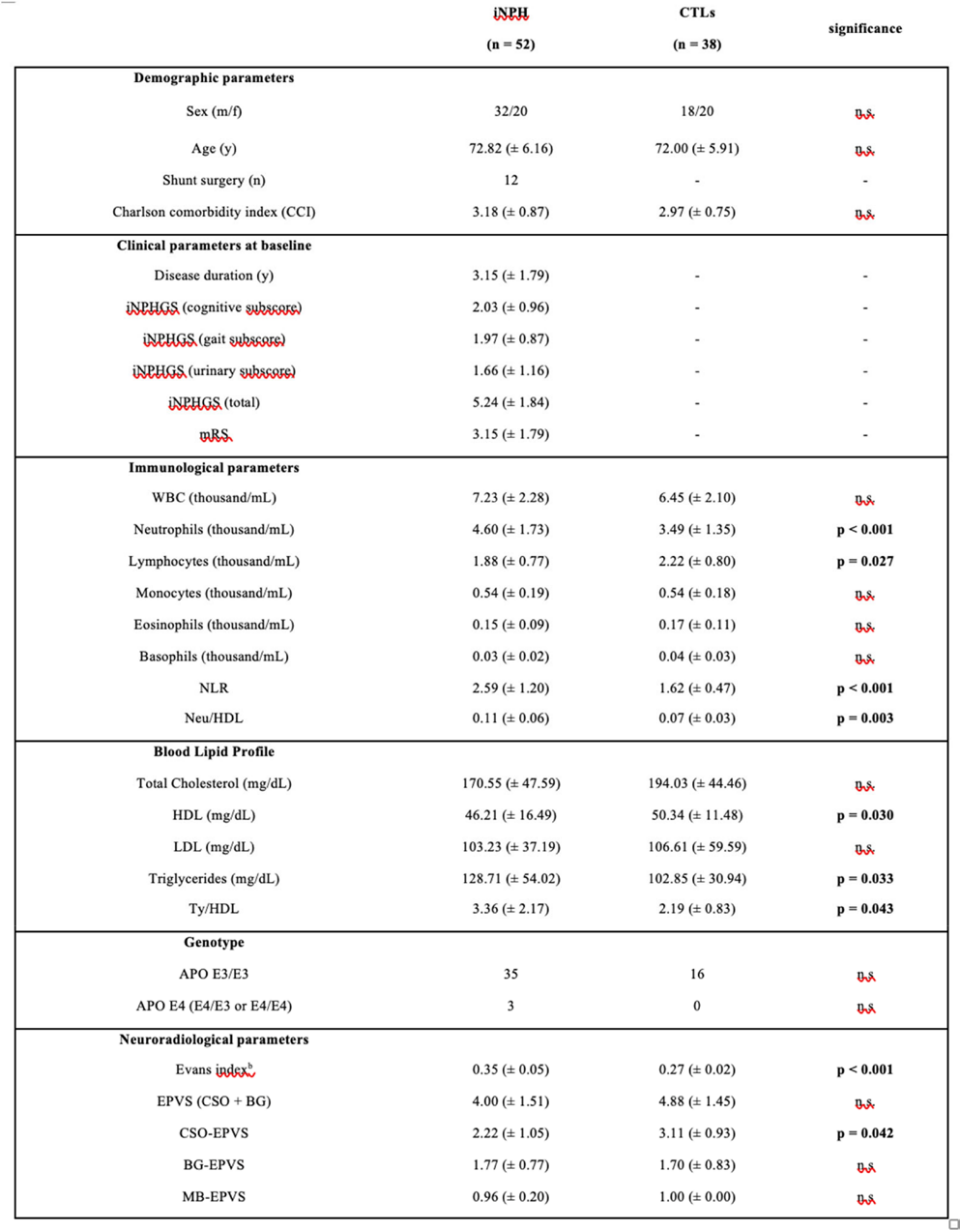
**TABLE 1** Demographic, clinical, biochemical and radiological features of iNPH and CTRLs.

**Results:** Compared with controls, iNPH patients showed higher triglycerides, triglyceride/high‐density lipoprotein (TG/HDL) and neutrophil/HDL ratios, and lower HDL. Within iNPH, patients with “high‐grade” CSO‐EPVS exhibited higher TG/HDL and neutrophil/HDL ratios than those with low‐grade. PO patients had higher TG and TG/HDL and lower HDL than FO patients. A TG/HDL cut‐off of 2.91 best predicted PO at 5 years.

**Figure:**
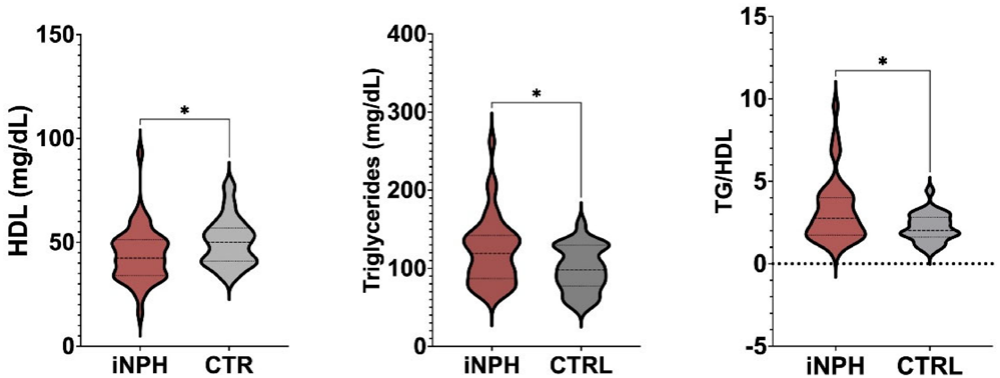
**FIGURE 1** Violin plots of HDL (mg/dL), triglycerides (mg/dL), and TG/HDL ratio in iNPH vs CTRL. Dashed lines show median and 25th–75th IQR. *p ≤ 0.05. Red violins: iNPH; grey violins: CTRL. Panels: (a) HDL, (b) TG, (c) TG/HDL. Data shown per group.

**Figure:**
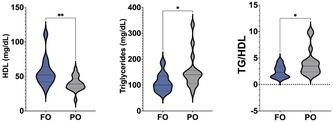
**FIGURE 2** Violin plots of HDL (mg/dL), triglycerides (mg/dL), and TG/HDL ratio in FO vs PO. Dashed lines show median and 25th–75th IQR. *p ≤ 0.05, **p ≤ 0.005. Blue violins: FO; grey violins: PO. Panels: (a) HDL, (b) TG, (c) TG/HDL.

**Conclusion:** iNPH patients exhibit a characteristic lipid profile within metabolic dysregulation and peripheral inflammation, tracked on a structural level by increased CSO‐EPVS, a marker of glymphatic impairment. TG/HDL index appears to predict long‐term clinical outcome, supporting its potential as prognostic marker in iNPH.

**Disclosure:** Nothing to disclose.

## EPO‐0897

### Deep stimulation in brain the Middle East: First UAE experience comparing subthalamic and pallidal targets for Parkinson's disease

#### S. Mittal; E. De Freitas; F. Roser; Y. Abdelmajid; M. Thomas; T. Maiti

##### Neurological Institute, Cleveland Clinic Abu Dhabi, United Arab Emirates

**Background and aims:** Deep brain stimulation (DBS) of the subthalamic nucleus (STN) and globus pallidus internus (GPi) is an established therapy for advanced Parkinson's disease (PD). However, comparative outcome data from the Middle East are limited, and it remains unclear whether target‐specific differences reported in randomized trials translate to regional real‐world practice. We report outcomes of STN and GPi DBS from a single tertiary center in the United Arab Emirates.

**Figure:**
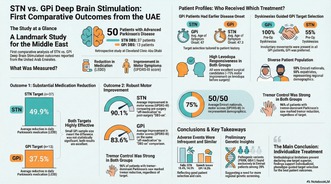
**FIGURE 1** STN vs. GPi deep brain stimulation: First comparative outcomes from the UAE.

**Methods:** We retrospectively reviewed 50 consecutive patients with PD who underwent bilateral STN or GPi DBS between 2020 and 2025 (37 STN, 13 GPi). Demographics, clinical phenotype, levodopa equivalent daily dose (LEDD), Unified Parkinson's Disease Rating Scale part III (UPDRS‐III) scores, and adverse events were collected. Outcomes were summarized by target, with exploratory comparisons acknowledging baseline heterogeneity and limited statistical power.

**Results:** GPi patients had earlier motor symptom onset and universal dyskinesias, while STN candidates demonstrated more heterogeneous phenotypes. Both targets were associated with substantial LEDD reduction (STN 49.9% ± 32.4; GPi 37.5% ± 22.0). Variability and unequal sample sizes limited definitive target comparisons. Mean OFF‐medication to DBS‐ON UPDRS‐III improvement exceeded 80% for both targets, likely influenced by high levodopa responsiveness, non‐blinded assessments, and variable postoperative timing.

**Conclusion:** In this first comparative DBS analysis from the UAE, both STN and GPi DBS produced meaningful medication reduction and robust motor improvement. Methodological limitations preclude conclusions on target superiority, supporting individualized, phenotype‐guided target selection and the need for larger prospective regional studies.

**Disclosure:** Nothing to disclose.

## EPO‐0898

### Spatial multi‐omics identifies early synaptic pruning and context‐specific dopaminergic vulnerability in Synucleinopathies

#### S. Rumpf^1^; F. Strübing^1^; K. Nalbach^1^; C. Vargi^2^; S. Lichtenthaler^1^; P. Parchi^2^; P. Gao^1^; W. Chen^1^; G. Berg^1^; M. Brendel^3^; J. Gnörich^3^; A. Bernhardt^1^; G. Höglinger^4^; J. Herms^1^; T. Koeglsperger ^1^

##### ^1^German Center for Neurodegenerative Diseases (DZNE), Munich, Germany Diseases; 81377 Munich, Germany; ^2^IRCCS Istituto delle Scienze Neurologiche, Ospedale Bellaria, Laboratorio di Neuropatologia; 40139 Bologna, Italy; ^3^Department of Nuclear Medicine, LMU University Hospital, LMU Munich, Munich, Germany; ^4^Department of Neurology, LMU University Hospital, Ludwig‐Maximilians‐Universität (LMU) München, Munich, Germany

**Background and aims:** The molecular events that precede overt neurodegeneration in Parkinson's disease (PD) remain poorly defined. Incidental Lewy body disease (iLBD), a prodromal state characterized by brainstem‐predominant Lewy pathology in the absence of clinical symptoms, provides a unique opportunity to investigate early pathogenic mechanisms underlying PD.

**Methods:** We applied a multi‐omic spatial profiling approach to post‐mortem midbrain tissue obtained from neurologically unaffected controls, iLBD cases, and patients with PD. This integrated strategy combined spatial transcriptomics, proteomics, and α‐synuclein seed amplification assays (SAA), along with proximity ligation assay (PLA), to assess molecular alterations and misfolded α‐synuclein across disease stages.

**Results:** We identified early and spatially distinct molecular changes in the substantia nigra pars compacta (SNpc) of iLBD cases, including a marked upregulation of the microglial synaptic pruning factor C1QC. These alterations occurred independently of overt Lewy pathology or detectable misfolded α‐synuclein, as determined by SAA and PLA, and were preferentially enriched at GABAergic synapses, indicating early vulnerability of inhibitory circuits. Notably, similar changes were absent in Alzheimer's disease cases with comorbid SNpc Lewy pathology, suggesting that α‐synuclein aggregation alone is insufficient to drive SNpc remodeling.

**Conclusion:** Our findings implicate microglia‐mediated synaptic dysfunction as an early, preclinical feature of PD and suggest that targeting microglial–synaptic interactions may represent a therapeutic strategy to halt disease progression prior to the onset of neurodegeneration.

**Disclosure:** Nothing to disclose.

## EPO‐0899

### Gastrointestinal autonomic dysfunction defines an axial–functional vulnerability phenotype in drug‐naive Parkinson's disease

#### Y. Hwang ^1^; M. Kim^2^; J. Park^3^; J. Seok^4^

##### ^1^Jeonbuk National Medical School and Hospital, Jeonju, Korea; ^2^Bobath Memorial Hospital, Seongnam, Republic of Korea; ^3^Director, Parkinson Neuropain Clinic, Neurologist and Movement Disorder Specialist, Cheonan, Republic of Korea; ^4^Department of Neurology, Ewha Womans University Seoul Hospital, Seoul, Republic of Korea

**Background and aims:** Autonomic dysfunction is common in Parkinson's disease (PD) from its prodromal stage, yet its heterogeneity and relationship with clinical features remain incompletely understood.

**Methods:** We analyzed a two‐center cohort of 88 newly diagnosed, drug‐naive PD patients who were assessed using the SCOPA‐AUT (The scales for autonomic dysfunction) questionnaire. Motor‐related ADL and severity were evaluated using UPDRS parts II and III, with predefined subscores for bradykinesia, rigidity, tremor, and postural instability/gait difficulty (PIGD). Autonomic burdens, especially gastrointestinal (GI) dysautonomia severity, were examined using a median split to define mild and moderate to severe subgroups, and the comparisons between the groups were performed.

**Results:** SCOPA‐AUT scores showed non‐normal, right‐skewed distributions, indicating substantial heterogeneity in autonomic burden. Among autonomic domains, GI symptoms showed the strongest correlation with activities of daily living (UPDRS II; *r* = 0.64, *p* < 0.001), exceeding other autonomic domains. GI dysautonomia correlated with overall motor severity (*r* = 0.38, *p* < 0.001), bradykinesia (*r* = 0.31, *p* = 0.003), and PIGD score (*r* = 0.28, *p* = 0.008), but not with tremor severity. Compared with GI‐mild patients, the GI‐moderate to severe subgroup demonstrated significantly worse UPDRS II and III scores, higher PIGD burden, lower MoCA‐K scores, and a higher proportion of PIGD motor subtype, without increased motor asymmetry. In multivariable analysis, functional disability (UPDRS II) and cognitive performance independently predicted GI‐moderate to severe status.

**Figure:**
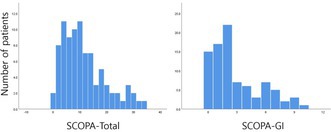
**FIGURE 1** The distribution of autonomic symptoms.

**Figure:**
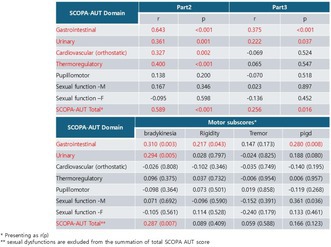
**TABLE 1** Correlation between autonomic symptoms and motor ADL and severity.

**Figure:**
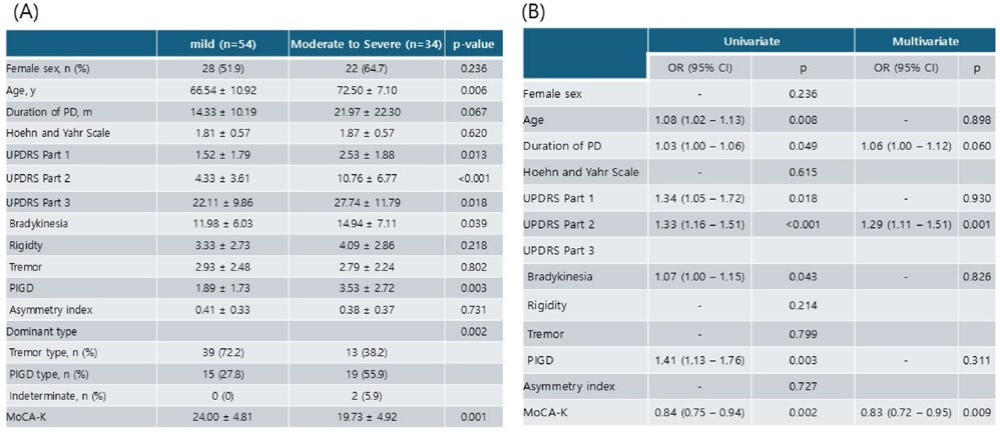
**TABLE 2** Comparison of characteristics of drug‐naive PD patients according to GI severity (A) and clinical indicators highly associated with moderate to severe GI severity calculated by logistic regression (B).

**Conclusion:** In drug‐naive PD, GI autonomic dysfunction identifies a distinct axial–functional vulnerability phenotype characterized by greater postural–gait impairment, functional disability, and cognitive vulnerability. Early GI dysautonomia may represent a key clinical marker of PIGD type with higher motor burden at diagnosis.

**Disclosure:** Nothing to disclose.

## EPO‐0900

### Parkinson's disease patients present with more pronounced impairment of vertical eye movements

#### Z. Popovic ^1^; T. Gilman Kuric^1^; E. Strujic^1^; S. Matosa^1^; T. Mirosevic Zubonja^1^; L. Kusic^2^; A. Sadikov^3^; D. Georgiev^4^; S. Tomic^1^

##### ^1^Faculty of Medicine, Josip Juraj Strossmayer University of Osijek, Osijek, Croatia; Department of Neurology, University Hospital Center Osijek, Osijek, Croatia; ^2^Faculty of Medicine Osijek, Josip Juraj Strossmayer University of Osijek, Osijek, Croatia; ^3^Faculty of Computer and Information Science, University of Ljubljana, Ljubljana, Slovenia; ^4^Faculty of Computer and Information Science, University of Ljubljana, Ljubljana, Slovenia; Department of Neurology, University Medical Centre Ljubljana, Ljubljana, Slovenia

**Background and aims:** Eye movement impairment is present in 75% of patients with Parkinson's disease (PD). Most pronaunced impairment is in smooth pursuit which become hypometric with prolonged latency. We aimed to investigate differences in smooth pursuit eye movements in PD patients, depending on the movement axis.

**Methods:** This is a cross‐sectional study with idiopathic PD patients. Velocity, phase, and number of fixations of horizontal and vertical smooth pursuit eye movements were evaluated using eye‐tracker analysis with a battery of tests. The results for pursuit gain, speed, and range of motion are expressed as mean square error (MSE), ranging from 0 to 1, with lower values indicating smaller deviation from expected values.

**Results:** Fifty subjects (36 men and 14 women) with early‐stage idiopathic PD were evaluated. Most patients (38%) were in Hoehn and Yahr stage II. Patients' characteristics can be seen in Table 1 PD patients showed significantly more impaired gain, velocity, and range of motion (Figure 1) of smooth pursuit eye movements, as well as an increased number of eye fixations (Figure 2) in the vertical axis compared to the horizontal axis. Differences were more pronounced during slow movement tasks.

**Figure:**
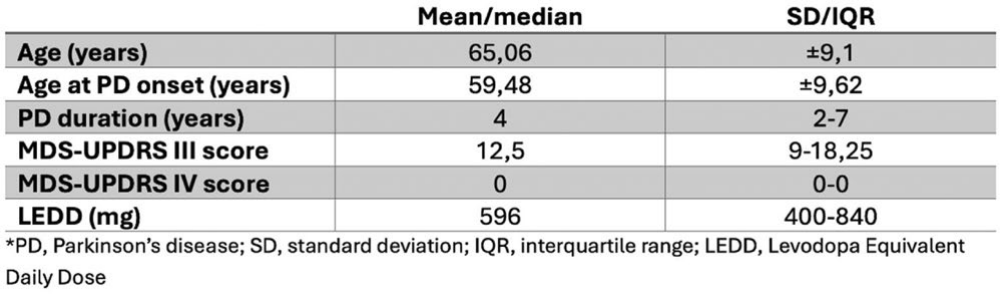
**TABLE 1** Characteristics of PD patients.

**Figure:**
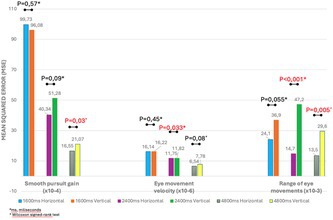
**FIGURE 1** Comparison of smooth pursuit eye movements with axis of movement.

**Figure:**
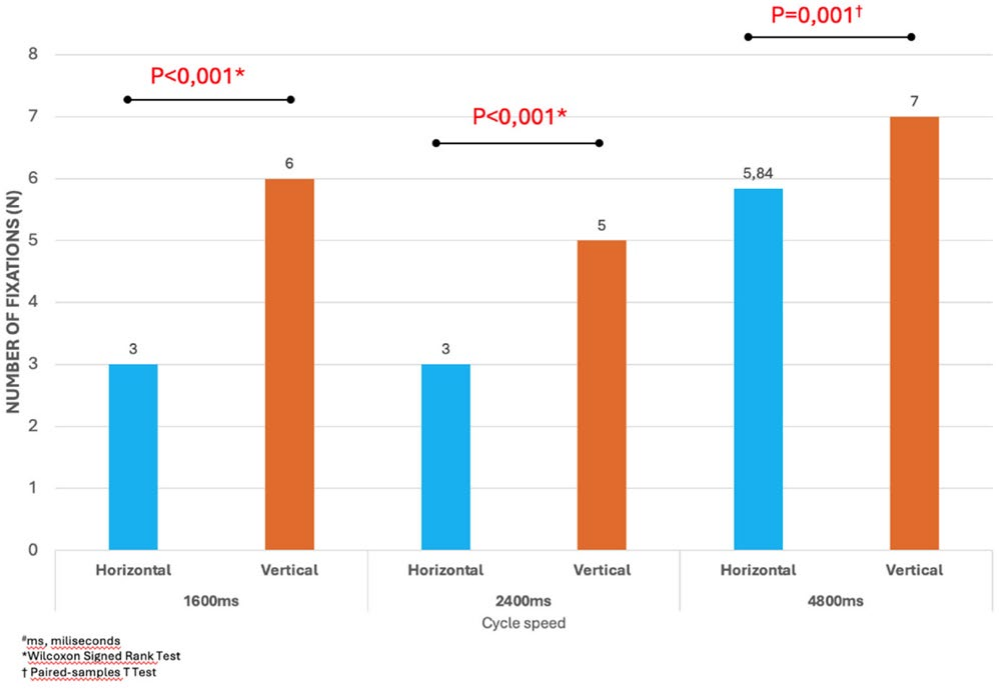
**FIGURE 2** Comparison of number of fixations during smooth pursuit eye movements with axis of movement.

**Conclusion:** Vertical smooth pursuit is more prominently impaired, with significantly lower gain and prolonged latencies. This axis‐specific deficit is present even in mild cases and is especially evident during slower cycles, when horizontal movement is still preserved. A possible cause is more complex interactions between the frontal and parietal eye fields and the basal ganglia, mainly the substantia nigra pars reticulata.

**Disclosure:** Nothing to disclose.

## Sleep‐Wake Disorders 2

## EPO‐0901

### Sleepwalking and risk of Parkinson's disease: A pooled analysis of 183,848 adults from the UK and USA

#### A. Otaiku

##### UK Dementia Research Institute, Imperial College London, London, UK

**Background and aims:** Sleepwalking is a non‐rapid eye movement (NREM) parasomnia that commonly occurs in Parkinson's disease (PD). Whether sleepwalking may precede the development of PD is unknown. This study investigated the association between sleepwalking and the risk of incident PD in the general population.

**Methods:** This prospective study used data from 183,848 community‐dwelling adults without PD (ages 18–96, 58% female) pooled from the UK Biobank Study (UK) and Parkinson's Progression Markers Initiative Online Study (USA). Sleepwalking (ever vs never) was self‐reported at baseline. Incident PD at follow‐up was defined by doctor‐diagnosis. Multivariable Firth logistic regression was used to estimate odds ratios (OR) for incident PD according to baseline sleepwalking status.

**Results:** Amongst 183,848 participants included at baseline, 10,316 (5·6%) had a history of sleepwalking. During a maximum follow‐up of 3.6 years, 284 participants were diagnosed with PD. After adjusting for demographic characteristics, participants who sleepwalked had a 2‐fold risk of developing PD during follow‐up (adjusted OR: 1.99; 95% CI 1.27–3.12; *p* = 0.003). The association between sleepwalking and incident PD was similar after adjusting for a wide range of possible confounders, including polygenic risk score for PD and idiopathic REM sleep behaviour disorder at baseline.

**Conclusion:** In this large prospective study spanning the UK and US, sleepwalking was independently associated with an increased risk of developing PD, suggesting it may represent a prodromal symptom of PD. These novel findings may help to identify individuals at risk of PD and could facilitate early prevention strategies.

**Disclosure:** Nothing to disclose.

## EPO‐0902

### Nightmares predict future cardiometabolic diseases: A pooled analysis of 113,073 participants across three continents

#### A. Otaiku

##### UK Dementia Research Institute, Imperial College London, London, UK

**Background and aims:** Nightmares are cross‐sectionally associated with cardiometabolic diseases (CMD), which are major contributors to neurodegeneration and premature mortality. Whether nightmares increase the risk of developing CMD is unknown. This prospective study investigated whether nightmares predict incident CMD in the general population.

**Methods:** Data from 113,073 CMD‐free participants (ages 31–96; 64% female) from seven population‐based cohort studies across three continents (UK Biobank; Midlife in Japan; Midlife in the United States; Wisconsin Sleep Cohort; The Osteoporotic Fractures in Men Study, Study of Osteoporotic Fractures, and Study of Women's Health Across the Nation) were used in this longitudinal analysis. Nightmare frequency was self‐reported at baseline. Incident CMD at follow‐up was defined as doctor‐diagnosed myocardial infarction, stroke, hypertension, or diabetes. Multivariable logistic regression was used to examine the association between nightmare frequency and incident CMD. Participants’ genetic risk for CMD was measured at baseline using polygenic risk scores.

**Results:** During a maximum follow‐up of 17 years, 2,786 participants developed a CMD. After adjustment for potential confounders, a higher frequency of nightmares was linearly associated with a greater risk of incident CMD (*p* for trend < 0.001). Compared with participants who had no nightmares at baseline, those with weekly nightmares had a 1.3‐fold risk of developing a CMD (adjusted odds ratio = 1.28; *p* = 0.002). Notably, there was no interaction between nightmare frequency and genetic risk (*p* = 0.32).

**Conclusion:** Nightmare frequency was independently associated with the development of CMD, regardless of genetic risk. Future studies are needed to determine whether treating nightmares could help reduce CMD risk in the general population.

**Disclosure:** Nothing to disclose.

## EPO‐0903

### REM sleep without atonia across dementia with Lewy body stages: Correspondence with clinical RBD and dopaminergic correlates

#### C. Bonomi ^1^; A. Castelli^2^; C. Motta^1^; F. Izzi^2^; A. Chiaravalloti^3^; L. Verdesca^3^; R. Ludovisi^2^; F. Placidi^2^; D. Centonze^4^; A. Martorana^1^

##### ^1^Memory Clinic and Neurodegenerative Dementia Research Unit, University of Rome Tor Vergata, Rome, Italy; ^2^Sleep Medicine Center, University of Rome Tor Vergata, Rome, Italy; ^3^Department of Biomedicine and Prevention, University of Rome Tor Vergata, Rome, Italy; ^4^Neurology Unit, University of Rome Tor Vergata, Rome, Italy

**Background and aims:** Dementia with Lewy bodies (DLB) and its prodromal stage with mild cognitive impairment (MCI‐LB), are frequently accompanied by REM sleep behavior disorder (RBD). REM sleep without atonia (RSWA), its neurophysiological correlate, is increasingly recognized as a biomarker of synucleinopathy; however, polysomnographic features across disease stages and their relationship with dopaminergic dysfunction remains incompletely elucidated.

**Methods:** Thirty‐three patients (17 MCI‐LB, 16 DLB) underwent overnight polysomnography (PSG) and dopamine transporter (DaT)‐SPECT. PSG parameters were compared using ANCOVA adjusted for age, sex, levodopa and acetylcholinesterase‐inhibitor doses. Concordance between clinical RBD and pathological RSWA was explored in patients with artifact‐free RSWA quantification. Associations between RSWA and regional DaT availability were assessed using voxel‐wise regressions.

**Results:** Groups were comparable for age, sex, and motor severity, while cognitive fluctuations were more prevalent in DLB. DLB also showed longer nighttime total sleep time and greater daytime sleepiness (Epworth Sleepiness Scale). Conversely, RSWA‐index values were higher in MCI‐LB than in DLB. Concordance between clinical RBD and pathological RSWA was only partial (Cohen's *k* = 0.23, see Fig. 1), indicating incomplete overlap between clinical and physiological definitions. Across the whole cohort, higher RSWA was associated with reduced DaT uptake in bilateral insulae, extending to the claustrum and right caudate (see Fig. 2).

**Figure:**
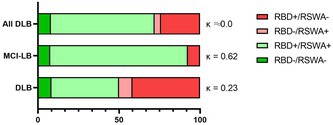
**FIGURE 1** Correspondence between clinical RBD and pathological RSWA. Bar plots show the distribution of concordant (dark green and light green) and discordant (dark red and light red) classifications in patients with MCI‐LB and DLB.

**Figure:**
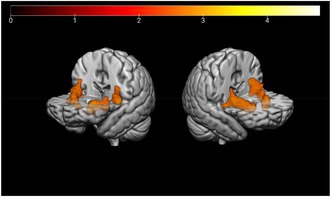
**FIGURE 2** Brain rendering showing clusters obtained in SPM regression analysis for the negative correlation between DaT uptake and RSWA index.

**Conclusion:** Sleep macrostructural alterations are equally present at the prodromal stage of DLB. Partial correspondence between clinical RBD and RSWA supports the added value of quantitative analysis, and the association between RSWA and extrastriatal dopaminergic dysfunction highlights RSWA as a translational biomarker linking sleep physiology with underlying neurodegenerative mechanisms.

**Disclosure:** Nothing to disclose.

## EPO‐0904

### Can early sleep disturbances predict cognitive impairment in amyotrophic lateral sclerosis patients?

#### D. Bottignole ^1^; V. Malanchini^2^; A. Nuredini^3^; L. Gambolò^4^; G. Balella^1^; L. Zinno^2^; C. Mutti^1^

##### ^1^"Mario Giovanni Terzano" Interdepartmental Center for Sleep Medicine, Department of Medicine and Surgery, University of Parma, Parma, Italy; ^2^Neurology Unit, Department of Medicine and Surgery, University of Parma, Parma, Italy; ^3^Department of Biomedical, Metabolic and Neural Sciences, University of Modena and Reggio Emilia, Modena, Italy; ^4^Psychiatric Unit, Local Health Agency of Piacenza, Piacenza, Italy

**Background and aims:** Amyotrophic lateral sclerosis (ALS) is a neurodegenerative disorder characterized by motor and non‐motor manifestations, including sleep disturbances and fronto‐temporal spectrum cognitive impairment. The relationship between sleep disorders and cognitive alterations in ALS remains insufficiently explored: this study aimed to assess the prevalence, characteristics, and cognitive impact of sleep disorders in a local ALS cohort.

**Methods:** Newly diagnosed ALS patients evaluated at our Neurology Unit in Parma (Italy) were prospectively enrolled. Participants underwent an overnight type III polysomnography and standardized questionnaires assessing disease severity, multidomain cognitive function, sleep quality, daytime sleepiness, and quality of life.

**Results:** Among 22 recruited ALS patients, 57.1% were affected by obstructive sleep apnea (OSA), with 23% deemed as moderate‐to‐severe forms. Numerous nocturnal respiratory parameters were strongly correlated (*p* = 0.01) with immediate and delayed memory impairment. Particularly, the hypoxic burden (HB) and the time spent with a peripheral blood oxygen saturation level below 90% were significantly predictive of memory compromise (Table 1). According to current guidelines, most patients with significant memory dysfunction were affected by mild OSA. Memory compromise is commonly described for patients with moderate‐to‐severe OSA. Our results suggest a higher prognostic relevance of the central hypoxic load than the apnoeic events count and support an increased sensitivity of ALS patients to the hypoxic damage.

**Figure:**
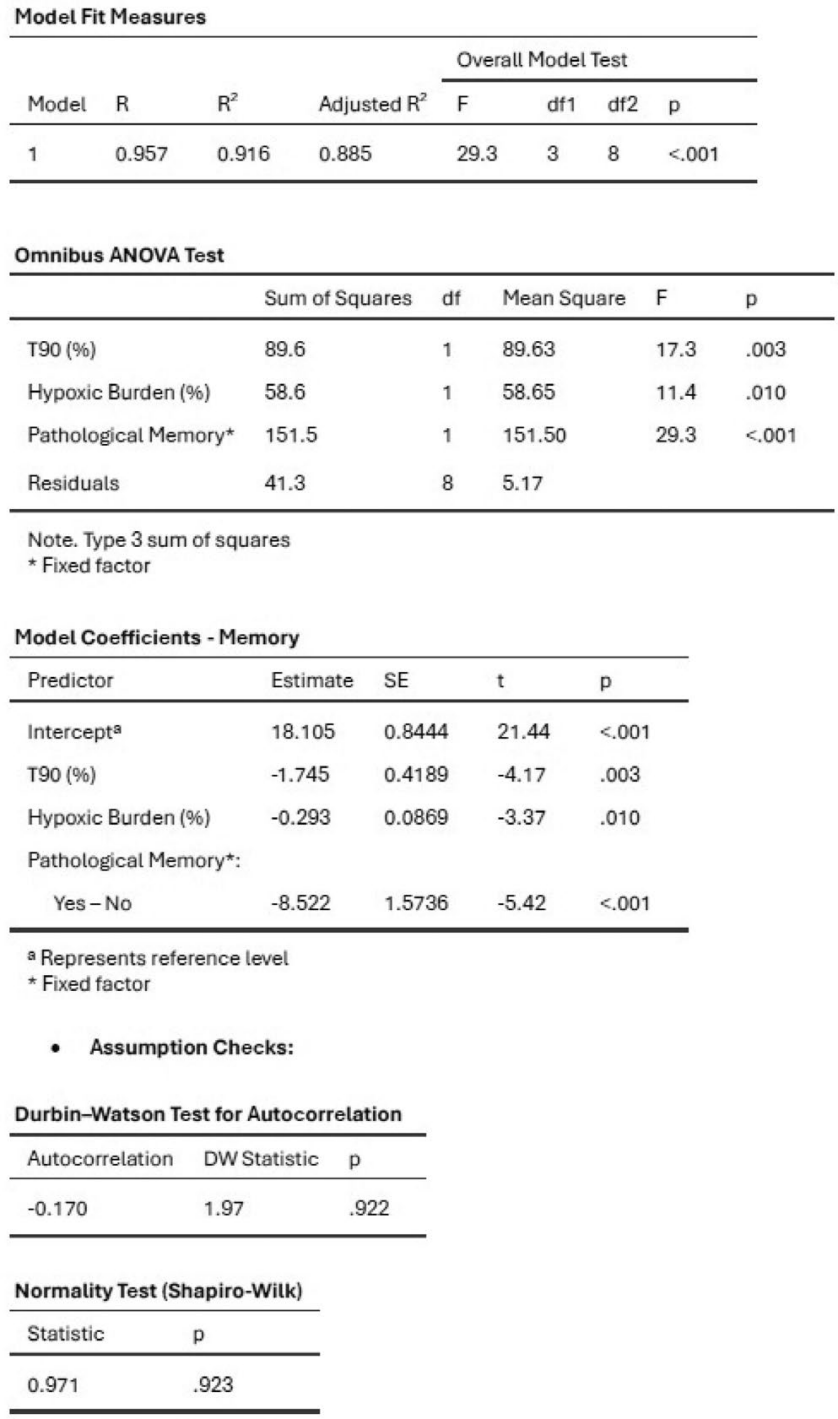
**TABLE 1** Linear regression model predicting memory performance from T90 (%), hypoxic burden (%). The overall model was significant (*p* < 0.001), explaining 91.6% of the variance in memory scores (adjusted *R* ^2^ = 0.885).

**Conclusion:** Sleep breathing disorders may adversely affect not only survival but also cognitive performance in ALS, particularly memory. A comprehensive sleep evaluation emphasizing hypoxic overload rather than apnea frequency may improve clinical management and outcomes in this population.

**Disclosure:** Nothing to disclose.

## EPO‐0905

### iSPHYNCS: Over five years of the international Swiss Primary HYpersomnolence and Narcolepsy Cohort Study – Where we are and where we are heading

#### E. Wenz ^1^; L. Fregolente^1^; J. van der Meer^1^; K. Zub^1^; J. Warncke^1^; R. Morand^2^; A. Helmy^1^; Z. Zhang^3^; R. Khatami^3^; S. von Manitius^4^; S. Miano^5^; M. Strub^6^; M. Tafti^7^; E. Lainey^8^; S. Knudsen^9^; A. Datta^10^; S. Bürki^10^; R. Rezaei^11^; U. Kallweit^11^; D. Bijlenga^12^; G. Lammers^12^; S. Mougiakakou^2^; A. Tzovara^13^; M. Schmidt^1^; C. Bassetti^1^

##### ^1^Sleep‐Wake Epilepsy Center, Department of Neurology, Inselspital, Bern University Hospital, University of Bern, Bern, Switzerland; ^2^ARTORG Center for Biomedical Engineering Research, University of Bern, Bern, Switzerland; ^3^Clinic Barmelweid, Center for Sleep Medicine and Sleep Research, Barmelweid, Switzerland; ^4^Department of Neurology, Kantonsspital St. Gallen St, Gallen, Switzerland; ^5^Sleep and Epilepsy Center, Neurocenter of Southern Switzerland, Regional Hospital (EOC) of Lugano, Lugano, Switzerland; ^6^Centre for Sleep Medicine Basel, Basel, Switzerland; ^7^Lausanne, Sleep Medicine Center, Lausanne, Switzerland; ^8^Department of Biomedical Science, Faculty of Biology and Medicine, University of Lausanne, Lausanne, Switzerland; ^9^Department of Rare Disorders and Disabilities, Oslo University Hospital, Oslo, Norway; ^10^Neuropaediatrics, University Children's Hospital Basel, Basel, Switzerland; ^11^Center for Narcolepsy and Hypersomnias, Professorship for Narcolepsy and Hypersomnolence Research, Department of Medicine, University Witten/Herdecke, Witten, Germany; ^12^Sleep Wake Center, Stichting Epilepsie Instellingen Nederland (SEIN), Heemstede, Netherlands; ^13^Institute of Computer Science, University of Bern, Bern, Switzerland

**Background and aims:** With the aim to discover new biomarkers for narcolepsy type 1 (NT1) and the narcolepsy borderland (NBL), and in order to facilitate diagnosis and find more individualized treatment options, the prospective, longitudinal Swiss Primary Hypersomnolence and Narcolepsy Cohort Study (SPHYNCS) was started in 2020 and expanded internationally in 2023 (iSPHYNCS). New technologies, hypotheseis‐ and data driven approaches have the potential to improve patient care and management.

**Methods:** The study has recently included new study sites in Norway and Austria and now counts 12 active study sites all over Europe. It plans to include 500 CDH patients and 60 healthy controls (HC) by the end of 2026. The multi‐modal approach includes questionnaires, clinical assessments, video‐polysomnography, the Multiple Sleep Latency Test (MSLT), vigilance tests, actigraphy, long‐term activity monitoring with Fitbit, immunological studies, quantitative hypocretin measurements, proteomics, gut microbiomics, and genetics/epigenetics. AI‐powered analyses, including unsupervised clustering, are used for data‐driven patient phenotyping.

**Results:** 366 participants, including 13 children, have been recruited. The study population comprises 81 individuals with NT1, 233 individuals of the NBL, as well as 52 HC. So far, 6 publications have resulted from the study and have revealed notable differences among the study groups by analyzing questionnaires, neuropsychiatric profiles, Actigraphy and Fitbit, more are currently in review.

**Conclusion:** The study is heading towards its inclusion goal of 500 participants. In the next phase, the focus will be on the longitudinal data, “omics”‐ analyses and comprehensive machine learning analyses of (a) different raw data types and (b) multimodal data integration.

**Disclosure:** The authors declare no conflict of interest. The study is supported by the Swiss National Science Foundation (SNF 320030_185362; SNF 32003B_215721).

## EPO‐0906

### Symptom severity in central disorders of hypersomnolence is associated with cerebrospinal fluid hypocretin‐1 concentration

#### J. Zhou^1^; J. Gool ^2^; Z. Zhang^3^; R. Fronczek^2^; G. Mayer^4^; M. Partinen^5^; S. Overeem^6^; J. Santamaria^7^; K. Šonka^8^; C. Bassetti^9^; J. van der Meer^9^; R. del Rio‐Villegas^10^; C. Veauthier^11^; S. Miano^12^; R. Peraita‐Adrados^13^; U. Kallweit^14^; E. Feketeova^15^; J. Bušková^16^; C. Donjacour^17^; R. Khatami^3^; G. Lammers^1^

##### ^1^Stichting Epilepsie Instellingen Nederlands (SEIN), Sleep‐wake Center, Heemstede, Netherlands; ^2^Leiden University Medical Center, Department of Neurology, Leiden, Netherlands; ^3^Center for Sleep Medicine, Sleep Research and Epileptology, Klinik Barmelweid AG, Barmelweid, Switzerland; ^4^Neurology Department, Hephata Klinik, Schwalmstadt, Germany; ^5^Department of Clinical Neurosciences, Clinicum, University of Helsinki, and Helsinki Sleep Clinic, Terveystalo Healthcare, Helsinki, Finland; ^6^Sleep Medicine Center Kempenhaeghe, Heeze, the Netherlands; ^7^Neurology Service, Institut de Neurociències Hospital Clínic, University of Barcelona, Barcelona, Spain; ^8^Neurology Department and Centre of Clinical Neurosciences, First Faculty of Medicine, Charles University and General University Hospital, Prague, Czech Republic; ^9^Department of Neurology, Inselspital, Bern University Hospital, and University of Bern, Bern, Switzerland; ^10^Neurophysiology and Sleep Disorders Unit, Hospital Universitario Vithas Madrid Arturo Soria, Universidad CEU San Pablo, CEU Universities, Madrid, Spain; ^11^Charité – Medical University Berlin, Interdisciplinary Center for Sleep Medicine, Berlin, Germany; ^12^Neurocenter of Southern Switzerland, Faculty of Biomedical Sciences, Università della Svizzera Italiana, Sleep Medicine Unit, EOC, Lugano, Switzerland; ^13^Sleep and Epilepsy Unit – Clinical Neurophysiology Service, University General Hospital Gregorio Marañón, Research InstituteGregorio Marañón, University Complutense of Madrid, Madrid, Spain; ^14^Center for Narcolepsy and Hypersomnias, Professorship for Narcolepsy and Hypersomnolence Research, Department of Medicine, University Witten/Herdecke, Witten, Germany; ^15^Neurology Department, Medical Faculty of P. J. Safarik University, University Hospital of L. Pasteur Kosice, Kosice, Slovak Republic; ^16^Department of Sleep Medicine, National Institute of Mental Health, Klecany and 3rd Faculty of Medicine, Charles University, Prague, Czech Republic; ^17^Sleep Wake Centre, Stichting Epilepsie Instellingen (SEIN), Zwolle, The Netherlands

**Background and aims:** Central disorders of hypersomnolence (CDH) are marked by excessive daytime sleepiness and/or increased sleep duration; narcolepsy type 1 is defined by hypocretin‐1 deficiency. This study aimed to identify symptoms beyond cataplexy that signal hypocretin‐1 deficiency and to examine how CDH symptom severity relates to hypocretin‐1 levels.

**Methods:** CDH patients from the European Narcolepsy Network database were included: 257 had low hypocretin‐1 (≤200 pg/mL; 163 ≤40, 59 at 41–110, 35 at 111–200 pg/mL) and 45 had levels >200 pg/mL. Stochastic gradient boosting identified non‐cataplexy predictors of hypocretin‐1 deficiency, while logistic and quantile regression assessed associations between hypocretin‐1 levels and 26 demographic, clinical, polysomnographic, MSLT variables, adjusted for age, sex, and BMI. Cataplexy features were analyzed only in hypocretin‐1 ≤200 pg/mL cases.

**Results:** The machine‐learning model predicted degrees of hypocretin‐1 deficiency with moderate accuracy. Across all CDH patients, lower hypocretin‐1 levels were associated with shorter REM and sleep latencies, more SOREMPs, higher Epworth scores, increased periodic limb movements, and more disturbed nocturnal sleep. In patients with cataplexy and hypocretin‐1 ≤200 pg/mL, lower levels were linked to earlier onset of excessive daytime sleepiness, younger age at diagnosis, greater certainty of hypnagogic hallucinations and sleep paralysis, and more frequent and complete cataplexy. In contrast, sleep drunkenness was more common at higher hypocretin‐1 levels and associated with longer daytime sleep duration.

**Figure:**
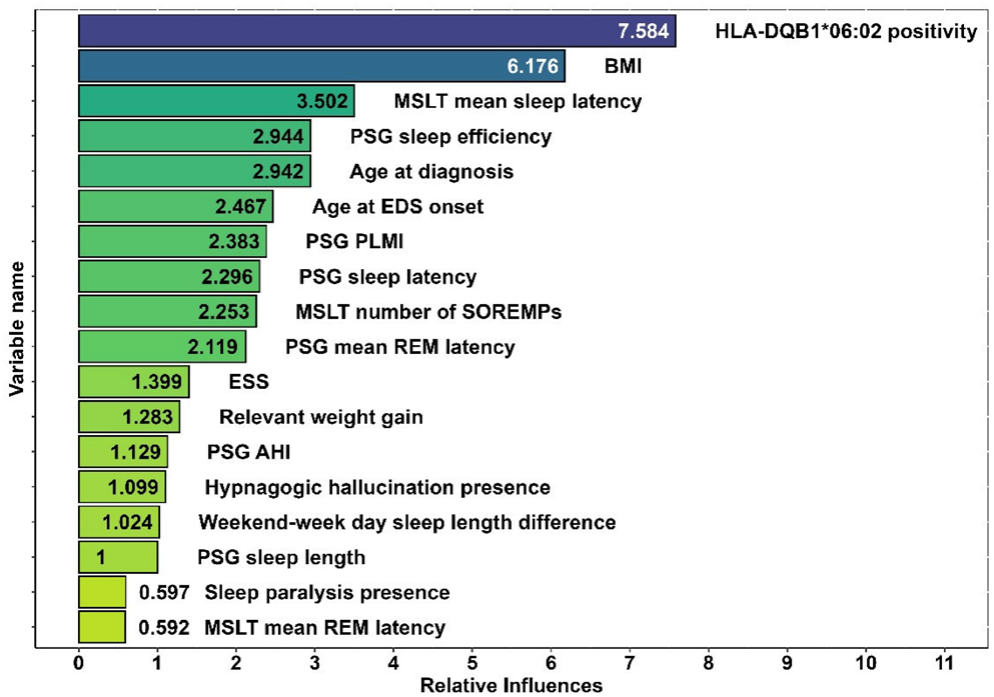
**FIGURE 1** Predictors with relative influence greater than 0.5 in machine learning.

**Figure:**
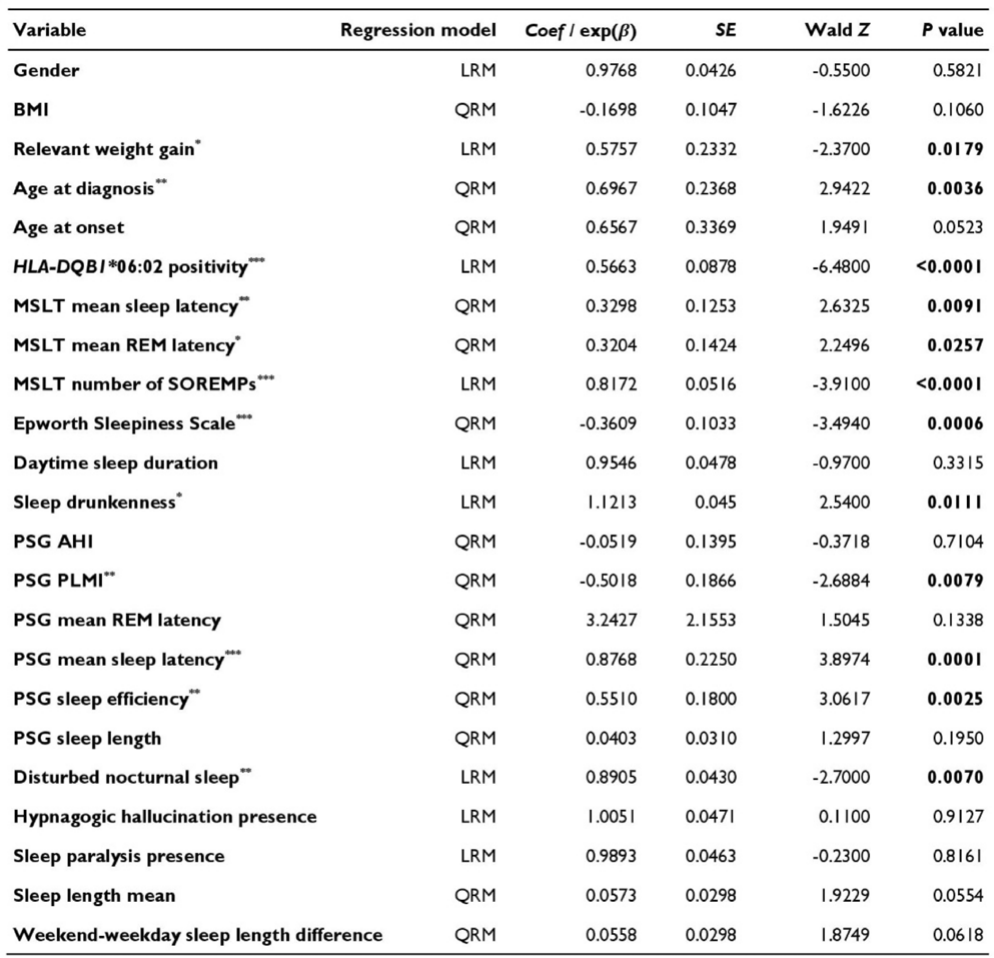
**TABLE 1** Logistic and quantile regression (Ʈ = 0.5) results for clinical variables using absolute hypocretin‐I levels in CDH individuals.

**Figure:**
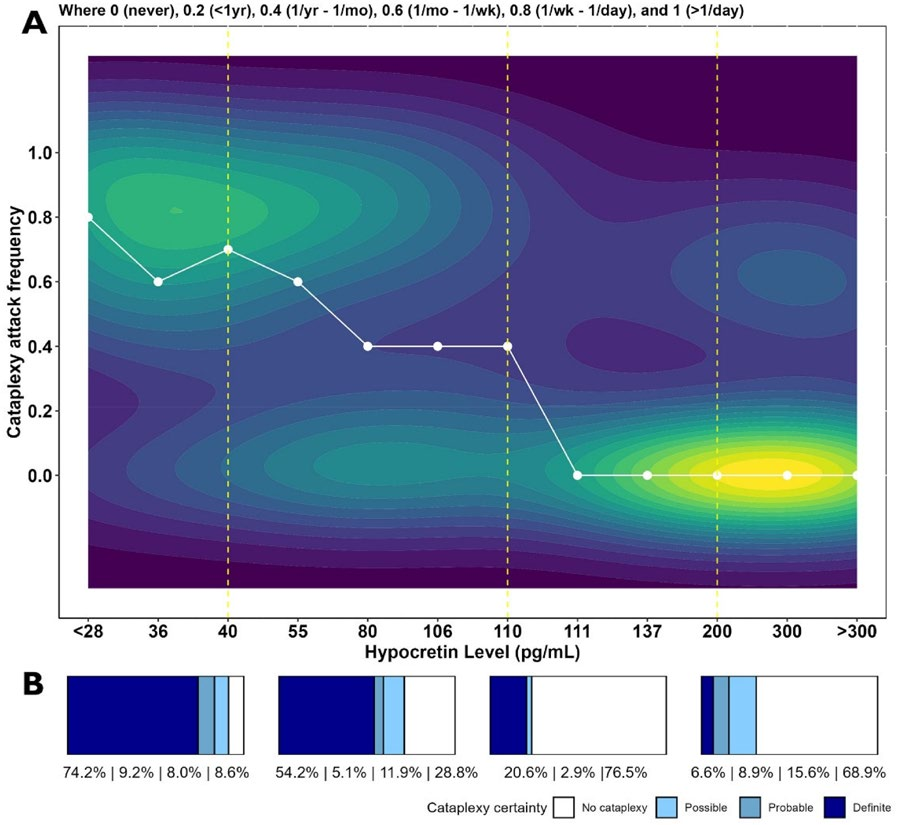
**FIGURE 2** Cataplexy frequency and certainty across hypocretin‐1 levels. (A) frequency coding: 0 = never, 0.2 = 1/day; (B) hypocretin groups: ≤40 pg/mL; 41–110 pg/mL; 111–200 pg/mL; >200 pg/mL.

**Conclusion:** Lower hypocretin‐1 levels were linked to greater symptom severity across CDH and within hypocretin‐deficient cataplexy patients (≤200 pg/mL). Even within this range, level differences explained clinical variability, highlighting a larger role for hypocretin‐1 in CDH heterogeneity than previously recognized.

**Disclosure:** Nothing to disclose.

## EPO‐0907

### Neurometabolic differentiation of central disorders of hypersomnolence: A harmonized spectroscopy study

#### N. de Joode^1^; J. Gool ^2^; N. Cross^3^; E. van Heese^1^; R. Fronczek^2^; G. Lammers^4^; C. Grova^5^; Y. van der Werf^1^; T. Dang‐Vu^6^

##### ^1^Amsterdam UMC, Anatomy and Neurosciences, Amsterdam, Netherlands; ^2^Department of Neurology, Leiden University Medical Centre, Leiden, Netherlands; ^3^The Brain and Mind Centre, School of Psychology, The University of Sydney, Sydney, Australia; ^4^Sleep‐Wake Center, Stichting Epilepsie Instellingen Nederland (SEIN), Heemstede, The Netherlands; ^5^Physics, Concordia University, Montreal, Canada; ^6^CIUSSS du Centre‐Sud‐de‐l’Île‐de‐Montréal, Institut Universitaire de Gériatrie de Montréal and CRIUGM, Montreal, Canada

**Background and aims:** Narcolepsy type 1 (NT1), narcolepsy type 2 (NT2), and idiopathic hypersomnia (IH) are central disorders of hypersomnolence (CDH); NT1 involves hypocretin deficiency, while NT2 and IH lack reliable biomarkers. We examined whether default mode network neurometabolites differ from controls, relate to symptoms and diagnostic differentiation.

**Methods:** Sixty‐two CDH patients (NT1 *n* = 20, NT2 *n* = 15, IH *n* = 27) and 18 well‐rested and sleep‐restricted healthy controls underwent GABA‐edited MEGA‐PRESS 3T MRI of the medial prefrontal (mPFC) and posterior cingulate (PCC) cortices. Water‐scaled, tissue‐corrected GABA^+^, glutamate‐glutamine, excitatory‐inhibitory ratio, and secondary metabolites were analyzed using linear mixed models adjusted for age, sex, site, and IQ. Correlations assessed links with clinical measures, and ROC analyses evaluated diagnostic discrimination.

**Figure:**
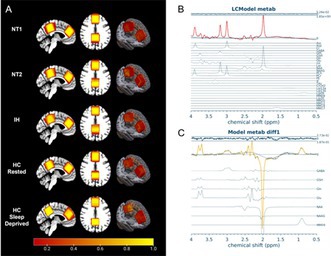
**FIGURE 1** Voxel placement and representative spectra. A. Group‐level voxel placement overlap maps for the medial prefrontal cortex and posterior cingulate cortex. Representative LCModel reconstruction for the standard MEGA‐PRESS editing scheme.

**Results:** No GABA^+^ or glutamate‐glutamine differences survived FDR‐correction between CDH and rested controls. Sleep‐restricted controls showed higher PCC excitatory‐inhibitory ratio and lower PCC myo‐Inositol than CDH. Across CDH, higher mPFC total‐N‐acetylaspartate correlated with poorer sleep quality, and higher PCC total‐Choline with longer MSLT sleep latency. Subtype‐specific associations emerged: in NT2, lower mPFC glutamate‐glutamine related to greater sleepiness; in IH, PCC glutamate‐glutamine and excitatory‐inhibitory ratio correlated with disease duration. ROC analyses showed good discrimination of NT1 vs NT2 (mPFC glutamate‐glutamine, myo‐Inositol) and NT2 vs IH (PCC total‐Choline, total‐N‐acetylaspartate).

**Figure:**
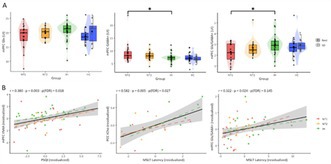
**FIGURE 2** Group differences in mPFC metabolites (A) and preregistered clinical associations (B).

**Conclusion:** Despite no differences versus rested controls, subgroup analyses revealed distinct frontal (NT1/NT2) and posterior (IH) default mode network neurometabolic profiles. Frontal metabolites differentiated NT1‐NT2 and tracked NT2 severity, while posterior markers distinguished IH‐NT2 and related to disease duration, suggesting biologically grounded signals for improved CDH subtype differentiation beyond current criteria.

**Disclosure:** Nothing to disclose.

## EPO‐0908

### Phenoconversion in isolated REM sleep behavior disorder: Long‐term data from the Innsbruck cohort

#### M. Aktan Süzgün ^1^; A. Ibrahim^1^; M. Wildt^1^; Q. Tang^1^; V. Anselmi^1^; E. Holzknecht^1^; E. Brandauer^1^; N. Campese^1^; M. Defrancesco^2^; M. Delazer^1^; R. De Marzi^1^; A. Djamshidian‐Tehrani^1^; A. Fanciulli^1^; R. Granata^1^; B. Heim^1^; F. Leys^1^; P. Mahlknecht^1^; K. Seppi^1^; H. Stockner^1^; L. Zamarian^1^; S. Kiechl^1^; B. Högl^1^; A. Stefani^1^

##### ^1^Department of Neurology, Medical University Innsbruck, Innsbruck, Austria; ^2^Department of Psychiatry, Psychotherapy, Psychosomatics and Medical Psychology, Medical University Innsbruck, Innsbruck, Austria

**Background and aims:** Polysomnographically‐proven isolated REM sleep behaviour disorder (iRBD) is a prodromal alpha‐synucleinopathy, with over 90% phenoconverting eventually to Parkinson's disease (PD), Lewy body dementia (LBD) or multiple system atrophy (MSA). This study aimed at analyzing phenoconversion characteristics along with clinical‐demographic features in the Innsbruck iRBD cohort.

**Methods:** We included 166 iRBD patients followed‐up longitudinally; those developing an overt alpha‐synucleinopathy were classified as “phenoconverters,” those remaining iRBD as “non‐converters”. Survival analyses assessed phenoconversion characteristics by demographic and clinical factors.

**Results:** Thirty‐nine (23.4%) subjects phenoconverted over a median of 62.1 (IQR 12.5–249.6) months follow‐up, mostly to PD (64.1%), followed by LBD (25.7%) and MSA (10.2%). Phenoconverters were older at diagnosis (68.3 vs 64.0y, *p* = 0.017), had longer follow‐up (80.6 vs 51.1 months, *p* = 0.008), higher mortality (51% vs 13%, *p* < 0.001) and more cardiovascular (44% vs 25%, *p* = 0.028) and cerebrovascular (28% vs 12%, *p* = 0.014) comorbidities. Cumulative phenoconversion risk (Kaplan–Meier analysis) was 13.4% at 3‐years, 17.6% at 5‐years, 26.5% at 10‐years, and 62.7% at 20‐years (annual phenoconversion rate: 3.2%, Figure1) without sex differences. We found a trend toward faster phenoconversion in MSA compared to PD and LBD (log‐rank‐*p* = 0.061, Figure2). LBD phenoconverters presented more depressive symptoms (LBD 70%, PD 20%, MSA 0%, *p* = 0.010).

**Figure:**
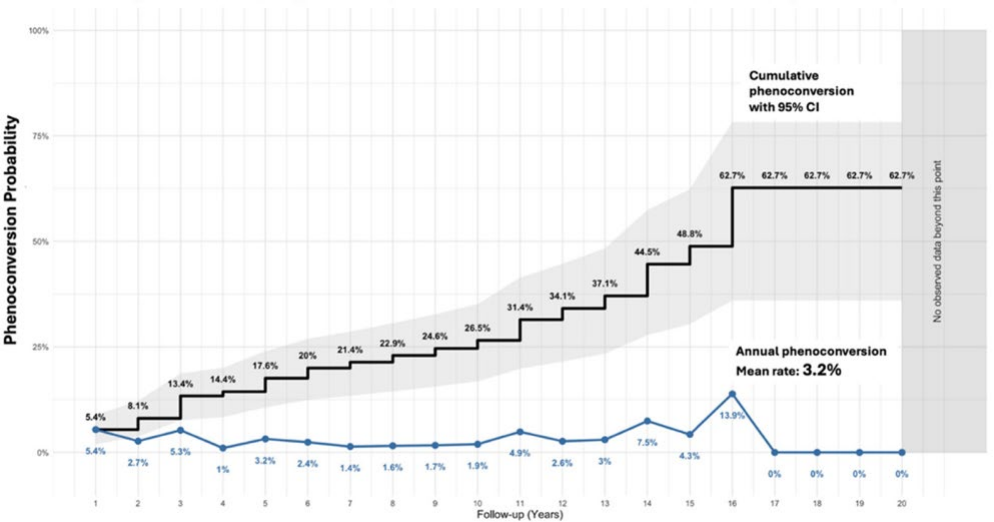
**FIGURE 1** Kaplan–Meier‐based cumulative and annual incremental phenoconversion plot.

**Figure:**
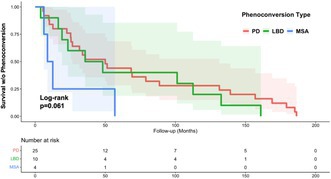
**FIGURE 2** Survival curves of the phenoconverters grouped by phenoconversion type, abbreviated as Parkinson's disease: PD, Lewy body dementia: LBD, and multiple system atrophy: MSA.

**Conclusion:** Our findings are consistent with prior reports, although with relatively more PD and MSA and fewer LBD cases compared to other cohorts. Beyond age and disease duration, our data suggest that cardio/cerebrovascular comorbidities may influence phenoconversion. Prospective studies are needed to determine whether targeted vascular risk management influences phenoconversion probability or timing.

**Disclosure:** Nothing to disclose.

## EPO‐0909

### Long‐term follow‐up of olfactory function in isolated REM‐sleep behaviour disorder

#### M. Aktan Süzgün ^1^; A. Ibrahim^1^; M. Wildt^1^; Q. Tang^1^; V. Anselmi^1^; E. Holzknecht^1^; E. Brandauer^1^; N. Campese^1^; M. Defrancesco^2^; M. Delazer^1^; R. De Marzi^1^; A. Djamshidian‐Tehrani^1^; A. Fanciulli^1^; R. Granata^1^; B. Heim^1^; F. Leys^1^; P. Mahlknecht^1^; K. Seppi^1^; H. Stockner^1^; L. Zamarian^1^; S. Kiechl^1^; B. Högl^1^; A. Stefani^1^

##### ^1^Department of Neurology, Medical University Innsbruck, Innsbruck, Austria; ^2^Department of Psychiatry, Psychotherapy, Psychosomatics and Medical Psychology, Medical University Innsbruck, Innsbruck, Austria

**Background and aims:** Olfactory dysfunction in isolated REM sleep behaviour disorder (iRBD) is a known risk factor of short‐term phenoconversion to overt alpha‐synucleinopathy. Yet data about longitudinal changes in olfactory function is scarce. This study aimed at investigating long‐term olfactory function changes in iRBD.

**Methods:** This long‐term observational study included iRBD with annual olfactory testing using the Sniffin’Stick 16‐item identification task. Those developing overt‐synucleinopathy were classified as “phenoconverters,” those remaining iRBD as “non‐converters”. Longitudinal olfaction scores were used to model the predictive power of olfactory function on phenoconversion.

**Figure:**
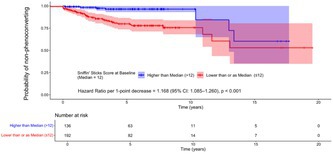
**FIGURE 1** Kaplan‐Meier analysis of phenoconversion‐free survival in iRBD stratified by baseline Sniffin’ Sticks scores. iRBD, isolated REM sleep behaviour disorder.

**Results:** 89 patients were included; 14 (15.7%) phenoconverted over 4.82 ± 3.87 years follow‐up (age at baseline: 65.02 ± 10.19 y, M/F:80/9). The baseline olfactory score was 11.3 ± 3.1 in non‐converters, 10.1 ± 3.5 in phenoconverters (*p* = 0.162). The olfactory score at last follow‐up was significantly higher in non‐converters compared to phenoconverters (11.0 ± 3.6 vs 8.5 ± 2.8, *p* = 0.019). Longitudinal mixed‐effects model (Figure1) showed lower olfactory scores in phenoconverters (*β* = −1.664, *p* = 0.047). Higher olfactory score reductions were associated with longer follow‐up time (*β* = −0.107/year, *p* = 0.017) and older age at diagnosis (*β* = −0.088/year, *p* = 0.002), with no sex effect (*p* = 0.802). In Kaplan–Meier analysis (Figure 2), baseline olfactory scores ≤12 were associated with higher phenoconversion risk (HR = 1.168 per 1‐point decrease; *p* < 0.001).

**Figure:**
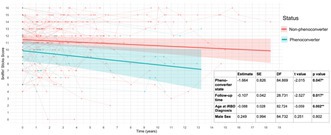
**FIGURE 2** Linear mixed model aims to predict Sniffin’ Sticks score over the follow‐up period. DF, degrees of freedom; iRBD, isolated REM sleep behaviour disorder, SE, standard error.

**Conclusion:** In our iRBD cohort, olfactory identification performance showed a gradual decline over time, underscoring its role as dynamic biomarker. Olfactory dysfunction was more severe at last follow‐up in phenoconverters, confirming the previously‐reported association with short‐term phenoconversion risk. The strong association between lower baseline olfactory scores and increased phenoconversion risk supports the prognostic value of quantitative olfactory testing in prodromal alpha‐synucleinopathies

**Disclosure:** Nothing to disclose.

## EPO‐0910

### Long‐term assessment of social jetlag by Fitbit smartwatch in patients with central disorder of hypersomnolence

#### Z. Zhang ^1^; R. Morand^2^; L. Fregolente^2^; J. van der Meer^2^; E. Wenz^2^; J. Warncke^2^; K. Zub^2^; S. von Manitius^3^; S. Miano^4^; J. Acker^5^; M. Strub^6^; U. Kallweit^7^; S. Knudsen‐Heier^8^; G. Lammers^9^; C. Bassetti^2^; M. Schmidt^2^; R. Khatami^2^

##### ^1^Center for Sleep Medicine, Sleep Research and Epileptology, Clinic Barmelweid AG, Barmelweid, Switzerland; ^2^Sleep‐Wake Epilepsy Center, NeuroTec, Department of Neurology, Inselspital, Bern University Hospital, University of Bern, Bern, Switzerland; ^3^Department of Neurology, Health Ostschweiz (HOCH), Kantonsspital St. Gallen St, Gallen, Switzerland; ^4^Sleep and Epilepsy Center, Neurocenter of Southern Switzerland, Regional Hospital (EOC) of Lugano, Lugano, Switzerland; ^5^ZurzachCare, Clinic for Sleep Medicine, Bad Zurzach, Switzerland; ^6^Centre for Sleep Medicine Basel, Basel, Switzerland; ^7^Center for Narcolepsy and Hypersomnias, Professorship for Narcolepsy and Hypersomnolence Research, Department of Medicine, University Witten/Herdecke, Witten, Germany; ^8^Norwegian Centre of Expertise for Neurodevelopmental Disorders and Hypersomnias (NevSom), Department of Rare Disorders, Oslo University Hospital, Ullevål, Norway, Norway; ^9^Sleep Wake Centre SEIN, Stichting Epilepsie Instellingen Nederland, Heemstede, The Netherlands

**Background and aims:** Central disorders of hypersomnolence (CDH) are characterized by excessive daytime sleepiness and altered sleep–wake patterns. Social jetlag (SJL), reflecting misalignment between circadian rhythm and social schedules, has rarely been examined in CDH. Using smartwatch data, we characterized SJL in CDH and hypothesized greater SJL compared to healthy controls (HC).

**Methods:** Sleep–wake data from Fitbit smartwatches were analyzed in 37 HC, 50 patients with narcolepsy type‐1 (NT1), and 116 narcolepsy borderline (NBL) patients enrolled in the Swiss Primary Hypersomnolence and Narcolepsy Cohort Study. Weekly SJL was defined as the difference between weekend and weekday mid‐sleep times; monthly SJL was averaged across weeks. Linear‐mixed‐models assessed effects of diagnosis on monthly SJL, adjusting for age, BMI, gender, season, daylight saving time (DST), and nocturnal sleep duration (NSD).

**Results:** NBL patients showed significantly larger SJL than HC (19.0 min, *p* = 0.0015), while NT1 showed a trend toward larger SJL (12.3 min, *p* = 0.074). Differences between NBL and NT1 were not significant. Findings were replicated during summer‐time DST, whereas in winter‐time only NBL differed from HC. HC exhibited greater SJL in winter versus summer, a seasonal effect absent in CDH. SJL was unrelated to BMI or gender but negatively associated with NSD. CDH patients showed greater weekend variability in NSD than HC.

**Conclusion:** Wearables enable longitudinal assessment of SJL in CDH. Patients exhibit increased SJL, particularly in summer‐time, with DST effects differing from HC. Persistent SJL in NBL may represent a disease marker, while reduced seasonal adaptability in CDH suggests limited compensation for circadian disruption or sleep debt.

**Disclosure:** Nothing to disclose.

## EPO‐0911

### Heterogeneous daily vigilance responses during supervised in‐lab CBT‐I in insomnia

#### Z. Zhang; R. Khatami

##### Center for Sleep Medicine, Sleep Research, and Epileptology, Clinic Barmelweid, Barmelweid, Switzerland

**Background and aims:** Cognitive behavioral therapy for insomnia (CBT‐I) is the first‐line treatment for chronic insomnia disorder (CID), with sleep restriction therapy (SRT) as a core component. Previous home‐based CBT‐I studies reported increased daytime sleepiness and impaired vigilance during SRT, findings that may be confounded by limited adherence and supervision. We therefore investigated changes in daytime sleepiness and vigilance during a tightly supervised, in‐lab CBT‐I program.

**Methods:** We retrospectively analyzed data from 56 CID patients who completed a standardized two‐week in‐house CBT‐I program including SRT (5–6 h/night). Daily Stanford Sleepiness Scale (SSS), subjective sleep quality (SQ), and afternoon psychomotor vigilance tests (PVT) were collected over 10 working days. Linear mixed‐effects models assessed changes in vigilance (inverse mean reaction time), SSS, and SQ, adjusting for age and sex. Subject‐level bootstrap analyses were used to evaluate robustness of individual vigilance trajectories.

**Results:** SQ improved significantly over time (*p* < 0.001), whereas vigilance and SSS showed no systematic change. Higher sleepiness was consistently associated with poorer vigilance (*p* < 0.001). Individual responses were heterogeneous: 19 patients showed improving and 26 declining vigilance. Subgroups did not differ in demographics or fatigue severity, but higher depressive symptom severity (Beck Depression Inventory score ≥16) tended to be more frequent in patients with declining vigilance (*p* = 0.092). SQ was positively associated with vigilance only in the improving subgroup (*p* = 0.032).

**Conclusion:** Unlike prior home‐based studies, supervised in‐lab CBT‐I did not produce consistent increases in daytime sleepiness or vigilance impairment during SRT. Improved sleep quality may mitigate adverse daytime effects despite restricted sleep opportunity.

**Disclosure:** Nothing to disclose.

## Movement Disorders 12

## EPO‐0912

### Redefining the accuracy of clinical diagnosis of Parkinson's disease: A systematic review and meta‐analysis

#### D. Urso^1^; S. Giannoni‐Luza ^2^; M. Guidetti^3^; F. Neri^4^; L. D'Amico^5^; S. Landolfo^6^; A. Sánchez Boluarte^7^; C. Santoro^8^; G. Volpe^2^; A. Priori^3^; G. Logroscino^1^

##### ^1^Center for Neurodegenerative Diseases and the Aging Brain, University of Bari 'Aldo Moro', "Pia Fondazione Cardinale G. Panico", Tricase, Lecce, Italy; ^2^Department of Translational Biomedicine and Neuroscience (DiBraiN), University of Bari ‘Aldo Moro’, Bari, Italy; ^3^Aldo Ravelli” Center for Neurotechnology and Experimental Brain Therapeutics, Department of Health Sciences, University of Milan, Italy; ^4^Humanitas University, Milan, Italy; ^5^Università degli Studi di Milano, Milan, Italy, Neurology Residency Program, San Giacomo – Monopoli, Italy; ^6^P.O. San Giacomo – Monopoli, Italy; ^7^Department of Global Health, University of Washington, Seattle, USA; ^8^Complex Structure of Neurology, SS Annunziata Hospital, Via Francesco Bruno, Taranto, Italy

**Background and aims:** Accurate diagnosis of Parkinson's disease (PD) remains challenging, particularly in early stages, with major implications for patient care, biomarker research, and clinical trials. Although modern diagnostic criteria such as the Movement Disorder Society (MDS) 2015 criteria were designed to improve diagnostic precision, their real‐world performance relative to earlier frameworks is incompletely defined.

**Methods:** We conducted a systematic review and meta‐analysis of studies evaluating the accuracy of clinical PD diagnosis against neuropathological confirmation. Studies using UK Parkinson's Disease Society Brain Bank (UKPDSBB), Gelb, and MDS‐PD 2015 criteria were included. Bayesian bivariate random‐effects models were used to estimate pooled sensitivity and specificity for early PD (first clinical assessment) and late PD (final diagnosis before death), and to explore between‐study heterogeneity and temporal trends.

**Results:** Twelve studies including 9,659 participants were analysed. In early PD, pooled sensitivity and specificity were 57% and 76%, respectively, with substantial heterogeneity across studies. Conversely, late PD showed markedly higher accuracy, with pooled sensitivity of 87% and specificity of 89%. Among late‐stage criteria, UKPDSBB provided the highest sensitivity (91%), whereas the MDS‐PD 2015 criteria achieved the highest specificity and overall diagnostic precision. Meta‐regression demonstrated progressive improvement in specificity over recent decades, with a modest decline in sensitivity, suggesting increasing diagnostic stringency.

**Conclusion:** Clinical diagnosis of PD is strongly stage‐dependent and suboptimal in early disease. Although modern criteria, particularly the MDS‐PD 2015, improved specificity and overall precision, misclassification in early PD remains common, underscoring the need for objective biomarkers to enhance diagnostic reliability in clinical practice and trials.

**Disclosure:** Nothing to disclose.

## EPO‐0913

### Evolving drivers of advanced therapy selection in essential tremor: European neurologists show a measurable shift toward patient‐centered priorities

#### A. Sanchez Fraga ^1^; C. Tengelin^1^; N. Kaempf^1^; P. Crivelli^1^; K. Gant^2^; C. Ferrer^1^; A. Grinspan^2^

##### ^1^Insightec Europe GmbH, Munich, Germany; ^2^Insightec. Inc, Miami, USA

**Background and aims:** The introduction of new advanced therapies reshaped the landscape for patients with essential tremor (ET). This study aimed to assess whether these developments influence the factors considered by neurologists when selecting advanced therapies.

**Methods:** A cross‐sectional survey was conducted among 270 general neurologists (GN) and movement disorder neurologists (MDN) across Europe. Prescribing drivers, perceptions of advanced therapies, and unmet educational needs were assessed. Outcomes were compared with the results of the same survey performed in 2023.

**Results:** Compared with 2023, improvement in quality of life became the leading driver of choice for advanced therapy selection (2023: 13.9 ± 2.9%. 2025:26.9 ± 4%). Patients’ willingness to undergo therapy ranked second in Italy (15%), Germany (13.3%) and UK (17.5%) (Figure 1). This factor had a greater influence on prescribing MRgFUS (22.9%) than DBS (4.1%) (Figure 2). Familiarity with MRgFUS increased across Europe (2023: 86.9 ± 6.5%. 2025: 96.5 ± 3.5%), becoming the most frequently cited advanced therapy for ET in Spain (23.6%) and Italy (16.7%). When asked about unmet educational needs, neurologists reported a marked increase, compared with 2023, in the need to improve patient information and education (2023: 14.2 ± 5.1%. 2025: 25.2 ± 9.1%) (Figure 3).

**Figure:**
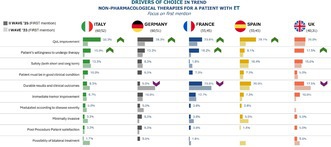
**FIGURE 1** Neurologists’ clinical priorities for advanced therapies in ET.

**Figure:**
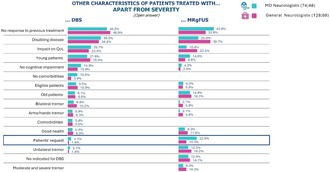
**FIGURE 2** Patient profile besides severity for advanced therapies.

**Figure:**
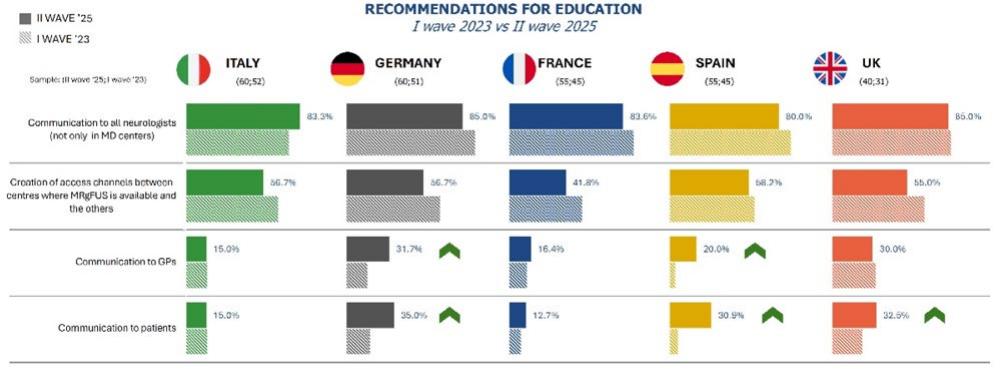
**FIGURE 3** Recommendations for education.

**Conclusion:** These findings demonstrate a shift in clinical decision‐making within the Neurology community towards patient‐centered priorities. Patient preferences and quality‐of‐life considerations are gaining importance alongside traditional efficacy and safety endpoints. Future efforts should enhance patient education, awareness and access to advanced therapies, ultimately enhancing their quality of life and treatment satisfaction.

**Disclosure:** All authors are Insightec employees.

## EPO‐0914

### Atypical late‐onset PRKN‐related Parkinson's disease due to compound heterozygosity: A case report

#### H. Rodrigues^1^; C. Oliveira^1^; B. Teixeira^2^; P. Almeida^3^; R. Rodrigues ^1^

##### ^1^Neurology Department, Local Health Unit Entre Douro Vouga, Santa Maria da Feira, Portugal; ^2^Neurology Department, Local Health Unit of the Aveiro Region, Aveiro, Portugal; ^3^Genetics Department, Local Health Unit Entre Douro Vouga, Santa Maria da Feira, Portugal

**Background and aims:** Approximately 5–10% of Parkinson's disease (PD) cases have a monogenic cause. PRKN is the commonest autosomal recessive gene. The typical phenotype involves early‐onset PD, limb dystonia and slow progression.

**Methods:** Case report

**Results:** A 58‐year‐old man (patient 1) presented with a 7‐year history of resting tremor, initially affecting the right lower limb and later the left upper limb, with gait impairment. Family history revealed a brother (patient 2) with similar symptoms beginning around 50s and a deceased father with PD. Both neurological examinations showed tremor‐predominant parkinsonism with more evident resting tremor in the right lower limb and bradykinesia and rigidity in the contralateral left upper limb. Cognitive functions were preserved, and no pyramidal, cerebellar, or autonomic signs were observed.They were investigated with brain MRI and laboratory panel, unremarkable. Both showed a good response to levodopa. Genetic testing started in patient 1, with LRRK2 single test that was negative. A NGS panel identified two heterozygous pathogenic variants in the PRKN gene, which confirmed to be present in the brother (patient 2), and in the descendant (son of patient 2) it was only found a single pathogenic PRKN variant, supporting a compound heterozygous configuration in trans in the affected cases.

**Conclusion:** We report two cases of genetic PD due to compound heterozygous PRKN variants, with an unusual phenotype: involvement of opposite limbs, absence of dystonia despite lower‐limb onset, and a later age‐at‐onset. The distinctive clinical presentation, positive family history, slow progression, good response to levodopa and confirmation of trans configuration allow a genotype–phenotype correlation.

**Disclosure:** Nothing to disclose.

## EPO‐0915

### Caregiver burden in Parkinson's disease and implications for a CBT/ACT‐based caregiver‐intervention (Care‐PD)

#### K. Bereswill; J. Reimer; A. Johannes; A. Kühn; P. Krause

##### Department of Neurology, Movement Disorder and Neuromodulation Unit, Charité‐Universitätsmedizin Berlin, Berlin, Germany

**Background and aims:** Parkinson's disease (PD) progressively increases patients´ needs for medical care and everyday support, most of which is provided by relatives without medical education. These informal caregivers frequently experience substantial physical and psychological strain summarized as caregiver burden (CB). The CARE‐PD study aims to quantify CB in caregivers of individuals with PD and identify patient‐ and caregiver‐related predictors to develop a targeted psychological caregiver intervention.

**Methods:** PD patients and their primary informal caregivers were recruited at the Charité movement disorders day clinic between December 2024 and May 2025. CB was measured using the Zarit Burden Interview (ZBI). Patient assessments included motor severity (MDS‐UPDRS‐III) and depressive symptoms (BDI‐II). Caregiver assessments included BDI‐II, mindfulness (MAAS), psychological inflexibility/experiential avoidance (AAQ‐II), and sociodemographic data. Multiple linear regression models were used to identify predictors of ZBI scores.

**Results:** 57 PD patients (age = 58.79 years, 19 female) and 57 caregivers (age = 63.71 years, 38 female) completed the study. Caregivers reported mild to moderate burden (ZBI = 21.30 ± 8.64). Higher UPDRS‐III predicted higher CB (*p* = 0.006), whereas BDI‐II did not (*p* = 0.838). Higher caregiver depressive symptoms (*p* = 0.002), lower psychological flexibility (AAQ‐II; *p* = 0.046), and female caregiver gender (*p* = 0.033) were significant predictors of CB; mindfulness was not (*p* = 0.549).

**Conclusion:** CB in PD is driven by both patient motor impairment and caregiver psychological vulnerability, particularly depressive symptoms and psychological inflexibility, with female caregivers at increased risk. These findings can support early identification of at‐risk caregivers and guide the development of targeted cognitive behavioral therapy (CBT)‐/Acceptance and Commitment Therapy (ACT)‐based interventions to mitigate CB and enhance care delivery.

**Disclosure:** Nothing to disclose.

## EPO‐0916

### Baseline dopaminergic treatment profiles of patients with advanced Parkinson's disease prior to Levodopa‐Entacapone‐Carbidopa intestinal gel infusion

#### J. Szasz ^1^; V. Constantin^2^; R. Szasz^1^; K. Orban Kis^1^; M. Ciorba^1^; R. Neagoe^1^; N. Craciun‐Ciorba^1^; J. Szederjesi^1^; K. Kelemen^1^; I. Mihaly^3^; S. Szatmari^1^

##### ^1^GE Palade University of Medicine, Pharmacy, Science and Technology Targu Mures, Targu Mures, Romania*;* ^2^Emergency Clinical County Hospital Targu Mures, Targu Mures, Romania; ^3^County Hospital Miercurea Ciuc, Romania

**Background and aims:** Continuous intra‐jejunal infusion of Levodopa‐Entacapone‐Carbidopa intestinal gel (LECIG) is an effective therapy for the treatment of advanced Parkinson's Disease (APD) associated with severe motor fluctuations with‐or‐without dyskinesias. The objective was to describe the characteristics of the last dopaminergic treatment in a large group of patients with APD in which current non‐invasive therapy alone was no longer effective.

**Methods:** We conducted a retrospective review of all APD patients treated in our department before the initiation of LECIG treatment between December 2021 and November 2025. We analyzed the use of dopaminergic agents based on the most recent treatment recommendations.

**Results:** During the four years that were included in the study, we initiated LECIG treatment in 50 patients with APD; mean age 65.2 ± 8.65 (mean ± SD), disease duration 11.2 ± 4.6 years. The average treatment duration with Levodopa was 10.0 ± 4.01 years. Patients were on relatively high‐dose levodopa regimens (mean: 857 ± 240 mg/day), administered at 5.34 ± 0.56 doses daily (5 to 7 times daily). Most patients also had treatment with dopamine agonists (82%; pramipexole *n* = 10, 2.35 ± 0.87 mg; ropinirole *n* = 11, 12.36 ± 5.5 mg; rotigotine patch *n* = 21, 8.48 ± 1.89 mg) as well as selective monoamine oxidase B inhibitors (MAO‐Bi, 60%, *n* = 30), apomorphine pen (14%, *n* = 7), Amantadine (28%, *n* = 14) and Entacapone (70%, *n* = 35).

**Conclusion:** The usage of dopaminergic treatments in patients with APD prior to LECIG treatment was more complex than those described in the literature. Such analyses help general neurologists identify the limits of conventional dopaminergic therapy and prompt timely referral to tertiary centers.

**Disclosure:** Nothing to disclose.

## EPO‐0917

### From scores to signals: A task‐level atlas of movement disorder rating scales

#### K. Sampson ^1^; A. Sadnicka^2^

##### ^1^Department of Clinical and Movement Neuroscience, University College London, London, UK; ^2^Gatsby Computational Neuroscience Unit, University College London, London, UK

**Background and aims:** Clinical observation and rating scales are the currency of movement‐disorder assessment, guiding diagnosis, tracking progression, and judging treatment response. Digital tools can quantify motor behaviour at scale in real‐world settings, but to translate this into clinically meaningful endpoints we must first understand what existing scales actually “sample.” We therefore mapped the content of widely used movement‐disorder rating scales to identify common anchors and syndrome‐defining phenomena.

**Methods:** Using a pragmatic, phenotype‐centred strategy, we selected scales spanning ataxia, chorea, dystonia, functional movement disorders, myoclonus, parkinsonism, tardive syndrome, tic disorders, and tremor. We analysed content at two levels: (i) scale composition (motor vs functional/ADL vs non‐motor) and (ii) item structure, classifying items by body region and task (Figure 1). Where available, structured video/examination protocols defined items; otherwise, items were extracted from text.

**Figure:**
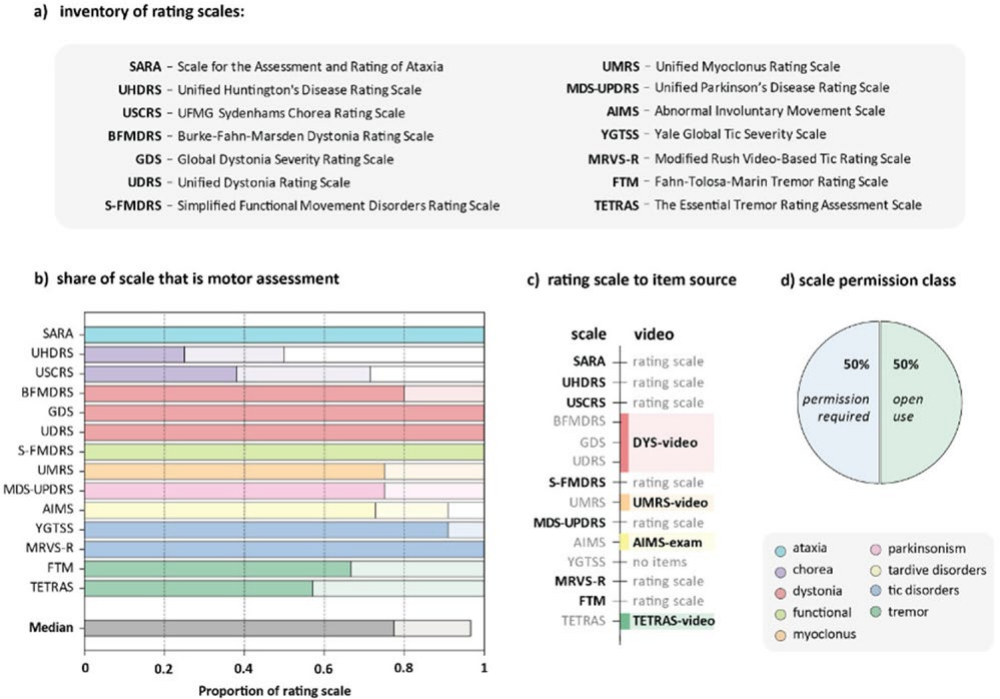
**FIGURE 1** Inventory of movement disorder rating scales. (a) Candidate scales for motor phenotypes. (b) Proportion devoted to motor (dark), function (pale), or non‐motor (white) assessment. (c) Source of motor‐assessment items. (d) Licensing status of scales.

**Results:** Across scales we collated 270 discrete items (Figure 2). Direct motor examination dominated (median ~73%), and each phenotype carved out a distinct “examination space,” emphasising different regions and tasks. Item‐frequency mapping revealed a shared backbone of cross‐phenotype tasks (including gait, speech, and bilateral finger–nose testing), supporting a feasible harmonised core for digital capture (Figure 3). Other scale‐specific items encode constructs clinicians use to distinguish syndromes, so a “core‐only” approach could risk reducing sensitivity. Scales for disorders with prominent non‐motor/behavioural/cognitive features allocated a smaller proportion to motor assessment.

**Figure:**
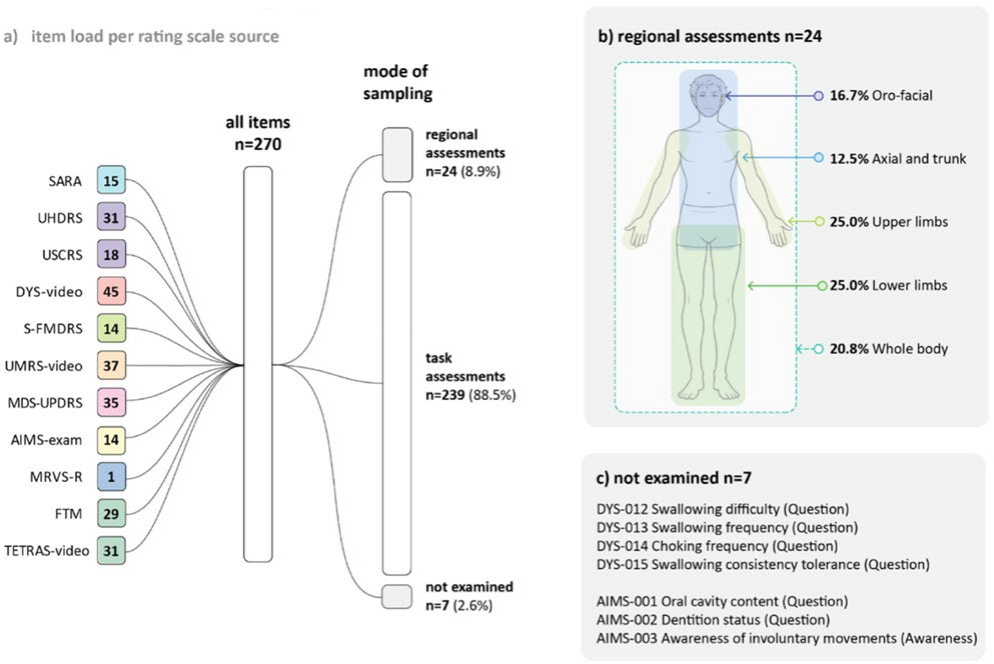
**FIGURE 2** Overview of rating‐scale items and regional assessments. (a) Classification of items by sampling mode. (b) Emphasis of body region(s) within region‐based assessments. (c) Not examined items: patient‐reported questions and qualitative judgements.

**Figure:**
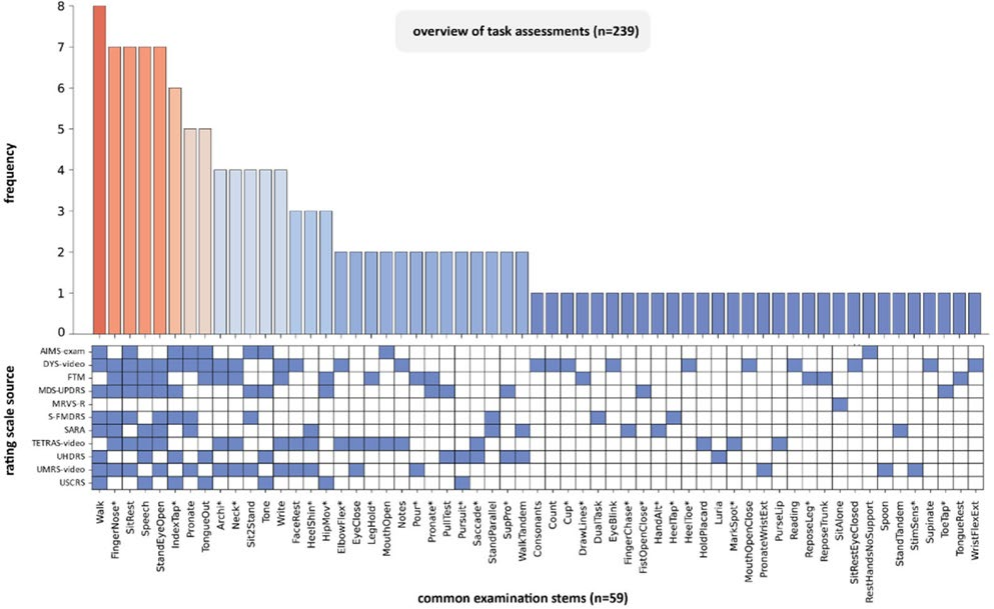
**FIGURE 3**: Overview of task‐based assessments across movement disorder rating scales. Frequency plot displaying each task's occurrence across included rating scales, identifying core and scale‐specific tasks. Grid plot indicates task inclusion by scale.

**Conclusion:** Digital measurement will not replace rating scales; it should be built on them. By using scales to define clinically grounded targets we can develop harmonised, interpretable, and more sensitive next‐generation outcomes.

**Disclosure:** Nothing to disclose.

## EPO‐0918

### Clinical and kinematic assessment of Parkinsonian features in young adults with 22q11.2 deletion syndrome

#### L. Angelini ^1^; A. Mazzeo^2^; E. Cerulli Irelli^2^; A. De Core^2^; D. Birreci^2^; A. Grandolfo^2^; M. Borioni^2^; S. Aloisio^2^; M. De Riggi^2^; F. Pulvirenti^3^; C. Di Bonaventura^2^; M. Bologna^2^

##### ^1^IRCCS Neuromed, Pozzilli (IS), Italy; ^2^Department of Human Neurosciences, Sapienza University of Rome, Rome, Italy; ^3^Reference Centre for Primary Immune Deficiencies, Sapienza University Hospital Policlinico Umberto I, Rome, Italy

**Background and aims:** 22q11.2 deletion syndrome (22q11.2DS) is associated with an increased risk of early‐onset parkinsonism, yet its motor phenotype in young adults remains poorly defined. We aimed to characterize parkinsonian features in young adults with 22q11.2DS using clinical and kinematic assessments, and to compare them with Parkinson's disease (PD) patients and healthy controls (HC).

**Methods:** Seventeen consecutive young adults with 22q11.2DS were enrolled and compared with 14 HC (age‐ and sex‐matched) and 35 PD patients. Motor assessment included UPDRS part III. Distinct bradykinesia features were objectively quantified using optoelectronic motion analysis of finger tapping. Cognition was assessed using the Montreal Cognitive Assessment (MoCA). Four patients were receiving antipsychotic treatment.

**Results:** All 22q11.2DS patients exhibited clinically detectable bradykinesia. Eight patients (47%) fulfilled criteria for parkinsonism, presenting with rigidity (*n* = 5), resting tremor (*n* = 2), or both (*n* = 1). UPDRS‐III total scores were comparable between 22q11.2DS and PD. Kinematic analysis showed that 22q11.2DS patients performed finger tapping more slowly than HC but similarly to PD. Compared with HC and PD groups, movements in 22q11.2DS patients were smaller in amplitude and less regular. MoCA scores were significantly lower in 22q11.2DS compared with PD. No correlations were found between UPDRS‐III or cognitive performance and any of the kinematic measures.

**Figure:**
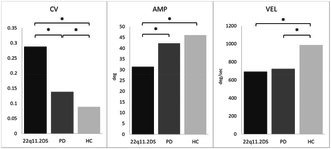
**FIGURE 1** Kinematic Features of Bradykinesia in 22q11.2DS, PD, and HC. Bar plots show coefficient of variation (CV), movement amplitude (AMP), and velocity (VEL) during finger tapping. Asterisks indicate significant between‐group differences.

**Conclusion:** Young adults with 22q11.2DS show a parkinsonian motor phenotype partially overlapping with PD but with distinct additional features (i.e, hypokinesia and dysrhythmia). Movement abnormalities are independent of cognitive impairment. These findings highlight kinematic analysis as a valuable tool for assessing motor function in young adults with 22q11.2DS.

**Disclosure:** Nothing to disclose.

## EPO‐0919

### Clinical subtypes of essential tremor: Neurological soft signs versus age at onset

#### L. Gattermeyer‐Kell; D. Kern; M. Suette; M. Zangl; P. Katschnig‐Winter; M. Kögl; C. Enzinger; P. Schwingenschuh

##### Department of Neurology, Medical University of Graz, Graz, Austria

**Background and aims:** Essential tremor (ET) subtypes may be defined by presence or absence of additional neurological signs (pure ET vs. ET‐plus) or by age at tremor onset (early‐onset ET, EOET vs. late‐onset ET, LOET). Prospective studies with detailed clinical phenotyping comparing these concepts are scarce.

**Methods:** We prospectively enrolled 79 ET‐patients (38.0% female) undergoing structured clinical assessment focused on predefined ET‐plus signs, including resting tremor, impaired tandem gait, questionable dystonia, questionable bradykinesia, mild parkinsonian signs (hypomimia, reduced arm swing) and marked action tremor asymmetry. Patients were classified as pure ET or ET‐plus and stratified by age at onset (40‐year cut‐off) into EOET and LOET. Tremor severity was assessed using TETRAS.

**Results:** Overall, 88.6% of patients were classified as ET‐plus and 11.4% as pure ET. ET‐plus‐patients were older at baseline, but groups did not differ in disease duration, tremor severity or alcohol responsiveness (Table 1). 70% of ET‐plus‐patients had more than one soft sign (Figure 1). EOET and LOET accounted for 62.0% and 38.0% of cases, respectively. LOET were older and had shorter disease duration than EOET (*p* < 0.001). Alcohol responsiveness was reported by 80.0% of EOET vs. 33.3% of LOET (*p* < 0.001). Despite shorter disease duration, tremor severity was higher in LOET than EOET (*p* < 0.001; Table 2).

**Figure:**
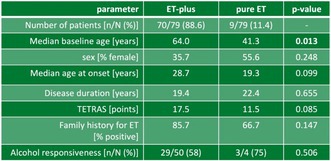
**TABLE 1** Demographic and clinical parameters in essential tremor (ET)‐plus vs. pure ET‐patients. Statistical significance was assessed at the *α* = 0.05 level. Values with *p* < 0.05 are displayed in bold.

**Figure:**
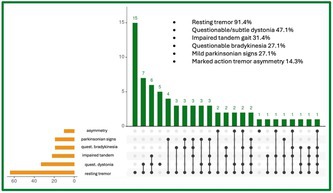
**FIGURE 1** UpSet plot visualizing the frequency and distribution of additional neurological signs in patients with essential tremor plus.

**Figure:**
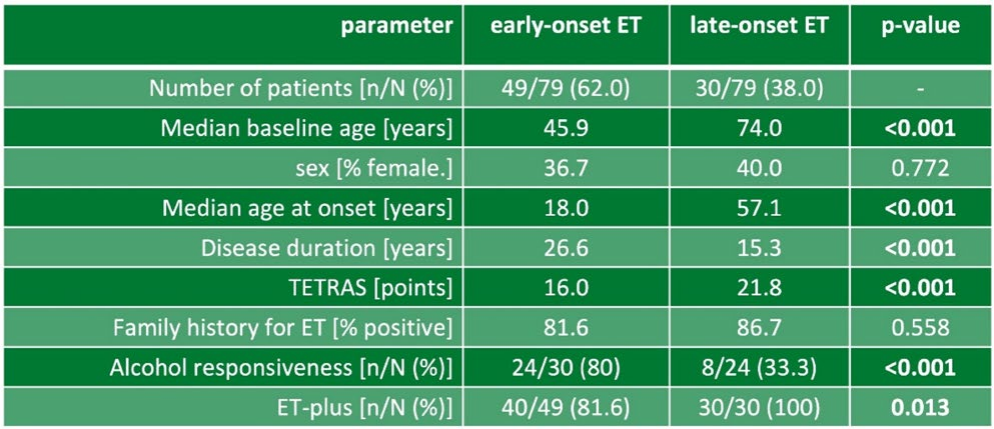
**TABLE 2** Demographic and clinical parameters in early‐onset (EOET) vs. late‐onset essential tremor (LOET)‐patients. Statistical significance was assessed at the *α* = 0.05 level. Values with *p* < 0.05 are displayed in bold.

**Conclusion:** Additional neurological signs are common in ET, but ET‐plus and pure ET do not differ in key aspects like alcohol responsiveness. In contrast, alcohol responsiveness is more frequent in EOET than LOET, while LOET appears characterized by faster tremor progression, suggesting different underlying pathophysiologies of these age‐defined ET‐subtypes.

**Disclosure:** Nothing to disclose.

## EPO‐0920

### Intrafamilial phenotypic heterogeneity in FBXO7‐related PARK15 with compound heterozygosity: A case report of two siblings

#### M. Vomero; C. Cabato; R. De Micco; M. Cirillo; O. Ciaramaglia; F. Ambrosio; A. Tessitore

##### Department of Advanced Medical and Surgical Sciences, University of Campania “Luigi Vanvitelli”, Naples, Italy

**Background and aims:** Biallelic pathogenic variants in the FBXO7 gene cause PARK15, a rare juvenile‐onset parkinsonism characterized by motor and nonmotor features, with limited reports of intrafamilial phenotypic variability. This case report describes two Italian siblings with the same compound heterozygosis in the FBXO7 gene (p.Val233GlufsTer8 and p.Ile74Met), showing different clinical presentations despite identical genotypes. We also identified a potential novel pathogenetic FBXO7 variant.

**Methods:** Patient 1 (male, 21‐years‐old) had childhood‐onset dysarthria and 2‐year history of progressive slowness of movement. He was diagnosed with juvenile‐onset parkinsonism and started dopaminergic medications with good levodopa response. Neurological examination showed mild left‐predominant parkinsonism, and fragmentation of saccadic movements. Patient 2 (female, 27‐years‐old) presented at age 20 with verbal aggression and visual hallucinations, followed by a 2‐year history of psychomotor slowing and gait impairment. Neurological examination showed mild‐to‐moderate left‐predominant parkinsonism, fragmentation of saccadic movements and mild oculomotor apraxia. She also presented dysarthria and stimulus‐induced myoclonus in the left hand. She shown poor levodopa tolerance due to behavioural side effects.

**Results:** Both patients exhibited MRI nigrosome‐1 bilateral loss. Next‐generation sequencing confirmed the presence of a compound FBXO7 heterozygosis with a pathogenic variant (p.Val233GlufsTer8) and a variant of unknown significance (p.Ile74Met). Mother and father carried the frameshift and missense variant respectively and were asymptomatic. A third sister was genetically wild‐type and clinically healthy.

**Conclusion:** These cases highlight the possible intrafamilial heterogeneity in FBXO7‐related PARK15 and support a potential pathogenic role for the p.Ile74Met variant (ACMG class 3), warranting functional studies for reclassification of variants in the FBXO7 gene.

**Disclosure:** Nothing to disclose.

## EPO‐0921

### Continuous subcutaneous foslevodopa/foscarbidopa improves nonmotor fluctuations in Parkinson's disease

#### O. Ciaramaglia ^1^; R. De Micco^1^; C. Cabato^1^; V. Sant'Elia^1^; M. Siciliano^1^; M. Sicialiano^2^; F. Ambrosio^1^; A. Tessitore^1^

##### ^1^Department of Advanced Medical and Surgical Sciences, University of Campania “Luigi Vanvitelli”, Naples, Italy; ^2^Neuropsychology Laboratory, Department of Psychology, University of Campania “Luigi Vanvitelli”, Caserta, Italy

**Background and aims:** Non‐motor fluctuations (NMFs) in Parkinson's disease (PD) are disabling features often related to pulsatile dopaminergic stimulation from oral levodopa. Continuous subcutaneous foslevodopa/foscarbidopa infusion (CSFLI) provides stable 24‐hour levodopa delivery, but real‐world data on NMFs remain limited.

**Methods:** We evaluated the effects on motor and nonmotor fluctuations after 3 months of treatment with CSFLI. Sixty‐one PD patients initiated CSFLI at our Center. Longitudinal data were available for 40 subjects. Motor fluctuations were assessed using the Unified Parkinson's Disease Rating Scale part IV (UPDRS‐IV), non‐motor fluctuations with the Nonmotor Fluctuation Assessment questionnaire (NoMoFa) and non‐motor symptom burden with the Non‐Motor Symptom Scale (NMSS). Sleep quality, behavioural features, autonomic dysfunction and quality of life (QoL) were also assessed using validated scales. Total levodopa equivalent daily dose (LEDD) was calculated at each timepoint. Within‐subject longitudinal differences on clinical variables were assessed using repeated‐measures ANOVA.

**Results:** After 3 months of treatment with CSFLI, significant changes were observed in the total NoMoFa score, mainly driven by a reduction in OFF‐related NMFs, particularly within the sleep/fatigue, depression/anxiety and miscellaneous domains. Significant improvement in NMSS scores, sleep quality and QoL were also found. As expected, wake time spent in the OFF state decreased, with a concomitant increase in good ON time due to reduced troublesome dyskinesias. No significant changes were observed in total LEDD after 3 months of treatment.

**Conclusion:** Our data reveal that, along with motor fluctuation stabilization, treatment with CSFLI markedly reduces NMFs severity, with broad non‐motor and motor benefits in PD.

**Disclosure:** Nothing to disclose.

## EPO‐0922

### Predicting atrophy in Parkinson's disease using the aggregation network diffusion model

#### R. Balestrino ^1^; S. Basaia^2^; T. Cusolito^2^; S. Pisano^1^; E. Sarasso^3^; E. Canu^1^; F. Valtorta^2^; E. Sibilla^4^; V. Kostic^5^; R. De Micco^6^; A. Tessitore^6^; F. Molinari^7^; F. Esposito^6^; F. Agosta^1^; M. Filippi^8^

##### ^1^Neuroimaging Research Unit, Division of Neuroscience, and Neurology Unit, IRCCS San Raffaele Scientific Institute, Milan, Italy; and Neurotech Hub, Vita‐Salute San Raffaele University, Milan, Italy; ^2^Neuroimaging Research Unit, Division of Neuroscience, IRCCS San Raffaele Scientific Institute, Milan, Italy; and Neurotech Hub, Vita‐Salute San Raffaele University, Milan, Italy; ^3^Neuroimaging Research Unit, Division of Neuroscience, IRCCS San Raffaele Scientific Institute, Milan, Italy; and Neurotech Hub, Vita‐Salute San Raffaele University, Milan, Italy; and DINOGMI, University of Genoa, Genoa, Italy; ^4^Neuroimaging Research Unit, Division of Neuroscience, IRCCS San Raffaele Scientific Institute, Milan, Italy; ^5^Clinic of Neurology, Faculty of Medicine, University of Belgrade, Belgrade, Serbia; ^6^Department of Advanced Medical and Surgical Sciences, University of Campania "Luigi Vanvitelli", Napoli, Italy; ^7^Biolab, PoliTo(BIO) Med Lab, Department of Electronics and Telecommunications, Politecnico di Torino, Torino, Italy; ^8^Neuroimaging Research Unit, Division of Neuroscience, Neurology Unit, Neurorehabilitation Unit, and Neurophysiology Service, IRCCS San Raffaele Scientific Institute, Milan, Italy; and Neurotech Hub, Vita‐Salute San Raffaele University, Milan, Italy

**Background and aims:** This study aimed to evaluate the Aggregation Network Diffusion (AND) model for predicting individualized atrophy progression by simulating the spatiotemporal dynamics of misfolded α‐synuclein spread and aggregation across functionally connected brain networks in PD.

**Methods:** Eighty‐seven PD patients and 60 controls underwent 3D T1‐weighted and resting‐state functional MRI at baseline, with PD patients followed longitudinally over 7 years. A cohort of 34 PD patients from the Parkinson's Progression Markers Initiative with 4‐year follow‐up was included. The AND model simulated alpha‐synuclein spread across the functional connectome through a system of ordinary differential equations modeling protein aggregation, and network diffusion based on parameters adaptable to individual patient dynamics. Patient‐specific parameters were optimized using surrogate optimization to reproduce observed atrophy at first follow‐up available. Model performance was assessed by Pearson's correlation between predicted and observed atrophy.

**Results:** The AND model successfully accurately predicted atrophy trajectories. Prefibrillar α‐synuclein species combined with intermediate‐weight protein aggregates most frequently yielded optimal atrophy predictions. Seed regions were predominantly localized to temporal and frontal lobes in PD patients. The model revealed two distinct atrophy progression patterns: one associated with low transmissibility, characterized by focal atrophy and gradual spread, and another linked to high transmissibility, marked by early, widespread bilateral involvement.

**Conclusion:** This study demonstrates the feasibility of using the AND model for personalized prediction of PD atrophy progression. By accounting for subject‐specific patterns of protein spread and optimizing key model parameters, this approach provides new insights into the mechanisms driving neurodegeneration in PD.

**Disclosure:** Supported by EU‐funding within NextGenerationEU ‐ Mission 4, Component 2, Investment .3 ‐ MUR‐PNRR NeuroDETECT, MNESYS ‐ A multiscale integrated approach to the study of the nervous system in health and disease, PE00000006, CUPB83C22004960002. R. Balestrino, S. Basaia, E.Sarasso, and E. Canu received research support from the Italian Ministry of Health. T. Cusolito, S. Pisano, F. Valtorta, E. Sibilla, V.S. Kostic, R. De Micco, A. Tessitore, F. Molinari, F. Esposito report no competing interests. M. Filippi is Editor‐in‐Chief of the Journal of Neurology, Associate Editor of Human Brain Mapping, Neurological Sciences, and Radiology; received compensation for consulting services from Almirall, Biogen, Bristol‐Myers Squibb, Eli Lilly, Merck, Novartis, Roche, Sanofi; speaking activities from Amgen, Bayer, Biogen, Bristol‐Myers Squibb, Celgene, Chiesi Italia SpA, Eisai, Eli Lilly, Fujirebio, Genzyme, Janssen, Merck, Neopharmed Gentili, Neuraxpharm, Novartis, Novo Nordisk, Roche, Sanofi, Takeda; participation in Advisory Boards for Alexion, Biogen, Bristol‐Myers Squibb, Eli Lilly, GE Healthcare Ltd, Merck, Neuraxpharm, Novartis, Roche, Sandoz, Sanofi, Takeda; scientific direction of educational events for Biogen, Merck, Roche, Celgene, Bristol‐Myers Squibb, Lilly, Novartis, Sanofi‐Genzyme; he receives research support from Biogen Idec, Merck‐Serono, Novartis, Roche, the Italian Ministry of Health, the Italian Ministry of University and Research, and Fondazione Italiana Sclerosi Multipla. F. Agosta is Associate Editor of NeuroImage: Clinical and the European Journal of Neurology; has received speaker honoraria from Biogen Idec, Bristol Myers Squibb, Eisai, Eli Lilly, GE Healthcare, Neuraxpharm, and Roche,; and receives or has received research supports from the Italian Ministry of Health, the Italian Ministry of University and Research, AriSLA (Fondazione Italiana di Ricerca per la SLA), the European Research Council (ERC), the EU Joint Programme – Neurodegenerative Disease Research (JPND), and Foundation Research on Alzheimer Disease (France).

## EPO‐0923

### CREXONT® (IPX203) significantly increases the duration of “Good on” intervals: Interim real‐world results from the first 100 patients in ELEVATE‐PD

#### S. Isaacson^1^; L. Cloud^2^; D. Kreitzman^3^; S. Brillman^4^; G. Banisadr^5^; S. Fisher ^5^; R. Hauser^6^

##### ^1^Parkinson's Disease and Movement Disorders Center of Boca Raton, Boca de Rotan, USA; ^2^Virginia Commonwealth University, Department of Neurology, USA; ^3^Movement Disorders Center of Long Island; ^4^Parkinson's Disease and Movement Disorders Center of Silicon Valley, USA; ^5^Amneal Pharmaceuticals; ^6^University of South Florida Parkinson's Disease and Movement Disorders Center, USA

**Background and aims:** CREXONT is a novel extended‐release carbidopa‐levodopa formulation containing a mucoadhesive polymer, designed to improve levodopa delivery and absorption.We evaluated the impact of CREXONT on the duration of “Good On” intervals and daily motor fluctuations in patients with PD after switching from other levodopa‐based therapies.

**Methods:** ELEVATE‐PD is an ongoing real‐world, open‐label phase 4 study where PD patients are converted from prior levodopa‐based therapies (IR CD/LD, IR+COMTinhibitors, Rytary) to CREXONT. Following a 5‐week dose‐optimization period and a 1‐week stable dosing, patients are followed for 12 months. The average duration of continuous “Good On” intervals and daily number of motor fluctuations were assessed using data from patient PD diaries. This interim analysis includes 111 patients who completed Day 42/Visit 4.

**Results:** At baseline, mean duration of continuous “Good On” intervals across all treatment groups was 2.92 ± 1.38 hrs and average number of daily motor fluctuations was 5.86 ± 1.99. CREXONT treatment increased mean duration of continuous “Good On” intervals to 6.65 hrs (+3.94 hrs increase) for patients switching from IR CD/LD (*n* = 74), to 3.46 hrs (+0.99 hrs increase) from IR+COMTinhibitor (*n* = 9), to 6.58 hrs (+3.09 hrs increase) from Rytary (*n* = 22). Moreover, switching to CREXONT resulted in a reduction in the average number of daily motor fluctuations; it decreased to 2.96 (−3.25 [52.85%] reduction) in subjects converting from IR CD/LD, 5.15 (−1.11 [17.73%] reduction) from IR+COMT inhibitor and 2.79 (−1.92 [40.42%] reduction) from Rytary.

**Conclusion:** The longer duration of continuous “Good On” intervals and reduction of motor fluctuations after switching to CREXONT provide greater time and predictability for patients with PD “Off” fluctuations to perform their daily activities.

**Disclosure:** Supported by Amneal Pharmaceuticals.

## MS and Related Disorders 6

## EPO‐0924

### The learning effect of smartphone‐derived cognitive processing speed assessments as a proxy of cognitive functioning in multiple sclerosis

#### D. de Jong ^1^; P. Molenaar^2^; V. Verhoef^2^; L. Novakova^3^; E. Colato^1^; T. Broeders^1^; K. Lam^2^; S. Cloosterman^4^; B. Moraal^5^; D. Nieuwkamp^6^; O. Gerlach^7^; J. Mostert^8^; E. Strijbis^2^; M. Schoonheim^1^; J. Killestein^2^; T. Fuchs^1^

##### ^1^MS Center Amsterdam, Amsterdam UMC location Vrije Universiteit Amsterdam, Anatomy & Neurosciences, De Boelelaan 1117, Amsterdam, the Netherlands; ^2^MS Center Amsterdam, Amsterdam UMC location Vrije Universiteit Amsterdam, Neurology, De Boelelaan 1117, Amsterdam, the Netherlands; ^3^Department of Clinical Neuroscience, Institute of Neuroscience and Physiology, Sahlgrenska Academy, University of Gothenburg, Gothenburg, Sweden; ^4^MS Sherpa B.V, Nijmegen, The Netherlands*;* ^5^MS Center Amsterdam, Amsterdam UMC location Vrije Universiteit Amsterdam, Radiology, De Boelelaan 1117, Amsterdam, the Netherlands; ^6^Department of Neurology, Jeroen Bosch Hospital, ‘s‐Hertogenbosch, the Netherlands; ^7^Academic MS Centrum Zuyd, Department of Neurology, Zuyderland MC, Sittard‐Geleen, the Netherlands; School for Mental Health and Neuroscience, Department of Neurology, Maastricht University Medical Center, Maastricht, the Netherlands; ^8^Department of Neurology, Rijnstate Hospital Arnhem, Arnhem, the Netherlands

**Background and aims:** The symbol digit modalities test (SDMT) score is crucial for assessing cognitive impairment in multiple sclerosis (MS), but is often influenced by a learning effect, regarded as unwanted variance. The goal of this study was to evaluate SDMT learning as a proxy of cognitive performance.

**Methods:** Learning of people with MS and healthy controls (HCs) was evaluated using a two‐part piecewise linear regression on daily smartphone SDMT (sSDMT) scores (see Figure 1). Cognitive impairment (CI) and preservation (CP) were defined using z‐scores of baseline sSDMT relative to HC (CI: *z* < 1.67, CP: *z* ≥ −1.67). sSDMT learning of HC/CP/CI was compared using ANCOVA and was correlated to baseline variables. Linear regression was performed to relate sSDMT learning to clinical outcomes and brain volumes.

**Figure:**
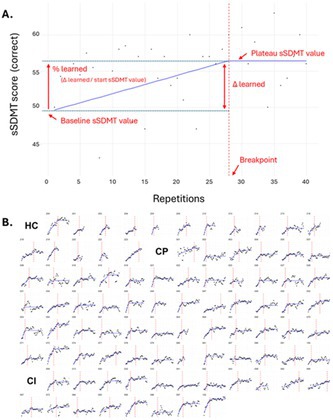
**FIGURE 1**: Depiction of variables extracted (A) from the sSDMT breakpoint analyses on the individual learning curves (B).

**Results:** CP/CI: 66/19 MS and 20 HCs were analysed. A learning effect plateau was identified in HC/CI/CP: 80%/82%/100%, Figure 1 A higher baseline sSDMT was related to a higher plateau sSDMT (*ρ* = 0.930, *p* < 0.001) and a lower %‐learned (*ρ* = −0.266, *p* = 0.013). The %‐learned was higher for CI than CP (CP/CI = 15.52%/22.04%, adj.*p* = 0.004), but the ∆‐learned and breakpoint were similar for HC/CI/CP, Figure 2 Relations of disability, cognition, and brain volumes with the plateau sSDMT were stronger when compared to the baseline sSDMT, Table 1.

**Figure:**
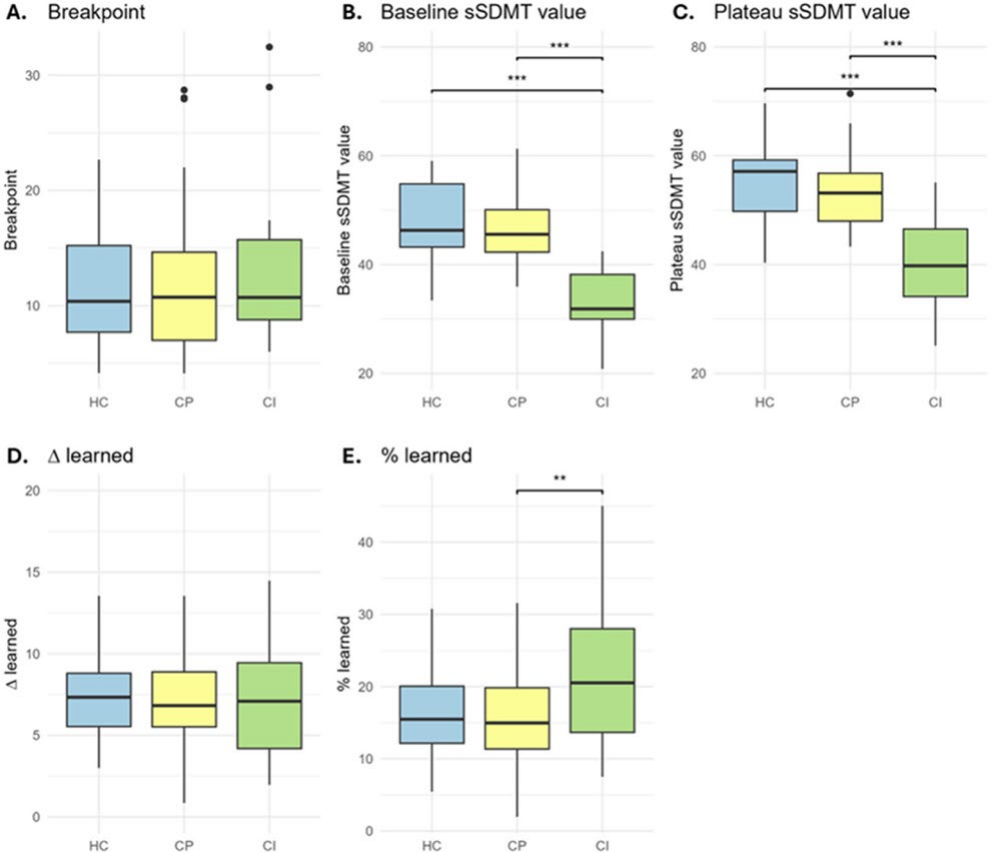
**FIGURE 2** Extracted variables from the individual learning curves.

**Figure:**
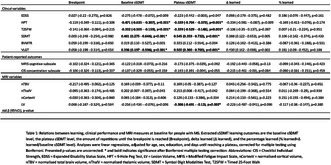
**TABLE 1** Relation of sSDMT learning to clinical outcomes and MRI measures.

**Conclusion:** Early‐phase sSDMT learning was related to cognitive performance in MS. However, learning was not unique to disease status, and was not associated with disease severity. Moreover, plateau sSDMT showed stronger associations with disability and brain volumes than baseline sSDMT, underscoring the need to account for learning effects.

**Disclosure:** The author(s) declared the following potential conflicts of interest with respect to the research, authorship, and/or publication of this article: D.J. de Jong declares no conflict of interest.; P.C.G. Molenaar declares no conflict of interest.; V. Verhoef declares no conflict of interest.; L. Novakova has received lecture honoraria from Biogen, Novartis, Teva, Sanofi and Merck, has served on advisory boards for Merck, Janssen, Argenx and Sanofi, and has received unconditional grants from Novartis and Sanofi.; E. Colato receives grant support from MAGNIMS and research support from EIP.; T.A.A. Broeders declares no conflict of interest.; S. Noteboom declares no conflict of interest.; K.H. Lam declares no conflict of interest.; S. Cloosterman is an employee of MS Sherpa.; B. Moraal declares no conflict of interest.; D. Nieuwkamp declares no conflict of interest.; O. Gerlach declares no conflict of interest.; J. Mostert declares has nothing to declare.; E.M.M. Strijbis reports speaker relations with Merck and Novartis (all payments to institution) and served on advisory board for Roche.; M.M. Schoonheim serves on the editorial board of Neurology and Frontiers in Neurology, receives research support from the Dutch MS Research Foundation, Eurostars‐EUREKA, ARSEP, Amsterdam Neuroscience, MAGNIMS and ZonMW (Vidi grant, project number 09150172010056) and has served as a consultant for or received research support from Atara Biotherapeutics, Biogen, Celgene/Bristol Meyers Squibb, EIP, Sanofi, MedDay and Merck.; J. Killestein received consulting fees for F. Hoffmann‐La Roche, Biogen, Teva, Merck, Novartis and Sanofi/Genzyme (payments to institution); reports speaker relationships with F. Hoffmann‐La Roche, Biogen, Teva, Merck, Novartis and Sanofi/Genzyme (payments to institution); adjudication committee of MS clinical trials of Immunic (payments to institution).; T.A. Fuchs received research support from the European Committee for Treatment and Research in Multiple Sclerosis, serves on the editorial board of Frontiers in Neurology, and received consulting fees for Click Therapeutics and Registry Multiple Sclerosis (ReMuS).

## EPO‐0925

### Prognostic biomarkers of 10‐year disease worsening in people with long‐lasting multiple sclerosis

#### D. de Jong ^1^; M. Wessels^1^; M. Barrantes‐Cepas^2^; L. Bos^3^; T. Fuchs^2^; J. Jelgerhuis^2^; P. Schipper^1^; T. Broeders^2^; F. Masi^2^; M. Steenwijk^2^; A. Wojdala^4^; A. Wink^3^; B. Jasperse^3^; B. Moraal^3^; H. Vrenken^3^; B. Uitdehaag^1^; E. de Vries^5^; F. Barkhof^3^; C. Teunissen^4^; J. Killestein^1^; E. Strijbis^1^; M. Schoonheim^2^

##### ^1^MS Center Amsterdam, Amsterdam UMC location Vrije Universiteit Amsterdam, Neurology, De Boelelaan 1117, Amsterdam, the Netherlands; ^2^MS Center Amsterdam, Amsterdam UMC location Vrije Universiteit Amsterdam, Anatomy & Neurosciences, De Boelelaan 1117, Amsterdam, the Netherlands; ^3^MS Center Amsterdam, Amsterdam UMC location Vrije Universiteit Amsterdam, Radiology, De Boelelaan 1117, Amsterdam, the Netherlands; ^4^MS Center Amsterdam, Amsterdam UMC location Vrije Universiteit Amsterdam, Clinical Chemistry, De Boelelaan 1117, Amsterdam, the Netherlands; ^5^MS Center Amsterdam, Amsterdam UMC location Vrije Universiteit Amsterdam, Molecular Cell Biology and Immunology, De Boelelaan 1117, Amsterdam, the Netherlands

**Background and aims:** Prognostication of people with multiple sclerosis (pwMS) is important to potentially delay clinical disease worsening. The goal of this study was to characterise pwMS that exhibited disability progression, cognitive decline or both using advanced MRI and blood‐based biomarkers.

**Methods:** PwMS (*N* = 162; Table 1) were evaluated three times with 5‐year intervals. MRI‐derived and blood‐based biomarkers (see Figure 1) were compared at each timepoint and over time in pwMS with and without worsening (disability progression, cognitive decline or both). Baseline predictors were identified using logistic regression.

**Figure:**
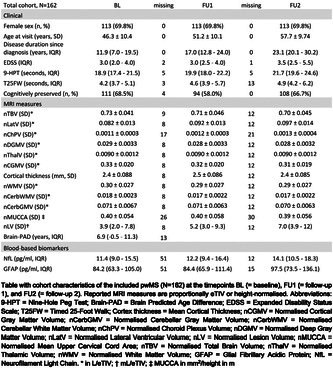
**TABLE 1** Participant characteristics.

**Results:** 50% exhibited disability progression, 26.3% declined cognitively, 12.4% experienced both. Disability progression was associated to lower baseline nChPV and lower brain age, with nChPV growth. See Figures 1‐2. Cognitive declining pwMS had lower nTBV and cortex thickness at all timepoints, with more nTBV atrophy over time. PwMS with combined worsening had lower cortical thicknesses at all timepoints and atrophy of nTBV and cortex, with an increase of nChPV and GFAP over follow‐up. For inter‐biomarker associations, see Figure 2 Baseline brain age (pooled OR = 0.95, *p* = 0.020) and high lesion volume (pooled OR = 1.08, *p* = 0.049) predicted disability progression (AUC = 0.68), high cortical thickness decreased predicted the odds of cognitive decline (pooled OR = 0.49, *p* < 0.001, AUC = 0.69) and combined worsening (pooled OR = 0.57, *p* = 0.022, AUC = 0.65).

**Figure:**
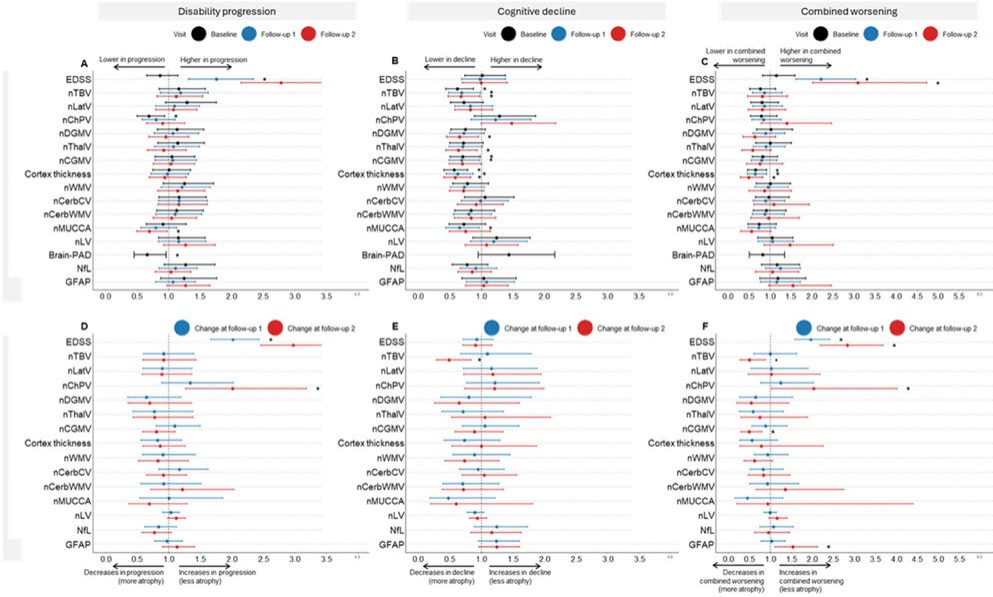
**FIGURE 1** Cross‐sectional scaled linear and longitudinal mixed‐model regression analyses of disability progression, cognitive decline, and combined worsening.

**Figure:**
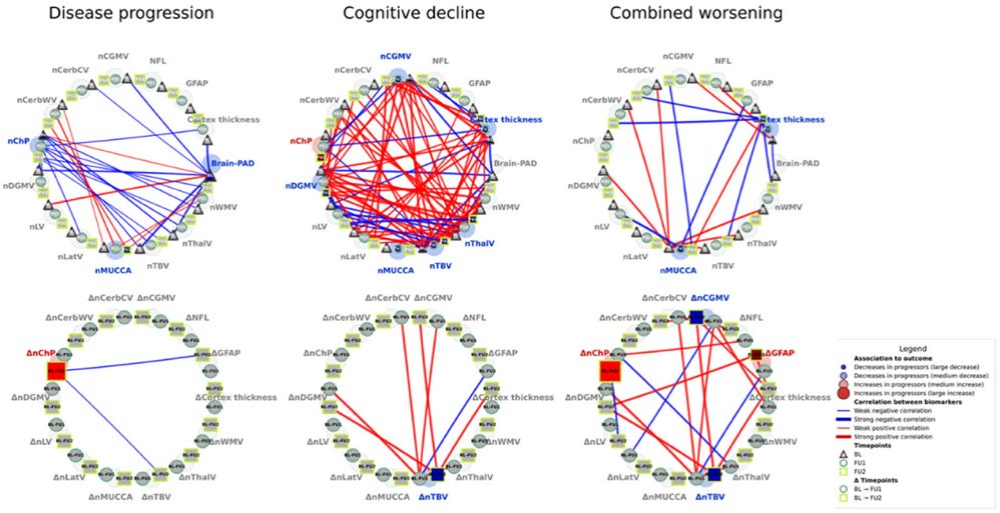
**FIGURE 2** Network plot of relations between biomarkers related to worsening.

**Conclusion:** Our results indicate that 10‐year disability progression and cognitively decline correlate with different biomarker patterns. Disability progression was associated inflammation (swelling of the choroid plexus), and the combination of low brain age with high lesional brain‐burden, whilst cognitive decline was associated to cortical grey matter atrophy.

**Disclosure:** D.J. de Jong, M.H.J. Wessels, L. Bos, J.R. Jelgerhuis, P. Schipper, T.A. Broeders, F. Masi declare no conflict of interest. M. Barrantes‐Cepas received research grants from Merck and Atara Biotherapeutics. T.A. Fuchs received research support from the European Committee for Treatment and Research in Multiple Sclerosis, serves on the editorial board of Frontiers in Neurology, and received consulting fees for Click Therapeutics and Registry Multiple Sclerosis (ReMuS). A. Wojdala reports project funding from the European Union's Horizon 2020 research and innovation programme under the Marie Skłodowska‐Curie grant agreement No 101210445. M.D. Steenwijk is supported by research grants from Merck, Atara Biotherapeutics, and Biogen. H. Vrenken has received research support from Merck, Novartis, Pfizer, and Teva, consulting fees from Merck, and speaker honoraria from Novartis; all funds were paid to his institution. B.M.J. Uitdehaag reports consultancy fees from Immunic Therapeutics. H.E. de Vries: nothing to disclose. B. Moraal: nothing to disclose. F. Barkhof is a steering committee or Data Safety Monitoring Board member for Biogen, Merck, ATRI/ACTC and Prothena. Consultant for Roche, Celltrion, Rewind Therapeutics, Merck, IXICO, Jansen, Combinostics. Research agreements with Merck, Biogen, GE Healthcare, Roche. Co‐founder and shareholder of Queen Square Analytics LTD. HV has received research support from Merck, Novartis, Pfizer, and Teva, consulting fees from Merck, and speaker honoraria from Novartis; all funds were paid to his institution. C.E. Teunissen: reports funding from National MS Society (Progressive MS alliance) and Innovative Medicines Initiatives 3TR (Horizon 2020, grant no 831434); has a research contract with Celgene; serves on editorial boards of Medidact Neurologie/Springer, Neurology: Neuroimmunology & Neuroinflammation, and is editor of a Neuromethods book Springer. J. Killestein received consulting fees for F. Hoffmann‐La Roche, Biogen, Teva, Merck, Novartis and Sanofi/Genzyme (payments to institution); reports speaker relationships with F. Hoffmann‐La Roche, Biogen, Teva, Merck, Novartis and Sanofi/Genzyme (payments to institution); adjudication committee of MS clinical trials of Immunic (payments to institution). E. M. M. Strijbis reports speaker relations with Merck and Novartis (all payments to institution) and served on advisory board for Roche. M. Schoonheim serves on the editorial board of Neurology and Frontiers in Neurology, receives research support from the Dutch MS Research Foundation, Eurostars‐EUREKA, ARSEP, Amsterdam Neuroscience, MAGNIMS and ZonMW (Vidi grant, project number 09150172010056) and has served as a consultant for or received research support from Atara Biotherapeutics, Biogen, Celgene/Bristol Meyers Squibb, EIP, Sanofi, MedDay and Merck.

## EPO‐0926

### Impact of OCT acquisition parameters on GCIPL thickness measurements in multiple sclerosis: A protocol comparison

#### N. Krajnc ^1^; M. Bertich^2^; F. Föttinger^1^; S. Macher^1^; F. Schwarz^1^; C. Stapf^2^; B. Pemp^2^; G. Bsteh^1^

##### ^1^Department of Neurology, Medical University of Vienna, Austria; ^2^Department of Ophthalmology, Medical University of Vienna, Vienna, Austria

**Background and aims:** Retinal layer thickness measured by optical coherence tomography (OCT), particularly the ganglion cell and inner plexiform layer (GCIPL), is an established marker of neuroaxonal integrity in multiple sclerosis (MS). However, small longitudinal changes at the individual level often approach inter‐scan variability, limiting clinical utility.

**Methods:** In this prospective study, participants with MS (pwMS) diagnosed according to the 2017 McDonald criteria and age‐ and sex‐matched healthy controls (HC) underwent spectral‐domain OCT at baseline and after two and four weeks. At each visit, five macular scan protocols were acquired: standard, high‐ART, high‐lines, high‐resolution, and maximum. Reliability was assessed using intraclass correlation coefficients (ICC; two‐way mixed‐effects model, absolute agreement). Absolute test‐retest reproducibility was quantified using mean absolute difference (MAD).

**Results:** Thirty‐eight eyes from 9 pwMS (mean age 34.1 years [SD: 8.0], 44.4% female) and 10 HC (31.7 years [SD: 11.1], 50.0% female) were included. Mean baseline GCIPL thickness ranged from 70.8 to 71.5 μm across protocols, demonstrating excellent inter‐protocol agreement (ICC 0.99; 95% CI: 0.98–0.99; *p* < 0.001). All protocols showed excellent test‐retest reliability. High‐ART and high‐lines protocols demonstrated marginally lower absolute measurement variability (MAD 0.26–0.27; 95% CI: 0.21–0.32), while temporal agreement remained comparable across protocols. Mean acquisition time was shortest for the standard protocol (10.6 s) and increased progressively for high‐resolution, high‐lines, high‐ART, and maximum protocols.

**Conclusion:** All OCT acquisition protocols demonstrated excellent inter‐protocol and test‐retest reliability for GCIPL measurements. The high‐lines protocol provides the most favorable balance between measurement reliability and acquisition time, supporting its use for longitudinal GCIPL monitoring.

**Disclosure:** The authors declare no conflicts of interest related to this study.

## EPO‐0927

### Exploring genetically determined leukocyte telomere length in relation to age at onset in multiple sclerosis

#### G. Visentin ^1^; F. Clarelli^2^; M. Sorosina^2^; A. Giordano^1^; E. Mascia^2^; M. Rocca^1^; S. D’Alfonso^3^; M. Filippi^1^; F. Esposito^2^

##### ^1^Vita‐Salute San Raffaele University, Milan, Italy; IRCCS San Raffaele Hospital, Milan, Italy; ^2^IRCCS San Raffaele Hospital, Milan, Italy; ^3^Department of Health Sciences, UPO University of Eastern Piedmont Novara, Italy. CAAD (Interdepartmental Research Center of Autoimmune and Allergic Diseases), University of Eastern Piedmont Novara, Italy

**Background and aims:** Telomere length is a marker of biological ageing and immune‐cell senescence. In multiple sclerosis (MS), telomere shortening has been linked to disease severity, but its relationship with age at onset (AAO), a major source of phenotypic heterogeneity, remains incompletely understood. Genetically determined leukocyte telomere length (gLTL) provides an opportunity to investigate telomere biology independently of disease‐related and environmental influences.

**Methods:** We analysed 2,824 relapsing–remitting MS patients with genome‐wide genotype data imputed to the Haplotype Reference Consortium panel. Polygenic risk scores for gLTL (gLTL‐PRS) were derived using summary statistics from a large genome‐wide association study of UK Biobank participants. Eight PRS were constructed using a clumping‐and‐thresholding approach across increasing p‐value thresholds. Associations between gLTL‐PRS and AAO were assessed using linear regression models adjusted for relevant covariates.

**Results:** Across all PRS models, higher genetic predisposition to longer telomere length was associated with later AAO in MS. The strongest association was observed using the most inclusive PRS (*p*‐value threshold <0.2; beta = 0.57, SE=0.21, *p* = 0.008), consistent with a polygenic contribution of telomere‐related variants to AAO variability.

**Conclusion:** These findings suggest that genetic variation underlying telomere biology may contribute to inter‐individual differences in AAO in MS. The progressive strengthening of associations across increasingly inclusive PRS models supports the complex polygenic nature of this relationship and highlights telomere‐related pathways as potentially relevant to MS phenotypic heterogeneity.

**Disclosure:** G. Visentin: nothing to disclose; F. Clarelli: nothing to disclose; M. Sorosina: nothing to disclose; A. Giordano: received consulting fees and travel grants from Novartis; E. Mascia: nothing to disclose; M. Rocca: received MS financial research support from Society of Canada, the Italian Ministry of Health, the Italian Ministry of University and Research, and Fondazione Italiana Sclerosi Multipla; consulting fees from Biogen, Bristol Myers Squibb, Eli Lilly, Janssen, Roche; speaking honoraria from AstraZaneca, Biogen, Bristol Myers Squibb, Bromatech, Celgene, Genzyme, Horizon Therapeutics Italy, Merck Serono SpA, Novartis, Roche, Sanofi and Teva; S. D’Alfonso: nothing to disclose; M. Filippi: received financial research support from Biogen Idec, Merck‐Serono, Novartis, Roche, the Italian Ministry of Health, the Italian Ministry of University and Research, and Fondazione Italiana Sclerosi Multipla; consulting fees from Alexion, Almirall, Biogen, Merck, Novartis, Roche, Sanofi; honoraria from Bayer, Biogen, Celgene, Chiesi Italia SpA, Eli Lilly, Genzyme, Janssen, Merck‐Serono, Neopharmed Gentili, Novartis, Novo Nordisk, Roche, Sanofi, Takeda, and TEVA, Biogen, Merck, Roche, Celgene, Bristol‐Myers Squibb, Lilly, Novartis, Sanofi‐Genzyme; F. Esposito: received consulting fees from Merck Serono and Genzyme.

## EPO‐0928

### Mitochondrial impact of fingolimod on peripheral immune cells in multiple sclerosis

#### I. Gómez‐Delgado ^1^; A. González‐Jiménez^1^; P. López‐Cotarelo^1^; A. Moreno‐Jerez^1^; J. Vela‐Artiza^1^; E. Mena‐Plaza^1^; Y. Aladro^2^; B. Pilo^2^; C. Oreja‐Guevara^3^; I. Gómez‐Estévez^3^; A. R.López‐Pastor^1^; E. Urcelay^1^

##### ^1^Laboratory of Genetics and Molecular Bases of Complex Diseases, Health Research Institute of Hospital Clínico San Carlos (IdISSC), Madrid, Spain; ^2^Department of Neurology, Hospital Universitario de Getafe, Getafe, Spain; ^3^Department of Neurology, Hospital Clínico San Carlos, Health Research Institute of Hospital Clínico San Carlos (IdISSC), Madrid, Spain

**Background and aims:** Fingolimod (FLM) limits lymphocyte egress from lymphoid organs. Mitochondrial dysfunction and metabolic reprogramming contribute to multiple sclerosis (MS) pathogenesis and disease progression. However, the impact of FLM treatment on mitochondrial activity and metabolism of peripheral immune cells (PBMCs) remains incompletely characterized. The aim of this study is to evaluate FLM effects on mitochondrial function of PBMCs from MS patients, compared with interferon (INF)‐treated patients and controls (HC).

**Methods:** Mitochondrial respiration and glycolytic activity were assessed by Seahorse XFp extracellular flux analyzer in MS patients treated with FLM (*n* = 24) or IFN (*n* = 45), and HC (*n* = 35). Mitochondrial mass, membrane potential, reactive oxygen species (ROS) production, and glycolytic mediators were evaluated by flow cytometry. Mitochondrial electron transport chain (ETC) complexes were analyzed by Western Blot.

**Results:** In RRMS patients with FLM therapy, a pre‐stimulated respiratory profile with significantly higher basal, maximal, and ATP‐linked respiratory values were detected compared to IFN‐treated patients and HC. Milder pre‐stimulation, but also precluding further PHA‐stimulation, was found in SPMS patients. In parallel, significant increases in unstimulated basal and maximal glycolytic capacities upon FLM‐treatment led to a lower glycolytic reserve upon PHA‐stimulation. FLM treatment produced lower fold‐changes after PHA and significantly decreased mitochondrial depolarization with lower increase of complex II, leaving ROS and coupling at basal levels. After PHA‐stimulation, decreased fold‐changes of mitochondrial mass and lactate transporters (MCT1/4) were observed in FLM‐treated compared to IFN‐treated patients and HC, with no changes in PFKB3.

**Figure:**
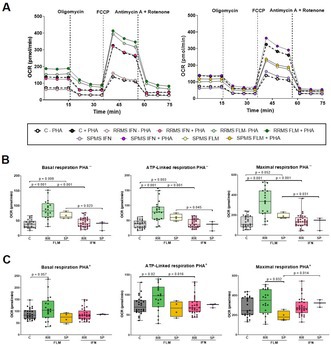
**FIGURE 1** Effects of fingolimod on mitochondrial respiration of PBMCs stratified by MS clinical form. Oxygen consumption rate profiles and parameters of PBMCs from healthy controls and MS patients treated with IFN or FLM.

**Figure:**
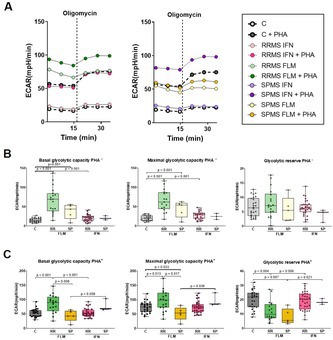
**FIGURE 2** Effect of fingolimod on glycolysis of PBMCs stratified by MS clinical forms. Extracellular acidification rate profiles and parameters of PBMCs from healthy controls and MS patients treated with IFN or FLM.

**Conclusion:** Fingolimod modulates mitochondrial activity and PBMCs metabolism, which probably contribute to its therapeutic response in MS patients.

**Disclosure:** C.O.G. has received speaking and/or consultancy fees from Alexion, Biogen Idec, Bristol‐Myers‐Squibb, Janssen, Merck, Novartis, Roche, Sanofi‐Genzyme, and Teva. Y.A. has received research grants, travel support, and lecturing and consulting fees from Bayer, Biogen, Roche, Merck, Novartis, Almirall, Sanofi‐Genzyme, Janssen, and Bristol Myers Squibb. B.P. has received speaking, travel and/or training fees from Novartis, Almirall, Merck and Sanofi‐Genzyme. The other authors nothing to disclose.

## EPO‐0929

### B cell activating factor gene variants and anti–JC virus antibody positivity in male patients with multiple sclerosis treated with natalizumab

#### E. Abed

##### Neurology Department, Al‐Azhar University, Cairo, Egypt

**Background and aims:** Progressive multifocal leukoencephalopathy is a serious complication of natalizumab‐treated multiple sclerosis, with anti–John Cunningham virus antibodies used for risk stratification. This study explores the role of the interferon‐β–B cell activating factor axis in anti–John Cunningham virus antibody development.

**Methods:** This prospective study was conducted at Al‐Azhar University Hospitals and Cairo Fatemic Hospital and included 186 patients with relapsing–remitting MS receiving natalizumab between October 2022 and November 2025. Serum anti‐JCV antibody levels were assessed at baseline and after 12 and 24 months of treatment. Expression of type I and II IFN‐inducible genes and BAFF was measured in peripheral blood using quantitative RT‐PCR. BAFF gene polymorphisms (rs9514828, rs1041569, rs9514827) were analyzed using RFLP‐PCR.

**Results:** No significant association was observed between type I or II IFN‐inducible gene expression and serum anti‐JCV antibody titers. In contrast, anti‐JCV levels showed a significant correlation with BAFF gene expression. The BAFF TTT haplotype was significantly more prevalent among male anti‐JCV–positive patients compared with anti‐JCV–negative males at baseline, 12 months, and 24 months of natalizumab therapy. The BAFF TTT haplotype demonstrated 88% specificity, 70% sensitivity, and a 45% positive predictive value for identifying anti‐JCV–positive male patients after 24 months of treatment.

**Figure:**
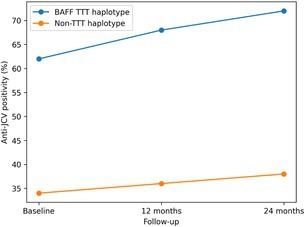
**FIGURE 1** Anti‐JCV seropositivity over time in male MS patients.

**Figure:**
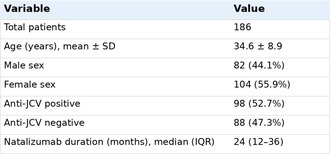
**TABLE 1** Baseline characteristics.

**Figure:**
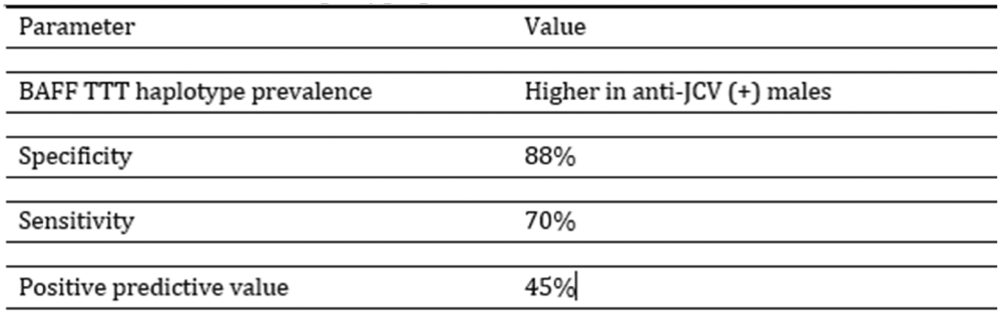
**TABLE 2** BAFF TTT Haplotype performance.

**Conclusion:** These findings support a role for the BAFF pathway in regulating anti‐JCV antibody production. The BAFF TTT haplotype may represent a novel genetic risk marker for anti‐JCV seropositivity and, indirectly, for PML development in male MS patients treated with natalizumab.

**Disclosure:** Nothing to disclose.

## EPO‐0930

### Abstract withdrawn

## EPO‐0931

### Confirmed disability accrual in multiple sclerosis patients with expanded disability status scale score 5.5

#### S. Degl'Innocenti ^1^; E. Portaccio^1^; M. Betti^1^; E. De Meo^1^; L. Pastò^2^; A. Lugaresi^3^; E. Cocco^4^; G. De Luca^5^; F. Patti^6^; V. Torri Clerici^7^; D. Ferraro^8^; V. Brescia Morra^9^; G. Salemi^10^; M. Vianello^11^; R. Cerqua^12^; M. Inglese^13^; M. Rovaris^14^; P. Perini^15^; S. Romano^16^; C. Pozzilli^17^; C. Tortorella^18^; A. Di Sapio^19^; P. Annovazzi^20^; M. Simone^21^; P. Iaffaldano^22^; M. Filippi^23^; M. Trojano^22^; M. Amato^1^

##### ^1^Department of Neurofarba, University of Florence, Florence, Italy; ^2^Careggi University Hospital, Florence, Italy; ^3^Dipartimento di Scienze Biomediche e Neuromotorie, Università di Bologna, Bologna, Italy; ^4^Department Medical Science and Public Health, University of Cagliari, Centro Sclerosi Multipla, Cagliari, Italy; ^5^University G. d”Annunzio di Chieti‐Pescara, Neuroscience, Imaging and Clinical Sciences, Chieti, Italy; ^6^University of Catania, Department of Medical and Surgical Sciences and Advanced Technologies "G.F. Ingrassia", Catania, Italy; ^7^Fondazione IRCCS Istituto Neurologico C. Besta, Neuroimmunology Unit, Milan, Italy; ^8^Dipartimento di Neuroscienze ‐ Ospedale Civile di Baggiovara ‐ Azienda Ospedaliero‐Universitaria, Modena, Italy; ^9^Department of Neuroscience (NSRO), Federico II University, Naples, Multiple Sclerosis Clinical Care and Research Center, Naples, Italy; ^10^Department of Biomedicine, Neuroscience and Advanced Diagnostics, University of Palermo, Palermo, Italy; ^11^Ca' Foncello Hospital, AULSS2, Neurology, Treviso, Italy; ^12^Clinica Neurologica ‐ AOU delle Marche, Ancona, Italy; ^13^IRCCS Ospedale Policlinico San Martino, Genova, Italy; ^14^Centro SM ‐ Fondazione Don Carlo Gnocchi IRCCS, Milan, Italy; ^15^Centro Specializzato Regionale per la Sclerosi Multipla (CeSMuV), Regione Veneto, Dipartimento di Neuroscienze DNS, Azienda Ospedaliera, Università degli Studi di Padova, Padova, Italy; ^16^Centro Neurologico Terapie Sperimentali – Università La Sapienza di Roma ‐ AO S. Andrea, Rome, Italy; ^17^Centro SM ‐ Policlinico S. Andrea ‐ Università La Sapienza di Roma, Rome, Italy; ^18^Centro Sclerosi Multipla ‐ AO S. Camillo Forlanini, Rome, Italy; ^19^Centro di riferimento Regionale SM (CRESM) ‐ SCDO Neurologia ‐ AOU San Luigi Gonzaga, Orbassano, Torino, Italy; ^20^Neurologia ad Indirizzo Neuroimmunologico ‐ Centro Sclerosi Multipla ‐ ASST della Valle Olona, Ospedale di Gallarate, Gallarate (VA), Italy; ^21^University ‘Aldo Moro’ of Bari, Child Neuropsychiatric Unit, Department of Biomedical Sciences and Human Oncology, Bari, Italy; ^22^University of Bari Aldo Moro, Department of Basic Medical Sciences, Neurosciences and Sense Organs, Bari, Italy; ^23^Vita‐Salute San Raffaele University, Milan, Italy

**Background and aims:** There is no consensus on the definition of disability accrual (DA) in patients with multiple sclerosis (MS) with a baseline Expanded Disability Status Scale (EDSS) score of 5.5. Indeed, progression has been variably defined as either a 1.0‐ or 0.5‐point increase. This study aims to compare the proportion of DA and its confirmation at the end of the follow‐up using the two definitions.

**Methods:** In this multicentric, retrospective, cohort study based on prospectively acquired data from the Italian Multiple Sclerosis Register, inclusion criteria were: diagnosis of Clinically Isolated Syndrome or relapsing‐remitting MS, first visit on or after January 1, 2000, ≥3 EDSS evaluations, ≥5 years of follow‐up. In patients with roving baseline EDSS of 5.5, confirmed DA (CDA) was defined as a 24‐week confirmed disability increase of either 0.5‐ or 1‐point. Proportions of CDA and sustained DA (absence of subsequent EDSS improvement until follow‐up end) were calculated and compared.

**Results:** The whole sample included 30203 relapsing MS patients followed‐up for 11.3 + −4.3 years. A total of 228 (0.8%) patients had roving baseline EDSS = 5.5. Among those, CDA with 0.5‐ or 1‐point EDSS increase occurred in 200 (87.7%) and 59 (25.9%) patients, respectively (*p* < 0.001). 104 out of 141 (73.8%) CDA with 0.5‐point EDSS increase and 51 out of 59 (86.4%) CDA with 1‐point EDSS increase were sustained at the end of the follow‐up (*p* = 0.05).

**Conclusion:** In patients with MS and baseline EDSS score of 5.5, the 0.5‐point CDA definition identifies a greater proportion of long‐term disability worsening than the 1.0‐point definition and should be preferred in this subgroup of patients.

**Disclosure:** E. Portaccio has received compensation for travel, advisory boards and/or speaking activities from Biogen, Merck Serono, Sanofi, Teva, Roche, BMS Celgene, Janssen, and Novartis, and serves on the editorial board of Frontiers in Neurology and Brain Sciences. L. Pastò received research support from Novartis and Biogen and speaker honoraria from Teva. A. Lugaresi has served on advisory boards and received honoraria or travel support from multiple companies including Biogen, Merck, Novartis, Roche, Sanofi/Genzyme, and others; her institutions received research grants from Novartis, Roche, and Sanofi/Genzyme. E. Cocco, F. Patti, V.L.A.M. Torri Clerici, D. Ferraro, V. Brescia Morra, G. Salemi, M. Vianello, R. Cerqua, P. Perini, S. Romano, C. Pozzilli, C. Tortorella, A. Di Sapio, P. Annovazzi, P. Iaffaldano, and M. Trojano received grants, honoraria, travel support, and/or served on advisory boards for pharmaceutical companies including Almirall, Alexion, Bayer, Biogen, BMS/Celgene, Janssen, Merck (Serono), Novartis, Roche, Sanofi/Genzyme, Teva, and others. M. Inglese received research grants from NIH, NMSS, and FISM and consultation fees from Roche, Genzyme, Merck, Biogen, and Novartis. M. Filippi is Editor‐in‐Chief of the Journal of Neurology and received consulting fees, speaker honoraria, and research support from multiple pharmaceutical companies and public institutions. M.P. Amato served on advisory boards and received speaker honoraria and research support from Biogen Idec, Merck Serono, Bayer Schering Pharma, and Sanofi Aventis and serves on editorial boards of Multiple Sclerosis Journal and BMC Neurology. All other authors report no relevant disclosures.

## EPO‐0932

### Satralizumab for the treatment of neuromyelitis optica spectrum disorders: A single center real life experience

#### V. Marino ^1^; I. Gattuso^1^; A. De Simone^1^; S. Guerrieri^1^; M. Margoni^2^; M. Rocca^3^; L. Moiola^1^; M. Filippi^4^

##### ^1^Neurology Unit, Multiple Sclerosis Center, IRCCS San Raffaele Scientific Institute, Milan, Italy; ^2^Neurology Unit, Multiple Sclerosis Center and Neuroimaging Research Unit, Division of Neuroscience IRCCS San Raffaele Scientific Institute, Milan, Italy; ^3^Neurology Unit, Multiple Sclerosis Center and Neuroimaging Research Unit, Division of Neuroscience IRCCS San Raffaele Scientific Institute, Milan, Italy. Vita‐Salute San Raffaele University, Milan, Italy; ^4^Neurology Unit, Multiple Sclerosis Center, Neurorehabilitation Unit, Neurophysiology Service and Neuroimaging Research Unit, Division of Neuroscience IRCCS San Raffaele Scientific Institute, Milan, Italy. Vita‐Salute San Raffaele University, Milan, Italy

**Background and aims:** Neuromyelitis optica spectrum disorders (NMOSD) is a rare, inflammatory, central nervous system disease historically treated with rituximab (RTX). Satralizumab (SATRA), an anti‐IL6 receptor monoclonal antibody, has recently been approved for AQP4‐IgG‐positive patients switching from RTX for inefficacy or adverse events. We report our real‐world experience.

**Methods:** We retrospectively collected demographic, clinical and MRI data from all patients starting SATRA at our center between February 2023 and July 2025.

**Results:** 11 NMOSD patients (10 females) were enrolled. At SATRA initiation, median age was 48.5 years (25.7–71.8), median disease duration was 16.3 years (range: 1.0–34.1), median EDSS was 3.5 (0–6.0). Prior therapy was tocilizumab (TOCI) in 5/11 patients and RTX in 6/11. Among TOCI‐treated patients, 3/5 had previously received RTX and 2/5 autologous hematopoietic stem‐cell transplantation. TOCI was started for disease activity and the switch to SATRA was to shift to an on‐label anti‐IL‐6R drug. All RTX switched for safety and among them 5/6 had hypogammaglobulinemia with recurrent infections. One started monthly intravenous immunoglobulin supplementation in February 2025. Over a median SATRA follow‐up of 2.2 years (0.6–3.0) all patients remained clinically stable (median EDSS 3.5, 0–6.0) and showed no radiological disease activity. For patients switching from TOCI, global follow‐up in anti‐IL‐6R therapy was 10.3 years (4.1–12.9).

**Conclusion:** In our small cohort, SATRA has proven to be effective and well tolerated, including long‐term anti‐IL‐6R treated patients. Those who switched to SATRA to change mechanism of action stopping B‐cell depletion because of worsening hypogammaglobulinemia all experienced a reduction of adverse events in both frequency and severity.

**Disclosure:** VM has nothing to disclose. IG received honoraria for speaking from Novartis, Roche and Merck. ADS has nothing to disclose. SG received compensation for speaking activities from Bristol Squibb Meyer, Novartis, Merck. MM received grants and personal fee from Sanofi, Genzyme, Merck, Roche, Biogen, Amgen and Novartis. MAR received consulting fees and/or speaker honoraria from Biogen, Bristol Myers Squibb, Eli Lilly, Janssen, Roche, AstraZeneca, Bromatech, Celgene, Genzyme, Horizon Therapeutics Italy, Merck Serono, Novartis, Sanofi and Teva. She receives research support from the MS Society of Canada, the Italian Ministry of Health and FISM. LM received compensations for speaking activities and/or for participating to advisory board from Merck, Celgene, Biogen, Sanofi, Novartis, Roche, Alexion, and Amgen. MF received compensation for consulting services and/or participation in Advisory Boards from Alexion, Almirall, Biogen, Merck, Novartis, Roche, Sanofi; speaking activities from Bayer, Biogen, Celgene, Chiesi Italia SpA, Eli Lilly, Genzyme, Janssen, Merck‐Serono, Neopharmed Gentili, Novartis, Novo Nordisk, Roche, Sanofi, Takeda, and TEVA; scientific direction of educational events for Biogen, Merck, Roche, Celgene, Bristol‐Myers Squibb, Lilly, Novartis, Sanofi‐Genzyme; he receives research support from Biogen Idec, Merck‐Serono, Novartis, Roche, the Italian Ministry.

## EPO‐0933

### Multiple sclerosis and outpatient physician care in the last years of life: A population‐based study

#### W. Kim ^1^; C. Watt^2^; S. Fung^2^; S. Yoo^1^; C. Webber^2^; D. Rotstein^3^; M. Howard^4^; P. Tanuseputro^5^; U. Ramanathan^3^

##### ^1^University of Ottawa, Ottawa, Canada; ^2^Bruyère Health Research Institute, Ottawa, Canada; ^3^University of Toronto, Toronto, Canada; ^4^McMaster University, Canada; ^5^University of Hong Kong, *Hong Kong*

**Background and aims:** Patients with MS require increasing healthcare support, particularly in the last year of life. Despite the benefits of palliative care in addressing symptom burden and improving quality of life, its integration into MS care remains inconsistent. This study aimed to characterize healthcare utilization and palliative care access among people with MS in the last years of life and to identify key factors associated with optimal care.

**Methods:** A retrospective cohort study was conducted using linked health administrative data. The cohort consisted of all adult decedents between 2016 and 2020, and we compared health care utilizations. Demographics, healthcare utilization, palliative care involvement, and medical interventions were analyzed. Predictors of palliative care receipt and hospital deaths were evaluated.

**Results:** MS decedents (*n* = 1975) were younger and had fewer comorbidities than non‐MS decedents (*n* = 500,904). High percentage of MS decedents had outpatient neurology visits in the last five years of life but a steep decline closer to death. Palliative care use was similar but occurred earlier in MS. Multivariable regression showed that rural residence was associated with increased odds of hospital death and lower odds of receiving palliative care, while receiving palliative care within the last five years of life reduced the odds of hospital death. Higher comorbidity and outpatient visits to urology were associated with a greater odd of receiving palliative care.

**Conclusion:** While palliative care receipt was comparable, rural disparities and high hospital deaths persist in MS. Earlier palliative integration, particularly through neuropalliative care, could improve quality of life and reduce hospitalizations.

**Disclosure:** Nothing to disclose.

## Sleep‐Wake Disorders 3

## EPO‐0934

### JU SLEEP WELL: A multiprofessional project designed to develop and test a digital CBT intervention to improve quality of life in patients with RLS

#### E. Odzakovic^1^; M. Ulander^2^; J. Lind^3^; F. Lundin^4^; A. Pakpour^1^; A. Hellström^5^; C. Fagerström^6^; C. Sandlund^7^; S. Öberg^1^; B. Fridlund^8^; M. Björk^1^; A. Broström ^1^

##### ^1^School of Health and Welfare, Jönköping University, Jönköping, Sweden; ^2^Department of Clinical Neurophysiology, Linköping University Hospital, Linköping, Sweden. Department of Biomedical and Clinical Sciences, Division of Neurobiology. Linköping University, Linköping, Sweden; ^3^Section of Neurology, Department of Internal Medicine, County Hospital Ryhov, Jönköping, Sweden; ^4^Department of Neurology, and Department of Biomedical and Clinical Sciences, Linköping University, Linköping, Sweden; ^5^Department of Health and Caring Sciences, Faculty of Health and Life Sciences, Linnaeus University, Kalmar, Sweden. 7Academic Primary Care, Öland, Kalmar County, Sweden. Kalmar County Region, Research Section; ^6^Kalmar County Region, Research Section; ^7^Department of Neurobiology, Care Sciences and Society, Karolinska Institute, Stockholm, Sweden, 10Academic Primary Health Care Centre, Region Stockholm, Stockholm, Sweden; ^8^Centre for Interprofessional Collaboration within Emergency Care (CICE), Linnaeus University, Växjö, Sweden

**Background and aims:** Restless Legs Syndrome (RLS) is a difficult‐to‐treat chronic neurological condition. Cognitive Behavioral Therapy (CBT) can support coping and self‐care in chronic diseases, yet no evidence‐based digital CBT programs exist to complement medication for RLS. Therefore, this project aims to investigate, from the perspectives of patients, relatives, and healthcare professionals, gender‐ and age‐related differences in experiences, symptom management, and treatment needs among patients with RLS, and to co‐create a digital CBT‐based intervention.

**Figure:**
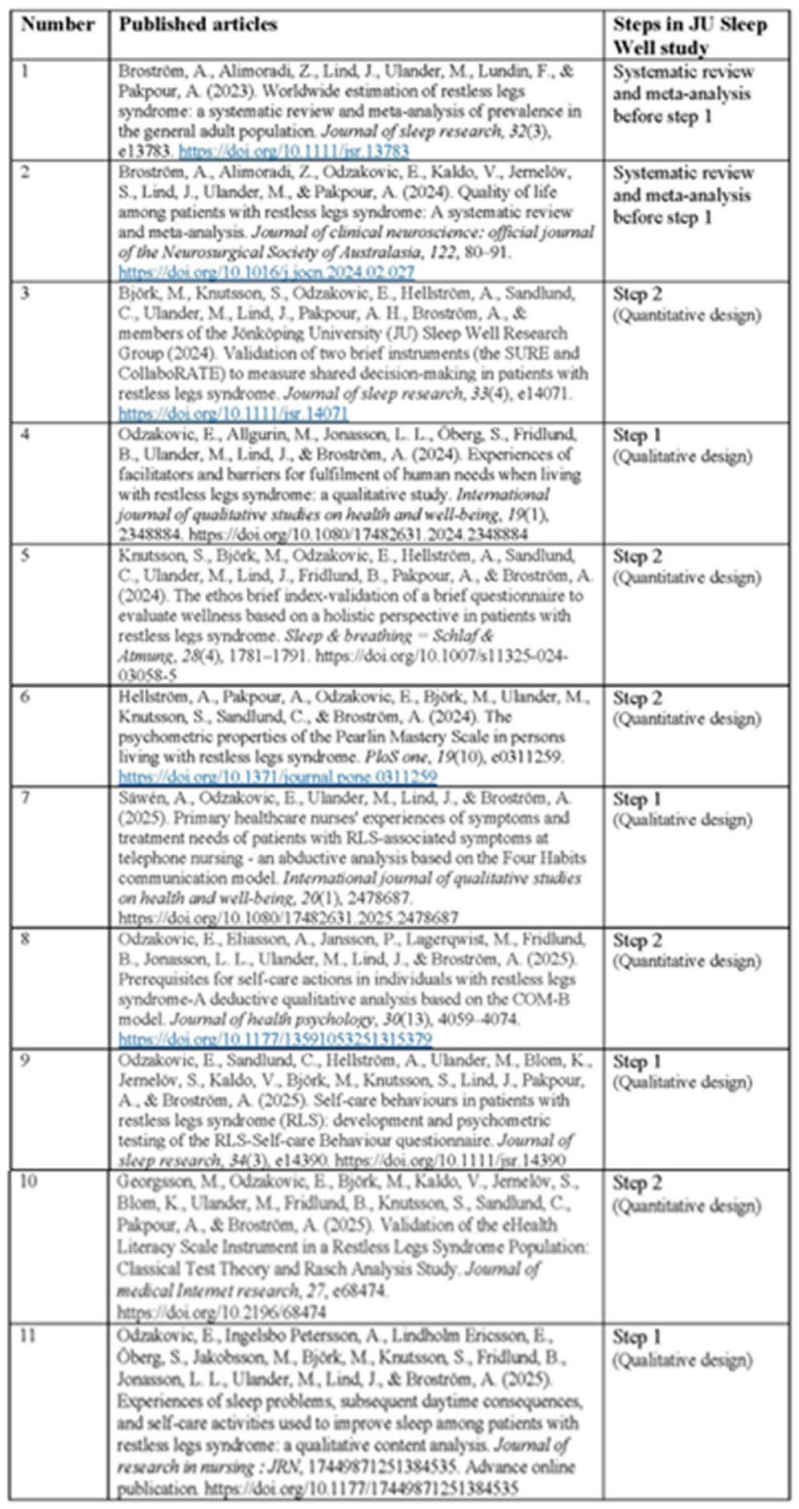
**TABLE 1** Overview of the steps in JU SLEEP WELL study.

**Methods:** A four‐step longitudinal multimethod design, including a randomized controlled trial (RCT) with long‐term follow‐up, will be used (Figure 1). Approximately 30 multiprofessional members, including people with RLS, researchers, and healthcare professionals (physicians, nurses, psychologists, and sociologists) specialized in RLS, sleep problems, and CBT, collaborate in the co‐design of the digital CBT‐based intervention.

**Figure:**
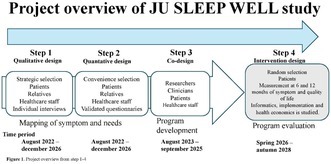
**FIGURE 1** Project overview from step 1–4.

**Results:** Step 1 (2022–2026) used various qualitative methods to explore and describe RLS‐related care needs as experienced by patients, relatives, and healthcare professionals. Step 2 (2022–2026) validated five instruments for shared decision‐making, mastery, wellness, e‐health literacy, and self‐care. Steps 1 and 2 have produced 11 publications to date (Table 1). In Step 3 (2026), the co‐designed digital CBT‐based intervention will be pilot‐tested at one primary care center. In Step 4 (2026–2028), a multicenter RCT conducted at eleven primary care centers will evaluate its long‐term (12‐month) effects.

**Conclusion:** The project's mapping of the experiences of patients, relatives, and healthcare professionals provides an evidence‐based foundation for the co‐created digital CBT‐based intervention, which, if successful, can complement pharmacological treatment and improve quality of life for patients with RLS.

**Disclosure:** Nothing to disclose.

## EPO‐0935

### Pupillometric biomarkers of autonomic dysfunction in isolated REM sleep behaviour disorder

#### B. Křupková ^1^; T. Serranová^1^; P. Dušek^1^; K. Šonka^1^; E. Růžička^1^; O. Slanař^2^; O. Bartošová^2^

##### ^1^Department of Neurology and Centre of Clinical Neuroscience, First Faculty of Medicine, Charles University and General University Hospital, Prague, Czech Republic; ^2^Department of Pharmacology, First Faculty of Medicine, Charles University and General University Hospital, Prague, Czech Republic

**Background and aims:** Pupillary abnormalities are common in neurodegenerative disorders. In isolated REM sleep behaviour disorder (iRBD), increased pupil size and reduced pupillary light reflex have been reported, suggesting parasympathetic dysfunction. This study aimed to assess pupillary abnormalities in prodromal synucleinopathy using a bedside portable pupillometer.

**Methods:** Sixty individuals with iRBD (mean age = 66.9 years, SD = 9.6; 5 females) and 48 controls (mean age = 67.0 years, SD = 9.7; 5 females) underwent pupillometry using a portable infrared pupillometer (Neuro‐Light, IDMed, France) after 5 minutes of dark adaptation. Five measurements were obtained from each eye following stimulation with a 320‐lux white light flash lasting 1 second. Median values of maximum and minimum pupil diameter, their difference and variation were calculated for each eye. Maximum constriction velocity and latency to onset of constriction were calculated as median values from ten measurements across both eyes. Between‐group comparisons were performed using ANCOVA with age as a covariate.

**Results:** After adjustment for age, the iRBD cohort showed significantly prolonged pupillary constriction latency compared with controls (239.3 ms, SD = 34.3 ms vs. 215.6 ms, SD = 37.9; *p* = 0.002). No significant group differences were found in other parameters. Age significantly influenced most pupil size–related measures.

**Conclusion:** Pupillometry identified prolonged pupillary constriction latency in iRBD, consistent with early parasympathetic dysfunction. However, latency alone is unlikely to represent a robust marker. Further studies exploring its relationship with other clinical or laboratory biomarkers are warranted.

**Disclosure:** Supported by NW24‐04‐00456 and NW25‐04‐0079.

## EPO‐0936

### Association of endoplasmic reticulum stress and BDNF levels with disease severity and sleep quality in patients with restless legs syndrome

#### C. Birimoglu Okuyan ^1^; H. Okuyan^2^; E. Çiçekli^3^; Ş. Ayçiçek Özen^2^; D. Kotan^3^

##### ^1^Department of Public Health Nursing, Sakarya University, Sakarya, Türkiye; ^2^Department of Physiotherapy and Rehabilitation, Sakarya University of Applied Sciences, Sakarya, Türkiye; ^3^Department of Neurology, Sakarya University, Sakarya, Türkiye

**Background and aims:** Restless legs syndrome (RLS) is a neurological condition associated with a significant socioeconomic burden and reduced quality of life. Endoplasmic reticulum (ER) stress can trigger adverse cellular processes, including inflammation and cell death, and has been implicated in various neurological disorders. Brain‐derived neurotrophic factor (BDNF) plays a crucial role in neuroplasticity, neuronal development, neuroprotection, and neuronal survival. However, the precise involvement of ER stress and BDNF in the molecular pathogenesis of RLS remains unclear. Therefore, this study aimed to evaluate the expression of ER stress–related proteins and BDNF in patients with RLS, assess their diagnostic potential, and examine their relationships with disease severity, quality of life, sleep quality, depression, and laboratory parameters.

**Methods:** This cross‐sectional study included 35 patients with RLS and 25 healthy controls. Serum concentrations of ER stress–related proteins (ATF6, GRP78, and SERP1) and BDNF were measured using ELISA. Disease severity, sleep quality, quality of life, and depressive symptoms were assessed using validated scales.

**Results:** Serum levels of ATF6, GRP78, SERP1, and BDNF were significantly higher in patients with RLS compared to healthy controls. Correlation analyses revealed positive associations between these biomarkers and disease severity, as well as poorer sleep quality. ROC analyses indicated that ATF6 levels above 302.05 pg/mL, GRP78 above 1.24 ng/mL, SERP1 above 155.14 pg/mL, and BDNF above 6.50 ng/mL could predict the presence of RLS.

**Figure:**
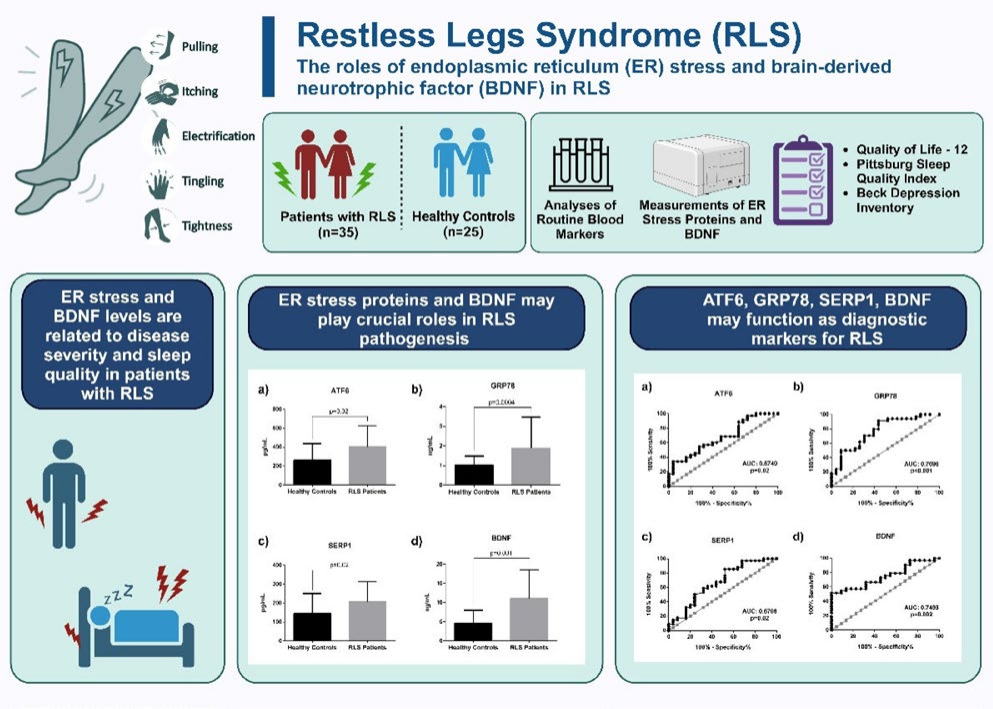
**FIGURE 1** Elevated ER stress proteins (ATF6, GRP78, SERP1) and BDNF in Restless Legs Syndrome and their associations with disease severity and sleep quality, highlighting their potential role in RLS pathogenesis and diagnostic value.

**Conclusion:** These findings provide novel evidence suggesting that ER stress and BDNF may play a significant role in the pathophysiology of RLS and may represent potential biomarkers for disease identification.

**Disclosure:** Nothing to disclose.

## EPO‐0937

### Interplay between diagnostic features and treatment biomarkers in narcolepsy type 1

#### B. Tracey ^1^; M. Olsen^1^; K. Galinsky^1^; R. Retnam^1^; Y. Gong^1^; P. Tierney^1^; E. Mignot^2^; D. Volfson^1^

##### ^1^Takeda Development Center Americas, Inc, Cambridge, USA; ^2^Stanford Center for Sleep Sciences and Medicine, Stanford University, Palo Alto, USA

**Background and aims:** Physiological features with diagnostic utility may also serve as disease‐specific prognostic and treatment‐response biomarkers. Here we study features emerging from recent work on multimodal diagnosis of narcolepsy type 1 (NT1), integrating sleep phenotyping, genetics, and artificial intelligence. We explore how these features change in individuals treated with oveporexton, an oral orexin receptor 2‐selective agonist, in one phase 2 trial (NCT05687903) and two phase 3 trials (The First Light [FL]: NCT06470828; The Radiant Light [RL]: NCT06505031).

**Methods:** Participants with NT1 were randomized to receive oveporexton (dose varied by trial) or placebo. Nocturnal polysomnography (nPSG) was collected at baseline and at end of treatment. Maintenance of Wakefulness Tests (MWTs) were performed after each PSG night. In addition to manual scoring, nPSG and MWT data were analyzed to compute hypnodensities from which overall sleep probability and NT1 diagnostic scores were calculated. Several NT1‐relevant sleep features were also evaluated. Treatment effects were evaluated with baseline‐adjusted models.

**Results:** Early‐night rapid eye movement (REM) features known to be elevated in NT1 were reduced with oveporexton versus placebo, including first‐quarter time in REM (*p* < 0.001 in all trials and treatment arms) and NT1‐specific transitions (REM to/from Wake or N1). The nPSG‐based NT1 diagnostic score was reduced in FL and RL 2 mg/2 mg arms versus placebo (both *p* < 0.05). In oveporexton‐treated participants, MWT sleep probability was reduced versus placebo (*p* < 0.05, Phase 2), consistent with reduced daytime sleepiness.

**Conclusion:** Diagnostic NT1 features responded significantly to oveporexton treatment, reflecting reduced daytime sleepiness and fewer sleep abnormalities.

**Disclosure:** This research was funded by Takeda Development Center Americas, Inc, who also provided funding to Envision Catalyst, an Envision Medical Communications agency, a part of Envision Pharma Group, for support in writing this abstract. BT, MO, KG, RR, YG, PT, and DV are employees of Takeda Development Center Americas, Inc, and stockholders in Takeda Pharmaceutical Company Limited. EM own stocks or received research grants/clinical trials, travel support or consultancy fees from Alkermes, Avadel, Centessa, Harmony Biosciences, Jazz Pharmaceuticals, Lundbeck, and Takeda.

## EPO‐0938

### Sleep biomarkers of stroke‐related delirium: The STORM study

#### E. Rollo ^1^; V. Brunetti^2^; F. Colò^3^; M. de Scisciolo^3^; R. Di Iorio^2^; M. Monforte^2^; A. Broccolini^2^; G. Frisullo^2^; P. Calabresi^2^; G. Della Marca^2^

##### ^1^Center for Neurodegenerative Diseases and the Aging Brain, University of Bari Aldo Moro at Pia Fondazione "Card. G. Panico", Tricase, Italy; ^2^Neurology Unit, Fondazione Policlinico Universitario A. Gemelli IRCCS, Rome, Italy; ^3^Department of Neurosciences, Università Cattolica del Sacro Cuore, Rome, Italy

**Background and aims:** Delirium is a frequent complication after acute stroke and is associated with worse outcomes. Sleep disturbances are common after stroke and may contribute to delirium, but evidence based on objective sleep measures is limited. The aim of this study is to investigate the association between sleep alterations and post‐stroke delirium, and to assess their impact on 90‐day functional outcome.

**Methods:** We retrospectively analyzed patients with acute ischemic stroke who underwent overnight bedside polysomnography during stroke unit hospitalization. Delirium was diagnosed using DSM‐5 criteria. Functional outcome at 90 days was assessed using the modified Rankin Scale (mRS).

**Results:** The cohort included 128 patients (mean age 70.8 ± 12.6 years, 53.1% females); delirium occurred in 28.9% of patients. Patients with delirium were older and had more severe strokes. Delirium was associated with lower sleep efficiency (SE) (*p* = 0.041), higher wake after sleep onset (*p* = 0.047), and lower REM% (*p* < 0.001). In multivariate analysis, lower SE independently predicted delirium (aOR 0.964, *p* = 0.010), along with higher stroke severity. At 90 days, delirium was strongly associated with poor functional outcome (mRS > 2). Unfavorable outcome was also linked with reduced SE (*p* = 0.032), shorter total sleep time (*p* = 0.015), reduced REM% (*p* < 0.001), higher nocturnal heart rate (*p* = 0.035), and increased daytime sleep (*p* = 0.002). Delirium, diabetes, and daytime sleep (aOR = 0.984; *p* = 0.037) independently predicted poorer recovery.

**Conclusion:** Reduced sleep efficiency is an independent risk factor for post‐stroke delirium, and sleep–wake disturbances are associated with worse long‐term outcomes. Sleep assessment in acute stroke may help identify patients at risk for delirium and represents a potential target for interventions to improve stroke prognosis.

**Disclosure:** Nothing to disclose.

## EPO‐0939

### Sleep disorders in dementia with Lewy bodies: A prospective cohort study

#### E. Rollo ^1^; L. Tamburrino^1^; D. Urso^1^; A. Valguarnera^1^; D. Vilella^1^; G. Volpe^1^; M. Sozzo^1^; A. Giugno^1^; A. Anastasia^2^; V. Gnoni^1^; G. Logroscino^1^

##### ^1^Center for Neurodegenerative Diseases and the Aging Brain, University of Bari Aldo Moro at Pia Fondazione "Card. G. Panico", Tricase, Italy; ^2^Department of Nuclear Medicine, Pia Fondazione "Card.G.Panico", Tricase, Italy

**Background and aims:** Sleep disturbances are highly prevalent in Dementia with Lewy Bodies (DLB), yet their role in the clinical phenotype remains under‐investigated. This study evaluates sleep disorders prevalence in DLB patients and their correlations with cognitive, and neuropsychiatric symptoms.

**Methods:** Consecutive patients with probable DLB or DLB‐Mild Cognitive Impairment (McKeith criteria 2017/2020) were enrolled in a tertiary neurodegenerative diseases clinic. All participants underwent a standardized diagnostic protocol, including neuropsychological assessment, and Dopamine Transporter (DAT) scan. Sleep was assessed in all patients both subjectively with questionnaires and objectively with overnight in‐lab videopolysomnography (VPSG).

**Results:** Thirty DLB patients (mean age 73.1 years; 60% males) were included. REM Behavior Disorder was diagnosed in 85.7% and insomnia in 50% of patients. Periodic limb movements (PLM > 15/h) were observed in 71.4%. Prevalence of Obstructive Sleep Apnea and Excessive Daytime Sleepiness was respectively 25% and 32.1%. Overall poor sleep quality was reported by 78.5% of patients. Worse performances in executive functions correlated with lower REM% (*p* = 0.008), and higher PLM index (*p* = 0.024), while worse performances in visuospatial functions correlated with higher N1% (*p* = 0.009). Lower mean saturation during sleep correlated with higher severity of visual hallucinations (*p* = 0.038). Lower DAT uptake in the caudate correlated with lower Sleep Efficiency (*p* = 0.022), lower N3% (*p* = 0.028), and higher PLM index (*p* = 0.045). Finally, Insomnia Severity Index (*p* = 0.021) and Epworth Sleepiness Scale (*p* = 0.018) inversely correlated with putamen uptake.

**Conclusion:** Sleep disorders are highly prevalent in DLB, being associated with worse cognitive functions and more severe neuropsychiatric symptoms. Reduced striatal uptake is associated with worse quality sleep, higher insomnia and daytime sleepiness.

**Disclosure:** Nothing to disclose.

## EPO‐0940

### Effect of oveporexton (TAK‐861) on cognitive symptoms in patients with narcolepsy type 1: Pooled data from two phase 3 trials

#### F. Pizza^1^; Y. Dauvilliers^2^; R. Del Rio Villegas^3^; G. Lammers ^4^; G. Plazzi^5^; T. Scammell^6^; T. Banerjee^7^; A. Cai^7^; P. Maruff^8^; H. Romero^7^; E. Stukalin^7^; B. Harel^7^

##### ^1^Department of Biomedical and Neuromotor Sciences, University of Bologna and IRCCS Istituto delle Scienze Neurologiche di Bologna, Bologna, Italy,^2^Sleep‐Wake Disorders Center, Department of Neurology, Gui‐de‐Chauliac Hospital, CHU, Institute of Neurosciences of Montpellier, INSERM, University of Montpellier and National Reference Network for Narcolepsy, Montpellier, France,^3^Neurophysiology and Sleep Disorders Unit, Vithas Hospitals and Department of Clinical Medical Sciences, Universidad Ceu San Pablo, CEU Universities, Madrid, Spain,^4^Sleep Wake Centre, Stichting Epilepsie Instellingen Nederland, Heemstede and Department of Neurology, Leiden University Medical Centre, Leiden, Netherlands,^5^Department of Biomedical, Metabolic and Neural Sciences, University of Modena and Reggio‐Emilia, Modena and IRCCS, Istituto delle Scienze Neurologiche, Bologna, Italy,^6^Department of Neurology, Beth Israel Deaconess Medical Center and Boston Children's Hospital, Boston, USA,^7^Takeda Development Center Americas, Inc, Cambridge, USA,^8^Cogstate, Melbourne, Victoria, Australia

**Background and aims:** Cognitive symptoms are frequent and disruptive in narcolepsy type 1 (NT1). Using pooled data from two randomized, double‐blind, phase 3 studies (The First Light: NCT06470828; The Radiant Light: NCT06505031) we report the effect of oveporexton (TAK‐861) on cognitive symptoms in participants with NT1.

**Methods:** Participants aged 16–70 years diagnosed with NT1 were randomized to twice daily oral oveporexton 1 mg (The First Light only), 2 mg, or placebo, ≥3 hours apart for 12 weeks. Cognitive impairment was assessed with neuropsychological tests of attention (Psychomotor Vigilance Test [PVT] from 2 intraday sessions [9 am, 3 pm]), memory (Continuous Paired Associate Learning, CPAL) and executive function (International‐Digit Symbol Substitution Test‐Symbols, IDSST‐S; One‐Back Test, ONB). Patient‐reported cognitive difficulties were assessed with the Functional Impacts of Narcolepsy Instrument Cognitive Function domain (FINI‐CF) and British Columbia Cognitive Complaints Inventory (BC‐CCI). For both trials, PVT and FINI‐CF were secondary endpoints while the remainder were exploratory.

**Results:** Overall, 273 participants were randomized to oveporexton 1 mg/1 mg (*n* = 61), 2 mg/2 mg (*n* = 136), or placebo (*n* = 76). Compared with placebo, 12‐week of treatment with oveporexton (1 mg/1 mg or 2 mg/2 mg) resulted in improvement from baseline for each objective (PVT, CPAL, iDSST‐s, ONB) and subjective (FINI‐CF; BC‐CCI) cognitive assessment. Group mean differences from placebo in change from baseline scores for each cognitive assessment are shown in Table 1.

**Figure:**
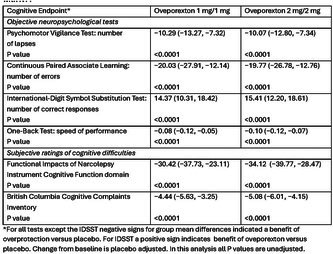
**TABLE 1** Group mean (least square and 95% CI) difference between oveporexton and placebo for endpoints from the objective and subjective cognitive assessments at week 12 in adults with NT1.

**Conclusion:** The benefit to cognition based on both objective neuropsychological tests and subjective ratings of cognition, following 12 weeks treatment, indicates that oveporexton reduces cognitive symptoms in NT1.

**Disclosure:** This research was funded by Takeda Development Center Americas, Inc, who also provided funding to Envision Catalyst, an Envision Medical Communications agency, a part of Envision Pharma Group, to provide writing assistance under the direction of the authors. Fabio Pizza participated in an advisory board for Takeda and received support for congress participation and funding from Bioprojet. Yves Dauvilliers received funds for seminars, board engagements, and travel to conferences from Avadel, Bioprojet, Idorsia, Jazz Pharmaceuticals, Centessa, Alkermes, Pharmanovia, and Takeda. Rafael Del Rio Villegas received consultancy fees from Alkermes, Bioprojet, and Takeda, and travel funds from Bioprojet, Jazz Pharmaceuticals, and Takeda. Gert J. Lammers has received consulting fees, grants, and travel funds from Alkermes, Bioprojet, Daiichi Sankyo, Eisai, Jazz Pharmaceuticals, and Takeda. Giuseppe Plazzi received consultancy fees from Bioprojet, Jazz Pharmaceuticals, Orexia, and Takeda. Thomas E Scammell received grants and personal fees from Takeda. Tathagata Banerjee, Alice Cai, Heather Romero, Ellie Stukalin, and Brian Harel are employees of Takeda Development Center Americas, Inc, and stockholders in Takeda Pharmaceuticals Company Limited. Paul Maruff is an employee of Cogstate Limited.

## EPO‐0941

### Improving automatic sleep stage scoring for narcolepsy through fine‐tuning with data from central disorders of hypersonmnolance

#### H. Thrainsson; Z. Zhang; K. Gevorgyan; J. Tepperberg; T. Lerchmüller; R. Khatami

##### Center of Sleep Medicine & Epileptology, Clinic Barmelweid, Barmelweid, Switzerland

**Background and aims:** Sleep stage classification is essential for narcolepsy diagnosis but remains time‐intensive. Current deep learning models, trained primarily on healthy controls and patients with common sleep disorders have, show reduced performance on narcolepsy patients due to the condition's low prevalence and distinct sleep patterns. We investigated whether fine‐tuning with data from central disorders of hypersomnolance (CDH) could improve automatic sleep stage classification in narcolepsy patients.

**Methods:** We employed a CNN‐Transformer network predicting sleep stages from one EOG and three EEG channels from overnight polysomnography. The baseline model was trained on 58 healthy controls, 18 OSA patients and 78 insomnia patients. Fine‐tuning used 15 insomnia patients, 7 OSA patients, 3 narcolepsy type 1 (NT1) patients, 4 narcolepsy type 2 (NT2) patients, 24 patients with other sleep disorders, and 23 narcolepsy borderland patients. Testing included 9 NT1, 6 NT2 and 8 narcolepsy borderland patients.

**Results:** The baseline model achieved Cohen's kappa of 0.72 and accuracy of 79%. Fine‐tuning improved kappa to 0.75 (*p* = 0.002) and accuracy to 81% (*p* = 0.002). N1 detection improved with kappa increasing from 0.46 to 0.54 (*p* < 0.001) and sensitivity from 45% to 56% (*p* < 0.001). REM stage detection showed kappa improvement from 0.77 to 0.79 (*p* < 0.001), and specificity from 93% to 97% (*p* < 0.001).

**Conclusion:** Fine‐tuning with rare sleep disorder data improved automatic sleep stage classification in narcolepsy patients. Enhanced N1 classification enables more precise sleep‐onset and fragmentation assessment, while improved REM stage detection facilitates SOREMP identification. These results demonstrate that targeted fine‐tuning can bridge performance gaps between common and rare sleep disorders in automated scoring.

**Disclosure:** Nothing to disclose.

## EPO‐0942

### Does age matter? Subjective and objective responses to hypoglossal nerve stimulation in older patients with obstructive sleep apnea

#### H. Moon ^1^; U. Cho^2^; C. Lee^3^; K. Im^4^

##### ^1^Department of Neurology, Soonchunhyang University Bucheon Hospital, Bucheon, Republic of Korea; ^2^Department of Psychiarty, Inha University Hospital, Incheon, Republic of Korea; ^3^Department of Internal Medicine and Lung Institute, Seoul National University College of Medicine, Seoul, Republic of Korea; ^4^Department of Clinical Psychiatry and Human Behavior, University of California, Irvine, USA

**Background and aims:** Obstructive sleep apnea (OSA) is common in elderly and is associated with higher CPAP failure rates due to comorbid sleep disorders and cognitive decline. Hypoglossal nerve stimulation (HNS) has emerged as an alternative treatment for OSA; however, data on age‐related treatment response remain limited.

**Methods:** We retrospectively analyzed 82 patients who underwent HNS implantation for OSA, stratified into ≤65 years (*n* = 29) and >65 years (*n* = 53). Subjective benefit was assessed at 12 months using a three‐point scale: very satisfied (2), somewhat satisfied (1), and dissatisfied (0). Treatment success was defined as a ≥50% reduction in apnea–hypopnea index (AHI) from baseline polysomnography to 12‐month home sleep test, with a final AHI ≤20 events/hour.

**Results:** Baseline polysomnographic parameters did not differ between age groups. During the HNS titration study, patients >65 years showed higher periodic limb movement index, longer sleep latency, and shorter total sleep time, while apnea–hypopnea index (AHI) was similar between groups. At 12‐month home sleep testing, total AHI did not differ by age; however, the >65‐year group demonstrated lower supine AHI and oxygen desaturation index, along with significantly greater subjective satisfaction. Overall treatment success was achieved in 42 patients (51.2%), with similar success rates in the >65‐year group (56.6%) and the ≤65‐year group (41.4%).

**Conclusion:** Despite less favorable sleep‐related findings during titration, older patients reported greater subjective satisfaction after HNS, with objective treatment success comparable to younger patients. Advanced age should not be considered a limiting factor for HNS therapy.

**Disclosure:** Nothing to disclose.

## EPO‐0943

### Telemedicine consultation as diagnostic triage for adult and children with suspected narcolepsy: The TENAR reliability and diagnostic accuracy study

#### L. Vignatelli ^1^; F. Pizza^2^; S. Vandi^2^; F. Baccari^1^; C. Zenesini^1^; F. Ingravallo^3^; G. Plazzi^4^

##### ^1^IRCCS Istituto Scienze Neurologiche, Bologna; ^2^Alma Mater Studiorum University of Bologna, Department of Biomedical and Neuromotor Sciences (DIBINEM), Bologna, Italy; ^3^Alma Mater Studiorum University of Bologna, Department of Medical and Surgical Sciences (DIMEC), Bologna, Italy; ^4^Department of Biomedical, Metabolic and Neural Sciences, University of Modena and Reggio‐Emilia, Modena, Italy

**Background and aims:** Sleep centres specialised in the care of narcolepsy are few and scattered in terms of countries and regions in countries distribution. Telemedicine consultation (TMC) could be important in guiding the diagnostic workup in cases where symptoms suggesting narcolepsy are present in people far from sleep centres. This study aimed at assessing the inter‐procedural reliability (compared to the in‐office consultation) and the diagnostic accuracy of the TMC when excessive daytime sleepiness (EDS) due to narcolepsy is suspected.

**Methods:** Cross‐sectional diagnostic study (2019–2023). Consecutive people of any age referred for EDS complaint to the Clinic for Narcolepsy in Bologna. The TMC was conducted via tablet for the patient, and via equipped personal computer in another room for the physician (interviewer). Interviewers expressed diagnostic orientation indicating narcolepsy as “probable”, “possible”, “excluded”. The final diagnosis was established by applying the ICSD‐3 criteria. Inter‐procedural reliability (Kappa statistics) and diagnostic accuracy measures were assessed.

**Results:** 221 participants were included in the reliability study, 198 in the diagnostic accuracy study. The agreement between the two consultation modalities on diagnostic orientation was “substantial” (Kappa 0.74, 95% CI 0.66–0.82). Agreement on the narcoleptic pentad symptoms was “substantial” (EDS, hypnagogic hallucinations, disrupted nocturnal sleep) or “almost perfect” (cataplexy, sleep paralysis). Telemedicine and in‐office consultation had identical sensitivity (100%, 95% CI 94–100) and similar specificity (43%, 95% CI 35–52, vs 40%, 95% CI 32–49).

**Figure:**
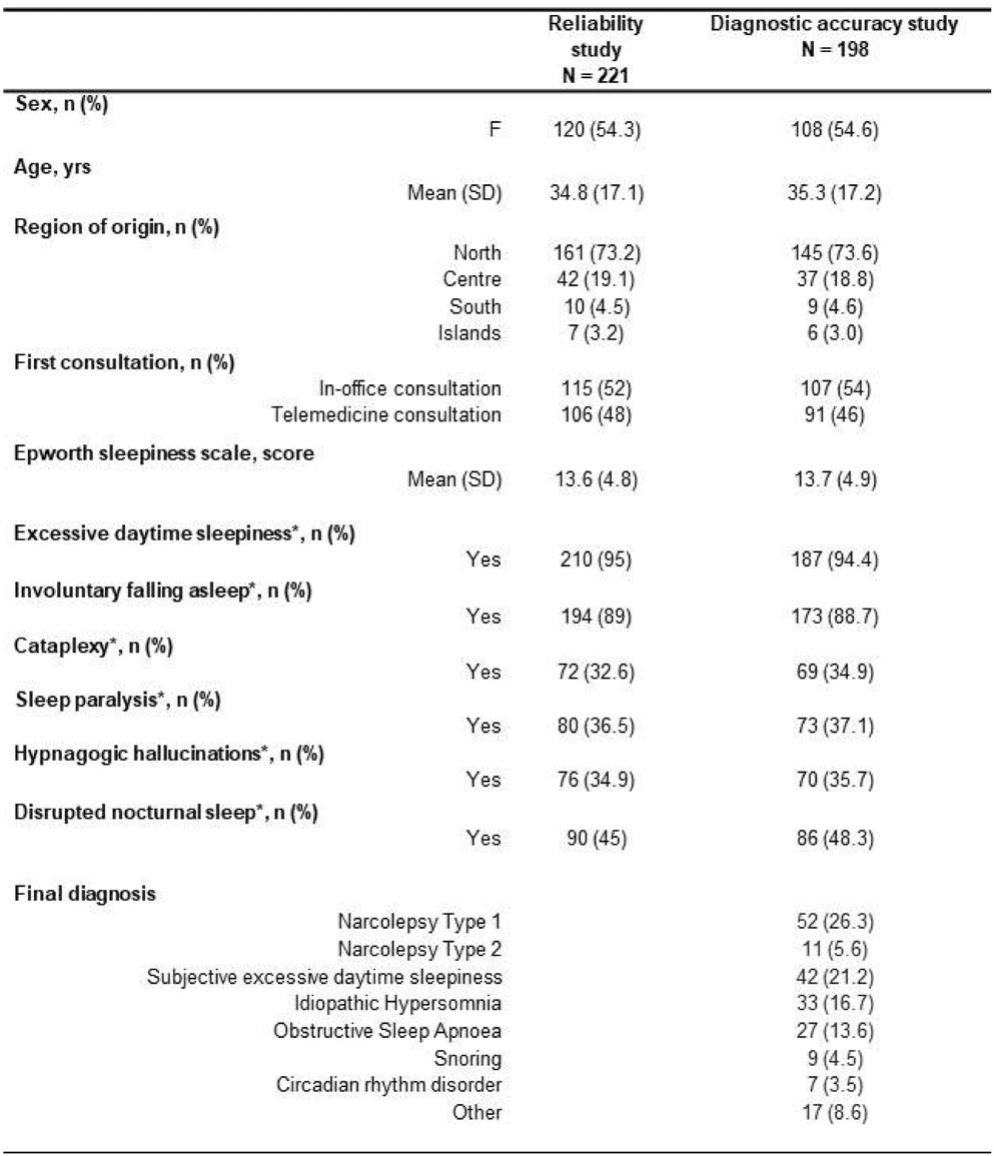
**TABLE 1** Baseline demographic and clinical characteristics of participants in the reliability and diagnostic accuracy studies.

**Figure:**
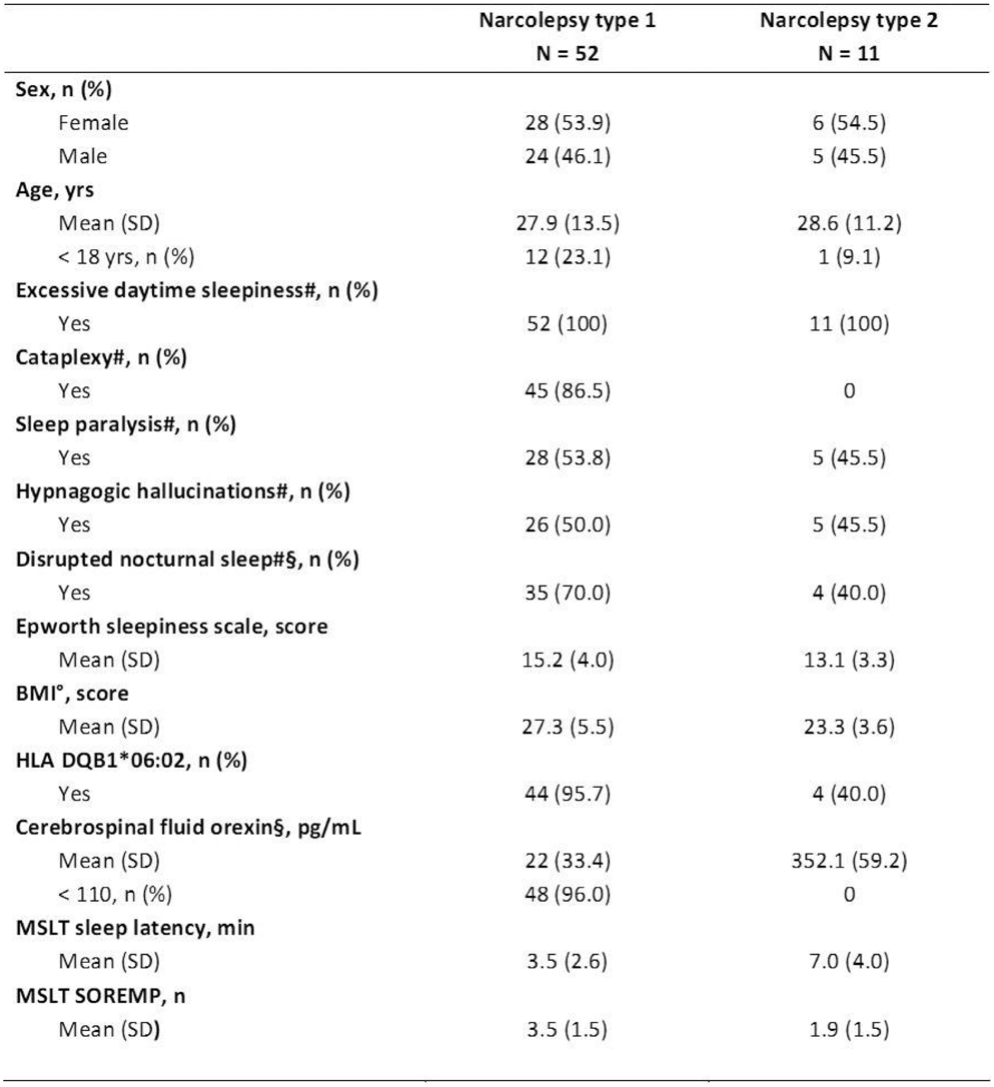
**TABLE 2** Clinical characteristics of participants with narcolepsy in the diagnostic accuracy study.

**Figure:**
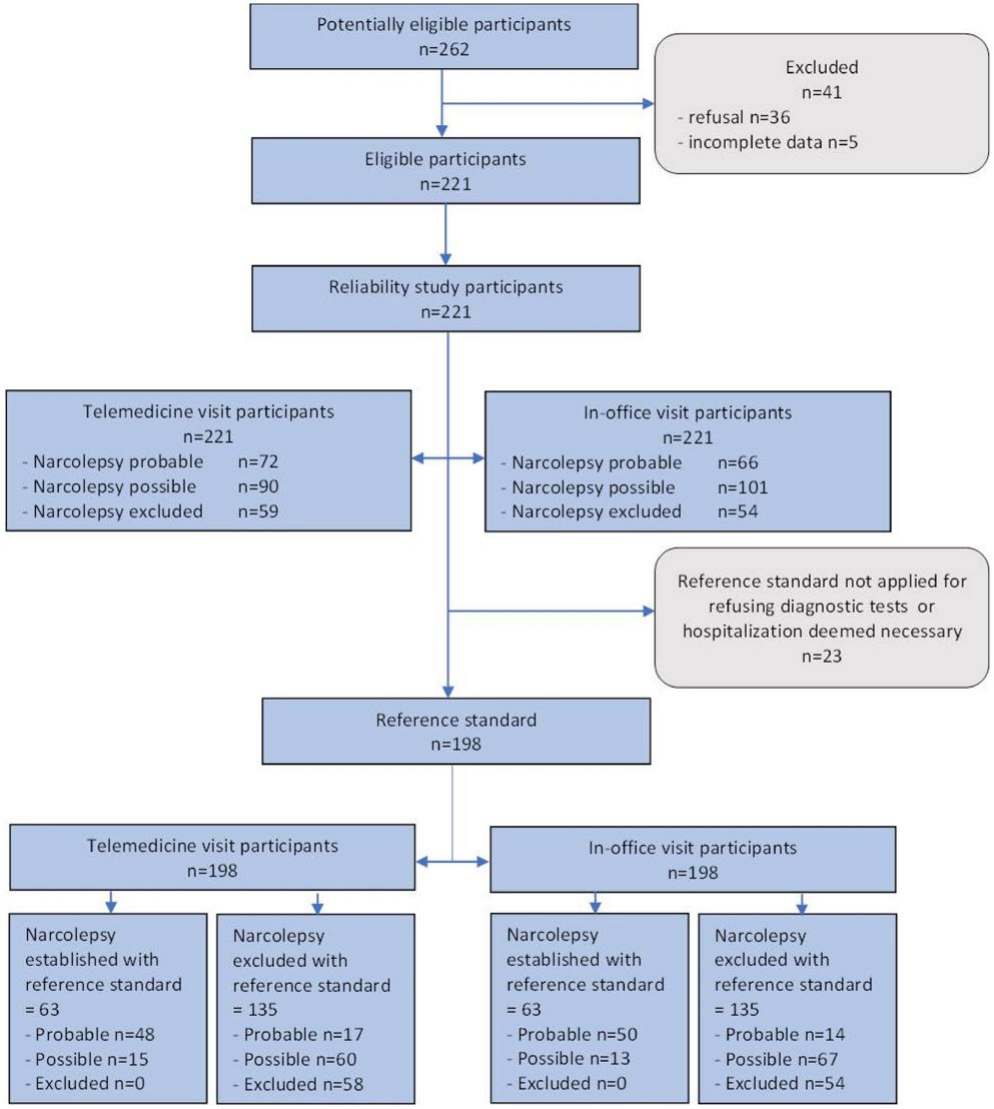
**FIGURE** Diagram of participant flow in the reliability and diagnostic accuracy studies.

**Conclusion:** TMC is a reliable diagnostic triage procedure when EDS possibly due to narcolepsy is suspected, in particularly to exclude people who do not suffer from narcolepsy without the risk of false negatives.

**Disclosure:** Nothing to disclose.

## EPO‐0944

### Incidence, prevalence and burden of narcolepsy in Germany using data from the Institute for Applied Health Research Berlin (InGef) database

#### M. Watt ^1^; S. Crawford^1^; I. Ubillos^2^; B. Podmore^2^; A. Azpeitia^2^; J. Knop^3^; A. Kitchin^2^; D. Obermueller^4^; R. Norris^4^; R. Almeida Schreck^5^

##### ^1^Takeda Development Center Americas, Inc, Cambridge, USA; ^2^OXON Epidemiology, Madrid, Spain; ^3^Takeda Pharma Vertrieb GmbH & Co. KG, Berlin, Germany; ^4^InGef – Institute for Applied Health Research Berlin GmbH, Berlin, Germany; ^5^Sleep Medicine Center, Clinic and Polyclinic for Psychiatry and Psychotherapy, Regensburg, Germany

**Background and aims:** Narcolepsy (types 1 and 2) is a neurologic hypersomnolence disorder defined by a range of symptoms including excessive daytime sleepiness (EDS) and cataplexy (type 1 only). The study evaluated the incidence, prevalence and burden of narcolepsy in Germany.

**Methods:** Retrospective cohort study using anonymized healthcare claims data involving 8.2 million people within the InGef database (2018–2023). Cohorts included pre‐existing narcolepsy cases (PCN), incident narcolepsy cases (ICN) and controls (Figure 1).

**Figure:**
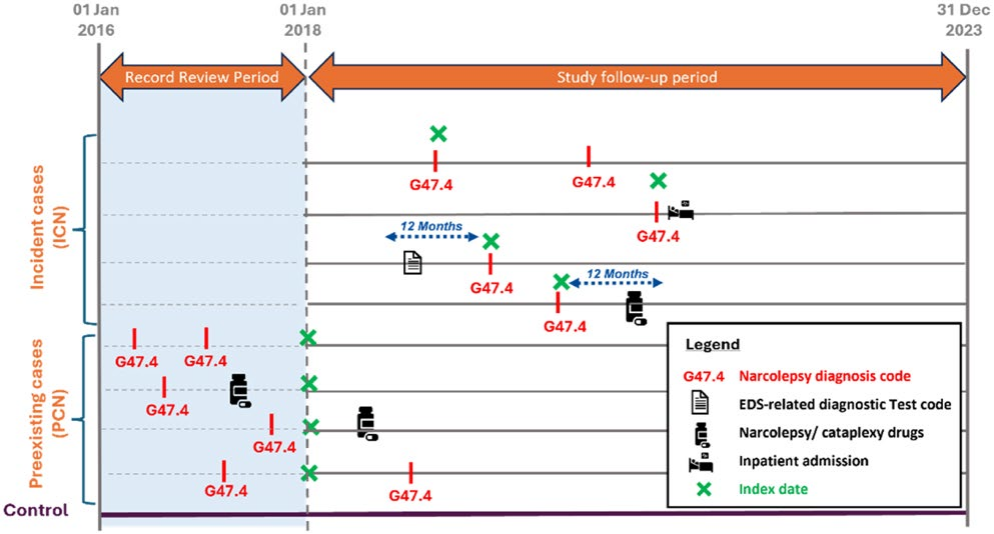
**FIGURE 1** Study design.

**Results:** A total of 1,757 narcolepsy cases were identified; 1,163 PCN and 594 ICN. Mean (SD) age was 40.13 (19.52) for ICN and 48.11 (18.1) for PCN cohorts. 13% of ICN and 4% of PCN were children/adolescents (<18 years). Overall (2018–2023) prevalence per 100,000 people was 21.29 (95% CI: 20.30–22.31); annual incidence decreased from 1.93 to 1.18 per 100,000 people between 2018 and 2023 (Figure 2). Overall prevalence and annual incidence were higher in adults (≥18 years) vs. children/adolescents (prevalence: 25.13 vs 7.10 per 100,000; annual incidence, 2018–2023: 1.27–2.08 vs 0.60–1.38 per 100,000, respectively), with comparable rates in males and females. Comorbidities were more frequent in ICN and PCN vs. controls, including non‐narcolepsy sleep disorders (42.6% and 45.4% vs. 7.4%, respectively), psychiatric disorders (43.9% and 58.5% vs. 20.6%), and nervous system diseases (50.3% and 47.7% vs. 22.0%). The most frequently prescribed narcolepsy medications in ICN were modafinil (122 [24.95%] patients) and methylphenidate (43 [8.8%] patients).

**Figure:**
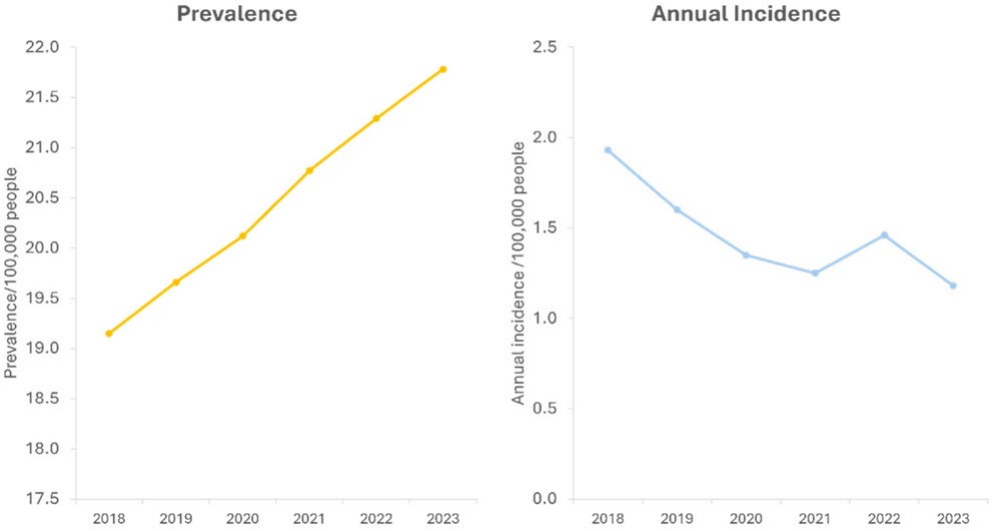
**FIGURE 2** Prevalence and incidence.

**Conclusion:** This study provides robust estimates of incidence, prevalence and comorbidity burden associated with narcolepsy within the German population relative to controls.

**Disclosure:** Funding by Takeda Development Center Americas, Inc, who also provided funding to Envision Catalyst, an Envision Medical Communications agency, a part of Envision Pharma Group, to provide writing assistance under the direction of the authors.. InGef acted as subcontractor and received funding from WIG2 for execution of the analysis. Maureen Watt and Stephen Crawford are employees of Takeda Development Center Americas, Inc and stockholders of Takeda Pharmaceuticals Company Ltd. Itziar Ubillos, Belene Podmore and Aguenda Azpeitia are employees of OXON epidemiology, contracted by Takeda for this work. Jana Knop is an employee of Takeda Pharma Vertrieb GmBH & Co. Dominik Obermueller and Raeleesha Norris are employees of InGef, contracted by Takeda for execution of the analysis. Renate Almeida Schreck has nothing to disclose.

## EPO‐0945

### Abstract withdrawn

## Neurological Manifestation of Systemic Diseases

## EPO‐0946

### Paroxysmal events in leptomeningeal carcinomatosis: The role of video‐EEG in differentiating seizures from intracranial hypertension

#### C. Baiata; R. Brusa; E. Verrengia; S. Leva; L. Politini; G. Toscano

##### Department of Neurology, ASST‐Ovest Milanese, Legnano, Italy

**Background and aims:** The objective of this study is to describe a case of leptomeningeal carcinomatosis (LC) presenting with recurrent transient neurological events (TNEs). We aim to emphasize the critical importance of a comprehensive diagnostic work‐up, including video‐electroencephalography (video‐EEG), to correctly differentiate these paroxysms from focal epilepsy.

**Methods:** We report the case of a 41‐year‐old woman with a remote history of breast cancer on negative follow‐up, presenting with recurrent episodes of intense headache and neck pain, tinnitus, upper limbs paraesthesia, right ptosis, and left hemiparesis with variable loss of consciousness (3–5 minutes). The work‐up included brain computed tomography angiography (CTA), magnetic resonance imaging (MRI), video‐EEG monitoring, and a lumbar puncture (LP).

**Results:** Brain imaging showed no pathological findings, while standard EEG showed focal slow anomalies. Thus, an initial suspicion of focal epilepsy was raised, and anti‐seizure medications (ASMs) were started, which were ineffective. Video‐EEG during an episode showed paroxysmal posterior bi‐hemispheric monomorphic delta activity and subsequently generalized rhythmic delta activity (GRDA), without electrographic seizures. Subsequent LP revealed a high opening pressure of 32 cmH2O and EpCAM‐positive malignant cells. Body CT scan confirmed breast cancer recurrence. A diagnosis of LC with transient elevations in intracranial pressure (ICP) was made. Events resolved following CSF drainage and the introduction of acetazolamide.

**Figure:**
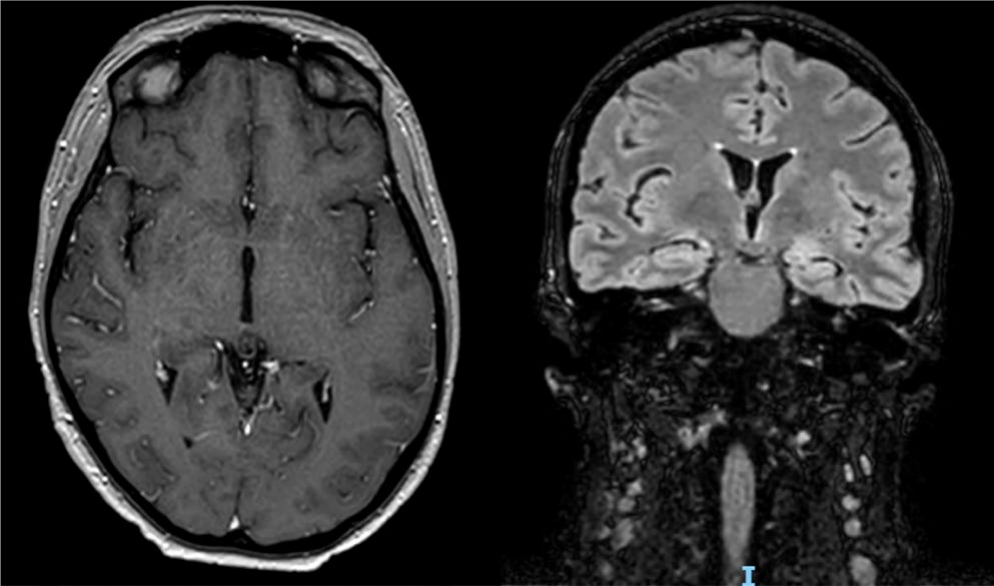
FIGURE 1

**Figure:**
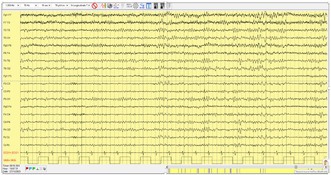
FIGURE 2

**Figure:**
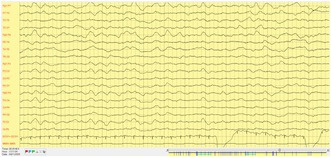
FIGURE 3

**Conclusion:** Paroxysmal events in LC are not always epileptic; they may result from transient ICP increases known as “plateau waves”. These may cause brainstem dysfunction by reducing cerebral perfusion pressure. Video‐EEG is essential to redefine the setting of recurrent, atypical TNEs, preventing inappropriate ASM use.

**Disclosure:** Nothing to disclose.

## EPO‐0947

### Presentation, treatment and outcome of a first stroke‐like episode in a carrier of the compound heterozygous variants c.695G>A and c.2209G>C in POLG

#### J. Finsterer ^1^; D. Zieglgänsberger^2^

##### ^1^Neurology and Neurophysiology Center, Vienna, Austria; ^2^Kantonsspital St. Gallen, *St. Gallen, Switzerland*

**Background and aims:** A patient with two heterozygous POLG variants in different exons, which manifested phenotypically as polyneuropathy and later with a first stroke‐like episode (SLE) and seizures, has not yet been reported.

**Methods:** Case report

**Results:** The patient is a 19‐year‐old female who has suffered from POLG‐related neuropathy since the age of 14 and was admitted for her first SLE, which manifested clinically as hemianopia and two days later led to impaired consciousness, hemiparesis, seizures and status epilepticus. Cerebral MRI showed a corresponding stroke‐like lesion (SLL) in the right occipital lobe. The seizures were treated with levetiracetam, lacosamide and perampanel, but the treatment of status epilepticus required anesthesia with thiopental, ketamine and midazolam. In addition, glucocorticoids, NO precursors and antioxidants were administered. Under this treatment, the SLL initially increased in size and intensity before regressing. After extubation, a recurrent focal motor status epilepticus occurred, which could be stopped with midazolam. Since then, she has remained seizure‐free and recovered completely

**Conclusion:** This case shows that POLG mutations can initially manifest phenotypically with polyneuropathy and SLE that SLE occurs before the onset of seizures, that status epilepticus due to POLG variants may require thiopental anesthesia and that levetiracetam, lacosamide and perampanel may have a favorable long‐term effect on seizure activity in carriers of POLG mutations

**Disclosure:** Nothing to disclose.

## EPO‐0948

### Beyond the classic triad: Sudden hearing loss and demyelinating polyneuropathy in wernicke encephalopathy

#### L. Caballero Sánchez; M. Vargas Cobos; A. De Luca; R. Alfonso González; M. Capra; C. Gómez López de San Román; I. Bermejo Casado; D. Cerdán Santacruz; A. Castrillo Sanz; A. Mendoza Rodriguez

##### General Hospital of Segovia, Segovia, Spain

**Background and aims:** Wernicke encephalopathy (WE) is an acute neurological disorder caused by thiamine deficiency, classically defined by the triad of encephalopathy, ocular signs, and gait ataxia. However, this triad is present in a minority of patients, and several atypical manifestations have been described. Among them, sudden sensorineural hearing loss and peripheral neuropathy are uncommon but increasingly recognized features.

**Methods:** We report a single case of WE presenting with atypical neurological manifestations. Clinical findings, laboratory results, neuroimaging, electrophysiological studies, and response to treatment were retrospectively reviewed.

**Results:** A 46‐year‐old man with chronic alcohol abuse and pre‐existing hypoacusis presented with sudden hearing loss of one‐week duration, associated with disorientation and progressive gait disturbance. Brain MRI showed characteristic lesions consistent with WE. Laboratory evaluation revealed hypertransaminasemia and multiple vitamin deficiencies. Paraneoplastic and autoimmune studies were negative. Electromyography demonstrated a demyelinating sensorimotor polyneuropathy, a finding that has been previously reported in WE and is likely related to severe thiamine deficiency and nutritional deficits. High‐dose intravenous thiamine was promptly initiated, leading to marked clinical improvement, particularly in cognitive function and gait, although mild to moderate ataxia persisted at discharge.

**Conclusion:** This case highlights sudden sensorineural hearing loss and demyelinating polyneuropathy as atypical but documented manifestations of Wernicke encephalopathy. Thiamine deficiency can affect both central and peripheral nervous systems, including auditory pathways and peripheral nerves. Awareness of these non‐classical features is essential to avoid diagnostic delay and to initiate timely thiamine treatment, thereby reducing the risk of permanent neurological damage.

**Disclosure:** Nothing to disclose.

## EPO‐0949

### Large vessel vasculitis and Ischemic stroke: A 10‐year experience in Seville, Spain

#### L. Manzano‐Hernandez; A. Luque Ambrosiani; L. Fernández Espigares; J. Román Rueda; P. Garcia Nuñez; A. Palomino Garcia; F. Hernández Ramos

##### Department of Neurology, Virgen del Rocío University Hospital, Sevilla, Spain

**Background and aims:** Ischemic stroke (IS) and transient ischemic attack (TIA) are rare but severe manifestations of large vessel vasculitis, including giant cell arteritis and Takayasu arteritis, with limited evidence guiding management.

**Methods:** Single‐center retrospective database review of patients with GCA or TAK presenting with IS/TIA between 2014 and 2024 at a tertiary stroke center in Seville, Spain.

**Results:** Fifteen patients with LVV‐related ischemic stroke were identified: 9 GCA‐related, 5 TAK‐related, and one with overlapping GCA/TAK. Median age was 64 years (range 40–86), and 66% (9/15) were female. In 13 cases, IS was the presenting manifestation leading to LVV diagnosis. Vertebral artery stenosis was observed in 67% of GCA patients, while all TAK patients had internal carotid artery (ICA) stenosis; the overlapping case showed both. Acute treatments included fibrinolysis (6.7%), thrombectomy (13%), and angioplasty with stenting (20%). For secondary prevention, 80% received dual antiplatelet therapy and 87% full‐dose prednisone, with adjunctive immunosuppressors in four cases; two required treatment switch due to refractory disease. Tocilizumab was initiated after the first IS in one patient with prior TAK. At 90 days, 14 patients had mRS ≤3, four experienced recurrent IS/TIA, and one TAK relapse was observed.

**Conclusion:** Although ischemic stroke secondary to LVV is uncommon, in our cohort, 87% of cases of GCA and TAK had IS as the initial presentation, highlighting the importance of having a high index of suspicion, which can guide clinical decision‐making.

**Disclosure:** Nothing to disclose.

## EPO‐0950

### Neurological complications of infective endocarditis: Retrospective study

#### M. Perdicoulis; N. Ribeiro; J. Pona‐Ferreira; S. Lima; M. Mendes

##### Neurology Department, Unidade Local de Saúde de Trás‐os‐montes e Alto Douro, Vila Real, Portugal

**Background and aims:** Infective endocarditis (IE) is an infectious disease of the endocardial surface, mostly involving heart valves and cardiac devices. It presents with a wide spectrum of clinical manifestations and may affect multiple organ systems. Neurological complications are the most frequent extra‐cardiac manifestations and are associated with significant morbidity and mortality.

**Methods:** We conducted a retrospective study reviewing electronic medical records of 158 patients diagnosed with IE at our hospital between 2017 and 2025.

**Results:** IE predominantly affected men (70.3%), the mean age of patients being 68.8 ± 14.6 years. The aortic valve was most frequently involved (46.2%), followed by the mitral valve (30.4%). Prosthetic valve involvement was observed in 30.4% of cases. Staphylococcus aureus was the most frequently identified pathogen (24%). Overall mortality rate was 28.5%. Neurological complications occurred in 24.1% of patients, most commonly ischemic stroke (57.9%), followed by encephalopathy (34.2%). Neurological symptoms were the initial manifestation in 50% of patients with neurological affection. A statistically significant association was found between the presence of neurological complications and mortality. Older age was associated with a higher frequency of neurological complications, whereas no association was observed with sex or the affected valve.

**Conclusion:** Neurological complications in the context of IE are fairly frequent, and often constitute the initial manifestation, with ischemic stroke being the most common manifestation. Their occurrence is significantly associated with mortality. Advanced age appears to be a predictive factor for neurological involvement. These findings emphasise the importance of early recognition and multidisciplinary management, particularly in older patients.

**Disclosure:** Nothing to disclose.

## EPO‐0951

### Cognitive function and sleep disturbances in rheumatoid arthritis: Clinical and inflammatory correlates

#### M. Yakubova; N. Abdurakhmanova; M. Abzalova; C. Rustamova

##### Department of Neurology and Medical Psychology, Tashkent State Medical University, Uzbekistan

**Background and aims:** This study aimed to evaluate cognitive function and sleep quality in RA and their associations with disease activity and inflammation.

**Methods:** This cross‐sectional study included 68 RA patients (ACR/EULAR 2010). Cognitive function was assessed with MMSE and MoCA, sleep with the Munich Chronotype Questionnaire (MCTQ) and Epworth Sleepiness Scale (ESS). Disease activity and inflammation were measured using DAS28 and CRP. Patients were divided into two equal groups: with cognitive impairment (*n* = 34, 50%) and without (*n* = 34, 50%). Sleep quality, cognitive scores, and inflammatory markers were compared between groups.

**Results:** Cognitive impairment was present in 50% of patients. Among these, 76% reported excessive daytime sleepiness (ESS > 10), 68% fatigue, 62% attention deficits, 59% memory complaints, and 53% poor sleep duration/quality (MCTQ midpoint >03:00). Patients without cognitive impairment reported lower rates: 29% excessive sleepiness, 35% fatigue, 24% attention deficits, and 21% memory complaints. DAS28 and CRP were higher in the cognitively impaired group (DAS28 5.4 ± 0.6 vs 3.8 ± 0.5; CRP 23.1 ± 8.9 mg/L vs 9.2 ± 4.7 mg/L; p <0.001). A significant positive correlation was observed between sleep disturbances (ESS, MCTQ) and cognitive impairment severity (MoCA *r* = −0.48, MMSE *r* = −0.43; *p* < 0.01) and with inflammatory activity.

**Conclusion:** Sleep disturbances are common in RA and closely associated with cognitive impairment and inflammation. Routine assessment may allow earlier identification of at‐risk patients and support multidisciplinary management.

**Disclosure:** Nothing to disclose.

## EPO‐0952

### Universal neurological involvement in triple‐A syndrome: Evidence from the largest genetically confirmed cohort

#### M. Oeztuerk ^1^; S. Peric^2^; M. Cehic^3^; D. Natera de Benito^4^; A. Codina^4^; K. Dumic Kubat^5^; A. Kosac^6^; Z. Stevic^7^; C. Ortez^4^; A. Nascimento^4^; J. Exposito Escudero^4^; S. Previtali^8^; V. Kammasandra Nanjundagowda^9^; A. Nalini^10^; A. Hentschel^11^; D. Muhmann^12^; U. Schara‐Schmidt^12^; S. Meuth^13^; M. Schuette^13^; N. Huntemann^13^; C. Nelke^1^; A. Schaenzer^14^; J. Weis^15^; S. Leonard‐Louis^16^; A. Mensch^17^; C. Stapf^17^; R. Fernández‐Torrón^18^; J. Reimann^19^; L. Kaluza^19^; J. Böhm^20^; A. Roos^12^; T. Ruck^1^

##### ^1^Department of Neurology, Ruhr University Bochum, BG University Hospital Bergmannsheil, Bochum, Germany; ^2^Department for Neuromuscular Disorders, Neurology Clinic, University Clinical Centre of Serbia, Faculty of Medicine, University of Belgrade; ^3^Department of Endocrinology, Mother and Child Health Care Institute of Serbia "Dr Vukan Čupić", Belgrade, Serbia; ^4^Neuromuscular Unit, Department of Neurology, Hospital Sant Joan de Déu, ‐ Barcelona, Spain; Applied Research in Neuromuscular Diseases, Institut de Recerca Sant Joan de Déu, Barcelona, Spain; ^5^Department of Pediatrics, University Hospital Centre Zagreb, Kispaticeva 12, Zagreb, Croatia; University of Zagreb, School of Medicine, Salata 3B, Zagreb, Croatia; ^6^Clinic for Neurology and Psychiatry for Children and Youth, Belgrade; ^7^Neurology Clinic, Clinical Center of Serbia, University of Belgrade, Belgrade, Serbia; ^8^Neuromuscular Repair Unit, InSpe (Institute of Experimental Neurology) and Division of Neuroscience, IRCCS San Raffaele Scientific Institute, Milan, Italy; ^9^Department of Pediatric Neurology, Indira Gandhi Institute of Child Health, Bangalore, India; ^10^National Institute of Mental Health and Neurosciences (NIMHANS), Bengaluru, India; ^11^Leibniz‐Institut für Analytische Wissenschaften ‐ ISAS ‐ e.V, Dortmund, Germany; ^12^Department of Pediatric Neurology, Centre for Neuromuscular Disorders, Centre for Translational Neuro‐ and Behavioral Sciences, University Duisburg‐Essen, Essen, Germany; ^13^Department of Neurology, Medical Faculty and University Hospital Düsseldorf, Heinrich Heine University, Düsseldorf, Germany; ^14^Institute of Neuropathology, Justus Liebig University, Gießen, Germany; ^15^Institute of Neuropathology, RWTH Aachen University Medical School, Pauwelsstr, Aachen, Germany; ^16^Sorbonne University ‐ AP‐HP, Pitié‐Salpêtrière Hospital, Department of Neuropathology, INSERM U974, Paris, France; ^17^Department of Neurology, University Medicine Halle, Halle (Saale), Germany; ^18^Neurology Department, Biodonostia Health Research Institute, Neuromuscular Area, Hospital Donostia, Basque Health Service, Doctor Begiristain, Donostia‐San Sebastian, Spain; ^19^Department of Neurology, University Hospital of Bonn, Neuromuscular Diseases Section, Bonn, Germany; ^20^IGBMC (Institut de Génétique et de Biologie Moléculaire et Cellulaire), Université de Strasbourg, Inserm, Illkirch, France

**Background and aims:** Triple‐A (AAAS, Allgrove) syndrome is a rare autosomal recessive disorder caused by biallelic mutations in the AAAS gene, which encodes ALADIN, a nuclear pore complex protein. It is classically defined by achalasia, alacrima, and adrenal insufficiency. While traditionally viewed as an endocrine disorder, increasing evidence suggests broader systemic involvement. Neurological symptoms such as neuropathy and dysautonomia have been described but remain poorly characterized and are often underrecognized. This study aimed to systematically define the neurological phenotype and underlying genetic landscape in the largest genetically confirmed AAAS cohort to date.

**Methods:** We analyzed 47 individuals with genetically (*n* = 43) or clinically (*n* = 4) confirmed AAAS from 13 international centers. All underwent standardized neurological assessment. Nerve conduction studies (*n* = 18), electromyography (*n* = 10), muscle MRI (*n* = 3), and skeletal muscle proteomics (*n* = 2) were performed. Genetic analysis identified variant types and frequencies; genotype‐phenotype correlations were explored.

**Results:** Neurological manifestations were universal. Motor weakness (84%), hyperreflexia (80%), autonomic dysfunction (91%), and gait instability (92%) were predominant. Electrophysiology showed length‐dependent axonal neuropathy; EMG confirmed chronic neurogenic changes. MRI revealed symmetric denervation. Proteomics identified consistent dysregulation of cytoskeletal, metabolic, and extracellular matrix pathways. Genetic testing revealed nine previously unreported AAAS variants. The most frequent variant, c.787T>C (p.Ser263Pro), was found in 18 individuals, 17 of Serbo‐Croatian origin. Truncating variants were associated with earlier and more severe phenotypes.

**Figure:**
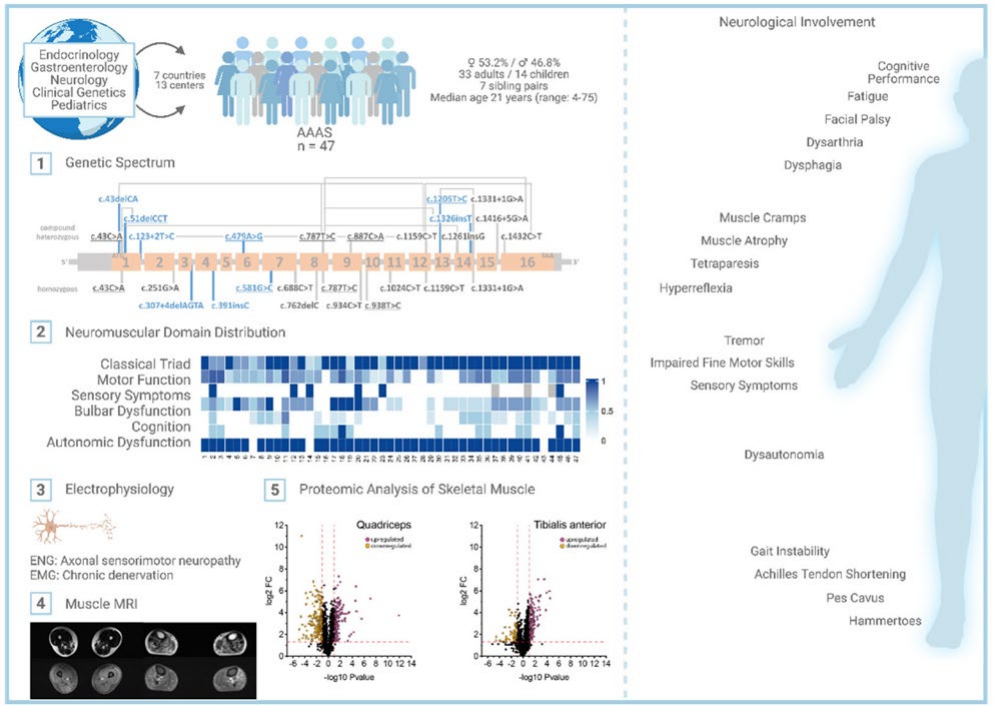
**FIGURE 1** Graphical Abstract. Multisystem neurological involvement and genetic spectrum in Triple‐A syndrome.

**Conclusion:** Neurological involvement in Triple‐A syndrome is early, universal, and clinically defining. Our data support reclassifying the disease as a neurogenetic multisystem disorder, warranting systematic neurological assessment and integrated care.

**Disclosure:** Nothing to disclose.

## EPO‐0953

### Inflammation‐mediated cognitive impairment following bone fracture

#### P. Fu

##### The Fourth Medical Center of the PLA General Hospital, Beijing, China

**Background and aims:** Bone fracture is increasingly recognized as a risk factor for cognitive dysfunction beyond local tissue injury. However, the temporal characteristics of fracture‐induced cognitive impairment and its underlying mechanisms remain unclear. This study investigated the effects of tibial fracture on hippocampus‐dependent cognitive function in mice and explored the role of inflammation.

**Methods:** Male C57BL/6J mice were randomly assigned to fracture (Fx), sham (S), or control (C) groups. Behavioral and molecular assessments were conducted immediately and on postoperative days 7, 14, and 28. Locomotor activity was evaluated using the open field test, while contextual fear conditioning assessed hippocampus‐dependent memory. Serum levels of IL‐1β, IL‐6, and TNF‐α were measured by ELISA. Hippocampal expression of BDNF and PSD95 was analyzed by Western blot, and microglial activation was assessed by Iba1 immunohistochemistry.

**Results:** Fx mice exhibited significant contextual memory impairment from day 7, peaking at day 14 and partially recovering by day 28, without motor deficits. These cognitive changes were accompanied by sustained peripheral inflammation, excessive hippocampal microglial activation, and downregulation of synaptic plasticity‐related proteins.

**Conclusion:** Bone fracture induces persistent systemic and central inflammation, leading to hippocampus‐dependent cognitive impairment. Inflammation may represent a key mechanistic link within the bone–brain axis and a potential therapeutic target.

**Disclosure:** Nothing to disclose.

## EPO‐0954

### Double trouble: Fungal brain abscess unmasking a lung malignancy

#### S. Shree ^1^; A. Agarwal^1^; D. Garg^1^; S. Gaikwad^2^; A. Garg^2^

##### ^1^Department of Neurology, AIIMS, New Delhi, India; ^2^Department of Neuroimaging and Interventional Neuroradiology

**Background and aims:** Ring‐enhancing brain lesions are commonly attributed to tuberculomas in tuberculosis‐endemic regions, often leading to empirical treatment. The objective of this report is to highlight diagnostic challenges and adverse consequences of misattributing ring‐enhancing lesions to tuberculosis when alternative etiologies coexist.

**Methods:** We describe the clinical presentation, neuroimaging findings, systemic evaluation, microbiological studies, and histopathological confirmation in a patient initially treated for presumed central nervous system tuberculosis and later reassessed at a tertiary care center.

**Results:** A 48‐year‐old woman presented with progressive headache followed by focal neurological deficits. She was empirically treated with antitubercular therapy and corticosteroids prior to referral. Magnetic resonance imaging of the brain demonstrated multiple heterogeneous ring‐enhancing lesions with diffusion restriction along the wall and intracavitary projections, suggestive of fungal abscess or metastasis. Systemic imaging revealed a left lower lobe lung mass with widespread metastatic disease. Endobronchial ultrasound‐guided biopsy confirmed lung adenocarcinoma with epidermal growth factor receptor exon 19 deletion and concomitant Aspergillus colonization. Cerebrospinal fluid analysis and infectious workup were negative. Antitubercular therapy and corticosteroids were discontinued, and antifungal therapy with liposomal amphotericin B followed by voriconazole was initiated. Neurological deficits stabilized at three‐month follow‐up under palliative care.

**Figure:**
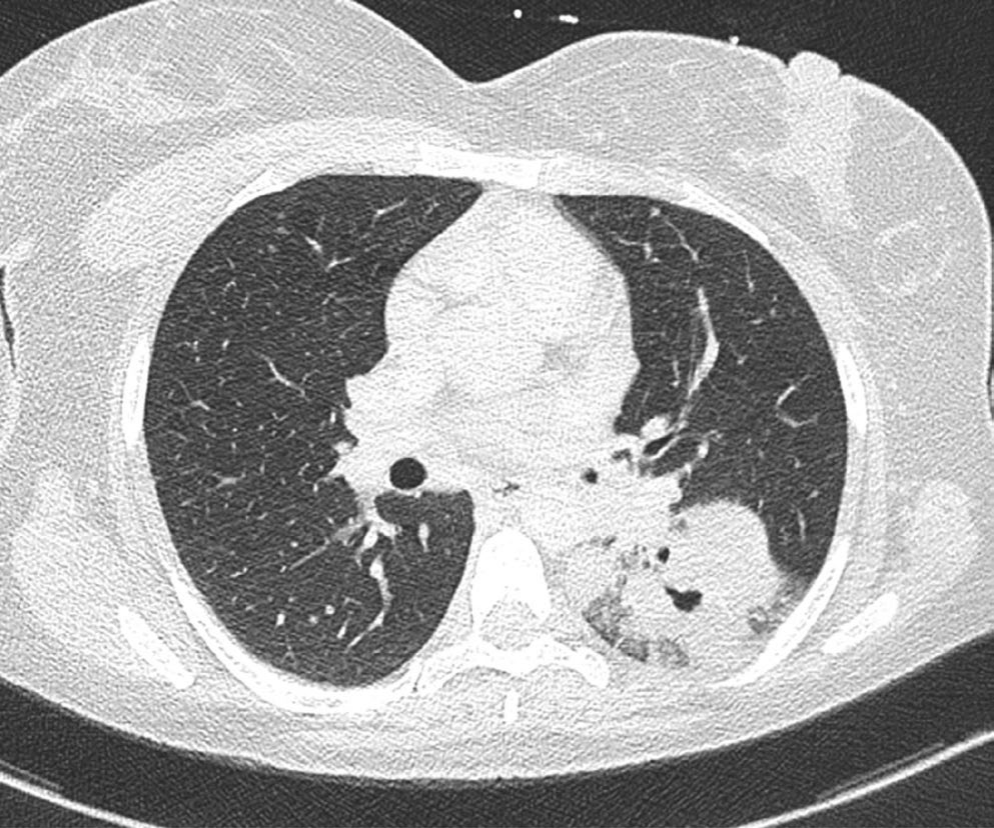
**FIGURE 1** CT Chest showing left lower lobe lung mass.

**Figure:**
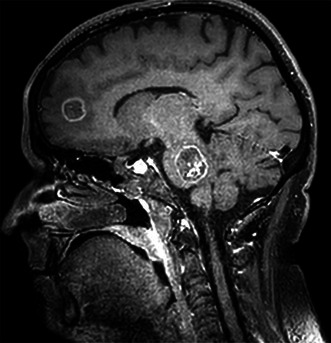
**FIGURE 2** MRI Brain ‐ T2 FLAIR sagittal section showing ring enhancing lesion with intracavitary projections in frontal lobe and brainstem.

**Figure:**
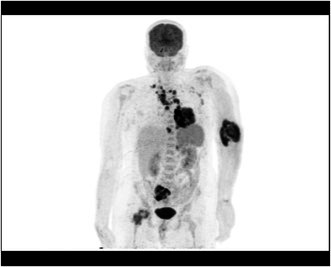
**FIGURE 3** PET scan depicting multiple site metastasis.

**Conclusion:** This case emphasizes the need for cautious evaluation of ring‐enhancing brain lesions in tuberculosis‐endemic settings. Early tissue diagnosis, multidisciplinary assessment, and careful radiological interpretation are essential to avoid diagnostic delay and treatment‐related harm.

**Disclosure:** Nothing to disclose.

## EPO‐0955

### Shunting, venous stenting, ONSF, and bariatric surgery for refractory IIH: A systematic review and meta‐analysis

#### T. Elboraay ^1^; Y. Nabil^1^; O. Rageh^2^; K. AbuElkair^3^; A. Aboali^4^; A. Alkheder^5^; M. Karam Allah Elkholy^6^; A. Samy Elboraay^7^; M. Alaa Hussein^1^; M. Maged^1^; M. Taha^8^

##### ^1^Faculty of Medicine Zagazig University; ^2^Faculty of Medicine Tanta University; ^3^Pediatric Surgery Department, Zagazig University; ^4^Radiology Department Damanhour Teaching Hospital, General Organization for Teaching Hospitals and Institutes; ^5^Faculty of Medicine Damascus University and Syrian Private University; ^6^Faculty of Pharmacy, Zagazig University; ^7^Faculty of Medicine East Port Said National University; ^8^Department of Neurosurgery Faculty of Medicine Zagazig University

**Background and aims:** Idiopathic intracranial hypertension (IIH) is characterized by elevated intracranial pressure (>25 cm H₂O) without an identifiable cause, mainly affecting obese women of reproductive age. Patients present with headaches, papilledema, tinnitus, and visual disturbances, with potential vision loss. First‐line treatment includes medical therapy and lifestyle modification, but optimal surgical management for medically refractory cases remains debated

**Methods:** A systematic review and meta‐analysis were conducted. PubMed, Web of Science, Cochrane Library, and Scopus were searched until March 2024. Studies evaluating surgical interventions for IIH, including cerebrospinal fluid (CSF) diversion, dural venous sinus stenting (VSS), optic nerve sheath fenestration (ONSF), and bariatric surgery, were included. Data on clinical outcomes, visual parameters, complications, and reintervention rates were pooled.

**Results:** A total of 119 studies were included: 2,561 CSF‐diversion procedures, 888 VSS placements, 1,189 ONSF procedures, and 249 bariatric surgeries. Improvement rates for headaches were 38%, 47%, 36%, and 14%; papilledema 39%, 25%, 10%, and 14%; tinnitus 27%, 27%, and 14%; and visual field defects 32%, 43%, 33%, and 72% for CSF diversion, VSS, ONSF, and bariatric surgery, respectively. Visual acuity improved most with bariatric surgery (33%, 57%, 31%, and 71%). Bariatric surgery had the lowest rates of subsequent procedures (0%) and lowest minor and major complications (1% each)

**Conclusion:** Dural venous sinus stenting and bariatric surgery showed favorable clinical and visual outcomes with lower complication and reintervention rates than traditional surgical approaches. These interventions may represent among the most effective options for medically refractory IIH.

**Disclosure:** Nothing to disclose.

## EPO‐0956

### Brain health awareness among solid organ transplant candidates and recipients: A single centre survey

#### V. Lo Re ^1^; E. Lo Gerfo^1^; M. Rizzo^2^; V. Agnese^2^; I. Colonna^3^; A. Toscano^4^; M. Pinzani^2^

##### ^1^Neurology Service, Istituto Mediterraneo per i Trapianti e Terapie ad Alta Specializzazione (ISMETT) ‐ Istituto di Ricovero e Cura a Carattere Scientifico (IRCCS); ^2^Department of Research, Istituto Mediterraneo per i Trapianti e Terapie ad Alta Specializzazione (ISMETT) ‐ Istituto di Ricovero e Cura a Carattere Scientifico (IRCCS), Palermo, Italy; ^3^Complex Operative Unit of Neurology, "F. Ferrari" Hospital, Casarano, Italy; ^4^ERN‐NMD Centre for Neuromuscular Disorders of Messina, Department of Clinical and Experimental Medicine, University of Messina, Messina, Italy

**Background and aims:** Solid organ transplant candidates and recipients are a highly vulnerable population for brain disorders variably alongside their transplant history. This population would greatly benefit from brain health programs. In order to set up brain health multidimensional plans targeting patients affected with peripheral organ disease, including solid organ transplant candidates and recipients, we explored their view on brain health

**Methods:** This study employed a descriptive qualitative methodology to review the answers received through a pencil and paper survey conducted in a single surgical centre. Gender, age, and clinical status differences in brain health awareness were included in our analysis.

**Results:** A little over half of our respondents, above all women, reported always or often thinking about their brain health. Just over half of our patients exercise regularly and perform cognitively stimulating activities, with this figure being even lower for older patients and men. There is low health literacy on neurology and brain diseases. We have found full recognition that peripheral organ functioning, above all heart, may have an impact on brain health. Lack of time, lack of information on what to do, and lack of motivation emerged as main barriers to enforcing lifestyle changes. Most of our respondents were willing to undergo a cognitive screening test and receive educational information on brain health.

**Figure:**
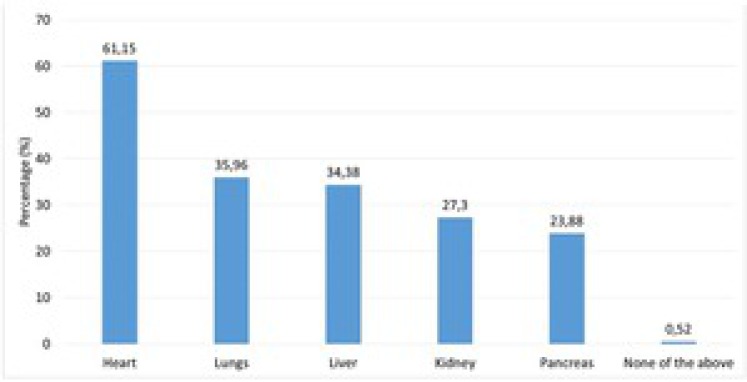
**FIGURE 1** Peripheal organs influencing brain health on the view of the survey’s participants.

**Figure:**
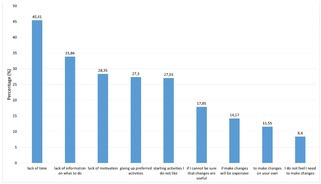
**FIGURE 1** Barriers to make changes in lifestyle.

**Conclusion:** Our survey provided insights on brain health topic previously unexplored among patients affected by non‐neurological disorders. The gathered results will be used to implement multidomain interventional brain health plans for transplant candidates and recipients.

**Disclosure:** This work was supported by a grant of the Italian Ministry of Health, Fondi 5x1000, year 2020 and Ricerca corrente‐Linea 2.

## EPO‐0957

### Comprehensive cognitive and blood based biomarkers assessment in kidney transplant candidates

#### V. Lo Re ^1^; M. Amato^2^; E. Lo Gerfo^1^; S. Rizzo^2^; F. Madonia^2^; M. D'Alessandro^2^; A. Mattina^3^; B. Buscemi^4^; F. Artale^4^; R. Tinnirello^5^; V. Agnese^5^; M. Pinzani^5^; V. Miceli^5^

##### ^1^Neurology Service, IRCCS ISMETT, Palermo, Italy; ^2^University of Pittsburgh Medical Center Italy (UPMCI); ^3^Diabetes Service, IRCCS‐ISMETT, Palermo, Italy; ^4^Nephrology Unit, IRCCS‐ISMETT, Palermo, Italy; ^5^Department of Research, IRCCS‐ISMETT, Palermo, Italy

**Background and aims:** Cognitive impairment (CI) is common in chronic kidney disease (CKD) above all in end‐stage phases as for kidney transplant (KTx) candidates. The pattern and etiology of CKD‐related CI are not well understood, mainly related to mechanisms involving pathogenetic pathways typical of Alzheimer Disease (AD). The aim of this study was to assess the association between cognitive dysfunction and AD‐related blood‐based biomarkers (BBBs) profile in KTx candidates.

**Methods:** 119 end‐stage CKD patients underwent a cognitive assessment through the Montreal Cognitive Assessment (MoCA) test, Trail Making Test (TMT) A and B and the Repeatable Battery for the Assessment of Neuropsychological Status (RBANS). Enrolled participants also underwent Aβ40, Aβ42 and p‐tau‐217 assessment

**Results:** demographic and clinical features of our cohort are reported in Table 1 According to equivalent scores of MoCA, 18.5% of our CKD patients resulted cognitively impaired. RBANS detected a greater number of cognitively impaired patients (63.9% with a mild and 12.6% with severe impairment) when a stratification based on severity was performed. Table 2 shows the number and the type of RBANS pathological domains. Plasma Aβ40, Aβ42 and p‐tau‐217 did not showed a significant association with CI as defined by either MoCA or RBANS. However, higher values for these biomarkers were found in cognitively impaired patients (Table 3).

**Figure:**
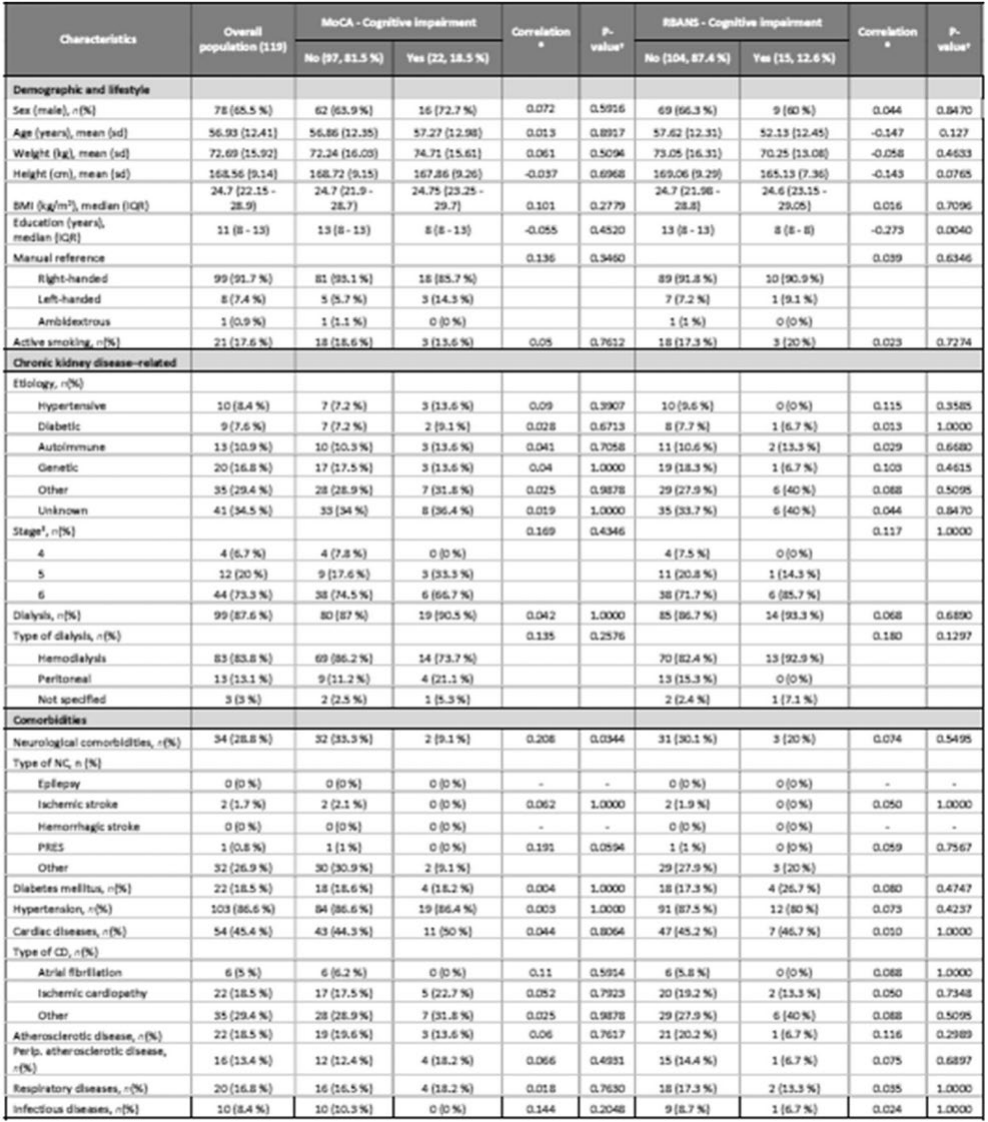
**TABLE** 1 Baseline characteristics of study participants overall and by cognitive impairment according to MoCA and RBANS.

**Figure:**
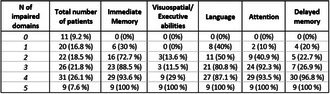
**TABLE 2** Frequencies of number and type of RBANS pathological domains.

**Figure:**
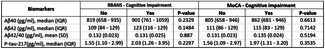
**TABLE 3** Biomarkers distribution by cognitive impairment according to RBANS and MoCA.

**Conclusion:** In this exploratory cross‐sectional study AD‐related BBBs did not reliably discriminate cognitively impaired from unimpaired patients in a cohort of end‐stage CKD patients, highlighting the strong influence of alternative pathways beyond amyloid pathology.

**Disclosure:** This work was supported by the Italian Ministry of University and Research under the National Recovery and Resilience Plan (NRRP), project HEAL ITALIA — Health Extended Alliance for Innovative Therapies, Advanced Lab research, and Integrated Approaches of Precision Medicine (Project code PE0000019, CUP B73D22000610004).

## Tuesday, June 30 2026

## Ageing and Dementia 5

## EPO‐0958

### Cataract surgery, cognitive trajectories, and blood–brain barrier integrity in ageing: A multimodal longitudinal study

#### A. Garcia Gallardo ^1^; A. McGlinchey^2^; K. Robb^2^; M. Campbell^2^; S. Hutchinson^1^

##### ^1^Neurology Department, St James's Hospital, Dublin, Ireland; ^2^Genetics Department, Trinity College Dublin, Ireland

**Background and aims:** Sensory impairment is a modifiable contributor to cognitive decline and dementia risk. Cataracts cause progressive visual deprivation and increased perceptual effort, potentially accelerating neurocognitive and neurovascular ageing. Cataract surgery provides a human model of sensory restoration without direct brain intervention. This study examined whether cataract‐related visual impairment and its correction are associated with changes in cognition, blood–brain barrier (BBB) integrity, brain structure, and brain‐age trajectories in older adults.

**Methods:** 18 older adults undergoing bilateral cataract surgery were assessed at baseline, 3‐months, and 9‐months post‐surgery. Each visit included assessments of visual function, cognition, neuroimaging, and blood sampling. Cognition was evaluated using the Repeatable Battery for Assessment of Neuropsychological Status‐Update, with mood and anxiety monitored for affective confounding. Neuroimaging included dynamic contrast‐enhanced MRI to quantify whole‐brain BBB permeability and structural imaging for volumetric, cortical thickness, and brain‐age analyses. Serum samples were collected for proteomic profiling.

**Results:** Cataract surgery induced significant visual improvement. Cognitive performance improved across domains, with greatest gains in immediate and delayed memory, independent of affective changes. BBB permeability decreased significantly by 9‐months, while structural brain measures remained stable. Brain‐age modelling demonstrated slowed ageing trajectories relative to chronological age. Proteomic analysis revealed modest changes in proteins related to cellular and metabolic regulation, without inflammatory activation or altered serum‐BBB markers.

**Conclusion:** Visual restoration after cataract surgery was associated with improved cognition, reduced BBB permeability, and decelerated brain ageing, without structural brain change. These findings support sensory integrity as contributor to cognitive and neurovascular resilience and suggest cataract surgery may modify brain ageing trajectories.

**Disclosure:** Nothing to disclose.

## EPO‐0959

### Anti‐amyloid drugs for patients with idiopathic normal pressure hydrocephalus and positive Alzheimer's disease biomarkers?

#### B. Pizzarotti ^1^; G. Bommarito^2^; P. Salvioni Chiabotti^2^; O. Rouaud^2^; M. Messerer^3^; G. Allali^2^

##### ^1^Neurology Service, Department of Clinical Neurosciences, University Hospital of Lausanne and University of Lausanne, Lausanne, Switzerland; ^2^Leenaards Memory Center, Department of Clinical Neurosciences, University Hospital of Lausanne and University of Lausanne, Lausanne, Switzerland; ^3^Neurosurgery Service, Department of Clinical Neurosciences, University Hospital of Lausanne and University of Lausanne, Lausanne, Switzerland

**Background and aims:** Anti‐amyloid therapy (AAT) has recently been approved for Alzheimer's disease (AD). Idiopathic normal pressure hydrocephalus (iNPH) frequently co‐occurs with AD, yet the presence of degenerative comorbidities is considered an exclusion criterion for anti‐amyloid therapy. Given the potential reversibility of iNPH and its frequent association with AD, a targeted evaluation of these patients is warranted. This study aimed to assess the prevalence and characteristics of iNPH patients with AD‐positive biomarkers potentially eligible for (AAT).

**Methods:** We retrospectively identified iNPH patients according to international diagnostic criteria. Demographic, clinical, cerebrospinal fluid (CSF), and MRI data were collected. AD positivity was defined as A+T+, and eligibility for AAT was evaluated based on established exclusion criteria.

**Results:** Eighty‐one iNPH patients (mean age 78.9 ± 5.1 years) referred from the Neurosurgery Service to the Leenaards Memory Center (Lausanne University Hospital, Switzerland) between May 2019 and December 2023 were included. Eleven patients (13.6%) showed AD‐positive biomarkers, and seven (8.6%) met eligibility criteria for AAT. Among ineligible patients, exclusion was due to >4 cerebral microbleeds on MRI (18%), Clinical Dementia Rating (CDR) >1 (9%), or active anticoagulant treatment (9%). No significant differences in clinical or radiological features were observed between eligible and ineligible patients.

**Figure:**
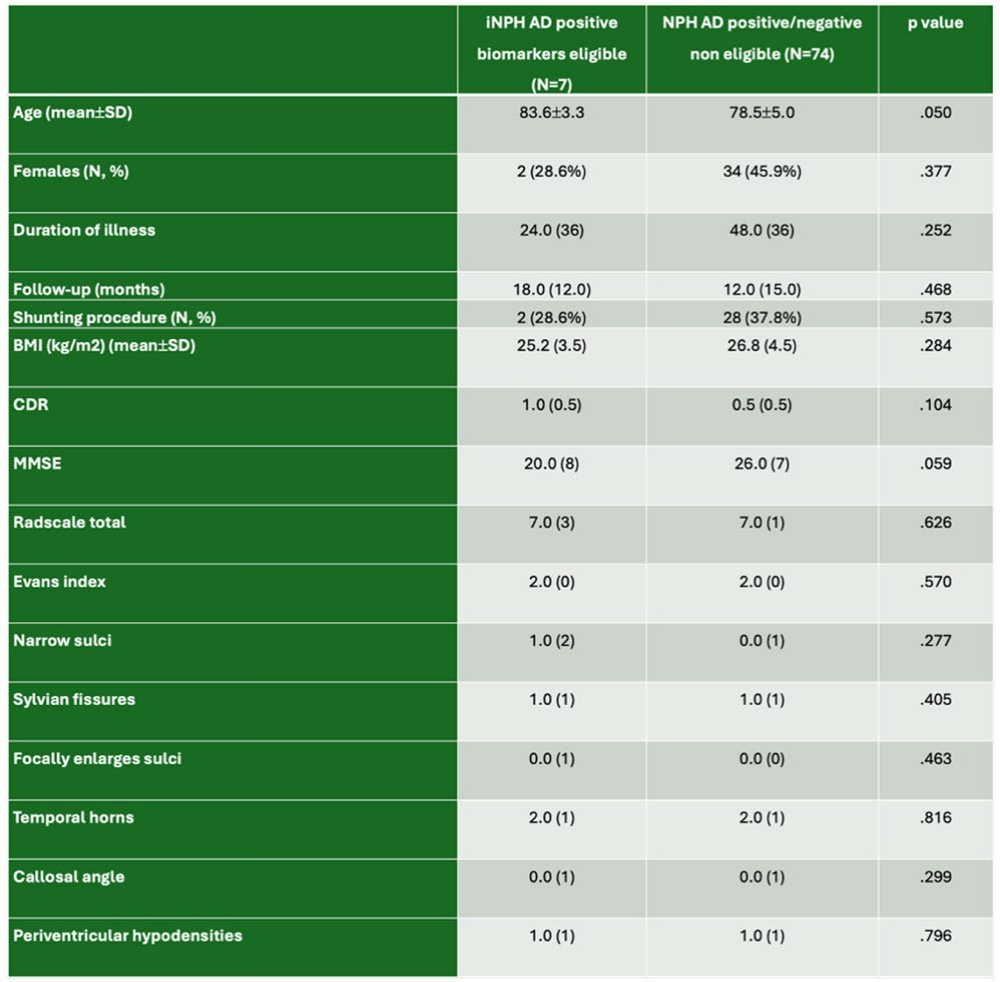
**TABLE 1** Demographic and clinical characteristics of iNPH patients with positive AD biomarkers eligible for AAT and the rest of iNPH patients, not eligible for AAT.

**Conclusion:** Most iNPH patients with AD biomarker positivity could be eligible for AAT according to Clarity AD criteria. Exclusion was mainly driven by imaging findings rather than clinical severity. No specific clinical or radiological markers predicted AD comorbidity in iNPH. These findings highlight the need for tailored clinical strategies to optimize therapeutic decision‐making in this population.

**Disclosure:** Funding declaration: This study was supported by a joint grant of the Lausanne University Hospital Foundation and the Private Foundation of the Geneva University Hospital (Project #ROMENS 22). GB received fundings from Novartis and the UNIL Faculty of Biology and Medicine. GA received fundings from the Faculty of Biology and Medicine of the University of Lausanne, the Swiss National Science Foundation (#214855), the Solis Foundation, the Empiris Foundation and the Marina Cuennet‐Mauvernay Foundation.

## EPO‐0960

### Multimodal characterization of subjective cognitive decline: Cognitive profiles, Alzheimer's disease blood biomarkers and brain connectivity

#### C. Imbimbo ^1^; G. Volpara^2^; M. Cotta Ramusino^3^; S. Leone^1^; G. Perini^3^; E. Minucchi^4^; C. Pullega^5^; G. Guicciardi^5^; C. Bonizzoni^5^; L. Farina^5^; S. Gagliardi^4^; A. Pichiecchio^5^; F. Conca^2^; V. Esposito^6^; E. Catricalà^2^; A. Costa^1^; S. Cappa^7^

##### ^1^Department of Brain and Behavioral Sciences, University of Pavia, Pavia, Italy*;* ^2^University Institute of Advanced Studies (IUSS), Pavia, Italy; ^3^Unit of Behavioral Neurology and Center for Cognitive Disorders and Dementias (CDCD), IRCCS Mondino Foundation, Pavia, Italy; ^4^Molecular Biology and Transcriptomics Section, IRCCS Mondino Foundation, Pavia, Italy; ^5^Advanced Imaging and Artificial Intelligence Center, IRCCS Mondino Foundation, Pavia, Italy; ^6^Istituti Clinici Scientifici Maugeri IRCCS, Milan, Italy; ^7^IRCCS Auxologico, Milan, Italy

**Background and aims:** Subjective cognitive decline (SCD) may represent a preclinical stage of Alzheimer's disease (AD), but its trajectory is highly heterogeneous. We compared cognitive profiles, AD blood biomarkers, and brain connectivity in SCD, amnestic mild cognitive impairment (aMCI) and healthy controls (HC).

**Methods:** Cognitive performance was assessed using the Italian Uniform Data Set Neuropsychological Battery, and metacognition (cognitive insight) with computerized tasks estimating metacognitive efficiency. Plasma p‐tau217, GFAP, and NfL were measured using the Fujirebio Lumipulse platform (*n* = 79), and resting‐state functional 3T MRI data were analyzed with independent component analysis (*n* = 51). Group comparisons and correlation analyses were performed.

**Figure:**
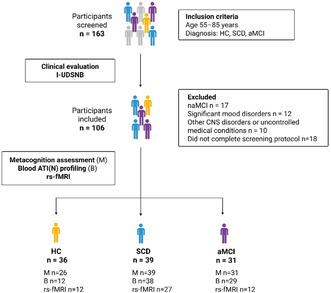
**FIGURE 1** Study flowchart. Abbreviations: I‐UDSNB, Uniform Data Set Neuropsychological Test Battery, Italian adaptation; ATI (N), amyloid, tau, neuroinflammation, neurodegeneration; rs‐fMRI, resting‐state functional MRI.

**Results:** We enrolled 106 participants (36 HC, 39 SCD, 31 aMCI; age 55–85 years). Compared with HC, SCD showed lower global cognition (*p* = 0.008) and metamemory efficiency (*p* = 0.002). GFAP was increased in SCD (*p* = 0.01) and aMCI (*p* = 0.001) versus HC and remained significant after adjustment for age and education (*p* = 0.0039). P‐tau217 was higher in aMCI than HC (*p* = 0.01), with a trend versus SCD (*p* = 0.078), while NfL did not differ between groups. Default mode network connectivity was reduced in aMCI (*p* = 0.021) and showed a trend toward reduction in SCD (*p* = 0.065) compared with HC. Across the sample, global cognition correlated negatively with p‐tau217 (*r* = −0.34, *p* = 0.007), GFAP (*r* = −0.23, *p* = 0.05), and NfL (*r* = −0.25, *p* = 0.04). Nominal associations were observed between metacognitive efficiency and default mode and dorsal attention network connectivity (*ρ* = 0.35; p≈0.02).

**Figure:**
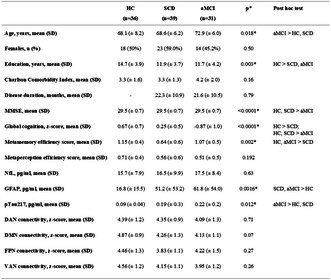
**TABLE 1** Participant characteristics. Abbreviations: aMCI, amnestic mild cognitive impairment; DAN, dorsal attention network; DMN, default mode network; FPN, frontoparietal network; HC, healthy controls; SCD, subjective cognitive decline; VAN, ventral att.

**Figure:**
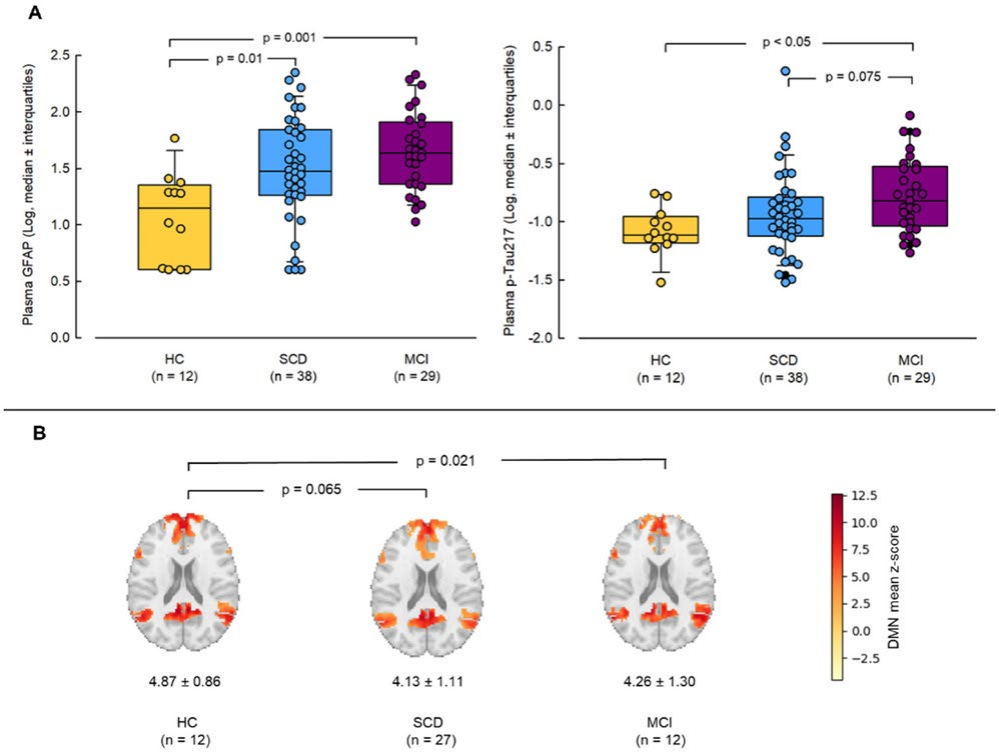
**FIGURE 2** Plasma GFAP and p‐Tau217 levels (A) and Default Mode Network connectivity (B) across diagnostic groups.

**Conclusion:** SCD showed subtle cognitive deficits, increased glial reactivity and connectivity changes, supporting its place along the AD continuum and the value of integrating multimodal biomarkers for early disease characterization.

**Disclosure:** Nothing to disclose.

## EPO‐0961

### Cerebrospinal fluid CCL25 as a biomarker for Alzheimer's disease: Associations with pathology, neurodegeneration, and cognitive decline

#### Y. Chen; S. Li

##### Hebei Medical University, Hebei, China

**Background and aims:** Neuroinflammation plays a crucial role in Alzheimer's disease (AD) pathogenesis. We investigated the relationship between cerebrospinal fluid (CSF) C–C chemokine ligand 25 (CCL25), an inflammatory regulator, and AD pathology and progression.

**Methods:** We analyzed CSF CCL25, AD biomarkers (CSF β‐amyloid [Aβ]42, phosphorylated tau [pTau]181, amyloid positron emission tomography [PET]), postmortem neuropathology, magnetic resonance imaging‐based neurodegeneration, and cognitive function from 703 participants in the Alzheimer's Disease Neuroimaging Initiative cohort.

**Results:** We found that elevated CSF CCL25 levels were associated with cognitive impairment, abnormal Aβ and tau pathology, greater brain atrophy, and worse cognitive performance (all *p* < 0.05). CSF CCL25 exhibited nonlinear relationships with Aβ and tau pathology, reaching a plateau as AD pathology increased. CSF CCL25 showed acceptable diagnostic accuracy in distinguishing amyloid‐positive/negative (A±) and tau‐positive/negative (T±) participants (area under the curve [AUC] = 0.71–0.77) and autopsy‐confirmed AD cases (AUC = 0.77), with optimal performance in differentiating A+T+ from A−T− participants (AUC = 0.82–0.85 with age and sex adjustment). Longitudinally, higher baseline CSF CCL25 predicted accelerated amyloid accumulation, hippocampal atrophy, and cognitive decline. Mediation analyses revealed that CCL25 partially mediated associations between Aβ pathology and tau pathology (mediating effect: 54.5%), neurodegeneration (18.2%), and cognitive decline (7.4%). Among 37 CSF CCL and CXCL chemokines examined, 28 were associated with at least one AD‐related outcome, with CCL25 demonstrating the strongest associations

**Figure:**
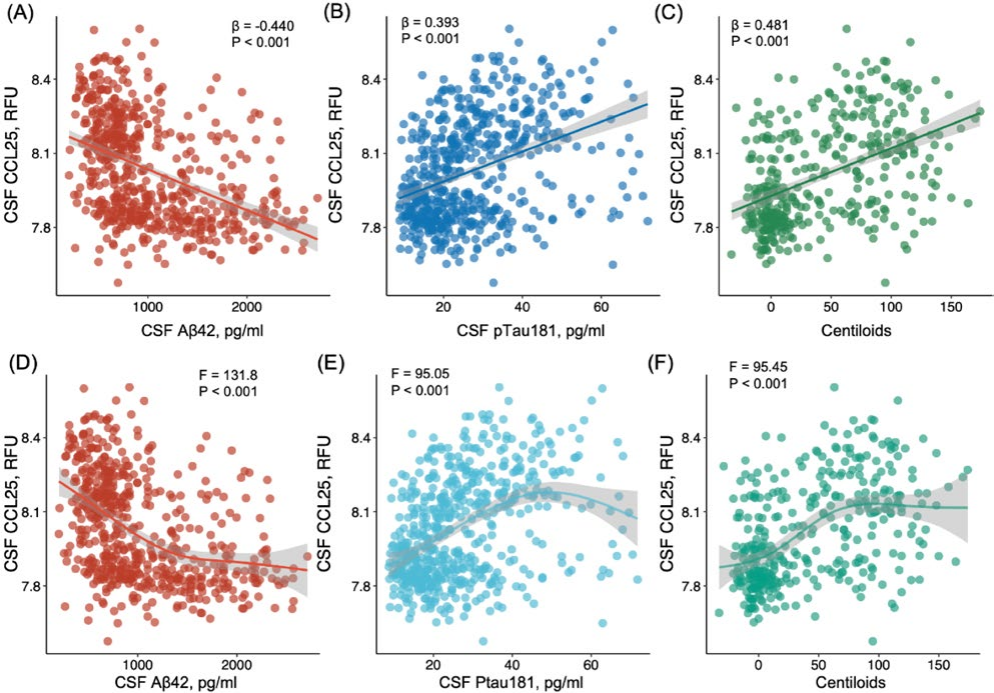
**FIGURE 1** Association between CSF CCL25 and AD core pathology.

**Figure:**
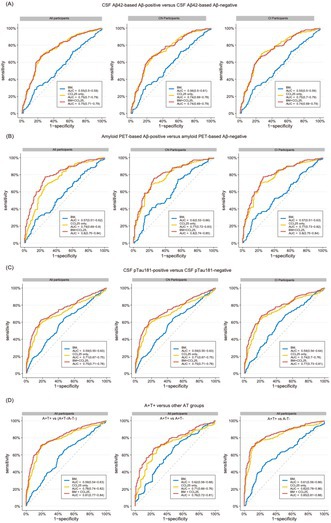
**FIGURE 2** Accuracy of CSF CCL25 in predicting AD pathology.

**Figure:**
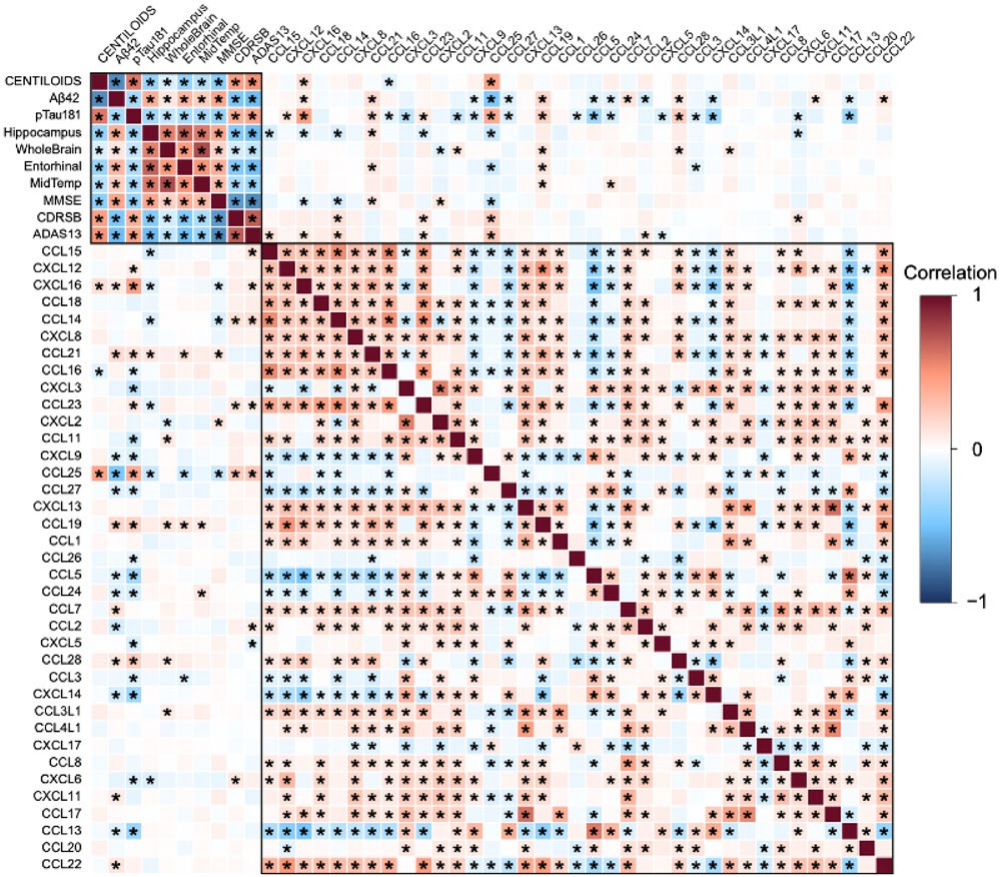
**FIGURE 3** Correlation between chemokines and AD biomarkers.

**Conclusion:** These findings suggest that CSF CCL25 is involved in early AD pathological progression and may serve as an inflammatory biomarker for diagnosis and monitoring disease progression in AD.

**Disclosure:** Nothing to disclose.

## EPO‐0962

### Beyond traditional screening: AI‐driven early detection of cognitive disorders and dementia

#### Z. Gelencsér^1^; E. Simonová^2^; M. Sátori^3^; D. Werner^4^; I. Boksay^5^; L. Varga ^1^

##### ^1^Netis Informatics Ltd, Hungary; ^2^ALSAD Medical, Hungary; ^3^S‐Medicon, Hungary; ^4^Kliniken am Goldenen Steig, Hospital Freyung, Germany; ^5^Department of Psychiatry, New York University Langone Health, New York, USA

**Background and aims:** Global aging drives increased dementia prevalence. While early detection is crucial for better prognosis and quality of life, diagnosis is often delayed because early symptoms are subtle. Since no widely available AI algorithm reliably identifies early dementia signs from digital behavioral patterns, our objective was to develop and validate such an AI‐based tool for timely diagnosis and treatment.

**Methods:** We utilized the PreDEM system, an AI‐based platform, for this investigation. Data was collected from 4,334 participants between September 2021 and March 2025, during which 171,577 cognitive tests were completed, resulting in 6,049,916 data points. A unified, normalized evaluation system was established for objective comparison across tests. The AI algorithm analyzed participants' digital behavioral patterns to identify early signs of dementia.

**Results:** Participants were categorized into "potentially treated" and "potentially healthy" groups. Analysis, including density function comparison and cluster analysis, showed clear differences between the groups. Within the affected group, a lower‐performing subgroup was identified and subsequent neurological/neuropsychological examinations confirmed cognitive impairment. In the higher‐performing affected subgroup, cognitive dysfunction was often linked to organic causes. Treating these underlying organic causes, combined with regular use of the PreDEM platform, led to significant cognitive improvement in these participants.

**Conclusion:** The study's platform is a promising new tool for the early detection and prevention of dementia. During pilot studies, the AI‐based system reliably identified dementia patients, significantly surpassing the accuracy of traditional diagnostic methods. This method is therefore suitable for detecting the first, often unnoticed signs of dementia through AI‐processed risk analysis.

**Disclosure:** Nothing to disclose.

## EPO‐0963

### When hearing loss is the first clue: An atypical onset of Creutzfeldt‐Jakob disease with an isolated thalamic lesion

#### M. Elguezabal; M. Gilot; M. Herrezuelo; P. Sánchez; N. Amazian; N. Juárez; N. Huertas

##### Department of Neurology, University Hospital Severo Ochoa, Madrid, Spain

**Background and aims:** Creutzfeldt–Jakob disease (CJD) is a rapidly progressive prion encephalopathy typically characterized by progressive dementia, myoclonus and ataxia. However, early presentations may be misleading, as atypical symptoms such as hearing loss and non‐suggestive neuroimaging can pose a diagnostic challenge.

**Methods:** We report the case of a 54‐year‐old woman with dyslipidaemia, ulcerative proctitis and chronic thrombocytopenia. In August 2025, she developed acute unilateral hearing loss, progressing to bilateral involvement within days, unresponsive to topical corticosteroids. In September 2025, she was assessed by otorhinolaryngology (ENT); audiometry showed normal hearing thresholds with impaired speech discrimination and topical and oral corticosteroids were prescribed without improvement. In October 2025, following language impairment, she presented to the emergency department for neurological evaluation. Neurological examination revealed scanning dysarthria, mixed aphasia with paraphasias, slow reading with aprosody, nystagmus, myoclonus and a wide‐based ataxic gait.

**Results:** Initial brain magnetic resonance imaging (MRI) in September 2025 showed an isolated unilateral hyperintense lesion in the right thalamus (T2/FLAIR) without diffusion restriction. Subsequent MRI in October 2025 demonstrated the evolution of classic “cortical ribboning” in frontoparietal regions and ipsilateral caudate nucleus involvement on diffusion‐weighted imaging (DWI). Cerebrospinal fluid analysis revealed positive 14‐3‐3 protein with otherwise normal biochemistry. Electroencephalography showed diffuse encephalopathy with characteristic periodic sharp wave complexes.

**Figure:**
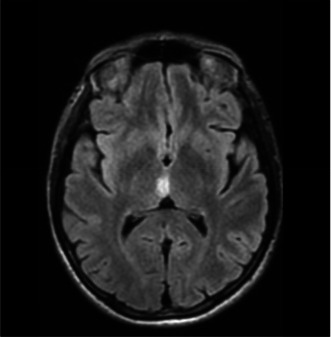
**FIGURE 1** Axial T2‐FLAIR MRI showing an isolated hyperintense lesion in the right thalamus.

**Figure:**
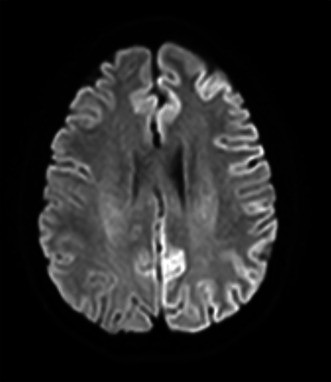
**FIGURE 2** Axial diffusion‐weighted MRI showing cortical ribboning in the frontoparietal regions.

**Conclusion:** This case highlights that CJD can masquerade as a primary ENT pathology. Hearing loss and atypical isolated thalamic lesions do not preclude a prion disease diagnosis. Recognizing these rare phenotypes is crucial for timely diagnosis in the absence of early “classic” imaging markers.

**Disclosure:** Nothing to disclose.

## EPO‐0964

### Acute and longitudinal effects of transcranial pulse stimulation on vascular, neuronal, and cognitive function in Alzheimer's models

#### M. Karakatsani ^1^; D. Nozdriukhin^1^; I. Gezginer^1^; C. Maschio^1^; S. Tiemann^1^; H. Yoshihara^1^; D. Kindler^1^; M. Reiss^1^; R. Storz^2^; M. Belau^2^; R. Ni^3^; X. Deán‐Ben^1^; D. Razansky^1^

##### ^1^Institute for Biomedical Engineering and Institute of Pharmacology and Toxicology, University of Zurich, Zurich, Switzerland; Institute for Biomedical Engineering, Department of Information Technology and Electrical Engineering, ETH Zurich, Zurich, Switzerland*;* ^2^Storz Medical AG, Switzerland*;* ^3^Institute for Regenerative Medicine, University of Zurich, Zurich, Switzerland

**Background and aims:** Transcranial Pulse Stimulation (TPS) is a non‐invasive neuromodulation technique with growing therapeutic relevance for neurodegenerative disease. This work investigated both the acute physiological effects of TPS on cerebrovascular, neuronal, and network‐level function, as well as the longitudinal impact of repeated TPS on Alzheimer's disease (AD) pathology and cognition.

**Methods:** Acute experiments were conducted in CD1, GCaMP‐expressing, 5xFAD, and 3xTg‐AD mice using multimodal imaging: real‐time optoacoustic tomography, contrast‐enhanced MRI, wide‐field calcium imaging, c‐Fos immunohistochemistry, and resting‐state fMRI. Longitudinal study followed a clinical‐analogous TPS protocol (six sessions over two weeks) to 3xTg‐AD mice with sham controls. BBB integrity, amyloid and tau pathology (targeted AOI987 imaging, immunohistochemistry), cognition (Novel Object Recognition, Fear Conditioning), and proteomic signatures were assessed.

**Results:** Acute TPS induced robust, energy‐dependent increases in cortical blood volume (~28–50%), with AD mice showing reduced vascular responsiveness consistent with vascular amyloidosis. BBB integrity remained intact across all modalities. TPS evoked strong neuronal activation, including immediate calcium influxes and ~2‐fold increases in c‐Fos expression, and transiently reorganized functional connectivity within hypothalamic–hippocampal–limbic circuits. Longitudinal TPS caused no BBB disruption and produced modest reductions in amyloid burden and attenuated tau accumulation. Behaviorally, TPS improved short‐term object recognition and enhanced forgetting of remote fear memory in 3xTg‐AD females. Proteomics revealed upregulation of pathways related to calcium handling, energy metabolism, ion transport, and neurotransmission.

**Conclusion:** TPS safely modulates vascular, neuronal, and network dynamics while mitigating aspects of AD pathology and improving cognitive and molecular outcomes. These findings support TPS as a mechanistically grounded therapeutic strategy for neurodegenerative disease.

**Disclosure:** Nothing to disclose.

## EPO‐0965

### Whole‐brain DTI‐ALPS: A novel voxel‐based biomarker for white matter dysfunction in Alzheimer's and Parkinson's disease

#### N. Schröter ^1^; M. Reisert^2^; H. Wiendl^3^; L. Schappe^1^; S. Groppa^1^; J. Hosp^3^; H. Urbach^4^; A. Rau^4^

##### ^1^Department of Neurology, Saarland University and Saarland University Medical Center, Homburg, Germany; ^2^Medical Physics, Department of Radiology, Medical Center – University of Freiburg, Faculty of Medicine, University of Freiburg, Germany; ^3^Department of Neurology and Neurophysiology, Medical Center‐University of Freiburg, Faculty of Medicine, University of Freiburg, Freiburg, Germany; ^4^Department of Neuroradiology, Medical Center – University of Freiburg, Faculty of Medicine, University of Freiburg, Freiburg, Germany

**Background and aims:** The glymphatic system's role in neurodegenerative waste clearance is established, but non‐invasive assessment remains challenging. This study compares the standard DTI‐ALPS method with a novel whole‐brain voxel‐based DTI‐ALPS approach to quantify perivascular flow in Alzheimer's (AD), Parkinson's disease (PD) and Dementia with Lewy Bodies (DLB).

**Methods:** Retrospective analysis included 25 PD patients with dementia (PDD), 58 with mild cognitive impairment (PD‐MCI), 50 cognitively intact PD (PD‐NC), 77 AD and 13 DLB patients, and 30 healthy controls (HC) (Table 1). Cognitive impairment was assessed by Mattis Dementia Rating Scale (DRS‐2). DTI‐ALPS was calculated using Frangi‐filter‐based perivascular space (PVS) segmentation on high‐resolution T2‐weighted MRI. A PVS orientation template enabled voxel‐wise diffusivity assessment along/perpendicular to PVS. Mean whole‐brain DTI‐ALPS was extracted from white matter (tissue probability > 0.5). Group comparisons and clinical associations were analyzed.

**Results:** Conventional DTI‐ALPS revealed no significant group differences. Voxel‐based‐DTI‐ALPS was significantly lower in PD‐NC (*p* = 0.040, Cohen's *d* = 0.635), DLB (*p* < 0.001, Cohen's *d* = 1.599) and AD (*p* < 0.001, Cohen's *d* = 0.971) compared with HC. Furthermore, DTI‐ALPS was lower in DLB than PD‐MCI (*p* = 0.026, Cohen's *d* = 1.027), Figure 1. A significant association was observed with initiation (*p* = 0.003), but not with DRS‐2 total‐ or subscores (*p* > 0.05).

**Figure:**
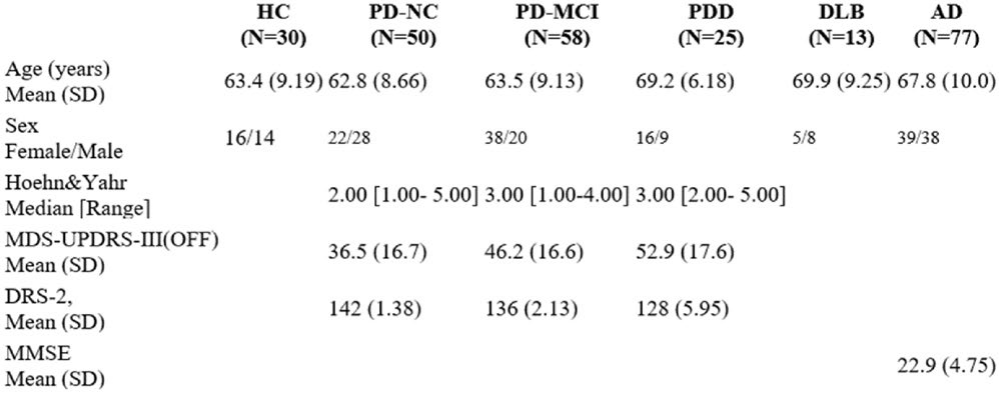
**TABLE 1** Demographics.

**Figure:**
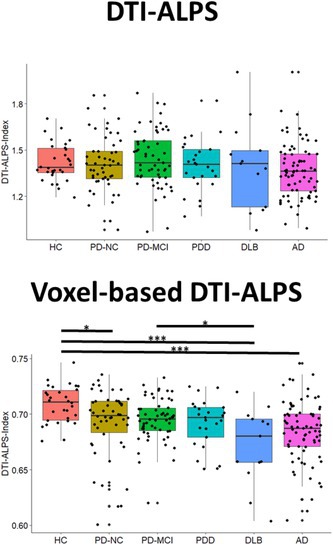
**FIGURE 1** DTI‐ALPS and voxel‐based DTI‐ALPS indices across HC, PD‐NC, PD‐MCI, PDD, DLB, and AD, highlighting group differences and statistical significance.

**Conclusion:** This novel whole‐brain voxel‐based approach, using a PVS template, demonstrates significant white matter dysfunction in AD, DLB, and cognitively intact PD, with the most pronounced impairment in DLB. The association with initiation deficits highlights its potential as a sensitive biomarker for early white matter dysfunction.

**Disclosure:** Nothing to disclose.

## Neuro‐Oncology 2

## EPO‐0966

### Development of the AO spinal metastasis staging (SMS) referral tool: An international multidisciplinary expert panel and survey study

#### H. Kuijten ^1^; R. Gal^1^; O. Groot^1^; M. Vial^2^; O. Barzilai^3^; C. Netzer^4^; R. Goodwin^5^; A. Gasbarrini^6^; N. Dea^7^; J. Reynolds^8^; I. Laufer^9^; J. van der Velden^1^; N. Kasperts^1^; J. Verlaan^1^

##### ^1^Imaging & Oncology, University Medical Center Utrecht, Utrecht, The Netherlands; ^2^AO Spine, AO Foundation, Davos, Switzerland; ^3^Neurosurgery, Memorial Sloan Kettering Cancer Center, New York, USA; ^4^Spine surgery, University Hospital Basel, Switzerland; ^5^Spine, Duke University, Durham, United States; ^6^Spine Surgery, Istituto Ortopedico Rizzoli, Bologna, Italy; ^7^Orthopedic Surgery, University of British Columbia, Vancouver, Canada; ^8^Spinal Surgery, Oxford University Hospitals NHS Foundation Trust, Oxford, UK; ^9^Neurosurgery, New York University Langone Health, New York, USA

**Background and aims:** Spinal metastases progress from asymptomatic disease to debilitating pain, instability, and neurological deficits. Although timely recognition is essential, referral delays are common because patients often first present to non‐spine clinicians and red flags rarely expedite referral. Existing guidelines target spine surgeons and radiation oncologists, leaving a gap for staging and referral tools for non‐spine clinicians. We aimed to developed the Spinal Metastasis Staging (SMS) referral tool.

**Methods:** We defined the SMS system as four stages following natural progression of most spinal metastases: SMS I, asymptomatic; SMS II, inflammatory pain; SMS III, mechanical pain and/or instability; and SMS IV, neurological deficits and/or high‐grade spinal cord compression. To aid clinical use, we translated the SMS stages into a referral algorithm, organized by urgency, presented in the form of a color‐coded pocket map. The four stages and pocket map were revised by regional and international multidisciplinary expert panels, including spine and non‐spine clinicians, and subsequently evaluated in an international online survey.

**Results:** Expert panels endorsed the four SMS stages. Among non‐spine clinician survey respondents (32/120), 94% found the pocket map easy to understand, 91% judged the format suitable, and 91% expected improved referrals. Overall, 88% indicated at least occasional use, including 61% who would use the pocket map frequently or always.

**Figure:**
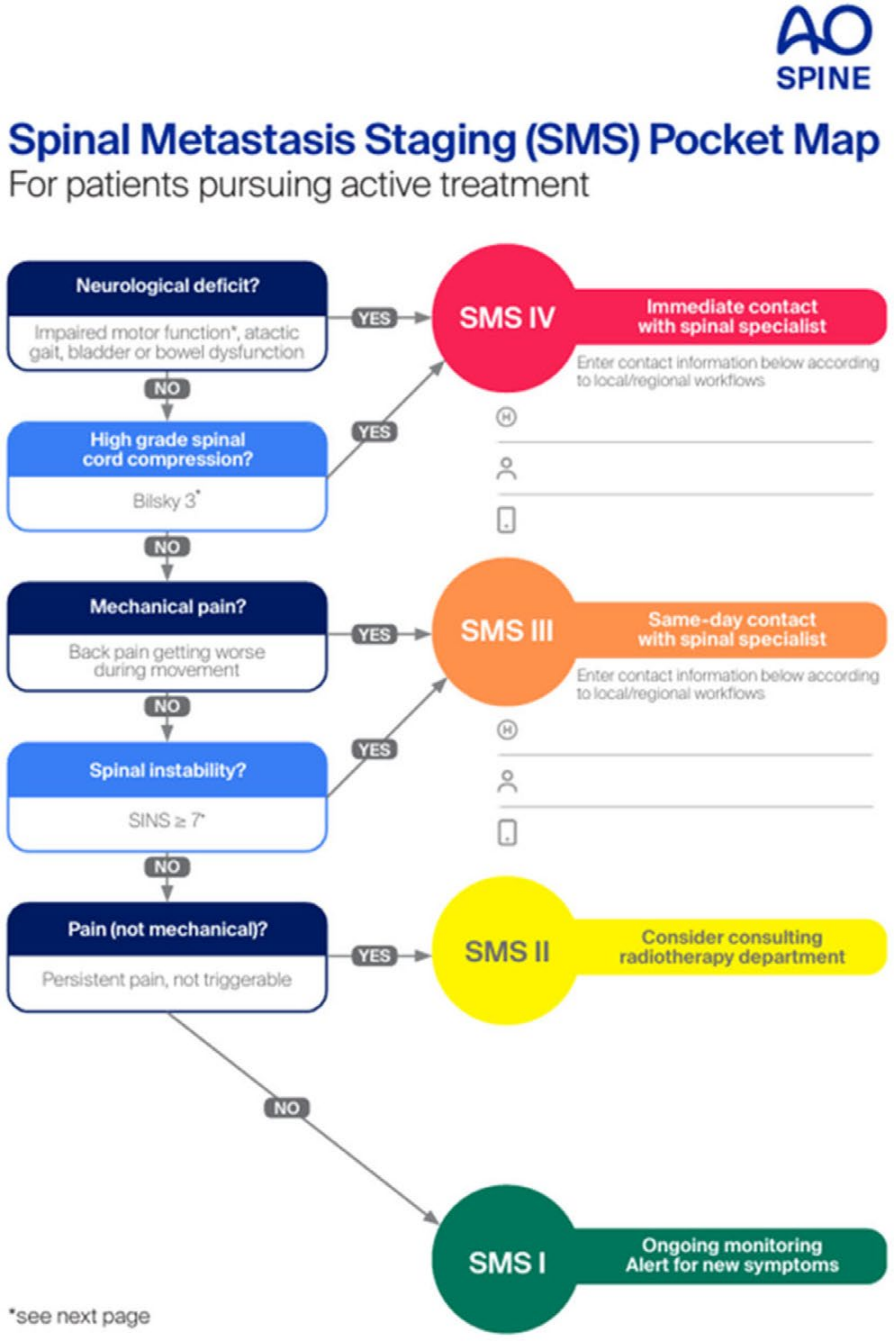
**FIGURE 1** Front page of the SMS pocket map with the referral algorithm for patients with spinal metastases.

**Figure:**
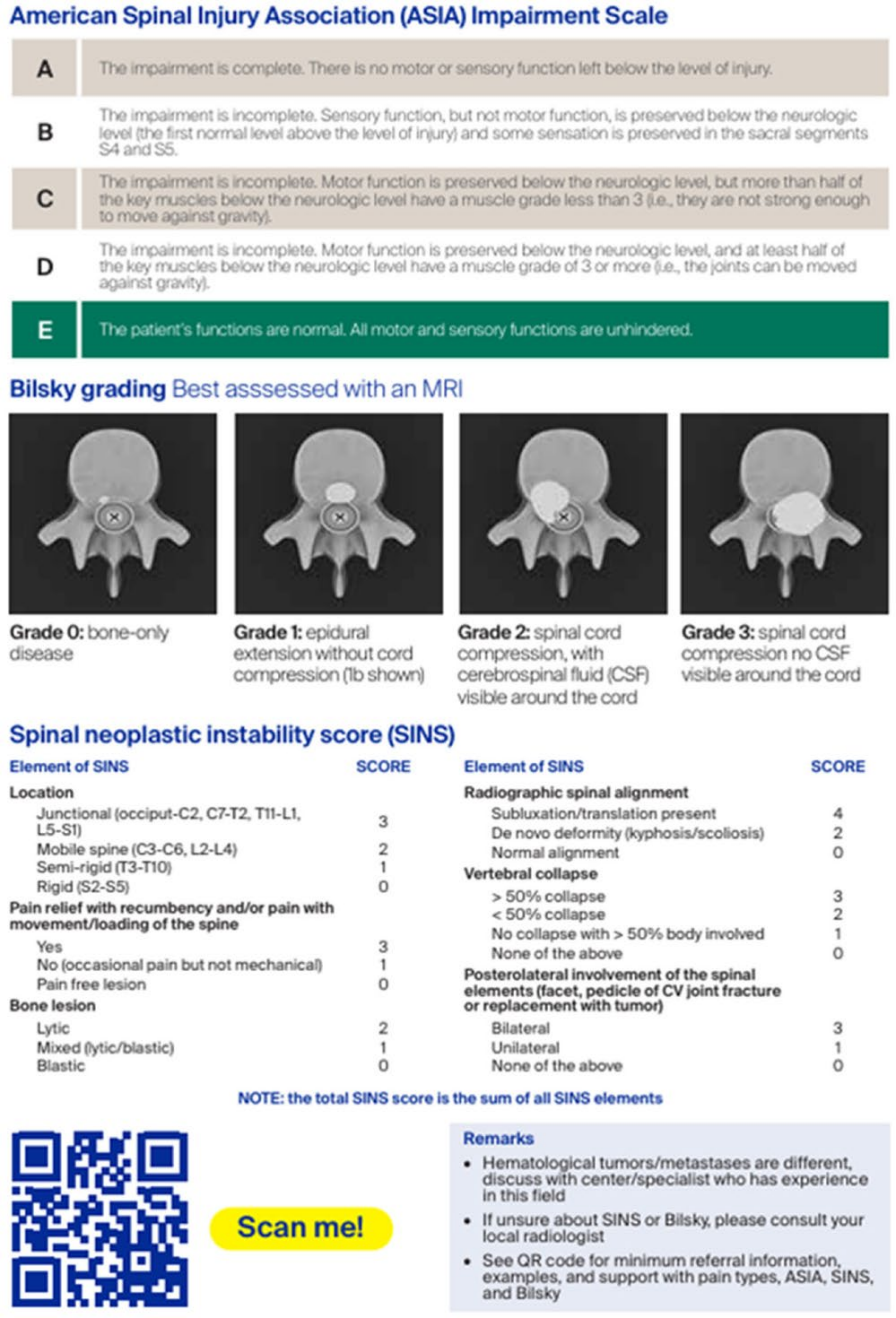
**FIGURE 2** Back page of the SMS pocket map with explanation of items on the front and a QR link to further educational material.

**Conclusion:** The SMS referral tool was well received by spine specialists and non‐spine clinicians, supporting that it is both expert‐driven and user‐friendly. A digital version with educational materials is provided for local implementation. Future work will focus on clinical validation to confirm the impact.

**Disclosure:** Nothing to disclose.

## EPO‐0967

### Late transient post‐radiotherapy enhancements in patients with high grade brain tumors – between pseudoprogression and radionecrosis: A case series

#### C. Pérez Prol; J. Gállego Pérez de Larraya; A. Atorrasagasti Villar; S. Urtasun Galmes; J. Ulloa Bravo; R. Villino Rodriguez; C. Espinoza Vinces

##### Department of Neurology, Clínica Universidad de Navarra, Pamplona, Spain

**Background and aims:** High‐grade malignant brain tumors have a poor prognosis despite treatment. Pseudoprogression and radionecrosis are contrast‐enhancing phenomena that usually appear before 3 months (post‐radiotherapy) in a transient manner or between 3–12 months permanently, respectively. Distinguising pseudoprogression and radionecrosis from true progression is of extreme importance, with variable treatment and prognostic implications. The aim of this work is to describe treatment related changes on MRI defined as late transient post‐radiotherapy enhancements (LTPE) which do not fit the definition of pseudoprogression or radionecrosis and can be misdiagnosed as tumor progression.

**Methods:** Retrospective description of a case series of 7 patients with malignant high‐grade brain tumors who showed late transient post‐radiotherapy contrast‐enhancing lesions that did not fit the definitions of pseudoprogression or radionecrosis and could be misdiagnosed as progressive disease. Demographic, tumor, treatment and LTPE characteristics (time of appearance and resolution, location), date of tumor progression, and MRI and methionine PET faetures were collected.

**Figure:**
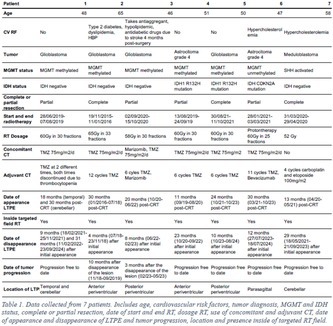
**TABLE 1** Data collected from 7 patients. CT, chemotheraphy; CV RF, cardiovascular risk factors; LTPE, late transient post‐radic enhancements.

**Results:** Seven patients aged 47–65 years were included: 4 had glioblastoma, 2 grade 4 IDH‐muted astrocytomas and 1 medulloblastoma. All patients underwent surgery followed by radio‐chemotherapy. LTPE were located within the radiotherapy field, appeared between 11–30 months post‐radiotherapy, and spontaneously resolved within 4–30 months after onset. Six LTPE showed frontotemporal distribution and four periventricular/ependimary.

**Figure:**
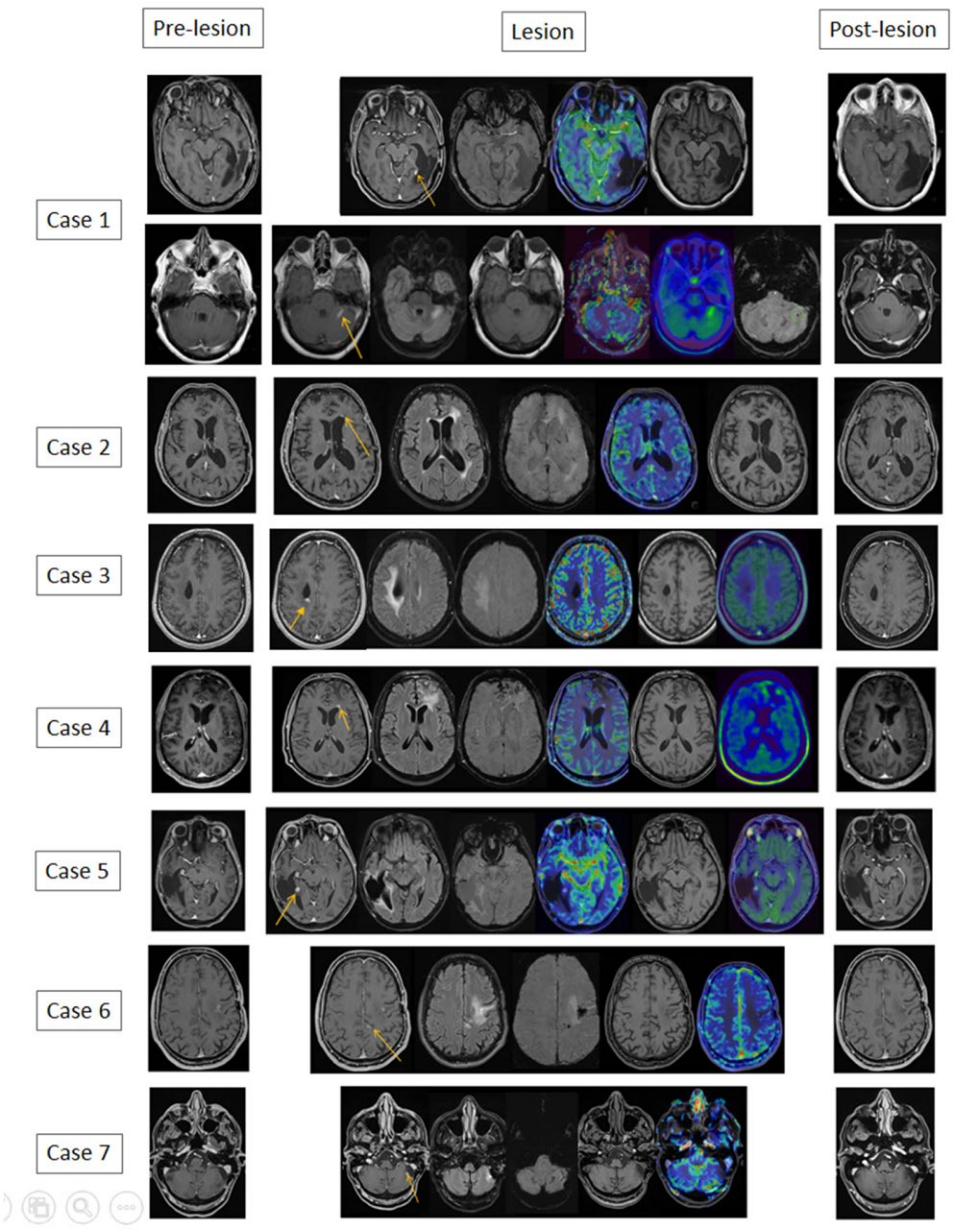
**FIGURE 1** Representative MRI/PET images of the seven reported cases before, during, and after LPTE (Late transient post‐radic enhancements).

**Conclusion:** The small size, late post‐radiotherapy onset, and reversibility of these contrast‐enhancing lesions highlight the overlap between LTPE, pseudoprogression and radionecrosis. Further studies are needed to better characterize this phenomenon and establish clear criterio to avoid misdiagnoses of tumor progression.

**Disclosure:** Nothing to disclose.

## EPO‐0968

### Survival impact and predictive factors of epilepsy and seizure control in glioblastoma patients

#### M. Dencker ^1^; B. Hasselbalch^1^; I. Christensen^1^; K. Grunnet^1^; A. Bjerrum^2^; V. Fougner^1^; D. Schou Nørøxe^1^; U. Lassen^1^; J. Weischenfeldt^3^; L. Pinborg^4^; T. Urup^1^

##### ^1^The DCCC Brain Tumor Center and Department of Cancer Treatment, Rigshospitalet, Copenhagen, Denmark; ^2^Department of Cancer Treatment, Rigshospitalet, Copenhagen, Denmark; ^3^Biotech Research & Innovation Centre (BRIC), The Finsen Laboratory, Rigshospitalet, University of Copenhagen, Copenhagen, Denmark; ^4^Neurobiology Research Unit, Copenhagen University Hospital and Epilepsy Clinic, Department of Neurology, Rigshospitalet, Copenhagen, Denmark

**Background and aims:** More than half of glioblastoma patients experience epileptic seizures. Epilepsy–glioma interactions are linked to poor prognosis, yet predictors of epilepsy remain unclear. We examined associations with prognosis, as well as predictors of epilepsy and seizure control.

**Methods:** From a prospective database, 595 glioblastoma IDH‐wildtype patients treated with standard therapy from 2016–2024 at Rigshospitalet were included and divided into training (2016–2021) and validation (2022–2024) datasets. Epilepsy diagnosis and medication use were extracted from medical records. As a surrogate marker of seizure control, we used dose burden of anti‐seizure medication calculated as prescribed daily dose relative to WHO defined daily dose. Candidate genetic factors comprised pathogenic and likely pathogenic variants from genomic profiling. We used Cox regression and generalized linear models for survival and prediction analysis.

**Results:** Pre‐ and postdiagnostic epilepsy (HR: 1.41 [95% CI 1.06–1.87], *p* = 0.018 and HR: 3.08 [2.34–4.05], *p* < 0.0001, respectively) and higher dose burden at time of tumour diagnosis (HR: 1.06 [1.03–1.10], *p* < 0.0001) were associated with poor overall survival. Multifocal disease and absence of RB1 alterations were associated with postdiagnostic epilepsy. Multifocal disease predicted higher dose burden in both pre‐ and postdiagnostic epilepsy (*p* = 0.009 and *p* = 0.016). In prediagnostic epilepsy, resection predicted lower dose burden than biopsy.

**Conclusion:** Epilepsy and lack of seizure control negatively impact prognosis in glioblastoma. They can be predicted using clinical and genomic factors. This can support clinical risk stratification and early epilepsy management if validated.

**Disclosure:** Benedikte Hasselbalch: paid member of an expert panel for Servier.

## EPO‐0969

### Continuous monitoring and prediction of fatigue in patients with glioblastoma

#### M. Touya ^1^; P. Ducouret^2^; S. Viguet‐Carrin^3^; N. Desbaillets^4^; P. Ryvlin^5^; A. Hottinger^3^

##### ^1^University of Lausanne, Lausanne, Switzerland; ^2^NeuroTech@NeuroDigital Lab, Lausanne University Hospital, Lausanne, Switzerland; ^3^Lundin Family Brain Tumor Research Center, Lausanne University Hospital, Lausanne, Switzerland; ^4^Department of Oncology, Lausanne University Hospital, Lausanne, Switzerland; ^5^Department of Neurology, Lausanne University Hospital, Lausanne, Switzerland

**Background and aims:** In glioblastoma (GBM), fatigue is a widely understudied and may particularly impact patients. Several hypotheses may explain the development of fatigue, including direct effect of the tumor and its treatments. Moreover, concomitant depressive state and dysregulation of the autonomic nervous system may be observed. We postulate that a combined measure of physical and autonomic activities, acquired through a wearable device will provide biomarkers of the clinical and physical evolution.

**Methods:** We used a wearable device (Embrace+ smartwatch, Empatica) and an electronic diary to monitor patient seizures as well as daily the fatigue using a Visual Analog Scale (VAS). Every 2 months, at time of planned MRI, patients complete questionnaires about medications and validated fatigue and quality of life questionnaires.

**Results:** As of January 2026, 15/20 patients were enrolled. Daily values of the VAS have been weakly linked standadized fatigue questionnaires. However, findings suggests that patients tend to be less active during days in which they end up feeling more fatigued. Furthermore, machine learning models show promising results regarding prediction of fatigue and disease progression, based on parameters acquired by the wearable device. More results regarding interactions between fatigue and mood, impact of therapies and life expectancy are under investigation.

**Conclusion:** Our preliminary data suggest that fatigue measured by standardized questionnaires might be anticipated based on the completion of a simple VAS question completed daily by the patient and correlated to continuous activity measures obtained by a wearable device.

**Disclosure:** Nothing to disclose.

## EPO‐0970

### Progressive ophthalmoparesis revealing an unknown lung adenocarcinoma. A new paraneoplastic syndrome?

#### P. Gómez Ramírez; M. El Harmochi Daoud; A. Sánchez Gómez; A. Herrera Ortega; V. Ros Escolar; M. Carrillo Carrillo; M. Usero Ruiz; M. Corrales Arroyo; J. Flores Barragán; A. Hernández González

##### Department of Neurology, Ciudad Real General University Hospital, Ciudad Real, Spain

**Background and aims:** Oculomotor disorders in oncology patients are usually caused by brainstem, orbital, metastases or meningeal infiltration, and may be the presentation of an occult tumor. Another etiology is paraneoplastic syndromes, such as myasthenia gravis or Eaton‐Lambert syndrome (LES), showing variable clinical patterns. We report a case of progressive ophthalmoparesis evolving into One and a Half Syndrome (OAHS) as the initial manifestation of a lung carcinoma.

**Methods:** A 56‐year‐old woman, ex‐smoker, presented with left ptosis and diplopia with limited bilateral abduction, unresponsive to pyridostigmine and corticosteroids. Diplopia progressively worsened over three weeks without fatigability. Examination only revealed a horizontal OAHS with left ptosis.

**Results:** TSH, antithyroid antibodies, anti‐AChR, anti‐MuSK, anti‐P/Q‐type VGCCs, ANA, onconeuronal antibodies were negative. Brain MRI and angio‐TC: normal. Jitter and repetitive stimulation: normal. CSF: normal. Body CT: pulmonary mass in the right middle lobe. Biopsy: lung adenocarcinoma. High‐dose methylprednisolone followed by tapering and intravenous immunoglobulins produced partial improvement. One month after surgery the patient was asymptomatic. Adjuvant chemotherapy was added.

**Conclusion:** The absence of structural lesions and resolution after surgery suggest a paraneoplastic mechanism. LES with isolated ophthalmoplegia is rare and may even precede cancer diagnosis, more commonly small‐cell lung carcinoma, but exceptionally other subtypes (including adenocarcinoma). Ant‐P/Q‐type VGCCs can be negative in 15% of LES associated with lung carcinoma, but the absence of neurophysiological abnormalities in our case suggests that progressive ophthalmoparesis may represent an independent paraneoplastic syndrome, already suggested in other cases with exophthalmos and non‐infiltrative ophthalmoparesis. This presentation should prompt awareness and thorough search for occult tumors.

**Disclosure:** Nothing to disclose.

## EPO‐0971

### Delayed acute motor axonal neuropathy following BCMA‐directed CAR‐T therapy in relapsed/refractory multiple myeloma

#### T. Jordà‐Baleri ^1^; A. Llauradó^4^; A. Rivas Delgado^2^; G. Iacoboni^2^; C. Carpio^2^; B. Rodriguez‐Acevedo^3^; Á. Cobo‐Calvo^3^; N. Raguer^5^; P. Barba^2^; X. Montalban^3^; H. Ariño^3^

##### ^1^Neurology Department, Vall d’Hebron University Hospital, Barcelona, Spain; ^2^Department of Haematology, Vall d’Hebron University Hospital, Barcelona, Spain; ^3^Centre d’Esclerosi Múltiple de Catalunya (Cemcat), Hospital Universitari de Vall d’Hebron, Barcelona, Spain; ^4^Clinic of Neuromuscular Disorders and Rare Diseases, Vall d’Hebron University Hospital, Barcelona, Spain; ^5^Department of Clinical Neurophysiology. Vall d’Hebron University Hospital, Spain

**Background and aims:** Chimeric antigen receptor T‐cell (CAR‐T) therapies targeting B‐cell maturation antigen (BCMA) have transformed the treatment of relapsed/refractory multiple myeloma (MM). However, compared with CD19‐directed constructs, BCMA‐directed CAR‐T therapies are associated with a distinct and still incompletely characterized spectrum of neurological complications.

**Methods:** A patient treated with BCMA‐directed CAR‐T therapy developed delayed neurological symptoms. Clinical, neurophysiological, neuroimaging, and cerebrospinal fluid (CSF) studies were performed.

**Results:** A 65‐year‐old woman with relapsed/refractory IgA kappa MM and high tumor burden received fourth‐line BCMA‐directed CAR‐T therapy (ciltacabtagene autoleucel). On day +25, she developed grade 2 cytokine release syndrome without immune effector cell–associated neurotoxicity syndrome (ICANS). One month after infusion, disease assessment showed a partial response, and she developed a sixth nerve palsy with normal CSF findings, which resolved after corticosteroid treatment. Two months post‐infusion, she presented with subacute progressive symmetric tetraparesis causing severe disability, without sensory loss or dysautonomia. Neurological examination revealed global areflexia and marked weakness, followed by encephalopathy. CSF analysis showed marked lymphocytic pleocytosis (685 cells/μL; 96% CAR‐T cells) and thiamine deficiency, without other abnormalities. Electromyography demonstrated features consistent with acute motor axonal neuropathy. Brain and spinal MRI excluded central nervous system involvement. Treatment included intravenous immunoglobulins, corticosteroids, thiamine supplementation, and anakinra. Encephalopathy resolved rapidly, but motor recovery was limited. The patient died four months after CAR‐T infusion due to respiratory infection.

**Figure:**
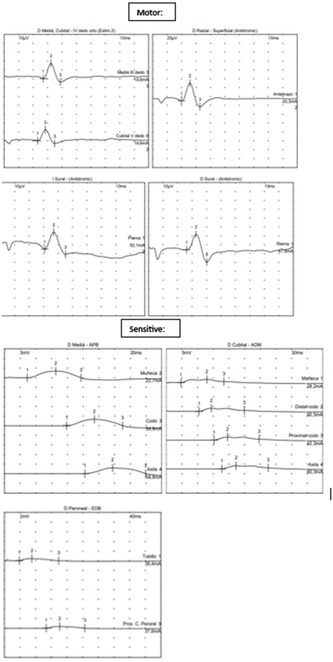
**FIGURE 1** EMG findings: Marked reduction in compound motor action potential amplitudes in the bilateral median and ulnar nerves, and the right common peroneal nerve. Below, preserved sensitive responses.

**Figure:**
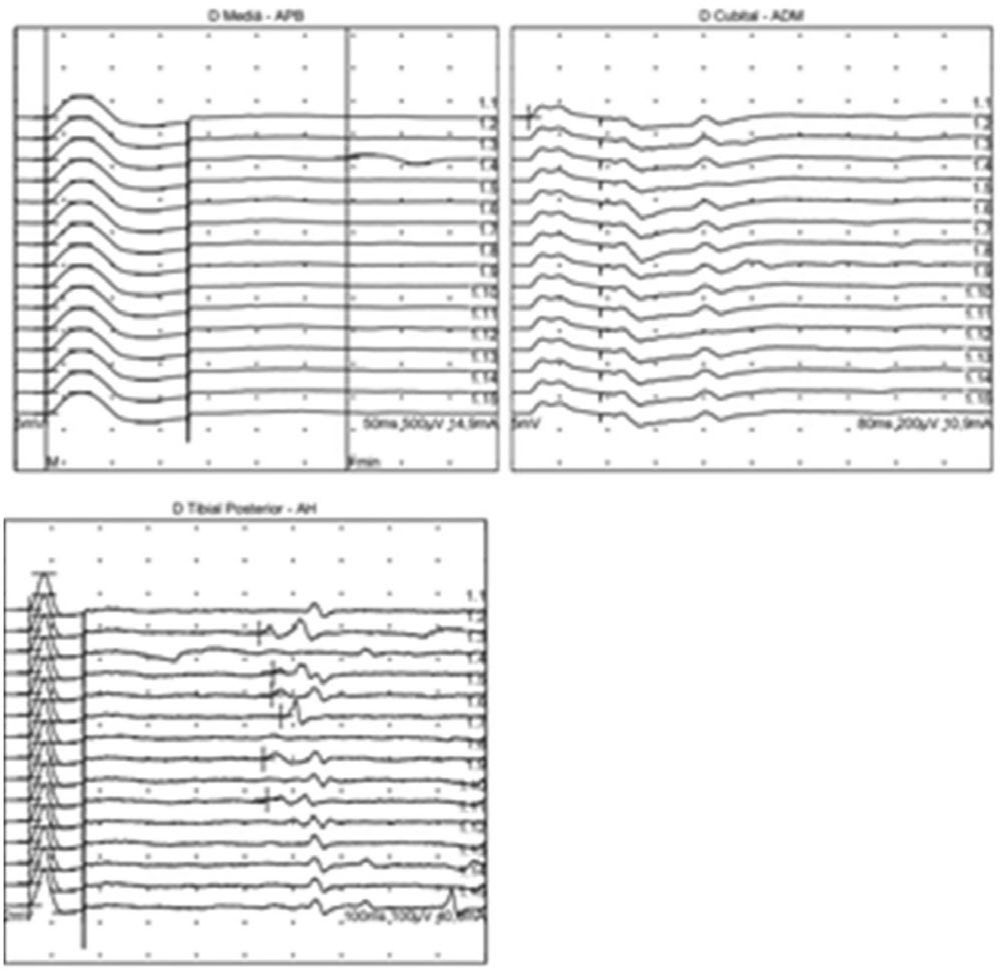
**FIGURE 2** EMG findings: normal F‐waves in the right median nerve, absent F‐waves in the ulnar nerve, and delayed F‐waves in the right posterior tibial nerve.

**Conclusion:** BCMA‐directed CAR‐T therapy may cause severe delayed peripheral neurotoxicity, highlighting the need for prolonged multidisciplinary neurological monitoring beyond the acute post‐infusion period.

**Disclosure:** Nothing to disclose.

## EPO‐0972

### Optimized parameters in image‐guided focused ultrasound enable safe and reproducible blood–brain barrier opening in preclinical glioma models

#### T. Kuhn ^1^; T. Knobloch^1^; M. Platten^2^; L. Bunse^2^

##### ^1^CCU Neuroimmunology and Brain Tumor Immunology, Deutsches Krebsforschungszentrum (DKFZ), Heidelberg, Germany; ^2^Department of Neurology, MCTN, Medical Faculty Mannheim, Heidelberg University, Mannheim, Germany

**Background and aims:** Focused ultrasound (FUS) in combination with systemically administered nanobubbles is an emerging, non‐invasive strategy to transiently disrupt the blood–brain barrier (BBB) and enhance drug delivery to the central nervous system. Despite its promise, defining acoustic parameters that ensure both efficacy and safety remains challenging, particularly in pathological settings such as brain tumors.

**Methods:** We systematically optimized key FUS parameters—including acoustic pressure, burst length, pulse repetition frequency, and number of bursts—to achieve consistent and safe BBB opening in healthy mice. The optimized protocol was then evaluated in several GL261 based glioma mouse models. BBB disruption was assessed using contrast‐enhanced MRI, Acoustic Feedback Mapping, and Evans Blue dye extravasation, with histological validation. Safety was evaluated using FLAIR and T2*‐weighted MRI to detect hemorrhage or tissue damage.

**Results:** An optimized set of FUS parameters (1 MPa, 10 ms bursts, 0.5 Hz, 60 bursts) reliably opened the BBB in healthy mice without parenchymal injury, confirmed by T1‐ and T2*‐weighted MRI (Figure 1). The same protocol effectively opened the BBB in tumor and peritumoral regions in GL261 based‐glioma models (Fig. 2). Higher pressures or increased burst number or frequency caused hemorrhage, establishing a safety threshold. Acoustic Feedback Mapping showed subharmonic and ultraharmonic emissions correlated with contrast enhancement, and histology confirmed Evans Blue extravasation supporting successful BBB disruption.

**Figure:**
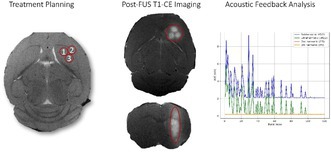
**FIGURE 1** Successful BBBO achieved using an optimized MRI‐guided FUS protocol, as evidenced by post‐FUS T1 contrast‐enhanced imaging and subharmonic/ultraharmonic frequency analysis.

**Figure:**
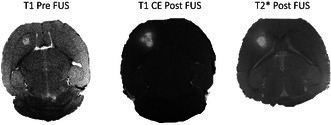
**FIGURE 2** Successful BBBO in GL261 tumor‐bearing mice demonstrated by T1 contrast‐enhanced MRI after FUS, with no evidence of hemorrhage on T2* post‐FUS Imaging.

**Conclusion:** We establish a reproducible and safe FUS protocol for BBB opening and demonstrate its successful application in preclinical glioma models. These findings provide an important foundation for the clinical translation of image‐guided, FUS‐mediated drug delivery strategies in neuro‐oncology.

**Disclosure:** Nothing to disclose.

## EPO‐0973

### Cognitive decline as a prognostic marker of disease activity in patients with glioblastoma

#### E. Konrath; B. Calabek‐Wohinz; S. Oberndorfer

##### Department of Neurology, University Hospital St. Pölten – NOE LGA, Karl Landsteiner University, St. Pölten, Austria

**Background and aims:** In patients with glioblastoma, cognitive impairment is influenced by treatment‐ and patient‐related factors, but is strongly driven by tumor‐related pathological processes. There is evidence that cognitive decline has predictive value for disease progression. However, existing studies are limited by small sample sizes, heterogeneous tumor entities, insufficient control of important covariates, and the lack of a direct comparison between neuropsychological outcomes and the Karnofsky Performance Status Scale (KPS).

**Methods:** The present study investigated the predictive value of a neuropsychological test battery comprising eight subtests (NeuroCogFX) in comparison with the KPS for predicting tumor progression according to the RANO criteria. A Cox regression model with time‐dependent covariates was applied, including the control variables age, education, sex, MGMT promoter methylation status, extent of resection, tumor hemisphere, and the use of steroids, anticonvulsants and psychotropic medication. The sample consisted of 99 patients with a total of 309 assessment time points.

**Results:** Cognitive decline, defined as deterioration in three tests relative to baseline, was associated with a fivefold increased risk of radiologically confirmed tumor progression (HR = 5.00; *p* < 0.001). In contrast, decline in KPS was not a significant predictor. Among the covariates, MGMT promoter methylation (HR = 0.42; *p* = 0.005) and a higher number of years of education (HR = 0.87; *p* = 0.019) emerged as protective factors.

**Conclusion:** .These results highlight the value of continuous neuropsychological monitoring as a sensitive approach for detecting disease progression. It provides clinically meaningful information, particularly in cases of equivocal radiological findings or for treatment‐related decision‐making.

**Disclosure:** Nothing to disclose.

## Cerebrovascular Diseases 8

## EPO‐0974

### Predicting intracranial aneurysm rupture risk from DSA images: Automated segmentation, multimodal prediction, and validation

#### H. Jin; R. Zhang; G. Liang

##### Department of Neurosurgery, General Hospital of Northern Theater Command, Shenyang, China

**Background and aims:** Subarachnoid hemorrhage (SAH) is a highly fatal acute cerebrovascular event, most commonly caused by the rupture of an intracranial aneurysm (IA). Current methods for predicting IA rupture are limited in accuracy and consistency. These methods often rely heavily on clinician experience and carry a non‐negligible risk of oversight, falling short of the requirements for precise clinical management.

**Methods:** To address these limitations, we conducted a retrospective study using digital subtraction angiography (DSA) images from 890 patients with IAs. Initially, the aneurysm regions of interest (ROIs) were annotated. We then developed a U‐KAMA segmentation algorithm to achieve fully automated IA segmentation. From these segmented regions, 1834 radiomic features were extracted. Following a feature selection process, 57 key imaging features were retained and subsequently integrated with clinical characteristics to build a multimodal model for predicting IA rupture.

**Results:** The model demonstrated robust performance. For automated IA segmentation, it achieved a mean Dice similarity coefficient of 0.815. The process was 92.62% faster than manual segmentation, reducing the average time per case from 8.36 minutes to 0.617 minutes. For rupture prediction, the multimodal model achieved an area under the curve (AUC) of 0.893, significantly outperforming models based on single data modalities.

**Conclusion:** This study demonstrates high performance in both fully automated IA segmentation and rupture prediction. Our work provides a reliable, artificial intelligence‐driven solution to advance the automated and precise assessment of IA rupture risk, showing significant potential for clinical translation and application.

**Disclosure:** Nothing to disclose.

## EPO‐0975

### Platelet function–defined clopidogrel inactivity as an independent contributor to recurrent ischemic stroke: A confounder‐adjusted analysis

#### R. Ramesh; B. Bagath Srinivasan; H. Arumugam; L. Narasimhan R; N. Gampa; S. Shanmugam; P. Hazeena; I. Tiruviru

##### Department of Neurology, Sri Ramachandra Institute of Higher Education and Research, Chennai, India

**Background and aims:** Recurrent ischemic stroke despite antiplatelet therapy remains a significant clinical challenge. Reduced or absent clopidogrel activity, reflected by reduced inhibition of platelet aggregation, may represent an under‐recognised and potentially modifiable contributor to recurrence. We aimed to evaluate clopidogrel antiplatelet activity as an independent factor in recurrent ischemic stroke, accounting for major confounders

**Methods:** Patients admitted with recurrent ischemic stroke between 2019 and 2025 were retrospectively analysed. Of 254 screened patients, 105 were included based on aggregometry test (assessed using adenosine diphosphate–induced platelet aggregation) being done, compliance to medication and availability of records. Platelet activity was categorized and clopidogrel inactivity was evaluated as an independent contributor to recurrence after excluding confounders, including atrial fibrillation, poorly controlled diabetes (HbA1c >8), uncontrolled LDL, large vessel occlusion, and coagulopathies.

**Figure:**
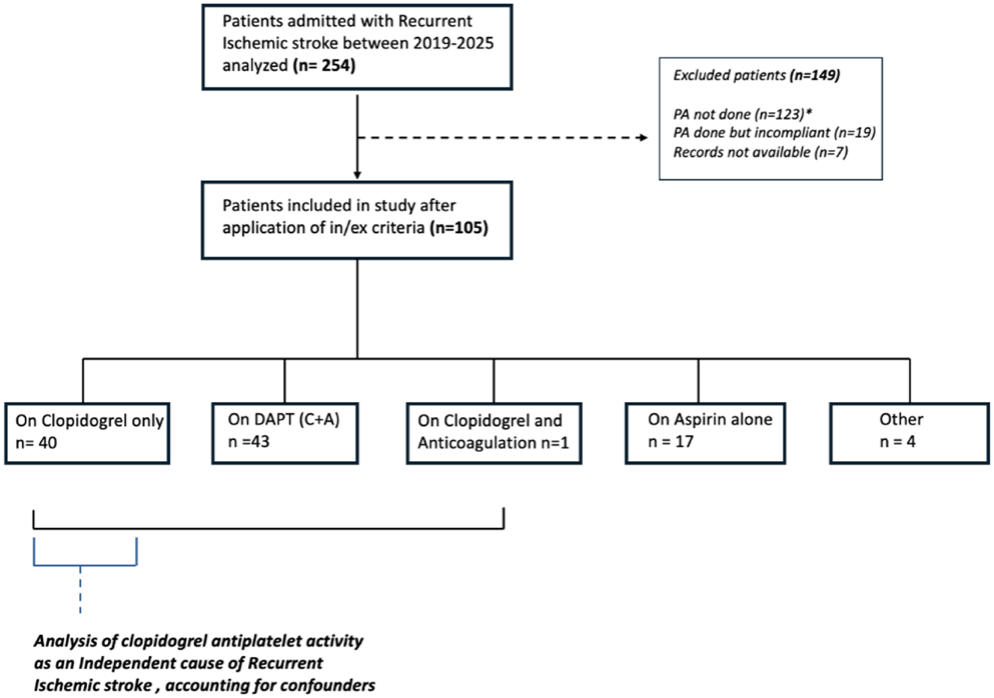
**FIGURE 1** Study design and patient allocation across anti‐thrombotic therapy groups.

**Results:** Among 105 patients, 40 were on clopidogrel alone and 43 on dual antiplatelet therapy. In the clopidogrel‐only group, suboptimal activity was observed in 31/40 patients (77.5 %) with poor activity in 16/40 patients (40%). After exclusion of all confounders, clopidogrel inactivity alone plausibly contributed to recurrence in 17 patients/105 (16.1%, 8.5 % [Poor activity]). Comparatively this cohort had only 17 patients on aspirin alone of which 4 patients developed recurrence (3.8%) after exclusion of all confounders.

**Figure:**
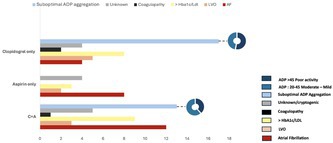
**FIGURE 2** Distribution of platelet inactivity and competing mechanisms of recurrent ischemic stroke across antiplatelet strategies.

**Conclusion:** Suboptimal clopidogrel antiplatelet activity is especially common in south Asians and Indians may independently contribute to recurrence in almost one‐fifth of the even after accounting for major confounders. Platelet function–guided tailoring of antiplatelets may represent an important strategy for secondary stroke prevention.

**Disclosure:** Nothing to disclose.

## EPO‐0976

### Prior stroke or transient ischaemic attack history and intensive care unit outcomes: A propensity‐matched cohort study

#### S. Biswas^1^; S. Vasireddy^2^; Y. Srivastava^1^; S. Arora ^3^; J. Rajamani^4^; N. Mally^5^

##### ^1^Department of Internal Medicine, Ivano‐Frankivsk National Medical University, Ivano‐Frankivsk, Ukraine; ^2^Department of Neurology, NMC Specialty Hospital, Abu Dhabi, United Arab Emirates; ^3^Department of Internal Medicine, University of Debrecen, Hungary; ^4^Department of Internal Medicine Medical College, Caucasus University, Tbilisi, Georgia; ^5^Department of Internal Medicine, Humanitas University, Milan, Italy

**Background and aims:** Prior cerebrovascular disease may increase vulnerability during critical illness through reduced cerebral reserve and impaired autoregulation. Whether prior stroke or transient ischaemic attack (TIA) independently predicts intensive care unit (ICU) mortality remains unclear. We evaluated whether prior stroke/TIA history affects ICU mortality and recurrent cerebrovascular events independent of cardiovascular comorbidity burden.

**Methods:** Retrospective propensity‐matched cohort study using the Medical Information Mart for Intensive Care IV (MIMIC‐IV) database (2008–2022). Adults with ICU stay ≥24 hours were included; those admitted for acute stroke or TIA were excluded. Prior stroke/TIA was identified using International Classification of Diseases codes. One‐to‐one propensity score matching balanced cardiovascular risk factors and comorbidities. Primary outcome was in‐hospital mortality; secondary outcomes included recurrent stroke, seizures, and delirium.

**Figure:**
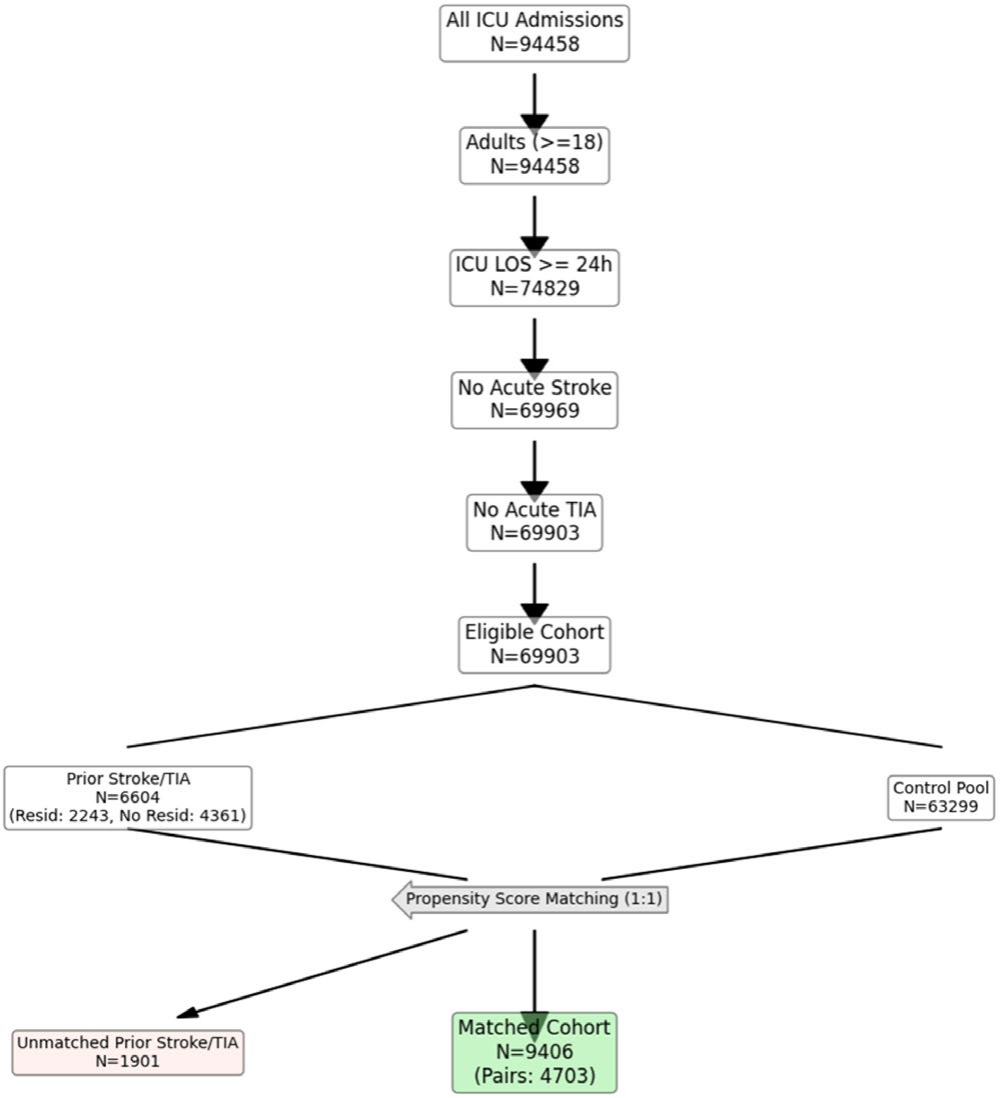
FIGURE 1

**Results:** Among 69,903 eligible ICU stays, 6,604 (9.4%) had prior stroke/TIA history. After matching (4,703 pairs), groups were well‐balanced (all standardized mean differences <0.1). In‐hospital mortality was similar between prior stroke/TIA patients (11.4%) and controls (11.5%; matched odds ratio 0.99, *p* = 0.95). Kaplan‐Meier analysis showed no survival difference (log‐rank *p* = 0.54). However, prior stroke/TIA patients experienced higher rates of recurrent ischemic stroke (4.3% vs 2.6%), seizures (16.2% vs 8.0%), and delirium (8.8% vs 6.3%). On multivariable analysis, prior stroke/TIA independently predicted recurrent stroke (odds ratio 1.36, 95% confidence interval 1.09–1.71, *p* = 0.007).

**Figure:**
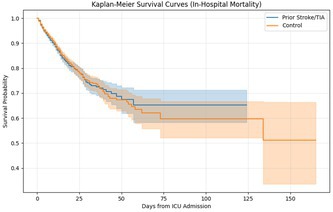
FIGURE 2

**Figure:**
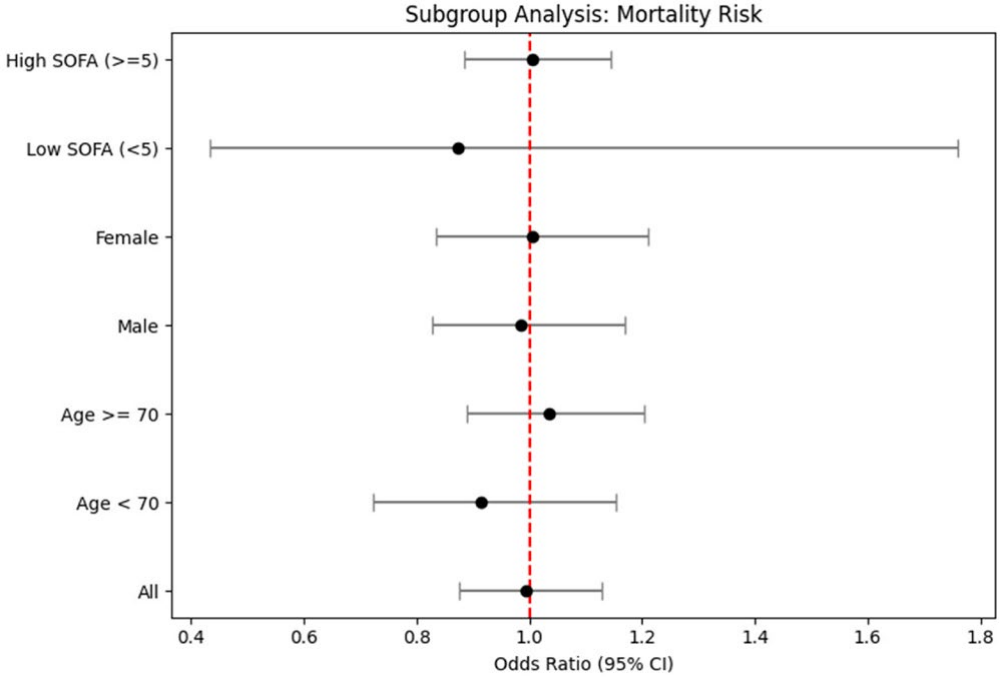
FIGURE 3

**Conclusion:** After controlling for cardiovascular comorbidities, prior stroke/TIA history does not independently increase ICU mortality. However, these patients experience significantly higher rates of recurrent cerebrovascular events and neurological complications, warranting enhanced neurovascular monitoring during critical illness.

**Disclosure:** Nothing to disclose.

## EPO‐0977

### Mortality in stroke survivors during a global health crisis: The Geneva population‐based study

#### S. Pandit; E. Dirren; E. Carrera

##### Stroke Research Lab, Division of Neurology, Department of Clinical Neuroscience, Geneva University Hospital, Geneva, Switzerland

**Background and aims:** We examined 5‐year mortality among stroke survivors and whether the impact of the COVID‐19 pandemic on mortality was higher in stroke patients compared to the general population.

**Methods:** We evaluated 5‐year mortality in all patients who suffered from a first‐ever stroke in the canton of Geneva (504’257 inhabitants) in 2018‐2019. Mortality was ascertained through official records. Predictors of mortality were identified using Cox proportional hazards models, and mortality among stroke survivors (alive ≥90 days post‐stroke) was compared with that of the general population using standardized mortality ratios (SMRs).

**Results:** In 2018 and 2019, there were 1123 first‐ever stroke patients (1,000 ischemic strokes [IS] and 123 intracerebral hemorrhages [ICH]). Five‐year mortality was 40.3% in IS and 56.1% in ICH. In IS, mortality was associated with age (HR = 1.06), premorbid disability (mRS, HR = 1.28), stroke severity (NIHSS, HR = 1.09), and coronary heart disease (HR = 1.54; all *p* < 0.001). In ICH, mortality was associated with age (HR = 1.07) and stroke severity (NIHSS, HR = 1.10; both *p* < 0.001). During follow‐up, mortality was consistently higher among stroke survivors than in the general population. However, no excess mortality was observed during the pandemic period (2020–2021) compared with the post‐pandemic years 2022–2024 (SMR 1.88 [1.56–2.25] vs 1.94 [1.62–2.30], *p* = 0.82).

**Figure:**
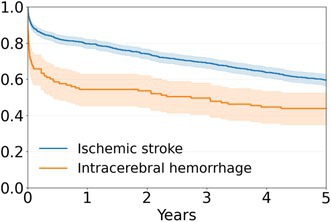
**FIGURE 1** Kaplan Meier curves.

**Figure:**
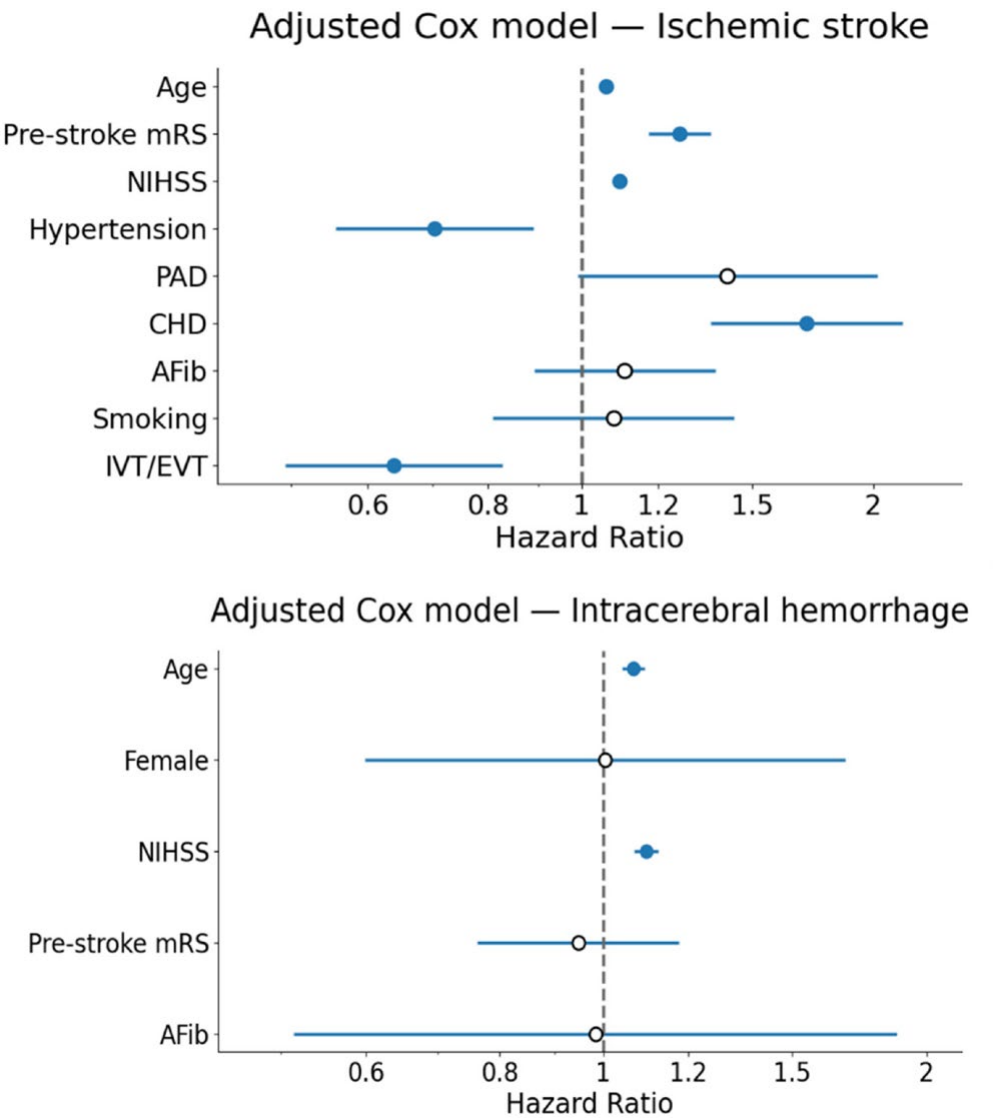
**FIGURE 2** Multivariable Cox proportional hazards models for 5‐year mortality after ischemic and hemorrhagic stroke. Variables with *p* < 0.05 in univariable Cox regression were entered into the multivariable model.

**Figure:**
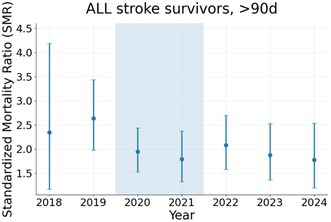
**FIGURE 3** Standardized mortality ratios (SMR) of stroke survivors. Ischemic and hemorrhagic strokes are combined.

**Conclusion:** While stroke survivors have sustained excess mortality compared with the general population, no increase was observed during the pandemic years (2020–2021) compared with the post‐pandemic years (2022–2024).

**Disclosure:** Nothing to disclose.

## EPO‐0978

### Epidemiological study, evaluation of clinical, genetics and neuroimaging aspects in a cohort of Brazilian patients with Cadasil

#### V. Neri ^1^; V. Neri^2^; V. Neri^3^; L. Wagner^2^; M. Barros^2^; M. Mussi^2^; A. Pereira^2^; T. Manhaes^2^; C. Cunha^2^; D. Viana^2^; A. Melo^4^; T. Louvain^5^

##### ^1^Neurology, Lagoa Hospital, Rio de Janeiro, Brazil; ^2^Neurology, Faculty of Medicine of Campos*,* Campos dos Goytacazes, Brazil; ^3^CADASIL Study Group; ^4^Neuroradiology Faculty of Medicine of Campos, Campos dos Goytacazes, Brazil; ^5^Genetics, Universidade Estadual do Norte Fluminense, Campos dos Goytacazes, Brazil

**Background and aims:** CADASIL is a dominant autosomal disease caused by mutations at the NOTCH 3 gene. This study aims to evaluate demographic aspects and clinical symptoms of patients with CADASIL in a region with a high prevalence of this disease in Rio de Janeiro/Brazil, analyze morphometrically white matter in cerebral MRI, and study variants of NOTCH3 gene.

**Methods:** Clinical evaluation and follow‐up of patients at a Specialized Center in CADASIL in the north of the State of Rio de Janeiro/Brazil (2018–2025). Research on mutations in NOTCH3 gene. Brain morphological evaluation by 1.5‐Tesla MRI.

**Results:** 33 patients from 27 different families. 19 (58%) men and 14 (42%) women; 70% white and 30% Afro‐descendant. Mean age at first symptom: 42.3 (±10.6); 19 (58%) presented migraine, 7 (21%) cognitive impairment. 32 patients heterozygous for the pathogenic allele variant c.457C>T from the ten probably unrelated kindreds. One patient is heterozygous for the pathogenic allele c.328C>T. All patients with pathogenic allele had MRI lesions; MRI analyses: 61% presented bilateral white matter lesions, 48% bilateral temporal pole lesions, 55% parietal lobe, 27% basal ganglia, and 24% cerebellar lesions.

**Figure:**
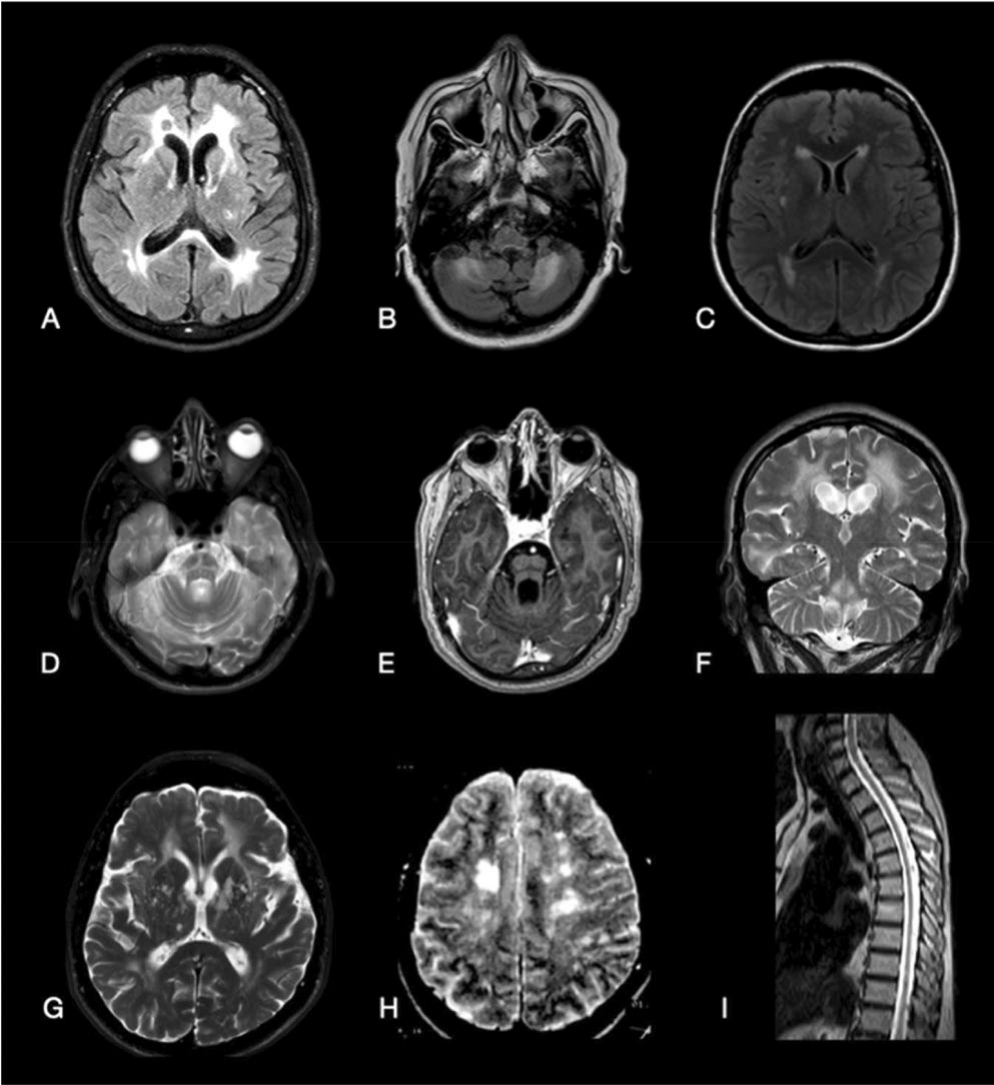
**FIGURE 1** MRI scans from 9 cases analyzed in this study.

**Figure:**
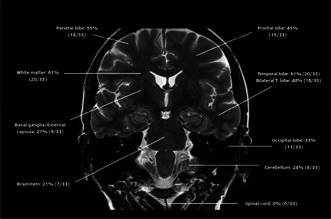
**FIGURE 2** Topography of brain lesions in the cohort of patients with CADASIL.

**Figure:**
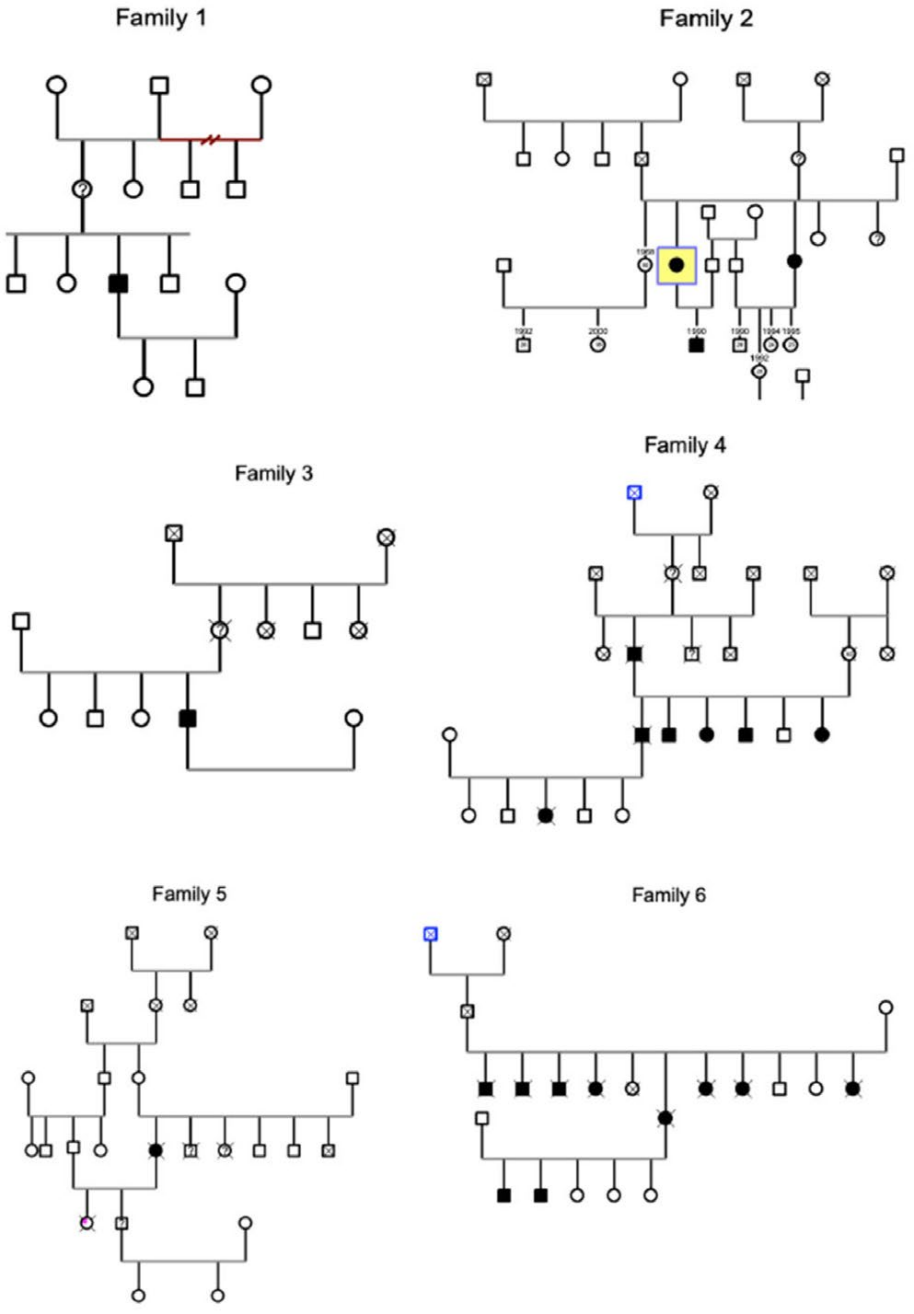
**FIGURE 3** Pedigree of six families with NOTCH3 gene mutations or variants. Square symbols, men; circular symbols, women; filled symbols, CADASIL affected.

**Conclusion:** CADASIL is a rare and neglected disease. In this study, conducted in a region of Rio de Janeiro with a high prevalence of the disease, there was a predominance of men and white individuals, and migraine was the main neurological symptom. Regarding the changes in MRI, the parietal and temporal lobes were the most affected regions; bilateral temporal lesion (a hallmark of the disease) was observed in 48% of cases.

**Disclosure:** Nothing to disclose.

## EPO‐0979

### Simulating and augmenting clinical workflows in emergency stroke triage: A multi‐center comparative study of three artificial intelligence paradigms

#### X. MD ^1^; S. Yang^2^; Y. Shi^2^; M. Li^2^; J. Wu^1^

##### ^1^Beijing Tsinghua Changgung Hospital, Beijing, China; ^2^Tsinghua University, Beijing, China

**Background and aims:** Early diagnosis and rapid identification of ischemic stroke and LVOs are demanding and challenging. This study aimed to simulate three typical emergency work paradigms using a large model in order to explore the robustness of different levels of information integration in real‐world multi‐center settings.

**Methods:** We developed three paradigms: Paradigm 1 (Clinical‐Only): Relies solely on medical records (simulating preliminary expressions after a clinical consultation and examination). Paradigm 2 (Report‐Guided): Medical records plus radiology reports (obtained from brain non‐contrast CT, simulating decisions based on the reports). Paradigm 3 (Holistic Review): Medical records, radiology reports, and original CT images (simulating high‐level physicians making comprehensive decisions by combining reports and personally reviewing the images).

**Results:** Paradigm 1 performs excellently in the central hospital but fails in external centers due to heterogeneity in medical record documentation (F1 score 93.6 vs. 57.4), revealing the limitations of relying solely on empirical inquiries. Paradigm 2 improves the stability of general diagnosis but is limited in identifying large vessel occlusion (LVO) due to missed descriptions of subtle signs in reports (Information Loss). Paradigm 3, by introducing direct interpretations of the original images, the model achieves the best performance in LVO tasks across all centers (F1 score 68.8), significantly outperforming Paradigm 2.

**Conclusion:** Although radiology reports provide important references, in high‐risk emergency tasks, AI (and doctors) must be able to read the original images directly (Paradigm 3) in order to overcome the heterogeneity of medical records and the limitations of report information, achieving a satisfactory diagnosis and treatment.

**Disclosure:** Nothing to disclose.

## EPO‐0980

### Long term outcomes of symptomatic intracranial aherosclerotic stenosis: A 3 year follow up study

#### X. Tan ^1^; I. Siow^1^; M. Zin^2^; K. Lee^3^; L. Ping^4^; W. Han^5^; C. Chiea^6^; W. Huang^7^; B. Tan^2^; C. Sia^8^; T. Andersson^9^; P. Bhogal^10^; B. Baxter^11^; K. Teo^2^; J. Yeo^12^; M. Jing^2^; L. Yeo^2^

##### ^1^Department of Medicine, Ministry of Health Holdings, Singapore; ^2^Division of Neurology, Department of Medicine, National University Health System, Singapore; ^3^Department of Neurosurgery, National Neuroscience Institute, Singapore; ^4^Department of Neurology, National Neuroscience Institute, Singapore; ^5^Laboratory of Metabolic Medicine, Singapore Bioimaging Consortium, A*STAR, Singapore; ^6^Genome Institute of Singapore, A*STAR, Singapore; ^7^Institute for Infocomm Research, A*Star, Singapore; ^8^Division of Cardiology, Department of Medicine, National University Health System, Singapore; ^9^Department of Neuroradiology, Karolinska University Hospital, Stockholm, Sweden; ^10^Department of Neuroradiology, St. Bartholomew's and the Royal London Hospital, London, UK; ^11^Department of Radiology, Erlanger Hospital, Chattanooga, USA; ^12^Department of Medicine, National University Health System, Singapore

**Background and aims:** Large artery intracranial atherosclerotic stenosis (ICAS) is often described to have high rates of complications such as stroke recurrence and mortality. Though latest guidelines recommend maximum medical therapy as the mainstay of treatment for symptomatic ICAS, there is insufficient analysis regarding the effectiveness of this in reducing rates of mortality, recurrent strokes and cardiovascular events.

**Methods:** This multicentre prospective cohort study included patients who were treated for symptomatic ICAS between 2020 and 2022. Patients were stratified into two groups; those who achieved maximal medical therapy and those who did not. The rates of adverse outcomes such as recurrent strokes, mortality and myocardial infarction were compared between the two groups via univariate and multivariate binary logistic regression.

**Results:** Among 399 patients, 33.2% achieved maximal medical therapy. Mortality occurred in 4.9% of patients, recurrent stroke in 17.0% and myocardial infarction in 2.3%. On multivariate analysis, achieving maximal medical therapy was associated with lower rates of mortality (OR 8.41; CI 1.08–65.36, *p* = 0.042) and a longer duration of survival (Chi‐square = 9.871; df = 1; *p* = 0.002). Achieving maximal medical therapy was not associated with a significant reduction in rates of recurrent strokes and myocardial infarction.

**Figure:**
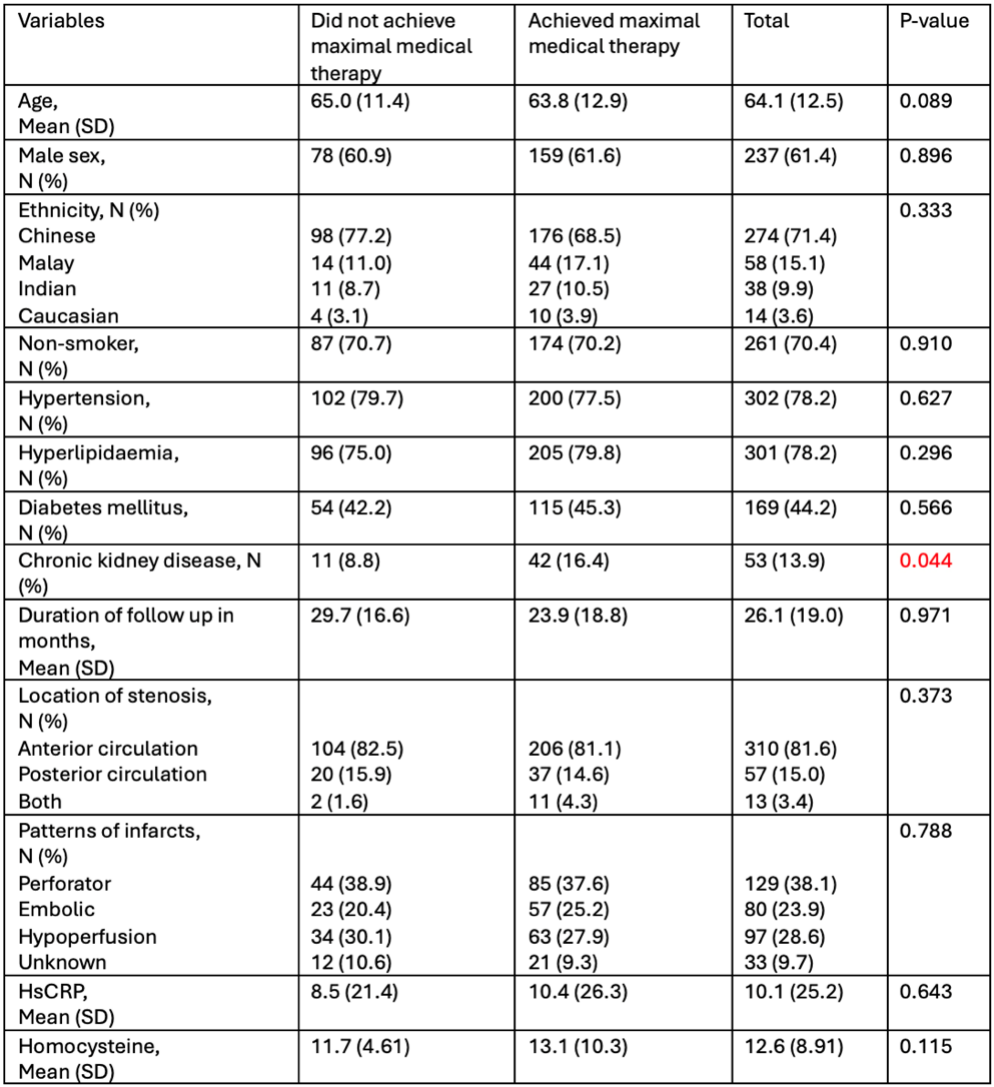
**TABLE 1** Baseline characteristics.

**Figure:**
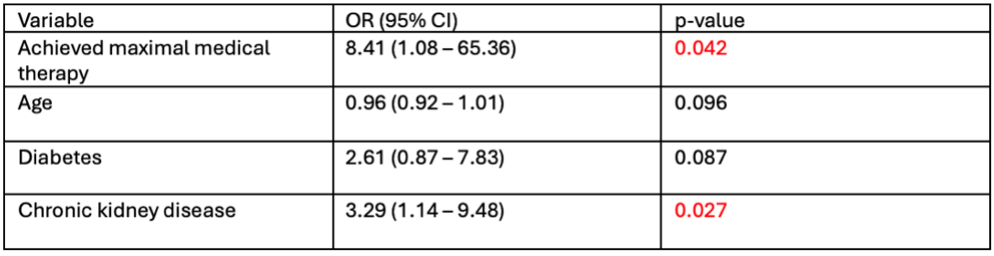
**TABLE 2** Multivariate analysis of factors affecting mortality rates in patients with ICAS.

**Conclusion:** ICAS is a serious condition which carries high rates of complications. Aggressive medical therapy to achieve optimum control of cardiovascular risk factors remains crucial to improving outcomes.

**Disclosure:** Nothing to disclose.

## EPO‐0981

### Is the mini‐cognitive evaluation superior to MMSE and MOCA to identify mild post stroke cognitive impairment?

#### Y. Lesnard; O. Godefroy; S. Wannepain; A. Routier‐Thorel; S. Canaple; C. Lamy; C. Leclercq; A. Courselle; M. Roussel

##### Departments of Neurology and Laboratory of Functional Neurosciences Jules Verne University of Picardie, Amiens, France

**Background and aims:** The diagnosis of post‐stroke cognitive impairment (PSCI) is imperative for its effective management. However, the sensitivity and specificity of screening tests are inadequate for the diagnosis of mild PSCI. The objective of this study was to assess the discriminatory capacity of a newly developed screening test, the Mini‐cognitive evaluation (MCE), in patients with at most mild PSCI (defined by an MMSE score ≥ 20). The primary objective was to ascertain the most accurate screening test (MMSE, MoCA, MCE) for discriminating stroke from controls. The secondary objectives were (1) to determine the most accurate test for identifying patients with PSCI, and (2) to examine the associations between MCE and the location of cerebral atrophy using VBM analysis.

**Methods:** We included 122 patients referred in the acute stroke unit (ASU) of Amiens University Hospital (62 at the ASU and 60 at post‐stroke visit) and 436 controls (comprehensive assessment performed in 89 patients, 44 with PSCI).

**Results:** The MCE demonstrated superiority (*p* = 0.0001) to the MMSE and MoCA in (1) discriminating between patients and controls (ROC AUC of 0.791 [0.731, 0.851]) and (2) discriminating patients with PSCI (ROC AUC = 0.896 [0.838–0.954]) (*p* = 0.001). MCE score was associated with overall brain atrophy (*p* = 0.01) and using VBM analysis with atrophy of numerous hemispheric structures, predominantly on the left side (*p* ≤ 0.001, uncorrected).

**Conclusion:** This study demonstrated that MCE is a promising diagnostic tool for identifying PSCI. The potential benefit of its use as a first line test in the management of stroke patients has now to be determined.

**Disclosure:** Y.Lesnard: Nothing to disclose O. Godefroy: during the last two years has served on scientific advisory boards and speaker (GE Healthcare, ESAI SAS), and did not received funding for travel and meetings from pharmaceutical companies S. Wannepain : Nothing to disclose A. Routier‐Thorel : Nothing to disclose S.Canaple: funding for travel from MEDTRONIK C.Lamy: Nothing to disclose C.Leclercq : Nothing to disclose A.Courselle: Nothing to disclose M. Roussel : Nothing to disclose.

## EPO‐0982

### Prominent vessel sign on susceptibility‐weighted imaging is associated with CTP perfusion‐core mismatch in anterior circulation ischemic stroke

#### Z. Wang; X. Hu; Q. Li

##### Department of Neurology, The Second Affiliated Hospital of Anhui Medical University, Hefei, China

**Background and aims:** Perfusion imaging can identify salvageable ischemic brain tissue but requires use of contrast agents and professional post‐processing software. This study aims to evaluate the role of prominent vessel sign (PVS) on acute susceptibility‐weighted imaging (SWI).

**Methods:** We included consecutive patients with anterior circulation acute ischemic stroke (AIS) admitted within 24 hours from onset or last known well from a prospective observational cohort between June 2023 and April 2025 in China. Emergency MRI was performed as the initial imaging modality for stroke diagnosis. PVS was defined as a focal increase in the number or diameter of hypointense vessels compared with the contralateral hemisphere. Perfusion‐core mismatch on CT perfusion was obtained according to the Extending the Time for Thrombolysis in Emergency Neurological Deficits (EXTEND) trial immediately after baseline MRI scan.

**Results:** Among 303 patients (median age 66 [IQR 57–75] years, 33.7% females), 102 (33.7%) had PVS on SWI at admission. The sensitivity of the PVS for identifying CTP mismatch was 66.9%, specificity 89.4%, positive predictive value 81.4%, and negative predictive value 79.6%. The presence of PVS on SWI remained associated with CTP mismatch (OR 10.25, 95% CI 5.32–19.74, *p* < 0.001) after adjusting for diabetes, atrial fibrillation, baseline stroke severity, and presence of large vessel occlusion.

**Figure:**
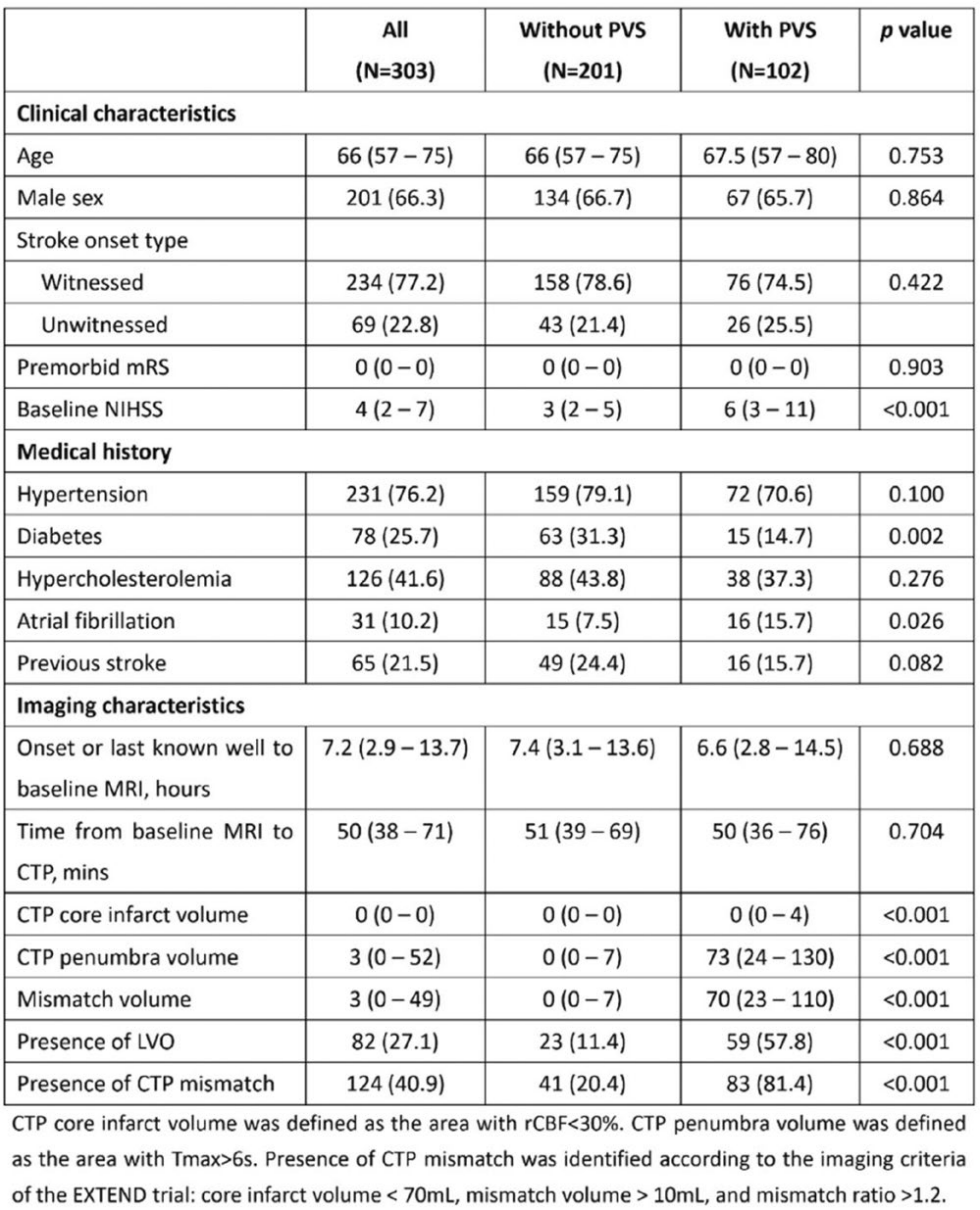
**TABLE 1** Baseline characteristics between patients with and without prominent vessel sign.

**Figure:**
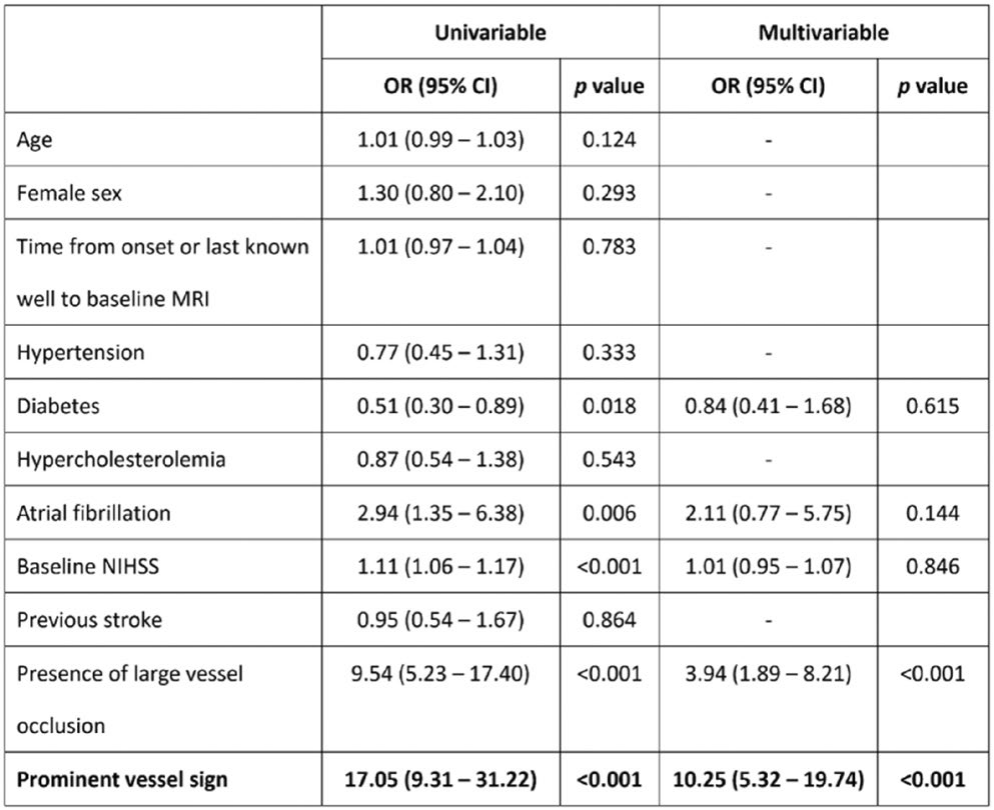
**TABLE 2** Univariable and multivariable logistic regression analyses of factors associated with presence of CTP perfusion‐core mismatch.

**Figure:**
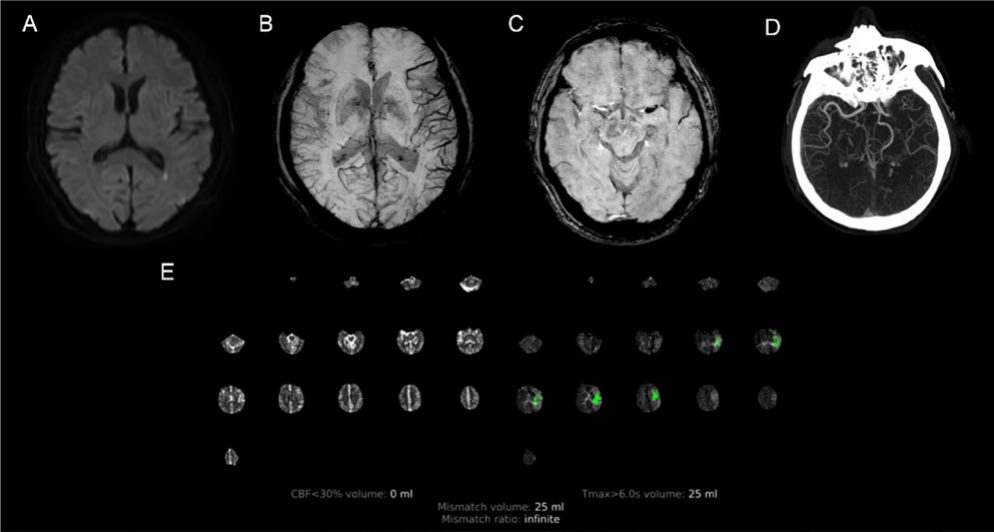
**FIGURE 1** Representative image of prominent vessel sign on SWI with MCA occlusion and perfusion‐core mismatch.

**Conclusion:** PVS on SWI is frequently observed and associated with CTP mismatch among patients with anterior circulation AIS within 24 hours from onset. The MRI marker may serve as a readily available tool for rapid identification of patients who may benefit from acute therapeutic intervention.

**Disclosure:** Nothing to disclose.

## MS and Related Disorders 7

## EPO‐0983

### Elevated serum BAFF in patients with neuromyelitis optica spectrum disorder after rituximab treatment

#### M. Deng; A. Lin

##### The First Affiliated Hospital of Fujian Medical University, Fuzhou, China

**Background and aims:** Despite achieving complete peripheral B cell deletion after over six months of rituximab therapy, a clinically significant minority of neuromyelitis optica spectrum disorder (NMOSD) patients experience breakthrough relapses, yet the underlying mechanism remains unclear.

**Methods:** From January 2024 to April 2025, this study enrolled 55 NMOSD patients and 15 healthy controls (all negative for autoimmune antibodies) at The First Affiliated Hospital of Fujian Medical University. Among the patients, 32 were in the remission phase under RTX treatment, and 23 were in the acute phase of the disease (16 were not receiving RTX treatment, and 7 were experiencing relapse despite RTX treatment). Serum BAFF protein concentrations were measured using ELISA. The non‐parametric Kruskal‐Wallis H test was used to assess overall differences among groups, followed by Dunn's test with Bonferroni correction for pairwise comparisons.

**Results:** Results showed that RTX‐treated patients in remission had significantly higher serum BAFF levels compared to healthy controls (*p* < 0.0001). In contrast, there was no significant difference in BAFF levels between the overall acute phase group and controls. Notably, one RTX‐treated patient in the stable group exhibited an exceptionally high BAFF level (1771.26 pg/mL) and experienced a clinical relapse one month later.

**Conclusion:** NMOSD patients treated with rituximab exhibit persistently high expression of BAFF. Extremely high BAFF levels may predict relapse in RTX‐treated patients, serving as a potential early biomarker for relapse.

**Disclosure:** Nothing to disclose.

## EPO‐0984

### Menopause in multiple sclerosis: Treatments and quality of life

#### C. Oreja‐Guevara ^1^; J. Casablanca Mezquita^1^; L. García‐Vasco^1^; J. Moarquech Rodriguez^1^; A. Paz Tamayo^1^; P. Coronado Martin^2^; I. Gömez Estévez^1^

##### ^1^Neurology, Hospital Clínico San Carlos, Madrid, Spain; ^2^Gynecology, Hospital Clinico San Carlos, Madrid, Spain

**Background and aims:** .Menopausal symptoms may overlap with multiple sclerosis (MS) manifestations and contribute to reduced quality of life (QoL). To characterize the use of menopausal hormone therapy (MHT) and symptomatic pharmacological treatments during menopause and to assess the effect of menopause on quality of life (QoL) in women with MS

**Methods:** .Cross‐sectional, observational study including postmenopausal women with MS. QoL was measured with the Short Cervantes Scale (SF‐16). Demographic and clinical data, as well as current MHT and symptomatic drug use, were recorded. Suitability for MHT was evaluated using a standardized algorithm derived from Spanish Society of Gynecology recommendations.

**Results:** .Ninety‐eight women were enrolled (mean age 52 years), with a mean interval of 4 years since the final menstrual period. Anxiety was present in 65%, fatigue in 76%, and urinary symptoms in 51%. Only 7.1% were on MHT, despite a large proportion fulfilling eligibility criteria (56.5% for combined oral MHT and 83% for combined transdermal MHT). Compared with the premenopausal phase, menopause was associated with increased prescriptions of symptomatic medications: antidepressants +33%, anxiolytics +42%, hypnotics +33%, and drugs for urinary symptoms +48%. Higher symptomatic drug use correlated with poorer baseline QoL. The worst scores were found in the menopause/health and psychological domains. The mean global Cervantes score was 37.6 (SD 19.0), and 50% of participants showed impaired QoL (29.6% mild impairment, 20.4% QoL could be improved).

**Conclusion:** .Menopause increases the QoL burden in women with MS and worsens shared “invisible” symptoms. Optimizing menopause care, including appropriate MHT, may reduce polypharmacy and improve well‐being

**Disclosure:** Celia Oreja‐Guevara has received speaker and consultation fees from Alexion, Amgen, Biogen Idec, BMS, Juvise, Merck, Novartis, Roche, Sanofi‐Genzyme, Sandoz, Viatris and Neuraxpharm. The rest of authors nothing to disclose.

## EPO‐0985

### Evaluation of anti‐NfL in multiple sclerosis

#### E. Dilsiz ^1^; V. Yılmaz^1^; E. Tüzün^1^; C. Ulusoy^1^; M. Kefeli^1^; S. Emekli^2^; T. Gündüz^2^; M. Kürtüncü^2^

##### ^1^Departmant of Neuroscience, Aziz Sancar Institute of Experimental Medicine, Istanbul University, Istanbul, Türkiye; ^2^Departmant of Neurology, Istanbul Faculty of Medicine, Istanbul University, Istanbul, Türkiye

**Background and aims:** Neurofilament light chain (NfL) is an important serological biomarker with prognostic relevance in multiple sclerosis (MS). This study aimed to investigate the presence of humoral immunity against NfL in patients with MS.

**Methods:** Patients fulfilling the 2017 MS diagnostic criteria with a minimum follow‐up of three years were included. Baseline cerebrospinal fluid (CSF) NfL levels, serum NfL levels at the last visit, anti‐NfL antibodies, and the levels of interleukin‐10, IL‐27, IL‐35, C‐X‐C motif chemokine ligand 13 (CXCL13) and transforming growth factor‐beta were measured. Associations between antibody and cytokine levels and disability accumulation were analyzed.

**Results:** The study included 47 patients with relapsing remitting MS, nine with primary progressive MS (PPMS), and 20 healthy controls. The median age of MS patients was 33 (IQR: 26–48), and the median disease duration was 5.4 years (IQR: 4.4–8.7). No association was found between CSF anti‐NfL antibody levels and the time to reach an Expanded Disability Status Scale (EDSS) score of 3 (*p* = 0.95) or the time to the second relapse (*p* = 0.6). Serum CXCL13 levels were lower in PPMS patients compared with healthy controls (*p* = 0.03). No correlation was observed between NfL levels and anti‐NfL antibodies in serum/CSF. Serum NfL was better at distinguishing MS subgroups than CSF NfL. CSF IL‐27 levels were lower in samples obtained during relapse compared with remission (*p* = 0.02).

**Conclusion:** Anti‐NfL antibody presence was not associated with prognostic outcomes in MS. Serum NfL levels were more sensitive than CSF NfL levels in differentiating MS subgroups.

**Disclosure:** The present work was supported by the Research Fund of Istanbul University and the “Gelecegimsin” Project Support Program of the Turkish Multiple Sclerosis Society. Project No: 41971.

## EPO‐0986

### Clinical and MRI correlates of overactive bladder symptoms in relapsing–remitting multiple sclerosis: A preliminary study

#### F. Sarıdaş; F. Hojjati; S. Albak; E. Koç

##### Department of Neurology, Faculty of Medicine, Bursa Uludağ University, Bursa, Türkiye

**Background and aims:** Overactive bladder (OAB) is a common but underrecognized condition in relapsing–remitting multiple sclerosis (RRMS). Its associations with clinical characteristics, disability, magnetic resonance imaging (MRI) findings, and symptom‐specific contributors remain unclear. This study evaluated factors associated with OAB symptoms and the contribution of individual OAB Questionnaire–V8 items.

**Methods:** Between February 2023 and February 2024, 197 patients assessed with the OAB‐V8 and with MRI performed within the preceding year were screened; after exclusions, 160 patients with RRMS were included. Patients were grouped by OAB‐V8 score (<8 vs. ≥8). Clinical and radiological characteristics were analyzed, and correlations between OAB‐V8 scores and clinical parameters were assessed.

**Results:** Patients with OAB‐V8 scores ≥8 were older and had higher Expanded Disability Status Scale (EDSS) scores than those with scores <8. OAB‐V8 scores correlated moderately with age (*ρ* = 0.327, *p* < 0.001) and weakly with EDSS (*ρ* = 0.177, *p* = 0.025). No significant associations were found with disease duration, annualized relapse rate, or MRI lesion localization. Sub‐question analysis showed that sudden urgency (Q3; *r* = 0.786), bothersome urgency (Q2; *r* = 0.773), and daytime frequency (Q1; *r* = 0.753) were the strongest contributors to the total OAB‐V8 score.

**Figure:**
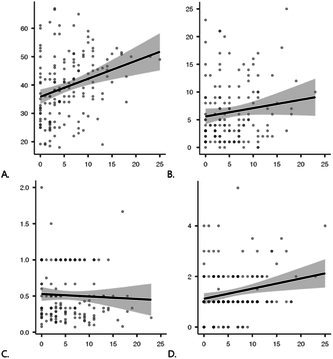
**FIGURE 1**: Correlation analyses between overactive bladder status and age, disease duration, annualized relapse rate, and EDSS.

**Figure:**
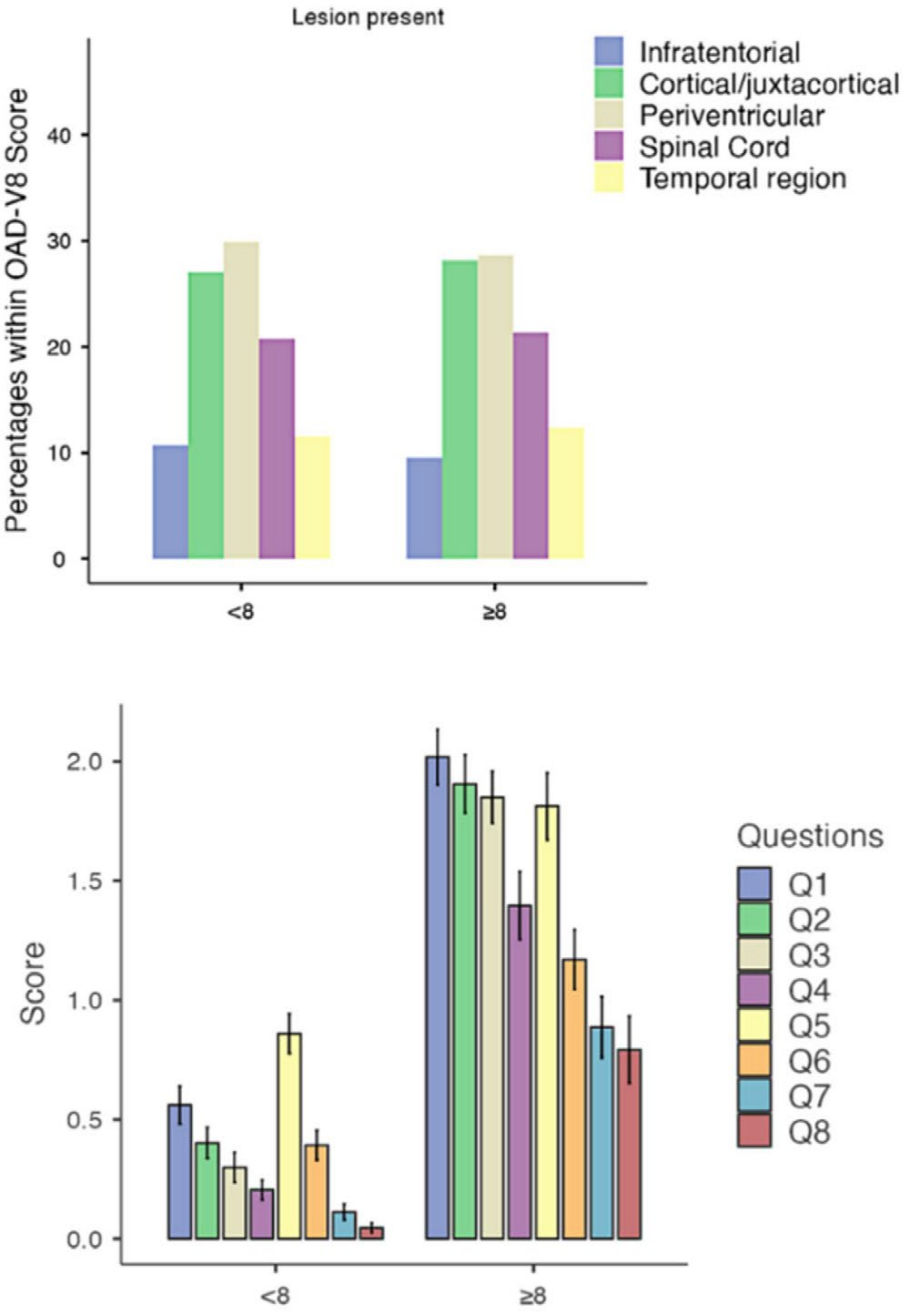
**FIGURE 2** Proportional distribution of lesion localization according to overactive bladder status and contribution of individual OAB‐V8 questionnaire items to the total score

**Figure:**
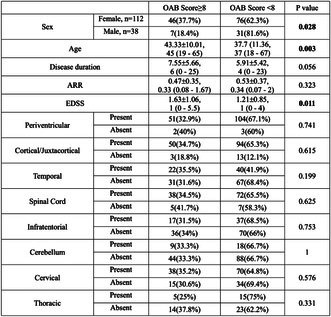
**TABLE 1** Comparison of demographic, clinical, and radiological characteristics according to OAB‐V8 score.

**Conclusion:** Although lesion localization—particularly spinal cord involvement—has been linked to urinary dysfunction in some studies, evidence remains inconsistent, with disability and overall disease burden emerging as stronger determinants of OAB symptoms. Urgency‐related symptoms primarily drive OAB severity, highlighting the importance of focused symptom assessment.

**Disclosure:** Nothing to disclose.

## EPO‐0987

### MS‐FLOWER, an ongoing U.S. multicenter pilot study to identify challenges to MSCopilot® 's integration into multiple sclerosis standard clinical care

#### L. Freeman^1^; G. Pardo^2^; J. Feng^3^; T. Brodu^4^; L. Carment ^4^; C. Gorin^4^; L. Klaeylé^4^; S. Zinaï^4^; J. Graves^5^; R. Naismith^6^

##### ^1^Department of Neurology, Dell Medical School, The University of Texas at Austin, Austin, USA; ^2^Arthritis and Clinical Immunology Research Program, Oklahoma Medical Research Foundation, Oklahoma City, USA; Multiple Sclerosis Center of Excellence, Oklahoma Medical Research Foundation, Oklahoma City, USA; ^3^The Desi Roth Harrison Center for Multiple Sclerosis, Ochsner Medical Center, New Orleans, USA; ^4^Ad Scientiam, Paris, France; ^5^Department of Neurosciences, University of California, San Diego, USA; ^6^Department of Neurology, Washington University School of Medicine, St. Louis, USA

**Background and aims:** MSCopilot®, a clinically validated software‐as‐medical‐device (SaMD), and its digital biomarkers measure Multiple Sclerosis (MS) impact on walking, vision, cognition, and dexterity enabling remote symptoms’ self‐assessment. While prior studies demonstrated that MSCopilot® scores correlate with the Extended Disability Scale Score (EDSS) and the 4‐item MS functional composite, integrating the tool into routine care remains challenging. MS‐FLOWER (NCT06922942) is a U.S. pilot study evaluating the feasibility, usability, and integration of MSCopilot® in routine care.

**Methods:** This 6‐month prospective study follows people with MS and their healthcare providers (HCPs). Patients remotely complete MSCopilot® monthly digital tests and questionnaires: Patient Health Questionnaire (PHQ‐8) and Modified Fatigue Impact Scale‐5 (MFIS‐5). HCPs review results through a web dashboard. Questionnaires evaluating training, onboarding process, ease of use of MSCopilot® at‐home and clinical workflows integration are administered to both patients and HCPs (Figure).

**Figure:**
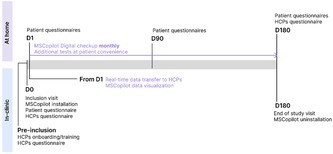
**FIGURE 1** MS‐FLOWER study design.

**Results:** Eight MS specialists recruited 62 patients (75.8% female, mean age 45.3 ± 11.2 years, median EDSS 2.0). The population included 92.0% relapsing‐remitting MS, 3.2% active secondary progressive MS (SPMS), 1.6% non‐active SPMS and 3.2% primary progressive MS. Following onboarding, all HCPs felt confident introducing MSCopilot® to patients with 75% doing so in 5 minutes or less. All patients surveyed after inclusion (*n* = 28) reported MSCopilot® was easy to use.

**Conclusion:** Clinical integration of MSCopilot® could enhance disease monitoring, optimize clinical workflows, and improve patient engagement. This study paves the way for broader adoption of digital biomarkers in routine care, supporting personalized, data‐driven disease management.

**Disclosure:** Freeman L.: Hoffmann La Roche, Genentech, EMD Serono, Sanofi, Horizon Therapeutics and TG Therapeutics‐consultant and/or advisory board member; Medscape, Inc and the MS Association of America━ speaking fees for participation in educational programs; MS Association of America, Genentech, and Merck━ speaking fees; NIH/NINDS, PCORI, Genentech, Sanofi and EMD Serono━ research support/grants. Pardo G.: Biogen Inc, EMD Serono, TG Therapeutics, Horizon Therapeutics, Novartis Pharmaceuticals, Sanofi Genzyme, BMS, Roche/Genentech, and Amgen━ speaking fees; Biogen Inc, Roche/Genentech, Sanofi Genzyme, Novartis Pharmaceuticals, EMD Serono, BMS, TG Therapeutics, ViewMind, Ad Scientiam, Contineum Therapeutics, and CorEvitas━ research support/grants. Feng J.: EMD Serono, Genentech, TG Therapeutics, Novartis, BMS, Horizon━advisory board member. Brochu T,Carment L, Gorin C, Klaeylé L, Zinaï S.: Ad Scientiam— employees; may hold shares or stock options. Graves J.: Sanofi, Genentech, Ad Scientium, Octave and EMD Serono━ research grants;Octave, TR1X and Google━ consulting fees. Naismith R.: Alexion Pharmaceuticals, Astoria, Biogen, Bristol Myers Squibb, Celltrion, Genentech, Genzyme, EMD Serono, Horizon Therapeutics, ImmPACT‐Bio, Kyverna, Novartis, Sandoz, TG Therapeutics━consultant and/or advisory board member and/or research grants.

## EPO‐0988

### Reduced hippocampus–amygdala transition area (HATA) volume in progressive multiple sclerosis: A cross‐sectional MRI study

#### F. Allahverdi ^1^; B. Celenk^1^; A. Unal^1^; B. Sunal^2^

##### ^1^Department of Neurology, Tekirdağ Namık Kemal University Hospital, Tekirdağ, Türkiye; ^2^Department of Radiology, Tekirdağ Namık Kemal University Hospital, Tekirdağ, Türkiye

**Background and aims:** MRI biomarkers of progression remain limited in multiple sclerosis. Progressive multiple sclerosis (PMS) is characterized by neurodegeneration and disability accumulation. Mesotemporal substructures, including the hippocampus–amygdala transition area (HATA), may undergo phenotype‐related atrophy. We examined whether bilateral HATA volume differs between PMS and relapsing–remitting MS (RRMS).

**Methods:** Cross‐sectional cohort of 106 MS patients (RRMS *n* = 68; PMS *n* = 38) underwent T1‐weighted MRI. HATA and amygdala nuclei volumes were derived with FreeSurfer. The primary endpoint was bilateral HATA volume (mm3). Phenotype effects were estimated using multivariable OLS with HC3‐robust standard errors, adjusting for age, sex, disease duration, and intracranial volume (ICV; per 100,000 mm3). Secondary ROI tests used Benjamini–Hochberg FDR.

**Results:** Bilateral HATA volume was lower in PMS than RRMS (mean [SD] 88.49 [17.85] vs 106.74 [18.49] mm^3^). In the adjusted model, PMS was associated with reduced bilateral HATA (beta = −10.11 mm3; 95% CI −17.62 to −2.60; *p* = 0.008). The phenotype effect was significant for left HATA (beta = −6.59; *p* = 0.003) but not right (beta = −3.52; *p* = 0.145). No secondary ROI survived FDR correction. An HATA‐by‐phenotype interaction suggested a stronger HATA–MSFC association in PMS (*p* = 0.035).

**Figure:**
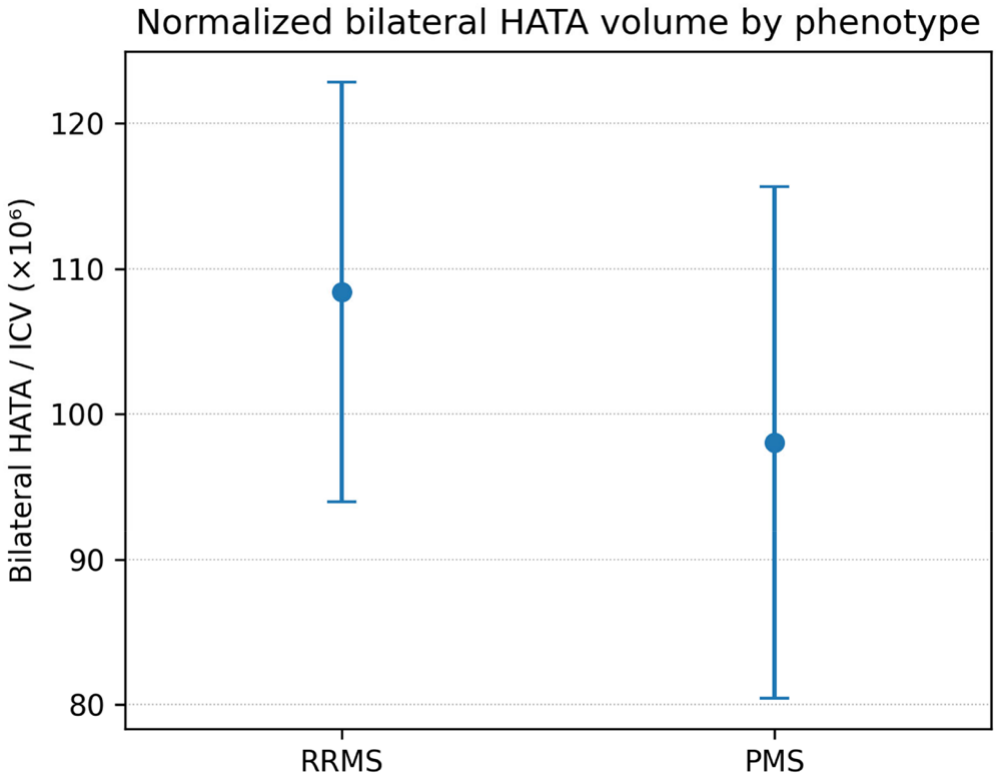
**FIGURE 1** Normalized bilateral HATA volume is lower in PMS than RRMS (normalization: (HATA/ICV) × 10^6^).

**Figure:**
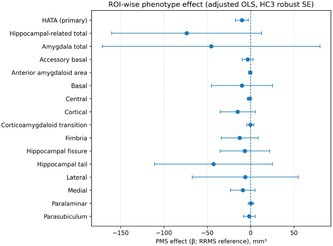
**FIGURE 2** ROI‐wise PMS effect (β, 95% CI) from HC3‐robust OLS models adjusted for age, sex, disease duration and ICV

**Figure:**
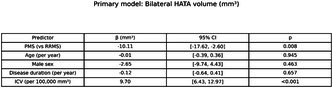
**TABLE 1** Primary model (bilateral HATA): PMS associated with lower HATA volume after adjustment (*β* = −10.11 mm^3^; 95% CI −17.62 to −2.60; *p* = 0.008)

**Conclusion:** Bilateral HATA volume is reduced in PMS after adjustment for intracranial volume and clinical covariates, consistent with HATA as a candidate mesotemporal marker of progressive phenotype. Longitudinal, lesion‐aware validation is warranted.

**Disclosure:** Nothing to disclose.

## EPO‐0989

### Lower lambda index in visual‐onset multiple sclerosis: A pivotal study

#### V. Ciampana ^1^; L. Paciolla^2^; A. Bianchi^3^; S. Asaro^1^; M. Limontini^1^; E. Virgilio^4^; R. Serino^5^; U. Dianzani^5^; A. Franceschini^1^; C. Comi^1^; D. Vecchio^1^

##### ^1^Neurology Unit, Maggiore della Carità University‐Hospital, University of Piemonte Orientale, Novara, Italy; ^2^Neurology Unit, Santa Croce Hospital, Moncalieri, Turin, Italy; ^3^Neurology Unit, Azienda Ospedale di Circolo e Fondazione Macchi di Varese, Varese, Italy; ^4^Neurology Unit, Department of Clinical and Biological Sciences, San Luigi Gonzaga University‐Hospital, University of Turin, Turin, Italy; ^5^Clinical Biochemistry, Department of Health Sciences, University of Piemonte Orientale, Novara, Italy; Interdisciplinary Research Center of Autoimmune Diseases (IRCAD), Department of Health Sciences, University of Piemonte Orientale, Novara, Italy

**Background and aims:** Visual onset (VO) is common in multiple sclerosis (MS) and is associated with a better prognosis. Cerebrospinal fluid (CSF) biomarkers of intrathecal immunoglobulin synthesis are increasingly investigated for early prognostic stratification; however, the prognostic role of lambda‐free‐light‐chain (L‐FLC, Lambda) index remains unclear. This pivotal study investigated whether VO is associated with a distinct CSF biomarker profile, focusing on Lambda index.

**Methods:** This monocentric retrospective observational study included 217 MS‐patients (2017 McDonald criteria) at diagnostic lumbar puncture, classified as VO (*n* = 50) or non‐VO (brainstem, cerebellar, sensory, motor, sphincteric, or spinal). Age at onset (AAO), disease duration (DD), and MRI features (lesion count, spinal cord involvement, and gadolinium‐enhancing lesions) were collected. CSF/serum IgG, kappa and LFLC were collected to calculate IgG, Kappa, and Lambda indexes. Group comparisons (Student's, Mann–Whitney U and Chi‐square test) and multivariable linear regression analyses were performed; sensitivity analyses used log‐transformed Lambda values.

**Results:** VO‐patients were younger than non‐VO (mean ± SD: 36.0 ± 11.3 vs 42.0 ± 12.2 years), with no differences in MRI burden. Lambda index was significantly lower in VO (median [IQR]: 4.9 [10.4]) than non‐VO (8.3 [17.0]; *p* = 0.019). This difference remained after adjustment for AAO and DD, which were not independently associated with Lambda index, and persisted in sensitivity analyses. No differences were observed for other biomarkers.

**Conclusion:** In this pivotal study, VO is associated with a lower Lambda index despite similar radiological burden, suggesting a distinct intrathecal humoral responses across onset phenotypes. The Lambda‐index may refine early prognostic stratification in MS and warrants validation in larger cohorts.

**Disclosure:** Nothing to disclose.

## MS and Related Disorders 8

## EPO‐0990

### Quantitative gait analysis for the detection and longitudinal monitoring of progressive multiple sclerosis: Relevance of the semiogram

#### C. Voisard; D. Ricard

##### Université Paris Saclay, Université Paris Cité, Ecole Normale Supérieure Paris Saclay, Centre National de la Recherche Scientifique, Service de Santé des Armées, INSERM, Centre Borelli, Gif‐sur‐Yvette, France

**Background and aims:** In progressive multiple sclerosis (pMS), gait deterioration is a key marker of disability. To detect progression, conventional clinical scales such as the EDSS remains limited in sensitivity and granularity. Inertial sensors are relevant tools for quantifying gait quality in clinical settings. It may enable the development of digital movement biomarkers for detection and follow‐up of progression, and to provide information for functional rehabilitation and therapeutic adaptation.

**Methods:** Twenty‐two patients with pMS (11 women, mean age 58 years, mean EDSS 5.5 [3.5–6]) and 19 control subjects (12 women, mean age 51 years) performed 10‐meter walking tests with u‐turn every 6 months over 1 to 2 years, equipped with 3 inertial sensors. Parameters computed from accelerometry and gyrometry time‐series were aggregated according to semiological criteria, compared to healthy population, and compiled into a visual radar named “semiogram” (see Figure). Correlations between these scores and established functional scales such as the EDSS were analyzed.

**Figure:**
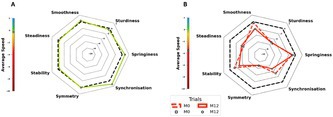
**FIGURE 1** Semiograms. Each criterion is compared with the healthy population (dark dashed lines). (A) healthy subject. (B) patient with progressive multiple sclerosis at M0 and M12 (EDSS 5.5 unchanged, however notable motor deterioration).

**Results:** The reproducibility of the parameters was excellent. All studied parameters, except stability, were able to discriminate patients from healthy subjects. A significant correlation was demonstrated between gait parameters and clinical scores, particularly the EDSS and motor subscores. It also support a better sensibility to progression compared with the EDSS.

**Conclusion:** Sensor‐based gait analysis through a multidimensional score is an objective and sensitive biomarker of motor disability in pMS detection and follow‐up. Sensors time‐series provide clinically relevant information for personalized monitoring and rehabilitation, which will be crucial in coming years to evaluate new treatments for progressive forms of multiple sclerosis.

**Disclosure:** Nothing to disclose.

## EPO‐0991

### Sleep disorders in multiple sclerosis

#### D. HadjKacem ^1^; S. Daoud^1^; F. Kchaou^1^; S. Msaad^2^; N. charfi^1^; K. Moalla^1^; N. Bouattour^1^; N. Farhat^1^; S. Sakka^1^; S. Kammoun^2^; M. Damak^1^

##### ^1^Department of Neurology, Habib Bourguiba University Hospital, Sfax, Tunisia; ^2^Department of Pulmonology, Hedi Chaker University Hospital, Sfax, Tunisia

**Background and aims:** Multiple sclerosis (MS) is an inflammatory demyelinating disease of the central nervous system, in which sleep disorders are frequent but often underrecognized, contributing to disability and reduced quality of life.

**Methods:** We conducted a cross‐sectional, descriptive, and analytical study including patients with MS at Habib Bourguiba University Hospital between January 2023 and September 2024. Sleep disorders were assessed using the Insomnia Severity Index (ISI), the Epworth Sleepiness Scale (ESS), the Pittsburgh Sleep Quality Index (PSQI), and the STOP‐BANG questionnaire. Restless legs syndrome and parasomnias were investigated through clinical interviews. Clinical, radiological, and therapeutic data were extracted from medical records.

**Results:** Eighty patients with MS were included; 63.8% had at least one sleep disorder. Poor sleep quality (PSQI > 5) was found in 57.5%, moderate to severe insomnia in 27.5%, parasomnias in 25%, excessive daytime sleepiness in 28%, and restless legs syndrome in 21.3%. Sleep disorders were significantly associated with unemployment (*p* = 0.037), longer diagnostic delay (*p* = 0.045), higher EDSS score (*p* = 0.048), and symptoms such as spasticity, neuropathic pain, and decreased libido (*p* = 0.026, *p* = 0.018, and *p* = 0.043, respectively). Radiologically, lesion localization in the corona radiata was associated with the presence of sleep disorders (*p* = 0.006). Therapeutically, baclofen (*p* = 0.004) and hypnotics (*p* = 0.045) were associated with these disorders.

**Conclusion:** Sleep disorders are commonly observed in patients with MS and are influenced by clinical, psychological, and radiological factors. Systematic screening and multidisciplinary management are essential to improve quality of life.

**Disclosure:** No

## EPO‐0992

### Clinical phenotype at onset predicts early functional outcomes in pediatric acquired demyelinating syndromes

#### E. Maida ^1^; M. Risi^1^; D. Iannaccone^2^; M. Gazzillo^1^; A. Varone^1^

##### ^1^Pediatric Neurology, Santobono‐Pausilipon Childrens Hospital, Naples, Italy; ^2^Department of Pediatric, Università degli Studi di Salerno, Salerno, Italy

**Background and aims:** We assessed whether onset phenotype predicts functional severity at presentation and outcome at discharge in pediatric acquired demyelinating syndromes (ADS).

**Methods:** We retrospectively analyzed pediatric ADS (*n* = 65), including myelin oligodendrocyte glycoprotein antibody‐associated disease, classified at onset as encephalitic (acute disseminated encephalomyelitis/cortical/cerebral deficits), optic neuritis, brainstem/cerebellar, or myelitis. Functional severity was measured with the Pediatric Cerebral Performance Category (PCPC). PCPC at onset was compared using Kruskal–Wallis with Dunn post‐hoc tests. Ordinal logistic regression assessed associations between phenotype and PCPC at onset and discharge, adjusting for age, sex, and preceding infection. Length of stay was analyzed using linear regression.

**Results:** PCPC at onset differed across phenotypes (*p* < 0.001); Dunn post‐hoc showed higher PCPC in encephalitic vs optic neuritis and myelitis vs optic neuritis (both *p* < 0.001). In adjusted ordinal models, encephalitic phenotype (OR 48.7, 95% CI 9.33–341; *p* < 0.001) and myelitis (OR 58.0, CI 9.49–474; *p* < 0.001) were associated with worse PCPC at onset; older age (OR 1.17, CI 1.02–1.37; *p* = 0.034) and preceding infection (OR 3.10, CI 1.03–9.89; *p* = 0.048) were also associated with higher PCPC. At discharge, encephalitic phenotype remained associated with worse PCPC (OR 9.01, CI 2.34–39.3; *p* = 0.002); myelitis showed a trend (OR 3.96, CI 0.92–18.4; *p* = 0.069); age remained associated (OR 1.17 per year, CI 1.02–1.34; *p* = 0.027). Encephalitic phenotype was associated with longer hospital stay (CI 1.67–19.7; *p* = 0.021).

**Figure:**
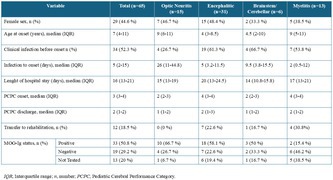
**TABLE 1** Demographic and clinical characteristics of the overall cohort and by onset phenotype in pediatric acquired demyelinating syndromes (ADS). Data are reported as n (%) or median (interquartile range).

**Figure:**
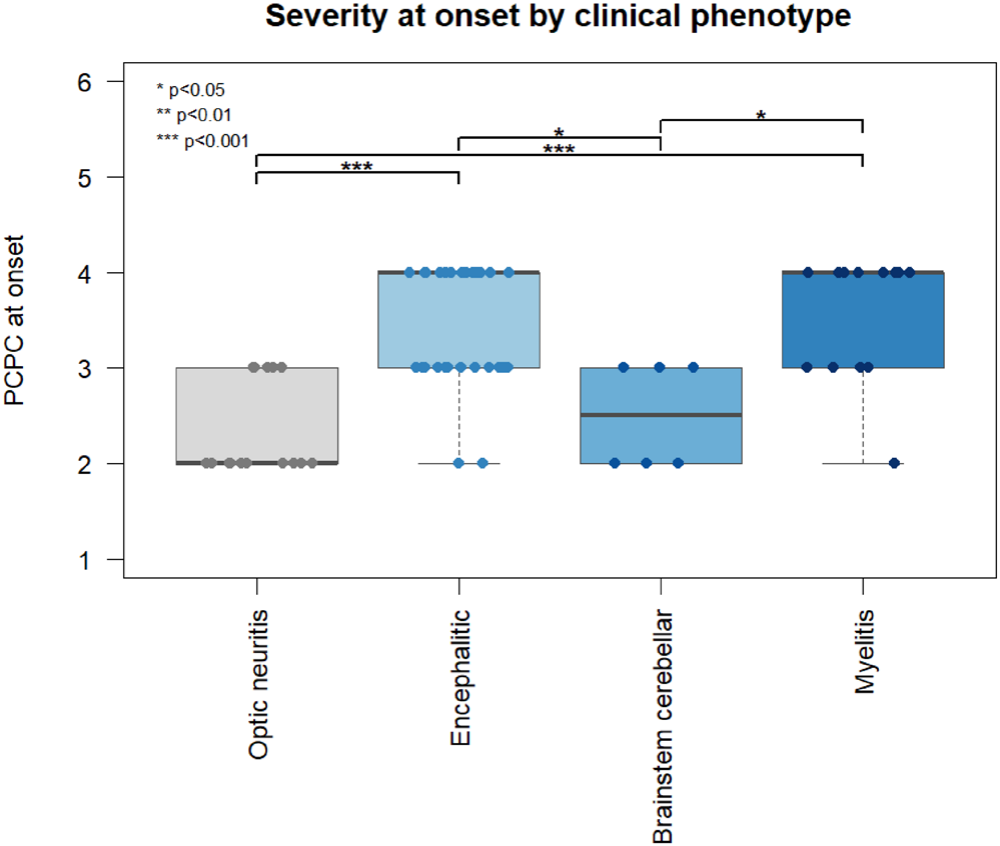
**FIGURE 1** PCPC severity at onset by clinical phenotype in pediatric acquired demyelinating syndromes (ADS). Boxplots show median and interquartile range. Pairwise differences are based on Dunn's post‐hoc test with Bonferroni correction.

**Conclusion:** In our cohort, onset phenotype strongly predicts functional severity and early outcome. Encephalitic presentations carry the highest acute burden, persistently worse discharge status, and longer hospitalization, supporting phenotype‐informed risk stratification at admission.

**Disclosure:** Nothing to disclose.

## EPO‐0993

### Optical coherence tomography (OCT)‐based retinal nerve fibre layer thickness associations with radiological data in lithuanian patients with MS

#### I. Vienažindytė ^1^; V. Danielius^1^; R. Balnytė^1^; A. Žygaitė^2^; B. Glebauskienė^3^

##### ^1^Department of Neurology, Medical Academy, Lithuanian University of Health Sciences, Kaunas, Lithuania; ^2^Medical Faculty, Medical Academy, Lithuanian University of Health Sciences, Kaunas, Lithuania; ^3^Department of Ophthalmology, Medical Academy, Lithuanian University of Health Sciences, Kaunas, Lithuania

**Background and aims:** Longitudinal monitoring of multiple sclerosis (MS) relies mainly on repeated magnetic resonance imaging (MRI), which can be limited by cost, availability, and the need for contrast agents. The 2024 McDonald criteria now include the optic nerve as a fifth location for dissemination in space, enabling assessment with optical coherence tomography (OCT), a non‐invasive and accessible alternative for tracking disease progression.

**Methods:** We conducted a retrospective medical documentation review of patients diagnosed with MS according to the 2010/2017 McDonald criteria, treated at the Department of Neurology of Kauno Klinikos, Lithuanian University of Health Sciences (LUHS) Hospital.

**Results:** A total of 84 MS patients were included, 85 % have relapsing‐remitting disease course. Spearman's bivariate correlation analyses and odds ratio estimations using ordinal logistic regression were performed (Table 1). A marginally significant association was observed between changes in pRNFL thickness over time and the appearance of new periventricular lesions. An ordinal logistic regression analysis was performed to determine whether a single measurement of pRNFL thickness correlated with MRI lesion load, adjusted for age and gender. pRNFL thickness was a significant prognostic factor for MRI lesion load, with each 1 μm reduction in pRNFL thickness associated with a 5 % higher probability of being classified into a higher lesion category (Table 2).

**Figure:**
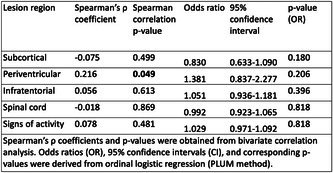
**TABLE 1** Correlation between MRI changes and peripapillary retinal nerve fiber layer (pRNFL) thickness changes over time in OCT measurements.

**Figure:**
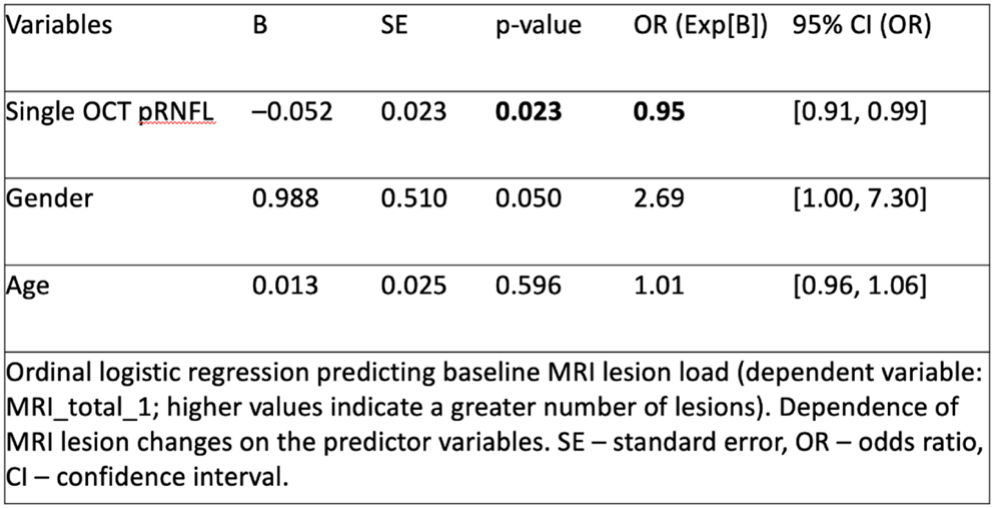
**TABLE 2** Correlation between MRI changes (number of lesions) and pRNFL variation in a single OCT measurement.

**Conclusion:** No significant associations were found between single‐timepoint pRNFL thickness and individual MRI lesion locations, while a weak borderline correlation with paraventricular lesion dynamics was considered a statistical artefact. Reduced pRNFL thickness was an independent predictor of higher overall MRI lesion burden.

**Disclosure:** Nothing to disclose.

## EPO‐0994

### sNfL and sGFAP as prognostic biomarkers in pediatric multiple sclerosis

#### M. Simone ^1^; F. Pennetta^2^; R. Palumbi^2^; M. Achille^2^; M. Ruggeri^2^; A. Frigeri^2^; L. Giuseppe^3^; D. Andrea^2^; M. Lucia^1^; G. Monte^4^; L. Papetti^4^; M. Ferilli^4^; F. Piemonte^5^; M. Valeriani^4^

##### ^1^Department of Precision and Regenerative Medicine, Jonic Area, Child Neuropsychiatric Unit, University of Bari, Bari, Italy; ^2^Department of Translational Biomedicine and Neuroscience, Child Neuropsychiatric Unit, University of Bari, Bari, Italy; ^3^Center for Outcomes Research and Clinical Epidemiology (CORESEARCH), Pescara, Italy; ^4^Developmental Neurology Unit, Ospedale Pediatrico Bambino Gesù, Rome, Italy; ^5^Unit of Muscular and Neurodegenerative Diseases, Ospedale Pediatrico Bambino Gesù, IRCCS, Rome, Italy

**Background and aims:** Pediatric multiple sclerosis (PedMS) is characterized by high inflammatory activity and neuroaxonal and glial damage. Serum biomarkers able to detect subclinical disease activity are relevant in this population. This study assessed the prognostic value of serum neurofilament light chain (sNfL) and glial fibrillary acidic protein (sGFAP) in predicting clinical, radiological, and cognitive outcomes in PedMS.

**Methods:** This study included PedMS followed at two Italian referral centers. All patients underwent cognitive assessment using the Symbol Digit Modalities Test (SDMT), physical disability evaluation using EDSS, as well as brain MRI scans and sNfL and sGFAP measurement by SIMOA and the Ella systems, at baseline and every 6 months for 24 months. Correlation analysis was performed using Spearman nonparametric test.

**Results:** Fifty‐three PedMS [71.7% females; median age at onset:14 (IQR 13.0–16.0) years; median EDSS: 2.0 (IQR 1.0–3.0); median SDMT scores: 49.0 (IQR 40.0–55.0)] were included. Baseline median levels of sNfL were 19.9 (IQR 7.5–37.0) pg/mL and those of sGFAP were 103.3 (IQR 86.6–131.3) pg/mL. Higher baseline sNfL levels were significantly (*p* < 0.05) associated with younger age at onset and, although not statistically significant (range *p* = 0.05–0.1), with increased physical and cognitive impairment and a treatment switch during follow‐up. Elevated baseline sGFAP levels were significantly (*p* < 0.05) associated with EDSS score at 24 months and higher number of clinical relapses (*p* < 0.05) during the follow‐up.

**Conclusion:** The integration of sNfL and sGFAP levels with clinical, radiological and cognitive assessments can improve early risk stratification and support personalised therapeutic strategies in PedMS.

**Disclosure:** Nothing to disclose.

## EPO‐0995

### Functional neurological symptoms in multiple sclerosis: A scoping review

#### Z. Soysal ^1^; S. Gluscevic^2^; S. Samadzadeh^3^; A. Dunalska^4^; K. Saramak^5^; A. Kulakowska^6^; M. Edwards^7^; S. Aybek^8^; N. Szejko^9^

##### ^1^Department of Neurology, Faculty of Medicine, Kocaeli University, Kocaeli, Türkiye; ^2^Department of Neurology, Clinical Centre of Montenegro, Podgorica, Montenegro; ^3^Experimental and Clinical Research Center, Charité‐Universitätsmedizin Berlin, Corporate Member of Freie Universität Berlin and Humboldt‐ Universität zu Berlin, Berlin, Germany; ^4^Department of Psychiatry, Faculty of Health Sciences, Medical University of Warsaw, Warsaw, Poland; ^5^Department of Neurology, Medical University of Innsbruck, Innsbruck, Austria; ^6^Department of Neurology, Medical University of Bialystok, Poland; ^7^Department of Basic & Clinical Neuroscience, Institute of Psychiatry, Psychology & Neuroscience, King's College London, London, UK. Department of Neuropsychiatry, South London and Maudsley NHS Foundation Trust, London, UK; ^8^Department of Neurosciences, Faculty of Science and Medicine, University of Fribourg, Fribourg, Switzerland; ^9^Department of Bioethics, Medical University of Warsaw, Warsaw, Poland. Department of Experimental and Clinical Pharmacology, Center for Preclinical Research and Technology CEPT, Medical University of Warsaw, Warsaw, Poland

**Background and aims:** Functional neurological symptoms (FNS), including functional motor and sensory disturbances or non‐epileptic seizures, may co‐occur with multiple sclerosis (MS) and complicate both diagnosis and management. This scoping review aimed to synthesize available literature on the prevalence, clinical features, diagnostic approaches, and management strategies for FNS and functional overlay in individuals with MS.

**Methods:** Following the PRISMA‐ScR guidelines, we searched PubMed, Embase, and PsycINFO from inception to July 2025. Studies were eligible if they reported FNS in people with MS. Two reviewers independently screened records, extracted data, and charted results. Data were summarized narratively across domains of prevalence, symptom types, diagnostic pathways, and management interventions.

**Results:** The search retrieved 1,042 records, of which 47 met inclusion criteria (15 observational studies, 20 case series, 12 single case reports). Reported prevalence of FNS varied widely, ranging from 3% to 21% across clinical samples. Functional motor symptoms (paresis, gait disturbance) and functional sensory loss were the most frequently described; psychogenic non‐epileptic seizures and functional tremor were less common but often led to diagnostic delay. Neuroimaging and neurophysiological testing were frequently used to distinguish functional from demyelinating symptoms, though misdiagnosis was reported in up to 15% of cases. Interventions most often involved multidisciplinary rehabilitation, psychological therapies, and patient education. Evidence for treatment effectiveness was limited, with most data from uncontrolled studies.

**Conclusion:** FNS are under‐recognized but clinically relevant in MS. They may contribute to increased disability, healthcare utilization, and patient distress if not appropriately identified. This review highlights the need for high‐quality prospective studies to evaluate targeted interventions.

**Disclosure:** Nothing to disclose.

## MS and Related Disorders 9

## EPO‐0996

### Reassessing PML risk in multiple sclerosis: The impact of anti‐JCV antibody Assay selection

#### J. Polovina; D. Landi; G. Mataluni; C. Nicoletti; F. Napoli; C. Dionisi; V. Di Gianvito; A. Miscioscia; M. Campobasso; G. Marfia

##### Department of Systems Medicine, MS Center, Tor Vergata University, Rome, Italy

**Background and aims:** Natalizumab (NTZ) treatment increases the risk of progressive multifocal leukoencephalopathy (PML) due to JC virus (JCV) reactivation in people with Multiple Sclerosis (pwMS). PML risk has been assessed for several years via the Stratify Test (ST), a reference anti‐JCV antibody assay. A new assay, Immunowell JCV Test (IT), has recently entered clinical use with the introduction of biosimilar NTZ. The aim of our study is to reassess PML risk using the IT in comparison with the established assay ST.

**Methods:** We enrolled 169 NTZ‐treated pwMS at Tor Vergata MS Center. Paired serum samples were collected and tested with both tests on the same day. Mean and median JCV index values were compared. Patients were categorized into four risk categories based on test‐specific cut‐offs, and frequency distributions were analyzed.

**Results:** Mean and median JCV index values were higher for IT (0.85 and 0.32) than for ST (0.63 and 0.00). Risk class distributions differed significantly between the two tests (McNemar test, *p* < 0.001), especially at risk extremes. A discordant risk class was observed in 113 patients (66.9%). Sign and Wilcoxon tests confirmed a significant statistical difference, with IT assigning higher risk categories than ST in 107 cases, compared with only 6 cases in which ST assigned a higher class (*p* < 0.001).

**Conclusion:** The new IT assay tends to reclassify patients into higher PML risk categories, which may influence clinical decisions. Further validation is needed to assess IT's specificity and correlation with JCV replication, to support accurate risk stratification and avoid unnecessary therapy changes.

**Disclosure:** Polovina J.C. received consultation fees for Sandoz. Landi D. received travel funding from Biogen, Merck Serono, Sanofi, Teva, Bristol Myers Squibb, Mylan, Neuraxapharm, speaking or consultation fees from Sanofi, Merck Serono, Teva, Biogen, Roche, Novartis, Bristol Myers Squibb, Janssen, Alexion, Amgen Nicoletti C.G. received travel funding from Biogen, Merck Serono, Sanofi‐Genzyme, Roche, Teva, Novartis, Bristol Mayer Squibb, Janssen, Almirall, Horizon, speaking and consultation fees from Sanofi, Almirall, Merck‐Serono, Roche, Novartis, Biogen. She is a sub‐investigator in clinical trials for Biogen, Merck Serono, Roche, Sanofi, Novartis, Teva, Bristol Mayer Squibb. Dionisi C. received travel fundings from Biogen, Merck Serono, Bristol Mayer Squibb, Almirall, Viatris. Mataluni G. is an Advisory Board member of Argenx. Received honoraria for speaking, consultation fees or travel fundings from Almirall, Biogen Idec, Merck, Novartis, Sanofi, Roche, CSL Behring, Horizon, Kedrion, She is a sub‐investigator in clinical trials for Biogen, Merck Serono, Novartis. Di Gianvito V: received travel fundings from Novartis, Biogen, Roche. G.A. Marfia is an Advisory Board member of Almirall, Amgen, Alexion, Neuraxpharm, Sandoz, Sanofi Genzyme, Merck‐Serono, Novartis, Roche and received honoraria for speaking or consultation fees from Almirall, Bayer Schering, Biogen, Merck Serono, Neuraxpharm, Novartis, Sanofi‐Genzyme, Roche, Amgen, Alexion and BMS. She is the principal investigator in clinical trials for Biogen Idec, Merck Serono, Novartis, Roche, Sanofi‐Genzyme, Merck Serono and BMS. Napoli F, Miscioscia A, Campobasso M: nothing to disclose.

## EPO‐0997

### Changing paradigms in the placement of autologous stem cell transplantation for multiple sclerosis: A single‐centre experience

#### I. Gattuso ^1^; G. Orofino^2^; A. Genchi^1^; M. Auriemma^2^; V. Marino^1^; M. Rocca^3^; L. Lazzari^2^; A. Bruno^2^; F. Ciceri^2^; R. Greco^2^; M. Filippi^4^; L. Moiola^1^

##### ^1^Neurology Unit, Multiple Sclerosis Center, IRCCS San Raffaele Scientific Institute, Milan, Italy; ^2^Hematology and BMT unit, IRCCS San Raffaele Hospital, Milan, Italy; ^3^Neurology Unit, Multiple Sclerosis Center; Neuroimaging Research Unit, Division of Neuroscience, IRCCS San Raffaele Scientific Institute; Vita‐Salute San Raffaele University, Milan, Italy,; ^4^Neurology Unit, Multiple Sclerosis Center; Neurorehabilitation Unit, Neurophysiology; Neuroimaging Research Unit, Division of Neuroscience, IRCCS San Raffaele Scientific Institute; Vita‐Salute San Raffaele University, Milan, Italy

**Background and aims:** Autologous haematopoietic stem cell transplantation (aHSCT) has historically been considered a rescue therapy for multiple sclerosis (MS) due to safety concerns. Improved transplant strategies and better patient selection are expanding its use, particularly in treatment‐naïve patients.

**Methods:** In this retrospective single‐centre observational study, all patients with MS treated with aHSCT per clinical practice (Muraro et al, Nature Reviews Neurology 2025) since 2022, were included.

**Results:** Fourteen patients (7F), all with ‘aggressive MS’, were included (Table 1): 13 with relapsing‐remitting and one with active‐progressive MS. Median age was 26.8 years (range 20.2–33.8), median EDSS 3.3 (range 1.0–6.0), and median disease duration 1.5 years (range 0.2–12.5). Patients were treatment‐naïve (6/14) or early switchers (7/14). Only one patient, with paediatric onset, displayed longer disease duration and several previous treatments. Follow‐up was more than 24 months in one patient, 12 months in six patients, six months in two, three months in two, while three were still completing the procedure (2 post‐apheresis, 1 post‐mobilization). Mobilization and conditioning regimens are listed in Table2. No clinical relapses or MRI activity were observed after transplant. EDSS improved in 9/11 evaluable patients, one patient remained stable, whereas the progressive one worsened without inflammatory activity. Febrile neutropenia occurred in 9/11 patients and mild anti‐thymocyte globulin reactions in 4/11. No late toxicities were reported.

**Figure:**
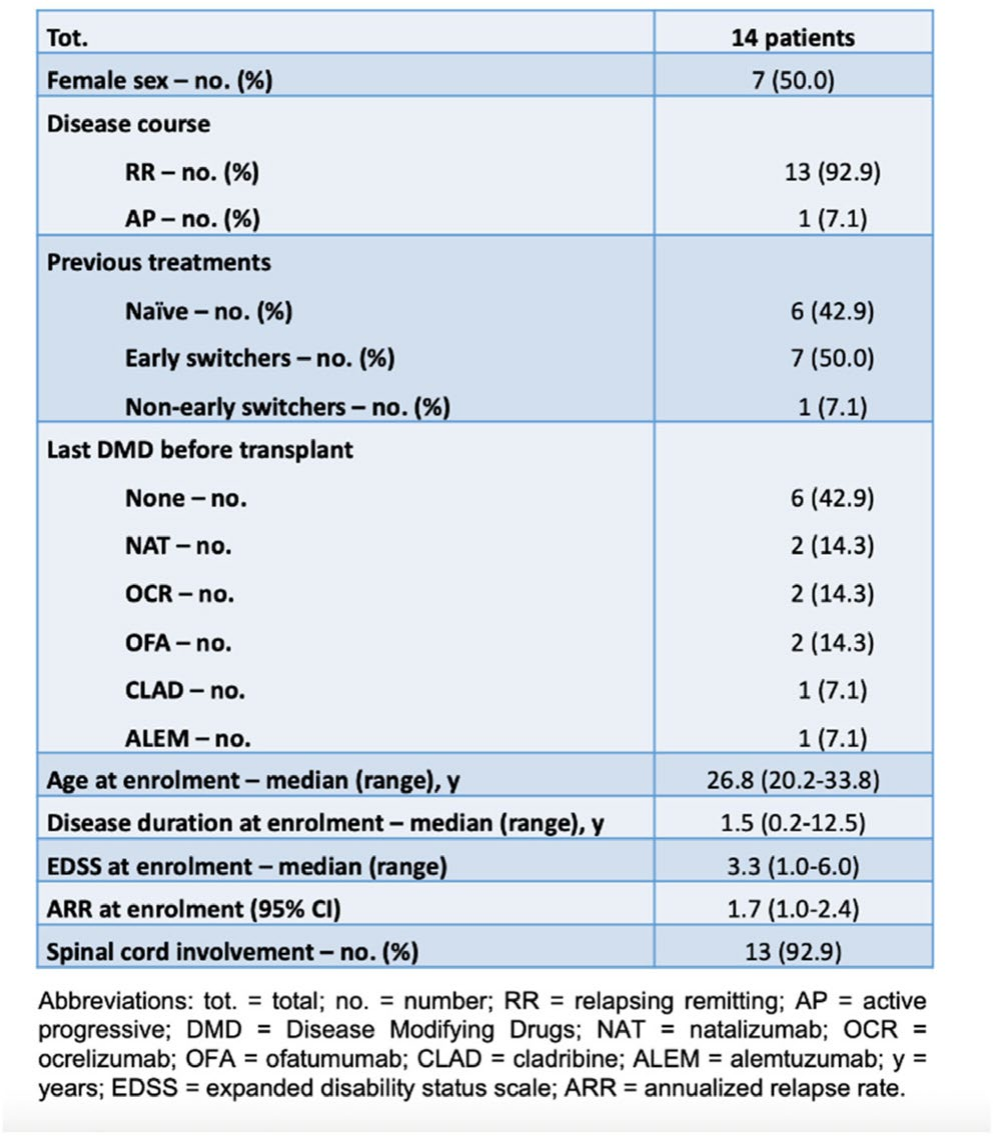
**TABLE 1** Baseline characteristics of patients.

**Figure:**
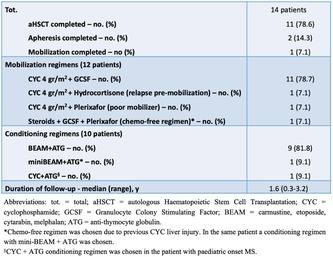
**TABLE 2** Mobilization and conditioning regimens.

**Conclusion:** Our findings support a paradigm shift toward earlier use of aHSCT in selected MS patients. Young age, short disease duration and low disability characterized our cohort. Larger studies with longer follow‐up are needed to confirm efficacy and long‐term safety.

**Disclosure:** IG received honoraria from Roche, Merck, Novartis, Biogen. GO has nothing to disclose. AG received compensation for speaking activities from Novartis. MCA and VM have nothing to disclose. MAR received honoraria from Biogen, Bristol Myers Squibb, Eli Lilly, Janssen, Roche, AstraZaneca, Bromatech, Celgene, Genzyme, Horizon Therapeutics Italy, Merck Serono, Novartis, Sanofi and Teva; she receives research support from the MS Society of Canada, the Italian Ministry of Health, the Italian Ministry of University and Research, and Fondazione Italiana Sclerosi Multipla; she is Associate Editor for Multiple Sclerosis and Related Disorders. LL and AB have nothing to disclose. FC has nothing to disclose. RG has nothing to disclose. MF is Editor‐in‐Chief of the Journal of Neurology, Associate Editor of Human Brain Mapping, Neurological Sciences, and Radiology; received honoraria from Alexion, Almirall, Biogen, Novartis, Roche, Sanofi Bayer, Celgene, Chiesi Italia SpA, Eli Lilly, Genzyme, Janssen, Merck‐Serono, Neopharmed Gentili, Novo Nordisk, Sanofi, Takeda, and TEVA, Bristol‐Myers Squibb; he receives research support from Biogen Idec, Merck‐Serono, Novartis, Roche, the Italian Ministry of Health, the Italian Ministry of University and Research, and Fondazione Italiana Sclerosi Multipla. LM received compensations for speaking activities and/or for participating to advisory board from Merck, Celgene, Biogen, Sanofi, Novartis, Roche, Alexion, and Amgen.

## EPO‐0998

### Clinical course, safety, and pregnancy outcomes in multiple sclerosis patients treated with cladribine: Single center real‐world experience

#### M. Koseoglu; M. Demir; U. Ozoglu; G. Yaman; K. Oguz

##### Neurology Clinic, University of Health Sciences, Kanuni Sultan Suleyman Training and Research Hospital, Istanbul, Türkiye

**Background and aims:** To evaluate clinical, hematological, and pregnancy outcomes of relapsing‐remitting multiple sclerosis (RRMS) patients treated with cladribine in a real‐world setting.

**Methods:** We retrospectively analyzed 145 patients (screened from 190 admissions) with active follow‐up and regular data. Demographics, EDSS scores, relapse rates, laboratory results (Years 1–2), MRI findings, adverse events, and pregnancy outcomes were evaluated.

**Results:** The mean age was 42.4 years (range 23–67), and 67% were female (*n* = 97). Mean disease duration was 13.6 years. Relapses occurred in 26 patients (18%) during treatment, observing a higher frequency in females. Clinical and radiological stabilization was achieved in the majority. Regarding safety, Grade 3 lymphopenia (<500/mm^3^) occurred in approximately 6–7% of patients, while Grade 4 (<200/mm^3^) severe lymphopenia was rare (1%). Common adverse events included fatigue, herpes zoster, and alopecia. A total of 14 pregnancies were recorded (1 paternal). Outcomes included healthy live births and some pregnancy losses (ectopic/chemical/stillbirth); no developmental issues were reported in infants.

**Conclusion:** Cladribine demonstrated high efficacy and a manageable safety profile in this expanded real‐world cohort (*n* = 145). The incidence of severe lymphopenia was consistent with current literature. While healthy live births were achieved, pregnancy planning and monitoring remain crucial due to potential risks.

**Disclosure:** Nothing to disclose.

## EPO‐0999

### Exploratory CSF metabolomic profiling across oligoclonal band types 1–5 using ^1^H‐NMR spectroscopy

#### P. Sengul ^1^; A. Baykal^2^; M. Serteser^3^

##### ^1^Department of Neuroscience, Graduate School of Health Sciences, Acibadem Mehmet Ali Aydinlar University, Istanbul, Türkiye; ^2^Department of Medical Biochemistry, School of Medicine, Acibadem Mehmet Ali Aydinlar University, Istanbul, Türkiye; ^3^Acibadem Labmed Clinical Laboratories, Istanbul, Türkiye

**Background and aims:** Oligoclonal band (OCB) patterns in cerebrospinal fluid (CSF) represent distinct intrathecal immunological states and are routinely classified into five types (OCB Types 1–5). While OCB classification provides important diagnostic and immunological information, the associated biochemical heterogeneity within the CSF has not been systematically explored. To investigate whether CSF metabolomic profiles differ across OCB Types 1–5 using proton nuclear magnetic resonance (^1^H‐NMR) spectroscopy, focusing on spectral‐level variability rather than definitive metabolite identification.

**Methods:** To investigate whether CSF metabolomic profiles differ across OCB Types 1–5 using proton nuclear magnetic resonance (^1^H‐NMR) spectroscopy, focusing on spectral‐level variability rather than definitive metabolite identification.

**Results:** Distinct differences in CSF spectral profiles were observed across OCB‐defined groups, with partial separation between specific OCB types. Several ppm regions demonstrated statistically significant or trend‐level differences across OCB classifications. Exploratory pairwise evaluations suggested that comparisons involving OCB Types 4 and 5 tended to display more pronounced spectral variability, with selected ppm regions contributing disproportionately to group‐wise patterns.

**Figure:**
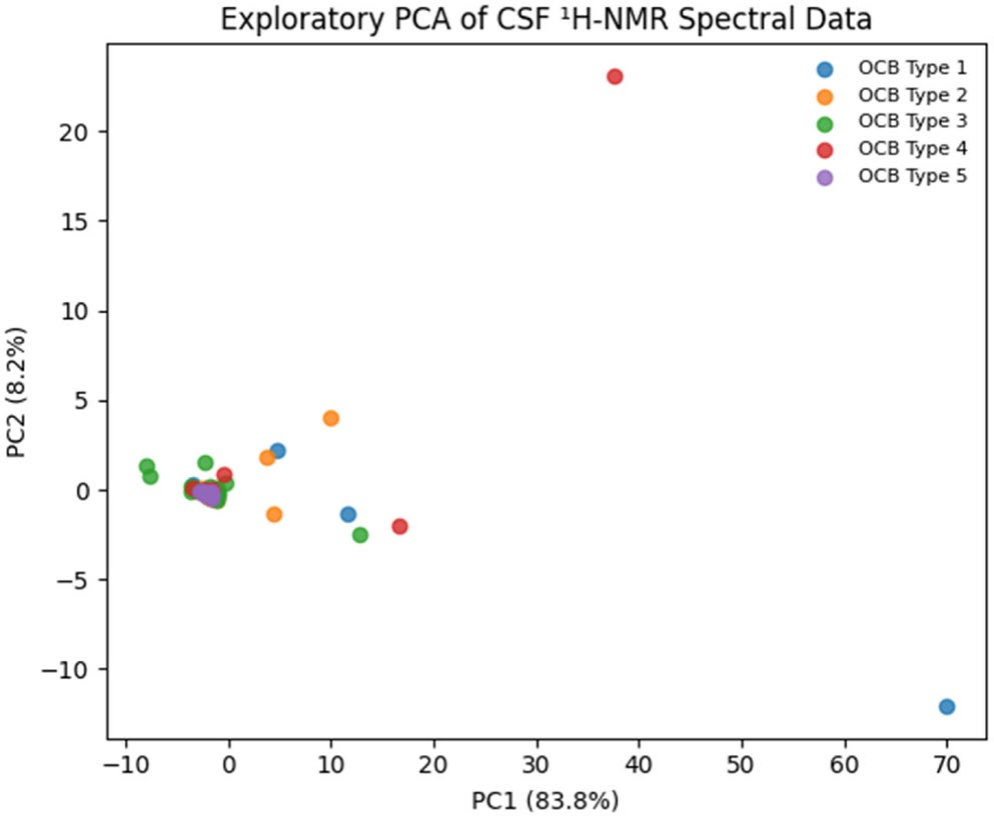
**FIGURE 1** Exploratory PCA score plot based on CSF ^1^H‐NMR spectral data, with samples colored according to OCB Types 1–5. PCA was performed at the ppm level without targeted metabolite annotation.

**Figure:**
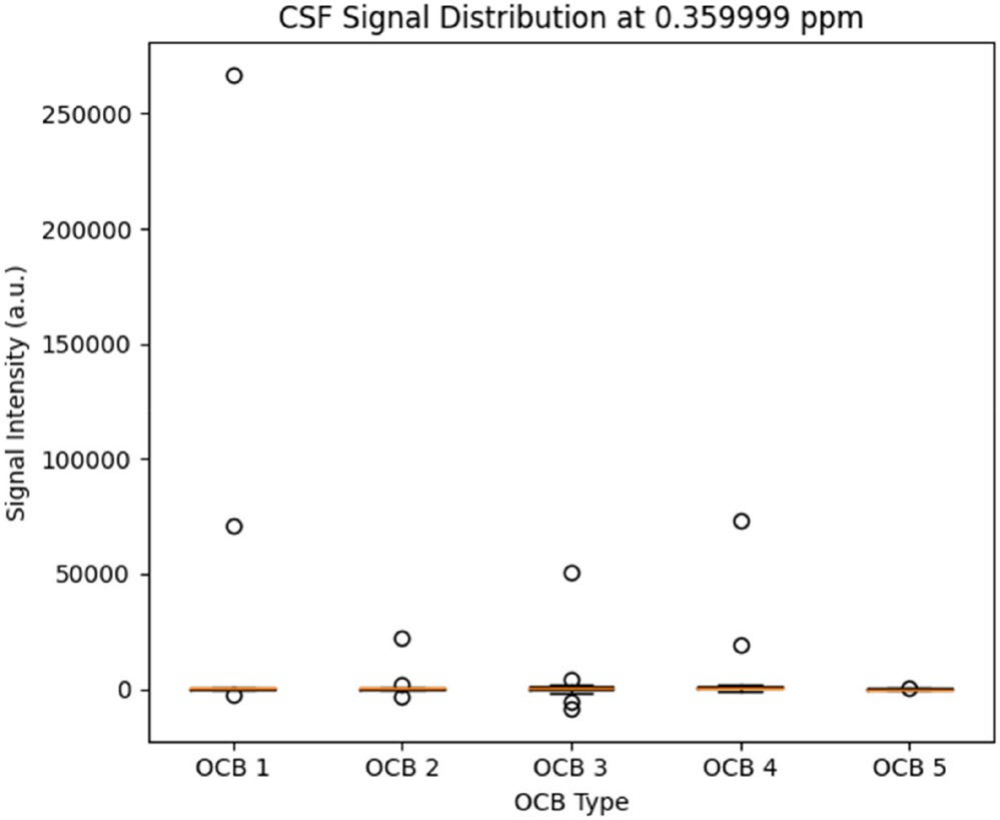
**FIGURE 2** Distribution of CSF signal intensity at a representative ppm region across OCB Types 1–5.

**Conclusion:** This exploratory analysis indicates that CSF metabolomic profiles vary across OCB Types 1–5, reflecting biochemical heterogeneity associated with distinct intrathecal immunological states. Although evaluated at the spectral level without definitive metabolite identification, these findings support the potential of CSF‐based ^1^H‐NMR metabolomics as a complementary approach to conventional OCB assessment. Further studies integrating targeted metabolite annotation and longitudinal clinical data are warranted.

**Disclosure:** Nothing to disclose.

## EPO‐1000

### From pregnancy to disability in multiple sclerosis: Can machine learning predict the course?

#### S. Alizada ^1^; M. Emec^2^; E. Kaya^1^; Y. Simsek^3^; M. Ozcanhan^4^; S. Ozakbas^3^

##### ^1^Department of Neurology, Dokuz Eylul University, *Izmir, Türkiye*; ^2^Department of Computer Science, Istanbul University, Istanbul*, Türkiye;* ^3^Neurology Clinic, Izmir University of Economics, *Izmir, Türkiye*; ^4^Faculty of Engineering, Dokuz Eylul University, *Türkiye*

**Background and aims:** Pregnancy reduces relapse activity in relapsing–remitting multiple sclerosis (RRMS), yet postpartum disability evolution is heterogeneous. We aimed to predict postpartum disability trajectories using pregnancy‐related clinical and obstetric variables with machine‐learning models.

**Methods:** We retrospectively analyzed 662 women with RRMS contributing 909 pregnancies. Predictors included pre‐/postpregnancy EDSS, disease duration, maternal age, postpartum relapse, pregnancy number, delivery type, and breastfeeding. After multiple imputation and standard preprocessing, regression (ΔEDSS) and 3‐class classification (improved/stable/worsened) models were trained with an 80/20 split and 5‐fold cross‐validation (Random Forest, XGBoost, elastic net, and support vector methods).

**Results:** Classification generalized better than regression (test accuracy 85–88%, F1 0.84–0.87 vs test *R* ^2^ 0.31–0.39; MAE 0.41–0.48 EDSS points). Postpartum relapse was the strongest predictor, followed by disease duration, age at pregnancy, and pre‐pregnancy EDSS; obstetric variables showed smaller but relevant contributions. Performance was highest in women with multiple pregnancies (classification accuracy > 92%).

**Figure:**
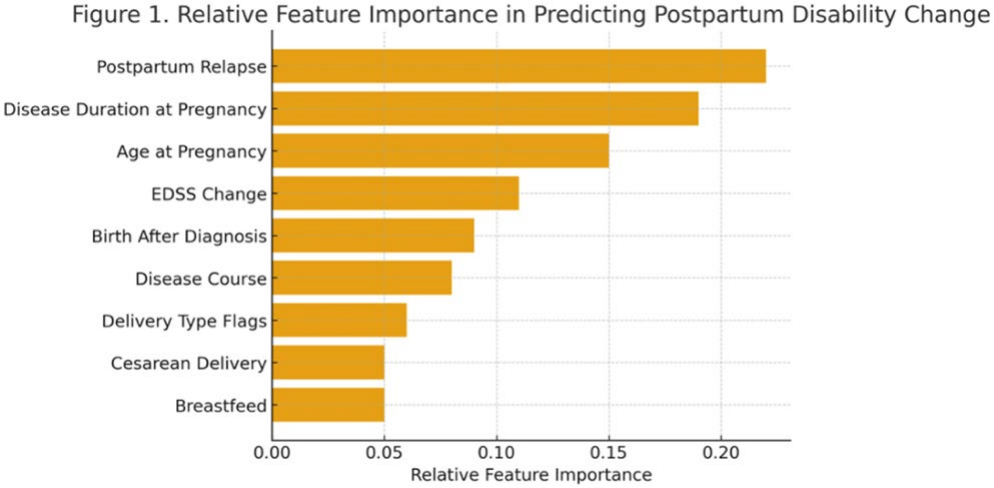
**FIGURE 1** Relative feature importance in predicting postpartum disability change top predictors of postpartum disability change (ΔEDSS) in women with multiple sclerosis. Feature importance values were averaged across Random Forest and XGBoost models.

**Figure:**
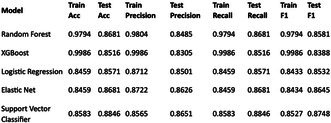
**TABLE 1** Performance of classification models for ΔEDSS categories.

**Figure:**
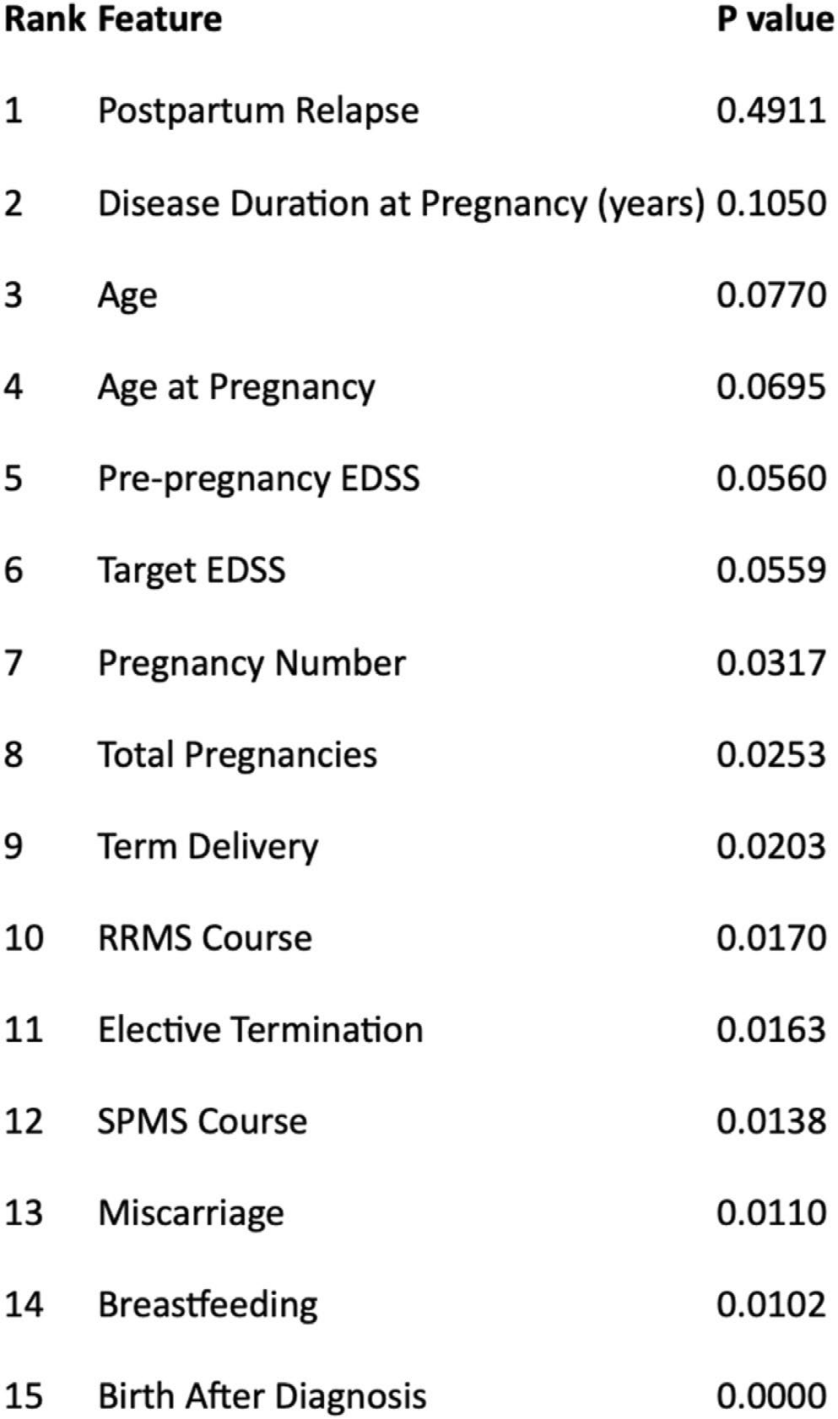
**TABLE 2** Top 15 predictors of ΔEDSS across models.

**Conclusion:** Machine learning can stratify postpartum disability risk in RRMS using routinely collected reproductive and clinical data, with categorical risk prediction outperforming continuous ΔEDSS prediction. Postpartum relapse remains the dominant driver of disability change, highlighting the postpartum period as a key therapeutic window; external validation is warranted.

**Disclosure:** Said Alizada: Nothing to disclose, Murat Emec: Nothing to disclose, Ergi Kaya: Nothing to disclose, Yasemin Simsek: Nothing to disclose, Mehmet Hilal Ozcanhan: Nothing to disclose, Serkan Ozakbas: Nothing to disclose.

## EPO‐1001

### Functional connectivity of sensorimotor network as function of EDSS: Preliminary resting state fMRI study in patients with secondary progressive MS

#### S. Gazdzinski ^1^; N. Hryniewicz^2^; E. Piątkowska‐Janko^2^; K. Lipiński^3^; B. Błażejewska‐Hyżorek^4^; A. Karlińska^1^; P. Bogorodzki^3^; P. Grieb^5^; M. Pawlak^6^; R. Rola^1^; I. Kurkowska‐Jastrzębska^4^

##### ^1^Military Institute of Aviation Medicine, Warsaw, Poland; ^2^Nałęcz Institute of Biocybernetics and Biomedical Engineering, Polish Academy of Sciences, Warsaw, Poland; ^3^Warsaw University of Technology, Warsaw, Poland; ^4^Institute of Psychiatry and Neurology, Warsaw, Poland; ^5^Mossakowski Medical Research Centre, Polish Academy of Sciences, Warsaw, Poland; ^6^Poznan University of Medical Sciences, Poznań, Poland

**Background and aims:** Demyelinating lesions in multiple sclerosis (MS) cause injury to neuronal paths and therefore lead to poorer communication between distant brain regions. This communication may be measured with functional magnetic resonance at rest (r‐fMRI) and visualized as synchronization of neuronal activity between these regions. Sensorimotor network links regions responsible for processing sensory input and executing motor output; these regions are interconnected and their activity is synchronized even at rest. This preliminary analysis evaluated if higher EDSS scores are associated with sensorimotor network connectivity with other brain regions.

**Methods:** Seventeen patients with a diagnosis of secondary progressive MS (EDSS: 6.47 ± 1.42 (range: 3.5–8.5), age: 54.1 ± 1.4, 7 males) without enhancing lesions on MRI were included. High resolution T1‐w images and functional magnetic resonance data at rest were obtained at 3T and processed with CONN software.

**Results:** Higher scores on EDSS were related with poorer connectivity of sensorimotor regions with multiple regions in occipital cortex and cuneal cortex that is associated with visual processing, visual attention, spatial processing and object recognition (blue color on FIGURE 1). However, higher EDSS scores demonstrated increased connectivity in the right crus of the cerebellum (contains motor tracts traveling from the cerebral cortex; red color on FIGURE 1).

**Figure:**
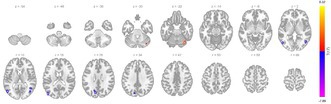
**FIGURE 1** Regions in blue depict decreased functional connectivity with sensorimotor network in patients with higher EDSS scores, whereas the red regions in cerebral crus demonstrate better connectivity in patients with higher EDSS scores.

**Conclusion:** Higher EDSS scores are associated with poorer connectivity with regions involved in visual processing. However, the increased connectivity with crus of the cerebellum suggests some compensatory mechanisms.

**Disclosure:** Nothing to disclose. This study was supported by Medical Research Agency of Poland, grant 2021/ABM/02/00002 – 00.

## MS and Related Disorders 10

## EPO‐1002

### Brain volumetric MRI abnormalities associated with dysphagia in multiple sclerosis

#### D. Ranucci ^1^; A. Esposito^1^; L. Corsaro^1^; L. Mollo^1^; C. Siniscalchi^1^; F. Falco^1^; G. Corsini^1^; M. Aiello^2^; R. Iandolo^2^; V. Nicolella^1^; A. Castiello^1^; S. Cocozza^3^; F. Briganti^3^; M. Moccia^4^; R. Iodice^1^; V. Brescia Morra^1^; R. Lanzillo^1^; M. Petracca^5^; A. Carotenuto^1^

##### ^1^Department of Neurosciences, Reproductive Sciences and Odontostomatology, University of Naples Federico II, Naples, Italy; ^2^IRCCS SYNLAB SDN, Naples, Italy; ^3^Department of Advanced Biomedical Sciences, University of Naples "Federico II", Naples, Italy; ^4^Department of Molecular Medicine and Medical Biotechnology, Federico II University of Naples, Naples, Italy; ^5^Department of Human Neurosciences, Sapienza University of Rome, Rome, Italy

**Background and aims:** Multiple sclerosis (MS) patients may present dysphagia. The neuroanatomical alterations underpinning dysphagia in MS are debated.

**Methods:** In this cross‐sectional study enrolling both MS patients and healthy controls (HCs), subjects underwent clinical assessment through Expanded Disability Status Scale (EDSS), Brief International Cognitive Assessment for MS (BICAMS), Swallowing Disturbance Questionnaire (SDQ), Beck Anxiety Inventory (BAI). Subjects performed brain MRI (T1/T2 sequences) at three sites. We assessed T2 lesions volume [lesion segmentation tool, SPM12, brain and gray matter volume (SIENAX), brainstem volume (Freesurfer), cerebellar volume (SUIT toolbox)]. We performed a multivariable linear regression using gray matter, T2 lesions, brainstem and cerebellar volume, BAI scores as factor of interest, SDQ as dependent variable and age, gender and sites as covariates.

**Results:** We enrolled 41 patients [30 females; age: 38.4 ± 10.4 years, 37 relapsing‐remitting; disease duration: 8.5 (0–26) years; EDSS: 2.5 (1–5.5)] and 20 HCs (8 females; mean age 38.7 ± 13.4 years). Compared with HCs, MS patients had higher T2 lesion volumes (coeff.6.97; *p* = 0.02), lower normalized brain volume (coeff. −87.56; *p* < 0.01) and reduced volumes of gray matter (coeff. −43.8; *p* = 0.001), midbrain (coeff. −0.49; *p* = 0.003), pons (coeff. −1.47; *p* = 0.002) and medulla (coeff. −0.60; *p* = 0.003) volume. Higher SDQ scores correlated with reduced volumes of pons (coeff. −0.93; *p* = 0.001) and anterior cerebellar lobules (coeff.−1.12; *p* = 0.006).

**Conclusion:** Dysphagia in MS may be caused by neurodegenerative processes affecting the infratentorial regions of the brain, leading to dysfunction of the cranial nerves that emerge from the pons and reduced motor coordination of the swallowing system related to anterior cerebellar damage.

**Disclosure:** Nothing to disclose.

## EPO‐1003

### Knowledge gaps in reproductive health among women with MS (KNOWwMS): An international survey

#### E. Baldin ^1^; C. De Santis^1^; R. Bove^2^; D. Jacobs^3^; J. McDonell^4^; N. Rijke^5^; D. Saylor^6^; S. Viswanathan^7^; F. Nonino^1^; KNOWwMS Working Group^8^

##### ^1^Epidemiology and Statistics Unit, IRCCS, Istituto delle Scienze Neurologiche di Bologna, Italy; ^2^UCSF Weill Institute for Neurosciences, University of California San Francisco, USA; ^3^Department of Neurology, University of Pennsylvania, Perelman School of Medicine, USA; ^4^MS Canada, Toronto, ON, Canada; ^5^Multiple Sclerosis International Federation, London, UK; ^6^Chapell Hill School of Medicine, University of North Carolina, USA; University Teaching Hospital, Lusaka, Zambia; ^7^Tunku Abdul Rahman Neuroscience institute, Kuala Lumpur Hospital, Department of Neurology, Malaysia; ^8^The KNOWwMS Working Group

**Background and aims:** Multiple sclerosis (MS) has typical onset in women during their reproductive years. Since reliable and consistent information about reproductive health is of pivotal importance for women with MS (WwMS), the KNOWwMS project was established to develop a core information set. As a first step, a global survey was conducted to assess access to information, knowledge gaps and misinformation concerning reproductive health in WwMS, focusing on limited resources settings.

**Methods:** The survey included 18 closed questions informed by a scoping review and refined by a multi‐stakeholder Scientific Advisory Group, and one open‐ended question. Target respondents of the online survey were WwMS aged ≥18 years. The survey was available from June to December 2025, in 9 languages.

**Results:** The questionnaire was completed by 480 WwMS (mean age 37.1 years, SD 9, 92% <50 years) from 31 countries (Figure 1: 23.5% from low‐ and middle‐ income countries), mostly from urban settings (91.9%). Gaps identified included: reproductive health issues never even discussed by their neurologist (42.6%), and absence of counselling about pregnancy while taking medications for MS (44.1%). Most women believed, or were not sure, that MS would affect their ability to became pregnant and have children (57.9%); responses to MS being passed on to children showed limited knowledge of familial risk (69% no, or unsure); 34.5% decided against having children due to MS‐related concerns (Tables 1 and 2).

**Figure:**
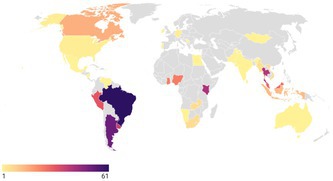
**FIGURE 1** Geographic distribution of survey responses.

**Figure:**
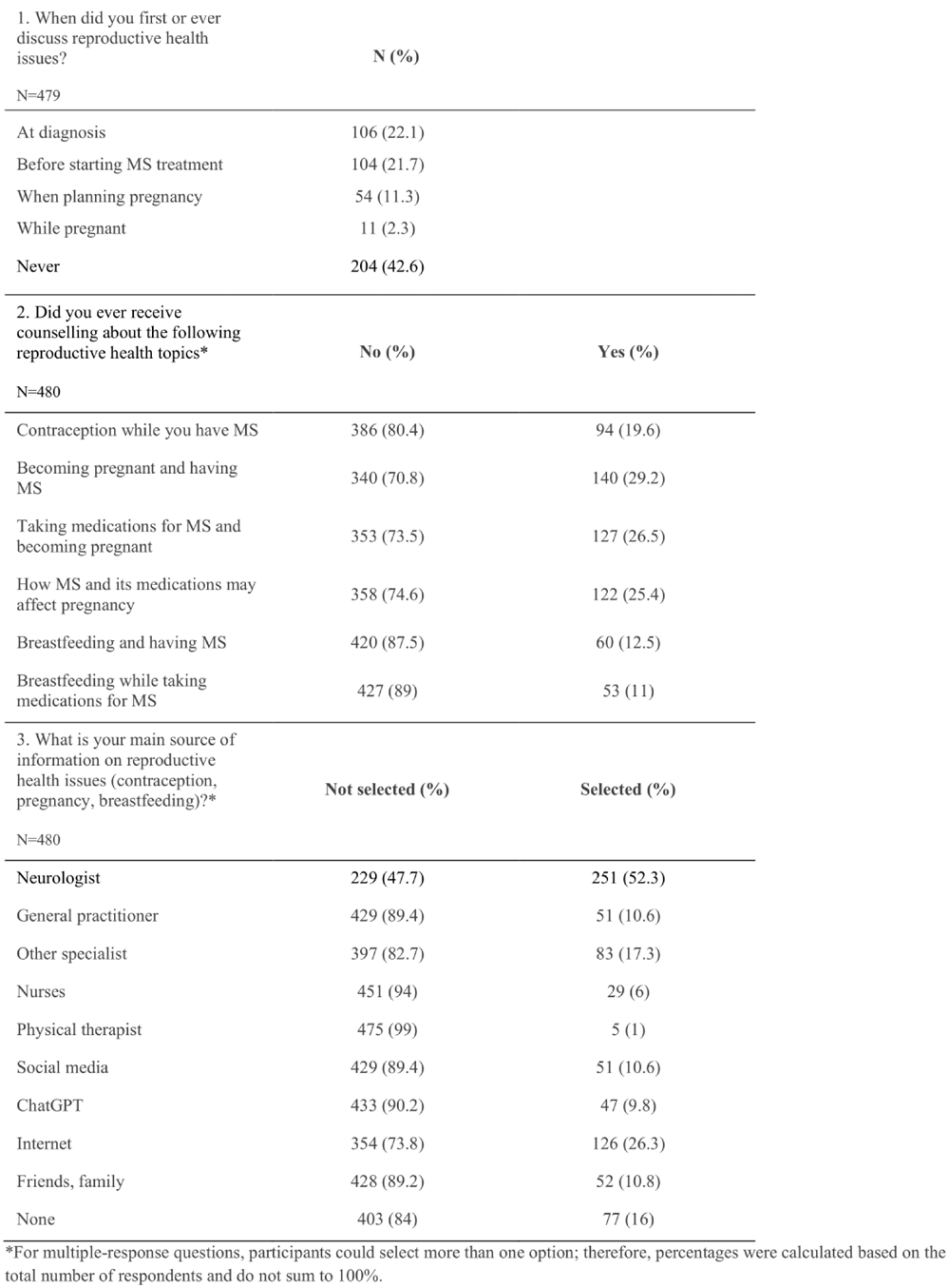
**TABLE 1** Survey responses‐ Items 1 to 3.

**Figure:**
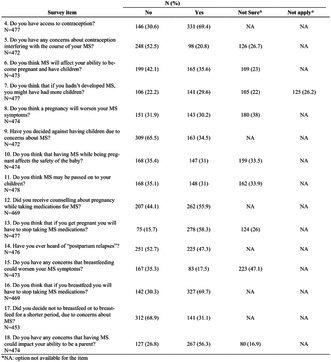
**TABLE 2** Survey responses‐ Items 4 to 18.

**Conclusion:** This global survey identified significant knowledge gaps among WwMS on key aspects of fertility, childbearing, and treatment safety. These findings will inform the core information set for WwMS.

**Disclosure:** Elisa Baldin, Claudia De Santis, Francesco Nonino, Deanna Saylor have nothing to disclose. Riley Bove reports funding grants from Biogen, Eli Lilly, Novartis, Roche Genentech; consulting or advisory for Alexion, Amgen, EMD Serono, TG Therapeutics, Cadenza, Sanofi. Dina Jacobs reports research funding from Roche Genentech, Novartis; consulting or advisory for Alexion, Amgen, EMD Serono, Roche Genentech, Novartis, TG Therapeutics, Sanofi. Nick Rijke reports past employment and consulting for Multiple Sclerosis International Federation. MSIF receives income from a wide range of sources, including health‐care and other companies, individuals, member organisations, campaigns, foundations, and trusts. During the past 5 years, MSIF received funding from the following companies: Bristol Myers Squibb, Sanofi, Merck, Viatris (formerly Mylan), Novartis, Biogen, and F. Hoffman‐La Roche—all of which is publicly disclosed. Shanthi Viswanathan is involved in industry/driven studies with different companies (i.e, Sanofi, Novartis and Alexion). Jennifer McDonell is employed by MS Canada.

## EPO‐1004

### Cancer unleashes multiple sclerosis activity: An Italian multicenter matched cohort study

#### V. Di Gianvito ^1^; M. Ponzano^2^; J. Frau^3^; M. Lucchini^4^; E. Signoriello^5^; L. Ventura^3^; E. Fedele^4^; F. Napoli^1^; C. Nicoletti^1^; G. Mataluni^1^; M. Mirabella^4^; G. Lus^5^; E. Cocco^6^; D. Centonze^1^; G. Marfia^1^; D. Landi^1^

##### ^1^Multiple Sclerosis Clinical and Research Unit, Department of Systems Medicine, Tor Vergata University, Rome, Italy; ^2^Department of Life Sciences, Health and Health Professions, Link Campus University, Rome, Italy; ^3^Multiple Sclerosis Center, Azienda Sanitaria Locale di Cagliari Department of Medical Science and Public Health, University of Cagliari, Cagliari, Italy; ^4^Fondazione Policlinico Universitario "A. Gemelli" IRCCS, Università Cattolica del Sacro Cuore, Centro di Ricerca Sclerosi Multipla "Anna Paola Batocchi, Rome, Italy; ^5^Multiple Sclerosis Center, II Neurological Clinic, University of Campania 'Luigi Vanvitelli', Napoli, Italy; ^6^Department of Medical Sciences and Public Health, Multiple Sclerosis Center, Binaghi Hospital, ASL Cagliari, University of Cagliari, Italy

**Background and aims:** Despite being an established autoimmunity driver, the potential impact of cancer on MS activity remains largely unexplored. This study aims to compare the risk of MS reactivation in the 12 months before and after cancer diagnosis with that observed in a matched cohort of MS patients without cancer.

**Methods:** We conducted a retrospective cohort study including MS patients with cancer from four Italian MS centers, matched 1:1 by sex and age to pwMS without cancer. Index date corresponded to cancer diagnosis date in cases and was aligned by MS disease duration in controls. MS activity was assessed longitudinally collecting relapses and new T2/Gd+ lesions during the 24 months peri‐index date. To account for repeated MRI scans within individuals, generalized estimating equation (GEE) negative binomial models were fitted, adjusting for DMT class at enrollment and MRI region, and including an interaction term between cancer status and time.

**Results:** We included 148 patients with cancer and 148 controls. During the peri‐index period at least one clinical relapse occurred in 20.7% (30) of cancer patients, while no relapses were observed among controls (*p* < 0.001). At least one new T2 and/or Gd+ lesion was detected in 29.7% (78/263) of MRI scans from cases, compared with 6.7% (17/254) from control (χ^2^=41.2,*p* < 0.001). Adjusted GEE models showed greater increase in T2 and/or Gd+ lesion accrual over time among cases (*p* = 0.019).

**Conclusion:** Cancer development appears to trigger disease reactivation, possibly reflecting interactions between immunosurveillance and autoimmunity, warranting tailored MS management strategies and oncological vigilance in unexpected MS reactivations.

**Disclosure:** V. DI GIANVITO received travel fundings from Novartis, Biogen, Roche. M. PONZANO nothing to disclose J. FRAU scientific advisory board and honoraria as a speaker and consultant from Alexion, Amgen, Biogen, Bristol, Novartis, Merck, Sanofi, Sandoz. M. LUCCHINI has received honoraria from Biogen, Merck Serono, Novartis, Roche, Sanofi‐Genzyme, Bristol‐Myers Squibb, Horizon for consulting ser‐ vices and/or speaking honoraria E. SIGNORIELLO received personal compensation from Almirall, Biogen, Sanofi, Novartis, Roche, Horizon, Alexion, Merck, Mylan and Teva for traveling and advisory boards. L. VENTURA nothing to disclose E. FEDELE nothing to disclose F. NAPOLI nothing to disclose C.G. NICOLETTI received travel funding from Biogen, Merck Serono, Sanofi‐Genzyme, Roche, Teva, Novartis, Bristol Mayer Squibb, Janssen, Almirall, Horizon, speaking and consultation fees from Sanofi, Almirall, Merck‐Serono, Roche, Novartis, Biogen. She is a sub‐investigator in clinical trials for Biogen, Merck Serono, Roche, Sanofi, Novartis, Teva, Bristol Mayer Squibb. G. MATALUNI is an Advisory Board member of Argenx. Received honoraria for speaking, consultation fees or travel fundings from Almirall, Biogen Idec, Merck, Novartis, Sanofi, Roche, CSL Behring, Horizon, Kedrion, She is a sub‐investigator in clinical trials for Biogen, Merck Serono, Novartis. M. MIRABELLA received honoraria for speaking, advisory board/consulting from Biogen, Novartis, Merck Serono, Roche, Almirall, Sanofi Genzyme, Janssen, Bristol‐Myers Squibb, Viatris, Alexion. He is principal investigator in clinical trials for Biogen, Merck‐Serono, Novartis, Roche, Sanofi Genzyme, Argenx. G. LUS received speaker honoraria and/or consultancy from Biogen, Teva, Genzyme, Merck, Novartis, Almirall and Roche E. COCCO serves on scientific advisory boards and received honoraria for speaking from Alexion, Biogen, BMS, Janssen, Merck, Novartis, Roche, and Sanofi Genzyme. D. CENTONZE acted as an Advisory Board member of Almirall, Bayer Schering, Biogen, GW Pharmaceuticals, Merck Serono, Novartis, Roche, Sanofi‐Genzyme, Teva, Protagon, Sandoz, Bristol‐Myers Squibb and Alexion and received honoraria for speaking or consultation fees from Almirall, Bayer Schering, Biogen G.A. MARFIA is an Advisory Board member of Almirall, Amgen, Alexion, Neuraxpharm, Sandoz, Sanofi Genzyme, Merck‐Serono, Novartis, Roche and received honoraria for speaking or consultation fees from Almirall, Bayer Schering, Biogen, Merck Serono, Neuraxpharm, Novartis, Sanofi‐Genzyme, Roche, Amgen, Alexion and BMS. She is the principal investigator in clinical trials for Biogen Idec, Merck Serono, Novartis, Roche, Sanofi‐Genzyme, Merck Serono and BMS. D. LANDI received travel funding from Biogen, Merck Serono, Sanofi, Teva, Bristol Myers Squibb, Mylan, Neuraxapharm, speaking or consultation fees from Sanofi, Merck Serono, Teva, Biogen, Roche, Novartis, Bristol Myers Squibb, Janssen, Alexion, Amgen

## EPO‐1005

### Dynamics of choroid plexus volume in multiple sclerosis patients treated with hematopoietic stem cell transplantation

#### G. Costantini ^1^; A. Mariottini^1^; L. Noferini^2^; F. Susini^2^; E. Simonetti^3^; S. Paolucci^2^; A. Repice^3^; A. Gozzini^4^; E. Carlesi^5^; A. Ginestroni^5^; F. Di Pasquale^5^; G. Belli^2^; E. Fainardi^5^; C. Nozzoli^4^; L. Massacesi^1^

##### ^1^Department of Neurosciences, Drug and Child Health, University of Florence, Florence, Italy; ^2^Health Physics Unit, Careggi University Hospital, Florence, Italy; ^3^Emergency Neurology Unit, Careggi University Hospital, Florence, Italy; ^4^Cell Therapy and Transfusion Medicine Unit, Careggi University Hospital, Florence, Italy; ^5^Neuroradiology Unit, Careggi University Hospital, Florence, Italy

**Background and aims:** Choroid plexus volume (ChPv) is a potential magnetic resonance imaging (MRI) biomarker of immune cell trafficking into the central nervous system (CNS). Increased ChPv is associated with focal and chronic inflammation in multiple sclerosis (MS). While some therapies reduce ChPv in patients with recent inflammation, data regarding autologous hematopoietic stem cell transplantation (AHSCT) are lacking. We therefore investigated longitudinal ChPv changes in MS patients after AHSCT.

**Methods:** Consecutive relapsing MS patients undergoing AHSCT (BEAM‐ATG conditioning) underwent standardized 3T MRI at baseline, 6–12 months post‐AHSCT, and then yearly. ChPv and total brain volume (TBV) were segmented using FreeSurfer, T2‐lesion volume (T2LV) manually by an expert operator.

**Results:** Twelve patients (92% female; 42% relapsing‐remitting MS) were included. At baseline, 92% lacked recent focal inflammatory activity. During a median 26‐month follow‐up, two patients relapsed (months 8 and 15) with new spinal cord lesions only. Post‐AHSCT, a median 7% ChPv reduction occurred at 6 months; the patients who relapsed both showed ChPv increase preceding disease activity. TBV decreased by 3% at 6 months, stabilizing at 12 months. No new brain lesions were detected after AHSCT, except for one case showing a mild increase in T2LV at month 6 without changes in ChPv.

**Conclusion:** ChPv decreased post‐AHSCT regardless of recent focal inflammation, suggesting an effect on CNS cell trafficking independent from this latter and supporting AHSCT effect on both peripheral and compartmentalized inflammation. ChPv increases preceded spinal cord‐only relapses, supporting the role of the choroid plexus as an entry point for pathogenic lymphocytes regardless of lesion location.

**Disclosure:** Nothing to disclose.

## EPO‐1006

### Multimodal assessment of optic nerve involvement in multiple sclerosis

#### V. Mauceri ^1^; E. Basili^1^; M. Passamonti^1^; M. Rozzi^1^; D. Di Mare^1^; A. Zolin^1^; F. Rinaldi^2^; M. Zoccarato^2^; P. Perini^2^; M. Puthenparampil^1^

##### ^1^University of Padua, Padua, Italy; ^2^Multiple Sclerosis Centre, Clinica Neurologica, Azienda Ospedale‐Università Padova, Padova, Italy

**Background and aims:** The 2024 McDonald criteria for multiple sclerosis (MS) have formally recognized the optic nerve as a fifth anatomical site for demonstrating dissemination in space. This study aimed to assess the diagnostic accuracy of visual evoked potentials (VEPs), magnetic resonance imaging (MRI), and optical coherence tomography (OCT) in detecting optic neuritis (ON), and to identify the most effective test combinations.

**Methods:** We performed a cross‐sectional analysis including 33 patients with relapsing–remitting MS (pwRRMS) and unilateral ON (ON+), 33 age‐ and sex‐matched patients with other inflammatory neurological diseases (ONIND), and 107 pwRRMS without a clinical history of ON. All participants underwent VEP, MRI, and OCT. OCT peripapillary and macular scans followed OSCAR‐IB QC4 and APOSTEL 2.0 standards. Diagnostic performance was evaluated using receiver operating characteristic (ROC) curve analysis.

**Figure:**
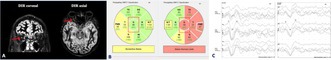
**FIGURE 1** Multimodal characterization of optic neuritis. DIR MRI demonstrates acute right optic nerve involvement (A, red arrow). OCT reveals peripapillary retinal nerve fiber layer thinning consistent with left optic neuritis sequelae (B), while VEPs show delayed.

**Results:** Compared with ONIND, VEPs showed the highest diagnostic accuracy in ON+ patients (AUC 0.9072, 93.94% sensitivity and 87.5% specificity). OCT demonstrated high specificity (92.19%) but lower sensitivity (69.7%; AUC 0.8094), while MRI reached 100% specificity with 63.64% sensitivity. The most accurate strategy was MRI or VEP plus OCT (AUC 0.931; sensitivity 84.85%; specificity 96.88%). The “one‐out‐of‐three” and “two‐out‐of‐three” rules also performed well (AUC 0.914 and 0.8935). Among pwRRMS without clinical ON, 9.8% fulfilled the MRI or (VEP + OCT) criterion, 8.4% were positive on at least two tests, and 37.8% on at least one.

**Figure:**
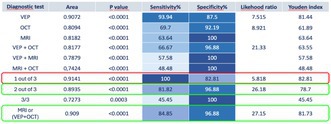
**FIGURE 2** ROC‐based comparison of diagnostic strategies for optic neuritis. ROC curves compare single‐modality and combined diagnostic approaches. Optimal trade‐offs between sensitivity and specificity are highlighted, with red indicating highest sensitivity.

**Figure:**
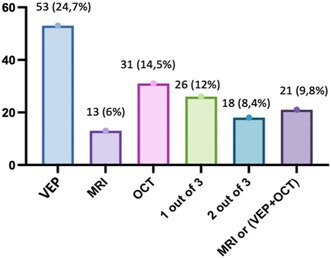
**FIGURE 3** Subclinical optic nerve involvement in RRMS patients without optic neuritis. Bar graph depicting the number and percentage of eyes testing positive on individual and combined diagnostic tests in 107 RRMS patients (214 eyes) with no prior optic neuritis.

**Conclusion:** A multimodal assessment improves detection of optic nerve involvement and may identify subclinical damage, with different criteria applicable according to clinical context.

**Disclosure:** V.A.M reports travel grants from Sanofi Genzyme, Biogen, Novartis, Roche and Viatris. E.B, D.DM, M.Pa, A.Z, M.R have nothing to disclose. P.P. reports grants from Almirall, Teva, Sanofi Genzyme, Merck Serono, Biogen Italy, Novartis, Roche, Alexion, Janssen, Brystol Mayer Squibb; consultancy for Novartis, Biogen Italy, Sanofi Genzyme, Roche, Janssen, Brystol Mayer Squibb. F.R. report grants from Almirall, Teva, Sanofi Genzyme, Merck Serono, Biogen Italy, Novartis, consultancy for Novartis, Biogen Italy, Sanofi Genzyme. M.Pu, report travel grants, consultancy, and board membership from Almirall, Teva, Sanofi Genzyme, Merck Serono, Biogen Italy, Novartis, Bristol Myers Squibb, Janssen, and Alexion.

## EPO‐1007

### Assessment of dietary habits in patients with multiple sclerosis in Opole and Silesian Province, Poland

#### V. Carbogno‐Barnabe^1^; M. Skrzypek^2^; P. Bednarczyk ^3^; I. Żabicka^3^; B. Łabuz‐Roszak^4^

##### ^1^Department of Nutrition Related Prevention, Faculty of Public Health in Bytom, Medical University of Silesia in Katowice, Poland; ^2^Department of Biostatistics, Department of Epidemiology, Faculty of Public Health in Bytom, Medical University of Silesia in Katowice, Poland; ^3^Department of Neurology, St. Jadwiga Provincial Specialist Hospital, Opole, Poland; ^4^Department of Neurology, St. Jadwiga Provincial Specialist Hospital, Institute of Medical Sciences, University of Opole, Opole, Poland

**Background and aims:** The aim of the study was to assess dietary habits in patients with multiple sclerosis (MS) in relation to psychosocial factors and quality of life.

**Methods:** The study included 91 patients with MS (68 women and 23 men) with a mean age of 49.8±11.0 years. Dietary patterns were assessed using the Pro‐Healthy Diet Index‐10 (pHDI‐10) and the Non‐Healthy Diet Index‐14 (nHDI‐14), derived from the Dietary Habits and Nutrition Beliefs Questionnaire (KomPAN). Additionally, a nutritional interview was conducted, and the Hospital Anxiety and Depression Scale (HADS), Visual Analogue Scale (VAS), and the EQ‐5D‐5L quality of life questionnaire were administered.

**Results:** The intensity of both health‐promoting (pHDI‐10) and health‐adverse (nHDI‐14) dietary characteristics was low in examined MS patients. In 60.4% of participants, the daily energy intake was lower than the total metabolic rate. The majority of respondents had a below‐average intake of dietary fiber (72.5%), vitamin D (93.4%), and iron (70.3%). Higher intakes of protein and saturated fatty acids were associated with higher nHDI‐14 scores, whereas higher intakes of dietary fiber, calcium, magnesium, and vitamin B12 were associated with higher pHDI‐10 scores. Men demonstrated significantly higher nHDI‐14 levels than women. Neither disease duration nor disability level had a significant effect on dietary habits. Dietary habits were associated with pain intensity and quality of life, but not with depressive or anxiety symptoms.

**Conclusion:** Nutritional education should be an integral component of the comprehensive management of MS patients, as it may contribute to improving dietary habits and quality of life.

**Disclosure:** Nothing to disclose.

## EPO‐1008

### Abstract withdrawn

## EPO‐1009

### Characterization of natural killer cell aging and terminal differentiation in multiple sclerosis

#### J. Nolte ^1^; H. Vietzen^2^; T. Zrzavy^1^; F. Föttinger^1^; N. Krajnc^1^; S. Zaic^1^; E. Puchhammer‐Stöckl^2^; T. Berger^1^; P. Rommer^1^; G. Bsteh^1^

##### ^1^Department of Neurology, Medical University of Vienna, Vienna, Austria; ^2^Center for Virology, Medical University of Vienna, Vienna, Austria

**Background and aims:** Insights into immune aging and related functional impairment are essential for advancing understanding of multiple sclerosis (MS) course and pathophysiology and may inform individualized treatment decisions. CD57 expression on CD56dim natural killer (NK) cells and its proportion within the total NK cell population is a well‐established indicator of mature NK cells. Here, we compared immunosenescence in patients with MS (pwMS) versus healthy controls (HCs) and evaluated influential covariates.

**Methods:** From an ongoing study, peripheral blood mononuclear cells from 69 pwMS and 10,437 HCs in Austria, Switzerland, and Germany were analyzed using flow cytometry, gated on CD3, CD56, CD57, NKG2D, DNAM‐1, NKG2A, and TIGIT. Age‐adjusted z‐scores for CD57+CD56dim NK‐cell proportions were estimated using a generalized additive model based on the HCs.

**Results:** Among 69 pwMS, 65.2% were female with a mean age of 40.5 years (SD 10.2), mean disease duration of 10.5 years (SD 9.47), and median EDSS of 2 (IQR 0–3). Age‐adjusted z‐scores for CD57 on CD56dim NK cells were comparable between pwMS and HCs. No differences were observed by sex or disease course. Notably, the proportion of the immunoregulatory CD56bright cells was significantly reduced in pwMS.

**Conclusion:** CD57+CD56dim NK cells are a robust marker for NK‐cell aging and may serve as a reliable surrogate of immunosenescence. Age‐adjusted z‐scores for the proportions of CD57+CD56dim NK cells can be estimated in pwMS to assess accelerated immune‐cell aging. In future studies, this may be useful to assess the impact of immunosenescence on disease activity and progression.

**Disclosure:** Nothing to disclose.

## Neurorehabilitation and Neurotraumatology

## EPO‐1010

### When copeptin and vasopressin diverge: Neuroendocrine dissociation and diffuse axonal injury in traumatic brain injury

#### A. Sacarescu ^1^; I. Plesca^2^; M. Turliuc^3^

##### ^1^Department of Medical Specialties III, “Grigore T. Popa” University of Medicine and Pharmacy, Iasi, Romania; ^2^Faculty of Mathematics, “Alexandru Ioan Cuza” University, Iasi, Romania; ^3^Department of Surgery II, “Grigore T. Popa” University of Medicine and Pharmacy, Iasi, Romania

**Background and aims:** Traumatic brain injury (TBI) can disrupt hypothalamic–pituitary regulation, leading to neuroendocrine alterations that may reflect injury severity. Copeptin and arginine vasopressin originate from the same precursor and are expected to show parallel regulation under physiological conditions. This study evaluated the relationship between copeptin and vasopressin levels in TBI and their association with injury severity and diffuse axonal injury (DAI).

**Methods:** Eighty‐six adult patients admitted with TBI to a tertiary neurotrauma center were included. Clinical severity was assessed using the Glasgow Coma Scale (GCS). Serum copeptin and vasopressin levels were measured, and neuroimaging data from computed tomography and magnetic resonance imaging were reviewed to identify traumatic lesions, including DAI.

**Results:** Copeptin and vasopressin levels demonstrated a significant inverse correlation (Spearman *ρ* = −0.45, *p* < 0.01), indicating dissociated regulation following TBI. DAI was identified in 9.3% of patients and was associated with significantly higher copeptin levels compared with those without DAI (*p* = 0.045). Higher copeptin concentrations were observed in patients with lower GCS scores. In contrast, vasopressin levels showed a positive correlation with GCS (*ρ* = 0.366, *p* = 0.003) and were significantly lower in patients with unfavorable outcomes, including mortality (*p* = 0.006) and the need for neurosurgical intervention (*p* = 0.048).

**Conclusion:** TBI is associated with altered copeptin and vasopressin regulation, resulting in a dissociated neuroendocrine profile. Within this context, elevated copeptin levels and their association with DAI suggest that copeptin may reflect axonal damage severity, supporting its further evaluation as a biomarker in neurotrauma research.

**Disclosure:** Nothing to disclose.

## EPO‐1011

### Dynamic EEG markers of confusional state and cognitive outcome after moderate–to‐severe traumatic brain injury

#### A. Comanducci; C. Derchi; T. Atzori; C. Valota; B. Mosconi; P. Arcuri; J. Navarro

##### IRCCS Fondazione Don Carlo Gnocchi, Milan, Italy

**Background and aims:** Cognitive impairment is a frequent and disabling consequence of moderate‐to‐severe traumatic brain injury. Post‐traumatic amnesia (PTA) duration is a well‐established prognostic marker of global disability, but its specificity for long‐term cognitive outcome is limited. After emergence from PTA, recently conceptualized as part of the post‐traumatic confusional state (PTCS), overt confusional symptoms typically resolve, while residual cognitive deficits persist. EEG may provide complementary prognostic information across this transition.

**Methods:** Forty‐two patients with moderate‐to‐severe TBI admitted to inpatient rehabilitation underwent resting‐state EEG at admission and discharge. EEG abnormalities were graded using the semi‐quantitative Grand Total EEG score (GTE), with exploratory analyses of slow wave activity and background activity subdomains. Cognitive outcome at six months was assessed using the Montreal Cognitive Assessment (MoCA), while disability was evaluated using the Disability Rating Scale (DRS).

**Results:** At admission, GTE was associated with PTA/PTCS severity. At discharge, SWA showed substantial normalization, consistent with resolution of PTCS, whereas Background Activity abnormalities persisted in a subset of patients, suggesting ongoing cortical disorganization. In multivariate logistic regression models predicting MoCA impairment at 6 months, discharge GTE score was an independent predictor (OR = 2.61, 95% CI 1.47–4.66, *p* = 0.002). In contrast, PTA/PTCS duration was strongly associated with DRS (*p* < 0.001).

**Conclusion:** EEG provides prognostic information complementary to PTA/PTCS duration after severe TBI. While PTA/PTCS duration remains a robust predictor of global disability, EEG—particularly background organization at T1—better captures vulnerability to persistent cognitive impairment. EEG thus emerges as a dynamic prognostic tool in the subacute phase of TBI.

**Disclosure:** Nothing to disclose.

## EPO‐1012

### Effects of localized muscle fatigue on dynamic balance in healthy young adults: A randomized crossover study

#### A. Patrício^1^; B. Carvalho^1^; R. Brandão^2^; D. Tomás ^2^

##### ^1^Department of Physiotherapy, Escola Superior de Saúde Atlântica, Barcarena, Portugal; ^2^Department of Physiotherapy, Escola Superior de Saúde do Alcoitão, Alcabideche, Portugal

**Background and aims:** Muscle fatigue is traditionally associated with impaired postural control; however, accumulating evidence suggests that exercise performed to the point of fatigue may also elicit short‐term adaptive responses within the neuromuscular system. The effects of exercise‐induced localized muscle fatigue, particularly when applied to different body segments, remain insufficiently understood. This study aimed to investigate the effects of exercise protocols designed to induce localized muscle fatigue of the upper and lower limbs on dynamic balance in healthy young adults.

**Methods:** A randomized, controlled crossover study was conducted in 32 healthy young adults. Dynamic balance was assessed using the Y Balance Test before and immediately after exercise protocols targeting either the upper or lower limbs and designed to induce localized muscle fatigue. Sessions were separated by at least 48 hours to minimize carry‐over effects. Heart rate and perceived exertion were monitored to ensure that the physiological response was predominantly muscular rather than cardiorespiratory.

**Results:** Following the exercise protocols, significant increases in reach distance were observed in the Y Balance Test across most directions, indicating short‐term changes in dynamic balance performance. No significant differences were found between upper‐ and lower‐limb exercise protocols. No significant associations were observed between self‐reported physical activity levels and balance changes.

**Conclusion:** Exercise protocols designed to induce localized muscle fatigue were associated with short‐term improvements in dynamic balance, regardless of the body segment involved. Although conducted in healthy individuals, these findings may inform the design of training and rehabilitation strategies incorporating controlled exposure to exercise‐induced fatigue.

**Disclosure:** Nothing to disclose.

## EPO‐1013

### SPInal CSF leaks in chronic subdural hematoma (SPICE): Interim results of a prospective observational trial

#### N. Lützen^1^; K. Wolf^2^; F. Volz^2^; A. El Rahal^2^; C. Zander^1^; M. Overstijns^2^; L. Krismer^2^; H. Urbach^1^; J. Beck ^2^

##### ^1^Department of Neuroradiology, CSF Center, Medical Center, University of Freiburg, *Freiburg, Germany,* ^2^Department of Neurosurgery, CSF Center, Medical Center, University of Freiburg, *Freiburg, Germany*

**Background and aims:** Chronic subdural hematoma (cSDH) is the most common intracranial hemorrhage, characterized by slowly expanding subdural fluid collections that ultimately cause brain compression and neurological impairment. The underlying pathophysiology remains incompletely understood. An increasingly recognized and directly treatable cause are spontaneous spinal cerebrospinal fluid (CSF) leaks. This study aimed to estimate the prevalence of spinal CSF leaks in patients presenting with cSDH.

**Methods:** This interim analysis derives from a prospective, monocentric observational study of patients admitted with cSDH to the Department of Neurosurgery, University Medical Center Freiburg (07/2024–12/2025; NCT06323434). Standard cSDH treatment was followed by MRI of the brain and spine according to standard‐workup in spontaneous intracranial hypotension. Patients with intermediate leak risk (Bern Score ≥3) or orthostatic headache underwent dynamic myelography. The primary outcome was the proportion of confirmed CSF leaks.

**Results:** Of 159 eligible patients, 132 consented; 22 withdrew. Six patients are awaiting myelography, and 20 were lost to follow‐up before myelography. Among the 84 patients who underwent myelography, a spinal CSF leak was identified in 16.7% (*n* = 14, 95% CI 10–26%), with a majority of CSF–venous fistulas (*n* = 12, 14.3%, 95% CI 8–23%). A more restrictive analysis yields a CSF leak prevalence of 13.5% (95% CI 8.2–21.3%) among the 104 primarily included patients.

**Figure:**
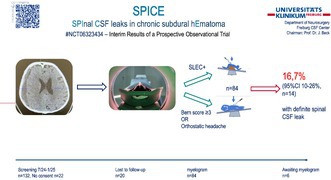
**FIGURE 1** Pipeline and interim results of the SPICE study: prevalence of underlying spontaneous spinal CSF leaks in patients with chronic subdural hematoma.

**Conclusion:** Spinal CSF leaks represent a relevant and directly curable cause in a substantial proportion of patients with cSDH. These findings challenge prevailing assumptions regarding the relationship between spinal CSF leaks and cSDH and have important implications for future diagnostic pathways.

**Disclosure:** none

## EPO‐1014

### Daytime dysfunction in military personnel following blast injury

#### K. Sarazhyna; A. Son

##### Odesa National Medical University, Odesa, Ukraine

**Background and aims:** Blast injuries (BI) are a leading cause of trauma among Ukrainian military personnel (MP), often resulting in mild traumatic brain injury (mTBI) and sleep disturbances, particularly daytime dysfunction (DD). Both mTBI and anxiety may contribute to DD, but their relative impact remains unclear.

**Methods:** 136 male MPs with BI (median 36.0; range 22–56) were enrolled. Participants were divided into two groups: BI with mTBI (*n* = 62; 45.6%) and without mTBI (*n* = 74; 54.4%). Only acute mTBI cases (≤21 days) were included. Anxiety was assessed using the Hospital Anxiety and Depression Scale. DD was measured using component 7 of the Pittsburgh Sleep Quality Index and dichotomized: 0 = no disturbance (score < 2), 1 = DD (score ≥ 2). Binomial logistic regression models evaluated associations between DD, mTBI and anxiety.

**Results:** The model demonstrated good fit (AIC = 151; McFadden *R* ^2^ = 0.201). Anxiety severity was significantly associated with DD (*β* = 0.209, SE = 0.047, OR = 1.23, 95% CI 1.13–1.35, *p* < 0.001). mTBI was was also linked to higher DD risk (*β* = 1.066, SE = 0.407, OR = 2.90, 95% CI 1.31–6.45, *p* = 0.009). In Model 2, the anxiety × mTBI interaction did not improve fit. Anxiety remained a significant predictor of DD (OR = 1.23, *p* = 0.002), whereas mTBI status (OR = 2.64, *p* = 0.327) was not statistically significant.

**Conclusion:** Final analyses indicated that anxiety was the only statistically significant predictor of DD irrespective of mTBI status.

**Disclosure:** Nothing to disclose.

## EPO‐1015

### Voice onset time contrast perception of hindi plosives in children with cochlear implant

#### M. Ansari ^1^; S. Chavan^2^

##### ^1^Ayjnishd (D), Mumbai, India; ^2^DMIHER, Wardha, India

**Background and aims:** Voice onset time (VOT) is an auditory‐based acoustic feature used to distinguish stop consonants in various languages. Children with sensorineural hearing impairment (HI) often struggle with discriminating between voiced and voiceless speech sounds. The role of this auditory ability in cochlear implant recipients has not been well understood. This study aimed to compare VOT contrast perception among Cochlear Implant (CI), Hearing Aid (HA) recipients, and Normal Hearing (NH) children.

**Methods:** A total of 60 subjects aged 6 to 12 years, divided into NH, HA, and CI groups, were selected. Test stimuli included 180 tokens of consonant‐vowel‐consonant combinations of nonsense Hindi stop phonemes with varying initial positions. These stimuli were presented in an ABX paradigm to assess identification scores.

**Results:** Statistical analysis revealed significant differences in VOT scores among all groups. There was no overlap in identification scores between NH & CI and HA & CI groups. CI children performed better than HA recipients but poorer than NH children. Significant differences were observed in scores between NH and both HA and CI groups, as well as between HA and CI groups. These findings align with previous research.

**Conclusion:** Children with CI and HA demonstrated different VOT discrimination abilities compared to NH children, likely due to limitations of CI and HA devices in processing speech signal characteristics. Older CI children with speech and language training and longer device use performed better than the HA group, suggesting that CI may enhance access to speech information and improve perception.

**Disclosure:** Nothing to disclose.

## EPO‐1016

### Neuroeducation and mindfulness‐based meditation as novel group‐based neurorehabilitation approach to improve cognition in multiple sclerosis

#### N. Gyger ^1^; T. Monschein^2^; T. Geiger^1^; F. Leutmezer^2^; G. Pusswald^2^; I. Penner^1^

##### ^1^Department of Neurology, Inselspital, Bern University Hospital, University of Bern, Switzerland; ^2^Department of Neurology, Medical University of Vienna, Vienna, Austria

**Background and aims:** To evaluate the efficacy of a novel group‐based neurorehabilitation approach, combining neuroeducation with mindfulness‐based meditation, in reducing self‐perceived but also objective cognitive deficits in people with relapsing‐remitting multiple sclerosis (pwRRMS).

**Methods:** In this ongoing study, pwRRMS aged 18–50 years with EDSS <4 and disease duration ≤10 years are enrolled. Participants attend a group‐based program (5–8 per group) consisting of seven MS‐specific neuroeducation sessions (90 minutes, weekly) led by a trained neuropsychologist, each followed by 10–15 minutes of mindfulness‐based meditation supported by home practice via an in‐house app. Objective neuropsychological and patient‐reported outcomes are assessed before and after the 7‐week intervention, with the Perceived Deficits Questionnaire (PDQ‐20) as the primary outcome measure.

**Results:** Currently, 57 participants have been included. The PDQ‐20 showed a significant reduction in perceived cognitive deficits (*d* = 0.39). Change scores showed a significant increase in information processing speed (IPS) with moderate effect size (SDMT, *d* = 0.63). More pronounced cognitive impairment at baseline tended to be associated with larger IPS gains. Moreover, significant improvements were observed in fatigue (FSMC, *d* = 0.47), anxiety (HADS‐A, *d* = 0.54), and depression (HADS‐D, *d* = 0.61), together with increased coping self‐efficacy (CSES, *d* = 0.61), general self‐efficacy expectation (SWE, *d* = 0.66), and reduced perceived stress (PSS, *d* = 0.53). Further, neuropsychological testing showed improved working memory.

**Conclusion:** These interims findings indicate that the combination of neuroeducation and mindfulness‐based meditation significantly enhances well‐being and additionally shows promising effects on cognitive functioning in pwRRMS.

**Disclosure:** Naomi Gyger: nothing to disclose. Tobias Monschein: has participated in meetings sponsored by, received speaker honoraria or travel funding from Biogen, BMS/Celgene, Merck, Novartis, Roche, Sanofi, Genzyme and Teva. Tania Geiger: nothing to disclose. Fritz Leutmezer: has participated in meetings sponsored by, received speaker honoraria or travel funding from Actelion, Almirall, Biogen, BMS/Celgene, Johnson&Johnson, MedDay, Merck, Novartis, Roche, Sanofi‐Genzyme and Teva, and received honoraria for consulting Biogen, BMS/Celgene, Merck, Novartis, Roche, Sanofi‐Genzyme and Teva. Gisela Pusswald: nothing to disclose. Iris‐Katharina Penner: Almirall, Biogen, BMS, Celgene, Genzyme, Janssen, Merck, Novartis, Roche, Teva // speakers bureau or advisory board, consulting fees; The German MS Society, Celgene, Novartis, Roche, Teva // research grants.

## EPO‐1017

### Effect of rTMS on neuropsychological outcomes in mild to moderate traumatic brain injury: A prospective cohort study

#### S. Singh

##### Department of Neurosurgery, AIIMS, Raebareli, India

**Background and aims:** Traumatic brain injury (TBI) is a major global public health problem and a leading cause of long‐ term cognitive, behavioural, and psychological disability, particularly among the productive age group. Disruption of fronto‐cortical networks and underlying white matter tracts has been strongly implicated in post‐TBI neuropsychological deficits. Recent advances and international evidence have highlighted repetitive transcranial magnetic stimulation (rTMS) as a promising non‐invasive neuromodulatory intervention in TBI rehabilitation.

**Methods:** Adult patients with mild to moderate TBI (GCS > 10) underwent MRI brain with Diffusion Tensor Imaging (DTI) and were classified based on white matter tract deviation and cerebral edema. Neuropsychological assessment using the Montreal Cognitive Assessment (MOCA) and Hamilton Depression Rating Scale (HDRS) was performed at baseline and follow‐up. Patients with persistent deficits received high‐frequency rTMS (10 Hz) over the right/left dorsolateral prefrontal cortex at 110% of individual resting motor threshold, delivered in 10 sessions over 5 days.

**Results:** Sixty patients (40 males, 20 females; mean age 36.24 years) were analyzed. Mean resting motor threshold reduced from 75.35% at baseline to 70.61% at follow‐up, with a corresponding reduction in stimulus intensity (110% RMT) from 82.81% to 77.84%, indicating enhanced cortical excitability. Cognitive outcomes improved significantly, with mean MOCA scores increasing from 19.22 at baseline to 23.82 post‐intervention. Mean HDRS scores decreased from 5.56 to 4.70, reflecting improvement in depressive symptoms.

**Conclusion:** rTMS resulted in sustained improvement in cortical excitability, cognitive performance, and psychological well‐being in mild to moderate TBI patients, particularly those with white matter tract involvement.

**Disclosure:** The Study was funded by UP Counsel for Science and Technology (UP CST). Nothing else to disclose

## EPO‐1018

### Predicting long‐term outcome in prolonged disorders of consciousness: Post‐acute prognostic factors from the nationwide DOCTOR study

#### D. Driessen^1^; C. Utens^2^; G. Ribbers^1^; W. van Erp ^2^; M. Heijenbrok ‐ Kal^1^

##### ^1^Rehabilitation Medicine, Erasmus MC, Rotterdam, the Netherlands; ^2^Primary Care, Radboudumc, Nijmegen, the Netherlands

**Background and aims:** Prolonged disorders of consciousness (PDOC) following acquired brain injury are characterised by major prognostic uncertainty, complicating clinical decision‐making and ethical deliberation. While acute‐phase predictors have been studied extensively, less is known about prognostic factors available at admission to specialised neurorehabilitation. This study aimed to identify demographic and clinical predictors for recovery of consciousness, level of functioning, and independence in PDOC.

**Methods:** Prospective cohort study including PDOC patients ≥16 years admitted to the nationwide Dutch PDOC chain of care (2019–2023). Potential predictors at rehabilitation admission: demographic and clinical characteristics, time post‐injury (TPI), level of consciousness, aetiology, Coma Recovery Scale–Revised (CRS‐R). Outcomes: recovery of consciousness, level of functioning, and functional independence measured with the Functional Independence Measure (FIM) up to 104 weeks. Multivariable Generalized Estimating Equations were used, applying Bonferroni correction.

**Results:** Of 129 enrolled patients (mean age 38 years; 52% male), 62% regained consciousness. A non‐smoking history was the only independent predictor for regaining consciousness (OR 2.46, 95% CI 1.33–4.52). Shorter TPI (B −0.26, *p* < 0.001) and higher CRS‐R at admission (B 3.15, *p* < 0.001) independently predicted higher functional outcome. Higher CRS‐R also predicted functional independence at 2 years (OR 1.38, 95% CI 1.13–1.67).

**Conclusion:** Prediction of long‐term recovery in PDOC at the start of specialised rehabilitation remains challenging. Simple clinical variables, particularly smoking history and CRS‐R score, provide limited but relevant prognostic information. These findings underline the need for integrated longitudinal prognostic models combining clinical, neurophysiological, and imaging data to better support personalised and ethically informed care pathways.

**Disclosure:** Nothing to disclose.

## Headache 6

## EPO‐1019

### Post‐traumatic headache: Evaluating repetitive transcranial magnetic stimulation (rTMS) as a novel treatment strategy

#### C. Nygaard ^1^; S. Futarmal Kothari^1^; H. Winter Schytz^2^; T. Skandsen^3^; L. Odgaard^1^; C. Bøcker Pedersen^1^; M. Møller Thastum^1^; H. Holm Stabel^1^; L. Hellemose^1^; S. Kristine Stage Pedersen^1^; A. Munch Lauridsen^1^; A. Bilgaard Nicolaisen^1^; J. Feldbæk Nielsen^1^

##### ^1^Hammel Neurorehabilitation Centre and University Research Clinic, Department of Clinical Medicine, Aarhus University, Hammel, Denmark; ^2^Danish Headache Center, Department of Neurology, Rigshospitalet Glostrup, Faculty of Health and Medical Sciences, University of Copenhagen, Denmark; ^3^Department of Neuromedicine and Movement Science, Norwegian University of Science and Technology (NTNU), Trondheim, Norway and Clinic of Rehabilitation, St. Olavs Hospital, Trondheim University Hospital, Trondheim, Norway

**Background and aims:** Post‐traumatic headache (PTH) is a disabling sequela of mild traumatic brain injury (mTBI), and currently there is no established effective treatment for PTH. Recently, repetitive transcranial magnetic stimulation (rTMS) has emerged as a promising treatment modality. Aim: To evaluate the effect of rTMS in adults with PTH 4–6 months after mTBI.

**Methods:** A randomised, sham‐controlled clinical trial in adults with PTH was conducted from August 2023 to August 2024. Participants received 5 sessions of active rTMS (*n* = 30) or sham rTMS (*n* = 31) over a 2‐week period, 4–6 months after mTBI. The primary outcome was defined as the mean change in the number of headache days of moderate to severe intensity per month before versus after intervention. Primary outcome data were collected via a prospective, daily headache diary. Follow‐up was conducted at 1‐month after end of treatment (EOT).

**Results:** At 1‐month EOT, preliminary results show no significant difference in the mean change of monthly headache days of moderate to severe intensity between the two groups. The mean reduction was 6.6 days in the active group versus 6.2 days in the sham group.

**Figure:**
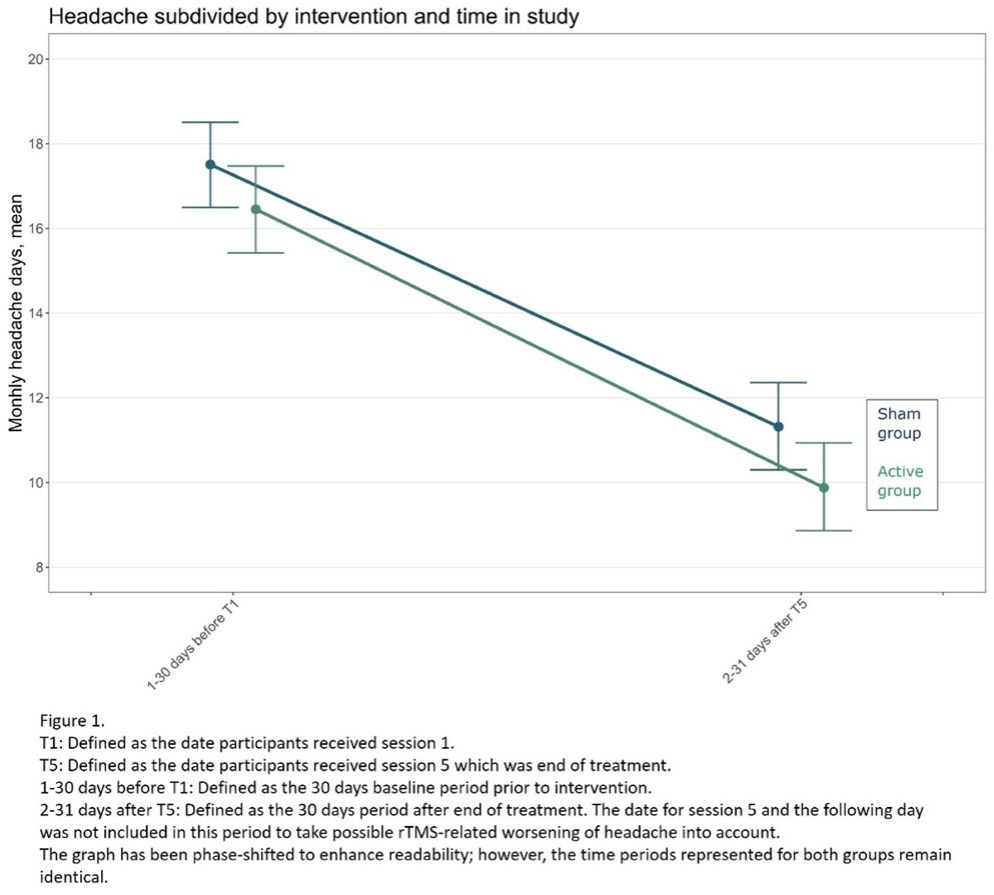
**FIGURE 1** Headache subdivided by intervention and time in study.

**Conclusion:** rTMS did not significantly reduce the number of monthly headache days of moderate to severe intensity compared with sham‐rTMS in adults with PTH 4–6 months post‐mTBI. Consequently, this study does not provide evidence to support rTMS as an effective treatment of PTH. Further research is needed to determine whether rTMS can improve long‐term outcomes in patients with PTH.

**Disclosure:** The authors declare no conflicts of interest related to the manuscript. The study was funded by The Health Research Fund of Central Denmark Region. The funder was not involved in the study design; collection, analysis, or interpretation of data; and in the writing of the article.

## EPO‐1020

### Distinguishing migraine with aura from acute ischemic events in emergency settings: A cross‐sectional study

#### M. Çakan Ercan^1^; M. Topçuoğlu^1^; R. Göçmen^2^; E. Arsava^1^; I. Ünal‐Çevik ^3^

##### ^1^Hacettepe University, Faculty of Medicine, Departments of Neurology, Ankara, Türkiye; ^2^Hacettepe University, Faculty of Medicine, Department of Radiology, Ankara, Türkiye; ^3^Hacettepe University, Faculty of Medicine, Department of Neurology, Headache and Pain Unit, Ankara, Türkiye

**Background and aims:** Differentiating migraine with aura (MA) from transient ischemic attack (TIA) is challenging because both conditions can present with negative neurological symptoms. Here, we aimed to evaluate two venous imaging markers: “the venous asymmetry index” and “index vein sign” on susceptibility‐weighted images (SWI) in MA and TIA groups, and to derive a simple, clinically applicable score to distinguish MA from TIA.

**Methods:** In this cross‐sectional study, patients presented with acute stroke‐like symptoms (speech disturbance, facial asymmetry, hemianopia, hemiparesis, or hypoesthesia) between January 2023 and December 2024, were prospectively evaluated. Primary analyses tested symptom‐concordant venous asymmetry index and index vein sign, evaluating their relationship to the final diagnosis. The Symptom‐concordant Venous Imaging (SVI) score was constructed.

**Results:** A total of 290 diffusion weighted image (DWI)‐negative patients were eligible (TIA *n* = 269; MA *n* = 21). The symptom‐concordant venous asymmetry index was present in 90.5% and the index vein sign in 85.7% of migraine with aura however these findings were observed in only 16.4% and 10.4% of the TIA group, respectively (Table‐1). On follow‐up MRIs, venous imaging signs frequently resolved in migraineurs. Multivariable analysis models revealed that symptom‐concordant venous imaging findings were associated with MA (Table‐2). The SVI score demonstrated a discriminatory performance with an AUC of 0.969 (95% CI: 0.949–0.990). At a cut‐off value of ≥8 points, sensitivity was 71.4% and specificity was 95.9%.

**Figure:**
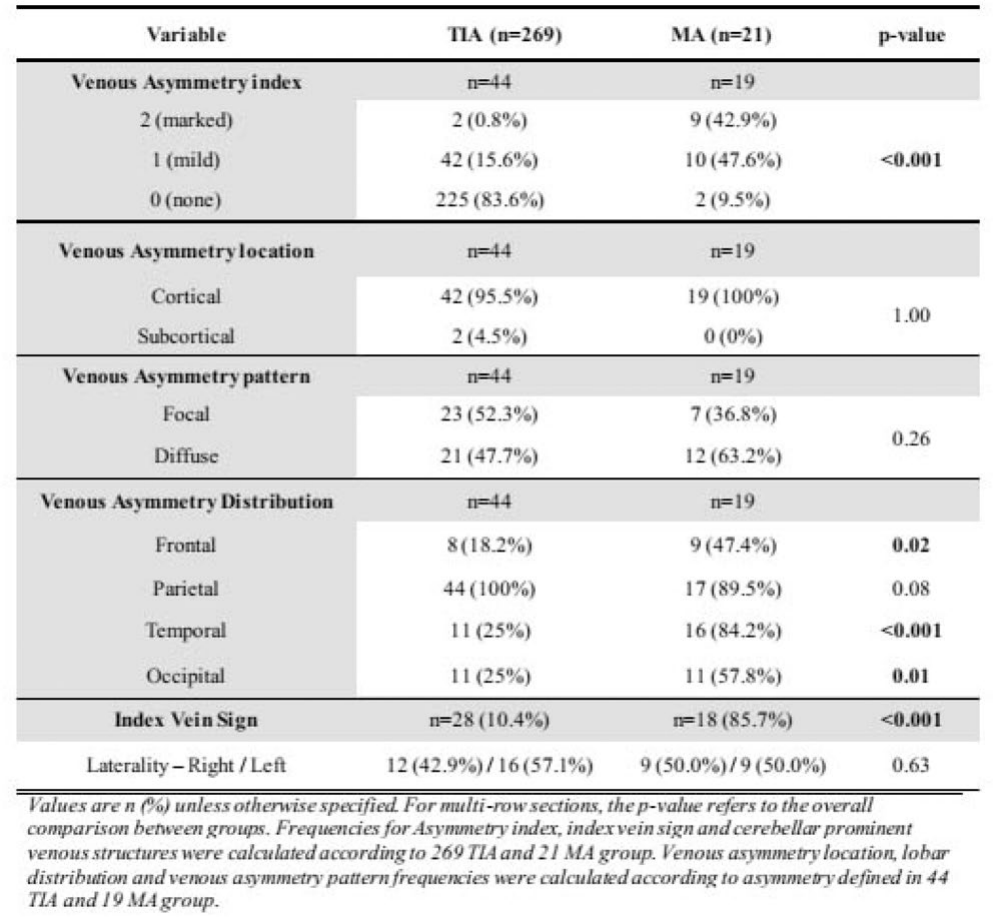
**TABLE 1** Venous imaging findings on acute SWI in patients with TIA and migraine with aura.

**Figure:**
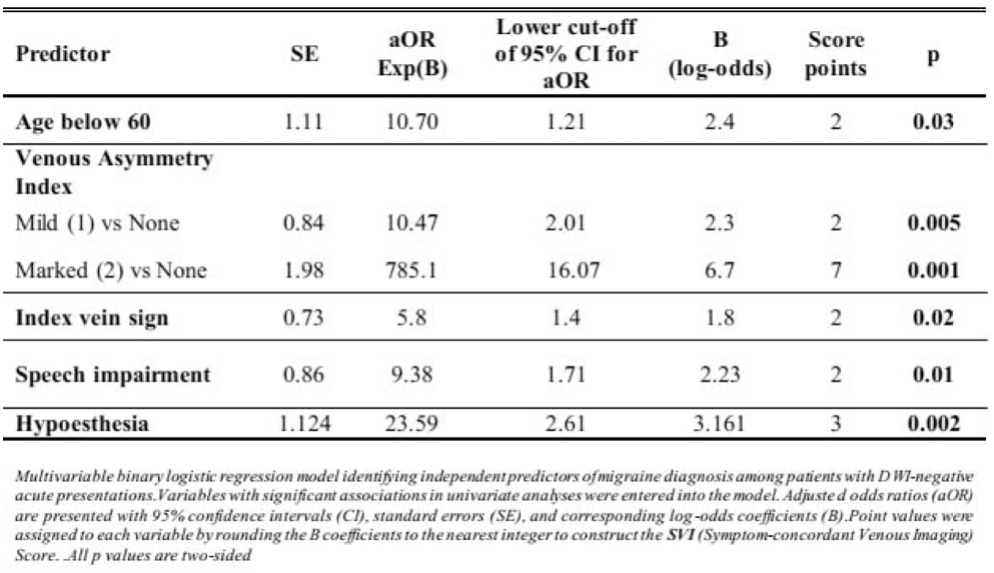
**TABLE 2** Multivariate logistic regression analysis results and symptom‐concordant venous imaging (SVI) scores.

**Conclusion:** In patients presented with acute stroke‐like symptoms, marked venous asymmetry and index vein sign concordant with symptoms and SVI score of >8 were strongly associated with MA.

**Disclosure:** Nothing to disclose.

## EPO‐1021

### Effective migraine prevention improves cognitive complaints

#### M. Costa Taveira ^1^; F. Dourado Sotero^1^; M. Dias da Costa^1^; A. Costa^2^; B. Martins^2^; R. Rato^2^; J. Nobre^3^; M. Roque^1^; I. Pavão Martins^1^

##### ^1^Neurology Service, Department of Neurosciences and Mental Health, ULS Santa Maria, Lisboa, Portugal; ^2^Neurology Service, ULS São João, Porto, Portugal; ^3^Centro de Estudos Egas Moniz, Faculty of Medicine, University of Lisbon, Lisboa, Portugal

**Background and aims:** Cognitive complaints, such as memory and concentration difficulties, are increasingly recognized as part of the clinical spectrum of migraine. We aimed to evaluate whether subjective cognitive complaints improve with effective preventive treatment for migraine acting outside the central nervous system.

**Methods:** We conducted a prospective observational study including adults with migraine diagnosed according to the ICHD‐3 criteria, initiating preventive treatment with either anti‐CGRP monoclonal antibodies (mAbs) or OnabotulinumtoxinA (BT). Responders were defined as those who achieved ≥50% reduction in monthly migraine days for episodic migraine and ≥30% reduction for chronic migraine from baseline to 3–6 months follow‐up. Cognitive complaints, assessed using the Subjective Memory Complaints scale (SMC) and depressive symptoms (Hospital Anxiety and Depression Scale (HADS‐D)), were compared between the two evaluations according to responder status.

**Results:** Participants (*N* = 100, mean age 42.27 years, 98% women, 71% with chronic migraine), received either mAbs (*N* = 66) or BT (34), and 57 responded to treatment. Responders showed a significant improvement in subjective memory complaints (*B* = −1.46, *p* = 0.037), regardless of the type of preventive treatment (*p* = 0.857). The improvement was also significant in patients without depressive symptoms (*p* = 0.027) but related to improvement of HADS scores in participants with higher scores.

**Conclusion:** Cognitive complaints are a frequent and burdensome manifestation of migraine, reflecting its broader ictal and interictal impact beyond pain. This study shows that effective preventive treatment is associated with significant improvement in these cognitive symptoms, reinforcing their role as part of the migraine spectrum.

**Disclosure:** Nothing to disclose.

## EPO‐1022

### Assessment of cortical excitability in migraine patients using the paired‐pulse transcranial magnetic stimulation technique

#### M. Kutusheva ^1^; E. Sokolov^1^; N. Grigoryan^1^; A. Pyatkov^1^; N. Vashchenko^2^; A. Sergeev^1^

##### ^*1*^ *I.M. Sechenov First Moscow State Medical University of the Ministry of Health of the Russian Federation (Sechenov University), Moscow, Russian Federation;* ^*2*^ *University Headache Clinic, Russian Federation*

**Background and aims:** Thalamocortical dysrhythmia, observed in migraine, is often linked to cortical hyperexcitability. This pattern also appears in fibromyalgia, etc. This study examines neurophysiological markers in migraine via short‐interval intracortical inhibition assessed with the threshold tracking technique (TT‐SICI).

**Methods:** Participants included 15 patients with chronic migraine (CM; mean age 37.8 years; 86.6% female), 9 with episodic migraine (EM; mean age 34.5 years; 77.3% female), and 16 healthy controls (C; mean age 28.3 years; 68.8%female). Bilateral paired‐pulse TMS targeted the primary motor cortex. Motorevoked potentials were recorded from hand muscles to evaluate threshold dynamics at interstimulus intervals (ISIs) of 2–7 ms using TT‐SICI. Validated tools included PHQ‐9, PDS (Personality Disorder Severity), clinical interviews for headache/psychiatric status, and the Edinburgh Handedness Inventory (EHI).

**Results:** A significant TT‐SICI reduction occurred in CM versus C only in the left hemisphere at ISIs of 3.5 and 4 ms (*p* < 0.05). No differences appeared at other ISIs, during right‐hemisphere stimulation, or in EM versus controls. All subjects were right‐handed (EHI). In CM, positive correlations emerged between TT‐SICI and headache intensity at ISIs 3, 3.5, and 7 ms (*r* = 0.415, 0.596, 0.438); with PHQ‐9 at 3.5 ms (*r* = 0.430); and with PDS at 5 ms (*r* = 0.420).

**Figure:**
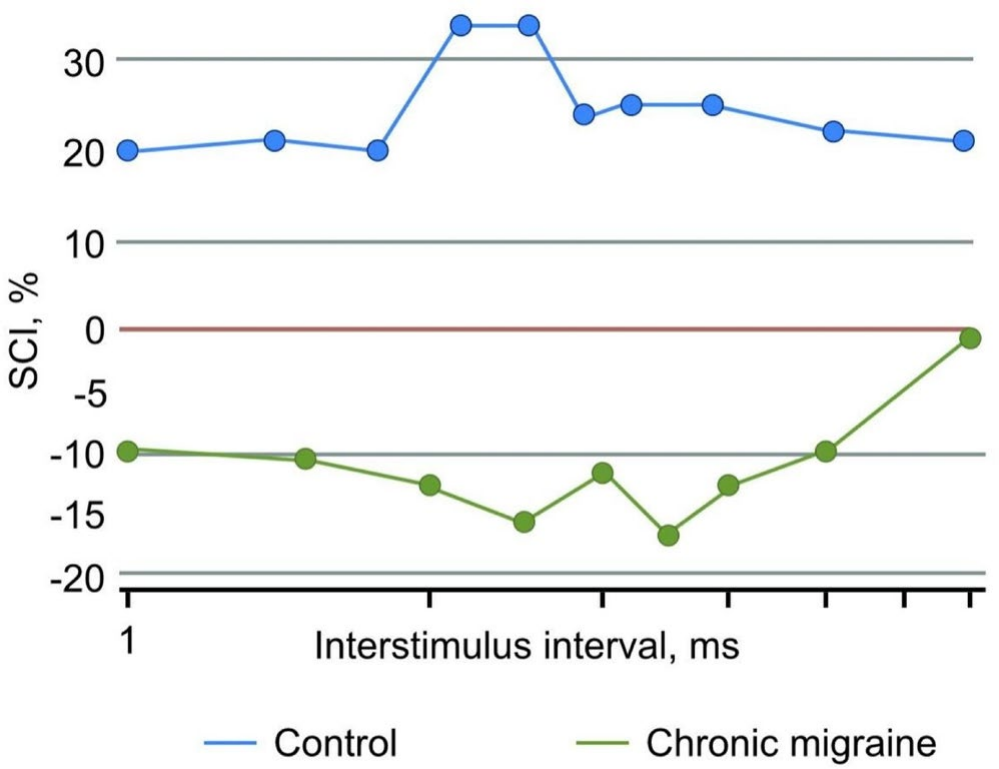
**FIGURE 1** Individual inhibition curves (CM vs C).

**Conclusion:** Reduced TT‐SICI at 3.5–4 ms in the left hemisphere CM patients indicates intracortical hyperexcitability and GABAergic inhibition deficit. Positive correlations with depression (PHQ‐9), personality disorder severity (PDS), and headache intensity suggest shared mechanisms linking emotional disturbances and chronic headache. These results reinforce central sensitization in CM and highlight TT‐SICI as a potential biomarker for personalized assessment and treatment monitoring.

**Disclosure:** Nothing to disclose.

## EPO‐1023

### Inflammatory markers and white matter lesions on brain MRI in patients with migraine

#### S. Jorquera‐Ortega^1^; A. Barbosa^2^; S. Sánchez^3^; M. Conversa^2^; P. Vera^4^; Á. Planchuelo‐Gómez^5^; Á. Sierra^6^; Y. González Osorio^6^; J. Matesanz^6^; Á. Guerrero‐Peral^6^; H. de la Fuente^4^; A. Gonzalez‐Martinez ^1^

##### ^1^Facultad de Medicina, Universidad Autónoma de Madrid, Madrid, Spain;Hospital Universitario de la Princesa, Madrid, Spain; Instituto de Investigación Sanitaria Princesa (IIS‐Princesa), Madrid, Spain; ^2^Neuroradiology Service, Hospital Universitario de la Princesa, Madrid, Spain; ^3^Laboratory Service, Hospital Universitario de la Princesa, Madrid, Spain; ^4^Immunology Service, Hospital Universitario de la Princesa, Madrid, Spain; ^5^Laboratorio de Procesado de Imagen, UVA, Valladolid, Spain; ^6^Headache Unit, Neurology Service, Hospital Clínico Universitario de Valladolid, Valladolid, Spain

**Background and aims:** Brain magnetic resonance imaging (MRI) frequently reveals white matter lesions (WML) in patients with migraine. Low grade systemic inflammation has been proposed as a contributing factor in migraine pathophysiology and may be associated with structural brain changes on MRI.

**Methods:** We performed a retrospective observational study including patients diagnosed with migraine with available brain MRI and blood analysis. Patients were classified as migraine with aura (MA) or migraine without aura (MWO), episodic migraine (EM) or chronic migraine (CM). Brain MRI were reviewed to evaluate the presence of white matter lesions. Laboratory parameters obtained from routine blood analysis included platelet count, neutrophils, lymphocytes, monocytes, C‐reactive protein (CRP), and erythrocyte sedimentation rate (ESR). Inflammatory ratios were calculated, including platelet‐to‐lymphocyte ratio (PLR), neutrophil‐to‐lymphocyte ratio (NLR), and lymphocyte‐to‐monocyte ratio (LMR). Descriptive and comparative analysis were performed.

**Results:** A total of 62 patients were included, 23/62 (37%) presented WML on brain MRI. WML were significantly more frequently present in patients with MA than in those with MWO [19/23 (83%) vs 15/39 (38%), *p* < 0.001]. Patients with WML showed significantly higher CRP levels compared to those without WML [0.28 (0.21–0.48) vs 0.10 (0.09–0.18), *p* < 0.001]. No significant differences were observed in other inflammatory parameters.

**Conclusion:** Our results show that WML are more prevalent in patients with MA and are associated with higher systemic inflammation, as reflected by elevated CRP levels. These findings may suggest the presence of inflammatory activity in patients with WML, highlighting the potential role of immune biomarkers in the underlying pathophysiology of WML which warrants further research.

**Disclosure:** ISCIII, EU and FSE funding through JR23/0005;PI24/01085.

## EPO‐1024

### Headache outcomes on the first day of fasting with once‐daily Rimegepant: A post‐hoc exploratory analysis

#### T. Alsaadi ^1^; G. Ifergane^2^; R. Suliman^1^; I. Al Qaisi^1^; J. Yang^3^; T. Fullerton^4^; D. Chou^5^; E. Whalen^4^; N. Vainstein^6^; D. Davidovici^7^; M. Tirosh^7^; A. Chan^8^; I. Rehman^9^; A. Hussain^10^

##### ^1^American Center for Psychiatry and Neurology, Abu Dhabi, UAE; ^2^Soroka Medical Center, Beer‐Sheva, Israel; ^3^Pfizer Inc, New York, USA; ^4^Pfizer Inc, Groton, USA; ^5^Pfizer Inc, Cambridge, USA; ^6^Pfizer, Buenos Aires, Argentina; ^7^Pfizer Pharmaceuticals Israel Ltd, Herzliya, Israel; ^8^Pfizer Healthcare Ireland, Dublin, Ireland; ^9^Rutgers University, New Brunswick, USA; ^10^Touro College of Pharmacy, New York, USA

**Background and aims:** Rimegepant was evaluated previously for short‐term prevention of fasting‐triggered headache during Ramadan, with outcomes assessed over weekly intervals. This post‐hoc analysis examined daily outcomes over the initial week of fasting, focusing on day 1, to evaluate rimegepant's potential for prevention of headache triggered by single‐day fasting for religious or medical reasons.

**Methods:** Participants were randomized to open‐label once‐daily rimegepant 75 mg from weeks 1–4 (Immediate Start; *n* = 52) or 2–4 (Staggered Start; *n* = 53) of the fast. The proportion of participants with headaches by pain intensity (none, mild, moderate, severe), the proportion with moderate‐to‐severe headaches, and the duration of moderate‐to‐severe headaches was compared between groups using nominal p‐values.

**Results:** Headache intensity on day 1 was lower in the Immediate Start arm than the Staggered Start arm (*p* = 0.015), with more participants reporting no headache (50.6% vs 27.8%) and fewer participants reporting moderate (21.0% vs 33.3%) or severe (8.0% vs 18.9%) headache. Fewer participants experienced moderate‐to‐severe headache on day 1 in the Immediate Start arm than the Staggered Start arm (difference [95% CI]: −28.8% [−49.1%, −8.5%]; *p* = 0.009). Moderate‐to‐severe headache duration on day 1 was shorter in the Immediate Start arm than the Staggered Start arm (difference [95% CI]: −1.9 [−4.3, 0.5] hours), although the difference was not significant (*p* = 0.117). Similar trends were observed across the initial week of fasting (not shown).

**Conclusion:** Rimegepant improved headache‐related outcomes on day 1 in individuals with fasting‐triggered headache. These exploratory findings support further evaluation of rimegepant's potential for prevention of headache triggered by single‐day fasting.

**Disclosure:** This study was conducted as a collaboration between the American Center for Psychiatry and Neurology (ACPN; Abu Dhabi, UAE) and Pfizer. The ACPN funded the study and is the study sponsor. Rimegepant was supplied by Pfizer. Tauofik Alsaadi has served on an advisory board for Pfizer. Reem Suliman and Ibrahim Al Qaisi have no conflicts of interest to declare. Gal Ifergane has received consulting fees and honoraria from Teva, Novartis, Eli Lilly, Pfizer, AbbVie, Organon, and Lundbeck; and has received research support from Teva and Pfizer. Jiyue Yang, Terence Fullerton, Denise Chou, Ed Whalen, Nora Vainstein, Danna Davidovici, Matanya Tirosh, and Anthony Chan are (or were at the time of the study) employees of Pfizer and may own stock/options in Pfizer. Ifra Rehman is a post‐doctoral fellow at Pfizer. Azhar Hussain is a student at Touro College of Pharmacy.

## Clinical Neurophysiology 1

## EPO‐1025

### Neurovascular reactivity during mental stress in narcolepsy with cataplexy: A microneurographic study

#### A. Furia; R. Liguori; V. Donadio

##### Dipartimento di Scienze Biomediche e Neuromotorie, Università di Bologna, Italy

**Background and aims:** Mental stress induces an arousal reaction and a subsequent response on various physiological parameters such as blood pressure (BP). Previous studies with microneurography, a technique allowing for in vivo nerve registration of muscle sympathetic nerve activity (MSNA), have shown that individuals with inhibited MSNA after arousal (“responders”) showed a lesser BP response when compared to “non‐responders”. In narcolepsy with cataplexy (NC), microneurography has shown a deranged sympathetic activation during REM sleep: the aim of the study is to assess whether a similar occurrence is found in wake patients.

**Methods:** 15 male patients with NC (aged 37 ± 11 years) and 15 male healthy controls (aged aged 36 ± 11 years) were subjected to microneurography of the peroneal nerve and concomitant recording of respiratory movements, blood pressure and electrodermal activity. Responders and non‐responders were defined by significant MSNA inhibition after electrical skin stimulation. All individuals were then recorded at rest, after mental arithmetic stress and after cold stimulation. BP variation was then analyzed between NC patients and healthy controls, as well as responders vs non‐responders between groups.

**Results:** 3 NC patients and 3 healthy controls could be classified as responders, thus showing no difference. As expected, stress induced a BP increase which was less elevated in responders, both in the NC and in the healthy control group.

**Conclusion:** In this study, wake NC patients showed no difference in sympathetic response to stress, which differed in responders vs non‐responders resulting in a different impact on blood pressure.

**Disclosure:** Nothing to disclose.

## EPO‐1026

### Direct current stimulation shapes metabolic phenotype of human i3 cortical neurons

#### S. Invernizzi^1^; M. Ghirimoldi^2^; V. Borrini^2^; E. Barberis^3^; E. Albi^4^; M. Piccoli^5^; A. De Grado ^6^; T. Bocci^6^; A. Priori^6^; M. Manfredi^2^; A. Ratti^1^; P. Signorelli^6^

##### ^1^Department of Medical Biotechnology and Translational Medicine, Università degli Studi di Milano, Milan, Italy; ^2^Department of Translational Medicine, University of Piemonte Orientale; Novara, Italy; ^3^Department of Sciences and Technological Innovation, University of Piemonte Orientale; Alessandria, Italy; ^4^Department of Pharmaceutical Sciences, University of Perugia, Perugia, Italy; ^5^School of Medicine, University Vita‐Salute San Raffaele, Via Olgettina, Milan, Italy; ^6^Aldo Ravelli Center for Neurotechnology and Experimental Brain Therapeutics, Department of Health Sciences, Università degli Studi di Milano, Milan, Italy

**Background and aims:** Transcranial direct current stimulation (DCS) is a promising disease‐modifying strategy in neurodegenerative disorders, offering a non‐invasive alternative to deep brain stimulation (DBS) and a complementary approach to pharmacological therapies. However, clinical outcomes remain heterogeneous, with responders and non‐responders, highlighting the need for mechanistic insight. At cellular level, we previously showed that DCS in neuroblastoma cells induces lipid metabolic remodeling, reduces inflammation, enhances mitochondrial oxidative metabolism and energy availability, and promotes extracellular vesicle release. We here aim at investigating the biological effects of DCS on human i^3^‐neurons using a multi‐omic approach.

**Methods:** Human i^3^‐neurons, generated from healthy donor iPSCs via doxycycline‐induced expression of an NGN2‐containing cassette, were stimulated with DCS (0.5 μA; ≈18 nA/cm^2^; 10 min), once or three times. Phenotypic modulation was assessed by mass spectrometry–based lipidomic and proteomic analyses and RNA sequencing.

**Results:** DCS induced a metabolic–nuclear response in i^3^‐neurons, affecting lipid mobilization and persistent modulation of the gene expression machinery, with proteomic enrichment of RNA processing, transcriptional control, chromatin remodeling, and genome stability pathways. A single stimulation elicited a transient response at 24 h, characterized by membrane lipid remodeling and stress‐metabolic proteins, that largely resolved by 72 h. In contrast, repeated stimulations stabilized a state marked by membrane reorganization, involving modulation of ether‐linked lipids and fatty acid unsaturation, mitochondrial–lipid metabolic coupling, and transcriptional regulation impacting cellular signaling activity.

**Conclusion:** Our integrated multi‐omics data show DCS induces biological changes on neuronal metabolism, signaling, and plasticity, supporting its clinical potential as a mechanism‐based neuromodulation strategy.

**Disclosure:** SI, nothing to declare MG, nothing to declare VB, nothing to declare EB, nothing to declare EA, nothing to declare MP, nothing to declare ADG, nothing to declare TB, nothing to declare AP, is founder and stakeholder of Newronika srl (Milan, Italy), a Company that develops and produces tDCS devices. MM, nothing to declare AR, nothing to declare PS, nothing to declare.

## EPO‐1027

### Towards a neurophysiological biomarker of neurotoxicity in Charcot‐Marie‐tooth disease

#### A. De Grado ^1^; V. Iacobelli^1^; C. Pisciotta^2^; C. Krarup^4^; H. Tankisi^5^; A. Priori^3^; P. Lanteri^1^; M. Moldovan^4^; D. Pareyson^2^

##### ^1^Neurophysiology Unit, Fondazione IRCCS Istituto Neurologico "Carlo Besta", Milan, Italy ‐ Università degli Studi di Milano, Milano, Italy; ^2^Unit of Rare Neurological Diseases, Fondazione IRCCS Istituto Neurologico “Carlo Besta”, Milan, Italy; ^3^Aldo Ravelli Center for Neurotechnology and Experimental Brain Therapeutics, Department of Health Sciences, University of Milan, Milan, Italy; ^4^Clinical Neurophysiology, Department of Clinical Neurophysiology, Rigshospitalet, Copenhagen, Denmark; ^5^Department of Clinical Neurophysiology, Aarhus University Hospital, Aarhus, Denmark

**Background and aims:** Demyelinating Charcot‐Marie‐Tooth (CMT) is characterized by progressive demyelination with secondary axonal degeneration. Although axonal loss is the primary cause of neurological disability, marked phenotypic variability exists and the underlying neurotoxic mechanisms remain poorly understood. We explored the relationship between CMT severity and motor axonal properties preceding degeneration.

**Methods:** Patients with CMT1A (*N* = 11), CMT1B (*N* = 5), and CMTX1 (*N* = 4) underwent conventional nerve conduction studies supplemented with median motor axon excitability testing at the wrist using threshold‐tracking (TRONDNF protocol). Axonal excitability parameters were correlated with the Charcot‐Marie‐Tooth Examination Score version 2 (CMTESv2) using Spearman correlation and linear regression.

**Results:** Across groups, several axonal excitability parameters correlated with CMTESv2, with subgroup‐specific regression patterns. For instance, refractoriness at 2 ms of the recovery cycle was positively associated with CMTESv2 in CMT1A and negatively in CMT1B, with regression slopes differing significantly (*t* = −2.906, *p* = 0.013). Temperature did not differ across subgroups (*p* = 0.46).

**Conclusion:** Refractoriness at 2 ms primarily reflects nodal voltage‐gated sodium channel (VGSC) function, which is independent of passive membrane properties. Translational studies in CMT1B show that dysmyelination is associated with VGSC dysregulation, increasing motor axon vulnerability to activity‐dependent degeneration. Notably, refractoriness changes occurred in opposite directions in CMT1A and CMT1B, enabling differentiation between these conditions, yet both indicate altered nodal VGSC function, supporting VGSC dysregulation as a shared pathophysiological mechanism across CMT subtypes. Recovery cycle refractoriness therefore reflects neuropathy severity in demyelinating and intermediate CMT and may serve as a neurophysiological biomarker of neurotoxicity, with implications for prognostication and assessment of therapeutic efficacy in future disease‐modifying trials.

**Disclosure:** Nothing to disclose.

## EPO‐1028

### Diagnostic ultrasound‐induced changes of motor cortical excitability in humans

#### E. Sartirana ^1^; S. Annaloro^2^; M. Guidetti^3^; A. De Grado^3^; S. Marceglia^3^; M. Bologna^4^; A. Priori^3^; T. Bocci^3^

##### ^1^Clinical Neurology Unit, “Azienda Socio‐Sanitaria Territoriale Santi Paolo E Carlo”, Department of Health Sciences, University of Milan, Milan, Italy; ^2^Faculty of Medicine, Department of Health Sciences, University of Milan, Milan, Italy; ^3^"Aldo Ravelli" Center for Neurotechnology and Experimental Brain Therapeutics, Department of Health Sciences, University of Milan, Milan, Italy; ^4^Department of Human Neurosciences, Sapienza University of Rome, Rome, Italy

**Background and aims:** This proof‐of‐concept study investigates whether certified diagnostic ultrasound effectively modulates cortical excitability. By targeting primary motor cortex, we demonstrate that conventional clinical protocols induce neuroplastic changes, potentially bypassing the experimental hardware hurdles and making transcranial ultrasound (TUS) immediately accessible for hospital applications.

**Methods:** Sixteen healthy volunteers (31.9 ± 11.0 years) participated in a sham‐controlled crossover study. Using a certified DWL Multi‐Dop X4 clinical system, 30 minutes sonication was delivered through the right transtemporal window via a 2.0 MHz linear probe, targeting the Abductor Pollicis Brevis (APB) hotspot at a depth of 45–58 mm. Cortical excitability was mapped via paired‐pulse Transcranial Magnetic Stimulation (TMS), measuring Short‐Interval Intracortical Inhibition (SICI), Intracortical Facilitation (ICF), and Long‐Interval Intracortical Inhibition (LICI). Resting Motor Threshold (RMT) and Breath‐Holding Index (BHI) monitored mechanical or vascular artifacts. Non‐parametric tests and Bayesian inference (Bayes Factor, BF) quantified the evidence.

**Results:** Post‐insonation, SICI was significantly reduced in the stimulated hemisphere (T0: 65.0%, T1: 67.5%; *p* = 0.016, BF₁₀ = 7.65), indicating increased local excitatory drive. Conversely, the control hemisphere showed a robust LICI increase (T0: 54.0%, T1: 86.0%; *p* = 0.010, BF = 7.88). Inter‐hemispheric comparisons confirmed that the reduction in inhibition was specific to the stimulated side (*p* = 0.046, BF = 3.90). No significant changes occurred in ICF, RMT, or BHI.

**Conclusion:** Diagnostic ultrasound elicits focal neuroplastic changes, likely via Gamma‐Aminobutyric Acid‐B (GABA‐B) pathways. This represents the first evidence that standard clinical Doppler system can selectively modulate the motor cortex, traditionally requiring high‐costly experimental hardware, establishing standard sonography as a viable neuromodulation tool ready for immediate clinical translation.

**Disclosure:** The authors declare that this research was conducted in the absence of any commercial or financial relationships that could be constructed as a potential conflict of interest.

## EPO‐1029

### Electrocortical changes due to vibro‐tactile stimulation in patients with cervical dystonia

#### G. Ruocco ^1^; G. Leodori^1^; M. Costanzo^1^; F. Martino^1^; D. Maccarrone^1^; B. Sellani^1^; G. Ferrazzano^1^; D. Belvisi^1^; G. Fabbrini^1^; J. Konczak^2^; A. Berardelli^1^; A. Conte^1^

##### ^1^Department of Human Neurosciences, Sapienza University of Rome, Italy; ^2^School of Kinesiology, Human Sensorimotor Control Laboratory, University of Minnesota, Minneapolis, USA

**Background and aims:** Cervical dystonia (CD) involves involuntary, sustained, or intermittent neck muscle movements. Although its pathophysiological remain unclear, increasing evidence points to altered sensory processing, particularly in the tactile and proprioceptive domains. Vibro‐tactile stimulation (VTS) has been proposed as a non‐invasive neuromodulatory intervention. This study investigated the clinical effects of VTS in CD and the associated cortical and cortico‐muscular correlates.

**Methods:** 10 CD patients underwent 64‐channel EEG and surface EMG recordings from bilateral sternocleidomastoid and trapezius muscles. Head kinematics were measured using a wireless inclinometer, with the head angle index (HAI) as outcome. During a preliminary session, 1‐minute VTS was applied to each of four muscles; the one producing maximal HAI improvement was selected. Patients then completed a randomized cross‐over protocol with three 5‐minute conditions: rest with inactive VTS, active VTS, and sham VTS (5 seconds per minute). EEG and EMG were analyzed for spectral power (theta, alpha, beta), cortico‐muscular coherence (CMC), and intermuscular coherence (IMC).

**Results:** Active VTS progressively reduced HAI relative to baseline, significantly more than sham. During active‐VTS EEG over the contralateral sensorimotor cortex showed increased alpha power and a trend toward reduced theta. Theta CMC decreased and alpha CMC increased during VTS. IMC between homologous muscles showed a non‐significant trend toward increased theta coherence.

**Conclusion:** VTS applied to cervical muscles improves dystonic posture in CD, associated with modulation of cortical alpha/theta rhythms and cortico‐muscular and intermuscular coupling. These findings support VTS as a promising non‐invasive neuromodulatory strategy targeting sensory dysfunction in cervical dystonia.

**Disclosure:** The authors declare no conflict of interest

## EPO‐1030

### Pupillary light reflex index in insomnia disorder: An index of daytime autonomic arousal

#### E. Fasiello^1^; E. Cini^2^; F. Bolengo^2^; S. Catena^2^; L. Dell'Acqua^2^; G. D'Este^1^; C. Leitner^2^; F. Casoni^2^; P. Proserpio^2^; V. Castronovo^2^; L. De Gennaro^3^; A. Galbiati^2^; L. Ferini‐Strambi ^2^

##### ^1^IUSS Cognitive Neuroscience (ICON) Center, Scuola Universitaria Superiore IUSS, Pavia, Italy; ^2^Department of Clinical Neurosciences, Neurology‐Sleep Disorders Center, IRCCS San Raffaele Scientific Institute, Milan, Italy; ^3^Department of Psychology, Sapienza University of Rome, Rome, Italy

**Background and aims:** The present study investigates whether dynamic Pupillary Light Reflex (PLR) metrics can serve as daytime physiological markers of arousal dysregulation in Insomnia Disorder (ID).

**Methods:** To evaluate diurnal autonomic nervous system activity, this study used the PLR as a noninvasive index of arousal. A Psychomotor Vigilance Test (PVT) was performed as a behavioral measure of objective daytime sleepiness, hypothesizing that, compared to Healthy Controls (*n* = 24; mean (*M*) = 49.87; standard deviation (SD) = 11.75; 15F), ID patients (*n* = 29; M = 49.03; SD = 11.07; 21F) would show objective signs of altered daytime arousal.

**Results:** The non‐parametric t‐test showed no significant group differences in PVT performance. Considering PLR metrics, ID patients showed a faster pupil re‐dilation after constriction, alongside a slower return of the pupil to 50% of its original diameter. Spearman's correlations were performed to evaluate the association between the PLR indices and clinical features. Results demonstrate positive associations between pupil re‐dilation after constriction and time for the pupil to return to 50% of its original diameter, more severe ID symptoms, higher pre‐sleep arousal, and more dysfunctional beliefs about sleep.

**Conclusion:** The results confirm increased daytime arousal state in individuals with insomnia, supporting the use of pupillometry to assess physiological alterations in this condition.

**Disclosure:** Nothing to disclose.

## EPO‐1031

### Propofol anesthesia induces a brain state of intermediate consciousness: Insights from EEG‐based time‐evolving functional brain networks

#### N. Dissouky ^1^; T. Bröhl^1^; A. Delis^2^; M. Thudium^2^; C. Helmstaedter^1^

##### ^1^Universität Bonn, Universitätsklinikum Bonn, Epileptologie, Bonn, Germany; ^2^Universität Bonn, Universitätsklinikum Bonn, Klinik für Anästhesiologie und Operative Intensivmedizin, Bonn, Germany

**Background and aims:** Consciousness depends on the brain's ability to balance excitability and stability [1, 2]. While non‐human primate studies suggest propofol disrupts cortical dynamics [3], its impact on dynamic network stability in humans remains unclear. We investigated how propofol alters cortical network stability and dynamics relative to sleep and wakefulness using EEG‐derived time‐evolving functional brain networks (TEFBNs), capturing dynamic changes in brain organization associated with consciousness [4].

**Methods:** We recorded EEG time‐series (10–20 system) from adults across three groups: healthy controls during wakefulness (*n* = 51), nighttime sleep (*n* = 14), and propofol anesthesia (*n* = 10). EEG in the anesthesia group was acquired prior electroconvulsive therapy (ECT) in depressed patients. Baseline network organization did not differ from controls, indicating no clinical influence. TEFBNs were constructed, and network‐based measures of stability and dynamics were analyzed.

**Results:** Under propofol anesthesia, subjects occupied an intermediate brain state that was neither fully awake nor asleep. Although slow‐wave activity resembled NREM sleep, TEFBNs revealed a distinct network organization. Bilateral cortical regions showed equal temporal importance, indicating increased local synchronization and reduced functional differentiation. Network metrics further demonstrated subtle destabilization of cortical dynamics not observed during natural sleep or wakefulness, suggesting that propofol induces a state of symmetric local coherence despite disrupted global integration.

**Conclusion:** These findings extend primate work showing that propofol impairs consciousness by destabilizing cortical networks. To our knowledge, this is the first human evidence that propofol produces an intermediate state that is awake‐like yet exhibits sleep‐like slow waves, reflecting a mechanism distinct from natural sleep, not merely a deeper stage of it.

**Disclosure**: This work was supported by the Marga und Walter Boll‐Stiftung Nr. 2020‐03.01–21.

## EPO‐1032

### Pathophysiology of fatigue in chronic inflammatory demyelinating polyradiculoneuropathy: The importance of proximal demyelination

#### N. Başcı ^1^; M. Tutuncu^1^; N. Uzun Adatepe^2^; A. Gündüz^2^

##### ^1^Department of Neurology, Cerrahpasa Faculty of Medicine, Istanbul University‐Cerahpasa, Istanbul, *Türkiye;* ^2^Department of Clinical Neurophysiology, Cerrahpasa Faculty of Medicine, Istanbul University‐Cerahpasa, Istanbul, *Türkiye*

**Background and aims:** Fatigue is a complex symptom that has multiple mechanisms from central nervous system to immunological processes. We hypothesize that fatigue's more pronounced in patients with proximal conduction blocks and proximal demyelinating and the involvement of cranial nerves.

**Methods:** CIDP patients were evaluated for fatigue and underwent electrophysiological investigations. Bilateral routine NCS, proximal assessments of median (wrist‐elbow‐axilla) and ulnar nerve (wrist‐elbow‐aboveelbow‐axilla), motor recordings on nasalis and trapezius muscles (stimulating from facial and spinal‐accessory nerve), blink‐reflex testing were performed. Conduction‐blocks, conduction‐slowing, F/A‐waves in proximal‐distal segments were evaluated. Fatigue was assessed with Krupp‐Fatigue‐Severity‐Scale (FSS) (<2.8:no fatigue, >6.1:chronic, 2,8‐6,1:mild).

**Results:** Ten CIDP patients were evaluated (mean age 49.8 ± 8.3). The mean FSS score was 4.32 ± 1.3. The mean INCAT score was 2.2 ± 1.8. Chronic fatigue was present in 20%, 10% had no fatigue. The mean number of conduction blocks was 3.9 ± 1.8 (min–max 1–6). In patients with chronic fatigue, conduction blocks in the median and ulnar nerves were more frequently located in proximal segments compared with the non‐fatigued/mildly fatigued groups. There were no significant differences in nasalis‐trapezius recordings. Blink reflex latencies tended to be longer in fatigued patients.

**Conclusion:** These findings suggest that fatigue in CIDP may be associated with the topographic distribution of conduction blocks. Although no marked differences were detected in cranial nerve studies, the tendency toward prolonged blink reflex latencies in fatigued patients is notable in terms of central‐peripheral interactions. These results highlight the need to consider the distribution of conduction blocks to better understand the pathophysiology of fatigue in CIDP.

**Disclosure:** This study is supported by Health Institutes of Türkiye (TÜSEB) (Project Number: 32685).

## EPO‐1033

### Association of CSF kappa free light chain index with evoked potentials and MRI parameters in patients with multiple sclerosis

#### M. Kolak^1^; M. Krbot Skorić^2^; I. Adamec ^1^; B. Barun^1^; I. Martinez^2^; M. Sredanović^2^; K. Tešija^2^; Ž. Vogrinc^3^; A. Hrkać Pustahija^4^; M. Habek^1^; T. Gabelic^1^

##### ^1^School of Medicine, University of Zagreb, Zagreb, Croatia; ^2^Department of Neurology, Referral Centre for Demyelinating Diseases of Central Nervous System, University Hospital Centre Zagreb, Zagreb, Croatia; ^3^Faculty of Pharmacy and Biochemistry, University of Zagreb, Zagreb, Croatia*;* ^4^Department of Diagnostic and Interventional Neuroradiology, University Hospital Centre Zagreb, Zagreb, Croatia

**Background and aims:** Novel 2024 McDonald criteria included the kappa free light chain (KFLC) index as an additional cerebrospinal fluid (CSF) biomarker representing intrathecal inflammation as well as evoked potentials (EPs) demonstrating functional central nervous system pathway dysfunction. Aim of this study was to evaluate association of these biomarkers reflecting inflammation and neurodegeneration in early relapsing–remitting multiple sclerosis (RRMS).

**Methods:** Analysis included 52 consecutive treatment‐naïve people with newly diagnosed RRMS (34 females; mean age 37.7 ± 11.1 years; median Expanded Disability Status Scale [EDSS] 2.0). All patients underwent standardized diagnostic work‐up including brain and cervical spinal cord MRI, CSF analysis with KFLC index calculation and multimodal EP testing (visual EP [VEP], brainstem auditory EP [BAER], and median and tibial somatosensory EP [mSSEP, tSSEP]). Pathological findings of EP were summerized calculating an EP score.

**Results:** Higher KFLC index correlated with shorter VEP P100 latency and higher VEP amplitude on left‐eye stimulation, shorter BAER wave V latencies bilaterally, and with higher tSSEP amplitudes. Consistent with these findings, KFLC index values were significantly higher in participants with lower EP pathological burden (EP score classification; *p* = 0.01), and similarly across VEP‐ and tSSEP‐based pathological groupings (*p* = 0.015 and *p* = 0.008, respectively). No significant associations were observed between KFLC index and clinical or neuroradiological parameters in our cohort.

**Conclusion:** In newly diagnosed RRMS, higher intrathecal inflammatory activity was paradoxically associated with less neurophysiological pathway dysfunction. These findings suggest that KFLC index and EP scores represent different aspects of early MS neuropathology emphasising their complementary utilisation in fulfilment of new MS diagnostic criteria.

**Disclosure:** Nothing to disclose.

## Clinical Neurophysiology 2

## EPO‐1034

### The effect of demyelination patterns on clinical course and prognosis in CIDP

#### S. Ozdemir; E. Ozbezen Kiziltan; S. Alpaydin Baslo; A. Soysal

##### Department of Neurology, Bakirkoy Prof. Dr. Mazhar Osman Research and Training Hospital for Psychiatry, Neurology, and Neurosurgery, Istanbul, Türkiye

**Background and aims:** Chronic inflammatory demyelinating polyneuropathy (CIDP) is a heterogeneous immune‐mediated neuropathy characterized by multifocal demyelination affecting different nerve segments. Our study aimed to investigate the association between demyelination distribution patterns, clinical features, and prognosis in CIDP.

**Methods:** We retrospectively evaluated initial and follow‐up electrophysiological studies of 42 patients with CIDP (2000–2024) according to the 2021 EFNS/PNS criteria. Patients were classified as distal (distal latencies >150% of the upper limit of normal), intermediate (conduction velocity <70% of the lower limit of normal and/or conduction block or abnormal temporal dispersion), or diffuse (combined distal and intermediate abnormalities). Patients were categorized according to electrophysiological patterns, and longitudinal changes were analyzed in relation to clinical prognosis.

**Results:** Forty‐two patients were analyzed longitudinally. At baseline, electrophysiological patterns were classified as intermediate in 61.9%, diffuse in 28.5%, and distal in 9.6% of patients; 12.5% showed normalization at follow‐up. Distal pattern was associated with shorter symptom duration, higher INCAT and mRS scores, more frequent triggering events, and a higher rate of subacute onset (all *p* ≤ 0.036). Treatment was initiated 24.1 months earlier, partial remission lasted 51 months longer, and total disease duration was 11.6 years shorter in distal compared with intermediate pattern (all *p* ≤ 0.03). Monophasic course was more frequent with temporal dispersion (*p* = 0.023), whereas axonal degeneration predicted lower remission rates (*p* = 0.011).

**Figure:**
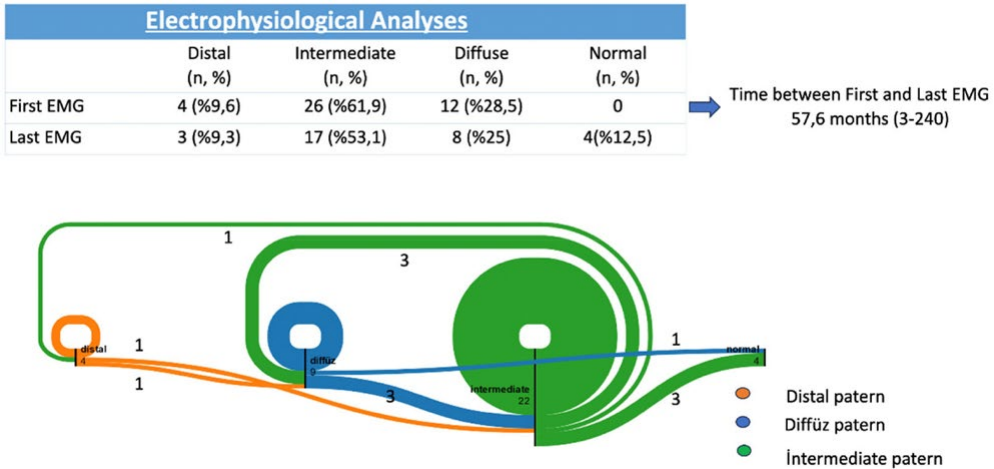
**FIGURE 1** Electrophysiological analysis: comparison between initial and final EMG results.

**Figure:**
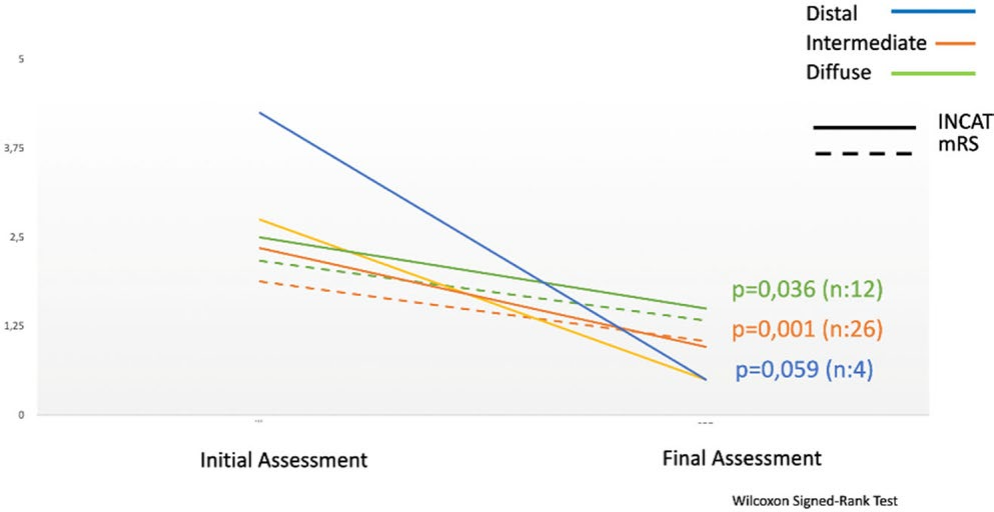
**FIGURE 2** Based on electrophysiological patterns after initial assessment, comparison of INCAT and mRS scores before and after treatment.

**Figure:**
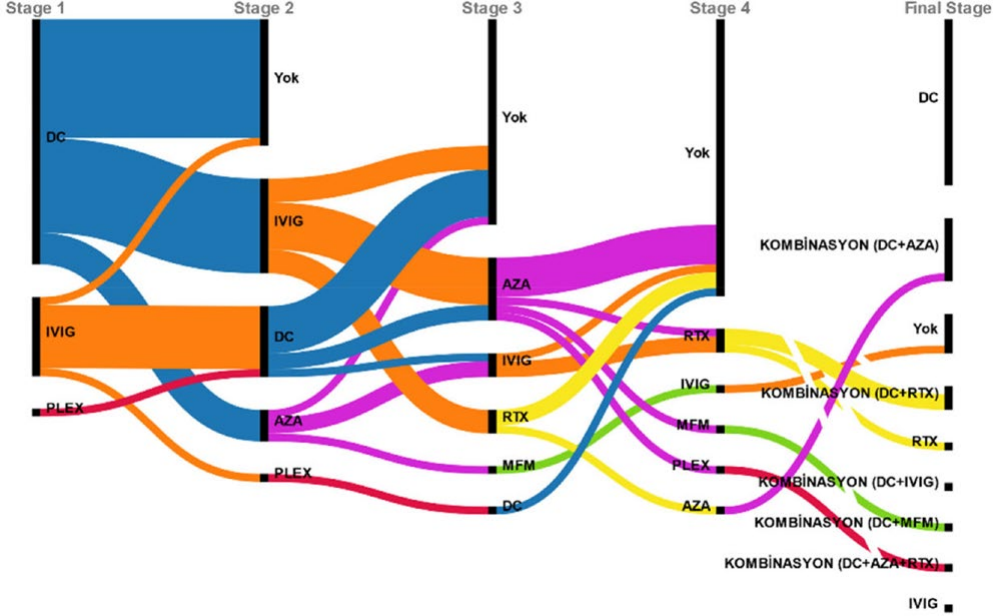
**FIGURE 3** Treatment records during initial treatment and follow‐up period.

**Conclusion:** Demyelination patterns significantly influence the clinical course and prognosis of CIDP, highlighting the prognostic value of electrophysiological pattern analysis.

**Disclosure:** Nothing to disclose.

## EPO‐1035

### Transient loss and recovery of dorsal rootlet excitability during selective dorsal rhizotomy

#### G. Zabrodzets ^1^; M. Talabaev^2^; Y. Rushkevich^1^

##### ^1^Department of Neurology, Republican Scientific and Practical Center of Neurology and Neurosurgery, Minsk, Belarus; ^2^Pediatric Neurosurgery Department, Republican Scientific and Practical Center of Neurology and Neurosurgery, Minsk, Belarus

**Background and aims:** Selective dorsal rhizotomy (SDR) is an established treatment for lower‐limb spasticity in children with cerebral palsy (CP). Intraoperative neurophysiological monitoring (IONM) guides the procedure, yet its protocols are debated. This study aimed to assess the dynamics of dorsal root (DR) excitability during SDR.

**Methods:** A retrospective analysis of IONM data from 15 children with CP (mean age 7.1 ± 4.3 years, GMFCS III‐V) was conducted. Surgery involved single‐level laminectomy (L1/L2) under total intravenous anesthesia. The IONM protocol determined the threshold stimulation intensity (TSI) using 3‐Hz pulses, followed by 50‐Hz train stimulation for each whole DR and its subsequently subdivided rootlets.

**Results:** Analysis of 150 DRs (L2‐S1) and 684 rootlets revealed significant inter‐root differences. The median TSI (in mA) for whole DRs was lowest at S1 (0.48, IQR 0.28–0.61) and highest at L4 (0.98, IQR 0.60–1.21). Subdivision into rootlets increased median TSI markedly, peaking at L4 (2.03, IQR 1.05–2.72). A key finding was transient loss of EMG response in 8 patients despite stimulation up to 5 mA, which was reversed by irrigation with warm saline during 5 min, leading to a significant decrease in recalculated TSI. Statistical analysis confirmed significant inter‐root differences at each procedural stage (Kruskal‐Wallis, *p* < 0.05) and a significant overall reduction in thresholds over time (Friedman test, *p* = 0.007), with the most pronounced decrease at L4 (from 2.03 to 1.22, *p* = 0.004).

**Conclusion:** The observed reduction in rootlet excitability may be attributed to specific surgical aspects of SDR. Thus, proper IONM interpretation is crucial to determine individual rootlet function at each segmental level.

**Disclosure:** Nothing to disclose

## EPO‐1036

### Ischemia resistance in pathophysiology of early diabetic motor and sensory polyneuropathy

#### M. Mroczek ^1^; S. Omidbakhsh^1^; A. Gramm Kristensen^2^; P. Karlsson^3^; P. Grafe^4^; H. Tankisi^5^

##### ^1^Department of Clinical Neurophysiology, Aarhus University Hospital, Aarhus, Denmark; ^2^Department of Clinical Neurophysiology, Aarhus University Hospital, Aarhus, Denmark; Danish Pain Research Center, Department of Clinical Medicine, Aarhus University, Aarhus, Denmark; ^3^Core Center for Molecular Morphology, Section for Stereology and Microscopy, Department of Clinical Medicine, Aarhus University, Aarhus, Denmark; Danish Pain Research Center, Department of Clinical Medicine, Aarhus University, Aarhus, Denmark; ^4^Institute of Physiology, Ludwig‐Maximilians University Munich, Germany; ^5^Department of Clinical Neurophysiology, Aarhus University Hospital, Aarhus, Denmark; Department of Clinical Medicine, Aarhus University, Aarhus, Denmark

**Background and aims:** Nerve excitability testing has shown resistance to ischemia in motor nerves after 10–30 minutes of compression as an early and specific finding in diabetic neuropathy. Sensory nerves have not previously been investigated. We hypothesize that resistance to ischemia is seen in motor and sensory nerves before clinical and electrophysiological signs of neuropathy.

**Methods:** We tested diabetic patients (10 sensory, 13 motor measurements, age 30–60 yrs) and compared them with healthy controls (12 sensory, 9 motor measurements). The following protocol was applied at two separate sessions, one for the motor and one for the sensory nerves: short TRONDNF including strength/duration time constant (SDTC), multitracking: 5 min pre‐ischemia, 5 min ischemia (induced with a pressure cuff), 10 min post‐ischemia. We have tracked 4 parameters: threshold decrease (in multitrack protocol), recovery cycle (RC) at 6 ms, TEd100, and TEh overshoot of threshold electrotonus.

**Results:** People with diabetes showed statistically highly significant less loss of axonal super‐excitability in both sensory and motor axons (SupEx isc motor −10.7 % vs. 1.3%, SupEx isch sensory −3.5 % vs. 13.9 %; *p* < 0.001). Preliminary results showed also that they differed from normal controls in ischemia‐induced changes in strength/duration time constant and in the time course of ischemia‐induced changes in threshold current.

**Conclusion:** Resistance to compression‐induced nerve ischemia in diabetic patients can be detected by the threshold current in the recovery cycle. The time course of ischemia‐induced changes in threshold current and an abnormal strength/duration time constant indicate that tissue acidification may contribute to nerve excitability parameters.

**Disclosure:** Nothing to disclose.

## EPO‐1037

### Functional relationship between large and small fibers in diabetic neuropathy

#### M. Baba ^1^; R. Haga^1^; I. Kinoshita^1^; M. Miura^1^; K. Hiyama^1^; Y. Murakami^1^; T. Ueno^1^; J. Matsui^2^; C. Suzuki^3^

##### ^1^Department of Neurology, Aomori Prefectural Central Hospital, Aomori City, Japan; ^2^Diabetes Centre, Aomori Prefectural Central Hospital, Aomori City, Japan; ^3^Department of Neurology, Hirosaki University School of Medicine, Hirosaki, Japan

**Background and aims:** Diabetic neuropathy (DN) has two different phenotypes, painful DN (PDN) and painless DN (PLDN). Small fiber (SF) damage has been suggested in pain production, however, role of large fibers (LF) remains unclear; We electrophysiologically investigated LF and SF functions in PDN and PLDN.

**Methods:** In 140 diabetics, we scaled SF and LF functions by electrodermal skin conductance (ESC) of SUDOSCAN and by nerve conduction study (NCS). NCS scale is defined as follows; NCS‐0: No NCS anomalies, NCS‐1: Prolonged F‐wave latency with normal SNAP & CMAP amplitudes, NCS‐2: Fall in SNAP less than 5 microvolts with normal CMAP, NCS‐3: Fall in CMAP amplitude to between 2mV and 5 mV, NCS‐4: Decrease in CMAP amplitudes less than 2mV.

**Results:** Fall in ESC and increase in the NCS‐scale showed positive correlation with coefficient 0.55. Among the cases with ESC lower than 60 micro‐siemens, 20% of the cases with NCS‐0 and NCS‐1 had pain, while 48% of the NCS‐2, ‐3, and ‐4 groups (*p* < 0.05) complained of pain. Among the NCS‐0 and ‐1 cases with lower ESC, PDN showed sural SNAP of 6.9 ± 1.2 μV, which was lower than 10.6 ± 4.1 μV of PLDN (*p* < 0.01).

**Conclusion:** In diabetes, electrophysiological functions of LF and SF generally decline in parallel. Even if the ESC decreases, pain is unlikely to occur if LF function is maintained normally. LF activate collateral inhibitory neurons in the dorsal horn of the spinal cord. Reduced impulse input from the LF might facilitate pain impulse transmission from the damaged SF.

**Disclosure:** None

## EPO‐1038

### Neurophysiological investigation of the bereitschaftspotential in healthy subjects during simple and complex, clonic and tonic movements

#### P. Antenucci ^1^; J. Moura^2^; K. Bhatia^3^; L. Rocchi^4^; A. Latorre^3^

##### ^1^Department of Neurosciences and Rehabilitation, University of Ferrara, Ferrara, Italy; ^2^Department of Neurology, Centro Hospitalar Universitário de Santo António, Unidade Local de Saúde de Santo António,Porto, Portugal; ^3^Department of Clinical and Movement Neurosciences, UCL Queen Square Institute of Neurology, London, UK; ^4^Department of Medical Sciences and Public Health, University of Cagliari, Cagliari, Italy

**Background and aims:** Recent evidence questions the clinical applicability of the Bereitschaftspotential (BP) due to limited study generalizability, multiple factors affecting BP morphology, and methodological heterogeneity, which collectively hinder definitive conclusions on BP onset timing and spatial distribution across body regions.

**Methods:** We cross‐sectionally examined the BP during voluntary hand, foot, and eye movements in healthy participants. Simple and complex hand movements, foot dorsiflexion, and eyelid contraction were recorded, including brisk (<0.5 s) and sustained (~1 s) actions. EMG onset at 50% maximal voluntary contraction served as the trigger, and BP was recorded via 64‐channel DC EEG. A 7‐second epoch window was applied, artifact‐free trials averaged, and BP morphology, distribution, and lateralization were qualitatively assessed using scalp maps and waveforms.

**Results:** Fifteen healthy participants (mean age 28.0 ± 4.5 years; 9 females) underwent BP recordings. A contralateral centro‐frontoparietal BP was observed during simple and complex clonic and tonic hand movements, whereas foot movements elicited a bilaterally distributed central BP with ipsilateral diffusion, and blink movements showed a bilateral frontal distribution (Figure 1; Table 1). Within‐subject analyses revealed an earlier onset of the early phase for complex hand and foot movements, and both earlier early phase onset and delayed late phase onset for tonic hand movements. No amplitude differences were observed (Table 2)

**Figure:**
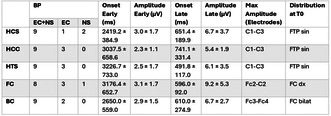
**TABLE 1** Morphological analysis of the BP from the recorded traces for each individual task. Data are presented as mean ± standard deviation and as counts.

**Figure:**
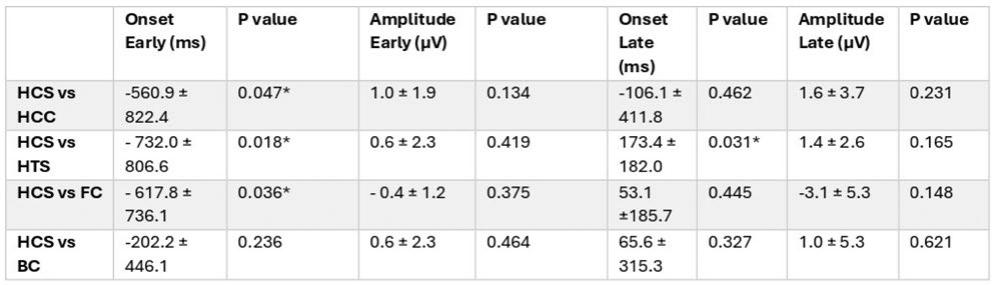
**TABLE 2** Results of the within‐subject comparison of recorded traces across trials versus the simple clonic hand movement. Data are presented as mean difference ± standard deviation.

**Figure:**
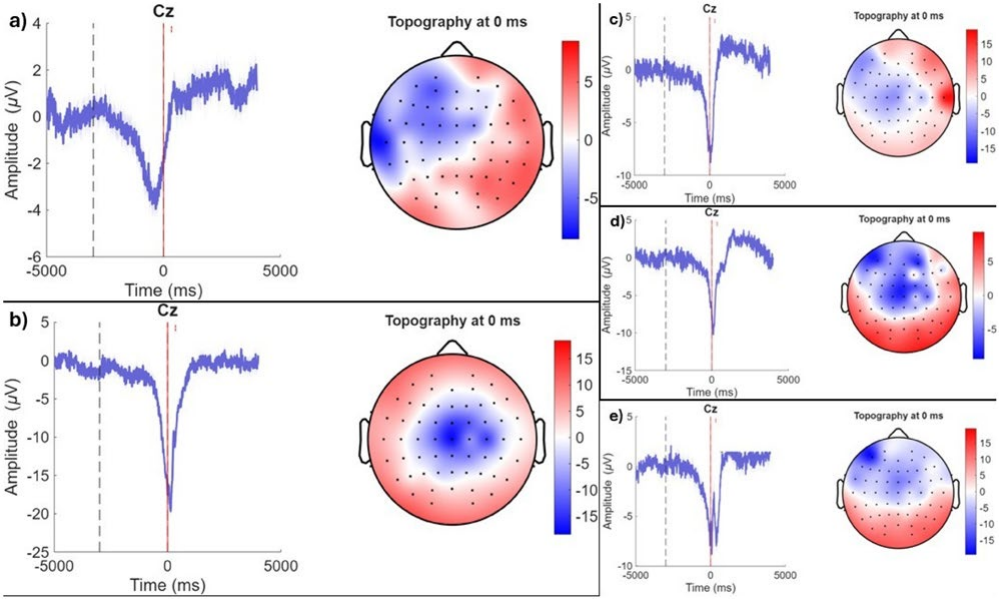
**FIGURE 1** BP recordings and colorimetric maps for: (a) simple clonic hand movement, (b) foot movement, (c) complex clonic hand movement, (d) tonic hand movement and (e) blink movement.

**Conclusion:** Several factors may influence differences in BP elicited by various movements. Studying normative models across healthy subjects is crucial to characterize BP topography, optimize electrode placement and analysis protocols, and provide a non‐invasive window into movement characteristics and potential pathological alterations.

**Disclosure:** Nothing to disclose.

## EPO‐1039

### Simulation of freezing of Gait in a virtual reality environment: An electrophysiological approach in Parkinson's disease

#### R. Unkun Altun ^1^; A. Geriş^2^; A. Gündüz^1^

##### ^1^Department of Neurology, Istanbul University‐Cerrahpaşa, Cerrahpaşa Faculty of Medicine, Istanbul, Türkiye; ^2^Department of Computer and Instructional Technologies Education, Faculty of Education, Manisa, Türkiye

**Background and aims:** This study investigated electrophysiological changes in motor and brainstem pathways under virtual reality (VR) conditions mimicking freezing of gait (FoG)‐triggering situations to better understand the pathophysiology of FoG in idiopathic Parkinson's disease (iPD).

**Methods:** In this prospective study (May 2024–August 2025), patients aged 40–80 years with iPD with FoG (iPD‐FoG), PD without FoG (iPD‐nFoG), and age‐ and sex‐matched healthy individuals were included. iPD diagnosis was based on Movement Disorder Society 2015 criteria, and FoG was assessed using established criteria. Clinical evaluation included the Unified Parkinson's Disease Rating Scale, Timed Up and Go test, auditory startle reflex, resting motor threshold, motor evoked potentials, and H‐reflex assessment. All evaluations were performed in the off‐medication state. Neurophysiological measurements were obtained at rest and during a VR condition designed to replicate the clinical setting of the Timed Up and Go test.

**Results:** Eight iPD‐FoG patients, ten iPD‐nFoG patients, and ten healthy individuals were included. Mean ages were comparable across groups. Resting motor threshold was lower in both iPD groups than in healthy individuals. During VR exposure, mean H/M ratios were higher in healthy individuals and iPD‐FoG patients than in iPD‐nFoG patients. Percentage increases in motor evoked potential amplitudes were greater in iPD‐FoG patients and healthy individuals than in iPD‐nFoG patients. Auditory startle reflex amplitudes decreased under VR conditions in all groups.

**Figure:**
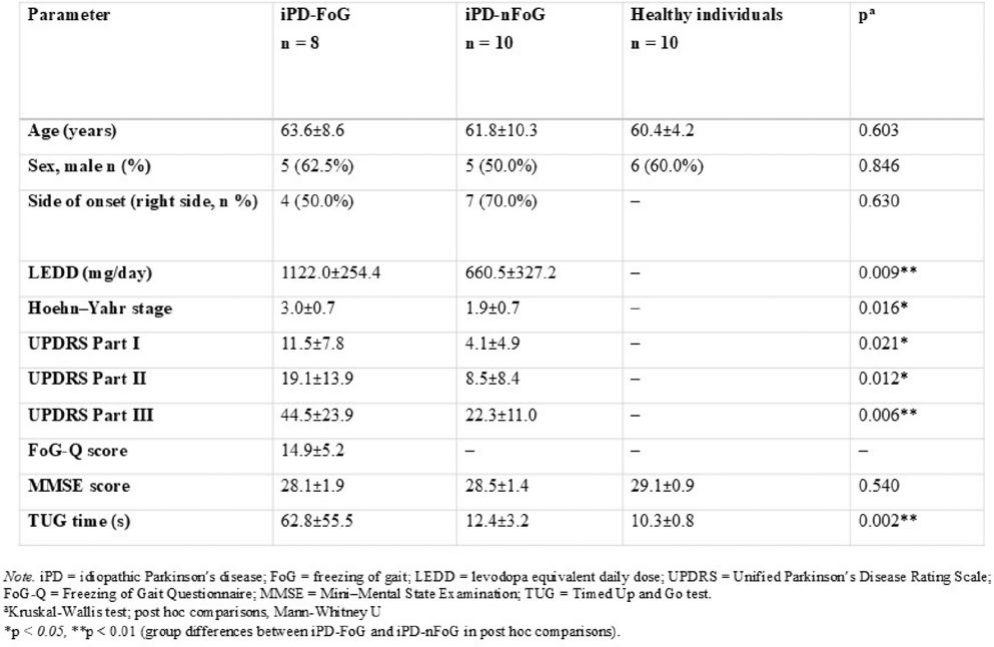
**TABLE 1** Demographic and clinical findings of the study groups: iPD‐FoG, iPD‐nFoG, and healthy individuals.

**Figure:**
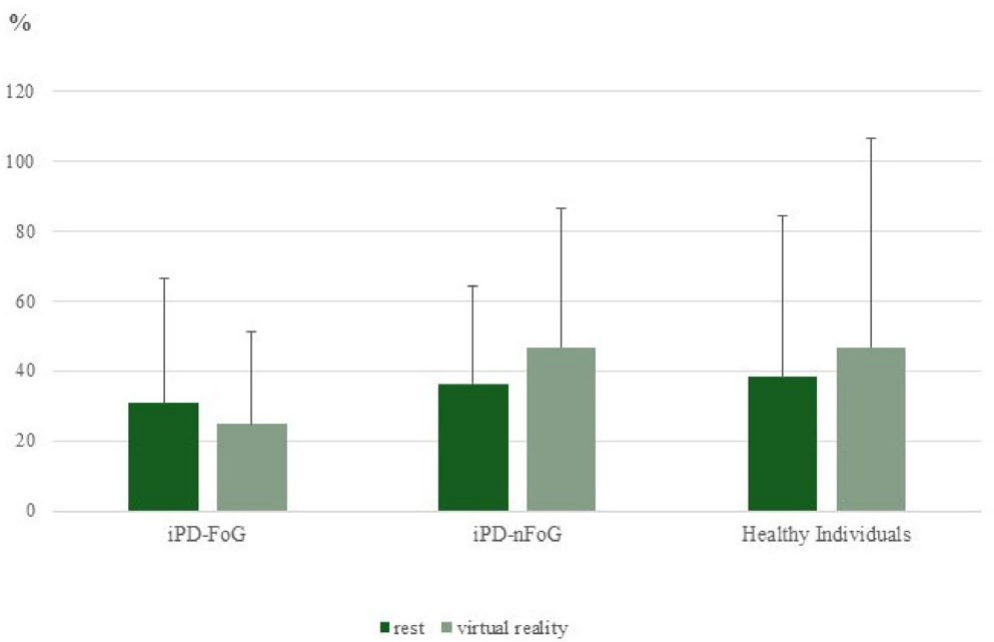
**FIGURE 1** Graphics of mean H/M ratios between rest and virtual reality conditions across the groups.

**Figure:**
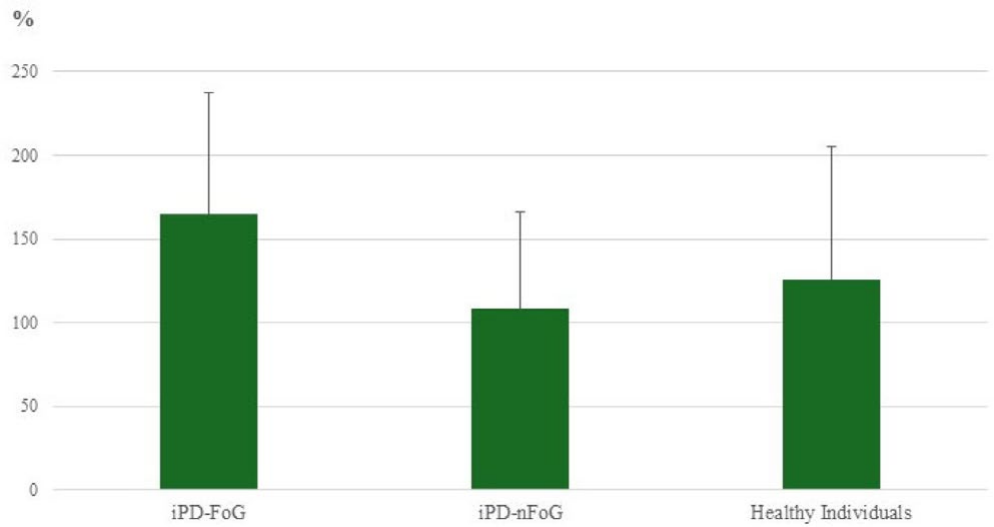
**FIGURE 2** MEP amplitude change (% of rest) in iPD‐FoG, iPD‐nFOG, and healthy individuals.

**Conclusion:** VR exposure induces corticospinal facilitation while suppressing brainstem pathways. Spinal excitability is facilitated in iPD‐nFoG patients, suppressed in iPD‐FoG patients, and remains unchanged in healthy individuals.

**Disclosure:** Nothing to disclose.

## EPO‐1040

### Improving indirect methods for calculating reference limits for nerve conduction studies using change point detection

#### T. Szczepanski ^1^; K. Nilsen^1^; Ø. Dunker^1^; A. Reiner^2^; T. Sand^3^; M. Tannemaat^4^; A. Yazidi^5^; K. Bach^6^; J. Zwart^7^; J. Jabre^8^; P. Omland^3^

##### ^1^Section for Clinical Neurophysiology, Department of Neurology, Oslo University Hospital, Oslo, Norway; ^2^Oslo Centre for Biostatistics and Epidemiology, Oslo University Hospital, Oslo, Norway; ^3^Department of Neurology and Clinical Neurophysiology, St. Olavs Hospital, Trondheim, Norway; ^4^Leiden University Medical Center, Netherlands; ^5^Oslo Metropolitan University, Oslo, Norway; ^6^Department of Computer Science, Norwegian University of Science and Technology, Trondheim, Norway; ^7^Faculty of Medicine, University of Oslo, Oslo, Norway; ^8^Department of Public Health and Community Medicine, Tufts University School of Medicine, Boston, USA

**Background and aims:** High‐quality reference limits for nerve conduction studies (NCS) are essential for accurate diagnosis of neuromuscular disorders. Traditionally, reference limits are derived from examinations of healthy individuals. This is expensive and time‐consuming, and it is challenging to include children and elderly. Alternative methods – extrapolated norms (E‐norms), extrapolated reference values (E‐Ref), and multivariate extrapolated reference values (MeRef) – estimate reference limits from historical hospital data. However, the performance of the current versions of these methods varies across different NCS measurements. We aimed to improve these methods by modifying them using changepoint detection.

**Methods:** The changepoint algorithm detects significant changes in a data sequence by evaluating different ways of segmenting the data based on segment mean and variance. The modified methods were used to calculate reference limits for common NCS measurements using a historical database of 24,816 patients. The results were compared with reference limits derived from 680 healthy subjects, and evaluated using the F1 score.

**Results:** The changepoint‐modified methods achieved greater F1 scores on average than the original methods for lower‐extremity NCS measurements (0.74 vs 0.64), but lower scores for upper‐extremity measurements (0.56 vs 0.66). In most cases, good performance required at least 500 measurements and datasets containing fewer than 20% abnormal values.

**Conclusion:** Changepoint detection improves the performance of E‐norms, E‐Ref, and MeRef methods for lower‐extremity nerves. These improvements are promising and may allow for more accurate diagnostics of neuromuscular disorders, in particular for children, elderly and people living in areas where obtaining local reference values from healthy subjects is unrealistic.

**Disclosure:** None of the authors has any conflict of interest to disclose. This work received financial support from the South‐Eastern Norway Regional Health Authority and the Norwegian Medical Association.

## EPO‐1041

### Identifying normality in historical datasets by multidimensional nerve conduction studies

#### T. Szczepanski ^1^; P. Omland^2^; A. Yazidi^3^; K. Bach^4^; J. Zwart^5^; K. Nilsen^1^

##### ^1^Section for Clinical Neurophysiology, Department of Neurology, Oslo University Hospital, Oslo, Norway; ^2^Department of Neurology and Clinical Neurophysiology, St. Olavs Hospital, Trondheim, Norway; ^3^Oslo Metropolitan University, Oslo, Norway; ^4^Department of Computer Science, Norwegian University of Science and Technology, Trondheim, Norway; ^5^Faculty of Medicine, University of Oslo, Oslo, Norway

**Background and aims:** Reference limits are essential for correctly interpreting nerve conduction studies (NCS). Traditionally, these limits are established using large groups of healthy individuals which is expensive, logistically demanding, and raises ethical concerns. Alternatively, reference limits can be estimated indirectly using historical hospital data containing a mixture of normal and abnormal measurements. Most current alternative methods assess each NCS measurement separately when trying to distinguish normal from abnormal results. In this study, we introduce a novel method – MultiNorm – that instead identifies healthy nerves.

**Methods:** We combined different types of historical NCS measurements from individual nerves into multidimensional maps and identified regions most likely to represent healthy nerves. Measurements from the presumed healthy nerves were then used to establish reference limits for commonly used NCS and compared with traditional reference limits (from *N* = 680 healthy individuals) and four recently published indirect methods (E‐norms, E‐Ref, MeRef, MMC) using F1 score.

**Results:** MultiNorm achieved a total average F1 score of 0.81, 0.13 higher than the second‐best performing method, extrapolated norms. Reliable performance required datasets containing at least 500 measurements. Unlike the other methods, the performance of MultiNorm remained stable until the proportion of abnormal measurements exceeded 50%, 30% higher than the tolerance of the other approaches.

**Conclusion:** The MultiNorm approach performs favorably compared with existing indirect methods and shows improved robustness in datasets containing more than 20% abnormal values. It allows accurate estimation of NCS reference limits using historical hospital data and represents a practical and reliable complement to traditional, directly derived reference limits.

**Disclosure:** None of the authors has any conflict of interest to disclose. This work received financial support from the South‐Eastern Norway Regional Health Authority and the Norwegian Medical Association.

## Movement Disorders 13

## EPO‐1042

### Change in functional impact of dyskinesias and motor fluctuations with foslevodopa/foscarbidopa in Parkinson's disease

#### E. Freire‐Álvarez ^1^; P. Odin^2^; R. Hauser^3^; J. Aldred^4^; L. Bergmann^5^; R. Gupta^5^; A. Saad^5^; A. Antonini^6^

##### ^*1*^ *General Universitario de Elche, Alicante, Spain;* ^*2*^ *University of Lund, Lund, Sweden;* ^*3*^ *University of South Florida, Tampa, USA;* ^*4*^ *Selkirk Neurology and Inland Northwest Research, Spokane, USA;* ^*5*^ *AbbVie Inc, North Chicago, USA;* ^*6*^ *University of Padua, Padua, Italy*

**Background and aims:** Foslevodopa/foscarbidopa (LDp/CDp), a continuous subcutaneous infusion of levodopa (LD) and carbidopa (CD) prodrugs, reduces dyskinesias and Off times in adults with advanced Parkinson's disease (aPD). Here, we investigated the functional impact of this reduction.

**Methods:** This post hoc analysis included two phase 3 aPD clinical trials: a 12‐week, double‐blind, double‐dummy, randomized controlled trial (RCT) of LDp/CDp vs oral LD/CD (NCT04380142) and a 52‐week open‐label safety trial (OLST) of LDp/CDp (NCT03781167). Change from baseline was analyzed for items 4.2 (functional impact of dyskinesias) and 4.4 (functional impact of motor fluctuations) on the Movement Disorder Society‐Unified Parkinson's Disease Rating Scale.

**Results:** In the RCT, the functional impact of dyskinesias significantly worsened from baseline to week 12 with oral LD/CD (mean = 0.2, standard deviation [SD] = 0.7, *p* = 0.045), whereas it significantly improved with LDp/CDp (mean = −0.5, SD = 1.0, *p* = 0.003, between‐group difference *p* = 0.001). In the OLST, this functional improvement for dyskinesias with LDp/CDp was −0.6 at week 52 (SD = 1.1, *p* ≤ 0.001). The functional impact of motor fluctuations in the RCT did not change significantly with oral LD/CD (*p* = 0.344), while it significantly improved with LDp/CDp (mean = −1.0, SD = 1.3, *p* ≤ 0.001, between‐group difference *p* = 0.003). In the OLST, motor fluctuation impairment remained improved at 52 weeks (mean = −1.2, SD = 1.4, *p* ≤ 0.001).

**Figure:**
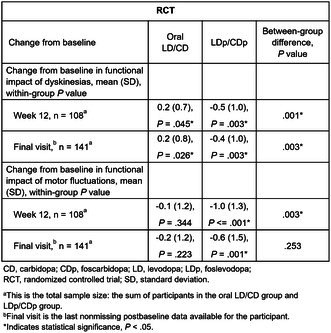
**TABLE 1** RCT.

**Figure:**
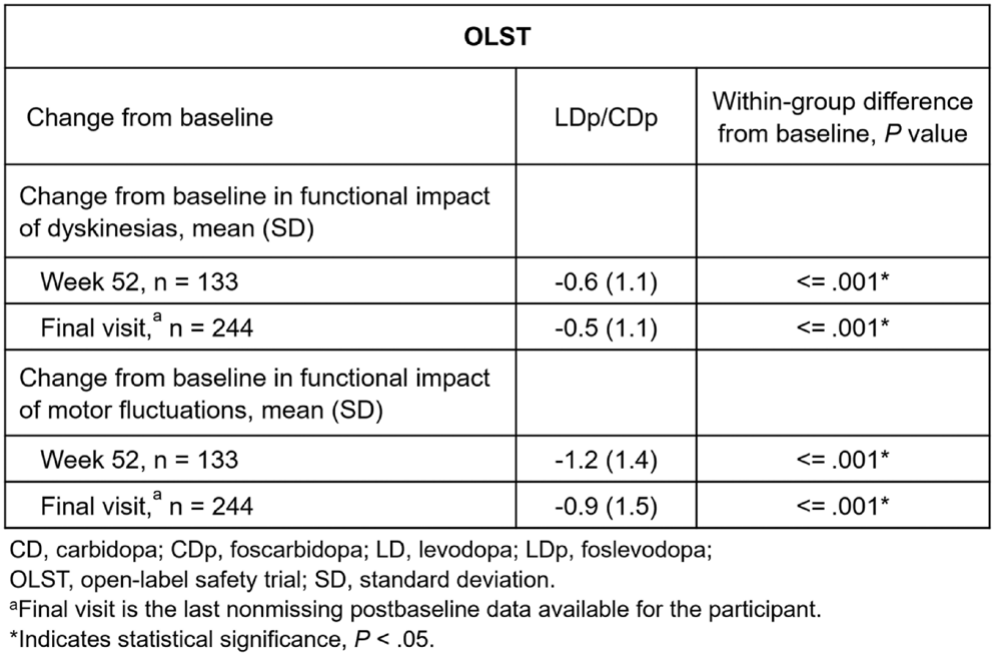
**TABLE 2** OLST.

**Conclusion:** Functional impairment from dyskinesias and motor fluctuations significantly improved after LDp/CDp treatment in patients with aPD.

**Disclosure:** AbbVie Inc. funded this study and participated in the study design, research, analysis, data collection, interpretation of data, reviewing, and approval of the abstract. All authors had access to relevant data and participated in the drafting, review, and approval of this abstract. No honoraria or payments were made for authorship. AS, RG, and LB are full‐time employees of AbbVie and may hold stock or stock options. EF‐A has received advisory, consulting, and lecture fees from AbbVie, Bial, Eisai, Esteve, Neuraxpharm, Medtronic, Orion, Pfizer, Stada, Teva, UCB, and Zambon, and support from AbbVie, Anavex, Bial, Cerevel, Ilisabio Foundation, Impax, IRLAB, Annovis, Neuroderm, Medtronic, Roche, TEVA and Zambon. PO received compensation for consultancy and speaker related activities from AbbVie, Bial, Britannia, Nordic Infucare, Stada, and Zambon. PO received personal compensation for serving on a scientific advisory or data safety monitoring board for AbbVie and Lobsor. PO received personal compensation for serving on a speaker's bureau for UCB. PO's institution has received research support from AbbVie, Lund University Medical Faculty, Parkinsonfonden, Swedish Research Council, Åhlens foundation, and Region Skåne. RAH has received speaking fees from AbbVie, Amneal Pharmaceuticals, Cerevel, Kyowa Kirin, Neurocrine Biosciences, and Supernus; has received consulting fees from AbbVie, Ameal, Avanex, Biogen, BlueRock, Cerevance, Cerevel, Forsee Pharmaceuticals, HanAll Biopharma, Inhibikase, Intrance (PSG), Jazz Pharmaceuticals, Kiefe RX, Kyowa Kirin, MDCE Suzhou, MedRythms, Merz, Mitsubishi Tanabe, Nano PharmaSolutions, Neurocrine, Neuroderm, NDP Pharmaceuticals, Pharma Two B, Photopharmics, Regenxbio, Revance, Serina Therapeutics, Stoparkinson, Supernus, Theravance, Tolmar, Tremor Research Group, Tris Pharma, Truebinding, UCB, Vivifi, and Zambon; serves on a scientific advisory board for Stoparkinson, Inhibikase, and PhotoPharmics; holds stock options in Enterin, Inhibikase, and Axial Therapeutics; has received intellectual property interests from a PD Diary through his University; acknowledges a Center of Excellence grant from the Parkinson Foundation; and his University has received research support from AbbVie, AEON Biopharma, Inc, Alexza Pharmaceuticals, Annovis Bio Inc, Biogen, Biogen MA, Inc, Bukwang Pharmaceuticals, Cavion, Inc, Cerevance Beta, Inc, Cerevel Therapeutics, Enterin, F. Hoffman La Roche Ltd, Genentech, Inc, Global Kinetics Corporation, Inhikibase Therapeutics, MJFF, Neuraly, NeuroDerm Ltd, Sage Therapeutics, Sanofi US Services Inc, Scion NeuroStim, UCB, UCB Biopharma SRL, National Parkinson Foundation, and Michael J Fox Foundation.

## EPO‐1043

### Cumulative symptoms improvement with foslevodopa/foscarbidopa vs oral medication in advanced Parkison's disease

#### A. Antonini^1^; A. Videnovic^2^; R. Hauser^3^; E. Freire‐Álvarez^4^; B. Bergmans^5^; L. Bergmann^6^; A. Saad^6^; M. Li^6^; K. Onuk^6^; J. Parra ^6^; J. Aldred^7^

##### ^1^Department of Neuroscience, University of Padova, Padova, Italy; ^2^Neurological Clinical Research Institute, Massachusetts General Hospital, Boston, USA; ^3^University of South Florida, Tampa, USA; ^4^General Universitario de Elche, Alicante, Spain; ^5^Department of Neurology, AZ St‐Jan Brugge, Bruges, Belgium and Department of Neurology, Ghent University Hospital, Ghent, Belgium; ^6^AbbVie Inc, North Chicago, USA; ^7^Selkirk Neurology and Inland Northwest Research, Spokane, USA

**Background and aims:** This post hoc analysis evaluated cumulative symptom improvements with foslevodopa/foscarbidopa (LDp/CDp) vs oral medication in advanced Parkinson's disease (aPD).

**Methods:** Analyses included data from a 12‐week, phase 3, double‐blind, randomized controlled trial (RCT, NCT04380142) of LDp/CDp vs oral levodopa/carbidopa (LD/CD), and a 52‐week open‐label trial (OLT, NCT03781167) plus 96‐week open‐label extension (OLE, NCT04379050) of LDp/CDp vs oral standard of care (SoC) from a 24‐month observational study (PROSPECT). Outcomes imputed between visits included change from baseline to week 12 in extrapolated cumulative hours in OFF, ON time with dyskinesia, “good” and “best” ON times from PD diaries (data normalized to 16‐h awake time), and number of motor fluctuations. Additional outcomes were assessed using area under the curve. Extrapolated cumulative outcomes were defined as the sum between visits and were reported as least squares mean differences between treatment groups using repeated‐measure analyses.

**Results:** In the RCT, over 85 days, participants receiving LDp/CDp experienced 5.9 fewer accumulative OFF days (*p* = 0.0219) and 7.4 more normalized “best ON” days (*p* = 0.0438) vs oral LD/CD. LDp/CDp also led to 163 fewer cumulative number of motor fluctuations (*p* ≤ 0.001) vs oral LD/CD (Figure 1). When comparing MDS‐UPDRS Part II scores, there were 3.5 times more cumulative improvements in motor activities of daily living (ADL) with LDp/CDp vs oral LD/CD (*p* = 0.3109). Similar results were obtained from the OLT/OLE vs SoC analysis.

**Figure:**
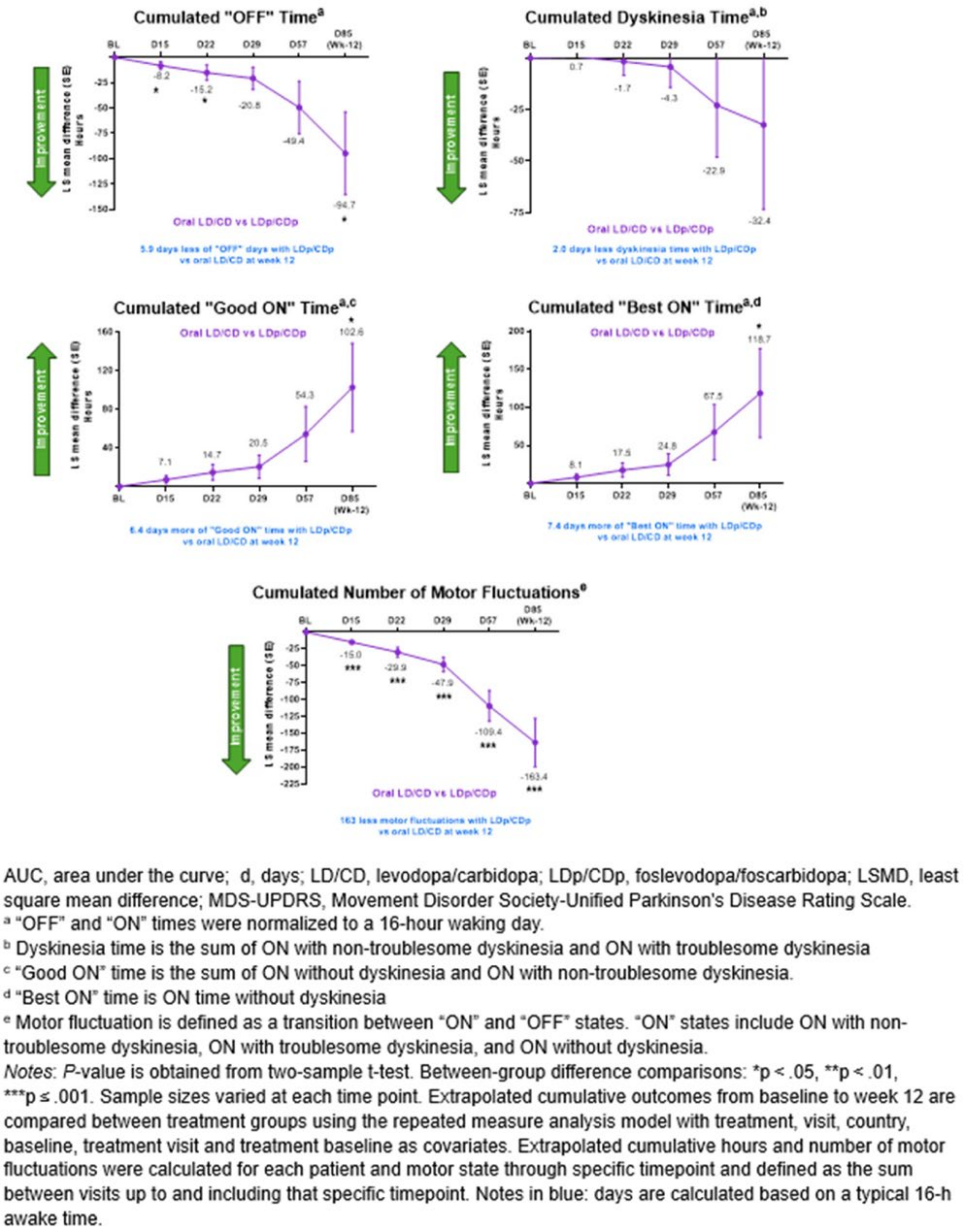
**FIGURE 1**: Least squares mean differences between treatment groups of cumulated outcomes in the randomized controlled trial.

**Conclusion:** These findings highlight that a sustained and consistent response to LDp/CDp provides meaningful benefits in people with aPD, resulting in improvements in motor symptoms and ADL compared to optimized oral medication.

**Disclosure:** Funding: AbbVie Inc. RAH: speaking fees: AbbVie,Amneal Pharmaceuticals,Cerevel,Kyowa Kirin,Neurocrine Biosciences,Supernus; consulting fees: AbbVie, Ameal,Avanex,Biogen, BlueRock,Cerevance, Cerevel, Forsee Pharmaceuticals, HanAll Biopharma,Inhibikase, Intrance, Jazz Pharmaceuticals,Kiefe RX,Kyowa Kirin,MDCE Suzhou, MedRythms, Merz, Mitsubishi Tanabe,Nano PharmaSolutions, Neurocrine,Neuroderm,NDP Pharmaceuticals,Pharma Two B,Photopharmics,Regenxbio,Revance,Serina Therapeutics, Stoparkinson, Supernus, Theravance, Tolmar, Tremor Research Group, Tris Pharma, Truebinding, UCB, Vivifi, Zambon; scientific advisory board: Stoparkinson, Inhibikase, PhotoPharmics; stock options: Enterin, Inhibikase, Axial Therapeutics; intellectual property: PD Diary; Center of Excellence grant: Parkinson Foundation; Research support: AbbVie,AEON Biopharma,Alexza Pharmaceuticals,Annovis Bio,Biogen,Bukwang Pharmaceuticals,Cavion,Cerevance Beta,Cerevel,Enterin,F. Hoffman La Roche Ltd, Genentech,Global Kinetics Corporation,Inhikibase Therapeutics, MJFF, Neuraly, NeuroDerm Ltd, Sage Therapeutics, Sanofi US Services, Scion NeuroStim, UCB Biopharma SRL, National Parkinson Foundation, Michael J Fox Foundation AV: research support: NIH, The Michael J. Fox Foundation for Parkinson's Research AA: consultancy and speaker activities: UCB, Bayer,Ever Pharma,Britannia,AbbVie,Zambon,Bial,Theravance Biopharma,Jazz Pharmaceuticals,Roche,Medscape; research support: Horizon 2020,Italian Ministry of Health,Next Generation EU‐National Center for Gene Therapy and Drugs based on RNA Technology and National Recovery and Resilience Plan, Investment PE8–Project Age‐It:“Ageing Well in an Ageing Society” EF‐Á: advisory, consulting, lecture fees: AbbVie, Bial, Eisai, Esteve, Neuraxpharm, Medtronic, Orion Pharma, Pfizer, Stada, Teva, UCB, and Zambon; support: AbbVie, Anavex, Annovis, Bial, Cerevel, Ilisabio Foundation, Impax, IRLAB, Neuroderm, Medtronic, Roche, Zambon BB: clinical practice: AZ St‐Jan Brugge; academic consultant: Ghent University Hospital; principal investigator: subcutaneous levodopa study; other clinical trials: Brugge; grants: AbbVie,Merz Pharma GmbH; speaker: AbbVie,EG,Merz GmbH,Teva,Zambon; advisory board member: Abbott,AbbVie,Boston Scientific,EG,Ipsen,Merz GmbH,Orion Pharma,Teva,UCB; grants BB's institution: Abbott,AbbVie, Boston Scientific,Elekta,GSK,Ipsen,Medtronic, Merck, Merz GmbH, Orion Pharma, Schwabe, Teva, EG for organization of MDS‐endorsed DBS symposium LB, AS, ML, JCP: AbbVie full‐time employees; may hold stock or stock options. JA: research funding: Biogen,Teva, AbbVie, Roche, Lundbeck, Parkinson's Foundation,Boston Scientific,Bial,UCB,Neurocrine,PhotoPharmics/Univ Rochester, Annovis, Sage,Aptinyx,Quadralynx,Takeda,Praxis; consulting fees:Supernus,AbbVie,Boston Scientific; honoraria: AbbVie, Teva, Neurocrine

## EPO‐1044

### EEG microstates as predictors for cognitive decline in prodromal DLB

#### D. Ondracek ^1^; D. Ondracek^2^; K. Mitterova^1^; L. Brabenec^1^; M. Lamos^1^; I. Rektorova^1^; I. Rektorova^2^

##### ^1^Brain and Mind Research Program, CEITEC, Masaryk University, Brno, Czech Republic; ^2^First Department of Neurology, Faculty of Medicine, Masaryk University and St. Anne's University Hospital, Brno, Czech Republic

**Background and aims:** EEG Microstates (MS) provide temporal and spatial characteristics of whole brain networks. We previously showed that changes in MS temporal parameters (occurrence, time coverage and mean duration) differ between healthy controls and subjects with mild cognitive impairment with Lewy bodies (MCI‐LB), such that MCI‐LB subjects display hyperexcitability, particularly in MS A (arousal network) and B (visual network). Here we studied whether baseline parameters of MS A and B predict cognitive decline in a prospective longitudinal study.

**Methods:** Altogether 85 participants on the prodromal spectrum of dementia with Lewy bodies (DLB) who were followed for up to 4 years were analysed: 38 cognitively normal subjects with core clinical features of DLB (CN‐CCF), and 26 subjects with mild cognitive impairment with Lewy bodies (MCI‐LB). The baseline MS topographies and their temporal parameters were calculated from 5‐minute, 204‐channel EEG recordings. Linear mixed models were used to predict cognitive decline both longitudinally and cross‐sectionally while accounting for age, sex, and the interval between the visits.

**Results:** Our results show that higher occurrence of MS A tended to predict deterioration of executive functions with time (*p* = 0.053). Higher occurrence of MS B predicted visual memory decline at baseline (*p* = 0.025).

**Conclusion:** We demonstrated that increased excitability of the MS representing the arousal network tends to predict changes (decline) in executive functions within up to 4 years of follow‐up. Higher excitability of the MS representing the visual network predicted visual memory impairment at baseline.

**Disclosure:** Nothing to disclose.

## EPO‐1045

### Beyond advanced therapies: Sublingual apomorphine as rescue treatment for refractory freezing of gait in LCIG‐treated Parkinson's disease

#### D. Cerdán Santacruz; M. Vargas Cobos; A. De Luca; R. Alfonso González; M. Capra; C. Gómez López de San Román; I. Bermejo Casado; L. Caballero Sánchez; J. Berrío; G. Suárez Fernández; A. Castrillo Sanz; A. Mendoza Rodríguez

##### General Hospital Complex of Segovia, Segovia, Spain

**Background and aims:** Freezing of gait (FoG) is a disabling motor symptom in advanced Parkinson's disease (PD) and may persist despite device‐aided therapies such as levodopa–carbidopa intestinal gel (LCIG). In some patients, FoG episodes remain refractory to additional LCIG bolus doses, representing an unmet therapeutic need. Sublingual apomorphine provides rapid dopaminergic stimulation through a non‐invasive route; however, evidence of its use in patients already receiving advanced therapies is scarce.

**Methods:** We report two male patients with advanced PD receiving stable LCIG therapy who presented recurrent FoG episodes not responding to additional LCIG boluses. The first patient was a 70‐year‐old man with 18 years of disease duration, treated with LCIG as first device‐aided therapy, who received sublingual apomorphine 10 mg as rescue treatment. The second patient was a 59‐year‐old man with 14 years of disease duration, previously treated with continuous subcutaneous apomorphine infusion and currently on LCIG due to suboptimal motor control. Sublingual apomorphine was introduced for persistent FoG. Clinical response, functional impact, and tolerability were assessed based on clinical evaluation and patient‐reported outcomes.

**Results:** Both patients experienced a clear clinical improvement in FoG episodes after sublingual apomorphine administration, allowing recovery from motor blocks refractory to LCIG boluses. Treatment was well tolerated, with no reported adverse effects.

**Conclusion:** Sublingual apomorphine may represent a useful and well‐tolerated rescue option for refractory FoG in complex PD patients treated with LCIG. These cases suggest that rescue strategies using different dopaminergic mechanisms should also be considered in advanced disease stages.

**Disclosure:** Nothing to disclose.

## EPO‐1046

### Seizures or Spells? What should the “S” stand for in FBDS associated with LGI1 encephalitis?

#### F. Brena ^2^; F. Duca^2^; A. Bettinelli^2^; E. Sartirana^2^; V. Chiesa^1^; C. Rosci^1^; J. Bottini^1^; G. Oggioni^1^; A. Priori^1^; E. Scelzo^1^

##### ^1^Dipartimento di Neuroscienze, ASST Santi Paolo e Carlo, Ospedale San Paolo, Milan, Italy; ^2^Dipartimento di Scienze Della Salute, Università Degli Studi di Milano, Milan, Italy

**Background and aims:** Anti‐leucine‐rich glioma‐inactivated 1 (LGI1) encephalitis is a rare antibody‐mediated neurological disease affecting the central nervous system. Its main feature is facio‐brachial dystonic seizures (FBDS), defined as brief, paroxysmal, stereotyped movements affecting facial, cervical and proximal upper limb muscles. The pathophysiological origin of these movements is still unclear.

**Methods:** We performed a narrative review of the available literature on LGI1 encephalitis, focusing on the clinical and instrumental characterization of FBDS. In parallel, we describe the clinical course, diagnostic work‐up, and therapeutic management of a 77‐year‐old man with LGI1 encephalitis, analyzing clinical and instrumental findings to assess which features supported an epileptic versus dystonic nature.

**Results:** In our patient, frequent FBDS‐like episodes occurred despite persistently normal prolonged video‐EEG recordings. The clinical course was atypical, with a prolonged isolated dystonic phase and no limbic seizures. These findings are in contrast to a purely epileptic genesis and suggest a prominent dystonic phenotype. In line with literature reports describing heterogeneous LGI1 presentations, our case is consistent with the existence of different phenotypes, with variable contributions of both epileptic and dystonic mechanisms.

**Conclusion:** Available evidence suggests that FBDS lie along a continuous spectrum, with one extreme representing a purely epileptic phenotype and the other a purely dystonic phenotype. This model may explain the heterogeneity observed in both clinical and instrumental findings, reflecting distinct patterns of cortical and subcortical network involvement. This perspective highlights why the meaning of the “S” in FBDS warrants reconsideration.

**Disclosure:** Nothing to disclose.

## EPO‐1047

### Frozen in Laughter: A curious case of paroxysmal facial pain

#### L. Basumatary ^1^; M. Bhoktiary^2^; D. Payeng^1^

##### ^1^Neurology, Apollo Excelcare Hospitals, Guwahati, India; ^2^Pediatrics, Diphu Medical College Hospitals, Diphu, India

**Background and aims:** Hemimasticatory spasm (HMS) is a rare movement disorder with paroxysmal, unilateral contractions of jaw‐closing muscles. Because it can mimic mandibular dystonia, temporomandibular joint disorders, trigeminal neuralgia, or hemifacial spasm, HMS is often under‐recognized. Most reported cases are associated with facial hemiatrophy; idiopathic HMS without hemiatrophy is uncommon and diagnostically challenging.

**Methods:** We report the clinical, neurophysiological, imaging, and treatment features of HMS without facial hemiatrophy. A single‐patient case report was conducted at a movement disorders clinic. Triggers and phenomenology were documented. Interictal and ictal needle EMG and trigeminal electrophysiology (blink reflex, masseter reflex, masseteric silent period) were performed, and brain/face MRI reviewed for structural lesions and masticatory asymmetry.

**Results:** A 34‐year‐old school teacher had 2 years of painful right masseter–temporalis spasms triggered by laughter/eating, sometimes causing brief jaw locking and teeth chattering, with occasional nocturnal episodes. Medications (clonazepam, haloperidol, cyclobenzaprine) were ineffective. Examination showed right masseter/temporalis hypertrophy with episodic contractions; cranial nerves and jaw jerk were normal and no sensory tricks were present. Autoimmune/inflammatory tests were normal. Interictal EMG was normal; ictal EMG showed clonic bursts (10–200 ms) with variable intervals and firing up to 200 Hz. Blink reflex was normal; during attacks, masseter reflex latency was delayed with reduced amplitude and an attenuated masseteric silent period. MRI showed right masseter enlargement only. Botulinum toxin produced marked improvement.

**Figure:**
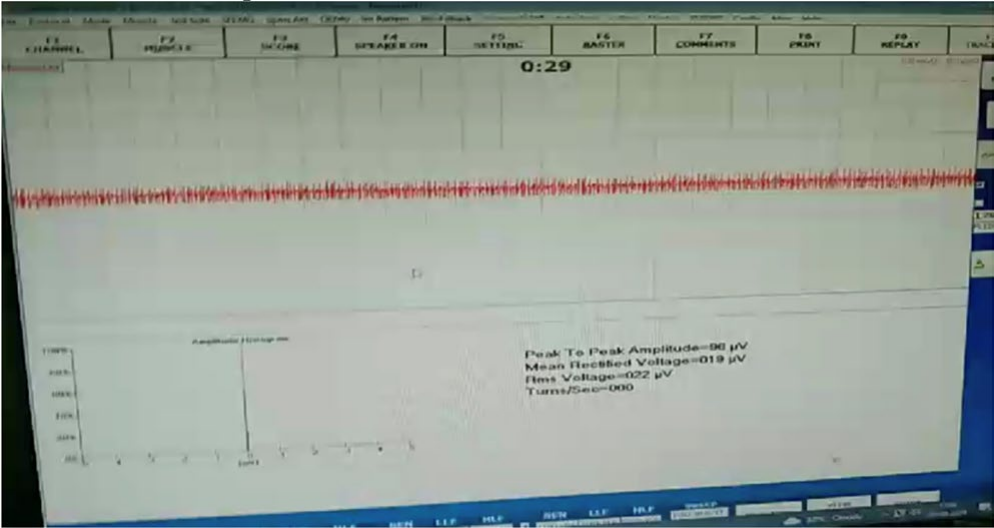
**FIGURE 1** EMG (Masseter): Normal at rest (no spontaneous activity; normal MUPs). During spasms, brief clonic bursts (10–200 ms) with variable inter‐burst intervals (20–200 ms), sometimes merging into continuous activity; peak firing up to ~200 Hz.

**Figure:**
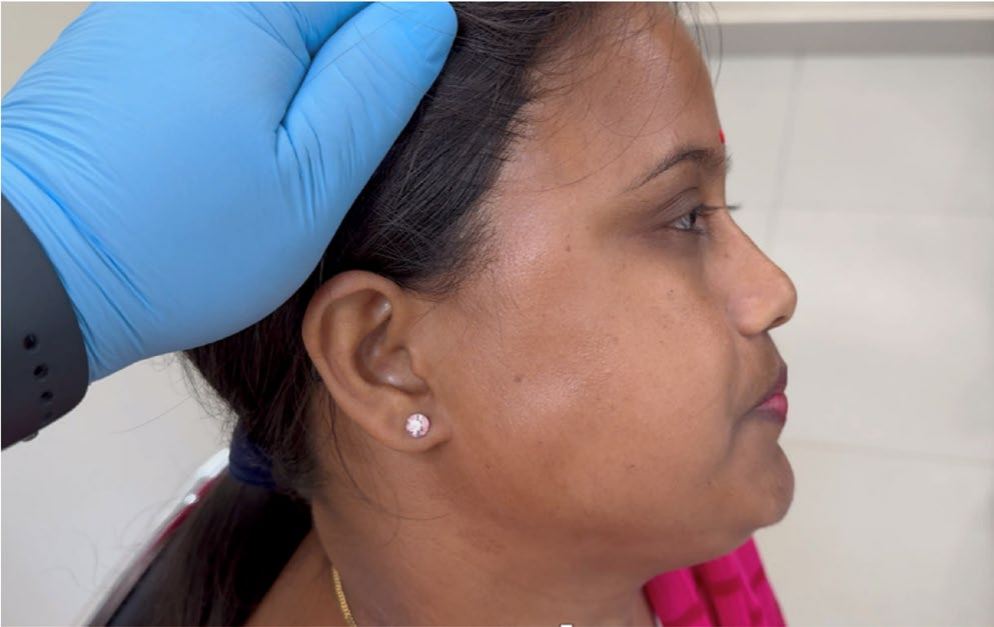
**FIGURE 2** Paroxysmal, painful involuntary spasms of the right masseter and temporalis muscles.

**Conclusion:** HMS should be considered in trigger‐sensitive unilateral jaw‐closing spasms with pain and hypertrophy even without facial hemiatrophy; attack‐related impairment of inhibitory silent periods supports diagnosis and botulinum toxin is an effective first‐line therapy.

**Disclosure:** Nothing to disclose.

## EPO‐1048

### Deep brain stimulation in KMT2B‐related dystonia: Electrophysiological and clinical parameters during a 12 month‐follow‐up period in two children

#### L. Feldmann ^1^; A. de Almeida Marcelino^1^; J. Kaplan^1^; P. Krause^1^; G. Brandt^1^; G. Schneider^2^; A. Kuehn^1^

##### ^1^Movement Disorders and Neuromodulation Section, Clinics of Neurology with Experimental Neurology, Charité University Medicine Berlin, Berlin, Germany; ^2^Clinics of Neurosurgery, Charité University Medicine Berlin, Berlin, Germany

**Background and aims:** Pathogenic variants in the KMT2B gene lead to childhood‐onset, generalized dystonia (DYT‐KMT2B). Deep brain stimulation (DBS) to the internal globus pallidus (GPi) is an effective therapy. With sensing‐enabled neurostimulators, electrophysiological biomarkers can be recorded chronically. Previously, pallidal theta activity correlated with symptom severity, and entrained gamma may indicate effective DBS. However, longitudinal dynamics of electrophysiological biomarkers in KMT2B‐related dystonia are unknown.

**Methods:** Clinical and electrophysiological data were recorded regularly in the first 12‐months follow‐ups after GPi‐DBS (COMEDD‐trial, NCT07244549) in two children with DYT‐KMT2B (age at surgery 5 and 9 years) with the Percept neurostimulator. Dystonia was quantified using the Burke‐Fahn‐Marsden‐Dystonia‐Rating‐Scale motor score (BFMDRS‐M). Pallidal electrophysiology during rest and movement was recorded ON/OFF DBS and analyzed using short‐fourier‐transformation and spectral parametrization. Peak activity in theta band and beta or gamma band was recorded out‐of‐hospital and analyzed for chronic biomarker changes and circadian periodicity.

**Results:** Both girls were DBS responders (54 ± 3% improvement in BMFDRS‐M at 12 months‐follow‐up). In both patients, entrained gamma occurred at clinically used amplitudes, and there was theta suppression over time. In one of the two patients, chronic recordings revealed increased peak biomarker activity especially around times of symptom deterioration. Circadian periodicity analysis suggests distinct patterns for theta, gamma and beta band activity.

**Conclusion:** DBS is an effective therapy in KMT2B‐related pediatric dystonia. Theta suppression and occurrence of entrained gamma seem to be associated with clinical improvement and may be used to guide electrophysiology‐based programming in future. Further, chronic biomarkers could be used to understand out‐of‐hospital symptom fluctuations.

**Disclosure:** This work is supported by the Dystonia Medical Research Foundation (award number DMRF‐PRF‐2024‐1), by the Deutsche Forschungsgemeinschaft (DFG, German Research Foundation) – Project ID 424778381 ‐ TRR 295 Grant and the Berlin Institute for Health (BIH) Clinician Scientist Program. LKF received honoraria for talks by Medtronic. P.K. has served on advisory boards for Medtronic, AbbVie and Gerresheimer and received lecture fees from Stadapharm and AbbVie. A.A.K. has served on advisory boards of Medtronic and has received honoraria and travel support from Medtronic, Boston Scientific, and Bial. A.A.K. reports a relationship with Medtronic that includes consulting or advisory, speaking and lecture fees, and travel reimbursement. A.A.K. reports a relationship with Boston Scientific Corporation that includes speaking and lecture fees, and travel reimbursement. All remaining authors have nothing to disclose.

## EPO‐1049

### Diagnostic sensitivity of a shortened accelerometry‐only functional tremor battery

#### M. Cibula; N. Prezelj; R. Berlot; M. Kojović

##### Department of Neurology, University Medical Centre Ljubljana, Ljubljana, Slovenia

**Background and aims:** Electrophysiologically supported identification of positive signs is important in confirming the diagnosis of functional tremor. In this respect, the functional tremor battery proposed by Schwingenschuh et al. is a valuable diagnostic tool but requires surface electromyography, specialised equipment and expertise, limiting its routine clinical use. As most battery tests rely on accelerometric data, we aimed to evaluate the diagnostic sensitivity of a shortened, accelerometry‐only version of the functional tremor battery.

**Methods:** Thirty patients with clinically diagnosed functional tremor were included. All patients underwent the full functional tremor battery (maximum score 10 points). A proposed cutoff score of ≥3 points was used as a standard to ensure a homogeneous cohort. Among patients meeting this cutoff, we examined whether the cutoff would still be met after excluding surface electromyography–based tests (coactivation sign and tremor coherence). Subsequently, the weight loading test was also excluded to evaluate its redundancy in the overall diagnostic sensitivity of the shortened test battery.

**Results:** Twenty‐four of 30 patients (80%) scored ≥3 points on the full battery and were included in further analyses. After exclusion of surface electromyography‐based tests, all 24 patients (100%) continued to score ≥3 points. Following additional exclusion of the weight loading test, 23 of 24 patients (95.8%) still met the cutoff.

**Conclusion:** A shortened accelerometry‐based functional tremor battery demonstrates diagnostic sensitivity comparable to the full battery that includes surface electromyography. This simplified approach reduces technical demands, examination time, and required expertise, potentially enabling wider clinical implementation.

**Disclosure:** Nothing to disclose.

## EPO‐1050

### Opicapone for management of sleep disturbances in Parkinson's disease: Post‐hoc analysis of the OASIS trial in patients with and without probable RBD

#### J. Ferreira^1^; M. Gago^2^; R. Costa ^3^; M. Fonseca^4^; H. Brigas^3^; J. Holenz^3^; C. Trenkwalder^5^

##### ^1^Laboratory of Clinical Pharmacology and Therapeutics, Faculty of Medicine, University of Lisbon, Lisbon, Portugal; ^2^Department of Neurology, ULS Alto Ave, Hospital da Senhora da Oliveira, Guimarães, Portugal; ^3^BIAL – Portela & Ca S.A, Coronado, Portugal; ^4^BIAL ‐ R&D Investments, S.A. Coronado, Portugal; ^5^Deptartment of Neurosurgery, University Medical Center, Goettingen, Germany

**Background and aims:** Rapid eye movement sleep behavior disorder (RBD) is prevalent in Parkinson's disease (PD). Opicapone improved sleep disturbances in fluctuating PD patients with PD‐related sleep disturbances in the exploratory Phase IV OpicApone in Sleep dISorder (OASIS) trial. This OASIS post‐hoc analysis evaluates opicapone's effect on sleep disturbances, motor/non‐motor symptoms and quality of life in PD patients with and without probable RBD, as determined by their medical history.

**Methods:** The 6‐week, single‐arm OASIS trial evaluated opicapone 50 mg in levodopa‐treated fluctuating PD patients with sleep disturbances. Assessments included PD Sleep Scale‐2 (PDSS‐2), Movement Disorder Society‐sponsored Non‐Motor Symptoms rating scale (MDS‐NMS), Patient and Clinician Global Impressions of Change, Parkinson's Fatigue Scale‐16 (PFS‐16), Parkinson's Disease Questionnaire‐8 (PDQ‐8).

**Results:** Of the 16 patients included, 8 (50%) had probable RBD. After 6 weeks with opicapone, similar reductions in PDSS‐2 total scores were reported in patients with (−8.4 ± 5.1) and without RBD (−7.5 ± 2.7; Figure 1a). Improvements were mostly seen in the disturbed sleep domain (Figure 1b), with >35% improvement in items assessing poor sleep quality in the previous week, sleep latency, sleep fragmentation, and non‐restorative sleep in both groups. No early‐morning dystonia was reported in patients with probable RBD. Similar improvements in OFF‐time/ON‐time without dyskinesia, MDS‐UPDRS Parts III and IV, PFS‐16, and PDQ‐8 scores were observed (Table 1). Opicapone was well‐tolerated in both groups.

**Figure:**
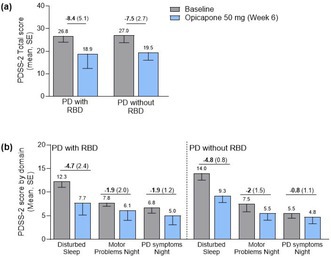
**FIGURE 1** Change in PDSS‐2 (a) total score and (b) by domain from baseline to week 6 of opicapone treatment in PD patients with or without probable RBD.

**Figure:**
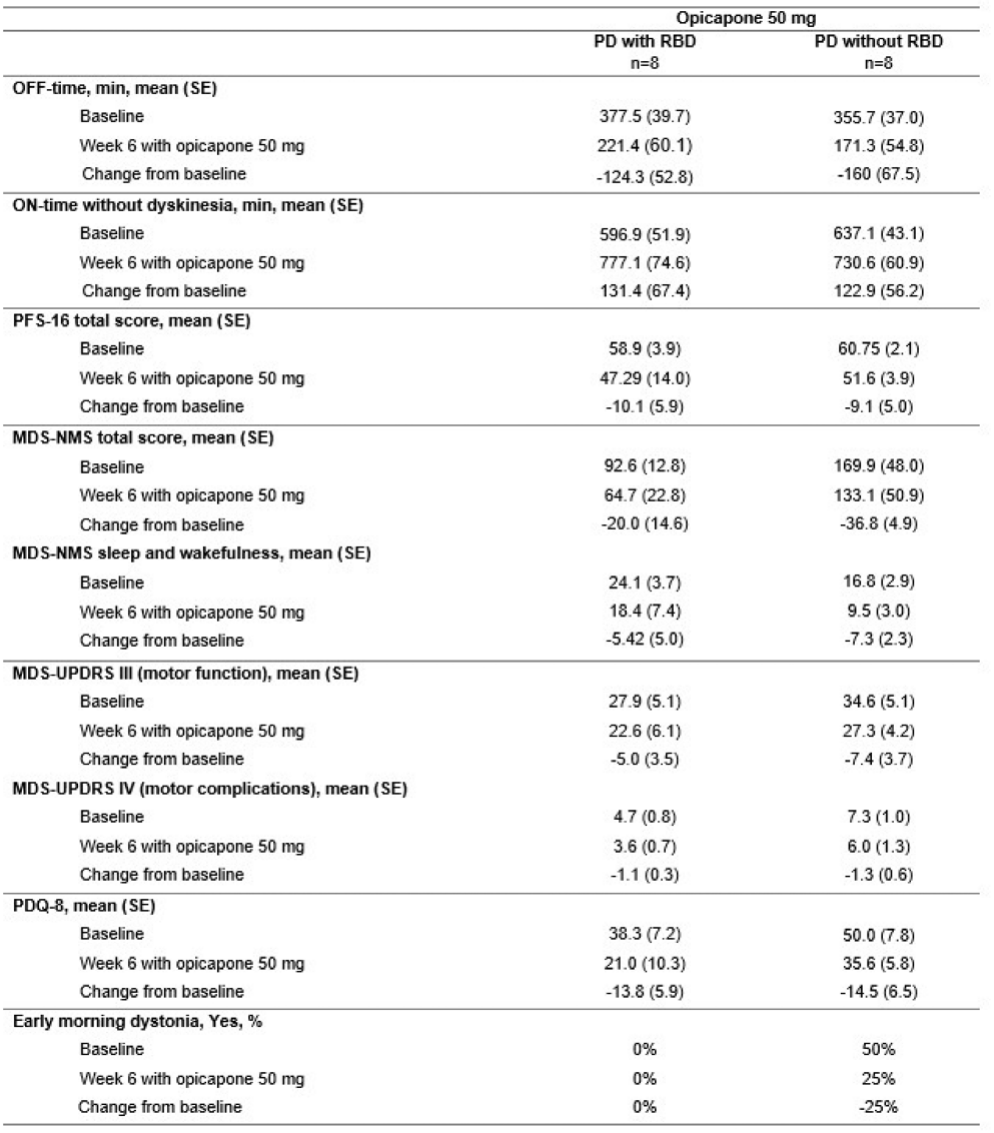
**TABLE 1** Change from baseline to week 6 in OFF and ON‐time without dyskinesia, PFS‐16, MDS‐NMS, MDS‐UPDRS, PDQ‐8 and early morning dystonia (full analysis set).

**Conclusion:** Adding opicapone to levodopa in patients with PD, wearing‐off and sleep disturbances improved sleep complaints similarly in patients with and without probable RBD, although the analysis is limited by the small sample size.

**Disclosure:** Supported by Bial.

## Muscle and Neuromuscular Junction Disorder 5

## EPO‐1051

### Efficacy of nipocalimab in patients with lower baseline score of myasthenia gravis activity of daily living in VIVACITY‐MG3 study

#### C. Antozzi^1^; W. Noel^2^; W. Karmous^3^; M. Fitzgibbon ^4^; K. Gandhi^5^; I. Turkoz^5^; M. Kutch^6^; S. Ramchandren^7^

##### ^1^Immunotherapy and Apheresis Unit, Neuroimmunology and Muscle Pathology Unit, Fondazione IRCCS Istituto Neurologico C. Besta, Milan, Italy; ^2^Johnson & Johnson, Diegem, Belgium; ^3^Johnson & Johnson, Paris, France; ^4^Johnson & Johnson, Raritan, USA; ^5^Johnson & Johnson, Horsham, USA; ^6^Cytel Inc, Cambridge, USA; ^7^Johnson & Johnson, Titusville, USA

**Background and aims:** Myasthenia gravis (MG) is a chronic autoimmune disease characterised by fluctuating muscle weakness and fatigability, which may worsen without effective treatment. In VIVACITY‐MG3 (NCT04496507) study, nipocalimab added to standard‐of‐care (SOC), demonstrated greater improvement in Myasthenia‐Gravis–Activities‐of‐Daily‐Living (MG‐ADL), and Quantitative‐Myasthenia‐Gravis (QMG) scores versus placebo (+SOC). This post hoc analysis evaluated whether efficacy could be achieved in patients with lower baseline symptom burden (MG‐ADL scores: 6–9).

**Methods:** Analysis included seropositive patients treated with nipocalimab or placebo with baseline MG‐ADL score below cohort median score of 9. Efficacy was evaluated by changes in MG‐ADL (meaningful clinical improvement [MCI]: ≥2‐point improvement; substantial clinical improvement [SCI]: ≥3‐point improvement), and QMG (MCI: ≥3‐point improvement; SCI: ≥4‐point improvement) scores at week (W) 24. Two‐sample *t*‐tests compared changes‐from‐baseline between groups.

**Results:** In this population, baseline demographics were generally balanced between nipocalimab and placebo‐treated patients (Table). At W24, MG‐ADL reductions (mean [SD]) from baseline were greater with nipocalimab (−4.5 [2.64]) versus placebo (−2.3 [2.37]); difference: −2.23 (standard error [SE]: 0.588); 95%CI (−3.41; −1.06); *p* < 0.001. QMG reductions (mean [SD]) were also greater with nipocalimab (−5.2 [4.45]) versus placebo (−1.9 [3.69]); difference: −3.38 (SE: 0.986); 95%CI (−5.34; −1.41); *p* = 0.001. A greater proportion of patients receiving nipocalimab met MCI and SCI criteria for MG‐ADL and QMG at W24 versus placebo (Table).

**Figure:**
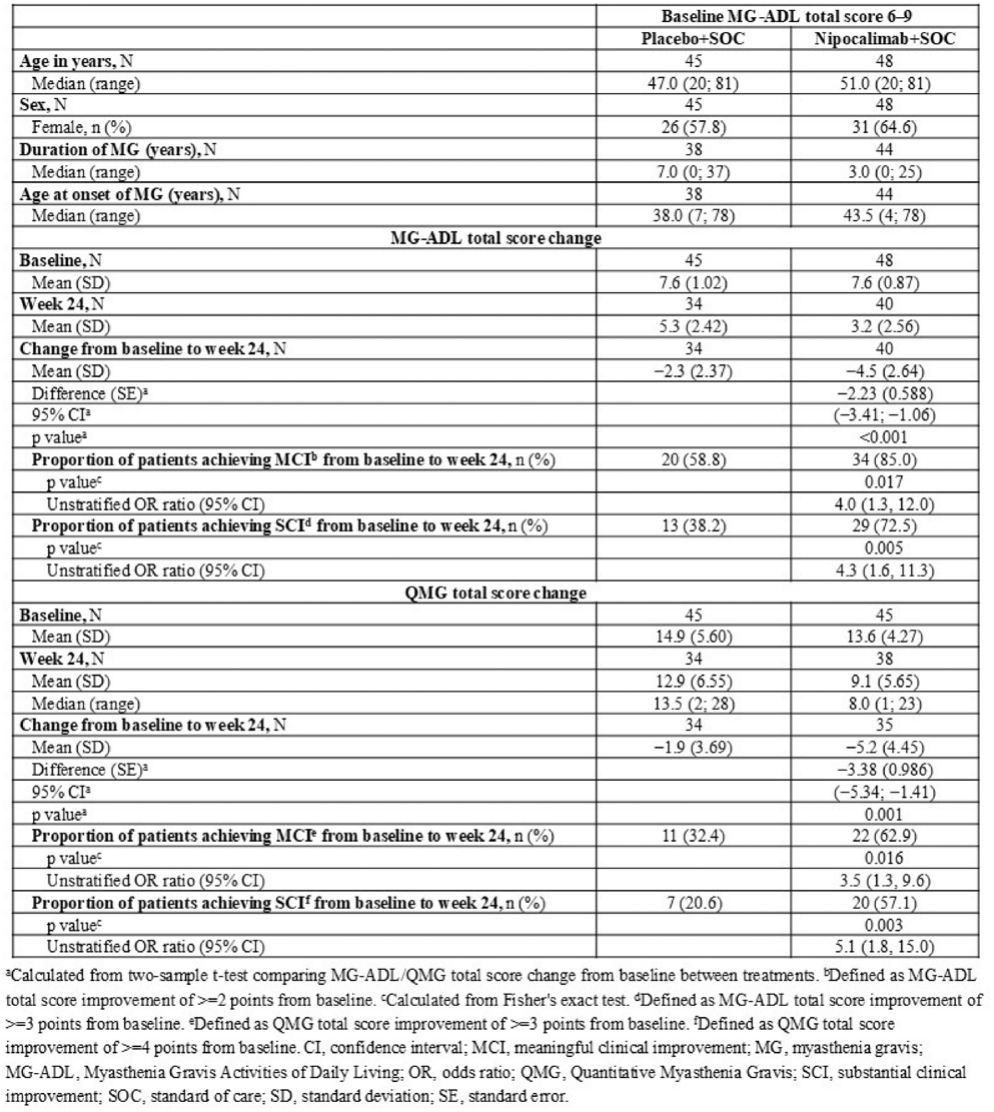
**TABLE** Baseline characteristics and symptom improvement in MG‐ADL and QMG scales.

**Conclusion:** In patients with baseline MG ADL scores 6–9, nipocalimab demonstrated greater efficacy versus placebo, achieving MCI and SCI in MG ADL and QMG at W24. These results support the benefit of nipocalimab for sustained disease control in patients with gMG with lower baseline MG‐ADL scores.

**Disclosure:** Carlo Antozzi: received funding for travel, meeting attendance, and advisory board participation from Alexion, Momenta, Sanofi, argenx, UCB, Janssen Pharmaceuticals and Johnson & Johnson. Wim Noel, Wissam Karmous, Marie Fitzgibbon, Kavita Gandhi, Ibrahim Turkoz, and Sindhu Ramchandren: are employees of Johnson & Johnson and may hold stock or stock options in the company. Michael Kutch: is an employee of Cytel Inc, which derives profits from interactions with pharmaceutical sponsors.

## EPO‐1052

### Corticosteroid use in nipocalimab‐treated Japanese patients with generalized myasthenia gravis: Phase 3 vivacity‐MG3 open‐label extension study

#### C. Antozzi^1^; K. Gandhi^2^; I. Turkoz^3^; M. Kutch^4^; M. Fitzgibbon ^5^; S. Ramchandren^3^; H. Murai^6^

##### ^1^Immunotherapy and Apheresis Departmental Unit, Fondazione IRCCS Istituto Neurologico C. Besta, Milan, Italy; ^2^Medical Affairs, Johnson & Johnson, Horsham, USA; ^3^Medical Affairs, Johnson & Johnson, Titusville, USA; ^4^Cytel, Inc, USA; ^5^Medical Affairs, Johnson & Johnson, Raritan, USA; ^6^Department of Neurology, International University of Health and Welfare, Narita, Japan

**Background and aims:** In seropositive adults with generalized myasthenia gravis (gMG), nipocalimab treatment (nipocalimab+standard‐of‐care [SOC]) vs placebo+SOC achieved sustained disease control during the 24‐week VIVACITY‐MG3 study (NCT04951622) double‐blind phase. Sustained disease control was maintained through open‐label extension (OLE) week 60. During OLE, 45% of patients receiving nipocalimab (*N* = 89) reduced/discontinued corticosteroids; a substantial proportion demonstrated minimum symptom expression (MSE; MG‐Activities of Daily Living [MG‐ADL] score = 0/1) and sustained for ≥8 weeks. In Japan, achieving minimal gMG symptoms with low corticosteroid doses (target: ≤5 mg/day) is an important treatment goal. We characterized the impact of nipocalimab on corticosteroid use in Japanese patients in the VIVACITY‐MG3 OLE phase.

**Methods:** Corticosteroid doses (mg/day prednisone‐equivalent) were evaluated through OLE data cut‐off (August 2024). Among patients who reduced corticosteroid dose (≤5 mg/day), the proportion achieving MSE was evaluated.

**Results:** Of 11 Japanese patients receiving corticosteroids at OLE baseline, 7 (64%) receiving nipocalimab in OLE reduced/discontinued corticosteroids at data cutoff; of these, 5 (71%) reached ≤5 mg/day. Among steroid reducers, mean (SD) steroid dose reduced from 10.4 (2.32) to 5.6 (2.82) mg/day prednisone‐equivalent in Japanese patients, while in remaining population dose reduced from 25.1 (20.53) to 11.2 (10.50) mg/day prednisone‐equivalent. Among those reducing corticosteroids, 4/7 (57%) patients achieved MSE within 48 weeks (Table 1).

**Figure:**
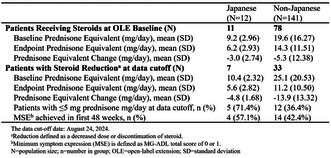
**TABLE 1** Patient baseline and efficacy endpoint results.

**Conclusion:** In OLE, over half of Japanese patients reduced/discontinued corticosteroids with substantial proportions tapering to low‐dose corticosteroid (≤5 mg/day) and achieving MSE within 48 weeks. These results are consistent with the overall population where almost half of patients receiving corticosteroids at baseline, reduced/discontinued steroids and showed sustained disease control with nipocalimab.

**Disclosure:** Carlo Antozzi: Travel funding, meeting attendance, and advisory board participation: Alexion, argenx, Johnson & Johnson, Momenta, Sanofi, and UCB. Kavita Gandhi, Ibrahim Turkoz, Marie Fitzgibbon and Sindhu Ramchandren: Employees of Johnson & Johnson, may hold stocks/stock options in Johnson & Johnson. Michael Kutch: Employee of Cytel, Inc, MA, may hold stocks/stock options in Cytel. Hiroyuki Murai: Scientific advisory board: Alexion, argenx, UCB. Speaker honoraria: Novartis, Johnson & Johnson, and Merck. Research Support: Ministry of Health, Labour and Welfare, Japan: Sciences Research Grant on Rare and Intractable Diseases.

## EPO‐1053

### Efficacy and cumulative safety of cemdisiran in adults with generalised myasthenia gravis (gMG): Results from the phase 3 NIMBLE trial

#### S. Jacob ^1^; T. Vu^2^; A. Habib^3^; R. Mantegazza^4^; H. Murai^5^; J. Vissing^6^; A. Shaibani^7^; T. Levine^8^; Y. Hussain^9^; A. Meisel^10^; R. Pavani^11^; U. Chaudhari^11^; N. Jalali^11^; M. DeVeaux^11^; K. Meagher^11^; S. Sherman^11^; D. Pawaskar^11^; A. Souttou^11^; L. Perlee^11^; J. Howard Jr^12^

##### ^1^University Hospitals Birmingham NHS Foundation Trust, Birmingham, UK; ^2^University of South Florida Morsani College of Medicine, Tampa, USA; ^3^University of California Irvine, Orange, USA; ^4^Fondazione IRCCS Istituto Neurologico Carlo Besta, Milan, Italy; ^5^International University of Health and Welfare, Narita, Japan; ^6^University of Copenhagen, Copenhagen, Denmark; ^7^Nerve and Muscle Center of Texas, Houston, USA; ^8^Bob Bové Neuroscience Institute, Scottsdale, USA; ^9^Austin Neuromuscular Center, Austin, USA; ^10^Charité‐Universitätsmedizin Berlin, Berlin, Germany; ^11^Regeneron Pharmaceuticals Inc, Tarrytown, USA; ^12^University of North Carolina, Chapel Hill, USA

**Background and aims:** Autoantibody‐mediated complement activation damages neuromuscular junctions in gMG, resulting in variable muscle weakness. The phase 3 NIMBLE trial evaluated subcutaneous cemdisiran (siRNA inhibiting hepatic C5 production) and subcutaneous pozelimab (monoclonal antibody targeting C5) as monotherapies or in combination in patients with gMG.

**Methods:** NIMBLE is a double‐blind, placebo‐controlled trial. A total of 288 patients with gMG were randomised to cemdisiran (600 mg Q12W), pozelimab (200 mg Q4W), combination (both 200 mg Q4W), or placebo. We present key results for the primary efficacy endpoint, change from baseline in MG‐ADL at Week 24, based on August 2025 database lock (*n* = 239), and the cumulative rate of TEAEs based on December 2025 database lock (*n* = 281).

**Results:** NIMBLE met its primary endpoint. In the cemdisiran (*n* = 64) and combination (*n* = 67) arms, the least squares mean (SE) of change from baseline at Week 24 in MG‐ADL total score was −4.5 (0.4) and −4.0 (0.4), with placebo‐adjusted differences of −2.3 (*p* = 0.0005) and −1.7 (*p* = 0.0086); achieving 76.6% (*n* = 75) and 99.6% (*n* = 75) terminal complement inhibition, respectively. TEAEs occurred in 74.7% (59/79), 81.6% (40/49), 81.5% (66/81), and 81.9% (59/72) in the cemdisiran, pozelimab, combination, and placebo arms, respectively. No meningococcal, serious infections or deaths occurred in the cemdisiran arm during the double‐blind treatment period (DBTP); SAEs of infections in other arms were low. There were 2 deaths (pneumonia [cemdisiran], septic shock [combination]) in patients on concomitant immuno‐suppressive therapies and comorbidities after the DBTP.

**Conclusion:** Quarterly, subcutaneous cemdisiran was generally well‐tolerated and achieved significant improvement in symptoms with partial complement inhibition among patients with gMG.

**Disclosure:** This study was funded by Regeneron Pharmaceuticals, Inc. SJ has served as an international advisory board member for Alexion, Alnylam, Argenx, Immunovant, Johnson and Johnson, Merck, Novartis, Regeneron and UCB pharmaceuticals, is currently an expert panel member of Myasthenia Gravis consortium for Argenx pharmaceuticals and has received speaker fees from Argenx, Eisai, Merck, Terumo BCT and UCB pharmaceuticals. He is also a board member (trustee) of the UK myasthenia patient charity, Myaware. TV has served as the USF Site Principal Investigator for MG clinical trials sponsored by Alexion/AstraZeneca Rare Disease, Amgen, argenx, Cartesian Therapeutics, COUR, Dianthus Therapeutics, EMD Serono, ImmunAbs, Immunovant, Johnson & Johnson, NMD Pharma, Regeneron Pharmaceuticals, Inc, VOR, and UCB. He has served as a speaker for Alexion/AstraZeneca Rare Disease, argenx, CSL Behring, and Johnson & Johnson. He served on advisory boards and/or performed consulting work for Alexion/AstraZeneca Rare Disease, argenx, Amgen, Dianthus Therapeutics, Johnson & Johnson, ImmunAbs, NMD Pharma, and VOR. He also participated in CME events sponsored by AcademicCME, CMEO, PER, and MedLive. HM has served as a consultant for Alexion, argenx, and UCB, and has received speaker honoraria from Novartis, Johnson & Johnson and Merck, and research support from the Ministry of Health, Labour and Welfare, Japan. AM received speaker or consultancy honoraria or financial research support from Alexion Pharmaceuticals, Argenx, Amgen, Axunio, Desitin, Grifols, Janssen, Merck, Novartis, Octapharma, Sanofi and UCB. He serves as chairmen of the Association for Research in Myasthenic Syndromes in Germany (VEMSID e.V.). RP, UC, NJ, MD, KAM, SS, DP, AS, and LP are employees and stockholders of Regeneron Pharmaceuticals, Inc. JFH Jr has received research funding from Ad Scientiam, Alexion AstraZeneca Rare Disease, argenx, Cartesian Therapeutics, Centers for Disease Control and Prevention, Merck EMD Serono, MGFA, Muscular Dystrophy Association, NIH, NMD Pharma, and UCB Bioscience; honoraria/consulting fees from AcademicCME, Alexion AstraZeneca Rare Disease, Amgen, argenx, Biohaven Ltd, Cartesian Therapeutics, CheckRare CME, CoreEvitas, Curie.bio, H. Lundbeck A/S, Japan Tobacco Company, Kyverna Therapeutics, Merck EMB Serono, NMD Pharma, Novartis Pharma, PeerView CME, Physicians' Education Resource (PER) CME, PlatformQ CME, Regeneron Pharmaceuticals, Inc, Seismic Therapeutics, TG Therapeutics, Toleranzia AB, UCB Bioscience and Vor Bio; non‐financial support from Alexion AstraZeneca Rare Disease, argenx, Biohaven Ltd, Cartesian Therapeutics, Toleranzia AB, and UCB Pharma. AH, RM, JV, AS, TL, and YH have nothing to declare.

## EPO‐1054

### Corticosteroid and biologic use in a real‐world population with generalized myasthenia gravis in the United States and Europe

#### J. Howard Jr^1^; S. Anderson^2^; B. Canales^2^; J. Park^2^; G. Gibson^3^; H. Connolly^3^; S. Birija^3^; B. Poirrette^3^; A. Foster^3^; A. Bhambri ^2^

##### ^1^Department of Neurology, The University of North Carolina, Chapel Hill, North Carolina; ^2^Amgen Inc. Thousand Oaks, USA; ^3^Adelphi Real World, Bollington, UK

**Background and aims:** Generalised myasthenia gravis (gMG) is a neuromuscular condition manifesting as fluctuating muscle weaknesses and fatigue. Treatments include corticosteroids and targeted biologic treatments. This study describes corticosteroid and biologic use in a multi‐country sample of gMG patients.

**Methods:** Data were drawn from the Adelphi MG Disease Specific Programme (DSP)™, a cross‐sectional survey of neurologists and their patients with gMG in the France, Germany, Italy, Spain, UK and United States, from January 2024 ‐ November 2025. Physicians provided patient‐level demographics, clinical characteristics and treatment.

**Results:** Overall, 146 neurologists reported on 917 gMG patients, patient demographics are shown by country in **TABLE 1** Physicians reported a mean (Standard Deviation; SD) Myasthenia Gravis ‐ Activities of Daily Living (MG‐ADL) score of 4.5 (3.3) and 59.8% of patients were in Myasthenia Gravis Foundation of America class II. At time of survey, 88.3% of patients were prescribed a gMG treatment. Of those, 40.4% were prescribed corticosteroids, most commonly prednisone (35.6%). Patients had been prescribed their current regimen of corticosteroids for a mean (SD) 1.8 (2.8) years and 43.1% were prescribed a dosage ≥20 mg. Among patients on treatment, 37.2% were prescribed biologics. Patients had been prescribed their current biologic treatment regimen, for a mean (SD) 1.1 (1.2) years at survey and the biologic was administered a mean (SD) 2.3 (4.1) times per month.

**Figure:**
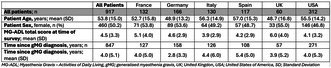
**TABLE 1** Patient age, sex, MG‐ADL score and time since gMG diagnosis, split by country.

**Figure:**
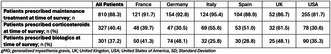
**TABLE 2** Proportion of gMG patients prescribed maintenance treatment of corticosteroid and biologic treatments, split by country.

**Conclusion:** Corticosteroid and biologic therapies were frequently prescribed in the six countries included in this analysis. Reducing steroid burden and frequent administrations of biologic therapies whilst maintaining efficacy could benefit this population.

**Disclosure:** James F Howard Jr receives research funding (paid to his institution) from Ad Scientiam, Alexion AstraZeneca Rare Disease, argenx, Cartesian, Centers for Disease Control and Prevention, MGFA, Muscular Dystrophy Association, NMD Pharma, NIH, PCORI, and UCB Pharma; honoraria/consulting fees from AcademicCME, Alexion AstraZeneca Rare Disease, Amgen, argenx, Biohaven Ltd, Biologix Pharma, Cartesian, CheckRare CME, CorEvitas, Curie.bio, Hansa Biopharma, Medscape CME, Merck EMB Serono, Novartis Pharma, PeerView CME, Physicians' Education Resource (PER) CME, PlatformQ CME, Regeneron Pharmaceuticals, Sanofi US, Seismic Therapeutics, TG Therapeutics and UCB Pharma; non‐financial support from Alexion, argenx, Biohaven Ltd, Cartesian Therapeutics, Toleranzia AB, and UCB Pharma. Seth Anderson is an employee of Amgen and receives salary and/or stocks/shares in Amgen. Blanca Canales is an employee of Amgen and receives salary and/or stocks/shares in Amgen. Jenny Y. Park is an employee of Amgen and receives salary and/or stocks/shares in Amgen. Gregor Gibson is an employee at Adelphi Real World, which received research funds from Amgen for this work. Hannah Connolly is an employee at Adelphi Real World, which received research funds from Amgen for this work. Shiva Lauretta Birija is an employee at Adelphi Real World, which received research funds from Amgen for this work. Beth Poirrette is an employee at Adelphi Real World, which received research funds from Amgen for this work. Amy Foster is an employee at Adelphi Real World, which received research funds from Amgen for this work. Ankur Bhambri is an employee of Amgen and receives salary and/or stocks/shares in Amgen.

## EPO‐1055

### Healthcare costs of generalized myasthenia gravis in Germany: A real‐world retrospective cohort study

#### C. Schneider‐Gold^1^; S. Anderson^2^; I. Bucholz^2^; V. Višković^3^; A. Marzà‐Florensa^4^; M. Ludwig^3^; A. Postema^4^; A. Bhambri ^2^

##### ^1^Department of Neurology, St. Josef‐Hospital, Bochum, Germany; ^2^Amgen Inc, Thousand Oaks, USA; ^3^InGef ‐ Institute for Applied Health Research Berlin GmbH, Berlin, Germany; ^4^PHARMO Institute for Drug Outcomes Research, Utrecht, The Netherlands

**Background and aims:** Generalized myasthenia gravis (gMG) is a chronic autoimmune neuromuscular disorder characterized by muscle weakness and fatigue affecting daily activities. This study describes patient characteristics and healthcare costs among patients with gMG in Germany.

**Methods:** We conducted a retrospective cohort study using anonymized secondary healthcare claims data from the InGef database from 2017 to 2024. Patients aged ≥18 years with ≥1 inpatient or ≥2 outpatient diagnoses and ≥12 months of continuous enrollment before and after the index date were included. Follow‐up was variable length with a minimum of 12 months. Annualized healthcare costs per patient per year (PPPY) were calculated across outpatient, inpatient, emergency department, and pharmacy services.

**Results:** A total of 4,025 patients with gMG were identified (mean age 61.2 years, standard deviation 16.7 years; 49.3 percent female). Median total healthcare costs were 6,115.06 euros (interquartile range 2,942.54 to 13,011.55 euros) PPPY, with gMG‐related costs of 1,279.06 euros (interquartile range 346.74 to 3,238.00 euros). Costs were higher among patients with ≥1 exacerbation (*N* = 2,144; median total costs 7,757.87 euros PPPY; median gMG‐related costs 2,262.18 euros PPPY) and among patients with ≥1 myasthenic crisis (*N* = 93; median total costs 34,605.52 euros PPPY; median gMG‐related costs 14,928.98 euros PPPY).

**Figure:**
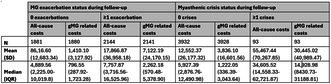
**TABLE 1** All‐cause and gMG‐related healthcare costs by disease severity.

**Conclusion:** This real‐world study demonstrates the substantial economic burden of gMG in Germany. Higher costs among patients with exacerbations and myasthenic crises emphasize the impact of disease severity and the need for improved disease management.

**Disclosure:** Christiane Schneider‐Gold receives research funding from Amgen. Seth Anderson is an employee of Amgen and receives salary and/or stocks/shares in Amgen. Ina Bucholz is an employee of Amgen and receives salary and/or stocks/shares in Amgen. Vukašin Višković is an employee at InGef, which received research funds from Amgen for this work. Anna Marzà‐Florensa is an employee at PHARMO, which received research funds from Amgen for this work. Marion Ludwig is an employee at InGef, which received research funds from Amgen for this work. Abigail Postema is an employee at PHARMO, which received research funds from Amgen for this work. Ankur Bhambr is an employee of Amgen and receives salary and/or stocks/shares in Amgen.

## EPO‐1056

### Diagnostic utility of anti‐SRP54 antibody ELISA in immune‐mediated necrotizing myopathy: Experience from a real‐world cohort

#### S. Kim

##### Department of Neurology, Gangnam Severance Hospital, Yonsei University College of Medicine, Seoul, Republic of Korea

**Background and aims:** Immune‐mediated necrotizing myopathy (IMNM) associated with anti‐signal recognition particle (SRP) antibodies typically presents with rapidly progressive proximal weakness and markedly elevated creatine kinase levels. In clinical practice, line‐blot immunoassays are commonly used to detect anti‐SRP antibodies, but their reliability and reproducibility are sometimes questioned. We examined the performance of an enzyme‐linked immunosorbent assay (ELISA) targeting anti‐SRP54 antibodies and explored its clinical relevance in a real‐world IMNM cohort.

**Methods:** Patients evaluated for idiopathic inflammatory myopathies between 2002 and 2023 were retrospectively reviewed. Eighty‐seven patients with inflammatory myopathies and 107 healthy controls were included. Anti‐SRP IMNM was defined based on line‐blot immunoassay results, and the diagnostic performance of anti‐SRP54 ELISA was assessed. Clinical features, laboratory findings, and coexisting autoantibodies were analyzed in ELISA‐positive patients.

**Results:** Among 32 patients diagnosed with anti‐SRP IMNM using line‐blot immunoassays, 28 (88%) tested positive on anti‐SRP54 ELISA. The ELISA demonstrated a sensitivity of 88% and a specificity of 99%, with high test–retest reliability. All ELISA‐positive patients showed proximal muscle weakness. Neck weakness, myalgia, dysphagia, or respiratory symptoms were observed in a subset of patients. Extra‐muscular involvement, including interstitial lung disease and myocarditis, was uncommon. Serum creatine kinase levels were markedly elevated overall and tended to be higher in patients with coexisting anti‐Ro‐52 antibodies.

**Conclusion:** Anti‐SRP54 antibody ELISA showed high diagnostic accuracy and good reproducibility in patients with anti‐SRP IMNM. In routine clinical practice, this assay can serve as a reliable complementary tool to support the diagnosis of IMNM and may help increase diagnostic confidence in patients with suspected disease.

**Disclosure:** Nothing to disclose.

## EPO‐1057

### Do we really need non‐steroidal immunosuppressants in generalized myasthenia gravis treated with new targeted therapies? A pilot study

#### V. Vera ^1^; E. Rossini^1^; L. Leonardi^2^; G. Antonini^1^; S. Morino^2^; M. Garibaldi^1^; A. Lauletta^1^; F. Forcina^1^; L. Tufano^1^; D. Marando^1^; L. Fionda^2^

##### ^1^Department of Neuroscience, Mental Health and Sensory Organs (NESMOS), SAPIENZA University of Rome, Rome, Italy; ^2^Neuromuscular and Rare Diseases Centre, Neurology Unit, Sant’Andrea Hospital, Rome, Italy

**Background and aims:** Targeted biologic therapies, including complement and neonatal Fc receptor (FcRn) inhibitors, have transformed the treatment of generalized myasthenia gravis (gMG). However, the role of concomitant non‐steroidal immunosuppressants (NSIST) remains uncertain. This study evaluated whether background NSIST influences clinical outcomes and corticosteroid reduction in gMG patients treated with targeted therapies.

**Methods:** We conducted a retrospective, observational study including adult gMG patients treated with complement inhibitors or anti‐FcRn agents (*n* = 41). Clinical outcomes were assessed at baseline and during follow‐up using Myasthenia Gravis Activities of Daily Living (MG‐ADL) and Quantitative Myasthenia Gravis (QMG) scales. Achievement of Minimal Symptom Expression (MSE), MG Foundation of America Post‐Intervention Status (MGFA‐PIS), and changes in corticosteroid dose were analyzed.

**Results:** Twenty patients received background NSIST (Group 1), while 21 did not (Group 2). Baseline MG‐ADL scores and corticosteroid doses were comparable between groups (9.10 ± 4.36 vs 8.24 ± 3.38) (22.17 ± 13.81 mg vs 22.06 ± 16.43). Baseline QMG scores were slightly higher in Group 1 (14.0 ± 5.1 vs 10.9 ± 4.8; *p* = 0.049). Both groups showed significant improvement in MG‐ADL and QMG scores at all follow‐up time points, without differences in clinical scale improvement comparing the two groups. Rates of MSE achievement, MGFA‐PIS improvement, and corticosteroid tapering did not differ between groups.

**Conclusion:** Our pilot analysis indicates that concomitant use of NSIST does not appear to be associated with greater improvement in clinical outcomes, enhance corticosteroid‐sparing effects or increased disease stability. Larger, prospective studies are needed to optimize treatment strategies in gMG.

**Disclosure:** LF received financial support from Alexion Pharmaceuticals, argenx, UCB, Dianthus, Johnson & Johnson and amgen. GA received conference honoraria, advisory board and travel grants from Kedrion, Alnylam, Alexion, Argenx, Takeda. ER received honoraria from UCB and Alexion.

## EPO‐1058

### Patient preferences and willingness‐to‐pay for therapy in generalized myasthenia Gravis: A large‐scale discrete choice experiment in China

#### Y. Xu ^1^; L. Feng^2^; M. Zhang^1^; J. Zhou^1^; H. Xiao^1^; Y. Ni^1^; R. Wang^2^; Y. He^2^; Q. Jiang^2^; J. Peng^2^; H. Chen^2^; H. Zhou^3^; D. He^1^; S. Luo^1^; J. Xi^1^; J. Song^1^; C. Yan^1^; J. Lu^1^; C. Zhao^1^

##### ^1^Huashan Rare Disease Center and Department of Neurology, National Center for Neurological Disorders, Huashan Hospital, Fudan University, Shanghai, China; ^2^Department of Neurology, West China Hospital, Sichuan University/West China School of Nursing, Sichuan University, Chengdu, China; ^3^Department of Neurology, West China Hospital, Sichuan University, Chengdu, China

**Background and aims:** Understanding patients' preferences for Generalised Myasthenia Gravis (gMG) is essential for informed treatment decisions. However, limited evidence is available regarding how Chinese patients weigh safety, efficacy, onset speed, and convenience when choosing treatments. This study aimed to assess treatment preferences and willingness to pay (WTP) among Chinese patients.

**Methods:** This multicenter, cross‐sectional survey was conducted from March to August 2025 among gMG patients across China. A discrete choice experiment was used to quantify patient preferences by presenting hypothetical treatment scenarios.

**Results:** Of 909 analysed patients, treatment safety was a key determinant of patient preferences. Oral administration was most preferred, followed by subcutaneous injection. Less frequent dosing was strongly favoured: once every 6 months, and once daily, compared to 2–3 times daily. Younger patients paid more attention to low‐frequency dosing, subcutaneous injection, and liver and kidney function impairment than older patients. Patients with MGFA class II were more concerned about oral administration, once‐weekly dosing, infection risk, and myelosuppression. High‐income patients (>¥100,000) favoured low risk of metabolic disease, infection, myelosuppression, and liver and kidney function impairment and infrequent (weekly, biweekly, or every‐6‐month), subcutaneous administration, and were less sensitive to cost compared to low‐ (< ¥50,000) and medium‐income groups (¥50,000–100,000).

**Figure:**
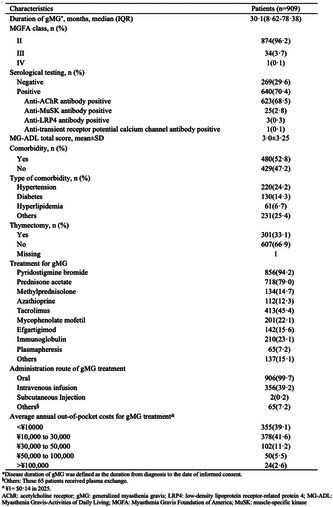
**TABLE 1** The disease characteristics and treatment of patients with gMG

**Figure:**
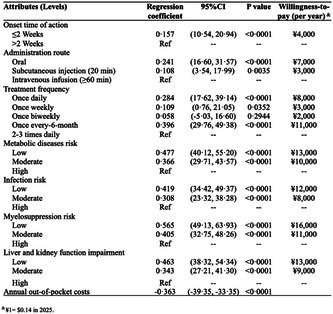
**TABLE 2** Analysis of patients' medication preferences and willingness to pay.

**Figure:**
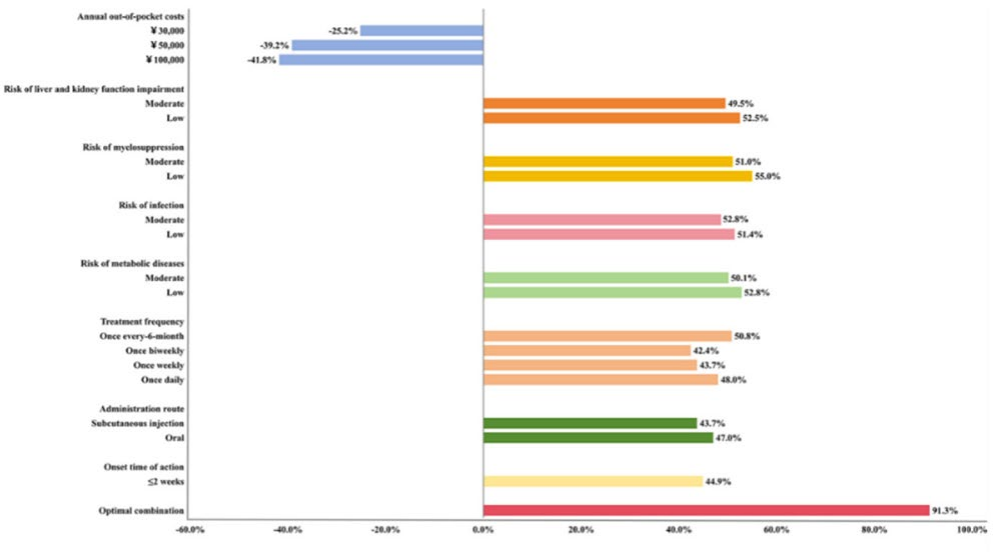
**FIGURE 1** The selection probability of gMG patients.

**Conclusion:** Safety, infrequent and convenient administration route (oral/subcutaneous), affordable cost, and rapid onset of action were prioritised. Patients were willing to invest financially in therapies that align with these preferences. Embedding patient preferences in clinical decision‐making and reimbursement policy can improve adherence, reduce disease burden, and enhance quality of life for individuals with gMG in China.

**Disclosure:** Nothing to disclose.

## Neurocritical Care

## EPO‐1059

### Aetiology, clinical features, and outcomes of paediatric TBI management in sub‐Saharan Africa: A systematic review and meta‐analysis of regional data

#### A. Moradeyo ^1^; E. Okoh^1^; M. Opabode^1^; E. Ibitunde^1^; B. Akinyemi^1^; S. Rath^2^; C. Orazulike^1^; H. Bukar^1^; D. Oladiran^1^; S. Olatokun^1^; D. Lawal^1^

##### ^1^Surgical Interest Group Lagos (SURGIL), Lagos, Nigeria; ^2^All India Institute of Medical Sciences Bhubaneswar, India

**Background and aims:** Despite the rising burden of paediatric traumatic brain injury (TBI) in sub‐Saharan Africa, there is still fragmented evidence on its clinical profile and outcomes. This review evaluated the aetiology, clinical presentation, management, and outcomes of paediatric TBI to establish pooled and regional estimates.

**Methods:** Articles published between January 2005 and August 2025 on pediatric TBI and neurosurgical management were identified from PubMed, ScienceDirect, Google Scholar, AJOL, and Cochrane Library. Pooled proportions and 95% confidence intervals were calculated using random‐effects meta‐analyses, with I^2^ statistic for heterogeneity.

**Figure:**
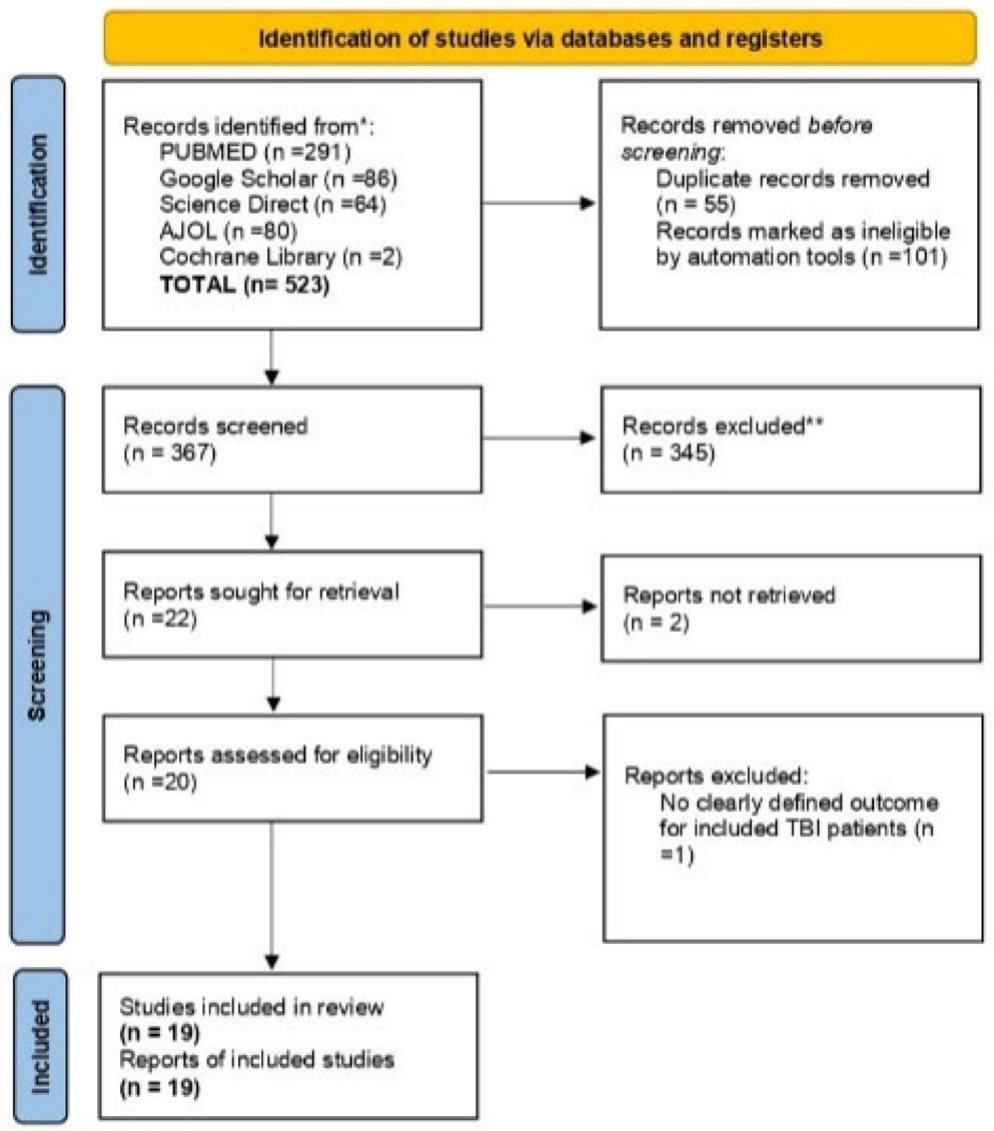
**FIGURE 1** PRISMA flow diagram for included studies.

**Results:** A total of 19 studies (*n* = 4452 patients) were included in this review. Road traffic accidents (RTA) were the primary aetiology (64%; 95% CI 57–70%), followed by falls (20%) and assaults (9%). Computed tomography scan was the dominant imaging modality. Common radiological findings included fracture‐related lesions (43.5%), intracranial hemorrhage (33.3%), cerebral contusions (13.70%), and cerebral edema (9.50%). On admission, the GCS (Glasgow Coma Scale) scores for mild, moderate, and severe TBI accounted for 56%, 26%, and 42% of cases, respectively. Neurosurgical intervention was reported in 15.4% of patients. The pooled favorable outcome was 81% (95% CI 67–90%), with the highest in West Africa (89%). The overall pooled mortality was 8% (95% CI 6–11%), with country‐specific rates varying from 3% to 13%.

**Figure:**
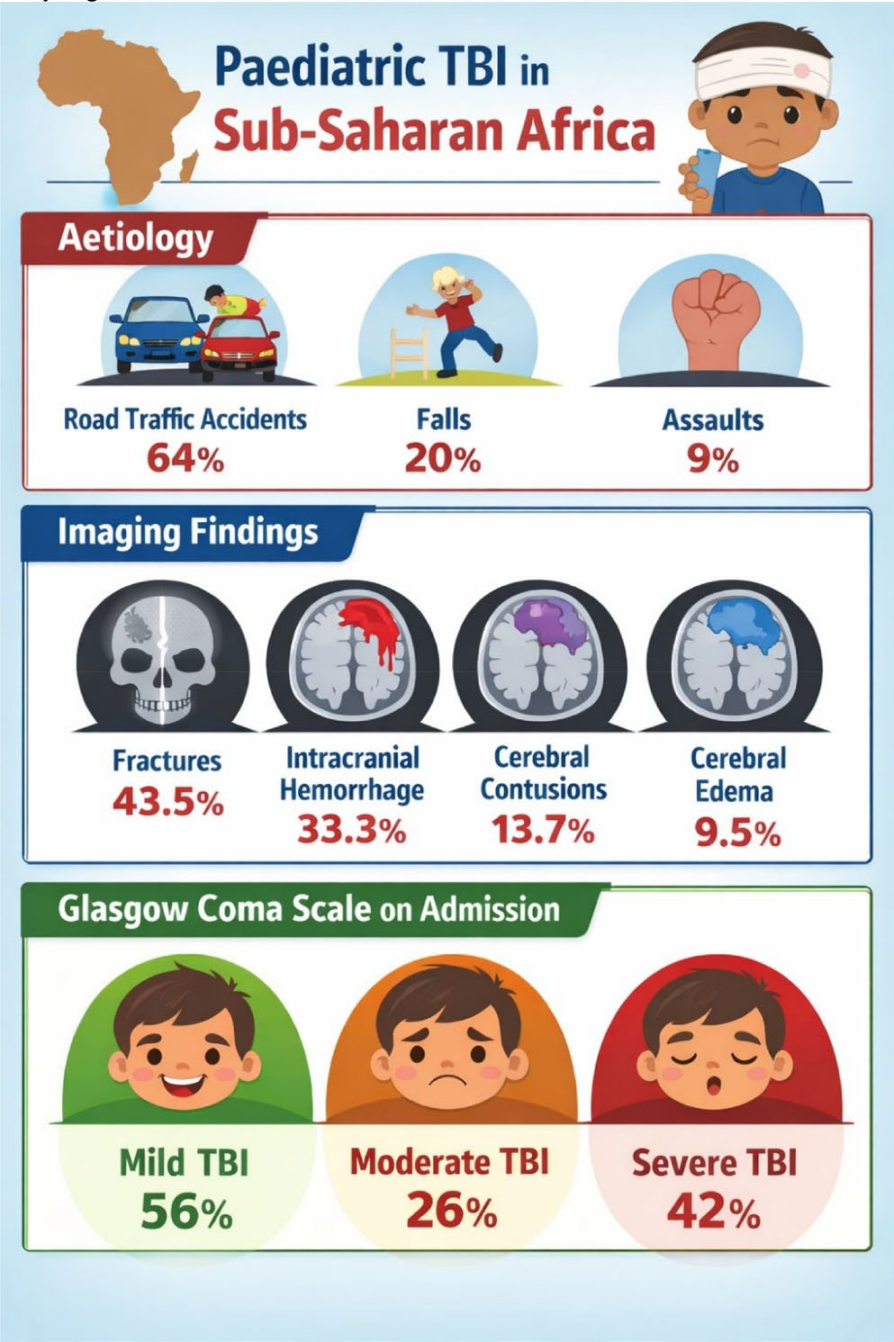
**FIGURE 2** Paediatric TBI in sub‐Saharan Africa.

**Conclusion:** RTAs are the major cause of TBIs, with a significant proportion presenting with a moderate to severe GCS score at admission. Despite relatively favourable functional outcomes among survivors, mortality remains considerably high and varies across regions.

**Disclosure:** Nothing to disclose.

## EPO‐1060

### Severe chronic inflammatory demyelinating polyradiculoneuropathy relapses in the ICU: Clinical characteristics, complications and outcome

#### A. Balcerac ^1^; C. Benoit^1^; C. Marois^2^; T. Maisonobe^3^; T. Lenglet^3^; R. Debs^3^; D. Psimaras^4^; B. Rohaut^5^; S. Demeret^1^; K. Viala^3^; N. Weiss^6^; L. Le Guennec^1^

##### ^1^Sorbonne Université et AP‐HP, Médecine Intensive Réanimation Neurologique et DMU Neuroscience, Hôpital de la Pitié‐Salpêtrière, Paris, France; ^2^Sorbonne Université et AP‐HP, Médecine Intensive Réanimation Neurologique et DMU Neuroscience, GRC‐RESPIRE, Hôpital de la Pitié‐Salpêtrière, Paris, France; ^3^Sorbonne Université et AP‐HP, Service de Neurophysiologie, Hôpital de la Pitié‐Salpêtrière, Paris, France; ^4^Sorbonne Université et AP‐HP, Service de Neuro‐oncologie et Service de Neurophysiologie, Hôpital de la Pitié‐Salpêtrière, Paris, France; ^5^Sorbonne Université et AP‐HP, Médecine Intensive Réanimation Neurologique, DMU Neuroscience, Brain Institute (ICM), Hôpital de la Pitié‐Salpêtrière, Paris, France; ^6^Sorbonne Université et AP‐HP, Médecine Intensive Réanimation Neurologique, DMU Neuroscience, GRC‐RESPIRE, BLIPS Study Group (INSERM UMR_S 938), Hôpital de la Pitié‐Salpêtrière, Paris, France

**Background and aims:** Severe relapses of chronic inflammatory demyelinating polyradiculoneuropathy (CIDP) requiring intensive care unit (ICU) admission are rare, and data on their presentation and prognosis are limited. This study aimed to describe their characteristics, management, and long‐term outcomes.

**Methods:** We conducted a single‐center retrospective study including all neurological ICU admissions for severe CIDP relapse between January 2004 and December 2024. CIDP was diagnosed according to EFNS criteria. Demographic data, indications for ICU admission, treatments, and 1‐year modified Rankin Scale (mRS) scores were analyzed. Patients were stratified by the need for invasive mechanical ventilation.

**Results:** Fifty‐two patients were included (median age 62.8 years). Main indications for ICU admission were plasma exchange for refractory disease (35%), acute respiratory failure (33%), and rapid motor worsening (33%). Twelve patients (23%) required invasive ventilation and more frequently presented with respiratory failure, bulbar dysfunction, and severe motor impairment. ICU stay was longer in ventilated patients (30 vs 7 days). Overall ICU mortality was low (3.8%). At ICU discharge, 58% of patients had significant disability (mRS 4–5). Pre‐relapse mRS independently predicted 1‐year functional outcome, whereas intubation did not.

**Figure:**
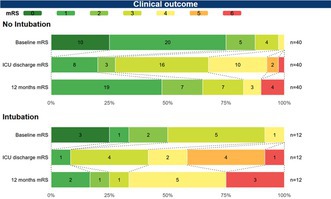
**FIGURE 1** Distribution of the modified Rankin Scale (mRS) in the population.

**Conclusion:** In this long‐term cohort, mortality from severe CIDP relapses was low, and invasive ventilation was not associated with worse 1‐year functional outcome. Respiratory and bulbar involvement were the main predictors of intubation.

**Disclosure:** Nicolas Weiss declares having perceived consultant fees from Alexion and Lucane Pharma. Clemence Marois declares having perceived consultant fees from Alexion, Johnson and Jonson and UCB Timothée Lenglet declares having perceived consultant fees from LFB.

## EPO‐1061

### Stimulating consciousness recovery after acute brain injury with psilocybin and apomorphine

#### A. Eigenbrodt ^1^; P. Laigaard^1^; S. Stückler^1^; M. Hassani^1^; K. Møller^2^; J. Kjærgaard^3^; D. Kondziella^1^

##### ^1^Department of Neurology, Copenhagen University Hospital ‐ Rigshospitalet, Copenhagen, Denmark; ^2^Department of Neuroanaesthesiology, Copenhagen University Hospital ‐ Rigshospitalet, Copenhagen, Denmark; ^3^Department of Cardiology, Copenhagen University Hospital ‐ Rigshospitalet, Copenhagen, Denmark

**Background and aims:** What can be done to promote recovery of consciousness after brain injury in the intensive care unit (ICU)? We hypothesize that simultaneously targeting the two components of consciousness—awareness with psilocybin and arousal with apomorphine—can promote consciousness after acute brain injury. Here, we assess the safety of this combination in comatose ICU patients.

**Methods:** Prospective, open‐label, phase 1b dose‐escalation study (*n* = 80). ICU coma patients receive psilocybin at doses of 1 mg (*n* = the first 26), 10 mg (*n* = next 27), or 25 mg (*n* = final 27), together with 2 mg apomorphine 1–2 hours after psilocybin administration (Figure 1).

**Figure:**
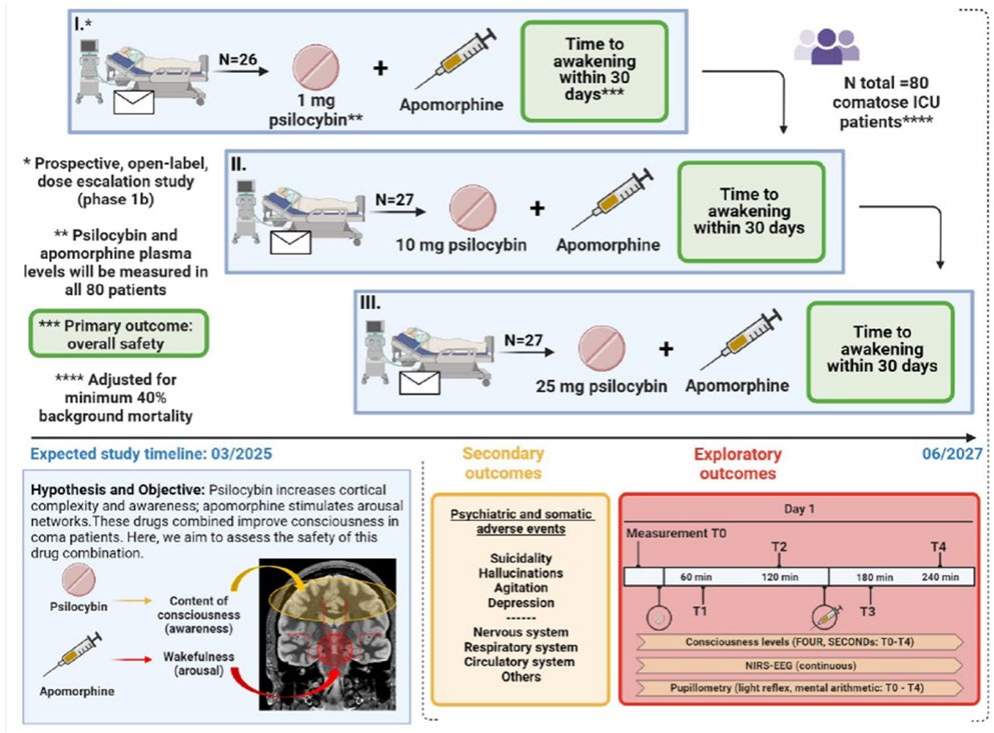
**FIGURE 1** Study design.

**Results:** The first participant was recruited in February 2025, and as of January 2026, 53 (66%) participants have been enrolled, i.e, enrollment of the 1 mg and the 10 mg groups has been completed. In total, 69 serious adverse events were observed as of mid‐January 2026 (Table 1). The most common events were pneumonia (*n* = 19), death after withdrawal of life‐sustaining support (*n* = 15), and urinary tract infection (*n* = 13). We identified no serious adverse events related to the study drugs. Occasionally, apparently beneficial effects were noted in the 10 mg group.

**Figure:**
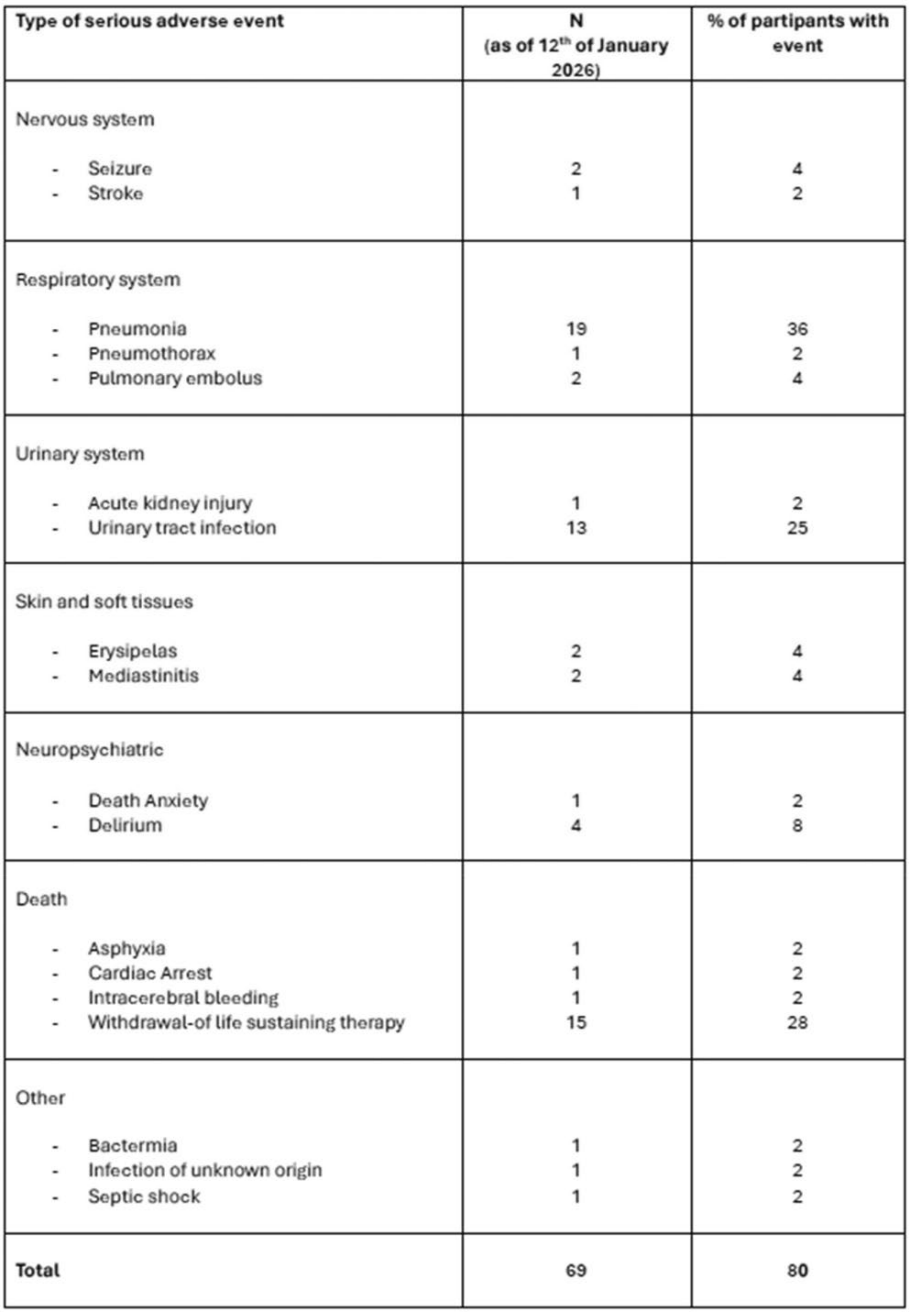
**TABLE 1** Serious adverse events.

**Conclusion:** No safety concerns have been identified following administration of 1 mg and 10 mg psilocybin in combination with 2 mg apomorphine so far. Pending replication of safety data in the 25 mg psilocybin group, we suggest that this combination appears safe to test in a randomized controlled trial‐design to boost arousal (apomorphine) and awareness (psilocybin) in comatose ICU patients.

**Disclosure:** Nothing to disclose.

## EPO‐1062

### Evaluation of CAPE rhythm and clinical outcomes: A single‐center study

#### B. Zeybek; K. Aslan‐Kara

##### Neurology Department, Cukurova University, Adana, Türkiye

**Background and aims:** Cyclic Alternating Pattern of Encephalopathy (CAPE) refers to cyclical fluctuations in background electroencephalography (EEG) activity lasting 10 to 60 seconds, characterized by a spontaneous and regular alternation between two distinct patterns occurring for at least six consecutive cycles. The clinical significance of CAPE remains unclear. In this study, we aimed to evaluate the electroencephalographic characteristics and clinical relevance of CAPE in critically ill patients.

**Methods:** We retrospectively reviewed EEG recordings obtained from patients hospitalized in the intensive care units of Cukurova University Hospital between 2022–2025. These patients were categorized according to age, sex, indication for intensive care admission, length of stay, clinical outcome. In addition, CAPE characteristics including the number of cycles, cycle durations, the proportions of pattern 1 and pattern 2 relative to the total CAPE duration and EEG recording time were analyzed.

**Results:** Among 371 patients with a total of 521 EEG recordings, CAPE rhythm was identified in 3.5% (*n* = 13). The mean age was 50.8 years (range 20–79), and 9 patients (69.2%) were female. Etiology for hospitalization were CVD (23.1%), CNS infection (15.4 %), metabolic encephalopathy (23.1%), intoxication (23.1 %), intracranial malignancy and NORCE (15.4%). Mortality rate was 53.8 %. During EEG recording, 53.8% (*n* = 7) of patients had a GCS score ≥11. The associations between CAPE ratio, pattern 1, and pattern 2 with mortality were not statistically significant (all *p* > 0.05).

**Conclusion:** CAPE is an increasingly recognized EEG pattern, particularly in critically ill patients. In this retrospective study, the mortality ratio was not affected according to CAPE findings.

**Disclosure:** Nothing to disclose.

## EPO‐1063

### Non‐convulsive status epilepticus: Treatment adequacy matters more than treatment sequence

#### F. Misirocchi

##### University Hospital of Parma, Parma, Italy

**Background and aims:** Non‐convulsive status epilepticus (NCSE) is often managed using algorithms derived from convulsive SE, despite major differences in etiological heterogeneity, diagnostic uncertainty, and patient vulnerability. Benzodiazepines (BZDs) and continuous intravenous anesthetic drugs (CIVADs) are frequently avoided due to comorbidities, respiratory risk, and benefit–risk balance. Although deviation from guideline‐recommended SE treatment is associated with adverse outcomes, its clinical relevance in NCSE remains unclear.

**Methods:** Adult NCSE episodes were extracted from a six‐year ambispective non‐anoxic SE registry and classified using Salzburg criteria. Etiology was categorized according to ILAE and grouped as potentially fatal or non‐potentially fatal; acute etiologies were further classified as primary or secondary CNS pathology. First‐line BZD use was defined as administration before any antiseizure medication (ASM). ASMs underdosing was defined as a loading dose <⅔ of the recommended weight‐adjusted dose. Outcomes included failure of NCSE termination, in‐hospital mortality, and worsening of the modified Rankin Scale (mRS).

**Results:** We analyzed 189 NCSE episodes (median age 74). Failure to NCSE cessation occurred in 12.1%, in‐hospital mortality in 31.7%, and mRS worsening in 63.0%. First‐line BZDs were used in 50.8% of episodes and were not associated with outcomes. ASM underdosing occurred in 38.6% and was independently associated with failure to NCSE cessation (OR6.56, *p* = 0.002) and in‐hospital mortality (OR 4.38, *p* < 0.001). CIVADs were used in 21.7% and were not independently associated with outcomes. The negative impact of ASMs underdosing was most evident in acute primary CNS and non‐potentially fatal etiologies.

**Figure:**
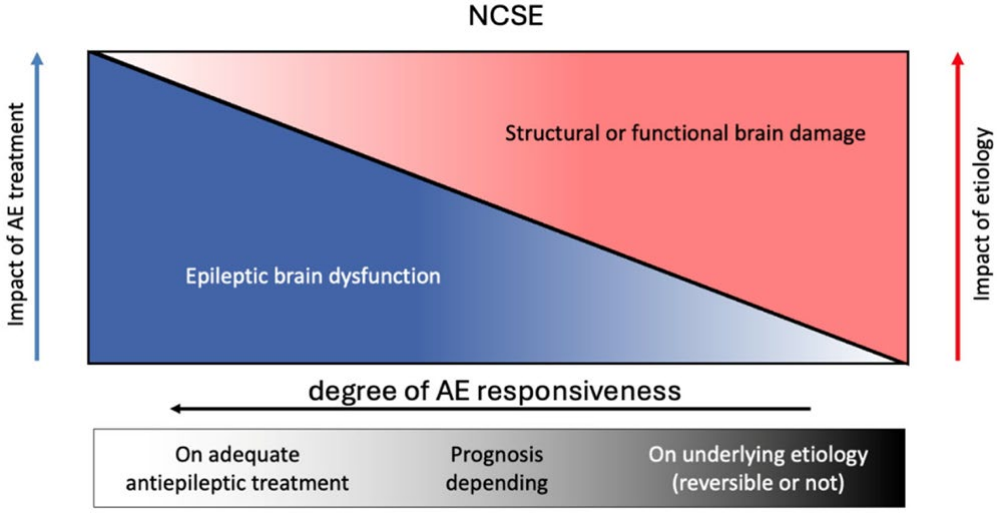
**FIGURE 1** Conceptual model of NCSE showing the inverse relationship between antiepileptic treatment responsiveness and etiological impact on outcome.

**Conclusion:** In NCSE, adequate ASM dosing is more strongly associated with outcome than adherence to treatment sequence, supporting a personalized, etiology‐driven treatment approach.

**Disclosure:** Nothing to disclose.

## EPO‐1064

### Altered level of consciousness in a tertiary emergency department: Etiologies, mortality, and predictors of outcome in a contemporary cohort

#### K. Kim; Y. Cho

##### Keimyung University School of Medicine, Daegu, Republic of Korea

**Background and aims:** Altered level of consciousness (ALC) is an emergency department (ED) presentation with diverse etiologies and substantial mortality. This study aimed to describe the incidence, etiologic distribution, and mortality of ALC in the ED and to identify predictors of death.

**Methods:** This is a retrospective single‐center cohort study of adults presenting with ALC to a tertiary ED between September 2023 and August 2025. ALC was defined as a Glasgow Coma Scale (GCS) score <15 or clearly abnormal orientation or behavior compared with baseline. Etiologies were categorized into ten predefined groups based on structured chart review. Logistic regression analyses were performed to identify baseline clinical predictors of overall mortality.

**Figure:**
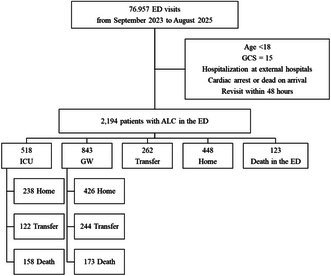
**FIGURE 1** Flowchart of patients including dispositions and destinations. ALC, altered level of consciousness; ED, emergency department; GCS, Glasgow Coma Scale; GW, general ward; ICU, intensive care unit; Transfer, transferred to another hospital.

**Results:** Among 76,957 ED visits, 2,194 (2.85%) involved ALC. Systemic Infection was the most frequent etiology (*n* = 516, 23.5%), followed by Metabolic Cause (*n* = 502, 22.9%) and Stroke (*n* = 325, 14.8%). Overall mortality of ALC was 20.7%, consisting of 5.6% and 24.3% in the ED and in‐hospital, respectively. The highest overall mortality occurred in Central Nervous System Infection (CNS‐i) (40.9%), Stroke (31.7%), and Systemic Infection (30.6%). In the multivariable model, older age, lower initial GCS, vasopressor use, and ICU admission were independently associated with increased odds of death, whereas ED length of stay was not.

**Figure:**
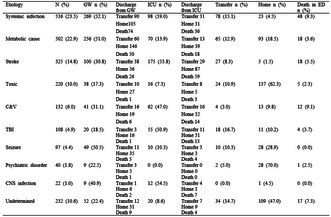
**TABLE 1** Disposition and post‐admission outcomes by etiology.

**Figure:**
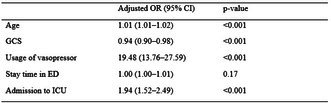
**TABLE 2** Independent and severity‐related factors associated with mortality.

**Conclusion:** Mortality varied markedly across etiologic categories, with the highest risk observed for CNS‐i and Stroke. These findings highlight the prognostic significance of age, initial neurologic status, and underlying etiology and may assist in risk stratification and early resource allocation.

**Disclosure:** Nothing to disclose.

## EPO‐1065

### Impact of extreme temperatures on emergency department visits among persons with neurological disorders

#### C. Zenesini^1^; L. Vignatelli ^1^; E. Baldin^1^; P. Pandolfi^2^; M. Musti^2^; V. Perlangeli^2^; G. Zanutto^2^; I. Frageri^1^; M. Marinelli^1^; A. Zini^1^; S. Forlivesi^1^; L. Muccioli^1^; L. Ferri^1^; F. Bisulli^1^; G. Calandra‐Buonaura^3^; V. Vacchiano^1^; V. Favoni^1^; S. Cevoli^1^; G. Loddo^1^; F. Tavalazzi^4^; F. Provini^3^; F. Nonino^1^

##### ^1^IRCCS Istituto delle Scienze Neurologiche di Bologna, Bologna, Italy; ^2^Azienda USL di Bologna, Bologna, Italy; ^3^DIBINEM, Università degli Studi di Bologna, Bologna, Italy; ^4^Azienda USL Romagna, Bologna, Italy

**Background and aims:** Climate change may crucially impact on various neurological diseases, which affect 40% of people globally. Socioeconomic status and green spaces could act as mitigating factors, promoting physical activity, social interaction, and psychological well‐being. Objectives: (1) to evaluate the impact of extreme temperatures on emergency department visits (EDVs) among cohorts of Bologna residents with Parkinson's disease, atypical parkinsonism, epilepsy, dementia, amyotrophic lateral sclerosis, obstructive sleep apnoea, migraine, stroke; (2) to investigate the moderating role of socioeconomic status and green spaces.

**Methods:** Case‐crossover design (Figure). Cohorts will be identified through record‐linkage systems (Table 1) and compared to the adult resident population of Bologna (2015–2024). The mean daily apparent temperature represents the exposure. The primary outcome is the number of EDVs. Effect modification will be assessed for age, sex, Deprivation Index and Normalized Difference Vegetation Index.

**Figure:**
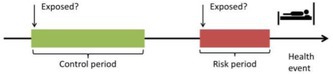
**FIGURE 1** Case cross‐over design: the control period is selected using a time‐stratified approach, in which each case is matched to control values recorded on the same subject in different time points.

**Results:** Sample sizes and data sources for the cohorts are reported in Table 1 Preliminary results on the association between mean daily apparent temperature and EDVs in the reference population (*N* = 190,971) are in Table 2 A one‐unit increase in mean apparent temperature was significantly associated with increased odds of EDVs (OR 1.0026, 95%CI 1.0015–1.0037) with a stronger effect observed for each 1°C increase above the 26 °C threshold (1.0044, 1.0015–1.0072). These associations will be further compared across the specific cohorts of patients.

**Figure:**
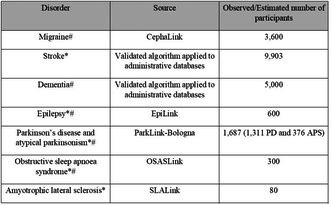
**TABLE 1** Sample sizes (observed and estimated) and data sources for the cohorts of patients included in the study.

**Figure:**
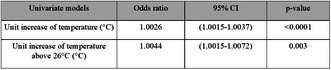
**TABLE 2** Odds ratios (OR) of emergency department visits (EDVs) per 1°C increase in mean apparent temperature, for the entire temperature range and above the 26°C threshold, with corresponding 95% confidence intervals (CI).

**Conclusion:** The project's findings may enhance our understanding of the role of extreme temperatures in the progression of neurological conditions and inform public health policies and preventive strategies to mitigate their impact on vulnerable populations.

**Disclosure:** Funded by the Fondazione del Monte di Bologna e Ravenna (grant number 2025.0163) and by IRCCS ISNB (Bando Ricerca Corrente 2025).

## EPO‐1066

### Endothelial activation and stress index (EASIX) as a predictor of delayed cerebral ischemia and functional outcome in subarachnoid hemorrhage

#### N. Yıldız Akbulut; A. Önalan; E. Gürkaş

##### Kartal Dr Lutfi Kırdar City Hospital*,* Istanbul, Türkiye

**Background and aims:** Endothelial dysfunction contributes to secondary brain injury and poor outcomes following subarachnoid hemorrhage (SAH). The Endothelial Activation and Stress Index (EASIX), derived from routine laboratory parameters, has recently emerged as a marker of endothelial stress. This study aimed to evaluate the relationship between admission EASIX values and clinical outcomes in both aneurysmal and non‐aneurysmal SAH.

**Methods:** We retrospectively analyzed 90 patients admitted to the neurointensive care unit with SAH between January 2020 and June 2025. EASIX was calculated within the first 2 h of admission as: EASIX = (LDH × Creatinine)/Platelet count. Clinical variables included age, sex, aneurysm presence, Glasgow Coma Scale (GCS), Fisher grade, Hunt–Hess, and WFNS scores. Delayed cerebral ischemia (DCI) was defined according to AHA/ASA guidelines. Logistic regression was used to identify independent predictors of DCI and 3‐month functional outcomes (mRS).

**Results:** DCI occurred in 37.7% of patients, and poor functional outcomes (mRS ≥ 3) in 35.6%. Patients with DCI had significantly higher EASIX values (median 1.01 [IQR 0.57] vs 0.64 [IQR 0.28], *p* = 0.001). Multivariable analysis identified higher EASIX (OR = 26.3, *p* = 0.004) and aneurysm presence (OR = 7.7, *p* = 0.005) as independent predictors of DCI. For functional outcome, both EASIX (OR = 2.83, *p* = 0.039) and age (OR = 1.05, *p* = 0.036) independently predicted poor prognosis.

**Conclusion:** EASIX independently predicts delayed cerebral ischemia and poor 3‐month functional outcome in SAH, supporting its potential as a simple, readily available biomarker of endothelial stress in neurocritical care.

**Disclosure:** Nothing to disclose.

## EPO‐1067

### Precision glycemic management for diabetic neuropathy in the ICU: The HYPO‐AWARE score in 96,129 admissions

#### S. Biswas^1^; S. Vasireddy ^2^; Y. Srivastava^1^; S. Arora^3^; J. Rajamani^4^; K. Jindal^5^; F. GOHIL^6^

##### ^1^Department of Internal Medicine, Ivano‐Frankivsk National Medical University, Ivano‐Frankivsk, Ukraine; ^2^Department of Neurology, NMC Specialty Hospital, Abu Dhabi, United Arab Emirates; ^3^Department of Internal Medicine, University of Debrecen, Hungary; ^4^Department of Internal Medicine Medical College, Caucasus University, Tbilisi, Georgia; ^5^Department of Internal Medicine Government Medical College, Patiala, India; ^6^Università Cattolica del Sacro Cuore, Rome, Italy

**Background and aims:** Diabetic neuropathy affects 50% of diabetic ICU patients, yet current glycemic protocols ignore neuropathy status despite impaired counterregulation and gastroparesis fundamentally altering glucose homeostasis. Existing hypoglycemia prediction tools achieve only modest discrimination (AUC 0.66–0.83) and exclude neuropathy‐specific factors.

**Methods:** We analyzed 96,129 ICU admissions with 3,093,411 glucose measurements from MIMIC‐IV v3.1 (2008–2022), comparing diabetic patients with neuropathy (*n* = 42,672), diabetic controls (*n* = 37,202), and non‐diabetics (*n* = 16,255). We developed the HYPO‐AWARE score incorporating gastroparesis, autonomic dysfunction, hypoglycemia frequency, and glucose variability, then evaluated prediction performance using gradient boosting models with 5‐fold cross‐validation.

**Results:** Neuropathy patients exhibited paradoxically lower mean glucose (148.76 vs 156.41 mg/dL, *p* < 0.001) but significantly higher coefficient of variation (26.20% vs 22.99%, *p* < 0.001). Feature importance analysis revealed glucose variability dominated hypoglycemia prediction (56.6%) over mean glucose (30.9%). The HYPO‐AWARE score achieved AUC 0.92 (95% CI: 0.92–0.93), substantially outperforming existing tools. Risk stratification effectively separated hypoglycemia incidence: low‐risk 8.2%, moderate‐risk 18.4%, high‐risk 41.2% (*p* < 0.001 for trend). Only 49.0% of neuropathy patients achieved adequate time‐in‐range (>70%) compared to 81.1% of non‐diabetics, representing systematic failure of universal protocols.

**Figure:**
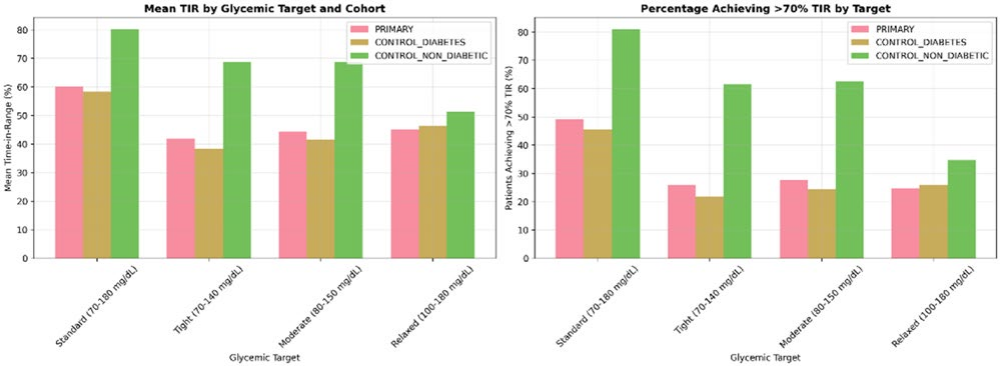
FIGURE 1

**Figure:**
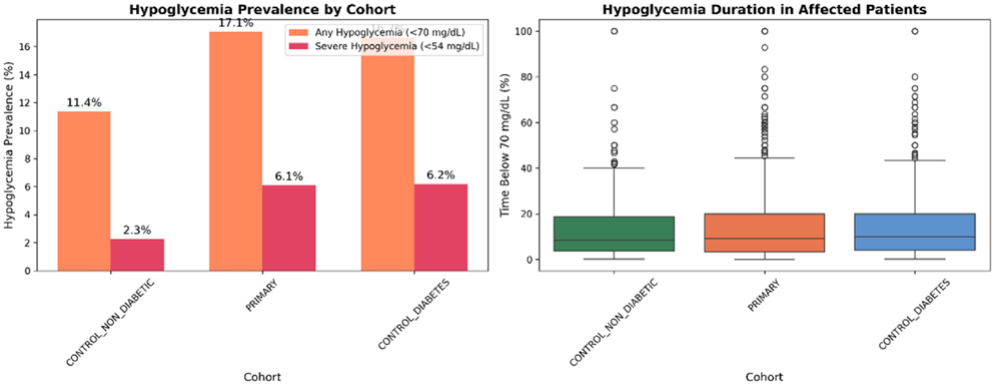
FIGURE 2

**Figure:**
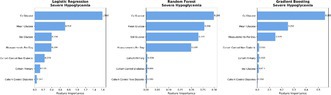
FIGURE 3

**Conclusion:** Diabetic neuropathy creates a distinct glycemic phenotype requiring targeted management strategies. The HYPO‐AWARE score provides validated risk stratification substantially superior to existing tools. Current one‐size‐fits‐all ICU glucose protocols fail half of neuropathy patients, demanding precision medicine approaches that prioritize variability reduction over mean glucose targets and incorporate neuropathy‐specific pathophysiology.

**Disclosure:** Nothing to disclose.

